# Global, regional, and national comparative risk assessment of 79 behavioural, environmental and occupational, and metabolic risks or clusters of risks, 1990–2015: a systematic analysis for the Global Burden of Disease Study 2015

**DOI:** 10.1016/S0140-6736(16)31679-8

**Published:** 2016-10-08

**Authors:** GBD 2015 Risk Factors Collaborators, Mohammad H Forouzanfar, Ashkan Afshin, Lily T Alexander, H Ross Anderson, Zulfiqar A Bhutta, Stan Biryukov, Michael Brauer, Richard Burnett, Kelly Cercy, Fiona J Charlson, Aaron J Cohen, Lalit Dandona, Kara Estep, Alize J Ferrari, Joseph J Frostad, Nancy Fullman, Peter W Gething, William W Godwin, Max Griswold, Simon I Hay, Yohannes Kinfu, Hmwe H Kyu, Heidi J Larson, Xiaofeng Liang, Stephen S Lim, Patrick Y Liu, Alan D Lopez, Rafael Lozano, Laurie Marczak, George A Mensah, Ali H Mokdad, Maziar Moradi-Lakeh, Mohsen Naghavi, Bruce Neal, Marissa B Reitsma, Gregory A Roth, Joshua A Salomon, Patrick J Sur, Theo Vos, Joseph A Wagner, Haidong Wang, Yi Zhao, Maigeng Zhou, Gunn Marit Aasvang, Amanuel Alemu Abajobir, Kalkidan Hassen Abate, Cristiana Abbafati, Kaja M Abbas, Foad Abd-Allah, Abdishakur M Abdulle, Semaw Ferede Abera, Biju Abraham, Laith J Abu-Raddad, Gebre Yitayih Abyu, Akindele Olupelumi Adebiyi, Isaac Akinkunmi Adedeji, Zanfina Ademi, Arsène Kouablan Adou, José C Adsuar, Emilie Elisabet Agardh, Arnav Agarwal, Anurag Agrawal, Aliasghar Ahmad Kiadaliri, Oluremi N Ajala, Tomi F Akinyemiju, Ziyad Al-Aly, Khurshid Alam, Noore K M Alam, Saleh Fahed Aldhahri, Robert William Aldridge, Zewdie Aderaw Alemu, Raghib Ali, Ala'a Alkerwi, François Alla, Peter Allebeck, Ubai Alsharif, Khalid A Altirkawi, Elena Alvarez Martin, Nelson Alvis-Guzman, Azmeraw T Amare, Alemayehu Amberbir, Adeladza Kofi Amegah, Heresh Amini, Walid Ammar, Stephen Marc Amrock, Hjalte H Andersen, Benjamin O Anderson, Carl Abelardo T Antonio, Palwasha Anwari, Johan Ärnlöv, Al Artaman, Hamid Asayesh, Rana Jawad Asghar, Reza Assadi, Suleman Atique, Euripide Frinel G Arthur Avokpaho, Ashish Awasthi, Beatriz Paulina Ayala Quintanilla, Peter Azzopardi, Umar Bacha, Alaa Badawi, Maria C Bahit, Kalpana Balakrishnan, Aleksandra Barac, Ryan M Barber, Suzanne L Barker-Collo, Till Bärnighausen, Simon Barquera, Lars Barregard, Lope H Barrero, Sanjay Basu, Carolina Batis, Shahrzad Bazargan-Hejazi, Justin Beardsley, Neeraj Bedi, Ettore Beghi, Brent Bell, Michelle L Bell, Aminu K Bello, Derrick A Bennett, Isabela M Bensenor, Adugnaw Berhane, Eduardo Bernabé, Balem Demtsu Betsu, Addisu Shunu Beyene, Neeraj Bhala, Anil Bhansali, Samir Bhatt, Sibhatu Biadgilign, Boris Bikbov, Donal Bisanzio, Espen Bjertness, Jed D Blore, Rohan Borschmann, Soufiane Boufous, Rupert R A Bourne, Michael Brainin, Alexandra Brazinova, Nicholas J K Breitborde, Hermann Brenner, David M Broday, Traolach S Brugha, Bert Brunekreef, Zahid A Butt, Leah E Cahill, Bianca Calabria, Ismael Ricardo Campos-Nonato, Rosario Cárdenas, David O Carpenter, Juan Jesus Carrero, Daniel C Casey, Carlos A Castañeda-Orjuela, Jacqueline Castillo Rivas, Ruben Estanislao Castro, Ferrán Catalá-López, Jung-Chen Chang, Peggy Pei-Chia Chiang, Mirriam Chibalabala, Odgerel Chimed-Ochir, Vesper Hichilombwe Chisumpa, Abdulaal A Chitheer, Jee-Young Jasmine Choi, Hanne Christensen, Devasahayam Jesudas Christopher, Liliana G Ciobanu, Matthew M Coates, Samantha M Colquhoun, Alejandra G Contreras Manzano, Leslie Trumbull Cooper, Kimberly Cooperrider, Leslie Cornaby, Monica Cortinovis, John A Crump, Lucia Cuevas-Nasu, Albertino Damasceno, Rakhi Dandona, Sarah C Darby, Paul I Dargan, José das Neves, Adrian C Davis, Kairat Davletov, E Filipa de Castro, Vanessa De la Cruz-Góngora, Diego De Leo, Louisa Degenhardt, Liana C Del Gobbo, Borja del Pozo-Cruz, Robert P Dellavalle, Amare Deribew, Don C Des Jarlais, Samath D Dharmaratne, Preet K Dhillon, Cesar Diaz-Torné, Daniel Dicker, Eric L Ding, E Ray Dorsey, Kerrie E Doyle, Tim R Driscoll, Leilei Duan, Manisha Dubey, Bruce Bartholow Duncan, Iqbal Elyazar, Aman Yesuf Endries, Sergey Petrovich Ermakov, Holly E Erskine, Babak Eshrati, Alireza Esteghamati, Saman Fahimi, Emerito Jose Aquino Faraon, Talha A Farid, Carla Sofia e Sa Farinha, André Faro, Maryam S Farvid, Farshad Farzadfar, Valery L Feigin, Seyed-Mohammad Fereshtehnejad, Jefferson G Fernandes, Florian Fischer, Joseph R A Fitchett, Tom Fleming, Nataliya Foigt, Kyle Foreman, F Gerry R Fowkes, Richard C Franklin, Thomas Fürst, Neal D Futran, Emmanuela Gakidou, Alberto L Garcia-Basteiro, Tsegaye Tewelde Gebrehiwot, Amanuel Tesfay Gebremedhin, Johanna M Geleijnse, Bradford D Gessner, Ababi Zergaw Giref, Maurice Giroud, Melkamu Dedefo Gishu, Giorgia Giussani, Shifalika Goenka, Mari Carmen Gomez-Cabrera, Hector Gomez-Dantes, Philimon Gona, Amador Goodridge, Sameer Vali Gopalani, Carolyn C Gotay, Atsushi Goto, Hebe N Gouda, Harish Chander Gugnani, Francis Guillemin, Yuming Guo, Rahul Gupta, Rajeev Gupta, Reyna A Gutiérrez, Juanita A Haagsma, Nima Hafezi-Nejad, Demewoz Haile, Gessessew Bugssa Hailu, Yara A Halasa, Randah Ribhi Hamadeh, Samer Hamidi, Alexis J Handal, Graeme J Hankey, Yuantao Hao, Hilda L Harb, Sivadasanpillai Harikrishnan, Josep Maria Haro, Mohammad Sadegh Hassanvand, Tahir Ahmed Hassen, Rasmus Havmoeller, Ileana Beatriz Heredia-Pi, Norberto Francisco Hernández-Llanes, Pouria Heydarpour, Hans W Hoek, Howard J Hoffman, Masako Horino, Nobuyuki Horita, H Dean Hosgood, Damian G Hoy, Mohamed Hsairi, Aung Soe Htet, Guoqing Hu, John J Huang, Abdullatif Husseini, Sally J Hutchings, Inge Huybrechts, Kim Moesgaard Iburg, Bulat T Idrisov, Bogdan Vasile Ileanu, Manami Inoue, Troy A Jacobs, Kathryn H Jacobsen, Nader Jahanmehr, Mihajlo B Jakovljevic, Henrica A F M Jansen, Simerjot K Jassal, Mehdi Javanbakht, Sudha P Jayaraman, Achala Upendra Jayatilleke, Sun Ha Jee, Panniyammakal Jeemon, Vivekanand Jha, Ying Jiang, Tariku Jibat, Ye Jin, Catherine O Johnson, Jost B Jonas, Zubair Kabir, Yogeshwar Kalkonde, Ritul Kamal, Haidong Kan, André Karch, Corine Kakizi Karema, Chante Karimkhani, Amir Kasaeian, Anil Kaul, Norito Kawakami, Dhruv S Kazi, Peter Njenga Keiyoro, Laura Kemmer, Andrew Haddon Kemp, Andre Pascal Kengne, Andre Keren, Chandrasekharan Nair Kesavachandran, Yousef Saleh Khader, Abdur Rahman Khan, Ejaz Ahmad Khan, Gulfaraz Khan, Young-Ho Khang, Shahab Khatibzadeh, Sahil Khera, Tawfik Ahmed Muthafer Khoja, Jagdish Khubchandani, Christian Kieling, Cho-il Kim, Daniel Kim, Ruth W Kimokoti, Niranjan Kissoon, Miia Kivipelto, Luke D Knibbs, Yoshihiro Kokubo, Jacek A Kopec, Parvaiz A Koul, Ai Koyanagi, Michael Kravchenko, Hans Kromhout, Hans Krueger, Tiffany Ku, Barthelemy Kuate Defo, Ricardo S Kuchenbecker, Burcu Kucuk Bicer, Ernst J Kuipers, G Anil Kumar, Gene F Kwan, Dharmesh Kumar Lal, Ratilal Lalloo, Tea Lallukka, Qing Lan, Anders Larsson, Asma Abdul Latif, Alicia Elena Beatriz Lawrynowicz, Janet L Leasher, James Leigh, Janni Leung, Miriam Levi, Xiaohong Li, Yichong Li, Juan Liang, Shiwei Liu, Belinda K Lloyd, Giancarlo Logroscino, Paulo A Lotufo, Raimundas Lunevicius, Michael MacIntyre, Mahdi Mahdavi, Marek Majdan, Azeem Majeed, Reza Malekzadeh, Deborah Carvalho Malta, Wondimu Ayele Ayele Manamo, Chabila C Mapoma, Wagner Marcenes, Randall V Martin, Jose Martinez-Raga, Felix Masiye, Kunihiro Matsushita, Richard Matzopoulos, Bongani M Mayosi, John J McGrath, Martin McKee, Peter A Meaney, Catalina Medina, Alem Mehari, Fabiola Mejia-Rodriguez, Alemayehu B Mekonnen, Yohannes Adama Melaku, Ziad A Memish, Walter Mendoza, Gert B M Mensink, Atte Meretoja, Tuomo J Meretoja, Yonatan Moges Mesfin, Francis Apolinary Mhimbira, Anoushka Millear, Ted R Miller, Edward J Mills, Mojde Mirarefin, Awoke Misganaw, Charles N Mock, Alireza Mohammadi, Shafiu Mohammed, Glen Liddell D Mola, Lorenzo Monasta, Julio Cesar Montañez Hernandez, Marcella Montico, Lidia Morawska, Rintaro Mori, Dariush Mozaffarian, Ulrich O Mueller, Erin Mullany, John Everett Mumford, Gudlavalleti Venkata Satyanarayana Murthy, Jean B Nachega, Aliya Naheed, Vinay Nangia, Nariman Nassiri, John N Newton, Marie Ng, Quyen Le Nguyen, Muhammad Imran Nisar, Patrick Martial Nkamedjie Pete, Ole F Norheim, Rosana E Norman, Bo Norrving, Luke Nyakarahuka, Carla Makhlouf Obermeyer, Felix Akpojene Ogbo, In-Hwan Oh, Olanrewaju Oladimeji, Pedro R Olivares, Helen Olsen, Bolajoko Olubukunola Olusanya, Jacob Olusegun Olusanya, John Nelson Opio, Eyal Oren, Ricardo Orozco, Alberto Ortiz, Erika Ota, Mahesh PA, Adrian Pana, Eun-Kee Park, Charles D Parry, Mahboubeh Parsaeian, Tejas Patel, Angel J Paternina Caicedo, Snehal T Patil, Scott B Patten, George C Patton, Neil Pearce, David M Pereira, Norberto Perico, Konrad Pesudovs, Max Petzold, Michael Robert Phillips, Frédéric B Piel, Julian David Pillay, Dietrich Plass, Suzanne Polinder, Constance D Pond, C Arden Pope, Daniel Pope, Svetlana Popova, Richie G Poulton, Farshad Pourmalek, Noela M Prasad, Mostafa Qorbani, Rynaz H S Rabiee, Amir Radfar, Anwar Rafay, Vafa Rahimi-Movaghar, Mahfuzar Rahman, Mohammad Hifz Ur Rahman, Sajjad Ur Rahman, Rajesh Kumar Rai, Sasa Rajsic, Murugesan Raju, Usha Ram, Saleem M Rana, Kavitha Ranganathan, Puja Rao, Christian Aspacia Razo García, Amany H Refaat, Colin D Rehm, Jürgen Rehm, Nikolas Reinig, Giuseppe Remuzzi, Serge Resnikoff, Antonio L Ribeiro, Juan A Rivera, Hirbo Shore Roba, Alina Rodriguez, Sonia Rodriguez-Ramirez, David Rojas-Rueda, Yesenia Roman, Luca Ronfani, Gholamreza Roshandel, Dietrich Rothenbacher, Ambuj Roy, Muhammad Muhammad Saleh, Juan R Sanabria, Lidia Sanchez-Riera, Maria Dolores Sanchez-Niño, Tania G Sánchez-Pimienta, Logan Sandar, Damian F Santomauro, Itamar S Santos, Rodrigo Sarmiento-Suarez, Benn Sartorius, Maheswar Satpathy, Miloje Savic, Monika Sawhney, Josef Schmidhuber, Maria Inês Schmidt, Ione J C Schneider, Ben Schöttker, Aletta E Schutte, David C Schwebel, James G Scott, Soraya Seedat, Sadaf G Sepanlou, Edson E Servan-Mori, Gavin Shaddick, Amira Shaheen, Saeid Shahraz, Masood Ali Shaikh, Teresa Shamah Levy, Rajesh Sharma, Jun She, Sara Sheikhbahaei, Jiabin Shen, Kevin N Sheth, Peilin Shi, Kenji Shibuya, Mika Shigematsu, Min-Jeong Shin, Rahman Shiri, Kawkab Shishani, Ivy Shiue, Mark G Shrime, Inga Dora Sigfusdottir, Diego Augusto Santos Silva, Dayane Gabriele Alves Silveira, Jonathan I Silverberg, Edgar P Simard, Shireen Sindi, Abhishek Singh, Jasvinder A Singh, Prashant Kumar Singh, Erica Leigh Slepak, Michael Soljak, Samir Soneji, Reed J D Sorensen, Luciano A Sposato, Chandrashekhar T Sreeramareddy, Vasiliki Stathopoulou, Nadine Steckling, Nicholas Steel, Dan J Stein, Murray B Stein, Heidi Stöckl, Saverio Stranges, Konstantinos Stroumpoulis, Bruno F Sunguya, Soumya Swaminathan, Bryan L Sykes, Cassandra E I Szoeke, Rafael Tabarés-Seisdedos, Ken Takahashi, Roberto Tchio Talongwa, Nikhil Tandon, David Tanne, Mohammad Tavakkoli, Belaynew Wasie Taye, Hugh R Taylor, Bemnet Amare Tedla, Worku Mekonnen Tefera, Teketo Kassaw Tegegne, Dejen Yemane Tekle, Abdullah Sulieman Terkawi, J S Thakur, Bernadette A Thomas, Matthew Lloyd Thomas, Alan J Thomson, Andrew L Thorne-Lyman, Amanda G Thrift, George D Thurston, Taavi Tillmann, Ruoyan Tobe-Gai, Myriam Tobollik, Roman Topor-Madry, Fotis Topouzis, Jeffrey Allen Towbin, Bach Xuan Tran, Zacharie Tsala Dimbuene, Nikolaos Tsilimparis, Abera Kenay Tura, Emin Murat Tuzcu, Stefanos Tyrovolas, Kingsley N Ukwaja, Eduardo A Undurraga, Chigozie Jesse Uneke, Olalekan A Uthman, Aaron van Donkelaar, Jim van Os, Yuri Y Varakin, Tommi Vasankari, J Lennert Veerman, Narayanaswamy Venketasubramanian, Francesco S Violante, Stein Emil Vollset, Gregory R Wagner, Stephen G Waller, Jian Li Wang, Linhong Wang, Yanping Wang, Scott Weichenthal, Elisabete Weiderpass, Robert G Weintraub, Andrea Werdecker, Ronny Westerman, Harvey A Whiteford, Tissa Wijeratne, Charles Shey Wiysonge, Charles D A Wolfe, Sungho Won, Anthony D Woolf, Mamo Wubshet, Denis Xavier, Gelin Xu, Ajit Kumar Yadav, Bereket Yakob, Ayalnesh Zemene Yalew, Yuichiro Yano, Mehdi Yaseri, Pengpeng Ye, Paul Yip, Naohiro Yonemoto, Seok-Jun Yoon, Mustafa Z Younis, Chuanhua Yu, Zoubida Zaidi, Maysaa El Sayed Zaki, Jun Zhu, Ben Zipkin, Sanjay Zodpey, Liesl Joanna Zuhlke, Christopher J L Murray

## Abstract

**Background:**

The Global Burden of Diseases, Injuries, and Risk Factors Study 2015 provides an up-to-date synthesis of the evidence for risk factor exposure and the attributable burden of disease. By providing national and subnational assessments spanning the past 25 years, this study can inform debates on the importance of addressing risks in context.

**Methods:**

We used the comparative risk assessment framework developed for previous iterations of the Global Burden of Disease Study to estimate attributable deaths, disability-adjusted life-years (DALYs), and trends in exposure by age group, sex, year, and geography for 79 behavioural, environmental and occupational, and metabolic risks or clusters of risks from 1990 to 2015. This study included 388 risk-outcome pairs that met World Cancer Research Fund-defined criteria for convincing or probable evidence. We extracted relative risk and exposure estimates from randomised controlled trials, cohorts, pooled cohorts, household surveys, census data, satellite data, and other sources. We used statistical models to pool data, adjust for bias, and incorporate covariates. We developed a metric that allows comparisons of exposure across risk factors—the summary exposure value. Using the counterfactual scenario of theoretical minimum risk level, we estimated the portion of deaths and DALYs that could be attributed to a given risk. We decomposed trends in attributable burden into contributions from population growth, population age structure, risk exposure, and risk-deleted cause-specific DALY rates. We characterised risk exposure in relation to a Socio-demographic Index (SDI).

**Findings:**

Between 1990 and 2015, global exposure to unsafe sanitation, household air pollution, childhood underweight, childhood stunting, and smoking each decreased by more than 25%. Global exposure for several occupational risks, high body-mass index (BMI), and drug use increased by more than 25% over the same period. All risks jointly evaluated in 2015 accounted for 57·8% (95% CI 56·6–58·8) of global deaths and 41·2% (39·8–42·8) of DALYs. In 2015, the ten largest contributors to global DALYs among Level 3 risks were high systolic blood pressure (211·8 million [192·7 million to 231·1 million] global DALYs), smoking (148·6 million [134·2 million to 163·1 million]), high fasting plasma glucose (143·1 million [125·1 million to 163·5 million]), high BMI (120·1 million [83·8 million to 158·4 million]), childhood undernutrition (113·3 million [103·9 million to 123·4 million]), ambient particulate matter (103·1 million [90·8 million to 115·1 million]), high total cholesterol (88·7 million [74·6 million to 105·7 million]), household air pollution (85·6 million [66·7 million to 106·1 million]), alcohol use (85·0 million [77·2 million to 93·0 million]), and diets high in sodium (83·0 million [49·3 million to 127·5 million]). From 1990 to 2015, attributable DALYs declined for micronutrient deficiencies, childhood undernutrition, unsafe sanitation and water, and household air pollution; reductions in risk-deleted DALY rates rather than reductions in exposure drove these declines. Rising exposure contributed to notable increases in attributable DALYs from high BMI, high fasting plasma glucose, occupational carcinogens, and drug use. Environmental risks and childhood undernutrition declined steadily with SDI; low physical activity, high BMI, and high fasting plasma glucose increased with SDI. In 119 countries, metabolic risks, such as high BMI and fasting plasma glucose, contributed the most attributable DALYs in 2015. Regionally, smoking still ranked among the leading five risk factors for attributable DALYs in 109 countries; childhood underweight and unsafe sex remained primary drivers of early death and disability in much of sub-Saharan Africa.

**Interpretation:**

Declines in some key environmental risks have contributed to declines in critical infectious diseases. Some risks appear to be invariant to SDI. Increasing risks, including high BMI, high fasting plasma glucose, drug use, and some occupational exposures, contribute to rising burden from some conditions, but also provide opportunities for intervention. Some highly preventable risks, such as smoking, remain major causes of attributable DALYs, even as exposure is declining. Public policy makers need to pay attention to the risks that are increasingly major contributors to global burden.

**Funding:**

Bill & Melinda Gates Foundation.

**Research in context**

**Evidence before this study**

The most recent assessment of attributable deaths and disability-adjusted life-years (DALYs) at the global, regional, and national level was the Global Burden of Diseases, Injuries, and Risk Factors Study 2013, which covered 79 risk factors or combinations of risks from 1990 to 2013 in 188 countries.

**Added value of this study**

This study (the Global Burden of Diseases, Injuries, and Risk Factors Study 2015) incorporates recently published studies, newly acquired data for exposure to relative risks, and new risk-outcome pairs meeting study inclusion criteria. To enhance transparency of the supporting evidence, we provided an assessment of the strength of evidence supporting causality for all 388 risk-outcome pairs. For the first time, we separately assessed trends in risk exposure by computing a summary exposure value, which allows comparisons over time and across place for dichotomous, polytomous, and continuous risks. Quantification of exposure trends allowed decomposition of trends in attributable DALYs into the portion contributed by changes in population growth, population structure, exposure, and risk-deleted DALY rates. We found that reductions in exposure have been key drivers of change for only a small set of environmental risks, including sanitation, household air pollution, and behavioural risks (eg, undernutrition and smoking). For many risks, trends in attributable DALYs have been driven by the interplay between population growth, ageing, and declines in risk-deleted DALY rates. For some risks, including body-mass index, fasting plasma glucose, occupational exposure to carcinogens, and drug use, exposure is increasing and driving up attributable burden. Although an average risk transition has occurred as countries move through the development continuum, many risks initially increase and then decline at the highest development levels. We document leading risks for each country and territory included in the study.

**Implications of all the available evidence**

Risk assessments allow identification of several groups of risk factors that deserve policy attention. Risks such as smoking, unsafe sanitation, and childhood undernutrition still cause many attributable DALYs, but recent trends show that exposure can be reduced. This assessment of risk also shows many large global risks for which changes in exposure are slow, such as high systolic blood pressure, ambient air pollution, diets high in sodium, high cholesterol, and alcohol intake, highlighting huge opportunities for intervention. Two large risks—high BMI and high fasting plasma glucose—have particularly large and concerning increases in exposure.

## Introduction

Analysis of the causes of poor health—specifically, the connections between risk factors and development of poor health—can provide insights into opportunities and priorities for prevention, research, policy, and development. One of the mainstays of modern epidemiology is quantification of elevated risks for particular diseases or injuries from exposure to a given risk factor for groups of individuals. Quantification of elevated risk for exposed groups of individuals from an array of risk-outcome pairs is important to inform decision making on individual health; however, public policy debates require the comprehensive metric of population-level risk, which is a function of elevated risk in the exposed population and the fraction of the population exposed to a given risk. Efforts to measure population risk have combined data for excess risk with the number of individuals exposed to provide comparative quantification of different health risks for populations that have been influential in establishment of policy priorities.^1^, ^2^

The comparative risk assessment (CRA) approach developed for the Global Burden of Diseases, Injuries, and Risk Factors (GBD) Study^3^, ^4^ provides an overarching conceptual framework for population risk assessment across risks and over time. The scale of the GBD Study required extensive work to develop exposure metrics, assess relationships, and compile health data from different parts of the world with differing levels of metadata and uncertainty, and the unique contribution of this work has been broadly recognised.^5^, ^6^, ^7^ A robust debate on specific risks and results emerged after publication of the Global Burden of Diseases, Injuries, and Risk Factors Study 2013 (GBD 2013).^8^ Inclusion and exclusion of particular risks and outcomes;^3^, ^4^, ^9^ the optimum targets for indicators such as high systolic blood pressure,^10^, ^11^ cholesterol,^11^, ^12^ diets high in sodium,^13^ and air pollution;^4^, ^14^ and the certainty of some dietary components of risk^8^, ^15^ were challenged, in addition to some details of methods. Underlying many of these discussions were heterogeneities in the strength of causal evidence for different risk-outcome pairs.^8^

The Global Burden of Diseases, Injuries, and Risk Factors Study 2015 (GBD 2015) CRA, in addition to updating data and methods, adds new transparency about the evidence supporting causal connections for each of the 388 risk-outcome pairs included in the analysis, allows the quantification and reporting of levels and trends in exposure, decomposes changes in attributable burden into population growth, ageing, risk exposure, and risk-deleted disability-adjusted life-year (DALY) rates, and examines how risks change with development. As with all iterations of the GBD Study, GBD 2015 results presented here supersede all previously published GBD CRA estimates.

## Methods

### Overview

The CRA conceptual framework was developed by Murray and Lopez,^16^ who established a causal web of hierarchically organised risks or causes that contribute to health outcomes (methods appendix p 161), which allows quantification of risks or causes at any level in the framework. In GBD 2015, as in previous iterations of the GBD Study, we evaluated a set of behavioural, environmental and occupational, and metabolic risks, where risk-outcome pairs were included based on evidence rules (methods appendix p 161). These risks were organised into four hierarchical levels, described in table 1. To date, we have not quantified the contribution of other classes of risk factors (methods appendix p 161); however, using an analysis of the relationship between risk exposures and development, measured with use of the Socio-demographic Index (SDI), we provide some insights into the potential magnitude of distal social, cultural, and economic factors.

Two types of risk assessments are possible within the CRA framework: attributable burden and avoidable burden. Attributable burden is the reduction in current disease burden that would have been possible if past population exposure had shifted to an alternative or counterfactual distribution of risk exposure. Avoidable burden is the potential reduction in future disease burden that could be achieved by changing the current distribution of exposure to a counterfactual distribution of exposure. Murray and Lopez^16^ identified four types of counterfactual exposure distributions: theoretical, plausible, feasible, and cost-effective minimum risk. In GBD studies to date and in this study, we focus on attributable burden using the theoretical minimum risk level (TMREL), which is the level of risk exposure that minimises risk at the population level, or the level of risk that captures the maximum attributable burden.

Overall, this analysis follows the CRA methods used in GBD 2013.^4^ The methods described in this study provide a high-level overview of the analytical logic, with a focus on areas of notable change from the methods used in GBD 2013, with details provided in the methods appendix. This study complies with the Guidelines for Accurate and Transparent Health Estimates Reporting statement (methods appendix pp 177–79).^17^

### Geographic units of analysis and years for estimation

In the GBD framework, geographies have been arranged as a set of hierarchical categories: seven super-regions, 21 regions nested within the seven super-regions, and 195 countries and territories nested in the 21 regions. Additionally, GBD collaborator interest and availability of data resulted in an expansion of countries for which we disaggregate our estimates at the subnational level. At the first level of subnational division, 256 geographic units are included in GBD 2015. For this study, we present results for the 195 national and territory-level geographies. We produced a complete set of age-specific, sex-specific, cause-specific, and location-specific estimates of risk factor exposure and attributable burden for 1990, 1995, 2000, 2005, 2010, and 2015 for included risk factors. Results presented in this study emphasise results for 1990, 2005, and 2015; online data visualisations provide access to results for all GBD metrics from 1990 to 2015.

### Attributable burden formula

Four key components are included in estimation of the burden attributable to a given risk factor: the metric of burden being assessed (number of deaths, years of life lost [YLLs], years lived with disability [YLDs], or DALYs [the sum of YLLs and YLDs]), the exposure levels for a risk factor, the relative risk of a given outcome due to exposure, and the counterfactual level of risk factor exposure. Estimates of attributable DALYs for a risk-outcome pair are equal to DALYs for the outcome multiplied by the population attributable fraction (PAF) for the risk-outcome pair for a given age, sex, location, and year. A similar logic applies for estimation of attributable deaths, YLLs, or YLDs. Risks are categorised on the basis of how exposure was measured: dichotomous, polytomous, and continuous. The PAF represents the proportion of risk that would be reduced in a given year if the exposure to a risk factor in the past were reduced to a counterfactual level of exposure (methods appendix p 27).

### Causal evidence for risk-outcome pairs

In this study, as in GBD 2013, we have included risk-outcome pairs that we have assessed as meeting the World Cancer Research Fund grades of convincing or probable evidence (methods appendix pp 12–13 contains definitions of these grades).^9^ Table 2 provides a summary of the evidence supporting a causal relationship between a risk and an outcome for each pair included in GBD 2015. For each risk-outcome pair, we used recent systematic reviews to identify independent prospective studies (randomised controlled trials, non-randomised interventions, and cohorts) that evaluated the putative relationship. For risk-outcome pairs for which no recent systematic review was available, we either updated reviews developed for GBD 2013 or did a new systematic search of literature (methods appendix pp 44–159). Table 2 summarises the evidence using multiple dimensions, which supports our assessment that each included risk-outcome pair meets the criteria of convincing or probable evidence (methods appendix [pp 12–13] contains a justification of the criteria presented to support causality). In this summary of evidence, we have focused on randomised controlled trials and prospective observational studies, along with supporting evidence, like dose-response relationships and biologically plausible mechanisms. Other evidence supporting causal connections, such as case-control studies, are not summarised in table 2.

### Estimation process

Information about the data sources, estimation methods, computational tools, and statistical analysis used in derivation of our estimates are provided in the methods appendix. The analytical steps for estimation of burden attributable to single or clusters of risk-outcome pairs are summarised in the methods appendix (p 162). Table 1 provides definitions of exposure for each risk factor, the TMREL used, and metrics of data availability. For each risk, we estimated effect size as a function of age and sex and exposure level, mean exposure, the distribution of exposure across individuals, and the TMREL. The approach taken is largely similar to GBD 2013 for each quantity for each risk. Some methodological improvements have been implemented and new data sources incorporated. The methods appendix (pp 44–159) provides details of each step by risk. Citation information for the data sources used for relative risks are provided in searchable form through a web tool. We estimate the joint effects of combinations of risk factors using the same methods as GBD 2013, namely using published studies to estimate the fraction of a risk that was mediated through the other risk (methods appendix pp 28–35). Relative risks by age and sex for each risk factor and outcome pair are provided in the methods appendix (pp 215–44).

All point estimates are reported with 95% uncertainty intervals (UIs). UIs include uncertainty from each relevant component, consisting of exposure, relative risks, TMREL, and burden rates. Where percentage change is reported (with 95% UIs), we computed it on the basis of the point estimates being compared. In this study, we provide further methodological detail on new extensions to the CRA analysis.

### Summary exposure value calculation

In previous GBD studies, we did not report comparable exposure metrics for the risk factors included because of the complexity of quantification of polytomous and continuous risks.^18^ Because of substantial interest in the trends in exposure, we developed a summary measure of exposure for each risk. This measure, called the summary exposure value (SEV), is the relative risk-weighted prevalence of exposure. Formally, it is defined as:

$$SEV=\frac{\sum_{i=1}^{n}{\mathrm{Pr}}_{i}RR_{i}-1}{RR_{\max}-1}$$

where Pr_i_ is prevalence of category i exposure, RR_i_ is relative risk of the category i, and RR_max_ is the maximum relative risk observed (between categories). This quantity is estimated for each age, sex, location, year, and outcome. For each risk factor, a single SEV is estimated by averaging of the outcome of specific SEV values for each age, sex, location, and year across outcomes. In the case of dichotomous exposure, SEV is equal to prevalence. For continuous risks:

$$SEV=\frac{\int_{x=l}^{u}RR(x)P(x)dx-1}{RR_{\max}-1}$$

where P(x) is the density of exposure at level x of exposure, RR(x) is relative risk of the level x, and RR_max_ is the highest relative risk that is supported by data and reflects a level where more than 1% of the global population are exposed to that level or a higher risk.

SEV takes the value zero when no excess risk for a population exists and the value one when the population is at the highest level of risk; we report SEV on a scale from 0% to 100% to emphasise that it is risk-weighted prevalence. We computed as the level for exposure with the highest relative risk supported by cohort or trial data and for which at least 1% or more of the global population is exposed. For comparison purposes, we have also computed age-standardised SEVs for every risk factor from the most detailed level using the GBD population standard.

### Decomposition of changes in deaths and DALYs into the contribution of population growth, ageing, risk exposure, and risk-deleted DALY rates

We did two related decomposition analyses of changes in DALYs from 1990 to 2015: decomposing changes in cause-specific DALYs due to changes in population growth, population age structure, exposure to all risks for a disease, and risk-deleted death and DALY rates; and decomposing changes in risk-attributable all-cause DALYs due to changes in population growth, population age structure, risk exposure to the single risk factor, and risk-deleted DALY rates. Risk-deleted rates are the rates after removal of the effect of a risk factor or combination of risk factors; in other words, observed DALY rates multiplied by one minus the PAF for the risk or set of risks. Our decomposition analyses draw from methods developed by Das Gupta^19^ to provide a computationally tractable solution to estimate the contribution of multiple factors to an outcome (methods appendix pp 36–37). For some risks where the PAF is 100%, such as fasting plasma glucose and diabetes, the methods have had to be further adapted. We were not able to include three outcomes in this analysis: cervical cancer, sexually transmitted diseases, and HIV/AIDS.

### Risk transition with development

We examined how changes in risk exposure were related to changes along the development spectrum. Drawing from methods used to construct the Human Development Index,^20^ we constructed the SDI, a summary measure of overall development based on estimates of lag-dependent income per capita, average educational attainment over the age of 15 years, and total fertility rate. In the SDI, we weighted each component equally and rescaled them from zero (for the lowest value observed during 1980–2015) to one (for the highest value observed) for income per capita and average years of schooling, and the reverse for the total fertility rate. We computed the final SDI score as the geometric mean of each of the components. For each risk, we calculated the average relationship between risk exposure, as measured by SEV, and SDI across all geography years by age and sex using spline regression (methods appendix pp 38–39). We then used this relationship to characterise how exposures to risk vary on the basis of SDI alone.

### Role of the funding source

The funder of the study had no role in study design, data collection, data analysis, data interpretation, or writing of the report. The authors had full access to all the data in the study and had final responsibility to submit for publication.

## Results

### Global exposure to risks

The SEV is a single, interpretable measure, which captures risk-weighted exposure for a population, or risk-weighted prevalence of an exposure. The scale for SEV spans 0% to 100%, such that an SEV of 0% reflects no risk exposure in a population and 100% indicates that an entire population has maximum possible risk. A decline in SEV indicates reduced exposure to a given risk factor, whereas an increase in SEV indicates increased exposure. Table 3 provides age-standardised SEVs for 61 risks at the global level, by sex, for 1990, 2005, and 2015 (results appendix pp 3619–4070 contains results for every geography). From 1990 to 2015, SEVs decreased by more than 30% for four risks: unsafe sanitation (38·3% [95% UI 36·1–40·5]), childhood underweight (34·2% [30·9–37·9]), childhood stunting (33·4% [30·3–37·4]), and household air pollution (30·2% [26·9–33·2]). The global SEV for smoking also decreased by 2015, decreasing by 27·5% (23·2–30·9) for men and 28·7% (20·2–34·1) for women; notably, smoking exposure among men still far exceeded that for women in 2015. Significant, although more moderate than for smoking reductions in global SEVs for both sexes occurred for second-hand smoke (12·2% [9·4–15·1]), unsafe water (9·4% [5·3–13·0]), and diet high in red meat (9·0% [7·6–10·3]) from 1990 to 2015. Risk exposure for high total cholesterol significantly declined for both men and women during this time, although this decrease was smaller among men (3·2% [2·2–4·4]) than among women (5·6% [4·6–6·7]). For a subset of occupational risk factors, such as ergonomic factors and asthmagens, global SEVs were reduced from 1990 to 2015.

For a subset of risks, minimal changes in exposure occurred between 1990 and 2015. This finding was particularly evident among various dietary risks (eg, diet low in fruits) and behaviours related to nutrition (eg, non-exclusive and discontinued breastfeeding). Discordant trends emerged by sex for some risk factors, such as low physical activity, where global SEVs for men increased by 2·4% (95% UI 1·8–2·9), whereas the SEV for women declined by 1·5% (1·0–2·0). Global SEVs significantly increased for 27 risk factors for both sexes combined from 1990 to 2015; significant increases occurred for 24 risks for men alone and 23 risks for women alone. We recorded the most pronounced rises for various occupational exposures, such as diesel engine exhaust, silica, and benzene. Global SEVs for high body-mass index (BMI) increased by 38·7% (29·9–55·6) for men and 34·4% (27·7–45·7) for women. For both sexes, other risks with large increases included drug use (30·2% [23·3–39·1]), ambient ozone pollution (24·6% [15·0–31·9]), and high fasting plasma glucose (23·8% [22·4–25·4]).

### Global attributable burden for all risk factors combined and their overlap

The proportion of deaths, YLLs, YLDs, and DALYs that could be jointly attributable to all risk factors combined differed by cause group and measure of health (table 4). Globally, 57·8% (95% UI 56·6–58·8) of deaths, 48·4% (47·4–49·3) of YLLs, 26·1% (25·0–27·1) of YLDs, and 41·2% (39·8–42·8) of DALYs could be attributed to the risk factors currently assessed as part of GBD 2015. Across health outcomes, attributable DALYs were highest for non-communicable diseases (NCDs), although the percentage of attributable burden ranged from 20·9% (19·7–22·0) for YLDs to 64·8% (63·3–66·2) for deaths in 2015. Among NCD cause groups, attributable DALYs were as high as 85·3% (84·0–86·6) for cardiovascular and circulatory diseases compared with low attributable DALYs, even among leading causes of disease burden (ie, 16·0% [13·9–18·2] for musculoskeletal disorders and 22·5% [20·1–25·4] for mental and substance use disorders). In 2015, approximately 40–60% of DALYs due to cancers, cirrhosis, and chronic respiratory diseases could be attributed to risk factors assessed in this study. Except for YLDs, less than 50% of disease burden for Group 1 causes—communicable, maternal, neonatal, and nutritional diseases—could be attributed to analysed risk factors. Risk factors accounted for less than 20% of early death and disability from maternal disorders (eg, 10·2% [4·1–16·8] of DALYs).

**Figure 1:**
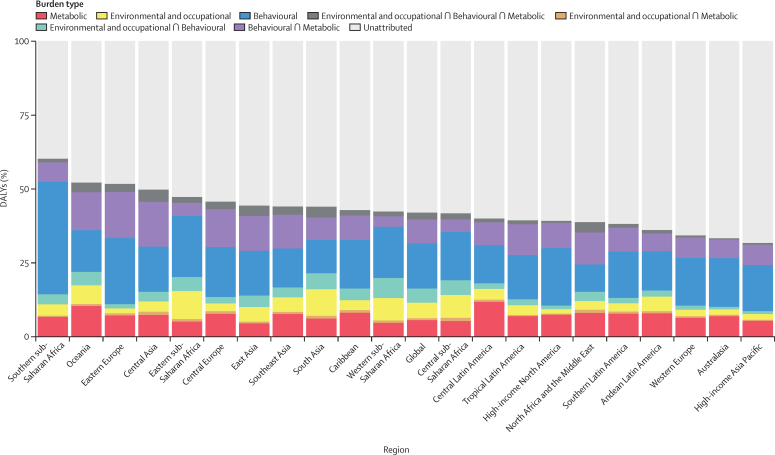
Global proportion of all-cause DALYs attributable to behavioural, environmental and occupational, and metabolic risk factors and their overlaps by region for both sexes combined in 2015 Locations are reported in order of total all-cause DALYs population attributable fraction. DALYs=disability-adjusted life-years. ∩=interaction.

Categories of risk factors—metabolic, environmental or occupational, and behavioural risks—often jointly contribute to disease burden. In 2015, 41·2% (95% UI 39·8–42·8) of global DALYs could be attributed to analysed risk factors, whereas 58·8% (57·2–60·2) of global disease burden could not be explicitly attributed to specific risk factors. In terms of individual risk categories, behavioural risk factors accounted for 30·3% (28·6–32·0) of attributable DALYs in 2015, followed by metabolic (15·5% [14·7–16·3]) and environmental or occupational risk factors (13·0 [11·9–14·0]). Regionally, total risk-attributable burden ranged from 59·0% (57·0–60·9) in southern sub-Saharan Africa to 33·5% (32·1–35·1) in north Africa and the Middle East; furthermore, ten regions had less than 40% of total DALYs attributable to risks being analysed (figure 1).

### Levels and trends in the burden attributable to risk factors

Table 4 reports all-cause deaths and DALYs attributable to all risk factors from 2005 to 2015, including detail on attributable deaths and DALYs by risk-outcome pair (results appendix pp 3–2488 contains results for every geography). Globally, 32·2 million (95% UI 31·5 million to 33·0 million) deaths were attributable to all risk factors in 2015, a 4·9% (3·2–6·7) increase since 2005; however, age-standardised attributable deaths declined from 2005 to 2015 (a 17·9% decrease [16·6–19·2] to 497·5 deaths per 100 000 [485·2–510·0]). By contrast, total DALYs attributable to all risks decreased since 2005 by 5·6% (3·8–7·5) to 1·0 billion DALYs (0·96–1·09 billion) in 2015 and age-standardised DALYs attributable to all risks decreased since 2005 by 20·9% (19·5–22·5) to 14 412·9 DALYs per 100 000 (13 553·0–15 360·1) in 2015. Deaths and burden attributable to environmental and occupational risks significantly fell across measures, with age-standardised deaths falling by 22·5% (20·6–24·4) to 142·6 deaths per 100 000 (130·1–155·6) in 2015 and age-standardised DALYs decreasing by 24·8% (22·1–27·1) to 4500 DALYs per 100 000 (4164·6–4853·9) in 2015.

Progress in environmental risks was mainly driven by sizeable reductions in mortality and disease burden attributable to unsafe water, sanitation, and hygiene, as well as to household air pollution. From 2005 to 2015, global deaths attributable to unsafe water and no handwashing with soap fell by more than 12%, whereas DALYs decreased by more than 20%. More rapid declines than in the above-mentioned risks in attributable deaths (27·5% [95% UI 22·3–32·7]) and burden (31·9% [25·7–37·4]) occurred for unsafe sanitation since 2005, to 807 904·2 deaths (727 439·6–895 462·5) in 2015 and to 46·3 million DALYs (41·1 million to 51·8 million) in 2015. Reductions in attributable mortality and DALYs due to diarrhoeal diseases (associated with unsafe water, sanitation, and hygiene) were particularly prominent. Attributable deaths due to household air pollution decreased by 13·0% (9·3–17·0) to 2·9 million deaths (2·2 million to 3·6 million) in 2015 and disease burden decreased by 20·3% (16·6–24·5) to 85·6 million DALYs (66·7 million to 106·1 million) in 2015; large declines also occurred in age-standardised rates of attributable mortality and DALYs. Occupational risk factors generally accounted for a smaller proportion of global deaths and disease burden than did environmental risk factors; nonetheless, attributable mortality and DALYS due to various occupational risk factors substantially increased from 2005 to 2015.

Behavioural risks can be grouped into four main categories: generally large reductions for risk-attributable mortality and disease burden for risk factors associated with child and maternal malnutrition, mixed results for risk factors pertaining to alcohol and drug use, rising attributable deaths and DALYs due to dietary risk factors, and considerably varied trends for other behavioural risks, which span from sexual abuse and intimate partner violence to low physical activity. Attributable deaths and disease burden due to metabolic risks have increased since 2005, particularly for high fasting plasma glucose, for which all measures of attributable mortality and DALYs increased by more than 15% from 2005 to 2015. These increases in attributable burden from high fasting plasma glucose were led by increased deaths and DALYs from ischaemic heart disease, haemorrhagic stroke, chronic kidney disease, and diabetes. Attributable deaths and DALYs for high BMI also increased substantially, with 645 244 (95% UI 457 647–862 412) more attributable deaths in 2015 than in 2005. Attributable mortality and DALYs due to low glomerular filtration rates also significantly increased from 2005 to 2015, with these increases primarily associated with rises in attributable deaths and burden due to cardiovascular and circulatory diseases and chronic kidney disease.

### Global risk patterns by sex

**Figure 2:**
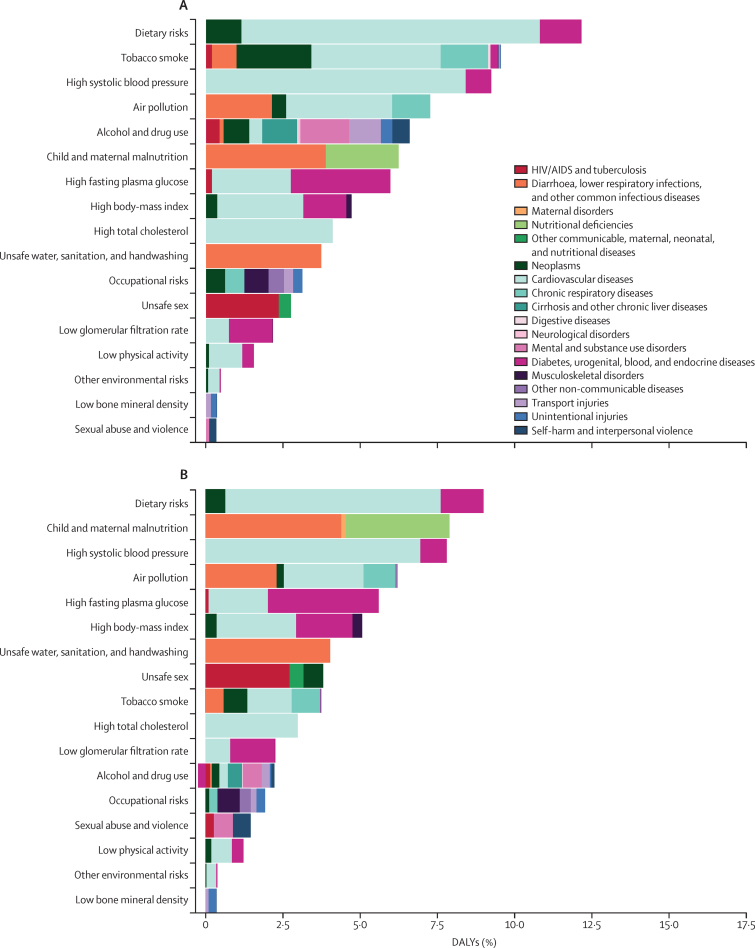
Global DALYs attributable to Level 2 risk factors for (A) men and (B) women in 2015 DALYs=disability-adjusted life-years.

In 2015, the relative ranks and attributable burden due to Level 2 risk factors varied between men and women (figure 2). As the leading risk factor for both sexes, dietary risks accounted for 12·2% (95% UI 10·8–13·6) of total DALYs for men and 9·0% (7·8–10·3%) of total DALYs for women. These risks, which include diet high in sodium and diet low in fruit, contributed most to DALYs associated with three cause groups: cardiovascular and circulatory diseases, cancers, and diabetes and urogenital, blood, and endocrine diseases. In 2015, high systolic blood pressure also ranked among the leading risks for both sexes, contributing to 9·2% (8·3–10·2) of DALYs for men and 7·8% (6·9–8·7) of DALYs for women. Air pollution was the fifth-leading risk for both sexes, largely contributing to DALYs associated with cardiovascular and circulatory diseases, as well as lower respiratory infections, diarrhoeal diseases, and other common infectious diseases. Child and maternal malnutrition, the leading global risk factor in 1990, was the second-leading risk for women and the sixth-leading risk for men in 2015.

Smoking was the second-leading risk factor for men in 2015, contributing to 9·6% (95% UI 8·5–10·7) of DALYs and a large proportion of male disease burden from cardiovascular and circulatory diseases, cancers, and chronic respiratory conditions. As the fifth-leading risk for men, alcohol and drug use was associated with 6·6% (6·1–7·1) of disease burden in 2015, primarily due to mental and substance use disorders, as well as cirrhosis and other chronic liver diseases; the burden attributable to these risk factors was far less for women (2·0% [1·8–2·2]) than for men. In 2015, high fasting plasma glucose was associated with 6·0% (5·4–6·6) of DALYs for men and 5·6% (5·1–6·2) for women. For women, 3·8% (3·4–4·3) of burden was attributable to unsafe sex, largely from HIV/AIDS and cervical cancer, whereas for men, 2·8% (2·5–3·0) was attributable to unsafe sex.

### Changes in leading risk factors in 1990, 2005, and 2015

**Figure 3:**
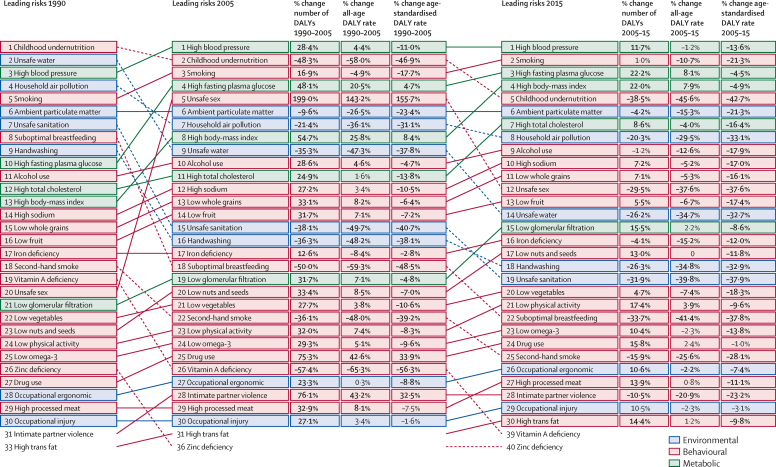
Leading 30 Level 3 global risk factors for DALYs for both sexes combined, 1990, 2005, and 2015, with percentage change in number of DALYs, and all-age, and age-standardised rates Risks are connected by lines between time periods. For the time period of 1990 to 2005 and for 2005 to 2015, three measures of change are shown: percent change in the number of DALYs, percent change in the all-age DALY rate, and percent change in the age-standardised DALY rate. Changes that are statistically significant are shown in bold. DALYs=disability-adjusted life-years.

Rising total attributable DALYs amid declines from 1990 to 2015 for age-standardised DALY rates were evident for various metabolic and behavioural risks, emphasising the need to parse out the effects of demographic and epidemiological factors on global risk profiles (figure 3). In 1990, childhood undernutrition, unsafe water, and high systolic blood pressure were the leading three risk factors for attributable DALYs. Of these risks, only high systolic blood pressure ranked among the leading three risks in 2015. Large reductions in both total attributable DALYs and age-standardised DALY rates from 1990 resulted in childhood undernutrition being ranked as the fifth-leading risk factor in 2015 and unsafe water being ranked as the 14th-leading risk factor.

Environmental risk factors, including household air pollution and unsafe sanitation, decreased in terms of total attributable DALYs, age-standardised DALY rates, and relative ranks from 1990 to 2015. Over the period 1990–2005, attributable total DALYs for occupational risk factors, such as ergonomic factors, rose by more than 20% from 1990 to 2005, although age-standardised rates decreased by 9·5% (95% UI 7·3–11·7) over the same time period. Similar patterns occurred for most behavioural risk factors from 1990 to 2005, with significant increases in total attributable DALYs occurring for many of these risks; at the same time, age-standardised DALY rates significantly fell (eg, smoking, low physical activity, and most dietary risks, including diet high in sodium). Unsafe sex and drug use were exceptions, with each measure of attributable burden significantly increasing since from 1990 to 2005. For unsafe sex and drug use in particular, this rapid rise corresponded with the global HIV/AIDS epidemic. For most risk factors, the time period of 2005–15 resulted in an extension of earlier trends, with continued gains in reductions of attributable DALYs due to various environmental risk factors and more varied patterns for many metabolic and behavioural risks than for environmental risks. Yet, some important changes occurred between 2005 and 2015, including large reductions in attributable total DALYs (29·5% [26·8–32·0]) and age-standardised DALY rates (37·6% [35·2–39·8]) for unsafe sex and in attributable total DALYs (10·5% [2·8–17·8]) and age-standardised DALY rates (23·2% [16·8–29·1]) for intimate partner violence.

### Contrasting global changes in risk exposure and attributable burden

**Figure 4:**
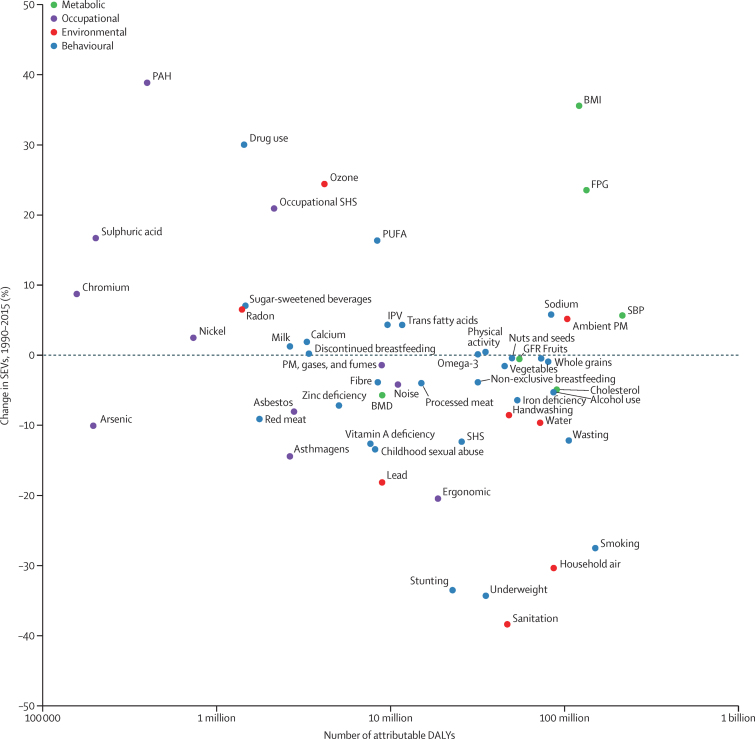
Global attributable DALYs in 2015 for each Level 3 risk factor versus percentage change in SEV from 1990 to 2015 for both sexes combined Risks with 100 000 DALYs or more are presented. DALYs are represented on a logarithmic scale. We have excluded occupational exposure to benzene, diesel engine exhaust, and occupational exposure to silica, which all had SEV increases of greater than 50%. Ambient PM=ambient particulate matter pollution. Arsenic=occupational exposure to arsenic. Asbestos=occupational exposure to asbestos. Asthmagens=occupational asthmagens. Beryllium=occupational exposure to beryllium. BMD=low bone mineral density. BMI=high body-mass index. Cadmium=occupational exposure to cadmium. Calcium=diet low in calcium. Cholesterol=high total cholesterol. Chromium=occupational exposure to chromium. DALYs=disability-adjusted life-years. Ergonomic=occupational ergonomic factors. Fibre=diet low in fibre. Formaldehyde=occupational exposure to formaldehyde. FPG=high fasting plasma glucose. Fruits=diet low in fruits. GFR=low glomerular filtration rate. Handwashing=no handwashing with soap. Household air=household air pollution. IPV=intimate partner violence. Lead=lead exposure. Milk=diet low in milk. Nickel=occupational exposure to nickel. Noise=occupational noise. Nuts and seeds=diet low in nuts and seeds. Occupational SHS=occupational exposure to second-hand smoke. Omega-3=diet low in seafood omega-3 fatty acids. Ozone=ambient ozone pollution. PAH=occupational exposure to polycyclic aromatic hydrocarbons. Physical activity=low physical activity. PM, gases, and fumes=occupational particulate matter, gases, and fumes. Processed meat=diet high in processed meat. PUFA=diet low in polyunsaturated fatty acids. Radon=residential radon. Red meat=diet high in red meat. Sanitation=unsafe sanitation. SBP=high systolic blood pressure. SEV=summary exposure value. SHS=second-hand smoke. Sodium=diet high in sodium. Sugar-sweetened beverages=diet high in sugar-sweetened beverages. Sulphuric acid=occupational exposure to sulphuric acid. Stunting=childhood stunting. Trans fatty acids=diet high in trans fatty acids. Trichloroethylene=occupational exposure to trichloroethylene. Underweight=childhood underweight. Vegetables=diet low in vegetables. Wasting=childhood wasting. Water=unsafe water. Whole grains=diet low in whole grains.

A comparison of percentage change in risk exposure from 1990 to 2015 with the level of attributable DALYs in 2015 helps to identify large risks for which a long-term increase in global exposure has occurred (figure 4). Although disease burden attributable to unsafe sanitation, household air pollution, stunting, and underweight caused more than 10 million DALYs in 2015, exposure to these risks decreased for both sexes from 1990 to 2015 by more than 30%. Conversely, two risks caused more than 100 million DALYs and increased by more than 20%: high fasting plasma glucose and high BMI. Other risks with large increases in exposure but which caused less than 10 million DALYs include various occupational exposures, drug use, ambient ozone pollution, second-hand smoke, and diets low in polyunsaturated fatty acids (PUFAs). For a large group of risks at the global scale, exposure increased or decreased by less than 10% from 1990 to 2015. These included many components of diet, high systolic blood pressure, ambient particulate matter pollution, and alcohol use.

### Decomposition of changes in risk-attributable DALYs to population growth, ageing, risk exposure, and risk-deleted DALY rates

**Figure 5:**
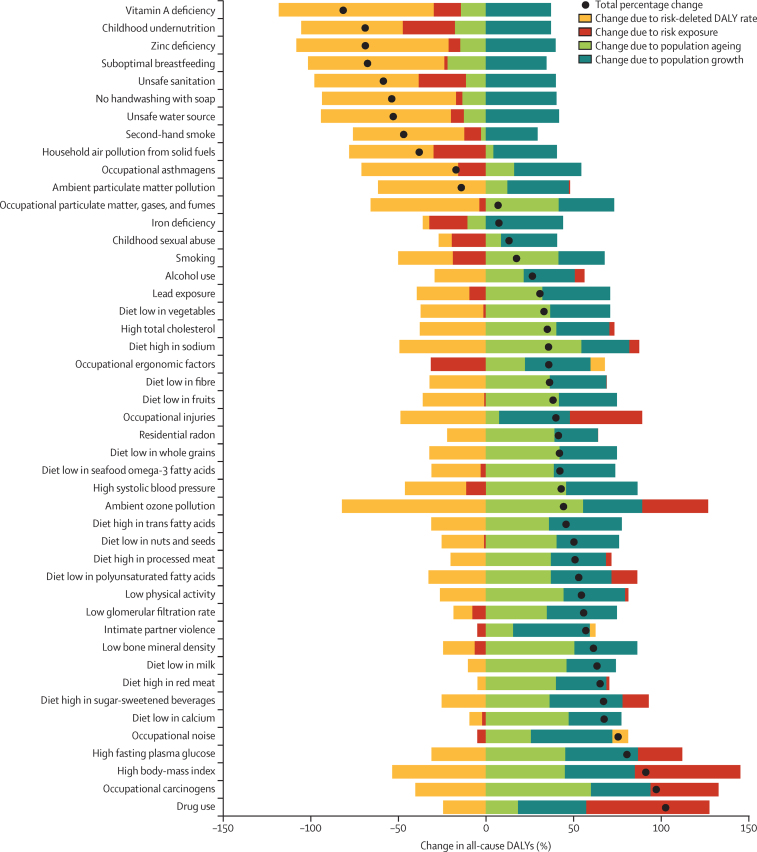
Global decomposition of changes in all-cause DALYs attributable to Level 3 risk factors from 1990 to 2015 Risks are reported in order of percentage change in the number of attributable DALYs from 1990 to 2015. We excluded DALYs attributable to unsafe sex because this risk factor is not estimated on the basis of exposure and relative risk. Changes due to population growth, population ageing, risk exposure, and the risk-deleted DALY rate are shown. DALYs=disability-adjusted life-years.

Drivers of global changes in overall DALYs attributable to risk factors varied (figure 5). Across Level 3 risk factors, overall changes in all-cause attributable DALYs ranged from declines exceeding 50% for seven risk factors, including childhood undernutrition, suboptimal breastfeeding, and unsafe sanitation, to increases near to or exceeding 100% (ie, high BMI, occupational carcinogens, and drug use). Of these 46 Level 3 risk factors, attributable all-cause DALYs decreased significantly for ten from 1990 to 2015, whereas 34 increased significantly; two did not significantly change. Population ageing led to increased attributable all-cause DALYs for most risk factors, with a relative contribution that spanned from lower than 10% (for household air pollution from solid fuels and occupational injuries) to greater than 60% (for occupational carcinogens). Population ageing contributed to reductions in all-cause DALYs attributable to eight risk factors, namely environmental risks (eg, a 13·5% [95% UI 6·0–19·9] decrease for no handwashing with soap), those associated with nutritional deficiencies (eg, a 17·6% [12·6–24·0] decline for childhood undernutrition), and behavioural risks (eg, a 22·0% [10·9–31·4] decline for suboptimal breastfeeding). Changes in risk-deleted DALY rates since 1990 were primary drivers of reductions in all-cause, risk-attributable burden, with decreases in underlying DALY rates exceeding 50% for 13 risks by 2015. By contrast, changes in risk exposure varied markedly, contributing to declines in all-cause DALYs for ten risks (eg, 30·0% [29·0–38·0] due to declines in risk exposure for household air pollution and 21·7% [17·5–30·4] due to declines in risk exposure for iron deficiency); at the same time, change attributable to risk exposure increased for 16 risk factors to more than 25%, including high fasting blood glucose (25·1% [19·7–27·9]), ambient ozone pollution (37·5% [31·5–42·7]), occupational carcinogens (40·1% [28·5–50·8]), occupational injuries (41·1% [37·0–48·1]), high BMI (60·0% [54·9–69·2]), and drug use (70·0% [65·5–73·6]).

Decreases in underlying cause-specific DALY rates—as opposed to other factors—were generally the main drivers of overarching reductions in cause-specific burden attributable to all risk factors (methods appendix pp 164–65). From 1990 to 2015, 36 causes decreased in terms of associated risk exposure, including a number of communicable causes, nutritional deficiencies, and chronic obstructive pulmonary disease. Notably, risk exposure was the only factor that improved from 1990 to 2015 for a subset of causes, with changes in population growth, ageing, and underlying cause-specific DALY rates all contributing to rising cause-specific disease burden; this finding was most evident for tracheal, bronchial, and lung cancer, as well as for cirrhosis and other chronic liver diseases due to alcohol use.

### The risk transition and development

**Figure 6:**
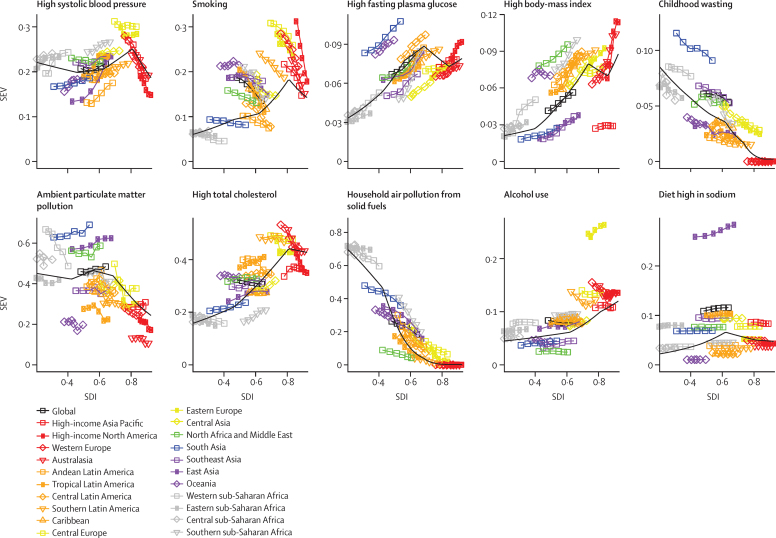
Coevolution of SEV and SDI for the top ten global risks in terms of attributable disability-adjusted life-years in 2015 Coloured points show SEVs for Global Burden of Disease regions. Each point represents 1 year in 5 year intervals from 1990 to 2015. The solid black line represents the expected SEV on the basis of SDI alone. SDI=Socio-demographic Index. SEV=summary exposure values.

Figure 6 shows the evolution of SEV by region for the ten leading global risk factors in terms of attributable DALYs as SDI changes and also provides the expected SEV level on the basis of SDI alone. Two main trends emerged: increasing and then levelling of SEVs for most metabolic and dietary risks and reductions in SEVs for environmental risks and those associated with childhood undernutrition as SDI approached mid-levels. For metabolic and dietary risks, above an SDI of approximately 0·8, expected levels of risk exposure either moderately dropped or remained fairly constant. An exception was alcohol use, for which SEVs increased with each increment of SDI. By contrast, SEVs for ambient particulate matter pollution did not substantially decline until above an SDI of 0·60, and the pace of SEV reductions for household air pollution accelerated above an SDI of about 0·40. These patterns reflect the complex shifts in risk exposure that accompany changes in development, which are further emphasised by regional SEV trends by risk factor.

Two risk factors related to nutrition or diet—childhood wasting and diet high in sodium—reflected the nuances of changing risk exposure and levels of development. Particularly among regions with an SDI below 0·8, SEVs for childhood wasting decreased over time and with increasing SDI; nonetheless, a subset of regions, including south Asia and western sub-Saharan Africa, had consistently higher than expected SEVs for childhood wasting on the basis of SDI. For diet high in sodium, most regions saw minimal changes in exposure over time, even amid increases in SDI. In east Asia, SEVs for diet high in sodium were consistently above expected levels of exposure given SDI, whereas the opposite trend was found for Oceania and central Latin America. Across the development spectrum—including high-income Asia Pacific, southeast Asia, and eastern sub-Saharan Africa—exposure for diet high in sodium was at least moderately higher than expected on the basis of the SDI for a given region. Heterogeneous risk patterns occurred for two leading environmental risks—household air pollution and ambient particulate matter pollution—particularly in terms of the relationship between SEVs and increasing SDI. For smoking and alcohol use, strikingly different trends for SEV and SDI occurred. Although nearly every region recorded declines in SEVs for smoking, the rate at which these reductions took place alongside changes in SDI varied.

### Regional and national risk profiles

**Figure 7:**
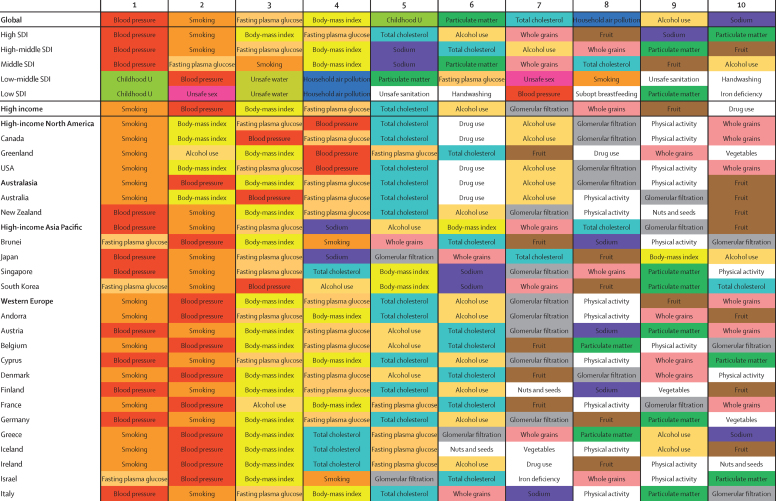

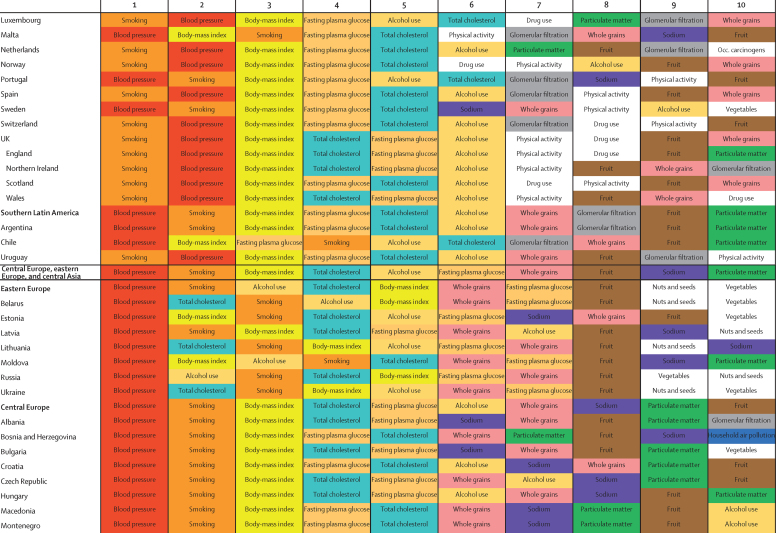

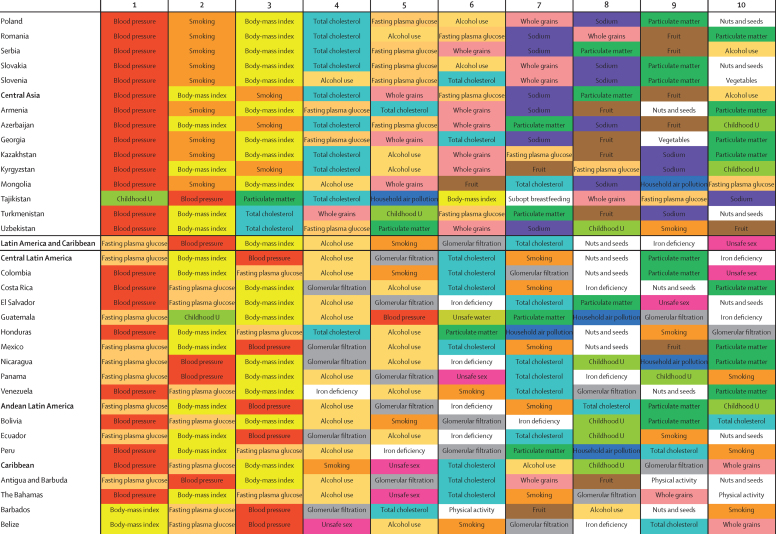

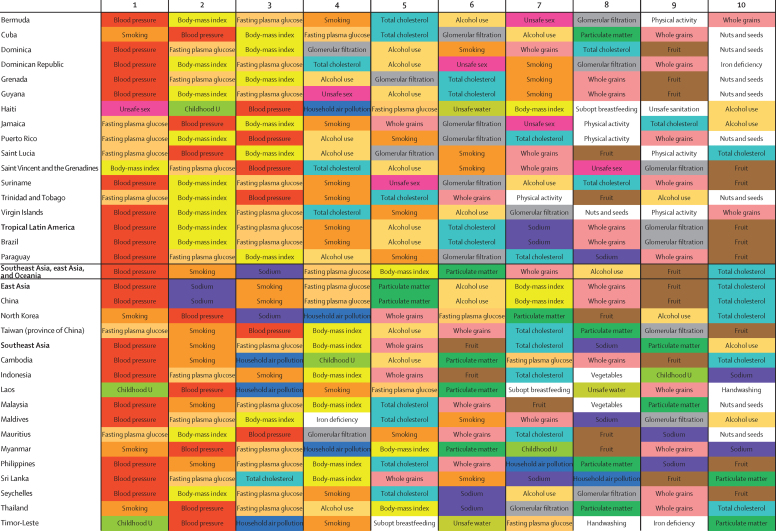

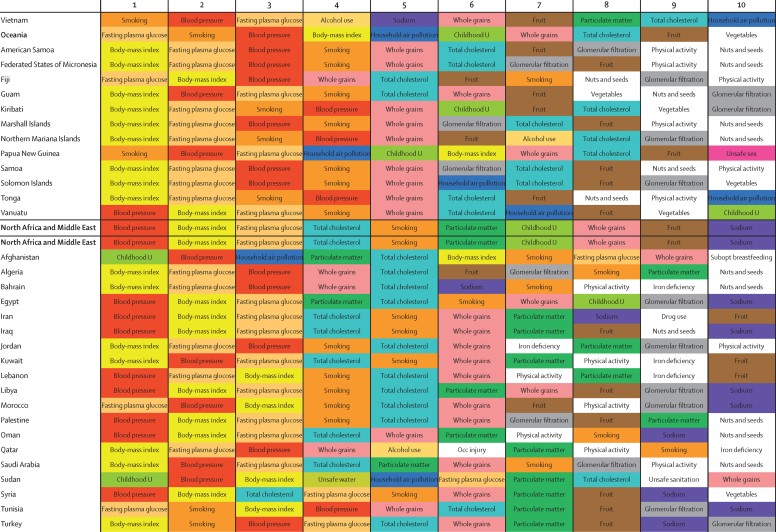

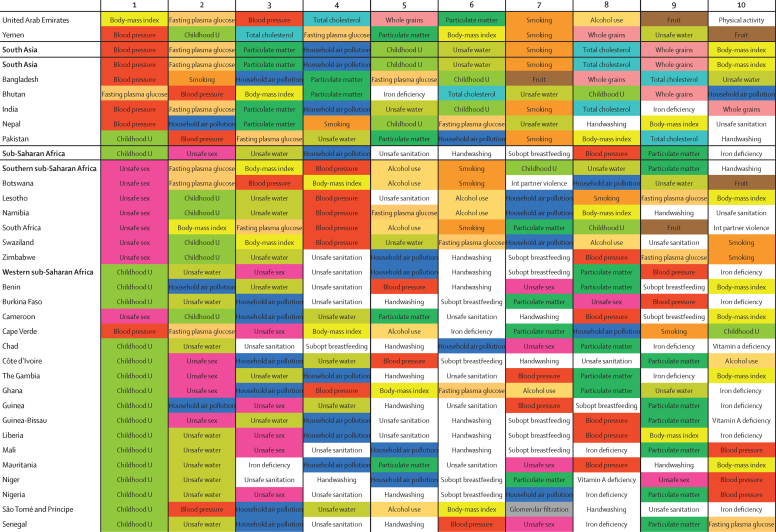

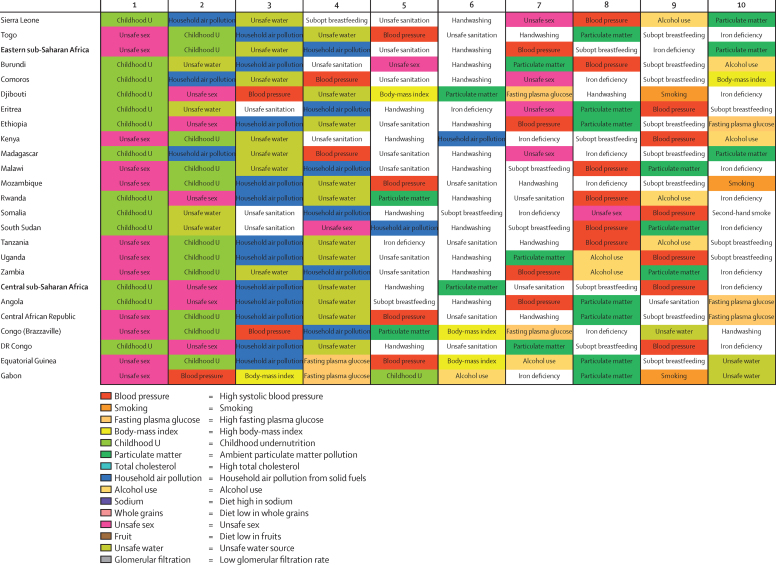
Leading ten Level 3 risk factors in terms of disability-adjusted life-years for both sexes combined in 2015, by location The 15 leading risk factors are coloured. Bone mineral density=low bone mineral density. Handwashing=no handwashing with soap. Int partner violence=intimate partner violence. Nuts and seeds=diet low in nuts and seeds. Occ=occupational. Particulate matter=ambient particulate matter pollution. Physical activity=low physical activity. Processed meat=diet high in processed meat. SDI=Socio-demographic Index. Subopt=suboptimal. Sweetened beverages=diet high in sugar-sweetened beverages. Vegetables=diet low in vegetables.

Leading risk factors for early death and disability, as measured by attributable DALYs, varied by region, level of SDI, and sex in 2015 (figure 7). In high-income North America and the UK, smoking was the leading risk for attributable DALYs among both men and women, but for most of western Europe, smoking was the leading risk factor only for men, whereas high systolic blood pressure was the leading risk factor for women. A similar pattern emerged in east and southeast Asia, with smoking ranked as the leading risk factors for men in China, Thailand, Vietnam, and the Philippines, whereas metabolic risk factors—namely high systolic blood pressure—was the leading risk factor for attributable DALYs for women in these countries. Childhood undernutrition ranked as the leading risk factor for early death and disability for both sexes throughout western and central sub-Saharan Africa, as well as in a few countries outside of sub-Saharan Africa (eg, Laos and Tajikistan).

In terms of the leading ten risk factors for both sexes, regional and country risk profiles showed both distinct patterns and heterogeneity. High systolic blood pressure was the leading risk for DALYs for 13 high-income countries and territories in 2015, high fasting plasma glucose was the leading risk for three, and smoking was the leading risk for 21. For a subset of geographies, including the USA, Canada, Australia, and the UK, drug use was a major risk for early death and disability in 2015. In 2015, high systolic blood pressure, high BMI, and high fasting plasma glucose were the leading risk factors for almost all geographies in Latin America and the Caribbean; Haiti was the primary exception, with unsafe sex as its leading risk for attributable DALYs in 2015. Across southeast Asia, east Asia, and Oceania, high systolic blood pressure was the leading risk factor for disease burden in nine countries and territories, ranging from China to Vanuatu. High BMI was the leading risk for DALYs in nine geographies, and high fasting plasma glucose ranked as the leading risk factor for three geographies, including Taiwan.

High systolic blood pressure was among the leading two risk factors for all geographies in south Asia and, except for Pakistan and Bhutan, household air pollution remained among the leading four risk factors for attributable DALYs across geographies in this region. Ambient particulate matter pollution ranked as the third-leading risk factor in India and Nepal, whereas smoking was the second-leading risk factor for attributable burden in Bangladesh. In central Europe, eastern Europe, and central Asia, 28 of 29 countries had high systolic blood pressure as their leading risk factor for attributable DALYs in 2015; Tajikistan, where childhood undernutrition was the leading risk factor, was the only exception. Across central Europe, smoking was the second-leading risk factor for early death and disability, whereas alcohol use was among the leading four risk factors for attributable DALYs in geographies including Belarus, Moldova, and Russia.

Throughout north Africa and the Middle East, except for Tunisia, high systolic blood pressure was among the three leading risk factors for disease burden in 2015, with nine geographies, including Egypt and Iran, recording this risk as the leading driver of early death and disability in that year. High BMI accounted for the highest attributable DALYs in eight countries, including Jordan and Saudi Arabia, and was also among the leading six risks for all countries in the region. For Afghanistan and Sudan, childhood undernutrition was the leading risk for DALYs in 2015, whereas it was the second-leading risk for DALYs in Yemen and the eighth-leading risk in Egypt. Unlike most of sub-Saharan Africa, several metabolic risks also emerged as leading drivers of attributable DALYs in southern sub-Saharan Africa by 2015; high BMI ranked as the second-leading risk factor in South Africa, and high systolic blood pressure was among the leading three risk factors for attributable DALYs in Botswana. In central sub-Saharan Africa, childhood undernutrition and unsafe sex were ranked first and second for attributable disease burden in all geographies except for Gabon. High systolic blood pressure ranked among the leading risk factors for attributable DALYs in most geographies in the region (eg, second in Gabon and third in the Congo).

## Discussion

### Overview

Drawing from 25 500 data sources, we estimated exposure to 79 metabolic, environmental and occupational, and behavioural risk factors or clusters of risks from 1990 to 2015 in 195 countries and territories and attributed deaths and overall disease burden to these risks. In 2015, all risks combined contributed to 57·8% (95% UI 56·6–58·8) of deaths and 41·2% (39·8–42·8) of DALYs worldwide. Since 1990, global risk exposure for both sexes combined increased significantly for 27 risks, did not significantly change for seven risks, and declined significantly for 27 risks. At the same time, that risk exposure increased for various leading risks, particularly metabolic risk factors associated with NCDs, and age-standardised risk-attributable deaths and DALYs declined for most risks. Globally, pronounced reductions in risk-deleted or underlying cause-specific DALY rates offset minimal changes in, or increased, risk exposure. These gains in risk-deleted DALY rates might not be large enough in the future to compensate for rising levels of risk exposure, such as high BMI or high fasting plasma glucose.

### Rethinking the risk transition

Societal processes of urbanisation, the so-called westernisation of diets and lifestyles, and changes in employment activities, have all been viewed as primary drivers of changes in human health.^21^, ^22^, ^23^, ^24^ Such shifts have been thought to lead to deteriorating diets, rising obesity, decreased physical activity, and, ultimately, to worsening levels of metabolic risks, with associated higher rates of cardiovascular diseases and cancers.^25^, ^26^ Results from the this study point to an ongoing risk transition, but with a trajectory complex and nuanced. The relationship between SEVs for most risks and SDI identified that poor water, poor sanitation, household air pollution, and micronutrient deficiencies and undernutrition decline steadily as countries develop. By contrast, some risks become worse as development proceeds, at least up to levels of SDI of about 0·8; these risks include low physical activity, high BMI, high total cholesterol, low PUFAs, partial breastfeeding, alcohol use, diet high in red meat, smoking, and diet high in sugar-sweetened beverages. Some risks that appear to worsen through early phases of development improved at the highest levels of SDI, such as smoking. As improvements in SDI continue and behavioural risks grow in dominance, an understanding of how to change behaviours effectively at both the individual level and for populations becomes increasingly relevant. Many other components of diet, occupational exposures, and some environmental risks do not show a marked relationship with development.

The effects of the risk transition, at least globally, are often mitigated by trends in risk-deleted death or DALY rates. In this study, we identify likely candidates for specific drivers of improvements. Unmeasured risk factors could be driving these trends. Unlike cardiovascular diseases and some neoplasms, for other causes such as mental disorders or neurological disorders, the set of risks included in this study account for comparatively little of the observed burden; these causes make up an increasing share of the burden for geographies at high SDI. To date, we have not identified risk factors that meet our criteria of convincing or probable evidence, suggesting that research into unquantified risks is needed. In accompanying GBD 2015 analyses,^27^, ^28^, ^29^ we documented widespread improvements in overall development as measured by SDI. Gains in SDI are likely to operate through many pathways, including improved access to health care, public health programmes, and social and welfare policy. Advances in treatment are well documented for various causes, including HIV/AIDS,^30^, ^31^, ^32^ ischaemic heart disease,^33^, ^34^, ^35^, ^36^, ^37^ and various cancers,^38^, ^39^, ^40^ including breast,^41^, ^42^, ^43^ testicular,^44^ and Hodgkin's,^45^ yet for other causes, such as oesophageal cancer and interpersonal violence, the policies, programmes, and interventions responsible for declining risk-deleted death and DALY rates are less clear than for the aforementioned causes. Improvement of understanding of the risk-deleted rates in cause-specific mortality and disease burden will strengthen the evidence base for intervention effectiveness, the role of medical care access in addressing disease burden, and the importance of other social and welfare policies.

### A global risk typology

We used our decomposition of drivers of attributable burden to identify four distinct groups of risks at the global level. First, for ten risks, attributable burden is declining, as is the exposure to the risk factor. This set of risks is dominated by the environmental risks, which are particularly common at low levels of SDI, and consist of vitamin A deficiency, undernutrition, zinc deficiency, suboptimal breastfeeding, poor sanitation, no handwashing, poor water, second-hand smoke, household air pollution, and occupational asthmagens. For these risks as a group, not only has exposure been declining, but risk-deleted DALY rates also declined, such as for diarrhoeal diseases. Furthermore, global shifts in population age structure contributed to decreases in both cause-specific and attributable burdens for risks that predominantly affect children. A second group of risks was characterised by declines in exposure exceeding 10% from 1990 to 2015, but increasing attributable burden, due in most cases to large increases driven by population growth and ageing. This group includes smoking, high systolic blood pressure, occupational ergonomic factors, childhood sexual abuse, and iron deficiency; for iron deficiency, the increase in attributable burden was quite small over the period 1990–2015. For a third group, attributable burden is increasing, and trends in exposure account for a less than 10% increase or decrease in attributable burden. This larger group of risks than the first two groups includes all components of diet except for low PUFA intake and diet high in sugar-sweetened beverages, some occupational exposures, residential radon, low glomerular filtration rate, alcohol use, high total cholesterol, intimate partner violence, lead exposure, low bone mineral density, and low physical activity. For these risks, attributable burden is increasing because of population growth and ageing. The degree of increase is driven by the extent to which declines in risk-deleted DALY rates compensate for the increases due to population growth and ageing. The final category is the risks that are perhaps the most concerning: those with increasing attributable burden and exposure contributing to an increase of at least 10% since 1990. This list includes occupational injuries, ambient ozone pollution, occupational carcinogens, diet low in PUFAs, diet high in sugar-sweetened beverages, drug use, high fasting plasma glucose, and high BMI. For these risks, declines in underlying rates were probably responsible for prevention of additional increases in attributable burden. Any risk for which attributable deaths and DALYs are increasing is a threat to both health systems and societies, but the risks that fall within the fourth group—risk factors with rising exposure and associated health loss—require immediate attention from policy makers and other stakeholders.

### Unsafe water, sanitation, and handwashing

The conventional approach to assessment of the contribution of unsafe water to health is to assess access to improved water sources as defined in the Millennium Development Goals.^46^, ^47^ WHO estimated that, in 2015, 91% of populations living in low-income and middle-income countries had access to improved water and 68% had access to improved sanitation since 1990.^48^, ^49^ Implicit in the focus on improved is the idea that the most important reductions in diarrhoea come from movement from an unimproved to an improved source of water or sanitation. However, findings from meta-analyses of intervention studies and retrospective cohorts show a wide variation of relative risks of diarrhoea within the category of improved water and improved sanitation. We found that the SEV for poor water was only 56·0% (95% UI 50·4–62·1) for men and 55·7% (50·2–61·8) for women in 2015, a small improvement of just 9·4% (5·3–13·0) since 1990. By contrast, for unsafe sanitation, the SEV decreased substantially, from 55·0% (53·8–56·5) in 1990 for men and 54·2% (52·8–55·7) for women to 33·7% (32·2–35·1) for both men and women in 2015. Our assessment of SEV for water showed that a substantial agenda is still needed across the world to achieve the overall goal of safe water and sanitation. The sixth Sustainable Development Goal includes targets for achievement of both universal and equitable access to safe drinking water and adequate and equitable sanitation for all by 2030.^50^, ^51^ Given SEVs in many of these areas of the world, a large gap remains between current levels of safe water and sanitation and universal access. Moreover, our analyses show that progress in provision of safe water lags behind that of safe sanitation. This shift in focus to the lowest risk categories raises the bar considerably for what is needed for investment to reduce the risk of diarrhoea in all regions of the world.

### Tobacco

The SEV for smoking has decreased in many countries as well as globally. Global tobacco-attributable deaths and DALYs, however, have continued to rise because of increases in population numbers and ageing, which overwhelm declines in both exposure and risk-deleted rates of related disease burden. Given a known highly effective set of intervention strategies to reduce tobacco consumption,^52^, ^53^, ^54^, ^55^, ^56^, ^57^, ^58^ the challenge for tobacco is one of political priority for tobacco control. Despite the important developments of the Framework Convention on Tobacco Control, in many countries, progress has been slow or consumption has even increased. Continued close monitoring of tobacco consumption and the deaths and DALYs attributable to tobacco remains an essential aid to promotion of policies to reduce tobacco consumption.

In GBD 2015, for long-term effects of smoking on lung cancer and chronic obstructive pulmonary disease, we used the Peto-Lopez method,^59^ which estimates the lifetime cumulative effect of cigarette smoking using a transformation of the observed lung cancer death rate. Although this method provides robust estimates of the burden of cancers related to tobacco, it is not fully consistent with the GBD approach of estimation of exposure independently of the outcomes affected by exposure. With a growing body of evidence for the association between smoking and other cancers and a good estimate of distribution of smoking, direct estimation of attributable burden is possible. Modelling of the direct relationship between smoking exposure in the past and present to cancers will also allow for exploration of counterfactual scenarios and other forms of CRA, such as estimation of avoidable burden. The set of outcomes that have been related to tobacco in pooled cohort studies^60^ includes many outcomes not quantified in this study, such as road traffic accidents, renal failure, and infectious diseases; careful assessment of which of these new outcomes meet the criteria of convincing or probable evidence is needed. Quantification of the full effects of tobacco will also require inclusion in future GBD studies of smokeless tobacco consumption. Regardless of the estimation method used or the scope of outcomes evaluated, tobacco remains a major global risk factor, despite more than 50 years of antitobacco efforts.

### Dietary assessment

Many aspects of dietary assessment remain controversial. Evidence for the effect of diet on NCDs mostly comes from prospective cohort studies using food frequency questionnaires (FFQs) with 1 year recall to establish diet at baseline. Findings from 24 h recalls or multiple-day diary records show a poor correlation with annual FFQs.^61^, ^62^ Proponents of FFQs argue that the rank order of levels of intake across individuals in the annual FFQ is robust.^62^ Some authors have argued that measurement error for each diet component in the statistical analysis of the cohort data will tend to bias the findings towards the null and thus underestimate the effects of a diet component.^63^ However, the direction of bias in settings with measurement error in multiple independent variables is unknown in the presence of correlation between different variables.^64^, ^65^ At the population level, single 24 h recall has been used in many nutrition surveys. 24 h recall probably underestimates, as shown in doubly labelled water^66^ and urinary sodium studies.^67^

Correlation between diet components is a crucial aspect of diet. Intake of beneficial dietary factors are generally, but not always, positively correlated with each other and inversely correlated with harmful dietary factors. This correlation could overestimate the relative risk of each dietary factor in cohorts as well as the total effect of dietary risks at the population level. Use of dietary pattern as the main exposure could potentially address this problem; however, several challenges exist in adoption of this approach for GBD. Concerns remain about the magnitude of the effect size of individual dietary risk factors on chronic diseases. Although many prospective cohorts have collected dietary data,^68^ published meta-analyses for most diet-disease pairs have included reports from only a fraction of these cohorts, indicating the potential for publication bias. Furthermore, most cohorts assessing the effect of diet on disease endpoints have adjusted for total energy intake in their statistical models. This practice emerged to address measurement error in dietary assessment tools and remove the effect of energy as a potential confounder. The adjustment for total energy intake means that diet components are defined as risks in terms of the share of diet and not as absolute levels of exposure. Because diet shares are analysed, increases in any component imply reductions in some other component, leading to the notion of replacement. Diet components are often analysed as pairs in which one component replaces another, further complicating analysis of cohort data. Given that many cohorts do not include the same dietary components in their analyses, the relative risks of dietary factors across cohorts might not be strictly comparable. This issue has been one of the main reasons for inconsistent findings in dietary meta-analyses.^69^, ^70^ Future work to encourage more pooled analyses than at present of diet components for all the major cohorts would be beneficial; release of more data than available from the present major cohorts would stimulate various alternative analyses of diet, which would help strengthen the evidence for diet and attributable burden.

### Sodium intake

Age-standardised DALYs attributable to diets high in sodium and exposure (as measured by SEV) for diets high in sodium increased slightly at the global level (7·2% [95% UI 0·7–19·0] for men and 4·0% [–0·3 to 12·1] for women) from 1990 to 2015. Reduction in sodium intake at the population level is one of WHO's nine global targets for NCDs.^71^ Many countries (eg, the UK, Finland, Japan, and Brazil) have already implemented or are considering implementing (eg, the USA) policies to reduce sodium intake.^71^, ^72^, ^73^, ^74^, ^75^ Reports from countries with high levels of sodium intake (eg, Japan) that have successfully implemented sodium reduction policies have argued for the beneficial effects of a lowering of sodium intake in these populations.^76^ Although multiple lines of epidemiological evidence support the harmful effects of very high levels of sodium intake, no scientific consensus has been reached on the optimal level of sodium intake. In GBD 2013, on the basis of findings of the Prospective Urban Rural Epidemiology (PURE) collaboration on sodium and cardiovascular mortality^13^, ^77^ and to incorporate the absence of scientific consensus on the optimal intake of sodium, we expanded the uncertainty for the TMREL for sodium to 1–5 g per day.^78^, ^79^, ^80^ PURE collaborators subsequently published a further analysis,^13^ which raised the possibility that, for those without hypertension, an inverse relationship might exist between sodium intake, all-cause mortality, and cardiovascular events. These findings challenge longstanding beliefs in the public health community with regard to the importance of modulation of sodium intake at the population level. Current policy is grounded in the evidence that links sodium intake and systolic blood pressure, which shows increases in systolic blood pressure with increases in sodium above 1 g per day.^81^, ^82^ However, we have identified no prospective cohort studies that directly link sodium to disease endpoints that support reductions in the risk of outcomes at levels of intake below 3 g per day. Proponents of a low TMREL for sodium have argued that the cohort studies that generally show rising mortality at levels below 3–5 g per day might have the issue of reverse causation because ill individuals reduce sodium consumption.^83^, ^84^ Given this continued debate, we chose not to change the uncertainty range of the TMREL from the current 1–5 g per day. If the findings from PURE and the pooled analysis are correct, an increased risk might exist for individuals without hypertension who consume less than 5 g per day. We have not included this potential increased risk in our quantification of uncertainty. Many studies have been completed or are underway examining the effects of sodium reduction in population groups or whole communities on systolic blood pressure, but none to date have been done that report the effects of sodium reduction on disease endpoints. Such studies, when and if they are done, could contribute to a resolution of outstanding questions about the TMREL for sodium. Regardless of the debate on the TMREL, we found that sodium accounted for at least 2·0% of global DALYs in 2015. The risk profile of increasing attributable numbers of DALYs, and no global progress in a reduction of exposure to diets high in sodium, places this risk among those of great concern for the management of health systems.

### Diet and policies

Much of the diet policy debate has focused on the importance of reductions of sodium, sugar, and fat.^85^, ^86^ Our assessment of the burden from diseases attributable to 14 dietary factors showed that, at the global scale, six factors each accounted for more than 1% of global DALYs, in order of importance: diets high in sodium, low in vegetables, low in fruit, low in whole grains, low in nuts and seeds, and low in seafood omega-3. Our findings suggest that, in addition to a policy focus on sodium, sugar, and fat, many other important components of diet should be minimised and promoted through education, subsidies, and other evidence-based programmes.^87^ If our findings are correct, a policy focus on the sugar and fat components of diets might have a comparatively smaller effect than that of promotion of increased uptake of vegetables, fruit, whole grains, nuts and seeds, and seafood omega-3. The disconnect between the diet policy debate and the evidence of which diet factors are most important globally can also be seen for red meat, where the harm of increased diabetes and colorectal cancer is smaller than that for all of the other 13 diet components considered under the GBD framework. Consideration of diet policy is made more complicated when cost-effectiveness, political feasibility, intensity of implementation, reactions of various stakeholders (eg, consumers and the food industry), and environmental effects, including climate change, are factored into national diet policy discussions. For example, promotion of some components of a healthy diet (eg, milk and dairy products) might have deleterious effects on environmental sustainability. These dimensions of diet policy should be added to the debates on how to transform national diets to be lower risk than at present.

### Cholesterol

On the basis of findings from studies^12^ that showed the benefits from the reduction of LDL and total cholesterol to very low values, for GBD 2015, we revised the TMREL for total cholesterol downwards from 3·80–4·00 mmol/L to 2·78–3·38 mmol/L. This revision increased our estimate of the burden of total cholesterol for the year 2010 from 2·7 million (95% UI 1·9 million to 3·8 million) deaths in GBD 2013 to 4·0 million (3·1 million to 5·1 million) deaths in GBD 2015 and shifted our placement of cholesterol among leading risks from tenth (GBD 2013 ranking for the year 2010) to fourth (GBD 2015 ranking for the year 2010). Effective intervention options to influence population total cholesterol are available, including efforts to increase physical activity and diet and pharmacological interventions. Although consumption of dietary cholesterol is not linked to serum cholesterol,^88^ dietary interventions, such as increased consumption of fruits, vegetables, and fibre, and increased physical activity can influence LDL and total cholesterol^89^ and should be encouraged.^90^, ^91^ Statins are highly effective, with few side-effects, justifying their use in many individuals, either alone or in combination with other medicines. In general, given the existence of proven and effective intervention strategies, the increase in the importance of cholesterol highlights a major opportunity for intervention.

### Systolic blood pressure

In this assessment, as in GBD 2013, we found that the most important Level 3 risk factor globally is elevated systolic blood pressure. The TMREL for this estimation, a systolic blood pressure of between 110 mm Hg and 115 mm Hg, is based on evidence from the pooling of prospective cohort studies that showed that individuals with a baseline blood pressure at this level have the lowest risk of future cardiovascular death.^92^, ^93^ In addition to this observational evidence, two randomised clinical trials of blood pressure lowering have added substantially to the evidence in this area. Findings from the SPRINT trial^10^ showed that adults in the USA with elevated vascular risk and pre-existing hypertension (mean systolic blood pressure at baseline 139·7 mm Hg and on a mean of 1·8 medications) benefited from antihypertensive therapy that targeted a systolic blood pressure of 120 mm Hg. Findings from the HOPE-3 trial,^77^ a multinational study, showed that older adults (aged ≥55 years) with elevated vascular risk but without susbtantially elevated systolic blood pressure (mean systolic blood pressure at baseline 138·2 mm Hg) did not have any benefit when they received hydrochlorothiazide 12·5 mg plus candesartan 16 mg daily. However, neither study directly addresses selection of the GBD TMREL of 110–115 mm Hg since observational studies necessarily reflect the benefits accrued through maintenance of a healthy blood pressure throughout life rather than through use of blood pressure-lowering medications. The GBD estimate necessarily represents the effect of primary prevention and lifestyle modification, as well as the possibility of pharmacotherapy for achievement of the TMREL. The large potential gain in global health that we estimate if an optimal blood pressure was to be achieved suggests that further studies of blood pressure in people younger than 60 years of age remains an important area for investigation. A wide array of clinical and population strategies are available to reduce systolic blood pressure, including lowering population salt intake, increasing physical activity, reducing or slowing the rise of high BMI, and providing access to effective antihypertensives, which merit considerable attention in many countries.

### Alcohol use

We report that the global SEV for alcohol decreased by 1·1% (95% UI −6·0 to 2·3) for men and 13·1% (−16·1 to −10·7) for women. Of note, however, because of the geographical distributions of alcohol consumption and background disease rates, alcohol consumption contributed to an increase in alcohol attributable DALYs over the same period. Assessment of the burden attributable to alcohol is complicated by potentially elevated risks in former drinkers compared with abstainers, potential protective effects of mild-to-moderate use of alcohol, and use of a TMREL of zero consumption for this study. Findings from meta-analyses have shown considerably elevated relative risks in former drinkers, equivalent to 30 g of pure alcohol per day or more for some outcomes,^94^, ^95^ although this finding could occur as a result of confounding by misclassification of former drinkers as lifetime abstainers.^96^, ^97^, ^98^, ^99^ Future GBD studies should carefully re-evaluate whether or not the excess risk in former drinkers is overestimated. If the relative risks for former drinkers have been overestimated, this overestimation will also affect our assessment of the alcohol SEV and the global trend in the SEV. The protective effect of mild-to-moderate alcohol use reported for ischaemic heart disease, diabetes in women, and reduced all-cause mortality is controversial;^100^, ^101^, ^102^, ^103^, ^104^, ^105^ some authors argue that this consistent finding is due to confounding.^106^, ^107^ However, investigators of studies^108^ of all-cause mortality with certain types of quality exclusions find no overall mortality benefit of mild-to-moderate use. Various mendelian randomisation studies have contradicted previously claimed benefits of moderate alcohol consumption for several outcomes.^109^, ^110^, ^111^ Marked differences in male and female patterns of relative risk, such as for diabetes,^104^, ^105^ raise many questions about the biological pathways through which these effects might act. Finally, our alcohol analysis uses a TMREL of zero consumption; use of a higher TMREL than zero for alcohol use would increase our estimate of the global burden attributed to alcohol. Given the large burden of alcohol and the availability of effective options to reduce consumption at the population level, such as increased alcohol taxes, controls on outlet location and density, establishment or maintenance of limits on days or hours of sale, and screening and advice from health-care givers,^112^, ^113^, ^114^, ^115^ narrowing of the uncertainty in the alcohol assessment is important.

### High BMI and fasting plasma glucose

Among the top five Level 3 risk factors, DALYs attributable to high BMI increased the most from 1990 to 2015 while SEV also increased. High BMI is mediated through increases in systolic blood pressure, cholesterol, and fasting plasma glucose (methods appendix pp 28–35). Our decomposition analysis suggests that the increase in risk-attributable burden due to high BMI is considerably smaller than it could have been if underlying rates, particularly for cardiovascular diseases, had not declined as much as they did over the past decade. The decline in the underlying rates is likely due to expanded access to preventive treatment and increased quality of care.^116^, ^117^, ^118^, ^119^, ^120^ If obesity continues to increase in the future, the consequences for health trends might be greater if the trend in underlying rates attenuates than if it continues to decrease. Although closely linked, we also estimate that the rate of increase in high fasting plasma glucose is slightly higher than that for obesity. The combined effect of rising obesity and rising fasting plasma glucose has consequences for various health outcomes and health-care delivery costs. For obesity, various policy options have been proposed,^121^, ^122^, ^123^ but few, if any, strategies have been proven to work at the population level. Analysis of obesity options needs to be closely linked to consideration of diet and physical activity. Although for most diet components we have reported on the effect of diet composition on health outcomes, a strong argument exists to explore the relationship of specific diet components with overall BMI.

### Methods strengths and challenges: uncertainty in causality

To move the GBD and CRA fields forward, we have reported, for the first time, details of the evidence that is available to support the causal relationship of each risk-outcome pair. The strength of the evidence of causality varies widely across risk factors. For the risk-outcome pairs where evidence from randomised controlled trials or high-quality prospective observational studies was not available, we considered the possibility of using other types of evidence, including mendelian randomisation, to establish causality.^124^ Although we have not used this approach in GBD 2015, this body of evidence seems to provide useful information that deserves a more detailed evaluation in future iterations of GBD than in this study. Likewise, the appropriate use of case-control data to support causality needs to be more clearly defined than in this study.

Being explicit about the supporting evidence also shows that some risks have a similar body of evidence supporting them, even though, in some debates, the strength of evidence is thought to be very different. For this study, we focused on evidence mainly from prospective studies of exposure and disease endpoints. In select cases, we had to use prospective studies of intermediate outcomes, including trials of the effect of sodium on systolic blood pressure, prospective cohort studies of the effect of lead on systolic blood pressure, and trials and cohort studies of sugar-sweetened beverages' effect on BMI.^125^, ^126^, ^127^, ^128^ We did not include the strength of evidence uncertainty in our estimation of attributable burden, which requires more consensus than we had available on how different types of evidence can be combined to support a causal connection.

### Integrated exposure response curve for particulates less than 2·5 μg

In this study, as for GBD 2013, we have estimated the relative risk of exposure to particulate matter with an aerodynamic diameter smaller than 2·5 μm (PM2·5) over a broad range of daily doses by integrating relative risks from diverse sources of PM2·5, consisting of ambient air pollution, household air pollution, second-hand smoke, and tobacco smoking, using a single integrated exposure response (IER) curve.^129^, ^130^ The premise behind this approach is that PM2·5 daily dose is a common indicator of risk from diverse sources of particulate pollution. Although assessments support an assumption of the equitoxicity of particulate matter mass from different sources for the purposes of burden estimation,^131^, ^132^ this issue remains an area of active research. We expect that the IER will continue to evolve in response to the latest evidence in the field of air pollution dose response; in keeping with the iterative nature of the GBD estimates, inclusion of additional cohort studies at lower levels of exposure to PM2·5 than those previously identified and included led to a revision of the TMREL distribution that is less than half of the level used in GBD 2013. Inclusion of new high-quality studies and the non-linear nature of the IER curve have increased our estimate of global burden. Methodological improvements to the curve-fitting algorithm that better capture the heterogeneity of component studies than do those in GBD 2013 produce UIs that are considerably wider than are those estimated in GBD 2013.

### Future directions for GBD CRA

To date, attributable burden has been the primary metric that was methodologically and computationally feasible for GBD risk assessments. As burden forecasts become routinely available, quantification of avoidable burden within the GBD analytic framework will become more tractable than at present. Development of avoidable burden estimates will require resolution of various important issues, such as the degree of reversibility for some risks and the type of counterfactual distribution most relevant to avoidable burden calculation. Arguably, feasible or cost-effective counterfactual distributions of exposure might be more relevant than is minimum risk.

In the next iterations of the GBD CRA, we plan to incorporate new risks into two areas: distal socioeconomic factors, such as educational attainment, and the absence of effective health interventions, such as vaccination or seat belts. We will assess the evidence supporting new risk factors for inclusion and expand the scope of the analysis on the basis of both the evidence for causality and the availability of evidence for estimation of exposure levels. For behavioural, environmental and occupational, and metabolic risks, other candidate risks have been proposed for inclusion in GBD, including climate change, smokeless tobacco, added sugar, and access to health care. For existing risks, we will also carefully assess inclusion of new risk-outcome pairs. Continued close examination of evidence supporting causality might lead to modifications of the criteria for inclusion. For many distal factors, such as educational attainment, highly consistent evidence exists from prospective cohorts for all-cause mortality, but the relationships for cause-specific mortality might vary by context; for example, where access to treatment might modulate the effects of education on an outcome. For GBD 2015, we have made progress clarifying the evidence base for each risk-outcome pair. This type of in-depth examination will, we hope, lead to a simpler and more transparent approach than has been used before to define which risk-outcome pairs at any level of the causal web should be considered convincing or probable.

### Limitations

Since estimation of the attributable burden of disease includes estimates of deaths, YLLs, YLDs, or DALYs, the limitations described elsewhere for those also apply to this analysis.^133^, ^134^ We have developed our modelling strategies to quantify uncertainty given the available data. New data—particularly from countries where we currently lack data—might reveal levels of exposure that are outside the UIs that we have estimated. We assume that the joint effect for risk factors where we do not correct for mediation can be estimated with the multiplicative risk model. Although plausible, this model might not accurately capture how all risks interact.^135^ Analyses of the National Health and Nutrition Examination Survey III cohort suggest submultiplicative effects for some risks.^136^ A shift from a multiplicative model to a submultiplicative model has profound implications for all aspects of risk factor epidemiology, beginning at the estimation of relative risks and extending to use of relative risks as a generalisable construct.

As a consequence of the absence of sufficient studies across all risk-outcome pairs, we did not systematically correct relative risks for publication bias. We did not correct relative risks for non-masking in studies; however, not all risks can be studied in a masked fashion (eg, tobacco smoking). Comparability would be compromised if we corrected some risks but not others. We generally assumed that relative risks were uniform across countries for a given age-sex group.^137^, ^138^ Differences in relative risks with geography might exist, as has been found for the BMI relative risk curve and TMREL, but with the exception of breast cancer, we find that insufficient evidence exists to date to identify statistically significant differences.

Development of robust models to estimate variation in SD is more difficult than for estimations of the mean. Because measurement error is necessarily included in SD from studies, they are an overestimate of the true SD. We did not correct SD for measurement error, with the exception of correction of observed systolic blood pressure to usual blood pressure. Measurement error also affects estimation of relative risks through regression dilution bias—the attenuation of the association between the level of risk and the incidence of disease outcomes. Because of the absence of detailed data for key cohorts, we have not corrected cholesterol or fasting plasma glucose relative risks for regression dilution bias. Our approach of estimation of the maximum relative risk on the basis of the reported cohorts and trials might underestimate burden if risk continues to rise at the highest levels of exposure beyond those reported. Although a log-linear function for relative risks and levels of exposure is adequate for the observed range of exposure, available data on the most extreme values are few. Given that a very small fraction of the population in any country is in these extreme exposure levels, the potential bias in our estimates is minimal. Additionally, estimation of burden for risks divided into polytomous risks might underestimate their burden compared with estimation of burden with a continuous risk variable.

Although use of a log-normal distribution is the best of the parametric distributions that we have assessed for fasting plasma glucose, important deviations from the log-normal exist in survey data such as from the National Health and Nutrition Examination Survey. We have explored different mixtures of distributions, including two log-normal or a normal and log-normal, some of which provided better overall fit than the one that we used. However, we have so far not been able to solve the optimisation problem of estimation of two distributions given only a mean and SD reported from particular studies.

We did not use the relative risk and exposure PAF calculation for unsafe sex, HIV risk from injecting drug use, and occupational injuries; we used direct evidence of the attributable fraction. Comparability of the results derived from these direct or categorical approaches to the risks estimated with the relative risk and exposure model is not certain.

Too few studies exist to allow estimation of the contribution of household air pollution to ambient air pollution or vice versa.^139^ As a consequence, we might have underestimated the burden of household air pollution as a single risk factor; we might also have overestimated the burden of air pollution combined. Furthermore, our analysis of ambient air pollution has focused on PM2·5; other pollutants, including larger particulates than PM2·5 and nitrogen dioxide, might also be important to quantify. For cholesterol, we established our estimates of burden using total cholesterol; however, increasing evidence supporting the effects of LDL cholesterol—and improvements in data availability for serum LDL in different populations—suggest future estimations of risk from high cholesterol should use LDL cholesterol as the unit of estimation.

Proxies for exposure to some risk factors are coarse, which is particularly the case for zinc deficiency, for which we estimated the balance between theoretical intake and physiological requirements from Food and Agriculture Organization food balance sheets for absorbable zinc. Although zinc deficiency can be estimated from the proportion of people estimated to have inadequate zinc intake, individual-level measurement of exposure truly needed to estimate the number of people at risk is not available. In a similar fashion, we used the proportion of the population in coarse occupational categories as a proxy for exposure to specific carcinogens and used fuel type as a proxy for household air pollution. Our ability to capture geographical variation and uncertainty in conversion of household solid fuel use to PM2·5 exposure improved the validity of our findings and UIs. Use of direct PM2·5 exposure measurement in households would be preferable to calibrate the widely available data for fuel use; however, such data have rarely been collected. As with all previous studies, we assessed the burden attributable to the availability of water and sanitation infrastructure, not the use of the infrastructure. Exposure for these risks has been defined in terms of availability and not use because that is what household surveys have collected. Our estimates are not biased, however, by this limitation because we derived relative risks from similar exposure definitions. Finally, we have not estimated intimate partner violence for men because of a scarcity of evidence of the health effects among men.

### Conclusion

Quantification of the health effects of a diverse set of largely avoidable risks is an important input into any national or global strategy to improve population health and make progress towards the Sustainable Development Goals. For the first time, we have been able to separately assess and report trends in risk exposure and decompose trends in attributable burden into the contribution of demographic change, risk exposure, and risk-deleted rates. Reductions in some risks strongly associated with low levels of development, such as poor water, poor sanitation, household air pollution, and undernutrition, have been important contributors to global progress. Some crucial risks for NCDs, particularly obesity, high fasting plasma glucose, and alcohol, are increasing. Deleterious trends have had a smaller effect than expected because of favourable declines in risk-deleted DALY rates for many causes, a component of which might be due to access to effective health care. In an era of rapid transition in societies and levels of health, tracking of and response to key risks will require constant monitoring at the local level. As the set of risk factors expands to encompass access to specific health-care interventions in future iterations of GBD, the GBD CRA will provide an even more comprehensive understanding than in this study of the drivers of health improvement and how they relate to the operation and priorities of health systems.

Correspondence to: Prof Christopher J L Murray, Institute for Health Metrics and Evaluation, Seattle, WA 98121, USA cjlm@uw.edu

**This online publication has been corrected. The corrected version first appeared at thelancet.com on January 5, 2017**

## Figures and Tables

**Table 1:** Global Burden of Disease 2015 risk factor hierarchy, exposure definitions, theoretical minimum risk exposure level, and data representativeness index for 1985–2015, pre-2005, and 2005–15

				**Exposure definition**	**Theoretical minimum risk exposure level**	**Data representativeness index**		
						<2005	2005–15	Total
**All risk factors**				**..**	**..**	**100·0%**	**100·0%**	**100·0%**
**Environmental and occupational risks**				**..**	**..**	**100·0%**	**100·0%**	**100·0%**
	**Unsafe water, sanitation, and handwashing**			**..**	**..**	**73·2%**	**60·6%**	**78·8%**
		Unsafe water source		Proportion of households with access to different water sources (unimproved, improved except piped, piped water supply) and reported use of household water treatment methods (boiling or filtering; chlorinating or solar filtering; no treatment)	All households have access to water from a piped water supply that is also boiled or filtered before drinking	83·5%	70·1%	88·4%
		Unsafe sanitation		Proportion of households with access to different sanitation facilities (unimproved, improved except sewer, sewer connection)	All households have access to toilets with sewer connection	83·5%	69·5%	88·4%
		No handwashing with soap		Proportion of individuals who wash their hands with soap and water after potential faecal contact	All individuals wash hands with soap and water after potential faecal contact	7·6%	24·2%	27·3%
	**Air pollution**			**..**	**..**	**100·0%**	**100·0%**	**100·0%**
		Ambient particulate matter pollution		Annual average daily exposure to outdoor air concentrations of PM with an aerodynamic diameter smaller than 2·5 μm, measured in μg/m^3^	Uniform distribution between 2·4 μg/m^3^ and 5·9 μg/m^3^	100·0%	100·0%	100·0%
		Household air pollution from solid fuels		Annual average daily exposure to household concentrations of PM with an aerodynamic diameter smaller than 2·5 μm, measured in μg/m^3^ from solid fuel use (coal, wood, charcoal, dung, and agricultural residues)	No households are exposed to excess indoor concentration of particles from solid fuel use (assuming concentration of particulate matters, aerodynamic diameter smaller than 2·5 μm, measured in μg/m^3^ in no fuel use is consistent with a theoretical minimum risk level of 2·4–5·9)	69·7%	60·6%	75·8%
		Ambient ozone pollution		Seasonal (3 month) hourly maximum ozone concentrations, measured in ppb	Uniform distribution between 33·3 μg/m^3^ and 41·9 μg/m^3^, according to minimum/5th percentile concentrations	100·0%	100·0%	100·0%
	**Other environmental risks**			**..**	**..**	**44·9%**	**40·9%**	**47·0%**
		Residential radon		Average daily exposure to indoor air radon levels measured in becquerels (radon disintegrations per s) per cubic metre (Bq/m^3^)	10 Bq/m^3^, corresponding to the outdoor concentration of radon	36·4%	36·4%	36·4%
		Lead exposure		Blood lead levels in μg/dL of blood, bone lead levels in μg/g of bone	2 μg/dL, corresponding to lead levels in preindustrial humans as natural sources of lead prevent the feasibility of zero exposure	33·3%	19·2%	36·9%
	**Occupational risks**			**..**	**..**	**94·4%**	**93·4%**	**94·4%**
		Occupational carcinogens				94·4%	93·4%	94·4%
			Occupational exposure to asbestos	Proportion of the population with cumulative exposure to asbestos	No occupational exposure to asbestos	94·4%	93·4%	94·4%
			Occupational exposure to arsenic	Proportion of the population ever exposed to arsenic at work/through their occupation	No occupational exposure to arsenic	94·4%	93·4%	94·4%
			Occupational exposure to benzene	Proportion of the population ever exposed to benzene at work/through their occupation	No occupational exposure to benzene	94·4%	93·4%	94·4%
			Occupational exposure to beryllium	Proportion of the population ever exposed to beryllium at work/through their occupation	No occupational exposure to beryllium	94·4%	93·4%	94·4%
			Occupational exposure to cadmium	Proportion of the population ever exposed to cadmium at work/through their occupation	No occupational exposure to cadmium	94·4%	93·4%	94·4%
			Occupational exposure to chromium	Proportion of the population ever exposed to chromium at work/through their occupation	No occupational exposure to chromium	94·4%	93·4%	94·4%
			Occupational exposure to diesel engine exhaust	Proportion of the population ever exposed to diesel engine exhaust at work/through their occupation	No occupational exposure to diesel engine exhaust	94·4%	93·4%	94·4%
			Occupational exposure to second-hand smoke	Proportion of the population ever exposed to second-hand smoke at work/through their occupation	No occupational exposure to second-hand smoke	94·4%	93·4%	94·4%
			Occupational exposure to formaldehyde	Proportion of the population ever exposed to formaldehyde at work/through their occupation	No occupational exposure to formaldehyde	94·4%	93·4%	94·4%
			Occupational exposure to nickel	Proportion of the population ever exposed to nickel at work/through their occupation	No occupational exposure to nickel	94·4%	93·4%	94·4%
			Occupational exposure to polycyclic aromatic hydrocarbons	Proportion of the population ever exposed to polycyclic aromatic hydrocarbons at work/through their occupation	No occupational exposure to polycyclic aromatic hydrocarbons	94·4%	93·4%	94·4%
			Occupational exposure to silica	Proportion of the population ever exposed to silica at work/through their occupation	No occupational exposure to silica	94·4%	93·4%	94·4%
			Occupational exposure to sulphuric acid	Proportion of the population ever exposed to sulphuric acid at work/through their occupation	No occupational exposure to sulphuric acid	94·4%	93·4%	94·4%
			Occupational exposure to trichloroethylene	Proportion of the population ever exposed to trichloroethylene at work/through their occupation	No occupational exposure to trichloroethylene	94·4%	93·4%	94·4%
		Occupational asthmagens		Proportion of the population currently exposed to asthmagens at work/through their occupation	Background asthmagen exposures	94·4%	93·4%	94·4%
		Occupational particulate matter, gases, and fumes		Proportion of the population ever exposed to particulates, gases, or fumes at work/through their occupation	No occupational exposure to particulates, gases, or fumes	94·4%	93·4%	94·4%
		Occupational noise		Proportion of the population ever exposed to noise greater than 85 decibels at work/through their occupation	Background noise exposure	94·4%	93·4%	94·4%
		Occupational injuries		Proportion of the population at risk of injuries related to work/through their occupation	The rate of injury deaths per 100 000 person-years is zero	24·2%	32·3%	35·4%
		Occupational ergonomic factors		Proportion of the population who are exposed to ergonomic risk factors for low back pain at work/through their occupation	All individuals have the ergonomic factors of clerical and related workers	94·4%	93·4%	94·4%
**Behavioural risks**				**..**	**..**	**100·0%**	**100·0%**	**100·0%**
	**Child and maternal malnutrition**			**..**	**..**	**93·9%**	**91·4%**	**93·9%**
		Suboptimal breastfeeding				70·7%	57·6%	77·8%
			Non-exclusive breastfeeding	Proportion of children younger than 6 months who receive predominant, partial, or no breastfeeding	All children are exclusively breastfed for first 6 months of life	70·7%	57·6%	77·8%
			Discontinued breastfeeding	Proportion of children aged 6–23 months who do not receive any breastmilk	All children continue to receive breastmilk until 2 years of age	68·1%	65·3%	79·2%
		Childhood undernutrition				77·8%	61·6%	79·3%
			Childhood underweight	Proportion of children less than −3 SDs, −3 to −2 SDs, and −2 to −1 SDs of the WHO 2006 standard weight-for-age curve	All children are above −1 SD of the WHO 2006 standard weight-for-age curve	77·3%	61·6%	78·8%
			Childhood wasting	Proportion of children less than −3 SDs, −3 to −2 SDs, and −2 to −1 SDs of the WHO 2006 standard weight-for-length curve	All children are above −1 SD of the WHO 2006 standard weight-for-height curve	75·8%	61·1%	79·3%
			Childhood stunting	Proportion of children less than −3 SDs, −3 to −2 SDs, and −2 to −1 SDs of the WHO 2006 standard height-for-age curve	All children are above −1 SD of the WHO 2006 standard height-for-height curve	92·3%	79·6%	93·7%
	Iron deficiency			Peripheral blood haemoglobin concentration in g/L	Country specific	66·8%	30·7%	68·3%
	Vitamin A deficiency			Proportion of children aged 28 days to 5 years with serum retinol concentration <0·7 μmol/L	No childhood vitamin A deficiency	38·9%	5·1%	40·9%
	Zinc deficiency			Proportion of the population with inadequate zinc intake versus loss	No inadequate zinc intake	84·3%	84·3%	84·3%
	**Tobacco smoke**			**..**	**..**	**87·9%**	**94·4%**	**97·0%**
		Smoking		Proportion of the population with cumulative exposure to tobacco smoking; proportion of the population who currently smoke	100% of population are lifelong non-smokers	84·8%	92·4%	95·5%
		Second-hand smoke		Average daily exposure to indoor air PM from second-hand smoke with an aerodynamic diameter smaller than 2·5 μg, measured in μg/m^3^	No second-hand smoke exposure	58·6%	79·8%	86·4%
	**Alcohol and drug use**			**..**	**..**	**100·0%**	**100·0%**	**100·0%**
		Alcohol use		Average daily alcohol consumption of pure alcohol (measured in g/day) in current drinkers who had consumed alcohol during the past 12 months; binge drinking: proportion of the population reporting binge consumption of at least 60 g for males and 48 g for females of pure alcohol on a single occasion	No alcohol consumption	100·0%	100·0%	100·0%
		Drug use		Proportion of the population dependent on opioids, cannabis, cocaine, or amphetamines; proportion of the population who have ever injected drugs	No use	26·3%	49·0%	50·0%
	**Dietary risks**			**..**	**..**	**90·4%**	**92·4%**	**92·9%**
		Diet low in fruits		Average daily consumption of fruits (fresh, frozen, cooked, canned, or dried, excluding fruit juices and salted or pickled fruits)	Consumption of fruit between 200 g and 300 g per day	88·9%	88·9%	88·9%
		Diet low in vegetables		Average daily consumption of vegetables (fresh, frozen, cooked, canned or dried vegetables including legumes but excluding salted or pickled vegetables, juices, nuts and seeds, and starchy vegetables such as potatoes or corn)	Consumption of vegetables between 340 g and 500 g per day	88·9%	88·9%	88·9%
		Diet low in whole grains		Average daily consumption of whole grains (bran, germ, and endosperm in their natural proportion) from breakfast cereals, bread, rice, pasta, biscuits, muffins, tortillas, pancakes, and other sources	Consumption of whole grains between 100 g and 150 g per day	10·6%	9·1%	16·2%
		Diet low in nuts and seeds		Average daily consumption of nut and seed foods	Consumption of nuts and seeds between 16 g and 25 g per day	88·9%	88·9%	88·9%
		Diet low in milk		Average daily consumption of milk, including non-fat, low-fat, and full-fat milk, excluding soy milk and other plant derivatives	Consumption of milk between 350 g and 520 g per day	88·9%	88·9%	88·9%
		Diet high in red meat		Average daily consumption of red meat (beef, pork, lamb, and goat but excluding poultry, fish, eggs, and all processed meats)	Consumption of red meat between 18 g and 27 g per day	88·9%	88·9%	88·9%
		Diet high in processed meat		Average daily consumption of meat preserved by smoking, curing, salting, or addition of chemical preservatives	Consumption of processed meat between 0 g and 4 g per day	22·2%	11·6%	27·3%
		Diet high in sugar-sweetened beverages		Average daily consumption of beverages with ≥50 kcal per 226·8 g serving, including carbonated beverages, sodas, energy drinks, and fruit drinks, but excluding 100% fruit and vegetable juices	Consumption of sugar-sweetened beverages between 0 g and 5 g per day	22·2%	12·6%	26·8%
		Diet low in fibre		Average daily intake of fibre from all sources, including fruits, vegetables, grains, legumes, and pulses	Consumption of fibre between 19 g and 28 g per day	88·9%	88·9%	88·9%
		Diet low in calcium		Average daily intake of calcium from all sources, including milk, yogurt, and cheese	Consumption of calcium between 1·00 g and 1·50 g per day	88·9%	88·9%	88·9%
		Diet low in seafood omega-3 fatty acids		Average daily intake of eicosapentaenoic acid and docosahexaenoic acid	Consumption of seafood omega-3 fatty acids between 200 mg and 300 mg per day	88·9%	88·9%	88·9%
		Diet low in polyunsaturated fatty acids		Average daily intake of omega-6 fatty acids from all sources, mainly liquid vegetable oils, including soybean oil, corn oil, and safflower oil	Consumption of polyunsaturated fatty acids between 9% and 13% of total daily energy	88·9%	88·9%	88·9%
		Diet high in trans fatty acids		Average daily intake of trans fat from all sources, mainly partially hydrogenated vegetable oils and ruminant products	Consumption of trans fatty acids between 0% and 1% of total daily energy	39·9%	39·4%	39·9%
		Diet high in sodium		24 h urinary sodium measured in g per day	24 h urinary sodium between 1 g and 5 g per day	28·8%	13·1%	32·3%
	**Sexual abuse and violence**			**..**	**..**	**43·9%**	**59·1%**	**66·2%**
		Childhood sexual abuse		Proportion of the population who have ever experienced one or more acts of childhood sexual abuse, defined as the experience with an older person of unwanted non-contact, contact abuse, or intercourse, when aged 15 years or younger	No childhood sexual abuse	27·8%	19·7%	34·3%
		Intimate partner violence		Proportion of the population who have ever experienced one or more acts of physical or sexual violence by a present or former intimate partner since age 15 years	No intimate partner violence	41·9%	56·6%	63·6%
	**Unsafe sex**			**Proportion of the population with exposure to sexual encounters that convey the risk of disease**	**No exposure to a disease agent through sex**	**17·7%**	**48·0%**	**48·0%**
	**Low physical activity**			**Average weekly physical activity at work, at home, transport related, and recreational measured by MET min per week**	**Highly active, ≥8000 MET min per week**	**45·5%**	**50·5%**	**66·7%**
**Metabolic risks**				**..**	**..**	**83·8%**	**88·4%**	**93·9%**
	High fasting plasma glucose			Serum fasting plasma glucose measured in mmol/L	4·8–5·4	46·0%	60·1%	71·2%
	High total cholesterol			Serum total cholesterol, measured in mmol/L	2·78–3·38	49·5%	48·5%	69·2%
	High systolic blood pressure			Systolic blood pressure, measured in mm Hg	110–115	55·1%	66·2%	79·3%
	High body-mass index			Body-mass index, measured in kg/m^2^	20–25	78·3%	83·3%	90·9%
	Low bone mineral density			Standardised mean bone mineral density values measured at the femoral neck in g/cm^2^	99th percentile of NHANES 2005–10 by age and sex	23·7%	11·1%	25·8%
	Low glomerular filtration rate			Proportion of the population with a glomerular filtration rate <60 mL/min per 1·73 m^2^, and excluding end-stage renal disease	>60 mL/min per 1·73 m^2^	9·1%	17·2%	20·2%

The percentage of available data is calculated out of a total of 519 subnational Level 2 geographies. PM=particulate matter. ppb=parts per billion. MET=metabolic equivalent. NHANES=National Health and Nutrition Examination Survey.

**Table 2:** Epidemiological evidence supporting causality for risk-outcome pairs included in the Global Burden of Disease study 2015

		**Outcome**	**RCTs (n)**	**RCTs with significant effect in the opposite direction (%)**	**RCTs with null findings (%)**	Prospective observational studies (n)*	**Prospective observational studies with significant association in the opposite direction (%)**	**Lower limit of RR >1·5**	**Dose-response relationship**	**Biological plausibility**†	**Analogy**‡
**Unsafe water, sanitation, and handwashing**											
Unsafe water source: chlorination or solar (point-of-use treatment)		Diarrhoeal diseases	24	0	42%	6	0	Yes	..	Yes	No
Unsafe water source: filter		Diarrhoeal diseases	11	0	45%	2	0	Yes	..	Yes	No
Unsafe water source: improved water		Diarrhoeal diseases	0	..	..	5	0	Yes	..	Yes	No
Unsafe water source: improved water		Typhoid fever	0	..	..	0	..	Yes	..	Yes	Yes
Unsafe water source: improved water		Paratyphoid fever	0	..	..	0	..	Yes	..	Yes	Yes
Unsafe water source: piped		Diarrhoeal diseases	1	0	0	9	11%	Yes	..	Yes	No
Unsafe water source: piped		Typhoid fever	0	..	..	0	..	Yes	..	Yes	Yes
Unsafe water source: piped		Paratyphoid fever	0	..	..	0	..	Yes	..	Yes	Yes
Unsafe sanitation: improved sanitation		Diarrhoeal diseases	0	..	..	9	0	Yes	..	Yes	No
Unsafe sanitation: improved sanitation		Typhoid fever	0	..	..	0	..	Yes	..	Yes	Yes
Unsafe sanitation: improved sanitation		Paratyphoid fever	0	..	..	0	..	Yes	..	Yes	Yes
Unsafe sanitation: piped		Diarrhoeal diseases	0	..	..	1	0	Yes	..	Yes	No
Unsafe sanitation: piped		Typhoid fever	0	..	..	0	..	Yes	..	Yes	Yes
Unsafe sanitation: piped		Paratyphoid fever	0	..	..	0	..	Yes	..	Yes	Yes
No handwashing with soap		Diarrhoeal diseases	19	0	42%	0	..	No	..	Yes	No
No handwashing with soap		Typhoid fever	0	..	..	0	..	No	..	Yes	Yes
No handwashing with soap		Paratyphoid fever	0	..	..	0	..	No	..	Yes	Yes
No handwashing with soap		Lower respiratory infections	1	0	0	6	0	No	..	Yes	No
**Air pollution**											
Ambient particulate matter pollution		Lower respiratory infections	0	..	..	13	0	No	Yes	Yes	No
Ambient particulate matter pollution		Ischaemic stroke	0	..	..	25	0	No	Yes	Yes	Yes
Ambient particulate matter pollution		Haemorrhagic stroke	0	..	..	25	0	No	Yes	Yes	Yes
Ambient particulate matter pollution		Ischaemic heart disease	0	..	..	16	0	No	Yes	Yes	Yes
Ambient particulate matter pollution		Chronic obstructive pulmonary disease	0	..	..	11	0	No	Yes	Yes	Yes
Ambient particulate matter pollution		Tracheal, bronchial, and lung cancer	0	..	..	27	0	No	Yes	Yes	Yes
Household air pollution from solid fuels		Lower respiratory infections	0	..	..	0	..	No	Yes	Yes	No
Household air pollution from solid fuels		Cataract	0	..	..	0	..	No	Yes	Yes	No
Household air pollution from solid fuels		Ischaemic stroke	0	..	..	25	0	No	Yes	Yes	Yes
Household air pollution from solid fuels		Haemorrhagic stroke	0	..	..	25	0	No	Yes	Yes	Yes
Household air pollution from solid fuels		Ischaemic heart disease	0	..	..	16	0	No	Yes	Yes	Yes
Household air pollution from solid fuels		Chronic obstructive pulmonary disease	0	..	..	0	..	No	Yes	Yes	Yes
Household air pollution from solid fuels		Tracheal, bronchial, and lung cancer	0	..	..	0	..	No	Yes	Yes	Yes
Ambient ozone pollution		Chronic obstructive pulmonary disease	0	..	..	4	0	No	Yes	Yes	No
**Other environmental risks**											
	Residential radon	Tracheal, bronchial, and lung cancer	0	..	..	3	0	No	Yes	Yes	No
	Lead exposure	Systolic blood pressure	0	..	..	3	0	..	Yes	Yes	..
	Lead exposure	Idiopathic intellectual disability	0	..	..	8	0	No	Yes	Yes	No
**Occupational risks**											
Occupational exposure to asbestos		Larynx cancer	0	..	..	27	..	No	..	Yes	Yes
	Occupational exposure to asbestos	Tracheal, bronchial, and lung cancer	0	..	..	18	0	Yes	..	Yes	Yes
	Occupational exposure to asbestos	Ovarian cancer	0	..	..	15	0	No	..	Yes	Yes
Occupational exposure to asbestos		Mesothelioma	0	..	..	5	0	Yes	..	Yes	Yes
Occupational exposure to arsenic		Tracheal, bronchial, and lung cancer	0	..	..	3	0	No	..	Yes	No
Occupational exposure to benzene		Leukaemia	0	..	..	12	0	Yes	..	Yes	No
Occupational exposure to beryllium		Tracheal, bronchial, and lung cancer	0	..	..	4	0	No	..	Yes	No
Occupational exposure to cadmium		Tracheal, bronchial, and lung cancer	0	..	..	7	0	No	..	Yes	No
Occupational exposure to chromium		Tracheal, bronchial, and lung cancer	0	..	..	26	0	No	..	Yes	No
Occupational exposure to diesel engine exhaust		Tracheal, bronchial, and lung cancer	0	..	..	17	0	No	..	Yes	No
Occupational exposure to second-hand smoke		Tracheal, bronchial, and lung cancer	0	..	..	25	0	No	..	Yes	No
Occupational exposure to formaldehyde		Nasopharyngeal cancer	0	..	..	1	0	No	..	Yes	Yes
Occupational exposure to formaldehyde		Leukaemia	0	..	..	12	0	No	..	Yes	Yes
Occupational exposure to nickel		Tracheal, bronchial, and lung cancer	0	..	..	1	0	No	..	Yes	No
Occupational exposure to polycyclic aromatic hydrocarbons		Tracheal, bronchial, and lung cancer	0	..	..	39	0	No	..	Yes	No
Occupational exposure to silica		Tracheal, bronchial, and lung cancer	0	..	..	17	0	No	..	Yes	No
Occupational exposure to sulphuric acid		Larynx cancer	0	..	..	3	0	Yes	..	Yes	No
Occupational exposure to trichloroethylene		Kidney cancer	0	..	..	20	0	No	..	Yes	No
Occupational asthmagens		Asthma	0	..	..	3	0	No	..	Yes	No
Occupational particulate matter, gases, and fumes		Chronic obstructive pulmonary disease	0	..	..	1	0	No	..	Yes	No
Occupational noise		Age-related and other hearing loss	0	..	..	4	0	Yes	..	Yes	No
Occupational ergonomic factors		Low back pain	0	..	..	10	0	No	..	Yes	No
**Child and maternal malnutrition**											
Non-exclusive breastfeeding		Diarrhoeal diseases	0	..	..	5	0	Yes	..	Yes	No
Non-exclusive breastfeeding		Lower respiratory infections	0	..	..	6	0	Yes	..	Yes	No
Discontinued breastfeeding		Diarrhoeal diseases	0	..	..	2	0	No	..	Yes	No
Childhood underweight		Diarrhoeal diseases	0	..	..	7	..	Yes	..	Yes	No
Childhood underweight		Lower respiratory infections	0	..	..	7	..	Yes	..	Yes	No
Childhood underweight		Measles	0	..	..	7	..	Yes	..	Yes	No
Childhood wasting		Diarrhoeal diseases	0	..	..	7	..	Yes	..	Yes	No
Childhood wasting		Lower respiratory infections	0	..	..	7	..	Yes	..	Yes	No
Childhood wasting		Measles	0	..	..	7	..	Yes	..	Yes	No
Childhood stunting		Diarrhoeal diseases	0	..	..	7	..	No	..	Yes	No
Childhood stunting		Lower respiratory infections	0	..	..	7	..	No	..	Yes	No
Childhood stunting		Measles	0	..	..	7	..	No	..	Yes	No
Iron deficiency		Maternal haemorrhage	0	..	..	0	..	No	..	Yes	Yes
Iron deficiency		Maternal sepsis and other pregnancy-related infections	0	..	..	0	..	No	..	Yes	Yes
Vitamin A deficiency		Diarrhoeal diseases	19	0	63%	0	..	No	..	Yes	No
Vitamin A deficiency		Measles	12	0	83%	0	..	Yes	..	Yes	No
Zinc deficiency		Diarrhoeal diseases	14	0	29%	0	..	No	..	Yes	No
Zinc deficiency		Lower respiratory infections	6	0	17%	0	..	No	..	Yes	No
**Tobacco smoke**											
Smoking		Tuberculosis	0	..	..	4	0	No	..	Yes	Yes
Smoking		Lower respiratory infections	0	..	..	0	..	No	..	Yes	Yes
Smoking		Lip and oral cavity cancer	0	..	..	3	0	Yes	..	Yes	Yes
Smoking		Nasopharyngeal cancer	0	..	..	3	0	Yes	..	Yes	Yes
Smoking		Oesophageal cancer	0	..	..	5	0	Yes	..	Yes	Yes
Smoking		Larynx cancer	0	..	..	4	0	Yes	..	Yes	Yes
Smoking		Stomach cancer	0	..	..	9	0	No	..	Yes	Yes
Smoking		Colon and rectum cancer	0	..	..	19	0	No	..	Yes	Yes
Smoking		Liver cancer	0	..	..	54	0	Yes	..	Yes	Yes
Smoking		Pancreatic cancer	0	..	..	13	0	Yes	..	Yes	Yes
Smoking		Tracheal, bronchial, and lung cancer	0	..	..	38	0	Yes	..	Yes	Yes
Smoking		Cervical cancer	0	..	..	15	0	No	..	Yes	Yes
Smoking		Kidney cancer	0	..	..	8	0	Yes	..	Yes	Yes
Smoking		Bladder cancer	0	..	..	17	0	Yes	..	Yes	Yes
Smoking		Leukaemia	0	..	..	14	0	No	..	Yes	Yes
Smoking		Ischaemic heart disease	0	..	..	86	..	No	..	Yes	Yes
Smoking		Ischemic stroke	0	..	..	60	..	No	..	Yes	Yes
Smoking		Haemorrhagic stroke	0	..	..	60	..	No	..	Yes	Yes
Smoking		Hypertensive heart disease	0	..	..	5	..	No	..	Yes	Yes
Smoking		Atrial fibrillation and flutter	0	..	..	16	0	No	..	Yes	Yes
Smoking		Aortic aneurysm	0	..	..	10	0	No	..	Yes	Yes
Smoking		Peripheral vascular disease	0	..	..	10	0	No	..	Yes	Yes
Smoking		Other cardiovascular and circulatory diseases	0	..	..	1	0	No	..	Yes	Yes
Smoking		Chronic obstructive pulmonary disease	0	..	..	42	0	Yes	..	Yes	Yes
Smoking		Silicosis	0	..	..	0	..	No	..	Yes	Yes
Smoking		Asbestosis	0	..	..	0	..	No	..	Yes	Yes
Smoking		Coal workers pneumoconiosis	0	..	..	0	..	Yes	..	Yes	Yes
Smoking		Other pneumoconiosis	0	..	..	0	..	Yes	..	Yes	Yes
Smoking		Asthma	0	..	..	6	0	No	..	Yes	Yes
Smoking		Interstitial lung disease and pulmonary sarcoidosis	0	..	..	0	..	Yes	..	Yes	Yes
Smoking		Other chronic respiratory diseases	0	..	..	1	0	Yes	..	Yes	Yes
Smoking		Peptic ulcer disease	0	..	..	7	0	No	..	Yes	No
Smoking		Diabetes mellitus	0	..	..	51	0	No	..	Yes	No
Smoking		Cataract	0	..	..	10	0	No	..	Yes	No
Smoking		Macular degeneration	0	..	..	5	20%	No	..	Yes	No
Smoking		Rheumatoid arthritis	0	..	..	5	0	No	..	Yes	No
Smoking		Hip fracture	0	..	..	15	20%	No	..	Yes	Yes
Smoking		Non-hip fracture	0	..	..	14	14%	No	..	Yes	Yes
Second-hand smoke		Otitis media	0	..	..	17	0	No	Yes	Yes	No
Second-hand smoke		Tracheal, bronchial, and lung cancer	0	..	..	4	0	No	Yes	Yes	Yes
Second-hand smoke		Ischaemic heart disease	0	..	..	4	25%	No	Yes	Yes	Yes
Second-hand smoke		Ischaemic stroke	0	..	..	10	0	Yes	Yes	Yes	Yes
Second-hand smoke		Haemorrhagic stroke	0	..	..	10	0	Yes	Yes	Yes	Yes
**Alcohol and drug use**											
Alcohol use		Tuberculosis	0	..	..	3	0	No	Yes	Yes	Yes
Alcohol use		Lower respiratory infections	0	..	..	2	0	No	Yes	Yes	Yes
Alcohol use		Lip and oral cavity cancer	0	..	..	1	0	No	Yes	Yes	Yes
Alcohol use		Nasopharyngeal cancer	0	..	..	1	0	No	Yes	Yes	Yes
Alcohol use		Other pharyngeal cancer	0	..	..	1	0	No	Yes	Yes	Yes
Alcohol use		Oesophageal cancer	0	..	..	1	0	No	Yes	Yes	Yes
Alcohol use		Colon and rectum cancer	0	..	..	6	0	No	Yes	Yes	Yes
Alcohol use		Liver cancer	0		..	3	0	No	Yes	Yes	Yes
Alcohol use		Laryngeal cancer	0	..	..	0	..	No	Yes	Yes	Yes
Alcohol use		Breast cancer	0	..	..	12	0	No	Yes	Yes	Yes
Alcohol use		Ischaemic heart disease	0	..	..	32	0	No	Yes	Yes	Yes
Alcohol use		Ischaemic stroke	0	..	..	20	0	No	Yes	Yes	Yes
Alcohol use		Haemorrhagic stroke	0	..	..	16	0	No	Yes	Yes	Yes
Alcohol use		Atrial fibrillation and flutter	0	..	..	10	0	No	Yes	Yes	Yes
Alcohol use		Hypertensive heart disease	0	..	..	2	0	No	Yes	Yes	Yes
Alcohol use		Pancreatitis	0	..	..	4	0	No	Yes	Yes	No
Alcohol use		Epilepsy	0	..	..	0	..	No	Yes	Yes	No
Alcohol use		Diabetes mellitus	0	..	..	9	0	No	Yes	Yes	No
Alcohol use		Cirrhosis	0	..	..	14	0	No	Yes	Yes	Yes
Alcohol use		Injuries	0	..	..	29	0	No	Yes	Yes	Yes
Alcohol use		Self-harm	0	..	..	0	..	No	Yes	Yes	Yes
Alcohol use		Interpersonal violence	0	..	..	11	0	No	Yes	Yes	Yes
Drug use		Hepatitis B	0	..	..	6	0	Yes	..	Yes	Yes
Drug use		Hepatitis C	0	..	..	16	0	Yes	..	Yes	Yes
Drug use		Self-harm	0	..	..	1	0	No	..	Yes	No
**Dietary risks**											
Diet low in fruits		Lip and oral cavity cancer	0	..	..	2	0	No	Yes	Yes	Yes
Diet low in fruits		Nasopharyngeal cancer	0	..	..	2	0	No	Yes	Yes	Yes
Diet low in fruits		Other pharyngeal cancer	0	..	..	2	0	No	Yes	Yes	Yes
Diet low in fruits		Larynx cancer	0	..	..	2	0	No	Yes	Yes	Yes
Diet low in fruits		Oesophageal cancer	0	..	..	5	0	No	Yes	Yes	Yes
Diet low in fruits		Tracheal, bronchial, and lung cancer	0	..	..	22	0	No	Yes	Yes	Yes
Diet low in fruits		Ischaemic heart disease	0	..	..	9	0	No	Yes	Yes	Yes
Diet low in fruits		Ischaemic stroke	0	..	..	9	0	No	Yes	Yes	Yes
Diet low in fruits		Haemorrhagic stroke	0	..	..	5	0	No	Yes	Yes	Yes
Diet low in fruits		Diabetes mellitus	0	..	..	9	0	No	Yes	Yes	No
Diet low in vegetables		Oesophageal cancer	0	..	..	5	0	No	Yes	Yes	No
Diet low in vegetables		Ischaemic heart disease	0	..	..	9	0	No	Yes	Yes	Yes
Diet low in vegetables		Ischaemic stroke	0	..	..	8	0	No	Yes	Yes	Yes
Diet low in vegetables		Haemorrhagic stroke	0	..	..	5	0	No	Yes	Yes	Yes
Diet low in whole grains		Ischaemic heart disease	0	..	..	7	0	No	Yes	Yes	Yes
Diet low in whole grains		Ischaemic stroke	0	..	..	6	0	No	Yes	Yes	Yes
Diet low in whole grains		Haemorrhagic stroke	0	..	..	6	0	No	Yes	Yes	Yes
Diet low in whole grains		Diabetes mellitus	0	..	..	10	0	No	Yes	Yes	No
Diet low in nuts and seeds		Ischaemic heart disease	1	0	100%	6	0	No	Yes	Yes	No
Diet low in nuts and seeds		Diabetes mellitus	1	0	100%	5	0	No	Yes	Yes	No
Diet low in milk		Colon and rectum cancer	0	..	..	7	0	No	Yes	Yes	No
Diet high in red meats		Colon and rectum cancer	0	..	..	8	0	No	Yes	Yes	No
Diet high in red meats		Diabetes mellitus	0	..	..	9	11%	No	Yes	Yes	No
Diet high in processed meats		Colon and rectum cancer	0	..	..	9	11%	No	Yes	Yes	No
Diet high in processed meats		Ischaemic heart disease	0	..	..	5	0	No	Yes	Yes	No
Diet high in processed meats		Diabetes mellitus	0	..	..	8	0	No	Yes	Yes	No
Diet high in sugar-sweetened beverages		Body-mass index	10	0	60%	22	0	..	Yes	Yes	..
Diet low in fibre		Colon and rectum cancer	0	..	..	15	0	No	Yes	Yes	No
Diet low in fibre		Ischaemic heart disease	0	..	..	12	0	No	Yes	Yes	No
Diet low in calcium		Colon and rectum cancer	0	..	..	13	0	No	Yes	Yes	No
Diet low in seafood omega-3 fatty acids		Ischaemic heart disease	17	0	88%	16	0	No	Yes	Yes	No
Diet low in polyunsaturated fatty acids		Ischaemic heart disease	8	0	75%	11	0	No	Yes	Yes	No
Diet high in trans fatty acids		Ischaemic heart disease	0	..	..	4	0	No	Yes	Yes	No
Diet high in sodium		Systolic blood pressure	45	0	73%	..	..	..	Yes	Yes	..
Diet high in sodium		Stomach cancer	0	..	..	3	0	No	Yes	Yes	No
**Sexual abuse and violence**											
Intimate partner violence		HIV/AIDS	0	..	..	2	0	No	..	Yes	No
Intimate partner violence		Maternal abortion, miscarriage, and ectopic pregnancy	0	..	..	1	0	Yes	..	Yes	No
Intimate partner violence		Depressive disorders	0	..	..	6	0	No	..	Yes	Yes
Intimate partner violence		Self-harm	0	..	..	2	0	Yes	..	Yes	Yes
Childhood sexual abuse		Alcohol use disorders	0	..	..	4	0	No	..	Yes	Yes
Childhood sexual abuse		Depressive disorders	0	..	..	5	0	No	..	Yes	Yes
Childhood sexual abuse		Self-harm	0	..	..	8	0	No	..	Yes	Yes
**Low physical activity**											
Low physical activity		Colon and rectum cancer	0	..	..	20	15%	No	Yes	Yes	Yes
Low physical activity		Breast cancer	0	..	..	35	0	No	Yes	Yes	Yes
Low physical activity		Ischaemic heart disease	0	..	..	45	9%	No	Yes	Yes	Yes
Low physical activity		Ischaemic stroke	0	..	..	27	11%	No	Yes	Yes	Yes
Low physical activity		Diabetes mellitus	0	..	..	57	7%	No	Yes	Yes	No
**Metabolic risks**											
High fasting plasma glucose		Ischaemic heart disease	8	0	100%	150	..	Yes	Yes	Yes	Yes
High fasting plasma glucose		Ischaemic stroke	9	0	100%	150	..	Yes	Yes	Yes	Yes
High fasting plasma glucose		Haemorrhagic stroke	9	0	100%	150	..	Yes	Yes	Yes	Yes
High fasting plasma glucose		Peripheral vascular disease	14	..	..	4	0	Yes	Yes	Yes	Yes
High fasting plasma glucose		Tuberculosis	0	..	..	17	0	Yes	Yes	Yes	No
High fasting plasma glucose		Chronic kidney disease	5	..	..	32	..	Yes	Yes	Yes	No
High total cholesterol		Ischaemic heart disease	21	0	57%	88	..	Yes	Yes	Yes	Yes
High total cholesterol		Ischaemic stroke	21	0	57%	88	..	Yes	Yes	Yes	Yes
High systolic blood pressure		Rheumatic heart disease	0	..	..	62	..	Yes	Yes	Yes	Yes
High systolic blood pressure		Ischaemic heart disease	56	0	..	88	..	Yes	Yes	Yes	Yes
High systolic blood pressure		Ischaemic stroke	54	0	..	150	..	Yes	Yes	Yes	Yes
High systolic blood pressure		Haemorrhagic stroke	54	0	..	150	..	Yes	Yes	Yes	Yes
High systolic blood pressure		Cardiomyopathy and myocarditis	0	..	..	62	..	Yes	Yes	Yes	Yes
High systolic blood pressure		Atrial fibrillation and flutter	20	5%	60%	88	..	Yes	Yes	Yes	Yes
High systolic blood pressure		Aortic aneurysm	0	..	..	62	..	Yes	Yes	Yes	Yes
High systolic blood pressure		Peripheral vascular disease	0	..	..	88	..	Yes	Yes	Yes	Yes
High systolic blood pressure		Endocarditis	0	..	..	62	..	Yes	Yes	Yes	Yes
High systolic blood pressure		Other cardiovascular and circulatory diseases	0	..	..	88	..	No	Yes	Yes	Yes
High systolic blood pressure		Chronic kidney disease	8	..	..	88	..	Yes	Yes	Yes	No
High body-mass index		Oesophageal cancer	0	..	..	8	0	No	Yes	Yes	Yes
High body-mass index		Colon and rectum cancer	0	..	..	38	0	No	Yes	Yes	Yes
High body-mass index		Liver cancer	0	..	..	34	0	No	Yes	Yes	Yes
High body-mass index		Gallbladder and biliary tract cancer	0	..	..	10	0	No	Yes	Yes	Yes
High body-mass index		Pancreatic cancer	0	..	..	20	0	No	Yes	Yes	Yes
High body-mass index		Breast cancer (after menopause)	0	..	..	44	2%	No	Yes	Yes	Yes
High body-mass index		Breast cancer (before menopause)	0	..	..	25	8%	No	Yes	Yes	No
High body-mass index		Uterine cancer	0	..	..	37	0	No	Yes	Yes	Yes
High body-mass index		Ovarian cancer	0	..	..	31	3%	No	Yes	Yes	Yes
High body-mass index		Kidney cancer	0	..	..	28	0	No	Yes	Yes	Yes
High body-mass index		Thyroid cancer	0	..	..	16	0	No	Yes	Yes	Yes
High body-mass index		Leukaemia	0	..	..	17	0	No	Yes	Yes	Yes
High body-mass index		Ischaemic heart disease	0	..	..	129	..	No	Yes	Yes	Yes
High body-mass index		Ischaemic stroke	0	..	..	102	..	No	Yes	Yes	Yes
High body-mass index		Haemorrhagic stroke	0	..	..	129	..	No	Yes	Yes	Yes
High body-mass index		Hypertensive heart disease	0	..	..	85	..	No	Yes	Yes	Yes
High body-mass index		Diabetes mellitus	0	..	..	85	..	Yes	Yes	Yes	No
High body-mass index		Chronic kidney disease	0	..	..	57	..	No	Yes	Yes	No
High body-mass index		Osteoarthritis	0	..	..	32	0	No	Yes	Yes	Yes
High body-mass index		Low back pain	0	..	..	5	0	No	Yes	Yes	Yes
Low bone mineral density		Injuries	0	..	..	12	..	No	Yes	Yes	Yes
Low glomerular filtration rate		Ischaemic heart disease	0	..	..	10	0	Yes	..	Yes	Yes
Low glomerular filtration rate		Ischaemic stroke	0	..	..	12	0	Yes	..	Yes	Yes
Low glomerular filtration rate		Haemorrhagic stroke	0	..	..	12	0	Yes	..	Yes	Yes
Low glomerular filtration rate		Peripheral vascular disease	0	..	..	1	0	Yes	..	Yes	Yes
Low glomerular filtration rate		Gout	0	..	..	3	0	Yes	..	Yes	No

If multiple reports existed from one study, we counted them as one study. We only assessed the dose-response relationship for continuous risks. To evaluate the magnitude of the effect size for continuous risks, we evaluated the RR comparing the 75th percentile with the 25th percentile of the exposure distribution at the global level. Additional information for this table is found in the methods appendix (pp 245–63). RCT=randomised controlled trial. RR=relative risk.

* Prospective cohort studies or non-randomised interventions.

† Whether or not any biological or mechanistic pathway exists that could potentially explain the relationship of the risk-outcome pair.

‡ Whether or not the risk is associated with another outcome from the same category and whether or not any evidence exists that it can cause the current outcome through the same pathway.

**Table 3:** Global age-standardised summary exposure values for all risk factors for 1990, 2005, and 2015

	**Men**						**Women**					
	1990 (%)	2005 (%)	2015 (%)	Percentage change 1990–2005	Percentage change 2005–15	Percentage change 1990–2015	1990 (%)	2005 (%)	2015 (%)	Percentage change 1990–2005	Percentage change 2005–15	Percentage change 1990–2015
Unsafe sanitation	55·0 (53·8 to 56·5)	42·6 (41·5 to 43·8)	33·7 (32·2 to 35·1)	−22·6 (−24·0 to −20·9)*	−26·5 (−23·5 to −18·5)*	−38·8 (−41·0 to −36·6)*	54·2 (52·8 to 55·7)	42·3 (41·2 to 43·6)	33·7 (32·2 to 35·1)	−21·9 (−23·4 to −20·2)*	−25·6 (−23·0 to −18·0)*	−37·8 (−40·0 to −35·6)*
Childhood underweight	13·2 (11·7 to 14·7)	11·3 (9·9 to 12·6)	8·7 (7·4 to 10·0)	−14·5 (−16·9 to −12·1)*	−30·0 (−26·5 to −20·1)*	−34·2 (−37·8 to −30·8)*	13·1 (11·6 to 14·5)	11·2 (9·8 to 12·5)	8·6 (7·3 to 9·9)	−14·7 (−17·1 to −12·3)*	−29·9 (−26·5 to −20·1)*	−34·3 (−37·9 to −31·0)*
Childhood stunting	27·0 (18·6 to 29·6)	22·5 (15·7 to 24·8)	18·0 (12·6 to 20·2)	−16·7 (−19·1 to −14·6)*	−25·2 (−23·5 to −17·7)*	−33·5 (−37·6 to −30·5)*	26·3 (18·1 to 28·8)	21·9 (15·2 to 24·2)	17·5 (12·2 to 19·8)	−16·6 (−19·0 to −14·5)*	−24·9 (−23·2 to −17·5)*	−33·2 (−37·3 to −30·2)*
Household air pollution from solid fuels	23·2 (15·7 to 32·1)	19·9 (13·5 to 27·6)	16·0 (10·8 to 22·2)	−14·1 (−16·1 to −12·1)*	−24·2 (−22·6 to −16·3)*	−30·8 (−33·9 to −27·4)*	29·3 (20·2 to 39·3)	25·4 (17·6 to 34·1)	20·6 (14·1 to 27·6)	−13·3 (−15·4 to −11·2)*	−23·2 (−21·9 to −15·8)*	−29·6 (−32·7 to −26·3)*
Smoking	29·0 (27·0 to 31·6)	24·7 (22·7 to 27·4)	21·0 (19·4 to 23·4)	−15·0 (−17·5 to −12·3)*	−17·2 (−17·9 to −11·5)*	−27·5 (−30·9 to −23·2)*	8·7 (7·7 to 10·6)	7·6 (6·6 to 9·5)	6·2 (5·5 to 7·9)	−12·9 (−16·0 to −8·2)*	−22·2 (−24·1 to −11·8)*	−28·7 (−34·1 to −20·2)*
Occupational ergonomic factors	36·5 (35·4 to 37·9)	30·5 (29·2 to 32·0)	26·8 (25·3 to 28·6)	−16·5 (−17·6 to −15·3)*	−13·7 (−13·5 to −10·6)*	−26·6 (−28·6 to −24·3)*	23·9 (23·3 to 24·7)	22·2 (21·4 to 23·0)	21·3 (20·4 to 22·4)	−7·4 (−8·5 to −6·3)*	−4·0 (−5·2 to −2·5)*	−11·0 (−12·9 to −9·0)*
Lead exposure	19·4 (7·6 to 36·3)	18·6 (7·3 to 35·2)	15·7 (5·7 to 31·5)	−4·1 (−6·3 to −2·6)*	−18·0 (−22·4 to −10·4)*	−18·8 (−26·3 to −13·1)*	17·5 (6·3 to 34·2)	17·1 (6·2 to 33·7)	14·5 (4·8 to 30·2)	−2·5 (−4·7 to −0·9)*	−17·9 (−22·7 to −10·3)*	−17·3 (−25·6 to −11·9)*
Occupational asthmagens	30·2 (23·1 to 38·1)	26·3 (20·4 to 32·9)	23·6 (18·7 to 29·2)	−12·9 (−14·7 to −10·6)*	−11·5 (−12·0 to −8·1)*	−21·8 (−24·7 to −18·1)*	17·3 (13·0 to 22·7)	16·9 (12·8 to 21·9)	17·1 (13·3 to 21·7)	−2·6 (−6·0 to 1·2)	1·4 (−1·1 to 4·2)	−1·3 (−5·8 to 4·9)
Childhood sexual abuse	8·8 (4·6 to 9·4)	7·9 (4·1 to 8·4)	7·5 (3·9 to 8·0)	−10·4 (−12·0 to −8·9)*	−4·9 (−6·2 to −3·2)*	−14·6 (−15·9 to −13·3)*	9·8 (5·1 to 10·4)	8·9 (4·6 to 9·5)	8·6 (4·5 to 9·2)	−9·0 (−10·6 to −7·5)*	−3·5 (−5·0 to −1·8)*	−12·1 (−13·5 to −10·6)*
Vitamin A deficiency	32·4 (30·6 to 34·6)	32·4 (30·4 to 34·7)	28·4 (26·7 to 30·4)	−0·3 (−3·1 to 2·5)	−13·8 (−14·6 to −9·5)*	−12·4 (−15·0 to −9·8)*	29·5 (27·8 to 31·7)	29·2 (27·4 to 31·5)	25·8 (24·2 to 27·8)	−1·0 (−3·8 to 1·9)	−13·2 (−14·2 to −9·1)*	−12·6 (−15·2 to −9·8)*
Second-hand smoke	21·0 (19·4 to 22·6)	19·2 (17·8 to 20·8)	18·5 (17·0 to 20·1)	−8·5 (−10·7 to −6·3)*	−3·5 (−5·1 to −1·6)*	−11·6 (−14·5 to −8·7)*	31·7 (29·8 to 33·8)	29·1 (27·6 to 30·7)	27·8 (26·3 to 29·3)	−8·1 (−10·9 to −5·5)*	−4·8 (−5·8 to −3·4)*	−12·3 (−15·6 to −9·3)*
Childhood wasting	6·2 (5·5 to 6·8)	6·0 (5·3 to 6·6)	5·4 (4·8 to 6·0)	−2·1 (−6·6 to 2·6)	−11·4 (−15·0 to −5·7)*	−12·1 (−16·7 to −7·0)*	6·0 (5·3 to 6·6)	5·9 (5·1 to 6·4)	5·3 (4·6 to 5·9)	−2·2 (−6·9 to 2·5)	−11·3 (−14·9 to −5·6)*	−12·1 (−16·7 to −6·9)*
Occupational exposure to arsenic	0·3 (0·3 to 0·3)	0·3 (0·3 to 0·3)	0·3 (0·3 to 0·3)	0·3 (−0·5 to 1·0)	−9·9 (−9·6 to −8·5)*	−8·8 (−9·6 to −7·9)*	0·1 (0·1 to 0·1)	0·1 (0·1 to 0·1)	0·1 (0·1 to 0·1)	2·5 (1·0 to 4·1)*	−20·5 (−18·1 to −15·8)*	−14·9 (−16·6 to −13·3)*
Unsafe water source	62·1 (57·8 to 66·8)	58·6 (53·6 to 64·1)	56·0 (50·4 to 62·1)	−5·7 (−8·2 to −3·2)*	−4·7 (−6·2 to −2·6)*	−10·0 (−13·5 to −5·9)*	61·1 (56·8 to 65·8)	58·0 (53·0 to 63·5)	55·7 (50·2 to 61·8)	−5·1 (−7·6 to −2·4)*	−4·3 (−5·8 to −2·2)*	−8·9 (−12·6 to −4·9)*
Diet high in red meat	10·5 (8·9 to 12·5)	9·8 (8·2 to 11·7)	9·7 (8·0 to 11·5)	−6·5 (−7·8 to −5·3)*	−2·0 (−3·3 to −0·8)*	−8·4 (−9·8 to −6·9)*	9·5 (7·9 to 11·4)	8·8 (7·3 to 10·6)	8·6 (7·1 to 10·4)	−7·2 (−8·5 to −5·9)*	−2·7 (−3·8 to −1·4)*	−9·6 (−11·0 to −8·1)*
No handwashing with soap	84·3 (81·3 to 87·2)	80·7 (76·7 to 84·6)	77·1 (72·4 to 81·8)	−4·4 (−5·9 to −2·9)*	−4·6 (−5·8 to −3·2)*	−8·6 (−11·3 to −6·0)*	83·9 (80·8 to 86·8)	80·3 (76·3 to 84·3)	76·9 (72·2 to 81·6)	−4·2 (−5·7 to −2·8)*	−4·5 (−5·6 to −3·1)*	−8·4 (−11·0 to −5·8)*
Occupational exposure to asbestos	2·5 (1·7 to 4·2)	2·3 (1·6 to 3·7)	2·4 (1·7 to 3·7)	−8·4 (−15·3 to 2·7)	4·9 (−0·4 to 11·8)	−3·7 (−14·3 to 10·6)	0·9 (0·7 to 1·5)	0·7 (0·5 to 1·2)	0·8 (0·6 to 1·2)	−21·5 (−30·4 to −9·1)*	3·0 (−3·2 to 10·5)	−19·1 (−27·4 to −8·5)*
Zinc deficiency	16·8 (9·8 to 20·3)	16·8 (9·8 to 20·2)	15·6 (9·1 to 18·8)	−0·0 (−2·6 to 2·9)	−7·8 (−9·6 to −4·7)*	−7·2 (−9·6 to −4·4)*	16·8 (9·8 to 20·2)	16·8 (9·8 to 20·3)	15·6 (9·1 to 18·9)	0·3 (−2·3 to 3·2)	−7·6 (−9·4 to −4·4)*	−6·8 (−9·2 to −4·0)*
Iron deficiency	..	..	..	..	..	..	17·6 (12·4 to 23·7)	17·5 (12·6 to 23·4)	16·5 (12·1 to 21·8)	−0·5 (−7·2 to 4·7)	−6·0 (−14·7 to 1·1)	−6·1 (−19·1 to 5·0)
Low bone mineral density	19·0 (15·8 to 22·9)	18·3 (15·3 to 22·0)	17·8 (14·4 to 21·6)	−3·8 (−8·4 to 1·6)	−2·8 (−8·3 to 2·8)	−6·4 (−11·5 to −0·9)*	22·1 (18·8 to 25·8)	21·5 (18·3 to 24·9)	21·1 (17·5 to 25·0)	−2·7 (−6·4 to 1·0)	−1·9 (−6·5 to 2·9)	−4·6 (−8·8 to −0·2)*
Alcohol use	10·9 (10·1 to 11·6)	10·5 (9·8 to 11·3)	10·7 (9·5 to 11·8)	−2·8 (−3·7 to −1·8)*	1·7 (−3·0 to 5·1)	−1·1 (−6·0 to 2·3)	5·9 (5·3 to 6·4)	5·3 (4·8 to 5·8)	5·1 (4·5 to 5·7)	−10·1 (−11·0 to −9·3)*	−3·4 (−6·4 to −0·9)*	−13·1 (−16·1 to −10·7)*
High total cholesterol	30·4 (24·2 to 37·2)	29·8 (23·7 to 36·6)	29·4 (23·3 to 36·2)	−1·8 (−2·7 to −1·0)*	−1·5 (−2·1 to −0·8)*	−3·2 (−4·4 to −2·2)*	33·7 (27·5 to 40·6)	32·4 (26·2 to 39·3)	31·8 (25·7 to 38·6)	−3·9 (−4·8 to −3·0)*	−1·8 (−2·5 to −1·2)*	−5·6 (−6·7 to −4·6)*
Occupational noise	42·5 (32·4 to 53·5)	40·6 (30·5 to 52·9)	40·5 (31·0 to 53·4)	−4·5 (−10·4 to 1·0)	−0·3 (−3·8 to 3·2)	−4·7 (−10·9 to 1·2)	25·2 (19·4 to 32·9)	24·1 (18·4 to 32·5)	24·4 (18·9 to 33·4)	−4·5 (−10·5 to 0·9)	1·2 (−4·1 to 6·1)	−3·3 (−9·8 to 3·7)
Diet high in processed meat	9·2 (7·3 to 11·3)	9·0 (7·1 to 11·1)	8·9 (7·0 to 11·0)	−2·6 (−3·9 to −1·2)*	−0·6 (−1·9 to 0·8)	−3·1 (−4·6 to −1·7)*	8·4 (6·6 to 10·5)	8·1 (6·3 to 10·1)	8·0 (6·3 to 10·0)	−3·6 (−5·0 to −2·3)*	−1·0 (−2·3 to 0·3)	−4·6 (−6·1 to −3·3)*
Diet low in fibre	15·6 (8·0 to 24·3)	15·2 (7·8 to 23·7)	15·0 (7·6 to 23·5)	−2·5 (−4·1 to −1·1)*	−1·2 (−3·5 to 0·9)	−3·7 (−5·3 to −2·5)*	14·4 (7·2 to 22·7)	14·0 (6·9 to 22·1)	13·8 (6·8 to 21·8)	−2·8 (−4·7 to −1·3)*	−1·1 (−3·4 to 1·2)	−3·9 (−5·4 to −2·6)*
Non-exclusive breastfeeding	16·5 (8·1 to 40·4)	15·8 (8·2 to 37·6)	15·9 (8·4 to 37·1)	−4·6 (−13·0 to 11·9)	1·2 (−7·9 to 18·6)	−3·5 (−14·2 to 23·2)	16·5 (8·0 to 40·4)	15·7 (8·1 to 37·6)	15·9 (8·3 to 37·1)	−5·0 (−13·0 to 10·5)	1·0 (−7·4 to 17·7)	−4·0 (−14·0 to 21·4)
Occupational exposure to beryllium	0·1 (0·1 to 0·1)	0·1 (0·1 to 0·1)	0·1 (0·1 to 0·1)	7·8 (6·3 to 9·1)*	−10·3 (−10·3 to −8·5)*	−2·3 (−3·8 to −0·8)*	0·1 (0·1 to 0·1)	0·1 (0·1 to 0·1)	0·0 (0·0 to 0·1)	12·9 (10·3 to 15·5)*	−20·2 (−18·4 to −15·2)*	−6·1 (−8·8 to −3·4)*
Diet low in vegetables	57·7 (39·1 to 77·0)	57·6 (39·0 to 77·1)	56·8 (38·5 to 76·1)	−0·1 (−0·7 to 0·5)	−1·3 (−1·8 to −0·8)*	−1·4 (−2·2 to −0·7)*	56·2 (37·9 to 75·5)	55·9 (37·6 to 75·2)	55·3 (37·2 to 74·4)	−0·4 (−1·0 to 0·2)	−1·1 (−1·8 to −0·6)*	−1·5 (−2·5 to −0·8)*
Occupational particulate matter, gases, and fumes	23·4 (17·7 to 30·5)	22·8 (17·5 to 29·5)	23·2 (18·0 to 29·7)	−2·6 (−4·6 to −0·6)*	1·6 (0·7 to 2·7)*	−1·1 (−3·8 to 2·0)	13·3 (10·0 to 19·2)	13·0 (9·9 to 18·4)	13·0 (9·9 to 18·5)	−2·6 (−5·5 to −0·0)*	0·2 (−0·7 to 1·1)	−2·4 (−5·3 to 0·2)
Diet low in whole grains	71·8 (52·1 to 89·1)	71·7 (51·9 to 89·2)	71·3 (51·5 to 89·1)	−0·1 (−0·5 to 0·2)	−0·5 (−0·9 to −0·1)*	−0·7 (−1·2 to 0·0)	71·7 (52·0 to 89·0)	71·5 (51·8 to 89·0)	71·1 (51·3 to 88·8)	−0·2 (−0·6 to 0·1)	−0·6 (−1·0 to −0·2)*	−0·9 (−1·4 to −0·1)*
Low glomerular filtration rate	3·5 (3·1 to 3·8)	3·4 (3·1 to 3·7)	3·5 (3·1 to 3·8)	−1·1 (−2·2 to −0·0)*	1·6 (0·8 to 2·5)*	0·5 (−0·5 to 1·4)	4·9 (4·4 to 5·3)	4·8 (4·4 to 5·3)	4·8 (4·4 to 5·3)	−0·7 (−1·9 to 0·3)	0·6 (−0·3 to 1·5)	−0·2 (−1·3 to 1·0)
Diet low in fruits	58·7 (41·1 to 75·6)	58·9 (41·3 to 75·8)	58·6 (40·9 to 75·4)	0·3 (0·1 to 0·6)*	−0·6 (−1·1 to −0·1)*	−0·3 (−0·7 to 0·1)	55·3 (38·3 to 71·7)	55·6 (38·5 to 72·0)	55·0 (38·0 to 71·5)	0·6 (0·3 to 0·9)*	−1·0 (−1·7 to −0·5)*	−0·4 (−1·1 to 0·1)
Diet low in nuts and seeds	96·3 (84·3 to 99·8)	96·1 (84·0 to 99·8)	96·0 (83·9 to 99·7)	−0·2 (−0·5 to −0·0)*	−0·1 (−0·2 to 0·1)	−0·3 (−0·6 to −0·1)*	96·1 (83·8 to 99·8)	95·9 (83·5 to 99·8)	95·8 (83·4 to 99·7)	−0·2 (−0·5 to −0·0)*	−0·1 (−0·2 to 0·0)	−0·3 (−0·6 to −0·1)*
Diet low in seafood omega-3 fatty acids	77·3 (57·6 to 94·0)	77·3 (57·6 to 94·0)	77·4 (57·8 to 94·1)	−0·0 (−0·3 to 0·2)	0·2 (0·0 to 0·5)*	0·2 (−0·1 to 0·6)	77·3 (57·5 to 94·0)	77·4 (57·7 to 94·1)	77·6 (57·9 to 94·3)	0·1 (−0·1 to 0·4)	0·3 (0·1 to 0·6)*	0·5 (0·1 to 0·9)*
Discontinued breastfeeding	13·5 (13·3 to 14·0)	12·9 (12·8 to 13·2)	13·7 (13·5 to 14·1)	−4·8 (−7·1 to −2·5)*	5·6 (3·9 to 8·0)*	0·8 (−1·8 to 3·9)	13·5 (13·3 to 13·9)	12·8 (12·7 to 13·1)	13·5 (13·4 to 13·9)	−5·4 (−7·5 to −3·2)*	5·4 (3·7 to 7·8)*	0·0 (−2·5 to 3·0)
Low physical activity	45·3 (40·9 to 49·2)	45·9 (41·5 to 49·8)	46·3 (42·0 to 50·3)	1·3 (0·7 to 2·0)*	1·0 (0·7 to 1·4)*	2·4 (1·8 to 2·9)*	39·9 (35·7 to 43·9)	39·4 (35·1 to 43·4)	39·4 (35·0 to 43·4)	−1·3 (−1·8 to −0·8)*	−0·2 (−0·6 to 0·2)	−1·5 (−2·0 to −1·0)*
Diet low in milk	81·0 (63·4 to 95·4)	81·6 (64·0 to 95·9)	81·9 (64·2 to 96·2)	0·8 (0·5 to 1·1)*	0·4 (0·2 to 0·5)*	1·2 (0·9 to 1·5)*	80·0 (62·4 to 94·7)	80·8 (63·1 to 95·2)	81·2 (63·5 to 95·7)	1·0 (0·6 to 1·3)*	0·5 (0·3 to 0·7)*	1·5 (1·1 to 1·9)*
Diet low in calcium	63·3 (34·2 to 94·0)	63·9 (34·6 to 94·8)	64·3 (35·1 to 95·2)	0·9 (0·5 to 1·4)*	0·7 (0·1 to 1·5)*	1·6 (0·8 to 2·7)*	60·2 (32·2 to 91·4)	61·0 (32·6 to 92·6)	61·7 (33·3 to 93·3)	1·3 (0·7 to 2·0)*	1·0 (0·3 to 2·0)*	2·4 (1·4 to 3·7)*
Occupational exposure to nickel	0·9 (0·9 to 1·0)	1·1 (1·1 to 1·1)	1·0 (1·0 to 1·0)	13·6 (12·1 to 14·9)*	−8·5 (−8·7 to −7·0)*	4·6 (3·1 to 6·2)*	0·4 (0·4 to 0·4)	0·5 (0·5 to 0·5)	0·4 (0·4 to 0·4)	14·9 (12·3 to 17·6)*	−19·4 (−17·7 to −14·7)*	−3·8 (−6·4 to −1·1)*
Diet high in trans fatty acids	7·8 (3·5 to 14·2)	8·0 (3·7 to 14·5)	8·1 (3·8 to 14·6)	2·4 (0·5 to 5·3)*	1·5 (−0·3 to 3·8)	4·0 (2·5 to 7·0)*	7·9 (3·6 to 14·3)	8·1 (3·7 to 14·6)	8·3 (3·9 to 14·8)	2·6 (0·9 to 5·4)*	2·3 (0·6 to 5·2)*	5·0 (3·2 to 8·8)*
Intimate partner violence	..	..	..	..	..	..	15·6 (13·2 to 17·8)	15·4 (13·2 to 17·4)	16·3 (14·0 to 18·5)	−1·4 (−3·7 to 1·1)	5·7 (4·4 to 7·6)*	4·5 (2·7 to 6·6)*
Ambient particulate matter pollution	46·4 (39·8 to 53·4)	47·7 (41·1 to 54·7)	48·9 (42·2 to 55·8)	2·8 (2·1 to 3·7)*	2·3 (1·8 to 3·1)*	5·3 (4·0 to 6·6)*	45·5 (39·0 to 52·5)	46·8 (40·2 to 53·7)	48·0 (41·4 to 54·9)	2·7 (2·0 to 3·6)*	2·6 (2·0 to 3·3)*	5·4 (4·1 to 6·8)*
High systolic blood pressure	18·2 (15·7 to 21·0)	18·9 (16·2 to 21·8)	20·7 (17·9 to 23·8)	3·3 (2·3 to 4·3)*	8·8 (8·7 to 10·8)*	13·3 (12·1 to 14·7)*	18·2 (15·8 to 20·8)	17·4 (15·0 to 20·1)	18·1 (15·6 to 20·8)	−4·6 (−5·9 to −3·3)*	3·7 (2·8 to 4·9)*	−0·9 (−2·3 to 0·5)
Diet high in sodium	12·2 (5·1 to 23·5)	12·8 (5·6 to 24·3)	13·1 (6·0 to 24·9)	4·9 (2·2 to 10·3)*	2·1 (−1·8 to 8·4)	7·2 (0·7 to 19·0)*	9·0 (3·4 to 19·0)	9·4 (3·7 to 19·4)	9·4 (3·7 to 19·4)	4·0 (1·8 to 8·7)*	−0·0 (−2·6 to 3·8)	4·0 (−0·3 to 12·1)
Residential radon	14·7 (12·6 to 16·8)	15·2 (13·0 to 17·4)	15·6 (13·5 to 17·9)	3·4 (2·3 to 4·4)*	3·0 (2·6 to 3·8)*	6·6 (5·0 to 8·2)*	14·8 (12·6 to 17·0)	15·3 (13·1 to 17·5)	15·8 (13·6 to 18·1)	3·4 (2·2 to 4·6)*	3·2 (2·7 to 3·9)*	6·8 (5·1 to 8·5)*
Diet high in sugar-sweetened beverages	8·3 (7·5 to 9·0)	8·8 (7·9 to 9·7)	8·7 (7·8 to 9·7)	6·6 (1·6 to 11·2)*	−1·0 (−2·9 to 1·0)	5·6 (−0·4 to 10·9)	6·4 (5·7 to 7·1)	7·0 (6·3 to 7·6)	7·0 (6·2 to 7·7)	8·8 (3·1 to 14·0)*	0·4 (−1·9 to 2·5)	9·2 (1·5 to 15·6)*
Occupational exposure to chromium	1·3 (1·3 to 1·3)	1·4 (1·4 to 1·5)	1·4 (1·4 to 1·4)	12·5 (11·2 to 13·8)*	−4·3 (−5·0 to −3·2)*	7·9 (6·4 to 9·4)*	0·6 (0·6 to 0·6)	0·7 (0·7 to 0·7)	0·6 (0·6 to 0·6)	19·7 (17·3 to 22·3)*	−10·3 (−10·7 to −8·0)*	8·5 (5·8 to 11·2)*
Occupational exposure to formaldehyde	1·1 (1·0 to 1·1)	1·1 (1·0 to 1·1)	1·1 (1·1 to 1·2)	5·9 (4·9 to 6·9)*	1·7 (0·8 to 2·4)*	7·7 (6·4 to 9·0)*	0·6 (0·5 to 0·6)	0·6 (0·6 to 0·6)	0·6 (0·6 to 0·6)	12·7 (11·0 to 14·6)*	−1·8 (−3·0 to −0·6)*	10·7 (8·6 to 12·8)*
Occupational exposure to trichloroethylene	0·5 (0·5 to 0·5)	0·5 (0·5 to 0·5)	0·5 (0·5 to 0·5)	13·5 (12·3 to 14·6)*	−1·2 (−2·0 to −0·3)*	12·2 (10·8 to 13·5)*	0·2 (0·2 to 0·2)	0·2 (0·2 to 0·2)	0·2 (0·2 to 0·2)	20·6 (18·5 to 22·8)*	−5·8 (−6·6 to −4·1)*	14·0 (11·6 to 16·4)*
Diet low in polyunsaturated fatty acids	34·1 (32·1 to 36·8)	35·7 (32·4 to 40·0)	39·8 (34·5 to 46·3)	4·7 (−1·4 to 11·9)	10·3 (4·1 to 19·5)*	16·7 (4·8 to 31·0)*	35·8 (33·6 to 38·5)	37·6 (33·8 to 42·1)	41·7 (35·9 to 48·4)	5·1 (−1·7 to 12·7)	9·8 (3·7 to 18·2)*	16·5 (4·4 to 29·8)*
Occupational exposure to sulphuric acid	1·4 (1·2 to 1·5)	1·6 (1·4 to 1·8)	1·6 (1·4 to 1·8)	16·7 (14·9 to 18·6)*	−0·4 (−2·0 to 0·9)	16·3 (14·5 to 18·1)*	0·5 (0·5 to 0·6)	0·6 (0·6 to 0·7)	0·6 (0·6 to 0·7)	22·9 (20·5 to 25·4)*	−6·8 (−8·1 to −4·9)*	15·1 (12·4 to 17·7)*
Occupational exposure to cadmium	0·3 (0·3 to 0·3)	0·4 (0·4 to 0·4)	0·4 (0·4 to 0·4)	17·1 (15·8 to 18·5)*	1·3 (0·4 to 2·3)*	18·7 (17·1 to 20·3)*	0·1 (0·1 to 0·1)	0·2 (0·2 to 0·2)	0·1 (0·1 to 0·2)	21·3 (18·9 to 23·8)*	−10·0 (−10·4 to −7·7)*	10·3 (7·6 to 12·9)*
Occupational exposure to second-hand smoke	14·3 (14·2 to 14·3)	16·2 (16·1 to 16·2)	16·5 (16·4 to 16·6)	13·3 (12·7 to 14·0)*	2·0 (1·5 to 2·7)*	15·6 (14·8 to 16·4)*	5·4 (5·3 to 5·4)	6·5 (6·5 to 6·6)	7·0 (7·0 to 7·1)	21·4 (20·8 to 22·1)*	7·4 (7·5 to 8·6)*	31·2 (30·3 to 32·0)*
High fasting plasma glucose	6·8 (5·7 to 8·2)	7·6 (6·4 to 9·1)	8·5 (7·2 to 10·1)	11·7 (10·8 to 12·7)*	10·6 (11·0 to 12·7)*	25·0 (23·3 to 26·7)*	6·6 (5·4 to 7·9)	7·2 (6·0 to 8·6)	8·0 (6·7 to 9·6)	9·4 (8·6 to 10·4)*	10·8 (11·2 to 13·1)*	22·7 (21·1 to 24·4)*
Ambient ozone pollution	38·5 (13·9 to 67·9)	42·8 (15·5 to 73·7)	48·2 (17·6 to 78·3)	11·2 (8·4 to 13·8)*	11·2 (6·3 to 16·8)*	25·2 (15·3 to 32·8)*	38·2 (13·8 to 67·4)	42·2 (15·3 to 72·8)	47·4 (17·2 to 77·4)	10·3 (7·9 to 12·7)*	11·0 (6·3 to 16·4)*	24·0 (14·8 to 31·0)*
Drug use	0·4 (0·2 to 0·7)	0·4 (0·2 to 0·8)	0·5 (0·2 to 0·9)	17·6 (12·7 to 24·4)*	9·5 (7·7 to 13·7)*	29·9 (22·4 to 40·3)*	0·2 (0·1 to 0·3)	0·2 (0·1 to 0·4)	0·2 (0·1 to 0·4)	15·6 (12·5 to 19·3)*	11·3 (9·9 to 15·6)*	30·3 (24·3 to 36·7)*
High body-mass index	3·6 (1·6 to 6·1)	4·4 (2·2 to 7·3)	5·0 (2·5 to 8·0)	23·7 (18·5 to 33·9)*	10·8 (9·6 to 16·5)*	38·7 (29·9 to 55·6)*	4·6 (2·5 to 7·3)	5·7 (3·2 to 8·7)	6·2 (3·7 to 9·4)	21·9 (17·8 to 28·8)*	9·3 (8·1 to 13·9)*	34·4 (27·7 to 45·7)*
Occupational exposure to polycyclic aromatic hydrocarbons	1·6 (1·6 to 1·6)	2·0 (2·0 to 2·0)	2·2 (2·2 to 2·2)	27·7 (26·4 to 28·9)*	6·4 (5·9 to 7·8)*	36·4 (34·9 to 38·0)*	0·6 (0·5 to 0·6)	0·8 (0·8 to 0·8)	0·8 (0·8 to 0·8)	37·6 (35·3 to 39·8)*	2·9 (1·7 to 4·2)*	41·7 (39·0 to 44·4)*
Occupational exposure to benzene	1·3 (1·2 to 1·4)	1·6 (1·5 to 1·8)	1·9 (1·7 to 2·1)	26·3 (24·4 to 28·2)*	15·0 (16·1 to 19·2)*	48·5 (46·0 to 51·2)*	0·6 (0·6 to 0·7)	0·9 (0·9 to 1·0)	1·2 (1·1 to 1·3)	52·6 (50·6 to 54·5)*	22·7 (28·2 to 30·8)*	97·5 (94·5 to 100·5)*
Occupational exposure to silica	6·6 (6·5 to 6·7)	9·6 (9·5 to 9·7)	11·3 (11·2 to 11·5)	46·5 (44·4 to 48·7)*	15·1 (16·3 to 19·3)*	72·6 (69·6 to 75·4)*	1·3 (1·2 to 1·3)	1·8 (1·7 to 1·8)	1·8 (1·8 to 1·8)	40·5 (38·6 to 42·4)*	3·1 (2·0 to 4·5)*	44·9 (42·6 to 47·4)*
Occupational exposure to diesel engine exhaust	6·7 (6·6 to 6·7)	9·9 (9·8 to 10·0)	11·5 (11·4 to 11·6)	47·9 (46·5 to 49·3)*	14·0 (15·3 to 17·4)*	72·1 (69·9 to 74·1)*	1·5 (1·5 to 1·6)	2·7 (2·6 to 2·7)	3·5 (3·5 to 3·6)	72·9 (70·7 to 75·0)*	24·7 (30·9 to 35·3)*	129·8 (125·0 to 134·7)*

Data in parentheses are 95% uncertainty intervals. Risks are reported in order of percentage change for both sexes combined, 1990–2015.

* Statistically significant increase or decrease.

**Table 4:** Global all-age deaths and DALYs attributable to each risk factor at each level of the risk factor hierarchy and outcome for both sexes combined in 2005 and 2015

			**2005 deaths(in thousands)**	**2015 deaths(in thousands)**	**Percentage change of 2005–15 deaths**	**Percentage change of 2005–15 age-standardised PAF**	**2005 DALYs(in thousands)**	**2015 DALYs(in thousands)**	**Percentage change of 2005–15 DALYs**	**Percentage change of 2005–15 age-standardised DALYs PAF**
**All risk factors: all causes**			**30 718 (30 074 to 31 360)**	**32 234 (31 456 to 33 035)**	4·9 (3·2 to 6·7)*	−1·1 (−1·7 to −0·4)*	**1 076 067 (1 022 161 to 1 137 872)**	**1 015 470 (953 300 to 1 084 249)**	−5·6 (−7·5 to −3·8)*	−4·6 (−5·8 to −3·5)*
**Environmental or occupational risks: all causes**			**9523 (8704 to 10 321)**	**9315 (8523 to 10 145)**	**−2·2 (−4·8 to 0·6)**	−6·7 (−8·3 to −5·1)*	**362 041 (335 616 to 387 712)**	**319 569 (295 706 to 344 884)**	−11·7 (−14·7 to −8·3)*	−9·2 (−11·8 to −6·5)*
	**Unsafe water, sanitation, and handwashing: all causes**		**2179 (1993 to 2386)**	**1766 (1586 to 1944)**	−18·9 (−23·6 to −14·2)*	−16·0 (−20·3 to −11·8)*	**129 221 (116 430 to 142 602)**	**95 305 (85 818 to 105 821)**	−26·2 (−31·4 to −20·5)*	−18·9 (−24·5 to −12·6)*
	Unsafe water source: all causes		1587 (1284 to 1812)	1251 (1008 to 1428)	−21·2 (−26·3 to −16·0)*	−18·0 (−22·9 to −13·0)*	97** **248 (78** **516 to 112** **113)	71** **745 (57** **707 to 83** **257)	−26·2 (−31·9 to −19·8)*	−18·8 (−25·0 to −12·1)*
		Diarrhoeal diseases	1411 (1147 to 1592)	1101 (887 to 1244)	−22·0 (−27·4 to −16·5)*	−1·4 (−1·9 to −0·9)*	84** **526 (68** **642 to 96** **429)	61** **104 (49** **352 to 69** **584)	−27·7 (−33·6 to −21·2)*	−0·6 (−1·0 to −0·4)*
		Typhoid fever	147 (77 to 245)	126 (67 to 215)	−14·6 (−21·0 to −8·8)*	−0·7 (−1·5 to −0·3)*	10** **688 (5712 to 17** **653)	8943 (4822 to 15 133)	−16·3 (−23·1 to −10·0)*	−0·7 (−1·5 to −0·3)*
		Paratyphoid fever	29 (13 to 57)	25 (11 to 48)	−14·8 (−22·2 to −7·1)*	−0·8 (−1·6 to −0·3)*	2034 (898 to 4009)	1699 (757 to 3318)	−16·5 (−24·8 to −8·1)*	−0·7 (−1·6 to −0·3)*
	Unsafe sanitation: all causes		1114 (1012 to 1230)	808 (727 to 895)	−27·5 (−32·7 to −22·3)*	−24·7 (−29·7 to −19·9)*	67** **949 (61** **085 to 75** **899)	46** **275 (41** **065 to 51** **818)	−31·9 (−37·4 to −25·7)*	−25·1 (−31·1 to −18·1)*
		Diarrhoeal diseases	997 (914 to 1087)	720 (657 to 792)	−27·8 (−33·5 to −22·3)*	−8·9 (−10·9 to −7·2)*	59** **454 (53** **638 to 65** **649)	40** **005 (36** **020 to 44** **351)	−32·7 (−38·8 to −26·1)*	−7·6 (−9·4 to −5·9)*
		Typhoid fever	98 (53 to 164)	74 (40 to 125)	−24·7 (−30·9 to −18·4)*	−12·4 (−15·2 to −10·0)*	7149 (3893 to 11 816)	5290 (2877 to 8857)	−26·0 (−32·6 to −19·3)*	−12·2 (−15·0 to −9·8)*
		Paratyphoid fever	19 (9 to 36)	14 (7 to 27)	−25·8 (−32·7 to −18·8)*	−13·6 (−16·5 to −10·9)*	1346 (627 to 2551)	981 (456 to 1902)	−27·1 (−34·7 to −19·7)*	−13·4 (−16·3 to −10·8)*
	No handwashing with soap: all causes		1116 (927 to 1296)	927 (760 to 1082)	−16·9 (−21·3 to −12·6)*	−14·3 (−18·3 to −10·4)*	64** **152 (53** **252 to 74** **634)	47** **271 (39** **034 to 55** **164)	−26·3 (−31·0 to −21·0)*	−19·0 (−24·0 to −13·4)*
		Diarrhoeal diseases	643 (514 to 763)	502 (402 to 599)	−21·9 (−27·4 to −16·5)*	−1·4 (−2·0 to −0·9)*	38** **311 (30** **403 to 45** **759)	27** **628 (22** **106 to 32** **796)	−27·9 (−33·9 to −21·3)*	−0·9 (−1·4 to −0·5)*
		Typhoid fever	67 (34 to 114)	56 (29 to 97)	−15·2 (−21·6 to −9·2)*	−1·4 (−2·1 to −0·8)*	4831 (2568 to 8256)	4019 (2135 to 6933)	−16·8 (−24·0 to −10·4)*	−1·3 (−2·1 to −0·7)*
		Paratyphoid fever	13 (6 to 26)	11 (5 to 22)	−15·2 (−22·7 to −7·5)*	−1·4 (−2·2 to −0·8)*	918 (422 to 1837)	763 (355 to 1543)	−16·9 (−25·0 to −8·5)*	−1·3 (−2·1 to −0·7)*
	**Air pollution: all causes**		**6466 (5675 to 7291)**	**6485 (5708 to 7292)**	**0·3 (−2·6 to 3·3)**	−6·3 (−8·2 to −4·4)*	**186 850 (164 716 to 209 142)**	**167 290 (148 167 to 185 780)**	−10·5 (−13·6 to −7·3)*	−10·5 (−13·1 to −7·9)*
	Ambient particulate matter pollution: all causes		3934 (3437 to 4448)	4241 (3698 to 4777)	7·8 (5·1 to 10·8)*	0·3 (−1·5 to 1·9)	107** **582 (94** **319 to 121** **177)	103** **066 (90** **830 to 115** **073)	−4·2 (−7·5 to −0·7)*	−4·9 (−7·7 to −2·3)*
		Lower respiratory infections	736 (549 to 957)	675 (492 to 889)	−8·3 (−13·2 to −3·6)*	−3·4 (−5·8 to −1·6)*	38** **632 (29** **407 to 48** **531)	28** **360 (21** **142 to 35** **797)	−26·6 (−31·4 to −21·5)*	−3·1 (−5·4 to −1·4)*
		Tracheal, bronchial, and lung cancer	225 (140 to 318)	283 (178 to 399)	25·7 (20·8 to 32·0)*	5·0 (3·2 to 7·7)*	5171 (3244 to 7263)	6209 (3935 to 8689)	20·1 (14·9 to 27·1)*	5·0 (3·2 to 7·6)*
		Ischaemic heart disease	1284 (1060 to 1530)	1521 (1232 to 1821)	18·5 (14·3 to 22·4)*	3·1 (1·7 to 4·5)*	28** **484 (24** **254 to 32** **699)	32** **406 (27** **078 to 37** **427)	13·8 (10·0 to 17·5)*	3·4 (2·4 to 4·7)*
		Ischaemic stroke	347 (260 to 432)	381 (283 to 483)	9·9 (3·9 to 15·2)*	3·2 (0·9 to 5·5)*	6271 (5039 to 7454)	6618 (5328 to 7905)	5·5 (0·7 to 10·1)*	3·5 (1·8 to 5·5)*
		Haemorrhagic stroke	505 (417 to 599)	517 (425 to 614)	2·3 (−2·2 to 7·1)	0·4 (−0·9 to 1·6)	12** **605 (10** **552 to 14** **743)	12** **625 (10** **570 to 14** **867)	0·2 (−3·9 to 4·6)	1·3 (0·3 to 2·5)*
		Chronic obstructive pulmonary disease	837 (522 to 1174)	864 (538 to 1213)	3·2 (−1·1 to 8·4)	0·2 (−0·9 to 1·9)	16** **418 (10** **295 to 23** **033)	16** **848 (10** **517 to 23** **590)	2·6 (−1·6 to 7·7)	1·2 (−0·1 to 3·3)
	Household air pollution from solid fuels: all causes		3280 (2505 to 4068)	2854 (2179 to 3587)	−13·0 (−17·0 to −9·3)*	−17·8 (−21·1 to −14·8)*	107** **509 (83** **554 to 132** **012)	85** **644 (66** **659 to 106** **136)	−20·3 (−24·5 to −16·6)*	−19·2 (−22·7 to −15·8)*
		Lower respiratory infections	905 (663 to 1160)	729 (523 to 949)	−19·5 (−24·2 to −14·5)*	−13·8 (−16·7 to −10·9)*	52** **898 (38** **845 to 67** **622)	36** **883 (26** **631 to 46** **916)	−30·3 (−35·1 to −25·0)*	−7·6 (−10·2 to −4·9)*
		Tracheal, bronchial, and lung cancer	159 (83 to 249)	149 (76 to 241)	−6·3 (−12·6 to 0·4)	−21·4 (−25·9 to −17·4)*	3806 (1996 to 5932)	3439 (1767 to 5534)	−9·6 (−15·8 to −3·2)*	−20·8 (−25·2 to −16·9)*
		Ischaemic heart disease	778 (621 to 964)	765 (598 to 965)	−1·6 (−7·0 to 3·7)	−13·8 (−18·0 to −10·1)*	18** **865 (15** **180 to 22** **902)	18** **200 (14** **417 to 22** **512)	−3·5 (−9·1 to 1·8)	−11·9 (−16·0 to −8·0)*
		Ischaemic stroke	245 (177 to 324)	214 (152 to 290)	−12·6 (−18·2 to −7·0)*	−17·5 (−21·5 to −13·6)*	4778 (3479 to 6282)	4104 (2931 to 5426)	−14·1 (−19·6 to −8·6)*	−15·6 (−19·6 to −12·0)*
		Haemorrhagic stroke	418 (306 to 542)	340 (252 to 446)	−18·6 (−23·5 to −13·7)*	−20·2 (−24·1 to −16·8)*	10** **857 (8159 to 13** **941)	8902 (6581 to 11 543)	−18·0 (−23·0 to −13·2)*	−17·2 (−21·2 to −13·7)*
		Chronic obstructive pulmonary disease	776 (422 to 1183)	657 (360 to 1014)	−15·3 (−21·2 to −9·8)*	−17·5 (−22·0 to −13·4)*	15** **600 (8513 to 23** **817)	13** **373 (7246 to 20** **608)	−14·3 (−19·9 to −8·8)*	−15·5 (−20·0 to −11·2)*
		Cataract	..	..	..	..	705 (484 to 972)	742 (512 to 1021)	5·3 (1·7 to 9·1)*	−15·9 (−18·6 to −13·2)*
	Ambient ozone pollution: all causes		207 (77 to 353)	254 (97 to 422)	22·7 (16·6 to 30·3)*	10·6 (5·5 to 17·0)*	3472 (1281 to 5913)	4116 (1577 to 6789)	18·5 (12·1 to 26·5)*	9·6 (3·7 to 17·3)*
		Chronic obstructive pulmonary disease	207 (77 to 353)	254 (97 to 422)	22·7 (16·6 to 30·3)*	19·1 (16·0 to 23·6)*	3472 (1281 to 5913)	4116 (1577 to 6789)	18·5 (12·1 to 26·5)*	16·6 (12·9 to 21·7)*
	**Other environmental risks: all causes**		**514 (273 to 804)**	**558 (293 to 883)**	8·6 (4·9 to 11·8)*	**−2·1 (−5·3 to 0·3)**	**10 400 (5470 to 16 412)**	**10 673 (5516 to 16 975)**	**2·6 (−0·8 to 5·4)**	−4·0 (−7·1 to −1·5)*
	Residential radon: all causes		53 (36 to 71)	64 (42 to 86)	19·7 (12·5 to 28·2)*	10·6 (5·8 to 17·6)*	1212 (841 to 1638)	1386 (941 to 1871)	14·3 (8·3 to 20·6)*	6·9 (3·1 to 12·0)*
		Tracheal, bronchial, and lung cancer	53 (36 to 71)	64 (42 to 86)	19·7 (12·5 to 28·2)*	−0·0 (−4·1 to 6·0)	1212 (841 to 1638)	1386 (941 to 1871)	14·3 (8·3 to 20·6)*	−0·1 (−3·2 to 4·6)
	Lead exposure: all causes		461 (227 to 745)	495 (237 to 815)	7·3 (2·6 to 10·7)*	−3·4 (−7·8 to −0·7)*	9188 (4357 to 15 216)	9287 (4200 to 15** **594)	1·1 (−3·7 to 4·4)	−5·3 (−9·5 to −2·3)*
		Rheumatic heart disease	4 (1 to 7)	3 (1 to 6)	−8·2 (−14·7 to −2·0)*	−6·1 (−10·8 to −3·1)*	97 (31 to 204)	83 (25 to 175)	−14·9 (−21·6 to −9·3)*	−9·9 (−16·4 to −6·2)*
		Ischaemic heart disease	220 (107 to 359)	240 (111 to 396)	8·8 (3·3 to 12·7)*	−7·0 (−11·4 to −4·3)*	3984 (1823 to 6675)	4123 (1807 to 7049)	3·5 (−1·8 to 7·1)	−7·6 (−11·9 to −5·0)*
		Ischaemic stroke	67 (32 to 115)	68 (31 to 118)	1·1 (−5·3 to 6·9)	−5·7 (−10·5 to −2·6)*	1092 (485 to 1882)	1055 (457 to 1828)	−3·4 (−9·3 to 1·0)	−5·9 (−10·5 to −3·3)*
		Haemorrhagic stroke	89 (38 to 155)	87 (37 to 154)	−1·4 (−6·7 to 3·6)	−4·7 (−8·7 to −2·1)*	1901 (750 to 3415)	1807 (695 to 3293)	−4·9 (−9·4 to −1·2)*	−5·2 (−9·7 to −2·7)*
		Hypertensive heart disease	40 (12 to 92)	47 (13 to 112)	18·1 (7·0 to 26·8)*	−7·9 (−14·2 to −4·0)*	710 (232 to 1561)	772 (240 to 1738)	8·8 (−0·1 to 15·4)	−9·1 (−15·1 to −5·4)*
		Cardiomyopathy and myocarditis	5 (2 to 8)	5 (2 to 8)	1·3 (−4·7 to 7·8)	−8·5 (−13·8 to −4·8)*	99 (34 to 202)	91 (31 to 183)	−8·5 (−13·2 to −3·1)*	−9·1 (−13·7 to −5·6)*
		Atrial fibrillation and flutter	3 (1 to 4)	3 (1 to 5)	22·1 (13·1 to 27·9)*	−9·7 (−15·8 to −6·3)*	65 (30 to 113)	72 (30 to 130)	10·1 (0·7 to 15·5)*	−13·3 (−20·5 to −9·2)*
		Aortic aneurysm	2 (1 to 4)	2 (1 to 4)	7·4 (−3·1 to 14·5)	−13·5 (−20·9 to −9·3)*	38 (16 to 67)	39 (15 to 73)	1·8 (−8·9 to 9·0)	−14·8 (−22·9 to −10·1)*
		Peripheral vascular disease	0 (0 to 1)	1 (0 to 1)	17·3 (3·0 to 28·6)*	−14·4 (−24·2 to −9·1)*	11 (4 to 20)	12 (4 to 23)	11·9 (−0·6 to 19·7)	−14·2 (−23·2 to −9·6)*
		Endocarditis	1 (0 to 2)	1 (0 to 2)	11·8 (2·8 to 18·6)*	−11·6 (−18·6 to −7·6)*	21 (8 to 39)	21 (8 to 42)	4·1 (−4·9 to 10·9)	−13·5 (−22·2 to −7·6)*
		Other cardiovascular and circulatory diseases	8 (4 to 14)	9 (4 to 15)	9·0 (2·6 to 14·3)*	−8·5 (−13·7 to −5·5)*	223 (98 to 388)	235 (98 to 422)	5·4 (−2·3 to 10·4)	−10·3 (−16·9 to −6·5)*
		Idiopathic developmental intellectual disability	..	..	..	..	471 (214 to 815)	423 (188 to 739)	−10·3 (−15·7 to −6·1)*	−13·7 (−19·2 to −9·8)*
		Chronic kidney disease due to diabetes mellitus	7 (3 to 12)	9 (4 to 17)	28·9 (20·2 to 34·6)*	−9·0 (−14·7 to −5·9)*	150 (58 to 293)	179 (65 to 356)	19·4 (10·5 to 24·7)*	−10·5 (−17·1 to −6·9)*
		Chronic kidney disease due to hypertension	11 (5 to 18)	14 (6 to 23)	28·0 (20·9 to 33·0)*	−7·2 (−11·5 to −4·7)*	196 (84 to 353)	231 (97 to 416)	17·8 (10·8 to 22·8)*	−8·4 (−13·5 to −5·4)*
		Chronic kidney disease due to glomerulonephritis	4 (2 to 7)	5 (2 to 8)	22·1 (16·9 to 27·8)*	0·1 (−2·1 to 2·0)	94 (34 to 180)	102 (37 to 197)	8·7 (4·5 to 12·9)*	−3·6 (−6·7 to −1·4)*
		Chronic kidney disease due to other causes	0 (0 to 1)	1 (0 to 1)	33·1 (24·5 to 42·3)*	−2·3 (−8·2 to 1·9)	36 (14 to 73)	41 (16 to 86)	13·7 (7·8 to 17·7)*	−9·4 (−15·1 to −5·6)*
	**Occupational risks: all causes**		**951 (889 to 1016)**	**1086 (1013 to 1165)**	14·2 (10·4 to 18·6)*	7·2 (4·2 to 10·5)*	**55 835 (47 024 to 65 679)**	**63 615 (53 616 to 75 415)**	13·9 (11·0 to 17·3)*	13·0 (10·2 to 16·2)*
	Occupational carcinogens: all causes		391 (370 to 410)	489 (461 to 517)	25·1 (20·4 to 30·2)*	15·9 (12·0 to 20·2)*	8109 (7708 to 8503)	9832 (9318 to 10** **385)	21·2 (16·5 to 26·7)*	12·9 (8·6 to 18·0)*
	Occupational exposure to asbestos: all causes		135 (118 to 154)	180 (158 to 205)	33·4 (27·3 to 40·1)*	21·5 (16·2 to 27·2)*	2210 (1919 to 2529)	2769 (2391 to 3185)	25·3 (19·4 to 31·6)*	16·1 (10·6 to 21·7)*
		Laryngeal cancer	1 (1 to 1)	1 (1 to 2)	38·5 (25·8 to 56·8)*	19·4 (9·0 to 35·4)*	17 (11 to 25)	22 (14 to 31)	27·8 (17·9 to 41·3)*	14·5 (6·5 to 25·6)*
		Tracheal, bronchial, and lung cancer	117 (101 to 135)	155 (132 to 179)	31·6 (25·6 to 38·6)*	8·3 (4·1 to 12·5)*	1875 (1590 to 2179)	2314 (1947 to 2732)	23·4 (17·3 to 29·9)*	6·8 (2·7 to 11·0)*
		Ovarian cancer	1 (1 to 2)	1 (1 to 2)	27·3 (18·6 to 37·3)*	3·7 (−2·6 to 10·4)	19 (9 to 31)	23 (11 to 38)	22·1 (12·3 to 33·8)*	0·7 (−6·0 to 8·2)
		Mesothelioma	16 (12 to 18)	23 (19 to 26)	47·2 (39·4 to 55·0)*	3·9 (1·9 to 7·1)*	298 (226 to 362)	410 (320 to 488)	37·3 (30·2 to 44·1)*	3·9 (1·8 to 6·8)*
	Occupational exposure to arsenic: all causes		9 (7 to 11)	9 (7 to 11)	−0·1 (−5·3 to 5·9)	−6·5 (−11·2 to −1·2)*	196 (152 to 242)	194 (150 to 242)	−0·9 (−6·2 to 5·4)	−7·6 (−12·4 to −1·7)*
		Tracheal, bronchial, and lung cancer	9 (7 to 11)	9 (7 to 11)	−0·1 (−5·3 to 5·9)	−15·5 (−18·6 to −12·2)*	196 (152 to 242)	194 (150 to 242)	−0·9 (−6·2 to 5·4)	−13·7 (−16·8 to −10·7)*
		Occupational exposure to benzene: all causes	4 (4 to 5)	6 (5 to 7)	31·6 (26·4 to 36·6)*	29·0 (24·9 to 33·2)*	162 (137 to 190)	211 (178 to 247)	30·8 (25·4 to 36·0)*	31·0 (25·9 to 36·5)*
		Leukaemia	4 (4 to 5)	6 (5 to 7)	31·6 (26·4 to 36·6)*	13·6 (10·7 to 16·4)*	162 (137 to 190)	211 (178 to 247)	30·8 (25·4 to 36·0)*	17·5 (14·4 to 20·7)*
	Occupational exposure to beryllium: all causes		1 (0 to 1)	1 (0 to 1)	−1·2 (−6·7 to 5·4)	−7·5 (−12·3 to −1·7)*	13 (10 to 15)	12 (10 to 15)	−2·1 (−7·7 to 4·7)	−8·7 (−14·0 to −2·7)*
		Tracheal, bronchial, and lung cancer	1 (0 to 1)	1 (0 to 1)	−1·2 (−6·7 to 5·4)	−16·4 (−19·7 to −12·8)*	13 (10 to 15)	12 (10 to 15)	−2·1 (−7·7 to 4·7)	−14·7 (−17·9 to −11·2)*
		Occupational exposure to cadmium: all causes	2 (2 to 2)	2 (2 to 3)	9·5 (3·6 to 16·2)*	2·4 (−2·6 to 8·2)	43 (36 to 51)	47 (38 to 56)	8·7 (2·8 to 15·5)*	1·2 (−4·2 to 7·5)
		Tracheal, bronchial, and lung cancer	2 (2 to 2)	2 (2 to 3)	9·5 (3·6 to 16·2)*	−7·4 (−10·8 to −3·7)*	43 (36 to 51)	47 (38 to 56)	8·7 (2·8 to 15·5)*	−5·5 (−8·6 to −2·0)*
	Occupational exposure to chromium: all causes		7 (6 to 8)	7 (6 to 8)	4·4 (−1·1 to 10·8)	−2·2 (−6·8 to 3·6)	151 (133 to 171)	157 (137 to 178)	3·6 (−2·1 to 10·1)	−3·5 (−8·3 to 2·4)
		Tracheal, bronchial, and lung cancer	7 (6 to 8)	7 (6 to 8)	4·4 (−1·1 to 10·8)	−11·7 (−14·6 to −8·5)*	151 (133 to 171)	157 (137 to 178)	3·6 (−2·1 to 10·1)	−9·8 (−12·6 to −6·8)*
	Occupational exposure to diesel engine exhaust: all causes		91 (81 to 103)	120 (106 to 136)	30·8 (24·6 to 37·8)*	22·2 (17·0 to 28·3)*	2048 (1815 to 2304)	2657 (2341 to 2994)	29·8 (23·6 to 37·1)*	20·8 (15·3 to 27·6)*
		Tracheal, bronchial, and lung cancer	91 (81 to 103)	120 (106 to 136)	30·8 (24·6 to 37·8)*	10·4 (7·4 to 13·7)*	2048 (1815 to 2304)	2657 (2341 to 2994)	29·8 (23·6 to 37·1)*	12·8 (10·0 to 15·7)*
	Occupational exposure to second-hand smoke: all causes		84 (79 to 89)	96 (90 to 103)	14·7 (9·9 to 20·1)*	7·3 (3·4 to 11·8)*	1860 (1742 to 1972)	2113 (1966 to 2262)	13·6 (8·9 to 18·9)*	5·7 (1·6 to 10·9)*
		Tracheal, bronchial, and lung cancer	84 (79 to 89)	96 (90 to 103)	14·7 (9·9 to 20·1)*	−3·0 (−5·1 to −0·7)*	1860 (1742 to 1972)	2113 (1966 to 2262)	13·6 (8·9 to 18·9)*	−1·2 (−3·1 to 0·8)
	Occupational exposure to formaldehyde: all causes		2 (2 to 2)	2 (2 to 2)	1·7 (−4·6 to 7·9)	−1·1 (−6·8 to 4·7)	71 (57 to 87)	71 (57 to 87)	−0·1 (−6·4 to 6·2)	−1·0 (−7·1 to 5·0)
		Nasopharyngeal cancer	1 (1 to 1)	1 (1 to 1)	−6·0 (−16·3 to 4·0)	−15·3 (−20·2 to −9·5)*	36 (23 to 51)	33 (21 to 47)	−8·8 (−19·4 to 1·8)	−13·4 (−18·5 to −7·1)*
	Occupational exposure to nickel: all causes		32 (24 to 42)	33 (24 to 43)	2·2 (−3·7 to 9·0)	−4·3 (−10·0 to 2·0)	720 (535 to 927)	729 (535 to 956)	1·3 (−4·9 to 8·5)	−5·6 (−11·3 to 1·2)
		Tracheal, bronchial, and lung cancer	32 (24 to 42)	33 (24 to 43)	2·2 (−3·7 to 9·0)	−13·6 (−17·5 to −9·5)*	720 (535 to 927)	729 (535 to 956)	1·3 (−4·9 to 8·5)	−11·8 (−15·7 to −7·9)*
	Occupational exposure to polycyclic aromatic hydrocarbons: all causes		15 (13 to 18)	18 (15 to 21)	16·9 (11·1 to 23·7)*	9·3 (4·2 to 15·5)*	342 (283 to 402)	397 (327 to 470)	16·1 (10·1 to 23·1)*	8·1 (2·6 to 14·4)*
		Tracheal, bronchial, and lung cancer	15 (13 to 18)	18 (15 to 21)	16·9 (11·1 to 23·7)*	−1·2 (−4·5 to 2·3)	342 (283 to 402)	397 (327 to 470)	16·1 (10·1 to 23·1)*	1·0 (−2·1 to 4·1)
	Occupational exposure to silica: all causes		65 (59 to 71)	86 (79 to 95)	32·9 (25·8 to 40·8)*	24·6 (18·6 to 31·3)*	1444 (1308 to 1578)	1894 (1724 to 2073)	31·1 (24·2 to 39·3)*	22·4 (16·2 to 29·8)*
		Tracheal, bronchial, and lung cancer	65 (59 to 71)	86 (79 to 95)	32·9 (25·8 to 40·8)*	12·6 (8·7 to 17·0)*	1444 (1308 to 1578)	1894 (1724 to 2073)	31·1 (24·2 to 39·3)*	14·4 (10·6 to 18·4)*
	Occupational exposure to sulphuric acid: all causes		7 (5 to 9)	8 (6 to 10)	13·9 (9·1 to 18·9)*	5·7 (1·5 to 10·2)*	175 (134 to 219)	199 (151 to 253)	13·8 (9·2 to 18·7)*	5·3 (1·0 to 10·0)*
		Laryngeal cancer	7 (5 to 9)	8 (6 to 10)	13·9 (9·1 to 18·9)*	0·7 (−2·4 to 3·7)	175 (134 to 219)	199 (151 to 253)	13·8 (9·2 to 18·7)*	2·0 (−0·8 to 5·0)
	Occupational exposure to trichloroethylene: all causes		0 (0 to 0)	0 (0 to 0)	25·3 (19·4 to 30·3)*	16·6 (11·3 to 20·5)*	3 (1 to 6)	4 (1 to 8)	26·3 (20·4 to 31·2)*	17·2 (11·6 to 21·8)*
		Kidney cancer	0 (0 to 0)	0 (0 to 0)	25·3 (19·4 to 30·3)*	−3·5 (−6·4 to −0·6)*	3 (1 to 6)	4 (1 to 8)	26·3 (20·4 to 31·2)*	−2·7 (−5·5 to 0·7)
	Occupational asthmagens: all causes		50 (39 to 61)	42 (35 to 48)	−16·9 (−26·0 to −2·4)*	−21·0 (−29·8 to −7·0)*	2754 (2190 to 3373)	2621 (2081 to 3223)	−4·8 (−12·0 to 3·6)	−5·3 (−12·2 to 3·8)
		Asthma	50 (39 to 61)	42 (35 to 48)	−16·9 (−26·0 to −2·4)*	−4·6 (−11·0 to 3·0)	2754 (2190 to 3373)	2621 (2081 to 3223)	−4·8 (−12·0 to 3·6)	−5·4 (−9·1 to −1·7)*
	Occupational particulate matter, gases, and fumes: all causes		337 (280 to 392)	357 (293 to 419)	5·9 (0·9 to 11·6)*	−3·7 (−7·4 to 0·6)	8405 (7325 to 9570)	8787 (7598 to 9987)	4·5 (0·5 to 9·2)*	−1·8 (−5·2 to 2·4)
		Chronic obstructive pulmonary disease	334 (278 to 389)	354 (290 to 417)	6·0 (1·0 to 11·8)*	3·8 (1·2 to 6·9)*	8346 (7264 to 9509)	8729 (7541 to 9926)	4·6 (0·6 to 9·3)*	4·5 (2·5 to 6·8)*
	Occupational noise: all causes		..	..	..	..	8659 (5925 to 11 948)	10 875 (7410 to 15 038)	25·6 (23·5 to 27·7)*	26·6 (24·1 to 29·2)*
		Age-related and other hearing loss	..	..	..	..	8659 (5925 to 11 948)	10 875 (7410 to 15 038)	25·6 (23·5 to 27·7)*	3·8 (2·8 to 4·8)*
	Occupational injuries: all causes		189 (168 to 207)	204 (191 to 232)	8·0 (−3·4 to 24·3)	12·4 (0·1 to 29·3)*	12 212 (10 736 to 13 682)	13 492 (12 006 to 15 545)	10·5 (−0·2 to 25·9)	16·9 (5·7 to 33·3)*
		Pedestrian road injuries	26 (22 to 30)	30 (27 to 37)	16·9 (−0·9 to 42·4)	23·3 (3·0 to 54·6)*	1320 (1110 to 1528)	1510 (1362 to 1842)	14·4 (−2·9 to 39·0)	26·8 (6·3 to 58·0)*
		Cyclist road injuries	3 (3 to 3)	3 (3 to 4)	21·9 (8·3 to 35·5)*	33·3 (18·7 to 50·2)*	190 (166 to 215)	243 (211 to 280)	27·8 (16·1 to 40·6)*	39·9 (26·6 to 55·3)*
		Motorcyclist road injuries	19 (17 to 21)	22 (20 to 26)	17·7 (3·2 to 36·2)*	13·3 (1·5 to 28·3)*	1101 (975 to 1222)	1287 (1158 to 1469)	16·9 (3·6 to 33·4)*	16·5 (4·8 to 31·0)*
		Motor vehicle road injuries	42 (38 to 47)	46 (43 to 51)	9·4 (−0·1 to 21·1)	15·2 (5·8 to 27·7)*	2419 (2206 to 2692)	2668 (2457 to 2977)	10·3 (1·2 to 21·5)*	18·2 (8·5 to 30·1)*
		Other road injuries	2 (1 to 3)	2 (1 to 3)	−6·0 (−32·1 to 41·6)	−14·8 (−39·3 to 28·1)	127 (86 to 169)	127 (95 to 182)	−0·1 (−26·0 to 42·7)	−9·4 (−32·9 to 29·2)
		Other transport injuries	5 (4 to 6)	6 (5 to 8)	23·5 (0·3 to 55·7)*	21·5 (1·2 to 52·9)*	275 (229 to 325)	332 (286 to 408)	20·8 (−0·6 to 51·1)	23·6 (3·7 to 53·3)*
		Falls	19 (17 to 22)	23 (21 to 26)	21·2 (7·1 to 39·4)*	6·2 (−7·0 to 21·7)	1847 (1520 to 2255)	2329 (1892 to 2877)	26·1 (13·2 to 41·2)*	17·9 (6·0 to 32·0)*
		Drowning	26 (20 to 31)	22 (19 to 31)	−15·0 (−33·5 to 15·9)	3·8 (−19·8 to 42·5)	1358 (1052 to 1625)	1137 (967 to 1591)	−16·3 (−33·9 to 14·8)	10·7 (−13·5 to 50·2)
		Fire, heat, and hot substances	12 (10 to 14)	12 (11 to 15)	7·7 (−6·0 to 25·5)	21·9 (7·2 to 42·1)*	815 (704 to 964)	909 (771 to 1084)	11·5 (−2·2 to 28·7)	26·6 (10·7 to 46·4)*
		Poisonings	4 (3 to 4)	3 (3 to 4)	−19·5 (−26·2 to −11·5)*	−4·9 (−18·9 to 9·0)	233 (193 to 262)	199 (168 to 225)	−14·6 (−22·0 to −6·1)*	0·2 (−20·3 to 20·8)
		Unintentional firearm injuries	2 (1 to 2)	2 (1 to 2)	−1·4 (−11·2 to 8·9)	3·8 (−6·2 to 14·3)	100 (71 to 113)	99 (73 to 112)	−1·5 (−10·9 to 8·4)	5·0 (−5·8 to 17·0)
		Unintentional suffocation	0 (0 to 0)	0 (0 to 0)	46·8 (7·5 to 75·3)*	39·4 (12·7 to 68·3)*	9 (8 to 11)	13 (10 to 15)	33·1 (11·7 to 54·0)*	34·6 (13·0 to 61·0)*
		Other exposure to mechanical forces	9 (8 to 10)	10 (8 to 12)	15·0 (−1·1 to 28·9)	18·1 (7·8 to 31·2)*	737 (625 to 859)	875 (704 to 1055)	18·7 (6·7 to 31·0)*	23·2 (11·9 to 35·9)*
		Venomous animal contact	3 (2 to 4)	3 (2 to 4)	−8·1 (−23·4 to 15·9)	2·2 (−12·2 to 18·8)	210 (152 to 247)	202 (143 to 247)	−3·8 (−17·2 to 15·6)	8·9 (−7·1 to 26·2)
		Non-venomous animal contact	0 (0 to 1)	0 (0 to 0)	−10·1 (−24·7 to 10·9)	0·6 (−14·0 to 17·7)	36 (29 to 45)	36 (29 to 46)	−0·1 (−14·3 to 16·5)	11·1 (−6·1 to 30·1)
		Pulmonary aspiration and foreign body in airway	3 (3 to 4)	4 (3 to 4)	28·0 (15·5 to 40·1)*	20·7 (10·3 to 32·0)*	192 (163 to 225)	239 (205 to 274)	24·4 (14·1 to 34·8)*	25·3 (11·8 to 40·9)*
		Foreign body in eyes	..	..	..	..	3 (1 to 6)	4 (2 to 8)	38·6 (28·8 to 49·0)*	20·1 (11·8 to 29·2)*
		Foreign body in other body part	2 (1 to 3)	2 (1 to 2)	6·1 (−12·7 to 24·0)	13·5 (−0·2 to 28·2)	138 (105 to 179)	157 (124 to 191)	13·8 (−2·8 to 29·5)	20·1 (4·4 to 36·1)*
		Other unintentional injuries	11 (10 to 13)	11 (9 to 14)	−5·8 (−22·6 to 23·4)	6·3 (−12·0 to 38·6)	1101 (882 to 1349)	1128 (895 to 1490)	2·4 (−12·1 to 24·5)	12·5 (−3·4 to 37·4)
	Occupational ergonomic factors: all causes		..	..	..	..	16 792 (11 730 to 23 448)	18 573 (12 979 to 25 759)	10·6 (9·2 to 12·0)*	11·7 (9·5 to 14·2)*
		Low back pain	..	..	..	..	16** **792 (11** **730 to 23** **448)	18** **573 (12** **979 to 25** **759)	10·6 (9·2 to 12·0)*	−4·9 (−5·6 to −4·1)*
**Behavioural risks: all causes**			**22 355 (21 183 to 23 567)**	**22 744 (21 408 to 24 126)**	**1·7 (−0·3 to 3·8)**	−3·1 (−4·2 to −2·0)*	**825 546 (781 724 to 872 847)**	**745 463 (698273 to 797 964)**	−9·7 (−11·8 to −7·7)*	−7·9 (−9·3 to −6·5)*
	**Child and maternal malnutrition: all causes**		**2280 (2113 to 2453)**	**1414 (1308 to 1533)**	−38·0 (−41·9 to −33·7)*	−30·4 (−34·9 to −25·6)*	**248 339 (226 327 to 275 262)**	**172 120 (151 792 to 196 176)**	−30·7 (−34·6 to −26·9)*	−22·2 (−26·4 to −18·3)*
	Suboptimal breastfeeding: all causes		592 (390 to 843)	391 (258 to 550)	−33·9 (−39·7 to −27·5)*	−25·3 (−31·8 to −18·1)*	51** **338 (33** **863 to 73** **062)	34** **030 (22** **497 to 47** **771)	−33·7 (−39·5 to −27·2)*	−24·9 (−31·2 to −17·6)*
	Non-exclusive breastfeeding: all causes		551 (347 to 789)	364 (227 to 520)	−34·0 (−39·5 to −27·6)*	−25·3 (−31·7 to −18·3)*	47** **654 (30** **141 to 68** **060)	31** **507 (19** **669 to 44** **929)	−33·9 (−39·4 to −27·5)*	−25·0 (−31·2 to −18·0)*
		Diarrhoeal diseases	228 (141 to 309)	156 (98 to 216)	−31·5 (−40·4 to −21·0)*	−5·0 (−15·2 to 7·9)	19** **768 (12** **250 to 26** **755)	13** **579 (8489 to 18** **758)	−31·3 (−40·1 to −20·8)*	−2·2 (−10·7 to 7·6)
		Lower respiratory infections	323 (137 to 555)	208 (86 to 355)	−35·7 (−41·6 to −30·2)*	−24·9 (−31·0 to −19·0)*	27** **886 (11** **846 to 47** **863)	17** **928 (7453 to 30** **580)	−35·7 (−41·5 to −30·2)*	−12·4 (−18·2 to −6·2)*
		Discontinued breastfeeding: all causes	55 (19 to 103)	37 (13 to 69)	−33·2 (−42·7 to −21·6)*	−25·2 (−35·9 to −12·3)*	4909 (1745 to 9248)	3356 (1147 to 6281)	−31·6 (−40·8 to −20·5)*	−23·2 (−33·3 to −10·8)*
		Diarrhoeal diseases	55 (19 to 103)	37 (13 to 69)	−33·2 (−42·7 to −21·6)*	−8·3 (−19·9 to 3·9)	4909 (1745 to 9248)	3356 (1147 to 6281)	−31·6 (−40·8 to −20·5)*	−3·6 (−12·1 to 5·5)
	Childhood undernutrition: all causes		2093 (1931 to 2261)	1265 (1160 to 1383)	−39·6 (−43·6 to −35·1)*	−32·1 (−36·9 to −27·0)*	184** **339 (170** **596 to 199** **097)	113** **280 (103** **941 to 123** **401)	−38·5 (−42·5 to −34·2)*	−30·8 (−35·2 to −26·0)*
	Childhood underweight: all causes		666 (531 to 857)	373 (304 to 468)	−44·0 (−52·0 to −34·8)*	−37·2 (−46·1 to −27·1)*	60** **146 (48** **823 to 76** **465)	34** **911 (28** **903 to 43** **357)	−42·0 (−49·6 to −33·2)*	−34·7 (−43·0 to −25·0)*
		Diarrhoeal diseases	124 (101 to 157)	69 (55 to 87)	−44·7 (−51·5 to −36·1)*	−23·8 (−31·6 to −15·2)*	10** **975 (8985 to 13** **785)	6162 (4984 to 7724)	−43·9 (−50·7 to −35·6)*	−20·6 (−26·2 to −14·9)*
		Lower respiratory infections	209 (139 to 359)	110 (70 to 197)	−47·5 (−52·7 to −42·3)*	−38·9 (−44·2 to −34·0)*	17 904 (11 962 to 30 872)	9416 (5994 to 16 919)	−47·4 (−52·6 to −42·2)*	−28·7 (−33·4 to −24·5)*
		Measles	100 (21 to 252)	20 (4 to 56)	−79·9 (−88·1 to −68·7)*	−20·2 (−37·5 to −9·6)*	8476 (1751 to 21 476)	1701 (311 to 4763)	−79·9 (−88·1 to −68·7)*	−20·2 (−37·3 to −9·9)*
		Protein-energy malnutrition	233 (180 to 301)	174 (131 to 227)	−25·3 (−42·5 to −3·6)*	−5·9 (−16·9 to 6·5)	22 791 (18 010 to 28 484)	17 633 (13 635 to 22 230)	−22·6 (−38·0 to −3·6)*	−0·8 (−4·3 to 2·8)
	Childhood wasting: all causes		1882 (1608 to 2125)	1169 (982 to 1306)	−37·9 (−42·9 to −32·8)*	−30·2 (−35·7 to −24·3)*	166 166 (142 764 to 186 926)	104 945 (88 636 to 117 151)	−36·8 (−41·7 to −32·0)*	−28·8 (−34·2 to −23·4)*
		Diarrhoeal diseases	663 (543 to 749)	431 (353 to 490)	−34·9 (−42·9 to −25·2)*	−10·4 (−19·4 to −1·0)*	58 779 (48 152 to 66 140)	38 905 (31 778 to 44 310)	−33·8 (−41·7 to −24·5)*	−6·5 (−11·4 to −1·3)*
		Lower respiratory infections	861 (623 to 1010)	535 (377 to 639)	−37·9 (−43·3 to −32·4)*	−27·8 (−32·6 to −22·6)*	73 987 (53 576 to 86 771)	45 989 (32 457 to 54 877)	−37·8 (−43·2 to −32·4)*	−15·7 (−19·7 to −12·0)*
		Measles	125 (30 to 374)	28 (6 to 92)	−77·2 (−86·2 to −63·8)*	−9·3 (−27·5 to −0·8)*	10 608 (2529 to 31 860)	2419 (483 to 7851)	−77·2 (−86·2 to −63·8)*	−9·3 (−27·4 to −0·9)*
		Protein-energy malnutrition	233 (180 to 301)	174 (131 to 227)	−25·3 (−42·5 to −3·6)*	−5·9 (−16·9 to 6·5)	22 791 (18 010 to 28 484)	17 633 (13 635 to 22 230)	−22·6 (−38·0 to −3·6)*	−0·8 (−4·3 to 2·8)
	Childhood stunting: all causes		508 (248 to 853)	257 (114 to 454)	−49·4 (−58·0 to −43·2)*	−43·1 (−53·0 to −36·2)*	43 961 (21 558 to 73 511)	22 420 (9964 to 39 448)	−49·0 (−57·4 to −42·9)*	−42·5 (−52·3 to −35·6)*
		Diarrhoeal diseases	154 (59 to 268)	87 (31 to 156)	−43·6 (−51·1 to −33·4)*	−22·3 (−30·5 to −13·0)*	13 638 (5210 to 23 658)	7824 (2807 to 13 947)	−42·6 (−50·1 to −32·8)*	−18·9 (−24·7 to −13·1)*
		Lower respiratory infections	282 (25 to 615)	155 (12 to 348)	−45·1 (−50·9 to −36·6)*	−36·1 (−42·0 to −26·4)*	24 248 (2170 to 52 767)	13 335 (1014 to 29 932)	−45·0 (−50·8 to −36·5)*	−25·4 (−31·1 to −16·1)*
		Measles	71 (7 to 202)	15 (1 to 45)	−79·2 (−87·9 to −61·9)*	−16·9 (−30·7 to −5·4)*	6074 (612 to 17 171)	1262 (108 to 3787)	−79·2 (−87·9 to −61·9)*	−16·9 (−30·6 to −5·3)*
	Iron deficiency: all causes		87 (59 to 116)	84 (58 to 114)	−3·5 (−14·8 to 10·0)	1·7 (−9·3 to 14·7)	55 120 (37 924 to 76 937)	52 870 (36 518 to 74 437)	−4·1 (−5·8 to −2·4)*	6·1 (3·9 to 8·5)*
		Maternal haemorrhage	30 (12 to 48)	24 (9 to 40)	−20·3 (−32·7 to −7·6)*	−4·4 (−11·2 to 2·2)	1694 (672 to 2705)	1338 (509 to 2236)	−21·0 (−32·8 to −8·8)*	−4·5 (−11·1 to 2·1)
		Maternal sepsis and other pregnancy-related infections	8 (3 to 13)	5 (2 to 9)	−34·1 (−47·7 to −17·6)*	−8·8 (−14·7 to −3·0)*	475 (187 to 776)	315 (118 to 543)	−33·7 (−46·9 to −17·2)*	−8·7 (−14·6 to −2·9)*
		Iron-deficiency anaemia	48 (32 to 63)	54 (35 to 73)	12·1 (−2·1 to 28·0)	..	52 951 (36 342 to 74 874)	51 217 (35 014 to 72 661)	−3·3 (−4·8 to −1·8)*	..
	Vitamin A deficiency: all causes		191 (116 to 291)	83 (50 to 119)	−56·7 (−66·6 to −47·3)*	−51·6 (−62·5 to −41·0)*	16 864 (10 421 to 25 457)	7611 (4693 to 10 841)	−54·9 (−64·7 to −45·6)*	−49·4 (−60·2 to −39·1)*
		Diarrhoeal diseases	107 (60 to 158)	64 (36 to 93)	−40·2 (−48·1 to −30·7)*	−17·9 (−26·8 to −8·5)*	9531 (5402 to 14 012)	5805 (3234 to 8402)	−39·1 (−46·7 to −29·8)*	−14·2 (−20·1 to −8·0)*
		Measles	84 (31 to 176)	19 (7 to 41)	−77·9 (−86·0 to −66·3)*	−12·1 (−23·1 to −2·7)*	7124 (2655 to 14 910)	1574 (556 to 3469)	−77·9 (−86·0 to −66·3)*	−12·0 (−22·8 to −2·8)*
		Vitamin A deficiency	..	..	..	..	209 (131 to 309)	232 (143 to 346)	10·9 (7·5 to 14·6)*	..
	Zinc deficiency: all causes		93 (5 to 208)	55 (3 to 125)	−40·5 (−49·1 to −28·8)*	−33·8 (−43·3 to −21·0)*	8162 (819 to 17 948)	4967 (600 to 10 853)	−39·1 (−47·3 to −25·0)*	−32·2 (−40·9 to −16·2)*
		Diarrhoeal diseases	51 (0 to 119)	31 (0 to 73)	−39·7 (−50·0 to 0·0)	−17·9 (−30·1 to 0·0)	4663 (301 to 10 330)	2911 (275 to 6369)	−37·6 (−47·4 to −8·3)*	−12·8 (−22·8 to 29·3)
		Lower respiratory infections	41 (0 to 143)	24 (0 to 83)	−41·4 (−49·4 to 0·0)	−32·8 (−40·8 to 0·0)	3498 (13 to 12 111)	2056 (11 to 7031)	−41·2 (−48·8 to −11·0)*	−21·4 (−29·4 to 21·5)
	**Tobacco smoke: all causes**		**6879 (6207 to 7522)**	**7165 (6544 to 7775)**	4·2 (0·9 to 7·6)*	−4·0 (−6·4 to −1·5)*	**174 309 (158 980 to 190 805)**	**170 889 (156 216 to 185 988)**	**−2·0 (−5·1 to 1·3)**	−6·4 (−9·0 to −3·6)*
	Smoking: all causes		6113 (5402 to 6780)	6402 (5749 to 7037)	4·7 (1·2 to 8·5)*	−3·9 (−6·7 to −0·8)*	147 153 (131 914 to 161 831)	148 623 (134 236 to 163 140)	1·0 (−2·4 to 4·6)	−5·0 (−8·1 to −1·9)*
		Tuberculosis	114 (57 to 180)	87 (41 to 140)	−23·9 (−32·0 to −16·9)*	−9·4 (−15·3 to −4·8)*	3717 (1859 to 5830)	2836 (1357 to 4514)	−23·7 (−31·0 to −17·4)*	−9·0 (−15·5 to −3·9)*
		Lower respiratory infections	332 (265 to 405)	350 (276 to 433)	5·4 (1·0 to 9·4)*	−1·3 (−4·6 to 2·0)	7119 (5724 to 8603)	7044 (5589 to 8640)	−1·1 (−5·4 to 3·3)	13·3 (7·0 to 19·9)*
		Lip and oral cavity cancer	48 (41 to 55)	60 (50 to 71)	25·8 (18·6 to 32·7)*	−4·9 (−8·8 to −1·6)*	1288 (1099 to 1487)	1548 (1284 to 1831)	20·2 (12·5 to 27·8)*	−6·8 (−10·9 to −3·1)*
		Nasopharyngeal cancer	21 (15 to 28)	23 (16 to 30)	8·3 (−2·0 to 20·0)	−4·5 (−9·9 to 2·3)	640 (431 to 887)	643 (451 to 867)	0·5 (−11·1 to 14·2)	−6·4 (−13·5 to 2·9)
		Oesophageal cancer	163 (101 to 236)	157 (104 to 216)	−4·0 (−13·8 to 8·6)	0·3 (−7·2 to 13·0)	3736 (2336 to 5384)	3418 (2286 to 4696)	−8·5 (−18·1 to 4·4)	−1·1 (−8·9 to 11·6)
		Stomach cancer	88 (51 to 138)	81 (47 to 126)	−8·0 (−14·7 to −1·5)*	−7·0 (−11·9 to −2·3)*	2030 (1162 to 3180)	1706 (958 to 2630)	−16·0 (−23·2 to −8·8)*	−10·0 (−15·9 to −4·6)*
		Colon and rectal cancer	45 (32 to 60)	50 (35 to 66)	10·2 (4·8 to 15·3)*	−10·3 (−13·7 to −7·4)*	978 (682 to 1293)	996 (696 to 1314)	1·9 (−3·4 to 6·8)	−14·0 (−17·5 to −11·1)*
		Liver cancer due to hepatitis B	37 (17 to 72)	35 (16 to 64)	−5·0 (−20·0 to 13·1)	−5·1 (−15·4 to 10·9)	1176 (512 to 2280)	1022 (467 to 1899)	−13·1 (−29·5 to 7·7)	−8·6 (−21·7 to 11·0)
		Liver cancer due to hepatitis C	20 (12 to 29)	24 (14 to 35)	20·3 (12·8 to 28·6)*	−0·4 (−6·0 to 5·0)	426 (248 to 634)	474 (275 to 712)	11·4 (2·1 to 21·4)*	−2·7 (−8·7 to 4·1)
		Liver cancer due to alcohol use	34 (17 to 58)	40 (20 to 69)	18·2 (6·8 to 31·0)*	−5·9 (−12·1 to −0·1)*	849 (426 to 1476)	958 (467 to 1652)	12·8 (−0·3 to 26·9)	−7·8 (−14·9 to −0·7)*
		Liver cancer due to other causes	16 (8 to 29)	16 (9 to 28)	0·5 (−12·5 to 18·5)	−0·1 (−10·4 to 15·6)	440 (204 to 817)	396 (202 to 684)	−9·8 (−24·7 to 11·0)	−4·0 (−16·4 to 15·6)
		Pancreatic cancer	62 (50 to 75)	73 (59 to 89)	18·0 (14·0 to 22·2)*	−9·3 (−11·8 to −6·9)*	1330 (1068 to 1630)	1467 (1159 to 1820)	10·3 (6·2 to 14·7)*	−12·4 (−15·3 to −9·6)*
		Tracheal, bronchial, and lung cancer	1014 (880 to 1127)	1175 (1012 to 1324)	15·8 (11·1 to 21·1)*	−3·6 (−5·3 to −2·1)*	22 060 (18 918 to 24 709)	24 140 (20 463 to 27 427)	9·4 (4·3 to 15·5)*	−4·7 (−6·7 to −2·8)*
		Cervical cancer	12 (4 to 21)	12 (4 to 21)	0·7 (−11·2 to 15·6)	−3·6 (−11·9 to 6·2)	355 (117 to 647)	330 (109 to 588)	−7·1 (−21·0 to 9·4)	−8·0 (−17·8 to 3·9)
		Kidney cancer	20 (13 to 26)	23 (15 to 31)	16·6 (10·1 to 22·4)*	−11·1 (−14·6 to −8·1)*	481 (326 to 625)	531 (349 to 709)	10·4 (3·9 to 16·1)*	−14·4 (−18·9 to −10·9)*
		Bladder cancer	44 (33 to 54)	51 (38 to 64)	16·8 (11·2 to 21·9)*	−6·2 (−9·0 to −3·6)*	835 (629 to 1034)	907 (674 to 1146)	8·6 (3·3 to 13·7)*	−8·8 (−11·8 to −6·1)*
		Ischaemic heart disease	1236 (1020 to 1455)	1280 (1049 to 1515)	3·5 (0·1 to 6·9)*	−8·9 (−11·1 to −6·6)*	32 669 (26 987 to 38 407)	33 161 (26 987 to 39 130)	1·5 (−2·1 to 4·9)	−7·4 (−9·4 to −5·4)*
		Ischaemic stroke	362 (301 to 420)	352 (293 to 416)	−2·7 (−6·8 to 1·3)	−7·6 (−10·6 to −4·5)*	7810 (6500 to 9146)	7520 (6258 to 8897)	−3·7 (−7·8 to 0·2)	−5·4 (−8·2 to −2·6)*
		Haemorrhagic stroke	609 (510 to 706)	573 (475 to 673)	−5·9 (−10·3 to −1·1)*	−7·1 (−10·1 to −3·9)*	16 619 (14 010 to 19 207)	15 512 (12 884 to 18 266)	−6·7 (−10·9 to −2·2)*	−5·5 (−8·2 to −2·6)*
		Hypertensive heart disease	99 (77 to 122)	114 (89 to 138)	14·9 (8·1 to 21·6)*	−6·8 (−11·2 to −1·4)*	2527 (1976 to 3091)	2771 (2168 to 3364)	9·7 (3·3 to 16·4)*	−5·9 (−9·9 to −1·3)*
		Atrial fibrillation and flutter	12 (8 to 15)	14 (10 to 18)	18·0 (13·9 to 22·2)*	−11·0 (−13·9 to −8·1)*	537 (379 to 732)	617 (433 to 846)	14·9 (12·7 to 16·9)*	−8·1 (−9·5 to −6·7)*
		Aortic aneurysm	22 (17 to 26)	23 (18 to 28)	6·3 (0·1 to 10·8)*	−12·9 (−15·0 to −10·6)*	542 (426 to 651)	572 (448 to 693)	5·6 (−1·1 to 10·4)	−10·3 (−12·4 to −8·1)*
		Peripheral vascular disease	4 (3 to 5)	5 (4 to 6)	15·3 (8·8 to 22·0)*	−13·4 (−17·4 to −9·5)*	144 (99 to 199)	167 (114 to 237)	16·3 (12·0 to 20·6)*	−10·6 (−12·9 to −8·4)*
		Other cardiovascular and circulatory diseases	58 (45 to 71)	61 (47 to 75)	5·9 (1·2 to 10·8)*	−8·8 (−11·7 to −5·5)*	2187 (1699 to 2688)	2348 (1816 to 2910)	7·4 (3·5 to 11·2)*	−6·7 (−9·2 to −4·2)*
		Chronic obstructive pulmonary disease	1355 (1010 to 1686)	1427 (1149 to 1699)	5·3 (−2·5 to 17·1)	1·8 (−4·5 to 13·4)	25 834 (20 021 to 31 596)	26 443 (21 842 to 31 310)	2·4 (−4·6 to 12·6)	0·6 (−5·4 to 10·8)
		Silicosis	2 (1 to 2)	1 (1 to 2)	−7·1 (−21·0 to 8·8)	−8·7 (−17·1 to −0·8)*	39 (22 to 66)	33 (18 to 56)	−15·2 (−29·6 to 1·8)	−14·6 (−24·6 to −4·8)*
		Asbestosis	1 (0 to 1)	1 (0 to 1)	18·5 (6·9 to 30·1)*	−8·5 (−13·2 to −3·3)*	12 (9 to 15)	13 (9 to 17)	7·8 (0·0 to 15·9)*	−12·6 (−16·9 to −7·8)*
		Coal workers pneumoconiosis	1 (0 to 1)	0 (0 to 1)	−18·5 (−30·4 to −5·7)*	−12·8 (−21·5 to −5·3)*	11 (8 to 15)	9 (6 to 13)	−17·0 (−29·5 to −2·5)*	−14·7 (−22·6 to −6·4)*
		Other pneumoconiosis	2 (1 to 2)	2 (2 to 3)	15·1 (4·9 to 27·8)*	−5·3 (−14·8 to 5·8)	49 (35 to 67)	52 (37 to 71)	6·3 (−2·8 to 15·5)	−13·5 (−20·2 to −6·6)*
		Asthma	55 (41 to 69)	45 (35 to 56)	−17·6 (−27·0 to −4·2)*	−8·2 (−17·3 to 2·6)	2226 (1692 to 2804)	2024 (1514 to 2579)	−9·0 (−15·9 to −0·7)*	−13·3 (−18·5 to −7·8)*
		Interstitial lung disease and pulmonary sarcoidosis	12 (9 to 16)	17 (12 to 21)	37·2 (26·4 to 45·4)*	−9·7 (−13·2 to −5·2)*	244 (179 to 302)	302 (224 to 377)	24·1 (15·2 to 31·7)*	−13·0 (−16·5 to −8·1)*
		Other chronic respiratory diseases	3 (2 to 4)	4 (3 to 6)	30·6 (16·5 to 49·4)*	6·3 (−4·5 to 20·5)	191 (140 to 248)	211 (150 to 278)	10·0 (0·6 to 20·1)*	3·2 (−6·7 to 13·2)
		Diabetes mellitus	56 (17 to 98)	68 (19 to 120)	21·2 (13·4 to 27·9)*	−7·6 (−13·1 to −3·5)*	2873 (831 to 5122)	3402 (949 to 6090)	18·4 (13·6 to 22·4)*	−8·5 (−11·7 to −6·1)*
	Second-hand smoke: all causes		884 (685 to 1093)	886 (695 to 1091)	0·2 (−3·5 to 3·9)	−4·8 (−7·4 to −2·4)*	29 996 (22 370 to 38 043)	25 212 (19 297 to 31 653)	−15·9 (−20·0 to −11·2)*	−13·3 (−16·5 to −9·7)*
		Lower respiratory infections	238 (151 to 330)	183 (114 to 259)	−23·0 (−27·4 to −19·0)*	−15·0 (−17·5 to −12·5)*	15 685 (9917 to 21 832)	10 103 (6305 to 14 390)	−35·6 (−39·9 to −31·4)*	−13·5 (−15·9 to −11·1)*
		Otitis media	0 (0 to 0)	0 (0 to 0)	−32·6 (−40·5 to −23·5)*	−11·7 (−25·4 to 2·6)	266 (149 to 426)	250 (138 to 406)	−5·8 (−9·4 to −3·0)*	−3·8 (−5·9 to −1·6)*
		Tracheal, bronchial, and lung cancer	22 (11 to 37)	29 (15 to 49)	32·6 (26·5 to 37·2)*	10·7 (6·1 to 14·9)*	536 (282 to 928)	691 (371 to 1189)	29·0 (21·4 to 35·0)*	13·5 (7·4 to 19·0)*
		Ischaemic heart disease	331 (252 to 422)	386 (291 to 494)	16·5 (12·1 to 20·5)*	0·9 (−0·8 to 2·6)	7187 (5734 to 8847)	8066 (6409 to 9894)	12·2 (7·9 to 16·2)*	1·9 (−0·0 to 3·9)
		Ischaemic stroke	69 (51 to 90)	72 (52 to 95)	5·4 (−0·7 to 10·9)	−1·5 (−4·2 to 0·9)	1229 (948 to 1561)	1248 (947 to 1589)	1·5 (−3·8 to 6·3)	−0·6 (−3·2 to 1·7)
		Haemorrhagic stroke	104 (81 to 132)	103 (80 to 132)	−0·6 (−5·2 to 4·7)	−2·5 (−5·1 to 0·1)	2637 (2076 to 3305)	2548 (1991 to 3201)	−3·4 (−8·2 to 2·2)	−2·1 (−4·9 to 0·9)
	**Alcohol and drug use: all causes**		**2595 (2314 to 2866)**	**2750 (2424 to 3051)**	6·0 (3·0 to 8·8)*	3·2 (1·0 to 5·4)*	**108 717 (100 094 to 117 134)**	**111 365 (102 247 to 120 352)**	2·4 (0·0 to 4·8)*	3·4 (1·2 to 5·5)*
	Alcohol use: all causes		2228 (1943 to 2500)	2306 (1986 to 2608)	3·5 (0·0 to 6·8)*	0·5 (−2·1 to 3·0)	86 048 (78 266 to 93 716)	84 990 (77 180 to 93 010)	−1·2 (−3·9 to 1·5)	−0·9 (−3·4 to 1·9)
		Tuberculosis	147 (117 to 187)	126 (94 to 169)	−13·9 (−23·0 to −5·2)*	4·7 (−3·7 to 11·1)	5498 (4497 to 6911)	4725 (3591 to 6198)	−14·1 (−22·4 to −6·2)*	4·7 (−3·3 to 11·0)
		Lower respiratory infections	93 (83 to 104)	106 (89 to 121)	13·6 (6·7 to 20·2)*	8·7 (2·9 to 14·5)*	2279 (2036 to 2495)	2355 (1995 to 2628)	3·3 (−4·2 to 10·9)	20·9 (11·7 to 30·3)*
		Lip and oral cavity cancer	29 (26 to 31)	38 (33 to 41)	31·9 (24·7 to 38·9)*	0·5 (−4·4 to 4·5)	820 (759 to 874)	1046 (929 to 1134)	27·5 (20·5 to 34·4)*	−0·3 (−4·8 to 3·6)
		Nasopharyngeal cancer	15 (11 to 16)	18 (13 to 20)	18·8 (7·8 to 28·3)*	5·2 (−0·6 to 9·6)	494 (367 to 538)	556 (407 to 623)	12·6 (1·9 to 22·3)*	5·6 (0·3 to 9·9)*
		Other pharyngeal cancer	16 (14 to 17)	20 (17 to 22)	26·3 (18·9 to 32·8)*	2·2 (−2·7 to 6·1)	454 (418 to 486)	560 (499 to 611)	23·4 (16·3 to 29·7)*	2·0 (−2·6 to 5·4)
		Oesophageal cancer	73 (66 to 80)	75 (64 to 84)	2·3 (−6·2 to 10·9)	7·6 (0·8 to 12·9)*	1847 (1673 to 2002)	1826 (1579 to 2027)	−1·1 (−9·3 to 7·3)	7·1 (0·9 to 11·9)*
		Colon and rectal cancer	27 (24 to 29)	32 (28 to 36)	20·8 (15·8 to 25·4)*	−1·4 (−5·2 to 1·6)	599 (548 to 650)	702 (626 to 771)	17·2 (12·3 to 21·5)*	−1·0 (−4·7 to 2·0)
		Liver cancer due to hepatitis B	1 (1 to 2)	1 (1 to 1)	−26·6 (−41·4 to −8·1)*	−17·6 (−33·3 to −2·5)*	75 (53 to 97)	56 (38 to 76)	−24·9 (−39·5 to −7·1)*	−12·8 (−28·3 to 1·5)
		Liver cancer due to hepatitis C	0 (0 to 0)	0 (0 to 0)	−10·0 (−26·7 to 8·8)	−14·5 (−30·3 to 2·0)	4 (3 to 6)	4 (2 to 5)	−12·0 (−27·8 to 5·2)	−12·2 (−27·1 to 3·5)
		Liver cancer due to alcohol use	195 (169 to 208)	245 (225 to 267)	26·1 (18·5 to 37·1)*	..	4787 (4075 to 5169)	5889 (5368 to 6441)	23·0 (14·8 to 36·1)*	..
		Liver cancer due to other causes	0 (0 to 0)	0 (0 to 0)	−30·2 (−43·3 to −13·8)*	−20·6 (−35·7 to −5·6)*	19 (13 to 25)	14 (9 to 19)	−28·7 (−41·6 to −12·5)*	−15·0 (−29·8 to −0·7)*
		Laryngeal cancer	18 (16 to 19)	20 (17 to 22)	9·9 (3·9 to 15·2)*	−3·0 (−7·9 to 0·7)	486 (443 to 523)	520 (456 to 572)	6·9 (1·6 to 11·9)*	−3·2 (−7·8 to 0·2)
		Breast cancer	27 (24 to 29)	30 (26 to 34)	13·8 (7·2 to 20·0)*	−6·1 (−10·7 to −1·7)*	801 (712 to 889)	899 (768 to 1023)	12·3 (5·4 to 18·7)*	−6·0 (−10·8 to −1·3)*
		Ischaemic heart disease	35 (−110 to 176)	10 (−130 to 150)	−71·4 (−400·6 to 326·2)	−85·9 (−325·6 to 241·9)	1760 (−648 to 4072)	1004 (−1092 to 2983)	−42·9 (−285·7 to 181·1)	−46·7 (−187·7 to 215·6)
		Ischaemic stroke	13 (−8 to 34)	8 (−11 to 27)	−38·1 (−206·5 to 204·3)	−31·8 (−162·0 to 138·1)	517 (210 to 814)	455 (213 to 691)	−12·0 (−21·3 to 10·0)	−11·9 (−21·3 to 16·5)
		Haemorrhagic stroke	237 (213 to 261)	248 (214 to 280)	4·7 (−2·1 to 11·3)	2·8 (−3·8 to 8·0)	5659 (5148 to 6143)	5754 (5004 to 6385)	1·7 (−4·8 to 7·3)	2·7 (−3·2 to 7·1)
		Hypertensive heart disease	40 (29 to 51)	51 (34 to 68)	30·0 (9·7 to 45·7)*	7·5 (−9·6 to 22·1)	1023 (840 to 1211)	1271 (970 to 1538)	24·3 (12·0 to 33·8)*	7·2 (−2·9 to 15·6)
		Atrial fibrillation and flutter	8 (6 to 9)	10 (8 to 13)	35·4 (26·8 to 43·1)*	0·5 (−5·9 to 5·8)	237 (184 to 303)	301 (233 to 391)	26·9 (20·6 to 32·3)*	0·5 (−4·6 to 4·5)
		Cirrhosis and other chronic liver diseases due to hepatitis B	82 (69 to 95)	87 (69 to 105)	6·9 (−3·0 to 14·9)	−1·5 (−10·2 to 4·4)	2456 (2076 to 2832)	2526 (1989 to 3019)	2·8 (−6·8 to 10·5)	−1·8 (−10·4 to 3·9)
		Cirrhosis and other chronic liver diseases due to hepatitis C	66 (54 to 77)	73 (57 to 88)	10·1 (−0·0 to 17·3)	−2·9 (−12·0 to 3·1)	1895 (1538 to 2203)	2017 (1542 to 2419)	6·4 (−3·5 to 13·4)	−2·6 (−11·8 to 3·3)
		Cirrhosis and other chronic liver diseases due to alcohol use	310 (289 to 333)	348 (323 to 375)	12·2 (8·4 to 16·7)*	..	10 093 (9424 to 10 841)	10 997 (10 197 to 11 875)	9·0 (5·0 to 13·7)*	..
		Cirrhosis and other chronic liver diseases due to other causes	48 (41 to 55)	52 (42 to 61)	7·9 (−0·5 to 14·8)	−0·7 (−8·5 to 4·5)	1399 (1197 to 1587)	1430 (1157 to 1681)	2·2 (−6·1 to 8·9)	1·1 (−7·0 to 6·7)
		Pancreatitis	16 (15 to 18)	19 (16 to 21)	13·3 (4·3 to 22·0)*	−4·4 (−11·1 to 1·5)	633 (567 to 691)	695 (600 to 778)	9·7 (1·4 to 18·1)*	−5·1 (−11·5 to 0·5)
		Epilepsy	12 (11 to 13)	14 (12 to 15)	10·9 (2·9 to 19·7)*	2·0 (−4·1 to 7·9)	1038 (866 to 1219)	1039 (859 to 1239)	0·1 (−6·5 to 6·5)	−0·7 (−6·4 to 4·1)
		Alcohol use disorders	157 (147 to 163)	138 (131 to 144)	−12·6 (−16·7 to −7·0)*	..	11 567 (9618 to 13 835)	11 194 (9136 to 13 871)	−3·2 (−7·0 to 0·6)	..
		Diabetes mellitus	−58 (−63 to −53)	−64 (−70 to −58)	10·5 (0·8 to 19·7)*	−18·4 (−25·9 to −10·3)*	−2324 (−2840 to −1866)	−2685 (−3321 to −2142)	15·5 (10·4 to 20·0)*	−11·7 (−15·4 to −8·2)*
		Pedestrian road injuries	107 (89 to 127)	100 (84 to 119)	−6·1 (−13·2 to −0·0)*	−0·4 (−3·1 to 2·1)	5360 (4475 to 6380)	4806 (4012 to 5678)	−10·3 (−16·8 to −4·6)*	0·3 (−2·5 to 2·9)
		Cyclist road injuries	13 (10 to 15)	11 (9 to 13)	−11·4 (−19·4 to −3·5)*	−2·4 (−5·7 to 0·7)	701 (578 to 838)	607 (511 to 720)	−13·4 (−19·8 to −6·7)*	−3·6 (−6·9 to −0·5)*
		Motorcyclist road injuries	77 (64 to 88)	81 (69 to 93)	4·3 (−5·1 to 16·4)	−0·0 (−3·3 to 3·5)	4299 (3632 to 4895)	4324 (3713 to 5003)	0·6 (−7·4 to 11·6)	−0·0 (−3·2 to 3·3)
		Motor vehicle road injuries	148 (130 to 167)	135 (120 to 152)	−8·8 (−14·7 to −2·9)*	−4·6 (−6·6 to −2·4)*	7917 (7015 to 8956)	7067 (6301 to 7927)	−10·7 (−15·9 to −5·6)*	−4·5 (−6·6 to −2·3)*
		Falls	42 (36 to 50)	50 (41 to 60)	17·8 (8·7 to 24·6)*	−1·2 (−5·0 to 2·2)	2416 (1951 to 2968)	2602 (2097 to 3230)	7·7 (2·2 to 12·2)*	−3·2 (−5·9 to −0·0)*
		Drowning	27 (24 to 32)	24 (21 to 28)	−13·2 (−17·0 to −9·1)*	3·0 (−2·9 to 8·4)	1225 (1057 to 1435)	1001 (871 to 1168)	−18·3 (−22·1 to −14·4)*	4·9 (−2·9 to 11·9)
		Fire, heat, and hot substances	14 (12 to 16)	12 (10 to 15)	−10·1 (−16·6 to −1·9)*	−3·6 (−10·3 to 1·7)	694 (586 to 832)	619 (511 to 755)	−10·8 (−16·2 to −4·5)*	−3·1 (−10·2 to 2·2)
		Poisonings	9 (7 to 11)	7 (5 to 9)	−15·8 (−21·5 to −8·5)*	−4·9 (−16·2 to 7·1)	386 (303 to 478)	319 (246 to 388)	−17·4 (−22·1 to −11·0)*	−7·0 (−23·3 to 11·0)
		Unintentional firearm injuries	3 (2 to 4)	3 (2 to 3)	−8·0 (−13·1 to −2·2)*	−6·7 (−9·8 to −3·0)*	145 (112 to 174)	129 (99 to 154)	−10·7 (−15·7 to −5·1)*	−6·9 (−10·6 to −2·5)*
		Unintentional suffocation	1 (1 to 1)	2 (1 to 2)	42·5 (16·3 to 57·0)*	27·3 (9·8 to 43·3)*	68 (55 to 84)	88 (68 to 108)	28·9 (13·2 to 38·9)*	25·8 (9·5 to 43·3)*
		Other exposure to mechanical forces	14 (11 to 17)	15 (10 to 18)	0·5 (−15·2 to 12·4)	−0·1 (−5·4 to 4·4)	913 (724 to 1127)	905 (684 to 1140)	−0·9 (−10·2 to 6·8)	−0·4 (−3·7 to 3·1)
		Self-harm	110 (95 to 129)	111 (96 to 130)	1·4 (−4·3 to 6·2)	0·6 (−3·6 to 3·8)	4649 (4032 to 5436)	4552 (3953 to 5303)	−2·1 (−7·5 to 2·7)	0·6 (−3·8 to 3·9)
		Assault by firearm	23 (21 to 26)	23 (20 to 26)	−0·6 (−4·2 to 4·2)	−5·3 (−6·9 to −3·6)*	1313 (1158 to 1481)	1285 (1128 to 1459)	−2·2 (−5·7 to 2·5)	−5·1 (−6·7 to −3·3)*
		Assault by sharp object	16 (14 to 18)	12 (11 to 14)	−19·7 (−24·0 to −14·5)*	−5·3 (−8·0 to −2·8)*	840 (744 to 968)	668 (590 to 769)	−20·5 (−24·7 to −15·2)*	−4·7 (−7·5 to −2·2)*
		Assault by other means	18 (16 to 22)	16 (14 to 19)	−13·9 (−18·4 to −7·3)*	−6·2 (−9·0 to −3·1)*	1007 (880 to 1181)	868 (745 to 1022)	−13·7 (−18·2 to −7·0)*	−5·0 (−7·9 to −1·8)*
	Drug use: all causes		407 (364 to 448)	489 (439 to 537)	20·2 (15·9 to 25·0)*	19·2 (15·2 to 24·2)*	24 036 (21 317 to 26 732)	27 831 (24 437 to 31 171)	15·8 (12·6 to 18·8)*	19·5 (16·1 to 23·0)*
		HIV/AIDS—tuberculosis	11 (9 to 14)	8 (7 to 10)	−25·7 (−33·4 to −15·9)*	22·1 (10·1 to 36·9)*	567 (465 to 698)	413 (333 to 513)	−27·1 (−33·9 to −18·2)*	19·4 (8·2 to 33·6)*
		HIV/AIDS resulting in other diseases	53 (46 to 65)	52 (44 to 63)	−3·6 (−12·0 to 7·2)	39·5 (28·9 to 51·1)*	2746 (2380 to 3317)	2594 (2216 to 3176)	−5·6 (−13·6 to 4·0)	34·4 (25·3 to 44·9)*
		Hepatitis B	1 (0 to 1)	0 (0 to 1)	−27·7 (−35·2 to −18·4)*	−21·3 (−29·3 to −10·3)*	18 (8 to 30)	13 (6 to 21)	−28·0 (−35·8 to −18·8)*	−22·3 (−30·6 to −12·2)*
		Hepatitis C	1 (0 to 2)	1 (0 to 2)	−12·0 (−30·1 to 11·0)	−1·4 (−13·0 to 11·5)	37 (9 to 83)	33 (8 to 72)	−12·3 (−30·1 to 10·0)	−3·8 (−14·3 to 7·5)
		Liver cancer due to hepatitis B	3 (1 to 5)	4 (2 to 6)	15·2 (1·3 to 38·4)*	15·3 (4·8 to 30·9)*	104 (43 to 171)	110 (49 to 179)	6·2 (−7·1 to 29·1)	11·7 (1·8 to 26·0)*
		Liver cancer due to hepatitis C	53 (38 to 68)	74 (57 to 91)	39·0 (28·8 to 54·2)*	16·6 (8·5 to 29·2)*	1316 (998 to 1627)	1678 (1329 to 2012)	27·5 (18·2 to 41·4)*	12·7 (5·5 to 24·1)*
		Cirrhosis and other chronic liver diseases due to hepatitis B	3 (1 to 5)	3 (1 to 5)	20·9 (10·3 to 35·7)*	12·6 (3·0 to 26·4)*	90 (38 to 149)	101 (43 to 162)	12·1 (2·3 to 24·9)*	8·1 (−1·3 to 20·6)
		Cirrhosis and other chronic liver diseases due to hepatitis C	124 (98 to 147)	147 (118 to 175)	19·0 (12·9 to 27·0)*	7·0 (2·2 to 14·1)*	4064 (3321 to 4750)	4556 (3727 to 5374)	12·1 (6·4 to 19·0)*	4·3 (0·2 to 10·2)*
		Opioid use disorders	94 (91 to 100)	122 (110 to 130)	29·6 (18·2 to 37·2)*	..	9864 (8127 to 11 517)	12 068 (9878 to 14 145)	22·3 (17·5 to 26·1)*	..
		Cocaine use disorders	7 (5 to 8)	11 (9 to 12)	49·7 (33·6 to 75·4)*	..	729 (558 to 902)	999 (773 to 1234)	37·0 (29·2 to 47·0)*	..
		Amphetamine use disorders	7 (4 to 8)	12 (8 to 14)	67·5 (25·6 to 118·9)*	..	1001 (706 to 1348)	1403 (1025 to 1847)	40·1 (26·1 to 55·2)*	..
		Cannabis use disorders	..	..	..	..	548 (352 to 781)	577 (372 to 818)	5·3 (3·7 to 7·1)*	..
		Other drug use disorders	20 (19 to 23)	25 (23 to 27)	23·0 (12·7 to 32·1)*	..	1529 (1245 to 1861)	1862 (1502 to 2274)	21·8 (15·7 to 27·5)*	..
		Self-harm	28 (19 to 41)	29 (20 to 41)	2·6 (−3·0 to 8·9)	5·6 (1·3 to 10·0)*	1423 (951 to 2030)	1425 (955 to 2037)	0·1 (−5·4 to 6·3)	5·8 (1·4 to 10·3)*
	**Dietary risks: all causes**		**10 738 (9473 to 12 010)**	**12 058 (10 615 to 13 538)**	12·3 (10·0 to 14·5)*	2·4 (1·1 to 3·6)*	**242 781 (217 378 to 269 659)**	**264411 (236 098 to 294 989)**	8·9 (6·3 to 11·3)*	2·1 (0·0 to 4·1)*
	Diet low in fruits: all causes		2713 (1806 to 3703)	2924 (1905 to 4018)	7·8 (4·3 to 10·9)*	−0·6 (−3·1 to 1·5)	68 838 (46 616 to 92 835)	72 590 (48 514 to 98 667)	5·5 (1·7 to 8·6)*	−0·3 (−3·4 to 2·5)
		Lip and oral cavity cancer	8 (0 to 17)	10 (0 to 22)	32·7 (0·0 to 37·5)*	0·1 (−0·6 to 0·7)	208 (0 to 451)	266 (0 to 583)	28·1 (0·0 to 33·1)*	−0·1 (−0·8 to 0·5)
		Nasopharyngeal cancer	4 (0 to 9)	5 (0 to 10)	11·3 (0·0 to 18·2)*	−1·7 (−2·7 to 0·0)	132 (0 to 281)	138 (0 to 293)	4·5 (−3·7 to 11·1)	−2·1 (−3·3 to 0·0)
		Other pharyngeal cancer	4 (0 to 8)	4 (0 to 10)	23·4 (0·0 to 29·4)*	−0·6 (−1·4 to 0·1)	99 (0 to 221)	119 (0 to 266)	20·2 (0·0 to 26·2)*	−0·6 (−1·4 to 0·1)
		Oesophageal cancer	106 (26 to 187)	99 (24 to 174)	−6·4 (−11·3 to −0·8)*	−2·0 (−3·2 to −1·2)*	2466 (618 to 4362)	2221 (542 to 3911)	−9·9 (−15·1 to −4·0)*	−2·5 (−3·9 to −1·6)*
		Laryngeal cancer	7 (0 to 15)	7 (0 to 17)	13·4 (0·0 to 17·4)*	−0·3 (−1·0 to 0·2)	168 (0 to 374)	184 (0 to 413)	9·8 (0·0 to 13·8)*	−0·5 (−1·3 to 0·1)
		Tracheal, bronchial, and lung cancer	172 (70 to 291)	207 (84 to 351)	20·1 (16·5 to 24·4)*	0·1 (−0·5 to 0·7)	3858 (1577 to 6495)	4391 (1780 to 7445)	13·8 (9·9 to 18·7)*	−0·5 (−1·3 to 0·1)
		Ischaemic heart disease	947 (340 to 1585)	1086 (388 to 1818)	14·7 (12·1 to 17·0)*	−0·3 (−0·9 to 0·3)	21 685 (7921 to 35 741)	23 777 (8598 to 39 451)	9·6 (6·3 to 12·4)*	−0·0 (−0·8 to 0·6)
		Ischaemic stroke	509 (271 to 776)	521 (274 to 805)	2·3 (−1·1 to 5·2)	−2·4 (−3·6 to −1·5)*	10 961 (5937 to 16 258)	11 001 (5940 to 16 513)	0·4 (−3·1 to 3·5)	−0·7 (−1·8 to 0·1)
		Haemorrhagic stroke	838 (465 to 1239)	831 (458 to 1241)	−0·9 (−4·5 to 2·9)	−1·7 (−2·8 to −0·8)*	22 973 (13 194 to 33 252)	22 476 (12 752 to 32 682)	−2·2 (−5·8 to 1·5)	−0·4 (−1·4 to 0·3)
	Diet low in vegetables: all causes		1842 (943 to 2869)	1994 (1024 to 3121)	8·2 (4·5 to 11·2)*	−1·1 (−3·7 to 0·7)	42 617 (22 635 to 65 749)	44 632 (23 572 to 68 817)	4·7 (1·5 to 7·9)*	−1·3 (−4·2 to 1·3)
		Ischaemic heart disease	1261 (489 to 2099)	1418 (548 to 2363)	12·5 (10·3 to 14·7)*	−2·8 (−3·7 to −2·1)*	27 791 (11 029 to 45 849)	30 048 (11 828 to 49 331)	8·1 (5·2 to 10·9)*	−1·9 (−2·8 to −1·1)*
		Ischaemic stroke	226 (51 to 412)	224 (51 to 412)	−0·8 (−3·8 to 2·0)	−5·7 (−7·1 to −4·4)*	4869 (1120 to 8768)	4762 (1097 to 8586)	−2·2 (−5·8 to 0·9)	−3·4 (−4·8 to −2·1)*
		Haemorrhagic stroke	355 (99 to 666)	352 (99 to 653)	−1·0 (−4·1 to 2·5)	−1·9 (−3·4 to −0·4)*	9957 (2799 to 18 328)	9823 (2800 to 18 053)	−1·3 (−4·6 to 2·3)	0·6 (−0·8 to 2·2)
	Diet low in whole grains: all causes		2879 (2056 to 3818)	3142 (2215 to 4189)	9·1 (6·3 to 11·8)*	0·4 (−1·5 to 2·0)	74 523 (54 213 to 96 519)	79 813 (57 571 to 104 238)	7·1 (3·7 to 9·8)*	1·3 (−1·3 to 3·5)
		Ischaemic heart disease	1497 (888 to 2168)	1713 (1013 to 2481)	14·4 (12·0 to 16·6)*	−0·6 (−1·2 to −0·2)*	33 870 (20 349 to 48 312)	36 933 (21 938 to 53 267)	9·0 (5·7 to 11·6)*	−0·7 (−1·4 to −0·2)*
		Ischaemic stroke	459 (311 to 620)	463 (311 to 631)	0·9 (−2·5 to 3·8)	−3·6 (−4·9 to −2·6)*	10 195 (6993 to 13 536)	10 143 (6849 to 13 505)	−0·5 (−4·0 to 2·7)	−1·5 (−2·6 to −0·6)*
		Haemorrhagic stroke	729 (515 to 982)	716 (497 to 962)	−1·8 (−5·4 to 2·2)	−2·6 (−3·7 to −1·6)*	20 024 (14 326 to 26 601)	19 443 (13 708 to 25 849)	−2·9 (−6·5 to 0·9)	−1·1 (−2·1 to −0·4)*
		Diabetes mellitus	195 (105 to 301)	250 (135 to 387)	28·5 (23·6 to 33·3)*	−1·5 (−2·4 to −0·7)*	10 434 (5659 to 16 438)	13 295 (7183 to 21 043)	27·4 (24·4 to 30·3)*	−0·8 (−1·4 to −0·3)*
	Diet low in nuts and seeds: all causes		1830 (1160 to 2583)	2131 (1354 to 3011)	16·4 (14·2 to 18·6)*	6·0 (4·5 to 7·5)*	43 725 (29 136 to 60 125)	49 411 (32 716 to 68 196)	13·0 (9·8 to 15·9)*	6·5 (3·9 to 9·0)*
		Ischaemic heart disease	1706 (1062 to 2438)	1970 (1226 to 2810)	15·5 (13·2 to 17·6)*	−0·0 (−0·4 to 0·3)	37 340 (23 613 to 52 283)	41 199 (26 175 to 57 706)	10·3 (7·3 to 12·9)*	0·3 (−0·2 to 0·8)
		Diabetes mellitus	123 (64 to 193)	160 (83 to 253)	29·9 (25·2 to 34·6)*	−0·5 (−1·1 to 0·1)	6384 (3038 to 10 211)	8212 (3923 to 13 200)	28·6 (25·6 to 31·5)*	0·1 (−0·2 to 0·5)
	Diet low in milk: all causes		101 (35 to 171)	126 (44 to 212)	24·7 (22·0 to 27·7)*	13·8 (11·6 to 15·9)*	2177 (750 to 3688)	2603 (897 to 4382)	19·6 (16·8 to 22·6)*	11·9 (9·3 to 14·7)*
		Colon and rectal cancer	101 (35 to 171)	126 (44 to 212)	24·7 (22·0 to 27·7)*	1·3 (0·9 to 1·8)*	2177 (750 to 3688)	2603 (897 to 4382)	19·6 (16·8 to 22·6)*	1·1 (0·7 to 1·6)*
		Diet high in red meat: all causes	37 (16 to 60)	43 (19 to 69)	14·8 (11·8 to 18·0)*	5·8 (3·4 to 8·3)*	1476 (549 to 2473)	1752 (638 to 2957)	18·7 (14·8 to 21·7)*	12·2 (8·2 to 15·1)*
		Colon and rectal cancer	20 (4 to 36)	24 (5 to 42)	17·1 (13·8 to 20·2)*	−4·5 (−6·2 to −2·7)*	442 (93 to 778)	504 (108 to 904)	14·2 (10·8 to 17·5)*	−3·8 (−5·5 to −1·9)*
		Diabetes mellitus	17 (2 to 31)	19 (2 to 35)	12·2 (9·0 to 15·2)*	−14·1 (−16·5 to −11·7)*	1034 (120 to 1958)	1247 (146 to 2359)	20·6 (17·2 to 23·5)*	−6·4 (−8·4 to −4·5)*
	Diet high in processed meat: all causes		476 (132 to 809)	541 (156 to 914)	13·8 (11·1 to 21·8)*	3·7 (1·4 to 11·6)*	13 088 (5240 to 20 672)	14 907 (6450 to 23 452)	13·9 (9·3 to 24·4)*	7·2 (3·2 to 18·2)*
		Colon and rectal cancer	31 (17 to 46)	37 (20 to 56)	20·7 (17·6 to 23·6)*	−2·5 (−3·8 to −1·3)*	644 (350 to 964)	745 (399 to 1123)	15·8 (12·8 to 18·7)*	−2·7 (−4·0 to −1·4)*
		Ischaemic heart disease	363 (16 to 680)	402 (17 to 759)	10·7 (7·5 to 12·8)*	−4·0 (−5·3 to −2·7)*	7931 (340 to 14 939)	8401 (357 to 15 858)	5·9 (1·8 to 8·5)*	−3·9 (−5·5 to −2·1)*
		Diabetes mellitus	82 (40 to 122)	102 (50 to 153)	24·8 (20·5 to 29·1)*	−4·9 (−6·5 to −3·5)*	4513 (2121 to 7031)	5761 (2708 to 8961)	27·6 (24·3 to 30·7)*	−0·9 (−2·1 to 0·3)
	Diet high in sugar-sweetened beverages: all causes		34 (23 to 47)	39 (27 to 55)	15·3 (12·5 to 18·0)*	7·3 (5·1 to 9·2)*	1234 (852 to 1748)	1449 (1004 to 2059)	17·4 (14·3 to 20·3)*	12·3 (9·9 to 14·7)*
		Oesophageal cancer	1 (0 to 1)	1 (0 to 1)	16·0 (9·5 to 21·3)*	21·7 (13·7 to 28·3)*	13 (4 to 24)	15 (4 to 27)	13·0 (6·3 to 18·6)*	22·7 (14·8 to 29·5)*
		Colon and rectum cancer	1 (0 to 1)	1 (0 to 1)	19·9 (16·4 to 23·5)*	−1·8 (−4·4 to 0·7)	14 (9 to 21)	17 (11 to 24)	17·8 (14·1 to 21·3)*	−0·1 (−3·0 to 2·8)
		Liver cancer due to hepatitis B	0 (0 to 0)	0 (0 to 0)	21·7 (13·0 to 30·4)*	21·8 (12·2 to 30·7)*	5 (2 to 9)	6 (3 to 10)	17·7 (8·5 to 27·3)*	24·4 (13·5 to 33·8)*
		Liver cancer due to hepatitis C	0 (0 to 0)	0 (0 to 0)	28·9 (23·8 to 33·9)*	6·7 (3·1 to 10·5)*	4 (2 to 6)	5 (2 to 8)	25·3 (19·8 to 31·0)*	9·2 (4·9 to 13·7)*
		Liver cancer due to alcohol use	0 (0 to 0)	0 (0 to 0)	30·5 (24·2 to 36·6)*	3·5 (−4·2 to 9·1)	5 (2 to 9)	7 (3 to 12)	28·1 (21·0 to 35·2)*	4·2 (−4·7 to 10·6)
		Liver cancer due to other causes	0 (0 to 0)	0 (0 to 0)	26·6 (19·3 to 33·4)*	25·0 (16·3 to 33·2)*	3 (1 to 5)	3 (2 to 5)	21·3 (13·4 to 28·3)*	28·3 (18·4 to 37·2)*
		Gallbladder and biliary tract cancer	0 (0 to 0)	0 (0 to 0)	8·2 (−0·3 to 14·6)	−3·4 (−7·5 to −0·3)*	4 (2 to 6)	4 (2 to 6)	4·9 (−2·7 to 11·0)	−1·5 (−6·1 to 2·0)
		Pancreatic cancer	0 (0 to 0)	0 (0 to 0)	25·2 (22·0 to 28·8)*	−3·7 (−6·1 to −1·5)*	4 (2 to 8)	5 (2 to 10)	22·0 (18·7 to 25·9)*	−3·0 (−5·4 to −0·4)*
		Breast cancer	0 (0 to 0)	0 (0 to 0)	25·6 (18·1 to 33·7)*	2·1 (−2·2 to 7·3)	8 (5 to 11)	10 (6 to 14)	26·6 (17·5 to 36·1)*	3·5 (−1·6 to 9·1)
		Uterine cancer	0 (0 to 0)	0 (0 to 0)	18·5 (11·9 to 25·7)*	7·7 (3·6 to 11·9)*	6 (4 to 9)	8 (5 to 11)	19·1 (11·7 to 26·8)*	10·5 (5·7 to 15·3)*
		Ovarian cancer	0 (0 to 0)	0 (0 to 0)	18·5 (0·0 to 22·9)*	−1·3 (−4·0 to 1·4)	1 (0 to 2)	1 (0 to 3)	17·9 (0·0 to 22·7)*	−0·4 (−3·4 to 2·5)
		Kidney cancer	0 (0 to 0)	0 (0 to 0)	25·6 (21·4 to 29·9)*	−4·1 (−6·6 to −1·7)*	6 (4 to 9)	7 (5 to 11)	23·2 (18·9 to 27·5)*	−3·9 (−7·4 to −0·5)*
		Thyroid cancer	0 (0 to 0)	0 (0 to 0)	25·9 (20·9 to 30·9)*	1·7 (−2·4 to 7·3)	1 (0 to 1)	1 (1 to 2)	46·0 (35·5 to 55·2)*	12·7 (5·5 to 20·1)*
		Ischaemic heart disease	12 (8 to 17)	13 (9 to 18)	8·4 (5·6 to 11·0)*	−4·4 (−6·4 to −2·5)*	326 (224 to 462)	348 (236 to 494)	6·7 (3·8 to 9·3)*	−1·9 (−4·1 to 0·2)
		Ischaemic stroke	2 (1 to 3)	2 (1 to 3)	2·7 (−1·5 to 6·7)	−1·1 (−4·0 to 1·9)	53 (37 to 75)	55 (39 to 78)	3·8 (0·3 to 7·2)*	3·5 (0·8 to 6·5)*
		Haemorrhagic stroke	5 (4 to 8)	6 (4 to 8)	7·5 (3·2 to 11·7)*	9·0 (5·1 to 12·2)*	186 (130 to 263)	198 (137 to 280)	6·6 (2·3 to 10·8)*	10·9 (7·3 to 14·2)*
		Hypertensive heart disease	2 (1 to 3)	3 (2 to 4)	20·9 (14·5 to 26·9)*	−2·4 (−6·8 to 3·2)	59 (39 to 86)	68 (45 to 100)	16·0 (10·8 to 21·0)*	0·2 (−3·6 to 4·5)
		Cardiomyopathy and myocarditis	..	..	..	..	..	..	..	..
		Atrial fibrillation and flutter	..	..	..	..	..	..	..	..
		Peripheral vascular disease	..	..	..	..	..	..	..	..
		Endocarditis	..	..	..	..	..	..	..	..
		Other cardiovascular and circulatory diseases	..	..	..	..	..	..	..	..
		Diabetes mellitus	6 (4 to 8)	7 (5 to 11)	23·2 (19·3 to 27·0)*	−5·1 (−7·7 to −2·3)*	378 (248 to 559)	488 (315 to 731)	28·9 (25·3 to 32·3)*	0·9 (−1·4 to 3·1)
		Chronic kidney disease due to diabetes mellitus	1 (1 to 2)	2 (1 to 4)	51·5 (44·8 to 58·8)*	8·7 (5·0 to 12·2)*	43 (19 to 75)	62 (28 to 107)	43·8 (37·8 to 51·5)*	8·5 (5·3 to 11·7)*
		Chronic kidney disease due to hypertension	1 (1 to 2)	2 (1 to 3)	28·4 (19·6 to 38·6)*	−4·5 (−8·4 to −0·5)*	33 (15 to 58)	42 (19 to 73)	24·7 (19·9 to 30·8)*	−1·4 (−4·9 to 1·8)
		Chronic kidney disease due to glomerulonephritis	0 (0 to 0)	0 (0 to 1)	27·5 (18·3 to 38·1)*	7·3 (2·5 to 12·1)*	12 (4 to 24)	15 (5 to 29)	24·6 (19·7 to 33·5)*	12·8 (8·9 to 16·9)*
		Chronic kidney disease due to other causes	0 (0 to 0)	0 (0 to 0)	36·1 (21·8 to 49·1)*	5·6 (−0·1 to 11·3)	8 (3 to 15)	10 (4 to 18)	28·3 (22·9 to 36·4)*	5·0 (2·0 to 8·0)*
	Diet low in fibre: all causes		334 (158 to 578)	387 (183 to 670)	15·8 (12·4 to 19·6)*	4·3 (1·6 to 7·1)*	7666 (3534 to 13 299)	8393 (3908 to 14 657)	9·5 (5·6 to 13·8)*	3·6 (0·1 to 7·1)*
		Colon and rectal cancer	38 (17 to 66)	47 (21 to 83)	24·4 (20·6 to 28·9)*	−0·5 (−2·8 to 2·1)	741 (327 to 1318)	870 (385 to 1545)	17·4 (13·5 to 21·5)*	−1·4 (−4·1 to 1·4)
		Ischaemic heart disease	297 (136 to 531)	340 (156 to 610)	14·7 (11·5 to 18·5)*	−1·6 (−4·0 to 0·7)	6925 (3137 to 12 333)	7523 (3410 to 13 333)	8·6 (4·7 to 12·9)*	−0·5 (−3·3 to 2·2)
		Diet low in calcium: all causes	127 (74 to 185)	160 (95 to 232)	26·1 (23·3 to 29·7)*	14·9 (12·4 to 17·6)*	2721 (1608 to 3966)	3284 (1966 to 4755)	20·7 (17·6 to 24·3)*	13·0 (10·1 to 16·2)*
		Colon and rectal cancer	127 (74 to 185)	160 (95 to 232)	26·1 (23·3 to 29·7)*	2·3 (1·3 to 3·9)*	2721 (1608 to 3966)	3284 (1966 to 4755)	20·7 (17·6 to 24·3)*	2·0 (1·0 to 3·5)*
	Diet low in seafood omega-3 fatty acids: all causes		1279 (537 to 2079)	1472 (616 to 2378)	15·0 (12·5 to 17·5)*	5·3 (3·7 to 6·8)*	28 350 (11 995 to 45 414)	31 310 (13 272 to 49 823)	10·4 (7·2 to 13·3)*	4·1 (1·2 to 6·8)*
		Ischaemic heart disease	1279 (537 to 2079)	1472 (616 to 2378)	15·0 (12·5 to 17·5)*	0·3 (−0·3 to 0·9)	28 350 (11 995 to 45 414)	31 310 (13 272 to 49 823)	10·4 (7·2 to 13·3)*	0·6 (−0·1 to 1·4)
	Diet low in polyunsaturated fatty acids: all causes		348 (145 to 548)	388 (158 to 610)	11·5 (3·6 to 20·8)*	1·2 (−6·1 to 10·3)	7535 (3243 to 11 727)	8270 (3534 to 12 996)	9·7 (2·0 to 18·7)*	2·9 (−4·4 to 11·3)
		Ischaemic heart disease	348 (145 to 548)	388 (158 to 610)	11·5 (3·6 to 20·8)*	−3·6 (−10·5 to 4·7)	7535 (3243 to 11 727)	8270 (3534 to 12 996)	9·7 (2·0 to 18·7)*	−0·6 (−7·3 to 6·7)
	Diet high in trans fatty acids: all causes		384 (158 to 713)	448 (190 to 827)	16·7 (13·1 to 22·8)*	7·8 (4·8 to 13·4)*	10 121 (4348 to 18 081)	11 578 (5138 to 20 322)	14·4 (9·9 to 21·4)*	8·9 (4·6 to 15·6)*
		Ischaemic heart disease	384 (158 to 713)	448 (190 to 827)	16·7 (13·1 to 22·8)*	2·7 (0·4 to 7·6)*	10 121 (4348 to 18 081)	11 578 (5138 to 20 322)	14·4 (9·9 to 21·4)*	5·2 (2·4 to 11·0)*
	Diet high in sodium: all causes		3668 (2145 to 5680)	4130 (2446 to 6419)	12·6 (8·3 to 16·9)*	2·8 (−0·1 to 6·3)	77 456 (45 739 to 118 672)	83 008 (49 326 to 127 452)	7·2 (2·7 to 11·3)*	0·2 (−3·4 to 3·8)
		Stomach cancer	413 (271 to 567)	397 (246 to 553)	−3·9 (−11·9 to 3·0)	−2·9 (−7·7 to 0·6)	9470 (6286 to 12 765)	8540 (5502 to 11 691)	−9·8 (−17·2 to −3·7)*	−3·4 (−7·8 to −0·4)*
		Rheumatic heart disease	30 (14 to 53)	28 (13 to 51)	−7·6 (−15·4 to −1·3)*	−4·9 (−11·3 to −0·1)*	873 (420 to 1603)	772 (359 to 1428)	−11·6 (−19·0 to −6·1)*	−6·6 (−13·1 to −2·1)*
		Ischaemic heart disease	1348 (682 to 2306)	1632 (852 to 2754)	21·1 (16·7 to 27·8)*	4·7 (2·0 to 9·7)*	26 945 (13 814 to 45 258)	31 088 (16 524 to 51 139)	15·4 (11·2 to 21·3)*	3·8 (1·6 to 8·1)*
		Ischaemic stroke	513 (278 to 848)	562 (308 to 916)	9·5 (3·7 to 17·1)*	3·4 (0·1 to 9·1)*	9239 (5141 to 14731)	9756 (5537 to 15 527)	5·6 (0·6 to 11·8)*	3·6 (0·6 to 8·6)*
		Haemorrhagic stroke	876 (516 to 1340)	875 (514 to 1347)	−0·0 (−6·1 to 6·0)	−2·7 (−7·0 to 1·4)	19 630 (11 721 to 29 698)	19 052 (11 138 to 28 916)	−2·9 (−9·2 to 2·6)	−3·1 (−7·5 to 0·8)
		Hypertensive heart disease	246 (88 to 484)	325 (116 to 634)	32·2 (18·7 to 45·3)*	4·3 (−0·6 to 11·9)	4756 (2134 to 8876)	5810 (2603 to 10 709)	22·2 (13·2 to 30·9)*	2·8 (−0·5 to 8·4)
		Cardiomyopathy and myocarditis	28 (12 to 51)	31 (14 to 57)	12·0 (5·8 to 20·4)*	3·5 (0·3 to 9·3)*	737 (312 to 1377)	764 (342 to 1396)	3·6 (−2·0 to 11·1)	4·3 (0·9 to 10·6)*
		Atrial fibrillation and flutter	14 (6 to 25)	19 (9 to 35)	39·1 (34·0 to 46·2)*	4·0 (1·6 to 9·2)*	449 (221 to 790)	592 (297 to 1033)	31·9 (28·8 to 37·4)*	4·8 (2·6 to 9·3)*
		Aortic aneurysm	13 (6 to 24)	16 (8 to 30)	25·9 (19·0 to 32·8)*	2·5 (0·3 to 6·2)*	262 (124 to 460)	319 (153 to 559)	21·8 (14·4 to 28·2)*	2·5 (0·4 to 5·9)*
		Peripheral vascular disease	2 (1 to 4)	3 (1 to 6)	38·4 (30·2 to 49·6)*	2·9 (−0·4 to 10·0)	70 (28 to 140)	97 (40 to 192)	37·7 (32·4 to 45·6)*	6·4 (3·3 to 12·7)*
		Endocarditis	6 (2 to 10)	7 (3 to 13)	28·6 (22·6 to 35·9)*	3·4 (0·3 to 8·1)*	133 (59 to 248)	163 (73 to 299)	22·6 (15·9 to 29·9)*	3·2 (−0·8 to 7·7)
		Other cardiovascular and circulatory diseases	50 (25 to 85)	59 (29 to 100)	18·1 (11·9 to 24·4)*	0·4 (−3·1 to 5·0)	1584 (804 to 2703)	1920 (993 to 3246)	21·3 (16·3 to 27·4)*	3·7 (1·0 to 8·2)*
		Chronic kidney disease due to diabetes mellitus	41 (18 to 74)	57 (25 to 103)	39·3 (33·0 to 44·8)*	−0·6 (−3·6 to 2·0)	1048 (467 to 1909)	1391 (635 to 2500)	32·7 (27·2 to 37·9)*	0·2 (−2·6 to 3·1)
		Chronic kidney disease due to hypertension	52 (22 to 99)	75 (32 to 139)	42·1 (36·0 to 49·9)*	4·4 (1·8 to 9·1)*	1107 (479 to 2079)	1467 (639 to 2717)	32·5 (27·3 to 38·8)*	4·1 (1·5 to 8·6)*
		Chronic kidney disease due to glomerulonephritis	35 (18 to 57)	40 (21 to 67)	15·1 (6·5 to 22·8)*	−2·9 (−8·9 to 2·1)	918 (471 to 1516)	971 (496 to 1648)	5·8 (−2·3 to 13·0)	−4·6 (−11·2 to 0·3)
		Chronic kidney disease due to other causes	2 (1 to 4)	3 (1 to 6)	43·8 (34·1 to 54·4)*	7·5 (3·0 to 12·7)*	236 (98 to 449)	306 (131 to 576)	29·9 (26·2 to 34·6)*	4·8 (2·6 to 8·4)*
	**Sexual abuse and violence: all causes**		**338 (244 to 444)**	**281 (199 to 378)**	−16·9 (−23·6 to −9·4)*	−15·2 (−21·9 to −8·3)*	**22 527 (17 002 to 28 715)**	**20 801 (15 633 to 26 810)**	−7·7 (−14·0 to −1·5)*	**−4·0 (−9·8 to 2·2)**
	Childhood sexual abuse: all causes		118 (34 to 229)	113 (31 to 221)	−4·9 (−11·8 to 1·1)	−3·1 (−10·2 to 2·6)	8200 (3468 to 14 180)	8107 (3472 to 14 062)	−1·1 (−8·5 to 5·8)	3·7 (−3·3 to 10·6)
		Alcohol use disorders	7 (0 to 16)	6 (0 to 13)	−22·0 (−26·6 to 0·0)	−10·1 (−12·5 to 0·0)	568 (0 to 1299)	512 (0 to 1183)	−9·9 (−14·6 to 0·0)	−6·3 (−8·3 to 0·0)
		Major depressive disorder	..	..	..	..	2062 (231 to 4518)	2321 (257 to 5097)	12·5 (10·7 to 14·0)*	−3·7 (−5·0 to −2·6)*
		Dysthymia	..	..	..	..	426 (46 to 943)	484 (53 to 1074)	13·8 (11·3 to 15·9)*	−4·1 (−5·4 to −2·8)*
		Self-harm	111 (27 to 222)	107 (27 to 213)	−3·8 (−10·5 to 2·1)	−2·5 (−4·5 to −0·7)*	5145 (1265 to 10 175)	4791 (1194 to 9542)	−6·9 (−13·6 to −0·9)*	−1·8 (−3·8 to 0·0)
	Intimate partner violence: all causes		237 (180 to 293)	186 (148 to 224)	−21·8 (−28·1 to −12·9)*	−20·1 (−26·5 to −11·9)*	15 431 (12 234 to 19 068)	13 803 (11 078 to 16 954)	−10·5 (−17·8 to −2·8)*	−7·4 (−14·0 to 0·1)
		HIV/AIDS—tuberculosis	20 (11 to 31)	10 (5 to 16)	−49·0 (−52·8 to −44·1)*	−18·0 (−23·3 to −13·0)*	937 (490 to 1432)	496 (251 to 775)	−47·1 (−50·6 to −42·5)*	−15·3 (−20·0 to −10·5)*
		HIV/AIDS resulting in other diseases	86 (45 to 128)	49 (25 to 72)	−43·5 (−47·8 to −38·9)*	−18·7 (−23·9 to −13·3)*	4301 (2252 to 6424)	2520 (1287 to 3716)	−41·4 (−45·9 to −36·8)*	−17·4 (−22·4 to −12·0)*
		Maternal abortion, miscarriage, and ectopic pregnancy	0 (0 to 0)	0 (0 to 0)	−22·8 (−36·5 to −5·4)*	0·2 (−11·4 to 12·9)	0 (0 to 0)	0 (0 to 0)	−23·2 (−36·6 to −6·0)*	0·1 (−10·5 to 13·3)
		Major depressive disorder	..	..	..	..	3562 (2279 to 5110)	4405 (2825 to 6361)	23·7 (21·3 to 26·0)*	2·8 (1·1 to 4·6)*
		Dysthymia	..	..	..	..	775 (496 to 1148)	980 (623 to 1438)	26·4 (23·3 to 29·8)*	4·0 (2·3 to 6·0)*
		Self-harm	104 (72 to 126)	103 (74 to 126)	−1·1 (−11·1 to 13·2)	−2·2 (−9·6 to 6·6)	4367 (2950 to 5417)	4107 (2944 to 5117)	−5·9 (−16·3 to 9·1)	−3·1 (−10·7 to 6·9)
		Assault by firearm	6 (5 to 7)	6 (5 to 7)	2·2 (−5·9 to 13·8)	−4·8 (−12·2 to 4·9)	315 (252 to 356)	312 (256 to 366)	−1·0 (−9·4 to 11·1)	−4·7 (−12·7 to 5·8)
		Assault by sharp object	7 (6 to 7)	5 (5 to 6)	−18·4 (−25·1 to −10·6)*	−4·8 (−12·0 to 2·4)	340 (305 to 379)	269 (238 to 302)	−21·0 (−28·1 to −12·6)*	−5·5 (−13·2 to 2·3)
		Assault by other means	14 (13 to 15)	12 (11 to 14)	−12·3 (−19·6 to −4·2)*	−3·8 (−10·7 to 3·3)	835 (760 to 913)	715 (624 to 805)	−14·3 (−21·5 to −6·0)*	−4·6 (−11·4 to 2·7)
	**Unsafe sex: all causes**		**2051 (1957 to 2156)**	**1452 (1381 to 1541)**	−29·2 (−31·6 to −26·1)*	−26·2 (−28·9 to −23·1)*	**112 703 (106 333 to 120 086)**	**79 451 (74 248 to 85 532)**	−29·5 (−32·0 to −26·8)*	−24·7 (−27·4 to −21·8)*
		HIV/AIDS—tuberculosis	334 (267 to 379)	199 (152 to 231)	−40·3 (−44·9 to −34·8)*	−0·8 (−1·4 to −0·4)*	18 403 (14 762 to 20 870)	10 990 (8439 to 12 703)	−40·3 (−44·7 to −34·7)*	−0·7 (−1·1 to −0·3)*
		HIV/AIDS resulting in other diseases	1356 (1271 to 1455)	906 (844 to 981)	−33·2 (−36·6 to −29·1)*	−1·8 (−2·5 to −1·4)*	75 102 (70 586 to 80 394)	51 167 (47 789 to 55 057)	−31·9 (−35·1 to −28·3)*	−1·5 (−2·1 to −1·1)*
		Syphilis	134 (80 to 206)	107 (63 to 165)	−20·3 (−28·5 to −12·4)*	..	11 190 (6607 to 17 281)	8957 (5273 to 13 970)	−20·0 (−28·4 to −11·8)*	..
		Chlamydial infection	0 (0 to 0)	0 (0 to 0)	−5·3 (−17·0 to 10·6)	..	337 (194 to 538)	370 (214 to 595)	9·7 (6·5 to 13·0)*	..
		Gonococcal infection	1 (1 to 1)	1 (1 to 1)	−15·7 (−26·7 to −4·7)*	..	383 (239 to 573)	470 (283 to 717)	22·7 (15·9 to 28·1)*	..
		Trichomoniasis	..	..	..	..	167 (67 to 355)	194 (78 to 412)	16·1 (15·0 to 17·2)*	..
		Genital herpes	..	..	..	..	198 (61 to 468)	236 (74 to 556)	19·5 (17·4 to 23·0)*	..
		Other sexually transmitted diseases	0 (0 to 0)	0 (0 to 0)	−16·8 (−27·6 to −6·1)*	..	103 (75 to 139)	104 (74 to 141)	0·3 (−3·1 to 3·2)	..
		Cervical cancer	225 (214 to 238)	239 (225 to 252)	5·8 (−0·5 to 13·8)	..	6819 (6393 to 7236)	6963 (6526 to 7408)	2·1 (−4·5 to 10·5)	..
	**Low physical activity: all causes**		**1351 (1047 to 1661)**	**1605 (1265 to 1956)**	18·9 (16·5 to 21·6)*	7·0 (5·3 to 9·1)*	**29 467 (22 694 to 36 268)**	**34 603 (26 905 to 42 282)**	17·4 (14·4 to 20·5)*	9·1 (6·8 to 11·8)*
		Colon and rectal cancer	95 (66 to 124)	119 (84 to 156)	25·2 (22·5 to 28·1)*	0·8 (0·2 to 1·6)*	1838 (1263 to 2441)	2209 (1529 to 2916)	20·2 (17·7 to 22·9)*	0·8 (0·1 to 1·8)*
		Breast cancer	39 (28 to 50)	48 (35 to 61)	22·1 (16·9 to 27·0)*	−0·3 (−1·5 to 1·3)	1064 (765 to 1369)	1276 (926 to 1644)	20·0 (14·2 to 25·5)*	−0·1 (−1·6 to 1·7)
		Ischaemic heart disease	727 (476 to 992)	859 (572 to 1161)	18·1 (15·8 to 20·8)*	1·3 (0·5 to 2·5)*	14 621 (9264 to 20 247)	16 673 (10 684 to 22 821)	14·0 (11·2 to 17·2)*	2·5 (1·6 to 4·1)*
		Ischaemic stroke	305 (169 to 449)	333 (188 to 485)	9·2 (6·1 to 12·8)*	1·3 (0·4 to 3·0)*	5049 (2688 to 7537)	5291 (2875 to 7911)	4·8 (1·4 to 8·4)*	2·1 (1·1 to 3·8)*
		Diabetes mellitus	184 (142 to 229)	247 (192 to 306)	34·2 (30·1 to 38·2)*	0·6 (−0·3 to 1·5)	6896 (4991 to 9242)	9154 (6647 to 12 251)	32·7 (29·8 to 35·5)*	1·4 (0·8 to 2·1)*
	**Metabolic risks: all causes**		**14 403 (13 691 to 15 063)**	**16 860 (16 021 to 17 697)**	17·1 (15·1 to 19·2)*	6·0 (5·1 to 7·0)*	**335 227 (315 739 to 357 389)**	**381 845 (357 846 to 409 436)**	13·9 (11·5 to 16·0)*	6·9 (5·2 to 8·4)*
	High fasting plasma glucose: all causes		4212 (3647 to 5047)	5240 (4547 to 6217)	24·4 (21·5 to 27·4)*	13·3 (11·5 to 15·3)*	117 101 (102 561 to 133 960)	143 076 (125 125 to 163 477)	22·2 (19·5 to 24·8)*	15·3 (13·1 to 17·5)*
		Tuberculosis	133 (83 to 189)	118 (73 to 169)	−11·1 (−19·7 to −3·5)*	5·4 (2·1 to 8·3)*	4313 (2737 to 5960)	3802 (2430 to 5304)	−11·9 (−19·5 to −5·2)*	4·2 (−0·6 to 8·1)
		Ischaemic heart disease	1415 (946 to 2016)	1761 (1175 to 2528)	24·4 (19·9 to 29·1)*	6·8 (5·3 to 8·4)*	27 891 (19 565 to 37 687)	33 535 (23 674 to 45 228)	20·2 (17·1 to 23·4)*	8·1 (6·9 to 9·5)*
		Ischaemic stroke	454 (257 to 803)	514 (291 to 913)	13·2 (7·9 to 20·3)*	6·0 (4·0 to 8·6)*	7750 (4771 to 12 343)	8524 (5350 to 13 291)	10·0 (6·0 to 14·3)*	7·6 (6·1 to 9·6)*
		Haemorrhagic stroke	529 (366 to 728)	585 (408 to 802)	10·5 (6·4 to 15·6)*	8·3 (6·8 to 10·2)*	12 434 (8510 to 17 327)	13 556 (9361 to 18 929)	9·0 (5·3 to 13·1)*	9·7 (8·3 to 11·5)*
		Diabetes mellitus	1150 (1121 to 1177)	1519 (1470 to 1576)	32·1 (27·7 to 36·3)*	..	49 725 (41 868 to 58 982)	64 135 (53 490 to 76 113)	29·0 (26·2 to 31·7)*	..
		Chronic kidney disease due to diabetes mellitus	299 (279 to 314)	418 (389 to 441)	39·5 (35·4 to 43·5)*	..	8713 (7991 to 9466)	11 258 (10 303 to 12 225)	29·2 (25·9 to 32·5)*	..
		Chronic kidney disease due to hypertension	149 (105 to 192)	216 (153 to 278)	44·9 (39·7 to 50·1)*	6·2 (5·1 to 7·4)*	3260 (2284 to 4222)	4467 (3172 to 5743)	37·0 (32·0 to 42·4)*	8·0 (6·6 to 9·4)*
		Chronic kidney disease due to glomerulonephritis	68 (48 to 89)	89 (63 to 114)	30·0 (24·2 to 36·0)*	9·2 (7·6 to 11·0)*	2106 (1466 to 2770)	2570 (1805 to 3358)	22·1 (17·1 to 27·1)*	10·5 (8·5 to 12·6)*
		Chronic kidney disease due to other causes	6 (4 to 8)	9 (6 to 12)	47·2 (39·6 to 55·6)*	10·3 (7·8 to 13·1)*	695 (453 to 983)	936 (615 to 1313)	34·6 (31·8 to 37·5)*	9·3 (7·6 to 11·2)*
	High total cholesterol: all causes		3816 (2973 to 4849)	4313 (3324 to 5512)	13·0 (9·5 to 16·2)*	1·4 (−0·6 to 3·2)	81 691 (68 219 to 96 877)	88 687 (74 558 to 105 681)	8·6 (5·8 to 11·0)*	0·9 (−1·5 to 3·1)
		Ischaemic heart disease	3279 (2594 to 4016)	3743 (2906 to 4650)	14·1 (10·2 to 17·7)*	−2·2 (−3·5 to −1·0)*	71 723 (60 172 to 84 381)	78 590 (65 999 to 92 772)	9·6 (6·6 to 12·1)*	−1·3 (−2·0 to −0·6)*
		Ischaemic stroke	537 (220 to 1058)	569 (221 to 1127)	6·0 (−1·8 to 11·5)	−2·7 (−6·2 to −0·5)*	9968 (5939 to 16 618)	10 098 (5961 to 16 912)	1·3 (−2·7 to 4·9)	−2·0 (−3·5 to −0·5)*
	High systolic blood pressure: all causes		9212 (8326 to 10 101)	10 704 (9601 to 11 787)	16·2 (13·9 to 18·6)*	5·2 (4·0 to 6·4)*	18 9579 (172 703 to 206 696)	211 816 (192 712 to 231 114)	11·7 (9·2 to 14·1)*	4·4 (2·2 to 6·4)*
		Rheumatic heart disease	79 (54 to 117)	80 (56 to 121)	1·7 (−3·2 to 7·0)	4·4 (2·9 to 6·3)*	2377 (1588 to 3481)	2363 (1615 to 3402)	−0·6 (−4·9 to 3·9)	5·4 (3·6 to 7·3)*
		Ischaemic heart disease	4135 (3408 to 4840)	4862 (3955 to 5740)	17·6 (14·7 to 20·2)*	0·9 (−0·0 to 1·7)	79 828 (68 710 to 90 328)	90 298 (77 837 to 102 138)	13·1 (10·4 to 15·6)*	1·4 (0·8 to 2·0)*
		Ischaemic stroke	1367 (1083 to 1656)	1489 (1167 to 1821)	8·9 (4·7 to 13·5)*	1·8 (0·4 to 3·4)*	22 995 (18 429 to 26 872)	24 198 (19 500 to 28 264)	5·2 (1·8 to 8·7)*	2·6 (1·8 to 3·7)*
		Haemorrhagic stroke	1819 (1486 to 2144)	1953 (1588 to 2313)	7·4 (3·6 to 12·1)*	4·4 (3·2 to 5·8)*	41 530 (34 162 to 47 708)	43 412 (36 092 to 49 999)	4·5 (1·0 to 8·7)*	4·7 (3·7 to 6·0)*
		Hypertensive heart disease	761 (712 to 824)	962 (874 to 1025)	26·5 (17·5 to 32·3)*	..	14 852 (13 919 to 16 053)	17 485 (16 287 to 18 594)	17·7 (11·6 to 22·9)*	..
		Cardiomyopathy and myocarditis	121 (98 to 145)	129 (104 to 157)	7·3 (1·6 to 13·0)*	−1·1 (−3·3 to 1·2)	3233 (2594 to 3859)	3188 (2570 to 3795)	−1·4 (−6·3 to 4·0)	−0·4 (−2·9 to 2·3)
		Atrial fibrillation and flutter	50 (38 to 65)	68 (50 to 90)	35·2 (31·4 to 39·1)*	0·0 (−1·2 to 1·3)	1409 (1087 to 1820)	1810 (1398 to 2347)	28·5 (26·8 to 30·2)*	1·2 (0·6 to 1·9)*
		Aortic aneurysm	49 (39 to 58)	60 (47 to 71)	22·7 (17·3 to 27·2)*	−1·1 (−2·0 to −0·2)*	928 (771 to 1080)	1110 (925 to 1291)	19·7 (13·6 to 23·7)*	0·3 (−0·4 to 1·1)
		Peripheral vascular disease	11 (8 to 14)	14 (10 to 19)	33·0 (25·1 to 42·0)*	−2·4 (−4·3 to −0·5)*	265 (184 to 386)	346 (235 to 505)	30·4 (26·2 to 34·9)*	0·0 (−1·2 to 1·1)
		Endocarditis	22 (17 to 28)	28 (22 to 36)	26·7 (21·8 to 31·6)*	0·8 (−0·5 to 2·0)	518 (403 to 644)	640 (496 to 792)	23·5 (18·0 to 29·1)*	3·7 (1·0 to 6·3)*
		Other cardiovascular and circulatory diseases	176 (154 to 199)	211 (182 to 240)	19·8 (15·0 to 24·4)*	0·8 (−0·2 to 1·7)	5165 (4411 to 6058)	6267 (5304 to 7424)	21·3 (17·6 to 25·1)*	3·5 (2·4 to 4·7)*
		Chronic kidney disease due to diabetes mellitus	132 (95 to 167)	190 (135 to 240)	43·5 (39·5 to 47·5)*	1·6 (0·9 to 2·4)*	3186 (2231 to 4120)	4365 (3085 to 5671)	37·0 (33·5 to 40·5)*	3·0 (2·3 to 3·8)*
		Chronic kidney disease due to hypertension	409 (377 to 428)	550 (502 to 576)	34·5 (30·0 to 38·7)*	..	10 366 (9401 to 10 985)	12 737 (11 489 to 13 554)	22·9 (18·6 to 27·4)*	..
		Chronic kidney disease due to glomerulonephritis	76 (54 to 97)	98 (71 to 125)	28·8 (23·9 to 34·0)*	7·1 (5·5 to 8·8)*	2151 (1481 to 2852)	2584 (1808 to 3384)	20·1 (15·9 to 24·5)*	7·5 (5·5 to 9·6)*
		Chronic kidney disease due to other causes	8 (6 to 11)	12 (8 to 15)	42·2 (34·0 to 50·6)*	5·3 (2·7 to 8·1)*	777 (513 to 1063)	1013 (668 to 1386)	30·4 (28·1 to 33·1)*	4·6 (3·2 to 6·2)*
	High body-mass index: all causes		3314 (2241 to 4504)	3960 (2728 to 5332)	19·5 (15·8 to 23·6)*	10·0 (7·2 to 13·5)*	98 478 (67 219 to 131 972)	120 132 (83 829 to 158 409)	22·0 (18·1 to 26·8)*	14·8 (11·7 to 19·0)*
		Oesophageal cancer	67 (20 to 123)	71 (22 to 130)	6·2 (−3·4 to 16·8)	10·7 (1·3 to 20·5)*	1573 (459 to 2901)	1644 (516 to 3015)	4·5 (−5·3 to 15·8)	12·3 (2·8 to 22·9)*
		Colon and rectal cancer	48 (29 to 70)	62 (38 to 91)	28·9 (25·5 to 32·9)*	4·7 (2·5 to 7·3)*	1050 (632 to 1527)	1329 (809 to 1924)	26·6 (23·3 to 30·6)*	6·2 (4·0 to 8·8)*
		Liver cancer due to hepatitis B	24 (9 to 48)	28 (11 to 54)	17·9 (8·3 to 35·0)*	16·0 (9·9 to 25·7)*	762 (268 to 1511)	870 (326 to 1678)	14·2 (3·9 to 33·3)*	18·5 (11·5 to 29·7)*
		Liver cancer due to hepatitis C	15 (6 to 26)	20 (9 to 35)	32·8 (25·8 to 40·8)*	9·2 (4·0 to 14·4)*	325 (141 to 574)	421 (186 to 733)	29·5 (21·7 to 38·8)*	12·0 (6·2 to 18·4)*
		Liver cancer due to alcohol use	21 (8 to 41)	30 (11 to 56)	38·0 (28·8 to 50·1)*	8·9 (3·6 to 13·9)*	529 (202 to 1034)	729 (282 to 1393)	37·7 (27·3 to 52·3)*	11·2 (5·8 to 17·3)*
		Liver cancer due to other causes	12 (5 to 22)	14 (6 to 25)	18·4 (9·2 to 31·4)*	16·3 (8·8 to 26·6)*	317 (127 to 583)	360 (150 to 646)	13·3 (3·6 to 28·1)*	18·7 (10·2 to 30·1)*
		Gallbladder and biliary tract cancer	18 (10 to 27)	21 (12 to 31)	14·5 (6·3 to 22·5)*	1·2 (−3·1 to 5·2)	362 (209 to 547)	402 (233 to 596)	11·0 (3·6 to 18·6)*	3·1 (−1·0 to 7·3)
		Pancreatic cancer	17 (6 to 29)	23 (8 to 40)	35·4 (31·6 to 39·5)*	3·3 (0·6 to 5·7)*	350 (122 to 614)	463 (163 to 812)	32·5 (28·8 to 36·7)*	4·4 (2·0 to 6·8)*
		Breast cancer	25 (14 to 39)	34 (20 to 52)	36·5 (25·1 to 48·0)*	7·9 (3·1 to 13·9)*	551 (299 to 887)	774 (439 to 1214)	40·4 (24·5 to 57·5)*	8·9 (1·7 to 17·3)*
		Uterine cancer	26 (17 to 35)	31 (21 to 41)	19·6 (11·4 to 28·9)*	8·3 (5·1 to 13·1)*	654 (441 to 891)	780 (538 to 1041)	19·2 (10·1 to 29·8)*	10·0 (6·2 to 15·5)*
		Ovarian cancer	4 (0 to 9)	5 (0 to 11)	26·3 (22·1 to 31·7)*	4·3 (2·3 to 7·5)*	98 (−3 to 221)	124 (−4 to 275)	26·0 (21·5 to 31·7)*	5·0 (2·8 to 8·6)*
		Kidney cancer	18 (12 to 24)	24 (16 to 33)	34·2 (29·8 to 38·8)*	1·5 (−0·6 to 3·7)	410 (272 to 565)	541 (364 to 741)	31·9 (27·7 to 36·3)*	1·8 (−0·8 to 4·9)
		Thyroid cancer	2 (1 to 4)	3 (2 to 5)	32·5 (22·6 to 41·1)*	5·9 (0·4 to 11·3)*	62 (35 to 96)	90 (50 to 141)	45·6 (32·8 to 57·8)*	10·6 (3·8 to 16·8)*
		Ischaemic heart disease	1228 (824 to 1687)	1436 (960 to 1965)	16·9 (13·2 to 20·9)*	1·9 (−0·5 to 4·5)	28 614 (19 490 to 38 838)	33 038 (22 659 to 44 539)	15·5 (12·1 to 19·1)*	4·4 (2·3 to 6·9)*
		Ischaemic stroke	313 (203 to 449)	320 (204 to 470)	2·3 (−4·1 to 8·1)	−1·9 (−6·4 to 2·5)	7020 (4693 to 9683)	7318 (4859 to 10 062)	4·2 (−0·1 to 8·9)	2·7 (−0·6 to 6·4)
		Haemorrhagic stroke	606 (390 to 850)	651 (431 to 894)	7·3 (1·9 to 13·9)*	6·9 (2·9 to 11·7)*	18 122 (11 921 to 24 775)	19 636 (13 414 to 26 434)	8·4 (3·3 to 15·0)*	10·3 (6·6 to 15·6)*
		Hypertensive heart disease	234 (144 to 341)	308 (182 to 455)	32·0 (20·8 to 42·0)*	4·9 (−0·5 to 10·3)	5010 (3364 to 6854)	6313 (4255 to 8539)	26·0 (19·4 to 33·6)*	6·8 (3·3 to 11·2)*
		Diabetes mellitus	408 (293 to 532)	555 (404 to 718)	36·0 (30·5 to 42·2)*	3·6 (0·9 to 6·8)*	21 809 (15 245 to 29 011)	30 396 (21 544 to 39 884)	39·4 (34·7 to 44·8)*	7·7 (5·3 to 11·4)*
		Chronic kidney disease due to diabetes mellitus	78 (40 to 124)	119 (62 to 187)	51·5 (41·5 to 62·0)*	8·0 (4·0 to 12·4)*	2205 (1075 to 3430)	3231 (1645 to 4932)	46·5 (40·2 to 55·0)*	10·0 (7·0 to 14·1)*
		Chronic kidney disease due to hypertension	94 (45 to 151)	134 (61 to 217)	42·5 (25·6 to 53·4)*	5·3 (−1·1 to 9·3)	2315 (1159 to 3656)	3193 (1634 to 4945)	37·9 (31·6 to 45·0)*	8·1 (5·1 to 11·7)*
		Chronic kidney disease due to glomerulonephritis	36 (15 to 61)	46 (19 to 78)	27·4 (11·2 to 42·7)*	7·0 (−2·9 to 14·7)	1275 (473 to 2206)	1583 (612 to 2662)	24·1 (16·8 to 35·7)*	10·9 (6·6 to 17·1)*
		Chronic kidney disease due to other causes	3 (1 to 6)	5 (1 to 9)	53·8 (10·0 to 69·1)*	16·2 (4·2 to 24·5)*	510 (223 to 850)	696 (308 to 1140)	36·3 (31·0 to 43·2)*	9·7 (6·8 to 13·5)*
		Osteoarthritis	..	..	..	..	1648 (949 to 2565)	2393 (1391 to 3716)	45·2 (42·1 to 49·6)*	7·4 (5·4 to 10·4)*
		Low back pain	..	..	..	..	2501 (1415 to 4001)	3299 (1896 to 5222)	31·9 (29·4 to 35·6)*	8·6 (6·8 to 11·4)*
	Low bone mineral density: all causes		283 (261 to 295)	361 (325 to 381)	27·7 (22·8 to 32·9)*	12·6 (8·4 to 17·2)*	7499 (6494 to 8674)	8810 (7565 to 10 270)	17·5 (13·9 to 21·1)*	7·8 (4·6 to 11·0)*
		Pedestrian road injuries	44 (41 to 48)	50 (46 to 56)	14·4 (6·5 to 21·6)*	5·5 (2·7 to 8·4)*	974 (882 to 1071)	1072 (948 to 1199)	10·0 (2·2 to 17·2)*	7·0 (3·7 to 10·7)*
		Cyclist road injuries	4 (4 to 5)	5 (4 to 5)	10·5 (1·0 to 20·0)*	8·0 (3·9 to 12·4)*	240 (193 to 298)	273 (215 to 343)	13·5 (7·3 to 19·1)*	10·9 (6·1 to 15·4)*
		Motorcyclist road injuries	11 (9 to 12)	14 (12 to 16)	31·4 (16·4 to 48·5)*	10·9 (4·3 to 17·5)*	439 (366 to 517)	559 (461 to 655)	27·2 (18·2 to 37·3)*	11·4 (5·2 to 16·5)*
		Motor vehicle road injuries	29 (26 to 32)	33 (29 to 37)	13·6 (6·1 to 21·7)*	4·8 (1·5 to 7·5)*	1029 (882 to 1196)	1163 (982 to 1357)	13·0 (7·1 to 18·6)*	6·4 (3·2 to 9·3)*
		Other road injuries	1 (1 to 2)	2 (1 to 2)	28·3 (12·6 to 47·9)*	0·6 (−10·6 to 11·8)	51 (40 to 64)	70 (54 to 88)	35·6 (26·3 to 45·0)*	7·2 (−5·5 to 19·7)
		Other transport injuries	8 (7 to 10)	10 (8 to 12)	19·6 (8·1 to 32·9)*	4·1 (0·4 to 7·4)*	219 (192 to 252)	247 (208 to 306)	13·0 (2·9 to 25·1)*	2·4 (−1·6 to 5·9)
		Falls	170 (151 to 179)	230 (199 to 245)	35·3 (29·0 to 42·3)*	2·3 (−1·2 to 6·1)	3987 (3362 to 4758)	4816 (4020 to 5788)	20·8 (17·2 to 24·7)*	−0·9 (−4·3 to 3·1)
		Other exposure to mechanical forces	8 (7 to 9)	10 (7 to 11)	21·1 (5·5 to 29·8)*	7·7 (0·0 to 13·4)*	316 (255 to 390)	361 (280 to 454)	14·2 (5·7 to 19·1)*	4·4 (−0·5 to 8·2)
		Non-venomous animal contact	1 (1 to 1)	1 (1 to 1)	6·4 (−2·5 to 24·9)	3·7 (−4·8 to 10·7)	20 (17 to 24)	20 (17 to 25)	1·1 (−5·9 to 15·2)	−1·1 (−9·2 to 5·5)
		Assault by other means	5 (5 to 6)	6 (5 to 6)	7·5 (1·4 to 14·1)*	2·4 (−2·8 to 7·3)	183 (154 to 215)	187 (156 to 222)	2·1 (−2·8 to 7·7)	−1·2 (−6·2 to 3·8)
		Exposure to forces of nature	..	..	..	..	40 (24 to 58)	42 (26 to 62)	6·3 (−21·5 to 40·4)	274·6 (178·4 to 423·0)*
	Low glomerular filtration rate: all causes		1991 (1881 to 2107)	2426 (2290 to 2559)	21·9 (19·2 to 24·5)*	10·7 (8·7 to 12·6)*	47 131 (44 090 to 50 123)	54 433 (50 890 to 57 912)	15·5 (12·7 to 18·2)*	10·4 (8·0 to 12·9)*
		Ischaemic heart disease	589 (526 to 656)	691 (618 to 770)	17·3 (14·7 to 20·1)*	0·0 (−1·8 to 2·0)	9659 (8602 to 10 739)	10 901 (9711 to 12 188)	12·9 (10·0 to 15·8)*	0·7 (−1·1 to 2·7)
		Ischaemic stroke	230 (197 to 267)	250 (215 to 288)	8·6 (5·0 to 12·6)*	0·4 (−1·9 to 3·0)	3323 (2845 to 3822)	3427 (2934 to 3932)	3·1 (−0·5 to 6·9)	0·2 (−2·3 to 3·0)
		Haemorrhagic stroke	228 (192 to 271)	242 (206 to 282)	6·1 (1·8 to 11·0)*	2·5 (−0·3 to 5·8)	4391 (3699 to 5126)	4492 (3791 to 5191)	2·3 (−2·0 to 7·1)	1·9 (−1·1 to 5·3)
		Peripheral vascular disease	6 (5 to 7)	8 (6 to 9)	36·1 (27·3 to 46·4)*	−0·4 (−3·8 to 3·2)	131 (96 to 187)	172 (125 to 244)	30·6 (26·4 to 35·7)*	0·2 (−1·7 to 2·4)
		Chronic kidney disease due to diabetes mellitus	299 (279 to 314)	418 (389 to 441)	39·5 (35·4 to 43·5)*	..	8713 (7991 to 9466)	11 258 (10 303 to 12 225)	29·2 (25·9 to 32·5)*	..
		Chronic kidney disease due to hypertension	409 (377 to 428)	550 (502 to 576)	34·5 (30·0 to 38·7)*	..	10 366 (9401 to 10 985)	12 737 (11 489 to 13 554)	22·9 (18·6 to 27·4)*	..
		Chronic kidney disease due to glomerulonephritis	206 (185 to 218)	238 (213 to 256)	15·6 (10·9 to 20·2)*	..	7720 (6930 to 8332)	8136 (7294 to 8861)	5·4 (1·3 to 9·3)*	..
		Chronic kidney disease due to other causes	24 (20 to 29)	30 (25 to 35)	23·9 (17·8 to 30·2)*	..	2689 (2209 to 3238)	3128 (2518 to 3803)	16·3 (13·1 to 19·2)*	..
		Gout	..	..	..	..	138 (91 to 191)	181 (120 to 250)	31·2 (29·3 to 33·1)*	0·9 (−0·0 to 1·8)

Data in parentheses are 95% uncertainty intervals. DALYs=disability-adjusted life-years. PAF=population attributable fraction.

* Statistically significant increase or decrease.
