# Department of Error

**DOI:** 10.1016/S0140-6736(16)32632-0

**Published:** 2017-01-07

*GBD 2015 Risk Factors Collaborators. Global, regional, and national comparative risk assessment of 79 behavioural, environmental and occupational, and metabolic risks or clusters of risks, 1990–2015: a systematic analysis for the Global Burden of Disease Study 2015.* Lancet *2016; **388:** 1659–724*—In this Article, Simon I Hay, Sudha P Jayaraman, Alejandra G Contreras Manzano, Anoushka Millear, Laura Kemmer, Brent Bell, Juan Jesus Carrero, Gavin Shaddick, Lidia Sanchez-Riera, and Giorgia Giussani should have been listed as authors. The affiliation details for Monica Cortinovis have been updated. The funding statement for Simon I Hay has been added. These corrections have been made to the online version as of Jan 5, 2017.

