# Supplementary material for: Global, regional, and national comparative risk assessment of 79 behavioural, environmental and occupational, and metabolic risks or clusters of risks, 1990–2015: a systematic analysis for the Global Burden of Disease Study 2015
Source: Lancet. 2016 Oct 8;388(10053):1659–724. doi: 10.1016/S0140-6736(16)31679-8 (PMC5388856; doi:10.1016/S0140-6736(16)31679-8)
Supplement: Supplementary appendix [file mmc2.pdf]

Results appendix for Global, regional, and national comparative risk assessment of 79 behavioural, environmental and occupational, and metabolic risks or clusters of risks, 1990-2015: a systematic analysis for the Global Burden of Disease Study 2015

Table of Contents

Supplemental Results Table 1. All-age deaths, death PAFs, DALYs, DALY PAFs, and median percent change for each risk factor and outcome for both sexes combined in 2005 and 2015, by geography…….. 3

Supplemental Results Table 2. Age-standardised PAFs attributable to the joint distribution of all risk factors for all ages, both sexes combined for each GBD Level 1 and Level 2 cause and all causes for 2015, by geography. Results are shown for deaths, YLLs, YLDs and DALYs………………………………………. 2489

Supplemental Results Table 3. Global age-standardised Summary Exposure Values (SEVs) for all risk factors and median percentage change, by sex, 1990, 2005, and 2015, by geography……………………… 3619

Supplemental Results Table 1. All-age deaths, death PAFs, DALYs, DALY PAFs, and percent change for each risk factor and outcome for both sexes combined in 2005 and 2015 for Global.

Data in parenthesis are 95% uncertainty intervals. PAFs=population attributable fractions. DALYs=disability-adjusted life-years.

	2005 Deaths (in thousands)	2005 Deaths PAF	2015 Deaths (in thousands)	2015 Deaths PAF	Percent change deaths 2005-2015	Percent change deaths PAF 2005-2015	2005 DALYs (in thousands)	2005 DALYs PAF	2015 DALYs (in thousands)	2015 DALYs PAF	Percent change DALYs 2005-2015	Percent change DALYs PAF 2005-2015
**All risk factors: All causes**	30,717·6 (30,074·3 to 31,360·2)	57·3% (56·2 to 58·3%)	32,234·4 (31,455·7 to 33,034·6)	57·8% (56·6 to 58·8%)	4·9% (3·2 to 6·7%)	0·8% (0·1 to 1·6%)	1,076,067·4 (1,022,161·1 to 1,137,872·5)	42·2% (40·9 to 43·5%)	1,015,469·6 (953,300·5 to 1,084,249·4)	41·2% (39·8 to 42·8%)	-5·6% (-7·5 to -3·8%)	-2·2% (-3·6 to -1·0%)
**Environmental/occupational risks: All causes**	9,523·5 (8,703·6 to 10,320·9)	17·8% (16·3 to 19·2%)	9,314·9 (8,523·3 to 10,145·3)	16·7% (15·3 to 18·1%)	-2·2% (-4·8 to 0·6%)	-6·0% (-8·0 to -4·1%)	362,041·3 (335,616·1 to 387,711·9)	14·2% (13·0 to 15·4%)	319,568·9 (295,705·5 to 344,883·5)	13·0% (11·9 to 14·0%)	-11·7% (-14·7 to -8·3%)	-8·5% (-11·4 to -5·4%)
**Unsafe water, sanitation, and handwashing: All causes**	2,179·1 (1,993·5 to 2,385·7)	4·1% (3·7 to 4·4%)	1,766·5 (1,585·7 to 1,943·6)	3·2% (2·8 to 3·5%)	-18·9% (-23·6 to -14·2%)	-22·1% (-26·4 to -17·7%)	129,221·2 (116,430·3 to 142,601·7)	5·1% (4·5 to 5·7%)	95,305·3 (85,818·4 to 105,820·6)	3·9% (3·4 to 4·4%)	-26·2% (-31·4 to -20·5%)	-23·6% (-29·0 to -17·5%)
**Unsafe water source: All causes**	1,587·0 (1,283·9 to 1,812·1)	3·0% (2·4 to 3·4%)	1,251·2 (1,007·9 to 1,428·4)	2·2% (1·8 to 2·6%)	-21·2% (-26·3 to -16·0%)	-24·2% (-29·0 to -19·4%)	97,247·8 (78,516·4 to 112,113·3)	3·8% (3·0 to 4·5%)	71,745·4 (57,707·1 to 83,257·0)	2·9% (2·3 to 3·4%)	-26·2% (-31·9 to -19·8%)	-23·6% (-29·5 to -17·0%)
Diarrhoeal diseases	1,410·9 (1,147·3 to 1,591·8)	85·1% (68·9 to 93·7%)	1,101·0 (886·9 to 1,244·1)	83·9% (67·6 to 92·6%)	-22·0% (-27·4 to -16·5%)	-1·5% (-2·0 to -1·0%)	84,526·4 (68,642·3 to 96,429·4)	85·9% (69·6 to 94·4%)	61,103·9 (49,351·8 to 69,584·3)	85·4% (69·0 to 94·0%)	-27·7% (-33·6 to -21·2%)	-0·6% (-1·0 to -0·4%)
Typhoid fever	147·2 (77·4 to 244·9)	85·2% (68·5 to 94·1%)	125·7 (66·7 to 215·1)	84·5% (67·5 to 93·8%)	-14·6% (-21·0 to -8·8%)	-0·7% (-1·5 to -0·3%)	10,687·5 (5,712·5 to 17,653·1)	85·2% (68·6 to 94·2%)	8,942·6 (4,822·4 to 15,132·5)	84·6% (67·6 to 93·9%)	-16·3% (-23·1 to -10·0%)	-0·7% (-1·5 to -0·3%)
Paratyphoid fever	28·8 (12·6 to 57·5)	85·0% (68·2 to 94·0%)	24·6 (10·8 to 47·7)	84·3% (67·2 to 93·7%)	-14·8% (-22·2 to -7·1%)	-0·8% (-1·6 to -0·3%)	2,033·9 (897·9 to 4,009·3)	85·0% (68·2 to 94·1%)	1,698·9 (757·3 to 3,318·0)	84·4% (67·3 to 93·7%)	-16·5% (-24·8 to -8·1%)	-0·7% (-1·6 to -0·3%)
**Unsafe sanitation: All causes**	1,114·5 (1,012·5 to 1,229·8)	2·1% (1·9 to 2·3%)	807·9 (727·4 to 895·5)	1·4% (1·3 to 1·6%)	-27·5% (-32·7 to -22·3%)	-30·3% (-35·0 to -25·7%)	67,948·8 (61,085·1 to 75,899·1)	2·7% (2·4 to 3·0%)	46,275·3 (41,064·9 to 51,817·5)	1·9% (1·6 to 2·2%)	-31·9% (-37·4 to -25·7%)	-29·4% (-35·1 to -22·7%)
Diarrhoeal diseases	997·2 (914·3 to 1,087·5)	60·2% (56·5 to 64·0%)	719·8 (657·2 to 792·2)	54·9% (51·0 to 58·8%)	-27·8% (-33·5 to -22·3%)	-8·8% (-10·8 to -7·2%)	59,454·2 (53,638·2 to 65,648·6)	60·4% (56·8 to 64·3%)	40,004·6 (36,020·3 to 44,350·8)	55·9% (52·1 to 59·8%)	-32·7% (-38·8 to -26·1%)	-7·5% (-9·4 to -5·9%)
Typhoid fever	98·2 (52·8 to 164·1)	56·8% (52·3 to 60·8%)	74·0 (39·6 to 124·6)	49·7% (45·3 to 53·6%)	-24·7% (-30·9 to -18·4%)	-12·5% (-15·3 to -10·1%)	7,148·9 (3,893·0 to 11,815·6)	57·0% (52·5 to 60·9%)	5,289·7 (2,877·0 to 8,857·0)	50·0% (45·6 to 53·9%)	-26·0% (-32·6 to -19·3%)	-12·3% (-15·0 to -9·8%)
Paratyphoid fever	19·0 (8·7 to 36·5)	56·1% (51·8 to 60·1%)	14·1 (6·5 to 27·5)	48·4% (44·0 to 52·6%)	-25·8% (-32·7 to -18·8%)	-13·6% (-16·6 to -11·0%)	1,345·8 (626·6 to 2,550·5)	56·2% (51·9 to 60·3%)	981·1 (455·5 to 1,901·7)	48·7% (44·3 to 52·9%)	-27·1% (-34·7 to -19·7%)	-13·4% (-16·4 to -10·8%)
**No handwashing with soap: All causes**	1,115·7 (926·6 to 1,295·8)	2·1% (1·7 to 2·4%)	927·1 (759·7 to 1,081·5)	1·7% (1·4 to 1·9%)	-16·9% (-21·3 to -12·6%)	-20·1% (-24·2 to -16·1%)	64,151·9 (53,252·3 to 74,633·6)	2·5% (2·1 to 3·0%)	47,271·3 (39,033·7 to 55,164·3)	1·9% (1·6 to 2·3%)	-26·3% (-31·0 to -21·0%)	-23·6% (-28·4 to -18·2%)
Diarrhoeal diseases	643·0 (514·1 to 763·0)	38·8% (31·4 to 45·7%)	501·9 (401·5 to 598·6)	38·3% (30·9 to 45·2%)	-21·9% (-27·4 to -16·5%)	-1·4% (-2·0 to -1·0%)	38,311·3 (30,403·4 to 45,758·7)	38·9% (31·6 to 45·9%)	27,628·4 (22,105·9 to 32,795·9)	38·6% (31·2 to 45·6%)	-27·9% (-33·9 to -21·3%)	-0·9% (-1·4 to -0·5%)
Typhoid fever	66·5 (34·4 to 114·4)	38·5% (30·4 to 45·2%)	56·4 (29·1 to 97·3)	37·9% (29·9 to 44·8%)	-15·2% (-21·6 to -9·2%)	-1·4% (-2·2 to -0·8%)	4,831·4 (2,567·8 to 8,255·9)	38·5% (30·4 to 45·3%)	4,018·8 (2,134·7 to 6,933·2)	38·0% (30·0 to 44·9%)	-16·8% (-24·0 to -10·4%)	-1·3% (-2·1 to -0·7%)
Paratyphoid fever	13·0 (5·9 to 26·3)	38·3% (30·3 to 45·4%)	11·0 (5·2 to 22·4)	37·8% (29·8 to 44·9%)	-15·2% (-22·7 to -7·5%)	-1·4% (-2·2 to -0·8%)	917·9 (422·4 to 1,836·9)	38·4% (30·3 to 45·5%)	762·6 (354·5 to 1,543·5)	37·8% (29·8 to 44·9%)	-16·9% (-25·0 to -8·5%)	-1·3% (-2·1 to -0·7%)
**Air pollution: All causes**	6,466·2 (5,675·4 to 7,290·7)	12·1% (10·6 to 13·6%)	6,485·2 (5,707·9 to 7,292·5)	11·6% (10·3 to 13·1%)	0·3% (-2·6 to 3·3%)	-3·6% (-5·8 to -1·4%)	186,850·3 (164,715·7 to 209,142·1)	7·3% (6·3 to 8·3%)	167,290·0 (148,166·8 to 185,779·6)	6·8% (5·9 to 7·7%)	-10·5% (-13·6 to -7·3%)	-7·2% (-10·4 to -4·0%)
**Ambient particulate matter pollution: All causes**	3,934·3 (3,437·0 to 4,448·3)	7·3% (6·4 to 8·3%)	4,241·1 (3,698·0 to 4,776·7)	7·6% (6·7 to 8·6%)	7·8% (5·1 to 10·8%)	3·6% (1·4 to 5·8%)	107,581·8 (94,319·1 to 121,177·4)	4·2% (3·6 to 4·8%)	103,066·2 (90,829·6 to 115,072·6)	4·2% (3·6 to 4·8%)	-4·2% (-7·5 to -0·7%)	-0·7% (-4·3 to 2·9%)
Lower respiratory infections	736·1 (548·6 to 956·8)	26·0% (19·4 to 33·4%)	675·0 (491·9 to 889·0)	24·7% (18·3 to 32·4%)	-8·3% (-13·2 to -3·6%)	-5·2% (-8·4 to -2·7%)	38,632·1 (29,407·4 to 48,530·7)	28·6% (21·8 to 35·6%)	28,359·9 (21,141·8 to 35,796·9)	27·5% (20·8 to 34·7%)	-26·6% (-31·4 to -21·5%)	-3·6% (-6·2 to -1·8%)
Tracheal, bronchus, and lung cancer	225·4 (140·5 to 318·4)	15·7% (9·8 to 22·2%)	283·3 (178·4 to 398·7)	16·4% (10·4 to 23·0%)	25·7% (20·8 to 32·0%)	4·7% (2·8 to 7·2%)	5,171·4 (3,243·6 to 7,262·9)	16·3% (10·2 to 22·9%)	6,209·1 (3,934·9 to 8,689·3)	17·0% (10·8 to 23·7%)	20·1% (14·9 to 27·1%)	4·8% (3·0 to 7·4%)
Ischaemic heart disease	1,283·8 (1,060·1 to 1,530·4)	16·8% (13·8 to 19·9%)	1,521·1 (1,231·7 to 1,821·2)	17·1% (13·9 to 20·3%)	18·5% (14·3 to 22·4%)	1·6% (-1·2 to 4·5%)	28,483·5 (24,253·6 to 32,699·2)	19·3% (16·4 to 22·1%)	32,406·0 (27,078·2 to 37,427·4)	19·8% (16·6 to 22·7%)	13·8% (10·0 to 17·5%)	2·5% (0·6 to 4·5%)
Ischaemic stroke	347·2 (259·8 to 431·6)	12·6% (9·5 to 15·7%)	381·5 (282·7 to 482·6)	12·8% (9·6 to 16·1%)	9·9% (3·9 to 15·2%)	1·9% (-2·6 to 5·8%)	6,271·2 (5,039·1 to 7,453·6)	14·2% (11·5 to 17·0%)	6,617·6 (5,327·6 to 7,904·8)	14·6% (11·9 to 17·4%)	5·5% (0·7 to 10·1%)	2·9% (-0·3 to 6·2%)
Hemorrhagic stroke	504·9 (417·5 to 598·9)	15·5% (12·7 to 18·2%)	516·6 (425·0 to 613·8)	15·4% (12·7 to 18·0%)	2·3% (-2·2 to 7·1%)	-0·4% (-3·0 to 2·3%)	12,605·4 (10,552·3 to 14,743·0)	16·9% (14·3 to 19·6%)	12,625·3 (10,570·4 to 14,866·6)	17·2% (14·5 to 19·9%)	0·2% (-3·9 to 4·6%)	1·6% (-0·3 to 3·7%)
Chronic obstructive pulmonary disease	837·0 (522·4 to 1,174·5)	27·0% (16·8 to 38·1%)	863·6 (538·5 to 1,212·8)	27·1% (16·9 to 38·0%)	3·2% (-1·1 to 8·4%)	0·3% (-0·7 to 2·0%)	16,418·2 (10,295·3 to 23,032·9)	25·7% (16·0 to 36·2%)	16,848·2 (10,517·4 to 23,590·0)	26·4% (16·6 to 37·0%)	2·6% (-1·6 to 7·7%)	2·5% (1·2 to 4·7%)
**Household air pollution from solid fuels: All causes**	3,280·1 (2,505·2 to 4,068·4)	6·1% (4·7 to 7·6%)	2,854·2 (2,178·5 to 3,586·5)	5·1% (3·9 to 6·4%)	-13·0% (-17·0 to -9·3%)	-16·4% (-19·7 to -13·4%)	107,508·6 (83,554·2 to 132,011·7)	4·2% (3·2 to 5·2%)	85,644·0 (66,659·3 to 106,136·2)	3·5% (2·6 to 4·4%)	-20·3% (-24·5 to -16·6%)	-17·4% (-21·5 to -13·7%)
Lower respiratory infections	905·1 (662·8 to 1,159·7)	32·0% (23·2 to 40·6%)	728·9 (522·9 to 948·7)	26·6% (19·0 to 34·6%)	-19·5% (-24·2 to -14·5%)	-16·8% (-19·6 to -14·1%)	52,898·0 (38,844·7 to 67,621·6)	39·1% (28·5 to 49·3%)	36,883·4 (26,631·2 to 46,916·4)	35·8% (25·8 to 45·5%)	-30·3% (-35·1 to -25·0%)	-8·5% (-11·0 to -5·8%)
Tracheal, bronchus, and lung cancer	159·0 (83·1 to 249·0)	11·1% (5·8 to 17·3%)	149·1 (76·3 to 241·2)	8·7% (4·5 to 13·9%)	-6·3% (-12·6 to 0·4%)	-21·9% (-26·4 to -17·9%)	3,805·6 (1,995·7 to 5,931·9)	12·0% (6·3 to 18·6%)	3,439·3 (1,767·0 to 5,534·0)	9·4% (4·9 to 15·1%)	-9·6% (-15·8 to -3·2%)	-21·1% (-25·5 to -17·2%)
Ischaemic heart disease	777·8 (621·0 to 964·4)	10·2% (8·1 to 12·6%)	765·1 (598·4 to 965·2)	8·6% (6·7 to 10·8%)	-1·6% (-7·0 to 3·7%)	-15·6% (-19·7 to -11·7%)	18,864·5 (15,179·6 to 22,901·9)	12·8% (10·3 to 15·5%)	18,199·8 (14,416·5 to 22,511·6)	11·1% (8·8 to 13·7%)	-3·5% (-9·1 to 1·8%)	-13·1% (-17·2 to -9·0%)
Ischaemic stroke	244·9 (176·8 to 324·5)	8·9% (6·4 to 11·9%)	214·0 (152·2 to 289·7)	7·2% (5·2 to 9·7%)	-12·6% (-18·2 to -7·0%)	-19·0% (-23·2 to -14·9%)	4,778·4 (3,478·6 to 6,282·5)	10·8% (7·9 to 14·2%)	4,103·8 (2,930·7 to 5,425·9)	9·1% (6·6 to 11·9%)	-14·1% (-19·6 to -8·6%)	-16·2% (-20·4 to -12·1%)
Hemorrhagic stroke	417·7 (306·1 to 541·7)	12·8% (9·5 to 16·6%)	339·8 (252·4 to 445·6)	10·1% (7·5 to 13·2%)	-18·6% (-23·5 to -13·7%)	-20·8% (-24·8 to -17·2%)	10,857·0 (8,159·5 to 13,940·8)	14·6% (10·9 to 18·8%)	8,902·0 (6,580·7 to 11,543·5)	12·1% (8·9 to 15·6%)	-18·0% (-23·0 to -13·2%)	-16·9% (-20·8 to -13·1%)
Chronic obstructive pulmonary disease	775·6 (422·5 to 1,182·7)	25·0% (13·6 to 37·6%)	657·3 (359·6 to 1,014·0)	20·6% (11·2 to 31·9%)	-15·3% (-21·2 to -9·8%)	-17·6% (-22·0 to -13·4%)	15,599·7 (8,513·0 to 23,817·0)	24·5% (13·4 to 36·9%)	13,373·2 (7,246·2 to 20,607·9)	20·9% (11·4 to 32·3%)	-14·3% (-19·9 to -8·8%)	-14·4% (-19·0 to -10·1%)
Cataract	-	-	-	-	-	-	705·4 (484·2 to 972·3)	22·8% (20·0 to 25·6%)	742·5 (511·8 to 1,021·4)	19·1% (16·5 to 21·6%)	5·3% (1·7 to 9·1%)	-16·1% (-18·6 to -13·1%)
**Ambient ozone pollution: All causes**	207·0 (76·7 to 353·3)	0·4% (0·1 to 0·7%)	254·0 (97·2 to 422·0)	0·5% (0·2 to 0·8%)	22·7% (16·6 to 30·3%)	17·9% (12·4 to 24·7%)	3,472·4 (1,280·9 to 5,913·2)	0·1% (0·1 to 0·2%)	4,116·2 (1,577·2 to 6,788·8)	0·2% (0·1 to 0·3%)	18·5% (12·1 to 26·5%)	22·9% (16·2 to 31·6%)
Chronic obstructive pulmonary disease	207·0 (76·7 to 353·3)	6·7% (2·5 to 11·4%)	254·0 (97·2 to 422·0)	8·0% (3·0 to 13·3%)	22·7% (16·6 to 30·3%)	19·4% (16·3 to 23·8%)	3,472·4 (1,280·9 to 5,913·2)	5·4% (2·1 to 9·2%)	4,116·2 (1,577·2 to 6,788·8)	6·4% (2·4 to 10·6%)	18·5% (12·1 to 26·5%)	18·4% (14·6 to 23·8%)
**Other environmental risks: All causes**	514·2 (273·2 to 804·3)	1·0% (0·5 to 1·5%)	558·3 (293·1 to 883·0)	1·0% (0·5 to 1·6%)	8·6% (4·9 to 11·8%)	4·3% (1·1 to 7·3%)	10,399·9 (5,470·3 to 16,412·5)	0·4% (0·2 to 0·6%)	10,672·7 (5,515·8 to 16,974·9)	0·4% (0·2 to 0·7%)	2·6% (-0·8 to 5·4%)	6·4% (2·6 to 9·4%)
**Residential radon: All causes**	53·2 (36·1 to 71·5)	0·1% (0·1 to 0·1%)	63·7 (42·3 to 86·0)	0·1% (0·1 to 0·2%)	19·7% (12·5 to 28·2%)	15·0% (8·3 to 23·1%)	1,212·0 (840·8 to 1,638·5)	0·0% (0·0 to 0·1%)	1,385·7 (940·6 to 1,871·0)	0·1% (0·0 to 0·1%)	14·3% (8·3 to 20·6%)	18·5% (12·4 to 24·8%)
Tracheal, bronchus, and lung cancer	53·2 (36·1 to 71·5)	3·7% (2·5 to 5·0%)	63·7 (42·3 to 86·0)	3·7% (2·5 to 5·1%)	19·7% (12·5 to 28·2%)	-0·3% (-5·9 to 6·8%)	1,212·0 (840·8 to 1,638·5)	3·8% (2·7 to 5·1%)	1,385·7 (940·6 to 1,871·0)	3·8% (2·6 to 5·2%)	14·3% (8·3 to 20·6%)	-0·1% (-5·2 to 5·1%)
**Lead exposure: All causes**	460·9 (227·4 to 745·1)	0·9% (0·4 to 1·4%)	494·6 (236·7 to 814·5)	0·9% (0·4 to 1·5%)	7·3% (2·6 to 10·7%)	3·1% (-1·2 to 6·1%)	9,188·0 (4,357·2 to 15,215·9)	0·4% (0·2 to 0·6%)	9,287·1 (4,199·9 to 15,594·4)	0·4% (0·2 to 0·6%)	1·1% (-3·7 to 4·4%)	4·8% (-0·3 to 8·2%)
Rheumatic heart disease	3·5 (1·3 to 6·8)	1·1% (0·4 to 2·0%)	3·2 (1·1 to 6·3)	1·0% (0·4 to 2·0%)	-8·2% (-14·7 to -2·0%)	-4·2% (-9·4 to 1·3%)	97·3 (30·6 to 204·5)	0·8% (0·3 to 1·8%)	82·8 (24·8 to 175·3)	0·8% (0·2 to 1·7%)	-14·9% (-21·6 to -9·3%)	-6·1% (-12·1 to -1·7%)
Ischaemic heart disease	220·2 (107·0 to 359·3)	2·9% (1·4 to 4·7%)	239·6 (110·5 to 395·8)	2·7% (1·3 to 4·5%)	8·8% (3·3 to 12·7%)	-6·7% (-11·2 to -3·6%)	3,983·7 (1,822·8 to 6,675·2)	2·7% (1·2 to 4·5%)	4,123·4 (1,807·1 to 7,048·5)	2·5% (1·1 to 4·3%)	3·5% (-1·8 to 7·1%)	-6·7% (-10·8 to -4·2%)
Ischaemic stroke	67·5 (31·6 to 115·4)	2·4% (1·1 to 4·2%)	68·2 (30·8 to 118·3)	2·3% (1·0 to 4·0%)	1·1% (-5·3 to 6·9%)	-6·3% (-11·4 to -1·4%)	1,091·6 (484·8 to 1,882·3)	2·5% (1·1 to 4·2%)	1,054·6 (456·9 to 1,827·9)	2·3% (1·0 to 4·1%)	-3·4% (-9·3 to 1·0%)	-5·8% (-10·6 to -2·5%)
Hemorrhagic stroke	88·7 (38·2 to 154·7)	2·7% (1·2 to 4·7%)	87·5 (36·7 to 154·3)	2·6% (1·1 to 4·6%)	-1·4% (-6·7 to 3·6%)	-4·0% (-8·4 to 0·0%)	1,900·6 (749·6 to 3,415·2)	2·6% (1·0 to 4·6%)	1,807·5 (694·6 to 3,292·8)	2·5% (0·9 to 4·5%)	-4·9% (-9·4 to -1·2%)	-3·5% (-7·6 to -1·0%)
Hypertensive heart disease	39·9 (11·6 to 91·9)	5·3% (1·6 to 12·3%)	47·2 (12·9 to 111·9)	4·9% (1·3 to 11·8%)	18·1% (7·0 to 26·8%)	-6·7% (-14·4 to -1·0%)	710·0 (232·2 to 1,561·0)	4·8% (1·6 to 10·7%)	772·2 (239·8 to 1,737·6)	4·4% (1·4 to 10·1%)	8·8% (-0·1 to 15·4%)	-7·7% (-13·7 to -3·6%)
Cardiomyopathy and myocarditis	4·6 (1·9 to 8·2)	1·4% (0·6 to 2·5%)	4·6 (1·9 to 8·3)	1·3% (0·5 to 2·4%)	1·3% (-4·7 to 7·8%)	-5·9% (-10·6 to -0·4%)	99·1 (34·3 to 201·7)	1·0% (0·4 to 2·1%)	90·7 (31·4 to 183·2)	1·0% (0·3 to 2·0%)	-8·5% (-13·2 to -3·1%)	-4·5% (-8·6 to 0·8%)

Supplemental Results Table 1. All-age deaths, death PAFs, DALYs, DALY PAFs, and percent change for each risk factor and outcome for both sexes combined in 2005 and 2015 for Global.

Data in parenthesis are 95% uncertainty intervals. PAFs=population attributable fractions. DALYs=disability-adjusted life-years.

	2005 Deaths (in thousands)	2005 Deaths PAF	2015 Deaths (in thousands)	2015 Deaths PAF	Percent change deaths 2005-2015	Percent change deaths PAF 2005-2015	2005 DALYs (in thousands)	2005 DALYs PAF	2015 DALYs (in thousands)	2015 DALYs PAF	Percent change DALYs 2005-2015	Percent change DALYs PAF 2005-2015
Atrial fibrillation and flutter	2·6 (1·2 to 4·3)	1·8% (0·9 to 2·9%)	3·1 (1·4 to 5·4)	1·6% (0·8 to 2·6%)	22·1% (13·1 to 27·9%)	-10·7% (-16·6 to -7·2%)	65·2 (29·8 to 113·1)	1·9% (0·9 to 3·1%)	71·8 (30·0 to 129·8)	1·6% (0·7 to 2·7%)	10·1% (0·7 to 15·5%)	-14·0% (-21·1 to -10·0%)
Aortic aneurysm	2·2 (1·0 to 3·7)	1·6% (0·7 to 2·7%)	2·4 (1·0 to 4·2)	1·4% (0·6 to 2·5%)	7·4% (-3·1 to 14·5%)	-14·0% (-21·4 to -9·3%)	38·5 (16·1 to 67·1)	1·6% (0·6 to 2·7%)	39·2 (14·9 to 72·5)	1·3% (0·5 to 2·5%)	1·8% (-8·9 to 9·0%)	-14·2% (-22·2 to -9·8%)
Peripheral vascular disease	0·5 (0·2 to 0·8)	1·2% (0·5 to 2·1%)	0·5 (0·2 to 1·0)	1·0% (0·4 to 1·9%)	17·3% (3·0 to 28·6%)	-14·9% (-24·6 to -8·9%)	10·8 (4·4 to 20·1)	1·2% (0·5 to 2·0%)	12·1 (4·5 to 23·5)	1·0% (0·4 to 1·8%)	11·9% (-0·6 to 19·7%)	-14·8% (-24·2 to -9·9%)
Endocarditis	1·0 (0·5 to 1·8)	1·5% (0·7 to 2·5%)	1·1 (0·5 to 2·0)	1·3% (0·6 to 2·4%)	11·8% (2·8 to 18·6%)	-9·3% (-16·4 to -4·5%)	20·5 (8·0 to 39·1)	1·0% (0·4 to 2·0%)	21·4 (7·7 to 42·4)	1·0% (0·3 to 1·9%)	4·1% (-4·9 to 10·9%)	-7·7% (-16·2 to -1·9%)
Other cardiovascular and circulatory diseases	8·3 (4·1 to 13·5)	1·8% (0·9 to 3·0%)	9·0 (4·3 to 15·0)	1·7% (0·8 to 2·8%)	9·0% (2·6 to 14·3%)	-7·9% (-12·7 to -4·8%)	223·3 (98·1 to 388·2)	1·5% (0·7 to 2·6%)	235·3 (97·9 to 421·7)	1·4% (0·6 to 2·4%)	5·4% (-2·3 to 10·4%)	-6·2% (-12·8 to -2·6%)
Idiopathic developmental intellectual disability	-	-	-	-	-	-	471·3 (214·4 to 814·7)	15·2% (11·1 to 18·7%)	422·9 (188·5 to 739·3)	12·4% (8·5 to 15·7%)	-10·3% (-15·7 to -6·1%)	-18·6% (-23·6 to -14·9%)
Chronic kidney disease due to diabetes mellitus	6·9 (3·1 to 12·5)	2·3% (1·0 to 4·2%)	9·0 (3·8 to 16·7)	2·1% (0·9 to 3·9%)	28·9% (20·2 to 34·6%)	-7·6% (-13·2 to -4·8%)	150·0 (58·5 to 293·0)	1·7% (0·7 to 3·2%)	179·1 (65·3 to 356·3)	1·6% (0·6 to 3·1%)	19·4% (10·5 to 24·7%)	-7·6% (-14·0 to -4·3%)
Chronic kidney disease due to hypertension	10·7 (5·0 to 18·0)	2·6% (1·2 to 4·4%)	13·7 (6·2 to 23·3)	2·5% (1·1 to 4·3%)	28·0% (20·9 to 33·0%)	-4·9% (-8·9 to -2·7%)	196·2 (84·2 to 353·0)	1·9% (0·8 to 3·4%)	231·1 (96·6 to 416·2)	1·8% (0·8 to 3·3%)	17·8% (10·8 to 22·8%)	-4·1% (-8·9 to -1·5%)
Chronic kidney disease due to glomerulonephritis	3·9 (1·6 to 6·7)	1·9% (0·8 to 3·3%)	4·7 (2·1 to 8·2)	2·0% (0·9 to 3·5%)	22·1% (16·9 to 27·8%)	5·6% (3·6 to 8·4%)	93·6 (33·8 to 180·0)	1·2% (0·4 to 2·3%)	101·7 (36·6 to 197·0)	1·2% (0·5 to 2·4%)	8·7% (4·5 to 12·9%)	3·2% (0·6 to 5·7%)
Chronic kidney disease due to other causes	0·5 (0·2 to 0·9)	2·1% (1·0 to 3·5%)	0·7 (0·3 to 1·2)	2·2% (1·1 to 3·8%)	33·1% (24·5 to 42·3%)	7·5% (1·8 to 12·5%)	36·4 (14·4 to 73·5)	1·4% (0·5 to 2·6%)	41·4 (16·0 to 85·9)	1·3% (0·5 to 2·6%)	13·7% (7·8 to 17·7%)	-2·2% (-7·5 to 1·6%)
**Occupational risks: All causes**	951·0 (889·2 to 1,015·7)	1·8% (1·7 to 1·9%)	1,085·8 (1,013·2 to 1,164·5)	1·9% (1·8 to 2·1%)	14·2% (10·4 to 18·6%)	9·7% (6·3 to 13·5%)	55,835·3 (47,024·0 to 65,679·3)	2·2% (2·0 to 2·4%)	63,614·9 (53,616·3 to 75,415·4)	2·6% (2·4 to 2·8%)	13·9% (11·0 to 17·3%)	18·0% (14·7 to 21·7%)
**Occupational carcinogens: All causes**	390·7 (370·3 to 410·1)	0·7% (0·7 to 0·8%)	488·7 (461·1 to 516·9)	0·9% (0·8 to 0·9%)	25·1% (20·4 to 30·2%)	20·2% (16·0 to 24·8%)	8,109·1 (7,708·2 to 8,502·5)	0·3% (0·3 to 0·3%)	9,831·7 (9,317·7 to 10,384·8)	0·4% (0·4 to 0·4%)	21·2% (16·5 to 26·7%)	25·7% (20·7 to 31·3%)
**Occupational exposure to asbestos: All causes**	135·1 (118·2 to 153·7)	0·3% (0·2 to 0·3%)	180·2 (157·9 to 204·8)	0·3% (0·3 to 0·4%)	33·4% (27·3 to 40·1%)	28·2% (22·7 to 34·2%)	2,210·2 (1,919·1 to 2,529·5)	0·1% (0·1 to 0·1%)	2,768·7 (2,390·9 to 3,185·3)	0·1% (0·1 to 0·1%)	25·3% (19·4 to 31·6%)	29·8% (23·6 to 36·3%)
Larynx cancer	1·0 (0·7 to 1·4)	1·1% (0·7 to 1·5%)	1·4 (1·0 to 1·9)	1·3% (0·9 to 1·8%)	38·5% (25·8 to 56·8%)	21·7% (11·5 to 37·6%)	17·1 (10·7 to 24·7)	0·7% (0·5 to 1·0%)	21·9 (14·2 to 30·8)	0·8% (0·5 to 1·2%)	27·8% (17·9 to 41·3%)	15·9% (7·7 to 26·9%)
Tracheal, bronchus, and lung cancer	117·5 (100·6 to 134·7)	8·2% (7·0 to 9·4%)	154·6 (132·5 to 179·1)	9·0% (7·7 to 10·3%)	31·6% (25·6 to 38·6%)	9·6% (5·4 to 13·9%)	1,875·4 (1,590·0 to 2,179·0)	5·9% (5·0 to 6·9%)	2,313·6 (1,947·5 to 2,731·5)	6·4% (5·4 to 7·4%)	23·4% (17·3 to 29·9%)	7·7% (3·5 to 12·0%)
Ovarian cancer	1·1 (0·5 to 1·7)	0·8% (0·4 to 1·3%)	1·4 (0·7 to 2·2)	0·9% (0·4 to 1·4%)	27·3% (18·6 to 37·3%)	5·7% (-0·8 to 12·8%)	19·2 (9·2 to 30·8)	0·5% (0·3 to 0·9%)	23·4 (11·1 to 38·3)	0·6% (0·3 to 0·9%)	22·1% (12·3 to 33·8%)	3·2% (-4·1 to 11·1%)
Mesothelioma	15·5 (12·5 to 18·0)	66·9% (53·7 to 77·7%)	22·8 (18·9 to 26·2)	70·5% (58·0 to 80·5%)	47·2% (39·4 to 55·0%)	5·4% (3·2 to 8·8%)	298·5 (226·3 to 362·2)	54·8% (41·6 to 66·4%)	409·8 (320·1 to 488·1)	58·4% (45·5 to 69·6%)	37·3% (30·2 to 44·1%)	6·6% (4·3 to 9·7%)
**Occupational exposure to arsenic: All causes**	8·8 (6·7 to 11·1)	0·0% (0·0 to 0·0%)	8·8 (6·7 to 11·2)	0·0% (0·0 to 0·0%)	-0·1% (-5·3 to 5·9%)	-4·0% (-8·5 to 1·5%)	195·7 (152·2 to 241·9)	0·0% (0·0 to 0·0%)	194·0 (150·0 to 242·4)	0·0% (0·0 to 0·0%)	-0·9% (-6·2 to 5·4%)	2·7% (-2·6 to 9·1%)
Tracheal, bronchus, and lung cancer	8·8 (6·7 to 11·1)	0·6% (0·5 to 0·8%)	8·8 (6·7 to 11·2)	0·5% (0·4 to 0·6%)	-0·1% (-5·3 to 5·9%)	-16·8% (-19·4 to -13·7%)	195·7 (152·2 to 241·9)	0·6% (0·5 to 0·8%)	194·0 (150·0 to 242·4)	0·5% (0·4 to 0·7%)	-0·9% (-6·2 to 5·4%)	-13·5% (-16·2 to -10·6%)
**Occupational exposure to benzene: All causes**	4·3 (3·7 to 5·0)	0·0% (0·0 to 0·0%)	5·7 (4·8 to 6·7)	0·0% (0·0 to 0·0%)	31·6% (26·4 to 36·6%)	26·4% (22·5 to 30·2%)	161·7 (136·5 to 190·2)	0·0% (0·0 to 0·0%)	211·4 (178·2 to 246·8)	0·0% (0·0 to 0·0%)	30·8% (25·4 to 36·0%)	35·6% (30·2 to 40·9%)
Leukaemia	4·3 (3·7 to 5·0)	1·4% (1·2 to 1·7%)	5·7 (4·8 to 6·7)	1·6% (1·4 to 1·9%)	31·6% (26·4 to 36·6%)	13·0% (10·1 to 15·7%)	161·7 (136·5 to 190·2)	1·4% (1·2 to 1·7%)	211·4 (178·2 to 246·8)	1·8% (1·5 to 2·0%)	30·8% (25·4 to 36·0%)	22·4% (18·9 to 26·1%)
**Occupational exposure to beryllium: All causes**	0·6 (0·5 to 0·7)	0·0% (0·0 to 0·0%)	0·6 (0·4 to 0·7)	0·0% (0·0 to 0·0%)	-1·2% (-6·7 to 5·4%)	-5·1% (-9·9 to 0·7%)	12·6 (10·2 to 15·2)	0·0% (0·0 to 0·0%)	12·3 (9·8 to 15·0)	0·0% (0·0 to 0·0%)	-2·1% (-7·7 to 4·7%)	1·5% (-4·0 to 8·3%)
Tracheal, bronchus, and lung cancer	0·6 (0·5 to 0·7)	0·0% (0·0 to 0·0%)	0·6 (0·4 to 0·7)	0·0% (0·0 to 0·0%)	-1·2% (-6·7 to 5·4%)	-17·7% (-20·8 to -14·2%)	12·6 (10·2 to 15·2)	0·0% (0·0 to 0·0%)	12·3 (9·8 to 15·0)	0·0% (0·0 to 0·0%)	-2·1% (-7·7 to 4·7%)	-14·5% (-17·4 to -11·1%)
**Occupational exposure to cadmium: All causes**	2·0 (1·6 to 2·3)	0·0% (0·0 to 0·0%)	2·1 (1·7 to 2·6)	0·0% (0·0 to 0·0%)	9·5% (3·6 to 16·2%)	5·2% (0·1 to 11·1%)	43·5 (35·8 to 51·5)	0·0% (0·0 to 0·0%)	47·2 (38·4 to 56·2)	0·0% (0·0 to 0·0%)	8·7% (2·8 to 15·5%)	12·7% (6·9 to 19·4%)
Tracheal, bronchus, and lung cancer	2·0 (1·6 to 2·3)	0·1% (0·1 to 0·2%)	2·1 (1·7 to 2·6)	0·1% (0·1 to 0·1%)	9·5% (3·6 to 16·2%)	-8·8% (-11·8 to -5·6%)	43·5 (35·8 to 51·5)	0·1% (0·1 to 0·2%)	47·2 (38·4 to 56·2)	0·1% (0·1 to 0·2%)	8·7% (2·8 to 15·5%)	-5·1% (-8·1 to -1·9%)
**Occupational exposure to chromium: All causes**	6·8 (6·0 to 7·7)	0·0% (0·0 to 0·0%)	7·1 (6·2 to 8·1)	0·0% (0·0 to 0·0%)	4·4% (-1·1 to 10·8%)	0·4% (-4·6 to 6·3%)	151·1 (133·5 to 170·6)	0·0% (0·0 to 0·0%)	156·6 (136·8 to 177·8)	0·0% (0·0 to 0·0%)	3·6% (-2·1 to 10·1%)	7·4% (1·9 to 14·2%)
Tracheal, bronchus, and lung cancer	6·8 (6·0 to 7·7)	0·5% (0·4 to 0·5%)	7·1 (6·2 to 8·1)	0·4% (0·4 to 0·5%)	4·4% (-1·1 to 10·8%)	-13·0% (-15·8 to -9·8%)	151·1 (133·5 to 170·6)	0·5% (0·4 to 0·5%)	156·6 (136·8 to 177·8)	0·4% (0·4 to 0·5%)	3·6% (-2·1 to 10·1%)	-9·5% (-12·3 to -6·5%)
**Occupational exposure to diesel engine exhaust: All causes**	91·5 (80·5 to 103·2)	0·2% (0·2 to 0·2%)	119·6 (105·9 to 135·6)	0·2% (0·2 to 0·2%)	30·8% (24·6 to 37·8%)	25·7% (20·2 to 31·9%)	2,047·8 (1,814·6 to 2,304·1)	0·1% (0·1 to 0·1%)	2,657·5 (2,341·1 to 2,993·7)	0·1% (0·1 to 0·1%)	29·8% (23·6 to 37·1%)	34·5% (28·1 to 41·9%)
Tracheal, bronchus, and lung cancer	91·5 (80·5 to 103·2)	6·4% (5·6 to 7·2%)	119·6 (105·9 to 135·6)	6·9% (6·2 to 7·8%)	30·8% (24·6 to 37·8%)	8·9% (5·9 to 12·2%)	2,047·8 (1,814·6 to 2,304·1)	6·4% (5·7 to 7·2%)	2,657·5 (2,341·1 to 2,993·7)	7·3% (6·5 to 8·2%)	29·8% (23·6 to 37·1%)	13·3% (10·4 to 16·3%)
**Occupational exposure to second-hand smoke: All causes**	83·9 (78·6 to 89·2)	0·2% (0·1 to 0·2%)	96·2 (89·6 to 103·1)	0·2% (0·2 to 0·2%)	14·7% (9·9 to 20·1%)	10·2% (6·2 to 14·8%)	1,860·4 (1,741·9 to 1,971·8)	0·1% (0·1 to 0·1%)	2,112·9 (1,965·9 to 2,262·4)	0·1% (0·1 to 0·1%)	13·6% (8·9 to 18·9%)	17·7% (12·9 to 23·5%)
Tracheal, bronchus, and lung cancer	83·9 (78·6 to 89·2)	5·9% (5·5 to 6·2%)	96·2 (89·6 to 103·1)	5·6% (5·3 to 5·9%)	14·7% (9·9 to 20·1%)	-4·5% (-6·5 to -2·3%)	1,860·4 (1,741·9 to 1,971·8)	5·8% (5·5 to 6·2%)	2,112·9 (1,965·9 to 2,262·4)	5·8% (5·5 to 6·1%)	13·6% (8·9 to 18·9%)	-0·8% (-2·7 to 1·2%)
**Occupational exposure to formaldehyde: All causes**	2·0 (1·6 to 2·4)	0·0% (0·0 to 0·0%)	2·0 (1·6 to 2·4)	0·0% (0·0 to 0·0%)	1·7% (-4·6 to 7·9%)	-2·3% (-7·9 to 3·4%)	70·6 (56·5 to 86·7)	0·0% (0·0 to 0·0%)	70·6 (57·2 to 86·7)	0·0% (0·0 to 0·0%)	-0·1% (-6·4 to 6·2%)	3·5% (-2·8 to 9·9%)
Nasopharynx cancer	1·0 (0·7 to 1·4)	1·8% (1·3 to 2·5%)	1·0 (0·6 to 1·4)	1·5% (1·0 to 2·1%)	-6·0% (-16·3 to 4·0%)	-16·6% (-22·4 to -10·7%)	35·9 (23·2 to 50·5)	2·0% (1·4 to 2·8%)	32·8 (21·0 to 47·1)	1·7% (1·2 to 2·4%)	-8·8% (-19·4 to 1·8%)	-13·8% (-19·4 to -7·4%)
**Occupational exposure to nickel: All causes**	32·3 (23·7 to 42·0)	0·1% (0·0 to 0·1%)	33·0 (23·8 to 43·4)	0·1% (0·0 to 0·1%)	2·2% (-3·7 to 9·0%)	-1·8% (-7·2 to 4·3%)	720·1 (535·1 to 927·5)	0·0% (0·0 to 0·0%)	729·2 (534·7 to 956·3)	0·0% (0·0 to 0·0%)	1·3% (-4·9 to 8·5%)	5·0% (-1·2 to 12·5%)
Tracheal, bronchus, and lung cancer	32·3 (23·7 to 42·0)	2·3% (1·7 to 2·9%)	33·0 (23·8 to 43·4)	1·9% (1·4 to 2·5%)	2·2% (-3·7 to 9·0%)	-14·9% (-18·2 to -11·0%)	720·1 (535·1 to 927·5)	2·3% (1·7 to 2·9%)	729·2 (534·7 to 956·3)	2·0% (1·5 to 2·6%)	1·3% (-4·9 to 8·5%)	-11·6% (-15·2 to -7·5%)
**Occupational exposure to polycyclic aromatic hydrocarbons: All causes**	15·4 (12·6 to 18·2)	0·0% (0·0 to 0·0%)	18·0 (14·7 to 21·3)	0·0% (0·0 to 0·0%)	16·9% (11·1 to 23·7%)	12·3% (7·2 to 18·6%)	341·8 (283·5 to 402·2)	0·0% (0·0 to 0·0%)	396·7 (327·0 to 470·4)	0·0% (0·0 to 0·0%)	16·1% (10·1 to 23·1%)	20·3% (14·5 to 27·4%)
Tracheal, bronchus, and lung cancer	15·4 (12·6 to 18·2)	1·1% (0·9 to 1·3%)	18·0 (14·7 to 21·3)	1·0% (0·9 to 1·2%)	16·9% (11·1 to 23·7%)	-2·6% (-5·7 to 0·7%)	341·8 (283·5 to 402·2)	1·1% (0·9 to 1·3%)	396·7 (327·0 to 470·4)	1·1% (0·9 to 1·3%)	16·1% (10·1 to 23·1%)	1·4% (-1·5 to 4·5%)
**Occupational exposure to silica: All causes**	65·1 (59·0 to 71·1)	0·1% (0·1 to 0·1%)	86·5 (78·5 to 94·7)	0·2% (0·1 to 0·2%)	32·9% (25·8 to 40·8%)	27·7% (21·5 to 34·6%)	1,444·4 (1,307·6 to 1,578·2)	0·1% (0·1 to 0·1%)	1,893·9 (1,723·6 to 2,073·3)	0·1% (0·1 to 0·1%)	31·1% (24·2 to 39·3%)	35·9% (28·9 to 44·1%)
Tracheal, bronchus, and lung cancer	65·1 (59·0 to 71·1)	4·5% (4·1 to 4·9%)	86·5 (78·5 to 94·7)	5·0% (4·6 to 5·5%)	32·9% (25·8 to 40·8%)	10·7% (6·9 to 14·8%)	1,444·4 (1,307·6 to 1,578·2)	4·5% (4·2 to 4·9%)	1,893·9 (1,723·6 to 2,073·3)	5·2% (4·8 to 5·6%)	31·1% (24·2 to 39·3%)	14·5% (10·7 to 18·5%)
**Occupational exposure to sulfphuric acid: All causes**	7·2 (5·4 to 9·1)	0·0% (0·0 to 0·0%)	8·1 (6·1 to 10·3)	0·0% (0·0 to 0·0%)	13·9% (9·1 to 18·9%)	9·4% (5·4 to 13·7%)	174·8 (133·9 to 219·2)	0·0% (0·0 to 0·0%)	198·9 (150·9 to 253·2)	0·0% (0·0 to 0·0%)	13·8% (9·2 to 18·7%)	18·0% (13·4 to 23·0%)
Larynx cancer	7·2 (5·4 to 9·1)	7·7% (5·8 to 9·7%)	8·1 (6·1 to 10·3)	7·7% (5·8 to 9·7%)	13·9% (9·1 to 18·9%)	0·1% (-2·3 to 2·8%)	174·8 (133·9 to 219·2)	7·4% (5·7 to 9·3%)	198·9 (150·9 to 253·2)	7·6% (5·8 to 9·7%)	13·8% (9·2 to 18·7%)	3·2% (0·7 to 6·1%)
**Occupational exposure to trichloroethylene: All causes**	0·1 (0·0 to 0·3)	0·0% (0·0 to 0·0%)	0·2 (0·0 to 0·3)	0·0% (0·0 to 0·0%)	25·3% (19·4 to 30·3%)	20·4% (14·9 to 24·4%)	3·3 (0·7 to 6·1)	0·0% (0·0 to 0·0%)	4·2 (0·9 to 7·9)	0·0% (0·0 to 0·0%)	26·3% (20·4 to 31·2%)	30·9% (24·8 to 36·1%)
Kidney cancer	0·1 (0·0 to 0·3)	0·1% (0·0 to 0·2%)	0·2 (0·0 to 0·3)	0·1% (0·0 to 0·2%)	25·3% (19·4 to 30·3%)	-4·4% (-7·1 to -1·4%)	3·3 (0·7 to 6·1)	0·1% (0·0 to 0·2%)	4·2 (0·9 to 7·9)	0·1% (0·0 to 0·2%)	26·3% (20·4 to 31·2%)	0·0% (-3·1 to 3·8%)

Supplemental Results Table 1. All-age deaths, death PAFs, DALYs, DALY PAFs, and percent change for each risk factor and outcome for both sexes combined in 2005 and 2015 for Global.

Data in parenthesis are 95% uncertainty intervals. PAFs=population attributable fractions. DALYs=disability-adjusted life-years.

	2005 Deaths (in thousands)	2005 Deaths PAF	2015 Deaths (in thousands)	2015 Deaths PAF	Percent change deaths 2005-2015	Percent change deaths PAF 2005-2015	2005 DALYs (in thousands)	2005 DALYs PAF	2015 DALYs (in thousands)	2015 DALYs PAF	Percent change DALYs 2005-2015	Percent change DALYs PAF 2005-2015
**Occupational asthmagens: All causes**	50·0<br>(38·8 to 61·5)	0·1%<br>(0·1 to 0·1%)	41·5<br>(35·0 to 48·4)	0·1%<br>(0·1 to 0·1%)	-16·9%<br>(-26·0 to -2·4%)	-20·1%<br>(-28·8 to -6·3%)	2,753·5<br>(2,189·8 to 3,373·1)	0·1%<br>(0·1 to 0·1%)	2,621·3<br>(2,080·6 to 3,223·1)	0·1%<br>(0·1 to 0·1%)	-4·8%<br>(-12·0 to 3·6%)	-1·4%<br>(-8·0 to 7·4%)
Asthma	50·0<br>(38·8 to 61·5)	11·1%<br>(9·4 to 12·9%)	41·5<br>(35·0 to 48·4)	10·5%<br>(9·1 to 11·9%)	-16·9%<br>(-26·0 to -2·4%)	-5·9%<br>(-11·7 to 1·0%)	2,753·5<br>(2,189·8 to 3,373·1)	10·3%<br>(9·0 to 11·7%)	2,621·3<br>(2,080·6 to 3,223·1)	10·0%<br>(8·8 to 11·4%)	-4·8%<br>(-12·0 to 3·6%)	-2·2%<br>(-5·6 to 1·1%)
**Occupational particulate matter, gases, and fumes: All causes**	336·6<br>(280·3 to 391·8)	0·6%<br>(0·5 to 0·7%)	356·6<br>(293·0 to 419·2)	0·6%<br>(0·5 to 0·7%)	5·9%<br>(0·9 to 11·6%)	1·8%<br>(-2·5 to 6·6%)	8,405·4<br>(7,324·7 to 9,570·0)	0·3%<br>(0·3 to 0·4%)	8,786·6<br>(7,597·9 to 9,986·8)	0·4%<br>(0·3 to 0·4%)	4·5%<br>(0·5 to 9·2%)	8·3%<br>(4·4 to 13·2%)
Chronic obstructive pulmonary disease	333·9<br>(277·6 to 389·1)	10·8%<br>(8·9 to 12·5%)	354·1<br>(290·3 to 416·7)	11·1%<br>(9·1 to 13·0%)	6·0%<br>(1·0 to 11·8%)	3·1%<br>(-0·4 to 6·8%)	8,345·7<br>(7,264·0 to 9,509·4)	13·1%<br>(11·4 to 14·7%)	8,729·1<br>(7,540·8 to 9,926·1)	13·7%<br>(11·9 to 15·3%)	4·6%<br>(0·6 to 9·3%)	4·5%<br>(2·1 to 7·1%)
**Occupational noise: All causes**	-	-	-	-	-	-	8,658·8<br>(5,924·6 to 11,947·7)	0·3%<br>(0·2 to 0·4%)	10,874·7<br>(7,410·1 to 15,037·7)	0·4%<br>(0·3 to 0·6%)	25·6%<br>(23·5 to 27·7%)	30·0%<br>(27·1 to 33·2%)
Age-related and other hearing loss	-	-	-	-	-	-	8,658·8<br>(5,924·6 to 11,947·7)	27·0%<br>(24·8 to 29·7%)	10,874·7<br>(7,410·1 to 15,037·7)	26·8%<br>(24·7 to 29·4%)	25·6%<br>(23·5 to 27·7%)	-0·7%<br>(-1·9 to 0·6%)
**Occupational injuries: All causes**	188·6<br>(168·5 to 207·3)	0·4%<br>(0·3 to 0·4%)	203·7<br>(190·9 to 231·9)	0·4%<br>(0·3 to 0·4%)	8·0%<br>(-3·4 to 24·3%)	3·8%<br>(-7·1 to 19·7%)	12,211·9<br>(10,736·3 to 13,681·5)	0·5%<br>(0·4 to 0·5%)	13,491·5<br>(12,006·4 to 15,545·0)	0·5%<br>(0·5 to 0·6%)	10·5%<br>(-0·2 to 25·9%)	14·4%<br>(3·4 to 30·5%)
Pedestrian road injuries	26·1<br>(21·8 to 30·3)	4·5%<br>(3·8 to 5·2%)	30·5<br>(27·4 to 36·8)	5·4%<br>(4·9 to 6·6%)	16·9%<br>(-0·9 to 42·4%)	21·3%<br>(1·6 to 51·8%)	1,320·4<br>(1,109·8 to 1,527·9)	4·9%<br>(4·1 to 5·6%)	1,510·2<br>(1,362·0 to 1,841·7)	6·2%<br>(5·6 to 7·5%)	14·4%<br>(-2·9 to 39·0%)	27·0%<br>(6·5 to 58·4%)
Cyclist road injuries	2·8<br>(2·5 to 3·1)	4·4%<br>(4·0 to 4·8%)	3·4<br>(3·1 to 3·8)	5·8%<br>(5·4 to 6·6%)	21·9%<br>(8·3 to 35·5%)	31·8%<br>(17·2 to 48·4%)	189·9<br>(166·5 to 215·2)	5·1%<br>(4·7 to 5·5%)	242·6<br>(211·0 to 279·7)	7·1%<br>(6·6 to 7·9%)	27·8%<br>(16·1 to 40·6%)	39·9%<br>(26·5 to 55·5%)
Motorcyclist road injuries	19·0<br>(16·8 to 21·4)	7·8%<br>(7·2 to 8·5%)	22·4<br>(20·0 to 25·8)	8·7%<br>(8·0 to 9·9%)	17·7%<br>(3·2 to 36·2%)	12·6%<br>(0·8 to 27·3%)	1,101·3<br>(975·2 to 1,222·0)	8·1%<br>(7·5 to 8·8%)	1,287·4<br>(1,158·0 to 1,469·3)	9·4%<br>(8·7 to 10·6%)	16·9%<br>(3·6 to 33·4%)	16·7%<br>(5·0 to 31·1%)
Motor vehicle road injuries	42·2<br>(38·4 to 47·0)	8·7%<br>(8·1 to 9·4%)	46·2<br>(42·7 to 51·4)	10·0%<br>(9·3 to 10·9%)	9·4%<br>(-0·1 to 21·1%)	13·9%<br>(4·6 to 26·2%)	2,419·1<br>(2,206·0 to 2,692·1)	9·2%<br>(8·6 to 9·8%)	2,667·7<br>(2,457·0 to 2,976·7)	10·9%<br>(10·2 to 11·9%)	10·3%<br>(1·2 to 21·5%)	18·4%<br>(8·6 to 30·3%)
Other road injuries	2·1<br>(1·4 to 2·9)	11·1%<br>(7·7 to 14·8%)	2·0<br>(1·4 to 3·0)	9·4%<br>(7·7 to 14·3%)	-6·0%<br>(-32·1 to 41·6%)	-15·3%<br>(-39·0 to 27·1%)	126·7<br>(85·7 to 168·7)	11·3%<br>(8·1 to 14·8%)	126·6<br>(94·5 to 181·7)	10·3%<br>(8·6 to 14·7%)	-0·1%<br>(-26·0 to 42·7%)	-9·0%<br>(-32·7 to 29·8%)
Other transport injuries	5·1<br>(4·2 to 6·0)	5·0%<br>(4·2 to 5·8%)	6·2<br>(5·4 to 7·7)	6·0%<br>(5·3 to 7·1%)	23·5%<br>(0·3 to 55·7%)	19·7%<br>(-0·1 to 50·2%)	274·6<br>(229·3 to 325·1)	5·3%<br>(4·5 to 6·1%)	331·6<br>(286·2 to 407·5)	6·5%<br>(5·8 to 7·7%)	20·8%<br>(-0·6 to 51·1%)	23·5%<br>(3·8 to 52·9%)
Falls	19·2<br>(17·0 to 21·6)	4·4%<br>(3·9 to 5·0%)	23·2<br>(21·2 to 26·1)	4·4%<br>(4·1 to 5·0%)	21·2%<br>(7·1 to 39·4%)	0·3%<br>(-11·3 to 14·6%)	1,847·2<br>(1,519·6 to 2,254·5)	7·7%<br>(7·0 to 8·5%)	2,328·7<br>(1,891·9 to 2,877·2)	8·9%<br>(8·1 to 10·0%)	26·1%<br>(13·2 to 41·2%)	15·5%<br>(3·9 to 29·3%)
Drowning	25·8<br>(19·8 to 31·3)	6·4%<br>(4·9 to 7·8%)	21·9<br>(18·7 to 30·7)	6·8%<br>(5·8 to 9·6%)	-15·0%<br>(-33·5 to 15·9%)	5·7%<br>(-17·6 to 44·7%)	1,357·7<br>(1,052·0 to 1,624·7)	5·6%<br>(4·3 to 6·8%)	1,137·0<br>(967·4 to 1,591·5)	6·4%<br>(5·5 to 9·0%)	-16·3%<br>(-33·9 to 14·8%)	13·4%<br>(-11·1 to 53·6%)
Fire, heat, and hot substances	11·6<br>(10·2 to 13·8)	5·9%<br>(5·3 to 7·0%)	12·4<br>(10·7 to 14·5)	7·1%<br>(6·4 to 8·1%)	7·7%<br>(-6·0 to 25·5%)	19·4%<br>(4·8 to 39·7%)	815·2<br>(703·6 to 963·8)	7·0%<br>(6·2 to 8·2%)	908·9<br>(771·2 to 1,083·9)	8·9%<br>(7·8 to 10·3%)	11·5%<br>(-2·2 to 28·7%)	27·3%<br>(11·1 to 47·1%)
Poisonings	4·0<br>(3·2 to 4·5)	4·0%<br>(3·4 to 4·7%)	3·2<br>(2·7 to 3·6)	3·8%<br>(3·2 to 4·6%)	-19·5%<br>(-26·2 to -11·5%)	-5·8%<br>(-20·1 to 8·5%)	233·3<br>(193·4 to 262·3)	4·1%<br>(3·3 to 5·0%)	199·2<br>(168·3 to 225·3)	4·2%<br>(3·3 to 5·4%)	-14·6%<br>(-22·0 to -6·1%)	1·2%<br>(-19·6 to 22·9%)
Unintentional firearm injuries	1·8<br>(1·2 to 2·0)	5·5%<br>(5·0 to 6·0%)	1·8<br>(1·3 to 2·0)	5·6%<br>(5·1 to 6·1%)	-1·4%<br>(-11·2 to 8·9%)	0·8%<br>(-9·2 to 11·5%)	100·4<br>(70·8 to 113·5)	6·1%<br>(5·4 to 6·7%)	98·9<br>(73·4 to 111·9)	6·4%<br>(5·8 to 7·1%)	-1·5%<br>(-10·9 to 8·4%)	5·6%<br>(-5·3 to 17·7%)
Unintentional suffocation	0·1<br>(0·1 to 0·1)	0·3%<br>(0·3 to 0·4%)	0·2<br>(0·1 to 0·2)	0·4%<br>(0·4 to 0·5%)	46·8%<br>(7·5 to 75·3%)	43·3%<br>(15·1 to 74·0%)	9·4<br>(7·8 to 11·4)	0·4%<br>(0·3 to 0·5%)	12·6<br>(10·0 to 15·4)	0·5%<br>(0·4 to 0·6%)	33·1%<br>(11·7 to 54·0%)	41·0%<br>(18·1 to 69·0%)
Other exposure to mechanical forces	9·0<br>(7·7 to 10·0)	6·7%<br>(6·2 to 7·3%)	10·4<br>(8·2 to 11·7)	7·8%<br>(7·3 to 8·6%)	15·0%<br>(-1·1 to 28·9%)	16·8%<br>(6·2 to 29·9%)	737·2<br>(624·6 to 858·7)	7·8%<br>(7·2 to 8·5%)	874·8<br>(704·3 to 1,054·6)	9·6%<br>(8·9 to 10·6%)	18·7%<br>(6·7 to 31·0%)	23·5%<br>(12·1 to 36·5%)
Venomous animal contact	3·3<br>(2·2 to 3·9)	3·8%<br>(3·3 to 4·4%)	3·1<br>(1·8 to 4·0)	3·9%<br>(3·3 to 4·5%)	-8·1%<br>(-23·4 to 15·9%)	2·2%<br>(-12·2 to 19·0%)	209·7<br>(152·2 to 247·3)	4·0%<br>(3·5 to 4·6%)	201·7<br>(143·1 to 247·5)	4·4%<br>(3·6 to 5·2%)	-3·8%<br>(-17·2 to 15·6%)	10·3%<br>(-5·9 to 28·0%)
Non-venomous animal contact	0·4<br>(0·3 to 0·5)	2·7%<br>(2·3 to 3·1%)	0·4<br>(0·3 to 0·5)	2·7%<br>(2·4 to 3·1%)	-10·1%<br>(-24·7 to 10·9%)	0·8%<br>(-14·1 to 18·3%)	36·0<br>(28·6 to 45·1)	3·1%<br>(2·7 to 3·8%)	36·0<br>(28·6 to 46·3)	3·5%<br>(3·0 to 4·3%)	-0·1%<br>(-14·3 to 16·5%)	12·8%<br>(-5·2 to 32·4%)
Pulmonary aspiration and foreign body in airway	3·1<br>(2·6 to 3·6)	2·6%<br>(2·3 to 3·1%)	3·9<br>(3·5 to 4·4)	3·2%<br>(2·9 to 3·6%)	28·0%<br>(15·5 to 40·1%)	19·7%<br>(7·8 to 31·8%)	192·1<br>(162·5 to 225·4)	3·0%<br>(2·3 to 3·8%)	239·0<br>(205·3 to 274·2)	3·8%<br>(3·2 to 4·6%)	24·4%<br>(14·1 to 34·8%)	29·0%<br>(14·1 to 46·2%)
Foreign body in eyes	-	-	-	-	-	-	3·2<br>(1·4 to 5·8)	6·6%<br>(5·7 to 7·4%)	4·4<br>(1·9 to 8·0)	7·9%<br>(6·9 to 8·9%)	38·6%<br>(28·8 to 49·0%)	20·2%<br>(11·8 to 29·3%)
Foreign body in other body part	1·8<br>(1·2 to 2·6)	6·3%<br>(5·4 to 7·4%)	1·9<br>(1·4 to 2·4)	7·1%<br>(6·1 to 8·0%)	6·1%<br>(-12·7 to 24·0%)	11·2%<br>(-3·3 to 26·1%)	137·6<br>(104·7 to 179·3)	7·0%<br>(5·7 to 8·3%)	156·7<br>(123·6 to 191·1)	8·4%<br>(7·1 to 9·7%)	13·8%<br>(-2·8 to 29·5%)	20·6%<br>(4·5 to 37·4%)
Other unintentional injuries	11·2<br>(9·7 to 13·1)	7·5%<br>(6·6 to 8·8%)	10·5<br>(9·0 to 14·2)	7·9%<br>(6·7 to 10·1%)	-5·8%<br>(-22·6 to 23·4%)	4·4%<br>(-13·4 to 36·7%)	1,100·8<br>(882·5 to 1,348·8)	8·2%<br>(7·2 to 9·4%)	1,127·6<br>(895·5 to 1,489·9)	9·2%<br>(8·1 to 11·1%)	2·4%<br>(-12·1 to 24·5%)	11·6%<br>(-4·3 to 36·3%)
**Occupational ergonomic factors: All causes**	-	-	-	-	-	-	16,791·6<br>(11,729·8 to 23,447·9)	0·7%<br>(0·5 to 0·9%)	18,573·5<br>(12,979·5 to 25,758·7)	0·8%<br>(0·6 to 1·0%)	10·6%<br>(9·2 to 12·0%)	14·5%<br>(12·0 to 17·3%)
Low back pain	-	-	-	-	-	-	16,791·6<br>(11,729·8 to 23,447·9)	32·8%<br>(30·9 to 34·6%)	18,573·5<br>(12,979·5 to 25,758·7)	30·9%<br>(29·2 to 32·5%)	10·6%<br>(9·2 to 12·0%)	-5·7%<br>(-6·5 to -4·7%)
**Behavioural risks: All causes**	22,354·9<br>(21,183·0 to 23,567·4)	41·7%<br>(39·6 to 43·9%)	22,744·5<br>(21,408·0 to 24,125·8)	40·8%<br>(38·5 to 43·2%)	1·7%<br>(-0·3 to 3·8%)	-2·2%<br>(-3·5 to -1·0%)	825,546·4<br>(781,724·1 to 872,847·5)	32·4%<br>(30·9 to 33·9%)	745,463·0<br>(698,273·2 to 797,964·2)	30·3%<br>(28·6 to 32·0%)	-9·7%<br>(-11·8 to -7·7%)	-6·4%<br>(-8·2 to -4·8%)
**Child and maternal malnutrition: All causes**	2,279·7<br>(2,112·8 to 2,453·4)	4·3%<br>(4·0 to 4·6%)	1,413·9<br>(1,307·6 to 1,532·7)	2·5%<br>(2·3 to 2·7%)	-38·0%<br>(-41·9 to -33·7%)	-40·4%<br>(-44·2 to -36·3%)	248,338·8<br>(226,327·2 to 275,262·0)	9·7%<br>(9·2 to 10·3%)	172,120·2<br>(151,792·3 to 196,175·5)	7·0%<br>(6·5 to 7·4%)	-30·7%<br>(-34·6 to -26·9%)	-28·2%<br>(-32·1 to -24·6%)
**Suboptimal breastfeeding: All causes**	592·0<br>(389·8 to 843·0)	1·1%<br>(0·7 to 1·6%)	391·1<br>(257·6 to 550·4)	0·7%<br>(0·5 to 1·0%)	-33·9%<br>(-39·7 to -27·5%)	-36·5%<br>(-42·0 to -30·4%)	51,337·7<br>(33,863·3 to 73,062·0)	2·0%<br>(1·3 to 2·9%)	34,029·9<br>(22,496·5 to 47,771·2)	1·4%<br>(0·9 to 1·9%)	-33·7%<br>(-39·5 to -27·2%)	-31·3%<br>(-37·1 to -24·7%)
**Non-exclusive breastfeeding : All causes**	551·4<br>(347·4 to 788·7)	1·0%<br>(0·6 to 1·5%)	364·1<br>(226·6 to 519·8)	0·7%<br>(0·4 to 0·9%)	-34·0%<br>(-39·5 to -27·6%)	-36·5%<br>(-42·0 to -30·7%)	47,654·4<br>(30,141·3 to 68,060·1)	1·9%<br>(1·2 to 2·7%)	31,507·3<br>(19,668·9 to 44,928·7)	1·3%<br>(0·8 to 1·8%)	-33·9%<br>(-39·4 to -27·5%)	-31·5%<br>(-37·1 to -25·1%)
Diarrhoeal diseases	228·2<br>(141·0 to 309·2)	13·8%<br>(8·5 to 18·4%)	156·3<br>(97·5 to 216·4)	11·9%<br>(7·5 to 16·1%)	-31·5%<br>(-40·4 to -21·0%)	-13·4%<br>(-22·6 to -2·1%)	19,768·4<br>(12,250·4 to 26,754·8)	20·1%<br>(12·6 to 26·6%)	13,579·4<br>(8,488·6 to 18,757·7)	19·0%<br>(12·0 to 25·4%)	-31·3%<br>(-40·1 to -20·8%)	-5·6%<br>(-13·7 to 3·8%)
Lower respiratory infections	323·2<br>(137·0 to 555·0)	11·4%<br>(4·9 to 19·6%)	207·8<br>(86·1 to 354·6)	7·6%<br>(3·1 to 13·0%)	-35·7%<br>(-41·6 to -30·2%)	-33·6%<br>(-38·9 to -28·4%)	27,886·1<br>(11,846·4 to 47,863·5)	20·6%<br>(8·7 to 35·4%)	17,927·9<br>(7,453·2 to 30,579·7)	17·4%<br>(7·2 to 29·6%)	-35·7%<br>(-41·5 to -30·2%)	-15·6%<br>(-21·3 to -9·6%)
**Discontinued breastfeeding : All causes**	54·8<br>(19·3 to 103·0)	0·1%<br>(0·0 to 0·2%)	36·6<br>(12·6 to 68·6)	0·1%<br>(0·0 to 0·1%)	-33·2%<br>(-42·7 to -21·6%)	-35·8%<br>(-44·9 to -24·7%)	4,908·9<br>(1,745·1 to 9,247·8)	0·2%<br>(0·1 to 0·4%)	3,355·7<br>(1,146·7 to 6,280·7)	0·1%<br>(0·0 to 0·3%)	-31·6%<br>(-40·8 to -20·5%)	-29·2%<br>(-38·3 to -17·7%)
Diarrhoeal diseases	54·8<br>(19·3 to 103·0)	3·3%<br>(1·2 to 6·2%)	36·6<br>(12·6 to 68·6)	2·8%<br>(1·0 to 5·2%)	-33·2%<br>(-42·7 to -21·6%)	-15·6%<br>(-25·7 to -5·1%)	4,908·9<br>(1,745·1 to 9,247·8)	5·0%<br>(1·7 to 9·3%)	3,355·7<br>(1,146·7 to 6,280·7)	4·7%<br>(1·6 to 8·7%)	-31·6%<br>(-40·8 to -20·5%)	-6·0%<br>(-14·0 to 2·9%)
**Childhood undernutrition: All causes**	2,093·3<br>(1,931·1 to 2,260·9)	3·9%<br>(3·6 to 4·2%)	1,264·9<br>(1,159·8 to 1,383·2)	2·3%<br>(2·1 to 2·5%)	-39·6%<br>(-43·6 to -35·1%)	-41·9%<br>(-46·0 to -37·6%)	184,339·2<br>(170,596·2 to 199,097·1)	7·2%<br>(6·5 to 8·0%)	113,280·5<br>(103,941·0 to 123,401·3)	4·6%<br>(4·1 to 5·1%)	-38·5%<br>(-42·5 to -34·2%)	-36·3%<br>(-40·4 to -32·0%)
**Childhood underweight: All causes**	665·7<br>(531·3 to 856·7)	1·2%<br>(1·0 to 1·6%)	372·6<br>(303·7 to 467·8)	0·7%<br>(0·5 to 0·8%)	-44·0%<br>(-52·0 to -34·8%)	-46·2%<br>(-53·9 to -37·6%)	60,145·9<br>(48,822·7 to 76,464·8)	2·4%<br>(1·9 to 3·0%)	34,911·4<br>(28,902·7 to 43,357·2)	1·4%<br>(1·2 to 1·8%)	-42·0%<br>(-49·6 to -33·2%)	-39·9%<br>(-47·4 to -30·9%)
Diarrhoeal diseases	124·1<br>(100·5 to 156·5)	7·5%<br>(6·3 to 9·3%)	68·6<br>(55·3 to 86·7)	5·2%<br>(4·3 to 6·5%)	-44·7%<br>(-51·5 to -36·1%)	-30·1%<br>(-36·8 to -22·6%)	10,974·5<br>(8,984·7 to 13,784·9)	11·1%<br>(9·4 to 13·8%)	6,161·5<br>(4,984·2 to 7,724·5)	8·6%<br>(7·1 to 10·7%)	-43·9%<br>(-50·7 to -35·6%)	-22·8%<br>(-28·1 to -17·4%)
Lower respiratory infections	208·5<br>(139·3 to 359·3)	7·4%<br>(4·9 to 12·5%)	109·5<br>(69·7 to 196·9)	4·0%<br>(2·6 to 7·1%)	-47·5%<br>(-52·7 to -42·3%)	-45·7%<br>(-50·3 to -41·5%)	17,904·3<br>(11,961·5 to 30,871·9)	13·2%<br>(8·9 to 22·6%)	9,416·4<br>(5,994·2 to 16,919·1)	9·1%<br>(5·9 to 16·2%)	-47·4%<br>(-52·6 to -42·2%)	-30·9%<br>(-35·3 to -27·0%)
Measles	99·6<br>(20·6 to 252·5)	33·9%<br>(10·7 to 59·3%)	20·0<br>(3·7 to 55·9)	27·1%<br>(7·6 to 52·3%)	-79·9%<br>(-88·1 to -68·7%)	-19·9%<br>(-37·3 to -9·3%)	8,476·0<br>(1,751·4 to 21,476·0)	34·4%<br>(10·8 to 60·2%)	1,700·7<br>(311·3 to 4,762·9)	27·6%<br>(7·7 to 53·1%)	-79·9%<br>(-88·1 to -68·7%)	-19·9%<br>(-37·1 to -9·6%)
Protein-energy malnutrition	233·5<br>(180·2 to 301·3)	61·3%<br>(56·0 to 67·1%)	174·4<br>(130·5 to 226·5)	53·8%<br>(48·4 to 59·6%)	-25·3%<br>(-42·5 to -3·6%)	-12·3%<br>(-21·1 to -2·2%)	22,791·1<br>(18,009·8 to 28,483·9)	85·4%<br>(82·8 to 88·1%)	17,632·7<br>(13,635·4 to 22,229·7)	83·5%<br>(80·5 to 86·3%)	-22·6%<br>(-38·0 to -3·6%)	-2·2%<br>(-5·3 to 1·1%)
**Childhood wasting: All causes**	1,882·1<br>(1,607·5 to 2,124·7)	3·5%<br>(3·0 to 4·0%)	1,168·8<br>(982·1 to 1,306·2)	2·1%<br>(1·8 to 2·3%)	-37·9%<br>(-42·9 to -32·8%)	-40·3%<br>(-45·0 to -35·4%)	166,165·9<br>(142,763·7 to 186,925·9)	6·5%<br>(5·5 to 7·4%)	104,944·8<br>(88,636·5 to 117,150·8)	4·3%<br>(3·6 to 4·9%)	-36·8%<br>(-41·7 to -32·0%)	-34·6%<br>(-39·4 to -29·6%)

Supplemental Results Table 1. All-age deaths, death PAFs, DALYs, DALY PAFs, and percent change for each risk factor and outcome for both sexes combined in 2005 and 2015 for Global.

Data in parenthesis are 95% uncertainty intervals. PAFs=population attributable fractions. DALYs=disability-adjusted life-years.

	2005 Deaths (in thousands)	2005 Deaths PAF	2015 Deaths (in thousands)	2015 Deaths PAF	Percent change deaths 2005-2015	Percent change deaths PAF 2005-2015	2005 DALYs (in thousands)	2005 DALYs PAF	2015 DALYs (in thousands)	2015 DALYs PAF	Percent change DALYs 2005-2015	Percent change DALYs PAF 2005-2015
Diarrhoeal diseases	662·8<br>(542·9 to 749·4)	40·0%<br>(33·4 to 43·4%)	431·3<br>(353·0 to 490·5)	32·9%<br>(27·2 to 36·8%)	-34·9%<br>(-42·9 to -25·2%)	-17·8%<br>(-25·5 to -9·9%)	58,779·3<br>(48,151·8 to 66,140·0)	59·7%<br>(50·1 to 63·9%)	38,905·0<br>(31,778·3 to 44,309·9)	54·3%<br>(45·4 to 58·9%)	-33·8%<br>(-41·7 to -24·5%)	-9·0%<br>(-13·5 to -4·3%)
Lower respiratory infections	861·2<br>(623·5 to 1,010·0)	30·5%<br>(21·8 to 35·8%)	534·7<br>(377·1 to 638·7)	19·6%<br>(13·8 to 23·5%)	-37·9%<br>(-43·3 to -32·4%)	-35·8%<br>(-40·0 to -31·6%)	73,987·1<br>(53,576·0 to 86,770·9)	54·7%<br>(39·5 to 63·4%)	45,988·5<br>(32,457·1 to 54,877·1)	44·6%<br>(31·7 to 52·7%)	-37·8%<br>(-43·2 to -32·4%)	-18·4%<br>(-22·1 to -14·8%)
Measles	124·7<br>(29·7 to 374·5)	42·5%<br>(16·2 to 81·0%)	28·4<br>(5·7 to 92·3)	38·7%<br>(13·0 to 79·4%)	-77·2%<br>(-86·2 to -63·8%)	-9·0%<br>(-27·3 to -0·4%)	10,608·4<br>(2,528·5 to 31,860·2)	43·2%<br>(16·5 to 82·1%)	2,418·5<br>(483·3 to 7,851·4)	39·3%<br>(13·2 to 80·6%)	-77·2%<br>(-86·2 to -63·8%)	-8·9%<br>(-27·2 to -0·5%)
Protein-energy malnutrition	233·5<br>(180·2 to 301·3)	61·3%<br>(56·0 to 67·1%)	174·4<br>(130·5 to 226·5)	53·8%<br>(48·4 to 59·6%)	-25·3%<br>(-42·5 to -3·6%)	-12·3%<br>(-21·1 to -2·2%)	22,791·1<br>(18,009·8 to 28,483·9)	85·4%<br>(82·8 to 88·1%)	17,632·7<br>(13,635·4 to 22,229·7)	83·5%<br>(80·5 to 86·3%)	-22·6%<br>(-38·0 to -3·6%)	-2·2%<br>(-5·3 to 1·1%)
**Childhood stunting: All causes**	507·7<br>(248·3 to 853·3)	0·9%<br>(0·5 to 1·6%)	256·8<br>(113·5 to 454·0)	0·5%<br>(0·2 to 0·8%)	-49·4%<br>(-58·0 to -43·2%)	-51·4%<br>(-59·8 to -45·6%)	43,960·6<br>(21,557·7 to 73,510·9)	1·7%<br>(0·9 to 2·9%)	22,420·0<br>(9,963·8 to 39,448·1)	0·9%<br>(0·4 to 1·6%)	-49·0%<br>(-57·4 to -42·9%)	-47·1%<br>(-56·1 to -40·9%)
Diarrhoeal diseases	154·1<br>(59·3 to 268·0)	9·3%<br>(3·6 to 16·1%)	86·9<br>(31·0 to 155·7)	6·6%<br>(2·4 to 11·7%)	-43·6%<br>(-51·1 to -33·4%)	-28·7%<br>(-35·9 to -20·8%)	13,638·3<br>(5,209·7 to 23,657·8)	13·8%<br>(5·4 to 23·8%)	7,823·5<br>(2,807·0 to 13,947·3)	10·9%<br>(4·0 to 19·3%)	-42·6%<br>(-50·1 to -32·8%)	-21·1%<br>(-26·6 to -15·8%)
Lower respiratory infections	282·3<br>(25·2 to 614·6)	10·0%<br>(0·9 to 21·7%)	155·1<br>(11·8 to 348·5)	5·7%<br>(0·4 to 12·9%)	-45·1%<br>(-50·9 to -36·6%)	-43·2%<br>(-48·2 to -34·5%)	24,248·5<br>(2,170·4 to 52,766·6)	17·9%<br>(1·6 to 39·2%)	13,334·9<br>(1,013·8 to 29,931·7)	12·9%<br>(1·0 to 29·5%)	-45·0%<br>(-50·8 to -36·5%)	-27·8%<br>(-33·2 to -18·8%)
Measles	71·4<br>(7·2 to 201·8)	24·5%<br>(3·0 to 57·6%)	14·8<br>(1·3 to 44·5)	20·4%<br>(2·3 to 52·0%)	-79·2%<br>(-87·9 to -61·9%)	-16·6%<br>(-30·4 to -5·1%)	6,073·9<br>(612·3 to 17,171·4)	24·8%<br>(3·0 to 58·5%)	1,261·5<br>(108·3 to 3,786·6)	20·7%<br>(2·3 to 52·8%)	-79·2%<br>(-87·9 to -61·9%)	-16·6%<br>(-30·3 to -4·9%)
**Iron deficiency: All causes**	86·5<br>(58·8 to 115·6)	0·2%<br>(0·1 to 0·2%)	83·5<br>(58·4 to 113·8)	0·1%<br>(0·1 to 0·2%)	-3·5%<br>(-14·8 to 10·0%)	-7·3%<br>(-17·8 to 5·5%)	55,120·1<br>(37,923·6 to 76,937·4)	2·1%<br>(1·6 to 2·8%)	52,870·1<br>(36,518·1 to 74,437·2)	2·1%<br>(1·6 to 2·8%)	-4·1%<br>(-5·8 to -2·4%)	-0·7%<br>(-2·8 to 1·7%)
Maternal haemorrhage	30·0<br>(12·0 to 48·2)	30·1%<br>(11·4 to 47·9%)	23·9<br>(9·1 to 39·9)	28·7%<br>(10·8 to 45·9%)	-20·3%<br>(-32·7 to -7·6%)	-4·5%<br>(-11·2 to 2·3%)	1,693·8<br>(672·2 to 2,704·9)	30·1%<br>(11·4 to 47·9%)	1,337·8<br>(508·8 to 2,235·6)	28·7%<br>(10·8 to 45·8%)	-21·0%<br>(-32·8 to -8·8%)	-4·6%<br>(-11·0 to 2·1%)
Maternal sepsis and other pregnancy related infections	8·2<br>(3·2 to 13·3)	33·0%<br>(12·7 to 52·3%)	5·4<br>(2·0 to 9·3)	30·0%<br>(11·3 to 48·0%)	-34·1%<br>(-47·7 to -17·6%)	-8·8%<br>(-14·7 to -3·1%)	475·0<br>(187·1 to 776·0)	32·9%<br>(12·7 to 52·3%)	315·1<br>(118·4 to 543·4)	30·0%<br>(11·3 to 47·8%)	-33·7%<br>(-46·9 to -17·2%)	-8·8%<br>(-14·7 to -2·9%)
Iron-deficiency anaemia	48·4<br>(31·9 to 63·1)	100·0%<br>(100·0 to 100·0%)	54·2<br>(35·1 to 72·9)	100·0%<br>(100·0 to 100·0%)	12·1%<br>(-2·1 to 28·0%)	-	52,951·3<br>(36,342·0 to 74,873·5)	100·0%<br>(100·0 to 100·0%)	51,217·1<br>(35,014·4 to 72,661·0)	100·0%<br>(100·0 to 100·0%)	-3·3%<br>(-4·8 to -1·8%)	-
**Vitamin A deficiency: All causes**	191·1<br>(116·1 to 290·6)	0·4%<br>(0·2 to 0·5%)	82·7<br>(50·1 to 119·2)	0·1%<br>(0·1 to 0·2%)	-56·7%<br>(-66·6 to -47·3%)	-58·4%<br>(-67·8 to -49·4%)	16,864·3<br>(10,421·0 to 25,456·8)	0·7%<br>(0·4 to 1·0%)	7,611·0<br>(4,693·1 to 10,840·7)	0·3%<br>(0·2 to 0·4%)	-54·9%<br>(-64·7 to -45·6%)	-53·2%<br>(-63·2 to -43·7%)
Diarrhoeal diseases	107·3<br>(60·4 to 157·7)	6·5%<br>(3·7 to 9·5%)	64·2<br>(36·1 to 93·3)	4·9%<br>(2·7 to 7·1%)	-40·2%<br>(-48·1 to -30·7%)	-24·4%<br>(-32·2 to -16·3%)	9,531·3<br>(5,401·7 to 14,011·9)	9·7%<br>(5·5 to 14·0%)	5,804·8<br>(3,233·8 to 8,402·2)	8·1%<br>(4·5 to 11·6%)	-39·1%<br>(-46·7 to -29·8%)	-16·3%<br>(-21·8 to -10·5%)
Measles	83·8<br>(31·3 to 175·6)	28·7%<br>(19·2 to 37·8%)	18·5<br>(6·5 to 40·8)	25·4%<br>(16·4 to 34·0%)	-77·9%<br>(-86·0 to -66·3%)	-11·6%<br>(-22·7 to -2·1%)	7,123·6<br>(2,654·9 to 14,909·7)	29·1%<br>(19·5 to 38·3%)	1,573·8<br>(556·2 to 3,469·2)	25·7%<br>(16·6 to 34·6%)	-77·9%<br>(-86·0 to -66·3%)	-11·6%<br>(-22·4 to -2·3%)
Vitamin A deficiency	-	-	-	-	-	-	209·5<br>(130·8 to 309·2)	100·0%<br>(100·0 to 100·0%)	232·4<br>(143·4 to 346·3)	100·0%<br>(100·0 to 100·0%)	10·9%<br>(7·5 to 14·6%)	-
**Zinc deficiency: All causes**	92·6<br>(5·3 to 208·3)	0·2%<br>(0·0 to 0·4%)	55·1<br>(3·0 to 124·8)	0·1%<br>(0·0 to 0·2%)	-40·5%<br>(-49·1 to -28·8%)	-42·8%<br>(-51·0 to -31·7%)	8,161·6<br>(819·3 to 17,948·4)	0·3%<br>(0·0 to 0·7%)	4,967·2<br>(600·3 to 10,852·6)	0·2%<br>(0·0 to 0·4%)	-39·1%<br>(-47·3 to -25·0%)	-36·9%<br>(-45·1 to -22·1%)
Diarrhoeal diseases	51·4<br>(0·0 to 118·6)	3·1%<br>(0·0 to 7·1%)	31·0<br>(0·0 to 72·8)	2·4%<br>(0·0 to 5·5%)	-39·7%<br>(-50·0 to 0·0%)	-23·9%<br>(-34·8 to 0·0%)	4,663·2<br>(301·0 to 10,330·3)	4·7%<br>(0·3 to 10·4%)	2,911·3<br>(274·6 to 6,368·8)	4·1%<br>(0·4 to 8·8%)	-37·6%<br>(-47·4 to -8·3%)	-14·3%<br>(-24·0 to 27·0%)
Lower respiratory infections	41·2<br>(0·0 to 143·1)	1·5%<br>(0·0 to 5·1%)	24·1<br>(0·0 to 83·0)	0·9%<br>(0·0 to 3·0%)	-41·4%<br>(-49·4 to 0·0%)	-39·4%<br>(-46·5 to 0·0%)	3,498·4<br>(13·0 to 12,111·4)	2·6%<br>(0·0 to 8·9%)	2,055·9<br>(11·3 to 7,031·4)	2·0%<br>(0·0 to 6·8%)	-41·2%<br>(-48·8 to -11·0%)	-22·8%<br>(-30·6 to 19·3%)
**Tobacco smoke: All causes**	6,879·0<br>(6,207·4 to 7,522·4)	12·8%<br>(11·6 to 14·0%)	7,164·5<br>(6,544·2 to 7,774·8)	12·8%<br>(11·7 to 13·9%)	4·2%<br>(0·9 to 7·6%)	0·1%<br>(-2·4 to 2·6%)	174,309·4<br>(158,980·3 to 190,805·5)	6·8%<br>(6·1 to 7·6%)	170,888·6<br>(156,215·6 to 185,987·6)	6·9%<br>(6·2 to 7·8%)	-2·0%<br>(-5·1 to 1·3%)	1·6%<br>(-1·4 to 4·8%)
**Smoking: All causes**	6,112·7<br>(5,402·2 to 6,779·8)	11·4%<br>(10·1 to 12·6%)	6,402·2<br>(5,748·7 to 7,036·6)	11·5%<br>(10·3 to 12·6%)	4·7%<br>(1·2 to 8·5%)	0·7%<br>(-2·2 to 3·7%)	147,152·6<br>(131,914·1 to 161,830·7)	5·8%<br>(5·1 to 6·5%)	148,623·2<br>(134,236·0 to 163,140·5)	6·0%<br>(5·3 to 6·8%)	1·0%<br>(-2·4 to 4·6%)	4·7%<br>(1·3 to 8·2%)
Tuberculosis	114·1<br>(56·8 to 179·9)	8·5%<br>(4·3 to 12·9%)	86·8<br>(41·3 to 140·2)	7·8%<br>(3·7 to 12·0%)	-23·9%<br>(-32·0 to -16·9%)	-7·8%<br>(-13·8 to -3·0%)	3,717·4<br>(1,859·5 to 5,830·4)	7·5%<br>(3·8 to 11·4%)	2,835·6<br>(1,357·0 to 4,514·2)	7·0%<br>(3·4 to 10·8%)	-23·7%<br>(-31·0 to -17·4%)	-5·8%<br>(-12·6 to -0·4%)
Lower respiratory infections	332·2<br>(264·6 to 404·5)	11·7%<br>(9·5 to 14·2%)	350·0<br>(276·2 to 432·7)	12·8%<br>(10·3 to 15·7%)	5·4%<br>(1·0 to 9·4%)	8·9%<br>(4·7 to 12·8%)	7,119·2<br>(5,724·4 to 8,602·9)	5·3%<br>(4·3 to 6·4%)	7,044·0<br>(5,589·2 to 8,639·6)	6·8%<br>(5·5 to 8·4%)	-1·1%<br>(-5·4 to 3·3%)	29·9%<br>(22·2 to 38·0%)
Lip and oral cavity cancer	47·8<br>(41·2 to 55·1)	43·4%<br>(37·5 to 49·8%)	60·1<br>(50·0 to 70·6)	41·2%<br>(34·7 to 48·1%)	25·8%<br>(18·6 to 32·7%)	-5·1%<br>(-9·0 to -1·7%)	1,287·8<br>(1,098·7 to 1,486·5)	43·6%<br>(37·6 to 50·0%)	1,547·8<br>(1,284·4 to 1,831·1)	40·9%<br>(34·4 to 48·0%)	20·2%<br>(12·5 to 27·8%)	-6·1%<br>(-10·2 to -2·3%)
Nasopharynx cancer	21·1<br>(14·5 to 28·4)	37·7%<br>(26·7 to 50·0%)	22·8<br>(16·2 to 30·4)	36·2%<br>(26·3 to 47·3%)	8·3%<br>(-2·0 to 20·0%)	-4·0%<br>(-10·2 to 4·2%)	640·0<br>(430·7 to 886·6)	35·4%<br>(24·3 to 48·0%)	642·9<br>(451·1 to 867·2)	33·6%<br>(24·1 to 44·6%)	0·5%<br>(-11·1 to 14·2%)	-5·1%<br>(-13·3 to 5·6%)
Oesophageal cancer	163·0<br>(101·2 to 235·9)	35·5%<br>(21·9 to 51·2%)	156·6<br>(103·7 to 215·7)	35·7%<br>(23·7 to 49·1%)	-4·0%<br>(-13·8 to 8·6%)	0·5%<br>(-7·1 to 13·0%)	3,736·1<br>(2,336·3 to 5,384·1)	35·0%<br>(21·9 to 50·4%)	3,418·1<br>(2,285·7 to 4,695·9)	34·7%<br>(23·1 to 47·7%)	-8·5%<br>(-18·1 to 4·4%)	-1·0%<br>(-8·9 to 12·0%)
Stomach cancer	88·5<br>(50·9 to 138·1)	10·7%<br>(6·2 to 16·7%)	81·4<br>(46·7 to 126·1)	9·9%<br>(5·7 to 15·4%)	-8·0%<br>(-14·7 to -1·5%)	-7·4%<br>(-12·5 to -2·2%)	2,030·4<br>(1,162·4 to 3,180·0)	10·9%<br>(6·3 to 17·1%)	1,706·2<br>(958·0 to 2,630·5)	9·8%<br>(5·5 to 15·1%)	-16·0%<br>(-23·2 to -8·8%)	-10·1%<br>(-16·4 to -3·8%)
Colon and rectum cancer	45·5<br>(32·0 to 59·7)	6·7%<br>(4·8 to 8·8%)	50·1<br>(35·0 to 66·2)	6·0%<br>(4·2 to 7·9%)	10·2%<br>(4·8 to 15·3%)	-10·6%<br>(-14·0 to -7·5%)	977·6<br>(682·2 to 1,293·3)	6·8%<br>(4·7 to 9·0%)	995·9<br>(695·7 to 1,314·2)	5·8%<br>(4·0 to 7·8%)	1·9%<br>(-3·4 to 6·8%)	-13·8%<br>(-17·4 to -10·5%)
Liver cancer due to hepatitis B	37·0<br>(16·6 to 72·1)	14·1%<br>(6·3 to 26·7%)	35·2<br>(16·2 to 63·9)	13·3%<br>(6·1 to 23·6%)	-5·0%<br>(-20·0 to 13·1%)	-5·8%<br>(-17·2 to 11·6%)	1,176·4<br>(512·3 to 2,280·2)	14·0%<br>(6·1 to 26·8%)	1,022·3<br>(467·0 to 1,898·9)	12·7%<br>(5·8 to 23·6%)	-13·1%<br>(-29·5 to 7·7%)	-8·9%<br>(-23·2 to 12·9%)
Liver cancer due to hepatitis C	19·7<br>(11·5 to 29·1)	14·3%<br>(8·3 to 21·1%)	23·7<br>(13·9 to 34·6)	14·2%<br>(8·4 to 20·9%)	20·3%<br>(12·8 to 28·6%)	-0·8%<br>(-6·6 to 5·2%)	426·0<br>(247·7 to 634·0)	14·7%<br>(8·5 to 22·0%)	474·4<br>(274·8 to 712·5)	14·3%<br>(8·2 to 21·3%)	11·4%<br>(2·1 to 21·4%)	-3·0%<br>(-9·7 to 4·5%)
Liver cancer due to alcohol use	34·1<br>(17·5 to 57·8)	17·5%<br>(9·0 to 29·4%)	40·2<br>(20·1 to 69·4)	16·4%<br>(8·1 to 27·7%)	18·2%<br>(6·8 to 31·0%)	-6·3%<br>(-12·9 to 0·1%)	849·5<br>(425·6 to 1,476·1)	17·8%<br>(9·0 to 30·5%)	957·9<br>(467·4 to 1,651·8)	16·3%<br>(8·0 to 27·7%)	12·8%<br>(-0·3 to 26·9%)	-8·3%<br>(-15·9 to -0·3%)
Liver cancer due to other causes	16·2<br>(7·7 to 29·4)	12·4%<br>(5·9 to 22·2%)	16·3<br>(8·5 to 27·9)	12·3%<br>(6·4 to 20·6%)	0·5%<br>(-12·5 to 18·5%)	-0·7%<br>(-11·7 to 16·6%)	439·7<br>(204·3 to 817·3)	12·4%<br>(5·9 to 22·6%)	396·5<br>(201·5 to 684·4)	11·9%<br>(6·1 to 20·5%)	-9·8%<br>(-24·7 to 11·0%)	-4·4%<br>(-18·2 to 17·7%)
Pancreatic cancer	61·7<br>(49·8 to 74·8)	19·6%<br>(15·9 to 23·8%)	72·9<br>(58·5 to 89·2)	17·7%<br>(14·3 to 21·6%)	18·0%<br>(14·0 to 22·2%)	-9·8%<br>(-12·3 to -7·4%)	1,329·7<br>(1,068·0 to 1,629·8)	20·4%<br>(16·4 to 25·0%)	1,466·9<br>(1,159·2 to 1,819·7)	17·8%<br>(14·2 to 22·0%)	10·3%<br>(6·2 to 14·7%)	-12·6%<br>(-15·6 to -9·5%)
Tracheal, bronchus, and lung cancer	1,014·2<br>(879·8 to 1,127·0)	70·7%<br>(61·6 to 78·1%)	1,174·7<br>(1,012·2 to 1,323·5)	68·2%<br>(59·2 to 76·2%)	15·8%<br>(11·1 to 21·1%)	-3·5%<br>(-5·4 to -2·0%)	22,060·1<br>(18,918·4 to 24,708·8)	69·4%<br>(59·4 to 77·4%)	24,139·9<br>(20,463·2 to 27,427·4)	66·3%<br>(56·7 to 74·9%)	9·4%<br>(4·3 to 15·5%)	-4·5%<br>(-6·7 to -2·4%)
Cervical cancer	11·6<br>(4·0 to 20·5)	5·2%<br>(1·8 to 9·3%)	11·7<br>(3·9 to 20·6)	4·9%<br>(1·7 to 8·7%)	0·7%<br>(-11·2 to 15·6%)	-4·8%<br>(-13·6 to 5·3%)	355·1<br>(117·4 to 646·9)	5·2%<br>(1·7 to 9·4%)	329·7<br>(109·0 to 588·1)	4·7%<br>(1·6 to 8·4%)	-7·1%<br>(-21·0 to 9·4%)	-9·0%<br>(-19·3 to 3·7%)
Kidney cancer	19·9<br>(13·4 to 26·1)	19·1%<br>(12·9 to 24·8%)	23·2<br>(15·4 to 31·2)	17·0%<br>(11·3 to 22·5%)	16·6%<br>(10·1 to 22·4%)	-11·0%<br>(-14·8 to -7·8%)	480·7<br>(325·8 to 624·6)	18·2%<br>(12·3 to 23·7%)	530·5<br>(349·4 to 708·5)	15·9%<br>(10·6 to 21·1%)	10·4%<br>(3·9 to 16·1%)	-12·5%<br>(-17·2 to -8·5%)
Bladder cancer	43·8<br>(32·6 to 54·5)	29·1%<br>(21·9 to 36·3%)	51·1<br>(38·0 to 64·4)	27·2%<br>(20·0 to 34·3%)	16·8%<br>(11·2 to 21·9%)	-6·5%<br>(-9·5 to -3·7%)	834·9<br>(629·2 to 1,033·6)	29·5%<br>(22·2 to 36·6%)	906·9<br>(674·3 to 1,145·9)	26·9%<br>(19·8 to 33·9%)	8·6%<br>(3·3 to 13·7%)	-8·7%<br>(-11·7 to -5·9%)
Ischaemic heart disease	1,236·2<br>(1,020·2 to 1,454·9)	16·2%<br>(13·4 to 19·0%)	1,279·7<br>(1,049·1 to 1,515·5)	14·4%<br>(11·8 to 16·9%)	3·5%<br>(0·1 to 6·9%)	-11·2%<br>(-13·3 to -9·0%)	32,668·8<br>(26,987·3 to 38,407·4)	22·1%<br>(18·2 to 25·9%)	33,160·5<br>(26,987·2 to 39,129·8)	20·2%<br>(16·5 to 23·8%)	1·5%<br>(-2·1 to 4·9%)	-8·5%<br>(-10·5 to -6·5%)
Ischaemic stroke	361·7<br>(301·1 to 420·1)	13·1%<br>(11·0 to 15·3%)	352·1<br>(293·1 to 415·7)	11·8%<br>(9·8 to 13·9%)	-2·7%<br>(-6·8 to 1·3%)	-9·8%<br>(-12·7 to -6·8%)	7,809·7<br>(6,499·8 to 9,145·9)	17·7%<br>(14·8 to 20·6%)	7,520·1<br>(6,258·0 to 8,896·8)	16·6%<br>(13·8 to 19·5%)	-3·7%<br>(-7·8 to 0·2%)	-6·1%<br>(-8·9 to -3·2%)
Hemorrhagic stroke	608·7<br>(510·3 to 706·3)	18·7%<br>(15·6 to 21·6%)	572·9<br>(475·0 to 673·4)	17·1%<br>(14·2 to 20·0%)	-5·9%<br>(-10·3 to -1·1%)	-8·4%<br>(-11·2 to -5·3%)	16,619·1<br>(14,009·7 to 19,207·3)	22·3%<br>(18·8 to 25·7%)	15,512·4<br>(12,883·8 to 18,265·6)	21·1%<br>(17·5 to 24·6%)	-6·7%<br>(-10·9 to -2·2%)	-5·3%<br>(-8·1 to -2·5%)
Hypertensive heart disease	99·1<br>(76·7 to 121·8)	13·0%<br>(10·1 to 16·0%)	113·8<br>(88·6 to 138·2)	11·8%<br>(9·2 to 14·4%)	14·9%<br>(8·1 to 21·6%)	-9·2%<br>(-13·3 to -4·1%)	2,526·9<br>(1,976·3 to 3,091·2)	17·0%<br>(13·2 to 20·7%)	2,771·1<br>(2,167·7 to 3,363·7)	15·9%<br>(12·3 to 19·2%)	9·7%<br>(3·3 to 16·4%)	-6·8%<br>(-10·8 to -2·4%)
Atrial fibrillation and flutter	11·6<br>(8·4 to 15·2)	8·1%<br>(6·1 to 10·0%)	13·6<br>(9·9 to 18·0)	7·0%<br>(5·3 to 8·6%)	18·0%<br>(13·9 to 22·2%)	-13·7%<br>(-16·3 to -11·2%)	537·3<br>(379·2 to 732·2)	15·5%<br>(12·0 to 18·9%)	617·1<br>(432·7 to 846·2)	13·9%<br>(10·7 to 17·1%)	14·9%<br>(12·7 to 16·9%)	-10·3%<br>(-11·7 to -9·0%)
Aortic aneurysm	21·8<br>(17·0 to 26·3)	16·1%<br>(12·6 to 19·4%)	23·1<br>(17·9 to 28·0)	13·8%<br>(10·7 to 16·7%)	6·3%<br>(0·1 to 10·8%)	-14·8%<br>(-16·9 to -12·7%)	541·8<br>(426·0 to 650·7)	22·0%<br>(17·4 to 26·2%)	572·0<br>(448·2 to 693·4)	19·5%<br>(15·3 to 23·7%)	5·6%<br>(-1·1 to 10·4%)	-11·1%<br>(-13·2 to -8·9%)

Supplemental Results Table 1. All-age deaths, death PAFs, DALYs, DALY PAFs, and percent change for each risk factor and outcome for both sexes combined in 2005 and 2015 for Global.

Data in parenthesis are 95% uncertainty intervals. PAFs=population attributable fractions. DALYs=disability-adjusted life-years.

	2005 Deaths (in thousands)	2005 Deaths PAF	2015 Deaths (in thousands)	2015 Deaths PAF	Percent change deaths 2005-2015	Percent change deaths PAF 2005-2015	2005 DALYs (in thousands)	2005 DALYs PAF	2015 DALYs (in thousands)	2015 DALYs PAF	Percent change DALYs 2005-2015	Percent change DALYs PAF 2005-2015
Peripheral vascular disease	4·3 (3·3 to 5·2)	11·2% (8·5 to 13·7%)	4·9 (3·7 to 6·1)	9·4% (7·1 to 11·5%)	15·3% (8·8 to 22·0%)	-16·3% (-20·1 to -12·5%)	143·7 (99·3 to 198·7)	15·8% (12·2 to 19·3%)	167·1 (114·4 to 236·7)	14·0% (10·7 to 17·1%)	16·3% (12·0 to 20·6%)	-11·5% (-13·8 to -9·2%)
Other cardiovascular and circulatory diseases	57·9 (44·7 to 70·6)	12·7% (9·8 to 15·3%)	61·3 (47·2 to 74·7)	11·3% (8·7 to 13·8%)	5·9% (1·2 to 10·8%)	-10·5% (-13·4 to -7·4%)	2,186·6 (1,699·0 to 2,687·6)	14·6% (11·4 to 17·6%)	2,347·5 (1,816·1 to 2,909·7)	13·9% (10·8 to 16·8%)	7·4% (3·5 to 11·2%)	-4·4% (-7·1 to -1·7%)
Chronic obstructive pulmonary disease	1,355·1 (1,009·9 to 1,686·4)	43·7% (32·5 to 54·2%)	1,427·1 (1,149·2 to 1,698·8)	44·8% (36·3 to 52·8%)	5·3% (-2·5 to 17·1%)	2·4% (-3·9 to 13·4%)	25,834·3 (20,021·0 to 31,596·3)	40·5% (31·4 to 49·4%)	26,442·6 (21,842·3 to 31,310·0)	41·4% (34·5 to 48·4%)	2·4% (-4·6 to 12·6%)	2·2% (-3·8 to 12·4%)
Silicosis	1·6 (0·9 to 2·5)	15·2% (9·3 to 23·5%)	1·4 (0·8 to 2·4)	13·8% (8·1 to 22·5%)	-7·1% (-21·0 to 8·8%)	-8·9% (-17·8 to -0·3%)	39·2 (22·0 to 65·8)	14·4% (8·4 to 23·4%)	33·2 (18·1 to 56·3)	12·3% (7·1 to 20·4%)	-15·2% (-29·6 to 1·8%)	-14·9% (-26·0 to -3·2%)
Asbestosis	0·5 (0·3 to 0·7)	18·3% (13·8 to 23·1%)	0·6 (0·4 to 0·8)	16·9% (12·8 to 21·4%)	18·5% (6·9 to 30·1%)	-7·7% (-12·7 to -2·0%)	11·9 (8·7 to 15·5)	15·7% (11·8 to 19·9%)	12·9 (9·3 to 16·9)	14·0% (10·6 to 17·8%)	7·8% (0·0 to 15·9%)	-10·6% (-15·6 to -5·3%)
Coal workers pneumoconiosis	0·5 (0·4 to 0·7)	19·8% (14·1 to 27·1%)	0·4 (0·3 to 0·6)	17·4% (11·8 to 25·0%)	-18·5% (-30·4 to -5·7%)	-12·2% (-21·1 to -4·3%)	11·0 (7·7 to 15·4)	18·5% (13·0 to 25·3%)	9·1 (6·1 to 13·5)	15·9% (11·1 to 22·4%)	-17·0% (-29·5 to -2·5%)	-14·0% (-22·5 to -4·8%)
Other pneumoconiosis	1·7 (1·4 to 2·2)	10·8% (8·1 to 13·7%)	2·0 (1·6 to 2·6)	10·2% (7·9 to 13·0%)	15·1% (4·9 to 27·8%)	-5·3% (-14·9 to 6·0%)	48·8 (35·0 to 67·1)	8·6% (6·4 to 11·5%)	51·9 (37·0 to 70·8)	7·7% (5·8 to 10·0%)	6·3% (-2·8 to 15·5%)	-11·1% (-18·7 to -3·4%)
Asthma	54·8 (40·9 to 69·2)	12·2% (9·4 to 15·4%)	45·2 (35·2 to 55·8)	11·4% (8·9 to 13·8%)	-17·6% (-27·0 to -4·2%)	-6·9% (-15·8 to 3·8%)	2,225·7 (1,692·5 to 2,804·2)	8·3% (6·6 to 10·1%)	2,024·3 (1,513·7 to 2,578·9)	7·8% (6·1 to 9·5%)	-9·0% (-15·9 to -0·7%)	-6·6% (-12·0 to -1·1%)
Interstitial lung disease and pulmonary sarcoidosis	12·5 (8·6 to 15·6)	15·5% (12·5 to 18·6%)	17·1 (11·9 to 21·5)	14·0% (11·3 to 17·0%)	37·2% (26·4 to 45·4%)	-9·6% (-13·1 to -4·8%)	243·6 (178·8 to 301·7)	14·6% (11·8 to 17·5%)	302·3 (223·6 to 376·7)	12·8% (10·3 to 15·5%)	24·1% (15·2 to 31·7%)	-11·9% (-15·7 to -6·5%)
Other chronic respiratory diseases	3·4 (2·5 to 4·2)	7·3% (5·6 to 9·0%)	4·4 (3·0 to 5·7)	8·4% (6·4 to 10·5%)	30·6% (16·5 to 49·4%)	16·1% (4·4 to 31·2%)	191·4 (139·9 to 248·4)	4·7% (3·6 to 6·2%)	210·6 (149·5 to 278·0)	5·3% (4·0 to 6·9%)	10·0% (0·6 to 20·1%)	12·4% (1·7 to 24·0%)
Diabetes mellitus	55·9 (16·5 to 98·5)	4·9% (1·4 to 8·5%)	67·7 (19·2 to 120·1)	4·5% (1·3 to 7·8%)	21·2% (13·4 to 27·9%)	-8·2% (-13·3 to -4·3%)	2,872·6 (831·0 to 5,121·9)	5·8% (1·7 to 10·1%)	3,401·7 (949·1 to 6,090·4)	5·3% (1·5 to 9·4%)	18·4% (13·6 to 22·4%)	-8·2% (-11·2 to -5·8%)
**Second-hand smoke: All causes**	884·1 (685·0 to 1,092·8)	1·6% (1·3 to 2·0%)	886·1 (694·7 to 1,091·0)	1·6% (1·3 to 2·0%)	0·2% (-3·5 to 3·9%)	-3·7% (-6·9 to -0·2%)	29,995·7 (22,370·4 to 38,043·0)	1·2% (0·9 to 1·5%)	25,212·1 (19,297·0 to 31,653·4)	1·0% (0·8 to 1·3%)	-15·9% (-20·0 to -11·2%)	-12·9% (-17·1 to -7·9%)
Lower respiratory infections	237·8 (150·9 to 330·2)	8·4% (5·3 to 11·6%)	183·0 (113·7 to 258·7)	6·7% (4·2 to 9·4%)	-23·0% (-27·4 to -19·0%)	-20·4% (-23·0 to -18·0%)	15,685·2 (9,916·5 to 21,832·2)	11·6% (7·3 to 16·1%)	10,103·2 (6,305·1 to 14,389·6)	9·8% (6·1 to 13·8%)	-35·6% (-39·9 to -31·4%)	-15·4% (-17·8 to -13·1%)
Otitis media	0·3 (0·2 to 0·3)	6·6% (4·5 to 8·7%)	0·2 (0·1 to 0·2)	5·4% (3·6 to 7·2%)	-32·6% (-40·5 to -23·5%)	-18·0% (-28·7 to -7·9%)	265·5 (149·2 to 426·5)	7·7% (5·2 to 10·2%)	250·2 (138·5 to 406·0)	7·2% (4·8 to 9·5%)	-5·8% (-9·4 to -3·0%)	-7·3% (-9·5 to -5·2%)
Tracheal, bronchus, and lung cancer	21·5 (11·4 to 37·2)	1·5% (0·8 to 2·6%)	28·6 (15·4 to 48·8)	1·7% (0·9 to 2·8%)	32·6% (26·5 to 37·2%)	10·4% (5·5 to 14·9%)	535·5 (282·3 to 927·8)	1·7% (0·9 to 2·9%)	690·7 (370·8 to 1,189·0)	1·9% (1·0 to 3·3%)	29·0% (21·4 to 35·0%)	12·6% (6·5 to 18·7%)
Ischaemic heart disease	331·0 (252·2 to 421·8)	4·3% (3·3 to 5·5%)	385·6 (290·8 to 494·2)	4·3% (3·3 to 5·6%)	16·5% (12·1 to 20·5%)	-0·1% (-3·1 to 2·7%)	7,187·3 (5,734·3 to 8,846·9)	4·9% (3·9 to 6·0%)	8,065·8 (6,409·5 to 9,893·8)	4·9% (3·9 to 6·1%)	12·2% (7·9 to 16·2%)	1·1% (-1·4 to 3·6%)
Ischaemic stroke	68·7 (51·1 to 90·2)	2·5% (1·8 to 3·3%)	72·4 (52·4 to 95·3)	2·4% (1·8 to 3·2%)	5·4% (-0·7 to 10·9%)	-2·3% (-6·5 to 1·7%)	1,229·3 (948·4 to 1,561·5)	2·8% (2·2 to 3·5%)	1,247·8 (946·7 to 1,589·1)	2·8% (2·1 to 3·5%)	1·5% (-3·8 to 6·3%)	-1·0% (-4·3 to 2·2%)
Hemorrhagic stroke	104·1 (80·8 to 131·6)	3·2% (2·5 to 4·0%)	103·5 (80·1 to 131·6)	3·1% (2·4 to 3·9%)	-0·6% (-5·2 to 4·7%)	-3·2% (-6·6 to 0·0%)	2,637·3 (2,075·7 to 3,304·8)	3·5% (2·8 to 4·4%)	2,548·4 (1,990·6 to 3,201·0)	3·5% (2·7 to 4·3%)	-3·4% (-8·2 to 2·2%)	-2·0% (-5·3 to 1·5%)
**Alcohol and drug use: All causes**	2,594·6 (2,314·1 to 2,865·6)	4·8% (4·3 to 5·3%)	2,750·3 (2,424·0 to 3,051·0)	4·9% (4·4 to 5·5%)	6·0% (3·0 to 8·8%)	1·9% (-0·4 to 4·0%)	108,716·8 (100,093·8 to 117,133·8)	4·3% (3·9 to 4·6%)	111,365·3 (102,247·0 to 120,351·8)	4·5% (4·1 to 4·9%)	2·4% (0·0 to 4·8%)	6·2% (3·9 to 8·4%)
**Alcohol use: All causes**	2,228·0 (1,943·0 to 2,499·6)	4·2% (3·6 to 4·7%)	2,306·5 (1,985·5 to 2,608·5)	4·1% (3·6 to 4·7%)	3·5% (0·0 to 6·8%)	-0·5% (-3·4 to 2·0%)	86,048·2 (78,265·8 to 93,716·5)	3·4% (3·0 to 3·7%)	84,990·0 (77,180·3 to 93,009·8)	3·5% (3·1 to 3·8%)	-1·2% (-3·9 to 1·5%)	2·4% (-0·3 to 5·1%)
Tuberculosis	146·8 (117·2 to 187·5)	10·9% (9·5 to 12·1%)	126·5 (94·1 to 168·7)	11·4% (9·3 to 13·0%)	-13·9% (-23·0 to -5·2%)	4·2% (-4·2 to 10·3%)	5,498·3 (4,496·9 to 6,911·4)	11·0% (9·8 to 12·1%)	4,724·6 (3,591·1 to 6,197·9)	11·7% (9·7 to 13·2%)	-14·1% (-22·4 to -6·2%)	6·0% (-2·1 to 12·4%)
Lower respiratory infections	93·3 (82·7 to 103·6)	3·3% (3·0 to 3·6%)	106·1 (89·4 to 120·6)	3·9% (3·4 to 4·3%)	13·6% (6·7 to 20·2%)	17·4% (10·9 to 24·0%)	2,279·3 (2,036·4 to 2,495·2)	1·7% (1·5 to 1·9%)	2,355·1 (1,994·5 to 2,628·4)	2·3% (2·0 to 2·5%)	3·3% (-4·2 to 10·9%)	35·6% (25·0 to 46·8%)
Lip and oral cavity cancer	28·5 (26·2 to 30·6)	25·9% (23·7 to 27·7%)	37·6 (33·0 to 41·1)	25·8% (22·8 to 28·1%)	31·9% (24·7 to 38·9%)	-0·4% (-5·2 to 3·5%)	820·5 (758·9 to 874·2)	27·8% (25·7 to 29·5%)	1,046·2 (928·7 to 1,133·7)	27·7% (24·8 to 29·9%)	27·5% (20·5 to 34·4%)	-0·3% (-4·9 to 3·4%)
Nasopharynx cancer	15·1 (11·2 to 16·4)	27·0% (24·2 to 28·8%)	17·9 (13·2 to 20·1)	28·4% (25·0 to 31·0%)	18·8% (7·8 to 28·3%)	5·3% (-0·5 to 9·6%)	493·5 (367·0 to 537·6)	27·3% (24·5 to 29·0%)	556·0 (407·0 to 623·0)	29·0% (25·8 to 31·5%)	12·6% (1·9 to 22·3%)	6·4% (1·0 to 10·8%)
Other pharynx cancer	15·6 (14·3 to 16·8)	30·1% (27·7 to 32·1%)	19·7 (17·5 to 21·5)	30·6% (27·4 to 33·1%)	26·3% (18·9 to 32·8%)	1·8% (-3·2 to 5·6%)	453·8 (418·2 to 486·0)	32·0% (29·6 to 33·8%)	560·1 (499·2 to 610·8)	32·6% (29·4 to 35·1%)	23·4% (16·3 to 29·7%)	2·1% (-2·5 to 5·5%)
Oesophageal cancer	73·3 (66·0 to 80·2)	16·0% (14·5 to 17·3%)	75·0 (64·4 to 84·0)	17·1% (14·9 to 18·9%)	2·3% (-6·2 to 10·9%)	7·0% (0·2 to 12·2%)	1,846·5 (1,673·0 to 2,002·3)	17·3% (15·8 to 18·6%)	1,825·9 (1,579·4 to 2,026·8)	18·5% (16·3 to 20·3%)	-1·1% (-9·3 to 7·3%)	7·0% (0·9 to 11·7%)
Colon and rectum cancer	26·7 (24·0 to 29·2)	4·0% (3·6 to 4·3%)	32·3 (28·5 to 35·8)	3·9% (3·4 to 4·3%)	20·8% (15·8 to 25·4%)	-1·9% (-5·7 to 1·2%)	599·5 (548·1 to 650·1)	4·2% (3·8 to 4·5%)	702·4 (625·9 to 770·9)	4·1% (3·7 to 4·5%)	17·2% (12·3 to 21·5%)	-0·8% (-4·6 to 2·1%)
Liver cancer due to hepatitis B	1·4 (0·9 to 1·8)	0·5% (0·4 to 0·7%)	1·0 (0·7 to 1·4)	0·4% (0·3 to 0·5%)	-26·6% (-41·4 to -8·1%)	-27·2% (-40·9 to -14·0%)	74·6 (52·9 to 97·3)	0·9% (0·7 to 1·1%)	56·0 (37·8 to 75·8)	0·7% (0·5 to 0·9%)	-24·9% (-39·5 to -7·1%)	-21·3% (-35·0 to -8·5%)
Liver cancer due to hepatitis C	0·1 (0·1 to 0·1)	0·1% (0·0 to 0·1%)	0·1 (0·0 to 0·1)	0·0% (0·0 to 0·1%)	-10·0% (-26·7 to 8·8%)	-25·8% (-39·7 to -10·7%)	4·3 (2·9 to 5·9)	0·1% (0·1 to 0·2%)	3·8 (2·5 to 5·3)	0·1% (0·1 to 0·2%)	-12·0% (-27·8 to 5·2%)	-23·3% (-36·9 to -9·4%)
Liver cancer due to alcohol use	194·5 (168·8 to 208·3)	100·0% (100·0 to 100·0%)	245·2 (225·1 to 266·6)	100·0% (100·0 to 100·0%)	26·1% (18·5 to 37·1%)	-	4,786·9 (4,075·0 to 5,168·7)	100·0% (100·0 to 100·0%)	5,888·5 (5,368·4 to 6,441·0)	100·0% (100·0 to 100·0%)	23·0% (14·8 to 36·1%)	-
Liver cancer due to other causes	0·3 (0·2 to 0·5)	0·3% (0·2 to 0·4%)	0·2 (0·2 to 0·3)	0·2% (0·1 to 0·3%)	-30·2% (-43·3 to -13·8%)	-31·0% (-44·0 to -18·2%)	19·3 (13·5 to 25·1)	0·5% (0·4 to 0·7%)	13·7 (9·4 to 19·1)	0·4% (0·3 to 0·6%)	-28·7% (-41·6 to -12·5%)	-24·4% (-37·4 to -11·9%)
Larynx cancer	17·9 (16·2 to 19·4)	19·3% (17·5 to 20·8%)	19·7 (17·2 to 21·8)	18·6% (16·3 to 20·5%)	9·9% (3·9 to 15·2%)	-3·4% (-8·2 to 0·2%)	486·3 (442·8 to 523·1)	20·6% (18·8 to 22·0%)	520·0 (455·8 to 572·2)	19·9% (17·6 to 21·8%)	6·9% (1·6 to 11·9%)	-3·1% (-7·7 to 0·2%)
Breast cancer	26·7 (23·8 to 29·4)	6·1% (5·4 to 6·7%)	30·4 (26·2 to 34·3)	5·7% (4·9 to 6·4%)	13·8% (7·2 to 20·0%)	-6·2% (-10·9 to -1·8%)	800·8 (711·6 to 888·8)	6·2% (5·5 to 6·8%)	899·0 (768·1 to 1,022·8)	5·8% (5·0 to 6·6%)	12·3% (5·4 to 18·7%)	-5·8% (-10·6 to -1·1%)
Ischaemic heart disease	34·9 (-110·4 to 176·3)	0·5% (-1·4 to 2·3%)	10·0 (-129·6 to 150·2)	0·1% (-1·5 to 1·7%)	-71·4% (-400·6 to 326·2%)	-75·5% (-359·1 to 266·9%)	1,759·7 (-647·6 to 4,071·8)	1·2% (-0·4 to 2·8%)	1,004·3 (-1,092·5 to 2,983·0)	0·6% (-0·7 to 1·8%)	-42·9% (-285·7 to 181·1%)	-48·6% (-267·8 to 152·6%)
Ischaemic stroke	13·2 (-7·5 to 33·7)	0·5% (-0·3 to 1·2%)	8·2 (-10·6 to 26·8)	0·3% (-0·4 to 0·9%)	-38·1% (-206·5 to 204·3%)	-42·5% (-198·8 to 176·0%)	516·7 (210·5 to 813·8)	1·2% (0·5 to 1·8%)	454·9 (212·9 to 691·2)	1·0% (0·5 to 1·5%)	-12·0% (-21·3 to 10·0%)	-14·1% (-22·8 to 5·3%)
Hemorrhagic stroke	236·9 (213·0 to 261·5)	7·3% (6·5 to 8·0%)	248·2 (214·0 to 280·3)	7·4% (6·4 to 8·3%)	4·7% (-2·1 to 11·3%)	2·0% (-4·6 to 7·1%)	5,658·9 (5,148·0 to 6,142·8)	7·6% (6·9 to 8·2%)	5,753·9 (5,004·1 to 6,385·3)	7·8% (6·8 to 8·6%)	1·7% (-4·8 to 7·3%)	3·1% (-2·9 to 7·4%)
Hypertensive heart disease	39·6 (29·4 to 50·7)	5·2% (3·9 to 6·6%)	51·5 (34·4 to 68·0)	5·4% (3·5 to 7·0%)	30·0% (9·7 to 45·7%)	2·7% (-12·9 to 15·7%)	1,022·7 (839·7 to 1,211·1)	6·9% (5·6 to 8·1%)	1,270·8 (970·2 to 1,537·9)	7·3% (5·6 to 8·8%)	24·3% (12·0 to 33·8%)	5·5% (-4·5 to 13·2%)
Atrial fibrillation and flutter	7·5 (6·1 to 9·3)	5·3% (4·6 to 6·1%)	10·2 (7·9 to 12·8)	5·2% (4·4 to 6·1%)	35·4% (26·8 to 43·1%)	-1·0% (-7·2 to 4·4%)	236·9 (184·0 to 303·3)	6·8% (6·1 to 7·5%)	300·6 (232·5 to 391·5)	6·8% (5·9 to 7·6%)	26·9% (20·6 to 32·3%)	-0·9% (-5·9 to 3·1%)
Cirrhosis and other chronic liver diseases due to hepatitis B	81·7 (69·0 to 94·7)	23·9% (20·6 to 26·8%)	87·3 (68·7 to 104·6)	23·5% (18·9 to 27·3%)	6·9% (-3·0 to 14·9%)	-1·6% (-10·4 to 4·2%)	2,456·2 (2,076·1 to 2,832·2)	23·8% (20·6 to 26·6%)	2,525·8 (1,988·9 to 3,019·0)	23·5% (18·8 to 27·1%)	2·8% (-6·8 to 10·5%)	-1·5% (-10·4 to 4·3%)
Cirrhosis and other chronic liver diseases due to hepatitis C	66·1 (54·4 to 76·8)	23·0% (19·6 to 26·0%)	72·8 (56·5 to 87·6)	22·3% (17·8 to 26·2%)	10·1% (0·0 to 17·3%)	-2·8% (-12·0 to 3·2%)	1,894·7 (1,538·4 to 2,203·1)	22·5% (19·1 to 25·4%)	2,016·7 (1,541·9 to 2,418·5)	22·0% (17·4 to 25·8%)	6·4% (-3·5 to 13·4%)	-2·3% (-11·5 to 3·6%)
Cirrhosis and other chronic liver diseases due to alcohol use	310·1 (289·4 to 333·4)	100·0% (100·0 to 100·0%)	347·9 (322·7 to 374·6)	100·0% (100·0 to 100·0%)	12·2% (8·4 to 16·7%)	-	10,093·5 (9,424·0 to 10,841·4)	100·0% (100·0 to 100·0%)	10,997·4 (10,197·4 to 11,875·1)	100·0% (100·0 to 100·0%)	9·0% (5·0 to 13·7%)	-
Cirrhosis and other chronic liver diseases due to other causes	47·8 (40·9 to 54·8)	20·5% (17·9 to 23·1%)	51·6 (41·7 to 61·1)	20·8% (17·2 to 24·2%)	7·9% (-0·5 to 14·8%)	1·5% (-6·4 to 6·9%)	1,399·2 (1,197·2 to 1,586·6)	16·9% (14·6 to 18·9%)	1,429·9 (1,156·6 to 1,680·8)	17·7% (14·6 to 20·5%)	2·2% (-6·1 to 8·9%)	5·3% (-3·1 to 11·0%)
Pancreatitis	16·4 (14·7 to 18·0)	15·0% (13·4 to 16·5%)	18·6 (15·8 to 21·0)	14·0% (12·1 to 15·6%)	13·3% (4·3 to 22·0%)	-6·4% (-13·1 to -0·6%)	633·0 (567·4 to 691·1)	16·8% (15·1 to 18·4%)	694·5 (600·3 to 778·2)	15·9% (14·0 to 17·5%)	9·7% (1·4 to 18·1%)	-5·5% (-11·8 to 0·1%)

Supplemental Results Table 1. All-age deaths, death PAFs, DALYs, DALY PAFs, and percent change for each risk factor and outcome for both sexes combined in 2005 and 2015 for Global.

Data in parenthesis are 95% uncertainty intervals. PAFs=population attributable fractions. DALYs=disability-adjusted life-years.

	2005 Deaths (in thousands)	2005 Deaths PAF	2015 Deaths (in thousands)	2015 Deaths PAF	Percent change deaths 2005-2015	Percent change deaths PAF 2005-2015	2005 DALYs (in thousands)	2005 DALYs PAF	2015 DALYs (in thousands)	2015 DALYs PAF	Percent change DALYs 2005-2015	Percent change DALYs PAF 2005-2015
Epilepsy	12·4 (11·4 to 13·3)	10·4% (9·5 to 11·2%)	13·7 (12·1 to 15·4)	11·0% (9·7 to 12·1%)	10·9% (2·9 to 19·7%)	5·5% (-0·9 to 11·7%)	1,038·2 (866·0 to 1,219·2)	8·1% (7·4 to 8·7%)	1,039·4 (858·8 to 1,239·0)	8·4% (7·3 to 9·3%)	0·1% (-6·5 to 6·5%)	3·9% (-2·1 to 9·1%)
Alcohol use disorders	157·4 (147·4 to 163·3)	100·0% (100·0 to 100·0%)	137·5 (131·5 to 144·0)	100·0% (100·0 to 100·0%)	-12·6% (-16·7 to -7·0%)	-	11,566·7 (9,617·6 to 13,834·9)	100·0% (100·0 to 100·0%)	11,194·3 (9,136·5 to 13,870·9)	100·0% (100·0 to 100·0%)	-3·2% (-7·0 to 0·6%)	-
Diabetes mellitus	-57·9 (-62·7 to -52·9)	-5·0% (-5·5 to -4·6%)	-64·0 (-70·0 to -58·0)	-4·2% (-4·7 to -3·8%)	10·5% (0·8 to 19·7%)	-16·3% (-23·5 to -8·5%)	-2,323·6 (-2,840·3 to -1,865·5)	-4·7% (-5·0 to -4·4%)	-2,684·6 (-3,321·0 to -2,142·0)	-4·2% (-4·5 to -3·9%)	15·5% (10·4 to 20·0%)	-10·5% (-14·0 to -7·2%)
Pedestrian road injuries	106·7 (88·6 to 127·3)	18·3% (15·6 to 21·5%)	100·2 (83·7 to 118·9)	17·9% (15·2 to 20·8%)	-6·1% (-13·2 to 0·0%)	-2·6% (-5·3 to -0·1%)	5,359·8 (4,474·5 to 6,380·2)	19·7% (16·7 to 23·1%)	4,805·9 (4,011·8 to 5,677·8)	19·6% (16·8 to 22·9%)	-10·3% (-16·8 to -4·6%)	-0·5% (-3·3 to 2·2%)
Cyclist road injuries	12·5 (10·3 to 15·0)	19·7% (16·7 to 23·1%)	11·1 (9·3 to 13·3)	18·9% (16·1 to 22·1%)	-11·4% (-19·4 to -3·5%)	-4·2% (-7·6 to -1·1%)	701·2 (578·2 to 838·0)	18·9% (15·9 to 22·1%)	607·3 (510·7 to 719·7)	17·9% (15·2 to 20·9%)	-13·4% (-19·8 to -6·7%)	-5·2% (-8·5 to -2·0%)
Motorcyclist road injuries	77·4 (64·5 to 88·3)	31·5% (28·0 to 35·2%)	80·7 (68·7 to 93·4)	31·4% (28·1 to 34·7%)	4·3% (-5·1 to 16·4%)	-0·3% (-3·5 to 3·3%)	4,298·9 (3,632·4 to 4,895·0)	31·5% (28·0 to 35·2%)	4,323·6 (3,712·5 to 5,002·5)	31·6% (28·4 to 34·8%)	0·6% (-7·4 to 11·6%)	0·4% (-2·9 to 3·8%)
Motor vehicle road injuries	147·5 (130·5 to 167·3)	30·5% (27·9 to 33·5%)	134·6 (119·6 to 152·0)	29·0% (26·5 to 31·8%)	-8·8% (-14·7 to -2·9%)	-5·0% (-7·0 to -2·8%)	7,916·7 (7,015·3 to 8,956·3)	30·2% (27·5 to 33·1%)	7,067·2 (6,301·3 to 7,926·6)	28·9% (26·4 to 31·6%)	-10·7% (-15·9 to -5·6%)	-4·2% (-6·3 to -1·8%)
Falls	42·4 (35·8 to 50·5)	9·7% (8·3 to 11·6%)	50·0 (40·9 to 59·9)	9·5% (8·0 to 11·3%)	17·8% (8·7 to 24·6%)	-2·6% (-6·4 to 0·8%)	2,415·6 (1,951·3 to 2,967·5)	10·1% (8·8 to 11·9%)	2,602·3 (2,096·9 to 3,230·0)	10·0% (8·7 to 11·7%)	7·7% (2·2 to 12·2%)	-1·3% (-4·2 to 2·0%)
Drowning	27·1 (23·5 to 31·7)	6·7% (5·7 to 8·2%)	23·5 (20·6 to 27·6)	7·3% (6·3 to 8·8%)	-13·2% (-17·0 to -9·1%)	8·1% (1·7 to 13·7%)	1,224·6 (1,056·6 to 1,435·0)	5·1% (4·3 to 6·2%)	1,000·5 (871·0 to 1,168·3)	5·6% (4·8 to 6·8%)	-18·3% (-22·1 to -14·4%)	10·6% (2·4 to 18·0%)
Fire, heat, and hot substances	13·9 (11·8 to 16·5)	7·1% (6·1 to 8·5%)	12·5 (10·4 to 15·0)	7·1% (6·0 to 8·5%)	-10·1% (-16·6 to -1·9%)	-0·3% (-7·7 to 5·3%)	694·1 (585·8 to 831·9)	5·9% (5·1 to 7·1%)	619·1 (510·5 to 755·4)	6·0% (5·2 to 7·1%)	-10·8% (-16·2 to -4·5%)	1·8% (-5·9 to 7·6%)
Poisonings	8·6 (6·6 to 10·6)	8·6% (7·0 to 11·1%)	7·2 (5·4 to 8·8)	8·4% (6·8 to 11·0%)	-15·8% (-21·5 to -8·5%)	-1·6% (-13·8 to 12·0%)	386·5 (302·9 to 478·3)	6·8% (5·3 to 9·1%)	319·3 (246·4 to 388·1)	6·7% (5·1 to 9·1%)	-17·4% (-22·1 to -11·0%)	-2·2% (-19·5 to 17·9%)
Unintentional firearm injuries	3·0 (2·3 to 3·6)	9·3% (8·0 to 10·9%)	2·8 (2·1 to 3·3)	8·7% (7·5 to 10·2%)	-8·0% (-13·1 to -2·2%)	-6·0% (-9·3 to -2·0%)	145·0 (111·7 to 174·2)	8·8% (7·5 to 10·3%)	129·4 (99·0 to 154·1)	8·4% (7·3 to 9·8%)	-10·7% (-15·7 to -5·1%)	-4·4% (-8·4 to 0·4%)
Unintentional suffocation	1·2 (1·0 to 1·5)	3·4% (2·8 to 4·2%)	1·7 (1·2 to 2·1)	4·8% (4·0 to 5·7%)	42·5% (16·3 to 57·0%)	39·3% (19·0 to 57·7%)	68·2 (55·1 to 83·6)	2·6% (2·1 to 3·3%)	87·9 (67·6 to 108·2)	3·5% (2·9 to 4·3%)	28·9% (13·2 to 38·9%)	36·5% (18·0 to 55·9%)
Other exposure to mechanical forces	14·5 (11·1 to 17·5)	10·7% (9·2 to 12·7%)	14·5 (9·9 to 18·2)	10·9% (9·3 to 12·9%)	0·5% (-15·2 to 12·4%)	1·8% (-3·3 to 6·0%)	913·3 (724·5 to 1,126·6)	9·7% (8·3 to 11·4%)	905·1 (684·4 to 1,139·7)	10·0% (8·6 to 11·7%)	-0·9% (-10·2 to 6·8%)	3·1% (-0·3 to 6·7%)
Self-harm	109·9 (94·7 to 128·8)	13·3% (11·5 to 15·7%)	111·3 (96·2 to 130·2)	13·5% (11·7 to 15·7%)	1·4% (-4·3 to 6·2%)	1·3% (-3·1 to 4·5%)	4,648·7 (4,032·0 to 5,435·6)	13·0% (11·3 to 15·3%)	4,552·3 (3,953·3 to 5,302·7)	13·3% (11·6 to 15·5%)	-2·1% (-7·5 to 2·7%)	2·3% (-2·2 to 5·8%)
Assault by firearm	23·1 (20·5 to 26·1)	14·2% (12·9 to 15·9%)	23·0 (20·2 to 26·2)	13·3% (12·0 to 14·9%)	-0·6% (-4·2 to 4·2%)	-6·5% (-8·1 to -4·7%)	1,312·8 (1,158·5 to 1,480·5)	14·8% (13·4 to 16·6%)	1,284·6 (1,127·7 to 1,458·9)	14·0% (12·7 to 15·7%)	-2·2% (-5·7 to 2·5%)	-5·7% (-7·2 to -3·9%)
Assault by sharp object	15·5 (13·8 to 17·8)	14·9% (13·3 to 17·0%)	12·5 (11·0 to 14·4)	13·9% (12·5 to 15·8%)	-19·7% (-24·0 to -14·5%)	-6·3% (-9·0 to -3·9%)	840·1 (744·5 to 967·7)	15·5% (13·8 to 17·7%)	667·8 (590·3 to 769·3)	14·7% (13·2 to 16·7%)	-20·5% (-24·7 to -15·2%)	-5·1% (-7·9 to -2·6%)
Assault by other means	18·5 (16·2 to 21·6)	11·7% (10·3 to 13·5%)	15·9 (13·7 to 18·9)	10·9% (9·7 to 12·5%)	-13·9% (-18·4 to -7·3%)	-6·8% (-9·5 to -3·8%)	1,006·6 (880·1 to 1,180·7)	11·8% (10·4 to 13·6%)	868·3 (745·1 to 1,021·7)	11·2% (9·9 to 12·9%)	-13·7% (-18·2 to -7·0%)	-4·9% (-7·8 to -1·6%)
**Drug use: All causes**	406·6 (364·1 to 447·8)	0·8% (0·7 to 0·8%)	488·8 (439·2 to 537·3)	0·9% (0·8 to 1·0%)	20·2% (15·9 to 25·0%)	15·5% (11·8 to 19·7%)	24,036·1 (21,317·1 to 26,732·2)	0·9% (0·8 to 1·0%)	27,831·0 (24,436·9 to 31,170·9)	1·1% (1·0 to 1·2%)	15·8% (12·6 to 18·8%)	20·0% (16·7 to 23·4%)
HIV/AIDS - Tuberculosis	11·4 (9·3 to 14·1)	3·2% (2·7 to 3·8%)	8·5 (6·7 to 10·5)	4·0% (3·3 to 4·9%)	-25·7% (-33·4 to -15·9%)	23·6% (11·5 to 38·5%)	566·6 (464·5 to 697·8)	2·9% (2·5 to 3·5%)	413·3 (332·7 to 512·8)	3·6% (3·0 to 4·3%)	-27·1% (-33·9 to -18·2%)	21·4% (10·0 to 35·7%)
HIV/AIDS resulting in other diseases	53·4 (46·1 to 65·4)	3·7% (3·2 to 4·4%)	51·5 (43·9 to 63·3)	5·3% (4·5 to 6·3%)	-3·6% (-12·0 to 7·2%)	41·6% (31·0 to 53·5%)	2,746·2 (2,380·2 to 3,316·8)	3·4% (3·0 to 4·1%)	2,593·7 (2,215·6 to 3,176·4)	4·7% (4·1 to 5·6%)	-5·6% (-13·6 to 4·0%)	36·5% (27·1 to 47·1%)
Hepatitis B	0·5 (0·2 to 0·8)	0·7% (0·3 to 1·1%)	0·4 (0·2 to 0·6)	0·6% (0·2 to 0·9%)	-27·7% (-35·2 to -18·4%)	-21·1% (-28·9 to -10·9%)	18·4 (7·8 to 30·1)	0·7% (0·3 to 1·1%)	13·2 (5·9 to 21·1)	0·5% (0·2 to 0·9%)	-28·0% (-35·8 to -18·8%)	-20·8% (-29·1 to -11·2%)
Hepatitis C	1·0 (0·2 to 2·4)	36·7% (28·4 to 44·4%)	0·9 (0·2 to 2·1)	35·8% (27·6 to 43·9%)	-12·0% (-30·1 to 11·0%)	-2·5% (-13·6 to 9·7%)	37·1 (8·8 to 83·3)	37·5% (29·9 to 45·0%)	32·5 (8·2 to 71·7)	36·3% (28·2 to 44·4%)	-12·3% (-30·1 to 10·0%)	-3·1% (-13·5 to 7·7%)
Liver cancer due to hepatitis B	3·1 (1·3 to 5·1)	1·2% (0·5 to 1·9%)	3·5 (1·5 to 5·7)	1·3% (0·6 to 2·1%)	15·2% (1·3 to 38·4%)	14·3% (4·4 to 28·6%)	103·5 (43·1 to 170·6)	1·2% (0·5 to 2·0%)	109·9 (48·7 to 178·7)	1·4% (0·6 to 2·2%)	6·2% (-7·1 to 29·1%)	11·4% (1·7 to 24·6%)
Liver cancer due to hepatitis C	53·5 (38·3 to 68·2)	38·8% (28·2 to 49·1%)	74·3 (56·9 to 91·3)	44·5% (34·4 to 53·8%)	39·0% (28·8 to 54·2%)	14·6% (7·1 to 26·4%)	1,316·2 (997·8 to 1,627·3)	45·4% (35·1 to 55·3%)	1,677·6 (1,328·5 to 2,012·1)	50·5% (40·7 to 59·4%)	27·5% (18·2 to 41·4%)	11·1% (4·4 to 21·3%)
Cirrhosis and other chronic liver diseases due to hepatitis B	2·7 (1·1 to 4·6)	0·8% (0·3 to 1·4%)	3·3 (1·4 to 5·4)	0·9% (0·4 to 1·5%)	20·9% (10·3 to 35·7%)	11·2% (2·1 to 24·1%)	90·0 (37·7 to 149·0)	0·9% (0·4 to 1·5%)	100·8 (43·4 to 162·4)	0·9% (0·4 to 1·5%)	12·1% (2·3 to 24·9%)	7·3% (-1·2 to 18·4%)
Cirrhosis and other chronic liver diseases due to hepatitis C	123·8 (98·0 to 147·5)	43·1% (34·4 to 50·8%)	147·3 (118·3 to 175·3)	45·2% (37·0 to 52·8%)	19·0% (12·9 to 27·0%)	5·1% (0·9 to 11·1%)	4,063·6 (3,320·5 to 4,749·7)	48·3% (39·7 to 55·8%)	4,555·9 (3,726·9 to 5,373·9)	49·7% (41·4 to 57·5%)	12·1% (6·4 to 19·0%)	2·9% (-0·8 to 8·0%)
Opioid use disorders	94·2 (90·5 to 99·7)	100·0% (100·0 to 100·0%)	122·1 (109·5 to 129·7)	100·0% (100·0 to 100·0%)	29·6% (18·2 to 37·2%)	-	9,864·0 (8,127·4 to 11,516·8)	100·0% (100·0 to 100·0%)	12,068·1 (9,878·0 to 14,145·1)	100·0% (100·0 to 100·0%)	22·3% (17·5 to 26·1%)	-
Cocaine use disorders	7·4 (5·0 to 7·9)	100·0% (100·0 to 100·0%)	11·1 (8·5 to 12·2)	100·0% (100·0 to 100·0%)	49·7% (33·6 to 75·4%)	-	729·3 (558·4 to 902·4)	100·0% (100·0 to 100·0%)	999·3 (773·5 to 1,233·9)	100·0% (100·0 to 100·0%)	37·0% (29·2 to 47·0%)	-
Amphetamine use disorders	7·3 (4·3 to 8·2)	100·0% (100·0 to 100·0%)	12·2 (8·4 to 14·2)	100·0% (100·0 to 100·0%)	67·5% (25·6 to 118·9%)	-	1,001·3 (706·2 to 1,348·3)	100·0% (100·0 to 100·0%)	1,402·6 (1,025·2 to 1,846·9)	100·0% (100·0 to 100·0%)	40·1% (26·1 to 55·2%)	-
Cannabis use disorders	-	-	-	-	-	-	548·1 (351·9 to 780·7)	100·0% (100·0 to 100·0%)	577·2 (371·8 to 817·6)	100·0% (100·0 to 100·0%)	5·3% (3·7 to 7·1%)	-
Other drug use disorders	19·9 (18·6 to 22·8)	100·0% (100·0 to 100·0%)	24·5 (22·7 to 27·3)	100·0% (100·0 to 100·0%)	23·0% (12·7 to 32·1%)	-	1,528·7 (1,245·5 to 1,861·2)	100·0% (100·0 to 100·0%)	1,862·2 (1,501·6 to 2,274·2)	100·0% (100·0 to 100·0%)	21·8% (15·7 to 27·5%)	-
Self-harm	28·4 (19·3 to 40·5)	3·4% (2·3 to 4·9%)	29·2 (19·7 to 41·4)	3·5% (2·4 to 5·0%)	2·6% (-3·0 to 8·9%)	2·6% (-1·5 to 6·5%)	1,423·2 (950·7 to 2,030·4)	4·0% (2·7 to 5·7%)	1,424·5 (954·9 to 2,037·3)	4·2% (2·8 to 5·9%)	0·1% (-5·4 to 6·3%)	4·6% (0·5 to 8·8%)
**Dietary risks: All causes**	10,737·7 (9,472·6 to 12,010·4)	20·0% (17·6 to 22·4%)	12,058·1 (10,615·0 to 13,538·4)	21·6% (19·0 to 24·2%)	12·3% (10·0 to 14·5%)	7·9% (6·5 to 9·3%)	242,780·7 (217,377·6 to 269,658·9)	9·5% (8·4 to 10·6%)	264,411·4 (236,098·3 to 294,989·0)	10·7% (9·5 to 12·1%)	8·9% (6·3 to 11·3%)	12·9% (10·4 to 15·2%)
**Diet low in fruits: All causes**	2,713·1 (1,806·1 to 3,703·3)	5·1% (3·4 to 6·9%)	2,924·2 (1,904·6 to 4,018·3)	5·2% (3·4 to 7·2%)	7·8% (4·3 to 10·9%)	3·6% (0·8 to 6·0%)	68,838·0 (46,615·8 to 92,835·2)	2·7% (1·8 to 3·7%)	72,590·5 (48,514·2 to 98,667·0)	2·9% (2·0 to 4·0%)	5·5% (1·7 to 8·6%)	9·3% (5·7 to 12·4%)
Lip and oral cavity cancer	7·7 (0·0 to 16·8)	7·0% (0·0 to 15·2%)	10·3 (0·0 to 22·4)	7·0% (0·0 to 15·3%)	32·7% (0·0 to 37·5%)	0·1% (-0·6 to 0·8%)	208·0 (0·0 to 450·8)	7·0% (0·0 to 15·2%)	266·5 (0·0 to 582·9)	7·0% (0·0 to 15·3%)	28·1% (0·0 to 33·1%)	0·1% (-0·7 to 0·8%)
Nasopharynx cancer	4·2 (0·0 to 8·8)	7·5% (0·0 to 15·8%)	4·6 (0·0 to 9·8)	7·4% (0·0 to 15·7%)	11·3% (0·0 to 18·2%)	-1·4% (-2·6 to 0·0%)	132·4 (0·0 to 280·9)	7·3% (0·0 to 15·5%)	138·4 (0·0 to 293·4)	7·2% (0·0 to 15·4%)	4·5% (-3·7 to 11·1%)	-1·3% (-2·7 to 0·0%)
Other pharynx cancer	3·6 (0·0 to 8·1)	7·0% (0·0 to 15·6%)	4·5 (0·0 to 10·0)	7·0% (0·0 to 15·6%)	23·4% (0·0 to 29·4%)	-0·5% (-1·3 to 0·2%)	99·3 (0·0 to 221·3)	7·0% (0·0 to 15·6%)	119·4 (0·0 to 265·9)	7·0% (0·0 to 15·6%)	20·2% (0·0 to 26·2%)	-0·4% (-1·4 to 0·3%)
Oesophageal cancer	105·8 (26·4 to 187·2)	23·0% (5·8 to 40·5%)	99·1 (24·2 to 174·0)	22·6% (5·6 to 39·8%)	-6·4% (-11·3 to -0·8%)	-2·1% (-3·4 to -1·2%)	2,465·6 (617·6 to 4,361·5)	23·1% (5·8 to 40·5%)	2,221·3 (542·1 to 3,910·6)	22·5% (5·6 to 39·7%)	-9·9% (-15·1 to -4·0%)	-2·5% (-4·0 to -1·5%)
Larynx cancer	6·6 (0·0 to 14·7)	7·1% (0·0 to 15·8%)	7·5 (0·0 to 16·7)	7·1% (0·0 to 15·8%)	13·4% (0·0 to 17·4%)	-0·3% (-1·1 to 0·3%)	167·7 (0·0 to 373·9)	7·1% (0·0 to 15·9%)	184·2 (0·0 to 413·4)	7·1% (0·0 to 15·8%)	9·8% (0·0 to 13·8%)	-0·5% (-1·4 to 0·2%)
Tracheal, bronchus, and lung cancer	172·3 (70·1 to 291·3)	12·0% (4·9 to 20·3%)	206·9 (83·7 to 351·0)	12·0% (4·8 to 20·3%)	20·1% (16·5 to 24·4%)	0·0% (-0·7 to 0·6%)	3,858·3 (1,577·4 to 6,494·7)	12·1% (4·9 to 20·5%)	4,391·5 (1,779·7 to 7,444·5)	12·1% (4·8 to 20·3%)	13·8% (9·9 to 18·7%)	-0·6% (-1·6 to 0·0%)
Ischaemic heart disease	946·8 (339·7 to 1,585·0)	12·4% (4·4 to 20·8%)	1,086·2 (388·2 to 1,817·5)	12·2% (4·4 to 20·4%)	14·7% (12·1 to 17·0%)	-1·6% (-2·3 to -1·0%)	21,684·8 (7,920·8 to 35,741·1)	14·7% (5·4 to 24·3%)	23,777·1 (8,597·9 to 39,450·8)	14·5% (5·3 to 24·0%)	9·6% (6·3 to 12·4%)	-1·2% (-2·1 to -0·5%)
Ischaemic stroke	509·4 (270·8 to 775·7)	18·5% (9·8 to 28·2%)	521·2 (274·3 to 805·1)	17·5% (9·3 to 26·8%)	2·3% (-1·1 to 5·2%)	-5·1% (-6·4 to -4·2%)	10,961·1 (5,936·9 to 16,257·5)	24·8% (13·7 to 36·9%)	11,001·1 (5,940·5 to 16,513·1)	24·3% (13·4 to 36·2%)	0·4% (-3·1 to 3·5%)	-2·1% (-3·3 to -1·2%)

Supplemental Results Table 1. All-age deaths, death PAFs, DALYs, DALY PAFs, and percent change for each risk factor and outcome for both sexes combined in 2005 and 2015 for Global.

Data in parenthesis are 95% uncertainty intervals. PAFs=population attributable fractions. DALYs=disability-adjusted life-years.

	2005 Deaths (in thousands)	2005 Deaths PAF	2015 Deaths (in thousands)	2015 Deaths PAF	Percent change deaths 2005-2015	Percent change deaths PAF 2005-2015	2005 DALYs (in thousands)	2005 DALYs PAF	2015 DALYs (in thousands)	2015 DALYs PAF	Percent change DALYs 2005-2015	Percent change DALYs PAF 2005-2015
Hemorrhagic stroke	838·1 (465·2 to 1,238·9)	25·7% (14·1 to 37·9%)	830·8 (458·2 to 1,241·4)	24·8% (13·6 to 36·8%)	-0·9% (-4·5 to 2·9%)	-3·5% (-4·7 to -2·6%)	22,973·3 (13,193·6 to 33,251·6)	30·9% (17·4 to 44·6%)	22,476·0 (12,752·3 to 32,681·8)	30·6% (17·2 to 44·4%)	-2·2% (-5·8 to 1·5%)	-0·8% (-1·9 to 0·0%)
**Diet low in vegetables: All causes**	1,842·4 (943·1 to 2,868·8)	3·4% (1·8 to 5·4%)	1,994·3 (1,024·4 to 3,120·5)	3·6% (1·8 to 5·6%)	8·2% (4·5 to 11·2%)	4·0% (0·9 to 6·4%)	42,617·5 (22,635·5 to 65,748·9)	1·7% (0·9 to 2·6%)	44,632·2 (23,572·0 to 68,816·8)	1·8% (1·0 to 2·8%)	4·7% (1·5 to 7·9%)	8·5% (5·0 to 11·6%)
Ischaemic heart disease	1,261·3 (488·8 to 2,098·6)	16·5% (6·4 to 27·4%)	1,418·5 (548·2 to 2,363·5)	15·9% (6·1 to 26·6%)	12·5% (10·3 to 14·7%)	-3·5% (-4·4 to -2·8%)	27,791·3 (11,029·0 to 45,849·1)	18·8% (7·4 to 30·9%)	30,047·6 (11,828·0 to 49,330·8)	18·3% (7·2 to 30·1%)	8·1% (5·2 to 10·9%)	-2·6% (-3·6 to -1·8%)
Ischaemic stroke	226·1 (51·0 to 411·7)	8·2% (1·9 to 15·0%)	224·3 (50·7 to 411·9)	7·5% (1·7 to 13·8%)	-0·8% (-3·8 to 2·0%)	-8·0% (-9·5 to -6·8%)	4,869·1 (1,119·6 to 8,768·3)	11·0% (2·6 to 20·0%)	4,761·8 (1,097·1 to 8,586·1)	10·5% (2·4 to 19·0%)	-2·2% (-5·8 to 0·9%)	-4·6% (-6·0 to -3·3%)
Hemorrhagic stroke	355·1 (99·4 to 666·0)	10·9% (3·0 to 20·3%)	351·5 (99·1 to 653·5)	10·5% (3·0 to 19·6%)	-1·0% (-4·1 to 2·5%)	-3·6% (-5·0 to -2·1%)	9,957·1 (2,798·9 to 18,328·5)	13·4% (3·8 to 24·6%)	9,822·8 (2,800·3 to 18,053·2)	13·4% (3·8 to 24·5%)	-1·3% (-4·6 to 2·3%)	0·1% (-1·3 to 1·7%)
**Diet low in whole grains: All causes**	2,879·5 (2,056·3 to 3,817·6)	5·4% (3·8 to 7·1%)	3,142·2 (2,215·3 to 4,188·6)	5·6% (4·0 to 7·5%)	9·1% (6·3 to 11·8%)	4·9% (2·7 to 6·7%)	74,523·1 (54,212·6 to 96,519·2)	2·9% (2·1 to 3·8%)	79,812·9 (57,570·7 to 104,238·4)	3·2% (2·3 to 4·3%)	7·1% (3·7 to 9·8%)	11·0% (8·0 to 13·6%)
Ischaemic heart disease	1,496·7 (888·5 to 2,167·9)	19·6% (11·7 to 28·4%)	1,712·6 (1,013·1 to 2,481·0)	19·2% (11·4 to 27·9%)	14·4% (12·0 to 16·6%)	-1·8% (-2·5 to -1·4%)	33,870·5 (20,348·9 to 48,312·0)	22·9% (13·8 to 32·9%)	36,932·6 (21,938·0 to 53,267·2)	22·5% (13·5 to 32·4%)	9·0% (5·7 to 11·6%)	-1·8% (-2·6 to -1·2%)
Ischaemic stroke	459·3 (311·2 to 619·8)	16·6% (11·3 to 22·5%)	463·4 (310·8 to 631·3)	15·6% (10·5 to 21·2%)	0·9% (-2·5 to 3·8%)	-6·5% (-7·7 to -5·4%)	10,194·9 (6,993·4 to 13,536·0)	23·1% (16·1 to 30·7%)	10,142·8 (6,848·7 to 13,504·9)	22·4% (15·5 to 29·9%)	-0·5% (-4·0 to 2·7%)	-2·9% (-4·1 to -2·0%)
Hemorrhagic stroke	728·5 (514·7 to 981·7)	22·3% (15·8 to 29·9%)	715·7 (497·4 to 962·1)	21·4% (15·0 to 28·8%)	-1·8% (-5·4 to 2·2%)	-4·3% (-5·5 to -3·4%)	20,023·6 (14,325·8 to 26,601·1)	26·9% (19·2 to 35·6%)	19,442·5 (13,707·5 to 25,849·5)	26·5% (18·8 to 35·2%)	-2·9% (-6·5 to 0·9%)	-1·5% (-2·6 to -0·7%)
Diabetes mellitus	194·9 (105·1 to 301·3)	16·9% (9·2 to 26·1%)	250·5 (135·2 to 387·5)	16·5% (8·9 to 25·5%)	28·5% (23·6 to 33·3%)	-2·7% (-3·6 to -1·9%)	10,434·2 (5,659·1 to 16,437·8)	21·0% (11·6 to 31·7%)	13,295·0 (7,183·0 to 21,043·4)	20·7% (11·5 to 31·4%)	27·4% (24·4 to 30·3%)	-1·2% (-1·9 to -0·7%)
**Diet low in nuts and seeds: All causes**	1,829·8 (1,159·5 to 2,582·5)	3·4% (2·2 to 4·8%)	2,130·6 (1,353·8 to 3,011·1)	3·8% (2·4 to 5·4%)	16·4% (14·2 to 18·6%)	11·9% (10·2 to 13·5%)	43,724·6 (29,136·3 to 60,125·3)	1·7% (1·1 to 2·4%)	49,411·2 (32,715·9 to 68,196·4)	2·0% (1·3 to 2·8%)	13·0% (9·8 to 15·9%)	17·1% (14·1 to 19·8%)
Ischaemic heart disease	1,706·3 (1,062·4 to 2,437·7)	22·3% (13·9 to 31·9%)	1,970·2 (1,225·8 to 2,810·2)	22·1% (13·8 to 31·6%)	15·5% (13·2 to 17·6%)	-1·0% (-1·4 to -0·7%)	37,340·4 (23,613·0 to 52,283·3)	25·3% (16·0 to 35·4%)	41,199·5 (26,174·9 to 57,705·6)	25·1% (15·9 to 35·0%)	10·3% (7·3 to 12·9%)	-0·6% (-1·2 to -0·1%)
Diabetes mellitus	123·5 (64·1 to 193·3)	10·7% (5·6 to 16·9%)	160·5 (83·3 to 252·6)	10·6% (5·5 to 16·6%)	29·9% (25·2 to 34·6%)	-1·6% (-2·2 to -1·0%)	6,384·2 (3,038·4 to 10,211·0)	12·8% (6·7 to 20·0%)	8,211·7 (3,923·3 to 13,199·6)	12·8% (6·7 to 20·0%)	28·6% (25·6 to 31·5%)	-0·3% (-0·6 to 0·1%)
**Diet low in milk: All causes**	100·9 (34·8 to 170·7)	0·2% (0·1 to 0·3%)	125·8 (43·5 to 211·5)	0·2% (0·1 to 0·4%)	24·7% (22·0 to 27·7%)	19·8% (17·5 to 22·0%)	2,176·7 (750·1 to 3,688·1)	0·1% (0·0 to 0·1%)	2,602·6 (897·4 to 4,382·0)	0·1% (0·0 to 0·2%)	19·6% (16·8 to 22·6%)	23·9% (20·9 to 27·1%)
Colon and rectum cancer	100·9 (34·8 to 170·7)	14·9% (5·2 to 25·3%)	125·8 (43·5 to 211·5)	15·1% (5·3 to 25·5%)	24·7% (22·0 to 27·7%)	1·2% (0·8 to 1·8%)	2,176·7 (750·1 to 3,688·1)	15·1% (5·3 to 25·5%)	2,602·6 (897·4 to 4,382·0)	15·3% (5·3 to 25·7%)	19·6% (16·8 to 22·6%)	1·2% (0·8 to 1·7%)
**Diet high in red meat: All causes**	37·2 (16·4 to 59·6)	0·1% (0·0 to 0·1%)	42·7 (18·8 to 69·2)	0·1% (0·0 to 0·1%)	14·8% (11·8 to 18·0%)	10·4% (7·6 to 13·1%)	1,475·6 (549·2 to 2,473·1)	0·1% (0·0 to 0·1%)	1,751·6 (638·2 to 2,957·1)	0·1% (0·0 to 0·1%)	18·7% (14·8 to 21·7%)	23·0% (19·4 to 26·1%)
Colon and rectum cancer	20·2 (4·3 to 35·5)	3·0% (0·6 to 5·3%)	23·7 (5·0 to 41·8)	2·8% (0·6 to 5·0%)	17·1% (13·8 to 20·2%)	-5·0% (-6·7 to -3·1%)	441·7 (93·3 to 777·6)	3·1% (0·7 to 5·5%)	504·4 (107·5 to 904·4)	3·0% (0·6 to 5·3%)	14·2% (10·8 to 17·5%)	-3·4% (-5·2 to -1·3%)
Diabetes mellitus	17·0 (2·1 to 31·3)	1·5% (0·2 to 2·7%)	19·0 (2·3 to 35·2)	1·3% (0·2 to 2·3%)	12·2% (9·0 to 15·2%)	-15·0% (-17·5 to -12·7%)	1,033·9 (119·8 to 1,957·7)	2·1% (0·2 to 3·8%)	1,247·2 (145·5 to 2,359·4)	1·9% (0·2 to 3·6%)	20·6% (17·2 to 23·5%)	-6·5% (-8·6 to -4·5%)
**Diet high in processed meat: All causes**	475·9 (132·0 to 808·6)	0·9% (0·2 to 1·5%)	541·4 (156·5 to 914·2)	1·0% (0·3 to 1·6%)	13·8% (11·1 to 21·8%)	9·3% (6·9 to 16·8%)	13,088·1 (5,239·8 to 20,671·7)	0·5% (0·2 to 0·8%)	14,907·2 (6,449·9 to 23,451·7)	0·6% (0·3 to 0·9%)	13·9% (9·3 to 24·4%)	18·0% (13·6 to 28·9%)
Colon and rectum cancer	30·9 (16·9 to 46·2)	4·6% (2·5 to 6·8%)	37·3 (20·2 to 55·7)	4·5% (2·4 to 6·7%)	20·7% (17·6 to 23·6%)	-2·0% (-3·5 to -0·7%)	643·7 (349·5 to 964·3)	4·5% (2·4 to 6·7%)	745·5 (399·5 to 1,122·6)	4·4% (2·4 to 6·5%)	15·8% (12·8 to 18·7%)	-2·0% (-3·5 to -0·5%)
Ischaemic heart disease	362·8 (15·7 to 679·8)	4·7% (0·2 to 8·9%)	401·6 (17·3 to 758·7)	4·5% (0·2 to 8·5%)	10·7% (7·5 to 12·8%)	-5·0% (-6·5 to -3·6%)	7,931·1 (340·1 to 14,938·6)	5·4% (0·2 to 10·1%)	8,401·1 (356·5 to 15,858·2)	5·1% (0·2 to 9·7%)	5·9% (1·8 to 8·5%)	-4·5% (-6·2 to -2·7%)
Diabetes mellitus	82·1 (40·4 to 122·1)	7·1% (3·6 to 10·6%)	102·5 (50·3 to 153·4)	6·7% (3·4 to 10·1%)	24·8% (20·5 to 29·1%)	-5·5% (-7·1 to -4·0%)	4,513·3 (2,120·8 to 7,030·8)	9·1% (4·5 to 13·4%)	5,760·6 (2,707·5 to 8,961·1)	9·0% (4·4 to 13·4%)	27·6% (24·3 to 30·7%)	-1·0% (-2·3 to 0·2%)
**Diet high in sugar-sweetened beverages: All causes**	33·6 (23·5 to 46·9)	0·1% (0·0 to 0·1%)	38·7 (27·0 to 54·6)	0·1% (0·0 to 0·1%)	15·3% (12·5 to 18·0%)	10·8% (8·3 to 13·1%)	1,233·6 (851·9 to 1,748·0)	0·0% (0·0 to 0·1%)	1,448·6 (1,004·2 to 2,059·2)	0·1% (0·0 to 0·1%)	17·4% (14·3 to 20·3%)	21·6% (18·7 to 24·5%)
Oesophageal cancer	0·5 (0·2 to 0·9)	0·1% (0·0 to 0·2%)	0·6 (0·2 to 1·1)	0·1% (0·0 to 0·2%)	16·0% (9·5 to 21·3%)	21·4% (13·5 to 28·2%)	13·1 (3·9 to 24·1)	0·1% (0·0 to 0·2%)	14·8 (4·3 to 27·3)	0·2% (0·0 to 0·3%)	13·0% (6·3 to 18·6%)	22·3% (14·2 to 29·1%)
Colon and rectum cancer	0·6 (0·4 to 0·8)	0·1% (0·1 to 0·1%)	0·7 (0·5 to 1·0)	0·1% (0·1 to 0·1%)	19·9% (16·4 to 23·5%)	-2·6% (-5·2 to -0·1%)	14·2 (9·4 to 20·8)	0·1% (0·1 to 0·1%)	16·8 (11·2 to 24·5)	0·1% (0·1 to 0·1%)	17·8% (14·1 to 21·3%)	-0·3% (-3·3 to 2·6%)
Liver cancer due to hepatitis B	0·1 (0·1 to 0·2)	0·1% (0·0 to 0·1%)	0·2 (0·1 to 0·3)	0·1% (0·0 to 0·1%)	21·7% (13·0 to 30·4%)	20·7% (10·9 to 29·6%)	5·1 (2·2 to 8·6)	0·1% (0·0 to 0·1%)	6·0 (2·6 to 10·2)	0·1% (0·0 to 0·1%)	17·7% (8·5 to 27·3%)	23·4% (12·5 to 33·0%)
Liver cancer due to hepatitis C	0·2 (0·1 to 0·3)	0·1% (0·1 to 0·2%)	0·2 (0·1 to 0·4)	0·1% (0·1 to 0·2%)	28·9% (23·8 to 33·9%)	6·3% (2·7 to 10·3%)	3·9 (1·8 to 6·5)	0·1% (0·1 to 0·2%)	4·9 (2·3 to 8·2)	0·1% (0·1 to 0·2%)	25·3% (19·8 to 31·0%)	9·2% (4·7 to 13·9%)
Liver cancer due to alcohol use	0·2 (0·1 to 0·3)	0·1% (0·0 to 0·2%)	0·3 (0·1 to 0·4)	0·1% (0·0 to 0·2%)	30·5% (24·2 to 36·6%)	3·5% (-4·6 to 9·5%)	5·2 (2·2 to 9·0)	0·1% (0·0 to 0·2%)	6·6 (2·8 to 11·5)	0·1% (0·0 to 0·2%)	28·1% (21·0 to 35·2%)	4·1% (-5·3 to 10·8%)
Liver cancer due to other causes	0·1 (0·0 to 0·2)	0·1% (0·0 to 0·1%)	0·1 (0·1 to 0·2)	0·1% (0·0 to 0·1%)	26·6% (19·3 to 33·4%)	25·0% (16·2 to 33·2%)	2·7 (1·3 to 4·6)	0·1% (0·0 to 0·1%)	3·3 (1·5 to 5·5)	0·1% (0·0 to 0·2%)	21·3% (13·4 to 28·3%)	28·6% (18·6 to 37·6%)
Gallbladder and biliary tract cancer	0·2 (0·1 to 0·3)	0·1% (0·1 to 0·2%)	0·2 (0·1 to 0·3)	0·1% (0·1 to 0·2%)	8·2% (-0·3 to 14·6%)	-4·2% (-8·3 to -1·1%)	3·9 (2·3 to 6·1)	0·2% (0·1 to 0·2%)	4·1 (2·4 to 6·4)	0·2% (0·1 to 0·2%)	4·9% (-2·7 to 11·0%)	-1·8% (-6·4 to 1·9%)
Pancreatic cancer	0·2 (0·1 to 0·3)	0·1% (0·0 to 0·1%)	0·2 (0·1 to 0·4)	0·1% (0·0 to 0·1%)	25·2% (22·0 to 28·8%)	-4·3% (-6·6 to -2·0%)	4·3 (1·6 to 8·0)	0·1% (0·0 to 0·1%)	5·3 (1·9 to 9·7)	0·1% (0·0 to 0·1%)	22·0% (18·7 to 25·9%)	-3·3% (-5·9 to -0·3%)
Breast cancer	0·3 (0·2 to 0·4)	0·1% (0·0 to 0·1%)	0·3 (0·2 to 0·5)	0·1% (0·0 to 0·1%)	25·6% (18·1 to 33·7%)	3·5% (-1·0 to 8·8%)	7·6 (4·9 to 11·1)	0·1% (0·0 to 0·1%)	9·6 (6·3 to 14·1)	0·1% (0·0 to 0·1%)	26·6% (17·5 to 36·1%)	6·3% (1·0 to 12·3%)
Uterine cancer	0·2 (0·2 to 0·3)	0·3% (0·2 to 0·4%)	0·3 (0·2 to 0·4)	0·3% (0·2 to 0·4%)	18·5% (11·9 to 25·7%)	7·7% (3·4 to 12·0%)	6·5 (4·4 to 9·1)	0·3% (0·2 to 0·4%)	7·7 (5·1 to 11·0)	0·3% (0·2 to 0·5%)	19·1% (11·7 to 26·8%)	10·9% (5·5 to 15·6%)
Ovarian cancer	0·0 (0·0 to 0·1)	0·0% (0·0 to 0·1%)	0·0 (0·0 to 0·1)	0·0% (0·0 to 0·1%)	18·5% (0·0 to 22·9%)	-1·6% (-4·5 to 1·1%)	1·1 (0·0 to 2·3)	0·0% (0·0 to 0·1%)	1·3 (0·0 to 2·8)	0·0% (0·0 to 0·1%)	17·9% (0·0 to 22·7%)	-0·3% (-3·4 to 2·8%)
Kidney cancer	0·2 (0·1 to 0·3)	0·2% (0·1 to 0·3%)	0·3 (0·2 to 0·4)	0·2% (0·1 to 0·3%)	25·6% (21·4 to 29·9%)	-4·1% (-6·9 to -1·5%)	5·9 (3·9 to 8·8)	0·2% (0·1 to 0·3%)	7·3 (4·8 to 10·8)	0·2% (0·1 to 0·3%)	23·2% (18·9 to 27·5%)	-2·3% (-6·2 to 1·8%)
Thyroid cancer	0·0 (0·0 to 0·0)	0·1% (0·1 to 0·2%)	0·0 (0·0 to 0·1)	0·1% (0·1 to 0·2%)	25·9% (20·9 to 30·9%)	0·9% (-3·1 to 6·5%)	0·9 (0·5 to 1·4)	0·1% (0·1 to 0·2%)	1·2 (0·6 to 2·1)	0·1% (0·1 to 0·2%)	46·0% (35·5 to 55·2%)	12·0% (4·9 to 19·1%)
Ischaemic heart disease	11·9 (8·1 to 16·9)	0·2% (0·1 to 0·2%)	12·9 (8·8 to 18·3)	0·1% (0·1 to 0·2%)	8·4% (5·6 to 11·0%)	-7·0% (-9·3 to -4·9%)	325·7 (223·8 to 461·7)	0·2% (0·2 to 0·3%)	347·7 (236·5 to 494·5)	0·2% (0·1 to 0·3%)	6·7% (3·8 to 9·3%)	-3·8% (-6·2 to -1·6%)
Ischaemic stroke	2·1 (1·4 to 3·0)	0·1% (0·1 to 0·1%)	2·1 (1·4 to 3·1)	0·1% (0·0 to 0·1%)	2·7% (-1·5 to 6·7%)	-4·8% (-8·5 to -1·4%)	53·4 (37·4 to 75·4)	0·1% (0·1 to 0·2%)	55·4 (38·7 to 78·4)	0·1% (0·1 to 0·2%)	3·8% (0·3 to 7·2%)	1·3% (-1·7 to 4·4%)
Hemorrhagic stroke	5·4 (3·7 to 7·6)	0·2% (0·1 to 0·2%)	5·8 (4·0 to 8·3)	0·2% (0·1 to 0·2%)	7·5% (3·2 to 11·7%)	4·7% (0·7 to 8·2%)	186·1 (129·8 to 263·0)	0·2% (0·2 to 0·4%)	198·3 (137·1 to 279·6)	0·3% (0·2 to 0·4%)	6·6% (2·3 to 10·8%)	8·1% (4·3 to 11·6%)
Hypertensive heart disease	2·2 (1·4 to 3·4)	0·3% (0·2 to 0·4%)	2·7 (1·7 to 4·1)	0·3% (0·2 to 0·4%)	20·9% (14·5 to 26·9%)	-4·4% (-10·1 to 1·3%)	58·9 (38·6 to 85·7)	0·4% (0·3 to 0·6%)	68·4 (44·5 to 99·8)	0·4% (0·3 to 0·6%)	16·0% (10·8 to 21·0%)	-1·5% (-5·8 to 3·0%)
Cardiomyopathy and myocarditis	-	-	-	-	-	-	-	-	-	-	-	-
Atrial fibrillation and flutter	-	-	-	-	-	-	-	-	-	-	-	-
Peripheral vascular disease	-	-	-	-	-	-	-	-	-	-	-	-

Supplemental Results Table 1. All-age deaths, death PAFs, DALYs, DALY PAFs, and percent change for each risk factor and outcome for both sexes combined in 2005 and 2015 for Global.

Data in parenthesis are 95% uncertainty intervals. PAFs=population attributable fractions. DALYs=disability-adjusted life-years.

	2005 Deaths (in thousands)	2005 Deaths PAF	2015 Deaths (in thousands)	2015 Deaths PAF	Percent change deaths 2005-2015	Percent change deaths PAF 2005-2015	2005 DALYs (in thousands)	2005 DALYs PAF	2015 DALYs (in thousands)	2015 DALYs PAF	Percent change DALYs 2005-2015	Percent change DALYs PAF 2005-2015
Endocarditis	-	-	-	-	-	-	-	-	-	-	-	-
Other cardiovascular and circulatory diseases	-	-	-	-	-	-	-	-	-	-	-	-
Diabetes mellitus	6·0 (4·2 to 8·5)	0·5% (0·4 to 0·7%)	7·4 (5·1 to 10·5)	0·5% (0·3 to 0·7%)	23·2% (19·3 to 27·0%)	-6·7% (-9·5 to -3·9%)	378·4 (248·3 to 558·9)	0·8% (0·5 to 1·1%)	487·7 (315·0 to 730·5)	0·8% (0·5 to 1·1%)	28·9% (25·3 to 32·3%)	-0·1% (-2·6 to 2·2%)
Chronic kidney disease due to diabetes mellitus	1·4 (0·7 to 2·4)	0·5% (0·2 to 0·8%)	2·2 (1·1 to 3·6)	0·5% (0·3 to 0·9%)	51·5% (44·8 to 58·8%)	8·6% (3·5 to 14·0%)	42·9 (19·5 to 74·9)	0·5% (0·2 to 0·9%)	61·7 (28·3 to 107·3)	0·5% (0·2 to 1·0%)	43·8% (37·8 to 51·5%)	11·3% (6·5 to 17·3%)
Chronic kidney disease due to hypertension	1·2 (0·6 to 2·1)	0·3% (0·1 to 0·5%)	1·6 (0·8 to 2·7)	0·3% (0·1 to 0·5%)	28·4% (19·6 to 38·6%)	-4·5% (-10·8 to 2·9%)	33·5 (15·0 to 57·8)	0·3% (0·1 to 0·6%)	41·8 (19·3 to 72·9)	0·3% (0·2 to 0·6%)	24·7% (19·9 to 30·8%)	1·5% (-2·4 to 6·4%)
Chronic kidney disease due to glomerulonephritis	0·2 (0·1 to 0·4)	0·1% (0·1 to 0·2%)	0·3 (0·1 to 0·5)	0·1% (0·1 to 0·2%)	27·5% (18·3 to 38·1%)	10·3% (2·2 to 20·4%)	12·3 (4·1 to 23·6)	0·2% (0·1 to 0·3%)	15·4 (5·5 to 29·4)	0·2% (0·1 to 0·3%)	24·6% (19·7 to 33·5%)	18·2% (13·0 to 26·8%)
Chronic kidney disease due to other causes	0·0 (0·0 to 0·1)	0·1% (0·1 to 0·3%)	0·0 (0·0 to 0·1)	0·2% (0·1 to 0·3%)	36·1% (21·8 to 49·1%)	9·8% (0·5 to 18·7%)	7·5 (2·9 to 14·6)	0·3% (0·1 to 0·5%)	9·6 (3·9 to 18·5)	0·3% (0·1 to 0·6%)	28·3% (22·9 to 36·4%)	10·3% (5·6 to 17·5%)
**Diet low in fibre: All causes**	334·3 (158·5 to 578·5)	0·6% (0·3 to 1·1%)	387·0 (183·5 to 670·1)	0·7% (0·3 to 1·2%)	15·8% (12·4 to 19·6%)	11·3% (8·1 to 14·7%)	7,666·3 (3,534·3 to 13,298·5)	0·3% (0·1 to 0·5%)	8,393·0 (3,908·0 to 14,657·5)	0·3% (0·2 to 0·6%)	9·5% (5·6 to 13·8%)	13·5% (9·3 to 17·8%)
Colon and rectum cancer	37·8 (16·7 to 66·4)	5·6% (2·5 to 9·8%)	47·0 (20·9 to 82·5)	5·6% (2·5 to 9·9%)	24·4% (20·6 to 28·9%)	1·0% (-1·4 to 3·9%)	741·2 (326·8 to 1,317·8)	5·1% (2·3 to 9·1%)	870·1 (384·9 to 1,545·4)	5·1% (2·2 to 9·0%)	17·4% (13·5 to 21·5%)	-0·7% (-3·6 to 2·2%)
Ischaemic heart disease	296·5 (135·8 to 531·4)	3·9% (1·8 to 6·9%)	340·0 (156·3 to 609·6)	3·8% (1·8 to 6·9%)	14·7% (11·5 to 18·5%)	-1·6% (-4·2 to 1·0%)	6,925·0 (3,136·8 to 12,332·8)	4·7% (2·1 to 8·3%)	7,523·0 (3,410·1 to 13,333·1)	4·6% (2·1 to 8·1%)	8·6% (4·7 to 12·9%)	-2·1% (-5·1 to 0·7%)
**Diet low in calcium: All causes**	126·6 (74·5 to 184·7)	0·2% (0·1 to 0·3%)	159·6 (94·8 to 231·8)	0·3% (0·2 to 0·4%)	26·1% (23·3 to 29·7%)	21·2% (18·6 to 24·1%)	2,721·4 (1,608·5 to 3,965·8)	0·1% (0·1 to 0·2%)	3,283·8 (1,966·4 to 4,754·8)	0·1% (0·1 to 0·2%)	20·7% (17·6 to 24·3%)	25·1% (21·7 to 28·7%)
Colon and rectum cancer	126·6 (74·5 to 184·7)	18·7% (11·0 to 27·4%)	159·6 (94·8 to 231·8)	19·2% (11·4 to 27·9%)	26·1% (23·3 to 29·7%)	2·4% (1·3 to 4·1%)	2,721·4 (1,608·5 to 3,965·8)	18·9% (11·2 to 27·5%)	3,283·8 (1,966·4 to 4,754·8)	19·3% (11·6 to 28·0%)	20·7% (17·6 to 24·3%)	2·1% (1·1 to 3·6%)
Prostate cancer	-	-	-	-	-	-	-	-	-	-	-	-
**Diet low in seafood omega-3 fatty acids: All causes**	1,279·5 (537·3 to 2,079·4)	2·4% (1·0 to 3·9%)	1,471·7 (616·4 to 2,377·7)	2·6% (1·1 to 4·3%)	15·0% (12·5 to 17·5%)	10·5% (8·8 to 12·2%)	28,349·9 (11,995·2 to 45,414·1)	1·1% (0·5 to 1·8%)	31,309·8 (13,272·3 to 49,823·0)	1·3% (0·5 to 2·1%)	10·4% (7·2 to 13·3%)	14·5% (11·0 to 17·5%)
Ischaemic heart disease	1,279·5 (537·3 to 2,079·4)	16·7% (7·0 to 27·0%)	1,471·7 (616·4 to 2,377·7)	16·5% (7·0 to 26·6%)	15·0% (12·5 to 17·5%)	-1·3% (-2·0 to -0·7%)	28,349·9 (11,995·2 to 45,414·1)	19·2% (8·2 to 30·4%)	31,309·8 (13,272·3 to 49,823·0)	19·1% (8·2 to 30·2%)	10·4% (7·2 to 13·3%)	-0·5% (-1·2 to 0·2%)
**Diet low in polyunsaturated fatty acids: All causes**	348·3 (144·8 to 547·7)	0·6% (0·3 to 1·0%)	388·5 (158·1 to 609·8)	0·7% (0·3 to 1·1%)	11·5% (3·6 to 20·8%)	7·2% (-0·3 to 15·9%)	7,535·0 (3,243·5 to 11,726·6)	0·3% (0·1 to 0·5%)	8,269·6 (3,533·7 to 12,995·9)	0·3% (0·1 to 0·5%)	9·7% (2·0 to 18·7%)	13·7% (5·8 to 22·9%)
Ischaemic heart disease	348·3 (144·8 to 547·7)	4·6% (1·9 to 7·2%)	388·5 (158·1 to 609·8)	4·4% (1·8 to 6·8%)	11·5% (3·6 to 20·8%)	-4·3% (-10·9 to 3·2%)	7,535·0 (3,243·5 to 11,726·6)	5·1% (2·2 to 8·0%)	8,269·6 (3,533·7 to 12,995·9)	5·0% (2·2 to 7·9%)	9·7% (2·0 to 18·7%)	-1·1% (-7·6 to 6·4%)
**Diet high in trans fatty acids: All causes**	383·8 (158·4 to 713·0)	0·7% (0·3 to 1·3%)	448·1 (189·8 to 827·0)	0·8% (0·3 to 1·5%)	16·7% (13·1 to 22·8%)	12·2% (9·0 to 17·7%)	10,120·8 (4,347·7 to 18,081·4)	0·4% (0·2 to 0·7%)	11,577·9 (5,137·8 to 20,321·6)	0·5% (0·2 to 0·8%)	14·4% (9·9 to 21·4%)	18·6% (13·9 to 25·7%)
Ischaemic heart disease	383·8 (158·4 to 713·0)	5·0% (2·1 to 9·3%)	448·1 (189·8 to 827·0)	5·0% (2·1 to 9·2%)	16·7% (13·1 to 22·8%)	0·1% (-2·2 to 4·4%)	10,120·8 (4,347·7 to 18,081·4)	6·8% (3·0 to 12·3%)	11,577·9 (5,137·8 to 20,321·6)	7·1% (3·1 to 12·5%)	14·4% (9·9 to 21·4%)	3·1% (0·4 to 8·4%)
**Diet high in sodium: All causes**	3,668·5 (2,145·2 to 5,680·2)	6·8% (4·0 to 10·6%)	4,129·7 (2,445·8 to 6,418·8)	7·4% (4·4 to 11·5%)	12·6% (8·3 to 16·9%)	8·2% (4·5 to 11·9%)	77,456·1 (45,738·9 to 118,671·8)	3·0% (1·8 to 4·7%)	83,007·7 (49,326·5 to 127,451·6)	3·4% (2·0 to 5·3%)	7·2% (2·7 to 11·3%)	11·1% (6·5 to 15·3%)
Stomach cancer	413·5 (271·0 to 566·7)	50·1% (33·0 to 68·9%)	397·2 (246·5 to 553·4)	48·5% (30·2 to 67·4%)	-3·9% (-11·9 to 3·0%)	-3·3% (-9·8 to 2·1%)	9,470·2 (6,286·5 to 12,764·7)	50·7% (33·7 to 68·2%)	8,539·7 (5,501·8 to 11,691·4)	49·0% (31·5 to 66·9%)	-9·8% (-17·2 to -3·7%)	-3·5% (-10·0 to 1·0%)
Rheumatic heart disease	30·0 (14·4 to 53·3)	9·0% (4·3 to 16·4%)	27·7 (12·9 to 50·7)	8·7% (4·1 to 16·6%)	-7·6% (-15·4 to -1·3%)	-3·7% (-10·6 to 2·2%)	873·1 (420·2 to 1,603·1)	7·5% (3·6 to 13·7%)	771·7 (359·1 to 1,427·8)	7·4% (3·5 to 13·6%)	-11·6% (-19·0 to -6·1%)	-2·5% (-9·4 to 3·1%)
Ischaemic heart disease	1,348·1 (681·8 to 2,305·6)	17·6% (8·9 to 30·2%)	1,632·2 (851·8 to 2,754·4)	18·3% (9·6 to 30·8%)	21·1% (16·7 to 27·8%)	3·9% (0·7 to 8·9%)	26,944·8 (13,814·0 to 45,257·7)	18·2% (9·4 to 30·6%)	31,088·5 (16,524·3 to 51,138·8)	19·0% (10·1 to 31·3%)	15·4% (11·2 to 21·3%)	4·0% (1·6 to 8·4%)
Ischaemic stroke	512·7 (278·4 to 848·3)	18·6% (10·0 to 30·7%)	561·7 (308·4 to 916·1)	18·9% (10·3 to 30·9%)	9·5% (3·7 to 17·1%)	1·5% (-2·7 to 7·8%)	9,239·3 (5,141·0 to 14,731·4)	20·9% (11·7 to 33·2%)	9,756·0 (5,536·8 to 15,526·5)	21·6% (12·4 to 34·0%)	5·6% (0·6 to 11·8%)	3·0% (-0·2 to 8·1%)
Hemorrhagic stroke	875·8 (516·3 to 1,339·6)	26·9% (15·8 to 40·9%)	875·4 (513·7 to 1,347·4)	26·1% (15·5 to 40·1%)	0·0% (-6·1 to 6·0%)	-2·7% (-7·5 to 2·0%)	19,629·7 (11,721·1 to 29,698·4)	26·4% (15·7 to 39·5%)	19,052·0 (11,138·5 to 28,915·7)	25·9% (15·3 to 39·2%)	-2·9% (-9·2 to 2·6%)	-1·6% (-6·2 to 2·6%)
Hypertensive heart disease	245·8 (88·0 to 484·3)	32·4% (11·7 to 63·5%)	324·8 (115·6 to 633·9)	33·8% (12·1 to 65·5%)	32·2% (18·7 to 45·3%)	4·3% (-2·1 to 12·8%)	4,756·0 (2,134·4 to 8,875·8)	32·1% (14·2 to 58·8%)	5,809·8 (2,603·0 to 10,708·9)	33·2% (14·7 to 60·5%)	22·2% (13·2 to 30·9%)	3·7% (-0·1 to 9·4%)
Cardiomyopathy and myocarditis	27·7 (12·1 to 50·9)	8·4% (3·7 to 15·5%)	31·0 (13·9 to 57·1)	8·8% (3·9 to 16·1%)	12·0% (5·8 to 20·4%)	4·1% (-0·1 to 10·6%)	737·1 (312·4 to 1,377·0)	7·7% (3·3 to 14·3%)	763·8 (341·5 to 1,395·9)	8·3% (3·7 to 15·1%)	3·6% (-2·0 to 11·1%)	8·1% (4·1 to 15·3%)
Atrial fibrillation and flutter	13·7 (6·3 to 24·9)	9·6% (4·7 to 16·8%)	19·1 (8·9 to 34·6)	9·8% (4·9 to 17·0%)	39·1% (34·0 to 46·2%)	1·7% (-1·3 to 6·5%)	448·6 (221·1 to 790·2)	12·9% (6·8 to 21·6%)	591·8 (297·0 to 1,032·9)	13·3% (7·2 to 21·9%)	31·9% (28·8 to 37·4%)	3·0% (0·8 to 7·2%)
Aortic aneurysm	12·9 (5·9 to 23·5)	9·6% (4·4 to 17·3%)	16·2 (7·6 to 29·5)	9·7% (4·5 to 17·5%)	25·9% (19·0 to 32·8%)	0·9% (-1·8 to 5·0%)	262·2 (124·3 to 459·8)	10·6% (5·1 to 18·8%)	319·4 (153·1 to 559·0)	10·9% (5·2 to 19·0%)	21·8% (14·4 to 28·2%)	2·6% (0·4 to 6·0%)
Peripheral vascular disease	2·0 (0·7 to 4·1)	5·2% (1·8 to 11·0%)	2·7 (1·0 to 5·8)	5·2% (1·9 to 11·0%)	38·4% (30·2 to 49·6%)	0·5% (-3·6 to 7·1%)	70·4 (27·6 to 139·5)	7·7% (3·4 to 13·9%)	97·0 (39·7 to 191·6)	8·0% (3·7 to 14·3%)	37·7% (32·4 to 45·6%)	4·8% (1·6 to 10·9%)
Endocarditis	5·6 (2·5 to 10·2)	8·1% (3·7 to 14·8%)	7·1 (3·2 to 13·1)	8·4% (3·9 to 15·4%)	28·6% (22·6 to 35·9%)	4·2% (0·4 to 9·3%)	132·9 (59·0 to 248·0)	6·8% (3·0 to 12·5%)	162·9 (73·0 to 298·8)	7·4% (3·3 to 13·5%)	22·6% (15·9 to 29·9%)	8·7% (3·5 to 14·4%)
Other cardiovascular and circulatory diseases	50·0 (24·8 to 84·7)	10·9% (5·4 to 18·5%)	59·1 (29·1 to 99·9)	10·9% (5·4 to 18·4%)	18·1% (11·9 to 24·4%)	-0·2% (-4·3 to 4·3%)	1,583·5 (804·1 to 2,702·9)	10·5% (5·4 to 17·6%)	1,920·5 (992·8 to 3,245·6)	11·4% (6·0 to 18·9%)	21·3% (16·3 to 27·4%)	7·9% (4·7 to 12·9%)
Chronic kidney disease due to diabetes mellitus	41·2 (17·7 to 74·4)	13·7% (5·9 to 24·5%)	57·3 (24·7 to 103·1)	13·7% (5·8 to 24·5%)	39·3% (33·0 to 44·8%)	-0·2% (-3·7 to 2·5%)	1,048·2 (467·5 to 1,908·9)	12·0% (5·3 to 21·1%)	1,390·5 (634·5 to 2,500·1)	12·3% (5·5 to 21·7%)	32·7% (27·2 to 37·9%)	2·7% (-0·7 to 5·6%)
Chronic kidney disease due to hypertension	52·4 (22·1 to 99·1)	12·8% (5·4 to 23·8%)	74·5 (31·8 to 138·9)	13·6% (5·8 to 24·8%)	42·1% (36·0 to 49·9%)	5·6% (3·0 to 10·3%)	1,106·7 (478·9 to 2,079·4)	10·7% (4·6 to 19·6%)	1,466·8 (638·8 to 2,716·8)	11·5% (5·1 to 20·8%)	32·5% (27·3 to 38·8%)	7·9% (4·7 to 12·2%)
Chronic kidney disease due to glomerulonephritis	34·8 (18·4 to 57·4)	16·9% (9·1 to 27·3%)	40·0 (21·0 to 67·4)	16·8% (9·0 to 27·7%)	15·1% (6·5 to 22·8%)	-0·4% (-7·1 to 5·1%)	917·8 (471·0 to 1,516·4)	11·9% (6·2 to 19·7%)	971·5 (495·7 to 1,648·1)	11·9% (6·1 to 20·3%)	5·8% (-2·3 to 13·0%)	0·4% (-6·7 to 6·4%)
Chronic kidney disease due to other causes	2·4 (1·0 to 4·5)	9·8% (4·2 to 18·2%)	3·4 (1·4 to 6·3)	11·3% (4·9 to 20·7%)	43·8% (34·1 to 54·4%)	16·1% (10·7 to 21·9%)	235·5 (97·8 to 448·6)	8·7% (3·9 to 16·2%)	306·0 (130·7 to 576·4)	9·8% (4·4 to 17·9%)	29·9% (26·2 to 34·6%)	11·7% (8·7 to 16·0%)
**Sexual abuse and violence: All causes**	337·8 (243·6 to 444·0)	0·6% (0·5 to 0·8%)	280·8 (199·1 to 378·4)	0·5% (0·4 to 0·7%)	-16·9% (-23·6 to -9·4%)	-20·1% (-26·5 to -13·3%)	22,526·9 (17,001·9 to 28,715·3)	0·9% (0·7 to 1·1%)	20,800·9 (15,633·3 to 26,809·5)	0·8% (0·7 to 1·1%)	-7·7% (-14·0 to -1·5%)	-4·4% (-10·5 to 1·9%)
**Childhood sexual abuse: All causes**	118·3 (33·7 to 228·9)	0·2% (0·1 to 0·4%)	112·5 (30·6 to 220·8)	0·2% (0·1 to 0·4%)	-4·9% (-11·8 to 1·1%)	-8·6% (-15·3 to -3·3%)	8,200·4 (3,468·2 to 14,180·2)	0·3% (0·1 to 0·5%)	8,107·3 (3,471·6 to 14,062·1)	0·3% (0·1 to 0·6%)	-1·1% (-8·5 to 5·8%)	2·4% (-4·8 to 9·5%)
Alcohol use disorders	7·2 (0·0 to 15·9)	4·6% (0·0 to 10·4%)	5·6 (0·0 to 12·7)	4·1% (0·0 to 9·2%)	-22·0% (-26·6 to 0·0%)	-10·7% (-13·2 to 0·0%)	567·6 (0·0 to 1,299·3)	4·9% (0·0 to 11·1%)	511·5 (0·0 to 1,183·2)	4·6% (0·0 to 10·3%)	-9·9% (-14·6 to 0·0%)	-6·9% (-8·9 to 0·0%)
Major depressive disorder	-	-	-	-	-	-	2,062·2 (231·2 to 4,518·0)	5·5% (0·7 to 11·4%)	2,320·5 (256·8 to 5,097·3)	5·3% (0·6 to 10·9%)	12·5% (10·7 to 14·0%)	-4·5% (-5·7 to -3·3%)
Dysthymia	-	-	-	-	-	-	425·6 (46·3 to 942·6)	5·1% (0·6 to 10·6%)	484·4 (52·7 to 1,073·9)	4·8% (0·6 to 10·1%)	13·8% (11·3 to 15·9%)	-5·0% (-6·3 to -3·7%)
Self-harm	111·1 (27·4 to 221·7)	13·4% (3·3 to 26·7%)	106·9 (26·6 to 213·2)	12·9% (3·1 to 25·8%)	-3·8% (-10·5 to 2·1%)	-3·9% (-5·9 to -2·0%)	5,145·1 (1,264·9 to 10,174·7)	14·4% (3·6 to 28·2%)	4,790·9 (1,193·6 to 9,541·7)	14·0% (3·4 to 27·5%)	-6·9% (-13·6 to -0·9%)	-2·6% (-4·7 to -0·8%)
**Intimate partner violence: All causes**	237·3 (180·2 to 292·7)	0·4% (0·3 to 0·5%)	185·7 (147·8 to 223·6)	0·3% (0·3 to 0·4%)	-21·8% (-28·1 to -12·9%)	-24·8% (-30·9 to -16·8%)	15,430·9 (12,234·1 to 19,068·0)	0·6% (0·5 to 0·7%)	13,803·0 (11,078·5 to 16,953·8)	0·6% (0·5 to 0·7%)	-10·5% (-17·8 to -2·8%)	-7·4% (-14·5 to 0·6%)

Supplemental Results Table 1. All-age deaths, death PAFs, DALYs, DALY PAFs, and percent change for each risk factor and outcome for both sexes combined in 2005 and 2015 for Global.

Data in parenthesis are 95% uncertainty intervals. PAFs=population attributable fractions. DALYs=disability-adjusted life-years.

	2005 Deaths (in thousands)	2005 Deaths PAF	2015 Deaths (in thousands)	2015 Deaths PAF	Percent change deaths 2005-2015	Percent change deaths PAF 2005-2015	2005 DALYs (in thousands)	2005 DALYs PAF	2015 DALYs (in thousands)	2015 DALYs PAF	Percent change DALYs 2005-2015	Percent change DALYs PAF 2005-2015
HIV/AIDS - Tuberculosis	20·4 (10·8 to 31·2)	5·8% (3·1 to 8·4%)	10·4 (5·3 to 16·2)	4·9% (2·6 to 7·2%)	-49·0% (-52·8 to -44·1%)	-15·3% (-20·2 to -10·6%)	936·5 (490·3 to 1,432·2)	4·8% (2·6 to 7·1%)	495·7 (251·4 to 775·3)	4·3% (2·2 to 6·3%)	-47·1% (-50·6 to -42·5%)	-12·0% (-16·7 to -7·3%)
HIV/AIDS resulting in other diseases	86·4 (45·3 to 128·3)	6·0% (3·2 to 8·9%)	48·8 (25·4 to 71·7)	5·0% (2·6 to 7·4%)	-43·5% (-47·8 to -38·9%)	-17·1% (-22·3 to -11·7%)	4,300·8 (2,252·5 to 6,423·9)	5·4% (2·8 to 8·0%)	2,520·0 (1,287·2 to 3,715·8)	4·6% (2·4 to 6·8%)	-41·4% (-45·9 to -36·8%)	-15·3% (-20·4 to -9·8%)
Maternal abortion, miscarriage, and ectopic pregnancy	0·0 (0·0 to 0·0)	0·0% (0·0 to 0·0%)	0·0 (0·0 to 0·0)	0·0% (0·0 to 0·0%)	-22·8% (-36·5 to -5·4%)	0·2% (-11·1 to 15·1%)	0·0 (0·0 to 0·0)	0·0% (0·0 to 0·0%)	0·0 (0·0 to 0·0)	0·0% (0·0 to 0·0%)	-23·2% (-36·6 to -6·0%)	0·3% (-10·9 to 15·7%)
Major depressive disorder	-	-	-	-	-	-	3,561·9 (2,279·4 to 5,110·2)	9·5% (7·7 to 11·2%)	4,404·9 (2,825·1 to 6,361·0)	10·0% (8·0 to 11·7%)	23·7% (21·3 to 26·0%)	5·0% (3·1 to 6·7%)
Dysthymia	-	-	-	-	-	-	775·0 (496·4 to 1,147·6)	9·3% (7·6 to 10·9%)	979·7 (623·4 to 1,438·2)	9·8% (8·0 to 11·5%)	26·4% (23·3 to 29·8%)	5·5% (3·4 to 7·8%)
Self-harm	103·7 (71·6 to 126·1)	12·5% (9·5 to 15·0%)	102·6 (73·6 to 126·3)	12·4% (9·4 to 15·0%)	-1·1% (-11·1 to 13·2%)	-1·2% (-8·8 to 8·5%)	4,366·7 (2,950·1 to 5,417·4)	12·2% (9·1 to 14·9%)	4,106·9 (2,944·1 to 5,116·8)	12·0% (9·0 to 14·7%)	-5·9% (-16·3 to 9·1%)	-1·6% (-9·7 to 9·4%)
Assault by firearm	5·9 (4·8 to 6·7)	3·6% (3·1 to 4·2%)	6·1 (5·0 to 7·2)	3·5% (3·0 to 4·2%)	2·2% (-5·9 to 13·8%)	-3·9% (-11·5 to 6·4%)	314·8 (252·3 to 356·0)	3·6% (3·0 to 4·1%)	311·6 (256·1 to 365·7)	3·4% (2·9 to 4·0%)	-1·0% (-9·4 to 11·1%)	-4·6% (-12·6 to 6·1%)
Assault by sharp object	6·6 (5·9 to 7·3)	6·3% (5·7 to 7·0%)	5·4 (4·8 to 6·0)	6·0% (5·4 to 6·7%)	-18·4% (-25·1 to -10·6%)	-4·8% (-12·2 to 2·8%)	340·4 (304·5 to 379·2)	6·3% (5·6 to 7·0%)	268·8 (237·9 to 302·1)	5·9% (5·3 to 6·6%)	-21·0% (-28·1 to -12·6%)	-5·8% (-13·6 to 2·3%)
Assault by other means	14·3 (13·0 to 15·5)	9·0% (8·3 to 9·9%)	12·5 (11·0 to 14·1)	8·6% (7·6 to 9·7%)	-12·3% (-19·6 to -4·2%)	-5·0% (-11·9 to 2·3%)	834·8 (760·3 to 913·1)	9·8% (9·0 to 10·7%)	715·4 (624·4 to 804·9)	9·3% (8·3 to 10·5%)	-14·3% (-21·5 to -6·0%)	-5·5% (-12·2 to 1·8%)
**Unsafe sex: All causes**	2,051·2 (1,956·6 to 2,156·0)	3·8% (3·6 to 4·0%)	1,452·4 (1,380·7 to 1,541·0)	2·6% (2·5 to 2·8%)	-29·2% (-31·6 to -26·1%)	-31·9% (-34·5 to -29·1%)	112,703·3 (106,333·1 to 120,086·2)	4·4% (4·0 to 4·8%)	79,450·8 (74,248·2 to 85,531·6)	3·2% (2·9 to 3·6%)	-29·5% (-32·0 to -26·8%)	-26·9% (-29·6 to -24·2%)
HIV/AIDS - Tuberculosis	334·2 (266·9 to 379·5)	95·0% (94·1 to 95·6%)	199·4 (151·9 to 230·9)	94·2% (93·2 to 95·0%)	-40·3% (-44·9 to -34·8%)	-0·9% (-1·4 to -0·4%)	18,403·1 (14,762·1 to 20,869·9)	95·2% (94·4 to 95·9%)	10,989·8 (8,439·4 to 12,702·5)	94·6% (93·6 to 95·3%)	-40·3% (-44·7 to -34·7%)	-0·7% (-1·2 to -0·3%)
HIV/AIDS resulting in other diseases	1,356·1 (1,270·6 to 1,454·6)	94·2% (93·4 to 94·8%)	906·3 (843·9 to 981·2)	92·4% (91·3 to 93·3%)	-33·2% (-36·6 to -29·1%)	-1·9% (-2·5 to -1·4%)	75,102·3 (70,586·0 to 80,393·7)	94·4% (93·6 to 95·0%)	51,167·2 (47,789·5 to 55,056·7)	92·9% (91·9 to 93·8%)	-31·9% (-35·1 to -28·3%)	-1·5% (-2·1 to -1·1%)
Syphilis	134·1 (79·9 to 205·7)	100·0% (100·0 to 100·0%)	106·8 (63·4 to 164·6)	100·0% (100·0 to 100·0%)	-20·3% (-28·5 to -12·4%)	-	11,190·5 (6,607·1 to 17,280·8)	100·0% (100·0 to 100·0%)	8,957·1 (5,273·2 to 13,969·7)	100·0% (100·0 to 100·0%)	-20·0% (-28·4 to -11·8%)	-
Chlamydial infection	0·2 (0·1 to 0·2)	100·0% (100·0 to 100·0%)	0·2 (0·1 to 0·2)	100·0% (100·0 to 100·0%)	-5·3% (-17·0 to 10·6%)	-	337·0 (194·2 to 537·7)	100·0% (100·0 to 100·0%)	369·8 (214·0 to 595·4)	100·0% (100·0 to 100·0%)	9·7% (6·5 to 13·0%)	-
Gonococcal infection	0·8 (0·7 to 0·9)	100·0% (100·0 to 100·0%)	0·7 (0·5 to 0·8)	100·0% (100·0 to 100·0%)	-15·7% (-26·7 to -4·7%)	-	382·8 (238·6 to 573·0)	100·0% (100·0 to 100·0%)	469·8 (283·4 to 716·8)	100·0% (100·0 to 100·0%)	22·7% (15·9 to 28·1%)	-
Trichomoniasis	-	-	-	-	-	-	167·4 (67·1 to 354·8)	100·0% (100·0 to 100·0%)	194·3 (77·8 to 412·1)	100·0% (100·0 to 100·0%)	16·1% (15·0 to 17·2%)	-
Genital herpes	-	-	-	-	-	-	197·8 (61·5 to 468·3)	100·0% (100·0 to 100·0%)	236·4 (74·3 to 555·8)	100·0% (100·0 to 100·0%)	19·5% (17·4 to 23·0%)	-
Other sexually transmitted diseases	0·4 (0·3 to 0·4)	100·0% (100·0 to 100·0%)	0·3 (0·2 to 0·4)	100·0% (100·0 to 100·0%)	-16·8% (-27·6 to -6·1%)	-	103·2 (74·9 to 139·2)	100·0% (100·0 to 100·0%)	103·5 (74·3 to 141·2)	100·0% (100·0 to 100·0%)	0·3% (-3·1 to 3·2%)	-
Cervical cancer	225·4 (213·9 to 237·8)	100·0% (100·0 to 100·0%)	238·6 (225·3 to 252·4)	100·0% (100·0 to 100·0%)	5·8% (-0·5 to 13·8%)	-	6,819·2 (6,393·4 to 7,236·4)	100·0% (100·0 to 100·0%)	6,963·0 (6,526·2 to 7,408·0)	100·0% (100·0 to 100·0%)	2·1% (-4·5 to 10·5%)	-
**Low physical activity: All causes**	1,350·6 (1,046·9 to 1,661·2)	2·5% (2·0 to 3·1%)	1,605·5 (1,265·2 to 1,955·7)	2·9% (2·3 to 3·5%)	18·9% (16·5 to 21·6%)	14·2% (12·4 to 16·6%)	29,467·1 (22,693·9 to 36,268·4)	1·2% (0·9 to 1·4%)	34,603·5 (26,904·9 to 42,282·1)	1·4% (1·1 to 1·7%)	17·4% (14·4 to 20·5%)	21·7% (19·1 to 24·8%)
Colon and rectum cancer	95·3 (66·4 to 124·2)	14·1% (9·8 to 18·4%)	119·4 (83·9 to 155·8)	14·3% (10·1 to 18·7%)	25·2% (22·5 to 28·1%)	1·6% (0·9 to 2·6%)	1,838·0 (1,262·5 to 2,441·2)	12·8% (8·8 to 16·9%)	2,209·2 (1,528·5 to 2,915·9)	13·0% (9·0 to 17·1%)	20·2% (17·7 to 22·9%)	1·7% (0·9 to 2·8%)
Breast cancer	39·0 (28·1 to 50·1)	8·9% (6·5 to 11·3%)	47·7 (34·8 to 60·6)	8·9% (6·5 to 11·3%)	22·1% (16·9 to 27·0%)	0·6% (-0·6 to 2·3%)	1,063·5 (764·9 to 1,369·0)	8·2% (6·0 to 10·5%)	1,276·2 (925·8 to 1,644·2)	8·3% (6·0 to 10·6%)	20·0% (14·2 to 25·5%)	0·8% (-0·7 to 2·7%)
Ischaemic heart disease	727·4 (475·5 to 992·3)	9·5% (6·3 to 13·0%)	858·7 (572·1 to 1,160·8)	9·6% (6·4 to 13·0%)	18·1% (15·8 to 20·8%)	1·3% (0·3 to 2·8%)	14,620·5 (9,263·6 to 20,246·7)	9·9% (6·3 to 13·6%)	16,673·1 (10,684·1 to 22,820·9)	10·2% (6·5 to 13·9%)	14·0% (11·2 to 17·2%)	2·7% (1·7 to 4·6%)
Ischaemic stroke	305·0 (169·0 to 448·6)	11·0% (6·1 to 16·2%)	332·9 (188·4 to 485·3)	11·2% (6·2 to 16·2%)	9·2% (6·1 to 12·8%)	1·2% (0·1 to 3·4%)	5,049·2 (2,687·8 to 7,537·4)	11·4% (6·1 to 17·0%)	5,291·0 (2,875·5 to 7,910·6)	11·7% (6·3 to 17·2%)	4·8% (1·4 to 8·4%)	2·3% (1·2 to 4·2%)
Diabetes mellitus	183·9 (142·4 to 228·9)	16·0% (12·3 to 19·9%)	246·8 (191·7 to 306·5)	16·3% (12·6 to 20·2%)	34·2% (30·1 to 38·2%)	1·6% (0·7 to 2·6%)	6,895·8 (4,990·7 to 9,241·9)	13·9% (10·5 to 17·4%)	9,153·9 (6,646·6 to 12,251·0)	14·3% (10·8 to 17·9%)	32·7% (29·8 to 35·5%)	2·9% (2·3 to 3·7%)
**Metabolic risks: All causes**	14,402·6 (13,691·3 to 15,063·2)	26·9% (25·6 to 28·1%)	16,860·3 (16,021·4 to 17,697·0)	30·2% (28·8 to 31·7%)	17·1% (15·1 to 19·2%)	12·5% (11·2 to 13·7%)	335,227·3 (315,739·0 to 357,389·2)	13·1% (12·5 to 13·8%)	381,844·9 (357,846·4 to 409,435·7)	15·5% (14·7 to 16·3%)	13·9% (11·5 to 16·0%)	18·0% (16·0 to 19·8%)
**High fasting plasma glucose: All causes**	4,212·0 (3,647·2 to 5,047·0)	7·9% (6·8 to 9·4%)	5,239·6 (4,546·6 to 6,216·9)	9·4% (8·2 to 11·2%)	24·4% (21·5 to 27·4%)	19·5% (17·3 to 21·9%)	117,100·5 (102,561·2 to 133,959·8)	4·6% (4·2 to 5·1%)	143,075·8 (125,124·6 to 163,477·4)	5·8% (5·3 to 6·4%)	22·2% (19·5 to 24·8%)	26·6% (24·0 to 29·0%)
Tuberculosis	133·1 (82·7 to 188·9)	9·9% (6·4 to 13·9%)	118·3 (73·1 to 169·3)	10·6% (6·8 to 14·8%)	-11·1% (-19·7 to -3·5%)	7·7% (4·0 to 10·9%)	4,313·4 (2,737·2 to 5,959·5)	8·7% (5·7 to 11·8%)	3,801·8 (2,430·3 to 5,303·5)	9·4% (6·2 to 12·8%)	-11·9% (-19·5 to -5·2%)	8·9% (3·7 to 13·3%)
Ischaemic heart disease	1,415·1 (946·0 to 2,015·9)	18·5% (12·3 to 26·3%)	1,760·9 (1,175·2 to 2,528·2)	19·7% (13·3 to 28·2%)	24·4% (19·9 to 29·1%)	6·7% (3·6 to 10·3%)	27,890·7 (19,564·5 to 37,687·0)	18·9% (13·3 to 25·5%)	33,535·3 (23,674·0 to 45,227·6)	20·4% (14·4 to 27·6%)	20·2% (17·1 to 23·4%)	8·3% (6·9 to 10·0%)
Ischaemic stroke	454·2 (257·3 to 803·4)	16·5% (9·3 to 29·2%)	514·4 (290·5 to 912·8)	17·3% (9·8 to 31·0%)	13·2% (7·9 to 20·3%)	5·0% (1·2 to 11·4%)	7,749·6 (4,770·6 to 12,342·6)	17·6% (10·8 to 28·2%)	8,524·1 (5,350·1 to 13,291·1)	18·9% (11·6 to 30·1%)	10·0% (6·0 to 14·3%)	7·3% (5·4 to 10·8%)
Hemorrhagic stroke	529·1 (366·1 to 728·4)	16·2% (11·2 to 22·3%)	584·8 (407·7 to 802·1)	17·5% (12·2 to 24·0%)	10·5% (6·4 to 15·6%)	7·6% (5·5 to 10·9%)	12,434·0 (8,510·2 to 17,327·3)	16·7% (11·4 to 23·4%)	13,556·4 (9,360·6 to 18,929·0)	18·5% (12·8 to 25·6%)	9·0% (5·3 to 13·1%)	10·6% (8·9 to 12·2%)
Diabetes mellitus	1,150·2 (1,120·9 to 1,176·8)	100·0% (100·0 to 100·0%)	1,519·0 (1,470·3 to 1,576·0)	100·0% (100·0 to 100·0%)	32·1% (27·7 to 36·3%)	-	49,724·5 (41,868·2 to 58,982·1)	100·0% (100·0 to 100·0%)	64,134·5 (53,489·7 to 76,112·8)	100·0% (100·0 to 100·0%)	29·0% (26·2 to 31·7%)	-
Chronic kidney disease due to diabetes mellitus	299·4 (278·7 to 314·2)	100·0% (100·0 to 100·0%)	417·8 (388·7 to 441·4)	100·0% (100·0 to 100·0%)	39·5% (35·4 to 43·5%)	-	8,713·0 (7,991·5 to 9,466·3)	100·0% (100·0 to 100·0%)	11,258·0 (10,302·9 to 12,225·2)	100·0% (100·0 to 100·0%)	29·2% (25·9 to 32·5%)	-
Chronic kidney disease due to hypertension	149·0 (105·2 to 192·5)	36·5% (25·9 to 46·5%)	215·9 (153·4 to 277·7)	39·3% (28·1 to 49·7%)	44·9% (39·7 to 50·1%)	7·7% (6·6 to 9·1%)	3,260·4 (2,284·4 to 4,221·9)	31·5% (22·3 to 40·3%)	4,467·3 (3,172·2 to 5,743·5)	35·1% (25·1 to 44·6%)	37·0% (32·0 to 42·4%)	11·5% (9·9 to 13·2%)
Chronic kidney disease due to glomerulonephritis	68·2 (48·2 to 88·6)	33·2% (23·3 to 42·7%)	88·6 (63·1 to 114·0)	37·3% (26·7 to 47·5%)	30·0% (24·2 to 36·0%)	12·5% (10·7 to 14·4%)	2,105·6 (1,466·0 to 2,769·6)	27·3% (19·2 to 35·4%)	2,570·2 (1,805·1 to 3,357·6)	31·6% (22·5 to 40·6%)	22·1% (17·1 to 27·1%)	15·8% (13·5 to 18·2%)
Chronic kidney disease due to other causes	6·2 (4·2 to 8·4)	25·5% (17·7 to 33·4%)	9·1 (6·2 to 12·2)	30·4% (21·3 to 39·3%)	47·2% (39·6 to 55·6%)	18·8% (15·4 to 22·8%)	695·3 (452·7 to 982·7)	25·8% (18·1 to 33·7%)	935·5 (615·3 to 1,312·8)	29·9% (21·0 to 38·7%)	34·6% (31·8 to 37·5%)	15·7% (13·4 to 18·5%)
**High total cholesterol: All causes**	3,816·2 (2,973·1 to 4,848·6)	7·1% (5·6 to 9·1%)	4,312·6 (3,323·6 to 5,512·3)	7·7% (6·0 to 9·9%)	13·0% (9·5 to 16·2%)	8·6% (5·4 to 11·5%)	81,690·5 (68,219·0 to 96,877·4)	3·2% (2·7 to 3·8%)	88,687·4 (74,557·7 to 105,680·6)	3·6% (3·0 to 4·3%)	8·6% (5·8 to 11·0%)	12·5% (9·7 to 15·1%)
Ischaemic heart disease	3,279·3 (2,593·8 to 4,015·7)	42·9% (33·9 to 52·5%)	3,743·3 (2,906·1 to 4,650·3)	42·0% (32·6 to 51·8%)	14·1% (10·2 to 17·7%)	-2·1% (-4·9 to 0·4%)	71,722·6 (60,171·6 to 84,380·7)	48·5% (40·9 to 56·8%)	78,589·5 (65,999·5 to 92,771·6)	47·9% (40·3 to 56·2%)	9·6% (6·6 to 12·1%)	-1·3% (-2·2 to -0·3%)
Ischaemic stroke	536·8 (219·8 to 1,058·3)	19·4% (8·0 to 37·9%)	569·2 (221·2 to 1,126·6)	19·1% (7·5 to 37·6%)	6·0% (-1·8 to 11·5%)	-1·7% (-7·9 to 2·7%)	9,967·9 (5,938·8 to 16,618·5)	22·6% (13·6 to 37·6%)	10,097·9 (5,961·4 to 16,912·4)	22·3% (13·3 to 37·2%)	1·3% (-2·7 to 4·9%)	-1·2% (-3·6 to 1·2%)
**High systolic blood pressure: All causes**	9,212·3 (8,326·3 to 10,101·1)	17·2% (15·5 to 18·9%)	10,703·8 (9,601·3 to 11,787·5)	19·2% (17·2 to 21·1%)	16·2% (13·9 to 18·6%)	11·7% (9·9 to 13·4%)	189,579·4 (172,702·6 to 206,695·6)	7·4% (6·7 to 8·2%)	211,816·4 (192,712·1 to 231,114·4)	8·6% (7·7 to 9·6%)	11·7% (9·2 to 14·1%)	15·8% (13·3 to 18·2%)
Rheumatic heart disease	78·7 (54·5 to 116·9)	23·7% (16·3 to 36·2%)	80·1 (55·6 to 121·5)	25·1% (17·4 to 38·5%)	1·7% (-3·2 to 7·0%)	6·1% (3·5 to 9·8%)	2,376·6 (1,588·5 to 3,480·9)	20·5% (13·9 to 30·7%)	2,363·5 (1,614·7 to 3,402·1)	22·5% (15·3 to 33·8%)	-0·6% (-4·9 to 3·9%)	9·7% (7·8 to 12·1%)
Ischaemic heart disease	4,134·8 (3,407·6 to 4,839·7)	54·1% (44·6 to 63·2%)	4,862·2 (3,954·6 to 5,740·4)	54·5% (44·4 to 64·2%)	17·6% (14·7 to 20·2%)	0·9% (-1·1 to 2·3%)	79,827·7 (68,710·2 to 90,328·4)	54·0% (46·4 to 61·1%)	90,298·4 (77,837·2 to 102,137·8)	55·1% (47·3 to 62·3%)	13·1% (10·4 to 15·6%)	1·9% (1·1 to 2·7%)
Ischaemic stroke	1,366·7 (1,083·3 to 1,655·6)	49·5% (39·1 to 59·5%)	1,488·5 (1,167·4 to 1,820·7)	50·0% (39·4 to 60·8%)	8·9% (4·7 to 13·5%)	1·0% (-1·4 to 4·2%)	22,994·7 (18,429·1 to 26,872·1)	52·1% (41·6 to 60·7%)	24,198·1 (19,500·3 to 28,264·1)	53·5% (43·6 to 61·9%)	5·2% (1·8 to 8·7%)	2·7% (1·4 to 4·6%)

Supplemental Results Table 1. All-age deaths, death PAFs, DALYs, DALY PAFs, and percent change for each risk factor and outcome for both sexes combined in 2005 and 2015 for Global.

Data in parenthesis are 95% uncertainty intervals. PAFs=population attributable fractions. DALYs=disability-adjusted life-years.

	2005 Deaths (in thousands)	2005 Deaths PAF	2015 Deaths (in thousands)	2015 Deaths PAF	Percent change deaths 2005-2015	Percent change deaths PAF 2005-2015	2005 DALYs (in thousands)	2005 DALYs PAF	2015 DALYs (in thousands)	2015 DALYs PAF	Percent change DALYs 2005-2015	Percent change DALYs PAF 2005-2015
Hemorrhagic stroke	1,819·0 (1,486·4 to 2,144·2)	55·8% (45·6 to 65·9%)	1,952·8 (1,587·6 to 2,312·6)	58·3% (47·9 to 68·5%)	7·4% (3·6 to 12·1%)	4·5% (2·5 to 7·0%)	41,530·5 (34,162·2 to 47,708·1)	55·8% (45·8 to 64·3%)	43,411·6 (36,091·7 to 49,999·4)	59·1% (49·0 to 67·4%)	4·5% (1·0 to 8·7%)	6·0% (4·8 to 7·6%)
Hypertensive heart disease	760·5 (711·9 to 823·7)	100·0% (100·0 to 100·0%)	962·4 (873·6 to 1,024·5)	100·0% (100·0 to 100·0%)	26·5% (17·5 to 32·3%)	-	14,852·4 (13,919·4 to 16,052·6)	100·0% (100·0 to 100·0%)	17,484·6 (16,286·9 to 18,593·6)	100·0% (100·0 to 100·0%)	17·7% (11·6 to 22·9%)	-
Cardiomyopathy and myocarditis	120·7 (98·0 to 145·0)	36·7% (29·7 to 44·1%)	129·4 (104·0 to 156·9)	36·6% (29·5 to 44·5%)	7·3% (1·6 to 13·0%)	-0·3% (-3·7 to 3·6%)	3,232·6 (2,594·5 to 3,858·5)	33·6% (26·8 to 40·0%)	3,187·9 (2,570·2 to 3,795·4)	34·6% (28·1 to 41·0%)	-1·4% (-6·3 to 4·0%)	2·9% (0·0 to 6·6%)
Atrial fibrillation and flutter	50·0 (37·7 to 65·3)	35·1% (28·0 to 42·4%)	67·6 (50·1 to 90·0)	34·6% (27·5 to 42·4%)	35·2% (31·4 to 39·1%)	-1·2% (-2·9 to 0·4%)	1,408·6 (1,086·8 to 1,820·4)	40·7% (35·7 to 45·6%)	1,810·1 (1,398·1 to 2,347·0)	40·8% (35·8 to 45·9%)	28·5% (26·8 to 30·2%)	0·4% (-0·5 to 1·2%)
Aortic aneurysm	48·5 (38·6 to 57·9)	36·0% (28·6 to 42·7%)	59·5 (47·2 to 70·9)	35·4% (28·0 to 42·3%)	22·7% (17·3 to 27·2%)	-1·7% (-3·3 to 0·3%)	927·8 (770·8 to 1,080·2)	37·6% (31·3 to 43·7%)	1,110·3 (924·7 to 1,291·0)	37·9% (31·6 to 44·0%)	19·7% (13·6 to 23·7%)	0·8% (0·0 to 1·6%)
Peripheral vascular disease	10·6 (7·8 to 13·8)	27·8% (20·2 to 35·9%)	14·1 (10·1 to 18·6)	26·8% (19·3 to 35·2%)	33·0% (25·1 to 42·0%)	-3·5% (-6·4 to -0·5%)	265·4 (184·4 to 385·6)	29·1% (22·7 to 35·7%)	346·2 (235·1 to 505·2)	28·9% (22·5 to 35·5%)	30·4% (26·2 to 34·9%)	-0·7% (-2·3 to 0·9%)
Endocarditis	22·1 (17·3 to 28·0)	32·2% (25·5 to 39·5%)	28·1 (21·9 to 35·7)	33·1% (26·2 to 40·9%)	26·7% (21·8 to 31·6%)	2·7% (0·7 to 4·8%)	518·1 (403·1 to 644·2)	26·4% (20·6 to 32·1%)	640·2 (495·5 to 792·2)	29·0% (22·7 to 34·8%)	23·5% (18·0 to 29·1%)	9·6% (6·3 to 12·8%)
Other cardiovascular and circulatory diseases	176·0 (154·5 to 199·2)	38·5% (33·7 to 43·3%)	210·7 (181·8 to 239·6)	38·9% (33·8 to 43·8%)	19·8% (15·0 to 24·4%)	1·2% (-0·4 to 2·7%)	5,164·6 (4,410·8 to 6,058·4)	34·4% (30·7 to 38·2%)	6,266·8 (5,304·0 to 7,423·5)	37·1% (33·3 to 41·2%)	21·3% (17·6 to 25·1%)	8·0% (6·6 to 9·5%)
Chronic kidney disease due to diabetes mellitus	132·0 (95·0 to 167·1)	44·1% (31·7 to 55·4%)	189·5 (135·3 to 239·7)	45·4% (32·8 to 56·8%)	43·5% (39·5 to 47·5%)	2·9% (2·1 to 3·7%)	3,186·2 (2,230·6 to 4,119·9)	36·6% (25·8 to 46·6%)	4,365·0 (3,085·2 to 5,670·7)	38·8% (27·5 to 49·1%)	37·0% (33·5 to 40·5%)	6·0% (5·1 to 7·0%)
Chronic kidney disease due to hypertension	408·5 (377·1 to 427·6)	100·0% (100·0 to 100·0%)	549·5 (501·6 to 575·6)	100·0% (100·0 to 100·0%)	34·5% (30·0 to 38·7%)	-	10,366·1 (9,400·8 to 10,985·1)	100·0% (100·0 to 100·0%)	12,737·4 (11,488·7 to 13,553·9)	100·0% (100·0 to 100·0%)	22·9% (18·6 to 27·4%)	-
Chronic kidney disease due to glomerulonephritis	75·9 (54·1 to 97·2)	36·9% (26·5 to 47·2%)	97·8 (71·1 to 124·7)	41·1% (29·8 to 52·0%)	28·8% (23·9 to 34·0%)	11·4% (9·4 to 13·5%)	2,151·5 (1,480·8 to 2,852·4)	27·9% (19·6 to 36·4%)	2,583·6 (1,807·7 to 3,384·3)	31·8% (22·7 to 41·2%)	20·1% (15·9 to 24·5%)	14·0% (11·5 to 16·5%)
Chronic kidney disease due to other causes	8·1 (5·6 to 10·7)	33·5% (23·8 to 42·9%)	11·5 (8·0 to 15·1)	38·5% (27·4 to 48·6%)	42·2% (34·0 to 50·6%)	14·8% (11·1 to 19·0%)	776·5 (512·9 to 1,063·0)	28·8% (20·1 to 37·2%)	1,012·9 (668·3 to 1,385·8)	32·3% (22·8 to 41·5%)	30·4% (28·1 to 33·1%)	12·2% (10·1 to 14·8%)
**High body-mass index: All causes**	3,314·4 (2,241·1 to 4,503·8)	6·2% (4·2 to 8·4%)	3,959·7 (2,728·4 to 5,331·6)	7·1% (4·9 to 9·6%)	19·5% (15·8 to 23·6%)	14·8% (11·8 to 18·5%)	98,478·3 (67,219·1 to 131,972·3)	3·9% (2·7 to 5·1%)	120,131·5 (83,828·9 to 158,408·7)	4·9% (3·5 to 6·4%)	22·0% (18·1 to 26·8%)	26·4% (22·9 to 31·1%)
Oesophageal cancer	66·9 (20·2 to 123·3)	14·6% (4·4 to 26·8%)	71·1 (22·1 to 130·2)	16·2% (5·1 to 29·5%)	6·2% (-3·4 to 16·8%)	11·0% (1·8 to 20·9%)	1,573·5 (459·2 to 2,901·0)	14·8% (4·4 to 27·2%)	1,643·8 (515·9 to 3,015·1)	16·7% (5·2 to 30·5%)	4·5% (-5·3 to 15·8%)	13·0% (3·5 to 23·7%)
Colon and rectum cancer	48·2 (28·9 to 70·4)	7·1% (4·3 to 10·4%)	62·2 (37·7 to 90·6)	7·5% (4·5 to 10·8%)	28·9% (25·5 to 32·9%)	4·7% (2·6 to 7·1%)	1,050·1 (632·2 to 1,527·5)	7·3% (4·4 to 10·6%)	1,329·5 (808·6 to 1,924·1)	7·8% (4·8 to 11·3%)	26·6% (23·3 to 30·6%)	7·1% (5·0 to 9·9%)
Liver cancer due to hepatitis B	24·0 (8·6 to 47·6)	9·1% (3·3 to 18·0%)	28·4 (10·6 to 54·3)	10·7% (4·1 to 20·2%)	17·9% (8·3 to 35·0%)	16·9% (10·8 to 26·9%)	762·1 (268·1 to 1,511·1)	9·0% (3·3 to 18·0%)	870·4 (325·8 to 1,677·8)	10·8% (4·1 to 20·6%)	14·2% (3·9 to 33·3%)	19·7% (12·8 to 31·2%)
Liver cancer due to hepatitis C	15·1 (6·4 to 26·2)	11·0% (4·8 to 19·1%)	20·1 (8·8 to 34·5)	12·0% (5·3 to 20·6%)	32·8% (25·8 to 40·8%)	9·5% (4·3 to 14·8%)	325·5 (141·1 to 574·4)	11·2% (4·9 to 19·5%)	421·5 (185·8 to 733·2)	12·7% (5·7 to 21·9%)	29·5% (21·7 to 38·8%)	12·8% (6·9 to 19·3%)
Liver cancer due to alcohol use	21·5 (8·2 to 41·3)	11·0% (4·2 to 21·1%)	29·6 (11·5 to 56·0)	12·1% (4·7 to 22·6%)	38·0% (28·8 to 50·1%)	9·4% (4·3 to 14·7%)	529·3 (202·0 to 1,033·8)	11·0% (4·2 to 21·1%)	728·7 (282·3 to 1,393·1)	12·4% (4·7 to 23·1%)	37·7% (27·3 to 52·3%)	11·9% (6·6 to 18·1%)
Liver cancer due to other causes	11·8 (4·8 to 21·5)	9·0% (3·6 to 16·3%)	14·0 (6·0 to 24·9)	10·5% (4·6 to 18·5%)	18·4% (9·2 to 31·4%)	17·0% (9·6 to 27·0%)	317·3 (127·1 to 583·0)	9·0% (3·6 to 16·4%)	359·6 (150·5 to 645·8)	10·8% (4·6 to 19·1%)	13·3% (3·6 to 28·1%)	20·2% (11·7 to 31·7%)
Gallbladder and biliary tract cancer	18·1 (10·4 to 27·5)	14·5% (8·3 to 22·1%)	20·7 (11·9 to 31·0)	14·7% (8·5 to 22·2%)	14·5% (6·3 to 22·5%)	1·4% (-2·7 to 5·3%)	362·1 (209·2 to 547·3)	14·8% (8·5 to 22·4%)	402·1 (232·8 to 595·5)	15·4% (8·9 to 22·9%)	11·0% (3·6 to 18·6%)	3·9% (-0·2 to 8·1%)
Pancreatic cancer	16·8 (6·0 to 29·5)	5·3% (1·9 to 9·3%)	22·7 (8·1 to 39·5)	5·5% (2·0 to 9·6%)	35·4% (31·6 to 39·5%)	3·5% (1·4 to 5·8%)	349·7 (121·5 to 614·0)	5·4% (1·8 to 9·4%)	463·5 (162·7 to 811·9)	5·6% (2·0 to 9·9%)	32·5% (28·8 to 36·7%)	5·0% (2·8 to 7·7%)
Breast cancer	25·3 (14·4 to 39·1)	5·7% (3·3 to 8·9%)	34·5 (20·4 to 52·3)	6·5% (3·9 to 9·7%)	36·5% (25·1 to 48·0%)	12·5% (6·8 to 19·5%)	551·1 (299·2 to 887·3)	4·3% (2·3 to 6·8%)	774·0 (438·9 to 1,214·4)	5·0% (2·9 to 7·7%)	40·4% (24·5 to 57·5%)	17·9% (8·8 to 28·9%)
Uterine cancer	25·9 (17·3 to 35·1)	31·7% (21·2 to 42·9%)	31·0 (21·4 to 41·4)	34·5% (23·9 to 45·7%)	19·6% (11·4 to 28·9%)	8·8% (5·4 to 13·7%)	654·4 (441·1 to 890·8)	31·5% (21·0 to 42·8%)	780·2 (537·6 to 1,040·9)	35·0% (24·4 to 46·2%)	19·2% (10·1 to 29·8%)	11·0% (7·0 to 17·1%)
Ovarian cancer	3·9 (-0·1 to 8·8)	2·9% (-0·1 to 6·5%)	4·9 (-0·2 to 10·9)	3·0% (-0·1 to 6·8%)	26·3% (22·1 to 31·7%)	4·9% (2·9 to 8·2%)	98·3 (-3·4 to 221·1)	2·8% (-0·1 to 6·4%)	123·8 (-4·4 to 275·3)	3·0% (-0·1 to 6·7%)	26·0% (21·5 to 31·7%)	6·5% (4·1 to 10·5%)
Kidney cancer	17·6 (11·7 to 24·4)	16·9% (11·1 to 23·2%)	23·7 (15·7 to 32·5)	17·3% (11·5 to 23·7%)	34·2% (29·8 to 38·8%)	2·4% (0·4 to 4·6%)	410·2 (272·2 to 565·2)	15·5% (10·3 to 21·3%)	541·2 (363·8 to 740·6)	16·2% (11·0 to 22·2%)	31·9% (27·7 to 36·3%)	4·6% (1·7 to 8·3%)
Thyroid cancer	2·4 (1·3 to 3·7)	9·2% (5·2 to 14·3%)	3·1 (1·7 to 4·9)	9·8% (5·5 to 15·1%)	32·5% (22·6 to 41·1%)	6·2% (0·8 to 11·3%)	62·0 (35·2 to 96·4)	9·6% (5·3 to 14·8%)	90·3 (50·3 to 140·7)	10·7% (5·9 to 16·4%)	45·6% (32·8 to 57·8%)	11·7% (5·0 to 17·9%)
Ischaemic heart disease	1,228·2 (823·6 to 1,687·3)	16·1% (10·8 to 21·9%)	1,435·7 (960·0 to 1,965·1)	16·1% (10·8 to 22·1%)	16·9% (13·2 to 20·9%)	0·3% (-2·7 to 3·3%)	28,614·4 (19,490·4 to 38,838·3)	19·4% (13·2 to 26·2%)	33,038·1 (22,658·6 to 44,538·9)	20·1% (14·0 to 27·0%)	15·5% (12·1 to 19·1%)	4·0% (1·8 to 6·7%)
Ischaemic stroke	313·2 (202·8 to 448·6)	11·3% (7·2 to 16·2%)	320·3 (203·5 to 469·6)	10·8% (6·8 to 15·6%)	2·3% (-4·1 to 8·1%)	-5·2% (-11·0 to -0·3%)	7,020·0 (4,693·1 to 9,683·5)	15·9% (10·7 to 21·8%)	7,318·1 (4,858·7 to 10,062·0)	16·2% (10·9 to 22·1%)	4·2% (-0·1 to 8·9%)	1·7% (-1·9 to 5·6%)
Hemorrhagic stroke	606·3 (389·8 to 850·0)	18·6% (12·0 to 26·1%)	650·6 (430·9 to 893·8)	19·4% (12·9 to 26·8%)	7·3% (1·9 to 13·9%)	4·5% (0·4 to 9·5%)	18,121·9 (11,920·9 to 24,774·8)	24·3% (15·9 to 33·3%)	19,635·8 (13,413·6 to 26,433·5)	26·7% (18·2 to 35·8%)	8·4% (3·3 to 15·0%)	9·9% (6·3 to 15·1%)
Hypertensive heart disease	233·7 (144·3 to 340·5)	30·7% (19·0 to 45·2%)	308·5 (182·1 to 455·2)	32·1% (19·4 to 47·7%)	32·0% (20·8 to 42·0%)	4·3% (-3·7 to 11·2%)	5,010·0 (3,364·5 to 6,853·6)	33·7% (22·7 to 46·0%)	6,313·2 (4,255·2 to 8,539·0)	36·1% (25·0 to 48·8%)	26·0% (19·4 to 33·6%)	7·0% (2·9 to 12·1%)
Diabetes mellitus	408·1 (293·2 to 531·6)	35·5% (25·6 to 46·2%)	554·8 (403·5 to 717·9)	36·5% (26·6 to 47·3%)	36·0% (30·5 to 42·2%)	3·0% (0·1 to 6·3%)	21,808·5 (15,245·2 to 29,010·6)	43·8% (32·7 to 55·0%)	30,395·6 (21,543·7 to 39,884·0)	47·4% (36·4 to 58·2%)	39·4% (34·7 to 44·8%)	8·0% (5·6 to 11·7%)
Chronic kidney disease due to diabetes mellitus	78·5 (39·7 to 124·2)	26·2% (13·6 to 40·9%)	118·9 (61·5 to 187·0)	28·5% (14·7 to 44·3%)	51·5% (41·5 to 62·0%)	8·6% (1·6 to 15·8%)	2,205·4 (1,075·0 to 3,430·0)	25·3% (12·6 to 39·9%)	3,230·6 (1,644·7 to 4,931·8)	28·7% (14·8 to 44·4%)	46·5% (40·2 to 55·0%)	13·4% (9·2 to 20·0%)
Chronic kidney disease due to hypertension	93·9 (44·9 to 150·9)	23·0% (10·9 to 37·0%)	133·9 (60·8 to 216·8)	24·4% (11·0 to 39·3%)	42·5% (25·6 to 53·4%)	5·9% (-5·6 to 13·5%)	2,315·0 (1,158·8 to 3,655·6)	22·3% (11·1 to 34·8%)	3,193·2 (1,633·7 to 4,944·6)	25·1% (12·6 to 38·7%)	37·9% (31·6 to 45·0%)	12·3% (7·6 to 17·9%)
Chronic kidney disease due to glomerulonephritis	36·0 (15·2 to 61·1)	17·5% (7·4 to 29·5%)	45·9 (18·6 to 77·7)	19·3% (8·0 to 32·5%)	27·4% (11·2 to 42·7%)	10·1% (-3·0 to 23·1%)	1,275·1 (472·6 to 2,205·7)	16·5% (6·1 to 28·4%)	1,582·9 (612·0 to 2,661·6)	19·4% (7·5 to 32·4%)	24·1% (16·8 to 35·7%)	17·7% (11·4 to 28·8%)
Chronic kidney disease due to other causes	3·2 (0·7 to 6·0)	13·4% (3·0 to 24·3%)	5·0 (0·7 to 9·3)	16·6% (2·5 to 30·8%)	53·8% (10·0 to 69·1%)	24·0% (-10·7 to 35·7%)	510·4 (223·4 to 849·8)	18·9% (8·9 to 30·3%)	695·5 (308·1 to 1,139·6)	22·2% (10·7 to 34·8%)	36·3% (31·0 to 43·2%)	17·1% (12·3 to 23·6%)
Osteoarthritis	-	-	-	-	-	-	1,647·9 (949·1 to 2,565·3)	17·2% (11·5 to 23·6%)	2,392·6 (1,390·7 to 3,716·2)	18·6% (12·6 to 25·1%)	45·2% (42·1 to 49·6%)	7·7% (5·7 to 10·8%)
Low back pain	-	-	-	-	-	-	2,501·3 (1,415·2 to 4,000·8)	4·9% (3·1 to 6·8%)	3,298·8 (1,895·8 to 5,221·9)	5·5% (3·6 to 7·6%)	31·9% (29·4 to 35·6%)	12·5% (10·4 to 15·7%)
**Low bone mineral density: All causes**	282·8 (261·3 to 295·3)	0·5% (0·5 to 0·6%)	361·1 (325·4 to 380·8)	0·6% (0·6 to 0·7%)	27·7% (22·8 to 32·9%)	22·7% (18·2 to 27·6%)	7,498·8 (6,493·8 to 8,674·1)	0·3% (0·3 to 0·3%)	8,809·9 (7,565·2 to 10,269·7)	0·4% (0·3 to 0·4%)	17·5% (13·9 to 21·1%)	21·7% (18·0 to 25·6%)
Pedestrian road injuries	43·8 (40·7 to 47·8)	7·5% (7·2 to 7·9%)	50·1 (45·8 to 55·6)	8·9% (8·4 to 9·5%)	14·4% (6·5 to 21·6%)	18·7% (15·4 to 22·2%)	974·3 (882·4 to 1,070·8)	3·6% (3·3 to 3·9%)	1,071·6 (948·0 to 1,199·2)	4·4% (4·0 to 4·7%)	10·0% (2·2 to 17·2%)	22·1% (18·2 to 26·6%)
Cyclist road injuries	4·4 (3·9 to 4·8)	6·9% (6·4 to 7·3%)	4·8 (4·3 to 5·4)	8·2% (7·6 to 8·8%)	10·5% (1·0 to 20·0%)	19·5% (14·7 to 24·5%)	240·5 (192·6 to 297·5)	6·4% (5·6 to 7·4%)	273·0 (215·4 to 343·2)	8·0% (6·9 to 9·2%)	13·5% (7·3 to 19·1%)	24·2% (18·8 to 29·4%)
Motorcyclist road injuries	10·9 (9·2 to 12·3)	4·4% (4·0 to 4·9%)	14·3 (12·3 to 16·1)	5·6% (5·0 to 6·1%)	31·4% (16·4 to 48·5%)	25·6% (18·0 to 33·0%)	439·5 (366·2 to 517·2)	3·2% (2·8 to 3·7%)	559·0 (461·1 to 655·3)	4·1% (3·5 to 4·7%)	27·2% (18·2 to 37·3%)	27·0% (19·7 to 33·0%)
Motor vehicle road injuries	29·5 (26·2 to 32·3)	6·1% (5·6 to 6·5%)	33·5 (29·4 to 36·8)	7·2% (6·7 to 7·7%)	13·6% (6·1 to 21·7%)	18·2% (14·2 to 21·3%)	1,028·6 (881·6 to 1,195·7)	3·9% (3·5 to 4·4%)	1,162·6 (982·1 to 1,357·3)	4·8% (4·2 to 5·4%)	13·0% (7·1 to 18·6%)	21·3% (17·5 to 24·8%)
Other road injuries	1·3 (1·1 to 1·6)	7·0% (6·0 to 8·7%)	1·7 (1·4 to 2·1)	8·1% (7·0 to 10·1%)	28·3% (12·6 to 47·9%)	15·3% (2·5 to 28·3%)	51·4 (40·3 to 64·0)	4·6% (3·7 to 6·0%)	69·7 (53·9 to 88·3)	5·7% (4·6 to 7·4%)	35·6% (26·3 to 45·0%)	23·2% (8·0 to 38·0%)

Supplemental Results Table 1. All-age deaths, death PAFs, DALYs, DALY PAFs, and percent change for each risk factor and outcome for both sexes combined in 2005 and 2015 for Global.

Data in parenthesis are 95% uncertainty intervals. PAFs=population attributable fractions. DALYs=disability-adjusted life-years.

	2005 Deaths (in thousands)	2005 Deaths PAF	2015 Deaths (in thousands)	2015 Deaths PAF	Percent change deaths 2005-2015	Percent change deaths PAF 2005-2015	2005 DALYs (in thousands)	2005 DALYs PAF	2015 DALYs (in thousands)	2015 DALYs PAF	Percent change DALYs 2005-2015	Percent change DALYs PAF 2005-2015
Other transport injuries	8·2<br>(7·4 to 9·6)	8·1%<br>(7·6 to 8·7%)	9·9<br>(8·4 to 12·4)	9·4%<br>(8·8 to 10·0%)	19·6%<br>(8·1 to 32·9%)	15·7%<br>(11·6 to 19·6%)	218·7<br>(192·4 to 251·6)	4·2%<br>(3·8 to 4·6%)	247·2<br>(208·3 to 305·9)	4·8%<br>(4·4 to 5·3%)	13·0%<br>(2·9 to 25·1%)	15·4%<br>(10·9 to 19·7%)
Falls	170·0<br>(151·1 to 178·7)	39·0%<br>(36·9 to 40·5%)	229·9<br>(199·1 to 244·7)	43·6%<br>(41·3 to 45·8%)	35·3%<br>(29·0 to 42·3%)	11·9%<br>(7·6 to 16·6%)	3,986·8<br>(3,361·7 to 4,757·8)	16·7%<br>(15·7 to 17·6%)	4,816·3<br>(4,019·9 to 5,788·4)	18·4%<br>(17·3 to 19·5%)	20·8%<br>(17·2 to 24·7%)	10·7%<br>(6·7 to 15·9%)
Other exposure to mechanical forces	8·4<br>(6·5 to 9·2)	6·2%<br>(5·3 to 6·8%)	10·2<br>(7·1 to 11·4)	7·6%<br>(6·5 to 8·3%)	21·1%<br>(5·5 to 29·8%)	22·7%<br>(13·9 to 29·4%)	315·8<br>(254·5 to 390·0)	3·3%<br>(2·9 to 3·8%)	360·8<br>(280·3 to 454·0)	4·0%<br>(3·5 to 4·4%)	14·2%<br>(5·7 to 19·1%)	18·9%<br>(13·2 to 23·5%)
Non-venomous animal contact	0·8<br>(0·7 to 0·9)	4·9%<br>(4·4 to 5·6%)	0·8<br>(0·7 to 1·0)	5·8%<br>(5·3 to 6·4%)	6·4%<br>(-2·5 to 24·9%)	19·1%<br>(8·7 to 27·8%)	20·2<br>(16·7 to 23·9)	1·8%<br>(1·5 to 2·1%)	20·4<br>(16·7 to 24·7)	2·0%<br>(1·8 to 2·3%)	1·1%<br>(-5·9 to 15·2%)	14·1%<br>(4·5 to 22·6%)
Assault by other means	5·5<br>(5·0 to 5·9)	3·5%<br>(3·2 to 3·7%)	5·9<br>(5·3 to 6·4)	4·0%<br>(3·7 to 4·4%)	7·5%<br>(1·4 to 14·1%)	16·4%<br>(10·3 to 22·3%)	183·4<br>(154·3 to 214·8)	2·2%<br>(1·9 to 2·5%)	187·3<br>(156·1 to 222·1)	2·4%<br>(2·1 to 2·8%)	2·1%<br>(-2·8 to 7·7%)	12·6%<br>(6·8 to 18·6%)
Exposure to forces of nature	-	-	-	-	-	-	39·6<br>(23·9 to 58·2)	0·7%<br>(0·4 to 1·0%)	42·1<br>(25·8 to 61·7)	2·9%<br>(2·0 to 4·2%)	6·3%<br>(-21·5 to 40·4%)	349·9%<br>(233·1 to 526·7%)
**Low glomerular filtration rate: All causes**	1,990·6<br>(1,881·3 to 2,106·8)	3·7%<br>(3·5 to 3·9%)	2,425·7<br>(2,290·3 to 2,558·9)	4·3%<br>(4·1 to 4·6%)	21·9%<br>(19·2 to 24·5%)	17·1%<br>(15·0 to 19·1%)	47,131·4<br>(44,089·7 to 50,123·0)	1·8%<br>(1·7 to 2·0%)	54,432·8<br>(50,890·2 to 57,911·6)	2·2%<br>(2·0 to 2·4%)	15·5%<br>(12·7 to 18·2%)	19·7%<br>(16·8 to 22·5%)
Ischaemic heart disease	589·3<br>(526·0 to 656·1)	7·7%<br>(6·9 to 8·6%)	691·3<br>(618·5 to 770·3)	7·8%<br>(6·9 to 8·6%)	17·3%<br>(14·7 to 20·1%)	0·6%<br>(-1·2 to 2·6%)	9,659·3<br>(8,601·6 to 10,739·0)	6·5%<br>(5·8 to 7·3%)	10,901·5<br>(9,710·7 to 12,188·1)	6·6%<br>(5·9 to 7·4%)	12·9%<br>(10·0 to 15·8%)	1·7%<br>(-0·3 to 3·6%)
Ischaemic stroke	230·3<br>(197·1 to 266·8)	8·3%<br>(7·1 to 9·6%)	250·2<br>(214·7 to 287·9)	8·4%<br>(7·2 to 9·6%)	8·6%<br>(5·0 to 12·6%)	0·7%<br>(-2·0 to 3·5%)	3,323·2<br>(2,845·4 to 3,821·6)	7·5%<br>(6·5 to 8·7%)	3,426·8<br>(2,933·7 to 3,932·2)	7·6%<br>(6·6 to 8·7%)	3·1%<br>(-0·5 to 6·9%)	0·6%<br>(-2·0 to 3·4%)
Hemorrhagic stroke	227·8<br>(191·7 to 270·7)	7·0%<br>(5·9 to 8·3%)	241·7<br>(205·7 to 281·8)	7·2%<br>(6·2 to 8·4%)	6·1%<br>(1·8 to 11·0%)	3·3%<br>(0·3 to 6·5%)	4,391·1<br>(3,698·5 to 5,125·8)	5·9%<br>(5·0 to 6·9%)	4,492·3<br>(3,790·9 to 5,191·1)	6·1%<br>(5·2 to 7·1%)	2·3%<br>(-2·0 to 7·1%)	3·8%<br>(0·5 to 7·2%)
Peripheral vascular disease	5·5<br>(4·6 to 6·5)	14·6%<br>(12·2 to 17·0%)	7·6<br>(6·3 to 9·0)	14·4%<br>(12·1 to 16·7%)	36·1%<br>(27·3 to 46·4%)	-1·1%<br>(-4·3 to 2·3%)	131·5<br>(95·7 to 187·1)	14·4%<br>(12·6 to 16·4%)	171·7<br>(125·5 to 244·3)	14·3%<br>(12·4 to 16·3%)	30·6%<br>(26·4 to 35·7%)	-0·5%<br>(-2·5 to 1·5%)
Chronic kidney disease due to diabetes mellitus	299·4<br>(278·7 to 314·2)	100·0%<br>(100·0 to 100·0%)	417·8<br>(388·7 to 441·4)	100·0%<br>(100·0 to 100·0%)	39·5%<br>(35·4 to 43·5%)	-	8,713·0<br>(7,991·5 to 9,466·3)	100·0%<br>(100·0 to 100·0%)	11,258·0<br>(10,302·9 to 12,225·2)	100·0%<br>(100·0 to 100·0%)	29·2%<br>(25·9 to 32·5%)	-
Chronic kidney disease due to hypertension	408·5<br>(377·1 to 427·6)	100·0%<br>(100·0 to 100·0%)	549·5<br>(501·6 to 575·6)	100·0%<br>(100·0 to 100·0%)	34·5%<br>(30·0 to 38·7%)	-	10,366·1<br>(9,400·8 to 10,985·1)	100·0%<br>(100·0 to 100·0%)	12,737·4<br>(11,488·7 to 13,553·9)	100·0%<br>(100·0 to 100·0%)	22·9%<br>(18·6 to 27·4%)	-
Chronic kidney disease due to glomerulonephritis	205·6<br>(184·9 to 217·9)	100·0%<br>(100·0 to 100·0%)	237·7<br>(212·6 to 255·9)	100·0%<br>(100·0 to 100·0%)	15·6%<br>(10·9 to 20·2%)	-	7,720·0<br>(6,930·5 to 8,332·1)	100·0%<br>(100·0 to 100·0%)	8,136·0<br>(7,294·2 to 8,861·4)	100·0%<br>(100·0 to 100·0%)	5·4%<br>(1·3 to 9·3%)	-
Chronic kidney disease due to other causes	24·2<br>(20·2 to 28·6)	100·0%<br>(100·0 to 100·0%)	30·0<br>(25·0 to 35·2)	100·0%<br>(100·0 to 100·0%)	23·9%<br>(17·8 to 30·2%)	-	2,689·3<br>(2,208·6 to 3,237·9)	100·0%<br>(100·0 to 100·0%)	3,128·3<br>(2,517·9 to 3,802·9)	100·0%<br>(100·0 to 100·0%)	16·3%<br>(13·1 to 19·2%)	-
Gout	-	-	-	-	-	-	137·9<br>(90·8 to 190·7)	13·0%<br>(11·7 to 14·4%)	180·9<br>(119·9 to 249·6)	13·5%<br>(12·2 to 14·9%)	31·2%<br>(29·3 to 33·1%)	3·9%<br>(2·6 to 5·1%)

Supplemental Results Table 1. All-age deaths, death PAFs, DALYs, DALY PAFs, and percent change for each risk factor and outcome for both sexes combined in 2005 and 2015 for High SDI.

Data in parenthesis are 95% uncertainty intervals. PAFs=population attributable fractions. DALYs=disability-adjusted life-years.

	2005 Deaths (in thousands)	2005 Deaths PAF	2015 Deaths (in thousands)	2015 Deaths PAF	Percent change deaths 2005-2015	Percent change deaths PAF 2005-2015	2005 DALYs (in thousands)	2005 DALYs PAF	2015 DALYs (in thousands)	2015 DALYs PAF	Percent change DALYs 2005-2015	Percent change DALYs PAF 2005-2015
**All risk factors: All causes**	6,749·3 (6,598·7 to 6,900·4)	60·4% (59·1 to 61·8%)	6,950·1 (6,749·6 to 7,125·5)	57·7% (56·1 to 59·1%)	3·0% (2·0 to 3·8%)	-4·5% (-5·1 to -4·0%)	155,927·6 (146,823·8 to 166,178·7)	44·5% (42·2 to 47·0%)	150,327·8 (139,873·2 to 161,590·3)	42·4% (40·2 to 44·9%)	-3·6% (-5·0 to -2·4%)	-4·7% (-5·2 to -4·2%)
**Environmental/occupational risks: All causes**	949·7 (822·1 to 1,090·7)	8·5% (7·4 to 9·8%)	959·3 (825·9 to 1,111·6)	8·0% (6·9 to 9·2%)	1·0% (-2·4 to 4·1%)	-6·3% (-9·4 to -3·6%)	21,145·3 (18,929·3 to 23,830·1)	6·0% (5·4 to 6·7%)	19,908·4 (17,765·8 to 22,418·7)	5·6% (5·0 to 6·3%)	-5·8% (-8·7 to -3·2%)	-6·9% (-9·5 to -4·4%)
**Unsafe water, sanitation, and handwashing: All causes**	42·4 (29·7 to 55·9)	0·4% (0·3 to 0·5%)	45·4 (31·1 to 61·4)	0·4% (0·3 to 0·5%)	7·3% (1·0 to 12·1%)	-0·5% (-6·5 to 4·0%)	739·6 (538·8 to 947·3)	0·2% (0·2 to 0·3%)	645·9 (458·2 to 852·2)	0·2% (0·1 to 0·2%)	-12·7% (-18·8 to -8·0%)	-13·6% (-19·5 to -8·9%)
**Unsafe water source: All causes**	1·1 (0·7 to 1·4)	0·0% (0·0 to 0·0%)	1·0 (0·7 to 1·3)	0·0% (0·0 to 0·0%)	-7·3% (-22·4 to 4·6%)	-14·1% (-28·1 to -3·0%)	78·4 (49·1 to 104·7)	0·0% (0·0 to 0·0%)	59·3 (36·0 to 81·1)	0·0% (0·0 to 0·0%)	-24·3% (-34·4 to -14·6%)	-25·2% (-35·1 to -15·8%)
Diarrhoeal diseases	1·1 (0·7 to 1·3)	6·2% (4·0 to 9·3%)	1·0 (0·6 to 1·2)	3·9% (2·6 to 6·3%)	-7·5% (-23·6 to 5·0%)	-36·0% (-43·2 to -28·6%)	74·3 (46·7 to 99·9)	20·4% (12·8 to 26·7%)	55·6 (32·9 to 76·2)	14·3% (8·7 to 19·7%)	-25·2% (-35·9 to -14·9%)	-30·0% (-39·6 to -18·2%)
Typhoid fever	0·1 (0·0 to 0·1)	57·6% (40·8 to 69·5%)	0·0 (0·0 to 0·1)	57·1% (39·9 to 69·1%)	-2·5% (-15·3 to 10·8%)	-1·0% (-7·0 to 5·0%)	3·2 (1·5 to 6·0)	61·2% (44·1 to 73·0%)	3·0 (1·3 to 5·8)	61·2% (43·1 to 73·6%)	-6·8% (-19·4 to 6·6%)	0·0% (-5·8 to 5·9%)
Paratyphoid fever	0·0 (0·0 to 0·0)	45·8% (31·2 to 57·2%)	0·0 (0·0 to 0·0)	44·0% (29·2 to 56·0%)	-10·0% (-21·6 to 1·3%)	-4·1% (-11·8 to 3·1%)	0·9 (0·3 to 2·0)	51·1% (35·6 to 62·6%)	0·8 (0·3 to 1·8)	49·7% (34·0 to 61·8%)	-13·6% (-25·0 to -2·7%)	-2·6% (-9·6 to 4·0%)
**Unsafe sanitation: All causes**	0·4 (0·3 to 0·5)	0·0% (0·0 to 0·0%)	0·2 (0·2 to 0·3)	0·0% (0·0 to 0·0%)	-42·1% (-56·7 to -26·9%)	-46·3% (-59·9 to -32·2%)	32·9 (27·1 to 40·0)	0·0% (0·0 to 0·0%)	17·8 (12·4 to 24·7)	0·0% (0·0 to 0·0%)	-45·8% (-60·7 to -31·1%)	-46·5% (-61·2 to -31·7%)
Diarrhoeal diseases	0·4 (0·3 to 0·5)	2·3% (1·9 to 4·2%)	0·2 (0·2 to 0·3)	0·9% (0·6 to 1·8%)	-42·5% (-57·2 to -27·0%)	-60·1% (-70·1 to -49·2%)	32·0 (26·2 to 39·0)	8·8% (7·4 to 11·2%)	17·2 (11·9 to 24·1)	4·4% (3·0 to 6·5%)	-46·1% (-61·1 to -31·1%)	-49·5% (-63·4 to -35·9%)
Typhoid fever	0·0 (0·0 to 0·0)	13·0% (10·4 to 16·6%)	0·0 (0·0 to 0·0)	8·9% (6·7 to 11·6%)	-32·7% (-44·9 to -18·7%)	-31·4% (-43·1 to -17·7%)	0·7 (0·3 to 1·2)	13·1% (10·4 to 16·9%)	0·4 (0·2 to 0·8)	9·0% (6·7 to 11·7%)	-36·4% (-48·1 to -23·3%)	-31·4% (-43·0 to -17·2%)
Paratyphoid fever	0·0 (0·0 to 0·0)	12·9% (10·8 to 15·9%)	0·0 (0·0 to 0·0)	9·4% (7·2 to 12·0%)	-31·4% (-46·4 to -17·0%)	-26·9% (-41·3 to -11·9%)	0·2 (0·1 to 0·5)	13·1% (10·9 to 16·3%)	0·1 (0·1 to 0·3)	9·5% (7·4 to 12·1%)	-35·8% (-48·5 to -22·3%)	-27·5% (-40·8 to -13·2%)
**No handwashing with soap: All causes**	41·5 (28·8 to 55·0)	0·4% (0·3 to 0·5%)	44·6 (30·2 to 60·6)	0·4% (0·3 to 0·5%)	7·6% (1·3 to 12·5%)	-0·2% (-5·9 to 4·4%)	675·6 (473·7 to 887·6)	0·2% (0·1 to 0·3%)	597·1 (410·7 to 801·3)	0·2% (0·1 to 0·2%)	-11·6% (-17·8 to -6·7%)	-12·6% (-18·6 to -7·6%)
Diarrhoeal diseases	4·1 (2·7 to 5·2)	24·0% (17·9 to 29·8%)	5·5 (3·1 to 7·0)	21·8% (16·0 to 27·2%)	32·1% (1·4 to 41·7%)	-9·3% (-12·2 to -7·0%)	92·9 (68·4 to 116·6)	25·5% (19·3 to 31·4%)	89·2 (58·6 to 113·9)	22·8% (17·0 to 28·5%)	-4·0% (-20·3 to 1·6%)	-10·4% (-13·3 to -8·1%)
Typhoid fever	0·0 (0·0 to 0·0)	29·6% (22·2 to 36·4%)	0·0 (0·0 to 0·0)	27·4% (20·3 to 34·1%)	-8·7% (-18·5 to 1·5%)	-7·3% (-10·2 to -4·9%)	1·5 (0·7 to 2·8)	29·9% (22·4 to 36·8%)	1·3 (0·6 to 2·6)	27·9% (20·6 to 34·7%)	-13·2% (-22·9 to -2·3%)	-6·8% (-9·7 to -4·5%)
Paratyphoid fever	0·0 (0·0 to 0·0)	28·3% (21·3 to 35·2%)	0·0 (0·0 to 0·0)	26·1% (19·2 to 32·8%)	-13·6% (-21·6 to -7·3%)	-8·0% (-10·9 to -5·7%)	0·5 (0·2 to 1·1)	28·8% (21·7 to 35·7%)	0·4 (0·2 to 0·9)	26·6% (19·7 to 33·4%)	-18·0% (-26·1 to -11·0%)	-7·5% (-10·5 to -5·3%)
**Air pollution: All causes**	653·4 (546·6 to 775·6)	5·9% (4·9 to 6·9%)	639·9 (524·5 to 770·1)	5·3% (4·4 to 6·4%)	-2·1% (-7·1 to 2·1%)	-9·2% (-13·7 to -5·2%)	11,602·4 (10,007·1 to 13,384·7)	3·3% (2·7 to 4·0%)	10,067·7 (8,609·9 to 11,637·9)	2·8% (2·3 to 3·5%)	-13·2% (-17·4 to -9·2%)	-14·2% (-18·5 to -10·1%)
**Ambient particulate matter pollution: All causes**	588·7 (485·8 to 703·7)	5·3% (4·3 to 6·3%)	594·5 (479·9 to 722·9)	4·9% (4·0 to 6·0%)	1·0% (-3·2 to 4·6%)	-6·4% (-10·1 to -3·1%)	10,410·9 (8,934·6 to 12,011·3)	3·0% (2·5 to 3·6%)	9,383·9 (7,998·2 to 10,943·8)	2·7% (2·1 to 3·2%)	-9·9% (-13·2 to -6·6%)	-10·8% (-14·3 to -7·6%)
Lower respiratory infections	52·3 (20·5 to 97·9)	12·1% (4·7 to 22·6%)	62·7 (26·1 to 116·6)	12·2% (5·1 to 22·5%)	19·9% (14·2 to 27·3%)	1·2% (-0·7 to 6·5%)	800·7 (334·4 to 1,457·8)	12·6% (5·3 to 23·0%)	796·7 (348·7 to 1,453·5)	12·6% (5·5 to 22·8%)	-0·5% (-6·7 to 6·0%)	0·0% (-1·7 to 4·1%)
Tracheal, bronchus, and lung cancer	51·3 (26·3 to 82·5)	8·6% (4·5 to 13·9%)	57·1 (29·7 to 91·2)	8·6% (4·5 to 13·8%)	11·4% (8·3 to 15·4%)	-0·3% (-1·8 to 2·2%)	1,039·2 (536·4 to 1,667·5)	8·8% (4·6 to 14·1%)	1,080·0 (563·0 to 1,721·9)	8·7% (4·6 to 13·9%)	3·9% (0·8 to 8·1%)	-0·8% (-2·6 to 1·7%)
Ischaemic heart disease	298·6 (222·3 to 374·4)	12·2% (9·1 to 15·1%)	292·7 (205·2 to 376·9)	11·7% (8·2 to 15·1%)	-2·0% (-9·5 to 4·0%)	-3·6% (-10·9 to 2·3%)	5,339·2 (4,364·8 to 6,308·3)	13·8% (11·3 to 16·4%)	4,654·2 (3,731·8 to 5,560·2)	13·3% (10·7 to 15·9%)	-12·8% (-18·0 to -8·2%)	-4·2% (-9·7 to 0·5%)
Ischaemic stroke	77·5 (49·3 to 111·3)	8·6% (5·5 to 12·3%)	72·1 (44·1 to 109·4)	8·5% (5·1 to 12·9%)	-7·0% (-18·6 to 2·0%)	-1·4% (-13·8 to 7·7%)	1,125·1 (817·9 to 1,442·9)	9·6% (6·9 to 12·3%)	934·1 (670·4 to 1,210·8)	9·4% (6·7 to 12·2%)	-17·0% (-24·7 to -8·9%)	-1·4% (-10·1 to 7·7%)
Hemorrhagic stroke	48·4 (35·4 to 63·3)	10·0% (7·3 to 13·0%)	43·8 (31·0 to 58·8)	9·6% (6·7 to 12·9%)	-9·6% (-18·4 to -1·1%)	-3·8% (-12·8 to 4·3%)	1,031·5 (796·8 to 1,264·0)	11·5% (8·9 to 14·1%)	835·8 (641·2 to 1,036·9)	11·2% (8·7 to 13·9%)	-19·0% (-23·9 to -13·5%)	-2·4% (-7·8 to 3·3%)
Chronic obstructive pulmonary disease	60·7 (27·0 to 103·4)	14·3% (6·4 to 24·1%)	66·2 (29·7 to 113·4)	13·7% (6·2 to 23·3%)	9·1% (5·5 to 12·2%)	-3·9% (-6·3 to -2·2%)	1,075·2 (482·0 to 1,826·0)	14·0% (6·3 to 23·6%)	1,083·0 (487·4 to 1,859·6)	13·3% (6·0 to 22·7%)	0·7% (-2·7 to 3·6%)	-4·6% (-7·3 to -2·8%)
**Household air pollution from solid fuels: All causes**	50·8 (31·8 to 78·2)	0·5% (0·3 to 0·7%)	24·2 (12·8 to 42·9)	0·2% (0·1 to 0·4%)	-52·3% (-72·3 to -11·6%)	-55·8% (-74·3 to -18·0%)	1,086·6 (681·9 to 1,700·9)	0·3% (0·2 to 0·5%)	459·6 (236·5 to 846·4)	0·1% (0·1 to 0·2%)	-57·7% (-76·2 to -19·4%)	-58·2% (-76·4 to -20·4%)
Lower respiratory infections	3·9 (2·1 to 6·9)	0·9% (0·5 to 1·6%)	2·2 (1·0 to 4·2)	0·4% (0·2 to 0·8%)	-43·9% (-66·0 to -1·1%)	-52·7% (-71·2 to -16·0%)	111·3 (57·6 to 205·6)	1·8% (0·9 to 3·2%)	49·0 (20·7 to 100·9)	0·8% (0·3 to 1·6%)	-55·9% (-76·8 to -11·0%)	-55·7% (-76·9 to -10·1%)
Tracheal, bronchus, and lung cancer	5·1 (2·4 to 9·4)	0·9% (0·4 to 1·6%)	2·9 (1·2 to 5·3)	0·4% (0·2 to 0·8%)	-43·7% (-62·0 to -9·6%)	-49·5% (-65·9 to -19·1%)	118·5 (54·9 to 216·6)	1·0% (0·5 to 1·8%)	63·1 (26·8 to 117·3)	0·5% (0·2 to 0·9%)	-46·7% (-64·5 to -13·1%)	-49·2% (-66·0 to -17·4%)
Ischaemic heart disease	24·0 (14·9 to 38·1)	1·0% (0·6 to 1·6%)	11·6 (5·9 to 21·6)	0·5% (0·2 to 0·9%)	-51·6% (-73·4 to -7·2%)	-52·4% (-73·8 to -9·1%)	499·3 (314·0 to 784·0)	1·3% (0·8 to 2·0%)	207·0 (103·6 to 384·7)	0·6% (0·3 to 1·1%)	-58·5% (-78·2 to -17·8%)	-54·5% (-76·0 to -9·7%)
Ischaemic stroke	8·7 (5·0 to 14·4)	1·0% (0·6 to 1·6%)	3·7 (1·7 to 7·0)	0·4% (0·2 to 0·8%)	-57·6% (-77·8 to -14·6%)	-55·1% (-76·7 to -10·0%)	144·3 (87·2 to 228·6)	1·2% (0·7 to 2·0%)	54·3 (25·4 to 105·0)	0·5% (0·3 to 1·1%)	-62·4% (-80·5 to -24·0%)	-55·3% (-76·9 to -9·2%)
Hemorrhagic stroke	4·6 (2·9 to 7·2)	1·0% (0·6 to 1·5%)	1·7 (0·9 to 3·2)	0·4% (0·2 to 0·7%)	-63·1% (-78·9 to -30·7%)	-60·8% (-77·5 to -26·9%)	114·2 (70·9 to 177·2)	1·3% (0·8 to 2·0%)	39·4 (20·1 to 72·3)	0·5% (0·3 to 1·0%)	-65·5% (-80·8 to -34·7%)	-58·5% (-76·7 to -21·7%)
Chronic obstructive pulmonary disease	4·5 (1·9 to 8·5)	1·1% (0·5 to 2·0%)	2·1 (0·9 to 4·5)	0·4% (0·2 to 0·9%)	-52·2% (-67·8 to -23·4%)	-57·9% (-71·6 to -32·2%)	88·9 (38·0 to 168·4)	1·2% (0·5 to 2·2%)	40·2 (15·7 to 84·7)	0·5% (0·2 to 1·1%)	-54·8% (-70·3 to -25·2%)	-57·2% (-72·1 to -28·8%)
Cataract	-	-	-	-	-	-	10·1 (5·8 to 15·8)	2·4% (1·6 to 3·5%)	6·6 (3·0 to 12·7)	1·3% (0·7 to 2·3%)	-35·0% (-63·1 to 16·7%)	-47·6% (-70·1 to -6·4%)
**Ambient ozone pollution: All causes**	25·9 (9·8 to 44·8)	0·2% (0·1 to 0·4%)	29·0 (11·0 to 49·9)	0·2% (0·1 to 0·4%)	12·1% (9·8 to 14·3%)	4·0% (1·9 to 6·1%)	341·7 (129·9 to 591·5)	0·1% (0·0 to 0·2%)	351·3 (133·0 to 606·0)	0·1% (0·0 to 0·2%)	2·8% (0·8 to 4·8%)	1·7% (-0·6 to 4·0%)
Chronic obstructive pulmonary disease	25·9 (9·8 to 44·8)	6·1% (2·3 to 10·6%)	29·0 (11·0 to 49·9)	6·0% (2·3 to 10·4%)	12·1% (9·8 to 14·3%)	-1·2% (-2·2 to -0·4%)	341·7 (129·9 to 591·5)	4·4% (1·7 to 7·6%)	351·3 (133·0 to 606·0)	4·3% (1·6 to 7·4%)	2·8% (0·8 to 4·8%)	-2·7% (-3·9 to -1·5%)
**Other environmental risks: All causes**	128·5 (65·2 to 202·5)	1·2% (0·6 to 1·8%)	120·2 (59·7 to 195·4)	1·0% (0·5 to 1·6%)	-6·5% (-13·4 to -1·2%)	-13·3% (-19·7 to -8·4%)	1,993·5 (986·5 to 3,252·7)	0·6% (0·3 to 0·9%)	1,659·6 (808·6 to 2,756·1)	0·5% (0·2 to 0·8%)	-16·7% (-21·6 to -13·0%)	-17·7% (-22·7 to -14·0%)
**Residential radon: All causes**	20·5 (13·1 to 28·3)	0·2% (0·1 to 0·3%)	22·5 (13·8 to 31·4)	0·2% (0·1 to 0·3%)	9·7% (1·6 to 18·0%)	1·8% (-5·9 to 9·2%)	424·4 (278·6 to 585·0)	0·1% (0·1 to 0·2%)	434·8 (278·3 to 603·8)	0·1% (0·1 to 0·2%)	2·5% (-4·2 to 8·8%)	1·4% (-5·2 to 7·9%)
Tracheal, bronchus, and lung cancer	20·5 (13·1 to 28·3)	3·4% (2·2 to 4·7%)	22·5 (13·8 to 31·4)	3·4% (2·1 to 4·7%)	9·7% (1·6 to 18·0%)	-1·7% (-8·9 to 5·4%)	424·4 (278·6 to 585·0)	3·6% (2·4 to 5·0%)	434·8 (278·3 to 603·8)	3·5% (2·2 to 4·9%)	2·5% (-4·2 to 8·8%)	-2·2% (-8·2 to 3·8%)
**Lead exposure: All causes**	108·1 (46·2 to 182·4)	1·0% (0·4 to 1·6%)	97·7 (39·2 to 171·3)	0·8% (0·3 to 1·4%)	-9·6% (-19·8 to -3·6%)	-16·2% (-25·6 to -10·5%)	1,569·1 (583·3 to 2,815·9)	0·4% (0·2 to 0·8%)	1,224·8 (416·3 to 2,309·8)	0·3% (0·1 to 0·7%)	-21·9% (-31·5 to -17·0%)	-22·8% (-32·4 to -17·9%)
Rheumatic heart disease	0·4 (0·1 to 0·9)	1·0% (0·3 to 2·3%)	0·3 (0·1 to 0·9)	0·9% (0·3 to 2·2%)	-17·9% (-30·0 to -6·5%)	-11·2% (-23·7 to 0·8%)	6·4 (1·9 to 13·3)	0·8% (0·2 to 1·8%)	4·2 (1·1 to 9·2)	0·7% (0·2 to 1·6%)	-33·4% (-42·8 to -25·1%)	-13·7% (-25·4 to -2·4%)
Ischaemic heart disease	59·2 (23·9 to 103·8)	2·4% (1·0 to 4·2%)	52·9 (19·5 to 96·1)	2·1% (0·8 to 3·9%)	-10·6% (-21·6 to -3·9%)	-12·0% (-22·9 to -5·4%)	818·5 (296·0 to 1,506·0)	2·1% (0·8 to 3·9%)	631·8 (201·8 to 1,220·9)	1·8% (0·6 to 3·5%)	-22·8% (-33·5 to -17·4%)	-15·2% (-27·1 to -9·4%)
Ischaemic stroke	18·2 (7·0 to 33·1)	2·0% (0·8 to 3·7%)	15·1 (5·3 to 28·3)	1·8% (0·6 to 3·4%)	-17·2% (-30·3 to -7·4%)	-12·3% (-26·2 to -2·0%)	234·5 (83·0 to 433·3)	2·0% (0·7 to 3·7%)	169·6 (54·1 to 326·1)	1·7% (0·6 to 3·3%)	-27·7% (-39·3 to -20·0%)	-14·1% (-28·2 to -5·8%)
Hemorrhagic stroke	11·8 (4·4 to 21·6)	2·4% (0·9 to 4·4%)	9·9 (3·4 to 19·4)	2·2% (0·7 to 4·2%)	-16·4% (-27·7 to -6·9%)	-11·1% (-22·3 to -1·7%)	190·2 (60·2 to 370·4)	2·1% (0·7 to 4·2%)	132·8 (38·5 to 272·9)	1·8% (0·5 to 3·6%)	-30·2% (-39·9 to -24·2%)	-15·9% (-27·1 to -9·1%)
Hypertensive heart disease	6·5 (1·4 to 16·9)	4·9% (1·1 to 12·8%)	7·1 (1·3 to 19·1)	4·4% (0·8 to 11·8%)	8·6% (-9·8 to 20·6%)	-10·5% (-25·4 to -1·4%)	80·8 (19·2 to 196·3)	4·1% (1·0 to 10·1%)	74·2 (14·8 to 197·3)	3·5% (0·7 to 9·2%)	-8·1% (-25·1 to 1·2%)	-15·1% (-30·5 to -7·1%)
Cardiomyopathy and myocarditis	2·0 (0·7 to 4·0)	1·2% (0·4 to 2·3%)	1·8 (0·6 to 3·6)	1·1% (0·3 to 2·1%)	-12·3% (-22·6 to -3·1%)	-11·6% (-21·4 to -2·1%)	40·8 (10·0 to 94·5)	0·9% (0·2 to 2·2%)	29·6 (6·9 to 68·7)	0·8% (0·2 to 1·9%)	-27·4% (-35·1 to -20·1%)	-13·6% (-22·8 to -6·1%)

Supplemental Results Table 1. All-age deaths, death PAFs, DALYs, DALY PAFs, and percent change for each risk factor and outcome for both sexes combined in 2005 and 2015 for High SDI.

Data in parenthesis are 95% uncertainty intervals. PAFs=population attributable fractions. DALYs=disability-adjusted life-years.

	2005 Deaths (in thousands)	2005 Deaths PAF	2015 Deaths (in thousands)	2015 Deaths PAF	Percent change deaths 2005-2015	Percent change deaths PAF 2005-2015	2005 DALYs (in thousands)	2005 DALYs PAF	2015 DALYs (in thousands)	2015 DALYs PAF	Percent change DALYs 2005-2015	Percent change DALYs PAF 2005-2015
Atrial fibrillation and flutter	1·1 (0·5 to 1·9)	1·6% (0·8 to 2·7%)	1·3 (0·5 to 2·3)	1·4% (0·6 to 2·4%)	13·6% (-0·2 to 21·8%)	-14·4% (-24·3 to -8·8%)	27·8 (11·5 to 49·3)	1·7% (0·7 to 2·8%)	27·2 (9·6 to 51·4)	1·4% (0·5 to 2·4%)	-2·2% (-17·4 to 5·7%)	-20·3% (-32·6 to -14·0%)
Aortic aneurysm	1·0 (0·4 to 1·8)	1·4% (0·6 to 2·5%)	0·9 (0·3 to 1·7)	1·1% (0·4 to 2·1%)	-8·8% (-23·2 to 0·1%)	-21·1% (-33·4 to -14·1%)	14·4 (5·4 to 26·6)	1·3% (0·5 to 2·5%)	11·6 (3·6 to 23·4)	1·0% (0·3 to 2·0%)	-19·7% (-34·9 to -11·2%)	-23·7% (-37·9 to -16·4%)
Peripheral vascular disease	0·3 (0·1 to 0·6)	1·1% (0·5 to 2·0%)	0·3 (0·1 to 0·7)	0·9% (0·3 to 1·7%)	8·1% (-9·2 to 20·6%)	-17·6% (-30·1 to -10·5%)	5·4 (2·0 to 10·0)	1·1% (0·4 to 1·9%)	5·2 (1·7 to 10·5)	0·8% (0·3 to 1·6%)	-2·4% (-20·2 to 8·6%)	-19·9% (-34·2 to -12·3%)
Endocarditis	0·3 (0·1 to 0·6)	1·4% (0·6 to 2·5%)	0·3 (0·1 to 0·7)	1·2% (0·4 to 2·2%)	1·6% (-12·2 to 11·0%)	-16·0% (-26·4 to -9·0%)	4·9 (1·7 to 9·3)	1·2% (0·4 to 2·2%)	4·2 (1·3 to 8·5)	0·9% (0·3 to 1·9%)	-13·8% (-27·5 to -5·5%)	-20·7% (-32·7 to -14·0%)
Other cardiovascular and circulatory diseases	2·5 (1·1 to 4·2)	1·6% (0·7 to 2·7%)	2·4 (1·0 to 4·3)	1·3% (0·5 to 2·4%)	-3·4% (-15·3 to 4·1%)	-16·7% (-26·6 to -11·2%)	50·7 (19·1 to 92·1)	1·5% (0·6 to 2·7%)	43·4 (13·9 to 84·3)	1·2% (0·4 to 2·3%)	-14·4% (-27·4 to -7·5%)	-20·5% (-32·4 to -14·7%)
Idiopathic developmental intellectual disability	-	-	-	-	-	-	16·4 (7·3 to 29·5)	5·2% (3·4 to 6·9%)	12·4 (5·5 to 22·4)	3·7% (2·3 to 5·2%)	-24·4% (-31·8 to -17·4%)	-28·2% (-35·4 to -21·5%)
Chronic kidney disease due to diabetes mellitus	1·6 (0·7 to 3·0)	2·2% (0·9 to 4·0%)	2·0 (0·8 to 3·8)	1·9% (0·8 to 3·7%)	22·4% (7·9 to 30·9%)	-12·6% (-22·5 to -7·8%)	28·2 (10·7 to 55·2)	1·7% (0·6 to 3·3%)	29·9 (9·9 to 62·0)	1·4% (0·5 to 2·9%)	5·9% (-8·3 to 13·6%)	-15·7% (-26·9 to -10·2%)
Chronic kidney disease due to hypertension	2·2 (1·0 to 3·8)	2·7% (1·2 to 4·6%)	2·5 (1·0 to 4·4)	2·3% (1·0 to 4·1%)	12·3% (0·2 to 20·2%)	-12·5% (-21·3 to -7·7%)	29·1 (11·7 to 52·7)	2·0% (0·8 to 3·7%)	27·9 (10·1 to 52·9)	1·7% (0·6 to 3·3%)	-4·1% (-15·2 to 2·4%)	-15·8% (-25·2 to -10·9%)
Chronic kidney disease due to glomerulonephritis	0·6 (0·3 to 1·0)	2·4% (1·1 to 4·1%)	0·7 (0·3 to 1·2)	2·3% (1·0 to 3·9%)	15·0% (5·3 to 21·4%)	-5·5% (-12·7 to -1·8%)	12·3 (4·5 to 23·7)	1·5% (0·5 to 2·8%)	11·8 (4·0 to 23·6)	1·3% (0·4 to 2·6%)	-4·1% (-13·0 to 0·7%)	-9·4% (-17·5 to -5·4%)
Chronic kidney disease due to other causes	0·1 (0·0 to 0·1)	2·6% (1·2 to 4·5%)	0·1 (0·0 to 0·2)	2·4% (1·0 to 4·3%)	31·4% (15·1 to 43·7%)	-6·5% (-17·4 to 0·6%)	9·0 (3·3 to 18·9)	1·5% (0·6 to 3·1%)	9·0 (3·1 to 19·8)	1·3% (0·5 to 2·7%)	0·6% (-11·4 to 7·3%)	-14·1% (-24·5 to -9·0%)
**Occupational risks: All causes**	159·7 (148·1 to 169·9)	1·4% (1·3 to 1·5%)	189·1 (174·7 to 203·2)	1·6% (1·5 to 1·7%)	18·4% (14·2 to 22·7%)	9·8% (5·9 to 13·8%)	7,429·6 (6,258·8 to 8,845·4)	2·1% (2·0 to 2·3%)	8,106·2 (6,833·6 to 9,673·0)	2·3% (2·1 to 2·5%)	9·1% (6·3 to 11·7%)	7·9% (5·0 to 10·8%)
**Occupational carcinogens: All causes**	122·1 (111·7 to 132·5)	1·1% (1·0 to 1·2%)	147·6 (134·3 to 161·0)	1·2% (1·1 to 1·3%)	20·9% (15·6 to 26·1%)	12·1% (7·1 to 16·8%)	2,075·4 (1,906·9 to 2,244·7)	0·6% (0·5 to 0·7%)	2,298·3 (2,096·4 to 2,503·1)	0·6% (0·6 to 0·7%)	10·7% (6·5 to 15·0%)	9·6% (5·2 to 13·8%)
**Occupational exposure to asbestos: All causes**	91·5 (80·5 to 102·5)	0·8% (0·7 to 0·9%)	117·8 (103·7 to 131·1)	1·0% (0·9 to 1·1%)	28·8% (21·9 to 35·6%)	19·4% (12·9 to 25·6%)	1,396·1 (1,219·3 to 1,574·1)	0·4% (0·3 to 0·5%)	1,642·6 (1,440·6 to 1,845·2)	0·5% (0·4 to 0·5%)	17·7% (11·6 to 23·6%)	16·4% (10·3 to 22·5%)
Larynx cancer	0·4 (0·3 to 0·6)	1·7% (1·3 to 2·2%)	0·6 (0·5 to 0·8)	2·7% (2·0 to 3·5%)	51·6% (24·3 to 90·9%)	59·0% (30·9 to 98·6%)	6·0 (4·2 to 8·1)	1·0% (0·7 to 1·4%)	8·0 (5·8 to 10·5)	1·5% (1·1 to 1·9%)	32·2% (11·1 to 60·7%)	46·9% (24·7 to 78·2%)
Tracheal, bronchus, and lung cancer	80·4 (69·6 to 90·9)	13·5% (11·7 to 15·3%)	101·4 (88·0 to 114·4)	15·3% (13·2 to 17·2%)	26·2% (19·5 to 32·9%)	13·1% (7·8 to 18·3%)	1,205·6 (1,030·9 to 1,378·4)	10·2% (8·7 to 11·6%)	1,385·9 (1,192·8 to 1,584·0)	11·2% (9·6 to 12·7%)	15·0% (9·1 to 20·6%)	9·8% (4·9 to 14·7%)
Ovarian cancer	0·4 (0·2 to 0·7)	0·7% (0·3 to 1·1%)	0·5 (0·2 to 0·8)	0·7% (0·3 to 1·1%)	21·9% (12·5 to 33·4%)	8·8% (1·7 to 16·6%)	5·8 (2·7 to 9·4)	0·4% (0·2 to 0·7%)	6·5 (3·2 to 10·7)	0·4% (0·2 to 0·7%)	12·7% (5·5 to 21·0%)	4·7% (-0·9 to 11·4%)
Mesothelioma	10·3 (8·5 to 11·7)	78·2% (64·7 to 88·4%)	15·3 (12·8 to 17·3)	81·3% (69·3 to 90·3%)	48·1% (37·6 to 57·6%)	4·0% (1·9 to 7·5%)	178·7 (141·8 to 210·0)	70·0% (55·6 to 81·9%)	242·2 (197·4 to 281·9)	73·3% (59·8 to 84·2%)	35·5% (26·8 to 43·3%)	4·7% (2·4 to 8·2%)
**Occupational exposure to arsenic: All causes**	0·9 (0·7 to 1·2)	0·0% (0·0 to 0·0%)	0·8 (0·6 to 1·0)	0·0% (0·0 to 0·0%)	-14·6% (-19·8 to -8·3%)	-20·8% (-25·4 to -15·2%)	20·1 (15·5 to 25·2)	0·0% (0·0 to 0·0%)	16·9 (12·9 to 21·3)	0·0% (0·0 to 0·0%)	-15·8% (-20·9 to -9·4%)	-16·7% (-21·8 to -10·7%)
Tracheal, bronchus, and lung cancer	0·9 (0·7 to 1·2)	0·2% (0·1 to 0·2%)	0·8 (0·6 to 1·0)	0·1% (0·1 to 0·2%)	-14·6% (-19·8 to -8·3%)	-23·5% (-27·7 to -18·5%)	20·1 (15·5 to 25·2)	0·2% (0·1 to 0·2%)	16·9 (12·9 to 21·3)	0·1% (0·1 to 0·2%)	-15·8% (-20·9 to -9·4%)	-19·6% (-23·9 to -14·5%)
**Occupational exposure to benzene: All causes**	0·5 (0·4 to 0·6)	0·0% (0·0 to 0·0%)	0·5 (0·4 to 0·6)	0·0% (0·0 to 0·0%)	11·1% (6·3 to 15·7%)	3·0% (-1·1 to 7·1%)	15·5 (13·3 to 17·9)	0·0% (0·0 to 0·0%)	17·1 (14·5 to 20·0)	0·0% (0·0 to 0·0%)	10·5% (5·2 to 15·9%)	9·3% (4·1 to 14·3%)
Leukaemia	0·5 (0·4 to 0·6)	0·5% (0·4 to 0·6%)	0·5 (0·4 to 0·6)	0·5% (0·4 to 0·5%)	11·1% (6·3 to 15·7%)	-4·9% (-8·6 to -0·9%)	15·5 (13·3 to 17·9)	0·7% (0·6 to 0·8%)	17·1 (14·5 to 20·0)	0·7% (0·6 to 0·8%)	10·5% (5·2 to 15·9%)	6·4% (2·3 to 10·8%)
**Occupational exposure to beryllium: All causes**	0·1 (0·1 to 0·1)	0·0% (0·0 to 0·0%)	0·1 (0·0 to 0·1)	0·0% (0·0 to 0·0%)	-17·1% (-22·5 to -10·5%)	-23·2% (-28·1 to -17·2%)	1·4 (1·1 to 1·7)	0·0% (0·0 to 0·0%)	1·2 (0·9 to 1·4)	0·0% (0·0 to 0·0%)	-18·3% (-23·9 to -11·7%)	-19·2% (-24·6 to -12·8%)
Tracheal, bronchus, and lung cancer	0·1 (0·1 to 0·1)	0·0% (0·0 to 0·0%)	0·1 (0·0 to 0·1)	0·0% (0·0 to 0·0%)	-17·1% (-22·5 to -10·5%)	-25·8% (-30·2 to -20·6%)	1·4 (1·1 to 1·7)	0·0% (0·0 to 0·0%)	1·2 (0·9 to 1·4)	0·0% (0·0 to 0·0%)	-18·3% (-23·9 to -11·7%)	-22·0% (-26·8 to -16·5%)
**Occupational exposure to cadmium: All causes**	0·2 (0·2 to 0·3)	0·0% (0·0 to 0·0%)	0·2 (0·2 to 0·3)	0·0% (0·0 to 0·0%)	-9·8% (-15·0 to -3·4%)	-16·4% (-21·0 to -10·7%)	5·2 (4·3 to 6·2)	0·0% (0·0 to 0·0%)	4·7 (3·8 to 5·6)	0·0% (0·0 to 0·0%)	-11·0% (-16·1 to -4·7%)	-12·0% (-17·2 to -5·8%)
Tracheal, bronchus, and lung cancer	0·2 (0·2 to 0·3)	0·0% (0·0 to 0·0%)	0·2 (0·2 to 0·3)	0·0% (0·0 to 0·0%)	-9·8% (-15·0 to -3·4%)	-19·2% (-23·4 to -14·3%)	5·2 (4·3 to 6·2)	0·0% (0·0 to 0·1%)	4·7 (3·8 to 5·6)	0·0% (0·0 to 0·0%)	-11·0% (-16·1 to -4·7%)	-15·0% (-19·4 to -9·9%)
**Occupational exposure to chromium: All causes**	0·8 (0·7 to 0·9)	0·0% (0·0 to 0·0%)	0·7 (0·6 to 0·8)	0·0% (0·0 to 0·0%)	-12·9% (-18·1 to -6·7%)	-19·3% (-23·9 to -13·7%)	17·8 (15·6 to 20·1)	0·0% (0·0 to 0·0%)	15·3 (13·3 to 17·6)	0·0% (0·0 to 0·0%)	-14·1% (-19·2 to -7·9%)	-15·0% (-20·3 to -8·9%)
Tracheal, bronchus, and lung cancer	0·8 (0·7 to 0·9)	0·1% (0·1 to 0·2%)	0·7 (0·6 to 0·8)	0·1% (0·1 to 0·1%)	-12·9% (-18·1 to -6·7%)	-22·0% (-26·1 to -16·9%)	17·8 (15·6 to 20·1)	0·2% (0·1 to 0·2%)	15·3 (13·3 to 17·6)	0·1% (0·1 to 0·1%)	-14·1% (-19·2 to -7·9%)	-18·0% (-22·2 to -12·8%)
**Occupational exposure to diesel engine exhaust: All causes**	10·6 (9·2 to 12·0)	0·1% (0·1 to 0·1%)	11·3 (9·9 to 13·0)	0·1% (0·1 to 0·1%)	7·3% (2·5 to 12·4%)	-0·6% (-4·7 to 4·2%)	226·5 (197·9 to 258·2)	0·1% (0·1 to 0·1%)	239·9 (209·1 to 273·8)	0·1% (0·1 to 0·1%)	5·9% (1·1 to 11·0%)	4·8% (0·1 to 10·0%)
Tracheal, bronchus, and lung cancer	10·6 (9·2 to 12·0)	1·8% (1·5 to 2·0%)	11·3 (9·9 to 13·0)	1·7% (1·5 to 2·0%)	7·3% (2·5 to 12·4%)	-3·9% (-7·6 to 0·1%)	226·5 (197·9 to 258·2)	1·9% (1·7 to 2·2%)	239·9 (209·1 to 273·8)	1·9% (1·7 to 2·2%)	5·9% (1·1 to 11·0%)	1·1% (-2·5 to 4·9%)
**Occupational exposure to second-hand smoke: All causes**	10·5 (9·7 to 11·2)	0·1% (0·1 to 0·1%)	10·5 (9·7 to 11·4)	0·1% (0·1 to 0·1%)	0·6% (-4·3 to 6·7%)	-6·7% (-11·0 to -1·4%)	222·8 (206·7 to 238·8)	0·1% (0·1 to 0·1%)	221·0 (204·2 to 238·6)	0·1% (0·1 to 0·1%)	-0·8% (-5·6 to 4·8%)	-1·9% (-6·7 to 3·6%)
Tracheal, bronchus, and lung cancer	10·5 (9·7 to 11·2)	1·8% (1·6 to 1·9%)	10·5 (9·7 to 11·4)	1·6% (1·5 to 1·7%)	0·6% (-4·3 to 6·7%)	-9·8% (-13·6 to -5·3%)	222·8 (206·7 to 238·8)	1·9% (1·7 to 2·0%)	221·0 (204·2 to 238·6)	1·8% (1·7 to 1·9%)	-0·8% (-5·6 to 4·8%)	-5·3% (-9·1 to -0·9%)
**Occupational exposure to formaldehyde: All causes**	0·1 (0·1 to 0·2)	0·0% (0·0 to 0·0%)	0·1 (0·1 to 0·2)	0·0% (0·0 to 0·0%)	-7·6% (-14·7 to 0·2%)	-14·4% (-20·8 to -7·3%)	4·6 (3·8 to 5·6)	0·0% (0·0 to 0·0%)	4·2 (3·4 to 5·2)	0·0% (0·0 to 0·0%)	-8·9% (-16·9 to -0·5%)	-9·9% (-17·5 to -1·6%)
Nasopharynx cancer	0·1 (0·0 to 0·1)	0·7% (0·5 to 1·0%)	0·0 (0·0 to 0·1)	0·5% (0·3 to 0·8%)	-14·5% (-26·8 to -0·6%)	-23·4% (-32·6 to -12·8%)	1·9 (1·3 to 2·7)	0·9% (0·6 to 1·2%)	1·6 (1·0 to 2·4)	0·7% (0·5 to 1·0%)	-16·4% (-29·0 to -1·5%)	-19·2% (-29·1 to -7·6%)
**Occupational exposure to nickel: All causes**	3·6 (2·5 to 4·8)	0·0% (0·0 to 0·0%)	3·0 (2·1 to 3·9)	0·0% (0·0 to 0·0%)	-17·3% (-22·6 to -11·1%)	-23·4% (-28·0 to -17·7%)	77·1 (55·7 to 101·6)	0·0% (0·0 to 0·0%)	62·9 (45·1 to 83·1)	0·0% (0·0 to 0·0%)	-18·4% (-23·8 to -12·1%)	-19·3% (-24·4 to -13·2%)
Tracheal, bronchus, and lung cancer	3·6 (2·5 to 4·8)	0·6% (0·4 to 0·8%)	3·0 (2·1 to 3·9)	0·4% (0·3 to 0·6%)	-17·3% (-22·6 to -11·1%)	-26·0% (-30·1 to -20·9%)	77·1 (55·7 to 101·6)	0·7% (0·5 to 0·9%)	62·9 (45·1 to 83·1)	0·5% (0·4 to 0·7%)	-18·4% (-23·8 to -12·1%)	-22·1% (-26·6 to -17·0%)
**Occupational exposure to polycyclic aromatic hydrocarbons: All causes**	1·9 (1·6 to 2·3)	0·0% (0·0 to 0·0%)	1·8 (1·5 to 2·2)	0·0% (0·0 to 0·0%)	-4·3% (-9·4 to 1·7%)	-11·3% (-15·7 to -6·0%)	41·1 (33·9 to 48·7)	0·0% (0·0 to 0·0%)	38·8 (31·5 to 45·9)	0·0% (0·0 to 0·0%)	-5·6% (-10·8 to 0·2%)	-6·6% (-11·7 to -0·9%)
Tracheal, bronchus, and lung cancer	1·9 (1·6 to 2·3)	0·3% (0·3 to 0·4%)	1·8 (1·5 to 2·2)	0·3% (0·2 to 0·3%)	-4·3% (-9·4 to 1·7%)	-14·3% (-18·2 to -9·9%)	41·1 (33·9 to 48·7)	0·3% (0·3 to 0·4%)	38·8 (31·5 to 45·9)	0·3% (0·3 to 0·4%)	-5·6% (-10·8 to 0·2%)	-9·9% (-13·8 to -5·3%)
**Occupational exposure to silica: All causes**	7·1 (6·4 to 7·9)	0·1% (0·1 to 0·1%)	7·5 (6·7 to 8·3)	0·1% (0·1 to 0·1%)	5·8% (-0·8 to 12·4%)	-1·9% (-7·8 to 4·1%)	151·4 (136·3 to 166·9)	0·0% (0·0 to 0·1%)	157·6 (141·1 to 175·1)	0·0% (0·0 to 0·1%)	4·1% (-2·1 to 10·5%)	3·0% (-2·9 to 9·4%)
Tracheal, bronchus, and lung cancer	7·1 (6·4 to 7·9)	1·2% (1·1 to 1·3%)	7·5 (6·7 to 8·3)	1·1% (1·0 to 1·3%)	5·8% (-0·8 to 12·4%)	-5·3% (-10·5 to 0·3%)	151·4 (136·3 to 166·9)	1·3% (1·2 to 1·4%)	157·6 (141·1 to 175·1)	1·3% (1·1 to 1·4%)	4·1% (-2·1 to 10·5%)	-0·6% (-6·0 to 4·8%)
**Occupational exposure to sulfphuric acid: All causes**	0·8 (0·6 to 1·0)	0·0% (0·0 to 0·0%)	0·6 (0·4 to 0·8)	0·0% (0·0 to 0·0%)	-21·5% (-25·7 to -16·8%)	-27·2% (-31·1 to -22·9%)	18·8 (14·0 to 24·7)	0·0% (0·0 to 0·0%)	14·7 (10·8 to 19·3)	0·0% (0·0 to 0·0%)	-22·0% (-26·2 to -17·5%)	-22·8% (-27·1 to -18·2%)
Larynx cancer	0·8 (0·6 to 1·0)	3·1% (2·3 to 4·0%)	0·6 (0·4 to 0·8)	2·5% (1·9 to 3·3%)	-21·5% (-25·7 to -16·8%)	-17·6% (-20·7 to -14·7%)	18·8 (14·0 to 24·7)	3·1% (2·3 to 4·1%)	14·7 (10·8 to 19·3)	2·7% (2·0 to 3·6%)	-22·0% (-26·2 to -17·5%)	-13·3% (-16·2 to -10·3%)
**Occupational exposure to trichloroethylene: All causes**	0·0 (0·0 to 0·1)	0·0% (0·0 to 0·0%)	0·0 (0·0 to 0·1)	0·0% (0·0 to 0·0%)	0·4% (-4·5 to 5·2%)	-6·9% (-11·3 to -2·4%)	0·7 (0·2 to 1·3)	0·0% (0·0 to 0·0%)	0·7 (0·2 to 1·3)	0·0% (0·0 to 0·0%)	0·8% (-4·3 to 5·7%)	-0·3% (-5·2 to 4·4%)
Kidney cancer	0·0 (0·0 to 0·1)	0·1% (0·0 to 0·1%)	0·0 (0·0 to 0·1)	0·0% (0·0 to 0·1%)	0·4% (-4·5 to 5·2%)	-18·6% (-21·6 to -15·4%)	0·7 (0·2 to 1·3)	0·1% (0·0 to 0·1%)	0·7 (0·2 to 1·3)	0·0% (0·0 to 0·1%)	0·8% (-4·3 to 5·7%)	-13·6% (-16·6 to -10·4%)

Supplemental Results Table 1. All-age deaths, death PAFs, DALYs, DALY PAFs, and percent change for each risk factor and outcome for both sexes combined in 2005 and 2015 for High SDI.

Data in parenthesis are 95% uncertainty intervals. PAFs=population attributable fractions. DALYs=disability-adjusted life-years.

	2005 Deaths (in thousands)	2005 Deaths PAF	2015 Deaths (in thousands)	2015 Deaths PAF	Percent change deaths 2005-2015	Percent change deaths PAF 2005-2015	2005 DALYs (in thousands)	2005 DALYs PAF	2015 DALYs (in thousands)	2015 DALYs PAF	Percent change DALYs 2005-2015	Percent change DALYs PAF 2005-2015
**Occupational asthmagens: All causes**	0·9<br>(0·8 to 0·9)	0·0%<br>(0·0 to 0·0%)	0·6<br>(0·6 to 0·7)	0·0%<br>(0·0 to 0·0%)	-30·4%<br>(-34·4 to -26·0%)	-35·5%<br>(-39·2 to -31·6%)	185·2<br>(132·0 to 247·7)	0·1%<br>(0·0 to 0·1%)	169·7<br>(118·5 to 227·9)	0·0%<br>(0·0 to 0·1%)	-8·4%<br>(-11·2 to -5·9%)	-9·4%<br>(-12·0 to -7·0%)
Asthma	0·9<br>(0·8 to 0·9)	3·3%<br>(3·1 to 3·5%)	0·6<br>(0·6 to 0·7)	2·8%<br>(2·6 to 3·0%)	-30·4%<br>(-34·4 to -26·0%)	-15·9%<br>(-20·9 to -10·7%)	185·2<br>(132·0 to 247·7)	5·4%<br>(5·1 to 5·7%)	169·7<br>(118·5 to 227·9)	5·3%<br>(5·0 to 5·6%)	-8·4%<br>(-11·2 to -5·9%)	-2·1%<br>(-3·9 to -0·3%)
**Occupational particulate matter, gases, and fumes: All causes**	16·9<br>(13·7 to 20·6)	0·2%<br>(0·1 to 0·2%)	19·2<br>(14·9 to 24·2)	0·2%<br>(0·1 to 0·2%)	13·8%<br>(6·6 to 20·3%)	5·5%<br>(-0·9 to 11·6%)	456·3<br>(387·7 to 526·2)	0·1%<br>(0·1 to 0·2%)	479·9<br>(405·4 to 557·9)	0·1%<br>(0·1 to 0·2%)	5·2%<br>(0·8 to 9·1%)	4·0%<br>(-0·2 to 8·1%)
Chronic obstructive pulmonary disease	15·7<br>(12·5 to 19·4)	3·7%<br>(2·9 to 4·5%)	18·2<br>(13·9 to 23·1)	3·8%<br>(2·9 to 4·8%)	15·9%<br>(8·2 to 22·8%)	2·1%<br>(-4·3 to 7·8%)	434·9<br>(366·7 to 503·8)	5·7%<br>(4·8 to 6·5%)	461·1<br>(386·7 to 538·9)	5·7%<br>(4·8 to 6·6%)	6·0%<br>(1·6 to 10·2%)	0·4%<br>(-3·3 to 3·8%)
**Occupational noise: All causes**	-	-	-	-	-	-	889·2<br>(602·8 to 1,251·9)	0·3%<br>(0·2 to 0·3%)	1,057·6<br>(713·1 to 1,490·4)	0·3%<br>(0·2 to 0·4%)	18·9%<br>(16·3 to 21·9%)	17·5%<br>(14·7 to 20·8%)
Age-related and other hearing loss	-	-	-	-	-	-	889·2<br>(602·8 to 1,251·9)	12·5%<br>(11·2 to 14·1%)	1,057·6<br>(713·1 to 1,490·4)	12·1%<br>(10·8 to 13·7%)	18·9%<br>(16·3 to 21·9%)	-3·1%<br>(-5·4 to -0·9%)
**Occupational injuries: All causes**	19·9<br>(18·4 to 21·9)	0·2%<br>(0·2 to 0·2%)	22·0<br>(20·4 to 24·1)	0·2%<br>(0·2 to 0·2%)	10·4%<br>(0·2 to 21·7%)	2·4%<br>(-7·2 to 12·8%)	1,546·1<br>(1,340·3 to 1,779·0)	0·4%<br>(0·4 to 0·5%)	1,792·7<br>(1,519·6 to 2,127·0)	0·5%<br>(0·5 to 0·6%)	15·9%<br>(5·0 to 27·3%)	14·6%<br>(4·2 to 25·9%)
Pedestrian road injuries	2·6<br>(2·3 to 2·9)	4·5%<br>(4·1 to 5·1%)	2·7<br>(2·5 to 3·1)	6·5%<br>(6·0 to 7·3%)	7·0%<br>(-6·5 to 21·2%)	43·9%<br>(26·8 to 63·1%)	135·8<br>(120·8 to 154·1)	5·8%<br>(5·2 to 6·5%)	143·8<br>(128·7 to 163·6)	9·1%<br>(8·4 to 10·2%)	5·9%<br>(-7·0 to 19·4%)	58·6%<br>(40·1 to 79·0%)
Cyclist road injuries	0·4<br>(0·4 to 0·5)	4·7%<br>(4·5 to 5·1%)	0·5<br>(0·5 to 0·6)	6·7%<br>(6·2 to 7·2%)	18·4%<br>(5·8 to 29·4%)	40·4%<br>(29·1 to 52·7%)	42·6<br>(35·1 to 51·8)	6·6%<br>(6·2 to 7·1%)	51·6<br>(41·7 to 64·3)	9·9%<br>(9·2 to 10·7%)	21·2%<br>(10·1 to 33·3%)	50·5%<br>(38·7 to 63·9%)
Motorcyclist road injuries	1·4<br>(1·3 to 1·5)	7·4%<br>(7·0 to 7·8%)	1·6<br>(1·4 to 1·8)	10·1%<br>(9·4 to 10·8%)	16·8%<br>(7·7 to 26·8%)	36·6%<br>(27·1 to 47·2%)	101·0<br>(89·8 to 114·3)	7·8%<br>(7·4 to 8·2%)	114·4<br>(99·2 to 131·1)	10·7%<br>(10·1 to 11·5%)	13·2%<br>(4·5 to 23·4%)	38·4%<br>(28·8 to 49·6%)
Motor vehicle road injuries	7·2<br>(6·6 to 8·0)	6·8%<br>(6·3 to 7·5%)	7·5<br>(7·0 to 8·3)	9·7%<br>(9·1 to 10·6%)	4·7%<br>(-4·2 to 15·3%)	42·7%<br>(29·2 to 58·1%)	450·2<br>(406·5 to 500·8)	7·7%<br>(7·1 to 8·5%)	483·1<br>(434·2 to 543·7)	11·4%<br>(10·6 to 12·4%)	7·3%<br>(-2·0 to 18·0%)	47·7%<br>(34·2 to 63·1%)
Other road injuries	0·1<br>(0·1 to 0·1)	7·5%<br>(6·9 to 8·1%)	0·1<br>(0·1 to 0·1)	9·9%<br>(9·0 to 10·8%)	17·7%<br>(4·3 to 35·8%)	30·9%<br>(20·1 to 44·1%)	6·8<br>(5·6 to 8·0)	9·1%<br>(8·4 to 9·7%)	8·7<br>(7·1 to 10·8)	12·6%<br>(11·6 to 13·8%)	29·2%<br>(16·6 to 44·2%)	39·1%<br>(27·8 to 52·8%)
Other transport injuries	0·8<br>(0·7 to 0·9)	5·1%<br>(4·7 to 5·8%)	1·0<br>(0·9 to 1·1)	7·7%<br>(7·0 to 8·6%)	18·8%<br>(4·9 to 33·9%)	49·2%<br>(32·7 to 68·0%)	44·4<br>(40·3 to 49·1)	5·8%<br>(5·3 to 6·5%)	52·5<br>(47·4 to 59·1)	9·0%<br>(8·3 to 10·0%)	18·3%<br>(5·3 to 32·7%)	54·9%<br>(38·8 to 74·0%)
Falls	2·6<br>(2·4 to 2·9)	2·1%<br>(2·0 to 2·3%)	3·4<br>(3·1 to 3·7)	2·4%<br>(2·2 to 2·6%)	29·3%<br>(16·8 to 43·7%)	10·9%<br>(0·2 to 23·8%)	379·8<br>(296·3 to 481·8)	6·0%<br>(5·5 to 6·5%)	491·7<br>(371·9 to 647·3)	7·9%<br>(7·3 to 8·7%)	29·5%<br>(16·5 to 44·2%)	33·3%<br>(22·0 to 45·4%)
Drowning	1·6<br>(1·4 to 1·8)	3·9%<br>(3·5 to 4·5%)	1·7<br>(1·5 to 1·9)	5·2%<br>(4·6 to 5·8%)	3·8%<br>(-10·7 to 19·8%)	32·4%<br>(14·5 to 52·7%)	78·0<br>(69·2 to 89·4)	4·5%<br>(4·0 to 5·1%)	79·3<br>(71·0 to 89·7)	6·8%<br>(6·1 to 7·7%)	1·7%<br>(-12·6 to 17·5%)	52·3%<br>(31·3 to 75·7%)
Fire, heat, and hot substances	1·0<br>(0·9 to 1·2)	3·2%<br>(2·8 to 3·8%)	1·1<br>(0·9 to 1·2)	4·3%<br>(3·7 to 5·0%)	1·8%<br>(-15·2 to 22·8%)	31·2%<br>(9·6 to 57·5%)	73·6<br>(61·0 to 88·6)	5·1%<br>(4·5 to 5·8%)	78·7<br>(63·5 to 99·7)	7·6%<br>(6·8 to 8·7%)	7·0%<br>(-8·7 to 24·3%)	50·6%<br>(30·2 to 75·4%)
Poisonings	0·2<br>(0·2 to 0·2)	1·7%<br>(1·5 to 2·0%)	0·2<br>(0·2 to 0·2)	1·8%<br>(1·6 to 2·1%)	-1·7%<br>(-16·5 to 14·3%)	8·5%<br>(-7·4 to 26·1%)	11·7<br>(10·1 to 13·9)	2·1%<br>(1·9 to 2·4%)	11·6<br>(10·2 to 13·5)	2·5%<br>(2·2 to 2·8%)	-0·7%<br>(-15·4 to 15·8%)	16·9%<br>(-0·3 to 36·5%)
Unintentional firearm injuries	0·1<br>(0·1 to 0·1)	1·4%<br>(1·2 to 1·6%)	0·1<br>(0·1 to 0·1)	1·7%<br>(1·4 to 2·0%)	15·6%<br>(-4·8 to 43·8%)	20·3%<br>(-1·4 to 48·7%)	4·1<br>(3·6 to 4·9)	2·3%<br>(2·0 to 2·7%)	4·8<br>(4·0 to 5·8)	3·3%<br>(2·9 to 4·0%)	15·6%<br>(-3·6 to 41·2%)	44·4%<br>(20·3 to 75·1%)
Unintentional suffocation	0·0<br>(0·0 to 0·0)	0·3%<br>(0·2 to 0·3%)	0·0<br>(0·0 to 0·0)	0·3%<br>(0·2 to 0·4%)	18·1%<br>(-9·9 to 58·4%)	6·6%<br>(-19·0 to 41·8%)	1·1<br>(0·9 to 1·4)	0·5%<br>(0·4 to 0·7%)	1·4<br>(1·0 to 1·8)	0·6%<br>(0·5 to 0·8%)	18·4%<br>(-5·7 to 49·9%)	17·9%<br>(-5·9 to 48·9%)
Other exposure to mechanical forces	0·8<br>(0·7 to 0·9)	4·8%<br>(4·4 to 5·4%)	0·8<br>(0·8 to 0·9)	6·6%<br>(6·0 to 7·3%)	8·4%<br>(-3·9 to 21·6%)	36·2%<br>(21·2 to 53·5%)	82·4<br>(66·8 to 102·7)	6·8%<br>(6·3 to 7·5%)	103·0<br>(79·6 to 135·0)	10·1%<br>(9·4 to 10·9%)	24·9%<br>(12·6 to 37·1%)	47·1%<br>(34·7 to 60·7%)
Venomous animal contact	0·0<br>(0·0 to 0·0)	0·7%<br>(0·6 to 0·9%)	0·0<br>(0·0 to 0·0)	0·7%<br>(0·6 to 0·9%)	-6·7%<br>(-29·4 to 24·2%)	2·8%<br>(-21·3 to 37·3%)	0·4<br>(0·3 to 0·5)	1·0%<br>(0·8 to 1·3%)	0·4<br>(0·3 to 0·6)	1·1%<br>(1·0 to 1·4%)	4·1%<br>(-17·2 to 30·8%)	11·8%<br>(-10·9 to 43·0%)
Non-venomous animal contact	0·0<br>(0·0 to 0·0)	0·5%<br>(0·4 to 0·6%)	0·0<br>(0·0 to 0·0)	0·5%<br>(0·4 to 0·5%)	1·1%<br>(-18·3 to 24·9%)	0·7%<br>(-18·4 to 24·2%)	0·5<br>(0·4 to 0·7)	0·9%<br>(0·8 to 1·0%)	0·6<br>(0·4 to 0·8)	1·1%<br>(1·0 to 1·2%)	16·0%<br>(-1·5 to 33·9%)	18·9%<br>(2·4 to 37·7%)
Pulmonary aspiration and foreign body in airway	0·6<br>(0·5 to 0·6)	1·7%<br>(1·5 to 1·9%)	0·7<br>(0·6 to 0·8)	1·9%<br>(1·6 to 2·2%)	21·9%<br>(4·0 to 40·6%)	10·7%<br>(-4·4 to 27·0%)	27·6<br>(24·3 to 31·4)	3·0%<br>(2·7 to 3·4%)	33·2<br>(28·7 to 38·0)	3·9%<br>(3·5 to 4·5%)	20·3%<br>(3·3 to 37·9%)	32·1%<br>(14·1 to 50·8%)
Foreign body in eyes	-	-	-	-	-	-	0·6<br>(0·3 to 1·1)	4·9%<br>(4·4 to 5·5%)	0·9<br>(0·4 to 1·7)	6·9%<br>(6·0 to 7·7%)	56·8%<br>(43·5 to 71·6%)	40·3%<br>(28·6 to 53·0%)
Foreign body in other body part	0·0<br>(0·0 to 0·0)	2·5%<br>(2·1 to 2·9%)	0·0<br>(0·0 to 0·1)	3·0%<br>(2·6 to 3·6%)	31·0%<br>(7·8 to 57·1%)	22·1%<br>(-1·5 to 47·5%)	3·5<br>(2·8 to 4·4)	4·4%<br>(4·0 to 5·1%)	4·6<br>(3·6 to 6·0)	5·9%<br>(5·2 to 6·8%)	31·6%<br>(10·0 to 53·8%)	33·0%<br>(12·8 to 55·9%)
Other unintentional injuries	0·5<br>(0·4 to 0·6)	4·8%<br>(4·2 to 5·7%)	0·5<br>(0·4 to 0·6)	7·2%<br>(6·1 to 8·6%)	3·9%<br>(-14·2 to 25·7%)	48·1%<br>(23·1 to 78·3%)	102·1<br>(74·7 to 137·2)	6·4%<br>(5·9 to 7·1%)	128·3<br>(89·5 to 176·5)	9·0%<br>(8·0 to 10·1%)	25·7%<br>(11·3 to 41·3%)	39·5%<br>(25·0 to 56·9%)
**Occupational ergonomic factors: All causes**	-	-	-	-	-	-	2,289·8<br>(1,598·1 to 3,187·0)	0·6%<br>(0·5 to 0·8%)	2,370·1<br>(1,662·8 to 3,280·6)	0·7%<br>(0·5 to 0·8%)	3·5%<br>(1·6 to 5·3%)	2·3%<br>(0·1 to 4·6%)
Low back pain	-	-	-	-	-	-	2,289·8<br>(1,598·1 to 3,187·0)	16·6%<br>(15·8 to 17·5%)	2,370·1<br>(1,662·8 to 3,280·6)	15·6%<br>(14·8 to 16·4%)	3·5%<br>(1·6 to 5·3%)	-6·2%<br>(-7·8 to -4·7%)
**Behavioural risks: All causes**	5,014·7<br>(4,718·8 to 5,316·8)	44·9%<br>(42·2 to 47·6%)	5,029·6<br>(4,708·6 to 5,361·7)	41·8%<br>(39·1 to 44·5%)	0·3%<br>(-0·8 to 1·3%)	-7·0%<br>(-7·8 to -6·3%)	120,579·9<br>(112,879·3 to 128,727·9)	34·4%<br>(31·9 to 37·1%)	112,897·8<br>(105,019·8 to 121,492·7)	31·9%<br>(29·4 to 34·5%)	-6·4%<br>(-7·6 to -5·2%)	-7·4%<br>(-8·1 to -6·8%)
**Child and maternal malnutrition: All causes**	4·9<br>(3·9 to 5·7)	0·0%<br>(0·0 to 0·1%)	3·1<br>(2·4 to 3·6)	0·0%<br>(0·0 to 0·0%)	-37·4%<br>(-43·0 to -31·3%)	-42·0%<br>(-47·2 to -36·2%)	5,118·8<br>(3,523·6 to 7,234·7)	1·4%<br>(1·1 to 1·9%)	5,125·5<br>(3,476·5 to 7,299·3)	1·4%<br>(1·1 to 1·9%)	0·1%<br>(-2·7 to 2·6%)	-1·1%<br>(-3·8 to 1·3%)
**Suboptimal breastfeeding: All causes**	2·6<br>(1·0 to 3·7)	0·0%<br>(0·0 to 0·0%)	1·6<br>(0·6 to 2·3)	0·0%<br>(0·0 to 0·0%)	-38·4%<br>(-44·2 to -31·8%)	-42·9%<br>(-48·4 to -36·8%)	230·5<br>(91·9 to 316·9)	0·1%<br>(0·0 to 0·1%)	143·1<br>(57·5 to 198·2)	0·0%<br>(0·0 to 0·1%)	-37·9%<br>(-43·6 to -31·2%)	-38·6%<br>(-44·5 to -32·1%)
**Non-exclusive breastfeeding : All causes**	2·6<br>(1·0 to 3·6)	0·0%<br>(0·0 to 0·0%)	1·6<br>(0·6 to 2·3)	0·0%<br>(0·0 to 0·0%)	-38·4%<br>(-44·2 to -31·7%)	-42·9%<br>(-48·4 to -36·7%)	228·3<br>(89·7 to 314·9)	0·1%<br>(0·0 to 0·1%)	141·1<br>(55·4 to 195·5)	0·0%<br>(0·0 to 0·1%)	-38·2%<br>(-44·1 to -31·5%)	-38·8%<br>(-44·8 to -32·3%)
Diarrhoeal diseases	0·1<br>(0·1 to 0·1)	0·5%<br>(0·3 to 0·8%)	0·0<br>(0·0 to 0·1)	0·2%<br>(0·1 to 0·4%)	-45·0%<br>(-56·7 to -29·5%)	-61·6%<br>(-70·7 to -49·2%)	7·7<br>(5·1 to 10·3)	2·1%<br>(1·4 to 2·9%)	4·5<br>(3·1 to 6·1)	1·2%<br>(0·8 to 1·8%)	-41·0%<br>(-52·9 to -25·7%)	-44·6%<br>(-55·9 to -29·4%)
Lower respiratory infections	2·5<br>(0·9 to 3·6)	0·6%<br>(0·2 to 0·8%)	1·6<br>(0·6 to 2·2)	0·3%<br>(0·1 to 0·4%)	-38·2%<br>(-44·2 to -31·2%)	-47·8%<br>(-53·2 to -41·8%)	220·6<br>(82·4 to 308·0)	3·5%<br>(1·3 to 4·8%)	136·6<br>(50·5 to 191·5)	2·2%<br>(0·8 to 3·1%)	-38·1%<br>(-44·0 to -31·1%)	-37·8%<br>(-44·2 to -29·8%)
**Discontinued breastfeeding : All causes**	0·0<br>(0·0 to 0·1)	0·0%<br>(0·0 to 0·0%)	0·0<br>(0·0 to 0·0)	0·0%<br>(0·0 to 0·0%)	-46·1%<br>(-57·6 to -31·7%)	-50·0%<br>(-60·7 to -36·8%)	3·8<br>(1·3 to 6·7)	0·0%<br>(0·0 to 0·0%)	2·9<br>(1·0 to 5·0)	0·0%<br>(0·0 to 0·0%)	-23·9%<br>(-39·8 to -4·7%)	-24·9%<br>(-40·3 to -5·8%)
Diarrhoeal diseases	0·0<br>(0·0 to 0·1)	0·2%<br>(0·0 to 0·3%)	0·0<br>(0·0 to 0·0)	0·1%<br>(0·0 to 0·1%)	-46·1%<br>(-57·6 to -31·7%)	-62·4%<br>(-71·1 to -50·2%)	3·8<br>(1·3 to 6·7)	1·0%<br>(0·4 to 1·8%)	2·9<br>(1·0 to 5·0)	0·7%<br>(0·3 to 1·4%)	-23·9%<br>(-39·8 to -4·7%)	-28·6%<br>(-44·0 to -8·5%)
**Childhood undernutrition: All causes**	3·0<br>(2·3 to 3·7)	0·0%<br>(0·0 to 0·0%)	1·7<br>(1·2 to 2·1)	0·0%<br>(0·0 to 0·0%)	-43·9%<br>(-52·7 to -34·7%)	-47·9%<br>(-56·1 to -39·4%)	303·4<br>(231·1 to 368·3)	0·1%<br>(0·1 to 0·1%)	191·5<br>(144·9 to 235·9)	0·1%<br>(0·0 to 0·1%)	-36·9%<br>(-45·4 to -26·4%)	-37·6%<br>(-45·8 to -27·4%)
**Childhood underweight: All causes**	0·3<br>(0·2 to 0·5)	0·0%<br>(0·0 to 0·0%)	0·2<br>(0·1 to 0·3)	0·0%<br>(0·0 to 0·0%)	-42·8%<br>(-49·7 to -34·9%)	-47·0%<br>(-53·3 to -39·7%)	53·2<br>(37·9 to 80·9)	0·0%<br>(0·0 to 0·0%)	44·6<br>(29·2 to 66·8)	0·0%<br>(0·0 to 0·0%)	-16·1%<br>(-39·8 to 19·6%)	-17·2%<br>(-40·4 to 17·9%)
Diarrhoeal diseases	0·0<br>(0·0 to 0·0)	0·1%<br>(0·1 to 0·1%)	0·0<br>(0·0 to 0·0)	0·0%<br>(0·0 to 0·0%)	-55·0%<br>(-63·9 to -45·3%)	-68·7%<br>(-75·5 to -60·6%)	2·0<br>(1·3 to 2·8)	0·5%<br>(0·4 to 0·8%)	1·2<br>(0·8 to 1·9)	0·3%<br>(0·2 to 0·5%)	-37·1%<br>(-47·7 to -26·5%)	-41·1%<br>(-50·7 to -30·3%)
Lower respiratory infections	0·2<br>(0·1 to 0·4)	0·0%<br>(0·0 to 0·1%)	0·1<br>(0·0 to 0·2)	0·0%<br>(0·0 to 0·0%)	-47·2%<br>(-56·8 to -38·3%)	-55·4%<br>(-63·5 to -47·5%)	14·2<br>(6·8 to 32·4)	0·2%<br>(0·1 to 0·5%)	7·5<br>(3·3 to 17·2)	0·1%<br>(0·1 to 0·3%)	-46·8%<br>(-56·4 to -37·9%)	-46·5%<br>(-56·2 to -37·1%)
Measles	0·0<br>(0·0 to 0·0)	2·4%<br>(0·0 to 11·2%)	0·0<br>(0·0 to 0·0)	3·6%<br>(0·0 to 18·2%)	-16·0%<br>(-98·1 to 866·2%)	49·5%<br>(-93·5 to 1,546·6%)	0·2<br>(0·0 to 1·1)	2·5%<br>(0·0 to 11·4%)	0·2<br>(0·0 to 1·1)	3·7%<br>(0·0 to 18·5%)	-15·7%<br>(-97·8 to 848·1%)	49·4%<br>(-93·2 to 1,511·0%)
Protein-energy malnutrition	0·1<br>(0·1 to 0·1)	1·0%<br>(0·9 to 1·2%)	0·1<br>(0·1 to 0·1)	0·6%<br>(0·5 to 0·7%)	-35·4%<br>(-45·1 to -23·4%)	-44·5%<br>(-53·3 to -33·8%)	36·8<br>(24·5 to 59·7)	21·9%<br>(15·9 to 31·5%)	35·6<br>(21·3 to 56·6)	20·4%<br>(13·4 to 29·5%)	-3·2%<br>(-38·1 to 51·4%)	-7·1%<br>(-33·8 to 30·9%)
**Childhood wasting: All causes**	2·8<br>(1·7 to 3·6)	0·0%<br>(0·0 to 0·0%)	1·6<br>(0·9 to 2·1)	0·0%<br>(0·0 to 0·0%)	-43·4%<br>(-53·6 to -32·3%)	-47·5%<br>(-57·0 to -37·3%)	280·4<br>(181·6 to 359·2)	0·1%<br>(0·1 to 0·1%)	179·3<br>(121·1 to 229·2)	0·1%<br>(0·0 to 0·1%)	-36·1%<br>(-45·7 to -23·6%)	-36·8%<br>(-46·1 to -24·4%)

Supplemental Results Table 1. All-age deaths, death PAFs, DALYs, DALY PAFs, and percent change for each risk factor and outcome for both sexes combined in 2005 and 2015 for High SDI.

Data in parenthesis are 95% uncertainty intervals. PAFs=population attributable fractions. DALYs=disability-adjusted life-years.

	2005 Deaths (in thousands)	2005 Deaths PAF	2015 Deaths (in thousands)	2015 Deaths PAF	Percent change deaths 2005-2015	Percent change deaths PAF 2005-2015	2005 DALYs (in thousands)	2005 DALYs PAF	2015 DALYs (in thousands)	2015 DALYs PAF	Percent change DALYs 2005-2015	Percent change DALYs PAF 2005-2015
Diarrhoeal diseases	0·4 (0·2 to 0·4)	2·1% (1·3 to 3·0%)	0·2 (0·1 to 0·2)	0·7% (0·4 to 1·2%)	-49·9% (-60·5 to -37·9%)	-65·1% (-73·1 to -55·7%)	42·3 (28·2 to 52·8)	11·6% (7·6 to 14·6%)	27·9 (17·3 to 36·3)	7·2% (4·4 to 9·9%)	-34·1% (-46·2 to -21·7%)	-38·3% (-50·1 to -26·2%)
Lower respiratory infections	2·3 (1·2 to 3·1)	0·5% (0·3 to 0·7%)	1·3 (0·6 to 1·8)	0·3% (0·1 to 0·4%)	-42·9% (-54·2 to -30·1%)	-51·8% (-61·4 to -40·7%)	200·7 (103·8 to 269·8)	3·2% (1·6 to 4·3%)	115·2 (56·0 to 156·9)	1·8% (0·9 to 2·5%)	-42·6% (-53·8 to -29·8%)	-42·3% (-53·9 to -29·0%)
Measles	0·0 (0·0 to 0·0)	7·5% (0·2 to 31·0%)	0·0 (0·0 to 0·0)	13·9% (0·6 to 51·7%)	-3·1% (-88·8 to 1,100·0%)	85·0% (-74·3 to 2,059·6%)	0·6 (0·0 to 3·0)	7·7% (0·2 to 31·5%)	0·6 (0·0 to 2·9)	14·1% (0·7 to 52·7%)	-2·6% (-88·6 to 1,086·8%)	84·7% (-73·6 to 1,999·4%)
Protein-energy malnutrition	0·1 (0·1 to 0·1)	1·0% (0·9 to 1·2%)	0·1 (0·1 to 0·1)	0·6% (0·5 to 0·7%)	-35·4% (-45·1 to -23·4%)	-44·5% (-53·3 to -33·8%)	36·8 (24·5 to 59·7)	21·9% (15·9 to 31·5%)	35·6 (21·3 to 56·6)	20·4% (13·4 to 29·5%)	-3·2% (-38·1 to 51·4%)	-7·1% (-33·8 to 30·9%)
**Childhood stunting: All causes**	0·5 (0·1 to 1·3)	0·0% (0·0 to 0·0%)	0·2 (0·0 to 0·6)	0·0% (0·0 to 0·0%)	-53·2% (-66·4 to -41·4%)	-56·6% (-68·9 to -45·7%)	41·7 (8·3 to 110·2)	0·0% (0·0 to 0·0%)	20·3 (4·4 to 57·9)	0·0% (0·0 to 0·0%)	-51·3% (-63·8 to -37·7%)	-51·9% (-64·4 to -38·8%)
Diarrhoeal diseases	0·0 (0·0 to 0·1)	0·2% (0·1 to 0·5%)	0·0 (0·0 to 0·0)	0·1% (0·0 to 0·2%)	-58·9% (-69·1 to -44·6%)	-71·4% (-79·2 to -61·2%)	4·9 (1·5 to 9·8)	1·4% (0·4 to 2·7%)	2·8 (0·8 to 5·7)	0·7% (0·2 to 1·5%)	-43·6% (-55·9 to -26·8%)	-47·2% (-59·1 to -32·1%)
Lower respiratory infections	0·4 (0·0 to 1·2)	0·1% (0·0 to 0·3%)	0·2 (0·0 to 0·6)	0·0% (0·0 to 0·1%)	-52·9% (-66·9 to -33·0%)	-60·2% (-72·0 to -43·6%)	36·5 (1·6 to 105·8)	0·6% (0·0 to 1·6%)	17·3 (0·5 to 54·8)	0·3% (0·0 to 0·9%)	-52·6% (-66·6 to -32·6%)	-52·3% (-67·0 to -32·3%)
Measles	0·0 (0·0 to 0·0)	2·9% (0·0 to 11·3%)	0·0 (0·0 to 0·0)	4·4% (0·0 to 17·2%)	-22·1% (-88·5 to 406·6%)	52·8% (-75·3 to 736·4%)	0·2 (0·0 to 1·0)	2·9% (0·0 to 11·4%)	0·2 (0·0 to 0·9)	4·5% (0·1 to 17·6%)	-21·8% (-88·3 to 397·8%)	52·5% (-74·8 to 719·2%)
**Iron deficiency: All causes**	0·4 (0·3 to 0·5)	0·0% (0·0 to 0·0%)	0·4 (0·3 to 0·5)	0·0% (0·0 to 0·0%)	-2·8% (-18·0 to 10·3%)	-9·9% (-23·7 to 2·3%)	4,685·8 (3,101·8 to 6,809·2)	1·3% (1·0 to 1·8%)	4,847·7 (3,220·3 to 7,012·1)	1·4% (1·0 to 1·8%)	3·5% (0·8 to 5·7%)	2·2% (-0·6 to 4·9%)
Maternal haemorrhage	0·1 (0·0 to 0·1)	22·5% (8·3 to 37·3%)	0·0 (0·0 to 0·1)	22·1% (8·3 to 36·8%)	-35·5% (-48·1 to -18·7%)	-2·0% (-7·4 to 3·6%)	6·5 (2·4 to 11·3)	21·0% (7·7 to 35·6%)	5·1 (1·8 to 8·9)	21·0% (7·8 to 35·5%)	-22·2% (-32·5 to -11·4%)	0·2% (-3·8 to 4·1%)
Maternal sepsis and other pregnancy related infections	0·0 (0·0 to 0·1)	21·6% (7·9 to 36·3%)	0·0 (0·0 to 0·0)	20·9% (7·8 to 35·1%)	-24·0% (-41·4 to -0·8%)	-2·9% (-9·2 to 3·3%)	3·0 (1·1 to 5·4)	22·2% (8·2 to 37·1%)	2·5 (0·8 to 4·8)	22·3% (8·2 to 37·3%)	-14·6% (-38·3 to 18·9%)	0·2% (-4·7 to 5·1%)
Iron-deficiency anaemia	0·3 (0·2 to 0·4)	100·0% (100·0 to 100·0%)	0·4 (0·2 to 0·4)	100·0% (100·0 to 100·0%)	5·5% (-10·2 to 19·6%)	-	4,676·3 (3,098·1 to 6,796·1)	100·0% (100·0 to 100·0%)	4,840·1 (3,212·1 to 7,003·8)	100·0% (100·0 to 100·0%)	3·5% (0·9 to 5·7%)	-
**Vitamin A deficiency: All causes**	0·0 (0·0 to 0·0)	0·0% (0·0 to 0·0%)	0·0 (0·0 to 0·0)	0·0% (0·0 to 0·0%)	-47·5% (-68·8 to -2·0%)	-51·4% (-71·0 to -9·3%)	2·9 (1·9 to 4·2)	0·0% (0·0 to 0·0%)	2·5 (1·6 to 3·5)	0·0% (0·0 to 0·0%)	-16·6% (-35·7 to 7·0%)	-17·7% (-36·4 to 5·1%)
Diarrhoeal diseases	0·0 (0·0 to 0·0)	0·1% (0·0 to 0·1%)	0·0 (0·0 to 0·0)	0·0% (0·0 to 0·0%)	-50·5% (-58·1 to -41·2%)	-65·5% (-71·6 to -56·7%)	1·4 (0·7 to 2·2)	0·4% (0·2 to 0·6%)	0·9 (0·5 to 1·4)	0·2% (0·1 to 0·4%)	-36·2% (-45·9 to -26·9%)	-40·2% (-50·1 to -27·7%)
Measles	0·0 (0·0 to 0·0)	4·5% (2·2 to 7·8%)	0·0 (0·0 to 0·0)	5·2% (2·8 to 8·5%)	-40·1% (-87·6 to 176·7%)	15·9% (-13·0 to 72·4%)	0·4 (0·1 to 1·2)	4·6% (2·2 to 7·9%)	0·2 (0·0 to 0·9)	5·3% (2·9 to 8·7%)	-39·8% (-87·2 to 173·9%)	15·9% (-12·1 to 70·9%)
Vitamin A deficiency	-	-	-	-	-	-	1·1 (0·7 to 1·7)	100·0% (100·0 to 100·0%)	1·3 (0·8 to 2·0)	100·0% (100·0 to 100·0%)	16·4% (6·9 to 26·4%)	-
**Zinc deficiency: All causes**	0·1 (0·0 to 0·3)	0·0% (0·0 to 0·0%)	0·0 (0·0 to 0·2)	0·0% (0·0 to 0·0%)	-40·4% (-49·4 to -28·3%)	-44·7% (-53·0 to -33·4%)	8·3 (1·5 to 24·4)	0·0% (0·0 to 0·0%)	5·6 (1·4 to 15·3)	0·0% (0·0 to 0·0%)	-33·0% (-43·8 to -0·3%)	-33·8% (-44·3 to -1·8%)
Diarrhoeal diseases	0·0 (0·0 to 0·0)	0·1% (0·0 to 0·2%)	0·0 (0·0 to 0·0)	0·0% (0·0 to 0·1%)	-38·8% (-47·7 to 0·0%)	-57·4% (-64·6 to 0·0%)	2·4 (1·0 to 4·3)	0·6% (0·3 to 1·2%)	1·9 (0·9 to 3·3)	0·5% (0·2 to 0·8%)	-17·4% (-32·7 to 8·5%)	-22·7% (-38·0 to 2·8%)
Lower respiratory infections	0·1 (0·0 to 0·3)	0·0% (0·0 to 0·1%)	0·0 (0·0 to 0·2)	0·0% (0·0 to 0·0%)	-40·7% (-50·3 to 0·0%)	-49·9% (-58·0 to 0·0%)	5·9 (0·1 to 22·1)	0·1% (0·0 to 0·3%)	3·6 (0·1 to 13·3)	0·1% (0·0 to 0·2%)	-39·1% (-48·3 to -0·1%)	-38·8% (-48·0 to 0·1%)
**Tobacco smoke: All causes**	1,902·7 (1,813·6 to 1,987·4)	17·0% (16·2 to 17·8%)	1,882·7 (1,796·1 to 1,970·5)	15·6% (14·9 to 16·4%)	-1·1% (-3·0 to 0·8%)	-8·3% (-9·9 to -6·7%)	41,211·9 (39,124·2 to 43,265·4)	11·8% (10·6 to 13·1%)	37,552·5 (35,654·4 to 39,499·8)	10·6% (9·4 to 11·9%)	-8·9% (-10·5 to -7·3%)	-9·9% (-11·6 to -8·2%)
**Smoking: All causes**	1,818·5 (1,733·9 to 1,901·5)	16·3% (15·5 to 17·0%)	1,802·2 (1,715·9 to 1,890·2)	15·0% (14·2 to 15·7%)	-0·9% (-2·9 to 1·0%)	-8·1% (-9·8 to -6·5%)	39,894·0 (37,814·8 to 41,943·5)	11·4% (10·2 to 12·7%)	36,445·4 (34,532·2 to 38,336·9)	10·3% (9·1 to 11·6%)	-8·6% (-10·3 to -7·0%)	-9·6% (-11·5 to -7·9%)
Tuberculosis	6·9 (3·5 to 10·2)	16·2% (8·3 to 24·1%)	3·3 (1·7 to 5·0)	13·1% (6·6 to 19·5%)	-51·4% (-55·9 to -46·4%)	-19·6% (-23·8 to -15·2%)	235·2 (120·2 to 345·3)	17·3% (8·9 to 25·5%)	107·2 (54·2 to 159·1)	15·3% (7·8 to 22·7%)	-54·4% (-58·9 to -49·2%)	-11·2% (-15·8 to -6·5%)
Lower respiratory infections	107·6 (87·4 to 129·0)	24·9% (20·2 to 29·9%)	105·0 (82·6 to 128·8)	20·5% (16·3 to 25·0%)	-2·3% (-7·6 to 2·7%)	-17·6% (-20·4 to -15·0%)	1,926·3 (1,594·1 to 2,275·1)	30·3% (25·2 to 35·7%)	1,686·7 (1,353·9 to 2,030·2)	26·7% (21·8 to 31·8%)	-12·4% (-19·2 to -6·2%)	-12·0% (-15·2 to -8·9%)
Lip and oral cavity cancer	20·2 (18·2 to 22·1)	63·3% (57·1 to 68·9%)	21·3 (18·8 to 23·7)	58·9% (52·1 to 65·1%)	5·5% (-0·2 to 12·2%)	-6·9% (-9·1 to -4·6%)	513·5 (463·2 to 559·5)	65·4% (59·6 to 71·0%)	503·4 (442·5 to 564·4)	60·7% (54·2 to 66·5%)	-2·0% (-8·0 to 5·6%)	-7·3% (-9·8 to -4·9%)
Nasopharynx cancer	4·7 (4·1 to 5·3)	57·9% (50·9 to 64·3%)	5·0 (4·3 to 5·7)	54·9% (47·5 to 61·2%)	5·7% (-1·2 to 12·8%)	-5·2% (-8·1 to -2·7%)	123·0 (106·8 to 138·4)	55·2% (48·2 to 61·8%)	119·6 (102·0 to 136·5)	51·9% (44·9 to 58·2%)	-2·7% (-10·0 to 4·9%)	-6·0% (-9·5 to -2·2%)
Oesophageal cancer	43·2 (35·9 to 49·6)	58·2% (48·4 to 66·9%)	46·3 (37·2 to 54·6)	55·1% (44·8 to 64·3%)	7·2% (2·1 to 12·4%)	-5·4% (-7·9 to -3·4%)	927·6 (774·5 to 1,068·2)	59·5% (49·9 to 68·2%)	929·8 (749·5 to 1,094·4)	55·7% (45·6 to 65·0%)	0·2% (-5·1 to 5·8%)	-6·3% (-9·0 to -4·1%)
Stomach cancer	34·3 (21·5 to 46·9)	16·2% (10·2 to 22·1%)	29·5 (18·3 to 41·1)	14·1% (8·8 to 19·6%)	-13·8% (-18·6 to -9·6%)	-13·1% (-17·2 to -9·4%)	712·8 (449·0 to 959·5)	17·6% (11·0 to 23·7%)	540·0 (332·6 to 747·0)	14·9% (9·3 to 20·6%)	-24·2% (-29·0 to -20·2%)	-15·4% (-19·7 to -11·7%)
Colon and rectum cancer	28·9 (21·0 to 36·7)	8·9% (6·5 to 11·3%)	29·6 (21·4 to 37·9)	8·0% (5·8 to 10·1%)	2·5% (-1·9 to 7·1%)	-10·8% (-14·0 to -7·7%)	574·4 (417·5 to 730·2)	9·6% (7·0 to 12·2%)	538·0 (384·2 to 688·6)	8·3% (6·0 to 10·6%)	-6·3% (-10·1 to -2·5%)	-13·4% (-16·2 to -10·6%)
Liver cancer due to hepatitis B	5·1 (2·9 to 7·5)	20·0% (10·9 to 29·0%)	5·3 (3·0 to 7·9)	17·9% (9·9 to 26·1%)	3·7% (-5·3 to 12·5%)	-10·8% (-17·0 to -5·0%)	133·7 (74·0 to 198·6)	19·4% (10·6 to 28·3%)	122·7 (68·3 to 184·3)	16·7% (9·3 to 24·9%)	-8·2% (-19·5 to 2·5%)	-13·7% (-22·1 to -5·6%)
Liver cancer due to hepatitis C	9·4 (5·6 to 13·3)	18·2% (10·8 to 25·7%)	11·2 (6·8 to 16·0)	17·5% (10·3 to 24·8%)	19·5% (13·2 to 25·9%)	-3·8% (-7·7 to -0·1%)	171·8 (100·6 to 243·6)	19·1% (11·2 to 27·0%)	186·0 (108·3 to 265·6)	18·2% (10·6 to 25·9%)	8·3% (2·7 to 13·6%)	-4·6% (-8·3 to -1·4%)
Liver cancer due to alcohol use	11·1 (6·4 to 15·6)	26·6% (15·5 to 37·3%)	11·7 (6·6 to 16·8)	24·0% (13·7 to 34·1%)	4·9% (-1·5 to 10·4%)	-9·8% (-13·6 to -6·6%)	245·7 (142·0 to 343·5)	27·6% (16·1 to 38·5%)	246·0 (139·6 to 354·3)	24·5% (14·0 to 34·9%)	0·1% (-6·5 to 5·9%)	-11·0% (-15·4 to -7·5%)
Liver cancer due to other causes	3·2 (2·0 to 4·4)	21·2% (13·2 to 28·9%)	3·8 (2·3 to 5·3)	20·3% (12·6 to 27·9%)	18·8% (11·6 to 26·0%)	-4·3% (-8·8 to -0·4%)	68·0 (41·2 to 94·2)	21·6% (13·6 to 29·5%)	72·6 (43·4 to 100·8)	20·3% (12·6 to 28·2%)	6·8% (-1·2 to 15·2%)	-5·6% (-11·4 to 0·1%)
Pancreatic cancer	39·5 (32·9 to 46·2)	25·4% (21·0 to 29·7%)	45·0 (36·9 to 53·3)	22·9% (18·8 to 26·9%)	13·8% (10·0 to 17·7%)	-9·8% (-12·2 to -7·7%)	792·6 (660·0 to 926·4)	27·4% (22·8 to 32·1%)	827·6 (676·9 to 975·7)	24·2% (19·8 to 28·4%)	4·4% (0·7 to 8·3%)	-11·8% (-14·2 to -9·6%)
Tracheal, bronchus, and lung cancer	483·0 (469·3 to 495·9)	81·3% (79·3 to 83·2%)	523·5 (502·3 to 544·4)	78·9% (76·7 to 81·1%)	8·4% (5·2 to 11·6%)	-2·9% (-3·7 to -2·2%)	9,679·1 (9,418·6 to 9,948·1)	81·7% (79·8 to 83·7%)	9,794·8 (9,405·2 to 10,187·0)	79·0% (76·8 to 81·1%)	1·2% (-1·7 to 4·2%)	-3·4% (-4·2 to -2·6%)
Cervical cancer	3·9 (1·4 to 6·4)	10·5% (3·7 to 17·3%)	3·7 (1·3 to 6·2)	10·0% (3·5 to 16·6%)	-4·8% (-11·8 to 3·1%)	-4·8% (-10·6 to 1·1%)	104·9 (38·6 to 174·3)	10·8% (3·9 to 18·0%)	88·7 (30·9 to 149·0)	9·7% (3·4 to 16·2%)	-15·5% (-22·5 to -7·0%)	-9·7% (-16·1 to -2·2%)
Kidney cancer	13·9 (9·6 to 17·9)	24·0% (16·6 to 30·5%)	15·1 (10·2 to 19·8)	21·1% (14·3 to 27·5%)	8·6% (2·3 to 14·8%)	-11·9% (-15·7 to -8·8%)	320·7 (223·6 to 408·2)	25·8% (17·9 to 32·6%)	324·1 (218·8 to 425·5)	22·3% (15·2 to 28·9%)	1·0% (-5·6 to 7·1%)	-13·4% (-17·4 to -10·3%)
Bladder cancer	26·1 (20·3 to 31·4)	38·1% (29·7 to 46·2%)	29·6 (22·5 to 36·5)	35·3% (26·8 to 43·4%)	13·1% (7·0 to 19·3%)	-7·5% (-10·6 to -4·9%)	459·8 (359·0 to 549·4)	40·9% (32·1 to 49·2%)	468·9 (359·3 to 574·4)	37·1% (28·4 to 45·5%)	2·0% (-3·7 to 7·5%)	-9·3% (-12·1 to -6·9%)
Ischaemic heart disease	362·6 (302·5 to 423·7)	14·8% (12·3 to 17·2%)	303·3 (253·1 to 355·5)	12·2% (10·2 to 14·2%)	-16·3% (-18·8 to -13·9%)	-17·7% (-19·9 to -15·6%)	8,959·3 (7,466·0 to 10,431·7)	23·2% (19·4 to 27·0%)	7,123·5 (5,920·0 to 8,314·2)	20·3% (17·0 to 23·7%)	-20·5% (-23·0 to -18·2%)	-12·7% (-14·6 to -10·8%)
Ischaemic stroke	95·9 (80·5 to 111·4)	10·7% (8·9 to 12·5%)	75·6 (63·1 to 89·1)	8·9% (7·4 to 10·5%)	-21·2% (-25·0 to -17·6%)	-16·4% (-19·9 to -13·1%)	1,940·2 (1,618·2 to 2,252·7)	16·5% (13·8 to 19·1%)	1,487·4 (1,245·9 to 1,741·4)	15·0% (12·6 to 17·5%)	-23·3% (-27·1 to -19·5%)	-9·0% (-12·3 to -5·9%)
Hemorrhagic stroke	82·7 (70·0 to 95·1)	17·0% (14·4 to 19·6%)	63·3 (53·4 to 73·6)	13·9% (11·6 to 16·1%)	-23·5% (-26·5 to -20·2%)	-18·6% (-21·1 to -16·1%)	2,272·5 (1,935·8 to 2,617·2)	25·4% (21·7 to 29·0%)	1,674·4 (1,416·1 to 1,941·2)	22·5% (19·0 to 26·0%)	-26·3% (-29·5 to -23·3%)	-11·3% (-13·6 to -9·2%)
Hypertensive heart disease	14·8 (11·1 to 18·3)	11·1% (8·4 to 13·8%)	14·5 (10·6 to 18·1)	9·0% (6·7 to 11·3%)	-1·8% (-7·0 to 3·4%)	-18·9% (-22·5 to -15·0%)	363·9 (277·9 to 447·3)	18·5% (14·2 to 22·5%)	335·3 (249·5 to 412·1)	15·8% (11·9 to 19·4%)	-7·8% (-12·1 to -3·1%)	-14·8% (-17·7 to -12·0%)
Atrial fibrillation and flutter	5·0 (3·6 to 6·5)	7·0% (5·1 to 8·9%)	5·5 (4·0 to 7·3)	5·9% (4·3 to 7·5%)	11·0% (6·3 to 16·3%)	-16·3% (-19·5 to -13·2%)	235·3 (163·1 to 322·0)	14·4% (10·9 to 17·7%)	253·3 (174·8 to 351·1)	12·6% (9·3 to 15·6%)	7·6% (5·4 to 9·8%)	-12·2% (-13·9 to -10·7%)
Aortic aneurysm	10·2 (7·8 to 12·3)	14·4% (11·1 to 17·5%)	9·5 (7·3 to 11·6)	11·6% (8·9 to 14·1%)	-6·9% (-11·3 to -3·5%)	-19·5% (-21·6 to -17·5%)	230·4 (179·3 to 277·0)	21·4% (16·8 to 25·7%)	209·6 (162·1 to 252·4)	18·5% (14·4 to 22·3%)	-9·0% (-13·6 to -5·4%)	-13·5% (-15·6 to -11·6%)

Supplemental Results Table 1. All-age deaths, death PAFs, DALYs, DALY PAFs, and percent change for each risk factor and outcome for both sexes combined in 2005 and 2015 for High SDI.

Data in parenthesis are 95% uncertainty intervals. PAFs=population attributable fractions. DALYs=disability-adjusted life-years.

	2005 Deaths (in thousands)	2005 Deaths PAF	2015 Deaths (in thousands)	2015 Deaths PAF	Percent change deaths 2005-2015	Percent change deaths PAF 2005-2015	2005 DALYs (in thousands)	2005 DALYs PAF	2015 DALYs (in thousands)	2015 DALYs PAF	Percent change DALYs 2005-2015	Percent change DALYs PAF 2005-2015
Peripheral vascular disease	3·1 (2·4 to 3·8)	10·9% (8·3 to 13·4%)	3·4 (2·5 to 4·2)	9·0% (6·7 to 11·2%)	8·8% (1·5 to 16·6%)	-17·0% (-21·9 to -12·2%)	81·0 (59·5 to 105·3)	16·0% (12·3 to 19·6%)	85·9 (62·6 to 112·5)	13·9% (10·5 to 17·2%)	6·1% (0·3 to 12·3%)	-12·9% (-16·5 to -9·5%)
Other cardiovascular and circulatory diseases	18·8 (14·2 to 23·1)	12·0% (9·0 to 14·8%)	17·5 (13·0 to 21·9)	9·7% (7·2 to 12·1%)	-6·8% (-10·6 to -2·8%)	-19·6% (-22·6 to -16·7%)	652·8 (497·7 to 816·0)	19·0% (14·4 to 23·1%)	607·7 (457·9 to 767·8)	16·4% (12·3 to 20·2%)	-6·9% (-10·1 to -3·9%)	-13·5% (-15·6 to -11·7%)
Chronic obstructive pulmonary disease	309·4 (292·0 to 327·9)	72·8% (69·1 to 76·3%)	348·1 (325·5 to 372·5)	72·2% (68·2 to 75·9%)	12·5% (9·6 to 15·3%)	-0·9% (-2·4 to 0·6%)	5,454·9 (5,126·5 to 5,793·8)	70·9% (67·5 to 74·2%)	5,637·8 (5,266·8 to 6,013·8)	69·4% (65·8 to 72·8%)	3·4% (1·1 to 5·5%)	-2·1% (-3·3 to -0·8%)
Silicosis	0·5 (0·4 to 0·7)	22·9% (17·3 to 29·0%)	0·4 (0·3 to 0·6)	21·5% (15·7 to 27·8%)	-15·6% (-29·4 to -0·9%)	-6·4% (-13·3 to 0·5%)	9·6 (7·2 to 12·6)	22·3% (17·6 to 27·9%)	7·2 (5·2 to 9·6)	19·4% (14·9 to 24·3%)	-25·2% (-32·8 to -17·4%)	-13·2% (-17·1 to -9·1%)
Asbestosis	0·4 (0·2 to 0·5)	25·1% (19·5 to 31·2%)	0·4 (0·3 to 0·6)	22·5% (17·1 to 28·5%)	14·8% (1·9 to 28·2%)	-10·2% (-14·5 to -5·6%)	7·6 (5·4 to 9·8)	25·4% (20·2 to 31·0%)	7·8 (5·4 to 10·2)	22·3% (17·5 to 27·6%)	2·5% (-5·2 to 10·0%)	-12·0% (-15·3 to -8·2%)
Coal workers pneumoconiosis	0·3 (0·3 to 0·4)	28·7% (22·3 to 35·6%)	0·2 (0·2 to 0·3)	24·1% (18·2 to 30·7%)	-28·0% (-36·2 to -18·7%)	-16·0% (-22·0 to -10·5%)	5·9 (4·5 to 7·4)	27·4% (21·5 to 33·7%)	4·1 (3·1 to 5·4)	22·0% (17·1 to 27·4%)	-29·3% (-35·5 to -22·9%)	-19·7% (-23·9 to -16·0%)
Other pneumoconiosis	0·8 (0·6 to 1·0)	17·9% (14·6 to 21·6%)	0·9 (0·7 to 1·1)	16·8% (13·5 to 20·5%)	7·9% (-0·2 to 16·7%)	-6·2% (-11·3 to -0·9%)	15·2 (12·0 to 19·3)	16·5% (13·4 to 19·8%)	13·8 (10·6 to 17·5)	14·8% (11·8 to 17·9%)	-9·4% (-15·1 to -3·4%)	-10·8% (-14·8 to -6·6%)
Asthma	3·9 (3·2 to 4·6)	14·7% (12·1 to 17·3%)	2·5 (2·1 to 3·0)	11·6% (9·6 to 13·6%)	-34·6% (-38·8 to -30·7%)	-21·0% (-24·5 to -17·6%)	392·1 (273·7 to 530·5)	11·5% (9·7 to 13·3%)	330·3 (225·9 to 453·5)	10·3% (8·7 to 12·0%)	-15·8% (-19·0 to -13·2%)	-10·0% (-12·1 to -8·1%)
Interstitial lung disease and pulmonary sarcoidosis	9·0 (5·7 to 11·2)	21·7% (18·1 to 25·3%)	11·5 (7·0 to 14·7)	19·9% (16·4 to 23·6%)	27·8% (16·3 to 35·9%)	-8·3% (-11·1 to -5·5%)	157·5 (111·3 to 192·5)	21·9% (18·3 to 25·5%)	178·4 (122·9 to 221·2)	19·6% (16·2 to 23·1%)	13·2% (5·2 to 19·4%)	-10·3% (-12·8 to -7·8%)
Other chronic respiratory diseases	1·1 (0·9 to 1·3)	22·6% (18·6 to 26·3%)	1·2 (0·9 to 1·4)	21·0% (17·2 to 24·8%)	6·2% (-0·4 to 12·7%)	-7·1% (-10·5 to -3·6%)	36·8 (30·4 to 43·4)	18·3% (15·3 to 21·3%)	34·8 (28·1 to 40·9)	17·2% (14·2 to 20·2%)	-5·6% (-11·4 to 0·5%)	-6·3% (-10·2 to -2·1%)
Diabetes mellitus	10·2 (3·3 to 17·8)	4·5% (1·5 to 7·8%)	9·2 (2·9 to 16·4)	3·8% (1·2 to 6·7%)	-9·6% (-14·0 to -5·7%)	-15·8% (-19·3 to -12·2%)	582·6 (179·1 to 1,038·4)	6·1% (1·9 to 10·5%)	582·5 (177·8 to 1,051·5)	5·2% (1·7 to 9·2%)	0·0% (-4·0 to 3·3%)	-14·2% (-16·6 to -12·3%)
**Second-hand smoke: All causes**	99·9 (76·7 to 127·2)	0·9% (0·7 to 1·1%)	95·2 (71·9 to 123·6)	0·8% (0·6 to 1·0%)	-4·7% (-9·7 to -0·3%)	-11·6% (-16·1 to -7·7%)	1,665·0 (1,326·3 to 2,049·4)	0·5% (0·4 to 0·6%)	1,409·4 (1,111·5 to 1,744·1)	0·4% (0·3 to 0·5%)	-15·4% (-19·1 to -11·8%)	-16·3% (-19·8 to -12·6%)
Lower respiratory infections	11·0 (6·9 to 16·0)	2·5% (1·6 to 3·7%)	12·6 (7·8 to 18·3)	2·5% (1·5 to 3·6%)	14·7% (8·0 to 20·3%)	-3·2% (-6·7 to 0·1%)	202·2 (129·8 to 290·9)	3·2% (2·0 to 4·6%)	173·2 (108·4 to 249·1)	2·7% (1·7 to 4·0%)	-14·3% (-19·9 to -9·7%)	-13·9% (-17·6 to -10·5%)
Otitis media	0·0 (0·0 to 0·0)	4·1% (2·7 to 5·4%)	0·0 (0·0 to 0·0)	3·2% (2·1 to 4·3%)	-22·6% (-33·2 to -9·8%)	-20·4% (-28·3 to -11·3%)	13·0 (7·1 to 21·6)	3·7% (2·4 to 5·0%)	11·6 (6·2 to 19·1)	3·3% (2·2 to 4·5%)	-11·1% (-14·3 to -8·1%)	-11·9% (-14·7 to -9·2%)
Tracheal, bronchus, and lung cancer	2·9 (1·7 to 4·6)	0·5% (0·3 to 0·8%)	3·5 (2·0 to 5·5)	0·5% (0·3 to 0·8%)	21·4% (17·7 to 25·3%)	8·7% (4·9 to 12·7%)	59·2 (34·3 to 93·7)	0·5% (0·3 to 0·8%)	70·1 (40·7 to 110·6)	0·6% (0·3 to 0·9%)	18·3% (13·5 to 23·1%)	12·9% (7·9 to 17·8%)
Ischaemic heart disease	60·2 (42·9 to 81·6)	2·5% (1·8 to 3·3%)	57·0 (38·9 to 78·9)	2·3% (1·6 to 3·2%)	-5·4% (-13·0 to 0·5%)	-6·9% (-14·3 to -1·5%)	968·7 (736·5 to 1,235·4)	2·5% (1·9 to 3·2%)	820·5 (622·3 to 1,054·2)	2·3% (1·8 to 3·0%)	-15·3% (-20·6 to -10·5%)	-7·0% (-12·3 to -2·1%)
Ischaemic stroke	14·2 (10·0 to 19·5)	1·6% (1·1 to 2·2%)	11·8 (7·7 to 16·5)	1·4% (0·9 to 2·0%)	-17·2% (-25·7 to -9·9%)	-12·3% (-20·9 to -5·1%)	193·9 (145·7 to 251·0)	1·7% (1·2 to 2·1%)	142·0 (103·8 to 185·6)	1·4% (1·0 to 1·9%)	-26·8% (-33·1 to -21·0%)	-13·1% (-19·8 to -6·6%)
Hemorrhagic stroke	7·6 (5·7 to 9·9)	1·6% (1·2 to 2·0%)	6·2 (4·5 to 8·3)	1·4% (1·0 to 1·8%)	-18·5% (-25·3 to -12·6%)	-13·3% (-19·7 to -7·4%)	154·6 (119·9 to 193·6)	1·7% (1·3 to 2·2%)	114·7 (88·1 to 146·2)	1·5% (1·2 to 2·0%)	-25·8% (-30·7 to -20·9%)	-10·6% (-15·4 to -5·8%)
**Alcohol and drug use: All causes**	759·8 (634·4 to 882·2)	6·8% (5·7 to 7·9%)	703·2 (573·8 to 828·5)	5·8% (4·8 to 6·9%)	-7·4% (-10·7 to -4·9%)	-14·2% (-17·1 to -11·8%)	31,583·8 (28,653·2 to 34,563·4)	9·0% (8·0 to 10·0%)	28,166·5 (25,457·9 to 30,920·2)	8·0% (7·0 to 8·9%)	-10·8% (-12·8 to -8·9%)	-11·8% (-13·5 to -10·3%)
**Alcohol use: All causes**	627·7 (499·5 to 749·4)	5·6% (4·5 to 6·7%)	542·7 (412·1 to 669·1)	4·5% (3·4 to 5·5%)	-13·5% (-18·8 to -9·8%)	-19·8% (-24·6 to -16·5%)	24,158·7 (21,418·5 to 26,752·7)	6·9% (6·0 to 7·8%)	19,476·4 (17,028·8 to 21,887·7)	5·5% (4·7 to 6·3%)	-19·4% (-21·9 to -17·2%)	-20·3% (-22·6 to -18·0%)
Tuberculosis	12·5 (10·7 to 14·1)	29·6% (25·7 to 32·6%)	6·3 (5·5 to 7·3)	24·7% (21·5 to 27·7%)	-49·5% (-54·8 to -43·7%)	-16·4% (-22·0 to -10·6%)	478·5 (412·4 to 534·4)	35·1% (30·9 to 38·3%)	229·1 (200·8 to 260·4)	32·7% (29·1 to 35·8%)	-52·1% (-57·2 to -46·5%)	-6·8% (-11·8 to -1·3%)
Lower respiratory infections	38·5 (34·2 to 42·9)	8·9% (7·9 to 9·9%)	39·8 (34·7 to 45·1)	7·8% (6·8 to 8·7%)	3·4% (-5·6 to 12·2%)	-12·7% (-18·7 to -6·8%)	876·2 (785·2 to 972·6)	13·8% (12·4 to 15·0%)	763·4 (676·5 to 843·9)	12·1% (10·9 to 13·1%)	-12·9% (-22·5 to -3·3%)	-12·4% (-19·4 to -5·8%)
Lip and oral cavity cancer	12·3 (11·3 to 13·2)	38·5% (35·6 to 40·9%)	13·1 (11·6 to 14·4)	36·1% (32·6 to 39·3%)	6·5% (0·2 to 13·9%)	-6·0% (-9·7 to -3·0%)	336·8 (312·7 to 357·9)	42·9% (40·0 to 45·2%)	341·6 (306·8 to 373·3)	41·2% (37·6 to 44·1%)	1·4% (-4·5 to 9·4%)	-4·1% (-7·1 to -1·6%)
Nasopharynx cancer	3·1 (2·8 to 3·3)	37·9% (35·0 to 40·3%)	3·4 (3·0 to 3·7)	37·3% (33·5 to 40·5%)	9·8% (1·3 to 16·6%)	-1·6% (-5·0 to 1·2%)	88·3 (80·1 to 94·3)	39·6% (36·6 to 41·8%)	91·7 (80·3 to 100·9)	39·7% (36·1 to 42·7%)	3·8% (-4·4 to 10·7%)	0·4% (-2·7 to 2·7%)
Other pharynx cancer	8·1 (7·5 to 8·6)	43·3% (40·5 to 45·7%)	9·4 (8·4 to 10·2)	41·8% (38·2 to 44·8%)	15·5% (8·3 to 23·2%)	-3·5% (-6·8 to -1·0%)	228·0 (210·8 to 243·0)	46·1% (43·3 to 48·4%)	254·7 (229·2 to 277·8)	44·9% (41·2 to 47·8%)	11·8% (4·3 to 19·6%)	-2·7% (-5·7 to -0·4%)
Oesophageal cancer	16·3 (14·7 to 17·8)	22·0% (19·8 to 24·0%)	17·8 (15·4 to 20·0)	21·2% (18·4 to 23·5%)	8·9% (2·0 to 15·6%)	-3·9% (-7·9 to -0·6%)	388·3 (351·4 to 419·7)	24·9% (22·6 to 26·8%)	405·0 (352·3 to 451·9)	24·3% (21·3 to 26·6%)	4·3% (-2·7 to 11·3%)	-2·5% (-6·2 to 0·3%)
Colon and rectum cancer	14·6 (13·1 to 16·0)	4·5% (4·0 to 4·9%)	16·1 (14·2 to 18·0)	4·3% (3·8 to 4·8%)	10·5% (5·5 to 15·7%)	-3·9% (-7·8 to -0·2%)	298·5 (271·8 to 325·3)	5·0% (4·5 to 5·4%)	314·2 (279·3 to 348·3)	4·9% (4·3 to 5·3%)	5·3% (0·6 to 9·9%)	-2·6% (-6·3 to 0·8%)
Liver cancer due to hepatitis B	0·1 (0·1 to 0·2)	0·4% (0·3 to 0·7%)	0·1 (0·1 to 0·2)	0·3% (0·2 to 0·5%)	-12·4% (-36·6 to 25·3%)	-24·7% (-45·1 to 4·6%)	6·0 (4·1 to 8·6)	0·9% (0·6 to 1·2%)	5·3 (3·5 to 7·9)	0·7% (0·5 to 1·0%)	-11·9% (-34·3 to 20·3%)	-17·3% (-37·2 to 10·9%)
Liver cancer due to hepatitis C	0·0 (0·0 to 0·0)	0·0% (0·0 to 0·1%)	0·0 (0·0 to 0·0)	0·0% (0·0 to 0·1%)	0·3% (-24·2 to 35·7%)	-19·3% (-39·5 to 8·8%)	1·0 (0·6 to 1·4)	0·1% (0·1 to 0·2%)	0·9 (0·6 to 1·3)	0·1% (0·1 to 0·1%)	-4·7% (-23·8 to 16·9%)	-16·0% (-32·7 to 3·4%)
Liver cancer due to alcohol use	41·8 (39·6 to 44·1)	100·0% (100·0 to 100·0%)	48·7 (45·7 to 51·6)	100·0% (100·0 to 100·0%)	16·3% (12·0 to 20·8%)	-	891·8 (841·6 to 944·8)	100·0% (100·0 to 100·0%)	1,003·0 (936·1 to 1,069·8)	100·0% (100·0 to 100·0%)	12·5% (7·8 to 17·5%)	-
Liver cancer due to other causes	0·0 (0·0 to 0·0)	0·2% (0·1 to 0·3%)	0·0 (0·0 to 0·0)	0·1% (0·1 to 0·2%)	-9·2% (-25·9 to 11·7%)	-26·9% (-39·9 to -11·0%)	1·6 (1·2 to 2·0)	0·5% (0·4 to 0·6%)	1·4 (1·0 to 1·9)	0·4% (0·3 to 0·5%)	-8·9% (-23·8 to 10·0%)	-19·5% (-31·8 to -4·1%)
Larynx cancer	7·0 (6·3 to 7·5)	28·2% (25·8 to 30·3%)	6·4 (5·6 to 7·1)	26·9% (23·9 to 29·5%)	-9·2% (-14·8 to -3·6%)	-4·8% (-8·4 to -1·6%)	184·1 (167·7 to 197·6)	30·7% (28·2 to 32·8%)	159·8 (141·8 to 176·5)	29·6% (26·5 to 32·1%)	-13·2% (-18·5 to -7·7%)	-3·5% (-6·8 to -0·7%)
Breast cancer	16·3 (14·6 to 18·0)	9·0% (8·1 to 9·8%)	17·4 (15·0 to 19·9)	8·6% (7·4 to 9·6%)	6·5% (-0·1 to 12·7%)	-5·1% (-9·7 to -1·2%)	467·8 (415·5 to 519·8)	10·1% (9·1 to 11·0%)	488·5 (415·5 to 562·7)	9·7% (8·4 to 10·8%)	4·4% (-2·4 to 10·6%)	-3·8% (-8·2 to -0·2%)
Ischaemic heart disease	17·0 (-76·8 to 113·1)	0·7% (-3·2 to 4·6%)	-4·0 (-94·7 to 89·0)	-0·2% (-3·8 to 3·6%)	-123·2% (-557·0 to 419·0%)	-122·9% (-552·5 to 413·2%)	1,376·2 (-237·5 to 2,923·2)	3·6% (-0·6 to 7·6%)	735·7 (-656·7 to 2,088·3)	2·1% (-1·9 to 5·9%)	-46·5% (-293·2 to 189·1%)	-41·3% (-313·9 to 217·9%)
Ischaemic stroke	4·6 (-12·8 to 20·7)	0·5% (-1·4 to 2·3%)	-3·6 (-19·4 to 9·9)	-0·4% (-2·3 to 1·2%)	-179·2% (-1,366·1 to 1,021·3%)	-183·8% (-1,452·8 to 1,093·9%)	280·2 (46·0 to 503·8)	2·4% (0·4 to 4·3%)	131·2 (-45·9 to 294·6)	1·3% (-0·5 to 3·0%)	-53·2% (-142·8 to -37·2%)	-44·4% (-150·9 to -26·0%)
Hemorrhagic stroke	53·6 (48·5 to 59·0)	11·0% (10·0 to 12·1%)	47·3 (41·1 to 53·8)	10·4% (9·0 to 11·7%)	-11·7% (-18·5 to -5·0%)	-6·1% (-12·7 to 0·5%)	1,112·0 (1,027·9 to 1,189·5)	12·4% (11·5 to 13·3%)	891·4 (793·8 to 980·2)	12·0% (10·7 to 13·1%)	-19·8% (-24·9 to -15·2%)	-3·5% (-8·7 to 1·2%)
Hypertensive heart disease	7·4 (3·8 to 11·3)	5·5% (2·8 to 8·5%)	6·5 (1·3 to 12·1)	4·0% (0·8 to 7·5%)	-12·0% (-73·6 to 38·4%)	-27·4% (-78·5 to 13·4%)	215·8 (168·7 to 262·3)	11·0% (8·6 to 13·3%)	207·4 (146·4 to 267·0)	9·8% (6·9 to 12·5%)	-3·9% (-19·8 to 9·8%)	-11·2% (-25·5 to 1·0%)
Atrial fibrillation and flutter	4·5 (3·7 to 5·5)	6·4% (5·6 to 7·3%)	5·8 (4·6 to 7·3)	6·2% (5·2 to 7·3%)	28·2% (19·4 to 37·1%)	-3·3% (-9·4 to 2·7%)	136·9 (107·4 to 174·9)	8·4% (7·5 to 9·1%)	162·9 (127·4 to 211·7)	8·1% (7·1 to 9·1%)	19·0% (12·7 to 24·7%)	-2·9% (-8·0 to 1·5%)
Cirrhosis and other chronic liver diseases due to hepatitis B	13·1 (10·7 to 15·3)	32·5% (27·7 to 36·5%)	14·2 (11·4 to 17·0)	32·8% (26·8 to 37·8%)	7·7% (-0·4 to 15·3%)	1·1% (-5·5 to 6·6%)	349·6 (281·6 to 412·3)	32·0% (26·7 to 36·3%)	352·3 (277·1 to 422·3)	32·9% (26·5 to 37·8%)	0·8% (-7·1 to 8·7%)	2·7% (-4·1 to 8·7%)
Cirrhosis and other chronic liver diseases due to hepatitis C	25·9 (21·7 to 29·7)	34·5% (30·3 to 38·4%)	28·4 (22·5 to 33·6)	34·2% (28·2 to 39·5%)	9·3% (0·1 to 16·4%)	-0·8% (-8·3 to 4·8%)	661·2 (540·8 to 772·3)	34·0% (29·3 to 38·1%)	688·7 (534·0 to 826·2)	34·3% (27·7 to 39·5%)	4·2% (-4·8 to 11·4%)	0·7% (-7·4 to 6·3%)
Cirrhosis and other chronic liver diseases due to alcohol use	81·9 (71·4 to 87·6)	100·0% (100·0 to 100·0%)	78·8 (66·5 to 85·6)	100·0% (100·0 to 100·0%)	-3·8% (-8·7 to 2·9%)	-	2,512·7 (2,129·5 to 2,692·4)	100·0% (100·0 to 100·0%)	2,314·3 (1,919·5 to 2,528·4)	100·0% (100·0 to 100·0%)	-7·9% (-13·2 to -1·0%)	-
Cirrhosis and other chronic liver diseases due to other causes	13·9 (11·9 to 15·7)	34·0% (30·0 to 37·6%)	15·3 (12·7 to 17·7)	34·7% (29·5 to 39·2%)	10·1% (2·3 to 17·1%)	1·9% (-3·9 to 6·8%)	362·7 (304·2 to 418·3)	33·4% (28·9 to 37·3%)	375·8 (304·6 to 441·3)	34·6% (29·0 to 39·2%)	3·6% (-3·6 to 11·3%)	3·8% (-2·2 to 8·9%)
Pancreatitis	8·0 (7·1 to 8·9)	26·9% (24·1 to 29·3%)	7·6 (6·6 to 8·4)	25·2% (22·2 to 27·8%)	-5·5% (-14·1 to 4·4%)	-6·6% (-12·8 to -0·4%)	312·2 (276·4 to 346·8)	33·4% (30·0 to 36·0%)	284·2 (247·6 to 316·5)	32·2% (29·1 to 35·0%)	-9·0% (-17·0 to 0·2%)	-3·4% (-8·7 to 1·9%)

Supplemental Results Table 1. All-age deaths, death PAFs, DALYs, DALY PAFs, and percent change for each risk factor and outcome for both sexes combined in 2005 and 2015 for High SDI.

Data in parenthesis are 95% uncertainty intervals. PAFs=population attributable fractions. DALYs=disability-adjusted life-years.

	2005 Deaths (in thousands)	2005 Deaths PAF	2015 Deaths (in thousands)	2015 Deaths PAF	Percent change deaths 2005-2015	Percent change deaths PAF 2005-2015	2005 DALYs (in thousands)	2005 DALYs PAF	2015 DALYs (in thousands)	2015 DALYs PAF	Percent change DALYs 2005-2015	Percent change DALYs PAF 2005-2015
Epilepsy	3·0 (2·8 to 3·2)	21·4% (19·9 to 22·6%)	2·9 (2·6 to 3·3)	20·1% (17·9 to 21·9%)	-2·6% (-8·4 to 3·9%)	-6·1% (-10·5 to -2·3%)	260·4 (212·2 to 315·9)	19·1% (17·8 to 20·4%)	215·8 (170·2 to 270·3)	18·9% (16·9 to 20·6%)	-17·1% (-24·2 to -10·5%)	-1·0% (-5·2 to 2·4%)
Alcohol use disorders	83·3 (74·5 to 87·8)	100·0% (100·0 to 100·0%)	60·6 (55·8 to 64·7)	100·0% (100·0 to 100·0%)	-27·3% (-32·1 to -20·2%)	-	4,784·9 (4,110·9 to 5,476·2)	100·0% (100·0 to 100·0%)	3,751·9 (3,176·7 to 4,446·3)	100·0% (100·0 to 100·0%)	-21·6% (-26·3 to -15·9%)	-
Diabetes mellitus	-32·6 (-36·2 to -29·2)	-14·3% (-15·9 to -12·9%)	-33·0 (-37·6 to -29·0)	-13·5% (-15·3 to -12·1%)	1·5% (-13·1 to 17·8%)	-5·5% (-17·6 to 9·6%)	-1,270·1 (-1,579·9 to -1,006·3)	-13·2% (-13·9 to -12·6%)	-1,397·9 (-1,759·2 to -1,082·7)	-12·5% (-13·1 to -11·8%)	10·1% (2·5 to 16·5%)	-5·6% (-10·8 to -0·3%)
Pedestrian road injuries	13·8 (11·6 to 16·0)	24·4% (21·1 to 27·9%)	8·9 (7·6 to 10·5)	21·2% (18·3 to 24·3%)	-35·3% (-40·3 to -31·3%)	-13·0% (-16·2 to -10·3%)	660·8 (554·7 to 764·4)	28·0% (24·3 to 31·8%)	400·9 (343·6 to 472·4)	25·4% (22·1 to 29·0%)	-39·3% (-44·6 to -35·1%)	-9·2% (-12·1 to -6·7%)
Cyclist road injuries	2·1 (1·7 to 2·4)	22·5% (19·2 to 25·9%)	1·6 (1·3 to 1·8)	20·2% (17·3 to 23·3%)	-24·3% (-31·1 to -19·6%)	-10·2% (-12·5 to -8·0%)	127·3 (106·1 to 151·1)	19·7% (16·9 to 22·8%)	93·0 (76·2 to 111·4)	17·9% (15·2 to 20·9%)	-27·0% (-32·6 to -23·3%)	-9·2% (-12·1 to -6·8%)
Motorcyclist road injuries	9·0 (7·7 to 10·0)	47·2% (44·0 to 50·5%)	7·5 (6·3 to 8·3)	45·8% (42·8 to 49·2%)	-16·8% (-23·1 to -12·1%)	-2·8% (-3·9 to -1·7%)	587·2 (510·1 to 666·4)	45·1% (42·1 to 48·4%)	468·7 (398·4 to 543·0)	44·0% (40·9 to 47·2%)	-20·2% (-25·1 to -16·3%)	-2·5% (-3·9 to -1·3%)
Motor vehicle road injuries	44·9 (39·5 to 51·0)	42·4% (39·6 to 45·3%)	31·1 (28·2 to 34·6)	40·1% (37·6 to 42·8%)	-30·7% (-37·0 to -23·5%)	-5·5% (-7·1 to -3·9%)	2,488·1 (2,212·5 to 2,792·9)	42·6% (39·8 to 45·5%)	1,717·5 (1,559·4 to 1,901·2)	40·5% (37·9 to 43·2%)	-31·0% (-36·6 to -24·8%)	-4·9% (-6·6 to -3·2%)
Falls	16·1 (13·8 to 19·1)	13·1% (11·2 to 15·4%)	16·5 (13·8 to 19·8)	11·5% (9·6 to 13·9%)	2·6% (-1·6 to 7·6%)	-12·0% (-15·2 to -9·0%)	976·7 (774·2 to 1,226·8)	15·4% (13·6 to 17·6%)	883·4 (686·8 to 1,143·8)	14·3% (12·6 to 16·5%)	-9·6% (-13·8 to -5·7%)	-6·9% (-8·1 to -5·6%)
Drowning	7·8 (6·9 to 8·8)	18·9% (17·0 to 21·4%)	5·6 (4·9 to 6·4)	17·3% (15·4 to 19·8%)	-28·2% (-32·7 to -23·9%)	-8·5% (-11·1 to -5·9%)	333·1 (299·7 to 375·5)	19·1% (17·3 to 21·3%)	218·7 (196·6 to 246·8)	18·8% (17·0 to 21·0%)	-34·4% (-39·1 to -30·1%)	-1·7% (-4·3 to 1·0%)
Fire, heat, and hot substances	5·5 (4·8 to 6·2)	16·9% (15·1 to 19·2%)	3·8 (3·3 to 4·5)	15·2% (13·4 to 17·5%)	-30·0% (-35·2 to -20·3%)	-9·8% (-13·6 to -5·4%)	257·7 (224·3 to 300·6)	17·7% (16·1 to 19·8%)	174·4 (147·9 to 213·0)	16·9% (15·2 to 19·0%)	-32·3% (-37·3 to -24·6%)	-4·6% (-7·2 to -1·6%)
Poisonings	2·1 (1·8 to 2·5)	16·9% (15·2 to 19·4%)	1·8 (1·6 to 2·1)	15·9% (14·3 to 18·0%)	-15·2% (-21·7 to -6·7%)	-6·3% (-9·4 to -2·9%)	97·3 (85·7 to 115·6)	17·5% (16·0 to 19·7%)	81·0 (71·5 to 93·9)	17·2% (15·8 to 19·3%)	-16·8% (-22·9 to -9·2%)	-2·0% (-4·9 to 1·1%)
Unintentional firearm injuries	0·8 (0·7 to 0·9)	14·7% (12·9 to 16·8%)	0·7 (0·6 to 0·8)	13·1% (11·3 to 15·3%)	-14·6% (-19·6 to -9·5%)	-11·1% (-14·8 to -7·8%)	33·0 (29·5 to 37·0)	18·4% (16·8 to 20·4%)	25·3 (22·6 to 28·8)	17·6% (16·0 to 19·6%)	-23·2% (-27·5 to -18·0%)	-4·2% (-6·3 to -2·0%)
Unintentional suffocation	0·4 (0·3 to 0·4)	8·0% (6·8 to 9·5%)	0·4 (0·3 to 0·5)	8·5% (7·1 to 10·1%)	17·4% (8·0 to 26·0%)	6·0% (1·5 to 10·3%)	16·5 (13·1 to 19·7)	7·8% (6·8 to 8·8%)	18·7 (14·0 to 22·5)	8·8% (7·7 to 9·9%)	13·7% (4·8 to 21·9%)	13·1% (8·3 to 17·7%)
Other exposure to mechanical forces	3·2 (2·8 to 3·6)	20·2% (18·2 to 22·9%)	2·3 (2·1 to 2·7)	18·4% (16·4 to 21·1%)	-27·6% (-31·7 to -23·3%)	-8·9% (-11·4 to -6·8%)	234·3 (191·9 to 289·4)	19·5% (17·5 to 22·0%)	186·6 (146·9 to 241·4)	18·3% (16·4 to 20·7%)	-20·3% (-25·2 to -16·0%)	-6·2% (-7·6 to -4·8%)
Self-harm	50·6 (45·2 to 57·5)	20·6% (18·5 to 23·4%)	46·2 (41·1 to 52·8)	19·6% (17·5 to 22·3%)	-8·7% (-12·9 to -3·8%)	-4·9% (-6·4 to -3·1%)	2,124·8 (1,906·9 to 2,393·6)	21·7% (19·6 to 24·3%)	1,856·3 (1,667·4 to 2,097·0)	21·1% (19·0 to 23·7%)	-12·6% (-16·9 to -7·7%)	-2·6% (-4·0 to -1·0%)
Assault by firearm	4·3 (4·0 to 4·6)	20·0% (18·9 to 21·3%)	3·5 (3·3 to 3·8)	18·7% (17·8 to 19·9%)	-17·3% (-21·0 to -10·1%)	-6·3% (-7·3 to -5·2%)	240·0 (225·5 to 257·1)	21·0% (19·9 to 22·3%)	195·1 (183·3 to 210·0)	19·9% (19·0 to 21·1%)	-18·7% (-22·4 to -11·5%)	-5·1% (-6·0 to -4·1%)
Assault by sharp object	5·3 (4·7 to 6·0)	21·1% (19·3 to 23·4%)	3·2 (2·8 to 3·6)	20·3% (18·6 to 22·4%)	-40·3% (-45·5 to -33·9%)	-3·7% (-6·0 to -1·2%)	267·9 (238·4 to 302·8)	22·7% (20·7 to 25·0%)	155·5 (139·8 to 177·8)	22·2% (20·4 to 24·4%)	-41·9% (-47·2 to -35·7%)	-2·0% (-4·3 to 0·4%)
Assault by other means	7·3 (6·6 to 8·2)	19·2% (17·5 to 21·3%)	4·5 (4·0 to 5·1)	18·5% (16·8 to 20·5%)	-39·0% (-43·8 to -33·2%)	-4·0% (-6·1 to -1·9%)	372·9 (335·8 to 420·2)	20·2% (18·4 to 22·3%)	228·8 (203·6 to 259·8)	19·4% (17·8 to 21·6%)	-38·6% (-43·5 to -33·1%)	-3·6% (-5·8 to -1·4%)
**Drug use: All causes**	151·8 (136·6 to 165·5)	1·4% (1·2 to 1·5%)	182·8 (166·5 to 198·0)	1·5% (1·4 to 1·6%)	20·4% (15·3 to 25·0%)	11·7% (7·0 to 15·8%)	8,062·9 (7,200·8 to 8,919·2)	2·3% (2·1 to 2·5%)	9,375·3 (8,350·6 to 10,381·1)	2·6% (2·4 to 2·9%)	16·3% (12·2 to 19·7%)	15·0% (11·1 to 18·3%)
HIV/AIDS - Tuberculosis	0·7 (0·6 to 0·8)	26·6% (23·8 to 29·6%)	0·4 (0·4 to 0·5)	24·9% (22·2 to 27·9%)	-40·7% (-46·5 to -34·1%)	-6·2% (-9·8 to -2·2%)	34·8 (30·7 to 39·7)	27·5% (24·5 to 30·8%)	20·7 (17·9 to 24·0)	26·5% (23·7 to 29·8%)	-40·5% (-46·2 to -33·9%)	-3·6% (-6·6 to -0·2%)
HIV/AIDS resulting in other diseases	6·1 (5·5 to 6·8)	19·8% (17·9 to 21·9%)	7·8 (6·9 to 8·9)	26·2% (23·5 to 29·4%)	29·0% (19·5 to 39·7%)	32·5% (28·0 to 36·7%)	318·2 (280·6 to 363·1)	20·4% (18·3 to 22·7%)	420·6 (362·5 to 487·7)	26·8% (23·7 to 30·0%)	32·2% (22·9 to 42·5%)	30·8% (26·8 to 34·9%)
Hepatitis B	0·2 (0·1 to 0·4)	3·4% (1·4 to 5·5%)	0·1 (0·0 to 0·2)	3·5% (1·5 to 5·5%)	-48·7% (-55·1 to -40·4%)	3·4% (-6·7 to 16·8%)	7·7 (3·3 to 12·4)	4·0% (1·7 to 6·3%)	4·0 (1·7 to 6·3)	3·7% (1·6 to 5·7%)	-48·2% (-55·1 to -39·3%)	-7·6% (-16·9 to 4·9%)
Hepatitis C	0·2 (0·0 to 0·5)	55·7% (46·4 to 64·2%)	0·1 (0·0 to 0·3)	62·0% (52·6 to 70·4%)	-44·0% (-55·6 to -28·7%)	11·2% (-1·6 to 25·6%)	6·9 (1·9 to 15·1)	63·8% (56·2 to 70·2%)	3·8 (1·3 to 8·0)	66·5% (59·2 to 72·3%)	-44·5% (-56·3 to -23·7%)	4·1% (-3·3 to 12·7%)
Liver cancer due to hepatitis B	0·4 (0·2 to 0·6)	1·5% (0·6 to 2·5%)	0·6 (0·2 to 0·9)	1·9% (0·8 to 3·0%)	45·1% (32·2 to 63·0%)	24·9% (14·6 to 39·7%)	11·7 (4·9 to 19·6)	1·7% (0·7 to 2·8%)	15·5 (6·8 to 24·7)	2·1% (0·9 to 3·3%)	32·5% (20·3 to 48·8%)	24·6% (14·7 to 38·8%)
Liver cancer due to hepatitis C	21·9 (15·5 to 28·0)	42·4% (30·4 to 54·0%)	32·0 (24·4 to 39·1)	49·8% (38·5 to 60·3%)	45·9% (35·6 to 62·1%)	17·5% (9·9 to 29·5%)	450·8 (331·0 to 559·6)	50·1% (37·5 to 61·9%)	598·5 (479·4 to 709·1)	58·6% (47·2 to 68·1%)	32·8% (23·0 to 48·0%)	17·0% (9·6 to 29·8%)
Cirrhosis and other chronic liver diseases due to hepatitis B	0·7 (0·3 to 1·2)	1·8% (0·8 to 3·0%)	0·9 (0·4 to 1·5)	2·2% (1·0 to 3·5%)	28·9% (15·7 to 45·8%)	21·0% (10·8 to 35·2%)	23·2 (10·0 to 38·4)	2·1% (0·9 to 3·5%)	27·8 (12·1 to 45·5)	2·6% (1·1 to 4·2%)	19·4% (5·8 to 35·3%)	21·7% (11·1 to 36·1%)
Cirrhosis and other chronic liver diseases due to hepatitis C	49·5 (41·1 to 56·1)	65·8% (56·4 to 73·2%)	57·6 (48·5 to 64·7)	69·5% (61·8 to 75·5%)	16·4% (9·6 to 25·0%)	5·6% (2·5 to 10·8%)	1,436·1 (1,220·6 to 1,599·8)	74·0% (65·6 to 79·9%)	1,555·7 (1,315·6 to 1,727·2)	77·5% (71·2 to 81·8%)	8·3% (1·3 to 16·4%)	4·7% (2·1 to 9·1%)
Opioid use disorders	41·0 (39·1 to 46·1)	100·0% (100·0 to 100·0%)	46·5 (41·3 to 49·9)	100·0% (100·0 to 100·0%)	13·3% (0·8 to 21·8%)	-	3,590·4 (3,036·3 to 4,121·8)	100·0% (100·0 to 100·0%)	4,117·9 (3,435·7 to 4,758·9)	100·0% (100·0 to 100·0%)	14·7% (7·9 to 19·5%)	-
Cocaine use disorders	5·3 (3·1 to 5·7)	100·0% (100·0 to 100·0%)	7·5 (4·9 to 8·3)	100·0% (100·0 to 100·0%)	42·7% (25·8 to 64·1%)	-	423·3 (310·6 to 510·2)	100·0% (100·0 to 100·0%)	576·4 (437·5 to 691·8)	100·0% (100·0 to 100·0%)	36·2% (26·2 to 47·2%)	-
Amphetamine use disorders	3·9 (1·4 to 4·7)	100·0% (100·0 to 100·0%)	5·7 (2·7 to 6·8)	100·0% (100·0 to 100·0%)	45·1% (6·7 to 119·4%)	-	349·2 (223·8 to 446·2)	100·0% (100·0 to 100·0%)	494·5 (353·2 to 634·7)	100·0% (100·0 to 100·0%)	41·6% (21·4 to 70·0%)	-
Cannabis use disorders	-	-	-	-	-	-	163·8 (105·0 to 231·6)	100·0% (100·0 to 100·0%)	165·4 (108·0 to 234·7)	100·0% (100·0 to 100·0%)	1·0% (-1·5 to 3·4%)	-
Other drug use disorders	8·5 (8·1 to 9·5)	100·0% (100·0 to 100·0%)	10·1 (8·9 to 10·7)	100·0% (100·0 to 100·0%)	18·1% (3·3 to 27·7%)	-	591·9 (501·8 to 699·1)	100·0% (100·0 to 100·0%)	724·1 (599·4 to 867·2)	100·0% (100·0 to 100·0%)	22·3% (12·6 to 29·7%)	-
Self-harm	13·4 (9·2 to 18·7)	5·5% (3·7 to 7·6%)	13·5 (9·4 to 18·9)	5·8% (4·0 to 8·0%)	1·3% (-6·3 to 9·8%)	5·5% (-0·3 to 12·2%)	654·9 (445·9 to 915·4)	6·7% (4·6 to 9·4%)	650·4 (445·9 to 906·9)	7·4% (5·1 to 10·3%)	-0·7% (-8·3 to 8·1%)	10·7% (4·6 to 17·9%)
**Dietary risks: All causes**	2,716·8 (2,366·5 to 3,087·1)	24·3% (21·2 to 27·6%)	2,701·7 (2,337·3 to 3,088·3)	22·4% (19·4 to 25·6%)	-0·6% (-2·0 to 0·8%)	-7·8% (-8·9 to -6·7%)	51,019·4 (45,024·0 to 57,511·9)	14·6% (12·6 to 16·7%)	46,937·8 (41,036·8 to 53,165·5)	13·2% (11·4 to 15·3%)	-8·0% (-9·8 to -6·5%)	-9·0% (-10·5 to -7·9%)
**Diet low in fruits: All causes**	591·3 (361·7 to 845·8)	5·3% (3·2 to 7·6%)	565·9 (340·8 to 813·9)	4·7% (2·8 to 6·8%)	-4·3% (-6·8 to -2·1%)	-11·3% (-13·6 to -9·3%)	12,384·8 (7,922·1 to 17,432·7)	3·5% (2·2 to 5·1%)	11,055·7 (6,982·7 to 15,687·7)	3·1% (1·9 to 4·5%)	-10·7% (-13·7 to -7·8%)	-11·7% (-14·6 to -9·0%)
Lip and oral cavity cancer	2·0 (0·0 to 4·5)	6·3% (0·0 to 13·9%)	2·3 (0·0 to 5·1)	6·3% (0·0 to 14·0%)	14·1% (0·0 to 20·1%)	0·7% (-0·1 to 1·7%)	49·6 (0·0 to 110·6)	6·3% (0·0 to 14·0%)	52·5 (0·0 to 116·2)	6·3% (0·0 to 14·0%)	5·9% (0·0 to 12·6%)	0·1% (-0·9 to 1·2%)
Nasopharynx cancer	0·5 (0·0 to 1·1)	6·3% (0·0 to 13·7%)	0·6 (0·0 to 1·3)	6·4% (0·0 to 13·9%)	12·7% (0·0 to 18·6%)	1·1% (0·0 to 2·2%)	13·9 (0·0 to 30·5)	6·3% (0·0 to 13·6%)	14·5 (0·0 to 31·5)	6·3% (0·0 to 13·7%)	4·1% (-3·1 to 9·8%)	0·7% (-0·5 to 1·9%)
Other pharynx cancer	1·2 (0·0 to 2·7)	6·4% (0·0 to 14·4%)	1·5 (0·0 to 3·3)	6·5% (0·0 to 14·7%)	21·1% (0·0 to 27·4%)	1·2% (0·0 to 2·2%)	31·9 (0·0 to 71·9)	6·5% (0·0 to 14·5%)	36·9 (0·0 to 83·7)	6·5% (0·0 to 14·7%)	15·6% (0·0 to 22·2%)	0·7% (-0·3 to 1·7%)
Oesophageal cancer	14·9 (3·6 to 27·0)	20·1% (4·8 to 36·4%)	17·1 (4·1 to 31·1)	20·4% (4·9 to 36·8%)	14·7% (9·0 to 20·1%)	1·2% (0·6 to 2·1%)	314·8 (75·2 to 570·3)	20·2% (4·8 to 36·6%)	339·5 (82·6 to 616·3)	20·3% (4·9 to 36·9%)	7·9% (1·9 to 13·8%)	0·8% (0·2 to 1·5%)
Larynx cancer	1·6 (0·0 to 3·6)	6·5% (0·0 to 14·5%)	1·5 (0·0 to 3·4)	6·5% (0·0 to 14·5%)	-4·6% (-8·8 to 0·1%)	0·1% (-0·8 to 1·0%)	39·0 (0·0 to 87·7)	6·5% (0·0 to 14·6%)	35·0 (0·0 to 79·1)	6·5% (0·0 to 14·6%)	-10·4% (-14·5 to 0·0%)	-0·4% (-1·4 to 0·7%)
Tracheal, bronchus, and lung cancer	64·7 (25·5 to 110·3)	10·9% (4·3 to 18·6%)	73·0 (29·0 to 125·4)	11·0% (4·3 to 18·8%)	12·9% (10·2 to 15·6%)	1·1% (0·5 to 1·8%)	1,291·8 (510·3 to 2,209·7)	10·9% (4·3 to 18·7%)	1,357·8 (533·7 to 2,345·3)	10·9% (4·3 to 18·8%)	5·1% (2·6 to 7·9%)	0·3% (-0·3 to 1·0%)
Ischaemic heart disease	258·9 (90·6 to 442·0)	10·6% (3·7 to 18·1%)	255·9 (89·6 to 437·2)	10·3% (3·6 to 17·6%)	-1·1% (-2·8 to 0·2%)	-2·8% (-3·6 to -1·9%)	4,824·5 (1,701·9 to 8,151·3)	12·5% (4·4 to 21·2%)	4,244·2 (1,492·8 to 7,149·9)	12·1% (4·2 to 20·5%)	-12·0% (-13·9 to -10·4%)	-3·4% (-4·4 to -2·6%)
Ischaemic stroke	130·8 (68·2 to 204·5)	14·6% (7·5 to 23·0%)	111·3 (57·3 to 174·8)	13·2% (6·8 to 20·7%)	-14·8% (-17·2 to -12·4%)	-9·7% (-11·3 to -8·5%)	2,368·9 (1,271·6 to 3,622·0)	20·2% (10·7 to 30·9%)	1,880·2 (995·7 to 2,887·5)	19·0% (10·0 to 29·1%)	-20·6% (-23·3 to -17·9%)	-5·8% (-7·3 to -4·4%)

Supplemental Results Table 1. All-age deaths, death PAFs, DALYs, DALY PAFs, and percent change for each risk factor and outcome for both sexes combined in 2005 and 2015 for High SDI.

Data in parenthesis are 95% uncertainty intervals. PAFs=population attributable fractions. DALYs=disability-adjusted life-years.

	2005 Deaths (in thousands)	2005 Deaths PAF	2015 Deaths (in thousands)	2015 Deaths PAF	Percent change deaths 2005-2015	Percent change deaths PAF 2005-2015	2005 DALYs (in thousands)	2005 DALYs PAF	2015 DALYs (in thousands)	2015 DALYs PAF	Percent change DALYs 2005-2015	Percent change DALYs PAF 2005-2015
Hemorrhagic stroke	98·8 (52·8 to 150·7)	20·3% (10·8 to 31·0%)	84·0 (44·5 to 130·2)	18·4% (9·8 to 28·2%)	-15·0% (-18·0 to -11·9%)	-9·6% (-11·0 to -8·3%)	2,439·1 (1,351·4 to 3,632·2)	27·3% (14·9 to 40·6%)	1,927·6 (1,070·8 to 2,883·3)	25·9% (14·2 to 38·8%)	-21·0% (-23·6 to -18·1%)	-4·8% (-6·0 to -3·8%)
**Diet low in vegetables: All causes**	526·5 (257·3 to 827·9)	4·7% (2·3 to 7·4%)	504·0 (240·8 to 796·2)	4·2% (2·0 to 6·6%)	-4·3% (-7·7 to -2·0%)	-11·2% (-14·2 to -9·2%)	9,770·1 (4,868·2 to 15,134·1)	2·8% (1·4 to 4·4%)	8,401·9 (4,164·3 to 12,996·8)	2·4% (1·1 to 3·7%)	-14·0% (-16·8 to -12·0%)	-14·9% (-17·5 to -12·8%)
Ischaemic heart disease	407·1 (157·4 to 677·8)	16·6% (6·4 to 27·5%)	402·8 (155·4 to 669·0)	16·2% (6·3 to 26·8%)	-1·1% (-2·6 to 0·4%)	-2·7% (-3·5 to -2·0%)	7,281·5 (2,872·2 to 11,897·2)	18·9% (7·4 to 31·1%)	6,437·2 (2,528·1 to 10,526·7)	18·3% (7·2 to 30·1%)	-11·6% (-13·3 to -10·0%)	-2·9% (-3·9 to -2·0%)
Ischaemic stroke	68·1 (15·5 to 123·8)	7·6% (1·7 to 13·8%)	57·7 (12·9 to 106·2)	6·8% (1·5 to 12·5%)	-15·4% (-17·9 to -12·9%)	-10·3% (-11·8 to -8·8%)	1,231·1 (284·4 to 2,217·8)	10·5% (2·4 to 18·9%)	971·9 (220·5 to 1,761·4)	9·8% (2·2 to 17·8%)	-21·1% (-23·9 to -18·2%)	-6·3% (-7·9 to -4·6%)
Hemorrhagic stroke	51·2 (14·2 to 94·6)	10·5% (2·9 to 19·5%)	43·5 (12·0 to 81·2)	9·5% (2·7 to 17·7%)	-15·0% (-17·8 to -12·1%)	-9·6% (-11·0 to -8·4%)	1,257·6 (353·5 to 2,299·8)	14·0% (4·0 to 25·5%)	992·8 (278·8 to 1,811·1)	13·4% (3·8 to 24·3%)	-21·1% (-23·7 to -18·2%)	-4·9% (-6·3 to -3·7%)
**Diet low in whole grains: All causes**	690·1 (470·7 to 937·3)	6·2% (4·2 to 8·4%)	645·4 (431·8 to 885·0)	5·4% (3·6 to 7·4%)	-6·5% (-8·7 to -4·8%)	-13·3% (-15·3 to -11·9%)	14,785·7 (10,422·5 to 19,494·7)	4·2% (3·0 to 5·7%)	12,905·5 (8,998·7 to 17,175·9)	3·6% (2·5 to 4·9%)	-12·7% (-15·5 to -10·3%)	-13·7% (-16·1 to -11·7%)
Ischaemic heart disease	437·1 (258·4 to 640·4)	17·8% (10·5 to 26·2%)	429·0 (250·5 to 629·0)	17·2% (10·0 to 25·3%)	-1·8% (-3·6 to -0·3%)	-3·4% (-4·5 to -2·6%)	8,210·6 (4,900·7 to 11,812·7)	21·3% (12·7 to 30·7%)	7,126·8 (4,160·2 to 10,330·8)	20·3% (12·0 to 29·5%)	-13·2% (-15·4 to -11·4%)	-4·7% (-6·0 to -3·7%)
Ischaemic stroke	127·3 (86·5 to 171·9)	14·2% (9·6 to 19·3%)	105·3 (71·0 to 144·9)	12·5% (8·4 to 17·1%)	-17·3% (-19·9 to -14·8%)	-12·4% (-13·9 to -11·0%)	2,405·9 (1,664·0 to 3,196·9)	20·5% (14·2 to 27·3%)	1,866·2 (1,277·7 to 2,527·9)	18·9% (13·0 to 25·3%)	-22·4% (-25·4 to -19·6%)	-7·9% (-9·5 to -6·5%)
Hemorrhagic stroke	93·3 (66·1 to 126·0)	19·2% (13·6 to 25·9%)	77·6 (54·3 to 104·5)	17·0% (11·9 to 23·0%)	-16·9% (-19·9 to -13·8%)	-11·6% (-13·1 to -10·2%)	2,328·9 (1,673·7 to 3,092·4)	26·0% (18·7 to 34·6%)	1,810·7 (1,290·8 to 2,421·9)	24·4% (17·4 to 32·5%)	-22·2% (-25·0 to -19·3%)	-6·4% (-7·6 to -5·2%)
Diabetes mellitus	32·5 (17·3 to 50·6)	14·3% (7·6 to 22·3%)	33·6 (17·8 to 52·0)	13·8% (7·3 to 21·5%)	3·5% (0·1 to 6·9%)	-3·6% (-5·0 to -2·5%)	1,840·4 (976·2 to 2,957·7)	19·1% (10·4 to 29·3%)	2,101·7 (1,109·2 to 3,413·8)	18·7% (10·2 to 28·8%)	14·2% (10·3 to 17·2%)	-2·0% (-3·2 to -1·1%)
**Diet low in nuts and seeds: All causes**	521·4 (322·2 to 748·0)	4·7% (2·9 to 6·7%)	517·7 (317·4 to 746·2)	4·3% (2·6 to 6·2%)	-0·7% (-2·1 to 0·7%)	-7·9% (-9·1 to -6·8%)	9,958·9 (6,429·1 to 13,906·9)	2·8% (1·8 to 4·0%)	9,059·8 (5,875·4 to 12,768·0)	2·6% (1·6 to 3·6%)	-9·0% (-11·4 to -6·1%)	-10·0% (-12·0 to -7·6%)
Ischaemic heart disease	501·6 (309·0 to 723·8)	20·4% (12·5 to 29·6%)	497·1 (306·5 to 719·2)	19·9% (12·2 to 28·8%)	-0·9% (-2·3 to 0·5%)	-2·5% (-3·0 to -2·2%)	8,884·4 (5,536·9 to 12,594·7)	23·0% (14·3 to 32·6%)	7,823·2 (4,884·4 to 11,100·5)	22·3% (13·9 to 31·6%)	-11·9% (-13·8 to -10·3%)	-3·3% (-4·3 to -2·3%)
Diabetes mellitus	19·8 (10·1 to 31·5)	8·7% (4·4 to 14·0%)	20·6 (10·5 to 32·9)	8·4% (4·3 to 13·5%)	4·2% (0·6 to 7·5%)	-3·0% (-3·7 to -2·3%)	1,074·5 (487·4 to 1,801·1)	11·2% (5·7 to 17·7%)	1,236·7 (559·3 to 2,086·5)	11·0% (5·6 to 17·5%)	15·1% (11·5 to 18·0%)	-1·2% (-1·7 to -0·8%)
**Diet low in milk: All causes**	43·9 (15·0 to 75·0)	0·4% (0·1 to 0·7%)	50·9 (17·3 to 86·5)	0·4% (0·1 to 0·7%)	16·0% (13·3 to 19·3%)	7·5% (5·0 to 10·6%)	820·6 (278·2 to 1,406·1)	0·2% (0·1 to 0·4%)	892·9 (299·5 to 1,526·8)	0·3% (0·1 to 0·4%)	8·8% (6·2 to 11·7%)	7·6% (5·1 to 10·6%)
Colon and rectum cancer	43·9 (15·0 to 75·0)	13·6% (4·7 to 23·3%)	50·9 (17·3 to 86·5)	13·7% (4·7 to 23·5%)	16·0% (13·3 to 19·3%)	0·9% (0·5 to 1·5%)	820·6 (278·2 to 1,406·1)	13·7% (4·7 to 23·5%)	892·9 (299·5 to 1,526·8)	13·8% (4·7 to 23·6%)	8·8% (6·2 to 11·7%)	0·7% (0·3 to 1·2%)
**Diet high in red meat: All causes**	19·5 (8·5 to 31·9)	0·2% (0·1 to 0·3%)	21·0 (8·9 to 34·7)	0·2% (0·1 to 0·3%)	7·7% (3·2 to 11·7%)	-0·1% (-4·2 to 3·8%)	683·0 (288·7 to 1,110·0)	0·2% (0·1 to 0·3%)	772·7 (320·7 to 1,274·9)	0·2% (0·1 to 0·3%)	13·1% (8·8 to 16·9%)	11·8% (7·8 to 15·1%)
Colon and rectum cancer	12·5 (2·7 to 22·0)	3·9% (0·8 to 6·8%)	13·9 (3·0 to 24·5)	3·7% (0·8 to 6·6%)	11·7% (8·2 to 14·9%)	-2·9% (-4·5 to -1·1%)	255·9 (54·7 to 448·8)	4·3% (0·9 to 7·6%)	277·3 (59·1 to 489·1)	4·3% (0·9 to 7·6%)	8·3% (4·7 to 11·5%)	0·2% (-1·5 to 2·0%)
Diabetes mellitus	7·0 (0·9 to 12·7)	3·1% (0·4 to 5·6%)	7·1 (0·9 to 12·8)	2·9% (0·4 to 5·2%)	0·7% (-3·2 to 4·1%)	-6·2% (-8·2 to -4·3%)	427·2 (49·7 to 792·8)	4·4% (0·5 to 8·0%)	495·4 (58·0 to 936·8)	4·4% (0·5 to 7·9%)	16·0% (11·5 to 19·7%)	-0·5% (-2·0 to 1·2%)
**Diet high in processed meat: All causes**	191·8 (51·0 to 315·0)	1·7% (0·5 to 2·8%)	189·7 (54·3 to 309·6)	1·6% (0·5 to 2·6%)	-1·1% (-2·9 to 4·8%)	-8·3% (-9·9 to -3·0%)	4,508·3 (1,771·9 to 6,959·7)	1·3% (0·5 to 2·0%)	4,353·7 (1,906·6 to 6,683·8)	1·2% (0·5 to 1·8%)	-3·4% (-7·8 to 9·4%)	-4·5% (-8·6 to 7·8%)
Colon and rectum cancer	18·9 (10·4 to 27·7)	5·8% (3·2 to 8·6%)	21·2 (11·6 to 31·6)	5·7% (3·1 to 8·4%)	12·6% (9·1 to 15·9%)	-2·1% (-3·5 to -0·5%)	359·5 (195·6 to 524·4)	6·0% (3·3 to 8·7%)	380·9 (206·3 to 561·5)	5·9% (3·2 to 8·6%)	6·0% (2·6 to 9·4%)	-2·0% (-3·5 to -0·3%)
Ischaemic heart disease	148·9 (6·8 to 270·9)	6·1% (0·3 to 11·1%)	144·2 (6·6 to 263·5)	5·8% (0·3 to 10·6%)	-3·1% (-4·9 to -0·7%)	-4·7% (-5·8 to -3·2%)	2,775·0 (128·4 to 5,014·1)	7·2% (0·3 to 13·0%)	2,424·9 (111·2 to 4,391·6)	6·9% (0·3 to 12·5%)	-12·6% (-14·6 to -10·2%)	-4·0% (-5·2 to -2·4%)
Diabetes mellitus	24·0 (12·2 to 34·5)	10·6% (5·3 to 15·1%)	24·3 (12·0 to 35·3)	9·9% (5·0 to 14·3%)	1·1% (-2·7 to 4·9%)	-5·8% (-7·6 to -4·2%)	1,373·7 (659·4 to 2,134·4)	14·3% (7·2 to 20·4%)	1,547·9 (743·6 to 2,439·9)	13·8% (6·9 to 19·8%)	12·7% (8·7 to 16·4%)	-3·3% (-5·0 to -1·5%)
**Diet high in sugar-sweetened beverages: All causes**	13·4 (9·1 to 19·4)	0·1% (0·1 to 0·2%)	13·3 (9·0 to 19·1)	0·1% (0·1 to 0·2%)	-0·9% (-3·6 to 2·1%)	-8·1% (-10·6 to -5·4%)	481·6 (325·5 to 697·5)	0·1% (0·1 to 0·2%)	488·8 (331·1 to 713·3)	0·1% (0·1 to 0·2%)	1·5% (-1·7 to 4·8%)	0·3% (-2·5 to 3·4%)
Oesophageal cancer	0·3 (0·1 to 0·5)	0·4% (0·1 to 0·7%)	0·3 (0·1 to 0·6)	0·4% (0·1 to 0·7%)	11·0% (5·3 to 16·7%)	-2·0% (-5·8 to 1·9%)	7·0 (1·8 to 13·5)	0·4% (0·1 to 0·9%)	7·5 (2·0 to 14·5)	0·4% (0·1 to 0·9%)	6·8% (0·8 to 12·7%)	-0·2% (-4·3 to 4·2%)
Colon and rectum cancer	0·4 (0·3 to 0·6)	0·1% (0·1 to 0·2%)	0·4 (0·3 to 0·6)	0·1% (0·1 to 0·2%)	10·0% (6·0 to 14·5%)	-4·3% (-7·7 to -1·0%)	9·4 (6·1 to 13·8)	0·2% (0·1 to 0·2%)	10·1 (6·5 to 14·8)	0·2% (0·1 to 0·2%)	7·4% (3·1 to 12·0%)	-0·6% (-4·3 to 3·1%)
Liver cancer due to hepatitis B	0·0 (0·0 to 0·1)	0·2% (0·1 to 0·3%)	0·0 (0·0 to 0·1)	0·2% (0·1 to 0·3%)	12·7% (5·1 to 21·0%)	-3·0% (-8·9 to 2·7%)	1·4 (0·6 to 2·4)	0·2% (0·1 to 0·3%)	1·4 (0·6 to 2·5)	0·2% (0·1 to 0·3%)	5·6% (-2·5 to 15·1%)	-0·7% (-7·5 to 6·1%)
Liver cancer due to hepatitis C	0·1 (0·0 to 0·1)	0·1% (0·1 to 0·2%)	0·1 (0·0 to 0·2)	0·1% (0·1 to 0·2%)	22·5% (17·6 to 27·5%)	-1·4% (-5·1 to 2·1%)	1·7 (0·8 to 2·9)	0·2% (0·1 to 0·3%)	2·0 (0·9 to 3·4)	0·2% (0·1 to 0·3%)	17·4% (12·0 to 22·9%)	3·5% (-0·6 to 7·9%)
Liver cancer due to alcohol use	0·1 (0·0 to 0·2)	0·2% (0·1 to 0·4%)	0·1 (0·0 to 0·2)	0·3% (0·1 to 0·4%)	21·4% (15·3 to 27·9%)	4·4% (-0·7 to 9·6%)	2·6 (1·0 to 4·5)	0·3% (0·1 to 0·5%)	3·0 (1·2 to 5·4)	0·3% (0·1 to 0·5%)	18·5% (12·1 to 25·3%)	5·4% (-0·7 to 11·3%)
Liver cancer due to other causes	0·0 (0·0 to 0·1)	0·3% (0·1 to 0·4%)	0·0 (0·0 to 0·1)	0·3% (0·1 to 0·4%)	26·4% (19·5 to 32·9%)	1·7% (-3·0 to 6·6%)	1·0 (0·5 to 1·7)	0·3% (0·1 to 0·5%)	1·2 (0·6 to 2·0)	0·3% (0·2 to 0·6%)	19·3% (12·2 to 26·4%)	5·5% (0·0 to 10·8%)
Gallbladder and biliary tract cancer	0·1 (0·0 to 0·1)	0·1% (0·1 to 0·2%)	0·1 (0·0 to 0·1)	0·1% (0·1 to 0·2%)	-0·9% (-9·3 to 6·1%)	-9·5% (-13·6 to -6·4%)	1·3 (0·7 to 2·0)	0·1% (0·1 to 0·2%)	1·2 (0·7 to 2·0)	0·1% (0·1 to 0·2%)	-5·9% (-13·3 to 0·4%)	-5·0% (-9·6 to -1·9%)
Pancreatic cancer	0·1 (0·0 to 0·2)	0·1% (0·0 to 0·1%)	0·1 (0·1 to 0·3)	0·1% (0·0 to 0·1%)	18·3% (14·1 to 23·0%)	-6·3% (-9·3 to -3·1%)	2·7 (0·9 to 5·1)	0·1% (0·0 to 0·2%)	3·1 (1·1 to 5·9)	0·1% (0·0 to 0·2%)	13·9% (9·8 to 18·6%)	-3·8% (-7·1 to 0·1%)
Breast cancer	0·1 (0·1 to 0·2)	0·1% (0·0 to 0·1%)	0·1 (0·1 to 0·2)	0·1% (0·0 to 0·1%)	13·1% (6·7 to 22·1%)	0·7% (-3·3 to 5·9%)	3·4 (2·0 to 5·4)	0·1% (0·0 to 0·1%)	3·9 (2·2 to 6·2)	0·1% (0·0 to 0·1%)	13·6% (6·7 to 22·6%)	4·6% (-0·2 to 9·6%)
Uterine cancer	0·1 (0·1 to 0·1)	0·4% (0·3 to 0·6%)	0·1 (0·1 to 0·2)	0·4% (0·3 to 0·6%)	17·0% (4·5 to 25·9%)	1·1% (-3·6 to 6·0%)	2·7 (1·8 to 3·9)	0·5% (0·3 to 0·7%)	3·3 (2·1 to 4·7)	0·5% (0·3 to 0·7%)	19·2% (6·0 to 29·7%)	4·2% (-1·0 to 9·4%)
Ovarian cancer	0·0 (0·0 to 0·0)	0·0% (0·0 to 0·1%)	0·0 (0·0 to 0·1)	0·0% (0·0 to 0·1%)	6·4% (0·0 to 11·4%)	-5·0% (-8·7 to 0·0%)	0·6 (0·0 to 1·2)	0·0% (0·0 to 0·1%)	0·6 (0·0 to 1·3)	0·0% (0·0 to 0·1%)	3·6% (-1·3 to 8·6%)	-3·7% (-7·6 to 0·2%)
Kidney cancer	0·2 (0·1 to 0·2)	0·3% (0·2 to 0·4%)	0·2 (0·1 to 0·3)	0·2% (0·2 to 0·4%)	16·2% (11·4 to 21·1%)	-5·7% (-8·9 to -2·4%)	3·9 (2·5 to 5·9)	0·3% (0·2 to 0·5%)	4·4 (2·8 to 6·6)	0·3% (0·2 to 0·5%)	12·9% (8·0 to 18·3%)	-3·3% (-7·0 to 0·6%)
Thyroid cancer	0·0 (0·0 to 0·0)	0·1% (0·1 to 0·2%)	0·0 (0·0 to 0·0)	0·1% (0·1 to 0·2%)	14·8% (8·8 to 20·7%)	-1·4% (-5·6 to 2·7%)	0·4 (0·2 to 0·7)	0·2% (0·1 to 0·3%)	0·7 (0·3 to 1·2)	0·2% (0·1 to 0·4%)	55·2% (40·3 to 69·2%)	16·7% (9·4 to 23·0%)
Ischaemic heart disease	5·7 (3·8 to 8·3)	0·2% (0·2 to 0·3%)	5·4 (3·6 to 7·8)	0·2% (0·1 to 0·3%)	-6·0% (-9·3 to -2·8%)	-7·5% (-10·7 to -4·5%)	146·8 (98·4 to 213·7)	0·4% (0·3 to 0·6%)	132·8 (88·8 to 193·6)	0·4% (0·3 to 0·6%)	-9·5% (-12·5 to -6·2%)	-0·6% (-3·8 to 2·9%)
Ischaemic stroke	0·8 (0·6 to 1·3)	0·1% (0·1 to 0·1%)	0·7 (0·5 to 1·1)	0·1% (0·1 to 0·1%)	-13·5% (-18·9 to -8·6%)	-8·3% (-13·7 to -3·5%)	20·5 (14·0 to 29·6)	0·2% (0·1 to 0·3%)	17·9 (12·1 to 26·2)	0·2% (0·1 to 0·3%)	-12·8% (-16·3 to -9·0%)	3·5% (-0·2 to 7·1%)
Hemorrhagic stroke	1·4 (0·9 to 2·0)	0·3% (0·2 to 0·4%)	1·2 (0·8 to 1·7)	0·3% (0·2 to 0·4%)	-14·0% (-17·5 to -9·8%)	-8·5% (-12·4 to -4·8%)	45·8 (30·9 to 66·6)	0·5% (0·3 to 0·7%)	38·6 (25·6 to 56·4)	0·5% (0·3 to 0·8%)	-15·8% (-18·8 to -12·2%)	1·5% (-2·1 to 5·4%)
Hypertensive heart disease	0·8 (0·5 to 1·2)	0·6% (0·4 to 0·9%)	0·9 (0·5 to 1·4)	0·6% (0·3 to 0·9%)	12·7% (4·2 to 19·6%)	-7·0% (-13·2 to -1·4%)	22·2 (14·2 to 33·2)	1·1% (0·7 to 1·7%)	23·7 (14·8 to 35·6)	1·1% (0·7 to 1·7%)	6·8% (-1·1 to 13·9%)	-1·3% (-7·0 to 4·4%)
Cardiomyopathy and myocarditis	-	-	-	-	-	-	-	-	-	-	-	-
Atrial fibrillation and flutter	-	-	-	-	-	-	-	-	-	-	-	-
Peripheral vascular disease	-	-	-	-	-	-	-	-	-	-	-	-

Supplemental Results Table 1. All-age deaths, death PAFs, DALYs, DALY PAFs, and percent change for each risk factor and outcome for both sexes combined in 2005 and 2015 for High SDI.

Data in parenthesis are 95% uncertainty intervals. PAFs=population attributable fractions. DALYs=disability-adjusted life-years.

	2005 Deaths (in thousands)	2005 Deaths PAF	2015 Deaths (in thousands)	2015 Deaths PAF	Percent change deaths 2005-2015	Percent change deaths PAF 2005-2015	2005 DALYs (in thousands)	2005 DALYs PAF	2015 DALYs (in thousands)	2015 DALYs PAF	Percent change DALYs 2005-2015	Percent change DALYs PAF 2005-2015
Endocarditis	-	-	-	-	-	-	-	-	-	-	-	-
Other cardiovascular and circulatory diseases	-	-	-	-	-	-	-	-	-	-	-	-
Diabetes mellitus	2·1 (1·4 to 3·0)	0·9% (0·6 to 1·3%)	2·0 (1·3 to 2·9)	0·8% (0·5 to 1·2%)	-2·8% (-7·1 to 2·1%)	-9·5% (-13·0 to -5·9%)	147·6 (94·3 to 225·2)	1·5% (1·0 to 2·2%)	164·1 (102·7 to 253·3)	1·5% (1·0 to 2·1%)	11·2% (6·0 to 15·7%)	-4·6% (-7·7 to -1·5%)
Chronic kidney disease due to diabetes mellitus	0·4 (0·2 to 0·7)	0·5% (0·3 to 0·9%)	0·6 (0·3 to 1·0)	0·6% (0·3 to 1·0%)	55·5% (45·6 to 67·0%)	11·1% (4·2 to 18·3%)	12·8 (5·8 to 23·0)	0·8% (0·3 to 1·4%)	17·7 (8·4 to 30·6)	0·8% (0·4 to 1·5%)	37·7% (27·5 to 53·9%)	9·7% (1·9 to 22·6%)
Chronic kidney disease due to hypertension	0·4 (0·2 to 0·8)	0·5% (0·3 to 0·9%)	0·5 (0·2 to 0·9)	0·5% (0·2 to 0·8%)	9·1% (-5·6 to 23·9%)	-14·9% (-26·3 to -4·5%)	11·5 (5·5 to 19·6)	0·8% (0·4 to 1·4%)	12·1 (5·9 to 20·8)	0·7% (0·4 to 1·3%)	5·4% (-1·5 to 14·1%)	-7·4% (-12·6 to -0·6%)
Chronic kidney disease due to glomerulonephritis	0·1 (0·0 to 0·1)	0·3% (0·1 to 0·6%)	0·1 (0·0 to 0·2)	0·3% (0·1 to 0·6%)	15·9% (-1·2 to 30·7%)	-4·8% (-19·5 to 6·9%)	4·1 (1·5 to 7·9)	0·5% (0·2 to 0·9%)	4·6 (1·8 to 8·5)	0·5% (0·2 to 0·9%)	9·9% (3·0 to 22·5%)	3·9% (-2·3 to 16·0%)
Chronic kidney disease due to other causes	0·0 (0·0 to 0·0)	0·2% (0·1 to 0·4%)	0·0 (0·0 to 0·0)	0·2% (0·0 to 0·4%)	28·7% (3·1 to 41·6%)	-8·4% (-26·8 to 0·4%)	3·2 (1·2 to 6·2)	0·6% (0·2 to 1·0%)	3·8 (1·6 to 7·1)	0·6% (0·2 to 1·0%)	17·0% (8·6 to 30·6%)	-0·2% (-6·6 to 10·9%)
**Diet low in fibre: All causes**	143·0 (71·8 to 241·2)	1·3% (0·6 to 2·2%)	150·2 (75·8 to 250·0)	1·2% (0·6 to 2·1%)	5·0% (2·0 to 9·1%)	-2·6% (-5·4 to 1·2%)	2,609·1 (1,277·2 to 4,429·6)	0·7% (0·4 to 1·3%)	2,398·5 (1,196·9 to 4,050·2)	0·7% (0·3 to 1·1%)	-8·1% (-11·5 to -3·9%)	-9·1% (-12·5 to -5·0%)
Colon and rectum cancer	24·1 (10·8 to 41·9)	7·5% (3·3 to 12·9%)	28·5 (12·8 to 49·1)	7·7% (3·4 to 13·2%)	18·0% (14·2 to 22·3%)	2·7% (0·5 to 5·5%)	427·2 (192·3 to 745·3)	7·1% (3·2 to 12·5%)	465·8 (210·6 to 813·7)	7·2% (3·2 to 12·5%)	9·0% (5·3 to 13·0%)	0·9% (-1·7 to 3·5%)
Ischaemic heart disease	118·9 (57·6 to 203·5)	4·8% (2·4 to 8·3%)	121·7 (59·0 to 209·5)	4·9% (2·4 to 8·4%)	2·4% (-0·6 to 6·4%)	0·7% (-2·1 to 4·2%)	2,181·9 (1,025·1 to 3,791·7)	5·7% (2·7 to 9·7%)	1,932·8 (915·7 to 3,328·4)	5·5% (2·6 to 9·5%)	-11·4% (-14·9 to -7·6%)	-2·7% (-6·0 to 1·3%)
**Diet low in calcium: All causes**	50·8 (27·3 to 78·0)	0·5% (0·2 to 0·7%)	59·5 (32·4 to 91·1)	0·5% (0·3 to 0·8%)	17·2% (14·0 to 21·0%)	8·7% (5·8 to 12·1%)	925·0 (497·3 to 1,429·2)	0·3% (0·1 to 0·4%)	1,008·4 (548·6 to 1,552·8)	0·3% (0·2 to 0·4%)	9·0% (6·0 to 12·4%)	7·8% (5·0 to 11·3%)
Colon and rectum cancer	50·8 (27·3 to 78·0)	15·7% (8·4 to 24·2%)	59·5 (32·4 to 91·1)	16·0% (8·8 to 24·6%)	17·2% (14·0 to 21·0%)	2·0% (0·6 to 3·9%)	925·0 (497·3 to 1,429·2)	15·4% (8·3 to 24·0%)	1,008·4 (548·6 to 1,552·8)	15·6% (8·4 to 24·2%)	9·0% (6·0 to 12·4%)	0·9% (-0·6 to 2·6%)
Prostate cancer	-	-	-	-	-	-	-	-	-	-	-	-
**Diet low in seafood omega-3 fatty acids: All causes**	335·3 (137·4 to 555·6)	3·0% (1·2 to 5·0%)	323·9 (132·0 to 542·3)	2·7% (1·1 to 4·5%)	-3·4% (-4·9 to -1·9%)	-10·4% (-11·7 to -9·2%)	6,009·5 (2,479·5 to 9,925·2)	1·7% (0·7 to 2·9%)	5,147·7 (2,129·1 to 8,576·3)	1·5% (0·6 to 2·5%)	-14·3% (-16·3 to -12·7%)	-15·3% (-17·5 to -13·4%)
Ischaemic heart disease	335·3 (137·4 to 555·6)	13·7% (5·6 to 22·7%)	323·9 (132·0 to 542·3)	13·0% (5·3 to 21·7%)	-3·4% (-4·9 to -1·9%)	-5·0% (-5·8 to -4·2%)	6,009·5 (2,479·5 to 9,925·2)	15·6% (6·4 to 25·5%)	5,147·7 (2,129·1 to 8,576·3)	14·7% (6·0 to 24·3%)	-14·3% (-16·3 to -12·7%)	-5·9% (-7·2 to -4·9%)
**Diet low in polyunsaturated fatty acids: All causes**	137·3 (56·3 to 217·2)	1·2% (0·5 to 1·9%)	135·3 (56·8 to 211·0)	1·1% (0·5 to 1·7%)	-1·5% (-17·8 to 15·8%)	-8·6% (-23·8 to 7·2%)	2,387·6 (988·7 to 3,716·6)	0·7% (0·3 to 1·1%)	1,990·1 (831·3 to 3,078·5)	0·6% (0·2 to 0·9%)	-16·6% (-23·7 to -9·9%)	-17·5% (-24·4 to -11·0%)
Ischaemic heart disease	137·3 (56·3 to 217·2)	5·6% (2·3 to 8·8%)	135·3 (56·8 to 211·0)	5·4% (2·3 to 8·4%)	-1·5% (-17·8 to 15·8%)	-3·0% (-19·0 to 14·1%)	2,387·6 (988·7 to 3,716·6)	6·2% (2·6 to 9·6%)	1,990·1 (831·3 to 3,078·5)	5·7% (2·4 to 8·7%)	-16·6% (-23·7 to -9·9%)	-8·4% (-16·1 to -1·5%)
**Diet high in trans fatty acids: All causes**	98·2 (35·2 to 192·8)	0·9% (0·3 to 1·7%)	93·5 (33·1 to 186·8)	0·8% (0·3 to 1·5%)	-4·8% (-8·3 to -2·1%)	-11·7% (-14·9 to -9·3%)	2,069·4 (776·1 to 3,916·3)	0·6% (0·2 to 1·2%)	1,815·7 (687·2 to 3,466·3)	0·5% (0·2 to 1·0%)	-12·3% (-14·8 to -8·5%)	-13·2% (-15·8 to -9·7%)
Ischaemic heart disease	98·2 (35·2 to 192·8)	4·0% (1·4 to 7·8%)	93·5 (33·1 to 186·8)	3·7% (1·3 to 7·4%)	-4·8% (-8·3 to -2·1%)	-6·3% (-9·4 to -3·8%)	2,069·4 (776·1 to 3,916·3)	5·4% (2·0 to 10·2%)	1,815·7 (687·2 to 3,466·3)	5·2% (2·0 to 9·9%)	-12·3% (-14·8 to -8·5%)	-3·6% (-6·1 to -0·2%)
**Diet high in sodium: All causes**	609·0 (246·3 to 1,135·6)	5·5% (2·2 to 10·2%)	615·1 (250·7 to 1,160·4)	5·1% (2·1 to 9·6%)	1·0% (-2·2 to 4·9%)	-6·4% (-9·2 to -2·8%)	10,852·6 (4,590·3 to 19,897·6)	3·1% (1·3 to 5·8%)	9,973·3 (4,237·7 to 18,346·4)	2·8% (1·2 to 5·3%)	-8·1% (-10·4 to -5·1%)	-9·1% (-11·4 to -6·1%)
Stomach cancer	70·4 (31·7 to 118·8)	33·4% (15·0 to 56·5%)	70·4 (29·3 to 122·8)	33·7% (13·9 to 58·6%)	0·1% (-13·8 to 11·8%)	0·9% (-12·8 to 11·9%)	1,361·7 (649·8 to 2,240·1)	33·7% (16·2 to 55·2%)	1,228·6 (593·1 to 2,030·1)	33·9% (16·3 to 55·8%)	-9·8% (-17·1 to -2·2%)	0·7% (-6·4 to 8·8%)
Rheumatic heart disease	2·1 (0·7 to 5·0)	5·1% (1·6 to 11·9%)	1·9 (0·6 to 4·8)	4·9% (1·5 to 12·3%)	-10·2% (-19·1 to 0·8%)	-3·0% (-11·9 to 8·8%)	41·5 (14·4 to 87·5)	5·5% (1·9 to 11·7%)	32·6 (11·7 to 68·1)	5·6% (2·0 to 11·7%)	-21·3% (-26·3 to -13·6%)	1·9% (-3·9 to 11·6%)
Ischaemic heart disease	286·1 (102·4 to 582·8)	11·7% (4·2 to 23·8%)	289·6 (103·1 to 587·2)	11·6% (4·2 to 23·5%)	1·2% (-3·8 to 6·2%)	-0·4% (-5·2 to 4·3%)	4,830·3 (1,841·0 to 9,472·8)	12·5% (4·7 to 24·6%)	4,425·1 (1,699·8 to 8,620·4)	12·6% (4·9 to 24·7%)	-8·4% (-11·2 to -5·0%)	0·6% (-1·9 to 4·4%)
Ischaemic stroke	96·7 (35·7 to 195·5)	10·8% (3·9 to 21·7%)	89·1 (32·9 to 180·5)	10·5% (3·9 to 21·4%)	-7·9% (-13·5 to 0·6%)	-2·4% (-8·0 to 6·7%)	1,469·5 (569·5 to 2,824·1)	12·5% (4·9 to 24·1%)	1,234·8 (500·4 to 2,366·8)	12·5% (5·0 to 23·9%)	-16·0% (-19·8 to -9·7%)	-0·3% (-3·8 to 6·6%)
Hemorrhagic stroke	70·0 (27·6 to 135·8)	14·4% (5·7 to 27·9%)	64·2 (25·4 to 127·1)	14·0% (5·6 to 27·7%)	-8·3% (-15·3 to -0·5%)	-2·5% (-9·2 to 5·2%)	1,418·0 (597·7 to 2,613·3)	15·8% (6·7 to 29·0%)	1,175·1 (512·3 to 2,169·7)	15·8% (6·8 to 28·9%)	-17·1% (-21·0 to -12·2%)	-0·2% (-3·5 to 4·6%)
Hypertensive heart disease	28·4 (5·8 to 75·6)	21·4% (4·3 to 56·3%)	34·3 (6·5 to 91·9)	21·3% (4·0 to 57·4%)	20·7% (8·9 to 29·6%)	-0·4% (-9·6 to 6·1%)	431·7 (120·4 to 1,018·5)	22·0% (6·1 to 51·2%)	465·0 (127·3 to 1,105·0)	21·9% (6·0 to 51·9%)	7·7% (0·7 to 14·4%)	-0·5% (-6·1 to 4·8%)
Cardiomyopathy and myocarditis	11·7 (4·0 to 23·9)	6·9% (2·3 to 14·2%)	11·4 (3·8 to 23·7)	6·8% (2·3 to 14·2%)	-2·6% (-9·9 to 4·9%)	-1·8% (-7·2 to 4·4%)	307·3 (101·7 to 636·3)	7·1% (2·4 to 14·5%)	264·0 (90·2 to 539·3)	7·2% (2·5 to 14·7%)	-14·1% (-20·2 to -7·0%)	2·2% (-1·7 to 8·3%)
Atrial fibrillation and flutter	4·4 (1·5 to 9·4)	6·2% (2·2 to 12·9%)	5·8 (2·0 to 12·4)	6·2% (2·2 to 12·8%)	31·7% (26·8 to 38·5%)	-0·7% (-3·4 to 3·6%)	134·0 (47·0 to 275·0)	8·2% (3·0 to 16·2%)	163·9 (58·2 to 335·6)	8·1% (3·1 to 16·0%)	22·3% (19·7 to 26·1%)	-0·3% (-2·1 to 2·5%)
Aortic aneurysm	5·1 (1·8 to 10·4)	7·2% (2·6 to 14·7%)	5·8 (2·1 to 11·8)	7·1% (2·5 to 14·4%)	13·9% (8·8 to 20·2%)	-1·4% (-5·0 to 3·4%)	86·5 (32·2 to 170·3)	8·0% (3·0 to 15·9%)	91·4 (34·2 to 180·6)	8·1% (3·0 to 15·8%)	5·6% (1·4 to 9·9%)	0·4% (-1·6 to 3·4%)
Peripheral vascular disease	1·3 (0·4 to 2·9)	4·5% (1·4 to 10·1%)	1·6 (0·5 to 3·7)	4·3% (1·3 to 9·9%)	26·6% (18·1 to 36·0%)	-3·5% (-8·1 to 0·5%)	27·3 (8·7 to 58·9)	5·4% (1·8 to 11·2%)	32·7 (10·5 to 70·2)	5·3% (1·8 to 11·1%)	20·1% (14·7 to 26·1%)	-1·5% (-3·9 to 1·9%)
Endocarditis	1·5 (0·5 to 3·3)	6·2% (2·0 to 13·2%)	1·8 (0·6 to 3·9)	6·1% (2·0 to 13·3%)	18·8% (12·6 to 25·5%)	-1·9% (-5·9 to 2·6%)	27·6 (9·5 to 56·4)	6·7% (2·3 to 13·5%)	30·2 (10·4 to 62·4)	6·7% (2·3 to 13·7%)	9·6% (3·5 to 15·0%)	0·7% (-1·8 to 3·5%)
Other cardiovascular and circulatory diseases	10·9 (3·7 to 22·1)	7·0% (2·3 to 14·1%)	12·2 (4·1 to 24·6)	6·7% (2·2 to 13·6%)	11·4% (6·3 to 16·3%)	-4·0% (-7·3 to -0·9%)	284·5 (100·6 to 571·0)	8·3% (3·0 to 16·3%)	308·6 (112·0 to 620·7)	8·3% (3·0 to 16·3%)	8·5% (5·1 to 12·8%)	0·7% (-1·2 to 3·8%)
Chronic kidney disease due to diabetes mellitus	8·4 (2·9 to 16·4)	11·4% (3·9 to 22·6%)	11·4 (3·8 to 22·7)	11·1% (3·8 to 22·4%)	37·0% (31·3 to 42·0%)	-2·1% (-5·3 to -0·3%)	168·7 (60·4 to 337·3)	10·1% (3·6 to 20·1%)	213·0 (74·8 to 430·3)	10·2% (3·6 to 20·3%)	26·3% (22·6 to 29·3%)	0·5% (-1·9 to 2·1%)
Chronic kidney disease due to hypertension	8·7 (2·9 to 18·2)	10·5% (3·6 to 21·8%)	11·4 (3·9 to 23·7)	10·7% (3·7 to 22·1%)	30·5% (26·0 to 36·3%)	1·8% (-0·1 to 5·1%)	137·3 (46·2 to 282·1)	9·6% (3·3 to 19·9%)	161·2 (55·4 to 330·4)	9·9% (3·5 to 20·4%)	17·4% (14·1 to 21·6%)	3·1% (1·4 to 6·3%)
Chronic kidney disease due to glomerulonephritis	2·9 (1·1 to 5·7)	11·6% (4·5 to 22·5%)	3·6 (1·4 to 7·1)	11·8% (4·6 to 23·1%)	23·9% (18·2 to 29·3%)	1·8% (-1·9 to 4·9%)	76·4 (28·0 to 154·0)	9·1% (3·5 to 17·9%)	84·7 (31·1 to 171·0)	9·5% (3·5 to 18·8%)	10·8% (6·5 to 14·6%)	4·7% (1·9 to 7·3%)
Chronic kidney disease due to other causes	0·3 (0·1 to 0·7)	9·5% (3·0 to 20·2%)	0·4 (0·1 to 1·0)	9·9% (3·1 to 21·2%)	46·9% (40·4 to 56·1%)	4·5% (2·5 to 7·2%)	50·6 (16·2 to 109·8)	8·7% (3·0 to 17·9%)	62·4 (20·6 to 134·3)	9·1% (3·2 to 18·8%)	23·4% (20·0 to 28·2%)	5·2% (3·1 to 8·6%)
**Sexual abuse and violence: All causes**	61·2 (40·0 to 89·3)	0·5% (0·4 to 0·8%)	54·5 (35·8 to 79·4)	0·5% (0·3 to 0·7%)	-11·0% (-14·5 to -6·9%)	-17·5% (-20·7 to -13·6%)	4,008·3 (2,792·6 to 5,428·8)	1·1% (0·8 to 1·5%)	3,715·1 (2,651·1 to 4,997·0)	1·0% (0·8 to 1·4%)	-7·3% (-11·3 to -2·7%)	-8·4% (-11·9 to -4·1%)
**Childhood sexual abuse: All causes**	35·1 (11·0 to 66·1)	0·3% (0·1 to 0·6%)	29·7 (8·6 to 57·5)	0·2% (0·1 to 0·5%)	-15·5% (-23·2 to -10·7%)	-21·6% (-28·7 to -17·2%)	2,089·3 (891·8 to 3,606·5)	0·6% (0·3 to 1·0%)	1,809·3 (786·6 to 3,114·4)	0·5% (0·2 to 0·9%)	-13·4% (-19·2 to -7·0%)	-14·4% (-20·0 to -8·0%)
Alcohol use disorders	4·2 (0·0 to 9·3)	5·1% (0·0 to 11·6%)	2·7 (0·0 to 6·2)	4·5% (0·0 to 10·1%)	-36·0% (-41·0 to 0·0%)	-11·9% (-15·4 to 0·0%)	254·3 (0·0 to 577·0)	5·3% (0·0 to 12·1%)	180·3 (0·0 to 408·8)	4·8% (0·0 to 10·8%)	-29·1% (-34·0 to 0·0%)	-9·6% (-12·6 to 0·0%)
Major depressive disorder	-	-	-	-	-	-	427·2 (46·9 to 942·6)	5·6% (0·7 to 11·6%)	440·5 (48·3 to 975·8)	5·2% (0·6 to 10·7%)	3·1% (0·5 to 5·2%)	-7·6% (-9·3 to -5·9%)
Dysthymia	-	-	-	-	-	-	97·1 (10·6 to 214·5)	5·4% (0·6 to 11·2%)	99·8 (10·9 to 221·1)	4·9% (0·6 to 10·3%)	2·8% (-0·1 to 5·6%)	-8·2% (-9·8 to -6·4%)
Self-harm	30·9 (7·3 to 62·0)	12·6% (3·0 to 25·4%)	27·0 (6·3 to 54·9)	11·5% (2·7 to 23·5%)	-12·7% (-17·0 to -9·0%)	-9·1% (-11·3 to -7·2%)	1,310·8 (313·5 to 2,613·9)	13·4% (3·2 to 26·6%)	1,088·7 (255·8 to 2,187·7)	12·4% (2·9 to 24·9%)	-16·9% (-20·8 to -13·3%)	-7·5% (-9·7 to -5·5%)
**Intimate partner violence: All causes**	30·1 (25·5 to 34·6)	0·3% (0·2 to 0·3%)	28·5 (23·6 to 33·2)	0·2% (0·2 to 0·3%)	-5·2% (-9·6 to -0·8%)	-12·1% (-16·2 to -8·1%)	2,149·5 (1,744·1 to 2,596·2)	0·6% (0·5 to 0·7%)	2,123·5 (1,694·3 to 2,608·4)	0·6% (0·5 to 0·7%)	-1·2% (-4·6 to 1·8%)	-2·3% (-5·4 to 0·6%)

Supplemental Results Table 1. All-age deaths, death PAFs, DALYs, DALY PAFs, and percent change for each risk factor and outcome for both sexes combined in 2005 and 2015 for High SDI.

Data in parenthesis are 95% uncertainty intervals. PAFs=population attributable fractions. DALYs=disability-adjusted life-years.

	2005 Deaths (in thousands)	2005 Deaths PAF	2015 Deaths (in thousands)	2015 Deaths PAF	Percent change deaths 2005-2015	Percent change deaths PAF 2005-2015	2005 DALYs (in thousands)	2005 DALYs PAF	2015 DALYs (in thousands)	2015 DALYs PAF	Percent change DALYs 2005-2015	Percent change DALYs PAF 2005-2015
HIV/AIDS - Tuberculosis	0·0 (0·0 to 0·1)	1·8% (0·9 to 2·7%)	0·0 (0·0 to 0·0)	1·9% (0·9 to 2·9%)	-32·3% (-37·3 to -26·6%)	7·2% (2·0 to 12·1%)	2·1 (1·0 to 3·2)	1·7% (0·8 to 2·6%)	1·4 (0·7 to 2·1)	1·8% (0·9 to 2·7%)	-34·1% (-39·3 to -28·0%)	6·9% (0·7 to 12·7%)
HIV/AIDS resulting in other diseases	0·7 (0·3 to 1·0)	2·2% (1·1 to 3·4%)	0·6 (0·3 to 0·9)	2·1% (1·0 to 3·2%)	-10·1% (-16·7 to -3·6%)	-7·6% (-14·4 to -0·3%)	32·5 (15·8 to 50·1)	2·1% (1·0 to 3·2%)	30·5 (14·6 to 47·6)	1·9% (0·9 to 3·0%)	-6·1% (-13·4 to 1·1%)	-7·0% (-14·0 to 0·4%)
Maternal abortion, miscarriage, and ectopic pregnancy	0·0 (0·0 to 0·0)	0·0% (0·0 to 0·0%)	0·0 (0·0 to 0·0)	0·0% (0·0 to 0·0%)	-23·7% (-40·4 to -4·7%)	-1·7% (-18·7 to 11·7%)	0·0 (0·0 to 0·0)	0·0% (0·0 to 0·0%)	0·0 (0·0 to 0·0)	0·0% (0·0 to 0·0%)	-18·2% (-39·6 to 1·3%)	-0·6% (-23·8 to 13·5%)
Major depressive disorder	-	-	-	-	-	-	746·8 (479·2 to 1,065·6)	9·8% (7·9 to 11·6%)	825·6 (538·8 to 1,173·7)	9·7% (7·8 to 11·5%)	10·6% (7·3 to 14·4%)	-0·9% (-3·6 to 2·0%)
Dysthymia	-	-	-	-	-	-	173·8 (109·7 to 261·0)	9·6% (7·8 to 11·4%)	192·9 (119·3 to 285·5)	9·5% (7·7 to 11·3%)	11·0% (7·2 to 15·0%)	-0·9% (-3·8 to 2·1%)
Self-harm	23·6 (19·1 to 28·0)	9·6% (7·8 to 11·4%)	23·5 (18·7 to 28·3)	10·0% (8·0 to 12·0%)	-0·1% (-5·5 to 4·9%)	4·0% (-2·9 to 9·9%)	889·8 (701·5 to 1,066·0)	9·1% (7·2 to 10·9%)	847·3 (674·7 to 1,019·5)	9·6% (7·6 to 11·6%)	-4·8% (-8·9 to -0·5%)	6·1% (-0·5 to 11·6%)
Assault by firearm	1·3 (1·1 to 1·4)	6·2% (5·2 to 6·7%)	1·3 (1·1 to 1·4)	6·8% (5·9 to 7·3%)	-3·0% (-7·9 to 2·7%)	9·9% (2·5 to 17·0%)	64·8 (53·6 to 70·1)	5·7% (4·8 to 6·2%)	60·2 (52·3 to 65·4)	6·1% (5·4 to 6·7%)	-7·0% (-12·2 to -0·9%)	8·6% (0·8 to 16·4%)
Assault by sharp object	1·6 (1·4 to 1·8)	6·5% (5·7 to 7·4%)	1·1 (1·0 to 1·3)	7·2% (6·4 to 8·1%)	-30·9% (-37·2 to -23·3%)	11·4% (1·0 to 23·9%)	75·9 (67·3 to 87·2)	6·4% (5·6 to 7·4%)	49·8 (44·5 to 56·3)	7·1% (6·4 to 8·1%)	-34·4% (-40·5 to -26·6%)	10·7% (-0·1 to 23·9%)
Assault by other means	2·8 (2·5 to 3·2)	7·5% (6·6 to 8·4%)	1·9 (1·8 to 2·2)	8·0% (7·2 to 8·9%)	-31·6% (-37·3 to -24·7%)	7·6% (-1·7 to 18·0%)	163·9 (145·2 to 185·3)	8·9% (8·0 to 9·9%)	115·7 (101·1 to 133·4)	9·8% (8·9 to 10·9%)	-29·4% (-34·4 to -23·2%)	10·9% (2·1 to 20·0%)
**Unsafe sex: All causes**	63·6 (62·4 to 65·0)	0·6% (0·6 to 0·6%)	60·4 (58·4 to 62·7)	0·5% (0·5 to 0·5%)	-5·1% (-8·3 to -1·8%)	-12·0% (-15·0 to -9·1%)	2,476·1 (2,354·5 to 2,632·8)	0·7% (0·7 to 0·8%)	2,310·1 (2,162·4 to 2,513·5)	0·7% (0·6 to 0·7%)	-6·7% (-9·6 to -3·5%)	-7·7% (-10·5 to -4·7%)
HIV/AIDS - Tuberculosis	1·8 (1·7 to 1·9)	71·4% (68·3 to 74·4%)	1·2 (1·1 to 1·3)	73·0% (69·9 to 75·7%)	-35·5% (-39·8 to -31·1%)	2·2% (0·7 to 3·6%)	89·2 (83·6 to 94·9)	70·4% (67·2 to 73·5%)	55·7 (51·9 to 59·8)	71·3% (68·1 to 74·3%)	-37·5% (-41·8 to -33·2%)	1·3% (0·0 to 2·5%)
HIV/AIDS resulting in other diseases	23·9 (23·1 to 24·8)	78·0% (75·8 to 79·9%)	21·4 (20·1 to 22·8)	71·6% (68·5 to 74·4%)	-10·6% (-14·4 to -6·1%)	-8·2% (-9·8 to -6·6%)	1,202·5 (1,143·3 to 1,278·0)	77·3% (75·1 to 79·5%)	1,116·9 (1,032·5 to 1,225·3)	71·1% (67·9 to 74·2%)	-7·1% (-11·3 to -2·5%)	-8·1% (-9·7 to -6·5%)
Syphilis	0·9 (0·7 to 1·0)	100·0% (100·0 to 100·0%)	0·8 (0·7 to 1·0)	100·0% (100·0 to 100·0%)	-5·1% (-14·4 to 5·0%)	-	68·3 (53·1 to 85·6)	100·0% (100·0 to 100·0%)	66·4 (51·4 to 83·2)	100·0% (100·0 to 100·0%)	-2·8% (-11·8 to 6·2%)	-
Chlamydial infection	0·0 (0·0 to 0·0)	100·0% (100·0 to 100·0%)	0·0 (0·0 to 0·0)	100·0% (100·0 to 100·0%)	10·2% (1·7 to 20·4%)	-	34·3 (20·2 to 57·4)	100·0% (100·0 to 100·0%)	42·8 (25·2 to 71·9)	100·0% (100·0 to 100·0%)	24·5% (19·8 to 29·3%)	-
Gonococcal infection	0·1 (0·1 to 0·1)	100·0% (100·0 to 100·0%)	0·1 (0·1 to 0·1)	100·0% (100·0 to 100·0%)	1·5% (-8·5 to 11·5%)	-	16·3 (10·4 to 24·9)	100·0% (100·0 to 100·0%)	20·8 (12·8 to 31·9)	100·0% (100·0 to 100·0%)	27·2% (14·4 to 43·3%)	-
Trichomoniasis	-	-	-	-	-	-	27·7 (11·0 to 58·6)	100·0% (100·0 to 100·0%)	29·8 (11·9 to 63·1)	100·0% (100·0 to 100·0%)	7·7% (6·0 to 9·5%)	-
Genital herpes	-	-	-	-	-	-	42·6 (12·9 to 103·4)	100·0% (100·0 to 100·0%)	46·2 (13·8 to 111·4)	100·0% (100·0 to 100·0%)	8·5% (6·4 to 10·3%)	-
Other sexually transmitted diseases	0·0 (0·0 to 0·0)	100·0% (100·0 to 100·0%)	0·0 (0·0 to 0·0)	100·0% (100·0 to 100·0%)	-1·6% (-11·5 to 8·4%)	-	20·4 (14·0 to 28·5)	100·0% (100·0 to 100·0%)	19·3 (13·2 to 27·1)	100·0% (100·0 to 100·0%)	-5·1% (-9·2 to -1·2%)	-
Cervical cancer	36·9 (36·0 to 37·9)	100·0% (100·0 to 100·0%)	36·9 (35·5 to 38·6)	100·0% (100·0 to 100·0%)	-0·1% (-4·7 to 4·5%)	-	974·8 (948·2 to 1,003·6)	100·0% (100·0 to 100·0%)	912·3 (876·0 to 951·3)	100·0% (100·0 to 100·0%)	-6·4% (-10·5 to -2·0%)	-
**Low physical activity: All causes**	451·5 (354·2 to 547·9)	4·0% (3·2 to 4·9%)	473·4 (377·3 to 568·2)	3·9% (3·1 to 4·7%)	4·8% (3·0 to 7·1%)	-2·8% (-4·4 to -0·7%)	8,067·7 (6,258·1 to 9,897·6)	2·3% (1·8 to 2·8%)	8,180·1 (6,486·5 to 9,892·5)	2·3% (1·9 to 2·8%)	1·4% (-1·4 to 4·7%)	0·2% (-2·1 to 3·5%)
Colon and rectum cancer	51·1 (36·4 to 66·0)	15·8% (11·2 to 20·3%)	60·1 (43·0 to 77·1)	16·2% (11·5 to 20·7%)	17·6% (14·5 to 21·1%)	2·3% (1·4 to 3·4%)	879·4 (608·2 to 1,141·6)	14·7% (10·2 to 19·1%)	973·0 (684·1 to 1,262·0)	15·0% (10·5 to 19·5%)	10·6% (7·9 to 13·8%)	2·4% (1·5 to 3·6%)
Breast cancer	19·2 (14·1 to 24·4)	10·6% (7·8 to 13·4%)	21·9 (16·2 to 27·7)	10·8% (7·9 to 13·5%)	13·7% (8·7 to 18·1%)	1·3% (0·5 to 2·2%)	472·8 (347·3 to 602·9)	10·2% (7·5 to 12·9%)	520·4 (385·9 to 666·6)	10·3% (7·6 to 13·0%)	10·1% (4·2 to 15·2%)	1·4% (0·6 to 2·3%)
Ischaemic heart disease	237·5 (158·5 to 319·7)	9·7% (6·5 to 13·1%)	246·9 (167·3 to 328·9)	9·9% (6·7 to 13·2%)	3·9% (2·0 to 6·9%)	2·2% (0·7 to 4·9%)	3,751·4 (2,345·4 to 5,178·7)	9·7% (6·1 to 13·5%)	3,577·4 (2,299·5 to 4,866·2)	10·2% (6·6 to 13·9%)	-4·6% (-6·8 to -0·8%)	4·7% (2·6 to 8·8%)
Ischaemic stroke	99·3 (55·7 to 145·0)	11·1% (6·2 to 16·1%)	96·3 (55·9 to 138·6)	11·4% (6·6 to 16·3%)	-3·0% (-6·0 to 1·3%)	2·8% (0·6 to 6·9%)	1,313·8 (677·2 to 1,977·9)	11·2% (5·8 to 16·7%)	1,154·5 (624·8 to 1,700·8)	11·7% (6·4 to 17·1%)	-12·1% (-15·3 to -7·4%)	4·3% (1·9 to 9·4%)
Diabetes mellitus	44·3 (35·4 to 53·8)	19·5% (15·5 to 23·7%)	48·2 (38·5 to 58·5)	19·7% (15·7 to 24·0%)	8·8% (4·7 to 12·8%)	1·3% (0·7 to 2·1%)	1,650·3 (1,207·8 to 2,227·3)	17·2% (13·4 to 21·3%)	1,954·7 (1,417·6 to 2,639·8)	17·5% (13·6 to 21·6%)	18·4% (14·8 to 21·7%)	1·7% (1·1 to 2·4%)
**Metabolic risks: All causes**	4,021·4 (3,798·0 to 4,245·8)	36·0% (34·0 to 38·0%)	4,111·7 (3,856·7 to 4,358·6)	34·1% (32·1 to 36·2%)	2·2% (0·9 to 3·4%)	-5·2% (-6·3 to -4·2%)	76,676·9 (71,669·8 to 82,275·9)	21·9% (20·5 to 23·3%)	73,910·2 (68,057·9 to 80,206·6)	20·8% (19·5 to 22·3%)	-3·6% (-5·4 to -2·0%)	-4·7% (-5·8 to -3·7%)
**High fasting plasma glucose: All causes**	1,058·8 (864·9 to 1,337·5)	9·5% (7·7 to 12·0%)	1,129·9 (914·4 to 1,427·3)	9·4% (7·6 to 11·8%)	6·7% (3·4 to 10·1%)	-1·1% (-4·0 to 2·2%)	23,968·0 (20,361·2 to 27,958·6)	6·8% (6·1 to 7·9%)	25,258·6 (21,222·7 to 29,507·3)	7·1% (6·4 to 8·2%)	5·4% (2·2 to 8·2%)	4·2% (1·8 to 6·5%)
Tuberculosis	3·1 (2·0 to 4·5)	7·4% (4·7 to 10·6%)	2·0 (1·3 to 2·9)	7·9% (5·0 to 11·5%)	-35·3% (-43·6 to -26·5%)	7·1% (-5·4 to 20·3%)	92·9 (58·2 to 129·7)	6·8% (4·3 to 9·7%)	53·1 (33·6 to 74·2)	7·6% (4·8 to 10·6%)	-42·8% (-49·3 to -35·8%)	11·3% (1·2 to 21·8%)
Ischaemic heart disease	472·4 (302·2 to 693·3)	19·3% (12·2 to 28·3%)	494·9 (298·6 to 749·2)	19·8% (11·9 to 30·1%)	4·8% (-2·2 to 11·7%)	3·1% (-3·6 to 9·8%)	7,634·0 (5,292·0 to 10,576·2)	19·8% (13·7 to 27·4%)	7,244·1 (5,037·3 to 10,080·4)	20·6% (14·3 to 28·7%)	-5·1% (-8·6 to -1·0%)	4·2% (0·7 to 8·7%)
Ischaemic stroke	149·8 (80·9 to 279·6)	16·7% (9·1 to 31·3%)	142·0 (73·6 to 276·7)	16·8% (8·7 to 32·9%)	-5·2% (-12·5 to 5·0%)	0·5% (-6·8 to 11·4%)	2,142·7 (1,267·1 to 3,577·2)	18·2% (10·7 to 30·5%)	1,849·4 (1,098·3 to 3,235·3)	18·7% (11·0 to 32·2%)	-13·7% (-18·0 to -6·5%)	2·4% (-1·7 to 11·2%)
Hemorrhagic stroke	81·8 (55·6 to 116·0)	16·8% (11·5 to 24·1%)	77·0 (50·6 to 114·3)	16·9% (11·2 to 25·0%)	-5·8% (-11·7 to 3·0%)	0·2% (-5·3 to 8·8%)	1,645·2 (1,145·0 to 2,288·3)	18·4% (12·9 to 25·2%)	1,419·5 (991·6 to 1,952·9)	19·1% (13·5 to 25·9%)	-13·7% (-17·4 to -8·9%)	3·9% (0·8 to 8·7%)
Diabetes mellitus	227·4 (222·9 to 232·2)	100·0% (100·0 to 100·0%)	244·2 (235·2 to 252·8)	100·0% (100·0 to 100·0%)	7·4% (3·4 to 11·1%)	-	9,616·3 (7,792·1 to 11,790·0)	100·0% (100·0 to 100·0%)	11,204·6 (8,841·7 to 13,942·6)	100·0% (100·0 to 100·0%)	16·5% (12·9 to 19·5%)	-
Chronic kidney disease due to diabetes mellitus	73·4 (71·1 to 75·9)	100·0% (100·0 to 100·0%)	102·7 (98·0 to 108·4)	100·0% (100·0 to 100·0%)	40·0% (35·8 to 44·8%)	-	1,666·3 (1,517·0 to 1,817·4)	100·0% (100·0 to 100·0%)	2,092·9 (1,918·7 to 2,278·9)	100·0% (100·0 to 100·0%)	25·6% (23·0 to 28·3%)	-
Chronic kidney disease due to hypertension	34·3 (24·6 to 43·9)	41·2% (29·6 to 52·0%)	45·1 (32·5 to 57·8)	42·2% (30·4 to 53·2%)	31·4% (26·5 to 35·9%)	2·4% (1·6 to 3·4%)	563·7 (399·4 to 721·6)	39·6% (28·3 to 50·2%)	665·6 (473·9 to 851·3)	41·1% (29·5 to 51·9%)	18·1% (14·9 to 21·0%)	3·7% (3·1 to 4·6%)
Chronic kidney disease due to glomerulonephritis	9·8 (7·0 to 12·6)	38·7% (27·5 to 49·2%)	12·5 (9·0 to 16·1)	40·6% (29·1 to 51·4%)	27·9% (23·2 to 32·0%)	5·0% (4·1 to 6·2%)	291·9 (202·5 to 387·1)	34·6% (24·4 to 44·5%)	332·5 (232·3 to 441·0)	37·2% (26·4 to 47·6%)	13·9% (10·8 to 17·0%)	7·6% (6·5 to 9·1%)
Chronic kidney disease due to other causes	1·2 (0·8 to 1·5)	36·6% (25·9 to 46·9%)	1·7 (1·2 to 2·3)	38·7% (27·4 to 49·3%)	48·4% (41·1 to 57·9%)	5·6% (3·9 to 7·5%)	203·2 (131·0 to 290·9)	34·8% (24·6 to 45·1%)	253·4 (165·2 to 360·5)	37·1% (26·3 to 47·5%)	24·7% (21·4 to 28·3%)	6·4% (5·3 to 7·6%)
**High total cholesterol: All causes**	1,329·7 (1,010·1 to 1,735·5)	11·9% (9·0 to 15·5%)	1,295·3 (947·2 to 1,705·5)	10·8% (7·9 to 14·2%)	-2·6% (-7·7 to 1·6%)	-9·7% (-14·4 to -6·1%)	23,189·8 (19,382·5 to 27,801·5)	6·6% (5·4 to 8·0%)	20,223·1 (16,697·8 to 24,339·0)	5·7% (4·6 to 7·1%)	-12·8% (-15·5 to -10·4%)	-13·7% (-16·5 to -11·4%)
Ischaemic heart disease	1,134·2 (853·7 to 1,416·7)	46·2% (34·8 to 57·5%)	1,114·5 (791·7 to 1,419·7)	44·7% (31·9 to 56·7%)	-1·7% (-7·6 to 2·8%)	-3·3% (-8·9 to 0·6%)	20,292·6 (17,152·8 to 23,674·6)	52·6% (44·5 to 61·1%)	17,828·0 (14,756·6 to 21,147·1)	50·8% (42·1 to 59·4%)	-12·1% (-15·1 to -9·7%)	-3·5% (-6·2 to -1·2%)
Ischaemic stroke	195·5 (64·6 to 407·3)	21·8% (7·2 to 45·3%)	180·8 (52·4 to 392·4)	21·4% (6·3 to 46·1%)	-7·5% (-19·9 to -0·4%)	-2·0% (-14·9 to 5·4%)	2,897·1 (1,534·6 to 5,188·8)	24·7% (13·1 to 44·3%)	2,395·1 (1,202·3 to 4,267·1)	24·2% (12·3 to 43·2%)	-17·3% (-23·5 to -12·0%)	-1·9% (-8·1 to 4·1%)
**High systolic blood pressure: All causes**	2,588·5 (2,273·9 to 2,878·8)	23·2% (20·4 to 25·8%)	2,573·0 (2,222·8 to 2,896·1)	21·4% (18·4 to 24·0%)	-0·6% (-3·1 to 1·6%)	-7·8% (-10·2 to -5·9%)	43,566·6 (39,269·8 to 47,473·9)	12·4% (10·9 to 14·0%)	39,221·2 (35,078·7 to 42,911·1)	11·1% (9·6 to 12·5%)	-10·0% (-12·0 to -8·2%)	-11·0% (-13·0 to -9·3%)
Rheumatic heart disease	11·4 (7·4 to 18·9)	27·4% (17·8 to 45·7%)	9·9 (6·0 to 17·8)	25·7% (15·7 to 46·4%)	-13·2% (-21·8 to -3·7%)	-6·1% (-14·7 to 3·9%)	210·7 (145·4 to 315·4)	28·0% (19·4 to 42·5%)	154·8 (107·7 to 235·2)	26·7% (18·5 to 40·7%)	-26·5% (-31·7 to -20·3%)	-4·7% (-10·4 to 2·8%)
Ischaemic heart disease	1,377·2 (1,098·6 to 1,643·4)	56·1% (44·9 to 67·0%)	1,356·9 (1,042·7 to 1,658·3)	54·4% (41·9 to 66·5%)	-1·5% (-5·8 to 1·4%)	-3·1% (-7·4 to -0·4%)	22,189·4 (18,999·9 to 25,143·0)	57·5% (49·2 to 65·0%)	19,679·7 (16,663·2 to 22,543·9)	56·0% (47·3 to 64·1%)	-11·3% (-13·9 to -9·2%)	-2·6% (-4·9 to -0·8%)
Ischaemic stroke	454·5 (350·3 to 566·6)	50·7% (39·0 to 63·2%)	406·1 (300·9 to 521·3)	48·0% (35·9 to 62·2%)	-10·6% (-15·8 to -4·9%)	-5·3% (-10·2 to 0·5%)	6,438·1 (5,158·2 to 7,539·0)	54·8% (43·7 to 63·9%)	5,212·1 (4,193·6 to 6,178·8)	52·7% (42·8 to 62·2%)	-19·0% (-22·5 to -15·1%)	-3·9% (-7·0 to 0·1%)

Supplemental Results Table 1. All-age deaths, death PAFs, DALYs, DALY PAFs, and percent change for each risk factor and outcome for both sexes combined in 2005 and 2015 for High SDI.

Data in parenthesis are 95% uncertainty intervals. PAFs=population attributable fractions. DALYs=disability-adjusted life-years.

	2005 Deaths (in thousands)	2005 Deaths PAF	2015 Deaths (in thousands)	2015 Deaths PAF	Percent change deaths 2005-2015	Percent change deaths PAF 2005-2015	2005 DALYs (in thousands)	2005 DALYs PAF	2015 DALYs (in thousands)	2015 DALYs PAF	Percent change DALYs 2005-2015	Percent change DALYs PAF 2005-2015
Hemorrhagic stroke	283·8 (226·5 to 341·5)	58·4% (46·0 to 70·0%)	255·8 (194·9 to 320·0)	56·0% (42·4 to 69·6%)	-9·9% (-15·9 to -4·1%)	-4·1% (-9·4 to 0·8%)	5,465·5 (4,624·6 to 6,278·2)	61·1% (51·7 to 69·7%)	4,411·8 (3,685·3 to 5,151·9)	59·4% (49·9 to 68·6%)	-19·3% (-22·8 to -15·8%)	-2·8% (-5·3 to -0·2%)
Hypertensive heart disease	132·9 (128·5 to 138·2)	100·0% (100·0 to 100·0%)	161·0 (153·9 to 168·7)	100·0% (100·0 to 100·0%)	21·2% (15·8 to 26·3%)	-	1,962·9 (1,896·6 to 2,033·8)	100·0% (100·0 to 100·0%)	2,123·6 (2,043·5 to 2,211·5)	100·0% (100·0 to 100·0%)	8·2% (3·9 to 12·1%)	-
Cardiomyopathy and myocarditis	66·8 (54·3 to 80·3)	39·4% (32·1 to 47·5%)	63·6 (50·9 to 78·5)	37·9% (30·6 to 46·7%)	-4·8% (-11·9 to 2·3%)	-3·9% (-9·1 to 1·6%)	1,788·5 (1,430·4 to 2,140·8)	41·1% (33·1 to 48·9%)	1,486·8 (1,217·2 to 1,762·4)	40·7% (33·3 to 48·0%)	-16·9% (-23·2 to -10·0%)	-1·1% (-5·2 to 4·2%)
Atrial fibrillation and flutter	25·5 (19·2 to 32·9)	35·9% (28·4 to 43·7%)	32·1 (23·5 to 42·0)	34·1% (26·7 to 42·1%)	25·9% (21·6 to 30·4%)	-5·0% (-7·3 to -2·8%)	672·6 (521·9 to 862·8)	41·1% (35·7 to 46·1%)	787·7 (607·1 to 1,015·4)	39·2% (34·0 to 44·4%)	17·1% (15·2 to 19·1%)	-4·5% (-5·7 to -3·4%)
Aortic aneurysm	25·3 (19·7 to 30·5)	35·8% (27·9 to 43·1%)	27·6 (21·1 to 33·8)	33·7% (25·8 to 41·4%)	8·9% (4·4 to 13·6%)	-5·8% (-8·5 to -2·7%)	415·1 (335·5 to 482·8)	38·6% (31·3 to 44·9%)	421·5 (342·5 to 494·1)	37·2% (30·4 to 43·6%)	1·5% (-2·4 to 4·8%)	-3·5% (-5·0 to -2·0%)
Peripheral vascular disease	8·0 (5·8 to 10·5)	27·9% (20·1 to 36·3%)	10·0 (7·1 to 13·4)	26·7% (19·1 to 35·3%)	25·5% (16·5 to 35·5%)	-4·4% (-8·0 to -0·9%)	148·9 (110·1 to 201·1)	29·4% (22·8 to 36·3%)	173·7 (125·5 to 235·6)	28·1% (21·8 to 34·9%)	16·6% (11·3 to 22·5%)	-4·2% (-6·7 to -1·8%)
Endocarditis	8·3 (6·4 to 10·8)	34·4% (26·3 to 44·5%)	9·6 (7·1 to 12·7)	32·7% (24·5 to 43·2%)	14·9% (9·2 to 21·1%)	-5·1% (-8·3 to -1·7%)	146·5 (116·7 to 175·1)	35·4% (28·3 to 42·1%)	155·9 (122·6 to 187·3)	34·7% (27·7 to 41·5%)	6·4% (0·8 to 12·4%)	-2·1% (-4·3 to 0·1%)
Other cardiovascular and circulatory diseases	60·9 (51·6 to 70·0)	39·0% (33·3 to 44·9%)	66·6 (55·0 to 78·3)	36·8% (30·6 to 43·2%)	9·3% (4·0 to 14·2%)	-5·8% (-8·6 to -3·3%)	1,419·6 (1,215·5 to 1,667·2)	41·3% (36·8 to 45·9%)	1,470·1 (1,241·8 to 1,744·2)	39·7% (35·2 to 44·5%)	3·6% (0·3 to 6·6%)	-3·8% (-5·1 to -2·5%)
Chronic kidney disease due to diabetes mellitus	36·6 (26·5 to 45·3)	49·9% (36·7 to 61·7%)	49·7 (35·8 to 62·0)	48·4% (35·3 to 60·0%)	35·8% (31·5 to 40·8%)	-3·0% (-4·1 to -2·0%)	711·7 (502·6 to 908·5)	42·7% (30·7 to 53·5%)	878·6 (617·9 to 1,131·2)	42·0% (29·9 to 52·9%)	23·4% (20·7 to 26·1%)	-1·7% (-2·7 to -0·9%)
Chronic kidney disease due to hypertension	83·3 (80·6 to 86·0)	100·0% (100·0 to 100·0%)	106·9 (101·5 to 111·7)	100·0% (100·0 to 100·0%)	28·3% (23·5 to 32·4%)	-	1,422·8 (1,331·1 to 1,518·3)	100·0% (100·0 to 100·0%)	1,619·7 (1,509·5 to 1,736·0)	100·0% (100·0 to 100·0%)	13·8% (11·0 to 16·4%)	-
Chronic kidney disease due to glomerulonephritis	12·3 (9·0 to 15·3)	48·7% (35·7 to 60·5%)	15·1 (11·0 to 18·9)	48·9% (36·0 to 60·9%)	22·2% (18·2 to 26·5%)	0·4% (-0·7 to 1·6%)	337·0 (234·3 to 442·1)	40·0% (28·7 to 51·0%)	366·0 (254·9 to 480·8)	41·0% (29·7 to 52·2%)	8·6% (5·5 to 11·7%)	2·6% (1·4 to 3·9%)
Chronic kidney disease due to other causes	1·6 (1·1 to 2·0)	49·6% (35·9 to 61·0%)	2·3 (1·6 to 2·9)	50·7% (36·7 to 62·4%)	43·6% (36·3 to 54·5%)	2·1% (-0·1 to 4·4%)	237·2 (156·4 to 326·6)	40·7% (29·0 to 51·5%)	279·0 (182·9 to 386·1)	40·8% (29·0 to 51·9%)	17·7% (14·4 to 21·0%)	0·4% (-0·8 to 1·5%)
**High body-mass index: All causes**	1,081·2 (785·6 to 1,400·4)	9·7% (7·0 to 12·5%)	1,088·5 (791·6 to 1,423·5)	9·0% (6·6 to 11·8%)	0·7% (-2·2 to 3·9%)	-6·6% (-9·3 to -3·8%)	28,164·6 (20,886·6 to 35,786·6)	8·0% (6·0 to 10·1%)	28,403·5 (21,337·9 to 35,730·6)	8·0% (6·1 to 9·9%)	0·8% (-1·9 to 4·1%)	-0·3% (-2·3 to 2·3%)
Oesophageal cancer	17·3 (5·7 to 30·0)	23·3% (7·7 to 40·4%)	20·1 (6·7 to 34·6)	24·0% (8·0 to 41·3%)	16·5% (10·3 to 23·0%)	2·8% (0·5 to 6·0%)	379·7 (119·4 to 661·1)	24·4% (7·6 to 42·6%)	424·0 (136·6 to 734·5)	25·4% (7·9 to 43·9%)	11·7% (5·3 to 18·5%)	4·4% (2·1 to 8·0%)
Colon and rectum cancer	28·6 (17·8 to 40·5)	8·8% (5·5 to 12·6%)	33·7 (21·3 to 47·8)	9·1% (5·7 to 12·8%)	18·0% (14·5 to 22·2%)	2·7% (0·6 to 5·4%)	578·9 (366·2 to 817·8)	9·7% (6·1 to 13·6%)	654·5 (419·0 to 914·8)	10·1% (6·5 to 14·1%)	13·0% (9·7 to 17·5%)	4·6% (2·5 to 7·5%)
Liver cancer due to hepatitis B	3·3 (1·3 to 6·1)	12·8% (5·2 to 23·6%)	4·1 (1·7 to 7·4)	13·7% (5·6 to 24·9%)	24·4% (17·3 to 33·0%)	6·9% (3·3 to 12·4%)	90·2 (34·8 to 168·6)	13·1% (5·1 to 24·1%)	105·6 (42·1 to 193·0)	14·4% (5·9 to 25·9%)	17·2% (9·4 to 27·4%)	10·1% (5·5 to 16·9%)
Liver cancer due to hepatitis C	6·4 (2·8 to 11·0)	12·4% (5·4 to 21·3%)	8·1 (3·6 to 13·9)	12·6% (5·6 to 21·4%)	26·2% (21·6 to 32·5%)	1·6% (-1·0 to 5·0%)	119·5 (51·9 to 209·8)	13·3% (5·8 to 23·1%)	142·7 (63·3 to 248·7)	14·0% (6·2 to 24·0%)	19·4% (14·7 to 25·5%)	5·2% (2·2 to 9·2%)
Liver cancer due to alcohol use	7·0 (3·0 to 12·0)	16·7% (7·1 to 29·1%)	8·6 (3·7 to 14·7)	17·6% (7·6 to 30·3%)	22·8% (16·4 to 29·6%)	5·5% (2·0 to 9·7%)	153·9 (65·2 to 267·8)	17·3% (7·2 to 30·2%)	185·6 (79·5 to 320·9)	18·5% (7·8 to 31·6%)	20·6% (14·2 to 27·9%)	7·3% (3·3 to 12·1%)
Liver cancer due to other causes	2·2 (1·0 to 3·6)	14·6% (6·7 to 24·2%)	2·9 (1·4 to 4·8)	15·6% (7·3 to 25·5%)	33·1% (26·9 to 40·7%)	7·1% (3·4 to 11·9%)	48·3 (22·4 to 79·7)	15·3% (7·0 to 25·3%)	60·6 (29·4 to 99·4)	17·0% (8·0 to 27·6%)	25·5% (19·2 to 33·4%)	10·9% (6·5 to 16·5%)
Gallbladder and biliary tract cancer	8·3 (4·8 to 12·6)	15·4% (8·9 to 23·4%)	8·6 (4·9 to 13·0)	14·6% (8·3 to 22·2%)	3·7% (-5·9 to 12·5%)	-5·3% (-10·3 to -1·2%)	142·1 (82·9 to 213·7)	16·3% (9·5 to 24·4%)	136·7 (78·4 to 201·8)	15·9% (9·2 to 23·7%)	-3·8% (-11·8 to 4·3%)	-2·8% (-8·2 to 1·2%)
Pancreatic cancer	10·0 (3·6 to 17·3)	6·4% (2·3 to 11·1%)	12·8 (4·6 to 21·8)	6·5% (2·3 to 11·2%)	27·4% (23·5 to 32·1%)	1·0% (-1·0 to 3·4%)	194·3 (69·2 to 335·0)	6·7% (2·4 to 11·6%)	237·2 (86·0 to 406·4)	6·9% (2·5 to 11·9%)	22·1% (18·4 to 26·6%)	3·1% (1·3 to 5·9%)
Breast cancer	11·5 (6·7 to 17·5)	6·3% (3·7 to 9·7%)	14·4 (8·5 to 21·5)	7·1% (4·2 to 10·5%)	25·1% (17·9 to 32·1%)	11·4% (7·9 to 15·7%)	215·4 (119·2 to 337·6)	4·6% (2·6 to 7·3%)	283·3 (158·6 to 434·2)	5·6% (3·2 to 8·6%)	31·5% (22·6 to 44·2%)	21·1% (14·4 to 30·3%)
Uterine cancer	11·2 (8·0 to 14·3)	41·7% (30·3 to 53·1%)	13·1 (9·4 to 17·0)	42·3% (31·2 to 53·4%)	17·5% (2·5 to 28·4%)	1·6% (-0·5 to 4·7%)	258·4 (191·3 to 329·4)	43·8% (32·5 to 55·1%)	304·2 (221·7 to 391·8)	45·1% (34·0 to 56·0%)	17·7% (1·0 to 30·6%)	3·0% (0·9 to 5·9%)
Ovarian cancer	2·2 (-0·1 to 4·9)	3·6% (-0·1 to 7·9%)	2·6 (-0·1 to 5·6)	3·7% (-0·1 to 8·0%)	15·0% (10·1 to 20·8%)	2·6% (0·6 to 5·7%)	50·8 (-1·8 to 111·2)	3·7% (-0·1 to 8·1%)	56·9 (-2·1 to 123·6)	3·8% (-0·1 to 8·3%)	11·9% (6·8 to 17·7%)	4·0% (1·9 to 7·3%)
Kidney cancer	11·6 (7·9 to 15·8)	20·0% (13·5 to 27·1%)	14·5 (9·9 to 19·5)	20·3% (13·8 to 27·4%)	25·3% (20·4 to 30·4%)	1·6% (-0·3 to 3·7%)	254·5 (174·6 to 344·7)	20·5% (14·0 to 27·5%)	307·4 (213·4 to 413·2)	21·2% (14·7 to 28·4%)	20·8% (15·8 to 25·8%)	3·5% (1·7 to 6·1%)
Thyroid cancer	1·1 (0·6 to 1·6)	12·1% (7·0 to 18·3%)	1·3 (0·7 to 1·9)	12·2% (6·9 to 18·4%)	17·2% (10·1 to 24·4%)	0·6% (-3·3 to 4·6%)	27·6 (15·8 to 41·5)	13·1% (7·5 to 19·6%)	39·1 (21·9 to 58·7)	14·0% (7·8 to 21·0%)	41·8% (29·2 to 54·8%)	6·7% (1·0 to 11·6%)
Ischaemic heart disease	501·6 (355·3 to 659·9)	20·4% (14·5 to 27·0%)	494·0 (346·4 to 655·3)	19·8% (13·9 to 26·5%)	-1·5% (-6·4 to 2·9%)	-3·1% (-7·7 to 1·2%)	10,256·4 (7,526·5 to 13,115·2)	26·6% (19·5 to 34·1%)	9,393·8 (6,989·8 to 11,942·0)	26·7% (19·9 to 33·9%)	-8·4% (-11·4 to -5·2%)	0·6% (-2·2 to 4·1%)
Ischaemic stroke	123·6 (83·2 to 175·8)	13·8% (9·3 to 19·7%)	101·5 (62·8 to 149·9)	12·0% (7·4 to 17·8%)	-17·9% (-28·6 to -10·5%)	-13·0% (-23·9 to -5·5%)	2,469·9 (1,793·3 to 3,206·8)	21·0% (15·3 to 27·2%)	1,990·1 (1,448·6 to 2,610·9)	20·1% (14·6 to 26·3%)	-19·4% (-24·1 to -14·6%)	-4·3% (-9·1 to 0·2%)
Hemorrhagic stroke	117·6 (84·3 to 156·9)	24·2% (17·3 to 32·4%)	96·0 (65·9 to 133·1)	21·0% (14·5 to 29·1%)	-18·4% (-26·6 to -12·0%)	-13·2% (-21·1 to -7·1%)	3,221·0 (2,424·8 to 4,041·1)	36·0% (27·2 to 45·0%)	2,587·8 (1,969·5 to 3,224·8)	34·8% (26·6 to 43·3%)	-19·7% (-23·3 to -15·7%)	-3·2% (-6·1 to 0·1%)
Hypertensive heart disease	57·1 (33·5 to 83·3)	43·0% (25·4 to 62·9%)	68·2 (36·4 to 102·8)	42·4% (22·8 to 63·4%)	19·5% (3·7 to 29·5%)	-1·4% (-13·7 to 5·2%)	998·7 (735·5 to 1,279·2)	50·9% (37·9 to 65·3%)	1,082·1 (782·3 to 1,400·8)	50·9% (36·9 to 65·9%)	8·4% (1·2 to 15·3%)	0·2% (-5·4 to 4·7%)
Diabetes mellitus	97·7 (73·0 to 125·0)	43·0% (31·9 to 55·0%)	102·7 (76·2 to 132·7)	42·0% (31·1 to 54·4%)	5·1% (0·1 to 9·8%)	-2·1% (-5·3 to 1·0%)	5,380·9 (3,921·6 to 6,926·7)	55·9% (45·4 to 66·0%)	6,403·9 (4,580·5 to 8,326·2)	57·1% (47·0 to 67·0%)	19·0% (14·8 to 22·6%)	2·1% (0·8 to 3·9%)
Chronic kidney disease due to diabetes mellitus	22·2 (9·5 to 36·1)	30·3% (12·7 to 49·2%)	31·9 (13·1 to 52·8)	31·1% (12·1 to 51·0%)	43·6% (23·9 to 56·0%)	2·6% (-11·8 to 10·5%)	577·2 (308·3 to 852·9)	34·7% (18·2 to 51·1%)	769·4 (434·1 to 1,132·9)	36·8% (20·5 to 53·4%)	33·3% (26·2 to 44·5%)	6·1% (0·6 to 14·7%)
Chronic kidney disease due to hypertension	24·7 (7·3 to 43·6)	29·7% (8·9 to 51·8%)	30·4 (4·8 to 57·5)	28·4% (4·5 to 53·4%)	22·8% (-29·3 to 39·9%)	-4·3% (-45·3 to 8·4%)	509·0 (268·9 to 754·6)	35·8% (19·0 to 52·4%)	579·8 (293·8 to 865·7)	35·8% (18·0 to 52·9%)	13·9% (2·4 to 23·4%)	0·0% (-9·8 to 7·9%)
Chronic kidney disease due to glomerulonephritis	7·1 (2·3 to 12·5)	28·1% (9·1 to 48·2%)	8·4 (1·7 to 15·8)	27·4% (5·6 to 51·0%)	18·7% (-29·8 to 37·3%)	-2·5% (-42·8 to 12·3%)	279·8 (125·8 to 438·0)	33·2% (15·2 to 49·5%)	309·6 (149·7 to 483·1)	34·7% (16·5 to 51·3%)	10·7% (2·3 to 24·4%)	4·5% (-3·1 to 17·9%)
Chronic kidney disease due to other causes	0·8 (-0·2 to 1·7)	23·5% (-7·2 to 51·9%)	1·1 (-0·5 to 2·5)	24·2% (-11·2 to 55·9%)	44·3% (-65·3 to 140·5%)	2·7% (-75·6 to 72·3%)	190·1 (90·1 to 300·3)	32·6% (16·7 to 48·1%)	230·0 (110·4 to 358·8)	33·6% (17·4 to 49·0%)	21·0% (12·1 to 30·4%)	3·2% (-4·0 to 11·0%)
Osteoarthritis	-	-	-	-	-	-	575·0 (340·6 to 874·9)	23·1% (16·4 to 29·8%)	724·9 (431·9 to 1,102·0)	23·6% (17·1 to 30·3%)	26·1% (24·1 to 28·9%)	2·5% (1·1 to 4·6%)
Low back pain	-	-	-	-	-	-	1,028·9 (608·0 to 1,592·6)	7·5% (5·1 to 10·0%)	1,210·5 (723·4 to 1,864·0)	8·0% (5·6 to 10·6%)	17·6% (15·4 to 21·0%)	6·6% (4·7 to 9·4%)
**Low bone mineral density: All causes**	87·3 (83·3 to 91·2)	0·8% (0·7 to 0·8%)	106·0 (100·5 to 111·4)	0·9% (0·8 to 0·9%)	21·4% (17·3 to 25·3%)	12·6% (8·8 to 16·0%)	2,446·8 (2,008·3 to 2,987·2)	0·7% (0·6 to 0·8%)	2,522·9 (2,048·2 to 3,125·5)	0·7% (0·6 to 0·8%)	3·1% (-0·5 to 6·8%)	1·9% (-1·6 to 5·4%)
Pedestrian road injuries	6·6 (6·2 to 7·1)	11·6% (10·9 to 12·4%)	6·4 (5·9 to 6·9)	15·1% (14·3 to 16·2%)	-3·1% (-7·6 to 1·7%)	30·3% (25·0 to 38·0%)	128·3 (114·9 to 142·6)	5·4% (4·9 to 6·0%)	108·4 (97·0 to 121·2)	6·9% (6·3 to 7·5%)	-15·5% (-18·9 to -12·1%)	26·4% (20·8 to 33·8%)
Cyclist road injuries	0·9 (0·9 to 1·0)	10·4% (9·8 to 11·0%)	1·0 (0·9 to 1·1)	12·5% (11·8 to 13·4%)	1·8% (-4·1 to 7·6%)	20·8% (16·6 to 25·6%)	73·3 (56·0 to 95·6)	11·3% (9·9 to 12·7%)	67·0 (50·0 to 88·9)	12·8% (11·3 to 14·3%)	-8·7% (-12·9 to -4·9%)	13·4% (10·3 to 17·5%)
Motorcyclist road injuries	0·8 (0·7 to 0·9)	4·1% (3·7 to 4·5%)	0·8 (0·7 to 0·9)	5·1% (4·5 to 5·6%)	6·1% (-1·1 to 12·8%)	24·1% (17·9 to 30·7%)	64·9 (49·8 to 83·4)	5·0% (4·1 to 5·9%)	61·9 (47·2 to 79·4)	5·8% (4·8 to 6·9%)	-4·6% (-9·3 to 0·2%)	16·6% (11·6 to 22·4%)
Motor vehicle road injuries	7·5 (6·6 to 8·4)	7·1% (6·6 to 7·6%)	6·6 (6·1 to 7·3)	8·6% (8·0 to 9·1%)	-11·7% (-18·7 to -3·3%)	20·5% (16·7 to 24·7%)	296·3 (243·9 to 357·7)	5·1% (4·4 to 5·8%)	265·9 (214·7 to 328·2)	6·3% (5·4 to 7·2%)	-10·3% (-15·7 to -4·9%)	23·5% (18·2 to 29·0%)
Other road injuries	0·1 (0·1 to 0·1)	11·0% (9·7 to 11·9%)	0·1 (0·1 to 0·1)	14·2% (12·0 to 15·8%)	15·9% (5·7 to 27·9%)	29·1% (17·7 to 40·8%)	6·7 (5·0 to 8·7)	8·9% (7·6 to 10·3%)	7·5 (5·5 to 10·1)	10·8% (9·2 to 12·5%)	12·9% (7·9 to 17·8%)	21·5% (14·8 to 27·9%)

Supplemental Results Table 1. All-age deaths, death PAFs, DALYs, DALY PAFs, and percent change for each risk factor and outcome for both sexes combined in 2005 and 2015 for High SDI.

Data in parenthesis are 95% uncertainty intervals. PAFs=population attributable fractions. DALYs=disability-adjusted life-years.

	2005 Deaths (in thousands)	2005 Deaths PAF	2015 Deaths (in thousands)	2015 Deaths PAF	Percent change deaths 2005-2015	Percent change deaths PAF 2005-2015	2005 DALYs (in thousands)	2005 DALYs PAF	2015 DALYs (in thousands)	2015 DALYs PAF	Percent change DALYs 2005-2015	Percent change DALYs PAF 2005-2015
Other transport injuries	1·4 (1·3 to 1·6)	9·3% (8·6 to 9·9%)	1·4 (1·3 to 1·5)	11·4% (10·7 to 12·1%)	-2·0% (-9·3 to 4·8%)	23·0% (17·2 to 29·0%)	42·7 (37·6 to 48·8)	5·6% (5·0 to 6·2%)	38·4 (33·5 to 44·1)	6·6% (5·9 to 7·2%)	-10·0% (-15·5 to -4·6%)	17·7% (12·3 to 23·7%)
Falls	66·6 (63·1 to 69·6)	54·3% (52·2 to 56·1%)	86·5 (81·6 to 91·3)	60·5% (57·8 to 62·7%)	29·9% (25·0 to 34·6%)	11·4% (9·0 to 13·4%)	1,696·3 (1,377·9 to 2,109·3)	26·6% (25·4 to 27·8%)	1,849·4 (1,477·2 to 2,316·8)	29·9% (28·5 to 31·3%)	9·0% (4·9 to 13·0%)	12·3% (9·9 to 14·8%)
Other exposure to mechanical forces	1·4 (1·3 to 1·5)	9·0% (8·4 to 9·6%)	1·5 (1·4 to 1·6)	11·8% (11·0 to 12·6%)	4·2% (-1·9 to 10·1%)	31·0% (23·8 to 37·5%)	61·3 (47·2 to 80·0)	5·1% (4·5 to 5·7%)	61·0 (45·8 to 81·9)	6·0% (5·3 to 6·6%)	-0·6% (-5·2 to 3·8%)	17·1% (12·7 to 21·8%)
Non-venomous animal contact	0·2 (0·2 to 0·2)	15·4% (14·5 to 16·3%)	0·2 (0·2 to 0·2)	18·0% (16·8 to 19·1%)	17·6% (7·0 to 28·9%)	17·1% (10·1 to 24·1%)	2·2 (2·0 to 2·5)	3·9% (3·2 to 4·6%)	2·3 (2·1 to 2·6)	4·2% (3·4 to 5·1%)	3·7% (-2·4 to 10·6%)	6·8% (-0·1 to 13·4%)
Assault by other means	1·8 (1·6 to 1·9)	4·6% (4·2 to 5·1%)	1·5 (1·4 to 1·6)	6·2% (5·6 to 6·7%)	-15·1% (-21·7 to -7·3%)	33·5% (22·7 to 44·9%)	65·4 (53·2 to 79·5)	3·5% (2·9 to 4·2%)	54·4 (43·3 to 67·9)	4·6% (3·8 to 5·5%)	-16·9% (-23·7 to -10·2%)	30·5% (19·5 to 41·1%)
Exposure to forces of nature	-	-	-	-	-	-	9·4 (2·5 to 23·2)	3·2% (1·0 to 6·4%)	6·7 (2·2 to 15·8)	6·9% (4·1 to 10·2%)	-28·1% (-67·6 to 41·3%)	116·1% (23·8 to 423·6%)
**Low glomerular filtration rate: All causes**	519·3 (479·1 to 562·1)	4·6% (4·3 to 5·0%)	578·7 (536·5 to 622·7)	4·8% (4·5 to 5·2%)	11·4% (9·1 to 13·9%)	3·3% (1·2 to 5·5%)	8,964·6 (8,185·1 to 9,694·9)	2·6% (2·3 to 2·8%)	9,313·0 (8,518·0 to 10,048·7)	2·6% (2·4 to 2·9%)	3·9% (1·6 to 6·0%)	2·7% (0·5 to 4·7%)
Ischaemic heart disease	208·9 (176·9 to 243·0)	8·5% (7·2 to 9·9%)	213·6 (180·9 to 249·0)	8·6% (7·3 to 10·0%)	2·3% (-0·5 to 5·0%)	0·6% (-1·6 to 2·9%)	2,737·8 (2,366·1 to 3,137·8)	7·1% (6·1 to 8·1%)	2,532·8 (2,191·2 to 2,902·4)	7·2% (6·3 to 8·2%)	-7·5% (-10·0 to -4·7%)	1·6% (-0·8 to 4·3%)
Ischaemic stroke	81·4 (63·2 to 100·0)	9·1% (7·1 to 11·1%)	76·5 (60·4 to 93·8)	9·1% (7·2 to 11·0%)	-6·0% (-10·7 to -0·9%)	-0·4% (-4·6 to 4·2%)	979·0 (767·1 to 1,207·9)	8·3% (6·6 to 10·3%)	819·8 (658·0 to 998·3)	8·3% (6·7 to 10·0%)	-16·3% (-20·5 to -11·4%)	-0·6% (-4·8 to 4·3%)
Hemorrhagic stroke	39·6 (33·1 to 46·5)	8·1% (6·8 to 9·6%)	38·2 (31·7 to 45·0)	8·4% (6·9 to 9·8%)	-3·6% (-9·1 to 2·2%)	2·6% (-2·0 to 7·7%)	609·4 (512·2 to 717·0)	6·8% (5·7 to 8·0%)	519·3 (438·9 to 602·7)	7·0% (6·0 to 8·1%)	-14·8% (-19·1 to -9·8%)	2·6% (-1·8 to 7·4%)
Peripheral vascular disease	4·2 (3·3 to 5·1)	14·7% (11·8 to 17·9%)	5·5 (4·3 to 6·7)	14·5% (11·6 to 17·6%)	30·1% (20·3 to 40·9%)	-0·8% (-4·8 to 3·2%)	74·2 (56·6 to 99·3)	14·6% (12·1 to 17·4%)	90·4 (68·6 to 121·2)	14·6% (12·1 to 17·4%)	21·9% (16·2 to 28·2%)	0·1% (-2·6 to 2·7%)
Chronic kidney disease due to diabetes mellitus	73·4 (71·1 to 75·9)	100·0% (100·0 to 100·0%)	102·7 (98·0 to 108·4)	100·0% (100·0 to 100·0%)	40·0% (35·8 to 44·8%)	-	1,666·3 (1,517·0 to 1,817·4)	100·0% (100·0 to 100·0%)	2,092·9 (1,918·7 to 2,278·9)	100·0% (100·0 to 100·0%)	25·6% (23·0 to 28·3%)	-
Chronic kidney disease due to hypertension	83·3 (80·6 to 86·0)	100·0% (100·0 to 100·0%)	106·9 (101·5 to 111·7)	100·0% (100·0 to 100·0%)	28·3% (23·5 to 32·4%)	-	1,422·8 (1,331·1 to 1,518·3)	100·0% (100·0 to 100·0%)	1,619·7 (1,509·5 to 1,736·0)	100·0% (100·0 to 100·0%)	13·8% (11·0 to 16·4%)	-
Chronic kidney disease due to glomerulonephritis	25·3 (24·0 to 26·6)	100·0% (100·0 to 100·0%)	30·8 (28·8 to 32·8)	100·0% (100·0 to 100·0%)	21·7% (17·7 to 25·7%)	-	843·2 (732·8 to 959·1)	100·0% (100·0 to 100·0%)	892·5 (762·9 to 1,032·1)	100·0% (100·0 to 100·0%)	5·8% (3·0 to 8·6%)	-
Chronic kidney disease due to other causes	3·2 (2·8 to 3·6)	100·0% (100·0 to 100·0%)	4·5 (3·9 to 5·2)	100·0% (100·0 to 100·0%)	40·6% (34·1 to 49·4%)	-	582·7 (440·6 to 731·3)	100·0% (100·0 to 100·0%)	683·2 (511·5 to 850·7)	100·0% (100·0 to 100·0%)	17·3% (14·2 to 20·3%)	-
Gout	-	-	-	-	-	-	49·2 (32·7 to 67·8)	15·1% (13·6 to 16·7%)	62·4 (41·5 to 85·6)	16·0% (14·5 to 17·6%)	26·7% (24·0 to 29·7%)	6·0% (4·3 to 7·8%)

Supplemental Results Table 1. All-age deaths, death PAFs, DALYs, DALY PAFs, and percent change for each risk factor and outcome for both sexes combined in 2005 and 2015 for High-middle SDI.

Data in parenthesis are 95% uncertainty intervals. PAFs=population attributable fractions. DALYs=disability-adjusted life-years.

	2005 Deaths (in thousands)	2005 Deaths PAF	2015 Deaths (in thousands)	2015 Deaths PAF	Percent change deaths 2005-2015	Percent change deaths PAF 2005-2015	2005 DALYs (in thousands)	2005 DALYs PAF	2015 DALYs (in thousands)	2015 DALYs PAF	Percent change DALYs 2005-2015	Percent change DALYs PAF 2005-2015
**All risk factors: All causes**	7,022·4 (6,837·7 to 7,207·1)	62·9% (61·5 to 64·2%)	7,411·9 (7,172·8 to 7,672·5)	62·9% (61·5 to 64·3%)	5·5% (2·4 to 8·8%)	0·0% (-0·7 to 0·6%)	206,559·1 (194,494·4 to 219,796·8)	44·1% (42·2 to 46·1%)	201,875·1 (187,615·9 to 218,181·3)	43·4% (41·4 to 45·6%)	-2·3% (-4·9 to 0·4%)	-1·6% (-2·7 to -0·4%)
**Environmental/occupational risks: All causes**	1,763·0 (1,583·7 to 1,941·9)	15·8% (14·2 to 17·5%)	1,691·3 (1,516·1 to 1,869·2)	14·4% (12·9 to 15·8%)	-4·1% (-8·4 to 0·4%)	-9·1% (-12·0 to -6·4%)	51,145·3 (46,635·0 to 55,856·7)	10·9% (10·0 to 11·9%)	46,614·8 (42,253·6 to 51,367·2)	10·0% (9·2 to 10·9%)	-8·9% (-12·3 to -5·5%)	-8·2% (-10·6 to -5·8%)
**Unsafe water, sanitation, and handwashing: All causes**	128·6 (107·7 to 148·3)	1·2% (1·0 to 1·3%)	107·4 (87·8 to 127·5)	0·9% (0·7 to 1·1%)	-16·4% (-21·5 to -12·0%)	-20·9% (-25·7 to -16·6%)	6,631·9 (5,712·9 to 7,603·6)	1·4% (1·2 to 1·6%)	4,371·2 (3,682·4 to 5,130·6)	0·9% (0·8 to 1·1%)	-34·1% (-38·1 to -30·2%)	-33·6% (-37·4 to -30·1%)
**Unsafe water source: All causes**	66·8 (51·2 to 80·1)	0·6% (0·5 to 0·7%)	48·2 (35·6 to 59·2)	0·4% (0·3 to 0·5%)	-27·9% (-32·9 to -23·6%)	-31·7% (-36·2 to -27·5%)	4,309·5 (3,279·2 to 5,263·6)	0·9% (0·7 to 1·1%)	2,749·6 (2,010·4 to 3,447·5)	0·6% (0·4 to 0·7%)	-36·2% (-41·4 to -31·9%)	-35·7% (-40·6 to -31·7%)
Diarrhoeal diseases	51·2 (40·1 to 58·4)	80·5% (62·3 to 91·0%)	35·6 (27·2 to 41·5)	77·7% (59·0 to 88·7%)	-30·5% (-35·4 to -25·7%)	-3·5% (-5·4 to -2·2%)	3,211·2 (2,481·5 to 3,753·2)	81·1% (62·6 to 91·8%)	1,902·7 (1,431·6 to 2,266·0)	79·1% (59·9 to 90·5%)	-40·7% (-45·3 to -36·5%)	-2·5% (-4·5 to -1·4%)
Typhoid fever	12·6 (6·7 to 21·0)	82·3% (64·1 to 92·6%)	10·1 (5·4 to 16·9)	80·5% (61·2 to 91·6%)	-19·5% (-27·5 to -12·2%)	-2·2% (-4·9 to -0·8%)	888·0 (481·4 to 1,470·6)	82·3% (64·1 to 92·6%)	684·9 (374·7 to 1,120·6)	80·5% (61·2 to 91·7%)	-22·9% (-31·1 to -15·3%)	-2·2% (-4·9 to -0·8%)
Paratyphoid fever	3·0 (1·3 to 5·9)	82·1% (63·8 to 92·5%)	2·4 (1·1 to 4·7)	80·3% (60·9 to 91·6%)	-19·7% (-28·4 to -10·8%)	-2·2% (-4·9 to -0·8%)	210·4 (90·6 to 408·0)	82·1% (63·8 to 92·5%)	162·1 (71·8 to 308·6)	80·3% (60·9 to 91·6%)	-23·0% (-32·0 to -13·8%)	-2·2% (-4·9 to -0·8%)
**Unsafe sanitation: All causes**	28·7 (24·6 to 34·1)	0·3% (0·2 to 0·3%)	16·1 (13·4 to 19·6)	0·1% (0·1 to 0·2%)	-43·9% (-50·1 to -36·8%)	-46·8% (-52·8 to -40·2%)	1,852·7 (1,587·8 to 2,197·9)	0·4% (0·3 to 0·5%)	933·0 (752·4 to 1,160·2)	0·2% (0·2 to 0·2%)	-49·6% (-55·6 to -43·3%)	-49·3% (-55·1 to -43·0%)
Diarrhoeal diseases	22·0 (19·5 to 24·8)	34·6% (31·3 to 38·0%)	11·8 (10·3 to 13·5)	25·7% (22·7 to 28·9%)	-46·5% (-52·1 to -40·0%)	-25·6% (-31·6 to -19·0%)	1,382·1 (1,226·9 to 1,567·2)	34·9% (31·8 to 38·2%)	640·9 (541·2 to 751·3)	26·6% (23·7 to 29·7%)	-53·6% (-58·6 to -48·5%)	-23·7% (-29·3 to -17·9%)
Typhoid fever	5·3 (2·9 to 8·8)	35·0% (30·2 to 40·3%)	3·5 (1·8 to 5·9)	27·5% (22·6 to 32·6%)	-35·3% (-46·5 to -23·0%)	-21·6% (-33·3 to -8·2%)	379·8 (212·4 to 612·0)	35·2% (30·3 to 40·5%)	235·6 (126·5 to 393·1)	27·6% (22·8 to 32·8%)	-38·0% (-48·9 to -25·6%)	-21·5% (-33·3 to -7·8%)
Paratyphoid fever	1·3 (0·6 to 2·6)	35·4% (30·6 to 40·3%)	0·9 (0·4 to 1·7)	27·9% (22·6 to 33·3%)	-35·2% (-46·7 to -21·4%)	-21·2% (-32·9 to -8·2%)	90·9 (42·4 to 173·5)	35·5% (30·7 to 40·4%)	56·5 (26·6 to 111·2)	28·0% (22·7 to 33·3%)	-37·8% (-49·3 to -23·8%)	-21·2% (-32·9 to -8·1%)
**No handwashing with soap: All causes**	83·0 (62·8 to 101·1)	0·7% (0·6 to 0·9%)	74·3 (54·8 to 93·5)	0·6% (0·5 to 0·8%)	-10·5% (-16·0 to -5·3%)	-15·2% (-20·6 to -10·6%)	3,680·4 (2,913·2 to 4,410·3)	0·8% (0·6 to 0·9%)	2,468·4 (1,944·2 to 3,016·3)	0·5% (0·4 to 0·7%)	-32·9% (-37·0 to -29·3%)	-32·4% (-36·3 to -28·9%)
Diarrhoeal diseases	23·0 (18·2 to 27·7)	36·2% (28·8 to 43·1%)	15·9 (12·5 to 19·2)	34·6% (27·3 to 41·4%)	-31·1% (-35·9 to -26·6%)	-4·3% (-5·9 to -3·1%)	1,428·1 (1,116·9 to 1,744·7)	36·1% (28·6 to 43·0%)	830·6 (637·4 to 1,025·7)	34·5% (27·1 to 41·3%)	-41·8% (-46·3 to -37·7%)	-4·3% (-5·9 to -3·0%)
Typhoid fever	5·7 (3·0 to 9·6)	37·4% (29·3 to 44·4%)	4·6 (2·4 to 7·8)	36·3% (28·2 to 43·4%)	-20·0% (-28·7 to -12·6%)	-3·0% (-4·7 to -1·7%)	404·7 (216·3 to 677·1)	37·5% (29·4 to 44·5%)	310·3 (164·3 to 522·6)	36·4% (28·3 to 43·4%)	-23·3% (-32·0 to -15·4%)	-2·9% (-4·6 to -1·6%)
Paratyphoid fever	1·4 (0·6 to 2·8)	37·0% (29·0 to 44·1%)	1·1 (0·5 to 2·2)	36·0% (28·2 to 43·1%)	-20·0% (-29·2 to -10·9%)	-2·6% (-4·2 to -1·4%)	94·9 (43·6 to 190·1)	37·0% (29·0 to 44·1%)	72·9 (34·1 to 143·6)	36·1% (28·2 to 43·2%)	-23·2% (-33·0 to -13·7%)	-2·5% (-4·2 to -1·3%)
**Air pollution: All causes**	1,352·1 (1,179·0 to 1,537·8)	12·1% (10·5 to 13·8%)	1,264·4 (1,094·0 to 1,440·9)	10·7% (9·4 to 12·2%)	-6·5% (-11·8 to -1·2%)	-11·4% (-15·1 to -7·7%)	30,059·8 (26,476·0 to 33,839·9)	6·4% (5·5 to 7·4%)	25,853·8 (22,754·9 to 29,004·3)	5·6% (4·7 to 6·5%)	-14·0% (-18·5 to -9·2%)	-13·3% (-17·4 to -9·3%)
**Ambient particulate matter pollution: All causes**	932·1 (811·4 to 1,052·6)	8·4% (7·3 to 9·4%)	986·5 (846·8 to 1,126·7)	8·4% (7·2 to 9·5%)	5·8% (1·5 to 10·7%)	0·2% (-2·5 to 3·3%)	20,725·0 (18,336·3 to 23,186·9)	4·4% (3·8 to 5·1%)	20,166·3 (17,780·1 to 22,643·4)	4·3% (3·7 to 5·0%)	-2·7% (-6·8 to 1·8%)	-1·9% (-5·3 to 1·6%)
Lower respiratory infections	93·2 (63·5 to 130·2)	22·5% (15·5 to 31·2%)	97·3 (64·9 to 139·0)	21·6% (14·7 to 30·4%)	4·4% (-1·3 to 9·9%)	-3·9% (-6·9 to -1·9%)	3,190·5 (2,254·8 to 4,323·2)	23·7% (16·9 to 32·2%)	2,395·5 (1,657·7 to 3,331·8)	22·9% (16·0 to 31·5%)	-24·9% (-29·9 to -20·7%)	-3·5% (-6·3 to -1·5%)
Tracheal, bronchus, and lung cancer	77·7 (49·1 to 107·6)	18·5% (11·8 to 25·7%)	96·1 (60·5 to 132·1)	19·4% (12·5 to 26·7%)	23·6% (15·1 to 34·2%)	4·7% (2·4 to 7·5%)	1,794·6 (1,132·5 to 2,486·9)	18·5% (11·7 to 25·7%)	2,116·9 (1,341·0 to 2,905·7)	19·5% (12·5 to 26·7%)	18·0% (9·4 to 28·7%)	5·2% (2·8 to 8·2%)
Ischaemic heart disease	351·7 (287·7 to 420·8)	16·6% (13·6 to 19·8%)	406·2 (322·5 to 488·9)	16·8% (13·4 to 20·2%)	15·5% (9·5 to 21·3%)	0·7% (-3·5 to 4·8%)	7,554·8 (6,391·0 to 8,737·8)	18·9% (16·0 to 21·8%)	8,148·0 (6,824·9 to 9,459·6)	19·2% (16·1 to 22·2%)	7·9% (2·4 to 13·0%)	1·4% (-1·6 to 4·2%)
Ischaemic stroke	96·3 (71·8 to 119·9)	12·9% (9·8 to 16·1%)	103·1 (75·8 to 131·8)	13·1% (9·7 to 16·5%)	7·0% (-0·7 to 14·5%)	1·3% (-4·5 to 6·5%)	1,706·3 (1,362·4 to 2,032·8)	14·4% (11·7 to 17·1%)	1,721·6 (1,378·1 to 2,064·2)	14·7% (11·9 to 17·5%)	0·9% (-5·4 to 7·8%)	2·4% (-1·6 to 6·6%)
Hemorrhagic stroke	132·5 (108·2 to 157·4)	15·2% (12·5 to 18·0%)	125·2 (101·1 to 151·2)	15·1% (12·2 to 17·9%)	-5·6% (-11·6 to 1·3%)	-0·7% (-4·9 to 3·3%)	3,168·0 (2,655·6 to 3,705·6)	16·9% (14·2 to 19·6%)	2,862·1 (2,366·6 to 3,380·4)	17·1% (14·3 to 19·9%)	-9·7% (-16·1 to -3·5%)	1·4% (-1·9 to 4·7%)
Chronic obstructive pulmonary disease	180·6 (109·5 to 256·3)	26·1% (15·8 to 37·2%)	158·7 (95·1 to 228·0)	25·4% (15·2 to 36·1%)	-12·1% (-17·8 to -6·8%)	-2·9% (-4·9 to -1·5%)	3,310·8 (2,020·3 to 4,688·1)	25·0% (15·1 to 35·6%)	2,922·1 (1,743·5 to 4,142·7)	25·1% (15·1 to 35·7%)	-11·7% (-16·2 to -7·3%)	0·2% (-1·1 to 1·5%)
**Household air pollution from solid fuels: All causes**	513·5 (367·4 to 676·4)	4·6% (3·3 to 6·0%)	325·8 (227·6 to 445·8)	2·8% (1·9 to 3·8%)	-36·6% (-43·7 to -29·1%)	-39·9% (-46·5 to -33·0%)	11,835·1 (8,565·7 to 15,491·6)	2·5% (1·8 to 3·3%)	7,010·0 (4,960·6 to 9,540·2)	1·5% (1·1 to 2·1%)	-40·8% (-47·5 to -33·7%)	-40·3% (-47·2 to -33·1%)
Lower respiratory infections	56·3 (35·0 to 82·5)	13·6% (8·5 to 20·0%)	37·8 (21·9 to 57·7)	8·4% (5·0 to 12·9%)	-32·8% (-42·4 to -23·8%)	-38·2% (-46·6 to -30·0%)	1,962·6 (1,235·2 to 2,846·4)	14·6% (9·3 to 21·2%)	952·8 (570·9 to 1,435·3)	9·1% (5·6 to 13·8%)	-51·4% (-58·3 to -44·5%)	-37·6% (-46·4 to -28·8%)
Tracheal, bronchus, and lung cancer	52·7 (26·3 to 86·6)	12·6% (6·2 to 20·5%)	38·2 (17·7 to 66·8)	7·7% (3·6 to 13·4%)	-27·6% (-37·4 to -16·4%)	-38·6% (-46·0 to -31·2%)	1,230·1 (613·7 to 2,019·3)	12·7% (6·3 to 20·6%)	847·3 (392·9 to 1,487·1)	7·8% (3·7 to 13·5%)	-31·1% (-40·6 to -20·4%)	-38·6% (-45·9 to -31·3%)
Ischaemic heart disease	152·6 (116·8 to 196·8)	7·2% (5·5 to 9·2%)	111·6 (82·0 to 148·8)	4·6% (3·4 to 6·1%)	-26·9% (-36·2 to -17·2%)	-36·3% (-44·1 to -28·5%)	3,421·1 (2,663·9 to 4,291·0)	8·6% (6·7 to 10·8%)	2,339·3 (1,743·4 to 3,066·9)	5·5% (4·1 to 7·2%)	-31·6% (-40·7 to -21·9%)	-35·7% (-43·8 to -27·1%)
Ischaemic stroke	55·1 (38·0 to 76·5)	7·4% (5·2 to 10·3%)	34·7 (22·8 to 49·5)	4·4% (3·0 to 6·2%)	-37·0% (-44·7 to -28·9%)	-40·3% (-47·1 to -33·3%)	1,014·7 (705·3 to 1,360·9)	8·6% (6·0 to 11·5%)	609·0 (406·0 to 854·2)	5·2% (3·5 to 7·3%)	-40·0% (-47·4 to -32·1%)	-39·1% (-46·0 to -32·1%)
Hemorrhagic stroke	80·6 (57·7 to 108·6)	9·3% (6·6 to 12·3%)	44·1 (30·5 to 61·6)	5·3% (3·7 to 7·4%)	-45·2% (-52·1 to -37·8%)	-42·4% (-48·8 to -35·8%)	1,973·5 (1,426·4 to 2,622·8)	10·5% (7·6 to 13·8%)	1,046·9 (708·2 to 1,461·0)	6·3% (4·3 to 8·7%)	-47·0% (-54·0 to -39·3%)	-40·5% (-47·1 to -33·5%)
Chronic obstructive pulmonary disease	116·1 (58·2 to 186·2)	16·8% (8·3 to 26·8%)	59·2 (27·1 to 100·8)	9·5% (4·4 to 16·2%)	-49·0% (-56·4 to -41·7%)	-43·6% (-51·1 to -36·3%)	2,124·8 (1,066·2 to 3,407·3)	16·0% (8·0 to 25·6%)	1,118·2 (509·4 to 1,923·9)	9·6% (4·4 to 16·5%)	-47·4% (-54·9 to -39·4%)	-40·2% (-48·3 to -32·2%)
Cataract	-	-	-	-	-	-	108·4 (73·0 to 150·5)	16·0% (13·5 to 18·4%)	96·4 (63·1 to 135·9)	10·9% (8·8 to 13·2%)	-11·0% (-19·3 to -1·9%)	-31·6% (-38·0 to -24·8%)
**Ambient ozone pollution: All causes**	43·2 (16·2 to 74·7)	0·4% (0·1 to 0·7%)	41·8 (16·0 to 71·8)	0·4% (0·1 to 0·6%)	-3·3% (-8·6 to 2·4%)	-8·4% (-12·4 to -4·0%)	666·9 (248·5 to 1,155·3)	0·1% (0·1 to 0·2%)	610·9 (233·4 to 1,048·2)	0·1% (0·1 to 0·2%)	-8·4% (-13·7 to -2·6%)	-7·7% (-12·5 to -2·3%)
Chronic obstructive pulmonary disease	43·2 (16·2 to 74·7)	6·2% (2·4 to 10·8%)	41·8 (16·0 to 71·8)	6·7% (2·5 to 11·4%)	-3·3% (-8·6 to 2·4%)	6·9% (5·3 to 9·2%)	666·9 (248·5 to 1,155·3)	5·0% (1·9 to 8·6%)	610·9 (233·4 to 1,048·2)	5·2% (2·0 to 8·7%)	-8·4% (-13·7 to -2·6%)	4·0% (1·1 to 7·6%)
**Other environmental risks: All causes**	131·4 (73·4 to 205·6)	1·2% (0·7 to 1·8%)	136·8 (74·4 to 216·8)	1·2% (0·6 to 1·8%)	4·1% (-0·8 to 8·1%)	-1·5% (-5·8 to 2·2%)	2,528·2 (1,369·8 to 3,990·7)	0·5% (0·3 to 0·9%)	2,433·3 (1,294·4 to 3,904·3)	0·5% (0·3 to 0·9%)	-3·8% (-8·3 to 0·0%)	-3·0% (-7·5 to 0·5%)
**Residential radon: All causes**	17·6 (12·2 to 23·7)	0·2% (0·1 to 0·2%)	20·0 (13·6 to 27·0)	0·2% (0·1 to 0·2%)	13·8% (6·2 to 22·2%)	7·8% (0·8 to 15·5%)	411·8 (286·1 to 548·8)	0·1% (0·1 to 0·1%)	449·8 (315·1 to 602·9)	0·1% (0·1 to 0·1%)	9·2% (2·4 to 16·8%)	10·1% (3·5 to 17·4%)
Tracheal, bronchus, and lung cancer	17·6 (12·2 to 23·7)	4·2% (2·9 to 5·6%)	20·0 (13·6 to 27·0)	4·0% (2·8 to 5·5%)	13·8% (6·2 to 22·2%)	-3·6% (-9·6 to 3·3%)	411·8 (286·1 to 548·8)	4·2% (3·0 to 5·7%)	449·8 (315·1 to 602·9)	4·1% (2·9 to 5·5%)	9·2% (2·4 to 16·8%)	-2·6% (-8·7 to 3·2%)
**Lead exposure: All causes**	113·8 (56·1 to 186·4)	1·0% (0·5 to 1·7%)	116·7 (55·8 to 195·2)	1·0% (0·5 to 1·7%)	2·6% (-3·9 to 6·9%)	-2·9% (-8·8 to 1·3%)	2,116·4 (982·1 to 3,569·6)	0·5% (0·2 to 0·8%)	1,983·5 (881·0 to 3,445·9)	0·4% (0·2 to 0·8%)	-6·3% (-13·4 to -1·8%)	-5·6% (-12·5 to -1·3%)
Rheumatic heart disease	0·6 (0·2 to 1·1)	1·0% (0·3 to 1·9%)	0·5 (0·2 to 1·0)	0·9% (0·3 to 2·0%)	-17·9% (-24·9 to -10·2%)	-2·5% (-9·9 to 7·1%)	13·9 (4·2 to 29·9)	0·7% (0·2 to 1·6%)	10·3 (3·0 to 22·4)	0·7% (0·2 to 1·5%)	-26·1% (-32·2 to -21·0%)	-4·6% (-11·5 to 0·4%)
Ischaemic heart disease	57·0 (27·9 to 93·4)	2·7% (1·3 to 4·4%)	59·4 (27·2 to 100·1)	2·5% (1·1 to 4·2%)	4·2% (-4·0 to 9·5%)	-9·2% (-16·0 to -4·6%)	989·9 (452·7 to 1,682·7)	2·5% (1·1 to 4·2%)	948·4 (401·7 to 1,671·3)	2·2% (0·9 to 3·9%)	-4·2% (-12·3 to 1·0%)	-10·0% (-17·2 to -5·8%)
Ischaemic stroke	16·0 (7·4 to 28·0)	2·1% (1·0 to 3·8%)	15·3 (6·6 to 27·5)	1·9% (0·8 to 3·5%)	-4·2% (-12·2 to 3·7%)	-9·3% (-16·3 to -2·0%)	248·0 (108·0 to 438·0)	2·1% (0·9 to 3·7%)	220·6 (90·6 to 401·3)	1·9% (0·8 to 3·4%)	-11·1% (-18·5 to -5·4%)	-9·8% (-17·1 to -4·6%)
Hemorrhagic stroke	20·0 (8·3 to 36·4)	2·3% (1·0 to 4·2%)	17·7 (6·9 to 32·8)	2·1% (0·8 to 3·9%)	-11·1% (-18·4 to -4·5%)	-6·6% (-14·0 to 0·2%)	392·7 (148·4 to 755·2)	2·1% (0·8 to 4·0%)	323·9 (113·7 to 630·4)	1·9% (0·7 to 3·8%)	-17·5% (-24·2 to -12·7%)	-7·5% (-14·8 to -3·2%)
Hypertensive heart disease	10·3 (2·9 to 24·2)	5·3% (1·5 to 12·6%)	12·2 (3·2 to 29·9)	4·9% (1·3 to 12·0%)	18·0% (4·0 to 29·5%)	-7·6% (-17·3 to 0·6%)	164·9 (54·5 to 369·5)	4·8% (1·5 to 10·9%)	176·4 (54·3 to 409·6)	4·4% (1·4 to 10·1%)	7·0% (-3·5 to 15·0%)	-8·0% (-15·7 to -2·1%)
Cardiomyopathy and myocarditis	1·2 (0·6 to 2·0)	1·6% (0·8 to 2·8%)	1·2 (0·5 to 2·1)	1·5% (0·7 to 2·7%)	1·2% (-7·1 to 10·0%)	-7·0% (-14·2 to -0·3%)	23·1 (9·6 to 43·1)	1·2% (0·5 to 2·2%)	20·8 (8·1 to 39·6)	1·1% (0·4 to 2·1%)	-10·1% (-17·8 to -3·9%)	-6·2% (-14·1 to -1·4%)

Supplemental Results Table 1. All-age deaths, death PAFs, DALYs, DALY PAFs, and percent change for each risk factor and outcome for both sexes combined in 2005 and 2015 for High-middle SDI.

Data in parenthesis are 95% uncertainty intervals. PAFs=population attributable fractions. DALYs=disability-adjusted life-years.

	2005 Deaths (in thousands)	2005 Deaths PAF	2015 Deaths (in thousands)	2015 Deaths PAF	Percent change deaths 2005-2015	Percent change deaths PAF 2005-2015	2005 DALYs (in thousands)	2005 DALYs PAF	2015 DALYs (in thousands)	2015 DALYs PAF	Percent change DALYs 2005-2015	Percent change DALYs PAF 2005-2015
Atrial fibrillation and flutter	0·6 (0·3 to 1·0)	1·9% (1·0 to 2·9%)	0·8 (0·4 to 1·3)	1·7% (0·8 to 2·7%)	24·8% (14·0 to 33·0%)	-11·1% (-18·2 to -6·2%)	15·5 (7·5 to 26·6)	1·9% (1·0 to 3·1%)	17·4 (7·7 to 30·9)	1·7% (0·8 to 2·8%)	12·3% (2·0 to 18·3%)	-14·0% (-21·6 to -9·8%)
Aortic aneurysm	0·5 (0·3 to 0·9)	1·8% (0·9 to 3·0%)	0·6 (0·3 to 1·0)	1·6% (0·7 to 2·7%)	9·1% (-2·4 to 17·3%)	-14·3% (-22·4 to -9·4%)	10·0 (4·5 to 17·1)	1·7% (0·7 to 2·9%)	10·0 (4·1 to 18·2)	1·4% (0·6 to 2·6%)	0·3% (-12·5 to 8·4%)	-15·5% (-25·0 to -10·4%)
Peripheral vascular disease	0·1 (0·0 to 0·2)	1·5% (0·7 to 2·5%)	0·1 (0·1 to 0·2)	1·3% (0·6 to 2·2%)	27·9% (10·3 to 44·6%)	-14·3% (-23·6 to -7·4%)	2·5 (1·1 to 4·5)	1·3% (0·6 to 2·2%)	2·9 (1·2 to 5·5)	1·1% (0·5 to 1·9%)	17·7% (4·0 to 27·5%)	-16·1% (-25·5 to -10·7%)
Endocarditis	0·2 (0·1 to 0·4)	1·5% (0·7 to 2·6%)	0·3 (0·1 to 0·5)	1·4% (0·6 to 2·5%)	9·7% (-0·6 to 19·0%)	-9·0% (-17·0 to -2·4%)	4·5 (1·8 to 8·7)	1·1% (0·4 to 2·0%)	4·4 (1·6 to 8·7)	1·0% (0·4 to 1·9%)	-3·2% (-12·4 to 3·8%)	-6·5% (-15·2 to -0·5%)
Other cardiovascular and circulatory diseases	2·2 (1·2 to 3·4)	2·0% (1·1 to 3·2%)	2·3 (1·2 to 3·7)	1·8% (1·0 to 2·9%)	5·2% (-4·4 to 13·5%)	-8·3% (-15·3 to -3·0%)	63·4 (30·3 to 108·7)	1·6% (0·8 to 2·6%)	64·1 (28·0 to 114·5)	1·5% (0·6 to 2·5%)	1·2% (-9·5 to 8·1%)	-7·5% (-16·3 to -2·3%)
Idiopathic developmental intellectual disability	-	-	-	-	-	-	78·8 (37·1 to 134·4)	10·7% (7·8 to 13·2%)	61·6 (27·8 to 105·9)	7·7% (5·1 to 10·2%)	-21·9% (-29·3 to -15·9%)	-27·7% (-34·6 to -22·3%)
Chronic kidney disease due to diabetes mellitus	1·9 (0·9 to 3·5)	2·3% (1·1 to 4·1%)	2·4 (1·1 to 4·4)	2·1% (0·9 to 3·9%)	24·2% (14·5 to 30·5%)	-8·0% (-14·8 to -4·7%)	40·4 (16·2 to 78·1)	1·7% (0·7 to 3·2%)	46·3 (17·1 to 91·7)	1·5% (0·6 to 3·0%)	14·6% (3·5 to 20·5%)	-8·8% (-17·0 to -5·0%)
Chronic kidney disease due to hypertension	2·2 (1·0 to 3·7)	2·5% (1·2 to 4·2%)	2·9 (1·3 to 5·0)	2·4% (1·1 to 4·1%)	30·0% (21·8 to 36·6%)	-4·5% (-9·8 to -1·5%)	38·6 (16·3 to 69·4)	1·8% (0·7 to 3·2%)	44·9 (17·7 to 83·2)	1·7% (0·7 to 3·1%)	16·2% (7·6 to 21·7%)	-3·9% (-9·9 to -0·7%)
Chronic kidney disease due to glomerulonephritis	0·8 (0·3 to 1·5)	1·6% (0·7 to 2·9%)	0·9 (0·4 to 1·6)	1·7% (0·7 to 3·2%)	13·3% (7·9 to 20·4%)	8·4% (4·4 to 14·8%)	20·4 (6·9 to 40·6)	1·1% (0·4 to 2·2%)	20·5 (7·1 to 41·4)	1·2% (0·4 to 2·3%)	0·6% (-2·9 to 4·6%)	4·7% (1·4 to 8·6%)
Chronic kidney disease due to other causes	0·1 (0·1 to 0·2)	2·5% (1·3 to 4·1%)	0·2 (0·1 to 0·3)	2·6% (1·3 to 4·4%)	38·1% (21·7 to 54·6%)	7·1% (-3·4 to 16·4%)	9·7 (4·1 to 19·1)	1·5% (0·6 to 2·8%)	11·1 (4·5 to 22·6)	1·5% (0·6 to 2·8%)	14·3% (5·5 to 19·7%)	-2·0% (-9·5 to 2·4%)
**Occupational risks: All causes**	264·1 (248·9 to 280·4)	2·4% (2·2 to 2·5%)	288·5 (271·2 to 308·9)	2·4% (2·3 to 2·6%)	9·3% (3·9 to 15·1%)	3·5% (-0·2 to 7·5%)	14,646·7 (12,442·8 to 17,150·3)	3·1% (2·9 to 3·4%)	16,285·7 (13,794·2 to 19,272·4)	3·5% (3·3 to 3·8%)	11·2% (8·1 to 14·1%)	12·0% (9·4 to 15·0%)
**Occupational carcinogens: All causes**	141·1 (133·5 to 148·5)	1·3% (1·2 to 1·3%)	164·2 (153·3 to 176·2)	1·4% (1·3 to 1·5%)	16·3% (8·7 to 25·1%)	10·2% (4·5 to 16·8%)	3,096·8 (2,938·5 to 3,261·0)	0·7% (0·6 to 0·7%)	3,516·9 (3,282·6 to 3,776·7)	0·8% (0·7 to 0·9%)	13·6% (6·0 to 22·4%)	14·4% (7·5 to 22·7%)
**Occupational exposure to asbestos: All causes**	27·8 (23·3 to 33·0)	0·2% (0·2 to 0·3%)	38·1 (31·3 to 45·7)	0·3% (0·3 to 0·4%)	37·1% (25·6 to 49·8%)	29·8% (20·6 to 40·6%)	510·8 (424·1 to 609·7)	0·1% (0·1 to 0·1%)	674·6 (551·2 to 820·3)	0·1% (0·1 to 0·2%)	32·1% (19·4 to 46·3%)	33·1% (21·2 to 46·2%)
Larynx cancer	0·3 (0·2 to 0·5)	1·2% (0·7 to 1·8%)	0·4 (0·3 to 0·6)	1·4% (0·9 to 2·1%)	30·3% (17·6 to 46·2%)	16·5% (6·7 to 28·5%)	6·1 (3·5 to 9·1)	0·9% (0·5 to 1·4%)	7·7 (4·6 to 11·4)	1·1% (0·6 to 1·6%)	26·2% (13·8 to 41·6%)	16·9% (7·1 to 28·3%)
Tracheal, bronchus, and lung cancer	24·2 (19·7 to 29·5)	5·8% (4·7 to 7·0%)	32·8 (26·1 to 40·1)	6·6% (5·3 to 8·0%)	35·7% (23·7 to 49·2%)	14·9% (7·2 to 22·9%)	432·2 (349·6 to 533·8)	4·5% (3·6 to 5·5%)	563·4 (444·2 to 703·5)	5·2% (4·1 to 6·3%)	30·4% (17·2 to 45·3%)	16·3% (7·6 to 26·1%)
Ovarian cancer	0·3 (0·2 to 0·5)	1·2% (0·6 to 1·8%)	0·5 (0·2 to 0·7)	1·3% (0·6 to 2·0%)	31·6% (15·8 to 50·0%)	8·7% (-1·8 to 22·1%)	6·3 (3·0 to 10·1)	0·8% (0·4 to 1·2%)	7·9 (3·8 to 12·8)	0·8% (0·4 to 1·3%)	26·1% (9·3 to 46·9%)	8·2% (-3·7 to 23·1%)
Mesothelioma	3·0 (2·2 to 3·6)	59·7% (45·0 to 72·0%)	4·4 (3·4 to 5·4)	63·5% (49·3 to 75·7%)	50·4% (40·1 to 62·0%)	6·4% (3·9 to 9·9%)	66·3 (47·3 to 83·9)	49·5% (35·5 to 62·4%)	95·6 (70·3 to 120·1)	53·9% (39·7 to 66·8%)	44·2% (32·9 to 57·9%)	8·8% (5·5 to 13·6%)
**Occupational exposure to arsenic: All causes**	4·2 (3·2 to 5·3)	0·0% (0·0 to 0·0%)	3·9 (2·9 to 5·0)	0·0% (0·0 to 0·0%)	-6·7% (-14·2 to 2·0%)	-11·6% (-17·7 to -4·5%)	92·0 (71·5 to 113·4)	0·0% (0·0 to 0·0%)	85·0 (65·2 to 107·9)	0·0% (0·0 to 0·0%)	-7·7% (-15·2 to 1·4%)	-6·9% (-14·2 to 1·4%)
Tracheal, bronchus, and lung cancer	4·2 (3·2 to 5·3)	1·0% (0·8 to 1·3%)	3·9 (2·9 to 5·0)	0·8% (0·6 to 1·0%)	-6·7% (-14·2 to 2·0%)	-21·0% (-24·3 to -17·4%)	92·0 (71·5 to 113·4)	0·9% (0·7 to 1·2%)	85·0 (65·2 to 107·9)	0·8% (0·6 to 1·0%)	-7·7% (-15·2 to 1·4%)	-17·7% (-21·1 to -14·3%)
**Occupational exposure to benzene: All causes**	1·6 (1·3 to 1·9)	0·0% (0·0 to 0·0%)	1·9 (1·6 to 2·3)	0·0% (0·0 to 0·0%)	21·1% (14·3 to 27·9%)	14·7% (9·8 to 19·2%)	59·3 (50·3 to 69·7)	0·0% (0·0 to 0·0%)	70·7 (59·5 to 82·8)	0·0% (0·0 to 0·0%)	19·1% (11·8 to 26·4%)	20·1% (13·5 to 26·3%)
Leukaemia	1·6 (1·3 to 1·9)	2·0% (1·7 to 2·3%)	1·9 (1·6 to 2·3)	2·1% (1·8 to 2·5%)	21·1% (14·3 to 27·9%)	7·4% (4·0 to 11·2%)	59·3 (50·3 to 69·7)	1·9% (1·6 to 2·2%)	70·7 (59·5 to 82·8)	2·2% (1·9 to 2·6%)	19·1% (11·8 to 26·4%)	19·4% (15·3 to 23·7%)
**Occupational exposure to beryllium: All causes**	0·3 (0·2 to 0·4)	0·0% (0·0 to 0·0%)	0·3 (0·2 to 0·3)	0·0% (0·0 to 0·0%)	-8·6% (-16·1 to 0·4%)	-13·4% (-19·4 to -5·9%)	6·5 (5·2 to 7·9)	0·0% (0·0 to 0·0%)	5·9 (4·6 to 7·2)	0·0% (0·0 to 0·0%)	-9·6% (-17·2 to -0·3%)	-8·9% (-16·1 to -0·1%)
Tracheal, bronchus, and lung cancer	0·3 (0·2 to 0·4)	0·1% (0·1 to 0·1%)	0·3 (0·2 to 0·3)	0·1% (0·0 to 0·1%)	-8·6% (-16·1 to 0·4%)	-22·6% (-26·0 to -19·0%)	6·5 (5·2 to 7·9)	0·1% (0·1 to 0·1%)	5·9 (4·6 to 7·2)	0·1% (0·0 to 0·1%)	-9·6% (-17·2 to -0·3%)	-19·4% (-22·8 to -16·0%)
**Occupational exposure to cadmium: All causes**	1·0 (0·8 to 1·2)	0·0% (0·0 to 0·0%)	1·0 (0·8 to 1·2)	0·0% (0·0 to 0·0%)	0·1% (-8·0 to 9·3%)	-5·2% (-11·5 to 2·4%)	21·6 (17·8 to 25·6)	0·0% (0·0 to 0·0%)	21·4 (17·3 to 25·7)	0·0% (0·0 to 0·0%)	-1·0% (-9·0 to 8·4%)	-0·2% (-7·6 to 8·6%)
Tracheal, bronchus, and lung cancer	1·0 (0·8 to 1·2)	0·2% (0·2 to 0·3%)	1·0 (0·8 to 1·2)	0·2% (0·2 to 0·2%)	0·1% (-8·0 to 9·3%)	-15·3% (-18·7 to -11·7%)	21·6 (17·8 to 25·6)	0·2% (0·2 to 0·3%)	21·4 (17·3 to 25·7)	0·2% (0·2 to 0·2%)	-1·0% (-9·0 to 8·4%)	-11·7% (-15·1 to -8·3%)
**Occupational exposure to chromium: All causes**	3·4 (3·0 to 3·9)	0·0% (0·0 to 0·0%)	3·3 (2·8 to 3·8)	0·0% (0·0 to 0·0%)	-4·4% (-12·3 to 4·5%)	-9·4% (-15·6 to -2·0%)	76·0 (66·8 to 86·0)	0·0% (0·0 to 0·0%)	71·9 (62·1 to 82·1)	0·0% (0·0 to 0·0%)	-5·4% (-13·2 to 3·8%)	-4·6% (-11·8 to 4·1%)
Tracheal, bronchus, and lung cancer	3·4 (3·0 to 3·9)	0·8% (0·7 to 0·9%)	3·3 (2·8 to 3·8)	0·7% (0·6 to 0·8%)	-4·4% (-12·3 to 4·5%)	-19·0% (-22·3 to -15·5%)	76·0 (66·8 to 86·0)	0·8% (0·7 to 0·9%)	71·9 (62·1 to 82·1)	0·7% (0·6 to 0·7%)	-5·4% (-13·2 to 3·8%)	-15·6% (-18·8 to -12·4%)
**Occupational exposure to diesel engine exhaust: All causes**	41·2 (36·4 to 46·2)	0·4% (0·3 to 0·4%)	49·4 (43·5 to 55·7)	0·4% (0·4 to 0·5%)	20·0% (11·4 to 29·8%)	13·6% (7·1 to 21·9%)	911·9 (809·3 to 1,021·7)	0·2% (0·2 to 0·2%)	1,081·2 (948·7 to 1,219·6)	0·2% (0·2 to 0·3%)	18·6% (9·7 to 28·6%)	19·5% (11·2 to 28·9%)
Tracheal, bronchus, and lung cancer	41·2 (36·4 to 46·2)	9·8% (8·7 to 10·9%)	49·4 (43·5 to 55·7)	10·0% (8·9 to 11·1%)	20·0% (11·4 to 29·8%)	1·6% (-2·2 to 5·4%)	911·9 (809·3 to 1,021·7)	9·4% (8·4 to 10·5%)	1,081·2 (948·7 to 1,219·6)	9·9% (8·9 to 11·1%)	18·6% (9·7 to 28·6%)	5·7% (2·1 to 9·4%)
**Occupational exposure to second-hand smoke: All causes**	36·9 (34·5 to 39·2)	0·3% (0·3 to 0·4%)	38·2 (35·2 to 41·2)	0·3% (0·3 to 0·3%)	3·5% (-3·2 to 11·5%)	-1·9% (-6·8 to 4·1%)	808·2 (755·0 to 860·5)	0·2% (0·2 to 0·2%)	826·1 (763·0 to 892·7)	0·2% (0·2 to 0·2%)	2·2% (-4·6 to 10·3%)	3·0% (-3·1 to 10·2%)
Tracheal, bronchus, and lung cancer	36·9 (34·5 to 39·2)	8·8% (8·3 to 9·3%)	38·2 (35·2 to 41·2)	7·7% (7·2 to 8·2%)	3·5% (-3·2 to 11·5%)	-12·3% (-14·7 to -9·5%)	808·2 (755·0 to 860·5)	8·3% (7·9 to 8·7%)	826·1 (763·0 to 892·7)	7·6% (7·1 to 8·0%)	2·2% (-4·6 to 10·3%)	-8·8% (-11·3 to -6·2%)
**Occupational exposure to formaldehyde: All causes**	0·8 (0·6 to 1·0)	0·0% (0·0 to 0·0%)	0·8 (0·6 to 1·0)	0·0% (0·0 to 0·0%)	-9·0% (-19·1 to 1·5%)	-13·8% (-22·2 to -4·9%)	30·6 (23·5 to 38·0)	0·0% (0·0 to 0·0%)	27·0 (21·1 to 33·7)	0·0% (0·0 to 0·0%)	-11·9% (-21·8 to -1·7%)	-11·2% (-20·4 to -1·5%)
Nasopharynx cancer	0·5 (0·3 to 0·7)	2·7% (1·8 to 3·8%)	0·4 (0·2 to 0·6)	2·1% (1·4 to 3·0%)	-18·2% (-32·8 to -2·5%)	-22·2% (-30·1 to -14·1%)	16·5 (9·8 to 23·7)	2·9% (2·0 to 4·1%)	12·8 (7·8 to 18·7)	2·4% (1·6 to 3·4%)	-22·3% (-37·1 to -6·6%)	-19·3% (-27·2 to -10·8%)
**Occupational exposure to nickel: All causes**	16·7 (12·3 to 21·5)	0·1% (0·1 to 0·2%)	15·8 (11·5 to 20·8)	0·1% (0·1 to 0·2%)	-5·2% (-13·2 to 4·4%)	-10·2% (-16·6 to -2·4%)	369·0 (276·4 to 471·1)	0·1% (0·1 to 0·1%)	346·1 (250·6 to 449·7)	0·1% (0·1 to 0·1%)	-6·2% (-14·3 to 3·7%)	-5·5% (-12·9 to 3·9%)
Tracheal, bronchus, and lung cancer	16·7 (12·3 to 21·5)	4·0% (2·9 to 5·2%)	15·8 (11·5 to 20·8)	3·2% (2·3 to 4·2%)	-5·2% (-13·2 to 4·4%)	-19·7% (-23·4 to -15·6%)	369·0 (276·4 to 471·1)	3·8% (2·9 to 4·9%)	346·1 (250·6 to 449·7)	3·2% (2·3 to 4·1%)	-6·2% (-14·3 to 3·7%)	-16·4% (-20·2 to -12·2%)
**Occupational exposure to polycyclic aromatic hydrocarbons: All causes**	7·5 (6·1 to 8·8)	0·1% (0·1 to 0·1%)	7·9 (6·5 to 9·5)	0·1% (0·1 to 0·1%)	6·0% (-2·1 to 15·3%)	0·4% (-6·1 to 8·1%)	164·6 (136·7 to 193·6)	0·0% (0·0 to 0·0%)	172·8 (142·8 to 205·2)	0·0% (0·0 to 0·0%)	4·9% (-3·0 to 14·7%)	5·7% (-1·6 to 14·6%)
Tracheal, bronchus, and lung cancer	7·5 (6·1 to 8·8)	1·8% (1·5 to 2·1%)	7·9 (6·5 to 9·5)	1·6% (1·3 to 1·9%)	6·0% (-2·1 to 15·3%)	-10·2% (-13·6 to -6·7%)	164·6 (136·7 to 193·6)	1·7% (1·4 to 2·0%)	172·8 (142·8 to 205·2)	1·6% (1·3 to 1·9%)	4·9% (-3·0 to 14·7%)	-6·4% (-9·7 to -3·1%)
**Occupational exposure to silica: All causes**	31·5 (28·5 to 34·4)	0·3% (0·3 to 0·3%)	39·4 (35·5 to 43·5)	0·3% (0·3 to 0·4%)	25·1% (15·1 to 36·8%)	18·5% (10·3 to 27·6%)	690·5 (627·3 to 752·5)	0·1% (0·1 to 0·2%)	846·5 (762·7 to 935·0)	0·2% (0·2 to 0·2%)	22·6% (12·6 to 34·3%)	23·5% (14·0 to 34·4%)
Tracheal, bronchus, and lung cancer	31·5 (28·5 to 34·4)	7·5% (6·9 to 8·1%)	39·4 (35·5 to 43·5)	7·9% (7·3 to 8·6%)	25·1% (15·1 to 36·8%)	5·9% (1·1 to 10·9%)	690·5 (627·3 to 752·5)	7·1% (6·5 to 7·7%)	846·5 (762·7 to 935·0)	7·8% (7·2 to 8·4%)	22·6% (12·6 to 34·3%)	9·3% (4·5 to 13·9%)
**Occupational exposure to sulfphuric acid: All causes**	2·7 (2·1 to 3·4)	0·0% (0·0 to 0·0%)	2·8 (2·1 to 3·6)	0·0% (0·0 to 0·0%)	4·2% (-1·9 to 10·4%)	-1·3% (-5·9 to 3·7%)	65·6 (50·7 to 82·2)	0·0% (0·0 to 0·0%)	68·3 (51·9 to 85·8)	0·0% (0·0 to 0·0%)	4·0% (-2·0 to 10·3%)	4·9% (-0·5 to 10·8%)
Larynx cancer	2·7 (2·1 to 3·4)	10·2% (7·8 to 12·8%)	2·8 (2·1 to 3·6)	9·5% (7·2 to 12·1%)	4·2% (-1·9 to 10·4%)	-6·8% (-9·9 to -3·7%)	65·6 (50·7 to 82·2)	9·8% (7·6 to 12·2%)	68·3 (51·9 to 85·8)	9·5% (7·3 to 11·9%)	4·0% (-2·0 to 10·3%)	-3·6% (-6·8 to -0·3%)
**Occupational exposure to trichloroethylene: All causes**	0·1 (0·0 to 0·1)	0·0% (0·0 to 0·0%)	0·1 (0·0 to 0·1)	0·0% (0·0 to 0·0%)	22·8% (15·1 to 29·9%)	16·3% (10·1 to 21·4%)	1·4 (0·3 to 2·6)	0·0% (0·0 to 0·0%)	1·7 (0·4 to 3·2)	0·0% (0·0 to 0·0%)	23·1% (15·6 to 30·2%)	24·1% (17·1 to 29·9%)
Kidney cancer	0·1 (0·0 to 0·1)	0·3% (0·1 to 0·5%)	0·1 (0·0 to 0·1)	0·2% (0·0 to 0·4%)	22·8% (15·1 to 29·9%)	-11·2% (-14·1 to -8·2%)	1·4 (0·3 to 2·6)	0·2% (0·0 to 0·4%)	1·7 (0·4 to 3·2)	0·2% (0·0 to 0·4%)	23·1% (15·6 to 30·2%)	-5·6% (-8·6 to -2·6%)

Supplemental Results Table 1. All-age deaths, death PAFs, DALYs, DALY PAFs, and percent change for each risk factor and outcome for both sexes combined in 2005 and 2015 for High-middle SDI.

Data in parenthesis are 95% uncertainty intervals. PAFs=population attributable fractions. DALYs=disability-adjusted life-years.

	2005 Deaths (in thousands)	2005 Deaths PAF	2015 Deaths (in thousands)	2015 Deaths PAF	Percent change deaths 2005-2015	Percent change deaths PAF 2005-2015	2005 DALYs (in thousands)	2005 DALYs PAF	2015 DALYs (in thousands)	2015 DALYs PAF	Percent change DALYs 2005-2015	Percent change DALYs PAF 2005-2015
**Occupational asthmagens: All causes**	4·9 (4·2 to 5·6)	0·0% (0·0 to 0·1%)	3·9 (3·4 to 4·6)	0·0% (0·0 to 0·0%)	-19·4% (-26·3 to -11·9%)	-23·7% (-29·7 to -16·6%)	427·1 (326·1 to 538·9)	0·1% (0·1 to 0·1%)	401·4 (298·3 to 514·9)	0·1% (0·1 to 0·1%)	-6·0% (-10·8 to -1·5%)	-5·4% (-9·4 to -1·5%)
Asthma	4·9 (4·2 to 5·6)	8·2% (7·3 to 9·2%)	3·9 (3·4 to 4·6)	7·3% (6·4 to 8·1%)	-19·4% (-26·3 to -11·9%)	-11·7% (-16·2 to -6·5%)	427·1 (326·1 to 538·9)	8·7% (7·7 to 9·8%)	401·4 (298·3 to 514·9)	8·4% (7·6 to 9·4%)	-6·0% (-10·8 to -1·5%)	-3·5% (-6·4 to -0·4%)
**Occupational particulate matter, gases, and fumes: All causes**	70·3 (57·8 to 82·9)	0·6% (0·5 to 0·7%)	65·1 (52·7 to 78·6)	0·6% (0·4 to 0·7%)	-7·4% (-14·1 to -0·8%)	-12·3% (-17·6 to -6·6%)	1,734·8 (1,516·9 to 1,969·4)	0·4% (0·3 to 0·4%)	1,596·0 (1,384·3 to 1,812·6)	0·3% (0·3 to 0·4%)	-8·0% (-12·8 to -3·1%)	-7·3% (-11·4 to -3·0%)
Chronic obstructive pulmonary disease	69·9 (57·4 to 82·5)	10·1% (8·3 to 11·9%)	64·8 (52·4 to 78·2)	10·3% (8·3 to 12·4%)	-7·4% (-14·2 to -0·8%)	2·4% (-2·9 to 7·3%)	1,724·0 (1,505·6 to 1,957·6)	13·0% (11·4 to 14·6%)	1,585·4 (1,374·0 to 1,801·5)	13·6% (11·8 to 15·3%)	-8·0% (-12·8 to -3·1%)	4·4% (1·4 to 7·4%)
**Occupational noise: All causes**	-	-	-	-	-	-	2,206·0 (1,507·8 to 3,070·7)	0·5% (0·3 to 0·6%)	2,757·9 (1,876·7 to 3,846·1)	0·6% (0·4 to 0·8%)	25·0% (21·7 to 28·1%)	25·8% (21·9 to 30·0%)
Age-related and other hearing loss	-	-	-	-	-	-	2,206·0 (1,507·8 to 3,070·7)	27·2% (25·1 to 29·9%)	2,757·9 (1,876·7 to 3,846·1)	26·7% (24·7 to 29·2%)	25·0% (21·7 to 28·1%)	-1·9% (-3·5 to -0·3%)
**Occupational injuries: All causes**	50·0 (47·5 to 53·5)	0·4% (0·4 to 0·5%)	57·0 (53·9 to 61·7)	0·5% (0·5 to 0·5%)	13·9% (5·8 to 24·3%)	7·9% (-0·4 to 18·3%)	3,265·3 (2,981·6 to 3,626·1)	0·7% (0·7 to 0·7%)	3,728·2 (3,349·4 to 4,170·8)	0·8% (0·7 to 0·9%)	14·2% (6·2 to 24·4%)	15·0% (6·6 to 25·4%)
Pedestrian road injuries	9·1 (8·5 to 10·0)	5·3% (4·9 to 5·8%)	11·4 (10·5 to 12·8)	7·2% (6·6 to 8·3%)	24·3% (11·9 to 39·9%)	36·7% (22·1 to 55·0%)	456·6 (423·5 to 496·9)	5·8% (5·4 to 6·4%)	554·9 (512·5 to 624·5)	8·4% (7·7 to 9·7%)	21·5% (9·8 to 36·5%)	45·9% (29·9 to 64·9%)
Cyclist road injuries	1·3 (1·2 to 1·5)	6·0% (5·5 to 6·7%)	1·6 (1·4 to 1·8)	8·3% (7·5 to 9·6%)	20·2% (6·8 to 33·0%)	38·1% (21·5 to 56·0%)	85·5 (75·3 to 97·0)	6·8% (6·2 to 7·5%)	109·7 (94·5 to 127·4)	9·9% (9·0 to 11·2%)	28·4% (14·8 to 42·2%)	46·4% (29·6 to 64·7%)
Motorcyclist road injuries	5·2 (4·5 to 5·7)	7·8% (7·2 to 8·7%)	6·1 (5·5 to 7·0)	10·1% (9·2 to 11·5%)	16·5% (4·4 to 35·5%)	29·7% (16·1 to 45·2%)	308·8 (269·5 to 337·1)	8·1% (7·6 to 9·0%)	361·2 (328·3 to 408·6)	11·0% (10·0 to 12·3%)	17·0% (5·8 to 33·7%)	34·8% (20·9 to 49·4%)
Motor vehicle road injuries	12·8 (11·8 to 14·1)	10·2% (9·5 to 11·0%)	14·3 (13·3 to 15·7)	12·2% (11·3 to 13·5%)	12·2% (4·2 to 21·7%)	20·1% (9·8 to 31·8%)	734·1 (678·7 to 810·2)	10·7% (10·0 to 11·5%)	830·5 (766·2 to 910·4)	13·5% (12·4 to 14·9%)	13·1% (5·3 to 22·5%)	26·0% (15·6 to 37·7%)
Other road injuries	0·4 (0·3 to 0·4)	9·8% (8·3 to 10·7%)	0·4 (0·4 to 0·5)	11·5% (10·1 to 12·8%)	23·7% (9·1 to 43·2%)	16·9% (4·2 to 32·7%)	24·4 (20·2 to 28·2)	10·9% (9·4 to 11·8%)	31·9 (26·8 to 37·7)	13·4% (12·3 to 14·7%)	30·5% (17·7 to 46·4%)	22·6% (11·3 to 37·5%)
Other transport injuries	1·7 (1·5 to 1·9)	6·7% (6·1 to 7·6%)	2·1 (1·9 to 2·6)	8·3% (7·3 to 10·0%)	27·4% (10·1 to 50·0%)	23·7% (7·3 to 45·9%)	89·5 (81·0 to 102·8)	7·3% (6·6 to 8·2%)	110·0 (98·0 to 132·2)	9·4% (8·4 to 11·2%)	22·8% (6·8 to 44·0%)	29·1% (12·5 to 51·1%)
Falls	4·4 (4·1 to 5·0)	5·4% (5·1 to 6·0%)	6·0 (5·5 to 6·6)	6·0% (5·4 to 6·8%)	36·0% (21·5 to 49·7%)	10·0% (0·1 to 21·2%)	483·8 (393·3 to 595·6)	10·0% (9·3 to 10·7%)	630·1 (500·4 to 787·7)	12·1% (11·1 to 13·4%)	30·2% (19·4 to 42·7%)	21·7% (11·8 to 33·5%)
Drowning	4·6 (4·3 to 5·1)	6·3% (5·8 to 6·9%)	4·4 (4·0 to 5·0)	8·2% (7·5 to 9·2%)	-4·2% (-13·7 to 7·1%)	30·6% (16·7 to 48·0%)	242·4 (224·1 to 267·1)	6·1% (5·6 to 6·6%)	224·2 (205·1 to 255·8)	8·6% (7·8 to 9·5%)	-7·5% (-16·5 to 3·4%)	41·2% (26·0 to 59·4%)
Fire, heat, and hot substances	3·0 (2·2 to 3·6)	7·2% (6·3 to 8·3%)	3·1 (2·2 to 4·1)	8·8% (7·5 to 11·0%)	4·7% (-12·9 to 31·5%)	23·1% (3·3 to 55·2%)	208·3 (166·5 to 252·4)	8·6% (7·6 to 10·0%)	219·9 (169·0 to 277·8)	11·5% (9·7 to 14·2%)	5·6% (-11·0 to 31·3%)	34·0% (13·0 to 66·9%)
Poisonings	1·1 (0·9 to 1·2)	4·5% (4·0 to 5·1%)	0·7 (0·6 to 0·9)	4·1% (3·6 to 4·7%)	-32·7% (-42·1 to -21·1%)	-7·5% (-21·5 to 7·5%)	60·9 (51·9 to 69·9)	5·0% (4·5 to 5·6%)	42·5 (36·1 to 49·6)	5·0% (4·4 to 5·8%)	-30·1% (-39·6 to -19·1%)	-0·3% (-15·3 to 15·8%)
Unintentional firearm injuries	0·8 (0·5 to 0·9)	8·0% (6·9 to 9·2%)	0·6 (0·4 to 0·7)	7·1% (6·0 to 8·5%)	-25·7% (-38·7 to -7·3%)	-11·2% (-26·1 to 10·1%)	42·4 (27·7 to 50·1)	8·5% (7·4 to 9·7%)	31·5 (22·4 to 38·5)	8·1% (6·8 to 9·7%)	-25·7% (-38·9 to -7·3%)	-4·5% (-21·1 to 18·6%)
Unintentional suffocation	0·0 (0·0 to 0·0)	0·5% (0·5 to 0·6%)	0·0 (0·0 to 0·1)	0·9% (0·7 to 1·0%)	52·6% (3·4 to 85·3%)	62·2% (32·7 to 97·6%)	3·3 (2·7 to 4·1)	0·7% (0·6 to 0·8%)	4·3 (3·3 to 5·3)	1·0% (0·8 to 1·2%)	28·2% (5·7 to 50·3%)	51·3% (25·2 to 82·7%)
Other exposure to mechanical forces	2·1 (1·7 to 2·3)	8·0% (7·4 to 8·8%)	2·5 (1·8 to 2·8)	10·3% (9·3 to 11·8%)	18·4% (-3·0 to 34·7%)	28·9% (14·3 to 42·7%)	180·6 (149·9 to 215·8)	9·6% (8·9 to 10·5%)	213·0 (165·4 to 260·9)	12·7% (11·5 to 14·3%)	17·9% (4·3 to 31·6%)	31·7% (18·1 to 45·5%)
Venomous animal contact	0·1 (0·1 to 0·2)	3·3% (2·6 to 4·2%)	0·1 (0·1 to 0·2)	3·5% (2·7 to 4·5%)	-8·3% (-31·9 to 28·6%)	6·9% (-18·0 to 41·6%)	11·3 (8·8 to 15·2)	3·8% (3·1 to 4·9%)	11·0 (8·1 to 15·3)	4·4% (3·4 to 5·7%)	-2·7% (-26·3 to 29·1%)	15·0% (-11·0 to 49·5%)
Non-venomous animal contact	0·0 (0·0 to 0·0)	1·8% (1·6 to 2·0%)	0·0 (0·0 to 0·0)	1·6% (1·3 to 1·9%)	-19·7% (-33·8 to -0·9%)	-10·2% (-25·7 to 7·3%)	2·9 (2·3 to 3·8)	2·5% (2·2 to 2·9%)	2·5 (1·9 to 3·3)	2·5% (2·1 to 3·1%)	-14·1% (-28·7 to 2·9%)	2·0% (-14·2 to 20·2%)
Pulmonary aspiration and foreign body in airway	0·9 (0·8 to 1·0)	3·6% (3·3 to 4·3%)	1·1 (1·0 to 1·3)	4·5% (4·0 to 5·3%)	28·0% (11·8 to 47·9%)	24·0% (7·7 to 42·8%)	52·6 (46·0 to 61·7)	3·9% (3·4 to 4·5%)	62·4 (53·9 to 72·5)	5·2% (4·6 to 6·1%)	18·7% (4·6 to 36·0%)	35·6% (17·9 to 56·3%)
Foreign body in eyes	-	-	-	-	-	-	0·9 (0·4 to 1·5)	7·9% (7·0 to 8·8%)	1·1 (0·5 to 2·0)	9·2% (8·0 to 10·5%)	30·0% (17·2 to 45·0%)	16·5% (4·6 to 29·2%)
Foreign body in other body part	0·4 (0·3 to 0·4)	8·5% (7·5 to 9·9%)	0·3 (0·3 to 0·4)	8·3% (6·8 to 10·2%)	-6·2% (-24·8 to 15·1%)	-2·9% (-22·0 to 17·6%)	26·9 (22·3 to 32·8)	10·5% (9·3 to 12·0%)	25·9 (20·7 to 32·6)	11·8% (10·0 to 14·1%)	-3·8% (-21·4 to 13·9%)	13·1% (-7·0 to 33·1%)
Other unintentional injuries	2·2 (2·1 to 2·5)	8·9% (8·2 to 9·7%)	2·2 (1·9 to 2·6)	10·6% (9·6 to 12·0%)	-3·8% (-14·5 to 9·7%)	19·4% (6·5 to 35·6%)	250·0 (203·0 to 308·8)	10·1% (9·4 to 10·8%)	261·6 (207·2 to 335·8)	12·5% (11·5 to 14·0%)	4·6% (-4·7 to 16·5%)	24·4% (13·4 to 37·6%)
**Occupational ergonomic factors: All causes**	-	-	-	-	-	-	4,113·5 (2,863·1 to 5,750·0)	0·9% (0·7 to 1·1%)	4,448·6 (3,095·2 to 6,188·2)	1·0% (0·7 to 1·2%)	8·1% (6·2 to 10·2%)	8·9% (5·6 to 12·3%)
Low back pain	-	-	-	-	-	-	4,113·5 (2,863·1 to 5,750·0)	32·4% (30·6 to 34·2%)	4,448·6 (3,095·2 to 6,188·2)	29·7% (28·0 to 31·3%)	8·1% (6·2 to 10·2%)	-8·3% (-9·5 to -7·0%)
**Behavioural risks: All causes**	5,345·9 (4,993·8 to 5,677·6)	47·9% (44·9 to 50·9%)	5,473·7 (5,101·7 to 5,876·4)	46·5% (43·5 to 49·6%)	2·4% (-1·1 to 5·9%)	-3·0% (-4·4 to -1·7%)	158,782·2 (148,401·5 to 169,706·3)	33·9% (31·8 to 36·2%)	149,167·3 (138,083·2 to 162,364·7)	32·1% (29·8 to 34·6%)	-6·1% (-8·9 to -3·1%)	-5·4% (-7·0 to -3·7%)
**Child and maternal malnutrition: All causes**	99·6 (89·3 to 109·7)	0·9% (0·8 to 1·0%)	44·7 (38·7 to 51·5)	0·4% (0·3 to 0·4%)	-55·1% (-59·4 to -50·8%)	-57·5% (-61·5 to -53·4%)	17,768·9 (14,681·7 to 22,042·0)	3·8% (3·3 to 4·3%)	12,801·5 (9,713·8 to 16,644·7)	2·7% (2·3 to 3·3%)	-28·0% (-34·2 to -22·9%)	-27·6% (-33·1 to -23·2%)
**Suboptimal breastfeeding: All causes**	38·8 (23·2 to 55·1)	0·3% (0·2 to 0·5%)	17·4 (9·9 to 25·4)	0·1% (0·1 to 0·2%)	-55·3% (-59·9 to -50·1%)	-57·6% (-62·3 to -52·8%)	3,397·1 (2,066·4 to 4,815·2)	0·7% (0·4 to 1·0%)	1,548·6 (921·8 to 2,239·3)	0·3% (0·2 to 0·5%)	-54·4% (-58·9 to -49·4%)	-54·1% (-58·7 to -49·1%)
**Non-exclusive breastfeeding : All causes**	36·9 (21·3 to 53·6)	0·3% (0·2 to 0·5%)	16·6 (9·2 to 24·7)	0·1% (0·1 to 0·2%)	-54·9% (-59·7 to -49·8%)	-57·3% (-62·0 to -52·4%)	3,201·6 (1,860·8 to 4,638·0)	0·7% (0·4 to 1·0%)	1,452·1 (814·3 to 2,145·1)	0·3% (0·2 to 0·5%)	-54·6% (-59·3 to -49·6%)	-54·3% (-59·0 to -49·2%)
Diarrhoeal diseases	10·0 (6·2 to 13·4)	15·7% (9·8 to 20·9%)	3·8 (2·3 to 5·1)	8·2% (5·1 to 11·2%)	-62·3% (-67·7 to -56·2%)	-47·7% (-54·5 to -39·8%)	877·2 (555·9 to 1,162·6)	22·2% (13·7 to 29·6%)	339·3 (217·1 to 455·8)	14·1% (9·0 to 19·2%)	-61·3% (-66·6 to -55·1%)	-36·3% (-43·0 to -28·6%)
Lower respiratory infections	26·9 (12·0 to 42·8)	6·5% (2·9 to 10·5%)	12·9 (5·6 to 20·6)	2·9% (1·3 to 4·7%)	-52·2% (-57·2 to -46·5%)	-55·9% (-60·5 to -50·9%)	2,324·3 (1,040·5 to 3,690·4)	17·3% (7·7 to 27·6%)	1,112·8 (487·8 to 1,776·2)	10·6% (4·6 to 17·1%)	-52·1% (-57·1 to -46·5%)	-38·4% (-44·1 to -31·7%)
**Discontinued breastfeeding : All causes**	3·1 (1·1 to 5·6)	0·0% (0·0 to 0·1%)	1·2 (0·4 to 2·1)	0·0% (0·0 to 0·0%)	-62·3% (-67·3 to -56·6%)	-64·3% (-69·2 to -58·9%)	305·9 (102·3 to 542·8)	0·1% (0·0 to 0·1%)	138·1 (49·6 to 244·0)	0·0% (0·0 to 0·1%)	-54·9% (-61·8 to -46·0%)	-54·6% (-61·2 to -45·8%)
Diarrhoeal diseases	3·1 (1·1 to 5·6)	4·9% (1·7 to 8·7%)	1·2 (0·4 to 2·1)	2·6% (0·9 to 4·6%)	-62·3% (-67·3 to -56·6%)	-47·7% (-54·4 to -39·9%)	305·9 (102·3 to 542·8)	7·7% (2·7 to 13·5%)	138·1 (49·6 to 244·0)	5·7% (2·1 to 10·2%)	-54·9% (-61·8 to -46·0%)	-25·8% (-35·3 to -13·5%)
**Childhood undernutrition: All causes**	86·7 (74·0 to 97·2)	0·8% (0·7 to 0·9%)	37·4 (30·3 to 44·2)	0·3% (0·3 to 0·4%)	-56·9% (-61·4 to -52·5%)	-59·1% (-63·5 to -54·7%)	7,970·5 (6,863·4 to 8,935·4)	1·7% (1·5 to 1·9%)	3,693·6 (3,038·7 to 4,288·0)	0·8% (0·7 to 0·9%)	-53·7% (-58·0 to -49·5%)	-53·3% (-57·5 to -49·1%)
**Childhood underweight: All causes**	16·9 (13·8 to 23·0)	0·2% (0·1 to 0·2%)	6·7 (5·4 to 9·4)	0·1% (0·0 to 0·1%)	-60·3% (-65·1 to -54·7%)	-62·4% (-66·9 to -57·2%)	1,759·4 (1,464·1 to 2,312·0)	0·4% (0·3 to 0·5%)	851·3 (697·5 to 1,080·3)	0·2% (0·2 to 0·2%)	-51·6% (-57·2 to -45·4%)	-51·3% (-56·4 to -45·4%)
Diarrhoeal diseases	2·2 (1·8 to 2·9)	3·5% (2·8 to 4·5%)	0·6 (0·4 to 0·8)	1·3% (1·0 to 1·8%)	-72·8% (-77·8 to -67·0%)	-62·2% (-68·8 to -54·6%)	214·2 (170·7 to 274·4)	5·4% (4·3 to 6·8%)	69·0 (51·4 to 91·2)	2·9% (2·2 to 3·7%)	-67·8% (-73·0 to -62·0%)	-47·1% (-53·8 to -39·9%)
Lower respiratory infections	6·4 (4·0 to 12·5)	1·5% (1·0 to 3·0%)	2·3 (1·4 to 4·9)	0·5% (0·3 to 1·1%)	-63·1% (-69·1 to -57·1%)	-66·0% (-71·3 to -60·4%)	548·1 (348·9 to 1,073·6)	4·1% (2·6 to 7·8%)	203·1 (117·3 to 419·5)	1·9% (1·1 to 4·1%)	-63·0% (-68·9 to -57·0%)	-52·3% (-59·2 to -45·5%)
Measles	1·4 (0·3 to 4·1)	23·6% (6·7 to 47·7%)	0·3 (0·0 to 1·0)	17·9% (4·2 to 40·6%)	-78·6% (-86·4 to -70·9%)	-24·4% (-50·1 to -10·2%)	120·0 (23·1 to 353·2)	24·0% (6·8 to 48·5%)	25·7 (4·1 to 83·0)	18·1% (4·3 to 41·2%)	-78·6% (-86·3 to -70·8%)	-24·4% (-50·1 to -10·3%)
Protein-energy malnutrition	6·9 (6·2 to 8·2)	27·4% (25·6 to 29·6%)	3·5 (2·9 to 4·3)	14·7% (12·7 to 17·0%)	-49·8% (-57·2 to -40·6%)	-46·5% (-54·0 to -38·0%)	877·2 (763·2 to 1,039·5)	70·7% (68·0 to 73·7%)	553·5 (445·0 to 679·8)	62·7% (57·6 to 67·7%)	-36·9% (-44·6 to -28·5%)	-11·3% (-16·1 to -6·7%)
**Childhood wasting: All causes**	78·3 (58·0 to 92·9)	0·7% (0·5 to 0·8%)	34·2 (24·2 to 42·1)	0·3% (0·2 to 0·4%)	-56·3% (-61·0 to -51·8%)	-58·6% (-63·1 to -54·3%)	7,236·4 (5,486·4 to 8,492·0)	1·5% (1·2 to 1·8%)	3,408·0 (2,514·8 to 4,096·0)	0·7% (0·5 to 0·9%)	-52·9% (-57·3 to -48·7%)	-52·6% (-56·9 to -48·4%)

Supplemental Results Table 1. All-age deaths, death PAFs, DALYs, DALY PAFs, and percent change for each risk factor and outcome for both sexes combined in 2005 and 2015 for High-middle SDI.

Data in parenthesis are 95% uncertainty intervals. PAFs=population attributable fractions. DALYs=disability-adjusted life-years.

	2005 Deaths (in thousands)	2005 Deaths PAF	2015 Deaths (in thousands)	2015 Deaths PAF	Percent change deaths 2005-2015	Percent change deaths PAF 2005-2015	2005 DALYs (in thousands)	2005 DALYs PAF	2015 DALYs (in thousands)	2015 DALYs PAF	Percent change DALYs 2005-2015	Percent change DALYs PAF 2005-2015
Diarrhoeal diseases	19·9 (14·7 to 22·7)	31·3% (23·7 to 35·2%)	7·3 (5·0 to 8·8)	15·8% (11·1 to 19·0%)	-63·5% (-69·0 to -58·1%)	-49·4% (-56·4 to -42·2%)	1,920·9 (1,425·6 to 2,183·9)	48·5% (36·4 to 54·1%)	820·7 (573·1 to 999·4)	34·1% (24·2 to 39·3%)	-57·3% (-63·2 to -51·5%)	-29·8% (-36·2 to -24·3%)
Lower respiratory infections	49·4 (29·1 to 62·6)	12·0% (7·0 to 15·5%)	23·0 (13·3 to 29·9)	5·1% (2·9 to 7·1%)	-53·5% (-58·8 to -48·4%)	-57·2% (-62·1 to -52·4%)	4,260·9 (2,505·4 to 5,393·5)	31·7% (18·8 to 40·3%)	1,987·3 (1,146·0 to 2,583·2)	19·0% (11·0 to 25·6%)	-53·4% (-58·6 to -48·3%)	-40·0% (-46·1 to -33·7%)
Measles	2·1 (0·4 to 6·5)	35·5% (10·3 to 77·6%)	0·5 (0·1 to 1·8)	32·8% (8·7 to 76·0%)	-73·9% (-82·4 to -64·8%)	-7·5% (-28·4 to 3·4%)	177·4 (35·9 to 557·5)	36·1% (10·4 to 78·8%)	46·4 (8·4 to 156·3)	33·4% (8·8 to 77·4%)	-73·9% (-82·3 to -64·8%)	-7·5% (-28·3 to 3·2%)
Protein-energy malnutrition	6·9 (6·2 to 8·2)	27·4% (25·6 to 29·6%)	3·5 (2·9 to 4·3)	14·7% (12·7 to 17·0%)	-49·8% (-57·2 to -40·6%)	-46·5% (-54·0 to -38·0%)	877·2 (763·2 to 1,039·5)	70·7% (68·0 to 73·7%)	553·5 (445·0 to 679·8)	62·7% (57·6 to 67·7%)	-36·9% (-44·6 to -28·5%)	-11·3% (-16·1 to -6·7%)
**Childhood stunting: All causes**	16·2 (6·0 to 34·2)	0·1% (0·1 to 0·3%)	5·6 (1·7 to 13·4)	0·0% (0·0 to 0·1%)	-65·4% (-72·6 to -59·2%)	-67·2% (-74·1 to -61·4%)	1,424·5 (540·4 to 2,971·2)	0·3% (0·1 to 0·6%)	509·4 (169·0 to 1,191·4)	0·1% (0·0 to 0·3%)	-64·2% (-70·5 to -58·7%)	-64·0% (-70·3 to -58·4%)
Diarrhoeal diseases	3·4 (1·2 to 6·2)	5·3% (1·9 to 9·8%)	1·0 (0·3 to 1·8)	2·1% (0·7 to 4·1%)	-71·3% (-76·0 to -65·9%)	-60·2% (-66·4 to -53·3%)	324·2 (116·1 to 601·1)	8·2% (2·9 to 15·1%)	109·2 (38·3 to 211·0)	4·5% (1·5 to 8·8%)	-66·3% (-71·7 to -60·6%)	-44·6% (-51·1 to -38·5%)
Lower respiratory infections	11·7 (0·8 to 29·8)	2·8% (0·2 to 7·4%)	4·4 (0·2 to 12·3)	1·0% (0·1 to 2·7%)	-62·4% (-69·0 to -53·4%)	-65·4% (-71·7 to -57·0%)	1,010·6 (68·6 to 2,570·5)	7·5% (0·5 to 19·2%)	381·3 (19·6 to 1,064·0)	3·6% (0·2 to 10·3%)	-62·3% (-68·9 to -53·2%)	-51·5% (-60·2 to -41·0%)
Measles	1·1 (0·1 to 3·4)	17·8% (1·7 to 49·1%)	0·2 (0·0 to 0·8)	13·4% (0·9 to 41·1%)	-78·9% (-86·5 to -68·5%)	-25·2% (-48·9 to -10·9%)	89·6 (7·8 to 290·0)	18·1% (1·7 to 50·0%)	18·9 (1·2 to 67·2)	13·6% (1·0 to 41·8%)	-78·9% (-86·4 to -68·4%)	-25·2% (-48·9 to -11·0%)
**Iron deficiency: All causes**	2·3 (1·5 to 3·3)	0·0% (0·0 to 0·0%)	1·9 (1·3 to 2·8)	0·0% (0·0 to 0·0%)	-17·3% (-28·1 to -1·8%)	-21·7% (-31·7 to -6·9%)	8,835·4 (5,929·9 to 12,752·2)	1·9% (1·4 to 2·5%)	8,587·0 (5,702·0 to 12,433·8)	1·8% (1·4 to 2·4%)	-2·8% (-4·3 to -1·3%)	-2·2% (-4·8 to 0·8%)
Maternal haemorrhage	0·7 (0·3 to 1·2)	23·8% (8·8 to 38·4%)	0·4 (0·2 to 0·7)	24·0% (8·8 to 39·0%)	-37·5% (-48·6 to -23·3%)	0·9% (-7·1 to 8·6%)	42·5 (16·2 to 71·2)	23·6% (8·7 to 38·2%)	27·5 (10·0 to 46·3)	23·6% (8·7 to 38·5%)	-35·4% (-45·7 to -22·3%)	0·1% (-7·7 to 7·1%)
Maternal sepsis and other pregnancy related infections	0·4 (0·1 to 0·6)	25·9% (9·6 to 40·8%)	0·2 (0·1 to 0·4)	23·6% (8·8 to 37·7%)	-41·0% (-50·6 to -30·7%)	-8·7% (-15·9 to -2·7%)	24·7 (9·1 to 40·4)	25·7% (9·6 to 40·6%)	15·1 (5·6 to 25·8)	23·6% (8·8 to 37·9%)	-38·9% (-48·6 to -27·9%)	-8·2% (-15·1 to -2·2%)
Iron-deficiency anaemia	1·2 (0·9 to 1·9)	100·0% (100·0 to 100·0%)	1·2 (0·9 to 2·0)	100·0% (100·0 to 100·0%)	2·2% (-12·1 to 21·6%)	-	8,768·1 (5,873·6 to 12,689·7)	100·0% (100·0 to 100·0%)	8,544·4 (5,662·5 to 12,385·5)	100·0% (100·0 to 100·0%)	-2·6% (-4·0 to -1·1%)	-
**Vitamin A deficiency: All causes**	4·5 (2·8 to 6·6)	0·0% (0·0 to 0·1%)	1·4 (0·8 to 2·2)	0·0% (0·0 to 0·0%)	-68·7% (-74·6 to -62·2%)	-70·3% (-76·1 to -64·0%)	446·8 (286·3 to 631·1)	0·1% (0·1 to 0·1%)	179·6 (119·8 to 254·2)	0·0% (0·0 to 0·1%)	-59·8% (-66·8 to -52·2%)	-59·6% (-66·3 to -52·0%)
Diarrhoeal diseases	2·9 (1·6 to 4·2)	4·5% (2·5 to 6·6%)	1·0 (0·5 to 1·5)	2·2% (1·2 to 3·3%)	-65·3% (-71·9 to -58·0%)	-51·8% (-60·2 to -42·5%)	271·2 (150·1 to 397·5)	6·9% (3·8 to 10·0%)	108·4 (60·1 to 160·5)	4·5% (2·5 to 6·7%)	-60·0% (-67·1 to -52·8%)	-34·3% (-42·9 to -25·5%)
Measles	1·7 (0·7 to 3·5)	28·8% (19·5 to 37·9%)	0·4 (0·2 to 0·9)	26·2% (17·1 to 35·4%)	-74·3% (-81·1 to -66·0%)	-8·9% (-25·2 to 5·3%)	144·2 (56·2 to 299·5)	29·2% (19·8 to 38·4%)	37·1 (14·3 to 80·0)	26·6% (17·4 to 35·8%)	-74·3% (-81·0 to -65·9%)	-9·0% (-25·0 to 5·0%)
Vitamin A deficiency	-	-	-	-	-	-	31·4 (19·6 to 47·0)	100·0% (100·0 to 100·0%)	34·1 (21·3 to 50·2)	100·0% (100·0 to 100·0%)	8·4% (3·4 to 13·5%)	-
**Zinc deficiency: All causes**	2·5 (0·1 to 6·8)	0·0% (0·0 to 0·1%)	1·0 (0·0 to 2·9)	0·0% (0·0 to 0·0%)	-59·3% (-68·0 to -52·3%)	-61·4% (-69·8 to -54·5%)	241·3 (38·0 to 598·1)	0·1% (0·0 to 0·1%)	112·3 (27·9 to 271·5)	0·0% (0·0 to 0·1%)	-53·5% (-60·8 to -23·2%)	-53·1% (-60·4 to -23·6%)
Diarrhoeal diseases	1·0 (0·0 to 2·4)	1·5% (0·0 to 3·8%)	0·4 (0·0 to 0·9)	0·8% (0·0 to 1·9%)	-63·4% (-70·3 to 0·0%)	-49·1% (-58·8 to 0·0%)	110·8 (25·7 to 227·0)	2·8% (0·7 to 5·9%)	54·8 (21·4 to 103·3)	2·3% (0·9 to 4·2%)	-50·6% (-60·8 to -12·0%)	-18·8% (-33·6 to 44·7%)
Lower respiratory infections	1·5 (0·0 to 5·6)	0·4% (0·0 to 1·3%)	0·7 (0·0 to 2·5)	0·1% (0·0 to 0·6%)	-56·7% (-62·9 to 0·0%)	-60·1% (-65·5 to 0·0%)	130·5 (1·2 to 470·6)	1·0% (0·0 to 3·5%)	57·5 (1·0 to 212·4)	0·5% (0·0 to 2·0%)	-55·9% (-61·8 to -11·1%)	-43·3% (-50·2 to 16·3%)
**Tobacco smoke: All causes**	1,830·5 (1,583·5 to 2,046·5)	16·4% (14·2 to 18·3%)	1,801·9 (1,575·2 to 2,018·7)	15·3% (13·4 to 17·1%)	-1·6% (-6·8 to 3·9%)	-6·8% (-9·9 to -3·4%)	43,708·3 (38,375·0 to 48,934·2)	9·3% (8·1 to 10·7%)	40,682·7 (35,914·6 to 45,580·7)	8·8% (7·6 to 10·2%)	-6·9% (-11·8 to -1·7%)	-6·2% (-10·1 to -2·0%)
**Smoking: All causes**	1,673·7 (1,419·2 to 1,902·8)	15·0% (12·7 to 17·0%)	1,640·9 (1,411·7 to 1,858·7)	13·9% (12·1 to 15·7%)	-2·0% (-7·4 to 4·0%)	-7·1% (-10·6 to -3·3%)	39,659·1 (34,044·8 to 44,901·1)	8·5% (7·2 to 9·9%)	37,213·4 (32,328·4 to 42,205·5)	8·0% (6·8 to 9·4%)	-6·2% (-11·5 to -0·6%)	-5·4% (-9·7 to -0·8%)
Tuberculosis	12·9 (6·3 to 20·2)	9·5% (4·7 to 14·6%)	8·6 (4·1 to 14·0)	8·5% (4·1 to 13·4%)	-33·6% (-39·0 to -28·5%)	-10·8% (-15·9 to -6·1%)	446·3 (221·6 to 695·8)	8·5% (4·3 to 13·2%)	295·2 (144·2 to 467·9)	7·8% (3·7 to 12·2%)	-33·9% (-39·0 to -28·4%)	-9·3% (-14·3 to -4·8%)
Lower respiratory infections	75·4 (59·8 to 92·3)	18·2% (14·8 to 22·0%)	82·2 (63·7 to 102·6)	18·3% (14·7 to 22·2%)	9·0% (1·3 to 17·3%)	0·3% (-4·5 to 5·3%)	1,607·2 (1,279·5 to 1,938·8)	11·9% (9·7 to 14·4%)	1,582·5 (1,212·2 to 1,954·4)	15·1% (12·2 to 18·2%)	-1·5% (-8·4 to 5·0%)	26·5% (20·3 to 33·3%)
Lip and oral cavity cancer	11·6 (9·6 to 13·5)	48·4% (40·6 to 56·3%)	14·4 (11·4 to 17·6)	44·3% (35·3 to 53·7%)	24·5% (14·3 to 36·0%)	-8·5% (-14·1 to -3·3%)	307·2 (255·5 to 360·6)	49·1% (41·2 to 57·1%)	355·6 (281·3 to 440·1)	43·9% (34·9 to 53·6%)	15·7% (5·9 to 27·1%)	-10·6% (-16·4 to -4·7%)
Nasopharynx cancer	6·7 (4·0 to 9·5)	39·8% (24·4 to 55·7%)	6·3 (4·0 to 9·1)	35·4% (22·7 to 50·4%)	-6·5% (-23·1 to 13·7%)	-11·1% (-21·6 to 1·2%)	207·6 (119·4 to 302·4)	37·1% (21·6 to 53·0%)	172·8 (105·5 to 258·7)	32·1% (20·2 to 46·4%)	-16·8% (-34·5 to 5·6%)	-13·5% (-27·0 to 3·5%)
Oesophageal cancer	53·4 (27·9 to 82·3)	36·3% (18·9 to 55·9%)	45·3 (25·5 to 68·5)	35·1% (19·7 to 52·3%)	-15·2% (-25·9 to 1·5%)	-3·4% (-11·0 to 11·2%)	1,196·7 (622·8 to 1,846·1)	35·9% (19·0 to 55·0%)	951·4 (537·9 to 1,451·7)	33·9% (19·2 to 51·1%)	-20·5% (-31·2 to -3·7%)	-5·6% (-13·7 to 9·3%)
Stomach cancer	28·6 (14·7 to 48·2)	10·9% (5·6 to 18·6%)	24·8 (13·2 to 41·6)	9·9% (5·3 to 16·8%)	-13·3% (-23·0 to -2·4%)	-9·4% (-16·5 to -2·2%)	670·7 (352·6 to 1,138·0)	11·2% (5·9 to 19·1%)	524·8 (284·6 to 883·5)	9·7% (5·2 to 16·2%)	-21·8% (-32·0 to -11·0%)	-12·9% (-20·9 to -4·2%)
Colon and rectum cancer	10·0 (6·6 to 14·5)	5·9% (3·9 to 8·6%)	11·1 (7·3 to 16·3)	5·3% (3·4 to 7·6%)	11·3% (2·7 to 19·8%)	-10·9% (-16·2 to -5·7%)	232·4 (151·7 to 337·2)	6·2% (4·0 to 9·0%)	233·1 (154·4 to 335·0)	5·3% (3·5 to 7·6%)	0·3% (-8·7 to 9·0%)	-15·0% (-20·9 to -9·0%)
Liver cancer due to hepatitis B	14·6 (5·7 to 29·0)	15·4% (6·0 to 30·3%)	12·1 (4·9 to 23·5)	13·6% (5·6 to 26·1%)	-17·0% (-34·6 to 7·9%)	-11·9% (-27·1 to 11·7%)	459·7 (174·9 to 933·0)	15·3% (5·9 to 30·4%)	336·4 (136·6 to 666·2)	12·8% (5·2 to 24·9%)	-26·8% (-44·8 to 0·1%)	-16·4% (-33·9 to 13·6%)
Liver cancer due to hepatitis C	5·1 (2·9 to 7·9)	16·1% (9·1 to 24·6%)	6·1 (3·3 to 9·3)	16·0% (8·9 to 24·4%)	20·0% (6·4 to 35·4%)	-0·5% (-9·5 to 9·7%)	113·4 (63·2 to 177·7)	16·9% (9·5 to 26·1%)	124·6 (67·7 to 194·9)	16·3% (8·9 to 25·2%)	9·8% (-5·2 to 26·4%)	-3·4% (-13·5 to 8·6%)
Liver cancer due to alcohol use	11·6 (5·5 to 21·0)	17·8% (8·4 to 31·9%)	13·6 (6·0 to 25·2)	16·3% (7·5 to 29·1%)	16·8% (-2·1 to 39·1%)	-8·0% (-18·5 to 5·1%)	292·7 (134·2 to 537·8)	18·0% (8·3 to 32·7%)	316·6 (138·6 to 603·8)	15·9% (7·1 to 28·8%)	8·2% (-11·5 to 31·8%)	-12·1% (-23·7 to 2·4%)
Liver cancer due to other causes	5·7 (2·3 to 11·1)	12·9% (5·1 to 25·0%)	4·9 (2·1 to 9·4)	12·0% (5·3 to 22·6%)	-14·2% (-29·9 to 9·0%)	-7·0% (-20·6 to 16·6%)	151·6 (58·6 to 308·4)	13·0% (5·0 to 25·7%)	111·0 (46·8 to 221·6)	11·3% (4·8 to 22·2%)	-26·8% (-43·3 to -2·0%)	-12·8% (-28·9 to 15·7%)
Pancreatic cancer	14·0 (10·6 to 18·8)	18·4% (13·9 to 24·7%)	16·1 (11·9 to 21·9)	16·1% (11·9 to 21·9%)	14·7% (7·4 to 22·5%)	-12·7% (-16·8 to -8·7%)	330·3 (249·6 to 439·8)	19·7% (14·9 to 26·2%)	350·4 (261·7 to 475·7)	16·5% (12·3 to 22·3%)	6·1% (-1·6 to 13·8%)	-16·1% (-20·8 to -11·1%)
Tracheal, bronchus, and lung cancer	288·7 (227·5 to 336·6)	68·8% (54·4 to 79·6%)	321·5 (245·0 to 384·5)	64·9% (49·8 to 77·0%)	11·4% (2·3 to 21·1%)	-5·7% (-9·6 to -2·6%)	6,556·6 (5,025·2 to 7,696·3)	67·6% (52·4 to 79·0%)	6,834·5 (5,124·0 to 8,283·3)	62·8% (47·9 to 75·7%)	4·2% (-5·1 to 15·1%)	-7·1% (-11·7 to -3·0%)
Cervical cancer	3·4 (1·2 to 6·3)	6·5% (2·2 to 11·8%)	3·2 (1·1 to 5·9)	5·8% (1·9 to 10·5%)	-6·0% (-20·4 to 9·7%)	-10·3% (-21·1 to 2·0%)	108·4 (36·3 to 198·6)	6·7% (2·2 to 12·2%)	88·8 (30·3 to 161·6)	5·6% (1·8 to 10·0%)	-18·0% (-32·8 to -0·3%)	-16·9% (-29·4 to -3·2%)
Kidney cancer	4·1 (2·7 to 5·6)	17·6% (11·5 to 24·0%)	5·2 (3·3 to 7·3)	16·1% (10·3 to 22·4%)	26·5% (16·2 to 36·3%)	-8·6% (-13·8 to -3·4%)	106·6 (70·7 to 144·6)	17·3% (11·5 to 23·4%)	126·0 (81·5 to 175·3)	15·7% (10·2 to 21·5%)	18·2% (8·1 to 28·4%)	-9·4% (-15·3 to -3·5%)
Bladder cancer	11·1 (8·0 to 14·3)	29·7% (21·3 to 38·1%)	12·5 (9·0 to 16·3)	27·2% (19·4 to 35·2%)	11·9% (4·5 to 19·2%)	-8·5% (-12·3 to -5·0%)	221·1 (163·6 to 282·5)	30·9% (22·8 to 39·4%)	229·4 (166·0 to 301·0)	27·7% (20·0 to 36·0%)	3·8% (-3·3 to 11·0%)	-10·6% (-14·5 to -7·1%)
Ischaemic heart disease	352·3 (292·1 to 413·1)	16·7% (13·8 to 19·5%)	355·2 (291·5 to 421·2)	14·7% (12·0 to 17·3%)	0·8% (-4·0 to 5·7%)	-12·1% (-15·3 to -9·2%)	9,097·7 (7,534·0 to 10,638·5)	22·8% (18·8 to 26·6%)	8,814·5 (7,180·6 to 10,473·1)	20·8% (17·0 to 24·4%)	-3·1% (-7·7 to 1·9%)	-8·9% (-11·7 to -6·3%)
Ischaemic stroke	111·8 (91·9 to 131·1)	15·0% (12·5 to 17·4%)	107·7 (87·7 to 127·1)	13·7% (11·4 to 16·2%)	-3·7% (-10·4 to 3·8%)	-8·8% (-13·7 to -3·8%)	2,360·9 (1,954·8 to 2,766·9)	19·9% (16·7 to 23·0%)	2,213·9 (1,803·6 to 2,632·4)	19·0% (15·8 to 22·2%)	-6·2% (-12·7 to 0·9%)	-4·8% (-8·9 to -0·5%)
Hemorrhagic stroke	175·4 (146·2 to 203·2)	20·1% (16·9 to 23·2%)	152·1 (126·4 to 179·3)	18·4% (15·3 to 21·5%)	-13·3% (-19·3 to -6·5%)	-8·8% (-12·9 to -4·7%)	4,680·9 (3,933·7 to 5,438·3)	24·9% (21·0 to 28·7%)	3,950·0 (3,285·7 to 4,656·8)	23·6% (19·7 to 27·4%)	-15·6% (-21·7 to -9·2%)	-5·3% (-9·0 to -1·5%)
Hypertensive heart disease	24·9 (19·2 to 30·3)	12·8% (9·9 to 15·7%)	29·0 (22·4 to 35·7)	11·7% (9·1 to 14·5%)	16·6% (7·1 to 26·7%)	-8·6% (-14·3 to -1·6%)	593·2 (460·0 to 713·5)	17·2% (13·5 to 20·9%)	648·3 (505·3 to 794·0)	16·2% (12·6 to 19·9%)	9·3% (0·8 to 18·2%)	-5·9% (-10·8 to 0·0%)
Atrial fibrillation and flutter	2·9 (2·1 to 3·9)	8·8% (6·8 to 10·8%)	3·6 (2·7 to 4·8)	7·7% (5·9 to 9·6%)	23·2% (15·5 to 31·6%)	-12·1% (-17·0 to -7·2%)	133·9 (94·5 to 182·3)	16·6% (12·9 to 20·1%)	159·9 (112·0 to 218·6)	15·2% (11·7 to 18·6%)	19·4% (16·0 to 22·9%)	-8·6% (-10·9 to -6·4%)
Aortic aneurysm	5·5 (4·3 to 6·6)	18·3% (14·5 to 21·9%)	6·0 (4·6 to 7·3)	15·5% (12·2 to 18·8%)	7·9% (-0·2 to 14·4%)	-15·2% (-18·2 to -12·2%)	143·5 (113·9 to 170·7)	24·0% (19·0 to 28·5%)	150·2 (115·5 to 183·8)	21·2% (16·7 to 25·5%)	4·7% (-4·0 to 11·4%)	-11·7% (-14·8 to -8·9%)

Supplemental Results Table 1. All-age deaths, death PAFs, DALYs, DALY PAFs, and percent change for each risk factor and outcome for both sexes combined in 2005 and 2015 for High-middle SDI.

Data in parenthesis are 95% uncertainty intervals. PAFs=population attributable fractions. DALYs=disability-adjusted life-years.

	2005 Deaths (in thousands)	2005 Deaths PAF	2015 Deaths (in thousands)	2015 Deaths PAF	Percent change deaths 2005-2015	Percent change deaths PAF 2005-2015	2005 DALYs (in thousands)	2005 DALYs PAF	2015 DALYs (in thousands)	2015 DALYs PAF	Percent change DALYs 2005-2015	Percent change DALYs PAF 2005-2015
Peripheral vascular disease	0·8 (0·6 to 0·9)	12·0% (9·2 to 14·6%)	0·9 (0·7 to 1·2)	10·0% (7·7 to 12·3%)	24·8% (15·8 to 35·1%)	-16·4% (-21·6 to -10·7%)	30·9 (20·7 to 44·9)	16·3% (12·7 to 19·9%)	39·2 (25·7 to 58·1)	14·7% (11·3 to 18·0%)	26·7% (20·7 to 32·2%)	-9·8% (-12·8 to -7·0%)
Other cardiovascular and circulatory diseases	16·0 (12·4 to 19·4)	14·7% (11·5 to 17·7%)	16·4 (12·7 to 20·0)	13·2% (10·2 to 16·1%)	2·6% (-3·7 to 10·1%)	-10·5% (-14·4 to -5·9%)	660·7 (515·5 to 822·5)	16·4% (12·9 to 19·7%)	709·3 (540·1 to 887·9)	16·1% (12·6 to 19·5%)	7·4% (2·4 to 12·9%)	-1·8% (-4·8 to 1·5%)
Chronic obstructive pulmonary disease	363·9 (251·7 to 457·5)	52·6% (36·2 to 65·8%)	316·7 (234·4 to 388·1)	50·6% (37·8 to 61·6%)	-13·0% (-20·1 to -2·0%)	-3·8% (-9·1 to 6·9%)	6,371·4 (4,564·2 to 7,948·7)	48·1% (34·3 to 59·7%)	5,258·3 (3,974·5 to 6,461·8)	45·1% (34·7 to 55·2%)	-17·5% (-23·8 to -8·6%)	-6·3% (-11·3 to 2·8%)
Silicosis	0·5 (0·3 to 0·8)	16·0% (8·9 to 26·2%)	0·4 (0·2 to 0·7)	14·0% (7·2 to 24·2%)	-17·2% (-34·3 to 2·0%)	-12·8% (-24·1 to -2·7%)	12·0 (6·6 to 19·9)	15·4% (8·7 to 25·3%)	8·8 (4·6 to 15·6)	12·2% (6·7 to 21·1%)	-26·6% (-40·7 to -9·0%)	-20·8% (-32·4 to -9·2%)
Asbestosis	0·1 (0·1 to 0·1)	15·4% (11·7 to 19·6%)	0·1 (0·1 to 0·1)	15·7% (12·1 to 19·9%)	26·9% (5·7 to 49·2%)	2·2% (-7·8 to 13·2%)	2·0 (1·5 to 2·8)	13·1% (9·6 to 17·5%)	2·2 (1·6 to 3·0)	12·5% (9·3 to 16·5%)	8·9% (-7·4 to 25·7%)	-4·3% (-14·1 to 7·1%)
Coal workers pneumoconiosis	0·1 (0·0 to 0·1)	15·5% (8·3 to 26·2%)	0·1 (0·0 to 0·1)	13·9% (7·5 to 24·2%)	-17·5% (-39·6 to 6·5%)	-10·5% (-20·7 to 1·0%)	1·7 (1·0 to 2·7)	16·0% (9·7 to 24·6%)	1·5 (0·9 to 2·3)	14·0% (8·9 to 21·2%)	-14·5% (-32·7 to 3·5%)	-12·6% (-22·3 to 1·3%)
Other pneumoconiosis	0·4 (0·3 to 0·6)	13·5% (10·4 to 17·3%)	0·4 (0·3 to 0·6)	11·9% (9·1 to 15·7%)	-5·3% (-16·6 to 9·5%)	-11·5% (-21·7 to -2·0%)	13·4 (9·4 to 19·3)	11·2% (8·2 to 15·1%)	11·5 (7·8 to 16·6)	8·8% (6·4 to 12·1%)	-14·3% (-25·0 to -3·4%)	-21·4% (-29·7 to -12·4%)
Asthma	7·5 (5·9 to 9·2)	12·7% (10·0 to 15·4%)	6·4 (4·9 to 8·0)	11·8% (9·1 to 14·5%)	-15·1% (-22·8 to -7·4%)	-7·0% (-13·2 to -1·0%)	403·5 (294·9 to 520·2)	8·3% (6·6 to 10·0%)	389·4 (277·0 to 513·1)	8·2% (6·5 to 10·0%)	-3·5% (-8·7 to 1·0%)	-0·9% (-4·5 to 2·7%)
Interstitial lung disease and pulmonary sarcoidosis	2·1 (1·6 to 2·6)	11·8% (9·2 to 14·4%)	3·1 (2·3 to 3·9)	11·1% (8·6 to 13·7%)	48·0% (25·2 to 65·1%)	-6·4% (-11·9 to -0·8%)	49·5 (38·0 to 62·6)	12·0% (9·4 to 14·7%)	62·5 (46·3 to 82·1)	10·8% (8·4 to 13·4%)	26·1% (8·6 to 38·9%)	-10·1% (-16·0 to -4·3%)
Other chronic respiratory diseases	0·8 (0·6 to 1·1)	10·1% (7·7 to 12·5%)	1·0 (0·7 to 1·3)	11·1% (8·4 to 14·1%)	18·8% (4·1 to 37·8%)	9·7% (-1·5 to 22·0%)	40·1 (30·0 to 52·5)	5·7% (4·3 to 7·3%)	36·3 (26·5 to 47·9)	6·0% (4·5 to 7·8%)	-9·6% (-20·3 to 1·9%)	5·8% (-8·1 to 20·6%)
Diabetes mellitus	13·4 (4·1 to 23·5)	4·8% (1·5 to 8·4%)	15·3 (4·5 to 27·3)	4·3% (1·3 to 7·7%)	13·6% (5·6 to 20·2%)	-10·5% (-15·7 to -6·2%)	768·6 (226·2 to 1,356·6)	6·0% (1·8 to 10·4%)	875·9 (246·4 to 1,579·5)	5·4% (1·6 to 9·5%)	14·0% (8·9 to 18·3%)	-9·5% (-13·0 to -6·9%)
**Second-hand smoke: All causes**	189·8 (149·0 to 235·7)	1·7% (1·3 to 2·1%)	193·1 (150·5 to 240·8)	1·6% (1·3 to 2·0%)	1·8% (-3·1 to 6·4%)	-3·6% (-7·4 to 0·1%)	4,820·2 (3,817·8 to 5,942·4)	1·0% (0·8 to 1·3%)	4,201·1 (3,315·3 to 5,136·2)	0·9% (0·7 to 1·1%)	-12·8% (-17·0 to -8·3%)	-12·2% (-16·3 to -8·2%)
Lower respiratory infections	27·5 (17·7 to 38·3)	6·6% (4·3 to 9·3%)	23·0 (14·4 to 32·6)	5·1% (3·2 to 7·2%)	-16·5% (-22·5 to -10·8%)	-23·1% (-27·2 to -19·7%)	1,350·6 (884·9 to 1,855·5)	10·0% (6·6 to 13·9%)	747·2 (467·3 to 1,041·9)	7·1% (4·5 to 10·0%)	-44·7% (-49·2 to -40·6%)	-28·9% (-33·0 to -25·3%)
Otitis media	0·0 (0·0 to 0·0)	6·7% (4·6 to 8·8%)	0·0 (0·0 to 0·0)	4·7% (3·2 to 6·3%)	-47·4% (-55·1 to -38·3%)	-29·6% (-37·3 to -21·3%)	45·0 (24·5 to 73·4)	6·5% (4·4 to 8·7%)	39·0 (21·1 to 64·1)	5·8% (3·9 to 7·8%)	-13·4% (-16·8 to -10·3%)	-10·2% (-13·3 to -7·4%)
Tracheal, bronchus, and lung cancer	7·6 (3·7 to 13·6)	1·8% (0·9 to 3·2%)	10·4 (5·1 to 18·6)	2·1% (1·0 to 3·7%)	35·5% (26·5 to 42·1%)	14·8% (6·9 to 23·4%)	194·4 (94·1 to 346·3)	2·0% (1·0 to 3·5%)	256·3 (126·5 to 459·5)	2·4% (1·2 to 4·2%)	31·8% (21·0 to 40·4%)	17·5% (8·0 to 28·0%)
Ischaemic heart disease	89·2 (67·4 to 114·9)	4·2% (3·2 to 5·4%)	99·7 (73·7 to 131·4)	4·1% (3·0 to 5·4%)	11·7% (5·2 to 18·1%)	-2·6% (-7·2 to 1·8%)	1,849·7 (1,462·0 to 2,291·8)	4·6% (3·7 to 5·7%)	1,930·2 (1,511·5 to 2,397·1)	4·5% (3·6 to 5·7%)	4·4% (-1·8 to 10·0%)	-1·9% (-5·9 to 1·9%)
Ischaemic stroke	18·3 (13·4 to 23·8)	2·5% (1·8 to 3·2%)	18·8 (13·4 to 25·0)	2·4% (1·7 to 3·2%)	2·3% (-5·3 to 9·2%)	-3·1% (-8·6 to 2·1%)	324·2 (247·9 to 411·9)	2·7% (2·1 to 3·5%)	312·5 (235·6 to 397·0)	2·7% (2·0 to 3·4%)	-3·6% (-10·4 to 2·4%)	-2·2% (-6·7 to 2·4%)
Hemorrhagic stroke	25·9 (19·9 to 33·2)	3·0% (2·3 to 3·8%)	23·3 (17·6 to 30·2)	2·8% (2·1 to 3·6%)	-10·0% (-16·4 to -3·5%)	-5·4% (-10·2 to -0·7%)	631·0 (501·7 to 793·3)	3·4% (2·7 to 4·2%)	537·2 (419·6 to 673·0)	3·2% (2·5 to 4·0%)	-14·9% (-21·2 to -8·1%)	-4·4% (-9·5 to 0·8%)
**Alcohol and drug use: All causes**	682·3 (600·7 to 763·9)	6·1% (5·4 to 6·8%)	712·9 (613·9 to 800·9)	6·1% (5·2 to 6·8%)	4·5% (0·1 to 9·1%)	-1·0% (-3·8 to 1·8%)	28,493·3 (26,189·1 to 30,876·5)	6·1% (5·5 to 6·6%)	28,590·4 (26,026·6 to 31,094·2)	6·2% (5·5 to 6·8%)	0·3% (-2·9 to 4·0%)	1·1% (-1·4 to 3·9%)
**Alcohol use: All causes**	595·2 (511·6 to 678·4)	5·3% (4·6 to 6·1%)	609·4 (512·9 to 699·5)	5·2% (4·4 to 5·9%)	2·4% (-2·5 to 7·5%)	-3·0% (-6·4 to 0·2%)	22,819·4 (20,718·5 to 25,006·6)	4·9% (4·3 to 5·4%)	22,131·5 (19,970·6 to 24,362·6)	4·8% (4·2 to 5·4%)	-3·0% (-6·7 to 1·0%)	-2·3% (-5·3 to 1·1%)
Tuberculosis	21·9 (17·1 to 26·2)	16·2% (14·3 to 18·4%)	17·2 (13·0 to 21·4)	17·0% (14·5 to 19·4%)	-21·5% (-28·5 to -14·3%)	5·4% (-2·0 to 11·2%)	855·8 (680·2 to 998·6)	16·4% (14·8 to 18·6%)	674·6 (519·1 to 807·8)	17·7% (15·4 to 19·9%)	-21·2% (-28·2 to -14·3%)	8·1% (1·0 to 13·9%)
Lower respiratory infections	19·0 (16·4 to 21·3)	4·6% (4·1 to 5·1%)	22·3 (18·1 to 25·8)	4·9% (4·3 to 5·6%)	17·3% (6·5 to 27·5%)	7·9% (-0·1 to 15·8%)	451·5 (398·7 to 492·6)	3·4% (3·1 to 3·6%)	466·6 (385·7 to 523·5)	4·5% (3·9 to 4·9%)	3·3% (-5·5 to 12·4%)	32·8% (22·8 to 43·5%)
Lip and oral cavity cancer	7·4 (6·8 to 8·0)	31·0% (28·6 to 33·1%)	10·2 (9·0 to 11·2)	31·5% (28·1 to 34·2%)	38·1% (28·0 to 48·2%)	1·4% (-3·7 to 5·5%)	212·1 (197·0 to 226·8)	33·9% (31·6 to 35·7%)	280·5 (249·9 to 306·9)	34·6% (31·4 to 37·1%)	32·2% (22·8 to 41·6%)	2·1% (-2·6 to 5·7%)
Nasopharynx cancer	4·9 (3·5 to 5·5)	29·2% (26·7 to 31·1%)	5·6 (4·2 to 6·6)	31·7% (28·1 to 34·4%)	14·3% (-1·6 to 31·2%)	8·6% (2·4 to 14·2%)	165·6 (117·4 to 184·2)	29·5% (27·1 to 31·4%)	176·5 (132·0 to 207·8)	32·7% (29·4 to 35·3%)	6·6% (-9·3 to 23·9%)	10·8% (4·7 to 16·8%)
Other pharynx cancer	3·7 (3·4 to 3·9)	34·3% (31·9 to 36·3%)	4·7 (4·2 to 5·2)	35·0% (31·8 to 37·8%)	29·6% (20·8 to 37·6%)	2·1% (-2·5 to 5·5%)	109·3 (101·2 to 116·5)	36·5% (34·2 to 38·5%)	138·5 (124·2 to 151·3)	37·6% (34·4 to 40·2%)	26·7% (18·0 to 34·5%)	2·8% (-1·5 to 6·2%)
Oesophageal cancer	24·7 (22·1 to 27·3)	16·8% (15·3 to 18·2%)	23·9 (20·3 to 27·2)	18·6% (16·1 to 20·5%)	-3·2% (-14·3 to 9·0%)	10·4% (2·8 to 17·3%)	618·9 (555·5 to 677·1)	18·6% (17·1 to 19·9%)	580·9 (496·4 to 655·6)	20·7% (18·3 to 22·5%)	-6·2% (-17·0 to 6·1%)	11·4% (4·2 to 17·4%)
Colon and rectum cancer	6·6 (6·0 to 7·3)	3·9% (3·6 to 4·3%)	8·4 (7·3 to 9·3)	4·0% (3·5 to 4·3%)	25·8% (18·3 to 32·9%)	0·8% (-4·6 to 5·1%)	156·1 (143·2 to 169·1)	4·2% (3·8 to 4·5%)	188·6 (167·9 to 207·7)	4·3% (3·8 to 4·6%)	20·8% (13·6 to 28·3%)	2·3% (-2·8 to 6·5%)
Liver cancer due to hepatitis B	0·5 (0·3 to 0·6)	0·5% (0·3 to 0·7%)	0·3 (0·2 to 0·4)	0·3% (0·2 to 0·5%)	-36·8% (-55·1 to -10·7%)	-33·0% (-50·7 to -10·8%)	24·6 (16·6 to 33·7)	0·8% (0·6 to 1·1%)	15·9 (9·8 to 23·4)	0·6% (0·4 to 0·9%)	-35·4% (-53·2 to -10·8%)	-26·2% (-44·1 to -4·4%)
Liver cancer due to hepatitis C	0·0 (0·0 to 0·0)	0·1% (0·0 to 0·1%)	0·0 (0·0 to 0·0)	0·0% (0·0 to 0·1%)	-22·7% (-44·1 to 4·2%)	-35·8% (-53·5 to -13·8%)	1·1 (0·7 to 1·6)	0·2% (0·1 to 0·2%)	0·9 (0·5 to 1·3)	0·1% (0·1 to 0·2%)	-22·8% (-44·2 to 2·2%)	-32·0% (-50·1 to -10·7%)
Liver cancer due to alcohol use	65·3 (52·4 to 70·5)	100·0% (100·0 to 100·0%)	82·9 (73·2 to 92·7)	100·0% (100·0 to 100·0%)	27·0% (16·1 to 45·4%)	-	1,623·1 (1,290·8 to 1,759·9)	100·0% (100·0 to 100·0%)	1,995·4 (1,767·8 to 2,251·6)	100·0% (100·0 to 100·0%)	22·9% (11·1 to 45·2%)	-
Liver cancer due to other causes	0·1 (0·1 to 0·2)	0·3% (0·2 to 0·4%)	0·1 (0·0 to 0·1)	0·2% (0·1 to 0·2%)	-42·1% (-59·1 to -18·7%)	-37·2% (-55·0 to -15·8%)	6·2 (4·1 to 8·5)	0·5% (0·4 to 0·7%)	3·6 (2·3 to 5·3)	0·4% (0·2 to 0·5%)	-41·0% (-57·3 to -18·2%)	-29·7% (-48·2 to -8·2%)
Larynx cancer	5·9 (5·3 to 6·4)	22·2% (20·2 to 24·0%)	6·4 (5·6 to 7·1)	21·6% (19·0 to 23·8%)	8·9% (1·8 to 15·9%)	-2·6% (-8·5 to 1·5%)	159·9 (146·1 to 171·2)	23·9% (21·9 to 25·5%)	169·5 (149·0 to 186·1)	23·5% (20·8 to 25·6%)	6·0% (-0·7 to 12·7%)	-1·7% (-6·8 to 2·0%)
Breast cancer	5·4 (4·7 to 6·0)	5·2% (4·6 to 5·8%)	6·4 (5·4 to 7·3)	5·0% (4·3 to 5·7%)	18·3% (8·9 to 26·8%)	-3·1% (-8·5 to 1·8%)	166·8 (146·3 to 186·2)	5·4% (4·7 to 5·9%)	191·7 (161·0 to 222·1)	5·2% (4·4 to 5·9%)	14·9% (4·8 to 24·0%)	-2·2% (-7·9 to 3·0%)
Ischaemic heart disease	13·4 (-36·6 to 62·1)	0·6% (-1·7 to 2·9%)	6·5 (-42·0 to 53·1)	0·3% (-1·7 to 2·2%)	-51·6% (-402·9 to 316·1%)	-57·9% (-364·8 to 264·1%)	409·3 (-406·3 to 1,176·8)	1·0% (-1·0 to 3·0%)	223·7 (-481·3 to 881·8)	0·5% (-1·1 to 2·1%)	-45·3% (-368·1 to 310·3%)	-48·6% (-353·0 to 286·9%)
Ischaemic stroke	4·8 (0·0 to 9·6)	0·6% (0·0 to 1·3%)	4·8 (0·2 to 9·8)	0·6% (0·0 to 1·3%)	-0·5% (-51·7 to 81·5%)	-5·7% (-54·6 to 70·2%)	129·4 (62·2 to 197·3)	1·1% (0·5 to 1·7%)	140·3 (80·7 to 199·9)	1·2% (0·7 to 1·7%)	8·4% (-6·8 to 41·4%)	10·1% (-4·3 to 42·5%)
Hemorrhagic stroke	72·6 (64·5 to 80·5)	8·3% (7·4 to 9·2%)	72·7 (62·3 to 82·6)	8·8% (7·6 to 9·9%)	0·2% (-8·6 to 8·5%)	5·3% (-2·5 to 11·6%)	1,716·3 (1,558·7 to 1,871·0)	9·1% (8·4 to 9·9%)	1,655·1 (1,436·7 to 1,852·0)	9·9% (8·7 to 10·9%)	-3·6% (-11·8 to 4·3%)	8·2% (1·3 to 14·0%)
Hypertensive heart disease	11·4 (8·6 to 14·6)	5·9% (4·4 to 7·4%)	15·2 (10·5 to 19·9)	6·1% (4·2 to 8·1%)	33·2% (12·7 to 52·1%)	4·4% (-10·5 to 19·2%)	263·3 (217·1 to 313·5)	7·7% (6·3 to 9·0%)	330·2 (253·7 to 401·3)	8·3% (6·3 to 9·9%)	25·4% (10·8 to 39·4%)	8·0% (-2·8 to 18·2%)
Atrial fibrillation and flutter	1·7 (1·3 to 2·1)	5·0% (4·3 to 5·9%)	2·4 (1·8 to 3·0)	5·0% (4·2 to 6·0%)	40·5% (28·6 to 51·5%)	0·2% (-7·5 to 7·7%)	53·9 (41·7 to 69·2)	6·7% (6·0 to 7·3%)	71·1 (54·7 to 93·1)	6·8% (5·9 to 7·6%)	32·1% (23·5 to 39·1%)	1·1% (-5·1 to 6·3%)
Cirrhosis and other chronic liver diseases due to hepatitis B	22·7 (19·4 to 26·2)	30·0% (26·4 to 33·3%)	22·6 (17·9 to 27·0)	30·0% (24·2 to 34·4%)	-0·2% (-11·2 to 8·9%)	-0·2% (-9·4 to 6·1%)	689·7 (591·6 to 789·7)	30·8% (27·3 to 33·9%)	658·6 (524·3 to 783·9)	31·1% (25·4 to 35·4%)	-4·5% (-15·0 to 4·5%)	1·0% (-7·8 to 7·2%)
Cirrhosis and other chronic liver diseases due to hepatitis C	15·7 (12·8 to 18·4)	27·3% (23·0 to 31·2%)	16·4 (12·0 to 19·9)	26·7% (20·4 to 31·6%)	4·2% (-8·6 to 13·3%)	-2·5% (-14·1 to 5·0%)	471·2 (383·2 to 554·6)	28·0% (23·7 to 31·7%)	471·9 (348·0 to 573·2)	27·6% (21·2 to 32·5%)	0·2% (-11·5 to 8·9%)	-1·3% (-12·2 to 5·8%)
Cirrhosis and other chronic liver diseases due to alcohol use	73·3 (68·4 to 78·8)	100·0% (100·0 to 100·0%)	81·6 (75·9 to 87·7)	100·0% (100·0 to 100·0%)	11·3% (7·3 to 16·8%)	-	2,359·7 (2,199·8 to 2,525·2)	100·0% (100·0 to 100·0%)	2,511·2 (2,335·7 to 2,707·2)	100·0% (100·0 to 100·0%)	6·4% (2·2 to 12·0%)	-
Cirrhosis and other chronic liver diseases due to other causes	13·7 (11·7 to 15·6)	27·0% (23·7 to 30·0%)	13·8 (11·1 to 16·4)	26·6% (21·6 to 30·8%)	1·2% (-8·2 to 9·1%)	-1·5% (-9·9 to 4·6%)	404·7 (350·8 to 456·3)	25·3% (22·6 to 27·7%)	383·9 (309·6 to 450·2)	25·9% (21·4 to 29·6%)	-5·1% (-13·9 to 2·8%)	2·5% (-6·2 to 8·8%)
Pancreatitis	3·5 (3·1 to 4·0)	16·9% (15·1 to 18·4%)	3·6 (3·1 to 4·3)	15·4% (13·3 to 17·2%)	2·8% (-6·9 to 13·3%)	-8·8% (-17·7 to -1·4%)	131·2 (116·0 to 149·0)	19·6% (17·7 to 21·2%)	129·3 (112·1 to 152·9)	18·3% (16·0 to 20·1%)	-1·4% (-10·8 to 8·3%)	-6·3% (-14·7 to 0·8%)

Supplemental Results Table 1. All-age deaths, death PAFs, DALYs, DALY PAFs, and percent change for each risk factor and outcome for both sexes combined in 2005 and 2015 for High-middle SDI.

Data in parenthesis are 95% uncertainty intervals. PAFs=population attributable fractions. DALYs=disability-adjusted life-years.

	2005 Deaths (in thousands)	2005 Deaths PAF	2015 Deaths (in thousands)	2015 Deaths PAF	Percent change deaths 2005-2015	Percent change deaths PAF 2005-2015	2005 DALYs (in thousands)	2005 DALYs PAF	2015 DALYs (in thousands)	2015 DALYs PAF	Percent change DALYs 2005-2015	Percent change DALYs PAF 2005-2015
Epilepsy	3·1 (2·9 to 3·4)	13·0% (12·2 to 13·8%)	3·2 (2·8 to 3·6)	14·2% (12·7 to 15·5%)	2·2% (-5·5 to 10·3%)	9·5% (2·8 to 15·0%)	289·9 (240·1 to 344·5)	10·2% (9·4 to 11·0%)	271·3 (220·2 to 327·7)	11·3% (9·9 to 12·4%)	-6·4% (-13·1 to 0·1%)	10·7% (4·0 to 15·8%)
Alcohol use disorders	27·8 (26·8 to 29·0)	100·0% (100·0 to 100·0%)	25·1 (23·7 to 26·9)	100·0% (100·0 to 100·0%)	-9·7% (-14·6 to -3·4%)	-	2,538·3 (2,035·5 to 3,151·6)	100·0% (100·0 to 100·0%)	2,570·2 (2,012·5 to 3,305·9)	100·0% (100·0 to 100·0%)	1·3% (-2·7 to 5·1%)	-
Diabetes mellitus	-13·2 (-14·3 to -12·0)	-4·7% (-5·1 to -4·3%)	-15·1 (-16·6 to -13·7)	-4·3% (-4·7 to -3·9%)	14·5% (6·1 to 22·6%)	-9·8% (-16·2 to -3·3%)	-566·2 (-689·7 to -458·0)	-4·4% (-4·7 to -4·1%)	-668·6 (-835·7 to -529·0)	-4·1% (-4·4 to -3·9%)	18·1% (12·3 to 23·5%)	-6·3% (-10·4 to -2·4%)
Pedestrian road injuries	35·1 (29·3 to 41·7)	20·2% (17·2 to 23·7%)	32·2 (26·5 to 38·3)	20·4% (17·3 to 23·6%)	-8·4% (-17·4 to -0·1%)	0·6% (-2·9 to 3·9%)	1,749·8 (1,464·7 to 2,079·2)	22·1% (18·9 to 25·9%)	1,517·4 (1,253·1 to 1,799·2)	23·0% (19·8 to 26·7%)	-13·3% (-21·5 to -5·6%)	4·0% (0·5 to 7·3%)
Cyclist road injuries	5·0 (4·1 to 6·1)	22·7% (19·3 to 26·7%)	4·2 (3·5 to 5·1)	22·2% (18·9 to 25·7%)	-15·0% (-25·1 to -4·0%)	-2·4% (-5·8 to 1·0%)	273·3 (225·3 to 331·5)	21·7% (18·5 to 25·4%)	228·9 (190·6 to 274·3)	20·7% (17·7 to 24·0%)	-16·2% (-24·6 to -7·2%)	-4·4% (-8·1 to -0·8%)
Motorcyclist road injuries	25·6 (19·3 to 29·2)	38·1% (34·2 to 42·0%)	23·3 (19·1 to 26·6)	38·7% (35·1 to 42·2%)	-8·7% (-18·9 to 9·7%)	1·7% (-1·7 to 5·5%)	1,433·0 (1,116·2 to 1,639·8)	37·7% (33·8 to 41·6%)	1,274·6 (1,072·7 to 1,453·3)	38·7% (35·2 to 42·0%)	-11·1% (-20·0 to 5·0%)	2·6% (-0·4 to 6·3%)
Motor vehicle road injuries	41·2 (36·5 to 47·2)	32·9% (30·0 to 36·0%)	37·8 (33·2 to 42·7)	32·3% (29·5 to 35·1%)	-8·1% (-15·5 to 0·0%)	-1·6% (-4·7 to 1·7%)	2,229·9 (1,973·7 to 2,550·9)	32·5% (29·6 to 35·7%)	2,004·8 (1,777·2 to 2,273·0)	32·6% (29·8 to 35·4%)	-10·1% (-16·4 to -3·1%)	0·1% (-2·7 to 3·3%)
Falls	9·6 (8·3 to 11·3)	11·8% (10·3 to 13·9%)	11·4 (9·5 to 13·5)	11·3% (9·8 to 13·3%)	18·6% (4·7 to 28·6%)	-4·2% (-8·6 to 0·2%)	600·3 (488·1 to 735·4)	12·4% (10·9 to 14·4%)	648·9 (525·5 to 808·0)	12·5% (11·1 to 14·4%)	8·1% (0·2 to 13·8%)	1·0% (-1·2 to 3·4%)
Drowning	7·7 (6·7 to 9·0)	10·5% (9·2 to 12·3%)	6·3 (5·6 to 7·3)	11·8% (10·5 to 13·6%)	-17·7% (-22·7 to -11·8%)	12·2% (8·3 to 16·5%)	350·1 (304·8 to 409·7)	8·8% (7·6 to 10·2%)	269·1 (235·6 to 310·9)	10·3% (9·1 to 11·7%)	-23·1% (-27·6 to -18·1%)	17·3% (12·3 to 22·5%)
Fire, heat, and hot substances	3·4 (2·7 to 4·1)	8·3% (6·9 to 10·4%)	3·1 (2·3 to 3·7)	8·7% (7·3 to 10·6%)	-10·1% (-19·2 to 0·8%)	5·5% (-4·5 to 14·3%)	173·5 (141·1 to 208·0)	7·2% (6·0 to 9·0%)	152·7 (120·6 to 185·4)	8·0% (6·8 to 9·8%)	-12·0% (-19·2 to -3·6%)	11·6% (1·4 to 20·3%)
Poisonings	2·8 (2·2 to 3·4)	11·4% (9·8 to 13·4%)	1·9 (1·5 to 2·5)	11·0% (9·4 to 13·3%)	-29·4% (-37·0 to -15·6%)	-3·2% (-9·4 to 4·0%)	121·2 (96·9 to 149·2)	10·0% (8·7 to 11·8%)	83·6 (65·2 to 105·7)	9·8% (8·4 to 11·8%)	-31·0% (-37·5 to -18·9%)	-1·7% (-8·7 to 6·4%)
Unintentional firearm injuries	1·0 (0·7 to 1·2)	10·6% (9·2 to 12·2%)	0·8 (0·6 to 1·0)	10·7% (9·3 to 12·4%)	-15·3% (-21·4 to -8·5%)	1·0% (-4·4 to 6·8%)	51·0 (37·3 to 60·1)	10·2% (8·9 to 11·9%)	42·1 (32·3 to 49·8)	10·9% (9·5 to 12·5%)	-17·4% (-23·5 to -10·1%)	5·9% (0·1 to 12·5%)
Unintentional suffocation	0·2 (0·2 to 0·3)	3·8% (3·1 to 4·5%)	0·3 (0·2 to 0·4)	6·1% (4·8 to 7·3%)	51·8% (9·7 to 76·5%)	61·3% (38·1 to 84·3%)	16·1 (12·8 to 19·9)	3·2% (2·6 to 4·0%)	20·5 (15·5 to 25·4)	4·9% (3·9 to 5·8%)	27·6% (6·5 to 41·8%)	50·4% (31·8 to 71·0%)
Other exposure to mechanical forces	3·7 (2·7 to 4·5)	14·0% (12·1 to 16·4%)	3·6 (2·2 to 4·5)	14·7% (12·6 to 17·2%)	-2·8% (-24·6 to 14·2%)	5·0% (-2·0 to 11·1%)	235·8 (184·9 to 292·3)	12·6% (10·9 to 14·7%)	227·5 (159·0 to 292·6)	13·5% (11·7 to 15·6%)	-3·5% (-17·4 to 7·7%)	7·4% (2·7 to 11·9%)
Self-harm	24·6 (21·6 to 28·8)	13·5% (11·9 to 15·6%)	24·8 (21·5 to 29·3)	14·4% (12·7 to 16·5%)	0·8% (-6·4 to 8·8%)	7·0% (1·5 to 11·0%)	1,043·2 (907·6 to 1,220·5)	13·3% (11·7 to 15·3%)	1,012·8 (877·4 to 1,192·4)	14·5% (12·8 to 16·6%)	-2·9% (-9·5 to 4·6%)	9·1% (3·5 to 13·3%)
Assault by firearm	10·6 (9·3 to 12·1)	15·2% (13·7 to 17·2%)	10·1 (8·9 to 11·5)	14·8% (13·4 to 16·7%)	-4·7% (-10·0 to 2·1%)	-2·8% (-4·1 to -1·4%)	604·7 (530·4 to 687·6)	15·9% (14·3 to 18·0%)	566·6 (497·3 to 646·8)	15·7% (14·1 to 17·6%)	-6·3% (-11·6 to 0·3%)	-1·6% (-2·9 to -0·3%)
Assault by sharp object	4·8 (4·2 to 5·6)	15·3% (13·6 to 17·4%)	4·4 (3·9 to 5·1)	15·4% (13·9 to 17·4%)	-8·2% (-14·8 to 0·2%)	1·0% (-1·2 to 3·3%)	265·9 (232·4 to 310·4)	16·1% (14·3 to 18·3%)	240·5 (211·6 to 279·1)	16·5% (14·9 to 18·6%)	-9·5% (-16·0 to -1·1%)	2·8% (0·7 to 5·2%)
Assault by other means	4·1 (3·6 to 4·9)	13·0% (11·5 to 14·9%)	3·8 (3·3 to 4·5)	13·0% (11·7 to 14·8%)	-7·7% (-13·9 to 1·1%)	0·3% (-1·9 to 2·6%)	230·2 (199·6 to 273·9)	13·4% (11·9 to 15·4%)	209·8 (181·0 to 248·5)	13·9% (12·5 to 15·7%)	-8·8% (-14·7 to -0·9%)	3·7% (1·3 to 6·0%)
**Drug use: All causes**	97·6 (86·6 to 108·2)	0·9% (0·8 to 1·0%)	115·0 (102·3 to 126·5)	1·0% (0·9 to 1·1%)	17·8% (10·9 to 24·3%)	11·5% (5·9 to 17·0%)	6,047·9 (5,308·8 to 6,776·0)	1·3% (1·1 to 1·4%)	6,838·0 (5,973·1 to 7,713·8)	1·5% (1·3 to 1·6%)	13·1% (8·1 to 17·5%)	13·9% (9·1 to 18·2%)
HIV/AIDS - Tuberculosis	3·0 (2·4 to 3·3)	4·4% (4·0 to 4·8%)	1·9 (1·5 to 2·1)	4·9% (4·3 to 5·7%)	-37·2% (-41·3 to -32·4%)	11·8% (-0·1 to 27·9%)	145·1 (117·5 to 163·4)	4·0% (3·7 to 4·5%)	88·0 (71·6 to 100·3)	4·4% (3·8 to 5·1%)	-39·3% (-43·0 to -34·9%)	8·6% (-2·1 to 23·3%)
HIV/AIDS resulting in other diseases	12·6 (11·3 to 14·5)	5·1% (4·5 to 6·0%)	11·2 (9·8 to 12·9)	7·2% (6·2 to 8·3%)	-11·5% (-19·4 to -3·0%)	40·3% (25·8 to 54·7%)	632·1 (561·7 to 714·7)	4·8% (4·2 to 5·5%)	530·2 (467·5 to 601·9)	6·3% (5·5 to 7·2%)	-16·1% (-22·8 to -9·1%)	31·8% (19·1 to 43·3%)
Hepatitis B	0·1 (0·0 to 0·2)	0·9% (0·4 to 1·5%)	0·1 (0·0 to 0·1)	0·9% (0·4 to 1·4%)	-27·7% (-37·1 to -15·7%)	-2·8% (-13·0 to 10·3%)	4·1 (1·8 to 6·7)	0·9% (0·4 to 1·5%)	2·9 (1·3 to 4·7)	0·9% (0·4 to 1·4%)	-28·1% (-37·2 to -16·8%)	-3·5% (-12·9 to 7·6%)
Hepatitis C	0·2 (0·0 to 0·6)	49·4% (39·7 to 57·7%)	0·2 (0·0 to 0·4)	49·2% (39·9 to 57·9%)	-24·2% (-39·9 to -3·1%)	-0·3% (-10·8 to 11·3%)	8·7 (2·2 to 19·7)	51·3% (42·8 to 58·6%)	6·5 (1·9 to 14·3)	51·1% (42·8 to 58·6%)	-25·2% (-40·3 to -4·2%)	-0·5% (-8·8 to 8·4%)
Liver cancer due to hepatitis B	1·3 (0·6 to 2·2)	1·4% (0·6 to 2·3%)	1·4 (0·6 to 2·4)	1·6% (0·7 to 2·6%)	7·0% (-9·6 to 38·7%)	13·7% (1·9 to 29·5%)	46·2 (19·3 to 75·8)	1·5% (0·7 to 2·5%)	45·6 (20·5 to 73·9)	1·7% (0·8 to 2·8%)	-1·4% (-17·6 to 28·5%)	12·9% (1·2 to 27·5%)
Liver cancer due to hepatitis C	13·2 (9·3 to 16·9)	41·9% (30·5 to 53·1%)	18·3 (13·8 to 22·6)	48·4% (37·4 to 58·5%)	38·9% (25·9 to 57·7%)	15·3% (7·3 to 28·0%)	339·5 (254·4 to 419·4)	50·6% (39·3 to 61·1%)	431·5 (343·6 to 522·1)	56·7% (46·4 to 66·2%)	27·1% (15·5 to 44·3%)	11·9% (5·1 to 22·2%)
Cirrhosis and other chronic liver diseases due to hepatitis B	0·9 (0·4 to 1·5)	1·2% (0·5 to 2·0%)	1·0 (0·4 to 1·6)	1·3% (0·6 to 2·1%)	10·2% (-1·1 to 24·7%)	10·1% (0·5 to 22·5%)	29·8 (12·8 to 49·7)	1·3% (0·6 to 2·2%)	30·3 (13·2 to 48·7)	1·4% (0·6 to 2·3%)	1·6% (-8·9 to 15·0%)	7·5% (-1·9 to 19·2%)
Cirrhosis and other chronic liver diseases due to hepatitis C	29·6 (23·4 to 35·5)	51·6% (40·9 to 60·6%)	33·3 (26·6 to 39·3)	54·3% (44·0 to 62·8%)	12·5% (6·4 to 21·4%)	5·3% (1·1 to 11·4%)	998·5 (822·2 to 1,156·1)	59·4% (49·4 to 67·5%)	1,042·5 (860·4 to 1,210·1)	61·1% (51·7 to 68·5%)	4·4% (-1·0 to 12·3%)	2·9% (-0·7 to 7·9%)
Opioid use disorders	22·7 (20·2 to 24·2)	100·0% (100·0 to 100·0%)	30·3 (23·9 to 33·2)	100·0% (100·0 to 100·0%)	33·4% (11·9 to 47·2%)	-	2,502·1 (2,036·5 to 2,933·5)	100·0% (100·0 to 100·0%)	3,071·6 (2,475·6 to 3,623·5)	100·0% (100·0 to 100·0%)	22·8% (14·2 to 28·8%)	-
Cocaine use disorders	1·2 (1·0 to 1·7)	100·0% (100·0 to 100·0%)	1·8 (1·4 to 2·9)	100·0% (100·0 to 100·0%)	57·4% (24·5 to 94·2%)	-	152·4 (112·8 to 200·8)	100·0% (100·0 to 100·0%)	201·9 (149·2 to 264·9)	100·0% (100·0 to 100·0%)	32·5% (20·8 to 49·6%)	-
Amphetamine use disorders	1·8 (1·4 to 2·2)	100·0% (100·0 to 100·0%)	3·7 (2·1 to 4·8)	100·0% (100·0 to 100·0%)	106·6% (38·9 to 156·3%)	-	314·9 (214·5 to 433·6)	100·0% (100·0 to 100·0%)	452·4 (320·9 to 603·8)	100·0% (100·0 to 100·0%)	43·7% (26·5 to 61·6%)	-
Cannabis use disorders	-	-	-	-	-	-	136·1 (87·2 to 194·0)	100·0% (100·0 to 100·0%)	139·8 (90·4 to 199·1)	100·0% (100·0 to 100·0%)	2·7% (0·1 to 5·3%)	-
Other drug use disorders	4·6 (4·1 to 5·4)	100·0% (100·0 to 100·0%)	5·6 (4·7 to 6·5)	100·0% (100·0 to 100·0%)	21·4% (0·4 to 44·7%)	-	414·0 (326·8 to 520·8)	100·0% (100·0 to 100·0%)	490·4 (382·8 to 619·2)	100·0% (100·0 to 100·0%)	18·4% (7·8 to 30·9%)	-
Self-harm	6·3 (4·3 to 9·1)	3·5% (2·3 to 5·0%)	6·1 (4·1 to 8·9)	3·6% (2·4 to 5·1%)	-3·2% (-10·6 to 5·9%)	2·7% (-1·5 to 7·3%)	324·4 (215·0 to 480·0)	4·1% (2·7 to 6·0%)	304·3 (199·8 to 449·2)	4·3% (2·9 to 6·3%)	-6·2% (-13·2 to 2·9%)	5·4% (1·1 to 10·1%)
**Dietary risks: All causes**	3,028·3 (2,691·5 to 3,372·3)	27·1% (24·1 to 30·2%)	3,283·5 (2,906·8 to 3,680·8)	27·9% (24·7 to 31·1%)	8·4% (4·8 to 12·2%)	2·7% (1·1 to 4·2%)	67,009·0 (60,057·9 to 74,143·3)	14·3% (12·6 to 16·1%)	69,300·5 (61,517·0 to 77,024·1)	14·9% (13·1 to 17·0%)	3·4% (-0·4 to 7·2%)	4·2% (1·5 to 6·8%)
**Diet low in fruits: All causes**	731·8 (483·6 to 1,007·5)	6·6% (4·3 to 9·0%)	743·0 (482·1 to 1,032·7)	6·3% (4·1 to 8·7%)	1·5% (-3·5 to 6·1%)	-3·9% (-7·4 to -1·0%)	18,153·0 (12,248·2 to 24,479·4)	3·9% (2·6 to 5·3%)	17,738·7 (11,617·9 to 24,414·5)	3·8% (2·5 to 5·3%)	-2·3% (-7·4 to 2·2%)	-1·5% (-5·8 to 2·1%)
Lip and oral cavity cancer	1·6 (0·0 to 3·5)	6·8% (0·0 to 14·8%)	2·2 (0·0 to 4·8)	6·8% (0·0 to 14·7%)	35·4% (0·0 to 42·5%)	-0·4% (-1·6 to 0·5%)	42·7 (0·0 to 92·9)	6·8% (0·0 to 14·8%)	55·0 (0·0 to 121·5)	6·8% (0·0 to 14·8%)	28·9% (0·0 to 36·0%)	-0·4% (-1·7 to 0·6%)
Nasopharynx cancer	1·3 (0·0 to 2·7)	7·4% (0·0 to 15·8%)	1·3 (0·0 to 2·8)	7·2% (0·0 to 15·5%)	2·7% (-9·8 to 14·2%)	-2·5% (-4·4 to 0·0%)	41·1 (0·0 to 88·4)	7·4% (0·0 to 15·6%)	38·7 (0·0 to 83·7)	7·2% (0·0 to 15·4%)	-5·8% (-18·4 to 6·3%)	-2·2% (-4·3 to 0·0%)
Other pharynx cancer	0·7 (0·0 to 1·6)	6·8% (0·0 to 15·3%)	0·9 (0·0 to 2·1)	6·8% (0·0 to 15·1%)	25·3% (0·0 to 31·4%)	-1·2% (-2·6 to 0·0%)	20·5 (0·0 to 45·3)	6·8% (0·0 to 15·3%)	25·0 (0·0 to 56·0)	6·8% (0·0 to 15·2%)	21·9% (0·0 to 28·2%)	-1·0% (-2·4 to 0·2%)
Oesophageal cancer	34·1 (8·4 to 59·7)	23·2% (5·8 to 40·6%)	29·1 (7·0 to 51·3)	22·6% (5·6 to 39·9%)	-14·5% (-21·6 to -6·3%)	-2·5% (-4·1 to -1·5%)	775·0 (192·5 to 1,365·2)	23·3% (5·8 to 40·7%)	632·1 (154·0 to 1,111·8)	22·5% (5·6 to 39·9%)	-18·4% (-25·9 to -9·9%)	-3·1% (-5·0 to -2·0%)
Larynx cancer	1·8 (0·0 to 4·0)	6·8% (0·0 to 15·2%)	2·0 (0·0 to 4·5)	6·8% (0·0 to 15·2%)	11·0% (0·0 to 15·9%)	-0·8% (-1·9 to 0·1%)	46·0 (0·0 to 102·8)	6·9% (0·0 to 15·3%)	49·1 (0·0 to 110·7)	6·8% (0·0 to 15·2%)	6·8% (0·0 to 11·6%)	-1·0% (-2·4 to 0·0%)
Tracheal, bronchus, and lung cancer	51·7 (21·1 to 87·7)	12·3% (5·0 to 20·8%)	60·0 (24·3 to 101·4)	12·1% (4·9 to 20·4%)	16·0% (9·5 to 23·8%)	-1·7% (-3·1 to -0·8%)	1,204·8 (491·1 to 2,038·5)	12·4% (5·0 to 20·9%)	1,319·2 (535·4 to 2,229·7)	12·1% (4·9 to 20·4%)	9·5% (2·9 to 17·8%)	-2·3% (-4·1 to -1·3%)
Ischaemic heart disease	260·1 (93·7 to 434·3)	12·3% (4·4 to 20·7%)	287·7 (103·1 to 481·8)	11·9% (4·2 to 19·9%)	10·6% (7·1 to 14·1%)	-3·6% (-4·6 to -2·8%)	5,769·4 (2,112·0 to 9,512·1)	14·4% (5·3 to 23·9%)	5,936·4 (2,159·0 to 9,887·8)	14·0% (5·1 to 23·2%)	2·9% (-1·5 to 6·7%)	-3·3% (-4·7 to -2·4%)
Ischaemic stroke	138·2 (72·1 to 209·0)	18·6% (10·0 to 28·4%)	134·6 (69·9 to 206·4)	17·1% (9·1 to 26·3%)	-2·6% (-7·5 to 2·0%)	-7·8% (-9·7 to -6·3%)	2,944·5 (1,584·2 to 4,343·0)	24·8% (13·8 to 36·8%)	2,778·0 (1,517·8 to 4,168·6)	23·8% (13·0 to 35·6%)	-5·7% (-10·5 to -0·9%)	-4·3% (-6·1 to -2·8%)

Supplemental Results Table 1. All-age deaths, death PAFs, DALYs, DALY PAFs, and percent change for each risk factor and outcome for both sexes combined in 2005 and 2015 for High-middle SDI.

Data in parenthesis are 95% uncertainty intervals. PAFs=population attributable fractions. DALYs=disability-adjusted life-years.

	2005 Deaths (in thousands)	2005 Deaths PAF	2015 Deaths (in thousands)	2015 Deaths PAF	Percent change deaths 2005-2015	Percent change deaths PAF 2005-2015	2005 DALYs (in thousands)	2005 DALYs PAF	2015 DALYs (in thousands)	2015 DALYs PAF	Percent change DALYs 2005-2015	Percent change DALYs PAF 2005-2015
Hemorrhagic stroke	215·2<br>(117·2 to 320·4)	24·7%<br>(13·4 to 36·5%)	192·2<br>(106·3 to 291·6)	23·2%<br>(12·7 to 34·8%)	-10·7%<br>(-15·7 to -5·4%)	-6·1%<br>(-8·2 to -4·6%)	5,719·6<br>(3,223·5 to 8,314·6)	30·5%<br>(17·0 to 44·2%)	4,950·1<br>(2,821·7 to 7,334·6)	29·6%<br>(16·6 to 43·4%)	-13·5%<br>(-18·6 to -8·8%)	-2·9%<br>(-4·7 to -1·6%)
**Diet low in vegetables: All causes**	459·0<br>(233·0 to 726·1)	4·1%<br>(2·1 to 6·5%)	473·8<br>(234·7 to 754·2)	4·0%<br>(2·0 to 6·4%)	3·2%<br>(-2·1 to 7·4%)	-2·2%<br>(-6·7 to 1·1%)	10,305·2<br>(5,312·6 to 16,115·6)	2·2%<br>(1·2 to 3·5%)	9,994·7<br>(5,052·0 to 15,774·9)	2·2%<br>(1·1 to 3·4%)	-3·0%<br>(-7·6 to 1·3%)	-2·3%<br>(-6·7 to 1·3%)
Ischaemic heart disease	321·9<br>(122·0 to 541·6)	15·2%<br>(5·8 to 25·6%)	351·0<br>(131·9 to 592·1)	14·5%<br>(5·5 to 24·5%)	9·0%<br>(6·2 to 11·8%)	-4·9%<br>(-6·2 to -4·0%)	6,912·3<br>(2,675·1 to 11,487·0)	17·3%<br>(6·7 to 28·9%)	7,047·2<br>(2,697·7 to 11,706·6)	16·6%<br>(6·4 to 27·9%)	2·0%<br>(-1·6 to 5·0%)	-4·1%<br>(-5·5 to -3·1%)
Ischaemic stroke	53·9<br>(12·2 to 101·0)	7·3%<br>(1·6 to 13·5%)	49·9<br>(11·4 to 93·6)	6·4%<br>(1·4 to 11·9%)	-7·5%<br>(-11·2 to -3·6%)	-12·4%<br>(-14·5 to -10·5%)	1,147·6<br>(266·5 to 2,130·6)	9·7%<br>(2·2 to 17·9%)	1,028·6<br>(240·9 to 1,907·8)	8·8%<br>(2·0 to 16·3%)	-10·4%<br>(-14·3 to -6·5%)	-9·0%<br>(-11·2 to -6·9%)
Hemorrhagic stroke	83·2<br>(22·5 to 157·9)	9·6%<br>(2·6 to 18·3%)	72·9<br>(19·6 to 138·7)	8·8%<br>(2·4 to 16·8%)	-12·4%<br>(-16·2 to -8·3%)	-7·9%<br>(-9·6 to -6·2%)	2,245·3<br>(613·3 to 4,211·0)	12·0%<br>(3·3 to 22·5%)	1,919·0<br>(525·6 to 3,601·6)	11·5%<br>(3·1 to 21·6%)	-14·5%<br>(-18·5 to -10·6%)	-4·1%<br>(-5·8 to -2·3%)
**Diet low in whole grains: All causes**	804·1<br>(574·5 to 1,062·1)	7·2%<br>(5·2 to 9·5%)	837·2<br>(593·6 to 1,115·5)	7·1%<br>(5·0 to 9·4%)	4·1%<br>(0·1 to 7·9%)	-1·4%<br>(-4·0 to 0·7%)	20,264·2<br>(14,801·7 to 26,137·3)	4·3%<br>(3·2 to 5·7%)	20,408·3<br>(14,722·6 to 26,363·4)	4·4%<br>(3·1 to 5·8%)	0·7%<br>(-3·5 to 4·8%)	1·5%<br>(-1·8 to 4·3%)
Ischaemic heart disease	430·4<br>(259·2 to 620·5)	20·4%<br>(12·2 to 29·4%)	478·3<br>(287·4 to 689·1)	19·7%<br>(11·8 to 28·5%)	11·1%<br>(7·7 to 14·7%)	-3·1%<br>(-4·0 to -2·4%)	9,425·1<br>(5,752·6 to 13,313·8)	23·6%<br>(14·4 to 33·6%)	9,748·5<br>(5,931·1 to 13,897·9)	22·9%<br>(13·9 to 32·7%)	3·4%<br>(-0·8 to 7·3%)	-2·8%<br>(-3·8 to -2·0%)
Ischaemic stroke	129·8<br>(88·3 to 174·9)	17·4%<br>(12·0 to 23·4%)	126·2<br>(85·2 to 171·6)	16·1%<br>(11·0 to 21·6%)	-2·8%<br>(-7·6 to 2·1%)	-7·9%<br>(-9·7 to -6·2%)	2,852·7<br>(1,983·2 to 3,807·0)	24·1%<br>(17·0 to 31·6%)	2,711·3<br>(1,855·1 to 3,584·3)	23·2%<br>(16·2 to 30·6%)	-5·0%<br>(-9·8 to 0·0%)	-3·5%<br>(-5·1 to -2·1%)
Hemorrhagic stroke	197·2<br>(140·2 to 264·3)	22·6%<br>(16·1 to 30·1%)	176·5<br>(123·7 to 235·9)	21·3%<br>(15·0 to 28·6%)	-10·5%<br>(-15·4 to -5·3%)	-5·9%<br>(-7·7 to -4·5%)	5,247·5<br>(3,779·6 to 6,905·1)	27·9%<br>(20·2 to 36·7%)	4,567·8<br>(3,225·5 to 6,032·9)	27·3%<br>(19·6 to 36·0%)	-13·0%<br>(-17·9 to -8·0%)	-2·3%<br>(-3·6 to -1·2%)
Diabetes mellitus	46·7<br>(25·4 to 70·5)	16·7%<br>(9·1 to 25·6%)	56·2<br>(30·5 to 86·7)	15·9%<br>(8·6 to 24·5%)	20·4%<br>(15·4 to 25·0%)	-5·1%<br>(-6·5 to -4·0%)	2,738·9<br>(1,504·2 to 4,303·1)	21·3%<br>(12·0 to 32·0%)	3,380·8<br>(1,840·2 to 5,360·9)	20·9%<br>(11·7 to 31·5%)	23·4%<br>(20·0 to 26·5%)	-2·1%<br>(-2·9 to -1·4%)
**Diet low in nuts and seeds: All causes**	497·2<br>(312·7 to 704·3)	4·5%<br>(2·8 to 6·3%)	563·6<br>(354·1 to 802·8)	4·8%<br>(3·0 to 6·8%)	13·4%<br>(10·3 to 16·6%)	7·4%<br>(5·5 to 9·0%)	11,630·4<br>(7,719·0 to 16,098·2)	2·5%<br>(1·6 to 3·5%)	12,523·8<br>(8,319·9 to 17,374·9)	2·7%<br>(1·8 to 3·8%)	7·7%<br>(3·9 to 11·5%)	8·5%<br>(5·5 to 11·2%)
Ischaemic heart disease	467·2<br>(290·1 to 670·1)	22·1%<br>(13·7 to 31·6%)	526·6<br>(324·2 to 751·5)	21·7%<br>(13·5 to 31·1%)	12·7%<br>(9·6 to 16·0%)	-1·7%<br>(-2·2 to -1·3%)	9,960·1<br>(6,292·0 to 13,996·5)	25·0%<br>(15·7 to 35·0%)	10,437·9<br>(6,617·4 to 14,707·0)	24·6%<br>(15·5 to 34·4%)	4·8%<br>(0·8 to 8·5%)	-1·5%<br>(-2·4 to -0·7%)
Diabetes mellitus	30·0<br>(15·3 to 46·7)	10·8%<br>(5·6 to 16·8%)	37·0<br>(19·2 to 58·1)	10·5%<br>(5·5 to 16·4%)	23·4%<br>(18·9 to 27·8%)	-2·7%<br>(-3·5 to -2·0%)	1,670·3<br>(791·5 to 2,689·3)	13·0%<br>(6·8 to 20·3%)	2,085·8<br>(989·3 to 3,379·9)	12·9%<br>(6·7 to 20·1%)	24·9%<br>(21·8 to 27·7%)	-0·9%<br>(-1·4 to -0·5%)
**Diet low in milk: All causes**	26·2<br>(9·1 to 44·1)	0·2%<br>(0·1 to 0·4%)	32·9<br>(11·6 to 55·4)	0·3%<br>(0·1 to 0·5%)	25·5%<br>(21·0 to 30·2%)	18·8%<br>(15·9 to 21·7%)	582·2<br>(201·3 to 978·9)	0·1%<br>(0·0 to 0·2%)	691·7<br>(243·7 to 1,168·6)	0·1%<br>(0·1 to 0·3%)	18·8%<br>(13·9 to 24·2%)	19·7%<br>(15·5 to 24·1%)
Colon and rectum cancer	26·2<br>(9·1 to 44·1)	15·5%<br>(5·4 to 26·0%)	32·9<br>(11·6 to 55·4)	15·5%<br>(5·5 to 26·1%)	25·5%<br>(21·0 to 30·2%)	0·5%<br>(-0·1 to 1·1%)	582·2<br>(201·3 to 978·9)	15·5%<br>(5·4 to 26·1%)	691·7<br>(243·7 to 1,168·6)	15·6%<br>(5·5 to 26·3%)	18·8%<br>(13·9 to 24·2%)	0·6%<br>(0·0 to 1·2%)
**Diet high in red meat: All causes**	10·2<br>(4·6 to 16·3)	0·1%<br>(0·0 to 0·1%)	12·0<br>(5·3 to 19·2)	0·1%<br>(0·0 to 0·2%)	17·6%<br>(12·9 to 22·8%)	11·4%<br>(7·7 to 15·4%)	438·4<br>(152·4 to 747·7)	0·1%<br>(0·0 to 0·2%)	530·3<br>(178·7 to 904·1)	0·1%<br>(0·0 to 0·2%)	21·0%<br>(16·4 to 24·6%)	21·8%<br>(17·9 to 25·2%)
Colon and rectum cancer	5·0<br>(1·1 to 8·9)	3·0%<br>(0·6 to 5·2%)	6·1<br>(1·3 to 10·9)	2·9%<br>(0·6 to 5·1%)	21·1%<br>(15·1 to 26·5%)	-3·0%<br>(-5·3 to -0·5%)	115·5<br>(24·5 to 202·5)	3·1%<br>(0·7 to 5·5%)	135·5<br>(28·7 to 244·9)	3·1%<br>(0·7 to 5·5%)	17·3%<br>(10·9 to 23·4%)	-0·7%<br>(-3·0 to 1·8%)
Diabetes mellitus	5·1<br>(0·6 to 9·5)	1·8%<br>(0·2 to 3·4%)	5·9<br>(0·7 to 10·8)	1·7%<br>(0·2 to 3·0%)	14·3%<br>(9·5 to 18·6%)	-9·9%<br>(-12·4 to -7·2%)	322·8<br>(37·5 to 613·0)	2·5%<br>(0·3 to 4·6%)	394·8<br>(46·2 to 747·9)	2·4%<br>(0·3 to 4·5%)	22·3%<br>(18·0 to 25·8%)	-3·0%<br>(-4·9 to -0·9%)
**Diet high in processed meat: All causes**	124·2<br>(31·8 to 213·9)	1·1%<br>(0·3 to 1·9%)	140·4<br>(38·2 to 238·9)	1·2%<br>(0·3 to 2·0%)	13·1%<br>(9·9 to 20·9%)	7·1%<br>(4·7 to 14·0%)	3,404·4<br>(1,315·3 to 5,438·2)	0·7%<br>(0·3 to 1·2%)	3,800·6<br>(1,618·1 to 6,051·2)	0·8%<br>(0·3 to 1·3%)	11·6%<br>(6·5 to 23·9%)	12·5%<br>(7·9 to 24·4%)
Colon and rectum cancer	7·0<br>(3·8 to 10·6)	4·1%<br>(2·2 to 6·2%)	8·8<br>(4·7 to 13·2)	4·2%<br>(2·3 to 6·2%)	26·4%<br>(22·4 to 30·7%)	1·3%<br>(-0·9 to 3·3%)	151·6<br>(82·4 to 228·8)	4·0%<br>(2·2 to 6·1%)	181·6<br>(99·2 to 275·0)	4·1%<br>(2·2 to 6·2%)	19·8%<br>(15·9 to 24·0%)	1·5%<br>(-0·5 to 3·7%)
Ischaemic heart disease	97·6<br>(4·2 to 184·2)	4·6%<br>(0·2 to 8·7%)	107·6<br>(4·7 to 204·4)	4·4%<br>(0·2 to 8·4%)	10·3%<br>(5·6 to 13·2%)	-3·8%<br>(-5·5 to -1·6%)	2,118·1<br>(90·4 to 3,987·7)	5·3%<br>(0·2 to 10·0%)	2,185·9<br>(94·2 to 4,126·3)	5·1%<br>(0·2 to 9·7%)	3·2%<br>(-0·5 to 6·5%)	-3·0%<br>(-4·6 to -0·7%)
Diabetes mellitus	19·7<br>(9·7 to 29·3)	7·1%<br>(3·5 to 10·5%)	24·0<br>(11·9 to 36·0)	6·8%<br>(3·4 to 10·1%)	21·9%<br>(17·1 to 26·7%)	-3·9%<br>(-5·7 to -2·1%)	1,134·8<br>(534·5 to 1,787·3)	8·8%<br>(4·3 to 13·1%)	1,433·0<br>(669·8 to 2,253·7)	8·9%<br>(4·4 to 13·3%)	26·3%<br>(22·3 to 29·8%)	0·2%<br>(-1·3 to 1·8%)
**Diet high in sugar-sweetened beverages: All causes**	9·7<br>(6·8 to 13·8)	0·1%<br>(0·1 to 0·1%)	11·7<br>(8·1 to 16·7)	0·1%<br>(0·1 to 0·1%)	20·3%<br>(16·6 to 23·6%)	14·0%<br>(10·2 to 17·7%)	346·7<br>(239·3 to 497·3)	0·1%<br>(0·1 to 0·1%)	422·7<br>(286·8 to 610·6)	0·1%<br>(0·1 to 0·1%)	21·9%<br>(17·6 to 25·6%)	22·8%<br>(18·7 to 26·6%)
Oesophageal cancer	0·1<br>(0·0 to 0·2)	0·1%<br>(0·0 to 0·1%)	0·1<br>(0·0 to 0·2)	0·1%<br>(0·0 to 0·2%)	19·3%<br>(10·0 to 26·3%)	35·9%<br>(22·6 to 47·6%)	2·9<br>(0·9 to 5·2)	0·1%<br>(0·0 to 0·2%)	3·3<br>(1·0 to 6·1)	0·1%<br>(0·0 to 0·2%)	15·3%<br>(5·3 to 22·5%)	37·0%<br>(22·4 to 49·4%)
Colon and rectum cancer	0·1<br>(0·1 to 0·2)	0·1%<br>(0·0 to 0·1%)	0·2<br>(0·1 to 0·2)	0·1%<br>(0·1 to 0·1%)	36·3%<br>(31·3 to 41·3%)	9·2%<br>(4·9 to 13·7%)	3·0<br>(2·0 to 4·4)	0·1%<br>(0·1 to 0·1%)	4·0<br>(2·7 to 5·9)	0·1%<br>(0·1 to 0·1%)	32·7%<br>(27·4 to 37·8%)	12·4%<br>(7·0 to 17·8%)
Liver cancer due to hepatitis B	0·0<br>(0·0 to 0·1)	0·0%<br>(0·0 to 0·1%)	0·1<br>(0·0 to 0·1)	0·1%<br>(0·0 to 0·1%)	22·3%<br>(10·9 to 34·7%)	29·7%<br>(13·3 to 45·0%)	1·4<br>(0·6 to 2·3)	0·0%<br>(0·0 to 0·1%)	1·6<br>(0·7 to 2·8)	0·1%<br>(0·0 to 0·1%)	14·8%<br>(2·8 to 27·8%)	31·2%<br>(13·2 to 48·2%)
Liver cancer due to hepatitis C	0·0<br>(0·0 to 0·1)	0·1%<br>(0·1 to 0·2%)	0·1<br>(0·0 to 0·1)	0·1%<br>(0·1 to 0·2%)	33·3%<br>(26·6 to 41·7%)	10·7%<br>(4·4 to 17·1%)	0·9<br>(0·4 to 1·5)	0·1%<br>(0·1 to 0·2%)	1·2<br>(0·6 to 2·0)	0·2%<br>(0·1 to 0·3%)	28·7%<br>(21·2 to 38·2%)	13·2%<br>(4·8 to 21·0%)
Liver cancer due to alcohol use	0·0<br>(0·0 to 0·1)	0·1%<br>(0·0 to 0·1%)	0·1<br>(0·0 to 0·1)	0·1%<br>(0·0 to 0·1%)	41·1%<br>(30·0 to 52·3%)	11·0%<br>(-3·0 to 21·4%)	1·2<br>(0·5 to 2·1)	0·1%<br>(0·0 to 0·1%)	1·7<br>(0·7 to 2·9)	0·1%<br>(0·0 to 0·1%)	37·4%<br>(25·6 to 48·8%)	11·6%<br>(-4·1 to 23·4%)
Liver cancer due to other causes	0·0<br>(0·0 to 0·0)	0·0%<br>(0·0 to 0·1%)	0·0<br>(0·0 to 0·0)	0·1%<br>(0·0 to 0·1%)	26·3%<br>(18·5 to 35·2%)	36·9%<br>(21·2 to 50·2%)	0·6<br>(0·3 to 1·0)	0·0%<br>(0·0 to 0·1%)	0·7<br>(0·3 to 1·1)	0·1%<br>(0·0 to 0·1%)	18·3%<br>(9·6 to 28·0%)	41·0%<br>(22·5 to 56·5%)
Gallbladder and biliary tract cancer	0·1<br>(0·0 to 0·1)	0·2%<br>(0·1 to 0·3%)	0·1<br>(0·0 to 0·1)	0·2%<br>(0·1 to 0·3%)	10·9%<br>(0·8 to 19·5%)	-2·3%<br>(-7·6 to 3·2%)	1·6<br>(0·9 to 2·5)	0·2%<br>(0·1 to 0·4%)	1·7<br>(1·0 to 2·7)	0·2%<br>(0·1 to 0·4%)	6·8%<br>(-2·4 to 14·5%)	-0·5%<br>(-6·2 to 5·4%)
Pancreatic cancer	0·0<br>(0·0 to 0·1)	0·1%<br>(0·0 to 0·1%)	0·1<br>(0·0 to 0·1)	0·1%<br>(0·0 to 0·1%)	33·5%<br>(29·1 to 38·1%)	1·6%<br>(-2·4 to 5·3%)	1·0<br>(0·4 to 1·8)	0·1%<br>(0·0 to 0·1%)	1·3<br>(0·5 to 2·4)	0·1%<br>(0·0 to 0·1%)	29·8%<br>(25·1 to 34·4%)	2·6%<br>(-1·6 to 6·8%)
Breast cancer	0·1<br>(0·0 to 0·1)	0·1%<br>(0·0 to 0·1%)	0·1<br>(0·1 to 0·1)	0·1%<br>(0·0 to 0·1%)	34·8%<br>(25·8 to 42·7%)	10·4%<br>(4·2 to 18·1%)	1·8<br>(1·2 to 2·6)	0·1%<br>(0·0 to 0·1%)	2·4<br>(1·6 to 3·5)	0·1%<br>(0·0 to 0·1%)	33·9%<br>(24·1 to 42·9%)	13·9%<br>(6·4 to 22·9%)
Uterine cancer	0·1<br>(0·0 to 0·1)	0·4%<br>(0·2 to 0·5%)	0·1<br>(0·1 to 0·1)	0·4%<br>(0·3 to 0·6%)	18·2%<br>(11·9 to 25·8%)	13·3%<br>(6·4 to 20·6%)	1·8<br>(1·2 to 2·6)	0·4%<br>(0·2 to 0·5%)	2·1<br>(1·4 to 3·0)	0·4%<br>(0·3 to 0·6%)	17·1%<br>(10·2 to 25·0%)	16·8%<br>(8·5 to 25·2%)
Ovarian cancer	0·0<br>(0·0 to 0·0)	0·0%<br>(0·0 to 0·1%)	0·0<br>(0·0 to 0·0)	0·0%<br>(0·0 to 0·1%)	28·7%<br>(0·0 to 33·9%)	6·3%<br>(0·0 to 11·0%)	0·3<br>(0·0 to 0·6)	0·0%<br>(0·0 to 0·1%)	0·4<br>(0·0 to 0·8)	0·0%<br>(0·0 to 0·1%)	26·4%<br>(0·0 to 31·9%)	8·4%<br>(0·0 to 13·6%)
Kidney cancer	0·0<br>(0·0 to 0·1)	0·2%<br>(0·1 to 0·3%)	0·1<br>(0·0 to 0·1)	0·2%<br>(0·1 to 0·3%)	43·3%<br>(37·2 to 49·6%)	3·6%<br>(-0·1 to 7·1%)	1·3<br>(0·9 to 1·9)	0·2%<br>(0·1 to 0·3%)	1·8<br>(1·2 to 2·7)	0·2%<br>(0·2 to 0·3%)	40·3%<br>(34·3 to 46·2%)	7·6%<br>(3·4 to 12·1%)
Thyroid cancer	0·0<br>(0·0 to 0·0)	0·1%<br>(0·1 to 0·2%)	0·0<br>(0·0 to 0·0)	0·1%<br>(0·1 to 0·2%)	34·6%<br>(27·2 to 41·0%)	4·2%<br>(-2·0 to 14·1%)	0·2<br>(0·1 to 0·4)	0·1%<br>(0·1 to 0·2%)	0·3<br>(0·2 to 0·5)	0·2%<br>(0·1 to 0·2%)	35·6%<br>(27·8 to 42·3%)	6·5%<br>(-0·1 to 16·8%)
Ischaemic heart disease	3·3<br>(2·3 to 4·7)	0·2%<br>(0·1 to 0·2%)	3·8<br>(2·6 to 5·4)	0·2%<br>(0·1 to 0·2%)	14·4%<br>(10·4 to 17·9%)	-0·3%<br>(-3·8 to 3·3%)	90·1<br>(61·6 to 128·0)	0·2%<br>(0·2 to 0·3%)	100·1<br>(68·3 to 144·0)	0·2%<br>(0·2 to 0·3%)	11·1%<br>(6·9 to 14·6%)	4·4%<br>(0·5 to 8·2%)
Ischaemic stroke	0·6<br>(0·4 to 0·8)	0·1%<br>(0·1 to 0·1%)	0·6<br>(0·4 to 0·8)	0·1%<br>(0·0 to 0·1%)	3·4%<br>(-2·2 to 8·6%)	-2·1%<br>(-7·6 to 3·2%)	14·0<br>(9·7 to 20·0)	0·1%<br>(0·1 to 0·2%)	14·4<br>(9·9 to 20·7)	0·1%<br>(0·1 to 0·2%)	2·9%<br>(-1·1 to 6·7%)	4·5%<br>(-0·5 to 9·1%)
Hemorrhagic stroke	1·5<br>(1·0 to 2·2)	0·2%<br>(0·1 to 0·3%)	1·6<br>(1·1 to 2·3)	0·2%<br>(0·1 to 0·3%)	3·8%<br>(-0·7 to 8·0%)	9·1%<br>(3·0 to 14·9%)	50·8<br>(34·7 to 73·0)	0·3%<br>(0·2 to 0·4%)	51·8<br>(35·0 to 74·7)	0·3%<br>(0·2 to 0·5%)	1·8%<br>(-2·6 to 5·8%)	14·3%<br>(7·9 to 20·5%)
Hypertensive heart disease	0·6<br>(0·4 to 1·0)	0·3%<br>(0·2 to 0·5%)	0·8<br>(0·5 to 1·3)	0·3%<br>(0·2 to 0·5%)	24·5%<br>(15·3 to 32·6%)	-2·3%<br>(-11·6 to 7·2%)	14·4<br>(9·4 to 21·3)	0·4%<br>(0·3 to 0·6%)	17·0<br>(10·8 to 25·2)	0·4%<br>(0·3 to 0·6%)	18·4%<br>(12·4 to 24·4%)	2·0%<br>(-4·4 to 9·2%)
Cardiomyopathy and myocarditis	-	-	-	-	-	-	-	-	-	-	-	-
Atrial fibrillation and flutter	-	-	-	-	-	-	-	-	-	-	-	-
Peripheral vascular disease	-	-	-	-	-	-	-	-	-	-	-	-

Supplemental Results Table 1. All-age deaths, death PAFs, DALYs, DALY PAFs, and percent change for each risk factor and outcome for both sexes combined in 2005 and 2015 for High-middle SDI.

Data in parenthesis are 95% uncertainty intervals. PAFs=population attributable fractions. DALYs=disability-adjusted life-years.

	2005 Deaths (in thousands)	2005 Deaths PAF	2015 Deaths (in thousands)	2015 Deaths PAF	Percent change deaths 2005-2015	Percent change deaths PAF 2005-2015	2005 DALYs (in thousands)	2005 DALYs PAF	2015 DALYs (in thousands)	2015 DALYs PAF	Percent change DALYs 2005-2015	Percent change DALYs PAF 2005-2015
Endocarditis	-	-	-	-	-	-	-	-	-	-	-	-
Other cardiovascular and circulatory diseases	-	-	-	-	-	-	-	-	-	-	-	-
Diabetes mellitus	1·8 (1·3 to 2·6)	0·7% (0·4 to 0·9%)	2·4 (1·6 to 3·5)	0·7% (0·5 to 1·0%)	32·4% (27·6 to 37·2%)	4·3% (0·6 to 8·3%)	108·2 (71·3 to 162·1)	0·8% (0·6 to 1·2%)	147·6 (95·0 to 222·6)	0·9% (0·6 to 1·3%)	36·4% (31·6 to 40·5%)	8·2% (4·9 to 11·1%)
Chronic kidney disease due to diabetes mellitus	0·6 (0·3 to 1·1)	0·8% (0·4 to 1·2%)	0·9 (0·5 to 1·5)	0·8% (0·4 to 1·4%)	45·6% (38·9 to 53·5%)	7·9% (2·7 to 13·6%)	17·6 (8·1 to 30·8)	0·7% (0·3 to 1·3%)	25·0 (11·4 to 44·3)	0·8% (0·4 to 1·5%)	42·1% (36·5 to 47·9%)	13·1% (8·9 to 17·6%)
Chronic kidney disease due to hypertension	0·4 (0·2 to 0·6)	0·4% (0·2 to 0·7%)	0·5 (0·3 to 0·9)	0·4% (0·2 to 0·7%)	40·2% (29·4 to 52·0%)	3·0% (-4·5 to 11·2%)	9·6 (4·4 to 16·5)	0·4% (0·2 to 0·8%)	12·9 (6·1 to 22·3)	0·5% (0·2 to 0·8%)	33·7% (27·7 to 41·8%)	10·7% (6·0 to 17·1%)
Chronic kidney disease due to glomerulonephritis	0·1 (0·0 to 0·1)	0·1% (0·1 to 0·3%)	0·1 (0·0 to 0·2)	0·2% (0·1 to 0·3%)	30·7% (19·8 to 46·4%)	25·1% (14·3 to 40·3%)	4·2 (1·4 to 8·1)	0·2% (0·1 to 0·4%)	5·5 (2·0 to 10·3)	0·3% (0·1 to 0·6%)	29·2% (22·8 to 39·7%)	34·4% (27·4 to 46·4%)
Chronic kidney disease due to other causes	0·0 (0·0 to 0·0)	0·2% (0·1 to 0·3%)	0·0 (0·0 to 0·0)	0·2% (0·1 to 0·3%)	43·4% (25·0 to 60·7%)	11·2% (-2·2 to 23·4%)	2·4 (0·9 to 4·5)	0·4% (0·1 to 0·7%)	3·2 (1·3 to 6·1)	0·4% (0·2 to 0·8%)	36·4% (30·8 to 43·6%)	17·0% (11·8 to 24·0%)
**Diet low in fibre: All causes**	74·1 (32·2 to 133·0)	0·7% (0·3 to 1·2%)	85·5 (37·7 to 154·2)	0·7% (0·3 to 1·3%)	15·3% (10·3 to 20·9%)	9·2% (4·8 to 14·6%)	1,705·8 (739·7 to 3,057·1)	0·4% (0·2 to 0·7%)	1,800·4 (787·4 to 3,247·4)	0·4% (0·2 to 0·7%)	5·5% (0·4 to 10·7%)	6·4% (1·4 to 11·0%)
Colon and rectum cancer	7·0 (2·9 to 13·0)	4·2% (1·7 to 7·6%)	9·2 (3·8 to 16·9)	4·3% (1·8 to 8·0%)	30·8% (24·6 to 38·0%)	4·8% (0·0 to 10·1%)	145·4 (59·7 to 268·2)	3·9% (1·6 to 7·2%)	176·5 (72·1 to 323·9)	4·0% (1·6 to 7·4%)	21·3% (15·3 to 27·8%)	2·8% (-2·3 to 8·3%)
Ischaemic heart disease	67·1 (28·6 to 122·7)	3·2% (1·4 to 5·9%)	76·3 (32·6 to 140·3)	3·1% (1·3 to 5·8%)	13·7% (8·7 to 19·2%)	-0·9% (-4·8 to 3·6%)	1,560·3 (665·5 to 2,843·9)	3·9% (1·7 to 7·1%)	1,623·9 (697·7 to 2,969·6)	3·8% (1·7 to 6·9%)	4·1% (-0·9 to 9·1%)	-2·2% (-6·2 to 1·8%)
**Diet low in calcium: All causes**	33·2 (19·8 to 47·8)	0·3% (0·2 to 0·4%)	42·0 (25·0 to 60·8)	0·4% (0·2 to 0·5%)	26·7% (22·1 to 31·6%)	20·0% (16·8 to 23·4%)	730·4 (438·6 to 1,055·6)	0·2% (0·1 to 0·2%)	872·3 (525·2 to 1,272·1)	0·2% (0·1 to 0·3%)	19·4% (14·5 to 24·9%)	20·4% (15·9 to 25·0%)
Colon and rectum cancer	33·2 (19·8 to 47·8)	19·6% (11·7 to 28·3%)	42·0 (25·0 to 60·8)	19·9% (11·9 to 28·7%)	26·7% (22·1 to 31·6%)	1·5% (0·3 to 2·9%)	730·4 (438·6 to 1,055·6)	19·5% (11·7 to 28·2%)	872·3 (525·2 to 1,272·1)	19·7% (11·8 to 28·5%)	19·4% (14·5 to 24·9%)	1·2% (-0·1 to 2·5%)
Prostate cancer	-	-	-	-	-	-	-	-	-	-	-	-
**Diet low in seafood omega-3 fatty acids: All causes**	346·1 (144·0 to 565·4)	3·1% (1·3 to 5·1%)	382·4 (157·8 to 621·1)	3·2% (1·4 to 5·3%)	10·5% (7·0 to 14·1%)	4·7% (2·5 to 6·5%)	7,450·5 (3,136·5 to 12,020·2)	1·6% (0·7 to 2·6%)	7,685·4 (3,222·9 to 12,319·7)	1·7% (0·7 to 2·7%)	3·2% (-1·2 to 7·2%)	4·0% (0·0 to 7·2%)
Ischaemic heart disease	346·1 (144·0 to 565·4)	16·4% (6·9 to 26·5%)	382·4 (157·8 to 621·1)	15·8% (6·6 to 25·6%)	10·5% (7·0 to 14·1%)	-3·6% (-4·5 to -2·9%)	7,450·5 (3,136·5 to 12,020·2)	18·7% (7·9 to 29·8%)	7,685·4 (3,222·9 to 12,319·7)	18·1% (7·6 to 29·0%)	3·2% (-1·2 to 7·2%)	-3·0% (-4·2 to -2·1%)
**Diet low in polyunsaturated fatty acids: All causes**	107·8 (43·7 to 170·7)	1·0% (0·4 to 1·5%)	115·8 (47·5 to 184·7)	1·0% (0·4 to 1·6%)	7·4% (-3·5 to 17·6%)	1·7% (-8·6 to 11·3%)	2,415·1 (1,020·9 to 3,875·0)	0·5% (0·2 to 0·9%)	2,583·1 (1,088·3 to 4,195·1)	0·6% (0·2 to 0·9%)	7·0% (-5·0 to 18·6%)	7·8% (-4·3 to 19·1%)
Ischaemic heart disease	107·8 (43·7 to 170·7)	5·1% (2·1 to 8·1%)	115·8 (47·5 to 184·7)	4·8% (1·9 to 7·7%)	7·4% (-3·5 to 17·6%)	-6·3% (-15·8 to 2·4%)	2,415·1 (1,020·9 to 3,875·0)	6·0% (2·5 to 9·7%)	2,583·1 (1,088·3 to 4,195·1)	6·1% (2·5 to 9·9%)	7·0% (-5·0 to 18·6%)	0·5% (-11·1 to 10·7%)
**Diet high in trans fatty acids: All causes**	88·1 (33·1 to 172·5)	0·8% (0·3 to 1·5%)	100·8 (39·3 to 196·4)	0·9% (0·3 to 1·7%)	14·5% (9·6 to 22·2%)	8·4% (4·7 to 15·3%)	2,343·5 (940·2 to 4,363·0)	0·5% (0·2 to 1·0%)	2,566·7 (1,071·5 to 4,785·8)	0·6% (0·2 to 1·0%)	9·5% (4·0 to 17·8%)	10·4% (5·4 to 18·2%)
Ischaemic heart disease	88·1 (33·1 to 172·5)	4·2% (1·6 to 8·2%)	100·8 (39·3 to 196·4)	4·2% (1·6 to 8·1%)	14·5% (9·6 to 22·2%)	-0·1% (-2·9 to 6·0%)	2,343·5 (940·2 to 4,363·0)	5·9% (2·4 to 11·0%)	2,566·7 (1,071·5 to 4,785·8)	6·0% (2·5 to 11·2%)	9·5% (4·0 to 17·8%)	3·0% (-0·2 to 9·9%)
**Diet high in sodium: All causes**	1,218·1 (784·1 to 1,763·9)	10·9% (7·0 to 15·8%)	1,327·4 (855·3 to 1,919·7)	11·3% (7·3 to 16·3%)	9·0% (3·0 to 15·8%)	3·2% (-1·1 to 8·1%)	25,354·3 (16,487·9 to 36,107·9)	5·4% (3·5 to 7·9%)	25,974·2 (16,946·3 to 37,153·9)	5·6% (3·6 to 8·2%)	2·4% (-3·3 to 9·0%)	3·2% (-1·6 to 8·8%)
Stomach cancer	148·5 (102·4 to 194·3)	56·8% (39·3 to 74·3%)	137·2 (90·3 to 181·3)	54·8% (36·2 to 72·8%)	-7·6% (-16·7 to 1·4%)	-3·5% (-10·2 to 2·5%)	3,426·2 (2,429·8 to 4,417·5)	57·1% (40·9 to 73·7%)	2,976·6 (2,006·2 to 3,920·7)	55·2% (37·4 to 72·4%)	-13·1% (-22·0 to -4·1%)	-3·3% (-10·3 to 1·9%)
Rheumatic heart disease	6·7 (3·5 to 11·9)	11·3% (5·9 to 20·0%)	5·6 (2·9 to 10·0)	11·2% (5·8 to 20·3%)	-16·7% (-24·6 to -7·4%)	-1·0% (-8·1 to 8·0%)	194·4 (101·5 to 336·7)	10·2% (5·4 to 17·6%)	153·3 (79·0 to 260·3)	10·4% (5·5 to 18·0%)	-21·2% (-27·6 to -14·2%)	1·9% (-4·3 to 8·8%)
Ischaemic heart disease	449·2 (247·3 to 721·5)	21·3% (11·8 to 33·9%)	533·2 (301·6 to 841·6)	22·0% (12·4 to 34·5%)	18·7% (12·3 to 27·5%)	3·5% (-0·7 to 9·9%)	8,816·5 (5,027·1 to 13,950·9)	22·1% (12·6 to 34·6%)	9,780·8 (5,750·6 to 15,046·7)	23·0% (13·7 to 35·5%)	10·9% (5·1 to 18·5%)	4·3% (1·1 to 10·0%)
Ischaemic stroke	184·3 (109·2 to 282·6)	24·8% (14·7 to 37·5%)	197·5 (118·4 to 299·7)	25·1% (15·4 to 37·9%)	7·2% (-0·7 to 17·2%)	1·5% (-3·7 to 9·2%)	3,304·1 (2,003·5 to 4,862·5)	27·9% (17·2 to 40·9%)	3,383·8 (2,086·9 to 4,946·5)	29·0% (18·5 to 41·7%)	2·4% (-4·5 to 11·2%)	3·9% (-0·2 to 10·3%)
Hemorrhagic stroke	286·5 (177·4 to 414·0)	32·9% (20·5 to 47·4%)	270·5 (168·7 to 395·5)	32·6% (20·6 to 47·1%)	-5·6% (-13·1 to 3·2%)	-0·8% (-5·6 to 4·9%)	6,307·0 (4,067·9 to 8,957·0)	33·6% (21·6 to 47·6%)	5,709·0 (3,641·0 to 8,039·4)	34·1% (22·0 to 47·9%)	-9·5% (-16·1 to -1·8%)	1·6% (-2·2 to 7·0%)
Hypertensive heart disease	71·8 (26·3 to 135·5)	37·0% (13·7 to 69·0%)	95·4 (34·9 to 178·0)	38·5% (14·3 to 71·1%)	32·8% (16·0 to 49·4%)	4·0% (-3·4 to 14·3%)	1,302·8 (613·2 to 2,262·0)	37·9% (18·0 to 66·4%)	1,571·2 (733·0 to 2,745·9)	39·3% (18·8 to 67·7%)	20·6% (9·4 to 32·8%)	3·8% (-1·3 to 11·0%)
Cardiomyopathy and myocarditis	7·2 (3·5 to 12·8)	10·1% (4·8 to 17·6%)	8·1 (3·9 to 14·3)	10·3% (5·0 to 18·1%)	11·8% (4·3 to 19·9%)	2·6% (-1·6 to 9·4%)	184·1 (89·0 to 318·2)	9·5% (4·6 to 16·5%)	192·2 (93·8 to 328·1)	10·4% (5·2 to 17·7%)	4·4% (-2·7 to 12·2%)	8·9% (5·4 to 15·3%)
Atrial fibrillation and flutter	4·3 (2·2 to 7·1)	12·7% (7·1 to 20·4%)	6·1 (3·2 to 10·0)	12·9% (7·4 to 20·5%)	42·4% (34·0 to 52·7%)	1·6% (-2·5 to 7·6%)	140·3 (77·6 to 228·1)	17·4% (10·3 to 26·6%)	189·7 (107·2 to 304·2)	18·0% (11·0 to 27·0%)	35·3% (30·3 to 42·1%)	3·6% (0·6 to 8·5%)
Aortic aneurysm	3·5 (1·8 to 5·9)	11·6% (5·9 to 19·5%)	4·4 (2·3 to 7·5)	11·6% (6·1 to 19·7%)	27·0% (17·1 to 36·6%)	-0·3% (-3·9 to 4·7%)	75·0 (38·7 to 124·4)	12·5% (6·6 to 20·9%)	90·8 (47·1 to 150·8)	12·8% (6·7 to 21·0%)	21·1% (11·1 to 30·4%)	2·1% (-1·3 to 6·7%)
Peripheral vascular disease	0·4 (0·2 to 0·8)	6·6% (2·8 to 12·7%)	0·6 (0·3 to 1·2)	6·6% (2·9 to 12·6%)	50·0% (38·1 to 66·5%)	0·5% (-5·3 to 8·5%)	20·1 (8·7 to 38·0)	10·5% (5·4 to 17·3%)	29·3 (13·1 to 55·2)	10·9% (5·7 to 17·7%)	46·2% (40·3 to 54·4%)	4·2% (0·5 to 9·5%)
Endocarditis	1·6 (0·9 to 2·8)	10·7% (5·6 to 18·0%)	2·1 (1·1 to 3·5)	11·2% (5·9 to 18·8%)	26·3% (17·2 to 37·3%)	4·7% (-1·0 to 11·7%)	40·1 (19·9 to 68·5)	9·4% (4·7 to 15·9%)	46·3 (23·2 to 77·5)	10·4% (5·4 to 17·5%)	15·4% (6·1 to 26·2%)	11·5% (5·2 to 19·3%)
Other cardiovascular and circulatory diseases	16·2 (9·1 to 25·2)	15·0% (8·4 to 23·1%)	18·2 (10·0 to 28·3)	14·6% (8·1 to 22·6%)	11·9% (2·9 to 21·1%)	-2·3% (-7·8 to 3·4%)	553·8 (310·5 to 871·3)	13·8% (7·8 to 21·1%)	666·4 (374·2 to 1,046·7)	15·1% (8·9 to 23·1%)	20·3% (13·3 to 27·9%)	10·0% (6·2 to 15·6%)
Chronic kidney disease due to diabetes mellitus	12·8 (6·0 to 21·7)	15·2% (7·2 to 25·8%)	16·8 (7·8 to 28·9)	14·9% (6·9 to 25·4%)	31·9% (23·2 to 40·2%)	-2·3% (-7·3 to 1·9%)	334·1 (162·9 to 573·6)	14·0% (6·8 to 23·2%)	422·9 (206·1 to 720·6)	14·1% (6·9 to 23·6%)	26·6% (19·6 to 33·8%)	0·8% (-3·6 to 4·9%)
Chronic kidney disease due to hypertension	13·6 (6·3 to 24·2)	15·4% (7·2 to 26·8%)	19·4 (9·1 to 33·5)	16·1% (7·5 to 27·8%)	42·3% (33·5 to 52·8%)	4·5% (1·0 to 9·3%)	298·0 (144·6 to 518·2)	13·6% (6·6 to 23·2%)	386·7 (190·3 to 663·8)	14·6% (7·1 to 24·9%)	29·7% (23·2 to 37·3%)	7·4% (3·9 to 12·4%)
Chronic kidney disease due to glomerulonephritis	10·7 (5·9 to 16·6)	21·5% (12·1 to 32·8%)	11·4 (6·5 to 17·6)	21·8% (12·6 to 33·2%)	6·1% (-2·8 to 15·8%)	1·6% (-4·2 to 8·0%)	285·5 (153·4 to 450·2)	15·5% (8·6 to 24·5%)	279·1 (153·9 to 450·4)	15·7% (8·8 to 25·0%)	-2·2% (-10·0 to 6·2%)	1·8% (-4·4 to 7·9%)
Chronic kidney disease due to other causes	0·7 (0·3 to 1·3)	12·6% (5·7 to 22·4%)	1·1 (0·5 to 2·0)	15·2% (6·9 to 26·8%)	55·4% (32·7 to 75·4%)	20·4% (11·1 to 29·9%)	72·3 (33·6 to 131·2)	11·1% (5·5 to 19·4%)	96·1 (45·3 to 173·0)	12·7% (6·4 to 21·9%)	33·0% (27·7 to 39·5%)	14·0% (10·2 to 20·0%)
**Sexual abuse and violence: All causes**	55·8 (40·0 to 74·3)	0·5% (0·4 to 0·7%)	44·4 (30·8 to 61·5)	0·4% (0·3 to 0·5%)	-20·5% (-28·0 to -11·9%)	-24·7% (-31·8 to -16·9%)	4,005·4 (3,030·0 to 5,155·0)	0·9% (0·7 to 1·1%)	3,643·8 (2,709·6 to 4,760·6)	0·8% (0·6 to 1·0%)	-9·0% (-15·4 to -2·2%)	-8·4% (-14·5 to -1·9%)
**Childhood sexual abuse: All causes**	19·7 (5·2 to 39·3)	0·2% (0·0 to 0·4%)	17·8 (4·6 to 36·2)	0·2% (0·0 to 0·3%)	-9·6% (-15·9 to -3·3%)	-14·4% (-20·0 to -9·0%)	1,406·7 (572·6 to 2,501·0)	0·3% (0·1 to 0·5%)	1,349·0 (559·0 to 2,334·9)	0·3% (0·1 to 0·5%)	-4·1% (-12·5 to 4·9%)	-3·4% (-11·6 to 5·7%)
Alcohol use disorders	1·0 (0·0 to 2·2)	3·5% (0·0 to 8·1%)	0·8 (0·0 to 1·9)	3·1% (0·0 to 7·2%)	-19·0% (-24·3 to 0·0%)	-10·4% (-13·7 to 0·0%)	96·4 (0·0 to 225·9)	3·8% (0·0 to 8·7%)	91·7 (0·0 to 214·4)	3·6% (0·0 to 8·2%)	-4·9% (-10·0 to 0·0%)	-6·2% (-8·7 to 0·0%)
Major depressive disorder	-	-	-	-	-	-	377·8 (41·3 to 846·6)	3·9% (0·5 to 8·2%)	422·3 (46·3 to 945·7)	3·8% (0·4 to 7·9%)	11·8% (9·0 to 14·1%)	-3·7% (-5·1 to -2·1%)
Dysthymia	-	-	-	-	-	-	80·2 (8·6 to 178·3)	3·6% (0·4 to 7·6%)	92·2 (9·9 to 205·1)	3·5% (0·4 to 7·3%)	15·0% (12·3 to 17·7%)	-4·4% (-6·0 to -2·5%)
Self-harm	18·7 (4·4 to 38·5)	10·2% (2·4 to 21·0%)	17·0 (4·0 to 35·4)	9·9% (2·3 to 20·4%)	-9·1% (-15·5 to -2·5%)	-3·4% (-6·2 to -0·9%)	852·2 (200·4 to 1,738·9)	10·8% (2·5 to 22·1%)	742·8 (176·0 to 1,531·4)	10·6% (2·5 to 21·7%)	-12·8% (-19·0 to -6·0%)	-2·0% (-4·5 to 0·5%)
**Intimate partner violence: All causes**	38·1 (30·1 to 46·4)	0·3% (0·3 to 0·4%)	28·4 (22·9 to 33·8)	0·2% (0·2 to 0·3%)	-25·5% (-33·0 to -15·6%)	-29·4% (-36·6 to -19·9%)	2,728·8 (2,194·7 to 3,358·0)	0·6% (0·5 to 0·7%)	2,422·5 (1,917·8 to 3,025·0)	0·5% (0·4 to 0·6%)	-11·2% (-19·2 to -2·8%)	-10·6% (-18·3 to -2·3%)

Supplemental Results Table 1. All-age deaths, death PAFs, DALYs, DALY PAFs, and percent change for each risk factor and outcome for both sexes combined in 2005 and 2015 for High-middle SDI.

Data in parenthesis are 95% uncertainty intervals. PAFs=population attributable fractions. DALYs=disability-adjusted life-years.

	2005 Deaths (in thousands)	2005 Deaths PAF	2015 Deaths (in thousands)	2015 Deaths PAF	Percent change deaths 2005-2015	Percent change deaths PAF 2005-2015	2005 DALYs (in thousands)	2005 DALYs PAF	2015 DALYs (in thousands)	2015 DALYs PAF	Percent change DALYs 2005-2015	Percent change DALYs PAF 2005-2015
HIV/AIDS - Tuberculosis	2·7 (1·4 to 4·3)	4·0% (2·0 to 6·1%)	1·3 (0·6 to 2·2)	3·5% (1·8 to 5·5%)	-50·8% (-57·3 to -43·2%)	-12·7% (-19·1 to -6·3%)	125·1 (61·6 to 196·0)	3·5% (1·8 to 5·3%)	62·5 (29·8 to 101·2)	3·1% (1·5 to 4·9%)	-50·1% (-55·8 to -43·1%)	-10·9% (-17·0 to -5·2%)
HIV/AIDS resulting in other diseases	10·5 (5·1 to 16·6)	4·3% (2·1 to 6·6%)	5·1 (2·5 to 8·0)	3·3% (1·6 to 5·1%)	-51·8% (-57·9 to -43·5%)	-23·6% (-29·6 to -17·4%)	523·0 (255·1 to 830·1)	3·9% (1·9 to 6·1%)	259·1 (125·7 to 410·0)	3·1% (1·5 to 4·8%)	-50·5% (-56·2 to -43·1%)	-22·1% (-27·7 to -16·6%)
Maternal abortion, miscarriage, and ectopic pregnancy	0·0 (0·0 to 0·0)	0·0% (0·0 to 0·0%)	0·0 (0·0 to 0·0)	0·0% (0·0 to 0·0%)	-32·4% (-43·2 to -18·4%)	0·0% (-12·2 to 15·2%)	0·0 (0·0 to 0·0)	0·0% (0·0 to 0·0%)	0·0 (0·0 to 0·0)	0·0% (0·0 to 0·0%)	-30·9% (-41·9 to -17·5%)	0·1% (-12·9 to 13·7%)
Major depressive disorder	-	-	-	-	-	-	791·4 (506·0 to 1,135·6)	8·2% (6·6 to 9·8%)	957·4 (618·1 to 1,371·7)	8·6% (6·8 to 10·2%)	21·0% (17·3 to 24·8%)	4·2% (1·4 to 7·2%)
Dysthymia	-	-	-	-	-	-	174·0 (109·9 to 255·0)	7·9% (6·4 to 9·4%)	219·3 (137·1 to 321·0)	8·2% (6·6 to 9·8%)	26·1% (22·0 to 30·1%)	4·8% (1·9 to 7·9%)
Self-harm	18·0 (13·7 to 22·0)	9·8% (7·7 to 11·9%)	16·3 (12·0 to 20·3)	9·4% (7·1 to 11·7%)	-9·6% (-21·1 to 8·3%)	-4·0% (-14·1 to 8·7%)	733·6 (546·7 to 914·6)	9·3% (7·0 to 11·7%)	620·6 (451·4 to 787·4)	8·9% (6·4 to 11·2%)	-15·4% (-26·3 to 2·1%)	-4·9% (-15·3 to 8·9%)
Assault by firearm	1·7 (1·4 to 2·0)	2·5% (2·0 to 2·9%)	1·6 (1·2 to 1·8)	2·3% (1·9 to 2·7%)	-11·0% (-20·7 to 2·7%)	-9·1% (-19·1 to 4·5%)	91·4 (72·0 to 103·4)	2·4% (2·0 to 2·8%)	77·3 (62·0 to 91·9)	2·1% (1·8 to 2·6%)	-15·4% (-25·0 to -1·9%)	-11·2% (-21·2 to 3·0%)
Assault by sharp object	2·1 (1·9 to 2·4)	6·8% (6·0 to 7·6%)	1·7 (1·5 to 2·0)	6·1% (5·2 to 6·8%)	-18·4% (-28·0 to -6·8%)	-10·3% (-21·2 to 0·2%)	111·6 (99·7 to 125·6)	6·8% (6·0 to 7·6%)	87·1 (73·3 to 100·8)	6·0% (5·1 to 6·8%)	-22·0% (-31·7 to -9·7%)	-11·3% (-22·9 to -0·6%)
Assault by other means	3·0 (2·7 to 3·4)	9·5% (8·5 to 10·6%)	2·4 (2·2 to 2·7)	8·4% (7·1 to 9·3%)	-18·7% (-27·1 to -7·8%)	-11·6% (-20·0 to -2·8%)	178·8 (162·4 to 204·9)	10·4% (9·4 to 11·5%)	139·3 (121·3 to 159·5)	9·2% (7·9 to 10·2%)	-22·1% (-30·4 to -11·9%)	-11·4% (-19·5 to -3·2%)
**Unsafe sex: All causes**	352·5 (322·5 to 388·5)	3·2% (2·9 to 3·5%)	235·6 (221·0 to 253·3)	2·0% (1·9 to 2·2%)	-33·2% (-37·3 to -27·6%)	-36·7% (-40·8 to -31·4%)	18,176·3 (16,634·6 to 20,039·4)	3·9% (3·5 to 4·4%)	11,812·0 (10,989·1 to 12,777·9)	2·5% (2·3 to 2·8%)	-35·0% (-38·6 to -30·3%)	-34·5% (-38·1 to -30·1%)
HIV/AIDS - Tuberculosis	63·9 (48·1 to 68·9)	94·1% (93·4 to 94·6%)	35·8 (25·6 to 42·1)	93·5% (92·5 to 94·3%)	-43·9% (-50·5 to -35·9%)	-0·6% (-1·4 to 0·0%)	3,397·7 (2,576·5 to 3,666·5)	94·3% (93·7 to 94·9%)	1,899·5 (1,373·7 to 2,218·5)	94·0% (93·0 to 94·7%)	-44·1% (-50·2 to -36·1%)	-0·4% (-1·1 to 0·1%)
HIV/AIDS resulting in other diseases	229·8 (200·4 to 267·5)	93·2% (92·3 to 94·1%)	141·1 (125·8 to 161·4)	90·8% (89·6 to 92·0%)	-38·6% (-44·1 to -30·6%)	-2·5% (-3·3 to -1·7%)	12,404·1 (10,903·9 to 14,368·0)	93·5% (92·7 to 94·3%)	7,732·8 (6,949·7 to 8,773·6)	91·8% (90·7 to 92·7%)	-37·7% (-42·7 to -30·9%)	-1·9% (-2·5 to -1·3%)
Syphilis	5·3 (3·4 to 8·1)	100·0% (100·0 to 100·0%)	2·7 (1·8 to 3·9)	100·0% (100·0 to 100·0%)	-49·5% (-57·0 to -40·4%)	-	448·4 (285·4 to 683·2)	100·0% (100·0 to 100·0%)	233·6 (159·0 to 339·5)	100·0% (100·0 to 100·0%)	-47·9% (-55·6 to -38·5%)	-
Chlamydial infection	0·0 (0·0 to 0·0)	100·0% (100·0 to 100·0%)	0·0 (0·0 to 0·0)	100·0% (100·0 to 100·0%)	2·1% (-9·2 to 17·2%)	-	97·9 (55·6 to 160·5)	100·0% (100·0 to 100·0%)	104·6 (59·1 to 169·9)	100·0% (100·0 to 100·0%)	6·9% (2·3 to 12·5%)	-
Gonococcal infection	0·1 (0·1 to 0·1)	100·0% (100·0 to 100·0%)	0·1 (0·1 to 0·1)	100·0% (100·0 to 100·0%)	-13·0% (-24·0 to -0·9%)	-	87·3 (51·7 to 134·8)	100·0% (100·0 to 100·0%)	106·6 (62·5 to 167·6)	100·0% (100·0 to 100·0%)	22·1% (16·9 to 27·9%)	-
Trichomoniasis	-	-	-	-	-	-	41·9 (16·7 to 89·4)	100·0% (100·0 to 100·0%)	47·0 (18·8 to 99·2)	100·0% (100·0 to 100·0%)	12·0% (10·2 to 14·0%)	-
Genital herpes	-	-	-	-	-	-	52·2 (16·2 to 123·9)	100·0% (100·0 to 100·0%)	65·2 (21·2 to 151·4)	100·0% (100·0 to 100·0%)	25·0% (20·0 to 39·2%)	-
Other sexually transmitted diseases	0·0 (0·0 to 0·0)	100·0% (100·0 to 100·0%)	0·0 (0·0 to 0·0)	100·0% (100·0 to 100·0%)	-14·9% (-26·1 to -2·6%)	-	25·9 (17·9 to 36·3)	100·0% (100·0 to 100·0%)	25·3 (17·3 to 36·1)	100·0% (100·0 to 100·0%)	-2·6% (-6·2 to 0·8%)	-
Cervical cancer	53·4 (51·5 to 55·2)	100·0% (100·0 to 100·0%)	55·9 (52·8 to 59·4)	100·0% (100·0 to 100·0%)	4·7% (-1·9 to 12·1%)	-	1,620·8 (1,561·1 to 1,685·1)	100·0% (100·0 to 100·0%)	1,597·5 (1,497·9 to 1,709·3)	100·0% (100·0 to 100·0%)	-1·4% (-8·4 to 5·9%)	-
**Low physical activity: All causes**	352·4 (268·6 to 437·3)	3·2% (2·4 to 3·9%)	415·4 (322·7 to 510·0)	3·5% (2·7 to 4·3%)	17·9% (14·4 to 22·0%)	11·7% (9·5 to 14·4%)	7,763·2 (5,932·7 to 9,594·9)	1·7% (1·3 to 2·0%)	8,907·6 (6,917·7 to 10,935·2)	1·9% (1·5 to 2·3%)	14·7% (10·7 to 19·1%)	15·6% (12·6 to 19·2%)
Colon and rectum cancer	22·8 (15·8 to 29·9)	13·4% (9·3 to 17·7%)	29·4 (20·5 to 38·5)	13·9% (9·7 to 18·2%)	29·2% (24·7 to 33·7%)	3·5% (2·3 to 5·4%)	460·9 (315·1 to 614·3)	12·3% (8·4 to 16·4%)	565·2 (386·0 to 750·5)	12·8% (8·8 to 16·9%)	22·6% (18·3 to 27·5%)	3·9% (2·7 to 5·6%)
Breast cancer	8·7 (6·3 to 11·1)	8·4% (6·1 to 10·7%)	10·9 (7·9 to 14·0)	8·6% (6·2 to 11·0%)	25·6% (17·1 to 32·5%)	2·8% (1·2 to 4·9%)	243·9 (175·6 to 315·7)	7·8% (5·6 to 10·0%)	296·0 (212·6 to 386·3)	8·1% (5·8 to 10·4%)	21·3% (12·4 to 29·1%)	3·3% (1·4 to 5·3%)
Ischaemic heart disease	193·6 (125·4 to 265·7)	9·2% (6·0 to 12·5%)	227·9 (149·9 to 310·2)	9·4% (6·3 to 12·8%)	17·7% (14·2 to 22·5%)	2·7% (1·0 to 5·6%)	3,909·9 (2,491·1 to 5,429·4)	9·8% (6·2 to 13·5%)	4,336·0 (2,772·0 to 5,949·6)	10·2% (6·6 to 14·0%)	10·9% (6·8 to 15·7%)	4·2% (2·5 to 7·4%)
Ischaemic stroke	82·3 (45·6 to 121·8)	11·1% (6·1 to 16·2%)	87·9 (51·0 to 129·0)	11·2% (6·3 to 16·2%)	6·8% (2·4 to 12·1%)	1·2% (-0·2 to 3·7%)	1,367·4 (725·9 to 2,050·8)	11·5% (6·1 to 17·1%)	1,375·4 (754·1 to 2,065·5)	11·8% (6·4 to 17·3%)	0·6% (-3·9 to 5·7%)	2·1% (0·9 to 4·3%)
Diabetes mellitus	45·0 (34·9 to 56·0)	16·2% (12·4 to 20·1%)	59·3 (46·2 to 73·1)	16·7% (13·0 to 20·7%)	31·5% (27·1 to 36·3%)	3·7% (2·6 to 5·1%)	1,781·1 (1,274·8 to 2,409·2)	13·9% (10·5 to 17·5%)	2,335·0 (1,675·4 to 3,148·4)	14·5% (11·0 to 18·2%)	31·1% (27·6 to 34·3%)	4·0% (3·3 to 5·1%)
**Metabolic risks: All causes**	3,824·9 (3,630·5 to 4,016·9)	34·3% (32·6 to 36·0%)	4,378·0 (4,147·2 to 4,625·0)	37·2% (35·3 to 39·0%)	14·5% (11·4 to 17·6%)	8·4% (7·2 to 9·6%)	87,557·8 (82,458·4 to 93,435·6)	18·7% (17·6 to 19·9%)	96,219·2 (89,516·4 to 103,741·3)	20·7% (19·4 to 22·1%)	9·9% (6·7 to 13·1%)	10·7% (8·8 to 12·5%)
**High fasting plasma glucose: All causes**	1,076·3 (920·7 to 1,298·0)	9·6% (8·3 to 11·7%)	1,307·6 (1,120·2 to 1,577·4)	11·1% (9·5 to 13·4%)	21·5% (17·4 to 25·9%)	15·1% (12·3 to 18·1%)	30,007·7 (26,128·4 to 34,413·2)	6·4% (5·8 to 7·2%)	35,677·4 (30,834·2 to 41,117·4)	7·7% (7·0 to 8·5%)	18·9% (15·5 to 22·3%)	19·8% (17·3 to 22·1%)
Tuberculosis	15·4 (9·5 to 21·8)	11·3% (7·4 to 15·8%)	12·1 (7·6 to 17·3)	12·0% (7·8 to 16·8%)	-21·0% (-27·6 to -14·0%)	6·2% (2·2 to 10·1%)	518·7 (322·9 to 710·2)	9·9% (6·5 to 13·5%)	408·8 (258·7 to 563·9)	10·7% (7·1 to 14·6%)	-21·2% (-27·6 to -14·2%)	8·1% (4·1 to 12·3%)
Ischaemic heart disease	382·1 (255·9 to 548·5)	18·1% (12·0 to 25·9%)	473·2 (312·2 to 686·7)	19·5% (12·9 to 28·3%)	23·9% (16·9 to 31·3%)	8·0% (2·8 to 13·6%)	7,446·6 (5,232·1 to 10,099·9)	18·7% (13·1 to 25·4%)	8,700·6 (6,153·7 to 11,789·3)	20·5% (14·4 to 27·8%)	16·8% (12·1 to 21·5%)	9·8% (7·5 to 12·5%)
Ischaemic stroke	120·4 (66·9 to 210·5)	16·2% (9·1 to 28·6%)	134·9 (75·5 to 239·4)	17·2% (9·6 to 31·4%)	12·0% (4·8 to 21·5%)	6·1% (1·1 to 14·5%)	2,063·8 (1,243·2 to 3,289·6)	17·4% (10·7 to 28·2%)	2,221·4 (1,367·2 to 3,550·0)	19·0% (11·7 to 30·8%)	7·6% (2·1 to 13·8%)	9·3% (6·8 to 13·5%)
Hemorrhagic stroke	142·9 (98·6 to 197·1)	16·4% (11·4 to 22·7%)	147·3 (100·8 to 207·6)	17·8% (12·3 to 24·5%)	3·0% (-3·0 to 9·7%)	8·3% (5·0 to 13·0%)	3,258·4 (2,236·4 to 4,530·3)	17·4% (11·9 to 24·2%)	3,260·3 (2,243·0 to 4,503·0)	19·5% (13·5 to 26·7%)	0·1% (-5·4 to 6·0%)	12·3% (9·5 to 14·8%)
Diabetes mellitus	278·8 (271·6 to 286·8)	100·0% (100·0 to 100·0%)	353·8 (342·6 to 367·0)	100·0% (100·0 to 100·0%)	26·9% (22·5 to 31·2%)	-	12,819·0 (10,568·8 to 15,417·2)	100·0% (100·0 to 100·0%)	16,153·8 (13,165·4 to 19,544·7)	100·0% (100·0 to 100·0%)	26·0% (22·8 to 28·9%)	-
Chronic kidney disease due to diabetes mellitus	83·9 (79·4 to 87·5)	100·0% (100·0 to 100·0%)	113·2 (105·8 to 119·6)	100·0% (100·0 to 100·0%)	35·0% (31·0 to 39·4%)	-	2,388·6 (2,216·3 to 2,577·6)	100·0% (100·0 to 100·0%)	2,999·2 (2,770·1 to 3,242·0)	100·0% (100·0 to 100·0%)	25·6% (22·3 to 28·9%)	-
Chronic kidney disease due to hypertension	33·0 (23·3 to 42·3)	37·3% (26·4 to 47·7%)	48·6 (34·5 to 62·3)	40·4% (28·9 to 51·0%)	47·2% (41·1 to 53·5%)	8·2% (6·8 to 9·7%)	738·0 (515·4 to 951·6)	33·6% (23·7 to 43·1%)	1,003·9 (710·1 to 1,289·5)	37·8% (27·0 to 47·8%)	36·0% (31·1 to 41·4%)	12·6% (11·0 to 14·4%)
Chronic kidney disease due to glomerulonephritis	16·9 (11·9 to 22·1)	34·0% (23·8 to 43·7%)	20·0 (14·0 to 25·8)	38·4% (27·4 to 48·6%)	17·9% (12·3 to 24·6%)	12·8% (11·0 to 14·9%)	545·6 (379·1 to 718·3)	29·5% (20·6 to 38·2%)	610·6 (428·4 to 796·1)	34·4% (24·4 to 43·8%)	11·9% (7·1 to 17·4%)	16·5% (14·4 to 18·9%)
Chronic kidney disease due to other causes	1·6 (1·1 to 2·1)	29·1% (20·4 to 37·8%)	2·5 (1·7 to 3·4)	35·1% (24·9 to 45·1%)	55·3% (40·7 to 68·8%)	20·4% (16·9 to 25·2%)	182·0 (119·2 to 258·5)	28·1% (19·5 to 36·7%)	251·3 (164·7 to 355·1)	33·2% (23·4 to 42·8%)	38·1% (34·3 to 41·5%)	18·4% (16·0 to 21·6%)
**High total cholesterol: All causes**	1,085·5 (848·5 to 1,376·2)	9·7% (7·6 to 12·4%)	1,208·9 (925·6 to 1,547·5)	10·3% (7·9 to 13·1%)	11·4% (6·1 to 16·5%)	5·5% (1·1 to 9·5%)	22,912·3 (19,163·4 to 27,169·9)	4·9% (4·0 to 5·9%)	23,867·7 (19,979·5 to 28,484·8)	5·1% (4·2 to 6·2%)	4·2% (0·4 to 7·9%)	5·0% (1·4 to 7·9%)
Ischaemic heart disease	936·5 (735·0 to 1,140·2)	44·3% (35·0 to 54·0%)	1,052·4 (806·0 to 1,309·8)	43·4% (33·5 to 53·6%)	12·4% (6·7 to 17·8%)	-2·0% (-6·3 to 1·7%)	20,123·2 (16,903·7 to 23,494·0)	50·4% (42·7 to 58·5%)	21,133·3 (17,707·0 to 24,840·0)	49·8% (41·9 to 58·0%)	5·0% (0·9 to 8·8%)	-1·3% (-2·8 to 0·2%)
Ischaemic stroke	148·9 (62·1 to 293·8)	20·0% (8·3 to 39·2%)	156·5 (59·5 to 314·5)	19·9% (7·6 to 39·6%)	5·1% (-5·9 to 13·1%)	-0·5% (-9·2 to 5·6%)	2,789·1 (1,654·3 to 4,636·3)	23·5% (14·1 to 39·2%)	2,734·4 (1,613·1 to 4,692·6)	23·4% (13·9 to 39·3%)	-2·0% (-8·0 to 3·2%)	-0·5% (-3·8 to 2·8%)
**High systolic blood pressure: All causes**	2,484·8 (2,236·0 to 2,728·8)	22·3% (20·0 to 24·5%)	2,844·5 (2,528·5 to 3,128·3)	24·1% (21·6 to 26·6%)	14·5% (11·0 to 18·2%)	8·4% (6·3 to 10·6%)	49,995·3 (45,433·5 to 54,567·2)	10·7% (9·5 to 12·0%)	53,865·0 (48,948·8 to 58,642·2)	11·6% (10·2 to 13·1%)	7·7% (4·3 to 11·2%)	8·6% (5·8 to 11·2%)
Rheumatic heart disease	15·2 (10·6 to 22·5)	25·6% (17·7 to 38·8%)	13·7 (9·5 to 20·6)	27·4% (19·0 to 42·0%)	-9·7% (-15·6 to -3·2%)	7·3% (2·5 to 13·6%)	444·9 (302·9 to 657·0)	23·4% (16·1 to 35·1%)	383·3 (263·3 to 566·9)	26·1% (18·3 to 38·6%)	-13·9% (-18·3 to -8·8%)	11·4% (8·4 to 15·2%)
Ischaemic heart disease	1,168·8 (972·7 to 1,365·3)	55·3% (45·9 to 64·6%)	1,366·1 (1,105·6 to 1,615·3)	56·4% (45·7 to 66·7%)	16·9% (12·8 to 20·6%)	1·9% (-1·1 to 4·0%)	22,324·1 (19,342·4 to 25,317·8)	55·9% (48·2 to 62·9%)	24,464·5 (21,042·0 to 27,586·6)	57·6% (49·6 to 64·8%)	9·6% (5·7 to 13·2%)	3·0% (1·8 to 4·3%)
Ischaemic stroke	370·6 (290·5 to 449·6)	49·8% (39·2 to 60·1%)	403·5 (312·1 to 494·8)	51·3% (40·5 to 62·6%)	8·9% (2·9 to 15·4%)	3·1% (-0·1 to 7·7%)	6,267·1 (4,945·1 to 7,321·4)	52·9% (42·3 to 61·5%)	6,481·3 (5,199·0 to 7,565·0)	55·5% (45·2 to 64·0%)	3·4% (-1·5 to 8·6%)	5·0% (3·1 to 7·6%)

Supplemental Results Table 1. All-age deaths, death PAFs, DALYs, DALY PAFs, and percent change for each risk factor and outcome for both sexes combined in 2005 and 2015 for High-middle SDI.

Data in parenthesis are 95% uncertainty intervals. PAFs=population attributable fractions. DALYs=disability-adjusted life-years.

	2005 Deaths (in thousands)	2005 Deaths PAF	2015 Deaths (in thousands)	2015 Deaths PAF	Percent change deaths 2005-2015	Percent change deaths PAF 2005-2015	2005 DALYs (in thousands)	2005 DALYs PAF	2015 DALYs (in thousands)	2015 DALYs PAF	Percent change DALYs 2005-2015	Percent change DALYs PAF 2005-2015
Hemorrhagic stroke	489·2 (397·3 to 574·7)	56·2% (45·7 to 66·7%)	493·9 (397·5 to 591·7)	59·6% (48·4 to 70·3%)	1·0% (-4·5 to 7·2%)	6·1% (3·2 to 9·6%)	10,832·4 (9,038·3 to 12,429·4)	57·7% (47·7 to 66·2%)	10,394·0 (8,797·7 to 11,974·7)	62·1% (52·6 to 70·5%)	-4·0% (-9·0 to 1·2%)	7·7% (5·8 to 9·7%)
Hypertensive heart disease	193·9 (180·8 to 202·3)	100·0% (100·0 to 100·0%)	247·4 (216·0 to 261·5)	100·0% (100·0 to 100·0%)	27·6% (15·9 to 35·5%)	-	3,441·4 (3,255·9 to 3,610·2)	100·0% (100·0 to 100·0%)	3,996·6 (3,613·2 to 4,214·4)	100·0% (100·0 to 100·0%)	16·1% (8·3 to 22·8%)	-
Cardiomyopathy and myocarditis	27·7 (22·5 to 33·3)	38·5% (31·1 to 46·4%)	30·3 (24·1 to 37·3)	38·7% (31·2 to 47·5%)	9·4% (3·3 to 15·3%)	0·4% (-2·6 to 4·2%)	686·5 (558·9 to 809·1)	35·5% (29·0 to 41·7%)	699·7 (568·1 to 826·7)	37·8% (30·7 to 44·2%)	1·9% (-3·8 to 7·1%)	6·3% (4·1 to 9·1%)
Atrial fibrillation and flutter	11·8 (9·0 to 15·6)	35·4% (28·5 to 42·5%)	16·8 (12·6 to 22·5)	35·8% (28·4 to 43·7%)	41·7% (36·0 to 47·5%)	1·1% (-1·6 to 3·7%)	336·8 (261·2 to 434·5)	41·8% (37·0 to 46·8%)	453·4 (348·6 to 586·9)	43·1% (38·1 to 48·2%)	34·6% (31·7 to 37·4%)	3·1% (1·8 to 4·5%)
Aortic aneurysm	11·4 (9·2 to 13·4)	37·8% (30·7 to 44·4%)	14·5 (11·6 to 17·3)	37·8% (30·2 to 44·8%)	27·3% (20·0 to 33·7%)	0·0% (-2·1 to 2·7%)	235·1 (197·9 to 273·2)	39·3% (33·1 to 45·6%)	285·6 (237·5 to 334·7)	40·2% (33·7 to 46·4%)	21·5% (13·8 to 27·3%)	2·4% (1·1 to 3·7%)
Peripheral vascular disease	1·8 (1·3 to 2·3)	28·0% (20·7 to 35·9%)	2·6 (1·9 to 3·4)	27·4% (19·9 to 35·7%)	46·2% (35·1 to 58·2%)	-2·1% (-5·5 to 1·0%)	56·0 (37·2 to 85·3)	29·5% (23·1 to 36·1%)	80·0 (51·9 to 122·4)	30·0% (23·5 to 36·9%)	42·9% (37·6 to 48·6%)	1·8% (-0·1 to 3·7%)
Endocarditis	5·0 (3·9 to 6·3)	33·0% (26·3 to 40·7%)	6·4 (4·9 to 8·1)	34·8% (27·7 to 43·1%)	27·0% (20·3 to 33·8%)	5·3% (2·0 to 8·9%)	121·8 (93·4 to 152·1)	28·5% (22·4 to 34·4%)	145·1 (112·2 to 180·2)	32·8% (26·1 to 39·2%)	19·1% (12·9 to 26·3%)	15·0% (11·7 to 18·7%)
Other cardiovascular and circulatory diseases	41·8 (36·7 to 47·3)	38·5% (33·9 to 43·3%)	49·4 (42·8 to 56·5)	39·7% (34·7 to 44·8%)	18·1% (12·3 to 23·4%)	3·1% (0·5 to 5·5%)	1,349·2 (1,138·1 to 1,605·5)	33·5% (29·9 to 37·5%)	1,663·3 (1,369·3 to 2,009·3)	37·8% (33·9 to 41·9%)	23·3% (18·5 to 27·6%)	12·7% (10·9 to 14·5%)
Chronic kidney disease due to diabetes mellitus	37·6 (26·8 to 47·4)	44·9% (32·3 to 56·4%)	53·1 (38·2 to 66·9)	46·9% (34·1 to 58·5%)	41·1% (37·0 to 45·9%)	4·5% (3·5 to 5·8%)	917·7 (648·0 to 1,179·7)	38·4% (27·3 to 48·8%)	1,240·0 (877·6 to 1,590·6)	41·3% (29·5 to 52·1%)	35·1% (31·6 to 39·0%)	7·6% (6·4 to 9·0%)
Chronic kidney disease due to hypertension	88·4 (82·4 to 92·4)	100·0% (100·0 to 100·0%)	120·4 (110·0 to 126·6)	100·0% (100·0 to 100·0%)	36·1% (30·9 to 41·6%)	-	2,198·9 (2,034·4 to 2,336·9)	100·0% (100·0 to 100·0%)	2,656·3 (2,420·0 to 2,827·4)	100·0% (100·0 to 100·0%)	20·8% (16·7 to 25·2%)	-
Chronic kidney disease due to glomerulonephritis	19·6 (14·0 to 25·1)	39·3% (28·2 to 50·2%)	23·5 (17·0 to 29·4)	45·0% (32·8 to 56·5%)	19·8% (13·8 to 26·6%)	14·7% (12·0 to 17·5%)	582·3 (406·4 to 770·2)	31·5% (22·2 to 40·9%)	649·8 (455·9 to 842·5)	36·6% (26·1 to 46·9%)	11·6% (6·7 to 16·5%)	16·2% (13·7 to 18·9%)
Chronic kidney disease due to other causes	2·0 (1·4 to 2·6)	35·7% (25·4 to 45·2%)	3·0 (2·1 to 3·9)	42·0% (29·8 to 52·8%)	51·7% (37·3 to 64·7%)	17·6% (13·4 to 22·6%)	201·1 (133·0 to 276·3)	31·0% (21·6 to 39·8%)	272·3 (180·3 to 373·1)	36·0% (25·3 to 45·7%)	35·4% (32·3 to 38·6%)	16·1% (13·6 to 19·4%)
**High body-mass index: All causes**	989·0 (677·5 to 1,326·7)	8·9% (6·1 to 11·9%)	1,134·1 (790·1 to 1,512·3)	9·6% (6·7 to 12·8%)	14·7% (10·0 to 20·0%)	8·6% (5·1 to 13·0%)	29,284·5 (20,187·1 to 38,847·3)	6·3% (4·3 to 8·3%)	34,123·5 (24,217·6 to 44,429·8)	7·3% (5·3 to 9·5%)	16·5% (11·9 to 22·1%)	17·4% (13·6 to 22·3%)
Oesophageal cancer	22·8 (7·0 to 42·1)	15·5% (4·8 to 28·7%)	22·3 (6·9 to 41·2)	17·3% (5·4 to 31·6%)	-2·0% (-14·0 to 11·7%)	11·6% (0·0 to 24·2%)	532·5 (160·5 to 984·3)	16·0% (4·7 to 29·6%)	512·2 (161·6 to 936·5)	18·3% (5·7 to 33·5%)	-3·8% (-16·8 to 10·6%)	14·2% (1·3 to 28·4%)
Colon and rectum cancer	12·6 (7·5 to 18·5)	7·4% (4·4 to 10·9%)	17·0 (10·2 to 24·8)	8·0% (4·8 to 11·6%)	35·4% (30·1 to 41·5%)	8·4% (5·5 to 12·5%)	283·8 (168·9 to 417·2)	7·6% (4·5 to 11·1%)	375·3 (227·2 to 545·8)	8·5% (5·1 to 12·3%)	32·2% (26·8 to 38·7%)	12·0% (8·7 to 16·6%)
Liver cancer due to hepatitis B	9·7 (3·4 to 19·1)	10·2% (3·8 to 19·9%)	10·7 (4·0 to 20·4)	12·0% (4·7 to 22·5%)	10·6% (-2·3 to 33·5%)	17·3% (10·2 to 29·9%)	308·4 (107·2 to 614·4)	10·3% (3·8 to 20·1%)	326·0 (123·7 to 633·0)	12·4% (4·8 to 23·3%)	5·7% (-7·9 to 30·9%)	20·8% (13·0 to 35·1%)
Liver cancer due to hepatitis C	4·0 (1·7 to 6·8)	12·6% (5·5 to 21·4%)	5·2 (2·3 to 9·0)	13·8% (6·2 to 23·2%)	32·7% (21·5 to 44·6%)	10·2% (2·6 to 18·0%)	85·9 (37·7 to 148·6)	12·8% (5·7 to 21·9%)	110·4 (49·2 to 191·7)	14·5% (6·5 to 24·4%)	28·5% (15·9 to 42·5%)	13·0% (3·8 to 22·9%)
Liver cancer due to alcohol use	7·4 (2·7 to 14·4)	11·3% (4·3 to 21·7%)	10·4 (4·0 to 19·9)	12·5% (4·8 to 23·5%)	41·0% (27·3 to 61·3%)	11·0% (4·9 to 19·0%)	185·7 (68·3 to 368·6)	11·4% (4·2 to 22·2%)	259·7 (97·5 to 501·2)	13·0% (4·9 to 24·4%)	39·9% (24·8 to 63·8%)	13·6% (7·2 to 23·1%)
Liver cancer due to other causes	4·3 (1·7 to 7·8)	9·8% (3·9 to 17·9%)	4·6 (1·9 to 8·3)	11·4% (4·8 to 20·0%)	7·0% (-4·6 to 25·6%)	16·0% (7·4 to 27·7%)	116·7 (45·8 to 215·1)	10·0% (4·0 to 18·4%)	117·6 (48·5 to 213·4)	12·0% (4·9 to 21·3%)	0·8% (-11·5 to 21·4%)	20·0% (10·2 to 33·4%)
Gallbladder and biliary tract cancer	5·7 (3·3 to 8·5)	17·0% (10·0 to 25·4%)	6·7 (3·9 to 9·8)	17·6% (10·5 to 25·8%)	17·5% (8·5 to 26·5%)	3·4% (-1·3 to 8·4%)	119·7 (70·7 to 176·9)	17·3% (10·2 to 25·7%)	134·9 (80·1 to 198·3)	18·2% (10·9 to 26·5%)	12·7% (4·7 to 21·7%)	5·0% (0·2 to 10·2%)
Pancreatic cancer	4·2 (1·5 to 7·4)	5·5% (1·9 to 9·8%)	5·8 (2·1 to 10·1)	5·8% (2·0 to 10·1%)	38·5% (32·7 to 44·8%)	5·4% (2·7 to 9·3%)	93·0 (31·8 to 165·2)	5·5% (1·9 to 9·8%)	125·9 (43·8 to 219·1)	5·9% (2·1 to 10·4%)	35·3% (29·0 to 41·7%)	7·0% (4·1 to 10·8%)
Breast cancer	6·8 (3·7 to 10·8)	6·6% (3·6 to 10·4%)	9·6 (5·4 to 15·1)	7·6% (4·3 to 11·6%)	40·3% (22·4 to 57·0%)	14·8% (7·1 to 24·1%)	152·7 (76·9 to 253·3)	4·9% (2·5 to 8·1%)	216·6 (113·7 to 354·7)	5·9% (3·1 to 9·5%)	41·9% (19·6 to 66·6%)	20·6% (9·2 to 35·7%)
Uterine cancer	6·7 (4·5 to 8·9)	35·4% (24·1 to 47·1%)	7·5 (5·2 to 9·8)	38·2% (26·8 to 49·8%)	12·8% (4·6 to 22·8%)	8·1% (4·7 to 13·7%)	169·1 (114·7 to 226·9)	34·8% (23·5 to 46·6%)	186·7 (129·7 to 247·6)	38·4% (27·0 to 49·9%)	10·4% (1·1 to 21·7%)	10·1% (6·1 to 16·7%)
Ovarian cancer	0·9 (0·0 to 2·1)	3·2% (-0·1 to 7·1%)	1·2 (0·0 to 2·8)	3·4% (-0·1 to 7·6%)	30·0% (24·0 to 37·6%)	7·4% (4·3 to 12·2%)	26·0 (-0·9 to 58·5)	3·1% (-0·1 to 7·0%)	33·2 (-1·2 to 73·8)	3·4% (-0·1 to 7·5%)	27·6% (21·0 to 35·8%)	9·5% (6·1 to 15·1%)
Kidney cancer	3·9 (2·6 to 5·5)	16·9% (11·1 to 23·4%)	5·7 (3·8 to 7·9)	17·6% (11·6 to 24·2%)	43·9% (38·2 to 50·0%)	4·0% (1·0 to 7·0%)	97·5 (64·4 to 135·0)	15·9% (10·4 to 22·0%)	137·7 (91·6 to 189·8)	17·2% (11·4 to 23·6%)	41·2% (35·3 to 47·3%)	8·3% (5·1 to 11·8%)
Thyroid cancer	0·7 (0·4 to 1·0)	10·4% (5·9 to 16·0%)	0·9 (0·5 to 1·4)	11·1% (6·2 to 17·1%)	38·2% (23·2 to 49·8%)	6·9% (0·2 to 13·0%)	17·8 (10·2 to 27·7)	10·6% (6·0 to 16·3%)	25·0 (13·9 to 39·2)	11·8% (6·5 to 18·2%)	40·9% (24·4 to 52·8%)	10·6% (3·2 to 16·9%)
Ischaemic heart disease	370·4 (248·6 to 506·9)	17·5% (11·8 to 23·8%)	420·1 (284·4 to 570·6)	17·3% (11·7 to 23·6%)	13·4% (8·3 to 18·6%)	-1·1% (-4·9 to 2·8%)	8,644·1 (5,923·6 to 11,675·9)	21·7% (14·8 to 29·1%)	9,538·7 (6,616·3 to 12,752·3)	22·5% (15·6 to 29·7%)	10·3% (5·8 to 15·1%)	3·7% (1·0 to 7·2%)
Ischaemic stroke	92·4 (58·2 to 132·1)	12·4% (7·9 to 17·7%)	92·8 (58·6 to 136·4)	11·8% (7·5 to 17·1%)	0·4% (-8·4 to 8·4%)	-4·9% (-11·8 to 1·4%)	2,069·8 (1,357·1 to 2,872·5)	17·5% (11·5 to 24·0%)	2,098·9 (1,391·2 to 2,881·2)	18·0% (12·1 to 24·5%)	1·4% (-4·5 to 8·0%)	2·9% (-1·5 to 8·0%)
Hemorrhagic stroke	182·8 (120·5 to 252·4)	21·0% (13·8 to 29·1%)	178·8 (121·7 to 245·4)	21·6% (14·7 to 29·5%)	-2·2% (-9·4 to 5·7%)	2·8% (-2·8 to 9·2%)	5,318·2 (3,551·8 to 7,163·3)	28·3% (19·1 to 37·9%)	5,186·6 (3,626·0 to 6,888·4)	31·0% (21·7 to 40·6%)	-2·5% (-8·9 to 5·2%)	9·5% (5·1 to 15·8%)
Hypertensive heart disease	70·1 (42·5 to 101·9)	36·2% (22·4 to 52·2%)	91·0 (53·4 to 133·9)	36·8% (21·8 to 54·0%)	29·7% (13·7 to 42·2%)	1·7% (-10·4 to 10·3%)	1,390·5 (962·5 to 1,852·6)	40·4% (28·3 to 54·0%)	1,683·1 (1,160·7 to 2,253·0)	42·1% (29·5 to 55·9%)	21·0% (12·0 to 29·8%)	4·2% (-1·3 to 10·2%)
Diabetes mellitus	115·6 (85·5 to 146·2)	41·5% (30·9 to 52·3%)	149·7 (112·7 to 189·6)	42·3% (32·1 to 53·4%)	29·5% (23·3 to 36·0%)	2·1% (-1·5 to 5·9%)	6,377·4 (4,563·6 to 8,394·5)	49·7% (38·1 to 60·9%)	8,580·5 (6,150·4 to 11,144·2)	53·1% (41·8 to 63·8%)	34·5% (29·6 to 40·0%)	6·8% (4·2 to 10·3%)
Chronic kidney disease due to diabetes mellitus	27·8 (15·0 to 42·2)	33·2% (18·0 to 49·9%)	39·9 (21·5 to 60·5)	35·3% (19·2 to 52·7%)	43·4% (32·2 to 55·3%)	6·2% (-1·7 to 14·7%)	767·4 (386·2 to 1,161·0)	32·2% (16·4 to 48·8%)	1,073·9 (563·8 to 1,589·9)	35·8% (19·1 to 53·3%)	39·9% (33·7 to 48·5%)	11·4% (6·9 to 18·1%)
Chronic kidney disease due to hypertension	24·1 (11·8 to 38·1)	27·2% (13·4 to 42·7%)	34·4 (15·6 to 55·6)	28·6% (12·9 to 45·8%)	43·2% (20·4 to 58·0%)	5·2% (-10·3 to 15·3%)	601·2 (307·5 to 925·4)	27·3% (14·1 to 41·6%)	816·8 (426·2 to 1,230·1)	30·8% (16·3 to 46·0%)	35·9% (27·8 to 45·5%)	12·5% (6·6 to 19·8%)
Chronic kidney disease due to glomerulonephritis	10·9 (4·8 to 18·0)	21·8% (9·5 to 35·7%)	12·5 (5·4 to 20·6)	24·0% (10·4 to 39·0%)	14·9% (-2·2 to 33·5%)	10·0% (-5·9 to 28·1%)	396·1 (143·5 to 677·2)	21·4% (7·6 to 36·0%)	454·4 (174·2 to 738·8)	25·6% (9·7 to 41·0%)	14·7% (6·7 to 27·3%)	19·4% (12·4 to 33·1%)
Chronic kidney disease due to other causes	1·1 (0·2 to 2·0)	19·4% (3·8 to 34·9%)	1·6 (0·2 to 3·1)	22·4% (2·8 to 41·0%)	48·9% (-10·2 to 69·0%)	15·4% (-28·1 to 28·6%)	151·8 (64·7 to 245·9)	23·4% (10·9 to 36·6%)	209·7 (92·2 to 337·3)	27·7% (13·0 to 42·3%)	38·1% (31·9 to 46·5%)	18·4% (12·9 to 26·1%)
Osteoarthritis	-	-	-	-	-	-	529·3 (305·2 to 827·1)	19·5% (13·0 to 26·7%)	792·8 (465·2 to 1,237·8)	20·8% (14·3 to 28·1%)	49·8% (46·3 to 55·2%)	6·8% (4·5 to 10·3%)
Low back pain	-	-	-	-	-	-	727·2 (411·6 to 1,163·2)	5·7% (3·7 to 8·0%)	972·7 (557·9 to 1,533·7)	6·5% (4·3 to 8·9%)	33·7% (30·7 to 38·2%)	13·4% (10·9 to 17·2%)
**Low bone mineral density: All causes**	59·0 (55·4 to 61·9)	0·5% (0·5 to 0·6%)	78·0 (69·3 to 83·6)	0·7% (0·6 to 0·7%)	32·1% (23·2 to 39·9%)	25·1% (17·4 to 32·0%)	1,730·9 (1,490·0 to 2,004·3)	0·4% (0·3 to 0·4%)	2,076·7 (1,753·1 to 2,441·1)	0·4% (0·4 to 0·5%)	20·0% (14·7 to 24·8%)	20·8% (16·4 to 25·4%)
Pedestrian road injuries	13·5 (12·5 to 14·5)	7·8% (7·4 to 8·2%)	15·3 (13·7 to 16·8)	9·7% (9·1 to 10·3%)	13·2% (4·6 to 21·1%)	24·3% (19·2 to 29·4%)	303·0 (271·7 to 331·8)	3·8% (3·5 to 4·1%)	328·9 (285·8 to 367·6)	5·0% (4·6 to 5·4%)	8·6% (0·1 to 16·3%)	30·2% (25·2 to 35·6%)
Cyclist road injuries	1·5 (1·3 to 1·6)	6·7% (6·1 to 7·1%)	1·6 (1·4 to 1·8)	8·3% (7·7 to 9·0%)	9·2% (-3·7 to 23·1%)	25·2% (18·9 to 32·1%)	83·3 (66·6 to 101·4)	6·6% (5·7 to 7·6%)	99·5 (77·3 to 126·0)	9·0% (7·8 to 10·3%)	19·5% (11·0 to 27·6%)	36·1% (30·1 to 42·9%)
Motorcyclist road injuries	2·5 (2·0 to 2·8)	3·7% (3·4 to 4·1%)	3·2 (2·6 to 3·7)	5·3% (4·7 to 5·9%)	28·5% (12·0 to 50·9%)	42·9% (30·9 to 55·1%)	120·0 (97·6 to 143·2)	3·2% (2·7 to 3·6%)	151·5 (121·0 to 183·2)	4·6% (3·9 to 5·4%)	26·2% (16·0 to 39·4%)	45·4% (34·4 to 55·0%)
Motor vehicle road injuries	7·0 (6·3 to 7·8)	5·6% (5·2 to 5·9%)	8·3 (7·4 to 9·4)	7·1% (6·6 to 7·6%)	19·0% (9·8 to 28·8%)	27·4% (21·4 to 33·2%)	272·9 (233·0 to 318·3)	4·0% (3·5 to 4·5%)	323·5 (268·3 to 382·2)	5·3% (4·6 to 6·0%)	18·5% (11·4 to 25·3%)	31·9% (25·7 to 37·8%)
Other road injuries	0·3 (0·3 to 0·4)	8·1% (7·4 to 8·9%)	0·4 (0·3 to 0·5)	10·4% (9·4 to 11·7%)	35·9% (16·5 to 61·9%)	28·3% (14·9 to 42·8%)	14·7 (11·3 to 18·9)	6·6% (5·3 to 7·9%)	20·8 (15·6 to 27·1)	8·7% (7·2 to 10·3%)	41·2% (32·3 to 49·2%)	32·5% (23·1 to 45·7%)

Supplemental Results Table 1. All-age deaths, death PAFs, DALYs, DALY PAFs, and percent change for each risk factor and outcome for both sexes combined in 2005 and 2015 for High-middle SDI.

Data in parenthesis are 95% uncertainty intervals. PAFs=population attributable fractions. DALYs=disability-adjusted life-years.

	2005 Deaths (in thousands)	2005 Deaths PAF	2015 Deaths (in thousands)	2015 Deaths PAF	Percent change deaths 2005-2015	Percent change deaths PAF 2005-2015	2005 DALYs (in thousands)	2005 DALYs PAF	2015 DALYs (in thousands)	2015 DALYs PAF	Percent change DALYs 2005-2015	Percent change DALYs PAF 2005-2015
Other transport injuries	2·1 (1·9 to 2·4)	8·5% (7·9 to 9·0%)	2·6 (2·2 to 3·2)	10·3% (9·6 to 11·1%)	24·8% (11·2 to 38·6%)	21·0% (15·3 to 26·8%)	52·9 (46·2 to 60·9)	4·3% (4·0 to 4·7%)	60·9 (51·0 to 74·3)	5·2% (4·7 to 5·7%)	15·2% (3·2 to 26·7%)	20·9% (15·3 to 26·4%)
Falls	29·4 (26·6 to 31·0)	36·2% (32·0 to 37·8%)	43·2 (36·3 to 47·4)	43·0% (38·7 to 45·6%)	47·2% (33·5 to 59·9%)	18·8% (11·9 to 25·7%)	762·0 (633·2 to 913·8)	15·7% (14·5 to 16·7%)	957·0 (788·5 to 1,166·1)	18·5% (17·3 to 19·6%)	25·6% (20·0 to 30·9%)	17·3% (13·2 to 22·0%)
Other exposure to mechanical forces	1·4 (1·1 to 1·5)	5·3% (4·8 to 5·8%)	1·7 (1·1 to 1·9)	6·9% (6·2 to 7·9%)	20·8% (0·5 to 33·9%)	31·1% (21·3 to 41·5%)	66·2 (52·0 to 83·8)	3·5% (3·1 to 4·0%)	75·2 (55·8 to 98·5)	4·5% (3·9 to 5·0%)	13·6% (3·3 to 21·4%)	26·6% (20·5 to 33·7%)
Non-venomous animal contact	0·1 (0·1 to 0·2)	6·8% (6·3 to 7·2%)	0·2 (0·1 to 0·2)	8·5% (7·8 to 9·1%)	11·9% (2·7 to 21·7%)	25·0% (17·1 to 31·9%)	3·1 (2·7 to 3·7)	2·6% (2·4 to 2·9%)	3·1 (2·6 to 3·9)	3·2% (2·9 to 3·5%)	2·2% (-4·8 to 11·3%)	21·3% (15·2 to 27·0%)
Assault by other means	1·3 (1·2 to 1·4)	4·1% (3·7 to 4·4%)	1·5 (1·3 to 1·6)	5·1% (4·6 to 5·6%)	15·2% (7·7 to 23·7%)	25·2% (16·8 to 33·4%)	45·3 (37·6 to 53·9)	2·6% (2·2 to 3·1%)	48·8 (40·4 to 58·6)	3·2% (2·7 to 3·8%)	7·8% (3·1 to 13·4%)	22·6% (15·7 to 28·8%)
Exposure to forces of nature	-	-	-	-	-	-	7·5 (4·8 to 10·7)	2·7% (1·9 to 3·7%)	7·5 (4·8 to 10·8)	5·2% (3·9 to 6·7%)	0·6% (-8·6 to 10·8%)	91·2% (58·5 to 139·5%)
**Low glomerular filtration rate: All causes**	498·5 (466·6 to 533·2)	4·5% (4·2 to 4·8%)	589·3 (552·6 to 629·4)	5·0% (4·7 to 5·3%)	18·2% (15·0 to 21·7%)	12·0% (9·7 to 14·3%)	11,553·2 (10,809·8 to 12,423·6)	2·5% (2·3 to 2·7%)	12,766·3 (11,897·7 to 13,723·1)	2·7% (2·5 to 3·0%)	10·5% (7·6 to 13·4%)	11·3% (8·9 to 13·7%)
Ischaemic heart disease	154·1 (135·8 to 171·8)	7·3% (6·5 to 8·1%)	177·2 (155·9 to 197·3)	7·3% (6·4 to 8·2%)	15·0% (11·6 to 18·8%)	0·3% (-2·3 to 2·6%)	2,514·8 (2,231·1 to 2,795·8)	6·3% (5·6 to 7·0%)	2,707·3 (2,403·6 to 3,014·4)	6·4% (5·7 to 7·1%)	7·7% (4·0 to 11·5%)	1·2% (-1·2 to 3·6%)
Ischaemic stroke	57·0 (46·3 to 69·3)	7·7% (6·2 to 9·3%)	60·3 (48·9 to 73·6)	7·7% (6·2 to 9·3%)	5·7% (1·2 to 10·4%)	0·1% (-2·8 to 3·2%)	819·7 (666·4 to 1,000·4)	6·9% (5·6 to 8·4%)	803·6 (657·2 to 980·5)	6·9% (5·6 to 8·3%)	-2·0% (-6·0 to 2·1%)	-0·5% (-2·9 to 2·1%)
Hemorrhagic stroke	58·7 (46·6 to 73·5)	6·7% (5·3 to 8·4%)	57·6 (46·6 to 72·2)	6·9% (5·6 to 8·5%)	-2·0% (-6·6 to 3·3%)	3·0% (-0·2 to 7·2%)	1,077·1 (852·3 to 1,333·1)	5·7% (4·6 to 7·1%)	989·6 (804·6 to 1,219·6)	5·9% (4·8 to 7·2%)	-8·1% (-13·0 to -3·1%)	3·1% (-0·1 to 7·0%)
Peripheral vascular disease	0·9 (0·7 to 1·1)	14·1% (11·9 to 16·6%)	1·3 (1·1 to 1·6)	13·9% (11·7 to 16·2%)	46·6% (34·3 to 59·9%)	-1·8% (-5·4 to 2·3%)	26·3 (17·9 to 38·9)	13·9% (12·0 to 16·1%)	36·2 (24·9 to 52·9)	13·6% (11·8 to 15·9%)	37·5% (31·8 to 43·7%)	-1·9% (-4·2 to 0·3%)
Chronic kidney disease due to diabetes mellitus	83·9 (79·4 to 87·5)	100·0% (100·0 to 100·0%)	113·2 (105·8 to 119·6)	100·0% (100·0 to 100·0%)	35·0% (31·0 to 39·4%)	-	2,388·6 (2,216·3 to 2,577·6)	100·0% (100·0 to 100·0%)	2,999·2 (2,770·1 to 3,242·0)	100·0% (100·0 to 100·0%)	25·6% (22·3 to 28·9%)	-
Chronic kidney disease due to hypertension	88·4 (82·4 to 92·4)	100·0% (100·0 to 100·0%)	120·4 (110·0 to 126·6)	100·0% (100·0 to 100·0%)	36·1% (30·9 to 41·6%)	-	2,198·9 (2,034·4 to 2,336·9)	100·0% (100·0 to 100·0%)	2,656·3 (2,420·0 to 2,827·4)	100·0% (100·0 to 100·0%)	20·8% (16·7 to 25·2%)	-
Chronic kidney disease due to glomerulonephritis	49·8 (46·0 to 53·7)	100·0% (100·0 to 100·0%)	52·1 (47·1 to 56·4)	100·0% (100·0 to 100·0%)	4·5% (-0·3 to 9·8%)	-	1,847·4 (1,689·2 to 2,021·2)	100·0% (100·0 to 100·0%)	1,775·1 (1,599·6 to 1,974·1)	100·0% (100·0 to 100·0%)	-3·9% (-7·9 to 0·0%)	-
Chronic kidney disease due to other causes	5·6 (5·1 to 6·1)	100·0% (100·0 to 100·0%)	7·2 (6·3 to 8·1)	100·0% (100·0 to 100·0%)	28·9% (18·2 to 38·8%)	-	647·6 (518·2 to 787·9)	100·0% (100·0 to 100·0%)	755·4 (594·0 to 928·3)	100·0% (100·0 to 100·0%)	16·7% (12·9 to 20·0%)	-
Gout	-	-	-	-	-	-	32·9 (21·7 to 45·7)	11·9% (10·6 to 13·2%)	43·5 (28·7 to 60·8)	12·1% (11·0 to 13·5%)	32·3% (29·3 to 35·3%)	2·5% (0·7 to 4·4%)

Supplemental Results Table 1. All-age deaths, death PAFs, DALYs, DALY PAFs, and percent change for each risk factor and outcome for both sexes combined in 2005 and 2015 for Middle SDI.

Data in parenthesis are 95% uncertainty intervals. PAFs=population attributable fractions. DALYs=disability-adjusted life-years.

	2005 Deaths (in thousands)	2005 Deaths PAF	2015 Deaths (in thousands)	2015 Deaths PAF	Percent change deaths 2005-2015	Percent change deaths PAF 2005-2015	2005 DALYs (in thousands)	2005 DALYs PAF	2015 DALYs (in thousands)	2015 DALYs PAF	Percent change DALYs 2005-2015	Percent change DALYs PAF 2005-2015
**All risk factors: All causes**	7,752·0 (7,478·9 to 8,025·0)	58·9% (57·2 to 60·4%)	8,505·8 (8,167·1 to 8,870·9)	60·9% (59·3 to 62·4%)	9·7% (5·8 to 13·9%)	3·4% (2·5 to 4·4%)	254,418·3 (239,033·4 to 271,539·8)	41·7% (40·3 to 43·2%)	253,988·8 (237,214·8 to 272,870·2)	42·3% (40·7 to 44·2%)	-0·2% (-3·2 to 2·9%)	1·3% (-0·2 to 3·0%)
**Environmental/occupational risks: All causes**	2,700·1 (2,413·9 to 2,975·6)	20·5% (18·4 to 22·6%)	2,585·6 (2,319·6 to 2,861·3)	18·5% (16·6 to 20·3%)	-4·2% (-8·2 to -0·1%)	-9·7% (-12·0 to -7·4%)	86,615·1 (79,108·8 to 94,142·2)	14·2% (12·9 to 15·5%)	76,287·0 (69,017·6 to 83,645·5)	12·7% (11·5 to 13·9%)	-11·9% (-15·5 to -8·4%)	-10·6% (-13·0 to -8·0%)
**Unsafe water, sanitation, and handwashing: All causes**	390·0 (347·4 to 435·7)	3·0% (2·6 to 3·3%)	294·5 (258·5 to 334·1)	2·1% (1·9 to 2·4%)	-24·5% (-29·0 to -20·0%)	-28·8% (-32·6 to -24·8%)	20,624·2 (18,077·1 to 23,353·0)	3·4% (2·9 to 3·9%)	13,378·5 (11,559·3 to 15,523·6)	2·2% (1·9 to 2·6%)	-35·1% (-39·5 to -30·3%)	-34·1% (-38·2 to -29·7%)
**Unsafe water source: All causes**	278·2 (214·4 to 329·5)	2·1% (1·6 to 2·5%)	202·2 (154·1 to 243·6)	1·4% (1·1 to 1·7%)	-27·3% (-32·5 to -22·4%)	-31·5% (-35·8 to -27·0%)	15,207·7 (11,724·5 to 18,330·7)	2·5% (1·9 to 3·0%)	9,874·2 (7,457·2 to 12,135·4)	1·6% (1·2 to 2·0%)	-35·1% (-39·9 to -30·0%)	-34·1% (-38·6 to -29·4%)
Diarrhoeal diseases	228·4 (177·7 to 262·2)	83·0% (64·4 to 93·2%)	162·6 (122·0 to 189·2)	82·2% (62·9 to 92·7%)	-28·8% (-34·2 to -23·4%)	-1·0% (-2·7 to -0·1%)	11,685·0 (9,170·9 to 13,575·3)	83·1% (64·8 to 93·3%)	7,165·3 (5,465·1 to 8,382·4)	82·3% (63·3 to 92·8%)	-38·7% (-43·5 to -33·6%)	-1·0% (-2·7 to -0·2%)
Typhoid fever	41·3 (21·6 to 69·3)	83·0% (64·6 to 93·2%)	32·6 (17·2 to 54·9)	82·1% (63·1 to 92·7%)	-20·9% (-27·5 to -14·3%)	-1·1% (-2·7 to -0·3%)	2,925·4 (1,551·8 to 4,850·2)	83·1% (64·7 to 93·3%)	2,242·2 (1,199·7 to 3,751·4)	82·2% (63·2 to 92·7%)	-23·4% (-30·0 to -16·4%)	-1·1% (-2·8 to -0·3%)
Paratyphoid fever	8·6 (3·6 to 17·2)	83·2% (64·9 to 93·3%)	6·9 (3·0 to 13·8)	82·4% (63·5 to 92·8%)	-19·6% (-27·0 to -12·0%)	-1·0% (-2·6 to -0·3%)	597·3 (258·8 to 1,193·6)	83·2% (64·9 to 93·3%)	466·7 (210·0 to 918·5)	82·3% (63·5 to 92·8%)	-21·9% (-30·1 to -13·8%)	-1·1% (-2·6 to -0·3%)
**Unsafe sanitation: All causes**	166·2 (146·7 to 189·6)	1·3% (1·1 to 1·4%)	97·3 (83·1 to 113·2)	0·7% (0·6 to 0·8%)	-41·4% (-47·0 to -35·8%)	-44·8% (-49·8 to -39·7%)	8,785·3 (7,605·5 to 10,241·5)	1·4% (1·2 to 1·7%)	4,591·2 (3,831·9 to 5,499·9)	0·8% (0·6 to 0·9%)	-47·7% (-52·8 to -42·2%)	-46·9% (-52·1 to -41·5%)
Diarrhoeal diseases	136·2 (122·2 to 150·5)	49·5% (45·3 to 53·6%)	78·4 (68·4 to 88·8)	39·6% (35·2 to 44·2%)	-42·4% (-48·2 to -36·4%)	-19·9% (-25·6 to -14·4%)	6,666·7 (5,943·1 to 7,470·2)	47·4% (43·4 to 51·5%)	3,296·3 (2,856·9 to 3,776·7)	37·9% (33·7 to 42·1%)	-50·6% (-55·5 to -45·1%)	-20·2% (-25·7 to -15·2%)
Typhoid fever	24·7 (13·2 to 41·4)	49·8% (45·2 to 53·9%)	15·5 (8·5 to 26·1)	39·1% (34·3 to 43·5%)	-37·2% (-44·4 to -29·8%)	-21·5% (-27·3 to -16·1%)	1,753·4 (959·7 to 2,906·9)	49·8% (45·3 to 54·0%)	1,067·1 (598·8 to 1,762·5)	39·1% (34·4 to 43·6%)	-39·1% (-46·5 to -31·5%)	-21·5% (-27·5 to -16·1%)
Paratyphoid fever	5·3 (2·4 to 10·2)	50·8% (46·5 to 55·0%)	3·4 (1·5 to 6·5)	40·2% (35·4 to 45·1%)	-35·8% (-43·3 to -27·3%)	-21·0% (-26·4 to -15·9%)	365·1 (170·3 to 700·6)	50·9% (46·6 to 55·1%)	227·8 (104·9 to 437·5)	40·2% (35·4 to 45·1%)	-37·6% (-45·7 to -28·9%)	-21·0% (-26·5 to -15·8%)
**No handwashing with soap: All causes**	197·7 (161·3 to 232·0)	1·5% (1·2 to 1·8%)	154·3 (124·4 to 183·2)	1·1% (0·9 to 1·3%)	-22·0% (-26·4 to -17·4%)	-26·4% (-30·3 to -22·4%)	10,140·1 (8,266·4 to 11,882·2)	1·7% (1·4 to 2·0%)	6,542·2 (5,304·2 to 7,807·4)	1·1% (0·9 to 1·3%)	-35·5% (-39·5 to -31·1%)	-34·5% (-38·4 to -30·4%)
Diarrhoeal diseases	102·4 (82·3 to 123·5)	37·2% (29·8 to 44·1%)	71·2 (56·3 to 85·9)	36·0% (28·6 to 42·8%)	-30·5% (-35·6 to -25·2%)	-3·3% (-4·7 to -2·0%)	5,215·7 (4,136·6 to 6,308·6)	37·1% (29·7 to 44·0%)	3,125·7 (2,467·4 to 3,788·2)	35·9% (28·5 to 42·7%)	-40·1% (-44·6 to -35·1%)	-3·3% (-4·7 to -2·0%)
Typhoid fever	18·5 (9·7 to 31·9)	37·2% (29·3 to 44·2%)	14·3 (7·5 to 24·4)	36·1% (28·1 to 43·2%)	-22·5% (-29·1 to -16·0%)	-3·1% (-4·6 to -1·8%)	1,312·5 (701·5 to 2,242·3)	37·3% (29·3 to 44·2%)	986·0 (530·3 to 1,655·7)	36·1% (28·1 to 43·2%)	-24·9% (-31·9 to -18·0%)	-3·1% (-4·6 to -1·8%)
Paratyphoid fever	3·9 (1·8 to 7·8)	37·5% (29·5 to 44·6%)	3·1 (1·4 to 6·2)	36·6% (28·5 to 43·6%)	-20·7% (-27·9 to -13·0%)	-2·5% (-3·9 to -1·4%)	269·1 (123·6 to 546·2)	37·5% (29·5 to 44·6%)	207·2 (97·3 to 421·2)	36·5% (28·5 to 43·5%)	-23·0% (-31·1 to -14·8%)	-2·6% (-3·9 to -1·5%)
**Air pollution: All causes**	2,053·8 (1,751·5 to 2,346·0)	15·6% (13·4 to 17·8%)	1,975·0 (1,718·4 to 2,256·1)	14·1% (12·2 to 16·1%)	-3·8% (-8·4 to 0·9%)	-9·3% (-12·2 to -6·4%)	50,779·9 (43,944·7 to 57,599·7)	8·3% (7·1 to 9·6%)	44,782·2 (39,158·3 to 50,741·5)	7·5% (6·3 to 8·6%)	-11·8% (-16·1 to -7·5%)	-10·4% (-13·8 to -6·9%)
**Ambient particulate matter pollution: All causes**	1,207·1 (1,044·1 to 1,383·5)	9·2% (7·9 to 10·5%)	1,295·3 (1,123·7 to 1,477·4)	9·3% (8·1 to 10·5%)	7·3% (2·9 to 12·3%)	1·2% (-1·4 to 4·1%)	29,932·6 (26,174·9 to 33,618·3)	4·9% (4·2 to 5·6%)	29,501·6 (25,902·1 to 32,897·6)	4·9% (4·2 to 5·6%)	-1·4% (-5·8 to 3·2%)	0·1% (-3·4 to 4·0%)
Lower respiratory infections	141·6 (104·6 to 182·0)	27·5% (20·5 to 34·8%)	132·3 (97·6 to 171·5)	27·2% (20·3 to 34·3%)	-6·6% (-10·4 to -2·3%)	-1·3% (-3·4 to 0·9%)	6,472·1 (4,835·6 to 8,265·9)	27·4% (20·3 to 34·8%)	4,544·5 (3,389·5 to 5,778·6)	27·6% (20·9 to 34·8%)	-29·8% (-33·9 to -25·0%)	0·6% (-2·2 to 4·6%)
Tracheal, bronchus, and lung cancer	70·8 (46·9 to 95·7)	22·7% (15·0 to 30·6%)	91·0 (59·4 to 124·5)	22·5% (14·9 to 30·3%)	28·5% (18·6 to 39·2%)	-1·0% (-2·6 to 0·6%)	1,688·4 (1,117·2 to 2,284·3)	22·6% (15·0 to 30·4%)	2,066·9 (1,352·6 to 2,826·1)	22·4% (14·8 to 30·1%)	22·4% (12·5 to 33·5%)	-1·0% (-2·7 to 0·7%)
Ischaemic heart disease	343·0 (289·2 to 402·3)	19·8% (16·8 to 23·3%)	429·4 (358·1 to 510·0)	19·5% (16·4 to 23·1%)	25·2% (20·0 to 30·5%)	-1·3% (-3·5 to 0·9%)	8,150·4 (6,941·7 to 9,330·0)	21·8% (18·7 to 24·8%)	9,729·4 (8,200·5 to 11,234·0)	21·6% (18·3 to 24·7%)	19·4% (14·1 to 25·1%)	-0·8% (-2·6 to 1·0%)
Ischaemic stroke	98·4 (76·8 to 119·6)	15·0% (11·7 to 17·9%)	113·6 (87·3 to 139·7)	14·6% (11·4 to 17·8%)	15·5% (8·3 to 22·6%)	-2·1% (-5·4 to 1·1%)	1,892·2 (1,549·7 to 2,254·8)	16·3% (13·4 to 19·2%)	2,091·4 (1,696·4 to 2,473·9)	16·1% (13·2 to 19·0%)	10·5% (4·1 to 17·0%)	-1·3% (-3·9 to 1·3%)
Hemorrhagic stroke	208·7 (173·3 to 244·3)	16·6% (13·8 to 19·3%)	208·4 (170·2 to 245·1)	16·1% (13·2 to 18·8%)	-0·1% (-6·1 to 6·2%)	-3·0% (-5·7 to -0·5%)	5,139·5 (4,311·8 to 5,968·8)	17·8% (15·1 to 20·6%)	5,002·8 (4,146·0 to 5,895·2)	17·5% (14·7 to 20·4%)	-2·7% (-8·3 to 3·6%)	-1·6% (-4·1 to 0·8%)
Chronic obstructive pulmonary disease	344·6 (218·4 to 487·1)	29·2% (18·3 to 41·3%)	320·5 (200·6 to 456·6)	28·9% (18·1 to 40·5%)	-7·0% (-12·6 to -1·0%)	-1·2% (-2·3 to -0·1%)	6,590·0 (4,156·6 to 9,315·2)	27·8% (17·4 to 39·3%)	6,066·5 (3,815·2 to 8,637·9)	27·8% (17·4 to 39·0%)	-7·9% (-12·9 to -2·7%)	0·0% (-1·0 to 1·3%)
**Household air pollution from solid fuels: All causes**	1,080·7 (775·5 to 1,394·2)	8·2% (5·9 to 10·6%)	837·6 (594·1 to 1,108·1)	6·0% (4·3 to 7·9%)	-22·5% (-29·4 to -16·2%)	-26·9% (-32·6 to -21·9%)	27,353·6 (19,864·8 to 34,863·5)	4·5% (3·2 to 5·8%)	19,520·5 (14,086·1 to 25,689·4)	3·3% (2·3 to 4·3%)	-28·6% (-35·0 to -22·3%)	-27·5% (-33·6 to -21·7%)
Lower respiratory infections	144·2 (96·0 to 202·0)	28·0% (18·6 to 38·9%)	101·9 (66·1 to 150·0)	20·9% (13·6 to 30·1%)	-29·3% (-35·9 to -22·8%)	-25·3% (-31·0 to -19·8%)	6,567·6 (4,369·8 to 9,263·6)	27·8% (18·4 to 38·5%)	3,442·3 (2,227·6 to 5,003·9)	20·9% (13·6 to 29·9%)	-47·6% (-52·8 to -42·1%)	-24·9% (-31·0 to -18·8%)
Tracheal, bronchus, and lung cancer	65·5 (33·0 to 103·1)	21·0% (10·7 to 33·4%)	59·8 (28·8 to 100·0)	14·8% (7·2 to 24·2%)	-8·7% (-18·7 to 1·3%)	-29·7% (-35·5 to -24·7%)	1,553·7 (782·7 to 2,453·8)	20·8% (10·6 to 33·0%)	1,354·4 (657·1 to 2,265·8)	14·7% (7·1 to 23·9%)	-12·8% (-22·6 to -2·6%)	-29·6% (-35·2 to -24·6%)
Ischaemic heart disease	268·9 (211·9 to 336·2)	15·5% (12·3 to 19·3%)	241·4 (184·3 to 313·8)	11·0% (8·4 to 14·0%)	-10·2% (-18·9 to -1·8%)	-29·2% (-35·0 to -23·5%)	6,448·3 (5,122·2 to 7,916·2)	17·2% (13·6 to 21·0%)	5,572·3 (4,294·1 to 7,108·0)	12·4% (9·5 to 15·7%)	-13·6% (-22·4 to -4·5%)	-28·2% (-34·2 to -21·9%)
Ischaemic stroke	84·4 (59·9 to 115·7)	12·8% (9·1 to 17·6%)	68·2 (46·9 to 94·1)	8·8% (6·1 to 12·2%)	-19·2% (-27·5 to -10·3%)	-31·6% (-37·5 to -25·7%)	1,617·8 (1,157·8 to 2,157·0)	13·9% (10·0 to 18·5%)	1,262·4 (885·6 to 1,715·8)	9·7% (6·8 to 13·2%)	-22·0% (-29·8 to -13·8%)	-30·3% (-36·4 to -24·4%)
Hemorrhagic stroke	181·4 (130·0 to 241·3)	14·4% (10·3 to 19·1%)	128·0 (90·6 to 171·9)	9·9% (7·1 to 13·3%)	-29·4% (-37·1 to -22·0%)	-31·5% (-37·3 to -25·9%)	4,478·7 (3,282·3 to 5,859·0)	15·5% (11·3 to 20·2%)	3,117·9 (2,209·5 to 4,183·4)	10·9% (7·7 to 14·4%)	-30·4% (-37·8 to -23·0%)	-29·7% (-36·1 to -23·6%)
Chronic obstructive pulmonary disease	336·5 (177·6 to 515·4)	28·5% (15·1 to 43·4%)	238·3 (119·5 to 386·4)	21·5% (10·9 to 34·4%)	-29·2% (-36·5 to -22·3%)	-24·7% (-31·4 to -19·1%)	6,434·0 (3,384·2 to 9,877·3)	27·1% (14·4 to 41·4%)	4,527·5 (2,271·3 to 7,313·1)	20·8% (10·5 to 33·4%)	-29·6% (-36·8 to -23·4%)	-23·5% (-30·2 to -17·7%)
Cataract	-	-	-	-	-	-	253·5 (173·1 to 350·2)	26·5% (23·0 to 29·8%)	243·8 (165·2 to 337·3)	20·6% (17·2 to 23·7%)	-3·8% (-10·4 to 2·3%)	-22·2% (-27·3 to -17·1%)
**Ambient ozone pollution: All causes**	75·5 (27·8 to 129·9)	0·6% (0·2 to 1·0%)	91·0 (35·1 to 150·8)	0·7% (0·3 to 1·1%)	20·5% (11·9 to 31·2%)	13·7% (6·6 to 22·4%)	1,274·0 (469·7 to 2,175·8)	0·2% (0·1 to 0·4%)	1,450·9 (556·8 to 2,401·7)	0·2% (0·1 to 0·4%)	13·9% (5·1 to 24·1%)	15·7% (7·3 to 25·4%)
Chronic obstructive pulmonary disease	75·5 (27·8 to 129·9)	6·4% (2·4 to 11·0%)	91·0 (35·1 to 150·8)	8·2% (3·1 to 13·7%)	20·5% (11·9 to 31·2%)	28·1% (23·6 to 34·8%)	1,274·0 (469·7 to 2,175·8)	5·4% (2·0 to 9·1%)	1,450·9 (556·8 to 2,401·7)	6·7% (2·5 to 11·0%)	13·9% (5·1 to 24·1%)	23·8% (18·6 to 30·7%)
**Other environmental risks: All causes**	132·3 (66·0 to 212·7)	1·0% (0·5 to 1·6%)	150·8 (73·8 to 244·9)	1·1% (0·5 to 1·8%)	14·0% (8·6 to 18·8%)	7·5% (3·4 to 11·0%)	2,868·5 (1,386·1 to 4,671·1)	0·5% (0·2 to 0·8%)	3,070·4 (1,435·7 to 5,070·9)	0·5% (0·2 to 0·9%)	7·0% (2·0 to 11·3%)	8·7% (3·9 to 12·1%)
**Residential radon: All causes**	8·3 (5·6 to 11·4)	0·1% (0·0 to 0·1%)	11·0 (7·3 to 15·0)	0·1% (0·1 to 0·1%)	31·8% (22·3 to 41·2%)	24·2% (16·5 to 32·3%)	201·1 (139·7 to 274·2)	0·0% (0·0 to 0·0%)	253·5 (173·7 to 346·8)	0·0% (0·0 to 0·1%)	26·1% (17·0 to 36·0%)	28·1% (19·6 to 37·7%)
Tracheal, bronchus, and lung cancer	8·3 (5·6 to 11·4)	2·7% (1·8 to 3·7%)	11·0 (7·3 to 15·0)	2·7% (1·8 to 3·7%)	31·8% (22·3 to 41·2%)	1·5% (-3·8 to 7·5%)	201·1 (139·7 to 274·2)	2·7% (1·9 to 3·7%)	253·5 (173·7 to 346·8)	2·7% (1·9 to 3·8%)	26·1% (17·0 to 36·0%)	2·0% (-3·7 to 7·8%)
**Lead exposure: All causes**	124·0 (58·9 to 203·2)	0·9% (0·4 to 1·6%)	139·8 (63·4 to 232·9)	1·0% (0·5 to 1·7%)	12·8% (7·1 to 17·8%)	6·4% (1·5 to 10·1%)	2,667·4 (1,198·8 to 4,486·3)	0·4% (0·2 to 0·7%)	2,816·9 (1,206·1 to 4,794·8)	0·5% (0·2 to 0·8%)	5·6% (-0·5 to 10·0%)	7·3% (1·2 to 11·0%)
Rheumatic heart disease	1·1 (0·3 to 2·2)	0·9% (0·3 to 1·9%)	0·9 (0·3 to 1·9)	0·9% (0·3 to 1·9%)	-13·2% (-18·9 to -6·5%)	-2·7% (-7·6 to 3·5%)	30·8 (8·1 to 69·0)	0·8% (0·2 to 1·7%)	24·6 (6·5 to 54·0)	0·7% (0·2 to 1·6%)	-20·1% (-25·6 to -14·2%)	-4·5% (-10·2 to 0·7%)
Ischaemic heart disease	52·0 (24·7 to 84·7)	3·0% (1·4 to 4·9%)	61·4 (27·8 to 101·3)	2·8% (1·3 to 4·6%)	18·0% (10·9 to 23·0%)	-6·9% (-11·9 to -3·8%)	1,031·9 (447·5 to 1,732·7)	2·8% (1·2 to 4·7%)	1,153·5 (475·0 to 1,984·7)	2·6% (1·1 to 4·4%)	11·8% (4·8 to 17·1%)	-7·1% (-12·5 to -4·2%)
Ischaemic stroke	16·7 (7·5 to 28·7)	2·5% (1·2 to 4·3%)	18·5 (8·2 to 32·1)	2·4% (1·0 to 4·2%)	11·1% (3·2 to 18·7%)	-5·8% (-11·4 to -1·3%)	292·7 (123·9 to 506·5)	2·5% (1·1 to 4·3%)	309·1 (126·6 to 539·7)	2·4% (1·0 to 4·1%)	5·6% (-1·5 to 11·5%)	-5·7% (-11·2 to -2·6%)
Hemorrhagic stroke	33·0 (13·3 to 58·5)	2·6% (1·1 to 4·6%)	32·7 (12·7 to 58·5)	2·5% (1·0 to 4·6%)	-1·0% (-7·5 to 5·5%)	-3·8% (-9·4 to 0·5%)	715·2 (258·5 to 1,328·5)	2·5% (0·9 to 4·6%)	683·7 (241·9 to 1,280·5)	2·4% (0·8 to 4·6%)	-4·4% (-10·9 to 0·7%)	-3·3% (-9·5 to -0·3%)
Hypertensive heart disease	11·2 (3·0 to 26·6)	4·8% (1·3 to 11·4%)	13·9 (3·6 to 34·2)	4·5% (1·2 to 11·1%)	24·0% (7·2 to 35·6%)	-5·8% (-14·0 to 0·4%)	207·6 (60·1 to 471·2)	4·4% (1·3 to 10·2%)	234·5 (64·1 to 553·3)	4·1% (1·1 to 9·8%)	13·0% (1·0 to 21·7%)	-6·5% (-13·7 to -2·0%)
Cardiomyopathy and myocarditis	0·8 (0·4 to 1·4)	1·5% (0·7 to 2·6%)	1·0 (0·5 to 1·8)	1·6% (0·7 to 2·6%)	23·0% (13·5 to 33·1%)	0·3% (-5·4 to 6·9%)	20·6 (8·1 to 38·3)	1·1% (0·4 to 2·0%)	23·5 (9·1 to 43·8)	1·1% (0·4 to 2·1%)	13·9% (4·6 to 23·4%)	5·0% (-2·9 to 12·0%)

Supplemental Results Table 1. All-age deaths, death PAFs, DALYs, DALY PAFs, and percent change for each risk factor and outcome for both sexes combined in 2005 and 2015 for Middle SDI.

Data in parenthesis are 95% uncertainty intervals. PAFs=population attributable fractions. DALYs=disability-adjusted life-years.

	2005 Deaths (in thousands)	2005 Deaths PAF	2015 Deaths (in thousands)	2015 Deaths PAF	Percent change deaths 2005-2015	Percent change deaths PAF 2005-2015	2005 DALYs (in thousands)	2005 DALYs PAF	2015 DALYs (in thousands)	2015 DALYs PAF	Percent change DALYs 2005-2015	Percent change DALYs PAF 2005-2015
Atrial fibrillation and flutter	0·4 (0·2 to 0·8)	1·8% (0·9 to 2·9%)	0·6 (0·3 to 1·0)	1·7% (0·8 to 2·7%)	32·1% (23·7 to 38·3%)	-6·9% (-11·8 to -3·9%)	12·1 (5·3 to 21·4)	1·9% (0·8 to 3·1%)	15·0 (6·2 to 27·0)	1·7% (0·7 to 2·9%)	23·6% (16·6 to 27·8%)	-8·1% (-13·1 to -5·5%)
Aortic aneurysm	0·4 (0·2 to 0·6)	1·8% (0·9 to 3·0%)	0·5 (0·2 to 0·8)	1·7% (0·7 to 2·8%)	24·9% (12·6 to 34·0%)	-8·8% (-15·1 to -5·3%)	7·6 (3·3 to 13·4)	1·7% (0·7 to 2·9%)	9·0 (3·6 to 16·4)	1·5% (0·6 to 2·7%)	18·7% (5·8 to 27·6%)	-9·4% (-16·8 to -5·8%)
Peripheral vascular disease	0·0 (0·0 to 0·0)	1·2% (0·6 to 2·1%)	0·0 (0·0 to 0·1)	1·1% (0·5 to 2·0%)	54·9% (37·7 to 70·1%)	-8·9% (-15·9 to -4·3%)	1·7 (0·6 to 3·3)	1·2% (0·5 to 2·1%)	2·2 (0·8 to 4·4)	1·1% (0·5 to 1·9%)	31·1% (20·2 to 38·7%)	-10·2% (-17·0 to -6·6%)
Endocarditis	0·2 (0·1 to 0·4)	1·6% (0·8 to 2·7%)	0·3 (0·1 to 0·5)	1·5% (0·7 to 2·6%)	15·8% (4·6 to 24·8%)	-5·7% (-13·9 to -0·9%)	5·7 (2·2 to 11·2)	1·1% (0·4 to 2·1%)	6·2 (2·2 to 12·5)	1·1% (0·4 to 2·1%)	8·7% (-3·4 to 17·9%)	-1·6% (-11·8 to 5·3%)
Other cardiovascular and circulatory diseases	1·7 (0·9 to 2·7)	2·0% (1·0 to 3·2%)	2·0 (1·0 to 3·2)	1·9% (0·9 to 3·1%)	14·4% (7·2 to 21·6%)	-4·0% (-9·4 to -0·2%)	50·0 (21·9 to 87·1)	1·5% (0·6 to 2·5%)	55·6 (23·4 to 98·2)	1·5% (0·6 to 2·6%)	11·3% (3·7 to 16·8%)	0·5% (-6·0 to 4·1%)
Idiopathic developmental intellectual disability	-	-	-	-	-	-	147·1 (69·7 to 254·4)	15·3% (11·0 to 18·8%)	130·0 (60·6 to 225·5)	12·8% (8·5 to 16·2%)	-11·6% (-17·8 to -6·4%)	-16·3% (-22·4 to -11·6%)
Chronic kidney disease due to diabetes mellitus	1·7 (0·7 to 3·2)	2·1% (0·8 to 3·8%)	2·3 (0·9 to 4·3)	1·9% (0·7 to 3·7%)	34·7% (23·9 to 42·1%)	-6·4% (-12·6 to -3·4%)	40·3 (14·0 to 81·8)	1·6% (0·6 to 3·1%)	51·0 (16·7 to 105·4)	1·5% (0·5 to 3·0%)	26·5% (16·1 to 33·0%)	-5·1% (-11·8 to -2·0%)
Chronic kidney disease due to hypertension	3·2 (1·5 to 5·4)	2·7% (1·2 to 4·5%)	4·1 (1·8 to 7·0)	2·5% (1·1 to 4·3%)	27·6% (17·9 to 35·3%)	-5·2% (-10·4 to -2·3%)	64·3 (27·6 to 115·2)	2·0% (0·8 to 3·5%)	76·1 (30·8 to 136·5)	1·9% (0·8 to 3·4%)	18·3% (8·8 to 25·3%)	-4·2% (-10·6 to -1·0%)
Chronic kidney disease due to glomerulonephritis	1·3 (0·5 to 2·3)	1·7% (0·7 to 3·2%)	1·5 (0·6 to 2·7)	1·9% (0·8 to 3·3%)	16·5% (10·0 to 23·9%)	6·0% (3·3 to 10·3%)	31·0 (10·2 to 61·1)	1·2% (0·4 to 2·3%)	32·5 (10·8 to 63·6)	1·2% (0·4 to 2·4%)	4·7% (-0·3 to 10·6%)	4·9% (2·1 to 8·8%)
Chronic kidney disease due to other causes	0·2 (0·1 to 0·3)	1·8% (0·8 to 3·2%)	0·2 (0·1 to 0·4)	1·9% (0·8 to 3·4%)	26·2% (9·2 to 44·3%)	7·0% (-3·8 to 19·4%)	8·8 (3·1 to 18·8)	1·2% (0·4 to 2·4%)	10·3 (3·7 to 22·4)	1·2% (0·4 to 2·5%)	17·4% (11·0 to 22·5%)	3·2% (-2·6 to 7·6%)
**Occupational risks: All causes**	296·7 (273·1 to 324·0)	2·3% (2·1 to 2·4%)	331·2 (303·6 to 362·9)	2·4% (2·2 to 2·6%)	11·6% (5·9 to 17·6%)	5·2% (1·2 to 9·3%)	17,098·4 (14,455·1 to 20,161·4)	2·8% (2·6 to 3·1%)	19,092·1 (16,082·3 to 22,525·7)	3·2% (2·9 to 3·5%)	11·7% (8·8 to 14·7%)	13·3% (10·4 to 16·4%)
**Occupational carcinogens: All causes**	96·9 (90·5 to 103·6)	0·7% (0·7 to 0·8%)	130·1 (120·1 to 140·3)	0·9% (0·9 to 1·0%)	34·3% (24·3 to 45·7%)	26·6% (19·2 to 35·0%)	2,208·9 (2,067·7 to 2,353·0)	0·4% (0·3 to 0·4%)	2,916·6 (2,695·0 to 3,152·9)	0·5% (0·4 to 0·5%)	32·0% (22·0 to 43·3%)	34·1% (24·7 to 44·2%)
**Occupational exposure to asbestos: All causes**	11·0 (8·6 to 13·8)	0·1% (0·1 to 0·1%)	18·3 (14·2 to 22·9)	0·1% (0·1 to 0·2%)	66·9% (44·1 to 93·6%)	57·3% (38·5 to 79·3%)	203·6 (158·7 to 258·7)	0·0% (0·0 to 0·0%)	334·5 (256·8 to 427·0)	0·1% (0·0 to 0·1%)	64·3% (39·5 to 92·4%)	66·8% (43·0 to 93·3%)
Larynx cancer	0·1 (0·1 to 0·2)	0·7% (0·4 to 1·1%)	0·2 (0·1 to 0·3)	0·8% (0·5 to 1·3%)	43·3% (24·6 to 64·9%)	14·5% (2·9 to 27·8%)	2·7 (1·5 to 4·2)	0·5% (0·3 to 0·8%)	3·9 (2·1 to 5·8)	0·6% (0·3 to 0·9%)	41·2% (21·2 to 63·3%)	16·3% (4·0 to 29·9%)
Tracheal, bronchus, and lung cancer	9·3 (7·0 to 11·9)	3·0% (2·3 to 3·8%)	15·7 (11·9 to 20·3)	3·9% (3·0 to 4·9%)	68·7% (44·0 to 98·0%)	29·9% (16·5 to 45·0%)	166·6 (124·3 to 216·9)	2·2% (1·7 to 2·9%)	277·0 (205·1 to 365·1)	3·0% (2·3 to 3·9%)	66·3% (39·6 to 97·5%)	34·4% (19·4 to 51·2%)
Ovarian cancer	0·2 (0·1 to 0·3)	0·8% (0·4 to 1·2%)	0·2 (0·1 to 0·4)	0·8% (0·4 to 1·4%)	41·3% (18·9 to 70·7%)	10·7% (-3·6 to 28·4%)	3·3 (1·6 to 5·5)	0·5% (0·2 to 0·8%)	4·5 (2·1 to 7·7)	0·5% (0·3 to 0·9%)	37·4% (13·0 to 70·2%)	10·2% (-6·2 to 30·9%)
Mesothelioma	1·3 (0·9 to 1·8)	44·7% (30·1 to 59·3%)	2·2 (1·5 to 2·8)	49·6% (33·9 to 64·4%)	60·4% (42·8 to 81·4%)	11·0% (6·0 to 17·3%)	31·0 (19·1 to 43·6)	34·2% (21·9 to 47·8%)	49·1 (31·6 to 67·2)	39·7% (26·0 to 53·9%)	58·1% (38·6 to 81·7%)	16·1% (9·3 to 24·9%)
**Occupational exposure to arsenic: All causes**	2·9 (2·2 to 3·7)	0·0% (0·0 to 0·0%)	3·1 (2·3 to 3·9)	0·0% (0·0 to 0·0%)	5·8% (-2·6 to 14·9%)	-0·3% (-6·6 to 6·5%)	65·1 (50·7 to 81·3)	0·0% (0·0 to 0·0%)	68·2 (52·4 to 85·4)	0·0% (0·0 to 0·0%)	4·7% (-3·9 to 14·0%)	6·3% (-1·3 to 14·9%)
Tracheal, bronchus, and lung cancer	2·9 (2·2 to 3·7)	0·9% (0·7 to 1·2%)	3·1 (2·3 to 3·9)	0·8% (0·6 to 1·0%)	5·8% (-2·6 to 14·9%)	-18·6% (-21·0 to -16·2%)	65·1 (50·7 to 81·3)	0·9% (0·7 to 1·1%)	68·2 (52·4 to 85·4)	0·7% (0·6 to 0·9%)	4·7% (-3·9 to 14·0%)	-15·4% (-18·0 to -13·0%)
**Occupational exposure to benzene: All causes**	1·5 (1·3 to 1·8)	0·0% (0·0 to 0·0%)	2·1 (1·8 to 2·4)	0·0% (0·0 to 0·0%)	35·5% (27·8 to 43·9%)	27·7% (22·4 to 33·0%)	59·0 (49·6 to 69·6)	0·0% (0·0 to 0·0%)	79·1 (66·4 to 93·0)	0·0% (0·0 to 0·0%)	34·1% (26·1 to 42·5%)	36·2% (29·3 to 43·4%)
Leukaemia	1·5 (1·3 to 1·8)	2·0% (1·7 to 2·4%)	2·1 (1·8 to 2·4)	2·5% (2·1 to 2·9%)	35·5% (27·8 to 43·9%)	21·4% (17·6 to 24·8%)	59·0 (49·6 to 69·6)	1·7% (1·4 to 2·0%)	79·1 (66·4 to 93·0)	2·3% (1·9 to 2·6%)	34·1% (26·1 to 42·5%)	32·1% (26·6 to 37·2%)
**Occupational exposure to beryllium: All causes**	0·2 (0·1 to 0·2)	0·0% (0·0 to 0·0%)	0·2 (0·1 to 0·2)	0·0% (0·0 to 0·0%)	11·4% (2·4 to 21·9%)	5·0% (-2·0 to 13·1%)	3·9 (3·1 to 4·7)	0·0% (0·0 to 0·0%)	4·2 (3·3 to 5·2)	0·0% (0·0 to 0·0%)	10·1% (1·1 to 20·6%)	11·8% (3·3 to 21·9%)
Tracheal, bronchus, and lung cancer	0·2 (0·1 to 0·2)	0·1% (0·0 to 0·1%)	0·2 (0·1 to 0·2)	0·0% (0·0 to 0·1%)	11·4% (2·4 to 21·9%)	-14·2% (-17·2 to -11·0%)	3·9 (3·1 to 4·7)	0·1% (0·0 to 0·1%)	4·2 (3·3 to 5·2)	0·0% (0·0 to 0·1%)	10·1% (1·1 to 20·6%)	-11·0% (-14·0 to -8·0%)
**Occupational exposure to cadmium: All causes**	0·6 (0·5 to 0·7)	0·0% (0·0 to 0·0%)	0·7 (0·6 to 0·9)	0·0% (0·0 to 0·0%)	23·8% (14·0 to 34·2%)	16·7% (9·4 to 24·8%)	13·5 (11·0 to 16·0)	0·0% (0·0 to 0·0%)	16·5 (13·3 to 19·8)	0·0% (0·0 to 0·0%)	22·5% (12·5 to 33·2%)	24·3% (15·4 to 34·1%)
Tracheal, bronchus, and lung cancer	0·6 (0·5 to 0·7)	0·2% (0·2 to 0·2%)	0·7 (0·6 to 0·9)	0·2% (0·1 to 0·2%)	23·8% (14·0 to 34·2%)	-4·7% (-7·6 to -1·5%)	13·5 (11·0 to 16·0)	0·2% (0·1 to 0·2%)	16·5 (13·3 to 19·8)	0·2% (0·1 to 0·2%)	22·5% (12·5 to 33·2%)	-1·0% (-4·0 to 2·0%)
**Occupational exposure to chromium: All causes**	2·1 (1·8 to 2·4)	0·0% (0·0 to 0·0%)	2·4 (2·1 to 2·8)	0·0% (0·0 to 0·0%)	17·8% (8·8 to 27·9%)	11·1% (3·9 to 18·7%)	46·7 (40·7 to 53·1)	0·0% (0·0 to 0·0%)	54·5 (46·8 to 62·6)	0·0% (0·0 to 0·0%)	16·5% (7·5 to 26·7%)	18·4% (9·6 to 27·6%)
Tracheal, bronchus, and lung cancer	2·1 (1·8 to 2·4)	0·7% (0·6 to 0·8%)	2·4 (2·1 to 2·8)	0·6% (0·5 to 0·7%)	17·8% (8·8 to 27·9%)	-9·3% (-11·9 to -6·4%)	46·7 (40·7 to 53·1)	0·6% (0·6 to 0·7%)	54·5 (46·8 to 62·6)	0·6% (0·5 to 0·7%)	16·5% (7·5 to 26·7%)	-5·8% (-8·4 to -3·1%)
**Occupational exposure to diesel engine exhaust: All causes**	31·0 (27·2 to 35·2)	0·2% (0·2 to 0·3%)	44·1 (38·6 to 50·8)	0·3% (0·3 to 0·4%)	42·2% (31·6 to 54·3%)	34·1% (26·3 to 43·3%)	704·1 (622·3 to 797·5)	0·1% (0·1 to 0·1%)	992·1 (872·4 to 1,131·9)	0·2% (0·1 to 0·2%)	40·9% (30·2 to 53·1%)	43·1% (33·5 to 54·3%)
Tracheal, bronchus, and lung cancer	31·0 (27·2 to 35·2)	10·0% (8·8 to 11·2%)	44·1 (38·6 to 50·8)	10·9% (9·7 to 12·3%)	42·2% (31·6 to 54·3%)	9·5% (6·5 to 12·8%)	704·1 (622·3 to 797·5)	9·4% (8·4 to 10·5%)	992·1 (872·4 to 1,131·9)	10·7% (9·5 to 12·1%)	40·9% (30·2 to 53·1%)	13·9% (10·9 to 17·0%)
**Occupational exposure to second-hand smoke: All causes**	27·3 (25·2 to 29·3)	0·2% (0·2 to 0·2%)	32·4 (29·8 to 35·3)	0·2% (0·2 to 0·3%)	18·9% (11·4 to 27·5%)	12·1% (6·7 to 17·9%)	612·0 (568·0 to 656·9)	0·1% (0·1 to 0·1%)	719·6 (660·5 to 786·2)	0·1% (0·1 to 0·1%)	17·6% (10·0 to 26·2%)	19·4% (12·6 to 26·8%)
Tracheal, bronchus, and lung cancer	27·3 (25·2 to 29·3)	8·8% (8·2 to 9·3%)	32·4 (29·8 to 35·3)	8·0% (7·5 to 8·6%)	18·9% (11·4 to 27·5%)	-8·5% (-10·8 to -6·2%)	612·0 (568·0 to 656·9)	8·2% (7·7 to 8·7%)	719·6 (660·5 to 786·2)	7·8% (7·3 to 8·3%)	17·6% (10·0 to 26·2%)	-4·9% (-7·5 to -2·5%)
**Occupational exposure to formaldehyde: All causes**	0·7 (0·6 to 0·9)	0·0% (0·0 to 0·0%)	0·8 (0·6 to 0·9)	0·0% (0·0 to 0·0%)	6·3% (-1·8 to 14·7%)	0·2% (-5·7 to 6·2%)	26·3 (21·0 to 32·3)	0·0% (0·0 to 0·0%)	27·5 (22·0 to 34·2)	0·0% (0·0 to 0·0%)	4·8% (-3·5 to 13·2%)	6·4% (-0·4 to 13·7%)
Nasopharynx cancer	0·4 (0·3 to 0·5)	1·8% (1·2 to 2·4%)	0·4 (0·3 to 0·6)	1·6% (1·1 to 2·2%)	1·1% (-9·8 to 11·8%)	-10·7% (-15·5 to -5·8%)	13·8 (9·0 to 19·4)	1·9% (1·3 to 2·6%)	13·7 (9·0 to 19·7)	1·8% (1·2 to 2·5%)	-1·1% (-11·7 to 9·6%)	-6·9% (-12·2 to -1·3%)
**Occupational exposure to nickel: All causes**	10·1 (7·4 to 13·1)	0·1% (0·1 to 0·1%)	11·7 (8·4 to 15·6)	0·1% (0·1 to 0·1%)	15·7% (5·7 to 26·4%)	9·1% (1·4 to 17·5%)	229·5 (170·0 to 295·2)	0·0% (0·0 to 0·0%)	262·5 (190·3 to 347·9)	0·0% (0·0 to 0·1%)	14·4% (4·5 to 25·5%)	16·1% (6·6 to 26·3%)
Tracheal, bronchus, and lung cancer	10·1 (7·4 to 13·1)	3·3% (2·4 to 4·2%)	11·7 (8·4 to 15·6)	2·9% (2·1 to 3·9%)	15·7% (5·7 to 26·4%)	-10·9% (-14·6 to -7·4%)	229·5 (170·0 to 295·2)	3·1% (2·3 to 4·0%)	262·5 (190·3 to 347·9)	2·8% (2·1 to 3·7%)	14·4% (4·5 to 25·5%)	-7·6% (-11·5 to -3·9%)
**Occupational exposure to polycyclic aromatic hydrocarbons: All causes**	4·8 (4·0 to 5·8)	0·0% (0·0 to 0·0%)	6·4 (5·2 to 7·6)	0·0% (0·0 to 0·1%)	31·8% (21·6 to 42·7%)	24·2% (16·6 to 32·6%)	109·4 (90·1 to 129·1)	0·0% (0·0 to 0·0%)	142·7 (116·8 to 170·2)	0·0% (0·0 to 0·0%)	30·5% (19·9 to 41·6%)	32·5% (23·1 to 42·4%)
Tracheal, bronchus, and lung cancer	4·8 (4·0 to 5·8)	1·6% (1·3 to 1·8%)	6·4 (5·2 to 7·6)	1·6% (1·3 to 1·9%)	31·8% (21·6 to 42·7%)	1·5% (-1·4 to 4·6%)	109·4 (90·1 to 129·1)	1·5% (1·2 to 1·7%)	142·7 (116·8 to 170·2)	1·5% (1·3 to 1·8%)	30·5% (19·9 to 41·6%)	5·5% (2·6 to 8·3%)
**Occupational exposure to silica: All causes**	21·5 (19·2 to 23·7)	0·2% (0·1 to 0·2%)	31·0 (27·3 to 34·6)	0·2% (0·2 to 0·2%)	44·5% (32·4 to 58·2%)	36·2% (26·9 to 46·9%)	484·3 (433·9 to 535·6)	0·1% (0·1 to 0·1%)	690·4 (610·4 to 772·0)	0·1% (0·1 to 0·1%)	42·6% (30·9 to 56·5%)	44·8% (33·8 to 57·2%)
Tracheal, bronchus, and lung cancer	21·5 (19·2 to 23·7)	6·9% (6·3 to 7·5%)	31·0 (27·3 to 34·6)	7·7% (7·0 to 8·4%)	44·5% (32·4 to 58·2%)	11·3% (7·3 to 15·5%)	484·3 (433·9 to 535·6)	6·5% (5·9 to 7·1%)	690·4 (610·4 to 772·0)	7·5% (6·8 to 8·1%)	42·6% (30·9 to 56·5%)	15·3% (11·1 to 19·4%)
**Occupational exposure to sulfphuric acid: All causes**	2·1 (1·6 to 2·6)	0·0% (0·0 to 0·0%)	2·5 (1·9 to 3·2)	0·0% (0·0 to 0·0%)	22·7% (14·8 to 31·0%)	15·7% (10·0 to 21·4%)	50·2 (38·7 to 63·0)	0·0% (0·0 to 0·0%)	61·5 (47·3 to 77·7)	0·0% (0·0 to 0·0%)	22·6% (14·8 to 30·8%)	24·5% (17·6 to 31·6%)
Larynx cancer	2·1 (1·6 to 2·6)	10·5% (8·0 to 13·2%)	2·5 (1·9 to 3·2)	10·3% (7·7 to 13·0%)	22·7% (14·8 to 31·0%)	-1·9% (-4·9 to 1·1%)	50·2 (38·7 to 63·0)	9·9% (7·7 to 12·4%)	61·5 (47·3 to 77·7)	10·0% (7·7 to 12·7%)	22·6% (14·8 to 30·8%)	1·0% (-1·9 to 4·1%)
**Occupational exposure to trichloroethylene: All causes**	0·0 (0·0 to 0·1)	0·0% (0·0 to 0·0%)	0·0 (0·0 to 0·1)	0·0% (0·0 to 0·0%)	37·0% (27·6 to 45·9%)	29·1% (21·6 to 35·0%)	0·8 (0·2 to 1·5)	0·0% (0·0 to 0·0%)	1·1 (0·2 to 2·1)	0·0% (0·0 to 0·0%)	38·0% (28·8 to 46·8%)	40·1% (31·5 to 47·1%)
Kidney cancer	0·0 (0·0 to 0·1)	0·3% (0·1 to 0·5%)	0·0 (0·0 to 0·1)	0·3% (0·1 to 0·5%)	37·0% (27·6 to 45·9%)	-3·4% (-6·1 to -0·7%)	0·8 (0·2 to 1·5)	0·2% (0·0 to 0·4%)	1·1 (0·2 to 2·1)	0·2% (0·0 to 0·4%)	38·0% (28·8 to 46·8%)	3·4% (-0·2 to 7·7%)

Supplemental Results Table 1. All-age deaths, death PAFs, DALYs, DALY PAFs, and percent change for each risk factor and outcome for both sexes combined in 2005 and 2015 for Middle SDI.

Data in parenthesis are 95% uncertainty intervals. PAFs=population attributable fractions. DALYs=disability-adjusted life-years.

	2005 Deaths (in thousands)	2005 Deaths PAF	2015 Deaths (in thousands)	2015 Deaths PAF	Percent change deaths 2005-2015	Percent change deaths PAF 2005-2015	2005 DALYs (in thousands)	2005 DALYs PAF	2015 DALYs (in thousands)	2015 DALYs PAF	Percent change DALYs 2005-2015	Percent change DALYs PAF 2005-2015
**Occupational asthmagens: All causes**	14·7 (12·4 to 17·3)	0·1% (0·1 to 0·1%)	12·5 (10·4 to 14·7)	0·1% (0·1 to 0·1%)	-15·3% (-25·7 to -3·6%)	-20·2% (-29·4 to -9·7%)	813·7 (658·2 to 990·7)	0·1% (0·1 to 0·2%)	760·4 (605·8 to 934·1)	0·1% (0·1 to 0·1%)	-6·6% (-13·7 to 0·5%)	-5·2% (-11·2 to 1·2%)
Asthma	14·7 (12·4 to 17·3)	11·3% (9·8 to 12·9%)	12·5 (10·4 to 14·7)	10·4% (9·0 to 11·8%)	-15·3% (-25·7 to -3·6%)	-8·6% (-14·3 to -1·9%)	813·7 (658·2 to 990·7)	11·6% (10·2 to 13·0%)	760·4 (605·8 to 934·1)	11·1% (9·8 to 12·5%)	-6·6% (-13·7 to 0·5%)	-4·0% (-7·6 to -0·4%)
**Occupational particulate matter, gases, and fumes: All causes**	137·7 (115·0 to 162·3)	1·0% (0·9 to 1·2%)	134·4 (109·7 to 159·3)	1·0% (0·8 to 1·1%)	-2·4% (-8·6 to 4·6%)	-8·0% (-13·2 to -2·7%)	3,357·9 (2,927·8 to 3,804·9)	0·6% (0·5 to 0·6%)	3,223·4 (2,781·3 to 3,709·1)	0·5% (0·5 to 0·6%)	-4·0% (-9·5 to 1·6%)	-2·5% (-7·2 to 2·0%)
Chronic obstructive pulmonary disease	136·8 (114·1 to 161·3)	11·6% (9·6 to 13·6%)	133·5 (108·9 to 158·3)	12·0% (9·9 to 14·1%)	-2·4% (-8·6 to 4·6%)	3·8% (-0·8 to 8·2%)	3,335·3 (2,906·3 to 3,783·5)	14·1% (12·2 to 15·9%)	3,201·4 (2,759·1 to 3,686·2)	14·7% (12·8 to 16·6%)	-4·0% (-9·5 to 1·6%)	4·3% (1·3 to 7·4%)
**Occupational noise: All causes**	-	-	-	-	-	-	2,718·4 (1,856·0 to 3,760·5)	0·4% (0·3 to 0·6%)	3,338·8 (2,275·5 to 4,651·0)	0·6% (0·4 to 0·7%)	22·8% (20·1 to 25·7%)	24·6% (20·7 to 28·8%)
Age-related and other hearing loss	-	-	-	-	-	-	2,718·4 (1,856·0 to 3,760·5)	31·3% (29·0 to 34·4%)	3,338·8 (2,275·5 to 4,651·0)	30·4% (28·2 to 33·3%)	22·8% (20·1 to 25·7%)	-2·7% (-4·2 to -1·1%)
**Occupational injuries: All causes**	47·6 (44·1 to 52·0)	0·4% (0·3 to 0·4%)	54·6 (50·7 to 61·1)	0·4% (0·4 to 0·4%)	14·7% (4·2 to 29·4%)	8·2% (-2·4 to 23·1%)	2,874·7 (2,611·0 to 3,184·9)	0·5% (0·4 to 0·5%)	3,269·8 (2,951·0 to 3,687·2)	0·5% (0·5 to 0·6%)	13·7% (2·6 to 28·8%)	15·5% (3·5 to 31·1%)
Pedestrian road injuries	6·4 (5·9 to 7·4)	3·0% (2·8 to 3·5%)	9·2 (8·4 to 10·8)	4·6% (4·2 to 5·4%)	44·0% (27·4 to 66·2%)	51·6% (31·1 to 77·2%)	320·8 (296·8 to 366·8)	3·3% (3·0 to 3·8%)	448·7 (409·5 to 522·6)	5·3% (4·8 to 6·3%)	39·9% (24·0 to 61·3%)	60·4% (38·4 to 86·2%)
Cyclist road injuries	0·6 (0·6 to 0·7)	2·6% (2·4 to 2·9%)	0·9 (0·8 to 1·0)	4·1% (3·6 to 4·8%)	41·1% (25·8 to 56·4%)	57·0% (36·0 to 79·3%)	37·7 (34·0 to 42·3)	2·8% (2·6 to 3·1%)	56·0 (49·4 to 63·7)	4·7% (4·1 to 5·4%)	48·6% (34·5 to 63·6%)	66·5% (45·0 to 88·9%)
Motorcyclist road injuries	7·2 (6·2 to 8·3)	7·0% (6·1 to 8·1%)	8·4 (7·2 to 10·0)	7·7% (6·6 to 9·1%)	17·1% (-0·6 to 41·9%)	9·6% (-8·7 to 32·9%)	399·7 (347·9 to 460·8)	7·3% (6·4 to 8·4%)	462·8 (400·1 to 546·2)	8·3% (7·1 to 9·7%)	15·8% (-1·2 to 39·7%)	13·2% (-5·3 to 36·6%)
Motor vehicle road injuries	9·6 (8·7 to 11·1)	7·8% (7·2 to 8·6%)	10·8 (9·7 to 12·5)	9·0% (8·2 to 10·2%)	12·3% (1·7 to 26·1%)	14·6% (2·2 to 29·6%)	537·7 (484·7 to 613·6)	8·3% (7·7 to 9·1%)	603·4 (543·0 to 688·4)	9·9% (9·0 to 11·1%)	12·2% (1·8 to 24·8%)	19·4% (6·6 to 35·0%)
Other road injuries	0·4 (0·3 to 0·5)	7·0% (6·0 to 8·1%)	0·5 (0·4 to 0·6)	7·9% (6·8 to 9·2%)	17·1% (-7·2 to 47·4%)	13·5% (-5·4 to 36·3%)	26·9 (20·0 to 32·6)	7·5% (6·5 to 8·7%)	32·7 (24·5 to 39·6)	8·9% (7·7 to 10·3%)	21·5% (-1·0 to 49·8%)	18·1% (-1·7 to 41·0%)
Other transport injuries	1·0 (0·9 to 1·4)	3·2% (2·8 to 4·0%)	1·7 (1·3 to 2·3)	4·8% (4·0 to 6·1%)	61·4% (25·1 to 116·0%)	50·9% (16·4 to 96·8%)	55·7 (47·8 to 72·2)	3·4% (3·0 to 4·2%)	86·5 (71·0 to 114·4)	5·3% (4·5 to 6·7%)	55·3% (21·8 to 103·4%)	56·1% (22·3 to 99·4%)
Falls	5·4 (4·6 to 6·0)	4·4% (3·9 to 5·1%)	7·0 (5·8 to 7·8)	4·6% (4·0 to 5·7%)	28·9% (12·4 to 48·7%)	4·8% (-9·2 to 20·5%)	391·3 (332·5 to 459·5)	6·7% (6·1 to 7·6%)	511·9 (425·8 to 621·4)	7·7% (6·9 to 9·0%)	30·8% (14·9 to 49·5%)	14·9% (0·8 to 32·2%)
Drowning	5·5 (4·8 to 6·1)	4·6% (4·2 to 5·1%)	5·0 (4·4 to 5·8)	5·7% (5·1 to 6·6%)	-8·5% (-19·5 to 5·8%)	24·2% (8·3 to 43·5%)	296·0 (258·2 to 331·1)	4·1% (3·7 to 4·6%)	260·4 (231·0 to 300·1)	5·4% (4·8 to 6·2%)	-12·0% (-22·1 to 1·8%)	31·9% (15·0 to 52·4%)
Fire, heat, and hot substances	2·1 (1·8 to 2·7)	5·5% (4·6 to 6·8%)	2·2 (1·8 to 2·9)	6·7% (5·6 to 8·6%)	3·5% (-15·2 to 31·1%)	23·3% (0·2 to 57·4%)	149·8 (125·3 to 186·7)	6·4% (5·5 to 7·8%)	159·4 (130·3 to 204·3)	8·5% (7·1 to 10·6%)	6·4% (-11·1 to 32·9%)	33·1% (10·7 to 65·9%)
Poisonings	1·4 (0·9 to 1·6)	4·9% (4·3 to 5·7%)	0·9 (0·6 to 1·1)	4·1% (3·6 to 4·7%)	-33·9% (-42·6 to -23·3%)	-17·9% (-29·8 to -4·1%)	75·0 (52·6 to 87·1)	5·3% (4·7 to 6·2%)	50·1 (35·3 to 58·3)	4·7% (4·1 to 5·5%)	-33·2% (-41·7 to -23·2%)	-12·1% (-25·2 to 3·0%)
Unintentional firearm injuries	0·3 (0·2 to 0·3)	4·6% (4·1 to 5·3%)	0·3 (0·2 to 0·3)	5·1% (4·3 to 5·9%)	6·0% (-10·0 to 25·9%)	9·8% (-5·8 to 28·0%)	15·0 (11·0 to 17·3)	5·0% (4·5 to 5·7%)	15·6 (10·8 to 19·0)	5·7% (4·9 to 6·7%)	4·0% (-11·1 to 22·9%)	14·0% (-2·0 to 33·3%)
Unintentional suffocation	0·0 (0·0 to 0·0)	0·2% (0·2 to 0·3%)	0·0 (0·0 to 0·1)	0·4% (0·3 to 0·5%)	73·9% (8·6 to 114·2%)	66·2% (29·4 to 108·2%)	2·2 (1·7 to 2·8)	0·2% (0·2 to 0·3%)	3·2 (2·0 to 4·0)	0·4% (0·3 to 0·5%)	45·2% (7·2 to 73·7%)	54·1% (24·4 to 90·1%)
Other exposure to mechanical forces	2·8 (2·2 to 3·3)	6·5% (5·9 to 7·3%)	3·2 (2·1 to 3·7)	7·5% (6·7 to 8·6%)	12·2% (-7·3 to 29·2%)	15·1% (0·4 to 32·5%)	190·5 (152·1 to 223·3)	7·1% (6·5 to 7·9%)	213·4 (156·7 to 255·9)	8·7% (7·8 to 9·9%)	12·0% (-3·7 to 28·7%)	22·8% (7·2 to 42·9%)
Venomous animal contact	0·9 (0·5 to 1·1)	3·5% (2·9 to 4·2%)	0·8 (0·4 to 1·1)	3·4% (2·8 to 4·2%)	-14·4% (-34·0 to 11·7%)	-0·9% (-21·2 to 21·5%)	56·7 (32·8 to 70·4)	3·7% (3·1 to 4·5%)	49·7 (28·4 to 67·1)	4·0% (3·2 to 4·8%)	-12·4% (-30·2 to 9·8%)	6·3% (-15·2 to 30·5%)
Non-venomous animal contact	0·1 (0·1 to 0·1)	2·8% (2·4 to 3·4%)	0·1 (0·1 to 0·1)	2·5% (2·1 to 3·0%)	-32·7% (-46·6 to -11·7%)	-12·9% (-29·8 to 8·2%)	7·7 (6·2 to 9·4)	3·2% (2·8 to 3·8%)	5·8 (4·5 to 7·6)	3·2% (2·7 to 3·8%)	-23·9% (-36·9 to -7·4%)	0·8% (-16·2 to 20·2%)
Pulmonary aspiration and foreign body in airway	0·5 (0·4 to 0·7)	2·8% (2·4 to 3·3%)	0·7 (0·5 to 0·8)	3·4% (2·9 to 3·9%)	23·0% (5·4 to 41·9%)	21·0% (2·8 to 43·1%)	38·4 (31·1 to 46·2)	2·8% (2·3 to 3·5%)	43·3 (35·3 to 52·0)	3·6% (3·0 to 4·1%)	12·8% (-2·0 to 30·3%)	26·3% (5·5 to 53·9%)
Foreign body in eyes	-	-	-	-	-	-	0·7 (0·3 to 1·2)	6·3% (5·4 to 7·4%)	0·8 (0·3 to 1·5)	6·9% (5·9 to 8·0%)	26·2% (11·2 to 43·0%)	10·4% (-2·2 to 24·1%)
Foreign body in other body part	0·3 (0·2 to 0·3)	5·3% (4·6 to 6·2%)	0·3 (0·2 to 0·3)	6·2% (5·2 to 7·3%)	1·5% (-17·9 to 23·7%)	15·3% (-5·2 to 39·2%)	19·9 (16·0 to 24·3)	6·0% (5·2 to 7·0%)	20·4 (16·7 to 25·6)	7·5% (6·4 to 8·7%)	2·6% (-14·6 to 21·7%)	23·6% (2·8 to 47·3%)
Other unintentional injuries	2·9 (2·6 to 3·3)	6·8% (6·2 to 7·7%)	2·7 (2·4 to 3·2)	7·5% (6·7 to 8·7%)	-8·0% (-21·5 to 8·0%)	10·0% (-5·1 to 30·4%)	253·1 (210·6 to 304·9)	7·2% (6·5 to 8·1%)	245·7 (200·0 to 305·7)	8·1% (7·3 to 9·3%)	-2·9% (-15·3 to 12·4%)	13·1% (-1·0 to 31·9%)
**Occupational ergonomic factors: All causes**	-	-	-	-	-	-	5,173·5 (3,588·4 to 7,210·6)	0·8% (0·6 to 1·1%)	5,630·2 (3,913·0 to 7,795·4)	0·9% (0·7 to 1·2%)	8·8% (6·6 to 11·0%)	10·4% (7·1 to 14·1%)
Low back pain	-	-	-	-	-	-	5,173·5 (3,588·4 to 7,210·6)	40·9% (38·5 to 43·2%)	5,630·2 (3,913·0 to 7,795·4)	38·0% (35·9 to 40·1%)	8·8% (6·6 to 11·0%)	-7·0% (-8·2 to -5·9%)
**Behavioural risks: All causes**	5,579·4 (5,173·2 to 6,018·5)	42·4% (39·4 to 45·4%)	6,061·6 (5,600·8 to 6,537·4)	43·4% (40·4 to 46·4%)	8·6% (3·6 to 13·6%)	2·4% (0·4 to 4·5%)	187,531·5 (174,720·7 to 202,317·8)	30·8% (29·1 to 32·6%)	182,595·8 (169,239·9 to 198,082·7)	30·4% (28·5 to 32·5%)	-2·6% (-6·3 to 0·9%)	-1·1% (-3·4 to 1·1%)
**Child and maternal malnutrition: All causes**	295·3 (269·2 to 321·4)	2·2% (2·0 to 2·4%)	144·6 (129·9 to 160·1)	1·0% (0·9 to 1·1%)	-51·0% (-55·7 to -46·0%)	-53·8% (-58·3 to -49·0%)	39,998·3 (34,875·1 to 46,746·6)	6·6% (6·0 to 7·1%)	25,577·9 (21,017·1 to 31,485·5)	4·2% (3·8 to 4·8%)	-36·1% (-41·2 to -31·3%)	-35·2% (-40·0 to -30·6%)
**Suboptimal breastfeeding: All causes**	103·3 (60·6 to 147·3)	0·8% (0·5 to 1·1%)	51·3 (31·0 to 72·9)	0·4% (0·2 to 0·5%)	-50·3% (-55·8 to -44·2%)	-53·2% (-58·3 to -47·3%)	8,994·4 (5,339·0 to 12,819·9)	1·5% (0·9 to 2·1%)	4,515·3 (2,760·5 to 6,370·1)	0·8% (0·4 to 1·1%)	-49·8% (-55·2 to -43·6%)	-49·0% (-54·1 to -42·9%)
**Non-exclusive breastfeeding : All causes**	98·1 (55·7 to 141·8)	0·7% (0·4 to 1·1%)	48·8 (28·5 to 70·9)	0·3% (0·2 to 0·5%)	-50·3% (-55·7 to -43·9%)	-53·1% (-58·2 to -47·1%)	8,485·7 (4,833·8 to 12,251·5)	1·4% (0·8 to 2·0%)	4,234·4 (2,493·8 to 6,137·9)	0·7% (0·4 to 1·0%)	-50·1% (-55·5 to -43·8%)	-49·3% (-54·5 to -43·1%)
Diarrhoeal diseases	33·3 (20·0 to 45·4)	12·1% (7·3 to 16·3%)	15·6 (9·5 to 21·4)	7·9% (4·8 to 10·7%)	-53·2% (-60·6 to -44·1%)	-34·8% (-44·4 to -22·8%)	2,899·4 (1,750·7 to 3,937·2)	20·6% (12·6 to 27·7%)	1,371·9 (844·5 to 1,879·2)	15·8% (9·7 to 21·2%)	-52·7% (-60·2 to -43·6%)	-23·6% (-31·7 to -13·3%)
Lower respiratory infections	64·8 (25·8 to 107·6)	12·6% (5·1 to 20·9%)	33·2 (13·5 to 53·3)	6·8% (2·8 to 11·1%)	-48·8% (-55·2 to -41·6%)	-45·9% (-52·0 to -38·9%)	5,586·3 (2,236·3 to 9,280·0)	23·7% (9·6 to 39·0%)	2,862·5 (1,171·9 to 4,595·7)	17·4% (7·1 to 28·1%)	-48·8% (-55·2 to -41·5%)	-26·5% (-33·0 to -18·6%)
**Discontinued breastfeeding : All causes**	8·4 (2·6 to 15·2)	0·1% (0·0 to 0·1%)	4·0 (1·3 to 7·3)	0·0% (0·0 to 0·1%)	-52·5% (-59·3 to -43·4%)	-55·2% (-61·7 to -46·7%)	785·1 (249·7 to 1,416·2)	0·1% (0·0 to 0·2%)	408·8 (138·7 to 734·5)	0·1% (0·0 to 0·1%)	-47·9% (-55·5 to -38·0%)	-47·2% (-54·8 to -37·2%)
Diarrhoeal diseases	8·4 (2·6 to 15·2)	3·0% (1·0 to 5·5%)	4·0 (1·3 to 7·3)	2·0% (0·7 to 3·6%)	-52·5% (-59·3 to -43·4%)	-33·9% (-43·0 to -22·9%)	785·1 (249·7 to 1,416·2)	5·6% (1·8 to 9·9%)	408·8 (138·7 to 734·5)	4·7% (1·6 to 8·4%)	-47·9% (-55·5 to -38·0%)	-16·0% (-25·5 to -3·9%)
**Childhood undernutrition: All causes**	266·2 (238·0 to 292·9)	2·0% (1·8 to 2·2%)	125·9 (109·7 to 142·2)	0·9% (0·8 to 1·0%)	-52·7% (-57·4 to -47·5%)	-55·4% (-60·2 to -50·4%)	24,066·6 (21,552·8 to 26,516·8)	4·0% (3·5 to 4·4%)	11,958·4 (10,469·8 to 13,448·4)	2·0% (1·7 to 2·3%)	-50·3% (-55·1 to -45·1%)	-49·6% (-54·3 to -44·4%)
**Childhood underweight: All causes**	64·2 (50·1 to 88·3)	0·5% (0·4 to 0·7%)	25·0 (19·4 to 35·5)	0·2% (0·1 to 0·3%)	-61·1% (-68·1 to -54·0%)	-63·3% (-70·0 to -56·1%)	6,273·9 (5,033·0 to 8,248·4)	1·0% (0·8 to 1·4%)	2,838·5 (2,295·5 to 3,760·1)	0·5% (0·4 to 0·6%)	-54·8% (-61·8 to -47·7%)	-54·1% (-61·1 to -46·9%)
Diarrhoeal diseases	11·7 (9·2 to 15·1)	4·3% (3·4 to 5·3%)	4·3 (3·3 to 5·5)	2·2% (1·7 to 2·8%)	-63·7% (-69·4 to -56·5%)	-49·4% (-56·9 to -40·3%)	1,074·3 (855·8 to 1,372·3)	7·6% (6·2 to 9·5%)	416·8 (325·6 to 530·4)	4·8% (3·8 to 6·1%)	-61·2% (-66·9 to -54·1%)	-37·3% (-43·6 to -30·4%)
Lower respiratory infections	25·4 (16·7 to 45·6)	4·9% (3·2 to 8·7%)	10·2 (6·5 to 19·9)	2·1% (1·3 to 4·0%)	-59·7% (-65·4 to -53·7%)	-57·4% (-62·7 to -51·9%)	2,182·4 (1,434·8 to 3,931·2)	9·2% (6·0 to 16·3%)	882·5 (562·9 to 1,714·7)	5·4% (3·5 to 10·1%)	-59·6% (-65·3 to -53·6%)	-42·0% (-48·1 to -36·4%)
Measles	11·0 (2·1 to 32·4)	30·4% (9·3 to 55·7%)	3·0 (0·4 to 10·3)	22·4% (5·6 to 46·8%)	-73·1% (-91·3 to -19·3%)	-26·4% (-53·3 to -8·3%)	937·1 (177·4 to 2,766·6)	31·0% (9·5 to 56·5%)	251·9 (33·0 to 876·7)	22·8% (5·7 to 47·6%)	-73·1% (-91·3 to -19·3%)	-26·4% (-52·8 to -9·2%)
Protein-energy malnutrition	16·1 (13·6 to 19·5)	27·7% (24·6 to 31·1%)	7·5 (6·3 to 9·2)	14·4% (12·2 to 17·2%)	-53·4% (-61·9 to -43·6%)	-47·8% (-57·2 to -37·7%)	2,080·1 (1,737·2 to 2,510·0)	69·8% (66·1 to 73·4%)	1,287·3 (1,031·5 to 1,610·5)	62·2% (56·9 to 67·6%)	-38·1% (-46·7 to -28·7%)	-10·9% (-16·5 to -6·0%)
**Childhood wasting: All causes**	236·9 (189·1 to 274·1)	1·8% (1·4 to 2·1%)	114·3 (87·9 to 134·8)	0·8% (0·6 to 1·0%)	-51·7% (-56·5 to -46·6%)	-54·5% (-59·4 to -49·5%)	21,521·1 (17,312·6 to 24,881·3)	3·5% (2·8 to 4·1%)	10,936·9 (8,620·9 to 12,776·8)	1·8% (1·4 to 2·2%)	-49·2% (-53·9 to -44·2%)	-48·4% (-53·0 to -43·4%)

Supplemental Results Table 1. All-age deaths, death PAFs, DALYs, DALY PAFs, and percent change for each risk factor and outcome for both sexes combined in 2005 and 2015 for Middle SDI.

Data in parenthesis are 95% uncertainty intervals. PAFs=population attributable fractions. DALYs=disability-adjusted life-years.

	2005 Deaths (in thousands)	2005 Deaths PAF	2015 Deaths (in thousands)	2015 Deaths PAF	Percent change deaths 2005-2015	Percent change deaths PAF 2005-2015	2005 DALYs (in thousands)	2005 DALYs PAF	2015 DALYs (in thousands)	2015 DALYs PAF	Percent change DALYs 2005-2015	Percent change DALYs PAF 2005-2015
Diarrhoeal diseases	74·4 (59·2 to 85·7)	27·0% (21·9 to 30·4%)	34·7 (26·6 to 41·7)	17·6% (13·7 to 20·9%)	-53·3% (-60·4 to -45·2%)	-35·0% (-43·9 to -25·0%)	6,849·5 (5,398·2 to 7,883·0)	48·7% (39·4 to 53·4%)	3,434·2 (2,633·7 to 4,064·3)	39·4% (31·2 to 44·3%)	-49·9% (-56·8 to -42·3%)	-19·1% (-26·0 to -12·3%)
Lower respiratory infections	132·2 (88·5 to 161·3)	25·7% (17·2 to 31·2%)	67·3 (43·7 to 82·7)	13·8% (9·0 to 17·3%)	-49·1% (-54·8 to -42·7%)	-46·2% (-51·7 to -40·5%)	11,385·6 (7,628·5 to 13,892·3)	48·2% (32·4 to 57·7%)	5,811·0 (3,765·6 to 7,145·5)	35·3% (22·9 to 43·1%)	-49·0% (-54·6 to -42·6%)	-26·8% (-31·9 to -21·8%)
Measles	14·2 (2·6 to 46·3)	39·0% (13·1 to 79·0%)	4·7 (0·7 to 19·6)	35·7% (9·2 to 78·1%)	-66·5% (-90·0 to 0·6%)	-8·5% (-34·8 to 5·7%)	1,205·8 (220·8 to 3,944·7)	39·7% (13·3 to 80·3%)	404·5 (55·5 to 1,667·0)	36·3% (9·3 to 79·4%)	-66·5% (-89·9 to 0·6%)	-8·4% (-34·5 to 5·3%)
Protein-energy malnutrition	16·1 (13·6 to 19·5)	27·7% (24·6 to 31·1%)	7·5 (6·3 to 9·2)	14·4% (12·2 to 17·2%)	-53·4% (-61·9 to -43·6%)	-47·8% (-57·2 to -37·7%)	2,080·1 (1,737·2 to 2,510·0)	69·8% (66·1 to 73·4%)	1,287·3 (1,031·5 to 1,610·5)	62·2% (56·9 to 67·6%)	-38·1% (-46·7 to -28·7%)	-10·9% (-16·5 to -6·0%)
**Childhood stunting: All causes**	60·9 (26·8 to 111·8)	0·5% (0·2 to 0·8%)	24·0 (9·8 to 48·7)	0·2% (0·1 to 0·3%)	-60·6% (-69·4 to -54·4%)	-62·8% (-71·1 to -56·5%)	5,325·5 (2,394·9 to 9,702·8)	0·9% (0·4 to 1·6%)	2,141·6 (900·8 to 4,293·9)	0·4% (0·1 to 0·7%)	-59·8% (-68·5 to -53·7%)	-59·2% (-67·8 to -53·0%)
Diarrhoeal diseases	15·1 (5·8 to 26·6)	5·5% (2·1 to 9·6%)	5·8 (2·0 to 10·7)	2·9% (1·1 to 5·4%)	-61·6% (-67·7 to -53·9%)	-46·6% (-54·5 to -37·6%)	1,388·6 (532·9 to 2,449·6)	9·9% (3·8 to 17·2%)	571·8 (202·0 to 1,064·8)	6·6% (2·3 to 12·1%)	-58·8% (-64·9 to -51·5%)	-33·5% (-40·2 to -26·8%)
Lower respiratory infections	37·9 (3·1 to 89·4)	7·4% (0·6 to 17·6%)	16·0 (1·1 to 40·6)	3·3% (0·2 to 8·2%)	-57·9% (-64·1 to -48·0%)	-55·5% (-61·4 to -46·4%)	3,267·6 (268·9 to 7,684·8)	13·8% (1·1 to 32·6%)	1,379·8 (92·3 to 3,504·6)	8·4% (0·6 to 20·9%)	-57·8% (-63·9 to -48·0%)	-39·5% (-46·3 to -28·8%)
Measles	7·9 (0·7 to 26·0)	21·6% (2·4 to 54·6%)	2·2 (0·1 to 8·6)	17·2% (1·4 to 48·8%)	-71·6% (-91·4 to 0·0%)	-20·3% (-37·8 to -5·2%)	669·2 (57·3 to 2,211·4)	22·0% (2·4 to 55·7%)	190·0 (10·8 to 736·3)	17·5% (1·5 to 49·6%)	-71·6% (-91·4 to 0·0%)	-20·3% (-37·5 to -5·5%)
**Iron deficiency: All causes**	7·7 (4·5 to 10·8)	0·1% (0·0 to 0·1%)	7·1 (3·8 to 9·5)	0·1% (0·0 to 0·1%)	-7·2% (-30·8 to 19·1%)	-12·5% (-34·6 to 12·4%)	13,997·4 (9,392·6 to 19,969·9)	2·3% (1·7 to 3·0%)	12,529·1 (8,467·2 to 17,917·2)	2·1% (1·5 to 2·7%)	-10·5% (-12·3 to -8·8%)	-9·2% (-11·8 to -6·2%)
Maternal haemorrhage	3·1 (1·2 to 5·1)	26·5% (10·2 to 42·0%)	1·9 (0·7 to 3·2)	25·7% (9·6 to 41·0%)	-40·0% (-51·5 to -27·2%)	-3·2% (-10·4 to 3·4%)	181·4 (69·4 to 295·7)	26·6% (10·2 to 42·2%)	109·6 (41·9 to 185·8)	25·7% (9·6 to 40·9%)	-39·6% (-50·6 to -27·4%)	-3·6% (-10·5 to 2·9%)
Maternal sepsis and other pregnancy related infections	1·1 (0·4 to 1·8)	31·4% (12·2 to 49·0%)	0·6 (0·2 to 1·1)	27·8% (10·5 to 43·8%)	-42·8% (-51·7 to -32·9%)	-11·6% (-18·5 to -5·0%)	67·8 (25·8 to 110·2)	31·4% (12·1 to 48·9%)	39·6 (14·4 to 66·8)	27·8% (10·5 to 43·7%)	-41·6% (-50·7 to -31·1%)	-11·4% (-18·4 to -4·9%)
Iron-deficiency anaemia	3·4 (1·3 to 4·4)	100·0% (100·0 to 100·0%)	4·6 (1·4 to 6·3)	100·0% (100·0 to 100·0%)	34·3% (-3·0 to 63·0%)	-	13,748·3 (9,225·6 to 19,637·2)	100·0% (100·0 to 100·0%)	12,380·0 (8,335·0 to 17,777·7)	100·0% (100·0 to 100·0%)	-10·0% (-11·6 to -8·3%)	-
**Vitamin A deficiency: All causes**	21·9 (12·4 to 36·3)	0·2% (0·1 to 0·3%)	8·1 (4·5 to 14·2)	0·1% (0·0 to 0·1%)	-63·1% (-77·0 to -41·2%)	-65·2% (-78·3 to -44·4%)	2,010·9 (1,178·0 to 3,243·8)	0·3% (0·2 to 0·5%)	828·6 (498·3 to 1,365·8)	0·1% (0·1 to 0·2%)	-58·8% (-73·1 to -37·9%)	-58·2% (-72·7 to -37·0%)
Diarrhoeal diseases	11·3 (6·1 to 16·9)	4·1% (2·2 to 6·1%)	4·9 (2·6 to 7·4)	2·5% (1·3 to 3·8%)	-56·7% (-63·9 to -48·2%)	-39·7% (-49·0 to -28·8%)	1,047·5 (577·6 to 1,560·7)	7·5% (4·1 to 10·8%)	487·3 (266·3 to 729·1)	5·6% (3·1 to 8·2%)	-53·5% (-60·5 to -45·3%)	-24·9% (-32·7 to -16·2%)
Measles	10·5 (3·3 to 25·6)	29·4% (19·2 to 39·4%)	3·2 (0·9 to 8·9)	24·8% (15·6 to 35·1%)	-69·9% (-89·2 to -13·4%)	-15·6% (-36·1 to 7·7%)	896·4 (283·5 to 2,172·9)	29·9% (19·6 to 40·0%)	269·8 (73·2 to 758·7)	25·2% (15·9 to 35·6%)	-69·9% (-89·2 to -13·4%)	-15·5% (-35·7 to 7·3%)
Vitamin A deficiency	-	-	-	-	-	-	67·0 (41·6 to 99·3)	100·0% (100·0 to 100·0%)	71·6 (43·7 to 106·5)	100·0% (100·0 to 100·0%)	6·8% (2·5 to 11·2%)	-
**Zinc deficiency: All causes**	8·3 (0·4 to 20·0)	0·1% (0·0 to 0·2%)	3·9 (0·2 to 9·6)	0·0% (0·0 to 0·1%)	-53·3% (-64·2 to -40·6%)	-56·0% (-66·1 to -44·7%)	770·7 (103·9 to 1,742·7)	0·1% (0·0 to 0·3%)	393·1 (81·3 to 873·4)	0·1% (0·0 to 0·2%)	-49·0% (-58·2 to -22·8%)	-48·2% (-57·4 to -21·5%)
Diarrhoeal diseases	3·9 (0·0 to 9·4)	1·4% (0·0 to 3·4%)	1·7 (0·0 to 4·2)	0·9% (0·0 to 2·2%)	-55·4% (-66·2 to 0·0%)	-37·9% (-52·6 to 0·0%)	395·6 (62·1 to 864·2)	2·8% (0·4 to 6·0%)	209·3 (57·1 to 428·5)	2·4% (0·7 to 4·8%)	-47·1% (-58·7 to -4·8%)	-14·7% (-30·9 to 54·6%)
Lower respiratory infections	4·4 (0·0 to 15·2)	0·8% (0·0 to 3·0%)	2·1 (0·0 to 7·4)	0·4% (0·0 to 1·5%)	-51·5% (-60·3 to 0·0%)	-48·7% (-57·8 to 0·0%)	375·1 (2·7 to 1,289·8)	1·6% (0·0 to 5·5%)	183·8 (2·3 to 633·7)	1·1% (0·0 to 3·8%)	-51·0% (-59·8 to -11·4%)	-29·7% (-40·6 to 30·4%)
**Tobacco smoke: All causes**	1,916·0 (1,597·8 to 2,283·2)	14·5% (12·1 to 17·2%)	2,052·5 (1,745·0 to 2,380·1)	14·7% (12·6 to 16·9%)	7·1% (-0·5 to 15·6%)	1·0% (-3·8 to 6·6%)	48,797·2 (42,124·6 to 56,650·2)	8·0% (6·8 to 9·4%)	49,800·0 (43,170·7 to 56,754·6)	8·3% (7·1 to 9·7%)	2·1% (-4·8 to 9·8%)	3·6% (-2·0 to 9·6%)
**Smoking: All causes**	1,661·4 (1,306·4 to 2,035·0)	12·6% (9·9 to 15·4%)	1,793·6 (1,465·5 to 2,134·1)	12·8% (10·5 to 15·2%)	8·0% (-0·7 to 18·1%)	1·8% (-4·1 to 9·2%)	40,829·9 (33,706·5 to 48,911·9)	6·7% (5·5 to 8·1%)	43,084·6 (36,444·4 to 50,291·1)	7·2% (6·0 to 8·5%)	5·5% (-2·6 to 14·8%)	7·2% (0·2 to 15·4%)
Tuberculosis	42·6 (20·9 to 70·4)	10·2% (5·1 to 15·4%)	32·5 (15·6 to 55·4)	10·4% (5·1 to 15·7%)	-23·7% (-35·7 to -11·7%)	1·3% (-7·0 to 9·0%)	1,348·6 (655·8 to 2,182·4)	9·3% (4·6 to 14·0%)	1,044·9 (501·4 to 1,718·7)	9·7% (4·7 to 14·7%)	-22·5% (-32·9 to -12·0%)	4·4% (-3·5 to 11·5%)
Lower respiratory infections	70·2 (56·1 to 89·7)	13·6% (11·1 to 16·5%)	80·6 (63·1 to 102·4)	16·5% (13·4 to 19·9%)	14·9% (7·9 to 23·2%)	21·4% (14·3 to 29·3%)	1,515·4 (1,218·7 to 1,931·7)	6·4% (5·2 to 7·8%)	1,635·6 (1,285·0 to 2,046·2)	9·9% (8·0 to 12·0%)	7·9% (1·5 to 15·3%)	54·7% (42·0 to 69·1%)
Lip and oral cavity cancer	7·0 (5·1 to 9·1)	30·8% (22·8 to 39·8%)	10·7 (7·6 to 14·0)	32·4% (23·2 to 42·3%)	52·6% (34·8 to 70·5%)	5·0% (-3·8 to 12·4%)	191·5 (140·7 to 248·9)	30·9% (22·8 to 39·9%)	276·2 (196·3 to 368·7)	32·0% (23·3 to 42·2%)	44·3% (26·8 to 61·8%)	3·5% (-5·2 to 11·1%)
Nasopharynx cancer	7·1 (4·0 to 10·9)	32·2% (18·3 to 48·7%)	8·1 (4·7 to 12·3)	32·3% (19·3 to 47·6%)	13·7% (-3·9 to 36·6%)	0·4% (-9·7 to 15·3%)	229·1 (127·9 to 355·5)	31·3% (17·4 to 47·3%)	242·3 (139·9 to 369·0)	31·2% (18·8 to 45·8%)	5·8% (-11·3 to 29·4%)	-0·4% (-12·1 to 16·1%)
Oesophageal cancer	52·8 (22·4 to 90·7)	29·6% (12·5 to 50·5%)	44·5 (21·0 to 73·0)	30·0% (14·2 to 48·5%)	-15·8% (-30·8 to 8·3%)	1·3% (-9·7 to 22·8%)	1,220·1 (527·4 to 2,076·2)	29·4% (13·0 to 49·1%)	965·7 (471·8 to 1,567·0)	29·2% (14·4 to 46·8%)	-20·9% (-36·1 to 3·1%)	-0·7% (-12·5 to 21·6%)
Stomach cancer	19·9 (8·3 to 40·6)	7·7% (3·3 to 15·6%)	19·6 (8·6 to 37·7)	7·8% (3·5 to 15·2%)	-1·8% (-17·9 to 21·5%)	1·8% (-11·1 to 21·3%)	487·0 (209·5 to 988·9)	7·8% (3·3 to 15·9%)	437·1 (191·7 to 839·8)	7·7% (3·4 to 14·7%)	-10·3% (-27·1 to 13·2%)	-1·6% (-16·3 to 20·2%)
Colon and rectum cancer	4·7 (2·6 to 8·1)	3·9% (2·2 to 6·9%)	6·3 (3·7 to 10·6)	4·0% (2·3 to 6·7%)	35·8% (13·9 to 66·9%)	2·2% (-12·4 to 21·5%)	113·8 (61·1 to 201·5)	3·9% (2·1 to 6·9%)	139·2 (80·4 to 229·6)	3·7% (2·2 to 6·2%)	22·3% (1·0 to 50·7%)	-3·7% (-18·4 to 15·6%)
Liver cancer due to hepatitis B	14·3 (5·1 to 31·1)	13·0% (4·6 to 27·3%)	13·7 (5·0 to 27·9)	12·8% (4·7 to 25·5%)	-4·2% (-27·1 to 24·1%)	-1·7% (-19·3 to 22·3%)	481·2 (169·3 to 1,041·2)	13·5% (4·8 to 28·4%)	427·6 (158·7 to 882·7)	13·0% (4·9 to 26·0%)	-11·2% (-34·7 to 18·7%)	-3·7% (-23·7 to 24·5%)
Liver cancer due to hepatitis C	3·7 (1·8 to 6·5)	10·8% (5·2 to 18·7%)	4·1 (2·1 to 7·3)	10·6% (5·3 to 17·8%)	12·8% (-6·5 to 38·1%)	-2·0% (-15·8 to 16·8%)	98·2 (47·7 to 174·7)	11·9% (5·7 to 20·9%)	103·3 (51·9 to 181·8)	11·2% (5·7 to 19·1%)	5·2% (-16·2 to 32·8%)	-5·4% (-20·4 to 15·9%)
Liver cancer due to alcohol use	9·3 (3·6 to 19·3)	14·2% (5·5 to 28·2%)	12·1 (4·6 to 24·5)	14·1% (5·5 to 27·6%)	29·5% (3·2 to 61·5%)	-1·3% (-15·1 to 16·9%)	255·7 (98·8 to 518·0)	15·0% (5·8 to 30·3%)	313·7 (120·5 to 636·9)	14·5% (5·7 to 28·7%)	22·7% (-4·8 to 56·0%)	-3·9% (-19·6 to 15·7%)
Liver cancer due to other causes	6·0 (2·4 to 12·1)	10·8% (4·4 to 21·5%)	5·9 (2·5 to 11·3)	10·8% (4·6 to 20·6%)	-2·2% (-21·7 to 24·8%)	0·5% (-15·1 to 24·9%)	179·6 (73·9 to 368·5)	11·4% (4·8 to 23·0%)	160·5 (68·1 to 306·4)	11·2% (4·8 to 21·4%)	-10·7% (-31·6 to 19·4%)	-2·3% (-20·8 to 26·3%)
Pancreatic cancer	5·3 (3·3 to 8·3)	10·9% (6·9 to 17·2%)	7·2 (4·6 to 11·4)	10·8% (6·9 to 17·0%)	36·9% (20·8 to 55·7%)	-0·9% (-10·5 to 10·0%)	129·5 (82·1 to 203·0)	11·3% (7·3 to 17·8%)	169·2 (109·3 to 268·1)	10·9% (7·1 to 17·1%)	30·7% (13·2 to 50·3%)	-3·7% (-14·2 to 7·6%)
Tracheal, bronchus, and lung cancer	179·9 (117·7 to 234·1)	57·8% (38·3 to 74·0%)	232·8 (156·2 to 302·0)	57·5% (39·1 to 73·2%)	29·4% (14·7 to 47·9%)	-0·4% (-5·6 to 6·7%)	4,220·3 (2,770·4 to 5,532·9)	56·5% (36·8 to 73·0%)	5,153·9 (3,429·1 to 6,803·0)	55·7% (37·7 to 71·4%)	22·1% (6·8 to 41·4%)	-1·3% (-7·6 to 6·4%)
Cervical cancer	2·1 (0·7 to 4·4)	3·6% (1·2 to 7·4%)	2·0 (0·7 to 4·1)	3·3% (1·1 to 6·5%)	-5·0% (-28·5 to 30·6%)	-9·5% (-28·1 to 22·0%)	68·3 (20·9 to 142·6)	3·7% (1·1 to 7·6%)	59·3 (18·3 to 121·4)	3·2% (1·0 to 6·2%)	-13·1% (-36·5 to 24·4%)	-14·0% (-34·5 to 19·4%)
Kidney cancer	1·1 (0·6 to 1·9)	8·9% (5·0 to 14·3%)	1·7 (0·9 to 2·8)	9·2% (5·1 to 15·0%)	46·5% (27·5 to 67·6%)	3·3% (-7·5 to 14·3%)	31·6 (17·9 to 51·0)	8·0% (4·5 to 13·0%)	44·0 (24·5 to 73·0)	8·3% (4·7 to 13·4%)	39·3% (19·7 to 60·5%)	4·4% (-8·1 to 17·8%)
Bladder cancer	3·7 (2·2 to 5·7)	15·6% (9·4 to 23·8%)	4·9 (2·9 to 7·4)	16·1% (9·8 to 24·2%)	32·4% (18·4 to 48·6%)	3·3% (-5·0 to 12·8%)	82·6 (51·4 to 124·2)	16·0% (10·1 to 24·0%)	104·3 (64·6 to 155·5)	16·2% (10·1 to 24·0%)	26·3% (11·9 to 41·7%)	1·3% (-7·4 to 10·5%)
Ischaemic heart disease	307·8 (249·4 to 362·9)	17·8% (14·5 to 21·0%)	370·2 (299·3 to 439·6)	16·8% (13·7 to 19·9%)	20·3% (12·2 to 28·1%)	-5·2% (-9·0 to -1·3%)	8,450·9 (6,881·5 to 9,985·2)	22·6% (18·5 to 26·7%)	9,883·2 (7,961·4 to 11,777·5)	21·9% (17·8 to 25·9%)	16·9% (9·3 to 24·5%)	-2·8% (-6·6 to 0·9%)
Ischaemic stroke	98·1 (81·5 to 115·9)	14·9% (12·5 to 17·5%)	109·5 (89·8 to 130·2)	14·1% (11·6 to 16·6%)	11·6% (3·0 to 21·4%)	-5·4% (-11·1 to -0·1%)	2,193·1 (1,818·7 to 2,585·4)	18·9% (15·8 to 22·1%)	2,398·8 (1,972·7 to 2,865·2)	18·4% (15·3 to 21·6%)	9·4% (1·4 to 17·8%)	-2·3% (-7·2 to 2·1%)
Hemorrhagic stroke	249·4 (207·5 to 290·5)	19·8% (16·6 to 23·0%)	249·1 (204·9 to 295·3)	19·2% (15·9 to 22·5%)	-0·1% (-8·1 to 8·3%)	-3·0% (-7·7 to 1·9%)	6,659·0 (5,559·1 to 7,741·7)	23·1% (19·5 to 26·7%)	6,597·2 (5,427·7 to 7,765·3)	23·1% (19·3 to 27·0%)	-0·9% (-8·6 to 7·3%)	0·1% (-4·4 to 4·9%)
Hypertensive heart disease	35·3 (27·9 to 42·8)	15·2% (12·0 to 18·4%)	44·1 (34·5 to 54·2)	14·5% (11·3 to 17·8%)	25·0% (13·2 to 37·1%)	-4·8% (-11·3 to 5·2%)	877·0 (695·9 to 1,060·3)	18·7% (14·8 to 22·4%)	1,031·2 (815·9 to 1,265·3)	18·2% (14·3 to 22·2%)	17·6% (7·2 to 28·6%)	-2·6% (-8·2 to 4·9%)
Atrial fibrillation and flutter	2·4 (1·7 to 3·3)	10·1% (7·7 to 12·5%)	3·1 (2·2 to 4·2)	9·1% (6·9 to 11·1%)	28·2% (19·0 to 38·3%)	-9·6% (-15·2 to -4·1%)	113·8 (79·5 to 154·0)	17·7% (13·9 to 21·3%)	141·4 (99·1 to 193·1)	16·4% (12·7 to 19·8%)	24·2% (19·7 to 28·3%)	-7·6% (-10·2 to -5·2%)
Aortic aneurysm	3·8 (3·0 to 4·6)	19·1% (15·2 to 23·1%)	4·8 (3·7 to 5·9)	17·5% (13·8 to 21·2%)	25·1% (11·7 to 37·0%)	-8·8% (-13·3 to -4·2%)	104·4 (82·3 to 126·4)	22·9% (18·3 to 27·5%)	128·6 (98·7 to 159·3)	21·5% (17·0 to 26·0%)	23·2% (9·8 to 35·3%)	-6·1% (-10·5 to -1·1%)

Supplemental Results Table 1. All-age deaths, death PAFs, DALYs, DALY PAFs, and percent change for each risk factor and outcome for both sexes combined in 2005 and 2015 for Middle SDI.

Data in parenthesis are 95% uncertainty intervals. PAFs=population attributable fractions. DALYs=disability-adjusted life-years.

	2005 Deaths<br>(in thousands)	2005 Deaths<br>PAF	2015 Deaths<br>(in thousands)	2015 Deaths<br>PAF	Percent change deaths<br>2005-2015	Percent change deaths<br>PAF<br>2005-2015	2005 DALYs<br>(in thousands)	2005 DALYs<br>PAF	2015 DALYs<br>(in thousands)	2015 DALYs<br>PAF	Percent change DALYs<br>2005-2015	Percent change DALYs<br>PAF<br>2005-2015
Peripheral vascular disease	0·3<br>(0·2 to 0·3)	13·2%<br>(10·3 to 16·2%)	0·4<br>(0·3 to 0·5)	11·2%<br>(8·7 to 13·9%)	44·8%<br>(32·4 to 59·2%)	-14·8%<br>(-20·5 to -7·5%)	21·5<br>(12·4 to 34·4)	16·1%<br>(12·6 to 19·5%)	29·0<br>(16·8 to 46·6)	14·9%<br>(11·6 to 18·2%)	35·0%<br>(29·3 to 41·0%)	-7·7%<br>(-11·0 to -4·5%)
Other cardiovascular and circulatory diseases	13·2<br>(10·4 to 15·9)	15·2%<br>(12·1 to 18·2%)	15·7<br>(12·5 to 19·0)	15·2%<br>(11·9 to 18·2%)	19·1%<br>(9·2 to 29·9%)	-0·1%<br>(-6·2 to 6·4%)	511·7<br>(402·2 to 631·0)	15·1%<br>(12·0 to 17·9%)	601·9<br>(471·2 to 746·4)	16·0%<br>(12·7 to 19·2%)	17·6%<br>(10·4 to 25·6%)	6·2%<br>(1·3 to 11·7%)
Chronic obstructive pulmonary disease	444·8<br>(232·8 to 656·0)	37·7%<br>(19·6 to 54·7%)	425·9<br>(261·0 to 584·7)	38·3%<br>(23·7 to 52·4%)	-4·3%<br>(-15·4 to 16·6%)	1·7%<br>(-7·0 to 22·4%)	8,278·0<br>(4,758·4 to 11,884·7)	34·9%<br>(20·3 to 49·7%)	7,722·5<br>(5,177·5 to 10,424·5)	35·4%<br>(24·0 to 47·3%)	-6·7%<br>(-17·6 to 11·9%)	1·4%<br>(-7·7 to 20·9%)
Silicosis	0·5<br>(0·2 to 1·0)	11·3%<br>(4·4 to 21·9%)	0·5<br>(0·2 to 1·1)	10·9%<br>(4·1 to 21·8%)	5·2%<br>(-23·8 to 41·4%)	-4·1%<br>(-23·4 to 15·8%)	15·5<br>(5·7 to 32·5)	11·8%<br>(4·4 to 22·9%)	14·5<br>(5·3 to 30·1)	10·6%<br>(4·0 to 21·1%)	-6·7%<br>(-35·2 to 30·5%)	-10·6%<br>(-32·5 to 14·5%)
Asbestosis	0·0<br>(0·0 to 0·1)	8·5%<br>(5·4 to 13·5%)	0·0<br>(0·0 to 0·1)	7·9%<br>(5·1 to 11·8%)	6·7%<br>(-17·3 to 35·5%)	-6·6%<br>(-21·0 to 10·0%)	1·5<br>(0·9 to 2·4)	8·1%<br>(4·8 to 13·2%)	1·6<br>(0·9 to 2·5)	7·4%<br>(4·5 to 11·6%)	3·4%<br>(-19·1 to 29·4%)	-9·2%<br>(-23·6 to 6·8%)
Coal workers pneumoconiosis	0·1<br>(0·0 to 0·2)	12·4%<br>(5·3 to 22·8%)	0·1<br>(0·0 to 0·2)	13·0%<br>(5·9 to 23·2%)	3·4%<br>(-32·5 to 58·7%)	5·4%<br>(-19·1 to 43·4%)	3·0<br>(1·3 to 5·5)	13·0%<br>(5·9 to 23·0%)	2·9<br>(1·2 to 5·6)	13·1%<br>(6·3 to 22·3%)	-1·0%<br>(-37·7 to 58·4%)	0·8%<br>(-25·2 to 43·6%)
Other pneumoconiosis	0·3<br>(0·2 to 0·5)	7·3%<br>(4·7 to 10·8%)	0·4<br>(0·2 to 0·6)	7·7%<br>(4·9 to 11·2%)	22·4%<br>(-17·0 to 83·1%)	5·8%<br>(-23·7 to 53·7%)	10·8<br>(6·1 to 18·3)	6·3%<br>(3·8 to 10·0%)	11·9<br>(7·3 to 18·7)	6·1%<br>(3·9 to 9·1%)	9·6%<br>(-9·3 to 37·4%)	-4·1%<br>(-19·0 to 17·1%)
Asthma	18·8<br>(14·4 to 23·3)	14·5%<br>(11·3 to 17·8%)	17·3<br>(13·1 to 21·7)	14·4%<br>(11·2 to 17·6%)	-8·1%<br>(-20·7 to 6·1%)	-0·8%<br>(-10·5 to 9·0%)	655·2<br>(495·0 to 813·0)	9·3%<br>(7·3 to 11·5%)	643·5<br>(481·0 to 819·3)	9·4%<br>(7·3 to 11·7%)	-1·8%<br>(-10·6 to 8·3%)	0·9%<br>(-6·1 to 8·2%)
Interstitial lung disease and pulmonary sarcoidosis	0·8<br>(0·5 to 1·2)	6·6%<br>(4·8 to 8·8%)	1·3<br>(0·9 to 1·8)	6·7%<br>(4·8 to 8·8%)	59·3%<br>(28·6 to 94·8%)	0·9%<br>(-10·3 to 13·0%)	20·2<br>(12·8 to 29·7)	6·6%<br>(4·7 to 8·9%)	29·6<br>(20·0 to 41·0)	6·5%<br>(4·7 to 8·8%)	46·5%<br>(20·9 to 74·4%)	-1·0%<br>(-10·8 to 9·4%)
Other chronic respiratory diseases	0·7<br>(0·4 to 1·0)	5·0%<br>(3·4 to 6·9%)	1·1<br>(0·5 to 1·8)	6·8%<br>(4·5 to 9·6%)	55·3%<br>(9·4 to 125·8%)	35·5%<br>(-1·4 to 83·0%)	45·4<br>(31·0 to 64·2)	3·6%<br>(2·6 to 4·9%)	52·6<br>(35·2 to 75·3)	4·4%<br>(3·2 to 6·0%)	15·9%<br>(-2·3 to 38·1%)	21·4%<br>(2·8 to 48·5%)
Diabetes mellitus	19·1<br>(5·3 to 33·4)	5·6%<br>(1·6 to 9·7%)	25·1<br>(6·8 to 44·4)	5·4%<br>(1·5 to 9·6%)	31·5%<br>(18·2 to 45·4%)	-2·7%<br>(-10·5 to 5·0%)	974·9<br>(275·1 to 1,733·2)	6·4%<br>(1·7 to 11·1%)	1,234·3<br>(353·6 to 2,163·4)	6·2%<br>(1·7 to 10·9%)	26·6%<br>(18·6 to 34·6%)	-2·4%<br>(-6·9 to 2·2%)
**Second-hand smoke: All causes**	298·1<br>(230·8 to 372·5)	2·3%<br>(1·8 to 2·8%)	305·5<br>(238·0 to 379·3)	2·2%<br>(1·7 to 2·7%)	2·5%<br>(-2·4 to 7·7%)	-3·4%<br>(-7·3 to 0·9%)	8,993·4<br>(6,929·7 to 11,194·8)	1·5%<br>(1·1 to 1·8%)	7,810·1<br>(6,077·4 to 9,639·5)	1·3%<br>(1·0 to 1·6%)	-13·2%<br>(-18·5 to -7·4%)	-11·8%<br>(-16·5 to -6·4%)
Lower respiratory infections	54·4<br>(34·9 to 75·3)	10·6%<br>(6·7 to 14·6%)	39·7<br>(24·7 to 56·2)	8·1%<br>(5·0 to 11·4%)	-27·1%<br>(-32·1 to -21·6%)	-23·0%<br>(-26·7 to -19·6%)	3,419·4<br>(2,193·5 to 4,691·9)	14·5%<br>(9·3 to 19·8%)	1,935·6<br>(1,213·6 to 2,694·4)	11·8%<br>(7·4 to 16·3%)	-43·4%<br>(-48·4 to -37·9%)	-18·8%<br>(-22·6 to -15·5%)
Otitis media	0·1<br>(0·0 to 0·1)	5·7%<br>(3·6 to 7·8%)	0·0<br>(0·0 to 0·0)	4·3%<br>(2·5 to 5·9%)	-42·8%<br>(-51·8 to -32·0%)	-24·9%<br>(-49·9 to 2·9%)	78·6<br>(43·8 to 126·9)	8·6%<br>(5·9 to 11·2%)	71·2<br>(39·5 to 116·5)	8·1%<br>(5·5 to 10·7%)	-9·4%<br>(-13·2 to -6·1%)	-5·2%<br>(-8·2 to -2·4%)
Tracheal, bronchus, and lung cancer	8·8<br>(4·3 to 15·6)	2·8%<br>(1·4 to 5·0%)	11·8<br>(5·8 to 20·7)	2·9%<br>(1·4 to 5·0%)	34·1%<br>(26·1 to 40·2%)	3·4%<br>(-3·8 to 11·7%)	225·9<br>(111·6 to 395·9)	3·0%<br>(1·5 to 5·3%)	292·6<br>(147·5 to 509·1)	3·2%<br>(1·6 to 5·4%)	29·5%<br>(20·6 to 37·1%)	4·8%<br>(-3·3 to 14·4%)
Ischaemic heart disease	106·1<br>(81·9 to 133·0)	6·1%<br>(4·7 to 7·7%)	129·4<br>(98·6 to 162·2)	5·9%<br>(4·5 to 7·4%)	21·9%<br>(15·7 to 28·1%)	-3·8%<br>(-7·2 to -0·5%)	2,453·2<br>(1,969·1 to 2,987·7)	6·6%<br>(5·3 to 8·0%)	2,842·7<br>(2,242·1 to 3,469·5)	6·3%<br>(5·0 to 7·7%)	15·9%<br>(9·4 to 22·9%)	-3·7%<br>(-7·4 to -0·1%)
Ischaemic stroke	22·9<br>(17·2 to 29·8)	3·5%<br>(2·6 to 4·5%)	26·0<br>(19·0 to 33·9)	3·4%<br>(2·5 to 4·4%)	13·9%<br>(5·8 to 22·3%)	-3·5%<br>(-7·9 to 0·8%)	437·2<br>(338·5 to 551·1)	3·8%<br>(2·9 to 4·7%)	474·8<br>(362·9 to 602·7)	3·7%<br>(2·8 to 4·6%)	8·6%<br>(1·0 to 15·9%)	-3·0%<br>(-7·4 to 1·0%)
Hemorrhagic stroke	49·6<br>(38·2 to 62·2)	3·9%<br>(3·0 to 4·9%)	49·6<br>(37·9 to 63·1)	3·8%<br>(2·9 to 4·8%)	-0·1%<br>(-7·0 to 7·5%)	-3·0%<br>(-7·5 to 1·5%)	1,242·6<br>(972·0 to 1,547·3)	4·3%<br>(3·4 to 5·3%)	1,198·2<br>(934·7 to 1,513·5)	4·2%<br>(3·3 to 5·3%)	-3·6%<br>(-10·8 to 4·4%)	-2·6%<br>(-7·4 to 2·6%)
**Alcohol and drug use: All causes**	672·5<br>(621·7 to 717·9)	5·1%<br>(4·8 to 5·4%)	757·3<br>(694·8 to 820·4)	5·4%<br>(5·0 to 5·8%)	12·6%<br>(6·7 to 18·9%)	6·2%<br>(2·4 to 9·9%)	27,634·1<br>(25,554·5 to 29,692·6)	4·5%<br>(4·1 to 4·9%)	29,652·6<br>(27,296·5 to 31,985·5)	4·9%<br>(4·5 to 5·4%)	7·3%<br>(2·6 to 12·1%)	9·0%<br>(5·3 to 12·6%)
**Alcohol use: All causes**	592·7<br>(545·9 to 639·4)	4·5%<br>(4·2 to 4·8%)	655·5<br>(592·8 to 715·7)	4·7%<br>(4·3 to 5·1%)	10·6%<br>(4·3 to 17·5%)	4·3%<br>(0·1 to 8·4%)	22,453·6<br>(20,683·6 to 24,233·2)	3·7%<br>(3·3 to 4·0%)	23,497·2<br>(21,599·6 to 25,494·9)	3·9%<br>(3·5 to 4·3%)	4·6%<br>(-0·5 to 10·4%)	6·3%<br>(2·0 to 10·6%)
Tuberculosis	41·7<br>(31·5 to 54·3)	10·0%<br>(8·6 to 11·2%)	33·5<br>(25·2 to 46·0)	10·7%<br>(8·8 to 12·3%)	-19·6%<br>(-28·7 to -9·7%)	7·0%<br>(-3·4 to 16·0%)	1,516·3<br>(1,174·0 to 1,916·2)	10·4%<br>(9·3 to 11·6%)	1,228·9<br>(939·9 to 1,596·5)	11·4%<br>(9·4 to 13·0%)	-19·0%<br>(-27·1 to -10·9%)	9·3%<br>(-0·5 to 17·3%)
Lower respiratory infections	12·9<br>(11·1 to 15·6)	2·5%<br>(2·2 to 2·8%)	16·1<br>(13·5 to 19·5)	3·3%<br>(2·8 to 3·8%)	24·8%<br>(15·3 to 34·9%)	31·9%<br>(21·4 to 42·2%)	308·3<br>(270·8 to 376·4)	1·3%<br>(1·2 to 1·5%)	359·5<br>(305·5 to 430·3)	2·2%<br>(1·9 to 2·5%)	16·6%<br>(7·2 to 26·3%)	67·2%<br>(50·5 to 83·9%)
Lip and oral cavity cancer	4·9<br>(4·4 to 5·3)	21·6%<br>(19·5 to 23·4%)	8·1<br>(7·0 to 9·1)	24·8%<br>(21·8 to 27·2%)	66·6%<br>(53·2 to 80·6%)	14·7%<br>(8·2 to 21·2%)	147·4<br>(133·0 to 160·8)	23·8%<br>(21·8 to 25·5%)	233·2<br>(203·8 to 259·1)	27·0%<br>(24·1 to 29·3%)	58·2%<br>(45·3 to 71·4%)	13·5%<br>(7·6 to 19·2%)
Nasopharynx cancer	5·7<br>(3·8 to 6·4)	25·8%<br>(21·3 to 27·9%)	7·0<br>(4·5 to 8·0)	27·8%<br>(23·5 to 30·6%)	22·0%<br>(7·9 to 37·1%)	7·7%<br>(1·1 to 14·6%)	193·3<br>(129·2 to 215·3)	26·4%<br>(21·9 to 28·4%)	222·8<br>(147·4 to 256·9)	28·6%<br>(24·4 to 31·4%)	15·3%<br>(1·5 to 29·5%)	8·6%<br>(1·9 to 15·8%)
Other pharynx cancer	2·2<br>(1·9 to 2·5)	21·9%<br>(19·6 to 24·0%)	3·2<br>(2·7 to 3·7)	24·7%<br>(21·3 to 27·6%)	45·4%<br>(29·5 to 62·8%)	12·7%<br>(4·7 to 20·4%)	66·5<br>(58·7 to 74·1)	23·6%<br>(21·2 to 25·5%)	94·7<br>(79·8 to 109·6)	26·5%<br>(23·2 to 29·5%)	42·5%<br>(26·5 to 61·0%)	12·6%<br>(5·2 to 19·9%)
Oesophageal cancer	26·5<br>(23·4 to 29·5)	14·9%<br>(13·4 to 16·2%)	25·0<br>(21·0 to 28·9)	16·9%<br>(14·6 to 18·8%)	-5·7%<br>(-18·8 to 9·8%)	13·4%<br>(4·1 to 22·1%)	675·3<br>(595·7 to 749·6)	16·3%<br>(14·7 to 17·6%)	606·9<br>(511·4 to 701·1)	18·4%<br>(16·0 to 20·2%)	-10·1%<br>(-22·5 to 4·5%)	12·8%<br>(4·4 to 21·0%)
Colon and rectum cancer	3·8<br>(3·4 to 4·2)	3·2%<br>(2·9 to 3·6%)	5·4<br>(4·7 to 6·0)	3·4%<br>(3·0 to 3·8%)	40·3%<br>(29·2 to 52·3%)	5·6%<br>(0·1 to 10·9%)	100·0<br>(90·0 to 109·4)	3·4%<br>(3·1 to 3·7%)	135·0<br>(119·2 to 149·2)	3·6%<br>(3·2 to 4·0%)	35·0%<br>(23·6 to 47·0%)	6·3%<br>(1·0 to 11·5%)
Liver cancer due to hepatitis B	0·6<br>(0·4 to 0·9)	0·6%<br>(0·4 to 0·8%)	0·4<br>(0·3 to 0·6)	0·4%<br>(0·2 to 0·6%)	-32·6%<br>(-49·6 to -11·5%)	-30·6%<br>(-47·0 to -11·4%)	34·7<br>(22·8 to 46·8)	1·0%<br>(0·7 to 1·3%)	23·9<br>(15·0 to 33·8)	0·7%<br>(0·5 to 1·0%)	-31·2%<br>(-47·2 to -10·6%)	-25·2%<br>(-41·6 to -6·4%)
Liver cancer due to hepatitis C	0·0<br>(0·0 to 0·0)	0·1%<br>(0·1 to 0·1%)	0·0<br>(0·0 to 0·0)	0·1%<br>(0·0 to 0·1%)	-17·1%<br>(-41·4 to 13·2%)	-27·9%<br>(-48·3 to -3·9%)	1·7<br>(1·0 to 2·5)	0·2%<br>(0·1 to 0·3%)	1·3<br>(0·8 to 2·0)	0·1%<br>(0·1 to 0·2%)	-20·0%<br>(-42·9 to 10·2%)	-27·8%<br>(-47·5 to -4·5%)
Liver cancer due to alcohol use	65·6<br>(55·3 to 72·0)	100·0%<br>(100·0 to 100·0%)	85·9<br>(77·0 to 97·5)	100·0%<br>(100·0 to 100·0%)	31·0%<br>(17·9 to 46·6%)	-	1,700·9<br>(1,403·0 to 1,871·5)	100·0%<br>(100·0 to 100·0%)	2,166·9<br>(1,928·5 to 2,451·5)	100·0%<br>(100·0 to 100·0%)	27·4%<br>(13·6 to 44·6%)	-
Liver cancer due to other causes	0·2<br>(0·1 to 0·2)	0·3%<br>(0·2 to 0·4%)	0·1<br>(0·1 to 0·2)	0·2%<br>(0·1 to 0·3%)	-34·9%<br>(-51·1 to -15·6%)	-32·9%<br>(-48·8 to -15·4%)	9·4<br>(6·2 to 12·7)	0·6%<br>(0·4 to 0·8%)	6·2<br>(3·9 to 8·9)	0·4%<br>(0·3 to 0·6%)	-33·8%<br>(-49·1 to -15·1%)	-27·5%<br>(-43·5 to -10·2%)
Larynx cancer	3·0<br>(2·7 to 3·3)	15·2%<br>(13·7 to 16·7%)	4·0<br>(3·5 to 4·5)	16·4%<br>(14·2 to 18·1%)	34·3%<br>(23·5 to 45·3%)	7·3%<br>(0·9 to 12·9%)	83·1<br>(74·5 to 91·1)	16·4%<br>(14·9 to 17·8%)	108·2<br>(94·0 to 120·9)	17·6%<br>(15·4 to 19·4%)	30·2%<br>(20·1 to 40·7%)	7·3%<br>(1·2 to 12·5%)
Breast cancer	2·5<br>(2·1 to 2·9)	3·1%<br>(2·7 to 3·5%)	3·2<br>(2·6 to 3·8)	3·1%<br>(2·7 to 3·5%)	28·0%<br>(9·1 to 44·4%)	1·1%<br>(-3·4 to 5·7%)	84·6<br>(70·6 to 98·0)	3·1%<br>(2·7 to 3·5%)	105·8<br>(85·4 to 125·2)	3·1%<br>(2·7 to 3·6%)	25·1%<br>(6·6 to 41·8%)	1·3%<br>(-3·4 to 5·9%)
Ischaemic heart disease	-0·1<br>(-2·9 to 3·0)	0·0%<br>(-0·2 to 0·2%)	0·9<br>(-3·5 to 5·3)	0·0%<br>(-0·2 to 0·2%)	-1,377·8%<br>(-1,047·1 to 1,391·1%)	-1,128·5%<br>(-854·2 to 1,057·2%)	-85·2<br>(-145·5 to -25·8)	-0·2%<br>(-0·4 to -0·1%)	-53·6<br>(-136·8 to 28·0)	-0·1%<br>(-0·3 to 0·1%)	-37·1%<br>(-192·6 to 10·3%)	-47·7%<br>(-177·5 to -8·5%)
Ischaemic stroke	2·5<br>(0·3 to 4·8)	0·4%<br>(0·1 to 0·7%)	4·8<br>(2·1 to 7·6)	0·6%<br>(0·3 to 1·0%)	92·0%<br>(39·9 to 351·4%)	62·6%<br>(18·9 to 279·9%)	72·2<br>(37·2 to 107·4)	0·6%<br>(0·3 to 0·9%)	123·9<br>(83·8 to 167·1)	1·0%<br>(0·6 to 1·3%)	71·6%<br>(44·2 to 137·0%)	53·2%<br>(29·6 to 109·5%)
Hemorrhagic stroke	78·6<br>(69·4 to 88·0)	6·2%<br>(5·5 to 6·9%)	87·9<br>(74·2 to 101·3)	6·8%<br>(5·8 to 7·7%)	11·8%<br>(2·1 to 22·5%)	8·5%<br>(0·7 to 16·2%)	1,922·1<br>(1,716·6 to 2,113·2)	6·7%<br>(6·0 to 7·3%)	2,082·0<br>(1,783·5 to 2,354·5)	7·3%<br>(6·3 to 8·1%)	8·3%<br>(-0·4 to 17·5%)	9·5%<br>(1·9 to 16·5%)
Hypertensive heart disease	12·3<br>(9·8 to 14·9)	5·3%<br>(4·2 to 6·4%)	18·4<br>(13·7 to 23·0)	6·0%<br>(4·5 to 7·5%)	49·0%<br>(29·4 to 67·5%)	13·5%<br>(-0·5 to 29·9%)	297·5<br>(249·5 to 344·9)	6·4%<br>(5·3 to 7·3%)	407·7<br>(321·9 to 485·1)	7·2%<br>(5·7 to 8·5%)	37·0%<br>(22·2 to 51·9%)	13·5%<br>(2·2 to 25·6%)
Atrial fibrillation and flutter	0·9<br>(0·7 to 1·2)	3·7%<br>(3·2 to 4·4%)	1·4<br>(1·0 to 1·9)	4·1%<br>(3·4 to 4·9%)	54·6%<br>(41·1 to 68·0%)	8·9%<br>(0·7 to 16·7%)	32·1<br>(24·6 to 41·7)	5·0%<br>(4·5 to 5·5%)	46·3<br>(35·2 to 60·7)	5·4%<br>(4·7 to 6·0%)	44·2%<br>(35·5 to 52·2%)	7·2%<br>(1·3 to 12·4%)
Cirrhosis and other chronic liver diseases due to hepatitis B	30·5<br>(25·9 to 35·3)	24·8%<br>(21·6 to 27·6%)	31·3<br>(24·5 to 37·5)	24·3%<br>(19·6 to 28·0%)	2·6%<br>(-9·3 to 13·2%)	-2·1%<br>(-11·5 to 5·1%)	931·2<br>(792·0 to 1,076·0)	25·2%<br>(22·1 to 27·9%)	912·5<br>(720·8 to 1,095·5)	24·6%<br>(20·1 to 28·3%)	-2·0%<br>(-13·2 to 8·4%)	-2·3%<br>(-11·8 to 5·2%)
Cirrhosis and other chronic liver diseases due to hepatitis C	15·3<br>(12·5 to 17·9)	17·1%<br>(14·4 to 19·5%)	16·5<br>(12·7 to 20·1)	16·2%<br>(12·7 to 19·2%)	8·1%<br>(-4·8 to 19·5%)	-5·6%<br>(-16·6 to 4·0%)	467·7<br>(384·8 to 542·5)	17·5%<br>(14·8 to 19·9%)	489·2<br>(370·5 to 592·1)	16·6%<br>(13·1 to 19·6%)	4·6%<br>(-7·4 to 14·8%)	-5·3%<br>(-15·7 to 3·5%)
Cirrhosis and other chronic liver diseases due to alcohol use	72·0<br>(65·7 to 78·7)	100·0%<br>(100·0 to 100·0%)	85·7<br>(78·4 to 93·5)	100·0%<br>(100·0 to 100·0%)	18·9%<br>(12·6 to 25·6%)	-	2,396·0<br>(2,195·7 to 2,618·1)	100·0%<br>(100·0 to 100·0%)	2,734·4<br>(2,503·9 to 2,999·7)	100·0%<br>(100·0 to 100·0%)	14·1%<br>(7·9 to 21·1%)	-
Cirrhosis and other chronic liver diseases due to other causes	12·4<br>(10·4 to 14·4)	17·9%<br>(15·4 to 20·3%)	13·0<br>(10·3 to 15·6)	17·9%<br>(14·5 to 21·0%)	5·1%<br>(-6·0 to 16·1%)	0·0%<br>(-10·3 to 8·8%)	364·4<br>(309·6 to 416·6)	15·5%<br>(13·6 to 17·4%)	359·3<br>(287·7 to 425·8)	16·1%<br>(13·2 to 18·7%)	-1·4%<br>(-11·9 to 9·2%)	4·1%<br>(-6·5 to 13·0%)
Pancreatitis	2·4<br>(2·1 to 2·8)	8·8%<br>(7·7 to 9·6%)	3·5<br>(2·8 to 4·1)	9·7%<br>(8·2 to 11·0%)	43·3%<br>(26·7 to 60·9%)	10·5%<br>(1·0 to 18·4%)	91·7<br>(79·8 to 105·3)	9·4%<br>(8·4 to 10·2%)	125·4<br>(103·5 to 149·9)	10·6%<br>(9·0 to 11·9%)	36·7%<br>(20·8 to 53·3%)	12·4%<br>(2·5 to 20·4%)

Supplemental Results Table 1. All-age deaths, death PAFs, DALYs, DALY PAFs, and percent change for each risk factor and outcome for both sexes combined in 2005 and 2015 for Middle SDI.

Data in parenthesis are 95% uncertainty intervals. PAFs=population attributable fractions. DALYs=disability-adjusted life-years.

	2005 Deaths (in thousands)	2005 Deaths PAF	2015 Deaths (in thousands)	2015 Deaths PAF	Percent change deaths 2005-2015	Percent change deaths PAF 2005-2015	2005 DALYs (in thousands)	2005 DALYs PAF	2015 DALYs (in thousands)	2015 DALYs PAF	Percent change DALYs 2005-2015	Percent change DALYs PAF 2005-2015
Epilepsy	2·9 (2·6 to 3·1)	9·5% (8·8 to 10·2%)	3·0 (2·6 to 3·4)	10·5% (9·2 to 11·6%)	5·8% (-3·7 to 16·1%)	9·8% (2·0 to 17·5%)	244·3 (205·0 to 287·0)	7·3% (6·7 to 7·9%)	237·8 (193·8 to 285·1)	8·0% (7·0 to 8·9%)	-2·7% (-10·0 to 4·7%)	10·2% (2·5 to 16·5%)
Alcohol use disorders	26·5 (25·2 to 28·0)	100·0% (100·0 to 100·0%)	27·7 (26·1 to 29·8)	100·0% (100·0 to 100·0%)	4·5% (-2·2 to 11·9%)	-	2,349·7 (1,882·3 to 2,905·8)	100·0% (100·0 to 100·0%)	2,516·3 (2,008·1 to 3,146·6)	100·0% (100·0 to 100·0%)	7·1% (2·8 to 10·8%)	-
Diabetes mellitus	-6·3 (-7·1 to -5·3)	-1·8% (-2·1 to -1·6%)	-7·7 (-8·8 to -6·6)	-1·7% (-1·9 to -1·4%)	23·2% (14·2 to 32·7%)	-8·9% (-15·0 to -2·3%)	-270·1 (-329·4 to -218·2)	-1·8% (-2·0 to -1·6%)	-323·7 (-396·9 to -256·1)	-1·6% (-1·8 to -1·4%)	19·8% (13·8 to 26·5%)	-7·7% (-12·2 to -3·1%)
Pedestrian road injuries	40·6 (33·3 to 49·5)	19·2% (16·2 to 22·7%)	38·8 (32·1 to 46·9)	19·4% (16·5 to 22·5%)	-4·2% (-13·9 to 4·8%)	0·8% (-2·4 to 4·3%)	2,033·7 (1,679·9 to 2,476·6)	20·9% (17·6 to 24·7%)	1,837·9 (1,518·5 to 2,220·0)	21·7% (18·5 to 25·2%)	-9·6% (-18·4 to -1·2%)	3·6% (0·3 to 7·2%)
Cyclist road injuries	4·4 (3·5 to 5·4)	18·0% (14·8 to 21·3%)	3·9 (3·2 to 4·9)	17·8% (14·8 to 21·2%)	-10·7% (-21·4 to 1·8%)	-0·7% (-7·3 to 5·2%)	238·2 (191·5 to 290·8)	17·7% (14·6 to 21·1%)	207·5 (170·5 to 251·1)	17·3% (14·4 to 20·6%)	-12·9% (-22·2 to -2·5%)	-2·5% (-8·5 to 3·3%)
Motorcyclist road injuries	29·0 (24·1 to 33·7)	28·3% (24·6 to 32·1%)	31·7 (26·6 to 37·8)	29·0% (25·4 to 32·7%)	9·4% (-2·6 to 24·0%)	2·4% (-3·4 to 8·6%)	1,551·2 (1,296·9 to 1,812·7)	28·3% (24·6 to 32·2%)	1,644·8 (1,390·6 to 1,958·1)	29·4% (25·7 to 33·0%)	6·0% (-4·2 to 19·3%)	3·6% (-2·3 to 9·5%)
Motor vehicle road injuries	36·0 (30·9 to 43·3)	29·2% (26·3 to 32·5%)	35·7 (30·2 to 41·9)	29·5% (26·7 to 32·5%)	-0·8% (-10·7 to 9·7%)	1·1% (-2·3 to 4·6%)	1,875·7 (1,611·9 to 2,248·0)	28·9% (26·0 to 32·2%)	1,808·1 (1,561·8 to 2,115·4)	29·7% (26·9 to 32·7%)	-3·6% (-12·3 to 5·3%)	2·5% (-0·9 to 6·0%)
Falls	10·6 (7·6 to 13·1)	8·6% (7·1 to 10·4%)	14·2 (9·3 to 17·6)	9·3% (7·4 to 11·1%)	33·1% (15·3 to 47·9%)	7·9% (0·5 to 16·5%)	506·8 (395·3 to 624·1)	8·7% (7·3 to 10·5%)	635·5 (474·5 to 779·3)	9·6% (8·1 to 11·3%)	25·4% (13·0 to 36·7%)	10·0% (4·3 to 16·3%)
Drowning	7·2 (6·0 to 8·6)	6·0% (5·0 to 7·5%)	6·7 (5·7 to 8·0)	7·6% (6·5 to 9·2%)	-7·5% (-16·5 to 2·2%)	25·5% (16·4 to 34·8%)	335·8 (274·6 to 407·6)	4·6% (3·7 to 5·8%)	286·6 (240·8 to 345·4)	5·9% (5·0 to 7·2%)	-14·6% (-22·8 to -5·8%)	27·8% (17·5 to 38·6%)
Fire, heat, and hot substances	2·1 (1·6 to 2·6)	5·4% (4·4 to 6·7%)	2·0 (1·6 to 2·5)	6·3% (5·0 to 7·9%)	-1·5% (-11·3 to 9·6%)	17·5% (1·7 to 30·3%)	110·3 (87·2 to 139·6)	4·7% (3·9 to 5·8%)	105·4 (84·7 to 130·1)	5·6% (4·5 to 6·9%)	-4·5% (-12·0 to 4·4%)	19·6% (5·0 to 30·7%)
Poisonings	2·5 (1·3 to 3·1)	8·7% (7·0 to 10·8%)	2·2 (1·2 to 2·7)	9·6% (7·8 to 11·8%)	-11·6% (-21·9 to 1·0%)	9·7% (1·1 to 19·2%)	106·3 (60·3 to 134·9)	7·5% (6·0 to 9·3%)	88·7 (50·9 to 112·5)	8·3% (6·7 to 10·2%)	-16·5% (-25·2 to -5·1%)	9·8% (-0·2 to 21·6%)
Unintentional firearm injuries	0·5 (0·3 to 0·6)	8·0% (6·8 to 9·7%)	0·4 (0·3 to 0·5)	7·3% (6·2 to 8·9%)	-11·9% (-21·1 to -3·0%)	-8·7% (-15·0 to -0·7%)	23·0 (15·6 to 28·3)	7·7% (6·6 to 9·2%)	19·8 (13·3 to 24·5)	7·3% (6·2 to 8·7%)	-14·1% (-22·3 to -5·8%)	-5·8% (-12·5 to 2·4%)
Unintentional suffocation	0·3 (0·2 to 0·4)	3·0% (2·3 to 3·8%)	0·5 (0·3 to 0·7)	4·6% (3·6 to 5·8%)	61·5% (9·3 to 89·4%)	54·7% (23·5 to 81·7%)	19·4 (14·4 to 24·5)	2·2% (1·7 to 2·8%)	26·3 (16·0 to 33·9)	3·1% (2·4 to 4·0%)	35·3% (5·3 to 53·2%)	43·7% (18·4 to 69·4%)
Other exposure to mechanical forces	4·6 (2·9 to 5·8)	10·5% (8·4 to 12·7%)	5·0 (2·7 to 6·6)	11·7% (9·2 to 14·1%)	9·0% (-15·8 to 26·8%)	11·0% (2·7 to 19·0%)	250·0 (171·0 to 321·9)	9·3% (7·5 to 11·3%)	258·6 (161·8 to 330·9)	10·5% (8·5 to 12·7%)	3·4% (-13·5 to 17·1%)	13·0% (6·2 to 19·8%)
Self-harm	21·6 (15·1 to 26·6)	9·7% (8·1 to 12·3%)	22·8 (18·1 to 28·2)	11·0% (9·3 to 13·4%)	5·5% (-6·4 to 20·2%)	13·3% (2·9 to 21·6%)	899·1 (644·1 to 1,109·9)	9·4% (7·7 to 11·8%)	909·4 (717·5 to 1,127·2)	10·8% (9·0 to 13·2%)	1·2% (-10·0 to 14·1%)	14·8% (4·0 to 23·5%)
Assault by firearm	6·0 (4·9 to 6·9)	13·3% (12·0 to 15·0%)	6·3 (5·0 to 7·2)	12·4% (11·1 to 14·0%)	4·9% (-2·5 to 12·2%)	-6·4% (-9·2 to -3·8%)	339·9 (274·3 to 389·0)	14·0% (12·6 to 15·8%)	350·1 (276·4 to 403·4)	13·2% (11·8 to 14·8%)	3·0% (-4·4 to 10·2%)	-5·7% (-8·5 to -3·0%)
Assault by sharp object	4·2 (3·6 to 5·1)	13·0% (11·5 to 15·0%)	3·5 (3·0 to 4·2)	12·7% (11·3 to 14·4%)	-15·7% (-23·5 to -6·8%)	-2·9% (-6·0 to 0·7%)	236·9 (203·6 to 284·6)	13·7% (12·1 to 15·9%)	195·5 (167·0 to 231·6)	13·5% (12·1 to 15·4%)	-17·5% (-25·2 to -8·7%)	-1·5% (-4·5 to 1·9%)
Assault by other means	3·4 (2·8 to 4·2)	10·1% (8·6 to 12·1%)	3·2 (2·6 to 3·8)	10·1% (8·6 to 12·0%)	-7·3% (-13·2 to 1·1%)	-0·3% (-3·5 to 3·2%)	192·0 (156·1 to 234·4)	10·5% (9·0 to 12·5%)	172·1 (142·8 to 208·2)	10·7% (9·3 to 12·7%)	-10·4% (-16·1 to -2·5%)	2·1% (-1·0 to 5·4%)
**Drug use: All causes**	87·5 (75·6 to 100·7)	0·7% (0·6 to 0·8%)	110·0 (95·7 to 124·9)	0·8% (0·7 to 0·9%)	25·8% (17·3 to 35·0%)	18·6% (12·2 to 26·2%)	5,451·3 (4,702·5 to 6,246·0)	0·9% (0·8 to 1·0%)	6,429·7 (5,509·6 to 7,399·0)	1·1% (0·9 to 1·2%)	17·9% (12·6 to 23·7%)	19·7% (15·2 to 25·2%)
HIV/AIDS - Tuberculosis	2·9 (2·1 to 4·0)	4·5% (3·4 to 5·9%)	3·0 (1·9 to 4·4)	6·5% (4·6 to 8·4%)	2·1% (-23·0 to 34·7%)	46·0% (20·0 to 75·4%)	144·4 (106·1 to 195·3)	4·1% (3·2 to 5·4%)	144·0 (95·3 to 208·3)	6·0% (4·4 to 7·7%)	-0·3% (-23·1 to 29·2%)	43·9% (19·6 to 71·8%)
HIV/AIDS resulting in other diseases	8·9 (6·8 to 17·2)	5·2% (4·1 to 9·1%)	12·3 (8·8 to 22·0)	8·1% (6·4 to 12·4%)	38·4% (14·2 to 90·1%)	55·7% (32·9 to 78·6%)	439·7 (338·2 to 836·7)	4·8% (3·8 to 8·3%)	571·5 (406·6 to 1,013·8)	7·2% (5·7 to 11·0%)	30·0% (7·8 to 78·9%)	49·5% (28·3 to 72·9%)
Hepatitis B	0·1 (0·0 to 0·2)	0·5% (0·2 to 0·8%)	0·1 (0·0 to 0·2)	0·5% (0·2 to 0·8%)	-11·7% (-24·7 to 4·7%)	-3·2% (-14·3 to 10·7%)	4·6 (1·9 to 7·6)	0·5% (0·2 to 0·8%)	3·8 (1·6 to 6·2)	0·5% (0·2 to 0·8%)	-16·0% (-27·6 to -0·8%)	-4·7% (-15·1 to 7·8%)
Hepatitis C	0·3 (0·1 to 0·8)	40·7% (31·8 to 48·6%)	0·3 (0·1 to 0·7)	40·7% (31·9 to 48·9%)	-6·7% (-27·4 to 20·8%)	-0·1% (-11·6 to 12·7%)	12·2 (2·8 to 27·8)	42·6% (34·1 to 50·2%)	10·9 (2·6 to 24·9)	42·2% (33·4 to 50·3%)	-10·4% (-29·5 to 15·6%)	-1·1% (-10·9 to 10·5%)
Liver cancer due to hepatitis B	1·2 (0·5 to 2·1)	1·1% (0·5 to 1·8%)	1·4 (0·6 to 2·3)	1·3% (0·5 to 2·1%)	13·2% (-3·3 to 37·9%)	16·5% (6·1 to 30·9%)	41·2 (17·3 to 69·2)	1·2% (0·5 to 1·9%)	43·2 (18·9 to 71·4)	1·3% (0·6 to 2·1%)	4·9% (-10·3 to 28·5%)	14·0% (4·2 to 26·9%)
Liver cancer due to hepatitis C	14·8 (10·8 to 18·3)	43·2% (32·5 to 52·5%)	18·5 (14·4 to 22·4)	47·2% (38·1 to 55·5%)	25·4% (10·7 to 45·6%)	9·1% (0·8 to 22·6%)	416·5 (315·5 to 499·3)	50·3% (40·7 to 58·9%)	485·2 (387·8 to 582·3)	52·8% (44·2 to 60·7%)	16·5% (2·7 to 34·8%)	4·9% (-2·0 to 15·4%)
Cirrhosis and other chronic liver diseases due to hepatitis B	0·8 (0·3 to 1·4)	0·7% (0·3 to 1·1%)	1·0 (0·4 to 1·6)	0·8% (0·3 to 1·3%)	18·2% (4·6 to 37·4%)	12·8% (2·7 to 27·1%)	27·3 (11·2 to 45·3)	0·7% (0·3 to 1·2%)	29·6 (12·7 to 47·8)	0·8% (0·3 to 1·3%)	8·7% (-4·1 to 25·8%)	8·4% (-1·0 to 21·3%)
Cirrhosis and other chronic liver diseases due to hepatitis C	29·7 (22·5 to 37·0)	33·3% (25·1 to 41·3%)	35·6 (26·8 to 44·7)	34·8% (26·3 to 43·0%)	19·8% (8·8 to 32·8%)	4·6% (-2·1 to 13·3%)	1,049·9 (820·7 to 1,275·7)	39·3% (31·0 to 47·4%)	1,184·3 (906·2 to 1,471·0)	40·1% (31·4 to 48·5%)	12·8% (2·3 to 25·3%)	2·1% (-4·0 to 9·7%)
Opioid use disorders	17·0 (14·1 to 18·3)	100·0% (100·0 to 100·0%)	24·1 (19·7 to 26·4)	100·0% (100·0 to 100·0%)	41·8% (27·7 to 54·9%)	-	2,106·1 (1,668·4 to 2,518·1)	100·0% (100·0 to 100·0%)	2,567·1 (2,057·2 to 3,047·5)	100·0% (100·0 to 100·0%)	21·9% (17·1 to 27·0%)	-
Cocaine use disorders	0·8 (0·5 to 0·9)	100·0% (100·0 to 100·0%)	1·2 (1·0 to 1·5)	100·0% (100·0 to 100·0%)	55·4% (26·7 to 99·8%)	-	113·5 (82·0 to 147·9)	100·0% (100·0 to 100·0%)	148·3 (108·3 to 193·2)	100·0% (100·0 to 100·0%)	30·6% (21·0 to 44·4%)	-
Amphetamine use disorders	1·3 (1·1 to 1·5)	100·0% (100·0 to 100·0%)	2·3 (1·8 to 2·7)	100·0% (100·0 to 100·0%)	70·6% (37·5 to 115·0%)	-	296·3 (196·5 to 421·7)	100·0% (100·0 to 100·0%)	390·9 (264·6 to 541·0)	100·0% (100·0 to 100·0%)	31·9% (23·4 to 43·3%)	-
Cannabis use disorders	-	-	-	-	-	-	149·9 (95·4 to 214·4)	100·0% (100·0 to 100·0%)	151·4 (96·9 to 216·6)	100·0% (100·0 to 100·0%)	1·0% (-1·7 to 4·2%)	-
Other drug use disorders	4·0 (3·4 to 5·2)	100·0% (100·0 to 100·0%)	4·8 (4·2 to 6·1)	100·0% (100·0 to 100·0%)	18·7% (4·0 to 39·1%)	-	369·9 (281·7 to 478·2)	100·0% (100·0 to 100·0%)	432·9 (327·2 to 561·9)	100·0% (100·0 to 100·0%)	17·0% (8·3 to 26·8%)	-
Self-harm	5·6 (3·6 to 8·2)	2·5% (1·7 to 3·7%)	5·5 (3·6 to 8·0)	2·7% (1·8 to 3·9%)	-1·0% (-10·4 to 12·3%)	6·5% (2·5 to 10·8%)	279·9 (180·2 to 413·6)	2·9% (1·9 to 4·3%)	266·6 (172·8 to 391·1)	3·2% (2·1 to 4·7%)	-4·8% (-13·5 to 8·1%)	8·1% (4·0 to 13·0%)
**Dietary risks: All causes**	3,094·7 (2,743·8 to 3,460·8)	23·5% (20·9 to 26·2%)	3,616·6 (3,183·8 to 4,056·3)	25·9% (23·0 to 28·7%)	16·9% (11·9 to 21·9%)	10·2% (8·2 to 12·2%)	74,795·4 (66,946·1 to 82,728·6)	12·3% (10·9 to 13·7%)	84,471·8 (75,130·0 to 93,795·7)	14·1% (12·4 to 15·9%)	12·9% (7·9 to 17·8%)	14·7% (11·5 to 17·8%)
**Diet low in fruits: All causes**	834·0 (574·8 to 1,114·9)	6·3% (4·4 to 8·4%)	917·1 (616·0 to 1,233·2)	6·6% (4·4 to 8·9%)	10·0% (3·8 to 15·9%)	3·7% (-0·3 to 7·3%)	22,319·6 (15,573·4 to 29,503·7)	3·7% (2·5 to 4·9%)	23,906·7 (16,285·6 to 32,097·8)	4·0% (2·7 to 5·4%)	7·1% (1·1 to 12·6%)	8·7% (3·8 to 13·1%)
Lip and oral cavity cancer	1·7 (0·0 to 3·6)	7·3% (0·0 to 15·8%)	2·4 (0·0 to 5·3)	7·3% (0·0 to 15·7%)	43·7% (0·0 to 51·7%)	-1·0% (-2·2 to 0·0%)	45·1 (0·0 to 98·1)	7·3% (0·0 to 15·8%)	62·3 (0·0 to 137·0)	7·2% (0·0 to 15·7%)	38·1% (0·0 to 46·2%)	-0·9% (-2·2 to 0·0%)
Nasopharynx cancer	1·7 (0·0 to 3·6)	7·8% (0·0 to 16·5%)	1·9 (0·0 to 4·1)	7·7% (0·0 to 16·3%)	11·3% (0·0 to 20·9%)	-1·8% (-3·2 to 0·0%)	56·0 (0·0 to 117·2)	7·7% (0·0 to 16·1%)	58·6 (0·0 to 125·2)	7·6% (0·0 to 16·0%)	4·6% (-5·2 to 13·7%)	-1·5% (-3·2 to 0·0%)
Other pharynx cancer	0·7 (0·0 to 1·7)	7·3% (0·0 to 16·2%)	0·9 (0·0 to 2·1)	7·2% (0·0 to 16·1%)	27·0% (0·0 to 37·8%)	-1·4% (-2·7 to 0·0%)	20·5 (0·0 to 45·8)	7·3% (0·0 to 16·1%)	25·6 (0·0 to 56·6)	7·2% (0·0 to 16·0%)	24·9% (0·0 to 36·0%)	-1·2% (-2·5 to 0·0%)
Oesophageal cancer	42·9 (10·9 to 76·1)	24·1% (6·1 to 41·9%)	34·9 (8·3 to 61·5)	23·5% (5·9 to 41·2%)	-18·7% (-26·9 to -9·3%)	-2·2% (-3·7 to -1·2%)	1,000·4 (253·8 to 1,774·5)	24·1% (6·1 to 42·0%)	774·2 (183·2 to 1,368·8)	23·4% (5·9 to 41·0%)	-22·6% (-31·0 to -13·3%)	-2·8% (-4·6 to -1·7%)
Larynx cancer	1·5 (0·0 to 3·2)	7·5% (0·0 to 16·6%)	1·8 (0·0 to 4·0)	7·3% (0·0 to 16·4%)	23·0% (0·0 to 30·2%)	-1·7% (-2·9 to 0·0%)	37·6 (0·0 to 83·3)	7·4% (0·0 to 16·5%)	44·9 (0·0 to 101·9)	7·3% (0·0 to 16·3%)	19·2% (0·0 to 26·0%)	-1·8% (-3·2 to 0·0%)
Tracheal, bronchus, and lung cancer	41·3 (17·0 to 68·6)	13·3% (5·4 to 22·1%)	52·7 (21·6 to 87·9)	13·0% (5·3 to 21·8%)	27·7% (18·6 to 37·2%)	-1·7% (-3·0 to -0·8%)	992·2 (411·9 to 1,645·9)	13·3% (5·4 to 22·2%)	1,200·4 (491·7 to 1,996·5)	13·0% (5·3 to 21·7%)	21·0% (11·9 to 30·9%)	-2·2% (-3·8 to -1·2%)
Ischaemic heart disease	233·6 (83·4 to 387·6)	13·5% (4·9 to 22·4%)	287·0 (102·4 to 482·1)	13·1% (4·7 to 21·8%)	22·9% (17·6 to 28·0%)	-3·1% (-4·3 to -2·2%)	5,869·7 (2,130·6 to 9,652·7)	15·7% (5·8 to 25·8%)	6,857·0 (2,475·1 to 11,409·2)	15·2% (5·6 to 25·2%)	16·8% (11·1 to 22·4%)	-3·0% (-4·3 to -1·9%)
Ischaemic stroke	136·0 (72·4 to 207·0)	20·7% (11·2 to 31·3%)	151·5 (78·8 to 230·3)	19·5% (10·4 to 29·7%)	11·4% (5·2 to 17·9%)	-5·6% (-7·5 to -4·1%)	3,127·0 (1,723·4 to 4,634·4)	26·9% (15·0 to 39·6%)	3,375·1 (1,822·9 to 5,031·0)	26·0% (14·3 to 38·4%)	7·9% (1·8 to 14·1%)	-3·6% (-5·5 to -2·3%)

Supplemental Results Table 1. All-age deaths, death PAFs, DALYs, DALY PAFs, and percent change for each risk factor and outcome for both sexes combined in 2005 and 2015 for Middle SDI.

Data in parenthesis are 95% uncertainty intervals. PAFs=population attributable fractions. DALYs=disability-adjusted life-years.

	2005 Deaths (in thousands)	2005 Deaths PAF	2015 Deaths (in thousands)	2015 Deaths PAF	Percent change deaths 2005-2015	Percent change deaths PAF 2005-2015	2005 DALYs (in thousands)	2005 DALYs PAF	2015 DALYs (in thousands)	2015 DALYs PAF	Percent change DALYs 2005-2015	Percent change DALYs PAF 2005-2015
Hemorrhagic stroke	336·2 (185·0 to 498·4)	26·7% (14·7 to 39·2%)	334·0 (184·7 to 500·1)	25·8% (14·2 to 38·2%)	-0·6% (-6·3 to 5·4%)	-3·5% (-5·3 to -2·2%)	9,106·0 (5,172·1 to 13,217·7)	31·6% (17·8 to 45·4%)	8,888·3 (5,026·0 to 13,043·8)	31·1% (17·5 to 45·2%)	-2·4% (-8·1 to 3·7%)	-1·4% (-3·0 to -0·1%)
**Diet low in vegetables: All causes**	452·8 (238·8 to 711·9)	3·4% (1·8 to 5·4%)	508·3 (266·1 to 809·4)	3·6% (1·9 to 5·8%)	12·2% (5·7 to 18·4%)	5·8% (1·1 to 10·2%)	11,584·3 (6,163·1 to 18,098·0)	1·9% (1·0 to 3·0%)	12,623·7 (6,706·9 to 19,995·8)	2·1% (1·1 to 3·3%)	9·0% (2·7 to 15·3%)	10·7% (5·1 to 16·2%)
Ischaemic heart disease	271·1 (104·4 to 455·1)	15·6% (6·0 to 26·2%)	323·9 (123·4 to 546·0)	14·7% (5·6 to 24·8%)	19·5% (14·2 to 24·6%)	-5·8% (-7·9 to -4·2%)	6,739·3 (2,655·4 to 11,106·4)	18·0% (7·0 to 29·7%)	7,767·5 (3,032·5 to 13,006·6)	17·2% (6·7 to 28·6%)	15·3% (9·6 to 21·1%)	-4·2% (-6·3 to -2·4%)
Ischaemic stroke	52·9 (12·0 to 98·4)	8·1% (1·8 to 14·9%)	56·9 (12·7 to 105·3)	7·3% (1·7 to 13·6%)	7·4% (1·2 to 13·8%)	-9·0% (-11·6 to -6·7%)	1,235·2 (286·7 to 2,264·7)	10·6% (2·5 to 19·5%)	1,295·0 (302·1 to 2,369·8)	10·0% (2·3 to 18·2%)	4·8% (-1·3 to 11·1%)	-6·4% (-9·0 to -4·1%)
Hemorrhagic stroke	128·8 (35·1 to 244·3)	10·2% (2·8 to 19·4%)	127·5 (35·5 to 243·5)	9·8% (2·8 to 18·5%)	-1·0% (-7·0 to 5·4%)	-3·9% (-6·7 to -0·8%)	3,609·7 (991·6 to 6,742·2)	12·5% (3·5 to 23·4%)	3,561·2 (1,010·6 to 6,653·7)	12·5% (3·5 to 23·2%)	-1·3% (-7·6 to 5·6%)	-0·3% (-2·9 to 2·9%)
**Diet low in whole grains: All causes**	852·5 (624·8 to 1,114·5)	6·5% (4·8 to 8·4%)	975·0 (709·2 to 1,274·3)	7·0% (5·1 to 9·2%)	14·4% (8·9 to 19·8%)	7·8% (4·8 to 10·5%)	23,628·3 (17,515·8 to 30,280·4)	3·9% (2·9 to 5·0%)	26,376·7 (19,381·5 to 34,171·8)	4·4% (3·2 to 5·7%)	11·6% (6·6 to 16·8%)	13·3% (9·4 to 17·3%)
Ischaemic heart disease	367·7 (220·7 to 527·4)	21·2% (12·8 to 30·5%)	457·0 (268·6 to 660·8)	20·8% (12·5 to 30·0%)	24·3% (19·5 to 29·5%)	-2·0% (-2·8 to -1·3%)	9,148·0 (5,520·7 to 12,999·7)	24·4% (14·9 to 34·7%)	10,824·4 (6,404·8 to 15,494·4)	24·0% (14·6 to 34·2%)	18·3% (12·8 to 23·8%)	-1·7% (-2·6 to -0·9%)
Ischaemic stroke	122·7 (83·8 to 164·4)	18·7% (12·8 to 25·1%)	136·9 (92·1 to 184·3)	17·6% (12·0 to 23·9%)	11·6% (5·2 to 18·2%)	-5·4% (-7·2 to -3·8%)	2,915·5 (2,019·5 to 3,820·9)	25·1% (17·6 to 33·2%)	3,167·7 (2,171·7 to 4,222·4)	24·4% (17·0 to 32·3%)	8·6% (2·5 to 15·0%)	-3·0% (-4·5 to -1·7%)
Hemorrhagic stroke	297·9 (209·2 to 400·7)	23·7% (16·8 to 31·5%)	296·7 (210·9 to 396·5)	22·9% (16·2 to 30·6%)	-0·4% (-6·1 to 5·8%)	-3·3% (-4·7 to -2·0%)	8,095·0 (5,757·1 to 10,759·4)	28·1% (20·2 to 36·9%)	7,938·2 (5,671·1 to 10,484·6)	27·8% (20·0 to 36·8%)	-1·9% (-7·7 to 4·2%)	-0·9% (-2·1 to 0·1%)
Diabetes mellitus	64·3 (35·4 to 98·4)	18·8% (10·3 to 28·6%)	84·4 (46·7 to 129·3)	18·2% (10·0 to 27·9%)	31·2% (22·6 to 39·6%)	-2·9% (-4·3 to -1·7%)	3,469·8 (1,896·0 to 5,342·5)	22·6% (12·7 to 33·9%)	4,446·5 (2,414·8 to 6,920·6)	22·4% (12·5 to 33·6%)	28·1% (23·4 to 33·2%)	-1·2% (-2·0 to -0·6%)
**Diet low in nuts and seeds: All causes**	451·2 (291·4 to 632·7)	3·4% (2·2 to 4·8%)	564·8 (363·8 to 792·9)	4·0% (2·6 to 5·7%)	25·2% (20·5 to 30·1%)	18·0% (15·5 to 20·6%)	12,078·4 (8,167·3 to 16,487·5)	2·0% (1·3 to 2·8%)	14,545·1 (9,695·7 to 19,887·5)	2·4% (1·6 to 3·4%)	20·4% (15·4 to 25·3%)	22·3% (18·7 to 25·9%)
Ischaemic heart disease	411·5 (257·7 to 587·9)	23·7% (15·0 to 33·6%)	512·4 (318·6 to 724·8)	23·3% (14·6 to 33·1%)	24·5% (19·8 to 29·5%)	-1·8% (-2·4 to -1·3%)	9,988·2 (6,398·5 to 13,996·7)	26·7% (17·0 to 37·1%)	11,849·1 (7,556·4 to 16,590·3)	26·3% (16·7 to 36·5%)	18·6% (13·4 to 24·2%)	-1·4% (-2·2 to -0·7%)
Diabetes mellitus	39·7 (20·5 to 62·3)	11·6% (6·1 to 18·2%)	52·4 (26·8 to 81·7)	11·3% (5·9 to 17·7%)	31·9% (23·5 to 40·0%)	-2·4% (-3·5 to -1·3%)	2,090·2 (1,006·6 to 3,325·3)	13·6% (7·2 to 21·1%)	2,696·1 (1,289·9 to 4,313·3)	13·6% (7·1 to 21·0%)	29·0% (24·2 to 33·8%)	-0·6% (-1·1 to -0·1%)
**Diet low in milk: All causes**	20·3 (7·2 to 33·9)	0·2% (0·1 to 0·3%)	27·0 (9·6 to 45·1)	0·2% (0·1 to 0·3%)	33·0% (25·7 to 41·1%)	25·4% (20·7 to 30·0%)	495·8 (175·2 to 828·1)	0·1% (0·0 to 0·1%)	631·6 (225·7 to 1,057·2)	0·1% (0·0 to 0·2%)	27·4% (19·5 to 35·8%)	29·4% (22·7 to 36·5%)
Colon and rectum cancer	20·3 (7·2 to 33·9)	17·1% (6·1 to 28·4%)	27·0 (9·6 to 45·1)	17·1% (6·1 to 28·4%)	33·0% (25·7 to 41·1%)	0·0% (-0·2 to 0·3%)	495·8 (175·2 to 828·1)	16·9% (6·1 to 28·1%)	631·6 (225·7 to 1,057·2)	17·0% (6·1 to 28·2%)	27·4% (19·5 to 35·8%)	0·3% (0·0 to 0·6%)
**Diet high in red meat: All causes**	5·9 (2·5 to 9·7)	0·0% (0·0 to 0·1%)	7·4 (3·3 to 12·1)	0·1% (0·0 to 0·1%)	26·4% (18·8 to 35·8%)	19·1% (13·2 to 26·6%)	279·3 (90·0 to 494·1)	0·0% (0·0 to 0·1%)	342·3 (110·1 to 600·0)	0·1% (0·0 to 0·1%)	22·6% (17·9 to 29·3%)	24·4% (20·0 to 30·3%)
Colon and rectum cancer	2·3 (0·5 to 4·2)	2·0% (0·4 to 3·6%)	3·1 (0·6 to 5·6)	2·0% (0·4 to 3·6%)	33·7% (22·0 to 45·1%)	0·5% (-3·4 to 4·8%)	60·2 (12·9 to 107·0)	2·1% (0·4 to 3·7%)	77·6 (16·3 to 140·5)	2·1% (0·4 to 3·8%)	28·9% (17·1 to 41·0%)	1·5% (-2·7 to 5·9%)
Diabetes mellitus	3·6 (0·4 to 6·7)	1·0% (0·1 to 2·0%)	4·3 (0·5 to 8·0)	0·9% (0·1 to 1·8%)	21·5% (14·4 to 29·2%)	-10·1% (-14·7 to -5·1%)	219·1 (25·3 to 420·7)	1·4% (0·2 to 2·7%)	264·7 (31·1 to 515·4)	1·3% (0·2 to 2·5%)	20·8% (16·5 to 25·0%)	-6·9% (-10·3 to -3·4%)
**Diet high in processed meat: All causes**	87·6 (26·1 to 152·5)	0·7% (0·2 to 1·2%)	113·3 (35·4 to 196·9)	0·8% (0·3 to 1·4%)	29·5% (23·5 to 38·9%)	22·0% (18·3 to 28·5%)	2,841·1 (1,207·5 to 4,630·0)	0·5% (0·2 to 0·8%)	3,605·0 (1,605·2 to 5,778·0)	0·6% (0·3 to 1·0%)	26·9% (20·7 to 35·5%)	28·8% (23·7 to 36·9%)
Colon and rectum cancer	3·4 (1·7 to 5·2)	2·8% (1·5 to 4·4%)	4·7 (2·4 to 7·3)	3·0% (1·6 to 4·6%)	41·0% (31·5 to 51·2%)	6·0% (2·8 to 9·5%)	84·1 (43·6 to 131·6)	2·9% (1·5 to 4·5%)	113·4 (58·7 to 177·9)	3·0% (1·6 to 4·7%)	35·0% (25·1 to 46·0%)	6·2% (2·9 to 10·0%)
Ischaemic heart disease	63·6 (2·6 to 123·8)	3·7% (0·1 to 7·2%)	80·7 (3·2 to 157·8)	3·7% (0·1 to 7·2%)	26·8% (18·8 to 32·9%)	0·0% (-2·0 to 2·8%)	1,616·1 (65·0 to 3,138·7)	4·3% (0·2 to 8·4%)	1,957·4 (78·2 to 3,805·0)	4·3% (0·2 to 8·5%)	21·1% (12·9 to 27·5%)	0·6% (-1·7 to 3·3%)
Diabetes mellitus	20·6 (9·7 to 31·7)	6·0% (2·9 to 9·3%)	27·9 (13·5 to 42·9)	6·0% (3·0 to 9·2%)	35·7% (26·3 to 45·9%)	0·4% (-2·3 to 3·5%)	1,140·9 (536·2 to 1,796·5)	7·5% (3·6 to 11·4%)	1,534·1 (715·5 to 2,411·2)	7·7% (3·8 to 11·8%)	34·5% (28·7 to 40·6%)	3·6% (1·6 to 6·2%)
**Diet high in sugar-sweetened beverages: All causes**	6·5 (4·6 to 9·2)	0·0% (0·0 to 0·1%)	8·4 (5·8 to 12·0)	0·1% (0·0 to 0·1%)	29·4% (21·0 to 37·5%)	22·0% (15·2 to 28·9%)	251·4 (175·0 to 353·7)	0·0% (0·0 to 0·1%)	327·3 (226·0 to 472·2)	0·1% (0·0 to 0·1%)	30·2% (22·3 to 38·3%)	32·2% (24·8 to 39·5%)
Oesophageal cancer	0·1 (0·0 to 0·1)	0·0% (0·0 to 0·1%)	0·1 (0·0 to 0·1)	0·0% (0·0 to 0·1%)	13·0% (-0·6 to 24·7%)	36·2% (17·3 to 52·6%)	1·7 (0·6 to 3·0)	0·0% (0·0 to 0·1%)	1·9 (0·6 to 3·4)	0·1% (0·0 to 0·1%)	10·3% (-3·3 to 22·0%)	38·7% (18·2 to 56·0%)
Colon and rectum cancer	0·0 (0·0 to 0·1)	0·0% (0·0 to 0·0%)	0·1 (0·0 to 0·1)	0·0% (0·0 to 0·1%)	47·8% (38·1 to 57·3%)	11·2% (4·0 to 19·0%)	1·1 (0·7 to 1·6)	0·0% (0·0 to 0·1%)	1·6 (1·1 to 2·3)	0·0% (0·0 to 0·1%)	43·6% (33·0 to 53·8%)	13·0% (4·9 to 21·3%)
Liver cancer due to hepatitis B	0·0 (0·0 to 0·1)	0·0% (0·0 to 0·0%)	0·0 (0·0 to 0·1)	0·0% (0·0 to 0·1%)	20·0% (7·2 to 34·3%)	23·5% (10·1 to 37·6%)	1·0 (0·4 to 1·8)	0·0% (0·0 to 0·1%)	1·2 (0·5 to 2·1)	0·0% (0·0 to 0·1%)	16·4% (2·5 to 31·6%)	26·4% (11·7 to 42·1%)
Liver cancer due to hepatitis C	0·0 (0·0 to 0·0)	0·1% (0·0 to 0·1%)	0·0 (0·0 to 0·1)	0·1% (0·0 to 0·2%)	33·9% (23·4 to 46·0%)	16·5% (7·3 to 26·1%)	0·7 (0·3 to 1·2)	0·1% (0·0 to 0·1%)	1·0 (0·4 to 1·6)	0·1% (0·0 to 0·2%)	31·1% (20·0 to 44·3%)	18·0% (7·6 to 28·8%)
Liver cancer due to alcohol use	0·0 (0·0 to 0·0)	0·0% (0·0 to 0·1%)	0·0 (0·0 to 0·1)	0·0% (0·0 to 0·1%)	42·9% (28·9 to 58·0%)	9·1% (-3·2 to 20·9%)	0·7 (0·3 to 1·2)	0·0% (0·0 to 0·1%)	0·9 (0·4 to 1·6)	0·0% (0·0 to 0·1%)	41·2% (25·3 to 58·2%)	10·8% (-2·3 to 24·3%)
Liver cancer due to other causes	0·0 (0·0 to 0·0)	0·0% (0·0 to 0·1%)	0·0 (0·0 to 0·0)	0·0% (0·0 to 0·1%)	22·6% (9·7 to 37·0%)	26·2% (13·2 to 40·8%)	0·7 (0·3 to 1·1)	0·0% (0·0 to 0·1%)	0·8 (0·4 to 1·3)	0·1% (0·0 to 0·1%)	18·5% (4·4 to 33·5%)	29·8% (15·1 to 46·5%)
Gallbladder and biliary tract cancer	0·0 (0·0 to 0·0)	0·1% (0·1 to 0·2%)	0·0 (0·0 to 0·0)	0·1% (0·1 to 0·2%)	16·4% (3·4 to 27·6%)	-0·3% (-8·9 to 7·1%)	0·6 (0·4 to 1·0)	0·1% (0·1 to 0·2%)	0·7 (0·4 to 1·1)	0·1% (0·1 to 0·2%)	13·3% (0·9 to 23·9%)	0·6% (-8·6 to 8·1%)
Pancreatic cancer	0·0 (0·0 to 0·0)	0·0% (0·0 to 0·1%)	0·0 (0·0 to 0·0)	0·0% (0·0 to 0·1%)	42·9% (33·4 to 51·1%)	3·4% (-3·4 to 9·1%)	0·4 (0·1 to 0·7)	0·0% (0·0 to 0·1%)	0·5 (0·2 to 0·9)	0·0% (0·0 to 0·1%)	41·1% (30·7 to 49·9%)	4·0% (-3·4 to 10·0%)
Breast cancer	0·0 (0·0 to 0·1)	0·1% (0·0 to 0·1%)	0·1 (0·0 to 0·1)	0·1% (0·0 to 0·1%)	38·0% (17·8 to 62·7%)	8·9% (-2·6 to 22·0%)	1·6 (1·0 to 2·5)	0·1% (0·0 to 0·1%)	2·2 (1·3 to 3·5)	0·1% (0·0 to 0·1%)	37·9% (16·2 to 65·1%)	11·7% (-0·8 to 26·1%)
Uterine cancer	0·0 (0·0 to 0·0)	0·2% (0·1 to 0·3%)	0·0 (0·0 to 0·1)	0·2% (0·1 to 0·3%)	19·1% (8·4 to 30·2%)	15·5% (5·5 to 25·5%)	1·0 (0·6 to 1·4)	0·2% (0·1 to 0·3%)	1·1 (0·8 to 1·6)	0·2% (0·1 to 0·3%)	18·7% (7·0 to 30·6%)	17·7% (6·9 to 28·8%)
Ovarian cancer	0·0 (0·0 to 0·0)	0·0% (0·0 to 0·0%)	0·0 (0·0 to 0·0)	0·0% (0·0 to 0·0%)	37·8% (0·0 to 51·7%)	8·0% (0·0 to 15·5%)	0·1 (0·0 to 0·3)	0·0% (0·0 to 0·0%)	0·2 (0·0 to 0·4)	0·0% (0·0 to 0·0%)	36·6% (0·0 to 51·6%)	9·6% (0·0 to 17·4%)
Kidney cancer	0·0 (0·0 to 0·0)	0·1% (0·1 to 0·2%)	0·0 (0·0 to 0·0)	0·1% (0·1 to 0·2%)	49·1% (39·9 to 58·2%)	5·2% (-0·7 to 10·4%)	0·4 (0·3 to 0·6)	0·1% (0·1 to 0·2%)	0·6 (0·4 to 0·9)	0·1% (0·1 to 0·2%)	47·2% (37·4 to 57·1%)	10·3% (3·7 to 16·7%)
Thyroid cancer	0·0 (0·0 to 0·0)	0·1% (0·0 to 0·1%)	0·0 (0·0 to 0·0)	0·1% (0·0 to 0·1%)	37·2% (25·9 to 47·3%)	3·3% (-4·2 to 15·4%)	0·1 (0·1 to 0·2)	0·1% (0·0 to 0·1%)	0·2 (0·1 to 0·3)	0·1% (0·0 to 0·1%)	38·9% (27·0 to 49·5%)	5·4% (-2·4 to 17·6%)
Ischaemic heart disease	1·8 (1·2 to 2·5)	0·1% (0·1 to 0·1%)	2·3 (1·6 to 3·3)	0·1% (0·1 to 0·1%)	29·0% (20·6 to 36·7%)	1·7% (-4·0 to 7·4%)	55·3 (38·3 to 78·1)	0·1% (0·1 to 0·2%)	70·1 (48·6 to 100·8)	0·2% (0·1 to 0·2%)	26·9% (17·7 to 35·8%)	5·4% (-0·5 to 11·7%)
Ischaemic stroke	0·4 (0·2 to 0·5)	0·1% (0·0 to 0·1%)	0·4 (0·3 to 0·6)	0·1% (0·0 to 0·1%)	20·3% (10·8 to 29·9%)	2·0% (-5·2 to 9·4%)	10·2 (7·1 to 14·8)	0·1% (0·1 to 0·1%)	12·1 (8·3 to 17·6)	0·1% (0·1 to 0·1%)	18·9% (9·7 to 28·2%)	6·2% (-0·7 to 13·6%)
Hemorrhagic stroke	1·5 (1·0 to 2·1)	0·1% (0·1 to 0·2%)	1·8 (1·2 to 2·6)	0·1% (0·1 to 0·2%)	18·5% (8·3 to 29·3%)	15·0% (6·3 to 24·4%)	52·8 (36·5 to 74·7)	0·2% (0·1 to 0·3%)	61·6 (42·2 to 88·0)	0·2% (0·1 to 0·3%)	16·8% (6·5 to 28·0%)	18·0% (9·1 to 27·5%)
Hypertensive heart disease	0·4 (0·3 to 0·6)	0·2% (0·1 to 0·3%)	0·5 (0·3 to 0·8)	0·2% (0·1 to 0·3%)	26·1% (15·9 to 35·8%)	-3·9% (-12·4 to 6·5%)	11·3 (7·4 to 16·2)	0·2% (0·2 to 0·3%)	13·6 (9·0 to 19·8)	0·2% (0·2 to 0·4%)	21·1% (11·5 to 30·4%)	0·3% (-6·8 to 8·9%)
Cardiomyopathy and myocarditis	-	-	-	-	-	-	-	-	-	-	-	-
Atrial fibrillation and flutter	-	-	-	-	-	-	-	-	-	-	-	-
Peripheral vascular disease	-	-	-	-	-	-	-	-	-	-	-	-

Supplemental Results Table 1. All-age deaths, death PAFs, DALYs, DALY PAFs, and percent change for each risk factor and outcome for both sexes combined in 2005 and 2015 for Middle SDI.

Data in parenthesis are 95% uncertainty intervals. PAFs=population attributable fractions. DALYs=disability-adjusted life-years.

	2005 Deaths (in thousands)	2005 Deaths PAF	2015 Deaths (in thousands)	2015 Deaths PAF	Percent change deaths 2005-2015	Percent change deaths PAF 2005-2015	2005 DALYs (in thousands)	2005 DALYs PAF	2015 DALYs (in thousands)	2015 DALYs PAF	Percent change DALYs 2005-2015	Percent change DALYs PAF 2005-2015
Endocarditis	-	-	-	-	-	-	-	-	-	-	-	-
Other cardiovascular and circulatory diseases	-	-	-	-	-	-	-	-	-	-	-	-
Diabetes mellitus	1·4 (1·0 to 1·9)	0·4% (0·3 to 0·6%)	1·9 (1·3 to 2·7)	0·4% (0·3 to 0·6%)	36·9% (27·3 to 47·5%)	1·3% (-3·6 to 6·7%)	81·4 (53·5 to 119·9)	0·5% (0·4 to 0·7%)	114·5 (74·8 to 170·4)	0·6% (0·4 to 0·8%)	40·6% (33·6 to 48·3%)	8·3% (3·8 to 12·9%)
Chronic kidney disease due to diabetes mellitus	0·3 (0·2 to 0·6)	0·4% (0·2 to 0·7%)	0·5 (0·3 to 0·9)	0·4% (0·2 to 0·7%)	55·8% (47·1 to 64·1%)	8·2% (2·8 to 14·5%)	9·7 (4·2 to 17·0)	0·4% (0·2 to 0·7%)	14·7 (6·4 to 25·6)	0·4% (0·2 to 0·8%)	52·1% (44·5 to 59·6%)	14·0% (9·1 to 19·4%)
Chronic kidney disease due to hypertension	0·3 (0·1 to 0·5)	0·2% (0·1 to 0·4%)	0·4 (0·2 to 0·7)	0·2% (0·1 to 0·4%)	36·5% (26·5 to 46·6%)	1·4% (-5·1 to 8·3%)	8·2 (3·5 to 14·9)	0·3% (0·1 to 0·5%)	11·0 (4·6 to 20·0)	0·3% (0·1 to 0·5%)	32·9% (25·1 to 41·1%)	7·6% (2·5 to 12·9%)
Chronic kidney disease due to glomerulonephritis	0·1 (0·0 to 0·1)	0·1% (0·0 to 0·1%)	0·1 (0·0 to 0·1)	0·1% (0·0 to 0·2%)	35·8% (24·9 to 48·0%)	23·6% (14·0 to 34·6%)	2·5 (0·8 to 4·9)	0·1% (0·0 to 0·2%)	3·4 (1·1 to 6·5)	0·1% (0·0 to 0·2%)	33·8% (24·9 to 42·8%)	33·9% (26·2 to 43·3%)
Chronic kidney disease due to other causes	0·0 (0·0 to 0·0)	0·1% (0·1 to 0·3%)	0·0 (0·0 to 0·0)	0·2% (0·1 to 0·3%)	32·5% (12·2 to 55·3%)	12·0% (0·6 to 24·6%)	1·2 (0·4 to 2·5)	0·2% (0·1 to 0·3%)	1·6 (0·6 to 3·4)	0·2% (0·1 to 0·4%)	35·9% (25·4 to 46·1%)	19·2% (12·4 to 27·1%)
**Diet low in fibre: All causes**	63·2 (29·1 to 113·3)	0·5% (0·2 to 0·9%)	79·5 (36·6 to 142·2)	0·6% (0·3 to 1·0%)	25·7% (17·3 to 32·9%)	18·5% (12·3 to 24·6%)	1,770·9 (814·5 to 3,126·1)	0·3% (0·1 to 0·5%)	2,137·8 (997·5 to 3,761·9)	0·4% (0·2 to 0·6%)	20·7% (12·3 to 29·2%)	22·6% (15·1 to 30·5%)
Colon and rectum cancer	4·0 (1·8 to 7·2)	3·4% (1·5 to 6·1%)	5·7 (2·5 to 10·1)	3·6% (1·6 to 6·4%)	41·6% (28·7 to 55·0%)	6·5% (-2·0 to 15·4%)	96·5 (42·4 to 177·0)	3·3% (1·4 to 6·0%)	130·8 (57·8 to 235·7)	3·5% (1·6 to 6·3%)	35·6% (23·6 to 49·1%)	6·7% (-1·3 to 16·1%)
Ischaemic heart disease	59·2 (27·2 to 107·1)	3·4% (1·6 to 6·1%)	73·8 (34·0 to 134·1)	3·4% (1·5 to 6·0%)	24·6% (16·6 to 31·9%)	-1·7% (-6·5 to 2·9%)	1,674·5 (767·6 to 2,973·5)	4·5% (2·1 to 7·9%)	2,007·0 (933·5 to 3,560·5)	4·5% (2·1 to 7·9%)	19·9% (11·4 to 28·4%)	-0·4% (-5·3 to 4·8%)
**Diet low in calcium: All causes**	27·3 (17·4 to 38·3)	0·2% (0·1 to 0·3%)	36·4 (23·4 to 50·5)	0·3% (0·2 to 0·4%)	33·0% (26·1 to 40·9%)	25·4% (20·6 to 29·9%)	665·4 (422·5 to 929·6)	0·1% (0·1 to 0·2%)	848·2 (547·9 to 1,181·9)	0·1% (0·1 to 0·2%)	27·5% (20·1 to 36·0%)	29·5% (23·2 to 36·4%)
Colon and rectum cancer	27·3 (17·4 to 38·3)	23·1% (14·8 to 32·2%)	36·4 (23·4 to 50·5)	23·1% (14·7 to 32·1%)	33·0% (26·1 to 40·9%)	0·1% (-0·9 to 1·0%)	665·4 (422·5 to 929·6)	22·7% (14·5 to 31·6%)	848·2 (547·9 to 1,181·9)	22·8% (14·5 to 31·7%)	27·5% (20·1 to 36·0%)	0·4% (-0·7 to 1·3%)
Prostate cancer	-	-	-	-	-	-	-	-	-	-	-	-
**Diet low in seafood omega-3 fatty acids: All causes**	324·6 (138·4 to 517·3)	2·5% (1·1 to 3·9%)	402·3 (171·9 to 650·0)	2·9% (1·2 to 4·6%)	24·0% (19·2 to 29·2%)	16·9% (14·0 to 19·5%)	7,836·5 (3,390·5 to 12,303·3)	1·3% (0·6 to 2·0%)	9,285·0 (4,045·1 to 14,771·4)	1·5% (0·7 to 2·5%)	18·5% (13·0 to 24·3%)	20·3% (15·8 to 24·4%)
Ischaemic heart disease	324·6 (138·4 to 517·3)	18·7% (8·0 to 29·9%)	402·3 (171·9 to 650·0)	18·3% (7·8 to 29·3%)	24·0% (19·2 to 29·2%)	-2·2% (-3·0 to -1·5%)	7,836·5 (3,390·5 to 12,303·3)	20·9% (9·1 to 32·9%)	9,285·0 (4,045·1 to 14,771·4)	20·6% (8·9 to 32·4%)	18·5% (13·0 to 24·3%)	-1·5% (-2·5 to -0·8%)
**Diet low in polyunsaturated fatty acids: All causes**	49·3 (20·8 to 81·9)	0·4% (0·2 to 0·6%)	69·4 (28·7 to 119·0)	0·5% (0·2 to 0·9%)	40·6% (22·2 to 61·8%)	32·5% (16·3 to 51·3%)	1,284·3 (553·1 to 2,117·5)	0·2% (0·1 to 0·4%)	1,853·5 (786·6 to 3,146·2)	0·3% (0·1 to 0·5%)	44·3% (23·5 to 70·5%)	46·5% (25·4 to 72·0%)
Ischaemic heart disease	49·3 (20·8 to 81·9)	2·8% (1·2 to 4·7%)	69·4 (28·7 to 119·0)	3·2% (1·3 to 5·4%)	40·6% (22·2 to 61·8%)	10·8% (-2·7 to 27·2%)	1,284·3 (553·1 to 2,117·5)	3·4% (1·5 to 5·6%)	1,853·5 (786·6 to 3,146·2)	4·1% (1·8 to 7·0%)	44·3% (23·5 to 70·5%)	19·8% (3·4 to 39·8%)
**Diet high in trans fatty acids: All causes**	87·1 (34·3 to 164·7)	0·7% (0·3 to 1·3%)	108·2 (42·8 to 205·4)	0·8% (0·3 to 1·5%)	24·2% (19·3 to 30·0%)	17·1% (13·2 to 22·2%)	2,499·5 (1,012·2 to 4,607·3)	0·4% (0·2 to 0·8%)	3,000·9 (1,230·0 to 5,536·1)	0·5% (0·2 to 0·9%)	20·1% (14·6 to 26·3%)	21·9% (17·5 to 27·8%)
Ischaemic heart disease	87·1 (34·3 to 164·7)	5·0% (2·0 to 9·5%)	108·2 (42·8 to 205·4)	4·9% (1·9 to 9·3%)	24·2% (19·3 to 30·0%)	-2·1% (-4·9 to 1·7%)	2,499·5 (1,012·2 to 4,607·3)	6·7% (2·7 to 12·3%)	3,000·9 (1,230·0 to 5,536·1)	6·7% (2·7 to 12·3%)	20·1% (14·6 to 26·3%)	-0·2% (-2·9 to 4·0%)
**Diet high in sodium: All causes**	1,350·3 (890·8 to 1,931·3)	10·3% (6·8 to 14·6%)	1,559·0 (1,028·9 to 2,241·4)	11·2% (7·4 to 16·0%)	15·5% (8·2 to 22·5%)	8·8% (3·5 to 13·7%)	29,466·7 (19,303·4 to 41,814·5)	4·8% (3·2 to 7·0%)	32,302·7 (21,233·5 to 46,257·4)	5·4% (3·5 to 7·8%)	9·6% (2·3 to 16·5%)	11·3% (4·8 to 17·1%)
Stomach cancer	165·0 (115·3 to 209·4)	63·7% (45·4 to 80·1%)	155·6 (104·6 to 198·7)	62·3% (42·5 to 79·2%)	-5·7% (-14·6 to 3·0%)	-2·2% (-7·7 to 2·1%)	3,938·7 (2,785·3 to 4,958·7)	63·2% (45·6 to 78·6%)	3,506·2 (2,418·2 to 4,477·2)	61·7% (42·6 to 77·9%)	-11·0% (-20·5 to -1·9%)	-2·4% (-9·3 to 2·6%)
Rheumatic heart disease	14·0 (7·4 to 24·7)	12·3% (6·4 to 21·6%)	12·3 (6·3 to 21·5)	12·1% (6·2 to 21·7%)	-12·1% (-20·4 to -3·9%)	-1·4% (-8·8 to 6·2%)	401·5 (211·2 to 707·3)	9·9% (5·2 to 17·0%)	332·5 (169·1 to 577·1)	9·8% (5·1 to 17·1%)	-17·2% (-24·8 to -10·0%)	-1·1% (-8·5 to 6·2%)
Ischaemic heart disease	417·4 (239·1 to 642·2)	24·1% (13·9 to 37·0%)	544·2 (319·2 to 830·0)	24·8% (14·6 to 37·7%)	30·4% (22·7 to 38·7%)	2·8% (-1·2 to 7·6%)	8,770·3 (5,090·3 to 13,640·1)	23·4% (13·7 to 36·2%)	10,856·3 (6,469·1 to 16,562·4)	24·1% (14·3 to 36·9%)	23·8% (15·7 to 31·8%)	2·8% (-1·4 to 7·7%)
Ischaemic stroke	167·6 (99·9 to 254·2)	25·5% (15·3 to 38·4%)	196·0 (117·5 to 293·9)	25·3% (15·2 to 38·0%)	16·9% (7·5 to 27·2%)	-0·9% (-6·3 to 5·2%)	3,170·6 (1,927·6 to 4,719·5)	27·3% (16·6 to 40·3%)	3,572·4 (2,207·6 to 5,312·3)	27·5% (16·9 to 40·4%)	12·7% (4·4 to 21·7%)	0·6% (-4·3 to 6·0%)
Hemorrhagic stroke	410·7 (253·3 to 595·2)	32·6% (20·1 to 47·4%)	412·7 (255·7 to 601·5)	31·8% (19·7 to 46·3%)	0·5% (-7·4 to 8·5%)	-2·5% (-7·9 to 2·7%)	9,108·9 (5,725·2 to 13,100·3)	31·6% (19·8 to 45·4%)	8,891·0 (5,521·6 to 13,006·1)	31·1% (19·2 to 44·9%)	-2·4% (-10·0 to 5·2%)	-1·4% (-7·0 to 3·6%)
Hypertensive heart disease	97·7 (39·2 to 172·4)	42·0% (16·9 to 73·7%)	134·8 (51·1 to 238·2)	44·1% (17·2 to 75·8%)	37·9% (18·3 to 53·6%)	4·9% (-2·1 to 13·1%)	1,907·5 (943·6 to 3,186·2)	40·8% (20·0 to 67·1%)	2,402·9 (1,162·1 to 4,016·8)	42·5% (20·7 to 70·1%)	26·0% (11·8 to 37·8%)	4·2% (-1·0 to 10·2%)
Cardiomyopathy and myocarditis	6·4 (3·5 to 10·5)	11·8% (6·5 to 19·1%)	8·4 (4·6 to 13·7)	12·6% (6·9 to 20·3%)	31·2% (20·9 to 41·7%)	6·9% (0·8 to 13·4%)	174·0 (91·4 to 283·6)	8·9% (4·7 to 14·6%)	214·4 (113·7 to 345·9)	10·1% (5·4 to 16·3%)	23·2% (12·8 to 34·2%)	13·4% (5·7 to 21·5%)
Atrial fibrillation and flutter	3·8 (2·0 to 6·2)	15·7% (9·3 to 23·9%)	5·5 (3·0 to 9·0)	15·9% (9·5 to 24·2%)	43·7% (35·6 to 53·1%)	1·3% (-2·6 to 6·2%)	132·8 (76·1 to 210·8)	20·7% (12·8 to 30·1%)	182·1 (105·4 to 284·2)	21·1% (13·4 to 30·4%)	37·1% (31·9 to 43·9%)	1·9% (-1·2 to 6·5%)
Aortic aneurysm	3·1 (1·7 to 4·9)	15·4% (8·7 to 24·2%)	4·1 (2·3 to 6·6)	15·1% (8·5 to 24·0%)	34·7% (23·4 to 44·4%)	-1·7% (-6·3 to 3·0%)	70·3 (39·4 to 110·6)	15·4% (8·8 to 24·1%)	92·2 (51·5 to 145·3)	15·5% (8·7 to 24·2%)	31·1% (19·2 to 41·6%)	0·0% (-5·0 to 4·8%)
Peripheral vascular disease	0·2 (0·1 to 0·3)	9·6% (4·9 to 16·5%)	0·3 (0·2 to 0·6)	10·0% (5·1 to 17·0%)	75·5% (59·6 to 94·6%)	3·3% (-2·7 to 11·0%)	17·3 (7·5 to 33·7)	12·9% (7·0 to 20·5%)	26·4 (11·8 to 50·3)	13·5% (7·5 to 21·0%)	52·4% (46·9 to 60·9%)	4·3% (1·1 to 9·5%)
Endocarditis	1·6 (0·8 to 2·7)	10·5% (5·5 to 17·7%)	2·1 (1·1 to 3·6)	11·5% (6·0 to 18·9%)	34·0% (24·4 to 44·8%)	9·0% (2·6 to 16·7%)	40·4 (19·9 to 70·0)	7·7% (3·8 to 13·2%)	51·7 (25·3 to 88·7)	8·9% (4·5 to 15·0%)	28·0% (18·3 to 38·8%)	15·8% (8·1 to 24·3%)
Other cardiovascular and circulatory diseases	14·6 (8·7 to 22·0)	16·8% (9·9 to 25·3%)	17·9 (10·5 to 26·9)	17·3% (10·2 to 26·0%)	23·1% (13·9 to 33·7%)	3·2% (-2·8 to 9·6%)	479·8 (280·0 to 728·1)	14·1% (8·5 to 21·4%)	600·0 (355·4 to 907·9)	16·0% (9·7 to 23·7%)	25·1% (17·3 to 32·9%)	12·8% (7·6 to 19·0%)
Chronic kidney disease due to diabetes mellitus	13·2 (6·3 to 22·3)	16·3% (7·8 to 27·2%)	19·3 (9·4 to 32·5)	16·5% (8·0 to 27·4%)	45·9% (36·4 to 55·3%)	1·4% (-3·6 to 5·9%)	364·8 (175·5 to 620·6)	14·2% (6·9 to 23·5%)	506·1 (245·8 to 868·5)	14·8% (7·1 to 24·3%)	38·7% (30·5 to 47·0%)	4·0% (-1·0 to 8·7%)
Chronic kidney disease due to hypertension	18·4 (8·4 to 32·1)	15·2% (7·0 to 26·5%)	26·9 (12·4 to 46·4)	16·5% (7·7 to 28·2%)	46·4% (35·7 to 57·9%)	8·7% (4·3 to 15·6%)	410·9 (192·7 to 721·7)	12·6% (6·0 to 22·1%)	560·8 (263·5 to 970·0)	13·9% (6·7 to 24·0%)	36·5% (28·2 to 45·6%)	10·5% (5·8 to 16·4%)
Chronic kidney disease due to glomerulonephritis	15·6 (8·6 to 24·5)	21·2% (12·0 to 32·4%)	17·4 (9·7 to 27·4)	21·5% (12·3 to 33·3%)	11·5% (2·1 to 22·2%)	1·5% (-5·1 to 8·0%)	403·8 (219·1 to 639·9)	15·5% (8·6 to 24·3%)	410·4 (223·5 to 660·9)	15·8% (8·8 to 25·2%)	1·6% (-7·0 to 10·8%)	1·8% (-5·3 to 8·8%)
Chronic kidney disease due to other causes	0·9 (0·4 to 1·8)	10·7% (4·8 to 19·2%)	1·3 (0·5 to 2·4)	12·3% (5·5 to 21·8%)	35·7% (20·3 to 51·6%)	14·9% (7·8 to 23·1%)	75·0 (34·4 to 132·5)	10·2% (4·9 to 17·4%)	97·4 (45·5 to 170·3)	11·6% (5·8 to 19·6%)	30·0% (23·5 to 36·9%)	14·1% (8·8 to 20·6%)
**Sexual abuse and violence: All causes**	74·3 (48·4 to 100·7)	0·6% (0·4 to 0·8%)	62·6 (41·2 to 86·0)	0·4% (0·3 to 0·6%)	-15·9% (-24·2 to -6·3%)	-20·7% (-28·0 to -11·6%)	4,868·3 (3,558·1 to 6,361·5)	0·8% (0·6 to 1·0%)	4,491·8 (3,338·9 to 5,889·8)	0·7% (0·6 to 1·0%)	-7·7% (-15·0 to 0·1%)	-6·4% (-13·3 to 1·4%)
**Childhood sexual abuse: All causes**	28·9 (7·6 to 57·4)	0·2% (0·1 to 0·4%)	25·8 (6·6 to 51·8)	0·2% (0·0 to 0·4%)	-10·8% (-19·8 to -0·6%)	-15·9% (-24·0 to -6·6%)	1,914·4 (760·3 to 3,359·8)	0·3% (0·1 to 0·6%)	1,775·7 (712·7 to 3,127·6)	0·3% (0·1 to 0·5%)	-7·2% (-16·9 to 3·0%)	-5·8% (-14·4 to 4·4%)
Alcohol use disorders	0·9 (0·0 to 2·1)	3·4% (0·0 to 7·9%)	0·9 (0·0 to 2·0)	3·2% (0·0 to 7·3%)	-4·0% (-11·8 to 5·2%)	-8·1% (-12·4 to 0·0%)	96·7 (0·0 to 223·6)	4·1% (0·0 to 9·3%)	97·7 (0·0 to 225·5)	3·9% (0·0 to 8·9%)	1·1% (-3·8 to 5·8%)	-5·7% (-8·5 to 0·0%)
Major depressive disorder	-	-	-	-	-	-	422·5 (47·0 to 935·6)	4·5% (0·5 to 9·3%)	463·8 (50·8 to 1,028·2)	4·2% (0·5 to 8·9%)	9·8% (6·9 to 12·4%)	-4·9% (-6·7 to -3·0%)
Dysthymia	-	-	-	-	-	-	98·1 (10·5 to 216·7)	4·1% (0·5 to 8·6%)	110·1 (11·9 to 244·5)	3·9% (0·5 to 8·2%)	12·2% (8·5 to 15·9%)	-5·4% (-7·3 to -2·7%)
Self-harm	28·0 (6·4 to 56·7)	12·6% (3·0 to 25·3%)	24·9 (5·8 to 51·1)	12·0% (2·9 to 24·2%)	-11·0% (-20·2 to -0·6%)	-4·2% (-7·8 to -1·0%)	1,297·2 (302·2 to 2,610·5)	13·5% (3·3 to 26·9%)	1,104·1 (262·2 to 2,249·4)	13·1% (3·2 to 26·1%)	-14·9% (-23·8 to -4·7%)	-3·3% (-6·7 to 0·0%)
**Intimate partner violence: All causes**	49·9 (34·3 to 60·7)	0·4% (0·3 to 0·5%)	40·8 (28·2 to 50·1)	0·3% (0·2 to 0·4%)	-18·4% (-27·7 to -6·8%)	-23·0% (-31·6 to -12·4%)	3,207·3 (2,525·5 to 3,931·9)	0·5% (0·4 to 0·6%)	2,947·5 (2,323·0 to 3,636·9)	0·5% (0·4 to 0·6%)	-8·1% (-16·4 to 1·2%)	-6·8% (-14·7 to 3·0%)

Supplemental Results Table 1. All-age deaths, death PAFs, DALYs, DALY PAFs, and percent change for each risk factor and outcome for both sexes combined in 2005 and 2015 for Middle SDI.

Data in parenthesis are 95% uncertainty intervals. PAFs=population attributable fractions. DALYs=disability-adjusted life-years.

	2005 Deaths (in thousands)	2005 Deaths PAF	2015 Deaths (in thousands)	2015 Deaths PAF	Percent change deaths 2005-2015	Percent change deaths PAF 2005-2015	2005 DALYs (in thousands)	2005 DALYs PAF	2015 DALYs (in thousands)	2015 DALYs PAF	Percent change DALYs 2005-2015	Percent change DALYs PAF 2005-2015
HIV/AIDS - Tuberculosis	4·0 (2·1 to 6·1)	6·1% (3·3 to 9·0%)	2·1 (1·1 to 3·2)	4·5% (2·4 to 6·8%)	-48·6% (-53·4 to -41·9%)	-25·8% (-34·1 to -17·7%)	187·4 (96·5 to 286·5)	5·4% (2·8 to 7·9%)	96·2 (48·3 to 147·1)	4·0% (2·1 to 6·0%)	-48·7% (-53·4 to -42·6%)	-25·4% (-32·4 to -17·7%)
HIV/AIDS resulting in other diseases	9·3 (4·8 to 14·0)	5·5% (2·8 to 8·1%)	6·0 (3·0 to 9·2)	4·0% (2·1 to 6·2%)	-34·9% (-42·4 to -25·3%)	-26·1% (-36·4 to -18·5%)	453·0 (232·7 to 684·6)	5·0% (2·6 to 7·5%)	298·5 (147·7 to 456·0)	3·8% (1·9 to 5·9%)	-34·1% (-41·6 to -24·9%)	-23·7% (-32·9 to -16·5%)
Maternal abortion, miscarriage, and ectopic pregnancy	0·0 (0·0 to 0·0)	0·0% (0·0 to 0·0%)	0·0 (0·0 to 0·0)	0·0% (0·0 to 0·0%)	-29·0% (-44·7 to -6·2%)	0·4% (-14·2 to 18·0%)	0·0 (0·0 to 0·0)	0·0% (0·0 to 0·0%)	0·0 (0·0 to 0·0)	0·0% (0·0 to 0·0%)	-28·9% (-44·3 to -5·9%)	0·4% (-13·9 to 18·7%)
Major depressive disorder	-	-	-	-	-	-	763·1 (484·4 to 1,097·6)	8·0% (6·5 to 9·6%)	955·3 (611·8 to 1,372·7)	8·7% (7·0 to 10·4%)	25·2% (21·7 to 28·7%)	8·5% (5·7 to 11·1%)
Dysthymia	-	-	-	-	-	-	182·8 (116·2 to 270·5)	7·7% (6·2 to 9·2%)	236·1 (149·0 to 350·0)	8·4% (6·8 to 10·0%)	29·1% (24·5 to 34·3%)	8·9% (5·4 to 12·3%)
Self-harm	29·6 (15·4 to 37·7)	13·2% (8·6 to 16·5%)	26·5 (15·2 to 34·7)	12·8% (8·5 to 16·2%)	-10·4% (-24·0 to 15·6%)	-3·5% (-15·4 to 12·6%)	1,232·0 (642·4 to 1,597·3)	12·8% (8·2 to 16·2%)	1,039·5 (612·3 to 1,379·9)	12·3% (8·1 to 15·9%)	-15·6% (-28·9 to 9·9%)	-4·0% (-16·3 to 13·4%)
Assault by firearm	1·5 (1·2 to 1·7)	3·3% (2·7 to 4·0%)	1·6 (1·3 to 2·0)	3·2% (2·6 to 4·0%)	7·5% (-4·7 to 22·4%)	-4·0% (-15·7 to 9·2%)	79·3 (61·5 to 92·9)	3·3% (2·7 to 4·0%)	82·5 (64·7 to 100·6)	3·1% (2·5 to 3·9%)	4·0% (-8·2 to 19·0%)	-4·8% (-16·9 to 9·2%)
Assault by sharp object	1·9 (1·7 to 2·3)	6·0% (5·2 to 7·0%)	1·5 (1·3 to 1·8)	5·3% (4·6 to 6·4%)	-22·9% (-32·7 to -11·9%)	-11·1% (-21·9 to 0·1%)	102·6 (88·5 to 121·1)	6·0% (5·2 to 6·9%)	75·4 (65·6 to 88·2)	5·2% (4·5 to 6·2%)	-26·5% (-36·1 to -15·8%)	-12·1% (-23·1 to -0·7%)
Assault by other means	3·6 (3·1 to 4·0)	10·8% (9·4 to 12·3%)	3·0 (2·5 to 3·4)	9·7% (8·5 to 11·2%)	-16·1% (-25·4 to -7·2%)	-9·8% (-17·6 to -1·5%)	207·0 (180·4 to 228·6)	11·4% (10·0 to 12·9%)	164·1 (137·5 to 184·6)	10·3% (9·1 to 11·7%)	-20·7% (-29·1 to -11·6%)	-9·7% (-17·3 to -1·7%)
**Unsafe sex: All causes**	290·8 (270·7 to 313·6)	2·2% (2·1 to 2·4%)	243·0 (222·7 to 287·8)	1·7% (1·6 to 2·1%)	-16·4% (-22·7 to -4·7%)	-21·2% (-27·2 to -10·2%)	14,988·2 (13,937·2 to 16,306·0)	2·5% (2·2 to 2·7%)	11,980·5 (10,958·8 to 14,183·8)	2·0% (1·8 to 2·4%)	-20·1% (-25·8 to -9·5%)	-18·8% (-24·7 to -7·9%)
HIV/AIDS - Tuberculosis	61·4 (48·5 to 69·2)	93·8% (92·4 to 95·0%)	41·7 (31·0 to 49·9)	91·8% (89·9 to 93·7%)	-32·0% (-40·5 to -21·4%)	-2·1% (-3·4 to -1·0%)	3,273·6 (2,597·7 to 3,677·8)	94·1% (92·7 to 95·1%)	2,216·0 (1,670·4 to 2,609·3)	92·4% (90·6 to 94·0%)	-32·3% (-39·9 to -23·2%)	-1·8% (-3·0 to -0·9%)
HIV/AIDS resulting in other diseases	157·1 (142·0 to 177·9)	92·8% (88·9 to 94·0%)	133·9 (118·7 to 171·8)	89·3% (85·3 to 91·1%)	-14·8% (-23·3 to 5·3%)	-3·8% (-5·2 to -2·8%)	8,429·4 (7,640·9 to 9,536·4)	93·2% (89·7 to 94·3%)	7,076·5 (6,297·7 to 8,974·2)	90·3% (86·7 to 91·9%)	-16·0% (-23·7 to 1·8%)	-3·1% (-4·3 to -2·2%)
Syphilis	13·2 (7·9 to 20·7)	100·0% (100·0 to 100·0%)	5·3 (3·5 to 8·2)	100·0% (100·0 to 100·0%)	-59·6% (-64·0 to -53·7%)	-	1,078·0 (630·3 to 1,716·0)	100·0% (100·0 to 100·0%)	432·0 (269·1 to 674·9)	100·0% (100·0 to 100·0%)	-59·9% (-64·4 to -54·1%)	-
Chlamydial infection	0·0 (0·0 to 0·0)	100·0% (100·0 to 100·0%)	0·0 (0·0 to 0·0)	100·0% (100·0 to 100·0%)	-13·6% (-26·4 to 3·3%)	-	129·3 (72·6 to 209·1)	100·0% (100·0 to 100·0%)	128·7 (72·2 to 206·9)	100·0% (100·0 to 100·0%)	-0·4% (-4·6 to 4·0%)	-
Gonococcal infection	0·2 (0·1 to 0·2)	100·0% (100·0 to 100·0%)	0·1 (0·1 to 0·1)	100·0% (100·0 to 100·0%)	-25·8% (-37·8 to -10·7%)	-	98·0 (58·4 to 151·5)	100·0% (100·0 to 100·0%)	114·4 (65·8 to 180·8)	100·0% (100·0 to 100·0%)	16·7% (11·0 to 22·5%)	-
Trichomoniasis	-	-	-	-	-	-	42·0 (16·8 to 89·5)	100·0% (100·0 to 100·0%)	46·5 (18·7 to 98·2)	100·0% (100·0 to 100·0%)	10·6% (8·7 to 12·7%)	-
Genital herpes	-	-	-	-	-	-	50·5 (15·7 to 119·5)	100·0% (100·0 to 100·0%)	58·3 (18·1 to 137·8)	100·0% (100·0 to 100·0%)	15·6% (12·0 to 18·7%)	-
Other sexually transmitted diseases	0·1 (0·0 to 0·1)	100·0% (100·0 to 100·0%)	0·1 (0·0 to 0·1)	100·0% (100·0 to 100·0%)	-27·8% (-38·5 to -13·5%)	-	26·6 (18·8 to 36·7)	100·0% (100·0 to 100·0%)	25·8 (17·8 to 36·4)	100·0% (100·0 to 100·0%)	-3·1% (-7·9 to 1·7%)	-
Cervical cancer	58·9 (54·3 to 63·2)	100·0% (100·0 to 100·0%)	61·9 (57·9 to 66·2)	100·0% (100·0 to 100·0%)	5·0% (-4·3 to 15·8%)	-	1,860·8 (1,700·9 to 2,006·0)	100·0% (100·0 to 100·0%)	1,882·2 (1,751·7 to 2,021·2)	100·0% (100·0 to 100·0%)	1·1% (-8·2 to 12·2%)	-
**Low physical activity: All causes**	328·2 (257·1 to 400·2)	2·5% (2·0 to 3·0%)	419·0 (332·4 to 510·3)	3·0% (2·4 to 3·7%)	27·7% (22·8 to 33·1%)	20·4% (17·5 to 23·5%)	8,164·5 (6,414·7 to 9,966·0)	1·3% (1·1 to 1·6%)	10,180·5 (8,090·8 to 12,375·8)	1·7% (1·4 to 2·0%)	24·7% (19·8 to 30·0%)	26·6% (22·9 to 30·6%)
Colon and rectum cancer	14·4 (9·8 to 19·4)	12·2% (8·3 to 16·2%)	19·6 (13·2 to 26·4)	12·5% (8·5 to 16·6%)	36·5% (29·6 to 43·8%)	2·6% (1·2 to 4·3%)	327·0 (220·5 to 442·0)	11·2% (7·5 to 15·0%)	429·7 (289·9 to 584·5)	11·6% (7·8 to 15·5%)	31·4% (24·3 to 39·2%)	3·5% (1·8 to 5·3%)
Breast cancer	6·0 (4·3 to 7·8)	7·4% (5·4 to 9·5%)	7·8 (5·5 to 10·2)	7·6% (5·5 to 9·8%)	30·1% (13·3 to 44·0%)	2·8% (1·0 to 4·8%)	189·9 (133·4 to 247·3)	7·0% (5·0 to 8·9%)	242·1 (170·1 to 319·6)	7·2% (5·1 to 9·2%)	27·5% (10·4 to 42·1%)	3·3% (1·4 to 5·4%)
Ischaemic heart disease	177·7 (120·0 to 239·7)	10·3% (6·9 to 13·8%)	225·2 (153·6 to 299·3)	10·2% (6·9 to 13·8%)	26·8% (21·6 to 32·3%)	0·0% (-1·5 to 1·6%)	4,112·7 (2,741·3 to 5,557·6)	11·0% (7·3 to 14·9%)	5,011·4 (3,346·1 to 6,657·0)	11·1% (7·4 to 15·0%)	21·9% (15·9 to 28·3%)	1·3% (-0·5 to 3·1%)
Ischaemic stroke	75·2 (42·6 to 110·5)	11·4% (6·5 to 16·6%)	89·1 (51·0 to 130·4)	11·5% (6·5 to 16·5%)	18·5% (12·4 to 25·7%)	0·5% (-0·9 to 2·4%)	1,408·3 (789·5 to 2,084·9)	12·1% (6·8 to 17·7%)	1,598·1 (899·9 to 2,368·1)	12·3% (6·8 to 17·8%)	13·5% (7·6 to 20·1%)	1·3% (-0·1 to 3·1%)
Diabetes mellitus	54·8 (42·4 to 68·1)	16·0% (12·3 to 19·9%)	77·2 (59·9 to 95·1)	16·7% (13·0 to 20·6%)	40·8% (32·2 to 49·3%)	4·1% (2·6 to 6·0%)	2,126·6 (1,554·4 to 2,815·6)	13·9% (10·6 to 17·4%)	2,899·3 (2,132·5 to 3,805·0)	14·6% (11·2 to 18·2%)	36·3% (30·6 to 41·9%)	5·1% (3·9 to 6·6%)
**Metabolic risks: All causes**	3,795·6 (3,591·0 to 3,993·1)	28·8% (27·5 to 30·2%)	4,715·2 (4,456·7 to 4,991·0)	33·8% (32·3 to 35·3%)	24·2% (19·7 to 29·0%)	17·1% (15·4 to 18·8%)	95,482·3 (89,892·1 to 101,989·4)	15·7% (14·7 to 16·6%)	114,340·1 (106,665·2 to 122,787·6)	19·0% (17·9 to 20·3%)	19·8% (15·4 to 24·5%)	21·6% (18·7 to 24·4%)
**High fasting plasma glucose: All causes**	1,165·5 (1,024·1 to 1,360·5)	8·9% (7·8 to 10·3%)	1,516·4 (1,333·8 to 1,778·5)	10·9% (9·6 to 12·6%)	30·1% (24·7 to 35·9%)	22·7% (19·6 to 25·8%)	35,063·0 (31,017·8 to 39,854·7)	5·7% (5·3 to 6·4%)	44,128·1 (38,753·3 to 50,159·9)	7·3% (6·7 to 8·1%)	25·9% (21·4 to 30·6%)	27·8% (24·6 to 31·0%)
Tuberculosis	47·4 (29·4 to 68·2)	11·4% (7·2 to 16·0%)	39·3 (23·7 to 58·0)	12·6% (8·1 to 17·6%)	-17·0% (-27·4 to -6·5%)	10·3% (6·7 to 14·0%)	1,522·2 (976·7 to 2,118·7)	10·5% (6·8 to 14·3%)	1,264·9 (793·5 to 1,812·5)	11·7% (7·7 to 16·1%)	-16·9% (-26·5 to -7·9%)	11·9% (7·4 to 16·5%)
Ischaemic heart disease	314·9 (214·6 to 440·2)	18·2% (12·4 to 25·4%)	435·3 (296·3 to 607·2)	19·8% (13·6 to 27·8%)	38·3% (31·9 to 44·5%)	9·0% (6·2 to 12·2%)	6,926·2 (4,863·7 to 9,419·6)	18·5% (13·0 to 24·9%)	9,212·2 (6,454·8 to 12,569·2)	20·5% (14·5 to 27·7%)	33·0% (27·1 to 39·2%)	10·5% (8·1 to 12·8%)
Ischaemic stroke	108·5 (62·6 to 185·4)	16·5% (9·4 to 28·6%)	137·6 (79·0 to 234·6)	17·7% (10·1 to 30·9%)	26·8% (19·5 to 35·3%)	7·6% (3·9 to 13·6%)	2,033·2 (1,262·2 to 3,226·0)	17·5% (10·7 to 27·5%)	2,496·2 (1,570·4 to 3,854·7)	19·2% (11·8 to 29·5%)	22·8% (16·1 to 29·8%)	9·7% (7·3 to 12·7%)
Hemorrhagic stroke	202·3 (138·4 to 281·2)	16·1% (11·0 to 22·0%)	228·2 (156·6 to 311·0)	17·6% (12·1 to 24·0%)	12·8% (6·7 to 20·2%)	9·6% (7·3 to 12·4%)	4,773·6 (3,271·8 to 6,677·3)	16·6% (11·3 to 23·2%)	5,319·9 (3,652·3 to 7,391·9)	18·6% (12·9 to 25·8%)	11·4% (5·3 to 18·1%)	12·6% (9·8 to 15·0%)
Diabetes mellitus	342·3 (328·1 to 356·9)	100·0% (100·0 to 100·0%)	462·7 (440·1 to 487·1)	100·0% (100·0 to 100·0%)	35·2% (27·4 to 43·0%)	-	15,306·4 (12,887·5 to 18,060·3)	100·0% (100·0 to 100·0%)	19,858·1 (16,639·1 to 23,414·4)	100·0% (100·0 to 100·0%)	29·7% (25·2 to 34·2%)	-
Chronic kidney disease due to diabetes mellitus	81·0 (73·4 to 87·9)	100·0% (100·0 to 100·0%)	116·6 (105·8 to 126·0)	100·0% (100·0 to 100·0%)	43·9% (37·2 to 50·4%)	-	2,566·9 (2,311·3 to 2,834·1)	100·0% (100·0 to 100·0%)	3,424·5 (3,080·5 to 3,749·9)	100·0% (100·0 to 100·0%)	33·4% (27·9 to 39·1%)	-
Chronic kidney disease due to hypertension	42·5 (29·6 to 55·2)	35·1% (24·7 to 45·2%)	62·7 (44·2 to 80·4)	38·5% (27·6 to 49·0%)	47·6% (39·7 to 55·3%)	9·7% (8·0 to 11·7%)	1,003·0 (698·9 to 1,310·7)	30·7% (21·6 to 39·7%)	1,409·2 (990·6 to 1,817·5)	35·0% (24·8 to 44·7%)	40·5% (33·6 to 47·9%)	13·7% (11·8 to 15·9%)
Chronic kidney disease due to glomerulonephritis	24·1 (16·8 to 31·8)	32·7% (22·8 to 42·2%)	30·1 (21·3 to 39·1)	37·3% (26·6 to 47·5%)	25·3% (18·2 to 33·4%)	14·0% (11·9 to 16·4%)	721·6 (500·9 to 966·0)	27·7% (19·3 to 36·0%)	852·1 (593·6 to 1,111·4)	32·7% (23·2 to 42·0%)	18·1% (11·8 to 25·0%)	18·3% (15·7 to 20·9%)
Chronic kidney disease due to other causes	2·2 (1·3 to 3·3)	25·2% (17·3 to 33·3%)	3·0 (1·9 to 4·4)	29·7% (20·7 to 38·8%)	39·2% (24·3 to 55·0%)	17·9% (12·5 to 24·5%)	174·4 (111·7 to 247·7)	23·7% (16·3 to 31·1%)	239·4 (155·0 to 336·2)	28·6% (20·0 to 37·0%)	37·3% (31·5 to 43·4%)	20·5% (16·8 to 25·2%)
**High total cholesterol: All causes**	818·7 (645·4 to 1,042·2)	6·2% (4·9 to 7·9%)	1,029·3 (812·2 to 1,307·6)	7·4% (5·8 to 9·4%)	25·7% (20·5 to 31·3%)	18·6% (15·0 to 21·9%)	20,015·6 (16,647·2 to 23,790·4)	3·3% (2·7 to 3·9%)	24,115·5 (20,251·4 to 28,681·5)	4·0% (3·4 to 4·8%)	20·5% (15·3 to 26·1%)	22·3% (18·4 to 26·3%)
Ischaemic heart disease	700·9 (552·5 to 858·5)	40·4% (32·2 to 49·4%)	890·7 (707·0 to 1,099·5)	40·5% (32·2 to 49·8%)	27·1% (21·5 to 32·8%)	0·2% (-2·3 to 2·3%)	17,499·1 (14,605·9 to 20,653·8)	46·8% (39·2 to 54·7%)	21,300·0 (17,901·7 to 25,100·9)	47·3% (39·9 to 55·4%)	21·7% (16·4 to 27·5%)	1·1% (0·0 to 2·2%)
Ischaemic stroke	117·7 (54·6 to 227·0)	17·9% (8·4 to 34·6%)	138·7 (61·5 to 269·1)	17·9% (8·0 to 34·7%)	17·8% (8·8 to 26·0%)	-0·1% (-5·6 to 4·4%)	2,516·5 (1,557·7 to 4,119·1)	21·7% (13·6 to 35·1%)	2,815·5 (1,730·8 to 4,596·7)	21·6% (13·5 to 35·3%)	11·9% (5·7 to 18·3%)	-0·1% (-2·9 to 2·1%)
**High systolic blood pressure: All causes**	2,439·2 (2,203·5 to 2,677·2)	18·5% (16·8 to 20·2%)	3,066·8 (2,759·2 to 3,367·2)	22·0% (19·9 to 23·9%)	25·7% (20·6 to 31·0%)	18·5% (16·1 to 21·0%)	54,181·0 (49,003·0 to 59,309·3)	8·9% (7·9 to 9·9%)	65,305·7 (59,390·3 to 71,660·6)	10·9% (9·6 to 12·1%)	20·5% (15·4 to 25·9%)	22·4% (18·6 to 25·9%)
Rheumatic heart disease	26·8 (18·5 to 39·6)	23·4% (16·0 to 35·6%)	26·9 (18·8 to 40·0)	26·4% (18·3 to 40·0%)	0·5% (-5·7 to 7·7%)	12·6% (8·9 to 17·6%)	833·1 (554·5 to 1,207·4)	20·6% (13·9 to 30·9%)	812·0 (557·9 to 1,178·9)	24·0% (16·6 to 35·7%)	-2·5% (-7·7 to 3·8%)	16·4% (13·1 to 20·5%)
Ischaemic heart disease	907·5 (752·0 to 1,049·0)	52·4% (43·8 to 60·6%)	1,211·9 (995·8 to 1,408·1)	55·1% (45·9 to 63·8%)	33·6% (27·7 to 39·4%)	5·3% (3·5 to 7·1%)	19,550·9 (16,621·2 to 22,226·1)	52·2% (44·8 to 59·1%)	25,074·9 (21,520·4 to 28,341·1)	55·7% (48·1 to 62·7%)	28·3% (22·2 to 34·0%)	6·6% (5·2 to 8·2%)
Ischaemic stroke	320·1 (250·9 to 386·9)	48·7% (38·5 to 58·2%)	400·1 (317·7 to 481·9)	51·6% (41·1 to 61·3%)	25·0% (17·6 to 33·2%)	5·9% (3·5 to 9·4%)	5,901·6 (4,726·1 to 6,981·3)	50·8% (40·8 to 59·3%)	7,099·1 (5,714·6 to 8,280·9)	54·6% (44·1 to 63·0%)	20·3% (13·9 to 27·1%)	7·4% (5·6 to 9·6%)

Supplemental Results Table 1. All-age deaths, death PAFs, DALYs, DALY PAFs, and percent change for each risk factor and outcome for both sexes combined in 2005 and 2015 for Middle SDI.

Data in parenthesis are 95% uncertainty intervals. PAFs=population attributable fractions. DALYs=disability-adjusted life-years.

	2005 Deaths (in thousands)	2005 Deaths PAF	2015 Deaths (in thousands)	2015 Deaths PAF	Percent change deaths 2005-2015	Percent change deaths PAF 2005-2015	2005 DALYs (in thousands)	2005 DALYs PAF	2015 DALYs (in thousands)	2015 DALYs PAF	Percent change DALYs 2005-2015	Percent change DALYs PAF 2005-2015
Hemorrhagic stroke	697·2 (562·5 to 823·4)	55·4% (45·2 to 65·4%)	773·8 (623·4 to 912·6)	59·7% (49·2 to 69·7%)	11·0% (4·7 to 18·4%)	7·7% (5·6 to 10·5%)	16,011·7 (13,072·2 to 18,529·8)	55·5% (45·6 to 64·2%)	17,334·0 (14,429·1 to 20,113·2)	60·7% (50·5 to 69·3%)	8·3% (2·1 to 15·4%)	9·4% (7·5 to 11·8%)
Hypertensive heart disease	232·7 (218·1 to 252·1)	100·0% (100·0 to 100·0%)	305·6 (265·6 to 323·5)	100·0% (100·0 to 100·0%)	31·3% (15·0 to 40·9%)	-	4,683·9 (4,409·5 to 5,066·3)	100·0% (100·0 to 100·0%)	5,655·2 (5,124·2 to 5,993·6)	100·0% (100·0 to 100·0%)	20·7% (9·8 to 28·5%)	-
Cardiomyopathy and myocarditis	16·6 (13·1 to 20·2)	30·5% (24·4 to 36·5%)	22·7 (17·8 to 27·6)	34·0% (27·4 to 40·8%)	37·0% (29·0 to 46·1%)	11·6% (8·5 to 14·9%)	457·9 (354·4 to 566·3)	23·5% (18·4 to 28·6%)	602·7 (469·1 to 738·3)	28·4% (22·6 to 33·8%)	31·6% (23·0 to 41·3%)	21·2% (15·8 to 26·5%)
Atrial fibrillation and flutter	8·3 (6·0 to 11·3)	34·0% (27·3 to 40·9%)	12·4 (9·0 to 16·8)	35·9% (28·8 to 43·3%)	49·7% (42·9 to 56·6%)	5·5% (3·3 to 7·6%)	256·2 (195·5 to 335·7)	39·9% (35·2 to 44·9%)	369·9 (284·3 to 483·7)	42·8% (38·0 to 48·0%)	44·4% (40·6 to 47·8%)	7·3% (5·8 to 9·0%)
Aortic aneurysm	7·1 (5·8 to 8·4)	35·7% (29·2 to 41·9%)	10·3 (8·3 to 12·3)	37·7% (31·0 to 44·2%)	44·9% (34·6 to 54·3%)	5·7% (3·9 to 7·8%)	163·3 (136·3 to 190·5)	35·9% (30·2 to 42·0%)	231·3 (190·4 to 271·1)	38·8% (33·0 to 44·9%)	41·6% (30·9 to 51·3%)	8·0% (6·5 to 9·9%)
Peripheral vascular disease	0·5 (0·4 to 0·7)	26·5% (20·1 to 33·5%)	0·9 (0·7 to 1·2)	27·3% (20·5 to 34·8%)	75·1% (59·7 to 91·5%)	3·0% (-0·2 to 6·4%)	38·0 (21·7 to 64·5)	28·4% (22·0 to 34·7%)	59·0 (34·7 to 98·9)	30·2% (23·6 to 36·8%)	55·3% (50·7 to 60·9%)	6·3% (4·7 to 8·2%)
Endocarditis	4·7 (3·6 to 6·1)	31·2% (24·9 to 37·5%)	6·4 (4·9 to 8·3)	34·6% (27·9 to 41·5%)	36·1% (27·3 to 46·3%)	10·8% (7·7 to 14·1%)	128·9 (96·0 to 168·7)	24·6% (18·9 to 30·3%)	170·5 (127·1 to 222·9)	29·4% (23·0 to 35·4%)	32·2% (21·9 to 43·7%)	19·7% (13·6 to 26·0%)
Other cardiovascular and circulatory diseases	32·5 (28·3 to 36·8)	37·5% (33·0 to 42·0%)	42·1 (36·7 to 47·5)	40·8% (36·0 to 45·4%)	29·7% (21·4 to 39·1%)	8·8% (6·4 to 11·0%)	1,050·7 (886·7 to 1,241·6)	31·0% (27·5 to 34·7%)	1,381·4 (1,167·6 to 1,636·0)	36·8% (33·0 to 40·8%)	31·5% (24·7 to 38·0%)	18·6% (15·5 to 21·7%)
Chronic kidney disease due to diabetes mellitus	34·6 (24·4 to 44·7)	42·7% (30·6 to 53·9%)	52·6 (37·2 to 67·7)	45·1% (32·7 to 56·6%)	52·2% (44·6 to 59·6%)	5·7% (4·4 to 7·3%)	925·4 (641·2 to 1,219·3)	36·0% (25·3 to 46·2%)	1,353·8 (952·2 to 1,771·5)	39·5% (28·0 to 50·1%)	46·3% (39·8 to 53·0%)	9·7% (8·1 to 11·6%)
Chronic kidney disease due to hypertension	120·9 (110·5 to 129·5)	100·0% (100·0 to 100·0%)	162·7 (145·0 to 173·1)	100·0% (100·0 to 100·0%)	34·6% (27·4 to 40·8%)	-	3,262·8 (2,944·0 to 3,474·0)	100·0% (100·0 to 100·0%)	4,031·0 (3,574·9 to 4,351·6)	100·0% (100·0 to 100·0%)	23·5% (17·8 to 29·8%)	-
Chronic kidney disease due to glomerulonephritis	26·7 (18·8 to 35·0)	36·3% (26·0 to 46·8%)	34·2 (24·3 to 44·0)	42·3% (30·7 to 53·5%)	27·8% (20·4 to 36·3%)	16·3% (13·4 to 19·4%)	725·9 (491·9 to 966·8)	27·9% (19·6 to 36·7%)	871·4 (612·4 to 1,146·2)	33·5% (23·8 to 43·5%)	20·0% (14·1 to 27·4%)	20·2% (16·9 to 23·8%)
Chronic kidney disease due to other causes	3·0 (1·8 to 4·4)	34·2% (23·6 to 44·0%)	4·0 (2·4 to 5·9)	39·0% (27·4 to 49·8%)	34·7% (19·4 to 51·3%)	14·0% (8·2 to 20·5%)	190·6 (122·0 to 268·3)	25·9% (17·6 to 33·5%)	259·5 (166·6 to 360·3)	30·9% (21·6 to 39·9%)	36·2% (29·6 to 42·7%)	19·6% (15·8 to 24·0%)
**High body-mass index: All causes**	751·8 (466·3 to 1,078·5)	5·7% (3·6 to 8·2%)	1,009·2 (659·6 to 1,399·2)	7·2% (4·7 to 10·1%)	34·2% (26·4 to 44·7%)	26·5% (20·6 to 35·5%)	24,615·3 (15,489·7 to 34,610·0)	4·0% (2·6 to 5·7%)	33,306·6 (22,397·4 to 45,150·8)	5·5% (3·7 to 7·5%)	35·3% (27·6 to 45·9%)	37·3% (30·6 to 47·8%)
Oesophageal cancer	20·6 (6·0 to 40·1)	11·6% (3·4 to 22·5%)	19·1 (5·4 to 37·2)	12·9% (3·8 to 25·0%)	-7·3% (-20·7 to 9·5%)	11·5% (-2·7 to 26·8%)	490·6 (141·0 to 951·4)	11·8% (3·4 to 22·9%)	448·2 (127·1 to 866·4)	13·6% (3·9 to 26·4%)	-8·6% (-22·9 to 9·3%)	14·7% (-0·9 to 31·5%)
Colon and rectum cancer	4·8 (2·4 to 7·8)	4·1% (2·0 to 6·6%)	7·6 (4·0 to 12·1)	4·8% (2·5 to 7·6%)	58·4% (48·5 to 71·9%)	19·1% (13·7 to 27·1%)	124·6 (63·2 to 201·6)	4·3% (2·2 to 6·9%)	194·9 (102·5 to 307·1)	5·2% (2·8 to 8·2%)	56·3% (45·7 to 71·0%)	23·1% (17·3 to 32·1%)
Liver cancer due to hepatitis B	8·5 (2·9 to 17·9)	7·7% (2·6 to 15·9%)	10·0 (3·5 to 20·0)	9·3% (3·4 to 18·5%)	17·0% (2·8 to 37·5%)	20·3% (12·0 to 32·9%)	276·6 (90·7 to 580·2)	7·7% (2·6 to 16·0%)	316·7 (113·0 to 636·8)	9·6% (3·5 to 18·9%)	14·5% (0·1 to 36·6%)	24·3% (15·2 to 38·7%)
Liver cancer due to hepatitis C	3·2 (1·3 to 5·8)	9·3% (3·9 to 16·5%)	4·4 (1·9 to 7·8)	11·2% (5·0 to 19·5%)	38·7% (26·1 to 54·0%)	20·6% (11·0 to 31·9%)	79·4 (33·2 to 146·1)	9·6% (4·0 to 17·2%)	108·9 (46·6 to 192·2)	11·8% (5·2 to 20·6%)	37·1% (23·8 to 53·6%)	23·4% (13·1 to 35·4%)
Liver cancer due to alcohol use	5·2 (1·7 to 11·2)	7·9% (2·7 to 16·5%)	7·9 (2·7 to 16·4)	9·2% (3·2 to 18·7%)	52·8% (35·5 to 76·5%)	16·5% (9·5 to 26·0%)	137·0 (44·6 to 295·6)	8·0% (2·7 to 16·9%)	209·1 (70·0 to 436·3)	9·6% (3·2 to 19·5%)	52·7% (34·4 to 78·6%)	19·7% (12·3 to 31·0%)
Liver cancer due to other causes	4·1 (1·5 to 7·9)	7·4% (2·8 to 13·9%)	4·8 (1·9 to 8·9)	8·8% (3·5 to 16·1%)	15·8% (3·2 to 33·1%)	19·2% (8·8 to 32·4%)	117·4 (42·6 to 227·1)	7·5% (2·8 to 14·2%)	132·4 (50·8 to 248·1)	9·2% (3·7 to 17·2%)	12·7% (-0·2 to 30·6%)	23·4% (12·4 to 37·7%)
Gallbladder and biliary tract cancer	2·4 (1·3 to 3·8)	11·3% (6·1 to 17·9%)	3·1 (1·7 to 4·9)	12·6% (7·1 to 19·6%)	30·8% (17·3 to 45·6%)	12·1% (3·9 to 21·0%)	56·7 (30·9 to 88·7)	11·7% (6·4 to 18·5%)	72·1 (40·1 to 110·9)	13·3% (7·5 to 20·3%)	27·3% (14·3 to 41·7%)	13·1% (4·6 to 22·4%)
Pancreatic cancer	1·6 (0·5 to 2·9)	3·2% (1·1 to 6·0%)	2·5 (0·8 to 4·6)	3·7% (1·2 to 6·9%)	59·5% (46·6 to 71·7%)	15·4% (7·0 to 23·0%)	38·0 (12·6 to 70·3)	3·3% (1·1 to 6·2%)	60·4 (20·0 to 111·3)	3·9% (1·3 to 7·2%)	58·8% (44·9 to 71·4%)	17·1% (7·6 to 25·7%)
Breast cancer	5·2 (2·3 to 9·5)	6·4% (2·8 to 11·7%)	7·7 (3·7 to 13·5)	7·5% (3·7 to 13·0%)	49·3% (21·9 to 84·8%)	17·8% (6·4 to 38·5%)	147·4 (62·2 to 278·2)	5·4% (2·3 to 10·1%)	220·4 (101·5 to 397·9)	6·5% (3·1 to 11·5%)	49·5% (20·0 to 91·8%)	20·9% (7·2 to 48·3%)
Uterine cancer	4·0 (2·2 to 6·0)	22·0% (12·4 to 33·3%)	4·8 (2·9 to 7·0)	26·1% (16·0 to 37·4%)	22·2% (9·2 to 39·1%)	18·4% (11·4 to 31·1%)	114·0 (63·5 to 174·0)	22·1% (12·4 to 33·5%)	138·3 (82·9 to 200·0)	26·6% (16·3 to 38·1%)	21·3% (7·8 to 39·6%)	20·3% (13·0 to 34·5%)
Ovarian cancer	0·4 (0·0 to 1·0)	1·8% (-0·1 to 4·4%)	0·6 (0·0 to 1·4)	2·1% (-0·1 to 5·0%)	52·2% (40·0 to 68·5%)	19·2% (12·9 to 30·2%)	12·2 (-0·4 to 29·9)	1·8% (-0·1 to 4·3%)	18·4 (-0·6 to 43·4)	2·2% (-0·1 to 5·1%)	51·1% (37·7 to 68·5%)	21·1% (14·5 to 33·0%)
Kidney cancer	1·3 (0·7 to 1·9)	9·8% (5·7 to 14·5%)	2·1 (1·3 to 3·0)	11·3% (6·8 to 16·5%)	64·5% (53·8 to 77·4%)	16·0% (9·6 to 23·4%)	34·6 (20·5 to 51·3)	8·8% (5·1 to 12·9%)	56·6 (34·7 to 82·5)	10·8% (6·5 to 15·7%)	63·6% (52·5 to 76·8%)	22·7% (15·8 to 31·0%)
Thyroid cancer	0·4 (0·2 to 0·6)	6·3% (3·2 to 10·2%)	0·6 (0·3 to 1·0)	7·4% (3·9 to 12·2%)	57·5% (36·1 to 75·6%)	18·6% (8·5 to 28·6%)	10·8 (5·6 to 17·9)	6·6% (3·4 to 10·7%)	17·2 (9·0 to 28·5)	7·9% (4·2 to 13·0%)	59·4% (36·1 to 78·2%)	20·9% (11·0 to 31·2%)
Ischaemic heart disease	210·8 (131·0 to 304·2)	12·2% (7·5 to 17·5%)	299·8 (193·2 to 425·3)	13·6% (8·8 to 19·1%)	42·2% (35·0 to 51·6%)	12·1% (7·2 to 18·8%)	5,657·5 (3,558·8 to 8,134·0)	15·1% (9·5 to 21·7%)	7,951·5 (5,140·2 to 11,149·8)	17·7% (11·7 to 24·5%)	40·5% (32·8 to 50·6%)	16·7% (11·7 to 24·2%)
Ischaemic stroke	57·1 (33·3 to 85·1)	8·7% (5·1 to 12·9%)	70·9 (42·6 to 104·2)	9·1% (5·5 to 13·5%)	24·2% (14·5 to 35·4%)	5·3% (-1·6 to 13·7%)	1,431·9 (867·6 to 2,101·1)	12·3% (7·5 to 18·0%)	1,783·9 (1,106·9 to 2,543·2)	13·7% (8·6 to 19·5%)	24·6% (15·7 to 35·2%)	11·2% (5·1 to 19·3%)
Hemorrhagic stroke	195·2 (114·5 to 287·3)	15·5% (9·2 to 22·8%)	225·3 (140·8 to 319·4)	17·4% (11·0 to 24·6%)	15·4% (6·7 to 27·5%)	12·1% (5·6 to 21·9%)	5,931·0 (3,561·9 to 8,483·3)	20·6% (12·4 to 29·5%)	6,885·1 (4,421·2 to 9,512·0)	24·1% (15·7 to 33·3%)	16·1% (7·3 to 28·2%)	17·3% (10·7 to 28·1%)
Hypertensive heart disease	57·3 (33·3 to 87·4)	24·6% (14·6 to 37·5%)	81·8 (46·8 to 126·1)	26·8% (15·8 to 40·9%)	42·7% (25·9 to 59·1%)	8·7% (-1·2 to 19·3%)	1,329·7 (829·4 to 1,897·1)	28·4% (17·8 to 40·6%)	1,801·2 (1,162·7 to 2,529·2)	31·8% (20·9 to 44·8%)	35·5% (23·3 to 48·2%)	12·2% (5·8 to 21·1%)
Diabetes mellitus	109·9 (75·8 to 146·9)	32·1% (22·2 to 43·1%)	165·4 (119·3 to 214·5)	35·8% (25·7 to 46·4%)	50·6% (39·4 to 63·8%)	11·4% (5·9 to 18·5%)	6,016·6 (4,075·0 to 8,200·3)	39·3% (27·8 to 51·1%)	8,997·4 (6,364·9 to 11,926·1)	45·3% (34·0 to 56·6%)	49·5% (41·2 to 60·7%)	15·3% (10·4 to 23·4%)
Chronic kidney disease due to diabetes mellitus	18·5 (9·0 to 30·1)	22·8% (11·3 to 36·4%)	30·8 (15·4 to 48·5)	26·4% (13·4 to 41·4%)	67·0% (56·2 to 81·1%)	16·0% (8·8 to 25·6%)	553·8 (246·0 to 921·8)	21·6% (9·6 to 35·7%)	898·1 (405·7 to 1,441·0)	26·2% (12·2 to 41·9%)	62·2% (52·1 to 75·8%)	21·5% (15·2 to 31·2%)
Chronic kidney disease due to hypertension	26·8 (13·5 to 41·9)	22·2% (11·1 to 34·4%)	40·3 (20·3 to 62·4)	24·8% (12·5 to 38·4%)	50·2% (37·4 to 62·7%)	11·6% (3·2 to 21·0%)	711·4 (333·3 to 1,141·0)	21·8% (10·3 to 34·5%)	1,039·1 (505·7 to 1,636·3)	25·8% (12·6 to 40·2%)	46·1% (36·8 to 57·0%)	18·2% (12·9 to 25·9%)
Chronic kidney disease due to glomerulonephritis	11·6 (4·6 to 20·4)	15·7% (6·3 to 27·6%)	14·8 (6·2 to 25·6)	18·4% (7·9 to 31·2%)	28·4% (12·7 to 48·3%)	16·8% (3·9 to 33·7%)	374·9 (125·0 to 700·1)	14·4% (4·9 to 26·8%)	476·0 (170·9 to 842·0)	18·3% (6·6 to 32·0%)	27·0% (15·8 to 45·0%)	27·1% (17·8 to 43·9%)
Chronic kidney disease due to other causes	0·8 (0·2 to 1·7)	9·5% (3·1 to 17·9%)	1·4 (0·4 to 2·7)	13·2% (4·3 to 24·2%)	64·4% (34·4 to 95·5%)	39·0% (15·9 to 62·2%)	97·0 (34·5 to 180·0)	13·1% (5·1 to 22·9%)	147·6 (54·7 to 263·1)	17·6% (7·3 to 29·6%)	52·2% (42·4 to 66·4%)	33·5% (25·5 to 46·5%)
Osteoarthritis	-	-	-	-	-	-	358·6 (187·7 to 611·4)	13·3% (8·0 to 19·8%)	579·2 (317·6 to 967·1)	15·5% (9·8 to 22·4%)	61·5% (55·3 to 71·0%)	16·4% (12·0 to 23·2%)
Low back pain	-	-	-	-	-	-	435·5 (226·3 to 733·0)	3·4% (2·0 to 5·1%)	643·4 (350·6 to 1,050·7)	4·3% (2·7 to 6·2%)	47·7% (42·0 to 57·4%)	26·2% (21·2 to 34·3%)
**Low bone mineral density: All causes**	81·0 (64·7 to 87·8)	0·6% (0·5 to 0·7%)	105·4 (80·3 to 116·1)	0·8% (0·6 to 0·8%)	30·1% (19·4 to 41·4%)	22·6% (13·3 to 32·2%)	1,886·2 (1,640·5 to 2,103·9)	0·3% (0·3 to 0·3%)	2,379·9 (2,011·6 to 2,681·8)	0·4% (0·3 to 0·4%)	26·2% (19·6 to 32·5%)	28·1% (22·1 to 33·6%)
Pedestrian road injuries	15·6 (14·3 to 17·4)	7·4% (7·0 to 7·8%)	18·3 (16·5 to 20·5)	9·1% (8·5 to 9·7%)	16·9% (6·1 to 26·5%)	23·0% (18·2 to 27·9%)	355·2 (317·9 to 396·7)	3·7% (3·4 to 3·9%)	403·7 (354·4 to 454·8)	4·8% (4·4 to 5·1%)	13·7% (3·3 to 23·1%)	30·2% (24·9 to 35·8%)
Cyclist road injuries	1·5 (1·3 to 1·8)	6·2% (5·7 to 6·8%)	1·7 (1·4 to 1·9)	7·6% (6·9 to 8·4%)	10·4% (-3·6 to 27·2%)	22·7% (13·6 to 32·2%)	68·0 (56·3 to 81·3)	5·0% (4·4 to 5·7%)	84·7 (68·3 to 103·6)	7·0% (6·0 to 8·1%)	24·6% (14·3 to 34·5%)	39·4% (28·7 to 49·5%)
Motorcyclist road injuries	4·9 (4·2 to 5·4)	4·8% (4·3 to 5·2%)	6·5 (5·5 to 7·5)	5·9% (5·3 to 6·6%)	33·7% (17·5 to 54·9%)	25·1% (15·3 to 35·6%)	168·2 (144·0 to 193·0)	3·1% (2·7 to 3·5%)	227·9 (191·4 to 263·1)	4·1% (3·5 to 4·7%)	35·5% (23·6 to 50·3%)	32·4% (21·9 to 41·6%)
Motor vehicle road injuries	7·6 (6·6 to 8·6)	6·2% (5·7 to 6·7%)	9·4 (7·8 to 10·6)	7·8% (7·1 to 8·4%)	23·1% (11·5 to 36·7%)	25·4% (19·1 to 31·2%)	247·8 (212·8 to 284·1)	3·8% (3·4 to 4·3%)	308·7 (256·9 to 360·0)	5·1% (4·5 to 5·7%)	24·6% (15·6 to 34·8%)	32·5% (26·7 to 37·5%)
Other road injuries	0·5 (0·4 to 0·6)	7·6% (6·6 to 9·8%)	0·6 (0·5 to 0·8)	9·0% (7·6 to 11·6%)	21·7% (-0·2 to 48·1%)	18·1% (0·1 to 36·5%)	17·2 (13·6 to 21·4)	4·8% (3·9 to 6·1%)	23·8 (18·4 to 29·9)	6·5% (5·1 to 8·3%)	38·7% (23·1 to 51·9%)	34·9% (16·4 to 54·1%)

Supplemental Results Table 1. All-age deaths, death PAFs, DALYs, DALY PAFs, and percent change for each risk factor and outcome for both sexes combined in 2005 and 2015 for Middle SDI.

Data in parenthesis are 95% uncertainty intervals. PAFs=population attributable fractions. DALYs=disability-adjusted life-years.

	2005 Deaths (in thousands)	2005 Deaths PAF	2015 Deaths (in thousands)	2015 Deaths PAF	Percent change deaths 2005-2015	Percent change deaths PAF 2005-2015	2005 DALYs (in thousands)	2005 DALYs PAF	2015 DALYs (in thousands)	2015 DALYs PAF	Percent change DALYs 2005-2015	Percent change DALYs PAF 2005-2015
Other transport injuries	2·8<br>(2·4 to 3·5)	8·5%<br>(7·9 to 9·2%)	3·5<br>(2·8 to 4·7)	9·9%<br>(9·3 to 10·7%)	25·0%<br>(9·1 to 42·7%)	16·7%<br>(10·7 to 22·9%)	71·3<br>(61·1 to 86·6)	4·3%<br>(3·9 to 4·7%)	85·0<br>(68·7 to 113·0)	5·2%<br>(4·7 to 5·7%)	19·2%<br>(5·5 to 35·0%)	19·6%<br>(13·0 to 26·4%)
Falls	44·0<br>(28·3 to 49·8)	35·5%<br>(29·7 to 38·1%)	60·4<br>(37·6 to 69·7)	39·5%<br>(34·3 to 43·6%)	37·3%<br>(21·8 to 56·0%)	11·4%<br>(2·7 to 20·5%)	821·2<br>(629·1 to 936·7)	14·1%<br>(12·5 to 14·9%)	1,088·0<br>(826·0 to 1,261·1)	16·4%<br>(14·8 to 17·8%)	32·5%<br>(24·0 to 41·5%)	16·3%<br>(9·3 to 23·7%)
Other exposure to mechanical forces	2·6<br>(1·7 to 2·9)	6·0%<br>(4·9 to 6·7%)	3·3<br>(1·9 to 3·9)	7·8%<br>(6·3 to 9·0%)	28·0%<br>(2·4 to 41·2%)	30·6%<br>(20·5 to 40·3%)	85·2<br>(66·6 to 103·1)	3·2%<br>(2·8 to 3·5%)	101·3<br>(72·0 to 124·6)	4·1%<br>(3·7 to 4·6%)	18·9%<br>(4·5 to 27·6%)	30·2%<br>(23·6 to 36·3%)
Non-venomous animal contact	0·2<br>(0·1 to 0·2)	5·5%<br>(4·8 to 6·0%)	0·2<br>(0·1 to 0·2)	6·7%<br>(5·8 to 7·3%)	-6·4%<br>(-17·9 to 12·9%)	21·3%<br>(13·2 to 28·9%)	5·7<br>(4·1 to 6·6)	2·4%<br>(2·1 to 2·6%)	5·2<br>(3·9 to 6·2)	2·9%<br>(2·5 to 3·2%)	-8·9%<br>(-17·7 to 4·4%)	20·9%<br>(13·6 to 28·3%)
Assault by other means	1·3<br>(1·0 to 1·4)	3·7%<br>(3·3 to 4·0%)	1·5<br>(1·2 to 1·7)	4·7%<br>(4·2 to 5·1%)	18·3%<br>(10·9 to 27·7%)	27·1%<br>(19·8 to 34·9%)	36·2<br>(30·1 to 41·7)	2·0%<br>(1·7 to 2·2%)	40·8<br>(33·5 to 47·2)	2·5%<br>(2·2 to 2·9%)	12·6%<br>(7·5 to 19·2%)	28·3%<br>(21·9 to 35·0%)
Exposure to forces of nature	-	-	-	-	-	-	10·1<br>(4·0 to 17·6)	1·7%<br>(0·8 to 2·9%)	10·7<br>(5·1 to 18·5)	4·6%<br>(3·1 to 6·2%)	6·3%<br>(-11·9 to 39·9%)	162·5%<br>(79·4 to 387·4%)
**Low glomerular filtration rate: All causes**	529·9<br>(488·0 to 575·6)	4·0%<br>(3·7 to 4·4%)	665·3<br>(614·9 to 716·6)	4·8%<br>(4·4 to 5·1%)	25·5%<br>(21·1 to 30·5%)	18·4%<br>(15·3 to 21·2%)	13,755·0<br>(12,725·9 to 14,801·9)	2·3%<br>(2·1 to 2·4%)	16,164·3<br>(14,951·2 to 17,303·1)	2·7%<br>(2·4 to 2·9%)	17·5%<br>(13·4 to 22·0%)	19·3%<br>(16·3 to 22·5%)
Ischaemic heart disease	118·4<br>(100·9 to 137·2)	6·8%<br>(5·8 to 7·9%)	152·6<br>(129·2 to 178·0)	6·9%<br>(5·9 to 8·1%)	28·9%<br>(24·5 to 33·3%)	1·7%<br>(-0·2 to 3·7%)	2,212·7<br>(1,884·0 to 2,571·3)	5·9%<br>(5·0 to 6·8%)	2,729·5<br>(2,305·5 to 3,179·2)	6·1%<br>(5·2 to 7·0%)	23·4%<br>(18·8 to 27·9%)	2·5%<br>(0·6 to 4·7%)
Ischaemic stroke	48·6<br>(38·4 to 60·9)	7·4%<br>(5·9 to 9·2%)	58·6<br>(46·2 to 72·7)	7·6%<br>(6·0 to 9·4%)	20·6%<br>(14·6 to 27·8%)	2·2%<br>(-0·2 to 5·4%)	775·6<br>(616·8 to 946·5)	6·7%<br>(5·3 to 8·2%)	886·6<br>(706·1 to 1,078·2)	6·8%<br>(5·5 to 8·3%)	14·3%<br>(8·8 to 20·3%)	2·1%<br>(-0·2 to 5·1%)
Hemorrhagic stroke	78·5<br>(59·8 to 99·7)	6·2%<br>(4·8 to 7·9%)	83·3<br>(65·1 to 103·8)	6·4%<br>(5·1 to 7·9%)	6·1%<br>(-0·2 to 13·9%)	3·0%<br>(-0·8 to 8·1%)	1,548·6<br>(1,187·4 to 1,915·6)	5·4%<br>(4·2 to 6·6%)	1,588·2<br>(1,248·0 to 1,928·4)	5·6%<br>(4·4 to 6·7%)	2·6%<br>(-3·8 to 9·8%)	3·7%<br>(-0·3 to 8·8%)
Peripheral vascular disease	0·3<br>(0·2 to 0·4)	14·0%<br>(11·0 to 17·0%)	0·5<br>(0·4 to 0·6)	13·6%<br>(10·6 to 16·7%)	65·2%<br>(49·8 to 81·2%)	-2·8%<br>(-7·0 to 1·1%)	17·4<br>(10·4 to 28·6)	13·0%<br>(10·5 to 15·7%)	25·0<br>(14·9 to 40·5)	12·9%<br>(10·3 to 15·6%)	44·2%<br>(39·3 to 50·3%)	-1·3%<br>(-3·2 to 0·6%)
Chronic kidney disease due to diabetes mellitus	81·0<br>(73·4 to 87·9)	100·0%<br>(100·0 to 100·0%)	116·6<br>(105·8 to 126·0)	100·0%<br>(100·0 to 100·0%)	43·9%<br>(37·2 to 50·4%)	-	2,566·9<br>(2,311·3 to 2,834·1)	100·0%<br>(100·0 to 100·0%)	3,424·5<br>(3,080·5 to 3,749·9)	100·0%<br>(100·0 to 100·0%)	33·4%<br>(27·9 to 39·1%)	-
Chronic kidney disease due to hypertension	120·9<br>(110·5 to 129·5)	100·0%<br>(100·0 to 100·0%)	162·7<br>(145·0 to 173·1)	100·0%<br>(100·0 to 100·0%)	34·6%<br>(27·4 to 40·8%)	-	3,262·8<br>(2,944·0 to 3,474·0)	100·0%<br>(100·0 to 100·0%)	4,031·0<br>(3,574·9 to 4,351·6)	100·0%<br>(100·0 to 100·0%)	23·5%<br>(17·8 to 29·8%)	-
Chronic kidney disease due to glomerulonephritis	73·6<br>(64·7 to 79·3)	100·0%<br>(100·0 to 100·0%)	80·8<br>(69·7 to 87·6)	100·0%<br>(100·0 to 100·0%)	9·9%<br>(4·0 to 16·1%)	-	2,605·2<br>(2,313·3 to 2,795·4)	100·0%<br>(100·0 to 100·0%)	2,601·5<br>(2,294·0 to 2,841·5)	100·0%<br>(100·0 to 100·0%)	-0·1%<br>(-4·9 to 4·8%)	-
Chronic kidney disease due to other causes	8·7<br>(6·0 to 11·6)	100·0%<br>(100·0 to 100·0%)	10·2<br>(7·1 to 13·7)	100·0%<br>(100·0 to 100·0%)	18·2%<br>(6·1 to 30·9%)	-	735·9<br>(586·0 to 905·8)	100·0%<br>(100·0 to 100·0%)	838·2<br>(655·7 to 1,044·1)	100·0%<br>(100·0 to 100·0%)	13·9%<br>(7·6 to 20·0%)	-
Gout	-	-	-	-	-	-	29·9<br>(19·8 to 41·6)	10·8%<br>(9·7 to 12·0%)	39·9<br>(26·6 to 56·1)	11·3%<br>(10·1 to 12·5%)	33·3%<br>(30·1 to 36·6%)	4·4%<br>(2·6 to 6·4%)

Supplemental Results Table 1. All-age deaths, death PAFs, DALYs, DALY PAFs, and percent change for each risk factor and outcome for both sexes combined in 2005 and 2015 for Low-middle SDI.

Data in parenthesis are 95% uncertainty intervals. PAFs=population attributable fractions. DALYs=disability-adjusted life-years.

	2005 Deaths (in thousands)	2005 Deaths PAF	2015 Deaths (in thousands)	2015 Deaths PAF	Percent change deaths 2005-2015	Percent change deaths PAF 2005-2015	2005 DALYs (in thousands)	2005 DALYs PAF	2015 DALYs (in thousands)	2015 DALYs PAF	Percent change DALYs 2005-2015	Percent change DALYs PAF 2005-2015
**All risk factors: All causes**	6,908·8<br>(6,664·0 to 7,146·6)	51·7%<br>(50·4 to 53·0%)	7,157·6<br>(6,829·5 to 7,495·5)	53·9%<br>(52·6 to 55·0%)	3·6%<br>(-0·8 to 8·2%)	4·2%<br>(2·5 to 5·9%)	326,955·7<br>(309,580·5 to 346,078·0)	40·8%<br>(39·6 to 42·2%)	291,552·0<br>(271,898·1 to 312,411·9)	39·7%<br>(38·4 to 41·0%)	-10·8%<br>(-14·4 to -7·3%)	-2·9%<br>(-5·4 to -0·3%)
**Environmental/occupational risks: All causes**	3,170·2<br>(2,911·6 to 3,404·0)	23·7%<br>(21·8 to 25·5%)	3,146·5<br>(2,872·3 to 3,419·6)	23·7%<br>(21·8 to 25·5%)	-0·7%<br>(-5·8 to 4·5%)	-0·2%<br>(-3·4 to 3·2%)	147,105·6<br>(134,563·2 to 158,251·8)	18·4%<br>(16·7 to 19·9%)	126,424·1<br>(116,430·9 to 136,818·3)	17·2%<br>(15·8 to 18·6%)	-14·1%<br>(-19·0 to -8·4%)	-6·4%<br>(-10·9 to -1·1%)
**Unsafe water, sanitation, and handwashing: All causes**	1,161·9<br>(1,056·6 to 1,276·1)	8·7%<br>(7·9 to 9·5%)	925·2<br>(829·9 to 1,024·3)	7·0%<br>(6·3 to 7·7%)	-20·4%<br>(-26·9 to -13·8%)	-19·9%<br>(-25·8 to -14·1%)	70,202·4<br>(62,587·3 to 78,610·4)	8·8%<br>(7·8 to 9·9%)	51,639·9<br>(45,942·6 to 57,689·7)	7·0%<br>(6·2 to 7·9%)	-26·4%<br>(-33·2 to -19·0%)	-19·9%<br>(-27·0 to -12·1%)
**Unsafe water source: All causes**	898·0<br>(731·3 to 1,031·2)	6·7%<br>(5·5 to 7·7%)	710·0<br>(574·9 to 819·1)	5·3%<br>(4·3 to 6·2%)	-20·9%<br>(-27·9 to -13·9%)	-20·5%<br>(-27·1 to -14·0%)	54,211·6<br>(43,555·1 to 62,944·7)	6·8%<br>(5·4 to 8·0%)	40,195·2<br>(32,552·4 to 46,919·7)	5·5%<br>(4·4 to 6·4%)	-25·9%<br>(-33·5 to -17·4%)	-19·2%<br>(-27·5 to -10·6%)
Diarrhoeal diseases	797·3<br>(656·5 to 909·4)	86·7%<br>(70·9 to 95·0%)	623·6<br>(501·9 to 721·0)	86·2%<br>(70·2 to 94·7%)	-21·8%<br>(-29·1 to -14·1%)	-0·6%<br>(-1·0 to -0·3%)	46,842·8<br>(37,778·7 to 54,009·9)	86·8%<br>(70·9 to 95·0%)	33,981·5<br>(27,536·0 to 39,264·8)	86·3%<br>(70·4 to 94·7%)	-27·5%<br>(-35·6 to -18·4%)	-0·5%<br>(-0·9 to -0·2%)
Typhoid fever	84·1<br>(44·5 to 140·4)	86·5%<br>(70·8 to 94·8%)	71·9<br>(39·0 to 121·1)	86·0%<br>(70·1 to 94·5%)	-14·6%<br>(-22·0 to -7·3%)	-0·6%<br>(-1·2 to -0·3%)	6,184·7<br>(3,330·5 to 10,286·6)	86·5%<br>(70·8 to 94·8%)	5,192·6<br>(2,845·9 to 8,695·8)	86·0%<br>(70·1 to 94·5%)	-16·0%<br>(-23·7 to -8·1%)	-0·6%<br>(-1·2 to -0·3%)
Paratyphoid fever	16·6<br>(7·3 to 33·0)	86·5%<br>(70·7 to 94·8%)	14·5<br>(6·4 to 28·6)	86·0%<br>(70·0 to 94·5%)	-12·4%<br>(-20·9 to -3·2%)	-0·6%<br>(-1·1 to -0·3%)	1,184·1<br>(526·4 to 2,335·5)	86·5%<br>(70·7 to 94·8%)	1,021·2<br>(460·6 to 2,011·9)	86·0%<br>(70·0 to 94·5%)	-13·8%<br>(-22·9 to -3·2%)	-0·6%<br>(-1·1 to -0·3%)
**Unsafe sanitation: All causes**	657·8<br>(592·8 to 732·9)	4·9%<br>(4·5 to 5·5%)	476·3<br>(421·0 to 535·6)	3·6%<br>(3·2 to 4·0%)	-27·6%<br>(-34·2 to -20·7%)	-27·2%<br>(-33·8 to -20·7%)	39,413·7<br>(34,863·9 to 44,561·7)	4·9%<br>(4·3 to 5·6%)	26,560·5<br>(23,152·8 to 30,243·0)	3·6%<br>(3·1 to 4·1%)	-32·6%<br>(-39·8 to -24·6%)	-26·6%<br>(-34·4 to -18·6%)
Diarrhoeal diseases	584·6<br>(529·6 to 643·2)	63·6%<br>(59·6 to 67·7%)	420·2<br>(373·0 to 471·6)	58·1%<br>(53·7 to 62·5%)	-28·1%<br>(-35·3 to -20·6%)	-8·6%<br>(-11·6 to -6·4%)	34,062·1<br>(30,423·6 to 38,003·5)	63·1%<br>(59·2 to 67·1%)	22,528·2<br>(19,796·4 to 25,378·0)	57·2%<br>(53·0 to 61·5%)	-33·9%<br>(-41·5 to -25·2%)	-9·3%<br>(-12·4 to -6·9%)
Typhoid fever	61·1<br>(33·0 to 101·9)	62·9%<br>(58·2 to 66·8%)	46·7<br>(25·3 to 79·3)	55·9%<br>(50·9 to 60·3%)	-23·6%<br>(-30·6 to -16·2%)	-11·1%<br>(-14·6 to -8·2%)	4,493·2<br>(2,448·7 to 7,488·1)	62·8%<br>(58·2 to 66·8%)	3,371·5<br>(1,852·7 to 5,698·2)	55·8%<br>(50·9 to 60·2%)	-25·0%<br>(-32·3 to -17·1%)	-11·2%<br>(-14·8 to -8·3%)
Paratyphoid fever	12·0<br>(5·5 to 23·1)	62·7%<br>(58·3 to 66·7%)	9·4<br>(4·4 to 18·3)	55·6%<br>(50·8 to 60·2%)	-21·8%<br>(-30·0 to -13·3%)	-11·2%<br>(-14·8 to -8·0%)	858·4<br>(401·8 to 1,630·8)	62·7%<br>(58·3 to 66·7%)	660·9<br>(309·6 to 1,288·1)	55·6%<br>(50·8 to 60·2%)	-23·0%<br>(-31·8 to -13·8%)	-11·3%<br>(-14·9 to -8·1%)
**No handwashing with soap: All causes**	564·0<br>(471·2 to 655·1)	4·2%<br>(3·5 to 4·9%)	452·1<br>(373·6 to 525·1)	3·4%<br>(2·8 to 3·9%)	-19·8%<br>(-25·5 to -13·7%)	-19·4%<br>(-24·7 to -13·9%)	34,125·2<br>(28,464·6 to 39,765·7)	4·3%<br>(3·5 to 5·0%)	24,895·2<br>(20,505·0 to 28,861·9)	3·4%<br>(2·8 to 4·0%)	-27·0%<br>(-33·1 to -20·1%)	-20·5%<br>(-27·0 to -13·4%)
Diarrhoeal diseases	361·6<br>(288·7 to 432·1)	39·3%<br>(31·9 to 46·3%)	282·2<br>(224·4 to 339·5)	39·0%<br>(31·6 to 46·0%)	-22·0%<br>(-29·3 to -14·3%)	-0·8%<br>(-1·3 to -0·4%)	21,227·4<br>(16,759·6 to 25,768·3)	39·3%<br>(31·9 to 46·3%)	15,358·2<br>(12,136·7 to 18,482·5)	39·0%<br>(31·6 to 46·0%)	-27·6%<br>(-35·8 to -18·6%)	-0·8%<br>(-1·3 to -0·4%)
Typhoid fever	38·1<br>(19·7 to 65·0)	39·2%<br>(31·1 to 46·0%)	32·5<br>(16·6 to 55·6)	38·8%<br>(30·7 to 45·6%)	-14·7%<br>(-22·2 to -7·4%)	-0·8%<br>(-1·4 to -0·4%)	2,801·2<br>(1,482·7 to 4,746·0)	39·2%<br>(31·1 to 46·0%)	2,347·9<br>(1,235·9 to 4,009·2)	38·8%<br>(30·7 to 45·6%)	-16·2%<br>(-24·0 to -8·1%)	-0·8%<br>(-1·4 to -0·4%)
Paratyphoid fever	7·5<br>(3·4 to 15·0)	39·0%<br>(30·9 to 46·2%)	6·6<br>(3·1 to 13·2)	38·7%<br>(30·6 to 45·8%)	-12·6%<br>(-21·0 to -3·4%)	-0·8%<br>(-1·4 to -0·4%)	534·7<br>(242·4 to 1,062·6)	39·0%<br>(30·9 to 46·2%)	460·3<br>(214·4 to 920·7)	38·7%<br>(30·6 to 45·8%)	-13·9%<br>(-23·0 to -3·3%)	-0·8%<br>(-1·4 to -0·4%)
**Air pollution: All causes**	1,909·5<br>(1,655·3 to 2,140·6)	14·3%<br>(12·4 to 16·0%)	2,068·3<br>(1,783·8 to 2,322·3)	15·6%<br>(13·5 to 17·4%)	8·3%<br>(2·6 to 14·4%)	8·9%<br>(5·2 to 12·9%)	69,405·1<br>(60,338·0 to 78,224·5)	8·7%<br>(7·5 to 10·0%)	63,124·6<br>(54,816·3 to 71,054·0)	8·6%<br>(7·4 to 9·8%)	-9·0%<br>(-14·3 to -3·3%)	-0·9%<br>(-6·0 to 4·5%)
**Ambient particulate matter pollution: All causes**	1,004·7<br>(874·9 to 1,136·1)	7·5%<br>(6·6 to 8·5%)	1,146·1<br>(999·1 to 1,292·4)	8·6%<br>(7·6 to 9·7%)	14·1%<br>(8·1 to 20·9%)	14·7%<br>(10·5 to 19·3%)	36,439·6<br>(31,151·7 to 41,752·5)	4·6%<br>(3·9 to 5·3%)	34,577·8<br>(30,113·1 to 39,023·1)	4·7%<br>(4·1 to 5·4%)	-5·1%<br>(-11·2 to 1·5%)	3·4%<br>(-2·8 to 9·8%)
Lower respiratory infections	330·0<br>(253·0 to 411·0)	32·8%<br>(25·4 to 39·5%)	272·8<br>(207·2 to 339·2)	32·1%<br>(24·9 to 39·0%)	-17·3%<br>(-23·7 to -10·9%)	-2·1%<br>(-4·7 to -0·2%)	20,249·2<br>(15,483·5 to 25,147·3)	33·0%<br>(25·6 to 39·8%)	14,000·2<br>(10,623·4 to 17,311·8)	32·1%<br>(24·8 to 38·9%)	-30·9%<br>(-36·8 to -24·5%)	-2·8%<br>(-5·5 to -0·7%)
Tracheal, bronchus, and lung cancer	23·5<br>(15·6 to 31·4)	24·2%<br>(16·3 to 31·9%)	36·0<br>(24·3 to 47·5)	25·4%<br>(17·4 to 33·3%)	53·0%<br>(40·8 to 65·6%)	5·3%<br>(3·2 to 8·0%)	595·0<br>(393·1 to 797·5)	24·0%<br>(16·2 to 31·7%)	864·9<br>(586·8 to 1,139·6)	25·1%<br>(17·1 to 32·9%)	45·4%<br>(33·8 to 57·6%)	4·8%<br>(2·8 to 7·6%)
Ischaemic heart disease	251·7<br>(215·2 to 292·6)	21·9%<br>(18·8 to 25·3%)	341·6<br>(288·6 to 402·6)	22·3%<br>(19·0 to 25·9%)	35·7%<br>(27·8 to 43·6%)	1·4%<br>(0·1 to 3·1%)	6,438·6<br>(5,494·7 to 7,385·3)	23·8%<br>(20·6 to 27·1%)	8,562·6<br>(7,259·3 to 9,957·7)	24·4%<br>(21·1 to 27·8%)	33·0%<br>(25·2 to 41·6%)	2·5%<br>(1·3 to 3·9%)
Ischaemic stroke	63·6<br>(49·8 to 77·4)	16·6%<br>(13·5 to 19·6%)	78·1<br>(61·8 to 94·8)	16·8%<br>(13·6 to 19·9%)	22·6%<br>(13·2 to 32·0%)	0·7%<br>(-1·9 to 3·2%)	1,300·2<br>(1,056·8 to 1,560·0)	17·9%<br>(15·0 to 20·9%)	1,557·1<br>(1,270·0 to 1,891·1)	18·2%<br>(15·4 to 21·2%)	19·8%<br>(10·2 to 28·9%)	1·8%<br>(0·0 to 3·8%)
Hemorrhagic stroke	95·7<br>(79·8 to 112·3)	18·4%<br>(15·6 to 21·2%)	114·1<br>(96·0 to 134·4)	18·9%<br>(16·0 to 21·6%)	19·3%<br>(11·6 to 28·3%)	2·4%<br>(0·6 to 4·3%)	2,688·4<br>(2,256·0 to 3,162·9)	18·9%<br>(16·1 to 21·8%)	3,185·4<br>(2,671·5 to 3,734·9)	19·8%<br>(16·9 to 22·8%)	18·5%<br>(10·7 to 27·0%)	4·8%<br>(3·0 to 7·2%)
Chronic obstructive pulmonary disease	240·2<br>(155·1 to 337·2)	31·7%<br>(20·6 to 44·4%)	303·6<br>(195·9 to 422·4)	33·5%<br>(22·2 to 46·6%)	26·4%<br>(15·2 to 39·1%)	5·4%<br>(3·4 to 9·1%)	5,168·2<br>(3,314·0 to 7,295·3)	29·2%<br>(18·9 to 40·9%)	6,407·6<br>(4,139·1 to 8,877·0)	31·4%<br>(20·8 to 43·8%)	24·0%<br>(13·2 to 36·2%)	7·6%<br>(4·9 to 11·8%)
**Household air pollution from solid fuels: All causes**	1,248·4<br>(972·9 to 1,529·0)	9·3%<br>(7·3 to 11·4%)	1,254·7<br>(963·5 to 1,555·8)	9·4%<br>(7·2 to 11·6%)	0·5%<br>(-6·1 to 7·0%)	1·1%<br>(-4·8 to 6·1%)	47,302·4<br>(36,635·0 to 58,040·5)	5·9%<br>(4·5 to 7·3%)	40,153·3<br>(31,284·0 to 49,667·7)	5·5%<br>(4·2 to 6·8%)	-15·1%<br>(-21·5 to -9·2%)	-7·5%<br>(-13·7 to -1·8%)
Lower respiratory infections	455·9<br>(332·3 to 583·2)	45·3%<br>(33·2 to 57·4%)	357·2<br>(253·4 to 470·7)	42·1%<br>(30·2 to 54·2%)	-21·6%<br>(-27·9 to -15·0%)	-7·2%<br>(-11·0 to -4·5%)	28,027·9<br>(20,357·1 to 35,879·4)	45·7%<br>(33·4 to 57·8%)	18,658·3<br>(13,364·8 to 24,224·8)	42·8%<br>(30·7 to 54·9%)	-33·4%<br>(-39·5 to -26·9%)	-6·4%<br>(-10·2 to -3·5%)
Tracheal, bronchus, and lung cancer	30·6<br>(18·0 to 43·5)	31·5%<br>(18·8 to 45·1%)	40·6<br>(23·5 to 59·3)	28·7%<br>(16·7 to 41·8%)	32·8%<br>(21·7 to 44·6%)	-8·6%<br>(-13·3 to -5·1%)	773·0<br>(455·4 to 1,098·8)	31·1%<br>(18·6 to 44·6%)	982·0<br>(565·2 to 1,428·2)	28·5%<br>(16·6 to 41·4%)	27·0%<br>(16·1 to 39·0%)	-8·4%<br>(-13·2 to -4·9%)
Ischaemic heart disease	274·1<br>(221·3 to 334·4)	23·9%<br>(19·4 to 29·0%)	326·3<br>(258·2 to 406·3)	21·3%<br>(17·0 to 26·0%)	19·1%<br>(9·5 to 27·8%)	-11·0%<br>(-16·2 to -7·0%)	7,017·6<br>(5,729·4 to 8,413·9)	26·0%<br>(21·2 to 31·1%)	8,216·1<br>(6,528·5 to 10,108·8)	23·4%<br>(18·7 to 28·5%)	17·1%<br>(7·5 to 26·3%)	-9·8%<br>(-14·8 to -5·9%)
Ischaemic stroke	75·9<br>(55·0 to 100·3)	19·8%<br>(14·8 to 26·1%)	81·1<br>(58·3 to 108·5)	17·4%<br>(12·8 to 23·1%)	6·9%<br>(-2·9 to 16·1%)	-12·3%<br>(-17·0 to -8·4%)	1,551·8<br>(1,129·7 to 2,015·9)	21·4%<br>(16·0 to 27·3%)	1,622·9<br>(1,169·2 to 2,138·6)	19·0%<br>(13·9 to 24·6%)	4·6%<br>(-4·9 to 13·6%)	-11·1%<br>(-15·6 to -7·6%)
Hemorrhagic stroke	115·1<br>(86·0 to 147·2)	22·2%<br>(16·6 to 28·1%)	120·7<br>(89·4 to 156·4)	19·9%<br>(14·8 to 25·4%)	4·8%<br>(-3·6 to 13·5%)	-10·1%<br>(-14·1 to -6·6%)	3,240·7<br>(2,458·9 to 4,087·7)	22·8%<br>(17·3 to 28·6%)	3,383·4<br>(2,505·1 to 4,342·4)	21·1%<br>(15·6 to 26·8%)	4·4%<br>(-4·0 to 12·9%)	-7·7%<br>(-11·8 to -4·1%)
Chronic obstructive pulmonary disease	296·9<br>(173·0 to 444·7)	39·2%<br>(23·2 to 57·2%)	328·8<br>(185·9 to 497·1)	36·2%<br>(21·0 to 54·7%)	10·8%<br>(-0·9 to 23·1%)	-7·6%<br>(-13·7 to -3·7%)	6,402·7<br>(3,710·3 to 9,597·7)	36·2%<br>(21·5 to 53·0%)	6,959·3<br>(3,965·4 to 10,498·8)	34·1%<br>(19·7 to 51·3%)	8·7%<br>(-2·6 to 20·4%)	-5·7%<br>(-11·9 to -1·3%)
Cataract	-	-	-	-	-	-	288·7<br>(201·0 to 396·5)	31·8%<br>(28·2 to 35·0%)	331·4<br>(230·3 to 454·3)	29·9%<br>(26·3 to 33·1%)	14·8%<br>(10·2 to 18·5%)	-6·0%<br>(-9·4 to -3·4%)
**Ambient ozone pollution: All causes**	60·2<br>(22·4 to 101·4)	0·5%<br>(0·2 to 0·8%)	89·1<br>(34·0 to 145·0)	0·7%<br>(0·3 to 1·1%)	48·0%<br>(34·1 to 64·3%)	48·9%<br>(36·8 to 63·9%)	1,143·6<br>(422·9 to 1,926·4)	0·1%<br>(0·1 to 0·2%)	1,639·5<br>(619·9 to 2,685·2)	0·2%<br>(0·1 to 0·4%)	43·4%<br>(28·7 to 60·9%)	56·3%<br>(40·9 to 73·9%)
Chronic obstructive pulmonary disease	60·2<br>(22·4 to 101·4)	8·0%<br>(3·0 to 13·3%)	89·1<br>(34·0 to 145·0)	9·8%<br>(3·8 to 16·1%)	48·0%<br>(34·1 to 64·3%)	23·6%<br>(20·3 to 28·2%)	1,143·6<br>(422·9 to 1,926·4)	6·5%<br>(2·4 to 10·8%)	1,639·5<br>(619·9 to 2,685·2)	8·0%<br>(3·1 to 13·1%)	43·4%<br>(28·7 to 60·9%)	24·6%<br>(19·8 to 30·6%)
**Other environmental risks: All causes**	103·1<br>(59·5 to 153·4)	0·8%<br>(0·4 to 1·1%)	125·7<br>(69·9 to 188·7)	0·9%<br>(0·5 to 1·4%)	22·0%<br>(15·1 to 29·6%)	22·7%<br>(17·1 to 28·5%)	2,482·7<br>(1,380·1 to 3,720·0)	0·3%<br>(0·2 to 0·5%)	2,843·3<br>(1,531·3 to 4,325·6)	0·4%<br>(0·2 to 0·6%)	14·5%<br>(7·0 to 21·4%)	24·8%<br>(17·1 to 31·6%)
**Residential radon: All causes**	6·0<br>(3·6 to 11·4)	0·0%<br>(0·0 to 0·1%)	9·0<br>(5·2 to 18·1)	0·1%<br>(0·0 to 0·1%)	49·5%<br>(31·3 to 77·6%)	50·4%<br>(33·1 to 79·3%)	152·2<br>(94·4 to 283·5)	0·0%<br>(0·0 to 0·0%)	213·6<br>(128·6 to 427·7)	0·0%<br>(0·0 to 0·1%)	40·4%<br>(25·9 to 58·1%)	53·0%<br>(38·1 to 71·1%)
Tracheal, bronchus, and lung cancer	6·0<br>(3·6 to 11·4)	6·2%<br>(3·7 to 11·5%)	9·0<br>(5·2 to 18·1)	6·3%<br>(3·7 to 12·9%)	49·5%<br>(31·3 to 77·6%)	2·9%<br>(-7·7 to 20·7%)	152·2<br>(94·4 to 283·5)	6·1%<br>(3·9 to 11·1%)	213·6<br>(128·6 to 427·7)	6·2%<br>(3·8 to 12·1%)	40·4%<br>(25·9 to 58·1%)	1·3%<br>(-6·9 to 11·8%)
**Lead exposure: All causes**	97·1<br>(53·8 to 146·7)	0·7%<br>(0·4 to 1·1%)	116·8<br>(62·8 to 178·4)	0·9%<br>(0·5 to 1·3%)	20·3%<br>(12·9 to 27·1%)	21·0%<br>(14·7 to 26·3%)	2,330·5<br>(1,256·7 to 3,541·7)	0·3%<br>(0·2 to 0·4%)	2,629·7<br>(1,348·0 to 4,118·0)	0·4%<br>(0·2 to 0·6%)	12·8%<br>(4·7 to 19·9%)	22·9%<br>(14·2 to 29·6%)
Rheumatic heart disease	1·3<br>(0·5 to 2·4)	1·3%<br>(0·5 to 2·4%)	1·3<br>(0·5 to 2·6)	1·2%<br>(0·4 to 2·2%)	1·0%<br>(-10·8 to 11·9%)	-7·2%<br>(-14·0 to -2·0%)	41·1<br>(13·7 to 84·6)	1·0%<br>(0·3 to 1·9%)	37·9<br>(11·8 to 80·8)	0·9%<br>(0·3 to 1·8%)	-7·8%<br>(-19·5 to 2·5%)	-9·4%<br>(-18·5 to -2·9%)
Ischaemic heart disease	44·6<br>(24·6 to 67·4)	3·9%<br>(2·2 to 5·9%)	56·3<br>(29·9 to 86·7)	3·7%<br>(2·0 to 5·6%)	26·2%<br>(17·3 to 34·4%)	-5·7%<br>(-10·3 to -2·4%)	963·3<br>(501·9 to 1,505·5)	3·6%<br>(1·9 to 5·5%)	1,160·6<br>(577·8 to 1,845·8)	3·3%<br>(1·7 to 5·3%)	20·5%<br>(11·2 to 28·7%)	-7·1%<br>(-12·5 to -3·9%)
Ischaemic stroke	14·1<br>(7·6 to 22·2)	3·7%<br>(2·0 to 5·7%)	16·1<br>(8·6 to 25·9)	3·5%<br>(1·8 to 5·4%)	14·2%<br>(3·9 to 23·8%)	-6·2%<br>(-11·7 to -1·5%)	263·0<br>(137·0 to 414·1)	3·6%<br>(1·9 to 5·6%)	288·5<br>(142·5 to 468·3)	3·4%<br>(1·7 to 5·4%)	9·7%<br>(0·0 to 18·9%)	-6·8%<br>(-12·2 to -3·1%)
Hemorrhagic stroke	19·8<br>(9·8 to 31·4)	3·8%<br>(1·9 to 6·0%)	21·8<br>(10·7 to 35·3)	3·6%<br>(1·8 to 5·8%)	10·4%<br>(1·8 to 18·9%)	-5·2%<br>(-11·1 to -1·3%)	485·4<br>(216·7 to 802·2)	3·4%<br>(1·6 to 5·7%)	520·4<br>(224·5 to 885·9)	3·2%<br>(1·4 to 5·5%)	7·2%<br>(-2·1 to 15·2%)	-5·2%<br>(-11·8 to -1·0%)
Hypertensive heart disease	9·6<br>(3·2 to 20·2)	6·2%<br>(2·1 to 13·8%)	11·1<br>(3·4 to 23·9)	5·9%<br>(1·9 to 13·2%)	15·4%<br>(1·1 to 29·1%)	-5·9%<br>(-13·7 to 0·0%)	206·0<br>(74·4 to 408·6)	5·6%<br>(2·1 to 11·7%)	223·6<br>(75·6 to 467·4)	5·1%<br>(1·7 to 11·1%)	8·5%<br>(-4·4 to 20·2%)	-8·3%<br>(-16·4 to -3·5%)
Cardiomyopathy and myocarditis	0·4<br>(0·2 to 0·6)	1·5%<br>(0·7 to 2·5%)	0·4<br>(0·2 to 0·8)	1·4%<br>(0·6 to 2·5%)	14·8%<br>(0·4 to 28·3%)	-4·3%<br>(-12·5 to 1·6%)	10·6<br>(3·9 to 21·1)	1·0%<br>(0·4 to 1·9%)	11·5<br>(4·1 to 23·5)	1·0%<br>(0·4 to 2·0%)	8·1%<br>(-7·3 to 22·6%)	-2·8%<br>(-14·5 to 5·7%)

Supplemental Results Table 1. All-age deaths, death PAFs, DALYs, DALY PAFs, and percent change for each risk factor and outcome for both sexes combined in 2005 and 2015 for Low-middle SDI.

Data in parenthesis are 95% uncertainty intervals. PAFs=population attributable fractions. DALYs=disability-adjusted life-years.

	2005 Deaths (in thousands)	2005 Deaths PAF	2015 Deaths (in thousands)	2015 Deaths PAF	Percent change deaths 2005-2015	Percent change deaths PAF 2005-2015	2005 DALYs (in thousands)	2005 DALYs PAF	2015 DALYs (in thousands)	2015 DALYs PAF	Percent change DALYs 2005-2015	Percent change DALYs PAF 2005-2015
Atrial fibrillation and flutter	0·3 (0·2 to 0·5)	2·5% (1·5 to 3·7%)	0·4 (0·2 to 0·7)	2·4% (1·3 to 3·5%)	32·1% (22·6 to 41·1%)	-6·2% (-10·7 to -2·9%)	8·8 (4·7 to 14·2)	2·7% (1·5 to 3·9%)	10·9 (5·5 to 17·8)	2·4% (1·3 to 3·7%)	23·6% (17·1 to 28·4%)	-8·0% (-12·2 to -5·4%)
Aortic aneurysm	0·2 (0·1 to 0·4)	2·1% (1·1 to 3·4%)	0·3 (0·2 to 0·5)	2·0% (0·9 to 3·2%)	36·4% (20·4 to 51·8%)	-8·3% (-13·7 to -4·7%)	5·4 (2·3 to 9·7)	2·0% (0·9 to 3·3%)	7·0 (3·0 to 12·3)	1·8% (0·8 to 3·1%)	29·9% (12·8 to 45·7%)	-10·1% (-17·3 to -5·9%)
Peripheral vascular disease	0·0 (0·0 to 0·0)	1·6% (0·8 to 2·5%)	0·0 (0·0 to 0·0)	1·5% (0·7 to 2·4%)	71·9% (48·9 to 100·2%)	-6·7% (-12·5 to -2·6%)	1·1 (0·5 to 2·2)	1·7% (0·9 to 2·6%)	1·5 (0·6 to 2·9)	1·5% (0·8 to 2·4%)	34·1% (24·7 to 43·6%)	-8·0% (-13·4 to -4·8%)
Endocarditis	0·1 (0·1 to 0·3)	1·5% (0·7 to 2·5%)	0·2 (0·1 to 0·3)	1·4% (0·6 to 2·5%)	21·6% (8·4 to 35·0%)	-4·9% (-13·7 to 2·6%)	3·8 (1·4 to 7·4)	1·0% (0·4 to 1·9%)	4·3 (1·6 to 8·8)	0·9% (0·3 to 1·8%)	14·3% (-0·7 to 28·1%)	-4·8% (-18·3 to 6·3%)
Other cardiovascular and circulatory diseases	1·0 (0·5 to 1·7)	1·9% (1·0 to 3·1%)	1·2 (0·6 to 2·0)	1·9% (0·9 to 3·1%)	17·9% (5·1 to 30·7%)	-2·7% (-11·1 to 3·9%)	32·4 (14·5 to 56·5)	1·4% (0·6 to 2·4%)	37·2 (15·6 to 66·5)	1·4% (0·6 to 2·5%)	14·5% (1·9 to 25·1%)	-0·8% (-10·3 to 5·8%)
Idiopathic developmental intellectual disability	-	-	-	-	-	-	181·8 (79·3 to 318·4)	20·6% (14·9 to 25·3%)	167·4 (74·0 to 295·7)	16·7% (11·4 to 21·0%)	-7·9% (-14·5 to -1·8%)	-18·8% (-25·1 to -13·6%)
Chronic kidney disease due to diabetes mellitus	1·5 (0·7 to 2·7)	2·8% (1·3 to 4·7%)	2·1 (0·9 to 3·7)	2·7% (1·3 to 4·6%)	33·8% (22·7 to 45·6%)	-4·1% (-7·9 to -2·0%)	37·2 (15·7 to 69·1)	2·1% (0·9 to 3·7%)	46·4 (19·2 to 87·4)	2·0% (0·8 to 3·5%)	24·9% (14·8 to 35·1%)	-4·6% (-9·2 to -1·7%)
Chronic kidney disease due to hypertension	2·8 (1·3 to 4·5)	2·7% (1·3 to 4·5%)	3·8 (1·8 to 6·3)	2·7% (1·3 to 4·5%)	37·0% (26·0 to 48·4%)	0·0% (-3·4 to 2·6%)	55·5 (24·3 to 97·4)	1·9% (0·8 to 3·3%)	69·9 (30·5 to 123·3)	1·9% (0·8 to 3·3%)	25·8% (15·6 to 35·9%)	0·5% (-4·3 to 3·9%)
Chronic kidney disease due to glomerulonephritis	1·1 (0·5 to 1·9)	2·1% (1·0 to 3·5%)	1·6 (0·7 to 2·6)	2·3% (1·1 to 3·8%)	37·1% (25·3 to 49·5%)	5·9% (2·5 to 10·1%)	27·6 (10·9 to 50·4)	1·3% (0·5 to 2·3%)	33·8 (13·6 to 61·4)	1·3% (0·5 to 2·4%)	22·3% (13·1 to 31·7%)	4·9% (-0·2 to 9·1%)
Chronic kidney disease due to other causes	0·1 (0·0 to 0·2)	1·8% (0·9 to 3·0%)	0·1 (0·1 to 0·2)	2·1% (1·1 to 3·4%)	35·4% (21·3 to 52·3%)	16·6% (7·8 to 26·1%)	7·4 (3·1 to 14·2)	1·3% (0·6 to 2·4%)	8·9 (3·7 to 17·4)	1·3% (0·6 to 2·5%)	19·9% (13·7 to 25·4%)	4·3% (-1·1 to 9·2%)
**Occupational risks: All causes**	202·0 (178·8 to 225·0)	1·5% (1·3 to 1·7%)	239·3 (215·2 to 270·9)	1·8% (1·6 to 2·0%)	18·4% (7·2 to 32·3%)	19·1% (7·9 to 32·9%)	13,680·6 (11,330·5 to 16,181·2)	1·7% (1·5 to 2·0%)	16,062·6 (13,364·1 to 19,171·3)	2·2% (1·9 to 2·5%)	17·4% (8·0 to 29·2%)	27·9% (17·3 to 41·5%)
**Occupational carcinogens: All causes**	27·3 (24·9 to 29·8)	0·2% (0·2 to 0·2%)	41·4 (37·5 to 45·6)	0·3% (0·3 to 0·3%)	51·8% (38·4 to 66·2%)	52·6% (41·6 to 65·7%)	646·7 (589·7 to 706·3)	0·1% (0·1 to 0·1%)	967·6 (872·8 to 1,073·6)	0·1% (0·1 to 0·1%)	49·6% (36·1 to 64·4%)	63·0% (50·5 to 78·1%)
**Occupational exposure to asbestos: All causes**	4·1 (3·0 to 5·4)	0·0% (0·0 to 0·0%)	5·0 (3·8 to 6·6)	0·0% (0·0 to 0·0%)	23·5% (2·8 to 55·1%)	24·2% (4·5 to 54·0%)	82·4 (60·2 to 110·9)	0·0% (0·0 to 0·0%)	95·9 (69·4 to 129·8)	0·0% (0·0 to 0·0%)	16·4% (-6·0 to 48·8%)	26·9% (2·9 to 61·3%)
Larynx cancer	0·1 (0·1 to 0·2)	0·5% (0·3 to 0·7%)	0·1 (0·1 to 0·2)	0·4% (0·2 to 0·6%)	5·0% (-12·1 to 34·8%)	-16·3% (-28·1 to 4·4%)	1·9 (1·1 to 3·0)	0·4% (0·2 to 0·6%)	1·9 (1·1 to 3·0)	0·3% (0·2 to 0·5%)	2·1% (-15·4 to 31·5%)	-16·9% (-29·2 to 5·8%)
Tracheal, bronchus, and lung cancer	3·2 (2·2 to 4·4)	3·2% (2·3 to 4·4%)	4·1 (2·9 to 5·5)	2·9% (2·1 to 3·8%)	28·5% (5·7 to 63·1%)	-11·6% (-24·1 to 6·3%)	61·2 (42·3 to 86·8)	2·5% (1·7 to 3·4%)	74·3 (51·5 to 104·8)	2·2% (1·5 to 2·9%)	21·3% (-3·0 to 57·9%)	-12·5% (-27·0 to 7·9%)
Ovarian cancer	0·1 (0·1 to 0·2)	0·9% (0·4 to 1·4%)	0·1 (0·1 to 0·3)	0·7% (0·3 to 1·2%)	13·7% (-17·1 to 60·0%)	-16·0% (-35·6 to 11·7%)	2·7 (1·2 to 4·7)	0·6% (0·3 to 1·0%)	3·0 (1·3 to 5·4)	0·5% (0·2 to 0·8%)	10·6% (-20·4 to 57·3%)	-17·8% (-37·5 to 11·5%)
Mesothelioma	0·7 (0·4 to 0·9)	42·7% (28·6 to 56·9%)	0·7 (0·5 to 1·0)	41·4% (28·0 to 54·5%)	5·2% (-11·3 to 29·5%)	-3·0% (-9·3 to 4·7%)	16·6 (10·0 to 23·8)	32·7% (20·3 to 45·9%)	16·7 (9·9 to 23·6)	31·2% (19·6 to 43·6%)	0·9% (-16·5 to 28·5%)	-4·6% (-13·1 to 6·0%)
**Occupational exposure to arsenic: All causes**	0·7 (0·5 to 0·9)	0·0% (0·0 to 0·0%)	0·9 (0·7 to 1·2)	0·0% (0·0 to 0·0%)	28·8% (17·8 to 40·0%)	29·5% (20·2 to 39·5%)	16·7 (12·8 to 21·0)	0·0% (0·0 to 0·0%)	21·2 (16·1 to 26·8)	0·0% (0·0 to 0·0%)	27·0% (16·4 to 38·2%)	38·4% (27·3 to 50·3%)
Tracheal, bronchus, and lung cancer	0·7 (0·5 to 0·9)	0·7% (0·6 to 0·9%)	0·9 (0·7 to 1·2)	0·6% (0·5 to 0·8%)	28·8% (17·8 to 40·0%)	-11·3% (-14·5 to -8·1%)	16·7 (12·8 to 21·0)	0·7% (0·5 to 0·8%)	21·2 (16·1 to 26·8)	0·6% (0·5 to 0·8%)	27·0% (16·4 to 38·2%)	-8·4% (-11·6 to -5·1%)
**Occupational exposure to benzene: All causes**	0·6 (0·5 to 0·8)	0·0% (0·0 to 0·0%)	1·0 (0·8 to 1·2)	0·0% (0·0 to 0·0%)	56·0% (42·6 to 70·8%)	56·8% (46·0 to 68·6%)	23·6 (19·1 to 28·7)	0·0% (0·0 to 0·0%)	36·9 (30·1 to 44·2)	0·0% (0·0 to 0·0%)	56·8% (42·6 to 72·1%)	70·9% (58·0 to 85·9%)
Leukaemia	0·6 (0·5 to 0·8)	1·5% (1·3 to 1·8%)	1·0 (0·8 to 1·2)	1·9% (1·6 to 2·2%)	56·0% (42·6 to 70·8%)	23·8% (17·1 to 31·8%)	23·6 (19·1 to 28·7)	1·2% (1·0 to 1·5%)	36·9 (30·1 to 44·2)	1·6% (1·3 to 1·8%)	56·8% (42·6 to 72·1%)	28·3% (18·6 to 40·2%)
**Occupational exposure to beryllium: All causes**	0·0 (0·0 to 0·0)	0·0% (0·0 to 0·0%)	0·0 (0·0 to 0·0)	0·0% (0·0 to 0·0%)	29·6% (16·5 to 43·6%)	30·4% (18·3 to 43·1%)	0·7 (0·6 to 0·9)	0·0% (0·0 to 0·0%)	0·9 (0·7 to 1·1)	0·0% (0·0 to 0·0%)	27·8% (14·7 to 41·6%)	39·3% (26·2 to 54·0%)
Tracheal, bronchus, and lung cancer	0·0 (0·0 to 0·0)	0·0% (0·0 to 0·0%)	0·0 (0·0 to 0·0)	0·0% (0·0 to 0·0%)	29·6% (16·5 to 43·6%)	-10·8% (-16·4 to -4·4%)	0·7 (0·6 to 0·9)	0·0% (0·0 to 0·0%)	0·9 (0·7 to 1·1)	0·0% (0·0 to 0·0%)	27·8% (14·7 to 41·6%)	-7·8% (-13·7 to -1·3%)
**Occupational exposure to cadmium: All causes**	0·1 (0·1 to 0·1)	0·0% (0·0 to 0·0%)	0·2 (0·1 to 0·2)	0·0% (0·0 to 0·0%)	49·7% (35·9 to 65·4%)	50·6% (37·8 to 64·6%)	2·9 (2·3 to 3·4)	0·0% (0·0 to 0·0%)	4·2 (3·3 to 5·1)	0·0% (0·0 to 0·0%)	47·7% (33·4 to 63·2%)	61·0% (46·1 to 76·9%)
Tracheal, bronchus, and lung cancer	0·1 (0·1 to 0·1)	0·1% (0·1 to 0·1%)	0·2 (0·1 to 0·2)	0·1% (0·1 to 0·2%)	49·7% (35·9 to 65·4%)	3·1% (-3·1 to 10·1%)	2·9 (2·3 to 3·4)	0·1% (0·1 to 0·1%)	4·2 (3·3 to 5·1)	0·1% (0·1 to 0·1%)	47·7% (33·4 to 63·2%)	6·6% (-0·1 to 14·1%)
**Occupational exposure to chromium: All causes**	0·4 (0·3 to 0·5)	0·0% (0·0 to 0·0%)	0·6 (0·5 to 0·7)	0·0% (0·0 to 0·0%)	41·3% (27·6 to 55·8%)	42·1% (30·0 to 55·9%)	9·5 (8·2 to 11·0)	0·0% (0·0 to 0·0%)	13·3 (11·3 to 15·3)	0·0% (0·0 to 0·0%)	39·4% (25·5 to 53·7%)	51·9% (38·2 to 66·8%)
Tracheal, bronchus, and lung cancer	0·4 (0·3 to 0·5)	0·4% (0·4 to 0·5%)	0·6 (0·5 to 0·7)	0·4% (0·3 to 0·5%)	41·3% (27·6 to 55·8%)	-2·7% (-8·5 to 3·5%)	9·5 (8·2 to 11·0)	0·4% (0·3 to 0·4%)	13·3 (11·3 to 15·3)	0·4% (0·3 to 0·4%)	39·4% (25·5 to 53·7%)	0·5% (-5·4 to 7·0%)
**Occupational exposure to diesel engine exhaust: All causes**	7·9 (6·8 to 9·1)	0·1% (0·1 to 0·1%)	13·2 (11·3 to 15·4)	0·1% (0·1 to 0·1%)	68·2% (52·8 to 84·1%)	69·2% (56·4 to 83·7%)	186·1 (159·2 to 215·3)	0·0% (0·0 to 0·0%)	308·5 (264·3 to 358·7)	0·0% (0·0 to 0·0%)	65·8% (50·1 to 82·2%)	80·7% (65·3 to 98·2%)
Tracheal, bronchus, and lung cancer	7·9 (6·8 to 9·1)	8·1% (7·0 to 9·3%)	13·2 (11·3 to 15·4)	9·4% (8·2 to 10·7%)	68·2% (52·8 to 84·1%)	15·8% (10·5 to 21·5%)	186·1 (159·2 to 215·3)	7·5% (6·5 to 8·5%)	308·5 (264·3 to 358·7)	9·0% (7·8 to 10·2%)	65·8% (50·1 to 82·2%)	19·6% (13·8 to 25·6%)
**Occupational exposure to second-hand smoke: All causes**	8·3 (7·5 to 9·1)	0·1% (0·1 to 0·1%)	13·2 (11·8 to 14·7)	0·1% (0·1 to 0·1%)	59·1% (44·5 to 75·6%)	60·0% (47·6 to 74·5%)	192·7 (174·9 to 211·8)	0·0% (0·0 to 0·0%)	300·8 (270·1 to 336·3)	0·0% (0·0 to 0·0%)	56·1% (41·6 to 72·2%)	70·1% (56·4 to 86·8%)
Tracheal, bronchus, and lung cancer	8·3 (7·5 to 9·1)	8·5% (7·9 to 9·1%)	13·2 (11·8 to 14·7)	9·3% (8·6 to 10·1%)	59·1% (44·5 to 75·6%)	9·5% (3·8 to 16·3%)	192·7 (174·9 to 211·8)	7·8% (7·2 to 8·3%)	300·8 (270·1 to 336·3)	8·7% (8·0 to 9·5%)	56·1% (41·6 to 72·2%)	12·6% (7·0 to 19·1%)
**Occupational exposure to formaldehyde: All causes**	0·2 (0·2 to 0·3)	0·0% (0·0 to 0·0%)	0·3 (0·2 to 0·3)	0·0% (0·0 to 0·0%)	26·3% (15·8 to 38·3%)	27·0% (18·9 to 36·7%)	7·9 (6·4 to 9·6)	0·0% (0·0 to 0·0%)	10·0 (8·2 to 12·1)	0·0% (0·0 to 0·0%)	27·2% (16·4 to 39·1%)	38·6% (28·4 to 50·8%)
Nasopharynx cancer	0·1 (0·1 to 0·1)	1·2% (0·8 to 1·6%)	0·1 (0·1 to 0·2)	1·2% (0·8 to 1·6%)	23·5% (10·4 to 38·0%)	-2·2% (-6·7 to 3·5%)	3·2 (2·2 to 4·6)	1·3% (0·9 to 1·7%)	4·0 (2·7 to 5·8)	1·3% (0·9 to 1·7%)	24·2% (11·0 to 39·2%)	0·7% (-4·1 to 6·5%)
**Occupational exposure to nickel: All causes**	1·7 (1·2 to 2·3)	0·0% (0·0 to 0·0%)	2·2 (1·6 to 3·0)	0·0% (0·0 to 0·0%)	29·9% (16·0 to 44·6%)	30·6% (18·3 to 44·5%)	40·2 (28·8 to 53·8)	0·0% (0·0 to 0·0%)	51·6 (37·0 to 69·2)	0·0% (0·0 to 0·0%)	28·2% (14·5 to 43·5%)	39·7% (26·2 to 55·5%)
Tracheal, bronchus, and lung cancer	1·7 (1·2 to 2·3)	1·8% (1·3 to 2·3%)	2·2 (1·6 to 3·0)	1·6% (1·1 to 2·1%)	29·9% (16·0 to 44·6%)	-10·6% (-16·6 to -3·7%)	40·2 (28·8 to 53·8)	1·6% (1·2 to 2·2%)	51·6 (37·0 to 69·2)	1·5% (1·1 to 2·0%)	28·2% (14·5 to 43·5%)	-7·5% (-13·7 to -0·8%)
**Occupational exposure to polycyclic aromatic hydrocarbons: All causes**	1·0 (0·8 to 1·2)	0·0% (0·0 to 0·0%)	1·6 (1·3 to 2·0)	0·0% (0·0 to 0·0%)	59·8% (44·7 to 76·8%)	60·7% (47·4 to 76·3%)	24·2 (19·6 to 29·0)	0·0% (0·0 to 0·0%)	38·1 (30·6 to 46·1)	0·0% (0·0 to 0·0%)	57·7% (42·8 to 74·4%)	71·8% (56·1 to 89·0%)
Tracheal, bronchus, and lung cancer	1·0 (0·8 to 1·2)	1·1% (0·9 to 1·3%)	1·6 (1·3 to 2·0)	1·2% (0·9 to 1·4%)	59·8% (44·7 to 76·8%)	10·0% (3·6 to 16·8%)	24·2 (19·6 to 29·0)	1·0% (0·8 to 1·2%)	38·1 (30·6 to 46·1)	1·1% (0·9 to 1·3%)	57·7% (42·8 to 74·4%)	13·7% (7·0 to 20·7%)
**Occupational exposure to silica: All causes**	4·6 (4·0 to 5·2)	0·0% (0·0 to 0·0%)	7·8 (6·8 to 9·0)	0·1% (0·1 to 0·1%)	69·6% (51·5 to 90·4%)	70·6% (54·3 to 88·8%)	108·4 (94·8 to 122·9)	0·0% (0·0 to 0·0%)	181·8 (157·7 to 209·3)	0·0% (0·0 to 0·0%)	67·7% (49·1 to 88·8%)	82·7% (63·6 to 104·1%)
Tracheal, bronchus, and lung cancer	4·6 (4·0 to 5·2)	4·7% (4·2 to 5·3%)	7·8 (6·8 to 9·0)	5·5% (4·9 to 6·2%)	69·6% (51·5 to 90·4%)	16·8% (8·5 to 26·6%)	108·4 (94·8 to 122·9)	4·4% (3·9 to 4·9%)	181·8 (157·7 to 209·3)	5·3% (4·7 to 5·9%)	67·7% (49·1 to 88·8%)	20·9% (12·1 to 30·8%)
**Occupational exposure to sulfphuric acid: All causes**	1·5 (1·1 to 1·9)	0·0% (0·0 to 0·0%)	1·9 (1·4 to 2·5)	0·0% (0·0 to 0·0%)	33·8% (20·9 to 47·4%)	34·6% (22·3 to 47·1%)	35·8 (27·0 to 46·3)	0·0% (0·0 to 0·0%)	48·0 (35·3 to 61·9)	0·0% (0·0 to 0·0%)	33·9% (21·3 to 47·7%)	45·9% (33·0 to 60·5%)
Larynx cancer	1·5 (1·1 to 1·9)	7·2% (5·4 to 9·3%)	1·9 (1·4 to 2·5)	7·7% (5·7 to 9·9%)	33·8% (20·9 to 47·4%)	6·7% (0·7 to 13·1%)	35·8 (27·0 to 46·3)	6·8% (5·2 to 8·6%)	48·0 (35·3 to 61·9)	7·4% (5·5 to 9·5%)	33·9% (21·3 to 47·7%)	8·9% (3·0 to 15·4%)
**Occupational exposure to trichloroethylene: All causes**	0·0 (0·0 to 0·0)	0·0% (0·0 to 0·0%)	0·0 (0·0 to 0·0)	0·0% (0·0 to 0·0%)	57·2% (39·2 to 77·2%)	58·1% (41·7 to 75·4%)	0·3 (0·1 to 0·6)	0·0% (0·0 to 0·0%)	0·5 (0·1 to 0·9)	0·0% (0·0 to 0·0%)	57·9% (40·1 to 78·7%)	72·0% (53·7 to 92·9%)
Kidney cancer	0·0 (0·0 to 0·0)	0·2% (0·0 to 0·3%)	0·0 (0·0 to 0·0)	0·2% (0·0 to 0·3%)	57·2% (39·2 to 77·2%)	9·2% (-0·1 to 22·5%)	0·3 (0·1 to 0·6)	0·1% (0·0 to 0·2%)	0·5 (0·1 to 0·9)	0·1% (0·0 to 0·2%)	57·9% (40·1 to 78·7%)	11·3% (-5·7 to 38·2%)

Supplemental Results Table 1. All-age deaths, death PAFs, DALYs, DALY PAFs, and percent change for each risk factor and outcome for both sexes combined in 2005 and 2015 for Low-middle SDI.

Data in parenthesis are 95% uncertainty intervals. PAFs=population attributable fractions. DALYs=disability-adjusted life-years.

	2005 Deaths (in thousands)	2005 Deaths PAF	2015 Deaths (in thousands)	2015 Deaths PAF	Percent change deaths 2005-2015	Percent change deaths PAF 2005-2015	2005 DALYs (in thousands)	2005 DALYs PAF	2015 DALYs (in thousands)	2015 DALYs PAF	Percent change DALYs 2005-2015	Percent change DALYs PAF 2005-2015
**Occupational asthmagens: All causes**	23·4 (15·8 to 30·9)	0·2% (0·1 to 0·2%)	17·6 (14·3 to 21·4)	0·1% (0·1 to 0·2%)	-25·1% (-38·4 to -0·2%)	-24·6% (-37·3 to 0·4%)	1,021·6 (762·9 to 1,281·5)	0·1% (0·1 to 0·2%)	898·9 (725·6 to 1,095·9)	0·1% (0·1 to 0·1%)	-12·0% (-24·2 to 5·9%)	-4·2% (-16·7 to 15·0%)
Asthma	23·4 (15·8 to 30·9)	11·9% (9·6 to 14·5%)	17·6 (14·3 to 21·4)	11·1% (9·4 to 12·8%)	-25·1% (-38·4 to -0·2%)	-7·5% (-18·2 to 5·4%)	1,021·6 (762·9 to 1,281·5)	11·3% (9·5 to 13·1%)	898·9 (725·6 to 1,095·9)	10·8% (9·3 to 12·4%)	-12·0% (-24·2 to 5·9%)	-4·3% (-10·4 to 1·9%)
**Occupational particulate matter, gases, and fumes: All causes**	103·7 (85·6 to 122·4)	0·8% (0·6 to 0·9%)	126·9 (105·1 to 147·5)	1·0% (0·8 to 1·1%)	22·4% (12·2 to 35·2%)	23·1% (14·0 to 34·4%)	2,629·6 (2,215·1 to 3,068·5)	0·3% (0·3 to 0·4%)	3,176·0 (2,723·4 to 3,623·4)	0·4% (0·4 to 0·5%)	20·8% (11·0 to 32·3%)	31·6% (21·4 to 43·1%)
Chronic obstructive pulmonary disease	103·6 (85·5 to 122·3)	13·7% (11·5 to 15·8%)	126·8 (104·9 to 147·3)	14·0% (11·6 to 16·2%)	22·4% (12·2 to 35·3%)	2·1% (-2·4 to 7·7%)	2,625·0 (2,211·2 to 3,063·6)	14·8% (12·8 to 16·8%)	3,170·4 (2,717·6 to 3,617·9)	15·5% (13·4 to 17·5%)	20·8% (11·0 to 32·3%)	4·8% (0·7 to 10·2%)
**Occupational noise: All causes**	-	-	-	-	-	-	2,206·1 (1,508·8 to 3,028·2)	0·3% (0·2 to 0·4%)	2,823·0 (1,928·7 to 3,884·2)	0·4% (0·3 to 0·5%)	28·0% (26·1 to 29·8%)	39·3% (35·3 to 44·2%)
Age-related and other hearing loss	-	-	-	-	-	-	2,206·1 (1,508·8 to 3,028·2)	33·9% (31·0 to 37·5%)	2,823·0 (1,928·7 to 3,884·2)	34·2% (31·5 to 37·7%)	28·0% (26·1 to 29·8%)	0·9% (-0·2 to 2·2%)
**Occupational injuries: All causes**	59·8 (40·9 to 76·2)	0·4% (0·3 to 0·6%)	55·0 (44·9 to 82·3)	0·4% (0·3 to 0·6%)	-8·0% (-34·6 to 41·1%)	-7·5% (-34·2 to 41·6%)	3,761·0 (2,648·0 to 4,743·9)	0·5% (0·3 to 0·6%)	3,618·7 (2,886·6 to 5,306·7)	0·5% (0·4 to 0·7%)	-3·8% (-29·6 to 43·0%)	4·8% (-23·8 to 55·4%)
Pedestrian road injuries	7·2 (3·4 to 11·0)	6·6% (3·1 to 10·1%)	5·9 (3·8 to 12·0)	4·8% (3·2 to 9·9%)	-18·4% (-55·8 to 79·7%)	-26·3% (-59·9 to 64·4%)	366·4 (169·6 to 553·6)	6·7% (3·2 to 10·1%)	293·8 (191·0 to 597·4)	5·1% (3·4 to 10·4%)	-19·8% (-56·3 to 75·3%)	-23·5% (-58·6 to 70·1%)
Cyclist road injuries	0·4 (0·2 to 0·5)	6·3% (3·2 to 9·6%)	0·3 (0·2 to 0·7)	4·8% (3·5 to 9·0%)	-8·3% (-45·9 to 84·6%)	-23·7% (-55·7 to 50·7%)	21·3 (11·0 to 31·9)	6·3% (3·3 to 9·3%)	20·2 (14·6 to 38·8)	5·1% (3·7 to 9·5%)	-4·9% (-42·6 to 87·4%)	-18·7% (-51·9 to 56·5%)
Motorcyclist road injuries	4·7 (3·3 to 6·2)	9·3% (7·1 to 11·5%)	5·5 (4·3 to 7·7)	8·8% (7·1 to 12·0%)	17·7% (-18·5 to 70·8%)	-4·6% (-29·3 to 34·1%)	256·6 (186·7 to 335·0)	9·6% (7·4 to 11·9%)	300·4 (238·1 to 419·6)	9·4% (7·6 to 12·7%)	17·1% (-17·2 to 67·8%)	-2·0% (-27·4 to 37·3%)
Motor vehicle road injuries	9·9 (7·0 to 12·8)	10·6% (7·8 to 13·1%)	9·8 (7·9 to 13·5)	9·4% (7·7 to 12·9%)	-1·0% (-27·7 to 43·5%)	-11·7% (-33·1 to 24·6%)	543·4 (390·5 to 695·0)	10·7% (7·9 to 13·3%)	538·5 (439·9 to 744·6)	9·8% (8·1 to 13·5%)	-0·9% (-26·9 to 42·8%)	-8·9% (-31·1 to 27·9%)
Other road injuries	1·2 (0·5 to 1·9)	16·5% (7·5 to 27·4%)	0·8 (0·5 to 1·9)	9·7% (6·1 to 21·6%)	-28·3% (-61·8 to 59·3%)	-41·4% (-69·4 to 29·8%)	64·6 (30·1 to 102·9)	15·8% (7·3 to 26·3%)	47·5 (28·4 to 103·5)	9·7% (6·2 to 20·9%)	-26·4% (-60·0 to 56·0%)	-38·3% (-67·6 to 35·9%)
Other transport injuries	1·4 (0·7 to 2·2)	6·1% (3·1 to 9·2%)	1·3 (0·8 to 2·4)	5·0% (3·4 to 9·0%)	-7·9% (-51·0 to 103·0%)	-17·6% (-54·3 to 72·6%)	77·6 (38·9 to 116·5)	6·2% (3·2 to 9·1%)	70·9 (45·7 to 129·9)	5·3% (3·6 to 9·3%)	-8·6% (-50·3 to 101·2%)	-15·1% (-52·3 to 75·6%)
Falls	5·7 (3·8 to 7·8)	7·1% (4·8 to 9·7%)	5·5 (4·4 to 7·9)	5·7% (4·6 to 8·1%)	-4·6% (-32·7 to 46·9%)	-19·8% (-43·9 to 24·2%)	491·5 (336·7 to 660·9)	9·4% (6·6 to 12·1%)	533·7 (400·0 to 778·6)	8·9% (7·1 to 12·5%)	8·6% (-20·1 to 58·3%)	-5·9% (-31·7 to 37·1%)
Drowning	12·8 (6·8 to 18·3)	9·7% (5·2 to 14·2%)	9·2 (6·2 to 17·9)	8·2% (5·7 to 16·5%)	-27·8% (-56·7 to 38·1%)	-15·5% (-50·8 to 60·0%)	668·6 (363·7 to 932·9)	7·8% (4·2 to 11·4%)	484·6 (330·0 to 930·7)	7·2% (5·0 to 14·4%)	-27·5% (-55·8 to 38·1%)	-7·4% (-46·0 to 74·0%)
Fire, heat, and hot substances	4·4 (3·4 to 6·0)	7·2% (5·6 to 9·7%)	4·7 (3·7 to 6·2)	7·9% (6·4 to 10·6%)	6·6% (-18·3 to 42·7%)	10·9% (-15·3 to 46·8%)	311·6 (242·1 to 418·3)	7·6% (5·9 to 10·3%)	347·0 (276·5 to 460·1)	8·9% (7·1 to 12·1%)	11·4% (-14·3 to 48·0%)	17·0% (-10·8 to 54·8%)
Poisonings	0·9 (0·7 to 1·1)	4·2% (2·7 to 5·7%)	0·9 (0·7 to 1·1)	4·4% (3·0 to 6·1%)	-3·4% (-21·7 to 16·4%)	6·3% (-26·7 to 58·4%)	58·5 (47·6 to 69·2)	3·9% (2·4 to 5·5%)	60·8 (49·1 to 74·2)	4·4% (2·7 to 6·4%)	4·0% (-15·7 to 24·8%)	14·6% (-24·4 to 81·4%)
Unintentional firearm injuries	0·4 (0·3 to 0·5)	5·6% (4·8 to 6·7%)	0·5 (0·3 to 0·6)	6·1% (5·2 to 7·5%)	14·0% (-2·6 to 33·1%)	9·6% (-8·3 to 30·8%)	23·2 (16·7 to 29·2)	5·6% (4·6 to 6·8%)	26·7 (20·1 to 32·3)	6·3% (5·2 to 7·6%)	14·9% (-1·6 to 34·3%)	11·6% (-7·7 to 35·2%)
Unintentional suffocation	0·0 (0·0 to 0·0)	0·3% (0·2 to 0·4%)	0·0 (0·0 to 0·1)	0·4% (0·2 to 0·6%)	29·6% (-17·5 to 116·7%)	32·2% (-19·4 to 131·5%)	2·1 (1·5 to 3·1)	0·3% (0·2 to 0·5%)	2·9 (1·9 to 4·7)	0·4% (0·2 to 0·7%)	34·4% (-12·5 to 117·1%)	44·9% (-12·1 to 150·0%)
Other exposure to mechanical forces	2·6 (2·1 to 3·3)	7·0% (5·7 to 8·5%)	2·9 (2·3 to 3·8)	7·4% (6·1 to 9·5%)	10·3% (-15·2 to 50·3%)	5·5% (-17·8 to 39·2%)	218·8 (170·4 to 274·5)	8·0% (6·5 to 9·7%)	249·3 (194·8 to 330·7)	9·0% (7·5 to 11·4%)	13·9% (-10·3 to 47·9%)	12·3% (-11·7 to 45·7%)
Venomous animal contact	1·8 (1·2 to 2·2)	3·9% (3·2 to 4·7%)	1·6 (0·9 to 2·3)	3·9% (3·1 to 4·9%)	-10·8% (-31·6 to 24·3%)	1·2% (-19·2 to 25·7%)	111·1 (80·8 to 136·3)	3·9% (3·2 to 4·8%)	103·4 (70·7 to 138·1)	4·3% (3·3 to 5·3%)	-6·9% (-26·1 to 21·5%)	8·9% (-13·1 to 35·0%)
Non-venomous animal contact	0·2 (0·1 to 0·2)	3·1% (2·6 to 3·8%)	0·2 (0·1 to 0·2)	3·3% (2·7 to 4·1%)	-8·5% (-29·4 to 23·7%)	4·6% (-17·9 to 31·1%)	15·1 (11·6 to 19·3)	3·4% (2·8 to 4·3%)	14·9 (11·4 to 19·6)	3·9% (3·1 to 5·0%)	-0·9% (-19·4 to 23·9%)	13·6% (-10·6 to 42·3%)
Pulmonary aspiration and foreign body in airway	0·8 (0·6 to 1·1)	3·0% (2·2 to 4·4%)	1·1 (0·9 to 1·4)	3·6% (2·9 to 4·7%)	31·5% (9·5 to 59·1%)	22·0% (-3·1 to 50·7%)	54·3 (41·3 to 71·7)	2·7% (1·8 to 4·2%)	72·2 (57·2 to 89·7)	3·4% (2·6 to 4·9%)	32·9% (10·8 to 58·0%)	28·3% (0·4 to 65·1%)
Foreign body in eyes	-	-	-	-	-	-	0·8 (0·3 to 1·5)	6·7% (5·4 to 8·1%)	1·1 (0·5 to 2·1)	7·8% (6·2 to 9·6%)	37·8% (14·8 to 65·3%)	16·5% (-2·5 to 40·5%)
Foreign body in other body part	0·6 (0·4 to 1·0)	6·3% (5·0 to 7·9%)	0·7 (0·4 to 0·9)	7·3% (5·8 to 9·2%)	4·3% (-22·5 to 35·9%)	16·2% (-11·8 to 47·9%)	48·5 (34·0 to 68·8)	6·6% (5·0 to 8·7%)	57·3 (43·5 to 74·1)	8·4% (6·4 to 10·5%)	18·1% (-7·1 to 50·4%)	25·9% (-4·2 to 60·2%)
Other unintentional injuries	4·9 (3·5 to 6·6)	9·2% (6·7 to 12·6%)	4·3 (3·0 to 7·6)	8·3% (5·7 to 14·5%)	-11·6% (-42·6 to 55·8%)	-9·3% (-42·1 to 53·3%)	427·1 (299·0 to 583·7)	10·1% (7·3 to 13·4%)	393·3 (272·8 to 654·7)	9·7% (7·0 to 15·0%)	-7·9% (-37·3 to 52·8%)	-3·5% (-34·7 to 56·2%)
**Occupational ergonomic factors: All causes**	-	-	-	-	-	-	4,216·8 (2,950·7 to 5,856·5)	0·5% (0·4 to 0·7%)	4,771·7 (3,319·8 to 6,602·1)	0·6% (0·5 to 0·9%)	13·2% (11·0 to 15·4%)	23·2% (19·0 to 27·7%)
Low back pain	-	-	-	-	-	-	4,216·8 (2,950·7 to 5,856·5)	41·7% (39·1 to 44·2%)	4,771·7 (3,319·8 to 6,602·1)	38·8% (36·5 to 41·0%)	13·2% (11·0 to 15·4%)	-7·0% (-8·3 to -5·7%)
**Behavioural risks: All causes**	4,686·1 (4,450·4 to 4,915·4)	35·1% (33·6 to 36·6%)	4,640·4 (4,341·5 to 4,950·0)	34·9% (33·1 to 36·7%)	-1·0% (-5·7 to 4·2%)	-0·4% (-3·2 to 2·5%)	247,970·8 (233,521·3 to 263,621·4)	31·0% (29·7 to 32·3%)	208,416·2 (193,452·0 to 223,242·4)	28·4% (27·2 to 29·6%)	-16·0% (-19·8 to -12·0%)	-8·4% (-11·6 to -5·3%)
**Child and maternal malnutrition: All causes**	1,194·7 (1,095·8 to 1,296·0)	8·9% (8·2 to 9·7%)	703·3 (640·1 to 771·1)	5·3% (4·8 to 5·8%)	-41·1% (-46·5 to -35·5%)	-40·8% (-46·2 to -35·1%)	122,851·5 (112,224·0 to 135,748·6)	15·3% (14·3 to 16·4%)	79,910·8 (71,223·0 to 90,268·6)	10·9% (10·0 to 11·7%)	-35·0% (-40·0 to -29·8%)	-29·2% (-34·4 to -23·8%)
**Suboptimal breastfeeding: All causes**	303·1 (202·0 to 427·4)	2·3% (1·5 to 3·2%)	203·8 (134·4 to 282·3)	1·5% (1·0 to 2·1%)	-32·8% (-40·5 to -23·8%)	-32·4% (-40·6 to -23·2%)	26,236·0 (17,501·9 to 36,990·6)	3·3% (2·1 to 4·6%)	17,681·6 (11,710·0 to 24,464·4)	2·4% (1·6 to 3·4%)	-32·6% (-40·3 to -23·7%)	-26·6% (-34·9 to -16·9%)
**Non-exclusive breastfeeding : All causes**	280·6 (178·4 to 402·1)	2·1% (1·3 to 3·0%)	188·1 (120·3 to 265·7)	1·4% (0·9 to 2·0%)	-33·0% (-40·4 to -23·8%)	-32·6% (-40·8 to -23·6%)	24,243·9 (15,461·5 to 34,716·2)	3·0% (1·9 to 4·3%)	16,269·4 (10,419·3 to 22,947·0)	2·2% (1·4 to 3·1%)	-32·9% (-40·4 to -23·8%)	-26·9% (-35·1 to -17·5%)
Diarrhoeal diseases	124·9 (76·2 to 171·2)	13·6% (8·5 to 18·5%)	90·8 (56·1 to 128·7)	12·6% (7·9 to 17·3%)	-27·3% (-40·8 to -10·4%)	-7·5% (-21·7 to 9·3%)	10,807·9 (6,611·3 to 14,799·1)	20·0% (12·6 to 27·1%)	7,872·0 (4,888·1 to 11,122·3)	20·0% (12·5 to 27·3%)	-27·2% (-40·6 to -10·4%)	-0·1% (-12·6 to 14·1%)
Lower respiratory infections	155·7 (65·5 to 269·8)	15·5% (6·5 to 26·6%)	97·3 (40·4 to 168·5)	11·5% (4·8 to 19·8%)	-37·5% (-45·3 to -28·8%)	-26·0% (-34·2 to -16·5%)	13,436·0 (5,654·6 to 23,258·5)	21·9% (9·3 to 37·7%)	8,397·4 (3,491·8 to 14,531·7)	19·3% (8·1 to 32·8%)	-37·5% (-45·2 to -28·8%)	-12·1% (-20·3 to -2·5%)
**Discontinued breastfeeding : All causes**	29·4 (10·5 to 56·8)	0·2% (0·1 to 0·4%)	21·1 (7·1 to 40·3)	0·2% (0·1 to 0·3%)	-28·2% (-43·5 to -8·9%)	-27·8% (-43·0 to -8·9%)	2,594·0 (932·0 to 5,016·6)	0·3% (0·1 to 0·6%)	1,887·8 (630·4 to 3,596·2)	0·3% (0·1 to 0·5%)	-27·2% (-41·8 to -8·6%)	-20·7% (-36·2 to -0·9%)
Diarrhoeal diseases	29·4 (10·5 to 56·8)	3·2% (1·1 to 6·1%)	21·1 (7·1 to 40·3)	2·9% (1·0 to 5·5%)	-28·2% (-43·5 to -8·9%)	-8·6% (-25·1 to 10·3%)	2,594·0 (932·0 to 5,016·6)	4·8% (1·7 to 9·1%)	1,887·8 (630·4 to 3,596·2)	4·8% (1·6 to 9·1%)	-27·2% (-41·8 to -8·6%)	-0·2% (-14·5 to 15·9%)
**Childhood undernutrition: All causes**	1,115·4 (1,020·2 to 1,218·0)	8·3% (7·6 to 9·1%)	643·9 (580·3 to 712·1)	4·8% (4·3 to 5·4%)	-42·3% (-47·7 to -36·5%)	-41·9% (-47·5 to -36·1%)	97,902·1 (89,713·9 to 106,629·8)	12·2% (11·1 to 13·5%)	57,462·3 (51,875·8 to 63,272·3)	7·8% (7·0 to 8·7%)	-41·3% (-46·6 to -35·7%)	-36·1% (-41·5 to -30·4%)
**Childhood underweight: All causes**	363·1 (282·6 to 478·9)	2·7% (2·1 to 3·6%)	182·9 (144·5 to 235·8)	1·4% (1·1 to 1·8%)	-49·6% (-59·2 to -37·4%)	-49·3% (-59·3 to -37·4%)	32,656·9 (25,714·8 to 42,596·9)	4·1% (3·2 to 5·4%)	17,142·9 (13,773·8 to 21,688·4)	2·3% (1·9 to 3·0%)	-47·5% (-56·9 to -35·5%)	-42·8% (-53·1 to -30·5%)
Diarrhoeal diseases	73·4 (58·7 to 94·1)	8·0% (6·6 to 10·0%)	40·9 (32·4 to 51·8)	5·7% (4·6 to 7·1%)	-44·2% (-53·7 to -33·3%)	-29·0% (-38·5 to -19·0%)	6,463·6 (5,219·5 to 8,250·8)	12·0% (10·1 to 14·9%)	3,655·5 (2,904·9 to 4,622·8)	9·3% (7·6 to 11·5%)	-43·4% (-52·6 to -32·7%)	-22·4% (-29·1 to -15·5%)
Lower respiratory infections	120·1 (80·8 to 204·6)	11·9% (8·1 to 20·3%)	59·3 (37·6 to 106·2)	7·0% (4·5 to 12·4%)	-50·6% (-56·4 to -44·3%)	-41·5% (-47·2 to -36·3%)	10,309·9 (6,936·9 to 17,572·3)	16·8% (11·5 to 28·3%)	5,101·2 (3,234·4 to 9,127·4)	11·7% (7·6 to 20·6%)	-50·5% (-56·4 to -44·2%)	-30·4% (-35·5 to -25·9%)
Measles	66·2 (13·6 to 168·9)	35·2% (11·2 to 60·6%)	10·2 (1·8 to 28·5)	29·3% (8·5 to 54·6%)	-84·7% (-90·0 to -78·0%)	-16·9% (-32·5 to -7·7%)	5,637·2 (1,159·2 to 14,377·8)	35·8% (11·4 to 61·6%)	864·6 (156·2 to 2,428·2)	29·7% (8·6 to 55·4%)	-84·7% (-90·0 to -78·0%)	-16·9% (-32·5 to -7·7%)
Protein-energy malnutrition	103·4 (73·1 to 154·3)	67·1% (59·6 to 75·9%)	72·5 (48·7 to 110·2)	61·2% (52·9 to 71·3%)	-29·9% (-54·8 to 9·3%)	-8·7% (-22·0 to 7·1%)	10,246·3 (7,617·5 to 14,481·9)	86·9% (83·5 to 90·8%)	7,521·7 (5,372·4 to 10,673·5)	85·4% (81·4 to 89·8%)	-26·6% (-49·5 to 6·1%)	-1·7% (-6·4 to 3·5%)
**Childhood wasting: All causes**	997·1 (855·8 to 1,129·3)	7·5% (6·4 to 8·5%)	596·2 (501·3 to 673·8)	4·5% (3·8 to 5·1%)	-40·2% (-46·7 to -33·5%)	-39·9% (-46·7 to -33·4%)	87,749·6 (75,817·4 to 99,451·7)	11·0% (9·5 to 12·5%)	53,334·9 (44,879·0 to 59,984·4)	7·3% (6·2 to 8·3%)	-39·2% (-45·7 to -32·7%)	-33·8% (-40·8 to -27·0%)

Supplemental Results Table 1. All-age deaths, death PAFs, DALYs, DALY PAFs, and percent change for each risk factor and outcome for both sexes combined in 2005 and 2015 for Low-middle SDI.

Data in parenthesis are 95% uncertainty intervals. PAFs=population attributable fractions. DALYs=disability-adjusted life-years.

	2005 Deaths (in thousands)	2005 Deaths PAF	2015 Deaths (in thousands)	2015 Deaths PAF	Percent change deaths 2005-2015	Percent change deaths PAF 2005-2015	2005 DALYs (in thousands)	2005 DALYs PAF	2015 DALYs (in thousands)	2015 DALYs PAF	Percent change DALYs 2005-2015	Percent change DALYs PAF 2005-2015
Diarrhoeal diseases	368·0 (301·8 to 427·0)	40·0% (33·9 to 44·0%)	245·5 (199·2 to 287·6)	34·0% (27·8 to 38·5%)	-33·3% (-44·2 to -20·2%)	-15·1% (-25·5 to -4·5%)	32,414·7 (26,744·0 to 37,463·5)	60·0% (51·0 to 64·3%)	21,901·6 (17,818·4 to 25,539·8)	55·6% (46·5 to 60·6%)	-32·4% (-43·1 to -19·6%)	-7·3% (-14·1 to -0·7%)
Lower respiratory infections	443·8 (326·5 to 521·3)	44·2% (32·4 to 52·3%)	264·1 (190·3 to 316·4)	31·2% (22·2 to 38·1%)	-40·5% (-47·1 to -33·7%)	-29·5% (-35·4 to -23·6%)	38,122·6 (28,054·1 to 44,743·4)	62·2% (46·0 to 71·3%)	22,717·4 (16,365·6 to 27,232·0)	52·1% (37·9 to 61·5%)	-40·4% (-47·0 to -33·6%)	-16·2% (-21·1 to -12·0%)
Measles	81·9 (20·3 to 239·0)	43·5% (16·8 to 81·4%)	14·0 (3·0 to 45·8)	40·3% (14·1 to 80·3%)	-82·9% (-89·4 to -74·6%)	-7·5% (-25·7 to 1·0%)	6,965·9 (1,729·3 to 20,346·1)	44·2% (17·1 to 82·6%)	1,194·2 (258·1 to 3,892·3)	40·9% (14·3 to 81·4%)	-82·9% (-89·4 to -74·6%)	-7·5% (-25·7 to 0·9%)
Protein-energy malnutrition	103·4 (73·1 to 154·3)	67·1% (59·6 to 75·9%)	72·5 (48·7 to 110·2)	61·2% (52·9 to 71·3%)	-29·9% (-54·8 to 9·3%)	-8·7% (-22·0 to 7·1%)	10,246·3 (7,617·5 to 14,481·9)	86·9% (83·5 to 90·8%)	7,521·7 (5,372·4 to 10,673·5)	85·4% (81·4 to 89·8%)	-26·6% (-49·5 to 6·1%)	-1·7% (-6·4 to 3·5%)
**Childhood stunting: All causes**	287·6 (144·3 to 477·0)	2·2% (1·1 to 3·6%)	136·5 (62·2 to 234·3)	1·0% (0·5 to 1·8%)	-52·6% (-62·2 to -44·4%)	-52·3% (-61·9 to -44·3%)	24,859·9 (12,559·4 to 41,174·3)	3·1% (1·6 to 5·2%)	11,895·6 (5,470·8 to 20,324·9)	1·6% (0·7 to 2·8%)	-52·1% (-61·7 to -44·0%)	-47·8% (-58·7 to -39·2%)
Diarrhoeal diseases	88·2 (34·0 to 151·7)	9·6% (3·8 to 16·6%)	50·0 (17·8 to 89·7)	6·9% (2·5 to 12·3%)	-43·3% (-53·2 to -30·4%)	-27·8% (-37·3 to -17·0%)	7,772·2 (2,994·6 to 13,342·0)	14·4% (5·6 to 24·6%)	4,469·8 (1,589·3 to 8,050·3)	11·3% (4·1 to 20·1%)	-42·5% (-52·3 to -30·0%)	-21·1% (-28·3 to -14·0%)
Lower respiratory infections	152·2 (14·3 to 320·8)	15·2% (1·4 to 32·1%)	79·1 (6·0 to 174·4)	9·3% (0·7 to 20·7%)	-48·0% (-55·0 to -37·9%)	-38·4% (-45·5 to -27·6%)	13,075·0 (1,225·3 to 27,537·8)	21·3% (2·0 to 45·1%)	6,804·4 (517·1 to 14,987·9)	15·6% (1·3 to 35·4%)	-48·0% (-54·9 to -37·9%)	-26·8% (-33·6 to -16·6%)
Measles	47·2 (5·1 to 132·2)	25·3% (3·1 to 57·9%)	7·3 (0·6 to 23·1)	21·1% (2·3 to 52·0%)	-84·5% (-90·1 to -76·4%)	-16·6% (-28·8 to -5·7%)	4,012·7 (438·0 to 11,244·7)	25·7% (3·2 to 58·9%)	621·4 (51·3 to 1,963·5)	21·4% (2·3 to 52·9%)	-84·5% (-90·1 to -76·5%)	-16·6% (-28·9 to -5·8%)
**Iron deficiency: All causes**	35·8 (22·8 to 49·9)	0·3% (0·2 to 0·4%)	30·5 (20·3 to 43·5)	0·2% (0·2 to 0·3%)	-14·9% (-29·3 to 3·9%)	-14·5% (-28·2 to 2·8%)	21,098·2 (14,673·4 to 29,265·7)	2·6% (1·9 to 3·5%)	19,826·2 (13,721·2 to 27,664·8)	2·7% (2·0 to 3·5%)	-6·0% (-8·9 to -3·2%)	2·3% (-1·4 to 6·3%)
Maternal haemorrhage	13·6 (5·3 to 22·8)	32·9% (12·7 to 53·6%)	9·6 (3·8 to 16·9)	30·5% (11·3 to 49·4%)	-29·2% (-46·5 to -8·5%)	-7·3% (-13·6 to -1·6%)	776·4 (308·1 to 1,303·3)	33·0% (12·8 to 53·8%)	544·2 (215·4 to 945·6)	30·6% (11·4 to 49·6%)	-29·9% (-46·7 to -10·0%)	-7·3% (-13·6 to -1·4%)
Maternal sepsis and other pregnancy related infections	5·4 (2·1 to 8·9)	35·2% (13·8 to 56·6%)	3·3 (1·2 to 5·8)	32·0% (12·1 to 51·2%)	-38·9% (-54·8 to -15·4%)	-9·1% (-15·2 to -2·1%)	308·4 (123·6 to 509·3)	35·3% (13·8 to 56·8%)	189·5 (67·9 to 334·4)	32·1% (12·1 to 51·3%)	-38·6% (-54·1 to -15·8%)	-9·0% (-15·1 to -2·1%)
Iron-deficiency anaemia	16·8 (10·6 to 22·4)	100·0% (100·0 to 100·0%)	17·5 (11·1 to 25·2)	100·0% (100·0 to 100·0%)	4·3% (-15·7 to 28·6%)	-	20,013·3 (13,783·1 to 28,042·7)	100·0% (100·0 to 100·0%)	19,092·6 (13,194·3 to 26,842·1)	100·0% (100·0 to 100·0%)	-4·6% (-7·2 to -2·0%)	-
**Vitamin A deficiency: All causes**	109·2 (65·6 to 168·3)	0·8% (0·5 to 1·3%)	43·7 (25·9 to 63·2)	0·3% (0·2 to 0·5%)	-60·0% (-70·0 to -48·7%)	-59·7% (-69·9 to -49·0%)	9,565·8 (5,775·1 to 14,606·1)	1·2% (0·7 to 1·8%)	3,981·5 (2,407·8 to 5,692·3)	0·5% (0·3 to 0·8%)	-58·4% (-68·5 to -47·5%)	-54·6% (-65·5 to -42·7%)
Diarrhoeal diseases	57·4 (32·1 to 83·6)	6·2% (3·5 to 9·0%)	34·7 (19·4 to 51·1)	4·8% (2·7 to 7·0%)	-39·5% (-50·1 to -27·5%)	-23·0% (-33·5 to -11·3%)	5,072·7 (2,837·2 to 7,356·0)	9·4% (5·3 to 13·6%)	3,118·0 (1,747·9 to 4,585·0)	7·9% (4·5 to 11·3%)	-38·5% (-48·9 to -26·8%)	-15·7% (-23·5 to -7·3%)
Measles	51·8 (18·5 to 108·8)	27·7% (18·6 to 37·0%)	9·0 (3·0 to 20·5)	26·1% (17·2 to 35·4%)	-82·6% (-88·0 to -75·8%)	-6·0% (-15·9 to 5·2%)	4,405·8 (1,571·8 to 9,238·6)	28·1% (18·9 to 37·5%)	765·9 (256·2 to 1,738·2)	26·4% (17·4 to 35·9%)	-82·6% (-88·0 to -75·9%)	-6·0% (-15·9 to 5·3%)
Vitamin A deficiency	-	-	-	-	-	-	87·2 (54·3 to 128·2)	100·0% (100·0 to 100·0%)	97·6 (60·7 to 144·6)	100·0% (100·0 to 100·0%)	11·9% (7·1 to 17·6%)	-
**Zinc deficiency: All causes**	51·7 (3·1 to 115·9)	0·4% (0·0 to 0·9%)	29·0 (1·6 to 65·1)	0·2% (0·0 to 0·5%)	-44·0% (-54·4 to -28·5%)	-43·6% (-54·2 to -27·0%)	4,531·8 (439·5 to 9,927·6)	0·6% (0·1 to 1·2%)	2,592·1 (299·9 to 5,670·0)	0·4% (0·0 to 0·8%)	-42·8% (-52·8 to -24·9%)	-37·7% (-48·5 to -17·6%)
Diarrhoeal diseases	29·1 (0·0 to 67·3)	3·2% (0·0 to 7·2%)	16·8 (0·0 to 40·6)	2·3% (0·0 to 5·7%)	-42·3% (-55·7 to 0·0%)	-26·6% (-40·8 to 0·0%)	2,608·8 (143·7 to 5,872·3)	4·8% (0·3 to 10·7%)	1,553·2 (127·6 to 3,543·8)	3·9% (0·3 to 9·1%)	-40·5% (-53·4 to -11·3%)	-18·5% (-31·1 to 22·9%)
Lower respiratory infections	22·6 (0·0 to 76·0)	2·2% (0·0 to 7·5%)	12·2 (0·0 to 41·1)	1·4% (0·0 to 4·9%)	-46·2% (-55·1 to 0·0%)	-36·2% (-45·6 to 0·0%)	1,923·0 (6·9 to 6,426·0)	3·1% (0·0 to 10·5%)	1,038·9 (5·8 to 3,478·3)	2·4% (0·0 to 8·0%)	-46·0% (-54·5 to -12·5%)	-24·0% (-33·9 to 27·8%)
**Tobacco smoke: All causes**	1,081·7 (971·2 to 1,183·7)	8·1% (7·4 to 8·8%)	1,245·0 (1,121·0 to 1,372·3)	9·4% (8·5 to 10·2%)	15·1% (6·8 to 24·7%)	15·7% (9·7 to 22·5%)	34,527·8 (30,324·9 to 38,856·9)	4·3% (3·8 to 4·9%)	35,972·4 (32,172·6 to 39,855·9)	4·9% (4·4 to 5·5%)	4·2% (-3·4 to 12·9%)	13·5% (6·5 to 21·8%)
**Smoking: All causes**	855·1 (764·3 to 943·8)	6·4% (5·8 to 7·0%)	1,029·0 (916·4 to 1,141·1)	7·7% (7·0 to 8·5%)	20·3% (10·5 to 31·4%)	21·0% (13·7 to 29·1%)	23,559·1 (21,139·6 to 25,995·5)	2·9% (2·6 to 3·3%)	27,688·8 (24,730·7 to 30,631·2)	3·8% (3·3 to 4·2%)	17·5% (8·2 to 28·1%)	28·1% (19·7 to 38·0%)
Tuberculosis	44·1 (22·4 to 70·2)	7·7% (3·9 to 11·8%)	34·8 (15·9 to 56·6)	7·1% (3·4 to 11·1%)	-20·9% (-35·7 to -8·4%)	-7·5% (-16·6 to -0·3%)	1,425·3 (717·2 to 2,265·3)	6·7% (3·4 to 10·3%)	1,122·4 (509·4 to 1,820·0)	6·4% (3·0 to 10·0%)	-21·3% (-35·1 to -9·4%)	-5·1% (-15·4 to 3·0%)
Lower respiratory infections	63·8 (45·7 to 79·8)	6·3% (4·9 to 7·9%)	63·7 (46·6 to 81·8)	7·5% (5·9 to 9·4%)	-0·1% (-11·7 to 11·3%)	18·3% (5·3 to 30·6%)	1,641·3 (1,176·7 to 2,061·4)	2·7% (2·0 to 3·4%)	1,620·2 (1,190·3 to 2,074·6)	3·7% (2·8 to 4·7%)	-1·3% (-12·9 to 10·4%)	38·8% (20·6 to 57·4%)
Lip and oral cavity cancer	8·6 (6·7 to 10·5)	29·7% (23·7 to 35·9%)	12·9 (10·1 to 15·8)	32·0% (26·0 to 38·1%)	50·4% (28·8 to 76·6%)	7·8% (-1·8 to 19·4%)	263·0 (203·7 to 324·3)	31·1% (24·5 to 37·7%)	389·4 (304·3 to 481·3)	33·5% (27·0 to 40·1%)	48·1% (25·7 to 74·0%)	7·8% (-1·9 to 18·9%)
Nasopharynx cancer	2·3 (1·7 to 3·1)	29·9% (23·3 to 36·2%)	3·2 (2·3 to 4·1)	32·0% (25·4 to 38·6%)	35·2% (15·2 to 58·8%)	7·1% (-3·5 to 17·9%)	74·7 (54·9 to 99·1)	28·9% (22·4 to 35·5%)	98·6 (72·5 to 128·2)	30·9% (24·6 to 37·5%)	32·0% (12·3 to 55·8%)	7·0% (-3·5 to 18·2%)
Oesophageal cancer	11·5 (8·3 to 14·7)	25·4% (18·7 to 32·3%)	16·6 (11·8 to 22·0)	28·5% (20·6 to 36·6%)	45·2% (24·2 to 70·2%)	12·5% (0·9 to 24·6%)	329·4 (241·5 to 420·7)	26·7% (20·0 to 34·0%)	460·8 (323·5 to 609·5)	29·6% (21·5 to 38·2%)	39·9% (18·7 to 66·2%)	10·7% (-1·1 to 23·2%)
Stomach cancer	4·7 (2·7 to 7·0)	6·4% (3·7 to 9·3%)	6·0 (3·5 to 8·9)	7·2% (4·3 to 10·5%)	27·3% (5·4 to 55·7%)	11·2% (-3·8 to 30·5%)	132·1 (75·1 to 197·3)	7·1% (4·1 to 10·3%)	160·0 (92·8 to 239·4)	7·8% (4·5 to 11·4%)	21·2% (-0·4 to 50·1%)	9·5% (-5·7 to 28·8%)
Colon and rectum cancer	1·7 (1·1 to 2·4)	3·2% (2·1 to 4·5%)	2·5 (1·6 to 3·7)	3·5% (2·3 to 4·9%)	52·2% (22·2 to 91·3%)	8·4% (-8·1 to 29·7%)	49·8 (30·9 to 74·0)	3·5% (2·3 to 5·0%)	72·1 (44·7 to 107·5)	3·8% (2·4 to 5·4%)	44·7% (15·1 to 83·0%)	6·7% (-9·8 to 28·3%)
Liver cancer due to hepatitis B	2·2 (1·1 to 3·7)	9·8% (5·1 to 15·8%)	2·8 (1·4 to 4·6)	10·6% (5·7 to 16·7%)	23·4% (-5·4 to 65·5%)	8·1% (-11·0 to 33·7%)	74·0 (35·8 to 124·7)	9·5% (4·8 to 15·4%)	88·8 (46·4 to 149·0)	10·2% (5·4 to 16·4%)	20·1% (-10·8 to 66·2%)	7·2% (-14·5 to 36·2%)
Liver cancer due to hepatitis C	1·3 (0·7 to 2·0)	9·2% (5·2 to 13·9%)	1·7 (1·0 to 2·6)	10·1% (5·8 to 15·1%)	33·4% (10·2 to 68·2%)	10·2% (-5·0 to 30·1%)	33·2 (18·4 to 51·5)	10·1% (5·8 to 15·2%)	43·3 (24·1 to 66·6)	11·1% (6·4 to 16·7%)	30·5% (6·1 to 67·3%)	9·4% (-7·3 to 31·3%)
Liver cancer due to alcohol use	1·5 (0·8 to 2·6)	10·2% (5·2 to 16·3%)	2·1 (1·0 to 3·6)	11·2% (5·8 to 18·2%)	37·6% (-1·7 to 96·5%)	10·1% (-11·4 to 39·7%)	41·5 (20·1 to 70·8)	10·7% (5·4 to 17·1%)	56·7 (27·0 to 102·4)	11·8% (6·1 to 19·1%)	36·7% (-5·6 to 104·0%)	10·4% (-13·3 to 45·3%)
Liver cancer due to other causes	1·1 (0·6 to 1·7)	9·3% (5·2 to 14·4%)	1·3 (0·7 to 2·1)	10·0% (5·7 to 15·0%)	21·5% (-3·2 to 58·0%)	7·8% (-9·1 to 30·9%)	31·4 (16·9 to 50·4)	9·5% (5·3 to 14·9%)	37·0 (20·1 to 58·7)	10·2% (5·9 to 15·5%)	17·8% (-7·9 to 55·5%)	7·6% (-11·5 to 32·5%)
Pancreatic cancer	2·5 (1·8 to 3·4)	9·3% (6·8 to 12·0%)	3·9 (2·8 to 5·2)	10·2% (7·5 to 13·4%)	52·7% (27·8 to 84·7%)	10·5% (-2·5 to 26·1%)	67·7 (48·1 to 90·1)	10·3% (7·6 to 13·5%)	100·8 (70·6 to 137·2)	11·2% (8·2 to 14·7%)	48·8% (23·3 to 81·3%)	8·8% (-4·3 to 24·6%)
Tracheal, bronchus, and lung cancer	57·3 (50·7 to 64·9)	58·8% (54·1 to 64·0%)	88·2 (77·4 to 99·6)	62·3% (57·5 to 66·9%)	53·9% (36·2 to 72·5%)	6·0% (1·2 to 10·9%)	1,469·3 (1,293·1 to 1,680·8)	59·1% (54·2 to 64·2%)	2,132·4 (1,850·5 to 2,433·1)	61·9% (57·1 to 66·6%)	45·1% (27·6 to 63·6%)	4·7% (0·0 to 9·6%)
Cervical cancer	1·8 (0·5 to 3·7)	3·5% (1·0 to 6·8%)	2·1 (0·6 to 4·2)	3·8% (1·1 to 7·1%)	17·6% (-22·4 to 84·8%)	10·3% (-20·3 to 53·6%)	60·6 (16·5 to 126·6)	3·7% (1·0 to 7·4%)	71·0 (19·5 to 143·5)	4·1% (1·2 to 7·9%)	17·2% (-24·9 to 86·6%)	12·2% (-20·8 to 59·4%)
Kidney cancer	0·6 (0·4 to 0·9)	7·9% (4·8 to 11·4%)	1·0 (0·6 to 1·5)	9·0% (5·5 to 12·9%)	64·3% (31·0 to 111·8%)	14·5% (-4·9 to 40·6%)	18·8 (11·3 to 27·3)	6·4% (3·8 to 9·9%)	30·5 (18·2 to 46·0)	7·3% (4·3 to 11·0%)	62·2% (27·4 to 111·7%)	15·0% (-12·0 to 52·0%)
Bladder cancer	2·4 (1·7 to 3·2)	15·0% (10·5 to 19·9%)	3·5 (2·4 to 4·8)	16·4% (11·4 to 21·8%)	44·9% (20·4 to 74·3%)	9·3% (-3·0 to 23·5%)	61·6 (43·0 to 81·5)	16·5% (11·6 to 21·6%)	86·7 (59·4 to 116·9)	17·9% (12·4 to 23·4%)	40·7% (15·5 to 70·7%)	8·4% (-4·5 to 23·4%)
Ischaemic heart disease	190·1 (155·0 to 228·2)	16·6% (13·6 to 19·7%)	221·2 (175·5 to 266·2)	14·4% (11·6 to 17·4%)	16·4% (7·1 to 26·2%)	-13·0% (-17·7 to -7·8%)	5,436·0 (4,414·7 to 6,518·8)	20·1% (16·3 to 23·9%)	6,415·6 (5,056·0 to 7,778·5)	18·3% (14·6 to 22·1%)	18·0% (8·7 to 28·0%)	-9·0% (-13·6 to -4·4%)
Ischaemic stroke	49·6 (40·1 to 59·6)	13·0% (10·5 to 15·5%)	51·8 (41·5 to 62·8)	11·1% (9·0 to 13·5%)	4·4% (-5·7 to 16·0%)	-14·3% (-20·7 to -7·1%)	1,147·9 (936·8 to 1,384·9)	15·8% (12·8 to 18·8%)	1,219·4 (982·2 to 1,487·0)	14·3% (11·6 to 17·2%)	6·2% (-3·6 to 17·1%)	-9·7% (-15·7 to -3·2%)
Hemorrhagic stroke	87·7 (71·1 to 104·3)	16·9% (14·0 to 19·9%)	91·8 (73·9 to 111·2)	15·2% (12·4 to 18·0%)	4·7% (-4·6 to 15·2%)	-10·1% (-16·0 to -4·1%)	2,563·6 (2,092·7 to 3,057·2)	18·1% (14·9 to 21·3%)	2,749·3 (2,211·5 to 3,339·1)	17·1% (13·9 to 20·2%)	7·2% (-2·0 to 18·2%)	-5·1% (-11·2 to 1·0%)
Hypertensive heart disease	20·5 (15·4 to 27·0)	13·3% (10·1 to 16·8%)	21·9 (16·1 to 28·3)	11·6% (8·8 to 14·5%)	6·9% (-6·1 to 21·7%)	-12·8% (-20·7 to -4·6%)	585·4 (437·5 to 768·6)	15·9% (12·2 to 19·8%)	629·7 (460·9 to 804·3)	14·5% (10·9 to 18·2%)	7·6% (-6·1 to 22·3%)	-9·1% (-17·1 to -1·2%)
Atrial fibrillation and flutter	1·1 (0·8 to 1·5)	9·0% (6·7 to 11·3%)	1·3 (0·9 to 1·7)	7·2% (5·4 to 9·0%)	12·2% (-0·7 to 26·7%)	-20·3% (-28·4 to -11·4%)	49·4 (33·9 to 67·4)	14·9% (11·4 to 18·4%)	56·3 (38·7 to 77·1)	12·7% (9·6 to 15·7%)	14·1% (8·3 to 20·5%)	-15·2% (-19·1 to -11·2%)
Aortic aneurysm	1·9 (1·4 to 2·6)	16·8% (13·0 to 21·0%)	2·4 (1·8 to 3·2)	14·6% (11·2 to 18·2%)	29·2% (12·9 to 46·5%)	-13·1% (-18·1 to -7·3%)	53·5 (38·5 to 73·6)	19·4% (15·1 to 24·3%)	69·6 (52·1 to 91·4)	17·5% (13·5 to 21·8%)	30·2% (13·2 to 47·8%)	-9·8% (-15·5 to -3·9%)

Supplemental Results Table 1. All-age deaths, death PAFs, DALYs, DALY PAFs, and percent change for each risk factor and outcome for both sexes combined in 2005 and 2015 for Low-middle SDI.

Data in parenthesis are 95% uncertainty intervals. PAFs=population attributable fractions. DALYs=disability-adjusted life-years.

	2005 Deaths (in thousands)	2005 Deaths PAF	2015 Deaths (in thousands)	2015 Deaths PAF	Percent change deaths 2005-2015	Percent change deaths PAF 2005-2015	2005 DALYs (in thousands)	2005 DALYs PAF	2015 DALYs (in thousands)	2015 DALYs PAF	Percent change DALYs 2005-2015	Percent change DALYs PAF 2005-2015
Peripheral vascular disease	0·1 (0·1 to 0·1)	12·2% (8·5 to 15·5%)	0·2 (0·1 to 0·2)	10·3% (7·5 to 13·2%)	55·8% (36·7 to 81·1%)	-15·8% (-22·7 to -6·1%)	9·1 (5·0 to 14·9)	13·5% (10·4 to 16·6%)	11·4 (6·7 to 18·4)	11·7% (8·9 to 14·5%)	26·0% (18·1 to 37·8%)	-13·5% (-18·1 to -8·6%)
Other cardiovascular and circulatory diseases	5·6 (4·2 to 7·1)	10·5% (8·0 to 13·0%)	6·3 (4·7 to 7·9)	9·7% (7·4 to 12·0%)	12·1% (-3·5 to 29·0%)	-7·3% (-16·3 to 2·5%)	207·5 (156·3 to 262·4)	9·1% (6·9 to 11·2%)	234·5 (175·3 to 299·3)	8·9% (6·8 to 10·9%)	13·0% (0·2 to 27·2%)	-2·0% (-10·2 to 7·7%)
Chronic obstructive pulmonary disease	226·6 (175·2 to 278·5)	29·9% (23·6 to 36·4%)	319·5 (258·9 to 381·0)	35·2% (29·1 to 41·5%)	41·0% (21·7 to 64·2%)	17·5% (5·3 to 31·3%)	5,447·8 (4,324·5 to 6,667·0)	30·8% (25·0 to 36·4%)	7,369·0 (6,040·0 to 8,700·6)	36·1% (30·1 to 41·8%)	35·3% (17·3 to 57·1%)	17·3% (6·7 to 29·2%)
Silicosis	0·1 (0·0 to 0·1)	10·4% (7·0 to 15·3%)	0·1 (0·1 to 0·1)	11·6% (7·8 to 16·2%)	38·6% (1·6 to 87·1%)	11·2% (-9·6 to 36·9%)	2·0 (1·2 to 3·1)	11·2% (7·5 to 16·2%)	2·7 (1·6 to 4·2)	11·8% (8·0 to 16·4%)	30·7% (-2·0 to 75·3%)	5·8% (-13·1 to 27·6%)
Asbestosis	0·0 (0·0 to 0·0)	7·3% (5·1 to 10·3%)	0·0 (0·0 to 0·1)	8·2% (5·8 to 11·2%)	74·8% (33·3 to 131·2%)	12·8% (-6·8 to 38·0%)	0·8 (0·4 to 1·2)	7·1% (4·7 to 9·9%)	1·2 (0·7 to 1·9)	7·7% (5·3 to 10·7%)	59·8% (27·2 to 105·5%)	9·3% (-9·8 to 33·8%)
Coal workers pneumoconiosis	0·0 (0·0 to 0·0)	9·7% (5·1 to 17·0%)	0·0 (0·0 to 0·0)	10·0% (5·8 to 16·7%)	19·1% (-24·9 to 103·1%)	3·3% (-23·9 to 39·1%)	0·4 (0·2 to 0·8)	9·7% (5·8 to 15·3%)	0·6 (0·3 to 0·9)	9·8% (6·3 to 14·9%)	25·6% (-15·4 to 91·3%)	1·7% (-21·3 to 30·5%)
Other pneumoconiosis	0·2 (0·1 to 0·3)	5·4% (3·6 to 7·7%)	0·3 (0·2 to 0·5)	6·3% (4·4 to 8·6%)	65·3% (34·1 to 110·3%)	15·6% (-6·8 to 41·3%)	8·3 (5·5 to 11·5)	5·5% (3·9 to 7·4%)	12·8 (8·5 to 17·9)	6·1% (4·4 to 8·1%)	54·9% (32·3 to 81·7%)	11·0% (-3·2 to 27·4%)
Asthma	22·2 (14·0 to 30·6)	11·3% (8·0 to 15·4%)	16·3 (12·3 to 21·1)	10·3% (7·9 to 12·7%)	-26·3% (-41·5 to -0·3%)	-9·1% (-25·5 to 13·2%)	680·6 (459·7 to 906·5)	7·5% (5·5 to 9·7%)	553·9 (418·8 to 713·3)	6·7% (5·1 to 8·3%)	-18·6% (-32·1 to 2·4%)	-11·4% (-23·1 to 3·0%)
Interstitial lung disease and pulmonary sarcoidosis	0·6 (0·2 to 1·2)	6·4% (4·4 to 9·1%)	1·2 (0·6 to 2·1)	7·4% (5·3 to 9·9%)	111·5% (59·8 to 191·1%)	15·7% (-4·6 to 37·5%)	15·4 (7·3 to 32·4)	6·9% (4·9 to 9·8%)	30·0 (16·9 to 51·9)	7·8% (5·6 to 10·3%)	94·2% (47·7 to 162·6%)	11·7% (-8·1 to 32·1%)
Other chronic respiratory diseases	0·6 (0·3 to 0·9)	3·9% (2·5 to 5·6%)	0·9 (0·5 to 1·4)	5·5% (3·8 to 7·7%)	52·9% (15·8 to 99·2%)	42·1% (12·0 to 81·0%)	61·7 (41·0 to 85·9)	4·0% (2·8 to 5·7%)	75·2 (50·2 to 103·5)	4·9% (3·6 to 6·7%)	21·9% (0·3 to 48·0%)	22·6% (1·5 to 46·4%)
Diabetes mellitus	11·8 (3·3 to 21·3)	4·6% (1·3 to 8·3%)	16·3 (4·5 to 29·2)	4·2% (1·1 to 7·4%)	37·6% (24·0 to 52·7%)	-9·5% (-18·3 to -0·8%)	484·9 (131·9 to 871·9)	4·8% (1·3 to 8·5%)	621·5 (167·7 to 1,132·7)	4·4% (1·2 to 7·9%)	28·2% (19·2 to 37·2%)	-8·1% (-13·9 to -2·5%)
**Second-hand smoke: All causes**	250·0 (186·6 to 314·8)	1·9% (1·4 to 2·4%)	243·5 (187·7 to 303·7)	1·8% (1·4 to 2·3%)	-2·6% (-8·7 to 4·7%)	-2·1% (-7·7 to 4·4%)	11,595·8 (8,149·3 to 15,129·0)	1·4% (1·0 to 1·9%)	9,014·4 (6,694·9 to 11,596·3)	1·2% (0·9 to 1·6%)	-22·3% (-28·2 to -14·9%)	-15·3% (-21·4 to -7·6%)
Lower respiratory infections	111·6 (69·9 to 156·2)	11·1% (7·1 to 15·3%)	76·6 (47·7 to 109·1)	9·0% (5·7 to 12·6%)	-31·3% (-37·0 to -25·3%)	-18·7% (-22·6 to -15·1%)	8,174·1 (5,106·1 to 11,413·8)	13·3% (8·5 to 18·4%)	4,981·3 (3,107·8 to 7,030·1)	11·4% (7·3 to 15·9%)	-39·1% (-44·7 to -32·5%)	-14·3% (-17·8 to -10·9%)
Otitis media	0·1 (0·1 to 0·1)	7·5% (5·1 to 9·8%)	0·1 (0·0 to 0·1)	6·2% (4·2 to 8·2%)	-36·2% (-45·7 to -23·9%)	-17·1% (-25·8 to -7·9%)	100·2 (56·7 to 159·3)	9·2% (6·3 to 12·2%)	95·1 (52·7 to 155·2)	8·5% (5·8 to 11·1%)	-5·1% (-9·6 to -1·3%)	-8·4% (-11·1 to -5·6%)
Tracheal, bronchus, and lung cancer	2·0 (1·2 to 3·2)	2·1% (1·2 to 3·2%)	2·6 (1·5 to 4·1)	1·9% (1·1 to 2·9%)	30·2% (24·8 to 35·2%)	-10·4% (-16·8 to -3·7%)	51·2 (30·2 to 81·3)	2·1% (1·2 to 3·2%)	65·1 (38·0 to 101·8)	1·9% (1·1 to 3·0%)	27·0% (21·1 to 32·3%)	-8·4% (-15·2 to -1·3%)
Ischaemic heart disease	68·3 (52·8 to 84·4)	6·0% (4·6 to 7·4%)	89·7 (68·7 to 112·4)	5·8% (4·5 to 7·3%)	31·5% (23·6 to 39·5%)	-1·7% (-4·9 to 1·3%)	1,722·1 (1,367·3 to 2,108·9)	6·4% (5·1 to 7·8%)	2,208·9 (1,723·6 to 2,737·8)	6·3% (5·0 to 7·8%)	28·3% (20·3 to 36·6%)	-1·1% (-4·6 to 2·1%)
Ischaemic stroke	11·8 (8·5 to 15·7)	3·1% (2·3 to 4·0%)	13·9 (10·0 to 18·4)	3·0% (2·2 to 3·9%)	17·6% (6·8 to 28·6%)	-3·4% (-8·2 to 1·2%)	240·5 (179·5 to 313·6)	3·3% (2·6 to 4·2%)	274·3 (204·9 to 354·0)	3·2% (2·4 to 4·1%)	14·1% (3·8 to 24·7%)	-3·0% (-7·8 to 1·2%)
Hemorrhagic stroke	18·2 (13·9 to 23·1)	3·5% (2·7 to 4·4%)	20·6 (15·8 to 26·3)	3·4% (2·6 to 4·3%)	13·1% (5·2 to 22·2%)	-2·9% (-7·0 to 1·1%)	521·0 (405·4 to 669·1)	3·7% (2·9 to 4·6%)	578·5 (448·1 to 741·2)	3·6% (2·8 to 4·5%)	11·1% (3·0 to 19·4%)	-1·8% (-5·9 to 2·3%)
**Alcohol and drug use: All causes**	374·5 (342·8 to 407·7)	2·8% (2·6 to 3·0%)	446·0 (396·3 to 502·1)	3·4% (3·0 to 3·7%)	19·1% (10·1 to 29·8%)	19·7% (13·2 to 27·1%)	16,662·3 (15,247·5 to 18,115·5)	2·1% (1·9 to 2·2%)	19,524·6 (17,617·1 to 21,704·0)	2·7% (2·4 to 2·9%)	17·2% (9·5 to 26·1%)	27·7% (20·9 to 35·3%)
**Alcohol use: All causes**	319·3 (287·4 to 352·5)	2·4% (2·2 to 2·6%)	382·4 (335·6 to 432·6)	2·9% (2·6 to 3·2%)	19·7% (10·4 to 31·1%)	20·4% (13·3 to 28·9%)	12,989·4 (11,774·8 to 14,255·8)	1·6% (1·5 to 1·8%)	15,288·2 (13,663·8 to 17,258·5)	2·1% (1·9 to 2·3%)	17·7% (9·2 to 27·7%)	28·2% (20·2 to 37·4%)
Tuberculosis	54·1 (42·4 to 73·5)	9·4% (8·0 to 10·8%)	51·7 (37·0 to 70·3)	10·5% (8·5 to 12·4%)	-4·3% (-20·5 to 10·9%)	11·8% (0·8 to 21·9%)	2,014·4 (1,602·5 to 2,683·2)	9·5% (8·3 to 10·6%)	1,910·6 (1,393·6 to 2,589·4)	10·8% (8·9 to 12·4%)	-5·2% (-20·1 to 8·9%)	14·2% (2·7 to 25·1%)
Lower respiratory infections	16·8 (12·5 to 20·3)	1·7% (1·4 to 1·9%)	20·2 (14·9 to 24·8)	2·4% (2·0 to 2·8%)	20·3% (2·7 to 41·6%)	42·3% (23·7 to 62·9%)	471·5 (356·2 to 570·4)	0·8% (0·6 to 0·9%)	551·3 (408·7 to 693·2)	1·3% (1·0 to 1·5%)	16·9% (-0·3 to 39·6%)	64·3% (38·6 to 95·7%)
Lip and oral cavity cancer	3·4 (3·0 to 3·9)	11·9% (10·3 to 13·3%)	5·4 (4·5 to 6·2)	13·5% (11·3 to 15·2%)	58·0% (42·5 to 76·0%)	13·3% (3·2 to 22·8%)	109·1 (95·6 to 122·0)	12·9% (11·4 to 14·2%)	168·2 (142·4 to 191·3)	14·5% (12·4 to 16·3%)	54·1% (38·9 to 71·3%)	12·3% (3·4 to 21·4%)
Nasopharynx cancer	1·1 (0·9 to 1·4)	14·5% (12·4 to 16·4%)	1·6 (1·2 to 1·9)	16·4% (13·6 to 18·6%)	42·2% (24·9 to 62·1%)	12·7% (2·3 to 23·1%)	39·3 (31·7 to 47·0)	15·2% (13·2 to 17·0%)	54·6 (40·6 to 64·8)	17·1% (14·4 to 19·3%)	38·9% (22·6 to 58·2%)	12·6% (3·0 to 22·8%)
Other pharynx cancer	1·5 (1·2 to 1·7)	12·9% (10·8 to 14·6%)	2·1 (1·8 to 2·5)	15·4% (12·8 to 17·6%)	46·6% (31·4 to 66·6%)	19·1% (8·7 to 29·3%)	44·7 (37·8 to 51·7)	14·2% (12·2 to 16·0%)	64·4 (53·1 to 74·8)	16·9% (14·2 to 19·2%)	44·1% (29·1 to 62·7%)	18·8% (8·8 to 28·7%)
Oesophageal cancer	4·0 (3·4 to 4·6)	8·9% (7·6 to 10·0%)	5·8 (4·8 to 6·9)	10·0% (8·4 to 11·4%)	46·1% (30·9 to 63·7%)	13·2% (3·5 to 22·3%)	115·6 (99·0 to 132·3)	9·4% (8·2 to 10·4%)	166·1 (137·5 to 196·5)	10·7% (9·1 to 12·1%)	43·7% (28·4 to 61·9%)	13·7% (3·8 to 23·3%)
Colon and rectum cancer	1·3 (1·1 to 1·4)	2·4% (2·1 to 2·7%)	1·8 (1·6 to 2·1)	2·5% (2·2 to 2·8%)	46·0% (33·1 to 60·4%)	4·1% (-1·9 to 10·3%)	34·6 (30·4 to 38·7)	2·4% (2·2 to 2·7%)	49·2 (42·6 to 56·5)	2·6% (2·3 to 2·9%)	42·2% (28·9 to 57·3%)	5·0% (-1·3 to 12·1%)
Liver cancer due to hepatitis B	0·1 (0·1 to 0·2)	0·5% (0·3 to 0·7%)	0·1 (0·1 to 0·2)	0·5% (0·3 to 0·7%)	10·5% (-20·4 to 58·3%)	-3·2% (-27·1 to 30·8%)	6·8 (4·3 to 10·9)	0·9% (0·6 to 1·2%)	7·6 (4·6 to 12·7)	0·9% (0·6 to 1·2%)	11·0% (-21·0 to 59·3%)	-0·9% (-25·4 to 32·0%)
Liver cancer due to hepatitis C	0·0 (0·0 to 0·0)	0·0% (0·0 to 0·1%)	0·0 (0·0 to 0·0)	0·0% (0·0 to 0·1%)	12·0% (-14·9 to 48·3%)	-7·5% (-28·5 to 19·8%)	0·3 (0·2 to 0·5)	0·1% (0·1 to 0·2%)	0·4 (0·2 to 0·6)	0·1% (0·1 to 0·2%)	12·3% (-14·4 to 47·7%)	-5·9% (-26·9 to 21·4%)
Liver cancer due to alcohol use	15·0 (12·3 to 18·2)	100·0% (100·0 to 100·0%)	18·7 (15·5 to 23·4)	100·0% (100·0 to 100·0%)	24·6% (6·6 to 47·1%)	-	386·7 (314·3 to 478·6)	100·0% (100·0 to 100·0%)	476·7 (386·2 to 613·7)	100·0% (100·0 to 100·0%)	23·3% (3·3 to 47·8%)	-
Liver cancer due to other causes	0·0 (0·0 to 0·0)	0·2% (0·1 to 0·3%)	0·0 (0·0 to 0·0)	0·2% (0·1 to 0·3%)	2·4% (-21·3 to 34·3%)	-9·2% (-29·1 to 15·9%)	1·4 (1·0 to 2·0)	0·4% (0·3 to 0·6%)	1·5 (1·0 to 2·1)	0·4% (0·3 to 0·6%)	3·2% (-20·8 to 35·6%)	-5·7% (-25·6 to 18·7%)
Larynx cancer	1·8 (1·5 to 2·1)	9·0% (7·6 to 10·2%)	2·6 (2·1 to 3·0)	10·2% (8·4 to 11·6%)	42·3% (26·9 to 58·5%)	13·5% (3·6 to 22·5%)	51·6 (44·1 to 58·3)	9·7% (8·4 to 10·8%)	71·8 (59·3 to 83·8)	11·0% (9·3 to 12·5%)	39·2% (24·7 to 54·4%)	13·2% (4·1 to 22·3%)
Breast cancer	1·8 (1·5 to 2·2)	2·9% (2·5 to 3·4%)	2·4 (1·9 to 3·0)	2·9% (2·4 to 3·5%)	34·4% (13·9 to 65·1%)	0·1% (-9·6 to 13·1%)	59·3 (48·0 to 73·8)	2·9% (2·5 to 3·4%)	79·6 (63·1 to 97·8)	3·0% (2·5 to 3·5%)	34·3% (13·6 to 65·9%)	0·6% (-8·9 to 13·4%)
Ischaemic heart disease	4·4 (2·5 to 6·4)	0·4% (0·2 to 0·6%)	6·6 (3·6 to 9·6)	0·4% (0·2 to 0·6%)	48·6% (18·9 to 83·9%)	11·1% (-10·4 to 36·8%)	61·6 (14·6 to 109·8)	0·2% (0·1 to 0·4%)	106·8 (36·3 to 175·1)	0·3% (0·1 to 0·5%)	73·5% (16·6 to 222·8%)	33·7% (-8·6 to 146·4%)
Ischaemic stroke	1·9 (0·9 to 3·0)	0·5% (0·2 to 0·8%)	3·0 (1·7 to 4·3)	0·6% (0·3 to 0·9%)	55·1% (26·2 to 114·4%)	27·3% (4·4 to 72·7%)	45·6 (26·1 to 65·1)	0·6% (0·4 to 0·9%)	70·6 (45·9 to 94·6)	0·8% (0·5 to 1·1%)	55·0% (30·9 to 95·2%)	31·6% (12·7 to 65·5%)
Hemorrhagic stroke	24·4 (21·3 to 27·8)	4·7% (4·1 to 5·3%)	30·5 (25·0 to 35·6)	5·0% (4·2 to 5·8%)	25·0% (10·3 to 39·9%)	7·3% (-2·5 to 16·5%)	692·9 (608·8 to 784·6)	4·9% (4·3 to 5·4%)	851·1 (712·3 to 986·0)	5·3% (4·5 to 6·0%)	22·8% (8·7 to 37·3%)	8·6% (-0·8 to 17·6%)
Hypertensive heart disease	6·2 (4·5 to 8·1)	4·0% (2·9 to 5·0%)	8·6 (5·8 to 11·3)	4·6% (3·2 to 5·8%)	39·2% (15·8 to 62·9%)	13·5% (-2·6 to 29·1%)	181·2 (142·2 to 226·7)	4·9% (3·9 to 5·8%)	245·0 (175·8 to 309·2)	5·6% (4·2 to 6·7%)	35·2% (14·7 to 57·0%)	14·2% (0·3 to 27·0%)
Atrial fibrillation and flutter	0·3 (0·2 to 0·4)	2·6% (2·1 to 3·1%)	0·5 (0·4 to 0·7)	2·9% (2·3 to 3·5%)	57·2% (42·3 to 72·2%)	11·6% (2·2 to 21·0%)	11·9 (8·9 to 15·5)	3·6% (3·1 to 4·0%)	17·2 (12·9 to 22·6)	3·9% (3·3 to 4·4%)	45·1% (36·3 to 53·8%)	8·0% (1·4 to 13·5%)
Cirrhosis and other chronic liver diseases due to hepatitis B	12·9 (10·0 to 15·8)	15·6% (12·6 to 18·5%)	15·9 (11·7 to 20·3)	16·4% (12·5 to 19·9%)	23·8% (4·6 to 42·3%)	5·0% (-8·6 to 17·2%)	410·6 (324·7 to 503·5)	15·5% (12·8 to 18·2%)	500·0 (370·9 to 637·4)	16·5% (12·9 to 19·9%)	21·8% (3·4 to 41·7%)	6·4% (-7·4 to 19·1%)
Cirrhosis and other chronic liver diseases due to hepatitis C	7·5 (5·8 to 9·4)	14·3% (11·4 to 17·2%)	9·3 (6·8 to 12·1)	15·0% (11·4 to 18·3%)	24·3% (5·4 to 41·3%)	5·2% (-9·6 to 17·5%)	246·5 (192·7 to 309·3)	14·4% (11·7 to 17·1%)	301·9 (222·5 to 391·0)	15·3% (11·8 to 18·5%)	22·4% (4·4 to 39·7%)	6·6% (-7·6 to 19·0%)
Cirrhosis and other chronic liver diseases due to alcohol use	61·8 (54·2 to 74·0)	100·0% (100·0 to 100·0%)	75·1 (64·7 to 91·6)	100·0% (100·0 to 100·0%)	21·4% (10·1 to 35·0%)	-	2,138·8 (1,869·2 to 2,553·8)	100·0% (100·0 to 100·0%)	2,555·0 (2,190·1 to 3,126·5)	100·0% (100·0 to 100·0%)	19·5% (7·7 to 34·3%)	-
Cirrhosis and other chronic liver diseases due to other causes	6·1 (4·8 to 7·4)	10·7% (8·6 to 12·9%)	7·2 (5·2 to 9·2)	12·0% (9·0 to 14·8%)	17·9% (-0·2 to 35·4%)	12·1% (-3·6 to 25·7%)	208·2 (166·9 to 253·2)	8·0% (6·5 to 9·5%)	234·3 (175·0 to 297·7)	9·5% (7·2 to 11·6%)	12·5% (-5·6 to 31·2%)	18·2% (0·6 to 35·8%)
Pancreatitis	2·2 (1·8 to 2·7)	7·8% (6·8 to 8·7%)	3·6 (2·7 to 4·6)	9·1% (7·6 to 10·5%)	63·3% (40·2 to 92·4%)	17·5% (5·1 to 30·1%)	88·8 (71·7 to 107·5)	8·3% (7·3 to 9·2%)	143·2 (108·1 to 183·4)	9·8% (8·4 to 11·2%)	61·3% (37·8 to 89·9%)	18·9% (6·8 to 31·5%)

Supplemental Results Table 1. All-age deaths, death PAFs, DALYs, DALY PAFs, and percent change for each risk factor and outcome for both sexes combined in 2005 and 2015 for Low-middle SDI.

Data in parenthesis are 95% uncertainty intervals. PAFs=population attributable fractions. DALYs=disability-adjusted life-years.

	2005 Deaths (in thousands)	2005 Deaths PAF	2015 Deaths (in thousands)	2015 Deaths PAF	Percent change deaths 2005-2015	Percent change deaths PAF 2005-2015	2005 DALYs (in thousands)	2005 DALYs PAF	2015 DALYs (in thousands)	2015 DALYs PAF	Percent change DALYs 2005-2015	Percent change DALYs PAF 2005-2015
Epilepsy	2·1 (1·8 to 2·4)	6·1% (5·4 to 7·0%)	2·8 (2·2 to 3·6)	7·5% (6·3 to 9·1%)	33·2% (9·9 to 68·0%)	21·5% (4·9 to 42·1%)	167·7 (140·4 to 198·5)	4·2% (3·7 to 4·7%)	209·6 (169·8 to 259·0)	5·0% (4·3 to 5·8%)	25·0% (9·1 to 46·7%)	18·8% (5·6 to 35·8%)
Alcohol use disorders	15·3 (13·6 to 17·0)	100·0% (100·0 to 100·0%)	18·2 (16·2 to 20·6)	100·0% (100·0 to 100·0%)	19·5% (7·1 to 34·1%)	-	1,498·1 (1,179·9 to 1,881·2)	100·0% (100·0 to 100·0%)	1,819·6 (1,431·2 to 2,301·7)	100·0% (100·0 to 100·0%)	21·5% (15·1 to 27·7%)	-
Diabetes mellitus	-4·1 (-5·0 to -3·4)	-1·6% (-1·9 to -1·3%)	-5·6 (-7·2 to -4·3)	-1·4% (-1·8 to -1·1%)	35·4% (14·0 to 64·0%)	-11·1% (-23·1 to 4·3%)	-145·4 (-174·4 to -118·3)	-1·4% (-1·7 to -1·3%)	-190·9 (-234·5 to -150·0)	-1·4% (-1·6 to -1·2%)	31·2% (15·5 to 51·5%)	-6·0% (-15·6 to 6·7%)
Pedestrian road injuries	14·0 (10·5 to 17·7)	12·8% (10·5 to 15·4%)	16·1 (12·9 to 20·0)	13·2% (11·1 to 15·7%)	14·7% (-0·6 to 41·8%)	3·6% (-1·9 to 9·7%)	736·8 (552·8 to 923·7)	13·4% (11·0 to 16·1%)	820·9 (659·7 to 1,012·6)	14·2% (11·9 to 16·9%)	11·4% (-3·5 to 37·6%)	6·2% (-0·6 to 13·0%)
Cyclist road injuries	0·8 (0·6 to 1·0)	13·8% (11·4 to 16·5%)	0·9 (0·7 to 1·2)	13·8% (11·5 to 16·4%)	20·6% (4·5 to 43·5%)	0·3% (-6·2 to 7·3%)	44·9 (34·2 to 55·5)	13·3% (11·0 to 15·8%)	53·4 (43·0 to 65·7)	13·5% (11·3 to 15·9%)	18·8% (4·2 to 38·7%)	1·6% (-5·6 to 9·7%)
Motorcyclist road injuries	12·5 (8·9 to 16·1)	24·7% (21·3 to 28·4%)	16·4 (13·0 to 20·1)	26·4% (23·2 to 29·8%)	31·6% (1·8 to 72·9%)	6·8% (-0·1 to 13·7%)	651·7 (472·1 to 835·1)	24·3% (21·1 to 28·1%)	838·7 (676·3 to 1,018·4)	26·2% (23·1 to 29·6%)	28·7% (2·3 to 66·4%)	7·8% (0·4 to 15·2%)
Motor vehicle road injuries	19·6 (15·8 to 25·5)	21·0% (18·2 to 24·2%)	22·6 (18·3 to 27·1)	21·6% (19·1 to 24·6%)	15·3% (-13·8 to 35·4%)	2·9% (-5·5 to 10·7%)	1,020·3 (831·7 to 1,305·7)	20·1% (17·4 to 23·4%)	1,156·8 (950·5 to 1,385·0)	21·0% (18·4 to 23·7%)	13·4% (-12·8 to 31·7%)	4·2% (-5·3 to 12·5%)
Falls	4·7 (3·8 to 5·8)	5·8% (4·7 to 7·2%)	6·1 (4·8 to 7·7)	6·3% (5·2 to 7·8%)	29·9% (12·2 to 54·6%)	9·0% (-2·7 to 22·4%)	268·5 (211·0 to 338·0)	5·2% (4·2 to 6·3%)	348·0 (274·6 to 434·6)	5·8% (4·8 to 7·0%)	29·6% (19·0 to 43·6%)	12·3% (2·0 to 24·6%)
Drowning	3·5 (2·9 to 4·3)	2·7% (2·1 to 3·7%)	3·9 (3·1 to 4·9)	3·4% (2·7 to 4·5%)	10·5% (-1·1 to 27·4%)	29·2% (11·9 to 46·7%)	163·8 (133·8 to 203·9)	1·9% (1·5 to 2·7%)	177·1 (143·2 to 221·7)	2·6% (2·0 to 3·4%)	8·1% (-3·0 to 24·4%)	37·8% (16·4 to 58·7%)
Fire, heat, and hot substances	2·1 (1·7 to 2·8)	3·5% (2·8 to 4·4%)	2·5 (1·9 to 3·3)	4·3% (3·5 to 5·6%)	19·2% (1·9 to 36·1%)	23·8% (11·1 to 38·5%)	118·6 (94·3 to 152·0)	2·9% (2·3 to 3·7%)	142·1 (111·4 to 177·9)	3·7% (3·0 to 4·6%)	19·8% (6·1 to 32·6%)	25·8% (14·5 to 38·2%)
Poisonings	0·9 (0·6 to 1·2)	4·0% (2·5 to 6·0%)	0·9 (0·6 to 1·2)	4·4% (2·8 to 6·5%)	-0·8% (-21·0 to 18·4%)	9·1% (-24·4 to 59·6%)	44·2 (31·6 to 58·8)	2·9% (1·7 to 4·5%)	44·7 (32·5 to 58·1)	3·2% (2·0 to 5·0%)	1·2% (-17·2 to 17·7%)	11·4% (-24·9 to 69·5%)
Unintentional firearm injuries	0·6 (0·4 to 0·8)	7·4% (5·9 to 9·2%)	0·6 (0·4 to 0·8)	7·5% (6·2 to 9·4%)	5·9% (-13·6 to 34·4%)	1·5% (-10·8 to 16·7%)	27·2 (18·4 to 38·5)	6·5% (5·2 to 8·4%)	29·0 (19·5 to 39·7)	6·7% (5·5 to 8·4%)	6·4% (-12·5 to 32·9%)	3·1% (-10·5 to 20·5%)
Unintentional suffocation	0·2 (0·2 to 0·3)	2·3% (1·6 to 3·4%)	0·3 (0·2 to 0·4)	3·5% (2·5 to 4·6%)	46·6% (19·3 to 74·9%)	49·3% (14·0 to 101·4%)	13·9 (10·7 to 18·1)	1·8% (1·2 to 2·8%)	19·2 (14·9 to 24·5)	2·7% (1·9 to 3·5%)	37·9% (20·4 to 55·8%)	48·5% (12·1 to 104·0%)
Other exposure to mechanical forces	2·4 (1·8 to 3·2)	6·4% (5·1 to 8·0%)	2·8 (2·1 to 3·7)	7·3% (6·0 to 8·9%)	19·5% (2·0 to 43·8%)	14·2% (4·4 to 26·1%)	154·6 (118·5 to 201·1)	5·6% (4·6 to 7·0%)	181·8 (139·3 to 231·9)	6·5% (5·5 to 7·9%)	17·6% (6·1 to 33·1%)	15·9% (7·8 to 25·1%)
Self-harm	10·9 (7·1 to 13·7)	7·2% (5·8 to 9·0%)	14·4 (9·8 to 18·6)	8·2% (6·7 to 10·3%)	33·0% (17·5 to 50·6%)	13·6% (5·0 to 22·2%)	493·1 (323·7 to 623·1)	6·6% (5·3 to 8·4%)	646·3 (436·1 to 828·6)	7·7% (6·3 to 9·7%)	31·1% (16·1 to 48·3%)	15·8% (6·4 to 25·0%)
Assault by firearm	1·9 (1·5 to 2·2)	9·2% (8·1 to 10·7%)	2·5 (1·9 to 3·0)	9·6% (8·3 to 11·0%)	32·3% (17·1 to 50·2%)	3·5% (-2·2 to 10·0%)	106·2 (87·3 to 125·5)	9·6% (8·5 to 11·1%)	138·5 (109·4 to 165·8)	10·0% (8·7 to 11·5%)	30·4% (15·1 to 47·1%)	4·2% (-1·9 to 10·7%)
Assault by sharp object	1·0 (0·8 to 1·2)	9·7% (8·4 to 11·4%)	1·0 (0·8 to 1·3)	9·8% (8·5 to 11·4%)	4·7% (-10·2 to 23·8%)	1·5% (-6·0 to 8·6%)	56·2 (45·9 to 67·6)	10·1% (8·7 to 12·0%)	57·7 (45·7 to 71·1)	10·3% (8·9 to 12·0%)	2·6% (-12·0 to 21·3%)	1·7% (-5·5 to 8·9%)
Assault by other means	2·6 (2·0 to 3·3)	6·4% (5·3 to 7·9%)	3·0 (2·3 to 4·0)	7·0% (5·9 to 8·5%)	16·1% (0·3 to 33·5%)	8·5% (1·0 to 17·0%)	147·3 (115·3 to 188·4)	6·5% (5·4 to 8·0%)	168·7 (128·1 to 221·3)	7·1% (6·0 to 8·7%)	14·5% (-0·6 to 31·3%)	9·8% (1·8 to 18·8%)
**Drug use: All causes**	57·1 (50·2 to 66·4)	0·4% (0·4 to 0·5%)	66·2 (57·3 to 77·3)	0·5% (0·4 to 0·6%)	16·0% (5·1 to 27·7%)	16·6% (6·7 to 27·2%)	3,744·5 (3,305·5 to 4,250·5)	0·5% (0·4 to 0·5%)	4,332·1 (3,739·6 to 4,972·2)	0·6% (0·5 to 0·7%)	15·7% (7·5 to 24·6%)	26·0% (18·1 to 34·7%)
HIV/AIDS - Tuberculosis	3·7 (2·7 to 5·2)	3·4% (2·7 to 4·6%)	2·5 (1·8 to 3·5)	4·0% (3·2 to 5·2%)	-31·4% (-39·0 to -20·8%)	17·8% (6·1 to 32·4%)	182·5 (137·9 to 254·5)	3·1% (2·5 to 4·1%)	123·9 (92·7 to 168·7)	3·6% (2·8 to 4·6%)	-32·1% (-39·4 to -23·0%)	16·3% (5·1 to 29·3%)
HIV/AIDS resulting in other diseases	21·1 (17·1 to 29·8)	3·5% (3·0 to 4·5%)	17·3 (13·8 to 23·8)	4·3% (3·6 to 5·5%)	-18·3% (-32·7 to -3·6%)	22·6% (7·2 to 39·1%)	1,107·2 (908·4 to 1,514·2)	3·3% (2·8 to 4·2%)	911·9 (735·8 to 1,203·2)	3·9% (3·3 to 4·9%)	-17·6% (-31·5 to -3·4%)	20·8% (6·3 to 36·3%)
Hepatitis B	0·0 (0·0 to 0·1)	0·2% (0·1 to 0·3%)	0·0 (0·0 to 0·1)	0·2% (0·1 to 0·3%)	17·1% (0·9 to 36·6%)	10·8% (-1·9 to 24·4%)	1·5 (0·6 to 2·5)	0·2% (0·1 to 0·3%)	1·7 (0·7 to 2·7)	0·2% (0·1 to 0·3%)	12·9% (-2·5 to 31·2%)	11·6% (-1·5 to 25·3%)
Hepatitis C	0·1 (0·0 to 0·3)	19·0% (13·1 to 26·0%)	0·2 (0·0 to 0·4)	21·2% (14·7 to 29·1%)	17·3% (-8·2 to 49·7%)	11·9% (-3·4 to 29·6%)	6·2 (1·3 to 14·2)	20·0% (13·7 to 27·3%)	7·0 (1·5 to 16·6)	22·4% (15·1 to 30·6%)	13·4% (-10·5 to 45·3%)	12·3% (-2·3 to 29·9%)
Liver cancer due to hepatitis B	0·1 (0·0 to 0·2)	0·4% (0·2 to 0·7%)	0·1 (0·0 to 0·2)	0·4% (0·2 to 0·7%)	22·4% (-3·5 to 59·1%)	7·3% (-10·2 to 31·8%)	3·2 (1·2 to 5·4)	0·4% (0·2 to 0·7%)	3·7 (1·5 to 6·2)	0·4% (0·2 to 0·7%)	16·8% (-8·4 to 51·6%)	4·4% (-12·0 to 26·1%)
Liver cancer due to hepatitis C	2·5 (1·6 to 3·5)	18·0% (11·8 to 25·2%)	3·6 (2·4 to 5·0)	21·6% (14·9 to 29·2%)	45·1% (25·9 to 69·0%)	19·8% (8·3 to 35·0%)	72·8 (48·3 to 101·8)	22·3% (15·1 to 30·8%)	102·0 (69·4 to 141·8)	26·1% (18·1 to 35·1%)	40·1% (21·7 to 63·9%)	17·5% (6·3 to 31·3%)
Cirrhosis and other chronic liver diseases due to hepatitis B	0·2 (0·1 to 0·4)	0·3% (0·1 to 0·4%)	0·3 (0·1 to 0·5)	0·3% (0·1 to 0·5%)	35·7% (12·1 to 69·3%)	15·2% (-1·4 to 36·6%)	7·6 (3·1 to 13·1)	0·3% (0·1 to 0·5%)	9·8 (4·1 to 16·3)	0·3% (0·1 to 0·5%)	29·1% (5·6 to 62·7%)	13·0% (-3·7 to 34·4%)
Cirrhosis and other chronic liver diseases due to hepatitis C	12·0 (7·9 to 16·5)	22·8% (15·7 to 30·5%)	16·0 (11·0 to 22·4)	25·8% (18·4 to 34·1%)	33·7% (17·7 to 54·5%)	13·3% (2·8 to 26·5%)	463·4 (310·0 to 633·9)	27·0% (18·9 to 35·5%)	594·1 (408·0 to 828·2)	30·1% (21·7 to 39·6%)	28·2% (12·4 to 48·9%)	11·6% (1·9 to 24·3%)
Opioid use disorders	11·4 (10·0 to 13·2)	100·0% (100·0 to 100·0%)	17·8 (15·1 to 21·9)	100·0% (100·0 to 100·0%)	55·9% (32·6 to 89·3%)	-	1,445·6 (1,158·7 to 1,728·4)	100·0% (100·0 to 100·0%)	1,966·5 (1,589·7 to 2,354·7)	100·0% (100·0 to 100·0%)	36·0% (26·6 to 49·4%)	-
Cocaine use disorders	0·2 (0·1 to 0·2)	100·0% (100·0 to 100·0%)	0·5 (0·3 to 0·7)	100·0% (100·0 to 100·0%)	181·6% (115·5 to 298·1%)	-	37·2 (25·1 to 51·2)	100·0% (100·0 to 100·0%)	67·2 (46·8 to 91·1)	100·0% (100·0 to 100·0%)	80·4% (63·2 to 107·5%)	-
Amphetamine use disorders	0·2 (0·1 to 0·5)	100·0% (100·0 to 100·0%)	0·5 (0·3 to 0·8)	100·0% (100·0 to 100·0%)	133·6% (68·2 to 252·5%)	-	39·5 (25·5 to 58·3)	100·0% (100·0 to 100·0%)	62·0 (41·6 to 89·3)	100·0% (100·0 to 100·0%)	57·0% (40·9 to 83·5%)	-
Cannabis use disorders	-	-	-	-	-	-	80·8 (51·7 to 115·5)	100·0% (100·0 to 100·0%)	96·3 (61·8 to 137·1)	100·0% (100·0 to 100·0%)	19·1% (14·3 to 24·1%)	-
Other drug use disorders	2·6 (2·1 to 3·2)	100·0% (100·0 to 100·0%)	3·8 (2·8 to 5·0)	100·0% (100·0 to 100·0%)	46·1% (2·9 to 98·1%)	-	145·1 (116·8 to 180·3)	100·0% (100·0 to 100·0%)	202·0 (151·3 to 260·1)	100·0% (100·0 to 100·0%)	39·2% (12·1 to 72·9%)	-
Self-harm	2·9 (1·8 to 4·3)	1·9% (1·2 to 2·8%)	3·6 (2·2 to 5·4)	2·0% (1·3 to 3·0%)	23·8% (11·8 to 37·2%)	5·9% (0·9 to 11·5%)	152·0 (92·3 to 226·6)	2·0% (1·3 to 3·0%)	184·1 (114·9 to 278·3)	2·2% (1·4 to 3·2%)	21·1% (9·2 to 34·7%)	7·1% (1·9 to 12·8%)
**Dietary risks: All causes**	1,609·5 (1,414·8 to 1,826·4)	12·0% (10·6 to 13·6%)	2,074·3 (1,806·9 to 2,353·5)	15·6% (13·7 to 17·6%)	28·9% (22·5 to 35·5%)	29·6% (26·0 to 33·2%)	42,025·7 (37,295·2 to 47,337·4)	5·2% (4·7 to 5·9%)	53,191·2 (47,083·8 to 59,990·5)	7·2% (6·4 to 8·2%)	26·6% (20·0 to 33·1%)	37·9% (32·4 to 43·6%)
**Diet low in fruits: All causes**	463·5 (311·5 to 627·6)	3·5% (2·3 to 4·7%)	576·6 (379·7 to 791·9)	4·3% (2·9 to 5·9%)	24·4% (17·2 to 31·6%)	25·1% (20·4 to 29·6%)	13,247·9 (9,102·9 to 17,733·0)	1·7% (1·1 to 2·2%)	16,292·1 (10,897·6 to 22,101·9)	2·2% (1·5 to 3·0%)	23·0% (16·0 to 30·1%)	34·0% (28·0 to 40·2%)
Lip and oral cavity cancer	2·2 (0·0 to 4·9)	7·8% (0·0 to 16·8%)	3·1 (0·0 to 6·7)	7·7% (0·0 to 16·6%)	38·1% (0·0 to 49·0%)	-1·1% (-2·1 to 0·0%)	65·0 (0·0 to 140·0)	7·7% (0·0 to 16·6%)	88·5 (0·0 to 190·7)	7·6% (0·0 to 16·5%)	36·1% (0·0 to 47·8%)	-0·9% (-2·0 to 0·0%)
Nasopharynx cancer	0·6 (0·0 to 1·3)	7·8% (0·0 to 16·5%)	0·8 (0·0 to 1·6)	7·7% (0·0 to 16·3%)	24·7% (0·0 to 37·7%)	-1·1% (-2·3 to 0·0%)	19·2 (0·0 to 42·2)	7·5% (0·0 to 15·8%)	23·6 (0·0 to 49·9)	7·4% (0·0 to 15·8%)	22·7% (0·0 to 36·0%)	-0·5% (-1·9 to 0·8%)
Other pharynx cancer	0·9 (0·0 to 2·0)	7·8% (0·0 to 17·2%)	1·1 (0·0 to 2·4)	7·6% (0·0 to 17·0%)	20·7% (0·0 to 35·5%)	-1·8% (-3·1 to 0·0%)	24·4 (0·0 to 53·9)	7·7% (0·0 to 17·1%)	29·1 (0·0 to 64·0)	7·6% (0·0 to 16·9%)	19·3% (0·0 to 33·3%)	-1·5% (-2·9 to 0·0%)
Oesophageal cancer	10·7 (2·6 to 18·9)	23·8% (6·0 to 41·4%)	13·6 (3·3 to 23·9)	23·4% (5·9 to 41·0%)	27·2% (18·2 to 37·6%)	-1·4% (-2·7 to -0·6%)	290·1 (71·4 to 508·5)	23·6% (5·9 to 41·0%)	362·3 (89·3 to 632·7)	23·3% (5·9 to 40·8%)	24·9% (14·9 to 35·5%)	-1·1% (-2·4 to -0·2%)
Larynx cancer	1·6 (0·0 to 3·4)	7·8% (0·0 to 17·2%)	1·9 (0·0 to 4·2)	7·6% (0·0 to 16·9%)	23·2% (0·0 to 34·1%)	-1·7% (-2·9 to 0·0%)	40·9 (0·0 to 90·4)	7·7% (0·0 to 17·0%)	49·5 (0·0 to 109·2)	7·6% (0·0 to 16·8%)	21·1% (0·0 to 31·5%)	-1·5% (-2·6 to 0·0%)
Tracheal, bronchus, and lung cancer	13·1 (5·4 to 21·9)	13·5% (5·5 to 22·4%)	19·0 (7·8 to 32·1)	13·4% (5·5 to 22·3%)	44·5% (33·6 to 55·8%)	-0·5% (-1·4 to 0·2%)	332·8 (137·0 to 554·3)	13·4% (5·5 to 22·3%)	458·6 (189·4 to 770·3)	13·3% (5·5 to 22·2%)	37·8% (26·5 to 49·2%)	-0·6% (-1·6 to 0·2%)
Ischaemic heart disease	165·9 (60·8 to 275·0)	14·5% (5·3 to 23·9%)	218·3 (78·2 to 362·4)	14·2% (5·2 to 23·6%)	31·6% (24·2 to 39·2%)	-1·6% (-2·7 to -0·6%)	4,453·4 (1,661·8 to 7,292·6)	16·5% (6·1 to 26·9%)	5,733·8 (2,080·5 to 9,399·2)	16·3% (6·0 to 26·7%)	28·7% (21·1 to 36·6%)	-0·8% (-1·9 to 0·3%)
Ischaemic stroke	86·2 (47·0 to 130·1)	22·5% (12·4 to 33·8%)	100·6 (54·0 to 153·4)	21·6% (11·8 to 32·4%)	16·6% (7·5 to 25·3%)	-4·2% (-6·2 to -2·6%)	2,061·6 (1,149·9 to 3,034·1)	28·4% (16·2 to 41·5%)	2,385·3 (1,313·2 to 3,505·4)	28·0% (15·8 to 40·9%)	15·7% (6·4 to 24·1%)	-1·7% (-3·4 to -0·3%)

Supplemental Results Table 1. All-age deaths, death PAFs, DALYs, DALY PAFs, and percent change for each risk factor and outcome for both sexes combined in 2005 and 2015 for Low-middle SDI.

Data in parenthesis are 95% uncertainty intervals. PAFs=population attributable fractions. DALYs=disability-adjusted life-years.

	2005 Deaths (in thousands)	2005 Deaths PAF	2015 Deaths (in thousands)	2015 Deaths PAF	Percent change deaths 2005-2015	Percent change deaths PAF 2005-2015	2005 DALYs (in thousands)	2005 DALYs PAF	2015 DALYs (in thousands)	2015 DALYs PAF	Percent change DALYs 2005-2015	Percent change DALYs PAF 2005-2015
Hemorrhagic stroke	152·4 (87·0 to 222·7)	29·4% (16·4 to 42·8%)	174·7 (98·4 to 255·8)	28·9% (16·1 to 42·2%)	14·7% (7·7 to 22·6%)	-1·6% (-3·1 to -0·3%)	4,589·6 (2,668·6 to 6,556·0)	32·3% (18·6 to 46·1%)	5,269·9 (3,059·8 to 7,570·2)	32·8% (18·8 to 46·9%)	14·8% (7·6 to 22·4%)	1·6% (0·0 to 3·1%)
**Diet low in vegetables: All causes**	334·2 (180·2 to 508·3)	2·5% (1·3 to 3·8%)	417·2 (219·8 to 641·1)	3·1% (1·6 to 4·8%)	24·8% (17·3 to 32·4%)	25·6% (20·0 to 30·6%)	9,009·9 (4,943·0 to 13,560·2)	1·1% (0·6 to 1·7%)	11,088·9 (6,005·8 to 16,793·8)	1·5% (0·8 to 2·3%)	23·1% (15·6 to 30·8%)	34·1% (27·2 to 41·1%)
Ischaemic heart disease	220·4 (88·9 to 355·1)	19·2% (7·6 to 31·4%)	287·1 (114·8 to 472·7)	18·7% (7·4 to 30·7%)	30·2% (23·1 to 37·8%)	-2·6% (-4·1 to -1·4%)	5,776·3 (2,375·5 to 9,244·7)	21·4% (8·6 to 34·5%)	7,379·6 (3,008·7 to 12,050·1)	21·0% (8·5 to 34·2%)	27·8% (20·2 to 35·7%)	-1·5% (-3·0 to -0·2%)
Ischaemic stroke	41·3 (9·0 to 73·9)	10·8% (2·5 to 19·4%)	47·4 (10·3 to 85·1)	10·2% (2·3 to 18·2%)	14·8% (5·8 to 23·4%)	-5·7% (-7·9 to -3·6%)	1,004·4 (226·6 to 1,768·7)	13·9% (3·3 to 24·5%)	1,148·9 (256·6 to 2,036·8)	13·5% (3·2 to 23·8%)	14·4% (4·9 to 23·0%)	-2·7% (-4·8 to -0·7%)
Hemorrhagic stroke	72·6 (20·9 to 132·6)	14·0% (4·0 to 25·3%)	82·8 (23·8 to 150·2)	13·7% (3·9 to 24·8%)	14·1% (7·0 to 22·1%)	-2·0% (-4·1 to 0·0%)	2,229·3 (648·2 to 4,006·3)	15·7% (4·6 to 28·1%)	2,560·4 (736·0 to 4,575·6)	16·0% (4·7 to 28·5%)	14·9% (7·4 to 22·6%)	1·7% (-0·5 to 3·9%)
**Diet low in whole grains: All causes**	458·9 (324·0 to 617·7)	3·4% (2·4 to 4·6%)	587·2 (409·5 to 788·4)	4·4% (3·1 to 5·9%)	28·0% (21·4 to 34·7%)	28·7% (24·5 to 33·0%)	13,547·3 (9,612·2 to 17,882·4)	1·7% (1·2 to 2·3%)	17,075·9 (12,038·8 to 22,593·3)	2·3% (1·6 to 3·1%)	26·0% (19·6 to 32·8%)	37·3% (31·9 to 42·9%)
Ischaemic heart disease	229·1 (132·1 to 336·0)	20·0% (11·7 to 29·2%)	305·3 (179·5 to 447·9)	19·9% (11·7 to 29·2%)	33·3% (26·2 to 40·9%)	-0·3% (-1·2 to 0·5%)	6,181·7 (3,613·8 to 8,961·0)	22·9% (13·6 to 33·0%)	8,044·2 (4,772·1 to 11,761·4)	22·9% (13·6 to 33·2%)	30·1% (22·8 to 38·1%)	0·3% (-0·6 to 1·3%)
Ischaemic stroke	67·6 (43·4 to 93·7)	17·7% (11·6 to 24·3%)	79·8 (51·8 to 109·8)	17·1% (11·3 to 23·6%)	18·1% (9·0 to 27·0%)	-3·0% (-4·8 to -1·1%)	1,696·9 (1,113·8 to 2,313·2)	23·4% (15·8 to 31·4%)	1,987·7 (1,318·1 to 2,699·4)	23·3% (15·7 to 31·4%)	17·1% (8·3 to 25·8%)	-0·4% (-2·0 to 1·3%)
Hemorrhagic stroke	117·2 (81·1 to 161·2)	22·6% (15·7 to 30·7%)	135·5 (93·1 to 186·6)	22·4% (15·5 to 30·6%)	15·6% (8·4 to 23·5%)	-0·8% (-2·3 to 0·6%)	3,600·3 (2,525·6 to 4,849·6)	25·4% (17·8 to 34·1%)	4,158·7 (2,902·9 to 5,648·4)	25·9% (18·1 to 34·9%)	15·5% (8·4 to 23·3%)	2·2% (0·7 to 3·8%)
Diabetes mellitus	45·0 (24·2 to 70·3)	17·5% (9·4 to 27·0%)	66·7 (35·8 to 104·7)	17·1% (9·2 to 26·4%)	48·2% (38·3 to 58·6%)	-2·6% (-3·7 to -1·5%)	2,068·5 (1,107·1 to 3,219·4)	20·5% (11·2 to 31·3%)	2,885·2 (1,564·7 to 4,489·5)	20·5% (11·2 to 31·3%)	39·5% (33·8 to 45·8%)	0·0% (-0·8 to 0·8%)
**Diet low in nuts and seeds: All causes**	308·6 (201·5 to 428·4)	2·3% (1·5 to 3·2%)	415·3 (266·3 to 576·5)	3·1% (2·0 to 4·4%)	34·6% (27·8 to 41·9%)	35·3% (30·6 to 40·3%)	8,605·7 (5,763·1 to 11,744·8)	1·1% (0·7 to 1·5%)	11,322·6 (7,388·4 to 15,467·3)	1·5% (1·0 to 2·1%)	31·6% (24·6 to 38·7%)	43·3% (37·0 to 50·0%)
Ischaemic heart disease	279·6 (174·6 to 394·0)	24·4% (15·4 to 34·5%)	372·4 (231·8 to 532·5)	24·3% (15·3 to 34·4%)	33·2% (26·0 to 40·6%)	-0·4% (-1·1 to 0·1%)	7,292·9 (4,660·9 to 10,101·2)	27·0% (17·3 to 37·5%)	9,488·9 (6,017·3 to 13,266·9)	27·0% (17·3 to 37·7%)	30·1% (22·7 to 38·0%)	0·3% (-0·5 to 1·0%)
Diabetes mellitus	29·0 (14·9 to 45·4)	11·3% (5·9 to 17·7%)	42·9 (22·0 to 68·0)	11·0% (5·7 to 17·3%)	48·0% (38·2 to 58·1%)	-2·7% (-3·8 to -1·6%)	1,312·8 (633·3 to 2,086·0)	13·0% (6·8 to 20·2%)	1,833·6 (888·0 to 2,890·9)	13·0% (6·8 to 20·3%)	39·7% (34·0 to 46·1%)	0·2% (-0·6 to 0·9%)
**Diet low in milk: All causes**	8·5 (2·9 to 14·3)	0·1% (0·0 to 0·1%)	11·9 (4·2 to 19·9)	0·1% (0·0 to 0·2%)	40·9% (30·7 to 52·1%)	41·7% (34·0 to 49·5%)	223·5 (77·0 to 377·3)	0·0% (0·0 to 0·0%)	305·0 (106·9 to 506·3)	0·0% (0·0 to 0·1%)	36·5% (26·2 to 47·6%)	48·7% (38·5 to 59·4%)
Colon and rectum cancer	8·5 (2·9 to 14·3)	16·3% (5·8 to 27·2%)	11·9 (4·2 to 19·9)	16·4% (5·8 to 27·2%)	40·9% (30·7 to 52·1%)	0·5% (-0·1 to 1·2%)	223·5 (77·0 to 377·3)	15·8% (5·6 to 26·3%)	305·0 (106·9 to 506·3)	15·9% (5·6 to 26·5%)	36·5% (26·2 to 47·6%)	0·8% (-0·2 to 1·9%)
**Diet high in red meat: All causes**	1·1 (0·4 to 2·0)	0·0% (0·0 to 0·0%)	1·5 (0·5 to 2·7)	0·0% (0·0 to 0·0%)	37·9% (23·1 to 52·7%)	38·6% (25·9 to 52·0%)	51·0 (12·5 to 93·9)	0·0% (0·0 to 0·0%)	70·3 (17·2 to 130·8)	0·0% (0·0 to 0·0%)	37·8% (28·1 to 48·4%)	50·1% (40·1 to 60·5%)
Colon and rectum cancer	0·3 (0·1 to 0·5)	0·5% (0·1 to 0·9%)	0·4 (0·1 to 0·7)	0·5% (0·1 to 0·9%)	39·3% (19·0 to 62·1%)	-0·7% (-11·2 to 13·4%)	7·1 (1·5 to 13·2)	0·5% (0·1 to 0·9%)	9·7 (2·0 to 17·9)	0·5% (0·1 to 1·0%)	36·7% (16·4 to 58·8%)	0·8% (-9·9 to 15·4%)
Diabetes mellitus	0·8 (0·1 to 1·7)	0·3% (0·0 to 0·7%)	1·2 (0·1 to 2·3)	0·3% (0·0 to 0·6%)	37·4% (22·0 to 53·2%)	-9·6% (-16·7 to -1·1%)	43·9 (5·1 to 87·2)	0·4% (0·1 to 0·9%)	60·7 (6·9 to 119·6)	0·4% (0·1 to 0·8%)	38·0% (28·6 to 48·2%)	-1·0% (-6·0 to 4·5%)
**Diet high in processed meat: All causes**	58·3 (16·3 to 102·8)	0·4% (0·1 to 0·8%)	78·3 (23·6 to 135·8)	0·6% (0·2 to 1·0%)	34·3% (25·6 to 46·5%)	35·1% (28·9 to 45·0%)	1,869·5 (721·8 to 3,124·5)	0·2% (0·1 to 0·4%)	2,488·9 (1,021·3 to 4,118·8)	0·3% (0·1 to 0·6%)	33·1% (25·0 to 43·1%)	45·0% (37·4 to 54·1%)
Colon and rectum cancer	1·3 (0·7 to 2·1)	2·6% (1·3 to 4·0%)	1·9 (1·0 to 3·0)	2·6% (1·4 to 4·1%)	43·3% (31·4 to 56·5%)	2·1% (-0·9 to 5·5%)	36·5 (18·5 to 58·8)	2·6% (1·3 to 4·1%)	51·1 (26·2 to 81·7)	2·7% (1·4 to 4·2%)	40·1% (27·8 to 54·3%)	3·4% (0·3 to 7·1%)
Ischaemic heart disease	43·0 (1·7 to 84·7)	3·7% (0·1 to 7·4%)	55·9 (2·3 to 111·2)	3·6% (0·1 to 7·2%)	30·2% (19·3 to 38·9%)	-2·6% (-5·2 to 0·0%)	1,158·5 (45·8 to 2,284·3)	4·3% (0·2 to 8·4%)	1,482·3 (60·1 to 2,915·7)	4·2% (0·2 to 8·3%)	27·9% (16·5 to 37·0%)	-1·3% (-4·0 to 1·5%)
Diabetes mellitus	14·0 (6·6 to 21·9)	5·5% (2·7 to 8·5%)	20·5 (9·5 to 32·1)	5·2% (2·5 to 8·3%)	45·9% (34·8 to 58·3%)	-4·0% (-7·4 to -0·3%)	674·5 (316·1 to 1,073·0)	6·7% (3·2 to 10·4%)	955·5 (444·5 to 1,502·8)	6·8% (3·3 to 10·6%)	41·7% (34·5 to 49·7%)	1·6% (-1·0 to 4·4%)
**Diet high in sugar-sweetened beverages: All causes**	2·7 (1·9 to 3·9)	0·0% (0·0 to 0·0%)	3·6 (2·5 to 5·1)	0·0% (0·0 to 0·0%)	29·6% (18·1 to 43·1%)	30·4% (20·6 to 40·4%)	106·6 (73·0 to 151·8)	0·0% (0·0 to 0·0%)	140·5 (96·3 to 197·1)	0·0% (0·0 to 0·0%)	31·8% (21·4 to 43·7%)	43·6% (33·4 to 54·7%)
Oesophageal cancer	0·0 (0·0 to 0·0)	0·1% (0·0 to 0·1%)	0·0 (0·0 to 0·1)	0·1% (0·0 to 0·1%)	37·7% (17·8 to 61·3%)	6·7% (-6·5 to 21·7%)	0·8 (0·3 to 1·5)	0·1% (0·0 to 0·1%)	1·1 (0·4 to 2·1)	0·1% (0·0 to 0·1%)	37·4% (16·7 to 63·3%)	8·7% (-5·9 to 25·3%)
Colon and rectum cancer	0·0 (0·0 to 0·0)	0·0% (0·0 to 0·0%)	0·0 (0·0 to 0·0)	0·0% (0·0 to 0·0%)	47·6% (30·6 to 69·0%)	5·2% (-4·2 to 16·2%)	0·5 (0·3 to 0·7)	0·0% (0·0 to 0·1%)	0·7 (0·5 to 1·1)	0·0% (0·0 to 0·1%)	46·2% (28·3 to 69·6%)	7·9% (-2·4 to 20·4%)
Liver cancer due to hepatitis B	0·0 (0·0 to 0·0)	0·1% (0·0 to 0·1%)	0·0 (0·0 to 0·0)	0·1% (0·0 to 0·2%)	25·0% (0·6 to 63·0%)	9·6% (-6·7 to 30·2%)	0·7 (0·3 to 1·3)	0·1% (0·0 to 0·2%)	0·9 (0·4 to 1·7)	0·1% (0·0 to 0·2%)	24·6% (-2·7 to 66·2%)	11·2% (-4·9 to 32·6%)
Liver cancer due to hepatitis C	0·0 (0·0 to 0·0)	0·1% (0·0 to 0·1%)	0·0 (0·0 to 0·0)	0·1% (0·0 to 0·2%)	34·8% (19·6 to 54·1%)	11·4% (1·1 to 22·3%)	0·3 (0·1 to 0·5)	0·1% (0·0 to 0·2%)	0·4 (0·2 to 0·7)	0·1% (0·0 to 0·2%)	32·3% (15·8 to 52·8%)	10·9% (-0·1 to 23·1%)
Liver cancer due to alcohol use	0·0 (0·0 to 0·0)	0·1% (0·0 to 0·2%)	0·0 (0·0 to 0·0)	0·1% (0·0 to 0·2%)	29·7% (3·3 to 67·3%)	4·0% (-8·1 to 17·5%)	0·5 (0·2 to 0·8)	0·1% (0·1 to 0·2%)	0·6 (0·3 to 1·1)	0·1% (0·1 to 0·2%)	29·3% (-0·6 to 72·3%)	4·8% (-7·7 to 19·2%)
Liver cancer due to other causes	0·0 (0·0 to 0·0)	0·1% (0·0 to 0·1%)	0·0 (0·0 to 0·0)	0·1% (0·0 to 0·1%)	27·0% (10·3 to 48·7%)	12·8% (2·1 to 25·6%)	0·3 (0·1 to 0·5)	0·1% (0·0 to 0·1%)	0·3 (0·2 to 0·6)	0·1% (0·0 to 0·2%)	24·9% (6·4 to 49·5%)	14·2% (2·7 to 28·4%)
Gallbladder and biliary tract cancer	0·0 (0·0 to 0·0)	0·1% (0·0 to 0·1%)	0·0 (0·0 to 0·0)	0·1% (0·0 to 0·1%)	15·8% (1·5 to 36·5%)	0·0% (-8·4 to 10·3%)	0·3 (0·2 to 0·5)	0·1% (0·1 to 0·1%)	0·3 (0·2 to 0·5)	0·1% (0·1 to 0·1%)	14·4% (-0·1 to 36·0%)	0·7% (-8·2 to 10·8%)
Pancreatic cancer	0·0 (0·0 to 0·0)	0·0% (0·0 to 0·0%)	0·0 (0·0 to 0·0)	0·0% (0·0 to 0·0%)	47·5% (30·2 to 70·2%)	6·9% (-2·6 to 16·8%)	0·2 (0·1 to 0·3)	0·0% (0·0 to 0·0%)	0·3 (0·1 to 0·5)	0·0% (0·0 to 0·1%)	47·7% (28·9 to 72·6%)	8·1% (-2·3 to 18·4%)
Breast cancer	0·0 (0·0 to 0·0)	0·0% (0·0 to 0·1%)	0·0 (0·0 to 0·1)	0·0% (0·0 to 0·1%)	40·6% (10·6 to 78·9%)	5·3% (-13·0 to 27·9%)	0·7 (0·3 to 1·3)	0·0% (0·0 to 0·1%)	0·9 (0·5 to 1·7)	0·0% (0·0 to 0·1%)	40·7% (8·4 to 81·1%)	6·2% (-12·5 to 30·4%)
Uterine cancer	0·0 (0·0 to 0·0)	0·2% (0·1 to 0·2%)	0·0 (0·0 to 0·0)	0·2% (0·1 to 0·2%)	19·2% (2·0 to 41·8%)	6·9% (-4·4 to 19·4%)	0·7 (0·4 to 0·9)	0·2% (0·1 to 0·3%)	0·8 (0·5 to 1·1)	0·2% (0·1 to 0·3%)	18·3% (0·1 to 41·6%)	7·6% (-3·9 to 20·1%)
Ovarian cancer	0·0 (0·0 to 0·0)	0·0% (0·0 to 0·0%)	0·0 (0·0 to 0·0)	0·0% (0·0 to 0·0%)	43·7% (0·0 to 70·2%)	6·3% (-4·5 to 17·5%)	0·1 (0·0 to 0·1)	0·0% (0·0 to 0·0%)	0·1 (0·0 to 0·2)	0·0% (0·0 to 0·0%)	43·6% (0·0 to 71·4%)	6·9% (-4·2 to 18·9%)
Kidney cancer	0·0 (0·0 to 0·0)	0·1% (0·0 to 0·1%)	0·0 (0·0 to 0·0)	0·1% (0·1 to 0·1%)	49·9% (32·3 to 72·7%)	4·6% (-7·4 to 18·3%)	0·2 (0·1 to 0·3)	0·1% (0·0 to 0·1%)	0·3 (0·2 to 0·4)	0·1% (0·0 to 0·1%)	49·4% (31·2 to 73·5%)	6·4% (-13·0 to 31·0%)
Thyroid cancer	0·0 (0·0 to 0·0)	0·0% (0·0 to 0·1%)	0·0 (0·0 to 0·0)	0·0% (0·0 to 0·1%)	32·3% (15·2 to 50·4%)	8·3% (-0·9 to 18·1%)	0·0 (0·0 to 0·1)	0·1% (0·0 to 0·1%)	0·1 (0·0 to 0·1)	0·1% (0·0 to 0·1%)	34·8% (16·4 to 52·4%)	7·8% (-0·8 to 17·4%)
Ischaemic heart disease	0·7 (0·5 to 1·1)	0·1% (0·0 to 0·1%)	0·9 (0·6 to 1·4)	0·1% (0·0 to 0·1%)	28·3% (17·9 to 40·1%)	-4·1% (-10·8 to 3·5%)	23·3 (15·9 to 33·6)	0·1% (0·1 to 0·1%)	29·9 (20·3 to 42·3)	0·1% (0·1 to 0·1%)	27·9% (17·0 to 40·3%)	-1·4% (-8·7 to 6·7%)
Ischaemic stroke	0·2 (0·1 to 0·3)	0·1% (0·0 to 0·1%)	0·2 (0·2 to 0·3)	0·1% (0·0 to 0·1%)	20·7% (8·3 to 34·3%)	-0·9% (-9·4 to 8·7%)	5·6 (3·9 to 8·1)	0·1% (0·1 to 0·1%)	6·8 (4·7 to 9·8)	0·1% (0·1 to 0·1%)	21·2% (8·9 to 35·2%)	3·0% (-5·4 to 12·5%)
Hemorrhagic stroke	0·7 (0·4 to 0·9)	0·1% (0·1 to 0·2%)	0·8 (0·5 to 1·1)	0·1% (0·1 to 0·2%)	19·6% (7·0 to 35·0%)	2·6% (-6·8 to 12·7%)	23·8 (15·9 to 34·1)	0·2% (0·1 to 0·2%)	28·5 (19·0 to 40·9)	0·2% (0·1 to 0·3%)	19·5% (6·9 to 35·6%)	5·7% (-3·4 to 15·8%)
Hypertensive heart disease	0·2 (0·1 to 0·3)	0·1% (0·1 to 0·2%)	0·3 (0·2 to 0·4)	0·1% (0·1 to 0·2%)	22·0% (7·7 to 39·3%)	-0·4% (-10·3 to 9·8%)	6·7 (4·3 to 9·9)	0·2% (0·1 to 0·3%)	8·1 (5·2 to 11·9)	0·2% (0·1 to 0·3%)	20·4% (6·2 to 38·3%)	1·9% (-8·4 to 12·5%)
Cardiomyopathy and myocarditis	-	-	-	-	-	-	-	-	-	-	-	-
Atrial fibrillation and flutter	-	-	-	-	-	-	-	-	-	-	-	-
Peripheral vascular disease	-	-	-	-	-	-	-	-	-	-	-	-

Supplemental Results Table 1. All-age deaths, death PAFs, DALYs, DALY PAFs, and percent change for each risk factor and outcome for both sexes combined in 2005 and 2015 for Low-middle SDI.

Data in parenthesis are 95% uncertainty intervals. PAFs=population attributable fractions. DALYs=disability-adjusted life-years.

	2005 Deaths (in thousands)	2005 Deaths PAF	2015 Deaths (in thousands)	2015 Deaths PAF	Percent change deaths 2005-2015	Percent change deaths PAF 2005-2015	2005 DALYs (in thousands)	2005 DALYs PAF	2015 DALYs (in thousands)	2015 DALYs PAF	Percent change DALYs 2005-2015	Percent change DALYs PAF 2005-2015
Endocarditis	-	-	-	-	-	-	-	-	-	-	-	-
Other cardiovascular and circulatory diseases	-	-	-	-	-	-	-	-	-	-	-	-
Diabetes mellitus	0·6 (0·4 to 0·8)	0·2% (0·2 to 0·3%)	0·8 (0·5 to 1·1)	0·2% (0·1 to 0·3%)	42·6% (29·3 to 58·2%)	-6·2% (-13·4 to 1·8%)	30·8 (20·1 to 44·6)	0·3% (0·2 to 0·4%)	44·8 (29·2 to 64·9)	0·3% (0·2 to 0·4%)	45·7% (36·3 to 56·0%)	4·4% (-1·2 to 10·1%)
Chronic kidney disease due to diabetes mellitus	0·1 (0·0 to 0·1)	0·1% (0·1 to 0·2%)	0·1 (0·1 to 0·2)	0·1% (0·1 to 0·2%)	60·7% (43·4 to 78·5%)	15·4% (3·5 to 28·3%)	2·3 (0·9 to 4·2)	0·1% (0·1 to 0·2%)	3·5 (1·5 to 6·3)	0·1% (0·1 to 0·3%)	54·3% (39·6 to 71·0%)	18·0% (7·8 to 30·9%)
Chronic kidney disease due to hypertension	0·1 (0·1 to 0·2)	0·1% (0·0 to 0·2%)	0·1 (0·1 to 0·2)	0·1% (0·1 to 0·2%)	38·7% (25·3 to 54·1%)	1·3% (-7·3 to 12·4%)	3·1 (1·3 to 5·7)	0·1% (0·0 to 0·2%)	4·2 (1·8 to 7·6)	0·1% (0·0 to 0·2%)	35·2% (22·7 to 49·9%)	8·0% (-0·8 to 18·8%)
Chronic kidney disease due to glomerulonephritis	0·0 (0·0 to 0·0)	0·0% (0·0 to 0·1%)	0·0 (0·0 to 0·1)	0·0% (0·0 to 0·1%)	32·9% (16·7 to 50·5%)	2·6% (-8·6 to 14·7%)	1·1 (0·3 to 2·1)	0·1% (0·0 to 0·1%)	1·5 (0·4 to 2·9)	0·1% (0·0 to 0·1%)	34·0% (21·8 to 47·7%)	14·8% (4·3 to 26·3%)
Chronic kidney disease due to other causes	0·0 (0·0 to 0·0)	0·1% (0·0 to 0·2%)	0·0 (0·0 to 0·0)	0·1% (0·0 to 0·2%)	34·8% (10·9 to 65·5%)	16·1% (-0·4 to 35·2%)	0·5 (0·2 to 1·0)	0·1% (0·0 to 0·2%)	0·7 (0·2 to 1·4)	0·1% (0·0 to 0·2%)	36·2% (25·4 to 47·7%)	18·4% (10·2 to 27·9%)
**Diet low in fibre: All causes**	50·1 (22·4 to 88·8)	0·4% (0·2 to 0·7%)	66·8 (30·2 to 117·4)	0·5% (0·2 to 0·9%)	33·3% (24·2 to 43·2%)	34·1% (26·5 to 42·5%)	1,461·1 (653·5 to 2,583·5)	0·2% (0·1 to 0·3%)	1,899·7 (863·7 to 3,314·8)	0·3% (0·1 to 0·5%)	30·0% (20·5 to 41·1%)	41·7% (31·7 to 53·3%)
Colon and rectum cancer	2·4 (1·0 to 4·4)	4·7% (2·0 to 8·3%)	3·4 (1·5 to 6·0)	4·6% (2·0 to 8·2%)	38·7% (25·4 to 53·3%)	-1·1% (-7·9 to 5·5%)	67·0 (28·9 to 120·3)	4·7% (2·1 to 8·5%)	89·3 (39·0 to 160·0)	4·7% (2·0 to 8·3%)	33·3% (19·9 to 48·0%)	-1·6% (-8·9 to 5·1%)
Ischaemic heart disease	47·7 (21·2 to 84·8)	4·2% (1·9 to 7·4%)	63·5 (28·3 to 112·3)	4·1% (1·9 to 7·3%)	33·0% (24·0 to 43·0%)	-0·5% (-4·8 to 4·5%)	1,394·1 (618·3 to 2,492·3)	5·2% (2·3 to 9·1%)	1,810·4 (823·1 to 3,191·2)	5·2% (2·3 to 9·0%)	29·9% (20·2 to 41·1%)	0·1% (-4·3 to 5·5%)
**Diet low in calcium: All causes**	12·4 (8·0 to 17·2)	0·1% (0·1 to 0·1%)	17·4 (11·1 to 24·1)	0·1% (0·1 to 0·2%)	40·4% (30·1 to 51·6%)	41·2% (33·5 to 49·2%)	325·3 (208·4 to 450·8)	0·0% (0·0 to 0·1%)	442·5 (283·4 to 610·3)	0·1% (0·0 to 0·1%)	36·0% (25·6 to 47·0%)	48·2% (38·1 to 59·0%)
Colon and rectum cancer	12·4 (8·0 to 17·2)	23·8% (15·5 to 32·7%)	17·4 (11·1 to 24·1)	23·9% (15·5 to 32·9%)	40·4% (30·1 to 51·6%)	0·1% (-0·8 to 1·0%)	325·3 (208·4 to 450·8)	23·0% (14·9 to 31·7%)	442·5 (283·4 to 610·3)	23·1% (15·0 to 31·8%)	36·0% (25·6 to 47·0%)	0·4% (-0·8 to 1·6%)
Prostate cancer	-	-	-	-	-	-	-	-	-	-	-	-
**Diet low in seafood omega-3 fatty acids: All causes**	231·6 (99·5 to 368·9)	1·7% (0·7 to 2·7%)	307·8 (130·4 to 483·7)	2·3% (1·0 to 3·7%)	32·9% (25·7 to 40·2%)	33·6% (29·0 to 38·4%)	5,972·9 (2,597·3 to 9,393·8)	0·7% (0·3 to 1·2%)	7,779·0 (3,328·8 to 12,124·8)	1·1% (0·5 to 1·7%)	30·2% (22·6 to 38·2%)	41·9% (35·1 to 49·2%)
Ischaemic heart disease	231·6 (99·5 to 368·9)	20·2% (8·7 to 31·9%)	307·8 (130·4 to 483·7)	20·1% (8·7 to 31·7%)	32·9% (25·7 to 40·2%)	-0·7% (-1·4 to 0·1%)	5,972·9 (2,597·3 to 9,393·8)	22·1% (9·7 to 34·3%)	7,779·0 (3,328·8 to 12,124·8)	22·2% (9·7 to 34·5%)	30·2% (22·6 to 38·2%)	0·4% (-0·4 to 1·2%)
**Diet low in polyunsaturated fatty acids: All causes**	43·7 (19·0 to 69·7)	0·3% (0·1 to 0·5%)	55·3 (23·1 to 89·0)	0·4% (0·2 to 0·7%)	26·5% (6·1 to 51·4%)	27·2% (7·3 to 51·4%)	1,149·5 (506·3 to 1,798·9)	0·1% (0·1 to 0·2%)	1,467·4 (645·4 to 2,316·9)	0·2% (0·1 to 0·3%)	27·7% (8·9 to 49·9%)	39·1% (19·3 to 63·1%)
Ischaemic heart disease	43·7 (19·0 to 69·7)	3·8% (1·6 to 6·0%)	55·3 (23·1 to 89·0)	3·6% (1·5 to 5·8%)	26·5% (6·1 to 51·4%)	-5·4% (-19·3 to 11·5%)	1,149·5 (506·3 to 1,798·9)	4·3% (1·8 to 6·7%)	1,467·4 (645·4 to 2,316·9)	4·2% (1·8 to 6·7%)	27·7% (8·9 to 49·9%)	-1·6% (-13·9 to 13·6%)
**Diet high in trans fatty acids: All causes**	100·0 (51·5 to 162·3)	0·7% (0·4 to 1·2%)	132·0 (66·9 to 215·4)	1·0% (0·5 to 1·6%)	31·9% (20·9 to 43·9%)	32·7% (22·7 to 42·8%)	2,892·2 (1,511·9 to 4,621·5)	0·4% (0·2 to 0·6%)	3,781·4 (1,970·2 to 6,008·5)	0·5% (0·3 to 0·8%)	30·7% (20·1 to 43·1%)	42·5% (30·9 to 54·9%)
Ischaemic heart disease	100·0 (51·5 to 162·3)	8·7% (4·5 to 14·2%)	132·0 (66·9 to 215·4)	8·6% (4·4 to 14·0%)	31·9% (20·9 to 43·9%)	-1·4% (-6·5 to 4·0%)	2,892·2 (1,511·9 to 4,621·5)	10·7% (5·6 to 17·2%)	3,781·4 (1,970·2 to 6,008·5)	10·8% (5·6 to 17·3%)	30·7% (20·1 to 43·1%)	0·8% (-4·5 to 6·9%)
**Diet high in sodium: All causes**	436·4 (206·6 to 761·9)	3·3% (1·6 to 5·7%)	556·5 (266·0 to 963·0)	4·2% (2·0 to 7·3%)	27·5% (19·6 to 36·1%)	28·3% (22·5 to 34·4%)	10,342·8 (4,876·2 to 18,090·8)	1·3% (0·6 to 2·3%)	12,884·0 (6,093·2 to 22,281·7)	1·8% (0·8 to 3·1%)	24·6% (16·8 to 33·2%)	35·7% (28·5 to 43·6%)
Stomach cancer	26·2 (14·0 to 40·8)	35·8% (19·3 to 55·6%)	29·7 (15·4 to 46·1)	35·4% (18·6 to 55·5%)	13·4% (3·3 to 24·1%)	-0·9% (-7·1 to 4·4%)	652·6 (347·2 to 1,018·7)	35·1% (18·8 to 54·6%)	714·2 (374·7 to 1,113·8)	34·7% (18·6 to 54·2%)	9·4% (-0·6 to 19·8%)	-1·1% (-7·2 to 3·9%)
Rheumatic heart disease	6·7 (2·7 to 13·1)	6·4% (2·6 to 12·5%)	7·4 (2·9 to 14·5)	6·5% (2·5 to 12·7%)	9·9% (-2·3 to 21·3%)	1·0% (-6·5 to 6·5%)	220·5 (84·7 to 448·7)	5·1% (2·0 to 10·4%)	235·0 (88·4 to 480·3)	5·3% (2·0 to 10·9%)	6·6% (-3·6 to 16·3%)	4·7% (-2·0 to 9·7%)
Ischaemic heart disease	175·6 (76·1 to 324·6)	15·3% (6·6 to 27·9%)	239·0 (104·3 to 433·9)	15·6% (6·9 to 28·2%)	36·1% (28·5 to 44·9%)	1·8% (-0·6 to 5·0%)	4,035·0 (1,764·5 to 7,356·6)	14·9% (6·5 to 27·1%)	5,377·3 (2,394·6 to 9,633·0)	15·3% (6·8 to 27·8%)	33·3% (25·4 to 42·5%)	2·8% (0·6 to 5·9%)
Ischaemic stroke	57·6 (25·7 to 105·2)	15·1% (6·7 to 27·2%)	70·6 (31·2 to 128·6)	15·2% (6·8 to 27·3%)	22·6% (12·1 to 33·9%)	0·7% (-4·2 to 6·9%)	1,146·8 (513·4 to 2,063·0)	15·8% (7·2 to 28·4%)	1,375·6 (626·6 to 2,461·3)	16·1% (7·5 to 28·6%)	20·0% (10·2 to 31·3%)	1·9% (-2·5 to 8·0%)
Hemorrhagic stroke	96·3 (46·3 to 170·3)	18·6% (8·9 to 32·1%)	112·1 (53·0 to 196·2)	18·5% (8·7 to 32·5%)	16·4% (6·8 to 26·9%)	-0·1% (-6·3 to 5·1%)	2,441·8 (1,141·4 to 4,320·3)	17·2% (8·0 to 29·8%)	2,820·1 (1,290·0 to 4,952·8)	17·6% (8·2 to 30·5%)	15·5% (6·2 to 26·0%)	2·2% (-3·8 to 7·4%)
Hypertensive heart disease	40·7 (13·7 to 87·5)	26·6% (8·8 to 57·4%)	51·3 (17·5 to 110·7)	27·3% (9·1 to 57·8%)	26·0% (12·9 to 41·0%)	2·9% (-1·8 to 9·3%)	941·2 (367·0 to 1,922·0)	25·7% (9·8 to 51·5%)	1,152·5 (449·4 to 2,313·3)	26·6% (10·3 to 52·8%)	22·5% (10·5 to 36·5%)	3·6% (-0·4 to 9·8%)
Cardiomyopathy and myocarditis	2·0 (0·9 to 3·6)	7·9% (3·6 to 14·3%)	2·6 (1·2 to 4·6)	8·7% (4·0 to 15·6%)	31·7% (16·6 to 50·4%)	9·8% (2·6 to 20·1%)	59·8 (25·2 to 114·1)	5·7% (2·5 to 10·7%)	76·4 (32·5 to 143·6)	6·5% (2·8 to 12·2%)	27·9% (13·0 to 46·1%)	14·9% (5·6 to 26·0%)
Atrial fibrillation and flutter	1·1 (0·5 to 2·2)	9·2% (4·1 to 16·8%)	1·6 (0·6 to 3·1)	9·1% (4·0 to 16·7%)	40·1% (31·4 to 49·4%)	-0·5% (-3·9 to 3·1%)	38·5 (16·7 to 71·7)	11·6% (5·4 to 20·5%)	51·9 (22·5 to 97·7)	11·6% (5·5 to 20·5%)	34·9% (30·8 to 39·4%)	0·4% (-1·5 to 3·0%)
Aortic aneurysm	1·1 (0·5 to 2·1)	9·8% (4·3 to 17·7%)	1·7 (0·7 to 3·1)	9·9% (4·3 to 17·9%)	50·0% (33·2 to 67·4%)	0·8% (-3·2 to 5·7%)	26·4 (11·0 to 50·0)	9·6% (4·2 to 17·5%)	39·0 (16·9 to 72·1)	9·8% (4·3 to 17·7%)	47·7% (30·7 to 65·6%)	2·2% (-2·3 to 7·9%)
Peripheral vascular disease	0·1 (0·0 to 0·1)	7·2% (3·0 to 13·5%)	0·1 (0·0 to 0·2)	7·2% (3·0 to 13·6%)	85·3% (62·8 to 111·1%)	0·3% (-5·0 to 6·0%)	5·2 (1·8 to 11·6)	7·7% (3·1 to 14·3%)	7·7 (2·7 to 16·6)	7·8% (3·2 to 14·3%)	47·9% (42·0 to 57·9%)	1·5% (-0·5 to 4·8%)
Endocarditis	0·6 (0·3 to 1·3)	6·8% (2·8 to 13·1%)	0·9 (0·4 to 1·7)	7·5% (3·1 to 14·1%)	39·5% (23·5 to 60·8%)	9·2% (0·5 to 21·6%)	18·9 (7·1 to 38·5)	4·9% (1·8 to 9·9%)	26·0 (10·0 to 50·7)	5·6% (2·2 to 11·2%)	37·8% (21·2 to 61·0%)	14·8% (2·2 to 31·1%)
Other cardiovascular and circulatory diseases	5·3 (2·4 to 9·4)	9·8% (4·5 to 17·3%)	6·8 (3·1 to 12·1)	10·5% (4·8 to 18·4%)	29·2% (9·3 to 56·6%)	6·7% (-4·9 to 22·8%)	171·2 (75·0 to 315·7)	7·5% (3·4 to 13·7%)	219·9 (97·6 to 401·4)	8·4% (3·7 to 15·0%)	28·4% (13·0 to 51·3%)	11·3% (1·1 to 27·1%)
Chronic kidney disease due to diabetes mellitus	6·5 (2·4 to 12·8)	11·8% (4·4 to 22·5%)	9·3 (3·5 to 17·8)	12·0% (4·5 to 22·9%)	42·8% (30·8 to 56·3%)	2·3% (-0·4 to 5·2%)	169·7 (63·9 to 335·6)	9·4% (3·5 to 18·0%)	233·1 (90·1 to 455·6)	9·8% (3·7 to 18·7%)	37·3% (27·4 to 48·5%)	4·9% (2·2 to 7·9%)
Chronic kidney disease due to hypertension	10·8 (3·9 to 22·2)	10·6% (4·0 to 21·0%)	15·5 (5·7 to 31·6)	11·1% (4·2 to 21·9%)	43·7% (32·2 to 56·5%)	4·9% (1·9 to 8·2%)	237·0 (86·3 to 488·6)	8·0% (3·0 to 15·9%)	324·1 (119·0 to 672·6)	8·7% (3·3 to 17·3%)	36·7% (25·9 to 48·5%)	9·2% (5·8 to 13·1%)
Chronic kidney disease due to glomerulonephritis	5·4 (2·2 to 10·3)	10·2% (4·2 to 19·1%)	7·4 (2·9 to 14·1)	10·8% (4·4 to 20·5%)	37·6% (23·6 to 50·3%)	6·2% (-0·9 to 11·6%)	145·1 (59·2 to 280·5)	6·7% (2·7 to 12·7%)	187·3 (73·3 to 362·3)	7·4% (2·9 to 14·3%)	29·1% (17·3 to 39·8%)	10·8% (3·1 to 16·7%)
Chronic kidney disease due to other causes	0·4 (0·1 to 0·7)	7·1% (2·8 to 13·8%)	0·5 (0·2 to 1·0)	8·5% (3·4 to 16·1%)	39·0% (21·4 to 57·2%)	19·8% (10·7 to 29·4%)	33·2 (12·2 to 67·0)	5·8% (2·2 to 11·5%)	43·8 (16·0 to 88·1)	6·6% (2·6 to 13·1%)	31·9% (25·6 to 38·3%)	14·8% (10·2 to 20·6%)
**Sexual abuse and violence: All causes**	101·9 (71·9 to 132·8)	0·8% (0·5 to 1·0%)	88·0 (60·5 to 116·7)	0·7% (0·5 to 0·9%)	-13·6% (-23·6 to -1·5%)	-13·1% (-22·9 to -1·7%)	6,944·5 (5,199·2 to 8,768·4)	0·9% (0·7 to 1·1%)	6,677·9 (4,980·1 to 8,623·4)	0·9% (0·7 to 1·2%)	-3·8% (-12·4 to 5·5%)	4·7% (-4·3 to 14·2%)
**Childhood sexual abuse: All causes**	29·6 (8·1 to 56·3)	0·2% (0·1 to 0·4%)	32·6 (8·9 to 62·6)	0·2% (0·1 to 0·5%)	10·1% (-1·5 to 22·3%)	10·7% (-0·1 to 21·2%)	2,346·3 (972·3 to 4,005·1)	0·3% (0·1 to 0·5%)	2,589·9 (1,064·2 to 4,472·9)	0·4% (0·1 to 0·6%)	10·4% (1·7 to 18·7%)	20·2% (10·8 to 28·6%)
Alcohol use disorders	0·8 (0·0 to 1·8)	5·1% (0·0 to 11·7%)	0·9 (0·0 to 2·0)	4·8% (0·0 to 11·0%)	12·3% (-0·3 to 29·0%)	-6·1% (-10·9 to 0·0%)	92·9 (0·0 to 209·7)	6·2% (0·0 to 13·8%)	107·3 (0·0 to 240·9)	5·9% (0·0 to 13·2%)	15·6% (0·0 to 22·3%)	-4·9% (-7·5 to 0·0%)
Major depressive disorder	-	-	-	-	-	-	655·0 (75·6 to 1,410·0)	7·9% (1·0 to 16·3%)	762·1 (85·4 to 1,644·6)	7·5% (0·9 to 15·6%)	16·4% (13·3 to 19·0%)	-5·1% (-7·1 to -3·2%)
Dysthymia	-	-	-	-	-	-	121·9 (13·5 to 264·6)	7·8% (0·9 to 15·9%)	145·3 (16·0 to 319·2)	7·4% (0·9 to 15·2%)	19·1% (15·0 to 22·5%)	-5·1% (-7·1 to -3·1%)
Self-harm	28·8 (7·1 to 55·7)	19·0% (4·9 to 36·2%)	31·7 (7·7 to 61·6)	17·9% (4·5 to 34·3%)	10·0% (-2·2 to 22·5%)	-5·9% (-9·4 to -3·3%)	1,476·6 (363·9 to 2,833·2)	19·8% (5·1 to 37·3%)	1,575·2 (385·0 to 3,030·7)	18·7% (4·8 to 35·4%)	6·7% (-5·2 to 19·7%)	-5·6% (-9·1 to -3·1%)
**Intimate partner violence: All causes**	79·0 (54·9 to 101·9)	0·6% (0·4 to 0·8%)	62·5 (45·4 to 78·2)	0·5% (0·3 to 0·6%)	-20·9% (-29·8 to -8·9%)	-20·4% (-28·7 to -8·6%)	5,034·2 (3,858·0 to 6,320·3)	0·6% (0·5 to 0·8%)	4,551·2 (3,583·4 to 5,683·0)	0·6% (0·5 to 0·8%)	-9·6% (-18·3 to 1·0%)	-1·6% (-10·8 to 10·0%)

Supplemental Results Table 1. All-age deaths, death PAFs, DALYs, DALY PAFs, and percent change for each risk factor and outcome for both sexes combined in 2005 and 2015 for Low-middle SDI.

Data in parenthesis are 95% uncertainty intervals. PAFs=population attributable fractions. DALYs=disability-adjusted life-years.

	2005 Deaths (in thousands)	2005 Deaths PAF	2015 Deaths (in thousands)	2015 Deaths PAF	Percent change deaths 2005-2015	Percent change deaths PAF 2005-2015	2005 DALYs (in thousands)	2005 DALYs PAF	2015 DALYs (in thousands)	2015 DALYs PAF	Percent change DALYs 2005-2015	Percent change DALYs PAF 2005-2015
HIV/AIDS - Tuberculosis	6·6 (3·5 to 10·2)	6·2% (3·4 to 9·0%)	3·4 (1·8 to 5·3)	5·5% (3·0 to 8·1%)	-48·2% (-52·5 to -42·7%)	-11·2% (-17·3 to -5·0%)	301·5 (157·4 to 467·5)	5·1% (2·7 to 7·4%)	165·0 (85·1 to 258·5)	4·8% (2·5 to 7·0%)	-45·3% (-49·3 to -39·9%)	-6·2% (-12·7 to -0·4%)
HIV/AIDS resulting in other diseases	38·3 (20·5 to 57·0)	6·3% (3·4 to 9·2%)	22·1 (11·4 to 32·8)	5·5% (2·9 to 8·1%)	-42·3% (-49·2 to -34·8%)	-13·3% (-21·1 to -4·4%)	1,903·9 (999·5 to 2,835·0)	5·6% (3·0 to 8·2%)	1,144·2 (588·4 to 1,691·5)	4·9% (2·6 to 7·3%)	-39·9% (-46·9 to -32·8%)	-11·9% (-19·7 to -3·3%)
Maternal abortion, miscarriage, and ectopic pregnancy	0·0 (0·0 to 0·0)	0·0% (0·0 to 0·0%)	0·0 (0·0 to 0·0)	0·0% (0·0 to 0·0%)	-27·2% (-44·0 to -2·7%)	0·3% (-14·9 to 19·5%)	0·0 (0·0 to 0·0)	0·0% (0·0 to 0·0%)	0·0 (0·0 to 0·0)	0·0% (0·0 to 0·0%)	-27·8% (-43·9 to -2·9%)	0·3% (-14·0 to 20·3%)
Major depressive disorder	-	-	-	-	-	-	969·3 (627·2 to 1,388·3)	11·7% (9·7 to 13·7%)	1,263·7 (823·2 to 1,811·5)	12·5% (10·3 to 14·5%)	30·4% (26·5 to 34·2%)	6·3% (3·6 to 9·1%)
Dysthymia	-	-	-	-	-	-	192·3 (123·3 to 282·7)	12·3% (10·3 to 14·3%)	258·5 (165·8 to 381·9)	13·1% (11·0 to 15·3%)	34·4% (28·5 to 40·6%)	7·0% (3·7 to 10·8%)
Self-harm	28·8 (15·8 to 36·2)	19·0% (12·8 to 23·4%)	31·4 (18·0 to 40·2)	17·8% (12·4 to 22·3%)	9·0% (-7·7 to 29·2%)	-6·7% (-18·1 to 5·4%)	1,365·5 (719·5 to 1,757·0)	18·2% (11·9 to 22·9%)	1,411·8 (796·1 to 1,855·8)	16·7% (11·3 to 21·3%)	3·4% (-13·5 to 25·6%)	-8·4% (-20·1 to 4·9%)
Assault by firearm	1·1 (0·8 to 1·4)	5·6% (4·4 to 7·1%)	1·3 (0·9 to 1·7)	5·0% (3·6 to 6·6%)	13·8% (-8·6 to 49·2%)	-11·0% (-28·2 to 12·1%)	63·3 (44·6 to 75·9)	5·7% (4·5 to 7·1%)	70·2 (45·2 to 92·9)	5·1% (3·7 to 6·6%)	10·9% (-11·7 to 42·7%)	-11·4% (-29·1 to 11·8%)
Assault by sharp object	0·6 (0·5 to 0·7)	5·6% (4·7 to 6·8%)	0·6 (0·5 to 0·7)	5·7% (4·7 to 7·1%)	4·7% (-11·4 to 23·7%)	1·6% (-15·4 to 21·0%)	30·9 (25·4 to 37·1)	5·6% (4·7 to 6·8%)	31·6 (25·1 to 39·8)	5·7% (4·6 to 7·1%)	2·3% (-13·4 to 21·4%)	1·6% (-14·9 to 20·5%)
Assault by other means	3·6 (3·0 to 4·1)	8·8% (7·7 to 10·5%)	3·7 (2·7 to 4·5)	8·4% (6·8 to 10·9%)	2·1% (-14·5 to 19·8%)	-4·4% (-18·7 to 11·2%)	207·3 (172·1 to 236·3)	9·2% (8·0 to 11·1%)	206·2 (160·8 to 250·1)	8·7% (7·2 to 11·2%)	-0·5% (-14·7 to 15·6%)	-4·5% (-18·0 to 9·9%)
**Unsafe sex: All causes**	785·8 (724·5 to 853·0)	5·9% (5·4 to 6·4%)	530·5 (486·8 to 581·4)	4·0% (3·7 to 4·3%)	-32·5% (-37·1 to -27·4%)	-32·1% (-36·6 to -27·4%)	44,670·0 (40,901·5 to 48,741·5)	5·6% (5·0 to 6·2%)	30,240·1 (27,681·1 to 33,280·3)	4·1% (3·7 to 4·6%)	-32·3% (-36·6 to -28·0%)	-26·2% (-30·5 to -21·8%)
HIV/AIDS - Tuberculosis	100·5 (80·3 to 120·3)	94·3% (93·1 to 95·2%)	58·1 (46·4 to 69·8)	93·5% (92·1 to 94·5%)	-42·2% (-45·5 to -36·8%)	-0·9% (-1·8 to -0·3%)	5,596·4 (4,450·5 to 6,667·4)	94·5% (93·5 to 95·4%)	3,241·6 (2,594·9 to 3,849·1)	93·8% (92·6 to 94·8%)	-42·1% (-45·0 to -36·9%)	-0·7% (-1·6 to -0·1%)
HIV/AIDS resulting in other diseases	568·3 (516·3 to 630·9)	93·7% (92·8 to 94·5%)	374·8 (332·5 to 419·6)	92·8% (91·4 to 93·8%)	-34·0% (-40·2 to -27·7%)	-1·0% (-2·2 to -0·2%)	31,860·9 (29,061·1 to 35,092·7)	93·9% (93·0 to 94·6%)	21,541·7 (19,325·7 to 23,905·2)	93·1% (91·7 to 94·1%)	-32·4% (-38·1 to -26·9%)	-0·9% (-2·0 to -0·1%)
Syphilis	63·9 (38·7 to 97·8)	100·0% (100·0 to 100·0%)	41·2 (24·3 to 63·7)	100·0% (100·0 to 100·0%)	-35·5% (-43·4 to -26·0%)	-	5,310·2 (3,164·4 to 8,192·8)	100·0% (100·0 to 100·0%)	3,429·6 (1,987·5 to 5,342·3)	100·0% (100·0 to 100·0%)	-35·4% (-43·3 to -25·8%)	-
Chlamydial infection	0·1 (0·1 to 0·1)	100·0% (100·0 to 100·0%)	0·1 (0·0 to 0·1)	100·0% (100·0 to 100·0%)	-10·5% (-29·7 to 11·5%)	-	49·8 (30·3 to 77·5)	100·0% (100·0 to 100·0%)	58·9 (35·7 to 91·2)	100·0% (100·0 to 100·0%)	18·3% (13·4 to 22·9%)	-
Gonococcal infection	0·4 (0·3 to 0·5)	100·0% (100·0 to 100·0%)	0·3 (0·2 to 0·4)	100·0% (100·0 to 100·0%)	-22·1% (-38·0 to -6·3%)	-	127·7 (81·5 to 189·6)	100·0% (100·0 to 100·0%)	153·9 (92·4 to 238·9)	100·0% (100·0 to 100·0%)	20·5% (10·5 to 29·4%)	-
Trichomoniasis	-	-	-	-	-	-	37·8 (15·2 to 80·6)	100·0% (100·0 to 100·0%)	46·2 (18·7 to 97·8)	100·0% (100·0 to 100·0%)	22·3% (19·9 to 24·5%)	-
Genital herpes	-	-	-	-	-	-	37·7 (12·0 to 88·4)	100·0% (100·0 to 100·0%)	46·3 (14·7 to 109·0)	100·0% (100·0 to 100·0%)	22·9% (19·4 to 26·8%)	-
Other sexually transmitted diseases	0·2 (0·2 to 0·2)	100·0% (100·0 to 100·0%)	0·1 (0·1 to 0·2)	100·0% (100·0 to 100·0%)	-23·5% (-38·3 to -8·3%)	-	22·8 (17·7 to 29·0)	100·0% (100·0 to 100·0%)	23·7 (17·6 to 31·2)	100·0% (100·0 to 100·0%)	4·0% (-4·1 to 10·6%)	-
Cervical cancer	52·5 (46·3 to 59·2)	100·0% (100·0 to 100·0%)	55·9 (48·1 to 64·4)	100·0% (100·0 to 100·0%)	6·5% (-10·3 to 24·2%)	-	1,626·8 (1,421·7 to 1,857·5)	100·0% (100·0 to 100·0%)	1,698·1 (1,441·8 to 1,980·7)	100·0% (100·0 to 100·0%)	4·4% (-11·7 to 22·7%)	-
**Low physical activity: All causes**	184·9 (139·0 to 232·0)	1·4% (1·0 to 1·7%)	250·7 (189·7 to 315·2)	1·9% (1·4 to 2·4%)	35·6% (28·3 to 43·2%)	36·4% (31·6 to 41·7%)	4,583·5 (3,396·0 to 5,793·4)	0·6% (0·4 to 0·7%)	6,091·5 (4,500·0 to 7,680·5)	0·8% (0·6 to 1·1%)	32·9% (26·2 to 40·2%)	44·8% (38·8 to 51·7%)
Colon and rectum cancer	5·7 (3·7 to 7·8)	11·0% (7·3 to 14·8%)	8·1 (5·2 to 11·1)	11·2% (7·4 to 15·0%)	42·9% (32·4 to 54·3%)	1·9% (0·4 to 3·9%)	138·7 (88·7 to 193·2)	9·8% (6·3 to 13·5%)	192·6 (121·9 to 267·1)	10·1% (6·5 to 13·7%)	38·9% (28·1 to 50·6%)	2·5% (0·7 to 4·8%)
Breast cancer	4·2 (2·8 to 5·8)	6·9% (4·9 to 8·9%)	5·7 (4·0 to 7·6)	7·0% (5·0 to 9·0%)	36·0% (19·4 to 54·2%)	1·4% (-0·9 to 4·0%)	129·1 (86·4 to 180·7)	6·4% (4·5 to 8·3%)	175·3 (119·6 to 236·3)	6·5% (4·6 to 8·5%)	35·8% (19·1 to 55·1%)	2·0% (-0·6 to 4·6%)
Ischaemic heart disease	100·8 (62·8 to 141·9)	8·8% (5·5 to 12·3%)	134·6 (82·6 to 187·7)	8·8% (5·5 to 12·2%)	33·5% (26·0 to 41·3%)	-0·3% (-2·2 to 1·7%)	2,401·2 (1,443·6 to 3,449·0)	8·9% (5·4 to 12·5%)	3,146·6 (1,894·3 to 4,480·5)	9·0% (5·5 to 12·7%)	31·0% (23·3 to 39·6%)	1·0% (-1·3 to 3·2%)
Ischaemic stroke	39·9 (20·9 to 60·6)	10·4% (5·4 to 15·7%)	48·7 (25·6 to 73·0)	10·5% (5·5 to 15·6%)	21·9% (14·0 to 31·2%)	0·2% (-1·6 to 2·5%)	782·3 (395·9 to 1,191·1)	10·8% (5·4 to 16·3%)	931·0 (477·7 to 1,422·8)	10·9% (5·5 to 16·6%)	19·0% (10·5 to 27·7%)	1·2% (-0·8 to 3·6%)
Diabetes mellitus	34·3 (25·8 to 44·1)	13·3% (10·0 to 17·0%)	53·6 (39·9 to 68·5)	13·7% (10·3 to 17·5%)	56·5% (45·5 to 67·4%)	2·9% (1·2 to 4·8%)	1,132·2 (810·5 to 1,524·2)	11·2% (8·2 to 14·5%)	1,646·0 (1,169·2 to 2,196·4)	11·7% (8·6 to 15·1%)	45·4% (38·7 to 52·2%)	4·2% (2·9 to 5·6%)
**Metabolic risks: All causes**	2,288·6 (2,162·5 to 2,418·8)	17·1% (16·3 to 17·9%)	3,024·4 (2,839·2 to 3,215·9)	22·8% (21·7 to 23·9%)	32·1% (25·9 to 38·8%)	32·9% (29·6 to 36·1%)	62,229·0 (58,359·2 to 66,619·0)	7·8% (7·4 to 8·2%)	79,641·4 (74,266·4 to 85,564·1)	10·8% (10·3 to 11·5%)	28·0% (22·2 to 34·3%)	39·4% (34·6 to 44·4%)
**High fasting plasma glucose: All causes**	800·9 (713·6 to 914·8)	6·0% (5·3 to 6·8%)	1,127·1 (997·0 to 1,293·8)	8·5% (7·6 to 9·7%)	40·7% (33·4 to 48·1%)	41·5% (36·5 to 46·2%)	24,299·3 (21,548·4 to 27,304·0)	3·0% (2·7 to 3·4%)	32,624·4 (28,804·8 to 36,720·6)	4·4% (4·0 to 4·9%)	34·3% (28·5 to 40·4%)	46·3% (40·9 to 51·7%)
Tuberculosis	55·4 (34·2 to 79·6)	9·7% (6·2 to 13·5%)	51·1 (31·6 to 74·1)	10·4% (6·6 to 14·6%)	-7·7% (-20·7 to 4·6%)	8·0% (3·5 to 12·1%)	1,802·2 (1,147·0 to 2,487·5)	8·5% (5·5 to 11·5%)	1,637·2 (1,029·7 to 2,287·5)	9·3% (6·1 to 12·6%)	-9·2% (-21·2 to 1·9%)	9·6% (3·3 to 15·1%)
Ischaemic heart disease	222·4 (154·5 to 308·8)	19·4% (13·5 to 26·7%)	323·7 (223·4 to 448·8)	21·1% (14·7 to 28·9%)	45·5% (37·2 to 54·2%)	8·8% (6·3 to 11·4%)	5,334·9 (3,738·0 to 7,188·1)	19·7% (13·8 to 26·3%)	7,593·8 (5,359·2 to 10,344·0)	21·6% (15·3 to 28·7%)	42·3% (34·3 to 51·1%)	9·7% (7·7 to 11·9%)
Ischaemic stroke	66·9 (39·6 to 111·8)	17·5% (10·2 to 29·4%)	87·6 (51·6 to 146·2)	18·8% (11·1 to 32·1%)	31·0% (21·5 to 42·3%)	7·7% (4·2 to 13·0%)	1,331·2 (844·5 to 2,141·8)	18·3% (11·5 to 28·4%)	1,707·5 (1,091·9 to 2,623·4)	20·0% (12·7 to 30·7%)	28·3% (18·9 to 38·2%)	9·1% (6·7 to 12·4%)
Hemorrhagic stroke	89·6 (61·1 to 122·8)	17·3% (11·9 to 23·5%)	114·7 (78·5 to 155·7)	19·0% (13·3 to 25·6%)	28·0% (20·0 to 37·8%)	9·9% (7·5 to 12·9%)	2,416·2 (1,603·7 to 3,424·2)	17·0% (11·3 to 24·2%)	3,077·7 (2,079·2 to 4,302·2)	19·2% (12·7 to 27·0%)	27·4% (20·0 to 36·0%)	12·7% (10·1 to 15·8%)
Diabetes mellitus	256·9 (240·7 to 271·5)	100·0% (100·0 to 100·0%)	390·9 (359·1 to 422·6)	100·0% (100·0 to 100·0%)	52·1% (42·1 to 62·5%)	-	10,078·6 (8,730·9 to 11,609·1)	100·0% (100·0 to 100·0%)	14,058·0 (12,091·8 to 16,186·3)	100·0% (100·0 to 100·0%)	39·5% (33·7 to 45·8%)	-
Chronic kidney disease due to diabetes mellitus	55·6 (47·8 to 61·8)	100·0% (100·0 to 100·0%)	77·5 (67·1 to 86·9)	100·0% (100·0 to 100·0%)	39·4% (28·2 to 51·4%)	-	1,808·7 (1,569·0 to 2,004·3)	100·0% (100·0 to 100·0%)	2,367·7 (2,077·3 to 2,635·1)	100·0% (100·0 to 100·0%)	30·9% (22·1 to 40·4%)	-
Chronic kidney disease due to hypertension	36·0 (25·5 to 47·5)	35·6% (25·4 to 45·4%)	54·6 (38·7 to 71·0)	39·3% (28·3 to 49·9%)	51·4% (38·7 to 65·3%)	10·6% (8·6 to 12·6%)	870·0 (611·3 to 1,145·1)	29·4% (20·9 to 37·9%)	1,253·8 (877·9 to 1,634·0)	33·8% (24·2 to 43·2%)	44·1% (32·6 to 57·0%)	15·1% (12·3 to 17·9%)
Chronic kidney disease due to glomerulonephritis	16·9 (11·8 to 22·2)	31·9% (22·7 to 41·1%)	25·2 (17·7 to 32·8)	36·8% (26·5 to 46·6%)	49·4% (35·7 to 63·4%)	15·3% (12·7 to 17·9%)	521·5 (359·0 to 693·5)	24·1% (17·0 to 31·5%)	736·2 (513·3 to 969·8)	29·2% (20·9 to 37·5%)	41·2% (29·8 to 52·7%)	21·0% (17·0 to 24·9%)
Chronic kidney disease due to other causes	1·0 (0·7 to 1·4)	19·5% (13·5 to 25·8%)	1·5 (1·0 to 2·0)	24·9% (17·4 to 32·3%)	48·3% (33·2 to 65·5%)	27·8% (20·3 to 35·7%)	119·5 (77·2 to 168·6)	20·9% (14·6 to 27·5%)	167·2 (109·6 to 233·9)	25·4% (17·9 to 32·8%)	39·8% (34·7 to 45·2%)	21·6% (17·3 to 26·6%)
**High total cholesterol: All causes**	498·7 (391·6 to 629·2)	3·7% (2·9 to 4·7%)	673·3 (532·8 to 841·4)	5·1% (4·0 to 6·3%)	35·0% (27·9 to 42·6%)	35·8% (30·9 to 41·3%)	13,286·2 (10,827·6 to 16,066·6)	1·7% (1·4 to 2·0%)	17,652·6 (14,364·6 to 21,287·9)	2·4% (2·0 to 2·9%)	32·9% (25·3 to 40·8%)	44·8% (38·0 to 52·3%)
Ischaemic heart disease	436·4 (347·2 to 537·1)	38·0% (30·4 to 46·8%)	595·5 (469·7 to 737·1)	38·8% (31·0 to 47·5%)	36·5% (29·0 to 44·2%)	2·0% (0·2 to 3·9%)	11,836·9 (9,799·0 to 14,192·7)	43·8% (36·5 to 51·8%)	15,880·0 (12,897·9 to 19,098·0)	45·2% (37·8 to 53·4%)	34·2% (26·5 to 42·2%)	3·4% (2·1 to 5·0%)
Ischaemic stroke	62·3 (30·4 to 119·8)	16·3% (8·2 to 30·9%)	77·7 (38·4 to 150·0)	16·7% (8·3 to 32·0%)	24·8% (14·2 to 35·1%)	2·5% (-2·0 to 6·5%)	1,449·2 (899·9 to 2,363·7)	20·0% (12·8 to 32·0%)	1,772·7 (1,120·1 to 2,896·1)	20·8% (13·3 to 32·9%)	22·3% (12·2 to 32·0%)	4·0% (1·3 to 7·0%)
**High systolic blood pressure: All causes**	1,378·7 (1,239·5 to 1,528·1)	10·3% (9·4 to 11·4%)	1,796·0 (1,616·4 to 1,994·8)	13·5% (12·2 to 14·8%)	30·3% (23·7 to 37·4%)	31·0% (27·4 to 34·7%)	33,621·2 (30,250·7 to 37,327·3)	4·2% (3·8 to 4·7%)	42,648·4 (38,473·4 to 47,093·3)	5·8% (5·2 to 6·5%)	26·8% (20·2 to 34·3%)	38·2% (32·8 to 44·1%)
Rheumatic heart disease	22·4 (15·6 to 32·5)	21·2% (14·5 to 33·0%)	26·0 (18·2 to 37·5)	22·7% (15·8 to 35·2%)	16·3% (5·0 to 27·6%)	6·8% (4·1 to 10·2%)	775·6 (506·6 to 1,143·8)	17·9% (11·6 to 26·8%)	875·5 (578·2 to 1,276·2)	19·9% (13·2 to 29·7%)	12·9% (3·2 to 22·5%)	10·8% (7·3 to 14·7%)
Ischaemic heart disease	569·8 (475·3 to 660·5)	49·7% (41·4 to 57·3%)	778·5 (648·8 to 904·9)	50·7% (42·6 to 58·5%)	36·6% (29·5 to 44·4%)	2·1% (0·5 to 3·7%)	13,127·2 (11,053·0 to 15,165·8)	48·5% (41·2 to 55·3%)	17,589·1 (14,906·2 to 20,120·1)	50·1% (42·7 to 56·9%)	34·0% (26·8 to 41·8%)	3·3% (1·8 to 4·8%)
Ischaemic stroke	178·9 (137·5 to 219·9)	46·8% (36·3 to 55·7%)	223·3 (174·3 to 274·9)	47·9% (37·9 to 57·3%)	24·8% (16·1 to 34·3%)	2·5% (0·3 to 5·5%)	3,499·4 (2,757·6 to 4,242·7)	48·3% (38·3 to 56·7%)	4,263·9 (3,401·3 to 5,124·6)	50·0% (40·1 to 58·4%)	21·8% (12·9 to 30·8%)	3·6% (2·0 to 5·7%)

Supplemental Results Table 1. All-age deaths, death PAFs, DALYs, DALY PAFs, and percent change for each risk factor and outcome for both sexes combined in 2005 and 2015 for Low-middle SDI.

Data in parenthesis are 95% uncertainty intervals. PAFs=population attributable fractions. DALYs=disability-adjusted life-years.

	2005 Deaths (in thousands)	2005 Deaths PAF	2015 Deaths (in thousands)	2015 Deaths PAF	Percent change deaths 2005-2015	Percent change deaths PAF 2005-2015	2005 DALYs (in thousands)	2005 DALYs PAF	2015 DALYs (in thousands)	2015 DALYs PAF	Percent change DALYs 2005-2015	Percent change DALYs PAF 2005-2015
Hemorrhagic stroke	276·1 (221·3 to 323·4)	53·2% (43·5 to 61·9%)	334·3 (268·0 to 395·3)	55·2% (45·7 to 64·1%)	21·1% (13·4 to 29·9%)	3·9% (2·2 to 6·1%)	7,203·5 (5,802·9 to 8,409·7)	50·7% (41·3 to 58·8%)	8,647·4 (6,980·6 to 10,226·3)	53·9% (44·1 to 62·2%)	20·0% (12·3 to 28·9%)	6·2% (4·5 to 8·2%)
Hypertensive heart disease	153·9 (136·0 to 186·1)	100·0% (100·0 to 100·0%)	188·6 (164·3 to 224·3)	100·0% (100·0 to 100·0%)	22·5% (10·8 to 35·0%)	-	3,680·4 (3,277·8 to 4,368·1)	100·0% (100·0 to 100·0%)	4,352·4 (3,831·9 to 5,059·1)	100·0% (100·0 to 100·0%)	18·3% (7·7 to 29·8%)	-
Cardiomyopathy and myocarditis	7·0 (5·3 to 8·8)	28·2% (22·5 to 34·1%)	9·2 (7·0 to 11·4)	30·9% (24·7 to 37·2%)	31·6% (18·0 to 47·3%)	9·7% (6·2 to 13·6%)	222·5 (163·5 to 289·2)	21·0% (15·6 to 26·6%)	290·7 (214·8 to 371·9)	24·7% (18·5 to 31·0%)	30·7% (17·3 to 47·2%)	17·4% (11·1 to 24·5%)
Atrial fibrillation and flutter	3·9 (2·8 to 5·4)	31·4% (25·1 to 37·9%)	5·6 (4·1 to 7·7)	32·0% (25·5 to 38·8%)	43·9% (35·8 to 52·7%)	2·2% (-0·5 to 5·0%)	122·2 (93·5 to 159·6)	36·9% (32·3 to 41·9%)	170·3 (130·3 to 223·9)	38·3% (33·4 to 43·4%)	39·4% (35·9 to 43·5%)	3·7% (2·2 to 5·3%)
Aortic aneurysm	3·7 (2·8 to 4·8)	32·8% (26·6 to 38·6%)	5·6 (4·4 to 6·9)	33·6% (27·3 to 39·6%)	52·8% (37·6 to 68·7%)	2·6% (0·5 to 4·9%)	89·5 (69·1 to 114·6)	32·7% (27·2 to 38·6%)	134·8 (109·3 to 165·9)	34·0% (28·8 to 40·0%)	50·7% (35·1 to 67·4%)	4·2% (2·0 to 6·4%)
Peripheral vascular disease	0·2 (0·2 to 0·3)	24·6% (18·6 to 31·1%)	0·4 (0·3 to 0·6)	25·0% (18·7 to 31·6%)	87·0% (64·0 to 115·1%)	1·4% (-1·7 to 4·4%)	17·9 (9·8 to 30·8)	26·5% (20·5 to 32·6%)	26·9 (15·4 to 45·3)	27·3% (21·2 to 33·5%)	50·2% (44·7 to 59·8%)	3·1% (1·3 to 5·1%)
Endocarditis	2·7 (1·9 to 3·5)	28·3% (21·9 to 34·4%)	3·7 (2·8 to 4·8)	30·6% (24·3 to 36·6%)	37·9% (23·4 to 55·3%)	7·9% (3·5 to 13·1%)	79·9 (56·1 to 107·0)	20·5% (14·8 to 26·8%)	109·4 (76·3 to 144·1)	23·5% (17·4 to 29·8%)	37·0% (22·3 to 55·5%)	14·2% (5·8 to 24·0%)
Other cardiovascular and circulatory diseases	20·1 (17·2 to 23·1)	37·2% (33·1 to 41·5%)	25·3 (20·8 to 30·0)	38·8% (34·3 to 43·1%)	26·3% (10·5 to 43·5%)	4·3% (1·1 to 7·4%)	700·9 (588·3 to 836·8)	30·7% (27·2 to 34·6%)	893·4 (730·6 to 1,084·4)	33·9% (30·2 to 38·0%)	27·5% (15·6 to 41·2%)	10·4% (6·0 to 15·4%)
Chronic kidney disease due to diabetes mellitus	21·3 (14·7 to 28·1)	38·3% (27·0 to 49·0%)	31·2 (21·6 to 41·1)	40·3% (28·5 to 51·3%)	46·7% (35·7 to 59·2%)	5·2% (3·5 to 7·3%)	565·2 (383·9 to 760·5)	31·2% (21·5 to 40·5%)	798·5 (553·7 to 1,068·8)	33·7% (23·4 to 43·4%)	41·3% (32·1 to 51·5%)	7·9% (6·0 to 10·1%)
Chronic kidney disease due to hypertension	101·3 (88·7 to 110·1)	100·0% (100·0 to 100·0%)	138·8 (121·1 to 153·4)	100·0% (100·0 to 100·0%)	36·9% (25·8 to 48·7%)	-	2,961·1 (2,567·1 to 3,210·5)	100·0% (100·0 to 100·0%)	3,707·6 (3,240·3 to 4,071·0)	100·0% (100·0 to 100·0%)	25·2% (15·6 to 35·4%)	-
Chronic kidney disease due to glomerulonephritis	16·3 (11·2 to 21·6)	30·9% (21·9 to 40·3%)	23·7 (16·3 to 31·2)	34·5% (24·7 to 44·8%)	45·0% (33·6 to 57·2%)	11·9% (9·0 to 15·0%)	458·9 (308·0 to 619·7)	21·2% (14·8 to 28·5%)	628·8 (426·2 to 837·4)	25·0% (17·2 to 33·3%)	37·0% (27·7 to 47·1%)	17·5% (13·6 to 21·6%)
Chronic kidney disease due to other causes	1·2 (0·8 to 1·7)	23·1% (15·9 to 30·2%)	1·7 (1·1 to 2·3)	28·4% (20·0 to 36·8%)	43·0% (27·8 to 59·8%)	23·1% (15·3 to 31·0%)	117·1 (74·6 to 163·9)	20·4% (13·8 to 27·1%)	159·7 (102·9 to 221·2)	24·2% (16·7 to 31·8%)	36·4% (30·8 to 42·1%)	18·6% (14·5 to 23·9%)
**High body-mass index: All causes**	398·0 (247·8 to 577·3)	3·0% (1·9 to 4·3%)	585·5 (376·2 to 830·1)	4·4% (2·8 to 6·2%)	47·1% (36·4 to 61·9%)	47·9% (39·6 to 60·0%)	13,269·8 (8,338·3 to 18,767·6)	1·7% (1·0 to 2·3%)	19,548·6 (12,766·7 to 27,186·1)	2·7% (1·8 to 3·7%)	47·3% (37·5 to 60·9%)	60·5% (51·1 to 73·7%)
Oesophageal cancer	4·7 (1·4 to 8·7)	10·4% (3·1 to 19·0%)	7·1 (2·2 to 13·2)	12·2% (4·0 to 22·5%)	52·5% (32·6 to 79·0%)	18·2% (5·2 to 35·3%)	129·1 (38·4 to 237·6)	10·5% (3·2 to 19·2%)	197·2 (62·4 to 364·3)	12·7% (4·1 to 23·1%)	52·8% (31·6 to 80·2%)	20·9% (6·8 to 38·8%)
Colon and rectum cancer	1·8 (1·0 to 3·0)	3·5% (1·9 to 5·7%)	3·1 (1·7 to 4·8)	4·2% (2·3 to 6·6%)	67·4% (52·7 to 87·6%)	19·3% (12·9 to 28·9%)	50·9 (27·3 to 82·5)	3·6% (1·9 to 5·8%)	84·2 (46·6 to 131·7)	4·4% (2·4 to 6·9%)	65·4% (49·5 to 86·6%)	22·1% (15·3 to 32·4%)
Liver cancer due to hepatitis B	1·8 (0·7 to 3·5)	7·7% (3·0 to 14·3%)	2·4 (0·9 to 4·5)	9·0% (3·6 to 16·4%)	33·7% (13·4 to 62·5%)	17·3% (7·2 to 30·1%)	59·5 (22·4 to 118·1)	7·6% (3·0 to 14·2%)	78·7 (31·0 to 149·9)	9·0% (3·6 to 16·5%)	32·2% (9·8 to 65·9%)	18·3% (7·2 to 31·7%)
Liver cancer due to hepatitis C	1·1 (0·4 to 2·1)	8·1% (3·2 to 14·3%)	1·6 (0·7 to 2·9)	9·7% (4·0 to 16·8%)	44·7% (28·5 to 68·0%)	19·6% (11·4 to 31·4%)	27·8 (11·1 to 51·6)	8·5% (3·4 to 15·0%)	40·0 (16·8 to 70·1)	10·2% (4·2 to 17·6%)	43·6% (26·4 to 68·5%)	20·5% (11·7 to 32·9%)
Liver cancer due to alcohol use	1·4 (0·5 to 2·8)	9·4% (3·7 to 17·3%)	2·0 (0·8 to 3·7)	10·5% (4·0 to 19·3%)	38·6% (12·5 to 75·2%)	11·4% (0·6 to 23·1%)	38·2 (13·9 to 75·3)	9·8% (3·8 to 18·3%)	52·4 (20·3 to 100·1)	10·9% (4·2 to 20·1%)	37·0% (9·3 to 77·2%)	11·3% (0·0 to 22·7%)
Liver cancer due to other causes	0·8 (0·3 to 1·6)	7·2% (2·8 to 13·1%)	1·1 (0·5 to 2·0)	8·7% (3·5 to 15·2%)	36·2% (20·4 to 58·9%)	20·9% (12·4 to 33·6%)	23·8 (9·1 to 44·8)	7·2% (2·8 to 13·1%)	32·0 (13·6 to 58·0)	8·9% (3·6 to 15·4%)	34·2% (17·1 to 59·1%)	22·8% (13·1 to 36·7%)
Gallbladder and biliary tract cancer	1·4 (0·7 to 2·2)	10·3% (5·6 to 16·6%)	1·8 (1·0 to 2·8)	11·8% (6·5 to 18·5%)	31·6% (15·9 to 54·9%)	13·6% (5·5 to 24·7%)	34·7 (18·6 to 57·0)	10·5% (5·7 to 16·9%)	45·4 (25·1 to 71·7)	12·1% (6·8 to 18·9%)	30·7% (14·3 to 54·9%)	15·0% (6·6 to 26·5%)
Pancreatic cancer	0·8 (0·3 to 1·5)	2·9% (1·0 to 5·3%)	1·3 (0·5 to 2·3)	3·4% (1·2 to 6·1%)	61·9% (45·2 to 86·8%)	17·4% (8·6 to 28·2%)	19·4 (6·7 to 35·7)	3·0% (1·1 to 5·4%)	31·4 (11·3 to 57·1)	3·5% (1·3 to 6·4%)	61·7% (43·9 to 89·1%)	18·4% (8·9 to 29·5%)
Breast cancer	1·6 (0·7 to 3·1)	2·6% (1·2 to 4·7%)	2·5 (1·2 to 4·6)	3·0% (1·5 to 5·5%)	57·4% (21·8 to 102·0%)	17·7% (-3·0 to 45·4%)	33·5 (12·5 to 75·1)	1·6% (0·6 to 3·3%)	51·0 (18·5 to 108·1)	1·9% (0·7 to 3·8%)	52·3% (1·2 to 126·6%)	15·1% (-21·2 to 64·9%)
Uterine cancer	3·1 (1·8 to 4·7)	22·6% (13·5 to 33·0%)	4·1 (2·5 to 6·0)	26·8% (16·9 to 37·5%)	31·9% (13·7 to 57·9%)	18·3% (10·5 to 31·2%)	86·3 (50·7 to 129·6)	23·3% (14·0 to 33·8%)	113·7 (70·1 to 163·6)	27·9% (17·8 to 38·7%)	31·8% (12·4 to 59·3%)	19·7% (11·6 to 33·6%)
Ovarian cancer	0·2 (0·0 to 0·6)	1·6% (0·0 to 3·8%)	0·4 (0·0 to 0·9)	1·9% (-0·1 to 4·4%)	62·0% (42·5 to 90·4%)	19·8% (12·6 to 31·2%)	7·4 (-0·2 to 18·6)	1·6% (0·0 to 3·8%)	12·1 (-0·4 to 29·1)	1·9% (-0·1 to 4·5%)	63·6% (42·6 to 94·7%)	21·7% (13·8 to 34·4%)
Kidney cancer	0·6 (0·4 to 1·0)	8·2% (4·8 to 12·4%)	1·1 (0·6 to 1·6)	9·6% (5·8 to 14·0%)	66·5% (46·8 to 91·3%)	16·2% (4·0 to 31·0%)	18·6 (11·0 to 28·1)	6·3% (3·6 to 10·0%)	31·1 (18·6 to 45·1)	7·5% (4·4 to 11·5%)	66·7% (46·1 to 91·4%)	18·6% (-1·6 to 48·0%)
Thyroid cancer	0·2 (0·1 to 0·3)	5·2% (2·6 to 8·6%)	0·3 (0·1 to 0·4)	6·3% (3·3 to 10·1%)	46·6% (30·8 to 65·2%)	19·9% (11·5 to 30·4%)	4·8 (2·5 to 7·9)	5·5% (2·8 to 8·9%)	7·2 (3·8 to 11·6)	6·6% (3·5 to 10·6%)	51·0% (34·4 to 69·6%)	20·8% (12·4 to 31·3%)
Ischaemic heart disease	120·8 (71·9 to 180·1)	10·5% (6·3 to 15·6%)	184·3 (113·5 to 267·6)	12·0% (7·4 to 17·3%)	52·5% (40·6 to 69·0%)	14·0% (7·4 to 22·9%)	3,362·1 (1,986·9 to 4,971·2)	12·4% (7·4 to 18·5%)	5,106·1 (3,175·9 to 7,437·3)	14·5% (9·0 to 20·9%)	51·9% (39·8 to 68·0%)	17·0% (10·6 to 26·1%)
Ischaemic stroke	31·6 (18·0 to 47·5)	8·3% (4·8 to 12·3%)	42·7 (25·6 to 63·7)	9·2% (5·6 to 13·4%)	35·4% (22·4 to 50·4%)	11·2% (3·9 to 20·8%)	821·1 (476·1 to 1,213·4)	11·3% (6·8 to 16·7%)	1,116·1 (681·2 to 1,620·9)	13·1% (8·1 to 18·7%)	35·9% (23·2 to 51·2%)	15·5% (8·6 to 24·7%)
Hemorrhagic stroke	86·0 (52·6 to 125·5)	16·6% (10·2 to 24·1%)	115·1 (73·8 to 162·6)	19·0% (12·3 to 26·7%)	33·8% (22·1 to 49·6%)	14·8% (7·5 to 24·8%)	2,825·5 (1,766·6 to 4,066·0)	19·9% (12·4 to 28·5%)	3,795·1 (2,485·8 to 5,281·2)	23·6% (15·5 to 32·4%)	34·3% (22·6 to 50·1%)	18·8% (11·8 to 28·3%)
Hypertensive heart disease	36·8 (22·0 to 55·4)	24·0% (14·4 to 35·9%)	50·3 (30·2 to 75·2)	26·7% (16·7 to 39·0%)	36·7% (22·1 to 55·5%)	11·6% (4·2 to 21·6%)	973·6 (594·5 to 1,420·5)	26·5% (16·4 to 37·9%)	1,312·8 (834·0 to 1,874·0)	30·2% (19·7 to 42·1%)	34·8% (20·4 to 53·7%)	14·0% (7·4 to 24·0%)
Diabetes mellitus	71·2 (46·8 to 98·2)	27·7% (18·2 to 38·5%)	114·0 (76·9 to 158·3)	29·2% (19·6 to 39·5%)	60·2% (46·1 to 77·0%)	5·3% (0·2 to 12·2%)	3,363·6 (2,201·7 to 4,671·8)	33·4% (22·6 to 44·7%)	5,299·7 (3,603·1 to 7,161·9)	37·7% (26·6 to 49·0%)	57·6% (48·2 to 70·1%)	13·0% (8·5 to 20·0%)
Chronic kidney disease due to diabetes mellitus	9·1 (4·0 to 16·0)	16·3% (7·2 to 28·0%)	14·8 (6·7 to 25·2)	19·1% (8·8 to 31·7%)	62·9% (47·5 to 82·8%)	16·8% (9·2 to 28·1%)	274·4 (111·2 to 488·2)	15·2% (6·2 to 26·8%)	437·5 (188·9 to 749·9)	18·5% (8·0 to 31·5%)	59·5% (46·9 to 77·4%)	21·8% (15·2 to 33·5%)
Chronic kidney disease due to hypertension	15·5 (7·0 to 26·1)	15·3% (6·8 to 25·8%)	24·0 (10·9 to 39·0)	17·3% (7·9 to 28·8%)	55·1% (39·6 to 73·8%)	13·3% (5·3 to 23·4%)	410·2 (173·5 to 721·1)	13·9% (5·9 to 24·5%)	621·0 (271·2 to 1,068·4)	16·8% (7·4 to 28·7%)	51·4% (38·1 to 69·0%)	20·9% (13·6 to 31·0%)
Chronic kidney disease due to glomerulonephritis	6·1 (2·3 to 11·1)	11·5% (4·5 to 20·4%)	9·4 (3·7 to 16·9)	13·8% (5·5 to 24·1%)	55·6% (38·3 to 76·2%)	20·0% (10·7 to 32·7%)	203·0 (68·3 to 389·5)	9·4% (3·3 to 17·8%)	309·3 (109·7 to 569·3)	12·3% (4·4 to 22·4%)	52·4% (40·2 to 69·7%)	30·5% (21·8 to 43·3%)
Chronic kidney disease due to other causes	0·4 (0·1 to 0·8)	7·9% (2·9 to 14·9%)	0·7 (0·2 to 1·2)	10·9% (3·9 to 19·6%)	59·0% (29·7 to 89·0%)	36·8% (16·4 to 56·5%)	57·2 (20·5 to 106·2)	10·0% (3·9 to 18·0%)	86·1 (31·6 to 156·4)	13·1% (5·2 to 22·9%)	50·4% (41·9 to 62·3%)	30·8% (23·3 to 41·8%)
Osteoarthritis	-	-	-	-	-	-	155·4 (83·4 to 257·4)	11·1% (6·6 to 16·5%)	248·3 (136·6 to 397·7)	13·0% (8·0 to 18·6%)	59·7% (52·9 to 70·0%)	17·3% (12·5 to 24·8%)
Low back pain	-	-	-	-	-	-	257·3 (131·4 to 439·8)	2·5% (1·4 to 3·9%)	389·7 (204·4 to 653·0)	3·2% (1·9 to 4·7%)	51·4% (45·3 to 61·0%)	24·4% (19·5 to 32·3%)
**Low bone mineral density: All causes**	42·2 (39·3 to 45·4)	0·3% (0·3 to 0·3%)	54·5 (49·7 to 59·6)	0·4% (0·4 to 0·4%)	29·1% (17·5 to 41·5%)	29·8% (19·9 to 40·4%)	1,128·1 (990·2 to 1,278·6)	0·1% (0·1 to 0·2%)	1,430·7 (1,247·9 to 1,634·4)	0·2% (0·2 to 0·2%)	26·8% (19·2 to 34·8%)	38·1% (31·1 to 45·7%)
Pedestrian road injuries	6·6 (5·6 to 7·8)	6·0% (5·6 to 6·6%)	8·2 (7·2 to 9·8)	6·8% (6·2 to 7·4%)	24·7% (10·0 to 48·4%)	12·5% (6·1 to 19·0%)	152·2 (127·5 to 178·3)	2·8% (2·5 to 3·1%)	184·4 (158·3 to 216·2)	3·2% (2·9 to 3·5%)	21·2% (6·8 to 44·1%)	15·4% (7·8 to 23·6%)
Cyclist road injuries	0·3 (0·3 to 0·4)	5·5% (4·9 to 6·1%)	0·4 (0·4 to 0·5)	6·1% (5·5 to 6·8%)	35·0% (17·1 to 56·7%)	12·2% (3·5 to 22·4%)	12·2 (10·0 to 14·6)	3·6% (3·1 to 4·2%)	16·9 (13·8 to 20·6)	4·3% (3·7 to 4·9%)	38·2% (27·6 to 50·8%)	18·0% (7·4 to 27·9%)
Motorcyclist road injuries	2·5 (1·9 to 3·5)	5·0% (4·4 to 6·0%)	3·5 (2·8 to 4·4)	5·6% (4·9 to 6·5%)	38·0% (9·0 to 74·4%)	12·0% (3·6 to 21·2%)	79·6 (62·4 to 103·6)	3·0% (2·5 to 3·5%)	109·0 (89·0 to 132·3)	3·4% (2·9 to 3·9%)	37·0% (13·3 to 64·8%)	14·7% (5·5 to 23·8%)
Motor vehicle road injuries	5·4 (4·2 to 6·7)	5·8% (5·1 to 6·6%)	6·7 (5·3 to 7·6)	6·4% (5·6 to 7·1%)	23·5% (-2·5 to 41·3%)	10·2% (3·2 to 16·7%)	159·3 (127·2 to 192·9)	3·1% (2·7 to 3·6%)	198·1 (161·8 to 230·5)	3·6% (3·1 to 4·1%)	24·4% (1·9 to 39·7%)	14·3% (6·4 to 22·0%)
Other road injuries	0·4 (0·3 to 0·5)	5·5% (4·1 to 7·4%)	0·5 (0·4 to 0·6)	6·0% (4·7 to 7·9%)	34·1% (11·7 to 71·2%)	9·1% (-9·8 to 33·8%)	11·7 (8·8 to 14·4)	2·9% (2·0 to 4·1%)	16·0 (12·0 to 19·8)	3·3% (2·4 to 4·7%)	36·9% (21·4 to 60·2%)	14·2% (-6·3 to 41·7%)

Supplemental Results Table 1. All-age deaths, death PAFs, DALYs, DALY PAFs, and percent change for each risk factor and outcome for both sexes combined in 2005 and 2015 for Low-middle SDI.

Data in parenthesis are 95% uncertainty intervals. PAFs=population attributable fractions. DALYs=disability-adjusted life-years.

	2005 Deaths (in thousands)	2005 Deaths PAF	2015 Deaths (in thousands)	2015 Deaths PAF	Percent change deaths 2005-2015	Percent change deaths PAF 2005-2015	2005 DALYs (in thousands)	2005 DALYs PAF	2015 DALYs (in thousands)	2015 DALYs PAF	Percent change DALYs 2005-2015	Percent change DALYs PAF 2005-2015
Other transport injuries	1·7 (1·4 to 2·1)	7·3% (6·6 to 8·2%)	2·1 (1·6 to 2·8)	8·0% (7·2 to 9·1%)	22·8% (3·8 to 45·0%)	9·8% (1·8 to 18·7%)	46·6 (38·2 to 55·6)	3·7% (3·3 to 4·2%)	56·7 (44·3 to 74·7)	4·2% (3·7 to 4·7%)	21·6% (3·9 to 42·5%)	12·9% (3·2 to 22·9%)
Falls	21·6 (19·8 to 23·9)	26·9% (24·8 to 29·2%)	28·6 (25·0 to 32·9)	30·0% (27·4 to 32·5%)	32·5% (16·0 to 51·2%)	11·2% (0·7 to 22·9%)	535·1 (457·7 to 627·3)	10·3% (9·3 to 11·3%)	694·2 (580·1 to 817·3)	11·6% (10·5 to 12·6%)	29·7% (21·2 to 39·1%)	12·4% (3·1 to 24·5%)
Other exposure to mechanical forces	2·5 (1·8 to 2·9)	6·7% (4·9 to 7·9%)	3·1 (2·1 to 3·5)	7·8% (5·9 to 8·9%)	22·9% (4·7 to 38·8%)	17·2% (2·9 to 30·2%)	82·8 (67·5 to 100·4)	3·0% (2·5 to 3·5%)	97·6 (78·2 to 118·5)	3·5% (3·0 to 4·0%)	17·9% (9·0 to 26·1%)	16·2% (7·2 to 25·0%)
Non-venomous animal contact	0·2 (0·1 to 0·2)	3·3% (2·8 to 3·9%)	0·2 (0·1 to 0·3)	3·8% (3·2 to 4·4%)	2·6% (-15·4 to 48·2%)	16·6% (2·5 to 30·9%)	6·3 (4·8 to 8·1)	1·4% (1·2 to 1·7%)	6·4 (4·8 to 8·6)	1·7% (1·4 to 2·0%)	1·9% (-9·7 to 28·5%)	16·6% (3·6 to 30·9%)
Assault by other means	1·0 (0·8 to 1·1)	2·4% (2·2 to 2·9%)	1·2 (0·9 to 1·4)	2·7% (2·4 to 3·2%)	20·0% (1·4 to 41·4%)	12·0% (0·8 to 23·3%)	29·9 (24·5 to 34·4)	1·3% (1·2 to 1·5%)	34·9 (26·8 to 42·0)	1·5% (1·3 to 1·7%)	16·5% (1·2 to 34·1%)	11·7% (0·7 to 22·5%)
Exposure to forces of nature	-	-	-	-	-	-	12·4 (5·7 to 20·3)	0·3% (0·1 to 0·5%)	16·5 (8·9 to 26·5)	1·8% (1·0 to 2·9%)	32·6% (-12·3 to 145·9%)	628·0% (374·1 to 1,255·2%)
**Low glomerular filtration rate: All causes**	382·8 (351·4 to 412·4)	2·9% (2·6 to 3·1%)	510·8 (464·2 to 553·2)	3·8% (3·5 to 4·1%)	33·4% (26·0 to 41·3%)	34·2% (28·6 to 39·9%)	10,923·3 (9,930·0 to 11,793·5)	1·4% (1·2 to 1·5%)	13,617·4 (12,352·1 to 14,762·7)	1·9% (1·7 to 2·0%)	24·7% (18·0 to 32·1%)	35·8% (29·5 to 42·7%)
Ischaemic heart disease	91·4 (76·0 to 107·0)	8·0% (6·7 to 9·4%)	125·8 (105·2 to 148·9)	8·2% (6·9 to 9·7%)	37·7% (30·2 to 45·2%)	2·9% (0·7 to 5·1%)	1,860·7 (1,537·4 to 2,199·4)	6·9% (5·7 to 8·1%)	2,494·0 (2,035·9 to 2,985·4)	7·1% (5·8 to 8·4%)	34·0% (26·6 to 41·5%)	3·3% (1·2 to 5·5%)
Ischaemic stroke	35·4 (27·4 to 44·3)	9·3% (7·4 to 11·3%)	44·4 (34·6 to 55·6)	9·5% (7·5 to 11·8%)	25·4% (16·6 to 35·2%)	3·0% (0·0 to 6·1%)	606·9 (467·8 to 752·2)	8·4% (6·7 to 10·1%)	733·6 (575·6 to 911·0)	8·6% (6·9 to 10·5%)	20·9% (12·0 to 29·7%)	2·8% (0·0 to 5·6%)
Hemorrhagic stroke	40·9 (32·8 to 49·2)	7·9% (6·5 to 9·4%)	49·6 (39·4 to 60·3)	8·2% (6·6 to 9·9%)	21·1% (12·9 to 30·1%)	3·9% (1·0 to 7·0%)	921·9 (736·6 to 1,116·1)	6·5% (5·2 to 7·8%)	1,092·8 (857·5 to 1,336·9)	6·8% (5·4 to 8·3%)	18·5% (10·8 to 27·2%)	4·9% (1·9 to 8·3%)
Peripheral vascular disease	0·1 (0·1 to 0·2)	15·0% (11·8 to 18·6%)	0·3 (0·2 to 0·3)	15·2% (11·6 to 19·2%)	86·8% (62·4 to 117·8%)	1·4% (-4·2 to 5·7%)	11·0 (5·9 to 18·5)	16·4% (13·0 to 20·2%)	16·3 (9·2 to 27·0)	16·6% (13·0 to 20·6%)	47·7% (41·4 to 57·0%)	1·4% (-0·7 to 3·3%)
Chronic kidney disease due to diabetes mellitus	55·6 (47·8 to 61·8)	100·0% (100·0 to 100·0%)	77·5 (67·1 to 86·9)	100·0% (100·0 to 100·0%)	39·4% (28·2 to 51·4%)	-	1,808·7 (1,569·0 to 2,004·3)	100·0% (100·0 to 100·0%)	2,367·7 (2,077·3 to 2,635·1)	100·0% (100·0 to 100·0%)	30·9% (22·1 to 40·4%)	-
Chronic kidney disease due to hypertension	101·3 (88·7 to 110·1)	100·0% (100·0 to 100·0%)	138·8 (121·1 to 153·4)	100·0% (100·0 to 100·0%)	36·9% (25·8 to 48·7%)	-	2,961·1 (2,567·1 to 3,210·5)	100·0% (100·0 to 100·0%)	3,707·6 (3,240·3 to 4,071·0)	100·0% (100·0 to 100·0%)	25·2% (15·6 to 35·4%)	-
Chronic kidney disease due to glomerulonephritis	52·9 (44·9 to 58·4)	100·0% (100·0 to 100·0%)	68·5 (59·0 to 77·5)	100·0% (100·0 to 100·0%)	29·5% (18·4 to 40·9%)	-	2,160·1 (1,844·4 to 2,369·4)	100·0% (100·0 to 100·0%)	2,519·1 (2,183·1 to 2,809·4)	100·0% (100·0 to 100·0%)	16·6% (7·6 to 26·1%)	-
Chronic kidney disease due to other causes	5·2 (4·2 to 6·3)	100·0% (100·0 to 100·0%)	6·0 (4·9 to 7·3)	100·0% (100·0 to 100·0%)	16·1% (6·6 to 28·3%)	-	572·1 (475·4 to 682·8)	100·0% (100·0 to 100·0%)	658·0 (539·7 to 790·4)	100·0% (100·0 to 100·0%)	15·0% (9·6 to 20·7%)	-
Gout	-	-	-	-	-	-	20·8 (14·0 to 28·9)	14·4% (13·0 to 15·9%)	28·3 (18·9 to 39·3)	14·9% (13·5 to 16·4%)	35·8% (31·8 to 39·7%)	3·7% (1·8 to 5·6%)

Supplemental Results Table 1. All-age deaths, death PAFs, DALYs, DALY PAFs, and percent change for each risk factor and outcome for both sexes combined in 2005 and 2015 for Low SDI.

Data in parenthesis are 95% uncertainty intervals. PAFs=population attributable fractions. DALYs=disability-adjusted life-years.

	2005 Deaths (in thousands)	2005 Deaths PAF	2015 Deaths (in thousands)	2015 Deaths PAF	Percent change deaths 2005-2015	Percent change deaths PAF 2005-2015	2005 DALYs (in thousands)	2005 DALYs PAF	2015 DALYs (in thousands)	2015 DALYs PAF	Percent change DALYs 2005-2015	Percent change DALYs PAF 2005-2015
**All risk factors: All causes**	2,263·2 (2,156·2 to 2,378·6)	47·9% (46·2 to 49·7%)	2,181·9 (2,007·2 to 2,378·0)	46·8% (45·1 to 48·5%)	-3·6% (-10·7 to 5·0%)	-2·4% (-5·3 to 0·4%)	131,508·0 (123,958·1 to 139,288·6)	41·0% (38·9 to 43·1%)	116,984·8 (108,544·1 to 126,017·2)	38·2% (36·1 to 40·1%)	-11·0% (-16·3 to -5·0%)	-6·8% (-10·7 to -3·1%)
**Environmental/occupational risks: All causes**	934·8 (849·4 to 1,015·4)	19·8% (18·1 to 21·4%)	925·8 (823·7 to 1,031·9)	19·8% (18·2 to 21·4%)	-1·0% (-9·8 to 9·1%)	0·3% (-5·1 to 5·9%)	55,833·7 (50,291·8 to 61,495·6)	17·4% (15·6 to 19·1%)	50,147·4 (45,600·4 to 54,898·7)	16·4% (14·8 to 17·9%)	-10·2% (-17·6 to -1·6%)	-5·9% (-12·7 to 1·3%)
**Unsafe water, sanitation, and handwashing: All causes**	454·9 (408·2 to 504·7)	9·6% (8·7 to 10·7%)	392·7 (349·7 to 440·0)	8·4% (7·6 to 9·2%)	-13·7% (-22·0 to -4·4%)	-12·6% (-19·4 to -5·3%)	30,960·7 (27,409·2 to 34,764·9)	9·6% (8·5 to 10·8%)	25,226·8 (22,514·2 to 28,213·7)	8·2% (7·3 to 9·2%)	-18·5% (-26·6 to -8·8%)	-14·6% (-22·7 to -5·6%)
**Unsafe water source: All causes**	342·0 (278·3 to 396·6)	7·2% (5·9 to 8·3%)	289·1 (233·5 to 337·1)	6·2% (5·1 to 7·0%)	-15·5% (-24·2 to -5·7%)	-14·4% (-21·9 to -6·1%)	23,396·5 (18,943·0 to 27,280·3)	7·3% (5·9 to 8·5%)	18,837·4 (15,236·5 to 21,856·6)	6·1% (5·0 to 7·1%)	-19·5% (-28·3 to -8·8%)	-15·6% (-24·3 to -5·9%)
Diarrhoeal diseases	332·3 (270·0 to 386·0)	87·3% (71·3 to 95·3%)	277·5 (225·1 to 324·0)	86·9% (70·9 to 95·1%)	-16·5% (-25·2 to -6·4%)	-0·4% (-0·8 to -0·2%)	22,672·0 (18,317·4 to 26,542·9)	87·3% (71·3 to 95·3%)	17,971·5 (14,626·9 to 20,875·0)	86·9% (70·9 to 95·1%)	-20·7% (-29·5 to -9·9%)	-0·4% (-0·7 to -0·2%)
Typhoid fever	9·2 (4·4 to 17·0)	87·1% (71·1 to 95·2%)	11·0 (5·3 to 20·5)	86·7% (70·8 to 95·0%)	19·6% (8·3 to 33·4%)	-0·5% (-0·8 to -0·2%)	683·8 (332·4 to 1,276·9)	87·1% (71·1 to 95·2%)	818·1 (401·4 to 1,523·0)	86·7% (70·8 to 95·0%)	19·6% (8·3 to 33·0%)	-0·5% (-0·8 to -0·2%)
Paratyphoid fever	0·5 (0·2 to 1·2)	87·4% (71·8 to 95·3%)	0·6 (0·2 to 1·4)	87·1% (71·6 to 95·2%)	17·4% (5·8 to 30·3%)	-0·3% (-0·5 to -0·2%)	40·7 (15·3 to 89·8)	87·4% (71·8 to 95·3%)	47·8 (17·9 to 105·5)	87·1% (71·6 to 95·2%)	17·5% (5·9 to 30·4%)	-0·3% (-0·5 to -0·2%)
**Unsafe sanitation: All causes**	260·8 (231·6 to 291·7)	5·5% (5·0 to 6·1%)	217·5 (192·5 to 248·2)	4·7% (4·2 to 5·2%)	-16·6% (-25·4 to -6·8%)	-15·6% (-23·1 to -7·5%)	17,833·6 (15,689·5 to 20,163·1)	5·6% (4·8 to 6·3%)	14,153·5 (12,547·3 to 16,086·8)	4·6% (4·1 to 5·2%)	-20·6% (-29·4 to -10·1%)	-16·8% (-25·5 to -7·1%)
Diarrhoeal diseases	253·5 (225·6 to 282·4)	66·6% (62·8 to 70·3%)	208·8 (184·7 to 237·5)	65·3% (61·8 to 68·8%)	-17·6% (-26·4 to -7·6%)	-1·8% (-2·5 to -1·1%)	17,282·6 (15,188·0 to 19,645·0)	66·5% (62·8 to 70·3%)	13,503·9 (11,977·5 to 15,397·1)	65·3% (61·8 to 68·7%)	-21·9% (-30·5 to -11·0%)	-1·9% (-2·6 to -1·2%)
Typhoid fever	7·0 (3·3 to 13·1)	66·3% (62·2 to 69·8%)	8·2 (4·0 to 15·3)	65·0% (61·4 to 68·3%)	18·0% (6·7 to 32·2%)	-1·8% (-2·6 to -1·0%)	520·2 (254·2 to 976·6)	66·3% (62·3 to 69·8%)	614·1 (305·7 to 1,143·8)	65·1% (61·4 to 68·3%)	18·0% (6·4 to 31·5%)	-1·8% (-2·6 to -1·1%)
Paratyphoid fever	0·4 (0·2 to 0·9)	66·1% (62·1 to 69·5%)	0·5 (0·2 to 1·0)	64·6% (60·9 to 68·0%)	15·2% (3·4 to 28·1%)	-2·2% (-3·2 to -1·3%)	30·8 (11·8 to 66·7)	66·1% (62·1 to 69·5%)	35·5 (13·4 to 76·2)	64·6% (60·9 to 68·0%)	15·3% (3·3 to 28·2%)	-2·2% (-3·2 to -1·3%)
**No handwashing with soap: All causes**	228·9 (187·7 to 270·2)	4·8% (4·0 to 5·7%)	201·2 (164·7 to 239·1)	4·3% (3·6 to 5·1%)	-12·1% (-20·2 to -3·3%)	-11·0% (-17·4 to -3·9%)	15,498·5 (12,553·9 to 18,467·6)	4·8% (3·9 to 5·8%)	12,746·0 (10,497·8 to 15,086·2)	4·2% (3·4 to 4·9%)	-17·8% (-25·3 to -8·8%)	-13·8% (-21·5 to -5·4%)
Diarrhoeal diseases	151·4 (119·5 to 183·5)	39·8% (32·4 to 46·6%)	126·9 (99·9 to 156·6)	39·7% (32·3 to 46·6%)	-16·2% (-25·0 to -6·2%)	-0·2% (-0·3 to -0·1%)	10,329·0 (8,036·7 to 12,631·7)	39·8% (32·3 to 46·6%)	8,212·6 (6,468·3 to 9,999·6)	39·7% (32·3 to 46·6%)	-20·5% (-29·2 to -9·7%)	-0·2% (-0·3 to -0·1%)
Typhoid fever	4·2 (2·0 to 8·0)	39·5% (31·3 to 46·4%)	5·0 (2·4 to 9·6)	39·5% (31·3 to 46·3%)	20·0% (8·6 to 33·9%)	-0·2% (-0·4 to -0·1%)	310·4 (148·1 to 591·8)	39·5% (31·3 to 46·4%)	372·5 (179·4 to 718·7)	39·5% (31·3 to 46·3%)	20·0% (8·3 to 33·2%)	-0·2% (-0·4 to -0·1%)
Paratyphoid fever	0·2 (0·1 to 0·5)	39·4% (31·3 to 46·7%)	0·3 (0·1 to 0·6)	39·4% (31·2 to 46·6%)	17·6% (5·9 to 30·5%)	-0·2% (-0·3 to -0·1%)	18·4 (6·8 to 41·4)	39·4% (31·3 to 46·7%)	21·7 (8·1 to 48·6)	39·4% (31·2 to 46·6%)	17·8% (6·0 to 30·6%)	-0·2% (-0·3 to -0·1%)
**Air pollution: All causes**	493·8 (422·5 to 560·9)	10·5% (9·0 to 11·8%)	533·4 (452·9 to 614·1)	11·4% (9·9 to 12·9%)	8·0% (-2·3 to 20·0%)	9·4% (3·6 to 15·8%)	24,903·1 (20,850·3 to 28,681·9)	7·8% (6·5 to 9·0%)	23,360·9 (19,657·2 to 26,874·1)	7·6% (6·3 to 8·8%)	-6·2% (-15·0 to 4·2%)	-1·7% (-9·3 to 7·2%)
**Ambient particulate matter pollution: All causes**	199·7 (164·3 to 237·6)	4·2% (3·5 to 5·0%)	216·3 (177·1 to 258·3)	4·6% (3·9 to 5·5%)	8·3% (-2·2 to 20·1%)	9·6% (3·3 to 16·6%)	10,024·5 (7,856·3 to 12,453·6)	3·1% (2·5 to 3·9%)	9,382·9 (7,467·1 to 11,557·1)	3·1% (2·4 to 3·8%)	-6·4% (-15·1 to 3·9%)	-1·9% (-10·0 to 7·0%)
Lower respiratory infections	118·5 (86·8 to 155·6)	25·8% (18·9 to 33·3%)	109·5 (79·7 to 144·1)	25·1% (18·3 to 32·8%)	-7·6% (-16·9 to 2·6%)	-2·5% (-5·9 to -0·4%)	7,903·9 (5,813·0 to 10,338·5)	25·9% (19·0 to 33·4%)	6,611·3 (4,802·1 to 8,815·2)	25·3% (18·5 to 33·0%)	-16·4% (-25·2 to -6·1%)	-2·4% (-5·8 to -0·3%)
Tracheal, bronchus, and lung cancer	2·0 (1·3 to 2·8)	18·7% (11·9 to 26·0%)	3·0 (1·9 to 4·3)	18·4% (11·7 to 25·8%)	49·2% (29·6 to 71·7%)	-1·5% (-5·6 to 2·0%)	52·1 (31·9 to 73·5)	18·5% (11·8 to 25·7%)	77·7 (48·5 to 111·0)	18·2% (11·6 to 25·6%)	49·0% (28·4 to 72·7%)	-1·4% (-5·6 to 2·4%)
Ischaemic heart disease	38·1 (31·2 to 45·6)	19·5% (16·4 to 22·6%)	50·2 (40·7 to 60·9)	19·4% (16·2 to 22·5%)	32·0% (15·1 to 50·3%)	-0·5% (-2·7 to 1·4%)	983·3 (806·2 to 1,179·5)	20·9% (17·5 to 24·2%)	1,290·3 (1,036·6 to 1,572·2)	21·0% (17·7 to 24·3%)	31·2% (12·8 to 51·5%)	0·2% (-2·0 to 2·4%)
Ischaemic stroke	11·2 (8·0 to 14·6)	14·2% (11·2 to 17·1%)	14·4 (10·4 to 18·8)	14·0% (11·0 to 16·8%)	28·8% (12·0 to 48·1%)	-1·4% (-4·2 to 1·0%)	244·5 (180·2 to 313·0)	15·1% (12·4 to 18·0%)	309·4 (228·5 to 393·2)	15·0% (12·3 to 17·8%)	26·6% (8·9 to 46·2%)	-1·0% (-3·8 to 1·5%)
Hemorrhagic stroke	19·3 (15·4 to 23·9)	15·8% (13·0 to 18·5%)	24·8 (19·3 to 31·1)	15·7% (13·0 to 18·5%)	28·5% (12·2 to 48·2%)	-0·3% (-2·9 to 2·3%)	571·6 (453·4 to 699·7)	15·7% (12·9 to 18·4%)	731·7 (573·1 to 919·1)	15·9% (13·2 to 18·6%)	28·0% (10·7 to 47·2%)	1·2% (-1·9 to 4·6%)
Chronic obstructive pulmonary disease	10·6 (5·8 to 15·8)	23·3% (13·5 to 33·6%)	14·3 (8·2 to 21·5)	23·5% (13·7 to 34·1%)	34·8% (17·0 to 56·1%)	1·0% (-2·5 to 4·5%)	269·1 (150·6 to 398·7)	19·4% (11·2 to 28·2%)	362·6 (209·3 to 541·1)	20·1% (11·8 to 29·1%)	34·7% (18·6 to 53·6%)	3·5% (-2·1 to 10·0%)
**Household air pollution from solid fuels: All causes**	384·8 (307·0 to 459·4)	8·1% (6·5 to 9·7%)	409·8 (325·4 to 490·3)	8·8% (7·0 to 10·4%)	6·5% (-3·7 to 18·5%)	7·8% (1·5 to 14·4%)	19,869·7 (15,454·6 to 24,054·7)	6·2% (4·8 to 7·5%)	18,444·8 (14,429·7 to 22,167·3)	6·0% (4·6 to 7·3%)	-7·2% (-15·9 to 3·5%)	-2·7% (-10·8 to 6·5%)
Lower respiratory infections	244·2 (185·6 to 299·7)	53·1% (40·1 to 63·5%)	229·2 (171·4 to 280·5)	52·6% (39·7 to 63·3%)	-6·2% (-14·5 to 4·0%)	-0·9% (-2·2 to 0·1%)	16,200·6 (12,328·0 to 19,885·1)	53·2% (40·2 to 63·5%)	13,761·6 (10,202·4 to 16,999·3)	52·7% (39·8 to 63·4%)	-15·1% (-23·8 to -4·5%)	-0·9% (-2·4 to 0·3%)
Tracheal, bronchus, and lung cancer	5·0 (3·2 to 6·7)	46·5% (29·6 to 61·4%)	7·5 (4·8 to 10·3)	45·7% (29·0 to 60·6%)	48·8% (30·5 to 71·7%)	-1·8% (-4·8 to 0·5%)	128·7 (81·4 to 172·1)	45·7% (29·1 to 60·5%)	190·7 (121·3 to 264·5)	44·9% (28·5 to 59·5%)	48·2% (28·4 to 72·3%)	-1·9% (-5·3 to 0·7%)
Ischaemic heart disease	57·7 (46·4 to 70·3)	29·5% (24·2 to 34·8%)	73·5 (57·6 to 91·5)	28·3% (23·1 to 33·9%)	27·5% (10·2 to 46·3%)	-3·9% (-8·9 to -0·6%)	1,464·7 (1,187·0 to 1,769·4)	31·2% (25·7 to 36·5%)	1,850·2 (1,444·5 to 2,311·7)	30·1% (24·6 to 35·7%)	26·3% (6·9 to 46·9%)	-3·6% (-9·1 to 0·2%)
Ischaemic stroke	20·7 (14·4 to 28·2)	26·3% (20·3 to 33·5%)	26·2 (18·3 to 35·8)	25·4% (19·5 to 32·5%)	26·1% (8·7 to 46·4%)	-3·5% (-6·9 to -0·8%)	446·6 (313·9 to 597·5)	27·6% (21·3 to 34·6%)	551·8 (394·8 to 740·0)	26·7% (20·4 to 33·6%)	23·5% (6·1 to 43·0%)	-3·3% (-7·4 to -0·5%)
Hemorrhagic stroke	35·7 (26·8 to 45·8)	29·1% (22·7 to 36·2%)	45·0 (33·3 to 58·3)	28·5% (22·0 to 35·5%)	26·0% (8·6 to 46·4%)	-2·2% (-5·4 to 0·4%)	1,042·7 (790·5 to 1,316·8)	28·6% (22·1 to 35·1%)	1,306·9 (975·7 to 1,674·8)	28·4% (21·9 to 35·1%)	25·3% (7·7 to 45·8%)	-0·9% (-4·8 to 2·5%)
Chronic obstructive pulmonary disease	21·4 (13·4 to 30·9)	47·0% (30·4 to 66·7%)	28·4 (17·9 to 41·8)	46·8% (30·4 to 67·1%)	32·8% (14·8 to 54·7%)	-0·4% (-3·7 to 2·5%)	542·2 (344·5 to 779·1)	39·1% (25·3 to 55·0%)	719·6 (462·8 to 1,046·0)	39·9% (25·7 to 56·6%)	32·7% (16·8 to 52·0%)	2·0% (-3·6 to 8·3%)
Cataract	-	-	-	-	-	-	44·3 (30·0 to 61·4)	35·9% (32·1 to 39·4%)	63·9 (43·8 to 88·9)	35·6% (31·8 to 38·9%)	44·4% (40·5 to 48·0%)	-0·8% (-2·3 to 0·8%)
**Ambient ozone pollution: All causes**	2·2 (0·7 to 3·8)	0·0% (0·0 to 0·1%)	3·0 (1·0 to 5·2)	0·1% (0·0 to 0·1%)	38·6% (18·2 to 63·6%)	40·2% (23·7 to 60·9%)	45·8 (15·7 to 80·9)	0·0% (0·0 to 0·0%)	62·8 (20·8 to 109·2)	0·0% (0·0 to 0·0%)	37·2% (16·5 to 62·5%)	43·7% (24·5 to 68·2%)
Chronic obstructive pulmonary disease	2·2 (0·7 to 3·8)	4·7% (1·7 to 8·3%)	3·0 (1·0 to 5·2)	4·9% (1·8 to 8·6%)	38·6% (18·2 to 63·6%)	4·0% (-1·9 to 10·6%)	45·8 (15·7 to 80·9)	3·3% (1·2 to 5·8%)	62·8 (20·8 to 109·2)	3·5% (1·2 to 6·0%)	37·2% (16·5 to 62·5%)	5·5% (-3·5 to 16·2%)
**Other environmental risks: All causes**	18·5 (10·3 to 28·9)	0·4% (0·2 to 0·6%)	24·3 (13·6 to 37·8)	0·5% (0·3 to 0·8%)	31·4% (14·1 to 51·5%)	33·0% (19·5 to 48·8%)	519·8 (299·4 to 796·6)	0·2% (0·1 to 0·2%)	657·2 (378·1 to 1,009·3)	0·2% (0·1 to 0·3%)	26·4% (8·8 to 46·7%)	32·5% (15·1 to 51·0%)
**Residential radon: All causes**	0·9 (0·6 to 1·1)	0·0% (0·0 to 0·0%)	1·3 (0·9 to 1·8)	0·0% (0·0 to 0·0%)	51·3% (32·9 to 73·9%)	53·1% (39·6 to 67·1%)	22·1 (15·6 to 29·3)	0·0% (0·0 to 0·0%)	33·4 (23·0 to 44·9)	0·0% (0·0 to 0·0%)	51·0% (31·5 to 74·3%)	58·1% (40·1 to 77·5%)
Tracheal, bronchus, and lung cancer	0·9 (0·6 to 1·1)	7·9% (5·5 to 10·4%)	1·3 (0·9 to 1·8)	7·9% (5·5 to 10·3%)	51·3% (32·9 to 73·9%)	-0·2% (-3·0 to 2·8%)	22·1 (15·6 to 29·3)	7·9% (5·6 to 10·1%)	33·4 (23·0 to 44·9)	7·8% (5·6 to 10·1%)	51·0% (31·5 to 74·3%)	-0·2% (-3·0 to 3·2%)
**Lead exposure: All causes**	17·6 (9·5 to 28·1)	0·4% (0·2 to 0·6%)	23·0 (12·5 to 36·4)	0·5% (0·3 to 0·8%)	30·5% (12·9 to 50·9%)	32·1% (17·7 to 48·1%)	497·6 (279·5 to 774·3)	0·2% (0·1 to 0·2%)	623·7 (349·0 to 971·5)	0·2% (0·1 to 0·3%)	25·3% (7·2 to 45·4%)	31·4% (13·2 to 50·3%)
Rheumatic heart disease	0·1 (0·1 to 0·3)	1·2% (0·5 to 2·2%)	0·2 (0·1 to 0·3)	1·2% (0·5 to 2·2%)	14·9% (-6·0 to 40·0%)	-0·6% (-13·3 to 13·6%)	5·1 (1·9 to 10·2)	0·9% (0·4 to 1·8%)	5·7 (2·1 to 11·2)	0·9% (0·3 to 1·8%)	13·1% (-8·1 to 35·0%)	-3·6% (-18·8 to 11·3%)
Ischaemic heart disease	7·1 (4·0 to 10·9)	3·6% (2·1 to 5·5%)	9·3 (5·3 to 14·4)	3·6% (2·0 to 5·5%)	31·0% (11·8 to 52·9%)	-1·1% (-11·7 to 10·3%)	177·0 (99·4 to 275·5)	3·8% (2·1 to 5·7%)	225·4 (127·2 to 352·6)	3·7% (2·1 to 5·6%)	27·3% (5·8 to 51·7%)	-2·6% (-15·2 to 11·2%)
Ischaemic stroke	2·4 (1·1 to 4·0)	3·1% (1·6 to 4·9%)	3·1 (1·5 to 5·2)	3·0% (1·6 to 4·9%)	29·6% (9·8 to 51·9%)	-0·7% (-9·2 to 9·3%)	52·6 (26·6 to 86·9)	3·2% (1·7 to 5·1%)	65·8 (32·8 to 109·0)	3·2% (1·7 to 5·1%)	25·2% (3·7 to 49·5%)	-1·9% (-12·6 to 9·7%)
Hemorrhagic stroke	4·1 (2·0 to 6·9)	3·3% (1·7 to 5·4%)	5·2 (2·5 to 8·7)	3·3% (1·6 to 5·4%)	28·2% (8·5 to 52·1%)	-0·3% (-11·1 to 11·9%)	115·9 (53·6 to 201·6)	3·2% (1·5 to 5·3%)	145·1 (68·3 to 246·6)	3·2% (1·5 to 5·2%)	25·2% (2·7 to 50·6%)	-0·8% (-13·8 to 13·4%)
Hypertensive heart disease	2·3 (0·7 to 5·2)	4·9% (1·6 to 11·1%)	2·9 (0·9 to 6·6)	5·0% (1·7 to 11·5%)	29·0% (7·8 to 54·8%)	1·7% (-7·9 to 13·7%)	50·1 (17·9 to 105·5)	4·7% (1·7 to 10·1%)	62·7 (22·2 to 135·9)	4·7% (1·7 to 10·4%)	25·2% (3·5 to 51·5%)	0·1% (-10·8 to 13·5%)
Cardiomyopathy and myocarditis	0·1 (0·1 to 0·2)	1·6% (0·8 to 2·8%)	0·2 (0·1 to 0·3)	1·7% (0·8 to 2·9%)	36·4% (9·4 to 65·6%)	3·0% (-9·9 to 16·5%)	4·0 (1·6 to 7·5)	1·2% (0·5 to 2·2%)	5·3 (2·2 to 9·8)	1·3% (0·6 to 2·3%)	33·2% (4·3 to 65·7%)	5·2% (-12·4 to 23·6%)

Supplemental Results Table 1. All-age deaths, death PAFs, DALYs, DALY PAFs, and percent change for each risk factor and outcome for both sexes combined in 2005 and 2015 for Low SDI.

Data in parenthesis are 95% uncertainty intervals. PAFs=population attributable fractions. DALYs=disability-adjusted life-years.

	2005 Deaths (in thousands)	2005 Deaths PAF	2015 Deaths (in thousands)	2015 Deaths PAF	Percent change deaths 2005-2015	Percent change deaths PAF 2005-2015	2005 DALYs (in thousands)	2005 DALYs PAF	2015 DALYs (in thousands)	2015 DALYs PAF	Percent change DALYs 2005-2015	Percent change DALYs PAF 2005-2015
Atrial fibrillation and flutter	0·0 (0·0 to 0·1)	1·9% (1·0 to 3·0%)	0·0 (0·0 to 0·1)	2·0% (1·0 to 3·0%)	41·3% (25·8 to 60·8%)	0·9% (-5·7 to 8·4%)	0·9 (0·4 to 1·6)	2·0% (1·0 to 3·2%)	1·2 (0·6 to 2·1)	2·0% (1·0 to 3·2%)	36·7% (28·8 to 46·9%)	-0·7% (-4·4 to 3·7%)
Aortic aneurysm	0·0 (0·0 to 0·1)	1·6% (0·7 to 2·8%)	0·1 (0·0 to 0·1)	1·7% (0·8 to 2·8%)	50·4% (20·5 to 92·0%)	0·9% (-8·9 to 14·0%)	1·0 (0·4 to 2·2)	1·6% (0·7 to 2·8%)	1·5 (0·6 to 3·0)	1·6% (0·7 to 2·8%)	47·9% (14·2 to 91·9%)	-0·2% (-13·5 to 14·8%)
Peripheral vascular disease	0·0 (0·0 to 0·0)	1·1% (0·5 to 1·9%)	0·0 (0·0 to 0·0)	1·1% (0·5 to 1·9%)	68·8% (34·4 to 128·5%)	0·7% (-7·5 to 11·4%)	0·2 (0·1 to 0·3)	1·1% (0·5 to 2·0%)	0·2 (0·1 to 0·5)	1·1% (0·5 to 1·9%)	42·4% (34·6 to 53·0%)	-0·9% (-4·6 to 3·7%)
Endocarditis	0·1 (0·0 to 0·1)	1·2% (0·5 to 2·1%)	0·1 (0·0 to 0·2)	1·2% (0·5 to 2·2%)	40·0% (17·8 to 68·6%)	2·4% (-9·2 to 17·5%)	1·6 (0·6 to 3·3)	0·8% (0·3 to 1·6%)	2·2 (0·8 to 4·6)	0·8% (0·3 to 1·6%)	37·9% (13·3 to 68·8%)	4·1% (-13·4 to 25·7%)
Other cardiovascular and circulatory diseases	0·9 (0·4 to 1·5)	1·7% (0·8 to 2·9%)	1·2 (0·5 to 2·0)	1·8% (0·8 to 2·9%)	32·1% (11·5 to 58·7%)	2·3% (-7·1 to 15·9%)	26·7 (11·4 to 47·5)	1·4% (0·6 to 2·4%)	34·8 (15·1 to 61·0)	1·5% (0·6 to 2·5%)	30·6% (11·3 to 52·7%)	3·3% (-7·9 to 17·4%)
Idiopathic developmental intellectual disability	-	-	-	-	-	-	46·9 (19·0 to 83·8)	24·9% (19·5 to 29·7%)	51·3 (21·1 to 94·2)	20·3% (14·9 to 25·0%)	9·3% (-8·0 to 27·3%)	-18·7% (-31·6 to -5·2%)
Chronic kidney disease due to diabetes mellitus	0·1 (0·1 to 0·2)	2·4% (1·2 to 3·9%)	0·2 (0·1 to 0·3)	2·5% (1·3 to 4·2%)	48·9% (24·2 to 78·8%)	7·6% (-3·7 to 21·6%)	3·8 (1·7 to 6·9)	1·4% (0·6 to 2·5%)	5·4 (2·5 to 9·7)	1·5% (0·7 to 2·6%)	42·0% (18·8 to 71·5%)	7·6% (-6·4 to 24·8%)
Chronic kidney disease due to hypertension	0·3 (0·1 to 0·5)	2·3% (1·1 to 3·8%)	0·5 (0·2 to 0·8)	2·3% (1·2 to 3·9%)	47·7% (24·2 to 76·7%)	3·4% (-7·4 to 17·6%)	8·4 (3·9 to 15·0)	1·7% (0·8 to 2·9%)	12·1 (5·6 to 21·1)	1·7% (0·8 to 3·0%)	43·7% (18·1 to 76·2%)	3·5% (-10·1 to 20·4%)
Chronic kidney disease due to glomerulonephritis	0·1 (0·0 to 0·1)	1·5% (0·7 to 2·4%)	0·1 (0·0 to 0·1)	1·6% (0·8 to 2·6%)	48·3% (24·5 to 78·5%)	9·0% (-4·2 to 25·2%)	2·2 (0·9 to 4·1)	0·8% (0·4 to 1·5%)	3·1 (1·3 to 5·7)	0·9% (0·4 to 1·6%)	40·2% (19·4 to 65·0%)	6·4% (-7·4 to 22·5%)
Chronic kidney disease due to other causes	0·0 (0·0 to 0·0)	1·7% (0·8 to 2·9%)	0·0 (0·0 to 0·1)	1·9% (1·0 to 3·2%)	45·6% (18·4 to 79·2%)	13·1% (-2·0 to 31·2%)	1·5 (0·6 to 3·1)	1·0% (0·4 to 1·9%)	2·0 (0·8 to 4·1)	1·1% (0·5 to 2·0%)	36·4% (20·0 to 56·2%)	6·3% (-4·1 to 18·6%)
**Occupational risks: All causes**	27·8 (25·1 to 31·0)	0·6% (0·5 to 0·7%)	36·9 (32·6 to 41·8)	0·8% (0·7 to 0·9%)	32·7% (17·9 to 48·6%)	34·3% (23·6 to 46·7%)	2,943·8 (2,379·6 to 3,595·2)	0·9% (0·8 to 1·1%)	4,024·5 (3,248·3 to 4,912·2)	1·3% (1·1 to 1·5%)	36·7% (29·8 to 43·4%)	43·3% (35·3 to 51·5%)
**Occupational carcinogens: All causes**	3·1 (2·8 to 3·5)	0·1% (0·1 to 0·1%)	5·2 (4·2 to 6·3)	0·1% (0·1 to 0·1%)	64·4% (38·9 to 97·8%)	66·3% (46·8 to 90·9%)	76·5 (66·7 to 86·7)	0·0% (0·0 to 0·0%)	125·5 (102·9 to 154·9)	0·0% (0·0 to 0·1%)	64·2% (37·6 to 99·5%)	72·0% (46·6 to 104·1%)
**Occupational exposure to asbestos: All causes**	0·7 (0·5 to 0·9)	0·0% (0·0 to 0·0%)	0·8 (0·6 to 1·2)	0·0% (0·0 to 0·0%)	24·1% (-5·2 to 64·8%)	25·5% (0·9 to 57·9%)	15·6 (10·4 to 21·5)	0·0% (0·0 to 0·0%)	19·0 (12·3 to 27·6)	0·0% (0·0 to 0·0%)	21·7% (-8·6 to 65·6%)	27·5% (-2·2 to 69·5%)
Larynx cancer	0·0 (0·0 to 0·0)	0·8% (0·4 to 1·3%)	0·0 (0·0 to 0·0)	0·7% (0·4 to 1·1%)	18·9% (-13·3 to 78·3%)	-14·4% (-31·5 to 10·7%)	0·4 (0·2 to 0·6)	0·6% (0·4 to 1·0%)	0·4 (0·2 to 0·7)	0·5% (0·3 to 0·9%)	16·8% (-16·6 to 75·9%)	-15·4% (-33·2 to 11·1%)
Tracheal, bronchus, and lung cancer	0·4 (0·3 to 0·6)	3·8% (2·5 to 5·3%)	0·5 (0·3 to 0·8)	3·3% (2·3 to 4·6%)	33·1% (-0·9 to 80·8%)	-12·5% (-27·5 to 7·6%)	8·6 (5·4 to 12·7)	3·1% (2·0 to 4·4%)	11·3 (6·8 to 17·4)	2·6% (1·8 to 3·8%)	31·3% (-5·4 to 82·6%)	-13·4% (-30·2 to 9·0%)
Ovarian cancer	0·0 (0·0 to 0·1)	1·2% (0·5 to 2·0%)	0·1 (0·0 to 0·1)	1·0% (0·4 to 1·7%)	29·1% (-17·8 to 104·3%)	-14·8% (-35·2 to 12·6%)	1·1 (0·5 to 2·0)	0·9% (0·4 to 1·6%)	1·4 (0·6 to 2·9)	0·8% (0·3 to 1·3%)	29·5% (-19·0 to 108·7%)	-15·2% (-36·7 to 15·5%)
Mesothelioma	0·2 (0·1 to 0·3)	47·0% (31·1 to 61·6%)	0·2 (0·1 to 0·3)	43·1% (27·4 to 57·9%)	5·7% (-19·2 to 37·2%)	-8·3% (-18·3 to 1·7%)	5·5 (3·1 to 8·2)	38·6% (24·2 to 52·5%)	5·8 (3·0 to 9·1)	34·8% (20·7 to 49·0%)	5·5% (-22·6 to 41·9%)	-9·8% (-22·3 to 3·1%)
**Occupational exposure to arsenic: All causes**	0·1 (0·1 to 0·1)	0·0% (0·0 to 0·0%)	0·1 (0·1 to 0·2)	0·0% (0·0 to 0·0%)	50·0% (27·9 to 76·8%)	51·7% (34·2 to 73·3%)	1·8 (1·3 to 2·2)	0·0% (0·0 to 0·0%)	2·6 (1·9 to 3·5)	0·0% (0·0 to 0·0%)	49·9% (26·9 to 78·4%)	57·1% (35·1 to 83·3%)
Tracheal, bronchus, and lung cancer	0·1 (0·1 to 0·1)	0·7% (0·5 to 0·9%)	0·1 (0·1 to 0·2)	0·7% (0·5 to 0·9%)	50·0% (27·9 to 76·8%)	-1·1% (-8·1 to 7·0%)	1·8 (1·3 to 2·2)	0·6% (0·5 to 0·8%)	2·6 (1·9 to 3·5)	0·6% (0·5 to 0·8%)	49·9% (26·9 to 78·4%)	-0·9% (-8·0 to 7·7%)
**Occupational exposure to benzene: All causes**	0·1 (0·1 to 0·1)	0·0% (0·0 to 0·0%)	0·2 (0·2 to 0·3)	0·0% (0·0 to 0·0%)	74·2% (43·4 to 114·5%)	76·1% (52·8 to 107·7%)	4·2 (3·4 to 5·2)	0·0% (0·0 to 0·0%)	7·5 (5·7 to 9·9)	0·0% (0·0 to 0·0%)	76·7% (46·1 to 117·5%)	85·0% (56·1 to 123·8%)
Leukaemia	0·1 (0·1 to 0·1)	1·5% (1·2 to 1·8%)	0·2 (0·2 to 0·3)	1·7% (1·4 to 2·2%)	74·2% (43·4 to 114·5%)	18·5% (3·4 to 38·9%)	4·2 (3·4 to 5·2)	1·0% (0·8 to 1·3%)	7·5 (5·7 to 9·9)	1·2% (0·9 to 1·5%)	76·7% (46·1 to 117·5%)	18·2% (-0·7 to 44·7%)
**Occupational exposure to beryllium: All causes**	0·0 (0·0 to 0·0)	0·0% (0·0 to 0·0%)	0·0 (0·0 to 0·0)	0·0% (0·0 to 0·0%)	46·8% (18·5 to 85·0%)	48·4% (23·3 to 81·1%)	0·1 (0·1 to 0·1)	0·0% (0·0 to 0·0%)	0·1 (0·1 to 0·1)	0·0% (0·0 to 0·0%)	46·6% (17·6 to 86·5%)	53·5% (25·3 to 91·7%)
Tracheal, bronchus, and lung cancer	0·0 (0·0 to 0·0)	0·0% (0·0 to 0·0%)	0·0 (0·0 to 0·0)	0·0% (0·0 to 0·0%)	46·8% (18·5 to 85·0%)	-3·3% (-16·2 to 13·1%)	0·1 (0·1 to 0·1)	0·0% (0·0 to 0·0%)	0·1 (0·1 to 0·1)	0·0% (0·0 to 0·0%)	46·6% (17·6 to 86·5%)	-3·2% (-16·8 to 14·0%)
**Occupational exposure to cadmium: All causes**	0·0 (0·0 to 0·0)	0·0% (0·0 to 0·0%)	0·0 (0·0 to 0·0)	0·0% (0·0 to 0·0%)	64·1% (32·8 to 107·6%)	65·9% (39·1 to 101·5%)	0·3 (0·2 to 0·3)	0·0% (0·0 to 0·0%)	0·4 (0·3 to 0·6)	0·0% (0·0 to 0·0%)	63·8% (31·9 to 108·0%)	71·6% (39·9 to 112·8%)
Tracheal, bronchus, and lung cancer	0·0 (0·0 to 0·0)	0·1% (0·1 to 0·1%)	0·0 (0·0 to 0·0)	0·1% (0·1 to 0·1%)	64·1% (32·8 to 107·6%)	8·1% (-5·7 to 26·1%)	0·3 (0·2 to 0·3)	0·1% (0·1 to 0·1%)	0·4 (0·3 to 0·6)	0·1% (0·1 to 0·1%)	63·8% (31·9 to 108·0%)	8·2% (-5·9 to 26·7%)
**Occupational exposure to chromium: All causes**	0·0 (0·0 to 0·0)	0·0% (0·0 to 0·0%)	0·1 (0·0 to 0·1)	0·0% (0·0 to 0·0%)	58·1% (28·7 to 98·4%)	59·8% (34·3 to 92·2%)	0·9 (0·8 to 1·1)	0·0% (0·0 to 0·0%)	1·5 (1·1 to 1·9)	0·0% (0·0 to 0·0%)	57·8% (27·4 to 99·7%)	65·3% (35·8 to 103·8%)
Tracheal, bronchus, and lung cancer	0·0 (0·0 to 0·0)	0·4% (0·3 to 0·4%)	0·1 (0·0 to 0·1)	0·4% (0·3 to 0·5%)	58·1% (28·7 to 98·4%)	4·1% (-8·5 to 20·0%)	0·9 (0·8 to 1·1)	0·3% (0·3 to 0·4%)	1·5 (1·1 to 1·9)	0·3% (0·3 to 0·4%)	57·8% (27·4 to 99·7%)	4·3% (-8·6 to 21·0%)
**Occupational exposure to diesel engine exhaust: All causes**	0·8 (0·6 to 0·9)	0·0% (0·0 to 0·0%)	1·4 (1·1 to 1·8)	0·0% (0·0 to 0·0%)	88·3% (58·9 to 125·5%)	90·5% (65·9 to 119·5%)	18·0 (14·9 to 20·9)	0·0% (0·0 to 0·0%)	33·8 (26·8 to 42·6)	0·0% (0·0 to 0·0%)	88·4% (57·8 to 127·5%)	97·4% (68·0 to 133·1%)
Tracheal, bronchus, and lung cancer	0·8 (0·6 to 0·9)	7·0% (6·1 to 8·1%)	1·4 (1·1 to 1·8)	8·7% (7·4 to 10·2%)	88·3% (58·9 to 125·5%)	24·1% (14·4 to 36·0%)	18·0 (14·9 to 20·9)	6·4% (5·4 to 7·3%)	33·8 (26·8 to 42·6)	7·9% (6·7 to 9·3%)	88·4% (57·8 to 127·5%)	24·5% (14·4 to 36·9%)
**Occupational exposure to second-hand smoke: All causes**	1·0 (0·9 to 1·1)	0·0% (0·0 to 0·0%)	1·9 (1·6 to 2·3)	0·0% (0·0 to 0·0%)	85·9% (56·3 to 125·6%)	88·0% (63·9 to 119·6%)	23·6 (20·6 to 26·6)	0·0% (0·0 to 0·0%)	43·6 (35·8 to 54·1)	0·0% (0·0 to 0·0%)	85·0% (54·8 to 126·2%)	93·8% (64·8 to 132·1%)
Tracheal, bronchus, and lung cancer	1·0 (0·9 to 1·1)	9·3% (8·4 to 10·3%)	1·9 (1·6 to 2·3)	11·4% (10·2 to 12·9%)	85·9% (56·3 to 125·6%)	22·5% (11·9 to 36·2%)	23·6 (20·6 to 26·6)	8·4% (7·6 to 9·2%)	43·6 (35·8 to 54·1)	10·2% (9·1 to 11·6%)	85·0% (54·8 to 126·2%)	22·3% (11·7 to 36·9%)
**Occupational exposure to formaldehyde: All causes**	0·0 (0·0 to 0·0)	0·0% (0·0 to 0·0%)	0·0 (0·0 to 0·1)	0·0% (0·0 to 0·0%)	47·5% (20·8 to 88·3%)	49·0% (28·0 to 81·5%)	1·2 (0·9 to 1·5)	0·0% (0·0 to 0·0%)	1·8 (1·3 to 2·4)	0·0% (0·0 to 0·0%)	49·5% (22·1 to 91·8%)	56·5% (31·3 to 95·8%)
Nasopharynx cancer	0·0 (0·0 to 0·0)	1·3% (0·9 to 1·8%)	0·0 (0·0 to 0·0)	1·4% (0·9 to 1·9%)	44·4% (10·6 to 99·7%)	3·1% (-8·4 to 20·6%)	0·4 (0·3 to 0·6)	1·3% (0·9 to 1·8%)	0·6 (0·4 to 1·0)	1·3% (0·9 to 1·9%)	45·9% (11·2 to 99·2%)	2·4% (-9·7 to 19·3%)
**Occupational exposure to nickel: All causes**	0·2 (0·1 to 0·2)	0·0% (0·0 to 0·0%)	0·2 (0·2 to 0·4)	0·0% (0·0 to 0·0%)	47·0% (19·9 to 81·6%)	48·7% (24·6 to 79·0%)	4·0 (2·8 to 5·4)	0·0% (0·0 to 0·0%)	5·9 (3·8 to 8·3)	0·0% (0·0 to 0·0%)	46·8% (19·2 to 84·4%)	53·7% (25·5 to 88·6%)
Tracheal, bronchus, and lung cancer	0·2 (0·1 to 0·2)	1·6% (1·1 to 2·1%)	0·2 (0·2 to 0·4)	1·5% (1·0 to 2·1%)	47·0% (19·9 to 81·6%)	-3·1% (-15·4 to 11·4%)	4·0 (2·8 to 5·4)	1·4% (1·0 to 1·9%)	5·9 (3·8 to 8·3)	1·4% (1·0 to 1·9%)	46·8% (19·2 to 84·4%)	-3·0% (-15·4 to 12·6%)
**Occupational exposure to polycyclic aromatic hydrocarbons: All causes**	0·1 (0·1 to 0·1)	0·0% (0·0 to 0·0%)	0·2 (0·1 to 0·2)	0·0% (0·0 to 0·0%)	76·7% (45·2 to 119·2%)	78·6% (52·2 to 114·3%)	2·3 (1·8 to 2·8)	0·0% (0·0 to 0·0%)	4·1 (3·0 to 5·4)	0·0% (0·0 to 0·0%)	76·4% (44·1 to 120·1%)	84·8% (53·2 to 126·6%)
Tracheal, bronchus, and lung cancer	0·1 (0·1 to 0·1)	0·9% (0·7 to 1·1%)	0·2 (0·1 to 0·2)	1·1% (0·8 to 1·3%)	76·7% (45·2 to 119·2%)	16·3% (3·7 to 32·2%)	2·3 (1·8 to 2·8)	0·8% (0·7 to 1·0%)	4·1 (3·0 to 5·4)	1·0% (0·8 to 1·2%)	76·4% (44·1 to 120·1%)	16·5% (3·8 to 33·1%)
**Occupational exposure to silica: All causes**	0·4 (0·3 to 0·4)	0·0% (0·0 to 0·0%)	0·7 (0·6 to 0·9)	0·0% (0·0 to 0·0%)	83·0% (46·8 to 132·3%)	85·1% (53·7 to 128·6%)	9·1 (7·7 to 10·6)	0·0% (0·0 to 0·0%)	16·7 (13·2 to 21·7)	0·0% (0·0 to 0·0%)	82·7% (46·4 to 133·3%)	91·4% (56·3 to 139·7%)
Tracheal, bronchus, and lung cancer	0·4 (0·3 to 0·4)	3·6% (3·1 to 4·1%)	0·7 (0·6 to 0·9)	4·3% (3·7 to 5·1%)	83·0% (46·8 to 132·3%)	20·5% (4·3 to 42·1%)	9·1 (7·7 to 10·6)	3·2% (2·8 to 3·7%)	16·7 (13·2 to 21·7)	3·9% (3·3 to 4·7%)	82·7% (46·4 to 133·3%)	20·7% (4·2 to 42·3%)
**Occupational exposure to sulfphuric acid: All causes**	0·2 (0·1 to 0·2)	0·0% (0·0 to 0·0%)	0·2 (0·2 to 0·4)	0·0% (0·0 to 0·0%)	51·5% (21·6 to 95·2%)	53·2% (26·6 to 93·4%)	4·2 (3·0 to 5·7)	0·0% (0·0 to 0·0%)	6·3 (4·2 to 9·2)	0·0% (0·0 to 0·0%)	51·1% (21·5 to 95·0%)	58·3% (29·0 to 102·4%)
Larynx cancer	0·2 (0·1 to 0·2)	8·0% (5·9 to 10·5%)	0·2 (0·2 to 0·4)	8·8% (6·3 to 11·7%)	51·5% (21·6 to 95·2%)	9·0% (-1·6 to 23·3%)	4·2 (3·0 to 5·7)	7·4% (5·5 to 9·8%)	6·3 (4·2 to 9·2)	8·1% (5·9 to 10·8%)	51·1% (21·5 to 95·0%)	9·5% (-1·6 to 24·7%)
**Occupational exposure to trichloroethylene: All causes**	0·0 (0·0 to 0·0)	0·0% (0·0 to 0·0%)	0·0 (0·0 to 0·0)	0·0% (0·0 to 0·0%)	70·1% (39·8 to 104·9%)	72·1% (46·7 to 99·6%)	0·1 (0·0 to 0·1)	0·0% (0·0 to 0·0%)	0·1 (0·0 to 0·2)	0·0% (0·0 to 0·0%)	69·5% (38·7 to 104·4%)	77·5% (48·3 to 110·3%)
Kidney cancer	0·0 (0·0 to 0·0)	0·1% (0·0 to 0·2%)	0·0 (0·0 to 0·0)	0·1% (0·0 to 0·3%)	70·1% (39·8 to 104·9%)	13·9% (0·0 to 31·2%)	0·1 (0·0 to 0·1)	0·1% (0·0 to 0·1%)	0·1 (0·0 to 0·2)	0·1% (0·0 to 0·2%)	69·5% (38·7 to 104·4%)	15·2% (-2·5 to 40·6%)

Supplemental Results Table 1. All-age deaths, death PAFs, DALYs, DALY PAFs, and percent change for each risk factor and outcome for both sexes combined in 2005 and 2015 for Low SDI.

Data in parenthesis are 95% uncertainty intervals. PAFs=population attributable fractions. DALYs=disability-adjusted life-years.

	2005 Deaths (in thousands)	2005 Deaths PAF	2015 Deaths (in thousands)	2015 Deaths PAF	Percent change deaths 2005-2015	Percent change deaths PAF 2005-2015	2005 DALYs (in thousands)	2005 DALYs PAF	2015 DALYs (in thousands)	2015 DALYs PAF	Percent change DALYs 2005-2015	Percent change DALYs PAF 2005-2015
**Occupational asthmagens: All causes**	6·0 (4·5 to 7·6)	0·1% (0·1 to 0·2%)	6·9 (5·2 to 8·9)	0·1% (0·1 to 0·2%)	14·7% (-2·6 to 37·3%)	16·1% (2·9 to 33·4%)	302·0 (238·4 to 370·5)	0·1% (0·1 to 0·1%)	386·6 (304·4 to 484·3)	0·1% (0·1 to 0·2%)	28·0% (13·5 to 43·0%)	34·1% (21·9 to 47·2%)
Asthma	6·0 (4·5 to 7·6)	16·3% (13·8 to 19·0%)	6·9 (5·2 to 8·9)	16·6% (14·2 to 19·1%)	14·7% (-2·6 to 37·3%)	1·8% (-3·9 to 9·0%)	302·0 (238·4 to 370·5)	12·6% (10·7 to 14·7%)	386·6 (304·4 to 484·3)	13·1% (11·0 to 15·4%)	28·0% (13·5 to 43·0%)	3·7% (-2·3 to 10·4%)
**Occupational particulate matter, gases, and fumes: All causes**	7·8 (6·3 to 9·6)	0·2% (0·1 to 0·2%)	10·6 (8·5 to 13·2)	0·2% (0·2 to 0·3%)	35·8% (16·7 to 58·5%)	37·4% (22·7 to 54·3%)	221·5 (187·1 to 261·6)	0·1% (0·1 to 0·1%)	304·4 (251·0 to 365·0)	0·1% (0·1 to 0·1%)	37·4% (21·4 to 56·7%)	44·0% (29·2 to 60·5%)
Chronic obstructive pulmonary disease	7·8 (6·3 to 9·6)	17·1% (14·5 to 19·6%)	10·6 (8·5 to 13·2)	17·4% (14·8 to 20·0%)	35·8% (16·7 to 58·5%)	1·8% (-3·5 to 7·4%)	221·2 (186·9 to 261·2)	15·9% (13·8 to 18·2%)	304·0 (250·5 to 364·6)	16·8% (14·7 to 19·1%)	37·4% (21·4 to 56·6%)	5·6% (-0·6 to 13·1%)
**Occupational noise: All causes**	-	-	-	-	-	-	633·7 (432·1 to 866·5)	0·2% (0·1 to 0·3%)	890·7 (608·1 to 1,216·0)	0·3% (0·2 to 0·4%)	40·6% (38·7 to 42·4%)	47·2% (40·3 to 54·2%)
Age-related and other hearing loss	-	-	-	-	-	-	633·7 (432·1 to 866·5)	38·6% (34·6 to 43·4%)	890·7 (608·1 to 1,216·0)	39·4% (35·6 to 44·3%)	40·6% (38·7 to 42·4%)	2·3% (1·2 to 3·4%)
**Occupational injuries: All causes**	11·1 (9·6 to 13·4)	0·2% (0·2 to 0·3%)	15·0 (13·1 to 17·5)	0·3% (0·3 to 0·4%)	34·6% (12·1 to 63·2%)	36·4% (10·9 to 64·9%)	756·3 (636·5 to 913·1)	0·2% (0·2 to 0·3%)	1,072·3 (920·6 to 1,275·0)	0·4% (0·3 to 0·4%)	41·8% (18·1 to 70·2%)	48·7% (21·8 to 80·1%)
Pedestrian road injuries	0·7 (0·6 to 0·9)	2·4% (2·0 to 3·0%)	1·3 (1·0 to 1·5)	3·3% (2·6 to 4·1%)	68·5% (36·7 to 112·6%)	34·8% (5·1 to 73·7%)	40·3 (32·9 to 48·8)	2·4% (1·9 to 2·9%)	68·4 (56·4 to 82·7)	3·3% (2·6 to 4·1%)	69·5% (36·6 to 114·0%)	39·7% (7·7 to 80·3%)
Cyclist road injuries	0·0 (0·0 to 0·1)	2·0% (1·6 to 2·4%)	0·1 (0·1 to 0·1)	2·6% (2·0 to 3·2%)	77·2% (49·1 to 114·3%)	29·3% (1·8 to 61·6%)	2·6 (2·1 to 3·1)	2·1% (1·7 to 2·5%)	4·8 (3·9 to 5·7)	2·8% (2·2 to 3·5%)	83·0% (53·9 to 118·8%)	34·6% (6·4 to 68·6%)
Motorcyclist road injuries	0·6 (0·5 to 0·8)	9·0% (7·1 to 11·7%)	0·8 (0·6 to 1·1)	9·5% (7·4 to 12·6%)	37·9% (6·0 to 81·3%)	5·9% (-21·0 to 40·5%)	34·5 (27·2 to 46·9)	8·8% (6·8 to 11·7%)	47·8 (38·2 to 62·2)	9·5% (7·4 to 12·5%)	38·5% (6·7 to 81·7%)	7·2% (-19·5 to 43·2%)
Motor vehicle road injuries	2·7 (2·2 to 3·3)	7·6% (6·3 to 9·3%)	3·7 (3·1 to 4·4)	8·4% (6·9 to 10·4%)	37·2% (13·0 to 66·2%)	10·5% (-11·7 to 38·9%)	151·1 (126·6 to 183·8)	7·6% (6·3 to 9·3%)	209·0 (179·3 to 248·9)	8·6% (7·1 to 10·5%)	38·3% (14·0 to 67·5%)	13·2% (-9·8 to 43·3%)
Other road injuries	0·1 (0·0 to 0·1)	9·4% (7·1 to 12·5%)	0·1 (0·1 to 0·1)	10·3% (7·9 to 13·4%)	42·1% (16·8 to 82·6%)	9·3% (-13·0 to 37·3%)	3·9 (3·0 to 5·1)	9·2% (6·6 to 12·8%)	5·7 (4·3 to 7·3)	10·3% (7·8 to 13·6%)	45·4% (21·0 to 82·8%)	12·2% (-10·7 to 44·4%)
Other transport injuries	0·1 (0·1 to 0·1)	2·5% (2·0 to 3·1%)	0·2 (0·1 to 0·2)	3·3% (2·5 to 4·2%)	51·9% (20·5 to 93·5%)	34·5% (3·0 to 72·9%)	7·2 (5·6 to 9·1)	2·3% (1·8 to 2·9%)	11·4 (8·8 to 14·1)	3·3% (2·5 to 4·1%)	58·5% (24·8 to 99·9%)	40·5% (6·4 to 82·5%)
Falls	1·0 (0·8 to 1·2)	3·6% (2·9 to 4·6%)	1·4 (1·2 to 1·7)	4·1% (3·3 to 5·1%)	41·2% (16·3 to 74·1%)	12·3% (-11·3 to 42·2%)	99·7 (76·6 to 126·9)	6·0% (4·6 to 7·8%)	159·9 (121·6 to 208·6)	7·7% (5·9 to 9·8%)	60·3% (30·7 to 95·1%)	28·0% (-3·0 to 66·0%)
Drowning	1·3 (1·0 to 1·7)	3·6% (2·8 to 4·8%)	1·6 (1·3 to 1·9)	4·4% (3·5 to 5·4%)	21·1% (-3·9 to 49·8%)	20·0% (-8·2 to 57·4%)	72·0 (58·5 to 91·9)	2·9% (2·2 to 3·8%)	87·7 (74·2 to 106·2)	3·6% (2·9 to 4·5%)	21·7% (-2·7 to 49·4%)	24·3% (-5·9 to 66·5%)
Fire, heat, and hot substances	1·1 (0·8 to 1·4)	5·0% (3·9 to 6·5%)	1·4 (1·1 to 1·8)	5·9% (4·8 to 7·3%)	34·4% (6·1 to 70·6%)	19·0% (-9·2 to 52·4%)	71·5 (55·9 to 92·5)	5·1% (4·0 to 6·6%)	103·4 (82·3 to 128·0)	6·7% (5·3 to 8·4%)	44·7% (15·6 to 81·1%)	30·9% (-0·6 to 70·3%)
Poisonings	0·4 (0·3 to 0·6)	3·5% (1·9 to 6·2%)	0·5 (0·4 to 0·7)	3·8% (2·1 to 6·8%)	17·3% (-10·2 to 56·7%)	10·0% (-37·6 to 90·5%)	27·2 (20·8 to 37·3)	3·0% (1·6 to 5·8%)	34·0 (26·0 to 44·9)	3·6% (1·9 to 6·9%)	25·4% (-4·2 to 64·5%)	18·5% (-36·4 to 117·0%)
Unintentional firearm injuries	0·3 (0·2 to 0·4)	6·3% (5·1 to 8·2%)	0·4 (0·2 to 0·5)	6·8% (5·5 to 8·5%)	29·4% (4·3 to 60·2%)	7·3% (-16·9 to 35·3%)	15·4 (10·7 to 21·3)	6·0% (4·7 to 7·9%)	20·1 (13·9 to 25·9)	6·5% (5·3 to 8·3%)	30·4% (5·3 to 61·6%)	9·5% (-14·9 to 39·1%)
Unintentional suffocation	0·0 (0·0 to 0·0)	0·2% (0·1 to 0·4%)	0·0 (0·0 to 0·0)	0·2% (0·1 to 0·4%)	28·4% (-5·3 to 76·8%)	10·8% (-26·7 to 78·6%)	0·6 (0·4 to 0·8)	0·2% (0·1 to 0·4%)	0·8 (0·6 to 1·2)	0·3% (0·2 to 0·5%)	39·2% (4·2 to 93·6%)	19·7% (-20·4 to 93·1%)
Other exposure to mechanical forces	0·7 (0·6 to 0·9)	6·1% (4·9 to 7·7%)	1·0 (0·8 to 1·2)	7·1% (5·8 to 8·7%)	39·9% (11·2 to 78·6%)	15·6% (-11·2 to 47·0%)	64·4 (51·1 to 82·1)	6·9% (5·5 to 8·8%)	95·5 (76·3 to 118·1)	8·4% (6·9 to 10·3%)	48·3% (20·2 to 81·0%)	22·4% (-5·1 to 55·9%)
Venomous animal contact	0·5 (0·3 to 0·7)	4·6% (3·5 to 6·4%)	0·5 (0·4 to 0·8)	4·9% (3·8 to 6·5%)	14·2% (-15·7 to 58·7%)	5·6% (-24·6 to 47·8%)	30·1 (20·9 to 43·1)	5·1% (3·7 to 7·1%)	37·2 (26·5 to 51·1)	5·8% (4·3 to 7·8%)	23·4% (-6·9 to 68·6%)	14·1% (-18·9 to 61·0%)
Non-venomous animal contact	0·1 (0·1 to 0·2)	3·1% (2·2 to 4·5%)	0·1 (0·1 to 0·2)	3·4% (2·5 to 4·8%)	11·8% (-20·8 to 60·4%)	11·2% (-23·8 to 67·5%)	9·8 (6·5 to 14·3)	3·4% (2·3 to 5·2%)	12·1 (8·2 to 17·3)	4·1% (2·8 to 5·9%)	23·0% (-10·4 to 74·2%)	21·2% (-16·7 to 75·7%)
Pulmonary aspiration and foreign body in airway	0·3 (0·2 to 0·4)	2·8% (1·8 to 4·6%)	0·4 (0·3 to 0·6)	3·3% (2·4 to 4·7%)	40·4% (14·2 to 74·4%)	18·5% (-9·8 to 59·5%)	19·1 (13·4 to 28·3)	2·6% (1·6 to 4·6%)	27·8 (20·2 to 38·5)	3·2% (2·2 to 5·0%)	45·4% (18·2 to 80·6%)	23·6% (-6·9 to 67·4%)
Foreign body in eyes	-	-	-	-	-	-	0·3 (0·1 to 0·5)	8·7% (6·4 to 11·5%)	0·4 (0·2 to 0·8)	10·3% (7·3 to 13·9%)	57·7% (20·2 to 102·6%)	17·4% (-10·7 to 51·5%)
Foreign body in other body part	0·5 (0·3 to 0·9)	6·8% (4·9 to 9·1%)	0·6 (0·4 to 0·9)	7·6% (5·7 to 9·7%)	16·6% (-12·5 to 56·2%)	12·2% (-14·2 to 44·0%)	38·7 (24·1 to 58·9)	6·9% (4·9 to 9·6%)	48·4 (34·0 to 66·0)	8·1% (6·2 to 10·7%)	24·9% (-3·0 to 57·9%)	18·2% (-8·1 to 53·2%)
Other unintentional injuries	0·7 (0·5 to 0·8)	3·8% (2·9 to 4·8%)	0·9 (0·7 to 1·1)	4·6% (3·6 to 5·8%)	32·1% (5·7 to 67·9%)	21·8% (-6·4 to 57·0%)	67·8 (52·7 to 86·2)	4·5% (3·6 to 5·6%)	97·8 (76·1 to 126·0)	6·0% (4·8 to 7·3%)	44·3% (18·6 to 74·6%)	32·3% (3·9 to 67·9%)
**Occupational ergonomic factors: All causes**	-	-	-	-	-	-	988·9 (691·2 to 1,381·2)	0·3% (0·2 to 0·4%)	1,342·3 (936·3 to 1,862·2)	0·4% (0·3 to 0·6%)	35·7% (33·0 to 38·4%)	42·2% (35·2 to 49·6%)
Low back pain	-	-	-	-	-	-	988·9 (691·2 to 1,381·2)	49·0% (46·1 to 51·8%)	1,342·3 (936·3 to 1,862·2)	48·3% (45·5 to 50·9%)	35·7% (33·0 to 38·4%)	-1·5% (-3·3 to 0·2%)
**Behavioural risks: All causes**	1,713·8 (1,621·6 to 1,814·2)	36·3% (34·5 to 38·2%)	1,521·4 (1,395·6 to 1,659·4)	32·6% (30·7 to 34·6%)	-11·2% (-17·4 to -4·1%)	-10·1% (-14·0 to -6·6%)	110,162·9 (103,288·4 to 117,204·2)	34·3% (32·3 to 36·4%)	91,867·4 (84,676·1 to 98,776·5)	30·0% (28·1 to 31·9%)	-16·6% (-21·9 to -11·0%)	-12·6% (-17·1 to -8·6%)
**Child and maternal malnutrition: All causes**	684·0 (618·5 to 756·2)	14·5% (13·1 to 16·1%)	517·6 (461·6 to 574·7)	11·1% (9·8 to 12·5%)	-24·3% (-31·3 to -16·6%)	-23·3% (-31·2 to -15·2%)	62,477·1 (56,666·4 to 68,848·2)	19·5% (17·8 to 21·4%)	48,624·1 (43,829·8 to 53,959·0)	15·9% (14·4 to 17·4%)	-22·2% (-29·0 to -14·9%)	-18·4% (-25·3 to -11·4%)
**Suboptimal breastfeeding: All causes**	143·9 (93·5 to 207·2)	3·0% (2·0 to 4·4%)	116·9 (73·4 to 170·8)	2·5% (1·6 to 3·7%)	-18·8% (-29·3 to -6·8%)	-17·7% (-29·3 to -4·7%)	12,452·2 (8,102·0 to 17,910·1)	3·9% (2·5 to 5·6%)	10,126·2 (6,376·6 to 14,778·4)	3·3% (2·1 to 4·8%)	-18·7% (-29·2 to -6·7%)	-14·7% (-25·9 to -2·1%)
**Non-exclusive breastfeeding : All causes**	132·8 (83·8 to 193·7)	2·8% (1·8 to 4·1%)	108·8 (65·4 to 160·6)	2·3% (1·4 to 3·4%)	-18·1% (-28·9 to -5·5%)	-17·0% (-29·0 to -3·5%)	11,469·5 (7,247·4 to 16,723·9)	3·6% (2·2 to 5·2%)	9,396·3 (5,661·2 to 13,863·0)	3·1% (1·8 to 4·6%)	-18·1% (-28·9 to -5·5%)	-14·1% (-25·6 to -1·1%)
Diarrhoeal diseases	59·7 (36·0 to 83·8)	15·7% (9·9 to 21·4%)	46·0 (27·9 to 64·8)	14·4% (8·9 to 20·0%)	-22·9% (-36·3 to -7·2%)	-8·0% (-20·2 to 6·1%)	5,165·8 (3,138·8 to 7,233·8)	19·9% (12·5 to 26·7%)	3,986·2 (2,428·6 to 5,606·9)	19·3% (12·2 to 26·5%)	-22·8% (-36·1 to -7·1%)	-3·1% (-14·3 to 9·9%)
Lower respiratory infections	73·1 (29·4 to 132·7)	15·9% (6·5 to 28·6%)	62·7 (23·3 to 111·5)	14·4% (5·3 to 25·2%)	-14·2% (-27·6 to 1·4%)	-9·4% (-23·0 to 4·7%)	6,303·7 (2,535·4 to 11,436·8)	20·7% (8·5 to 36·8%)	5,410·1 (2,009·5 to 9,604·5)	20·7% (7·7 to 36·9%)	-14·2% (-27·5 to 1·4%)	0·1% (-13·2 to 14·3%)
**Discontinued breastfeeding : All causes**	13·8 (4·6 to 27·7)	0·3% (0·1 to 0·6%)	10·3 (3·5 to 20·8)	0·2% (0·1 to 0·4%)	-25·6% (-37·3 to -9·8%)	-24·6% (-37·2 to -7·7%)	1,217·3 (406·3 to 2,438·2)	0·4% (0·1 to 0·8%)	916·7 (307·7 to 1,837·8)	0·3% (0·1 to 0·6%)	-24·7% (-36·2 to -9·4%)	-21·0% (-33·4 to -4·3%)
Diarrhoeal diseases	13·8 (4·6 to 27·7)	3·6% (1·2 to 7·1%)	10·3 (3·5 to 20·8)	3·2% (1·1 to 6·4%)	-25·6% (-37·3 to -9·8%)	-11·1% (-22·5 to 2·3%)	1,217·3 (406·3 to 2,438·2)	4·7% (1·5 to 9·1%)	916·7 (307·7 to 1,837·8)	4·4% (1·5 to 8·7%)	-24·7% (-36·2 to -9·4%)	-5·2% (-14·7 to 6·0%)
**Childhood undernutrition: All causes**	620·9 (554·5 to 691·4)	13·1% (11·7 to 14·7%)	455·4 (405·8 to 512·4)	9·8% (8·6 to 11·1%)	-26·7% (-33·8 to -18·9%)	-25·7% (-34·0 to -17·1%)	54,009·3 (48,396·6 to 60,018·0)	16·8% (15·1 to 18·9%)	39,928·0 (35,645·4 to 44,714·6)	13·0% (11·5 to 14·7%)	-26·1% (-33·1 to -18·4%)	-22·5% (-29·8 to -14·9%)
**Childhood underweight: All causes**	220·9 (176·9 to 279·4)	4·7% (3·7 to 5·9%)	157·7 (126·4 to 200·7)	3·4% (2·7 to 4·3%)	-28·6% (-40·3 to -14·3%)	-27·7% (-40·2 to -12·9%)	19,378·4 (15,596·5 to 24,462·8)	6·0% (4·8 to 7·7%)	14,021·8 (11,332·7 to 17,709·9)	4·6% (3·6 to 5·7%)	-27·6% (-39·2 to -13·7%)	-24·1% (-36·1 to -9·4%)
Diarrhoeal diseases	36·7 (28·8 to 47·4)	9·6% (8·0 to 12·1%)	22·8 (18·1 to 29·4)	7·1% (5·8 to 9·2%)	-37·8% (-47·4 to -26·4%)	-25·8% (-34·0 to -16·8%)	3,216·8 (2,556·7 to 4,148·5)	12·4% (10·4 to 15·3%)	2,017·4 (1,604·4 to 2,606·8)	9·8% (8·1 to 12·4%)	-37·3% (-46·6 to -26·0%)	-21·2% (-27·4 to -14·7%)
Lower respiratory infections	56·5 (36·4 to 98·2)	12·3% (8·2 to 21·0%)	37·5 (23·9 to 67·0)	8·6% (5·5 to 15·2%)	-33·6% (-42·9 to -22·7%)	-29·8% (-37·5 to -22·3%)	4,841·9 (3,115·9 to 8,421·4)	15·9% (10·6 to 27·4%)	3,218·7 (2,047·9 to 5,753·2)	12·3% (8·0 to 21·9%)	-33·5% (-42·9 to -22·7%)	-22·4% (-28·5 to -16·9%)
Measles	20·9 (3·2 to 67·5)	33·3% (10·4 to 59·2%)	6·6 (0·9 to 21·7)	28·0% (7·5 to 53·4%)	-68·7% (-91·3 to -6·4%)	-16·1% (-34·3 to -3·6%)	1,779·0 (272·2 to 5,743·5)	33·8% (10·5 to 59·9%)	557·4 (80·1 to 1,847·3)	28·4% (7·6 to 54·3%)	-68·7% (-91·3 to -6·6%)	-16·0% (-34·1 to -3·6%)
Protein-energy malnutrition	106·8 (77·2 to 140·8)	80·9% (74·5 to 85·6%)	90·8 (66·0 to 127·6)	77·6% (70·6 to 83·0%)	-15·0% (-36·0 to 15·3%)	-4·1% (-10·5 to 2·3%)	9,540·8 (7,033·8 to 12,365·7)	90·8% (87·6 to 93·1%)	8,228·4 (6,141·9 to 11,289·9)	89·5% (85·9 to 92·2%)	-13·8% (-34·1 to 15·0%)	-1·4% (-4·5 to 1·5%)
**Childhood wasting: All causes**	566·1 (484·2 to 647·4)	12·0% (10·2 to 13·7%)	422·0 (358·3 to 482·4)	9·1% (7·5 to 10·5%)	-25·5% (-32·8 to -17·3%)	-24·4% (-33·0 to -15·2%)	49,300·6 (42,212·7 to 56,235·0)	15·4% (13·2 to 17·5%)	37,043·8 (31,629·1 to 42,258·3)	12·1% (10·2 to 14·0%)	-24·9% (-32·0 to -16·9%)	-21·2% (-28·7 to -12·9%)

Supplemental Results Table 1. All-age deaths, death PAFs, DALYs, DALY PAFs, and percent change for each risk factor and outcome for both sexes combined in 2005 and 2015 for Low SDI.

Data in parenthesis are 95% uncertainty intervals. PAFs=population attributable fractions. DALYs=disability-adjusted life-years.

	2005 Deaths (in thousands)	2005 Deaths PAF	2015 Deaths (in thousands)	2015 Deaths PAF	Percent change deaths 2005-2015	Percent change deaths PAF 2005-2015	2005 DALYs (in thousands)	2005 DALYs PAF	2015 DALYs (in thousands)	2015 DALYs PAF	Percent change DALYs 2005-2015	Percent change DALYs PAF 2005-2015
Diarrhoeal diseases	199·8 (161·4 to 233·3)	52·4% (43·7 to 57·8%)	143·5 (115·5 to 167·2)	44·9% (36·7 to 50·8%)	-28·2% (-38·6 to -16·2%)	-14·3% (-22·8 to -5·9%)	17,525·7 (14,178·9 to 20,434·4)	67·4% (57·0 to 72·4%)	12,707·1 (10,212·6 to 14,754·8)	61·4% (50·9 to 67·2%)	-27·5% (-37·7 to -15·8%)	-8·9% (-14·2 to -4·0%)
Lower respiratory infections	232·9 (167·9 to 280·7)	50·7% (37·3 to 59·0%)	178·7 (126·0 to 218·3)	41·1% (29·2 to 49·4%)	-23·3% (-32·9 to -12·3%)	-19·0% (-26·2 to -12·0%)	19,978·6 (14,413·1 to 24,062·2)	65·6% (48·0 to 75·1%)	15,336·8 (10,822·3 to 18,734·1)	58·7% (41·9 to 68·6%)	-23·2% (-32·8 to -12·3%)	-10·4% (-15·2 to -6·2%)
Measles	26·5 (4·9 to 88·2)	42·7% (16·0 to 82·2%)	9·1 (1·5 to 34·9)	39·1% (11·6 to 80·3%)	-65·8% (-90·3 to 2·4%)	-8·6% (-32·8 to 9·2%)	2,255·6 (420·2 to 7,497·0)	43·3% (16·3 to 83·2%)	771·5 (126·0 to 2,968·9)	39·6% (11·8 to 81·4%)	-65·8% (-90·3 to 2·3%)	-8·5% (-32·6 to 9·3%)
Protein-energy malnutrition	106·8 (77·2 to 140·8)	80·9% (74·5 to 85·6%)	90·8 (66·0 to 127·6)	77·6% (70·6 to 83·0%)	-15·0% (-36·0 to 15·3%)	-4·1% (-10·5 to 2·3%)	9,540·8 (7,033·8 to 12,365·7)	90·8% (87·6 to 93·1%)	8,228·4 (6,141·9 to 11,289·9)	89·5% (85·9 to 92·2%)	-13·8% (-34·1 to 15·0%)	-1·4% (-4·5 to 1·5%)
**Childhood stunting: All causes**	142·3 (66·5 to 239·7)	3·0% (1·4 to 5·1%)	90·5 (39·3 to 160·6)	1·9% (0·9 to 3·4%)	-36·5% (-48·2 to -26·9%)	-35·7% (-47·7 to -25·6%)	12,289·4 (5,775·2 to 20,612·6)	3·8% (1·8 to 6·4%)	7,844·1 (3,414·1 to 13,857·6)	2·6% (1·1 to 4·5%)	-36·2% (-47·8 to -26·8%)	-33·1% (-45·2 to -23·5%)
Diarrhoeal diseases	47·2 (17·9 to 84·1)	12·4% (4·8 to 21·5%)	30·1 (11·0 to 54·1)	9·4% (3·4 to 16·8%)	-36·3% (-46·0 to -23·5%)	-24·0% (-32·1 to -14·8%)	4,143·0 (1,560·2 to 7,356·8)	15·9% (6·2 to 27·3%)	2,667·7 (984·0 to 4,779·4)	12·9% (4·8 to 22·5%)	-35·6% (-45·0 to -23·1%)	-19·2% (-25·2 to -12·9%)
Lower respiratory infections	79·8 (7·0 to 171·7)	17·4% (1·6 to 37·6%)	55·3 (4·5 to 124·1)	12·7% (1·0 to 27·9%)	-30·7% (-40·4 to -11·2%)	-26·9% (-34·6 to -15·7%)	6,846·5 (604·9 to 14,716·8)	22·5% (2·1 to 48·3%)	4,746·3 (384·0 to 10,640·1)	18·2% (1·5 to 40·0%)	-30·7% (-40·3 to -11·3%)	-19·1% (-25·8 to -10·0%)
Measles	15·3 (1·1 to 51·9)	24·4% (2·9 to 57·6%)	5·1 (0·3 to 17·5)	21·9% (2·5 to 54·6%)	-66·9% (-90·9 to 3·9%)	-10·3% (-23·6 to 0·0%)	1,300·0 (93·0 to 4,415·6)	24·7% (2·9 to 58·3%)	430·1 (26·9 to 1,487·6)	22·2% (2·6 to 55·4%)	-66·9% (-90·9 to 3·8%)	-10·3% (-23·4 to 0·0%)
**Iron deficiency: All causes**	40·2 (28·6 to 54·3)	0·9% (0·6 to 1·1%)	43·5 (30·4 to 59·7)	0·9% (0·7 to 1·3%)	8·2% (-8·7 to 26·3%)	9·4% (-3·3 to 23·6%)	6,471·9 (4,881·5 to 8,449·1)	2·0% (1·6 to 2·5%)	7,049·6 (5,244·3 to 9,282·2)	2·3% (1·8 to 2·8%)	8·9% (2·3 to 15·6%)	14·1% (7·6 to 21·0%)
Maternal haemorrhage	12·5 (4·8 to 20·7)	28·8% (10·9 to 45·5%)	11·9 (4·4 to 20·4)	28·2% (10·5 to 45·1%)	-4·7% (-24·2 to 18·8%)	-2·1% (-13·9 to 8·8%)	685·1 (265·2 to 1,141·3)	28·8% (11·0 to 45·5%)	650·4 (241·1 to 1,116·2)	28·2% (10·5 to 45·3%)	-5·1% (-24·2 to 17·7%)	-2·2% (-14·0 to 8·5%)
Maternal sepsis and other pregnancy related infections	1·2 (0·5 to 2·2)	29·1% (10·9 to 46·0%)	1·2 (0·4 to 2·3)	28·3% (10·6 to 45·5%)	-3·5% (-25·7 to 21·1%)	-2·7% (-14·7 to 7·5%)	70·9 (26·4 to 125·7)	29·2% (10·9 to 46·0%)	68·3 (24·0 to 126·8)	28·4% (10·6 to 45·6%)	-3·7% (-25·6 to 19·6%)	-2·9% (-14·7 to 7·3%)
Iron-deficiency anaemia	26·5 (18·1 to 35·4)	100·0% (100·0 to 100·0%)	30·5 (20·6 to 42·2)	100·0% (100·0 to 100·0%)	14·7% (-3·8 to 36·2%)	-	5,715·9 (4,250·7 to 7,569·5)	100·0% (100·0 to 100·0%)	6,331·0 (4,687·6 to 8,429·2)	100·0% (100·0 to 100·0%)	10·8% (4·8 to 17·3%)	-
**Vitamin A deficiency: All causes**	55·4 (32·6 to 87·6)	1·2% (0·7 to 1·9%)	29·4 (17·2 to 43·5)	0·6% (0·4 to 1·0%)	-46·9% (-63·9 to -29·0%)	-46·2% (-63·7 to -27·5%)	4,833·0 (2,851·1 to 7,584·7)	1·5% (0·9 to 2·3%)	2,616·7 (1,543·3 to 3,836·0)	0·9% (0·5 to 1·3%)	-45·9% (-62·9 to -28·4%)	-43·2% (-61·1 to -24·5%)
Diarrhoeal diseases	35·7 (20·2 to 53·6)	9·4% (5·4 to 13·7%)	23·5 (13·0 to 35·0)	7·4% (4·0 to 10·8%)	-34·1% (-44·4 to -21·0%)	-21·3% (-30·0 to -10·9%)	3,135·3 (1,779·5 to 4,682·7)	12·1% (6·9 to 17·5%)	2,088·8 (1,158·3 to 3,081·9)	10·1% (5·6 to 14·7%)	-33·4% (-43·3 to -20·6%)	-16·3% (-23·2 to -8·6%)
Measles	19·7 (5·7 to 52·0)	32·0% (21·0 to 41·7%)	5·9 (1·5 to 16·0)	25·6% (15·5 to 35·7%)	-70·1% (-90·7 to -12·0%)	-19·8% (-41·0 to -7·0%)	1,675·0 (481·4 to 4,418·0)	32·3% (21·3 to 42·2%)	500·1 (127·8 to 1,357·6)	25·9% (15·8 to 36·1%)	-70·1% (-90·7 to -12·1%)	-19·8% (-40·9 to -7·0%)
Vitamin A deficiency	-	-	-	-	-	-	22·7 (14·0 to 33·7)	100·0% (100·0 to 100·0%)	27·8 (17·3 to 42·3)	100·0% (100·0 to 100·0%)	22·7% (16·0 to 29·5%)	-
**Zinc deficiency: All causes**	30·0 (1·6 to 67·2)	0·6% (0·0 to 1·4%)	21·2 (1·2 to 48·1)	0·5% (0·0 to 1·0%)	-29·3% (-41·6 to -11·5%)	-28·3% (-41·4 to -10·9%)	2,605·8 (220·7 to 5,755·1)	0·8% (0·1 to 1·8%)	1,862·4 (177·1 to 4,142·7)	0·6% (0·1 to 1·4%)	-28·5% (-40·0 to -11·1%)	-25·1% (-37·2 to -7·7%)
Diarrhoeal diseases	17·4 (0·0 to 41·8)	4·6% (0·0 to 10·6%)	12·1 (0·0 to 28·8)	3·8% (0·0 to 8·8%)	-30·5% (-43·2 to 0·0%)	-17·2% (-29·5 to 0·0%)	1,543·7 (68·1 to 3,604·8)	5·9% (0·3 to 13·4%)	1,091·1 (65·0 to 2,494·7)	5·3% (0·3 to 11·7%)	-29·3% (-41·2 to -4·6%)	-11·3% (-22·5 to 20·3%)
Lower respiratory infections	12·5 (0·0 to 44·8)	2·7% (0·0 to 9·5%)	9·1 (0·0 to 31·8)	2·1% (0·0 to 7·4%)	-27·5% (-40·5 to 0·0%)	-23·4% (-34·8 to 0·0%)	1,062·1 (2·1 to 3,781·9)	3·5% (0·0 to 12·0%)	771·3 (2·1 to 2,691·2)	2·9% (0·0 to 10·4%)	-27·4% (-40·3 to 2·8%)	-15·3% (-26·3 to 24·1%)
**Tobacco smoke: All causes**	143·0 (122·3 to 166·3)	3·0% (2·6 to 3·5%)	176·2 (145·7 to 211·0)	3·8% (3·2 to 4·4%)	23·2% (6·8 to 45·2%)	24·6% (12·4 to 39·2%)	5,936·7 (4,759·9 to 7,275·4)	1·8% (1·5 to 2·3%)	6,732·0 (5,398·9 to 8,174·2)	2·2% (1·8 to 2·6%)	13·4% (0·2 to 31·2%)	18·8% (7·4 to 34·1%)
**Smoking: All causes**	99·3 (85·5 to 113·8)	2·1% (1·8 to 2·4%)	130·7 (105·7 to 161·9)	2·8% (2·4 to 3·3%)	31·6% (9·3 to 59·9%)	33·1% (15·0 to 53·5%)	3,096·9 (2,656·8 to 3,550·5)	1·0% (0·8 to 1·1%)	4,054·7 (3,288·2 to 5,033·0)	1·3% (1·1 to 1·6%)	30·9% (9·1 to 59·4%)	37·1% (15·9 to 61·9%)
Tuberculosis	7·5 (3·2 to 12·9)	4·2% (2·0 to 6·7%)	7·5 (3·2 to 13·3)	4·1% (2·0 to 6·8%)	0·3% (-23·2 to 29·5%)	-1·6% (-14·9 to 13·0%)	259·9 (112·8 to 444·0)	3·5% (1·7 to 5·6%)	264·1 (112·7 to 464·5)	3·6% (1·7 to 5·8%)	1·6% (-22·2 to 31·9%)	1·6% (-14·2 to 18·6%)
Lower respiratory infections	14·9 (11·0 to 19·1)	3·2% (2·5 to 4·1%)	18·0 (12·8 to 24·4)	4·1% (3·1 to 5·5%)	21·1% (0·3 to 45·6%)	27·6% (8·1 to 50·4%)	421·6 (312·7 to 548·8)	1·4% (1·0 to 1·8%)	510·8 (360·8 to 697·4)	2·0% (1·4 to 2·6%)	21·2% (-0·6 to 48·7%)	41·2% (15·1 to 73·3%)
Lip and oral cavity cancer	0·4 (0·3 to 0·5)	14·3% (10·0 to 19·0%)	0·7 (0·5 to 1·1)	18·5% (12·3 to 25·5%)	89·3% (30·9 to 177·3%)	28·9% (-2·1 to 67·7%)	11·6 (7·6 to 15·6)	14·8% (10·2 to 19·4%)	21·8 (13·6 to 33·3)	18·9% (12·7 to 25·5%)	88·4% (32·0 to 176·5%)	27·6% (-3·2 to 65·5%)
Nasopharynx cancer	0·2 (0·1 to 0·2)	17·2% (11·2 to 24·2%)	0·3 (0·1 to 0·4)	21·0% (13·2 to 29·8%)	71·0% (10·2 to 180·0%)	22·1% (-8·5 to 71·8%)	5·3 (3·1 to 8·4)	15·9% (10·1 to 22·4%)	9·1 (4·9 to 15·3)	19·2% (11·9 to 27·2%)	72·4% (10·4 to 186·8%)	21·1% (-10·3 to 71·7%)
Oesophageal cancer	2·1 (1·3 to 3·2)	14·5% (9·0 to 21·0%)	3·8 (2·0 to 6·0)	19·3% (11·4 to 27·9%)	78·7% (11·7 to 181·3%)	33·2% (-3·6 to 80·7%)	60·8 (36·4 to 90·8)	15·5% (9·6 to 22·7%)	108·5 (58·1 to 173·3)	20·8% (12·6 to 29·9%)	78·3% (10·1 to 187·8%)	33·7% (-3·3 to 82·4%)
Stomach cancer	0·9 (0·4 to 1·4)	4·4% (2·3 to 6·9%)	1·4 (0·7 to 2·3)	5·5% (2·9 to 8·7%)	64·4% (18·1 to 140·4%)	25·4% (-4·7 to 69·8%)	26·7 (12·2 to 45·0)	5·1% (2·5 to 8·1%)	43·0 (19·5 to 72·1)	6·3% (3·1 to 9·9%)	60·9% (13·0 to 139·8%)	23·9% (-6·2 to 72·0%)
Colon and rectum cancer	0·2 (0·1 to 0·3)	1·6% (0·9 to 2·5%)	0·4 (0·2 to 0·7)	2·1% (1·1 to 3·3%)	99·3% (29·6 to 212·1%)	32·1% (-5·9 to 88·3%)	6·4 (3·0 to 10·8)	2·0% (1·0 to 3·2%)	12·6 (5·5 to 21·4)	2·6% (1·3 to 4·1%)	96·6% (26·8 to 219·4%)	30·9% (-7·8 to 94·5%)
Liver cancer due to hepatitis B	0·7 (0·3 to 1·3)	7·3% (3·5 to 12·1%)	1·2 (0·5 to 2·2)	9·2% (4·4 to 15·0%)	71·5% (10·2 to 177·5%)	26·6% (-9·8 to 85·5%)	27·3 (12·0 to 48·3)	7·3% (3·3 to 12·3%)	46·3 (19·6 to 85·3)	9·0% (4·3 to 15·0%)	69·4% (6·7 to 188·3%)	24·1% (-13·1 to 89·7%)
Liver cancer due to hepatitis C	0·3 (0·1 to 0·5)	4·7% (2·3 to 7·8%)	0·6 (0·3 to 1·1)	6·4% (3·2 to 11·1%)	86·5% (17·5 to 201·9%)	37·4% (-5·1 to 96·7%)	9·1 (4·3 to 15·8)	5·3% (2·6 to 8·8%)	16·8 (7·4 to 31·9)	7·2% (3·6 to 12·5%)	84·7% (15·9 to 198·8%)	36·3% (-5·2 to 95·7%)
Liver cancer due to alcohol use	0·5 (0·2 to 0·8)	6·7% (3·1 to 11·3%)	0·8 (0·3 to 1·6)	8·9% (4·2 to 15·4%)	78·4% (11·2 to 185·5%)	33·1% (-4·2 to 84·5%)	13·5 (6·2 to 23·6)	7·4% (3·3 to 12·6%)	24·3 (10·1 to 47·0)	9·8% (4·5 to 17·1%)	80·0% (10·5 to 201·1%)	33·3% (-3·7 to 87·0%)
Liver cancer due to other causes	0·3 (0·1 to 0·4)	5·4% (2·7 to 8·6%)	0·4 (0·2 to 0·8)	7·1% (3·5 to 11·6%)	74·8% (17·6 to 167·2%)	32·0% (-5·2 to 84·6%)	8·7 (4·0 to 14·8)	5·7% (2·8 to 9·4%)	15·1 (6·9 to 26·8)	7·4% (3·5 to 12·0%)	72·3% (13·3 to 174·2%)	29·6% (-8·3 to 88·8%)
Pancreatic cancer	0·3 (0·2 to 0·4)	4·8% (3·2 to 6·7%)	0·6 (0·4 to 1·0)	6·5% (4·3 to 9·2%)	102·7% (41·7 to 190·8%)	33·9% (1·8 to 75·5%)	8·7 (5·6 to 12·2)	5·5% (3·6 to 7·5%)	17·7 (10·5 to 27·2)	7·3% (4·6 to 10·3%)	103·2% (39·3 to 195·4%)	33·4% (0·7 to 78·9%)
Tracheal, bronchus, and lung cancer	4·5 (3·4 to 5·7)	41·5% (33·6 to 48·2%)	7·7 (5·5 to 10·3)	46·9% (38·4 to 55·4%)	71·4% (30·0 to 124·2%)	12·8% (-2·3 to 31·2%)	120·3 (87·8 to 151·8)	42·6% (34·5 to 49·1%)	205·1 (142·0 to 278·9)	47·9% (39·1 to 56·3%)	70·6% (28·8 to 122·7%)	12·6% (-2·7 to 30·5%)
Cervical cancer	0·4 (0·1 to 0·8)	1·5% (0·4 to 3·2%)	0·6 (0·1 to 1·4)	2·2% (0·5 to 4·8%)	72·8% (-20·0 to 300·3%)	45·5% (-23·4 to 189·0%)	12·5 (2·4 to 29·0)	1·7% (0·4 to 3·8%)	21·3 (4·1 to 48·9)	2·4% (0·5 to 5·3%)	70·7% (-21·8 to 327·2%)	43·4% (-23·9 to 203·3%)
Kidney cancer	0·1 (0·0 to 0·1)	3·9% (2·2 to 5·9%)	0·2 (0·1 to 0·3)	5·4% (2·9 to 8·5%)	104·7% (35·1 to 220·6%)	37·2% (-4·3 to 100·5%)	2·8 (1·4 to 4·4)	2·9% (1·6 to 4·4%)	5·6 (2·7 to 9·5)	4·0% (2·1 to 6·5%)	103·1% (31·9 to 221·4%)	39·0% (-7·5 to 111·6%)
Bladder cancer	0·3 (0·2 to 0·5)	7·9% (4·8 to 11·4%)	0·6 (0·4 to 1·0)	10·2% (6·2 to 14·7%)	83·3% (31·7 to 158·6%)	28·9% (1·1 to 67·3%)	9·1 (5·4 to 13·3)	8·7% (5·3 to 12·7%)	16·7 (9·4 to 25·7)	11·3% (6·8 to 16·4%)	83·2% (31·2 to 162·4%)	29·3% (0·8 to 68·4%)
Ischaemic heart disease	22·5 (17·5 to 28·1)	11·5% (9·1 to 14·0%)	28·6 (21·9 to 36·8)	11·0% (8·6 to 13·8%)	27·4% (7·1 to 51·5%)	-3·9% (-14·6 to 8·2%)	697·8 (540·2 to 883·1)	14·8% (11·8 to 18·1%)	892·6 (675·2 to 1,158·4)	14·5% (11·3 to 18·0%)	27·9% (6·7 to 53·6%)	-2·3% (-12·7 to 9·0%)
Ischaemic stroke	6·0 (4·5 to 7·7)	7·7% (6·0 to 9·5%)	7·3 (5·4 to 9·4)	7·1% (5·4 to 8·8%)	20·4% (1·1 to 41·6%)	-7·9% (-18·9 to 3·9%)	162·3 (123·3 to 205·8)	10·1% (7·9 to 12·3%)	194·6 (144·7 to 252·6)	9·4% (7·2 to 11·7%)	19·9% (0·5 to 42·3%)	-6·2% (-17·1 to 5·4%)
Hemorrhagic stroke	13·1 (10·4 to 16·0)	10·7% (8·5 to 13·0%)	16·0 (12·2 to 20·6)	10·2% (8·0 to 12·7%)	22·1% (2·2 to 47·0%)	-5·3% (-15·9 to 7·0%)	430·6 (339·1 to 524·9)	11·8% (9·5 to 14·2%)	527·9 (396·5 to 682·6)	11·5% (9·1 to 14·2%)	22·6% (1·2 to 48·4%)	-3·1% (-13·9 to 9·7%)
Hypertensive heart disease	3·5 (2·4 to 5·5)	7·6% (5·1 to 10·3%)	4·1 (2·7 to 6·4)	7·0% (4·4 to 9·7%)	17·4% (-3·6 to 45·0%)	-7·2% (-21·5 to 11·1%)	105·1 (73·4 to 159·3)	9·9% (6·8 to 13·2%)	124·0 (83·5 to 187·7)	9·3% (6·2 to 12·6%)	18·0% (-4·0 to 48·1%)	-5·5% (-19·8 to 12·6%)
Atrial fibrillation and flutter	0·1 (0·1 to 0·1)	5·0% (3·6 to 6·5%)	0·1 (0·1 to 0·1)	4·6% (3·3 to 6·0%)	27·6% (8·7 to 50·3%)	-8·9% (-19·7 to 2·3%)	4·5 (3·1 to 6·3)	9·8% (7·2 to 12·4%)	5·7 (3·8 to 8·0)	9·0% (6·7 to 11·5%)	27·2% (18·1 to 37·5%)	-7·6% (-12·9 to -1·8%)
Aortic aneurysm	0·3 (0·2 to 0·5)	12·5% (9·3 to 15·8%)	0·4 (0·3 to 0·7)	11·6% (8·5 to 15·0%)	38·7% (7·3 to 84·1%)	-7·2% (-17·8 to 5·7%)	9·6 (5·9 to 15·8)	15·3% (11·3 to 19·6%)	13·4 (8·3 to 21·1)	14·3% (10·6 to 18·7%)	39·8% (6·5 to 89·4%)	-6·0% (-16·7 to 6·9%)

Supplemental Results Table 1. All-age deaths, death PAFs, DALYs, DALY PAFs, and percent change for each risk factor and outcome for both sexes combined in 2005 and 2015 for Low SDI.

Data in parenthesis are 95% uncertainty intervals. PAFs=population attributable fractions. DALYs=disability-adjusted life-years.

	2005 Deaths (in thousands)	2005 Deaths PAF	2015 Deaths (in thousands)	2015 Deaths PAF	Percent change deaths 2005-2015	Percent change deaths PAF 2005-2015	2005 DALYs (in thousands)	2005 DALYs PAF	2015 DALYs (in thousands)	2015 DALYs PAF	Percent change DALYs 2005-2015	Percent change DALYs PAF 2005-2015
Peripheral vascular disease	0·0 (0·0 to 0·0)	6·9% (4·4 to 9·4%)	0·0 (0·0 to 0·0)	6·0% (3·9 to 8·6%)	47·6% (14·5 to 97·9%)	-13·3% (-29·1 to 7·3%)	1·1 (0·6 to 2·0)	8·1% (5·9 to 10·2%)	1·5 (0·8 to 2·6)	7·4% (5·3 to 9·5%)	30·7% (19·4 to 46·3%)	-9·1% (-15·8 to -1·6%)
Other cardiovascular and circulatory diseases	4·2 (3·1 to 5·4)	8·2% (6·1 to 10·3%)	5·3 (3·6 to 7·1)	7·9% (5·7 to 10·3%)	25·0% (3·1 to 52·7%)	-3·2% (-16·4 to 13·3%)	151·6 (112·0 to 196·3)	8·0% (5·9 to 10·1%)	191·5 (134·1 to 254·8)	8·0% (5·8 to 10·2%)	26·3% (5·6 to 53·3%)	-0·2% (-12·5 to 14·4%)
Chronic obstructive pulmonary disease	9·4 (6·5 to 13·0)	20·7% (15·2 to 26·9%)	15·7 (10·8 to 22·0)	25·7% (19·2 to 32·7%)	66·4% (20·9 to 126·8%)	24·5% (0·0 to 55·0%)	263·5 (182·1 to 355·3)	19·0% (13·8 to 24·2%)	430·3 (304·0 to 588·0)	23·7% (18·0 to 30·1%)	63·3% (20·7 to 119·5%)	25·3% (0·0 to 56·7%)
Silicosis	0·0 (0·0 to 0·0)	3·5% (1·9 to 5·6%)	0·0 (0·0 to 0·0)	4·7% (2·3 to 7·7%)	97·7% (-0·6 to 311·8%)	32·1% (-16·4 to 120·1%)	0·1 (0·0 to 0·1)	4·1% (2·3 to 6·2%)	0·1 (0·1 to 0·2)	5·1% (2·9 to 7·8%)	87·5% (16·2 to 228·0%)	25·1% (-11·9 to 92·2%)
Asbestosis	0·0 (0·0 to 0·0)	3·1% (1·7 to 5·5%)	0·0 (0·0 to 0·0)	4·1% (2·2 to 6·6%)	130·4% (32·1 to 329·7%)	29·9% (-13·1 to 113·4%)	0·0 (0·0 to 0·1)	2·9% (1·4 to 5·7%)	0·1 (0·0 to 0·2)	3·8% (1·9 to 6·6%)	123·5% (29·2 to 342·2%)	28·7% (-14·7 to 130·0%)
Coal workers pneumoconiosis	0·0 (0·0 to 0·0)	3·9% (2·2 to 6·5%)	0·0 (0·0 to 0·0)	5·1% (2·8 to 8·3%)	79·4% (-9·3 to 272·4%)	29·8% (-17·3 to 111·7%)	0·0 (0·0 to 0·0)	4·7% (2·6 to 7·9%)	0·0 (0·0 to 0·1)	6·0% (3·4 to 9·8%)	76·0% (-10·3 to 260·4%)	27·1% (-17·4 to 100·6%)
Other pneumoconiosis	0·0 (0·0 to 0·0)	3·0% (1·6 to 5·1%)	0·0 (0·0 to 0·1)	3·9% (2·2 to 6·5%)	95·4% (23·1 to 223·8%)	33·0% (-10·6 to 98·1%)	1·0 (0·4 to 1·7)	2·9% (1·5 to 4·7%)	1·9 (0·9 to 3·2)	3·7% (2·1 to 5·9%)	81·7% (26·4 to 184·6%)	26·5% (-6·4 to 81·3%)
Asthma	2·4 (1·7 to 3·3)	6·5% (4·8 to 8·5%)	2·6 (1·7 to 3·6)	6·2% (4·5 to 8·2%)	6·8% (-11·1 to 29·4%)	-5·2% (-17·2 to 9·3%)	90·3 (64·2 to 120·1)	3·8% (2·8 to 4·9%)	103·0 (73·2 to 140·2)	3·5% (2·5 to 4·7%)	14·0% (-2·2 to 32·4%)	-7·5% (-18·5 to 4·7%)
Interstitial lung disease and pulmonary sarcoidosis	0·0 (0·0 to 0·1)	4·2% (2·5 to 7·6%)	0·1 (0·0 to 0·1)	5·4% (3·3 to 8·7%)	119·3% (39·9 to 267·1%)	27·8% (-5·4 to 75·8%)	0·8 (0·3 to 2·0)	4·4% (2·5 to 7·7%)	1·7 (0·7 to 3·8)	5·6% (3·4 to 9·0%)	115·8% (41·3 to 246·0%)	27·6% (-5·9 to 77·1%)
Other chronic respiratory diseases	0·1 (0·1 to 0·2)	2·8% (1·6 to 4·4%)	0·2 (0·1 to 0·4)	4·0% (2·2 to 6·3%)	86·2% (20·5 to 186·8%)	43·6% (-3·6 to 108·0%)	6·8 (3·5 to 11·7)	1·9% (1·0 to 3·2%)	11·0 (5·6 to 18·3)	2·5% (1·4 to 4·0%)	61·8% (20·8 to 129·0%)	31·7% (-3·3 to 90·4%)
Diabetes mellitus	1·3 (0·4 to 2·4)	3·0% (0·8 to 5·5%)	1·8 (0·5 to 3·4)	2·8% (0·7 to 5·1%)	40·5% (15·4 to 72·9%)	-7·0% (-20·1 to 8·2%)	58·4 (15·5 to 109·6)	3·2% (0·9 to 5·8%)	83·2 (21·8 to 155·6)	3·0% (0·8 to 5·5%)	42·4% (24·3 to 62·9%)	-5·5% (-14·4 to 4·9%)
**Second-hand smoke: All causes**	45·8 (30·2 to 63·0)	1·0% (0·6 to 1·3%)	48·1 (33·0 to 64·5)	1·0% (0·7 to 1·4%)	5·1% (-4·5 to 16·5%)	6·5% (-3·1 to 17·1%)	2,905·1 (1,767·8 to 4,185·1)	0·9% (0·6 to 1·3%)	2,761·9 (1,769·0 to 3,886·0)	0·9% (0·6 to 1·3%)	-4·9% (-14·3 to 5·9%)	-0·3% (-9·6 to 10·2%)
Lower respiratory infections	33·2 (19·0 to 49·3)	7·2% (4·2 to 10·6%)	31·1 (18·2 to 45·7)	7·1% (4·3 to 10·5%)	-6·3% (-15·2 to 4·0%)	-1·1% (-7·0 to 5·4%)	2,530·8 (1,438·1 to 3,769·5)	8·3% (4·7 to 12·3%)	2,260·9 (1,321·0 to 3,365·5)	8·7% (5·2 to 12·5%)	-10·7% (-19·9 to -0·3%)	4·2% (-1·0 to 10·7%)
Otitis media	0·1 (0·0 to 0·1)	7·1% (4·8 to 9·5%)	0·1 (0·0 to 0·1)	6·6% (4·3 to 9·1%)	-9·2% (-29·9 to 19·3%)	-7·7% (-23·5 to 8·9%)	28·6 (16·3 to 44·8)	7·2% (4·8 to 9·7%)	33·2 (18·6 to 52·3)	7·0% (4·7 to 9·4%)	16·1% (9·6 to 21·8%)	-2·7% (-5·4 to -0·2%)
Tracheal, bronchus, and lung cancer	0·2 (0·1 to 0·3)	1·6% (0·9 to 2·6%)	0·2 (0·1 to 0·4)	1·5% (0·8 to 2·4%)	39·9% (32·1 to 48·0%)	-7·4% (-18·7 to 3·6%)	4·6 (2·6 to 7·6)	1·7% (0·9 to 2·7%)	6·5 (3·6 to 10·7)	1·5% (0·8 to 2·5%)	40·4% (31·9 to 51·2%)	-6·8% (-18·8 to 4·9%)
Ischaemic heart disease	7·0 (5·2 to 8·9)	3·6% (2·8 to 4·6%)	9·6 (7·2 to 12·2)	3·7% (2·8 to 4·7%)	36·2% (20·0 to 54·1%)	2·8% (-5·0 to 10·2%)	188·8 (141·9 to 244·9)	4·0% (3·1 to 5·1%)	257·3 (191·2 to 330·9)	4·2% (3·2 to 5·4%)	36·2% (18·5 to 56·4%)	4·2% (-4·2 to 12·2%)
Ischaemic stroke	1·4 (1·0 to 1·9)	1·8% (1·3 to 2·3%)	1·9 (1·3 to 2·5)	1·8% (1·3 to 2·4%)	33·3% (16·0 to 53·8%)	2·2% (-5·5 to 9·2%)	32·8 (22·6 to 44·8)	2·0% (1·5 to 2·6%)	43·3 (30·8 to 58·6)	2·1% (1·6 to 2·7%)	32·0% (13·5 to 52·4%)	3·4% (-4·5 to 10·7%)
Hemorrhagic stroke	2·7 (2·0 to 3·6)	2·2% (1·7 to 2·9%)	3·7 (2·8 to 5·0)	2·4% (1·8 to 3·0%)	36·0% (17·1 to 58·4%)	5·6% (-3·1 to 14·0%)	86·7 (62·8 to 114·9)	2·4% (1·8 to 3·1%)	117·8 (86·4 to 156·7)	2·6% (1·9 to 3·3%)	35·9% (16·4 to 59·1%)	7·6% (-1·2 to 16·6%)
**Alcohol and drug use: All causes**	104·3 (89·7 to 118·1)	2·2% (1·9 to 2·5%)	129·3 (104·2 to 159·4)	2·8% (2·3 to 3·2%)	23·9% (5·3 to 47·0%)	25·3% (11·7 to 42·0%)	4,291·7 (3,750·1 to 4,806·7)	1·3% (1·2 to 1·5%)	5,373·7 (4,434·1 to 6,523·4)	1·8% (1·5 to 2·1%)	25·2% (8·2 to 46·5%)	31·1% (15·1 to 50·4%)
**Alcohol use: All causes**	92·1 (78·4 to 105·5)	1·9% (1·7 to 2·2%)	115·3 (91·7 to 143·2)	2·5% (2·0 to 2·9%)	25·1% (5·7 to 49·2%)	26·5% (12·4 to 44·2%)	3,586·4 (3,096·0 to 4,078·6)	1·1% (1·0 to 1·3%)	4,552·1 (3,708·9 to 5,565·8)	1·5% (1·2 to 1·8%)	26·9% (8·0 to 49·5%)	32·9% (15·5 to 53·7%)
Tuberculosis	16·5 (11·8 to 23·1)	9·3% (7·3 to 10·9%)	17·6 (10·8 to 25·8)	9·7% (6·8 to 12·1%)	6·5% (-20·2 to 40·8%)	4·2% (-11·3 to 20·1%)	631·6 (456·2 to 867·6)	8·6% (6·9 to 10·0%)	679·9 (426·2 to 982·6)	9·2% (6·6 to 11·4%)	7·6% (-19·3 to 41·7%)	7·4% (-9·6 to 24·5%)
Lower respiratory infections	6·1 (5·0 to 7·3)	1·3% (1·1 to 1·6%)	7·6 (5·8 to 9·6)	1·7% (1·4 to 2·1%)	24·8% (2·9 to 47·9%)	31·5% (11·4 to 52·4%)	170·4 (139·8 to 203·2)	0·6% (0·5 to 0·7%)	212·7 (159·7 to 268·6)	0·8% (0·6 to 1·0%)	24·8% (1·4 to 50·6%)	45·5% (18·2 to 75·8%)
Lip and oral cavity cancer	0·5 (0·4 to 0·6)	17·8% (15·1 to 20·6%)	0·7 (0·5 to 0·9)	18·2% (14·2 to 21·6%)	49·5% (19·1 to 87·0%)	2·2% (-8·4 to 12·2%)	14·6 (12·1 to 17·3)	18·7% (16·0 to 21·2%)	22·1 (16·4 to 28·8)	19·2% (15·3 to 22·6%)	51·3% (21·0 to 89·0%)	2·8% (-7·8 to 12·9%)
Nasopharynx cancer	0·2 (0·1 to 0·2)	21·1% (17·6 to 24·2%)	0·3 (0·2 to 0·4)	21·7% (17·1 to 25·9%)	43·3% (8·9 to 90·1%)	2·5% (-9·7 to 16·2%)	6·9 (5·0 to 8·4)	20·8% (17·3 to 23·7%)	10·1 (6·6 to 14·1)	21·4% (16·9 to 25·6%)	46·5% (10·2 to 95·2%)	2·9% (-9·5 to 17·0%)
Other pharynx cancer	0·2 (0·1 to 0·2)	17·4% (14·3 to 20·2%)	0·2 (0·2 to 0·3)	17·9% (13·8 to 21·6%)	42·2% (15·3 to 75·5%)	2·9% (-8·6 to 14·2%)	5·1 (4·1 to 6·1)	17·9% (15·0 to 20·9%)	7·3 (5·4 to 9·4)	18·6% (14·5 to 22·4%)	44·4% (17·5 to 77·4%)	3·8% (-7·6 to 15·7%)
Oesophageal cancer	1·7 (1·4 to 2·1)	11·7% (9·9 to 13·4%)	2·3 (1·7 to 3·2)	12·0% (9·2 to 14·6%)	37·6% (6·7 to 80·6%)	3·0% (-9·8 to 16·5%)	47·9 (38·4 to 58·3)	12·3% (10·4 to 14·1%)	66·3 (47·0 to 91·6)	12·8% (9·9 to 15·4%)	38·5% (6·5 to 83·1%)	4·2% (-8·8 to 18·4%)
Colon and rectum cancer	0·4 (0·3 to 0·4)	3·0% (2·7 to 3·3%)	0·5 (0·4 to 0·7)	3·0% (2·6 to 3·4%)	50·5% (25·5 to 83·7%)	0·2% (-6·8 to 7·2%)	9·9 (8·6 to 11·5)	3·1% (2·8 to 3·4%)	15·0 (11·7 to 18·8)	3·1% (2·6 to 3·5%)	50·7% (24·3 to 85·9%)	0·7% (-6·8 to 8·5%)
Liver cancer due to hepatitis B	0·0 (0·0 to 0·1)	0·4% (0·3 to 0·6%)	0·1 (0·0 to 0·1)	0·4% (0·3 to 0·6%)	37·7% (0·7 to 78·2%)	2·4% (-20·2 to 26·2%)	2·4 (1·6 to 3·5)	0·6% (0·4 to 0·9%)	3·3 (2·1 to 5·0)	0·7% (0·4 to 0·9%)	37·9% (1·5 to 77·2%)	1·8% (-19·3 to 23·6%)
Liver cancer due to hepatitis C	0·0 (0·0 to 0·0)	0·0% (0·0 to 0·1%)	0·0 (0·0 to 0·0)	0·1% (0·0 to 0·1%)	40·2% (2·2 to 86·8%)	4·2% (-21·9 to 34·0%)	0·2 (0·1 to 0·3)	0·1% (0·1 to 0·2%)	0·3 (0·1 to 0·4)	0·1% (0·1 to 0·2%)	40·3% (3·4 to 85·2%)	4·5% (-20·8 to 32·2%)
Liver cancer due to alcohol use	6·8 (5·3 to 7·9)	100·0% (100·0 to 100·0%)	9·0 (6·9 to 11·2)	100·0% (100·0 to 100·0%)	32·7% (10·6 to 60·1%)	-	182·6 (144·0 to 213·1)	100·0% (100·0 to 100·0%)	243·8 (187·2 to 308·6)	100·0% (100·0 to 100·0%)	33·5% (10·5 to 64·1%)	-
Liver cancer due to other causes	0·0 (0·0 to 0·0)	0·3% (0·2 to 0·3%)	0·0 (0·0 to 0·0)	0·3% (0·2 to 0·4%)	35·3% (3·8 to 69·8%)	2·7% (-17·8 to 22·2%)	0·7 (0·5 to 1·0)	0·5% (0·3 to 0·6%)	1·0 (0·6 to 1·4)	0·5% (0·3 to 0·7%)	35·4% (4·7 to 68·3%)	2·5% (-16·8 to 20·8%)
Larynx cancer	0·3 (0·2 to 0·3)	12·6% (10·3 to 14·8%)	0·4 (0·2 to 0·5)	12·7% (9·5 to 15·4%)	40·2% (12·9 to 80·0%)	0·9% (-10·6 to 12·1%)	7·3 (5·6 to 9·0)	12·9% (10·6 to 15·1%)	10·2 (7·1 to 14·1)	13·1% (9·9 to 15·8%)	40·3% (13·9 to 78·9%)	1·6% (-9·7 to 12·8%)
Breast cancer	0·6 (0·5 to 0·8)	5·1% (4·3 to 5·8%)	0·9 (0·7 to 1·3)	5·1% (4·1 to 6·0%)	48·3% (10·0 to 104·3%)	-0·3% (-10·6 to 11·1%)	21·7 (17·4 to 28·5)	5·1% (4·4 to 5·9%)	32·7 (23·5 to 45·9)	5·1% (4·1 to 6·2%)	50·4% (12·8 to 106·3%)	0·1% (-9·8 to 10·9%)
Ischaemic heart disease	0·2 (-0·6 to 0·9)	0·1% (-0·3 to 0·5%)	0·0 (-1·2 to 1·1)	0·0% (-0·5 to 0·4%)	-96·3% (-820·8 to 1,507·6%)	-93·2% (-658·8 to 1,081·8%)	-0·9 (-18·4 to 16·1)	0·0% (-0·4 to 0·3%)	-7·0 (-36·1 to 17·3)	-0·1% (-0·6 to 0·3%)	685·0% (-1,696·1 to 2,259·6%)	476·3% (-1,351·7 to 1,668·0%)
Ischaemic stroke	-0·7 (-1·3 to -0·1)	-0·8% (-1·6 to -0·2%)	-0·8 (-1·6 to -0·1)	-0·7% (-1·5 to -0·1%)	17·4% (-49·3 to 101·2%)	-10·1% (-61·2 to 45·4%)	-10·4 (-21·0 to -1·1)	-0·6% (-1·3 to -0·1%)	-11·0 (-24·4 to 1·1)	-0·5% (-1·1 to 0·1%)	5·5% (-106·5 to 99·9%)	-17·5% (-104·9 to 53·3%)
Hemorrhagic stroke	7·6 (6·0 to 9·1)	6·2% (5·1 to 7·2%)	9·6 (7·1 to 12·4)	6·1% (4·7 to 7·3%)	27·0% (4·9 to 53·3%)	-1·5% (-12·6 to 9·5%)	212·7 (173·1 to 253·9)	5·8% (4·9 to 6·7%)	270·7 (202·8 to 343·7)	5·9% (4·6 to 7·0%)	27·2% (5·1 to 52·7%)	0·5% (-10·9 to 11·7%)
Hypertensive heart disease	2·3 (1·3 to 4·1)	5·0% (2·9 to 7·1%)	2·8 (1·2 to 5·2)	4·8% (2·2 to 7·2%)	21·9% (-22·1 to 63·9%)	-3·8% (-36·9 to 23·4%)	64·2 (39·0 to 103·8)	6·0% (3·9 to 8·0%)	79·7 (41·5 to 135·3)	5·9% (3·3 to 8·4%)	24·1% (-12·5 to 60·7%)	-0·8% (-28·4 to 21·5%)
Atrial fibrillation and flutter	0·1 (0·0 to 0·1)	3·5% (2·9 to 4·2%)	0·1 (0·1 to 0·1)	3·5% (2·8 to 4·3%)	41·1% (20·4 to 64·5%)	0·7% (-8·5 to 10·2%)	2·1 (1·5 to 2·8)	4·5% (3·9 to 5·2%)	2·9 (2·1 to 4·0)	4·6% (3·7 to 5·3%)	39·6% (27·1 to 51·7%)	1·4% (-5·8 to 7·5%)
Cirrhosis and other chronic liver diseases due to hepatitis B	2·4 (1·6 to 3·5)	12·4% (8·6 to 16·6%)	3·3 (1·9 to 4·8)	12·5% (7·8 to 17·4%)	34·6% (7·5 to 63·1%)	1·6% (-15·9 to 16·6%)	74·2 (46·5 to 106·8)	11·9% (8·3 to 16·0%)	101·5 (58·4 to 152·4)	12·2% (7·6 to 17·0%)	36·8% (8·7 to 67·3%)	2·4% (-16·3 to 18·3%)
Cirrhosis and other chronic liver diseases due to hepatitis C	1·6 (0·9 to 2·4)	12·2% (8·1 to 16·7%)	2·1 (1·1 to 3·3)	12·4% (7·1 to 18·0%)	33·7% (4·1 to 67·3%)	1·8% (-18·9 to 19·6%)	47·2 (27·4 to 71·3)	12·0% (7·8 to 16·6%)	64·1 (32·1 to 102·6)	12·3% (7·0 to 17·9%)	35·7% (3·9 to 71·2%)	2·6% (-19·3 to 20·6%)
Cirrhosis and other chronic liver diseases due to alcohol use	20·8 (18·0 to 23·7)	100·0% (100·0 to 100·0%)	26·4 (21·8 to 32·5)	100·0% (100·0 to 100·0%)	27·3% (7·5 to 51·8%)	-	678·9 (586·5 to 777·0)	100·0% (100·0 to 100·0%)	874·1 (710·3 to 1,075·6)	100·0% (100·0 to 100·0%)	28·8% (8·2 to 54·8%)	-
Cirrhosis and other chronic liver diseases due to other causes	1·8 (1·3 to 2·3)	11·5% (8·8 to 14·5%)	2·3 (1·5 to 3·1)	12·1% (8·4 to 15·5%)	29·1% (5·3 to 55·3%)	4·7% (-12·4 to 19·9%)	58·6 (43·8 to 74·1)	8·8% (6·8 to 11·0%)	75·8 (51·4 to 100·8)	9·6% (6·6 to 12·2%)	29·3% (4·1 to 54·1%)	8·5% (-8·8 to 24·7%)
Pancreatitis	0·2 (0·2 to 0·3)	8·0% (6·7 to 9·4%)	0·3 (0·2 to 0·4)	8·1% (6·3 to 9·9%)	36·3% (5·2 to 72·8%)	1·6% (-11·0 to 15·7%)	8·9 (6·7 to 11·7)	8·4% (7·0 to 9·8%)	12·3 (8·6 to 17·2)	8·6% (6·7 to 10·4%)	37·1% (5·3 to 75·3%)	2·4% (-10·4 to 16·4%)

Supplemental Results Table 1. All-age deaths, death PAFs, DALYs, DALY PAFs, and percent change for each risk factor and outcome for both sexes combined in 2005 and 2015 for Low SDI.

Data in parenthesis are 95% uncertainty intervals. PAFs=population attributable fractions. DALYs=disability-adjusted life-years.

	2005 Deaths (in thousands)	2005 Deaths PAF	2015 Deaths (in thousands)	2015 Deaths PAF	Percent change deaths 2005-2015	Percent change deaths PAF 2005-2015	2005 DALYs (in thousands)	2005 DALYs PAF	2015 DALYs (in thousands)	2015 DALYs PAF	Percent change DALYs 2005-2015	Percent change DALYs PAF 2005-2015
Epilepsy	1·3 (1·1 to 1·5)	7·6% (6·1 to 9·3%)	1·8 (1·3 to 2·4)	8·1% (6·4 to 10·1%)	39·1% (10·0 to 77·3%)	6·4% (-8·7 to 22·7%)	75·2 (63·2 to 88·5)	5·7% (4·6 to 7·0%)	104·1 (81·6 to 134·5)	6·1% (4·8 to 7·6%)	38·5% (13·2 to 71·1%)	7·5% (-7·5 to 23·1%)
Alcohol use disorders	4·4 (3·6 to 5·5)	100·0% (100·0 to 100·0%)	5·8 (4·4 to 7·8)	100·0% (100·0 to 100·0%)	31·1% (7·4 to 62·5%)	-	388·9 (307·9 to 485·3)	100·0% (100·0 to 100·0%)	529·0 (404·3 to 686·6)	100·0% (100·0 to 100·0%)	36·0% (22·9 to 51·6%)	-
Diabetes mellitus	-1·7 (-2·1 to -1·4)	-4·1% (-4·7 to -3·5%)	-2·5 (-3·2 to -1·9)	-3·9% (-4·8 to -3·0%)	43·4% (5·3 to 93·4%)	-5·0% (-26·3 to 20·0%)	-69·6 (-83·8 to -57·4)	-3·8% (-4·2 to -3·4%)	-100·8 (-124·3 to -78·3)	-3·6% (-4·2 to -3·1%)	44·9% (18·3 to 75·2%)	-3·7% (-18·7 to 12·5%)
Pedestrian road injuries	3·2 (2·4 to 4·1)	10·4% (8·3 to 12·7%)	4·1 (3·1 to 5·3)	10·7% (8·6 to 13·1%)	29·2% (6·3 to 60·3%)	3·0% (-6·7 to 14·4%)	177·3 (134·3 to 225·2)	10·4% (8·2 to 12·8%)	227·5 (169·8 to 289·9)	11·0% (8·8 to 13·4%)	28·3% (5·8 to 59·5%)	5·5% (-5·9 to 19·1%)
Cyclist road injuries	0·3 (0·2 to 0·4)	14·1% (11·3 to 17·2%)	0·4 (0·3 to 0·6)	14·4% (11·1 to 18·2%)	41·4% (3·1 to 96·5%)	2·3% (-14·1 to 23·4%)	17·2 (12·7 to 22·2)	13·7% (10·8 to 16·9%)	24·3 (16·8 to 34·6)	14·2% (10·8 to 18·2%)	41·7% (4·9 to 93·9%)	3·6% (-14·4 to 25·9%)
Motorcyclist road injuries	1·3 (1·0 to 1·8)	20·3% (16·8 to 24·2%)	1·7 (1·3 to 2·3)	19·9% (16·6 to 23·5%)	28·5% (2·1 to 64·9%)	-1·7% (-11·8 to 9·3%)	73·7 (55·2 to 98·1)	18·8% (15·2 to 22·9%)	94·8 (69·9 to 128·4)	18·6% (15·3 to 22·2%)	28·6% (3·4 to 63·5%)	-0·8% (-12·3 to 11·8%)
Motor vehicle road injuries	5·8 (4·5 to 7·0)	16·4% (13·8 to 19·3%)	7·2 (5·6 to 9·4)	16·5% (13·7 to 19·7%)	25·8% (3·0 to 55·9%)	0·9% (-10·2 to 14·2%)	297·3 (236·3 to 363·5)	15·0% (12·5 to 17·7%)	374·7 (290·6 to 477·5)	15·4% (12·7 to 18·4%)	26·1% (4·1 to 54·7%)	2·9% (-9·1 to 17·2%)
Falls	1·4 (1·1 to 1·8)	5·0% (3·9 to 6·4%)	1·8 (1·4 to 2·4)	5·2% (4·0 to 6·7%)	31·3% (8·2 to 62·8%)	4·2% (-10·9 to 24·1%)	62·1 (48·4 to 81·0)	3·8% (2·8 to 4·8%)	85·4 (65·7 to 110·3)	4·1% (3·3 to 5·2%)	37·4% (20·3 to 59·9%)	9·5% (-8·8 to 35·4%)
Drowning	0·9 (0·7 to 1·2)	2·6% (1·9 to 3·5%)	1·1 (0·8 to 1·4)	3·0% (2·3 to 3·9%)	16·9% (-6·4 to 49·2%)	15·8% (-7·3 to 46·7%)	41·0 (31·1 to 55·8)	1·6% (1·2 to 2·3%)	48·2 (36·4 to 65·0)	2·0% (1·5 to 2·7%)	17·7% (-6·2 to 50·4%)	20·2% (-7·4 to 56·9%)
Fire, heat, and hot substances	0·8 (0·6 to 1·0)	3·7% (2·9 to 4·6%)	1·0 (0·7 to 1·4)	4·1% (3·2 to 5·3%)	27·1% (5·2 to 57·3%)	12·2% (-4·5 to 32·2%)	33·7 (26·7 to 42·7)	2·4% (1·9 to 3·1%)	44·3 (33·9 to 58·3)	2·9% (2·3 to 3·6%)	31·3% (12·3 to 57·7%)	18·5% (0·5 to 40·9%)
Poisonings	0·4 (0·3 to 0·5)	3·0% (1·7 to 5·0%)	0·5 (0·3 to 0·7)	3·4% (1·8 to 5·7%)	17·9% (-4·2 to 45·5%)	10·5% (-34·9 to 79·4%)	17·3 (12·8 to 23·6)	1·9% (1·0 to 3·5%)	21·1 (14·8 to 29·5)	2·2% (1·1 to 4·1%)	21·6% (0·9 to 46·3%)	14·9% (-36·9 to 100·7%)
Unintentional firearm injuries	0·2 (0·1 to 0·3)	4·8% (3·8 to 6·2%)	0·3 (0·2 to 0·4)	4·9% (3·9 to 6·1%)	23·0% (-2·9 to 55·6%)	1·5% (-11·3 to 17·1%)	10·5 (6·5 to 15·5)	4·1% (3·1 to 5·3%)	13·1 (7·9 to 18·6)	4·2% (3·3 to 5·3%)	23·8% (-2·1 to 57·8%)	3·5% (-11·3 to 21·3%)
Unintentional suffocation	0·0 (0·0 to 0·0)	1·1% (0·7 to 1·8%)	0·0 (0·0 to 0·1)	1·3% (0·8 to 2·0%)	36·5% (7·3 to 75·9%)	17·8% (-21·5 to 73·1%)	2·3 (1·8 to 3·0)	0·9% (0·6 to 1·5%)	3·2 (2·4 to 4·2)	1·1% (0·7 to 1·7%)	38·2% (19·2 to 62·3%)	19·0% (-17·1 to 69·1%)
Other exposure to mechanical forces	0·6 (0·5 to 0·8)	5·1% (4·1 to 6·5%)	0·8 (0·6 to 1·1)	5·4% (4·3 to 6·8%)	28·7% (1·9 to 68·3%)	6·0% (-9·1 to 25·2%)	38·3 (29·0 to 50·3)	4·1% (3·3 to 5·2%)	50·3 (37·7 to 65·4)	4·4% (3·6 to 5·5%)	31·2% (12·2 to 59·3%)	8·1% (-5·1 to 24·0%)
Self-harm	2·1 (1·6 to 2·8)	8·3% (6·9 to 10·2%)	3·0 (2·3 to 4·1)	8·3% (6·8 to 10·1%)	42·9% (12·3 to 80·7%)	0·0% (-9·8 to 11·9%)	86·5 (67·1 to 112·4)	7·8% (6·5 to 9·6%)	125·3 (94·4 to 168·8)	7·8% (6·5 to 9·5%)	44·8% (12·0 to 84·5%)	0·7% (-9·5 to 13·0%)
Assault by firearm	0·4 (0·3 to 0·5)	5·7% (4·2 to 7·4%)	0·6 (0·4 to 0·8)	6·0% (4·6 to 7·7%)	58·2% (22·1 to 112·6%)	6·6% (-10·0 to 29·8%)	21·2 (15·4 to 30·3)	5·8% (4·4 to 7·6%)	33·4 (23·6 to 47·8)	6·2% (4·8 to 7·9%)	57·8% (21·3 to 113·0%)	6·8% (-9·5 to 29·6%)
Assault by sharp object	0·2 (0·2 to 0·3)	4·1% (2·8 to 5·9%)	0·3 (0·2 to 0·4)	4·6% (3·3 to 6·3%)	42·0% (11·7 to 83·0%)	12·6% (-8·6 to 41·1%)	12·8 (9·3 to 18·2)	4·2% (2·9 to 6·1%)	18·1 (13·0 to 25·7)	4·8% (3·4 to 6·6%)	41·8% (11·0 to 83·0%)	13·0% (-7·7 to 41·0%)
Assault by other means	1·1 (0·8 to 1·4)	7·7% (6·4 to 9·3%)	1·5 (1·0 to 2·2)	8·4% (6·8 to 10·4%)	39·3% (0·7 to 92·5%)	9·1% (-7·0 to 28·7%)	63·8 (50·1 to 84·1)	7·6% (6·3 to 9·2%)	88·5 (61·2 to 129·2)	8·4% (6·8 to 10·4%)	38·8% (1·3 to 90·3%)	10·3% (-6·2 to 30·4%)
**Drug use: All causes**	12·5 (9·9 to 15·6)	0·3% (0·2 to 0·3%)	14·6 (11·2 to 18·7)	0·3% (0·2 to 0·4%)	16·4% (-0·7 to 40·0%)	17·8% (2·9 to 35·1%)	717·8 (586·6 to 867·8)	0·2% (0·2 to 0·3%)	842·4 (681·7 to 1,015·9)	0·3% (0·2 to 0·3%)	17·4% (3·5 to 34·4%)	23·0% (9·3 to 38·1%)
HIV/AIDS - Tuberculosis	1·1 (0·7 to 1·7)	1·0% (0·7 to 1·5%)	0·7 (0·4 to 1·0)	1·1% (0·7 to 1·5%)	-39·9% (-45·9 to -34·0%)	2·4% (-1·2 to 7·3%)	59·5 (37·7 to 91·4)	1·0% (0·7 to 1·4%)	36·5 (22·8 to 55·8)	1·0% (0·7 to 1·5%)	-38·7% (-44·7 to -32·9%)	3·7% (0·5 to 8·4%)
HIV/AIDS resulting in other diseases	4·6 (3·2 to 6·7)	1·2% (0·8 to 1·7%)	2·9 (2·0 to 4·1)	1·2% (0·8 to 1·6%)	-38·0% (-42·2 to -33·4%)	-0·7% (-4·9 to 3·8%)	247·6 (172·7 to 360·8)	1·1% (0·8 to 1·6%)	158·4 (110·3 to 228·8)	1·1% (0·8 to 1·6%)	-36·0% (-40·2 to -31·6%)	-1·0% (-4·9 to 3·0%)
Hepatitis B	0·0 (0·0 to 0·0)	0·3% (0·1 to 0·5%)	0·0 (0·0 to 0·0)	0·3% (0·1 to 0·5%)	44·4% (16·5 to 83·7%)	19·8% (6·7 to 34·9%)	0·6 (0·2 to 1·0)	0·3% (0·1 to 0·4%)	0·8 (0·3 to 1·4)	0·3% (0·1 to 0·5%)	41·9% (15·9 to 78·7%)	16·7% (4·2 to 30·5%)
Hepatitis C	0·1 (0·0 to 0·2)	25·3% (16·0 to 35·3%)	0·1 (0·0 to 0·2)	29·6% (19·4 to 40·6%)	41·2% (3·9 to 95·3%)	17·2% (-0·7 to 40·1%)	3·1 (0·6 to 7·2)	26·2% (16·8 to 36·6%)	4·2 (0·9 to 10·2)	29·8% (19·2 to 41·4%)	37·9% (2·6 to 87·2%)	13·6% (-2·9 to 35·5%)
Liver cancer due to hepatitis B	0·0 (0·0 to 0·1)	0·3% (0·1 to 0·6%)	0·1 (0·0 to 0·1)	0·4% (0·2 to 0·6%)	58·6% (31·7 to 95·7%)	17·8% (6·8 to 30·9%)	1·2 (0·5 to 2·1)	0·3% (0·1 to 0·6%)	1·9 (0·8 to 3·2)	0·4% (0·2 to 0·6%)	55·5% (27·4 to 91·8%)	14·7% (4·4 to 26·4%)
Liver cancer due to hepatitis C	1·1 (0·6 to 1·7)	16·5% (10·0 to 24·5%)	1·9 (1·1 to 2·9)	21·0% (13·0 to 30·0%)	71·1% (38·2 to 115·7%)	26·8% (11·4 to 46·3%)	36·1 (21·2 to 55·0)	21·2% (13·3 to 30·2%)	59·5 (35·0 to 92·7)	25·9% (16·2 to 36·8%)	64·7% (32·6 to 108·6%)	22·4% (7·4 to 39·6%)
Cirrhosis and other chronic liver diseases due to hepatitis B	0·1 (0·0 to 0·1)	0·3% (0·1 to 0·5%)	0·1 (0·0 to 0·2)	0·4% (0·2 to 0·6%)	63·9% (34·3 to 105·1%)	23·3% (10·0 to 40·5%)	2·0 (0·8 to 3·5)	0·3% (0·1 to 0·6%)	3·3 (1·3 to 5·7)	0·4% (0·2 to 0·6%)	61·3% (31·3 to 101·4%)	20·3% (8·1 to 35·4%)
Cirrhosis and other chronic liver diseases due to hepatitis C	3·0 (1·7 to 4·6)	23·1% (14·4 to 33·4%)	4·7 (2·8 to 7·5)	28·1% (17·8 to 40·8%)	60·0% (30·4 to 100·5%)	21·5% (8·5 to 38·1%)	112·9 (66·2 to 170·4)	28·8% (18·2 to 40·7%)	176·0 (104·3 to 275·2)	33·9% (21·4 to 48·4%)	55·9% (27·0 to 95·7%)	17·6% (6·4 to 31·7%)
Opioid use disorders	2·1 (1·3 to 2·8)	100·0% (100·0 to 100·0%)	3·4 (2·3 to 4·7)	100·0% (100·0 to 100·0%)	63·8% (33·9 to 100·5%)	-	215·5 (165·1 to 264·0)	100·0% (100·0 to 100·0%)	339·8 (259·7 to 421·7)	100·0% (100·0 to 100·0%)	57·7% (42·9 to 75·1%)	-
Cocaine use disorders	0·0 (0·0 to 0·0)	100·0% (100·0 to 100·0%)	0·0 (0·0 to 0·1)	100·0% (100·0 to 100·0%)	163·3% (71·5 to 285·5%)	-	2·4 (1·5 to 3·6)	100·0% (100·0 to 100·0%)	4·9 (3·0 to 8·0)	100·0% (100·0 to 100·0%)	109·0% (76·3 to 160·1%)	-
Amphetamine use disorders	0·0 (0·0 to 0·0)	100·0% (100·0 to 100·0%)	0·0 (0·0 to 0·1)	100·0% (100·0 to 100·0%)	181·3% (73·5 to 348·6%)	-	1·0 (0·5 to 2·1)	100·0% (100·0 to 100·0%)	2·4 (1·2 to 4·4)	100·0% (100·0 to 100·0%)	131·4% (77·7 to 231·7%)	-
Cannabis use disorders	-	-	-	-	-	-	17·1 (10·6 to 25·5)	100·0% (100·0 to 100·0%)	24·0 (14·7 to 35·8)	100·0% (100·0 to 100·0%)	39·8% (33·4 to 46·4%)	-
Other drug use disorders	0·1 (0·1 to 0·3)	100·0% (100·0 to 100·0%)	0·2 (0·1 to 0·5)	100·0% (100·0 to 100·0%)	58·3% (18·5 to 115·7%)	-	7·2 (3·9 to 12·4)	100·0% (100·0 to 100·0%)	12·0 (7·1 to 21·3)	100·0% (100·0 to 100·0%)	68·0% (31·6 to 115·4%)	-
Self-harm	0·2 (0·1 to 0·4)	0·9% (0·6 to 1·4%)	0·4 (0·2 to 0·6)	1·1% (0·6 to 1·6%)	60·4% (30·9 to 92·7%)	12·5% (0·8 to 25·6%)	11·6 (6·7 to 17·9)	1·0% (0·6 to 1·6%)	18·7 (10·9 to 28·7)	1·2% (0·7 to 1·8%)	61·2% (30·2 to 95·9%)	12·4% (1·0 to 25·7%)
**Dietary risks: All causes**	281·3 (236·9 to 336·1)	6·0% (5·1 to 7·0%)	372·6 (304·0 to 459·4)	8·0% (6·7 to 9·6%)	32·5% (16·2 to 50·4%)	34·0% (24·6 to 43·6%)	7,767·6 (6,568·4 to 9,124·2)	2·4% (2·1 to 2·8%)	10,299·5 (8,479·0 to 12,630·6)	3·4% (2·8 to 4·0%)	32·6% (16·1 to 50·5%)	38·9% (25·4 to 54·0%)
**Diet low in fruits: All causes**	90·8 (61·8 to 124·3)	1·9% (1·3 to 2·6%)	119·3 (79·0 to 165·5)	2·6% (1·7 to 3·5%)	31·4% (14·1 to 52·0%)	33·0% (22·1 to 43·9%)	2,686·5 (1,852·0 to 3,640·4)	0·8% (0·6 to 1·1%)	3,539·9 (2,389·2 to 4,873·3)	1·2% (0·8 to 1·6%)	31·8% (14·1 to 51·1%)	38·1% (23·0 to 54·1%)
Lip and oral cavity cancer	0·2 (0·0 to 0·4)	7·2% (0·0 to 15·5%)	0·3 (0·0 to 0·6)	7·2% (0·0 to 15·4%)	45·9% (0·0 to 78·1%)	-0·1% (-4·4 to 4·2%)	5·4 (0·0 to 11·9)	7·0% (0·0 to 15·1%)	7·9 (0·0 to 18·1)	6·9% (0·0 to 14·8%)	46·2% (0·0 to 79·3%)	-0·5% (-4·8 to 3·9%)
Nasopharynx cancer	0·1 (0·0 to 0·1)	6·7% (0·0 to 14·3%)	0·1 (0·0 to 0·2)	6·7% (0·0 to 14·1%)	39·0% (0·0 to 78·2%)	-0·3% (-7·6 to 7·0%)	2·1 (0·0 to 4·5)	6·3% (0·0 to 13·2%)	2·9 (0·0 to 6·6)	6·2% (0·0 to 13·1%)	40·9% (0·0 to 81·3%)	-0·7% (-8·7 to 7·2%)
Other pharynx cancer	0·1 (0·0 to 0·2)	7·3% (0·0 to 16·2%)	0·1 (0·0 to 0·2)	7·3% (0·0 to 16·2%)	38·3% (0·0 to 65·4%)	0·4% (-3·6 to 4·7%)	2·0 (0·0 to 4·6)	7·2% (0·0 to 16·0%)	2·8 (0·0 to 6·4)	7·2% (0·0 to 16·0%)	39·0% (0·0 to 66·2%)	0·2% (-3·9 to 4·4%)
Oesophageal cancer	3·2 (0·8 to 5·8)	21·7% (5·4 to 38·2%)	4·2 (1·0 to 7·8)	21·8% (5·5 to 38·5%)	33·7% (8·2 to 67·4%)	0·3% (-5·5 to 5·5%)	84·8 (20·7 to 153·4)	21·7% (5·4 to 38·2%)	112·4 (26·0 to 208·5)	21·7% (5·4 to 38·3%)	32·6% (6·6 to 68·0%)	0·1% (-5·8 to 5·5%)
Larynx cancer	0·1 (0·0 to 0·3)	7·2% (0·0 to 15·9%)	0·2 (0·0 to 0·5)	7·3% (0·0 to 15·9%)	39·4% (0·0 to 69·8%)	0·5% (-3·6 to 4·5%)	4·1 (0·0 to 9·1)	7·2% (0·0 to 15·8%)	5·6 (0·0 to 13·1)	7·2% (0·0 to 15·7%)	38·3% (0·0 to 67·4%)	0·3% (-3·8 to 4·3%)
Tracheal, bronchus, and lung cancer	1·3 (0·5 to 2·3)	12·4% (5·1 to 20·8%)	2·1 (0·8 to 3·5)	12·5% (5·1 to 20·9%)	52·6% (33·5 to 77·3%)	0·7% (-3·1 to 4·3%)	34·7 (14·1 to 58·7)	12·3% (5·1 to 20·6%)	52·8 (21·2 to 89·5)	12·4% (5·1 to 20·7%)	52·0% (31·3 to 78·3%)	0·6% (-3·6 to 4·5%)
Ischaemic heart disease	27·7 (10·4 to 47·3)	14·2% (5·2 to 23·4%)	36·6 (13·6 to 62·1)	14·1% (5·1 to 23·3%)	32·0% (13·4 to 51·5%)	-0·5% (-3·6 to 2·6%)	753·0 (284·2 to 1,266·1)	16·0% (6·0 to 26·2%)	987·4 (369·9 to 1,664·5)	16·0% (5·9 to 26·3%)	31·1% (11·4 to 51·8%)	0·1% (-2·9 to 3·3%)
Ischaemic stroke	17·9 (9·4 to 27·6)	22·7% (12·5 to 33·7%)	22·9 (12·2 to 35·5)	22·3% (12·3 to 33·3%)	27·9% (9·4 to 48·9%)	-2·0% (-5·7 to 1·7%)	452·6 (246·5 to 693·0)	28·0% (15·9 to 40·8%)	574·5 (316·1 to 867·5)	27·8% (15·7 to 40·6%)	26·9% (8·4 to 47·2%)	-0·7% (-4·1 to 2·8%)

Supplemental Results Table 1. All-age deaths, death PAFs, DALYs, DALY PAFs, and percent change for each risk factor and outcome for both sexes combined in 2005 and 2015 for Low SDI.

Data in parenthesis are 95% uncertainty intervals. PAFs=population attributable fractions. DALYs=disability-adjusted life-years.

	2005 Deaths (in thousands)	2005 Deaths PAF	2015 Deaths (in thousands)	2015 Deaths PAF	Percent change deaths 2005-2015	Percent change deaths PAF 2005-2015	2005 DALYs (in thousands)	2005 DALYs PAF	2015 DALYs (in thousands)	2015 DALYs PAF	Percent change DALYs 2005-2015	Percent change DALYs PAF 2005-2015
Hemorrhagic stroke	35·1 (19·4 to 52·4)	28·6% (15·9 to 41·8%)	45·2 (24·5 to 68·9)	28·6% (15·7 to 42·0%)	28·8% (10·4 to 50·6%)	-0·1% (-3·6 to 3·3%)	1,104·2 (629·2 to 1,608·2)	30·3% (17·3 to 43·4%)	1,422·5 (793·6 to 2,120·3)	30·9% (17·3 to 44·2%)	28·8% (11·0 to 49·6%)	1·8% (-1·9 to 5·5%)
**Diet low in vegetables: All causes**	68·3 (36·3 to 104·2)	1·4% (0·8 to 2·2%)	89·1 (48·4 to 137·1)	1·9% (1·0 to 2·9%)	30·3% (13·8 to 47·6%)	31·9% (21·9 to 42·5%)	1,911·9 (1,043·2 to 2,906·2)	0·6% (0·3 to 0·9%)	2,478·7 (1,333·3 to 3,808·0)	0·8% (0·4 to 1·2%)	29·6% (12·3 to 48·7%)	35·9% (21·3 to 52·2%)
Ischaemic heart disease	39·7 (16·2 to 66·3)	20·3% (8·0 to 33·1%)	52·4 (20·8 to 87·5)	20·2% (8·0 to 33·0%)	32·0% (15·0 to 50·1%)	-0·5% (-2·3 to 1·2%)	1,058·5 (432·9 to 1,768·2)	22·5% (9·1 to 36·4%)	1,387·0 (555·3 to 2,322·6)	22·5% (9·1 to 36·4%)	31·0% (12·1 to 50·8%)	0·0% (-1·8 to 2·0%)
Ischaemic stroke	9·6 (2·0 to 18·0)	12·2% (2·8 to 21·7%)	12·2 (2·5 to 22·4)	11·9% (2·7 to 21·1%)	27·4% (8·6 to 47·8%)	-2·5% (-5·8 to 0·8%)	247·0 (52·1 to 450·4)	15·3% (3·6 to 26·6%)	312·6 (67·0 to 558·5)	15·1% (3·6 to 26·2%)	26·6% (8·1 to 46·2%)	-1·0% (-4·2 to 2·2%)
Hemorrhagic stroke	19·1 (5·6 to 34·5)	15·6% (4·5 to 27·6%)	24·5 (7·0 to 44·3)	15·5% (4·5 to 27·7%)	28·3% (10·1 to 48·3%)	-0·4% (-2·9 to 2·3%)	606·4 (183·5 to 1,086·1)	16·6% (4·9 to 29·2%)	779·1 (228·3 to 1,393·8)	16·9% (5·0 to 29·8%)	28·5% (10·4 to 48·3%)	1·6% (-1·5 to 4·8%)
**Diet low in whole grains: All causes**	71·8 (47·6 to 99·6)	1·5% (1·0 to 2·1%)	94·6 (60·7 to 133·9)	2·0% (1·3 to 2·8%)	31·8% (15·0 to 49·6%)	33·4% (23·3 to 43·8%)	2,241·8 (1,517·9 to 3,034·8)	0·7% (0·5 to 0·9%)	2,976·4 (1,987·1 to 4,163·5)	1·0% (0·7 to 1·3%)	32·8% (15·8 to 50·6%)	39·1% (24·2 to 54·3%)
Ischaemic heart disease	31·5 (17·4 to 47·6)	16·1% (8·8 to 24·5%)	41·7 (22·6 to 65·1)	16·0% (8·8 to 24·3%)	32·2% (14·2 to 50·4%)	-0·4% (-3·1 to 2·5%)	881·4 (496·4 to 1,316·5)	18·8% (10·4 to 28·0%)	1,159·2 (629·6 to 1,768·4)	18·8% (10·5 to 28·1%)	31·5% (12·1 to 51·8%)	0·3% (-2·5 to 3·4%)
Ischaemic stroke	11·7 (6·7 to 17·7)	14·8% (9·1 to 21·2%)	14·8 (8·6 to 22·9)	14·4% (8·7 to 20·6%)	27·1% (9·2 to 47·3%)	-2·7% (-6·8 to 1·5%)	317·8 (188·3 to 472·3)	19·6% (12·3 to 27·5%)	402·3 (241·9 to 606·6)	19·5% (12·2 to 27·3%)	26·6% (8·0 to 46·9%)	-1·0% (-5·3 to 3·3%)
Hemorrhagic stroke	22·5 (14·5 to 32·1)	18·4% (11·9 to 26·1%)	28·9 (18·1 to 42·9)	18·3% (11·8 to 26·2%)	28·6% (11·5 to 48·1%)	-0·3% (-3·1 to 2·5%)	738·7 (479·7 to 1,041·3)	20·3% (13·3 to 28·4%)	951·6 (606·2 to 1,395·8)	20·7% (13·5 to 29·0%)	28·8% (11·2 to 48·4%)	1·8% (-1·5 to 5·2%)
Diabetes mellitus	6·1 (3·0 to 9·9)	14·2% (7·2 to 22·9%)	9·2 (4·5 to 15·0)	14·2% (7·2 to 22·8%)	50·7% (29·1 to 76·4%)	-0·2% (-2·0 to 1·8%)	303·9 (154·6 to 493·1)	16·5% (8·5 to 26·0%)	463·3 (233·7 to 754·9)	16·7% (8·6 to 26·4%)	52·4% (38·7 to 68·0%)	1·3% (0·3 to 2·5%)
**Diet low in nuts and seeds: All causes**	49·9 (31·3 to 70·0)	1·1% (0·7 to 1·5%)	67·1 (40·8 to 96·6)	1·4% (0·9 to 2·0%)	34·6% (17·3 to 53·3%)	36·1% (25·4 to 46·9%)	1,415·3 (925·3 to 1,962·9)	0·4% (0·3 to 0·6%)	1,914·0 (1,226·7 to 2,714·7)	0·6% (0·4 to 0·9%)	35·2% (17·9 to 54·0%)	41·7% (26·5 to 57·5%)
Ischaemic heart disease	45·1 (27·4 to 64·2)	23·1% (14·2 to 33·0%)	59·9 (35·5 to 88·3)	23·1% (14·2 to 33·3%)	32·8% (15·7 to 51·3%)	0·1% (-1·3 to 1·5%)	1,186·6 (740·6 to 1,671·9)	25·3% (15·6 to 35·5%)	1,565·1 (941·7 to 2,292·5)	25·4% (15·9 to 36·0%)	31·9% (13·9 to 51·5%)	0·6% (-0·9 to 2·3%)
Diabetes mellitus	4·8 (2·4 to 7·7)	11·1% (5·7 to 17·5%)	7·2 (3·6 to 11·9)	11·1% (5·7 to 17·5%)	51·2% (29·7 to 77·5%)	0·1% (-1·8 to 2·3%)	228·7 (108·2 to 365·6)	12·4% (6·4 to 19·4%)	348·9 (168·0 to 564·4)	12·5% (6·5 to 19·7%)	52·5% (38·3 to 68·5%)	1·3% (0·2 to 2·6%)
**Diet low in milk: All causes**	2·0 (0·7 to 3·4)	0·0% (0·0 to 0·1%)	3·1 (1·0 to 5·2)	0·1% (0·0 to 0·1%)	50·2% (29·7 to 76·4%)	51·9% (38·2 to 67·6%)	53·1 (18·4 to 89·4)	0·0% (0·0 to 0·0%)	79·3 (26·9 to 136·1)	0·0% (0·0 to 0·0%)	49·4% (28·4 to 75·9%)	56·4% (36·9 to 79·5%)
Colon and rectum cancer	2·0 (0·7 to 3·4)	16·9% (6·0 to 28·0%)	3·1 (1·0 to 5·2)	16·9% (6·1 to 28·0%)	50·2% (29·7 to 76·4%)	0·0% (-0·8 to 0·7%)	53·1 (18·4 to 89·4)	16·4% (5·8 to 27·2%)	79·3 (26·9 to 136·1)	16·3% (5·9 to 27·2%)	49·4% (28·4 to 75·9%)	-0·1% (-1·4 to 0·9%)
**Diet high in red meat: All causes**	0·5 (0·1 to 0·9)	0·0% (0·0 to 0·0%)	0·7 (0·2 to 1·3)	0·0% (0·0 to 0·0%)	49·5% (25·1 to 76·5%)	51·4% (31·8 to 71·8%)	22·8 (5·6 to 42·9)	0·0% (0·0 to 0·0%)	34·6 (8·5 to 65·9)	0·0% (0·0 to 0·0%)	52·0% (33·0 to 69·8%)	59·3% (42·4 to 75·3%)
Colon and rectum cancer	0·1 (0·0 to 0·2)	0·8% (0·2 to 1·5%)	0·1 (0·0 to 0·3)	0·8% (0·2 to 1·5%)	47·5% (21·2 to 71·7%)	-1·5% (-11·8 to 9·4%)	2·8 (0·5 to 5·3)	0·9% (0·2 to 1·6%)	4·1 (0·8 to 7·8)	0·8% (0·2 to 1·6%)	48·2% (21·0 to 74·9%)	-0·5% (-11·2 to 11·2%)
Diabetes mellitus	0·4 (0·0 to 0·8)	0·9% (0·1 to 1·7%)	0·6 (0·1 to 1·1)	0·9% (0·1 to 1·8%)	50·1% (23·4 to 77·9%)	-0·2% (-12·0 to 12·4%)	20·0 (2·4 to 40·1)	1·1% (0·1 to 2·1%)	30·5 (3·5 to 61·5)	1·1% (0·1 to 2·2%)	52·6% (33·2 to 69·4%)	1·6% (-6·3 to 9·5%)
**Diet high in processed meat: All causes**	13·6 (4·4 to 23·0)	0·3% (0·1 to 0·5%)	19·0 (6·4 to 32·7)	0·4% (0·1 to 0·7%)	39·4% (20·1 to 61·7%)	41·0% (28·5 to 56·0%)	452·1 (192·6 to 724·2)	0·1% (0·1 to 0·2%)	642·4 (287·6 to 1,017·2)	0·2% (0·1 to 0·3%)	42·1% (24·3 to 61·7%)	48·9% (33·5 to 66·1%)
Colon and rectum cancer	0·4 (0·2 to 0·7)	3·6% (1·9 to 5·5%)	0·7 (0·4 to 1·0)	3·7% (2·0 to 5·6%)	53·2% (31·4 to 81·7%)	2·0% (-0·5 to 4·7%)	11·6 (6·2 to 18·0)	3·6% (1·9 to 5·4%)	17·7 (9·6 to 28·1)	3·7% (2·0 to 5·6%)	52·6% (29·5 to 82·6%)	2·1% (-0·9 to 5·2%)
Ischaemic heart disease	9·5 (0·4 to 18·2)	4·9% (0·2 to 9·3%)	12·7 (0·5 to 24·9)	4·9% (0·2 to 9·4%)	33·9% (10·9 to 53·9%)	0·9% (-1·8 to 4·0%)	257·1 (10·2 to 492·6)	5·5% (0·2 to 10·4%)	342·7 (12·5 to 668·5)	5·6% (0·2 to 10·6%)	33·3% (8·6 to 54·3%)	1·7% (-1·1 to 5·0%)
Diabetes mellitus	3·7 (1·7 to 5·6)	8·5% (4·2 to 12·7%)	5·6 (2·8 to 8·7)	8·6% (4·2 to 12·8%)	51·8% (29·1 to 80·4%)	0·6% (-2·8 to 4·4%)	183·3 (86·1 to 282·3)	9·9% (4·9 to 14·7%)	281·9 (131·9 to 430·0)	10·1% (5·0 to 15·0%)	53·8% (38·6 to 71·2%)	2·2% (-0·1 to 4·7%)
**Diet high in sugar-sweetened beverages: All causes**	1·2 (0·8 to 1·7)	0·0% (0·0 to 0·0%)	1·7 (1·1 to 2·4)	0·0% (0·0 to 0·1%)	44·5% (24·9 to 66·8%)	46·2% (33·9 to 59·4%)	44·9 (30·2 to 64·2)	0·0% (0·0 to 0·0%)	66·1 (44·7 to 92·8)	0·0% (0·0 to 0·0%)	47·2% (29·6 to 67·5%)	54·3% (39·4 to 70·6%)
Oesophageal cancer	0·0 (0·0 to 0·0)	0·2% (0·1 to 0·3%)	0·0 (0·0 to 0·1)	0·2% (0·1 to 0·3%)	42·5% (13·8 to 83·2%)	7·2% (-5·5 to 21·1%)	0·7 (0·2 to 1·3)	0·2% (0·1 to 0·3%)	1·0 (0·3 to 1·8)	0·2% (0·1 to 0·3%)	42·8% (12·5 to 82·9%)	8·1% (-5·3 to 21·7%)
Colon and rectum cancer	0·0 (0·0 to 0·0)	0·1% (0·0 to 0·1%)	0·0 (0·0 to 0·0)	0·1% (0·0 to 0·1%)	65·2% (39·1 to 98·4%)	10·1% (0·2 to 21·5%)	0·2 (0·1 to 0·3)	0·1% (0·0 to 0·1%)	0·3 (0·2 to 0·5)	0·1% (0·0 to 0·1%)	66·0% (39·2 to 99·5%)	11·0% (1·2 to 22·3%)
Liver cancer due to hepatitis B	0·0 (0·0 to 0·0)	0·1% (0·1 to 0·2%)	0·0 (0·0 to 0·0)	0·2% (0·1 to 0·3%)	45·7% (21·5 to 76·2%)	8·2% (1·9 to 15·6%)	0·6 (0·3 to 1·0)	0·2% (0·1 to 0·3%)	0·9 (0·4 to 1·5)	0·2% (0·1 to 0·3%)	46·7% (21·4 to 78·3%)	8·1% (1·9 to 15·4%)
Liver cancer due to hepatitis C	0·0 (0·0 to 0·0)	0·1% (0·0 to 0·2%)	0·0 (0·0 to 0·0)	0·1% (0·1 to 0·2%)	44·0% (19·3 to 74·6%)	6·9% (-1·3 to 16·3%)	0·2 (0·1 to 0·4)	0·1% (0·1 to 0·2%)	0·3 (0·1 to 0·5)	0·1% (0·1 to 0·2%)	44·6% (19·5 to 77·5%)	7·6% (-0·2 to 17·2%)
Liver cancer due to alcohol use	0·0 (0·0 to 0·0)	0·1% (0·1 to 0·2%)	0·0 (0·0 to 0·0)	0·1% (0·1 to 0·2%)	43·0% (17·2 to 77·1%)	7·8% (2·0 to 14·6%)	0·3 (0·1 to 0·5)	0·1% (0·1 to 0·2%)	0·4 (0·2 to 0·7)	0·2% (0·1 to 0·3%)	44·6% (18·3 to 80·7%)	8·4% (2·5 to 15·0%)
Liver cancer due to other causes	0·0 (0·0 to 0·0)	0·1% (0·1 to 0·2%)	0·0 (0·0 to 0·0)	0·1% (0·1 to 0·2%)	42·6% (21·0 to 69·8%)	8·1% (0·7 to 17·7%)	0·2 (0·1 to 0·3)	0·1% (0·1 to 0·2%)	0·3 (0·1 to 0·5)	0·1% (0·1 to 0·2%)	43·8% (21·3 to 73·0%)	8·7% (1·6 to 18·0%)
Gallbladder and biliary tract cancer	0·0 (0·0 to 0·0)	0·1% (0·1 to 0·2%)	0·0 (0·0 to 0·0)	0·1% (0·1 to 0·2%)	36·3% (14·0 to 62·5%)	7·2% (1·0 to 13·7%)	0·1 (0·0 to 0·2)	0·1% (0·1 to 0·2%)	0·1 (0·1 to 0·2)	0·1% (0·1 to 0·2%)	36·7% (13·5 to 64·7%)	7·9% (1·5 to 14·6%)
Pancreatic cancer	0·0 (0·0 to 0·0)	0·0% (0·0 to 0·1%)	0·0 (0·0 to 0·0)	0·0% (0·0 to 0·1%)	61·8% (39·0 to 88·8%)	7·3% (-0·1 to 14·9%)	0·1 (0·0 to 0·1)	0·0% (0·0 to 0·1%)	0·1 (0·0 to 0·2)	0·0% (0·0 to 0·1%)	63·4% (39·9 to 91·6%)	7·8% (-0·3 to 15·4%)
Breast cancer	0·0 (0·0 to 0·0)	0·0% (0·0 to 0·0%)	0·0 (0·0 to 0·0)	0·0% (0·0 to 0·0%)	57·2% (23·1 to 97·4%)	5·8% (-4·2 to 15·9%)	0·1 (0·1 to 0·1)	0·0% (0·0 to 0·0%)	0·1 (0·1 to 0·2)	0·0% (0·0 to 0·0%)	58·6% (25·0 to 97·0%)	5·5% (-5·3 to 16·4%)
Uterine cancer	0·0 (0·0 to 0·0)	0·2% (0·2 to 0·3%)	0·0 (0·0 to 0·0)	0·2% (0·2 to 0·3%)	32·7% (5·1 to 70·8%)	8·1% (2·4 to 15·2%)	0·3 (0·2 to 0·4)	0·2% (0·2 to 0·4%)	0·4 (0·2 to 0·6)	0·3% (0·2 to 0·4%)	32·7% (5·1 to 71·6%)	9·0% (3·1 to 16·2%)
Ovarian cancer	0·0 (0·0 to 0·0)	0·0% (0·0 to 0·0%)	0·0 (0·0 to 0·0)	0·0% (0·0 to 0·0%)	62·6% (0·0 to 111·9%)	8·1% (0·0 to 15·0%)	0·0 (0·0 to 0·1)	0·0% (0·0 to 0·0%)	0·0 (0·0 to 0·1)	0·0% (0·0 to 0·0%)	64·7% (0·0 to 119·0%)	8·6% (0·0 to 15·5%)
Kidney cancer	0·0 (0·0 to 0·0)	0·1% (0·1 to 0·1%)	0·0 (0·0 to 0·0)	0·1% (0·1 to 0·2%)	62·8% (39·4 to 89·6%)	9·2% (-0·2 to 20·3%)	0·1 (0·0 to 0·1)	0·1% (0·0 to 0·1%)	0·1 (0·1 to 0·2)	0·1% (0·1 to 0·1%)	64·0% (39·2 to 92·0%)	11·9% (-3·5 to 32·0%)
Thyroid cancer	0·0 (0·0 to 0·0)	0·1% (0·0 to 0·1%)	0·0 (0·0 to 0·0)	0·1% (0·0 to 0·1%)	45·0% (23·8 to 71·4%)	9·0% (2·3 to 17·7%)	0·0 (0·0 to 0·0)	0·1% (0·0 to 0·1%)	0·0 (0·0 to 0·0)	0·1% (0·0 to 0·1%)	50·1% (29·2 to 75·6%)	8·9% (2·4 to 17·5%)
Ischaemic heart disease	0·3 (0·2 to 0·4)	0·2% (0·1 to 0·2%)	0·4 (0·3 to 0·6)	0·2% (0·1 to 0·2%)	45·6% (24·8 to 69·7%)	9·8% (3·6 to 16·8%)	9·6 (6·5 to 13·9)	0·2% (0·1 to 0·3%)	14·1 (9·4 to 20·1)	0·2% (0·2 to 0·3%)	46·3% (24·2 to 71·2%)	11·8% (6·2 to 18·1%)
Ischaemic stroke	0·1 (0·1 to 0·1)	0·1% (0·1 to 0·2%)	0·1 (0·1 to 0·2)	0·1% (0·1 to 0·2%)	37·9% (18·0 to 61·5%)	5·6% (-1·0 to 12·8%)	2·8 (1·9 to 4·1)	0·2% (0·1 to 0·2%)	3·9 (2·6 to 5·6)	0·2% (0·1 to 0·3%)	38·7% (18·8 to 61·8%)	8·5% (2·4 to 15·1%)
Hemorrhagic stroke	0·3 (0·2 to 0·5)	0·3% (0·2 to 0·4%)	0·5 (0·3 to 0·7)	0·3% (0·2 to 0·4%)	39·4% (19·9 to 62·3%)	8·2% (2·2 to 15·0%)	12·4 (8·3 to 18·0)	0·3% (0·2 to 0·5%)	17·4 (11·5 to 25·5)	0·4% (0·3 to 0·5%)	40·4% (20·9 to 63·5%)	11·1% (5·2 to 17·6%)
Hypertensive heart disease	0·1 (0·1 to 0·2)	0·3% (0·2 to 0·5%)	0·2 (0·1 to 0·3)	0·3% (0·2 to 0·5%)	34·4% (13·8 to 60·9%)	6·1% (0·2 to 13·0%)	4·2 (2·6 to 6·6)	0·4% (0·3 to 0·6%)	5·7 (3·5 to 8·9)	0·4% (0·3 to 0·6%)	34·9% (13·2 to 62·1%)	7·9% (2·5 to 14·4%)
Cardiomyopathy and myocarditis	-	-	-	-	-	-	-	-	-	-	-	-
Atrial fibrillation and flutter	-	-	-	-	-	-	-	-	-	-	-	-
Peripheral vascular disease	-	-	-	-	-	-	-	-	-	-	-	-

Supplemental Results Table 1. All-age deaths, death PAFs, DALYs, DALY PAFs, and percent change for each risk factor and outcome for both sexes combined in 2005 and 2015 for Low SDI.

Data in parenthesis are 95% uncertainty intervals. PAFs=population attributable fractions. DALYs=disability-adjusted life-years.

	2005 Deaths (in thousands)	2005 Deaths PAF	2015 Deaths (in thousands)	2015 Deaths PAF	Percent change deaths 2005-2015	Percent change deaths PAF 2005-2015	2005 DALYs (in thousands)	2005 DALYs PAF	2015 DALYs (in thousands)	2015 DALYs PAF	Percent change DALYs 2005-2015	Percent change DALYs PAF 2005-2015
Endocarditis	-	-	-	-	-	-	-	-	-	-	-	-
Other cardiovascular and circulatory diseases	-	-	-	-	-	-	-	-	-	-	-	-
Diabetes mellitus	0·2 (0·1 to 0·2)	0·4% (0·3 to 0·5%)	0·3 (0·2 to 0·4)	0·4% (0·3 to 0·6%)	60·6% (36·1 to 89·3%)	6·4% (0·8 to 12·5%)	9·6 (6·3 to 14·2)	0·5% (0·4 to 0·7%)	15·7 (10·4 to 23·2)	0·6% (0·4 to 0·8%)	63·4% (47·5 to 81·1%)	8·6% (4·5 to 13·0%)
Chronic kidney disease due to diabetes mellitus	0·0 (0·0 to 0·0)	0·2% (0·1 to 0·3%)	0·0 (0·0 to 0·0)	0·2% (0·1 to 0·3%)	57·6% (34·7 to 86·1%)	13·8% (6·5 to 23·6%)	0·4 (0·1 to 0·8)	0·1% (0·1 to 0·3%)	0·6 (0·2 to 1·2)	0·2% (0·1 to 0·3%)	52·6% (36·6 to 72·1%)	15·6% (8·5 to 24·4%)
Chronic kidney disease due to hypertension	0·0 (0·0 to 0·1)	0·2% (0·1 to 0·4%)	0·0 (0·0 to 0·1)	0·2% (0·1 to 0·4%)	59·7% (37·7 to 84·5%)	11·7% (5·6 to 18·6%)	1·0 (0·4 to 1·8)	0·2% (0·1 to 0·3%)	1·5 (0·6 to 2·8)	0·2% (0·1 to 0·4%)	59·0% (37·6 to 82·9%)	14·4% (7·6 to 21·9%)
Chronic kidney disease due to glomerulonephritis	0·0 (0·0 to 0·0)	0·1% (0·0 to 0·2%)	0·0 (0·0 to 0·0)	0·1% (0·1 to 0·3%)	57·1% (33·0 to 83·8%)	15·4% (6·4 to 25·9%)	0·3 (0·1 to 0·6)	0·1% (0·0 to 0·2%)	0·5 (0·1 to 0·9)	0·1% (0·0 to 0·3%)	51·9% (37·7 to 68·5%)	15·3% (7·2 to 24·0%)
Chronic kidney disease due to other causes	0·0 (0·0 to 0·0)	0·1% (0·0 to 0·2%)	0·0 (0·0 to 0·0)	0·1% (0·1 to 0·2%)	54·1% (25·2 to 91·7%)	19·4% (6·8 to 34·6%)	0·2 (0·1 to 0·4)	0·1% (0·0 to 0·2%)	0·3 (0·1 to 0·6)	0·1% (0·0 to 0·3%)	48·0% (37·5 to 61·2%)	15·3% (7·6 to 23·2%)
**Diet low in fibre: All causes**	3·6 (1·3 to 7·1)	0·1% (0·0 to 0·1%)	4·7 (1·7 to 9·5)	0·1% (0·0 to 0·2%)	32·8% (10·1 to 58·2%)	34·5% (12·1 to 59·2%)	114·0 (41·2 to 224·4)	0·0% (0·0 to 0·1%)	150·2 (54·5 to 295·9)	0·0% (0·0 to 0·1%)	31·8% (6·5 to 59·3%)	38·2% (11·7 to 65·3%)
Colon and rectum cancer	0·2 (0·1 to 0·3)	1·3% (0·4 to 2·8%)	0·2 (0·1 to 0·5)	1·4% (0·4 to 2·9%)	52·3% (28·8 to 78·7%)	1·7% (-15·5 to 18·6%)	4·6 (1·4 to 9·6)	1·4% (0·4 to 3·0%)	7·0 (2·1 to 15·2)	1·4% (0·4 to 3·1%)	51·5% (26·7 to 80·1%)	1·7% (-15·7 to 19·0%)
Ischaemic heart disease	3·4 (1·2 to 6·8)	1·7% (0·6 to 3·5%)	4·5 (1·6 to 9·1)	1·7% (0·6 to 3·5%)	31·9% (8·8 to 57·0%)	-0·4% (-16·0 to 15·3%)	109·3 (39·2 to 216·0)	2·3% (0·8 to 4·6%)	143·2 (52·1 to 286·6)	2·3% (0·8 to 4·6%)	31·0% (5·4 to 58·2%)	0·2% (-15·9 to 17·0%)
**Diet low in calcium: All causes**	2·8 (1·8 to 3·9)	0·1% (0·0 to 0·1%)	4·2 (2·7 to 6·1)	0·1% (0·1 to 0·1%)	50·5% (30·7 to 77·0%)	52·2% (38·5 to 68·3%)	73·4 (47·0 to 102·0)	0·0% (0·0 to 0·0%)	109·7 (69·4 to 157·4)	0·0% (0·0 to 0·1%)	49·4% (28·7 to 77·0%)	56·5% (37·2 to 79·7%)
Colon and rectum cancer	2·8 (1·8 to 3·9)	23·4% (15·1 to 32·2%)	4·2 (2·7 to 6·1)	23·4% (15·1 to 32·3%)	50·5% (30·7 to 77·0%)	0·3% (-1·2 to 1·6%)	73·4 (47·0 to 102·0)	22·6% (14·5 to 31·2%)	109·7 (69·4 to 157·4)	22·6% (14·6 to 31·1%)	49·4% (28·7 to 77·0%)	0·0% (-1·7 to 1·6%)
Prostate cancer	-	-	-	-	-	-	-	-	-	-	-	-
**Diet low in seafood omega-3 fatty acids: All causes**	41·0 (18·0 to 65·0)	0·9% (0·4 to 1·4%)	54·0 (23·2 to 87·4)	1·2% (0·5 to 1·8%)	31·7% (14·8 to 49·9%)	33·3% (22·0 to 44·4%)	1,059·5 (469·6 to 1,665·5)	0·3% (0·1 to 0·5%)	1,386·6 (599·3 to 2,232·4)	0·5% (0·2 to 0·7%)	30·9% (12·0 to 51·0%)	37·1% (20·5 to 54·2%)
Ischaemic heart disease	41·0 (18·0 to 65·0)	20·9% (9·1 to 33·0%)	54·0 (23·2 to 87·4)	20·8% (9·1 to 32·8%)	31·7% (14·8 to 49·9%)	-0·7% (-2·1 to 0·7%)	1,059·5 (469·6 to 1,665·5)	22·5% (9·9 to 35·2%)	1,386·6 (599·3 to 2,232·4)	22·5% (10·0 to 35·3%)	30·9% (12·0 to 51·0%)	-0·1% (-1·7 to 1·5%)
**Diet low in polyunsaturated fatty acids: All causes**	9·9 (4·2 to 15·8)	0·2% (0·1 to 0·3%)	12·4 (5·4 to 20·5)	0·3% (0·1 to 0·4%)	25·8% (0·8 to 55·1%)	27·4% (2·7 to 55·9%)	293·4 (124·3 to 468·0)	0·1% (0·0 to 0·1%)	369·0 (158·9 to 605·2)	0·1% (0·1 to 0·2%)	25·8% (-1·2 to 57·4%)	31·8% (4·0 to 66·5%)
Ischaemic heart disease	9·9 (4·2 to 15·8)	5·1% (2·2 to 8·2%)	12·4 (5·4 to 20·5)	4·8% (2·0 to 7·8%)	25·8% (0·8 to 55·1%)	-5·0% (-23·5 to 15·1%)	293·4 (124·3 to 468·0)	6·2% (2·7 to 10·1%)	369·0 (158·9 to 605·2)	6·0% (2·5 to 9·9%)	25·8% (-1·2 to 57·4%)	-3·8% (-22·5 to 17·4%)
**Diet high in trans fatty acids: All causes**	10·2 (3·5 to 20·3)	0·2% (0·1 to 0·4%)	13·3 (4·5 to 26·8)	0·3% (0·1 to 0·6%)	30·9% (10·4 to 52·4%)	32·3% (17·3 to 46·9%)	310·6 (111·2 to 607·0)	0·1% (0·0 to 0·2%)	406·3 (145·5 to 799·7)	0·1% (0·0 to 0·3%)	30·8% (9·8 to 53·8%)	37·0% (17·9 to 57·3%)
Ischaemic heart disease	10·2 (3·5 to 20·3)	5·2% (1·8 to 10·2%)	13·3 (4·5 to 26·8)	5·1% (1·7 to 10·0%)	30·9% (10·4 to 52·4%)	-1·4% (-7·1 to 3·7%)	310·6 (111·2 to 607·0)	6·6% (2·4 to 12·6%)	406·3 (145·5 to 799·7)	6·6% (2·4 to 12·5%)	30·8% (9·8 to 53·8%)	-0·2% (-5·4 to 4·9%)
**Diet high in sodium: All causes**	53·7 (13·3 to 127·8)	1·1% (0·3 to 2·7%)	70·3 (17·5 to 169·9)	1·5% (0·4 to 3·6%)	30·9% (12·2 to 50·5%)	32·3% (18·9 to 46·0%)	1,418·0 (364·4 to 3,276·5)	0·4% (0·1 to 1·0%)	1,844·2 (468·5 to 4,369·8)	0·6% (0·2 to 1·4%)	30·1% (10·7 to 50·8%)	36·2% (19·5 to 55·0%)
Stomach cancer	3·3 (0·9 to 7·2)	16·9% (4·9 to 36·4%)	4·2 (1·2 to 9·1)	16·3% (4·7 to 35·9%)	26·7% (6·2 to 46·1%)	-3·4% (-13·6 to 3·8%)	89·4 (26·1 to 191·6)	17·1% (5·2 to 36·0%)	112·1 (33·0 to 241·6)	16·5% (4·9 to 35·3%)	25·5% (3·4 to 46·4%)	-3·5% (-14·7 to 4·5%)
Rheumatic heart disease	0·4 (0·1 to 1·0)	3·3% (0·6 to 8·8%)	0·4 (0·1 to 1·2)	3·4% (0·6 to 9·0%)	17·8% (-2·2 to 40·9%)	1·5% (-8·7 to 11·4%)	15·0 (2·6 to 40·8)	2·8% (0·5 to 7·4%)	18·1 (3·0 to 49·6)	2·8% (0·5 to 7·7%)	20·5% (2·3 to 39·1%)	2·4% (-7·4 to 11·3%)
Ischaemic heart disease	19·3 (5·1 to 46·2)	9·8% (2·6 to 23·4%)	25·6 (6·8 to 61·4)	9·8% (2·7 to 23·4%)	32·8% (13·0 to 53·8%)	0·0% (-7·5 to 8·1%)	484·1 (130·9 to 1,122·9)	10·3% (2·8 to 23·5%)	637·1 (172·6 to 1,501·9)	10·3% (2·9 to 23·6%)	31·6% (11·5 to 54·6%)	0·4% (-7·2 to 8·6%)
Ischaemic stroke	6·5 (1·4 to 17·0)	8·3% (1·8 to 20·8%)	8·4 (1·8 to 21·7)	8·1% (1·8 to 20·5%)	29·3% (9·7 to 49·9%)	-1·2% (-9·5 to 7·0%)	146·2 (34·0 to 369·1)	9·0% (2·1 to 22·1%)	186·5 (42·5 to 468·3)	9·0% (2·1 to 21·9%)	27·6% (8·1 to 49·2%)	-0·4% (-8·3 to 7·6%)
Hemorrhagic stroke	12·1 (2·8 to 29·7)	9·9% (2·3 to 25·0%)	15·7 (3·5 to 38·6)	9·9% (2·3 to 25·0%)	29·6% (10·3 to 53·0%)	0·4% (-8·0 to 8·8%)	349·3 (79·8 to 851·5)	9·6% (2·2 to 23·8%)	450·6 (102·8 to 1,134·7)	9·8% (2·3 to 23·9%)	29·0% (8·9 to 52·5%)	1·9% (-6·7 to 10·4%)
Hypertensive heart disease	7·0 (1·1 to 21·5)	15·2% (2·6 to 45·6%)	8·9 (1·4 to 27·9)	15·2% (2·5 to 46·2%)	27·3% (5·3 to 53·3%)	0·0% (-9·9 to 11·0%)	171·1 (32·1 to 475·9)	16·1% (3·1 to 44·2%)	216·0 (39·8 to 619·5)	16·1% (3·2 to 43·7%)	26·2% (3·7 to 53·3%)	0·5% (-8·8 to 11·3%)
Cardiomyopathy and myocarditis	0·4 (0·1 to 0·9)	4·8% (1·1 to 12·0%)	0·5 (0·1 to 1·3)	5·0% (1·1 to 12·5%)	39·7% (15·3 to 72·9%)	4·8% (-4·5 to 16·4%)	11·7 (2·6 to 29·5)	3·7% (0·8 to 9·4%)	16·4 (3·5 to 43·9)	4·0% (0·9 to 10·2%)	40·1% (15·1 to 73·6%)	9·8% (-2·7 to 25·1%)
Atrial fibrillation and flutter	0·1 (0·0 to 0·2)	4·9% (1·0 to 13·1%)	0·1 (0·0 to 0·3)	4·8% (1·0 to 13·0%)	37·2% (20·3 to 55·2%)	-2·2% (-9·4 to 3·9%)	2·9 (0·6 to 7·6)	6·3% (1·3 to 16·6%)	4·0 (0·8 to 10·5)	6·3% (1·3 to 16·4%)	36·6% (28·4 to 45·5%)	-0·8% (-4·2 to 2·5%)
Aortic aneurysm	0·1 (0·0 to 0·4)	5·9% (1·2 to 15·2%)	0·2 (0·0 to 0·6)	5·9% (1·2 to 15·1%)	49·7% (19·2 to 95·5%)	0·0% (-8·1 to 9·6%)	3·8 (0·7 to 10·1)	6·1% (1·3 to 15·2%)	5·7 (1·1 to 14·9)	6·1% (1·3 to 15·2%)	49·8% (16·9 to 98·3%)	0·5% (-7·7 to 11·2%)
Peripheral vascular disease	0·0 (0·0 to 0·0)	3·4% (0·5 to 9·7%)	0·0 (0·0 to 0·0)	3·3% (0·5 to 9·8%)	67·8% (33·2 to 123·1%)	-0·9% (-10·9 to 10·8%)	0·6 (0·1 to 1·9)	3·9% (0·7 to 11·0%)	0·8 (0·1 to 2·7)	3·9% (0·7 to 10·9%)	43·9% (37·0 to 54·6%)	0·1% (-2·2 to 3·5%)
Endocarditis	0·2 (0·0 to 0·5)	4·0% (0·8 to 10·9%)	0·3 (0·0 to 0·8)	4·2% (0·8 to 11·4%)	44·1% (20·7 to 76·9%)	4·8% (-5·5 to 17·9%)	5·9 (1·1 to 16·2)	2·9% (0·5 to 8·4%)	8·6 (1·5 to 25·2)	3·2% (0·6 to 9·2%)	45·7% (19·5 to 81·9%)	9·1% (-5·4 to 27·7%)
Other cardiovascular and circulatory diseases	3·0 (0·6 to 7·7)	5·7% (1·1 to 14·9%)	3·9 (0·8 to 10·2)	5·8% (1·2 to 15·0%)	32·3% (9·9 to 60·9%)	2·2% (-6·7 to 13·4%)	93·6 (18·6 to 240·8)	4·9% (1·0 to 12·7%)	124·6 (24·7 to 330·6)	5·2% (1·0 to 13·2%)	33·1% (13·5 to 56·9%)	5·1% (-4·1 to 15·9%)
Chronic kidney disease due to diabetes mellitus	0·3 (0·1 to 0·8)	5·8% (1·4 to 13·7%)	0·5 (0·1 to 1·1)	6·2% (1·5 to 14·5%)	47·7% (24·0 to 75·2%)	6·4% (-2·8 to 17·3%)	10·4 (2·3 to 26·6)	3·8% (0·9 to 9·3%)	14·8 (3·5 to 37·4)	4·1% (1·0 to 10·1%)	42·8% (23·5 to 65·7%)	8·0% (-0·4 to 19·4%)
Chronic kidney disease due to hypertension	0·9 (0·2 to 2·1)	6·1% (1·4 to 15·1%)	1·3 (0·3 to 3·1)	6·3% (1·5 to 15·3%)	47·1% (25·4 to 71·6%)	2·8% (-4·7 to 13·3%)	22·9 (5·2 to 57·9)	4·5% (1·0 to 11·3%)	33·1 (7·6 to 83·6)	4·7% (1·1 to 11·6%)	44·9% (23·2 to 69·9%)	4·2% (-4·2 to 14·7%)
Chronic kidney disease due to glomerulonephritis	0·1 (0·0 to 0·4)	3·8% (0·9 to 9·3%)	0·2 (0·0 to 0·5)	4·1% (1·0 to 10·2%)	48·0% (24·5 to 74·0%)	8·5% (-1·8 to 20·3%)	6·8 (1·4 to 18·1)	2·6% (0·5 to 6·9%)	9·7 (1·9 to 26·4)	2·8% (0·6 to 7·3%)	42·5% (26·1 to 61·4%)	8·0% (-0·3 to 17·8%)
Chronic kidney disease due to other causes	0·1 (0·0 to 0·2)	3·7% (0·9 to 9·3%)	0·1 (0·0 to 0·2)	4·2% (1·0 to 10·2%)	45·4% (19·5 to 78·4%)	12·6% (0·4 to 29·2%)	4·4 (0·9 to 12·1)	3·0% (0·6 to 7·8%)	6·1 (1·3 to 16·7)	3·2% (0·7 to 8·4%)	38·8% (27·5 to 53·4%)	8·1% (1·8 to 17·9%)
**Sexual abuse and violence: All causes**	44·3 (27·3 to 61·1)	0·9% (0·6 to 1·3%)	31·1 (21·1 to 41·8)	0·7% (0·5 to 0·9%)	-29·7% (-38·7 to -15·6%)	-28·8% (-37·9 to -16·2%)	2,686·1 (1,857·0 to 3,555·4)	0·8% (0·6 to 1·1%)	2,259·4 (1,654·3 to 2,906·5)	0·7% (0·6 to 0·9%)	-15·9% (-25·9 to -0·4%)	-11·9% (-21·9 to 2·1%)
**Childhood sexual abuse: All causes**	4·9 (1·5 to 9·0)	0·1% (0·0 to 0·2%)	6·6 (1·8 to 13·0)	0·1% (0·0 to 0·3%)	33·4% (9·5 to 61·1%)	34·9% (15·3 to 53·6%)	438·9 (184·0 to 745·0)	0·1% (0·1 to 0·2%)	578·7 (238·4 to 988·5)	0·2% (0·1 to 0·3%)	31·9% (18·6 to 47·7%)	38·1% (26·0 to 51·0%)
Alcohol use disorders	0·3 (0·0 to 0·7)	6·6% (0·0 to 14·8%)	0·4 (0·0 to 0·8)	6·1% (0·0 to 13·8%)	21·7% (-1·6 to 51·3%)	-7·0% (-11·2 to 0·0%)	27·0 (0·0 to 60·8)	7·0% (0·0 to 15·6%)	34·2 (0·0 to 77·6)	6·5% (0·0 to 14·5%)	26·7% (0·0 to 40·5%)	-6·8% (-10·5 to 0·0%)
Major depressive disorder	-	-	-	-	-	-	178·4 (20·3 to 387·9)	7·3% (0·9 to 15·0%)	230·5 (26·1 to 506·1)	6·9% (0·8 to 14·3%)	29·2% (25·3 to 32·7%)	-5·6% (-8·0 to -3·3%)
Dysthymia	-	-	-	-	-	-	28·1 (3·0 to 61·9)	7·2% (0·9 to 15·0%)	36·7 (3·9 to 81·5)	6·8% (0·8 to 14·2%)	31·0% (26·9 to 34·2%)	-5·5% (-7·7 to -3·3%)
Self-harm	4·6 (1·2 to 8·8)	18·1% (4·6 to 34·8%)	6·2 (1·5 to 12·4)	17·0% (4·1 to 33·1%)	34·2% (9·9 to 61·9%)	-6·1% (-9·6 to -3·2%)	205·4 (51·9 to 393·3)	18·5% (4·7 to 35·3%)	277·2 (66·9 to 551·7)	17·4% (4·3 to 33·7%)	35·0% (10·1 to 63·2%)	-6·1% (-9·6 to -3·2%)
**Intimate partner violence: All causes**	40·1 (24·2 to 57·1)	0·8% (0·5 to 1·2%)	25·4 (16·8 to 34·7)	0·5% (0·4 to 0·8%)	-36·6% (-43·6 to -26·0%)	-35·7% (-42·9 to -26·1%)	2,301·2 (1,498·7 to 3,145·9)	0·7% (0·5 to 1·0%)	1,749·9 (1,258·3 to 2,286·5)	0·6% (0·4 to 0·7%)	-24·0% (-32·5 to -10·6%)	-20·3% (-29·0 to -7·7%)

Supplemental Results Table 1. All-age deaths, death PAFs, DALYs, DALY PAFs, and percent change for each risk factor and outcome for both sexes combined in 2005 and 2015 for Low SDI.

Data in parenthesis are 95% uncertainty intervals. PAFs=population attributable fractions. DALYs=disability-adjusted life-years.

	2005 Deaths (in thousands)	2005 Deaths PAF	2015 Deaths (in thousands)	2015 Deaths PAF	Percent change deaths 2005-2015	Percent change deaths PAF 2005-2015	2005 DALYs (in thousands)	2005 DALYs PAF	2015 DALYs (in thousands)	2015 DALYs PAF	Percent change DALYs 2005-2015	Percent change DALYs PAF 2005-2015
HIV/AIDS - Tuberculosis	7·0<br>(3·6 to 10·9)	6·4%<br>(3·5 to 9·2%)	3·5<br>(1·8 to 5·8)	5·5%<br>(3·0 to 8·0%)	-49·5%<br>(-55·5 to -43·7%)	-13·9%<br>(-18·8 to -9·2%)	320·2<br>(164·4 to 502·8)	5·2%<br>(2·8 to 7·5%)	170·5<br>(86·2 to 278·9)	4·6%<br>(2·5 to 6·8%)	-46·7%<br>(-52·8 to -41·1%)	-9·8%<br>(-15·2 to -4·9%)
HIV/AIDS resulting in other diseases	27·6<br>(15·0 to 42·1)	7·2%<br>(3·9 to 10·6%)	15·0<br>(8·0 to 22·6)	6·2%<br>(3·3 to 9·1%)	-45·8%<br>(-52·7 to -38·2%)	-13·3%<br>(-20·3 to -5·9%)	1,385·0<br>(744·2 to 2,126·3)	6·4%<br>(3·4 to 9·5%)	786·3<br>(415·8 to 1,201·0)	5·6%<br>(3·0 to 8·2%)	-43·2%<br>(-50·2 to -35·2%)	-12·2%<br>(-19·7 to -4·7%)
Maternal abortion, miscarriage, and ectopic pregnancy	0·0<br>(0·0 to 0·0)	0·0%<br>(0·0 to 0·0%)	0·0<br>(0·0 to 0·0)	0·0%<br>(0·0 to 0·0%)	0·9%<br>(-20·0 to 27·2%)	0·5%<br>(-9·2 to 11·7%)	0·0<br>(0·0 to 0·0)	0·0%<br>(0·0 to 0·0%)	0·0<br>(0·0 to 0·0)	0·0%<br>(0·0 to 0·0%)	0·3%<br>(-19·9 to 26·4%)	0·4%<br>(-8·6 to 12·4%)
Major depressive disorder	-	-	-	-	-	-	288·8<br>(184·7 to 413·4)	11·8%<br>(9·6 to 13·9%)	400·0<br>(256·3 to 568·6)	11·9%<br>(9·7 to 14·0%)	38·5%<br>(34·3 to 43·3%)	1·1%<br>(-1·6 to 4·1%)
Dysthymia	-	-	-	-	-	-	51·5<br>(33·1 to 76·3)	13·3%<br>(11·0 to 15·6%)	72·2<br>(46·2 to 106·2)	13·5%<br>(11·2 to 15·8%)	40·3%<br>(36·0 to 44·8%)	1·2%<br>(-1·4 to 4·0%)
Self-harm	3·7<br>(2·7 to 4·9)	14·4%<br>(10·8 to 18·1%)	4·7<br>(3·4 to 6·4)	13·0%<br>(9·2 to 17·4%)	28·4%<br>(-1·5 to 73·9%)	-9·8%<br>(-31·9 to 18·4%)	144·0<br>(104·4 to 192·1)	13·0%<br>(9·4 to 16·5%)	185·9<br>(132·9 to 253·5)	11·7%<br>(8·2 to 15·7%)	29·1%<br>(-3·1 to 75·3%)	-9·8%<br>(-33·2 to 20·0%)
Assault by firearm	0·3<br>(0·2 to 0·4)	4·2%<br>(2·6 to 6·7%)	0·4<br>(0·2 to 0·6)	3·8%<br>(2·3 to 6·1%)	34·7%<br>(-2·8 to 99·9%)	-9·0%<br>(-38·9 to 42·5%)	15·7<br>(10·0 to 23·9)	4·4%<br>(2·7 to 6·7%)	21·0<br>(13·5 to 33·1)	4·0%<br>(2·4 to 6·2%)	34·0%<br>(-3·9 to 99·2%)	-9·1%<br>(-39·3 to 43·3%)
Assault by sharp object	0·3<br>(0·2 to 0·5)	6·1%<br>(4·0 to 9·2%)	0·4<br>(0·3 to 0·6)	6·3%<br>(4·2 to 9·2%)	30·2%<br>(-3·9 to 71·6%)	3·2%<br>(-27·1 to 50·3%)	18·9<br>(12·7 to 27·0)	6·2%<br>(4·1 to 9·4%)	24·4<br>(16·0 to 36·8)	6·4%<br>(4·3 to 9·5%)	29·4%<br>(-3·8 to 70·6%)	3·1%<br>(-27·2 to 47·6%)
Assault by other means	1·2<br>(1·0 to 1·5)	8·7%<br>(6·5 to 10·8%)	1·4<br>(1·0 to 2·1)	8·0%<br>(5·5 to 11·1%)	17·4%<br>(-12·1 to 61·0%)	-7·5%<br>(-33·4 to 22·0%)	77·1<br>(61·8 to 98·6)	9·2%<br>(7·0 to 11·4%)	89·5<br>(64·0 to 129·7)	8·5%<br>(5·9 to 11·6%)	16·1%<br>(-11·7 to 55·1%)	-7·4%<br>(-31·9 to 19·8%)
**Unsafe sex: All causes**	556·7<br>(514·4 to 607·4)	11·8%<br>(10·9 to 12·8%)	381·9<br>(346·6 to 423·5)	8·2%<br>(7·3 to 9·2%)	-31·4%<br>(-35·3 to -27·0%)	-30·5%<br>(-35·9 to -24·7%)	32,309·1<br>(29,676·1 to 35,567·6)	10·1%<br>(9·1 to 11·1%)	23,055·8<br>(20,568·2 to 26,123·4)	7·5%<br>(6·6 to 8·6%)	-28·6%<br>(-32·8 to -24·0%)	-25·2%<br>(-30·0 to -19·5%)
HIV/AIDS - Tuberculosis	106·5<br>(77·7 to 130·5)	97·5%<br>(96·5 to 98·2%)	62·6<br>(42·6 to 81·9)	97·5%<br>(96·5 to 98·2%)	-41·3%<br>(-48·2 to -35·0%)	0·0%<br>(-0·1 to 0·1%)	6,039·4<br>(4,414·3 to 7,341·9)	97·5%<br>(96·4 to 98·3%)	3,572·8<br>(2,441·7 to 4,644·6)	97·5%<br>(96·4 to 98·2%)	-40·8%<br>(-47·8 to -34·7%)	0·0%<br>(-0·1 to 0·1%)
HIV/AIDS resulting in other diseases	375·7<br>(331·9 to 428·6)	97·3%<br>(96·4 to 98·0%)	234·5<br>(204·0 to 266·4)	97·3%<br>(96·3 to 97·9%)	-37·6%<br>(-42·5 to -31·9%)	0·0%<br>(-0·1 to 0·1%)	21,143·3<br>(18,792·9 to 23,891·9)	97·3%<br>(96·3 to 98·0%)	13,665·4<br>(11,950·1 to 15,467·1)	97·3%<br>(96·3 to 98·0%)	-35·4%<br>(-39·9 to -30·4%)	0·0%<br>(-0·1 to 0·1%)
Syphilis	50·7<br>(29·5 to 78·6)	100·0%<br>(100·0 to 100·0%)	56·7<br>(32·6 to 89·4)	100·0%<br>(100·0 to 100·0%)	11·8%<br>(-2·3 to 27·3%)	-	4,278·7<br>(2,474·1 to 6,662·0)	100·0%<br>(100·0 to 100·0%)	4,790·1<br>(2,728·8 to 7,575·0)	100·0%<br>(100·0 to 100·0%)	12·0%<br>(-2·5 to 27·9%)	-
Chlamydial infection	0·0<br>(0·0 to 0·0)	100·0%<br>(100·0 to 100·0%)	0·0<br>(0·0 to 0·0)	100·0%<br>(100·0 to 100·0%)	1·6%<br>(-26·4 to 46·1%)	-	25·5<br>(14·9 to 40·8)	100·0%<br>(100·0 to 100·0%)	34·5<br>(19·8 to 55·9)	100·0%<br>(100·0 to 100·0%)	35·3%<br>(27·8 to 42·5%)	-
Gonococcal infection	0·1<br>(0·1 to 0·2)	100·0%<br>(100·0 to 100·0%)	0·2<br>(0·1 to 0·2)	100·0%<br>(100·0 to 100·0%)	4·7%<br>(-19·1 to 40·0%)	-	53·2<br>(31·6 to 83·4)	100·0%<br>(100·0 to 100·0%)	73·8<br>(41·2 to 122·0)	100·0%<br>(100·0 to 100·0%)	38·8%<br>(22·4 to 52·8%)	-
Trichomoniasis	-	-	-	-	-	-	17·9<br>(7·2 to 38·7)	100·0%<br>(100·0 to 100·0%)	24·7<br>(10·0 to 53·1)	100·0%<br>(100·0 to 100·0%)	38·0%<br>(35·4 to 40·5%)	-
Genital herpes	-	-	-	-	-	-	14·7<br>(4·7 to 34·7)	100·0%<br>(100·0 to 100·0%)	20·1<br>(6·3 to 47·0)	100·0%<br>(100·0 to 100·0%)	36·6%<br>(32·5 to 40·4%)	-
Other sexually transmitted diseases	0·1<br>(0·1 to 0·1)	100·0%<br>(100·0 to 100·0%)	0·1<br>(0·1 to 0·1)	100·0%<br>(100·0 to 100·0%)	4·1%<br>(-20·4 to 38·3%)	-	7·4<br>(5·9 to 9·3)	100·0%<br>(100·0 to 100·0%)	9·4<br>(7·2 to 12·0)	100·0%<br>(100·0 to 100·0%)	25·8%<br>(9·7 to 44·2%)	-
Cervical cancer	23·5<br>(17·7 to 27·8)	100·0%<br>(100·0 to 100·0%)	27·8<br>(18·8 to 36·3)	100·0%<br>(100·0 to 100·0%)	18·1%<br>(-8·8 to 50·0%)	-	728·9<br>(540·5 to 866·6)	100·0%<br>(100·0 to 100·0%)	865·0<br>(577·5 to 1,126·4)	100·0%<br>(100·0 to 100·0%)	18·7%<br>(-8·4 to 51·2%)	-
**Low physical activity: All causes**	32·3<br>(24·1 to 41·5)	0·7%<br>(0·5 to 0·9%)	45·1<br>(33·8 to 57·6)	1·0%<br>(0·7 to 1·2%)	39·5%<br>(23·5 to 57·3%)	41·2%<br>(31·3 to 52·4%)	857·5<br>(641·6 to 1,092·3)	0·3%<br>(0·2 to 0·3%)	1,202·3<br>(903·3 to 1,522·6)	0·4%<br>(0·3 to 0·5%)	40·2%<br>(24·1 to 57·9%)	46·9%<br>(33·4 to 61·5%)
Colon and rectum cancer	1·2<br>(0·8 to 1·7)	10·3%<br>(6·7 to 14·1%)	1·9<br>(1·2 to 2·7)	10·5%<br>(6·8 to 14·5%)	53·0%<br>(34·5 to 77·1%)	2·0%<br>(-2·7 to 6·3%)	30·6<br>(19·1 to 42·8)	9·4%<br>(6·0 to 13·0%)	46·6<br>(28·0 to 67·3)	9·6%<br>(6·1 to 13·3%)	52·3%<br>(32·7 to 77·0%)	1·9%<br>(-3·4 to 7·0%)
Breast cancer	0·8<br>(0·5 to 1·3)	6·6%<br>(4·7 to 8·6%)	1·2<br>(0·8 to 1·9)	6·8%<br>(4·7 to 9·0%)	51·4%<br>(20·6 to 87·0%)	2·1%<br>(-7·9 to 10·1%)	26·7<br>(16·9 to 42·6)	6·3%<br>(4·3 to 8·2%)	41·0<br>(24·7 to 65·2)	6·4%<br>(4·4 to 8·6%)	53·5%<br>(22·8 to 88·4%)	2·3%<br>(-8·2 to 11·0%)
Ischaemic heart disease	17·0<br>(10·2 to 23·8)	8·7%<br>(5·4 to 12·2%)	23·2<br>(13·9 to 32·9)	8·9%<br>(5·5 to 12·5%)	36·0%<br>(19·5 to 54·4%)	2·6%<br>(-2·9 to 8·4%)	431·5<br>(246·2 to 607·9)	9·2%<br>(5·5 to 13·0%)	583·5<br>(340·0 to 832·0)	9·5%<br>(5·7 to 13·4%)	35·2%<br>(16·9 to 55·4%)	3·3%<br>(-2·5 to 9·7%)
Ischaemic stroke	8·0<br>(4·0 to 12·7)	10·1%<br>(5·0 to 15·6%)	10·6<br>(5·4 to 16·7)	10·3%<br>(5·2 to 15·9%)	33·0%<br>(16·4 to 52·8%)	2·0%<br>(-2·1 to 6·9%)	173·3<br>(87·0 to 273·4)	10·7%<br>(5·2 to 16·4%)	226·8<br>(114·3 to 358·5)	11·0%<br>(5·4 to 16·9%)	30·9%<br>(13·5 to 51·5%)	2·5%<br>(-1·9 to 7·7%)
Diabetes mellitus	5·2<br>(3·7 to 6·8)	12·1%<br>(8·8 to 15·7%)	8·1<br>(5·8 to 10·8)	12·5%<br>(9·1 to 16·0%)	55·7%<br>(36·8 to 78·1%)	3·3%<br>(-1·1 to 7·1%)	195·5<br>(136·5 to 267·9)	10·6%<br>(7·6 to 13·8%)	304·4<br>(210·7 to 418·9)	11·0%<br>(8·0 to 14·3%)	55·7%<br>(42·3 to 70·6%)	3·5%<br>(0·6 to 6·2%)
**Metabolic risks: All causes**	460·3<br>(421·2 to 501·1)	9·7%<br>(9·1 to 10·4%)	615·0<br>(541·6 to 697·8)	13·2%<br>(12·2 to 14·1%)	33·6%<br>(18·1 to 50·9%)	35·2%<br>(26·6 to 44·0%)	13,010·4<br>(11,816·8 to 14,294·6)	4·1%<br>(3·7 to 4·4%)	17,376·6<br>(15,237·2 to 19,676·9)	5·7%<br>(5·1 to 6·2%)	33·6%<br>(18·9 to 49·5%)	39·9%<br>(28·4 to 52·7%)
**High fasting plasma glucose: All causes**	106·4<br>(91·9 to 122·7)	2·3%<br>(2·0 to 2·6%)	152·9<br>(128·0 to 181·5)	3·3%<br>(2·9 to 3·8%)	43·7%<br>(25·8 to 64·4%)	45·4%<br>(34·8 to 57·0%)	3,653·1<br>(3,175·4 to 4,162·6)	1·1%<br>(1·0 to 1·3%)	5,237·5<br>(4,436·8 to 6,119·8)	1·7%<br>(1·5 to 1·9%)	43·4%<br>(28·6 to 59·4%)	50·2%<br>(38·8 to 62·8%)
Tuberculosis	11·8<br>(6·5 to 18·8)	6·6%<br>(4·1 to 9·4%)	13·6<br>(7·6 to 22·7)	7·5%<br>(4·7 to 10·6%)	15·9%<br>(-7·0 to 43·9%)	13·8%<br>(5·8 to 20·5%)	375·3<br>(216·4 to 563·7)	5·1%<br>(3·2 to 7·0%)	435·7<br>(253·1 to 686·4)	5·9%<br>(3·8 to 8·1%)	16·1%<br>(-7·6 to 44·5%)	16·0%<br>(4·7 to 25·9%)
Ischaemic heart disease	22·3<br>(14·9 to 32·2)	11·4%<br>(7·7 to 16·3%)	32·3<br>(21·0 to 47·8)	12·4%<br>(8·3 to 17·5%)	44·9%<br>(24·6 to 66·0%)	9·2%<br>(4·8 to 14·0%)	528·7<br>(348·5 to 737·3)	11·3%<br>(7·6 to 15·5%)	757·7<br>(498·1 to 1,082·5)	12·3%<br>(8·3 to 16·9%)	43·3%<br>(21·6 to 65·9%)	9·4%<br>(4·9 to 14·4%)
Ischaemic stroke	8·4<br>(4·8 to 15·2)	10·6%<br>(6·1 to 18·2%)	11·9<br>(6·7 to 21·2)	11·6%<br>(6·6 to 19·9%)	42·4%<br>(23·4 to 65·3%)	9·1%<br>(3·8 to 15·1%)	173·8<br>(103·1 to 292·4)	10·7%<br>(6·6 to 17·3%)	243·0<br>(144·4 to 393·9)	11·7%<br>(7·1 to 19·0%)	39·8%<br>(20·4 to 63·0%)	9·5%<br>(4·8 to 15·1%)
Hemorrhagic stroke	12·1<br>(7·8 to 17·4)	9·8%<br>(6·6 to 14·0%)	17·1<br>(11·1 to 24·4)	10·8%<br>(7·4 to 15·3%)	41·5%<br>(22·1 to 64·8%)	9·8%<br>(4·5 to 15·8%)	331·9<br>(207·3 to 497·5)	9·1%<br>(5·8 to 13·5%)	467·9<br>(299·7 to 696·0)	10·2%<br>(6·5 to 15·1%)	41·0%<br>(20·7 to 64·0%)	11·5%<br>(5·7 to 18·0%)
Diabetes mellitus	43·0<br>(38·5 to 48·2)	100·0%<br>(100·0 to 100·0%)	64·8<br>(56·4 to 75·1)	100·0%<br>(100·0 to 100·0%)	50·9%<br>(30·7 to 75·1%)	-	1,845·2<br>(1,580·1 to 2,153·8)	100·0%<br>(100·0 to 100·0%)	2,777·0<br>(2,326·6 to 3,285·2)	100·0%<br>(100·0 to 100·0%)	50·5%<br>(37·2 to 65·8%)	-
Chronic kidney disease due to diabetes mellitus	5·3<br>(4·5 to 6·3)	100·0%<br>(100·0 to 100·0%)	7·4<br>(6·1 to 8·9)	100·0%<br>(100·0 to 100·0%)	38·6%<br>(23·1 to 58·1%)	-	274·4<br>(236·4 to 317·0)	100·0%<br>(100·0 to 100·0%)	362·2<br>(312·8 to 423·1)	100·0%<br>(100·0 to 100·0%)	32·0%<br>(21·5 to 44·5%)	-
Chronic kidney disease due to hypertension	2·9<br>(1·9 to 4·0)	20·8%<br>(13·9 to 28·3%)	4·6<br>(3·0 to 6·4)	23·2%<br>(15·6 to 31·0%)	58·8%<br>(38·8 to 80·2%)	11·1%<br>(7·4 to 15·3%)	81·5<br>(53·5 to 111·8)	16·0%<br>(10·7 to 22·1%)	128·5<br>(82·8 to 180·6)	18·2%<br>(12·1 to 24·6%)	57·8%<br>(37·9 to 80·1%)	13·5%<br>(8·4 to 19·0%)
Chronic kidney disease due to glomerulonephritis	0·5<br>(0·3 to 0·7)	12·1%<br>(8·0 to 17·1%)	0·8<br>(0·5 to 1·1)	14·3%<br>(9·4 to 19·9%)	60·8%<br>(38·0 to 87·6%)	18·0%<br>(10·0 to 26·8%)	23·9<br>(15·4 to 34·0)	9·2%<br>(6·1 to 13·2%)	37·3<br>(23·8 to 53·1)	10·9%<br>(7·1 to 15·3%)	55·7%<br>(40·5 to 72·0%)	18·0%<br>(11·0 to 26·0%)
Chronic kidney disease due to other causes	0·2<br>(0·1 to 0·3)	11·7%<br>(7·6 to 16·3%)	0·3<br>(0·2 to 0·4)	14·2%<br>(9·1 to 19·7%)	56·5%<br>(27·8 to 92·7%)	21·3%<br>(9·2 to 35·6%)	15·6<br>(9·7 to 22·8)	10·4%<br>(6·7 to 14·6%)	23·5<br>(14·7 to 34·9)	12·3%<br>(8·1 to 16·9%)	51·2%<br>(40·6 to 64·3%)	17·8%<br>(11·6 to 25·2%)
**High total cholesterol: All causes**	80·8<br>(62·1 to 105·1)	1·7%<br>(1·3 to 2·2%)	102·0<br>(75·5 to 136·2)	2·2%<br>(1·7 to 2·9%)	26·3%<br>(9·2 to 43·6%)	27·8%<br>(15·5 to 41·1%)	2,224·2<br>(1,748·0 to 2,782·9)	0·7%<br>(0·5 to 0·9%)	2,750·6<br>(2,092·6 to 3,513·7)	0·9%<br>(0·7 to 1·1%)	23·7%<br>(6·3 to 42·1%)	29·6%<br>(13·7 to 46·7%)
Ischaemic heart disease	68·7<br>(52·8 to 87·4)	35·1%<br>(27·8 to 43·7%)	87·0<br>(65·1 to 112·7)	33·5%<br>(26·0 to 42·1%)	26·6%<br>(9·2 to 44·8%)	-4·6%<br>(-9·5 to 0·0%)	1,915·3<br>(1,504·5 to 2,383·6)	40·8%<br>(33·4 to 49·3%)	2,379·0<br>(1,819·6 to 3,025·9)	38·7%<br>(31·0 to 47·0%)	24·2%<br>(6·1 to 43·8%)	-5·1%<br>(-10·1 to -0·5%)
Ischaemic stroke	12·1<br>(6·0 to 23·3)	15·3%<br>(8·4 to 27·6%)	15·1<br>(7·3 to 29·3)	14·6%<br>(7·7 to 27·2%)	24·7%<br>(4·6 to 43·9%)	-4·5%<br>(-13·9 to 2·0%)	308·9<br>(185·6 to 503·6)	19·1%<br>(12·6 to 29·5%)	371·6<br>(223·1 to 615·6)	18·0%<br>(11·4 to 28·1%)	20·3%<br>(0·9 to 40·7%)	-5·9%<br>(-13·3 to 0·2%)
**High systolic blood pressure: All causes**	314·0<br>(279·3 to 350·0)	6·6%<br>(6·0 to 7·3%)	414·2<br>(358·9 to 478·6)	8·9%<br>(8·0 to 9·8%)	31·9%<br>(16·8 to 48·7%)	33·5%<br>(25·1 to 42·3%)	8,074·7<br>(7,153·2 to 9,048·5)	2·5%<br>(2·2 to 2·8%)	10,592·9<br>(9,172·3 to 12,237·3)	3·5%<br>(3·0 to 3·9%)	31·2%<br>(16·2 to 48·1%)	37·5%<br>(24·9 to 51·2%)
Rheumatic heart disease	2·8<br>(1·9 to 4·2)	25·1%<br>(17·3 to 38·2%)	3·4<br>(2·3 to 4·9)	25·9%<br>(18·1 to 39·1%)	19·5%<br>(1·2 to 41·1%)	3·2%<br>(-0·3 to 6·9%)	111·0<br>(72·3 to 161·2)	20·4%<br>(13·7 to 29·5%)	136·5<br>(87·8 to 196·4)	21·3%<br>(14·1 to 30·6%)	23·0%<br>(7·3 to 40·2%)	4·7%<br>(-0·2 to 9·9%)
Ischaemic heart disease	108·5<br>(91·6 to 127·3)	55·5%<br>(47·4 to 62·9%)	144·8<br>(117·0 to 174·3)	55·8%<br>(47·5 to 63·2%)	33·4%<br>(16·9 to 50·3%)	0·6%<br>(-1·2 to 2·4%)	2,579·0<br>(2,173·4 to 3,018·0)	54·9%<br>(47·5 to 61·5%)	3,415·8<br>(2,759·8 to 4,083·6)	55·5%<br>(47·8 to 62·2%)	32·4%<br>(15·2 to 50·4%)	1·1%<br>(-0·8 to 3·1%)
Ischaemic stroke	41·8<br>(30·4 to 52·4)	53·0%<br>(41·7 to 62·0%)	54·4<br>(40·7 to 69·4)	52·8%<br>(41·6 to 61·8%)	30·2%<br>(13·3 to 49·5%)	-0·3%<br>(-2·4 to 1·9%)	874·2<br>(650·5 to 1,087·3)	54·1%<br>(44·2 to 62·4%)	1,123·1<br>(855·3 to 1,404·2)	54·3%<br>(44·6 to 62·7%)	28·5%<br>(11·7 to 46·6%)	0·5%<br>(-1·2 to 2·3%)

Supplemental Results Table 1. All-age deaths, death PAFs, DALYs, DALY PAFs, and percent change for each risk factor and outcome for both sexes combined in 2005 and 2015 for Low SDI.

Data in parenthesis are 95% uncertainty intervals. PAFs=population attributable fractions. DALYs=disability-adjusted life-years.

	2005 Deaths (in thousands)	2005 Deaths PAF	2015 Deaths (in thousands)	2015 Deaths PAF	Percent change deaths 2005-2015	Percent change deaths PAF 2005-2015	2005 DALYs (in thousands)	2005 DALYs PAF	2015 DALYs (in thousands)	2015 DALYs PAF	Percent change DALYs 2005-2015	Percent change DALYs PAF 2005-2015
Hemorrhagic stroke	71·5 (57·0 to 84·9)	58·3% (48·8 to 66·8%)	93·4 (73·4 to 114·3)	59·1% (49·8 to 67·2%)	30·7% (14·3 to 51·1%)	1·4% (-0·5 to 3·3%)	1,988·7 (1,597·0 to 2,348·9)	54·6% (45·1 to 62·1%)	2,588·6 (2,081·6 to 3,203·2)	56·2% (46·8 to 64·0%)	30·2% (13·2 to 49·5%)	3·0% (0·4 to 5·5%)
Hypertensive heart disease	46·4 (33·3 to 69·8)	100·0% (100·0 to 100·0%)	58·9 (43·0 to 92·0)	100·0% (100·0 to 100·0%)	26·8% (8·1 to 51·6%)	-	1,071·5 (818·2 to 1,494·0)	100·0% (100·0 to 100·0%)	1,340·3 (1,020·0 to 1,927·1)	100·0% (100·0 to 100·0%)	25·1% (6·6 to 49·5%)	-
Cardiomyopathy and myocarditis	2·5 (1·9 to 3·1)	31·6% (25·7 to 37·5%)	3·4 (2·5 to 4·4)	33·1% (26·6 to 39·4%)	39·4% (17·6 to 66·7%)	4·7% (0·0 to 9·5%)	75·3 (56·8 to 95·3)	23·6% (18·4 to 28·8%)	105·6 (77·0 to 135·1)	26·0% (20·2 to 31·7%)	40·3% (17·6 to 69·1%)	10·2% (1·0 to 20·3%)
Atrial fibrillation and flutter	0·6 (0·4 to 0·8)	36·7% (29·7 to 43·6%)	0·8 (0·6 to 1·1)	36·5% (29·7 to 43·5%)	39·4% (25·4 to 56·6%)	-0·5% (-3·1 to 2·0%)	19·9 (15·1 to 25·9)	43·2% (38·5 to 48·3%)	27·5 (20·9 to 36·0)	43·4% (38·5 to 48·4%)	38·2% (31·7 to 45·6%)	0·4% (-1·0 to 1·7%)
Aortic aneurysm	0·9 (0·6 to 1·4)	38·6% (31·9 to 44·9%)	1·4 (1·0 to 2·1)	38·7% (32·0 to 45·2%)	50·2% (23·8 to 84·1%)	0·2% (-2·1 to 2·5%)	23·9 (16·4 to 36·3)	38·5% (32·9 to 44·7%)	36·0 (25·9 to 51·6)	38·8% (32·9 to 45·2%)	50·5% (21·8 to 88·6%)	0·9% (-1·9 to 3·8%)
Peripheral vascular disease	0·0 (0·0 to 0·1)	28·4% (21·8 to 35·4%)	0·1 (0·0 to 0·1)	28·2% (21·4 to 35·3%)	66·6% (34·7 to 118·6%)	-0·9% (-4·0 to 2·1%)	4·3 (2·3 to 7·5)	30·4% (23·7 to 37·0%)	6·2 (3·3 to 10·9)	30·4% (23·9 to 37·1%)	44·1% (37·3 to 53·5%)	0·2% (-1·4 to 1·7%)
Endocarditis	1·4 (0·8 to 2·0)	28·9% (22·9 to 35·4%)	2·0 (1·2 to 2·8)	30·1% (23·8 to 36·3%)	43·2% (22·9 to 67·8%)	4·4% (-1·0 to 11·3%)	40·3 (24·1 to 55·6)	20·1% (14·4 to 26·9%)	58·4 (37·0 to 82·6)	21·8% (15·8 to 28·3%)	45·0% (22·5 to 73·1%)	8·9% (-1·3 to 21·9%)
Other cardiovascular and circulatory diseases	20·5 (17·6 to 23·7)	39·6% (35·5 to 44·0%)	27·1 (21·7 to 32·7)	40·5% (36·0 to 44·9%)	32·0% (13·3 to 54·8%)	2·1% (-0·8 to 5·1%)	639·5 (543·3 to 751·0)	33·8% (30·1 to 37·8%)	852·2 (698·2 to 1,026·2)	35·7% (31·7 to 39·9%)	33·3% (17·5 to 52·0%)	5·4% (1·3 to 10·0%)
Chronic kidney disease due to diabetes mellitus	1·8 (1·2 to 2·5)	34·1% (24·3 to 43·6%)	2·7 (1·8 to 3·7)	36·0% (25·9 to 45·7%)	46·5% (28·2 to 68·2%)	5·7% (2·1 to 9·7%)	63·1 (42·0 to 87·1)	23·0% (15·6 to 30·1%)	89·5 (59·3 to 122·9)	24·7% (17·2 to 32·2%)	41·9% (28·0 to 56·8%)	7·5% (2·6 to 12·9%)
Chronic kidney disease due to hypertension	14·0 (12·3 to 15·7)	100·0% (100·0 to 100·0%)	20·0 (17·1 to 23·0)	100·0% (100·0 to 100·0%)	42·9% (26·5 to 60·3%)	-	507·9 (444·4 to 566·9)	100·0% (100·0 to 100·0%)	705·7 (603·3 to 812·5)	100·0% (100·0 to 100·0%)	38·9% (23·6 to 54·9%)	-
Chronic kidney disease due to glomerulonephritis	0·9 (0·6 to 1·2)	23·8% (17·0 to 30·9%)	1·4 (0·9 to 1·9)	25·8% (18·5 to 33·5%)	47·8% (28·6 to 70·5%)	8·5% (2·9 to 14·3%)	46·2 (31·2 to 62·5)	17·7% (12·3 to 23·6%)	66·1 (44·9 to 90·1)	19·3% (13·4 to 25·8%)	43·1% (30·7 to 57·4%)	8·5% (2·8 to 14·6%)
Chronic kidney disease due to other causes	0·4 (0·2 to 0·5)	22·8% (15·9 to 29·9%)	0·5 (0·3 to 0·7)	25·2% (17·7 to 33·1%)	42·7% (21·0 to 71·0%)	10·6% (2·5 to 20·8%)	29·9 (19·3 to 41·2)	20·1% (13·9 to 26·6%)	41·4 (27·1 to 57·3)	21·7% (15·1 to 28·8%)	38·4% (30·8 to 47·1%)	7·8% (2·7 to 13·5%)
**High body-mass index: All causes**	91·3 (57·9 to 129·2)	1·9% (1·2 to 2·7%)	137·8 (90·2 to 190·5)	3·0% (2·0 to 4·0%)	51·0% (31·4 to 72·7%)	52·8% (39·1 to 68·6%)	3,045·0 (1,965·4 to 4,263·5)	0·9% (0·6 to 1·3%)	4,613·6 (3,045·5 to 6,323·5)	1·5% (1·0 to 2·0%)	51·5% (33·6 to 71·7%)	58·7% (43·0 to 76·3%)
Oesophageal cancer	1·5 (0·4 to 2·9)	10·4% (3·1 to 19·9%)	2·3 (0·7 to 4·4)	11·9% (3·7 to 22·2%)	51·1% (20·1 to 99·6%)	13·8% (-2·5 to 34·9%)	41·0 (12·0 to 78·8)	10·5% (3·1 to 20·0%)	61·3 (18·5 to 118·8)	11·9% (3·8 to 22·3%)	49·3% (18·1 to 96·5%)	13·1% (-4·1 to 32·8%)
Colon and rectum cancer	0·4 (0·2 to 0·6)	3·4% (1·8 to 5·4%)	0·7 (0·4 to 1·1)	4·0% (2·2 to 6·3%)	79·5% (54·0 to 113·5%)	19·6% (9·0 to 33·1%)	11·0 (5·8 to 17·7)	3·4% (1·8 to 5·5%)	19·6 (10·4 to 31·0)	4·0% (2·2 to 6·3%)	77·4% (51·8 to 111·7%)	18·7% (8·4 to 31·5%)
Liver cancer due to hepatitis B	0·8 (0·3 to 1·5)	7·7% (3·1 to 14·4%)	1·2 (0·5 to 2·2)	9·0% (3·7 to 16·6%)	57·2% (34·0 to 90·7%)	16·9% (7·7 to 28·9%)	27·0 (10·2 to 52·7)	7·2% (2·8 to 13·8%)	42·9 (16·8 to 80·8)	8·5% (3·4 to 15·6%)	58·5% (34·4 to 93·9%)	16·9% (7·6 to 29·8%)
Liver cancer due to hepatitis C	0·5 (0·2 to 0·9)	7·3% (2·9 to 13·3%)	0·7 (0·3 to 1·4)	8·3% (3·3 to 14·8%)	53·2% (27·9 to 83·9%)	13·9% (4·4 to 25·9%)	12·6 (4·8 to 23·9)	7·4% (2·9 to 13·4%)	19·2 (7·4 to 35·8)	8·4% (3·3 to 14·9%)	52·5% (27·2 to 84·5%)	13·7% (3·8 to 26·2%)
Liver cancer due to alcohol use	0·5 (0·2 to 1·0)	7·7% (2·9 to 14·9%)	0·8 (0·3 to 1·5)	8·7% (3·3 to 16·8%)	50·9% (26·7 to 82·3%)	13·9% (3·8 to 25·9%)	14·2 (5·1 to 28·5)	7·8% (3·0 to 15·2%)	21·5 (8·0 to 42·6)	8·8% (3·4 to 17·0%)	51·3% (26·2 to 85·7%)	13·5% (3·0 to 26·1%)
Liver cancer due to other causes	0·4 (0·1 to 0·6)	7·5% (3·0 to 13·6%)	0·5 (0·2 to 1·0)	8·8% (3·6 to 15·4%)	54·0% (32·3 to 83·8%)	16·9% (7·8 to 28·9%)	10·8 (4·3 to 19·9)	7·0% (2·8 to 12·7%)	16·8 (6·9 to 29·9)	8·3% (3·4 to 14·5%)	54·8% (32·0 to 87·4%)	17·1% (7·5 to 30·7%)
Gallbladder and biliary tract cancer	0·3 (0·2 to 0·6)	11·9% (6·4 to 19·3%)	0·5 (0·2 to 0·8)	13·6% (7·5 to 21·9%)	46·0% (22·6 to 77·5%)	14·9% (5·6 to 26·5%)	8·8 (4·0 to 15·8)	12·1% (6·5 to 19·5%)	12·7 (6·1 to 21·6)	13·8% (7·6 to 22·2%)	44·6% (20·7 to 78·0%)	14·4% (4·8 to 26·2%)
Pancreatic cancer	0·2 (0·1 to 0·3)	2·9% (1·0 to 5·3%)	0·3 (0·1 to 0·6)	3·4% (1·2 to 6·1%)	74·9% (49·3 to 103·4%)	16·0% (5·8 to 27·9%)	4·6 (1·7 to 8·5)	2·9% (1·0 to 5·4%)	8·1 (2·9 to 14·4)	3·4% (1·3 to 6·1%)	74·5% (48·9 to 102·8%)	15·1% (4·0 to 27·2%)
Breast cancer	0·2 (0·1 to 0·3)	1·3% (0·5 to 2·3%)	0·3 (0·1 to 0·5)	1·4% (0·6 to 2·5%)	60·8% (13·3 to 125·0%)	8·2% (-22·3 to 48·9%)	1·5 (-2·1 to 4·1)	0·4% (-0·4 to 1·0%)	1·7 (-4·4 to 5·8)	0·3% (-0·6 to 1·0%)	13·7% (-467·3 to 638·6%)	-23·0% (-361·1 to 430·6%)
Uterine cancer	0·9 (0·5 to 1·4)	22·5% (13·4 to 32·8%)	1·3 (0·8 to 2·0)	26·0% (16·0 to 36·8%)	41·6% (14·8 to 82·5%)	15·6% (6·7 to 29·6%)	25·7 (14·5 to 40·2)	22·5% (13·4 to 32·9%)	36·1 (20·9 to 55·3)	26·0% (16·1 to 36·9%)	40·2% (13·2 to 82·9%)	15·5% (6·0 to 29·5%)
Ovarian cancer	0·1 (0·0 to 0·1)	1·5% (0·0 to 3·7%)	0·1 (0·0 to 0·3)	1·8% (-0·1 to 4·3%)	74·8% (39·3 to 124·7%)	16·7% (5·9 to 30·6%)	1·8 (-0·1 to 4·4)	1·4% (0·0 to 3·6%)	3·2 (-0·1 to 7·7)	1·7% (-0·1 to 4·1%)	75·7% (38·4 to 126·6%)	16·3% (5·2 to 30·9%)
Kidney cancer	0·2 (0·1 to 0·2)	7·4% (4·4 to 11·3%)	0·3 (0·2 to 0·4)	8·4% (5·1 to 12·3%)	69·7% (42·3 to 100·5%)	13·9% (0·7 to 28·7%)	4·7 (2·7 to 7·1)	5·0% (2·9 to 8·1%)	8·0 (4·8 to 11·7)	5·8% (3·5 to 8·8%)	70·2% (42·1 to 102·0%)	16·2% (-1·0 to 38·6%)
Thyroid cancer	0·0 (0·0 to 0·1)	5·5% (2·8 to 8·9%)	0·1 (0·0 to 0·1)	6·4% (3·4 to 10·3%)	54·9% (32·2 to 83·7%)	16·6% (7·2 to 28·5%)	1·0 (0·5 to 1·8)	5·4% (2·8 to 8·9%)	1·6 (0·8 to 2·8)	6·3% (3·3 to 10·0%)	58·6% (35·1 to 87·1%)	15·3% (6·4 to 26·6%)
Ischaemic heart disease	23·6 (14·2 to 34·5)	12·1% (7·4 to 17·6%)	36·2 (22·8 to 51·9)	14·0% (8·9 to 19·7%)	53·3% (32·2 to 79·1%)	15·7% (7·2 to 26·3%)	669·7 (405·3 to 983·4)	14·3% (8·9 to 20·5%)	1,015·5 (634·3 to 1,454·6)	16·5% (10·5 to 23·2%)	51·6% (29·9 to 78·9%)	15·9% (6·7 to 26·6%)
Ischaemic stroke	8·3 (4·8 to 12·6)	10·5% (6·5 to 15·2%)	12·1 (7·2 to 17·9)	11·8% (7·4 to 16·6%)	46·5% (22·9 to 73·3%)	12·3% (3·4 to 23·9%)	222·2 (131·0 to 335·4)	13·7% (8·7 to 19·5%)	322·2 (195·2 to 459·7)	15·6% (10·1 to 21·6%)	45·0% (22·4 to 70·3%)	13·5% (5·2 to 25·1%)
Hemorrhagic stroke	24·2 (15·3 to 34·6)	19·8% (12·7 to 27·6%)	34·8 (22·3 to 48·6)	22·0% (14·7 to 30·1%)	43·6% (23·4 to 70·4%)	11·5% (4·0 to 21·1%)	810·2 (519·7 to 1,141·1)	22·2% (14·4 to 30·9%)	1,161·3 (762·0 to 1,602·8)	25·2% (17·2 to 34·1%)	43·3% (23·0 to 70·0%)	13·4% (5·9 to 23·2%)
Hypertensive heart disease	12·1 (6·8 to 20·2)	26·3% (16·1 to 38·5%)	16·8 (9·6 to 28·0)	28·8% (17·9 to 41·4%)	38·7% (16·5 to 67·7%)	9·4% (0·9 to 19·8%)	312·3 (184·0 to 478·9)	29·3% (18·4 to 41·5%)	426·8 (256·4 to 656·1)	32·0% (20·7 to 44·6%)	36·7% (15·0 to 64·8%)	9·3% (1·9 to 19·1%)
Diabetes mellitus	13·0 (8·6 to 18·3)	30·1% (20·5 to 41·5%)	21·9 (14·6 to 29·8)	33·7% (23·2 to 44·7%)	68·6% (43·8 to 99·1%)	11·8% (4·5 to 20·8%)	638·2 (418·4 to 891·1)	34·6% (23·9 to 45·9%)	1,068·7 (718·9 to 1,457·3)	38·5% (27·3 to 49·4%)	67·5% (50·5 to 85·9%)	11·3% (5·6 to 18·5%)
Chronic kidney disease due to diabetes mellitus	0·8 (0·3 to 1·4)	14·4% (6·5 to 25·0%)	1·3 (0·6 to 2·2)	17·2% (8·1 to 28·4%)	65·3% (38·3 to 97·7%)	19·4% (8·3 to 34·5%)	29·9 (11·6 to 54·3)	10·9% (4·4 to 19·7%)	47·6 (19·7 to 83·6)	13·1% (5·5 to 22·8%)	59·1% (40·0 to 83·9%)	20·6% (10·9 to 34·5%)
Chronic kidney disease due to hypertension	2·7 (1·2 to 4·5)	19·4% (9·0 to 31·6%)	4·5 (2·1 to 7·5)	22·7% (10·9 to 35·9%)	67·1% (44·2 to 95·2%)	16·9% (8·9 to 28·3%)	79·4 (33·1 to 138·0)	15·6% (6·5 to 27·2%)	130·9 (53·7 to 224·7)	18·6% (8·1 to 31·2%)	64·8% (42·3 to 92·9%)	18·6% (10·3 to 30·9%)
Chronic kidney disease due to glomerulonephritis	0·4 (0·1 to 0·7)	9·6% (3·7 to 17·1%)	0·6 (0·2 to 1·1)	11·6% (4·7 to 19·8%)	64·3% (38·8 to 95·7%)	20·7% (8·2 to 37·5%)	20·4 (6·0 to 39·8)	7·8% (2·4 to 14·8%)	32·2 (9·8 to 59·4)	9·4% (2·9 to 17·0%)	57·8% (42·0 to 78·3%)	19·7% (10·2 to 33·4%)
Chronic kidney disease due to other causes	0·2 (0·1 to 0·3)	9·7% (3·9 to 17·8%)	0·2 (0·1 to 0·5)	12·3% (4·9 to 21·7%)	63·1% (30·0 to 105·5%)	26·5% (9·4 to 49·0%)	13·9 (5·0 to 26·2)	9·3% (3·3 to 17·2%)	21·5 (7·5 to 40·0)	11·2% (4·2 to 20·1%)	54·8% (41·4 to 71·5%)	20·6% (11·9 to 32·6%)
Osteoarthritis	-	-	-	-	-	-	28·1 (15·2 to 45·0)	11·6% (7·1 to 16·8%)	45·1 (24·8 to 72·0)	13·3% (8·4 to 18·7%)	60·6% (53·8 to 70·1%)	13·8% (9·2 to 20·2%)
Low back pain	-	-	-	-	-	-	50·2 (25·7 to 85·5)	2·5% (1·4 to 3·7%)	80·0 (42·0 to 134·0)	2·9% (1·7 to 4·3%)	59·2% (53·1 to 68·1%)	15·5% (11·2 to 21·9%)
**Low bone mineral density: All causes**	12·9 (11·2 to 14·7)	0·3% (0·2 to 0·3%)	16·9 (14·3 to 19·5)	0·4% (0·3 to 0·4%)	30·7% (12·9 to 51·5%)	32·3% (19·4 to 48·2%)	301·7 (262·0 to 340·7)	0·1% (0·1 to 0·1%)	392·7 (333·4 to 454·7)	0·1% (0·1 to 0·1%)	30·2% (16·7 to 46·5%)	36·4% (24·7 to 49·4%)
Pedestrian road injuries	1·5 (1·3 to 1·8)	4·9% (4·4 to 5·5%)	2·0 (1·7 to 2·4)	5·2% (4·6 to 5·8%)	31·2% (10·4 to 60·4%)	4·6% (-5·2 to 15·9%)	35·2 (30·0 to 41·2)	2·1% (1·8 to 2·4%)	45·5 (37·4 to 55·7)	2·2% (1·9 to 2·5%)	29·3% (7·6 to 58·7%)	6·3% (-6·8 to 21·4%)
Cyclist road injuries	0·1 (0·1 to 0·1)	5·2% (4·4 to 6·1%)	0·2 (0·1 to 0·2)	5·1% (4·4 to 6·0%)	35·6% (9·0 to 71·2%)	-1·6% (-12·9 to 12·7%)	3·5 (2·8 to 4·2)	2·8% (2·3 to 3·3%)	4·8 (3·8 to 5·9)	2·8% (2·3 to 3·3%)	37·5% (17·5 to 65·3%)	0·7% (-10·9 to 13·8%)
Motorcyclist road injuries	0·2 (0·2 to 0·3)	3·1% (2·6 to 3·8%)	0·3 (0·2 to 0·3)	3·1% (2·6 to 3·6%)	28·2% (8·2 to 53·7%)	-1·8% (-13·7 to 11·1%)	6·5 (5·3 to 8·1)	1·7% (1·4 to 2·1%)	8·4 (6·7 to 10·5)	1·7% (1·4 to 2·0%)	29·1% (10·6 to 52·1%)	-0·4% (-13·4 to 14·1%)
Motor vehicle road injuries	1·9 (1·5 to 2·2)	5·3% (4·7 to 5·9%)	2·4 (1·9 to 2·8)	5·4% (4·8 to 6·0%)	26·8% (9·0 to 48·0%)	1·8% (-6·5 to 11·0%)	51·4 (41·6 to 60·2)	2·6% (2·2 to 2·9%)	65·3 (52·9 to 78·4)	2·7% (2·3 to 3·0%)	27·1% (10·0 to 47·4%)	3·8% (-6·1 to 15·1%)
Other road injuries	0·0 (0·0 to 0·0)	5·3% (4·1 to 7·0%)	0·0 (0·0 to 0·1)	5·3% (4·3 to 6·4%)	29·3% (6·2 to 65·8%)	-0·8% (-16·9 to 19·3%)	1·1 (0·9 to 1·4)	2·6% (2·0 to 3·7%)	1·5 (1·1 to 1·9)	2·7% (2·2 to 3·5%)	34·7% (17·9 to 58·7%)	3·8% (-15·7 to 27·8%)

Supplemental Results Table 1. All-age deaths, death PAFs, DALYs, DALY PAFs, and percent change for each risk factor and outcome for both sexes combined in 2005 and 2015 for Low SDI.

Data in parenthesis are 95% uncertainty intervals. PAFs=population attributable fractions. DALYs=disability-adjusted life-years.

	2005 Deaths (in thousands)	2005 Deaths PAF	2015 Deaths (in thousands)	2015 Deaths PAF	Percent change deaths 2005-2015	Percent change deaths PAF 2005-2015	2005 DALYs (in thousands)	2005 DALYs PAF	2015 DALYs (in thousands)	2015 DALYs PAF	Percent change DALYs 2005-2015	Percent change DALYs PAF 2005-2015
Other transport injuries	0·2<br>(0·1 to 0·2)	3·8%<br>(3·2 to 4·9%)	0·2<br>(0·2 to 0·3)	4·0%<br>(3·3 to 5·0%)	16·9%<br>(-1·7 to 42·2%)	3·0%<br>(-10·7 to 18·9%)	5·1<br>(3·9 to 6·2)	1·6%<br>(1·3 to 2·1%)	5·9<br>(4·4 to 7·7)	1·7%<br>(1·4 to 2·2%)	16·9%<br>(-2·1 to 41·9%)	3·2%<br>(-12·5 to 24·3%)
Falls	8·2<br>(7·0 to 9·7)	29·6%<br>(25·1 to 33·4%)	10·9<br>(9·0 to 12·8)	31·1%<br>(27·6 to 34·4%)	32·4%<br>(13·0 to 57·3%)	5·2%<br>(-8·9 to 22·9%)	169·2<br>(143·3 to 196·2)	10·3%<br>(8·3 to 11·8%)	223·8<br>(191·1 to 262·7)	10·8%<br>(9·3 to 12·2%)	32·2%<br>(18·8 to 48·4%)	5·4%<br>(-11·6 to 29·9%)
Other exposure to mechanical forces	0·5<br>(0·4 to 0·6)	4·2%<br>(3·6 to 5·2%)	0·6<br>(0·5 to 0·8)	4·4%<br>(3·7 to 5·1%)	24·6%<br>(5·2 to 46·9%)	2·7%<br>(-10·8 to 17·9%)	20·2<br>(15·9 to 24·9)	2·2%<br>(1·8 to 2·6%)	25·5<br>(20·1 to 31·9)	2·2%<br>(1·9 to 2·6%)	26·4%<br>(14·9 to 39·7%)	4·2%<br>(-6·8 to 15·6%)
Non-venomous animal contact	0·1<br>(0·0 to 0·1)	2·4%<br>(1·9 to 3·9%)	0·1<br>(0·1 to 0·1)	2·7%<br>(2·2 to 4·1%)	13·9%<br>(-8·8 to 61·4%)	13·1%<br>(-6·4 to 35·5%)	2·9<br>(1·9 to 3·8)	1·0%<br>(0·8 to 1·6%)	3·3<br>(2·4 to 4·4)	1·1%<br>(0·9 to 1·7%)	15·4%<br>(-1·4 to 43·0%)	13·8%<br>(-5·0 to 35·2%)
Assault by other means	0·2<br>(0·1 to 0·3)	1·3%<br>(1·1 to 1·5%)	0·2<br>(0·2 to 0·3)	1·3%<br>(1·1 to 1·6%)	29·3%<br>(2·8 to 64·8%)	2·0%<br>(-11·7 to 17·2%)	6·4<br>(5·0 to 8·7)	0·8%<br>(0·6 to 0·9%)	8·3<br>(6·2 to 11·2)	0·8%<br>(0·6 to 1·0%)	29·2%<br>(4·7 to 61·6%)	3·5%<br>(-10·8 to 19·8%)
Exposure to forces of nature	-	-	-	-	-	-	0·2<br>(0·1 to 0·5)	0·4%<br>(0·2 to 0·8%)	0·3<br>(0·2 to 0·7)	0·6%<br>(0·3 to 1·1%)	44·2%<br>(20·5 to 74·4%)	51·1%<br>(8·8 to 106·6%)
**Low glomerular filtration rate: All causes**	58·4<br>(53·0 to 64·9)	1·2%<br>(1·1 to 1·4%)	79·1<br>(68·7 to 90·5)	1·7%<br>(1·5 to 1·9%)	35·6%<br>(21·0 to 50·9%)	37·2%<br>(29·4 to 45·1%)	1,895·3<br>(1,717·5 to 2,079·3)	0·6%<br>(0·5 to 0·6%)	2,518·7<br>(2,239·7 to 2,838·2)	0·8%<br>(0·7 to 0·9%)	32·9%<br>(20·7 to 45·9%)	39·3%<br>(29·4 to 49·1%)
Ischaemic heart disease	16·1<br>(13·8 to 18·5)	8·2%<br>(7·2 to 9·2%)	21·4<br>(18·0 to 25·7)	8·3%<br>(7·3 to 9·2%)	33·4%<br>(17·9 to 49·8%)	0·6%<br>(-2·6 to 3·7%)	325·9<br>(279·1 to 378·1)	6·9%<br>(6·1 to 7·8%)	428·1<br>(355·6 to 518·3)	7·0%<br>(6·1 to 7·8%)	31·4%<br>(15·4 to 48·7%)	0·3%<br>(-3·3 to 3·7%)
Ischaemic stroke	7·7<br>(5·7 to 9·7)	9·8%<br>(8·3 to 11·3%)	10·2<br>(7·7 to 12·8)	9·9%<br>(8·4 to 11·4%)	31·8%<br>(15·0 to 51·4%)	0·9%<br>(-2·4 to 4·3%)	139·7<br>(106·4 to 174·1)	8·6%<br>(7·4 to 10·0%)	180·1<br>(137·4 to 224·0)	8·7%<br>(7·5 to 10·0%)	28·9%<br>(12·2 to 47·7%)	0·8%<br>(-2·5 to 4·4%)
Hemorrhagic stroke	9·8<br>(8·0 to 11·9)	8·0%<br>(6·9 to 9·3%)	12·9<br>(10·4 to 15·8)	8·1%<br>(7·1 to 9·3%)	30·8%<br>(14·1 to 51·3%)	1·5%<br>(-2·4 to 5·5%)	230·9<br>(191·2 to 278·6)	6·3%<br>(5·4 to 7·3%)	298·3<br>(239·3 to 365·7)	6·5%<br>(5·6 to 7·4%)	29·2%<br>(12·6 to 49·5%)	2·2%<br>(-2·2 to 7·0%)
Peripheral vascular disease	0·0<br>(0·0 to 0·0)	15·9%<br>(13·3 to 18·4%)	0·0<br>(0·0 to 0·1)	15·8%<br>(13·2 to 18·3%)	66·2%<br>(32·1 to 118·0%)	-1·1%<br>(-5·6 to 3·9%)	2·4<br>(1·3 to 4·2)	17·2%<br>(14·7 to 19·8%)	3·5<br>(1·9 to 6·0)	17·2%<br>(14·7 to 19·9%)	43·6%<br>(36·7 to 53·4%)	-0·1%<br>(-2·0 to 1·7%)
Chronic kidney disease due to diabetes mellitus	5·3<br>(4·5 to 6·3)	100·0%<br>(100·0 to 100·0%)	7·4<br>(6·1 to 8·9)	100·0%<br>(100·0 to 100·0%)	38·6%<br>(23·1 to 58·1%)	-	274·4<br>(236·4 to 317·0)	100·0%<br>(100·0 to 100·0%)	362·2<br>(312·8 to 423·1)	100·0%<br>(100·0 to 100·0%)	32·0%<br>(21·5 to 44·5%)	-
Chronic kidney disease due to hypertension	14·0<br>(12·3 to 15·7)	100·0%<br>(100·0 to 100·0%)	20·0<br>(17·1 to 23·0)	100·0%<br>(100·0 to 100·0%)	42·9%<br>(26·5 to 60·3%)	-	507·9<br>(444·4 to 566·9)	100·0%<br>(100·0 to 100·0%)	705·7<br>(603·3 to 812·5)	100·0%<br>(100·0 to 100·0%)	38·9%<br>(23·6 to 54·9%)	-
Chronic kidney disease due to glomerulonephritis	3·9<br>(3·3 to 4·5)	100·0%<br>(100·0 to 100·0%)	5·3<br>(4·4 to 6·3)	100·0%<br>(100·0 to 100·0%)	36·2%<br>(20·9 to 54·6%)	-	260·4<br>(224·3 to 297·8)	100·0%<br>(100·0 to 100·0%)	343·3<br>(293·2 to 400·1)	100·0%<br>(100·0 to 100·0%)	31·8%<br>(20·6 to 45·1%)	-
Chronic kidney disease due to other causes	1·5<br>(1·2 to 1·9)	100·0%<br>(100·0 to 100·0%)	2·0<br>(1·5 to 2·5)	100·0%<br>(100·0 to 100·0%)	28·8%<br>(13·2 to 47·1%)	-	148·8<br>(123·2 to 178·2)	100·0%<br>(100·0 to 100·0%)	190·9<br>(158·3 to 227·3)	100·0%<br>(100·0 to 100·0%)	28·3%<br>(18·8 to 37·3%)	-
Gout	-	-	-	-	-	-	4·9<br>(3·2 to 6·7)	13·7%<br>(12·2 to 15·2%)	6·7<br>(4·5 to 9·3)	13·6%<br>(12·2 to 15·1%)	37·6%<br>(33·4 to 41·8%)	-0·5%<br>(-2·7 to 1·9%)

Supplemental Results Table 1. All-age deaths, death PAFs, DALYs, DALY PAFs, and percent change for each risk factor and outcome for both sexes combined in 2005 and 2015 for Central Europe, Eastern Europe, and Central Asia.

Data in parenthesis are 95% uncertainty intervals. PAFs=population attributable fractions. DALYs=disability-adjusted life-years.

	2005 Deaths (in thousands)	2005 Deaths PAF	2015 Deaths (in thousands)	2015 Deaths PAF	Percent change deaths 2005-2015	Percent change deaths PAF 2005-2015	2005 DALYs (in thousands)	2005 DALYs PAF	2015 DALYs (in thousands)	2015 DALYs PAF	Percent change DALYs 2005-2015	Percent change DALYs PAF 2005-2015
**All risk factors: All causes**	3,769·1 (3,702·4 to 3,833·0)	71·4% (70·1 to 72·6%)	3,413·3 (3,334·2 to 3,492·3)	70·2% (68·7 to 71·5%)	-9·4% (-10·7 to -8·2%)	-1·7% (-2·5 to -1·1%)	94,985·3 (91,257·8 to 99,140·4)	54·4% (52·3 to 56·6%)	79,753·6 (75,604·3 to 84,181·9)	52·2% (49·8 to 54·6%)	-16·0% (-17·4 to -14·7%)	-4·2% (-5·1 to -3·3%)
**Environmental/occupational risks: All causes**	606·6 (525·3 to 693·6)	11·5% (9·9 to 13·2%)	527·5 (448·7 to 608·8)	10·8% (9·2 to 12·5%)	-13·1% (-17·9 to -8·0%)	-5·6% (-10·9 to 0·0%)	15,745·8 (14,010·2 to 17,643·6)	9·0% (8·1 to 10·1%)	12,718·9 (11,313·6 to 14,175·7)	8·3% (7·4 to 9·2%)	-19·2% (-22·9 to -15·2%)	-7·8% (-11·9 to -3·4%)
**Unsafe water, sanitation, and handwashing: All causes**	18·7 (13·8 to 23·6)	0·4% (0·3 to 0·4%)	13·4 (9·7 to 17·6)	0·3% (0·2 to 0·4%)	-28·1% (-33·8 to -22·8%)	-22·0% (-28·2 to -16·4%)	1,012·5 (798·4 to 1,238·3)	0·6% (0·5 to 0·7%)	611·2 (470·4 to 760·9)	0·4% (0·3 to 0·5%)	-39·6% (-44·9 to -34·3%)	-31·1% (-36·9 to -25·1%)
**Unsafe water source: All causes**	3·8 (2·6 to 4·8)	0·1% (0·0 to 0·1%)	2·1 (1·4 to 2·8)	0·0% (0·0 to 0·1%)	-43·7% (-52·2 to -33·7%)	-38·9% (-48·3 to -28·3%)	340·4 (232·7 to 429·0)	0·2% (0·1 to 0·2%)	187·9 (119·5 to 249·7)	0·1% (0·1 to 0·2%)	-44·8% (-53·4 to -35·0%)	-37·0% (-46·5 to -26·0%)
Diarrhoeal diseases	3·8 (2·6 to 4·7)	72·3% (49·2 to 86·9%)	2·1 (1·4 to 2·8)	66·4% (44·8 to 82·2%)	-44·0% (-52·4 to -34·0%)	-8·2% (-13·7 to -3·8%)	338·5 (231·6 to 426·9)	72·6% (49·4 to 87·3%)	186·2 (118·6 to 247·5)	67·8% (44·6 to 84·2%)	-45·0% (-53·6 to -35·2%)	-6·7% (-12·7 to -2·2%)
Typhoid fever	0·0 (0·0 to 0·0)	70·1% (46·5 to 85·7%)	0·0 (0·0 to 0·0)	66·0% (42·8 to 83·1%)	-14·1% (-21·8 to -6·8%)	-5·9% (-12·7 to -0·4%)	1·4 (0·7 to 2·6)	70·5% (46·6 to 85·9%)	1·2 (0·5 to 2·2)	66·5% (43·3 to 83·4%)	-16·2% (-24·4 to -8·5%)	-5·6% (-12·1 to -0·5%)
Paratyphoid fever	0·0 (0·0 to 0·0)	66·8% (43·7 to 83·3%)	0·0 (0·0 to 0·0)	62·9% (39·8 to 81·1%)	-4·2% (-14·7 to 5·7%)	-5·7% (-14·4 to 1·5%)	0·5 (0·2 to 1·1)	67·5% (44·0 to 83·8%)	0·5 (0·2 to 1·1)	63·9% (40·8 to 81·7%)	-6·3% (-17·4 to 4·3%)	-5·3% (-13·1 to 1·2%)
**Unsafe sanitation: All causes**	2·5 (2·2 to 2·8)	0·0% (0·0 to 0·1%)	1·3 (1·0 to 1·6)	0·0% (0·0 to 0·0%)	-48·4% (-58·1 to -37·0%)	-44·0% (-54·5 to -31·6%)	225·2 (197·9 to 254·4)	0·1% (0·1 to 0·1%)	120·0 (96·8 to 145·5)	0·1% (0·1 to 0·1%)	-46·7% (-56·3 to -35·6%)	-39·2% (-49·9 to -26·7%)
Diarrhoeal diseases	2·5 (2·2 to 2·8)	47·2% (44·3 to 50·1%)	1·3 (1·0 to 1·6)	39·7% (35·8 to 43·6%)	-48·6% (-58·4 to -37·2%)	-15·9% (-23·4 to -8·0%)	223·9 (196·4 to 253·2)	48·0% (45·3 to 51·0%)	118·9 (95·4 to 144·4)	43·2% (39·5 to 47·0%)	-46·9% (-56·5 to -35·8%)	-10·0% (-16·7 to -3·2%)
Typhoid fever	0·0 (0·0 to 0·0)	48·3% (45·0 to 51·3%)	0·0 (0·0 to 0·0)	41·6% (36·9 to 46·0%)	-21·4% (-29·4 to -13·3%)	-13·8% (-21·2 to -7·4%)	1·0 (0·5 to 1·7)	49·1% (46·0 to 52·0%)	0·7 (0·4 to 1·3)	42·7% (38·1 to 46·9%)	-22·7% (-30·9 to -14·1%)	-13·0% (-20·0 to -7·0%)
Paratyphoid fever	0·0 (0·0 to 0·0)	43·3% (40·0 to 46·7%)	0·0 (0·0 to 0·0)	37·5% (32·0 to 42·5%)	-12·0% (-23·3 to -0·6%)	-13·5% (-23·3 to -5·2%)	0·3 (0·1 to 0·7)	44·6% (41·4 to 48·0%)	0·3 (0·1 to 0·6)	39·1% (33·9 to 43·9%)	-13·2% (-24·4 to -1·5%)	-12·5% (-21·1 to -4·9%)
**No handwashing with soap: All causes**	15·6 (10·7 to 20·7)	0·3% (0·2 to 0·4%)	11·6 (7·9 to 15·7)	0·2% (0·2 to 0·3%)	-25·5% (-31·1 to -20·0%)	-19·1% (-25·3 to -13·2%)	738·4 (530·6 to 960·2)	0·4% (0·3 to 0·6%)	452·7 (318·9 to 604·0)	0·3% (0·2 to 0·4%)	-38·7% (-43·8 to -33·7%)	-30·0% (-35·7 to -24·5%)
Diarrhoeal diseases	1·6 (1·3 to 2·1)	31·5% (24·3 to 38·3%)	0·9 (0·7 to 1·2)	29·5% (22·4 to 36·2%)	-42·9% (-51·1 to -32·9%)	-6·4% (-9·1 to -4·1%)	146·9 (112·2 to 184·1)	31·5% (24·3 to 38·3%)	80·6 (57·5 to 106·1)	29·3% (22·3 to 36·1%)	-45·1% (-53·5 to -35·4%)	-6·9% (-9·5 to -4·6%)
Typhoid fever	0·0 (0·0 to 0·0)	30·9% (23·0 to 38·1%)	0·0 (0·0 to 0·0)	28·3% (20·8 to 35·5%)	-16·3% (-22·6 to -10·8%)	-8·3% (-11·2 to -5·9%)	0·6 (0·3 to 1·1)	30·9% (23·1 to 38·2%)	0·5 (0·2 to 0·9)	28·4% (20·8 to 35·6%)	-18·5% (-25·9 to -11·8%)	-8·3% (-11·2 to -5·9%)
Paratyphoid fever	0·0 (0·0 to 0·0)	30·7% (23·0 to 37·9%)	0·0 (0·0 to 0·0)	28·2% (20·9 to 35·3%)	-6·5% (-13·8 to -0·2%)	-8·1% (-11·1 to -5·8%)	0·2 (0·1 to 0·5)	30·8% (23·1 to 38·0%)	0·2 (0·1 to 0·5)	28·3% (21·0 to 35·3%)	-8·9% (-16·8 to -1·0%)	-8·1% (-11·0 to -5·8%)
**Air pollution: All causes**	487·4 (416·4 to 564·6)	9·2% (7·9 to 10·7%)	419·7 (352·3 to 493·8)	8·6% (7·2 to 10·1%)	-13·9% (-20·1 to -7·8%)	-6·5% (-13·1 to 0·2%)	10,484·7 (9,128·4 to 11,936·0)	6·0% (5·1 to 7·0%)	8,044·1 (6,982·1 to 9,111·4)	5·3% (4·4 to 6·2%)	-23·3% (-28·1 to -17·8%)	-12·4% (-18·2 to -6·4%)
**Ambient particulate matter pollution: All causes**	386·3 (327·2 to 448·4)	7·3% (6·2 to 8·5%)	361·1 (297·1 to 425·5)	7·4% (6·1 to 8·7%)	-6·5% (-12·1 to -1·0%)	1·5% (-4·5 to 7·4%)	8,185·3 (7,102·8 to 9,259·3)	4·7% (4·0 to 5·5%)	6,847·4 (5,903·9 to 7,793·2)	4·5% (3·8 to 5·3%)	-16·3% (-20·8 to -12·1%)	-4·5% (-9·8 to 0·5%)
Lower respiratory infections	19·6 (10·4 to 31·9)	16·0% (8·5 to 25·7%)	18·2 (10·3 to 28·4)	17·6% (10·0 to 27·0%)	-7·2% (-14·7 to 3·2%)	10·0% (4·3 to 19·8%)	873·6 (491·7 to 1,377·4)	16·8% (9·5 to 26·3%)	683·1 (407·6 to 1,023·5)	18·9% (11·5 to 28·1%)	-21·8% (-29·5 to -10·2%)	12·1% (4·9 to 25·3%)
Tracheal, bronchus, and lung cancer	17·2 (9·2 to 26·9)	10·6% (5·7 to 16·5%)	18·4 (10·1 to 28·3)	11·7% (6·4 to 17·9%)	7·2% (2·6 to 12·4%)	10·2% (7·8 to 14·0%)	413·4 (219·9 to 646·4)	10·6% (5·7 to 16·5%)	425·9 (234·0 to 656·1)	11·7% (6·5 to 17·9%)	3·0% (-1·5 to 8·5%)	10·6% (8·1 to 14·6%)
Ischaemic heart disease	234·2 (184·8 to 284·2)	13·7% (10·7 to 16·6%)	222·6 (167·5 to 276·7)	13·6% (10·2 to 16·9%)	-5·0% (-14·5 to 3·0%)	-0·4% (-10·1 to 7·8%)	4,671·0 (3,834·6 to 5,499·2)	15·2% (12·5 to 17·9%)	3,927·2 (3,195·7 to 4,666·5)	15·3% (12·4 to 18·2%)	-15·9% (-22·4 to -9·8%)	0·4% (-6·9 to 7·5%)
Ischaemic stroke	60·0 (40·6 to 81·5)	9·4% (6·3 to 12·6%)	52·7 (34·4 to 72·9)	9·6% (6·3 to 13·2%)	-12·2% (-24·3 to -1·3%)	2·3% (-11·6 to 14·8%)	986·3 (716·3 to 1,261·9)	10·2% (7·4 to 13·0%)	782·7 (576·8 to 996·9)	10·5% (7·8 to 13·4%)	-20·6% (-29·7 to -10·9%)	3·3% (-7·7 to 15·4%)
Hemorrhagic stroke	34·6 (26·1 to 43·3)	10·9% (8·2 to 13·6%)	29·0 (21·7 to 36·6)	11·3% (8·5 to 14·2%)	-16·3% (-24·4 to -8·0%)	3·5% (-6·0 to 12·9%)	807·6 (627·7 to 999·6)	12·0% (9·3 to 14·8%)	633·6 (493·2 to 783·8)	12·6% (9·8 to 15·5%)	-21·6% (-28·0 to -14·6%)	5·2% (-2·6 to 13·1%)
Chronic obstructive pulmonary disease	20·7 (9·6 to 33·6)	16·5% (7·6 to 27·0%)	20·2 (9·8 to 32·1)	18·0% (8·7 to 28·6%)	-2·3% (-6·5 to 5·8%)	9·1% (5·8 to 18·6%)	433·5 (200·1 to 709·3)	16·1% (7·4 to 26·3%)	394·9 (192·8 to 623·3)	17·7% (8·5 to 28·1%)	-8·9% (-12·8 to -1·0%)	9·8% (6·3 to 19·5%)
**Household air pollution from solid fuels: All causes**	113·2 (80·4 to 156·7)	2·1% (1·5 to 3·0%)	64·8 (43·7 to 92·5)	1·3% (0·9 to 1·9%)	-42·7% (-55·7 to -23·1%)	-37·8% (-52·0 to -17·1%)	2,657·6 (1,887·0 to 3,631·2)	1·5% (1·1 to 2·1%)	1,380·0 (917·7 to 1,993·7)	0·9% (0·6 to 1·3%)	-48·1% (-59·9 to -30·2%)	-40·7% (-53·9 to -20·0%)
Lower respiratory infections	11·1 (6·6 to 17·5)	9·0% (5·4 to 14·0%)	6·2 (3·5 to 10·0)	6·0% (3·4 to 9·8%)	-44·3% (-56·7 to -26·7%)	-34·0% (-48·9 to -13·1%)	551·0 (332·5 to 853·4)	10·6% (6·3 to 16·4%)	262·0 (146·0 to 428·8)	7·2% (4·0 to 11·7%)	-52·4% (-65·1 to -34·5%)	-31·8% (-49·6 to -7·9%)
Tracheal, bronchus, and lung cancer	8·8 (4·4 to 15·5)	5·4% (2·7 to 9·7%)	5·9 (2·8 to 10·4)	3·8% (1·8 to 6·6%)	-32·4% (-45·0 to -12·3%)	-30·5% (-43·5 to -9·2%)	212·4 (105·5 to 375·3)	5·4% (2·7 to 9·7%)	137·7 (65·9 to 239·6)	3·8% (1·8 to 6·7%)	-35·2% (-47·2 to -16·0%)	-30·4% (-43·5 to -9·4%)
Ischaemic heart disease	55·5 (40·0 to 77·7)	3·2% (2·3 to 4·5%)	31·6 (20·9 to 45·7)	1·9% (1·3 to 2·8%)	-42·9% (-57·4 to -21·7%)	-40·2% (-55·5 to -18·7%)	1,130·8 (825·7 to 1,558·5)	3·7% (2·7 to 5·1%)	582·6 (393·0 to 828·6)	2·3% (1·5 to 3·2%)	-48·5% (-61·5 to -29·1%)	-38·5% (-54·1 to -16·2%)
Ischaemic stroke	16·4 (10·6 to 24·4)	2·6% (1·7 to 3·8%)	8·9 (5·5 to 13·8)	1·6% (1·0 to 2·5%)	-45·8% (-60·9 to -22·8%)	-36·8% (-54·5 to -10·1%)	274·2 (184·4 to 406·9)	2·8% (1·9 to 4·2%)	134·8 (86·5 to 203·4)	1·8% (1·2 to 2·7%)	-50·8% (-64·0 to -30·2%)	-36·0% (-53·4 to -9·1%)
Hemorrhagic stroke	12·1 (8·5 to 17·0)	3·8% (2·7 to 5·4%)	6·4 (4·3 to 9·2)	2·5% (1·7 to 3·5%)	-47·1% (-58·5 to -32·4%)	-34·6% (-48·6 to -17·0%)	281·6 (195·9 to 395·4)	4·2% (2·9 to 5·8%)	141·0 (93·8 to 200·9)	2·8% (1·9 to 4·0%)	-49·9% (-60·8 to -34·9%)	-32·9% (-47·2 to -13·4%)
Chronic obstructive pulmonary disease	9·3 (4·4 to 16·8)	7·5% (3·5 to 13·5%)	5·7 (2·5 to 10·2)	5·1% (2·3 to 9·2%)	-38·4% (-50·4 to -22·8%)	-31·2% (-44·0 to -13·7%)	191·8 (90·7 to 348·2)	7·1% (3·4 to 12·9%)	110·2 (49·0 to 194·5)	4·9% (2·2 to 8·8%)	-42·6% (-54·5 to -26·8%)	-30·8% (-44·3 to -11·9%)
Cataract	-	-	-	-	-	-	15·8 (9·6 to 23·4)	8·4% (6·2 to 11·3%)	11·6 (6·6 to 19·3)	5·3% (3·5 to 8·0%)	-26·3% (-47·5 to 7·4%)	-37·0% (-54·9 to -7·8%)
**Ambient ozone pollution: All causes**	6·1 (2·3 to 10·9)	0·1% (0·0 to 0·2%)	5·5 (2·0 to 9·8)	0·1% (0·0 to 0·2%)	-10·0% (-12·5 to -7·5%)	-2·3% (-4·9 to 0·3%)	110·5 (40·2 to 195·4)	0·1% (0·0 to 0·1%)	89·5 (32·7 to 158·8)	0·1% (0·0 to 0·1%)	-19·0% (-21·4 to -16·3%)	-7·5% (-10·5 to -4·1%)
Chronic obstructive pulmonary disease	6·1 (2·3 to 10·9)	4·9% (1·8 to 8·7%)	5·5 (2·0 to 9·8)	4·9% (1·8 to 8·7%)	-10·0% (-12·5 to -7·5%)	0·5% (-0·6 to 1·5%)	110·5 (40·2 to 195·4)	4·1% (1·5 to 7·2%)	89·5 (32·7 to 158·8)	4·0% (1·4 to 7·0%)	-19·0% (-21·4 to -16·3%)	-2·4% (-3·8 to -1·0%)
**Other environmental risks: All causes**	78·9 (38·1 to 127·7)	1·5% (0·7 to 2·4%)	70·4 (34·1 to 115·3)	1·4% (0·7 to 2·4%)	-10·8% (-18·2 to -2·8%)	-3·2% (-11·2 to 5·4%)	1,444·4 (698·1 to 2,375·1)	0·8% (0·4 to 1·4%)	1,168·0 (569·0 to 1,926·1)	0·8% (0·4 to 1·3%)	-19·1% (-23·7 to -13·5%)	-7·7% (-12·9 to -0·8%)
**Residential radon: All causes**	11·7 (7·6 to 16·5)	0·2% (0·1 to 0·3%)	11·5 (7·6 to 16·4)	0·2% (0·2 to 0·3%)	-1·9% (-10·6 to 7·7%)	6·5% (-2·7 to 17·1%)	282·5 (188·2 to 394·9)	0·2% (0·1 to 0·2%)	265·3 (176·1 to 370·5)	0·2% (0·1 to 0·2%)	-6·1% (-15·1 to 3·6%)	7·2% (-3·1 to 18·6%)
Tracheal, bronchus, and lung cancer	11·7 (7·6 to 16·5)	7·3% (4·7 to 10·2%)	11·5 (7·6 to 16·4)	7·3% (4·7 to 10·3%)	-1·9% (-10·6 to 7·7%)	0·9% (-7·6 to 10·8%)	282·5 (188·2 to 394·9)	7·2% (4·8 to 10·1%)	265·3 (176·1 to 370·5)	7·3% (4·9 to 10·2%)	-6·1% (-15·1 to 3·6%)	0·8% (-8·0 to 10·6%)
**Lead exposure: All causes**	67·2 (27·5 to 116·2)	1·3% (0·5 to 2·2%)	58·9 (23·7 to 103·2)	1·2% (0·5 to 2·1%)	-12·3% (-22·0 to -3·3%)	-4·8% (-15·3 to 5·1%)	1,162·0 (436·3 to 2,094·4)	0·7% (0·2 to 1·2%)	902·7 (331·1 to 1,665·4)	0·6% (0·2 to 1·1%)	-22·3% (-28·8 to -17·5%)	-11·3% (-18·8 to -6·1%)
Rheumatic heart disease	0·2 (0·1 to 0·4)	0·9% (0·3 to 1·9%)	0·1 (0·0 to 0·2)	0·9% (0·3 to 1·9%)	-34·4% (-39·5 to -26·0%)	0·6% (-6·7 to 13·8%)	5·2 (1·5 to 11·1)	0·9% (0·2 to 1·8%)	3·3 (0·9 to 6·8)	0·9% (0·2 to 1·9%)	-37·7% (-41·7 to -32·4%)	1·8% (-3·2 to 9·6%)
Ischaemic heart disease	38·6 (15·3 to 68·6)	2·2% (0·9 to 4·0%)	34·8 (13·2 to 63·4)	2·1% (0·8 to 3·9%)	-9·7% (-20·3 to 1·3%)	-5·3% (-16·7 to 6·0%)	632·1 (228·5 to 1,169·9)	2·1% (0·7 to 3·8%)	501·0 (174·5 to 942·2)	2·0% (0·7 to 3·7%)	-20·7% (-27·9 to -15·3%)	-5·4% (-13·7 to 0·9%)
Ischaemic stroke	12·9 (4·8 to 24·0)	2·0% (0·8 to 3·8%)	10·2 (3·7 to 19·2)	1·9% (0·7 to 3·5%)	-21·1% (-34·1 to -6·0%)	-8·1% (-22·7 to 9·3%)	195·8 (69·8 to 364·5)	2·0% (0·7 to 3·8%)	138·6 (48·4 to 259·5)	1·9% (0·6 to 3·5%)	-29·2% (-38·7 to -20·5%)	-7·9% (-20·5 to 3·0%)
Hemorrhagic stroke	8·2 (3·3 to 14·7)	2·6% (1·0 to 4·7%)	6·4 (2·5 to 11·6)	2·5% (1·0 to 4·5%)	-22·2% (-29·6 to -12·4%)	-3·9% (-12·4 to 7·8%)	163·0 (58·0 to 307·1)	2·4% (0·9 to 4·5%)	116·1 (42·2 to 219·4)	2·3% (0·8 to 4·4%)	-28·8% (-33·9 to -23·7%)	-4·5% (-10·7 to 2·0%)
Hypertensive heart disease	3·9 (1·0 to 9·4)	5·2% (1·4 to 12·3%)	4·1 (1·0 to 10·3)	5·0% (1·3 to 12·4%)	5·2% (-7·4 to 21·0%)	-2·3% (-13·6 to 10·7%)	66·0 (19·7 to 154·1)	4·8% (1·5 to 11·1%)	61·6 (18·3 to 145·3)	4·7% (1·4 to 10·9%)	-6·6% (-15·1 to 3·7%)	-2·1% (-9·5 to 7·1%)
Cardiomyopathy and myocarditis	1·3 (0·4 to 2·8)	1·1% (0·3 to 2·3%)	1·2 (0·4 to 2·5)	1·1% (0·4 to 2·2%)	-7·8% (-17·5 to 10·4%)	-2·0% (-11·2 to 16·1%)	32·7 (7·1 to 79·9)	0·9% (0·2 to 2·2%)	24·6 (5·8 to 58·7)	0·9% (0·2 to 2·0%)	-24·9% (-31·7 to -12·5%)	-5·3% (-13·1 to 11·5%)

Supplemental Results Table 1. All-age deaths, death PAFs, DALYs, DALY PAFs, and percent change for each risk factor and outcome for both sexes combined in 2005 and 2015 for Central Europe, Eastern Europe, and Central Asia.

Data in parenthesis are 95% uncertainty intervals. PAFs=population attributable fractions. DALYs=disability-adjusted life-years.

	2005 Deaths (in thousands)	2005 Deaths PAF	2015 Deaths (in thousands)	2015 Deaths PAF	Percent change deaths 2005-2015	Percent change deaths PAF 2005-2015	2005 DALYs (in thousands)	2005 DALYs PAF	2015 DALYs (in thousands)	2015 DALYs PAF	Percent change DALYs 2005-2015	Percent change DALYs PAF 2005-2015
Atrial fibrillation and flutter	0·3 (0·1 to 0·5)	1·6% (0·7 to 2·7%)	0·3 (0·1 to 0·6)	1·4% (0·6 to 2·5%)	10·9% (-4·7 to 24·0%)	-11·7% (-24·1 to -1·6%)	7·5 (3·0 to 13·4)	1·7% (0·7 to 2·9%)	7·5 (2·7 to 14·0)	1·5% (0·6 to 2·6%)	0·3% (-12·1 to 7·1%)	-12·7% (-22·9 to -7·1%)
Aortic aneurysm	0·2 (0·1 to 0·4)	1·3% (0·5 to 2·5%)	0·2 (0·1 to 0·4)	1·2% (0·4 to 2·3%)	1·4% (-11·9 to 10·3%)	-12·3% (-23·3 to -5·6%)	4·0 (1·3 to 7·7)	1·2% (0·4 to 2·4%)	3·6 (1·0 to 7·4)	1·1% (0·3 to 2·2%)	-7·8% (-20·1 to -0·8%)	-11·7% (-23·1 to -6·3%)
Peripheral vascular disease	0·1 (0·0 to 0·1)	1·0% (0·3 to 1·8%)	0·1 (0·0 to 0·2)	0·9% (0·3 to 1·7%)	19·3% (-5·3 to 45·5%)	-10·1% (-27·4 to 6·8%)	1·7 (0·6 to 3·3)	1·0% (0·3 to 1·8%)	1·8 (0·5 to 3·6)	0·9% (0·3 to 1·6%)	3·9% (-13·6 to 17·8%)	-10·9% (-26·1 to -0·7%)
Endocarditis	0·0 (0·0 to 0·1)	1·1% (0·4 to 2·2%)	0·0 (0·0 to 0·1)	1·0% (0·3 to 2·0%)	-8·9% (-20·0 to 1·3%)	-11·7% (-22·8 to -4·7%)	1·1 (0·3 to 2·5)	0·9% (0·2 to 2·1%)	0·9 (0·2 to 2·2)	0·8% (0·2 to 1·9%)	-15·6% (-27·2 to -4·2%)	-13·0% (-25·3 to -5·5%)
Other cardiovascular and circulatory diseases	0·7 (0·3 to 1·2)	1·6% (0·6 to 2·8%)	0·6 (0·2 to 1·0)	1·4% (0·5 to 2·6%)	-12·2% (-21·0 to -5·7%)	-12·5% (-21·1 to -7·3%)	16·4 (5·8 to 30·4)	1·5% (0·5 to 2·9%)	13·7 (4·4 to 26·5)	1·3% (0·4 to 2·6%)	-16·7% (-25·5 to -11·9%)	-11·5% (-20·4 to -7·3%)
Idiopathic developmental intellectual disability	-	-	-	-	-	-	13·9 (5·5 to 25·3)	9·6% (7·1 to 11·9%)	9·0 (3·5 to 16·3)	6·3% (4·2 to 8·4%)	-35·7% (-47·5 to -22·9%)	-34·6% (-46·8 to -21·3%)
Chronic kidney disease due to diabetes mellitus	0·2 (0·1 to 0·4)	2·1% (0·9 to 3·9%)	0·2 (0·1 to 0·4)	2·0% (0·8 to 3·8%)	-6·2% (-15·3 to 0·1%)	-4·2% (-12·7 to 0·4%)	5·1 (1·9 to 10·1)	1·6% (0·6 to 3·1%)	4·5 (1·6 to 9·3)	1·5% (0·5 to 3·0%)	-10·2% (-17·8 to -5·6%)	-6·2% (-14·3 to -2·4%)
Chronic kidney disease due to hypertension	0·3 (0·1 to 0·6)	2·3% (1·0 to 4·1%)	0·3 (0·1 to 0·6)	2·2% (0·9 to 4·0%)	1·1% (-7·2 to 7·2%)	-4·3% (-11·5 to 0·0%)	6·8 (2·6 to 12·6)	1·8% (0·7 to 3·4%)	6·3 (2·2 to 11·9)	1·7% (0·6 to 3·3%)	-8·0% (-15·7 to -3·5%)	-5·6% (-13·1 to -2·0%)
Chronic kidney disease due to glomerulonephritis	0·2 (0·1 to 0·4)	1·7% (0·7 to 3·1%)	0·2 (0·1 to 0·4)	1·8% (0·7 to 3·2%)	-0·9% (-6·3 to 5·2%)	3·7% (-0·6 to 8·7%)	7·6 (2·5 to 15·3)	1·2% (0·4 to 2·4%)	7·0 (2·3 to 14·2)	1·2% (0·4 to 2·4%)	-7·7% (-13·5 to -3·4%)	-1·0% (-6·3 to 3·0%)
Chronic kidney disease due to other causes	0·0 (0·0 to 0·1)	1·9% (0·8 to 3·5%)	0·0 (0·0 to 0·1)	2·1% (0·9 to 3·9%)	36·3% (17·9 to 59·4%)	10·1% (-3·2 to 24·1%)	3·1 (1·2 to 6·7)	1·4% (0·5 to 2·9%)	3·3 (1·2 to 7·0)	1·4% (0·5 to 2·9%)	3·8% (-5·5 to 11·3%)	-0·5% (-8·8 to 5·0%)
**Occupational risks: All causes**	42·2 (39·5 to 45·1)	0·8% (0·7 to 0·9%)	41·4 (38·5 to 44·6)	0·8% (0·8 to 0·9%)	-2·0% (-8·3 to 4·2%)	6·4% (-0·5 to 13·2%)	3,323·2 (2,726·3 to 4,019·6)	1·9% (1·7 to 2·2%)	3,290·3 (2,696·4 to 3,973·0)	2·1% (1·9 to 2·4%)	-1·0% (-5·9 to 4·3%)	12·9% (7·3 to 19·5%)
**Occupational carcinogens: All causes**	20·0 (18·4 to 21·8)	0·4% (0·3 to 0·4%)	20·1 (18·4 to 22·0)	0·4% (0·4 to 0·5%)	0·8% (-5·1 to 5·9%)	9·4% (3·2 to 14·7%)	460·8 (423·6 to 500·0)	0·3% (0·2 to 0·3%)	450·2 (411·7 to 492·5)	0·3% (0·3 to 0·3%)	-2·3% (-8·3 to 2·6%)	11·5% (4·6 to 17·2%)
**Occupational exposure to asbestos: All causes**	9·4 (7·7 to 11·0)	0·2% (0·1 to 0·2%)	10·0 (8·3 to 11·8)	0·2% (0·2 to 0·2%)	6·7% (-3·8 to 15·6%)	15·8% (4·9 to 25·6%)	201·7 (166·9 to 237·9)	0·1% (0·1 to 0·1%)	205·4 (169·5 to 245·0)	0·1% (0·1 to 0·2%)	1·8% (-9·2 to 11·1%)	16·2% (4·3 to 26·6%)
Larynx cancer	0·1 (0·0 to 0·1)	0·3% (0·2 to 0·5%)	0·1 (0·0 to 0·1)	0·4% (0·2 to 0·6%)	0·9% (-10·7 to 12·4%)	13·9% (2·8 to 25·0%)	1·2 (0·6 to 2·0)	0·3% (0·1 to 0·5%)	1·2 (0·6 to 2·0)	0·3% (0·2 to 0·5%)	-3·2% (-14·7 to 9·0%)	13·7% (1·6 to 25·3%)
Tracheal, bronchus, and lung cancer	8·2 (6·6 to 9·9)	5·1% (4·1 to 6·1%)	8·4 (6·8 to 10·1)	5·4% (4·4 to 6·4%)	2·9% (-7·1 to 12·0%)	5·9% (-3·0 to 14·2%)	174·0 (140·5 to 210·2)	4·5% (3·6 to 5·4%)	170·3 (137·3 to 206·0)	4·7% (3·8 to 5·7%)	-2·1% (-12·6 to 6·7%)	5·1% (-4·1 to 14·3%)
Ovarian cancer	0·1 (0·0 to 0·1)	0·4% (0·2 to 0·6%)	0·1 (0·0 to 0·2)	0·4% (0·2 to 0·7%)	20·3% (7·1 to 34·6%)	8·0% (-1·5 to 18·9%)	1·7 (0·8 to 2·8)	0·3% (0·1 to 0·5%)	2·0 (1·0 to 3·2)	0·3% (0·2 to 0·5%)	15·9% (2·8 to 30·7%)	7·9% (-1·9 to 19·7%)
Mesothelioma	1·1 (0·7 to 1·4)	54·4% (34·5 to 73·1%)	1·4 (0·9 to 1·9)	58·2% (38·3 to 75·9%)	35·2% (20·9 to 50·6%)	6·9% (2·9 to 13·5%)	24·8 (15·0 to 34·7)	47·3% (28·7 to 65·8%)	32·0 (20·1 to 43·9)	51·2% (32·5 to 69·0%)	29·0% (13·4 to 44·5%)	8·2% (3·3 to 15·3%)
**Occupational exposure to arsenic: All causes**	0·3 (0·2 to 0·4)	0·0% (0·0 to 0·0%)	0·2 (0·2 to 0·3)	0·0% (0·0 to 0·0%)	-16·8% (-21·2 to -12·4%)	-9·7% (-14·6 to -4·9%)	6·7 (5·1 to 8·5)	0·0% (0·0 to 0·0%)	5·5 (4·2 to 7·0)	0·0% (0·0 to 0·0%)	-18·1% (-22·6 to -13·6%)	-6·5% (-11·8 to -1·3%)
Tracheal, bronchus, and lung cancer	0·3 (0·2 to 0·4)	0·2% (0·1 to 0·2%)	0·2 (0·2 to 0·3)	0·1% (0·1 to 0·2%)	-16·8% (-21·2 to -12·4%)	-14·4% (-17·5 to -11·0%)	6·7 (5·1 to 8·5)	0·2% (0·1 to 0·2%)	5·5 (4·2 to 7·0)	0·2% (0·1 to 0·2%)	-18·1% (-22·6 to -13·6%)	-12·0% (-15·2 to -8·6%)
**Occupational exposure to benzene: All causes**	0·1 (0·1 to 0·2)	0·0% (0·0 to 0·0%)	0·2 (0·1 to 0·2)	0·0% (0·0 to 0·0%)	13·1% (6·0 to 20·1%)	22·8% (15·1 to 30·6%)	5·0 (4·2 to 5·8)	0·0% (0·0 to 0·0%)	5·6 (4·8 to 6·6)	0·0% (0·0 to 0·0%)	13·5% (6·1 to 20·9%)	29·6% (21·4 to 38·1%)
Leukaemia	0·1 (0·1 to 0·2)	0·5% (0·4 to 0·6%)	0·2 (0·1 to 0·2)	0·6% (0·5 to 0·7%)	13·1% (6·0 to 20·1%)	11·5% (6·9 to 16·0%)	5·0 (4·2 to 5·8)	0·6% (0·5 to 0·7%)	5·6 (4·8 to 6·6)	0·7% (0·6 to 0·9%)	13·5% (6·1 to 20·9%)	22·7% (17·8 to 27·7%)
**Occupational exposure to beryllium: All causes**	0·0 (0·0 to 0·0)	0·0% (0·0 to 0·0%)	0·0 (0·0 to 0·0)	0·0% (0·0 to 0·0%)	-20·2% (-25·4 to -15·3%)	-13·4% (-19·0 to -8·3%)	0·4 (0·3 to 0·5)	0·0% (0·0 to 0·0%)	0·3 (0·3 to 0·4)	0·0% (0·0 to 0·0%)	-21·6% (-26·6 to -16·9%)	-10·5% (-16·5 to -5·2%)
Tracheal, bronchus, and lung cancer	0·0 (0·0 to 0·0)	0·0% (0·0 to 0·0%)	0·0 (0·0 to 0·0)	0·0% (0·0 to 0·0%)	-20·2% (-25·4 to -15·3%)	-17·9% (-21·6 to -14·1%)	0·4 (0·3 to 0·5)	0·0% (0·0 to 0·0%)	0·3 (0·3 to 0·4)	0·0% (0·0 to 0·0%)	-21·6% (-26·6 to -16·9%)	-15·8% (-19·6 to -11·9%)
**Occupational exposure to cadmium: All causes**	0·1 (0·1 to 0·1)	0·0% (0·0 to 0·0%)	0·1 (0·0 to 0·1)	0·0% (0·0 to 0·0%)	-10·2% (-15·3 to -5·5%)	-2·5% (-7·9 to 2·8%)	1·6 (1·2 to 1·9)	0·0% (0·0 to 0·0%)	1·4 (1·1 to 1·7)	0·0% (0·0 to 0·0%)	-11·6% (-16·6 to -6·7%)	0·9% (-5·0 to 6·6%)
Tracheal, bronchus, and lung cancer	0·1 (0·1 to 0·1)	0·0% (0·0 to 0·0%)	0·1 (0·0 to 0·1)	0·0% (0·0 to 0·0%)	-10·2% (-15·3 to -5·5%)	-7·6% (-11·6 to -4·1%)	1·6 (1·2 to 1·9)	0·0% (0·0 to 0·0%)	1·4 (1·1 to 1·7)	0·0% (0·0 to 0·0%)	-11·6% (-16·6 to -6·7%)	-5·1% (-8·9 to -1·6%)
**Occupational exposure to chromium: All causes**	0·2 (0·2 to 0·3)	0·0% (0·0 to 0·0%)	0·2 (0·2 to 0·2)	0·0% (0·0 to 0·0%)	-15·5% (-20·3 to -11·1%)	-8·3% (-13·4 to -3·6%)	5·2 (4·5 to 5·9)	0·0% (0·0 to 0·0%)	4·3 (3·7 to 5·0)	0·0% (0·0 to 0·0%)	-16·8% (-21·5 to -12·6%)	-5·1% (-10·4 to 0·2%)
Tracheal, bronchus, and lung cancer	0·2 (0·2 to 0·3)	0·1% (0·1 to 0·2%)	0·2 (0·2 to 0·2)	0·1% (0·1 to 0·1%)	-15·5% (-20·3 to -11·1%)	-13·1% (-16·2 to -10·1%)	5·2 (4·5 to 5·9)	0·1% (0·1 to 0·2%)	4·3 (3·7 to 5·0)	0·1% (0·1 to 0·1%)	-16·8% (-21·5 to -12·6%)	-10·7% (-13·9 to -7·7%)
**Occupational exposure to diesel engine exhaust: All causes**	3·7 (3·2 to 4·3)	0·1% (0·1 to 0·1%)	3·8 (3·3 to 4·4)	0·1% (0·1 to 0·1%)	2·7% (-2·7 to 8·3%)	11·4% (6·1 to 17·3%)	88·6 (75·5 to 102·1)	0·1% (0·0 to 0·1%)	89·6 (77·0 to 103·3)	0·1% (0·0 to 0·1%)	1·1% (-4·2 to 6·5%)	15·4% (9·3 to 21·7%)
Tracheal, bronchus, and lung cancer	3·7 (3·2 to 4·3)	2·3% (2·0 to 2·6%)	3·8 (3·3 to 4·4)	2·4% (2·1 to 2·8%)	2·7% (-2·7 to 8·3%)	5·6% (1·8 to 9·7%)	88·6 (75·5 to 102·1)	2·3% (2·0 to 2·6%)	89·6 (77·0 to 103·3)	2·5% (2·1 to 2·8%)	1·1% (-4·2 to 6·5%)	8·6% (4·7 to 12·5%)
**Occupational exposure to second-hand smoke: All causes**	3·0 (2·8 to 3·3)	0·1% (0·1 to 0·1%)	3·0 (2·7 to 3·2)	0·1% (0·1 to 0·1%)	-2·7% (-7·2 to 2·2%)	5·7% (0·8 to 10·8%)	72·8 (67·4 to 78·4)	0·0% (0·0 to 0·0%)	69·2 (63·4 to 74·9)	0·0% (0·0 to 0·1%)	-5·0% (-9·6 to -0·3%)	8·4% (3·0 to 13·8%)
Tracheal, bronchus, and lung cancer	3·0 (2·8 to 3·3)	1·9% (1·7 to 2·0%)	3·0 (2·7 to 3·2)	1·9% (1·7 to 2·0%)	-2·7% (-7·2 to 2·2%)	0·1% (-2·9 to 3·6%)	72·8 (67·4 to 78·4)	1·9% (1·7 to 2·0%)	69·2 (63·4 to 74·9)	1·9% (1·8 to 2·0%)	-5·0% (-9·6 to -0·3%)	2·0% (-1·2 to 5·1%)
**Occupational exposure to formaldehyde: All causes**	0·0 (0·0 to 0·0)	0·0% (0·0 to 0·0%)	0·0 (0·0 to 0·0)	0·0% (0·0 to 0·0%)	-3·1% (-9·9 to 2·6%)	5·2% (-1·8 to 11·3%)	1·1 (0·9 to 1·3)	0·0% (0·0 to 0·0%)	1·1 (0·9 to 1·3)	0·0% (0·0 to 0·0%)	-2·8% (-9·7 to 2·8%)	11·0% (3·2 to 17·6%)
Nasopharynx cancer	0·0 (0·0 to 0·0)	0·4% (0·2 to 0·5%)	0·0 (0·0 to 0·0)	0·3% (0·2 to 0·5%)	-0·1% (-12·9 to 9·0%)	-4·9% (-10·0 to 0·6%)	0·3 (0·2 to 0·4)	0·4% (0·3 to 0·6%)	0·3 (0·2 to 0·4)	0·4% (0·3 to 0·6%)	0·1% (-13·4 to 9·3%)	-1·6% (-6·7 to 4·2%)
**Occupational exposure to nickel: All causes**	1·0 (0·7 to 1·4)	0·0% (0·0 to 0·0%)	0·8 (0·6 to 1·1)	0·0% (0·0 to 0·0%)	-20·1% (-25·1 to -15·0%)	-13·3% (-18·5 to -7·8%)	24·7 (17·2 to 33·4)	0·0% (0·0 to 0·0%)	19·4 (13·5 to 26·3)	0·0% (0·0 to 0·0%)	-21·5% (-26·4 to -16·6%)	-10·3% (-16·1 to -4·5%)
Tracheal, bronchus, and lung cancer	1·0 (0·7 to 1·4)	0·6% (0·4 to 0·9%)	0·8 (0·6 to 1·1)	0·5% (0·4 to 0·7%)	-20·1% (-25·1 to -15·0%)	-17·8% (-21·8 to -13·9%)	24·7 (17·2 to 33·4)	0·6% (0·4 to 0·9%)	19·4 (13·5 to 26·3)	0·5% (0·4 to 0·7%)	-21·5% (-26·4 to -16·6%)	-15·7% (-19·6 to -11·5%)
**Occupational exposure to polycyclic aromatic hydrocarbons: All causes**	0·6 (0·5 to 0·7)	0·0% (0·0 to 0·0%)	0·5 (0·4 to 0·6)	0·0% (0·0 to 0·0%)	-5·4% (-10·4 to -0·4%)	2·7% (-2·5 to 8·2%)	13·4 (10·9 to 16·2)	0·0% (0·0 to 0·0%)	12·5 (10·1 to 15·1)	0·0% (0·0 to 0·0%)	-6·7% (-11·7 to -1·7%)	6·5% (0·6 to 12·3%)
Tracheal, bronchus, and lung cancer	0·6 (0·5 to 0·7)	0·3% (0·3 to 0·4%)	0·5 (0·4 to 0·6)	0·3% (0·3 to 0·4%)	-5·4% (-10·4 to -0·4%)	-2·6% (-6·2 to 0·8%)	13·4 (10·9 to 16·2)	0·3% (0·3 to 0·4%)	12·5 (10·1 to 15·1)	0·3% (0·3 to 0·4%)	-6·7% (-11·7 to -1·7%)	0·2% (-3·5 to 3·6%)
**Occupational exposure to silica: All causes**	2·3 (2·1 to 2·6)	0·0% (0·0 to 0·0%)	2·4 (2·1 to 2·7)	0·0% (0·0 to 0·1%)	4·7% (-2·4 to 12·2%)	13·7% (6·3 to 22·0%)	55·2 (49·6 to 61·5)	0·0% (0·0 to 0·0%)	56·9 (50·2 to 63·9)	0·0% (0·0 to 0·0%)	3·0% (-4·1 to 10·3%)	17·6% (9·1 to 26·4%)
Tracheal, bronchus, and lung cancer	2·3 (2·1 to 2·6)	1·4% (1·3 to 1·6%)	2·4 (2·1 to 2·7)	1·5% (1·4 to 1·7%)	4·7% (-2·4 to 12·2%)	7·7% (1·4 to 14·2%)	55·2 (49·6 to 61·5)	1·4% (1·3 to 1·6%)	56·9 (50·2 to 63·9)	1·6% (1·4 to 1·7%)	3·0% (-4·1 to 10·3%)	10·6% (4·1 to 17·3%)
**Occupational exposure to sulfphuric acid: All causes**	0·5 (0·4 to 0·7)	0·0% (0·0 to 0·0%)	0·4 (0·3 to 0·6)	0·0% (0·0 to 0·0%)	-16·6% (-22·0 to -10·8%)	-9·5% (-15·2 to -3·1%)	13·1 (9·7 to 17·5)	0·0% (0·0 to 0·0%)	10·8 (7·9 to 14·3)	0·0% (0·0 to 0·0%)	-17·6% (-22·9 to -11·9%)	-6·0% (-12·1 to 0·4%)
Larynx cancer	0·5 (0·4 to 0·7)	3·2% (2·3 to 4·2%)	0·4 (0·3 to 0·6)	3·0% (2·2 to 3·9%)	-16·6% (-22·0 to -10·8%)	-5·9% (-9·9 to -1·9%)	13·1 (9·7 to 17·5)	3·1% (2·3 to 4·0%)	10·8 (7·9 to 14·3)	3·0% (2·2 to 3·9%)	-17·6% (-22·9 to -11·9%)	-3·3% (-7·0 to 0·9%)
**Occupational exposure to trichloroethylene: All causes**	0·0 (0·0 to 0·0)	0·0% (0·0 to 0·0%)	0·0 (0·0 to 0·0)	0·0% (0·0 to 0·0%)	0·2% (-7·5 to 7·7%)	8·8% (0·0 to 16·8%)	0·3 (0·1 to 0·5)	0·0% (0·0 to 0·0%)	0·3 (0·1 to 0·5)	0·0% (0·0 to 0·0%)	-0·4% (-8·2 to 7·4%)	13·6% (4·3 to 22·8%)
Kidney cancer	0·0 (0·0 to 0·0)	0·1% (0·0 to 0·1%)	0·0 (0·0 to 0·0)	0·0% (0·0 to 0·1%)	0·2% (-7·5 to 7·7%)	-11·1% (-14·8 to -6·7%)	0·3 (0·1 to 0·5)	0·1% (0·0 to 0·1%)	0·3 (0·1 to 0·5)	0·0% (0·0 to 0·1%)	-0·4% (-8·2 to 7·4%)	-7·3% (-11·0 to -3·0%)

Supplemental Results Table 1. All-age deaths, death PAFs, DALYs, DALY PAFs, and percent change for each risk factor and outcome for both sexes combined in 2005 and 2015 for Central Europe, Eastern Europe, and Central Asia.

Data in parenthesis are 95% uncertainty intervals. PAFs=population attributable fractions. DALYs=disability-adjusted life-years.

	2005 Deaths (in thousands)	2005 Deaths PAF	2015 Deaths (in thousands)	2015 Deaths PAF	Percent change deaths 2005-2015	Percent change deaths PAF 2005-2015	2005 DALYs (in thousands)	2005 DALYs PAF	2015 DALYs (in thousands)	2015 DALYs PAF	Percent change DALYs 2005-2015	Percent change DALYs PAF 2005-2015
**Occupational asthmagens: All causes**	0·7 (0·6 to 0·8)	0·0% (0·0 to 0·0%)	0·4 (0·4 to 0·5)	0·0% (0·0 to 0·0%)	-40·3% (-45·6 to -34·7%)	-35·2% (-41·0 to -29·1%)	76·3 (56·7 to 98·2)	0·0% (0·0 to 0·1%)	62·5 (45·1 to 82·7)	0·0% (0·0 to 0·1%)	-18·2% (-23·3 to -13·7%)	-6·7% (-12·0 to -1·9%)
Asthma	0·7 (0·6 to 0·8)	5·5% (4·9 to 6·1%)	0·4 (0·4 to 0·5)	4·9% (4·4 to 5·5%)	-40·3% (-45·6 to -34·7%)	-10·7% (-16·7 to -4·6%)	76·3 (56·7 to 98·2)	7·3% (6·6 to 8·1%)	62·5 (45·1 to 82·7)	6·9% (6·2 to 7·6%)	-18·2% (-23·3 to -13·7%)	-6·2% (-9·4 to -3·0%)
**Occupational particulate matter, gases, and fumes: All causes**	7·5 (6·3 to 8·8)	0·1% (0·1 to 0·2%)	6·5 (5·3 to 7·7)	0·1% (0·1 to 0·2%)	-14·2% (-20·6 to -7·8%)	-6·8% (-13·8 to 0·1%)	231·5 (194·2 to 269·1)	0·1% (0·1 to 0·2%)	189·6 (157·7 to 220·6)	0·1% (0·1 to 0·1%)	-18·1% (-23·3 to -12·7%)	-6·5% (-12·4 to -0·1%)
Chronic obstructive pulmonary disease	7·3 (6·1 to 8·6)	5·8% (4·9 to 6·8%)	6·3 (5·2 to 7·5)	5·7% (4·7 to 6·7%)	-13·2% (-19·8 to -6·7%)	-3·1% (-9·5 to 3·5%)	226·2 (188·8 to 263·7)	8·4% (7·0 to 9·7%)	185·9 (153·9 to 216·8)	8·3% (6·9 to 9·7%)	-17·8% (-23·0 to -12·3%)	-1·0% (-6·4 to 4·2%)
**Occupational noise: All causes**	-	-	-	-	-	-	447·1 (304·1 to 629·0)	0·3% (0·2 to 0·3%)	489·1 (334·5 to 688·1)	0·3% (0·2 to 0·4%)	9·4% (6·8 to 12·0%)	24·7% (21·1 to 28·6%)
Age-related and other hearing loss	-	-	-	-	-	-	447·1 (304·1 to 629·0)	16·5% (14·7 to 18·7%)	489·1 (334·5 to 688·1)	15·9% (14·2 to 17·9%)	9·4% (6·8 to 12·0%)	-3·4% (-5·4 to -1·5%)
**Occupational injuries: All causes**	16·0 (14·1 to 18·4)	0·3% (0·3 to 0·3%)	15·2 (13·2 to 17·8)	0·3% (0·3 to 0·4%)	-5·5% (-20·0 to 11·7%)	2·6% (-13·2 to 21·7%)	1,067·3 (915·6 to 1,245·7)	0·6% (0·5 to 0·7%)	1,059·0 (879·3 to 1,266·1)	0·7% (0·6 to 0·8%)	-0·8% (-15·7 to 16·5%)	13·2% (-3·6 to 32·4%)
Pedestrian road injuries	1·7 (1·4 to 2·1)	4·9% (4·1 to 5·9%)	1·5 (1·3 to 1·9)	7·3% (6·1 to 9·2%)	-9·3% (-29·6 to 16·2%)	49·4% (17·2 to 89·9%)	85·1 (69·8 to 104·7)	5·5% (4·6 to 6·7%)	76·3 (63·2 to 95·8)	8·7% (7·2 to 10·8%)	-10·3% (-30·3 to 15·1%)	56·9% (24·2 to 98·9%)
Cyclist road injuries	0·2 (0·2 to 0·3)	5·8% (4·8 to 7·0%)	0·2 (0·2 to 0·3)	7·6% (6·5 to 9·2%)	-5·3% (-27·6 to 18·9%)	32·8% (6·9 to 64·1%)	19·3 (15·6 to 24·2)	7·0% (6·0 to 8·1%)	20·7 (16·3 to 26·2)	9·8% (8·5 to 11·6%)	7·3% (-12·5 to 30·2%)	39·9% (16·4 to 69·5%)
Motorcyclist road injuries	0·7 (0·6 to 0·8)	10·1% (8·8 to 11·8%)	0·7 (0·6 to 0·8)	12·7% (11·0 to 14·8%)	-1·7% (-21·5 to 17·3%)	26·1% (5·6 to 51·0%)	42·3 (36·4 to 50·3)	10·0% (8·8 to 11·5%)	42·8 (36·3 to 50·8)	12·9% (11·2 to 14·8%)	1·2% (-17·2 to 19·7%)	28·0% (8·3 to 52·1%)
Motor vehicle road injuries	4·3 (3·7 to 5·1)	8·0% (6·7 to 9·6%)	4·1 (3·5 to 4·9)	12·3% (10·5 to 14·5%)	-5·5% (-21·3 to 14·6%)	52·6% (26·3 to 86·9%)	268·1 (226·8 to 315·6)	8·8% (7·5 to 10·4%)	271·7 (228·1 to 323·9)	13·5% (11·7 to 15·8%)	1·4% (-14·8 to 21·5%)	53·3% (27·9 to 85·7%)
Other road injuries	0·1 (0·1 to 0·1)	10·6% (9·1 to 12·0%)	0·1 (0·1 to 0·1)	13·3% (11·4 to 15·3%)	2·5% (-14·0 to 22·9%)	25·2% (7·0 to 45·9%)	6·2 (5·1 to 7·4)	12·2% (10·9 to 13·3%)	7·1 (5·7 to 8·9)	15·7% (14·0 to 17·7%)	14·4% (-0·7 to 30·7%)	29·1% (13·6 to 46·3%)
Other transport injuries	0·6 (0·5 to 0·8)	6·2% (5·0 to 7·5%)	0·6 (0·5 to 0·7)	8·3% (7·0 to 10·2%)	-7·3% (-26·4 to 19·0%)	35·6% (8·0 to 72·0%)	33·9 (27·7 to 41·6)	6·9% (5·6 to 8·4%)	31·0 (26·0 to 38·3)	9·5% (8·0 to 11·5%)	-8·6% (-27·4 to 17·1%)	37·8% (9·9 to 73·5%)
Falls	2·0 (1·7 to 2·2)	4·5% (4·0 to 5·2%)	2·2 (1·9 to 2·5)	5·9% (5·1 to 6·8%)	11·2% (-5·7 to 31·5%)	30·1% (10·9 to 53·3%)	195·9 (154·3 to 242·8)	7·8% (6·8 to 8·9%)	220·0 (170·4 to 281·2)	10·9% (9·4 to 12·6%)	12·3% (-3·9 to 31·6%)	39·6% (18·3 to 63·8%)
Drowning	2·3 (2·0 to 2·6)	6·1% (5·3 to 7·1%)	2·0 (1·7 to 2·3)	8·6% (7·4 to 10·0%)	-13·2% (-27·8 to 4·3%)	40·7% (16·8 to 70·8%)	115·1 (99·7 to 133·1)	6·2% (5·3 to 7·2%)	97·2 (83·4 to 114·6)	8·8% (7·6 to 10·3%)	-15·6% (-29·8 to 1·9%)	42·6% (18·3 to 74·2%)
Fire, heat, and hot substances	1·5 (1·3 to 1·7)	5·6% (4·8 to 6·6%)	1·3 (1·1 to 1·6)	7·9% (6·6 to 9·3%)	-9·8% (-25·8 to 10·1%)	40·0% (16·1 to 68·5%)	95·1 (79·3 to 112·9)	7·5% (6·4 to 8·7%)	87·9 (71·7 to 107·8)	10·8% (9·1 to 12·6%)	-7·6% (-23·6 to 10·9%)	44·8% (19·3 to 73·0%)
Poisonings	0·4 (0·3 to 0·5)	3·4% (2·7 to 4·4%)	0·3 (0·2 to 0·4)	3·6% (2·8 to 4·7%)	-35·2% (-51·6 to -10·8%)	6·0% (-21·2 to 45·3%)	21·6 (17·2 to 28·3)	3·9% (3·2 to 5·0%)	14·5 (11·0 to 19·7)	4·4% (3·4 to 5·7%)	-33·0% (-49·6 to -7·6%)	11·1% (-16·7 to 51·0%)
Unintentional firearm injuries	0·2 (0·2 to 0·3)	9·0% (7·4 to 11·3%)	0·1 (0·1 to 0·2)	8·0% (6·1 to 10·5%)	-32·7% (-49·5 to -10·8%)	-11·7% (-33·3 to 17·5%)	11·4 (9·2 to 15·2)	10·1% (8·4 to 12·6%)	7·6 (5·7 to 10·1)	9·5% (7·3 to 12·4%)	-33·8% (-50·7 to -12·4%)	-6·3% (-30·0 to 25·4%)
Unintentional suffocation	0·0 (0·0 to 0·0)	0·7% (0·5 to 1·0%)	0·0 (0·0 to 0·0)	0·9% (0·6 to 1·3%)	28·7% (-14·9 to 99·9%)	30·2% (-14·9 to 105·3%)	0·7 (0·5 to 1·0)	0·7% (0·5 to 1·0%)	0·9 (0·6 to 1·3)	0·9% (0·6 to 1·3%)	27·3% (-10·8 to 84·4%)	29·9% (-10·6 to 91·3%)
Other exposure to mechanical forces	0·6 (0·5 to 0·6)	5·5% (4·8 to 6·4%)	0·6 (0·5 to 0·7)	8·7% (7·5 to 10·2%)	-0·3% (-17·0 to 19·3%)	57·2% (31·6 to 90·0%)	50·1 (40·7 to 60·8)	7·3% (6·4 to 8·4%)	52·2 (40·9 to 66·4)	11·4% (9·9 to 13·1%)	4·1% (-11·6 to 22·5%)	54·5% (32·9 to 82·7%)
Venomous animal contact	0·0 (0·0 to 0·0)	1·8% (1·5 to 2·3%)	0·0 (0·0 to 0·0)	1·9% (1·5 to 2·5%)	-20·4% (-39·6 to 7·1%)	7·3% (-18·7 to 42·6%)	0·9 (0·7 to 1·2)	2·8% (2·3 to 3·5%)	0·8 (0·5 to 1·1)	3·0% (2·3 to 3·9%)	-18·5% (-37·2 to 8·2%)	8·1% (-16·8 to 42·7%)
Non-venomous animal contact	0·0 (0·0 to 0·0)	2·2% (1·8 to 2·8%)	0·0 (0·0 to 0·0)	2·2% (1·7 to 2·9%)	-21·3% (-41·3 to 9·0%)	-1·9% (-25·5 to 32·6%)	1·1 (0·9 to 1·4)	3·1% (2·5 to 3·8%)	0·8 (0·6 to 1·1)	3·0% (2·3 to 3·9%)	-24·7% (-42·8 to 2·4%)	-2·3% (-26·2 to 31·3%)
Pulmonary aspiration and foreign body in airway	0·5 (0·4 to 0·6)	3·7% (3·2 to 4·6%)	0·6 (0·5 to 0·7)	5·1% (4·3 to 6·2%)	17·4% (-5·0 to 44·2%)	35·8% (10·6 to 65·4%)	27·2 (22·4 to 32·4)	4·2% (3·6 to 5·2%)	30·6 (25·4 to 36·0)	5·8% (5·0 to 7·0%)	12·2% (-8·4 to 36·2%)	37·0% (13·2 to 65·9%)
Foreign body in eyes	-	-	-	-	-	-	0·3 (0·1 to 0·5)	8·1% (7·0 to 9·5%)	0·3 (0·1 to 0·6)	9·7% (8·0 to 11·6%)	23·5% (3·9 to 45·4%)	19·5% (1·2 to 40·3%)
Foreign body in other body part	0·3 (0·2 to 0·3)	18·0% (14·7 to 21·8%)	0·3 (0·2 to 0·3)	17·7% (13·4 to 22·9%)	-6·6% (-30·4 to 22·1%)	-1·9% (-26·2 to 27·1%)	19·9 (15·9 to 25·3)	19·5% (16·4 to 23·5%)	19·3 (14·5 to 25·2)	20·5% (16·3 to 26·0%)	-3·4% (-25·8 to 22·3%)	5·5% (-18·3 to 34·0%)
Other unintentional injuries	0·7 (0·6 to 0·8)	6·6% (5·8 to 7·7%)	0·6 (0·5 to 0·8)	9·4% (8·0 to 11·2%)	-6·1% (-21·8 to 13·8%)	41·2% (18·1 to 71·3%)	72·9 (57·3 to 90·8)	7·6% (6·8 to 8·8%)	77·3 (58·5 to 100·7)	10·8% (9·3 to 12·6%)	6·1% (-10·7 to 25·2%)	41·0% (19·3 to 68·4%)
**Occupational ergonomic factors: All causes**	-	-	-	-	-	-	1,194·6 (831·9 to 1,665·9)	0·7% (0·5 to 0·9%)	1,121·2 (786·7 to 1,562·8)	0·7% (0·6 to 0·9%)	-6·1% (-8·4 to -3·9%)	7·0% (3·9 to 10·4%)
Low back pain	-	-	-	-	-	-	1,194·6 (831·9 to 1,665·9)	22·0% (20·8 to 23·3%)	1,121·2 (786·7 to 1,562·8)	19·5% (18·4 to 20·6%)	-6·1% (-8·4 to -3·9%)	-11·4% (-13·2 to -9·4%)
**Behavioural risks: All causes**	2,936·5 (2,756·9 to 3,109·1)	55·6% (52·2 to 58·8%)	2,595·2 (2,419·0 to 2,767·4)	53·3% (49·8 to 56·7%)	-11·6% (-12·8 to -10·3%)	-4·1% (-5·0 to -3·2%)	76,741·8 (72,858·7 to 80,781·8)	44·0% (41·3 to 46·8%)	62,946·0 (59,231·8 to 66,871·2)	41·2% (38·5 to 44·1%)	-18·0% (-19·3 to -16·6%)	-6·4% (-7·4 to -5·4%)
**Child and maternal malnutrition: All causes**	27·5 (23·7 to 31·3)	0·5% (0·4 to 0·6%)	15·3 (12·7 to 17·9)	0·3% (0·3 to 0·4%)	-44·5% (-52·5 to -35·5%)	-39·7% (-48·7 to -30·0%)	4,678·1 (3,830·1 to 5,767·8)	2·7% (2·3 to 3·1%)	3,644·0 (2,824·7 to 4,669·9)	2·4% (2·0 to 2·8%)	-22·1% (-29·7 to -15·5%)	-11·2% (-19·2 to -4·5%)
**Suboptimal breastfeeding: All causes**	11·0 (6·2 to 16·3)	0·2% (0·1 to 0·3%)	6·1 (3·4 to 9·4)	0·1% (0·1 to 0·2%)	-44·3% (-52·7 to -34·8%)	-39·5% (-48·6 to -29·2%)	953·4 (536·1 to 1,408·8)	0·5% (0·3 to 0·8%)	533·3 (300·1 to 813·3)	0·3% (0·2 to 0·5%)	-44·1% (-52·5 to -34·6%)	-36·1% (-45·7 to -25·6%)
**Non-exclusive breastfeeding : All causes**	10·8 (6·0 to 16·0)	0·2% (0·1 to 0·3%)	6·0 (3·3 to 9·2)	0·1% (0·1 to 0·2%)	-44·2% (-52·7 to -34·6%)	-39·4% (-48·7 to -29·2%)	931·6 (519·1 to 1,380·3)	0·5% (0·3 to 0·8%)	520·6 (288·0 to 792·8)	0·3% (0·2 to 0·5%)	-44·1% (-52·6 to -34·6%)	-36·2% (-45·9 to -25·7%)
Diarrhoeal diseases	1·3 (0·8 to 1·9)	25·4% (15·8 to 33·9%)	0·7 (0·4 to 1·0)	20·9% (12·7 to 29·9%)	-49·6% (-63·3 to -32·8%)	-17·7% (-32·6 to 0·7%)	115·5 (72·3 to 161·0)	24·8% (15·6 to 33·3%)	58·8 (34·4 to 90·3)	21·3% (13·3 to 30·2%)	-49·1% (-62·7 to -32·3%)	-13·9% (-28·4 to 2·6%)
Lower respiratory infections	9·5 (4·7 to 14·7)	7·7% (3·8 to 11·9%)	5·3 (2·7 to 8·5)	5·2% (2·6 to 8·2%)	-43·5% (-52·4 to -33·5%)	-33·0% (-43·4 to -20·4%)	816·1 (402·9 to 1,269·1)	15·7% (7·9 to 24·1%)	461·8 (230·1 to 729·3)	12·7% (6·3 to 19·9%)	-43·4% (-52·4 to -33·5%)	-18·9% (-29·5 to -6·7%)
**Discontinued breastfeeding : All causes**	0·4 (0·1 to 0·7)	0·0% (0·0 to 0·0%)	0·2 (0·1 to 0·4)	0·0% (0·0 to 0·0%)	-48·5% (-61·8 to -31·7%)	-44·1% (-58·4 to -26·0%)	33·4 (11·2 to 62·1)	0·0% (0·0 to 0·0%)	18·7 (6·4 to 35·3)	0·0% (0·0 to 0·0%)	-44·0% (-57·8 to -27·2%)	-36·1% (-51·6 to -17·2%)
Diarrhoeal diseases	0·4 (0·1 to 0·7)	6·8% (2·3 to 12·7%)	0·2 (0·1 to 0·4)	5·7% (1·9 to 10·7%)	-48·5% (-61·8 to -31·7%)	-15·8% (-31·3 to 3·1%)	33·4 (11·2 to 62·1)	7·2% (2·4 to 13·1%)	18·7 (6·4 to 35·3)	6·8% (2·4 to 12·2%)	-44·0% (-57·8 to -27·2%)	-5·1% (-20·3 to 11·6%)
**Childhood undernutrition: All causes**	24·6 (20·0 to 28·7)	0·5% (0·4 to 0·5%)	13·4 (10·5 to 16·2)	0·3% (0·2 to 0·3%)	-45·5% (-54·0 to -36·4%)	-40·9% (-50·1 to -30·8%)	2,205·6 (1,815·0 to 2,560·0)	1·3% (1·0 to 1·5%)	1,240·0 (981·9 to 1,480·6)	0·8% (0·6 to 1·0%)	-43·8% (-51·8 to -34·9%)	-35·8% (-45·1 to -25·8%)
**Childhood underweight: All causes**	2·6 (1·7 to 4·6)	0·0% (0·0 to 0·1%)	1·2 (0·8 to 2·3)	0·0% (0·0 to 0·0%)	-52·3% (-63·3 to -39·6%)	-48·2% (-60·0 to -34·5%)	275·1 (199·5 to 450·8)	0·2% (0·1 to 0·3%)	160·5 (113·6 to 245·8)	0·1% (0·1 to 0·2%)	-41·7% (-52·8 to -27·6%)	-33·5% (-45·7 to -18·1%)
Diarrhoeal diseases	0·3 (0·2 to 0·4)	5·1% (3·8 to 6·7%)	0·1 (0·1 to 0·2)	3·5% (2·4 to 5·1%)	-58·0% (-72·0 to -38·4%)	-31·4% (-49·9 to -6·2%)	25·1 (18·5 to 33·7)	5·4% (4·0 to 7·0%)	11·4 (7·4 to 16·8)	4·1% (2·8 to 5·8%)	-54·8% (-68·6 to -36·2%)	-23·4% (-40·5 to 0·2%)
Lower respiratory infections	2·0 (1·2 to 4·0)	1·6% (1·0 to 3·3%)	0·9 (0·5 to 2·0)	0·9% (0·5 to 2·0%)	-52·3% (-63·8 to -40·0%)	-43·5% (-57·0 to -28·3%)	171·2 (103·2 to 342·7)	3·3% (2·0 to 6·7%)	81·8 (44·6 to 175·9)	2·3% (1·2 to 4·9%)	-52·2% (-63·8 to -39·9%)	-31·6% (-46·4 to -15·2%)
Measles	0·0 (0·0 to 0·0)	8·2% (0·1 to 27·7%)	0·0 (0·0 to 0·0)	4·9% (0·0 to 22·9%)	-96·6% (-100·0 to -90·5%)	-40·9% (-99·1 to 41·3%)	0·7 (0·0 to 2·6)	8·3% (0·1 to 28·1%)	0·0 (0·0 to 0·1)	5·2% (0·0 to 23·6%)	-95·6% (-99·9 to -89·3%)	-38·1% (-99·0 to 36·6%)
Protein-energy malnutrition	0·3 (0·2 to 0·4)	28·4% (19·7 to 37·9%)	0·2 (0·1 to 0·3)	19·3% (12·3 to 33·8%)	-45·4% (-68·4 to 12·4%)	-32·0% (-54·7 to 16·6%)	78·1 (54·8 to 110·7)	76·0% (68·7 to 82·3%)	67·4 (42·9 to 102·1)	78·8% (70·6 to 85·4%)	-13·8% (-35·2 to 19·1%)	3·6% (-3·0 to 11·5%)
**Childhood wasting: All causes**	22·4 (15·0 to 27·8)	0·4% (0·3 to 0·5%)	12·3 (8·1 to 15·6)	0·3% (0·2 to 0·3%)	-45·1% (-53·8 to -35·8%)	-40·4% (-49·7 to -30·2%)	2,011·7 (1,368·8 to 2,483·7)	1·2% (0·8 to 1·4%)	1,141·2 (778·3 to 1,431·6)	0·7% (0·5 to 1·0%)	-43·3% (-51·7 to -34·2%)	-35·2% (-44·7 to -25·0%)

Supplemental Results Table 1. All-age deaths, death PAFs, DALYs, DALY PAFs, and percent change for each risk factor and outcome for both sexes combined in 2005 and 2015 for Central Europe, Eastern Europe, and Central Asia.

Data in parenthesis are 95% uncertainty intervals. PAFs=population attributable fractions. DALYs=disability-adjusted life-years.

	2005 Deaths (in thousands)	2005 Deaths PAF	2015 Deaths (in thousands)	2015 Deaths PAF	Percent change deaths 2005-2015	Percent change deaths PAF 2005-2015	2005 DALYs (in thousands)	2005 DALYs PAF	2015 DALYs (in thousands)	2015 DALYs PAF	Percent change DALYs 2005-2015	Percent change DALYs PAF 2005-2015
Diarrhoeal diseases	3·1 (2·4 to 3·7)	60·3% (45·9 to 67·0%)	1·6 (1·0 to 2·0)	49·0% (34·5 to 56·9%)	-50·4% (-61·4 to -36·7%)	-18·9% (-28·7 to -7·7%)	300·4 (227·5 to 351·8)	64·5% (48·9 to 71·1%)	162·4 (109·9 to 204·0)	59·0% (42·3 to 66·9%)	-46·0% (-56·5 to -33·4%)	-8·5% (-16·3 to -1·1%)
Lower respiratory infections	18·9 (11·8 to 24·1)	15·4% (9·6 to 19·5%)	10·6 (6·3 to 13·8)	10·2% (6·1 to 13·2%)	-44·2% (-53·6 to -33·8%)	-33·9% (-45·0 to -21·1%)	1,631·1 (1,014·8 to 2,075·5)	31·4% (19·4 to 39·0%)	911·4 (544·5 to 1,190·0)	25·2% (15·0 to 32·1%)	-44·1% (-53·5 to -33·7%)	-19·9% (-31·3 to -7·6%)
Measles	0·0 (0·0 to 0·1)	26·2% (2·0 to 73·3%)	0·0 (0·0 to 0·0)	22·7% (0·9 to 71·0%)	-95·0% (-98·4 to -89·3%)	-13·3% (-67·7 to 18·3%)	2·0 (0·2 to 6·9)	26·6% (2·1 to 74·6%)	0·1 (0·0 to 0·5)	22·5% (0·8 to 71·7%)	-93·9% (-98·1 to -87·9%)	-15·3% (-70·8 to 7·7%)
Protein-energy malnutrition	0·3 (0·2 to 0·4)	28·4% (19·7 to 37·9%)	0·2 (0·1 to 0·3)	19·3% (12·3 to 33·8%)	-45·4% (-68·4 to 12·4%)	-32·0% (-54·7 to 16·6%)	78·1 (54·8 to 110·7)	76·0% (68·7 to 82·3%)	67·4 (42·9 to 102·1)	78·8% (70·6 to 85·4%)	-13·8% (-35·2 to 19·1%)	3·6% (-3·0 to 11·5%)
**Childhood stunting: All causes**	5·1 (1·0 to 11·9)	0·1% (0·0 to 0·2%)	2·3 (0·4 to 6·0)	0·0% (0·0 to 0·1%)	-54·4% (-65·0 to -45·3%)	-50·5% (-61·9 to -40·7%)	440·8 (94·2 to 1,029·3)	0·3% (0·1 to 0·6%)	203·0 (37·3 to 525·4)	0·1% (0·0 to 0·3%)	-54·0% (-64·1 to -45·0%)	-47·4% (-58·7 to -37·4%)
Diarrhoeal diseases	0·5 (0·2 to 1·0)	10·1% (3·7 to 18·7%)	0·2 (0·1 to 0·4)	6·8% (2·5 to 13·3%)	-58·8% (-69·6 to -44·7%)	-32·6% (-43·5 to -20·4%)	50·2 (18·1 to 91·3)	10·8% (3·9 to 19·7%)	22·4 (8·5 to 43·6)	8·1% (2·9 to 15·7%)	-55·4% (-65·8 to -42·3%)	-24·4% (-34·2 to -13·5%)
Lower respiratory infections	4·5 (0·3 to 11·4)	3·7% (0·3 to 9·3%)	2·1 (0·1 to 5·8)	2·0% (0·1 to 5·5%)	-53·7% (-64·0 to -37·1%)	-45·2% (-57·3 to -26·2%)	389·7 (27·4 to 982·2)	7·5% (0·5 to 18·9%)	180·6 (10·3 to 502·1)	5·0% (0·3 to 13·6%)	-53·7% (-64·0 to -37·1%)	-33·7% (-46·9 to -14·7%)
Measles	0·0 (0·0 to 0·0)	12·6% (0·8 to 40·4%)	0·0 (0·0 to 0·0)	9·1% (0·4 to 33·3%)	-95·8% (-98·6 to -90·7%)	-27·6% (-69·2 to -9·5%)	1·0 (0·1 to 2·9)	12·8% (0·8 to 41·1%)	0·1 (0·0 to 0·2)	9·7% (0·4 to 35·3%)	-94·5% (-97·8 to -88·5%)	-24·5% (-63·8 to -7·7%)
**Iron deficiency: All causes**	0·2 (0·1 to 0·3)	0·0% (0·0 to 0·0%)	0·2 (0·1 to 0·3)	0·0% (0·0 to 0·0%)	-18·4% (-26·2 to -8·0%)	-11·4% (-20·0 to -0·7%)	2,244·3 (1,495·2 to 3,228·1)	1·3% (0·9 to 1·7%)	2,258·7 (1,513·9 to 3,240·8)	1·5% (1·1 to 2·0%)	0·6% (-4·2 to 4·8%)	14·7% (9·2 to 20·1%)
Maternal haemorrhage	0·1 (0·0 to 0·2)	25·2% (9·4 to 40·9%)	0·1 (0·0 to 0·1)	25·0% (9·2 to 40·9%)	-32·2% (-41·7 to -21·7%)	-1·0% (-5·2 to 3·4%)	6·7 (2·5 to 11·1)	24·8% (9·2 to 40·5%)	5·0 (1·9 to 8·6)	24·8% (9·2 to 40·9%)	-24·8% (-33·8 to -15·5%)	-0·2% (-4·2 to 4·0%)
Maternal sepsis and other pregnancy related infections	0·0 (0·0 to 0·0)	25·1% (9·3 to 41·3%)	0·0 (0·0 to 0·0)	25·2% (9·4 to 41·8%)	-31·5% (-45·0 to -15·7%)	0·5% (-4·7 to 5·6%)	2·6 (0·9 to 4·5)	25·3% (9·4 to 41·4%)	2·1 (0·7 to 3·8)	25·9% (9·6 to 42·6%)	-19·0% (-40·2 to 10·9%)	2·3% (-2·5 to 7·3%)
Iron-deficiency anaemia	0·1 (0·1 to 0·2)	100·0% (100·0 to 100·0%)	0·1 (0·1 to 0·2)	100·0% (100·0 to 100·0%)	-3·2% (-16·3 to 9·8%)	-	2,235·0 (1,489·5 to 3,220·2)	100·0% (100·0 to 100·0%)	2,251·6 (1,509·3 to 3,230·5)	100·0% (100·0 to 100·0%)	0·7% (-4·1 to 4·9%)	-
**Vitamin A deficiency: All causes**	0·3 (0·2 to 0·5)	0·0% (0·0 to 0·0%)	0·1 (0·1 to 0·2)	0·0% (0·0 to 0·0%)	-60·6% (-70·8 to -49·0%)	-57·2% (-68·2 to -44·5%)	30·6 (16·7 to 45·6)	0·0% (0·0 to 0·0%)	13·8 (7·6 to 21·1)	0·0% (0·0 to 0·0%)	-55·0% (-64·0 to -44·1%)	-48·6% (-58·7 to -36·3%)
Diarrhoeal diseases	0·3 (0·1 to 0·5)	5·7% (2·8 to 8·7%)	0·1 (0·1 to 0·2)	3·8% (1·9 to 6·0%)	-58·5% (-68·8 to -46·1%)	-32·1% (-42·6 to -21·4%)	28·2 (14·6 to 43·1)	6·0% (3·1 to 9·2%)	12·7 (6·5 to 20·0)	4·6% (2·4 to 7·0%)	-54·9% (-64·6 to -43·5%)	-23·6% (-31·9 to -15·5%)
Measles	0·0 (0·0 to 0·0)	18·0% (10·9 to 25·2%)	0·0 (0·0 to 0·0)	7·6% (4·0 to 12·0%)	-97·6% (-98·8 to -94·9%)	-57·7% (-70·3 to -43·9%)	1·4 (0·6 to 2·6)	18·3% (11·1 to 25·5%)	0·0 (0·0 to 0·1)	8·5% (4·6 to 13·0%)	-96·7% (-98·2 to -93·5%)	-53·3% (-65·2 to -41·5%)
Vitamin A deficiency	-	-	-	-	-	-	1·0 (0·6 to 1·6)	100·0% (100·0 to 100·0%)	1·0 (0·6 to 1·6)	100·0% (100·0 to 100·0%)	1·4% (-7·0 to 10·3%)	-
**Zinc deficiency: All causes**	0·5 (0·0 to 1·6)	0·0% (0·0 to 0·0%)	0·3 (0·0 to 0·9)	0·0% (0·0 to 0·0%)	-48·1% (-63·3 to -34·3%)	-43·6% (-60·1 to -28·3%)	44·5 (3·8 to 141·7)	0·0% (0·0 to 0·1%)	24·0 (2·7 to 77·0)	0·0% (0·0 to 0·1%)	-46·0% (-56·7 to -21·6%)	-38·3% (-50·6 to -10·7%)
Diarrhoeal diseases	0·1 (0·0 to 0·2)	1·8% (0·0 to 4·4%)	0·0 (0·0 to 0·1)	1·4% (0·0 to 3·6%)	-52·8% (-66·9 to 0·0%)	-22·7% (-42·6 to 3·5%)	10·2 (2·2 to 21·9)	2·2% (0·5 to 4·7%)	5·8 (1·9 to 11·9)	2·1% (0·7 to 4·3%)	-43·7% (-57·0 to -12·6%)	-4·5% (-24·9 to 49·9%)
Lower respiratory infections	0·4 (0·0 to 1·6)	0·3% (0·0 to 1·3%)	0·2 (0·0 to 0·8)	0·2% (0·0 to 0·8%)	-47·0% (-57·8 to 0·0%)	-37·2% (-50·4 to 0·0%)	34·2 (0·2 to 133·6)	0·7% (0·0 to 2·5%)	18·3 (0·2 to 71·2)	0·5% (0·0 to 2·0%)	-46·6% (-57·1 to -8·7%)	-23·4% (-38·0 to 31·6%)
**Tobacco smoke: All causes**	889·1 (830·3 to 946·4)	16·8% (15·7 to 17·9%)	746·6 (696·6 to 795·8)	15·3% (14·3 to 16·3%)	-16·0% (-18·2 to -13·9%)	-8·8% (-11·0 to -7·0%)	23,161·5 (21,619·1 to 24,553·9)	13·3% (12·1 to 14·5%)	18,521·6 (17,312·1 to 19,695·6)	12·1% (10·9 to 13·4%)	-20·0% (-22·3 to -18·2%)	-8·7% (-11·4 to -6·5%)
**Smoking: All causes**	815·2 (757·9 to 869·7)	15·4% (14·3 to 16·5%)	684·3 (636·9 to 733·4)	14·1% (13·1 to 15·0%)	-16·1% (-18·5 to -13·9%)	-8·9% (-11·3 to -6·9%)	21,726·5 (20,189·4 to 23,153·0)	12·5% (11·3 to 13·7%)	17,486·8 (16,309·1 to 18,649·3)	11·4% (10·3 to 12·7%)	-19·5% (-21·8 to -17·5%)	-8·1% (-10·9 to -5·8%)
Tuberculosis	8·5 (4·2 to 12·5)	17·4% (8·9 to 26·0%)	4·0 (2·0 to 6·0)	16·4% (8·3 to 24·4%)	-52·5% (-56·7 to -47·5%)	-5·7% (-9·3 to -1·9%)	326·5 (164·6 to 482·8)	16·6% (8·5 to 24·8%)	152·4 (76·6 to 228·2)	15·7% (7·9 to 23·2%)	-53·3% (-57·3 to -48·4%)	-5·8% (-9·7 to -1·6%)
Lower respiratory infections	33·5 (27·8 to 39·6)	27·3% (22·9 to 31·6%)	28·5 (23·2 to 33·8)	27·5% (22·8 to 32·5%)	-14·8% (-24·0 to -4·7%)	1·0% (-5·7 to 7·9%)	1,107·4 (920·5 to 1,312·2)	21·3% (17·9 to 24·9%)	868·3 (716·4 to 1,020·0)	24·0% (19·9 to 28·3%)	-21·6% (-30·9 to -11·8%)	12·5% (0·6 to 25·1%)
Lip and oral cavity cancer	10·1 (9·2 to 10·9)	68·1% (62·8 to 72·8%)	9·4 (8·3 to 10·6)	62·3% (56·2 to 67·8%)	-6·3% (-13·8 to 4·5%)	-8·5% (-11·5 to -5·2%)	288·9 (264·0 to 313·5)	71·7% (66·5 to 76·4%)	261·7 (231·0 to 294·7)	66·1% (60·1 to 71·6%)	-9·4% (-17·0 to 1·5%)	-7·8% (-10·5 to -4·9%)
Nasopharynx cancer	1·3 (1·2 to 1·4)	62·7% (56·9 to 67·1%)	1·3 (1·2 to 1·5)	60·9% (54·4 to 65·9%)	2·0% (-11·3 to 10·7%)	-2·9% (-6·2 to 0·2%)	39·6 (35·5 to 43·4)	61·3% (55·6 to 65·9%)	39·3 (34·1 to 43·8)	59·9% (53·6 to 65·1%)	-0·7% (-14·3 to 8·2%)	-2·3% (-5·9 to 1·1%)
Oesophageal cancer	12·4 (10·7 to 14·0)	59·8% (51·6 to 66·7%)	11·8 (9·7 to 13·7)	56·1% (46·5 to 64·0%)	-5·4% (-13·3 to 3·6%)	-6·2% (-10·1 to -3·0%)	325·8 (283·4 to 364·8)	64·7% (56·3 to 71·4%)	300·4 (251·0 to 348·2)	60·9% (51·3 to 68·6%)	-7·8% (-16·0 to 1·6%)	-5·9% (-9·4 to -3·0%)
Stomach cancer	19·5 (12·2 to 25·9)	20·8% (13·2 to 27·5%)	13·3 (8·2 to 18·3)	17·2% (10·6 to 23·5%)	-31·6% (-37·0 to -26·7%)	-17·4% (-23·1 to -12·8%)	508·8 (320·9 to 670·4)	23·7% (15·1 to 31·0%)	334·7 (205·4 to 455·0)	19·9% (12·3 to 27·0%)	-34·2% (-40·1 to -29·1%)	-15·9% (-21·7 to -11·2%)
Colon and rectum cancer	9·3 (6·5 to 12·1)	9·0% (6·3 to 11·7%)	8·0 (5·5 to 10·6)	7·4% (5·0 to 9·7%)	-14·2% (-20·1 to -8·8%)	-18·4% (-23·9 to -13·8%)	230·9 (161·2 to 297·9)	10·6% (7·4 to 13·7%)	190·0 (131·2 to 250·4)	8·8% (6·1 to 11·7%)	-17·7% (-23·4 to -12·4%)	-16·7% (-22·1 to -12·1%)
Liver cancer due to hepatitis B	1·4 (0·8 to 1·9)	28·2% (17·6 to 37·3%)	1·4 (0·8 to 2·0)	25·9% (15·7 to 35·4%)	3·6% (-6·5 to 12·0%)	-8·2% (-14·5 to -3·1%)	40·1 (24·9 to 55·0)	28·8% (18·1 to 38·0%)	40·6 (24·0 to 57·0)	26·8% (16·1 to 36·5%)	1·2% (-9·4 to 10·2%)	-6·9% (-13·8 to -1·2%)
Liver cancer due to hepatitis C	1·5 (0·9 to 2·1)	20·5% (12·7 to 27·5%)	1·6 (0·9 to 2·2)	18·7% (11·4 to 25·9%)	3·8% (-4·8 to 11·3%)	-8·8% (-14·9 to -3·2%)	36·3 (22·6 to 49·4)	23·9% (15·1 to 31·5%)	37·1 (22·3 to 52·2)	22·3% (13·8 to 30·2%)	2·2% (-6·6 to 9·7%)	-6·6% (-12·9 to -1·1%)
Liver cancer due to alcohol use	3·6 (2·2 to 4·8)	31·0% (19·2 to 41·0%)	3·6 (2·1 to 4·9)	27·6% (16·6 to 37·8%)	-0·2% (-10·1 to 8·1%)	-10·9% (-17·1 to -5·7%)	90·5 (56·2 to 119·2)	34·7% (21·9 to 45·3%)	89·2 (53·5 to 120·6)	31·3% (19·0 to 42·3%)	-1·4% (-11·9 to 7·0%)	-9·7% (-16·0 to -4·4%)
Liver cancer due to other causes	0·5 (0·3 to 0·7)	20·0% (12·4 to 26·7%)	0·5 (0·3 to 0·7)	18·3% (11·2 to 24·9%)	1·8% (-6·8 to 8·8%)	-8·6% (-15·0 to -2·9%)	13·1 (8·2 to 17·6)	22·2% (14·0 to 29·2%)	12·9 (7·9 to 17·6)	20·9% (12·9 to 28·1%)	-0·9% (-9·9 to 6·5%)	-5·7% (-12·4 to 0·4%)
Pancreatic cancer	11·2 (9·2 to 13·1)	27·8% (23·0 to 32·5%)	10·8 (8·6 to 12·8)	23·4% (18·8 to 27·7%)	-3·8% (-9·8 to 3·2%)	-16·0% (-20·0 to -11·9%)	292·8 (239·9 to 341·4)	32·5% (26·8 to 37·8%)	268·8 (215·2 to 318·7)	27·6% (22·2 to 32·4%)	-8·2% (-14·1 to -0·8%)	-15·1% (-18·9 to -11·0%)
Tracheal, bronchus, and lung cancer	135·4 (131·4 to 139·4)	83·8% (82·3 to 85·4%)	126·3 (120·1 to 132·4)	80·4% (78·5 to 82·3%)	-6·7% (-11·4 to -2·3%)	-4·0% (-5·1 to -3·0%)	3,344·2 (3,242·0 to 3,441·2)	85·7% (84·0 to 87·2%)	3,013·1 (2,862·1 to 3,159·6)	82·9% (81·2 to 84·7%)	-9·9% (-14·7 to -5·5%)	-3·3% (-4·2 to -2·3%)
Cervical cancer	1·6 (0·6 to 2·8)	7·1% (2·4 to 12·4%)	1·2 (0·4 to 2·1)	5·9% (2·0 to 10·3%)	-27·0% (-36·3 to -13·6%)	-17·2% (-26·8 to -3·9%)	53·5 (18·8 to 93·4)	7·9% (2·8 to 13·9%)	36·6 (12·2 to 65·1)	6·4% (2·1 to 11·5%)	-31·5% (-41·2 to -18·2%)	-18·8% (-28·8 to -5·2%)
Kidney cancer	5·7 (4·0 to 7·2)	28·5% (19·9 to 35·8%)	5·4 (3·6 to 7·1)	23·8% (16·0 to 30·9%)	-5·7% (-15·7 to 4·3%)	-16·4% (-22·1 to -11·1%)	155·9 (110·9 to 194·8)	31·3% (22·1 to 38·7%)	142·1 (95·7 to 185·8)	26·6% (18·0 to 34·1%)	-8·8% (-18·8 to 1·4%)	-15·2% (-21·0 to -10·0%)
Bladder cancer	8·8 (6·9 to 10·5)	41·8% (32·9 to 50·0%)	8·2 (6·2 to 10·1)	36·2% (27·3 to 44·6%)	-7·2% (-16·1 to 2·5%)	-13·3% (-17·6 to -9·7%)	197·5 (157·3 to 232·5)	46·9% (37·6 to 55·2%)	176·4 (135·2 to 215·0)	41·8% (32·1 to 50·3%)	-10·7% (-19·1 to -1·1%)	-10·9% (-14·9 to -7·7%)
Ischaemic heart disease	287·3 (238·6 to 336·6)	16·7% (13·9 to 19·5%)	235·0 (193·2 to 276·2)	14·4% (11·8 to 16·9%)	-18·2% (-21·8 to -15·1%)	-14·2% (-17·4 to -11·3%)	7,625·7 (6,350·7 to 8,889·9)	24·9% (20·7 to 28·9%)	5,903·8 (4,895·3 to 6,878·1)	23·0% (19·1 to 26·8%)	-22·6% (-26·1 to -19·6%)	-7·6% (-10·2 to -5·2%)
Ischaemic stroke	73·7 (61·0 to 85·5)	11·5% (9·6 to 13·4%)	57·3 (47·6 to 67·2)	10·4% (8·6 to 12·2%)	-22·3% (-26·7 to -18·1%)	-9·4% (-14·1 to -4·7%)	1,649·8 (1,375·3 to 1,920·1)	17·0% (14·2 to 19·7%)	1,251·3 (1,049·8 to 1,459·9)	16·8% (14·1 to 19·5%)	-24·2% (-28·6 to -19·8%)	-1·3% (-5·5 to 3·0%)
Hemorrhagic stroke	57·6 (48·5 to 66·2)	18·1% (15·3 to 20·8%)	43·3 (36·3 to 50·2)	16·8% (14·1 to 19·4%)	-24·8% (-28·8 to -20·9%)	-7·1% (-10·9 to -3·5%)	1,698·2 (1,436·6 to 1,952·3)	25·3% (21·5 to 28·9%)	1,245·2 (1,051·1 to 1,428·0)	24·8% (21·0 to 28·5%)	-26·7% (-30·9 to -22·7%)	-1·7% (-4·8 to 1·4%)
Hypertensive heart disease	10·0 (7·7 to 12·2)	13·2% (10·2 to 16·0%)	9·5 (7·1 to 11·5)	11·6% (8·9 to 14·2%)	-5·4% (-11·5 to 2·4%)	-12·0% (-17·0 to -6·6%)	263·0 (204·2 to 316·6)	19·2% (15·1 to 23·0%)	234·8 (179·6 to 281·6)	17·9% (13·9 to 21·7%)	-10·7% (-17·0 to -2·7%)	-6·3% (-10·8 to -2·0%)
Atrial fibrillation and flutter	1·3 (1·0 to 1·7)	7·7% (5·8 to 9·5%)	1·5 (1·1 to 1·9)	6·8% (5·1 to 8·5%)	11·8% (4·6 to 19·5%)	-11·0% (-16·0 to -5·4%)	71·2 (51·0 to 96·2)	16·0% (12·4 to 19·4%)	81·0 (57·5 to 110·2)	15·8% (12·2 to 19·3%)	13·7% (9·3 to 18·3%)	-1·0% (-4·4 to 2·3%)
Aortic aneurysm	3·4 (2·6 to 4·1)	23·1% (18·5 to 27·7%)	3·5 (2·8 to 4·2)	20·9% (16·5 to 24·8%)	4·2% (-3·8 to 10·8%)	-9·9% (-13·8 to -6·3%)	94·4 (74·7 to 112·8)	29·6% (23·9 to 35·1%)	93·7 (74·2 to 112·5)	28·1% (22·5 to 33·1%)	-0·7% (-9·1 to 6·1%)	-5·0% (-8·6 to -1·7%)

Supplemental Results Table 1. All-age deaths, death PAFs, DALYs, DALY PAFs, and percent change for each risk factor and outcome for both sexes combined in 2005 and 2015 for Central Europe, Eastern Europe, and Central Asia.

Data in parenthesis are 95% uncertainty intervals. PAFs=population attributable fractions. DALYs=disability-adjusted life-years.

	2005 Deaths (in thousands)	2005 Deaths PAF	2015 Deaths (in thousands)	2015 Deaths PAF	Percent change deaths 2005-2015	Percent change deaths PAF 2005-2015	2005 DALYs (in thousands)	2005 DALYs PAF	2015 DALYs (in thousands)	2015 DALYs PAF	Percent change DALYs 2005-2015	Percent change DALYs PAF 2005-2015
Peripheral vascular disease	1·3 (1·1 to 1·6)	16·7% (13·2 to 20·6%)	1·5 (1·1 to 1·8)	13·9% (10·6 to 17·3%)	10·4% (-2·4 to 23·5%)	-16·7% (-24·9 to -7·6%)	36·7 (28·2 to 45·7)	20·6% (16·3 to 25·2%)	39·2 (29·3 to 49·3)	18·9% (14·7 to 23·3%)	6·9% (-3·4 to 17·9%)	-8·4% (-14·6 to -1·4%)
Other cardiovascular and circulatory diseases	7·7 (6·1 to 9·1)	19·1% (15·1 to 22·7%)	7·0 (5·4 to 8·4)	17·3% (13·6 to 20·6%)	-9·1% (-14·5 to -3·9%)	-9·5% (-13·3 to -5·7%)	267·3 (212·3 to 319·2)	24·8% (19·8 to 29·5%)	245·9 (195·4 to 295·5)	24·2% (19·1 to 28·9%)	-8·0% (-13·2 to -3·2%)	-2·4% (-5·5 to 0·8%)
Chronic obstructive pulmonary disease	77·7 (72·4 to 82·8)	62·1% (58·2 to 66·0%)	64·6 (58·7 to 70·3)	57·7% (52·7 to 62·4%)	-16·8% (-20·8 to -12·8%)	-7·1% (-10·5 to -4·2%)	1,770·6 (1,657·5 to 1,876·8)	65·7% (62·0 to 69·0%)	1,380·8 (1,269·8 to 1,483·6)	61·8% (57·3 to 65·8%)	-22·0% (-25·3 to -18·5%)	-6·0% (-8·5 to -3·4%)
Silicosis	0·1 (0·1 to 0·1)	32·1% (25·9 to 38·8%)	0·0 (0·0 to 0·0)	26·6% (20·9 to 33·3%)	-52·8% (-62·4 to -42·7%)	-17·1% (-22·8 to -12·1%)	2·7 (2·0 to 3·6)	31·5% (25·5 to 37·6%)	1·8 (1·2 to 2·5)	25·3% (20·3 to 30·8%)	-33·4% (-42·8 to -25·3%)	-19·7% (-23·7 to -16·2%)
Asbestosis	0·0 (0·0 to 0·0)	23·1% (17·6 to 29·0%)	0·0 (0·0 to 0·0)	21·0% (15·1 to 27·0%)	-24·2% (-47·1 to 1·8%)	-9·1% (-27·3 to 10·8%)	1·0 (0·8 to 1·4)	23·2% (17·7 to 28·7%)	0·8 (0·6 to 1·1)	20·7% (15·7 to 25·9%)	-21·7% (-40·0 to -2·9%)	-11·0% (-24·8 to 4·3%)
Coal workers pneumoconiosis	0·1 (0·1 to 0·1)	34·4% (27·5 to 41·7%)	0·0 (0·0 to 0·0)	27·1% (20·8 to 33·7%)	-59·1% (-68·0 to -49·6%)	-21·2% (-27·3 to -15·9%)	1·9 (1·5 to 2·4)	36·0% (29·4 to 43·0%)	1·1 (0·8 to 1·4)	28·4% (22·3 to 34·9%)	-44·9% (-54·9 to -33·6%)	-21·2% (-25·9 to -16·6%)
Other pneumoconiosis	0·3 (0·2 to 0·4)	16·2% (12·8 to 19·8%)	0·2 (0·2 to 0·3)	14·4% (10·8 to 18·4%)	-17·2% (-33·4 to -0·5%)	-11·5% (-26·7 to 5·4%)	8·1 (6·5 to 10·1)	19·1% (15·5 to 23·0%)	6·0 (4·5 to 7·8)	16·4% (12·9 to 20·4%)	-25·8% (-39·4 to -13·0%)	-14·1% (-24·4 to -2·5%)
Asthma	2·0 (1·6 to 2·6)	15·8% (12·5 to 19·1%)	1·3 (1·0 to 1·6)	14·8% (11·6 to 18·1%)	-37·2% (-42·6 to -31·6%)	-6·1% (-11·7 to 0·0%)	123·4 (89·1 to 160·3)	11·9% (9·7 to 14·1%)	110·1 (77·6 to 148·3)	12·1% (9·9 to 14·3%)	-10·8% (-17·9 to -4·6%)	2·2% (-2·3 to 6·9%)
Interstitial lung disease and pulmonary sarcoidosis	0·8 (0·6 to 1·0)	19·5% (15·6 to 23·5%)	0·8 (0·6 to 1·1)	16·7% (13·1 to 20·6%)	2·4% (-15·0 to 18·7%)	-14·5% (-22·5 to -7·7%)	28·2 (21·3 to 35·2)	21·0% (17·0 to 25·0%)	27·4 (20·4 to 35·2)	18·4% (14·6 to 22·3%)	-2·7% (-15·7 to 10·9%)	-12·5% (-19·6 to -6·0%)
Other chronic respiratory diseases	0·4 (0·3 to 0·5)	23·4% (18·7 to 28·4%)	0·3 (0·2 to 0·4)	19·3% (15·2 to 23·7%)	-24·8% (-36·7 to -11·8%)	-17·6% (-24·4 to -9·8%)	13·6 (10·6 to 16·8)	19·5% (15·5 to 23·8%)	9·7 (7·3 to 12·4)	16·7% (13·1 to 20·8%)	-28·3% (-38·7 to -16·7%)	-14·5% (-22·5 to -5·3%)
Diabetes mellitus	2·4 (0·7 to 4·1)	5·1% (1·5 to 8·8%)	2·5 (0·8 to 4·3)	5·0% (1·5 to 8·8%)	5·3% (-0·6 to 11·3%)	-1·4% (-7·5 to 4·3%)	166·1 (49·3 to 294·4)	6·4% (1·9 to 10·9%)	189·8 (57·3 to 336·3)	6·4% (2·0 to 11·0%)	14·3% (8·3 to 21·0%)	0·0% (-4·0 to 4·0%)
**Second-hand smoke: All causes**	84·2 (65·2 to 106·4)	1·6% (1·2 to 2·0%)	70·9 (53·7 to 91·9)	1·5% (1·1 to 1·9%)	-15·8% (-21·7 to -10·6%)	-8·6% (-14·7 to -3·0%)	1,704·6 (1,355·2 to 2,105·7)	1·0% (0·8 to 1·2%)	1,251·5 (983·6 to 1,547·1)	0·8% (0·6 to 1·0%)	-26·6% (-30·8 to -22·7%)	-16·2% (-20·8 to -11·7%)
Lower respiratory infections	5·8 (3·6 to 8·2)	4·7% (2·9 to 6·7%)	4·1 (2·5 to 5·9)	4·0% (2·4 to 5·7%)	-28·6% (-33·6 to -23·6%)	-15·4% (-20·5 to -10·1%)	328·8 (204·2 to 470·4)	6·3% (4·0 to 9·0%)	195·1 (118·9 to 282·3)	5·4% (3·3 to 7·8%)	-40·7% (-46·2 to -34·8%)	-15·0% (-20·4 to -9·1%)
Otitis media	0·0 (0·0 to 0·0)	5·7% (3·8 to 7·6%)	0·0 (0·0 to 0·0)	5·0% (3·4 to 6·7%)	-25·0% (-39·4 to -5·3%)	-11·9% (-23·0 to 1·7%)	7·7 (4·2 to 12·7)	4·7% (3·0 to 6·3%)	6·8 (3·7 to 11·1)	4·5% (3·0 to 6·1%)	-11·8% (-16·4 to -7·4%)	-2·9% (-6·9 to 1·0%)
Tracheal, bronchus, and lung cancer	0·9 (0·5 to 1·4)	0·6% (0·3 to 0·9%)	1·0 (0·6 to 1·6)	0·6% (0·4 to 1·0%)	7·5% (3·8 to 10·8%)	10·6% (5·4 to 15·7%)	19·4 (11·5 to 30·5)	0·5% (0·3 to 0·8%)	20·1 (11·8 to 31·8)	0·6% (0·3 to 0·9%)	3·8% (0·0 to 6·8%)	11·5% (6·0 to 16·8%)
Ischaemic heart disease	56·3 (40·8 to 74·5)	3·3% (2·4 to 4·4%)	49·5 (34·4 to 67·3)	3·0% (2·1 to 4·1%)	-12·1% (-20·0 to -5·3%)	-7·9% (-16·1 to -0·8%)	980·6 (750·4 to 1,244·8)	3·2% (2·4 to 4·1%)	769·4 (584·3 to 979·7)	3·0% (2·3 to 3·8%)	-21·5% (-27·6 to -16·2%)	-6·3% (-13·1 to -0·3%)
Ischaemic stroke	12·5 (9·0 to 16·7)	2·0% (1·4 to 2·6%)	9·5 (6·5 to 13·1)	1·7% (1·2 to 2·4%)	-24·0% (-32·4 to -16·0%)	-11·5% (-20·5 to -3·1%)	186·6 (139·4 to 241·5)	1·9% (1·4 to 2·5%)	126·5 (92·5 to 165·5)	1·7% (1·2 to 2·2%)	-32·2% (-38·6 to -26·1%)	-11·8% (-19·4 to -4·3%)
Hemorrhagic stroke	6·5 (4·9 to 8·3)	2·0% (1·6 to 2·6%)	4·8 (3·5 to 6·2)	1·9% (1·4 to 2·4%)	-26·6% (-32·8 to -20·8%)	-9·3% (-16·2 to -2·9%)	141·1 (109·5 to 177·7)	2·1% (1·6 to 2·7%)	97·5 (73·4 to 123·2)	1·9% (1·5 to 2·5%)	-30·9% (-35·9 to -25·8%)	-7·4% (-13·1 to -1·5%)
**Alcohol and drug use: All causes**	586·1 (423·1 to 743·7)	11·1% (8·0 to 14·1%)	471·1 (317·2 to 625·9)	9·7% (6·5 to 12·8%)	-19·6% (-26·6 to -15·4%)	-12·7% (-20·1 to -8·3%)	22,866·2 (19,761·0 to 25,861·9)	13·1% (11·2 to 14·9%)	17,886·5 (15,260·7 to 20,491·3)	11·7% (9·9 to 13·5%)	-21·8% (-24·3 to -19·4%)	-10·7% (-13·3 to -8·1%)
**Alcohol use: All causes**	519·6 (356·5 to 677·8)	9·8% (6·8 to 12·8%)	401·8 (246·6 to 556·4)	8·3% (5·1 to 11·4%)	-22·7% (-31·7 to -17·5%)	-16·1% (-25·9 to -10·7%)	19,125·0 (16,082·9 to 22,052·6)	11·0% (9·1 to 12·7%)	14,013·5 (11,387·4 to 16,594·4)	9·2% (7·4 to 10·9%)	-26·7% (-30·2 to -23·7%)	-16·4% (-19·9 to -13·1%)
Tuberculosis	16·8 (14·8 to 18·6)	34·6% (30·7 to 37·4%)	8·2 (7·1 to 9·3)	33·5% (29·5 to 36·6%)	-51·3% (-55·6 to -46·3%)	-3·2% (-7·6 to 1·3%)	689·2 (612·2 to 758·5)	35·0% (31·5 to 37·9%)	332·6 (292·1 to 376·8)	34·2% (30·3 to 37·3%)	-51·7% (-55·8 to -47·1%)	-2·6% (-6·8 to 1·8%)
Lower respiratory infections	22·1 (19·7 to 24·6)	18·0% (16·2 to 19·7%)	18·8 (16·5 to 20·8)	18·2% (16·3 to 19·8%)	-15·0% (-25·2 to -4·1%)	0·7% (-8·1 to 9·8%)	752·1 (668·9 to 840·5)	14·5% (12·9 to 15·9%)	579·6 (512·1 to 640·3)	16·0% (14·2 to 17·6%)	-22·9% (-32·9 to -12·3%)	10·5% (-2·2 to 24·2%)
Lip and oral cavity cancer	6·5 (6·0 to 7·0)	44·1% (41·1 to 46·6%)	6·5 (5·8 to 7·2)	43·1% (39·5 to 46·0%)	0·0% (-7·9 to 10·9%)	-2·3% (-5·3 to 0·7%)	190·4 (176·1 to 202·9)	47·2% (44·4 to 49·5%)	184·4 (166·1 to 203·6)	46·6% (43·0 to 49·2%)	-3·2% (-11·0 to 8·1%)	-1·4% (-3·9 to 1·2%)
Nasopharynx cancer	0·9 (0·8 to 0·9)	41·7% (38·8 to 44·0%)	0·9 (0·8 to 1·0)	43·2% (39·7 to 45·9%)	8·8% (-5·4 to 17·6%)	3·6% (0·4 to 6·3%)	27·6 (25·5 to 29·6)	42·8% (40·0 to 44·9%)	29·2 (25·6 to 32·1)	44·6% (41·3 to 47·2%)	6·0% (-8·7 to 14·7%)	4·2% (1·2 to 6·8%)
Other pharynx cancer	4·3 (4·0 to 4·6)	48·9% (45·9 to 51·2%)	5·0 (4·5 to 5·4)	49·3% (45·7 to 51·9%)	16·1% (5·9 to 25·2%)	0·8% (-1·3 to 2·8%)	128·0 (119·1 to 137·5)	50·6% (47·8 to 52·8%)	145·1 (130·2 to 158·5)	51·0% (47·6 to 53·5%)	13·3% (2·6 to 22·6%)	0·7% (-1·4 to 2·7%)
Oesophageal cancer	5·2 (4·6 to 5·6)	24·9% (22·2 to 27·0%)	5·3 (4·6 to 6·0)	25·3% (22·3 to 27·8%)	2·7% (-6·3 to 13·4%)	1·8% (-1·7 to 5·1%)	138·1 (124·1 to 149·9)	27·4% (24·8 to 29·4%)	137·9 (121·1 to 154·3)	28·0% (24·9 to 30·3%)	-0·1% (-9·2 to 11·1%)	1·9% (-1·3 to 4·8%)
Colon and rectum cancer	5·0 (4·5 to 5·5)	4·9% (4·3 to 5·3%)	5·3 (4·7 to 5·9)	4·8% (4·3 to 5·3%)	4·7% (-2·1 to 12·3%)	-0·4% (-5·8 to 5·5%)	115·1 (104·0 to 125·7)	5·3% (4·8 to 5·8%)	115·0 (103·0 to 127·2)	5·3% (4·8 to 5·9%)	-0·1% (-6·8 to 7·1%)	1·1% (-4·4 to 6·9%)
Liver cancer due to hepatitis B	0·0 (0·0 to 0·0)	0·5% (0·3 to 0·6%)	0·0 (0·0 to 0·0)	0·4% (0·3 to 0·6%)	5·3% (-14·6 to 28·6%)	-6·7% (-23·4 to 13·5%)	1·2 (0·9 to 1·6)	0·9% (0·7 to 1·1%)	1·3 (0·9 to 1·7)	0·8% (0·6 to 1·1%)	3·5% (-14·3 to 23·4%)	-4·8% (-20·5 to 13·0%)
Liver cancer due to hepatitis C	0·0 (0·0 to 0·0)	0·1% (0·0 to 0·1%)	0·0 (0·0 to 0·0)	0·1% (0·0 to 0·1%)	5·8% (-23·6 to 46·1%)	-7·0% (-32·6 to 29·1%)	0·2 (0·1 to 0·3)	0·1% (0·1 to 0·2%)	0·2 (0·1 to 0·4)	0·1% (0·1 to 0·2%)	4·7% (-20·9 to 36·7%)	-4·3% (-27·2 to 24·7%)
Liver cancer due to alcohol use	11·7 (11·0 to 12·4)	100·0% (100·0 to 100·0%)	13·1 (12·1 to 14·0)	100·0% (100·0 to 100·0%)	12·1% (3·3 to 18·9%)	-	260·8 (245·0 to 277·6)	100·0% (100·0 to 100·0%)	284·7 (261·1 to 306·3)	100·0% (100·0 to 100·0%)	9·2% (0·1 to 15·9%)	-
Liver cancer due to other causes	0·0 (0·0 to 0·0)	0·2% (0·2 to 0·3%)	0·0 (0·0 to 0·0)	0·2% (0·1 to 0·3%)	-3·1% (-19·8 to 16·4%)	-13·0% (-27·5 to 4·2%)	0·3 (0·3 to 0·4)	0·6% (0·4 to 0·7%)	0·3 (0·2 to 0·4)	0·5% (0·4 to 0·7%)	-5·1% (-19·8 to 11·7%)	-9·7% (-22·7 to 4·6%)
Larynx cancer	5·1 (4·7 to 5·5)	32·3% (29·5 to 34·7%)	4·5 (4·0 to 5·0)	32·0% (28·7 to 34·5%)	-12·3% (-17·9 to -6·4%)	-0·9% (-3·8 to 1·9%)	145·7 (133·0 to 157·0)	33·9% (31·2 to 36·0%)	123·3 (110·8 to 135·5)	33·7% (30·6 to 36·1%)	-15·3% (-21·0 to -9·3%)	-0·6% (-3·5 to 2·1%)
Breast cancer	4·7 (4·0 to 5·3)	7·8% (6·7 to 8·8%)	4·9 (4·1 to 5·7)	7·7% (6·5 to 8·8%)	5·9% (-6·5 to 13·9%)	-1·2% (-6·1 to 3·8%)	141·9 (120·6 to 162·9)	8·7% (7·5 to 9·8%)	145·3 (120·2 to 168·6)	8·7% (7·4 to 10·0%)	2·4% (-10·4 to 10·4%)	-0·1% (-5·2 to 4·8%)
Ischaemic heart disease	90·4 (-52·0 to 231·5)	5·3% (-3·1 to 13·5%)	67·0 (-69·7 to 204·4)	4·1% (-4·3 to 12·5%)	-25·9% (-287·0 to 144·0%)	-22·3% (-298·9 to 155·9%)	2,816·3 (408·2 to 5,137·2)	9·2% (1·3 to 16·8%)	1,972·0 (-118·2 to 3,989·6)	7·7% (-0·5 to 15·5%)	-30·0% (-92·7 to -21·3%)	-16·4% (-91·2 to -6·8%)
Ischaemic stroke	39·3 (21·4 to 57·2)	6·1% (3·4 to 8·9%)	31·4 (16·8 to 46·0)	5·7% (3·0 to 8·4%)	-20·2% (-25·4 to -16·3%)	-7·1% (-12·2 to -4·2%)	646·3 (369·5 to 933·2)	6·7% (3·8 to 9·6%)	464·0 (267·9 to 675·9)	6·2% (3·5 to 9·0%)	-28·2% (-31·6 to -25·1%)	-6·5% (-9·6 to -4·2%)
Hemorrhagic stroke	34·3 (31·5 to 37·1)	10·8% (9·9 to 11·6%)	27·2 (24·5 to 30·1)	10·6% (9·6 to 11·6%)	-20·7% (-27·0 to -13·8%)	-2·0% (-9·1 to 5·1%)	794·7 (738·4 to 850·9)	11·8% (11·1 to 12·6%)	589·8 (537·0 to 642·2)	11·8% (10·8 to 12·6%)	-25·8% (-30·9 to -20·8%)	-0·5% (-6·2 to 5·2%)
Hypertensive heart disease	6·6 (4·7 to 8·5)	8·7% (6·3 to 11·1%)	6·5 (4·4 to 8·8)	7·9% (5·3 to 10·7%)	-1·9% (-17·7 to 14·6%)	-8·8% (-23·8 to 5·5%)	162·2 (128·5 to 196·6)	11·8% (9·4 to 14·1%)	150·9 (111·7 to 185·7)	11·5% (8·7 to 14·3%)	-7·0% (-17·6 to 4·7%)	-2·4% (-12·6 to 7·2%)
Atrial fibrillation and flutter	1·0 (0·8 to 1·3)	6·1% (5·2 to 7·0%)	1·3 (1·0 to 1·6)	6·0% (5·0 to 7·1%)	24·0% (13·8 to 35·3%)	-1·2% (-8·6 to 6·5%)	37·3 (29·2 to 47·8)	8·4% (7·5 to 9·3%)	43·3 (33·5 to 55·0)	8·5% (7·5 to 9·5%)	15·8% (9·0 to 22·7%)	0·9% (-4·7 to 6·7%)
Cirrhosis and other chronic liver diseases due to hepatitis B	9·4 (7·7 to 10·9)	33·2% (28·7 to 37·2%)	9·5 (7·6 to 11·3)	34·6% (29·0 to 39·2%)	0·9% (-7·1 to 10·6%)	4·5% (-2·5 to 10·8%)	299·0 (242·0 to 347·5)	33·5% (29·0 to 37·5%)	296·8 (234·4 to 353·4)	35·5% (29·7 to 40·2%)	-0·7% (-8·9 to 9·1%)	6·0% (-1·2 to 12·8%)
Cirrhosis and other chronic liver diseases due to hepatitis C	9·7 (7·7 to 11·7)	32·8% (28·1 to 37·2%)	9·3 (7·1 to 11·3)	33·9% (27·9 to 38·9%)	-3·7% (-11·2 to 5·8%)	3·2% (-3·5 to 9·7%)	311·2 (245·4 to 374·8)	33·0% (28·2 to 37·5%)	293·8 (228·1 to 356·2)	34·5% (28·7 to 39·5%)	-5·6% (-13·2 to 4·2%)	4·6% (-2·4 to 11·8%)
Cirrhosis and other chronic liver diseases due to alcohol use	46·1 (39·1 to 50·3)	100·0% (100·0 to 100·0%)	41·5 (34·0 to 46·4)	100·0% (100·0 to 100·0%)	-9·9% (-18·2 to 0·5%)	-	1,560·6 (1,292·1 to 1,703·7)	100·0% (100·0 to 100·0%)	1,358·2 (1,071·5 to 1,520·1)	100·0% (100·0 to 100·0%)	-13·0% (-21·8 to -2·4%)	-
Cirrhosis and other chronic liver diseases due to other causes	12·6 (10·6 to 14·5)	33·8% (29·6 to 37·3%)	12·3 (10·1 to 14·4)	34·7% (29·6 to 38·9%)	-2·4% (-9·9 to 7·1%)	2·8% (-3·5 to 8·4%)	408·2 (343·1 to 467·2)	33·8% (29·8 to 37·4%)	385·9 (315·7 to 450·0)	35·3% (30·4 to 39·5%)	-5·5% (-12·6 to 3·8%)	4·6% (-1·4 to 10·0%)
Pancreatitis	8·3 (7·3 to 9·2)	38·4% (34·7 to 41·2%)	7·6 (6·7 to 8·4)	37·3% (33·8 to 40·3%)	-9·1% (-18·0 to 2·3%)	-2·8% (-7·8 to 2·9%)	334·7 (295·2 to 370·8)	42·9% (39·4 to 45·7%)	294·7 (261·3 to 324·0)	42·6% (39·1 to 45·5%)	-11·9% (-20·3 to -1·7%)	-0·8% (-5·2 to 3·8%)

Supplemental Results Table 1. All-age deaths, death PAFs, DALYs, DALY PAFs, and percent change for each risk factor and outcome for both sexes combined in 2005 and 2015 for Central Europe, Eastern Europe, and Central Asia.

Data in parenthesis are 95% uncertainty intervals. PAFs=population attributable fractions. DALYs=disability-adjusted life-years.

	2005 Deaths (in thousands)	2005 Deaths PAF	2015 Deaths (in thousands)	2015 Deaths PAF	Percent change deaths 2005-2015	Percent change deaths PAF 2005-2015	2005 DALYs (in thousands)	2005 DALYs PAF	2015 DALYs (in thousands)	2015 DALYs PAF	Percent change DALYs 2005-2015	Percent change DALYs PAF 2005-2015
Epilepsy	1·6 (1·5 to 1·7)	20·9% (19·7 to 22·0%)	1·4 (1·2 to 1·5)	21·2% (19·2 to 23·0%)	-13·0% (-19·8 to -3·5%)	1·2% (-4·4 to 6·3%)	130·4 (110·6 to 153·5)	17·6% (16·4 to 18·7%)	107·9 (89·4 to 128·7)	18·3% (16·5 to 19·9%)	-17·3% (-25·0 to -8·6%)	4·0% (-1·7 to 9·1%)
Alcohol use disorders	66·8 (60·2 to 71·1)	100·0% (100·0 to 100·0%)	40·2 (37·1 to 44·3)	100·0% (100·0 to 100·0%)	-39·8% (-45·2 to -30·8%)	-	3,566·3 (3,127·1 to 4,012·6)	100·0% (100·0 to 100·0%)	2,412·0 (2,078·6 to 2,824·4)	100·0% (100·0 to 100·0%)	-32·4% (-37·9 to -24·9%)	-
Diabetes mellitus	-4·8 (-5·2 to -4·4)	-10·2% (-11·0 to -9·4%)	-4·8 (-5·4 to -4·3)	-9·6% (-10·6 to -8·8%)	0·8% (-10·1 to 14·0%)	-5·7% (-14·2 to 4·6%)	-286·7 (-358·4 to -224·8)	-10·9% (-11·8 to -10·2%)	-314·4 (-403·6 to -241·8)	-10·5% (-11·4 to -9·7%)	9·7% (-0·4 to 20·8%)	-4·1% (-12·2 to 4·7%)
Pedestrian road injuries	10·3 (8·6 to 12·0)	29·7% (25·7 to 33·8%)	5·8 (4·9 to 6·9)	27·5% (23·9 to 31·4%)	-43·7% (-50·8 to -36·6%)	-7·3% (-10·6 to -4·3%)	500·9 (417·0 to 585·9)	32·5% (28·3 to 36·8%)	269·4 (229·5 to 319·8)	30·6% (26·7 to 34·7%)	-46·2% (-53·4 to -38·9%)	-5·9% (-8·9 to -3·1%)
Cyclist road injuries	1·2 (1·0 to 1·5)	28·8% (25·2 to 32·8%)	0·8 (0·7 to 1·0)	27·5% (23·9 to 31·4%)	-31·9% (-43·0 to -24·4%)	-4·5% (-7·3 to -1·7%)	70·4 (59·3 to 82·7)	25·5% (22·2 to 29·2%)	49·0 (40·3 to 58·7)	23·2% (20·2 to 26·8%)	-30·4% (-39·4 to -23·7%)	-9·2% (-13·4 to -5·7%)
Motorcyclist road injuries	3·5 (3·0 to 4·0)	52·2% (49·1 to 55·3%)	2·8 (2·4 to 3·3)	53·0% (50·0 to 56·1%)	-20·7% (-33·7 to -10·3%)	1·6% (-0·3 to 3·2%)	212·0 (181·3 to 241·4)	50·3% (47·2 to 53·4%)	169·1 (147·2 to 194·6)	50·7% (47·8 to 53·7%)	-20·2% (-32·3 to -10·6%)	0·8% (-1·4 to 2·7%)
Motor vehicle road injuries	27·1 (22·7 to 32·1)	49·8% (46·9 to 53·0%)	16·8 (15·2 to 18·5)	50·0% (47·3 to 52·9%)	-38·1% (-47·0 to -26·7%)	0·4% (-1·2 to 1·8%)	1,473·4 (1,253·3 to 1,719·1)	48·4% (45·4 to 51·4%)	945·8 (858·0 to 1,047·2)	47·1% (44·4 to 50·0%)	-35·8% (-44·3 to -25·4%)	-2·6% (-4·5 to -0·8%)
Falls	8·8 (7·9 to 9·9)	20·2% (18·2 to 22·8%)	7·0 (6·2 to 8·0)	18·8% (16·8 to 21·2%)	-20·6% (-25·0 to -12·7%)	-7·1% (-9·9 to -4·1%)	502·6 (426·4 to 594·8)	20·0% (18·2 to 22·4%)	396·5 (332·3 to 478·3)	19·7% (17·8 to 22·0%)	-21·1% (-24·8 to -16·1%)	-1·9% (-3·6 to -0·1%)
Drowning	7·9 (7·1 to 8·8)	21·5% (19·5 to 23·9%)	4·9 (4·4 to 5·5)	21·6% (19·7 to 24·0%)	-37·8% (-42·1 to -33·5%)	0·8% (-2·1 to 3·2%)	362·2 (327·2 to 406·6)	19·5% (17·7 to 21·7%)	217·9 (195·7 to 245·1)	19·8% (18·0 to 22·0%)	-39·8% (-44·2 to -35·4%)	1·6% (-2·4 to 4·8%)
Fire, heat, and hot substances	5·3 (4·6 to 6·0)	20·0% (18·1 to 22·5%)	3·3 (2·9 to 3·8)	19·3% (17·3 to 21·7%)	-37·8% (-42·9 to -26·9%)	-3·5% (-7·1 to 0·2%)	242·1 (213·1 to 276·6)	19·0% (17·2 to 21·2%)	150·0 (131·6 to 174·7)	18·4% (16·6 to 20·6%)	-38·0% (-42·7 to -29·2%)	-2·9% (-6·4 to 0·5%)
Poisonings	2·4 (2·0 to 2·9)	20·1% (18·2 to 22·7%)	1·4 (1·2 to 1·9)	19·9% (17·9 to 22·4%)	-39·5% (-46·0 to -28·7%)	-1·4% (-4·8 to 2·6%)	104·5 (90·5 to 126·9)	18·9% (17·2 to 21·3%)	63·0 (53·9 to 82·2)	18·9% (17·1 to 21·3%)	-39·7% (-45·7 to -30·3%)	-0·2% (-3·7 to 3·6%)
Unintentional firearm injuries	0·5 (0·4 to 0·6)	20·6% (18·5 to 23·0%)	0·4 (0·3 to 0·4)	20·0% (18·0 to 22·5%)	-25·6% (-31·6 to -16·9%)	-2·5% (-5·6 to 0·6%)	23·4 (20·4 to 26·9)	20·7% (18·6 to 23·0%)	16·7 (14·7 to 19·3)	20·9% (18·9 to 23·3%)	-28·4% (-34·2 to -20·5%)	1·1% (-2·1 to 4·3%)
Unintentional suffocation	0·1 (0·1 to 0·2)	11·8% (9·7 to 14·5%)	0·2 (0·1 to 0·2)	14·5% (11·0 to 17·6%)	22·1% (1·3 to 41·2%)	23·4% (5·0 to 44·0%)	9·7 (6·8 to 11·4)	9·8% (8·2 to 12·2%)	11·8 (7·3 to 14·1)	12·1% (9·7 to 14·8%)	21·2% (8·6 to 35·7%)	23·6% (5·1 to 43·8%)
Other exposure to mechanical forces	2·5 (2·2 to 2·8)	24·5% (22·3 to 27·3%)	1·5 (1·4 to 1·7)	24·0% (21·9 to 26·8%)	-37·8% (-43·2 to -32·0%)	-1·9% (-4·4 to 0·5%)	152·2 (130·0 to 179·5)	22·3% (20·3 to 24·9%)	101·4 (84·9 to 122·3)	22·1% (20·1 to 24·6%)	-33·4% (-38·2 to -28·9%)	-1·0% (-3·1 to 0·9%)
Self-harm	32·3 (29·1 to 36·2)	24·3% (22·1 to 27·1%)	25·8 (23·2 to 28·9)	24·4% (22·3 to 27·0%)	-20·0% (-25·8 to -12·8%)	0·4% (-1·4 to 2·7%)	1,411·4 (1,272·6 to 1,576·8)	24·9% (22·7 to 27·5%)	1,096·1 (984·0 to 1,223·8)	25·4% (23·3 to 28·0%)	-22·3% (-28·2 to -14·8%)	2·3% (0·5 to 4·3%)
Assault by firearm	1·2 (1·0 to 1·4)	21·9% (19·8 to 24·5%)	0·7 (0·6 to 0·9)	21·5% (19·5 to 24·1%)	-39·2% (-46·5 to -23·7%)	-1·7% (-4·5 to 1·3%)	63·7 (54·2 to 73·9)	22·9% (20·7 to 25·6%)	38·0 (32·8 to 45·1)	22·6% (20·5 to 25·3%)	-40·4% (-48·0 to -25·4%)	-1·3% (-4·1 to 1·9%)
Assault by sharp object	5·3 (4·7 to 6·0)	21·7% (19·7 to 24·0%)	3·1 (2·7 to 3·5)	21·5% (19·6 to 23·8%)	-42·6% (-48·0 to -36·0%)	-1·0% (-3·1 to 1·4%)	267·3 (236·9 to 303·3)	23·2% (21·1 to 25·6%)	149·3 (132·0 to 171·5)	23·1% (21·1 to 25·6%)	-44·2% (-49·5 to -37·8%)	-0·4% (-2·5 to 2·1%)
Assault by other means	7·5 (6·7 to 8·4)	20·2% (18·3 to 22·4%)	4·4 (3·9 to 5·0)	20·0% (18·3 to 22·2%)	-41·1% (-46·1 to -34·9%)	-0·8% (-2·9 to 1·3%)	358·1 (321·6 to 403·1)	21·7% (19·8 to 24·1%)	205·5 (182·6 to 232·6)	21·8% (20·0 to 24·2%)	-42·6% (-47·6 to -36·6%)	0·4% (-1·6 to 2·5%)
**Drug use: All causes**	75·4 (68·9 to 81·9)	1·4% (1·3 to 1·5%)	78·3 (70·5 to 85·2)	1·6% (1·4 to 1·7%)	3·8% (-5·4 to 10·7%)	12·7% (3·1 to 19·9%)	4,096·5 (3,732·9 to 4,488·3)	2·3% (2·1 to 2·6%)	4,215·7 (3,775·9 to 4,654·9)	2·8% (2·5 to 3·0%)	2·9% (-4·5 to 8·6%)	17·4% (9·4 to 23·7%)
HIV/AIDS - Tuberculosis	1·7 (1·5 to 1·8)	42·1% (39·2 to 44·9%)	0·8 (0·7 to 0·9)	39·3% (36·8 to 42·1%)	-50·2% (-54·7 to -45·1%)	-6·6% (-9·6 to -3·3%)	84·1 (77·3 to 91·5)	41·5% (38·6 to 44·2%)	41·4 (36·9 to 46·4)	38·8% (36·2 to 41·7%)	-50·8% (-55·1 to -45·8%)	-6·4% (-9·3 to -3·1%)
HIV/AIDS resulting in other diseases	8·6 (7·9 to 9·3)	41·6% (38·5 to 44·7%)	10·3 (9·3 to 11·5)	39·3% (36·1 to 42·7%)	20·5% (11·8 to 29·3%)	-5·6% (-8·4 to -2·8%)	458·1 (412·6 to 508·8)	41·1% (38·0 to 44·3%)	548·8 (482·9 to 615·9)	38·9% (35·7 to 42·2%)	19·8% (11·1 to 28·4%)	-5·4% (-8·3 to -2·6%)
Hepatitis B	0·0 (0·0 to 0·0)	1·7% (0·7 to 2·7%)	0·0 (0·0 to 0·0)	2·0% (0·8 to 3·2%)	-5·6% (-16·2 to 6·3%)	18·7% (8·9 to 29·8%)	0·9 (0·4 to 1·4)	1·6% (0·7 to 2·7%)	0·8 (0·4 to 1·4)	1·9% (0·8 to 3·1%)	-4·8% (-15·8 to 7·9%)	16·0% (6·2 to 27·6%)
Hepatitis C	0·0 (0·0 to 0·1)	59·8% (50·0 to 68·3%)	0·0 (0·0 to 0·1)	66·5% (57·5 to 73·6%)	-8·4% (-26·4 to 16·8%)	11·2% (3·5 to 21·2%)	1·6 (0·5 to 3·2)	53·9% (45·3 to 61·2%)	1·4 (0·5 to 2·8)	57·9% (49·0 to 65·6%)	-10·8% (-25·2 to 9·8%)	7·4% (-1·5 to 18·2%)
Liver cancer due to hepatitis B	0·1 (0·0 to 0·1)	1·6% (0·7 to 2·7%)	0·1 (0·0 to 0·2)	2·0% (0·9 to 3·4%)	42·4% (25·5 to 63·2%)	26·1% (14·2 to 43·4%)	2·5 (1·0 to 4·3)	1·8% (0·8 to 3·1%)	3·4 (1·5 to 5·7)	2·2% (1·0 to 3·6%)	34·7% (19·1 to 52·3%)	23·8% (13·2 to 38·3%)
Liver cancer due to hepatitis C	3·3 (2·1 to 4·4)	44·1% (29·2 to 58·2%)	4·5 (3·3 to 5·7)	53·6% (39·9 to 66·1%)	38·3% (24·8 to 59·7%)	21·5% (12·7 to 38·1%)	79·6 (56·2 to 101·2)	52·4% (37·6 to 65·4%)	102·2 (78·5 to 124·9)	61·5% (47·8 to 72·3%)	28·4% (16·0 to 45·7%)	17·4% (9·4 to 32·0%)
Cirrhosis and other chronic liver diseases due to hepatitis B	0·6 (0·2 to 1·0)	2·0% (0·8 to 3·4%)	0·6 (0·3 to 1·1)	2·4% (1·0 to 3·9%)	14·6% (0·5 to 34·5%)	18·6% (8·5 to 33·4%)	20·1 (8·5 to 34·1)	2·2% (1·0 to 3·8%)	21·8 (9·3 to 36·2)	2·6% (1·1 to 4·3%)	8·5% (-5·6 to 27·0%)	15·8% (6·2 to 29·3%)
Cirrhosis and other chronic liver diseases due to hepatitis C	20·2 (16·1 to 23·6)	68·7% (57·2 to 76·9%)	20·1 (16·4 to 23·4)	73·4% (63·9 to 80·2%)	-0·3% (-9·9 to 12·5%)	6·8% (3·2 to 13·4%)	707·8 (572·6 to 816·9)	75·1% (65·7 to 81·7%)	668·9 (541·6 to 771·5)	78·6% (70·8 to 83·7%)	-5·5% (-15·0 to 6·1%)	4·7% (2·0 to 9·6%)
Opioid use disorders	24·9 (23·0 to 28·1)	100·0% (100·0 to 100·0%)	25·0 (18·7 to 27·8)	100·0% (100·0 to 100·0%)	0·6% (-22·7 to 15·1%)	-	1,769·8 (1,535·2 to 1,999·0)	100·0% (100·0 to 100·0%)	1,802·6 (1,474·9 to 2,072·1)	100·0% (100·0 to 100·0%)	1·9% (-12·7 to 11·1%)	-
Cocaine use disorders	1·2 (0·8 to 1·5)	100·0% (100·0 to 100·0%)	1·5 (1·0 to 2·6)	100·0% (100·0 to 100·0%)	29·8% (-4·8 to 84·0%)	-	87·4 (66·7 to 111·7)	100·0% (100·0 to 100·0%)	106·9 (79·2 to 155·5)	100·0% (100·0 to 100·0%)	22·3% (-0·4 to 53·9%)	-
Amphetamine use disorders	2·9 (0·9 to 3·6)	100·0% (100·0 to 100·0%)	4·2 (2·0 to 5·4)	100·0% (100·0 to 100·0%)	47·2% (-15·7 to 152·7%)	-	200·9 (112·8 to 253·3)	100·0% (100·0 to 100·0%)	283·9 (174·6 to 362·9)	100·0% (100·0 to 100·0%)	41·3% (0·6 to 94·5%)	-
Cannabis use disorders	-	-	-	-	-	-	38·2 (24·4 to 54·3)	100·0% (100·0 to 100·0%)	35·2 (22·3 to 50·3)	100·0% (100·0 to 100·0%)	-7·9% (-12·2 to -3·0%)	-
Other drug use disorders	2·9 (2·6 to 3·4)	100·0% (100·0 to 100·0%)	3·0 (2·3 to 3·4)	100·0% (100·0 to 100·0%)	5·3% (-21·9 to 25·4%)	-	184·5 (152·8 to 226·1)	100·0% (100·0 to 100·0%)	206·9 (162·8 to 253·8)	100·0% (100·0 to 100·0%)	12·1% (-9·7 to 29·1%)	-
Self-harm	9·3 (6·2 to 13·3)	7·0% (4·7 to 9·9%)	7·9 (5·4 to 11·3)	7·5% (5·1 to 10·6%)	-14·7% (-23·4 to -3·6%)	7·0% (-1·5 to 17·6%)	461·2 (310·8 to 653·0)	8·1% (5·5 to 11·5%)	391·4 (265·4 to 562·7)	9·1% (6·1 to 12·8%)	-15·1% (-24·3 to -3·9%)	11·8% (2·8 to 23·0%)
**Dietary risks: All causes**	1,875·2 (1,655·5 to 2,090·6)	35·5% (31·3 to 39·6%)	1,696·0 (1,487·7 to 1,903·4)	34·9% (30·6 to 39·0%)	-9·6% (-11·4 to -7·8%)	-1·8% (-3·5 to -0·5%)	37,623·6 (33,545·8 to 41,666·4)	21·6% (19·0 to 24·2%)	31,002·1 (27,566·4 to 34,550·1)	20·3% (17·7 to 23·1%)	-17·6% (-19·7 to -15·9%)	-5·9% (-8·5 to -4·2%)
**Diet low in fruits: All causes**	438·1 (269·2 to 623·5)	8·3% (5·1 to 11·8%)	374·7 (223·5 to 538·8)	7·7% (4·6 to 11·1%)	-14·5% (-18·0 to -11·8%)	-7·1% (-10·6 to -4·4%)	9,661·1 (6,111·3 to 13,498·3)	5·5% (3·4 to 7·9%)	7,677·0 (4,793·0 to 10,737·0)	5·0% (3·1 to 7·1%)	-20·5% (-23·3 to -18·2%)	-9·3% (-12·6 to -6·8%)
Lip and oral cavity cancer	1·1 (0·0 to 2·3)	7·2% (0·0 to 15·5%)	1·1 (0·0 to 2·4)	7·2% (0·0 to 15·5%)	2·1% (-4·5 to 10·5%)	-0·2% (-1·2 to 0·7%)	29·0 (0·0 to 63·9)	7·2% (0·0 to 15·6%)	28·5 (0·0 to 62·7)	7·2% (0·0 to 15·6%)	-2·0% (-8·7 to 7·2%)	-0·2% (-1·2 to 0·8%)
Nasopharynx cancer	0·1 (0·0 to 0·3)	7·3% (0·0 to 15·5%)	0·2 (0·0 to 0·3)	7·4% (0·0 to 15·7%)	6·5% (-5·4 to 14·6%)	1·4% (0·0 to 2·4%)	4·5 (0·0 to 9·8)	7·0% (0·0 to 15·0%)	4·7 (0·0 to 10·2)	7·2% (0·0 to 15·4%)	4·3% (-8·5 to 12·1%)	2·6% (0·0 to 3·9%)
Other pharynx cancer	0·6 (0·0 to 1·4)	7·3% (0·0 to 16·2%)	0·7 (0·0 to 1·7)	7·3% (0·0 to 16·3%)	15·2% (0·0 to 23·1%)	0·1% (-1·0 to 1·1%)	18·4 (0·0 to 41·1)	7·3% (0·0 to 16·2%)	20·7 (0·0 to 46·9)	7·3% (0·0 to 16·3%)	12·7% (0·0 to 20·9%)	0·2% (-0·9 to 1·3%)
Oesophageal cancer	4·7 (1·2 to 8·4)	22·8% (5·7 to 40·1%)	4·8 (1·2 to 8·5)	22·8% (5·7 to 40·2%)	1·2% (-6·5 to 9·9%)	0·3% (-0·5 to 1·1%)	114·6 (28·6 to 203·2)	22·8% (5·7 to 40·1%)	112·6 (27·7 to 199·5)	22·8% (5·7 to 40·3%)	-1·8% (-9·6 to 7·8%)	0·2% (-0·7 to 1·1%)
Larynx cancer	1·1 (0·0 to 2·5)	7·2% (0·0 to 16·2%)	1·0 (0·0 to 2·3)	7·3% (0·0 to 16·3%)	-11·1% (-16·1 to 0·0%)	0·4% (-0·5 to 1·4%)	31·2 (0·0 to 69·1)	7·3% (0·0 to 16·2%)	26·6 (0·0 to 59·3)	7·3% (0·0 to 16·3%)	-14·6% (-19·4 to 0·0%)	0·3% (-0·6 to 1·4%)
Tracheal, bronchus, and lung cancer	19·8 (7·9 to 33·4)	12·2% (4·9 to 20·7%)	19·2 (7·7 to 32·6)	12·2% (4·9 to 20·7%)	-2·9% (-7·1 to 1·1%)	-0·1% (-1·1 to 0·9%)	479·6 (192·5 to 807·8)	12·3% (5·0 to 20·8%)	445·7 (177·3 to 754·6)	12·3% (4·9 to 20·7%)	-7·1% (-11·5 to -2·9%)	-0·2% (-1·3 to 0·8%)
Ischaemic heart disease	212·0 (76·2 to 355·9)	12·4% (4·4 to 20·7%)	194·3 (69·7 to 326·6)	11·9% (4·3 to 19·9%)	-8·3% (-10·4 to -6·5%)	-3·9% (-4·8 to -3·1%)	4,373·2 (1,593·2 to 7,262·3)	14·3% (5·2 to 23·7%)	3,537·6 (1,286·5 to 5,878·7)	13·8% (5·0 to 22·9%)	-19·1% (-21·8 to -17·1%)	-3·4% (-4·5 to -2·6%)
Ischaemic stroke	114·8 (61·6 to 176·7)	17·9% (9·5 to 27·7%)	88·8 (46·8 to 137·0)	16·1% (8·5 to 24·8%)	-22·7% (-25·3 to -20·3%)	-9·9% (-11·4 to -8·5%)	2,241·0 (1,226·3 to 3,364·0)	23·1% (12·6 to 34·9%)	1,638·9 (879·8 to 2,474·9)	22·0% (11·9 to 33·2%)	-26·9% (-29·7 to -24·3%)	-4·8% (-6·3 to -3·4%)

Supplemental Results Table 1. All-age deaths, death PAFs, DALYs, DALY PAFs, and percent change for each risk factor and outcome for both sexes combined in 2005 and 2015 for Central Europe, Eastern Europe, and Central Asia.

Data in parenthesis are 95% uncertainty intervals. PAFs=population attributable fractions. DALYs=disability-adjusted life-years.

	2005 Deaths (in thousands)	2005 Deaths PAF	2015 Deaths (in thousands)	2015 Deaths PAF	Percent change deaths 2005-2015	Percent change deaths PAF 2005-2015	2005 DALYs (in thousands)	2005 DALYs PAF	2015 DALYs (in thousands)	2015 DALYs PAF	Percent change DALYs 2005-2015	Percent change DALYs PAF 2005-2015
Hemorrhagic stroke	78·9 (42·6 to 118·1)	24·8% (13·5 to 37·0%)	59·6 (32·3 to 89·8)	23·2% (12·6 to 34·7%)	-24·4% (-27·5 to -21·5%)	-6·6% (-8·0 to -5·3%)	2,047·5 (1,149·9 to 2,998·7)	30·5% (17·0 to 44·5%)	1,496·0 (841·6 to 2,205·2)	29·8% (16·6 to 43·7%)	-26·9% (-30·1 to -23·9%)	-2·0% (-3·1 to -0·9%)
**Diet low in vegetables: All causes**	385·1 (188·8 to 604·4)	7·3% (3·6 to 11·5%)	339·8 (163·5 to 536·0)	7·0% (3·4 to 11·0%)	-11·8% (-15·9 to -8·8%)	-4·2% (-8·6 to -1·2%)	7,985·7 (3,995·2 to 12,323·7)	4·6% (2·3 to 7·1%)	6,275·1 (3,097·3 to 9,699·3)	4·1% (2·0 to 6·4%)	-21·4% (-24·4 to -19·0%)	-10·3% (-13·8 to -7·4%)
Ischaemic heart disease	290·4 (113·2 to 481·4)	16·9% (6·6 to 28·1%)	267·7 (104·0 to 441·0)	16·4% (6·3 to 27·1%)	-7·8% (-10·0 to -5·8%)	-3·3% (-4·4 to -2·3%)	5,865·9 (2,320·5 to 9,571·3)	19·1% (7·6 to 31·5%)	4,741·6 (1,857·1 to 7,712·6)	18·5% (7·2 to 30·4%)	-19·2% (-21·9 to -17·0%)	-3·5% (-4·7 to -2·4%)
Ischaemic stroke	56·6 (12·9 to 102·2)	8·8% (2·0 to 16·0%)	43·6 (9·8 to 79·4)	7·9% (1·8 to 14·4%)	-23·0% (-25·9 to -20·4%)	-10·3% (-12·1 to -8·5%)	1,113·6 (258·9 to 2,009·5)	11·5% (2·7 to 20·7%)	808·4 (183·1 to 1,452·5)	10·9% (2·5 to 19·6%)	-27·4% (-30·5 to -24·4%)	-5·5% (-7·4 to -3·6%)
Hemorrhagic stroke	38·2 (10·7 to 70·6)	12·0% (3·4 to 22·1%)	28·5 (7·8 to 52·5)	11·1% (3·1 to 20·4%)	-25·4% (-28·7 to -22·3%)	-7·8% (-9·6 to -6·2%)	1,006·3 (284·3 to 1,835·0)	15·0% (4·3 to 27·0%)	725·1 (203·6 to 1,316·6)	14·5% (4·1 to 26·2%)	-27·9% (-31·3 to -24·6%)	-3·4% (-5·1 to -1·9%)
**Diet low in whole grains: All causes**	585·8 (406·9 to 784·0)	11·1% (7·7 to 14·8%)	508·3 (347·2 to 684·4)	10·4% (7·1 to 14·0%)	-13·2% (-15·8 to -11·3%)	-5·8% (-8·4 to -4·1%)	12,743·5 (9,037·3 to 16,620·5)	7·3% (5·2 to 9·7%)	10,164·6 (7,209·4 to 13,170·2)	6·6% (4·7 to 8·9%)	-20·2% (-22·8 to -18·3%)	-9·0% (-11·8 to -7·0%)
Ischaemic heart disease	383·2 (234·3 to 546·3)	22·3% (13·6 to 31·8%)	352·4 (212·9 to 499·0)	21·5% (13·1 to 30·7%)	-8·0% (-9·9 to -6·2%)	-3·6% (-4·3 to -3·0%)	7,796·8 (4,822·3 to 10,988·6)	25·4% (15·7 to 35·8%)	6,312·9 (3,861·7 to 8,884·4)	24·6% (15·1 to 34·7%)	-19·0% (-21·6 to -17·0%)	-3·3% (-4·2 to -2·7%)
Ischaemic stroke	116·1 (80·4 to 155·2)	18·1% (12·5 to 24·2%)	88·4 (61·1 to 119·1)	16·1% (11·0 to 21·5%)	-23·8% (-26·5 to -21·4%)	-11·2% (-12·7 to -9·9%)	2,316·6 (1,618·9 to 3,050·6)	23·9% (16·8 to 31·4%)	1,679·5 (1,177·5 to 2,234·1)	22·6% (15·8 to 29·8%)	-27·5% (-30·5 to -25·0%)	-5·6% (-7·0 to -4·4%)
Hemorrhagic stroke	76·6 (55·3 to 101·4)	24·1% (17·4 to 32·0%)	57·3 (41·2 to 75·9)	22·3% (16·0 to 29·6%)	-25·2% (-28·3 to -22·4%)	-7·6% (-8·9 to -6·3%)	1,988·4 (1,459·0 to 2,589·7)	29·6% (21·6 to 38·8%)	1,443·7 (1,051·1 to 1,887·6)	28·8% (21·0 to 37·9%)	-27·4% (-30·7 to -24·5%)	-2·6% (-3·6 to -1·7%)
Diabetes mellitus	9·9 (5·6 to 14·7)	21·2% (12·1 to 31·5%)	10·1 (5·7 to 15·1)	20·4% (11·6 to 30·2%)	2·4% (-2·2 to 6·8%)	-4·2% (-5·4 to -3·1%)	641·7 (359·0 to 988·7)	24·5% (14·1 to 36·0%)	728·5 (400·2 to 1,134·5)	24·3% (14·0 to 35·8%)	13·5% (8·7 to 17·8%)	-0·7% (-1·4 to -0·1%)
**Diet low in nuts and seeds: All causes**	387·8 (243·8 to 550·7)	7·3% (4·6 to 10·4%)	358·7 (224·1 to 508·0)	7·4% (4·6 to 10·5%)	-7·5% (-9·4 to -5·8%)	0·4% (-1·3 to 1·9%)	7,963·5 (5,096·6 to 11,077·6)	4·6% (2·9 to 6·5%)	6,569·3 (4,248·0 to 9,146·4)	4·3% (2·7 to 6·1%)	-17·5% (-19·9 to -15·1%)	-5·8% (-8·7 to -3·5%)
Ischaemic heart disease	382·7 (240·0 to 544·8)	22·3% (13·9 to 31·8%)	353·5 (220·2 to 500·8)	21·6% (13·5 to 30·8%)	-7·6% (-9·5 to -5·9%)	-3·1% (-3·7 to -2·8%)	7,631·3 (4,816·7 to 10,675·0)	24·9% (15·7 to 35·0%)	6,192·8 (3,916·5 to 8,637·0)	24·1% (15·2 to 33·7%)	-18·8% (-21·4 to -16·8%)	-3·1% (-4·0 to -2·3%)
Diabetes mellitus	5·1 (2·7 to 8·0)	10·9% (5·7 to 17·1%)	5·2 (2·7 to 8·2)	10·5% (5·5 to 16·5%)	2·4% (-2·1 to 7·0%)	-4·1% (-5·2 to -3·2%)	332·2 (158·3 to 543·1)	12·7% (6·6 to 19·7%)	376·5 (176·5 to 628·3)	12·5% (6·6 to 19·6%)	13·3% (8·6 to 17·6%)	-0·9% (-1·4 to -0·3%)
**Diet low in milk: All causes**	14·1 (4·8 to 24·2)	0·3% (0·1 to 0·5%)	14·8 (5·0 to 25·4)	0·3% (0·1 to 0·5%)	5·0% (0·7 to 10·5%)	14·0% (9·3 to 20·1%)	297·3 (100·5 to 511·8)	0·2% (0·1 to 0·3%)	293·3 (98·9 to 505·7)	0·2% (0·1 to 0·3%)	-1·3% (-5·5 to 3·8%)	12·6% (7·8 to 18·3%)
Colon and rectum cancer	14·1 (4·8 to 24·2)	13·6% (4·6 to 23·4%)	14·8 (5·0 to 25·4)	13·6% (4·6 to 23·4%)	5·0% (0·7 to 10·5%)	-0·1% (-0·9 to 0·6%)	297·3 (100·5 to 511·8)	13·7% (4·7 to 23·5%)	293·3 (98·9 to 505·7)	13·6% (4·7 to 23·5%)	-1·3% (-5·5 to 3·8%)	-0·1% (-0·9 to 0·6%)
**Diet high in red meat: All causes**	4·5 (1·7 to 7·4)	0·1% (0·0 to 0·1%)	4·7 (1·8 to 7·8)	0·1% (0·0 to 0·2%)	4·8% (0·4 to 9·7%)	13·8% (9·1 to 18·9%)	159·5 (69·8 to 257·1)	0·1% (0·0 to 0·1%)	175·4 (77·0 to 285·5)	0·1% (0·1 to 0·2%)	9·9% (3·0 to 17·1%)	25·4% (18·0 to 32·7%)
Colon and rectum cancer	3·2 (0·7 to 5·8)	3·1% (0·7 to 5·6%)	3·4 (0·7 to 6·1)	3·1% (0·7 to 5·5%)	5·6% (0·2 to 11·6%)	0·4% (-2·9 to 3·7%)	72·9 (15·4 to 128·2)	3·3% (0·7 to 6·0%)	74·4 (15·6 to 132·7)	3·5% (0·7 to 6·1%)	2·1% (-3·1 to 7·9%)	3·3% (0·0 to 6·8%)
Diabetes mellitus	1·2 (0·1 to 2·2)	2·6% (0·3 to 4·8%)	1·2 (0·2 to 2·3)	2·5% (0·3 to 4·6%)	2·7% (-2·5 to 8·1%)	-3·8% (-6·9 to -0·3%)	86·7 (10·0 to 162·9)	3·3% (0·4 to 6·1%)	100·9 (11·5 to 191·8)	3·4% (0·4 to 6·2%)	16·5% (10·8 to 22·3%)	1·9% (-1·0 to 5·0%)
**Diet high in processed meat: All causes**	125·3 (19·3 to 216·6)	2·4% (0·4 to 4·1%)	117·5 (19·7 to 202·5)	2·4% (0·4 to 4·2%)	-6·2% (-8·5 to 3·2%)	1·8% (-0·5 to 12·3%)	2,907·4 (727·1 to 4,793·6)	1·7% (0·4 to 2·8%)	2,569·5 (775·0 to 4,135·2)	1·7% (0·5 to 2·7%)	-11·6% (-15·6 to 6·6%)	0·8% (-3·5 to 21·5%)
Colon and rectum cancer	7·2 (4·0 to 10·5)	6·9% (3·9 to 10·1%)	7·8 (4·3 to 11·5)	7·2% (4·0 to 10·5%)	8·6% (3·5 to 14·7%)	3·3% (1·2 to 5·6%)	155·4 (86·0 to 225·8)	7·1% (4·0 to 10·4%)	158·6 (87·1 to 232·2)	7·4% (4·1 to 10·8%)	2·1% (-2·8 to 8·1%)	3·3% (1·2 to 5·5%)
Ischaemic heart disease	111·7 (5·2 to 203·7)	6·5% (0·3 to 11·9%)	103·0 (4·8 to 186·7)	6·3% (0·3 to 11·4%)	-7·8% (-10·0 to -4·6%)	-3·3% (-4·8 to -1·2%)	2,308·7 (107·7 to 4,176·1)	7·5% (0·4 to 13·6%)	1,894·8 (87·7 to 3,410·9)	7·4% (0·3 to 13·3%)	-17·9% (-20·6 to -15·0%)	-2·0% (-3·4 to -0·1%)
Diabetes mellitus	6·4 (3·2 to 9·2)	13·7% (7·0 to 19·6%)	6·7 (3·4 to 9·7)	13·6% (6·9 to 19·4%)	5·4% (0·5 to 10·4%)	-1·4% (-3·4 to 0·8%)	443·4 (216·1 to 689·6)	16·9% (8·6 to 24·0%)	516·1 (255·7 to 814·2)	17·2% (8·8 to 24·6%)	16·4% (11·4 to 21·4%)	1·8% (0·1 to 3·7%)
**Diet high in sugar-sweetened beverages: All causes**	3·3 (2·3 to 4·8)	0·1% (0·0 to 0·1%)	2·8 (1·9 to 4·1)	0·1% (0·0 to 0·1%)	-15·5% (-19·0 to -11·6%)	-8·2% (-12·1 to -4·3%)	103·6 (70·1 to 149·3)	0·1% (0·0 to 0·1%)	89·2 (59·3 to 129·9)	0·1% (0·0 to 0·1%)	-13·8% (-17·7 to -10·0%)	-1·7% (-6·0 to 2·4%)
Oesophageal cancer	0·0 (0·0 to 0·1)	0·1% (0·0 to 0·3%)	0·0 (0·0 to 0·1)	0·1% (0·0 to 0·3%)	1·5% (-6·8 to 11·6%)	0·6% (-4·3 to 5·6%)	0·8 (0·2 to 1·6)	0·2% (0·0 to 0·3%)	0·8 (0·2 to 1·6)	0·2% (0·0 to 0·3%)	-1·3% (-9·5 to 9·1%)	0·7% (-4·2 to 5·9%)
Colon and rectum cancer	0·0 (0·0 to 0·1)	0·0% (0·0 to 0·1%)	0·1 (0·0 to 0·1)	0·0% (0·0 to 0·1%)	4·2% (-0·9 to 9·5%)	-0·8% (-5·2 to 3·7%)	1·3 (0·8 to 1·8)	0·1% (0·0 to 0·1%)	1·3 (0·8 to 1·9)	0·1% (0·0 to 0·1%)	0·3% (-4·9 to 5·6%)	1·6% (-3·3 to 6·3%)
Liver cancer due to hepatitis B	0·0 (0·0 to 0·0)	0·1% (0·1 to 0·2%)	0·0 (0·0 to 0·0)	0·1% (0·1 to 0·2%)	15·5% (6·1 to 24·2%)	2·3% (-3·2 to 7·4%)	0·2 (0·1 to 0·3)	0·1% (0·1 to 0·2%)	0·2 (0·1 to 0·4)	0·1% (0·1 to 0·2%)	13·7% (4·3 to 22·4%)	4·5% (-1·5 to 9·9%)
Liver cancer due to hepatitis C	0·0 (0·0 to 0·0)	0·1% (0·0 to 0·2%)	0·0 (0·0 to 0·0)	0·1% (0·0 to 0·2%)	13·7% (4·6 to 22·6%)	-0·1% (-5·3 to 5·5%)	0·2 (0·1 to 0·3)	0·1% (0·0 to 0·2%)	0·2 (0·1 to 0·3)	0·1% (0·0 to 0·2%)	12·2% (3·0 to 20·9%)	2·6% (-3·1 to 8·7%)
Liver cancer due to alcohol use	0·0 (0·0 to 0·0)	0·1% (0·0 to 0·2%)	0·0 (0·0 to 0·0)	0·1% (0·0 to 0·2%)	12·0% (2·5 to 20·4%)	-0·1% (-5·2 to 4·7%)	0·3 (0·1 to 0·6)	0·1% (0·1 to 0·2%)	0·4 (0·2 to 0·6)	0·1% (0·1 to 0·2%)	10·3% (0·9 to 18·7%)	1·1% (-4·5 to 6·1%)
Liver cancer due to other causes	0·0 (0·0 to 0·0)	0·1% (0·0 to 0·2%)	0·0 (0·0 to 0·0)	0·1% (0·0 to 0·2%)	11·9% (3·1 to 20·3%)	0·4% (-4·6 to 6·1%)	0·1 (0·0 to 0·1)	0·1% (0·1 to 0·2%)	0·1 (0·0 to 0·1)	0·1% (0·1 to 0·2%)	9·6% (0·9 to 18·0%)	4·3% (-1·1 to 10·1%)
Gallbladder and biliary tract cancer	0·0 (0·0 to 0·0)	0·1% (0·1 to 0·2%)	0·0 (0·0 to 0·0)	0·1% (0·1 to 0·2%)	-11·1% (-17·3 to -3·5%)	-5·8% (-10·0 to -1·7%)	0·3 (0·2 to 0·5)	0·1% (0·1 to 0·2%)	0·3 (0·1 to 0·4)	0·1% (0·1 to 0·2%)	-13·8% (-19·5 to -6·6%)	-3·4% (-7·6 to 0·9%)
Pancreatic cancer	0·0 (0·0 to 0·0)	0·0% (0·0 to 0·1%)	0·0 (0·0 to 0·0)	0·0% (0·0 to 0·1%)	11·9% (6·4 to 18·0%)	-2·2% (-6·1 to 2·6%)	0·4 (0·1 to 0·7)	0·0% (0·0 to 0·1%)	0·4 (0·1 to 0·7)	0·0% (0·0 to 0·1%)	7·5% (1·9 to 14·3%)	-0·6% (-5·1 to 5·7%)
Breast cancer	0·0 (0·0 to 0·0)	0·0% (0·0 to 0·0%)	0·0 (0·0 to 0·0)	0·0% (0·0 to 0·0%)	9·5% (-1·6 to 17·5%)	2·1% (-2·7 to 7·1%)	0·4 (0·3 to 0·7)	0·0% (0·0 to 0·0%)	0·5 (0·3 to 0·7)	0·0% (0·0 to 0·0%)	9·5% (-1·9 to 18·0%)	6·8% (1·6 to 12·5%)
Uterine cancer	0·0 (0·0 to 0·0)	0·2% (0·1 to 0·3%)	0·0 (0·0 to 0·0)	0·2% (0·1 to 0·3%)	0·3% (-13·2 to 15·1%)	-3·1% (-7·9 to 1·6%)	0·7 (0·4 to 1·0)	0·2% (0·1 to 0·3%)	0·7 (0·4 to 1·0)	0·2% (0·1 to 0·3%)	1·4% (-12·8 to 17·3%)	-1·1% (-6·0 to 3·5%)
Ovarian cancer	0·0 (0·0 to 0·0)	0·0% (0·0 to 0·0%)	0·0 (0·0 to 0·0)	0·0% (0·0 to 0·0%)	8·5% (0·0 to 15·3%)	-2·6% (-6·5 to 1·3%)	0·1 (0·0 to 0·2)	0·0% (0·0 to 0·0%)	0·1 (0·0 to 0·2)	0·0% (0·0 to 0·0%)	6·1% (-0·7 to 12·9%)	-1·2% (-5·2 to 2·9%)
Kidney cancer	0·0 (0·0 to 0·0)	0·1% (0·1 to 0·2%)	0·0 (0·0 to 0·0)	0·1% (0·1 to 0·2%)	10·1% (3·0 to 17·4%)	-2·3% (-6·4 to 1·7%)	0·6 (0·4 to 0·9)	0·1% (0·1 to 0·2%)	0·6 (0·4 to 0·9)	0·1% (0·1 to 0·2%)	7·1% (-0·3 to 14·8%)	-0·3% (-4·7 to 3·8%)
Thyroid cancer	0·0 (0·0 to 0·0)	0·1% (0·0 to 0·1%)	0·0 (0·0 to 0·0)	0·1% (0·0 to 0·1%)	-5·0% (-12·0 to 3·5%)	0·0% (-5·6 to 6·4%)	0·1 (0·0 to 0·1)	0·1% (0·0 to 0·1%)	0·1 (0·0 to 0·1)	0·1% (0·0 to 0·1%)	-5·4% (-13·0 to 3·4%)	2·9% (-2·8 to 8·8%)
Ischaemic heart disease	1·7 (1·1 to 2·4)	0·1% (0·1 to 0·1%)	1·4 (0·9 to 2·1)	0·1% (0·1 to 0·1%)	-15·7% (-20·3 to -11·1%)	-11·6% (-16·0 to -7·1%)	44·8 (30·3 to 65·2)	0·1% (0·1 to 0·2%)	35·8 (23·8 to 52·4)	0·1% (0·1 to 0·2%)	-20·2% (-24·5 to -15·9%)	-4·7% (-9·2 to -0·2%)
Ischaemic stroke	0·4 (0·3 to 0·6)	0·1% (0·0 to 0·1%)	0·3 (0·2 to 0·5)	0·1% (0·0 to 0·1%)	-25·6% (-30·8 to -20·6%)	-13·2% (-19·5 to -7·8%)	10·7 (7·1 to 15·3)	0·1% (0·1 to 0·2%)	8·0 (5·3 to 11·5)	0·1% (0·1 to 0·2%)	-25·2% (-29·6 to -20·7%)	-2·6% (-7·6 to 2·1%)
Hemorrhagic stroke	0·6 (0·4 to 0·8)	0·2% (0·1 to 0·3%)	0·4 (0·3 to 0·6)	0·2% (0·1 to 0·2%)	-25·8% (-30·2 to -21·1%)	-8·2% (-13·0 to -3·4%)	18·3 (12·2 to 26·4)	0·3% (0·2 to 0·4%)	13·6 (9·0 to 19·8)	0·3% (0·2 to 0·4%)	-25·5% (-29·9 to -21·1%)	-0·1% (-4·7 to 4·6%)
Hypertensive heart disease	0·2 (0·1 to 0·3)	0·2% (0·2 to 0·4%)	0·2 (0·1 to 0·3)	0·2% (0·1 to 0·4%)	0·1% (-10·0 to 10·2%)	-6·9% (-15·7 to 0·7%)	4·5 (2·9 to 6·9)	0·3% (0·2 to 0·5%)	4·2 (2·7 to 6·5)	0·3% (0·2 to 0·5%)	-5·9% (-12·6 to 2·4%)	-1·3% (-7·5 to 4·9%)
Cardiomyopathy and myocarditis	-	-	-	-	-	-	-	-	-	-	-	-
Atrial fibrillation and flutter	-	-	-	-	-	-	-	-	-	-	-	-
Peripheral vascular disease	-	-	-	-	-	-	-	-	-	-	-	-

Supplemental Results Table 1. All-age deaths, death PAFs, DALYs, DALY PAFs, and percent change for each risk factor and outcome for both sexes combined in 2005 and 2015 for Central Europe, Eastern Europe, and Central Asia.

Data in parenthesis are 95% uncertainty intervals. PAFs=population attributable fractions. DALYs=disability-adjusted life-years.

	**2005 Deaths (in thousands)**	**2005 Deaths PAF**	**2015 Deaths (in thousands)**	**2015 Deaths PAF**	**Percent change deaths 2005-2015**	**Percent change deaths PAF 2005-2015**	**2005 DALYs (in thousands)**	**2005 DALYs PAF**	**2015 DALYs (in thousands)**	**2015 DALYs PAF**	**Percent change DALYs 2005-2015**	**Percent change DALYs PAF 2005-2015**
Endocarditis	-	-	-	-	-	-	-	-	-	-	-	-
Other cardiovascular and circulatory diseases	-	-	-	-	-	-	-	-	-	-	-	-
Diabetes mellitus	0·2 (0·1 to 0·2)	0·3% (0·2 to 0·5%)	0·2 (0·1 to 0·2)	0·3% (0·2 to 0·5%)	0·6% (-4·5 to 6·3%)	-5·8% (-10·3 to -0·8%)	11·9 (7·5 to 18·3)	0·5% (0·3 to 0·7%)	13·7 (8·4 to 21·4)	0·5% (0·3 to 0·7%)	14·6% (8·7 to 20·2%)	0·3% (-3·5 to 3·9%)
Chronic kidney disease due to diabetes mellitus	0·0 (0·0 to 0·0)	0·2% (0·1 to 0·4%)	0·0 (0·0 to 0·0)	0·2% (0·1 to 0·4%)	-7·1% (-15·0 to 2·5%)	-5·1% (-12·9 to 4·2%)	0·8 (0·3 to 1·4)	0·3% (0·1 to 0·4%)	0·8 (0·3 to 1·4)	0·3% (0·1 to 0·5%)	-3·1% (-8·8 to 4·9%)	1·2% (-4·1 to 8·7%)
Chronic kidney disease due to hypertension	0·0 (0·0 to 0·1)	0·2% (0·1 to 0·4%)	0·0 (0·0 to 0·1)	0·2% (0·1 to 0·4%)	-2·4% (-10·9 to 9·6%)	-7·6% (-15·0 to 3·0%)	1·0 (0·5 to 1·8)	0·3% (0·1 to 0·5%)	0·9 (0·4 to 1·7)	0·3% (0·1 to 0·5%)	-3·7% (-9·4 to 4·6%)	-1·2% (-6·5 to 6·1%)
Chronic kidney disease due to glomerulonephritis	0·0 (0·0 to 0·0)	0·2% (0·1 to 0·4%)	0·0 (0·0 to 0·0)	0·2% (0·1 to 0·4%)	-5·1% (-12·4 to 7·9%)	-0·7% (-7·7 to 12·5%)	1·3 (0·4 to 2·6)	0·2% (0·1 to 0·4%)	1·3 (0·4 to 2·5)	0·2% (0·1 to 0·4%)	-2·7% (-8·9 to 9·5%)	4·5% (-1·2 to 18·5%)
Chronic kidney disease due to other causes	0·0 (0·0 to 0·0)	0·2% (0·1 to 0·3%)	0·0 (0·0 to 0·0)	0·1% (0·1 to 0·3%)	11·9% (-9·8 to 40·8%)	-9·4% (-28·2 to 11·8%)	0·5 (0·2 to 0·9)	0·2% (0·1 to 0·4%)	0·5 (0·2 to 1·0)	0·2% (0·1 to 0·4%)	7·8% (0·7 to 17·6%)	3·2% (-2·2 to 11·9%)
**Diet low in fibre: All causes**	70·6 (30·7 to 126·8)	1·3% (0·6 to 2·4%)	67·7 (29·5 to 120·3)	1·4% (0·6 to 2·5%)	-4·2% (-10·4 to 2·7%)	4·0% (-2·3 to 11·3%)	1,549·1 (678·3 to 2,786·9)	0·9% (0·4 to 1·6%)	1,255·9 (550·3 to 2,235·5)	0·8% (0·4 to 1·5%)	-18·9% (-24·6 to -12·9%)	-7·4% (-14·0 to -0·4%)
Colon and rectum cancer	5·3 (2·1 to 9·9)	5·2% (2·0 to 9·6%)	5·8 (2·2 to 10·7)	5·3% (2·1 to 9·8%)	7·9% (0·1 to 17·3%)	2·7% (-3·5 to 10·5%)	110·6 (43·5 to 206·3)	5·1% (2·0 to 9·5%)	109·8 (42·7 to 205·9)	5·1% (2·0 to 9·5%)	-0·8% (-8·6 to 8·5%)	0·4% (-6·2 to 7·7%)
Ischaemic heart disease	65·3 (27·8 to 119·3)	3·8% (1·6 to 6·9%)	61·9 (26·6 to 112·3)	3·8% (1·6 to 6·9%)	-5·2% (-11·3 to 1·8%)	-0·6% (-6·9 to 6·2%)	1,438·5 (614·8 to 2,607·3)	4·7% (2·0 to 8·5%)	1,146·1 (491·1 to 2,061·5)	4·5% (1·9 to 8·1%)	-20·3% (-25·8 to -14·7%)	-4·9% (-10·6 to 1·2%)
**Diet low in calcium: All causes**	16·5 (8·9 to 25·3)	0·3% (0·2 to 0·5%)	17·5 (9·6 to 26·8)	0·4% (0·2 to 0·6%)	6·4% (1·0 to 12·7%)	15·5% (9·8 to 22·2%)	343·8 (184·6 to 530·5)	0·2% (0·1 to 0·3%)	341·7 (185·6 to 527·6)	0·2% (0·1 to 0·3%)	-0·6% (-5·9 to 5·2%)	13·5% (7·5 to 19·7%)
Colon and rectum cancer	16·5 (8·9 to 25·3)	15·9% (8·6 to 24·5%)	17·5 (9·6 to 26·8)	16·1% (8·8 to 24·8%)	6·4% (1·0 to 12·7%)	1·2% (-1·5 to 4·2%)	343·8 (184·6 to 530·5)	15·8% (8·5 to 24·4%)	341·7 (185·6 to 527·6)	15·9% (8·7 to 24·5%)	-0·6% (-5·9 to 5·2%)	0·6% (-2·4 to 3·6%)
Prostate cancer	-	-	-	-	-	-	-	-	-	-	-	-
**Diet low in seafood omega-3 fatty acids: All causes**	267·1 (110·7 to 438·4)	5·1% (2·1 to 8·3%)	238·7 (98·3 to 396·0)	4·9% (2·0 to 8·1%)	-10·6% (-12·8 to -8·8%)	-3·0% (-5·0 to -1·3%)	5,382·5 (2,245·2 to 8,775·6)	3·1% (1·3 to 5·0%)	4,256·4 (1,773·4 to 7,006·6)	2·8% (1·2 to 4·6%)	-20·9% (-23·5 to -18·9%)	-9·7% (-13·0 to -7·1%)
Ischaemic heart disease	267·1 (110·7 to 438·4)	15·6% (6·4 to 25·5%)	238·7 (98·3 to 396·0)	14·6% (6·0 to 24·0%)	-10·6% (-12·8 to -8·8%)	-6·3% (-7·4 to -5·3%)	5,382·5 (2,245·2 to 8,775·6)	17·6% (7·4 to 28·4%)	4,256·4 (1,773·4 to 7,006·6)	16·6% (6·9 to 27·0%)	-20·9% (-23·5 to -18·9%)	-5·6% (-6·9 to -4·5%)
**Diet low in polyunsaturated fatty acids: All causes**	145·0 (58·6 to 227·4)	2·7% (1·1 to 4·3%)	131·8 (54·7 to 203·6)	2·7% (1·1 to 4·2%)	-9·1% (-24·9 to 5·2%)	-1·3% (-18·8 to 14·7%)	2,961·6 (1,215·5 to 4,601·6)	1·7% (0·7 to 2·7%)	2,346·1 (973·0 to 3,595·3)	1·5% (0·6 to 2·4%)	-20·8% (-27·2 to -15·0%)	-9·6% (-16·9 to -3·2%)
Ischaemic heart disease	145·0 (58·6 to 227·4)	8·5% (3·4 to 13·2%)	131·8 (54·7 to 203·6)	8·1% (3·4 to 12·4%)	-9·1% (-24·9 to 5·2%)	-4·7% (-21·4 to 10·8%)	2,961·6 (1,215·5 to 4,601·6)	9·7% (3·9 to 15·0%)	2,346·1 (973·0 to 3,595·3)	9·1% (3·8 to 14·1%)	-20·8% (-27·2 to -15·0%)	-5·4% (-12·8 to 0·6%)
**Diet high in trans fatty acids: All causes**	49·5 (11·6 to 114·6)	0·9% (0·2 to 2·2%)	42·1 (9·5 to 99·9)	0·9% (0·2 to 2·0%)	-15·0% (-20·7 to -11·5%)	-7·7% (-13·6 to -4·0%)	1,198·8 (302·8 to 2,634·3)	0·7% (0·2 to 1·5%)	932·9 (231·9 to 2,091·7)	0·6% (0·2 to 1·4%)	-22·2% (-26·4 to -18·6%)	-11·2% (-16·1 to -7·2%)
Ischaemic heart disease	49·5 (11·6 to 114·6)	2·9% (0·7 to 6·7%)	42·1 (9·5 to 99·9)	2·6% (0·6 to 6·1%)	-15·0% (-20·7 to -11·5%)	-10·8% (-16·3 to -7·7%)	1,198·8 (302·8 to 2,634·3)	3·9% (1·0 to 8·6%)	932·9 (231·9 to 2,091·7)	3·6% (0·9 to 8·1%)	-22·2% (-26·4 to -18·6%)	-7·1% (-11·0 to -3·6%)
**Diet high in sodium: All causes**	430·0 (174·3 to 789·9)	8·1% (3·3 to 15·0%)	388·2 (155·9 to 721·1)	8·0% (3·2 to 14·8%)	-9·7% (-13·5 to -6·2%)	-2·0% (-5·9 to 2·0%)	8,491·5 (3,541·9 to 15,259·8)	4·9% (2·0 to 8·9%)	7,043·9 (2,962·7 to 12,724·8)	4·6% (1·9 to 8·4%)	-17·0% (-19·5 to -14·2%)	-5·3% (-8·4 to -1·9%)
Stomach cancer	29·8 (12·9 to 51·1)	31·8% (13·8 to 54·5%)	24·8 (10·8 to 42·6)	32·1% (13·9 to 54·9%)	-16·6% (-27·7 to -3·7%)	0·8% (-11·9 to 16·7%)	685·9 (301·8 to 1,156·3)	31·9% (14·2 to 54·1%)	544·7 (242·1 to 922·0)	32·4% (14·4 to 54·8%)	-20·6% (-30·6 to -8·7%)	1·5% (-11·1 to 16·0%)
Rheumatic heart disease	1·2 (0·4 to 2·5)	6·1% (2·1 to 13·0%)	0·8 (0·3 to 1·7)	6·3% (2·2 to 13·2%)	-32·0% (-36·3 to -24·8%)	4·2% (-0·7 to 14·0%)	35·8 (12·0 to 74·8)	5·9% (2·0 to 12·4%)	23·4 (8·3 to 48·0)	6·3% (2·2 to 12·9%)	-34·6% (-38·2 to -29·6%)	6·9% (2·8 to 14·1%)
Ischaemic heart disease	234·3 (89·1 to 450·2)	13·7% (5·2 to 26·1%)	218·5 (82·4 to 423·6)	13·4% (5·0 to 25·8%)	-6·8% (-12·5 to -2·3%)	-2·2% (-8·0 to 2·5%)	4,396·2 (1,772·3 to 8,270·6)	14·3% (5·8 to 26·9%)	3,706·8 (1,483·1 to 6,897·7)	14·4% (5·9 to 26·9%)	-15·7% (-18·6 to -12·8%)	0·7% (-1·8 to 3·6%)
Ischaemic stroke	72·9 (25·4 to 147·3)	11·4% (4·0 to 23·0%)	59·9 (20·6 to 120·8)	10·9% (3·7 to 22·0%)	-17·8% (-22·8 to -9·5%)	-4·3% (-9·5 to 4·9%)	1,242·2 (448·8 to 2,430·5)	12·8% (4·6 to 25·1%)	947·4 (349·4 to 1,841·4)	12·7% (4·6 to 24·7%)	-23·7% (-26·9 to -18·9%)	-0·8% (-3·5 to 5·2%)
Hemorrhagic stroke	49·1 (18·6 to 93·5)	15·4% (5·9 to 29·5%)	39·8 (15·1 to 76·4)	15·5% (6·0 to 29·8%)	-18·8% (-23·7 to -12·2%)	0·4% (-4·7 to 7·9%)	1,099·0 (429·3 to 2,043·0)	16·3% (6·4 to 30·4%)	844·2 (333·4 to 1,557·5)	16·8% (6·6 to 31·0%)	-23·2% (-26·4 to -19·1%)	3·0% (0·5 to 7·4%)
Hypertensive heart disease	20·5 (5·2 to 48·9)	26·9% (7·0 to 62·5%)	22·3 (5·6 to 54·0)	27·1% (6·9 to 64·7%)	8·5% (-2·1 to 20·8%)	0·8% (-7·7 to 11·3%)	383·5 (123·1 to 851·3)	27·9% (9·0 to 61·4%)	374·9 (123·7 to 842·1)	28·6% (9·4 to 62·3%)	-2·2% (-8·3 to 5·6%)	2·4% (-1·6 to 9·3%)
Cardiomyopathy and myocarditis	9·7 (3·5 to 19·2)	8·0% (2·9 to 16·0%)	9·0 (3·3 to 17·9)	8·0% (2·9 to 15·6%)	-6·6% (-14·3 to 2·4%)	-0·9% (-6·9 to 6·9%)	285·3 (99·1 to 577·8)	7·8% (2·8 to 15·8%)	234·6 (84·6 to 465·9)	8·1% (2·9 to 16·2%)	-17·8% (-24·6 to -10·3%)	3·6% (-0·4 to 10·2%)
Atrial fibrillation and flutter	1·4 (0·5 to 2·8)	8·3% (3·2 to 15·8%)	1·7 (0·6 to 3·3)	7·7% (2·9 to 15·0%)	17·8% (11·4 to 23·6%)	-6·3% (-10·5 to -3·0%)	46·9 (17·7 to 90·5)	10·5% (4·2 to 19·3%)	52·6 (19·9 to 102·0)	10·3% (4·1 to 18·9%)	12·2% (9·0 to 15·2%)	-2·4% (-4·4 to -0·5%)
Aortic aneurysm	1·4 (0·5 to 2·6)	9·5% (3·7 to 18·1%)	1·5 (0·6 to 2·9)	9·0% (3·5 to 17·1%)	9·3% (2·3 to 15·4%)	-5·4% (-9·4 to -1·8%)	31·0 (12·2 to 58·0)	9·7% (3·8 to 18·4%)	32·0 (12·6 to 61·1)	9·6% (3·8 to 18·2%)	3·1% (-3·7 to 8·0%)	-1·4% (-4·3 to 0·9%)
Peripheral vascular disease	0·5 (0·2 to 1·0)	6·0% (2·1 to 12·1%)	0·6 (0·2 to 1·3)	5·5% (1·8 to 11·6%)	22·6% (8·9 to 37·0%)	-7·6% (-15·6 to -0·8%)	11·5 (3·9 to 23·7)	6·4% (2·2 to 12·6%)	13·0 (4·4 to 26·8)	6·2% (2·2 to 12·3%)	12·6% (4·2 to 21·2%)	-3·6% (-7·6 to 0·1%)
Endocarditis	0·3 (0·1 to 0·6)	8·5% (3·3 to 16·2%)	0·3 (0·1 to 0·7)	8·4% (3·2 to 16·2%)	2·5% (-6·5 to 12·7%)	-0·6% (-4·0 to 2·7%)	9·4 (3·5 to 19·0)	7·7% (2·9 to 15·2%)	9·4 (3·5 to 18·6)	7·9% (3·0 to 15·7%)	-0·6% (-10·7 to 12·1%)	2·6% (-1·3 to 5·9%)
Other cardiovascular and circulatory diseases	4·3 (1·8 to 7·8)	10·8% (4·4 to 19·4%)	4·1 (1·7 to 7·5)	10·2% (4·1 to 18·4%)	-5·1% (-9·7 to -0·7%)	-5·5% (-9·1 to -2·7%)	121·1 (49·3 to 220·4)	11·2% (4·6 to 20·3%)	114·3 (46·3 to 209·7)	11·3% (4·6 to 20·4%)	-5·6% (-9·6 to -2·0%)	0·2% (-1·9 to 2·1%)
Chronic kidney disease due to diabetes mellitus	1·2 (0·4 to 2·3)	12·3% (4·3 to 23·4%)	1·2 (0·4 to 2·3)	12·6% (4·5 to 24·0%)	0·7% (-3·8 to 5·2%)	2·9% (0·5 to 5·3%)	32·5 (11·5 to 64·0)	10·3% (3·6 to 19·6%)	32·4 (11·5 to 64·4)	10·7% (3·8 to 20·4%)	-0·3% (-3·9 to 3·5%)	4·2% (2·1 to 6·5%)
Chronic kidney disease due to hypertension	1·9 (0·7 to 3·7)	12·9% (4·9 to 25·0%)	2·1 (0·8 to 4·0)	13·2% (5·0 to 25·5%)	7·8% (3·7 to 12·0%)	2·0% (-0·1 to 3·8%)	41·7 (15·7 to 81·1)	11·2% (4·3 to 21·8%)	42·3 (15·7 to 82·4)	11·7% (4·4 to 22·6%)	1·3% (-2·2 to 4·9%)	3·9% (2·2 to 5·8%)
Chronic kidney disease due to glomerulonephritis	1·3 (0·5 to 2·5)	10·3% (3·8 to 19·9%)	1·3 (0·5 to 2·6)	11·1% (4·1 to 21·5%)	3·2% (-1·1 to 7·8%)	8·0% (5·9 to 10·9%)	50·0 (17·5 to 101·5)	8·2% (2·9 to 16·2%)	50·2 (17·6 to 101·2)	8·8% (3·1 to 17·4%)	0·5% (-3·0 to 4·4%)	7·8% (6·0 to 10·6%)
Chronic kidney disease due to other causes	0·2 (0·1 to 0·3)	10·0% (3·8 to 19·3%)	0·2 (0·1 to 0·5)	11·1% (4·0 to 21·6%)	36·9% (24·1 to 52·5%)	10·6% (4·8 to 14·3%)	19·3 (6·6 to 40·2)	8·9% (3·1 to 17·8%)	21·7 (7·4 to 44·6)	9·6% (3·4 to 19·0%)	12·7% (8·5 to 17·3%)	8·0% (5·6 to 10·7%)
**Sexual abuse and violence: All causes**	33·8 (20·6 to 50·4)	0·6% (0·4 to 1·0%)	24·7 (15·3 to 37·1)	0·5% (0·3 to 0·8%)	-27·1% (-31·8 to -21·6%)	-20·8% (-25·9 to -15·1%)	1,979·1 (1,299·3 to 2,764·8)	1·1% (0·8 to 1·6%)	1,563·8 (1,058·5 to 2,156·1)	1·0% (0·7 to 1·4%)	-21·0% (-25·8 to -15·0%)	-9·9% (-14·9 to -3·3%)
**Childhood sexual abuse: All causes**	21·4 (7·3 to 39·5)	0·4% (0·1 to 0·7%)	15·0 (4·7 to 28·6)	0·3% (0·1 to 0·6%)	-30·0% (-37·6 to -23·3%)	-24·0% (-32·3 to -16·8%)	1,158·1 (484·1 to 1,963·9)	0·7% (0·3 to 1·1%)	842·9 (367·4 to 1,441·4)	0·6% (0·2 to 0·9%)	-27·2% (-32·8 to -19·9%)	-17·0% (-23·2 to -8·6%)
Alcohol use disorders	3·6 (0·0 to 7·8)	5·3% (0·0 to 12·1%)	1·9 (0·0 to 4·4)	4·8% (0·0 to 10·9%)	-45·7% (-51·1 to 0·0%)	-9·7% (-14·3 to 0·0%)	196·3 (0·0 to 440·2)	5·5% (0·0 to 12·5%)	121·4 (0·0 to 275·0)	5·0% (0·0 to 11·3%)	-38·2% (-44·1 to 0·0%)	-8·6% (-13·0 to 0·0%)
Major depressive disorder	-	-	-	-	-	-	140·4 (15·3 to 307·6)	4·9% (0·6 to 10·2%)	135·1 (14·7 to 297·8)	4·5% (0·5 to 9·5%)	-3·8% (-7·9 to 0·6%)	-7·3% (-10·2 to -4·0%)
Dysthymia	-	-	-	-	-	-	29·4 (3·2 to 64·8)	4·9% (0·6 to 10·2%)	29·2 (3·1 to 64·4)	4·5% (0·5 to 9·4%)	-0·7% (-4·4 to 2·9%)	-7·2% (-10·0 to -4·0%)
Self-harm	17·9 (4·2 to 35·8)	13·5% (3·2 to 26·8%)	13·1 (3·1 to 26·5)	12·4% (2·9 to 25·0%)	-26·9% (-32·3 to -21·2%)	-8·3% (-12·0 to -4·8%)	792·0 (189·9 to 1,582·5)	14·0% (3·4 to 27·7%)	557·2 (133·5 to 1,112·6)	12·9% (3·1 to 25·8%)	-29·7% (-35·0 to -23·9%)	-7·4% (-11·2 to -3·7%)
**Intimate partner violence: All causes**	14·1 (12·0 to 16·1)	0·3% (0·2 to 0·3%)	10·9 (9·1 to 12·7)	0·2% (0·2 to 0·3%)	-22·4% (-27·9 to -16·3%)	-15·7% (-21·8 to -9·1%)	908·9 (746·6 to 1,084·4)	0·5% (0·4 to 0·6%)	791·3 (636·1 to 972·4)	0·5% (0·4 to 0·6%)	-12·9% (-17·8 to -8·1%)	-0·7% (-6·1 to 4·4%)

Supplemental Results Table 1. All-age deaths, death PAFs, DALYs, DALY PAFs, and percent change for each risk factor and outcome for both sexes combined in 2005 and 2015 for Central Europe, Eastern Europe, and Central Asia.

Data in parenthesis are 95% uncertainty intervals. PAFs=population attributable fractions. DALYs=disability-adjusted life-years.

	2005 Deaths (in thousands)	2005 Deaths PAF	2015 Deaths (in thousands)	2015 Deaths PAF	Percent change deaths 2005-2015	Percent change deaths PAF 2005-2015	2005 DALYs (in thousands)	2005 DALYs PAF	2015 DALYs (in thousands)	2015 DALYs PAF	Percent change DALYs 2005-2015	Percent change DALYs PAF 2005-2015
HIV/AIDS - Tuberculosis	0·1 (0·0 to 0·1)	1·7% (0·8 to 2·6%)	0·0 (0·0 to 0·1)	1·7% (0·8 to 2·7%)	-45·7% (-51·3 to -39·9%)	1·8% (-4·0 to 7·2%)	3·2 (1·6 to 4·9)	1·6% (0·8 to 2·4%)	1·7 (0·8 to 2·7)	1·6% (0·8 to 2·5%)	-46·3% (-51·7 to -40·6%)	2·3% (-3·8 to 8·2%)
HIV/AIDS resulting in other diseases	0·3 (0·2 to 0·5)	1·6% (0·8 to 2·5%)	0·5 (0·2 to 0·7)	1·7% (0·8 to 2·7%)	37·8% (23·5 to 53·8%)	8·0% (-2·6 to 21·3%)	16·8 (8·3 to 26·1)	1·5% (0·7 to 2·3%)	23·0 (10·9 to 36·5)	1·6% (0·8 to 2·6%)	36·8% (22·2 to 53·1%)	8·0% (-3·0 to 21·1%)
Maternal abortion, miscarriage, and ectopic pregnancy	0·0 (0·0 to 0·0)	0·0% (0·0 to 0·0%)	0·0 (0·0 to 0·0)	0·0% (0·0 to 0·0%)	-38·6% (-54·3 to -21·9%)	0·1% (-22·6 to 15·9%)	0·0 (0·0 to 0·0)	0·0% (0·0 to 0·0%)	0·0 (0·0 to 0·0)	0·0% (0·0 to 0·0%)	-32·5% (-51·2 to -12·7%)	0·3% (-27·6 to 17·7%)
Major depressive disorder	-	-	-	-	-	-	288·7 (189·5 to 408·8)	10·1% (8·3 to 11·9%)	311·5 (205·0 to 440·4)	10·4% (8·5 to 12·5%)	7·9% (2·2 to 13·8%)	3·9% (-0·7 to 8·6%)
Dysthymia	-	-	-	-	-	-	62·1 (39·1 to 91·8)	10·3% (8·5 to 12·2%)	68·4 (43·0 to 101·5)	10·6% (8·6 to 12·7%)	10·1% (5·3 to 15·4%)	2·9% (-1·7 to 7·5%)
Self-harm	10·1 (8·2 to 12·1)	7·6% (6·2 to 9·1%)	8·4 (6·6 to 10·1)	7·9% (6·2 to 9·5%)	-17·6% (-25·2 to -9·9%)	3·4% (-9·2 to 13·7%)	371·4 (295·7 to 448·4)	6·5% (5·3 to 7·9%)	292·4 (228·5 to 356·0)	6·8% (5·3 to 8·3%)	-21·3% (-27·7 to -14·1%)	3·7% (-8·4 to 13·8%)
Assault by firearm	0·2 (0·1 to 0·3)	3·8% (2·7 to 4·8%)	0·2 (0·1 to 0·2)	4·4% (3·5 to 5·4%)	-28·5% (-36·9 to -15·4%)	15·3% (-3·4 to 37·5%)	9·8 (7·2 to 12·1)	3·6% (2·6 to 4·6%)	6·6 (5·4 to 8·1)	4·0% (3·2 to 4·9%)	-32·6% (-40·6 to -19·6%)	11·4% (-7·9 to 34·0%)
Assault by sharp object	1·2 (1·0 to 1·4)	4·9% (4·1 to 5·9%)	0·7 (0·6 to 0·8)	4·9% (4·2 to 5·9%)	-41·7% (-48·8 to -31·9%)	0·6% (-11·6 to 15·7%)	56·2 (47·3 to 67·7)	4·9% (4·1 to 5·9%)	31·4 (26·5 to 37·6)	4·9% (4·1 to 5·8%)	-44·1% (-51·3 to -34·6%)	-0·3% (-13·2 to 15·6%)
Assault by other means	2·1 (1·8 to 2·5)	5·7% (4·8 to 6·7%)	1·2 (1·1 to 1·4)	5·5% (4·7 to 6·5%)	-42·2% (-49·0 to -33·4%)	-2·5% (-13·4 to 9·8%)	100·6 (85·2 to 119·0)	6·1% (5·2 to 7·2%)	56·3 (48·1 to 66·0)	6·0% (5·1 to 7·0%)	-44·1% (-50·3 to -35·8%)	-2·1% (-12·8 to 9·9%)
**Unsafe sex: All causes**	38·0 (36·9 to 39·3)	0·7% (0·7 to 0·7%)	37·9 (36·0 to 39·5)	0·8% (0·7 to 0·8%)	-0·5% (-4·9 to 4·2%)	8·0% (3·4 to 12·9%)	1,593·1 (1,512·2 to 1,691·1)	0·9% (0·8 to 1·0%)	1,613·8 (1,513·1 to 1,723·0)	1·1% (1·0 to 1·2%)	1·3% (-3·1 to 6·1%)	15·6% (10·6 to 20·9%)
HIV/AIDS - Tuberculosis	2·2 (2·1 to 2·3)	56·1% (53·3 to 59·0%)	1·2 (1·1 to 1·4)	58·9% (56·1 to 61·5%)	-44·0% (-48·8 to -38·4%)	5·0% (2·4 to 7·5%)	115·0 (107·6 to 122·4)	56·7% (53·9 to 59·5%)	63·2 (57·8 to 69·5)	59·4% (56·5 to 62·0%)	-45·0% (-49·6 to -39·7%)	4·7% (2·2 to 7·1%)
HIV/AIDS resulting in other diseases	11·7 (10·9 to 12·4)	56·6% (53·4 to 59·7%)	15·5 (14·3 to 16·8)	58·9% (55·5 to 62·1%)	33·0% (23·9 to 42·9%)	4·2% (2·1 to 6·1%)	636·3 (584·5 to 695·3)	57·1% (53·9 to 60·3%)	837·8 (762·4 to 922·4)	59·3% (55·8 to 62·5%)	31·7% (23·1 to 40·8%)	3·9% (1·9 to 6·0%)
Syphilis	1·3 (0·8 to 2·0)	100·0% (100·0 to 100·0%)	0·9 (0·6 to 1·5)	100·0% (100·0 to 100·0%)	-26·6% (-40·2 to -11·2%)	-	120·2 (76·8 to 182·3)	100·0% (100·0 to 100·0%)	97·4 (64·0 to 143·1)	100·0% (100·0 to 100·0%)	-19·0% (-33·2 to -4·0%)	-
Chlamydial infection	0·0 (0·0 to 0·0)	100·0% (100·0 to 100·0%)	0·0 (0·0 to 0·0)	100·0% (100·0 to 100·0%)	-5·5% (-18·5 to 10·3%)	-	7·5 (4·7 to 11·5)	100·0% (100·0 to 100·0%)	7·0 (4·4 to 11·0)	100·0% (100·0 to 100·0%)	-5·6% (-11·7 to 0·4%)	-
Gonococcal infection	0·0 (0·0 to 0·0)	100·0% (100·0 to 100·0%)	0·0 (0·0 to 0·0)	100·0% (100·0 to 100·0%)	-22·3% (-31·8 to -9·9%)	-	3·5 (2·5 to 4·9)	100·0% (100·0 to 100·0%)	3·2 (2·2 to 4·5)	100·0% (100·0 to 100·0%)	-8·4% (-16·6 to 0·2%)	-
Trichomoniasis	-	-	-	-	-	-	9·4 (3·7 to 19·8)	100·0% (100·0 to 100·0%)	9·9 (3·9 to 21·2)	100·0% (100·0 to 100·0%)	5·3% (2·4 to 8·2%)	-
Genital herpes	-	-	-	-	-	-	12·4 (3·8 to 29·4)	100·0% (100·0 to 100·0%)	13·4 (4·1 to 32·1)	100·0% (100·0 to 100·0%)	7·8% (5·0 to 9·8%)	-
Other sexually transmitted diseases	0·0 (0·0 to 0·0)	100·0% (100·0 to 100·0%)	0·0 (0·0 to 0·0)	100·0% (100·0 to 100·0%)	-22·5% (-32·2 to -9·9%)	-	13·0 (8·8 to 18·8)	100·0% (100·0 to 100·0%)	12·0 (8·1 to 17·5)	100·0% (100·0 to 100·0%)	-8·1% (-14·2 to -2·5%)	-
Cervical cancer	22·9 (22·1 to 23·7)	100·0% (100·0 to 100·0%)	20·2 (19·2 to 21·2)	100·0% (100·0 to 100·0%)	-11·9% (-16·9 to -7·0%)	-	675·8 (653·2 to 699·7)	100·0% (100·0 to 100·0%)	569·8 (543·1 to 598·8)	100·0% (100·0 to 100·0%)	-15·7% (-20·5 to -11·1%)	-
**Low physical activity: All causes**	198·8 (134·1 to 261·2)	3·8% (2·5 to 4·9%)	192·4 (133·0 to 250·5)	4·0% (2·7 to 5·1%)	-3·2% (-6·1 to 0·7%)	5·0% (2·3 to 9·1%)	3,622·5 (2,387·2 to 4,809·7)	2·1% (1·4 to 2·8%)	3,240·0 (2,214·2 to 4,248·6)	2·1% (1·5 to 2·8%)	-10·6% (-13·7 to -6·1%)	2·1% (-1·4 to 7·1%)
Colon and rectum cancer	11·7 (7·6 to 16·1)	11·3% (7·3 to 15·5%)	12·8 (8·4 to 17·6)	11·8% (7·7 to 16·0%)	9·4% (4·9 to 15·3%)	4·1% (2·6 to 6·8%)	223·7 (137·9 to 314·4)	10·3% (6·3 to 14·4%)	228·6 (143·4 to 319·4)	10·6% (6·6 to 14·8%)	2·2% (-1·9 to 7·6%)	3·5% (2·1 to 5·9%)
Breast cancer	4·5 (3·2 to 5·8)	7·5% (5·3 to 9·7%)	4·9 (3·5 to 6·4)	7·7% (5·4 to 9·9%)	9·9% (-1·0 to 15·6%)	2·4% (1·1 to 3·9%)	112·6 (79·2 to 146·5)	6·9% (4·9 to 9·0%)	117·9 (82·3 to 155·9)	7·1% (5·0 to 9·2%)	4·7% (-6·2 to 10·4%)	2·1% (0·8 to 3·5%)
Ischaemic heart disease	118·2 (65·0 to 171·6)	6·9% (3·8 to 10·0%)	116·6 (67·9 to 166·2)	7·1% (4·2 to 10·2%)	-1·3% (-4·5 to 4·8%)	3·5% (0·8 to 9·5%)	2,089·8 (1,025·3 to 3,163·8)	6·8% (3·4 to 10·3%)	1,831·1 (961·8 to 2,676·1)	7·1% (3·8 to 10·5%)	-12·4% (-15·5 to -6·0%)	4·6% (1·7 to 12·0%)
Ischaemic stroke	58·4 (27·2 to 90·9)	9·1% (4·2 to 14·1%)	51·2 (25·4 to 78·1)	9·3% (4·6 to 14·2%)	-12·4% (-15·8 to -7·0%)	2·1% (-0·4 to 7·9%)	904·4 (390·6 to 1,434·2)	9·3% (4·1 to 14·7%)	714·2 (322·3 to 1,126·0)	9·6% (4·4 to 15·0%)	-21·0% (-24·3 to -16·4%)	2·8% (0·6 to 8·5%)
Diabetes mellitus	6·1 (4·4 to 7·9)	13·0% (9·5 to 16·9%)	6·9 (5·1 to 8·8)	13·8% (10·2 to 17·6%)	13·0% (7·2 to 18·5%)	5·7% (4·2 to 8·1%)	292·0 (192·7 to 410·3)	11·2% (7·8 to 14·8%)	348·2 (229·3 to 494·3)	11·6% (8·3 to 15·3%)	19·3% (14·6 to 23·9%)	4·3% (3·0 to 6·0%)
**Metabolic risks: All causes**	2,535·0 (2,412·2 to 2,651·8)	48·0% (45·7 to 50·2%)	2,346·6 (2,209·6 to 2,471·3)	48·2% (45·4 to 50·7%)	-7·4% (-9·3 to -5·7%)	0·5% (-1·3 to 1·9%)	50,814·6 (48,468·6 to 53,231·7)	29·1% (27·4 to 30·8%)	43,267·7 (40,954·1 to 45,710·7)	28·3% (26·4 to 30·2%)	-14·9% (-16·8 to -13·1%)	-2·8% (-5·0 to -1·3%)
**High fasting plasma glucose: All causes**	530·0 (410·1 to 706·4)	10·0% (7·8 to 13·4%)	500·5 (379·1 to 671·5)	10·3% (7·8 to 13·8%)	-5·6% (-11·4 to 1·1%)	2·5% (-3·6 to 9·4%)	11,847·9 (9,835·5 to 14,481·5)	6·8% (5·7 to 8·3%)	10,818·5 (8,987·4 to 13,180·3)	7·1% (6·0 to 8·6%)	-8·7% (-12·1 to -5·3%)	4·2% (0·9 to 7·9%)
Tuberculosis	3·3 (2·1 to 4·7)	6·8% (4·3 to 9·7%)	1·9 (1·2 to 2·7)	7·7% (4·9 to 10·8%)	-43·7% (-49·4 to -37·5%)	11·8% (3·4 to 20·5%)	120·2 (75·6 to 167·2)	6·1% (3·8 to 8·6%)	65·9 (41·5 to 91·9)	6·8% (4·3 to 9·5%)	-45·2% (-50·7 to -39·1%)	10·6% (2·2 to 19·2%)
Ischaemic heart disease	302·0 (196·7 to 438·1)	17·6% (11·5 to 25·5%)	295·7 (186·3 to 438·5)	18·1% (11·3 to 26·9%)	-2·1% (-10·3 to 7·7%)	2·7% (-6·1 to 12·9%)	5,527·5 (3,780·2 to 7,693·7)	18·0% (12·3 to 25·2%)	4,812·7 (3,326·9 to 6,694·3)	18·7% (12·8 to 26·1%)	-12·9% (-17·4 to -7·6%)	4·0% (-0·6 to 10·1%)
Ischaemic stroke	105·1 (57·7 to 187·2)	16·4% (9·1 to 29·2%)	89·3 (47·9 to 165·2)	16·2% (8·8 to 29·9%)	-15·0% (-22·5 to -2·7%)	-1·0% (-8·8 to 13·4%)	1,704·1 (999·9 to 2,778·8)	17·6% (10·4 to 28·7%)	1,330·8 (794·0 to 2,209·4)	17·9% (10·6 to 29·8%)	-21·9% (-26·2 to -14·8%)	1·7% (-2·5 to 10·7%)
Hemorrhagic stroke	52·3 (36·0 to 71·8)	16·4% (11·3 to 22·6%)	42·0 (28·5 to 59·7)	16·3% (11·2 to 23·0%)	-19·6% (-24·4 to -11·9%)	-0·6% (-5·3 to 7·6%)	1,164·6 (792·5 to 1,609·9)	17·3% (11·8 to 23·8%)	894·3 (614·8 to 1,235·8)	17·8% (12·4 to 24·5%)	-23·2% (-26·8 to -18·9%)	3·0% (0·5 to 7·0%)
Diabetes mellitus	46·5 (45·4 to 47·6)	100·0% (100·0 to 100·0%)	49·7 (47·6 to 51·9)	100·0% (100·0 to 100·0%)	6·9% (2·0 to 11·7%)	-	2,620·1 (2,099·2 to 3,206·5)	100·0% (100·0 to 100·0%)	2,995·4 (2,338·7 to 3,748·4)	100·0% (100·0 to 100·0%)	14·3% (9·8 to 18·6%)	-
Chronic kidney disease due to diabetes mellitus	9·7 (9·2 to 10·1)	100·0% (100·0 to 100·0%)	9·5 (8·9 to 10·1)	100·0% (100·0 to 100·0%)	-2·1% (-6·4 to 2·4%)	-	314·9 (284·5 to 349·5)	100·0% (100·0 to 100·0%)	301·6 (267·8 to 337·8)	100·0% (100·0 to 100·0%)	-4·2% (-7·8 to -0·6%)	-
Chronic kidney disease due to hypertension	5·3 (3·7 to 6·8)	35·7% (25·1 to 46·1%)	5·7 (4·0 to 7·5)	36·8% (25·9 to 47·1%)	8·8% (4·2 to 13·7%)	2·9% (1·3 to 4·7%)	123·8 (86·3 to 161·2)	33·3% (23·3 to 43·1%)	126·7 (87·2 to 166·3)	34·9% (24·6 to 45·0%)	2·3% (-1·4 to 6·1%)	4·9% (3·6 to 6·5%)
Chronic kidney disease due to glomerulonephritis	3·9 (2·7 to 5·2)	31·3% (21·8 to 40·7%)	4·0 (2·8 to 5·3)	33·5% (23·5 to 43·2%)	2·2% (-2·3 to 6·5%)	6·9% (5·4 to 8·8%)	176·0 (120·1 to 235·3)	28·8% (19·9 to 37·8%)	177·8 (122·0 to 236·3)	31·2% (21·8 to 40·7%)	1·0% (-2·7 to 4·9%)	8·4% (6·8 to 10·5%)
Chronic kidney disease due to other causes	0·5 (0·3 to 0·6)	28·5% (19·7 to 37·6%)	0·7 (0·5 to 1·0)	32·4% (22·5 to 42·4%)	41·0% (28·4 to 61·4%)	13·7% (10·1 to 17·8%)	62·9 (40·6 to 89·9)	29·1% (20·2 to 38·2%)	71·7 (46·6 to 102·0)	31·7% (22·2 to 41·4%)	14·0% (8·9 to 19·4%)	9·2% (7·2 to 11·5%)
**High total cholesterol: All causes**	981·9 (767·2 to 1,246·9)	18·6% (14·5 to 23·6%)	912·3 (688·0 to 1,168·7)	18·8% (14·2 to 23·9%)	-7·1% (-13·1 to -2·0%)	0·9% (-5·5 to 6·3%)	19,075·4 (15,997·7 to 22,712·4)	10·9% (9·1 to 13·1%)	15,568·9 (12,907·5 to 18,690·4)	10·2% (8·4 to 12·3%)	-18·4% (-21·6 to -15·6%)	-6·8% (-10·6 to -3·4%)
Ischaemic heart disease	833·5 (655·8 to 1,011·5)	48·6% (38·2 to 58·6%)	783·6 (585·9 to 974·7)	47·9% (35·9 to 59·3%)	-6·0% (-13·0 to 0·0%)	-1·4% (-8·6 to 4·5%)	16,564·6 (14,055·7 to 19,255·5)	54·0% (45·8 to 62·7%)	13,625·8 (11,351·9 to 15,916·6)	53·0% (44·7 to 61·6%)	-17·7% (-21·3 to -14·6%)	-1·8% (-5·0 to 1·0%)
Ischaemic stroke	148·4 (57·9 to 294·2)	23·1% (9·0 to 45·7%)	128·8 (44·3 to 261·2)	23·4% (8·0 to 47·4%)	-13·2% (-25·2 to -4·4%)	1·1% (-12·4 to 10·7%)	2,510·8 (1,419·4 to 4,341·5)	25·9% (14·6 to 44·5%)	1,943·0 (1,050·5 to 3,419·7)	26·1% (14·1 to 45·9%)	-22·6% (-28·5 to -17·0%)	0·7% (-5·4 to 7·2%)
**High systolic blood pressure: All causes**	1,833·2 (1,634·2 to 2,011·9)	34·7% (30·9 to 38·1%)	1,673·4 (1,455·1 to 1,856·4)	34·4% (29·8 to 38·1%)	-8·7% (-12·2 to -5·9%)	-0·9% (-4·6 to 2·0%)	34,896·5 (31,907·8 to 37,635·0)	20·0% (18·0 to 22·1%)	28,713·5 (26,016·3 to 31,134·1)	18·8% (16·7 to 20·9%)	-17·7% (-20·3 to -15·5%)	-6·1% (-9·2 to -3·7%)
Rheumatic heart disease	6·5 (4·4 to 9·7)	32·8% (22·7 to 48·7%)	4·2 (3·0 to 6·1)	32·7% (22·9 to 47·8%)	-35·1% (-39·2 to -29·8%)	-0·5% (-5·2 to 6·4%)	192·8 (134·6 to 282·2)	31·6% (22·2 to 45·8%)	118·6 (83·0 to 166·4)	31·8% (22·2 to 45·6%)	-38·5% (-42·0 to -35·0%)	0·5% (-2·8 to 4·7%)
Ischaemic heart disease	1,061·9 (891·9 to 1,220·5)	61·9% (52·0 to 71·1%)	1,001·5 (807·2 to 1,176·6)	61·2% (49·6 to 71·6%)	-5·7% (-10·6 to -2·2%)	-1·1% (-5·6 to 2·2%)	19,415·5 (16,917·9 to 21,549·5)	63·3% (55·1 to 70·2%)	16,252·8 (14,034·2 to 18,138·3)	63·3% (54·8 to 70·6%)	-16·3% (-19·4 to -13·8%)	-0·1% (-2·5 to 2·0%)
Ischaemic stroke	362·9 (291·4 to 430·0)	56·6% (45·5 to 67·3%)	301·4 (235·6 to 367·2)	54·8% (43·0 to 67·1%)	-16·9% (-22·1 to -10·8%)	-3·2% (-8·6 to 3·2%)	5,831·2 (4,684·2 to 6,699·7)	60·2% (48·2 to 69·0%)	4,413·0 (3,606·5 to 5,094·4)	59·3% (48·1 to 68·1%)	-24·3% (-28·1 to -20·5%)	-1·5% (-4·5 to 2·3%)

Supplemental Results Table 1. All-age deaths, death PAFs, DALYs, DALY PAFs, and percent change for each risk factor and outcome for both sexes combined in 2005 and 2015 for Central Europe, Eastern Europe, and Central Asia.

Data in parenthesis are 95% uncertainty intervals. PAFs=population attributable fractions. DALYs=disability-adjusted life-years.

	2005 Deaths (in thousands)	2005 Deaths PAF	2015 Deaths (in thousands)	2015 Deaths PAF	Percent change deaths 2005-2015	Percent change deaths PAF 2005-2015	2005 DALYs (in thousands)	2005 DALYs PAF	2015 DALYs (in thousands)	2015 DALYs PAF	Percent change DALYs 2005-2015	Percent change DALYs PAF 2005-2015
Hemorrhagic stroke	206·7 (171·5 to 239·0)	65·0% (53·9 to 75·1%)	165·2 (134·2 to 194·7)	64·2% (52·3 to 75·2%)	-20·1% (-24·8 to -15·1%)	-1·2% (-5·4 to 3·4%)	4,539·2 (3,834·1 to 5,120·3)	67·5% (57·6 to 75·6%)	3,383·9 (2,899·9 to 3,846·0)	67·5% (58·3 to 75·6%)	-25·5% (-28·9 to -22·0%)	0·0% (-1·9 to 2·2%)
Hypertensive heart disease	76·2 (70·9 to 79·0)	100·0% (100·0 to 100·0%)	82·0 (75·2 to 86·0)	100·0% (100·0 to 100·0%)	7·6% (2·6 to 13·0%)	-	1,372·7 (1,288·6 to 1,421·9)	100·0% (100·0 to 100·0%)	1,308·1 (1,218·3 to 1,369·5)	100·0% (100·0 to 100·0%)	-4·7% (-8·9 to 1·0%)	-
Cardiomyopathy and myocarditis	54·3 (44·6 to 63·7)	45·1% (37·0 to 52·8%)	50·2 (41·0 to 59·4)	44·3% (36·6 to 52·6%)	-7·5% (-15·4 to 0·5%)	-1·7% (-7·4 to 5·1%)	1,663·2 (1,329·7 to 1,982·8)	45·8% (36·6 to 54·1%)	1,338·4 (1,098·1 to 1,581·4)	46·4% (38·2 to 54·3%)	-19·5% (-26·3 to -12·4%)	1·5% (-2·7 to 7·1%)
Atrial fibrillation and flutter	7·5 (5·9 to 9·5)	43·3% (36·2 to 50·3%)	9·1 (7·0 to 11·6)	41·8% (34·4 to 49·7%)	21·2% (14·7 to 27·8%)	-3·5% (-7·2 to 0·0%)	222·4 (176·2 to 281·0)	49·9% (45·0 to 54·7%)	252·2 (197·8 to 320·5)	49·3% (44·2 to 54·3%)	13·4% (9·8 to 16·6%)	-1·3% (-3·2 to 0·5%)
Aortic aneurysm	6·6 (5·4 to 7·5)	45·2% (37·0 to 51·8%)	7·4 (6·0 to 8·6)	44·2% (36·4 to 51·0%)	13·0% (5·9 to 19·5%)	-2·3% (-5·5 to 1·4%)	148·0 (124·7 to 169·5)	46·5% (39·5 to 53·0%)	155·2 (129·3 to 177·7)	46·6% (39·2 to 53·2%)	4·8% (-1·8 to 10·3%)	0·3% (-1·8 to 2·6%)
Peripheral vascular disease	2·7 (2·1 to 3·3)	33·5% (26·2 to 41·1%)	3·5 (2·6 to 4·5)	32·5% (24·5 to 40·6%)	28·5% (14·1 to 45·3%)	-3·1% (-9·8 to 3·5%)	61·9 (46·2 to 82·2)	34·7% (27·5 to 41·7%)	71·6 (53·3 to 95·2)	34·4% (27·3 to 41·7%)	15·6% (7·0 to 24·9%)	-0·9% (-4·6 to 3·3%)
Endocarditis	1·7 (1·4 to 2·0)	44·0% (36·0 to 51·3%)	1·8 (1·5 to 2·1)	44·5% (36·7 to 51·9%)	4·2% (-5·5 to 16·7%)	1·1% (-2·2 to 4·4%)	51·5 (39·9 to 63·1)	42·4% (33·4 to 50·4%)	52·1 (40·7 to 63·7)	44·2% (35·1 to 52·7%)	1·1% (-10·6 to 16·6%)	4·2% (0·4 to 8·4%)
Other cardiovascular and circulatory diseases	19·9 (17·9 to 21·8)	49·4% (44·8 to 54·1%)	19·4 (17·3 to 21·6)	48·1% (43·1 to 52·9%)	-2·2% (-6·8 to 2·0%)	-2·6% (-5·2 to -0·2%)	544·4 (484·9 to 608·8)	50·5% (46·3 to 55·0%)	516·7 (457·3 to 586·1)	50·9% (46·5 to 55·5%)	-5·1% (-9·3 to -1·4%)	0·8% (-0·7 to 2·4%)
Chronic kidney disease due to diabetes mellitus	5·2 (3·9 to 6·4)	53·9% (40·2 to 65·5%)	5·3 (4·0 to 6·5)	55·6% (41·9 to 67·2%)	1·0% (-3·7 to 5·7%)	3·1% (1·6 to 4·9%)	147·6 (107·1 to 188·3)	46·9% (34·2 to 57·9%)	146·4 (105·3 to 186·5)	48·5% (35·7 to 59·7%)	-0·8% (-4·8 to 3·0%)	3·5% (2·0 to 5·4%)
Chronic kidney disease due to hypertension	14·8 (14·1 to 15·3)	100·0% (100·0 to 100·0%)	15·6 (14·7 to 16·3)	100·0% (100·0 to 100·0%)	5·7% (1·3 to 9·9%)	-	371·8 (348·8 to 394·3)	100·0% (100·0 to 100·0%)	362·5 (335·6 to 388·1)	100·0% (100·0 to 100·0%)	-2·5% (-5·9 to 0·8%)	-
Chronic kidney disease due to glomerulonephritis	5·8 (4·2 to 7·2)	45·9% (33·8 to 57·0%)	5·8 (4·3 to 7·3)	48·6% (36·3 to 60·0%)	1·3% (-2·9 to 5·2%)	5·9% (4·4 to 7·8%)	243·8 (172·7 to 313·8)	39·9% (28·9 to 50·2%)	240·6 (170·2 to 310·4)	42·3% (30·9 to 52·9%)	-1·3% (-4·9 to 2·6%)	5·9% (4·1 to 8·1%)
Chronic kidney disease due to other causes	0·7 (0·5 to 1·0)	43·8% (31·6 to 54·4%)	1·0 (0·7 to 1·4)	49·6% (36·1 to 60·7%)	40·3% (27·8 to 62·3%)	13·2% (10·1 to 16·9%)	90·5 (60·8 to 121·8)	41·8% (30·1 to 52·0%)	101·4 (68·5 to 136·6)	44·9% (32·7 to 55·7%)	12·1% (7·1 to 17·2%)	7·3% (5·3 to 9·5%)
**High body-mass index: All causes**	717·8 (530·0 to 914·3)	13·6% (10·0 to 17·3%)	657·2 (491·5 to 836·5)	13·5% (10·1 to 17·2%)	-8·4% (-12·4 to -4·1%)	-0·6% (-4·6 to 4·1%)	17,872·5 (13,278·7 to 22,465·3)	10·2% (7·7 to 12·8%)	16,068·6 (12,239·5 to 19,909·0)	10·5% (8·0 to 13·0%)	-10·1% (-13·4 to -6·2%)	2·6% (-0·6 to 6·5%)
Oesophageal cancer	5·3 (1·8 to 8·9)	25·3% (8·6 to 43·3%)	5·8 (2·0 to 9·7)	27·5% (9·4 to 46·1%)	9·8% (0·8 to 21·3%)	8·8% (4·3 to 15·0%)	128·2 (42·5 to 219·2)	25·5% (8·5 to 43·9%)	137·4 (46·4 to 232·6)	27·9% (9·4 to 47·0%)	7·2% (-1·9 to 18·8%)	9·4% (5·4 to 15·6%)
Colon and rectum cancer	10·0 (6·4 to 14·0)	9·7% (6·2 to 13·6%)	11·4 (7·4 to 15·9)	10·5% (6·8 to 14·6%)	14·2% (8·3 to 21·3%)	8·6% (5·0 to 13·7%)	218·8 (140·2 to 304·9)	10·0% (6·4 to 14·0%)	237·4 (154·6 to 330·5)	11·0% (7·2 to 15·3%)	8·5% (3·0 to 15·6%)	9·9% (6·1 to 14·8%)
Liver cancer due to hepatitis B	0·8 (0·4 to 1·4)	17·4% (8·1 to 28·6%)	1·1 (0·5 to 1·8)	19·5% (9·2 to 31·5%)	26·3% (16·0 to 37·5%)	11·9% (6·5 to 18·3%)	23·5 (10·5 to 39·6)	16·9% (7·9 to 28·0%)	29·2 (13·5 to 48·6)	19·3% (9·1 to 31·1%)	24·0% (13·1 to 35·7%)	14·1% (7·9 to 21·2%)
Liver cancer due to hepatitis C	1·3 (0·6 to 2·1)	17·2% (7·8 to 28·1%)	1·6 (0·7 to 2·5)	18·8% (8·7 to 30·2%)	24·4% (14·5 to 35·0%)	9·3% (5·0 to 15·3%)	26·9 (12·2 to 43·9)	17·7% (8·2 to 28·7%)	32·5 (15·4 to 52·4)	19·6% (9·3 to 30·9%)	20·9% (10·8 to 32·2%)	10·6% (4·9 to 17·5%)
Liver cancer due to alcohol use	2·2 (1·0 to 3·6)	19·2% (9·0 to 31·3%)	2·7 (1·3 to 4·4)	20·7% (9·9 to 33·4%)	21·1% (10·2 to 31·2%)	8·1% (3·0 to 13·8%)	51·2 (23·8 to 83·5)	19·6% (9·1 to 31·8%)	60·8 (29·4 to 98·7)	21·3% (10·1 to 34·5%)	18·7% (7·4 to 29·3%)	8·8% (3·1 to 14·8%)
Liver cancer due to other causes	0·4 (0·2 to 0·7)	16·7% (7·5 to 27·4%)	0·5 (0·2 to 0·8)	18·4% (8·5 to 29·6%)	22·7% (12·9 to 33·2%)	10·2% (5·7 to 16·3%)	9·7 (4·5 to 15·6)	16·5% (7·5 to 26·8%)	11·5 (5·6 to 18·3)	18·6% (8·8 to 29·4%)	18·6% (8·9 to 29·2%)	12·8% (6·9 to 20·2%)
Gallbladder and biliary tract cancer	3·0 (1·9 to 4·4)	23·3% (14·5 to 32·9%)	3·0 (1·9 to 4·2)	24·1% (15·3 to 33·6%)	-2·0% (-10·2 to 7·4%)	3·7% (-0·5 to 8·4%)	60·7 (38·5 to 85·9)	23·4% (14·8 to 32·9%)	56·5 (35·7 to 78·9)	24·4% (15·6 to 33·9%)	-6·9% (-14·4 to 1·8%)	4·3% (0·1 to 8·7%)
Pancreatic cancer	3·0 (1·1 to 5·0)	7·4% (2·8 to 12·5%)	3·7 (1·4 to 6·2)	8·1% (3·1 to 13·3%)	24·1% (17·8 to 32·3%)	8·5% (4·8 to 14·1%)	66·5 (24·1 to 114·1)	7·4% (2·6 to 12·6%)	78·8 (29·8 to 132·1)	8·1% (3·0 to 13·6%)	18·5% (12·3 to 26·5%)	9·6% (5·9 to 15·8%)
Breast cancer	3·4 (1·9 to 5·4)	5·7% (3·2 to 8·9%)	4·6 (2·6 to 7·0)	7·1% (4·1 to 10·9%)	32·9% (17·9 to 48·3%)	23·9% (15·8 to 36·5%)	56·9 (23·9 to 95·5)	3·5% (1·5 to 5·9%)	86·1 (46·3 to 137·0)	5·2% (2·8 to 8·1%)	51·4% (28·0 to 107·1%)	47·6% (28·3 to 98·8%)
Uterine cancer	5·8 (4·3 to 7·2)	47·3% (35·7 to 58·5%)	6·2 (4·6 to 7·9)	49·1% (37·5 to 60·0%)	7·5% (-9·7 to 25·0%)	3·9% (0·9 to 8·3%)	145·6 (110·0 to 181·8)	48·2% (36·7 to 59·3%)	155·9 (115·4 to 198·3)	50·3% (39·0 to 61·0%)	7·1% (-10·5 to 26·0%)	4·5% (1·7 to 8·7%)
Ovarian cancer	0·9 (0·0 to 1·9)	4·2% (-0·2 to 9·1%)	1·0 (0·0 to 2·2)	4·5% (-0·2 to 9·6%)	18·2% (10·2 to 27·4%)	6·1% (2·7 to 11·1%)	23·0 (-0·8 to 49·7)	4·2% (-0·2 to 9·1%)	26·5 (-1·0 to 56·4)	4·5% (-0·2 to 9·7%)	14·8% (6·7 to 24·1%)	6·9% (3·4 to 12·0%)
Kidney cancer	4·2 (2·9 to 5·7)	21·3% (14·8 to 28·6%)	5·1 (3·6 to 6·8)	22·9% (16·0 to 30·2%)	20·9% (12·6 to 30·7%)	7·3% (3·9 to 12·1%)	103·5 (71·0 to 139·0)	20·8% (14·4 to 27·9%)	120·8 (85·1 to 160·4)	22·6% (15·9 to 29·9%)	16·8% (7·9 to 26·0%)	8·7% (5·2 to 13·8%)
Thyroid cancer	0·4 (0·3 to 0·6)	14·3% (8·7 to 20·7%)	0·4 (0·3 to 0·6)	15·3% (9·4 to 22·0%)	1·4% (-7·0 to 11·2%)	6·8% (1·5 to 12·0%)	10·7 (6·5 to 15·5)	14·5% (8·7 to 21·2%)	10·6 (6·4 to 15·4)	15·7% (9·5 to 22·7%)	-0·6% (-8·5 to 9·5%)	8·2% (2·1 to 13·8%)
Ischaemic heart disease	386·0 (276·7 to 502·0)	22·5% (16·2 to 29·2%)	362·2 (257·8 to 475·0)	22·1% (15·8 to 28·9%)	-6·1% (-12·0 to -0·2%)	-1·6% (-7·5 to 4·4%)	8,318·0 (6,053·9 to 10,686·9)	27·1% (19·7 to 34·8%)	7,159·5 (5,293·5 to 9,076·2)	27·9% (20·7 to 35·2%)	-13·9% (-18·0 to -9·4%)	2·8% (-1·0 to 7·5%)
Ischaemic stroke	115·3 (81·2 to 154·9)	18·0% (12·7 to 24·2%)	89·8 (61·2 to 124·9)	16·3% (11·2 to 22·8%)	-22·2% (-30·9 to -14·8%)	-9·3% (-19·3 to -0·9%)	2,362·9 (1,743·6 to 3,018·4)	24·4% (17·9 to 31·1%)	1,801·3 (1,347·1 to 2,296·7)	24·2% (18·1 to 30·7%)	-23·8% (-28·3 to -18·9%)	-0·8% (-5·7 to 4·6%)
Hemorrhagic stroke	99·2 (74·4 to 126·1)	31·2% (23·3 to 39·7%)	75·8 (56·1 to 98·0)	29·5% (22·1 to 38·1%)	-23·7% (-29·7 to -17·8%)	-5·6% (-12·2 to 0·6%)	2,727·1 (2,090·3 to 3,363·3)	40·6% (31·1 to 49·8%)	2,072·9 (1,620·2 to 2,539·9)	41·3% (32·4 to 50·5%)	-24·0% (-28·5 to -19·3%)	1·9% (-1·6 to 6·4%)
Hypertensive heart disease	36·0 (24·1 to 48·1)	47·3% (32·3 to 63·3%)	39·3 (24·9 to 53·7)	47·9% (31·2 to 65·6%)	9·1% (-7·1 to 21·3%)	1·4% (-13·0 to 10·7%)	710·0 (532·0 to 897·1)	51·7% (39·1 to 65·2%)	698·0 (517·7 to 878·2)	53·4% (39·9 to 66·9%)	-1·7% (-9·5 to 7·1%)	3·2% (-3·5 to 9·9%)
Diabetes mellitus	24·2 (19·3 to 29·2)	52·1% (41·5 to 62·7%)	26·0 (20·7 to 31·7)	52·3% (41·7 to 63·2%)	7·3% (1·1 to 14·1%)	0·4% (-3·2 to 4·4%)	1,559·0 (1,153·5 to 2,028·4)	59·5% (48·8 to 69·3%)	1,853·1 (1,351·3 to 2,429·4)	61·8% (51·6 to 71·1%)	18·9% (13·4 to 24·4%)	4·0% (1·7 to 6·8%)
Chronic kidney disease due to diabetes mellitus	3·8 (2·0 to 5·5)	38·9% (21·2 to 56·5%)	3·7 (1·9 to 5·6)	39·6% (20·6 to 58·5%)	-0·4% (-16·8 to 13·8%)	1·6% (-14·5 to 14·1%)	118·9 (61·8 to 174·2)	37·8% (20·2 to 54·6%)	120·9 (67·0 to 177·2)	40·1% (22·3 to 57·3%)	1·6% (-5·4 to 11·8%)	6·1% (-0·2 to 15·7%)
Chronic kidney disease due to hypertension	5·5 (3·0 to 8·2)	37·1% (20·3 to 54·6%)	5·9 (2·8 to 8·9)	37·5% (18·2 to 55·9%)	6·8% (-14·4 to 21·8%)	1·1% (-19·6 to 14·2%)	141·1 (78·3 to 206·7)	38·0% (21·2 to 54·6%)	145·2 (82·0 to 206·2)	40·1% (22·6 to 56·4%)	2·9% (-5·7 to 12·3%)	5·6% (-2·3 to 14·5%)
Chronic kidney disease due to glomerulonephritis	4·3 (2·0 to 6·4)	34·3% (16·0 to 51·1%)	4·4 (2·1 to 6·5)	36·4% (17·2 to 54·0%)	1·3% (-11·0 to 17·7%)	5·9% (-6·6 to 23·4%)	201·2 (73·6 to 316·7)	32·9% (12·2 to 50·4%)	205·9 (82·4 to 313·9)	36·2% (14·9 to 53·2%)	2·3% (-4·5 to 14·6%)	9·9% (3·6 to 24·2%)
Chronic kidney disease due to other causes	0·4 (0·1 to 0·7)	24·5% (5·1 to 41·7%)	0·6 (0·0 to 1·1)	27·1% (-1·4 to 50·8%)	37·4% (-94·6 to 77·1%)	10·9% (-95·5 to 35·4%)	69·0 (32·7 to 106·6)	31·8% (16·3 to 46·6%)	78·1 (37·7 to 120·9)	34·5% (17·3 to 49·9%)	13·2% (-0·7 to 25·7%)	8·4% (-3·7 to 19·7%)
Osteoarthritis	-	-	-	-	-	-	262·6 (159·7 to 396·0)	26·1% (19·0 to 33·8%)	330·0 (202·8 to 493·5)	27·7% (20·5 to 35·6%)	25·7% (22·1 to 30·4%)	6·1% (3·8 to 9·7%)
Low back pain	-	-	-	-	-	-	422·7 (250·8 to 647·4)	7·8% (5·4 to 10·4%)	501·6 (305·0 to 759·5)	8·7% (6·1 to 11·5%)	18·7% (14·5 to 24·4%)	12·0% (8·7 to 16·9%)
**Low bone mineral density: All causes**	20·5 (18·8 to 22·0)	0·4% (0·4 to 0·4%)	18·9 (17·5 to 20·3)	0·4% (0·4 to 0·4%)	-7·7% (-13·5 to -2·1%)	0·2% (-5·9 to 6·2%)	667·5 (555·3 to 800·1)	0·4% (0·3 to 0·4%)	578·4 (475·6 to 702·7)	0·4% (0·3 to 0·4%)	-13·4% (-19·0 to -7·9%)	-1·2% (-7·2 to 4·9%)
Pedestrian road injuries	2·5 (2·3 to 2·8)	7·4% (6·6 to 8·1%)	1·9 (1·7 to 2·1)	9·2% (8·3 to 10·0%)	-24·4% (-30·2 to -17·9%)	24·5% (15·9 to 36·1%)	62·3 (53·4 to 71·5)	4·0% (3·4 to 4·6%)	43·3 (37·3 to 50·0)	4·9% (4·3 to 5·5%)	-30·4% (-36·6 to -24·0%)	21·6% (11·9 to 33·4%)
Cyclist road injuries	0·3 (0·2 to 0·3)	6·5% (5·7 to 7·2%)	0·2 (0·2 to 0·3)	7·3% (6·4 to 8·1%)	-19·7% (-29·5 to -11·3%)	12·4% (4·9 to 20·1%)	22·2 (16·8 to 29·0)	8·0% (6·6 to 9·4%)	20·6 (15·2 to 27·6)	9·7% (8·1 to 11·3%)	-7·2% (-13·4 to -1·6%)	20·8% (13·1 to 31·1%)
Motorcyclist road injuries	0·2 (0·1 to 0·2)	2·5% (2·0 to 3·0%)	0·1 (0·1 to 0·2)	2·5% (2·0 to 3·0%)	-20·5% (-31·1 to -8·9%)	2·0% (-9·8 to 16·0%)	14·1 (10·4 to 18·3)	3·3% (2·5 to 4·2%)	12·8 (9·2 to 16·9)	3·8% (2·9 to 4·9%)	-9·3% (-17·3 to -0·8%)	14·6% (2·1 to 30·8%)
Motor vehicle road injuries	2·7 (2·1 to 3·5)	5·0% (4·3 to 5·6%)	1·7 (1·5 to 2·0)	5·2% (4·5 to 5·8%)	-35·9% (-48·3 to -18·1%)	4·1% (-5·0 to 15·2%)	130·8 (103·4 to 164·5)	4·3% (3·5 to 5·1%)	109·2 (83·9 to 138·6)	5·4% (4·5 to 6·5%)	-16·5% (-26·7 to -5·5%)	26·3% (16·5 to 36·9%)
Other road injuries	0·1 (0·0 to 0·1)	7·7% (6·9 to 8·5%)	0·1 (0·0 to 0·1)	8·8% (7·8 to 9·7%)	-6·6% (-16·8 to 8·5%)	14·0% (4·6 to 23·6%)	4·7 (3·4 to 6·2)	9·1% (7·5 to 10·9%)	5·0 (3·6 to 6·7)	11·0% (9·0 to 13·1%)	6·7% (0·5 to 12·7%)	20·4% (12·6 to 28·4%)

Supplemental Results Table 1. All-age deaths, death PAFs, DALYs, DALY PAFs, and percent change for each risk factor and outcome for both sexes combined in 2005 and 2015 for Central Europe, Eastern Europe, and Central Asia.

Data in parenthesis are 95% uncertainty intervals. PAFs=population attributable fractions. DALYs=disability-adjusted life-years.

	2005 Deaths (in thousands)	2005 Deaths PAF	2015 Deaths (in thousands)	2015 Deaths PAF	Percent change deaths 2005-2015	Percent change deaths PAF 2005-2015	2005 DALYs (in thousands)	2005 DALYs PAF	2015 DALYs (in thousands)	2015 DALYs PAF	Percent change DALYs 2005-2015	Percent change DALYs PAF 2005-2015
Other transport injuries	0·8 (0·7 to 0·9)	7·4% (6·6 to 8·2%)	0·6 (0·5 to 0·7)	8·9% (7·9 to 9·8%)	-18·2% (-27·3 to -8·1%)	19·8% (10·7 to 31·7%)	18·8 (16·0 to 21·9)	3·8% (3·2 to 4·4%)	14·0 (12·1 to 15·8)	4·3% (3·7 to 4·8%)	-25·6% (-34·1 to -16·5%)	12·2% (2·9 to 23·2%)
Falls	11·9 (11·2 to 12·6)	27·4% (25·8 to 28·9%)	12·7 (11·7 to 13·6)	34·1% (31·8 to 36·2%)	6·5% (0·0 to 13·4%)	24·5% (17·6 to 30·6%)	346·0 (285·6 to 417·2)	13·8% (12·6 to 14·9%)	325·1 (266·4 to 396·2)	16·1% (14·8 to 17·4%)	-6·0% (-10·5 to -1·3%)	16·8% (11·6 to 22·1%)
Other exposure to mechanical forces	0·5 (0·4 to 0·5)	4·7% (4·0 to 5·3%)	0·4 (0·3 to 0·4)	6·1% (5·4 to 6·8%)	-17·2% (-25·1 to -8·4%)	30·6% (20·4 to 42·3%)	25·3 (19·4 to 32·5)	3·7% (3·1 to 4·3%)	20·3 (15·4 to 26·9)	4·4% (3·8 to 5·1%)	-19·5% (-26·3 to -12·0%)	19·5% (11·0 to 29·5%)
Non-venomous animal contact	0·0 (0·0 to 0·1)	6·8% (6·1 to 7·5%)	0·0 (0·0 to 0·0)	7·6% (6·8 to 8·3%)	-10·7% (-19·2 to 0·2%)	11·4% (3·2 to 19·9%)	1·2 (1·0 to 1·4)	3·4% (2·9 to 4·0%)	1·0 (0·8 to 1·2)	3·7% (3·1 to 4·2%)	-18·2% (-26·2 to -6·8%)	6·3% (-2·4 to 14·7%)
Assault by other means	1·5 (1·3 to 1·7)	4·1% (3·5 to 4·6%)	1·1 (0·9 to 1·2)	4·9% (4·3 to 5·5%)	-28·4% (-35·5 to -20·3%)	20·6% (9·4 to 33·1%)	41·0 (33·3 to 48·7)	2·5% (2·0 to 2·9%)	25·9 (21·3 to 30·4)	2·8% (2·3 to 3·2%)	-36·7% (-43·9 to -28·5%)	10·7% (-1·8 to 24·1%)
Exposure to forces of nature	-	-	-	-	-	-	1·3 (0·6 to 2·1)	4·0% (2·3 to 6·1%)	1·2 (0·5 to 2·0)	7·5% (5·0 to 9·8%)	-8·1% (-18·6 to 4·2%)	84·9% (38·7 to 157·8%)
**Low glomerular filtration rate: All causes**	250·5 (218·2 to 284·4)	4·7% (4·1 to 5·4%)	228·4 (200·5 to 257·3)	4·7% (4·1 to 5·3%)	-8·8% (-11·6 to -5·9%)	-1·0% (-3·8 to 1·8%)	4,805·6 (4,300·3 to 5,325·9)	2·8% (2·5 to 3·1%)	4,083·6 (3,677·6 to 4,488·8)	2·7% (2·4 to 3·0%)	-15·0% (-17·4 to -12·5%)	-3·0% (-5·7 to -0·4%)
Ischaemic heart disease	128·8 (107·2 to 151·6)	7·5% (6·2 to 8·9%)	120·2 (99·5 to 141·3)	7·3% (6·1 to 8·6%)	-6·7% (-10·0 to -3·4%)	-2·2% (-5·2 to 0·7%)	2,006·8 (1,670·2 to 2,361·1)	6·5% (5·4 to 7·7%)	1,657·1 (1,385·5 to 1,935·1)	6·5% (5·4 to 7·5%)	-17·4% (-20·0 to -14·8%)	-1·4% (-3·9 to 1·2%)
Ischaemic stroke	56·7 (40·9 to 73·4)	8·8% (6·4 to 11·4%)	47·5 (34·8 to 61·4)	8·6% (6·4 to 11·1%)	-16·2% (-20·4 to -11·6%)	-2·3% (-6·6 to 2·5%)	792·0 (591·4 to 1,002·5)	8·2% (6·1 to 10·3%)	591·7 (448·9 to 745·4)	8·0% (6·0 to 10·0%)	-25·3% (-28·8 to -21·5%)	-2·7% (-6·2 to 1·4%)
Hemorrhagic stroke	25·2 (20·0 to 30·3)	7·9% (6·3 to 9·6%)	20·2 (16·4 to 24·3)	7·9% (6·3 to 9·4%)	-19·6% (-23·8 to -14·9%)	-0·6% (-4·4 to 3·7%)	461·3 (372·4 to 558·8)	6·9% (5·6 to 8·3%)	340·3 (279·4 to 406·7)	6·8% (5·6 to 8·1%)	-26·2% (-30·0 to -22·3%)	-1·1% (-4·4 to 2·7%)
Peripheral vascular disease	1·0 (0·7 to 1·3)	12·6% (9·1 to 16·4%)	1·3 (0·9 to 1·7)	11·9% (8·5 to 15·8%)	25·0% (10·2 to 40·4%)	-5·8% (-10·8 to -0·7%)	23·0 (16·2 to 31·8)	12·9% (9·7 to 16·2%)	25·7 (17·9 to 35·3)	12·3% (9·4 to 15·5%)	11·4% (3·0 to 20·6%)	-4·5% (-8·1 to -0·7%)
Chronic kidney disease due to diabetes mellitus	9·7 (9·2 to 10·1)	100·0% (100·0 to 100·0%)	9·5 (8·9 to 10·1)	100·0% (100·0 to 100·0%)	-2·1% (-6·4 to 2·4%)	-	314·9 (284·5 to 349·5)	100·0% (100·0 to 100·0%)	301·6 (267·8 to 337·8)	100·0% (100·0 to 100·0%)	-4·2% (-7·8 to -0·6%)	-
Chronic kidney disease due to hypertension	14·8 (14·1 to 15·3)	100·0% (100·0 to 100·0%)	15·6 (14·7 to 16·3)	100·0% (100·0 to 100·0%)	5·7% (1·3 to 9·9%)	-	371·8 (348·8 to 394·3)	100·0% (100·0 to 100·0%)	362·5 (335·6 to 388·1)	100·0% (100·0 to 100·0%)	-2·5% (-5·9 to 0·8%)	-
Chronic kidney disease due to glomerulonephritis	12·6 (11·9 to 13·1)	100·0% (100·0 to 100·0%)	12·0 (11·3 to 12·7)	100·0% (100·0 to 100·0%)	-4·4% (-8·4 to -0·7%)	-	610·7 (551·3 to 674·4)	100·0% (100·0 to 100·0%)	569·0 (507·1 to 635·0)	100·0% (100·0 to 100·0%)	-6·8% (-10·2 to -3·6%)	-
Chronic kidney disease due to other causes	1·7 (1·5 to 2·0)	100·0% (100·0 to 100·0%)	2·1 (1·8 to 2·6)	100·0% (100·0 to 100·0%)	23·8% (13·8 to 40·6%)	-	216·2 (171·8 to 262·8)	100·0% (100·0 to 100·0%)	225·8 (177·2 to 274·2)	100·0% (100·0 to 100·0%)	4·4% (0·4 to 8·7%)	-
Gout	-	-	-	-	-	-	8·9 (5·9 to 12·6)	13·5% (12·1 to 15·2%)	10·0 (6·6 to 14·0)	13·3% (11·9 to 14·8%)	11·8% (5·3 to 18·4%)	-1·8% (-5·5 to 2·0%)

Supplemental Results Table 1. All-age deaths, death PAFs, DALYs, DALY PAFs, and percent change for each risk factor and outcome for both sexes combined in 2005 and 2015 for High-income.

Data in parenthesis are 95% uncertainty intervals. PAFs=population attributable fractions. DALYs=disability-adjusted life-years.

	2005 Deaths (in thousands)	2005 Deaths PAF	2015 Deaths (in thousands)	2015 Deaths PAF	Percent change deaths 2005-2015	Percent change deaths PAF 2005-2015	2005 DALYs (in thousands)	2005 DALYs PAF	2015 DALYs (in thousands)	2015 DALYs PAF	Percent change DALYs 2005-2015	Percent change DALYs PAF 2005-2015
**All risk factors: All causes**	4,694·7 (4,568·5 to 4,817·7)	55·7% (54·2 to 57·1%)	5,154·5 (5,002·4 to 5,304·4)	53·4% (51·8 to 55·0%)	9·8% (9·1 to 10·4%)	-4·1% (-4·6 to -3·6%)	101,589·9 (94,477·9 to 109,684·4)	39·6% (37·5 to 42·0%)	105,612·3 (97,608·7 to 114,397·9)	38·7% (36·6 to 41·0%)	4·0% (3·1 to 4·6%)	-2·4% (-2·8 to -2·1%)
**Environmental/occupational risks: All causes**	636·1 (545·9 to 731·7)	7·5% (6·5 to 8·7%)	690·1 (591·1 to 799·7)	7·2% (6·1 to 8·3%)	8·5% (6·5 to 10·5%)	-5·2% (-7·0 to -3·5%)	13,276·6 (11,778·2 to 15,000·6)	5·2% (4·6 to 5·8%)	13,475·3 (11,931·3 to 15,232·8)	4·9% (4·4 to 5·5%)	1·5% (0·0 to 3·0%)	-4·8% (-6·0 to -3·5%)
**Unsafe water, sanitation, and handwashing: All causes**	37·8 (26·5 to 50·1)	0·4% (0·3 to 0·6%)	41·8 (28·9 to 56·7)	0·4% (0·3 to 0·6%)	10·6% (5·4 to 15·2%)	-3·4% (-8·0 to 0·6%)	479·5 (342·9 to 627·0)	0·2% (0·1 to 0·3%)	464·5 (325·8 to 620·4)	0·2% (0·1 to 0·2%)	-3·1% (-7·8 to 0·7%)	-9·1% (-13·6 to -5·3%)
**Unsafe water source: All causes**	0·5 (0·2 to 0·8)	0·0% (0·0 to 0·0%)	0·5 (0·2 to 0·8)	0·0% (0·0 to 0·0%)	-11·1% (-39·1 to 14·0%)	-22·3% (-46·7 to -0·5%)	19·7 (9·3 to 28·7)	0·0% (0·0 to 0·0%)	13·3 (5·5 to 21·5)	0·0% (0·0 to 0·0%)	-32·6% (-54·1 to -13·1%)	-36·8% (-56·9 to -18·5%)
Diarrhoeal diseases	0·5 (0·2 to 0·8)	3·1% (1·4 to 4·7%)	0·5 (0·2 to 0·8)	1·9% (0·8 to 3·2%)	-11·1% (-39·0 to 14·0%)	-39·5% (-57·7 to -22·7%)	19·6 (9·2 to 28·5)	6·6% (3·1 to 9·7%)	13·2 (5·4 to 21·3)	3·9% (1·6 to 6·2%)	-32·7% (-54·2 to -13·1%)	-41·5% (-60·0 to -25·3%)
Typhoid fever	0·0 (0·0 to 0·0)	10·5% (4·5 to 17·6%)	0·0 (0·0 to 0·0)	7·7% (2·8 to 13·9%)	-24·3% (-44·7 to -6·9%)	-27·1% (-46·9 to -10·2%)	0·1 (0·0 to 0·2)	12·7% (5·5 to 20·5%)	0·1 (0·0 to 0·2)	9·3% (3·5 to 16·2%)	-28·6% (-47·7 to -11·7%)	-26·9% (-46·6 to -9·3%)
Paratyphoid fever	0·0 (0·0 to 0·0)	3·9% (1·6 to 6·7%)	0·0 (0·0 to 0·0)	3·6% (1·3 to 6·4%)	-9·4% (-33·8 to 13·8%)	-7·7% (-32·9 to 15·7%)	0·0 (0·0 to 0·1)	4·8% (2·0 to 8·0%)	0·0 (0·0 to 0·1)	4·5% (1·6 to 8·0%)	-13·3% (-36·5 to 8·4%)	-6·7% (-32·1 to 16·9%)
**Unsafe sanitation: All causes**	0·2 (0·1 to 0·3)	0·0% (0·0 to 0·0%)	0·1 (0·1 to 0·2)	0·0% (0·0 to 0·0%)	-30·6% (-53·0 to -3·2%)	-39·4% (-58·9 to -15·4%)	7·5 (5·6 to 9·8)	0·0% (0·0 to 0·0%)	3·9 (2·4 to 5·9)	0·0% (0·0 to 0·0%)	-47·7% (-65·4 to -24·1%)	-50·9% (-67·5 to -28·9%)
Diarrhoeal diseases	0·2 (0·1 to 0·3)	1·1% (0·8 to 1·7%)	0·1 (0·1 to 0·2)	0·5% (0·3 to 1·0%)	-30·6% (-53·0 to -3·2%)	-52·6% (-67·6 to -33·5%)	7·4 (5·5 to 9·8)	2·5% (1·9 to 3·5%)	3·9 (2·3 to 5·9)	1·1% (0·7 to 1·9%)	-47·7% (-65·6 to -24·1%)	-54·3% (-69·9 to -34·1%)
Typhoid fever	0·0 (0·0 to 0·0)	3·7% (2·4 to 5·6%)	0·0 (0·0 to 0·0)	2·2% (1·3 to 3·6%)	-38·2% (-55·4 to -15·6%)	-40·4% (-57·3 to -18·7%)	0·0 (0·0 to 0·1)	4·4% (3·0 to 6·5%)	0·0 (0·0 to 0·0)	2·7% (1·6 to 4·2%)	-41·6% (-57·8 to -20·6%)	-40·1% (-57·3 to -18·6%)
Paratyphoid fever	0·0 (0·0 to 0·0)	1·3% (0·9 to 2·1%)	0·0 (0·0 to 0·0)	1·0% (0·6 to 1·7%)	-25·5% (-45·8 to 1·9%)	-24·2% (-44·7 to 3·0%)	0·0 (0·0 to 0·0)	1·7% (1·1 to 2·5%)	0·0 (0·0 to 0·0)	1·3% (0·8 to 2·0%)	-28·7% (-48·4 to -3·5%)	-23·3% (-44·5 to 3·6%)
**No handwashing with soap: All causes**	37·3 (26·1 to 49·7)	0·4% (0·3 to 0·6%)	41·3 (28·6 to 56·4)	0·4% (0·3 to 0·6%)	10·8% (5·7 to 15·5%)	-3·2% (-7·6 to 0·8%)	462·8 (325·1 to 608·6)	0·2% (0·1 to 0·2%)	452·9 (315·8 to 610·6)	0·2% (0·1 to 0·2%)	-2·1% (-6·7 to 1·7%)	-8·1% (-12·5 to -4·4%)
Diarrhoeal diseases	4·2 (2·7 to 5·3)	23·9% (17·7 to 29·7%)	5·6 (3·1 to 7·2)	21·7% (15·9 to 27·2%)	34·4% (5·0 to 44·2%)	-9·0% (-11·9 to -6·7%)	71·6 (50·9 to 90·1)	24·2% (17·9 to 30·0%)	74·8 (45·9 to 96·2)	21·9% (16·0 to 27·3%)	4·5% (-15·0 to 10·4%)	-9·5% (-12·4 to -7·0%)
Typhoid fever	0·0 (0·0 to 0·0)	25·7% (19·3 to 31·7%)	0·0 (0·0 to 0·0)	23·1% (17·0 to 29·0%)	-6·7% (-10·6 to -3·0%)	-10·2% (-13·1 to -7·7%)	0·2 (0·1 to 0·5)	25·7% (19·3 to 31·7%)	0·2 (0·1 to 0·4)	23·1% (17·0 to 29·0%)	-12·2% (-16·0 to -8·4%)	-10·3% (-13·1 to -7·7%)
Paratyphoid fever	0·0 (0·0 to 0·0)	24·3% (18·2 to 30·4%)	0·0 (0·0 to 0·0)	21·9% (16·1 to 28·1%)	-11·4% (-14·9 to -8·3%)	-9·7% (-12·6 to -7·2%)	0·1 (0·0 to 0·3)	24·2% (18·1 to 30·4%)	0·1 (0·0 to 0·3)	21·9% (16·0 to 28·0%)	-16·2% (-19·7 to -12·8%)	-9·7% (-12·6 to -7·2%)
**Air pollution: All causes**	382·2 (304·3 to 469·1)	4·5% (3·6 to 5·6%)	407·3 (319·3 to 507·1)	4·2% (3·3 to 5·3%)	6·6% (3·6 to 9·3%)	-6·9% (-9·4 to -4·6%)	5,843·3 (4,884·5 to 6,912·7)	2·3% (1·8 to 2·8%)	5,658·8 (4,672·0 to 6,783·7)	2·1% (1·6 to 2·6%)	-3·2% (-5·3 to -0·9%)	-9·1% (-11·3 to -7·1%)
**Ambient particulate matter pollution: All causes**	359·0 (282·0 to 449·9)	4·3% (3·3 to 5·3%)	382·2 (293·6 to 485·9)	4·0% (3·0 to 5·0%)	6·5% (3·4 to 9·2%)	-7·0% (-9·6 to -4·6%)	5,543·3 (4,606·6 to 6,608·6)	2·2% (1·7 to 2·7%)	5,363·7 (4,382·4 to 6,466·3)	2·0% (1·5 to 2·5%)	-3·2% (-5·5 to -1·1%)	-9·2% (-11·4 to -7·1%)
Lower respiratory infections	43·5 (14·2 to 86·9)	10·9% (3·5 to 21·7%)	52·7 (17·7 to 105·5)	10·8% (3·6 to 21·5%)	21·0% (17·4 to 26·8%)	-0·5% (-2·0 to 3·6%)	511·1 (167·5 to 1,023·8)	10·9% (3·6 to 21·7%)	541·3 (180·9 to 1,088·1)	10·6% (3·5 to 21·3%)	5·9% (2·3 to 9·5%)	-2·8% (-4·8 to -0·3%)
Tracheal, bronchus, and lung cancer	35·8 (17·0 to 60·2)	7·1% (3·4 to 12·0%)	39·9 (19·1 to 66·6)	7·0% (3·3 to 11·7%)	11·3% (8·3 to 14·3%)	-2·3% (-3·9 to -0·9%)	685·4 (325·0 to 1,152·4)	7·1% (3·4 to 12·0%)	710·0 (339·5 to 1,193·0)	6·9% (3·3 to 11·5%)	3·6% (0·8 to 6·3%)	-3·5% (-5·4 to -2·1%)
Ischaemic heart disease	169·2 (116·8 to 221·4)	10·9% (7·5 to 14·2%)	171·4 (112·7 to 231·9)	10·4% (6·8 to 14·0%)	1·3% (-4·6 to 5·9%)	-4·3% (-9·9 to -0·2%)	2,637·9 (2,129·9 to 3,165·0)	12·2% (9·9 to 14·7%)	2,449·4 (1,925·5 to 2,976·9)	11·5% (9·1 to 14·0%)	-7·1% (-10·5 to -4·2%)	-5·6% (-8·8 to -2·9%)
Ischaemic stroke	35·5 (18·7 to 57·2)	7·4% (3·9 to 12·1%)	36·1 (18·0 to 60·4)	7·3% (3·6 to 12·1%)	1·9% (-7·7 to 7·6%)	-2·1% (-10·8 to 2·9%)	416·6 (277·5 to 575·0)	8·0% (5·2 to 10·9%)	386·4 (248·9 to 548·9)	7·6% (4·9 to 10·8%)	-7·3% (-12·9 to -1·9%)	-3·9% (-9·5 to 1·2%)
Hemorrhagic stroke	27·7 (18·4 to 38·9)	8·4% (5·6 to 11·8%)	28·2 (17·9 to 41·5)	8·2% (5·2 to 12·0%)	2·0% (-7·5 to 10·0%)	-3·2% (-11·7 to 3·4%)	511·8 (377·5 to 645·2)	9·8% (7·2 to 12·3%)	459·5 (338·8 to 587·7)	9·4% (6·9 to 11·9%)	-10·2% (-14·6 to -5·5%)	-4·3% (-8·4 to -0·1%)
Chronic obstructive pulmonary disease	47·3 (17·8 to 85·2)	12·3% (4·6 to 22·1%)	54·0 (20·5 to 97·0)	11·9% (4·5 to 21·5%)	14·1% (10·6 to 17·1%)	-3·4% (-5·4 to -1·8%)	780·5 (293·6 to 1,402·7)	12·0% (4·5 to 21·5%)	817·1 (305·0 to 1,490·3)	11·4% (4·2 to 20·7%)	4·7% (0·9 to 7·6%)	-5·2% (-8·2 to -3·1%)
**Household air pollution from solid fuels: All causes**	2·5 (0·8 to 5·8)	0·0% (0·0 to 0·1%)	1·2 (0·2 to 3·9)	0·0% (0·0 to 0·0%)	-50·6% (-87·3 to 53·7%)	-56·8% (-88·9 to 34·3%)	43·8 (14·9 to 102·9)	0·0% (0·0 to 0·0%)	19·5 (3·7 to 62·8)	0·0% (0·0 to 0·0%)	-55·4% (-88·6 to 41·2%)	-58·2% (-89·3 to 32·9%)
Lower respiratory infections	0·7 (0·2 to 1·8)	0·2% (0·0 to 0·5%)	0·4 (0·0 to 1·3)	0·1% (0·0 to 0·3%)	-46·6% (-86·7 to 71·4%)	-56·1% (-89·1 to 40·4%)	10·9 (2·3 to 29·6)	0·2% (0·1 to 0·6%)	5·0 (0·6 to 18·4)	0·1% (0·0 to 0·4%)	-54·6% (-89·0 to 51·7%)	-58·3% (-89·8 to 37·1%)
Tracheal, bronchus, and lung cancer	0·2 (0·0 to 0·5)	0·0% (0·0 to 0·1%)	0·1 (0·0 to 0·4)	0·0% (0·0 to 0·1%)	-47·8% (-85·9 to 52·1%)	-54·2% (-87·5 to 32·8%)	4·1 (0·8 to 11·0)	0·0% (0·0 to 0·1%)	2·0 (0·3 to 6·9)	0·0% (0·0 to 0·1%)	-51·0% (-86·9 to 47·4%)	-54·3% (-87·8 to 38·6%)
Ischaemic heart disease	0·8 (0·3 to 1·8)	0·1% (0·0 to 0·1%)	0·4 (0·1 to 1·2)	0·0% (0·0 to 0·1%)	-52·6% (-88·1 to 53·8%)	-55·2% (-88·7 to 45·1%)	14·4 (5·5 to 32·1)	0·1% (0·0 to 0·1%)	6·4 (1·3 to 20·2)	0·0% (0·0 to 0·1%)	-55·8% (-88·8 to 43·1%)	-55·1% (-88·6 to 45·5%)
Ischaemic stroke	0·2 (0·1 to 0·4)	0·0% (0·0 to 0·1%)	0·1 (0·0 to 0·3)	0·0% (0·0 to 0·1%)	-55·2% (-87·3 to 28·4%)	-56·9% (-87·9 to 23·0%)	2·7 (1·0 to 5·8)	0·1% (0·0 to 0·1%)	1·1 (0·2 to 3·5)	0·0% (0·0 to 0·1%)	-59·4% (-88·7 to 16·2%)	-57·9% (-88·4 to 19·4%)
Hemorrhagic stroke	0·2 (0·1 to 0·4)	0·1% (0·0 to 0·1%)	0·1 (0·0 to 0·3)	0·0% (0·0 to 0·1%)	-57·7% (-88·3 to 21·5%)	-59·8% (-89·1 to 17·2%)	4·7 (1·7 to 10·1)	0·1% (0·0 to 0·2%)	1·8 (0·4 to 5·9)	0·0% (0·0 to 0·1%)	-61·0% (-89·5 to 13·9%)	-58·5% (-88·9 to 22·4%)
Chronic obstructive pulmonary disease	0·4 (0·1 to 1·0)	0·1% (0·0 to 0·3%)	0·2 (0·0 to 0·7)	0·0% (0·0 to 0·2%)	-49·1% (-86·5 to 49·1%)	-56·9% (-88·6 to 27·0%)	6·4 (1·6 to 15·8)	0·1% (0·0 to 0·2%)	3·0 (0·4 to 10·6)	0·0% (0·0 to 0·1%)	-53·2% (-87·8 to 43·6%)	-57·7% (-88·9 to 30·1%)
Cataract	-	-	-	-	-	-	0·5 (0·2 to 1·1)	0·2% (0·1 to 0·4%)	0·3 (0·1 to 0·8)	0·1% (0·0 to 0·2%)	-50·7% (-86·4 to 41·3%)	-60·3% (-89·0 to 12·8%)
**Ambient ozone pollution: All causes**	24·1 (9·2 to 41·2)	0·3% (0·1 to 0·5%)	27·4 (10·3 to 46·8)	0·3% (0·1 to 0·5%)	13·6% (10·7 to 16·1%)	-0·8% (-3·3 to 1·3%)	300·1 (114·5 to 514·2)	0·1% (0·0 to 0·2%)	315·8 (119·5 to 544·8)	0·1% (0·0 to 0·2%)	5·2% (2·7 to 7·4%)	-1·2% (-3·6 to 0·8%)
Chronic obstructive pulmonary disease	24·1 (9·2 to 41·2)	6·3% (2·4 to 10·9%)	27·4 (10·3 to 46·8)	6·1% (2·3 to 10·5%)	13·6% (10·7 to 16·1%)	-3·8% (-5·1 to -2·9%)	300·1 (114·5 to 514·2)	4·6% (1·7 to 7·8%)	315·8 (119·5 to 544·8)	4·4% (1·7 to 7·5%)	5·2% (2·7 to 7·4%)	-4·7% (-6·4 to -3·3%)
**Other environmental risks: All causes**	100·6 (57·6 to 152·2)	1·2% (0·7 to 1·8%)	97·1 (53·0 to 151·6)	1·0% (0·5 to 1·6%)	-3·5% (-9·8 to 0·8%)	-15·7% (-21·2 to -12·1%)	1,438·6 (808·7 to 2,178·0)	0·6% (0·3 to 0·9%)	1,249·0 (683·0 to 1,947·2)	0·5% (0·2 to 0·7%)	-13·2% (-18·1 to -9·8%)	-18·5% (-23·1 to -15·3%)
**Residential radon: All causes**	16·2 (10·4 to 22·8)	0·2% (0·1 to 0·3%)	18·4 (11·4 to 26·2)	0·2% (0·1 to 0·3%)	13·8% (6·1 to 20·9%)	-0·6% (-7·3 to 5·6%)	318·3 (206·2 to 437·5)	0·1% (0·1 to 0·2%)	340·1 (218·0 to 475·3)	0·1% (0·1 to 0·2%)	6·9% (2·4 to 11·2%)	0·3% (-4·1 to 4·4%)
Tracheal, bronchus, and lung cancer	16·2 (10·4 to 22·8)	3·2% (2·0 to 4·5%)	18·4 (11·4 to 26·2)	3·2% (2·0 to 4·6%)	13·8% (6·1 to 20·9%)	-0·1% (-6·5 to 6·1%)	318·3 (206·2 to 437·5)	3·3% (2·1 to 4·5%)	340·1 (218·0 to 475·3)	3·3% (2·1 to 4·6%)	6·9% (2·4 to 11·2%)	-0·5% (-4·2 to 3·3%)
**Lead exposure: All causes**	84·4 (42·3 to 134·6)	1·0% (0·5 to 1·6%)	78·7 (36·8 to 130·9)	0·8% (0·4 to 1·4%)	-6·8% (-16·1 to -1·2%)	-18·6% (-26·7 to -13·7%)	1,120·3 (525·9 to 1,849·7)	0·4% (0·2 to 0·7%)	908·9 (378·0 to 1,591·2)	0·3% (0·1 to 0·6%)	-18·9% (-28·3 to -13·1%)	-23·9% (-32·7 to -18·4%)
Rheumatic heart disease	0·5 (0·2 to 1·1)	1·3% (0·5 to 2·9%)	0·4 (0·1 to 1·0)	1·1% (0·4 to 2·7%)	-11·1% (-23·2 to -0·7%)	-13·8% (-25·4 to -4·6%)	6·0 (2·3 to 12·0)	1·2% (0·4 to 2·3%)	4·5 (1·6 to 9·8)	0·9% (0·3 to 2·1%)	-25·3% (-35·7 to -16·4%)	-18·4% (-29·6 to -9·3%)
Ischaemic heart disease	44·8 (21·3 to 74·2)	2·9% (1·4 to 4·8%)	40·1 (17·0 to 69·7)	2·4% (1·0 to 4·3%)	-10·5% (-20·3 to -4·7%)	-15·4% (-24·6 to -10·2%)	565·5 (251·3 to 955·3)	2·6% (1·2 to 4·4%)	441·6 (171·5 to 797·9)	2·1% (0·8 to 3·7%)	-21·9% (-32·2 to -15·8%)	-20·6% (-31·1 to -14·5%)
Ischaemic stroke	11·5 (5·2 to 20·2)	2·4% (1·1 to 4·2%)	10·1 (4·1 to 18·7)	2·0% (0·8 to 3·8%)	-12·3% (-23·1 to -5·7%)	-15·7% (-26·0 to -9·2%)	127·9 (57·0 to 220·3)	2·4% (1·1 to 4·2%)	99·4 (39·0 to 180·8)	2·0% (0·8 to 3·6%)	-22·3% (-33·1 to -15·7%)	-19·5% (-30·7 to -12·8%)
Hemorrhagic stroke	9·7 (4·3 to 17·0)	3·0% (1·3 to 5·2%)	8·7 (3·4 to 16·5)	2·5% (1·0 to 4·7%)	-10·6% (-22·5 to -1·7%)	-15·1% (-26·3 to -6·7%)	137·5 (56·8 to 243·9)	2·6% (1·1 to 4·7%)	102·1 (37·6 to 195·1)	2·1% (0·8 to 4·0%)	-25·8% (-36·6 to -18·7%)	-20·9% (-31·8 to -13·7%)
Hypertensive heart disease	6·1 (1·4 to 15·7)	5·7% (1·3 to 14·4%)	6·8 (1·3 to 17·9)	5·0% (0·9 to 13·2%)	11·5% (-7·0 to 23·1%)	-12·5% (-26·4 to -4·3%)	68·9 (18·6 to 162·6)	4·9% (1·3 to 11·5%)	65·8 (14·6 to 167·9)	4·0% (0·9 to 10·2%)	-4·6% (-22·7 to 5·1%)	-18·3% (-33·4 to -10·4%)
Cardiomyopathy and myocarditis	1·5 (0·7 to 2·6)	1·6% (0·8 to 2·9%)	1·3 (0·5 to 2·4)	1·4% (0·6 to 2·5%)	-11·8% (-24·3 to -3·0%)	-17·7% (-28·5 to -10·4%)	21·4 (9·1 to 38·3)	1·4% (0·6 to 2·4%)	16·3 (6·1 to 30·8)	1·1% (0·4 to 2·0%)	-24·2% (-35·0 to -17·2%)	-22·0% (-33·1 to -15·5%)

Supplemental Results Table 1. All-age deaths, death PAFs, DALYs, DALY PAFs, and percent change for each risk factor and outcome for both sexes combined in 2005 and 2015 for High-income.

Data in parenthesis are 95% uncertainty intervals. PAFs=population attributable fractions. DALYs=disability-adjusted life-years.

	2005 Deaths (in thousands)	2005 Deaths PAF	2015 Deaths (in thousands)	2015 Deaths PAF	Percent change deaths 2005-2015	Percent change deaths PAF 2005-2015	2005 DALYs (in thousands)	2005 DALYs PAF	2015 DALYs (in thousands)	2015 DALYs PAF	Percent change DALYs 2005-2015	Percent change DALYs PAF 2005-2015
Atrial fibrillation and flutter	1·2 (0·6 to 1·9)	1·8% (0·9 to 2·8%)	1·3 (0·6 to 2·3)	1·5% (0·7 to 2·5%)	14·4% (1·3 to 22·0%)	-14·3% (-23·6 to -9·0%)	27·5 (12·6 to 47·3)	1·9% (0·9 to 3·0%)	26·8 (10·6 to 48·9)	1·5% (0·6 to 2·5%)	-2·7% (-16·4 to 5·1%)	-21·1% (-32·3 to -15·0%)
Aortic aneurysm	1·1 (0·5 to 1·8)	1·6% (0·7 to 2·7%)	1·0 (0·4 to 1·7)	1·2% (0·5 to 2·3%)	-9·7% (-22·2 to -1·2%)	-21·1% (-31·9 to -14·3%)	14·8 (6·4 to 25·5)	1·6% (0·7 to 2·7%)	11·6 (4·3 to 21·8)	1·2% (0·4 to 2·2%)	-21·2% (-33·5 to -13·5%)	-24·3% (-36·0 to -17·4%)
Peripheral vascular disease	0·3 (0·1 to 0·5)	1·2% (0·5 to 2·2%)	0·3 (0·1 to 0·6)	1·0% (0·4 to 1·9%)	5·5% (-10·9 to 18·1%)	-19·1% (-30·5 to -11·9%)	4·7 (2·0 to 8·4)	1·2% (0·5 to 2·1%)	4·5 (1·7 to 8·7)	0·9% (0·4 to 1·7%)	-4·7% (-20·9 to 6·1%)	-22·6% (-35·4 to -15·0%)
Endocarditis	0·4 (0·2 to 0·7)	1·6% (0·8 to 2·8%)	0·4 (0·2 to 0·7)	1·4% (0·6 to 2·5%)	2·4% (-10·4 to 11·6%)	-15·8% (-25·8 to -8·7%)	5·4 (2·3 to 9·4)	1·5% (0·6 to 2·6%)	4·7 (1·8 to 8·6)	1·2% (0·4 to 2·1%)	-13·1% (-24·9 to -5·3%)	-20·7% (-31·1 to -14·3%)
Other cardiovascular and circulatory diseases	2·6 (1·3 to 4·3)	1·8% (0·9 to 2·9%)	2·6 (1·2 to 4·5)	1·5% (0·7 to 2·6%)	-0·7% (-10·8 to 6·2%)	-16·1% (-24·2 to -10·9%)	51·9 (22·7 to 88·7)	1·7% (0·8 to 2·9%)	44·5 (17·3 to 81·1)	1·4% (0·5 to 2·4%)	-14·2% (-24·9 to -7·5%)	-21·4% (-31·3 to -15·8%)
Idiopathic developmental intellectual disability	-	-	-	-	-	-	11·0 (5·0 to 19·1)	4·4% (2·8 to 6·2%)	9·4 (4·2 to 16·4)	3·7% (2·3 to 5·3%)	-14·1% (-18·9 to -10·4%)	-16·7% (-20·2 to -13·1%)
Chronic kidney disease due to diabetes mellitus	1·8 (0·8 to 3·1)	2·5% (1·1 to 4·3%)	2·1 (0·9 to 3·9)	2·1% (0·9 to 3·9%)	20·9% (6·1 to 29·9%)	-14·7% (-24·6 to -9·4%)	28·3 (11·6 to 53·3)	1·9% (0·8 to 3·5%)	29·5 (10·6 to 59·3)	1·5% (0·6 to 3·1%)	4·4% (-10·6 to 12·5%)	-18·5% (-30·0 to -12·4%)
Chronic kidney disease due to hypertension	2·4 (1·2 to 4·0)	3·0% (1·4 to 5·0%)	2·8 (1·2 to 4·7)	2·6% (1·2 to 4·5%)	13·0% (1·3 to 21·0%)	-12·8% (-21·2 to -8·1%)	30·8 (13·8 to 53·4)	2·4% (1·1 to 4·2%)	29·7 (12·1 to 54·3)	2·0% (0·8 to 3·6%)	-3·6% (-13·6 to 2·7%)	-16·4% (-24·8 to -11·5%)
Chronic kidney disease due to glomerulonephritis	0·6 (0·3 to 0·9)	2·9% (1·4 to 4·7%)	0·7 (0·3 to 1·1)	2·6% (1·2 to 4·4%)	18·6% (6·5 to 26·9%)	-10·8% (-19·5 to -6·0%)	9·7 (4·0 to 17·4)	1·9% (0·8 to 3·3%)	9·5 (3·7 to 18·0)	1·6% (0·6 to 2·9%)	-1·8% (-12·0 to 4·4%)	-14·9% (-23·6 to -10·2%)
Chronic kidney disease due to other causes	0·1 (0·1 to 0·2)	3·2% (1·6 to 5·3%)	0·1 (0·1 to 0·2)	3·0% (1·5 to 5·0%)	28·6% (12·4 to 43·2%)	-7·4% (-17·9 to 1·3%)	9·1 (3·7 to 18·3)	1·8% (0·7 to 3·4%)	9·1 (3·4 to 19·0)	1·5% (0·6 to 3·0%)	-0·2% (-11·0 to 6·5%)	-16·1% (-25·3 to -11·1%)
**Occupational risks: All causes**	139·0 (128·2 to 149·1)	1·6% (1·5 to 1·8%)	169·4 (155·4 to 182·6)	1·8% (1·6 to 1·9%)	21·8% (17·5 to 26·3%)	6·4% (2·6 to 10·4%)	5,885·8 (4,983·5 to 6,975·9)	2·3% (2·1 to 2·5%)	6,468·6 (5,480·6 to 7,687·2)	2·4% (2·2 to 2·5%)	9·9% (7·7 to 12·0%)	3·1% (1·0 to 5·3%)
**Occupational carcinogens: All causes**	111·5 (101·2 to 121·7)	1·3% (1·2 to 1·4%)	138·0 (125·1 to 150·8)	1·4% (1·3 to 1·6%)	23·7% (17·9 to 29·1%)	8·1% (3·1 to 12·8%)	1,811·9 (1,657·6 to 1,969·1)	0·7% (0·6 to 0·8%)	2,056·8 (1,870·5 to 2,245·7)	0·8% (0·6 to 0·9%)	13·5% (8·9 to 17·9%)	6·6% (2·3 to 10·7%)
**Occupational exposure to asbestos: All causes**	91·3 (80·6 to 101·8)	1·1% (1·0 to 1·2%)	118·5 (104·7 to 131·6)	1·2% (1·1 to 1·4%)	29·7% (22·7 to 36·7%)	13·3% (7·3 to 19·4%)	1,366·3 (1,199·0 to 1,534·9)	0·5% (0·4 to 0·6%)	1,626·9 (1,430·9 to 1,817·1)	0·6% (0·5 to 0·7%)	19·1% (13·0 to 25·1%)	11·8% (6·0 to 17·3%)
Larynx cancer	0·5 (0·3 to 0·6)	2·6% (1·9 to 3·3%)	0·7 (0·5 to 0·9)	3·9% (3·0 to 5·0%)	50·5% (25·0 to 87·8%)	52·8% (27·8 to 90·2%)	6·3 (4·5 to 8·4)	1·6% (1·1 to 2·1%)	8·4 (6·3 to 11·0)	2·2% (1·7 to 2·9%)	32·9% (12·5 to 59·8%)	42·5% (21·2 to 71·5%)
Tracheal, bronchus, and lung cancer	80·1 (69·5 to 90·2)	15·9% (13·8 to 17·9%)	101·8 (88·5 to 114·5)	17·8% (15·5 to 19·9%)	27·1% (20·4 to 34·0%)	11·7% (6·4 to 16·9%)	1,176·6 (1,010·3 to 1,339·7)	12·2% (10·5 to 13·9%)	1,369·3 (1,184·7 to 1,557·1)	13·3% (11·4 to 15·0%)	16·4% (10·5 to 22·2%)	8·4% (3·3 to 13·2%)
Ovarian cancer	0·4 (0·2 to 0·7)	0·8% (0·4 to 1·3%)	0·5 (0·2 to 0·8)	0·9% (0·4 to 1·4%)	23·9% (14·5 to 35·9%)	10·5% (3·4 to 19·0%)	5·4 (2·6 to 8·9)	0·5% (0·2 to 0·8%)	6·3 (3·0 to 10·2)	0·5% (0·3 to 0·9%)	15·7% (8·2 to 25·2%)	7·4% (1·5 to 14·5%)
Mesothelioma	10·4 (8·7 to 11·7)	80·4% (67·4 to 90·0%)	15·5 (13·3 to 17·3)	83·3% (71·8 to 91·6%)	48·9% (38·3 to 58·2%)	3·6% (1·7 to 6·7%)	178·0 (144·4 to 206·1)	73·1% (59·1 to 84·3%)	242·9 (201·9 to 279·4)	76·1% (63·1 to 86·3%)	36·5% (27·5 to 44·5%)	4·1% (2·1 to 7·3%)
**Occupational exposure to arsenic: All causes**	0·6 (0·4 to 0·7)	0·0% (0·0 to 0·0%)	0·5 (0·4 to 0·6)	0·0% (0·0 to 0·0%)	-17·1% (-19·9 to -14·5%)	-27·6% (-30·0 to -25·2%)	11·9 (9·1 to 15·1)	0·0% (0·0 to 0·0%)	9·8 (7·5 to 12·4)	0·0% (0·0 to 0·0%)	-17·6% (-20·3 to -14·9%)	-22·6% (-25·2 to -20·0%)
Tracheal, bronchus, and lung cancer	0·6 (0·4 to 0·7)	0·1% (0·1 to 0·1%)	0·5 (0·4 to 0·6)	0·1% (0·1 to 0·1%)	-17·1% (-19·9 to -14·5%)	-27·2% (-28·9 to -25·5%)	11·9 (9·1 to 15·1)	0·1% (0·1 to 0·2%)	9·8 (7·5 to 12·4)	0·1% (0·1 to 0·1%)	-17·6% (-20·3 to -14·9%)	-23·2% (-25·0 to -21·4%)
**Occupational exposure to benzene: All causes**	0·4 (0·3 to 0·5)	0·0% (0·0 to 0·0%)	0·4 (0·3 to 0·5)	0·0% (0·0 to 0·0%)	8·9% (5·1 to 12·4%)	-4·9% (-8·2 to -1·8%)	11·6 (9·8 to 13·5)	0·0% (0·0 to 0·0%)	12·6 (10·7 to 14·7)	0·0% (0·0 to 0·0%)	8·6% (4·9 to 12·2%)	2·0% (-1·5 to 5·2%)
Leukaemia	0·4 (0·3 to 0·5)	0·4% (0·4 to 0·5%)	0·4 (0·3 to 0·5)	0·4% (0·3 to 0·5%)	8·9% (5·1 to 12·4%)	-8·4% (-11·1 to -5·8%)	11·6 (9·8 to 13·5)	0·6% (0·5 to 0·7%)	12·6 (10·7 to 14·7)	0·6% (0·5 to 0·7%)	8·6% (4·9 to 12·2%)	2·9% (0·5 to 5·5%)
**Occupational exposure to beryllium: All causes**	0·0 (0·0 to 0·0)	0·0% (0·0 to 0·0%)	0·0 (0·0 to 0·0)	0·0% (0·0 to 0·0%)	-19·9% (-22·8 to -17·1%)	-30·0% (-32·5 to -27·6%)	0·8 (0·6 to 1·0)	0·0% (0·0 to 0·0%)	0·7 (0·5 to 0·8)	0·0% (0·0 to 0·0%)	-20·4% (-23·3 to -17·4%)	-25·2% (-28·1 to -22·5%)
Tracheal, bronchus, and lung cancer	0·0 (0·0 to 0·0)	0·0% (0·0 to 0·0%)	0·0 (0·0 to 0·0)	0·0% (0·0 to 0·0%)	-19·9% (-22·8 to -17·1%)	-29·7% (-31·4 to -27·8%)	0·8 (0·6 to 1·0)	0·0% (0·0 to 0·0%)	0·7 (0·5 to 0·8)	0·0% (0·0 to 0·0%)	-20·4% (-23·3 to -17·4%)	-25·8% (-27·8 to -23·8%)
**Occupational exposure to cadmium: All causes**	0·2 (0·1 to 0·2)	0·0% (0·0 to 0·0%)	0·1 (0·1 to 0·2)	0·0% (0·0 to 0·0%)	-12·4% (-15·3 to -9·7%)	-23·5% (-26·0 to -21·1%)	3·2 (2·6 to 3·8)	0·0% (0·0 to 0·0%)	2·8 (2·3 to 3·3)	0·0% (0·0 to 0·0%)	-12·9% (-15·7 to -10·2%)	-18·3% (-21·0 to -15·6%)
Tracheal, bronchus, and lung cancer	0·2 (0·1 to 0·2)	0·0% (0·0 to 0·0%)	0·1 (0·1 to 0·2)	0·0% (0·0 to 0·0%)	-12·4% (-15·3 to -9·7%)	-23·1% (-24·8 to -21·4%)	3·2 (2·6 to 3·8)	0·0% (0·0 to 0·0%)	2·8 (2·3 to 3·3)	0·0% (0·0 to 0·0%)	-12·9% (-15·7 to -10·2%)	-18·9% (-20·6 to -17·1%)
**Occupational exposure to chromium: All causes**	0·5 (0·4 to 0·6)	0·0% (0·0 to 0·0%)	0·4 (0·4 to 0·5)	0·0% (0·0 to 0·0%)	-14·6% (-17·3 to -12·1%)	-25·4% (-27·7 to -23·1%)	10·7 (9·4 to 12·2)	0·0% (0·0 to 0·0%)	9·1 (8·0 to 10·4)	0·0% (0·0 to 0·0%)	-15·1% (-17·6 to -12·5%)	-20·3% (-22·9 to -17·9%)
Tracheal, bronchus, and lung cancer	0·5 (0·4 to 0·6)	0·1% (0·1 to 0·1%)	0·4 (0·4 to 0·5)	0·1% (0·1 to 0·1%)	-14·6% (-17·3 to -12·1%)	-25·0% (-26·5 to -23·4%)	10·7 (9·4 to 12·2)	0·1% (0·1 to 0·1%)	9·1 (8·0 to 10·4)	0·1% (0·1 to 0·1%)	-15·1% (-17·6 to -12·5%)	-20·9% (-22·5 to -19·3%)
**Occupational exposure to diesel engine exhaust: All causes**	7·3 (6·3 to 8·4)	0·1% (0·1 to 0·1%)	7·8 (6·8 to 9·0)	0·1% (0·1 to 0·1%)	6·7% (3·4 to 10·0%)	-6·8% (-9·7 to -3·9%)	154·2 (134·3 to 176·4)	0·1% (0·1 to 0·1%)	163·4 (142·3 to 187·2)	0·1% (0·0 to 0·1%)	5·9% (2·7 to 9·1%)	-0·6% (-3·8 to 2·4%)
Tracheal, bronchus, and lung cancer	7·3 (6·3 to 8·4)	1·5% (1·3 to 1·7%)	7·8 (6·8 to 9·0)	1·4% (1·2 to 1·6%)	6·7% (3·4 to 10·0%)	-6·3% (-8·1 to -4·3%)	154·2 (134·3 to 176·4)	1·6% (1·4 to 1·8%)	163·4 (142·3 to 187·2)	1·6% (1·4 to 1·8%)	5·9% (2·7 to 9·1%)	-1·3% (-3·4 to 0·8%)
**Occupational exposure to second-hand smoke: All causes**	7·4 (6·9 to 8·0)	0·1% (0·1 to 0·1%)	7·3 (6·7 to 7·9)	0·1% (0·1 to 0·1%)	-1·5% (-4·2 to 1·1%)	-14·0% (-16·3 to -11·8%)	156·3 (144·9 to 167·7)	0·1% (0·1 to 0·1%)	153·0 (141·1 to 165·4)	0·1% (0·0 to 0·1%)	-2·1% (-4·7 to 0·5%)	-8·1% (-10·7 to -5·8%)
Tracheal, bronchus, and lung cancer	7·4 (6·9 to 8·0)	1·5% (1·4 to 1·6%)	7·3 (6·7 to 7·9)	1·3% (1·2 to 1·4%)	-1·5% (-4·2 to 1·1%)	-13·5% (-14·8 to -12·3%)	156·3 (144·9 to 167·7)	1·6% (1·5 to 1·7%)	153·0 (141·1 to 165·4)	1·5% (1·4 to 1·6%)	-2·1% (-4·7 to 0·5%)	-8·8% (-10·1 to -7·5%)
**Occupational exposure to formaldehyde: All causes**	0·1 (0·1 to 0·1)	0·0% (0·0 to 0·0%)	0·1 (0·1 to 0·1)	0·0% (0·0 to 0·0%)	-7·3% (-10·2 to -4·3%)	-19·0% (-21·6 to -16·3%)	2·2 (1·9 to 2·5)	0·0% (0·0 to 0·0%)	2·0 (1·7 to 2·4)	0·0% (0·0 to 0·0%)	-7·4% (-10·4 to -4·5%)	-13·1% (-15·9 to -10·4%)
Nasopharynx cancer	0·0 (0·0 to 0·0)	0·2% (0·2 to 0·4%)	0·0 (0·0 to 0·0)	0·2% (0·1 to 0·3%)	-8·9% (-14·8 to -3·5%)	-20·4% (-23·2 to -17·9%)	0·4 (0·3 to 0·7)	0·3% (0·2 to 0·5%)	0·4 (0·3 to 0·6)	0·3% (0·2 to 0·4%)	-8·9% (-14·7 to -3·5%)	-14·6% (-17·1 to -12·2%)
**Occupational exposure to nickel: All causes**	2·1 (1·5 to 2·9)	0·0% (0·0 to 0·0%)	1·7 (1·2 to 2·3)	0·0% (0·0 to 0·0%)	-19·6% (-22·6 to -16·6%)	-29·8% (-32·3 to -27·2%)	44·6 (31·8 to 59·6)	0·0% (0·0 to 0·0%)	35·7 (25·5 to 47·5)	0·0% (0·0 to 0·0%)	-20·1% (-23·0 to -17·0%)	-25·0% (-27·8 to -22·2%)
Tracheal, bronchus, and lung cancer	2·1 (1·5 to 2·9)	0·4% (0·3 to 0·6%)	1·7 (1·2 to 2·3)	0·3% (0·2 to 0·4%)	-19·6% (-22·6 to -16·6%)	-29·4% (-31·4 to -27·4%)	44·6 (31·8 to 59·6)	0·5% (0·3 to 0·6%)	35·7 (25·5 to 47·5)	0·3% (0·2 to 0·5%)	-20·1% (-23·0 to -17·0%)	-25·5% (-27·7 to -23·4%)
**Occupational exposure to polycyclic aromatic hydrocarbons: All causes**	1·2 (1·0 to 1·4)	0·0% (0·0 to 0·0%)	1·1 (0·9 to 1·4)	0·0% (0·0 to 0·0%)	-5·8% (-8·6 to -3·1%)	-17·7% (-20·2 to -15·4%)	25·7 (21·1 to 30·5)	0·0% (0·0 to 0·0%)	24·1 (19·9 to 28·4)	0·0% (0·0 to 0·0%)	-6·4% (-9·2 to -3·7%)	-12·1% (-14·8 to -9·6%)
Tracheal, bronchus, and lung cancer	1·2 (1·0 to 1·4)	0·2% (0·2 to 0·3%)	1·1 (0·9 to 1·4)	0·2% (0·2 to 0·2%)	-5·8% (-8·6 to -3·1%)	-17·3% (-18·8 to -15·8%)	25·7 (21·1 to 30·5)	0·3% (0·2 to 0·3%)	24·1 (19·9 to 28·4)	0·2% (0·2 to 0·3%)	-6·4% (-9·2 to -3·7%)	-12·8% (-14·3 to -11·1%)
**Occupational exposure to silica: All causes**	4·7 (4·2 to 5·3)	0·1% (0·1 to 0·1%)	4·6 (4·1 to 5·1)	0·0% (0·0 to 0·1%)	-2·7% (-6·2 to 0·8%)	-15·0% (-18·1 to -12·0%)	99·3 (89·6 to 110·2)	0·0% (0·0 to 0·0%)	96·0 (86·3 to 106·9)	0·0% (0·0 to 0·0%)	-3·3% (-6·9 to 0·1%)	-9·2% (-12·6 to -6·0%)
Tracheal, bronchus, and lung cancer	4·7 (4·2 to 5·3)	0·9% (0·8 to 1·0%)	4·6 (4·1 to 5·1)	0·8% (0·7 to 0·9%)	-2·7% (-6·2 to 0·8%)	-14·5% (-16·8 to -12·4%)	99·3 (89·6 to 110·2)	1·0% (0·9 to 1·1%)	96·0 (86·3 to 106·9)	0·9% (0·8 to 1·0%)	-3·3% (-6·9 to 0·1%)	-9·9% (-12·4 to -7·6%)
**Occupational exposure to sulfphuric acid: All causes**	0·6 (0·4 to 0·7)	0·0% (0·0 to 0·0%)	0·4 (0·3 to 0·6)	0·0% (0·0 to 0·0%)	-19·7% (-24·4 to -14·7%)	-29·8% (-34·0 to -25·5%)	13·4 (9·9 to 17·6)	0·0% (0·0 to 0·0%)	10·7 (7·9 to 14·1)	0·0% (0·0 to 0·0%)	-20·2% (-24·8 to -15·3%)	-25·1% (-29·5 to -20·5%)
Larynx cancer	0·6 (0·4 to 0·7)	3·1% (2·3 to 4·1%)	0·4 (0·3 to 0·6)	2·6% (1·9 to 3·4%)	-19·7% (-24·4 to -14·7%)	-18·4% (-21·5 to -15·3%)	13·4 (9·9 to 17·6)	3·4% (2·5 to 4·4%)	10·7 (7·9 to 14·1)	2·9% (2·1 to 3·8%)	-20·2% (-24·8 to -15·3%)	-14·4% (-17·5 to -11·4%)
**Occupational exposure to trichloroethylene: All causes**	0·0 (0·0 to 0·0)	0·0% (0·0 to 0·0%)	0·0 (0·0 to 0·0)	0·0% (0·0 to 0·0%)	3·5% (-1·4 to 9·1%)	-9·6% (-13·8 to -4·5%)	0·6 (0·1 to 1·1)	0·0% (0·0 to 0·0%)	0·6 (0·1 to 1·1)	0·0% (0·0 to 0·0%)	4·5% (-0·6 to 10·1%)	-1·9% (-6·5 to 3·4%)
Kidney cancer	0·0 (0·0 to 0·0)	0·1% (0·0 to 0·1%)	0·0 (0·0 to 0·0)	0·0% (0·0 to 0·1%)	3·5% (-1·4 to 9·1%)	-18·3% (-21·3 to -15·1%)	0·6 (0·1 to 1·1)	0·1% (0·0 to 0·1%)	0·6 (0·1 to 1·1)	0·1% (0·0 to 0·1%)	4·5% (-0·6 to 10·1%)	-13·3% (-16·2 to -10·1%)

Supplemental Results Table 1. All-age deaths, death PAFs, DALYs, DALY PAFs, and percent change for each risk factor and outcome for both sexes combined in 2005 and 2015 for High-income.

Data in parenthesis are 95% uncertainty intervals. PAFs=population attributable fractions. DALYs=disability-adjusted life-years.

	2005 Deaths (in thousands)	2005 Deaths PAF	2015 Deaths (in thousands)	2015 Deaths PAF	Percent change deaths 2005-2015	Percent change deaths PAF 2005-2015	2005 DALYs (in thousands)	2005 DALYs PAF	2015 DALYs (in thousands)	2015 DALYs PAF	Percent change DALYs 2005-2015	Percent change DALYs PAF 2005-2015
**Occupational asthmagens: All causes**	0·6 (0·6 to 0·7)	0·0% (0·0 to 0·0%)	0·5 (0·4 to 0·5)	0·0% (0·0 to 0·0%)	-25·7% (-30·0 to -21·7%)	-35·1% (-38·8 to -31·6%)	161·6 (114·3 to 217·9)	0·1% (0·0 to 0·1%)	153·1 (106·4 to 207·6)	0·1% (0·0 to 0·1%)	-5·3% (-7·8 to -3·0%)	-11·1% (-13·2 to -9·0%)
Asthma	0·6 (0·6 to 0·7)	2·9% (2·7 to 3·0%)	0·5 (0·4 to 0·5)	2·4% (2·2 to 2·6%)	-25·7% (-30·0 to -21·7%)	-16·9% (-21·4 to -12·5%)	161·6 (114·3 to 217·9)	5·2% (4·8 to 5·5%)	153·1 (106·4 to 207·6)	5·1% (4·8 to 5·4%)	-5·3% (-7·8 to -3·0%)	-0·6% (-2·4 to 1·1%)
**Occupational particulate matter, gases, and fumes: All causes**	12·7 (10·1 to 15·8)	0·2% (0·1 to 0·2%)	15·7 (12·1 to 20·0)	0·2% (0·1 to 0·2%)	23·1% (16·6 to 28·4%)	7·6% (1·8 to 12·3%)	329·1 (277·4 to 382·0)	0·1% (0·1 to 0·2%)	372·5 (312·8 to 435·5)	0·1% (0·1 to 0·2%)	13·2% (9·8 to 16·5%)	6·2% (2·9 to 9·4%)
Chronic obstructive pulmonary disease	11·8 (9·2 to 14·8)	3·1% (2·4 to 3·9%)	14·9 (11·3 to 19·2)	3·3% (2·5 to 4·2%)	26·3% (20·2 to 31·7%)	7·0% (2·0 to 11·2%)	312·8 (262·0 to 365·7)	4·8% (4·1 to 5·6%)	357·7 (298·1 to 420·3)	5·0% (4·2 to 5·8%)	14·3% (10·9 to 17·8%)	3·6% (0·7 to 6·4%)
**Occupational noise: All causes**	-	-	-	-	-	-	599·9 (405·5 to 846·7)	0·2% (0·2 to 0·3%)	716·0 (482·0 to 1,013·7)	0·3% (0·2 to 0·3%)	19·3% (16·4 to 22·5%)	12·0% (9·2 to 15·0%)
Age-related and other hearing loss	-	-	-	-	-	-	599·9 (405·5 to 846·7)	10·4% (9·2 to 11·9%)	716·0 (482·0 to 1,013·7)	10·1% (8·9 to 11·6%)	19·3% (16·4 to 22·5%)	-3·2% (-5·8 to -0·8%)
**Occupational injuries: All causes**	14·2 (13·8 to 14·6)	0·2% (0·2 to 0·2%)	15·4 (14·7 to 16·1)	0·2% (0·2 to 0·2%)	8·7% (3·5 to 14·7%)	-5·0% (-9·6 to 0·2%)	1,235·8 (1,062·2 to 1,449·5)	0·5% (0·5 to 0·5%)	1,370·2 (1,142·3 to 1,661·9)	0·5% (0·5 to 0·6%)	10·9% (3·2 to 19·1%)	4·0% (-3·2 to 11·8%)
Pedestrian road injuries	1·5 (1·3 to 1·6)	5·0% (4·7 to 5·2%)	1·5 (1·3 to 1·6)	5·8% (5·3 to 6·1%)	-0·1% (-8·4 to 7·1%)	16·0% (8·2 to 23·6%)	84·1 (74·5 to 91·7)	6·9% (6·7 to 7·2%)	82·4 (69·8 to 91·6)	8·9% (8·3 to 9·4%)	-2·0% (-9·6 to 5·3%)	28·2% (20·0 to 36·8%)
Cyclist road injuries	0·3 (0·2 to 0·3)	4·6% (4·3 to 4·9%)	0·3 (0·2 to 0·3)	5·6% (5·1 to 6·0%)	8·7% (-2·1 to 19·0%)	21·0% (11·0 to 31·6%)	30·5 (24·5 to 37·8)	6·8% (6·4 to 7·3%)	30·9 (24·0 to 39·0)	8·6% (7·9 to 9·4%)	1·3% (-9·1 to 13·0%)	26·1% (15·2 to 38·4%)
Motorcyclist road injuries	1·3 (1·1 to 1·4)	8·5% (8·2 to 8·8%)	1·4 (1·2 to 1·6)	11·1% (10·5 to 11·8%)	12·6% (2·6 to 22·1%)	30·4% (22·1 to 39·5%)	94·8 (82·3 to 108·6)	8·9% (8·5 to 9·3%)	99·6 (82·2 to 114·6)	11·5% (10·8 to 12·3%)	5·1% (-4·0 to 14·5%)	29·8% (20·9 to 39·6%)
Motor vehicle road injuries	5·9 (5·6 to 6·4)	8·3% (8·1 to 8·6%)	6·1 (5·8 to 6·8)	10·5% (10·1 to 11·0%)	3·6% (-1·6 to 9·2%)	26·5% (20·4 to 32·9%)	367·2 (340·2 to 398·7)	9·4% (9·1 to 9·7%)	375·6 (342·9 to 414·4)	12·4% (11·8 to 13·0%)	2·3% (-2·8 to 8·0%)	31·9% (25·3 to 39·2%)
Other road injuries	0·1 (0·1 to 0·1)	9·0% (8·4 to 9·4%)	0·1 (0·1 to 0·1)	10·7% (9·9 to 11·5%)	9·5% (-1·4 to 28·3%)	19·0% (10·4 to 28·6%)	5·8 (4·8 to 6·8)	10·4% (9·9 to 11·1%)	6·4 (5·2 to 7·7)	13·1% (12·1 to 14·2%)	11·1% (1·6 to 23·6%)	25·5% (16·4 to 36·3%)
Other transport injuries	0·6 (0·6 to 0·7)	6·9% (6·6 to 7·1%)	0·7 (0·7 to 0·8)	8·6% (8·2 to 9·1%)	12·8% (5·0 to 20·8%)	25·5% (17·2 to 33·6%)	36·0 (34·0 to 38·2)	7·8% (7·5 to 8·1%)	40·2 (37·2 to 43·7)	10·2% (9·7 to 10·8%)	11·5% (4·2 to 19·5%)	30·8% (23·0 to 38·6%)
Falls	1·7 (1·6 to 1·8)	1·8% (1·7 to 1·9%)	2·1 (2·0 to 2·2)	1·8% (1·7 to 1·9%)	25·7% (18·0 to 33·9%)	-0·5% (-6·6 to 6·5%)	321·1 (243·0 to 416·9)	6·5% (6·1 to 7·0%)	383·3 (278·9 to 517·0)	7·7% (7·0 to 8·5%)	19·4% (7·1 to 31·7%)	17·9% (8·7 to 28·0%)
Drowning	0·8 (0·8 to 0·8)	4·2% (4·1 to 4·3%)	0·9 (0·9 to 1·0)	4·7% (4·4 to 4·9%)	12·1% (5·9 to 19·1%)	11·0% (4·6 to 17·8%)	38·9 (37·8 to 40·2)	5·6% (5·4 to 5·8%)	42·8 (40·6 to 45·3)	7·3% (6·9 to 7·7%)	9·8% (3·4 to 16·6%)	30·5% (23·3 to 38·2%)
Fire, heat, and hot substances	0·6 (0·5 to 0·6)	3·6% (3·5 to 3·7%)	0·6 (0·6 to 0·7)	4·0% (3·8 to 4·2%)	10·5% (4·1 to 18·2%)	12·1% (5·8 to 18·7%)	45·4 (37·1 to 57·8)	6·7% (6·4 to 7·0%)	49·9 (38·9 to 65·0)	8·6% (8·1 to 9·1%)	10·0% (2·6 to 17·3%)	28·0% (20·2 to 35·4%)
Poisonings	0·1 (0·1 to 0·1)	1·9% (1·8 to 1·9%)	0·1 (0·1 to 0·1)	1·8% (1·8 to 2·0%)	7·8% (1·3 to 15·0%)	-1·0% (-6·1 to 4·6%)	7·5 (6·9 to 8·3)	2·5% (2·4 to 2·6%)	8·0 (7·3 to 8·9)	2·7% (2·5 to 2·8%)	7·0% (0·1 to 14·3%)	5·4% (-0·5 to 11·9%)
Unintentional firearm injuries	0·1 (0·1 to 0·1)	1·7% (1·6 to 1·8%)	0·1 (0·1 to 0·1)	1·8% (1·6 to 2·0%)	7·6% (-4·0 to 20·9%)	6·9% (-3·8 to 20·1%)	4·5 (4·1 to 4·9)	3·0% (2·9 to 3·2%)	4·8 (4·2 to 5·5)	3·9% (3·5 to 4·3%)	7·4% (-3·9 to 21·0%)	27·8% (14·8 to 43·5%)
Unintentional suffocation	0·0 (0·0 to 0·0)	0·2% (0·2 to 0·3%)	0·0 (0·0 to 0·0)	0·2% (0·2 to 0·2%)	-0·5% (-13·5 to 13·4%)	-10·2% (-22·1 to 3·3%)	0·8 (0·7 to 1·0)	0·5% (0·4 to 0·6%)	0·8 (0·6 to 1·0)	0·5% (0·4 to 0·6%)	-7·3% (-19·6 to 6·7%)	-5·5% (-18·7 to 9·5%)
Other exposure to mechanical forces	0·5 (0·5 to 0·5)	5·8% (5·7 to 6·0%)	0·6 (0·5 to 0·6)	6·5% (6·2 to 6·9%)	6·0% (-0·6 to 12·9%)	12·1% (5·8 to 19·0%)	70·3 (54·5 to 92·4)	8·9% (8·6 to 9·3%)	84·8 (62·9 to 115·7)	11·2% (10·5 to 11·9%)	20·7% (11·4 to 29·9%)	25·3% (17·5 to 33·5%)
Venomous animal contact	0·0 (0·0 to 0·0)	0·7% (0·6 to 0·7%)	0·0 (0·0 to 0·0)	0·7% (0·6 to 0·8%)	16·6% (2·9 to 32·6%)	8·6% (-3·9 to 22·7%)	0·2 (0·2 to 0·3)	1·1% (1·0 to 1·2%)	0·3 (0·2 to 0·4)	1·3% (1·2 to 1·4%)	25·3% (12·9 to 39·4%)	19·1% (7·7 to 31·5%)
Non-venomous animal contact	0·0 (0·0 to 0·0)	0·4% (0·4 to 0·4%)	0·0 (0·0 to 0·0)	0·4% (0·4 to 0·5%)	19·5% (6·6 to 34·6%)	8·9% (-3·2 to 22·4%)	0·4 (0·3 to 0·6)	1·0% (0·9 to 1·1%)	0·6 (0·4 to 0·8)	1·2% (1·1 to 1·3%)	29·4% (16·7 to 42·5%)	22·9% (12·1 to 35·4%)
Pulmonary aspiration and foreign body in airway	0·4 (0·4 to 0·4)	1·5% (1·5 to 1·7%)	0·5 (0·5 to 0·6)	1·7% (1·5 to 1·9%)	27·8% (16·6 to 40·9%)	7·3% (-2·4 to 19·6%)	18·6 (17·6 to 19·9)	3·2% (3·0 to 3·4%)	22·9 (20·7 to 25·4)	3·9% (3·6 to 4·4%)	23·5% (12·3 to 36·3%)	23·6% (12·4 to 37·4%)
Foreign body in eyes	-	-	-	-	-	-	0·5 (0·2 to 0·9)	5·0% (4·4 to 5·6%)	0·7 (0·3 to 1·2)	6·1% (5·4 to 7·0%)	33·2% (23·9 to 42·9%)	23·0% (14·9 to 32·0%)
Foreign body in other body part	0·0 (0·0 to 0·0)	2·7% (2·5 to 2·9%)	0·0 (0·0 to 0·0)	3·0% (2·6 to 3·4%)	25·8% (11·8 to 39·6%)	9·6% (-6·3 to 27·6%)	2·7 (2·2 to 3·5)	5·7% (5·4 to 6·0%)	3·4 (2·7 to 4·6)	6·9% (6·2 to 7·6%)	27·2% (13·9 to 40·7%)	21·1% (8·1 to 34·2%)
Other unintentional injuries	0·3 (0·2 to 0·3)	8·4% (8·1 to 8·8%)	0·2 (0·2 to 0·3)	9·8% (9·1 to 10·6%)	-2·4% (-10·4 to 7·6%)	16·2% (7·5 to 25·6%)	106·4 (72·5 to 148·7)	9·3% (8·7 to 10·0%)	132·8 (88·0 to 189·9)	11·6% (10·5 to 12·8%)	24·8% (13·1 to 36·7%)	24·7% (13·8 to 36·5%)
**Occupational ergonomic factors: All causes**	-	-	-	-	-	-	1,754·2 (1,221·7 to 2,447·8)	0·7% (0·5 to 0·9%)	1,832·3 (1,283·0 to 2,535·9)	0·7% (0·5 to 0·8%)	4·5% (2·7 to 6·1%)	-2·0% (-3·8 to -0·3%)
Low back pain	-	-	-	-	-	-	1,754·2 (1,221·7 to 2,447·8)	15·9% (15·2 to 16·7%)	1,832·3 (1,283·0 to 2,535·9)	15·2% (14·4 to 16·0%)	4·5% (2·7 to 6·1%)	-4·9% (-6·4 to -3·6%)
**Behavioural risks: All causes**	3,360·3 (3,149·8 to 3,589·5)	39·9% (37·4 to 42·6%)	3,613·9 (3,378·1 to 3,869·6)	37·4% (35·0 to 40·1%)	7·5% (6·6 to 8·4%)	-6·0% (-6·8 to -5·3%)	75,694·6 (70,355·1 to 81,656·1)	29·5% (27·2 to 32·1%)	76,950·2 (71,122·1 to 83,368·3)	28·2% (25·9 to 30·7%)	1·7% (0·8 to 2·4%)	-4·6% (-5·1 to -4·0%)
**Child and maternal malnutrition: All causes**	2·5 (1·8 to 3·0)	0·0% (0·0 to 0·0%)	1·8 (1·3 to 2·1)	0·0% (0·0 to 0·0%)	-29·7% (-35·7 to -23·4%)	-38·6% (-43·8 to -33·2%)	3,668·0 (2,505·6 to 5,249·8)	1·4% (1·1 to 1·9%)	3,739·3 (2,512·1 to 5,393·2)	1·4% (1·0 to 1·8%)	1·9% (0·0 to 3·7%)	-4·4% (-6·0 to -2·8%)
**Suboptimal breastfeeding: All causes**	1·5 (0·7 to 2·0)	0·0% (0·0 to 0·0%)	1·0 (0·5 to 1·3)	0·0% (0·0 to 0·0%)	-33·9% (-39·9 to -27·6%)	-42·3% (-47·6 to -36·6%)	127·0 (60·6 to 170·3)	0·0% (0·0 to 0·1%)	84·1 (41·5 to 113·8)	0·0% (0·0 to 0·0%)	-33·7% (-39·6 to -27·2%)	-37·8% (-43·3 to -31·6%)
**Non-exclusive breastfeeding : All causes**	1·4 (0·7 to 1·9)	0·0% (0·0 to 0·0%)	0·9 (0·5 to 1·3)	0·0% (0·0 to 0·0%)	-33·9% (-39·9 to -27·2%)	-42·2% (-47·6 to -36·4%)	124·5 (58·2 to 167·9)	0·0% (0·0 to 0·1%)	82·5 (40·0 to 112·4)	0·0% (0·0 to 0·0%)	-33·8% (-39·8 to -27·1%)	-37·8% (-43·4 to -31·5%)
Diarrhoeal diseases	0·1 (0·1 to 0·1)	0·6% (0·4 to 1·0%)	0·1 (0·0 to 0·1)	0·3% (0·2 to 0·6%)	-32·1% (-46·8 to -11·4%)	-53·4% (-64·8 to -38·8%)	9·4 (5·8 to 12·8)	3·2% (1·9 to 4·6%)	6·4 (4·0 to 9·0)	1·9% (1·2 to 3·1%)	-31·7% (-46·4 to -11·1%)	-40·3% (-54·3 to -21·9%)
Lower respiratory infections	1·3 (0·6 to 1·8)	0·3% (0·1 to 0·5%)	0·9 (0·4 to 1·2)	0·2% (0·1 to 0·3%)	-34·0% (-40·2 to -26·6%)	-45·7% (-50·9 to -39·2%)	115·2 (50·2 to 158·2)	2·5% (1·1 to 3·4%)	76·1 (34·4 to 106·2)	1·5% (0·7 to 2·1%)	-33·9% (-40·1 to -26·6%)	-39·4% (-45·0 to -32·0%)
**Discontinued breastfeeding : All causes**	0·0 (0·0 to 0·1)	0·0% (0·0 to 0·0%)	0·0 (0·0 to 0·1)	0·0% (0·0 to 0·0%)	-36·6% (-53·6 to -11·7%)	-44·6% (-59·5 to -22·9%)	4·4 (1·3 to 8·1)	0·0% (0·0 to 0·0%)	2·9 (0·9 to 5·3)	0·0% (0·0 to 0·0%)	-33·9% (-50·8 to -10·4%)	-38·0% (-53·8 to -15·6%)
Diarrhoeal diseases	0·0 (0·0 to 0·1)	0·3% (0·1 to 0·6%)	0·0 (0·0 to 0·1)	0·1% (0·0 to 0·3%)	-36·6% (-53·6 to -11·7%)	-56·5% (-68·7 to -38·8%)	4·4 (1·3 to 8·1)	1·5% (0·4 to 2·8%)	2·9 (0·9 to 5·3)	0·9% (0·3 to 1·6%)	-33·9% (-50·8 to -10·4%)	-42·2% (-57·6 to -20·5%)
**Childhood undernutrition: All causes**	1·0 (0·8 to 1·3)	0·0% (0·0 to 0·0%)	0·6 (0·5 to 0·8)	0·0% (0·0 to 0·0%)	-38·1% (-46·3 to -29·1%)	-45·9% (-53·0 to -38·0%)	91·5 (71·5 to 109·9)	0·0% (0·0 to 0·0%)	57·3 (42·6 to 71·4)	0·0% (0·0 to 0·0%)	-37·4% (-45·5 to -28·4%)	-41·2% (-48·9 to -32·8%)
**Childhood underweight: All causes**	0·3 (0·2 to 0·4)	0·0% (0·0 to 0·0%)	0·2 (0·1 to 0·2)	0·0% (0·0 to 0·0%)	-44·3% (-53·5 to -34·1%)	-51·3% (-59·4 to -42·5%)	25·1 (21·3 to 31·7)	0·0% (0·0 to 0·0%)	14·0 (11·5 to 18·1)	0·0% (0·0 to 0·0%)	-44·2% (-53·4 to -33·9%)	-47·6% (-56·2 to -38·1%)
Diarrhoeal diseases	0·0 (0·0 to 0·0)	0·1% (0·0 to 0·1%)	0·0 (0·0 to 0·0)	0·0% (0·0 to 0·0%)	-45·2% (-58·9 to -26·7%)	-62·3% (-72·6 to -49·4%)	0·8 (0·6 to 1·2)	0·3% (0·2 to 0·4%)	0·5 (0·3 to 0·7)	0·1% (0·1 to 0·2%)	-42·3% (-56·1 to -24·1%)	-49·6% (-62·2 to -33·6%)
Lower respiratory infections	0·1 (0·0 to 0·1)	0·0% (0·0 to 0·0%)	0·0 (0·0 to 0·1)	0·0% (0·0 to 0·0%)	-41·2% (-55·3 to -27·0%)	-51·6% (-62·9 to -39·7%)	4·6 (2·3 to 10·1)	0·1% (0·0 to 0·2%)	2·7 (1·2 to 6·2)	0·1% (0·0 to 0·1%)	-40·8% (-54·9 to -26·7%)	-45·7% (-58·2 to -32·8%)
Measles	0·0 (0·0 to 0·0)	0·0% (0·0 to 0·1%)	0·0 (0·0 to 0·0)	0·0% (0·0 to 0·0%)	-97·8% (-100·0 to 52·8%)	-85·1% (-100·0 to 960·0%)	0·0 (0·0 to 0·0)	0·0% (0·0 to 0·1%)	0·0 (0·0 to 0·0)	0·0% (0·0 to 0·0%)	-97·6% (-100·0 to 1,045·7%)	-83·9% (-100·0 to 7,684·5%)
Protein-energy malnutrition	0·2 (0·2 to 0·3)	1·9% (1·6 to 2·3%)	0·1 (0·1 to 0·2)	0·9% (0·7 to 1·1%)	-45·0% (-55·3 to -32·7%)	-52·1% (-61·6 to -40·5%)	19·6 (16·7 to 23·0)	12·6% (10·8 to 15·0%)	10·8 (8·9 to 12·9)	6·9% (5·7 to 8·5%)	-45·0% (-55·4 to -32·7%)	-45·6% (-55·0 to -34·2%)
**Childhood wasting: All causes**	1·0 (0·6 to 1·2)	0·0% (0·0 to 0·0%)	0·6 (0·4 to 0·8)	0·0% (0·0 to 0·0%)	-37·8% (-46·7 to -27·8%)	-45·7% (-53·4 to -36·9%)	84·2 (56·3 to 106·7)	0·0% (0·0 to 0·0%)	52·9 (33·2 to 69·3)	0·0% (0·0 to 0·0%)	-37·2% (-45·9 to -27·3%)	-41·0% (-49·2 to -31·6%)

Supplemental Results Table 1. All-age deaths, death PAFs, DALYs, DALY PAFs, and percent change for each risk factor and outcome for both sexes combined in 2005 and 2015 for High-income.

Data in parenthesis are 95% uncertainty intervals. PAFs=population attributable fractions. DALYs=disability-adjusted life-years.

	2005 Deaths (in thousands)	2005 Deaths PAF	2015 Deaths (in thousands)	2015 Deaths PAF	Percent change deaths 2005-2015	Percent change deaths PAF 2005-2015	2005 DALYs (in thousands)	2005 DALYs PAF	2015 DALYs (in thousands)	2015 DALYs PAF	Percent change DALYs 2005-2015	Percent change DALYs PAF 2005-2015
Diarrhoeal diseases	0·2 (0·1 to 0·2)	0·9% (0·5 to 1·3%)	0·1 (0·1 to 0·1)	0·4% (0·2 to 0·7%)	-36·6% (-51·1 to -19·0%)	-56·5% (-67·2 to -43·4%)	14·4 (8·0 to 18·5)	4·9% (2·7 to 6·7%)	9·5 (5·1 to 12·8)	2·8% (1·4 to 4·3%)	-33·9% (-48·2 to -17·1%)	-42·3% (-55·6 to -26·6%)
Lower respiratory infections	0·6 (0·3 to 0·8)	0·1% (0·1 to 0·2%)	0·4 (0·2 to 0·6)	0·1% (0·0 to 0·1%)	-35·3% (-47·0 to -21·9%)	-46·8% (-56·7 to -35·8%)	50·2 (22·6 to 71·5)	1·1% (0·5 to 1·5%)	32·6 (13·3 to 47·9)	0·6% (0·3 to 0·9%)	-35·0% (-46·7 to -21·6%)	-40·4% (-51·3 to -28·4%)
Measles	0·0 (0·0 to 0·0)	0·0% (0·0 to 0·3%)	0·0 (0·0 to 0·0)	0·0% (0·0 to 0·0%)	-97·8% (-100·0 to 80·0%)	-85·3% (-100·0 to 1,076·9%)	0·0 (0·0 to 0·0)	0·0% (0·0 to 0·3%)	0·0 (0·0 to 0·0)	0·0% (0·0 to 0·0%)	-97·6% (-100·0 to 2,811·6%)	-83·8% (-100·0 to 18,051·9%)
Protein-energy malnutrition	0·2 (0·2 to 0·3)	1·9% (1·6 to 2·3%)	0·1 (0·1 to 0·2)	0·9% (0·7 to 1·1%)	-45·0% (-55·3 to -32·7%)	-52·1% (-61·6 to -40·5%)	19·6 (16·7 to 23·0)	12·6% (10·8 to 15·0%)	10·8 (8·9 to 12·9)	6·9% (5·7 to 8·5%)	-45·0% (-55·4 to -32·7%)	-45·6% (-55·0 to -34·2%)
**Childhood stunting: All causes**	0·1 (0·0 to 0·3)	0·0% (0·0 to 0·0%)	0·1 (0·0 to 0·2)	0·0% (0·0 to 0·0%)	-43·4% (-58·2 to -26·2%)	-50·5% (-63·4 to -35·4%)	10·1 (2·3 to 27·7)	0·0% (0·0 to 0·0%)	5·8 (1·2 to 15·8)	0·0% (0·0 to 0·0%)	-42·6% (-57·4 to -25·4%)	-46·2% (-60·1 to -30·1%)
Diarrhoeal diseases	0·0 (0·0 to 0·0)	0·1% (0·0 to 0·2%)	0·0 (0·0 to 0·0)	0·0% (0·0 to 0·1%)	-44·9% (-59·8 to -22·4%)	-62·1% (-73·3 to -46·7%)	1·6 (0·5 to 3·3)	0·5% (0·2 to 1·1%)	0·9 (0·3 to 1·9)	0·3% (0·1 to 0·6%)	-42·1% (-56·9 to -20·3%)	-49·4% (-63·0 to -30·1%)
Lower respiratory infections	0·1 (0·0 to 0·3)	0·0% (0·0 to 0·1%)	0·1 (0·0 to 0·2)	0·0% (0·0 to 0·0%)	-43·1% (-59·4 to -7·7%)	-53·2% (-67·2 to -24·0%)	8·5 (0·3 to 26·1)	0·2% (0·0 to 0·6%)	4·9 (0·1 to 15·2)	0·1% (0·0 to 0·3%)	-42·7% (-59·1 to -7·2%)	-47·4% (-63·1 to -14·8%)
Measles	0·0 (0·0 to 0·0)	0·0% (0·0 to 0·1%)	0·0 (0·0 to 0·0)	0·0% (0·0 to 0·0%)	-97·5% (-100·0 to 56·5%)	-83·5% (-100·0 to 1,034·3%)	0·0 (0·0 to 0·0)	0·0% (0·0 to 0·1%)	0·0 (0·0 to 0·0)	0·0% (0·0 to 0·0%)	-97·3% (-100·0 to 1,730·3%)	-82·3% (-99·9 to 12,538·2%)
**Iron deficiency: All causes**	0·3 (0·2 to 0·4)	0·0% (0·0 to 0·0%)	0·4 (0·2 to 0·5)	0·0% (0·0 to 0·0%)	15·2% (-3·6 to 31·6%)	0·6% (-15·7 to 15·1%)	3,476·9 (2,305·0 to 5,079·9)	1·3% (1·0 to 1·8%)	3,616·2 (2,386·1 to 5,278·2)	1·3% (1·0 to 1·7%)	4·0% (2·4 to 5·5%)	-2·4% (-4·0 to -0·9%)
Maternal haemorrhage	0·0 (0·0 to 0·1)	18·1% (6·5 to 31·4%)	0·0 (0·0 to 0·1)	18·1% (6·5 to 31·2%)	-18·2% (-36·1 to 7·0%)	0·2% (-3·5 to 4·2%)	4·6 (1·6 to 8·5)	18·0% (6·4 to 31·7%)	3·9 (1·3 to 7·2)	18·3% (6·5 to 31·9%)	-14·1% (-24·6 to -1·9%)	1·7% (-1·4 to 4·8%)
Maternal sepsis and other pregnancy related infections	0·0 (0·0 to 0·1)	17·5% (6·3 to 30·5%)	0·0 (0·0 to 0·1)	17·7% (6·3 to 30·4%)	2·3% (-20·6 to 32·8%)	0·7% (-4·9 to 6·9%)	2·3 (0·8 to 4·2)	17·8% (6·4 to 31·0%)	2·3 (0·8 to 4·3)	18·0% (6·5 to 31·3%)	0·8% (-22·0 to 31·0%)	1·2% (-3·0 to 6·0%)
Iron-deficiency anaemia	0·2 (0·1 to 0·3)	100·0% (100·0 to 100·0%)	0·3 (0·1 to 0·4)	100·0% (100·0 to 100·0%)	21·6% (0·8 to 40·7%)	-	3,470·1 (2,300·4 to 5,072·2)	100·0% (100·0 to 100·0%)	3,609·9 (2,381·3 to 5,268·1)	100·0% (100·0 to 100·0%)	4·0% (2·4 to 5·5%)	-
**Vitamin A deficiency: All causes**	0·0 (0·0 to 0·0)	0·0% (0·0 to 0·0%)	0·0 (0·0 to 0·0)	0·0% (0·0 to 0·0%)	-46·0% (-52·9 to -39·0%)	-52·9% (-58·8 to -46·6%)	0·5 (0·3 to 0·8)	0·0% (0·0 to 0·0%)	0·3 (0·2 to 0·5)	0·0% (0·0 to 0·0%)	-43·2% (-49·1 to -37·0%)	-46·7% (-52·2 to -40·8%)
Diarrhoeal diseases	0·0 (0·0 to 0·0)	0·0% (0·0 to 0·1%)	0·0 (0·0 to 0·0)	0·0% (0·0 to 0·0%)	-42·4% (-48·7 to -35·0%)	-60·4% (-66·2 to -52·2%)	0·5 (0·2 to 0·8)	0·2% (0·1 to 0·3%)	0·3 (0·1 to 0·5)	0·1% (0·0 to 0·2%)	-39·7% (-45·8 to -33·2%)	-47·3% (-53·1 to -37·4%)
Measles	0·0 (0·0 to 0·0)	1·3% (0·7 to 2·1%)	0·0 (0·0 to 0·0)	1·1% (0·6 to 1·7%)	-87·8% (-90·1 to -84·7%)	-20·0% (-26·1 to -14·3%)	0·0 (0·0 to 0·1)	1·3% (0·7 to 2·2%)	0·0 (0·0 to 0·0)	1·1% (0·6 to 1·7%)	-87·6% (-89·8 to -84·6%)	-20·0% (-26·0 to -14·4%)
Vitamin A deficiency	-	-	-	-	-	-	-	-	-	-	-	-
**Zinc deficiency: All causes**	0·0 (0·0 to 0·1)	0·0% (0·0 to 0·0%)	0·0 (0·0 to 0·1)	0·0% (0·0 to 0·0%)	-37·1% (-42·2 to -24·7%)	-45·0% (-49·5 to -34·1%)	3·9 (0·5 to 11·8)	0·0% (0·0 to 0·0%)	2·6 (0·4 to 7·6)	0·0% (0·0 to 0·0%)	-34·1% (-40·2 to -12·6%)	-38·2% (-43·7 to -17·9%)
Diarrhoeal diseases	0·0 (0·0 to 0·0)	0·0% (0·0 to 0·1%)	0·0 (0·0 to 0·0)	0·0% (0·0 to 0·1%)	-30·3% (-36·6 to 0·0%)	-52·2% (-58·1 to 0·0%)	0·9 (0·2 to 1·9)	0·3% (0·1 to 0·7%)	0·7 (0·2 to 1·4)	0·2% (0·1 to 0·4%)	-23·9% (-31·3 to -6·3%)	-33·6% (-41·4 to -14·9%)
Lower respiratory infections	0·0 (0·0 to 0·1)	0·0% (0·0 to 0·0%)	0·0 (0·0 to 0·1)	0·0% (0·0 to 0·0%)	-38·6% (-43·8 to 0·0%)	-49·4% (-53·9 to 0·0%)	3·0 (0·1 to 11·2)	0·1% (0·0 to 0·2%)	1·9 (0·1 to 7·0)	0·0% (0·0 to 0·1%)	-37·0% (-41·9 to -4·6%)	-42·2% (-47·1 to -12·5%)
**Tobacco smoke: All causes**	1,404·3 (1,343·3 to 1,460·8)	16·7% (15·9 to 17·3%)	1,481·3 (1,411·8 to 1,547·9)	15·3% (14·6 to 16·0%)	5·5% (3·7 to 7·3%)	-7·8% (-9·5 to -6·3%)	27,743·6 (26,418·0 to 29,040·6)	10·8% (9·7 to 12·1%)	27,256·6 (25,932·1 to 28,568·3)	10·0% (8·8 to 11·2%)	-1·8% (-3·1 to -0·3%)	-7·8% (-9·1 to -6·4%)
**Smoking: All causes**	1,362·6 (1,302·6 to 1,418·0)	16·2% (15·4 to 16·8%)	1,436·6 (1,369·3 to 1,503·7)	14·9% (14·2 to 15·6%)	5·4% (3·5 to 7·3%)	-7·9% (-9·6 to -6·3%)	27,198·1 (25,868·6 to 28,523·1)	10·6% (9·5 to 11·9%)	26,720·9 (25,407·1 to 28,054·5)	9·8% (8·7 to 11·0%)	-1·8% (-3·1 to -0·3%)	-7·8% (-9·1 to -6·4%)
Tuberculosis	1·7 (0·9 to 2·5)	10·9% (5·9 to 16·2%)	1·0 (0·6 to 1·6)	8·0% (4·3 to 11·9%)	-37·1% (-41·0 to -33·2%)	-26·8% (-30·2 to -23·6%)	37·6 (20·4 to 55·1)	12·8% (6·9 to 18·8%)	22·0 (11·8 to 32·8)	10·1% (5·4 to 15·0%)	-41·5% (-44·6 to -38·2%)	-21·1% (-24·1 to -18·4%)
Lower respiratory infections	88·3 (70·7 to 107·7)	22·1% (17·8 to 27·0%)	88·4 (69·4 to 110·0)	18·2% (14·3 to 22·5%)	0·1% (-3·8 to 3·9%)	-17·6% (-20·2 to -15·3%)	1,195·4 (971·9 to 1,435·8)	25·5% (20·7 to 30·6%)	1,126·9 (896·1 to 1,381·7)	22·1% (17·6 to 26·9%)	-5·7% (-9·1 to -2·2%)	-13·5% (-15·7 to -11·5%)
Lip and oral cavity cancer	14·0 (12·4 to 15·4)	60·4% (53·9 to 66·4%)	15·4 (13·4 to 17·2)	57·4% (50·5 to 63·8%)	10·2% (5·3 to 15·7%)	-5·1% (-6·9 to -3·4%)	326·8 (290·3 to 358·9)	62·2% (55·8 to 68·0%)	333·8 (291·6 to 371·8)	58·7% (51·9 to 64·9%)	2·1% (-2·8 to 7·6%)	-5·6% (-7·5 to -3·9%)
Nasopharynx cancer	3·6 (3·2 to 4·0)	61·3% (53·7 to 67·9%)	4·0 (3·4 to 4·5)	58·5% (50·2 to 65·3%)	9·4% (3·2 to 15·7%)	-4·5% (-6·5 to -2·8%)	85·8 (75·0 to 95·1)	59·2% (51·8 to 65·4%)	87·1 (75·1 to 98·5)	56·3% (48·5 to 62·8%)	1·5% (-4·4 to 7·4%)	-4·8% (-6·9 to -2·7%)
Oesophageal cancer	36·2 (29·8 to 41·9)	58·0% (47·9 to 67·2%)	40·1 (32·5 to 47·2)	55·3% (44·7 to 64·9%)	10·8% (5·8 to 15·4%)	-4·7% (-7·1 to -2·8%)	742·5 (612·9 to 856·8)	59·0% (48·8 to 68·2%)	769·6 (621·0 to 909·8)	55·7% (45·2 to 65·3%)	3·7% (-1·2 to 8·3%)	-5·6% (-8·0 to -3·7%)
Stomach cancer	22·7 (14·0 to 31·7)	14·4% (8·9 to 20·0%)	22·4 (13·9 to 31·5)	13·3% (8·1 to 18·6%)	-1·6% (-6·0 to 2·3%)	-7·6% (-11·3 to -4·2%)	413·0 (256·5 to 569·8)	14·8% (9·2 to 20·4%)	362·2 (222·9 to 508·4)	13·4% (8·2 to 18·7%)	-12·3% (-15·9 to -8·9%)	-9·6% (-12·9 to -6·8%)
Colon and rectum cancer	23·6 (17·1 to 29·7)	8·7% (6·4 to 11·0%)	25·4 (18·2 to 32·3)	8·0% (5·8 to 10·2%)	7·6% (3·2 to 12·1%)	-8·5% (-11·6 to -5·5%)	443·6 (321·0 to 561·3)	9·3% (6·8 to 11·8%)	438·8 (316·4 to 561·1)	8·3% (6·0 to 10·5%)	-1·1% (-4·5 to 2·4%)	-11·1% (-13·6 to -8·7%)
Liver cancer due to hepatitis B	3·5 (1·9 to 5·1)	20·5% (11·5 to 29·6%)	4·0 (2·2 to 5·8)	18·7% (10·3 to 27·2%)	13·5% (5·3 to 21·3%)	-8·8% (-13·2 to -4·7%)	83·7 (45·4 to 124·2)	19·9% (11·0 to 29·0%)	85·5 (47·0 to 127·0)	17·7% (9·8 to 26·3%)	2·2% (-7·0 to 10·5%)	-10·7% (-16·5 to -5·3%)
Liver cancer due to hepatitis C	8·8 (5·2 to 12·5)	17·9% (10·5 to 25·4%)	10·8 (6·5 to 15·4)	17·4% (10·1 to 24·8%)	22·8% (16·2 to 29·4%)	-2·7% (-6·6 to 1·2%)	155·5 (90·9 to 223·7)	18·8% (10·9 to 26·7%)	174·6 (102·3 to 252·7)	18·2% (10·6 to 25·9%)	12·3% (6·2 to 17·7%)	-3·1% (-6·5 to 0·1%)
Liver cancer due to alcohol use	8·4 (4·8 to 12·1)	26·7% (15·3 to 37·9%)	9·2 (5·1 to 13·4)	24·7% (13·9 to 35·3%)	9·1% (3·6 to 14·0%)	-7·4% (-10·5 to -4·9%)	176·9 (100·2 to 251·9)	27·6% (15·8 to 39·0%)	185·9 (102·9 to 269·6)	25·4% (14·3 to 36·2%)	5·1% (-0·6 to 10·2%)	-8·1% (-11·3 to -5·6%)
Liver cancer due to other causes	2·4 (1·5 to 3·2)	22·6% (14·3 to 30·4%)	3·1 (1·9 to 4·3)	21·6% (13·3 to 29·4%)	29·5% (23·5 to 35·2%)	-4·4% (-7·6 to -1·7%)	47·7 (29·3 to 64·8)	23·7% (14·8 to 32·0%)	57·5 (34·4 to 79·1)	22·4% (13·8 to 30·7%)	20·6% (14·6 to 26·2%)	-5·3% (-8·4 to -2·6%)
Pancreatic cancer	33·9 (28·1 to 39·7)	24·7% (20·5 to 29·0%)	39·9 (32·7 to 47·2)	22·8% (18·7 to 26·8%)	17·4% (13·5 to 21·5%)	-7·6% (-10·2 to -5·3%)	640·3 (530·5 to 751·7)	26·4% (21·9 to 31·0%)	695·3 (570·3 to 819·8)	23·9% (19·6 to 28·2%)	8·6% (5·1 to 12·3%)	-9·2% (-11·4 to -7·2%)
Tracheal, bronchus, and lung cancer	409·9 (398·3 to 420·1)	81·5% (79·5 to 83·3%)	456·5 (438·8 to 474·0)	79·8% (77·6 to 81·8%)	11·4% (8·3 to 14·4%)	-2·2% (-2·9 to -1·6%)	7,878·3 (7,674·9 to 8,066·4)	82·0% (80·2 to 83·7%)	8,248·1 (7,965·3 to 8,541·0)	79·9% (78·0 to 81·8%)	4·7% (1·9 to 7·4%)	-2·5% (-3·1 to -1·8%)
Cervical cancer	3·1 (1·1 to 5·1)	11·4% (4·1 to 18·7%)	3·2 (1·2 to 5·4)	11·2% (4·0 to 18·5%)	5·7% (-1·0 to 13·4%)	-1·6% (-6·7 to 2·9%)	80·0 (29·0 to 133·5)	11·9% (4·3 to 19·7%)	75·9 (26·8 to 126·5)	11·2% (3·9 to 18·6%)	-5·1% (-11·6 to 2·0%)	-5·7% (-11·3 to -0·1%)
Kidney cancer	10·5 (7·1 to 13·6)	22·5% (15·3 to 29·0%)	12·1 (8·1 to 15·9)	20·5% (13·7 to 26·8%)	15·4% (9·8 to 21·5%)	-8·9% (-12·1 to -6·1%)	224·4 (152·9 to 291·9)	23·6% (16·1 to 30·2%)	242·3 (162·1 to 320·3)	21·1% (14·2 to 27·5%)	8·0% (2·9 to 13·3%)	-10·4% (-13·4 to -7·9%)
Bladder cancer	22·7 (17·4 to 27·6)	36·9% (28·4 to 45·2%)	26·7 (20·2 to 33·1)	34·8% (26·3 to 43·1%)	17·5% (11·7 to 23·4%)	-5·8% (-8·7 to -3·1%)	373·8 (290·3 to 450·4)	39·2% (30·0 to 47·6%)	397·2 (302·3 to 492·8)	36·1% (27·4 to 44·4%)	6·2% (1·3 to 11·2%)	-7·8% (-10·5 to -5·5%)
Ischaemic heart disease	197·2 (165·5 to 230·1)	12·7% (10·6 to 14·7%)	169·9 (142·2 to 199·4)	10·3% (8·6 to 12·1%)	-13·9% (-16·0 to -11·7%)	-18·6% (-20·3 to -16·9%)	4,358·1 (3,650·2 to 5,087·2)	20·1% (16·8 to 23·5%)	3,664·8 (3,040·8 to 4,303·9)	17·2% (14·3 to 20·2%)	-15·9% (-17·5 to -14·4%)	-14·5% (-15·7 to -13·3%)
Ischaemic stroke	44·9 (37·8 to 52·3)	9·4% (7·9 to 11·0%)	36·3 (30·5 to 42·5)	7·3% (6·1 to 8·5%)	-19·2% (-22·1 to -16·0%)	-22·3% (-24·8 to -19·8%)	768·2 (644·3 to 908·1)	14·7% (12·3 to 17·1%)	610·5 (507·3 to 728·8)	12·1% (10·1 to 14·3%)	-20·5% (-22·9 to -17·9%)	-17·7% (-19·3 to -16·0%)
Hemorrhagic stroke	48·5 (41·1 to 56·0)	14·8% (12·5 to 17·2%)	39·8 (33·6 to 46·4)	11·5% (9·7 to 13·4%)	-18·0% (-20·7 to -15·0%)	-22·2% (-24·0 to -20·4%)	1,219·2 (1,038·2 to 1,408·8)	23·3% (19·9 to 26·9%)	958·7 (810·9 to 1,118·8)	19·5% (16·4 to 22·7%)	-21·4% (-23·4 to -19·2%)	-16·2% (-17·7 to -14·8%)
Hypertensive heart disease	10·1 (7·5 to 12·8)	9·4% (6·9 to 11·9%)	10·5 (7·6 to 13·5)	7·7% (5·6 to 9·7%)	4·1% (-1·9 to 9·7%)	-18·2% (-22·1 to -14·1%)	232·4 (173·4 to 287·3)	16·4% (12·3 to 20·2%)	229·2 (167·4 to 286·2)	13·9% (10·2 to 17·3%)	-1·4% (-6·5 to 2·4%)	-15·6% (-18·1 to -13·3%)
Atrial fibrillation and flutter	4·5 (3·2 to 5·9)	6·7% (4·9 to 8·6%)	4·9 (3·4 to 6·5)	5·5% (4·0 to 7·2%)	9·3% (4·3 to 14·6%)	-18·0% (-21·4 to -14·9%)	203·8 (141·1 to 280·3)	13·8% (10·4 to 17·1%)	214·4 (147·0 to 297·7)	11·8% (8·7 to 14·7%)	5·2% (2·8 to 7·4%)	-14·7% (-16·4 to -13·2%)
Aortic aneurysm	8·7 (6·7 to 10·6)	13·0% (10·0 to 15·8%)	7·8 (5·9 to 9·5)	10·1% (7·6 to 12·4%)	-10·8% (-14·3 to -7·6%)	-22·0% (-24·1 to -20·2%)	182·4 (140·8 to 219·6)	19·2% (14·9 to 23·2%)	159·2 (121·4 to 195·0)	16·1% (12·4 to 19·7%)	-12·7% (-15·9 to -9·8%)	-16·1% (-17·9 to -14·6%)

Supplemental Results Table 1. All-age deaths, death PAFs, DALYs, DALY PAFs, and percent change for each risk factor and outcome for both sexes combined in 2005 and 2015 for High-income.

Data in parenthesis are 95% uncertainty intervals. PAFs=population attributable fractions. DALYs=disability-adjusted life-years.

	2005 Deaths (in thousands)	2005 Deaths PAF	2015 Deaths (in thousands)	2015 Deaths PAF	Percent change deaths 2005-2015	Percent change deaths PAF 2005-2015	2005 DALYs (in thousands)	2005 DALYs PAF	2015 DALYs (in thousands)	2015 DALYs PAF	Percent change DALYs 2005-2015	Percent change DALYs PAF 2005-2015
Peripheral vascular disease	2·1 (1·5 to 2·6)	8·8% (6·5 to 10·9%)	2·2 (1·6 to 2·8)	7·2% (5·2 to 9·1%)	7·1% (0·1 to 15·2%)	-17·8% (-21·8 to -13·8%)	52·9 (36·8 to 71·6)	13·6% (10·1 to 17·1%)	55·8 (38·6 to 76·0)	11·6% (8·4 to 14·6%)	5·5% (0·8 to 10·6%)	-14·1% (-17·0 to -11·7%)
Other cardiovascular and circulatory diseases	15·3 (11·4 to 19·2)	10·5% (7·8 to 13·1%)	14·4 (10·5 to 18·3)	8·3% (6·0 to 10·5%)	-5·9% (-9·9 to -2·1%)	-20·6% (-23·7 to -17·7%)	522·9 (389·8 to 664·8)	17·4% (13·0 to 21·3%)	485·9 (354·8 to 622·6)	14·8% (10·9 to 18·3%)	-7·1% (-9·9 to -4·7%)	-14·9% (-16·9 to -13·3%)
Chronic obstructive pulmonary disease	278·3 (262·0 to 295·8)	72·6% (68·7 to 76·1%)	328·2 (305·7 to 350·1)	72·5% (68·4 to 76·4%)	18·0% (15·2 to 20·7%)	-0·1% (-1·7 to 1·5%)	4,659·6 (4,383·3 to 4,930·6)	71·7% (68·3 to 74·7%)	5,092·8 (4,759·6 to 5,416·2)	70·9% (67·4 to 74·3%)	9·3% (7·2 to 11·4%)	-1·0% (-2·1 to 0·0%)
Silicosis	0·6 (0·4 to 0·7)	23·1% (17·5 to 29·5%)	0·5 (0·3 to 0·7)	21·7% (16·0 to 28·0%)	-17·1% (-29·7 to -2·4%)	-6·3% (-13·1 to 0·5%)	9·6 (7·3 to 12·5)	23·2% (18·1 to 29·0%)	7·1 (5·2 to 9·6)	20·7% (15·8 to 25·9%)	-26·1% (-33·6 to -18·3%)	-10·9% (-14·8 to -6·8%)
Asbestosis	0·4 (0·2 to 0·5)	25·2% (19·5 to 31·5%)	0·4 (0·3 to 0·6)	22·6% (17·2 to 28·6%)	16·2% (3·7 to 29·4%)	-10·5% (-14·7 to -6·2%)	7·2 (5·0 to 9·3)	25·7% (20·5 to 31·5%)	7·5 (5·1 to 9·8)	22·8% (17·8 to 28·3%)	4·3% (-3·1 to 11·9%)	-11·5% (-14·7 to -8·1%)
Coal workers pneumoconiosis	0·3 (0·2 to 0·3)	29·0% (22·1 to 36·0%)	0·2 (0·1 to 0·3)	25·8% (19·4 to 32·7%)	-26·9% (-35·3 to -17·9%)	-10·8% (-15·4 to -6·4%)	4·3 (3·3 to 5·5)	26·4% (20·5 to 32·7%)	3·3 (2·4 to 4·4)	22·2% (17·0 to 28·0%)	-23·1% (-30·4 to -16·4%)	-15·7% (-19·0 to -12·2%)
Other pneumoconiosis	0·7 (0·6 to 0·9)	18·2% (14·6 to 22·2%)	0·8 (0·6 to 1·0)	17·1% (13·6 to 20·9%)	8·8% (0·4 to 17·5%)	-6·2% (-10·4 to -1·8%)	12·1 (9·4 to 15·3)	16·5% (13·2 to 20·0%)	11·1 (8·5 to 14·2)	15·1% (12·1 to 18·3%)	-7·9% (-13·1 to -2·5%)	-8·6% (-12·2 to -4·8%)
Asthma	2·8 (2·4 to 3·3)	13·3% (11·1 to 15·5%)	1·9 (1·6 to 2·3)	10·1% (8·4 to 11·8%)	-32·1% (-36·7 to -27·0%)	-24·1% (-27·7 to -20·5%)	352·4 (241·0 to 482·9)	11·3% (9·5 to 13·0%)	302·4 (203·6 to 418·9)	10·1% (8·5 to 11·7%)	-14·2% (-16·9 to -11·8%)	-10·1% (-12·0 to -8·3%)
Interstitial lung disease and pulmonary sarcoidosis	9·2 (5·8 to 11·5)	21·1% (17·5 to 24·8%)	11·9 (7·3 to 15·4)	19·6% (16·0 to 23·3%)	29·7% (17·8 to 38·5%)	-7·4% (-10·4 to -4·4%)	155·0 (109·1 to 189·5)	21·4% (17·9 to 25·1%)	179·2 (122·7 to 223·5)	19·5% (16·2 to 23·0%)	15·6% (7·6 to 22·0%)	-8·9% (-11·5 to -6·5%)
Other chronic respiratory diseases	1·1 (0·9 to 1·3)	21·2% (17·2 to 25·0%)	1·3 (1·0 to 1·5)	20·7% (16·8 to 24·7%)	14·8% (8·3 to 21·4%)	-2·2% (-5·9 to 1·4%)	34·5 (28·2 to 40·6)	17·0% (14·1 to 19·9%)	34·8 (28·2 to 41·1)	17·1% (14·0 to 20·1%)	1·0% (-4·0 to 6·1%)	0·3% (-3·2 to 4·0%)
Diabetes mellitus	8·6 (2·8 to 15·1)	4·2% (1·4 to 7·3%)	7·6 (2·3 to 13·5)	3·5% (1·1 to 6·3%)	-11·9% (-16·4 to -8·0%)	-16·5% (-20·0 to -13·3%)	465·9 (141·2 to 832·4)	5·8% (1·9 to 10·1%)	448·7 (137·8 to 809·2)	4·9% (1·6 to 8·7%)	-3·7% (-7·5 to -0·6%)	-15·7% (-18·2 to -13·9%)
**Second-hand smoke: All causes**	51·0 (38·5 to 66·1)	0·6% (0·5 to 0·8%)	54·1 (40·0 to 71·1)	0·6% (0·4 to 0·7%)	6·1% (3·2 to 8·7%)	-7·3% (-9·9 to -5·0%)	720·0 (566·4 to 891·1)	0·3% (0·2 to 0·4%)	701·2 (548·1 to 874·7)	0·3% (0·2 to 0·3%)	-2·6% (-4·7 to -0·7%)	-8·6% (-10·5 to -6·7%)
Lower respiratory infections	10·1 (6·4 to 14·7)	2·5% (1·6 to 3·7%)	11·7 (7·3 to 17·1)	2·4% (1·5 to 3·5%)	16·2% (11·1 to 21·2%)	-4·4% (-7·7 to -1·0%)	133·8 (86·3 to 193·9)	2·9% (1·8 to 4·2%)	129·7 (82·0 to 190·1)	2·5% (1·6 to 3·7%)	-3·0% (-6·8 to 0·9%)	-11·0% (-14·1 to -8·1%)
Otitis media	0·0 (0·0 to 0·0)	3·7% (2·5 to 4·8%)	0·0 (0·0 to 0·0)	2·8% (1·9 to 3·8%)	-23·0% (-30·7 to -14·3%)	-22·7% (-28·3 to -16·8%)	9·6 (5·2 to 15·9)	3·4% (2·2 to 4·7%)	8·5 (4·6 to 14·0)	2·9% (1·9 to 4·0%)	-12·2% (-14·9 to -9·6%)	-14·7% (-17·0 to -12·4%)
Tracheal, bronchus, and lung cancer	1·9 (1·1 to 2·9)	0·4% (0·2 to 0·6%)	2·1 (1·2 to 3·4)	0·4% (0·2 to 0·6%)	14·0% (11·5 to 16·6%)	0·2% (-3·1 to 3·2%)	34·5 (20·3 to 54·4)	0·4% (0·2 to 0·6%)	37·7 (22·1 to 60·0)	0·4% (0·2 to 0·6%)	9·2% (6·4 to 11·5%)	1·8% (-1·6 to 4·8%)
Ischaemic heart disease	27·5 (18·7 to 38·7)	1·8% (1·2 to 2·5%)	28·7 (19·1 to 41·0)	1·7% (1·2 to 2·5%)	4·3% (-0·2 to 7·9%)	-1·4% (-5·5 to 1·5%)	385·8 (289·0 to 500·8)	1·8% (1·3 to 2·3%)	377·5 (281·6 to 490·8)	1·8% (1·3 to 2·3%)	-2·2% (-5·1 to 0·6%)	-0·6% (-3·2 to 1·9%)
Ischaemic stroke	4·9 (3·0 to 7·0)	1·0% (0·6 to 1·5%)	4·8 (2·9 to 7·0)	1·0% (0·6 to 1·4%)	-1·3% (-6·7 to 3·1%)	-5·2% (-10·0 to -1·5%)	50·6 (36·7 to 67·9)	1·0% (0·7 to 1·3%)	46·5 (32·6 to 63·5)	0·9% (0·7 to 1·3%)	-8·1% (-12·1 to -4·1%)	-4·8% (-8·7 to -1·2%)
Hemorrhagic stroke	3·6 (2·5 to 4·8)	1·1% (0·8 to 1·5%)	3·5 (2·4 to 4·8)	1·0% (0·7 to 1·4%)	-2·2% (-8·0 to 3·2%)	-7·1% (-12·0 to -2·8%)	59·7 (45·6 to 75·7)	1·1% (0·9 to 1·5%)	53·1 (39·9 to 68·4)	1·1% (0·8 to 1·4%)	-11·1% (-14·4 to -7·6%)	-5·2% (-8·3 to -2·2%)
**Alcohol and drug use: All causes**	354·0 (320·9 to 384·3)	4·2% (3·8 to 4·6%)	390·4 (349·4 to 428·5)	4·0% (3·6 to 4·4%)	10·3% (6·3 to 13·8%)	-3·7% (-7·0 to -0·6%)	15,630·4 (14,465·6 to 16,847·3)	6·1% (5·6 to 6·6%)	16,139·0 (14,886·1 to 17,470·6)	5·9% (5·3 to 6·4%)	3·3% (1·5 to 4·8%)	-3·1% (-4·8 to -1·5%)
**Alcohol use: All causes**	263·5 (233·5 to 293·8)	3·1% (2·8 to 3·5%)	270·5 (229·7 to 308·2)	2·8% (2·4 to 3·2%)	2·7% (-3·1 to 8·0%)	-10·3% (-15·3 to -5·7%)	10,631·5 (9,781·9 to 11,538·7)	4·2% (3·8 to 4·6%)	9,938·7 (9,026·5 to 10,942·4)	3·6% (3·2 to 4·1%)	-6·5% (-9·3 to -4·0%)	-12·2% (-15·0 to -9·8%)
Tuberculosis	2·0 (1·7 to 2·5)	13·3% (10·8 to 16·1%)	1·5 (1·1 to 1·9)	11·6% (8·7 to 14·7%)	-25·5% (-34·8 to -16·1%)	-13·3% (-23·8 to -3·0%)	51·8 (44·9 to 58·4)	17·6% (15·3 to 19·8%)	35·9 (29·4 to 42·1)	16·5% (13·5 to 19·2%)	-30·7% (-37·8 to -24·5%)	-6·7% (-15·5 to 0·7%)
Lower respiratory infections	23·2 (20·0 to 26·6)	5·8% (5·0 to 6·7%)	27·1 (22·8 to 31·6)	5·6% (4·7 to 6·5%)	16·8% (8·1 to 26·3%)	-3·9% (-10·9 to 3·4%)	308·5 (276·9 to 340·4)	6·6% (5·9 to 7·3%)	330·2 (285·1 to 373·3)	6·5% (5·6 to 7·3%)	7·0% (-0·7 to 14·7%)	-1·8% (-7·9 to 4·6%)
Lip and oral cavity cancer	8·3 (7·7 to 8·8)	35·8% (33·2 to 38·1%)	8·9 (7·8 to 9·9)	33·2% (29·5 to 36·4%)	7·9% (0·9 to 14·5%)	-7·1% (-11·9 to -3·0%)	211·8 (197·5 to 224·5)	40·3% (37·7 to 42·5%)	217·7 (193·2 to 238·7)	38·3% (34·4 to 41·4%)	2·8% (-3·8 to 9·0%)	-5·0% (-9·6 to -1·6%)
Nasopharynx cancer	2·3 (2·1 to 2·4)	38·5% (35·6 to 41·0%)	2·5 (2·2 to 2·8)	36·9% (32·8 to 40·4%)	10·0% (2·1 to 17·3%)	-4·0% (-8·6 to -0·5%)	58·7 (54·7 to 62·6)	40·5% (37·8 to 42·7%)	61·3 (54·2 to 67·6)	39·6% (35·6 to 42·8%)	4·3% (-3·2 to 10·9%)	-2·3% (-6·6 to 0·8%)
Other pharynx cancer	5·5 (5·1 to 5·9)	40·8% (38·1 to 43·1%)	6·3 (5·6 to 7·0)	38·7% (34·9 to 41·9%)	14·6% (6·8 to 22·7%)	-5·1% (-9·7 to -1·5%)	149·5 (139·5 to 159·2)	43·6% (41·1 to 45·8%)	165·8 (148·1 to 183·2)	41·8% (37·9 to 45·0%)	10·9% (3·3 to 18·8%)	-4·1% (-8·5 to -0·8%)
Oesophageal cancer	13·2 (11·9 to 14·5)	21·2% (19·0 to 23·2%)	14·6 (12·6 to 16·6)	20·1% (17·3 to 22·6%)	10·4% (3·0 to 17·3%)	-5·0% (-10·1 to -1·0%)	300·8 (272·0 to 325·7)	23·9% (21·7 to 25·9%)	317·8 (275·7 to 357·8)	23·0% (20·0 to 25·5%)	5·7% (-1·4 to 12·2%)	-3·8% (-8·8 to 0·0%)
Colon and rectum cancer	12·0 (10·8 to 13·2)	4·5% (4·0 to 4·9%)	13·5 (11·7 to 15·2)	4·3% (3·7 to 4·8%)	12·2% (6·1 to 18·3%)	-4·6% (-9·6 to -0·2%)	236·7 (216·1 to 257·8)	5·0% (4·5 to 5·4%)	253·8 (224·1 to 283·0)	4·8% (4·2 to 5·3%)	7·2% (1·6 to 12·5%)	-3·6% (-8·5 to 0·5%)
Liver cancer due to hepatitis B	0·1 (0·0 to 0·1)	0·4% (0·2 to 0·7%)	0·1 (0·0 to 0·1)	0·3% (0·2 to 0·5%)	-8·9% (-41·3 to 46·2%)	-26·8% (-52·6 to 15·2%)	3·5 (2·3 to 5·4)	0·8% (0·5 to 1·3%)	3·2 (2·0 to 5·2)	0·7% (0·4 to 1·0%)	-9·2% (-38·0 to 35·9%)	-20·6% (-46·0 to 18·5%)
Liver cancer due to hepatitis C	0·0 (0·0 to 0·0)	0·0% (0·0 to 0·1%)	0·0 (0·0 to 0·0)	0·0% (0·0 to 0·1%)	5·0% (-23·3 to 47·3%)	-16·8% (-39·4 to 16·3%)	0·8 (0·6 to 1·1)	0·1% (0·1 to 0·1%)	0·8 (0·5 to 1·3)	0·1% (0·1 to 0·1%)	0·5% (-20·8 to 25·6%)	-13·3% (-31·0 to 8·4%)
Liver cancer due to alcohol use	31·6 (29·4 to 33·5)	100·0% (100·0 to 100·0%)	37·2 (34·4 to 39·7)	100·0% (100·0 to 100·0%)	17·8% (13·3 to 22·0%)	-	640·2 (595·2 to 681·2)	100·0% (100·0 to 100·0%)	732·7 (674·8 to 788·0)	100·0% (100·0 to 100·0%)	14·4% (9·7 to 18·6%)	-
Liver cancer due to other causes	0·0 (0·0 to 0·0)	0·1% (0·1 to 0·2%)	0·0 (0·0 to 0·0)	0·1% (0·1 to 0·2%)	5·2% (-10·3 to 20·2%)	-22·3% (-33·2 to -11·4%)	0·9 (0·7 to 1·0)	0·4% (0·4 to 0·5%)	0·9 (0·7 to 1·1)	0·4% (0·3 to 0·5%)	4·6% (-8·8 to 17·2%)	-17·8% (-27·4 to -8·9%)
Larynx cancer	4·6 (4·2 to 5·0)	25·9% (23·8 to 27·8%)	4·2 (3·7 to 4·8)	24·3% (21·2 to 27·0%)	-7·7% (-14·9 to -0·3%)	-6·2% (-12·6 to -1·4%)	112·8 (103·7 to 120·8)	28·3% (26·1 to 30·0%)	100·2 (87·6 to 112·1)	26·9% (23·7 to 29·6%)	-11·1% (-17·9 to -4·8%)	-4·7% (-10·7 to -0·3%)
Breast cancer	14·5 (13·1 to 15·9)	9·3% (8·4 to 10·0%)	15·5 (13·4 to 17·6)	8·7% (7·5 to 9·8%)	6·8% (0·0 to 13·4%)	-5·9% (-11·1 to -1·6%)	409·4 (368·2 to 451·9)	10·4% (9·5 to 11·2%)	430·5 (367·6 to 498·0)	9·9% (8·6 to 11·1%)	5·2% (-1·7 to 11·7%)	-4·6% (-9·8 to -0·5%)
Ischaemic heart disease	-56·3 (-71·5 to -43·0)	-3·6% (-4·6 to -2·8%)	-60·5 (-77·7 to -46·2)	-3·7% (-4·7 to -2·8%)	7·4% (2·1 to 12·4%)	1·5% (-3·2 to 6·2%)	-891·0 (-1,021·1 to -764·7)	-4·1% (-4·7 to -3·5%)	-882·3 (-1,019·5 to -756·7)	-4·1% (-4·8 to -3·6%)	-1·0% (-4·3 to 2·5%)	0·7% (-2·4 to 3·8%)
Ischaemic stroke	-31·7 (-40·5 to -23·5)	-6·6% (-8·5 to -5·0%)	-33·5 (-42·8 to -24·9)	-6·7% (-8·6 to -5·0%)	5·5% (-0·1 to 11·4%)	1·4% (-3·3 to 6·4%)	-298·8 (-360·4 to -243·4)	-5·7% (-6·9 to -4·6%)	-293·3 (-354·4 to -236·9)	-5·8% (-7·0 to -4·7%)	-1·8% (-6·5 to 2·9%)	1·7% (-2·4 to 6·0%)
Hemorrhagic stroke	37·2 (32·8 to 42·0)	11·4% (10·0 to 12·8%)	35·7 (29·8 to 42·0)	10·4% (8·6 to 12·1%)	-3·9% (-11·8 to 4·0%)	-8·8% (-16·1 to -2·0%)	693·2 (629·5 to 754·4)	13·3% (12·0 to 14·4%)	608·3 (524·1 to 684·4)	12·4% (10·7 to 13·9%)	-12·2% (-18·0 to -6·8%)	-6·5% (-12·8 to -1·7%)
Hypertensive heart disease	4·1 (0·9 to 7·5)	3·8% (0·9 to 6·9%)	3·4 (-1·3 to 8·6)	2·5% (-0·9 to 6·3%)	-16·2% (-174·5 to 85·1%)	-34·2% (-158·2 to 47·6%)	131·7 (99·9 to 164·6)	9·3% (7·2 to 11·6%)	135·1 (84·9 to 183·4)	8·2% (5·2 to 11·2%)	2·6% (-23·2 to 23·8%)	-12·1% (-33·8 to 5·7%)
Atrial fibrillation and flutter	4·3 (3·5 to 5·2)	6·5% (5·6 to 7·4%)	5·5 (4·3 to 6·9)	6·2% (5·2 to 7·3%)	28·2% (18·6 to 37·6%)	-3·8% (-10·7 to 3·2%)	123·0 (96·5 to 157·4)	8·4% (7·6 to 9·1%)	145·9 (113·5 to 190·5)	8·0% (7·0 to 9·1%)	18·6% (11·2 to 25·1%)	-3·8% (-9·9 to 1·3%)
Cirrhosis and other chronic liver diseases due to hepatitis B	8·7 (7·0 to 10·3)	33·5% (28·1 to 38·2%)	9·7 (7·5 to 11·8)	33·4% (26·8 to 39·2%)	11·2% (0·9 to 20·7%)	-0·5% (-8·8 to 6·5%)	202·8 (158·5 to 246·1)	33·0% (26·9 to 37·8%)	209·1 (156·9 to 258·0)	33·2% (26·0 to 38·8%)	3·1% (-7·5 to 12·0%)	0·4% (-8·9 to 8·0%)
Cirrhosis and other chronic liver diseases due to hepatitis C	23·5 (19·6 to 27·0)	35·0% (30·6 to 39·1%)	26·3 (20·8 to 31·5)	34·6% (28·0 to 40·2%)	12·2% (0·6 to 20·6%)	-1·1% (-10·3 to 5·4%)	558·9 (455·1 to 652·4)	35·1% (30·2 to 39·1%)	599·6 (461·1 to 723·6)	35·0% (27·7 to 40·7%)	7·3% (-3·9 to 15·6%)	-0·2% (-10·0 to 6·4%)
Cirrhosis and other chronic liver diseases due to alcohol use	57·9 (51·1 to 62·1)	100·0% (100·0 to 100·0%)	58·1 (50·7 to 63·2)	100·0% (100·0 to 100·0%)	0·3% (-2·8 to 5·2%)	-	1,619·5 (1,430·8 to 1,729·6)	100·0% (100·0 to 100·0%)	1,570·8 (1,372·5 to 1,710·3)	100·0% (100·0 to 100·0%)	-3·0% (-6·2 to 1·9%)	-
Cirrhosis and other chronic liver diseases due to other causes	8·0 (6·9 to 9·1)	37·6% (33·8 to 41·3%)	9·5 (7·9 to 11·1)	37·3% (31·9 to 42·3%)	18·7% (10·1 to 25·8%)	-0·7% (-7·3 to 4·3%)	168·4 (144·5 to 192·0)	37·0% (33·0 to 40·4%)	191·7 (156·9 to 226·7)	37·1% (31·2 to 42·0%)	13·9% (4·8 to 20·9%)	0·5% (-7·0 to 5·5%)
Pancreatitis	1·8 (1·6 to 2·1)	11·4% (10·0 to 12·8%)	1·8 (1·4 to 2·1)	10·4% (8·4 to 12·4%)	-2·9% (-13·8 to 6·8%)	-8·3% (-17·5 to -1·3%)	58·1 (51·1 to 66·4)	14·4% (12·9 to 15·8%)	55·1 (45·0 to 66·1)	13·6% (11·2 to 15·8%)	-5·2% (-14·7 to 2·7%)	-5·4% (-14·2 to 0·9%)

Supplemental Results Table 1. All-age deaths, death PAFs, DALYs, DALY PAFs, and percent change for each risk factor and outcome for both sexes combined in 2005 and 2015 for High-income.

Data in parenthesis are 95% uncertainty intervals. PAFs=population attributable fractions. DALYs=disability-adjusted life-years.

	2005 Deaths (in thousands)	2005 Deaths PAF	2015 Deaths (in thousands)	2015 Deaths PAF	Percent change deaths 2005-2015	Percent change deaths PAF 2005-2015	2005 DALYs (in thousands)	2005 DALYs PAF	2015 DALYs (in thousands)	2015 DALYs PAF	Percent change DALYs 2005-2015	Percent change DALYs PAF 2005-2015
Epilepsy	2·3 (2·1 to 2·4)	20·3% (18·9 to 21·7%)	2·4 (2·1 to 2·6)	19·1% (16·8 to 21·1%)	3·4% (-3·2 to 10·2%)	-6·3% (-11·5 to -1·5%)	202·7 (161·4 to 248·6)	18·6% (17·3 to 19·8%)	173·1 (133·9 to 220·3)	18·5% (16·4 to 20·2%)	-14·6% (-22·6 to -7·5%)	-0·9% (-6·0 to 3·3%)
Alcohol use disorders	27·1 (24·7 to 27·9)	100·0% (100·0 to 100·0%)	28·5 (24·7 to 30·0)	100·0% (100·0 to 100·0%)	5·1% (0·5 to 9·9%)	-	1,911·8 (1,560·9 to 2,341·7)	100·0% (100·0 to 100·0%)	1,923·0 (1,568·2 to 2,369·3)	100·0% (100·0 to 100·0%)	0·6% (-2·2 to 3·4%)	-
Diabetes mellitus	-32·4 (-36·2 to -29·2)	-15·8% (-17·5 to -14·3%)	-32·9 (-37·5 to -28·9)	-15·2% (-17·1 to -13·6%)	1·3% (-13·2 to 17·6%)	-4·0% (-16·4 to 11·5%)	-1,180·8 (-1,459·5 to -937·9)	-14·7% (-15·5 to -14·0%)	-1,295·7 (-1,634·8 to -996·2)	-14·1% (-14·9 to -13·3%)	9·7% (2·3 to 16·2%)	-4·1% (-9·5 to 1·3%)
Pedestrian road injuries	5·7 (4·7 to 6·7)	18·5% (15·8 to 21·4%)	4·4 (3·5 to 5·2)	16·6% (14·0 to 19·4%)	-22·9% (-27·1 to -19·9%)	-10·5% (-12·6 to -8·7%)	263·6 (220·5 to 310·8)	21·7% (18·6 to 24·8%)	188·5 (152·9 to 223·2)	20·3% (17·4 to 23·2%)	-28·5% (-32·8 to -25·7%)	-6·4% (-8·2 to -5·0%)
Cyclist road injuries	1·1 (0·9 to 1·3)	18·9% (16·1 to 22·1%)	0·9 (0·7 to 1·1)	17·0% (14·2 to 19·9%)	-19·5% (-25·1 to -15·5%)	-10·4% (-12·7 to -8·5%)	74·3 (60·4 to 90·8)	16·7% (14·2 to 19·5%)	54·5 (43·6 to 66·9)	15·3% (12·9 to 18·0%)	-26·6% (-30·8 to -23·6%)	-8·6% (-10·8 to -6·4%)
Motorcyclist road injuries	6·9 (5·8 to 7·6)	46·0% (42·8 to 49·5%)	5·7 (4·5 to 6·4)	44·4% (41·2 to 47·9%)	-16·7% (-22·4 to -12·7%)	-3·6% (-4·5 to -2·8%)	470·5 (399·5 to 544·5)	44·1% (40·9 to 47·5%)	369·0 (296·9 to 426·1)	42·7% (39·4 to 46·1%)	-21·6% (-26·2 to -18·3%)	-3·1% (-4·2 to -2·2%)
Motor vehicle road injuries	27·1 (24·5 to 30·0)	38·3% (35·6 to 41·0%)	21·1 (19·1 to 24·1)	36·3% (33·7 to 39·2%)	-22·3% (-25·0 to -18·1%)	-5·2% (-6·0 to -4·3%)	1,533·0 (1,389·2 to 1,693·1)	39·2% (36·6 to 42·1%)	1,142·5 (1,037·0 to 1,290·1)	37·7% (35·0 to 40·5%)	-25·5% (-27·7 to -21·9%)	-3·9% (-4·8 to -2·9%)
Falls	10·2 (8·4 to 12·5)	10·9% (9·0 to 13·3%)	11·8 (9·4 to 14·6)	9·9% (8·0 to 12·3%)	15·3% (10·8 to 20·1%)	-8·7% (-11·7 to -6·0%)	673·3 (510·4 to 879·9)	13·7% (11·9 to 16·0%)	635·8 (474·5 to 856·7)	12·8% (11·0 to 15·0%)	-5·6% (-10·1 to -1·3%)	-6·6% (-7·8 to -5·7%)
Drowning	2·6 (2·2 to 3·1)	13·5% (11·5 to 16·0%)	2·5 (2·1 to 3·0)	12·8% (10·7 to 15·3%)	-4·5% (-7·7 to -1·3%)	-5·4% (-7·6 to -3·3%)	94·9 (83·8 to 109·4)	13·6% (12·0 to 15·7%)	81·9 (71·8 to 94·9)	14·0% (12·3 to 16·1%)	-13·7% (-16·2 to -11·3%)	2·5% (1·2 to 3·9%)
Fire, heat, and hot substances	1·7 (1·5 to 2·1)	11·2% (9·6 to 13·2%)	1·6 (1·3 to 2·0)	10·7% (9·0 to 12·8%)	-6·0% (-9·9 to 1·0%)	-4·6% (-7·6 to -1·5%)	88·3 (70·4 to 113·9)	13·0% (11·6 to 14·9%)	75·5 (58·5 to 99·5)	13·0% (11·5 to 14·9%)	-14·5% (-17·9 to -10·1%)	-0·5% (-1·8 to 0·8%)
Poisonings	0·9 (0·8 to 1·1)	13·6% (12·2 to 15·4%)	1·0 (0·9 to 1·1)	12·9% (11·6 to 14·6%)	3·7% (-2·4 to 11·0%)	-4·8% (-6·8 to -3·1%)	45·0 (39·9 to 51·5)	15·1% (13·8 to 16·8%)	45·3 (40·0 to 51·6)	15·0% (13·8 to 16·6%)	0·6% (-5·1 to 6·8%)	-0·9% (-2·6 to 0·5%)
Unintentional firearm injuries	0·6 (0·5 to 0·7)	12·5% (10·8 to 14·6%)	0·5 (0·5 to 0·6)	11·4% (9·6 to 13·7%)	-7·8% (-12·7 to -2·3%)	-8·4% (-11·9 to -5·5%)	23·6 (20·9 to 27·0)	16·0% (14·4 to 18·0%)	19·4 (17·0 to 22·6)	15·6% (14·0 to 17·7%)	-17·8% (-21·4 to -13·7%)	-2·2% (-3·8 to -0·6%)
Unintentional suffocation	0·3 (0·2 to 0·3)	6·6% (5·4 to 7·9%)	0·3 (0·2 to 0·4)	6·7% (5·4 to 8·3%)	13·4% (7·7 to 19·6%)	2·3% (-1·7 to 5·6%)	9·7 (7·9 to 12·0)	5·8% (5·0 to 6·7%)	10·1 (8·2 to 12·6)	6·1% (5·3 to 7·2%)	4·1% (1·1 to 6·4%)	6·0% (0·5 to 10·1%)
Other exposure to mechanical forces	1·5 (1·3 to 1·7)	16·3% (14·3 to 18·9%)	1·3 (1·1 to 1·5)	15·3% (13·2 to 17·9%)	-11·2% (-14·1 to -8·2%)	-6·2% (-8·0 to -4·4%)	134·9 (103·5 to 178·9)	17·2% (15·2 to 19·8%)	124·2 (92·7 to 169·6)	16·4% (14·5 to 19·0%)	-8·0% (-11·2 to -5·2%)	-4·3% (-5·0 to -3·7%)
Self-harm	27·5 (24·1 to 31·7)	18·0% (15·8 to 20·8%)	28·8 (25·2 to 33·6)	17·2% (15·1 to 20·1%)	4·8% (1·8 to 7·4%)	-3·9% (-4·9 to -3·1%)	1,104·5 (978·2 to 1,258·7)	18·9% (16·7 to 21·6%)	1,114·2 (987·1 to 1,281·6)	18·5% (16·4 to 21·2%)	0·9% (-2·0 to 3·4%)	-2·1% (-2·6 to -1·4%)
Assault by firearm	3·5 (3·3 to 3·7)	19·6% (18·7 to 20·9%)	3·1 (2·9 to 3·4)	18·8% (17·8 to 20·1%)	-10·3% (-14·2 to -5·2%)	-4·4% (-5·1 to -3·7%)	195·7 (185·4 to 209·3)	20·6% (19·7 to 22·0%)	171·7 (161·2 to 185·8)	20·0% (19·1 to 21·3%)	-12·3% (-16·2 to -6·8%)	-3·0% (-3·7 to -2·4%)
Assault by sharp object	1·1 (1·0 to 1·2)	17·1% (15·7 to 19·1%)	1·0 (0·9 to 1·1)	16·4% (15·0 to 18·3%)	-11·0% (-15·1 to -6·9%)	-4·4% (-5·5 to -3·4%)	57·4 (52·1 to 63·9)	18·9% (17·4 to 20·9%)	49·7 (44·8 to 55·8)	18·6% (17·1 to 20·6%)	-13·4% (-17·5 to -9·0%)	-1·9% (-2·8 to -1·0%)
Assault by other means	1·1 (1·0 to 1·3)	14·0% (12·7 to 15·8%)	1·0 (0·9 to 1·1)	13·1% (11·8 to 14·8%)	-14·8% (-17·7 to -11·5%)	-6·4% (-7·6 to -5·2%)	77·8 (66·9 to 91·4)	15·0% (13·6 to 16·8%)	65·1 (55·0 to 77·6)	14·5% (13·2 to 16·2%)	-16·3% (-19·4 to -13·3%)	-3·4% (-4·4 to -2·4%)
**Drug use: All causes**	106·0 (92·9 to 118·2)	1·3% (1·1 to 1·4%)	138·4 (123·6 to 151·9)	1·4% (1·3 to 1·6%)	30·6% (25·8 to 36·1%)	14·1% (9·9 to 19·0%)	5,450·0 (4,817·6 to 6,075·8)	2·1% (1·9 to 2·3%)	6,718·3 (5,952·4 to 7,463·3)	2·5% (2·2 to 2·7%)	23·3% (19·8 to 26·4%)	15·7% (12·4 to 18·9%)
HIV/AIDS - Tuberculosis	0·1 (0·1 to 0·1)	6·8% (6·2 to 7·4%)	0·1 (0·1 to 0·1)	6·6% (5·9 to 7·4%)	-21·2% (-27·2 to -14·4%)	-3·6% (-10·3 to 4·0%)	6·1 (5·5 to 6·7)	6·7% (6·1 to 7·3%)	4·5 (4·0 to 5·1)	6·4% (5·7 to 7·2%)	-25·8% (-31·6 to -19·1%)	-3·9% (-10·6 to 3·8%)
HIV/AIDS resulting in other diseases	1·9 (1·7 to 2·1)	9·3% (8·5 to 10·3%)	1·1 (1·0 to 1·2)	9·1% (8·3 to 10·1%)	-40·1% (-42·4 to -37·5%)	-2·2% (-5·9 to 1·6%)	90·4 (81·1 to 101·0)	9·1% (8·3 to 10·1%)	55·7 (49·3 to 63·8)	8·9% (8·1 to 9·9%)	-38·3% (-41·7 to -34·9%)	-2·4% (-6·2 to 1·4%)
Hepatitis B	0·2 (0·1 to 0·3)	3·4% (1·5 to 5·6%)	0·1 (0·0 to 0·2)	3·5% (1·5 to 5·5%)	-49·6% (-56·0 to -41·2%)	2·3% (-7·7 to 16·0%)	7·2 (3·1 to 11·7)	4·2% (1·8 to 6·8%)	3·6 (1·6 to 5·7)	4·0% (1·7 to 6·2%)	-50·3% (-57·2 to -41·6%)	-5·9% (-15·4 to 7·1%)
Hepatitis C	0·2 (0·0 to 0·5)	53·9% (44·7 to 62·4%)	0·1 (0·0 to 0·3)	58·4% (49·1 to 67·0%)	-45·2% (-56·9 to -29·7%)	8·4% (-5·3 to 24·4%)	6·2 (1·6 to 13·7)	62·4% (54·7 to 68·9%)	3·3 (1·0 to 7·0)	64·5% (57·6 to 70·4%)	-46·7% (-58·2 to -27·7%)	3·3% (-5·1 to 12·6%)
Liver cancer due to hepatitis B	0·3 (0·1 to 0·4)	1·6% (0·7 to 2·6%)	0·4 (0·2 to 0·7)	1·9% (0·8 to 3·1%)	55·0% (41·1 to 74·1%)	24·6% (13·3 to 39·9%)	7·7 (3·2 to 12·8)	1·8% (0·8 to 3·0%)	10·9 (4·8 to 17·5)	2·3% (1·0 to 3·6%)	42·7% (28·9 to 60·6%)	24·7% (13·5 to 39·7%)
Liver cancer due to hepatitis C	19·7 (13·7 to 25·5)	40·1% (28·4 to 51·6%)	29·5 (22·4 to 36·3)	47·6% (36·4 to 58·0%)	49·9% (39·1 to 66·2%)	18·7% (10·9 to 30·5%)	392·1 (283·9 to 494·5)	47·3% (34·8 to 59·2%)	539·8 (427·6 to 644·7)	56·2% (44·7 to 65·8%)	37·7% (26·8 to 54·2%)	18·8% (10·6 to 31·5%)
Cirrhosis and other chronic liver diseases due to hepatitis B	0·4 (0·2 to 0·8)	1·7% (0·7 to 2·9%)	0·6 (0·3 to 1·0)	2·1% (0·9 to 3·4%)	36·9% (24·6 to 53·9%)	22·5% (11·6 to 37·3%)	12·8 (5·5 to 21·7)	2·1% (0·9 to 3·5%)	16·3 (7·0 to 26·4)	2·6% (1·1 to 4·2%)	27·4% (15·5 to 43·4%)	24·1% (12·2 to 39·8%)
Cirrhosis and other chronic liver diseases due to hepatitis C	41·6 (34·4 to 47·7)	62·1% (52·8 to 70·1%)	50·6 (42·6 to 57·1)	66·5% (58·7 to 72·9%)	21·6% (15·3 to 30·5%)	7·2% (3·5 to 12·7%)	1,131·3 (967·9 to 1,267·0)	71·2% (62·8 to 77·3%)	1,288·3 (1,111·3 to 1,430·2)	75·4% (69·0 to 79·8%)	13·9% (7·9 to 21·9%)	5·9% (2·8 to 10·7%)
Opioid use disorders	22·9 (22·1 to 25·3)	100·0% (100·0 to 100·0%)	30·7 (27·4 to 33·4)	100·0% (100·0 to 100·0%)	33·9% (20·5 to 41·1%)	-	2,329·6 (1,927·9 to 2,702·9)	100·0% (100·0 to 100·0%)	2,909·0 (2,414·8 to 3,361·9)	100·0% (100·0 to 100·0%)	24·9% (19·5 to 28·5%)	-
Cocaine use disorders	4·4 (2·4 to 4·8)	100·0% (100·0 to 100·0%)	6·5 (3·6 to 7·2)	100·0% (100·0 to 100·0%)	48·9% (33·4 to 63·9%)	-	365·6 (257·3 to 444·6)	100·0% (100·0 to 100·0%)	509·6 (362·1 to 615·2)	100·0% (100·0 to 100·0%)	39·4% (30·8 to 47·7%)	-
Amphetamine use disorders	1·8 (0·8 to 2·0)	100·0% (100·0 to 100·0%)	3·2 (1·4 to 3·6)	100·0% (100·0 to 100·0%)	76·3% (41·2 to 106·6%)	-	202·0 (144·5 to 265·6)	100·0% (100·0 to 100·0%)	300·2 (214·5 to 384·4)	100·0% (100·0 to 100·0%)	48·7% (34·0 to 62·2%)	-
Cannabis use disorders	-	-	-	-	-	-	145·1 (93·0 to 204·9)	100·0% (100·0 to 100·0%)	146·6 (95·5 to 207·5)	100·0% (100·0 to 100·0%)	1·1% (-1·3 to 3·4%)	-
Other drug use disorders	6·4 (6·2 to 7·0)	100·0% (100·0 to 100·0%)	8·0 (7·2 to 8·5)	100·0% (100·0 to 100·0%)	25·0% (12·2 to 33·2%)	-	463·8 (392·6 to 547·5)	100·0% (100·0 to 100·0%)	578·8 (481·2 to 694·2)	100·0% (100·0 to 100·0%)	24·8% (17·4 to 30·7%)	-
Self-harm	6·0 (4·1 to 8·5)	3·9% (2·7 to 5·6%)	7·5 (5·1 to 10·6)	4·5% (3·1 to 6·3%)	24·3% (19·0 to 29·0%)	13·9% (10·9 to 17·7%)	290·1 (199·8 to 407·6)	5·0% (3·4 to 7·0%)	351·5 (240·0 to 492·5)	5·8% (4·0 to 8·1%)	21·2% (16·2 to 25·9%)	17·6% (14·5 to 21·3%)
**Dietary risks: All causes**	1,669·6 (1,425·1 to 1,928·1)	19·8% (16·9 to 22·9%)	1,776·8 (1,510·5 to 2,063·0)	18·4% (15·7 to 21·4%)	6·4% (5·1 to 7·6%)	-7·0% (-8·1 to -6·1%)	28,931·9 (25,049·7 to 33,147·8)	11·3% (9·6 to 13·2%)	29,181·3 (25,105·3 to 33,630·9)	10·7% (9·1 to 12·5%)	0·9% (-0·4 to 2·0%)	-5·4% (-6·3 to -4·5%)
**Diet low in fruits: All causes**	346·6 (208·8 to 500·9)	4·1% (2·5 to 5·9%)	365·3 (221·3 to 529·1)	3·8% (2·3 to 5·5%)	5·4% (3·8 to 7·0%)	-7·9% (-9·3 to -6·6%)	6,746·2 (4,202·5 to 9,630·6)	2·6% (1·6 to 3·8%)	6,750·4 (4,166·7 to 9,675·5)	2·5% (1·5 to 3·6%)	0·1% (-2·2 to 2·3%)	-6·1% (-8·1 to -4·1%)
Lip and oral cavity cancer	1·4 (0·0 to 3·2)	6·1% (0·0 to 13·6%)	1·7 (0·0 to 3·8)	6·2% (0·0 to 13·9%)	18·1% (0·0 to 23·0%)	1·7% (0·0 to 2·7%)	32·2 (0·0 to 71·7)	6·1% (0·0 to 13·6%)	35·2 (0·0 to 78·6)	6·2% (0·0 to 13·8%)	9·3% (0·0 to 14·1%)	1·0% (0·0 to 1·9%)
Nasopharynx cancer	0·4 (0·0 to 0·8)	6·3% (0·0 to 13·6%)	0·4 (0·0 to 0·9)	6·4% (0·0 to 13·8%)	16·5% (0·0 to 22·4%)	1·7% (0·0 to 2·7%)	9·0 (0·0 to 19·5)	6·2% (0·0 to 13·4%)	9·7 (0·0 to 21·0)	6·3% (0·0 to 13·6%)	8·0% (0·0 to 13·3%)	1·2% (0·0 to 2·0%)
Other pharynx cancer	0·8 (0·0 to 1·9)	6·2% (0·0 to 14·0%)	1·0 (0·0 to 2·4)	6·3% (0·0 to 14·3%)	23·1% (0·0 to 28·9%)	2·0% (0·0 to 3·2%)	21·4 (0·0 to 48·5)	6·2% (0·0 to 14·1%)	25·1 (0·0 to 56·8)	6·3% (0·0 to 14·3%)	17·1% (0·0 to 23·0%)	1·3% (0·0 to 2·3%)
Oesophageal cancer	12·4 (2·9 to 22·5)	19·8% (4·7 to 36·0%)	14·7 (3·5 to 26·6)	20·2% (4·8 to 36·5%)	18·6% (13·3 to 23·3%)	2·0% (1·3 to 3·1%)	249·1 (59·2 to 453·7)	19·8% (4·7 to 36·0%)	278·1 (67·6 to 504·7)	20·1% (4·8 to 36·6%)	11·7% (6·7 to 16·4%)	1·6% (1·0 to 2·6%)
Larynx cancer	1·1 (0·0 to 2·4)	6·1% (0·0 to 13·7%)	1·1 (0·0 to 2·4)	6·2% (0·0 to 13·9%)	-0·3% (-4·4 to 4·5%)	1·2% (0·0 to 2·2%)	24·3 (0·0 to 55·0)	6·1% (0·0 to 13·8%)	22·9 (0·0 to 51·8)	6·1% (0·0 to 13·8%)	-6·0% (-9·9 to 0·0%)	0·8% (-0·1 to 1·7%)
Tracheal, bronchus, and lung cancer	53·3 (21·0 to 91·3)	10·6% (4·2 to 18·2%)	61·8 (24·6 to 106·3)	10·8% (4·3 to 18·5%)	15·9% (13·2 to 18·6%)	1·7% (1·1 to 2·7%)	1,015·3 (399·6 to 1,746·7)	10·6% (4·2 to 18·2%)	1,102·0 (431·8 to 1,904·4)	10·7% (4·2 to 18·4%)	8·5% (6·1 to 10·9%)	1·1% (0·5 to 1·9%)
Ischaemic heart disease	149·5 (52·1 to 258·1)	9·6% (3·3 to 16·7%)	157·6 (54·9 to 271·9)	9·6% (3·3 to 16·6%)	5·4% (3·4 to 6·9%)	-0·4% (-1·1 to 0·3%)	2,435·1 (854·2 to 4,167·6)	11·3% (3·9 to 19·3%)	2,379·7 (831·4 to 4,058·3)	11·2% (3·9 to 19·2%)	-2·3% (-3·7 to -0·9%)	-0·7% (-1·2 to -0·1%)
Ischaemic stroke	55·2 (27·9 to 87·9)	11·5% (5·8 to 18·5%)	55·0 (27·9 to 88·3)	11·1% (5·6 to 17·8%)	-0·4% (-2·7 to 2·3%)	-4·2% (-5·2 to -3·2%)	863·5 (446·3 to 1,350·7)	16·5% (8·6 to 25·6%)	813·3 (418·9 to 1,266·7)	16·1% (8·4 to 25·0%)	-5·8% (-8·0 to -3·3%)	-2·4% (-3·3 to -1·5%)

Supplemental Results Table 1. All-age deaths, death PAFs, DALYs, DALY PAFs, and percent change for each risk factor and outcome for both sexes combined in 2005 and 2015 for High-income.

Data in parenthesis are 95% uncertainty intervals. PAFs=population attributable fractions. DALYs=disability-adjusted life-years.

	2005 Deaths (in thousands)	2005 Deaths PAF	2015 Deaths (in thousands)	2015 Deaths PAF	Percent change deaths 2005-2015	Percent change deaths PAF 2005-2015	2005 DALYs (in thousands)	2005 DALYs PAF	2015 DALYs (in thousands)	2015 DALYs PAF	Percent change DALYs 2005-2015	Percent change DALYs PAF 2005-2015
Hemorrhagic stroke	56·8 (30·1 to 87·8)	17·4% (9·1 to 26·9%)	55·9 (29·2 to 86·7)	16·2% (8·5 to 25·1%)	-1·6% (-4·3 to 1·7%)	-6·6% (-7·7 to -5·6%)	1,276·1 (698·4 to 1,924·4)	24·4% (13·2 to 36·8%)	1,152·7 (622·2 to 1,737·2)	23·5% (12·7 to 35·5%)	-9·7% (-11·7 to -7·4%)	-3·8% (-4·6 to -3·0%)
**Diet low in vegetables: All causes**	309·0 (146·7 to 491·5)	3·7% (1·7 to 5·8%)	319·5 (150·6 to 508·5)	3·3% (1·6 to 5·3%)	3·4% (1·7 to 4·8%)	-9·7% (-11·1 to -8·4%)	5,043·9 (2,501·3 to 7,863·4)	2·0% (0·9 to 3·1%)	4,853·6 (2,378·0 to 7,564·8)	1·8% (0·8 to 2·8%)	-3·8% (-5·3 to -2·4%)	-9·7% (-11·1 to -8·4%)
Ischaemic heart disease	248·6 (95·3 to 414·8)	16·0% (6·2 to 26·5%)	260·0 (99·9 to 433·2)	15·8% (6·1 to 26·2%)	4·6% (2·7 to 6·1%)	-1·1% (-1·7 to -0·6%)	3,898·7 (1,527·4 to 6,436·7)	18·0% (7·0 to 29·7%)	3,801·4 (1,489·6 to 6,253·1)	17·9% (7·0 to 29·5%)	-2·5% (-3·9 to -1·2%)	-0·9% (-1·4 to -0·4%)
Ischaemic stroke	29·9 (6·7 to 55·0)	6·2% (1·4 to 11·5%)	29·5 (6·6 to 54·8)	5·9% (1·3 to 10·9%)	-1·3% (-3·6 to 1·4%)	-5·1% (-6·0 to -4·2%)	464·9 (105·4 to 850·7)	8·9% (2·0 to 16·1%)	437·3 (99·8 to 803·6)	8·7% (2·0 to 15·7%)	-5·9% (-8·1 to -3·2%)	-2·5% (-3·4 to -1·5%)
Hemorrhagic stroke	30·6 (8·5 to 56·8)	9·3% (2·6 to 17·4%)	30·0 (8·2 to 56·3)	8·7% (2·4 to 16·2%)	-2·0% (-4·5 to 1·2%)	-7·0% (-8·0 to -5·9%)	680·3 (192·5 to 1,247·4)	13·0% (3·7 to 23·8%)	615·0 (172·8 to 1,127·5)	12·5% (3·5 to 22·9%)	-9·6% (-11·7 to -7·2%)	-3·7% (-4·6 to -2·8%)
**Diet low in whole grains: All causes**	345·1 (225·1 to 485·7)	4·1% (2·7 to 5·8%)	355·5 (230·8 to 502·5)	3·7% (2·4 to 5·2%)	3·0% (1·5 to 4·6%)	-10·0% (-11·3 to -8·7%)	6,950·1 (4,667·7 to 9,515·2)	2·7% (1·8 to 3·7%)	6,819·2 (4,539·3 to 9,390·6)	2·5% (1·6 to 3·5%)	-1·9% (-4·0 to 0·1%)	-7·9% (-9·7 to -6·2%)
Ischaemic heart disease	223·1 (124·9 to 338·6)	14·3% (8·0 to 21·8%)	235·3 (130·8 to 356·0)	14·3% (7·9 to 21·6%)	5·5% (3·5 to 7·4%)	-0·3% (-1·4 to 0·9%)	3,654·3 (2,039·1 to 5,453·5)	16·9% (9·5 to 25·5%)	3,546·6 (1,968·6 to 5,311·8)	16·7% (9·3 to 25·2%)	-2·9% (-4·6 to -1·5%)	-1·4% (-2·3 to -0·4%)
Ischaemic stroke	46·1 (29·7 to 65·0)	9·6% (6·2 to 13·6%)	45·2 (28·7 to 64·1)	9·1% (5·8 to 12·8%)	-2·1% (-4·5 to 0·7%)	-5·9% (-7·0 to -4·8%)	765·0 (492·7 to 1,071·6)	14·6% (9·6 to 20·2%)	703·3 (452·7 to 988·9)	13·9% (9·1 to 19·3%)	-8·1% (-10·3 to -5·6%)	-4·8% (-5·9 to -3·7%)
Hemorrhagic stroke	48·9 (33·3 to 68·2)	14·9% (10·2 to 20·8%)	47·5 (32·4 to 66·0)	13·8% (9·3 to 19·2%)	-2·9% (-5·5 to 0·3%)	-7·9% (-9·1 to -6·8%)	1,117·8 (774·9 to 1,522·2)	21·4% (14·8 to 29·2%)	994·3 (684·7 to 1,360·2)	20·3% (14·0 to 27·8%)	-11·0% (-13·1 to -8·8%)	-5·2% (-6·3 to -4·4%)
Diabetes mellitus	26·9 (14·1 to 42·7)	13·1% (6·8 to 20·9%)	27·6 (14·4 to 43·5)	12·8% (6·7 to 20·2%)	2·5% (-1·2 to 6·2%)	-2·9% (-4·2 to -1·4%)	1,413·0 (732·3 to 2,288·9)	17·6% (9·4 to 27·4%)	1,575·0 (820·2 to 2,587·4)	17·2% (9·1 to 26·7%)	11·5% (7·8 to 14·4%)	-2·5% (-3·9 to -1·4%)
**Diet low in nuts and seeds: All causes**	310·2 (190·4 to 450·5)	3·7% (2·3 to 5·3%)	326·0 (197·6 to 475·8)	3·4% (2·0 to 4·9%)	5·1% (3·1 to 6·7%)	-8·2% (-9·9 to -6·9%)	5,353·6 (3,422·4 to 7,563·7)	2·1% (1·3 to 3·0%)	5,355·8 (3,449·5 to 7,569·5)	2·0% (1·2 to 2·8%)	0·0% (-1·9 to 2·4%)	-6·1% (-7·8 to -4·1%)
Ischaemic heart disease	293·2 (176·9 to 429·0)	18·8% (11·3 to 27·6%)	308·5 (186·5 to 453·1)	18·7% (11·3 to 27·4%)	5·2% (3·3 to 6·8%)	-0·6% (-0·8 to -0·2%)	4,500·9 (2,753·8 to 6,499·5)	20·8% (12·7 to 30·0%)	4,390·4 (2,705·8 to 6,361·4)	20·6% (12·6 to 29·6%)	-2·5% (-3·9 to -1·1%)	-0·9% (-1·4 to -0·2%)
Diabetes mellitus	17·0 (8·6 to 27·1)	8·3% (4·2 to 13·3%)	17·5 (8·9 to 27·9)	8·1% (4·1 to 13·0%)	3·2% (-0·8 to 6·5%)	-2·2% (-3·0 to -1·5%)	852·7 (383·3 to 1,437·5)	10·6% (5·3 to 17·0%)	965·4 (432·6 to 1,630·2)	10·5% (5·3 to 16·8%)	13·2% (9·8 to 16·1%)	-1·0% (-1·5 to -0·6%)
**Diet low in milk: All causes**	36·0 (12·3 to 62·0)	0·4% (0·1 to 0·7%)	42·8 (14·6 to 73·1)	0·4% (0·2 to 0·8%)	18·7% (16·0 to 21·9%)	3·7% (1·3 to 6·5%)	644·4 (218·6 to 1,109·7)	0·3% (0·1 to 0·4%)	722·3 (242·7 to 1,240·3)	0·3% (0·1 to 0·5%)	12·1% (9·4 to 15·0%)	5·2% (2·8 to 7·8%)
Colon and rectum cancer	36·0 (12·3 to 62·0)	13·4% (4·6 to 23·0%)	42·8 (14·6 to 73·1)	13·5% (4·6 to 23·2%)	18·7% (16·0 to 21·9%)	1·0% (0·6 to 1·6%)	644·4 (218·6 to 1,109·7)	13·5% (4·6 to 23·2%)	722·3 (242·7 to 1,240·3)	13·6% (4·7 to 23·4%)	12·1% (9·4 to 15·0%)	0·8% (0·5 to 1·3%)
**Diet high in red meat: All causes**	17·6 (7·8 to 28·7)	0·2% (0·1 to 0·3%)	19·0 (8·2 to 31·2)	0·2% (0·1 to 0·3%)	7·8% (2·4 to 12·5%)	-5·8% (-10·4 to -1·8%)	591·9 (249·1 to 959·4)	0·2% (0·1 to 0·4%)	662·0 (276·5 to 1,086·5)	0·2% (0·1 to 0·4%)	11·9% (8·4 to 15·1%)	4·9% (2·0 to 7·8%)
Colon and rectum cancer	11·1 (2·4 to 19·6)	4·1% (0·9 to 7·2%)	12·5 (2·7 to 21·9)	3·9% (0·8 to 6·9%)	12·7% (9·0 to 16·0%)	-4·2% (-6·0 to -2·5%)	219·7 (47·3 to 385·4)	4·6% (1·0 to 8·1%)	240·7 (51·5 to 424·7)	4·5% (1·0 to 8·0%)	9·5% (5·7 to 12·7%)	-1·5% (-3·2 to 0·1%)
Diabetes mellitus	6·5 (0·8 to 11·8)	3·2% (0·4 to 5·7%)	6·5 (0·8 to 11·7)	3·0% (0·4 to 5·4%)	-0·5% (-4·4 to 3·0%)	-5·7% (-7·7 to -3·8%)	372·1 (43·5 to 685·6)	4·6% (0·6 to 8·3%)	421·3 (49·6 to 795·4)	4·6% (0·6 to 8·2%)	13·2% (8·7 to 16·7%)	-1·0% (-2·6 to 0·6%)
**Diet high in processed meat: All causes**	123·4 (42·0 to 197·0)	1·5% (0·5 to 2·3%)	127·2 (43·9 to 202·5)	1·3% (0·5 to 2·1%)	3·1% (0·9 to 5·7%)	-9·9% (-11·8 to -7·6%)	2,827·3 (1,372·5 to 4,259·3)	1·1% (0·5 to 1·6%)	2,896·1 (1,477·8 to 4,310·4)	1·1% (0·5 to 1·6%)	2·4% (-0·8 to 8·8%)	-3·9% (-6·9 to 2·1%)
Colon and rectum cancer	15·1 (8·3 to 22·2)	5·6% (3·1 to 8·2%)	17·3 (9·4 to 25·8)	5·5% (3·0 to 8·1%)	14·5% (10·8 to 17·8%)	-2·6% (-4·1 to -1·1%)	272·4 (147·7 to 399·0)	5·7% (3·1 to 8·3%)	294·9 (159·7 to 433·0)	5·5% (3·0 to 8·2%)	8·2% (4·9 to 11·2%)	-2·7% (-4·2 to -1·1%)
Ischaemic heart disease	86·8 (3·9 to 158·9)	5·6% (0·2 to 10·2%)	88·5 (4·0 to 162·6)	5·4% (0·2 to 9·9%)	2·0% (-0·2 to 4·0%)	-3·5% (-4·8 to -2·0%)	1,424·5 (64·6 to 2,595·5)	6·6% (0·3 to 12·0%)	1,359·1 (62·0 to 2,488·5)	6·4% (0·3 to 11·7%)	-4·6% (-6·4 to -2·4%)	-3·0% (-4·2 to -1·2%)
Diabetes mellitus	21·5 (10·9 to 31·0)	10·5% (5·3 to 15·1%)	21·4 (10·5 to 31·0)	9·9% (4·9 to 14·3%)	-0·7% (-4·6 to 3·1%)	-5·9% (-7·6 to -4·2%)	1,130·5 (543·1 to 1,754·2)	14·1% (7·1 to 20·2%)	1,242·1 (593·3 to 1,950·6)	13·5% (6·8 to 19·5%)	9·9% (6·1 to 13·4%)	-3·9% (-5·8 to -1·9%)
**Diet high in sugar-sweetened beverages: All causes**	11·7 (7·9 to 16·8)	0·1% (0·1 to 0·2%)	11·7 (7·9 to 16·9)	0·1% (0·1 to 0·2%)	0·7% (-2·2 to 3·8%)	-12·0% (-14·5 to -9·3%)	423·6 (286·0 to 615·6)	0·2% (0·1 to 0·2%)	435·0 (294·9 to 636·4)	0·2% (0·1 to 0·2%)	2·7% (-0·6 to 6·1%)	-3·7% (-6·5 to -0·6%)
Oesophageal cancer	0·3 (0·1 to 0·5)	0·4% (0·1 to 0·8%)	0·3 (0·1 to 0·6)	0·4% (0·1 to 0·8%)	10·9% (5·1 to 16·8%)	-4·5% (-8·1 to -0·9%)	6·6 (1·8 to 12·7)	0·5% (0·1 to 1·0%)	7·1 (1·9 to 13·8)	0·5% (0·1 to 1·0%)	6·8% (0·9 to 12·8%)	-2·8% (-6·4 to 1·0%)
Colon and rectum cancer	0·4 (0·2 to 0·5)	0·1% (0·1 to 0·2%)	0·4 (0·3 to 0·6)	0·1% (0·1 to 0·2%)	10·0% (5·8 to 14·4%)	-6·4% (-9·6 to -3·2%)	8·9 (5·8 to 13·1)	0·2% (0·1 to 0·3%)	9·6 (6·2 to 14·2)	0·2% (0·1 to 0·3%)	7·6% (3·4 to 12·3%)	-3·2% (-6·7 to 0·4%)
Liver cancer due to hepatitis B	0·0 (0·0 to 0·1)	0·2% (0·1 to 0·3%)	0·0 (0·0 to 0·1)	0·2% (0·1 to 0·3%)	20·7% (14·7 to 26·4%)	-3·0% (-7·6 to 1·7%)	0·9 (0·4 to 1·6)	0·2% (0·1 to 0·4%)	1·1 (0·4 to 1·9)	0·2% (0·1 to 0·4%)	14·5% (8·4 to 20·7%)	0·1% (-5·3 to 6·0%)
Liver cancer due to hepatitis C	0·1 (0·0 to 0·1)	0·1% (0·1 to 0·2%)	0·1 (0·0 to 0·1)	0·1% (0·1 to 0·2%)	23·8% (18·8 to 28·9%)	-2·0% (-5·4 to 1·4%)	1·6 (0·7 to 2·7)	0·2% (0·1 to 0·3%)	1·9 (0·8 to 3·2)	0·2% (0·1 to 0·3%)	19·6% (14·2 to 25·2%)	3·2% (-0·7 to 7·2%)
Liver cancer due to alcohol use	0·1 (0·0 to 0·2)	0·3% (0·1 to 0·5%)	0·1 (0·0 to 0·2)	0·3% (0·1 to 0·5%)	24·3% (17·5 to 30·9%)	5·5% (0·5 to 10·5%)	2·2 (0·9 to 3·9)	0·3% (0·1 to 0·6%)	2·7 (1·1 to 4·8)	0·4% (0·1 to 0·7%)	22·0% (14·4 to 29·2%)	6·6% (1·0 to 12·3%)
Liver cancer due to other causes	0·0 (0·0 to 0·1)	0·3% (0·1 to 0·5%)	0·0 (0·0 to 0·1)	0·3% (0·1 to 0·5%)	32·1% (25·2 to 38·9%)	-2·5% (-6·7 to 1·9%)	0·8 (0·4 to 1·4)	0·4% (0·2 to 0·7%)	1·0 (0·5 to 1·8)	0·4% (0·2 to 0·7%)	26·6% (19·7 to 33·5%)	-0·6% (-4·7 to 4·2%)
Gallbladder and biliary tract cancer	0·1 (0·0 to 0·1)	0·1% (0·1 to 0·2%)	0·1 (0·0 to 0·1)	0·1% (0·1 to 0·2%)	1·4% (-7·6 to 8·9%)	-9·4% (-13·4 to -6·3%)	1·4 (0·8 to 2·2)	0·2% (0·1 to 0·3%)	1·4 (0·8 to 2·2)	0·2% (0·1 to 0·3%)	-3·5% (-11·6 to 3·8%)	-5·0% (-9·1 to -1·5%)
Pancreatic cancer	0·1 (0·0 to 0·2)	0·1% (0·0 to 0·2%)	0·1 (0·1 to 0·3)	0·1% (0·0 to 0·1%)	18·1% (13·8 to 23·0%)	-7·1% (-10·0 to -4·0%)	2·6 (0·9 to 4·9)	0·1% (0·0 to 0·2%)	3·0 (1·0 to 5·7)	0·1% (0·0 to 0·2%)	13·8% (9·7 to 18·7%)	-4·9% (-8·0 to -1·3%)
Breast cancer	0·1 (0·1 to 0·2)	0·1% (0·0 to 0·1%)	0·1 (0·1 to 0·2)	0·1% (0·0 to 0·1%)	12·2% (5·9 to 21·9%)	-1·1% (-5·2 to 4·0%)	3·2 (1·8 to 5·0)	0·1% (0·0 to 0·1%)	3·6 (2·0 to 5·9)	0·1% (0·0 to 0·1%)	12·8% (6·0 to 22·5%)	2·3% (-2·4 to 7·5%)
Uterine cancer	0·1 (0·1 to 0·1)	0·5% (0·3 to 0·7%)	0·1 (0·1 to 0·2)	0·5% (0·3 to 0·7%)	18·3% (7·4 to 27·0%)	-1·4% (-5·5 to 2·9%)	2·4 (1·6 to 3·5)	0·6% (0·4 to 0·9%)	3·0 (1·9 to 4·2)	0·6% (0·4 to 0·9%)	20·7% (8·5 to 30·6%)	1·3% (-3·1 to 5·7%)
Ovarian cancer	0·0 (0·0 to 0·0)	0·0% (0·0 to 0·1%)	0·0 (0·0 to 0·0)	0·0% (0·0 to 0·1%)	5·8% (0·0 to 11·1%)	-5·6% (-9·3 to 0·0%)	0·5 (0·0 to 1·1)	0·0% (0·0 to 0·1%)	0·5 (0·0 to 1·2)	0·0% (0·0 to 0·1%)	3·1% (-2·1 to 8·4%)	-4·3% (-8·2 to 0·0%)
Kidney cancer	0·1 (0·1 to 0·2)	0·3% (0·2 to 0·5%)	0·2 (0·1 to 0·2)	0·3% (0·2 to 0·4%)	16·8% (11·9 to 22·1%)	-7·8% (-10·8 to -4·5%)	3·7 (2·4 to 5·6)	0·4% (0·3 to 0·6%)	4·2 (2·7 to 6·2)	0·4% (0·2 to 0·5%)	13·6% (8·6 to 19·3%)	-5·7% (-8·7 to -2·0%)
Thyroid cancer	0·0 (0·0 to 0·0)	0·2% (0·1 to 0·3%)	0·0 (0·0 to 0·0)	0·2% (0·1 to 0·3%)	15·5% (9·4 to 21·6%)	-5·0% (-8·8 to -1·2%)	0·4 (0·2 to 0·7)	0·2% (0·1 to 0·4%)	0·6 (0·3 to 1·1)	0·3% (0·1 to 0·4%)	58·8% (43·9 to 72·9%)	10·0% (3·7 to 15·5%)
Ischaemic heart disease	5·0 (3·3 to 7·2)	0·3% (0·2 to 0·5%)	4·7 (3·1 to 6·9)	0·3% (0·2 to 0·4%)	-4·5% (-7·8 to -1·2%)	-9·7% (-12·8 to -6·7%)	124·6 (83·0 to 181·7)	0·6% (0·4 to 0·8%)	115·1 (76·9 to 166·7)	0·5% (0·4 to 0·8%)	-7·6% (-10·7 to -4·1%)	-6·1% (-9·0 to -2·6%)
Ischaemic stroke	0·6 (0·4 to 0·9)	0·1% (0·1 to 0·2%)	0·6 (0·3 to 0·8)	0·1% (0·1 to 0·2%)	-7·5% (-12·8 to -2·6%)	-11·0% (-15·9 to -6·9%)	14·6 (10·0 to 21·2)	0·3% (0·2 to 0·4%)	13·6 (9·2 to 19·9)	0·3% (0·2 to 0·4%)	-6·8% (-10·5 to -2·7%)	-3·4% (-6·6 to -0·2%)
Hemorrhagic stroke	1·1 (0·8 to 1·7)	0·4% (0·2 to 0·5%)	1·0 (0·7 to 1·5)	0·3% (0·2 to 0·4%)	-11·1% (-15·0 to -6·8%)	-15·6% (-19·2 to -11·9%)	38·1 (25·7 to 55·1)	0·7% (0·5 to 1·1%)	33·0 (22·1 to 47·9)	0·7% (0·4 to 1·0%)	-13·3% (-16·5 to -9·6%)	-7·6% (-10·8 to -4·3%)
Hypertensive heart disease	0·8 (0·5 to 1·1)	0·7% (0·4 to 1·1%)	0·9 (0·5 to 1·4)	0·6% (0·4 to 1·0%)	14·6% (6·1 to 21·5%)	-10·0% (-16·0 to -4·6%)	20·3 (12·9 to 30·4)	1·4% (0·9 to 2·2%)	22·1 (13·8 to 33·3)	1·3% (0·8 to 2·0%)	8·5% (0·0 to 15·7%)	-7·1% (-12·5 to -1·5%)
Cardiomyopathy and myocarditis	-	-	-	-	-	-	-	-	-	-	-	-
Atrial fibrillation and flutter	-	-	-	-	-	-	-	-	-	-	-	-
Peripheral vascular disease	-	-	-	-	-	-	-	-	-	-	-	-

Supplemental Results Table 1. All-age deaths, death PAFs, DALYs, DALY PAFs, and percent change for each risk factor and outcome for both sexes combined in 2005 and 2015 for High-income.

Data in parenthesis are 95% uncertainty intervals. PAFs=population attributable fractions. DALYs=disability-adjusted life-years.

	2005 Deaths (in thousands)	2005 Deaths PAF	2015 Deaths (in thousands)	2015 Deaths PAF	Percent change deaths 2005-2015	Percent change deaths PAF 2005-2015	2005 DALYs (in thousands)	2005 DALYs PAF	2015 DALYs (in thousands)	2015 DALYs PAF	Percent change DALYs 2005-2015	Percent change DALYs PAF 2005-2015
Endocarditis	-	-	-	-	-	-	-	-	-	-	-	-
Other cardiovascular and circulatory diseases	-	-	-	-	-	-	-	-	-	-	-	-
Diabetes mellitus	1·8 (1·2 to 2·6)	0·9% (0·6 to 1·3%)	1·7 (1·1 to 2·5)	0·8% (0·5 to 1·2%)	-5·4% (-9·9 to 0·0%)	-10·3% (-14·1 to -6·4%)	134·5 (86·0 to 205·3)	1·7% (1·1 to 2·4%)	147·6 (91·7 to 229·0)	1·6% (1·1 to 2·4%)	9·7% (4·5 to 14·5%)	-4·1% (-7·4 to -0·7%)
Chronic kidney disease due to diabetes mellitus	0·3 (0·2 to 0·6)	0·5% (0·2 to 0·8%)	0·5 (0·3 to 0·9)	0·5% (0·3 to 0·9%)	58·3% (47·8 to 70·2%)	11·7% (4·4 to 19·0%)	11·4 (5·1 to 20·4)	0·8% (0·3 to 1·3%)	15·8 (7·5 to 27·4)	0·8% (0·4 to 1·5%)	38·7% (27·4 to 56·4%)	8·3% (0·1 to 21·7%)
Chronic kidney disease due to hypertension	0·4 (0·2 to 0·7)	0·5% (0·3 to 0·9%)	0·5 (0·2 to 0·8)	0·4% (0·2 to 0·8%)	8·8% (-6·8 to 23·7%)	-16·0% (-27·9 to -5·0%)	10·9 (5·2 to 18·7)	0·8% (0·4 to 1·5%)	11·4 (5·6 to 19·6)	0·8% (0·4 to 1·4%)	4·8% (-2·5 to 13·9%)	-9·0% (-14·4 to -2·1%)
Chronic kidney disease due to glomerulonephritis	0·1 (0·0 to 0·1)	0·4% (0·1 to 0·7%)	0·1 (0·0 to 0·2)	0·3% (0·1 to 0·6%)	18·9% (0·6 to 32·2%)	-10·5% (-24·5 to -1·2%)	3·5 (1·3 to 6·6)	0·7% (0·3 to 1·2%)	3·8 (1·5 to 7·1)	0·6% (0·3 to 1·1%)	11·4% (4·3 to 23·0%)	-3·5% (-8·9 to 6·4%)
Chronic kidney disease due to other causes	0·0 (0·0 to 0·0)	0·2% (0·0 to 0·5%)	0·0 (0·0 to 0·0)	0·2% (0·0 to 0·4%)	26·4% (-0·7 to 37·8%)	-9·0% (-28·8 to -1·6%)	3·1 (1·2 to 6·0)	0·6% (0·2 to 1·1%)	3·7 (1·5 to 6·9)	0·6% (0·3 to 1·1%)	17·2% (8·8 to 30·2%)	-1·6% (-7·9 to 8·9%)
**Diet low in fibre: All causes**	106·6 (56·2 to 174·9)	1·3% (0·7 to 2·1%)	117·2 (61·3 to 191·6)	1·2% (0·6 to 2·0%)	9·9% (7·5 to 12·7%)	-4·0% (-6·0 to -1·6%)	1,742·7 (914·2 to 2,869·7)	0·7% (0·3 to 1·1%)	1,747·0 (913·4 to 2,876·5)	0·6% (0·3 to 1·1%)	0·2% (-2·2 to 2·6%)	-5·9% (-8·3 to -3·7%)
Colon and rectum cancer	21·8 (10·0 to 37·4)	8·1% (3·7 to 13·9%)	26·2 (12·0 to 44·9)	8·3% (3·8 to 14·1%)	20·0% (16·7 to 24·0%)	2·1% (0·4 to 4·1%)	373·3 (170·7 to 643·6)	7·8% (3·5 to 13·4%)	416·4 (191·9 to 717·8)	7·8% (3·6 to 13·5%)	11·5% (8·2 to 15·0%)	0·3% (-1·7 to 2·2%)
Ischaemic heart disease	84·8 (42·7 to 142·4)	5·4% (2·7 to 9·1%)	91·0 (45·9 to 153·2)	5·5% (2·8 to 9·3%)	7·3% (4·8 to 10·1%)	1·4% (-0·3 to 3·5%)	1,369·4 (684·4 to 2,312·5)	6·3% (3·2 to 10·6%)	1,330·6 (665·8 to 2,246·5)	6·3% (3·1 to 10·5%)	-2·8% (-5·1 to -0·8%)	-1·2% (-3·1 to 0·4%)
**Diet low in calcium: All causes**	41·8 (22·2 to 64·5)	0·5% (0·3 to 0·8%)	50·2 (27·2 to 77·3)	0·5% (0·3 to 0·8%)	20·2% (17·0 to 24·0%)	5·0% (2·2 to 8·3%)	723·4 (386·1 to 1,124·0)	0·3% (0·1 to 0·4%)	814·3 (439·9 to 1,267·5)	0·3% (0·2 to 0·5%)	12·6% (9·6 to 15·7%)	5·6% (2·8 to 8·6%)
Colon and rectum cancer	41·8 (22·2 to 64·5)	15·5% (8·3 to 24·0%)	50·2 (27·2 to 77·3)	15·9% (8·6 to 24·4%)	20·2% (17·0 to 24·0%)	2·2% (1·0 to 4·2%)	723·4 (386·1 to 1,124·0)	15·1% (8·0 to 23·7%)	814·3 (439·9 to 1,267·5)	15·3% (8·1 to 23·9%)	12·6% (9·6 to 15·7%)	1·2% (0·1 to 2·7%)
Prostate cancer	-	-	-	-	-	-	-	-	-	-	-	-
**Diet low in seafood omega-3 fatty acids: All causes**	194·6 (79·3 to 326·3)	2·3% (0·9 to 3·9%)	200·7 (81·6 to 336·4)	2·1% (0·8 to 3·5%)	3·1% (1·2 to 4·6%)	-9·9% (-11·5 to -8·7%)	3,033·6 (1,248·2 to 5,063·6)	1·2% (0·5 to 2·0%)	2,884·8 (1,183·7 to 4,836·1)	1·1% (0·4 to 1·8%)	-4·9% (-6·4 to -3·7%)	-10·7% (-12·2 to -9·5%)
Ischaemic heart disease	194·6 (79·3 to 326·3)	12·5% (5·1 to 20·9%)	200·7 (81·6 to 336·4)	12·2% (4·9 to 20·4%)	3·1% (1·2 to 4·6%)	-2·5% (-3·0 to -2·1%)	3,033·6 (1,248·2 to 5,063·6)	14·0% (5·7 to 23·3%)	2,884·8 (1,183·7 to 4,836·1)	13·6% (5·5 to 22·6%)	-4·9% (-6·4 to -3·7%)	-3·3% (-4·1 to -2·7%)
**Diet low in polyunsaturated fatty acids: All causes**	52·5 (21·4 to 83·0)	0·6% (0·3 to 1·0%)	57·1 (23·0 to 90·2)	0·6% (0·2 to 0·9%)	8·8% (3·9 to 13·1%)	-4·9% (-9·1 to -1·2%)	630·0 (261·5 to 1,003·0)	0·2% (0·1 to 0·4%)	637·0 (261·6 to 1,010·5)	0·2% (0·1 to 0·4%)	1·1% (-3·0 to 5·2%)	-5·1% (-9·0 to -1·1%)
Ischaemic heart disease	52·5 (21·4 to 83·0)	3·4% (1·4 to 5·3%)	57·1 (23·0 to 90·2)	3·5% (1·4 to 5·5%)	8·8% (3·9 to 13·1%)	2·8% (-1·4 to 6·5%)	630·0 (261·5 to 1,003·0)	2·9% (1·2 to 4·6%)	637·0 (261·6 to 1,010·5)	3·0% (1·2 to 4·7%)	1·1% (-3·0 to 5·2%)	2·8% (-1·0 to 7·0%)
**Diet high in trans fatty acids: All causes**	68·1 (27·0 to 126·9)	0·8% (0·3 to 1·5%)	68·1 (26·8 to 130·2)	0·7% (0·3 to 1·3%)	0·1% (-4·9 to 3·4%)	-12·6% (-17·0 to -9·7%)	1,304·4 (557·2 to 2,333·0)	0·5% (0·2 to 0·9%)	1,225·4 (515·5 to 2,234·0)	0·4% (0·2 to 0·8%)	-6·1% (-10·6 to -3·0%)	-11·8% (-16·1 to -9·1%)
Ischaemic heart disease	68·1 (27·0 to 126·9)	4·4% (1·7 to 8·2%)	68·1 (26·8 to 130·2)	4·1% (1·6 to 7·9%)	0·1% (-4·9 to 3·4%)	-5·4% (-10·0 to -2·8%)	1,304·4 (557·2 to 2,333·0)	6·0% (2·6 to 10·8%)	1,225·4 (515·5 to 2,234·0)	5·8% (2·4 to 10·5%)	-6·1% (-10·6 to -3·0%)	-4·5% (-9·0 to -1·7%)
**Diet high in sodium: All causes**	362·5 (134·5 to 708·9)	4·3% (1·6 to 8·4%)	391·3 (144·2 to 769·9)	4·1% (1·5 to 8·0%)	7·9% (5·1 to 11·2%)	-5·7% (-8·2 to -2·9%)	5,774·4 (2,211·1 to 10,897·4)	2·3% (0·9 to 4·4%)	5,763·6 (2,192·8 to 10,980·7)	2·1% (0·8 to 4·1%)	-0·2% (-2·5 to 1·8%)	-6·3% (-8·5 to -4·5%)
Stomach cancer	50·7 (20·8 to 89·5)	32·0% (13·1 to 56·9%)	54·1 (20·2 to 98·3)	32·2% (12·0 to 58·9%)	6·8% (-6·9 to 17·1%)	0·3% (-12·3 to 9·9%)	909·0 (409·1 to 1,516·2)	32·6% (14·8 to 54·4%)	879·9 (390·5 to 1,485·9)	32·5% (14·4 to 54·8%)	-3·2% (-9·6 to 2·9%)	-0·2% (-6·4 to 5·5%)
Rheumatic heart disease	1·6 (0·4 to 4·1)	4·3% (1·1 to 11·1%)	1·6 (0·4 to 4·2)	4·2% (1·1 to 11·1%)	1·0% (-6·5 to 9·4%)	-2·1% (-8·8 to 5·9%)	23·1 (6·6 to 54·1)	4·4% (1·3 to 10·4%)	20·8 (5·8 to 48·8)	4·4% (1·2 to 10·3%)	-9·7% (-14·8 to -4·0%)	-1·4% (-6·1 to 4·4%)
Ischaemic heart disease	162·0 (51·0 to 344·2)	10·4% (3·3 to 22·2%)	169·8 (53·2 to 362·2)	10·3% (3·2 to 22·1%)	4·8% (1·0 to 8·5%)	-0·9% (-4·6 to 2·4%)	2,416·1 (827·0 to 4,974·7)	11·2% (3·8 to 23·3%)	2,357·6 (799·0 to 4,912·3)	11·1% (3·7 to 23·3%)	-2·4% (-5·1 to -0·1%)	-0·8% (-3·3 to 1·3%)
Ischaemic stroke	44·4 (14·1 to 95·6)	9·3% (2·9 to 19·8%)	44·9 (14·1 to 98·0)	9·0% (2·8 to 19·7%)	1·1% (-3·9 to 6·7%)	-2·8% (-6·9 to 2·4%)	564·0 (194·8 to 1,159·6)	10·8% (3·7 to 21·9%)	527·6 (178·6 to 1,095·2)	10·4% (3·6 to 21·3%)	-6·5% (-10·3 to -2·2%)	-3·1% (-6·3 to 1·2%)
Hemorrhagic stroke	39·7 (13·2 to 83·5)	12·1% (4·0 to 25·6%)	41·0 (13·4 to 89·5)	11·9% (3·8 to 25·7%)	3·4% (-3·9 to 10·9%)	-2·0% (-8·3 to 4·0%)	697·0 (247·9 to 1,373·6)	13·3% (4·7 to 26·2%)	644·1 (226·4 to 1,311·6)	13·1% (4·6 to 26·2%)	-7·6% (-11·8 to -3·1%)	-1·6% (-5·1 to 2·2%)
Hypertensive heart disease	21·3 (3·6 to 60·1)	19·9% (3·4 to 56·0%)	26·9 (4·3 to 76·5)	19·7% (3·2 to 56·1%)	26·4% (16·7 to 33·6%)	-0·7% (-7·5 to 3·3%)	284·8 (69·6 to 706·8)	20·2% (5·0 to 50·0%)	329·8 (78·1 to 823·6)	20·0% (4·8 to 50·0%)	15·8% (8·8 to 21·0%)	-0·9% (-5·4 to 2·4%)
Cardiomyopathy and myocarditis	5·3 (1·7 to 11·7)	6·0% (1·9 to 13·1%)	5·6 (1·7 to 12·5)	5·8% (1·8 to 13·0%)	4·7% (-0·8 to 10·3%)	-2·3% (-6·1 to 1·6%)	98·5 (32·0 to 205·5)	6·2% (2·0 to 13·0%)	96·3 (31·3 to 204·8)	6·3% (2·1 to 13·2%)	-2·3% (-6·3 to 2·0%)	0·5% (-2·2 to 3·1%)
Atrial fibrillation and flutter	3·7 (1·1 to 8·1)	5·6% (1·7 to 12·1%)	4·9 (1·5 to 10·8)	5·5% (1·7 to 12·0%)	31·5% (26·8 to 36·6%)	-1·4% (-4·0 to 1·7%)	107·8 (33·9 to 229·7)	7·3% (2·4 to 15·1%)	130·8 (40·9 to 280·5)	7·2% (2·4 to 15·0%)	21·3% (18·5 to 24·1%)	-1·6% (-3·7 to 0·5%)
Aortic aneurysm	4·4 (1·5 to 9·3)	6·6% (2·2 to 13·9%)	5·0 (1·7 to 10·5)	6·5% (2·2 to 13·6%)	12·6% (7·4 to 18·9%)	-1·6% (-5·4 to 3·6%)	70·3 (24·3 to 143·0)	7·4% (2·6 to 15·0%)	72·9 (25·4 to 149·0)	7·4% (2·6 to 15·0%)	3·8% (-0·2 to 8·0%)	-0·3% (-2·6 to 2·9%)
Peripheral vascular disease	1·0 (0·3 to 2·3)	4·1% (1·1 to 9·5%)	1·2 (0·3 to 3·0)	4·0% (1·1 to 9·3%)	27·2% (18·5 to 37·1%)	-2·4% (-6·4 to 1·2%)	18·8 (5·4 to 42·6)	4·8% (1·5 to 10·6%)	22·7 (6·5 to 51·3)	4·7% (1·4 to 10·5%)	20·3% (15·2 to 26·8%)	-2·1% (-4·9 to 0·3%)
Endocarditis	1·4 (0·4 to 3·2)	5·7% (1·7 to 12·8%)	1·7 (0·5 to 3·8)	5·6% (1·7 to 12·6%)	18·8% (12·4 to 25·4%)	-2·4% (-6·7 to 2·1%)	22·8 (7·3 to 48·7)	6·2% (2·0 to 13·1%)	24·9 (7·9 to 53·3)	6·2% (2·0 to 13·2%)	9·1% (4·0 to 13·6%)	-0·4% (-3·3 to 2·2%)
Other cardiovascular and circulatory diseases	9·2 (2·8 to 19·2)	6·2% (1·9 to 13·1%)	10·5 (3·1 to 22·0)	6·1% (1·8 to 12·7%)	14·8% (9·7 to 19·9%)	-3·1% (-6·3 to 0·1%)	222·2 (69·6 to 465·6)	7·4% (2·3 to 15·1%)	240·0 (75·5 to 505·9)	7·3% (2·3 to 15·0%)	8·0% (4·9 to 11·1%)	-1·1% (-3·2 to 1·0%)
Chronic kidney disease due to diabetes mellitus	7·6 (2·4 to 15·5)	10·6% (3·3 to 22·0%)	10·6 (3·2 to 21·7)	10·5% (3·2 to 21·8%)	39·5% (34·0 to 44·9%)	-1·6% (-4·3 to 0·1%)	140·6 (44·1 to 294·2)	9·4% (3·0 to 19·5%)	180·3 (55·5 to 379·5)	9·4% (3·0 to 19·6%)	28·2% (23·9 to 31·7%)	0·1% (-2·6 to 1·8%)
Chronic kidney disease due to hypertension	8·0 (2·5 to 17·1)	9·8% (3·1 to 21·0%)	10·4 (3·3 to 22·2)	9·9% (3·1 to 21·2%)	30·5% (25·3 to 36·1%)	0·7% (-1·2 to 3·4%)	115·6 (36·3 to 247·1)	9·0% (2·8 to 19·2%)	135·0 (42·5 to 289·7)	9·1% (2·9 to 19·4%)	16·8% (13·4 to 20·6%)	1·4% (-0·4 to 3·5%)
Chronic kidney disease due to glomerulonephritis	2·0 (0·6 to 4·2)	10·2% (3·2 to 21·5%)	2·6 (0·8 to 5·5)	10·2% (3·1 to 21·5%)	32·8% (27·3 to 38·4%)	-0·1% (-2·4 to 1·9%)	42·8 (13·1 to 92·2)	8·2% (2·5 to 17·3%)	50·8 (15·5 to 109·8)	8·4% (2·5 to 17·9%)	18·7% (15·3 to 21·9%)	2·9% (0·8 to 4·7%)
Chronic kidney disease due to other causes	0·3 (0·1 to 0·7)	9·3% (2·7 to 20·4%)	0·4 (0·1 to 0·9)	9·5% (2·8 to 20·9%)	41·9% (34·9 to 49·5%)	2·2% (-0·6 to 5·0%)	41·2 (12·2 to 91·8)	8·1% (2·5 to 17·3%)	50·4 (15·1 to 112·7)	8·3% (2·6 to 17·9%)	22·5% (18·6 to 26·3%)	2·9% (0·6 to 5·0%)
**Sexual abuse and violence: All causes**	36·0 (24·2 to 51·8)	0·4% (0·3 to 0·6%)	37·1 (25·2 to 53·0)	0·4% (0·3 to 0·5%)	3·0% (0·2 to 6·0%)	-10·0% (-12·4 to -7·3%)	2,640·1 (1,898·8 to 3,498·9)	1·0% (0·8 to 1·3%)	2,695·8 (1,959·9 to 3,552·0)	1·0% (0·7 to 1·3%)	2·1% (0·0 to 4·6%)	-4·2% (-6·1 to -2·0%)
**Childhood sexual abuse: All causes**	18·7 (5·1 to 36·7)	0·2% (0·1 to 0·4%)	18·7 (5·1 to 37·4)	0·2% (0·1 to 0·4%)	0·3% (-3·3 to 2·8%)	-12·3% (-15·5 to -10·2%)	1,233·2 (519·9 to 2,120·9)	0·5% (0·2 to 0·8%)	1,214·9 (514·0 to 2,118·8)	0·4% (0·2 to 0·7%)	-1·5% (-4·6 to 1·3%)	-7·5% (-10·4 to -4·9%)
Alcohol use disorders	1·1 (0·0 to 2·6)	4·2% (0·0 to 9·6%)	1·1 (0·0 to 2·6)	3·8% (0·0 to 8·8%)	-3·7% (-8·3 to 1·2%)	-8·3% (-11·4 to 0·0%)	88·5 (0·0 to 205·6)	4·6% (0·0 to 10·5%)	81·8 (0·0 to 190·1)	4·3% (0·0 to 9·8%)	-7·5% (-10·8 to 0·0%)	-8·0% (-10·5 to 0·0%)
Major depressive disorder	-	-	-	-	-	-	353·7 (38·9 to 784·1)	5·5% (0·7 to 11·4%)	365·2 (40·1 to 813·6)	5·1% (0·6 to 10·6%)	3·3% (0·6 to 5·3%)	-7·3% (-9·2 to -5·6%)
Dysthymia	-	-	-	-	-	-	80·0 (8·8 to 176·9)	5·3% (0·6 to 11·2%)	81·6 (8·9 to 179·7)	4·9% (0·6 to 10·3%)	2·0% (-1·3 to 5·1%)	-8·0% (-10·0 to -6·0%)
Self-harm	17·5 (4·1 to 36·0)	11·4% (2·7 to 23·5%)	17·6 (4·1 to 36·3)	10·5% (2·4 to 21·8%)	0·6% (-2·9 to 3·0%)	-7·8% (-9·7 to -6·4%)	711·0 (165·7 to 1,439·7)	12·1% (2·8 to 24·6%)	686·2 (158·3 to 1,395·5)	11·4% (2·6 to 23·2%)	-3·5% (-6·6 to -1·3%)	-6·3% (-8·2 to -4·8%)
**Intimate partner violence: All causes**	20·0 (16·8 to 23·2)	0·2% (0·2 to 0·3%)	21·1 (17·4 to 24·6)	0·2% (0·2 to 0·3%)	5·5% (1·3 to 9·7%)	-7·8% (-11·5 to -4·1%)	1,571·6 (1,255·9 to 1,923·9)	0·6% (0·5 to 0·7%)	1,647·6 (1,306·0 to 2,039·0)	0·6% (0·5 to 0·7%)	4·8% (2·0 to 7·6%)	-1·6% (-4·2 to 1·0%)

Supplemental Results Table 1. All-age deaths, death PAFs, DALYs, DALY PAFs, and percent change for each risk factor and outcome for both sexes combined in 2005 and 2015 for High-income.

Data in parenthesis are 95% uncertainty intervals. PAFs=population attributable fractions. DALYs=disability-adjusted life-years.

	2005 Deaths (in thousands)	2005 Deaths PAF	2015 Deaths (in thousands)	2015 Deaths PAF	Percent change deaths 2005-2015	Percent change deaths PAF 2005-2015	2005 DALYs (in thousands)	2005 DALYs PAF	2015 DALYs (in thousands)	2015 DALYs PAF	Percent change DALYs 2005-2015	Percent change DALYs PAF 2005-2015
HIV/AIDS - Tuberculosis	0·0 (0·0 to 0·1)	2·1% (1·0 to 3·2%)	0·0 (0·0 to 0·1)	2·1% (1·0 to 3·2%)	-17·4% (-21·3 to -13·5%)	1·2% (-2·7 to 4·9%)	1·8 (0·9 to 2·8)	1·9% (0·9 to 3·1%)	1·4 (0·7 to 2·2)	2·0% (0·9 to 3·1%)	-21·8% (-26·1 to -17·9%)	1·3% (-3·3 to 5·2%)
HIV/AIDS resulting in other diseases	0·5 (0·2 to 0·8)	2·5% (1·2 to 3·8%)	0·3 (0·1 to 0·5)	2·4% (1·2 to 3·7%)	-40·8% (-43·1 to -38·8%)	-3·3% (-7·1 to 0·0%)	23·3 (11·5 to 35·9)	2·4% (1·2 to 3·6%)	14·3 (7·1 to 22·1)	2·3% (1·1 to 3·5%)	-38·8% (-42·1 to -35·6%)	-3·2% (-7·2 to 0·2%)
Maternal abortion, miscarriage, and ectopic pregnancy	0·0 (0·0 to 0·0)	0·0% (0·0 to 0·0%)	0·0 (0·0 to 0·0)	0·0% (0·0 to 0·0%)	-14·2% (-27·6 to 0·2%)	-1·7% (-12·7 to 11·0%)	0·0 (0·0 to 0·0)	0·0% (0·0 to 0·0%)	0·0 (0·0 to 0·0)	0·0% (0·0 to 0·0%)	-11·0% (-26·0 to 6·2%)	-0·7% (-11·9 to 11·3%)
Major depressive disorder	-	-	-	-	-	-	599·1 (382·8 to 859·3)	9·3% (7·4 to 11·2%)	663·4 (426·6 to 951·2)	9·3% (7·4 to 11·1%)	10·7% (7·3 to 14·5%)	-0·6% (-3·5 to 2·7%)
Dysthymia	-	-	-	-	-	-	138·9 (87·2 to 207·5)	9·3% (7·6 to 11·1%)	153·5 (95·0 to 226·8)	9·2% (7·4 to 11·0%)	10·5% (6·2 to 15·1%)	-0·3% (-3·5 to 3·1%)
Self-harm	16·2 (13·0 to 19·4)	10·6% (8·5 to 12·6%)	17·7 (14·1 to 21·2)	10·6% (8·5 to 12·7%)	9·2% (4·2 to 14·2%)	0·1% (-4·2 to 4·8%)	623·9 (490·7 to 751·1)	10·7% (8·4 to 12·8%)	649·3 (515·2 to 780·9)	10·8% (8·5 to 12·9%)	4·1% (0·2 to 8·3%)	1·1% (-3·0 to 5·4%)
Assault by firearm	1·3 (1·0 to 1·4)	7·2% (6·1 to 7·7%)	1·3 (1·1 to 1·4)	7·7% (6·8 to 8·3%)	0·5% (-4·6 to 6·1%)	7·1% (0·9 to 13·5%)	62·2 (52·2 to 67·1)	6·6% (5·6 to 7·1%)	59·8 (52·5 to 65·1)	7·0% (6·2 to 7·6%)	-3·8% (-9·1 to 2·6%)	6·3% (-0·4 to 13·3%)
Assault by sharp object	0·7 (0·7 to 0·8)	11·5% (10·8 to 12·6%)	0·7 (0·6 to 0·8)	11·6% (10·6 to 12·6%)	-6·6% (-11·8 to -1·4%)	0·2% (-5·2 to 5·7%)	34·4 (31·9 to 38·2)	11·3% (10·6 to 12·3%)	30·3 (27·6 to 33·7)	11·3% (10·4 to 12·4%)	-11·9% (-16·8 to -6·6%)	-0·2% (-5·8 to 5·5%)
Assault by other means	1·2 (1·2 to 1·3)	15·2% (14·5 to 16·2%)	1·1 (1·0 to 1·2)	14·8% (13·9 to 15·9%)	-11·3% (-16·0 to -6·2%)	-2·5% (-6·4 to 1·5%)	88·1 (77·8 to 101·3)	16·9% (16·1 to 17·8%)	75·6 (64·7 to 89·3)	16·8% (15·9 to 17·7%)	-14·2% (-18·4 to -9·7%)	-0·9% (-3·7 to 1·9%)
**Unsafe sex: All causes**	47·4 (46·6 to 48·1)	0·6% (0·6 to 0·6%)	42·1 (40·9 to 43·4)	0·4% (0·4 to 0·4%)	-11·1% (-13·8 to -8·2%)	-22·3% (-24·7 to -19·8%)	1,789·0 (1,696·7 to 1,914·3)	0·7% (0·6 to 0·8%)	1,456·7 (1,359·4 to 1,591·8)	0·5% (0·5 to 0·6%)	-18·6% (-20·8 to -16·0%)	-23·6% (-25·5 to -21·4%)
HIV/AIDS - Tuberculosis	1·8 (1·7 to 1·8)	90·2% (89·5 to 90·9%)	1·4 (1·4 to 1·5)	90·5% (89·4 to 91·4%)	-18·1% (-21·1 to -14·9%)	0·3% (-0·5 to 1·0%)	82·2 (80·1 to 84·4)	90·1% (89·3 to 90·8%)	63·7 (61·4 to 66·0)	90·4% (89·3 to 91·4%)	-22·6% (-25·3 to -19·7%)	0·3% (-0·5 to 1·1%)
HIV/AIDS resulting in other diseases	17·8 (17·6 to 18·1)	88·3% (87·3 to 89·2%)	11·0 (10·8 to 11·1)	88·6% (87·6 to 89·5%)	-38·5% (-39·4 to -37·7%)	0·3% (-0·1 to 0·7%)	874·8 (834·6 to 920·7)	88·5% (87·5 to 89·4%)	555·2 (515·3 to 606·2)	88·8% (87·8 to 89·7%)	-36·5% (-38·6 to -34·2%)	0·4% (-0·1 to 0·8%)
Syphilis	0·7 (0·6 to 0·9)	100·0% (100·0 to 100·0%)	0·6 (0·5 to 0·8)	100·0% (100·0 to 100·0%)	-11·4% (-25·7 to 3·6%)	-	54·9 (41·6 to 70·3)	100·0% (100·0 to 100·0%)	47·7 (36·2 to 61·8)	100·0% (100·0 to 100·0%)	-13·2% (-29·3 to 2·4%)	-
Chlamydial infection	0·0 (0·0 to 0·0)	100·0% (100·0 to 100·0%)	0·0 (0·0 to 0·0)	100·0% (100·0 to 100·0%)	11·0% (-0·1 to 19·8%)	-	22·7 (13·3 to 38·6)	100·0% (100·0 to 100·0%)	27·8 (16·1 to 47·3)	100·0% (100·0 to 100·0%)	22·4% (17·5 to 26·7%)	-
Gonococcal infection	0·0 (0·0 to 0·1)	100·0% (100·0 to 100·0%)	0·1 (0·0 to 0·1)	100·0% (100·0 to 100·0%)	3·7% (-7·8 to 13·4%)	-	10·2 (6·3 to 16·0)	100·0% (100·0 to 100·0%)	11·0 (6·9 to 17·8)	100·0% (100·0 to 100·0%)	8·8% (-7·4 to 35·2%)	-
Trichomoniasis	-	-	-	-	-	-	22·6 (9·0 to 47·9)	100·0% (100·0 to 100·0%)	23·9 (9·5 to 50·3)	100·0% (100·0 to 100·0%)	5·8% (4·1 to 7·4%)	-
Genital herpes	-	-	-	-	-	-	34·5 (10·3 to 84·0)	100·0% (100·0 to 100·0%)	36·7 (10·9 to 88·5)	100·0% (100·0 to 100·0%)	6·5% (4·2 to 8·5%)	-
Other sexually transmitted diseases	0·0 (0·0 to 0·0)	100·0% (100·0 to 100·0%)	0·0 (0·0 to 0·0)	100·0% (100·0 to 100·0%)	0·4% (-10·7 to 9·8%)	-	13·9 (9·6 to 19·5)	100·0% (100·0 to 100·0%)	13·4 (9·2 to 18·6)	100·0% (100·0 to 100·0%)	-4·2% (-7·7 to -0·3%)	-
Cervical cancer	27·0 (26·3 to 27·6)	100·0% (100·0 to 100·0%)	29·0 (27·7 to 30·2)	100·0% (100·0 to 100·0%)	7·5% (2·7 to 12·6%)	-	673·2 (654·6 to 692·2)	100·0% (100·0 to 100·0%)	677·4 (649·8 to 705·7)	100·0% (100·0 to 100·0%)	0·6% (-3·6 to 5·0%)	-
**Low physical activity: All causes**	348·9 (287·1 to 411·7)	4·1% (3·4 to 4·9%)	374·6 (311·7 to 438·8)	3·9% (3·2 to 4·5%)	7·4% (6·1 to 8·7%)	-6·2% (-7·4 to -5·0%)	6,092·6 (4,993·4 to 7,234·1)	2·4% (2·0 to 2·8%)	6,395·2 (5,283·0 to 7,567·9)	2·3% (2·0 to 2·8%)	5·0% (3·0 to 7·2%)	-1·5% (-3·0 to 0·4%)
Colon and rectum cancer	46·1 (33·3 to 58·9)	17·1% (12·3 to 21·8%)	54·9 (39·8 to 69·9)	17·3% (12·5 to 22·0%)	18·9% (16·0 to 22·3%)	1·1% (0·6 to 1·8%)	774·5 (548·1 to 992·7)	16·2% (11·4 to 20·8%)	869·4 (623·2 to 1,115·6)	16·4% (11·6 to 21·0%)	12·2% (9·5 to 15·2%)	0·9% (0·4 to 1·6%)
Breast cancer	17·9 (13·2 to 22·8)	11·4% (8·5 to 14·4%)	20·5 (15·1 to 26·0)	11·5% (8·5 to 14·5%)	14·3% (9·6 to 19·3%)	0·7% (0·3 to 1·2%)	436·7 (322·0 to 556·4)	11·1% (8·2 to 14·0%)	484·2 (361·9 to 619·8)	11·2% (8·2 to 14·1%)	10·9% (5·9 to 16·6%)	0·6% (0·2 to 1·0%)
Ischaemic heart disease	179·5 (126·0 to 234·0)	11·5% (8·1 to 15·0%)	188·9 (133·0 to 246·8)	11·5% (8·1 to 14·9%)	5·2% (3·3 to 6·9%)	-0·5% (-1·0 to 0·3%)	2,662·9 (1,820·0 to 3,516·3)	12·3% (8·4 to 16·3%)	2,614·0 (1,795·3 to 3,447·9)	12·3% (8·5 to 16·2%)	-1·8% (-3·3 to -0·4%)	-0·2% (-0·7 to 0·6%)
Ischaemic stroke	63·2 (39·5 to 88·0)	13·2% (8·3 to 18·5%)	65·6 (41·1 to 91·0)	13·2% (8·3 to 18·4%)	3·7% (1·3 to 6·8%)	-0·3% (-0·9 to 0·8%)	730·5 (443·8 to 1,030·6)	13·9% (8·4 to 19·7%)	705·1 (435·6 to 992·2)	13·9% (8·5 to 19·7%)	-3·5% (-5·6 to -0·8%)	0·0% (-0·7 to 1·3%)
Diabetes mellitus	42·1 (33·9 to 50·9)	20·5% (16·4 to 24·8%)	44·8 (35·9 to 54·3)	20·7% (16·6 to 25·0%)	6·4% (2·1 to 10·6%)	0·9% (0·4 to 1·4%)	1,488·0 (1,101·4 to 1,989·9)	18·6% (14·6 to 22·8%)	1,722·5 (1,257·1 to 2,306·2)	18·8% (14·8 to 23·0%)	15·8% (12·1 to 19·0%)	1·2% (0·8 to 1·8%)
**Metabolic risks: All causes**	2,660·9 (2,504·3 to 2,820·9)	31·6% (29·7 to 33·5%)	2,888·7 (2,706·3 to 3,078·1)	29·9% (28·1 to 31·9%)	8·6% (7·5 to 9·5%)	-5·2% (-6·0 to -4·3%)	47,626·9 (43,790·0 to 51,889·1)	18·6% (17·4 to 19·8%)	49,668·3 (45,148·2 to 54,602·0)	18·2% (17·1 to 19·4%)	4·3% (2·9 to 5·5%)	-2·2% (-3·0 to -1·4%)
**High fasting plasma glucose: All causes**	763·1 (626·8 to 945·0)	9·1% (7·4 to 11·2%)	854·1 (701·8 to 1,058·0)	8·9% (7·3 to 11·0%)	11·9% (9·8 to 14·2%)	-2·2% (-4·1 to -0·3%)	16,766·9 (14,276·9 to 19,392·7)	6·5% (5·9 to 7·4%)	18,557·2 (15,623·0 to 21,649·1)	6·8% (6·1 to 7·6%)	10·7% (8·4 to 12·7%)	3·8% (2·2 to 5·5%)
Tuberculosis	1·3 (0·7 to 1·9)	8·2% (4·4 to 12·8%)	1·1 (0·6 to 1·7)	8·1% (4·2 to 12·6%)	-15·7% (-23·3 to -6·6%)	-1·9% (-10·2 to 7·1%)	25·1 (15·2 to 36·1)	8·5% (5·2 to 12·4%)	19·0 (11·6 to 27·8)	8·7% (5·3 to 12·8%)	-24·2% (-29·0 to -19·3%)	2·2% (-3·1 to 7·4%)
Ischaemic heart disease	306·6 (182·7 to 469·6)	19·7% (11·7 to 30·1%)	333·6 (192·4 to 518·2)	20·2% (11·6 to 31·3%)	8·8% (3·9 to 13·4%)	2·8% (-1·6 to 6·6%)	4,377·5 (3,015·1 to 6,137·1)	20·2% (13·9 to 28·3%)	4,449·9 (3,032·5 to 6,240·3)	20·9% (14·3 to 29·2%)	1·7% (-0·9 to 4·2%)	3·3% (1·2 to 5·7%)
Ischaemic stroke	78·4 (39·5 to 156·9)	16·4% (8·3 to 32·8%)	82·7 (40·9 to 171·5)	16·6% (8·2 to 34·1%)	5·5% (0·0 to 10·9%)	1·4% (-3·1 to 6·7%)	939·8 (536·1 to 1,662·4)	17·9% (10·4 to 31·5%)	926·6 (530·5 to 1,686·0)	18·3% (10·6 to 32·8%)	-1·4% (-5·1 to 3·1%)	2·1% (-0·6 to 6·8%)
Hemorrhagic stroke	53·1 (35·0 to 79·8)	16·2% (10·8 to 24·3%)	56·0 (35·4 to 86·1)	16·2% (10·4 to 25·3%)	5·4% (-0·3 to 13·1%)	0·1% (-4·6 to 7·0%)	944·6 (661·5 to 1,289·6)	18·1% (12·6 to 24·6%)	907·1 (639·7 to 1,241·2)	18·5% (13·0 to 25·1%)	-4·0% (-7·5 to 1·0%)	2·3% (-1·0 to 6·8%)
Diabetes mellitus	205·2 (200·8 to 210·4)	100·0% (100·0 to 100·0%)	216·5 (207·6 to 225·0)	100·0% (100·0 to 100·0%)	5·5% (1·2 to 9·4%)	-	8,016·9 (6,527·8 to 9,831·4)	100·0% (100·0 to 100·0%)	9,166·5 (7,277·6 to 11,361·2)	100·0% (100·0 to 100·0%)	14·3% (10·9 to 17·4%)	-
Chronic kidney disease due to diabetes mellitus	71·3 (69·0 to 73·9)	100·0% (100·0 to 100·0%)	101·2 (96·4 to 106·9)	100·0% (100·0 to 100·0%)	41·8% (37·3 to 46·8%)	-	1,490·0 (1,360·8 to 1,619·6)	100·0% (100·0 to 100·0%)	1,907·9 (1,757·9 to 2,072·9)	100·0% (100·0 to 100·0%)	28·0% (25·4 to 31·0%)	-
Chronic kidney disease due to hypertension	33·6 (24·0 to 42·9)	41·4% (29·7 to 52·1%)	44·3 (32·0 to 56·5)	42·2% (30·3 to 53·1%)	32·0% (26·7 to 37·0%)	1·9% (1·1 to 2·8%)	515·6 (366·1 to 658·6)	40·0% (28·6 to 50·7%)	610·8 (434·3 to 781·4)	41·2% (29·5 to 51·9%)	18·5% (15·3 to 21·6%)	2·8% (2·2 to 3·5%)
Chronic kidney disease due to glomerulonephritis	7·8 (5·6 to 10·1)	39·9% (28·5 to 50·5%)	10·6 (7·6 to 13·7)	41·0% (29·4 to 51·9%)	36·6% (31·4 to 41·5%)	2·8% (1·9 to 3·8%)	187·5 (129·7 to 249·7)	35·8% (25·2 to 45·9%)	228·0 (158·7 to 303·8)	37·7% (26·8 to 48·2%)	21·6% (18·8 to 24·6%)	5·3% (4·6 to 6·3%)
Chronic kidney disease due to other causes	1·2 (0·8 to 1·6)	37·7% (26·7 to 48·1%)	1·7 (1·2 to 2·3)	39·3% (27·8 to 50·2%)	44·5% (37·4 to 51·5%)	4·0% (2·2 to 6·1%)	180·1 (116·3 to 257·7)	35·3% (24·9 to 45·6%)	226·0 (147·1 to 322·5)	37·2% (26·4 to 47·7%)	25·5% (22·2 to 29·2%)	5·5% (4·6 to 6·6%)
**High total cholesterol: All causes**	775·3 (565·6 to 1,026·2)	9·2% (6·7 to 12·2%)	790·8 (555·4 to 1,069·0)	8·2% (5·8 to 11·1%)	2·0% (-1·9 to 5·2%)	-10·9% (-14·4 to -8·2%)	11,889·2 (9,782·6 to 14,397·3)	4·6% (3·8 to 5·7%)	11,287·2 (9,162·6 to 13,807·1)	4·1% (3·3 to 5·2%)	-5·1% (-7·1 to -3·3%)	-10·9% (-12·9 to -9·2%)
Ischaemic heart disease	678·8 (480·0 to 870·1)	43·6% (30·9 to 55·6%)	693·4 (472·4 to 908·5)	42·0% (29·0 to 54·7%)	2·1% (-2·3 to 5·5%)	-3·5% (-7·2 to -0·8%)	10,724·6 (8,881·8 to 12,732·1)	49·6% (41·1 to 58·4%)	10,194·9 (8,334·7 to 12,195·3)	47·9% (39·2 to 56·8%)	-4·9% (-7·1 to -3·0%)	-3·4% (-5·2 to -1·8%)
Ischaemic stroke	96·5 (24·0 to 216·5)	20·2% (5·0 to 45·3%)	97·4 (22·6 to 225·0)	19·6% (4·6 to 44·9%)	0·9% (-7·8 to 5·6%)	-3·0% (-11·0 to 0·6%)	1,164·6 (530·2 to 2,180·0)	22·2% (10·1 to 41·4%)	1,092·3 (478·4 to 2,073·6)	21·6% (9·5 to 40·8%)	-6·2% (-10·9 to -2·2%)	-2·8% (-7·1 to 0·5%)
**High systolic blood pressure: All causes**	1,617·8 (1,402·2 to 1,826·4)	19·2% (16·6 to 21·7%)	1,714·0 (1,480·5 to 1,955·1)	17·8% (15·3 to 20·3%)	5·9% (4·4 to 7·5%)	-7·4% (-8·8 to -6·2%)	23,891·5 (21,328·6 to 26,321·7)	9·3% (8·1 to 10·6%)	23,530·5 (20,866·9 to 25,980·5)	8·6% (7·4 to 9·8%)	-1·5% (-2·7 to -0·4%)	-7·6% (-8·6 to -6·6%)
Rheumatic heart disease	9·4 (5·7 to 17·2)	25·9% (15·4 to 47·2%)	9·2 (5·2 to 17·8)	24·6% (14·0 to 46·8%)	-2·1% (-9·2 to 5·3%)	-5·0% (-10·9 to 1·3%)	133·1 (88·8 to 207·8)	25·6% (17·2 to 40·4%)	116·5 (76·3 to 186·5)	24·5% (16·1 to 39·7%)	-12·5% (-16·9 to -7·2%)	-4·3% (-8·2 to 0·8%)
Ischaemic heart disease	825·7 (631·9 to 1,012·2)	53·0% (40·7 to 65·2%)	838·4 (621·6 to 1,051·3)	50·9% (37·8 to 64·1%)	1·5% (-1·8 to 4·2%)	-4·0% (-7·2 to -1·9%)	11,557·9 (9,670·3 to 13,430·2)	53·4% (44·5 to 62·0%)	10,926·9 (8,994·3 to 12,834·7)	51·3% (42·2 to 60·2%)	-5·5% (-7·5 to -3·7%)	-3·9% (-5·5 to -2·6%)
Ischaemic stroke	220·2 (159·5 to 290·9)	46·0% (33·3 to 61·0%)	215·8 (153·7 to 292·0)	43·4% (30·5 to 58·5%)	-2·0% (-6·2 to 2·1%)	-5·8% (-9·0 to -2·3%)	2,571·0 (2,030·7 to 3,129·2)	49·1% (38·9 to 59·1%)	2,349·3 (1,836·6 to 2,887·9)	46·5% (36·6 to 57·0%)	-8·6% (-11·6 to -5·4%)	-5·3% (-7·5 to -2·5%)

Supplemental Results Table 1. All-age deaths, death PAFs, DALYs, DALY PAFs, and percent change for each risk factor and outcome for both sexes combined in 2005 and 2015 for High-income.

Data in parenthesis are 95% uncertainty intervals. PAFs=population attributable fractions. DALYs=disability-adjusted life-years.

	2005 Deaths (in thousands)	2005 Deaths PAF	2015 Deaths (in thousands)	2015 Deaths PAF	Percent change deaths 2005-2015	Percent change deaths PAF 2005-2015	2005 DALYs (in thousands)	2005 DALYs PAF	2015 DALYs (in thousands)	2015 DALYs PAF	Percent change DALYs 2005-2015	Percent change DALYs PAF 2005-2015
Hemorrhagic stroke	180·9 (135·3 to 224·5)	55·2% (41·6 to 68·5%)	181·8 (131·6 to 235·7)	52·7% (38·0 to 67·5%)	0·5% (-5·3 to 6·6%)	-4·6% (-9·1 to -0·5%)	2,975·7 (2,458·1 to 3,457·1)	56·9% (47·1 to 66·1%)	2,688·0 (2,183·9 to 3,217·3)	54·7% (45·0 to 64·7%)	-9·7% (-12·8 to -5·8%)	-3·8% (-6·2 to -1·5%)
Hypertensive heart disease	107·2 (102·9 to 111·2)	100·0% (100·0 to 100·0%)	136·5 (128·9 to 143·6)	100·0% (100·0 to 100·0%)	27·3% (21·1 to 33·1%)	-	1,412·7 (1,364·9 to 1,464·9)	100·0% (100·0 to 100·0%)	1,649·9 (1,579·9 to 1,719·6)	100·0% (100·0 to 100·0%)	16·8% (11·1 to 20·6%)	-
Cardiomyopathy and myocarditis	30·0 (22·8 to 39·4)	33·6% (25·5 to 43·9%)	30·5 (22·6 to 40·9)	31·8% (23·6 to 43·0%)	1·5% (-3·5 to 6·9%)	-5·4% (-8·6 to -1·6%)	525·4 (422·5 to 630·6)	33·3% (26·8 to 39·9%)	495·5 (393·0 to 600·4)	32·3% (25·7 to 39·3%)	-5·7% (-9·0 to -2·1%)	-3·0% (-5·0 to -0·7%)
Atrial fibrillation and flutter	23·1 (17·3 to 30·1)	34·9% (27·2 to 42·9%)	29·1 (21·4 to 38·3)	32·9% (25·5 to 40·8%)	25·9% (21·7 to 30·5%)	-5·6% (-8·0 to -3·3%)	583·0 (449·3 to 750·8)	39·6% (34·2 to 44·8%)	679·6 (522·7 to 877·6)	37·5% (32·2 to 42·8%)	16·6% (14·5 to 18·6%)	-5·4% (-6·8 to -4·2%)
Aortic aneurysm	23·3 (17·8 to 28·4)	34·7% (26·7 to 42·3%)	24·9 (18·9 to 31·0)	32·4% (24·5 to 40·5%)	6·9% (2·4 to 11·7%)	-6·6% (-9·4 to -3·6%)	352·5 (283·5 to 412·8)	37·2% (30·2 to 43·8%)	349·1 (279·9 to 410·7)	35·4% (28·4 to 41·8%)	-1·0% (-4·4 to 2·4%)	-4·9% (-6·4 to -3·2%)
Peripheral vascular disease	6·2 (4·3 to 8·4)	26·2% (18·3 to 35·2%)	7·7 (5·2 to 10·6)	24·8% (17·0 to 33·9%)	23·7% (14·3 to 34·3%)	-5·1% (-8·3 to -2·1%)	107·6 (76·5 to 149·1)	27·6% (20·9 to 34·5%)	124·6 (86·8 to 171·6)	26·0% (19·5 to 32·7%)	15·8% (10·8 to 21·9%)	-5·8% (-7·8 to -3·8%)
Endocarditis	8·2 (6·2 to 11·0)	33·6% (25·2 to 44·7%)	9·5 (6·9 to 12·8)	31·8% (23·4 to 43·4%)	15·1% (8·7 to 21·2%)	-5·4% (-8·7 to -1·8%)	125·5 (100·6 to 152·3)	34·1% (27·2 to 41·3%)	132·6 (103·4 to 161·7)	32·9% (26·0 to 40·3%)	5·7% (1·4 to 9·7%)	-3·6% (-5·4 to -1·4%)
Other cardiovascular and circulatory diseases	54·9 (45·8 to 64·1)	37·4% (31·2 to 43·7%)	61·2 (49·8 to 72·7)	35·2% (28·8 to 41·9%)	11·5% (6·1 to 16·6%)	-5·9% (-8·6 to -3·4%)	1,196·8 (1,009·7 to 1,414·9)	39·7% (35·3 to 44·5%)	1,244·9 (1,037·7 to 1,483·5)	37·8% (33·2 to 42·5%)	4·0% (1·1 to 6·8%)	-4·8% (-6·2 to -3·5%)
Chronic kidney disease due to diabetes mellitus	36·0 (26·2 to 44·5)	50·5% (37·2 to 62·5%)	49·3 (35·6 to 61·4)	48·7% (35·5 to 60·7%)	36·8% (32·2 to 42·2%)	-3·5% (-4·8 to -2·5%)	646·2 (458·7 to 823·7)	43·4% (31·3 to 54·4%)	805·9 (567·5 to 1,031·9)	42·2% (30·2 to 53·3%)	24·7% (21·9 to 27·7%)	-2·6% (-3·7 to -1·7%)
Chronic kidney disease due to hypertension	81·1 (78·5 to 83·9)	100·0% (100·0 to 100·0%)	105·1 (99·8 to 110·3)	100·0% (100·0 to 100·0%)	29·6% (24·7 to 34·1%)	-	1,287·8 (1,205·8 to 1,371·0)	100·0% (100·0 to 100·0%)	1,483·9 (1,384·2 to 1,587·9)	100·0% (100·0 to 100·0%)	15·2% (12·1 to 18·1%)	-
Chronic kidney disease due to glomerulonephritis	9·9 (7·3 to 12·2)	50·7% (37·3 to 62·7%)	12·7 (9·2 to 16·0)	49·2% (36·1 to 61·2%)	29·0% (23·8 to 34·1%)	-3·0% (-4·6 to -1·6%)	210·9 (146·4 to 277·9)	40·3% (29·2 to 51·4%)	241·6 (167·5 to 318·9)	40·0% (28·8 to 51·3%)	14·5% (11·6 to 17·2%)	-0·8% (-1·9 to 0·2%)
Chronic kidney disease due to other causes	1·6 (1·1 to 2·0)	50·1% (36·3 to 61·8%)	2·2 (1·6 to 2·8)	49·7% (35·9 to 61·4%)	37·7% (30·8 to 45·5%)	-0·8% (-3·8 to 2·1%)	205·4 (134·8 to 283·6)	40·3% (28·6 to 51·1%)	242·1 (159·6 to 336·9)	39·9% (28·2 to 50·9%)	17·8% (14·7 to 21·0%)	-1·0% (-2·3 to 0·2%)
**High body-mass index: All causes**	692·2 (496·4 to 911·3)	8·2% (5·9 to 10·8%)	748·5 (533·5 to 987·2)	7·8% (5·5 to 10·2%)	8·1% (5·5 to 10·9%)	-5·5% (-7·9 to -3·2%)	17,938·1 (13,346·4 to 22,732·9)	7·0% (5·2 to 8·8%)	19,411·5 (14,549·1 to 24,481·7)	7·1% (5·4 to 8·8%)	8·2% (6·1 to 10·6%)	1·5% (-0·1 to 3·5%)
Oesophageal cancer	14·9 (5·0 to 25·7)	23·9% (8·0 to 41·1%)	17·6 (5·9 to 30·5)	24·3% (8·2 to 41·5%)	18·1% (12·5 to 23·2%)	1·6% (-0·2 to 4·0%)	315·6 (100·1 to 543·8)	25·1% (7·9 to 43·4%)	356·9 (116·0 to 612·5)	25·8% (8·1 to 44·2%)	13·1% (7·7 to 18·6%)	2·9% (1·2 to 5·6%)
Colon and rectum cancer	24·2 (15·2 to 34·4)	9·0% (5·6 to 12·7%)	28·8 (18·0 to 40·8)	9·1% (5·7 to 12·8%)	18·8% (15·4 to 22·8%)	1·0% (-0·9 to 3·4%)	473·6 (300·6 to 671·0)	9·9% (6·3 to 13·9%)	541·1 (345·3 to 755·1)	10·2% (6·5 to 14·2%)	14·3% (11·0 to 17·9%)	2·7% (1·0 to 5·0%)
Liver cancer due to hepatitis B	2·2 (0·9 to 4·1)	13·2% (5·4 to 24·1%)	2·9 (1·2 to 5·2)	13·9% (5·8 to 24·9%)	30·9% (23·8 to 38·3%)	5·2% (1·9 to 9·4%)	56·4 (22·1 to 103·9)	13·4% (5·3 to 24·6%)	70·1 (28·2 to 124·9)	14·5% (6·0 to 26·1%)	24·2% (16·0 to 32·5%)	8·4% (4·5 to 14·1%)
Liver cancer due to hepatitis C	6·2 (2·8 to 10·7)	12·7% (5·7 to 21·8%)	7·9 (3·5 to 13·6)	12·8% (5·7 to 21·7%)	26·7% (21·7 to 32·5%)	0·3% (-2·1 to 3·1%)	113·0 (49·3 to 195·7)	13·6% (5·9 to 23·6%)	135·8 (60·8 to 236·1)	14·1% (6·3 to 24·2%)	20·2% (15·0 to 25·9%)	3·7% (1·0 to 7·2%)
Liver cancer due to alcohol use	5·5 (2·3 to 9·6)	17·4% (7·2 to 30·3%)	6·7 (2·9 to 11·6)	18·1% (7·6 to 31·1%)	22·7% (17·1 to 28·7%)	4·2% (1·3 to 7·7%)	116·4 (48·5 to 203·6)	18·2% (7·5 to 31·3%)	141·2 (59·1 to 244·1)	19·3% (8·0 to 32·8%)	21·3% (15·9 to 27·9%)	6·0% (2·9 to 10·1%)
Liver cancer due to other causes	1·7 (0·8 to 2·7)	16·2% (7·6 to 26·3%)	2·4 (1·1 to 3·9)	16·8% (8·0 to 27·1%)	41·1% (34·8 to 47·9%)	4·1% (1·0 to 7·7%)	35·1 (16·9 to 55·9)	17·4% (8·3 to 28·2%)	47·7 (23·0 to 75·8)	18·6% (9·1 to 29·7%)	35·9% (29·9 to 42·6%)	6·7% (3·6 to 10·2%)
Gallbladder and biliary tract cancer	7·6 (4·4 to 11·7)	15·3% (8·7 to 23·3%)	8·1 (4·5 to 12·3)	14·5% (8·2 to 22·0%)	6·2% (-4·4 to 15·4%)	-5·2% (-10·2 to -1·3%)	126·9 (73·0 to 190·3)	16·1% (9·3 to 24·2%)	125·5 (71·0 to 186·9)	15·7% (9·0 to 23·5%)	-1·1% (-10·0 to 7·0%)	-2·6% (-8·2 to 1·3%)
Pancreatic cancer	8·8 (3·1 to 15·2)	6·4% (2·3 to 11·1%)	11·2 (4·0 to 19·2)	6·4% (2·3 to 11·0%)	26·9% (23·2 to 31·1%)	-0·2% (-2·2 to 1·9%)	163·3 (56·9 to 282·4)	6·7% (2·4 to 11·6%)	198·6 (70·7 to 343·2)	6·8% (2·4 to 11·8%)	21·6% (18·5 to 25·8%)	1·7% (0·0 to 3·9%)
Breast cancer	9·7 (5·4 to 14·9)	6·2% (3·5 to 9·6%)	11·8 (6·7 to 18·1)	6·6% (3·8 to 10·2%)	21·6% (15·5 to 28·2%)	7·1% (3·8 to 11·1%)	175·7 (93·1 to 284·0)	4·5% (2·4 to 7·2%)	219·9 (118·1 to 352·5)	5·1% (2·7 to 8·1%)	25·2% (17·8 to 35·3%)	13·6% (8·0 to 21·0%)
Uterine cancer	7·9 (5·6 to 10·4)	39·5% (28·1 to 51·1%)	9·6 (6·8 to 12·6)	40·0% (28·7 to 51·2%)	21·3% (11·2 to 29·1%)	1·1% (-0·6 to 3·8%)	171·2 (123·3 to 222·7)	41·7% (30·4 to 53·2%)	209·2 (149·6 to 274·3)	42·8% (31·7 to 53·8%)	22·2% (10·9 to 31·0%)	2·6% (1·1 to 5·1%)
Ovarian cancer	1·8 (-0·1 to 3·9)	3·4% (-0·1 to 7·6%)	2·0 (-0·1 to 4·4)	3·5% (-0·1 to 7·7%)	13·4% (9·1 to 18·7%)	1·2% (-0·6 to 3·8%)	37·9 (-1·4 to 83·8)	3·6% (-0·1 to 7·8%)	41·8 (-1·5 to 92·1)	3·6% (-0·1 to 8·0%)	10·4% (6·2 to 15·7%)	2·5% (0·8 to 5·0%)
Kidney cancer	9·3 (6·2 to 12·7)	19·8% (13·4 to 26·9%)	11·8 (7·9 to 16·0)	19·8% (13·5 to 27·0%)	26·9% (22·4 to 32·0%)	0·2% (-1·7 to 2·0%)	194·9 (133·5 to 266·0)	20·5% (14·0 to 27·6%)	239·1 (163·7 to 324·9)	20·8% (14·4 to 28·1%)	22·7% (18·5 to 27·4%)	1·8% (0·3 to 3·5%)
Thyroid cancer	0·9 (0·5 to 1·3)	11·9% (6·6 to 18·1%)	1·1 (0·6 to 1·6)	11·9% (6·6 to 18·1%)	21·8% (15·2 to 27·9%)	0·1% (-3·0 to 3·3%)	22·3 (12·4 to 34·0)	12·9% (7·2 to 19·6%)	34·4 (19·0 to 52·3)	13·8% (7·6 to 20·9%)	54·4% (41·6 to 68·1%)	7·0% (2·7 to 11·2%)
Ischaemic heart disease	290·5 (201·7 to 390·7)	18·6% (12·9 to 25·1%)	299·4 (206·0 to 405·5)	18·2% (12·5 to 24·6%)	3·0% (-0·6 to 6·6%)	-2·6% (-5·7 to 0·4%)	5,488·1 (4,009·8 to 7,053·5)	25·4% (18·6 to 32·5%)	5,403·9 (3,996·0 to 6,920·3)	25·4% (18·7 to 32·4%)	-1·5% (-3·5 to 0·9%)	0·1% (-1·6 to 2·1%)
Ischaemic stroke	45·5 (25·8 to 71·3)	9·5% (5·4 to 14·8%)	43·3 (22·6 to 70·0)	8·7% (4·5 to 14·0%)	-4·8% (-15·3 to 1·0%)	-8·5% (-18·3 to -3·1%)	847·8 (582·1 to 1,158·9)	16·2% (11·1 to 22·0%)	800·4 (555·2 to 1,094·9)	15·8% (11·0 to 21·5%)	-5·6% (-9·1 to -1·8%)	-2·2% (-5·4 to 1·0%)
Hemorrhagic stroke	64·4 (42·9 to 91·0)	19·7% (13·0 to 27·7%)	59·5 (37·7 to 88·1)	17·3% (10·9 to 25·6%)	-7·5% (-17·1 to -1·2%)	-12·2% (-20·9 to -6·7%)	1,676·0 (1,252·9 to 2,124·1)	32·0% (24·0 to 40·6%)	1,508·1 (1,133·7 to 1,912·6)	30·7% (23·1 to 38·9%)	-10·0% (-13·0 to -7·0%)	-4·1% (-6·4 to -1·8%)
Hypertensive heart disease	44·5 (22·6 to 68·1)	41·5% (21·6 to 63·3%)	56·3 (27·3 to 88·2)	41·2% (20·2 to 63·7%)	26·5% (13·5 to 35·7%)	-0·6% (-9·9 to 4·7%)	702·8 (496·5 to 920·0)	49·7% (35·6 to 64·9%)	825·5 (583·4 to 1,089·4)	50·0% (35·5 to 66·0%)	17·5% (10·5 to 23·3%)	0·6% (-3·4 to 4·1%)
Diabetes mellitus	87·8 (65·3 to 113·4)	42·8% (31·6 to 55·3%)	90·6 (66·8 to 118·1)	41·8% (30·7 to 54·6%)	3·1% (-1·8 to 8·0%)	-2·3% (-5·5 to 0·7%)	4,500·7 (3,292·7 to 5,774·4)	56·1% (45·8 to 66·2%)	5,231·2 (3,767·1 to 6,771·0)	57·0% (46·9 to 66·8%)	16·2% (12·3 to 19·7%)	1·6% (0·5 to 3·2%)
Chronic kidney disease due to diabetes mellitus	21·6 (8·5 to 35·7)	30·3% (11·9 to 50·3%)	31·3 (11·1 to 52·9)	30·9% (11·1 to 51·9%)	44·7% (26·0 to 56·8%)	2·1% (-12·0 to 9·2%)	522·1 (278·2 to 778·5)	35·1% (18·8 to 51·8%)	704·8 (390·0 to 1,042·3)	37·0% (20·8 to 54·0%)	35·0% (28·1 to 45·1%)	5·4% (0·5 to 12·6%)
Chronic kidney disease due to hypertension	24·0 (5·8 to 43·8)	29·6% (7·2 to 53·1%)	29·8 (2·8 to 57·9)	28·3% (2·6 to 54·8%)	24·0% (-37·9 to 40·8%)	-4·3% (-51·8 to 7·8%)	461·7 (244·0 to 689·6)	35·8% (18·9 to 52·9%)	528·9 (262·6 to 802·8)	35·6% (17·6 to 53·6%)	14·6% (1·7 to 24·5%)	-0·6% (-11·6 to 7·4%)
Chronic kidney disease due to glomerulonephritis	5·2 (0·7 to 10·1)	26·8% (3·4 to 51·9%)	6·8 (0·0 to 14·0)	26·3% (0·1 to 53·7%)	30·5% (-36·6 to 44·5%)	-1·9% (-52·2 to 6·8%)	172·0 (79·4 to 270·8)	32·9% (14·9 to 49·4%)	203·8 (93·1 to 321·7)	33·7% (15·4 to 50·8%)	18·5% (9·9 to 28·4%)	2·6% (-4·4 to 11·0%)
Chronic kidney disease due to other causes	0·7 (-0·4 to 1·8)	23·0% (-12·3 to 54·6%)	1·0 (-0·6 to 2·6)	23·1% (-14·2 to 56·5%)	39·4% (-4·9 to 109·8%)	0·5% (-31·2 to 51·2%)	166·6 (79·3 to 262·4)	32·7% (16·4 to 48·1%)	204·3 (97·8 to 316·9)	33·6% (17·6 to 48·9%)	22·6% (15·5 to 31·2%)	3·0% (-2·6 to 9·6%)
Osteoarthritis	-	-	-	-	-	-	429·2 (246·1 to 663·6)	22·6% (15·9 to 29·4%)	526·2 (302·3 to 810·6)	22·9% (16·3 to 29·5%)	22·6% (21·0 to 24·6%)	1·3% (0·0 to 3·0%)
Low back pain	-	-	-	-	-	-	828·6 (493·3 to 1,284·5)	7·5% (5·2 to 10·1%)	955·5 (571·8 to 1,466·7)	7·9% (5·5 to 10·5%)	15·3% (13·5 to 18·0%)	5·0% (3·4 to 7·1%)
**Low bone mineral density: All causes**	74·0 (70·6 to 77·1)	0·9% (0·8 to 0·9%)	94·3 (89·3 to 99·4)	1·0% (0·9 to 1·0%)	27·5% (23·1 to 31·8%)	11·4% (7·6 to 15·1%)	2,088·2 (1,694·4 to 2,578·5)	0·8% (0·7 to 0·9%)	2,210·1 (1,780·5 to 2,762·1)	0·8% (0·7 to 0·9%)	5·8% (2·0 to 9·5%)	-0·7% (-4·3 to 2·7%)
Pedestrian road injuries	4·9 (4·6 to 5·3)	15·8% (15·1 to 17·0%)	5·0 (4·7 to 5·5)	19·1% (18·1 to 20·9%)	3·9% (-1·1 to 10·1%)	20·7% (16·6 to 25·6%)	85·0 (76·1 to 95·4)	7·0% (6·5 to 7·5%)	77·4 (69·3 to 86·7)	8·3% (7·7 to 9·1%)	-9·0% (-12·1 to -5·7%)	19·2% (15·7 to 23·6%)
Cyclist road injuries	0·7 (0·7 to 0·8)	12·2% (11·6 to 13·0%)	0·7 (0·7 to 0·8)	14·3% (13·5 to 15·6%)	5·4% (-0·3 to 12·0%)	17·4% (13·5 to 22·0%)	58·7 (44·2 to 77·1)	13·1% (11·7 to 14·5%)	51·6 (38·1 to 68·9)	14·3% (12·8 to 16·0%)	-12·1% (-16·5 to -8·0%)	9·4% (6·8 to 12·6%)
Motorcyclist road injuries	0·6 (0·6 to 0·7)	4·3% (3·9 to 4·7%)	0·7 (0·6 to 0·8)	5·3% (4·7 to 5·9%)	5·9% (-1·0 to 12·2%)	22·6% (17·4 to 28·1%)	56·0 (42·7 to 72·2)	5·2% (4·4 to 6·2%)	51·1 (38·7 to 66·1)	5·9% (4·9 to 7·0%)	-8·7% (-13·4 to -4·1%)	12·7% (8·1 to 18·4%)
Motor vehicle road injuries	5·9 (5·3 to 6·4)	8·3% (7·9 to 8·8%)	5·8 (5·4 to 6·4)	10·1% (9·5 to 10·7%)	-1·1% (-5·2 to 3·9%)	20·8% (17·8 to 24·0%)	222·5 (184·5 to 266·8)	5·7% (5·0 to 6·4%)	204·2 (168·7 to 249·3)	6·7% (5·9 to 7·6%)	-8·2% (-11·6 to -5·1%)	18·3% (15·0 to 21·5%)
Other road injuries	0·1 (0·1 to 0·1)	13·1% (11·3 to 14·3%)	0·1 (0·1 to 0·1)	16·7% (13·8 to 19·1%)	17·2% (6·6 to 29·7%)	27·6% (15·8 to 40·3%)	4·8 (3·6 to 6·1)	8·6% (7·5 to 9·8%)	4·9 (3·7 to 6·5)	10·1% (8·7 to 11·5%)	3·3% (-0·9 to 7·4%)	16·7% (9·9 to 22·8%)

Supplemental Results Table 1. All-age deaths, death PAFs, DALYs, DALY PAFs, and percent change for each risk factor and outcome for both sexes combined in 2005 and 2015 for High-income.

Data in parenthesis are 95% uncertainty intervals. PAFs=population attributable fractions. DALYs=disability-adjusted life-years.

	2005 Deaths (in thousands)	2005 Deaths PAF	2015 Deaths (in thousands)	2015 Deaths PAF	Percent change deaths 2005-2015	Percent change deaths PAF 2005-2015	2005 DALYs (in thousands)	2005 DALYs PAF	2015 DALYs (in thousands)	2015 DALYs PAF	Percent change DALYs 2005-2015	Percent change DALYs PAF 2005-2015
Other transport injuries	1·0 (1·0 to 1·1)	11·1% (10·6 to 11·7%)	1·1 (1·0 to 1·2)	13·0% (12·3 to 13·7%)	5·3% (-1·2 to 11·7%)	17·1% (13·4 to 21·1%)	33·2 (28·9 to 38·5)	7·2% (6·5 to 7·9%)	31·9 (27·6 to 37·2)	8·1% (7·3 to 8·9%)	-3·8% (-8·3 to 0·9%)	12·8% (9·3 to 16·2%)
Falls	58·8 (55·9 to 61·6)	62·8% (60·5 to 64·9%)	78·6 (74·0 to 83·2)	66·4% (63·6 to 68·8%)	33·7% (28·3 to 39·0%)	5·8% (4·2 to 7·2%)	1,535·0 (1,234·0 to 1,925·0)	31·2% (29·8 to 32·6%)	1,694·1 (1,341·5 to 2,141·8)	34·1% (32·3 to 35·9%)	10·4% (6·1 to 14·4%)	9·2% (7·1 to 11·2%)
Other exposure to mechanical forces	1·1 (1·1 to 1·2)	12·8% (12·1 to 13·4%)	1·3 (1·2 to 1·4)	15·0% (14·0 to 15·9%)	10·8% (3·7 to 17·4%)	17·2% (11·4 to 22·3%)	47·8 (36·1 to 63·8)	6·1% (5·5 to 6·6%)	50·3 (37·1 to 68·8)	6·6% (6·1 to 7·2%)	5·4% (1·9 to 8·8%)	9·6% (6·8 to 12·5%)
Non-venomous animal contact	0·2 (0·2 to 0·2)	18·7% (17·7 to 19·6%)	0·2 (0·2 to 0·2)	21·0% (19·6 to 22·1%)	23·0% (11·8 to 35·3%)	12·1% (6·1 to 18·0%)	1·7 (1·6 to 1·9)	3·8% (3·0 to 4·6%)	1·9 (1·8 to 2·1)	4·1% (3·1 to 5·1%)	13·0% (6·7 to 19·9%)	7·8% (1·4 to 14·2%)
Assault by other means	0·6 (0·6 to 0·7)	7·7% (7·3 to 8·1%)	0·8 (0·7 to 0·8)	10·3% (9·5 to 11·2%)	22·4% (12·2 to 35·0%)	34·5% (25·5 to 44·6%)	34·5 (26·2 to 44·8)	6·6% (5·5 to 7·8%)	35·8 (26·6 to 47·5)	7·9% (6·6 to 9·3%)	3·8% (-1·0 to 8·4%)	19·7% (14·9 to 24·6%)
Exposure to forces of nature	-	-	-	-	-	-	9·1 (2·4 to 23·0)	3·4% (0·9 to 6·8%)	6·8 (2·2 to 16·0)	6·2% (3·3 to 9·5%)	-25·1% (-66·2 to 50·7%)	84·0% (3·2 to 358·9%)
**Low glomerular filtration rate: All causes**	397·3 (367·7 to 431·3)	4·7% (4·4 to 5·1%)	475·3 (441·6 to 511·8)	4·9% (4·6 to 5·3%)	19·6% (17·6 to 21·7%)	4·5% (2·8 to 6·4%)	6,428·7 (5,905·5 to 6,973·5)	2·5% (2·3 to 2·7%)	7,242·1 (6,636·2 to 7,842·8)	2·7% (2·4 to 2·9%)	12·7% (11·3 to 14·1%)	5·7% (4·4 to 7·1%)
Ischaemic heart disease	145·0 (121·4 to 170·6)	9·3% (7·8 to 11·0%)	155·8 (130·2 to 183·8)	9·4% (7·9 to 11·1%)	7·4% (4·8 to 9·7%)	1·5% (-0·2 to 3·1%)	1,677·3 (1,442·8 to 1,946·5)	7·8% (6·7 to 8·9%)	1,688·2 (1,442·3 to 1,963·7)	7·9% (6·8 to 9·2%)	0·6% (-1·4 to 2·6%)	2·3% (0·7 to 3·8%)
Ischaemic stroke	45·4 (35·6 to 55·6)	9·5% (7·5 to 11·7%)	47·7 (36·9 to 58·6)	9·6% (7·4 to 11·9%)	5·1% (1·6 to 8·8%)	1·1% (-1·3 to 3·5%)	461·2 (374·1 to 556·3)	8·8% (7·2 to 10·6%)	451·9 (364·9 to 544·5)	8·9% (7·2 to 10·8%)	-2·0% (-4·8 to 0·9%)	1·5% (-0·5 to 3·4%)
Hemorrhagic stroke	28·2 (23·5 to 33·2)	8·6% (7·1 to 10·1%)	30·5 (25·0 to 36·3)	8·8% (7·2 to 10·5%)	8·3% (3·6 to 13·4%)	2·8% (-0·3 to 5·8%)	372·4 (314·2 to 431·5)	7·1% (6·0 to 8·2%)	365·3 (305·6 to 429·3)	7·4% (6·2 to 8·7%)	-1·9% (-5·2 to 1·8%)	4·5% (2·1 to 6·9%)
Peripheral vascular disease	3·6 (2·8 to 4·5)	15·2% (11·9 to 18·8%)	4·7 (3·6 to 6·0)	15·3% (11·9 to 18·8%)	30·9% (20·3 to 43·3%)	0·5% (-4·0 to 5·1%)	60·1 (44·7 to 82·2)	15·4% (12·5 to 18·4%)	74·8 (55·4 to 102·8)	15·6% (12·5 to 18·7%)	24·4% (18·5 to 31·3%)	1·2% (-1·6 to 3·8%)
Chronic kidney disease due to diabetes mellitus	71·3 (69·0 to 73·9)	100·0% (100·0 to 100·0%)	101·2 (96·4 to 106·9)	100·0% (100·0 to 100·0%)	41·8% (37·3 to 46·8%)	-	1,490·0 (1,360·8 to 1,619·6)	100·0% (100·0 to 100·0%)	1,907·9 (1,757·9 to 2,072·9)	100·0% (100·0 to 100·0%)	28·0% (25·4 to 31·0%)	-
Chronic kidney disease due to hypertension	81·1 (78·5 to 83·9)	100·0% (100·0 to 100·0%)	105·1 (99·8 to 110·3)	100·0% (100·0 to 100·0%)	29·6% (24·7 to 34·1%)	-	1,287·8 (1,205·8 to 1,371·0)	100·0% (100·0 to 100·0%)	1,483·9 (1,384·2 to 1,587·9)	100·0% (100·0 to 100·0%)	15·2% (12·1 to 18·1%)	-
Chronic kidney disease due to glomerulonephritis	19·5 (18·2 to 20·7)	100·0% (100·0 to 100·0%)	25·9 (23·9 to 27·9)	100·0% (100·0 to 100·0%)	33·0% (28·0 to 37·7%)	-	523·3 (446·7 to 603·3)	100·0% (100·0 to 100·0%)	604·2 (513·3 to 699·7)	100·0% (100·0 to 100·0%)	15·5% (12·9 to 18·1%)	-
Chronic kidney disease due to other causes	3·2 (2·8 to 3·6)	100·0% (100·0 to 100·0%)	4·4 (3·9 to 5·0)	100·0% (100·0 to 100·0%)	38·9% (32·6 to 45·2%)	-	509·7 (387·0 to 636·0)	100·0% (100·0 to 100·0%)	606·5 (453·6 to 755·1)	100·0% (100·0 to 100·0%)	19·0% (16·0 to 22·0%)	-
Gout	-	-	-	-	-	-	46·8 (31·0 to 64·3)	15·7% (14·1 to 17·3%)	59·3 (39·4 to 81·5)	16·8% (15·2 to 18·4%)	26·8% (24·0 to 29·6%)	7·0% (5·3 to 8·8%)

Supplemental Results Table 1. All-age deaths, death PAFs, DALYs, DALY PAFs, and percent change for each risk factor and outcome for both sexes combined in 2005 and 2015 for Latin America and Caribbean.

Data in parenthesis are 95% uncertainty intervals. PAFs=population attributable fractions. DALYs=disability-adjusted life-years.

	2005 Deaths (in thousands)	2005 Deaths PAF	2015 Deaths (in thousands)	2015 Deaths PAF	Percent change deaths 2005-2015	Percent change deaths PAF 2005-2015	2005 DALYs (in thousands)	2005 DALYs PAF	2015 DALYs (in thousands)	2015 DALYs PAF	Percent change DALYs 2005-2015	Percent change DALYs PAF 2005-2015
**All risk factors: All causes**	1,460·9 (1,433·9 to 1,488·2)	54·2% (53·2 to 55·1%)	1,852·6 (1,807·7 to 1,898·5)	55·4% (54·3 to 56·4%)	26·8% (24·4 to 29·1%)	2·3% (1·7 to 2·8%)	50,941·6 (47,534·9 to 54,832·5)	37·5% (36·3 to 38·9%)	56,030·9 (51,909·2 to 60,525·3)	38·3% (36·8 to 39·8%)	10·0% (7·9 to 11·9%)	2·0% (1·1 to 2·9%)
**Environmental/occupational risks: All causes**	318·8 (288·5 to 350·2)	11·8% (10·7 to 13·0%)	336·3 (300·8 to 374·5)	10·1% (9·0 to 11·2%)	5·5% (1·8 to 9·0%)	-14·9% (-17·4 to -12·5%)	12,267·5 (11,170·7 to 13,429·2)	9·0% (8·3 to 9·7%)	11,078·4 (9,901·5 to 12,336·4)	7·6% (7·0 to 8·2%)	-9·7% (-12·9 to -6·6%)	-16·3% (-18·6 to -14·0%)
**Unsafe water, sanitation, and handwashing: All causes**	55·8 (47·7 to 63·3)	2·1% (1·8 to 2·3%)	44·9 (36·5 to 53·7)	1·3% (1·1 to 1·6%)	-19·5% (-26·1 to -13·8%)	-35·1% (-40·0 to -30·8%)	3,349·6 (2,972·9 to 3,726·7)	2·5% (2·2 to 2·8%)	2,003·3 (1,721·9 to 2,316·2)	1·4% (1·2 to 1·6%)	-40·2% (-45·1 to -35·5%)	-44·6% (-48·6 to -40·5%)
**Unsafe water source: All causes**	31·0 (24·0 to 35·4)	1·1% (0·9 to 1·3%)	19·8 (15·2 to 23·5)	0·6% (0·5 to 0·7%)	-36·0% (-40·7 to -30·7%)	-48·4% (-52·0 to -44·2%)	2,289·6 (1,753·8 to 2,666·9)	1·7% (1·3 to 2·0%)	1,268·0 (956·7 to 1,527·8)	0·9% (0·7 to 1·0%)	-44·6% (-49·8 to -38·9%)	-48·7% (-53·0 to -43·5%)
Diarrhoeal diseases	30·8 (23·9 to 35·1)	82·7% (64·4 to 92·9%)	19·6 (15·1 to 23·3)	81·6% (62·6 to 92·2%)	-36·2% (-41·0 to -30·9%)	-1·3% (-2·8 to -0·6%)	2,279·9 (1,745·6 to 2,655·2)	82·7% (64·5 to 92·9%)	1,258·4 (949·9 to 1,519·0)	81·6% (62·6 to 92·3%)	-44·8% (-49·9 to -39·1%)	-1·3% (-2·8 to -0·5%)
Typhoid fever	0·2 (0·1 to 0·3)	81·9% (63·4 to 92·4%)	0·2 (0·1 to 0·4)	81·1% (62·4 to 91·9%)	9·8% (6·8 to 12·6%)	-1·0% (-2·1 to -0·4%)	9·4 (4·6 to 17·0)	82·0% (63·5 to 92·4%)	9·4 (4·6 to 16·7)	81·2% (62·5 to 92·0%)	-0·5% (-3·7 to 2·7%)	-1·0% (-2·1 to -0·4%)
Paratyphoid fever	0·0 (0·0 to 0·0)	81·7% (63·2 to 92·3%)	0·0 (0·0 to 0·0)	80·8% (61·8 to 91·8%)	5·9% (-0·7 to 12·1%)	-1·1% (-2·3 to -0·4%)	0·3 (0·1 to 0·6)	81·8% (63·4 to 92·4%)	0·3 (0·1 to 0·6)	80·9% (62·0 to 91·9%)	-4·8% (-11·1 to 1·8%)	-1·1% (-2·2 to -0·4%)
**Unsafe sanitation: All causes**	15·0 (13·5 to 16·7)	0·6% (0·5 to 0·6%)	7·7 (6·7 to 9·1)	0·2% (0·2 to 0·3%)	-48·8% (-54·6 to -41·7%)	-58·7% (-63·2 to -53·0%)	1,128·2 (1,007·7 to 1,267·2)	0·8% (0·7 to 1·0%)	519·3 (441·0 to 625·4)	0·4% (0·3 to 0·4%)	-54·0% (-60·1 to -45·9%)	-57·3% (-62·7 to -49·9%)
Diarrhoeal diseases	14·9 (13·4 to 16·6)	40·0% (36·9 to 43·1%)	7·6 (6·6 to 9·0)	31·5% (28·5 to 34·9%)	-49·0% (-54·8 to -41·8%)	-21·1% (-26·1 to -15·3%)	1,124·2 (1,004·8 to 1,262·7)	40·8% (37·7 to 44·0%)	516·1 (438·2 to 622·1)	33·4% (30·2 to 37·2%)	-54·1% (-60·3 to -46·0%)	-18·0% (-23·1 to -11·6%)
Typhoid fever	0·1 (0·0 to 0·1)	33·0% (29·6 to 36·2%)	0·1 (0·0 to 0·1)	26·3% (23·2 to 29·4%)	-11·6% (-17·1 to -6·0%)	-20·2% (-24·7 to -15·9%)	3·8 (2·0 to 6·8)	33·6% (30·1 to 36·8%)	3·1 (1·6 to 5·4)	27·1% (23·9 to 30·1%)	-19·0% (-24·5 to -13·8%)	-19·4% (-23·9 to -14·7%)
Paratyphoid fever	0·0 (0·0 to 0·0)	31·0% (27·9 to 34·2%)	0·0 (0·0 to 0·0)	25·4% (22·5 to 28·5%)	-12·2% (-19·1 to -5·2%)	-18·0% (-22·0 to -14·0%)	0·1 (0·0 to 0·2)	31·9% (28·6 to 35·3%)	0·1 (0·0 to 0·2)	26·6% (23·4 to 29·9%)	-19·7% (-26·5 to -12·5%)	-16·6% (-20·6 to -12·1%)
**No handwashing with soap: All causes**	34·2 (26·1 to 41·7)	1·3% (1·0 to 1·5%)	30·9 (22·5 to 38·8)	0·9% (0·7 to 1·2%)	-9·7% (-17·3 to -3·6%)	-27·2% (-33·0 to -22·2%)	1,752·2 (1,402·9 to 2,098·3)	1·3% (1·0 to 1·5%)	1,105·3 (861·0 to 1,348·7)	0·8% (0·6 to 0·9%)	-36·9% (-41·6 to -32·4%)	-41·5% (-45·6 to -37·6%)
Diarrhoeal diseases	13·1 (10·4 to 15·9)	35·3% (28·0 to 42·1%)	8·0 (6·3 to 9·9)	33·4% (26·1 to 40·0%)	-38·9% (-43·6 to -33·3%)	-5·4% (-7·1 to -3·9%)	977·3 (777·6 to 1,180·2)	35·4% (28·2 to 42·2%)	520·6 (397·9 to 648·5)	33·7% (26·4 to 40·3%)	-46·7% (-52·0 to -40·9%)	-4·8% (-6·6 to -3·3%)
Typhoid fever	0·1 (0·0 to 0·1)	34·0% (26·3 to 40·8%)	0·1 (0·0 to 0·1)	32·2% (24·7 to 38·9%)	5·0% (2·0 to 8·1%)	-5·3% (-6·8 to -4·0%)	3·9 (1·9 to 6·9)	34·0% (26·3 to 40·8%)	3·7 (1·8 to 6·6)	32·3% (24·8 to 39·0%)	-4·7% (-7·9 to -1·4%)	-5·1% (-6·6 to -3·9%)
Paratyphoid fever	0·0 (0·0 to 0·0)	33·7% (26·1 to 40·6%)	0·0 (0·0 to 0·0)	32·1% (24·9 to 39·0%)	1·9% (-4·4 to 8·2%)	-4·8% (-6·5 to -3·3%)	0·1 (0·0 to 0·2)	33·8% (26·2 to 40·8%)	0·1 (0·0 to 0·2)	32·3% (25·1 to 39·2%)	-8·1% (-14·2 to -1·6%)	-4·5% (-6·2 to -3·0%)
**Air pollution: All causes**	199·6 (171·3 to 230·7)	7·4% (6·4 to 8·6%)	208·6 (176·6 to 246·0)	6·2% (5·3 to 7·3%)	4·5% (-0·6 to 9·2%)	-15·7% (-19·6 to -12·1%)	5,156·2 (4,403·2 to 5,966·5)	3·8% (3·2 to 4·5%)	4,432·6 (3,826·0 to 5,138·6)	3·0% (2·5 to 3·6%)	-14·0% (-18·4 to -9·5%)	-20·3% (-24·2 to -16·3%)
**Ambient particulate matter pollution: All causes**	133·1 (112·1 to 156·1)	4·9% (4·2 to 5·8%)	151·2 (125·1 to 181·7)	4·5% (3·7 to 5·4%)	13·7% (8·8 to 18·1%)	-8·4% (-11·7 to -5·2%)	3,286·1 (2,769·6 to 3,887·0)	2·4% (2·0 to 2·9%)	3,129·7 (2,631·4 to 3,702·8)	2·1% (1·7 to 2·6%)	-4·8% (-9·1 to -0·5%)	-11·7% (-15·2 to -8·0%)
Lower respiratory infections	29·3 (17·1 to 44·8)	18·3% (10·7 to 27·8%)	28·1 (14·1 to 46·8)	15·0% (7·6 to 24·7%)	-4·1% (-20·2 to 8·8%)	-18·0% (-31·0 to -9·1%)	1,099·0 (662·0 to 1,640·5)	18·9% (11·4 to 28·2%)	738·8 (385·8 to 1,203·3)	15·7% (8·3 to 25·2%)	-32·8% (-43·1 to -24·7%)	-17·1% (-28·9 to -8·6%)
Tracheal, bronchus, and lung cancer	5·7 (3·1 to 8·7)	11·2% (6·2 to 17·2%)	6·6 (3·4 to 10·4)	9·4% (4·9 to 14·9%)	15·1% (7·2 to 21·3%)	-16·5% (-21·4 to -13·0%)	125·0 (68·9 to 191·8)	11·1% (6·1 to 17·0%)	136·2 (71·0 to 215·5)	9·3% (4·8 to 14·8%)	9·0% (1·8 to 14·9%)	-16·2% (-21·2 to -12·8%)
Ischaemic heart disease	55·0 (44·0 to 66·3)	14·8% (11·9 to 17·7%)	67·3 (51·5 to 82·7)	13·4% (10·3 to 16·3%)	22·4% (16·1 to 28·0%)	-9·8% (-14·1 to -6·1%)	1,187·7 (987·0 to 1,392·0)	16·3% (13·5 to 19·1%)	1,347·2 (1,100·8 to 1,593·3)	14·8% (12·1 to 17·5%)	13·4% (9·3 to 17·4%)	-9·5% (-11·8 to -7·3%)
Ischaemic stroke	8·6 (5·9 to 11·6)	10·0% (6·8 to 13·4%)	10·3 (6·6 to 15·0)	8·9% (5·7 to 12·8%)	19·9% (9·0 to 28·7%)	-11·1% (-18·5 to -4·9%)	138·8 (105·3 to 171·2)	11·0% (8·4 to 13·6%)	153·1 (113·5 to 195·4)	9·8% (7·3 to 12·5%)	10·3% (4·3 to 16·3%)	-10·9% (-15·3 to -6·7%)
Hemorrhagic stroke	15·9 (12·3 to 19·8)	11·1% (8·6 to 13·7%)	17·1 (12·8 to 21·9)	9·8% (7·3 to 12·5%)	7·6% (0·6 to 15·0%)	-11·7% (-16·8 to -6·5%)	404·3 (317·0 to 495·0)	11·9% (9·3 to 14·5%)	400·0 (306·1 to 504·3)	10·8% (8·4 to 13·4%)	-1·1% (-5·6 to 3·8%)	-8·7% (-12·2 to -5·2%)
Chronic obstructive pulmonary disease	18·6 (8·8 to 30·1)	16·9% (8·0 to 27·3%)	21·8 (9·8 to 36·7)	14·9% (6·8 to 24·9%)	17·7% (5·1 to 24·1%)	-11·6% (-20·4 to -7·4%)	331·3 (156·9 to 539·2)	15·8% (7·5 to 25·3%)	354·3 (158·4 to 597·8)	14·3% (6·4 to 23·8%)	7·0% (-4·2 to 12·7%)	-9·8% (-18·9 to -5·4%)
**Household air pollution from solid fuels: All causes**	77·9 (56·5 to 101·5)	2·9% (2·1 to 3·8%)	62·2 (44·5 to 84·5)	1·9% (1·3 to 2·5%)	-20·1% (-27·2 to -13·4%)	-35·6% (-41·2 to -30·2%)	2,269·4 (1,639·9 to 2,962·5)	1·7% (1·2 to 2·2%)	1,486·8 (1,076·2 to 1,991·1)	1·0% (0·7 to 1·4%)	-34·5% (-40·7 to -28·5%)	-39·2% (-44·6 to -33·7%)
Lower respiratory infections	26·9 (17·6 to 39·2)	16·8% (11·0 to 24·2%)	19·5 (12·3 to 29·5)	10·4% (6·5 to 15·5%)	-27·6% (-35·1 to -20·3%)	-38·1% (-44·0 to -32·7%)	1,144·9 (752·0 to 1,633·0)	19·7% (13·1 to 27·8%)	600·2 (385·0 to 890·2)	12·7% (8·1 to 18·7%)	-47·6% (-54·0 to -41·0%)	-35·4% (-42·8 to -29·0%)
Tracheal, bronchus, and lung cancer	4·3 (2·2 to 7·1)	8·5% (4·4 to 14·0%)	3·7 (1·9 to 6·2)	5·2% (2·7 to 8·9%)	-15·2% (-23·9 to -6·4%)	-38·5% (-44·4 to -32·4%)	95·8 (49·8 to 157·6)	8·5% (4·4 to 13·9%)	77·4 (39·4 to 130·4)	5·3% (2·7 to 8·9%)	-19·2% (-27·3 to -11·2%)	-37·9% (-43·8 to -32·1%)
Ischaemic heart disease	21·8 (16·6 to 28·9)	5·9% (4·4 to 7·7%)	18·5 (13·7 to 25·0)	3·7% (2·7 to 5·0%)	-15·5% (-22·9 to -7·1%)	-37·7% (-43·2 to -31·9%)	489·6 (377·9 to 617·0)	6·7% (5·2 to 8·6%)	390·2 (296·8 to 498·6)	4·3% (3·3 to 5·5%)	-20·3% (-27·2 to -12·8%)	-36·4% (-41·5 to -30·8%)
Ischaemic stroke	4·5 (3·1 to 6·4)	5·2% (3·6 to 7·4%)	3·9 (2·7 to 5·6)	3·4% (2·3 to 4·8%)	-12·8% (-20·8 to -4·3%)	-35·3% (-40·9 to -29·4%)	77·2 (55·6 to 106·0)	6·1% (4·4 to 8·3%)	64·2 (45·7 to 89·1)	4·1% (2·9 to 5·7%)	-16·8% (-24·8 to -8·8%)	-32·8% (-38·6 to -26·8%)
Hemorrhagic stroke	8·3 (6·0 to 11·2)	5·8% (4·2 to 7·8%)	6·4 (4·6 to 8·8)	3·7% (2·6 to 5·1%)	-22·6% (-29·5 to -15·6%)	-36·5% (-41·7 to -31·0%)	217·8 (159·2 to 293·7)	6·4% (4·7 to 8·6%)	159·7 (113·3 to 217·9)	4·3% (3·1 to 5·9%)	-26·7% (-33·2 to -20·2%)	-32·3% (-37·7 to -26·4%)
Chronic obstructive pulmonary disease	12·0 (5·7 to 21·0)	11·0% (5·2 to 19·2%)	10·3 (4·9 to 18·3)	7·0% (3·3 to 12·5%)	-14·5% (-23·2 to -5·5%)	-35·8% (-42·4 to -29·2%)	215·3 (104·4 to 372·6)	10·3% (4·9 to 17·9%)	168·8 (79·3 to 296·2)	6·8% (3·2 to 12·0%)	-21·6% (-29·1 to -13·8%)	-33·8% (-40·2 to -27·7%)
Cataract	-	-	-	-	-	-	28·8 (19·5 to 40·1)	11·9% (9·8 to 14·0%)	26·3 (17·2 to 37·4)	7·9% (6·2 to 9·7%)	-8·7% (-17·5 to 0·4%)	-33·2% (-39·6 to -26·9%)
**Ambient ozone pollution: All causes**	3·5 (1·2 to 6·5)	0·1% (0·0 to 0·2%)	6·3 (2·2 to 11·3)	0·2% (0·1 to 0·3%)	77·7% (65·9 to 94·0%)	43·3% (34·4 to 56·0%)	53·8 (18·4 to 99·1)	0·0% (0·0 to 0·1%)	87·3 (31·1 to 156·7)	0·1% (0·0 to 0·1%)	62·3% (51·0 to 78·2%)	50·6% (40·5 to 65·1%)
Chronic obstructive pulmonary disease	3·5 (1·2 to 6·5)	3·2% (1·1 to 5·9%)	6·3 (2·2 to 11·3)	4·3% (1·5 to 7·7%)	77·7% (65·9 to 94·0%)	33·5% (26·0 to 44·3%)	53·8 (18·4 to 99·1)	2·6% (0·9 to 4·7%)	87·3 (31·1 to 156·7)	3·5% (1·3 to 6·2%)	62·3% (51·0 to 78·2%)	37·0% (28·6 to 49·5%)
**Other environmental risks: All causes**	26·6 (14·2 to 41·2)	1·0% (0·5 to 1·5%)	32·8 (16·8 to 52·1)	1·0% (0·5 to 1·5%)	23·3% (17·3 to 27·9%)	-0·6% (-5·0 to 2·4%)	506·2 (249·8 to 815·8)	0·4% (0·2 to 0·6%)	557·3 (267·0 to 918·3)	0·4% (0·2 to 0·6%)	10·1% (4·3 to 14·1%)	2·1% (-3·1 to 5·8%)
**Residential radon: All causes**	2·5 (1·8 to 3·3)	0·1% (0·1 to 0·1%)	3·5 (2·4 to 4·6)	0·1% (0·1 to 0·1%)	36·7% (27·8 to 44·5%)	10·2% (4·0 to 16·0%)	56·5 (40·6 to 73·5)	0·0% (0·0 to 0·1%)	72·8 (51·9 to 94·9)	0·0% (0·0 to 0·1%)	28·7% (22·5 to 34·1%)	19·4% (14·0 to 24·3%)
Tracheal, bronchus, and lung cancer	2·5 (1·8 to 3·3)	5·0% (3·5 to 6·6%)	3·5 (2·4 to 4·6)	5·0% (3·4 to 6·6%)	36·7% (27·8 to 44·5%)	-0·8% (-6·0 to 3·5%)	56·5 (40·6 to 73·5)	5·0% (3·6 to 6·5%)	72·8 (51·9 to 94·9)	5·0% (3·6 to 6·5%)	28·7% (22·5 to 34·1%)	-1·1% (-4·0 to 1·5%)
**Lead exposure: All causes**	24·1 (11·6 to 38·7)	0·9% (0·4 to 1·4%)	29·4 (13·5 to 48·5)	0·9% (0·4 to 1·4%)	21·9% (14·0 to 27·0%)	-1·7% (-7·6 to 1·9%)	449·7 (195·5 to 754·6)	0·3% (0·1 to 0·6%)	484·6 (197·9 to 844·6)	0·3% (0·1 to 0·6%)	7·8% (-0·6 to 12·7%)	-0·1% (-7·7 to 4·7%)
Rheumatic heart disease	0·1 (0·0 to 0·1)	1·0% (0·4 to 2·0%)	0·1 (0·0 to 0·1)	1·0% (0·3 to 2·0%)	-8·4% (-17·6 to 2·3%)	-4·6% (-12·6 to 5·6%)	1·8 (0·5 to 3·8)	0·7% (0·2 to 1·6%)	1·4 (0·4 to 3·1)	0·7% (0·2 to 1·5%)	-19·4% (-26·9 to -12·5%)	-4·7% (-11·9 to 2·9%)
Ischaemic heart disease	11·2 (5·3 to 18·2)	3·0% (1·4 to 4·9%)	13·6 (6·0 to 22·8)	2·7% (1·2 to 4·6%)	21·4% (11·5 to 27·7%)	-10·5% (-17·3 to -6·6%)	192·1 (80·6 to 328·6)	2·6% (1·1 to 4·5%)	208·1 (80·1 to 371·4)	2·3% (0·9 to 4·1%)	8·3% (-1·5 to 14·5%)	-13·5% (-21·1 to -9·5%)
Ischaemic stroke	2·5 (1·2 to 4·2)	2·9% (1·4 to 4·8%)	3·0 (1·4 to 5·2)	2·6% (1·2 to 4·5%)	20·7% (10·9 to 29·0%)	-10·5% (-16·8 to -5·0%)	36·3 (16·9 to 59·7)	2·9% (1·3 to 4·7%)	39·8 (17·3 to 67·3)	2·5% (1·1 to 4·3%)	9·4% (0·2 to 16·3%)	-11·6% (-18·3 to -6·8%)
Hemorrhagic stroke	4·3 (1·8 to 7·4)	3·0% (1·2 to 5·1%)	4·7 (1·8 to 8·3)	2·7% (1·0 to 4·7%)	9·2% (-1·2 to 18·4%)	-10·4% (-18·6 to -3·9%)	84·8 (30·5 to 156·5)	2·5% (0·9 to 4·6%)	81·5 (27·1 to 156·6)	2·2% (0·7 to 4·2%)	-4·0% (-13·1 to 2·6%)	-11·4% (-19·1 to -6·3%)
Hypertensive heart disease	2·5 (0·6 to 5·9)	5·7% (1·5 to 13·7%)	3·1 (0·7 to 7·7)	5·3% (1·2 to 13·2%)	27·9% (11·4 to 38·2%)	-7·4% (-19·0 to -0·7%)	37·7 (10·7 to 86·4)	5·0% (1·4 to 11·5%)	42·6 (11·2 to 101·1)	4·4% (1·2 to 10·6%)	13·0% (-1·8 to 21·6%)	-10·3% (-21·5 to -3·9%)
Cardiomyopathy and myocarditis	0·3 (0·1 to 0·6)	1·5% (0·6 to 2·6%)	0·4 (0·1 to 0·7)	1·3% (0·5 to 2·4%)	8·8% (-3·1 to 20·5%)	-10·3% (-18·4 to -4·3%)	6·9 (2·3 to 13·4)	1·0% (0·3 to 2·0%)	6·7 (2·1 to 13·5)	0·9% (0·3 to 1·9%)	-3·1% (-14·4 to 6·5%)	-8·1% (-17·8 to -2·2%)

Supplemental Results Table 1. All-age deaths, death PAFs, DALYs, DALY PAFs, and percent change for each risk factor and outcome for both sexes combined in 2005 and 2015 for Latin America and Caribbean.

Data in parenthesis are 95% uncertainty intervals. PAFs=population attributable fractions. DALYs=disability-adjusted life-years.

	2005 Deaths (in thousands)	2005 Deaths PAF	2015 Deaths (in thousands)	2015 Deaths PAF	Percent change deaths 2005-2015	Percent change deaths PAF 2005-2015	2005 DALYs (in thousands)	2005 DALYs PAF	2015 DALYs (in thousands)	2015 DALYs PAF	Percent change DALYs 2005-2015	Percent change DALYs PAF 2005-2015
Atrial fibrillation and flutter	0·1 (0·1 to 0·2)	2·0% (1·0 to 3·0%)	0·2 (0·1 to 0·3)	1·8% (0·9 to 2·8%)	41·3% (31·3 to 48·1%)	-9·3% (-15·0 to -6·0%)	2·9 (1·3 to 4·8)	2·0% (1·0 to 3·2%)	3·6 (1·6 to 6·3)	1·8% (0·8 to 2·9%)	26·7% (16·1 to 32·6%)	-13·0% (-19·6 to -9·4%)
Aortic aneurysm	0·2 (0·1 to 0·3)	1·7% (0·8 to 2·9%)	0·2 (0·1 to 0·4)	1·5% (0·6 to 2·6%)	23·3% (10·0 to 33·7%)	-14·7% (-22·7 to -9·6%)	3·5 (1·3 to 6·2)	1·5% (0·6 to 2·7%)	3·8 (1·3 to 7·3)	1·3% (0·4 to 2·4%)	10·8% (-3·5 to 20·2%)	-16·9% (-26·6 to -11·7%)
Peripheral vascular disease	0·0 (0·0 to 0·1)	1·3% (0·6 to 2·2%)	0·1 (0·0 to 0·1)	1·2% (0·5 to 2·1%)	47·4% (29·3 to 63·2%)	-11·2% (-19·3 to -5·5%)	0·7 (0·3 to 1·2)	1·3% (0·6 to 2·2%)	0·9 (0·3 to 1·6)	1·1% (0·4 to 1·9%)	29·5% (13·8 to 41·0%)	-14·3% (-23·3 to -8·6%)
Endocarditis	0·1 (0·0 to 0·1)	1·3% (0·5 to 2·3%)	0·1 (0·0 to 0·1)	1·3% (0·5 to 2·3%)	21·6% (10·7 to 31·8%)	-4·6% (-11·4 to 1·8%)	1·3 (0·4 to 2·6)	0·8% (0·3 to 1·6%)	1·4 (0·4 to 2·9)	0·8% (0·3 to 1·7%)	7·1% (-2·6 to 15·3%)	0·3% (-7·8 to 7·4%)
Other cardiovascular and circulatory diseases	0·5 (0·2 to 0·8)	1·8% (0·8 to 3·0%)	0·6 (0·3 to 1·0)	1·6% (0·7 to 2·8%)	23·7% (15·0 to 30·0%)	-9·4% (-15·2 to -5·7%)	15·1 (5·5 to 27·8)	1·2% (0·5 to 2·3%)	16·3 (5·5 to 31·4)	1·2% (0·4 to 2·2%)	8·4% (-1·3 to 14·0%)	-6·0% (-14·2 to -1·4%)
Idiopathic developmental intellectual disability	-	-	-	-	-	-	17·8 (7·6 to 31·4)	12·4% (8·5 to 16·3%)	16·5 (7·3 to 29·8)	10·3% (6·9 to 13·7%)	-7·1% (-15·5 to 1·4%)	-17·0% (-24·6 to -9·6%)
Chronic kidney disease due to diabetes mellitus	1·3 (0·5 to 2·4)	2·2% (0·9 to 4·0%)	1·8 (0·7 to 3·5)	2·0% (0·8 to 3·7%)	40·9% (29·8 to 47·7%)	-9·1% (-16·0 to -5·8%)	26·7 (9·9 to 52·1)	1·7% (0·6 to 3·3%)	34·2 (11·6 to 68·3)	1·5% (0·5 to 3·1%)	28·0% (15·6 to 35·0%)	-10·7% (-19·1 to -6·5%)
Chronic kidney disease due to hypertension	0·9 (0·4 to 1·5)	2·5% (1·1 to 4·3%)	1·3 (0·6 to 2·2)	2·4% (1·1 to 4·2%)	46·0% (37·4 to 52·1%)	-2·5% (-7·4 to 0·1%)	14·9 (5·9 to 27·4)	1·7% (0·6 to 3·0%)	18·9 (7·3 to 35·0)	1·6% (0·6 to 3·0%)	26·7% (19·9 to 31·5%)	-1·7% (-6·3 to 0·8%)
Chronic kidney disease due to glomerulonephritis	0·1 (0·0 to 0·2)	1·9% (0·9 to 3·2%)	0·2 (0·1 to 0·3)	2·0% (0·9 to 3·4%)	51·5% (42·9 to 57·9%)	3·8% (-1·5 to 6·9%)	4·3 (1·5 to 8·8)	1·1% (0·4 to 2·2%)	5·3 (1·7 to 10·9)	1·1% (0·4 to 2·2%)	22·4% (14·7 to 27·0%)	-2·7% (-8·9 to 1·1%)
Chronic kidney disease due to other causes	0·0 (0·0 to 0·0)	2·3% (1·2 to 3·9%)	0·0 (0·0 to 0·0)	2·5% (1·2 to 4·3%)	55·6% (44·6 to 65·6%)	9·3% (2·8 to 14·5%)	3·0 (1·1 to 6·2)	1·5% (0·6 to 2·9%)	3·6 (1·3 to 7·8)	1·4% (0·5 to 2·8%)	21·9% (13·8 to 27·1%)	-6·5% (-12·9 to -3·1%)
**Occupational risks: All causes**	54·5 (51·9 to 57·4)	2·0% (1·9 to 2·1%)	66·9 (63·0 to 71·1)	2·0% (1·9 to 2·1%)	22·7% (18·6 to 27·0%)	-1·1% (-3·9 to 2·1%)	3,754·2 (3,192·2 to 4,428·2)	2·8% (2·6 to 3·0%)	4,462·5 (3,790·5 to 5,263·4)	3·0% (2·8 to 3·3%)	18·9% (16·1 to 21·9%)	10·2% (7·5 to 13·3%)
**Occupational carcinogens: All causes**	20·1 (19·4 to 20·9)	0·7% (0·7 to 0·8%)	24·6 (23·3 to 26·0)	0·7% (0·7 to 0·8%)	22·0% (16·7 to 27·0%)	-1·6% (-5·3 to 1·6%)	434·4 (418·7 to 451·5)	0·3% (0·3 to 0·4%)	516·3 (489·8 to 543·5)	0·4% (0·3 to 0·4%)	18·9% (14·0 to 23·7%)	10·3% (6·1 to 14·0%)
**Occupational exposure to asbestos: All causes**	3·4 (2·8 to 4·2)	0·1% (0·1 to 0·2%)	4·6 (3·7 to 5·6)	0·1% (0·1 to 0·2%)	33·0% (23·1 to 44·7%)	7·3% (-0·2 to 15·8%)	59·7 (48·4 to 72·6)	0·0% (0·0 to 0·1%)	73·4 (59·1 to 89·3)	0·1% (0·0 to 0·1%)	22·8% (13·9 to 33·0%)	13·9% (6·5 to 22·7%)
Larynx cancer	0·1 (0·0 to 0·1)	1·2% (0·7 to 1·9%)	0·1 (0·1 to 0·2)	1·2% (0·7 to 1·9%)	25·4% (14·5 to 38·3%)	0·8% (-5·6 to 8·5%)	1·6 (0·9 to 2·5)	0·9% (0·5 to 1·4%)	1·8 (1·0 to 2·9)	0·9% (0·5 to 1·4%)	16·7% (5·9 to 29·2%)	-2·8% (-9·4 to 4·3%)
Tracheal, bronchus, and lung cancer	2·7 (2·1 to 3·4)	5·2% (4·1 to 6·6%)	3·6 (2·7 to 4·5)	5·1% (3·9 to 6·4%)	34·5% (23·5 to 46·4%)	-2·5% (-7·8 to 4·3%)	43·3 (33·1 to 55·2)	3·8% (2·9 to 4·9%)	53·2 (40·4 to 67·8)	3·6% (2·7 to 4·6%)	22·8% (13·0 to 33·3%)	-5·6% (-10·8 to 0·6%)
Ovarian cancer	0·1 (0·1 to 0·2)	1·3% (0·6 to 2·1%)	0·1 (0·1 to 0·2)	1·2% (0·6 to 2·0%)	25·9% (11·5 to 49·4%)	-9·1% (-17·9 to 8·6%)	1·8 (0·9 to 3·0)	0·8% (0·4 to 1·3%)	2·2 (1·1 to 3·6)	0·7% (0·3 to 1·2%)	20·1% (7·7 to 38·8%)	-9·6% (-17·0 to 4·1%)
Mesothelioma	0·6 (0·4 to 0·8)	53·1% (37·3 to 67·9%)	0·8 (0·5 to 1·0)	53·2% (36·8 to 68·0%)	29·0% (21·1 to 41·0%)	0·3% (-2·1 to 3·5%)	13·0 (8·5 to 17·5)	42·0% (27·6 to 56·6%)	16·2 (10·4 to 21·7)	42·3% (27·5 to 56·8%)	24·2% (15·6 to 33·9%)	0·6% (-2·5 to 3·8%)
**Occupational exposure to arsenic: All causes**	0·4 (0·3 to 0·6)	0·0% (0·0 to 0·0%)	0·4 (0·3 to 0·6)	0·0% (0·0 to 0·0%)	1·1% (-3·6 to 5·4%)	-18·5% (-21·8 to -15·7%)	9·5 (7·4 to 11·9)	0·0% (0·0 to 0·0%)	9·5 (7·4 to 11·9)	0·0% (0·0 to 0·0%)	0·0% (-4·4 to 4·3%)	-7·2% (-11·1 to -3·7%)
Tracheal, bronchus, and lung cancer	0·4 (0·3 to 0·6)	0·9% (0·7 to 1·1%)	0·4 (0·3 to 0·6)	0·6% (0·5 to 0·8%)	1·1% (-3·6 to 5·4%)	-26·7% (-28·4 to -25·0%)	9·5 (7·4 to 11·9)	0·8% (0·7 to 1·1%)	9·5 (7·4 to 11·9)	0·6% (0·5 to 0·8%)	0·0% (-4·4 to 4·3%)	-23·1% (-24·8 to -21·4%)
**Occupational exposure to benzene: All causes**	0·5 (0·4 to 0·6)	0·0% (0·0 to 0·0%)	0·6 (0·5 to 0·7)	0·0% (0·0 to 0·0%)	26·3% (21·8 to 30·9%)	1·8% (-1·2 to 5·0%)	17·4 (14·8 to 20·3)	0·0% (0·0 to 0·0%)	21·9 (18·7 to 25·5)	0·0% (0·0 to 0·0%)	26·1% (21·6 to 31·1%)	17·0% (13·2 to 21·1%)
Leukaemia	0·5 (0·4 to 0·6)	2·3% (1·9 to 2·6%)	0·6 (0·5 to 0·7)	2·4% (2·0 to 2·8%)	26·3% (21·8 to 30·9%)	5·3% (2·9 to 7·7%)	17·4 (14·8 to 20·3)	1·8% (1·5 to 2·1%)	21·9 (18·7 to 25·5)	2·2% (1·9 to 2·5%)	26·1% (21·6 to 31·1%)	20·2% (16·8 to 23·5%)
**Occupational exposure to beryllium: All causes**	0·0 (0·0 to 0·0)	0·0% (0·0 to 0·0%)	0·0 (0·0 to 0·0)	0·0% (0·0 to 0·0%)	-3·0% (-7·5 to 1·2%)	-21·8% (-25·0 to -18·8%)	0·6 (0·5 to 0·8)	0·0% (0·0 to 0·0%)	0·6 (0·5 to 0·7)	0·0% (0·0 to 0·0%)	-4·0% (-8·4 to 0·1%)	-10·9% (-14·7 to -7·3%)
Tracheal, bronchus, and lung cancer	0·0 (0·0 to 0·0)	0·1% (0·0 to 0·1%)	0·0 (0·0 to 0·0)	0·0% (0·0 to 0·0%)	-3·0% (-7·5 to 1·2%)	-29·6% (-31·4 to -28·0%)	0·6 (0·5 to 0·8)	0·1% (0·0 to 0·1%)	0·6 (0·5 to 0·7)	0·0% (0·0 to 0·0%)	-4·0% (-8·4 to 0·1%)	-26·2% (-28·0 to -24·5%)
**Occupational exposure to cadmium: All causes**	0·1 (0·1 to 0·1)	0·0% (0·0 to 0·0%)	0·1 (0·1 to 0·1)	0·0% (0·0 to 0·0%)	7·4% (2·7 to 12·1%)	-13·4% (-16·9 to -10·2%)	2·4 (1·9 to 2·8)	0·0% (0·0 to 0·0%)	2·5 (2·0 to 3·0)	0·0% (0·0 to 0·0%)	6·3% (1·7 to 10·8%)	-1·4% (-5·5 to 2·4%)
Tracheal, bronchus, and lung cancer	0·1 (0·1 to 0·1)	0·2% (0·2 to 0·3%)	0·1 (0·1 to 0·1)	0·2% (0·1 to 0·2%)	7·4% (2·7 to 12·1%)	-22·1% (-23·9 to -20·2%)	2·4 (1·9 to 2·8)	0·2% (0·2 to 0·3%)	2·5 (2·0 to 3·0)	0·2% (0·1 to 0·2%)	6·3% (1·7 to 10·8%)	-18·3% (-20·1 to -16·6%)
**Occupational exposure to chromium: All causes**	0·4 (0·3 to 0·4)	0·0% (0·0 to 0·0%)	0·4 (0·3 to 0·4)	0·0% (0·0 to 0·0%)	3·7% (-0·9 to 7·9%)	-16·3% (-19·7 to -13·5%)	7·9 (6·9 to 8·9)	0·0% (0·0 to 0·0%)	8·1 (7·1 to 9·2)	0·0% (0·0 to 0·0%)	2·7% (-1·9 to 6·9%)	-4·7% (-8·7 to -1·1%)
Tracheal, bronchus, and lung cancer	0·4 (0·3 to 0·4)	0·7% (0·6 to 0·8%)	0·4 (0·3 to 0·4)	0·5% (0·5 to 0·6%)	3·7% (-0·9 to 7·9%)	-24·7% (-26·4 to -23·1%)	7·9 (6·9 to 8·9)	0·7% (0·6 to 0·8%)	8·1 (7·1 to 9·2)	0·6% (0·5 to 0·6%)	2·7% (-1·9 to 6·9%)	-21·0% (-22·7 to -19·5%)
**Occupational exposure to diesel engine exhaust: All causes**	5·4 (4·8 to 6·1)	0·2% (0·2 to 0·2%)	7·2 (6·3 to 8·0)	0·2% (0·2 to 0·2%)	32·1% (25·4 to 38·4%)	6·5% (1·7 to 10·6%)	116·4 (103·8 to 130·5)	0·1% (0·1 to 0·1%)	152·1 (134·0 to 170·1)	0·1% (0·1 to 0·1%)	30·7% (24·2 to 36·7%)	21·2% (15·9 to 26·3%)
Tracheal, bronchus, and lung cancer	5·4 (4·8 to 6·1)	10·7% (9·5 to 12·0%)	7·2 (6·3 to 8·0)	10·3% (9·2 to 11·5%)	32·1% (25·4 to 38·4%)	-4·2% (-6·9 to -1·4%)	116·4 (103·8 to 130·5)	10·3% (9·2 to 11·5%)	152·1 (134·0 to 170·1)	10·4% (9·2 to 11·6%)	30·7% (24·2 to 36·7%)	0·5% (-2·3 to 3·3%)
**Occupational exposure to second-hand smoke: All causes**	8·3 (8·0 to 8·6)	0·3% (0·3 to 0·3%)	9·0 (8·4 to 9·4)	0·3% (0·3 to 0·3%)	8·2% (3·3 to 12·7%)	-12·7% (-16·5 to -9·8%)	176·0 (169·8 to 182·5)	0·1% (0·1 to 0·1%)	187·4 (176·7 to 197·6)	0·1% (0·1 to 0·1%)	6·5% (1·8 to 10·9%)	-1·2% (-5·5 to 2·3%)
Tracheal, bronchus, and lung cancer	8·3 (8·0 to 8·6)	16·4% (15·9 to 16·8%)	9·0 (8·4 to 9·4)	12·8% (12·3 to 13·3%)	8·2% (3·3 to 12·7%)	-21·5% (-23·2 to -19·7%)	176·0 (169·8 to 182·5)	15·6% (15·1 to 16·1%)	187·4 (176·7 to 197·6)	12·8% (12·2 to 13·3%)	6·5% (1·8 to 10·9%)	-18·1% (-20·0 to -16·3%)
**Occupational exposure to formaldehyde: All causes**	0·1 (0·1 to 0·1)	0·0% (0·0 to 0·0%)	0·1 (0·1 to 0·1)	0·0% (0·0 to 0·0%)	8·6% (4·6 to 12·7%)	-12·4% (-15·0 to -9·9%)	3·7 (3·2 to 4·3)	0·0% (0·0 to 0·0%)	4·0 (3·4 to 4·7)	0·0% (0·0 to 0·0%)	8·7% (4·7 to 12·9%)	0·8% (-2·4 to 4·3%)
Nasopharynx cancer	0·0 (0·0 to 0·0)	1·6% (1·1 to 2·2%)	0·0 (0·0 to 0·0)	1·3% (0·9 to 1·9%)	5·1% (-0·6 to 10·2%)	-16·2% (-18·3 to -13·9%)	0·6 (0·4 to 0·9)	1·6% (1·1 to 2·2%)	0·7 (0·5 to 0·9)	1·4% (1·0 to 2·0%)	5·6% (-0·2 to 11·0%)	-10·3% (-12·6 to -8·0%)
**Occupational exposure to nickel: All causes**	1·5 (1·1 to 2·0)	0·1% (0·0 to 0·1%)	1·5 (1·0 to 1·9)	0·0% (0·0 to 0·1%)	-2·2% (-6·8 to 2·2%)	-21·1% (-24·6 to -18·2%)	32·1 (23·4 to 41·5)	0·0% (0·0 to 0·0%)	31·1 (22·8 to 40·3)	0·0% (0·0 to 0·0%)	-3·3% (-7·6 to 1·1%)	-10·3% (-14·3 to -6·7%)
Tracheal, bronchus, and lung cancer	1·5 (1·1 to 2·0)	3·0% (2·1 to 3·9%)	1·5 (1·0 to 1·9)	2·1% (1·5 to 2·7%)	-2·2% (-6·8 to 2·2%)	-29·0% (-30·8 to -27·2%)	32·1 (23·4 to 41·5)	2·8% (2·1 to 3·7%)	31·1 (22·8 to 40·3)	2·1% (1·6 to 2·7%)	-3·3% (-7·6 to 1·1%)	-25·6% (-27·5 to -23·8%)
**Occupational exposure to polycyclic aromatic hydrocarbons: All causes**	0·9 (0·7 to 1·0)	0·0% (0·0 to 0·0%)	1·0 (0·8 to 1·2)	0·0% (0·0 to 0·0%)	15·0% (10·0 to 19·7%)	-7·3% (-11·1 to -3·9%)	18·9 (15·6 to 22·3)	0·0% (0·0 to 0·0%)	21·6 (17·8 to 25·4)	0·0% (0·0 to 0·0%)	13·9% (8·9 to 18·6%)	5·6% (1·1 to 9·6%)
Tracheal, bronchus, and lung cancer	0·9 (0·7 to 1·0)	1·7% (1·4 to 2·1%)	1·0 (0·8 to 1·2)	1·5% (1·2 to 1·7%)	15·0% (10·0 to 19·7%)	-16·5% (-18·4 to -14·7%)	18·9 (15·6 to 22·3)	1·7% (1·4 to 2·0%)	21·6 (17·8 to 25·4)	1·5% (1·2 to 1·7%)	13·9% (8·9 to 18·6%)	-12·5% (-14·3 to -10·7%)
**Occupational exposure to silica: All causes**	3·6 (3·3 to 3·9)	0·1% (0·1 to 0·1%)	4·4 (3·9 to 4·8)	0·1% (0·1 to 0·1%)	21·7% (15·6 to 28·3%)	-1·9% (-6·5 to 3·0%)	76·4 (69·7 to 83·4)	0·1% (0·0 to 0·1%)	92·0 (83·2 to 101·4)	0·1% (0·1 to 0·1%)	20·5% (14·5 to 27·0%)	11·7% (6·4 to 17·3%)
Tracheal, bronchus, and lung cancer	3·6 (3·3 to 3·9)	7·1% (6·5 to 7·7%)	4·4 (3·9 to 4·8)	6·2% (5·7 to 6·9%)	21·7% (15·6 to 28·3%)	-11·7% (-14·9 to -8·1%)	76·4 (69·7 to 83·4)	6·8% (6·2 to 7·4%)	92·0 (83·2 to 101·4)	6·3% (5·7 to 6·9%)	20·5% (14·5 to 27·0%)	-7·4% (-10·5 to -3·9%)
**Occupational exposure to sulfphuric acid: All causes**	1·0 (0·8 to 1·3)	0·0% (0·0 to 0·0%)	1·1 (0·8 to 1·4)	0·0% (0·0 to 0·0%)	5·7% (0·1 to 11·6%)	-14·8% (-18·9 to -10·5%)	24·5 (18·9 to 30·6)	0·0% (0·0 to 0·0%)	25·9 (19·6 to 32·8)	0·0% (0·0 to 0·0%)	5·4% (-0·5 to 11·3%)	-2·3% (-7·4 to 2·9%)
Larynx cancer	1·0 (0·8 to 1·3)	14·5% (11·2 to 18·1%)	1·1 (0·8 to 1·4)	12·3% (9·4 to 15·5%)	5·7% (0·1 to 11·6%)	-15·0% (-16·8 to -13·0%)	24·5 (18·9 to 30·6)	14·1% (11·0 to 17·7%)	25·9 (19·6 to 32·8)	12·4% (9·6 to 15·6%)	5·4% (-0·5 to 11·3%)	-12·2% (-13·9 to -10·3%)
**Occupational exposure to trichloroethylene: All causes**	0·0 (0·0 to 0·0)	0·0% (0·0 to 0·0%)	0·0 (0·0 to 0·1)	0·0% (0·0 to 0·0%)	28·2% (21·1 to 34·6%)	3·4% (-1·7 to 8·0%)	0·5 (0·1 to 1·0)	0·0% (0·0 to 0·0%)	0·7 (0·1 to 1·2)	0·0% (0·0 to 0·0%)	28·2% (21·3 to 34·5%)	18·9% (12·6 to 24·2%)
Kidney cancer	0·0 (0·0 to 0·0)	0·4% (0·1 to 0·6%)	0·0 (0·0 to 0·1)	0·3% (0·1 to 0·6%)	28·2% (21·1 to 34·6%)	-14·1% (-16·1 to -12·1%)	0·5 (0·1 to 1·0)	0·3% (0·1 to 0·5%)	0·7 (0·1 to 1·2)	0·3% (0·1 to 0·5%)	28·2% (21·3 to 34·5%)	-6·5% (-8·9 to -4·1%)

Supplemental Results Table 1. All-age deaths, death PAFs, DALYs, DALY PAFs, and percent change for each risk factor and outcome for both sexes combined in 2005 and 2015 for Latin America and Caribbean.

Data in parenthesis are 95% uncertainty intervals. PAFs=population attributable fractions. DALYs=disability-adjusted life-years.

	2005 Deaths (in thousands)	2005 Deaths PAF	2015 Deaths (in thousands)	2015 Deaths PAF	Percent change deaths 2005-2015	Percent change deaths PAF 2005-2015	2005 DALYs (in thousands)	2005 DALYs PAF	2015 DALYs (in thousands)	2015 DALYs PAF	Percent change DALYs 2005-2015	Percent change DALYs PAF 2005-2015
**Occupational asthmagens: All causes**	0·7 (0·6 to 0·8)	0·0% (0·0 to 0·0%)	0·6 (0·6 to 0·7)	0·0% (0·0 to 0·0%)	-10·9% (-16·0 to -5·3%)	-28·1% (-31·8 to -24·1%)	131·9 (93·5 to 176·3)	0·1% (0·1 to 0·1%)	139·1 (97·7 to 186·7)	0·1% (0·1 to 0·1%)	5·5% (1·9 to 8·9%)	-2·3% (-5·6 to 0·8%)
Asthma	0·7 (0·6 to 0·8)	6·6% (6·0 to 7·3%)	0·6 (0·6 to 0·7)	6·2% (5·6 to 6·9%)	-10·9% (-16·0 to -5·3%)	-6·3% (-10·4 to -1·9%)	131·9 (93·5 to 176·3)	6·0% (5·2 to 6·8%)	139·1 (97·7 to 186·7)	6·5% (5·7 to 7·3%)	5·5% (1·9 to 8·9%)	7·8% (4·4 to 11·1%)
**Occupational particulate matter, gases, and fumes: All causes**	11·6 (9·4 to 13·8)	0·4% (0·3 to 0·5%)	15·3 (12·1 to 18·7)	0·5% (0·4 to 0·6%)	32·0% (25·7 to 37·9%)	6·4% (1·7 to 10·6%)	263·4 (230·1 to 298·9)	0·2% (0·2 to 0·2%)	317·2 (275·2 to 362·8)	0·2% (0·2 to 0·3%)	20·4% (16·4 to 24·7%)	11·7% (8·2 to 15·2%)
Chronic obstructive pulmonary disease	11·5 (9·3 to 13·7)	10·5% (8·5 to 12·4%)	15·2 (12·0 to 18·6)	10·4% (8·3 to 12·6%)	32·0% (25·6 to 37·9%)	-0·9% (-4·8 to 2·8%)	261·1 (228·0 to 296·7)	12·5% (10·9 to 14·0%)	314·4 (272·5 to 360·0)	12·7% (11·1 to 14·3%)	20·4% (16·4 to 24·8%)	1·6% (-0·7 to 3·7%)
**Occupational noise: All causes**	-	-	-	-	-	-	640·4 (439·6 to 885·0)	0·5% (0·4 to 0·6%)	833·0 (569·2 to 1,159·3)	0·6% (0·4 to 0·7%)	30·1% (26·9 to 33·0%)	20·6% (17·4 to 24·2%)
Age-related and other hearing loss	-	-	-	-	-	-	640·4 (439·6 to 885·0)	27·7% (25·5 to 30·6%)	833·0 (569·2 to 1,159·3)	27·4% (25·3 to 30·0%)	30·1% (26·9 to 33·0%)	-1·1% (-2·8 to 0·6%)
**Occupational injuries: All causes**	22·3 (21·3 to 23·8)	0·8% (0·8 to 0·9%)	27·3 (26·0 to 29·6)	0·8% (0·8 to 0·9%)	22·5% (14·6 to 32·6%)	-1·2% (-7·8 to 7·0%)	1,330·2 (1,242·6 to 1,443·8)	1·0% (0·9 to 1·1%)	1,585·1 (1,475·5 to 1,731·4)	1·1% (1·0 to 1·2%)	19·2% (11·5 to 28·7%)	10·5% (3·1 to 19·6%)
Pedestrian road injuries	5·3 (4·9 to 5·7)	10·9% (10·1 to 11·8%)	6·0 (5·5 to 6·7)	12·5% (11·5 to 13·8%)	14·9% (4·9 to 27·3%)	15·5% (5·5 to 27·7%)	261·6 (241·1 to 285·8)	11·4% (10·6 to 12·4%)	290·1 (264·0 to 319·5)	14·1% (12·9 to 15·5%)	10·9% (1·3 to 22·7%)	23·6% (13·0 to 36·1%)
Cyclist road injuries	0·5 (0·5 to 0·5)	12·1% (11·3 to 13·0%)	0·6 (0·6 to 0·7)	14·1% (12·9 to 15·5%)	24·4% (13·1 to 38·5%)	16·1% (5·9 to 27·2%)	28·7 (26·3 to 31·7)	12·5% (11·6 to 13·4%)	34·5 (31·2 to 38·8)	15·1% (13·9 to 16·6%)	20·5% (9·6 to 33·3%)	21·4% (10·8 to 33·0%)
Motorcyclist road injuries	2·7 (2·3 to 2·9)	16·8% (15·7 to 18·0%)	3·9 (3·4 to 4·4)	18·1% (16·7 to 20·0%)	47·6% (33·2 to 65·4%)	7·7% (-0·5 to 18·2%)	155·4 (136·7 to 167·9)	16·8% (15·6 to 18·0%)	225·9 (194·5 to 247·2)	18·3% (16·8 to 20·0%)	45·4% (32·1 to 62·4%)	8·8% (0·5 to 18·9%)
Motor vehicle road injuries	5·9 (5·5 to 6·7)	17·3% (16·3 to 18·5%)	7·2 (6·7 to 8·1)	19·6% (18·3 to 21·1%)	21·3% (12·3 to 31·2%)	13·0% (5·4 to 22·2%)	330·0 (309·9 to 374·0)	17·6% (16·5 to 18·8%)	395·1 (369·8 to 444·2)	20·5% (19·2 to 22·1%)	19·7% (11·0 to 29·2%)	16·6% (8·8 to 26·1%)
Other road injuries	0·2 (0·2 to 0·3)	14·0% (13·1 to 15·3%)	0·3 (0·2 to 0·4)	15·8% (14·3 to 17·8%)	35·5% (20·7 to 60·6%)	12·4% (2·6 to 23·4%)	13·8 (10·7 to 15·6)	14·8% (13·7 to 16·0%)	18·5 (15·0 to 21·2)	17·4% (15·9 to 19·3%)	34·3% (20·6 to 55·4%)	17·5% (7·5 to 29·5%)
Other transport injuries	0·6 (0·6 to 0·7)	12·5% (11·6 to 13·7%)	0·8 (0·7 to 0·9)	14·4% (13·2 to 15·9%)	35·8% (20·4 to 52·6%)	14·9% (3·7 to 27·7%)	33·1 (30·4 to 36·3)	12·7% (11·7 to 13·9%)	43·4 (38·8 to 48·7)	15·0% (13·8 to 16·6%)	31·1% (16·3 to 47·1%)	18·6% (6·6 to 31·7%)
Falls	1·9 (1·7 to 2·0)	7·7% (7·2 to 8·2%)	2·5 (2·3 to 2·7)	7·5% (7·0 to 8·2%)	36·8% (26·3 to 48·0%)	-1·7% (-9·6 to 7·4%)	166·0 (138·0 to 200·6)	13·7% (12·9 to 14·7%)	206·0 (170·5 to 249·2)	15·1% (14·0 to 16·3%)	24·1% (15·4 to 34·2%)	10·3% (2·4 to 18·8%)
Drowning	2·2 (2·0 to 2·3)	10·3% (9·6 to 11·1%)	2·3 (2·1 to 2·5)	12·4% (11·5 to 13·6%)	5·5% (-2·8 to 16·1%)	20·4% (10·6 to 32·2%)	115·6 (108·8 to 124·7)	9·4% (8·8 to 10·1%)	117·6 (109·1 to 129·8)	12·0% (11·0 to 13·2%)	1·7% (-6·1 to 11·8%)	27·3% (16·4 to 39·7%)
Fire, heat, and hot substances	0·6 (0·6 to 0·7)	9·6% (8·9 to 10·5%)	0·7 (0·6 to 0·8)	11·0% (10·0 to 12·0%)	13·4% (3·3 to 25·9%)	14·0% (5·1 to 24·9%)	42·0 (37·0 to 48·9)	11·6% (10·4 to 12·6%)	45·2 (39·5 to 52·9)	15·0% (13·4 to 16·4%)	7·7% (-2·0 to 18·7%)	29·5% (19·6 to 41·7%)
Poisonings	0·1 (0·1 to 0·1)	3·8% (3·2 to 4·4%)	0·1 (0·1 to 0·1)	4·0% (3·3 to 4·7%)	-1·4% (-14·5 to 12·7%)	4·8% (-13·8 to 28·2%)	7·2 (6·3 to 8·5)	4·0% (3·2 to 4·7%)	7·1 (6·1 to 8·4)	4·6% (3·7 to 5·5%)	-1·6% (-14·2 to 11·5%)	15·5% (-9·1 to 45·4%)
Unintentional firearm injuries	0·3 (0·2 to 0·4)	7·0% (6·2 to 7·9%)	0·4 (0·3 to 0·4)	7·6% (6·8 to 8·6%)	15·9% (1·2 to 33·5%)	8·5% (-5·1 to 24·6%)	18·6 (14·4 to 21·2)	7·4% (6·5 to 8·4%)	21·1 (17·9 to 24·4)	8·4% (7·4 to 9·7%)	13·6% (-1·3 to 31·1%)	13·9% (-0·8 to 32·2%)
Unintentional suffocation	0·0 (0·0 to 0·0)	0·5% (0·4 to 0·5%)	0·0 (0·0 to 0·0)	0·5% (0·4 to 0·6%)	-4·0% (-16·2 to 9·9%)	18·7% (-1·6 to 42·8%)	0·6 (0·5 to 0·7)	0·5% (0·4 to 0·6%)	0·5 (0·4 to 0·6)	0·6% (0·4 to 0·7%)	-7·3% (-19·6 to 6·7%)	15·6% (-4·5 to 40·6%)
Other exposure to mechanical forces	0·6 (0·6 to 0·7)	13·1% (12·3 to 14·4%)	0·8 (0·7 to 0·9)	15·3% (14·2 to 17·0%)	17·7% (5·9 to 30·5%)	16·7% (5·1 to 30·1%)	49·7 (42·9 to 58·3)	14·1% (13·1 to 15·6%)	56·9 (49·1 to 67·1)	17·0% (15·7 to 19·0%)	14·6% (3·3 to 27·5%)	20·8% (8·7 to 34·7%)
Venomous animal contact	0·0 (0·0 to 0·0)	1·8% (1·6 to 2·0%)	0·0 (0·0 to 0·0)	1·8% (1·6 to 2·1%)	-1·2% (-15·4 to 14·3%)	2·5% (-11·5 to 19·0%)	1·5 (1·3 to 1·8)	2·1% (1·9 to 2·5%)	1·5 (1·2 to 1·8)	2·5% (2·2 to 3·0%)	-1·2% (-16·3 to 15·8%)	19·4% (1·2 to 39·6%)
Non-venomous animal contact	0·0 (0·0 to 0·0)	1·6% (1·5 to 1·8%)	0·0 (0·0 to 0·0)	1·7% (1·5 to 2·0%)	11·2% (-2·4 to 26·7%)	4·4% (-7·9 to 18·1%)	0·6 (0·5 to 0·7)	1·8% (1·6 to 2·0%)	0·6 (0·5 to 0·8)	2·1% (1·9 to 2·5%)	7·5% (-6·3 to 22·8%)	19·0% (3·7 to 36·7%)
Pulmonary aspiration and foreign body in airway	0·4 (0·4 to 0·5)	3·6% (3·2 to 4·5%)	0·6 (0·5 to 0·7)	4·7% (4·1 to 6·0%)	34·4% (10·1 to 67·0%)	30·3% (6·1 to 61·9%)	24·8 (21·4 to 30·1)	3·2% (2·8 to 4·1%)	30·5 (26·4 to 38·7)	4·6% (4·0 to 5·8%)	23·2% (1·0 to 55·3%)	42·0% (14·9 to 78·4%)
Foreign body in eyes	-	-	-	-	-	-	0·2 (0·1 to 0·4)	8·8% (7·2 to 10·5%)	0·2 (0·1 to 0·4)	10·2% (8·1 to 12·6%)	-1·7% (-14·9 to 16·0%)	16·1% (1·2 to 36·5%)
Foreign body in other body part	0·1 (0·1 to 0·1)	6·2% (5·6 to 7·1%)	0·1 (0·1 to 0·1)	6·5% (5·6 to 8·1%)	15·2% (-3·5 to 43·7%)	4·3% (-12·0 to 28·0%)	4·5 (3·7 to 5·3)	7·5% (6·8 to 8·7%)	4·9 (4·2 to 6·4)	9·3% (8·0 to 11·7%)	11·1% (-7·3 to 38·9%)	24·6% (4·0 to 54·0%)
Other unintentional injuries	0·9 (0·9 to 1·0)	13·9% (13·1 to 14·7%)	1·0 (0·9 to 1·1)	15·5% (14·5 to 16·9%)	9·2% (0·2 to 20·2%)	11·7% (3·1 to 21·2%)	76·5 (66·1 to 89·7)	14·4% (13·5 to 15·3%)	85·3 (72·3 to 101·3)	16·8% (15·7 to 18·2%)	11·4% (3·0 to 21·2%)	16·7% (8·1 to 26·5%)
**Occupational ergonomic factors: All causes**	-	-	-	-	-	-	971·6 (673·7 to 1,355·9)	0·7% (0·5 to 0·9%)	1,139·1 (796·8 to 1,584·3)	0·8% (0·6 to 1·0%)	17·2% (14·8 to 19·6%)	8·7% (6·1 to 11·5%)
Low back pain	-	-	-	-	-	-	971·6 (673·7 to 1,355·9)	29·1% (27·4 to 30·7%)	1,139·1 (796·8 to 1,584·3)	27·6% (26·1 to 29·0%)	17·2% (14·8 to 19·6%)	-5·1% (-6·6 to -3·7%)
**Behavioural risks: All causes**	990·2 (937·1 to 1,047·9)	36·7% (34·8 to 38·8%)	1,196·7 (1,118·8 to 1,281·2)	35·8% (33·6 to 38·2%)	20·9% (17·9 to 23·8%)	-2·5% (-3·7 to -1·4%)	36,733·1 (34,211·0 to 39,404·6)	27·1% (25·6 to 28·6%)	38,172·8 (35,269·2 to 41,486·4)	26·1% (24·5 to 27·9%)	3·9% (1·5 to 6·4%)	-3·6% (-5·1 to -2·2%)
**Child and maternal malnutrition: All causes**	62·0 (56·3 to 67·0)	2·3% (2·1 to 2·5%)	30·4 (26·5 to 34·5)	0·9% (0·8 to 1·0%)	-51·0% (-55·2 to -46·1%)	-60·5% (-63·9 to -56·7%)	8,643·4 (7,471·2 to 10,145·0)	6·4% (5·8 to 6·9%)	5,992·4 (4,810·2 to 7,459·6)	4·1% (3·6 to 4·7%)	-30·7% (-36·6 to -25·6%)	-35·8% (-40·5 to -31·5%)
**Suboptimal breastfeeding: All causes**	26·1 (16·2 to 36·0)	1·0% (0·6 to 1·3%)	12·4 (7·3 to 17·7)	0·4% (0·2 to 0·5%)	-52·6% (-58·2 to -47·2%)	-61·8% (-66·3 to -57·7%)	2,293·1 (1,444·7 to 3,145·3)	1·7% (1·1 to 2·4%)	1,103·8 (665·4 to 1,568·5)	0·8% (0·4 to 1·1%)	-51·9% (-57·1 to -46·8%)	-55·4% (-60·1 to -50·7%)
**Non-exclusive breastfeeding : All causes**	24·1 (14·3 to 33·9)	0·9% (0·5 to 1·3%)	11·6 (6·4 to 16·9)	0·3% (0·2 to 0·5%)	-51·9% (-57·7 to -46·6%)	-61·2% (-66·0 to -56·9%)	2,091·5 (1,250·5 to 2,940·3)	1·5% (0·9 to 2·2%)	1,010·5 (571·2 to 1,470·2)	0·7% (0·4 to 1·0%)	-51·7% (-57·4 to -46·4%)	-55·2% (-60·5 to -50·4%)
Diarrhoeal diseases	9·3 (5·8 to 12·3)	24·9% (15·3 to 32·8%)	3·6 (2·3 to 5·0)	15·2% (9·1 to 20·4%)	-60·6% (-66·6 to -53·0%)	-39·0% (-45·9 to -30·8%)	810·1 (514·1 to 1,070·4)	29·4% (18·2 to 38·8%)	324·7 (206·6 to 443·0)	21·1% (13·1 to 28·6%)	-59·9% (-65·8 to -52·2%)	-28·3% (-35·3 to -20·7%)
Lower respiratory infections	14·8 (5·5 to 23·9)	9·3% (3·4 to 15·2%)	7·9 (3·0 to 12·9)	4·3% (1·6 to 7·1%)	-46·5% (-51·5 to -41·1%)	-54·2% (-58·7 to -49·3%)	1,281·4 (476·3 to 2,059·0)	22·1% (8·2 to 35·8%)	685·8 (263·7 to 1,111·6)	14·6% (5·6 to 23·9%)	-46·5% (-51·5 to -41·0%)	-34·0% (-39·0 to -27·7%)
**Discontinued breastfeeding : All causes**	3·6 (1·2 to 6·4)	0·1% (0·0 to 0·2%)	1·3 (0·4 to 2·3)	0·0% (0·0 to 0·1%)	-62·7% (-67·5 to -56·8%)	-69·9% (-73·9 to -65·1%)	341·6 (114·8 to 601·9)	0·3% (0·1 to 0·4%)	143·3 (50·5 to 249·4)	0·1% (0·0 to 0·2%)	-58·1% (-64·0 to -50·1%)	-61·1% (-66·5 to -53·8%)
Diarrhoeal diseases	3·6 (1·2 to 6·4)	9·6% (3·2 to 16·9%)	1·3 (0·4 to 2·3)	5·6% (1·9 to 9·8%)	-62·7% (-67·5 to -56·8%)	-42·2% (-48·2 to -34·8%)	341·6 (114·8 to 601·9)	12·4% (4·2 to 21·5%)	143·3 (50·5 to 249·4)	9·3% (3·3 to 16·2%)	-58·1% (-64·0 to -50·1%)	-25·1% (-33·1 to -12·3%)
**Childhood undernutrition: All causes**	53·6 (46·9 to 59·3)	2·0% (1·7 to 2·2%)	25·7 (21·5 to 30·1)	0·8% (0·6 to 0·9%)	-52·0% (-56·5 to -46·7%)	-61·3% (-65·0 to -57·1%)	4,824·7 (4,213·7 to 5,328·1)	3·6% (3·0 to 4·0%)	2,390·0 (2,000·2 to 2,769·8)	1·6% (1·4 to 1·9%)	-50·5% (-54·8 to -45·4%)	-54·1% (-58·0 to -49·5%)
**Childhood underweight: All causes**	13·2 (11·5 to 16·5)	0·5% (0·4 to 0·6%)	6·3 (5·2 to 8·0)	0·2% (0·2 to 0·2%)	-52·3% (-58·6 to -44·9%)	-61·5% (-66·4 to -55·6%)	1,204·7 (1,056·8 to 1,490·5)	0·9% (0·8 to 1·1%)	597·8 (506·4 to 744·7)	0·4% (0·3 to 0·5%)	-50·4% (-56·5 to -43·3%)	-54·0% (-59·3 to -47·7%)
Diarrhoeal diseases	1·5 (1·1 to 2·0)	4·1% (3·1 to 5·2%)	0·5 (0·4 to 0·8)	2·3% (1·6 to 3·0%)	-64·2% (-71·2 to -53·9%)	-44·6% (-53·8 to -32·5%)	141·1 (106·3 to 184·3)	5·1% (3·9 to 6·5%)	54·7 (38·5 to 73·9)	3·5% (2·5 to 4·7%)	-61·2% (-68·2 to -51·7%)	-30·7% (-39·9 to -20·0%)
Lower respiratory infections	3·1 (1·9 to 6·0)	1·9% (1·2 to 3·8%)	1·4 (0·8 to 2·9)	0·8% (0·4 to 1·6%)	-53·7% (-60·1 to -47·4%)	-60·3% (-65·7 to -54·6%)	266·0 (164·8 to 518·7)	4·6% (2·9 to 9·0%)	123·4 (70·4 to 253·2)	2·6% (1·5 to 5·5%)	-53·6% (-60·0 to -47·3%)	-42·8% (-50·0 to -35·8%)
Measles	0·0 (0·0 to 0·0)	10·2% (0·5 to 32·8%)	0·0 (0·0 to 0·0)	5·9% (0·0 to 24·2%)	827·2% (-83·9 to 3,530·1%)	-42·6% (-99·1 to 1·6%)	0·0 (0·0 to 0·0)	10·4% (0·5 to 33·3%)	0·0 (0·0 to 0·1)	6·0% (0·0 to 24·7%)	829·2% (-83·9 to 3,527·8%)	-42·4% (-99·1 to 1·2%)
Protein-energy malnutrition	8·6 (7·6 to 10·1)	27·5% (25·3 to 30·2%)	4·3 (3·7 to 5·5)	14·1% (12·2 to 16·7%)	-49·6% (-57·9 to -39·5%)	-48·8% (-55·9 to -40·2%)	797·7 (710·8 to 928·9)	64·8% (62·4 to 67·4%)	419·6 (359·2 to 520·9)	49·5% (45·7 to 53·9%)	-47·4% (-55·5 to -38·0%)	-23·6% (-29·1 to -17·3%)
**Childhood wasting: All causes**	48·4 (36·5 to 56·8)	1·8% (1·3 to 2·1%)	23·3 (16·7 to 28·7)	0·7% (0·5 to 0·9%)	-51·8% (-56·7 to -46·2%)	-61·1% (-65·1 to -56·7%)	4,363·2 (3,317·3 to 5,094·0)	3·2% (2·4 to 3·8%)	2,173·7 (1,578·2 to 2,647·7)	1·5% (1·1 to 1·8%)	-50·2% (-54·8 to -44·8%)	-53·8% (-57·9 to -49·1%)

Supplemental Results Table 1. All-age deaths, death PAFs, DALYs, DALY PAFs, and percent change for each risk factor and outcome for both sexes combined in 2005 and 2015 for Latin America and Caribbean.

Data in parenthesis are 95% uncertainty intervals. PAFs=population attributable fractions. DALYs=disability-adjusted life-years.

	2005 Deaths (in thousands)	2005 Deaths PAF	2015 Deaths (in thousands)	2015 Deaths PAF	Percent change deaths 2005-2015	Percent change deaths PAF 2005-2015	2005 DALYs (in thousands)	2005 DALYs PAF	2015 DALYs (in thousands)	2015 DALYs PAF	Percent change DALYs 2005-2015	Percent change DALYs PAF 2005-2015
Diarrhoeal diseases	16·4 (11·2 to 19·1)	44·0% (30·2 to 50·7%)	6·6 (4·2 to 8·3)	27·4% (17·8 to 33·3%)	-59·8% (-65·9 to -51·6%)	-37·8% (-44·8 to -28·5%)	1,547·4 (1,037·4 to 1,822·7)	56·1% (37·9 to 64·4%)	681·4 (440·2 to 848·6)	44·2% (28·9 to 51·9%)	-56·0% (-62·1 to -48·2%)	-21·3% (-27·0 to -14·9%)
Lower respiratory infections	23·4 (12·4 to 30·8)	14·7% (7·7 to 19·9%)	12·4 (6·1 to 16·9)	6·7% (3·3 to 9·6%)	-47·0% (-52·7 to -41·4%)	-54·6% (-59·5 to -49·2%)	2,018·1 (1,070·0 to 2,661·4)	34·7% (18·4 to 46·0%)	1,072·6 (529·6 to 1,462·5)	22·8% (11·5 to 31·3%)	-46·9% (-52·6 to -41·3%)	-34·4% (-39·9 to -28·5%)
Measles	0·0 (0·0 to 0·0)	19·1% (0·4 to 66·9%)	0·0 (0·0 to 0·0)	20·9% (0·6 to 67·9%)	1,847·6% (664·6 to 6,128·8%)	9·0% (-20·0 to 165·3%)	0·0 (0·0 to 0·0)	19·4% (0·4 to 67·9%)	0·1 (0·0 to 0·3)	21·2% (0·7 to 69·2%)	1,850·6% (666·8 to 6,092·5%)	9·3% (-19·7 to 165·3%)
Protein-energy malnutrition	8·6 (7·6 to 10·1)	27·5% (25·3 to 30·2%)	4·3 (3·7 to 5·5)	14·1% (12·2 to 16·7%)	-49·6% (-57·9 to -39·5%)	-48·8% (-55·9 to -40·2%)	797·7 (710·8 to 928·9)	64·8% (62·4 to 67·4%)	419·6 (359·2 to 520·9)	49·5% (45·7 to 53·9%)	-47·4% (-55·5 to -38·0%)	-23·6% (-29·1 to -17·3%)
**Childhood stunting: All causes**	9·6 (3·5 to 19·5)	0·4% (0·1 to 0·7%)	4·1 (1·3 to 8·9)	0·1% (0·0 to 0·3%)	-57·4% (-64·6 to -51·3%)	-65·7% (-71·4 to -60·8%)	852·4 (325·7 to 1,697·7)	0·6% (0·2 to 1·3%)	370·8 (128·5 to 795·1)	0·3% (0·1 to 0·5%)	-56·5% (-62·4 to -50·8%)	-59·7% (-64·9 to -54·4%)
Diarrhoeal diseases	3·0 (1·1 to 5·6)	8·0% (2·9 to 14·7%)	1·1 (0·4 to 2·0)	4·4% (1·5 to 8·3%)	-64·4% (-69·9 to -56·9%)	-44·9% (-51·4 to -36·5%)	279·0 (99·4 to 526·2)	10·1% (3·7 to 18·9%)	108·2 (37·0 to 205·6)	7·0% (2·4 to 13·2%)	-61·2% (-66·6 to -54·0%)	-30·7% (-36·6 to -23·9%)
Lower respiratory infections	6·7 (0·5 to 16·3)	4·2% (0·3 to 10·2%)	3·0 (0·2 to 7·7)	1·6% (0·1 to 4·2%)	-54·3% (-60·3 to -44·0%)	-60·9% (-65·8 to -51·3%)	573·4 (40·4 to 1,407·5)	9·9% (0·7 to 24·3%)	262·6 (15·5 to 666·1)	5·6% (0·3 to 14·4%)	-54·2% (-60·2 to -44·0%)	-43·5% (-50·0 to -33·2%)
Measles	0·0 (0·0 to 0·0)	14·4% (1·3 to 43·4%)	0·0 (0·0 to 0·0)	9·6% (0·4 to 35·4%)	994·1% (27·9 to 3,448·9%)	-33·0% (-75·9 to 0·0%)	0·0 (0·0 to 0·0)	14·6% (1·3 to 44·1%)	0·0 (0·0 to 0·1)	9·8% (0·4 to 36·0%)	995·9% (28·4 to 3,439·2%)	-32·9% (-75·9 to 0·0%)
**Iron deficiency: All causes**	0·8 (0·3 to 1·2)	0·0% (0·0 to 0·0%)	0·6 (0·3 to 1·0)	0·0% (0·0 to 0·0%)	-20·4% (-29·7 to -8·3%)	-35·8% (-43·1 to -26·1%)	3,131·5 (2,098·8 to 4,524·7)	2·3% (1·7 to 3·0%)	3,226·3 (2,158·1 to 4,663·0)	2·2% (1·6 to 2·9%)	3·0% (1·4 to 4·6%)	-4·5% (-6·5 to -2·3%)
Maternal haemorrhage	0·4 (0·2 to 0·7)	24·4% (9·2 to 39·3%)	0·3 (0·1 to 0·5)	24·3% (9·2 to 39·6%)	-27·2% (-37·6 to -14·6%)	-0·4% (-5·4 to 4·2%)	25·9 (9·8 to 43·0)	24·2% (9·0 to 39·0%)	18·9 (7·0 to 31·4)	24·0% (9·1 to 39·4%)	-26·9% (-36·9 to -14·7%)	-0·5% (-5·4 to 3·9%)
Maternal sepsis and other pregnancy related infections	0·2 (0·1 to 0·4)	24·4% (9·1 to 39·0%)	0·2 (0·1 to 0·3)	24·2% (9·1 to 38·9%)	-22·1% (-34·9 to -4·5%)	-1·1% (-5·8 to 3·3%)	14·7 (5·4 to 24·7)	24·3% (9·1 to 38·7%)	11·5 (4·3 to 19·2)	24·0% (9·1 to 38·8%)	-21·9% (-35·1 to -5·6%)	-1·1% (-5·8 to 3·3%)
Iron-deficiency anaemia	0·1 (0·1 to 0·1)	100·0% (100·0 to 100·0%)	0·1 (0·1 to 0·1)	100·0% (100·0 to 100·0%)	24·0% (6·2 to 36·4%)	-	3,091·0 (2,072·8 to 4,479·4)	100·0% (100·0 to 100·0%)	3,195·9 (2,126·9 to 4,633·4)	100·0% (100·0 to 100·0%)	3·4% (1·8 to 5·0%)	-
**Vitamin A deficiency: All causes**	1·3 (0·7 to 2·1)	0·0% (0·0 to 0·1%)	0·5 (0·2 to 0·7)	0·0% (0·0 to 0·0%)	-64·3% (-70·9 to -55·2%)	-71·2% (-76·4 to -63·8%)	140·3 (80·3 to 206·6)	0·1% (0·1 to 0·2%)	62·6 (37·3 to 90·8)	0·0% (0·0 to 0·1%)	-55·4% (-62·3 to -46·2%)	-58·7% (-64·9 to -50·3%)
Diarrhoeal diseases	1·3 (0·7 to 2·1)	3·6% (1·8 to 5·5%)	0·5 (0·2 to 0·7)	2·0% (1·0 to 3·0%)	-64·4% (-70·9 to -55·3%)	-44·8% (-53·1 to -34·2%)	127·2 (65·4 to 193·7)	4·6% (2·4 to 7·0%)	50·0 (25·6 to 77·0)	3·2% (1·7 to 4·8%)	-60·7% (-67·1 to -51·9%)	-29·8% (-37·5 to -20·7%)
Measles	0·0 (0·0 to 0·0)	13·1% (7·3 to 19·7%)	0·0 (0·0 to 0·0)	8·8% (4·7 to 13·5%)	1,068·3% (399·4 to 3,392·3%)	-32·8% (-52·0 to -13·6%)	0·0 (0·0 to 0·0)	13·3% (7·5 to 19·9%)	0·0 (0·0 to 0·1)	8·9% (4·8 to 13·7%)	1,069·6% (401·3 to 3,382·4%)	-32·7% (-51·8 to -13·8%)
Vitamin A deficiency	-	-	-	-	-	-	13·2 (7·9 to 20·2)	100·0% (100·0 to 100·0%)	12·6 (7·7 to 19·1)	100·0% (100·0 to 100·0%)	-4·3% (-8·5 to 0·2%)	-
**Zinc deficiency: All causes**	2·0 (0·1 to 4·6)	0·1% (0·0 to 0·2%)	1·0 (0·0 to 2·4)	0·0% (0·0 to 0·1%)	-51·8% (-64·5 to -39·2%)	-61·1% (-71·2 to -50·9%)	184·6 (26·4 to 404·0)	0·1% (0·0 to 0·3%)	95·0 (17·6 to 213·7)	0·1% (0·0 to 0·1%)	-48·6% (-58·2 to -29·5%)	-52·3% (-61·2 to -34·4%)
Diarrhoeal diseases	1·0 (0·0 to 2·5)	2·8% (0·0 to 6·6%)	0·4 (0·0 to 1·0)	1·8% (0·0 to 4·3%)	-57·3% (-66·3 to 0·0%)	-33·7% (-45·8 to 0·0%)	103·4 (15·6 to 223·7)	3·7% (0·6 to 8·1%)	50·7 (12·6 to 99·5)	3·3% (0·8 to 6·6%)	-50·9% (-60·7 to -17·5%)	-12·2% (-27·1 to 47·4%)
Lower respiratory infections	1·0 (0·0 to 3·3)	0·6% (0·0 to 2·1%)	0·5 (0·0 to 1·8)	0·3% (0·0 to 1·0%)	-45·9% (-55·3 to 0·0%)	-53·7% (-61·4 to 0·0%)	81·2 (0·5 to 280·8)	1·4% (0·0 to 4·8%)	44·2 (0·4 to 156·9)	0·9% (0·0 to 3·3%)	-45·6% (-54·4 to -11·1%)	-33·0% (-42·9 to 9·6%)
**Tobacco smoke: All causes**	266·6 (250·4 to 282·7)	9·9% (9·3 to 10·5%)	313·7 (291·2 to 335·0)	9·4% (8·8 to 10·0%)	17·6% (12·8 to 22·4%)	-5·1% (-8·1 to -2·6%)	6,703·1 (6,241·3 to 7,187·0)	4·9% (4·4 to 5·5%)	7,013·0 (6,495·9 to 7,537·2)	4·8% (4·3 to 5·4%)	4·6% (0·7 to 8·7%)	-3·0% (-6·0 to 0·0%)
**Smoking: All causes**	246·4 (230·0 to 262·0)	9·1% (8·5 to 9·7%)	288·4 (267·2 to 308·1)	8·6% (8·0 to 9·2%)	17·0% (11·9 to 22·1%)	-5·6% (-8·9 to -2·7%)	6,042·1 (5,611·4 to 6,499·9)	4·5% (4·0 to 5·0%)	6,421·2 (5,933·2 to 6,937·5)	4·4% (3·9 to 4·9%)	6·3% (2·1 to 10·5%)	-1·4% (-4·7 to 1·9%)
Tuberculosis	1·5 (0·7 to 2·3)	7·4% (3·9 to 11·2%)	1·0 (0·5 to 1·6)	6·0% (3·1 to 9·2%)	-30·6% (-36·0 to -23·4%)	-19·2% (-23·3 to -15·2%)	47·2 (24·2 to 73·1)	6·6% (3·5 to 10·0%)	31·5 (15·6 to 49·9)	5·5% (2·8 to 8·5%)	-33·3% (-38·8 to -26·3%)	-16·5% (-21·4 to -11·9%)
Lower respiratory infections	22·5 (16·9 to 28·2)	14·1% (11·1 to 17·4%)	24·1 (17·5 to 30·8)	12·9% (10·0 to 16·1%)	7·0% (-1·3 to 16·0%)	-8·5% (-14·7 to -2·4%)	480·5 (360·6 to 594·2)	8·3% (6·4 to 10·2%)	467·0 (343·2 to 586·1)	9·9% (7·6 to 12·4%)	-2·8% (-9·3 to 3·6%)	19·8% (12·9 to 26·7%)
Lip and oral cavity cancer	2·8 (2·3 to 3·2)	43·1% (35·9 to 50·2%)	3·6 (3·0 to 4·3)	41·3% (34·4 to 48·4%)	30·7% (21·3 to 40·8%)	-4·2% (-8·5 to -0·5%)	71·8 (59·0 to 83·8)	43·1% (35·7 to 50·2%)	86·2 (69·8 to 102·6)	40·1% (33·1 to 47·1%)	20·1% (10·2 to 30·2%)	-6·8% (-11·4 to -2·6%)
Nasopharynx cancer	0·4 (0·3 to 0·5)	36·0% (29·0 to 42·2%)	0·5 (0·4 to 0·6)	35·0% (28·0 to 41·3%)	21·6% (14·1 to 29·1%)	-2·9% (-7·2 to 0·7%)	12·3 (9·8 to 14·8)	31·2% (25·1 to 36·6%)	14·1 (11·1 to 17·2)	30·4% (24·1 to 36·0%)	14·5% (6·8 to 22·8%)	-2·6% (-7·5 to 2·0%)
Oesophageal cancer	5·3 (4·0 to 6·6)	42·5% (32·4 to 52·2%)	6·7 (5·0 to 8·4)	40·3% (30·4 to 49·9%)	25·7% (15·4 to 37·1%)	-5·1% (-9·7 to -1·1%)	129·8 (98·8 to 160·4)	43·2% (33·2 to 53·0%)	150·7 (111·3 to 190·3)	40·0% (29·8 to 49·8%)	16·1% (5·5 to 27·3%)	-7·5% (-12·3 to -3·1%)
Stomach cancer	3·5 (2·2 to 5·1)	8·0% (4·9 to 11·4%)	4·2 (2·5 to 6·1)	7·6% (4·6 to 10·9%)	17·6% (8·6 to 27·7%)	-4·8% (-11·2 to 1·0%)	80·0 (48·9 to 115·2)	8·0% (4·9 to 11·6%)	85·1 (50·8 to 124·1)	7·3% (4·4 to 10·6%)	6·4% (-2·3 to 15·3%)	-8·7% (-14·6 to -2·7%)
Colon and rectum cancer	1·5 (1·0 to 1·9)	4·4% (3·2 to 5·7%)	2·1 (1·5 to 2·7)	4·3% (3·1 to 5·5%)	42·9% (30·1 to 56·5%)	-2·7% (-9·6 to 4·1%)	32·6 (23·0 to 42·7)	4·4% (3·1 to 5·8%)	41·4 (29·3 to 53·8)	4·0% (2·9 to 5·3%)	27·0% (15·4 to 39·3%)	-8·2% (-14·5 to -1·6%)
Liver cancer due to hepatitis B	0·5 (0·3 to 0·7)	11·1% (6·3 to 16·5%)	0·6 (0·3 to 0·8)	10·7% (6·0 to 15·8%)	24·8% (14·6 to 36·5%)	-4·1% (-10·7 to 2·2%)	12·4 (6·9 to 19·0)	10·5% (5·9 to 16·1%)	14·2 (7·8 to 21·7)	9·9% (5·5 to 15·0%)	14·4% (4·0 to 26·0%)	-6·1% (-13·2 to 0·9%)
Liver cancer due to hepatitis C	0·7 (0·4 to 1·0)	9·0% (5·1 to 13·0%)	0·9 (0·5 to 1·4)	8·6% (4·9 to 12·4%)	35·7% (24·8 to 46·9%)	-3·7% (-10·8 to 2·7%)	14·3 (8·3 to 20·9)	9·2% (5·3 to 13·4%)	17·5 (10·1 to 25·6)	8·5% (4·9 to 12·4%)	22·7% (12·4 to 32·5%)	-7·6% (-14·2 to -1·9%)
Liver cancer due to alcohol use	0·7 (0·4 to 1·1)	12·5% (6·8 to 19·0%)	1·0 (0·5 to 1·5)	11·6% (6·3 to 17·4%)	35·0% (24·4 to 46·1%)	-7·5% (-13·1 to -2·0%)	16·1 (8·6 to 24·2)	12·9% (6·9 to 19·4%)	20·4 (11·1 to 30·9)	11·6% (6·3 to 17·4%)	26·7% (15·6 to 38·0%)	-10·1% (-15·8 to -4·4%)
Liver cancer due to other causes	0·3 (0·2 to 0·4)	8·3% (4·8 to 12·0%)	0·4 (0·2 to 0·6)	8·2% (4·7 to 11·9%)	32·3% (22·2 to 42·9%)	-1·7% (-8·6 to 4·7%)	6·8 (3·9 to 10·1)	7·8% (4·6 to 11·4%)	8·1 (4·5 to 11·9)	7·5% (4·3 to 11·0%)	18·2% (9·1 to 27·3%)	-4·4% (-11·1 to 1·4%)
Pancreatic cancer	2·2 (1·7 to 2·7)	12·9% (10·1 to 15·8%)	3·2 (2·5 to 3·9)	12·5% (9·8 to 15·3%)	41·7% (30·5 to 53·4%)	-3·6% (-9·6 to 1·9%)	49·1 (38·0 to 60·2)	13·2% (10·3 to 16·2%)	63·5 (49·5 to 77·9)	12·2% (9·5 to 15·0%)	29·4% (19·1 to 40·5%)	-7·7% (-13·2 to -2·5%)
Tracheal, bronchus, and lung cancer	33·5 (31·5 to 35·3)	66·1% (62·4 to 69·2%)	45·3 (41·8 to 48·5)	64·9% (61·0 to 68·3%)	35·4% (27·0 to 42·1%)	-1·8% (-4·0 to -0·1%)	734·2 (691·6 to 774·6)	65·1% (61·6 to 68·3%)	927·0 (856·3 to 993·0)	63·2% (59·6 to 66·6%)	26·3% (18·8 to 33·0%)	-3·0% (-5·2 to -1·0%)
Cervical cancer	1·3 (0·4 to 2·4)	5·5% (1·7 to 9·7%)	1·5 (0·5 to 2·8)	5·5% (1·8 to 9·8%)	15·7% (1·3 to 29·3%)	0·2% (-9·6 to 10·2%)	41·3 (13·0 to 74·5)	5·4% (1·7 to 10·0%)	42·9 (13·8 to 79·4)	5·2% (1·7 to 9·5%)	3·8% (-10·1 to 17·9%)	-4·7% (-15·0 to 6·3%)
Kidney cancer	0·6 (0·4 to 0·9)	10·1% (6·4 to 13·8%)	0·9 (0·6 to 1·3)	9·5% (5·9 to 13·3%)	40·5% (27·0 to 54·2%)	-5·9% (-12·5 to 0·5%)	16·0 (10·2 to 22·2)	8·9% (5·6 to 12·2%)	20·8 (13·0 to 29·2)	8·4% (5·3 to 11·7%)	29·9% (17·0 to 43·4%)	-5·3% (-12·6 to 1·8%)
Bladder cancer	1·4 (1·0 to 1·8)	21·4% (15·6 to 27·5%)	2·0 (1·4 to 2·6)	20·8% (15·1 to 26·8%)	39·9% (27·4 to 51·0%)	-2·9% (-8·8 to 2·2%)	26·9 (19·7 to 34·2)	21·5% (15·8 to 27·5%)	34·0 (24·9 to 43·6)	20·1% (14·7 to 25·5%)	26·7% (15·9 to 37·0%)	-6·7% (-11·8 to -2·1%)
Ischaemic heart disease	54·1 (45·2 to 63·4)	14·6% (12·1 to 17·1%)	56·1 (46·2 to 66·7)	11·1% (9·2 to 13·2%)	3·6% (-1·5 to 8·6%)	-23·6% (-26·7 to -20·5%)	1,482·7 (1,233·7 to 1,735·0)	20·4% (16·9 to 23·9%)	1,476·2 (1,215·1 to 1,758·0)	16·2% (13·4 to 19·2%)	-0·4% (-4·9 to 4·1%)	-20·5% (-23·6 to -17·7%)
Ischaemic stroke	8·5 (7·0 to 10·1)	9·8% (8·2 to 11·7%)	8·8 (7·2 to 10·5)	7·5% (6·2 to 8·9%)	3·0% (-3·2 to 9·3%)	-23·6% (-27·9 to -19·0%)	177·6 (147·4 to 212·1)	14·1% (11·7 to 16·7%)	174·0 (142·4 to 209·3)	11·1% (9·2 to 13·2%)	-2·0% (-6·5 to 2·8%)	-20·9% (-24·1 to -17·7%)
Hemorrhagic stroke	23·0 (19·3 to 26·9)	16·0% (13·4 to 18·8%)	21·1 (17·4 to 25·1)	12·1% (9·9 to 14·3%)	-8·0% (-12·8 to -2·8%)	-24·5% (-27·6 to -21·6%)	674·9 (566·0 to 793·3)	19·8% (16·6 to 23·2%)	589·3 (485·7 to 707·4)	16·0% (13·2 to 18·9%)	-12·7% (-17·4 to -7·8%)	-19·4% (-22·7 to -16·3%)
Hypertensive heart disease	4·5 (3·3 to 5·7)	10·4% (7·7 to 13·2%)	4·7 (3·4 to 5·9)	7·9% (5·7 to 10·0%)	3·9% (-2·4 to 9·9%)	-24·7% (-28·6 to -21·2%)	114·7 (84·3 to 144·4)	15·1% (11·1 to 19·0%)	112·7 (82·4 to 142·5)	11·8% (8·6 to 14·8%)	-1·7% (-7·2 to 3·6%)	-21·9% (-25·2 to -18·9%)
Atrial fibrillation and flutter	0·5 (0·3 to 0·6)	6·6% (4·8 to 8·2%)	0·6 (0·4 to 0·8)	5·2% (3·8 to 6·6%)	23·2% (12·8 to 34·4%)	-20·8% (-27·3 to -14·2%)	17·4 (11·8 to 24·1)	12·4% (9·1 to 15·6%)	20·1 (13·7 to 27·6)	9·9% (7·2 to 12·5%)	15·6% (10·6 to 20·4%)	-20·6% (-23·6 to -17·3%)
Aortic aneurysm	1·7 (1·3 to 2·1)	15·7% (11·9 to 19·3%)	1·9 (1·4 to 2·4)	12·0% (9·0 to 14·9%)	10·9% (1·4 to 20·3%)	-23·3% (-27·7 to -18·9%)	45·9 (34·8 to 56·2)	20·3% (15·5 to 24·9%)	48·4 (35·8 to 60·4)	16·1% (12·1 to 19·9%)	5·5% (-3·4 to 14·5%)	-20·8% (-25·1 to -16·6%)

Supplemental Results Table 1. All-age deaths, death PAFs, DALYs, DALY PAFs, and percent change for each risk factor and outcome for both sexes combined in 2005 and 2015 for Latin America and Caribbean.

Data in parenthesis are 95% uncertainty intervals. PAFs=population attributable fractions. DALYs=disability-adjusted life-years.

	2005 Deaths (in thousands)	2005 Deaths PAF	2015 Deaths (in thousands)	2015 Deaths PAF	Percent change deaths 2005-2015	Percent change deaths PAF 2005-2015	2005 DALYs (in thousands)	2005 DALYs PAF	2015 DALYs (in thousands)	2015 DALYs PAF	Percent change DALYs 2005-2015	Percent change DALYs PAF 2005-2015
Peripheral vascular disease	0·3 (0·2 to 0·4)	11·0% (8·2 to 13·8%)	0·4 (0·3 to 0·5)	8·4% (6·2 to 10·5%)	26·6% (13·8 to 40·9%)	-23·7% (-30·1 to -17·1%)	7·9 (5·7 to 10·3)	15·1% (11·3 to 18·8%)	9·5 (6·8 to 12·5)	12·0% (8·8 to 15·0%)	19·8% (10·6 to 29·7%)	-20·7% (-25·1 to -16·0%)
Other cardiovascular and circulatory diseases	3·4 (2·5 to 4·3)	12·3% (9·1 to 15·4%)	3·7 (2·7 to 4·6)	9·7% (7·1 to 12·2%)	8·1% (1·7 to 14·3%)	-20·9% (-24·9 to -17·4%)	161·8 (119·0 to 209·1)	13·4% (9·9 to 16·8%)	164·8 (120·4 to 213·0)	11·8% (8·6 to 14·9%)	1·8% (-3·1 to 6·6%)	-11·7% (-15·4 to -8·0%)
Chronic obstructive pulmonary disease	56·7 (50·5 to 62·9)	51·7% (46·3 to 56·9%)	76·0 (67·0 to 85·4)	52·0% (46·1 to 57·9%)	34·0% (27·1 to 40·9%)	0·7% (-3·2 to 4·0%)	1,005·0 (900·2 to 1,106·9)	47·9% (43·2 to 52·5%)	1,186·6 (1,051·4 to 1,324·6)	47·7% (42·7 to 52·6%)	18·1% (12·6 to 24·1%)	-0·4% (-3·9 to 2·7%)
Silicosis	0·0 (0·0 to 0·1)	11·7% (8·5 to 15·4%)	0·0 (0·0 to 0·1)	10·8% (7·7 to 14·3%)	2·2% (-11·8 to 17·0%)	-7·7% (-16·1 to 0·8%)	1·3 (0·9 to 1·8)	11·4% (7·9 to 15·5%)	1·2 (0·8 to 1·6)	10·1% (6·8 to 13·8%)	-7·5% (-20·8 to 6·1%)	-11·8% (-20·9 to -2·7%)
Asbestosis	0·0 (0·0 to 0·0)	9·8% (7·4 to 12·4%)	0·0 (0·0 to 0·0)	9·5% (7·2 to 12·2%)	30·8% (11·0 to 53·7%)	-2·2% (-10·6 to 6·0%)	0·3 (0·2 to 0·4)	9·5% (6·8 to 12·5%)	0·3 (0·2 to 0·5)	9·0% (6·4 to 11·8%)	19·0% (4·4 to 34·4%)	-5·4% (-13·2 to 2·4%)
Coal workers pneumoconiosis	0·0 (0·0 to 0·0)	11·1% (8·5 to 14·2%)	0·0 (0·0 to 0·0)	10·5% (8·0 to 13·5%)	25·1% (8·8 to 43·8%)	-5·5% (-13·6 to 3·7%)	0·3 (0·2 to 0·4)	12·1% (8·9 to 15·7%)	0·3 (0·2 to 0·4)	10·8% (7·8 to 14·2%)	10·1% (-4·2 to 25·1%)	-10·8% (-19·4 to -2·1%)
Other pneumoconiosis	0·0 (0·0 to 0·1)	9·5% (7·4 to 11·7%)	0·1 (0·0 to 0·1)	10·0% (7·8 to 12·6%)	31·6% (14·2 to 54·9%)	5·7% (-5·8 to 18·9%)	1·4 (1·0 to 1·9)	7·2% (5·4 to 9·1%)	1·7 (1·2 to 2·3)	7·1% (5·4 to 8·9%)	17·2% (6·9 to 28·3%)	-2·2% (-9·3 to 5·6%)
Asthma	1·0 (0·8 to 1·2)	9·1% (7·5 to 10·7%)	0·8 (0·6 to 0·9)	7·4% (6·0 to 8·8%)	-22·6% (-27·8 to -17·4%)	-18·6% (-22·5 to -14·5%)	88·9 (62·7 to 118·6)	4·0% (3·3 to 4·9%)	81·6 (56·2 to 111·7)	3·8% (3·0 to 4·7%)	-8·1% (-13·1 to -3·4%)	-6·1% (-11·4 to -1·1%)
Interstitial lung disease and pulmonary sarcoidosis	0·5 (0·4 to 0·7)	7·8% (6·0 to 9·8%)	1·0 (0·7 to 1·3)	7·8% (5·9 to 10·0%)	79·8% (51·3 to 105·4%)	0·1% (-9·8 to 9·9%)	10·9 (8·1 to 14·3)	7·4% (5·7 to 9·5%)	16·4 (11·6 to 22·1)	7·0% (5·4 to 9·0%)	49·8% (29·2 to 67·6%)	-5·1% (-12·4 to 2·2%)
Other chronic respiratory diseases	0·2 (0·1 to 0·2)	5·0% (3·8 to 6·3%)	0·2 (0·2 to 0·3)	6·5% (4·9 to 8·2%)	35·2% (21·9 to 49·8%)	29·2% (17·1 to 42·6%)	6·2 (4·4 to 8·6)	2·6% (1·9 to 3·5%)	7·1 (5·0 to 9·7)	3·4% (2·5 to 4·6%)	13·6% (4·0 to 23·2%)	30·1% (17·2 to 43·9%)
Diabetes mellitus	4·6 (1·5 to 8·1)	3·7% (1·2 to 6·5%)	5·4 (1·7 to 9·8)	3·0% (0·9 to 5·3%)	17·9% (9·8 to 24·9%)	-19·3% (-24·3 to -15·1%)	201·3 (60·7 to 357·9)	4·3% (1·4 to 7·5%)	224·3 (66·7 to 404·0)	3·4% (1·1 to 6·2%)	11·4% (5·2 to 16·5%)	-19·4% (-23·2 to -16·2%)
**Second-hand smoke: All causes**	24·0 (18·5 to 30·1)	0·9% (0·7 to 1·1%)	29·8 (23·0 to 37·4)	0·9% (0·7 to 1·1%)	24·0% (18·4 to 29·0%)	0·0% (-4·0 to 3·6%)	752·5 (565·0 to 969·1)	0·6% (0·4 to 0·7%)	689·7 (531·8 to 864·0)	0·5% (0·4 to 0·6%)	-8·3% (-13·8 to -2·4%)	-15·0% (-19·8 to -9·6%)
Lower respiratory infections	7·4 (4·6 to 10·4)	4·6% (2·9 to 6·6%)	7·3 (4·5 to 10·4)	3·9% (2·4 to 5·6%)	-0·7% (-8·9 to 6·3%)	-15·1% (-19·4 to -12·2%)	395·8 (249·6 to 561·7)	6·8% (4·3 to 9·6%)	252·0 (154·2 to 357·4)	5·3% (3·3 to 7·7%)	-36·3% (-41·0 to -32·1%)	-21·5% (-25·4 to -18·3%)
Otitis media	0·0 (0·0 to 0·0)	5·6% (3·7 to 7·5%)	0·0 (0·0 to 0·0)	4·3% (2·8 to 5·7%)	-31·7% (-39·4 to -22·2%)	-23·9% (-28·1 to -19·1%)	13·3 (7·2 to 21·8)	5·6% (3·7 to 7·5%)	11·7 (6·2 to 19·4)	4·8% (3·2 to 6·5%)	-12·5% (-15·2 to -9·8%)	-12·9% (-15·1 to -10·7%)
Tracheal, bronchus, and lung cancer	0·5 (0·3 to 0·7)	0·9% (0·5 to 1·4%)	0·7 (0·4 to 1·0)	0·9% (0·6 to 1·5%)	40·4% (38·2 to 42·5%)	1·9% (-1·8 to 6·3%)	10·5 (6·2 to 16·6)	0·9% (0·6 to 1·5%)	14·0 (8·2 to 22·1)	1·0% (0·6 to 1·5%)	33·7% (31·0 to 36·0%)	2·8% (-1·0 to 6·8%)
Ischaemic heart disease	10·5 (7·9 to 13·7)	2·8% (2·1 to 3·7%)	14·4 (10·6 to 19·1)	2·9% (2·1 to 3·8%)	37·2% (30·5 to 43·5%)	1·1% (-3·4 to 5·1%)	218·5 (171·5 to 272·2)	3·0% (2·3 to 3·8%)	276·9 (215·2 to 346·5)	3·0% (2·4 to 3·8%)	26·7% (21·9 to 31·3%)	1·1% (-1·7 to 4·0%)
Ischaemic stroke	1·3 (0·9 to 1·7)	1·5% (1·0 to 2·0%)	1·8 (1·2 to 2·4)	1·5% (1·0 to 2·1%)	39·4% (30·9 to 47·8%)	3·4% (-1·9 to 8·1%)	18·9 (14·3 to 24·2)	1·5% (1·1 to 1·9%)	24·0 (17·9 to 31·3)	1·5% (1·2 to 2·0%)	27·0% (21·0 to 33·5%)	2·6% (-1·2 to 6·2%)
Hemorrhagic stroke	2·5 (1·9 to 3·2)	1·7% (1·3 to 2·2%)	3·1 (2·4 to 4·0)	1·8% (1·4 to 2·3%)	24·5% (17·4 to 32·1%)	2·2% (-2·3 to 7·2%)	61·2 (48·0 to 76·8)	1·8% (1·4 to 2·2%)	69·2 (54·1 to 87·9)	1·9% (1·5 to 2·3%)	13·1% (8·1 to 19·0%)	4·4% (1·0 to 8·0%)
**Alcohol and drug use: All causes**	173·6 (163·9 to 182·6)	6·4% (6·1 to 6·8%)	204·4 (191·6 to 216·9)	6·1% (5·7 to 6·5%)	17·7% (14·0 to 21·6%)	-5·1% (-7·3 to -2·9%)	7,844·4 (7,411·8 to 8,307·5)	5·8% (5·3 to 6·3%)	8,748·4 (8,226·7 to 9,306·1)	6·0% (5·4 to 6·6%)	11·5% (8·4 to 14·6%)	3·4% (1·1 to 5·9%)
**Alcohol use: All causes**	156·9 (148·0 to 165·7)	5·8% (5·5 to 6·1%)	180·7 (168·4 to 192·1)	5·4% (5·0 to 5·7%)	15·2% (11·3 to 19·1%)	-7·1% (-9·5 to -4·8%)	6,788·9 (6,421·1 to 7,203·7)	5·0% (4·6 to 5·5%)	7,394·3 (6,963·7 to 7,876·6)	5·1% (4·6 to 5·6%)	8·9% (5·6 to 12·2%)	1·0% (-1·5 to 3·7%)
Tuberculosis	3·3 (2·8 to 4·6)	16·9% (14·7 to 18·5%)	2·9 (2·3 to 4·1)	16·9% (14·4 to 19·1%)	-13·8% (-21·1 to -4·5%)	0·4% (-5·4 to 5·9%)	123·5 (104·4 to 168·2)	17·3% (15·2 to 18·8%)	104·0 (85·2 to 145·4)	18·3% (15·7 to 20·2%)	-15·7% (-22·9 to -6·3%)	5·5% (-0·4 to 10·7%)
Lower respiratory infections	5·8 (4·7 to 6·7)	3·6% (3·2 to 4·1%)	7·7 (6·0 to 9·0)	4·1% (3·5 to 4·7%)	32·4% (21·4 to 44·2%)	13·2% (5·5 to 21·3%)	134·4 (108·1 to 148·2)	2·3% (2·0 to 2·5%)	161·6 (125·6 to 182·4)	3·4% (2·9 to 3·8%)	20·2% (10·8 to 30·6%)	48·2% (37·5 to 59·8%)
Lip and oral cavity cancer	1·9 (1·7 to 2·1)	29·8% (27·2 to 32·0%)	2·5 (2·2 to 2·7)	28·5% (25·4 to 31·1%)	30·2% (21·4 to 38·0%)	-4·6% (-8·6 to -1·1%)	55·8 (51·3 to 59·6)	33·5% (31·0 to 35·5%)	69·8 (62·8 to 76·3)	32·5% (29·5 to 34·8%)	25·2% (17·0 to 32·8%)	-2·9% (-6·4 to 0·1%)
Nasopharynx cancer	0·4 (0·3 to 0·4)	29·2% (26·5 to 31·2%)	0·4 (0·4 to 0·5)	29·2% (26·1 to 31·8%)	25·6% (17·2 to 32·7%)	0·2% (-4·0 to 3·6%)	11·5 (9·4 to 12·4)	29·1% (26·7 to 30·7%)	13·9 (11·3 to 15·3)	29·9% (27·1 to 32·1%)	21·1% (13·2 to 28·1%)	2·9% (-1·1 to 6·3%)
Other pharynx cancer	1·2 (1·1 to 1·3)	32·9% (30·0 to 35·2%)	1·6 (1·5 to 1·8)	32·6% (29·4 to 35·2%)	31·6% (22·0 to 42·8%)	-1·1% (-4·8 to 2·4%)	37·4 (34·0 to 40·3)	35·9% (33·2 to 38·0%)	48·1 (42·7 to 53·0)	35·9% (32·9 to 38·4%)	28·6% (19·2 to 39·4%)	0·1% (-3·2 to 3·1%)
Oesophageal cancer	2·4 (2·1 to 2·6)	19·1% (17·1 to 20·7%)	3·0 (2·7 to 3·4)	18·4% (16·1 to 20·2%)	27·5% (18·7 to 36·8%)	-3·7% (-7·9 to 0·5%)	64·8 (58·1 to 70·3)	21·6% (19·5 to 23·1%)	79·9 (70·7 to 87·7)	21·2% (18·9 to 23·0%)	23·2% (14·4 to 32·5%)	-1·8% (-5·8 to 1·6%)
Colon and rectum cancer	1·2 (1·0 to 1·3)	3·6% (3·2 to 3·9%)	1·7 (1·5 to 1·9)	3·5% (3·1 to 3·9%)	44·0% (36·9 to 51·1%)	-2·0% (-5·6 to 1·4%)	29·0 (26·2 to 31·5)	3·9% (3·5 to 4·2%)	39·7 (35·6 to 43·7)	3·9% (3·5 to 4·2%)	37·1% (30·0 to 44·0%)	-0·8% (-4·7 to 2·6%)
Liver cancer due to hepatitis B	0·0 (0·0 to 0·0)	0·7% (0·5 to 0·8%)	0·0 (0·0 to 0·0)	0·5% (0·4 to 0·7%)	2·5% (-12·9 to 16·8%)	-21·3% (-32·9 to -9·6%)	1·6 (1·3 to 1·9)	1·3% (1·1 to 1·6%)	1·6 (1·2 to 2·1)	1·1% (0·9 to 1·4%)	2·1% (-12·6 to 15·9%)	-16·2% (-27·5 to -4·9%)
Liver cancer due to hepatitis C	0·0 (0·0 to 0·0)	0·1% (0·0 to 0·1%)	0·0 (0·0 to 0·0)	0·1% (0·0 to 0·1%)	25·7% (-4·7 to 61·0%)	-10·9% (-32·4 to 14·4%)	0·3 (0·2 to 0·4)	0·2% (0·1 to 0·2%)	0·3 (0·2 to 0·5)	0·2% (0·1 to 0·2%)	15·2% (-4·5 to 38·9%)	-13·3% (-27·9 to 4·6%)
Liver cancer due to alcohol use	5·7 (5·4 to 6·2)	100·0% (100·0 to 100·0%)	8·4 (7·8 to 9·1)	100·0% (100·0 to 100·0%)	45·9% (40·4 to 51·1%)	-	125·0 (116·7 to 136·3)	100·0% (100·0 to 100·0%)	176·1 (163·3 to 192·7)	100·0% (100·0 to 100·0%)	40·9% (35·4 to 46·6%)	-
Liver cancer due to other causes	0·0 (0·0 to 0·0)	0·3% (0·2 to 0·3%)	0·0 (0·0 to 0·0)	0·2% (0·1 to 0·3%)	4·9% (-7·0 to 17·0%)	-22·1% (-31·4 to -13·3%)	0·5 (0·4 to 0·6)	0·6% (0·5 to 0·7%)	0·5 (0·4 to 0·7)	0·5% (0·4 to 0·6%)	2·2% (-8·6 to 12·3%)	-17·4% (-26·0 to -9·5%)
Larynx cancer	1·5 (1·4 to 1·7)	21·9% (19·5 to 23·9%)	1·9 (1·6 to 2·1)	21·2% (18·6 to 23·5%)	20·6% (12·5 to 28·8%)	-3·0% (-7·0 to 0·7%)	42·3 (37·9 to 45·9)	24·3% (22·0 to 26·2%)	49·9 (44·1 to 55·1)	23·9% (21·3 to 26·1%)	18·1% (9·9 to 25·8%)	-1·6% (-5·3 to 1·6%)
Breast cancer	1·1 (1·0 to 1·3)	4·0% (3·5 to 4·6%)	1·6 (1·3 to 1·8)	4·0% (3·4 to 4·5%)	37·1% (29·4 to 45·3%)	-1·9% (-5·5 to 1·6%)	35·9 (30·5 to 41·5)	4·1% (3·5 to 4·7%)	47·5 (39·6 to 55·1)	4·0% (3·4 to 4·6%)	32·2% (24·5 to 40·1%)	-1·4% (-4·9 to 2·0%)
Ischaemic heart disease	-3·0 (-4·3 to -1·6)	-0·8% (-1·2 to -0·4%)	-4·1 (-5·9 to -2·3)	-0·8% (-1·2 to -0·5%)	37·5% (22·0 to 58·0%)	1·4% (-9·9 to 16·0%)	-86·3 (-113·0 to -60·3)	-1·2% (-1·6 to -0·8%)	-109·9 (-141·0 to -78·2)	-1·2% (-1·6 to -0·9%)	27·4% (18·6 to 38·1%)	1·7% (-4·7 to 9·6%)
Ischaemic stroke	-0·6 (-1·1 to -0·1)	-0·7% (-1·3 to -0·1%)	-0·9 (-1·6 to -0·2)	-0·8% (-1·3 to -0·2%)	50·9% (17·3 to 182·4%)	11·9% (-12·6 to 113·7%)	-3·9 (-9·8 to 2·1)	-0·3% (-0·8 to 0·2%)	-4·9 (-12·1 to 2·5)	-0·3% (-0·8 to 0·2%)	23·8% (-146·0 to 184·4%)	0·0% (-137·1 to 132·1%)
Hemorrhagic stroke	10·9 (9·6 to 12·3)	7·6% (6·7 to 8·5%)	12·8 (10·9 to 14·6)	7·3% (6·2 to 8·3%)	16·8% (9·3 to 24·4%)	-4·2% (-9·7 to 1·1%)	283·0 (253·2 to 310·5)	8·3% (7·5 to 9·1%)	310·3 (268·9 to 347·5)	8·4% (7·4 to 9·3%)	9·6% (3·4 to 16·0%)	1·2% (-3·7 to 5·9%)
Hypertensive heart disease	2·1 (1·5 to 2·9)	5·0% (3·4 to 6·8%)	2·6 (1·6 to 3·8)	4·5% (2·8 to 6·4%)	23·6% (4·1 to 42·7%)	-10·5% (-24·7 to 3·5%)	50·3 (38·7 to 62·0)	6·6% (5·1 to 8·2%)	60·7 (44·7 to 75·6)	6·3% (4·7 to 8·0%)	20·5% (8·2 to 32·1%)	-4·3% (-13·7 to 4·5%)
Atrial fibrillation and flutter	0·3 (0·2 to 0·4)	4·6% (3·8 to 5·6%)	0·5 (0·4 to 0·7)	4·4% (3·6 to 5·4%)	48·9% (39·2 to 58·5%)	-4·4% (-10·2 to 0·8%)	8·9 (6·9 to 11·5)	6·3% (5·6 to 7·1%)	12·4 (9·6 to 16·3)	6·1% (5·3 to 6·9%)	40·4% (33·7 to 46·7%)	-3·6% (-8·1 to 0·5%)
Cirrhosis and other chronic liver diseases due to hepatitis B	1·5 (1·0 to 1·9)	14·5% (10·4 to 18·2%)	1·6 (1·0 to 2·2)	12·7% (8·3 to 17·0%)	8·2% (-8·2 to 22·6%)	-12·0% (-25·2 to -1·0%)	43·6 (30·3 to 56·9)	15·4% (11·1 to 19·1%)	44·7 (28·2 to 61·1)	13·7% (9·0 to 18·0%)	2·6% (-14·0 to 16·3%)	-10·7% (-25·2 to 0·5%)
Cirrhosis and other chronic liver diseases due to hepatitis C	4·8 (3·5 to 5·9)	18·5% (14·1 to 22·4%)	5·2 (3·4 to 6·8)	16·0% (10·8 to 20·7%)	8·0% (-8·1 to 20·7%)	-13·3% (-26·2 to -3·8%)	139·1 (100·8 to 173·6)	18·7% (14·1 to 22·5%)	142·8 (92·1 to 188·2)	16·3% (10·8 to 21·1%)	2·6% (-13·9 to 15·4%)	-12·6% (-26·4 to -3·1%)
Cirrhosis and other chronic liver diseases due to alcohol use	39·1 (35·7 to 40·9)	100·0% (100·0 to 100·0%)	48·9 (45·5 to 51·3)	100·0% (100·0 to 100·0%)	25·0% (20·6 to 30·3%)	-	1,253·5 (1,148·9 to 1,313·7)	100·0% (100·0 to 100·0%)	1,489·9 (1,398·4 to 1,567·5)	100·0% (100·0 to 100·0%)	18·9% (14·4 to 24·7%)	-
Cirrhosis and other chronic liver diseases due to other causes	2·5 (1·9 to 3·1)	15·8% (12·4 to 19·2%)	2·9 (2·0 to 3·8)	14·5% (10·4 to 18·6%)	15·3% (1·3 to 27·4%)	-8·4% (-19·5 to 0·8%)	66·7 (52·1 to 80·1)	14·3% (11·5 to 16·9%)	69·2 (48·1 to 88·2)	13·4% (9·6 to 16·9%)	3·6% (-10·3 to 14·4%)	-6·3% (-19·0 to 2·8%)
Pancreatitis	1·0 (0·8 to 1·0)	12·7% (11·5 to 13·7%)	1·2 (1·0 to 1·4)	12·0% (10·4 to 13·3%)	28·2% (18·7 to 38·3%)	-5·4% (-11·0 to -0·2%)	34·8 (31·2 to 37·9)	14·3% (13·2 to 15·2%)	42·6 (36·8 to 48·0)	14·1% (12·5 to 15·4%)	22·4% (13·2 to 31·7%)	-1·3% (-6·9 to 3·9%)

Supplemental Results Table 1. All-age deaths, death PAFs, DALYs, DALY PAFs, and percent change for each risk factor and outcome for both sexes combined in 2005 and 2015 for Latin America and Caribbean.

Data in parenthesis are 95% uncertainty intervals. PAFs=population attributable fractions. DALYs=disability-adjusted life-years.

	2005 Deaths (in thousands)	2005 Deaths PAF	2015 Deaths (in thousands)	2015 Deaths PAF	Percent change deaths 2005-2015	Percent change deaths PAF 2005-2015	2005 DALYs (in thousands)	2005 DALYs PAF	2015 DALYs (in thousands)	2015 DALYs PAF	Percent change DALYs 2005-2015	Percent change DALYs PAF 2005-2015
Epilepsy	1·0 (0·9 to 1·0)	14·1% (13·3 to 14·8%)	1·1 (1·0 to 1·2)	14·6% (13·2 to 15·7%)	13·9% (7·7 to 19·8%)	3·5% (-1·2 to 8·1%)	109·5 (87·3 to 132·1)	11·1% (10·3 to 11·8%)	109·5 (88·5 to 133·3)	11·8% (10·6 to 12·8%)	-0·1% (-7·1 to 7·3%)	6·3% (1·6 to 10·8%)
Alcohol use disorders	16·5 (16·0 to 17·2)	100·0% (100·0 to 100·0%)	18·2 (17·3 to 19·0)	100·0% (100·0 to 100·0%)	10·2% (4·8 to 16·5%)	-	1,060·9 (907·2 to 1,245·0)	100·0% (100·0 to 100·0%)	1,174·2 (996·3 to 1,400·2)	100·0% (100·0 to 100·0%)	10·7% (6·6 to 14·5%)	-
Diabetes mellitus	-6·4 (-7·0 to -5·8)	-5·1% (-5·6 to -4·7%)	-8·8 (-9·6 to -7·9)	-4·8% (-5·2 to -4·3%)	37·4% (28·3 to 47·1%)	-6·0% (-11·8 to -0·4%)	-259·4 (-311·8 to -216·2)	-5·5% (-5·9 to -5·1%)	-342·2 (-411·7 to -281·0)	-5·2% (-5·6 to -4·9%)	31·9% (24·9 to 38·6%)	-4·6% (-9·1 to -0·3%)
Pedestrian road injuries	10·6 (9·1 to 12·3)	21·9% (19·0 to 24·9%)	9·9 (8·4 to 11·6)	20·6% (17·9 to 23·5%)	-6·6% (-11·1 to -0·8%)	-6·1% (-7·9 to -4·3%)	548·1 (469·2 to 635·8)	23·8% (20·8 to 27·2%)	486·1 (413·4 to 564·5)	23·5% (20·4 to 26·7%)	-11·3% (-15·7 to -5·5%)	-1·2% (-2·9 to 0·6%)
Cyclist road injuries	1·0 (0·8 to 1·1)	23·5% (20·4 to 26·7%)	1·0 (0·8 to 1·2)	22·2% (19·3 to 25·4%)	1·5% (-4·7 to 9·8%)	-5·3% (-7·2 to -3·6%)	54·1 (46·4 to 62·5)	23·5% (20·5 to 26·8%)	52·2 (44·4 to 62·0)	22·9% (20·0 to 26·1%)	-3·5% (-9·1 to 3·8%)	-2·7% (-4·5 to -0·8%)
Motorcyclist road injuries	7·1 (5·9 to 7·8)	45·0% (42·2 to 47·8%)	9·8 (7·4 to 11·3)	45·0% (42·0 to 47·8%)	37·0% (16·3 to 55·7%)	-0·2% (-1·7 to 1·3%)	410·6 (338·6 to 449·3)	44·4% (41·5 to 47·2%)	550·4 (428·1 to 631·4)	44·5% (41·5 to 47·3%)	34·1% (15·9 to 51·5%)	0·2% (-1·5 to 1·9%)
Motor vehicle road injuries	13·3 (12·2 to 16·0)	39·0% (36·6 to 41·7%)	14·1 (12·7 to 16·7)	38·6% (36·1 to 41·2%)	6·2% (-1·3 to 12·5%)	-1·1% (-2·9 to 0·4%)	721·1 (655·4 to 867·8)	38·4% (35·9 to 41·2%)	745·9 (669·7 to 882·9)	38·7% (36·1 to 41·3%)	3·4% (-3·5 to 9·3%)	0·8% (-1·2 to 2·6%)
Falls	2·5 (2·2 to 2·9)	10·5% (9·3 to 11·8%)	3·1 (2·7 to 3·6)	9·3% (8·1 to 10·7%)	23·8% (17·0 to 30·8%)	-11·1% (-14·4 to -7·8%)	149·4 (126·9 to 176·5)	12·3% (11·3 to 13·6%)	164·1 (137·9 to 195·4)	12·0% (11·0 to 13·3%)	9·8% (5·7 to 14·3%)	-2·4% (-4·0 to -0·7%)
Drowning	2·3 (2·1 to 2·6)	11·0% (10·0 to 12·1%)	2·2 (2·0 to 2·4)	12·0% (10·9 to 13·2%)	-4·6% (-10·0 to 3·2%)	8·9% (5·2 to 13·1%)	113·8 (102·5 to 127·3)	9·3% (8·3 to 10·3%)	104·2 (92·5 to 117·0)	10·6% (9·6 to 11·8%)	-8·5% (-13·9 to -0·6%)	14·5% (9·1 to 20·4%)
Fire, heat, and hot substances	0·5 (0·4 to 0·6)	7·5% (6·6 to 8·4%)	0·5 (0·4 to 0·6)	8·0% (6·9 to 9·1%)	5·6% (0·6 to 11·4%)	6·2% (1·4 to 10·9%)	24·0 (21·1 to 28·6)	6·6% (5·9 to 7·4%)	23·7 (20·7 to 28·1)	7·8% (6·9 to 8·8%)	-1·6% (-6·1 to 3·4%)	18·3% (12·3 to 24·5%)
Poisonings	0·2 (0·2 to 0·3)	7·5% (6·1 to 9·3%)	0·2 (0·2 to 0·3)	7·9% (6·6 to 9·7%)	-0·2% (-7·7 to 6·3%)	6·0% (-7·1 to 24·7%)	11·5 (9·9 to 15·8)	6·4% (4·9 to 8·0%)	11·2 (9·5 to 15·3)	7·3% (5·8 to 9·0%)	-2·8% (-9·1 to 2·8%)	14·0% (-6·0 to 40·7%)
Unintentional firearm injuries	0·6 (0·4 to 0·7)	12·5% (11·0 to 14·0%)	0·6 (0·5 to 0·7)	12·1% (10·6 to 13·6%)	3·1% (-5·6 to 12·5%)	-3·5% (-6·4 to -0·3%)	31·8 (24·2 to 37·1)	12·7% (11·2 to 14·2%)	32·2 (25·5 to 37·7)	12·9% (11·4 to 14·4%)	1·2% (-7·6 to 10·8%)	1·5% (-1·7 to 4·9%)
Unintentional suffocation	0·0 (0·0 to 0·0)	2·3% (2·0 to 2·7%)	0·0 (0·0 to 0·0)	3·4% (2·8 to 4·0%)	18·8% (11·0 to 26·2%)	46·8% (25·3 to 68·4%)	2·3 (2·0 to 2·8)	2·0% (1·7 to 2·4%)	2·7 (2·2 to 3·1)	2·9% (2·4 to 3·4%)	14·7% (9·0 to 20·0%)	43·0% (22·8 to 63·1%)
Other exposure to mechanical forces	0·7 (0·6 to 0·8)	13·6% (12·5 to 14·9%)	0·7 (0·6 to 0·8)	13·7% (12·6 to 15·2%)	2·1% (-3·8 to 9·2%)	1·2% (-1·0 to 3·3%)	44·5 (38·0 to 52·1)	12·6% (11·6 to 13·9%)	44·3 (37·7 to 52·3)	13·2% (12·2 to 14·5%)	-0·4% (-4·8 to 4·9%)	4·9% (2·9 to 7·0%)
Self-harm	4·8 (4·4 to 5·4)	14·5% (13·4 to 16·0%)	5·7 (5·1 to 6·5)	14·5% (13·3 to 16·0%)	19·1% (13·1 to 24·7%)	-0·2% (-2·0 to 1·7%)	229·5 (207·8 to 259·8)	14·4% (13·2 to 16·0%)	264·9 (237·1 to 300·5)	14·6% (13·2 to 16·1%)	15·4% (9·4 to 21·1%)	0·9% (-1·0 to 3·0%)
Assault by firearm	14·0 (11·9 to 15·9)	15·3% (13·8 to 17·2%)	14·8 (12·7 to 16·8)	14·7% (13·3 to 16·4%)	5·0% (0·1 to 10·6%)	-4·2% (-5·3 to -3·2%)	814·1 (683·0 to 922·8)	15·9% (14·4 to 17·9%)	839·7 (717·9 to 954·9)	15·4% (14·0 to 17·3%)	3·1% (-1·7 to 8·7%)	-3·3% (-4·3 to -2·3%)
Assault by sharp object	2·9 (2·6 to 3·3)	14·5% (13·1 to 16·2%)	3·1 (2·7 to 3·6)	13·9% (12·6 to 15·5%)	7·5% (1·3 to 15·4%)	-3·9% (-5·1 to -2·5%)	162·4 (145·2 to 187·2)	15·3% (13·9 to 17·1%)	169·5 (150·2 to 196·4)	14·9% (13·5 to 16·7%)	4·4% (-1·9 to 12·4%)	-2·2% (-3·4 to -0·9%)
Assault by other means	1·9 (1·7 to 2·3)	12·1% (11·0 to 13·6%)	2·0 (1·7 to 2·4)	12·1% (11·0 to 13·5%)	5·5% (-2·4 to 13·1%)	-0·3% (-2·4 to 1·9%)	108·8 (96·0 to 130·7)	12·5% (11·3 to 14·0%)	110·8 (95·3 to 130·4)	12·8% (11·6 to 14·4%)	1·8% (-5·7 to 9·1%)	2·8% (0·5 to 5·1%)
**Drug use: All causes**	19·5 (15·2 to 23·7)	0·7% (0·6 to 0·9%)	27·0 (21·6 to 32·3)	0·8% (0·6 to 1·0%)	38·4% (32·3 to 46·9%)	11·6% (7·1 to 17·9%)	1,156·4 (980·6 to 1,321·2)	0·9% (0·7 to 1·0%)	1,465·8 (1,250·7 to 1,665·1)	1·0% (0·9 to 1·1%)	26·8% (23·1 to 31·2%)	17·5% (14·2 to 21·8%)
HIV/AIDS - Tuberculosis	0·1 (0·0 to 0·1)	1·1% (0·9 to 1·3%)	0·0 (0·0 to 0·1)	1·2% (1·0 to 1·5%)	-5·2% (-12·6 to 6·1%)	12·1% (8·9 to 15·9%)	2·4 (1·8 to 3·1)	1·0% (0·8 to 1·2%)	2·1 (1·6 to 2·9)	1·1% (0·9 to 1·4%)	-10·5% (-17·1 to -0·1%)	12·6% (9·3 to 16·5%)
HIV/AIDS resulting in other diseases	0·4 (0·3 to 0·5)	0·9% (0·8 to 1·1%)	0·5 (0·4 to 0·6)	1·1% (0·9 to 1·4%)	13·9% (1·7 to 26·1%)	19·7% (13·3 to 26·1%)	19·9 (16·3 to 25·0)	0·9% (0·7 to 1·1%)	21·0 (16·6 to 26·7)	1·0% (0·8 to 1·3%)	5·5% (-4·6 to 15·7%)	18·5% (12·3 to 24·8%)
Hepatitis B	0·0 (0·0 to 0·0)	0·8% (0·3 to 1·4%)	0·0 (0·0 to 0·0)	0·9% (0·4 to 1·5%)	-0·2% (-10·0 to 12·3%)	11·5% (3·0 to 22·4%)	0·8 (0·3 to 1·3)	0·9% (0·4 to 1·4%)	0·7 (0·3 to 1·2)	1·0% (0·4 to 1·6%)	-3·3% (-12·7 to 8·2%)	10·4% (1·8 to 20·6%)
Hepatitis C	0·1 (0·0 to 0·2)	38·2% (26·0 to 49·1%)	0·1 (0·0 to 0·2)	44·7% (32·8 to 56·2%)	-2·0% (-23·0 to 25·2%)	17·0% (1·3 to 36·5%)	3·0 (0·8 to 6·6)	41·6% (29·9 to 51·6%)	2·8 (0·8 to 5·9)	48·0% (36·6 to 58·4%)	-7·0% (-25·5 to 17·8%)	15·4% (2·7 to 31·5%)
Liver cancer due to hepatitis B	0·0 (0·0 to 0·0)	0·6% (0·3 to 1·0%)	0·0 (0·0 to 0·1)	0·7% (0·3 to 1·2%)	58·0% (44·7 to 75·2%)	21·4% (12·6 to 34·4%)	0·8 (0·3 to 1·4)	0·7% (0·3 to 1·2%)	1·2 (0·5 to 2·0)	0·8% (0·4 to 1·4%)	45·7% (33·4 to 60·1%)	19·6% (11·0 to 31·6%)
Liver cancer due to hepatitis C	1·8 (1·1 to 2·7)	23·3% (14·0 to 35·1%)	3·2 (2·0 to 4·6)	29·2% (18·7 to 41·7%)	76·1% (61·4 to 98·6%)	24·9% (15·3 to 40·5%)	47·4 (29·4 to 67·4)	30·5% (19·1 to 43·5%)	75·4 (50·5 to 103·3)	36·4% (24·5 to 49·0%)	58·9% (45·9 to 78·1%)	19·6% (10·7 to 33·6%)
Cirrhosis and other chronic liver diseases due to hepatitis B	0·1 (0·0 to 0·1)	0·8% (0·3 to 1·3%)	0·1 (0·0 to 0·2)	0·9% (0·4 to 1·5%)	47·4% (34·9 to 64·7%)	19·9% (10·8 to 33·4%)	2·6 (1·0 to 4·4)	0·9% (0·4 to 1·6%)	3·5 (1·5 to 5·9)	1·1% (0·5 to 1·8%)	36·4% (24·6 to 52·5%)	18·7% (9·5 to 32·1%)
Cirrhosis and other chronic liver diseases due to hepatitis C	12·7 (9·0 to 15·9)	49·0% (35·2 to 61·4%)	17·3 (13·0 to 21·3)	53·9% (40·7 to 65·4%)	36·8% (29·0 to 48·2%)	9·8% (5·0 to 17·4%)	449·0 (343·0 to 535·8)	60·3% (46·2 to 71·6%)	565·6 (449·0 to 661·5)	64·7% (51·5 to 74·4%)	26·0% (18·9 to 36·4%)	7·3% (3·1 to 13·9%)
Opioid use disorders	2·3 (2·2 to 2·5)	100·0% (100·0 to 100·0%)	2·8 (2·6 to 3·2)	100·0% (100·0 to 100·0%)	24·4% (16·1 to 32·5%)	-	362·8 (282·1 to 441·9)	100·0% (100·0 to 100·0%)	439·9 (340·9 to 537·8)	100·0% (100·0 to 100·0%)	21·2% (17·6 to 25·0%)	-
Cocaine use disorders	0·4 (0·3 to 0·5)	100·0% (100·0 to 100·0%)	0·6 (0·5 to 0·8)	100·0% (100·0 to 100·0%)	58·5% (38·9 to 98·6%)	-	60·2 (43·1 to 79·1)	100·0% (100·0 to 100·0%)	85·5 (62·4 to 110·6)	100·0% (100·0 to 100·0%)	41·9% (35·0 to 53·8%)	-
Amphetamine use disorders	0·1 (0·1 to 0·1)	100·0% (100·0 to 100·0%)	0·2 (0·1 to 0·3)	100·0% (100·0 to 100·0%)	83·2% (50·0 to 147·4%)	-	47·5 (29·0 to 70·7)	100·0% (100·0 to 100·0%)	65·7 (40·7 to 95·5)	100·0% (100·0 to 100·0%)	38·2% (30·4 to 48·3%)	-
Cannabis use disorders	-	-	-	-	-	-	27·3 (17·3 to 38·7)	100·0% (100·0 to 100·0%)	30·3 (19·3 to 43·0)	100·0% (100·0 to 100·0%)	11·0% (7·3 to 15·0%)	-
Other drug use disorders	0·7 (0·7 to 0·8)	100·0% (100·0 to 100·0%)	0·9 (0·8 to 1·0)	100·0% (100·0 to 100·0%)	30·3% (18·3 to 40·4%)	-	84·0 (62·6 to 109·8)	100·0% (100·0 to 100·0%)	110·9 (82·7 to 146·2)	100·0% (100·0 to 100·0%)	32·0% (25·4 to 38·6%)	-
Self-harm	0·9 (0·6 to 1·3)	2·7% (1·8 to 4·0%)	1·2 (0·8 to 1·7)	2·9% (1·9 to 4·3%)	28·3% (21·6 to 35·2%)	7·6% (3·6 to 11·2%)	48·7 (32·0 to 72·1)	3·1% (2·0 to 4·5%)	61·2 (39·8 to 91·5)	3·4% (2·2 to 5·0%)	25·8% (18·8 to 32·8%)	10·0% (5·7 to 13·8%)
**Dietary risks: All causes**	463·1 (401·5 to 529·1)	17·2% (14·9 to 19·6%)	610·9 (526·6 to 701·3)	18·3% (15·8 to 20·9%)	31·9% (28·7 to 34·9%)	6·4% (5·1 to 7·7%)	11,135·0 (9,743·5 to 12,675·2)	8·2% (7·1 to 9·4%)	13,833·4 (12,098·1 to 15,847·5)	9·5% (8·1 to 10·9%)	24·2% (21·2 to 27·1%)	15·2% (13·3 to 17·0%)
**Diet low in fruits: All causes**	98·8 (61·6 to 140·7)	3·7% (2·3 to 5·2%)	124·1 (76·7 to 178·9)	3·7% (2·3 to 5·4%)	25·5% (21·2 to 29·4%)	1·2% (-1·3 to 3·5%)	2,626·5 (1,628·7 to 3,718·4)	1·9% (1·2 to 2·8%)	3,134·9 (1,905·2 to 4,509·1)	2·1% (1·3 to 3·1%)	19·4% (14·6 to 23·8%)	10·7% (6·9 to 14·0%)
Lip and oral cavity cancer	0·4 (0·0 to 0·8)	5·7% (0·0 to 12·8%)	0·5 (0·0 to 1·1)	5·6% (0·0 to 12·7%)	34·9% (0·0 to 40·7%)	-1·1% (-2·7 to 0·1%)	9·3 (0·0 to 21·0)	5·6% (0·0 to 12·5%)	11·9 (0·0 to 27·2)	5·6% (0·0 to 12·5%)	28·3% (0·0 to 34·0%)	-0·4% (-2·1 to 0·9%)
Nasopharynx cancer	0·1 (0·0 to 0·1)	5·5% (0·0 to 11·9%)	0·1 (0·0 to 0·2)	5·5% (0·0 to 12·1%)	27·1% (0·0 to 32·5%)	1·5% (0·0 to 2·5%)	1·9 (0·0 to 4·3)	4·9% (0·0 to 10·8%)	2·4 (0·0 to 5·3)	5·1% (0·0 to 11·3%)	21·9% (0·0 to 27·2%)	3·7% (0·0 to 5·0%)
Other pharynx cancer	0·2 (0·0 to 0·5)	5·7% (0·0 to 13·0%)	0·3 (0·0 to 0·6)	5·6% (0·0 to 12·8%)	31·4% (0·0 to 39·3%)	-1·2% (-3·2 to 0·3%)	5·8 (0·0 to 13·4)	5·6% (0·0 to 12·8%)	7·5 (0·0 to 17·0)	5·6% (0·0 to 12·8%)	28·1% (0·0 to 36·1%)	-0·2% (-2·3 to 1·5%)
Oesophageal cancer	2·3 (0·5 to 4·3)	18·6% (4·3 to 34·0%)	3·0 (0·7 to 5·6)	18·3% (4·3 to 33·8%)	30·7% (24·0 to 37·6%)	-1·2% (-3·0 to 0·1%)	54·6 (12·7 to 101·8)	18·2% (4·2 to 33·6%)	68·1 (15·8 to 124·2)	18·1% (4·2 to 33·5%)	24·8% (18·0 to 31·8%)	-0·6% (-2·5 to 0·9%)
Larynx cancer	0·4 (0·0 to 0·9)	5·8% (0·0 to 13·1%)	0·5 (0·0 to 1·2)	5·7% (0·0 to 13·0%)	23·5% (0·0 to 29·7%)	-0·7% (-2·4 to 0·6%)	9·8 (0·0 to 22·5)	5·6% (0·0 to 12·9%)	11·7 (0·0 to 26·9)	5·6% (0·0 to 12·9%)	19·7% (0·0 to 25·9%)	-0·2% (-2·1 to 1·3%)
Tracheal, bronchus, and lung cancer	5·1 (2·0 to 8·7)	10·0% (3·9 to 17·2%)	6·9 (2·7 to 11·9)	9·8% (3·8 to 17·0%)	35·9% (30·0 to 40·5%)	-1·4% (-2·9 to -0·3%)	109·9 (43·2 to 190·5)	9·7% (3·8 to 16·9%)	141·2 (55·8 to 246·5)	9·6% (3·8 to 16·7%)	28·5% (23·3 to 33·1%)	-1·2% (-2·8 to -0·1%)
Ischaemic heart disease	37·9 (13·3 to 65·2)	10·2% (3·6 to 17·7%)	49·4 (17·2 to 85·7)	9·8% (3·4 to 17·0%)	30·3% (26·9 to 33·3%)	-4·0% (-5·2 to -3·1%)	866·1 (307·4 to 1,478·8)	11·9% (4·2 to 20·4%)	1,052·6 (370·8 to 1,805·0)	11·6% (4·1 to 19·8%)	21·5% (18·1 to 24·8%)	-3·0% (-4·2 to -2·1%)
Ischaemic stroke	12·0 (6·2 to 19·4)	13·9% (7·1 to 22·0%)	14·9 (7·5 to 23·9)	12·8% (6·5 to 20·4%)	23·6% (19·3 to 27·6%)	-8·3% (-10·0 to -7·0%)	242·4 (127·8 to 378·4)	19·2% (10·0 to 29·6%)	282·0 (146·7 to 444·7)	18·1% (9·4 to 28·2%)	16·3% (12·3 to 20·2%)	-6·0% (-7·7 to -4·7%)

Supplemental Results Table 1. All-age deaths, death PAFs, DALYs, DALY PAFs, and percent change for each risk factor and outcome for both sexes combined in 2005 and 2015 for Latin America and Caribbean.

Data in parenthesis are 95% uncertainty intervals. PAFs=population attributable fractions. DALYs=disability-adjusted life-years.

	2005 Deaths (in thousands)	2005 Deaths PAF	2015 Deaths (in thousands)	2015 Deaths PAF	Percent change deaths 2005-2015	Percent change deaths PAF 2005-2015	2005 DALYs (in thousands)	2005 DALYs PAF	2015 DALYs (in thousands)	2015 DALYs PAF	Percent change DALYs 2005-2015	Percent change DALYs PAF 2005-2015
Hemorrhagic stroke	30·0 (16·0 to 46·7)	20·8% (11·0 to 32·2%)	33·9 (18·0 to 53·1)	19·3% (10·2 to 30·0%)	12·9% (9·1 to 17·1%)	-7·3% (-9·0 to -6·1%)	843·7 (461·7 to 1,283·1)	24·8% (13·5 to 37·6%)	898·9 (488·6 to 1,370·4)	24·4% (13·1 to 37·1%)	6·5% (2·8 to 10·5%)	-1·7% (-3·2 to -0·6%)
**Diet low in vegetables: All causes**	98·0 (50·7 to 151·5)	3·6% (1·9 to 5·6%)	124·5 (64·2 to 193·1)	3·7% (1·9 to 5·8%)	27·0% (22·3 to 30·9%)	2·4% (-0·7 to 4·8%)	2,309·0 (1,224·5 to 3,517·1)	1·7% (0·9 to 2·6%)	2,710·4 (1,445·9 to 4,163·2)	1·9% (1·0 to 2·9%)	17·4% (12·9 to 21·4%)	8·9% (5·2 to 12·0%)
Ischaemic heart disease	70·3 (28·2 to 114·5)	19·0% (7·5 to 30·8%)	92·3 (36·5 to 150·9)	18·3% (7·2 to 29·9%)	31·3% (28·1 to 34·3%)	-3·3% (-4·0 to -2·7%)	1,584·0 (650·7 to 2,540·8)	21·8% (8·8 to 35·0%)	1,925·7 (782·1 to 3,106·6)	21·2% (8·5 to 34·1%)	21·6% (18·3 to 24·7%)	-2·9% (-3·8 to -2·3%)
Ischaemic stroke	7·9 (1·8 to 14·2)	9·1% (2·0 to 16·2%)	9·8 (2·2 to 17·6)	8·4% (1·9 to 15·0%)	24·8% (21·0 to 28·6%)	-7·4% (-8·6 to -6·5%)	161·7 (37·2 to 283·3)	12·8% (3·0 to 22·4%)	188·5 (44·1 to 332·8)	12·1% (2·8 to 21·3%)	16·5% (12·7 to 20·1%)	-5·8% (-7·1 to -4·8%)
Hemorrhagic stroke	19·8 (5·8 to 35·5)	13·8% (4·0 to 24·5%)	22·4 (6·5 to 40·1)	12·8% (3·7 to 22·9%)	12·9% (9·0 to 16·9%)	-7·4% (-8·7 to -6·3%)	563·2 (166·1 to 993·7)	16·5% (4·9 to 28·9%)	596·2 (174·6 to 1,048·0)	16·2% (4·8 to 28·5%)	5·9% (2·0 to 9·8%)	-2·3% (-3·6 to -1·4%)
**Diet low in whole grains: All causes**	96·4 (63·5 to 133·7)	3·6% (2·4 to 4·9%)	121·5 (79·7 to 168·9)	3·6% (2·4 to 5·1%)	26·0% (21·6 to 29·9%)	1·6% (-0·8 to 3·6%)	2,646·6 (1,769·7 to 3,642·7)	2·0% (1·3 to 2·7%)	3,153·2 (2,077·7 to 4,363·1)	2·2% (1·4 to 3·0%)	19·1% (14·9 to 22·9%)	10·5% (7·4 to 13·3%)
Ischaemic heart disease	50·2 (28·0 to 76·2)	13·5% (7·6 to 20·5%)	64·6 (35·8 to 98·7)	12·8% (7·1 to 19·5%)	28·6% (24·3 to 32·7%)	-5·2% (-6·8 to -3·7%)	1,175·7 (665·2 to 1,753·4)	16·2% (9·1 to 24·2%)	1,402·0 (790·2 to 2,099·4)	15·4% (8·6 to 23·1%)	19·2% (15·1 to 23·2%)	-4·8% (-6·5 to -3·3%)
Ischaemic stroke	8·8 (5·4 to 12·6)	10·2% (6·3 to 14·5%)	11·0 (6·8 to 15·9)	9·5% (5·8 to 13·5%)	25·2% (20·5 to 30·2%)	-7·1% (-8·9 to -5·2%)	189·0 (117·5 to 266·5)	15·0% (9·3 to 21·1%)	221·4 (138·9 to 314·6)	14·2% (8·9 to 20·0%)	17·2% (12·8 to 21·7%)	-5·3% (-7·1 to -3·6%)
Hemorrhagic stroke	24·0 (16·2 to 33·7)	16·7% (11·3 to 23·3%)	26·9 (18·1 to 38·1)	15·3% (10·3 to 21·7%)	12·2% (7·7 to 17·7%)	-7·9% (-9·6 to -6·4%)	680·5 (460·6 to 937·6)	20·0% (13·5 to 27·6%)	714·7 (481·4 to 1,000·0)	19·4% (13·0 to 27·0%)	5·0% (0·7 to 10·1%)	-3·1% (-4·8 to -1·5%)
Diabetes mellitus	13·5 (7·0 to 21·7)	10·8% (5·7 to 17·4%)	19·0 (9·9 to 30·5)	10·4% (5·4 to 16·8%)	41·0% (36·0 to 45·8%)	-3·6% (-5·4 to -1·8%)	601·4 (306·3 to 987·9)	12·7% (6·6 to 20·6%)	815·2 (416·1 to 1,343·1)	12·5% (6·4 to 20·3%)	35·5% (31·6 to 39·2%)	-1·9% (-3·4 to -0·7%)
**Diet low in nuts and seeds: All causes**	102·6 (68·4 to 141·7)	3·8% (2·5 to 5·2%)	137·2 (91·4 to 188·8)	4·1% (2·7 to 5·7%)	33·7% (30·8 to 36·6%)	7·8% (6·3 to 9·2%)	2,599·9 (1,791·1 to 3,517·1)	1·9% (1·3 to 2·6%)	3,287·0 (2,270·2 to 4,489·3)	2·2% (1·5 to 3·1%)	26·4% (23·4 to 29·6%)	17·2% (15·0 to 19·4%)
Ischaemic heart disease	88·6 (56·1 to 124·9)	23·9% (15·1 to 33·6%)	117·4 (74·4 to 165·1)	23·3% (14·7 to 32·9%)	32·5% (29·3 to 35·4%)	-2·3% (-2·7 to -2·0%)	1,962·6 (1,272·1 to 2,699·3)	27·0% (17·4 to 37·1%)	2,412·2 (1,557·1 to 3,332·7)	26·5% (17·0 to 36·5%)	22·9% (19·7 to 25·9%)	-1·9% (-2·4 to -1·5%)
Diabetes mellitus	14·0 (7·4 to 21·8)	11·2% (5·9 to 17·5%)	19·8 (10·3 to 31·0)	10·9% (5·7 to 17·0%)	41·3% (37·6 to 45·0%)	-3·3% (-3·8 to -2·9%)	637·3 (308·9 to 1,013·9)	13·5% (7·1 to 20·8%)	874·8 (423·0 to 1,379·2)	13·4% (7·0 to 20·7%)	37·3% (34·1 to 40·4%)	-0·7% (-1·1 to -0·4%)
**Diet low in milk: All causes**	5·0 (1·7 to 8·4)	0·2% (0·1 to 0·3%)	7·3 (2·5 to 12·4)	0·2% (0·1 to 0·4%)	46·5% (41·3 to 51·5%)	18·1% (14·6 to 21·4%)	110·5 (37·9 to 186·6)	0·1% (0·0 to 0·1%)	153·2 (52·4 to 261·3)	0·1% (0·0 to 0·2%)	38·6% (33·9 to 43·2%)	28·5% (24·6 to 32·1%)
Colon and rectum cancer	5·0 (1·7 to 8·4)	15·1% (5·2 to 25·4%)	7·3 (2·5 to 12·4)	15·0% (5·2 to 25·4%)	46·5% (41·3 to 51·5%)	-0·3% (-0·7 to 0·1%)	110·5 (37·9 to 186·6)	14·9% (5·2 to 25·2%)	153·2 (52·4 to 261·3)	14·9% (5·2 to 25·3%)	38·6% (33·9 to 43·2%)	0·2% (-0·2 to 0·6%)
**Diet high in red meat: All causes**	3·9 (1·3 to 6·6)	0·1% (0·0 to 0·2%)	5·6 (1·9 to 9·4)	0·2% (0·1 to 0·3%)	41·2% (36·3 to 46·2%)	13·8% (10·8 to 17·4%)	170·1 (44·6 to 297·0)	0·1% (0·0 to 0·2%)	240·6 (63·4 to 423·2)	0·2% (0·0 to 0·3%)	41·4% (37·6 to 45·5%)	31·1% (27·8 to 34·6%)
Colon and rectum cancer	1·0 (0·2 to 1·7)	2·9% (0·6 to 5·1%)	1·4 (0·3 to 2·5)	2·9% (0·6 to 5·1%)	46·1% (38·7 to 52·3%)	-0·6% (-2·6 to 1·5%)	23·3 (4·9 to 41·3)	3·1% (0·7 to 5·6%)	33·1 (7·0 to 59·5)	3·2% (0·7 to 5·8%)	42·2% (35·1 to 48·3%)	2·8% (0·4 to 5·0%)
Diabetes mellitus	3·0 (0·4 to 5·5)	2·4% (0·3 to 4·4%)	4·2 (0·5 to 7·7)	2·3% (0·3 to 4·2%)	39·6% (34·7 to 44·3%)	-4·5% (-6·3 to -2·7%)	146·8 (17·1 to 272·9)	3·1% (0·4 to 5·7%)	207·5 (24·0 to 386·9)	3·2% (0·4 to 5·9%)	41·3% (37·3 to 45·6%)	2·2% (0·4 to 4·0%)
**Diet high in processed meat: All causes**	27·9 (11·0 to 45·1)	1·0% (0·4 to 1·7%)	37·3 (15·5 to 59·5)	1·1% (0·5 to 1·8%)	33·8% (29·9 to 39·7%)	7·9% (5·9 to 12·2%)	887·2 (440·8 to 1,348·9)	0·7% (0·3 to 1·0%)	1,153·6 (592·7 to 1,751·4)	0·8% (0·4 to 1·2%)	30·0% (25·6 to 37·1%)	20·5% (17·2 to 26·8%)
Colon and rectum cancer	1·3 (0·7 to 1·9)	3·9% (2·1 to 5·8%)	1·9 (1·0 to 2·9)	3·9% (2·1 to 5·9%)	47·2% (41·2 to 52·7%)	0·2% (-1·2 to 1·4%)	29·6 (16·1 to 44·7)	4·0% (2·2 to 6·0%)	41·2 (22·2 to 62·7)	4·0% (2·2 to 6·0%)	39·1% (33·9 to 44·3%)	0·6% (-0·9 to 1·8%)
Ischaemic heart disease	17·5 (0·8 to 32·8)	4·7% (0·2 to 8·9%)	22·7 (1·0 to 43·0)	4·5% (0·2 to 8·5%)	30·0% (25·3 to 33·3%)	-4·2% (-5·3 to -3·1%)	413·7 (18·0 to 768·6)	5·7% (0·2 to 10·6%)	505·0 (22·0 to 937·9)	5·5% (0·2 to 10·4%)	22·1% (17·5 to 25·5%)	-2·6% (-3·6 to -1·5%)
Diabetes mellitus	9·2 (4·5 to 13·6)	7·3% (3·6 to 10·8%)	12·8 (6·3 to 18·9)	7·0% (3·4 to 10·3%)	39·2% (34·9 to 43·6%)	-4·8% (-6·2 to -3·5%)	443·9 (210·4 to 682·8)	9·4% (4·6 to 13·9%)	607·4 (289·6 to 933·0)	9·3% (4·6 to 13·8%)	36·8% (33·2 to 40·3%)	-1·0% (-2·1 to 0·0%)
**Diet high in sugar-sweetened beverages: All causes**	8·8 (6·1 to 12·5)	0·3% (0·2 to 0·5%)	11·7 (8·0 to 16·8)	0·4% (0·2 to 0·5%)	33·6% (29·5 to 37·2%)	7·7% (4·8 to 10·3%)	311·3 (214·3 to 445·4)	0·2% (0·2 to 0·3%)	412·9 (280·8 to 596·6)	0·3% (0·2 to 0·4%)	32·7% (28·4 to 36·2%)	23·0% (19·2 to 26·1%)
Oesophageal cancer	0·1 (0·0 to 0·1)	0·6% (0·2 to 1·1%)	0·1 (0·0 to 0·2)	0·6% (0·2 to 1·2%)	35·4% (27·0 to 42·2%)	2·3% (-3·0 to 6·0%)	1·9 (0·6 to 3·6)	0·6% (0·2 to 1·2%)	2·5 (0·7 to 4·7)	0·7% (0·2 to 1·2%)	31·4% (22·4 to 38·3%)	4·7% (-1·3 to 8·7%)
Colon and rectum cancer	0·1 (0·1 to 0·1)	0·2% (0·2 to 0·4%)	0·1 (0·1 to 0·2)	0·3% (0·2 to 0·4%)	51·4% (45·0 to 57·4%)	3·1% (-0·5 to 7·0%)	2·1 (1·4 to 3·0)	0·3% (0·2 to 0·4%)	3·0 (2·0 to 4·4)	0·3% (0·2 to 0·4%)	46·7% (40·0 to 52·9%)	6·1% (2·1 to 10·2%)
Liver cancer due to hepatitis B	0·0 (0·0 to 0·0)	0·5% (0·2 to 0·8%)	0·0 (0·0 to 0·0)	0·5% (0·2 to 0·8%)	34·9% (27·3 to 43·2%)	3·6% (-0·7 to 7·6%)	0·6 (0·3 to 1·0)	0·5% (0·2 to 0·8%)	0·8 (0·4 to 1·3)	0·5% (0·3 to 0·9%)	29·8% (21·8 to 37·9%)	6·6% (1·8 to 11·2%)
Liver cancer due to hepatitis C	0·0 (0·0 to 0·1)	0·5% (0·2 to 0·8%)	0·1 (0·0 to 0·1)	0·5% (0·2 to 0·8%)	38·4% (32·5 to 45·3%)	-1·8% (-4·8 to 2·2%)	0·9 (0·4 to 1·4)	0·6% (0·3 to 0·9%)	1·2 (0·5 to 1·9)	0·6% (0·3 to 0·9%)	33·8% (27·3 to 40·9%)	0·7% (-2·7 to 5·5%)
Liver cancer due to alcohol use	0·0 (0·0 to 0·1)	0·6% (0·3 to 0·9%)	0·0 (0·0 to 0·1)	0·6% (0·3 to 0·9%)	47·0% (40·0 to 54·2%)	0·7% (-2·8 to 4·4%)	0·7 (0·3 to 1·3)	0·6% (0·3 to 1·0%)	1·1 (0·5 to 1·8)	0·6% (0·3 to 1·0%)	43·9% (36·5 to 51·6%)	2·1% (-1·6 to 5·9%)
Liver cancer due to other causes	0·0 (0·0 to 0·0)	0·5% (0·2 to 0·8%)	0·0 (0·0 to 0·0)	0·5% (0·2 to 0·8%)	35·5% (29·4 to 42·2%)	0·7% (-2·3 to 4·8%)	0·4 (0·2 to 0·7)	0·5% (0·2 to 0·9%)	0·6 (0·3 to 1·0)	0·5% (0·3 to 0·9%)	30·1% (23·7 to 36·9%)	5·2% (1·5 to 9·9%)
Gallbladder and biliary tract cancer	0·1 (0·0 to 0·1)	0·6% (0·4 to 0·9%)	0·1 (0·0 to 0·1)	0·6% (0·3 to 0·9%)	15·0% (3·9 to 24·2%)	-4·4% (-8·1 to -1·0%)	1·4 (0·8 to 2·2)	0·7% (0·4 to 1·0%)	1·5 (0·9 to 2·4)	0·6% (0·4 to 1·0%)	10·9% (0·5 to 19·7%)	-2·3% (-6·2 to 1·4%)
Pancreatic cancer	0·0 (0·0 to 0·1)	0·2% (0·1 to 0·3%)	0·0 (0·0 to 0·1)	0·2% (0·1 to 0·3%)	45·1% (39·0 to 50·3%)	-1·2% (-4·7 to 1·7%)	0·7 (0·3 to 1·3)	0·2% (0·1 to 0·4%)	1·1 (0·4 to 1·9)	0·2% (0·1 to 0·4%)	41·6% (35·6 to 46·8%)	0·9% (-2·9 to 3·9%)
Breast cancer	0·0 (0·0 to 0·1)	0·1% (0·1 to 0·2%)	0·1 (0·0 to 0·1)	0·2% (0·1 to 0·2%)	46·4% (39·2 to 54·1%)	4·8% (0·7 to 8·5%)	1·1 (0·7 to 1·6)	0·1% (0·1 to 0·2%)	1·6 (1·0 to 2·4)	0·1% (0·1 to 0·2%)	46·8% (39·0 to 54·9%)	9·4% (5·0 to 13·7%)
Uterine cancer	0·1 (0·0 to 0·1)	1·2% (0·8 to 1·7%)	0·1 (0·1 to 0·1)	1·2% (0·8 to 1·7%)	27·8% (21·0 to 35·2%)	0·3% (-3·1 to 3·5%)	1·6 (1·1 to 2·3)	1·3% (0·9 to 1·8%)	2·1 (1·4 to 3·0)	1·3% (0·9 to 1·9%)	27·2% (20·1 to 34·7%)	2·0% (-1·6 to 5·2%)
Ovarian cancer	0·0 (0·0 to 0·0)	0·1% (0·0 to 0·2%)	0·0 (0·0 to 0·0)	0·1% (0·0 to 0·2%)	39·1% (0·0 to 45·3%)	0·5% (-2·7 to 3·3%)	0·2 (0·0 to 0·5)	0·1% (0·0 to 0·2%)	0·3 (0·0 to 0·7)	0·1% (0·0 to 0·2%)	36·8% (0·0 to 43·2%)	3·0% (-0·5 to 6·2%)
Kidney cancer	0·0 (0·0 to 0·1)	0·6% (0·4 to 0·9%)	0·1 (0·0 to 0·1)	0·6% (0·4 to 0·9%)	52·7% (45·8 to 59·5%)	2·4% (-0·6 to 5·3%)	1·0 (0·7 to 1·5)	0·5% (0·4 to 0·8%)	1·5 (1·0 to 2·2)	0·6% (0·4 to 0·9%)	49·7% (42·4 to 56·5%)	9·2% (5·7 to 12·5%)
Thyroid cancer	0·0 (0·0 to 0·0)	0·4% (0·2 to 0·6%)	0·0 (0·0 to 0·0)	0·4% (0·2 to 0·6%)	42·0% (35·0 to 48·4%)	-0·7% (-4·5 to 2·6%)	0·2 (0·1 to 0·3)	0·4% (0·2 to 0·6%)	0·3 (0·2 to 0·5)	0·4% (0·2 to 0·7%)	42·2% (34·8 to 48·6%)	2·2% (-1·8 to 5·9%)
Ischaemic heart disease	2·4 (1·7 to 3·4)	0·7% (0·5 to 0·9%)	3·2 (2·2 to 4·5)	0·6% (0·4 to 0·9%)	30·9% (26·2 to 35·1%)	-3·5% (-6·6 to -0·9%)	64·8 (44·4 to 92·6)	0·9% (0·6 to 1·3%)	82·3 (56·3 to 118·3)	0·9% (0·6 to 1·3%)	27·1% (22·1 to 31·4%)	1·4% (-1·8 to 4·1%)
Ischaemic stroke	0·4 (0·2 to 0·5)	0·4% (0·3 to 0·6%)	0·4 (0·3 to 0·6)	0·4% (0·3 to 0·6%)	21·8% (15·3 to 27·5%)	-9·7% (-14·2 to -6·0%)	8·8 (6·1 to 12·3)	0·7% (0·5 to 1·0%)	10·4 (7·1 to 14·7)	0·7% (0·5 to 1·0%)	18·5% (12·9 to 23·6%)	-4·3% (-7·7 to -1·3%)
Hemorrhagic stroke	1·3 (0·9 to 1·9)	0·9% (0·6 to 1·3%)	1·5 (1·0 to 2·1)	0·8% (0·6 to 1·2%)	15·5% (10·1 to 20·3%)	-5·3% (-9·1 to -2·3%)	41·5 (28·2 to 59·6)	1·2% (0·8 to 1·8%)	46·6 (31·3 to 67·2)	1·3% (0·9 to 1·8%)	12·3% (7·3 to 16·8%)	3·6% (-0·3 to 6·7%)
Hypertensive heart disease	0·6 (0·3 to 0·9)	1·3% (0·8 to 2·0%)	0·8 (0·4 to 1·2)	1·3% (0·7 to 2·0%)	32·8% (23·5 to 40·8%)	-3·8% (-10·2 to 0·9%)	12·4 (8·1 to 18·0)	1·6% (1·0 to 2·3%)	15·7 (10·0 to 22·8)	1·6% (1·0 to 2·4%)	26·0% (19·9 to 31·9%)	0·1% (-4·0 to 3·6%)
Cardiomyopathy and myocarditis	-	-	-	-	-	-	-	-	-	-	-	-
Atrial fibrillation and flutter	-	-	-	-	-	-	-	-	-	-	-	-
Peripheral vascular disease	-	-	-	-	-	-	-	-	-	-	-	-

Supplemental Results Table 1. All-age deaths, death PAFs, DALYs, DALY PAFs, and percent change for each risk factor and outcome for both sexes combined in 2005 and 2015 for Latin America and Caribbean.

Data in parenthesis are 95% uncertainty intervals. PAFs=population attributable fractions. DALYs=disability-adjusted life-years.

	2005 Deaths (in thousands)	2005 Deaths PAF	2015 Deaths (in thousands)	2015 Deaths PAF	Percent change deaths 2005-2015	Percent change deaths PAF 2005-2015	2005 DALYs (in thousands)	2005 DALYs PAF	2015 DALYs (in thousands)	2015 DALYs PAF	Percent change DALYs 2005-2015	Percent change DALYs PAF 2005-2015
Endocarditis	-	-	-	-	-	-	-	-	-	-	-	-
Other cardiovascular and circulatory diseases	-	-	-	-	-	-	-	-	-	-	-	-
Diabetes mellitus	2·2 (1·5 to 3·2)	1·8% (1·2 to 2·6%)	3·1 (2·1 to 4·4)	1·7% (1·1 to 2·4%)	38·0% (33·2 to 42·7%)	-5·6% (-7·9 to -3·3%)	116·5 (76·7 to 173·3)	2·5% (1·7 to 3·5%)	162·4 (105·6 to 244·5)	2·5% (1·7 to 3·6%)	39·5% (35·0 to 43·4%)	0·9% (-1·5 to 2·8%)
Chronic kidney disease due to diabetes mellitus	0·9 (0·5 to 1·5)	1·5% (0·8 to 2·5%)	1·3 (0·7 to 2·3)	1·5% (0·7 to 2·4%)	51·6% (44·8 to 59·1%)	-2·2% (-5·8 to 2·0%)	24·2 (11·1 to 42·4)	1·6% (0·7 to 2·7%)	35·8 (16·3 to 63·2)	1·6% (0·7 to 2·8%)	48·3% (43·0 to 53·6%)	3·4% (0·7 to 6·1%)
Chronic kidney disease due to hypertension	0·4 (0·2 to 0·7)	1·1% (0·6 to 1·9%)	0·6 (0·3 to 1·0)	1·1% (0·5 to 1·9%)	45·9% (35·0 to 57·7%)	-2·6% (-9·6 to 5·1%)	10·5 (4·8 to 17·9)	1·2% (0·5 to 2·0%)	14·5 (6·8 to 25·0)	1·2% (0·6 to 2·2%)	38·8% (33·2 to 45·8%)	7·6% (4·1 to 12·5%)
Chronic kidney disease due to glomerulonephritis	0·0 (0·0 to 0·1)	0·9% (0·4 to 1·6%)	0·1 (0·0 to 0·1)	1·0% (0·4 to 1·7%)	56·2% (45·5 to 68·7%)	7·0% (-0·1 to 15·4%)	3·7 (1·3 to 7·0)	1·0% (0·3 to 1·8%)	5·3 (1·9 to 10·0)	1·1% (0·4 to 2·1%)	43·3% (37·4 to 52·3%)	13·9% (9·7 to 21·5%)
Chronic kidney disease due to other causes	0·0 (0·0 to 0·0)	0·6% (0·3 to 1·1%)	0·0 (0·0 to 0·0)	0·7% (0·3 to 1·3%)	57·7% (38·7 to 72·9%)	10·8% (-2·3 to 21·6%)	2·2 (0·9 to 4·1)	1·1% (0·4 to 1·9%)	3·1 (1·3 to 5·8)	1·2% (0·5 to 2·1%)	41·9% (36·3 to 48·5%)	8·8% (4·7 to 13·9%)
**Diet low in fibre: All causes**	16·4 (7·5 to 29·1)	0·6% (0·3 to 1·1%)	21·1 (9·4 to 37·9)	0·6% (0·3 to 1·1%)	28·6% (21·3 to 33·9%)	3·7% (-1·4 to 7·3%)	403·4 (181·4 to 705·4)	0·3% (0·1 to 0·5%)	471·6 (208·2 to 840·7)	0·3% (0·1 to 0·6%)	16·9% (9·7 to 22·0%)	8·4% (2·1 to 12·6%)
Colon and rectum cancer	1·8 (0·7 to 3·2)	5·3% (2·2 to 9·6%)	2·5 (1·0 to 4·7)	5·2% (2·1 to 9·5%)	42·9% (33·6 to 49·8%)	-2·7% (-7·6 to 0·2%)	38·7 (16·1 to 70·6)	5·2% (2·1 to 9·5%)	51·2 (20·7 to 94·7)	5·0% (2·0 to 9·2%)	32·2% (22·9 to 38·9%)	-4·5% (-9·6 to -1·3%)
Ischaemic heart disease	14·7 (6·5 to 26·2)	4·0% (1·8 to 7·2%)	18·6 (8·1 to 33·9)	3·7% (1·6 to 6·8%)	26·9% (19·8 to 32·1%)	-6·5% (-10·9 to -3·8%)	364·7 (161·3 to 644·6)	5·0% (2·2 to 9·0%)	420·5 (182·6 to 760·7)	4·6% (2·0 to 8·3%)	15·3% (8·3 to 20·4%)	-8·0% (-12·7 to -5·1%)
**Diet low in calcium: All causes**	6·5 (3·9 to 9·4)	0·2% (0·1 to 0·3%)	9·6 (5·6 to 13·9)	0·3% (0·2 to 0·4%)	46·3% (40·7 to 51·7%)	17·9% (14·3 to 21·5%)	143·6 (85·7 to 208·0)	0·1% (0·1 to 0·2%)	197·7 (115·8 to 289·0)	0·1% (0·1 to 0·2%)	37·6% (32·5 to 42·5%)	27·6% (23·4 to 31·8%)
Colon and rectum cancer	6·5 (3·9 to 9·4)	19·8% (11·8 to 28·6%)	9·6 (5·6 to 13·9)	19·7% (11·7 to 28·6%)	46·3% (40·7 to 51·7%)	-0·4% (-2·0 to 0·6%)	143·6 (85·7 to 208·0)	19·4% (11·5 to 28·1%)	197·7 (115·8 to 289·0)	19·3% (11·4 to 28·0%)	37·6% (32·5 to 42·5%)	-0·5% (-2·1 to 0·7%)
Prostate cancer	-	-	-	-	-	-	-	-	-	-	-	-
**Diet low in seafood omega-3 fatty acids: All causes**	50·3 (20·3 to 84·6)	1·9% (0·8 to 3·1%)	66·0 (26·2 to 111·3)	2·0% (0·8 to 3·3%)	31·3% (28·2 to 34·1%)	5·8% (4·2 to 7·6%)	1,100·8 (451·0 to 1,836·3)	0·8% (0·3 to 1·4%)	1,339·5 (539·1 to 2,242·6)	0·9% (0·4 to 1·5%)	21·7% (18·3 to 24·9%)	12·9% (10·3 to 15·2%)
Ischaemic heart disease	50·3 (20·3 to 84·6)	13·6% (5·5 to 22·8%)	66·0 (26·2 to 111·3)	13·1% (5·3 to 22·0%)	31·3% (28·2 to 34·1%)	-3·3% (-4·0 to -2·6%)	1,100·8 (451·0 to 1,836·3)	15·1% (6·2 to 25·2%)	1,339·5 (539·1 to 2,242·6)	14·7% (6·0 to 24·5%)	21·7% (18·3 to 24·9%)	-2·9% (-3·7 to -2·1%)
**Diet low in polyunsaturated fatty acids: All causes**	14·3 (5·6 to 22·4)	0·5% (0·2 to 0·8%)	19·2 (7·6 to 30·3)	0·6% (0·2 to 0·9%)	34·8% (29·0 to 41·0%)	8·7% (4·0 to 13·7%)	291·1 (116·7 to 457·5)	0·2% (0·1 to 0·3%)	370·4 (149·6 to 585·2)	0·3% (0·1 to 0·4%)	27·2% (22·0 to 32·8%)	18·0% (13·0 to 23·0%)
Ischaemic heart disease	14·3 (5·6 to 22·4)	3·8% (1·5 to 6·1%)	19·2 (7·6 to 30·3)	3·8% (1·5 to 6·1%)	34·8% (29·0 to 41·0%)	-0·6% (-4·7 to 3·8%)	291·1 (116·7 to 457·5)	4·0% (1·6 to 6·3%)	370·4 (149·6 to 585·2)	4·1% (1·6 to 6·4%)	27·2% (22·0 to 32·8%)	1·6% (-2·4 to 5·8%)
**Diet high in trans fatty acids: All causes**	44·5 (27·2 to 64·7)	1·6% (1·0 to 2·4%)	57·3 (34·8 to 84·1)	1·7% (1·0 to 2·5%)	28·9% (24·3 to 33·1%)	4·0% (1·3 to 6·4%)	1,189·7 (750·2 to 1,676·0)	0·9% (0·6 to 1·3%)	1,431·2 (893·8 to 2,035·3)	1·0% (0·6 to 1·4%)	20·3% (16·2 to 24·5%)	11·6% (8·3 to 14·8%)
Ischaemic heart disease	44·5 (27·2 to 64·7)	12·0% (7·4 to 17·4%)	57·3 (34·8 to 84·1)	11·4% (6·9 to 16·7%)	28·9% (24·3 to 33·1%)	-5·0% (-7·1 to -3·4%)	1,189·7 (750·2 to 1,676·0)	16·4% (10·5 to 23·1%)	1,431·2 (893·8 to 2,035·3)	15·7% (9·9 to 22·4%)	20·3% (16·2 to 24·5%)	-4·0% (-5·8 to -2·5%)
**Diet high in sodium: All causes**	111·0 (50·6 to 204·6)	4·1% (1·9 to 7·6%)	145·5 (65·2 to 270·0)	4·3% (2·0 to 8·0%)	31·1% (25·3 to 35·9%)	5·7% (2·0 to 8·4%)	2,432·7 (1,137·0 to 4,387·4)	1·8% (0·8 to 3·3%)	2,960·9 (1,353·1 to 5,379·7)	2·0% (0·9 to 3·7%)	21·7% (16·1 to 26·1%)	12·9% (8·4 to 16·1%)
Stomach cancer	12·1 (5·5 to 21·2)	27·3% (12·5 to 48·0%)	15·1 (6·8 to 26·3)	27·6% (12·5 to 48·3%)	24·7% (15·0 to 33·1%)	0·9% (-6·0 to 6·4%)	273·8 (127·2 to 469·1)	27·5% (12·8 to 47·3%)	321·8 (151·9 to 548·3)	27·7% (13·0 to 47·4%)	17·5% (10·6 to 23·9%)	0·9% (-3·4 to 4·7%)
Rheumatic heart disease	0·3 (0·1 to 0·7)	4·5% (1·6 to 9·4%)	0·3 (0·1 to 0·7)	4·8% (1·7 to 9·9%)	2·4% (-5·9 to 12·8%)	6·7% (-0·2 to 15·2%)	9·4 (3·3 to 19·9)	3·9% (1·4 to 8·3%)	8·8 (3·2 to 18·8)	4·3% (1·6 to 9·2%)	-5·5% (-12·7 to 3·1%)	11·8% (5·5 to 18·9%)
Ischaemic heart disease	44·2 (18·7 to 86·3)	11·9% (5·0 to 23·0%)	58·4 (24·1 to 114·4)	11·6% (4·8 to 22·6%)	32·2% (25·2 to 37·9%)	-2·6% (-7·2 to 0·5%)	925·8 (405·7 to 1,730·9)	12·7% (5·6 to 23·9%)	1,145·4 (500·6 to 2,154·7)	12·6% (5·5 to 23·7%)	23·7% (17·9 to 28·6%)	-1·2% (-4·6 to 1·2%)
Ischaemic stroke	8·7 (3·4 to 17·3)	10·0% (4·0 to 19·9%)	11·6 (4·5 to 23·0)	9·9% (3·9 to 19·8%)	33·4% (26·0 to 41·6%)	-1·1% (-5·3 to 4·0%)	141·7 (57·6 to 273·4)	11·2% (4·6 to 21·7%)	175·8 (73·3 to 340·2)	11·3% (4·6 to 21·8%)	24·1% (18·6 to 30·3%)	0·2% (-2·9 to 3·8%)
Hemorrhagic stroke	22·5 (9·9 to 40·3)	15·6% (7·0 to 28·1%)	26·8 (11·6 to 49·2)	15·3% (6·6 to 27·9%)	19·3% (11·5 to 27·7%)	-2·2% (-7·0 to 2·6%)	524·3 (234·7 to 932·6)	15·4% (6·9 to 27·4%)	575·2 (252·8 to 1,030·6)	15·6% (6·9 to 27·7%)	9·7% (3·9 to 15·8%)	1·2% (-2·5 to 4·8%)
Hypertensive heart disease	9·1 (2·5 to 21·6)	21·2% (5·8 to 50·5%)	12·4 (3·3 to 30·7)	20·9% (5·4 to 50·5%)	36·6% (22·6 to 45·9%)	-1·1% (-10·4 to 4·7%)	172·9 (60·6 to 376·0)	22·7% (7·9 to 49·6%)	214·6 (72·6 to 474·6)	22·4% (7·5 to 49·1%)	24·1% (14·4 to 31·2%)	-1·4% (-8·0 to 2·6%)
Cardiomyopathy and myocarditis	2·2 (1·0 to 3·9)	9·6% (4·5 to 16·9%)	2·7 (1·2 to 4·8)	9·7% (4·5 to 17·3%)	22·8% (12·9 to 36·8%)	1·3% (-2·7 to 5·5%)	54·0 (24·5 to 96·8)	8·0% (3·7 to 14·2%)	62·4 (28·1 to 111·4)	8·8% (4·0 to 15·4%)	15·4% (6·6 to 27·7%)	9·5% (5·4 to 13·9%)
Atrial fibrillation and flutter	0·4 (0·1 to 0·9)	6·0% (2·4 to 12·0%)	0·7 (0·2 to 1·4)	6·2% (2·4 to 12·2%)	59·5% (53·0 to 67·7%)	2·4% (-0·3 to 6·5%)	11·4 (4·4 to 22·5)	8·1% (3·4 to 15·5%)	16·7 (6·5 to 32·8)	8·1% (3·4 to 15·6%)	46·6% (43·2 to 50·7%)	0·7% (-1·1 to 3·0%)
Aortic aneurysm	1·1 (0·5 to 1·9)	9·6% (4·4 to 17·1%)	1·5 (0·7 to 2·8)	9·6% (4·3 to 17·2%)	44·3% (34·2 to 55·3%)	-0·2% (-3·5 to 3·7%)	23·3 (10·7 to 41·4)	10·3% (4·7 to 18·3%)	31·5 (14·5 to 56·6)	10·5% (4·8 to 18·5%)	35·4% (25·7 to 45·6%)	1·6% (-1·2 to 4·6%)
Peripheral vascular disease	0·2 (0·1 to 0·3)	6·4% (2·7 to 12·3%)	0·3 (0·1 to 0·5)	6·2% (2·6 to 12·1%)	59·3% (43·8 to 75·2%)	-4·0% (-9·9 to 0·9%)	3·7 (1·5 to 6·9)	7·0% (3·0 to 12·7%)	5·5 (2·3 to 10·4)	6·8% (2·9 to 12·6%)	48·2% (38·1 to 59·1%)	-2·0% (-5·4 to 1·0%)
Endocarditis	0·3 (0·1 to 0·6)	6·4% (2·7 to 12·1%)	0·4 (0·2 to 0·8)	7·0% (3·0 to 13·3%)	40·7% (31·2 to 50·2%)	10·2% (5·7 to 15·6%)	7·8 (3·1 to 15·1)	4·8% (2·0 to 9·3%)	10·2 (4·0 to 19·6)	5·9% (2·4 to 11·3%)	30·8% (22·3 to 39·7%)	22·4% (16·5 to 29·3%)
Other cardiovascular and circulatory diseases	2·4 (1·0 to 4·3)	8·5% (3·6 to 15·5%)	3·3 (1·4 to 5·9)	8·7% (3·7 to 15·8%)	39·3% (32·0 to 46·7%)	2·0% (-1·4 to 5·3%)	88·5 (37·2 to 162·7)	7·3% (3·2 to 13·4%)	110·5 (46·3 to 204·6)	7·9% (3·4 to 14·6%)	24·9% (19·6 to 29·5%)	8·3% (4·6 to 11·1%)
Chronic kidney disease due to diabetes mellitus	4·7 (1·5 to 10·0)	7·9% (2·5 to 16·8%)	7·3 (2·3 to 15·8)	8·0% (2·5 to 17·1%)	56·4% (49·0 to 63·0%)	0·9% (-2·8 to 3·8%)	107·9 (33·9 to 233·1)	6·9% (2·2 to 14·8%)	158·4 (49·0 to 344·1)	7·1% (2·2 to 15·2%)	46·9% (40·3 to 52·7%)	2·5% (-1·3 to 5·5%)
Chronic kidney disease due to hypertension	2·6 (0·9 to 5·6)	7·5% (2·5 to 16·0%)	4·1 (1·4 to 8·8)	7·8% (2·6 to 16·7%)	56·4% (48·7 to 63·0%)	4·4% (1·1 to 7·4%)	55·3 (19·0 to 117·8)	6·1% (2·1 to 12·8%)	77·2 (26·5 to 164·6)	6·6% (2·3 to 13·9%)	39·5% (33·0 to 44·8%)	8·2% (4·7 to 10·7%)
Chronic kidney disease due to glomerulonephritis	0·3 (0·1 to 0·7)	5·8% (1·8 to 12·7%)	0·5 (0·2 to 1·1)	6·4% (2·0 to 14·1%)	62·1% (53·8 to 69·0%)	11·0% (6·7 to 14·4%)	19·5 (6·6 to 41·9)	5·1% (1·8 to 10·8%)	27·6 (9·3 to 59·0)	5·8% (2·0 to 12·1%)	41·3% (36·5 to 45·4%)	12·3% (8·9 to 15·4%)
Chronic kidney disease due to other causes	0·0 (0·0 to 0·1)	6·5% (2·2 to 13·8%)	0·0 (0·0 to 0·1)	7·5% (2·5 to 15·9%)	63·7% (55·9 to 72·2%)	15·0% (11·1 to 19·0%)	13·5 (4·6 to 29·4)	6·6% (2·4 to 13·7%)	19·2 (6·6 to 41·7)	7·2% (2·7 to 14·9%)	42·3% (38·2 to 46·8%)	9·0% (6·6 to 11·9%)
**Sexual abuse and violence: All causes**	9·5 (7·3 to 12·3)	0·4% (0·3 to 0·5%)	9·7 (7·3 to 12·7)	0·3% (0·2 to 0·4%)	1·9% (-3·8 to 7·0%)	-17·9% (-22·1 to -13·9%)	953·3 (723·8 to 1,229·8)	0·7% (0·5 to 0·9%)	995·9 (746·7 to 1,306·9)	0·7% (0·5 to 0·9%)	4·5% (0·9 to 7·5%)	-3·2% (-6·3 to -0·1%)
**Childhood sexual abuse: All causes**	3·2 (1·0 to 6·2)	0·1% (0·0 to 0·2%)	3·4 (1·0 to 6·8)	0·1% (0·0 to 0·2%)	7·8% (0·7 to 13·0%)	-13·1% (-18·5 to -8·8%)	310·4 (130·7 to 539·6)	0·2% (0·1 to 0·4%)	332·5 (137·7 to 584·2)	0·2% (0·1 to 0·4%)	7·1% (2·6 to 10·6%)	-0·7% (-4·5 to 2·6%)
Alcohol use disorders	0·4 (0·0 to 0·9)	2·3% (0·0 to 5·4%)	0·4 (0·0 to 0·8)	2·0% (0·0 to 4·7%)	-5·2% (-10·8 to 1·3%)	-14·1% (-18·0 to 0·0%)	29·9 (0·0 to 69·1)	2·8% (0·0 to 6·5%)	30·0 (0·0 to 69·8)	2·5% (0·0 to 6·0%)	0·4% (-5·1 to 5·4%)	-9·4% (-13·0 to 0·0%)
Major depressive disorder	-	-	-	-	-	-	118·7 (13·1 to 267·9)	3·3% (0·4 to 7·1%)	129·2 (14·0 to 291·3)	3·2% (0·4 to 6·7%)	8·9% (5·9 to 11·7%)	-4·9% (-6·9 to -2·9%)
Dysthymia	-	-	-	-	-	-	21·1 (2·3 to 46·6)	3·4% (0·4 to 7·2%)	24·1 (2·6 to 53·2)	3·2% (0·4 to 6·7%)	14·2% (10·8 to 17·4%)	-6·7% (-8·8 to -4·5%)
Self-harm	2·8 (0·7 to 5·8)	8·6% (2·0 to 17·4%)	3·1 (0·7 to 6·4)	7·9% (1·8 to 16·2%)	9·5% (3·4 to 14·5%)	-8·2% (-11·8 to -4·8%)	140·9 (32·9 to 288·1)	8·9% (2·1 to 18·1%)	149·3 (34·8 to 307·0)	8·2% (1·9 to 16·9%)	6·0% (-0·3 to 11·1%)	-7·3% (-11·0 to -3·9%)
**Intimate partner violence: All causes**	6·6 (5·6 to 7·6)	0·2% (0·2 to 0·3%)	6·6 (5·7 to 7·5)	0·2% (0·2 to 0·2%)	-1·0% (-7·0 to 5·3%)	-20·2% (-24·7 to -15·1%)	673·5 (540·0 to 832·9)	0·5% (0·4 to 0·6%)	694·0 (551·3 to 867·1)	0·5% (0·4 to 0·6%)	3·0% (-1·2 to 7·2%)	-4·5% (-8·2 to -0·7%)

Supplemental Results Table 1. All-age deaths, death PAFs, DALYs, DALY PAFs, and percent change for each risk factor and outcome for both sexes combined in 2005 and 2015 for Latin America and Caribbean.

Data in parenthesis are 95% uncertainty intervals. PAFs=population attributable fractions. DALYs=disability-adjusted life-years.

	2005 Deaths (in thousands)	2005 Deaths PAF	2015 Deaths (in thousands)	2015 Deaths PAF	Percent change deaths 2005-2015	Percent change deaths PAF 2005-2015	2005 DALYs (in thousands)	2005 DALYs PAF	2015 DALYs (in thousands)	2015 DALYs PAF	Percent change DALYs 2005-2015	Percent change DALYs PAF 2005-2015
HIV/AIDS - Tuberculosis	0·1 (0·1 to 0·2)	2·2% (1·1 to 3·4%)	0·1 (0·0 to 0·1)	1·9% (0·9 to 3·0%)	-27·6% (-32·3 to -21·6%)	-14·3% (-17·7 to -11·2%)	4·9 (2·4 to 7·9)	2·1% (1·0 to 3·2%)	3·5 (1·8 to 5·8)	1·9% (0·9 to 2·9%)	-28·6% (-33·1 to -22·8%)	-10·1% (-13·6 to -6·9%)
HIV/AIDS resulting in other diseases	1·4 (0·7 to 2·1)	3·1% (1·5 to 4·7%)	0·9 (0·5 to 1·4)	2·2% (1·1 to 3·3%)	-32·9% (-38·6 to -25·9%)	-29·4% (-34·3 to -23·5%)	65·8 (32·3 to 101·5)	2·8% (1·4 to 4·4%)	43·4 (21·2 to 68·0)	2·1% (1·1 to 3·2%)	-34·1% (-39·3 to -28·0%)	-25·8% (-30·7 to -20·2%)
Maternal abortion, miscarriage, and ectopic pregnancy	0·0 (0·0 to 0·0)	0·0% (0·0 to 0·0%)	0·0 (0·0 to 0·0)	0·0% (0·0 to 0·0%)	-20·9% (-35·7 to -0·4%)	0·0% (-15·5 to 15·5%)	0·0 (0·0 to 0·0)	0·0% (0·0 to 0·0%)	0·0 (0·0 to 0·0)	0·0% (0·0 to 0·0%)	-21·4% (-35·3 to -1·2%)	0·1% (-13·9 to 16·9%)
Major depressive disorder	-	-	-	-	-	-	283·8 (180·4 to 409·5)	8·0% (6·4 to 9·5%)	317·3 (204·3 to 455·9)	7·8% (6·2 to 9·3%)	11·8% (7·2 to 16·7%)	-2·3% (-5·9 to 1·5%)
Dysthymia	-	-	-	-	-	-	52·9 (33·6 to 78·8)	8·6% (7·1 to 10·2%)	62·3 (39·6 to 91·9)	8·3% (6·8 to 9·9%)	17·9% (12·9 to 22·9%)	-3·7% (-7·1 to -0·4%)
Self-harm	2·3 (1·8 to 2·9)	7·1% (5·5 to 8·7%)	2·6 (2·0 to 3·2)	6·7% (5·2 to 8·2%)	11·9% (3·4 to 20·0%)	-6·1% (-12·8 to 1·3%)	104·7 (77·4 to 132·1)	6·6% (4·9 to 8·3%)	107·8 (80·3 to 136·2)	5·9% (4·4 to 7·5%)	2·9% (-5·6 to 11·1%)	-10·0% (-16·9 to -2·3%)
Assault by firearm	1·4 (1·0 to 1·6)	1·5% (1·2 to 1·7%)	1·5 (1·1 to 1·7)	1·5% (1·1 to 1·7%)	6·0% (-0·7 to 13·0%)	-3·4% (-10·1 to 3·9%)	77·3 (58·4 to 87·6)	1·5% (1·2 to 1·7%)	78·2 (58·5 to 90·1)	1·4% (1·1 to 1·6%)	1·2% (-5·2 to 7·7%)	-5·1% (-11·9 to 2·0%)
Assault by sharp object	0·7 (0·6 to 0·8)	3·4% (3·0 to 4·1%)	0·7 (0·6 to 0·9)	3·3% (2·8 to 4·0%)	8·2% (0·9 to 16·2%)	-3·1% (-11·0 to 5·6%)	37·7 (33·3 to 44·7)	3·6% (3·1 to 4·2%)	38·4 (33·0 to 46·2)	3·4% (2·9 to 4·1%)	1·9% (-5·1 to 9·1%)	-4·5% (-12·2 to 4·3%)
Assault by other means	0·7 (0·7 to 0·9)	4·8% (4·0 to 5·6%)	0·7 (0·6 to 0·9)	4·5% (3·8 to 5·3%)	-0·5% (-8·0 to 7·1%)	-5·9% (-12·5 to 1·8%)	46·3 (40·5 to 54·3)	5·3% (4·5 to 6·1%)	43·0 (37·0 to 49·9)	5·0% (4·2 to 5·8%)	-7·1% (-14·0 to -0·2%)	-6·1% (-12·4 to 1·0%)
**Unsafe sex: All causes**	75·9 (72·5 to 79·8)	2·8% (2·7 to 3·0%)	75·1 (71·3 to 79·3)	2·2% (2·1 to 2·4%)	-1·1% (-5·3 to 3·6%)	-20·3% (-23·6 to -16·6%)	3,578·7 (3,365·9 to 3,826·3)	2·6% (2·4 to 2·9%)	3,214·6 (3,020·7 to 3,432·1)	2·2% (2·0 to 2·4%)	-10·2% (-14·4 to -5·9%)	-16·7% (-20·6 to -12·8%)
HIV/AIDS - Tuberculosis	4·6 (4·0 to 5·4)	96·0% (95·3 to 96·5%)	3·9 (3·2 to 4·9)	96·0% (95·4 to 96·5%)	-15·4% (-20·6 to -7·5%)	0·1% (0·0 to 0·2%)	229·8 (198·3 to 268·6)	95·9% (95·2 to 96·5%)	182·9 (152·8 to 226·5)	96·0% (95·3 to 96·5%)	-20·4% (-25·0 to -13·1%)	0·1% (0·0 to 0·2%)
HIV/AIDS resulting in other diseases	43·3 (40·2 to 46·8)	96·3% (95·6 to 96·8%)	41·1 (37·6 to 45·3)	96·2% (95·6 to 96·7%)	-5·0% (-11·5 to 2·3%)	-0·1% (-0·3 to 0·0%)	2,222·9 (2,061·2 to 2,395·0)	96·2% (95·5 to 96·8%)	1,974·0 (1,815·2 to 2,161·4)	96·1% (95·4 to 96·6%)	-11·2% (-16·6 to -5·4%)	-0·1% (-0·2 to 0·0%)
Syphilis	3·5 (2·1 to 5·4)	100·0% (100·0 to 100·0%)	1·8 (1·2 to 2·7)	100·0% (100·0 to 100·0%)	-48·6% (-55·0 to -39·4%)	-	280·7 (166·1 to 444·4)	100·0% (100·0 to 100·0%)	134·8 (85·2 to 208·3)	100·0% (100·0 to 100·0%)	-52·0% (-58·3 to -44·2%)	-
Chlamydial infection	0·0 (0·0 to 0·0)	100·0% (100·0 to 100·0%)	0·0 (0·0 to 0·0)	100·0% (100·0 to 100·0%)	17·9% (5·6 to 33·0%)	-	25·5 (14·3 to 42·6)	100·0% (100·0 to 100·0%)	27·8 (15·6 to 45·8)	100·0% (100·0 to 100·0%)	9·1% (5·1 to 13·3%)	-
Gonococcal infection	0·0 (0·0 to 0·0)	100·0% (100·0 to 100·0%)	0·0 (0·0 to 0·0)	100·0% (100·0 to 100·0%)	11·3% (-2·9 to 23·6%)	-	12·0 (7·3 to 19·2)	100·0% (100·0 to 100·0%)	13·2 (8·0 to 20·7)	100·0% (100·0 to 100·0%)	10·1% (2·3 to 20·5%)	-
Trichomoniasis	-	-	-	-	-	-	19·5 (7·8 to 41·3)	100·0% (100·0 to 100·0%)	22·7 (9·1 to 48·1)	100·0% (100·0 to 100·0%)	16·7% (14·7 to 18·8%)	-
Genital herpes	-	-	-	-	-	-	21·9 (6·9 to 51·8)	100·0% (100·0 to 100·0%)	26·2 (8·1 to 62·2)	100·0% (100·0 to 100·0%)	19·6% (15·7 to 22·8%)	-
Other sexually transmitted diseases	0·0 (0·0 to 0·0)	100·0% (100·0 to 100·0%)	0·0 (0·0 to 0·0)	100·0% (100·0 to 100·0%)	6·6% (-6·3 to 19·2%)	-	7·5 (5·4 to 10·2)	100·0% (100·0 to 100·0%)	7·5 (5·3 to 10·2)	100·0% (100·0 to 100·0%)	-0·2% (-4·5 to 4·2%)	-
Cervical cancer	24·5 (23·7 to 25·1)	100·0% (100·0 to 100·0%)	28·2 (27·0 to 29·5)	100·0% (100·0 to 100·0%)	15·3% (10·6 to 20·1%)	-	758·9 (733·8 to 782·1)	100·0% (100·0 to 100·0%)	825·5 (787·7 to 865·8)	100·0% (100·0 to 100·0%)	8·8% (4·2 to 13·5%)	-
**Low physical activity: All causes**	87·2 (71·8 to 103·0)	3·2% (2·7 to 3·8%)	121·3 (100·8 to 142·9)	3·6% (3·0 to 4·3%)	39·1% (36·0 to 42·5%)	12·2% (10·6 to 14·0%)	2,123·7 (1,753·9 to 2,538·2)	1·6% (1·3 to 1·9%)	2,809·9 (2,325·8 to 3,358·4)	1·9% (1·6 to 2·3%)	32·3% (29·1 to 35·6%)	22·7% (20·5 to 25·0%)
Colon and rectum cancer	5·4 (3·9 to 6·9)	16·3% (11·7 to 20·8%)	8·0 (5·8 to 10·2)	16·6% (12·0 to 21·1%)	49·4% (43·8 to 55·3%)	1·7% (0·9 to 2·6%)	114·4 (82·0 to 146·7)	15·4% (11·0 to 19·8%)	161·0 (115·5 to 204·9)	15·7% (11·2 to 20·1%)	40·8% (35·7 to 46·1%)	1·8% (1·0 to 2·8%)
Breast cancer	3·0 (2·2 to 3·8)	10·4% (7·7 to 13·1%)	4·2 (3·1 to 5·3)	10·5% (7·8 to 13·2%)	41·2% (34·5 to 48·3%)	1·1% (0·2 to 2·1%)	88·7 (65·3 to 113·6)	10·1% (7·4 to 12·8%)	120·0 (88·9 to 153·2)	10·2% (7·6 to 13·0%)	35·3% (29·0 to 42·2%)	0·9% (0·0 to 1·8%)
Ischaemic heart disease	44·7 (31·2 to 58·1)	12·1% (8·4 to 15·7%)	59·8 (42·3 to 77·5)	11·9% (8·4 to 15·5%)	33·7% (30·3 to 37·2%)	-1·5% (-2·3 to -0·4%)	973·7 (667·2 to 1,273·5)	13·4% (9·3 to 17·6%)	1,215·0 (842·5 to 1,582·8)	13·3% (9·3 to 17·5%)	24·8% (21·0 to 28·5%)	-0·4% (-1·3 to 0·7%)
Ischaemic stroke	11·6 (7·2 to 16·3)	13·4% (8·3 to 18·8%)	15·6 (9·8 to 21·8)	13·4% (8·4 to 18·7%)	34·6% (30·5 to 39·2%)	-0·2% (-1·1 to 1·2%)	183·3 (110·6 to 256·5)	14·5% (8·8 to 20·5%)	228·2 (141·0 to 320·0)	14·6% (9·0 to 20·5%)	24·5% (20·8 to 29·0%)	0·6% (-0·4 to 2·1%)
Diabetes mellitus	22·5 (17·6 to 27·7)	18·0% (14·1 to 22·2%)	33·7 (26·6 to 41·2)	18·4% (14·5 to 22·6%)	49·5% (45·3 to 53·9%)	2·2% (1·4 to 3·3%)	763·6 (566·2 to 999·6)	16·2% (12·4 to 20·2%)	1,085·7 (805·0 to 1,421·5)	16·7% (12·9 to 20·7%)	42·2% (38·8 to 45·6%)	2·9% (2·1 to 3·8%)
**Metabolic risks: All causes**	802·5 (769·9 to 831·0)	29·7% (28·6 to 30·8%)	1,114·8 (1,067·3 to 1,161·4)	33·3% (32·0 to 34·6%)	38·9% (36·1 to 41·4%)	12·0% (11·0 to 13·1%)	19,994·8 (18,642·5 to 21,492·7)	14·7% (14·0 to 15·5%)	26,005·0 (24,131·8 to 28,057·5)	17·8% (16·8 to 18·7%)	30·1% (27·6 to 32·3%)	20·6% (19·2 to 22·1%)
**High fasting plasma glucose: All causes**	309·7 (282·8 to 347·6)	11·5% (10·5 to 12·9%)	451·7 (412·5 to 506·1)	13·5% (12·3 to 15·1%)	45·8% (42·8 to 49·0%)	17·6% (15·7 to 19·6%)	8,978·1 (8,034·9 to 10,088·6)	6·6% (6·2 to 7·1%)	12,279·9 (10,988·9 to 13,841·6)	8·4% (7·9 to 9·0%)	36·8% (34·3 to 39·3%)	26·8% (24·9 to 28·9%)
Tuberculosis	2·0 (1·3 to 2·8)	10·3% (6·6 to 14·3%)	1·9 (1·2 to 2·7)	11·5% (7·3 to 16·2%)	-4·1% (-10·6 to 4·3%)	11·8% (6·9 to 16·7%)	61·1 (39·4 to 84·4)	8·6% (5·7 to 11·8%)	56·3 (36·0 to 77·4)	9·9% (6·6 to 13·5%)	-7·9% (-13·9 to 0·0%)	15·4% (10·9 to 20·8%)
Ischaemic heart disease	69·6 (46·6 to 98·8)	18·8% (12·6 to 26·7%)	99·5 (65·5 to 143·9)	19·8% (13·0 to 28·5%)	43·0% (35·8 to 50·4%)	5·4% (0·6 to 10·4%)	1,387·3 (968·2 to 1,882·7)	19·1% (13·4 to 25·7%)	1,840·2 (1,288·7 to 2,475·7)	20·2% (14·2 to 27·2%)	32·6% (28·2 to 37·4%)	5·9% (3·4 to 9·2%)
Ischaemic stroke	14·3 (7·9 to 26·5)	16·5% (9·2 to 30·1%)	19·8 (10·6 to 37·0)	17·0% (9·3 to 31·8%)	38·8% (31·0 to 48·2%)	3·0% (-1·4 to 9·6%)	223·4 (137·6 to 360·9)	17·7% (11·0 to 29·0%)	287·8 (178·8 to 475·3)	18·4% (11·3 to 30·5%)	28·8% (23·1 to 36·1%)	4·1% (0·9 to 9·4%)
Hemorrhagic stroke	23·8 (16·3 to 32·6)	16·5% (11·5 to 22·6%)	30·1 (20·7 to 41·9)	17·2% (11·9 to 23·9%)	26·8% (20·5 to 34·9%)	4·0% (-0·3 to 10·0%)	566·5 (385·7 to 804·0)	16·6% (11·3 to 23·6%)	666·4 (456·4 to 929·3)	18·1% (12·5 to 25·2%)	17·6% (12·1 to 24·0%)	8·6% (4·3 to 13·0%)
Diabetes mellitus	125·0 (122·9 to 127·2)	100·0% (100·0 to 100·0%)	182·8 (177·9 to 187·6)	100·0% (100·0 to 100·0%)	46·2% (42·4 to 50·0%)	-	4,717·2 (4,037·3 to 5,516·9)	100·0% (100·0 to 100·0%)	6,521·1 (5,555·4 to 7,673·1)	100·0% (100·0 to 100·0%)	38·2% (35·2 to 41·3%)	-
Chronic kidney disease due to diabetes mellitus	59·5 (57·5 to 61·6)	100·0% (100·0 to 100·0%)	92·3 (88·7 to 96·1)	100·0% (100·0 to 100·0%)	55·1% (51·2 to 59·0%)	-	1,559·2 (1,490·4 to 1,642·4)	100·0% (100·0 to 100·0%)	2,235·4 (2,135·8 to 2,344·5)	100·0% (100·0 to 100·0%)	43·4% (40·0 to 46·7%)	-
Chronic kidney disease due to hypertension	13·2 (9·3 to 16·8)	37·2% (26·6 to 47·3%)	21·2 (15·1 to 26·9)	39·9% (28·7 to 50·5%)	60·9% (56·4 to 65·8%)	7·4% (6·5 to 8·5%)	284·7 (200·3 to 368·4)	31·5% (22·4 to 40·3%)	412·3 (292·7 to 529·3)	35·3% (25·3 to 45·0%)	44·8% (41·2 to 48·8%)	12·3% (11·1 to 13·6%)
Chronic kidney disease due to glomerulonephritis	1·7 (1·2 to 2·2)	31·8% (22·7 to 40·7%)	2·8 (2·0 to 3·6)	36·3% (26·2 to 46·0%)	66·5% (61·4 to 71·9%)	14·1% (12·8 to 15·6%)	102·7 (69·1 to 139·6)	27·1% (19·1 to 35·2%)	148·8 (100·5 to 200·9)	31·2% (22·3 to 40·1%)	44·9% (42·0 to 47·8%)	15·2% (13·4 to 17·3%)
Chronic kidney disease due to other causes	0·1 (0·1 to 0·2)	27·8% (19·4 to 36·0%)	0·2 (0·1 to 0·3)	33·0% (23·4 to 42·5%)	68·8% (62·0 to 75·9%)	18·5% (16·0 to 21·7%)	62·2 (39·7 to 90·3)	30·6% (21·4 to 39·8%)	89·6 (57·5 to 129·5)	33·8% (23·9 to 43·6%)	44·1% (41·1 to 47·2%)	10·5% (9·2 to 12·0%)
**High total cholesterol: All causes**	183·6 (145·4 to 228·7)	6·8% (5·4 to 8·5%)	249·8 (192·9 to 317·3)	7·5% (5·8 to 9·5%)	36·1% (30·5 to 41·1%)	9·7% (5·7 to 13·2%)	4,102·8 (3,512·8 to 4,767·7)	3·0% (2·5 to 3·6%)	5,121·3 (4,341·5 to 6,009·5)	3·5% (2·9 to 4·2%)	24·8% (21·2 to 28·5%)	15·8% (13·0 to 18·5%)
Ischaemic heart disease	166·5 (133·0 to 202·5)	44·9% (35·9 to 54·4%)	226·3 (175·8 to 279·1)	45·0% (35·1 to 55·1%)	35·9% (29·7 to 41·3%)	0·1% (-3·8 to 3·2%)	3,805·9 (3,264·3 to 4,402·0)	52·4% (45·2 to 60·4%)	4,753·5 (4,062·6 to 5,527·8)	52·2% (44·7 to 60·3%)	24·9% (21·2 to 28·6%)	-0·3% (-1·9 to 1·1%)
Ischaemic stroke	17·1 (6·4 to 34·1)	19·7% (7·5 to 39·8%)	23·6 (8·0 to 48·9)	20·2% (6·8 to 42·3%)	37·9% (23·1 to 46·5%)	2·3% (-8·3 to 7·6%)	296·9 (176·0 to 503·1)	23·5% (14·1 to 39·9%)	367·8 (205·4 to 641·2)	23·6% (13·2 to 41·1%)	23·9% (15·1 to 31·2%)	0·1% (-6·5 to 5·0%)
**High systolic blood pressure: All causes**	459·3 (413·5 to 501·7)	17·0% (15·3 to 18·6%)	625·2 (560·7 to 689·2)	18·7% (16·8 to 20·6%)	36·1% (32·6 to 39·4%)	9·8% (8·1 to 11·5%)	9,722·1 (8,847·5 to 10,575·2)	7·2% (6·4 to 8·0%)	12,218·3 (11,078·5 to 13,307·3)	8·4% (7·4 to 9·4%)	25·7% (22·7 to 28·6%)	16·6% (14·2 to 18·6%)
Rheumatic heart disease	1·7 (1·1 to 2·5)	23·4% (16·3 to 35·6%)	1·7 (1·2 to 2·5)	24·8% (17·1 to 37·7%)	1·9% (-5·0 to 9·8%)	6·1% (1·0 to 12·5%)	47·1 (31·8 to 70·6)	19·4% (13·2 to 29·0%)	44·5 (30·5 to 63·8)	21·7% (15·0 to 32·4%)	-5·6% (-10·7 to 0·8%)	11·7% (8·1 to 16·8%)
Ischaemic heart disease	200·3 (165·1 to 237·1)	54·0% (44·5 to 63·4%)	272·4 (220·1 to 326·0)	54·1% (43·7 to 64·6%)	36·0% (31·4 to 40·1%)	0·2% (-2·6 to 2·1%)	3,956·2 (3,416·2 to 4,490·8)	54·4% (46·8 to 61·7%)	5,010·4 (4,310·5 to 5,712·5)	55·0% (47·2 to 62·5%)	26·6% (23·0 to 30·3%)	1·1% (-0·3 to 2·3%)
Ischaemic stroke	41·4 (32·1 to 51·5)	47·9% (37·2 to 59·1%)	55·5 (42·1 to 70·0)	47·6% (36·4 to 60·1%)	34·0% (28·8 to 39·8%)	-0·6% (-3·1 to 2·7%)	636·7 (513·5 to 763·8)	50·5% (40·7 to 59·1%)	794·7 (639·1 to 953·6)	50·9% (41·3 to 59·6%)	24·8% (20·6 to 29·6%)	0·9% (-0·9 to 3·2%)

Supplemental Results Table 1. All-age deaths, death PAFs, DALYs, DALY PAFs, and percent change for each risk factor and outcome for both sexes combined in 2005 and 2015 for Latin America and Caribbean.

Data in parenthesis are 95% uncertainty intervals. PAFs=population attributable fractions. DALYs=disability-adjusted life-years.

	2005 Deaths (in thousands)	2005 Deaths PAF	2015 Deaths (in thousands)	2015 Deaths PAF	Percent change deaths 2005-2015	Percent change deaths PAF 2005-2015	2005 DALYs (in thousands)	2005 DALYs PAF	2015 DALYs (in thousands)	2015 DALYs PAF	Percent change DALYs 2005-2015	Percent change DALYs PAF 2005-2015
Hemorrhagic stroke	82·1 (67·9 to 96·3)	57·1% (47·2 to 66·8%)	100·6 (80·6 to 120·0)	57·4% (46·1 to 68·1%)	22·6% (16·9 to 29·2%)	0·5% (-2·6 to 3·7%)	1,872·9 (1,539·3 to 2,147·0)	55·0% (45·3 to 63·1%)	2,111·8 (1,750·4 to 2,462·1)	57·2% (47·5 to 65·5%)	12·8% (8·5 to 17·5%)	4·0% (2·3 to 6·1%)
Hypertensive heart disease	42·9 (40·2 to 44·8)	100·0% (100·0 to 100·0%)	59·3 (55·2 to 61·8)	100·0% (100·0 to 100·0%)	38·0% (33·2 to 43·1%)	-	760·9 (720·7 to 800·4)	100·0% (100·0 to 100·0%)	957·7 (900·6 to 1,006·4)	100·0% (100·0 to 100·0%)	25·9% (21·9 to 30·0%)	-
Cardiomyopathy and myocarditis	7·9 (6·1 to 9·5)	35·0% (28·4 to 42·1%)	9·9 (7·8 to 12·2)	36·1% (28·7 to 43·8%)	25·0% (16·5 to 36·1%)	3·1% (0·4 to 6·4%)	196·9 (154·7 to 236·2)	29·2% (23·5 to 34·5%)	232·4 (183·8 to 279·5)	32·7% (26·5 to 38·6%)	18·0% (10·3 to 28·1%)	11·9% (8·9 to 14·9%)
Atrial fibrillation and flutter	2·4 (1·7 to 3·4)	34·1% (26·9 to 41·6%)	3·7 (2·7 to 5·3)	34·2% (26·7 to 41·9%)	56·1% (51·0 to 61·2%)	0·3% (-1·7 to 1·9%)	56·4 (43·8 to 72·9)	40·2% (34·9 to 45·5%)	82·2 (63·4 to 106·5)	40·3% (34·9 to 45·6%)	45·9% (43·2 to 48·8%)	0·2% (-1·1 to 1·4%)
Aortic aneurysm	4·2 (3·3 to 4·9)	37·8% (31·0 to 44·4%)	6·0 (4·8 to 7·1)	37·5% (29·9 to 44·4%)	43·6% (35·1 to 52·5%)	-0·7% (-3·1 to 1·7%)	88·3 (73·5 to 102·4)	39·1% (32·8 to 45·3%)	118·9 (98·7 to 139·5)	39·5% (32·7 to 45·7%)	34·7% (26·6 to 42·9%)	1·1% (-0·6 to 2·9%)
Peripheral vascular disease	0·7 (0·5 to 0·9)	27·1% (20·3 to 34·6%)	1·2 (0·9 to 1·5)	26·8% (19·7 to 34·7%)	64·0% (50·3 to 78·5%)	-1·2% (-4·9 to 2·3%)	15·1 (11·2 to 20·6)	28·6% (22·3 to 35·0%)	22·9 (16·9 to 30·8)	28·6% (22·5 to 35·0%)	51·1% (42·5 to 60·0%)	0·0% (-2·5 to 2·4%)
Endocarditis	1·4 (1·0 to 1·7)	30·1% (24·2 to 36·2%)	1·9 (1·4 to 2·4)	33·0% (26·5 to 40·1%)	39·7% (31·8 to 47·1%)	9·6% (6·8 to 12·9%)	35·7 (26·5 to 44·7)	22·2% (17·5 to 26·8%)	46·5 (34·1 to 57·9)	27·1% (21·6 to 32·8%)	30·3% (23·6 to 36·9%)	22·0% (17·7 to 27·0%)
Other cardiovascular and circulatory diseases	10·5 (9·1 to 11·7)	37·8% (33·3 to 42·4%)	14·7 (12·8 to 16·7)	39·0% (34·1 to 44·0%)	40·9% (35·4 to 46·6%)	3·2% (1·2 to 5·2%)	375·5 (314·0 to 451·8)	31·1% (27·7 to 34·6%)	486·5 (404·6 to 583·8)	34·9% (31·2 to 38·9%)	29·6% (26·4 to 33·2%)	12·3% (10·6 to 14·2%)
Chronic kidney disease due to diabetes mellitus	26·4 (18·9 to 33·3)	44·3% (31·9 to 55·7%)	41·9 (30·2 to 52·6)	45·4% (32·8 to 56·9%)	58·9% (54·6 to 63·4%)	2·5% (1·8 to 3·3%)	607·3 (424·8 to 775·8)	38·9% (27·7 to 49·4%)	903·2 (639·7 to 1,150·9)	40·4% (28·7 to 51·1%)	48·7% (45·1 to 52·6%)	3·7% (3·0 to 4·6%)
Chronic kidney disease due to hypertension	35·4 (33·6 to 37·1)	100·0% (100·0 to 100·0%)	53·0 (50·0 to 56·0)	100·0% (100·0 to 100·0%)	49·8% (45·5 to 54·2%)	-	904·5 (850·5 to 956·7)	100·0% (100·0 to 100·0%)	1,166·3 (1,097·0 to 1,241·1)	100·0% (100·0 to 100·0%)	28·9% (25·8 to 32·2%)	-
Chronic kidney disease due to glomerulonephritis	1·9 (1·3 to 2·4)	35·3% (25·4 to 44·9%)	3·1 (2·2 to 4·0)	40·0% (29·1 to 50·5%)	65·4% (59·8 to 71·0%)	13·3% (11·8 to 15·2%)	102·5 (68·4 to 140·3)	27·0% (18·9 to 35·2%)	146·5 (99·2 to 200·4)	30·7% (21·6 to 39·6%)	42·9% (39·8 to 46·3%)	13·6% (11·7 to 16·0%)
Chronic kidney disease due to other causes	0·2 (0·1 to 0·2)	35·2% (25·3 to 44·1%)	0·3 (0·2 to 0·3)	41·2% (29·9 to 51·6%)	66·7% (59·9 to 74·3%)	17·1% (14·6 to 20·2%)	66·1 (42·3 to 93·6)	32·6% (22·6 to 41·8%)	93·9 (60·0 to 132·1)	35·5% (24·9 to 45·3%)	42·1% (39·2 to 45·5%)	9·0% (7·4 to 10·7%)
**High body-mass index: All causes**	258·6 (190·3 to 328·7)	9·6% (7·1 to 12·2%)	370·0 (279·0 to 465·6)	11·1% (8·3 to 13·9%)	43·1% (38·5 to 48·3%)	15·4% (12·5 to 19·1%)	8,434·9 (6,366·1 to 10,516·6)	6·2% (4·8 to 7·7%)	11,655·7 (9,041·3 to 14,304·3)	8·0% (6·3 to 9·6%)	38·2% (34·2 to 43·0%)	28·1% (25·0 to 32·4%)
Oesophageal cancer	2·8 (0·9 to 5·0)	22·6% (7·3 to 39·8%)	4·1 (1·4 to 7·1)	24·9% (8·2 to 42·6%)	45·8% (36·8 to 57·3%)	10·1% (6·2 to 16·8%)	71·0 (22·9 to 123·6)	23·6% (7·7 to 41·4%)	99·0 (32·5 to 169·1)	26·3% (8·6 to 44·8%)	39·5% (30·1 to 51·7%)	11·1% (6·7 to 18·5%)
Colon and rectum cancer	2·6 (1·6 to 3·8)	8·0% (5·0 to 11·6%)	4·3 (2·7 to 6·1)	8·9% (5·7 to 12·7%)	64·6% (57·5 to 73·2%)	12·1% (8·7 to 17·0%)	63·8 (40·0 to 90·8)	8·6% (5·4 to 12·4%)	100·4 (64·8 to 139·9)	9·8% (6·4 to 13·7%)	57·3% (50·3 to 65·7%)	13·8% (10·2 to 19·0%)
Liver cancer due to hepatitis B	0·6 (0·3 to 1·1)	15·5% (7·1 to 25·4%)	0·9 (0·4 to 1·6)	17·6% (8·3 to 28·1%)	47·8% (39·7 to 58·8%)	13·5% (9·6 to 19·8%)	18·3 (8·6 to 30·7)	15·5% (7·1 to 25·5%)	25·9 (12·4 to 42·6)	18·0% (8·6 to 28·6%)	41·2% (32·9 to 52·3%)	15·9% (11·6 to 22·8%)
Liver cancer due to hepatitis C	1·2 (0·5 to 2·0)	15·2% (6·6 to 25·2%)	1·8 (0·8 to 3·0)	16·4% (7·2 to 26·9%)	52·5% (46·4 to 60·2%)	8·2% (5·4 to 12·4%)	25·3 (11·1 to 43·2)	16·3% (7·2 to 26·6%)	36·8 (16·6 to 61·7)	17·8% (8·0 to 28·7%)	45·3% (39·3 to 53·4%)	9·4% (6·5 to 14·2%)
Liver cancer due to alcohol use	1·0 (0·4 to 1·6)	16·8% (7·7 to 28·1%)	1·5 (0·7 to 2·5)	18·4% (8·6 to 30·0%)	59·4% (52·4 to 69·0%)	9·3% (6·3 to 13·8%)	22·2 (10·3 to 36·9)	17·8% (8·2 to 29·3%)	34·5 (16·5 to 56·2)	19·6% (9·2 to 31·7%)	55·0% (47·9 to 65·4%)	10·1% (6·9 to 15·0%)
Liver cancer due to other causes	0·5 (0·2 to 0·9)	14·6% (6·1 to 24·6%)	0·8 (0·3 to 1·3)	16·0% (7·0 to 26·8%)	48·3% (41·9 to 56·1%)	10·2% (7·2 to 14·4%)	12·8 (5·4 to 21·6)	14·7% (6·4 to 24·4%)	17·9 (8·0 to 29·8)	16·6% (7·4 to 27·2%)	40·3% (34·0 to 48·4%)	13·4% (10·1 to 18·6%)
Gallbladder and biliary tract cancer	2·1 (1·3 to 3·1)	20·9% (12·7 to 30·4%)	2·7 (1·7 to 4·0)	22·5% (14·1 to 32·1%)	29·5% (15·6 to 41·8%)	7·6% (2·9 to 12·7%)	47·2 (29·1 to 67·7)	22·1% (13·5 to 31·7%)	57·9 (36·4 to 83·0)	23·9% (15·1 to 33·7%)	22·7% (10·3 to 34·1%)	8·1% (3·4 to 13·4%)
Pancreatic cancer	1·1 (0·4 to 1·9)	6·5% (2·5 to 11·1%)	1·8 (0·7 to 3·1)	7·2% (2·9 to 12·1%)	62·4% (55·5 to 71·2%)	10·5% (7·5 to 15·3%)	25·3 (9·8 to 43·1)	6·8% (2·6 to 11·7%)	39·5 (15·3 to 66·5)	7·6% (2·9 to 12·7%)	56·0% (49·2 to 64·4%)	11·3% (8·0 to 16·0%)
Breast cancer	0·7 (0·2 to 1·4)	2·6% (0·9 to 4·9%)	1·5 (0·6 to 2·6)	3·7% (1·5 to 6·4%)	96·6% (76·0 to 153·9%)	40·7% (26·9 to 82·4%)	-0·5 (-15·6 to 13·2)	-0·1% (-1·8 to 1·5%)	10·3 (-11·3 to 33·7)	0·9% (-1·0 to 2·9%)	-2,067·8% (-2,234·8 to 2,854·8%)	-1,545·0% (-1,721·2 to 2,133·5%)
Uterine cancer	2·1 (1·5 to 2·8)	39·8% (28·1 to 51·4%)	2·9 (2·1 to 3·7)	43·2% (31·5 to 54·4%)	38·2% (30·6 to 47·8%)	8·5% (5·6 to 13·3%)	52·2 (37·6 to 67·0)	41·7% (30·0 to 53·1%)	71·0 (52·8 to 89·1)	45·5% (33·9 to 56·6%)	36·0% (28·3 to 45·4%)	9·1% (6·1 to 13·9%)
Ovarian cancer	0·3 (0·0 to 0·6)	3·6% (-0·1 to 7·9%)	0·4 (0·0 to 1·0)	4·0% (-0·1 to 8·7%)	54·8% (46·8 to 65·0%)	11·8% (8·5 to 17·5%)	8·2 (-0·3 to 18·2)	3·6% (-0·1 to 8·0%)	12·4 (-0·5 to 27·2)	4·1% (-0·1 to 8·9%)	50·8% (43·0 to 61·4%)	13·6% (10·0 to 19·5%)
Kidney cancer	1·2 (0·8 to 1·6)	18·4% (12·4 to 24·8%)	1·9 (1·3 to 2·6)	20·4% (14·1 to 27·2%)	65·3% (57·3 to 74·5%)	10·8% (7·7 to 14·9%)	30·4 (20·8 to 40·9)	16·8% (11·5 to 22·5%)	48·6 (34·3 to 64·6)	19·6% (13·8 to 25·9%)	60·0% (52·0 to 68·9%)	16·7% (13·2 to 21·2%)
Thyroid cancer	0·3 (0·1 to 0·4)	12·2% (7·1 to 18·2%)	0·4 (0·2 to 0·6)	13·4% (8·0 to 19·7%)	57·5% (49·5 to 66·3%)	10·2% (7·0 to 14·4%)	6·9 (4·1 to 10·1)	12·7% (7·5 to 18·9%)	10·6 (6·3 to 15·5)	14·2% (8·5 to 20·8%)	55·3% (47·1 to 64·3%)	11·7% (8·4 to 15·9%)
Ischaemic heart disease	74·9 (52·5 to 99·4)	20·2% (14·2 to 26·8%)	106·0 (76·2 to 138·8)	21·1% (15·2 to 27·7%)	41·6% (36·0 to 48·5%)	4·3% (0·7 to 8·9%)	1,915·9 (1,378·4 to 2,469·4)	26·4% (19·0 to 34·2%)	2,574·1 (1,923·6 to 3,254·7)	28·3% (21·2 to 35·7%)	34·4% (29·3 to 40·6%)	7·2% (4·3 to 11·6%)
Ischaemic stroke	11·2 (7·5 to 15·8)	12·9% (8·6 to 18·3%)	15·1 (10·1 to 21·8)	13·0% (8·6 to 18·6%)	35·0% (24·9 to 43·8%)	0·1% (-6·8 to 6·1%)	256·5 (184·3 to 335·4)	20·3% (14·6 to 26·5%)	329·7 (241·2 to 426·9)	21·1% (15·5 to 27·3%)	28·5% (23·0 to 34·9%)	3·8% (0·5 to 8·0%)
Hemorrhagic stroke	41·7 (31·1 to 53·0)	29·0% (21·5 to 36·9%)	50·5 (37·9 to 64·4)	28·8% (21·8 to 36·6%)	21·2% (14·1 to 28·0%)	-0·6% (-5·3 to 4·3%)	1,284·0 (994·0 to 1,578·7)	37·7% (29·0 to 46·4%)	1,489·6 (1,187·2 to 1,815·0)	40·4% (32·1 to 48·9%)	16·0% (10·9 to 22·0%)	7·0% (4·0 to 11·3%)
Hypertensive heart disease	17·4 (11·0 to 25·1)	40·5% (26·0 to 57·8%)	25·4 (15·3 to 36·9)	42·8% (26·4 to 61·7%)	45·8% (31·1 to 56·4%)	5·6% (-4·5 to 12·1%)	359·4 (263·1 to 461·6)	47·2% (35·4 to 60·6%)	479·3 (351·0 to 613·5)	50·0% (37·4 to 63·9%)	33·4% (26·4 to 40·4%)	6·0% (1·4 to 10·6%)
Diabetes mellitus	60·1 (46·9 to 73·7)	48·1% (37·7 to 58·9%)	89·3 (69·7 to 109·6)	48·8% (38·2 to 59·9%)	48·4% (42·8 to 54·3%)	1·5% (-1·2 to 4·5%)	2,772·9 (2,146·9 to 3,448·0)	58·8% (48·4 to 68·4%)	4,001·6 (3,107·0 to 4,937·6)	61·3% (51·4 to 70·4%)	44·3% (40·3 to 48·7%)	4·4% (2·7 to 6·7%)
Chronic kidney disease due to diabetes mellitus	23·4 (13·1 to 34·1)	39·4% (22·0 to 57·3%)	37·6 (21·1 to 54·6)	40·7% (23·0 to 58·8%)	60·4% (47·9 to 73·0%)	3·4% (-4·0 to 11·1%)	622·4 (317·0 to 901·0)	39·9% (20·1 to 58·6%)	960·7 (508·8 to 1,370·6)	43·0% (22·6 to 61·8%)	54·4% (48·2 to 63·0%)	7·7% (4·2 to 12·8%)
Chronic kidney disease due to hypertension	10·6 (5·0 to 16·7)	30·0% (14·2 to 46·7%)	16·7 (7·0 to 27·0)	31·5% (13·4 to 50·0%)	57·5% (26·3 to 74·4%)	5·1% (-14·7 to 16·2%)	269·7 (135·2 to 400·5)	29·8% (14·9 to 43·8%)	392·6 (206·2 to 563·1)	33·7% (17·5 to 48·5%)	45·6% (36·5 to 57·2%)	12·9% (6·1 to 21·6%)
Chronic kidney disease due to glomerulonephritis	1·3 (0·5 to 2·1)	24·3% (9·8 to 39·0%)	2·2 (0·8 to 3·6)	27·7% (10·5 to 44·9%)	66·3% (40·7 to 85·2%)	13·9% (-3·5 to 26·5%)	97·1 (33·7 to 157·9)	25·6% (9·5 to 40·0%)	145·4 (55·1 to 228·4)	30·5% (12·0 to 45·6%)	49·8% (42·6 to 63·6%)	19·1% (13·6 to 29·3%)
Chronic kidney disease due to other causes	0·1 (0·0 to 0·2)	18·7% (4·2 to 33·5%)	0·1 (0·0 to 0·3)	22·5% (2·2 to 41·7%)	71·3% (-12·0 to 91·8%)	20·2% (-38·9 to 34·6%)	59·0 (24·5 to 94·0)	29·1% (13·9 to 44·1%)	88·4 (38·8 to 139·1)	33·4% (16·4 to 49·2%)	49·8% (42·6 to 60·0%)	14·8% (9·8 to 22·6%)
Osteoarthritis	-	-	-	-	-	-	154·5 (93·7 to 239·5)	23·9% (16·8 to 31·7%)	251·5 (154·5 to 386·3)	26·1% (18·6 to 34·0%)	62·8% (58·1 to 69·4%)	9·3% (6·6 to 13·5%)
Low back pain	-	-	-	-	-	-	222·7 (129·7 to 351·2)	6·7% (4·5 to 9·0%)	325·4 (195·9 to 501·6)	7·9% (5·5 to 10·3%)	46·1% (41·7 to 53·1%)	18·3% (14·8 to 23·7%)
**Low bone mineral density: All causes**	16·5 (15·7 to 17·3)	0·6% (0·6 to 0·6%)	25·0 (23·0 to 26·6)	0·7% (0·7 to 0·8%)	51·3% (43·1 to 57·3%)	22·0% (15·2 to 26·5%)	390·7 (344·5 to 442·9)	0·3% (0·3 to 0·3%)	523·4 (462·5 to 596·1)	0·4% (0·3 to 0·4%)	34·0% (29·7 to 38·3%)	24·2% (20·3 to 28·1%)
Pedestrian road injuries	3·5 (3·2 to 3·7)	7·1% (6·7 to 7·5%)	4·4 (4·1 to 4·7)	9·1% (8·5 to 9·6%)	26·5% (21·4 to 32·5%)	27·2% (23·4 to 31·4%)	72·5 (66·0 to 78·7)	3·2% (2·9 to 3·4%)	83·8 (76·5 to 91·7)	4·1% (3·7 to 4·4%)	15·6% (10·6 to 21·2%)	28·8% (24·3 to 33·7%)
Cyclist road injuries	0·2 (0·2 to 0·2)	4·8% (4·3 to 5·1%)	0·3 (0·2 to 0·3)	6·0% (5·5 to 6·5%)	34·5% (26·3 to 43·7%)	25·6% (21·1 to 30·3%)	8·3 (7·1 to 9·7)	3·6% (3·2 to 4·1%)	10·4 (8·8 to 12·4)	4·6% (4·0 to 5·2%)	25·9% (19·8 to 32·3%)	26·9% (22·2 to 31·8%)
Motorcyclist road injuries	0·3 (0·3 to 0·4)	2·2% (1·9 to 2·5%)	0·6 (0·4 to 0·7)	2·7% (2·3 to 3·0%)	66·7% (36·1 to 85·8%)	21·4% (13·7 to 29·5%)	16·8 (14·1 to 19·8)	1·8% (1·5 to 2·1%)	27·7 (22·5 to 33·4)	2·2% (1·9 to 2·6%)	65·1% (44·8 to 80·2%)	23·5% (15·6 to 31·8%)
Motor vehicle road injuries	1·6 (1·4 to 1·8)	4·6% (4·2 to 4·9%)	2·1 (1·9 to 2·4)	5·6% (5·2 to 6·1%)	31·9% (24·7 to 39·4%)	22·9% (18·6 to 27·6%)	52·9 (46·2 to 61·3)	2·8% (2·5 to 3·2%)	67·4 (58·6 to 77·5)	3·5% (3·1 to 3·9%)	27·5% (20·8 to 34·1%)	24·2% (19·5 to 28·9%)
Other road injuries	0·1 (0·1 to 0·1)	5·8% (5·4 to 6·5%)	0·1 (0·1 to 0·2)	7·3% (6·7 to 8·3%)	52·5% (38·0 to 81·8%)	26·3% (20·2 to 33·3%)	3·7 (2·9 to 4·6)	4·0% (3·3 to 4·7%)	5·3 (4·2 to 6·6)	4·9% (4·1 to 5·9%)	42·4% (34·3 to 57·2%)	24·4% (15·8 to 31·2%)

Supplemental Results Table 1. All-age deaths, death PAFs, DALYs, DALY PAFs, and percent change for each risk factor and outcome for both sexes combined in 2005 and 2015 for Latin America and Caribbean.

Data in parenthesis are 95% uncertainty intervals. PAFs=population attributable fractions. DALYs=disability-adjusted life-years.

	2005 Deaths (in thousands)	2005 Deaths PAF	2015 Deaths (in thousands)	2015 Deaths PAF	Percent change deaths 2005-2015	Percent change deaths PAF 2005-2015	2005 DALYs (in thousands)	2005 DALYs PAF	2015 DALYs (in thousands)	2015 DALYs PAF	Percent change DALYs 2005-2015	Percent change DALYs PAF 2005-2015
Other transport injuries	0·3 (0·3 to 0·3)	6·3% (5·9 to 6·7%)	0·4 (0·4 to 0·5)	7·7% (7·2 to 8·2%)	43·7% (31·4 to 54·5%)	21·6% (16·4 to 26·8%)	7·5 (6·8 to 8·3)	2·9% (2·6 to 3·1%)	10·0 (8·7 to 11·2)	3·5% (3·1 to 3·8%)	33·3% (21·6 to 44·3%)	20·6% (14·5 to 27·0%)
Falls	9·8 (9·1 to 10·3)	40·6% (38·8 to 42·2%)	16·1 (14·0 to 17·3)	47·8% (45·2 to 50·0%)	64·1% (52·1 to 72·8%)	17·9% (14·3 to 21·3%)	200·6 (172·5 to 235·1)	16·6% (15·8 to 17·3%)	278·9 (237·7 to 327·6)	20·5% (19·4 to 21·6%)	39·0% (33·2 to 43·9%)	23·6% (19·7 to 27·7%)
Other exposure to mechanical forces	0·2 (0·2 to 0·3)	4·9% (4·5 to 5·2%)	0·3 (0·3 to 0·3)	6·2% (5·7 to 6·6%)	28·8% (21·6 to 35·3%)	27·7% (22·6 to 32·3%)	10·8 (8·7 to 13·5)	3·1% (2·7 to 3·5%)	12·6 (10·0 to 15·9)	3·8% (3·3 to 4·3%)	16·5% (12·1 to 21·1%)	22·7% (18·3 to 26·4%)
Non-venomous animal contact	0·0 (0·0 to 0·0)	6·7% (6·3 to 7·2%)	0·1 (0·1 to 0·1)	9·0% (8·2 to 9·8%)	42·4% (29·1 to 56·5%)	33·6% (23·5 to 43·6%)	0·8 (0·7 to 0·9)	2·3% (2·1 to 2·5%)	1·0 (0·9 to 1·1)	3·2% (3·0 to 3·5%)	26·3% (18·2 to 34·9%)	39·8% (30·8 to 48·1%)
Assault by other means	0·5 (0·4 to 0·5)	3·0% (2·8 to 3·2%)	0·7 (0·6 to 0·7)	4·0% (3·6 to 4·4%)	40·8% (31·7 to 51·2%)	33·2% (26·1 to 40·6%)	15·0 (12·7 to 17·5)	1·7% (1·5 to 2·0%)	19·0 (16·0 to 22·1)	2·2% (1·9 to 2·5%)	26·6% (20·0 to 32·8%)	27·9% (22·1 to 34·2%)
Exposure to forces of nature	-	-	-	-	-	-	1·8 (1·0 to 2·8)	1·5% (0·8 to 2·2%)	7·2 (2·1 to 13·5)	5·1% (3·3 to 6·8%)	296·3% (45·1 to 597·6%)	249·0% (138·5 to 450·5%)
**Low glomerular filtration rate: All causes**	162·9 (154·5 to 172·9)	6·0% (5·7 to 6·4%)	238·7 (226·3 to 253·9)	7·1% (6·8 to 7·5%)	46·5% (43·4 to 49·8%)	18·1% (16·2 to 20·1%)	4,096·9 (3,842·8 to 4,373·4)	3·0% (2·8 to 3·2%)	5,476·2 (5,149·1 to 5,819·0)	3·7% (3·4 to 4·1%)	33·7% (31·2 to 36·2%)	24·0% (21·7 to 26·1%)
Ischaemic heart disease	36·8 (31·6 to 42·2)	9·9% (8·6 to 11·4%)	51·3 (44·1 to 59·0)	10·2% (8·8 to 11·7%)	39·5% (35·4 to 43·6%)	2·8% (0·8 to 4·9%)	623·4 (534·4 to 713·6)	8·6% (7·4 to 9·8%)	811·1 (695·8 to 930·7)	8·9% (7·7 to 10·2%)	30·1% (26·8 to 33·7%)	3·9% (2·3 to 5·6%)
Ischaemic stroke	10·3 (8·2 to 12·8)	11·9% (9·5 to 14·8%)	14·2 (11·1 to 17·8)	12·1% (9·5 to 15·1%)	37·0% (32·0 to 42·5%)	1·6% (-0·7 to 3·9%)	135·5 (112·2 to 163·9)	10·7% (8·9 to 12·8%)	172·8 (141·0 to 209·5)	11·1% (9·1 to 13·3%)	27·6% (23·5 to 31·9%)	3·1% (1·3 to 5·1%)
Hemorrhagic stroke	14·7 (11·6 to 18·3)	10·2% (8·0 to 12·7%)	18·8 (14·6 to 23·6)	10·7% (8·3 to 13·4%)	27·9% (22·7 to 33·8%)	5·0% (2·0 to 8·2%)	274·8 (215·6 to 342·5)	8·1% (6·3 to 9·9%)	323·1 (257·4 to 400·3)	8·8% (6·9 to 10·8%)	17·6% (12·9 to 22·5%)	8·5% (5·8 to 11·6%)
Peripheral vascular disease	0·4 (0·3 to 0·5)	15·3% (10·9 to 19·5%)	0·7 (0·5 to 0·9)	15·1% (10·9 to 19·3%)	64·0% (50·9 to 77·6%)	-1·2% (-4·4 to 1·4%)	8·7 (6·1 to 12·0)	16·5% (12·2 to 20·6%)	13·2 (9·3 to 18·3)	16·6% (12·3 to 20·7%)	51·6% (43·7 to 60·3%)	0·3% (-1·5 to 2·3%)
Chronic kidney disease due to diabetes mellitus	59·5 (57·5 to 61·6)	100·0% (100·0 to 100·0%)	92·3 (88·7 to 96·1)	100·0% (100·0 to 100·0%)	55·1% (51·2 to 59·0%)	-	1,559·2 (1,490·4 to 1,642·4)	100·0% (100·0 to 100·0%)	2,235·4 (2,135·8 to 2,344·5)	100·0% (100·0 to 100·0%)	43·4% (40·0 to 46·7%)	-
Chronic kidney disease due to hypertension	35·4 (33·6 to 37·1)	100·0% (100·0 to 100·0%)	53·0 (50·0 to 56·0)	100·0% (100·0 to 100·0%)	49·8% (45·5 to 54·2%)	-	904·5 (850·5 to 956·7)	100·0% (100·0 to 100·0%)	1,166·3 (1,097·0 to 1,241·1)	100·0% (100·0 to 100·0%)	28·9% (25·8 to 32·2%)	-
Chronic kidney disease due to glomerulonephritis	5·3 (5·0 to 5·7)	100·0% (100·0 to 100·0%)	7·8 (7·2 to 8·4)	100·0% (100·0 to 100·0%)	46·0% (41·3 to 50·4%)	-	378·7 (319·6 to 438·0)	100·0% (100·0 to 100·0%)	476·4 (400·2 to 552·9)	100·0% (100·0 to 100·0%)	25·8% (23·3 to 28·3%)	-
Chronic kidney disease due to other causes	0·4 (0·4 to 0·5)	100·0% (100·0 to 100·0%)	0·6 (0·6 to 0·7)	100·0% (100·0 to 100·0%)	42·4% (36·5 to 48·7%)	-	202·9 (151·0 to 258·9)	100·0% (100·0 to 100·0%)	264·6 (197·4 to 335·5)	100·0% (100·0 to 100·0%)	30·4% (27·6 to 33·2%)	-
Gout	-	-	-	-	-	-	9·3 (6·2 to 12·9)	14·7% (13·3 to 16·2%)	13·2 (8·8 to 18·3)	15·8% (14·2 to 17·4%)	43·1% (40·1 to 46·2%)	7·3% (5·7 to 9·0%)

Supplemental Results Table 1. All-age deaths, death PAFs, DALYs, DALY PAFs, and percent change for each risk factor and outcome for both sexes combined in 2005 and 2015 for North Africa and Middle East.

Data in parenthesis are 95% uncertainty intervals. PAFs=population attributable fractions. DALYs=disability-adjusted life-years.

	2005 Deaths (in thousands)	2005 Deaths PAF	2015 Deaths (in thousands)	2015 Deaths PAF	Percent change deaths 2005-2015	Percent change deaths PAF 2005-2015	2005 DALYs (in thousands)	2005 DALYs PAF	2015 DALYs (in thousands)	2015 DALYs PAF	Percent change DALYs 2005-2015	Percent change DALYs PAF 2005-2015
**All risk factors: All causes**	1,316·8 (1,257·2 to 1,379·3)	51·6% (50·4 to 53·0%)	1,540·5 (1,464·8 to 1,614·3)	51·6% (49·8 to 53·3%)	17·0% (10·9 to 22·6%)	-0·1% (-2·9 to 2·7%)	51,352·7 (47,222·2 to 55,858·8)	34·7% (33·2 to 36·3%)	55,458·9 (50,540·5 to 60,967·5)	33·5% (32·1 to 35·1%)	8·0% (2·8 to 13·0%)	-3·4% (-7·2 to 0·4%)
**Environmental/occupational risks: All causes**	403·7 (364·6 to 443·1)	15·8% (14·5 to 17·2%)	431·2 (388·9 to 471·3)	14·4% (13·2 to 15·7%)	6·8% (0·5 to 13·2%)	-8·8% (-12·5 to -4·6%)	17,806·7 (15,950·6 to 19,705·7)	12·0% (10·8 to 13·4%)	16,377·5 (14,823·4 to 18,085·2)	9·9% (9·0 to 10·9%)	-8·0% (-14·6 to -0·9%)	-17·7% (-22·7 to -11·9%)
**Unsafe water, sanitation, and handwashing: All causes**	74·0 (61·8 to 87·6)	2·9% (2·4 to 3·4%)	49·0 (40·1 to 58·4)	1·6% (1·3 to 2·0%)	-33·8% (-43·4 to -23·5%)	-43·5% (-51·5 to -35·0%)	5,845·5 (4,872·5 to 6,929·4)	4·0% (3·3 to 4·8%)	3,599·5 (2,950·8 to 4,306·4)	2·2% (1·8 to 2·6%)	-38·4% (-48·2 to -27·8%)	-44·9% (-53·1 to -35·9%)
**Unsafe water source: All causes**	48·1 (35·9 to 60·1)	1·9% (1·4 to 2·4%)	28·0 (20·7 to 35·4)	0·9% (0·7 to 1·2%)	-41·8% (-52·3 to -29·5%)	-50·3% (-59·4 to -39·7%)	4,129·4 (3,107·1 to 5,119·4)	2·8% (2·1 to 3·5%)	2,440·8 (1,800·1 to 3,068·4)	1·5% (1·1 to 1·9%)	-40·9% (-51·5 to -28·5%)	-47·1% (-56·7 to -36·4%)
Diarrhoeal diseases	45·6 (34·0 to 56·9)	86·6% (70·7 to 94·9%)	25·9 (19·0 to 33·1)	86·0% (70·0 to 94·6%)	-43·2% (-54·2 to -30·4%)	-0·6% (-1·3 to -0·2%)	3,949·5 (2,953·4 to 4,917·5)	86·6% (70·7 to 94·9%)	2,294·5 (1,689·1 to 2,906·7)	86·0% (70·0 to 94·6%)	-41·9% (-53·0 to -28·9%)	-0·7% (-1·3 to -0·3%)
Typhoid fever	2·1 (1·1 to 3·8)	85·7% (69·6 to 94·3%)	1·8 (0·9 to 3·2)	85·2% (69·1 to 94·1%)	-17·5% (-23·8 to -11·3%)	-0·5% (-1·1 to -0·1%)	151·9 (76·2 to 274·2)	85·7% (69·6 to 94·3%)	123·5 (61·9 to 219·0)	85·3% (69·2 to 94·1%)	-18·7% (-25·3 to -12·1%)	-0·5% (-1·0 to -0·1%)
Paratyphoid fever	0·4 (0·2 to 0·8)	85·8% (69·8 to 94·4%)	0·3 (0·1 to 0·7)	85·3% (69·3 to 94·2%)	-17·5% (-27·0 to -7·3%)	-0·5% (-1·0 to -0·2%)	28·0 (11·5 to 59·7)	85·8% (69·8 to 94·4%)	22·8 (9·5 to 48·2)	85·4% (69·3 to 94·2%)	-18·7% (-28·7 to -7·8%)	-0·5% (-1·0 to -0·2%)
**Unsafe sanitation: All causes**	26·9 (20·9 to 34·0)	1·1% (0·8 to 1·3%)	14·1 (10·4 to 18·3)	0·5% (0·3 to 0·6%)	-47·7% (-59·3 to -33·1%)	-55·4% (-65·4 to -42·2%)	2,307·0 (1,783·4 to 2,905·1)	1·6% (1·2 to 2·0%)	1,217·7 (924·1 to 1,578·6)	0·7% (0·5 to 1·0%)	-47·2% (-58·9 to -32·3%)	-52·8% (-63·2 to -39·0%)
Diarrhoeal diseases	25·9 (19·9 to 32·9)	49·0% (44·7 to 53·4%)	13·3 (9·8 to 17·6)	44·1% (38·6 to 49·4%)	-48·6% (-60·4 to -33·4%)	-10·1% (-18·8 to -1·1%)	2,232·4 (1,721·4 to 2,825·9)	48·8% (44·4 to 53·3%)	1,163·3 (864·3 to 1,518·7)	43·5% (38·0 to 48·9%)	-47·9% (-59·8 to -32·6%)	-11·0% (-19·5 to -2·3%)
Typhoid fever	0·9 (0·4 to 1·6)	35·2% (31·4 to 39·4%)	0·6 (0·3 to 1·2)	31·1% (27·1 to 35·2%)	-26·7% (-35·1 to -17·2%)	-11·5% (-18·7 to -4·0%)	62·9 (32·6 to 111·5)	35·5% (31·7 to 39·7%)	45·8 (23·5 to 81·2)	31·6% (27·6 to 35·7%)	-27·3% (-35·6 to -17·5%)	-11·0% (-18·0 to -3·5%)
Paratyphoid fever	0·2 (0·1 to 0·3)	35·7% (32·1 to 39·5%)	0·1 (0·1 to 0·3)	31·8% (28·0 to 35·9%)	-26·1% (-36·9 to -14·2%)	-10·8% (-17·3 to -3·9%)	11·7 (4·9 to 24·7)	36·0% (32·2 to 39·8%)	8·6 (3·7 to 17·9)	32·2% (28·3 to 36·4%)	-26·8% (-37·9 to -14·0%)	-10·4% (-17·1 to -3·5%)
**No handwashing with soap: All causes**	42·1 (33·2 to 51·5)	1·7% (1·3 to 2·0%)	30·3 (23·2 to 37·3)	1·0% (0·8 to 1·3%)	-28·1% (-37·5 to -19·3%)	-38·6% (-46·5 to -31·2%)	3,112·9 (2,447·0 to 3,809·0)	2·1% (1·6 to 2·6%)	1,972·9 (1,533·2 to 2,422·2)	1·2% (0·9 to 1·5%)	-36·6% (-45·3 to -27·2%)	-43·3% (-50·8 to -35·4%)
Diarrhoeal diseases	20·2 (15·0 to 25·8)	38·3% (30·9 to 45·4%)	11·3 (8·4 to 14·9)	37·6% (30·2 to 44·6%)	-43·9% (-54·8 to -30·8%)	-1·8% (-2·9 to -1·0%)	1,746·1 (1,304·8 to 2,254·0)	38·3% (30·9 to 45·3%)	1,001·7 (746·6 to 1,306·6)	37·6% (30·2 to 44·6%)	-42·6% (-53·7 to -29·7%)	-1·9% (-3·0 to -1·0%)
Typhoid fever	0·9 (0·5 to 1·7)	37·1% (29·2 to 44·2%)	0·7 (0·4 to 1·3)	36·3% (28·3 to 43·6%)	-18·9% (-25·1 to -12·9%)	-2·2% (-3·2 to -1·2%)	65·9 (32·2 to 117·8)	37·2% (29·2 to 44·2%)	52·7 (26·1 to 93·1)	36·4% (28·4 to 43·6%)	-20·0% (-26·7 to -13·3%)	-2·1% (-3·1 to -1·2%)
Paratyphoid fever	0·2 (0·1 to 0·4)	37·2% (29·2 to 44·3%)	0·1 (0·1 to 0·3)	36·4% (28·4 to 43·5%)	-18·8% (-28·5 to -8·8%)	-2·1% (-3·1 to -1·2%)	12·1 (5·1 to 25·5)	37·2% (29·2 to 44·3%)	9·7 (4·2 to 20·5)	36·4% (28·4 to 43·5%)	-19·9% (-30·0 to -9·5%)	-2·0% (-3·0 to -1·2%)
**Air pollution: All causes**	262·5 (232·3 to 294·1)	10·3% (9·2 to 11·5%)	300·1 (266·8 to 335·3)	10·0% (9·0 to 11·1%)	14·3% (6·8 to 22·1%)	-2·4% (-6·9 to 2·6%)	8,965·2 (7,778·4 to 10,206·4)	6·1% (5·2 to 7·0%)	8,912·7 (7,810·0 to 10,051·0)	5·4% (4·7 to 6·2%)	-0·6% (-9·3 to 8·9%)	-11·0% (-17·7 to -3·4%)
**Ambient particulate matter pollution: All causes**	204·3 (181·5 to 229·1)	8·0% (7·2 to 8·9%)	249·8 (222·1 to 278·5)	8·4% (7·5 to 9·3%)	22·3% (15·9 to 28·9%)	4·4% (1·0 to 8·4%)	6,519·5 (5,687·8 to 7,397·4)	4·4% (3·8 to 5·1%)	7,095·7 (6,278·0 to 7,955·9)	4·3% (3·7 to 5·0%)	8·8% (0·9 to 17·2%)	-2·6% (-8·7 to 3·7%)
Lower respiratory infections	41·1 (30·6 to 52·5)	29·3% (22·0 to 36·4%)	39·6 (30·1 to 49·2)	31·4% (24·0 to 38·2%)	-3·7% (-13·3 to 7·0%)	7·3% (3·3 to 12·8%)	2,553·8 (1,887·9 to 3,265·1)	29·7% (22·4 to 36·8%)	1,980·8 (1,485·4 to 2,501·1)	31·9% (24·5 to 38·7%)	-22·4% (-32·5 to -10·6%)	7·2% (3·1 to 13·1%)
Tracheal, bronchus, and lung cancer	7·8 (4·8 to 10·9)	17·8% (11·0 to 25·0%)	11·8 (7·7 to 16·3)	20·8% (13·5 to 28·5%)	52·4% (42·1 to 64·8%)	16·8% (12·9 to 22·4%)	194·2 (120·5 to 272·2)	17·7% (11·0 to 24·9%)	296·6 (192·2 to 407·3)	20·8% (13·5 to 28·4%)	52·7% (42·3 to 65·2%)	17·1% (13·1 to 22·7%)
Ischaemic heart disease	99·0 (84·0 to 117·1)	20·1% (17·0 to 23·5%)	126·9 (106·4 to 149·1)	21·2% (18·0 to 24·7%)	28·1% (20·2 to 35·7%)	5·4% (3·4 to 7·8%)	2,411·0 (2,040·9 to 2,806·7)	22·1% (18·9 to 25·3%)	3,096·5 (2,617·1 to 3,614·6)	23·5% (20·1 to 26·9%)	28·4% (19·6 to 36·9%)	6·2% (4·5 to 8·1%)
Ischaemic stroke	18·0 (14·0 to 22·1)	14·7% (11·6 to 17·7%)	23·0 (18·1 to 27·9)	15·4% (12·3 to 18·5%)	27·7% (17·7 to 37·4%)	4·8% (1·3 to 8·1%)	368·0 (300·6 to 445·2)	16·2% (13·5 to 19·0%)	462·3 (381·3 to 551·0)	17·2% (14·5 to 20·0%)	25·6% (15·4 to 35·7%)	6·4% (3·8 to 9·4%)
Hemorrhagic stroke	21·4 (17·7 to 25·5)	16·3% (13·7 to 18·8%)	27·2 (22·7 to 32·4)	17·7% (15·1 to 20·3%)	27·1% (17·5 to 37·8%)	8·9% (6·0 to 12·0%)	602·0 (500·3 to 709·2)	15·9% (13·5 to 18·3%)	765·5 (636·5 to 912·4)	18·2% (15·6 to 20·8%)	27·2% (17·2 to 37·9%)	14·8% (11·4 to 18·5%)
Chronic obstructive pulmonary disease	16·9 (10·1 to 24·1)	25·8% (15·6 to 36·6%)	21·3 (13·3 to 30·1)	28·4% (17·7 to 40·0%)	25·7% (16·3 to 36·0%)	10·1% (6·8 to 16·4%)	390·6 (235·3 to 559·9)	23·0% (14·0 to 32·4%)	494·0 (311·0 to 700·3)	26·2% (16·3 to 37·0%)	26·5% (18·4 to 36·3%)	14·0% (9·9 to 21·1%)
**Household air pollution from solid fuels: All causes**	73·6 (54·3 to 95·0)	2·9% (2·2 to 3·7%)	62·5 (45·1 to 82·5)	2·1% (1·5 to 2·7%)	-15·0% (-30·3 to 2·4%)	-27·5% (-39·8 to -14·0%)	3,298·2 (2,348·3 to 4,372·3)	2·2% (1·6 to 3·0%)	2,430·0 (1,701·3 to 3,261·7)	1·5% (1·0 to 2·0%)	-26·3% (-39·7 to -10·9%)	-34·1% (-45·3 to -21·0%)
Lower respiratory infections	27·4 (17·9 to 38·7)	19·5% (13·1 to 27·0%)	17·3 (10·8 to 25·1)	13·7% (8·7 to 19·5%)	-37·0% (-50·2 to -21·3%)	-29·7% (-40·2 to -17·8%)	2,018·2 (1,320·5 to 2,883·5)	23·4% (16·0 to 32·1%)	1,161·3 (721·4 to 1,697·8)	18·6% (11·9 to 26·2%)	-42·5% (-55·2 to -25·7%)	-20·4% (-33·3 to -6·0%)
Tracheal, bronchus, and lung cancer	2·0 (1·0 to 3·6)	4·5% (2·2 to 8·1%)	1·7 (0·9 to 2·9)	3·0% (1·5 to 5·0%)	-12·6% (-42·1 to 26·9%)	-33·0% (-55·1 to -3·3%)	50·9 (24·4 to 91·4)	4·6% (2·3 to 8·4%)	45·7 (22·6 to 77·6)	3·2% (1·6 to 5·4%)	-10·2% (-40·2 to 28·4%)	-31·2% (-53·0 to -3·1%)
Ischaemic heart disease	25·9 (19·8 to 33·7)	5·3% (4·0 to 6·8%)	25·4 (18·5 to 34·0)	4·2% (3·1 to 5·6%)	-1·9% (-21·2 to 18·1%)	-19·3% (-34·2 to -4·4%)	713·1 (541·3 to 933·9)	6·5% (5·0 to 8·3%)	707·2 (505·3 to 963·8)	5·4% (3·9 to 7·1%)	-0·8% (-21·2 to 20·8%)	-18·0% (-33·8 to -2·5%)
Ischaemic stroke	5·7 (3·8 to 8·0)	4·6% (3·2 to 6·3%)	5·6 (3·7 to 8·0)	3·7% (2·5 to 5·2%)	-1·7% (-21·1 to 19·5%)	-19·3% (-33·8 to -3·9%)	135·1 (91·2 to 189·3)	5·9% (4·2 to 8·0%)	133·3 (86·9 to 188·3)	5·0% (3·4 to 6·8%)	-1·3% (-22·5 to 22·8%)	-16·3% (-32·4 to 0·9%)
Hemorrhagic stroke	7·2 (5·0 to 9·9)	5·4% (3·9 to 7·3%)	7·2 (4·7 to 10·1)	4·7% (3·2 to 6·4%)	0·5% (-21·7 to 26·6%)	-13·9% (-31·0 to 4·3%)	231·7 (162·7 to 324·7)	6·1% (4·4 to 8·2%)	234·1 (151·8 to 332·1)	5·6% (3·8 to 7·6%)	1·0% (-22·5 to 28·9%)	-8·8% (-27·4 to 10·4%)
Chronic obstructive pulmonary disease	5·5 (2·7 to 9·3)	8·4% (4·2 to 13·6%)	5·3 (2·6 to 9·1)	7·1% (3·6 to 11·7%)	-3·1% (-24·0 to 24·2%)	-15·0% (-32·8 to 8·2%)	139·0 (70·0 to 230·6)	8·2% (4·2 to 13·2%)	138·7 (69·7 to 231·9)	7·3% (3·7 to 11·8%)	-0·2% (-20·4 to 24·1%)	-10·0% (-27·1 to 10·7%)
Cataract	-	-	-	-	-	-	10·2 (6·7 to 14·7)	6·0% (4·9 to 7·5%)	9·7 (6·5 to 13·9)	4·4% (3·6 to 5·6%)	-5·1% (-22·0 to 14·3%)	-26·9% (-39·6 to -12·3%)
**Ambient ozone pollution: All causes**	5·1 (1·9 to 8·4)	0·2% (0·1 to 0·3%)	5·9 (2·2 to 9·8)	0·2% (0·1 to 0·3%)	16·0% (7·7 to 24·8%)	-0·9% (-7·4 to 5·9%)	95·2 (35·4 to 160·0)	0·1% (0·0 to 0·1%)	109·6 (41·2 to 182·2)	0·1% (0·0 to 0·1%)	15·1% (6·9 to 24·2%)	2·9% (-3·7 to 10·6%)
Chronic obstructive pulmonary disease	5·1 (1·9 to 8·4)	7·7% (2·9 to 12·9%)	5·9 (2·2 to 9·8)	7·8% (3·0 to 13·2%)	16·0% (7·7 to 24·8%)	1·7% (-0·2 to 3·8%)	95·2 (35·4 to 160·0)	5·6% (2·1 to 9·3%)	109·6 (41·2 to 182·2)	5·8% (2·2 to 9·7%)	15·1% (6·9 to 24·2%)	3·7% (0·3 to 7·6%)
**Other environmental risks: All causes**	59·1 (41·5 to 78·2)	2·3% (1·6 to 3·0%)	67·3 (46·3 to 90·5)	2·3% (1·6 to 3·0%)	14·0% (5·9 to 21·8%)	-2·7% (-6·9 to 1·7%)	1,411·7 (975·5 to 1,868·6)	1·0% (0·7 to 1·3%)	1,554·4 (1,031·4 to 2,090·2)	0·9% (0·6 to 1·3%)	10·1% (1·2 to 18·8%)	-1·5% (-8·1 to 5·1%)
**Residential radon: All causes**	3·3 (2·1 to 4·7)	0·1% (0·1 to 0·2%)	4·2 (2·7 to 5·9)	0·1% (0·1 to 0·2%)	27·3% (14·5 to 42·5%)	8·7% (-2·0 to 20·4%)	80·6 (52·3 to 114·1)	0·1% (0·0 to 0·1%)	102·7 (67·4 to 143·3)	0·1% (0·0 to 0·1%)	27·5% (13·5 to 42·6%)	14·1% (1·3 to 27·0%)
Tracheal, bronchus, and lung cancer	3·3 (2·1 to 4·7)	7·5% (4·9 to 10·8%)	4·2 (2·7 to 5·9)	7·3% (4·8 to 10·3%)	27·3% (14·5 to 42·5%)	-2·4% (-9·5 to 5·8%)	80·6 (52·3 to 114·1)	7·4% (4·8 to 10·3%)	102·7 (67·4 to 143·3)	7·2% (4·8 to 9·8%)	27·5% (13·5 to 42·6%)	-2·2% (-9·3 to 5·5%)
**Lead exposure: All causes**	55·8 (38·7 to 74·7)	2·2% (1·5 to 2·9%)	63·2 (42·6 to 86·4)	2·1% (1·4 to 2·9%)	13·2% (4·9 to 21·3%)	-3·3% (-8·1 to 1·1%)	1,331·1 (901·4 to 1,792·4)	0·9% (0·6 to 1·2%)	1,451·7 (937·5 to 1,979·3)	0·9% (0·6 to 1·2%)	9·1% (-0·2 to 18·1%)	-2·4% (-9·7 to 4·4%)
Rheumatic heart disease	0·2 (0·1 to 0·3)	2·3% (1·2 to 3·6%)	0·2 (0·1 to 0·3)	2·1% (1·1 to 3·5%)	-3·4% (-16·7 to 10·1%)	-6·2% (-15·1 to 0·6%)	7·5 (3·5 to 12·9)	1·8% (0·9 to 3·1%)	6·8 (3·1 to 12·3)	1·7% (0·8 to 3·0%)	-8·4% (-22·3 to 5·1%)	-5·9% (-17·6 to 2·9%)
Ischaemic heart disease	29·9 (20·1 to 40·7)	6·1% (4·0 to 8·2%)	33·6 (21·5 to 46·4)	5·6% (3·6 to 7·7%)	12·3% (3·8 to 20·6%)	-7·6% (-11·3 to -4·9%)	661·0 (432·7 to 906·2)	6·1% (4·0 to 8·2%)	725·2 (454·8 to 1,011·6)	5·5% (3·5 to 7·6%)	9·7% (-0·1 to 19·3%)	-9·3% (-13·8 to -6·1%)
Ischaemic stroke	6·7 (4·3 to 9·6)	5·5% (3·6 to 7·8%)	7·5 (4·7 to 10·9)	5·0% (3·2 to 7·3%)	12·7% (1·9 to 22·1%)	-7·6% (-11·8 to -4·0%)	131·4 (85·4 to 185·9)	5·8% (3·9 to 8·1%)	143·0 (89·8 to 205·5)	5·3% (3·4 to 7·6%)	8·8% (-2·3 to 18·9%)	-7·8% (-12·3 to -4·3%)
Hemorrhagic stroke	8·4 (5·5 to 11·9)	6·4% (4·3 to 8·8%)	9·2 (5·8 to 13·3)	6·0% (3·8 to 8·4%)	9·6% (-0·9 to 20·0%)	-6·1% (-11·1 to -2·2%)	220·2 (135·4 to 318·1)	5·8% (3·7 to 8·2%)	233·7 (140·4 to 343·4)	5·6% (3·4 to 8·0%)	6·1% (-6·1 to 18·5%)	-4·2% (-11·4 to 1·5%)
Hypertensive heart disease	4·7 (1·9 to 9·7)	10·5% (4·2 to 21·9%)	5·4 (2·2 to 11·2)	9·9% (4·0 to 20·7%)	15·5% (2·3 to 29·8%)	-6·0% (-11·7 to -0·4%)	91·2 (45·6 to 172·2)	10·5% (5·4 to 19·7%)	101·4 (49·7 to 193·4)	9·7% (4·7 to 18·4%)	11·1% (-2·0 to 25·1%)	-8·0% (-13·7 to -3·8%)
Cardiomyopathy and myocarditis	0·6 (0·4 to 0·9)	2·7% (1·7 to 3·9%)	0·8 (0·4 to 1·1)	2·7% (1·6 to 3·9%)	29·9% (14·9 to 44·2%)	-2·5% (-9·2 to 3·7%)	15·8 (8·7 to 24·3)	1·8% (1·0 to 2·7%)	19·2 (10·3 to 30·1)	1·8% (1·0 to 2·8%)	21·8% (6·7 to 36·8%)	2·3% (-9·4 to 13·0%)

Supplemental Results Table 1. All-age deaths, death PAFs, DALYs, DALY PAFs, and percent change for each risk factor and outcome for both sexes combined in 2005 and 2015 for North Africa and Middle East.

Data in parenthesis are 95% uncertainty intervals. PAFs=population attributable fractions. DALYs=disability-adjusted life-years.

	2005 Deaths (in thousands)	2005 Deaths PAF	2015 Deaths (in thousands)	2015 Deaths PAF	Percent change deaths 2005-2015	Percent change deaths PAF 2005-2015	2005 DALYs (in thousands)	2005 DALYs PAF	2015 DALYs (in thousands)	2015 DALYs PAF	Percent change DALYs 2005-2015	Percent change DALYs PAF 2005-2015
Atrial fibrillation and flutter	0·2 (0·1 to 0·3)	3·3% (2·2 to 4·6%)	0·2 (0·1 to 0·4)	3·1% (1·9 to 4·3%)	23·8% (13·4 to 32·8%)	-8·0% (-13·6 to -3·4%)	4·5 (2·9 to 6·6)	3·9% (2·6 to 5·2%)	5·4 (3·4 to 7·9)	3·5% (2·2 to 4·8%)	18·1% (11·1 to 23·6%)	-10·8% (-15·4 to -7·5%)
Aortic aneurysm	0·2 (0·1 to 0·3)	3·4% (2·2 to 4·8%)	0·2 (0·1 to 0·3)	3·1% (1·9 to 4·4%)	15·1% (3·2 to 26·9%)	-11·4% (-16·2 to -8·0%)	4·0 (2·4 to 5·8)	3·4% (2·1 to 4·8%)	4·4 (2·6 to 6·5)	2·9% (1·7 to 4·2%)	11·3% (-1·2 to 23·5%)	-13·7% (-19·2 to -9·6%)
Peripheral vascular disease	0·0 (0·0 to 0·0)	2·3% (1·4 to 3·4%)	0·0 (0·0 to 0·0)	2·0% (1·1 to 3·1%)	29·6% (6·1 to 58·2%)	-12·9% (-22·1 to -4·9%)	0·7 (0·3 to 1·2)	2·5% (1·6 to 3·6%)	0·8 (0·4 to 1·5)	2·2% (1·3 to 3·2%)	20·4% (10·5 to 30·0%)	-12·4% (-18·7 to -7·9%)
Endocarditis	0·2 (0·1 to 0·4)	2·7% (1·6 to 3·9%)	0·3 (0·1 to 0·4)	2·5% (1·5 to 3·8%)	15·6% (4·1 to 27·2%)	-7·1% (-13·1 to -1·3%)	5·3 (2·7 to 8·7)	1·9% (1·1 to 3·0%)	5·8 (2·9 to 10·0)	1·9% (1·0 to 3·0%)	10·3% (-1·5 to 21·9%)	-4·3% (-14·1 to 4·7%)
Other cardiovascular and circulatory diseases	1·9 (1·2 to 2·5)	3·8% (2·6 to 5·1%)	2·1 (1·4 to 2·9)	3·6% (2·4 to 5·0%)	15·1% (3·4 to 27·6%)	-3·9% (-8·7 to 0·0%)	62·6 (38·2 to 90·8)	2·8% (1·7 to 3·9%)	70·1 (41·1 to 103·9)	2·7% (1·6 to 4·0%)	12·0% (1·4 to 21·6%)	-0·8% (-7·8 to 5·1%)
Idiopathic developmental intellectual disability	-	-	-	-	-	-	57·9 (26·0 to 102·4)	19·3% (15·8 to 22·4%)	53·6 (23·6 to 94·9)	14·9% (11·1 to 18·4%)	-7·4% (-23·6 to 9·8%)	-22·5% (-36·2 to -8·4%)
Chronic kidney disease due to diabetes mellitus	0·5 (0·3 to 0·8)	4·2% (2·4 to 6·5%)	0·7 (0·4 to 1·1)	3·8% (2·2 to 6·1%)	29·2% (17·4 to 40·6%)	-8·0% (-13·3 to -4·1%)	13·3 (7·1 to 21·6)	3·3% (1·8 to 5·3%)	16·2 (8·2 to 26·6)	3·0% (1·6 to 4·9%)	21·7% (10·7 to 32·2%)	-9·5% (-15·2 to -5·1%)
Chronic kidney disease due to hypertension	1·9 (1·1 to 2·9)	4·5% (2·5 to 6·7%)	2·4 (1·4 to 3·7)	4·2% (2·4 to 6·3%)	24·8% (14·9 to 34·8%)	-6·3% (-10·2 to -3·5%)	43·8 (24·2 to 67·5)	3·6% (2·0 to 5·5%)	51·9 (27·9 to 81·7)	3·3% (1·8 to 5·2%)	18·4% (7·9 to 28·1%)	-6·7% (-11·8 to -3·3%)
Chronic kidney disease due to glomerulonephritis	0·2 (0·1 to 0·3)	4·0% (2·3 to 5·9%)	0·3 (0·2 to 0·4)	3·9% (2·2 to 5·7%)	25·0% (12·9 to 36·8%)	-3·2% (-7·9 to 0·2%)	6·6 (3·5 to 10·2)	2·7% (1·5 to 4·1%)	7·7 (3·9 to 12·4)	2·6% (1·4 to 4·0%)	17·1% (6·6 to 27·8%)	-3·9% (-10·3 to 0·9%)
Chronic kidney disease due to other causes	0·1 (0·1 to 0·2)	3·3% (1·9 to 4·9%)	0·2 (0·1 to 0·3)	3·6% (2·1 to 5·5%)	36·6% (20·7 to 53·5%)	10·3% (1·8 to 20·7%)	5·5 (2·9 to 9·1)	2·1% (1·1 to 3·4%)	6·5 (3·3 to 11·1)	2·2% (1·2 to 3·6%)	18·9% (9·5 to 26·6%)	4·0% (-3·8 to 12·0%)
**Occupational risks: All causes**	35·2 (33·1 to 37·5)	1·4% (1·3 to 1·5%)	43·7 (40·7 to 46·9)	1·5% (1·4 to 1·6%)	24·1% (16·9 to 32·0%)	5·9% (-0·2 to 13·1%)	2,647·9 (2,205·1 to 3,157·8)	1·8% (1·6 to 2·0%)	3,261·9 (2,726·2 to 3,884·2)	2·0% (1·8 to 2·2%)	23·2% (18·7 to 28·1%)	10·2% (4·5 to 16·5%)
**Occupational carcinogens: All causes**	15·9 (14·5 to 17·4)	0·6% (0·6 to 0·7%)	20·4 (18·4 to 22·6)	0·7% (0·6 to 0·8%)	28·4% (17·8 to 40·9%)	9·6% (1·3 to 19·0%)	368·6 (336·9 to 403·6)	0·2% (0·2 to 0·3%)	476·3 (427·6 to 527·2)	0·3% (0·2 to 0·3%)	29·2% (19·0 to 41·9%)	15·6% (6·4 to 26·0%)
**Occupational exposure to asbestos: All causes**	6·0 (4·6 to 7·5)	0·2% (0·2 to 0·3%)	6·5 (4·9 to 8·2)	0·2% (0·2 to 0·3%)	8·1% (-7·7 to 26·2%)	-7·7% (-20·8 to 7·1%)	122·1 (94·5 to 155·0)	0·1% (0·1 to 0·1%)	129·1 (96·8 to 163·7)	0·1% (0·1 to 0·1%)	5·7% (-10·4 to 25·3%)	-5·4% (-19·3 to 11·1%)
Larynx cancer	0·1 (0·1 to 0·2)	2·3% (1·2 to 3·6%)	0·1 (0·1 to 0·2)	1·9% (0·9 to 2·9%)	0·1% (-15·7 to 21·0%)	-19·7% (-31·1 to -6·4%)	1·9 (1·0 to 3·1)	1·7% (0·9 to 2·7%)	1·9 (0·9 to 3·0)	1·4% (0·7 to 2·2%)	-3·4% (-18·7 to 16·4%)	-22·1% (-32·9 to -9·5%)
Tracheal, bronchus, and lung cancer	4·9 (3·6 to 6·4)	11·2% (8·3 to 14·4%)	5·3 (3·9 to 7·0)	9·3% (6·9 to 12·0%)	8·1% (-9·2 to 28·0%)	-17·1% (-27·7 to -5·1%)	96·2 (70·7 to 128·3)	8·8% (6·5 to 11·6%)	102·2 (73·6 to 137·3)	7·2% (5·3 to 9·4%)	6·2% (-11·3 to 27·9%)	-18·6% (-29·9 to -6·1%)
Ovarian cancer	0·1 (0·0 to 0·1)	1·7% (0·8 to 2·8%)	0·1 (0·0 to 0·1)	1·3% (0·6 to 2·1%)	-2·5% (-23·4 to 23·9%)	-23·4% (-36·4 to -6·7%)	1·6 (0·7 to 2·7)	1·2% (0·5 to 2·0%)	1·4 (0·7 to 2·5)	0·9% (0·4 to 1·4%)	-8·0% (-27·6 to 18·2%)	-27·5% (-40·1 to -9·9%)
Mesothelioma	0·9 (0·7 to 1·1)	64·4% (49·8 to 76·5%)	1·0 (0·7 to 1·2)	61·5% (46·7 to 74·2%)	9·6% (-3·4 to 23·3%)	-4·5% (-9·3 to -0·8%)	22·4 (16·1 to 28·5)	54·8% (40·6 to 67·7%)	23·6 (16·0 to 30·6)	51·5% (36·8 to 64·9%)	5·4% (-8·2 to 20·3%)	-6·0% (-12·1 to -0·9%)
**Occupational exposure to arsenic: All causes**	0·3 (0·2 to 0·4)	0·0% (0·0 to 0·0%)	0·4 (0·3 to 0·5)	0·0% (0·0 to 0·0%)	18·4% (8·2 to 29·6%)	1·1% (-7·0 to 9·7%)	7·7 (5·9 to 9·8)	0·0% (0·0 to 0·0%)	9·2 (6·9 to 11·7)	0·0% (0·0 to 0·0%)	18·6% (8·3 to 29·6%)	6·2% (-3·2 to 15·7%)
Tracheal, bronchus, and lung cancer	0·3 (0·2 to 0·4)	0·7% (0·6 to 1·0%)	0·4 (0·3 to 0·5)	0·7% (0·5 to 0·9%)	18·4% (8·2 to 29·6%)	-9·1% (-13·8 to -4·7%)	7·7 (5·9 to 9·8)	0·7% (0·5 to 0·9%)	9·2 (6·9 to 11·7)	0·6% (0·5 to 0·8%)	18·6% (8·3 to 29·6%)	-9·0% (-13·5 to -4·6%)
**Occupational exposure to benzene: All causes**	0·4 (0·3 to 0·5)	0·0% (0·0 to 0·0%)	0·6 (0·5 to 0·7)	0·0% (0·0 to 0·0%)	45·2% (32·7 to 58·9%)	24·0% (15·0 to 33·7%)	14·5 (12·0 to 17·6)	0·0% (0·0 to 0·0%)	21·0 (17·4 to 25·1)	0·0% (0·0 to 0·0%)	44·8% (31·9 to 58·6%)	29·6% (18·8 to 41·1%)
Leukaemia	0·4 (0·3 to 0·5)	1·9% (1·6 to 2·3%)	0·6 (0·5 to 0·7)	2·2% (1·8 to 2·6%)	45·2% (32·7 to 58·9%)	16·8% (10·3 to 23·9%)	14·5 (12·0 to 17·6)	1·6% (1·3 to 1·9%)	21·0 (17·4 to 25·1)	1·9% (1·6 to 2·3%)	44·8% (31·9 to 58·6%)	24·7% (16·4 to 33·8%)
**Occupational exposure to beryllium: All causes**	0·0 (0·0 to 0·0)	0·0% (0·0 to 0·0%)	0·0 (0·0 to 0·0)	0·0% (0·0 to 0·0%)	13·9% (3·0 to 26·0%)	-2·8% (-12·1 to 7·6%)	0·4 (0·3 to 0·5)	0·0% (0·0 to 0·0%)	0·5 (0·4 to 0·6)	0·0% (0·0 to 0·0%)	14·1% (2·9 to 26·2%)	2·1% (-8·0 to 12·9%)
Tracheal, bronchus, and lung cancer	0·0 (0·0 to 0·0)	0·0% (0·0 to 0·1%)	0·0 (0·0 to 0·0)	0·0% (0·0 to 0·0%)	13·9% (3·0 to 26·0%)	-12·6% (-19·0 to -5·8%)	0·4 (0·3 to 0·5)	0·0% (0·0 to 0·0%)	0·5 (0·4 to 0·6)	0·0% (0·0 to 0·0%)	14·1% (2·9 to 26·2%)	-12·5% (-19·1 to -5·5%)
**Occupational exposure to cadmium: All causes**	0·1 (0·1 to 0·1)	0·0% (0·0 to 0·0%)	0·1 (0·1 to 0·1)	0·0% (0·0 to 0·0%)	30·9% (19·7 to 43·2%)	11·7% (2·0 to 21·4%)	1·7 (1·3 to 2·0)	0·0% (0·0 to 0·0%)	2·2 (1·7 to 2·7)	0·0% (0·0 to 0·0%)	30·9% (19·3 to 43·2%)	17·1% (7·0 to 28·3%)
Tracheal, bronchus, and lung cancer	0·1 (0·1 to 0·1)	0·2% (0·1 to 0·2%)	0·1 (0·1 to 0·1)	0·2% (0·1 to 0·2%)	30·9% (19·7 to 43·2%)	0·4% (-5·6 to 6·2%)	1·7 (1·3 to 2·0)	0·2% (0·1 to 0·2%)	2·2 (1·7 to 2·7)	0·2% (0·1 to 0·2%)	30·9% (19·3 to 43·2%)	0·4% (-5·9 to 6·2%)
**Occupational exposure to chromium: All causes**	0·2 (0·2 to 0·3)	0·0% (0·0 to 0·0%)	0·3 (0·2 to 0·3)	0·0% (0·0 to 0·0%)	21·9% (11·1 to 34·1%)	4·1% (-5·1 to 13·9%)	5·4 (4·6 to 6·2)	0·0% (0·0 to 0·0%)	6·6 (5·6 to 7·7)	0·0% (0·0 to 0·0%)	22·1% (10·9 to 34·6%)	9·3% (-0·8 to 19·9%)
Tracheal, bronchus, and lung cancer	0·2 (0·2 to 0·3)	0·5% (0·4 to 0·6%)	0·3 (0·2 to 0·3)	0·5% (0·4 to 0·6%)	21·9% (11·1 to 34·1%)	-6·5% (-12·4 to -0·5%)	5·4 (4·6 to 6·2)	0·5% (0·4 to 0·6%)	6·6 (5·6 to 7·7)	0·5% (0·4 to 0·5%)	22·1% (10·9 to 34·6%)	-6·4% (-12·3 to -0·3%)
**Occupational exposure to diesel engine exhaust: All causes**	3·5 (3·0 to 4·0)	0·1% (0·1 to 0·2%)	5·1 (4·4 to 6·0)	0·2% (0·1 to 0·2%)	47·3% (35·2 to 61·0%)	25·8% (16·0 to 36·4%)	82·6 (71·2 to 94·9)	0·1% (0·0 to 0·1%)	122·2 (104·0 to 142·2)	0·1% (0·1 to 0·1%)	47·9% (36·0 to 61·6%)	32·4% (21·4 to 44·3%)
Tracheal, bronchus, and lung cancer	3·5 (3·0 to 4·0)	8·0% (6·9 to 9·1%)	5·1 (4·4 to 6·0)	9·0% (7·9 to 10·2%)	47·3% (35·2 to 61·0%)	13·0% (7·3 to 19·0%)	82·6 (71·2 to 94·9)	7·5% (6·6 to 8·6%)	122·2 (104·0 to 142·2)	8·6% (7·4 to 9·8%)	47·9% (36·0 to 61·6%)	13·4% (7·8 to 19·3%)
**Occupational exposure to second-hand smoke: All causes**	4·1 (3·7 to 4·4)	0·2% (0·1 to 0·2%)	5·7 (5·1 to 6·3)	0·2% (0·2 to 0·2%)	41·2% (29·6 to 53·7%)	20·6% (11·0 to 29·8%)	95·5 (87·1 to 103·9)	0·1% (0·1 to 0·1%)	133·4 (119·1 to 147·0)	0·1% (0·1 to 0·1%)	39·6% (28·0 to 51·8%)	24·9% (14·4 to 35·4%)
Tracheal, bronchus, and lung cancer	4·1 (3·7 to 4·4)	9·3% (8·7 to 9·9%)	5·7 (5·1 to 6·3)	10·1% (9·4 to 10·8%)	41·2% (29·6 to 53·7%)	8·3% (3·1 to 13·8%)	95·5 (87·1 to 103·9)	8·7% (8·1 to 9·3%)	133·4 (119·1 to 147·0)	9·3% (8·7 to 10·0%)	39·6% (28·0 to 51·8%)	7·0% (2·1 to 12·2%)
**Occupational exposure to formaldehyde: All causes**	0·1 (0·1 to 0·1)	0·0% (0·0 to 0·0%)	0·1 (0·1 to 0·1)	0·0% (0·0 to 0·0%)	24·6% (14·0 to 35·8%)	6·4% (-1·0 to 14·4%)	3·4 (2·8 to 4·0)	0·0% (0·0 to 0·0%)	4·2 (3·5 to 5·0)	0·0% (0·0 to 0·0%)	24·1% (13·1 to 35·5%)	11·1% (1·9 to 20·7%)
Nasopharynx cancer	0·0 (0·0 to 0·0)	1·4% (0·9 to 1·9%)	0·0 (0·0 to 0·0)	1·3% (0·8 to 1·8%)	14·1% (2·8 to 26·4%)	-6·6% (-11·6 to -1·4%)	0·8 (0·5 to 1·2)	1·4% (0·9 to 1·9%)	0·9 (0·6 to 1·4)	1·3% (0·8 to 1·9%)	13·9% (2·1 to 26·5%)	-4·0% (-9·6 to 1·5%)
**Occupational exposure to nickel: All causes**	1·0 (0·7 to 1·3)	0·0% (0·0 to 0·1%)	1·1 (0·8 to 1·5)	0·0% (0·0 to 0·1%)	14·6% (3·2 to 26·9%)	-2·1% (-11·0 to 8·1%)	22·8 (16·4 to 30·4)	0·0% (0·0 to 0·0%)	26·2 (18·4 to 35·5)	0·0% (0·0 to 0·0%)	15·0% (3·7 to 27·1%)	2·9% (-7·1 to 13·6%)
Tracheal, bronchus, and lung cancer	1·0 (0·7 to 1·3)	2·2% (1·6 to 3·0%)	1·1 (0·8 to 1·5)	1·9% (1·3 to 2·6%)	14·6% (3·2 to 26·9%)	-12·1% (-18·5 to -5·8%)	22·8 (16·4 to 30·4)	2·1% (1·5 to 2·8%)	26·2 (18·4 to 35·5)	1·8% (1·3 to 2·5%)	15·0% (3·7 to 27·1%)	-11·8% (-18·1 to -5·3%)
**Occupational exposure to polycyclic aromatic hydrocarbons: All causes**	0·6 (0·5 to 0·7)	0·0% (0·0 to 0·0%)	0·8 (0·6 to 0·9)	0·0% (0·0 to 0·0%)	39·1% (27·5 to 52·5%)	18·8% (9·3 to 28·9%)	13·3 (10·7 to 16·0)	0·0% (0·0 to 0·0%)	18·4 (14·9 to 22·5)	0·0% (0·0 to 0·0%)	38·9% (27·0 to 52·1%)	24·3% (14·2 to 35·4%)
Tracheal, bronchus, and lung cancer	0·6 (0·5 to 0·7)	1·3% (1·0 to 1·5%)	0·8 (0·6 to 0·9)	1·4% (1·1 to 1·6%)	39·1% (27·5 to 52·5%)	6·7% (1·6 to 12·7%)	13·3 (10·7 to 16·0)	1·2% (1·0 to 1·4%)	18·4 (14·9 to 22·5)	1·3% (1·1 to 1·5%)	38·9% (27·0 to 52·1%)	6·5% (1·3 to 12·7%)
**Occupational exposure to silica: All causes**	2·5 (2·2 to 2·8)	0·1% (0·1 to 0·1%)	3·8 (3·4 to 4·4)	0·1% (0·1 to 0·1%)	54·2% (39·5 to 70·0%)	31·6% (20·3 to 43·4%)	58·9 (52·7 to 65·7)	0·0% (0·0 to 0·0%)	90·9 (79·6 to 103·1)	0·1% (0·0 to 0·1%)	54·2% (39·5 to 69·5%)	38·0% (25·1 to 51·6%)
Tracheal, bronchus, and lung cancer	2·5 (2·2 to 2·8)	5·7% (5·1 to 6·3%)	3·8 (3·4 to 4·4)	6·8% (6·1 to 7·5%)	54·2% (39·5 to 70·0%)	18·2% (10·2 to 26·6%)	58·9 (52·7 to 65·7)	5·4% (4·9 to 5·9%)	90·9 (79·6 to 103·1)	6·4% (5·7 to 7·0%)	54·2% (39·5 to 69·5%)	18·2% (10·3 to 26·4%)
**Occupational exposure to sulfphuric acid: All causes**	0·4 (0·3 to 0·6)	0·0% (0·0 to 0·0%)	0·5 (0·4 to 0·7)	0·0% (0·0 to 0·0%)	24·4% (11·0 to 39·7%)	6·2% (-3·6 to 17·4%)	10·7 (8·0 to 13·6)	0·0% (0·0 to 0·0%)	13·3 (10·1 to 17·4)	0·0% (0·0 to 0·0%)	25·1% (11·8 to 40·6%)	11·9% (0·4 to 24·7%)
Larynx cancer	0·4 (0·3 to 0·6)	10·1% (7·5 to 12·9%)	0·5 (0·4 to 0·7)	10·1% (7·5 to 12·9%)	24·4% (11·0 to 39·7%)	-0·1% (-5·4 to 5·8%)	10·7 (8·0 to 13·6)	9·6% (7·2 to 12·2%)	13·3 (10·1 to 17·4)	9·6% (7·3 to 12·3%)	25·1% (11·8 to 40·6%)	0·8% (-4·5 to 6·8%)
**Occupational exposure to trichloroethylene: All causes**	0·0 (0·0 to 0·0)	0·0% (0·0 to 0·0%)	0·0 (0·0 to 0·0)	0·0% (0·0 to 0·0%)	37·5% (21·2 to 53·8%)	17·5% (5·2 to 30·6%)	0·2 (0·0 to 0·4)	0·0% (0·0 to 0·0%)	0·3 (0·1 to 0·5)	0·0% (0·0 to 0·0%)	38·3% (22·3 to 54·4%)	23·8% (10·5 to 37·9%)
Kidney cancer	0·0 (0·0 to 0·0)	0·2% (0·0 to 0·4%)	0·0 (0·0 to 0·0)	0·2% (0·1 to 0·4%)	37·5% (21·2 to 53·8%)	2·1% (-4·6 to 9·4%)	0·2 (0·0 to 0·4)	0·2% (0·0 to 0·3%)	0·3 (0·1 to 0·5)	0·2% (0·0 to 0·3%)	38·3% (22·3 to 54·4%)	6·3% (-1·8 to 15·7%)

Supplemental Results Table 1. All-age deaths, death PAFs, DALYs, DALY PAFs, and percent change for each risk factor and outcome for both sexes combined in 2005 and 2015 for North Africa and Middle East.

Data in parenthesis are 95% uncertainty intervals. PAFs=population attributable fractions. DALYs=disability-adjusted life-years.

	2005 Deaths (in thousands)	2005 Deaths PAF	2015 Deaths (in thousands)	2015 Deaths PAF	Percent change deaths 2005-2015	Percent change deaths PAF 2005-2015	2005 DALYs (in thousands)	2005 DALYs PAF	2015 DALYs (in thousands)	2015 DALYs PAF	Percent change DALYs 2005-2015	Percent change DALYs PAF 2005-2015
**Occupational asthmagens: All causes**	1·8 (1·4 to 2·3)	0·1% (0·1 to 0·1%)	1·8 (1·4 to 2·2)	0·1% (0·0 to 0·1%)	-3·7% (-16·3 to 11·5%)	-17·8% (-27·3 to -6·1%)	124·6 (94·5 to 158·1)	0·1% (0·1 to 0·1%)	139·6 (105·2 to 179·4)	0·1% (0·1 to 0·1%)	12·0% (2·9 to 20·9%)	0·1% (-7·5 to 7·1%)
Asthma	1·8 (1·4 to 2·3)	7·6% (6·3 to 9·2%)	1·8 (1·4 to 2·2)	7·1% (5·9 to 8·5%)	-3·7% (-16·3 to 11·5%)	-6·5% (-15·4 to 3·4%)	124·6 (94·5 to 158·1)	6·6% (5·6 to 7·6%)	139·6 (105·2 to 179·4)	6·6% (5·7 to 7·6%)	12·0% (2·9 to 20·9%)	-0·3% (-5·9 to 5·1%)
**Occupational particulate matter, gases, and fumes: All causes**	5·8 (4·7 to 6·9)	0·2% (0·2 to 0·3%)	6·7 (5·5 to 8·1)	0·2% (0·2 to 0·3%)	15·8% (5·8 to 27·4%)	-1·1% (-8·7 to 7·3%)	175·2 (149·4 to 204·8)	0·1% (0·1 to 0·1%)	206·4 (174·4 to 240·1)	0·1% (0·1 to 0·1%)	17·8% (8·5 to 27·0%)	5·4% (-2·0 to 13·0%)
Chronic obstructive pulmonary disease	5·8 (4·7 to 6·8)	8·8% (7·3 to 10·4%)	6·7 (5·5 to 8·0)	8·9% (7·3 to 10·5%)	15·9% (5·9 to 27·4%)	1·5% (-4·0 to 8·2%)	174·6 (148·9 to 204·3)	10·3% (8·9 to 11·8%)	205·7 (173·7 to 239·4)	10·9% (9·4 to 12·5%)	17·8% (8·5 to 27·0%)	6·1% (1·2 to 12·0%)
**Occupational noise: All causes**	-	-	-	-	-	-	314·7 (210·2 to 440·6)	0·2% (0·2 to 0·3%)	426·5 (284·0 to 593·2)	0·3% (0·2 to 0·3%)	35·5% (32·5 to 38·4%)	21·2% (15·9 to 27·0%)
Age-related and other hearing loss	-	-	-	-	-	-	314·7 (210·2 to 440·6)	23·4% (21·3 to 25·9%)	426·5 (284·0 to 593·2)	23·9% (21·8 to 26·4%)	35·5% (32·5 to 38·4%)	2·2% (0·8 to 3·8%)
**Occupational injuries: All causes**	11·7 (10·8 to 12·8)	0·5% (0·4 to 0·5%)	14·9 (13·6 to 16·4)	0·5% (0·4 to 0·6%)	26·8% (13·4 to 41·6%)	8·3% (-4·3 to 23·4%)	801·8 (711·2 to 900·7)	0·5% (0·5 to 0·6%)	985·0 (868·1 to 1,125·1)	0·6% (0·5 to 0·7%)	22·9% (9·4 to 38·2%)	9·9% (-3·0 to 25·3%)
Pedestrian road injuries	1·3 (1·0 to 1·4)	3·4% (3·1 to 3·9%)	1·8 (1·5 to 2·1)	4·7% (4·2 to 5·2%)	44·2% (27·8 to 61·9%)	36·5% (17·9 to 58·3%)	63·7 (52·9 to 71·4)	3·3% (2·9 to 3·7%)	89·8 (77·1 to 101·8)	4·8% (4·2 to 5·3%)	41·0% (25·0 to 58·7%)	43·5% (23·7 to 65·4%)
Cyclist road injuries	0·1 (0·1 to 0·1)	3·8% (3·2 to 4·5%)	0·1 (0·1 to 0·2)	5·4% (4·5 to 6·3%)	48·8% (26·4 to 73·7%)	39·8% (16·9 to 67·8%)	5·6 (4·6 to 6·5)	3·7% (3·2 to 4·4%)	8·2 (6·9 to 9·7)	5·4% (4·6 to 6·3%)	46·7% (26·4 to 69·2%)	43·7% (19·8 to 71·5%)
Motorcyclist road injuries	0·7 (0·6 to 0·9)	6·6% (5·4 to 8·0%)	0·8 (0·7 to 1·1)	8·0% (6·6 to 9·7%)	17·2% (-2·7 to 40·0%)	20·8% (-4·8 to 54·3%)	43·0 (35·8 to 54·7)	6·5% (5·4 to 7·8%)	49·8 (41·4 to 64·6)	8·0% (6·6 to 9·7%)	15·8% (-2·9 to 38·2%)	22·8% (-2·0 to 55·2%)
Motor vehicle road injuries	4·9 (4·5 to 5·5)	7·0% (6·2 to 7·9%)	6·4 (5·7 to 7·3)	8·2% (7·2 to 9·4%)	30·2% (15·5 to 47·8%)	16·7% (0·9 to 36·2%)	269·6 (244·5 to 300·5)	6·8% (6·0 to 7·7%)	345·7 (308·9 to 390·6)	8·2% (7·3 to 9·4%)	28·2% (13·8 to 45·2%)	20·3% (3·8 to 40·4%)
Other road injuries	0·2 (0·1 to 0·2)	5·0% (3·2 to 7·3%)	0·2 (0·1 to 0·3)	5·7% (3·7 to 8·2%)	36·7% (-4·3 to 91·4%)	13·3% (-21·1 to 65·5%)	9·5 (4·7 to 13·4)	4·8% (2·9 to 7·2%)	12·9 (6·7 to 18·5)	5·6% (3·6 to 8·2%)	36·2% (-0·5 to 83·9%)	15·8% (-19·8 to 69·8%)
Other transport injuries	0·3 (0·2 to 0·3)	4·3% (3·8 to 4·9%)	0·5 (0·4 to 0·6)	5·9% (5·1 to 6·8%)	81·3% (52·7 to 113·9%)	36·4% (16·5 to 62·8%)	16·1 (14·0 to 18·1)	4·3% (3·7 to 4·9%)	27·2 (23·3 to 31·7)	6·0% (5·2 to 6·9%)	69·4% (44·8 to 98·0%)	39·5% (19·6 to 64·6%)
Falls	0·9 (0·8 to 1·1)	4·8% (3·9 to 6·2%)	1·3 (1·1 to 1·5)	5·4% (4·5 to 6·9%)	43·2% (21·3 to 65·9%)	12·1% (-8·9 to 38·8%)	101·7 (80·9 to 126·0)	6·6% (5·3 to 7·9%)	133·8 (106·6 to 170·4)	7·5% (6·2 to 9·0%)	31·6% (14·0 to 52·7%)	14·0% (-8·5 to 41·6%)
Drowning	0·7 (0·6 to 0·8)	3·8% (3·1 to 4·5%)	0·8 (0·7 to 0·9)	4·7% (4·0 to 5·7%)	11·9% (-2·4 to 29·0%)	26·2% (2·1 to 55·0%)	41·0 (36·6 to 46·0)	3·1% (2·5 to 3·8%)	44·6 (39·0 to 51·4)	4·0% (3·3 to 4·9%)	8·9% (-5·4 to 25·9%)	28·7% (1·1 to 62·0%)
Fire, heat, and hot substances	0·5 (0·4 to 0·6)	4·2% (3·6 to 5·0%)	0·6 (0·5 to 0·7)	5·3% (4·6 to 6·1%)	21·4% (4·9 to 42·7%)	26·5% (6·0 to 52·2%)	39·6 (33·3 to 47·8)	4·8% (4·1 to 5·6%)	46·2 (38·0 to 56·3)	6·3% (5·5 to 7·2%)	16·7% (1·2 to 35·8%)	29·4% (9·2 to 54·6%)
Poisonings	0·2 (0·1 to 0·2)	2·6% (1·8 to 3·5%)	0·2 (0·1 to 0·2)	2·5% (1·7 to 3·5%)	-11·2% (-30·8 to 14·5%)	-3·5% (-32·9 to 38·2%)	12·4 (9·8 to 16·1)	2·7% (1·8 to 3·6%)	11·7 (9·1 to 15·2)	2·8% (1·9 to 3·8%)	-6·3% (-26·4 to 18·6%)	3·7% (-28·3 to 55·3%)
Unintentional firearm injuries	0·4 (0·2 to 0·6)	6·4% (4·6 to 8·2%)	0·3 (0·2 to 0·5)	5·4% (4·1 to 7·3%)	-22·2% (-43·7 to 8·7%)	-15·5% (-40·0 to 20·0%)	24·4 (12·6 to 31·4)	6·4% (4·6 to 8·1%)	18·8 (10·8 to 25·8)	5·6% (4·2 to 7·5%)	-22·7% (-43·6 to 7·3%)	-12·3% (-37·6 to 25·7%)
Unintentional suffocation	0·0 (0·0 to 0·0)	0·1% (0·1 to 0·2%)	0·0 (0·0 to 0·0)	0·2% (0·1 to 0·3%)	34·0% (-0·7 to 83·0%)	55·1% (0·6 to 149·0%)	0·4 (0·3 to 0·6)	0·3% (0·2 to 0·4%)	0·6 (0·4 to 0·8)	0·4% (0·3 to 0·6%)	38·6% (10·1 to 80·0%)	53·8% (5·7 to 132·1%)
Other exposure to mechanical forces	0·6 (0·5 to 0·8)	6·1% (5·1 to 7·3%)	0·8 (0·6 to 0·9)	7·6% (6·4 to 8·9%)	26·2% (5·2 to 48·5%)	24·2% (4·3 to 48·2%)	61·6 (49·5 to 74·9)	7·3% (6·2 to 8·5%)	74·0 (59·2 to 91·3)	8·7% (7·5 to 10·2%)	20·1% (5·1 to 38·4%)	20·2% (2·7 to 41·2%)
Venomous animal contact	0·0 (0·0 to 0·0)	1·7% (1·3 to 2·3%)	0·0 (0·0 to 0·0)	1·6% (1·2 to 2·2%)	-16·4% (-39·4 to 15·6%)	-3·9% (-31·7 to 32·7%)	2·1 (1·5 to 2·9)	2·1% (1·5 to 2·8%)	1·9 (1·3 to 2·6)	2·1% (1·5 to 2·8%)	-11·9% (-35·0 to 18·7%)	0·6% (-27·6 to 38·6%)
Non-venomous animal contact	0·0 (0·0 to 0·0)	1·6% (1·1 to 2·3%)	0·0 (0·0 to 0·0)	1·7% (1·2 to 2·3%)	-0·5% (-28·6 to 37·6%)	3·4% (-27·4 to 44·5%)	1·5 (1·0 to 2·0)	1·8% (1·3 to 2·5%)	1·4 (1·0 to 1·9)	1·9% (1·3 to 2·7%)	-1·7% (-27·4 to 34·4%)	5·3% (-24·2 to 44·2%)
Pulmonary aspiration and foreign body in airway	0·1 (0·1 to 0·1)	3·0% (2·1 to 3·9%)	0·1 (0·1 to 0·2)	3·6% (2·7 to 4·6%)	20·9% (-1·4 to 48·2%)	20·7% (-3·5 to 49·7%)	7·5 (6·1 to 9·2)	2·9% (1·9 to 3·9%)	8·4 (6·8 to 10·3)	3·5% (2·5 to 4·4%)	13·0% (-7·2 to 38·0%)	21·2% (-4·0 to 52·2%)
Foreign body in eyes	-	-	-	-	-	-	0·3 (0·1 to 0·5)	7·4% (6·2 to 8·9%)	0·4 (0·2 to 0·7)	8·1% (6·6 to 9·9%)	33·7% (11·3 to 62·2%)	8·9% (-9·0 to 32·0%)
Foreign body in other body part	0·1 (0·1 to 0·2)	5·0% (4·0 to 6·3%)	0·1 (0·1 to 0·2)	5·4% (4·4 to 6·6%)	5·5% (-15·9 to 31·0%)	7·5% (-14·6 to 32·7%)	11·7 (9·3 to 14·8)	5·5% (4·5 to 6·8%)	12·5 (9·9 to 16·0)	6·1% (5·1 to 7·4%)	7·0% (-11·8 to 28·8%)	10·8% (-9·9 to 34·6%)
Other unintentional injuries	0·7 (0·6 to 0·9)	4·0% (3·3 to 4·8%)	0·8 (0·6 to 0·9)	4·2% (3·3 to 5·3%)	1·4% (-17·5 to 27·0%)	5·9% (-18·0 to 37·7%)	90·1 (70·6 to 114·4)	5·0% (4·1 to 5·8%)	97·0 (75·1 to 125·2)	5·5% (4·5 to 6·6%)	7·6% (-7·8 to 25·0%)	10·4% (-9·2 to 34·5%)
**Occupational ergonomic factors: All causes**	-	-	-	-	-	-	868·4 (600·3 to 1,219·9)	0·6% (0·4 to 0·8%)	1,036·5 (716·6 to 1,453·1)	0·6% (0·5 to 0·8%)	19·4% (15·9 to 22·8%)	6·7% (1·5 to 12·4%)
Low back pain	-	-	-	-	-	-	868·4 (600·3 to 1,219·9)	26·4% (24·5 to 28·3%)	1,036·5 (716·6 to 1,453·1)	24·7% (23·0 to 26·5%)	19·4% (15·9 to 22·8%)	-6·4% (-8·8 to -3·9%)
**Behavioural risks: All causes**	935·5 (860·8 to 1,013·5)	36·7% (34·1 to 39·4%)	1,063·3 (973·8 to 1,163·0)	35·6% (32·9 to 38·6%)	13·7% (7·2 to 19·6%)	-3·0% (-6·1 to 0·2%)	38,595·7 (35,095·8 to 42,121·6)	26·1% (24·4 to 27·8%)	39,894·1 (36,241·6 to 44,198·1)	24·1% (22·5 to 25·8%)	3·4% (-2·2 to 8·9%)	-7·6% (-11·5 to -3·4%)
**Child and maternal malnutrition: All causes**	132·3 (112·3 to 151·3)	5·2% (4·4 to 6·0%)	75·8 (63·0 to 90·2)	2·5% (2·1 to 3·0%)	-42·7% (-50·7 to -32·8%)	-51·1% (-57·9 to -42·7%)	14,634·1 (12,520·3 to 16,860·8)	9·9% (8·7 to 11·1%)	10,195·1 (8,504·9 to 12,173·7)	6·2% (5·4 to 7·0%)	-30·3% (-38·1 to -21·3%)	-37·8% (-44·1 to -30·6%)
**Suboptimal breastfeeding: All causes**	41·3 (25·4 to 61·4)	1·6% (1·0 to 2·4%)	24·0 (13·9 to 37·0)	0·8% (0·5 to 1·2%)	-41·8% (-51·6 to -30·4%)	-50·3% (-58·7 to -40·9%)	3,602·3 (2,224·9 to 5,340·4)	2·4% (1·5 to 3·6%)	2,119·7 (1,254·0 to 3,249·4)	1·3% (0·7 to 1·9%)	-41·2% (-50·9 to -30·0%)	-47·4% (-55·9 to -38·0%)
**Non-exclusive breastfeeding : All causes**	39·0 (22·7 to 58·7)	1·5% (0·9 to 2·3%)	22·9 (12·9 to 35·9)	0·8% (0·4 to 1·2%)	-41·3% (-51·1 to -30·0%)	-49·9% (-58·3 to -40·2%)	3,374·2 (1,970·7 to 5,063·7)	2·3% (1·3 to 3·4%)	1,987·1 (1,125·1 to 3,107·0)	1·2% (0·7 to 1·8%)	-41·1% (-50·9 to -29·8%)	-47·3% (-55·8 to -37·5%)
Diarrhoeal diseases	15·8 (9·6 to 23·3)	29·9% (19·0 to 40·9%)	8·3 (5·0 to 12·7)	27·6% (17·5 to 37·5%)	-47·1% (-60·2 to -30·1%)	-7·6% (-19·2 to 5·5%)	1,370·5 (844·8 to 2,015·2)	30·0% (18·9 to 40·8%)	731·7 (446·3 to 1,103·9)	27·4% (17·5 to 37·7%)	-46·6% (-59·7 to -29·7%)	-8·7% (-20·6 to 4·1%)
Lower respiratory infections	23·2 (8·9 to 41·6)	16·5% (6·6 to 29·8%)	14·5 (5·5 to 26·3)	11·5% (4·4 to 20·5%)	-37·4% (-49·2 to -23·4%)	-30·2% (-39·5 to -18·9%)	2,003·7 (770·3 to 3,583·1)	23·3% (9·4 to 41·9%)	1,255·4 (471·6 to 2,270·5)	20·2% (7·9 to 35·4%)	-37·3% (-49·1 to -23·3%)	-13·3% (-23·4 to -1·5%)
**Discontinued breastfeeding : All causes**	3·4 (1·1 to 6·6)	0·1% (0·0 to 0·3%)	1·7 (0·6 to 3·4)	0·1% (0·0 to 0·1%)	-48·8% (-61·1 to -34·0%)	-56·3% (-66·8 to -43·3%)	321·4 (106·3 to 605·3)	0·2% (0·1 to 0·4%)	182·7 (62·4 to 351·4)	0·1% (0·0 to 0·2%)	-43·2% (-55·0 to -28·8%)	-49·2% (-59·8 to -36·1%)
Diarrhoeal diseases	3·4 (1·1 to 6·6)	6·4% (2·1 to 11·8%)	1·7 (0·6 to 3·4)	5·8% (1·9 to 10·6%)	-48·8% (-61·1 to -34·0%)	-10·2% (-22·3 to 2·2%)	321·4 (106·3 to 605·3)	7·0% (2·3 to 12·9%)	182·7 (62·4 to 351·4)	6·9% (2·4 to 12·6%)	-43·2% (-55·0 to -28·8%)	-2·6% (-13·0 to 9·1%)
**Childhood undernutrition: All causes**	122·1 (102·5 to 141·3)	4·8% (4·0 to 5·6%)	69·3 (57·2 to 83·3)	2·3% (1·9 to 2·8%)	-43·3% (-51·5 to -33·2%)	-51·6% (-58·6 to -43·0%)	10,933·8 (9,253·0 to 12,598·4)	7·4% (6·2 to 8·6%)	6,446·2 (5,375·7 to 7,702·7)	3·9% (3·2 to 4·7%)	-41·0% (-49·1 to -31·2%)	-47·3% (-54·0 to -38·7%)
**Childhood underweight: All causes**	28·2 (20·2 to 42·5)	1·1% (0·8 to 1·7%)	14·8 (10·2 to 23·1)	0·5% (0·3 to 0·8%)	-47·6% (-61·6 to -29·6%)	-55·2% (-67·5 to -40·2%)	2,665·3 (1,966·0 to 3,864·4)	1·8% (1·4 to 2·7%)	1,549·0 (1,147·8 to 2,253·4)	0·9% (0·7 to 1·4%)	-41·9% (-56·1 to -25·3%)	-48·0% (-60·6 to -33·8%)
Diarrhoeal diseases	6·5 (4·6 to 9·0)	12·3% (10·0 to 15·4%)	3·3 (2·2 to 4·8)	10·8% (8·3 to 13·7%)	-49·8% (-63·7 to -31·1%)	-12·5% (-25·5 to 1·5%)	585·3 (421·1 to 799·7)	12·8% (10·4 to 15·9%)	306·2 (211·0 to 444·5)	11·4% (8·8 to 14·7%)	-47·7% (-60·7 to -29·6%)	-10·7% (-22·7 to 2·4%)
Lower respiratory infections	13·0 (7·7 to 23·0)	9·2% (5·8 to 16·4%)	7·7 (4·6 to 14·1)	6·1% (3·7 to 11·0%)	-41·0% (-54·1 to -23·2%)	-34·2% (-46·2 to -19·7%)	1,114·5 (663·5 to 1,970·8)	12·9% (8·2 to 23·0%)	659·0 (394·5 to 1,209·0)	10·6% (6·6 to 19·3%)	-40·9% (-53·9 to -23·1%)	-18·3% (-31·0 to -3·3%)
Measles	4·3 (0·6 to 15·6)	31·7% (9·5 to 56·8%)	1·4 (0·1 to 6·1)	27·9% (7·6 to 52·9%)	-68·4% (-94·0 to 74·2%)	-12·0% (-44·0 to 23·0%)	367·4 (48·3 to 1,326·9)	32·3% (9·7 to 57·9%)	116·0 (11·9 to 522·3)	28·3% (7·7 to 53·8%)	-68·4% (-94·0 to 74·1%)	-12·2% (-43·9 to 22·6%)
Protein-energy malnutrition	4·3 (2·9 to 6·9)	63·8% (54·9 to 74·1%)	2·5 (1·5 to 4·0)	48·3% (38·5 to 60·7%)	-43·1% (-65·1 to -6·5%)	-24·2% (-39·1 to -5·3%)	598·1 (435·9 to 838·0)	88·6% (85·2 to 91·7%)	467·9 (339·6 to 638·7)	87·4% (83·4 to 90·7%)	-21·8% (-42·6 to 2·4%)	-1·4% (-4·8 to 1·6%)
**Childhood wasting: All causes**	109·6 (86·0 to 130·8)	4·3% (3·4 to 5·1%)	63·2 (48·1 to 78·3)	2·1% (1·6 to 2·6%)	-42·3% (-50·6 to -32·6%)	-50·8% (-57·8 to -42·4%)	9,844·5 (7,773·0 to 11,651·4)	6·7% (5·3 to 8·0%)	5,914·8 (4,606·2 to 7,251·0)	3·6% (2·8 to 4·4%)	-39·9% (-48·2 to -30·3%)	-46·3% (-53·3 to -38·0%)

Supplemental Results Table 1. All-age deaths, death PAFs, DALYs, DALY PAFs, and percent change for each risk factor and outcome for both sexes combined in 2005 and 2015 for North Africa and Middle East.

Data in parenthesis are 95% uncertainty intervals. PAFs=population attributable fractions. DALYs=disability-adjusted life-years.

	2005 Deaths (in thousands)	2005 Deaths PAF	2015 Deaths (in thousands)	2015 Deaths PAF	Percent change deaths 2005-2015	Percent change deaths PAF 2005-2015	2005 DALYs (in thousands)	2005 DALYs PAF	2015 DALYs (in thousands)	2015 DALYs PAF	Percent change DALYs 2005-2015	Percent change DALYs PAF 2005-2015
Diarrhoeal diseases	39·1 (29·4 to 49·5)	74·2% (61·4 to 79·8%)	20·6 (14·8 to 27·1)	68·1% (55·7 to 74·8%)	-47·4% (-59·6 to -32·5%)	-8·2% (-14·3 to -2·4%)	3,570·3 (2,716·2 to 4,457·5)	78·2% (64·7 to 83·9%)	1,991·0 (1,444·2 to 2,569·3)	74·5% (61·4 to 80·5%)	-44·2% (-55·9 to -30·0%)	-4·7% (-9·1 to -0·4%)
Lower respiratory infections	60·8 (41·0 to 76·4)	43·3% (30·0 to 52·4%)	38·3 (25·5 to 50·1)	30·4% (21·0 to 37·6%)	-37·0% (-47·5 to -23·8%)	-29·8% (-37·2 to -20·8%)	5,227·5 (3,523·5 to 6,570·3)	60·8% (42·1 to 71·6%)	3,299·8 (2,192·6 to 4,312·1)	53·0% (37·2 to 63·3%)	-36·9% (-47·4 to -23·7%)	-12·8% (-18·5 to -5·9%)
Measles	5·3 (0·8 to 18·0)	40·4% (14·1 to 79·4%)	1·8 (0·2 to 7·9)	38·1% (11·5 to 78·9%)	-65·2% (-92·9 to 65·9%)	-5·6% (-33·5 to 13·6%)	448·5 (68·4 to 1,535·8)	41·1% (14·3 to 80·5%)	156·1 (19·4 to 675·0)	38·7% (11·7 to 80·0%)	-65·2% (-92·9 to 65·9%)	-5·7% (-33·6 to 13·2%)
Protein-energy malnutrition	4·3 (2·9 to 6·9)	63·8% (54·9 to 74·1%)	2·5 (1·5 to 4·0)	48·3% (38·5 to 60·7%)	-43·1% (-65·1 to -6·5%)	-24·2% (-39·1 to -5·3%)	598·1 (435·9 to 838·0)	88·6% (85·2 to 91·7%)	467·9 (339·6 to 638·7)	87·4% (83·4 to 90·7%)	-21·8% (-42·6 to 2·4%)	-1·4% (-4·8 to 1·6%)
**Childhood stunting: All causes**	30·9 (14·3 to 55·7)	1·2% (0·6 to 2·2%)	15·9 (6·3 to 31·0)	0·5% (0·2 to 1·0%)	-48·7% (-61·6 to -35·3%)	-56·2% (-67·7 to -45·1%)	2,695·9 (1,257·5 to 4,819·1)	1·8% (0·9 to 3·2%)	1,400·8 (571·8 to 2,710·8)	0·8% (0·3 to 1·6%)	-48·0% (-60·8 to -35·1%)	-53·5% (-64·9 to -42·5%)
Diarrhoeal diseases	8·6 (3·3 to 15·9)	16·3% (6·4 to 28·5%)	4·0 (1·3 to 7·5)	13·1% (4·7 to 23·4%)	-54·0% (-66·1 to -37·4%)	-19·6% (-29·3 to -9·3%)	778·9 (289·4 to 1,428·5)	17·0% (6·7 to 29·9%)	376·8 (127·1 to 706·4)	14·1% (5·1 to 25·1%)	-51·6% (-63·5 to -35·6%)	-17·3% (-26·3 to -8·1%)
Lower respiratory infections	19·2 (1·6 to 42·6)	13·7% (1·2 to 30·6%)	10·9 (0·9 to 25·8)	8·6% (0·7 to 19·9%)	-43·4% (-54·5 to -15·6%)	-37·0% (-46·6 to -19·1%)	1,649·8 (136·6 to 3,657·0)	19·2% (1·6 to 42·3%)	934·6 (77·3 to 2,223·3)	15·0% (1·2 to 34·5%)	-43·4% (-54·3 to -15·7%)	-21·8% (-31·7 to -6·3%)
Measles	3·1 (0·2 to 10·2)	23·5% (2·7 to 56·1%)	1·1 (0·1 to 5·1)	20·6% (2·2 to 52·0%)	-66·5% (-94·2 to 72·8%)	-12·1% (-38·3 to 13·1%)	267·1 (20·7 to 871·0)	23·9% (2·8 to 56·9%)	89·3 (4·6 to 431·6)	21·0% (2·2 to 52·9%)	-66·6% (-94·2 to 72·8%)	-12·2% (-38·4 to 12·8%)
**Iron deficiency: All causes**	3·3 (1·5 to 5·4)	0·1% (0·1 to 0·2%)	2·9 (1·4 to 4·7)	0·1% (0·0 to 0·2%)	-12·7% (-32·5 to 13·2%)	-25·4% (-41·7 to -4·4%)	3,083·6 (2,091·5 to 4,428·8)	2·1% (1·5 to 2·7%)	3,398·4 (2,312·2 to 4,873·8)	2·0% (1·5 to 2·7%)	10·2% (6·0 to 14·0%)	-1·5% (-6·2 to 3·9%)
Maternal haemorrhage	2·1 (0·8 to 3·9)	30·3% (11·2 to 49·7%)	1·8 (0·7 to 3·4)	28·6% (10·6 to 47·3%)	-14·0% (-38·3 to 19·1%)	-5·7% (-13·5 to 0·8%)	120·6 (44·4 to 218·6)	30·3% (11·2 to 49·6%)	103·4 (38·5 to 189·3)	28·6% (10·6 to 47·0%)	-14·3% (-38·2 to 18·4%)	-5·6% (-13·4 to 0·8%)
Maternal sepsis and other pregnancy related infections	0·5 (0·2 to 0·9)	28·6% (10·5 to 47·4%)	0·5 (0·2 to 0·8)	26·7% (9·8 to 43·9%)	-16·1% (-36·5 to 11·2%)	-6·7% (-14·9 to 0·0%)	31·0 (11·6 to 54·9)	28·6% (10·5 to 47·2%)	26·0 (9·5 to 46·1)	26·8% (9·8 to 44·1%)	-16·3% (-35·4 to 9·6%)	-6·3% (-13·8 to -0·1%)
Iron-deficiency anaemia	0·6 (0·4 to 0·9)	100·0% (100·0 to 100·0%)	0·6 (0·4 to 1·0)	100·0% (100·0 to 100·0%)	-5·1% (-24·5 to 20·9%)	-	2,931·9 (1,956·9 to 4,228·3)	100·0% (100·0 to 100·0%)	3,269·0 (2,194·6 to 4,722·4)	100·0% (100·0 to 100·0%)	11·5% (7·4 to 15·1%)	-
**Vitamin A deficiency: All causes**	6·4 (3·0 to 12·4)	0·2% (0·1 to 0·5%)	2·2 (1·0 to 4·3)	0·1% (0·0 to 0·1%)	-65·2% (-84·7 to -25·9%)	-70·3% (-86·9 to -35·0%)	580·5 (285·9 to 1,089·3)	0·4% (0·2 to 0·7%)	229·4 (122·2 to 407·2)	0·1% (0·1 to 0·2%)	-60·5% (-80·9 to -23·0%)	-64·7% (-82·8 to -30·0%)
Diarrhoeal diseases	3·0 (1·5 to 5·3)	5·7% (2·9 to 9·1%)	1·5 (0·7 to 2·6)	5·0% (2·5 to 8·0%)	-50·5% (-65·0 to -30·5%)	-13·1% (-29·6 to 8·1%)	280·1 (136·6 to 477·1)	6·1% (3·1 to 9·6%)	148·7 (75·0 to 242·8)	5·6% (2·9 to 8·7%)	-46·9% (-61·3 to -27·8%)	-8·9% (-24·4 to 11·0%)
Measles	3·3 (0·6 to 9·4)	24·4% (12·8 to 36·1%)	0·7 (0·1 to 2·8)	15·0% (7·0 to 28·0%)	-78·7% (-96·1 to 35·7%)	-38·4% (-66·4 to 20·8%)	282·0 (48·0 to 804·0)	24·8% (13·0 to 36·7%)	59·8 (9·9 to 233·9)	15·2% (7·1 to 28·3%)	-78·8% (-96·1 to 35·6%)	-38·5% (-66·4 to 20·6%)
Vitamin A deficiency	-	-	-	-	-	-	18·4 (11·2 to 28·5)	100·0% (100·0 to 100·0%)	20·9 (12·9 to 31·8)	100·0% (100·0 to 100·0%)	13·3% (7·6 to 19·9%)	-
**Zinc deficiency: All causes**	4·1 (0·2 to 11·0)	0·2% (0·0 to 0·4%)	2·2 (0·1 to 6·2)	0·1% (0·0 to 0·2%)	-47·7% (-67·0 to -27·1%)	-55·3% (-71·8 to -37·1%)	376·1 (41·3 to 956·4)	0·3% (0·0 to 0·7%)	209·8 (32·1 to 549·4)	0·1% (0·0 to 0·3%)	-44·2% (-60·2 to -17·0%)	-50·1% (-64·4 to -26·5%)
Diarrhoeal diseases	1·6 (0·0 to 4·3)	3·1% (0·0 to 7·7%)	0·7 (0·0 to 1·8)	2·4% (0·0 to 6·1%)	-55·5% (-71·4 to 0·0%)	-22·4% (-44·9 to 8·6%)	164·4 (23·3 to 389·3)	3·6% (0·5 to 8·1%)	87·2 (23·1 to 184·0)	3·3% (0·9 to 6·8%)	-47·0% (-63·6 to 0·0%)	-9·4% (-32·7 to 76·4%)
Lower respiratory infections	2·5 (0·0 to 9·4)	1·8% (0·0 to 6·6%)	1·4 (0·0 to 5·4)	1·1% (0·0 to 4·3%)	-42·4% (-57·7 to 0·0%)	-35·9% (-50·5 to 0·0%)	211·7 (1·0 to 796·7)	2·5% (0·0 to 9·1%)	122·6 (1·0 to 453·3)	2·0% (0·0 to 7·4%)	-42·1% (-57·3 to 6·0%)	-20·0% (-36·5 to 53·6%)
**Tobacco smoke: All causes**	272·5 (248·8 to 295·7)	10·7% (9·9 to 11·5%)	321·7 (292·7 to 351·2)	10·8% (9·9 to 11·7%)	18·1% (10·0 to 26·3%)	0·8% (-3·6 to 5·5%)	8,324·4 (7,513·8 to 9,117·1)	5·6% (5·0 to 6·3%)	9,497·9 (8,615·4 to 10,434·8)	5·7% (5·1 to 6·4%)	14·1% (6·4 to 22·0%)	2·1% (-3·5 to 8·3%)
**Smoking: All causes**	212·6 (191·1 to 232·6)	8·3% (7·6 to 9·0%)	260·1 (233·9 to 288·0)	8·7% (7·9 to 9·5%)	22·3% (13·0 to 32·0%)	4·5% (-1·1 to 10·6%)	6,123·3 (5,448·8 to 6,726·3)	4·1% (3·6 to 4·6%)	7,555·6 (6,751·5 to 8,423·1)	4·6% (4·0 to 5·1%)	23·4% (14·1 to 33·1%)	10·4% (3·4 to 17·9%)
Tuberculosis	1·3 (0·7 to 2·1)	6·5% (3·3 to 9·9%)	1·2 (0·6 to 2·0)	6·3% (3·2 to 9·7%)	-9·6% (-22·4 to 3·3%)	-3·0% (-12·0 to 6·4%)	44·6 (22·3 to 72·1)	5·6% (2·9 to 8·7%)	40·5 (20·2 to 67·6)	5·7% (2·9 to 8·8%)	-9·2% (-22·3 to 4·7%)	1·3% (-8·7 to 12·9%)
Lower respiratory infections	10·3 (7·9 to 13·7)	7·3% (5·7 to 9·4%)	13·1 (10·0 to 17·0)	10·4% (8·2 to 13·1%)	27·3% (16·1 to 40·4%)	41·8% (26·5 to 57·7%)	254·3 (196·4 to 325·9)	3·0% (2·3 to 3·9%)	313·6 (238·2 to 396·5)	5·1% (3·9 to 6·5%)	23·3% (12·8 to 35·3%)	70·5% (45·3 to 96·1%)
Lip and oral cavity cancer	0·8 (0·7 to 1·0)	36·0% (30·1 to 42·3%)	1·1 (0·9 to 1·3)	36·2% (29·7 to 42·7%)	29·8% (14·1 to 46·0%)	0·4% (-6·2 to 7·3%)	23·5 (18·9 to 28·2)	36·1% (30·1 to 42·5%)	30·8 (24·6 to 37·2)	36·7% (30·1 to 43·3%)	31·2% (16·2 to 48·3%)	1·7% (-4·8 to 8·3%)
Nasopharynx cancer	0·7 (0·5 to 0·9)	40·5% (33·9 to 47·1%)	0·8 (0·6 to 1·1)	41·0% (33·7 to 47·8%)	23·8% (8·6 to 40·3%)	1·3% (-5·3 to 8·0%)	22·1 (17·3 to 28·6)	36·2% (29·8 to 42·7%)	27·2 (21·0 to 35·1)	37·5% (30·9 to 44·4%)	23·1% (7·6 to 40·2%)	3·7% (-3·9 to 11·8%)
Oesophageal cancer	2·1 (1·5 to 2·8)	31·0% (23·3 to 39·3%)	2·7 (1·9 to 3·5)	32·9% (23·9 to 41·5%)	27·4% (0·7 to 56·4%)	6·2% (-5·8 to 18·5%)	55·2 (40·0 to 71·9)	32·4% (24·5 to 40·9%)	70·3 (50·0 to 92·3)	34·4% (25·5 to 43·1%)	27·3% (1·7 to 56·1%)	6·3% (-5·0 to 18·3%)
Stomach cancer	2·8 (1·7 to 4·0)	10·7% (6·5 to 15·0%)	3·2 (1·9 to 4·8)	10·4% (6·1 to 15·0%)	15·9% (-2·5 to 36·0%)	-3·2% (-14·6 to 8·2%)	78·3 (46·5 to 112·3)	11·6% (7·1 to 16·3%)	91·1 (51·8 to 133·9)	11·4% (6·7 to 16·3%)	16·4% (-2·8 to 38·7%)	-1·8% (-13·8 to 10·6%)
Colon and rectum cancer	0·9 (0·6 to 1·2)	4·9% (3·3 to 6·5%)	1·3 (0·8 to 1·8)	4·8% (3·2 to 6·6%)	38·8% (18·2 to 61·1%)	-1·6% (-12·2 to 10·4%)	26·0 (17·4 to 36·1)	5·3% (3·6 to 7·2%)	36·5 (23·0 to 52·5)	5·3% (3·5 to 7·3%)	40·2% (19·4 to 64·3%)	0·0% (-11·5 to 13·0%)
Liver cancer due to hepatitis B	0·7 (0·4 to 1·0)	14·3% (7·9 to 21·1%)	1·0 (0·5 to 1·5)	14·8% (8·2 to 22·0%)	39·0% (17·1 to 63·0%)	3·6% (-7·1 to 14·9%)	21·5 (11·8 to 32·4)	14·3% (8·0 to 21·0%)	29·6 (15·9 to 44·1)	15·0% (8·3 to 22·2%)	37·9% (14·9 to 62·8%)	5·1% (-6·7 to 16·8%)
Liver cancer due to hepatitis C	0·8 (0·5 to 1·3)	10·4% (5·7 to 15·6%)	1·1 (0·6 to 1·7)	10·6% (5·8 to 16·1%)	35·9% (19·1 to 53·9%)	2·3% (-8·1 to 13·7%)	21·2 (11·9 to 32·5)	11·5% (6·3 to 17·4%)	28·7 (15·6 to 44·3)	11·8% (6·6 to 18·0%)	35·8% (17·5 to 54·3%)	3·1% (-7·5 to 14·5%)
Liver cancer due to alcohol use	0·4 (0·2 to 0·6)	17·0% (9·0 to 25·6%)	0·5 (0·3 to 0·8)	16·5% (8·8 to 24·8%)	30·9% (12·2 to 52·4%)	-3·0% (-13·1 to 8·0%)	9·9 (5·0 to 15·7)	18·2% (9·7 to 27·0%)	13·0 (6·5 to 21·1)	17·7% (9·5 to 26·3%)	31·8% (12·3 to 52·0%)	-2·7% (-12·9 to 7·8%)
Liver cancer due to other causes	0·3 (0·2 to 0·5)	9·9% (5·6 to 15·0%)	0·4 (0·2 to 0·6)	10·7% (6·0 to 16·3%)	41·2% (18·8 to 64·8%)	7·7% (-4·8 to 20·1%)	8·3 (4·5 to 13·0)	10·4% (5·8 to 15·6%)	11·7 (6·4 to 18·2)	11·4% (6·4 to 17·1%)	40·0% (17·0 to 64·1%)	9·6% (-4·0 to 22·8%)
Pancreatic cancer	2·0 (1·5 to 2·5)	18·7% (14·3 to 22·8%)	2·3 (1·7 to 2·9)	16·6% (12·5 to 20·7%)	14·7% (0·7 to 31·7%)	-11·5% (-19·6 to -2·0%)	52·4 (40·0 to 64·3)	20·5% (15·8 to 24·9%)	61·4 (45·2 to 77·9)	18·4% (13·8 to 22·9%)	17·2% (2·3 to 34·9%)	-10·2% (-18·7 to -1·4%)
Tracheal, bronchus, and lung cancer	30·9 (28·4 to 33·6)	70·7% (67·3 to 73·9%)	39·6 (35·1 to 43·9)	69·4% (65·3 to 72·9%)	28·0% (15·9 to 42·0%)	-1·9% (-4·8 to 1·2%)	771·2 (700·1 to 845·3)	70·4% (66·6 to 74·0%)	994·0 (881·5 to 1,111·1)	69·6% (65·4 to 73·3%)	28·9% (17·0 to 43·2%)	-1·2% (-4·2 to 1·7%)
Cervical cancer	0·3 (0·1 to 0·7)	5·2% (1·4 to 10·8%)	0·4 (0·1 to 0·8)	5·4% (1·5 to 12·0%)	22·4% (-20·8 to 90·0%)	3·9% (-25·6 to 48·2%)	10·9 (2·4 to 25·9)	6·1% (1·6 to 13·0%)	13·3 (2·7 to 33·3)	6·5% (1·7 to 14·4%)	21·9% (-21·1 to 94·2%)	5·3% (-24·9 to 51·0%)
Kidney cancer	0·5 (0·3 to 0·7)	14·6% (9·4 to 19·6%)	0·7 (0·4 to 1·0)	14·3% (8·9 to 19·5%)	32·3% (11·9 to 55·6%)	-1·9% (-11·6 to 9·8%)	14·5 (9·5 to 20·0)	12·9% (8·4 to 17·4%)	19·4 (12·3 to 27·1)	13·2% (8·6 to 18·0%)	33·5% (13·3 to 56·3%)	2·4% (-8·8 to 16·3%)
Bladder cancer	2·1 (1·5 to 2·8)	23·3% (16·9 to 30·0%)	2·5 (1·7 to 3·3)	22·7% (16·1 to 29·3%)	16·5% (1·4 to 34·3%)	-2·7% (-11·5 to 6·7%)	50·2 (36·2 to 64·4)	24·9% (17·9 to 31·7%)	59·3 (41·1 to 77·1)	24·6% (17·6 to 31·6%)	18·1% (3·7 to 34·8%)	-0·9% (-8·8 to 8·1%)
Ischaemic heart disease	73·9 (59·6 to 89·2)	15·0% (12·1 to 17·9%)	90·1 (72·1 to 109·0)	15·0% (12·2 to 18·0%)	21·9% (11·9 to 31·9%)	0·3% (-5·1 to 6·2%)	2,153·8 (1,741·5 to 2,587·5)	19·8% (16·1 to 23·6%)	2,636·8 (2,096·0 to 3,210·8)	20·0% (16·3 to 23·9%)	22·4% (11·7 to 32·8%)	1·2% (-4·0 to 6·3%)
Ischaemic stroke	12·1 (9·8 to 14·6)	9·9% (8·1 to 11·7%)	14·4 (11·6 to 17·5)	9·6% (7·9 to 11·5%)	19·2% (8·1 to 30·5%)	-2·3% (-9·3 to 5·4%)	305·2 (246·6 to 367·7)	13·4% (11·1 to 15·7%)	364·0 (297·3 to 438·3)	13·6% (11·2 to 16·0%)	19·3% (7·6 to 30·2%)	1·0% (-5·8 to 7·7%)
Hemorrhagic stroke	17·6 (14·4 to 21·2)	13·4% (11·1 to 15·8%)	21·3 (17·1 to 26·0)	13·8% (11·4 to 16·4%)	20·6% (9·4 to 32·7%)	3·3% (-3·8 to 10·1%)	555·4 (455·4 to 670·3)	14·7% (12·1 to 17·3%)	671·7 (541·1 to 828·5)	16·0% (13·3 to 18·8%)	20·9% (8·9 to 33·4%)	9·1% (1·6 to 16·9%)
Hypertensive heart disease	4·0 (3·0 to 5·0)	8·9% (6·6 to 11·2%)	5·0 (3·8 to 6·3)	9·1% (6·9 to 11·5%)	25·1% (10·5 to 42·5%)	1·9% (-8·5 to 13·0%)	104·9 (79·3 to 131·5)	12·1% (9·0 to 15·1%)	132·9 (100·9 to 167·3)	12·7% (9·5 to 16·0%)	26·7% (11·3 to 43·5%)	4·8% (-4·7 to 15·4%)
Atrial fibrillation and flutter	0·4 (0·3 to 0·5)	6·2% (4·6 to 7·8%)	0·5 (0·3 to 0·6)	5·8% (4·3 to 7·3%)	26·3% (13·1 to 42·2%)	-6·1% (-15·7 to 4·3%)	14·2 (10·0 to 19·1)	12·2% (9·3 to 15·1%)	18·4 (12·9 to 24·9)	11·9% (9·0 to 14·9%)	30·0% (22·8 to 37·6%)	-1·8% (-6·8 to 3·1%)
Aortic aneurysm	0·9 (0·7 to 1·1)	16·6% (12·9 to 20·2%)	1·1 (0·9 to 1·4)	16·3% (12·6 to 19·9%)	27·4% (10·9 to 44·7%)	-1·8% (-8·9 to 5·5%)	25·5 (19·7 to 32·0)	21·6% (16·9 to 26·1%)	32·7 (25·4 to 39·9)	21·4% (16·7 to 26·0%)	27·9% (10·2 to 46·8%)	-0·8% (-7·7 to 6·2%)

Supplemental Results Table 1. All-age deaths, death PAFs, DALYs, DALY PAFs, and percent change for each risk factor and outcome for both sexes combined in 2005 and 2015 for North Africa and Middle East.

Data in parenthesis are 95% uncertainty intervals. PAFs=population attributable fractions. DALYs=disability-adjusted life-years.

	2005 Deaths (in thousands)	2005 Deaths PAF	2015 Deaths (in thousands)	2015 Deaths PAF	Percent change deaths 2005-2015	Percent change deaths PAF 2005-2015	2005 DALYs (in thousands)	2005 DALYs PAF	2015 DALYs (in thousands)	2015 DALYs PAF	Percent change DALYs 2005-2015	Percent change DALYs PAF 2005-2015
Peripheral vascular disease	0·1 (0·1 to 0·1)	15·4% (11·9 to 19·2%)	0·1 (0·1 to 0·1)	13·1% (10·0 to 16·2%)	26·7% (4·0 to 61·4%)	-14·7% (-26·8 to -1·1%)	4·0 (2·5 to 6·1)	14·9% (11·3 to 18·6%)	5·2 (3·3 to 8·0)	14·0% (10·8 to 17·4%)	29·3% (15·7 to 44·2%)	-6·1% (-13·2 to 1·3%)
Other cardiovascular and circulatory diseases	5·4 (4·1 to 6·7)	11·0% (8·3 to 13·5%)	7·1 (5·5 to 8·8)	12·0% (9·3 to 14·9%)	31·5% (17·9 to 47·0%)	9·9% (1·4 to 19·6%)	235·6 (179·8 to 296·4)	10·4% (7·9 to 12·7%)	310·3 (234·6 to 387·8)	12·1% (9·4 to 14·9%)	31·7% (20·8 to 43·2%)	16·7% (9·1 to 25·7%)
Chronic obstructive pulmonary disease	28·5 (24·5 to 32·9)	43·5% (37·7 to 49·4%)	33·0 (27·5 to 38·7)	44·0% (37·2 to 50·7%)	15·5% (2·1 to 29·9%)	1·2% (-6·8 to 8·7%)	680·6 (587·7 to 778·8)	40·1% (34·6 to 45·5%)	789·0 (661·0 to 916·2)	41·9% (35·7 to 47·9%)	15·9% (3·8 to 28·8%)	4·5% (-2·6 to 11·5%)
Silicosis	0·0 (0·0 to 0·0)	19·1% (12·3 to 27·0%)	0·0 (0·0 to 0·1)	20·1% (12·5 to 28·9%)	13·0% (-33·9 to 94·5%)	5·0% (-23·5 to 47·3%)	1·0 (0·6 to 1·5)	17·4% (10·7 to 24·7%)	1·1 (0·6 to 1·8)	18·1% (11·8 to 25·4%)	7·7% (-29·7 to 68·2%)	3·8% (-22·3 to 48·1%)
Asbestosis	0·0 (0·0 to 0·0)	12·6% (8·6 to 17·0%)	0·0 (0·0 to 0·0)	11·5% (8·3 to 15·8%)	20·8% (-22·9 to 89·7%)	-8·1% (-30·2 to 20·9%)	0·2 (0·1 to 0·2)	8·4% (5·6 to 11·4%)	0·2 (0·1 to 0·3)	8·6% (6·0 to 11·8%)	28·4% (-4·9 to 66·4%)	2·8% (-18·5 to 28·9%)
Coal workers pneumoconiosis	0·0 (0·0 to 0·0)	23·3% (16·9 to 30·7%)	0·0 (0·0 to 0·0)	19·8% (14·5 to 26·3%)	-16·5% (-59·8 to 76·3%)	-14·7% (-30·4 to 6·2%)	0·1 (0·1 to 0·2)	19·6% (14·9 to 25·4%)	0·1 (0·1 to 0·2)	17·2% (13·1 to 22·2%)	4·5% (-28·6 to 48·1%)	-12·4% (-26·1 to 2·1%)
Other pneumoconiosis	0·1 (0·1 to 0·2)	10·1% (7·3 to 13·7%)	0·1 (0·1 to 0·2)	9·5% (6·7 to 13·3%)	18·8% (-5·1 to 45·8%)	-6·4% (-23·9 to 9·2%)	4·3 (2·9 to 6·0)	9·6% (7·0 to 12·5%)	5·3 (3·5 to 7·4)	9·4% (6·8 to 12·6%)	20·7% (2·5 to 41·3%)	-1·8% (-14·8 to 11·6%)
Asthma	2·4 (1·8 to 3·1)	10·2% (7·9 to 12·6%)	2·6 (1·9 to 3·3)	10·4% (8·0 to 13·0%)	5·7% (-7·2 to 20·7%)	2·5% (-6·6 to 13·3%)	116·2 (85·4 to 148·8)	6·2% (4·8 to 7·7%)	139·7 (100·5 to 183·0)	6·6% (5·1 to 8·2%)	20·2% (9·7 to 31·0%)	7·0% (-0·1 to 14·5%)
Interstitial lung disease and pulmonary sarcoidosis	0·2 (0·2 to 0·3)	10·8% (8·2 to 13·9%)	0·3 (0·2 to 0·5)	10·3% (7·7 to 13·2%)	43·5% (13·2 to 75·6%)	-4·9% (-17·0 to 8·5%)	6·6 (4·8 to 9·4)	10·9% (8·3 to 14·0%)	9·2 (6·6 to 12·5)	10·6% (7·9 to 13·5%)	39·1% (11·7 to 67·2%)	-3·3% (-14·7 to 9·9%)
Other chronic respiratory diseases	0·5 (0·2 to 0·7)	9·0% (6·4 to 11·9%)	0·6 (0·4 to 0·9)	10·2% (7·3 to 13·6%)	33·3% (9·7 to 63·8%)	12·8% (-2·0 to 29·6%)	19·5 (12·2 to 26·9)	7·6% (5·6 to 9·9%)	22·6 (13·7 to 32·0)	8·7% (6·3 to 11·6%)	16·1% (-2·7 to 37·0%)	14·9% (-1·1 to 33·4%)
Diabetes mellitus	3·4 (0·9 to 6·2)	4·2% (1·1 to 7·5%)	4·8 (1·3 to 8·7)	4·3% (1·2 to 7·6%)	39·6% (26·5 to 53·8%)	1·7% (-6·4 to 10·4%)	209·5 (57·4 to 375·1)	4·9% (1·4 to 8·7%)	307·6 (84·4 to 562·4)	5·0% (1·4 to 9·0%)	46·8% (37·1 to 55·7%)	2·7% (-3·0 to 8·1%)
**Second-hand smoke: All causes**	66·9 (53·5 to 80·9)	2·6% (2·1 to 3·2%)	69·7 (55·7 to 85·2)	2·3% (1·9 to 2·8%)	4·2% (-1·9 to 11·3%)	-11·0% (-15·5 to -6·7%)	2,401·1 (1,868·5 to 2,956·3)	1·6% (1·2 to 2·0%)	2,173·9 (1,740·2 to 2,651·9)	1·3% (1·0 to 1·6%)	-9·5% (-16·5 to -0·9%)	-19·0% (-24·8 to -12·4%)
Lower respiratory infections	16·5 (11·3 to 22·8)	11·8% (8·3 to 15·8%)	12·4 (8·5 to 17·0)	9·9% (6·8 to 13·4%)	-24·7% (-33·2 to -15·1%)	-16·0% (-22·0 to -10·3%)	1,151·0 (778·5 to 1,579·4)	13·4% (9·5 to 18·1%)	743·2 (499·1 to 1,023·3)	12·0% (8·3 to 16·1%)	-35·4% (-44·0 to -24·7%)	-10·7% (-18·0 to -3·5%)
Otitis media	0·0 (0·0 to 0·0)	7·5% (4·9 to 9·9%)	0·0 (0·0 to 0·0)	6·0% (4·0 to 8·1%)	-32·6% (-48·7 to -12·0%)	-20·1% (-30·3 to -7·9%)	19·8 (10·8 to 31·8)	7·7% (5·2 to 10·3%)	20·6 (11·2 to 33·5)	7·3% (4·9 to 9·8%)	4·5% (0·1 to 8·2%)	-5·3% (-8·1 to -2·7%)
Tracheal, bronchus, and lung cancer	0·9 (0·6 to 1·4)	2·1% (1·3 to 3·2%)	1·2 (0·7 to 1·8)	2·0% (1·2 to 3·1%)	24·1% (20·4 to 27·6%)	-4·8% (-11·5 to 2·3%)	23·6 (14·8 to 35·7)	2·2% (1·3 to 3·3%)	29·5 (18·0 to 44·4)	2·1% (1·3 to 3·1%)	24·6% (20·5 to 28·8%)	-4·4% (-11·2 to 2·7%)
Ischaemic heart disease	35·5 (27·6 to 44·2)	7·2% (5·6 to 8·9%)	40·9 (31·6 to 51·3)	6·8% (5·3 to 8·5%)	15·2% (8·2 to 23·4%)	-5·2% (-8·7 to -1·7%)	855·6 (679·4 to 1,041·1)	7·8% (6·3 to 9·5%)	997·3 (790·5 to 1,224·8)	7·6% (6·1 to 9·2%)	16·6% (8·1 to 25·6%)	-3·6% (-7·7 to 0·5%)
Ischaemic stroke	4·8 (3·7 to 6·1)	3·9% (3·0 to 5·0%)	5·4 (4·1 to 6·9)	3·6% (2·8 to 4·6%)	12·0% (2·7 to 20·8%)	-8·1% (-12·2 to -4·0%)	101·8 (81·1 to 127·3)	4·5% (3·6 to 5·5%)	112·6 (88·8 to 142·5)	4·2% (3·3 to 5·2%)	10·6% (1·0 to 20·1%)	-6·3% (-10·6 to -1·4%)
Hemorrhagic stroke	5·8 (4·6 to 7·2)	4·4% (3·5 to 5·4%)	6·3 (4·9 to 8·0)	4·1% (3·3 to 5·1%)	10·1% (1·4 to 19·7%)	-5·7% (-10·3 to -1·2%)	168·4 (134·1 to 211·6)	4·4% (3·6 to 5·4%)	185·5 (145·3 to 236·4)	4·4% (3·5 to 5·5%)	10·2% (0·8 to 19·9%)	-0·6% (-6·3 to 5·1%)
**Alcohol and drug use: All causes**	54·5 (48·1 to 60·4)	2·1% (1·9 to 2·3%)	69·3 (60·3 to 78·1)	2·3% (2·0 to 2·6%)	27·2% (17·5 to 36·3%)	8·6% (2·4 to 14·7%)	2,987·7 (2,590·2 to 3,351·8)	2·0% (1·8 to 2·2%)	3,794·2 (3,277·1 to 4,304·7)	2·3% (2·0 to 2·6%)	27·0% (19·7 to 34·0%)	13·6% (7·9 to 19·7%)
**Alcohol use: All causes**	38·5 (34·1 to 43·3)	1·5% (1·3 to 1·7%)	46·0 (39·5 to 52·3)	1·5% (1·3 to 1·7%)	19·4% (9·6 to 28·5%)	1·9% (-4·6 to 8·7%)	1,462·3 (1,289·0 to 1,642·6)	1·0% (0·9 to 1·1%)	1,685·8 (1,459·1 to 1,909·4)	1·0% (0·9 to 1·2%)	15·3% (6·9 to 23·1%)	3·1% (-3·5 to 9·8%)
Tuberculosis	0·6 (0·5 to 0·9)	3·1% (2·6 to 3·8%)	0·6 (0·4 to 0·8)	3·1% (2·4 to 3·8%)	-8·5% (-21·4 to 4·2%)	-1·7% (-13·0 to 8·2%)	23·8 (18·1 to 29·9)	3·0% (2·5 to 3·6%)	21·7 (15·3 to 28·8)	3·1% (2·4 to 3·7%)	-8·8% (-22·1 to 4·1%)	1·7% (-10·3 to 12·6%)
Lower respiratory infections	1·1 (0·9 to 1·4)	0·8% (0·7 to 1·0%)	1·4 (1·2 to 1·7)	1·1% (0·9 to 1·4%)	27·4% (14·2 to 40·3%)	41·9% (25·8 to 58·5%)	26·9 (22·6 to 32·1)	0·3% (0·3 to 0·4%)	32·7 (26·3 to 38·5)	0·5% (0·4 to 0·6%)	21·5% (9·6 to 33·1%)	67·9% (43·3 to 95·4%)
Lip and oral cavity cancer	0·2 (0·1 to 0·2)	7·0% (5·6 to 8·4%)	0·2 (0·1 to 0·2)	6·5% (4·8 to 8·1%)	19·7% (4·5 to 33·9%)	-7·5% (-17·1 to 0·4%)	4·9 (4·0 to 5·8)	7·6% (6·3 to 8·9%)	6·0 (4·5 to 7·4)	7·2% (5·4 to 8·7%)	21·6% (5·8 to 35·3%)	-5·8% (-15·5 to 1·6%)
Nasopharynx cancer	0·1 (0·1 to 0·2)	8·5% (7·1 to 10·0%)	0·2 (0·1 to 0·2)	7·8% (5·8 to 9·7%)	12·0% (-5·3 to 28·8%)	-8·4% (-19·9 to 1·0%)	5·3 (4·3 to 6·5)	8·7% (7·4 to 10·1%)	5·9 (4·3 to 7·5)	8·1% (6·1 to 9·9%)	10·1% (-7·0 to 27·0%)	-7·3% (-18·9 to 1·8%)
Other pharynx cancer	0·1 (0·1 to 0·1)	6·2% (5·0 to 7·4%)	0·1 (0·1 to 0·1)	5·9% (4·4 to 7·3%)	19·4% (5·0 to 32·0%)	-4·4% (-13·8 to 3·0%)	2·2 (1·8 to 2·7)	6·5% (5·3 to 7·7%)	2·7 (2·0 to 3·3)	6·3% (4·9 to 7·7%)	20·7% (5·9 to 33·4%)	-3·0% (-12·4 to 4·6%)
Oesophageal cancer	0·3 (0·2 to 0·3)	4·0% (3·1 to 5·0%)	0·3 (0·2 to 0·4)	3·8% (2·8 to 4·9%)	14·7% (-0·2 to 31·3%)	-4·4% (-11·5 to 2·6%)	7·4 (5·7 to 9·2)	4·4% (3·5 to 5·3%)	8·6 (6·3 to 10·8)	4·2% (3·2 to 5·2%)	15·4% (-0·4 to 32·2%)	-3·6% (-11·2 to 3·3%)
Colon and rectum cancer	0·4 (0·3 to 0·4)	2·0% (1·7 to 2·2%)	0·5 (0·4 to 0·6)	1·9% (1·6 to 2·2%)	36·2% (26·6 to 47·1%)	-3·4% (-7·1 to 0·5%)	9·6 (8·2 to 11·1)	2·0% (1·7 to 2·2%)	13·1 (11·0 to 15·4)	1·9% (1·7 to 2·2%)	36·0% (26·5 to 46·5%)	-2·9% (-6·7 to 0·7%)
Liver cancer due to hepatitis B	0·0 (0·0 to 0·0)	0·2% (0·1 to 0·3%)	0·0 (0·0 to 0·0)	0·2% (0·1 to 0·3%)	10·9% (-13·4 to 35·3%)	-17·4% (-34·7 to 0·2%)	0·5 (0·3 to 0·8)	0·3% (0·2 to 0·5%)	0·5 (0·3 to 0·9)	0·3% (0·1 to 0·4%)	10·1% (-12·7 to 32·1%)	-16·1% (-32·6 to -1·2%)
Liver cancer due to hepatitis C	0·0 (0·0 to 0·0)	0·0% (0·0 to 0·1%)	0·0 (0·0 to 0·0)	0·0% (0·0 to 0·1%)	14·1% (-29·0 to 77·9%)	-14·1% (-46·9 to 33·3%)	0·1 (0·1 to 0·2)	0·1% (0·0 to 0·1%)	0·1 (0·0 to 0·3)	0·1% (0·0 to 0·1%)	15·4% (-18·9 to 62·6%)	-12·3% (-38·7 to 24·7%)
Liver cancer due to alcohol use	2·3 (1·7 to 3·0)	100·0% (100·0 to 100·0%)	3·1 (2·3 to 4·1)	100·0% (100·0 to 100·0%)	34·9% (23·4 to 47·5%)	-	54·6 (40·2 to 74·5)	100·0% (100·0 to 100·0%)	73·9 (52·6 to 102·0)	100·0% (100·0 to 100·0%)	35·3% (23·0 to 48·6%)	-
Liver cancer due to other causes	0·0 (0·0 to 0·0)	0·1% (0·1 to 0·2%)	0·0 (0·0 to 0·0)	0·1% (0·1 to 0·2%)	9·8% (-16·8 to 40·3%)	-16·3% (-36·4 to 4·7%)	0·2 (0·1 to 0·3)	0·2% (0·1 to 0·4%)	0·2 (0·1 to 0·4)	0·2% (0·1 to 0·3%)	9·8% (-13·2 to 35·2%)	-14·1% (-30·7 to 3·4%)
Larynx cancer	0·2 (0·2 to 0·3)	5·2% (4·1 to 6·4%)	0·2 (0·2 to 0·3)	4·6% (3·3 to 5·9%)	9·3% (-6·1 to 26·0%)	-12·3% (-23·8 to -2·9%)	6·3 (5·1 to 7·6)	5·7% (4·6 to 6·8%)	7·0 (5·0 to 8·8)	5·0% (3·7 to 6·3%)	10·0% (-5·8 to 26·1%)	-11·4% (-22·7 to -2·6%)
Breast cancer	0·6 (0·4 to 0·7)	2·9% (2·5 to 3·3%)	0·7 (0·6 to 0·9)	2·7% (2·3 to 3·2%)	29·7% (16·7 to 43·8%)	-4·5% (-10·3 to 0·7%)	19·4 (15·3 to 25·2)	2·9% (2·5 to 3·3%)	25·4 (20·2 to 32·6)	2·8% (2·3 to 3·2%)	31·1% (18·2 to 44·8%)	-4·4% (-10·1 to 1·1%)
Ischaemic heart disease	4·5 (3·8 to 5·4)	0·9% (0·8 to 1·1%)	5·4 (4·5 to 6·3)	0·9% (0·8 to 1·0%)	19·2% (10·6 to 28·9%)	-1·9% (-7·3 to 4·3%)	90·4 (75·2 to 105·8)	0·8% (0·7 to 1·0%)	106·5 (89·3 to 124·7)	0·8% (0·7 to 0·9%)	17·8% (7·9 to 27·7%)	-2·6% (-8·7 to 4·2%)
Ischaemic stroke	0·8 (0·4 to 1·2)	0·6% (0·3 to 1·0%)	0·9 (0·4 to 1·5)	0·6% (0·3 to 1·0%)	18·3% (-10·1 to 43·2%)	-3·0% (-26·5 to 16·5%)	16·0 (10·0 to 22·2)	0·7% (0·4 to 1·0%)	18·7 (11·5 to 26·1)	0·7% (0·4 to 1·0%)	17·0% (1·1 to 34·7%)	-0·9% (-13·4 to 13·2%)
Hemorrhagic stroke	3·1 (2·4 to 3·7)	2·3% (1·9 to 2·8%)	3·6 (2·8 to 4·6)	2·4% (1·8 to 2·9%)	18·7% (1·0 to 39·6%)	1·7% (-12·5 to 17·3%)	83·1 (68·2 to 98·3)	2·2% (1·8 to 2·5%)	97·9 (78·6 to 119·4)	2·3% (1·9 to 2·8%)	17·8% (2·4 to 35·1%)	6·3% (-6·1 to 20·0%)
Hypertensive heart disease	0·4 (0·1 to 0·9)	0·9% (0·1 to 1·9%)	0·4 (0·0 to 0·9)	0·7% (-0·1 to 1·7%)	-4·4% (-130·3 to 48·6%)	-22·1% (-125·0 to 19·3%)	10·8 (4·2 to 18·8)	1·2% (0·5 to 2·2%)	11·8 (3·1 to 22·3)	1·1% (0·3 to 2·1%)	9·1% (-35·7 to 38·7%)	-9·8% (-46·3 to 11·3%)
Atrial fibrillation and flutter	0·1 (0·1 to 0·2)	2·2% (1·7 to 2·7%)	0·2 (0·1 to 0·2)	2·1% (1·6 to 2·6%)	29·8% (16·5 to 44·8%)	-3·6% (-12·0 to 5·2%)	2·7 (2·1 to 3·6)	2·3% (1·9 to 2·7%)	3·5 (2·6 to 4·6)	2·3% (1·8 to 2·7%)	27·4% (19·2 to 36·0%)	-3·8% (-9·6 to 2·0%)
Cirrhosis and other chronic liver diseases due to hepatitis B	2·4 (1·8 to 3·0)	12·4% (10·0 to 14·9%)	2·9 (2·1 to 3·8)	11·7% (8·9 to 14·5%)	22·1% (1·1 to 42·4%)	-5·5% (-19·9 to 8·9%)	64·0 (49·6 to 81·5)	12·0% (9·7 to 14·3%)	77·3 (55·8 to 100·5)	11·4% (8·6 to 14·0%)	20·8% (0·0 to 39·8%)	-4·8% (-19·5 to 9·1%)
Cirrhosis and other chronic liver diseases due to hepatitis C	3·5 (2·5 to 4·6)	10·1% (7·7 to 13·0%)	4·3 (2·9 to 5·7)	9·7% (7·0 to 12·7%)	23·6% (-8·2 to 65·8%)	-3·8% (-28·2 to 29·1%)	87·7 (63·5 to 116·8)	9·4% (7·1 to 12·1%)	107·4 (75·7 to 143·3)	9·1% (6·6 to 11·8%)	22·5% (-7·8 to 64·3%)	-2·9% (-26·1 to 29·6%)
Cirrhosis and other chronic liver diseases due to alcohol use	3·8 (3·4 to 4·3)	100·0% (100·0 to 100·0%)	4·9 (4·3 to 5·6)	100·0% (100·0 to 100·0%)	27·8% (21·2 to 34·8%)	-	119·0 (104·1 to 135·0)	100·0% (100·0 to 100·0%)	149·8 (130·2 to 171·1)	100·0% (100·0 to 100·0%)	25·9% (19·2 to 33·3%)	-
Cirrhosis and other chronic liver diseases due to other causes	2·5 (2·0 to 3·1)	13·4% (10·9 to 16·2%)	3·1 (2·3 to 3·8)	13·5% (10·4 to 16·4%)	22·2% (-0·7 to 48·6%)	0·3% (-17·6 to 22·0%)	67·2 (53·9 to 81·2)	10·6% (8·9 to 12·5%)	79·3 (61·2 to 98·6)	11·3% (8·9 to 13·5%)	18·0% (-2·1 to 39·8%)	5·9% (-11·4 to 24·1%)
Pancreatitis	0·1 (0·1 to 0·1)	2·8% (2·4 to 3·2%)	0·1 (0·1 to 0·1)	2·7% (2·1 to 3·2%)	27·9% (13·6 to 42·7%)	-5·0% (-13·0 to 2·8%)	2·6 (2·0 to 3·3)	2·9% (2·5 to 3·3%)	3·2 (2·4 to 4·2)	2·8% (2·3 to 3·4%)	26·7% (11·0 to 40·9%)	-2·5% (-11·6 to 6·0%)

Supplemental Results Table 1. All-age deaths, death PAFs, DALYs, DALY PAFs, and percent change for each risk factor and outcome for both sexes combined in 2005 and 2015 for North Africa and Middle East.

Data in parenthesis are 95% uncertainty intervals. PAFs=population attributable fractions. DALYs=disability-adjusted life-years.

	2005 Deaths (in thousands)	2005 Deaths PAF	2015 Deaths (in thousands)	2015 Deaths PAF	Percent change deaths 2005-2015	Percent change deaths PAF 2005-2015	2005 DALYs (in thousands)	2005 DALYs PAF	2015 DALYs (in thousands)	2015 DALYs PAF	Percent change DALYs 2005-2015	Percent change DALYs PAF 2005-2015
Epilepsy	0·2 (0·2 to 0·3)	2·9% (2·5 to 3·3%)	0·3 (0·2 to 0·3)	3·0% (2·5 to 3·5%)	15·1% (2·8 to 27·8%)	4·5% (-5·6 to 14·8%)	30·2 (23·3 to 37·8)	2·4% (2·1 to 2·7%)	30·5 (22·9 to 39·0)	2·4% (2·0 to 2·8%)	1·2% (-9·6 to 11·9%)	1·1% (-7·8 to 9·7%)
Alcohol use disorders	1·5 (1·3 to 1·6)	100·0% (100·0 to 100·0%)	1·8 (1·6 to 2·0)	100·0% (100·0 to 100·0%)	20·6% (9·0 to 33·5%)	-	172·2 (132·6 to 222·4)	100·0% (100·0 to 100·0%)	214·3 (162·7 to 279·0)	100·0% (100·0 to 100·0%)	24·5% (17·1 to 31·7%)	-
Diabetes mellitus	-0·9 (-1·1 to -0·7)	-1·1% (-1·4 to -0·9%)	-1·3 (-1·5 to -1·0)	-1·1% (-1·4 to -0·9%)	35·4% (13·3 to 62·1%)	-1·2% (-16·8 to 17·7%)	-48·8 (-63·6 to -37·5)	-1·1% (-1·3 to -1·0%)	-69·4 (-90·9 to -51·5)	-1·1% (-1·3 to -1·0%)	42·3% (28·4 to 57·8%)	-0·4% (-9·4 to 10·2%)
Pedestrian road injuries	1·4 (1·1 to 1·9)	4·0% (3·1 to 5·0%)	1·6 (1·2 to 2·1)	4·1% (3·2 to 5·2%)	10·1% (-3·6 to 26·2%)	4·2% (-3·0 to 11·6%)	79·0 (58·0 to 102·4)	4·1% (3·2 to 5·2%)	82·4 (62·3 to 105·5)	4·4% (3·4 to 5·5%)	4·3% (-8·3 to 19·7%)	6·3% (-1·9 to 15·2%)
Cyclist road injuries	0·1 (0·1 to 0·2)	4·9% (3·8 to 6·0%)	0·1 (0·1 to 0·2)	5·2% (4·0 to 6·4%)	12·8% (-6·2 to 37·6%)	6·1% (-3·8 to 18·3%)	6·8 (4·8 to 8·9)	4·5% (3·6 to 5·7%)	7·3 (5·3 to 9·7)	4·8% (3·8 to 6·0%)	7·4% (-9·6 to 29·6%)	5·3% (-5·7 to 19·0%)
Motorcyclist road injuries	1·1 (0·8 to 1·6)	10·5% (8·5 to 12·8%)	1·2 (0·8 to 1·6)	11·0% (8·9 to 13·3%)	1·5% (-19·1 to 26·9%)	4·5% (-2·2 to 12·6%)	67·8 (49·2 to 92·0)	10·2% (8·2 to 12·5%)	67·1 (47·1 to 93·3)	10·7% (8·6 to 13·0%)	-1·0% (-20·3 to 22·4%)	4·8% (-2·6 to 13·8%)
Motor vehicle road injuries	5·8 (4·6 to 7·2)	8·2% (6·7 to 9·9%)	6·8 (5·3 to 8·4)	8·6% (7·1 to 10·4%)	17·3% (3·5 to 32·2%)	5·1% (-1·0 to 11·8%)	310·3 (246·1 to 391·1)	7·9% (6·4 to 9·6%)	352·0 (277·3 to 437·6)	8·4% (6·9 to 10·2%)	13·5% (0·6 to 26·9%)	6·5% (-0·2 to 14·0%)
Falls	0·3 (0·2 to 0·4)	1·6% (1·2 to 2·1%)	0·4 (0·3 to 0·5)	1·7% (1·3 to 2·3%)	39·3% (17·8 to 58·0%)	8·8% (-7·2 to 29·9%)	24·0 (18·0 to 31·3)	1·6% (1·2 to 2·0%)	29·4 (22·2 to 38·6)	1·7% (1·3 to 2·1%)	22·6% (13·1 to 31·9%)	6·2% (-9·1 to 27·7%)
Drowning	0·2 (0·2 to 0·3)	1·1% (0·8 to 1·4%)	0·2 (0·2 to 0·3)	1·2% (0·9 to 1·6%)	4·7% (-8·8 to 20·8%)	18·1% (-1·5 to 40·1%)	11·0 (8·2 to 14·5)	0·8% (0·6 to 1·1%)	11·1 (8·2 to 14·5)	1·0% (0·7 to 1·3%)	0·9% (-12·3 to 16·6%)	19·2% (-4·0 to 46·2%)
Fire, heat, and hot substances	0·2 (0·1 to 0·2)	1·4% (1·1 to 1·8%)	0·2 (0·1 to 0·2)	1·6% (1·3 to 2·1%)	7·7% (-7·0 to 24·2%)	12·3% (1·1 to 24·9%)	10·9 (8·3 to 14·5)	1·3% (1·1 to 1·7%)	11·1 (8·6 to 14·6)	1·5% (1·2 to 1·9%)	1·4% (-9·0 to 13·1%)	12·5% (2·9 to 23·0%)
Poisonings	0·1 (0·1 to 0·1)	1·5% (1·1 to 2·0%)	0·1 (0·1 to 0·1)	1·6% (1·1 to 2·1%)	-3·4% (-21·2 to 19·3%)	4·9% (-18·9 to 37·8%)	6·0 (4·4 to 8·3)	1·3% (0·9 to 1·7%)	5·7 (4·2 to 7·8)	1·4% (0·9 to 1·9%)	-3·8% (-18·8 to 13·9%)	6·6% (-20·4 to 48·4%)
Unintentional firearm injuries	0·2 (0·1 to 0·3)	2·8% (2·0 to 3·6%)	0·1 (0·1 to 0·2)	2·2% (1·8 to 2·9%)	-27·7% (-41·3 to -8·0%)	-21·5% (-31·0 to -4·0%)	10·5 (4·9 to 13·8)	2·7% (1·9 to 3·5%)	7·3 (4·1 to 9·9)	2·2% (1·7 to 2·8%)	-30·1% (-43·3 to -10·5%)	-20·7% (-30·3 to -2·5%)
Unintentional suffocation	0·0 (0·0 to 0·0)	0·3% (0·2 to 0·4%)	0·0 (0·0 to 0·0)	0·3% (0·2 to 0·5%)	1·1% (-16·1 to 20·1%)	16·6% (-19·3 to 72·4%)	0·7 (0·5 to 0·9)	0·4% (0·2 to 0·6%)	0·7 (0·5 to 1·0)	0·5% (0·3 to 0·7%)	7·5% (-1·1 to 15·0%)	19·2% (-11·6 to 66·7%)
Other exposure to mechanical forces	0·2 (0·1 to 0·2)	1·8% (1·4 to 2·4%)	0·2 (0·1 to 0·3)	2·1% (1·7 to 2·7%)	16·4% (-1·7 to 35·4%)	14·4% (4·4 to 26·0%)	15·9 (11·9 to 21·1)	1·9% (1·5 to 2·3%)	17·4 (12·8 to 23·5)	2·1% (1·7 to 2·6%)	9·8% (-0·7 to 21·1%)	9·8% (3·2 to 17·5%)
Self-harm	0·7 (0·5 to 0·8)	2·8% (2·3 to 3·4%)	0·7 (0·5 to 0·9)	2·5% (2·0 to 3·1%)	1·9% (-10·2 to 14·7%)	-9·1% (-14·8 to -3·1%)	33·8 (26·8 to 42·8)	2·8% (2·2 to 3·4%)	33·2 (26·0 to 42·7)	2·5% (2·0 to 3·1%)	-2·0% (-14·1 to 11·3%)	-8·7% (-14·8 to -2·3%)
Assault by firearm	0·2 (0·2 to 0·3)	2·5% (1·9 to 3·2%)	0·3 (0·2 to 0·4)	2·3% (1·8 to 3·0%)	19·6% (-0·9 to 39·2%)	-8·1% (-15·8 to 0·9%)	14·0 (9·2 to 18·9)	2·6% (2·0 to 3·4%)	16·4 (9·7 to 23·0)	2·4% (1·9 to 3·1%)	17·4% (-3·6 to 36·7%)	-8·0% (-15·6 to 1·2%)
Assault by sharp object	0·1 (0·1 to 0·2)	2·1% (1·5 to 2·8%)	0·2 (0·1 to 0·2)	2·2% (1·7 to 2·9%)	24·9% (2·6 to 51·8%)	5·7% (-6·3 to 20·2%)	7·5 (4·6 to 10·6)	2·2% (1·6 to 3·0%)	9·2 (5·1 to 13·5)	2·4% (1·8 to 3·1%)	22·6% (0·2 to 49·8%)	6·0% (-6·6 to 20·5%)
Assault by other means	0·1 (0·1 to 0·1)	1·4% (1·1 to 1·9%)	0·1 (0·1 to 0·2)	1·6% (1·2 to 2·0%)	19·7% (0·3 to 42·9%)	8·2% (0·3 to 17·3%)	5·6 (3·7 to 8·0)	1·5% (1·1 to 2·0%)	6·5 (4·1 to 9·3)	1·6% (1·2 to 2·1%)	16·6% (-2·0 to 38·5%)	9·4% (0·7 to 20·1%)
**Drug use: All causes**	16·3 (12·8 to 20·0)	0·6% (0·5 to 0·8%)	23·8 (18·8 to 29·4)	0·8% (0·6 to 1·0%)	46·1% (30·7 to 62·9%)	24·8% (13·7 to 36·8%)	1,535·5 (1,246·2 to 1,837·7)	1·0% (0·9 to 1·2%)	2,122·1 (1,703·9 to 2,564·5)	1·3% (1·1 to 1·5%)	38·2% (29·6 to 47·1%)	23·6% (15·9 to 31·6%)
HIV/AIDS - Tuberculosis	0·2 (0·1 to 0·2)	27·2% (21·3 to 33·5%)	0·3 (0·2 to 0·4)	33·6% (27·3 to 40·9%)	72·3% (36·4 to 129·0%)	23·5% (5·7 to 50·4%)	8·3 (6·6 to 10·4)	27·0% (21·2 to 33·0%)	13·4 (10·9 to 17·0)	33·3% (27·4 to 39·4%)	62·9% (33·6 to 108·2%)	23·3% (7·8 to 46·3%)
HIV/AIDS resulting in other diseases	0·7 (0·5 to 1·2)	16·9% (11·3 to 23·7%)	1·2 (0·8 to 1·7)	17·8% (12·2 to 24·3%)	64·7% (28·1 to 104·0%)	5·5% (-1·6 to 21·0%)	36·6 (22·5 to 57·4)	16·2% (10·7 to 23·1%)	58·1 (38·3 to 83·9)	17·1% (11·7 to 23·8%)	59·0% (23·8 to 96·4%)	5·1% (-1·6 to 19·7%)
Hepatitis B	0·0 (0·0 to 0·0)	0·1% (0·0 to 0·2%)	0·0 (0·0 to 0·0)	0·2% (0·1 to 0·3%)	57·1% (28·9 to 94·3%)	27·5% (9·6 to 50·5%)	0·2 (0·1 to 0·3)	0·1% (0·0 to 0·2%)	0·2 (0·1 to 0·5)	0·2% (0·1 to 0·3%)	48·2% (22·6 to 81·3%)	24·6% (8·6 to 45·6%)
Hepatitis C	0·0 (0·0 to 0·0)	16·4% (9·5 to 25·1%)	0·0 (0·0 to 0·1)	20·3% (12·3 to 30·6%)	56·5% (13·9 to 115·4%)	23·8% (2·0 to 52·5%)	0·7 (0·2 to 1·6)	16·5% (9·7 to 25·6%)	1·0 (0·2 to 2·3)	19·7% (11·4 to 30·3%)	44·5% (8·5 to 94·6%)	18·8% (-1·1 to 45·4%)
Liver cancer due to hepatitis B	0·0 (0·0 to 0·0)	0·1% (0·0 to 0·2%)	0·0 (0·0 to 0·0)	0·2% (0·1 to 0·3%)	78·2% (45·5 to 116·7%)	32·7% (14·3 to 56·2%)	0·2 (0·1 to 0·4)	0·1% (0·0 to 0·2%)	0·3 (0·1 to 0·6)	0·2% (0·1 to 0·3%)	70·5% (41·1 to 107·0%)	29·8% (12·5 to 51·5%)
Liver cancer due to hepatitis C	0·8 (0·4 to 1·4)	10·3% (5·3 to 17·4%)	1·4 (0·7 to 2·3)	13·6% (7·4 to 22·0%)	75·6% (50·6 to 108·2%)	32·1% (17·0 to 53·6%)	23·3 (11·9 to 38·8)	12·7% (6·6 to 21·1%)	39·0 (20·8 to 63·5)	16·1% (8·8 to 26·2%)	67·3% (45·2 to 97·4%)	27·0% (13·8 to 45·4%)
Cirrhosis and other chronic liver diseases due to hepatitis B	0·0 (0·0 to 0·0)	0·1% (0·0 to 0·2%)	0·0 (0·0 to 0·1)	0·1% (0·1 to 0·3%)	62·4% (41·9 to 87·2%)	25·7% (12·4 to 44·1%)	0·7 (0·2 to 1·3)	0·1% (0·0 to 0·2%)	1·0 (0·4 to 2·1)	0·2% (0·1 to 0·3%)	55·6% (37·0 to 78·6%)	22·6% (10·3 to 39·4%)
Cirrhosis and other chronic liver diseases due to hepatitis C	4·4 (2·2 to 7·5)	12·8% (6·3 to 21·6%)	6·8 (3·4 to 11·3)	15·5% (8·1 to 25·7%)	55·4% (40·2 to 76·5%)	21·0% (10·6 to 35·8%)	147·2 (72·9 to 254·7)	15·8% (8·1 to 26·7%)	216·2 (111·7 to 361·5)	18·4% (9·5 to 30·8%)	46·8% (32·9 to 65·6%)	16·5% (7·8 to 29·2%)
Opioid use disorders	7·8 (5·2 to 9·0)	100·0% (100·0 to 100·0%)	11·0 (7·7 to 12·7)	100·0% (100·0 to 100·0%)	39·9% (19·0 to 64·1%)	-	1,144·2 (886·3 to 1,394·2)	100·0% (100·0 to 100·0%)	1,573·4 (1,212·2 to 1,938·7)	100·0% (100·0 to 100·0%)	37·5% (29·0 to 46·3%)	-
Cocaine use disorders	0·1 (0·0 to 0·1)	100·0% (100·0 to 100·0%)	0·1 (0·1 to 0·2)	100·0% (100·0 to 100·0%)	84·4% (47·3 to 131·1%)	-	12·1 (8·3 to 16·7)	100·0% (100·0 to 100·0%)	17·8 (12·6 to 23·7)	100·0% (100·0 to 100·0%)	47·0% (32·2 to 64·7%)	-
Amphetamine use disorders	0·2 (0·1 to 0·2)	100·0% (100·0 to 100·0%)	0·4 (0·3 to 0·5)	100·0% (100·0 to 100·0%)	99·1% (56·8 to 154·5%)	-	21·2 (14·9 to 29·2)	100·0% (100·0 to 100·0%)	33·0 (24·4 to 44·2)	100·0% (100·0 to 100·0%)	55·7% (36·2 to 79·9%)	-
Cannabis use disorders	-	-	-	-	-	-	24·8 (15·4 to 35·9)	100·0% (100·0 to 100·0%)	29·4 (18·3 to 42·8)	100·0% (100·0 to 100·0%)	18·7% (12·7 to 25·4%)	-
Other drug use disorders	0·8 (0·6 to 1·0)	100·0% (100·0 to 100·0%)	1·1 (0·8 to 1·4)	100·0% (100·0 to 100·0%)	26·4% (-4·9 to 70·2%)	-	51·6 (39·0 to 65·4)	100·0% (100·0 to 100·0%)	63·0 (48·4 to 81·6)	100·0% (100·0 to 100·0%)	22·0% (-4·0 to 55·8%)	-
Self-harm	1·2 (0·7 to 1·8)	5·1% (3·1 to 7·6%)	1·5 (0·9 to 2·2)	5·6% (3·4 to 8·2%)	21·8% (3·6 to 41·9%)	8·6% (-1·1 to 18·7%)	64·6 (39·5 to 96·8)	5·3% (3·2 to 7·8%)	76·2 (46·6 to 113·9)	5·8% (3·5 to 8·5%)	17·9% (-0·3 to 38·7%)	9·8% (-0·2 to 20·6%)
**Dietary risks: All causes**	550·4 (474·1 to 634·5)	21·6% (18·7 to 24·7%)	679·1 (584·3 to 785·0)	22·7% (19·6 to 26·0%)	23·4% (16·4 to 30·0%)	5·3% (2·2 to 8·8%)	14,308·7 (12,463·4 to 16,312·0)	9·7% (8·4 to 11·0%)	18,006·1 (15,591·1 to 20,675·7)	10·9% (9·4 to 12·5%)	25·8% (18·5 to 32·9%)	12·6% (7·5 to 17·7%)
**Diet low in fruits: All causes**	129·5 (79·6 to 185·7)	5·1% (3·1 to 7·3%)	160·1 (97·2 to 229·6)	5·4% (3·3 to 7·6%)	23·6% (16·4 to 30·6%)	5·5% (1·7 to 9·6%)	3,829·4 (2,434·1 to 5,404·7)	2·6% (1·6 to 3·7%)	4,821·5 (3,041·2 to 6,789·0)	2·9% (1·8 to 4·1%)	25·9% (17·6 to 33·4%)	12·6% (6·2 to 18·6%)
Lip and oral cavity cancer	0·1 (0·0 to 0·3)	6·4% (0·0 to 14·0%)	0·2 (0·0 to 0·4)	6·5% (0·0 to 14·2%)	31·7% (0·0 to 40·6%)	1·8% (0·0 to 3·1%)	4·1 (0·0 to 9·1)	6·3% (0·0 to 13·8%)	5·4 (0·0 to 12·0)	6·4% (0·0 to 14·1%)	32·1% (0·0 to 41·3%)	2·3% (0·0 to 3·6%)
Nasopharynx cancer	0·1 (0·0 to 0·2)	6·5% (0·0 to 13·9%)	0·1 (0·0 to 0·3)	6·7% (0·0 to 14·4%)	26·1% (0·0 to 36·7%)	3·3% (0·0 to 5·0%)	3·7 (0·0 to 8·0)	6·0% (0·0 to 13·0%)	4·6 (0·0 to 10·0)	6·3% (0·0 to 13·6%)	24·6% (0·0 to 35·7%)	5·1% (0·0 to 7·6%)
Other pharynx cancer	0·1 (0·0 to 0·2)	6·5% (0·0 to 14·7%)	0·1 (0·0 to 0·2)	6·6% (0·0 to 14·9%)	27·1% (0·0 to 37·1%)	1·6% (0·0 to 2·8%)	2·2 (0·0 to 5·0)	6·5% (0·0 to 14·4%)	2·8 (0·0 to 6·5)	6·6% (0·0 to 14·8%)	27·0% (0·0 to 37·6%)	2·0% (0·0 to 3·4%)
Oesophageal cancer	1·3 (0·3 to 2·5)	19·6% (4·6 to 35·7%)	1·6 (0·4 to 3·0)	20·0% (4·8 to 36·3%)	22·6% (8·5 to 37·2%)	2·2% (0·4 to 4·3%)	33·6 (7·9 to 63·3)	19·7% (4·7 to 35·9%)	41·2 (9·8 to 76·6)	20·2% (4·9 to 36·6%)	22·5% (8·6 to 37·7%)	2·3% (0·6 to 4·3%)
Larynx cancer	0·3 (0·0 to 0·6)	6·4% (0·0 to 14·4%)	0·3 (0·0 to 0·8)	6·5% (0·0 to 14·5%)	26·1% (0·0 to 36·9%)	1·2% (-0·5 to 2·9%)	7·2 (0·0 to 16·0)	6·4% (0·0 to 14·5%)	9·0 (0·0 to 20·2)	6·5% (0·0 to 14·6%)	25·6% (0·0 to 36·8%)	1·2% (-0·4 to 2·8%)
Tracheal, bronchus, and lung cancer	4·8 (1·9 to 8·3)	11·1% (4·4 to 18·9%)	6·4 (2·6 to 10·9)	11·2% (4·5 to 19·2%)	32·4% (23·6 to 43·0%)	1·6% (0·1 to 3·1%)	122·1 (48·9 to 209·9)	11·2% (4·5 to 19·0%)	161·8 (66·1 to 275·1)	11·3% (4·5 to 19·3%)	32·5% (23·6 to 42·8%)	1·6% (0·2 to 3·2%)
Ischaemic heart disease	59·5 (21·3 to 101·0)	12·1% (4·3 to 20·6%)	73·7 (26·3 to 126·1)	12·3% (4·4 to 20·8%)	23·8% (16·0 to 31·4%)	1·8% (0·2 to 3·4%)	1,543·9 (560·6 to 2,606·5)	14·2% (5·2 to 23·8%)	1,913·3 (688·6 to 3,226·9)	14·5% (5·3 to 24·3%)	23·9% (15·1 to 32·3%)	2·4% (0·8 to 4·1%)
Ischaemic stroke	23·3 (12·3 to 36·4)	19·0% (10·1 to 29·4%)	28·2 (15·1 to 43·2)	18·9% (10·1 to 28·9%)	20·8% (10·9 to 29·5%)	-0·9% (-3·6 to 2·0%)	587·6 (323·8 to 885·0)	25·8% (14·3 to 38·2%)	707·7 (393·1 to 1,056·8)	26·3% (14·6 to 38·7%)	20·4% (9·9 to 30·0%)	2·0% (-0·5 to 4·8%)

Supplemental Results Table 1. All-age deaths, death PAFs, DALYs, DALY PAFs, and percent change for each risk factor and outcome for both sexes combined in 2005 and 2015 for North Africa and Middle East.

Data in parenthesis are 95% uncertainty intervals. PAFs=population attributable fractions. DALYs=disability-adjusted life-years.

	2005 Deaths (in thousands)	2005 Deaths PAF	2015 Deaths (in thousands)	2015 Deaths PAF	Percent change deaths 2005-2015	Percent change deaths PAF 2005-2015	2005 DALYs (in thousands)	2005 DALYs PAF	2015 DALYs (in thousands)	2015 DALYs PAF	Percent change DALYs 2005-2015	Percent change DALYs PAF 2005-2015
Hemorrhagic stroke	32·0 (17·6 to 47·6)	24·3% (13·3 to 36·2%)	38·5 (20·5 to 58·0)	25·1% (13·5 to 37·3%)	20·4% (11·2 to 30·5%)	3·1% (0·3 to 6·0%)	1,008·3 (570·1 to 1,472·2)	26·6% (14·9 to 38·7%)	1,216·8 (657·0 to 1,788·4)	29·0% (16·2 to 41·9%)	20·7% (10·8 to 31·3%)	8·9% (5·3 to 12·5%)
**Diet low in vegetables: All causes**	94·8 (46·8 to 153·7)	3·7% (1·8 to 6·0%)	117·4 (56·8 to 188·2)	3·9% (1·9 to 6·3%)	23·8% (14·9 to 32·4%)	5·7% (1·1 to 10·9%)	2,541·2 (1,304·6 to 4,077·9)	1·7% (0·9 to 2·8%)	3,144·9 (1,580·4 to 4,908·5)	1·9% (1·0 to 3·0%)	23·8% (13·8 to 33·4%)	10·7% (3·5 to 17·8%)
Ischaemic heart disease	70·6 (27·3 to 120·0)	14·3% (5·5 to 24·2%)	88·1 (33·3 to 151·0)	14·7% (5·6 to 24·8%)	24·7% (15·9 to 33·3%)	2·6% (0·2 to 5·4%)	1,818·3 (712·1 to 3,051·8)	16·7% (6·4 to 27·8%)	2,268·5 (869·8 to 3,829·1)	17·2% (6·6 to 28·6%)	24·8% (14·7 to 34·5%)	3·1% (0·5 to 6·2%)
Ischaemic stroke	10·1 (2·2 to 19·0)	8·3% (1·9 to 15·2%)	12·3 (2·7 to 22·7)	8·2% (1·9 to 15·1%)	21·5% (9·3 to 32·3%)	-0·4% (-4·4 to 3·8%)	264·9 (60·4 to 481·9)	11·7% (2·7 to 21·0%)	320·8 (73·7 to 579·3)	12·0% (2·8 to 21·4%)	21·1% (8·5 to 33·5%)	2·6% (-1·7 to 7·0%)
Hemorrhagic stroke	14·0 (3·8 to 26·0)	10·7% (2·9 to 19·9%)	17·0 (4·6 to 31·7)	11·1% (3·1 to 20·8%)	21·1% (10·0 to 32·9%)	3·8% (-0·4 to 8·2%)	458·1 (124·9 to 835·2)	12·1% (3·4 to 22·1%)	555·5 (151·7 to 1,021·2)	13·2% (3·7 to 24·2%)	21·3% (8·9 to 33·9%)	9·4% (4·0 to 14·6%)
**Diet low in whole grains: All causes**	160·5 (110·1 to 219·0)	6·3% (4·3 to 8·6%)	197·1 (134·8 to 270·1)	6·6% (4·5 to 9·0%)	22·8% (15·6 to 29·7%)	4·8% (1·6 to 8·4%)	4,859·9 (3,398·1 to 6,413·0)	3·3% (2·3 to 4·3%)	6,137·5 (4,295·6 to 8,184·0)	3·7% (2·6 to 4·9%)	26·3% (18·7 to 33·5%)	12·9% (7·6 to 18·4%)
Ischaemic heart disease	97·5 (57·3 to 142·8)	19·8% (11·5 to 29·0%)	119·5 (69·2 to 176·4)	19·9% (11·6 to 29·2%)	22·6% (15·0 to 29·7%)	0·8% (0·0 to 1·8%)	2,500·8 (1,498·4 to 3,607·1)	22·9% (13·6 to 33·2%)	3,071·5 (1,813·6 to 4,462·4)	23·3% (13·8 to 33·6%)	22·8% (14·3 to 30·8%)	1·5% (0·6 to 2·4%)
Ischaemic stroke	21·1 (13·7 to 29·4)	17·2% (11·4 to 23·6%)	25·1 (16·3 to 35·2)	16·8% (11·2 to 23·0%)	19·3% (9·7 to 27·8%)	-2·2% (-4·7 to 0·3%)	551·5 (368·2 to 748·0)	24·2% (16·6 to 32·2%)	658·4 (440·9 to 900·9)	24·5% (16·8 to 32·6%)	19·4% (9·0 to 28·8%)	1·1% (-1·2 to 3·5%)
Hemorrhagic stroke	27·9 (19·3 to 37·9)	21·1% (14·7 to 28·6%)	33·2 (22·8 to 45·4)	21·6% (15·0 to 29·4%)	19·2% (10·4 to 28·8%)	2·1% (-0·3 to 4·5%)	887·3 (626·3 to 1,180·9)	23·4% (16·5 to 31·1%)	1,062·6 (740·5 to 1,437·1)	25·3% (17·9 to 33·8%)	19·8% (10·2 to 30·1%)	8·1% (4·9 to 11·2%)
Diabetes mellitus	14·1 (7·5 to 22·1)	17·3% (9·4 to 26·9%)	19·3 (10·5 to 29·7)	17·3% (9·3 to 26·8%)	36·6% (28·2 to 45·0%)	-0·4% (-1·6 to 0·8%)	920·3 (494·6 to 1,446·6)	21·5% (11·9 to 32·6%)	1,345·0 (717·5 to 2,122·8)	22·0% (12·1 to 33·4%)	46·2% (41·0 to 50·7%)	2·3% (1·7 to 2·9%)
**Diet low in nuts and seeds: All causes**	113·0 (70·3 to 161·8)	4·4% (2·7 to 6·3%)	139·7 (87·3 to 201·8)	4·7% (2·9 to 6·7%)	23·7% (16·9 to 30·2%)	5·6% (2·1 to 9·3%)	3,072·1 (2,009·9 to 4,267·5)	2·1% (1·3 to 2·9%)	3,888·4 (2,536·5 to 5,418·5)	2·4% (1·5 to 3·3%)	26·6% (18·9 to 33·9%)	13·2% (8·0 to 18·9%)
Ischaemic heart disease	104·8 (63·5 to 152·4)	21·3% (12·9 to 30·7%)	128·6 (76·8 to 188·2)	21·5% (13·1 to 31·1%)	22·7% (15·7 to 29·4%)	0·9% (-0·4 to 2·4%)	2,571·1 (1,569·0 to 3,698·1)	23·6% (14·5 to 33·6%)	3,161·4 (1,938·8 to 4,584·5)	24·0% (14·8 to 34·1%)	23·0% (15·0 to 30·7%)	1·6% (0·0 to 3·4%)
Diabetes mellitus	8·1 (4·1 to 12·9)	9·9% (5·1 to 15·9%)	11·0 (5·7 to 17·5)	9·9% (5·1 to 15·7%)	36·1% (27·8 to 44·9%)	-0·8% (-2·3 to 0·7%)	501·1 (231·9 to 824·7)	11·7% (6·0 to 18·6%)	727·0 (335·6 to 1,208·3)	11·9% (6·1 to 18·9%)	45·1% (39·9 to 49·8%)	1·6% (0·6 to 2·4%)
**Diet low in milk: All causes**	2·9 (1·0 to 4·9)	0·1% (0·0 to 0·2%)	4·1 (1·4 to 7·0)	0·1% (0·0 to 0·2%)	41·2% (32·4 to 50·0%)	20·6% (14·1 to 26·7%)	75·3 (26·5 to 126·6)	0·1% (0·0 to 0·1%)	106·1 (36·6 to 180·6)	0·1% (0·0 to 0·1%)	40·9% (32·1 to 49·6%)	26·1% (18·7 to 33·9%)
Colon and rectum cancer	2·9 (1·0 to 4·9)	15·7% (5·5 to 26·4%)	4·1 (1·4 to 7·0)	15·7% (5·5 to 26·4%)	41·2% (32·4 to 50·0%)	0·2% (-0·3 to 0·7%)	75·3 (26·5 to 126·6)	15·5% (5·4 to 25·9%)	106·1 (36·6 to 180·6)	15·6% (5·5 to 26·1%)	40·9% (32·1 to 49·6%)	0·6% (0·1 to 1·2%)
**Diet high in red meat: All causes**	0·4 (0·1 to 0·8)	0·0% (0·0 to 0·0%)	0·7 (0·2 to 1·2)	0·0% (0·0 to 0·0%)	46·3% (32·9 to 60·2%)	25·0% (15·2 to 34·5%)	30·6 (6·1 to 58·3)	0·0% (0·0 to 0·0%)	48·1 (9·7 to 91·7)	0·0% (0·0 to 0·1%)	57·2% (48·4 to 64·8%)	40·6% (33·1 to 48·0%)
Colon and rectum cancer	0·1 (0·0 to 0·2)	0·5% (0·1 to 1·0%)	0·1 (0·0 to 0·3)	0·5% (0·1 to 1·0%)	50·0% (33·7 to 65·0%)	6·3% (-1·2 to 14·2%)	2·7 (0·5 to 5·3)	0·6% (0·1 to 1·1%)	4·2 (0·8 to 7·9)	0·6% (0·1 to 1·2%)	51·6% (35·1 to 68·1%)	8·2% (0·0 to 16·7%)
Diabetes mellitus	0·4 (0·0 to 0·7)	0·4% (0·1 to 0·9%)	0·5 (0·1 to 1·0)	0·5% (0·1 to 0·9%)	45·4% (31·6 to 59·7%)	6·1% (-1·8 to 14·4%)	27·8 (3·2 to 55·3)	0·7% (0·1 to 1·3%)	43·9 (4·9 to 87·3)	0·7% (0·1 to 1·4%)	57·8% (49·3 to 65·0%)	10·5% (6·0 to 15·0%)
**Diet high in processed meat: All causes**	17·0 (3·8 to 32·5)	0·7% (0·1 to 1·3%)	22·2 (5·4 to 41·6)	0·7% (0·2 to 1·4%)	30·8% (23·0 to 43·2%)	11·7% (6·9 to 22·0%)	567·1 (208·2 to 1,003·0)	0·4% (0·1 to 0·7%)	780·8 (311·9 to 1,322·4)	0·5% (0·2 to 0·8%)	37·7% (28·5 to 53·9%)	23·1% (15·4 to 37·9%)
Colon and rectum cancer	0·3 (0·2 to 0·5)	1·7% (0·9 to 2·9%)	0·5 (0·2 to 0·8)	1·8% (0·9 to 3·0%)	50·7% (41·1 to 61·2%)	6·8% (4·1 to 10·4%)	8·6 (4·3 to 14·3)	1·8% (0·9 to 2·9%)	12·9 (6·5 to 21·4)	1·9% (0·9 to 3·1%)	50·3% (40·4 to 61·0%)	7·3% (4·4 to 10·8%)
Ischaemic heart disease	13·8 (0·5 to 28·8)	2·8% (0·1 to 5·8%)	17·6 (0·7 to 35·8)	2·9% (0·1 to 6·0%)	27·2% (17·1 to 36·0%)	4·7% (1·5 to 8·2%)	360·9 (13·7 to 743·6)	3·3% (0·1 to 6·8%)	459·5 (16·8 to 924·7)	3·5% (0·1 to 7·1%)	27·3% (15·9 to 36·9%)	5·3% (1·6 to 8·8%)
Diabetes mellitus	2·9 (1·3 to 4·8)	3·5% (1·6 to 5·8%)	4·2 (1·9 to 6·9)	3·7% (1·7 to 6·2%)	46·1% (36·5 to 56·1%)	6·6% (3·5 to 10·6%)	197·6 (87·7 to 334·0)	4·6% (2·1 to 7·6%)	308·4 (136·0 to 524·7)	5·0% (2·3 to 8·3%)	56·1% (49·9 to 62·0%)	9·2% (7·0 to 12·3%)
**Diet high in sugar-sweetened beverages: All causes**	2·4 (1·6 to 3·4)	0·1% (0·1 to 0·1%)	3·1 (2·0 to 4·5)	0·1% (0·1 to 0·1%)	28·6% (21·3 to 35·6%)	9·8% (5·0 to 14·7%)	102·1 (66·6 to 153·6)	0·1% (0·0 to 0·1%)	137·4 (88·4 to 206·0)	0·1% (0·1 to 0·1%)	34·5% (27·6 to 40·9%)	20·3% (14·5 to 26·2%)
Oesophageal cancer	0·0 (0·0 to 0·0)	0·2% (0·1 to 0·3%)	0·0 (0·0 to 0·0)	0·2% (0·1 to 0·3%)	25·9% (12·2 to 40·7%)	5·0% (-4·0 to 13·8%)	0·4 (0·1 to 0·7)	0·2% (0·1 to 0·4%)	0·5 (0·2 to 0·8)	0·2% (0·1 to 0·4%)	26·0% (11·9 to 40·7%)	5·3% (-3·6 to 14·5%)
Colon and rectum cancer	0·0 (0·0 to 0·0)	0·1% (0·1 to 0·1%)	0·0 (0·0 to 0·0)	0·1% (0·1 to 0·1%)	50·4% (38·7 to 61·2%)	6·7% (1·3 to 12·7%)	0·5 (0·3 to 0·7)	0·1% (0·1 to 0·1%)	0·7 (0·5 to 1·1)	0·1% (0·1 to 0·2%)	50·6% (38·7 to 61·7%)	7·6% (2·0 to 13·5%)
Liver cancer due to hepatitis B	0·0 (0·0 to 0·0)	0·2% (0·1 to 0·3%)	0·0 (0·0 to 0·0)	0·2% (0·1 to 0·3%)	38·9% (25·6 to 53·2%)	3·5% (-2·4 to 9·8%)	0·3 (0·1 to 0·5)	0·2% (0·1 to 0·3%)	0·4 (0·2 to 0·6)	0·2% (0·1 to 0·3%)	37·4% (24·1 to 51·5%)	4·7% (-1·5 to 11·5%)
Liver cancer due to hepatitis C	0·0 (0·0 to 0·0)	0·1% (0·1 to 0·3%)	0·0 (0·0 to 0·0)	0·2% (0·1 to 0·3%)	37·2% (23·1 to 54·4%)	3·2% (-3·4 to 10·7%)	0·3 (0·1 to 0·6)	0·2% (0·1 to 0·3%)	0·4 (0·2 to 0·8)	0·2% (0·1 to 0·3%)	36·1% (22·2 to 53·4%)	3·3% (-3·6 to 11·5%)
Liver cancer due to alcohol use	0·0 (0·0 to 0·0)	0·2% (0·1 to 0·3%)	0·0 (0·0 to 0·0)	0·2% (0·1 to 0·3%)	38·1% (24·1 to 54·4%)	2·4% (-3·5 to 8·8%)	0·1 (0·0 to 0·2)	0·2% (0·1 to 0·3%)	0·1 (0·1 to 0·3)	0·2% (0·1 to 0·4%)	38·5% (24·3 to 54·8%)	2·3% (-3·9 to 9·0%)
Liver cancer due to other causes	0·0 (0·0 to 0·0)	0·1% (0·1 to 0·2%)	0·0 (0·0 to 0·0)	0·1% (0·1 to 0·2%)	36·8% (24·5 to 51·2%)	4·2% (-1·8 to 11·4%)	0·1 (0·1 to 0·2)	0·1% (0·1 to 0·3%)	0·2 (0·1 to 0·3)	0·2% (0·1 to 0·3%)	35·5% (23·1 to 49·2%)	5·9% (-0·5 to 14·1%)
Gallbladder and biliary tract cancer	0·0 (0·0 to 0·0)	0·2% (0·1 to 0·2%)	0·0 (0·0 to 0·0)	0·2% (0·1 to 0·2%)	19·7% (4·2 to 35·4%)	-0·6% (-5·8 to 4·9%)	0·2 (0·1 to 0·3)	0·2% (0·1 to 0·3%)	0·2 (0·1 to 0·4)	0·2% (0·1 to 0·3%)	19·7% (4·5 to 35·8%)	-0·2% (-5·4 to 5·4%)
Pancreatic cancer	0·0 (0·0 to 0·0)	0·1% (0·0 to 0·1%)	0·0 (0·0 to 0·0)	0·1% (0·0 to 0·1%)	30·0% (19·3 to 41·2%)	0·4% (-4·8 to 7·7%)	0·2 (0·1 to 0·3)	0·1% (0·0 to 0·1%)	0·2 (0·1 to 0·4)	0·1% (0·0 to 0·1%)	31·1% (20·6 to 42·1%)	0·5% (-4·5 to 6·9%)
Breast cancer	0·0 (0·0 to 0·0)	0·0% (0·0 to 0·1%)	0·0 (0·0 to 0·0)	0·0% (0·0 to 0·1%)	39·0% (25·0 to 54·1%)	2·3% (-5·2 to 9·4%)	0·2 (0·1 to 0·3)	0·0% (0·0 to 0·0%)	0·3 (0·2 to 0·4)	0·0% (0·0 to 0·0%)	41·6% (27·6 to 56·8%)	3·3% (-4·9 to 11·2%)
Uterine cancer	0·0 (0·0 to 0·0)	0·3% (0·2 to 0·5%)	0·0 (0·0 to 0·0)	0·3% (0·2 to 0·5%)	17·5% (4·6 to 31·5%)	-0·5% (-5·7 to 4·7%)	0·4 (0·2 to 0·6)	0·4% (0·2 to 0·6%)	0·4 (0·3 to 0·7)	0·4% (0·2 to 0·6%)	18·7% (6·1 to 32·6%)	0·1% (-4·9 to 5·3%)
Ovarian cancer	0·0 (0·0 to 0·0)	0·0% (0·0 to 0·1%)	0·0 (0·0 to 0·0)	0·0% (0·0 to 0·1%)	26·5% (0·0 to 39·6%)	-0·6% (-5·5 to 4·6%)	0·0 (0·0 to 0·1)	0·0% (0·0 to 0·1%)	0·1 (0·0 to 0·1)	0·0% (0·0 to 0·1%)	25·9% (0·0 to 38·5%)	-0·8% (-5·8 to 4·5%)
Kidney cancer	0·0 (0·0 to 0·0)	0·2% (0·1 to 0·3%)	0·0 (0·0 to 0·0)	0·2% (0·1 to 0·3%)	38·0% (25·9 to 50·1%)	2·4% (-2·3 to 6·9%)	0·2 (0·1 to 0·3)	0·2% (0·1 to 0·3%)	0·3 (0·2 to 0·4)	0·2% (0·1 to 0·3%)	38·5% (26·3 to 50·6%)	6·3% (0·5 to 12·8%)
Thyroid cancer	0·0 (0·0 to 0·0)	0·1% (0·1 to 0·2%)	0·0 (0·0 to 0·0)	0·1% (0·1 to 0·2%)	32·7% (22·5 to 44·7%)	3·7% (-1·5 to 8·9%)	0·0 (0·0 to 0·1)	0·1% (0·1 to 0·2%)	0·1 (0·0 to 0·1)	0·1% (0·1 to 0·2%)	35·3% (24·2 to 47·3%)	3·2% (-2·2 to 8·5%)
Ischaemic heart disease	1·0 (0·6 to 1·4)	0·2% (0·1 to 0·3%)	1·2 (0·8 to 1·8)	0·2% (0·1 to 0·3%)	24·6% (17·2 to 31·9%)	2·5% (-1·2 to 6·5%)	30·7 (20·1 to 45·8)	0·3% (0·2 to 0·4%)	38·5 (25·2 to 57·2)	0·3% (0·2 to 0·4%)	25·1% (17·1 to 33·3%)	3·4% (-0·5 to 7·4%)
Ischaemic stroke	0·2 (0·1 to 0·2)	0·1% (0·1 to 0·2%)	0·2 (0·1 to 0·3)	0·1% (0·1 to 0·2%)	23·5% (14·1 to 33·3%)	1·3% (-3·7 to 6·4%)	5·3 (3·5 to 7·8)	0·2% (0·2 to 0·3%)	6·6 (4·3 to 9·7)	0·2% (0·2 to 0·4%)	24·3% (14·5 to 33·7%)	5·2% (1·0 to 9·7%)
Hemorrhagic stroke	0·4 (0·3 to 0·6)	0·3% (0·2 to 0·5%)	0·5 (0·3 to 0·8)	0·3% (0·2 to 0·5%)	24·8% (15·6 to 34·3%)	6·9% (1·8 to 12·4%)	16·2 (10·4 to 23·9)	0·4% (0·3 to 0·6%)	20·3 (12·8 to 30·3)	0·5% (0·3 to 0·7%)	25·1% (15·3 to 35·1%)	12·9% (7·7 to 18·4%)
Hypertensive heart disease	0·2 (0·1 to 0·2)	0·3% (0·2 to 0·5%)	0·2 (0·1 to 0·3)	0·3% (0·2 to 0·5%)	27·4% (16·3 to 39·2%)	3·7% (-2·8 to 10·1%)	4·0 (2·5 to 6·2)	0·5% (0·3 to 0·7%)	5·1 (3·2 to 7·9)	0·5% (0·3 to 0·8%)	27·3% (17·3 to 38·6%)	5·4% (-0·2 to 10·9%)
Cardiomyopathy and myocarditis	-	-	-	-	-	-	-	-	-	-	-	-
Atrial fibrillation and flutter	-	-	-	-	-	-	-	-	-	-	-	-
Peripheral vascular disease	-	-	-	-	-	-	-	-	-	-	-	-

Supplemental Results Table 1. All-age deaths, death PAFs, DALYs, DALY PAFs, and percent change for each risk factor and outcome for both sexes combined in 2005 and 2015 for North Africa and Middle East.

Data in parenthesis are 95% uncertainty intervals. PAFs=population attributable fractions. DALYs=disability-adjusted life-years.

	2005 Deaths (in thousands)	2005 Deaths PAF	2015 Deaths (in thousands)	2015 Deaths PAF	Percent change deaths 2005-2015	Percent change deaths PAF 2005-2015	2005 DALYs (in thousands)	2005 DALYs PAF	2015 DALYs (in thousands)	2015 DALYs PAF	Percent change DALYs 2005-2015	Percent change DALYs PAF 2005-2015
Endocarditis	-	-	-	-	-	-	-	-	-	-	-	-
Other cardiovascular and circulatory diseases	-	-	-	-	-	-	-	-	-	-	-	-
Diabetes mellitus	0·4 (0·2 to 0·6)	0·5% (0·3 to 0·7%)	0·5 (0·3 to 0·8)	0·5% (0·3 to 0·7%)	39·0% (29·4 to 48·1%)	1·3% (-2·8 to 5·6%)	31·9 (19·4 to 50·6)	0·7% (0·5 to 1·1%)	47·6 (28·5 to 76·3)	0·8% (0·5 to 1·2%)	49·4% (42·9 to 55·4%)	4·5% (1·2 to 7·8%)
Chronic kidney disease due to diabetes mellitus	0·0 (0·0 to 0·1)	0·3% (0·2 to 0·5%)	0·1 (0·0 to 0·1)	0·3% (0·2 to 0·6%)	46·3% (33·8 to 59·9%)	4·2% (-1·9 to 10·5%)	1·3 (0·6 to 2·5)	0·3% (0·1 to 0·6%)	1·9 (0·8 to 3·6)	0·4% (0·1 to 0·7%)	44·3% (35·1 to 54·6%)	7·4% (2·7 to 12·6%)
Chronic kidney disease due to hypertension	0·1 (0·1 to 0·2)	0·3% (0·2 to 0·6%)	0·2 (0·1 to 0·3)	0·3% (0·2 to 0·6%)	37·5% (26·1 to 49·8%)	3·2% (-1·8 to 9·5%)	4·1 (1·6 to 7·5)	0·3% (0·1 to 0·6%)	5·5 (2·2 to 10·2)	0·4% (0·1 to 0·7%)	35·7% (25·6 to 46·6%)	6·9% (1·9 to 12·5%)
Chronic kidney disease due to glomerulonephritis	0·0 (0·0 to 0·0)	0·3% (0·1 to 0·5%)	0·0 (0·0 to 0·0)	0·3% (0·1 to 0·5%)	36·6% (24·7 to 49·7%)	5·6% (-0·5 to 12·7%)	0·6 (0·2 to 1·2)	0·3% (0·1 to 0·5%)	0·9 (0·3 to 1·7)	0·3% (0·1 to 0·6%)	37·4% (28·3 to 47·9%)	12·6% (7·0 to 19·1%)
Chronic kidney disease due to other causes	0·0 (0·0 to 0·0)	0·2% (0·1 to 0·3%)	0·0 (0·0 to 0·0)	0·2% (0·1 to 0·3%)	51·5% (32·6 to 69·9%)	22·0% (10·5 to 35·5%)	0·5 (0·2 to 1·0)	0·2% (0·1 to 0·4%)	0·7 (0·2 to 1·4)	0·2% (0·1 to 0·5%)	43·7% (34·5 to 53·0%)	25·5% (17·7 to 36·5%)
**Diet low in fibre: All causes**	13·7 (5·0 to 26·7)	0·5% (0·2 to 1·0%)	17·3 (6·3 to 34·0)	0·6% (0·2 to 1·1%)	25·8% (14·5 to 37·9%)	7·4% (-0·1 to 15·4%)	393·0 (147·7 to 756·0)	0·3% (0·1 to 0·5%)	489·3 (180·7 to 962·4)	0·3% (0·1 to 0·6%)	24·5% (12·9 to 37·6%)	11·4% (1·3 to 21·9%)
Colon and rectum cancer	0·6 (0·2 to 1·2)	3·0% (1·0 to 6·2%)	0·8 (0·3 to 1·7)	3·1% (1·0 to 6·3%)	45·5% (33·4 to 58·8%)	3·2% (-3·7 to 10·5%)	15·1 (5·1 to 31·0)	3·1% (1·0 to 6·3%)	21·7 (7·0 to 44·5)	3·2% (1·1 to 6·5%)	43·6% (31·3 to 57·0%)	2·6% (-4·4 to 9·3%)
Ischaemic heart disease	13·2 (4·7 to 25·7)	2·7% (1·0 to 5·2%)	16·5 (6·0 to 32·6)	2·7% (1·0 to 5·4%)	24·9% (13·7 to 37·3%)	2·8% (-3·7 to 9·2%)	377·9 (139·4 to 732·7)	3·5% (1·3 to 6·7%)	467·6 (170·3 to 924·1)	3·5% (1·3 to 6·9%)	23·7% (12·1 to 37·0%)	2·3% (-4·9 to 9·3%)
**Diet low in calcium: All causes**	3·9 (2·4 to 5·6)	0·2% (0·1 to 0·2%)	5·6 (3·4 to 7·9)	0·2% (0·1 to 0·3%)	41·5% (31·7 to 50·4%)	20·8% (14·2 to 27·0%)	102·3 (63·1 to 145·5)	0·1% (0·0 to 0·1%)	144·2 (88·0 to 204·6)	0·1% (0·1 to 0·1%)	40·9% (31·3 to 50·1%)	26·1% (18·6 to 33·9%)
Colon and rectum cancer	3·9 (2·4 to 5·6)	21·3% (13·2 to 30·2%)	5·6 (3·4 to 7·9)	21·4% (13·3 to 30·3%)	41·5% (31·7 to 50·4%)	0·4% (-1·0 to 1·7%)	102·3 (63·1 to 145·5)	21·0% (13·1 to 29·6%)	144·2 (88·0 to 204·6)	21·1% (13·2 to 29·9%)	40·9% (31·3 to 50·1%)	0·6% (-0·8 to 1·9%)
Prostate cancer	-	-	-	-	-	-	-	-	-	-	-	-
**Diet low in seafood omega-3 fatty acids: All causes**	91·0 (38·7 to 145·6)	3·6% (1·5 to 5·7%)	110·7 (47·5 to 178·6)	3·7% (1·6 to 5·9%)	21·7% (14·0 to 28·8%)	3·9% (0·5 to 7·7%)	2,235·6 (963·8 to 3,561·5)	1·5% (0·6 to 2·4%)	2,719·9 (1,182·7 to 4,351·7)	1·6% (0·7 to 2·7%)	21·7% (13·1 to 30·2%)	8·9% (2·7 to 15·2%)
Ischaemic heart disease	91·0 (38·7 to 145·6)	18·4% (7·8 to 29·5%)	110·7 (47·5 to 178·6)	18·5% (7·8 to 29·5%)	21·7% (14·0 to 28·8%)	0·1% (-0·9 to 1·2%)	2,235·6 (963·8 to 3,561·5)	20·5% (8·8 to 32·4%)	2,719·9 (1,182·7 to 4,351·7)	20·6% (8·9 to 32·4%)	21·7% (13·1 to 30·2%)	0·6% (-0·6 to 1·8%)
**Diet low in polyunsaturated fatty acids: All causes**	21·7 (9·4 to 34·5)	0·8% (0·4 to 1·4%)	25·5 (11·0 to 41·5)	0·9% (0·4 to 1·4%)	17·9% (3·1 to 33·1%)	0·7% (-11·4 to 12·6%)	615·8 (274·3 to 970·9)	0·4% (0·2 to 0·7%)	723·9 (316·5 to 1,175·6)	0·4% (0·2 to 0·7%)	17·6% (1·9 to 34·0%)	5·2% (-9·1 to 19·4%)
Ischaemic heart disease	21·7 (9·4 to 34·5)	4·4% (1·9 to 6·9%)	25·5 (11·0 to 41·5)	4·3% (1·8 to 6·8%)	17·9% (3·1 to 33·1%)	-3·0% (-14·5 to 7·5%)	615·8 (274·3 to 970·9)	5·6% (2·5 to 8·9%)	723·9 (316·5 to 1,175·6)	5·5% (2·4 to 8·7%)	17·6% (1·9 to 34·0%)	-2·8% (-14·3 to 8·3%)
**Diet high in trans fatty acids: All causes**	35·1 (16·0 to 61·1)	1·4% (0·6 to 2·4%)	42·9 (19·5 to 75·3)	1·4% (0·7 to 2·5%)	22·3% (12·7 to 32·2%)	4·5% (-1·2 to 10·4%)	994·4 (460·3 to 1,689·2)	0·7% (0·3 to 1·2%)	1,235·8 (578·3 to 2,090·4)	0·7% (0·4 to 1·3%)	24·3% (14·2 to 35·2%)	11·2% (3·7 to 19·5%)
Ischaemic heart disease	35·1 (16·0 to 61·1)	7·1% (3·2 to 12·3%)	42·9 (19·5 to 75·3)	7·2% (3·3 to 12·3%)	22·3% (12·7 to 32·2%)	0·7% (-3·2 to 4·6%)	994·4 (460·3 to 1,689·2)	9·1% (4·3 to 15·4%)	1,235·8 (578·3 to 2,090·4)	9·4% (4·4 to 15·9%)	24·3% (14·2 to 35·2%)	2·8% (-0·8 to 7·0%)
**Diet high in sodium: All causes**	154·9 (63·8 to 281·4)	6·1% (2·5 to 11·0%)	190·9 (79·3 to 343·1)	6·4% (2·7 to 11·5%)	23·3% (15·8 to 30·5%)	5·2% (1·3 to 9·5%)	3,541·5 (1,500·4 to 6,308·7)	2·4% (1·0 to 4·3%)	4,391·5 (1,859·4 to 7,797·5)	2·7% (1·1 to 4·7%)	24·0% (16·0 to 31·4%)	10·9% (5·4 to 16·8%)
Stomach cancer	9·7 (4·7 to 15·4)	37·1% (17·9 to 58·8%)	11·6 (5·6 to 18·5)	37·2% (17·9 to 59·2%)	19·9% (8·1 to 31·4%)	0·2% (-5·4 to 5·6%)	243·4 (119·2 to 383·0)	36·3% (18·0 to 56·9%)	291·3 (139·4 to 463·7)	36·7% (18·2 to 57·6%)	19·7% (8·1 to 31·6%)	0·9% (-3·5 to 5·2%)
Rheumatic heart disease	0·5 (0·1 to 0·9)	5·4% (1·8 to 11·0%)	0·5 (0·2 to 1·0)	5·9% (2·0 to 11·9%)	12·0% (3·3 to 21·7%)	8·7% (5·0 to 13·3%)	17·1 (5·5 to 36·6)	4·2% (1·4 to 8·9%)	19·1 (6·1 to 39·8)	4·8% (1·5 to 10·1%)	11·6% (3·5 to 20·6%)	14·7% (9·6 to 20·4%)
Ischaemic heart disease	78·4 (31·8 to 146·2)	15·9% (6·4 to 29·7%)	95·9 (38·5 to 177·7)	16·0% (6·5 to 29·8%)	22·3% (14·8 to 29·8%)	0·6% (-2·1 to 3·6%)	1,729·3 (723·0 to 3,147·9)	15·9% (6·6 to 29·2%)	2,136·4 (883·0 to 3,920·6)	16·2% (6·8 to 29·8%)	23·5% (15·4 to 32·1%)	2·1% (-0·5 to 5·1%)
Ischaemic stroke	17·4 (6·4 to 32·9)	14·2% (5·2 to 26·7%)	21·0 (7·9 to 40·0)	14·1% (5·2 to 26·9%)	21·0% (11·8 to 30·3%)	-0·8% (-4·1 to 2·9%)	341·0 (130·0 to 631·4)	15·0% (5·8 to 27·7%)	409·6 (158·6 to 758·6)	15·2% (5·8 to 28·3%)	20·1% (11·1 to 29·0%)	1·7% (-1·0 to 4·5%)
Hemorrhagic stroke	21·1 (8·5 to 38·9)	16·1% (6·5 to 29·4%)	25·6 (10·4 to 47·7)	16·6% (6·7 to 30·3%)	20·9% (12·0 to 30·9%)	3·6% (0·8 to 6·8%)	537·8 (217·0 to 990·8)	14·2% (5·7 to 26·0%)	653·1 (259·5 to 1,214·0)	15·6% (6·2 to 28·3%)	21·4% (12·8 to 31·4%)	9·6% (6·5 to 13·4%)
Hypertensive heart disease	12·0 (3·1 to 27·7)	26·8% (7·1 to 61·5%)	14·9 (4·0 to 34·4)	27·1% (7·5 to 61·6%)	24·4% (12·5 to 39·5%)	1·3% (-2·7 to 7·9%)	231·3 (77·4 to 485·7)	26·8% (9·1 to 56·8%)	286·9 (98·5 to 609·7)	27·5% (9·6 to 57·5%)	24·0% (12·2 to 38·1%)	2·6% (-1·3 to 8·3%)
Cardiomyopathy and myocarditis	1·6 (0·6 to 3·1)	7·4% (2·8 to 13·8%)	2·4 (0·9 to 4·4)	8·2% (3·1 to 15·2%)	48·7% (34·2 to 63·4%)	11·5% (6·1 to 17·7%)	42·6 (16·2 to 83·1)	4·8% (1·9 to 9·2%)	63·0 (24·5 to 119·6)	6·0% (2·3 to 11·4%)	47·8% (33·3 to 63·5%)	23·9% (13·4 to 34·8%)
Atrial fibrillation and flutter	0·5 (0·2 to 1·0)	8·8% (3·3 to 16·9%)	0·7 (0·2 to 1·3)	8·5% (3·2 to 16·6%)	30·9% (22·6 to 38·9%)	-2·7% (-7·0 to 0·1%)	12·9 (4·8 to 24·5)	11·1% (4·3 to 20·4%)	17·0 (6·4 to 32·3)	11·0% (4·3 to 20·2%)	31·3% (25·4 to 36·8%)	-0·9% (-4·4 to 2·0%)
Aortic aneurysm	0·5 (0·2 to 1·0)	9·8% (3·8 to 18·5%)	0·7 (0·3 to 1·3)	9·9% (3·8 to 18·5%)	30·3% (17·3 to 43·4%)	0·2% (-3·7 to 4·1%)	11·7 (4·6 to 21·5)	9·9% (3·9 to 18·2%)	15·5 (6·2 to 28·9)	10·1% (4·0 to 18·6%)	32·0% (18·0 to 46·9%)	2·2% (-1·9 to 6·1%)
Peripheral vascular disease	0·0 (0·0 to 0·1)	7·0% (2·6 to 13·5%)	0·0 (0·0 to 0·1)	6·7% (2·5 to 13·3%)	42·7% (18·2 to 73·5%)	-4·1% (-14·4 to 2·5%)	2·1 (0·6 to 4·6)	7·6% (2·9 to 14·5%)	2·8 (0·9 to 6·1)	7·5% (2·8 to 14·1%)	34·5% (24·1 to 46·1%)	-2·1% (-8·0 to 2·2%)
Endocarditis	0·6 (0·2 to 1·3)	7·7% (2·8 to 14·8%)	0·8 (0·3 to 1·7)	8·1% (3·0 to 15·5%)	31·5% (20·5 to 43·8%)	5·6% (1·6 to 10·4%)	15·5 (5·7 to 30·5)	5·7% (2·1 to 11·1%)	20·5 (7·6 to 40·2)	6·6% (2·5 to 12·6%)	32·3% (22·1 to 44·8%)	14·7% (6·9 to 22·9%)
Other cardiovascular and circulatory diseases	4·7 (1·8 to 8·7)	9·6% (3·7 to 17·6%)	6·0 (2·3 to 11·1)	10·2% (4·0 to 18·6%)	27·0% (16·5 to 39·0%)	6·0% (1·7 to 10·5%)	162·9 (64·4 to 308·2)	7·2% (2·9 to 13·4%)	213·7 (84·1 to 409·7)	8·3% (3·4 to 15·3%)	31·1% (22·6 to 39·9%)	16·1% (11·1 to 21·8%)
Chronic kidney disease due to diabetes mellitus	1·6 (0·5 to 3·1)	12·7% (4·6 to 24·4%)	2·2 (0·8 to 4·4)	12·8% (4·6 to 24·5%)	41·6% (30·7 to 51·3%)	0·8% (-3·5 to 3·9%)	40·3 (14·0 to 80·0)	10·1% (3·5 to 19·1%)	56·2 (19·2 to 111·1)	10·4% (3·6 to 19·9%)	39·5% (30·0 to 48·0%)	3·8% (-0·2 to 7·3%)
Chronic kidney disease due to hypertension	5·3 (1·9 to 10·4)	12·2% (4·3 to 23·6%)	7·3 (2·6 to 14·1)	12·5% (4·5 to 24·0%)	36·7% (27·5 to 46·3%)	2·6% (-0·1 to 5·5%)	120·5 (42·8 to 238·3)	9·8% (3·6 to 19·0%)	161·7 (58·0 to 315·2)	10·4% (3·8 to 20·0%)	34·2% (25·4 to 43·3%)	5·7% (2·8 to 8·9%)
Chronic kidney disease due to glomerulonephritis	0·6 (0·2 to 1·2)	10·7% (3·8 to 20·6%)	0·8 (0·3 to 1·6)	11·3% (4·0 to 21·8%)	36·0% (25·7 to 46·3%)	5·2% (1·6 to 8·7%)	17·8 (6·2 to 35·0)	7·3% (2·6 to 14·3%)	24·0 (8·3 to 47·4)	8·1% (2·9 to 15·8%)	34·6% (26·2 to 43·0%)	10·3% (6·3 to 14·6%)
Chronic kidney disease due to other causes	0·3 (0·1 to 0·6)	8·3% (3·0 to 16·5%)	0·4 (0·2 to 0·9)	9·8% (3·6 to 19·2%)	47·2% (32·5 to 62·5%)	18·8% (11·7 to 27·9%)	15·1 (5·0 to 30·4)	5·8% (2·1 to 11·6%)	20·8 (7·1 to 42·6)	7·0% (2·5 to 13·7%)	38·1% (30·4 to 45·3%)	20·8% (14·3 to 29·1%)
**Sexual abuse and violence: All causes**	8·2 (6·0 to 10·9)	0·3% (0·2 to 0·4%)	9·1 (6·8 to 12·1)	0·3% (0·2 to 0·4%)	10·9% (-0·9 to 24·4%)	-5·3% (-13·7 to 4·3%)	929·2 (697·5 to 1,209·8)	0·6% (0·5 to 0·8%)	1,103·5 (838·3 to 1,431·8)	0·7% (0·5 to 0·8%)	18·8% (11·0 to 25·8%)	6·2% (-0·2 to 12·7%)
**Childhood sexual abuse: All causes**	3·0 (0·7 to 5·9)	0·1% (0·0 to 0·2%)	3·1 (0·8 to 6·5)	0·1% (0·0 to 0·2%)	5·8% (-5·9 to 18·4%)	-9·6% (-18·6 to 0·0%)	274·7 (109·8 to 482·8)	0·2% (0·1 to 0·3%)	299·9 (118·1 to 536·7)	0·2% (0·1 to 0·3%)	9·2% (-1·7 to 17·9%)	-2·3% (-10·9 to 5·9%)
Alcohol use disorders	0·1 (0·0 to 0·1)	4·3% (0·0 to 9·8%)	0·1 (0·0 to 0·2)	4·0% (0·0 to 9·3%)	12·2% (0·0 to 26·1%)	-7·0% (-10·9 to 0·0%)	7·5 (0·0 to 17·7)	4·4% (0·0 to 10·1%)	8·8 (0·0 to 20·6)	4·1% (0·0 to 9·5%)	16·5% (0·0 to 25·7%)	-6·5% (-10·4 to 0·0%)
Major depressive disorder	-	-	-	-	-	-	97·0 (10·3 to 218·3)	3·7% (0·4 to 7·8%)	114·8 (12·2 to 254·7)	3·4% (0·4 to 7·3%)	18·4% (12·6 to 24·0%)	-6·7% (-10·1 to -3·2%)
Dysthymia	-	-	-	-	-	-	17·4 (1·8 to 39·2)	3·7% (0·4 to 7·8%)	20·9 (2·2 to 47·6)	3·4% (0·4 to 7·3%)	20·3% (15·1 to 24·9%)	-6·9% (-10·0 to -3·4%)
Self-harm	2·9 (0·7 to 5·9)	12·2% (2·9 to 24·9%)	3·1 (0·7 to 6·5)	11·5% (2·7 to 23·9%)	5·7% (-6·2 to 18·4%)	-5·6% (-9·9 to -1·4%)	152·8 (35·1 to 307·8)	12·4% (3·0 to 25·3%)	155·3 (35·4 to 329·7)	11·8% (2·7 to 24·3%)	1·7% (-10·0 to 14·5%)	-5·2% (-9·6 to -0·7%)
**Intimate partner violence: All causes**	5·5 (4·5 to 6·6)	0·2% (0·2 to 0·3%)	6·3 (5·2 to 7·4)	0·2% (0·2 to 0·2%)	13·1% (-1·7 to 30·1%)	-3·5% (-14·5 to 9·8%)	678·2 (531·6 to 851·3)	0·5% (0·4 to 0·6%)	829·1 (637·6 to 1,051·6)	0·5% (0·4 to 0·6%)	22·2% (13·4 to 30·1%)	9·3% (2·1 to 16·9%)

Supplemental Results Table 1. All-age deaths, death PAFs, DALYs, DALY PAFs, and percent change for each risk factor and outcome for both sexes combined in 2005 and 2015 for North Africa and Middle East.

Data in parenthesis are 95% uncertainty intervals. PAFs=population attributable fractions. DALYs=disability-adjusted life-years.

	2005 Deaths (in thousands)	2005 Deaths PAF	2015 Deaths (in thousands)	2015 Deaths PAF	Percent change deaths 2005-2015	Percent change deaths PAF 2005-2015	2005 DALYs (in thousands)	2005 DALYs PAF	2015 DALYs (in thousands)	2015 DALYs PAF	Percent change DALYs 2005-2015	Percent change DALYs PAF 2005-2015
HIV/AIDS - Tuberculosis	0·0 (0·0 to 0·0)	3·0% (1·6 to 4·5%)	0·0 (0·0 to 0·0)	2·8% (1·4 to 4·1%)	25·6% (3·7 to 50·6%)	-9·0% (-20·8 to 1·9%)	0·9 (0·4 to 1·4)	2·8% (1·5 to 4·2%)	1·1 (0·5 to 1·6)	2·6% (1·4 to 3·9%)	22·6% (2·0 to 45·3%)	-6·5% (-17·0 to 3·3%)
HIV/AIDS resulting in other diseases	0·1 (0·1 to 0·2)	3·2% (1·7 to 4·9%)	0·2 (0·1 to 0·3)	3·1% (1·6 to 4·7%)	47·9% (12·6 to 84·2%)	-4·8% (-15·5 to 7·8%)	6·8 (3·2 to 11·4)	3·0% (1·6 to 4·5%)	9·8 (5·0 to 15·3)	2·9% (1·5 to 4·4%)	44·9% (11·6 to 80·7%)	-3·8% (-14·1 to 8·4%)
Maternal abortion, miscarriage, and ectopic pregnancy	0·0 (0·0 to 0·0)	0·0% (0·0 to 0·0%)	0·0 (0·0 to 0·0)	0·0% (0·0 to 0·0%)	-7·0% (-33·2 to 26·8%)	1·1% (-18·0 to 21·5%)	0·0 (0·0 to 0·0)	0·0% (0·0 to 0·0%)	0·0 (0·0 to 0·0)	0·0% (0·0 to 0·0%)	-7·6% (-32·6 to 27·3%)	1·2% (-16·8 to 21·7%)
Major depressive disorder	-	-	-	-	-	-	329·8 (212·2 to 472·9)	12·5% (10·0 to 15·0%)	434·2 (280·4 to 618·4)	12·9% (10·4 to 15·4%)	31·7% (27·0 to 36·4%)	3·8% (0·5 to 7·2%)
Dysthymia	-	-	-	-	-	-	60·6 (38·6 to 89·4)	12·7% (10·5 to 15·2%)	80·8 (51·1 to 119·2)	13·1% (10·7 to 15·5%)	33·4% (29·4 to 37·3%)	3·2% (0·5 to 6·1%)
Self-harm	3·3 (2·4 to 4·2)	13·8% (10·3 to 17·4%)	3·6 (2·7 to 4·6)	13·4% (10·2 to 16·6%)	8·2% (-8·6 to 29·0%)	-3·4% (-16·1 to 12·1%)	158·2 (113·2 to 207·6)	12·9% (9·2 to 16·6%)	164·1 (120·8 to 219·4)	12·4% (9·2 to 15·9%)	3·7% (-13·4 to 25·4%)	-3·4% (-17·2 to 13·4%)
Assault by firearm	0·6 (0·4 to 0·8)	6·4% (4·5 to 9·9%)	0·8 (0·5 to 1·1)	6·3% (4·4 to 10·1%)	25·7% (2·1 to 57·5%)	-2·9% (-24·8 to 28·6%)	34·7 (23·6 to 46·9)	6·5% (4·5 to 10·0%)	42·5 (28·6 to 58·9)	6·3% (4·4 to 10·0%)	22·4% (-1·2 to 54·5%)	-3·5% (-25·1 to 28·3%)
Assault by sharp object	0·5 (0·3 to 0·6)	7·8% (5·4 to 10·8%)	0·6 (0·4 to 0·8)	8·0% (5·6 to 11·6%)	20·1% (-2·5 to 45·6%)	2·1% (-23·6 to 34·6%)	26·4 (17·8 to 35·6)	7·9% (5·4 to 11·0%)	30·7 (19·6 to 43·9)	7·9% (5·6 to 11·3%)	16·4% (-5·7 to 41·8%)	0·9% (-24·4 to 33·9%)
Assault by other means	1·0 (0·7 to 1·3)	15·9% (12·2 to 21·1%)	1·1 (0·8 to 1·5)	16·2% (12·6 to 21·1%)	12·7% (-7·1 to 36·9%)	1·7% (-15·9 to 23·0%)	60·9 (45·9 to 77·5)	16·3% (12·5 to 21·3%)	65·9 (45·2 to 88·0)	16·5% (13·0 to 21·0%)	8·1% (-11·5 to 31·8%)	1·3% (-16·2 to 22·2%)
**Unsafe sex: All causes**	15·0 (11·8 to 19·3)	0·6% (0·5 to 0·8%)	15·8 (13·1 to 18·8)	0·5% (0·4 to 0·6%)	5·1% (-8·1 to 19·7%)	-10·3% (-21·6 to 2·1%)	1,046·5 (781·6 to 1,374·6)	0·7% (0·5 to 0·9%)	1,042·0 (818·6 to 1,298·6)	0·6% (0·5 to 0·8%)	-0·4% (-13·6 to 13·4%)	-11·0% (-23·0 to 2·1%)
HIV/AIDS - Tuberculosis	0·4 (0·3 to 0·5)	63·5% (57·3 to 69·3%)	0·5 (0·4 to 0·6)	57·3% (50·5 to 63·6%)	24·6% (5·0 to 46·7%)	-9·7% (-18·1 to -2·6%)	19·6 (15·1 to 25·4)	63·3% (57·3 to 69·0%)	23·2 (19·1 to 28·1)	57·3% (51·4 to 63·2%)	18·7% (1·4 to 38·1%)	-9·5% (-16·5 to -3·6%)
HIV/AIDS resulting in other diseases	3·2 (2·2 to 5·1)	73·4% (66·5 to 79·3%)	4·9 (3·8 to 6·3)	72·6% (66·0 to 78·1%)	53·3% (12·5 to 94·2%)	-1·1% (-4·1 to 0·6%)	167·4 (114·6 to 259·3)	73·6% (67·1 to 79·2%)	249·3 (193·9 to 325·1)	72·9% (66·4 to 78·3%)	48·9% (10·4 to 87·8%)	-0·9% (-3·6 to 0·6%)
Syphilis	6·1 (3·4 to 10·0)	100·0% (100·0 to 100·0%)	4·1 (2·2 to 6·5)	100·0% (100·0 to 100·0%)	-32·9% (-46·0 to -16·9%)	-	513·0 (281·7 to 848·2)	100·0% (100·0 to 100·0%)	348·4 (190·0 to 552·6)	100·0% (100·0 to 100·0%)	-32·1% (-45·2 to -15·8%)	-
Chlamydial infection	0·0 (0·0 to 0·0)	100·0% (100·0 to 100·0%)	0·0 (0·0 to 0·0)	100·0% (100·0 to 100·0%)	-4·1% (-24·7 to 25·2%)	-	16·0 (8·9 to 27·1)	100·0% (100·0 to 100·0%)	19·4 (10·7 to 32·2)	100·0% (100·0 to 100·0%)	21·3% (14·8 to 27·9%)	-
Gonococcal infection	0·0 (0·0 to 0·0)	100·0% (100·0 to 100·0%)	0·0 (0·0 to 0·0)	100·0% (100·0 to 100·0%)	-13·6% (-27·9 to 5·3%)	-	134·8 (72·4 to 213·8)	100·0% (100·0 to 100·0%)	172·1 (92·3 to 273·7)	100·0% (100·0 to 100·0%)	27·7% (22·6 to 32·7%)	-
Trichomoniasis	-	-	-	-	-	-	7·7 (3·1 to 16·3)	100·0% (100·0 to 100·0%)	9·7 (3·9 to 20·3)	100·0% (100·0 to 100·0%)	26·5% (22·8 to 30·3%)	-
Genital herpes	-	-	-	-	-	-	11·0 (3·4 to 26·0)	100·0% (100·0 to 100·0%)	14·3 (4·4 to 34·4)	100·0% (100·0 to 100·0%)	30·0% (24·3 to 34·0%)	-
Other sexually transmitted diseases	0·0 (0·0 to 0·0)	100·0% (100·0 to 100·0%)	0·0 (0·0 to 0·0)	100·0% (100·0 to 100·0%)	-12·0% (-28·1 to 8·5%)	-	4·8 (3·4 to 6·7)	100·0% (100·0 to 100·0%)	5·6 (3·9 to 7·9)	100·0% (100·0 to 100·0%)	16·7% (8·8 to 24·4%)	-
Cervical cancer	5·3 (4·3 to 6·5)	100·0% (100·0 to 100·0%)	6·3 (5·2 to 7·5)	100·0% (100·0 to 100·0%)	18·2% (3·6 to 33·5%)	-	172·3 (134·1 to 215·6)	100·0% (100·0 to 100·0%)	199·9 (158·7 to 244·8)	100·0% (100·0 to 100·0%)	16·0% (0·5 to 32·5%)	-
**Low physical activity: All causes**	103·4 (81·2 to 126·7)	4·1% (3·2 to 4·9%)	133·1 (106·8 to 160·0)	4·5% (3·6 to 5·4%)	28·7% (22·0 to 35·5%)	9·9% (6·5 to 13·8%)	2,787·8 (2,209·6 to 3,411·5)	1·9% (1·5 to 2·3%)	3,712·3 (2,991·1 to 4,483·4)	2·2% (1·8 to 2·7%)	33·2% (26·2 to 40·1%)	19·1% (14·2 to 24·6%)
Colon and rectum cancer	3·0 (2·1 to 3·9)	16·2% (11·6 to 20·7%)	4·3 (3·1 to 5·6)	16·6% (12·0 to 21·2%)	44·3% (35·1 to 53·1%)	2·3% (1·4 to 3·7%)	74·6 (52·8 to 96·9)	15·3% (10·8 to 19·8%)	107·6 (75·8 to 141·1)	15·8% (11·2 to 20·3%)	44·1% (35·1 to 52·7%)	2·9% (1·9 to 4·4%)
Breast cancer	2·0 (1·4 to 2·7)	10·3% (7·5 to 13·1%)	2·7 (1·9 to 3·6)	10·4% (7·6 to 13·3%)	37·8% (25·1 to 50·1%)	1·4% (0·5 to 2·6%)	67·1 (46·9 to 92·1)	10·0% (7·2 to 12·7%)	93·5 (64·6 to 126·6)	10·2% (7·4 to 12·9%)	39·5% (26·2 to 52·3%)	1·8% (0·7 to 2·9%)
Ischaemic heart disease	64·7 (45·3 to 86·4)	13·1% (9·3 to 17·2%)	81·4 (56·9 to 105·7)	13·6% (9·6 to 17·7%)	25·8% (18·7 to 33·0%)	3·5% (2·0 to 5·6%)	1,537·5 (1,059·2 to 2,047·2)	14·1% (9·8 to 18·7%)	1,951·2 (1,343·4 to 2,557·5)	14·8% (10·4 to 19·4%)	26·9% (18·8 to 35·3%)	4·9% (3·3 to 7·4%)
Ischaemic stroke	17·9 (11·0 to 25·2)	14·6% (8·8 to 20·6%)	22·3 (13·8 to 31·2)	14·9% (9·2 to 20·9%)	24·2% (15·1 to 33·0%)	1·9% (0·5 to 4·3%)	356·5 (210·8 to 501·5)	15·7% (9·3 to 22·1%)	437·0 (263·5 to 618·7)	16·3% (9·8 to 22·8%)	22·6% (13·1 to 31·6%)	3·9% (2·3 to 6·5%)
Diabetes mellitus	15·8 (12·4 to 19·4)	19·4% (15·3 to 23·5%)	22·4 (17·6 to 27·3)	20·0% (16·0 to 24·3%)	41·8% (32·8 to 51·0%)	3·4% (2·3 to 4·8%)	752·1 (551·3 to 1,004·8)	17·6% (13·7 to 21·6%)	1,123·0 (818·3 to 1,503·4)	18·4% (14·4 to 22·5%)	49·3% (43·7 to 54·4%)	4·5% (3·6 to 5·7%)
**Metabolic risks: All causes**	825·5 (774·6 to 872·7)	32·4% (31·0 to 33·7%)	1,036·8 (970·9 to 1,096·2)	34·7% (33·0 to 36·4%)	25·6% (19·0 to 31·6%)	7·2% (4·2 to 10·3%)	22,404·5 (20,575·6 to 24,283·2)	15·1% (14·3 to 15·9%)	28,683·6 (26,223·4 to 31,544·6)	17·3% (16·3 to 18·4%)	28·0% (21·8 to 33·9%)	14·5% (10·3 to 19·0%)
**High fasting plasma glucose: All causes**	232·4 (202·0 to 273·5)	9·1% (8·0 to 10·7%)	313·5 (271·7 to 369·8)	10·5% (9·2 to 12·3%)	34·9% (27·6 to 41·4%)	15·1% (11·2 to 19·1%)	7,881·3 (6,719·8 to 9,262·8)	5·3% (4·8 to 5·9%)	10,887·4 (9,251·6 to 12,869·4)	6·6% (5·9 to 7·3%)	38·1% (32·2 to 43·3%)	23·5% (19·1 to 28·3%)
Tuberculosis	2·6 (1·7 to 3·8)	13·0% (8·5 to 17·9%)	2·8 (1·8 to 4·1)	14·7% (9·7 to 20·2%)	5·8% (-6·8 to 19·7%)	13·6% (8·9 to 18·3%)	83·8 (55·0 to 118·2)	10·6% (6·9 to 14·2%)	88·1 (57·1 to 123·6)	12·4% (8·3 to 16·5%)	5·1% (-8·0 to 19·8%)	17·2% (10·6 to 24·4%)
Ischaemic heart disease	81·7 (55·7 to 115·6)	16·6% (11·2 to 23·5%)	108·2 (73·4 to 153·6)	18·1% (12·4 to 25·5%)	32·5% (24·4 to 40·7%)	9·0% (5·9 to 12·4%)	1,814·9 (1,262·2 to 2,489·7)	16·7% (11·6 to 22·6%)	2,402·5 (1,677·9 to 3,302·0)	18·2% (12·8 to 24·7%)	32·4% (24·1 to 40·7%)	9·4% (7·0 to 11·9%)
Ischaemic stroke	19·0 (11·1 to 32·5)	15·5% (8·9 to 26·8%)	25·0 (14·9 to 42·5)	16·7% (9·8 to 28·9%)	31·6% (20·7 to 42·9%)	8·0% (4·1 to 13·6%)	365·3 (230·3 to 568·6)	16·1% (10·3 to 25·2%)	472·1 (303·0 to 711·3)	17·6% (11·4 to 27·1%)	29·3% (19·3 to 38·8%)	9·6% (7·0 to 12·8%)
Hemorrhagic stroke	19·0 (12·9 to 26·9)	14·4% (9·9 to 19·9%)	24·6 (16·8 to 34·7)	16·0% (11·2 to 22·0%)	29·5% (20·0 to 40·1%)	11·0% (7·9 to 14·4%)	500·4 (325·3 to 727·3)	13·2% (8·6 to 19·2%)	646·7 (425·3 to 939·8)	15·4% (10·1 to 22·4%)	29·3% (19·6 to 40·1%)	16·6% (12·7 to 21·0%)
Diabetes mellitus	81·6 (76·9 to 87·2)	100·0% (100·0 to 100·0%)	111·8 (104·7 to 119·2)	100·0% (100·0 to 100·0%)	37·1% (28·5 to 45·9%)	-	4,276·4 (3,484·3 to 5,217·1)	100·0% (100·0 to 100·0%)	6,109·8 (4,907·6 to 7,560·3)	100·0% (100·0 to 100·0%)	42·9% (37·7 to 47·4%)	-
Chronic kidney disease due to diabetes mellitus	12·4 (10·7 to 14·2)	100·0% (100·0 to 100·0%)	17·4 (15·0 to 20·0)	100·0% (100·0 to 100·0%)	40·5% (31·5 to 49·2%)	-	399·8 (348·6 to 456·7)	100·0% (100·0 to 100·0%)	537·3 (467·1 to 616·7)	100·0% (100·0 to 100·0%)	34·4% (27·0 to 41·5%)	-
Chronic kidney disease due to hypertension	13·7 (9·4 to 18·1)	31·3% (21·7 to 40·9%)	19·8 (13·6 to 26·2)	34·1% (23·9 to 44·2%)	45·2% (35·5 to 54·7%)	9·0% (6·7 to 11·4%)	329·1 (226·6 to 439·2)	26·8% (18·4 to 35·3%)	468·1 (319·0 to 618·3)	30·0% (20·9 to 39·2%)	42·2% (33·3 to 51·6%)	12·0% (9·6 to 14·8%)
Chronic kidney disease due to glomerulonephritis	1·6 (1·1 to 2·2)	28·6% (20·0 to 37·7%)	2·3 (1·6 to 3·1)	32·4% (22·9 to 42·2%)	46·3% (35·6 to 57·2%)	13·2% (10·5 to 16·2%)	54·0 (36·8 to 73·8)	22·3% (15·4 to 29·9%)	78·2 (53·1 to 106·7)	26·4% (18·5 to 34·7%)	44·7% (36·5 to 53·3%)	18·6% (15·1 to 22·6%)
Chronic kidney disease due to other causes	0·8 (0·6 to 1·1)	23·3% (15·9 to 30·7%)	1·3 (0·9 to 1·8)	29·7% (20·7 to 38·5%)	57·9% (42·7 to 72·9%)	27·5% (21·3 to 34·8%)	48·7 (32·2 to 67·8)	18·7% (12·8 to 25·3%)	71·3 (47·2 to 99·1)	24·0% (16·7 to 31·6%)	46·7% (38·5 to 54·1%)	28·3% (22·9 to 35·2%)
**High total cholesterol: All causes**	255·6 (206·6 to 312·8)	10·0% (8·2 to 12·2%)	309·1 (250·4 to 374·6)	10·3% (8·5 to 12·6%)	20·9% (13·3 to 28·4%)	3·2% (-0·4 to 7·5%)	6,318·5 (5,326·8 to 7,360·7)	4·3% (3·6 to 5·0%)	7,726·9 (6,577·4 to 8,986·5)	4·7% (3·9 to 5·5%)	22·3% (13·9 to 30·7%)	9·4% (3·5 to 15·3%)
Ischaemic heart disease	228·9 (185·3 to 274·7)	46·4% (38·1 to 55·2%)	277·0 (223·9 to 333·0)	46·2% (37·9 to 55·1%)	21·0% (13·2 to 28·6%)	-0·4% (-2·2 to 1·6%)	5,712·2 (4,825·3 to 6,664·2)	52·4% (44·9 to 60·2%)	7,002·8 (5,952·0 to 8,171·1)	53·1% (45·8 to 61·0%)	22·6% (13·9 to 31·0%)	1·3% (0·0 to 3·0%)
Ischaemic stroke	26·8 (12·8 to 48·8)	21·8% (10·9 to 40·3%)	32·1 (15·6 to 59·9)	21·5% (10·7 to 39·1%)	20·0% (9·7 to 30·1%)	-1·6% (-5·7 to 2·1%)	606·3 (395·4 to 928·4)	26·6% (17·9 to 40·5%)	724·1 (474·9 to 1,100·2)	26·9% (18·1 to 41·0%)	19·4% (8·9 to 30·2%)	1·1% (-1·7 to 4·7%)
**High systolic blood pressure: All causes**	521·5 (468·6 to 573·4)	20·5% (18·6 to 22·3%)	650·0 (582·9 to 717·2)	21·8% (19·6 to 23·8%)	24·6% (18·1 to 30·8%)	6·4% (3·2 to 9·6%)	12,198·8 (10,968·4 to 13,415·0)	8·2% (7·3 to 9·2%)	15,215·0 (13,688·5 to 16,846·8)	9·2% (8·1 to 10·4%)	24·7% (17·5 to 31·4%)	11·6% (6·5 to 16·7%)
Rheumatic heart disease	2·0 (1·4 to 2·8)	23·4% (16·4 to 33·4%)	2·2 (1·6 to 3·2)	25·8% (18·1 to 37·4%)	13·4% (3·7 to 23·9%)	10·1% (5·9 to 14·2%)	79·4 (50·0 to 114·1)	19·3% (12·4 to 27·9%)	89·7 (57·4 to 128·5)	22·4% (14·0 to 33·0%)	13·0% (4·0 to 22·9%)	16·0% (10·2 to 22·1%)
Ischaemic heart disease	261·6 (220·8 to 303·2)	53·1% (44·7 to 60·9%)	322·8 (273·2 to 374·7)	53·9% (45·2 to 61·9%)	23·4% (16·4 to 29·9%)	1·5% (-0·3 to 3·4%)	5,809·7 (4,946·5 to 6,636·3)	53·3% (45·8 to 60·0%)	7,223·9 (6,157·3 to 8,297·3)	54·8% (47·2 to 61·8%)	24·3% (16·6 to 31·9%)	2·8% (1·2 to 4·5%)
Ischaemic stroke	60·1 (47·5 to 72·6)	49·1% (39·7 to 58·4%)	73·5 (57·9 to 89·1)	49·2% (40·0 to 58·7%)	22·2% (13·3 to 30·8%)	0·2% (-2·2 to 2·9%)	1,162·3 (932·2 to 1,380·4)	51·1% (42·2 to 59·2%)	1,401·3 (1,129·6 to 1,642·4)	52·2% (43·4 to 60·3%)	20·6% (11·5 to 28·7%)	2·1% (0·3 to 4·0%)

Supplemental Results Table 1. All-age deaths, death PAFs, DALYs, DALY PAFs, and percent change for each risk factor and outcome for both sexes combined in 2005 and 2015 for North Africa and Middle East.

Data in parenthesis are 95% uncertainty intervals. PAFs=population attributable fractions. DALYs=disability-adjusted life-years.

	2005 Deaths (in thousands)	2005 Deaths PAF	2015 Deaths (in thousands)	2015 Deaths PAF	Percent change deaths 2005-2015	Percent change deaths PAF 2005-2015	2005 DALYs (in thousands)	2005 DALYs PAF	2015 DALYs (in thousands)	2015 DALYs PAF	Percent change DALYs 2005-2015	Percent change DALYs PAF 2005-2015
Hemorrhagic stroke	70·4 (58·2 to 84·0)	53·4% (44·3 to 61·7%)	85·6 (69·9 to 104·0)	55·7% (46·2 to 64·6%)	21·6% (13·1 to 31·2%)	4·2% (2·2 to 6·5%)	1,832·0 (1,500·4 to 2,174·1)	48·3% (40·0 to 55·5%)	2,231·4 (1,857·2 to 2,681·0)	53·1% (44·1 to 60·6%)	21·8% (12·8 to 31·2%)	9·9% (7·1 to 12·9%)
Hypertensive heart disease	44·9 (40·1 to 50·6)	100·0% (100·0 to 100·0%)	55·1 (49·4 to 61·0)	100·0% (100·0 to 100·0%)	22·8% (11·2 to 35·4%)	-	866·5 (780·7 to 997·0)	100·0% (100·0 to 100·0%)	1,047·0 (942·4 to 1,184·0)	100·0% (100·0 to 100·0%)	20·8% (9·7 to 32·7%)	-
Cardiomyopathy and myocarditis	6·0 (4·7 to 7·5)	27·4% (21·7 to 32·9%)	9·0 (7·1 to 11·1)	31·1% (24·8 to 36·9%)	51·1% (37·8 to 64·4%)	13·4% (7·9 to 18·5%)	166·4 (126·4 to 213·4)	19·0% (14·6 to 23·5%)	249·5 (193·9 to 310·6)	23·8% (18·7 to 29·0%)	49·9% (36·3 to 64·7%)	25·7% (15·1 to 36·2%)
Atrial fibrillation and flutter	2·0 (1·5 to 2·8)	34·7% (27·6 to 41·5%)	2·7 (1·9 to 3·7)	34·1% (27·1 to 41·4%)	32·5% (24·7 to 40·8%)	-1·5% (-4·8 to 1·7%)	47·1 (36·7 to 60·2)	40·4% (35·4 to 45·4%)	62·4 (48·7 to 80·2)	40·4% (35·3 to 45·6%)	32·5% (27·7 to 36·8%)	0·1% (-1·9 to 1·8%)
Aortic aneurysm	1·9 (1·6 to 2·3)	37·0% (30·3 to 43·4%)	2·5 (2·1 to 3·0)	37·4% (30·6 to 43·8%)	31·4% (19·8 to 43·4%)	1·1% (-1·3 to 3·4%)	44·7 (37·4 to 53·5)	37·8% (32·1 to 43·9%)	59·4 (49·8 to 69·6)	38·9% (33·2 to 45·3%)	32·9% (19·9 to 46·4%)	2·9% (0·6 to 5·3%)
Peripheral vascular disease	0·1 (0·1 to 0·2)	28·2% (21·5 to 35·4%)	0·2 (0·1 to 0·3)	26·9% (20·2 to 34·4%)	41·7% (19·5 to 71·5%)	-4·7% (-10·2 to 0·2%)	8·0 (4·6 to 13·4)	29·3% (22·9 to 35·7%)	10·8 (6·3 to 18·0)	28·8% (22·5 to 35·1%)	34·9% (27·1 to 44·5%)	-1·9% (-4·8 to 0·7%)
Endocarditis	2·6 (2·0 to 3·8)	32·1% (25·7 to 38·3%)	3·4 (2·6 to 5·1)	34·1% (27·4 to 40·9%)	31·9% (20·8 to 44·4%)	6·0% (2·1 to 9·7%)	66·4 (50·1 to 93·5)	24·5% (19·2 to 29·8%)	88·1 (66·0 to 124·9)	28·2% (22·0 to 34·0%)	32·7% (22·2 to 45·8%)	15·1% (7·5 to 22·7%)
Other cardiovascular and circulatory diseases	17·5 (15·2 to 20·0)	35·5% (31·5 to 39·8%)	22·4 (19·4 to 25·5)	38·0% (33·6 to 42·4%)	28·2% (18·5 to 38·9%)	7·1% (3·9 to 10·0%)	615·7 (514·5 to 730·0)	27·2% (23·9 to 30·8%)	816·4 (679·7 to 966·5)	31·9% (28·2 to 35·9%)	32·6% (25·1 to 40·0%)	17·5% (13·4 to 21·8%)
Chronic kidney disease due to diabetes mellitus	5·6 (3·9 to 7·3)	44·9% (32·2 to 56·5%)	7·9 (5·5 to 10·4)	45·3% (32·8 to 56·8%)	41·7% (32·6 to 51·0%)	0·9% (-1·2 to 3·1%)	147·9 (102·5 to 196·0)	37·0% (25·8 to 47·3%)	205·2 (140·6 to 270·7)	38·2% (27·0 to 48·6%)	38·7% (30·7 to 46·2%)	3·2% (1·2 to 6·0%)
Chronic kidney disease due to hypertension	43·7 (40·3 to 48·0)	100·0% (100·0 to 100·0%)	58·2 (52·9 to 64·0)	100·0% (100·0 to 100·0%)	33·2% (25·0 to 41·7%)	-	1,229·7 (1,115·6 to 1,347·2)	100·0% (100·0 to 100·0%)	1,561·2 (1,406·9 to 1,716·9)	100·0% (100·0 to 100·0%)	27·0% (18·9 to 35·1%)	-
Chronic kidney disease due to glomerulonephritis	2·1 (1·5 to 2·7)	37·5% (26·9 to 47·9%)	2·9 (2·0 to 3·8)	40·0% (28·8 to 50·9%)	37·6% (27·9 to 47·4%)	6·5% (3·8 to 9·4%)	66·3 (45·3 to 88·4)	27·4% (19·1 to 36·0%)	90·1 (61·3 to 122·0)	30·5% (21·6 to 39·8%)	35·8% (28·1 to 43·6%)	11·3% (7·8 to 15·4%)
Chronic kidney disease due to other causes	1·0 (0·7 to 1·4)	28·6% (19·8 to 37·0%)	1·5 (1·0 to 2·1)	34·4% (24·0 to 44·2%)	49·2% (36·1 to 63·5%)	20·5% (13·6 to 28·7%)	56·7 (37·5 to 77·4)	21·8% (14·9 to 28·8%)	78·7 (51·6 to 107·9)	26·5% (18·2 to 35·0%)	39·0% (32·7 to 45·2%)	21·6% (16·0 to 29·4%)
**High body-mass index: All causes**	284·9 (215·4 to 355·8)	11·2% (8·5 to 13·9%)	375·6 (287·8 to 463·9)	12·6% (9·7 to 15·5%)	31·8% (24·3 to 39·0%)	12·6% (8·3 to 17·1%)	9,659·2 (7,489·0 to 11,834·6)	6·5% (5·2 to 7·9%)	13,257·1 (10,382·0 to 16,215·4)	8·0% (6·4 to 9·6%)	37·2% (30·1 to 43·5%)	22·7% (17·7 to 28·2%)
Oesophageal cancer	1·6 (0·6 to 2·7)	24·3% (8·4 to 40·2%)	2·1 (0·8 to 3·4)	25·5% (9·3 to 41·9%)	26·0% (9·4 to 47·2%)	5·2% (-4·6 to 15·8%)	42·2 (14·8 to 69·2)	24·8% (8·6 to 40·6%)	53·3 (19·7 to 86·5)	26·2% (9·5 to 42·5%)	26·4% (9·5 to 47·7%)	5·6% (-4·4 to 16·7%)
Colon and rectum cancer	1·7 (1·1 to 2·5)	9·4% (6·1 to 13·2%)	2·7 (1·8 to 3·8)	10·5% (6·8 to 14·5%)	56·1% (44·9 to 66·2%)	10·8% (7·2 to 15·5%)	46·9 (30·2 to 65·2)	9·6% (6·3 to 13·4%)	73·3 (47·9 to 101·0)	10·7% (7·1 to 14·7%)	56·3% (45·7 to 66·7%)	11·6% (8·0 to 16·7%)
Liver cancer due to hepatitis B	0·8 (0·4 to 1·4)	17·5% (8·3 to 28·5%)	1·2 (0·6 to 2·1)	19·2% (9·1 to 30·8%)	46·8% (32·6 to 63·3%)	9·4% (3·9 to 15·7%)	25·6 (12·1 to 42·7)	17·1% (8·2 to 27·5%)	37·3 (16·8 to 61·5)	18·9% (9·1 to 30·1%)	45·3% (31·2 to 61·9%)	10·7% (5·0 to 17·8%)
Liver cancer due to hepatitis C	1·5 (0·7 to 2·5)	19·5% (9·3 to 31·0%)	2·2 (1·1 to 3·5)	21·0% (10·0 to 33·1%)	42·9% (28·0 to 60·4%)	7·6% (2·2 to 14·4%)	37·5 (17·8 to 61·1)	20·4% (9·7 to 32·2%)	53·2 (25·3 to 85·3)	21·9% (10·6 to 34·3%)	41·7% (27·6 to 60·4%)	7·6% (2·1 to 14·8%)
Liver cancer due to alcohol use	0·4 (0·2 to 0·8)	19·5% (8·9 to 32·8%)	0·7 (0·3 to 1·2)	21·2% (9·5 to 35·0%)	46·5% (33·4 to 63·4%)	8·6% (4·1 to 14·2%)	11·1 (4·6 to 20·4)	20·3% (9·3 to 33·8%)	16·3 (6·7 to 30·0)	22·0% (9·9 to 35·9%)	47·0% (33·3 to 64·6%)	8·6% (3·8 to 14·4%)
Liver cancer due to other causes	0·5 (0·2 to 0·9)	17·4% (8·2 to 28·3%)	0·7 (0·3 to 1·2)	18·9% (9·1 to 29·9%)	42·3% (28·4 to 58·5%)	8·5% (2·6 to 16·7%)	13·7 (6·4 to 23·2)	17·1% (8·0 to 27·6%)	19·3 (9·3 to 31·6)	18·8% (9·1 to 29·5%)	41·0% (27·0 to 57·4%)	10·3% (4·2 to 19·4%)
Gallbladder and biliary tract cancer	1·0 (0·6 to 1·5)	22·2% (13·9 to 31·6%)	1·3 (0·8 to 1·9)	24·0% (15·4 to 33·7%)	30·1% (12·5 to 50·4%)	7·9% (2·2 to 14·3%)	25·6 (16·1 to 37·3)	22·9% (14·3 to 32·5%)	33·3 (20·1 to 48·3)	24·8% (16·0 to 34·6%)	30·0% (12·1 to 50·4%)	8·4% (2·4 to 15·0%)
Pancreatic cancer	0·8 (0·3 to 1·3)	7·1% (2·6 to 12·3%)	1·1 (0·4 to 1·8)	7·8% (2·9 to 13·1%)	41·0% (31·7 to 51·9%)	8·9% (5·1 to 15·2%)	18·8 (6·7 to 32·7)	7·4% (2·6 to 12·7%)	26·8 (9·7 to 45·3)	8·0% (2·9 to 13·6%)	42·1% (32·3 to 52·6%)	8·9% (5·1 to 14·6%)
Breast cancer	0·2 (-0·1 to 0·5)	1·0% (-0·3 to 2·6%)	0·4 (0·0 to 0·8)	1·4% (0·0 to 3·0%)	87·7% (-290·3 to 631·0%)	37·7% (-247·0 to 445·7%)	-13·6 (-27·5 to -3·6)	-2·0% (-3·9 to -0·6%)	-14·9 (-31·6 to -1·6)	-1·6% (-3·4 to -0·2%)	10·1% (-65·6 to 56·4%)	-19·8% (-73·8 to 11·1%)
Uterine cancer	1·6 (1·2 to 2·1)	44·2% (32·6 to 55·0%)	2·0 (1·5 to 2·6)	46·3% (34·9 to 57·2%)	23·4% (11·3 to 36·6%)	4·6% (1·6 to 8·4%)	43·8 (31·7 to 57·9)	44·8% (33·3 to 55·6%)	54·4 (39·9 to 70·7)	47·0% (35·5 to 57·6%)	24·2% (11·9 to 37·7%)	4·8% (1·8 to 8·9%)
Ovarian cancer	0·2 (0·0 to 0·4)	4·3% (-0·2 to 9·2%)	0·2 (0·0 to 0·5)	4·5% (-0·2 to 9·6%)	35·6% (22·8 to 48·6%)	6·6% (3·3 to 10·9%)	5·6 (-0·2 to 12·1)	4·2% (-0·2 to 9·1%)	7·5 (-0·3 to 15·8)	4·5% (-0·2 to 9·6%)	35·1% (21·8 to 48·3%)	6·5% (3·1 to 10·9%)
Kidney cancer	0·7 (0·5 to 0·9)	19·3% (13·4 to 25·8%)	1·0 (0·7 to 1·3)	20·8% (14·7 to 27·6%)	45·8% (33·8 to 57·1%)	8·2% (4·2 to 12·3%)	18·7 (13·0 to 24·9)	16·7% (11·6 to 22·1%)	27·5 (19·3 to 36·8)	18·7% (13·4 to 24·6%)	46·5% (34·8 to 57·9%)	12·4% (7·3 to 19·0%)
Thyroid cancer	0·2 (0·1 to 0·3)	13·8% (8·3 to 20·2%)	0·2 (0·1 to 0·4)	15·0% (9·2 to 21·8%)	39·5% (28·5 to 51·8%)	9·0% (5·1 to 13·6%)	5·0 (2·9 to 7·4)	13·9% (8·5 to 20·4%)	7·2 (4·3 to 10·4)	15·3% (9·3 to 22·1%)	43·7% (31·1 to 56·6%)	9·5% (5·1 to 14·7%)
Ischaemic heart disease	119·8 (87·5 to 155·1)	24·3% (17·9 to 31·0%)	155·1 (114·6 to 197·5)	25·9% (19·2 to 32·7%)	29·4% (21·7 to 37·1%)	6·5% (3·4 to 10·0%)	3,156·6 (2,350·5 to 4,068·7)	29·0% (21·8 to 36·6%)	4,126·3 (3,106·0 to 5,200·3)	31·3% (23·9 to 39·0%)	30·7% (22·4 to 38·9%)	8·1% (5·2 to 11·5%)
Ischaemic stroke	23·2 (16·7 to 30·6)	19·0% (13·8 to 24·7%)	29·1 (21·0 to 38·3)	19·5% (14·3 to 25·3%)	25·2% (15·2 to 35·1%)	2·7% (-1·7 to 7·2%)	600·2 (447·6 to 767·7)	26·4% (20·0 to 32·9%)	757·4 (573·7 to 955·5)	28·2% (21·7 to 34·9%)	26·2% (16·2 to 35·8%)	6·9% (3·6 to 10·7%)
Hemorrhagic stroke	42·4 (32·6 to 53·3)	32·2% (24·8 to 39·9%)	53·0 (41·1 to 67·1)	34·5% (27·1 to 42·4%)	25·2% (15·8 to 35·2%)	7·3% (3·4 to 11·4%)	1,371·5 (1,080·2 to 1,697·3)	36·2% (28·7 to 43·7%)	1,729·8 (1,389·3 to 2,143·5)	41·2% (33·2 to 49·1%)	26·1% (16·5 to 36·4%)	13·8% (10·1 to 18·2%)
Hypertensive heart disease	21·1 (14·3 to 28·7)	47·1% (32·9 to 62·7%)	27·1 (18·2 to 36·7)	49·2% (34·0 to 65·3%)	28·5% (16·1 to 42·9%)	4·7% (-1·2 to 10·3%)	453·0 (344·7 to 571·9)	52·3% (40·9 to 64·4%)	579·7 (442·5 to 731·4)	55·4% (43·8 to 67·4%)	28·0% (16·4 to 40·9%)	5·9% (2·3 to 10·0%)
Diabetes mellitus	41·7 (33·3 to 50·9)	51·2% (41·0 to 61·4%)	59·2 (47·5 to 71·1)	53·0% (42·9 to 63·4%)	42·0% (32·7 to 51·2%)	3·6% (0·7 to 6·6%)	2,645·5 (1,981·9 to 3,375·9)	61·8% (52·5 to 70·2%)	4,008·2 (2,999·6 to 5,148·0)	65·5% (56·4 to 73·6%)	51·5% (45·7 to 57·0%)	6·1% (4·4 to 8·1%)
Chronic kidney disease due to diabetes mellitus	4·6 (2·5 to 7·1)	37·3% (20·4 to 54·8%)	6·9 (3·7 to 10·3)	40·0% (22·4 to 57·6%)	50·5% (37·1 to 65·0%)	7·2% (-0·1 to 14·3%)	139·4 (68·6 to 213·0)	34·9% (17·5 to 51·2%)	207·7 (103·1 to 309·8)	38·7% (19·7 to 56·1%)	49·1% (39·1 to 59·1%)	10·9% (6·9 to 16·2%)
Chronic kidney disease due to hypertension	16·7 (9·5 to 24·0)	38·3% (21·9 to 54·3%)	23·6 (13·3 to 33·3)	40·5% (22·7 to 57·1%)	40·6% (28·9 to 52·9%)	5·6% (0·0 to 11·7%)	438·2 (231·0 to 631·6)	35·7% (18·8 to 51·5%)	608·1 (325·2 to 873·2)	39·0% (21·1 to 55·1%)	38·8% (29·3 to 48·8%)	9·3% (5·6 to 14·3%)
Chronic kidney disease due to glomerulonephritis	1·8 (0·9 to 2·7)	31·9% (15·5 to 47·3%)	2·5 (1·2 to 3·7)	35·0% (16·9 to 51·4%)	41·8% (28·2 to 55·3%)	9·7% (3·0 to 16·9%)	63·9 (23·5 to 97·4)	26·4% (9·8 to 39·6%)	90·6 (33·4 to 139·2)	30·7% (11·2 to 45·4%)	41·8% (32·3 to 51·7%)	16·2% (11·5 to 22·3%)
Chronic kidney disease due to other causes	0·7 (0·3 to 1·2)	20·0% (8·6 to 32·4%)	1·1 (0·4 to 1·8)	25·0% (9·3 to 40·2%)	55·2% (25·7 to 75·0%)	25·2% (3·8 to 40·2%)	51·5 (21·6 to 81·9)	19·8% (8·7 to 30·6%)	75·8 (32·8 to 120·4)	25·5% (11·4 to 38·2%)	47·1% (39·0 to 56·2%)	28·7% (21·5 to 39·9%)
Osteoarthritis	-	-	-	-	-	-	172·6 (107·6 to 255·6)	28·0% (20·7 to 35·6%)	266·9 (167·7 to 391·3)	29·8% (22·4 to 37·3%)	54·7% (51·0 to 59·0%)	6·4% (4·2 to 9·2%)
Low back pain	-	-	-	-	-	-	241·1 (146·0 to 372·0)	7·3% (5·2 to 9·6%)	348·4 (213·1 to 534·6)	8·3% (6·0 to 10·7%)	44·5% (41·0 to 48·8%)	13·3% (10·8 to 16·5%)
**Low bone mineral density: All causes**	10·1 (8·9 to 11·0)	0·4% (0·4 to 0·4%)	14·2 (11·7 to 15·6)	0·5% (0·4 to 0·5%)	40·3% (26·1 to 54·2%)	19·8% (9·4 to 29·3%)	327·6 (280·3 to 376·5)	0·2% (0·2 to 0·2%)	429·7 (365·0 to 497·0)	0·3% (0·2 to 0·3%)	31·2% (22·7 to 39·8%)	17·3% (10·4 to 24·6%)
Pedestrian road injuries	2·0 (1·8 to 2·3)	5·6% (5·1 to 6·0%)	2·5 (2·2 to 2·9)	6·6% (6·0 to 7·2%)	25·0% (11·4 to 39·3%)	18·3% (9·4 to 27·3%)	44·6 (38·5 to 50·6)	2·3% (2·1 to 2·6%)	54·8 (47·5 to 62·8)	2·9% (2·6 to 3·2%)	22·8% (8·9 to 38·6%)	25·0% (14·0 to 37·0%)
Cyclist road injuries	0·1 (0·1 to 0·1)	3·7% (3·2 to 4·3%)	0·1 (0·1 to 0·1)	4·4% (3·8 to 5·0%)	25·7% (3·9 to 50·5%)	18·0% (3·9 to 35·5%)	4·5 (3·5 to 5·6)	3·0% (2·4 to 3·7%)	5·9 (4·6 to 7·4)	3·9% (3·1 to 4·6%)	31·0% (17·1 to 45·1%)	28·2% (13·6 to 44·7%)
Motorcyclist road injuries	0·2 (0·1 to 0·2)	1·8% (1·2 to 2·2%)	0·2 (0·1 to 0·3)	2·2% (1·5 to 2·7%)	16·9% (-14·8 to 52·7%)	20·4% (-1·8 to 43·7%)	8·9 (6·7 to 10·9)	1·3% (1·0 to 1·7%)	10·7 (8·1 to 13·5)	1·7% (1·3 to 2·1%)	20·1% (-2·2 to 43·1%)	27·4% (10·1 to 47·0%)
Motor vehicle road injuries	3·3 (2·8 to 3·7)	4·7% (4·2 to 5·1%)	4·3 (3·6 to 4·8)	5·4% (4·9 to 5·8%)	28·2% (15·8 to 42·0%)	14·9% (7·7 to 22·4%)	103·4 (88·2 to 117·2)	2·6% (2·3 to 2·9%)	134·5 (114·0 to 154·4)	3·2% (2·8 to 3·6%)	30·1% (18·0 to 43·0%)	22·1% (13·0 to 31·4%)
Other road injuries	0·1 (0·1 to 0·2)	3·4% (1·9 to 6·0%)	0·1 (0·1 to 0·2)	3·9% (2·4 to 6·9%)	38·2% (7·7 to 81·6%)	14·4% (-15·0 to 63·3%)	3·7 (2·6 to 5·1)	2·0% (1·1 to 3·8%)	5·3 (3·8 to 7·2)	2·4% (1·4 to 4·5%)	41·2% (20·3 to 66·1%)	20·0% (-13·0 to 74·0%)

Supplemental Results Table 1. All-age deaths, death PAFs, DALYs, DALY PAFs, and percent change for each risk factor and outcome for both sexes combined in 2005 and 2015 for North Africa and Middle East.

Data in parenthesis are 95% uncertainty intervals. PAFs=population attributable fractions. DALYs=disability-adjusted life-years.

	2005 Deaths (in thousands)	2005 Deaths PAF	2015 Deaths (in thousands)	2015 Deaths PAF	Percent change deaths 2005-2015	Percent change deaths PAF 2005-2015	2005 DALYs (in thousands)	2005 DALYs PAF	2015 DALYs (in thousands)	2015 DALYs PAF	Percent change DALYs 2005-2015	Percent change DALYs PAF 2005-2015
Other transport injuries	0·4 (0·3 to 0·4)	5·9% (5·2 to 6·7%)	0·6 (0·5 to 0·7)	7·0% (6·2 to 7·9%)	58·0% (31·2 to 88·8%)	18·8% (6·5 to 33·0%)	9·2 (7·9 to 10·6)	2·4% (2·2 to 2·8%)	14·1 (11·6 to 16·8)	3·1% (2·7 to 3·4%)	52·9% (28·1 to 80·9%)	25·8% (12·3 to 41·5%)
Falls	3·5 (2·8 to 3·8)	18·8% (15·3 to 21·5%)	5·7 (3·7 to 6·6)	23·9% (18·3 to 27·1%)	62·7% (29·3 to 87·8%)	26·9% (5·7 to 56·1%)	122·0 (98·3 to 149·4)	7·9% (6·4 to 9·0%)	167·1 (134·1 to 204·8)	9·4% (8·0 to 10·5%)	36·9% (29·1 to 44·2%)	18·6% (1·3 to 42·9%)
Other exposure to mechanical forces	0·3 (0·3 to 0·3)	3·0% (2·6 to 3·5%)	0·4 (0·3 to 0·4)	3·7% (3·1 to 4·2%)	22·6% (8·3 to 35·2%)	20·6% (9·3 to 32·2%)	22·5 (16·7 to 29·3)	2·6% (2·2 to 3·2%)	27·1 (19·8 to 35·9)	3·2% (2·6 to 3·8%)	20·3% (13·2 to 27·4%)	20·2% (11·6 to 28·7%)
Non-venomous animal contact	0·0 (0·0 to 0·0)	3·6% (2·8 to 4·7%)	0·0 (0·0 to 0·1)	4·5% (3·7 to 5·6%)	20·7% (5·4 to 38·9%)	25·2% (9·1 to 44·5%)	0·9 (0·7 to 1·1)	1·1% (0·9 to 1·4%)	1·1 (0·8 to 1·3)	1·4% (1·1 to 1·7%)	15·6% (5·1 to 27·1%)	23·8% (10·0 to 41·9%)
Assault by other means	0·2 (0·1 to 0·2)	3·1% (2·2 to 3·7%)	0·3 (0·1 to 0·3)	3·7% (2·6 to 4·6%)	32·7% (7·5 to 66·0%)	19·7% (-2·2 to 46·0%)	5·0 (3·7 to 6·0)	1·3% (1·1 to 1·6%)	6·4 (4·5 to 7·6)	1·6% (1·3 to 1·9%)	27·1% (9·5 to 46·7%)	19·5% (2·3 to 39·0%)
Exposure to forces of nature	-	-	-	-	-	-	2·9 (1·2 to 4·9)	2·1% (0·9 to 3·5%)	2·9 (1·2 to 5·0)	4·0% (2·3 to 5·8%)	0·6% (-9·3 to 9·9%)	93·4% (60·2 to 150·2%)
**Low glomerular filtration rate: All causes**	122·2 (112·9 to 131·4)	4·8% (4·5 to 5·1%)	156·2 (144·2 to 168·7)	5·2% (4·8 to 5·6%)	27·8% (21·4 to 33·8%)	9·1% (5·5 to 13·0%)	3,204·1 (2,950·6 to 3,466·8)	2·2% (2·0 to 2·4%)	3,984·3 (3,664·4 to 4,317·7)	2·4% (2·2 to 2·7%)	24·3% (18·0 to 30·3%)	11·3% (6·6 to 16·3%)
Ischaemic heart disease	37·2 (32·2 to 42·4)	7·5% (6·6 to 8·5%)	45·0 (39·2 to 51·2)	7·5% (6·6 to 8·5%)	21·0% (14·3 to 27·4%)	-0·5% (-2·6 to 1·8%)	695·3 (602·8 to 794·7)	6·4% (5·6 to 7·2%)	841·4 (731·9 to 953·3)	6·4% (5·6 to 7·2%)	21·0% (13·7 to 28·1%)	0·0% (-2·3 to 2·3%)
Ischaemic stroke	10·4 (8·6 to 12·3)	8·5% (7·1 to 9·9%)	12·7 (10·4 to 15·0)	8·5% (7·1 to 9·9%)	22·6% (13·4 to 31·7%)	0·5% (-2·3 to 3·6%)	169·3 (140·7 to 199·8)	7·4% (6·3 to 8·6%)	202·3 (168·7 to 237·9)	7·5% (6·4 to 8·7%)	19·4% (10·5 to 28·0%)	1·2% (-1·2 to 3·9%)
Hemorrhagic stroke	9·3 (7·8 to 11·1)	7·1% (6·0 to 8·3%)	11·2 (9·4 to 13·4)	7·3% (6·2 to 8·5%)	19·9% (11·1 to 30·1%)	2·7% (-0·2 to 5·7%)	197·0 (164·5 to 236·6)	5·2% (4·4 to 6·2%)	235·3 (196·0 to 283·0)	5·6% (4·8 to 6·6%)	19·4% (10·7 to 29·7%)	7·8% (4·0 to 11·7%)
Peripheral vascular disease	0·1 (0·0 to 0·1)	13·4% (9·3 to 17·9%)	0·1 (0·1 to 0·1)	13·2% (9·3 to 17·4%)	46·7% (25·2 to 76·8%)	-1·3% (-5·6 to 2·8%)	4·2 (2·4 to 7·3)	15·3% (12·4 to 18·8%)	5·7 (3·3 to 9·9)	15·2% (12·4 to 18·5%)	36·1% (29·7 to 45·1%)	-0·9% (-3·4 to 1·3%)
Chronic kidney disease due to diabetes mellitus	12·4 (10·7 to 14·2)	100·0% (100·0 to 100·0%)	17·4 (15·0 to 20·0)	100·0% (100·0 to 100·0%)	40·5% (31·5 to 49·2%)	-	399·8 (348·6 to 456·7)	100·0% (100·0 to 100·0%)	537·3 (467·1 to 616·7)	100·0% (100·0 to 100·0%)	34·4% (27·0 to 41·5%)	-
Chronic kidney disease due to hypertension	43·7 (40·3 to 48·0)	100·0% (100·0 to 100·0%)	58·2 (52·9 to 64·0)	100·0% (100·0 to 100·0%)	33·2% (25·0 to 41·7%)	-	1,229·7 (1,115·6 to 1,347·2)	100·0% (100·0 to 100·0%)	1,561·2 (1,406·9 to 1,716·9)	100·0% (100·0 to 100·0%)	27·0% (18·9 to 35·1%)	-
Chronic kidney disease due to glomerulonephritis	5·6 (4·9 to 6·4)	100·0% (100·0 to 100·0%)	7·2 (6·2 to 8·3)	100·0% (100·0 to 100·0%)	29·2% (20·0 to 38·6%)	-	242·5 (209·8 to 276·9)	100·0% (100·0 to 100·0%)	295·5 (253·9 to 341·8)	100·0% (100·0 to 100·0%)	21·9% (13·9 to 29·4%)	-
Chronic kidney disease due to other causes	3·6 (2·9 to 4·3)	100·0% (100·0 to 100·0%)	4·5 (3·6 to 5·3)	100·0% (100·0 to 100·0%)	23·7% (11·3 to 36·6%)	-	259·9 (214·3 to 313·2)	100·0% (100·0 to 100·0%)	296·8 (244·7 to 354·3)	100·0% (100·0 to 100·0%)	14·2% (4·9 to 22·3%)	-
Gout	-	-	-	-	-	-	6·4 (4·2 to 8·8)	12·7% (11·3 to 14·1%)	8·9 (5·9 to 12·2)	12·6% (11·3 to 14·0%)	39·1% (33·7 to 44·8%)	-0·3% (-3·0 to 2·6%)

Supplemental Results Table 1. All-age deaths, death PAFs, DALYs, DALY PAFs, and percent change for each risk factor and outcome for both sexes combined in 2005 and 2015 for South Asia.

Data in parenthesis are 95% uncertainty intervals. PAFs=population attributable fractions. DALYs=disability-adjusted life-years.

	2005 Deaths (in thousands)	2005 Deaths PAF	2015 Deaths (in thousands)	2015 Deaths PAF	Percent change deaths 2005-2015	Percent change deaths PAF 2005-2015	2005 DALYs (in thousands)	2005 DALYs PAF	2015 DALYs (in thousands)	2015 DALYs PAF	Percent change DALYs 2005-2015	Percent change DALYs PAF 2005-2015
**All risk factors: All causes**	6,473·7<br>(6,238·4 to 6,680·5)	52·5%<br>(51·1 to 53·8%)	7,272·4<br>(6,983·9 to 7,562·6)	56·5%<br>(55·1 to 57·8%)	12·3%<br>(8·4 to 16·5%)	7·6%<br>(6·2 to 9·2%)	274,538·3<br>(257,194·4 to 293,580·5)	40·0%<br>(38·7 to 41·2%)	261,852·7<br>(243,380·6 to 281,112·5)	40·5%<br>(39·3 to 41·9%)	-4·6%<br>(-7·4 to -1·6%)	1·4%<br>(-0·7 to 3·8%)
**Environmental/occupational risks: All causes**	3,145·1<br>(2,877·1 to 3,389·3)	25·5%<br>(23·4 to 27·5%)	3,248·7<br>(2,964·2 to 3,519·2)	25·2%<br>(23·1 to 27·3%)	3·3%<br>(-0·9 to 7·9%)	-1·1%<br>(-3·9 to 2·0%)	129,548·4<br>(119,109·3 to 139,405·7)	18·9%<br>(17·2 to 20·5%)	114,870·2<br>(105,188·6 to 124,288·3)	17·8%<br>(16·2 to 19·2%)	-11·3%<br>(-15·3 to -6·5%)	-5·8%<br>(-9·3 to -1·5%)
**Unsafe water, sanitation, and handwashing: All causes**	977·5<br>(887·9 to 1,080·2)	7·9%<br>(7·2 to 8·7%)	771·0<br>(685·3 to 869·2)	6·0%<br>(5·4 to 6·7%)	-21·1%<br>(-26·6 to -15·1%)	-24·5%<br>(-29·7 to -19·2%)	54,078·4<br>(48,000·8 to 61,162·0)	7·9%<br>(6·9 to 9·0%)	38,164·8<br>(33,351·2 to 43,804·5)	5·9%<br>(5·1 to 6·8%)	-29·4%<br>(-34·8 to -23·3%)	-25·0%<br>(-30·6 to -19·1%)
**Unsafe water source: All causes**	766·6<br>(617·9 to 889·7)	6·2%<br>(5·0 to 7·2%)	600·1<br>(471·0 to 707·9)	4·7%<br>(3·7 to 5·5%)	-21·7%<br>(-28·0 to -15·2%)	-25·1%<br>(-30·7 to -19·2%)	42,145·5<br>(33,511·2 to 49,791·0)	6·1%<br>(4·8 to 7·3%)	30,008·3<br>(23,778·3 to 36,007·7)	4·6%<br>(3·7 to 5·7%)	-28·8%<br>(-34·8 to -22·4%)	-24·3%<br>(-30·4 to -18·1%)
Diarrhoeal diseases	632·9<br>(514·7 to 710·7)	86·3%<br>(70·3 to 94·7%)	490·0<br>(383·9 to 569·9)	85·7%<br>(69·4 to 94·4%)	-22·6%<br>(-29·7 to -14·8%)	-0·7%<br>(-1·4 to -0·3%)	32,439·2<br>(26,276·5 to 36,898·3)	86·2%<br>(70·3 to 94·7%)	22,223·3<br>(17,581·3 to 25,331·5)	85·7%<br>(69·5 to 94·4%)	-31·5%<br>(-38·4 to -23·9%)	-0·7%<br>(-1·3 to -0·3%)
Typhoid fever	110·8<br>(59·5 to 182·6)	85·9%<br>(69·7 to 94·5%)	90·4<br>(49·6 to 150·3)	85·1%<br>(68·6 to 94·1%)	-18·3%<br>(-24·9 to -12·3%)	-0·9%<br>(-1·8 to -0·4%)	8,086·9<br>(4,403·3 to 13,281·9)	85·9%<br>(69·7 to 94·5%)	6,434·4<br>(3,565·7 to 10,572·6)	85·1%<br>(68·7 to 94·1%)	-20·4%<br>(-27·6 to -13·7%)	-0·9%<br>(-1·8 to -0·4%)
Paratyphoid fever	23·0<br>(10·1 to 44·9)	85·8%<br>(69·6 to 94·4%)	19·6<br>(8·7 to 38·5)	85·0%<br>(68·4 to 94·0%)	-14·5%<br>(-22·7 to -5·7%)	-0·9%<br>(-1·9 to -0·4%)	1,619·3<br>(716·6 to 3,181·8)	85·8%<br>(69·6 to 94·4%)	1,350·6<br>(605·5 to 2,650·1)	85·0%<br>(68·5 to 94·0%)	-16·6%<br>(-25·5 to -6·9%)	-0·9%<br>(-1·8 to -0·4%)
**Unsafe sanitation: All causes**	537·7<br>(482·2 to 606·5)	4·4%<br>(3·9 to 4·9%)	372·8<br>(323·7 to 425·4)	2·9%<br>(2·5 to 3·3%)	-30·7%<br>(-36·8 to -24·1%)	-33·6%<br>(-39·2 to -27·7%)	29,308·6<br>(25,729·8 to 33,844·1)	4·3%<br>(3·7 to 5·0%)	18,245·7<br>(15,547·3 to 21,257·8)	2·8%<br>(2·4 to 3·3%)	-37·7%<br>(-43·3 to -31·3%)	-33·8%<br>(-39·9 to -27·3%)
Diarrhoeal diseases	447·8<br>(406·8 to 495·3)	61·0%<br>(56·7 to 65·5%)	308·5<br>(270·5 to 350·0)	53·9%<br>(49·1 to 58·7%)	-31·1%<br>(-38·0 to -23·4%)	-11·6%<br>(-15·2 to -8·7%)	22,772·1<br>(20,303·8 to 25,416·8)	60·5%<br>(56·1 to 65·0%)	13,691·6<br>(11,991·7 to 15,448·8)	52·8%<br>(47·9 to 57·7%)	-39·9%<br>(-46·3 to -32·9%)	-12·8%<br>(-16·7 to -9·4%)
Typhoid fever	74·6<br>(40·5 to 124·1)	57·8%<br>(53·1 to 62·0%)	52·9<br>(29·1 to 88·8)	49·7%<br>(45·0 to 54·1%)	-29·1%<br>(-35·5 to -22·8%)	-14·0%<br>(-17·6 to -11·1%)	5,453·2<br>(3,010·5 to 9,039·7)	57·9%<br>(53·2 to 62·1%)	3,769·3<br>(2,107·3 to 6,256·0)	49·8%<br>(45·1 to 54·2%)	-30·9%<br>(-37·6 to -24·0%)	-13·9%<br>(-17·5 to -11·0%)
Paratyphoid fever	15·3<br>(7·0 to 29·6)	57·2%<br>(52·6 to 61·6%)	11·4<br>(5·3 to 22·0)	49·2%<br>(44·6 to 53·8%)	-25·8%<br>(-33·7 to -17·5%)	-14·1%<br>(-17·5 to -11·0%)	1,083·2<br>(498·4 to 2,081·2)	57·4%<br>(52·8 to 61·7%)	784·8<br>(369·2 to 1,524·5)	49·3%<br>(44·7 to 54·0%)	-27·5%<br>(-35·8 to -18·7%)	-14·0%<br>(-17·4 to -10·8%)
**No handwashing with soap: All causes**	467·7<br>(394·2 to 540·3)	3·8%<br>(3·2 to 4·4%)	372·2<br>(308·0 to 433·7)	2·9%<br>(2·4 to 3·4%)	-20·4%<br>(-25·6 to -14·7%)	-23·8%<br>(-28·5 to -18·8%)	26,058·4<br>(21,543·2 to 30,442·1)	3·8%<br>(3·1 to 4·5%)	18,251·4<br>(14,991·6 to 21,369·3)	2·8%<br>(2·3 to 3·4%)	-30·0%<br>(-34·6 to -24·2%)	-25·6%<br>(-30·7 to -20·1%)
Diarrhoeal diseases	288·8<br>(233·0 to 344·8)	39·4%<br>(32·0 to 46·3%)	223·0<br>(176·9 to 270·6)	39·0%<br>(31·6 to 46·0%)	-22·8%<br>(-29·8 to -15·0%)	-0·9%<br>(-1·6 to -0·5%)	14,809·3<br>(11,842·4 to 17,745·5)	39·4%<br>(32·0 to 46·3%)	10,122·8<br>(8,045·0 to 12,233·9)	39·0%<br>(31·6 to 46·0%)	-31·6%<br>(-38·5 to -24·2%)	-0·9%<br>(-1·5 to -0·4%)
Typhoid fever	50·4<br>(26·1 to 85·4)	39·1%<br>(31·0 to 45·8%)	41·1<br>(21·3 to 70·2)	38·7%<br>(30·5 to 45·5%)	-18·4%<br>(-25·0 to -12·2%)	-1·1%<br>(-1·8 to -0·5%)	3,682·1<br>(1,950·0 to 6,192·6)	39·1%<br>(31·0 to 45·9%)	2,926·1<br>(1,550·8 to 4,885·9)	38·7%<br>(30·5 to 45·5%)	-20·5%<br>(-27·5 to -13·9%)	-1·1%<br>(-1·8 to -0·5%)
Paratyphoid fever	10·4<br>(4·8 to 20·9)	39·0%<br>(30·9 to 46·1%)	8·9<br>(4·2 to 18·2)	38·6%<br>(30·4 to 45·6%)	-14·7%<br>(-22·9 to -6·0%)	-1·1%<br>(-1·9 to -0·6%)	736·0<br>(342·6 to 1,458·2)	39·0%<br>(30·9 to 46·1%)	613·1<br>(289·1 to 1,233·9)	38·6%<br>(30·4 to 45·6%)	-16·7%<br>(-25·5 to -7·1%)	-1·1%<br>(-1·9 to -0·5%)
**Air pollution: All causes**	2,021·6<br>(1,745·6 to 2,276·6)	16·4%<br>(14·2 to 18·4%)	2,274·8<br>(1,968·3 to 2,546·2)	17·7%<br>(15·4 to 19·8%)	12·5%<br>(7·3 to 18·0%)	7·7%<br>(4·2 to 11·5%)	64,969·3<br>(56,581·7 to 73,001·7)	9·5%<br>(8·1 to 10·8%)	62,395·9<br>(54,461·3 to 69,705·3)	9·7%<br>(8·3 to 11·0%)	-4·0%<br>(-9·1 to 1·8%)	2·1%<br>(-2·8 to 7·1%)
**Ambient particulate matter pollution: All causes**	1,128·2<br>(974·4 to 1,282·6)	9·2%<br>(8·0 to 10·4%)	1,366·6<br>(1,187·6 to 1,555·2)	10·6%<br>(9·3 to 12·0%)	21·1%<br>(15·6 to 27·0%)	16·0%<br>(12·6 to 19·9%)	36,660·0<br>(31,881·0 to 41,604·6)	5·3%<br>(4·6 to 6·1%)	37,771·9<br>(33,228·1 to 42,549·4)	5·9%<br>(5·1 to 6·7%)	3·0%<br>(-3·0 to 9·5%)	9·5%<br>(4·0 to 15·4%)
Lower respiratory infections	275·0<br>(210·5 to 334·2)	36·5%<br>(28·5 to 43·8%)	243·2<br>(185·3 to 299·0)	37·8%<br>(29·9 to 45·2%)	-11·5%<br>(-18·0 to -4·5%)	3·7%<br>(1·9 to 5·9%)	16,019·8<br>(12,424·7 to 19,537·8)	36·8%<br>(28·8 to 44·0%)	11,293·2<br>(8,717·1 to 13,819·2)	38·1%<br>(30·1 to 45·4%)	-29·5%<br>(-35·8 to -22·1%)	3·6%<br>(1·9 to 5·7%)
Tracheal, bronchus, and lung cancer	21·8<br>(14·8 to 28·6)	26·5%<br>(18·2 to 34·7%)	35·5<br>(24·0 to 46·4)	28·4%<br>(19·9 to 36·7%)	62·7%<br>(50·4 to 74·7%)	7·2%<br>(5·3 to 9·8%)	551·9<br>(374·1 to 729·5)	26·2%<br>(18·0 to 34·4%)	849·2<br>(574·0 to 1,112·4)	28·1%<br>(19·6 to 36·3%)	53·9%<br>(42·1 to 65·3%)	7·0%<br>(5·1 to 9·5%)
Ischaemic heart disease	347·4<br>(296·1 to 404·1)	22·4%<br>(19·3 to 25·9%)	473·3<br>(398·9 to 556·1)	22·8%<br>(19·6 to 26·5%)	36·3%<br>(30·0 to 42·9%)	1·8%<br>(0·3 to 3·5%)	8,911·1<br>(7,652·1 to 10,230·6)	24·4%<br>(21·1 to 27·7%)	11,788·3<br>(9,979·6 to 13,524·2)	25·1%<br>(21·6 to 28·6%)	32·3%<br>(25·7 to 39·1%)	3·0%<br>(1·7 to 4·3%)
Ischaemic stroke	70·4<br>(55·8 to 85·0)	17·4%<br>(14·2 to 20·4%)	88·9<br>(71·3 to 107·5)	17·8%<br>(14·5 to 21·0%)	26·3%<br>(18·3 to 35·9%)	2·4%<br>(-0·4 to 5·2%)	1,441·0<br>(1,170·2 to 1,736·4)	18·7%<br>(15·9 to 21·7%)	1,768·7<br>(1,437·4 to 2,124·6)	19·4%<br>(16·5 to 22·4%)	22·7%<br>(14·8 to 31·5%)	3·7%<br>(1·8 to 5·9%)
Hemorrhagic stroke	99·2<br>(83·3 to 117·0)	19·8%<br>(16·9 to 22·6%)	122·4<br>(103·1 to 143·6)	20·6%<br>(17·6 to 23·5%)	23·3%<br>(16·1 to 32·6%)	3·9%<br>(2·0 to 5·9%)	2,847·0<br>(2,390·0 to 3,331·9)	20·6%<br>(17·6 to 23·6%)	3,458·5<br>(2,917·6 to 4,048·1)	21·9%<br>(18·9 to 25·0%)	21·5%<br>(14·6 to 30·1%)	6·4%<br>(4·7 to 8·5%)
Chronic obstructive pulmonary disease	314·4<br>(202·5 to 442·5)	31·8%<br>(20·6 to 44·4%)	403·3<br>(262·1 to 561·0)	33·6%<br>(22·2 to 47·0%)	28·3%<br>(18·9 to 39·4%)	5·8%<br>(3·8 to 9·3%)	6,889·3<br>(4,442·6 to 9,729·7)	29·5%<br>(19·1 to 41·2%)	8,613·9<br>(5,599·9 to 11,997·1)	32·0%<br>(21·1 to 44·6%)	25·0%<br>(16·4 to 35·2%)	8·5%<br>(5·9 to 12·3%)
**Household air pollution from solid fuels: All causes**	1,232·7<br>(934·9 to 1,534·5)	10·0%<br>(7·6 to 12·5%)	1,239·5<br>(918·9 to 1,575·1)	9·6%<br>(7·2 to 12·2%)	0·6%<br>(-6·8 to 7·3%)	-3·7%<br>(-10·1 to 1·7%)	41,114·4<br>(31,298·1 to 50,795·6)	6·0%<br>(4·5 to 7·5%)	35,279·3<br>(26,718·4 to 44,233·2)	5·5%<br>(4·1 to 6·9%)	-14·2%<br>(-21·2 to -7·3%)	-8·8%<br>(-15·8 to -2·5%)
Lower respiratory infections	320·6<br>(231·6 to 420·4)	42·6%<br>(30·9 to 55·4%)	241·1<br>(166·6 to 327·5)	37·5%<br>(26·3 to 50·1%)	-24·8%<br>(-32·2 to -18·3%)	-11·8%<br>(-17·1 to -7·9%)	18,944·1<br>(13,524·4 to 24,864·6)	43·5%<br>(31·7 to 56·6%)	11,556·8<br>(8,116·0 to 15,559·2)	39·0%<br>(27·4 to 51·7%)	-39·0%<br>(-45·3 to -32·6%)	-10·4%<br>(-15·4 to -6·6%)
Tracheal, bronchus, and lung cancer	26·3<br>(15·4 to 37·5)	31·9%<br>(19·0 to 45·7%)	35·3<br>(20·7 to 51·5)	28·2%<br>(16·6 to 41·2%)	34·2%<br>(21·3 to 45·7%)	-11·7%<br>(-16·6 to -7·8%)	664·4<br>(386·7 to 949·2)	31·6%<br>(18·7 to 45·2%)	845·6<br>(494·5 to 1,241·1)	27·9%<br>(16·4 to 40·8%)	27·3%<br>(15·1 to 38·2%)	-11·6%<br>(-16·5 to -7·6%)
Ischaemic heart disease	335·3<br>(268·0 to 413·8)	21·7%<br>(17·4 to 26·5%)	378·0<br>(296·9 to 473·2)	18·2%<br>(14·3 to 22·6%)	12·7%<br>(4·0 to 21·1%)	-15·8%<br>(-21·4 to -10·7%)	8,616·6<br>(6,932·2 to 10,416·8)	23·6%<br>(18·9 to 28·5%)	9,493·6<br>(7,467·9 to 11,759·3)	20·2%<br>(15·9 to 24·9%)	10·2%<br>(1·5 to 18·7%)	-14·3%<br>(-19·6 to -9·2%)
Ischaemic stroke	75·7<br>(54·5 to 100·2)	18·7%<br>(13·7 to 24·7%)	79·0<br>(56·2 to 107·1)	15·8%<br>(11·4 to 20·9%)	4·5%<br>(-5·0 to 14·6%)	-15·4%<br>(-20·6 to -10·2%)	1,543·3<br>(1,108·8 to 2,030·5)	20·1%<br>(14·8 to 26·1%)	1,570·5<br>(1,125·8 to 2,081·4)	17·3%<br>(12·6 to 22·6%)	1·8%<br>(-7·4 to 10·8%)	-14·0%<br>(-19·2 to -9·2%)
Hemorrhagic stroke	106·8<br>(79·8 to 138·8)	21·3%<br>(16·1 to 27·3%)	109·9<br>(78·8 to 143·3)	18·5%<br>(13·5 to 23·8%)	2·9%<br>(-5·5 to 12·0%)	-13·3%<br>(-18·5 to -8·6%)	3,043·6<br>(2,299·1 to 3,883·4)	22·0%<br>(16·6 to 27·8%)	3,095·5<br>(2,247·2 to 4,016·0)	19·6%<br>(14·4 to 25·2%)	1·7%<br>(-6·5 to 10·0%)	-10·9%<br>(-16·3 to -6·1%)
Chronic obstructive pulmonary disease	368·0<br>(213·5 to 557·0)	37·2%<br>(21·9 to 55·5%)	396·2<br>(221·5 to 609·7)	33·1%<br>(18·9 to 51·1%)	7·7%<br>(-2·6 to 18·0%)	-11·1%<br>(-16·7 to -6·7%)	7,957·8<br>(4,547·5 to 12,032·8)	34·0%<br>(19·9 to 50·9%)	8,333·7<br>(4,679·1 to 12,801·0)	30·9%<br>(17·7 to 47·9%)	4·7%<br>(-5·0 to 14·1%)	-9·1%<br>(-14·9 to -4·2%)
Cataract	-	-	-	-	-	-	344·5<br>(240·0 to 473·4)	29·4%<br>(25·9 to 32·6%)	383·5<br>(267·4 to 523·5)	26·2%<br>(22·8 to 29·5%)	11·3%<br>(5·6 to 16·9%)	-10·6%<br>(-14·7 to -6·5%)
**Ambient ozone pollution: All causes**	82·0<br>(30·8 to 137·8)	0·7%<br>(0·3 to 1·1%)	122·4<br>(46·5 to 200·3)	1·0%<br>(0·4 to 1·6%)	49·4%<br>(38·2 to 63·0%)	43·1%<br>(33·7 to 54·9%)	1,575·0<br>(593·2 to 2,647·1)	0·2%<br>(0·1 to 0·4%)	2,260·4<br>(848·4 to 3,720·9)	0·4%<br>(0·1 to 0·6%)	43·5%<br>(32·2 to 57·4%)	52·6%<br>(41·1 to 66·5%)
Chronic obstructive pulmonary disease	82·0<br>(30·8 to 137·8)	8·3%<br>(3·2 to 13·9%)	122·4<br>(46·5 to 200·3)	10·2%<br>(4·0 to 16·8%)	49·4%<br>(38·2 to 63·0%)	23·3%<br>(20·3 to 27·7%)	1,575·0<br>(593·2 to 2,647·1)	6·7%<br>(2·5 to 11·2%)	2,260·4<br>(848·4 to 3,720·9)	8·4%<br>(3·3 to 13·7%)	43·5%<br>(32·2 to 57·4%)	24·6%<br>(20·5 to 30·1%)
**Other environmental risks: All causes**	117·9<br>(64·5 to 177·2)	1·0%<br>(0·5 to 1·4%)	145·4<br>(77·7 to 220·7)	1·1%<br>(0·6 to 1·7%)	23·3%<br>(17·1 to 29·5%)	18·1%<br>(13·5 to 22·9%)	2,758·6<br>(1,451·9 to 4,226·0)	0·4%<br>(0·2 to 0·6%)	3,159·3<br>(1,593·2 to 4,927·6)	0·5%<br>(0·2 to 0·8%)	14·5%<br>(7·5 to 20·5%)	21·7%<br>(14·7 to 27·2%)
**Residential radon: All causes**	5·3<br>(3·2 to 10·3)	0·0%<br>(0·0 to 0·1%)	8·3<br>(4·8 to 17·5)	0·1%<br>(0·0 to 0·1%)	56·6%<br>(39·0 to 85·9%)	50·0%<br>(34·2 to 77·0%)	134·8<br>(82·0 to 256·9)	0·0%<br>(0·0 to 0·0%)	196·7<br>(117·9 to 407·7)	0·0%<br>(0·0 to 0·1%)	45·9%<br>(33·0 to 61·5%)	55·1%<br>(41·7 to 71·5%)
Tracheal, bronchus, and lung cancer	5·3<br>(3·2 to 10·3)	6·4%<br>(3·9 to 12·5%)	8·3<br>(4·8 to 17·5)	6·6%<br>(3·8 to 13·7%)	56·6%<br>(39·0 to 85·9%)	3·1%<br>(-5·9 to 18·9%)	134·8<br>(82·0 to 256·9)	6·4%<br>(3·9 to 12·2%)	196·7<br>(117·9 to 407·7)	6·5%<br>(3·9 to 13·1%)	45·9%<br>(33·0 to 61·5%)	1·4%<br>(-4·1 to 10·4%)
**Lead exposure: All causes**	112·6<br>(59·9 to 171·7)	0·9%<br>(0·5 to 1·4%)	137·1<br>(70·5 to 211·3)	1·1%<br>(0·6 to 1·6%)	21·7%<br>(14·9 to 27·6%)	16·6%<br>(11·1 to 20·6%)	2,623·8<br>(1,336·1 to 4,076·8)	0·4%<br>(0·2 to 0·6%)	2,962·6<br>(1,421·2 to 4,720·8)	0·5%<br>(0·2 to 0·7%)	12·9%<br>(5·1 to 19·2%)	20·0%<br>(12·0 to 25·5%)
Rheumatic heart disease	1·6<br>(0·6 to 3·0)	1·3%<br>(0·5 to 2·4%)	1·7<br>(0·6 to 3·2)	1·2%<br>(0·4 to 2·2%)	2·4%<br>(-9·0 to 13·0%)	-7·3%<br>(-13·7 to -1·9%)	49·9<br>(15·9 to 104·5)	0·9%<br>(0·3 to 1·9%)	46·3<br>(13·9 to 99·8)	0·8%<br>(0·2 to 1·8%)	-7·1%<br>(-17·9 to 2·4%)	-9·1%<br>(-17·7 to -2·9%)
Ischaemic heart disease	57·2<br>(29·8 to 87·7)	3·7%<br>(1·9 to 5·7%)	72·2<br>(36·1 to 112·8)	3·5%<br>(1·8 to 5·5%)	26·3%<br>(18·8 to 33·3%)	-5·7%<br>(-9·7 to -2·9%)	1,205·6<br>(570·3 to 1,936·1)	3·3%<br>(1·6 to 5·3%)	1,440·2<br>(648·8 to 2,347·1)	3·1%<br>(1·4 to 5·0%)	19·5%<br>(11·1 to 26·2%)	-7·0%<br>(-11·8 to -4·4%)
Ischaemic stroke	15·2<br>(8·1 to 23·8)	3·7%<br>(2·0 to 5·8%)	17·4<br>(9·1 to 28·1)	3·5%<br>(1·8 to 5·5%)	14·7%<br>(5·0 to 24·3%)	-7·1%<br>(-12·0 to -2·0%)	278·6<br>(140·0 to 446·4)	3·6%<br>(1·8 to 5·7%)	303·9<br>(145·1 to 496·8)	3·3%<br>(1·6 to 5·4%)	9·1%<br>(-0·5 to 17·5%)	-7·9%<br>(-13·3 to -4·2%)
Hemorrhagic stroke	20·0<br>(9·7 to 31·8)	4·0%<br>(2·0 to 6·3%)	22·2<br>(10·5 to 36·1)	3·7%<br>(1·8 to 6·0%)	11·0%<br>(2·0 to 20·5%)	-6·5%<br>(-11·9 to -2·6%)	489·6<br>(211·6 to 819·7)	3·5%<br>(1·5 to 5·9%)	522·1<br>(216·7 to 903·9)	3·3%<br>(1·4 to 5·7%)	6·6%<br>(-2·6 to 14·5%)	-6·6%<br>(-12·4 to -3·0%)
Hypertensive heart disease	10·7<br>(3·5 to 22·9)	6·4%<br>(2·1 to 14·2%)	12·6<br>(3·8 to 27·8)	6·0%<br>(1·8 to 13·5%)	18·2%<br>(3·7 to 31·4%)	-6·1%<br>(-14·1 to -0·2%)	229·7<br>(80·7 to 458·8)	5·6%<br>(2·0 to 11·8%)	252·0<br>(82·9 to 537·5)	5·1%<br>(1·6 to 11·2%)	9·7%<br>(-3·4 to 20·4%)	-8·7%<br>(-16·4 to -3·9%)
Cardiomyopathy and myocarditis	0·2<br>(0·1 to 0·4)	1·4%<br>(0·5 to 2·5%)	0·2<br>(0·1 to 0·4)	1·3%<br>(0·5 to 2·4%)	12·1%<br>(-3·6 to 25·8%)	-7·1%<br>(-14·0 to -1·8%)	6·7<br>(1·9 to 14·9)	0·9%<br>(0·3 to 2·0%)	6·9<br>(1·9 to 15·9)	0·9%<br>(0·2 to 2·0%)	3·1%<br>(-14·2 to 17·6%)	-8·6%<br>(-19·7 to -0·9%)

Supplemental Results Table 1. All-age deaths, death PAFs, DALYs, DALY PAFs, and percent change for each risk factor and outcome for both sexes combined in 2005 and 2015 for South Asia.

Data in parenthesis are 95% uncertainty intervals. PAFs=population attributable fractions. DALYs=disability-adjusted life-years.

	2005 Deaths (in thousands)	2005 Deaths PAF	2015 Deaths (in thousands)	2015 Deaths PAF	Percent change deaths 2005-2015	Percent change deaths PAF 2005-2015	2005 DALYs (in thousands)	2005 DALYs PAF	2015 DALYs (in thousands)	2015 DALYs PAF	Percent change DALYs 2005-2015	Percent change DALYs PAF 2005-2015
Atrial fibrillation and flutter	0·3 (0·2 to 0·5)	2·5% (1·4 to 3·7%)	0·5 (0·3 to 0·8)	2·3% (1·3 to 3·5%)	41·7% (33·0 to 49·6%)	-5·9% (-10·2 to -3·0%)	10·0 (5·1 to 16·3)	2·7% (1·4 to 4·0%)	12·8 (6·3 to 21·1)	2·4% (1·3 to 3·7%)	28·2% (22·0 to 32·5%)	-8·1% (-12·1 to -5·7%)
Aortic aneurysm	0·3 (0·1 to 0·5)	2·1% (1·0 to 3·4%)	0·4 (0·2 to 0·6)	2·0% (0·9 to 3·2%)	41·3% (24·6 to 56·7%)	-8·6% (-13·6 to -5·5%)	6·2 (2·6 to 11·0)	1·9% (0·8 to 3·3%)	8·3 (3·4 to 14·5)	1·7% (0·7 to 3·0%)	32·6% (15·6 to 47·7%)	-10·2% (-16·1 to -6·7%)
Peripheral vascular disease	0·0 (0·0 to 0·0)	1·7% (0·9 to 2·7%)	0·0 (0·0 to 0·1)	1·6% (0·8 to 2·5%)	86·4% (57·8 to 123·7%)	-7·3% (-12·5 to -3·3%)	1·2 (0·6 to 2·4)	1·8% (0·9 to 2·7%)	1·7 (0·8 to 3·2)	1·6% (0·8 to 2·6%)	39·4% (28·7 to 51·3%)	-8·4% (-13·9 to -5·0%)
Endocarditis	0·1 (0·0 to 0·1)	1·5% (0·6 to 2·7%)	0·1 (0·0 to 0·2)	1·4% (0·6 to 2·6%)	21·0% (7·6 to 37·7%)	-6·9% (-13·5 to -1·0%)	2·0 (0·6 to 4·4)	1·0% (0·3 to 2·2%)	2·3 (0·7 to 5·1)	0·9% (0·3 to 2·1%)	11·5% (-3·7 to 26·5%)	-8·3% (-18·8 to -0·1%)
Other cardiovascular and circulatory diseases	0·4 (0·2 to 0·6)	2·6% (1·3 to 3·9%)	0·5 (0·3 to 0·8)	2·4% (1·2 to 3·6%)	33·8% (21·7 to 45·3%)	-7·8% (-12·3 to -5·1%)	8·6 (4·1 to 13·8)	2·4% (1·1 to 3·9%)	10·7 (4·8 to 17·5)	2·2% (1·0 to 3·6%)	24·2% (14·2 to 33·0%)	-8·3% (-13·0 to -5·5%)
Idiopathic developmental intellectual disability	-	-	-	-	-	-	185·9 (80·1 to 327·7)	18·7% (13·0 to 23·3%)	165·4 (71·4 to 295·8)	15·0% (10·0 to 19·1%)	-11·0% (-16·1 to -6·3%)	-19·6% (-24·3 to -15·5%)
Chronic kidney disease due to diabetes mellitus	1·9 (0·9 to 3·3)	2·8% (1·3 to 4·7%)	2·5 (1·1 to 4·5)	2·7% (1·2 to 4·6%)	35·7% (24·1 to 47·2%)	-3·5% (-6·5 to -1·8%)	43·7 (17·8 to 81·7)	2·1% (0·9 to 3·8%)	55·4 (22·1 to 105·5)	2·0% (0·8 to 3·7%)	26·8% (16·6 to 36·5%)	-4·3% (-8·3 to -2·1%)
Chronic kidney disease due to hypertension	3·2 (1·5 to 5·4)	2·7% (1·3 to 4·6%)	4·5 (2·1 to 7·5)	2·7% (1·3 to 4·6%)	40·4% (28·7 to 51·9%)	-0·2% (-2·5 to 1·5%)	62·4 (26·2 to 110·7)	1·9% (0·8 to 3·4%)	80·2 (32·8 to 141·6)	1·9% (0·8 to 3·5%)	28·4% (17·8 to 38·3%)	0·5% (-3·1 to 2·9%)
Chronic kidney disease due to glomerulonephritis	1·5 (0·7 to 2·6)	2·2% (1·0 to 3·7%)	2·1 (1·0 to 3·6)	2·3% (1·1 to 3·9%)	40·5% (28·1 to 51·8%)	4·4% (1·6 to 6·9%)	36·1 (13·8 to 65·9)	1·4% (0·5 to 2·4%)	45·2 (17·6 to 83·5)	1·4% (0·5 to 2·6%)	25·0% (15·4 to 33·9%)	4·8% (0·3 to 8·1%)
Chronic kidney disease due to other causes	0·1 (0·0 to 0·1)	1·6% (0·8 to 2·7%)	0·1 (0·0 to 0·2)	2·1% (1·0 to 3·5%)	56·4% (42·6 to 71·6%)	28·6% (22·0 to 36·4%)	7·5 (3·1 to 14·6)	1·4% (0·6 to 2·6%)	9·3 (3·8 to 18·4)	1·5% (0·6 to 2·8%)	23·9% (19·0 to 28·2%)	4·9% (0·2 to 9·7%)
**Occupational risks: All causes**	231·7 (205·7 to 259·5)	1·9% (1·7 to 2·1%)	276·2 (244·4 to 313·7)	2·1% (1·9 to 2·4%)	19·2% (8·8 to 32·2%)	14·1% (4·7 to 25·6%)	15,246·8 (12,611·9 to 18,034·5)	2·2% (1·9 to 2·5%)	17,734·1 (14,784·9 to 21,091·0)	2·7% (2·4 to 3·1%)	16·3% (7·5 to 27·4%)	23·6% (14·2 to 35·2%)
**Occupational carcinogens: All causes**	21·2 (19·6 to 23·0)	0·2% (0·2 to 0·2%)	33·6 (30·7 to 36·7)	0·3% (0·2 to 0·3%)	58·2% (44·8 to 71·8%)	51·5% (40·0 to 63·2%)	509·6 (472·0 to 552·0)	0·1% (0·1 to 0·1%)	792·9 (722·3 to 867·0)	0·1% (0·1 to 0·1%)	55·6% (42·5 to 68·8%)	65·4% (51·8 to 78·7%)
**Occupational exposure to asbestos: All causes**	1·4 (1·0 to 1·8)	0·0% (0·0 to 0·0%)	1·6 (1·2 to 2·2)	0·0% (0·0 to 0·0%)	17·6% (-6·4 to 61·9%)	12·5% (-9·7 to 54·9%)	25·7 (18·2 to 34·2)	0·0% (0·0 to 0·0%)	26·5 (18·8 to 35·9)	0·0% (0·0 to 0·0%)	3·0% (-18·5 to 47·5%)	9·5% (-12·6 to 57·6%)
Larynx cancer	0·1 (0·0 to 0·1)	0·2% (0·1 to 0·4%)	0·1 (0·0 to 0·1)	0·2% (0·1 to 0·3%)	-3·4% (-22·9 to 36·3%)	-23·5% (-36·6 to 7·2%)	1·1 (0·6 to 1·6)	0·2% (0·1 to 0·3%)	1·0 (0·5 to 1·5)	0·1% (0·1 to 0·2%)	-8·7% (-27·9 to 32·9%)	-25·7% (-39·5 to 6·1%)
Tracheal, bronchus, and lung cancer	1·1 (0·7 to 1·5)	1·3% (0·9 to 1·8%)	1·4 (0·9 to 1·9)	1·1% (0·8 to 1·5%)	25·7% (-0·5 to 70·4%)	-17·3% (-31·5 to 9·4%)	19·7 (13·2 to 27·7)	0·9% (0·6 to 1·3%)	21·6 (14·3 to 30·3)	0·7% (0·5 to 1·0%)	9·8% (-12·8 to 56·0%)	-23·7% (-37·0 to 4·8%)
Ovarian cancer	0·0 (0·0 to 0·0)	0·2% (0·1 to 0·3%)	0·0 (0·0 to 0·0)	0·1% (0·1 to 0·2%)	4·3% (-23·8 to 41·6%)	-23·7% (-41·5 to -1·7%)	0·5 (0·2 to 1·0)	0·1% (0·1 to 0·2%)	0·5 (0·2 to 0·9)	0·1% (0·0 to 0·1%)	-1·8% (-29·7 to 37·2%)	-27·2% (-45·9 to -2·7%)
Mesothelioma	0·2 (0·1 to 0·3)	23·1% (12·9 to 35·6%)	0·2 (0·1 to 0·3)	18·2% (9·5 to 29·5%)	-18·7% (-37·2 to 29·7%)	-21·1% (-33·2 to 3·4%)	4·4 (2·2 to 7·5)	15·6% (8·0 to 25·4%)	3·4 (1·6 to 5·9)	11·6% (5·6 to 20·3%)	-23·6% (-42·5 to 29·8%)	-25·3% (-39·1 to 3·9%)
**Occupational exposure to arsenic: All causes**	0·6 (0·4 to 0·7)	0·0% (0·0 to 0·0%)	0·8 (0·6 to 1·0)	0·0% (0·0 to 0·0%)	34·1% (22·5 to 44·6%)	28·3% (18·4 to 38·3%)	13·8 (10·5 to 17·5)	0·0% (0·0 to 0·0%)	18·2 (13·9 to 23·0)	0·0% (0·0 to 0·0%)	31·8% (20·0 to 42·5%)	40·0% (28·2 to 51·2%)
Tracheal, bronchus, and lung cancer	0·6 (0·4 to 0·7)	0·7% (0·5 to 0·9%)	0·8 (0·6 to 1·0)	0·6% (0·5 to 0·8%)	34·1% (22·5 to 44·6%)	-11·7% (-15·1 to -8·0%)	13·8 (10·5 to 17·5)	0·7% (0·5 to 0·8%)	18·2 (13·9 to 23·0)	0·6% (0·5 to 0·8%)	31·8% (20·0 to 42·5%)	-8·4% (-12·1 to -4·6%)
**Occupational exposure to benzene: All causes**	0·5 (0·4 to 0·7)	0·0% (0·0 to 0·0%)	0·9 (0·7 to 1·0)	0·0% (0·0 to 0·0%)	58·0% (46·3 to 71·9%)	51·3% (41·6 to 62·4%)	20·5 (16·6 to 25·2)	0·0% (0·0 to 0·0%)	32·6 (26·7 to 39·3)	0·0% (0·0 to 0·0%)	59·0% (46·4 to 73·5%)	69·0% (57·3 to 82·6%)
Leukaemia	0·5 (0·4 to 0·7)	1·5% (1·2 to 1·8%)	0·9 (0·7 to 1·0)	1·9% (1·5 to 2·2%)	58·0% (46·3 to 71·9%)	25·3% (18·6 to 32·6%)	20·5 (16·6 to 25·2)	1·2% (1·0 to 1·5%)	32·6 (26·7 to 39·3)	1·6% (1·3 to 1·9%)	59·0% (46·4 to 73·5%)	32·2% (23·0 to 43·2%)
**Occupational exposure to beryllium: All causes**	0·0 (0·0 to 0·0)	0·0% (0·0 to 0·0%)	0·0 (0·0 to 0·0)	0·0% (0·0 to 0·0%)	33·0% (18·1 to 47·2%)	27·4% (13·4 to 40·7%)	0·5 (0·4 to 0·7)	0·0% (0·0 to 0·0%)	0·7 (0·6 to 0·9)	0·0% (0·0 to 0·0%)	30·6% (15·6 to 45·0%)	38·9% (23·1 to 53·8%)
Tracheal, bronchus, and lung cancer	0·0 (0·0 to 0·0)	0·0% (0·0 to 0·0%)	0·0 (0·0 to 0·0)	0·0% (0·0 to 0·0%)	33·0% (18·1 to 47·2%)	-12·4% (-19·0 to -4·7%)	0·5 (0·4 to 0·7)	0·0% (0·0 to 0·0%)	0·7 (0·6 to 0·9)	0·0% (0·0 to 0·0%)	30·6% (15·6 to 45·0%)	-9·2% (-16·0 to -1·6%)
**Occupational exposure to cadmium: All causes**	0·1 (0·1 to 0·1)	0·0% (0·0 to 0·0%)	0·2 (0·1 to 0·2)	0·0% (0·0 to 0·0%)	56·6% (40·8 to 72·4%)	49·9% (35·5 to 64·9%)	2·3 (1·9 to 2·8)	0·0% (0·0 to 0·0%)	3·6 (2·8 to 4·3)	0·0% (0·0 to 0·0%)	54·0% (38·6 to 70·4%)	63·7% (47·3 to 80·3%)
Tracheal, bronchus, and lung cancer	0·1 (0·1 to 0·1)	0·1% (0·1 to 0·1%)	0·2 (0·1 to 0·2)	0·1% (0·1 to 0·1%)	56·6% (40·8 to 72·4%)	3·1% (-4·1 to 11·1%)	2·3 (1·9 to 2·8)	0·1% (0·1 to 0·1%)	3·6 (2·8 to 4·3)	0·1% (0·1 to 0·1%)	54·0% (38·6 to 70·4%)	7·1% (-0·6 to 15·2%)
**Occupational exposure to chromium: All causes**	0·3 (0·3 to 0·4)	0·0% (0·0 to 0·0%)	0·5 (0·4 to 0·5)	0·0% (0·0 to 0·0%)	46·7% (32·2 to 61·5%)	40·5% (26·4 to 54·2%)	7·5 (6·4 to 8·7)	0·0% (0·0 to 0·0%)	10·8 (9·1 to 12·5)	0·0% (0·0 to 0·0%)	44·1% (29·6 to 59·1%)	53·2% (37·5 to 68·9%)
Tracheal, bronchus, and lung cancer	0·3 (0·3 to 0·4)	0·4% (0·3 to 0·4%)	0·5 (0·4 to 0·5)	0·4% (0·3 to 0·4%)	46·7% (32·2 to 61·5%)	-3·3% (-10·0 to 4·3%)	7·5 (6·4 to 8·7)	0·4% (0·3 to 0·4%)	10·8 (9·1 to 12·5)	0·4% (0·3 to 0·4%)	44·1% (29·6 to 59·1%)	0·2% (-6·6 to 7·8%)
**Occupational exposure to diesel engine exhaust: All causes**	6·6 (5·7 to 7·6)	0·1% (0·0 to 0·1%)	11·4 (9·8 to 13·3)	0·1% (0·1 to 0·1%)	74·4% (58·7 to 89·9%)	66·9% (53·2 to 80·4%)	155·9 (133·6 to 181·1)	0·0% (0·0 to 0·0%)	267·0 (229·9 to 311·2)	0·0% (0·0 to 0·0%)	71·3% (55·8 to 87·2%)	82·1% (66·0 to 98·1%)
Tracheal, bronchus, and lung cancer	6·6 (5·7 to 7·6)	8·0% (6·9 to 9·1%)	11·4 (9·8 to 13·3)	9·1% (7·9 to 10·5%)	74·4% (58·7 to 89·9%)	14·9% (9·0 to 20·9%)	155·9 (133·6 to 181·1)	7·4% (6·4 to 8·5%)	267·0 (229·9 to 311·2)	8·8% (7·7 to 10·0%)	71·3% (55·8 to 87·2%)	19·1% (13·1 to 25·1%)
**Occupational exposure to second-hand smoke: All causes**	6·8 (6·2 to 7·4)	0·1% (0·1 to 0·1%)	11·0 (9·9 to 12·3)	0·1% (0·1 to 0·1%)	62·2% (46·5 to 79·4%)	55·3% (41·2 to 70·5%)	158·1 (144·1 to 172·7)	0·0% (0·0 to 0·0%)	251·2 (225·4 to 281·3)	0·0% (0·0 to 0·0%)	58·9% (43·6 to 75·6%)	68·9% (53·4 to 85·5%)
Tracheal, bronchus, and lung cancer	6·8 (6·2 to 7·4)	8·2% (7·6 to 8·9%)	11·0 (9·9 to 12·3)	8·8% (8·1 to 9·6%)	62·2% (46·5 to 79·4%)	6·9% (0·2 to 14·2%)	158·1 (144·1 to 172·7)	7·5% (6·9 to 8·1%)	251·2 (225·4 to 281·3)	8·3% (7·6 to 9·1%)	58·9% (43·6 to 75·6%)	10·5% (4·0 to 18·1%)
**Occupational exposure to formaldehyde: All causes**	0·2 (0·2 to 0·2)	0·0% (0·0 to 0·0%)	0·3 (0·2 to 0·3)	0·0% (0·0 to 0·0%)	31·6% (21·8 to 42·9%)	26·0% (18·2 to 36·0%)	7·1 (5·7 to 8·6)	0·0% (0·0 to 0·0%)	9·4 (7·6 to 11·5)	0·0% (0·0 to 0·0%)	32·6% (23·0 to 44·1%)	40·9% (31·3 to 52·6%)
Nasopharynx cancer	0·1 (0·1 to 0·1)	1·1% (0·7 to 1·5%)	0·1 (0·1 to 0·2)	1·1% (0·7 to 1·5%)	32·5% (20·0 to 46·8%)	-1·0% (-5·5 to 4·3%)	3·0 (2·0 to 4·2)	1·2% (0·8 to 1·6%)	3·9 (2·6 to 5·7)	1·2% (0·8 to 1·7%)	33·4% (20·7 to 47·9%)	2·6% (-1·9 to 8·0%)
**Occupational exposure to nickel: All causes**	1·2 (0·9 to 1·7)	0·0% (0·0 to 0·0%)	1·6 (1·1 to 2·2)	0·0% (0·0 to 0·0%)	32·0% (17·7 to 46·5%)	26·4% (12·8 to 39·5%)	28·8 (20·4 to 38·8)	0·0% (0·0 to 0·0%)	37·3 (26·2 to 49·8)	0·0% (0·0 to 0·0%)	29·6% (15·4 to 44·1%)	37·8% (23·1 to 51·9%)
Tracheal, bronchus, and lung cancer	1·2 (0·9 to 1·7)	1·5% (1·0 to 2·0%)	1·6 (1·1 to 2·2)	1·3% (0·9 to 1·7%)	32·0% (17·7 to 46·5%)	-13·0% (-19·6 to -5·6%)	28·8 (20·4 to 38·8)	1·4% (1·0 to 1·9%)	37·3 (26·2 to 49·8)	1·2% (0·9 to 1·6%)	29·6% (15·4 to 44·1%)	-9·8% (-16·7 to -2·4%)
**Occupational exposure to polycyclic aromatic hydrocarbons: All causes**	0·8 (0·7 to 1·0)	0·0% (0·0 to 0·0%)	1·4 (1·1 to 1·7)	0·0% (0·0 to 0·0%)	66·7% (50·6 to 83·4%)	59·6% (45·0 to 74·6%)	19·7 (15·8 to 23·9)	0·0% (0·0 to 0·0%)	32·3 (25·9 to 39·1)	0·0% (0·0 to 0·0%)	64·0% (47·9 to 80·8%)	74·3% (56·9 to 90·5%)
Tracheal, bronchus, and lung cancer	0·8 (0·7 to 1·0)	1·0% (0·8 to 1·2%)	1·4 (1·1 to 1·7)	1·1% (0·9 to 1·3%)	66·7% (50·6 to 83·4%)	9·8% (2·6 to 17·8%)	19·7 (15·8 to 23·9)	0·9% (0·8 to 1·1%)	32·3 (25·9 to 39·1)	1·1% (0·9 to 1·3%)	64·0% (47·9 to 80·8%)	14·0% (6·5 to 22·0%)
**Occupational exposure to silica: All causes**	3·9 (3·4 to 4·4)	0·0% (0·0 to 0·0%)	6·8 (6·0 to 7·8)	0·1% (0·0 to 0·1%)	77·1% (57·7 to 97·2%)	69·5% (52·1 to 87·5%)	91·6 (80·4 to 103·9)	0·0% (0·0 to 0·0%)	159·9 (139·5 to 182·2)	0·0% (0·0 to 0·0%)	74·6% (55·1 to 95·1%)	85·6% (65·5 to 106·3%)
Tracheal, bronchus, and lung cancer	3·9 (3·4 to 4·4)	4·7% (4·1 to 5·3%)	6·8 (6·0 to 7·8)	5·5% (4·8 to 6·1%)	77·1% (57·7 to 97·2%)	16·7% (7·2 to 27·0%)	91·6 (80·4 to 103·9)	4·4% (3·9 to 4·9%)	159·9 (139·5 to 182·2)	5·3% (4·7 to 5·9%)	74·6% (55·1 to 95·1%)	21·4% (11·3 to 32·3%)
**Occupational exposure to sulfphuric acid: All causes**	1·6 (1·2 to 2·1)	0·0% (0·0 to 0·0%)	2·1 (1·5 to 2·8)	0·0% (0·0 to 0·0%)	33·6% (21·8 to 46·4%)	27·9% (17·5 to 38·5%)	38·7 (28·8 to 49·9)	0·0% (0·0 to 0·0%)	51·7 (38·1 to 66·8)	0·0% (0·0 to 0·0%)	33·7% (21·7 to 46·6%)	42·1% (29·6 to 54·8%)
Larynx cancer	1·6 (1·2 to 2·1)	6·8% (5·1 to 8·8%)	2·1 (1·5 to 2·8)	7·2% (5·3 to 9·3%)	33·6% (21·8 to 46·4%)	5·9% (0·3 to 11·9%)	38·7 (28·8 to 49·9)	6·3% (4·8 to 8·1%)	51·7 (38·1 to 66·8)	6·9% (5·2 to 8·9%)	33·7% (21·7 to 46·6%)	8·8% (3·1 to 14·8%)
**Occupational exposure to trichloroethylene: All causes**	0·0 (0·0 to 0·0)	0·0% (0·0 to 0·0%)	0·0 (0·0 to 0·0)	0·0% (0·0 to 0·0%)	59·2% (38·0 to 80·1%)	52·4% (32·8 to 71·4%)	0·2 (0·1 to 0·5)	0·0% (0·0 to 0·0%)	0·4 (0·1 to 0·8)	0·0% (0·0 to 0·0%)	60·1% (38·8 to 81·9%)	70·1% (47·9 to 92·0%)
Kidney cancer	0·0 (0·0 to 0·0)	0·2% (0·0 to 0·3%)	0·0 (0·0 to 0·0)	0·2% (0·0 to 0·3%)	59·2% (38·0 to 80·1%)	8·9% (1·6 to 16·4%)	0·2 (0·1 to 0·5)	0·1% (0·0 to 0·3%)	0·4 (0·1 to 0·8)	0·2% (0·0 to 0·3%)	60·1% (38·8 to 81·9%)	13·0% (4·3 to 21·9%)

Supplemental Results Table 1. All-age deaths, death PAFs, DALYs, DALY PAFs, and percent change for each risk factor and outcome for both sexes combined in 2005 and 2015 for South Asia.

Data in parenthesis are 95% uncertainty intervals. PAFs=population attributable fractions. DALYs=disability-adjusted life-years.

	2005 Deaths (in thousands)	2005 Deaths PAF	2015 Deaths (in thousands)	2015 Deaths PAF	Percent change deaths 2005-2015	Percent change deaths PAF 2005-2015	2005 DALYs (in thousands)	2005 DALYs PAF	2015 DALYs (in thousands)	2015 DALYs PAF	Percent change DALYs 2005-2015	Percent change DALYs PAF 2005-2015
**Occupational asthmagens: All causes**	23·3 (15·6 to 30·8)	0·2% (0·1 to 0·2%)	16·3 (13·1 to 20·4)	0·1% (0·1 to 0·2%)	-29·9% (-43·1 to -6·0%)	-32·9% (-45·8 to -10·2%)	981·3 (725·8 to 1,235·9)	0·1% (0·1 to 0·2%)	791·7 (634·9 to 967·4)	0·1% (0·1 to 0·1%)	-19·3% (-31·1 to -2·1%)	-14·3% (-25·7 to 3·9%)
Asthma	23·3 (15·6 to 30·8)	11·4% (9·0 to 13·9%)	16·3 (13·1 to 20·4)	10·0% (8·5 to 11·6%)	-29·9% (-43·1 to -6·0%)	-12·5% (-23·0 to -0·7%)	981·3 (725·8 to 1,235·9)	11·5% (9·7 to 13·4%)	791·7 (634·9 to 967·4)	10·6% (9·1 to 12·2%)	-19·3% (-31·1 to -2·1%)	-7·5% (-13·8 to -1·3%)
**Occupational particulate matter, gases, and fumes: All causes**	138·1 (115·1 to 162·9)	1·1% (0·9 to 1·3%)	170·6 (140·9 to 198·7)	1·3% (1·1 to 1·5%)	23·5% (14·9 to 34·4%)	18·2% (11·0 to 27·1%)	3,578·1 (3,053·0 to 4,137·8)	0·5% (0·4 to 0·6%)	4,320·6 (3,720·8 to 4,936·3)	0·7% (0·6 to 0·8%)	20·8% (13·1 to 30·9%)	28·4% (20·6 to 38·2%)
Chronic obstructive pulmonary disease	138·1 (115·0 to 162·9)	14·0% (11·7 to 16·2%)	170·5 (140·9 to 198·6)	14·2% (11·8 to 16·5%)	23·5% (14·9 to 34·4%)	1·9% (-2·0 to 7·1%)	3,573·9 (3,049·5 to 4,134·3)	15·3% (13·3 to 17·2%)	4,314·9 (3,715·2 to 4,929·9)	16·0% (13·9 to 18·0%)	20·7% (13·0 to 30·9%)	4·7% (1·3 to 9·3%)
**Occupational noise: All causes**	-	-	-	-	-	-	2,456·6 (1,676·3 to 3,380·9)	0·4% (0·3 to 0·5%)	3,143·0 (2,146·4 to 4,340·8)	0·5% (0·4 to 0·6%)	27·9% (26·1 to 29·7%)	35·8% (32·3 to 40·0%)
Age-related and other hearing loss	-	-	-	-	-	-	2,456·6 (1,676·3 to 3,380·9)	34·3% (31·5 to 37·9%)	3,143·0 (2,146·4 to 4,340·8)	34·3% (31·7 to 37·8%)	27·9% (26·1 to 29·7%)	0·2% (-1·0 to 1·6%)
**Occupational injuries: All causes**	61·2 (42·5 to 79·2)	0·5% (0·3 to 0·6%)	57·0 (45·4 to 84·3)	0·4% (0·4 to 0·7%)	-6·8% (-33·3 to 45·7%)	-10·8% (-36·5 to 40·2%)	3,821·7 (2,684·2 to 4,878·9)	0·6% (0·4 to 0·7%)	3,627·2 (2,843·7 to 5,310·5)	0·6% (0·4 to 0·8%)	-5·1% (-31·0 to 46·3%)	0·8% (-26·8 to 54·6%)
Pedestrian road injuries	7·6 (3·7 to 11·8)	5·5% (2·6 to 8·5%)	6·7 (4·2 to 12·9)	4·5% (2·9 to 8·9%)	-12·3% (-52·0 to 89·4%)	-17·3% (-54·7 to 80·7%)	384·4 (182·6 to 583·0)	6·0% (2·9 to 9·1%)	328·4 (206·1 to 638·0)	5·2% (3·4 to 10·1%)	-14·6% (-53·2 to 84·2%)	-13·8% (-53·1 to 89·9%)
Cyclist road injuries	0·3 (0·1 to 0·5)	6·6% (2·6 to 11·3%)	0·2 (0·1 to 0·6)	4·4% (2·7 to 9·9%)	-19·1% (-61·4 to 102·5%)	-34·1% (-68·7 to 67·5%)	17·4 (7·0 to 28·1)	7·0% (2·8 to 11·4%)	14·5 (8·5 to 33·5)	5·1% (3·1 to 11·2%)	-16·7% (-58·4 to 101·4%)	-27·7% (-64·3 to 76·8%)
Motorcyclist road injuries	5·9 (4·4 to 7·6)	7·9% (6·3 to 9·6%)	7·4 (5·6 to 10·1)	8·0% (6·5 to 10·5%)	25·9% (-9·9 to 74·4%)	1·1% (-22·3 to 36·7%)	313·8 (238·5 to 406·0)	8·7% (7·0 to 10·6%)	389·4 (298·6 to 530·5)	9·1% (7·4 to 12·0%)	24·1% (-10·3 to 71·1%)	4·8% (-19·6 to 41·8%)
Motor vehicle road injuries	8·8 (6·1 to 11·8)	9·9% (7·0 to 12·5%)	8·7 (6·3 to 12·6)	8·6% (6·8 to 12·4%)	-1·7% (-35·3 to 56·9%)	-12·9% (-36·9 to 32·3%)	476·9 (326·3 to 630·3)	10·8% (7·6 to 13·6%)	462·7 (342·1 to 669·9)	9·8% (7·7 to 14·2%)	-3·0% (-35·3 to 53·8%)	-9·4% (-34·2 to 36·9%)
Other road injuries	1·1 (0·5 to 1·8)	20·1% (8·9 to 31·6%)	0·7 (0·4 to 1·7)	11·2% (6·9 to 26·6%)	-33·2% (-67·0 to 56·6%)	-44·5% (-72·0 to 28·9%)	60·5 (26·5 to 97·9)	21·1% (9·6 to 32·4%)	41·5 (25·0 to 97·0)	12·4% (7·9 to 28·6%)	-31·4% (-65·4 to 54·4%)	-41·1% (-69·6 to 30·8%)
Other transport injuries	1·7 (0·9 to 2·6)	4·8% (2·7 to 7·3%)	1·9 (1·2 to 3·3)	4·7% (3·3 to 7·6%)	10·6% (-38·8 to 117·3%)	-2·6% (-42·7 to 94·2%)	90·0 (47·4 to 135·4)	5·3% (2·9 to 7·9%)	96·5 (59·7 to 168·3)	5·2% (3·6 to 8·4%)	7·2% (-40·2 to 114·1%)	-0·8% (-41·4 to 96·8%)
Falls	5·3 (3·4 to 7·6)	7·0% (4·4 to 9·8%)	5·1 (3·9 to 7·6)	5·6% (4·3 to 8·2%)	-3·8% (-35·5 to 56·6%)	-20·0% (-46·4 to 30·2%)	473·9 (310·8 to 644·0)	9·0% (6·1 to 11·6%)	504·6 (369·6 to 755·4)	8·5% (6·6 to 12·4%)	6·5% (-23·5 to 59·4%)	-5·3% (-32·7 to 43·7%)
Drowning	12·5 (6·5 to 18·1)	10·8% (5·6 to 17·1%)	9·0 (5·7 to 17·7)	9·2% (6·3 to 19·2%)	-28·4% (-58·0 to 41·4%)	-14·6% (-51·9 to 68·2%)	651·7 (346·4 to 918·6)	9·1% (4·8 to 14·6%)	466·0 (300·7 to 917·5)	8·7% (5·8 to 17·8%)	-28·5% (-57·5 to 39·1%)	-4·9% (-46·6 to 85·7%)
Fire, heat, and hot substances	5·7 (4·5 to 7·3)	7·4% (5·9 to 10·3%)	6·0 (4·5 to 7·8)	8·9% (7·1 to 11·8%)	4·8% (-18·3 to 39·5%)	19·1% (-7·9 to 58·6%)	402·1 (322·5 to 510·7)	8·1% (6·4 to 11·2%)	434·2 (344·9 to 569·0)	10·0% (8·0 to 13·6%)	8·0% (-15·2 to 41·7%)	24·0% (-3·8 to 64·6%)
Poisonings	0·7 (0·4 to 0·8)	4·0% (3·1 to 5·2%)	0·6 (0·4 to 0·8)	4·6% (3·5 to 6·2%)	-5·2% (-28·3 to 20·9%)	12·9% (-14·2 to 52·4%)	42·6 (28·7 to 52·7)	3·9% (2·9 to 5·3%)	42·6 (29·2 to 54·3)	4·8% (3·5 to 6·7%)	0·0% (-24·2 to 27·1%)	21·0% (-10·3 to 64·9%)
Unintentional firearm injuries	0·2 (0·1 to 0·2)	4·9% (4·0 to 6·0%)	0·1 (0·1 to 0·2)	4·9% (4·0 to 6·0%)	-8·6% (-29·5 to 13·1%)	0·0% (-17·8 to 20·8%)	8·6 (4·2 to 11·0)	5·4% (4·5 to 6·6%)	7·8 (3·8 to 10·0)	5·7% (4·8 to 6·8%)	-9·4% (-29·9 to 12·1%)	4·8% (-13·2 to 26·4%)
Unintentional suffocation	0·0 (0·0 to 0·0)	0·3% (0·2 to 0·5%)	0·0 (0·0 to 0·1)	0·4% (0·3 to 0·7%)	20·9% (-26·9 to 109·5%)	22·2% (-25·7 to 113·3%)	1·9 (1·3 to 2·9)	0·3% (0·2 to 0·5%)	2·3 (1·5 to 3·9)	0·5% (0·3 to 0·8%)	21·0% (-26·0 to 112·0%)	38·0% (-19·8 to 157·3%)
Other exposure to mechanical forces	2·7 (2·2 to 3·5)	6·7% (5·5 to 8·2%)	3·0 (2·3 to 3·9)	7·2% (5·9 to 9·5%)	7·9% (-18·6 to 47·4%)	7·1% (-16·9 to 43·8%)	225·1 (172·0 to 285·9)	7·9% (6·4 to 9·6%)	242·8 (188·0 to 328·4)	9·0% (7·4 to 11·5%)	7·9% (-17·3 to 44·6%)	13·3% (-11·4 to 48·8%)
Venomous animal contact	2·4 (1·4 to 2·9)	3·8% (3·2 to 4·5%)	2·1 (1·1 to 3·1)	3·8% (3·1 to 4·5%)	-12·1% (-31·3 to 16·8%)	0·5% (-16·8 to 22·6%)	149·6 (103·0 to 180·0)	3·8% (3·2 to 4·6%)	134·9 (85·9 to 181·7)	4·1% (3·3 to 5·0%)	-9·8% (-26·6 to 14·5%)	6·9% (-11·8 to 28·9%)
Non-venomous animal contact	0·1 (0·1 to 0·1)	2·9% (2·3 to 3·5%)	0·1 (0·0 to 0·1)	3·1% (2·5 to 3·8%)	-9·8% (-34·2 to 45·4%)	7·4% (-13·6 to 36·0%)	10·9 (8·1 to 14·9)	3·6% (2·9 to 4·4%)	10·5 (7·6 to 14·5)	4·1% (3·1 to 5·1%)	-4·3% (-22·6 to 19·1%)	13·4% (-9·3 to 38·8%)
Pulmonary aspiration and foreign body in airway	0·7 (0·5 to 1·0)	3·0% (2·3 to 4·1%)	0·9 (0·6 to 1·2)	3·7% (3·1 to 4·6%)	27·9% (5·2 to 56·0%)	23·5% (-3·7 to 52·1%)	44·2 (32·8 to 59·8)	2·8% (2·0 to 4·1%)	55·2 (40·4 to 71·4)	3·6% (2·9 to 4·8%)	24·9% (3·4 to 50·4%)	30·5% (1·0 to 65·3%)
Foreign body in eyes	-	-	-	-	-	-	0·8 (0·3 to 1·4)	5·9% (4·8 to 7·1%)	1·0 (0·5 to 1·9)	6·6% (5·5 to 8·0%)	30·8% (11·0 to 52·6%)	12·8% (-4·4 to 32·0%)
Foreign body in other body part	0·3 (0·1 to 0·5)	5·6% (4·4 to 7·4%)	0·2 (0·2 to 0·3)	6·3% (5·0 to 8·2%)	-14·4% (-51·3 to 31·3%)	12·3% (-16·1 to 45·0%)	23·8 (15·9 to 35·3)	6·1% (4·7 to 7·9%)	23·2 (17·0 to 29·8)	7·3% (5·8 to 9·3%)	-2·6% (-33·0 to 29·7%)	19·6% (-8·5 to 52·6%)
Other unintentional injuries	5·2 (3·9 to 7·1)	8·9% (6·6 to 12·0%)	4·3 (2·9 to 7·6)	8·2% (5·5 to 14·0%)	-17·4% (-47·0 to 45·6%)	-7·5% (-41·3 to 55·9%)	443·5 (314·6 to 604·2)	9·8% (7·2 to 12·9%)	369·1 (250·0 to 622·4)	9·5% (6·6 to 15·2%)	-16·8% (-44·4 to 40·7%)	-3·1% (-36·3 to 64·4%)
**Occupational ergonomic factors: All causes**	-	-	-	-	-	-	4,693·9 (3,275·7 to 6,538·3)	0·7% (0·5 to 0·9%)	5,193·7 (3,627·8 to 7,186·1)	0·8% (0·6 to 1·0%)	10·6% (8·2 to 13·0%)	17·5% (13·9 to 21·3%)
Low back pain	-	-	-	-	-	-	4,693·9 (3,275·7 to 6,538·3)	41·6% (39·0 to 44·1%)	5,193·7 (3,627·8 to 7,186·1)	37·9% (35·7 to 40·0%)	10·6% (8·2 to 13·0%)	-8·9% (-9·8 to -7·9%)
**Behavioural risks: All causes**	4,012·1 (3,771·6 to 4,246·7)	32·6% (30·8 to 34·3%)	4,424·9 (4,113·4 to 4,742·1)	34·4% (32·3 to 36·6%)	10·3% (5·7 to 15·0%)	5·6% (3·2 to 7·9%)	190,228·2 (176,086·0 to 205,628·6)	27·7% (26·5 to 28·8%)	172,877·0 (159,315·3 to 187,858·1)	26·8% (25·6 to 28·0%)	-9·1% (-12·5 to -5·3%)	-3·4% (-6·2 to -0·2%)
**Child and maternal malnutrition: All causes**	731·9 (666·6 to 799·4)	5·9% (5·4 to 6·5%)	378·3 (342·0 to 414·9)	2·9% (2·7 to 3·2%)	-48·3% (-52·8 to -42·7%)	-50·5% (-55·2 to -45·3%)	86,028·5 (76,698·9 to 97,357·1)	12·5% (11·6 to 13·5%)	53,221·0 (45,447·5 to 61,728·8)	8·2% (7·5 to 9·0%)	-38·1% (-42·8 to -33·0%)	-34·3% (-38·8 to -29·4%)
**Suboptimal breastfeeding: All causes**	193·8 (124·4 to 284·3)	1·6% (1·0 to 2·3%)	115·9 (74·7 to 167·1)	0·9% (0·6 to 1·3%)	-40·2% (-46·8 to -32·2%)	-42·8% (-49·4 to -35·1%)	16,790·6 (10,817·8 to 24,615·1)	2·4% (1·6 to 3·6%)	10,078·8 (6,552·9 to 14,512·0)	1·6% (1·0 to 2·3%)	-40·0% (-46·5 to -32·0%)	-36·2% (-43·1 to -28·0%)
**Non-exclusive breastfeeding : All causes**	183·5 (114·4 to 271·9)	1·5% (0·9 to 2·2%)	109·4 (68·4 to 159·1)	0·8% (0·5 to 1·2%)	-40·4% (-46·9 to -32·5%)	-42·9% (-49·6 to -35·3%)	15,857·4 (9,909·7 to 23,460·9)	2·3% (1·4 to 3·4%)	9,465·9 (5,929·2 to 13,767·1)	1·5% (0·9 to 2·2%)	-40·3% (-46·8 to -32·4%)	-36·6% (-43·5 to -28·5%)
Diarrhoeal diseases	76·9 (45·7 to 107·0)	10·5% (6·2 to 14·5%)	48·5 (29·1 to 70·4)	8·5% (5·1 to 12·3%)	-36·9% (-48·4 to -23·4%)	-18·9% (-32·4 to -3·2%)	6,653·3 (3,968·8 to 9,248·2)	17·7% (10·6 to 24·3%)	4,211·8 (2,543·6 to 6,100·7)	16·2% (9·9 to 22·9%)	-36·7% (-48·1 to -23·2%)	-8·2% (-20·2 to 5·3%)
Lower respiratory infections	106·7 (45·0 to 190·6)	14·2% (5·9 to 25·2%)	60·9 (25·4 to 108·6)	9·5% (4·0 to 16·7%)	-42·9% (-50·8 to -33·2%)	-33·1% (-40·6 to -23·9%)	9,204·1 (3,883·7 to 16,428·6)	21·1% (8·9 to 37·2%)	5,254·1 (2,197·1 to 9,376·0)	17·7% (7·4 to 30·7%)	-42·9% (-50·7 to -33·2%)	-16·1% (-24·2 to -7·1%)
**Discontinued breastfeeding : All causes**	14·7 (4·9 to 28·4)	0·1% (0·0 to 0·2%)	9·6 (3·1 to 18·5)	0·1% (0·0 to 0·1%)	-35·0% (-47·3 to -19·7%)	-37·8% (-49·5 to -22·7%)	1,312·5 (436·3 to 2,529·2)	0·2% (0·1 to 0·4%)	875·6 (285·8 to 1,674·3)	0·1% (0·0 to 0·3%)	-33·3% (-45·0 to -18·7%)	-29·1% (-41·5 to -12·9%)
Diarrhoeal diseases	14·7 (4·9 to 28·4)	2·0% (0·7 to 3·8%)	9·6 (3·1 to 18·5)	1·7% (0·5 to 3·2%)	-35·0% (-47·3 to -19·7%)	-16·4% (-30·8 to 1·9%)	1,312·5 (436·3 to 2,529·2)	3·5% (1·1 to 6·5%)	875·6 (285·8 to 1,674·3)	3·4% (1·1 to 6·4%)	-33·3% (-45·0 to -18·7%)	-3·3% (-15·2 to 11·1%)
**Childhood undernutrition: All causes**	690·4 (628·9 to 754·9)	5·6% (5·1 to 6·1%)	350·1 (314·0 to 387·5)	2·7% (2·4 to 3·0%)	-49·3% (-53·8 to -43·7%)	-51·4% (-56·2 to -46·0%)	61,390·8 (56,131·4 to 66,953·3)	8·9% (8·1 to 9·9%)	32,040·7 (28,855·1 to 35,116·1)	5·0% (4·4 to 5·5%)	-47·8% (-52·4 to -42·4%)	-44·5% (-49·5 to -38·6%)
**Childhood underweight: All causes**	226·8 (173·2 to 303·9)	1·8% (1·4 to 2·5%)	93·5 (73·6 to 125·8)	0·7% (0·6 to 1·0%)	-58·8% (-65·1 to -51·9%)	-60·5% (-66·7 to -53·7%)	21,017·5 (16,391·5 to 27,712·0)	3·1% (2·4 to 4·0%)	9,393·4 (7,645·1 to 12,257·9)	1·5% (1·2 to 1·9%)	-55·3% (-61·7 to -48·4%)	-52·5% (-59·4 to -45·3%)
Diarrhoeal diseases	51·4 (41·0 to 64·9)	7·0% (5·7 to 8·7%)	25·3 (20·0 to 32·0)	4·4% (3·5 to 5·6%)	-50·7% (-59·2 to -40·7%)	-36·6% (-46·8 to -25·6%)	4,569·9 (3,670·5 to 5,789·3)	12·1% (10·2 to 15·0%)	2,302·8 (1,827·3 to 2,910·9)	8·9% (7·3 to 11·0%)	-49·6% (-58·0 to -39·9%)	-26·9% (-34·6 to -19·2%)
Lower respiratory infections	94·3 (63·5 to 157·3)	12·5% (8·6 to 20·8%)	43·0 (27·6 to 75·1)	6·7% (4·4 to 11·7%)	-54·4% (-60·3 to -47·0%)	-46·4% (-51·9 to -40·7%)	8,105·0 (5,460·8 to 13,498·9)	18·6% (12·9 to 30·8%)	3,706·5 (2,380·0 to 6,474·6)	12·5% (8·2 to 21·6%)	-54·3% (-60·2 to -47·0%)	-32·8% (-37·9 to -27·7%)
Measles	45·2 (9·5 to 117·6)	39·4% (13·5 to 63·9%)	6·7 (1·3 to 17·4)	33·8% (10·4 to 58·8%)	-85·2% (-89·2 to -80·4%)	-14·1% (-25·3 to -6·7%)	3,844·7 (811·7 to 10,009·4)	40·0% (13·7 to 64·9%)	567·5 (107·6 to 1,479·5)	34·4% (10·6 to 59·9%)	-85·2% (-89·2 to -80·4%)	-14·0% (-25·2 to -6·6%)
Protein-energy malnutrition	35·9 (28·4 to 46·5)	46·0% (40·8 to 52·0%)	18·4 (13·4 to 27·8)	32·7% (26·9 to 39·9%)	-48·6% (-62·1 to -29·6%)	-29·0% (-40·7 to -13·7%)	4,497·9 (3,641·2 to 5,449·2)	78·7% (75·2 to 82·2%)	2,816·5 (2,194·3 to 3,707·0)	74·4% (69·2 to 78·9%)	-37·4% (-48·2 to -24·0%)	-5·6% (-10·7 to -1·1%)
**Childhood wasting: All causes**	618·0 (531·7 to 702·4)	5·0% (4·3 to 5·7%)	323·4 (270·6 to 362·4)	2·5% (2·1 to 2·8%)	-47·7% (-53·2 to -41·8%)	-49·9% (-55·6 to -44·2%)	55,163·3 (47,787·6 to 62,455·1)	8·0% (6·9 to 9·2%)	29,712·8 (25,093·5 to 33,216·5)	4·6% (3·9 to 5·2%)	-46·1% (-51·5 to -40·4%)	-42·8% (-48·6 to -37·1%)

Supplemental Results Table 1. All-age deaths, death PAFs, DALYs, DALY PAFs, and percent change for each risk factor and outcome for both sexes combined in 2005 and 2015 for South Asia.

Data in parenthesis are 95% uncertainty intervals. PAFs=population attributable fractions. DALYs=disability-adjusted life-years.

	**2005 Deaths (in thousands)**	**2005 Deaths PAF**	**2015 Deaths (in thousands)**	**2015 Deaths PAF**	**Percent change deaths 2005-2015**	**Percent change deaths PAF 2005-2015**	**2005 DALYs (in thousands)**	**2005 DALYs PAF**	**2015 DALYs (in thousands)**	**2015 DALYs PAF**	**Percent change DALYs 2005-2015**	**Percent change DALYs PAF 2005-2015**
Diarrhoeal diseases	218·7 (180·3 to 248·7)	29·8% (25·6 to 32·8%)	128·3 (102·7 to 152·5)	22·5% (18·2 to 26·6%)	-41·3% (-51·2 to -30·0%)	-24·6% (-35·9 to -12·2%)	19,470·4 (16,070·1 to 22,022·4)	51·7% (45·2 to 55·4%)	11,691·6 (9,467·6 to 13,781·6)	45·0% (37·9 to 50·3%)	-40·0% (-49·6 to -28·9%)	-12·9% (-21·1 to -4·3%)
Lower respiratory infections	311·6 (239·5 to 362·8)	41·4% (31·8 to 48·5%)	168·1 (124·2 to 199·9)	26·2% (19·4 to 31·7%)	-46·0% (-52·4 to -38·6%)	-36·7% (-42·4 to -30·9%)	26,786·5 (20,590·9 to 31,181·0)	61·5% (47·7 to 69·5%)	14,482·2 (10,706·4 to 17,215·6)	48·8% (36·5 to 56·8%)	-45·9% (-52·2 to -38·5%)	-20·6% (-25·4 to -16·1%)
Measles	51·8 (13·0 to 145·9)	45·2% (18·7 to 81·6%)	8·5 (2·0 to 25·5)	43·1% (16·6 to 80·8%)	-83·6% (-87·8 to -79·0%)	-4·5% (-15·8 to 1·1%)	4,408·6 (1,106·1 to 12,430·6)	45·9% (19·0 to 82·9%)	722·5 (169·8 to 2,177·6)	43·9% (16·9 to 82·1%)	-83·6% (-87·8 to -79·0%)	-4·4% (-15·8 to 1·0%)
Protein-energy malnutrition	35·9 (28·4 to 46·5)	46·0% (40·8 to 52·0%)	18·4 (13·4 to 27·8)	32·7% (26·9 to 39·9%)	-48·6% (-62·1 to -29·6%)	-29·0% (-40·7 to -13·7%)	4,497·9 (3,641·2 to 5,449·2)	78·7% (75·2 to 82·2%)	2,816·5 (2,194·3 to 3,707·0)	74·4% (69·2 to 78·9%)	-37·4% (-48·2 to -24·0%)	-5·6% (-10·7 to -1·1%)
**Childhood stunting: All causes**	190·0 (94·3 to 313·3)	1·5% (0·8 to 2·5%)	82·9 (37·8 to 144·5)	0·6% (0·3 to 1·1%)	-56·4% (-65·9 to -49·3%)	-58·2% (-67·3 to -51·5%)	16,471·0 (8,291·2 to 27,073·8)	2·4% (1·2 to 4·0%)	7,268·7 (3,378·7 to 12,553·0)	1·1% (0·5 to 2·0%)	-55·9% (-65·3 to -48·8%)	-53·1% (-63·2 to -45·8%)
Diarrhoeal diseases	53·6 (20·9 to 91·4)	7·3% (2·9 to 12·3%)	27·6 (10·4 to 48·5)	4·8% (1·8 to 8·5%)	-48·6% (-57·3 to -37·6%)	-34·0% (-44·4 to -22·3%)	4,767·6 (1,880·0 to 8,067·6)	12·7% (5·1 to 21·2%)	2,506·8 (945·0 to 4,426·6)	9·7% (3·7 to 16·8%)	-47·4% (-55·9 to -36·8%)	-23·7% (-31·7 to -15·6%)
Lower respiratory infections	106·7 (10·6 to 223·5)	14·2% (1·4 to 29·7%)	51·0 (4·1 to 112·7)	8·0% (0·7 to 17·4%)	-52·2% (-58·8 to -41·8%)	-43·9% (-50·5 to -34·7%)	9,169·3 (913·8 to 19,212·7)	21·1% (2·1 to 44·1%)	4,393·6 (357·4 to 9,694·4)	14·8% (1·3 to 32·7%)	-52·1% (-58·7 to -41·8%)	-29·6% (-35·9 to -20·0%)
Measles	29·8 (3·2 to 82·7)	26·1% (3·3 to 58·8%)	4·3 (0·4 to 13·0)	22·1% (2·6 to 53·4%)	-85·5% (-89·4 to -79·7%)	-15·3% (-25·6 to -5·4%)	2,534·0 (268·9 to 7,043·4)	26·5% (3·4 to 59·8%)	368·4 (34·0 to 1,107·5)	22·4% (2·7 to 54·5%)	-85·5% (-89·3 to -79·7%)	-15·3% (-25·6 to -5·3%)
**Iron deficiency: All causes**	18·9 (10·8 to 27·2)	0·2% (0·1 to 0·2%)	14·7 (8·2 to 21·0)	0·1% (0·1 to 0·2%)	-22·1% (-36·5 to -2·2%)	-25·4% (-39·0 to -6·3%)	22,578·3 (15,508·1 to 31,608·7)	3·3% (2·4 to 4·3%)	19,887·4 (13,597·6 to 27,993·4)	3·1% (2·3 to 4·0%)	-11·9% (-14·2 to -9·7%)	-6·5% (-9·3 to -3·5%)
Maternal haemorrhage	7·9 (3·2 to 12·8)	37·0% (14·7 to 59·8%)	4·8 (2·0 to 8·3)	34·3% (13·2 to 54·5%)	-38·4% (-51·2 to -23·2%)	-7·4% (-14·7 to 3·9%)	459·5 (188·4 to 749·6)	37·1% (14·7 to 60·1%)	282·3 (113·7 to 485·9)	34·4% (13·2 to 54·5%)	-38·6% (-51·4 to -23·8%)	-7·4% (-14·6 to 4·8%)
Maternal sepsis and other pregnancy related infections	4·0 (1·6 to 6·5)	38·4% (15·3 to 60·9%)	2·2 (0·8 to 3·6)	34·5% (13·4 to 54·3%)	-46·8% (-56·6 to -34·9%)	-10·1% (-16·4 to 0·6%)	236·6 (95·0 to 384·3)	38·5% (15·4 to 60·9%)	127·8 (48·9 to 217·3)	34·6% (13·4 to 54·4%)	-46·0% (-55·8 to -34·4%)	-10·1% (-16·4 to 1·4%)
Iron-deficiency anaemia	7·0 (2·4 to 9·8)	100·0% (100·0 to 100·0%)	7·7 (2·3 to 11·3)	100·0% (100·0 to 100·0%)	10·3% (-15·2 to 41·0%)	-	21,882·2 (14,974·5 to 30,927·9)	100·0% (100·0 to 100·0%)	19,477·3 (13,294·2 to 27,613·9)	100·0% (100·0 to 100·0%)	-11·0% (-13·1 to -8·9%)	-
**Vitamin A deficiency: All causes**	77·9 (46·8 to 121·0)	0·6% (0·4 to 1·0%)	29·4 (17·8 to 42·7)	0·2% (0·1 to 0·3%)	-62·3% (-71·5 to -51·5%)	-63·9% (-72·7 to -53·3%)	6,912·7 (4,177·8 to 10,677·2)	1·0% (0·6 to 1·5%)	2,772·1 (1,730·8 to 3,944·6)	0·4% (0·3 to 0·6%)	-59·9% (-69·6 to -49·1%)	-57·4% (-67·7 to -46·0%)
Diarrhoeal diseases	40·4 (22·8 to 58·4)	5·5% (3·1 to 7·9%)	22·6 (12·4 to 33·8)	4·0% (2·2 to 5·9%)	-43·9% (-53·9 to -32·7%)	-27·9% (-39·9 to -14·5%)	3,612·4 (2,048·9 to 5,205·2)	9·6% (5·5 to 13·6%)	2,080·9 (1,164·4 to 3,077·6)	8·0% (4·6 to 11·6%)	-42·4% (-51·8 to -31·5%)	-16·5% (-25·9 to -6·2%)
Measles	37·6 (12·9 to 78·6)	33·0% (22·9 to 43·1%)	6·7 (2·3 to 14·9)	34·3% (24·1 to 44·3%)	-82·1% (-85·9 to -77·5%)	3·9% (-7·3 to 18·7%)	3,194·8 (1,100·4 to 6,697·7)	33·5% (23·3 to 43·7%)	571·7 (194·9 to 1,268·6)	34·8% (24·5 to 45·0%)	-82·1% (-85·8 to -77·5%)	4·0% (-7·1 to 18·9%)
Vitamin A deficiency	-	-	-	-	-	-	105·4 (66·1 to 155·7)	100·0% (100·0 to 100·0%)	119·6 (74·5 to 174·6)	100·0% (100·0 to 100·0%)	13·4% (7·5 to 19·6%)	-
**Zinc deficiency: All causes**	31·9 (2·0 to 73·2)	0·3% (0·0 to 0·6%)	16·1 (1·0 to 36·5)	0·1% (0·0 to 0·3%)	-49·7% (-58·7 to -35·2%)	-51·8% (-60·8 to -38·0%)	2,833·5 (319·1 to 6,313·1)	0·4% (0·0 to 0·9%)	1,476·0 (208·0 to 3,216·9)	0·2% (0·0 to 0·5%)	-47·9% (-56·2 to -29·7%)	-44·6% (-53·6 to -24·8%)
Diarrhoeal diseases	15·9 (0·0 to 36·0)	2·2% (0·0 to 4·9%)	8·3 (0·0 to 18·7)	1·4% (0·0 to 3·3%)	-48·1% (-59·3 to 0·0%)	-33·4% (-47·3 to 0·0%)	1,471·9 (120·2 to 3,207·3)	3·9% (0·3 to 8·4%)	808·4 (104·7 to 1,695·3)	3·1% (0·4 to 6·6%)	-45·1% (-55·5 to -12·7%)	-20·5% (-33·5 to 27·0%)
Lower respiratory infections	16·0 (0·0 to 53·5)	2·1% (0·0 to 7·0%)	7·8 (0·0 to 25·8)	1·2% (0·0 to 4·1%)	-51·3% (-59·8 to 0·0%)	-42·8% (-51·3 to 0·0%)	1,361·6 (6·7 to 4,524·3)	3·1% (0·0 to 10·2%)	667·6 (5·3 to 2,187·7)	2·2% (0·0 to 7·6%)	-51·0% (-59·5 to -16·2%)	-28·0% (-37·5 to 28·0%)
**Tobacco smoke: All causes**	1,069·1 (961·7 to 1,179·6)	8·7% (7·9 to 9·5%)	1,263·6 (1,123·6 to 1,396·5)	9·8% (8·8 to 10·8%)	18·2% (10·2 to 26·6%)	13·2% (8·0 to 18·6%)	33,488·4 (29,544·8 to 37,643·9)	4·9% (4·3 to 5·5%)	35,866·4 (31,925·8 to 39,858·3)	5·6% (4·9 to 6·2%)	7·1% (0·3 to 15·0%)	13·8% (7·4 to 20·8%)
**Smoking: All causes**	844·1 (749·5 to 944·4)	6·8% (6·1 to 7·6%)	1,040·8 (916·7 to 1,168·3)	8·1% (7·2 to 8·9%)	23·3% (13·9 to 33·5%)	18·0% (11·5 to 25·2%)	23,997·1 (21,490·8 to 26,666·5)	3·5% (3·1 to 3·9%)	28,525·3 (25,268·2 to 31,795·0)	4·4% (3·8 to 5·0%)	18·9% (10·4 to 27·9%)	26·3% (19·0 to 34·2%)
Tuberculosis	45·6 (22·2 to 72·9)	6·8% (3·4 to 10·5%)	35·4 (15·9 to 57·5)	6·4% (3·0 to 10·2%)	-22·3% (-36·5 to -11·7%)	-5·2% (-14·4 to 1·7%)	1,514·4 (715·2 to 2,450·9)	6·1% (3·0 to 9·5%)	1,160·2 (516·4 to 1,891·3)	5·9% (2·8 to 9·4%)	-23·4% (-36·8 to -13·4%)	-3·1% (-13·2 to 4·6%)
Lower respiratory infections	48·2 (35·3 to 60·5)	6·4% (5·0 to 7·9%)	49·1 (36·6 to 62·3)	7·6% (6·0 to 9·6%)	2·0% (-8·9 to 11·7%)	19·5% (8·0 to 31·3%)	1,294·5 (950·7 to 1,622·0)	3·0% (2·3 to 3·7%)	1,289·4 (960·0 to 1,629·5)	4·3% (3·3 to 5·6%)	-0·4% (-10·3 to 8·8%)	46·3% (29·9 to 64·5%)
Lip and oral cavity cancer	8·9 (6·7 to 11·2)	25·8% (20·0 to 31·9%)	13·4 (10·3 to 16·6)	28·2% (22·3 to 34·1%)	51·2% (29·5 to 76·8%)	9·4% (-1·6 to 21·5%)	278·0 (208·8 to 351·5)	27·7% (21·4 to 34·2%)	410·9 (313·9 to 513·0)	30·3% (24·0 to 36·7%)	47·8% (26·1 to 73·5%)	9·4% (-1·3 to 21·6%)
Nasopharynx cancer	1·8 (1·3 to 2·4)	23·3% (17·3 to 29·3%)	2·7 (2·0 to 3·6)	26·1% (19·8 to 32·4%)	49·7% (29·7 to 72·0%)	11·8% (0·9 to 22·7%)	58·6 (42·5 to 76·5)	23·2% (17·2 to 28·9%)	85·0 (60·9 to 111·6)	25·9% (19·6 to 32·4%)	45·0% (25·1 to 67·6%)	11·4% (0·7 to 22·9%)
Oesophageal cancer	10·9 (7·7 to 14·4)	21·9% (15·6 to 28·6%)	16·0 (11·1 to 21·5)	25·2% (17·5 to 33·0%)	46·8% (24·9 to 72·1%)	14·8% (2·1 to 27·7%)	318·8 (227·3 to 418·9)	23·6% (17·1 to 30·7%)	446·1 (309·1 to 598·7)	26·5% (18·8 to 34·7%)	39·9% (18·5 to 64·4%)	12·6% (-0·5 to 25·3%)
Stomach cancer	2·7 (1·4 to 4·0)	4·9% (2·7 to 7·4%)	3·7 (2·1 to 5·7)	5·9% (3·3 to 8·8%)	40·5% (18·7 to 66·1%)	19·7% (4·2 to 37·0%)	75·8 (41·2 to 114·7)	5·7% (3·1 to 8·6%)	99·2 (55·0 to 149·7)	6·5% (3·6 to 9·8%)	30·8% (10·3 to 56·6%)	15·5% (0·1 to 33·5%)
Colon and rectum cancer	1·0 (0·6 to 1·4)	2·1% (1·4 to 3·1%)	1·6 (1·0 to 2·3)	2·5% (1·6 to 3·6%)	67·1% (34·7 to 106·4%)	16·1% (-1·8 to 36·5%)	30·6 (18·6 to 46·5)	2·5% (1·5 to 3·9%)	46·6 (28·3 to 69·2)	2·8% (1·7 to 4·1%)	52·3% (18·3 to 92·6%)	10·4% (-9·2 to 32·1%)
Liver cancer due to hepatitis B	1·1 (0·5 to 1·8)	8·1% (4·0 to 13·1%)	1·5 (0·7 to 2·5)	9·1% (4·5 to 14·4%)	40·3% (15·2 to 74·6%)	12·8% (-1·8 to 30·6%)	34·8 (16·9 to 56·6)	8·4% (4·1 to 13·7%)	46·8 (23·2 to 76·6)	9·4% (4·7 to 15·0%)	34·8% (10·5 to 68·5%)	11·6% (-3·4 to 30·6%)
Liver cancer due to hepatitis C	0·7 (0·4 to 1·1)	7·7% (4·0 to 12·2%)	1·0 (0·5 to 1·5)	8·7% (4·6 to 13·4%)	39·4% (9·1 to 81·0%)	12·9% (-7·7 to 37·3%)	18·8 (9·6 to 29·5)	9·1% (4·7 to 14·0%)	25·1 (13·3 to 39·0)	10·0% (5·3 to 15·5%)	33·7% (2·0 to 78·8%)	10·2% (-11·4 to 36·9%)
Liver cancer due to alcohol use	0·5 (0·2 to 0·8)	8·6% (4·2 to 14·0%)	0·8 (0·4 to 1·3)	9·6% (4·6 to 15·3%)	55·6% (27·6 to 94·5%)	12·0% (-2·6 to 28·2%)	13·1 (6·3 to 21·2)	9·6% (4·7 to 15·4%)	19·8 (9·3 to 32·5)	10·6% (5·0 to 16·7%)	51·2% (22·9 to 90·3%)	10·0% (-4·3 to 26·1%)
Liver cancer due to other causes	0·5 (0·2 to 0·7)	6·8% (3·5 to 10·8%)	0·7 (0·3 to 1·0)	7·8% (4·2 to 12·1%)	40·6% (16·0 to 73·6%)	14·8% (-2·9 to 35·0%)	13·4 (7·0 to 20·8)	7·5% (3·9 to 11·7%)	17·9 (9·6 to 27·5)	8·5% (4·6 to 13·1%)	33·8% (8·5 to 68·0%)	13·0% (-6·2 to 34·5%)
Pancreatic cancer	1·7 (1·2 to 2·3)	7·0% (4·8 to 9·3%)	2·7 (1·9 to 3·6)	7·8% (5·5 to 10·4%)	54·2% (33·0 to 79·3%)	12·3% (-0·8 to 27·4%)	48·5 (33·7 to 65·6)	8·1% (5·6 to 10·8%)	71·4 (50·6 to 95·9)	8·8% (6·3 to 11·8%)	47·1% (26·1 to 71·8%)	9·6% (-3·2 to 24·4%)
Tracheal, bronchus, and lung cancer	42·8 (37·9 to 48·0)	52·0% (46·6 to 57·0%)	70·8 (61·4 to 79·3)	56·6% (51·6 to 61·0%)	65·3% (45·0 to 85·9%)	8·9% (3·0 to 14·8%)	1,120·3 (989·9 to 1,256·6)	53·2% (48·0 to 58·3%)	1,714·0 (1,471·7 to 1,941·6)	56·6% (51·5 to 61·1%)	53·0% (34·0 to 72·4%)	6·4% (0·5 to 12·4%)
Cervical cancer	0·6 (0·1 to 1·3)	1·4% (0·4 to 3·0%)	0·8 (0·2 to 1·6)	1·9% (0·6 to 3·8%)	42·6% (-9·4 to 117·3%)	37·7% (-0·1 to 98·6%)	19·4 (4·9 to 43·4)	1·5% (0·4 to 3·4%)	27·1 (7·8 to 54·0)	2·1% (0·6 to 4·2%)	40·1% (-10·8 to 119·0%)	40·1% (0·0 to 104·4%)
Kidney cancer	0·4 (0·3 to 0·7)	7·5% (4·5 to 10·9%)	0·7 (0·4 to 1·1)	8·5% (5·1 to 12·5%)	66·2% (26·9 to 111·9%)	13·6% (-6·0 to 35·5%)	13·7 (8·1 to 20·4)	7·5% (4·5 to 10·9%)	22·4 (12·8 to 33·9)	8·6% (5·1 to 12·8%)	63·8% (23·4 to 112·3%)	15·6% (-6·2 to 41·3%)
Bladder cancer	1·8 (1·2 to 2·5)	11·6% (7·9 to 15·8%)	2·7 (1·8 to 3·8)	12·9% (8·7 to 17·7%)	51·4% (23·8 to 83·1%)	10·8% (-4·3 to 27·0%)	47·0 (31·5 to 64·4)	13·5% (9·2 to 18·3%)	67·8 (45·7 to 93·8)	14·7% (9·9 to 19·9%)	44·1% (17·6 to 75·8%)	8·8% (-6·2 to 25·5%)
Ischaemic heart disease	225·1 (179·0 to 274·3)	14·5% (11·6 to 17·5%)	261·9 (204·5 to 320·8)	12·6% (9·9 to 15·4%)	16·3% (8·1 to 24·9%)	-13·1% (-17·7 to -8·5%)	6,594·5 (5,215·7 to 8,047·1)	18·0% (14·3 to 21·8%)	7,708·5 (5,992·1 to 9,506·4)	16·4% (12·9 to 20·0%)	16·9% (8·5 to 25·2%)	-9·0% (-13·3 to -4·6%)
Ischaemic stroke	45·6 (36·2 to 56·1)	11·3% (8·9 to 13·6%)	48·6 (38·3 to 60·1)	9·7% (7·7 to 12·0%)	6·5% (-2·7 to 17·1%)	-13·7% (-20·2 to -7·0%)	1,090·4 (862·1 to 1,344·2)	14·2% (11·3 to 17·2%)	1,176·9 (922·9 to 1,458·9)	12·9% (10·3 to 15·9%)	7·9% (-1·2 to 18·1%)	-8·9% (-14·5 to -3·0%)
Hemorrhagic stroke	72·7 (58·2 to 88·4)	14·5% (11·8 to 17·4%)	79·3 (62·4 to 97·0)	13·3% (10·7 to 16·2%)	9·1% (-0·3 to 20·0%)	-8·2% (-14·1 to -2·1%)	2,239·0 (1,789·8 to 2,707·2)	16·2% (13·1 to 19·4%)	2,481·9 (1,954·1 to 3,032·4)	15·7% (12·6 to 19·0%)	10·8% (1·8 to 21·6%)	-2·9% (-8·8 to 3·2%)
Hypertensive heart disease	20·5 (15·3 to 26·8)	12·3% (9·2 to 15·4%)	22·2 (16·3 to 28·7)	10·6% (8·0 to 13·5%)	8·4% (-5·3 to 22·2%)	-13·8% (-21·4 to -5·5%)	610·8 (458·4 to 792·4)	14·9% (11·3 to 18·6%)	662·7 (483·3 to 852·3)	13·5% (10·2 to 17·1%)	8·5% (-5·4 to 21·9%)	-9·7% (-17·4 to -2·2%)
Atrial fibrillation and flutter	1·0 (0·7 to 1·4)	7·3% (5·3 to 9·4%)	1·2 (0·8 to 1·7)	5·8% (4·4 to 7·5%)	19·8% (7·4 to 33·4%)	-20·4% (-28·6 to -11·7%)	48·4 (33·1 to 67·0)	12·9% (9·7 to 16·1%)	56·6 (38·7 to 78·4)	10·8% (8·1 to 13·6%)	17·0% (11·5 to 22·9%)	-16·1% (-19·9 to -12·5%)
Aortic aneurysm	1·9 (1·4 to 2·6)	15·1% (11·7 to 19·0%)	2·6 (1·9 to 3·3)	13·1% (10·0 to 16·4%)	33·1% (15·2 to 51·0%)	-13·8% (-18·6 to -8·6%)	57·2 (41·8 to 76·6)	17·6% (13·6 to 22·2%)	75·9 (56·9 to 96·8)	15·8% (12·2 to 19·8%)	32·8% (14·1 to 51·1%)	-10·0% (-15·4 to -4·2%)

Supplemental Results Table 1. All-age deaths, death PAFs, DALYs, DALY PAFs, and percent change for each risk factor and outcome for both sexes combined in 2005 and 2015 for South Asia.

Data in parenthesis are 95% uncertainty intervals. PAFs=population attributable fractions. DALYs=disability-adjusted life-years.

	2005 Deaths (in thousands)	2005 Deaths PAF	2015 Deaths (in thousands)	2015 Deaths PAF	Percent change deaths 2005-2015	Percent change deaths PAF 2005-2015	2005 DALYs (in thousands)	2005 DALYs PAF	2015 DALYs (in thousands)	2015 DALYs PAF	Percent change DALYs 2005-2015	Percent change DALYs PAF 2005-2015
Peripheral vascular disease	0·1 (0·1 to 0·1)	11·0% (7·1 to 14·4%)	0·2 (0·1 to 0·3)	9·2% (6·3 to 12·1%)	68·8% (45·2 to 106·1%)	-16·4% (-23·8 to -5·4%)	8·1 (4·6 to 13·2)	11·6% (8·8 to 14·5%)	10·7 (6·4 to 16·8)	10·1% (7·6 to 12·7%)	32·0% (21·4 to 47·4%)	-13·2% (-18·3 to -8·0%)
Other cardiovascular and circulatory diseases	1·6 (1·2 to 2·1)	10·9% (8·4 to 13·4%)	2·0 (1·5 to 2·6)	9·3% (7·1 to 11·6%)	24·1% (11·8 to 38·3%)	-14·6% (-21·5 to -7·6%)	49·4 (37·8 to 62·4)	13·8% (10·7 to 17·0%)	60·5 (45·6 to 75·9)	12·5% (9·6 to 15·5%)	22·6% (11·5 to 34·7%)	-9·6% (-15·4 to -3·5%)
Chronic obstructive pulmonary disease	245·1 (184·5 to 310·5)	24·8% (18·7 to 30·5%)	355·5 (280·3 to 433·1)	29·7% (23·7 to 35·6%)	45·0% (25·9 to 67·4%)	19·6% (7·3 to 34·6%)	6,150·2 (4,739·1 to 7,590·9)	26·3% (20·4 to 31·8%)	8,421·7 (6,719·5 to 10,129·9)	31·2% (25·4 to 37·0%)	36·9% (21·1 to 56·3%)	18·8% (8·4 to 32·1%)
Silicosis	0·0 (0·0 to 0·1)	6·7% (4·5 to 9·1%)	0·1 (0·0 to 0·1)	8·2% (5·5 to 11·2%)	51·9% (15·5 to 103·3%)	22·7% (2·0 to 47·3%)	1·1 (0·5 to 2·0)	7·5% (5·2 to 10·0%)	1·6 (0·8 to 2·9)	8·4% (5·9 to 11·3%)	43·6% (17·9 to 77·1%)	12·9% (-2·3 to 30·8%)
Asbestosis	0·0 (0·0 to 0·0)	5·6% (3·7 to 7·9%)	0·0 (0·0 to 0·1)	6·5% (4·4 to 9·1%)	78·8% (35·8 to 142·6%)	15·6% (-2·7 to 37·1%)	0·9 (0·5 to 1·5)	5·7% (3·6 to 8·1%)	1·4 (0·8 to 2·3)	6·4% (4·2 to 9·0%)	61·4% (28·7 to 104·9%)	12·2% (-5·3 to 34·0%)
Coal workers pneumoconiosis	0·0 (0·0 to 0·0)	6·4% (4·3 to 9·2%)	0·0 (0·0 to 0·0)	8·0% (5·2 to 11·5%)	50·2% (1·9 to 121·2%)	25·1% (-3·8 to 63·8%)	0·3 (0·1 to 0·5)	6·8% (4·6 to 9·3%)	0·4 (0·2 to 0·7)	7·5% (5·0 to 10·2%)	53·5% (24·9 to 84·8%)	10·6% (-4·0 to 27·7%)
Other pneumoconiosis	0·2 (0·1 to 0·2)	3·9% (2·3 to 5·8%)	0·3 (0·2 to 0·4)	4·8% (3·0 to 6·9%)	74·7% (41·2 to 117·4%)	23·7% (-1·0 to 52·4%)	7·3 (4·5 to 10·6)	4·4% (2·9 to 6·1%)	11·9 (7·5 to 17·4)	5·0% (3·4 to 6·9%)	63·3% (41·7 to 90·1%)	14·3% (-2·1 to 32·4%)
Asthma	19·7 (11·8 to 27·8)	9·7% (6·6 to 13·3%)	13·9 (10·1 to 18·5)	8·5% (6·4 to 10·8%)	-29·4% (-45·0 to -0·8%)	-11·8% (-28·8 to 10·2%)	621·7 (407·1 to 843·7)	7·3% (5·2 to 9·6%)	488·6 (360·6 to 644·0)	6·6% (4·9 to 8·3%)	-21·4% (-35·5 to 0·3%)	-9·7% (-22·4 to 6·0%)
Interstitial lung disease and pulmonary sarcoidosis	0·6 (0·3 to 1·4)	4·3% (2·8 to 6·4%)	1·3 (0·7 to 2·3)	5·2% (3·5 to 7·2%)	107·2% (56·8 to 189·5%)	19·0% (-0·2 to 40·2%)	17·8 (8·6 to 36·6)	4·8% (3·2 to 7·1%)	33·8 (19·7 to 57·9)	5·6% (3·8 to 7·7%)	89·4% (44·6 to 157·3%)	14·8% (-2·5 to 35·2%)
Other chronic respiratory diseases	0·4 (0·2 to 0·7)	2·7% (1·5 to 4·0%)	0·6 (0·3 to 1·0)	4·1% (2·6 to 5·9%)	49·7% (13·1 to 91·6%)	55·3% (19·8 to 99·1%)	58·9 (36·9 to 86·8)	3·0% (1·9 to 4·5%)	67·7 (41·6 to 97·4)	3·8% (2·5 to 5·3%)	15·0% (-4·5 to 37·6%)	25·0% (4·4 to 50·4%)
Diabetes mellitus	12·3 (3·3 to 22·8)	4·3% (1·1 to 7·8%)	17·5 (4·5 to 32·2)	3·9% (1·0 to 7·1%)	41·9% (28·8 to 55·5%)	-9·9% (-16·9 to -2·7%)	513·3 (139·4 to 949·0)	4·5% (1·2 to 8·2%)	659·8 (175·3 to 1,216·8)	4·1% (1·1 to 7·6%)	28·5% (20·1 to 37·3%)	-8·2% (-13·6 to -3·3%)
**Second-hand smoke: All causes**	248·5 (187·4 to 313·9)	2·0% (1·5 to 2·5%)	250·6 (191·4 to 316·9)	1·9% (1·5 to 2·5%)	0·8% (-5·0 to 7·4%)	-3·5% (-8·5 to 2·1%)	10,136·1 (7,205·4 to 13,149·4)	1·5% (1·1 to 1·9%)	8,091·7 (5,972·3 to 10,450·9)	1·3% (0·9 to 1·6%)	-20·2% (-26·6 to -12·5%)	-15·2% (-21·5 to -7·4%)
Lower respiratory infections	87·8 (53·6 to 125·7)	11·7% (7·2 to 16·4%)	58·4 (34·9 to 85·4)	9·1% (5·4 to 13·3%)	-33·5% (-40·1 to -26·8%)	-22·0% (-26·8 to -18·3%)	6,213·6 (3,770·2 to 8,879·2)	14·3% (8·9 to 20·0%)	3,533·8 (2,099·2 to 5,149·5)	11·9% (7·2 to 17·2%)	-43·1% (-49·5 to -36·0%)	-16·5% (-20·4 to -13·1%)
Otitis media	0·1 (0·1 to 0·1)	5·1% (3·3 to 6·9%)	0·0 (0·0 to 0·1)	3·8% (2·4 to 5·3%)	-43·9% (-52·8 to -33·6%)	-25·6% (-46·3 to -3·1%)	99·8 (56·1 to 158·8)	9·5% (6·5 to 12·5%)	93·3 (51·2 to 153·4)	8·8% (5·9 to 11·5%)	-6·6% (-12·0 to -1·5%)	-7·8% (-11·4 to -4·2%)
Tracheal, bronchus, and lung cancer	1·7 (1·0 to 2·8)	2·1% (1·2 to 3·3%)	2·2 (1·3 to 3·5)	1·8% (1·0 to 2·8%)	27·2% (23·0 to 30·9%)	-16·2% (-21·3 to -10·5%)	42·9 (24·6 to 68·3)	2·0% (1·2 to 3·3%)	53·4 (30·3 to 85·7)	1·8% (1·0 to 2·9%)	24·5% (20·0 to 28·3%)	-13·5% (-19·0 to -7·5%)
Ischaemic heart disease	81·9 (62·9 to 102·1)	5·3% (4·1 to 6·6%)	106·1 (81·4 to 133·9)	5·1% (3·9 to 6·5%)	29·5% (22·6 to 36·7%)	-3·3% (-6·1 to -0·3%)	2,051·2 (1,601·5 to 2,543·9)	5·6% (4·4 to 6·9%)	2,572·4 (2,001·9 to 3,232·9)	5·5% (4·3 to 6·8%)	25·4% (18·9 to 32·7%)	-2·4% (-5·4 to 0·6%)
Ischaemic stroke	11·8 (8·4 to 15·9)	2·9% (2·1 to 3·9%)	13·8 (9·6 to 18·5)	2·8% (2·0 to 3·7%)	16·7% (7·0 to 28·0%)	-5·4% (-10·5 to -0·5%)	235·8 (170·9 to 310·5)	3·1% (2·3 to 4·0%)	265·6 (190·2 to 350·0)	2·9% (2·2 to 3·7%)	12·7% (3·6 to 22·6%)	-4·8% (-9·2 to -0·8%)
Hemorrhagic stroke	16·9 (12·6 to 21·8)	3·4% (2·6 to 4·3%)	19·2 (14·4 to 24·7)	3·2% (2·5 to 4·1%)	13·3% (5·7 to 23·2%)	-4·6% (-8·3 to -1·1%)	486·7 (373·3 to 623·4)	3·5% (2·7 to 4·5%)	539·0 (408·0 to 700·7)	3·4% (2·6 to 4·4%)	10·7% (3·9 to 19·0%)	-3·0% (-6·7 to 0·6%)
**Alcohol and drug use: All causes**	352·3 (319·0 to 387·3)	2·9% (2·6 to 3·1%)	431·0 (384·6 to 480·4)	3·3% (3·0 to 3·7%)	22·3% (15·5 to 29·8%)	17·1% (12·1 to 22·1%)	16,267·9 (14,817·1 to 17,756·9)	2·4% (2·2 to 2·6%)	19,284·3 (17,413·0 to 21,261·2)	3·0% (2·7 to 3·3%)	18·5% (13·1 to 24·4%)	26·0% (21·1 to 31·0%)
**Alcohol use: All causes**	312·1 (279·6 to 346·5)	2·5% (2·3 to 2·8%)	382·8 (336·5 to 426·8)	3·0% (2·6 to 3·3%)	22·7% (15·4 to 30·4%)	17·4% (11·8 to 23·0%)	13,177·9 (11,929·6 to 14,622·7)	1·9% (1·7 to 2·1%)	15,654·9 (14,027·2 to 17,410·4)	2·4% (2·2 to 2·7%)	18·8% (12·7 to 25·5%)	26·3% (20·7 to 32·2%)
Tuberculosis	64·0 (48·0 to 84·5)	9·6% (8·0 to 10·9%)	59·0 (40·8 to 80·3)	10·7% (8·6 to 12·7%)	-7·9% (-23·3 to 5·4%)	12·4% (2·3 to 21·6%)	2,409·4 (1,905·3 to 3,124·9)	9·8% (8·4 to 11·0%)	2,189·7 (1,590·5 to 2,930·2)	11·2% (9·1 to 12·9%)	-9·1% (-23·1 to 3·0%)	14·9% (4·0 to 24·3%)
Lower respiratory infections	11·3 (9·0 to 13·1)	1·5% (1·3 to 1·7%)	14·5 (11·4 to 17·4)	2·3% (1·9 to 2·6%)	29·2% (17·3 to 42·2%)	51·4% (38·7 to 66·6%)	324·4 (258·6 to 373·2)	0·7% (0·6 to 0·9%)	400·0 (312·7 to 469·2)	1·3% (1·1 to 1·6%)	23·3% (12·7 to 35·5%)	81·0% (60·7 to 104·2%)
Lip and oral cavity cancer	4·4 (3·7 to 5·0)	12·7% (10·8 to 14·3%)	6·8 (5·6 to 7·8)	14·4% (12·0 to 16·2%)	56·4% (41·2 to 71·3%)	13·2% (4·6 to 21·2%)	142·0 (123·1 to 159·2)	14·2% (12·3 to 15·6%)	215·3 (181·8 to 242·6)	15·9% (13·5 to 17·7%)	51·6% (37·7 to 65·2%)	12·3% (4·4 to 19·7%)
Nasopharynx cancer	1·1 (0·9 to 1·4)	14·7% (12·6 to 16·6%)	1·8 (1·4 to 2·1)	17·0% (14·2 to 19·1%)	54·7% (36·1 to 74·1%)	15·5% (6·7 to 25·0%)	39·7 (33·2 to 47·8)	15·7% (13·7 to 17·6%)	59·7 (47·2 to 70·2)	18·2% (15·4 to 20·3%)	50·5% (31·8 to 69·3%)	15·7% (7·2 to 24·9%)
Other pharynx cancer	1·9 (1·6 to 2·3)	13·7% (11·4 to 15·5%)	2·9 (2·3 to 3·4)	16·4% (13·6 to 18·7%)	49·3% (35·2 to 67·0%)	20·1% (10·4 to 29·6%)	59·6 (50·5 to 68·3)	15·3% (13·0 to 17·1%)	87·0 (71·2 to 101·0)	18·3% (15·4 to 20·6%)	46·0% (32·6 to 62·7%)	19·8% (10·7 to 28·4%)
Oesophageal cancer	3·9 (3·2 to 4·4)	7·8% (6·5 to 8·9%)	5·8 (4·7 to 6·7)	9·1% (7·5 to 10·4%)	48·9% (34·8 to 62·7%)	16·5% (7·4 to 24·5%)	114·1 (97·3 to 129·6)	8·4% (7·2 to 9·5%)	165·2 (137·6 to 191·9)	9·8% (8·3 to 11·3%)	44·8% (31·1 to 57·8%)	16·5% (7·1 to 24·2%)
Colon and rectum cancer	1·0 (0·8 to 1·1)	2·2% (1·9 to 2·5%)	1·5 (1·3 to 1·7)	2·3% (2·0 to 2·6%)	52·5% (40·2 to 65·5%)	6·0% (1·6 to 10·4%)	27·1 (23·7 to 30·3)	2·2% (2·0 to 2·5%)	40·0 (34·9 to 44·9)	2·4% (2·1 to 2·7%)	47·7% (36·0 to 59·4%)	7·2% (2·4 to 12·3%)
Liver cancer due to hepatitis B	0·1 (0·0 to 0·1)	0·4% (0·3 to 0·5%)	0·1 (0·0 to 0·1)	0·4% (0·3 to 0·5%)	26·4% (2·4 to 51·9%)	1·7% (-14·7 to 18·7%)	2·7 (2·0 to 3·7)	0·7% (0·5 to 0·8%)	3·5 (2·5 to 4·6)	0·7% (0·5 to 0·9%)	26·2% (3·1 to 50·7%)	4·6% (-11·1 to 20·5%)
Liver cancer due to hepatitis C	0·0 (0·0 to 0·0)	0·0% (0·0 to 0·1%)	0·0 (0·0 to 0·0)	0·0% (0·0 to 0·1%)	19·9% (-8·3 to 53·1%)	-2·9% (-25·1 to 23·5%)	0·2 (0·1 to 0·3)	0·1% (0·1 to 0·1%)	0·2 (0·1 to 0·3)	0·1% (0·1 to 0·1%)	19·4% (-5·3 to 45·9%)	-1·6% (-20·8 to 21·9%)
Liver cancer due to alcohol use	5·9 (5·1 to 6·8)	100·0% (100·0 to 100·0%)	8·1 (7·2 to 9·4)	100·0% (100·0 to 100·0%)	38·8% (22·3 to 56·8%)	-	136·2 (117·5 to 159·2)	100·0% (100·0 to 100·0%)	186·9 (164·2 to 217·7)	100·0% (100·0 to 100·0%)	37·2% (20·9 to 55·0%)	-
Liver cancer due to other causes	0·0 (0·0 to 0·0)	0·1% (0·1 to 0·2%)	0·0 (0·0 to 0·0)	0·1% (0·1 to 0·2%)	19·1% (-1·2 to 41·6%)	-2·7% (-17·6 to 14·1%)	0·6 (0·4 to 0·7)	0·3% (0·2 to 0·4%)	0·7 (0·5 to 0·9)	0·3% (0·2 to 0·4%)	18·4% (-0·8 to 39·2%)	0·0% (-14·2 to 16·1%)
Larynx cancer	2·0 (1·6 to 2·3)	8·5% (7·1 to 9·8%)	2·9 (2·3 to 3·4)	9·8% (8·0 to 11·4%)	45·8% (31·3 to 60·4%)	15·6% (6·9 to 23·9%)	57·3 (48·0 to 65·5)	9·4% (8·0 to 10·6%)	81·3 (66·2 to 95·6)	10·9% (9·1 to 12·4%)	41·8% (28·6 to 55·7%)	15·4% (7·0 to 23·3%)
Breast cancer	1·2 (1·0 to 1·4)	2·3% (1·9 to 2·6%)	1·7 (1·4 to 2·0)	2·3% (1·9 to 2·7%)	40·6% (22·2 to 56·0%)	3·1% (-2·2 to 8·2%)	37·9 (30·6 to 46·0)	2·2% (1·9 to 2·6%)	52·7 (42·5 to 62·7)	2·3% (1·9 to 2·7%)	39·1% (21·1 to 55·1%)	3·5% (-1·9 to 9·0%)
Ischaemic heart disease	3·9 (1·8 to 6·1)	0·3% (0·1 to 0·4%)	7·6 (4·2 to 11·0)	0·4% (0·2 to 0·5%)	93·2% (54·9 to 172·7%)	44·3% (15·8 to 103·1%)	20·3 (-33·3 to 72·9)	0·1% (-0·1 to 0·2%)	101·3 (14·1 to 181·7)	0·2% (0·0 to 0·4%)	399·3% (-2,765·1 to 3,476·4%)	288·5% (-2,200·8 to 2,693·8%)
Ischaemic stroke	2·9 (1·9 to 4·0)	0·7% (0·5 to 1·0%)	4·4 (3·0 to 6·0)	0·9% (0·6 to 1·2%)	52·4% (34·5 to 79·0%)	23·4% (9·9 to 43·3%)	65·6 (45·9 to 87·1)	0·9% (0·6 to 1·1%)	99·4 (73·6 to 126·7)	1·1% (0·8 to 1·4%)	51·6% (35·2 to 73·6%)	28·0% (16·0 to 44·6%)
Hemorrhagic stroke	19·4 (16·2 to 22·6)	3·9% (3·3 to 4·5%)	26·3 (21·3 to 31·1)	4·4% (3·7 to 5·2%)	35·4% (22·4 to 50·5%)	13·9% (4·1 to 23·5%)	587·2 (502·8 to 667·1)	4·3% (3·7 to 4·8%)	771·2 (635·8 to 896·4)	4·9% (4·1 to 5·6%)	31·3% (19·5 to 44·5%)	15·0% (6·1 to 23·6%)
Hypertensive heart disease	5·7 (4·1 to 7·2)	3·4% (2·5 to 4·2%)	8·7 (6·0 to 11·3)	4·2% (3·0 to 5·2%)	54·1% (30·5 to 75·8%)	22·4% (6·7 to 37·9%)	178·4 (137·0 to 218·1)	4·4% (3·5 to 5·2%)	260·7 (191·3 to 322·1)	5·3% (4·1 to 6·4%)	46·1% (26·2 to 65·2%)	21·5% (8·8 to 33·4%)
Atrial fibrillation and flutter	0·4 (0·3 to 0·5)	2·6% (2·1 to 3·1%)	0·6 (0·4 to 0·8)	2·9% (2·4 to 3·6%)	72·5% (56·1 to 88·6%)	14·6% (5·1 to 24·0%)	13·2 (9·8 to 17·1)	3·5% (3·0 to 4·0%)	20·3 (15·1 to 26·6)	3·9% (3·3 to 4·4%)	53·8% (44·1 to 62·8%)	10·3% (3·7 to 16·1%)
Cirrhosis and other chronic liver diseases due to hepatitis B	12·8 (9·7 to 16·1)	15·0% (11·7 to 18·3%)	17·2 (12·1 to 22·0)	17·0% (12·4 to 20·8%)	34·1% (17·0 to 48·6%)	13·2% (0·3 to 24·2%)	429·9 (333·1 to 532·9)	15·6% (12·5 to 18·5%)	564·1 (415·1 to 710·7)	17·9% (13·4 to 21·5%)	31·2% (15·9 to 44·7%)	14·6% (2·6 to 25·1%)
Cirrhosis and other chronic liver diseases due to hepatitis C	9·5 (7·2 to 11·7)	15·9% (12·4 to 19·2%)	12·3 (8·9 to 15·5)	17·8% (13·2 to 21·5%)	29·7% (12·7 to 44·3%)	11·5% (-1·2 to 23·3%)	324·8 (253·6 to 400·6)	16·5% (13·3 to 19·5%)	412·0 (310·6 to 515·2)	18·6% (14·3 to 22·1%)	26·9% (11·5 to 40·5%)	12·6% (0·9 to 23·4%)
Cirrhosis and other chronic liver diseases due to alcohol use	55·8 (50·2 to 66·4)	100·0% (100·0 to 100·0%)	68·8 (62·0 to 83·5)	100·0% (100·0 to 100·0%)	23·4% (17·1 to 30·4%)	-	1,941·8 (1,755·3 to 2,348·3)	100·0% (100·0 to 100·0%)	2,340·7 (2,110·7 to 2,856·9)	100·0% (100·0 to 100·0%)	20·5% (14·1 to 27·6%)	-
Cirrhosis and other chronic liver diseases due to other causes	5·6 (4·2 to 7·0)	9·9% (7·7 to 12·2%)	7·1 (5·1 to 9·2)	12·2% (8·9 to 15·4%)	26·7% (8·7 to 42·3%)	23·5% (7·5 to 37·4%)	192·4 (151·4 to 239·9)	7·5% (6·0 to 9·1%)	229·9 (169·9 to 296·3)	10·0% (7·3 to 12·4%)	19·5% (2·4 to 35·1%)	32·7% (14·1 to 49·5%)
Pancreatitis	2·9 (2·4 to 3·5)	7·8% (6·7 to 8·7%)	4·8 (3·7 to 6·2)	9·4% (7·8 to 10·7%)	67·5% (46·2 to 93·9%)	20·7% (9·9 to 30·0%)	116·1 (95·7 to 138·3)	8·4% (7·3 to 9·3%)	189·8 (146·7 to 240·9)	10·2% (8·6 to 11·6%)	63·5% (42·9 to 88·7%)	22·1% (11·6 to 31·5%)

Supplemental Results Table 1. All-age deaths, death PAFs, DALYs, DALY PAFs, and percent change for each risk factor and outcome for both sexes combined in 2005 and 2015 for South Asia.

Data in parenthesis are 95% uncertainty intervals. PAFs=population attributable fractions. DALYs=disability-adjusted life-years.

	2005 Deaths (in thousands)	2005 Deaths PAF	2015 Deaths (in thousands)	2015 Deaths PAF	Percent change deaths 2005-2015	Percent change deaths PAF 2005-2015	2005 DALYs (in thousands)	2005 DALYs PAF	2015 DALYs (in thousands)	2015 DALYs PAF	Percent change DALYs 2005-2015	Percent change DALYs PAF 2005-2015
Epilepsy	1·1 (1·0 to 1·2)	4·2% (3·8 to 4·7%)	1·5 (1·3 to 1·7)	5·4% (4·6 to 6·4%)	34·4% (22·2 to 47·7%)	28·7% (14·5 to 44·7%)	120·0 (96·1 to 144·9)	3·4% (3·1 to 3·8%)	141·4 (112·4 to 175·2)	4·1% (3·5 to 4·7%)	17·9% (8·6 to 26·9%)	19·5% (8·6 to 29·8%)
Alcohol use disorders	8·2 (7·6 to 8·8)	100·0% (100·0 to 100·0%)	10·8 (10·0 to 11·8)	100·0% (100·0 to 100·0%)	32·5% (20·3 to 44·5%)	-	1,361·4 (1,003·3 to 1,824·2)	100·0% (100·0 to 100·0%)	1,678·5 (1,247·1 to 2,241·0)	100·0% (100·0 to 100·0%)	23·3% (19·2 to 27·4%)	-
Diabetes mellitus	-2·5 (-3·0 to -1·9)	-0·9% (-1·0 to -0·7%)	-3·7 (-4·7 to -2·8)	-0·8% (-1·0 to -0·6%)	51·5% (34·5 to 68·4%)	-3·9% (-13·1 to 5·2%)	-78·9 (-97·2 to -61·6)	-0·7% (-0·8 to -0·6%)	-109·9 (-135·8 to -85·1)	-0·7% (-0·8 to -0·6%)	39·2% (28·0 to 50·7%)	-0·6% (-7·7 to 6·2%)
Pedestrian road injuries	18·4 (14·1 to 23·5)	13·3% (10·9 to 16·0%)	20·6 (16·4 to 26·2)	14·0% (11·7 to 16·7%)	12·3% (-1·3 to 32·4%)	5·7% (1·5 to 10·8%)	930·9 (722·2 to 1,178·7)	14·6% (11·9 to 17·8%)	1,005·1 (800·0 to 1,278·2)	15·9% (13·2 to 18·9%)	8·0% (-4·9 to 27·6%)	8·7% (4·0 to 14·3%)
Cyclist road injuries	0·6 (0·4 to 0·7)	12·8% (10·4 to 15·3%)	0·7 (0·5 to 1·0)	13·4% (11·1 to 16·1%)	28·2% (9·1 to 51·6%)	4·6% (-0·3 to 9·9%)	32·7 (22·9 to 42·6)	13·2% (10·8 to 15·8%)	40·3 (29·5 to 53·8)	14·1% (11·7 to 16·9%)	23·3% (6·3 to 42·8%)	7·0% (1·5 to 12·9%)
Motorcyclist road injuries	18·3 (14·0 to 22·7)	24·6% (21·2 to 28·2%)	24·8 (19·0 to 30·9)	26·7% (23·4 to 30·3%)	35·1% (5·7 to 71·1%)	8·4% (1·9 to 14·2%)	904·2 (704·6 to 1,113·5)	25·1% (21·6 to 28·8%)	1,183·1 (923·4 to 1,480·7)	27·7% (24·2 to 31·4%)	30·8% (4·7 to 64·2%)	10·5% (4·0 to 16·6%)
Motor vehicle road injuries	20·9 (16·9 to 26·7)	23·3% (20·2 to 26·7%)	25·1 (18·7 to 31·1)	24·8% (21·6 to 28·2%)	20·4% (-12·9 to 42·7%)	6·6% (-0·9 to 12·7%)	1,031·5 (835·3 to 1,309·5)	23·4% (20·1 to 26·8%)	1,203·4 (912·1 to 1,480·4)	25·4% (22·1 to 28·9%)	16·7% (-13·4 to 37·3%)	8·8% (1·5 to 15·2%)
Falls	4·0 (3·1 to 5·2)	5·2% (4·2 to 6·6%)	5·5 (4·2 to 7·5)	6·0% (4·8 to 7·5%)	37·4% (20·4 to 59·0%)	14·1% (3·2 to 25·0%)	266·3 (202·0 to 342·9)	5·0% (4·1 to 6·3%)	354·2 (270·7 to 454·7)	6·0% (4·9 to 7·3%)	33·0% (23·2 to 45·2%)	18·3% (9·3 to 28·5%)
Drowning	3·4 (2·6 to 4·3)	2·9% (2·2 to 4·4%)	3·7 (2·9 to 4·9)	3·8% (2·9 to 5·3%)	10·3% (-0·9 to 28·2%)	31·5% (13·8 to 49·5%)	152·6 (118·3 to 194·2)	2·1% (1·6 to 3·3%)	163·9 (127·2 to 215·8)	3·1% (2·3 to 4·3%)	7·5% (-3·4 to 25·3%)	42·7% (20·3 to 66·3%)
Fire, heat, and hot substances	2·7 (2·0 to 3·5)	3·5% (2·7 to 4·8%)	3·0 (2·0 to 4·0)	4·5% (3·5 to 6·3%)	12·2% (-3·4 to 24·6%)	27·8% (11·6 to 41·5%)	162·8 (123·0 to 209·6)	3·3% (2·6 to 4·3%)	183·1 (133·9 to 231·7)	4·2% (3·3 to 5·5%)	12·5% (1·4 to 22·4%)	29·2% (15·0 to 41·0%)
Poisonings	0·8 (0·3 to 1·1)	4·7% (3·1 to 6·2%)	0·8 (0·3 to 1·0)	5·4% (3·5 to 7·0%)	-3·0% (-22·1 to 12·3%)	15·4% (-7·4 to 39·6%)	39·7 (18·0 to 53·4)	3·6% (2·6 to 4·8%)	39·1 (18·0 to 52·5)	4·3% (2·9 to 5·6%)	-1·6% (-18·0 to 12·0%)	19·2% (-7·2 to 50·1%)
Unintentional firearm injuries	0·2 (0·1 to 0·3)	7·2% (5·7 to 10·0%)	0·2 (0·1 to 0·3)	7·7% (6·0 to 10·8%)	-2·7% (-25·8 to 16·3%)	6·3% (-5·0 to 17·2%)	11·4 (5·0 to 15·2)	7·1% (5·6 to 9·4%)	10·9 (4·4 to 14·9)	7·9% (6·3 to 10·5%)	-4·2% (-25·5 to 13·9%)	10·7% (0·1 to 20·6%)
Unintentional suffocation	0·4 (0·3 to 0·5)	4·5% (3·4 to 5·9%)	0·5 (0·4 to 0·7)	6·4% (4·9 to 8·2%)	40·9% (16·8 to 63·0%)	42·5% (20·5 to 73·2%)	20·1 (15·0 to 26·5)	3·6% (2·6 to 4·7%)	27·3 (19·5 to 35·4)	5·5% (4·2 to 7·0%)	35·9% (19·0 to 52·1%)	55·5% (24·8 to 99·0%)
Other exposure to mechanical forces	2·6 (2·0 to 3·6)	6·5% (5·1 to 8·3%)	3·1 (2·3 to 4·1)	7·5% (6·0 to 9·3%)	16·0% (-2·6 to 39·3%)	15·1% (6·0 to 26·8%)	169·4 (127·5 to 225·1)	5·9% (4·7 to 7·5%)	192·9 (143·0 to 250·7)	7·1% (5·8 to 8·7%)	13·8% (2·1 to 28·8%)	19·6% (12·3 to 27·4%)
Self-harm	14·3 (6·4 to 18·9)	7·2% (5·5 to 9·7%)	19·5 (9·4 to 25·8)	8·5% (6·5 to 11·0%)	35·7% (19·8 to 51·4%)	17·7% (8·5 to 27·0%)	660·6 (294·6 to 874·3)	6·7% (5·1 to 9·1%)	872·3 (422·6 to 1,160·5)	8·0% (6·2 to 10·5%)	32·0% (16·8 to 47·0%)	20·0% (9·7 to 30·1%)
Assault by firearm	0·5 (0·3 to 0·7)	5·4% (4·3 to 7·0%)	0·7 (0·4 to 1·0)	5·9% (4·8 to 7·5%)	30·6% (-3·8 to 63·2%)	9·8% (0·2 to 20·1%)	25·8 (16·2 to 34·8)	5·4% (4·2 to 6·9%)	32·8 (19·4 to 47·9)	6·0% (4·7 to 7·7%)	26·9% (-6·4 to 59·2%)	11·3% (-0·6 to 24·2%)
Assault by sharp object	0·2 (0·1 to 0·3)	5·4% (4·3 to 6·9%)	0·2 (0·1 to 0·3)	6·2% (5·0 to 7·8%)	15·9% (-9·8 to 63·8%)	15·3% (3·9 to 27·4%)	9·4 (5·3 to 12·6)	5·5% (4·3 to 7·1%)	10·5 (6·0 to 16·7)	6·4% (5·1 to 7·9%)	12·6% (-11·9 to 59·5%)	16·5% (4·3 to 29·8%)
Assault by other means	2·5 (1·7 to 3·4)	6·2% (4·9 to 7·8%)	2·9 (1·9 to 4·0)	6·7% (5·4 to 8·2%)	15·1% (0·5 to 30·0%)	7·9% (1·3 to 14·7%)	137·6 (95·7 to 182·6)	6·3% (5·1 to 8·0%)	154·8 (103·6 to 210·3)	6·9% (5·6 to 8·6%)	12·5% (-0·8 to 27·1%)	9·2% (1·8 to 16·4%)
**Drug use: All causes**	41·9 (37·0 to 47·6)	0·3% (0·3 to 0·4%)	50·7 (44·3 to 59·4)	0·4% (0·3 to 0·5%)	21·1% (12·8 to 31·6%)	15·9% (9·2 to 25·4%)	3,159·6 (2,725·4 to 3,615·3)	0·5% (0·4 to 0·5%)	3,730·1 (3,174·5 to 4,343·0)	0·6% (0·5 to 0·7%)	18·1% (13·4 to 25·8%)	25·4% (20·6 to 33·4%)
HIV/AIDS - Tuberculosis	3·1 (2·4 to 3·9)	6·0% (5·1 to 7·1%)	1·8 (1·3 to 2·2)	6·4% (5·4 to 7·5%)	-44·3% (-50·1 to -38·7%)	6·5% (3·2 to 9·6%)	152·1 (119·7 to 186·6)	5·6% (4·8 to 6·7%)	83·6 (64·4 to 104·4)	6·0% (5·1 to 7·1%)	-45·0% (-50·6 to -40·0%)	6·8% (3·4 to 9·8%)
HIV/AIDS resulting in other diseases	8·7 (7·2 to 10·5)	5·9% (5·0 to 7·1%)	6·7 (5·5 to 8·3)	6·3% (5·3 to 7·6%)	-22·1% (-29·4 to -11·1%)	5·3% (1·3 to 16·4%)	431·6 (355·5 to 522·8)	5·7% (4·8 to 6·7%)	334·3 (273·3 to 411·4)	5·9% (5·0 to 7·2%)	-22·5% (-29·6 to -11·7%)	5·1% (1·1 to 16·1%)
Hepatitis B	0·0 (0·0 to 0·0)	0·1% (0·0 to 0·1%)	0·0 (0·0 to 0·0)	0·1% (0·0 to 0·1%)	28·6% (13·1 to 46·6%)	22·2% (12·8 to 34·7%)	0·8 (0·3 to 1·4)	0·1% (0·0 to 0·1%)	1·0 (0·4 to 1·7)	0·1% (0·0 to 0·1%)	21·5% (5·7 to 39·7%)	21·8% (10·8 to 34·4%)
Hepatitis C	0·1 (0·0 to 0·2)	11·5% (7·0 to 16·8%)	0·1 (0·0 to 0·3)	13·8% (8·7 to 20·0%)	30·4% (3·6 to 62·6%)	20·3% (5·5 to 39·2%)	3·6 (0·7 to 8·9)	13·4% (8·1 to 19·6%)	4·4 (0·9 to 10·8)	16·1% (10·2 to 23·0%)	22·2% (-2·5 to 52·9%)	19·9% (5·4 to 37·9%)
Liver cancer due to hepatitis B	0·0 (0·0 to 0·0)	0·1% (0·0 to 0·1%)	0·0 (0·0 to 0·0)	0·1% (0·0 to 0·1%)	53·0% (30·2 to 82·3%)	23·2% (11·4 to 40·4%)	0·3 (0·1 to 0·6)	0·1% (0·0 to 0·1%)	0·5 (0·2 to 0·8)	0·1% (0·0 to 0·2%)	46·9% (25·3 to 74·0%)	21·8% (10·4 to 36·8%)
Liver cancer due to hepatitis C	0·9 (0·5 to 1·4)	10·3% (6·0 to 15·5%)	1·5 (0·9 to 2·3)	13·5% (8·3 to 19·6%)	61·6% (32·9 to 101·7%)	30·9% (13·5 to 56·6%)	28·1 (16·4 to 41·5)	13·6% (8·2 to 20·1%)	43·8 (26·0 to 63·8)	17·4% (10·8 to 25·1%)	55·8% (27·1 to 94·5%)	28·3% (11·3 to 51·8%)
Cirrhosis and other chronic liver diseases due to hepatitis B	0·1 (0·0 to 0·1)	0·1% (0·0 to 0·1%)	0·1 (0·0 to 0·1)	0·1% (0·0 to 0·1%)	45·5% (31·2 to 63·6%)	22·8% (13·3 to 36·1%)	2·1 (0·9 to 3·6)	0·1% (0·0 to 0·1%)	2·8 (1·2 to 4·9)	0·1% (0·0 to 0·2%)	38·5% (25·0 to 54·4%)	20·9% (12·4 to 33·0%)
Cirrhosis and other chronic liver diseases due to hepatitis C	7·8 (4·7 to 11·8)	13·1% (8·1 to 19·5%)	10·9 (6·6 to 16·4)	15·7% (10·0 to 22·8%)	39·5% (27·1 to 53·7%)	19·9% (12·7 to 30·0%)	314·9 (190·5 to 472·3)	16·0% (10·0 to 23·6%)	417·6 (253·4 to 631·7)	18·9% (11·9 to 27·3%)	32·6% (21·2 to 45·4%)	17·7% (11·5 to 26·2%)
Opioid use disorders	12·8 (11·6 to 15·2)	100·0% (100·0 to 100·0%)	18·2 (16·2 to 23·0)	100·0% (100·0 to 100·0%)	42·7% (28·6 to 59·1%)	-	1,643·3 (1,316·2 to 1,973·0)	100·0% (100·0 to 100·0%)	2,083·4 (1,676·8 to 2,502·9)	100·0% (100·0 to 100·0%)	26·8% (21·1 to 35·2%)	-
Cocaine use disorders	0·3 (0·2 to 0·5)	100·0% (100·0 to 100·0%)	0·7 (0·5 to 1·1)	100·0% (100·0 to 100·0%)	123·8% (85·3 to 179·6%)	-	56·7 (39·0 to 76·9)	100·0% (100·0 to 100·0%)	95·6 (67·0 to 130·0)	100·0% (100·0 to 100·0%)	68·6% (56·2 to 86·6%)	-
Amphetamine use disorders	0·3 (0·2 to 0·7)	100·0% (100·0 to 100·0%)	0·8 (0·6 to 1·2)	100·0% (100·0 to 100·0%)	140·9% (60·6 to 259·3%)	-	35·2 (23·7 to 53·7)	100·0% (100·0 to 100·0%)	63·3 (46·2 to 87·6)	100·0% (100·0 to 100·0%)	79·6% (44·9 to 129·8%)	-
Cannabis use disorders	-	-	-	-	-	-	78·5 (50·7 to 111·8)	100·0% (100·0 to 100·0%)	93·5 (60·9 to 132·4)	100·0% (100·0 to 100·0%)	19·1% (12·8 to 25·6%)	-
Other drug use disorders	3·9 (3·2 to 4·8)	100·0% (100·0 to 100·0%)	5·3 (3·9 to 6·8)	100·0% (100·0 to 100·0%)	35·0% (6·0 to 62·7%)	-	209·1 (166·2 to 269·0)	100·0% (100·0 to 100·0%)	271·1 (203·6 to 359·3)	100·0% (100·0 to 100·0%)	29·7% (12·1 to 51·1%)	-
Self-harm	3·9 (2·1 to 5·8)	1·9% (1·2 to 2·9%)	4·6 (2·5 to 6·9)	2·0% (1·3 to 2·9%)	19·4% (7·0 to 31·8%)	3·7% (-0·7 to 8·5%)	203·2 (112·2 to 308·0)	2·1% (1·3 to 3·1%)	235·3 (129·0 to 352·8)	2·2% (1·4 to 3·2%)	15·8% (3·8 to 27·8%)	5·3% (0·5 to 10·3%)
**Dietary risks: All causes**	1,909·3 (1,676·7 to 2,149·1)	15·5% (13·6 to 17·4%)	2,489·7 (2,175·6 to 2,818·2)	19·3% (16·9 to 21·7%)	30·4% (25·3 to 35·6%)	24·8% (22·1 to 27·5%)	49,882·5 (44,350·4 to 55,752·8)	7·3% (6·4 to 8·2%)	63,234·5 (56,044·9 to 70,858·2)	9·8% (8·6 to 11·1%)	26·8% (21·8 to 32·1%)	34·7% (30·5 to 39·0%)
**Diet low in fruits: All causes**	534·1 (343·6 to 732·0)	4·3% (2·8 to 5·9%)	668·0 (423·4 to 930·7)	5·2% (3·3 to 7·2%)	25·1% (18·7 to 30·8%)	19·8% (15·7 to 23·4%)	15,359·5 (10,213·9 to 20,788·6)	2·2% (1·5 to 3·0%)	18,800·5 (12,254·9 to 25,798·4)	2·9% (1·9 to 4·0%)	22·4% (16·6 to 28·0%)	30·1% (25·0 to 34·9%)
Lip and oral cavity cancer	2·7 (0·0 to 5·8)	7·8% (0·0 to 16·8%)	3·6 (0·0 to 8·0)	7·7% (0·0 to 16·5%)	35·9% (0·0 to 45·7%)	-1·7% (-2·8 to 0·0%)	77·4 (0·0 to 167·7)	7·7% (0·0 to 16·6%)	103·1 (0·0 to 224·8)	7·6% (0·0 to 16·4%)	33·2% (0·0 to 43·6%)	-1·3% (-2·5 to 0·0%)
Nasopharynx cancer	0·6 (0·0 to 1·3)	7·8% (0·0 to 16·5%)	0·8 (0·0 to 1·7)	7·7% (0·0 to 16·3%)	31·5% (0·0 to 45·3%)	-1·8% (-3·2 to 0·0%)	19·0 (0·0 to 41·3)	7·5% (0·0 to 15·9%)	24·5 (0·0 to 52·8)	7·5% (0·0 to 15·8%)	28·9% (0·0 to 43·0%)	-0·8% (-2·3 to 0·3%)
Other pharynx cancer	1·1 (0·0 to 2·5)	7·8% (0·0 to 17·3%)	1·3 (0·0 to 3·0)	7·6% (0·0 to 17·0%)	21·3% (0·0 to 35·1%)	-2·3% (-3·7 to 0·0%)	30·4 (0·0 to 67·0)	7·8% (0·0 to 17·2%)	36·2 (0·0 to 79·8)	7·6% (0·0 to 17·0%)	19·3% (0·0 to 32·8%)	-2·0% (-3·5 to 0·0%)
Oesophageal cancer	11·9 (2·9 to 20·9)	23·9% (6·0 to 41·6%)	14·9 (3·7 to 26·3)	23·5% (5·9 to 41·1%)	25·7% (17·5 to 34·2%)	-1·7% (-3·0 to -0·9%)	320·4 (79·4 to 561·1)	23·7% (6·0 to 41·2%)	393·1 (97·2 to 691·0)	23·4% (5·9 to 40·8%)	22·7% (14·1 to 31·2%)	-1·3% (-2·7 to -0·5%)
Larynx cancer	1·8 (0·0 to 4·0)	7·8% (0·0 to 17·2%)	2·2 (0·0 to 5·0)	7·6% (0·0 to 16·9%)	23·3% (0·0 to 32·3%)	-2·2% (-3·5 to 0·0%)	47·1 (0·0 to 104·3)	7·7% (0·0 to 17·1%)	56·8 (0·0 to 127·1)	7·6% (0·0 to 16·8%)	20·6% (0·0 to 30·1%)	-1·9% (-3·2 to 0·0%)
Tracheal, bronchus, and lung cancer	11·2 (4·6 to 18·5)	13·6% (5·6 to 22·7%)	16·8 (6·8 to 28·4)	13·5% (5·5 to 22·4%)	49·9% (38·5 to 60·3%)	-1·3% (-2·3 to -0·6%)	285·4 (117·4 to 471·0)	13·6% (5·6 to 22·5%)	405·1 (163·9 to 684·6)	13·4% (5·5 to 22·3%)	41·9% (30·7 to 52·3%)	-1·3% (-2·5 to -0·5%)
Ischaemic heart disease	225·5 (82·3 to 373·9)	14·6% (5·3 to 24·1%)	292·9 (104·1 to 489·7)	14·1% (5·1 to 23·4%)	29·9% (23·5 to 36·1%)	-3·0% (-4·4 to -2·0%)	6,069·0 (2,245·5 to 9,958·5)	16·6% (6·2 to 27·1%)	7,645·6 (2,735·1 to 12,647·0)	16·3% (6·0 to 26·6%)	26·0% (19·6 to 32·6%)	-2·0% (-3·4 to -0·9%)
Ischaemic stroke	92·9 (50·4 to 139·6)	22·9% (12·6 to 34·3%)	108·4 (58·4 to 165·0)	21·7% (11·8 to 32·6%)	16·7% (9·4 to 24·7%)	-5·4% (-7·4 to -3·7%)	2,233·0 (1,243·1 to 3,256·7)	29·1% (16·6 to 42·2%)	2,572·6 (1,429·7 to 3,800·6)	28·3% (15·9 to 41·3%)	15·2% (7·8 to 22·7%)	-2·7% (-4·4 to -1·2%)

Supplemental Results Table 1. All-age deaths, death PAFs, DALYs, DALY PAFs, and percent change for each risk factor and outcome for both sexes combined in 2005 and 2015 for South Asia.

Data in parenthesis are 95% uncertainty intervals. PAFs=population attributable fractions. DALYs=disability-adjusted life-years.

	2005 Deaths (in thousands)	2005 Deaths PAF	2015 Deaths (in thousands)	2015 Deaths PAF	Percent change deaths 2005-2015	Percent change deaths PAF 2005-2015	2005 DALYs (in thousands)	2005 DALYs PAF	2015 DALYs (in thousands)	2015 DALYs PAF	Percent change DALYs 2005-2015	Percent change DALYs PAF 2005-2015
Hemorrhagic stroke	152·3<br>(87·0 to 222·8)	30·4%<br>(17·1 to 44·2%)	176·1<br>(98·9 to 259·6)	29·6%<br>(16·6 to 43·1%)	15·6%<br>(9·0 to 23·8%)	-2·7%<br>(-4·2 to -1·4%)	4,684·2<br>(2,743·3 to 6,697·6)	33·9%<br>(19·5 to 48·1%)	5,388·6<br>(3,126·7 to 7,767·9)	34·2%<br>(19·6 to 48·6%)	15·0%<br>(8·6 to 22·4%)	0·7%<br>(-0·6 to 1·8%)
**Diet low in vegetables: All causes**	409·6<br>(212·4 to 632·5)	3·3%<br>(1·7 to 5·1%)	509·2<br>(258·7 to 793·8)	4·0%<br>(2·0 to 6·2%)	24·3%<br>(18·0 to 30·5%)	19·0%<br>(14·2 to 23·2%)	11,028·3<br>(5,856·5 to 16,839·3)	1·6%<br>(0·8 to 2·5%)	13,391·0<br>(6,990·5 to 20,557·6)	2·1%<br>(1·1 to 3·2%)	21·4%<br>(15·0 to 27·7%)	29·1%<br>(23·1 to 34·4%)
Ischaemic heart disease	292·1<br>(116·2 to 471·6)	18·9%<br>(7·4 to 30·9%)	375·1<br>(148·8 to 623·9)	18·1%<br>(7·1 to 30·0%)	28·4%<br>(22·2 to 34·7%)	-4·1%<br>(-5·8 to -2·9%)	7,663·9<br>(3,106·8 to 12,262·3)	21·0%<br>(8·4 to 34·1%)	9,563·8<br>(3,873·4 to 15,735·8)	20·4%<br>(8·1 to 33·3%)	24·8%<br>(18·4 to 31·5%)	-2·9%<br>(-4·7 to -1·6%)
Ischaemic stroke	44·3<br>(9·9 to 79·6)	10·9%<br>(2·5 to 19·6%)	50·7<br>(11·3 to 91·6)	10·1%<br>(2·3 to 18·1%)	14·4%<br>(6·8 to 22·3%)	-7·3%<br>(-9·7 to -5·3%)	1,084·2<br>(248·7 to 1,924·6)	14·1%<br>(3·3 to 25·0%)	1,228·5<br>(280·3 to 2,204·1)	13·5%<br>(3·2 to 23·9%)	13·3%<br>(5·4 to 21·0%)	-4·3%<br>(-6·5 to -2·4%)
Hemorrhagic stroke	73·2<br>(21·1 to 132·9)	14·6%<br>(4·2 to 26·5%)	83·4<br>(23·1 to 151·2)	14·0%<br>(4·0 to 25·5%)	14·0%<br>(7·2 to 21·9%)	-4·0%<br>(-6·0 to -2·3%)	2,280·3<br>(662·0 to 4,089·3)	16·5%<br>(4·8 to 29·6%)	2,598·7<br>(729·8 to 4,640·3)	16·5%<br>(4·8 to 29·5%)	14·0%<br>(7·3 to 21·4%)	-0·2%<br>(-2·0 to 1·5%)
**Diet low in whole grains: All causes**	561·6<br>(388·5 to 759·9)	4·6%<br>(3·2 to 6·2%)	727·3<br>(498·3 to 981·4)	5·7%<br>(3·9 to 7·6%)	29·5%<br>(24·3 to 34·9%)	24·0%<br>(20·9 to 27·1%)	16,604·3<br>(11,727·1 to 22,029·4)	2·4%<br>(1·7 to 3·2%)	20,912·6<br>(14,653·2 to 27,993·0)	3·2%<br>(2·3 to 4·3%)	25·9%<br>(20·9 to 31·0%)	33·9%<br>(29·6 to 38·3%)
Ischaemic heart disease	317·9<br>(184·3 to 460·9)	20·5%<br>(12·1 to 29·8%)	421·1<br>(247·4 to 616·8)	20·3%<br>(12·0 to 29·6%)	32·5%<br>(26·7 to 38·5%)	-1·0%<br>(-1·6 to -0·5%)	8,579·7<br>(5,063·4 to 12,245·5)	23·5%<br>(14·0 to 33·6%)	10,990·1<br>(6,540·3 to 15,874·2)	23·4%<br>(14·0 to 33·7%)	28·1%<br>(22·2 to 34·5%)	-0·3%<br>(-0·9 to 0·3%)
Ischaemic stroke	73·4<br>(47·0 to 101·3)	18·1%<br>(12·0 to 24·8%)	87·3<br>(56·8 to 119·9)	17·5%<br>(11·6 to 23·8%)	18·9%<br>(11·4 to 27·1%)	-3·6%<br>(-5·2 to -2·0%)	1,854·2<br>(1,218·5 to 2,516·1)	24·1%<br>(16·3 to 32·4%)	2,175·9<br>(1,447·3 to 2,944·5)	23·9%<br>(16·2 to 32·0%)	17·4%<br>(10·1 to 25·2%)	-0·8%<br>(-2·3 to 0·6%)
Hemorrhagic stroke	117·6<br>(81·7 to 163·4)	23·5%<br>(16·3 to 31·9%)	138·1<br>(94·0 to 191·1)	23·2%<br>(16·1 to 31·6%)	17·5%<br>(11·1 to 25·9%)	-1·1%<br>(-2·4 to 0·1%)	3,702·0<br>(2,612·5 to 5,019·8)	26·8%<br>(18·8 to 35·8%)	4,310·7<br>(2,980·8 to 5,838·8)	27·3%<br>(19·2 to 36·5%)	16·4%<br>(10·1 to 23·8%)	2·0%<br>(1·0 to 2·9%)
Diabetes mellitus	52·8<br>(28·5 to 80·8)	18·4%<br>(9·9 to 28·1%)	80·7<br>(43·5 to 124·9)	17·8%<br>(9·6 to 27·3%)	52·9%<br>(44·2 to 62·0%)	-3·0%<br>(-3·9 to -2·2%)	2,468·5<br>(1,326·7 to 3,797·1)	21·6%<br>(12·0 to 32·8%)	3,435·8<br>(1,852·9 to 5,336·6)	21·5%<br>(11·9 to 32·6%)	39·2%<br>(34·4 to 44·6%)	-0·6%<br>(-1·2 to -0·1%)
**Diet low in nuts and seeds: All causes**	412·4<br>(268·6 to 575·0)	3·3%<br>(2·2 to 4·7%)	552·6<br>(356·5 to 768·3)	4·3%<br>(2·8 to 6·0%)	34·0%<br>(28·3 to 40·0%)	28·3%<br>(24·8 to 31·9%)	11,452·1<br>(7,679·0 to 15,686·6)	1·7%<br>(1·1 to 2·3%)	14,825·5<br>(9,716·5 to 20,245·0)	2·3%<br>(1·5 to 3·2%)	29·5%<br>(24·2 to 35·4%)	37·6%<br>(32·7 to 42·7%)
Ischaemic heart disease	379·5<br>(236·9 to 537·3)	24·5%<br>(15·5 to 34·6%)	502·4<br>(311·8 to 713·7)	24·2%<br>(15·3 to 34·3%)	32·4%<br>(26·5 to 38·3%)	-1·1%<br>(-1·6 to -0·7%)	9,925·0<br>(6,371·5 to 13,818·5)	27·1%<br>(17·4 to 37·7%)	12,706·0<br>(8,056·6 to 17,741·3)	27·0%<br>(17·3 to 37·6%)	28·0%<br>(22·1 to 34·4%)	-0·3%<br>(-0·9 to 0·2%)
Diabetes mellitus	33·0<br>(17·0 to 51·6)	11·5%<br>(6·0 to 18·0%)	50·2<br>(25·7 to 78·5)	11·1%<br>(5·8 to 17·3%)	52·3%<br>(43·7 to 61·4%)	-3·3%<br>(-4·1 to -2·6%)	1,527·1<br>(741·2 to 2,439·3)	13·4%<br>(7·0 to 20·7%)	2,119·5<br>(1,043·8 to 3,364·4)	13·3%<br>(6·9 to 20·5%)	38·8%<br>(34·1 to 44·2%)	-0·9%<br>(-1·4 to -0·4%)
**Diet low in milk: All causes**	6·9<br>(2·3 to 11·7)	0·1%<br>(0·0 to 0·1%)	10·0<br>(3·5 to 16·9)	0·1%<br>(0·0 to 0·1%)	45·6%<br>(34·9 to 56·5%)	39·4%<br>(30·7 to 47·5%)	182·5<br>(62·2 to 307·5)	0·0%<br>(0·0 to 0·0%)	255·3<br>(89·1 to 429·0)	0·0%<br>(0·0 to 0·1%)	39·9%<br>(30·0 to 50·4%)	48·7%<br>(39·1 to 58·5%)
Colon and rectum cancer	6·9<br>(2·3 to 11·7)	15·5%<br>(5·4 to 26·0%)	10·0<br>(3·5 to 16·9)	15·6%<br>(5·5 to 26·2%)	45·6%<br>(34·9 to 56·5%)	1·2%<br>(0·7 to 1·8%)	182·5<br>(62·2 to 307·5)	15·1%<br>(5·3 to 25·3%)	255·3<br>(89·1 to 429·0)	15·3%<br>(5·3 to 25·7%)	39·9%<br>(30·0 to 50·4%)	1·5%<br>(0·5 to 2·5%)
**Diet high in red meat: All causes**	0·2<br>(0·0 to 0·4)	0·0%<br>(0·0 to 0·0%)	0·2<br>(0·1 to 0·5)	0·0%<br>(0·0 to 0·0%)	38·2%<br>(15·8 to 63·8%)	32·3%<br>(11·1 to 55·8%)	8·9<br>(1·7 to 18·5)	0·0%<br>(0·0 to 0·0%)	12·4<br>(2·3 to 25·7)	0·0%<br>(0·0 to 0·0%)	38·8%<br>(23·0 to 54·5%)	47·5%<br>(31·7 to 63·7%)
Colon and rectum cancer	0·0<br>(0·0 to 0·1)	0·1%<br>(0·0 to 0·1%)	0·0<br>(0·0 to 0·1)	0·1%<br>(0·0 to 0·1%)	32·6%<br>(6·6 to 57·3%)	-8·0%<br>(-22·4 to 7·5%)	0·9<br>(0·2 to 1·7)	0·1%<br>(0·0 to 0·1%)	1·1<br>(0·2 to 2·4)	0·1%<br>(0·0 to 0·1%)	34·2%<br>(6·1 to 62·0%)	-2·8%<br>(-18·6 to 13·3%)
Diabetes mellitus	0·2<br>(0·0 to 0·3)	0·1%<br>(0·0 to 0·1%)	0·2<br>(0·0 to 0·5)	0·0%<br>(0·0 to 0·1%)	39·2%<br>(15·4 to 65·6%)	-11·7%<br>(-24·4 to 3·2%)	8·1<br>(0·9 to 17·2)	0·1%<br>(0·0 to 0·1%)	11·2<br>(1·2 to 24·1)	0·1%<br>(0·0 to 0·1%)	39·3%<br>(24·0 to 54·0%)	-0·6%<br>(-9·6 to 8·5%)
**Diet high in processed meat: All causes**	62·9<br>(14·8 to 115·9)	0·5%<br>(0·1 to 0·9%)	83·1<br>(20·9 to 150·3)	0·6%<br>(0·2 to 1·2%)	32·1%<br>(23·8 to 44·2%)	26·5%<br>(19·9 to 37·2%)	1,988·6<br>(662·9 to 3,471·7)	0·3%<br>(0·1 to 0·5%)	2,549·0<br>(881·3 to 4,442·9)	0·4%<br>(0·1 to 0·7%)	28·2%<br>(20·7 to 36·3%)	36·2%<br>(29·1 to 44·8%)
Colon and rectum cancer	0·8<br>(0·4 to 1·4)	1·9%<br>(1·0 to 3·1%)	1·2<br>(0·6 to 1·9)	1·8%<br>(0·9 to 3·0%)	40·0%<br>(28·5 to 52·6%)	-2·7%<br>(-6·4 to 0·7%)	23·7<br>(12·0 to 37·8)	2·0%<br>(1·0 to 3·2%)	32·2<br>(16·3 to 52·2)	1·9%<br>(1·0 to 3·1%)	35·9%<br>(24·2 to 48·9%)	-1·5%<br>(-5·4 to 2·5%)
Ischaemic heart disease	49·9<br>(1·9 to 100·2)	3·2%<br>(0·1 to 6·5%)	64·0<br>(2·4 to 129·5)	3·1%<br>(0·1 to 6·3%)	28·2%<br>(17·7 to 36·0%)	-4·2%<br>(-8·1 to 0·0%)	1,363·1<br>(51·8 to 2,727·7)	3·7%<br>(0·1 to 7·5%)	1,700·7<br>(65·3 to 3,433·2)	3·6%<br>(0·1 to 7·3%)	24·8%<br>(13·7 to 33·0%)	-2·8%<br>(-7·0 to 1·0%)
Diabetes mellitus	12·1<br>(5·4 to 19·6)	4·2%<br>(2·0 to 6·8%)	17·9<br>(7·8 to 29·0)	3·9%<br>(1·8 to 6·5%)	47·6%<br>(36·6 to 57·4%)	-6·4%<br>(-10·4 to -2·8%)	601·8<br>(272·8 to 984·7)	5·3%<br>(2·4 to 8·5%)	816·1<br>(363·9 to 1,332·6)	5·1%<br>(2·3 to 8·3%)	35·6%<br>(28·8 to 42·8%)	-3·2%<br>(-6·8 to 0·1%)
**Diet high in sugar-sweetened beverages: All causes**	0·8<br>(0·5 to 1·3)	0·0%<br>(0·0 to 0·0%)	1·1<br>(0·7 to 1·7)	0·0%<br>(0·0 to 0·0%)	32·9%<br>(24·8 to 41·9%)	27·2%<br>(20·7 to 34·5%)	33·4<br>(21·3 to 51·1)	0·0%<br>(0·0 to 0·0%)	43·7<br>(27·9 to 66·2)	0·0%<br>(0·0 to 0·0%)	31·0%<br>(23·4 to 39·3%)	39·2%<br>(31·4 to 47·6%)
Oesophageal cancer	0·0<br>(0·0 to 0·0)	0·0%<br>(0·0 to 0·0%)	0·0<br>(0·0 to 0·0)	0·0%<br>(0·0 to 0·0%)	31·9%<br>(15·3 to 49·0%)	3·2%<br>(-8·0 to 14·9%)	0·3<br>(0·1 to 0·6)	0·0%<br>(0·0 to 0·0%)	0·4<br>(0·1 to 0·7)	0·0%<br>(0·0 to 0·0%)	30·5%<br>(13·7 to 48·3%)	5·0%<br>(-6·6 to 17·4%)
Colon and rectum cancer	0·0<br>(0·0 to 0·0)	0·0%<br>(0·0 to 0·0%)	0·0<br>(0·0 to 0·0)	0·0%<br>(0·0 to 0·0%)	48·0%<br>(34·2 to 62·2%)	2·9%<br>(-2·9 to 9·7%)	0·1<br>(0·1 to 0·2)	0·0%<br>(0·0 to 0·0%)	0·1<br>(0·1 to 0·2)	0·0%<br>(0·0 to 0·0%)	44·8%<br>(30·7 to 59·6%)	5·0%<br>(-1·5 to 12·6%)
Liver cancer due to hepatitis B	0·0<br>(0·0 to 0·0)	0·0%<br>(0·0 to 0·0%)	0·0<br>(0·0 to 0·0)	0·0%<br>(0·0 to 0·0%)	30·3%<br>(15·9 to 47·5%)	4·8%<br>(-1·8 to 12·7%)	0·1<br>(0·0 to 0·1)	0·0%<br>(0·0 to 0·0%)	0·1<br>(0·0 to 0·2)	0·0%<br>(0·0 to 0·0%)	28·7%<br>(14·0 to 46·5%)	6·6%<br>(-0·1 to 15·0%)
Liver cancer due to hepatitis C	0·0<br>(0·0 to 0·0)	0·0%<br>(0·0 to 0·0%)	0·0<br>(0·0 to 0·0)	0·0%<br>(0·0 to 0·0%)	29·4%<br>(13·8 to 48·4%)	4·8%<br>(-2·7 to 13·8%)	0·0<br>(0·0 to 0·0)	0·0%<br>(0·0 to 0·0%)	0·0<br>(0·0 to 0·1)	0·0%<br>(0·0 to 0·0%)	29·2%<br>(12·5 to 50·3%)	6·5%<br>(-1·8 to 16·2%)
Liver cancer due to alcohol use	0·0<br>(0·0 to 0·0)	0·0%<br>(0·0 to 0·0%)	0·0<br>(0·0 to 0·0)	0·0%<br>(0·0 to 0·0%)	45·7%<br>(28·6 to 65·7%)	4·9%<br>(-1·4 to 12·5%)	0·0<br>(0·0 to 0·0)	0·0%<br>(0·0 to 0·0%)	0·0<br>(0·0 to 0·1)	0·0%<br>(0·0 to 0·0%)	45·2%<br>(27·8 to 65·5%)	5·7%<br>(-0·7 to 13·5%)
Liver cancer due to other causes	0·0<br>(0·0 to 0·0)	0·0%<br>(0·0 to 0·0%)	0·0<br>(0·0 to 0·0)	0·0%<br>(0·0 to 0·0%)	27·8%<br>(14·3 to 44·1%)	4·4%<br>(-2·5 to 13·1%)	0·0<br>(0·0 to 0·0)	0·0%<br>(0·0 to 0·0%)	0·0<br>(0·0 to 0·1)	0·0%<br>(0·0 to 0·0%)	26·3%<br>(12·3 to 44·1%)	6·7%<br>(-0·6 to 16·6%)
Gallbladder and biliary tract cancer	0·0<br>(0·0 to 0·0)	0·0%<br>(0·0 to 0·0%)	0·0<br>(0·0 to 0·0)	0·0%<br>(0·0 to 0·0%)	18·1%<br>(6·7 to 32·1%)	-0·3%<br>(-5·6 to 5·2%)	0·0<br>(0·0 to 0·1)	0·0%<br>(0·0 to 0·0%)	0·1<br>(0·0 to 0·1)	0·0%<br>(0·0 to 0·0%)	16·2%<br>(4·4 to 29·8%)	0·8%<br>(-4·9 to 6·7%)
Pancreatic cancer	0·0<br>(0·0 to 0·0)	0·0%<br>(0·0 to 0·0%)	0·0<br>(0·0 to 0·0)	0·0%<br>(0·0 to 0·0%)	41·3%<br>(32·4 to 51·7%)	3·0%<br>(-1·9 to 8·2%)	0·0<br>(0·0 to 0·1)	0·0%<br>(0·0 to 0·0%)	0·0<br>(0·0 to 0·1)	0·0%<br>(0·0 to 0·0%)	40·0%<br>(30·4 to 50·7%)	4·3%<br>(-0·8 to 10·0%)
Breast cancer	0·0<br>(0·0 to 0·0)	0·0%<br>(0·0 to 0·0%)	0·0<br>(0·0 to 0·0)	0·0%<br>(0·0 to 0·0%)	42·6%<br>(22·9 to 61·4%)	4·5%<br>(-2·1 to 11·5%)	0·1<br>(0·0 to 0·1)	0·0%<br>(0·0 to 0·0%)	0·1<br>(0·0 to 0·1)	0·0%<br>(0·0 to 0·0%)	44·4%<br>(24·8 to 63·8%)	7·1%<br>(0·1 to 15·3%)
Uterine cancer	0·0<br>(0·0 to 0·0)	0·0%<br>(0·0 to 0·0%)	0·0<br>(0·0 to 0·0)	0·0%<br>(0·0 to 0·0%)	15·6%<br>(-0·8 to 35·3%)	2·4%<br>(-4·5 to 10·4%)	0·1<br>(0·1 to 0·1)	0·0%<br>(0·0 to 0·1%)	0·1<br>(0·1 to 0·2)	0·0%<br>(0·0 to 0·1%)	16·2%<br>(-0·9 to 37·6%)	4·7%<br>(-2·1 to 12·8%)
Ovarian cancer	0·0<br>(0·0 to 0·0)	0·0%<br>(0·0 to 0·0%)	0·0<br>(0·0 to 0·0)	0·0%<br>(0·0 to 0·0%)	40·1%<br>(0·0 to 54·4%)	2·6%<br>(-3·7 to 8·8%)	0·0<br>(0·0 to 0·0)	0·0%<br>(0·0 to 0·0%)	0·0<br>(0·0 to 0·0)	0·0%<br>(0·0 to 0·0%)	40·6%<br>(0·0 to 56·6%)	4·2%<br>(-2·3 to 10·9%)
Kidney cancer	0·0<br>(0·0 to 0·0)	0·0%<br>(0·0 to 0·0%)	0·0<br>(0·0 to 0·0)	0·0%<br>(0·0 to 0·0%)	52·6%<br>(35·7 to 70·6%)	4·4%<br>(-1·9 to 11·0%)	0·0<br>(0·0 to 0·0)	0·0%<br>(0·0 to 0·0%)	0·0<br>(0·0 to 0·1)	0·0%<br>(0·0 to 0·0%)	52·6%<br>(36·2 to 70·9%)	7·8%<br>(0·9 to 15·3%)
Thyroid cancer	0·0<br>(0·0 to 0·0)	0·0%<br>(0·0 to 0·0%)	0·0<br>(0·0 to 0·0)	0·0%<br>(0·0 to 0·0%)	25·8%<br>(15·2 to 38·6%)	3·7%<br>(-2·1 to 10·5%)	0·0<br>(0·0 to 0·0)	0·0%<br>(0·0 to 0·0%)	0·0<br>(0·0 to 0·0)	0·0%<br>(0·0 to 0·0%)	29·6%<br>(18·2 to 43·1%)	4·6%<br>(-1·5 to 11·9%)
Ischaemic heart disease	0·3<br>(0·2 to 0·5)	0·0%<br>(0·0 to 0·0%)	0·4<br>(0·3 to 0·6)	0·0%<br>(0·0 to 0·0%)	33·4%<br>(24·3 to 43·3%)	-0·4%<br>(-5·4 to 5·1%)	10·6<br>(6·6 to 16·3)	0·0%<br>(0·0 to 0·0%)	13·8<br>(8·7 to 20·9)	0·0%<br>(0·0 to 0·0%)	31·1%<br>(21·8 to 41·3%)	2·0%<br>(-3·4 to 8·0%)
Ischaemic stroke	0·1<br>(0·0 to 0·1)	0·0%<br>(0·0 to 0·0%)	0·1<br>(0·0 to 0·1)	0·0%<br>(0·0 to 0·0%)	21·7%<br>(12·3 to 32·6%)	-1·4%<br>(-6·5 to 4·3%)	1·7<br>(1·0 to 2·6)	0·0%<br>(0·0 to 0·0%)	2·0<br>(1·3 to 3·1)	0·0%<br>(0·0 to 0·0%)	21·6%<br>(11·8 to 32·2%)	2·7%<br>(-2·5 to 9·0%)
Hemorrhagic stroke	0·2<br>(0·1 to 0·3)	0·0%<br>(0·0 to 0·1%)	0·2<br>(0·1 to 0·3)	0·0%<br>(0·0 to 0·1%)	20·5%<br>(12·1 to 30·2%)	1·4%<br>(-3·8 to 7·5%)	6·6<br>(4·1 to 10·1)	0·0%<br>(0·0 to 0·1%)	7·9<br>(5·0 to 11·7)	0·1%<br>(0·0 to 0·1%)	20·1%<br>(11·8 to 29·9%)	5·2%<br>(0·1 to 11·3%)
Hypertensive heart disease	0·1<br>(0·0 to 0·1)	0·0%<br>(0·0 to 0·1%)	0·1<br>(0·1 to 0·1)	0·0%<br>(0·0 to 0·1%)	25·5%<br>(14·3 to 36·6%)	-0·2%<br>(-6·3 to 6·3%)	2·2<br>(1·4 to 3·4)	0·1%<br>(0·0 to 0·1%)	2·7<br>(1·7 to 4·2)	0·1%<br>(0·0 to 0·1%)	23·5%<br>(12·7 to 34·7%)	2·8%<br>(-3·1 to 9·1%)
Cardiomyopathy and myocarditis	-	-	-	-	-	-	-	-	-	-	-	-
Atrial fibrillation and flutter	-	-	-	-	-	-	-	-	-	-	-	-
Peripheral vascular disease	-	-	-	-	-	-	-	-	-	-	-	-

Supplemental Results Table 1. All-age deaths, death PAFs, DALYs, DALY PAFs, and percent change for each risk factor and outcome for both sexes combined in 2005 and 2015 for South Asia.

Data in parenthesis are 95% uncertainty intervals. PAFs=population attributable fractions. DALYs=disability-adjusted life-years.

	2005 Deaths (in thousands)	2005 Deaths PAF	2015 Deaths (in thousands)	2015 Deaths PAF	Percent change deaths 2005-2015	Percent change deaths PAF 2005-2015	2005 DALYs (in thousands)	2005 DALYs PAF	2015 DALYs (in thousands)	2015 DALYs PAF	Percent change DALYs 2005-2015	Percent change DALYs PAF 2005-2015
Endocarditis	-	-	-	-	-	-	-	-	-	-	-	-
Other cardiovascular and circulatory diseases	-	-	-	-	-	-	-	-	-	-	-	-
Diabetes mellitus	0·1 (0·1 to 0·2)	0·0% (0·0 to 0·1%)	0·2 (0·1 to 0·3)	0·0% (0·0 to 0·1%)	51·5% (40·0 to 63·0%)	-3·9% (-8·5 to 1·3%)	8·1 (5·0 to 12·8)	0·1% (0·0 to 0·1%)	11·4 (7·2 to 17·5)	0·1% (0·0 to 0·1%)	40·5% (32·8 to 48·6%)	0·3% (-4·3 to 5·3%)
Chronic kidney disease due to diabetes mellitus	0·0 (0·0 to 0·0)	0·0% (0·0 to 0·1%)	0·0 (0·0 to 0·0)	0·0% (0·0 to 0·1%)	44·3% (31·1 to 58·8%)	2·6% (-2·4 to 8·7%)	0·6 (0·2 to 1·3)	0·0% (0·0 to 0·1%)	0·9 (0·3 to 1·8)	0·0% (0·0 to 0·1%)	41·4% (30·4 to 53·9%)	6·8% (1·8 to 13·1%)
Chronic kidney disease due to hypertension	0·0 (0·0 to 0·0)	0·0% (0·0 to 0·0%)	0·0 (0·0 to 0·1)	0·0% (0·0 to 0·0%)	44·2% (31·1 to 58·3%)	2·5% (-2·6 to 8·7%)	0·8 (0·3 to 1·5)	0·0% (0·0 to 0·0%)	1·2 (0·5 to 2·2)	0·0% (0·0 to 0·1%)	40·8% (29·0 to 54·3%)	10·2% (5·0 to 16·8%)
Chronic kidney disease due to glomerulonephritis	0·0 (0·0 to 0·0)	0·0% (0·0 to 0·0%)	0·0 (0·0 to 0·0)	0·0% (0·0 to 0·0%)	43·9% (30·0 to 58·9%)	6·8% (1·3 to 14·2%)	0·6 (0·2 to 1·2)	0·0% (0·0 to 0·0%)	0·8 (0·2 to 1·6)	0·0% (0·0 to 0·1%)	39·9% (28·8 to 52·3%)	17·1% (11·1 to 25·5%)
Chronic kidney disease due to other causes	0·0 (0·0 to 0·0)	0·0% (0·0 to 0·0%)	0·0 (0·0 to 0·0)	0·0% (0·0 to 0·0%)	47·5% (29·7 to 67·2%)	21·3% (9·8 to 34·8%)	0·1 (0·0 to 0·2)	0·0% (0·0 to 0·0%)	0·2 (0·0 to 0·3)	0·0% (0·0 to 0·0%)	38·0% (30·5 to 47·2%)	16·8% (10·7 to 24·8%)
**Diet low in fibre: All causes**	70·7 (30·0 to 127·8)	0·6% (0·2 to 1·0%)	92·5 (40·3 to 165·4)	0·7% (0·3 to 1·3%)	30·7% (24·0 to 38·3%)	25·2% (20·1 to 31·1%)	2,067·7 (879·2 to 3,715·6)	0·3% (0·1 to 0·5%)	2,609·1 (1,125·1 to 4,681·5)	0·4% (0·2 to 0·7%)	26·2% (18·7 to 34·3%)	34·1% (27·1 to 41·9%)
Colon and rectum cancer	2·4 (0·9 to 4·3)	5·3% (2·2 to 9·7%)	3·3 (1·4 to 6·1)	5·2% (2·1 to 9·5%)	40·5% (29·1 to 52·0%)	-2·4% (-5·9 to 1·3%)	65·4 (26·5 to 118·2)	5·4% (2·2 to 9·8%)	87·3 (35·6 to 158·0)	5·2% (2·1 to 9·5%)	33·5% (21·9 to 44·8%)	-3·2% (-7·7 to 0·5%)
Ischaemic heart disease	68·4 (28·9 to 123·7)	4·4% (1·9 to 8·0%)	89·1 (38·5 to 160·3)	4·3% (1·8 to 7·8%)	30·4% (23·5 to 38·0%)	-2·6% (-5·6 to 0·9%)	2,002·3 (847·2 to 3,604·8)	5·5% (2·3 to 9·8%)	2,521·8 (1,077·8 to 4,567·6)	5·4% (2·3 to 9·6%)	25·9% (18·5 to 34·1%)	-1·9% (-5·2 to 1·7%)
**Diet low in calcium: All causes**	10·3 (6·6 to 14·2)	0·1% (0·1 to 0·1%)	14·9 (9·6 to 20·9)	0·1% (0·1 to 0·2%)	44·5% (34·0 to 55·4%)	38·3% (29·4 to 46·5%)	270·8 (174·1 to 375·0)	0·0% (0·0 to 0·1%)	376·5 (242·9 to 526·9)	0·1% (0·0 to 0·1%)	39·1% (29·1 to 49·6%)	47·8% (38·3 to 57·7%)
Colon and rectum cancer	10·3 (6·6 to 14·2)	23·2% (14·9 to 32·1%)	14·9 (9·6 to 20·9)	23·3% (14·9 to 32·3%)	44·5% (34·0 to 55·4%)	0·4% (-0·4 to 1·2%)	270·8 (174·1 to 375·0)	22·4% (14·3 to 31·0%)	376·5 (242·9 to 526·9)	22·6% (14·4 to 31·3%)	39·1% (29·1 to 49·6%)	0·9% (-0·4 to 2·1%)
Prostate cancer	-	-	-	-	-	-	-	-	-	-	-	-
**Diet low in seafood omega-3 fatty acids: All causes**	318·5 (138·9 to 502·8)	2·6% (1·1 to 4·1%)	421·1 (179·3 to 660·8)	3·3% (1·4 to 5·2%)	32·2% (26·5 to 38·1%)	26·6% (23·0 to 30·3%)	8,240·8 (3,657·2 to 12,935·6)	1·2% (0·5 to 1·9%)	10,579·5 (4,540·3 to 16,437·9)	1·6% (0·7 to 2·6%)	28·4% (22·3 to 34·8%)	36·5% (31·0 to 42·3%)
Ischaemic heart disease	318·5 (138·9 to 502·8)	20·6% (8·9 to 32·4%)	421·1 (179·3 to 660·8)	20·3% (8·8 to 32·0%)	32·2% (26·5 to 38·1%)	-1·2% (-1·9 to -0·7%)	8,240·8 (3,657·2 to 12,935·6)	22·5% (9·9 to 35·0%)	10,579·5 (4,540·3 to 16,437·9)	22·5% (9·9 to 35·1%)	28·4% (22·3 to 34·8%)	0·0% (-0·7 to 0·6%)
**Diet low in polyunsaturated fatty acids: All causes**	48·5 (19·9 to 85·0)	0·4% (0·2 to 0·7%)	67·5 (27·4 to 117·9)	0·5% (0·2 to 0·9%)	39·2% (13·2 to 70·8%)	33·2% (8·7 to 63·1%)	1,242·5 (518·3 to 2,143·1)	0·2% (0·1 to 0·3%)	1,864·8 (804·7 to 3,247·0)	0·3% (0·1 to 0·5%)	50·1% (21·2 to 83·1%)	59·5% (29·2 to 93·7%)
Ischaemic heart disease	48·5 (19·9 to 85·0)	3·1% (1·3 to 5·4%)	67·5 (27·4 to 117·9)	3·3% (1·3 to 5·7%)	39·2% (13·2 to 70·8%)	3·9% (-14·3 to 27·2%)	1,242·5 (518·3 to 2,143·1)	3·4% (1·4 to 5·8%)	1,864·8 (804·7 to 3,247·0)	4·0% (1·7 to 6·8%)	50·1% (21·2 to 83·1%)	16·8% (-4·3 to 42·1%)
**Diet high in trans fatty acids: All causes**	126·5 (60·8 to 212·2)	1·0% (0·5 to 1·7%)	166·7 (79·5 to 278·7)	1·3% (0·6 to 2·2%)	31·8% (21·7 to 42·2%)	26·1% (18·3 to 34·8%)	3,704·6 (1,826·2 to 6,132·8)	0·5% (0·3 to 0·9%)	4,796·9 (2,363·1 to 7,845·6)	0·7% (0·4 to 1·2%)	29·5% (19·6 to 40·9%)	37·6% (28·0 to 48·4%)
Ischaemic heart disease	126·5 (60·8 to 212·2)	8·2% (3·9 to 13·6%)	166·7 (79·5 to 278·7)	8·0% (3·9 to 13·4%)	31·8% (21·7 to 42·2%)	-1·6% (-6·6 to 3·7%)	3,704·6 (1,826·2 to 6,132·8)	10·1% (5·0 to 16·7%)	4,796·9 (2,363·1 to 7,845·6)	10·2% (5·1 to 16·6%)	29·5% (19·6 to 40·9%)	0·8% (-4·0 to 7·2%)
**Diet high in sodium: All causes**	468·6 (198·8 to 858·1)	3·8% (1·6 to 6·9%)	612·1 (258·3 to 1,116·6)	4·8% (2·0 to 8·7%)	30·6% (25·5 to 36·4%)	25·0% (22·0 to 28·6%)	11,118·6 (4,729·2 to 20,347·3)	1·6% (0·7 to 3·0%)	14,126·5 (6,006·2 to 25,588·4)	2·2% (0·9 to 4·0%)	27·1% (22·0 to 33·0%)	35·0% (30·6 to 40·0%)
Stomach cancer	19·5 (9·1 to 31·3)	36·2% (17·1 to 58·2%)	22·9 (10·8 to 37·4)	36·3% (17·3 to 58·5%)	17·8% (9·7 to 25·8%)	0·2% (-3·8 to 4·3%)	474·5 (222·7 to 763·2)	35·4% (16·7 to 56·7%)	541·3 (257·3 to 877·3)	35·7% (16·9 to 57·1%)	14·1% (7·2 to 22·1%)	0·7% (-1·8 to 3·4%)
Rheumatic heart disease	7·2 (2·5 to 14·9)	5·5% (1·9 to 11·6%)	8·3 (2·9 to 17·1)	5·8% (2·0 to 12·2%)	16·0% (6·2 to 26·3%)	5·1% (2·5 to 8·9%)	246·1 (81·0 to 537·8)	4·6% (1·5 to 9·9%)	274·1 (91·9 to 581·6)	5·0% (1·7 to 10·6%)	11·4% (3·6 to 20·4%)	9·1% (6·0 to 13·1%)
Ischaemic heart disease	230·9 (94·2 to 431·8)	14·9% (6·1 to 28·0%)	312·3 (127·3 to 582·0)	15·1% (6·2 to 28·1%)	35·3% (29·2 to 41·9%)	1·1% (-0·8 to 3·2%)	5,323·2 (2,204·7 to 9,944·8)	14·6% (6·0 to 27·2%)	7,020·1 (2,918·8 to 12,815·6)	14·9% (6·2 to 27·8%)	31·9% (25·8 to 38·8%)	2·7% (1·4 to 4·7%)
Ischaemic stroke	55·6 (21·3 to 107·9)	13·7% (5·3 to 26·0%)	68·6 (26·7 to 130·5)	13·7% (5·3 to 26·1%)	23·3% (15·6 to 32·4%)	-0·1% (-2·6 to 3·8%)	1,110·3 (426·8 to 2,117·6)	14·4% (5·6 to 27·3%)	1,336·9 (526·3 to 2,556·7)	14·7% (5·8 to 27·6%)	20·4% (13·1 to 28·5%)	1·7% (-0·1 to 4·6%)
Hemorrhagic stroke	80·1 (31·5 to 154·4)	16·0% (6·2 to 29·8%)	97·3 (37·7 to 184·3)	16·4% (6·4 to 30·2%)	21·4% (14·3 to 31·0%)	2·2% (0·1 to 5·4%)	2,115·6 (809·3 to 4,073·9)	15·3% (5·8 to 28·7%)	2,532·2 (985·3 to 4,739·7)	16·1% (6·3 to 29·9%)	19·7% (13·1 to 28·5%)	4·8% (3·1 to 7·8%)
Hypertensive heart disease	43·2 (13·4 to 98·4)	25·9% (7·8 to 57·6%)	55·4 (17·0 to 123·4)	26·4% (8·0 to 58·3%)	28·2% (16·0 to 40·2%)	1·9% (-2·3 to 6·3%)	1,022·6 (360·9 to 2,123·8)	25·1% (8·7 to 51·4%)	1,267·7 (446·9 to 2,611·4)	25·9% (8·9 to 52·8%)	24·0% (13·1 to 34·9%)	3·2% (0·4 to 6·5%)
Cardiomyopathy and myocarditis	1·0 (0·3 to 1·9)	6·5% (2·3 to 12·9%)	1·3 (0·5 to 2·5)	7·1% (2·6 to 13·8%)	31·2% (15·4 to 45·0%)	8·8% (5·7 to 13·0%)	35·6 (11·4 to 75·1)	5·0% (1·7 to 10·6%)	45·8 (15·5 to 95·0)	5·7% (2·0 to 11·9%)	28·5% (14·2 to 41·4%)	13·9% (9·5 to 19·1%)
Atrial fibrillation and flutter	1·2 (0·4 to 2·3)	8·4% (3·3 to 16·3%)	1·7 (0·6 to 3·4)	8·3% (3·3 to 16·1%)	49·1% (42·1 to 57·3%)	-0·9% (-3·6 to 2·0%)	40·1 (15·1 to 79·1)	10·6% (4·3 to 19·8%)	55·8 (21·1 to 109·3)	10·6% (4·3 to 19·7%)	39·1% (35·8 to 43·2%)	-0·3% (-2·0 to 1·9%)
Aortic aneurysm	1·2 (0·5 to 2·3)	9·2% (3·7 to 17·5%)	1·8 (0·7 to 3·5)	9·3% (3·7 to 17·5%)	55·3% (37·9 to 71·5%)	0·5% (-1·5 to 3·0%)	29·3 (11·2 to 55·9)	9·0% (3·6 to 17·3%)	44·3 (17·6 to 84·0)	9·2% (3·7 to 17·4%)	51·3% (34·5 to 67·6%)	2·4% (0·4 to 4·8%)
Peripheral vascular disease	0·1 (0·0 to 0·1)	6·5% (2·4 to 13·0%)	0·1 (0·0 to 0·3)	6·5% (2·4 to 13·0%)	100·6% (72·8 to 141·8%)	-0·3% (-3·4 to 2·9%)	5·0 (1·5 to 11·3)	7·0% (2·5 to 13·7%)	7·5 (2·4 to 16·7)	7·0% (2·6 to 13·6%)	51·9% (44·3 to 65·2%)	-0·1% (-2·0 to 2·4%)
Endocarditis	0·3 (0·1 to 0·7)	6·8% (2·4 to 13·4%)	0·4 (0·2 to 0·9)	7·3% (2·7 to 14·3%)	39·6% (29·0 to 56·4%)	7·3% (3·1 to 13·3%)	10·3 (3·3 to 22·0)	5·2% (1·7 to 10·7%)	14·1 (4·5 to 29·7)	5·9% (1·9 to 12·0%)	36·9% (27·1 to 51·8%)	12·3% (6·4 to 20·2%)
Other cardiovascular and circulatory diseases	1·5 (0·6 to 2·9)	10·1% (3·8 to 18·8%)	2·2 (0·8 to 4·2)	10·0% (3·9 to 18·8%)	44·9% (34·4 to 57·2%)	-0·3% (-3·1 to 2·7%)	36·6 (14·1 to 70·7)	10·3% (4·0 to 19·6%)	50·9 (20·2 to 96·3)	10·5% (4·1 to 19·9%)	38·9% (30·2 to 49·0%)	2·5% (0·6 to 5·1%)
Chronic kidney disease due to diabetes mellitus	7·7 (2·6 to 15·8)	11·4% (3·9 to 22·3%)	11·2 (3·9 to 22·4)	11·8% (4·1 to 22·8%)	45·2% (32·4 to 58·7%)	3·2% (1·6 to 5·5%)	196·3 (66·6 to 398·7)	9·4% (3·2 to 18·4%)	274·8 (94·9 to 553·5)	9·9% (3·4 to 19·3%)	40·0% (29·3 to 51·4%)	5·6% (4·0 to 8·0%)
Chronic kidney disease due to hypertension	12·8 (4·4 to 26·3)	10·9% (3·9 to 21·7%)	18·9 (6·5 to 39·0)	11·4% (4·1 to 22·8%)	48·0% (34·8 to 61·5%)	5·2% (3·3 to 7·7%)	274·0 (96·0 to 564·7)	8·4% (3·0 to 16·8%)	385·2 (136·1 to 806·7)	9·2% (3·3 to 18·3%)	40·6% (28·7 to 53·1%)	10·0% (7·7 to 12·9%)
Chronic kidney disease due to glomerulonephritis	6·2 (2·2 to 12·6)	9·1% (3·2 to 18·3%)	9·2 (3·1 to 18·4)	10·0% (3·5 to 20·0%)	48·1% (34·1 to 61·9%)	10·0% (7·7 to 12·9%)	166·2 (58·4 to 343·3)	6·3% (2·2 to 12·7%)	230·7 (80·4 to 467·6)	7·3% (2·6 to 14·5%)	38·8% (27·7 to 50·2%)	16·2% (12·6 to 19·8%)
Chronic kidney disease due to other causes	0·2 (0·1 to 0·4)	5·9% (2·1 to 12·0%)	0·3 (0·1 to 0·7)	7·8% (2·7 to 15·6%)	59·6% (43·7 to 74·0%)	31·2% (24·7 to 37·5%)	32·9 (10·8 to 70·4)	6·1% (2·1 to 12·3%)	45·0 (14·8 to 94·7)	7·0% (2·5 to 14·4%)	36·7% (32·7 to 41·7%)	15·7% (11·7 to 20·5%)
**Sexual abuse and violence: All causes**	88·5 (51·5 to 120·8)	0·7% (0·4 to 1·0%)	91·0 (52·5 to 126·6)	0·7% (0·4 to 1·0%)	2·9% (-8·3 to 13·2%)	-1·5% (-11·4 to 7·7%)	6,475·6 (4,404·0 to 8,535·9)	0·9% (0·7 to 1·2%)	6,924·2 (4,801·5 to 9,260·9)	1·1% (0·8 to 1·4%)	6·9% (-1·0 to 14·8%)	13·6% (5·3 to 21·8%)
**Childhood sexual abuse: All causes**	39·7 (9·9 to 78·1)	0·3% (0·1 to 0·6%)	42·5 (10·8 to 83·8)	0·3% (0·1 to 0·7%)	7·1% (-4·4 to 17·3%)	2·5% (-8·0 to 12·0%)	2,979·9 (1,182·3 to 5,227·6)	0·4% (0·2 to 0·8%)	3,187·4 (1,286·8 to 5,560·4)	0·5% (0·2 to 0·8%)	7·0% (-2·4 to 14·4%)	13·6% (4·3 to 22·0%)
Alcohol use disorders	0·5 (0·0 to 1·0)	5·6% (0·0 to 12·7%)	0·6 (0·0 to 1·3)	5·3% (0·0 to 12·0%)	23·3% (0·0 to 33·6%)	-6·9% (-10·0 to 0·0%)	88·7 (0·0 to 198·2)	6·5% (0·0 to 14·4%)	103·2 (0·0 to 234·1)	6·1% (0·0 to 13·7%)	16·3% (0·0 to 21·0%)	-5·7% (-8·4 to 0·0%)
Major depressive disorder	-	-	-	-	-	-	745·5 (86·3 to 1,608·0)	8·5% (1·1 to 17·5%)	859·3 (96·5 to 1,846·8)	8·1% (1·0 to 16·7%)	15·3% (11·3 to 18·0%)	-4·8% (-7·5 to -2·3%)
Dysthymia	-	-	-	-	-	-	139·8 (15·4 to 301·1)	8·5% (1·0 to 17·3%)	167·0 (18·3 to 361·1)	8·1% (1·0 to 16·6%)	19·4% (13·1 to 25·3%)	-4·9% (-7·7 to -2·4%)
Self-harm	39·2 (9·4 to 77·6)	19·6% (5·1 to 37·1%)	41·9 (9·9 to 83·4)	18·2% (4·6 to 34·8%)	6·9% (-4·9 to 17·2%)	-7·2% (-10·8 to -4·3%)	2,005·8 (484·3 to 3,953·6)	20·3% (5·3 to 38·2%)	2,057·9 (487·1 to 4,042·2)	19·0% (4·8 to 36·1%)	2·6% (-8·9 to 13·3%)	-6·7% (-10·2 to -3·7%)
**Intimate partner violence: All causes**	58·9 (35·1 to 72·5)	0·5% (0·3 to 0·6%)	58·9 (34·6 to 73·8)	0·5% (0·3 to 0·6%)	0·1% (-12·0 to 13·0%)	-4·2% (-15·5 to 7·7%)	4,111·9 (2,904·7 to 5,069·9)	0·6% (0·4 to 0·7%)	4,375·2 (3,163·0 to 5,446·8)	0·7% (0·5 to 0·8%)	6·4% (-3·0 to 16·3%)	13·0% (2·9 to 23·7%)

Supplemental Results Table 1. All-age deaths, death PAFs, DALYs, DALY PAFs, and percent change for each risk factor and outcome for both sexes combined in 2005 and 2015 for South Asia.

Data in parenthesis are 95% uncertainty intervals. PAFs=population attributable fractions. DALYs=disability-adjusted life-years.

	2005 Deaths (in thousands)	2005 Deaths PAF	2015 Deaths (in thousands)	2015 Deaths PAF	Percent change deaths 2005-2015	Percent change deaths PAF 2005-2015	2005 DALYs (in thousands)	2005 DALYs PAF	2015 DALYs (in thousands)	2015 DALYs PAF	Percent change DALYs 2005-2015	Percent change DALYs PAF 2005-2015
HIV/AIDS - Tuberculosis	3·5 (1·9 to 5·3)	6·7% (3·6 to 9·8%)	2·1 (1·1 to 3·2)	7·5% (4·0 to 11·1%)	-41·4% (-49·1 to -34·2%)	12·0% (5·8 to 17·8%)	162·9 (86·5 to 243·7)	6·1% (3·3 to 8·9%)	92·6 (48·0 to 143·7)	6·7% (3·5 to 9·9%)	-43·2% (-50·6 to -36·5%)	10·3% (3·7 to 16·3%)
HIV/AIDS resulting in other diseases	9·8 (5·2 to 14·4)	6·7% (3·6 to 9·8%)	8·1 (4·2 to 11·8)	7·5% (3·9 to 11·0%)	-17·9% (-26·7 to -8·5%)	11·1% (3·4 to 18·8%)	470·0 (249·9 to 696·7)	6·2% (3·3 to 9·0%)	377·8 (195·4 to 556·1)	6·7% (3·5 to 9·9%)	-19·6% (-28·0 to -11·1%)	9·2% (1·4 to 17·0%)
Maternal abortion, miscarriage, and ectopic pregnancy	0·0 (0·0 to 0·0)	0·0% (0·0 to 0·0%)	0·0 (0·0 to 0·0)	0·0% (0·0 to 0·0%)	-27·3% (-41·6 to -5·8%)	0·4% (-12·9 to 21·0%)	0·0 (0·0 to 0·0)	0·0% (0·0 to 0·0%)	0·0 (0·0 to 0·0)	0·0% (0·0 to 0·0%)	-27·8% (-41·4 to -6·1%)	0·4% (-12·6 to 20·8%)
Major depressive disorder	-	-	-	-	-	-	1,082·5 (696·9 to 1,540·2)	12·4% (10·2 to 14·5%)	1,416·0 (924·4 to 2,024·4)	13·4% (11·0 to 15·5%)	30·8% (26·2 to 35·1%)	8·0% (4·5 to 11·0%)
Dysthymia	-	-	-	-	-	-	209·9 (134·6 to 307·6)	12·7% (10·7 to 14·8%)	288·7 (185·9 to 426·3)	13·9% (11·7 to 16·1%)	37·5% (28·7 to 47·6%)	9·6% (4·9 to 14·9%)
Self-harm	40·5 (17·8 to 51·5)	20·2% (11·7 to 24·9%)	43·6 (20·0 to 57·9)	18·9% (11·2 to 24·1%)	7·5% (-8·7 to 26·5%)	-6·5% (-17·6 to 5·5%)	1,916·3 (842·0 to 2,522·8)	19·4% (11·3 to 24·4%)	1,928·2 (903·3 to 2,628·3)	17·7% (10·6 to 23·0%)	0·6% (-15·5 to 21·3%)	-8·4% (-19·8 to 5·4%)
Assault by firearm	1·0 (0·7 to 1·2)	9·7% (7·8 to 12·9%)	1·1 (0·7 to 1·6)	9·3% (6·3 to 13·4%)	12·5% (-12·9 to 50·3%)	-4·3% (-28·0 to 29·5%)	51·4 (33·6 to 63·2)	10·7% (8·6 to 14·0%)	55·7 (32·8 to 80·3)	10·3% (6·9 to 14·7%)	8·3% (-18·2 to 43·5%)	-4·0% (-28·0 to 28·7%)
Assault by sharp object	0·2 (0·2 to 0·3)	6·1% (4·9 to 9·0%)	0·2 (0·2 to 0·3)	6·5% (3·9 to 10·8%)	5·4% (-25·2 to 39·9%)	6·5% (-32·3 to 45·8%)	10·2 (7·0 to 12·3)	6·0% (4·7 to 8·5%)	10·2 (7·1 to 13·3)	6·4% (3·9 to 10·4%)	-0·6% (-27·4 to 29·9%)	5·2% (-33·4 to 44·6%)
Assault by other means	3·8 (2·9 to 4·4)	9·3% (8·1 to 11·6%)	3·9 (2·7 to 4·8)	9·0% (7·2 to 12·9%)	2·6% (-13·3 to 19·3%)	-3·4% (-18·0 to 13·2%)	208·7 (159·1 to 243·7)	9·6% (8·4 to 12·5%)	206·1 (147·8 to 252·1)	9·3% (7·5 to 13·7%)	-1·2% (-16·0 to 13·7%)	-3·7% (-18·3 to 12·2%)
**Unsafe sex: All causes**	267·5 (246·8 to 292·3)	2·2% (2·0 to 2·4%)	187·0 (174·6 to 200·3)	1·5% (1·4 to 1·6%)	-30·1% (-34·7 to -25·5%)	-33·1% (-37·4 to -28·8%)	14,540·4 (13,023·4 to 16,518·9)	2·1% (1·9 to 2·4%)	9,571·5 (8,776·3 to 10,477·3)	1·5% (1·3 to 1·7%)	-34·2% (-39·1 to -29·6%)	-30·0% (-35·1 to -25·2%)
HIV/AIDS - Tuberculosis	48·6 (39·0 to 53·7)	92·3% (91·1 to 93·4%)	25·3 (20·3 to 30·0)	91·9% (90·6 to 93·0%)	-47·9% (-53·4 to -43·0%)	-0·5% (-0·7 to -0·3%)	2,494·0 (2,019·0 to 2,763·9)	92·6% (91·4 to 93·7%)	1,278·4 (1,028·9 to 1,513·9)	92·2% (90·9 to 93·3%)	-48·7% (-53·9 to -44·2%)	-0·5% (-0·6 to -0·2%)
HIV/AIDS resulting in other diseases	134·5 (122·4 to 148·8)	92·4% (91·1 to 93·4%)	99·1 (90·3 to 108·1)	92·0% (90·6 to 93·2%)	-26·3% (-32·3 to -19·6%)	-0·4% (-1·1 to -0·1%)	7,065·0 (6,473·1 to 7,771·7)	92·6% (91·3 to 93·7%)	5,186·7 (4,728·7 to 5,679·7)	92·3% (90·9 to 93·4%)	-26·6% (-32·1 to -20·5%)	-0·4% (-1·0 to -0·1%)
Syphilis	42·1 (25·6 to 64·1)	100·0% (100·0 to 100·0%)	19·0 (11·7 to 28·1)	100·0% (100·0 to 100·0%)	-55·0% (-62·3 to -46·1%)	-	3,474·8 (2,048·5 to 5,328·7)	100·0% (100·0 to 100·0%)	1,560·8 (931·2 to 2,375·7)	100·0% (100·0 to 100·0%)	-55·1% (-62·4 to -45·9%)	-
Chlamydial infection	0·1 (0·0 to 0·1)	100·0% (100·0 to 100·0%)	0·0 (0·0 to 0·1)	100·0% (100·0 to 100·0%)	-11·9% (-33·4 to 12·1%)	-	29·0 (18·3 to 44·5)	100·0% (100·0 to 100·0%)	33·1 (20·2 to 51·8)	100·0% (100·0 to 100·0%)	13·9% (6·7 to 21·0%)	-
Gonococcal infection	0·4 (0·3 to 0·5)	100·0% (100·0 to 100·0%)	0·3 (0·2 to 0·4)	100·0% (100·0 to 100·0%)	-28·8% (-44·2 to -12·4%)	-	124·2 (74·9 to 193·3)	100·0% (100·0 to 100·0%)	143·3 (83·6 to 231·1)	100·0% (100·0 to 100·0%)	15·4% (6·3 to 24·4%)	-
Trichomoniasis	-	-	-	-	-	-	29·6 (11·9 to 63·1)	100·0% (100·0 to 100·0%)	35·0 (14·1 to 74·3)	100·0% (100·0 to 100·0%)	18·1% (15·8 to 20·4%)	-
Genital herpes	-	-	-	-	-	-	35·0 (11·1 to 82·4)	100·0% (100·0 to 100·0%)	45·7 (15·6 to 104·2)	100·0% (100·0 to 100·0%)	30·6% (20·3 to 55·4%)	-
Other sexually transmitted diseases	0·2 (0·1 to 0·2)	100·0% (100·0 to 100·0%)	0·1 (0·1 to 0·2)	100·0% (100·0 to 100·0%)	-30·8% (-44·9 to -15·3%)	-	21·7 (16·7 to 28·1)	100·0% (100·0 to 100·0%)	21·8 (16·0 to 29·0)	100·0% (100·0 to 100·0%)	0·7% (-6·6 to 7·7%)	-
Cervical cancer	41·7 (37·9 to 47·4)	100·0% (100·0 to 100·0%)	43·2 (39·5 to 47·1)	100·0% (100·0 to 100·0%)	3·6% (-13·8 to 16·2%)	-	1,267·1 (1,154·1 to 1,425·3)	100·0% (100·0 to 100·0%)	1,266·7 (1,143·6 to 1,381·1)	100·0% (100·0 to 100·0%)	0·0% (-16·2 to 12·5%)	-
**Low physical activity: All causes**	237·1 (180·6 to 298·7)	1·9% (1·5 to 2·4%)	323·3 (243·7 to 405·3)	2·5% (1·9 to 3·2%)	36·3% (30·8 to 42·5%)	30·5% (26·5 to 34·7%)	5,921·5 (4,483·9 to 7,490·7)	0·9% (0·7 to 1·1%)	7,791·0 (5,838·8 to 9,877·1)	1·2% (0·9 to 1·5%)	31·6% (26·3 to 37·2%)	39·8% (34·9 to 45·0%)
Colon and rectum cancer	5·0 (3·3 to 6·8)	11·3% (7·6 to 15·1%)	7·3 (4·9 to 9·9)	11·5% (7·7 to 15·4%)	46·4% (35·0 to 57·6%)	1·8% (0·3 to 3·4%)	121·3 (79·3 to 165·7)	10·0% (6·6 to 13·7%)	171·0 (110·3 to 234·5)	10·3% (6·7 to 13·9%)	41·0% (30·1 to 52·0%)	2·3% (0·4 to 4·3%)
Breast cancer	3·4 (2·4 to 4·5)	6·5% (4·6 to 8·4%)	4·7 (3·3 to 6·1)	6·6% (4·7 to 8·5%)	37·8% (21·1 to 54·6%)	1·0% (-1·4 to 3·6%)	100·0 (69·4 to 133·2)	5·9% (4·2 to 7·7%)	136·2 (95·0 to 178·2)	6·0% (4·2 to 7·7%)	36·3% (20·2 to 53·4%)	1·3% (-1·4 to 4·0%)
Ischaemic heart disease	145·1 (95·4 to 199·2)	9·4% (6·1 to 12·8%)	192·5 (125·0 to 265·1)	9·3% (6·0 to 12·7%)	32·7% (26·6 to 39·2%)	-0·9% (-2·7 to 0·8%)	3,508·5 (2,251·0 to 4,872·6)	9·6% (6·1 to 13·2%)	4,510·2 (2,875·9 to 6,281·5)	9·6% (6·1 to 13·3%)	28·6% (22·1 to 35·4%)	0·1% (-2·1 to 2·0%)
Ischaemic stroke	43·5 (23·8 to 65·8)	10·7% (5·8 to 16·0%)	53·4 (29·0 to 80·2)	10·7% (5·8 to 15·8%)	22·7% (16·1 to 30·7%)	-0·5% (-2·5 to 1·7%)	858·5 (455·2 to 1,317·5)	11·2% (5·9 to 16·8%)	1,020·2 (545·3 to 1,550·0)	11·2% (6·0 to 16·8%)	18·8% (12·1 to 26·1%)	0·4% (-1·9 to 2·6%)
Diabetes mellitus	40·0 (30·5 to 50·2)	13·9% (10·6 to 17·5%)	65·3 (49·4 to 81·8)	14·4% (11·0 to 18·1%)	63·1% (53·6 to 72·4%)	3·5% (1·7 to 5·3%)	1,333·1 (963·2 to 1,770·8)	11·7% (8·7 to 14·9%)	1,953·3 (1,430·8 to 2,562·4)	12·2% (9·1 to 15·5%)	46·5% (40·7 to 52·6%)	4·6% (3·2 to 6·1%)
**Metabolic risks: All causes**	2,690·8 (2,548·1 to 2,833·1)	21·8% (20·8 to 22·8%)	3,621·2 (3,420·1 to 3,817·8)	28·1% (26·9 to 29·5%)	34·6% (29·8 to 39·8%)	28·8% (26·5 to 31·2%)	72,789·6 (68,648·9 to 77,394·4)	10·6% (10·1 to 11·2%)	93,617·2 (87,826·8 to 99,661·0)	14·5% (13·6 to 15·4%)	28·6% (24·4 to 33·5%)	36·7% (33·0 to 40·4%)
**High fasting plasma glucose: All causes**	1,023·8 (901·4 to 1,175·4)	8·3% (7·3 to 9·5%)	1,451·4 (1,277·6 to 1,681·4)	11·3% (10·0 to 12·9%)	41·8% (36·1 to 47·7%)	35·7% (31·9 to 39·3%)	30,488·3 (26,793·8 to 34,426·1)	4·4% (4·0 to 5·0%)	40,730·2 (35,828·0 to 46,014·4)	6·3% (5·7 to 7·1%)	33·6% (28·9 to 38·5%)	42·0% (37·8 to 45·9%)
Tuberculosis	69·9 (43·9 to 99·6)	10·5% (6·7 to 14·6%)	60·0 (37·1 to 87·1)	11·0% (7·0 to 15·3%)	-14·1% (-25·9 to -4·1%)	5·0% (1·1 to 8·7%)	2,309·4 (1,472·9 to 3,173·8)	9·4% (6·1 to 12·8%)	1,948·0 (1,228·0 to 2,740·5)	10·0% (6·6 to 13·8%)	-15·6% (-26·5 to -7·0%)	6·8% (1·9 to 11·4%)
Ischaemic heart disease	340·2 (237·2 to 466·8)	22·0% (15·4 to 30·1%)	489·9 (344·0 to 669·5)	23·6% (16·5 to 32·3%)	44·0% (37·0 to 51·5%)	7·6% (5·3 to 10·3%)	8,149·7 (5,699·7 to 10,974·4)	22·3% (15·7 to 29·3%)	11,365·7 (8,074·0 to 15,203·3)	24·2% (17·2 to 31·8%)	39·5% (32·8 to 46·2%)	8·6% (6·7 to 10·5%)
Ischaemic stroke	82·3 (49·3 to 138·2)	20·3% (12·0 to 33·6%)	108·4 (64·8 to 178·3)	21·7% (13·0 to 36·6%)	31·8% (23·5 to 42·1%)	6·9% (3·7 to 12·3%)	1,637·4 (1,047·9 to 2,597·8)	21·3% (13·6 to 32·3%)	2,098·7 (1,334·5 to 3,217·1)	23·1% (14·9 to 34·7%)	28·2% (20·5 to 36·8%)	8·3% (6·4 to 11·5%)
Hemorrhagic stroke	104·0 (71·9 to 141·0)	20·8% (14·6 to 27·9%)	133·7 (94·2 to 182·0)	22·5% (16·0 to 30·1%)	28·6% (21·3 to 38·4%)	8·3% (6·3 to 11·0%)	2,864·2 (1,896·9 to 4,073·5)	20·7% (13·8 to 29·3%)	3,629·8 (2,438·5 to 5,136·0)	23·0% (15·4 to 32·0%)	26·7% (20·0 to 35·2%)	11·0% (8·7 to 13·1%)
Diabetes mellitus	287·5 (271·1 to 303·2)	100·0% (100·0 to 100·0%)	453·1 (419·3 to 485·9)	100·0% (100·0 to 100·0%)	57·6% (48·5 to 66·7%)	-	11,397·9 (9,846·5 to 13,157·2)	100·0% (100·0 to 100·0%)	15,967·6 (13,760·4 to 18,410·5)	100·0% (100·0 to 100·0%)	40·1% (35·1 to 45·6%)	-
Chronic kidney disease due to diabetes mellitus	67·6 (57·3 to 74·6)	100·0% (100·0 to 100·0%)	95·0 (80·6 to 106·1)	100·0% (100·0 to 100·0%)	40·6% (28·7 to 53·3%)	-	2,095·8 (1,807·6 to 2,306·9)	100·0% (100·0 to 100·0%)	2,774·8 (2,361·1 to 3,091·0)	100·0% (100·0 to 100·0%)	32·4% (22·6 to 42·1%)	-
Chronic kidney disease due to hypertension	47·1 (33·3 to 61·1)	40·1% (29·0 to 50·4%)	72·3 (51·3 to 93·2)	43·8% (32·0 to 54·5%)	53·6% (40·5 to 67·0%)	9·2% (7·8 to 10·8%)	1,120·7 (789·2 to 1,455·0)	34·3% (24·7 to 43·6%)	1,633·2 (1,149·6 to 2,099·6)	39·1% (28·5 to 49·1%)	45·7% (33·5 to 58·2%)	14·1% (12·1 to 16·2%)
Chronic kidney disease due to glomerulonephritis	24·3 (17·0 to 31·7)	35·7% (25·7 to 45·4%)	37·1 (26·3 to 48·1)	40·6% (29·6 to 50·9%)	52·9% (39·1 to 66·2%)	13·6% (11·6 to 15·7%)	747·9 (518·8 to 986·7)	28·2% (20·1 to 36·2%)	1,074·6 (743·3 to 1,398·4)	33·9% (24·5 to 42·7%)	43·7% (32·3 to 54·9%)	20·3% (17·1 to 23·5%)
Chronic kidney disease due to other causes	0·9 (0·6 to 1·1)	23·8% (17·0 to 30·4%)	1·4 (1·0 to 1·8)	31·7% (22·9 to 39·8%)	61·8% (47·1 to 75·9%)	33·1% (27·4 to 39·1%)	147·1 (96·1 to 206·5)	27·1% (19·3 to 35·2%)	209·5 (137·7 to 292·3)	32·6% (23·5 to 41·7%)	42·4% (38·1 to 46·9%)	20·5% (16·8 to 24·9%)
**High total cholesterol: All causes**	648·0 (511·8 to 809·8)	5·3% (4·2 to 6·6%)	882·4 (704·8 to 1,103·1)	6·9% (5·5 to 8·5%)	36·2% (30·1 to 43·2%)	30·4% (26·4 to 34·9%)	17,502·8 (14,295·9 to 21,147·8)	2·6% (2·1 to 3·1%)	23,275·7 (19,225·6 to 28,028·5)	3·6% (3·0 to 4·3%)	33·0% (27·0 to 40·1%)	41·3% (36·0 to 47·4%)
Ischaemic heart disease	583·5 (459·0 to 715·8)	37·7% (29·9 to 46·4%)	800·1 (634·3 to 990·2)	38·6% (30·9 to 47·4%)	37·1% (30·7 to 44·1%)	2·4% (0·7 to 4·4%)	15,979·0 (13,160·1 to 19,221·6)	43·7% (36·4 to 51·7%)	21,371·8 (17,617·8 to 25,619·4)	45·5% (38·0 to 53·6%)	33·7% (27·6 to 40·9%)	4·1% (2·9 to 5·8%)
Ischaemic stroke	64·5 (32·0 to 124·1)	15·9% (8·1 to 30·1%)	82·3 (40·9 to 158·2)	16·4% (8·3 to 31·5%)	27·6% (18·2 to 38·0%)	3·5% (-0·9 to 7·6%)	1,523·8 (948·7 to 2,497·5)	19·8% (12·8 to 31·6%)	1,903·9 (1,201·6 to 3,098·9)	20·9% (13·5 to 33·0%)	24·9% (16·2 to 34·4%)	5·6% (2·8 to 8·6%)
**High systolic blood pressure: All causes**	1,571·1 (1,401·9 to 1,740·2)	12·8% (11·5 to 14·1%)	2,097·0 (1,879·6 to 2,319·9)	16·3% (14·6 to 18·0%)	33·5% (28·3 to 38·9%)	27·8% (25·0 to 30·7%)	38,279·7 (34,298·0 to 42,395·9)	5·6% (5·0 to 6·2%)	49,198·4 (44,123·9 to 54,512·3)	7·6% (6·7 to 8·5%)	28·5% (23·4 to 33·7%)	36·6% (32·2 to 40·9%)
Rheumatic heart disease	27·4 (19·1 to 39·9)	21·2% (14·6 to 32·6%)	32·5 (23·0 to 46·7)	22·7% (15·8 to 35·1%)	18·3% (8·3 to 28·3%)	7·2% (4·5 to 10·6%)	978·2 (633·6 to 1,438·4)	18·3% (11·8 to 27·4%)	1,110·7 (730·7 to 1,593·5)	20·3% (13·3 to 30·5%)	13·5% (5·1 to 22·3%)	11·1% (8·0 to 15·0%)
Ischaemic heart disease	753·3 (625·6 to 877·8)	48·6% (40·5 to 56·1%)	1,037·9 (855·8 to 1,202·0)	50·1% (41·8 to 57·8%)	37·8% (31·9 to 44·1%)	2·9% (1·4 to 4·5%)	17,518·0 (14,712·2 to 20,238·4)	47·9% (40·4 to 54·5%)	23,405·4 (19,728·0 to 26,856·6)	49·8% (42·3 to 56·5%)	33·6% (27·7 to 40·0%)	4·0% (2·9 to 5·5%)
Ischaemic stroke	181·7 (140·0 to 227·0)	44·8% (34·8 to 53·6%)	231·6 (178·1 to 287·6)	46·3% (36·6 to 55·6%)	27·4% (19·5 to 36·4%)	3·3% (1·0 to 6·5%)	3,566·2 (2,792·2 to 4,362·3)	46·4% (36·8 to 54·9%)	4,402·3 (3,455·4 to 5,312·8)	48·4% (39·1 to 56·7%)	23·4% (15·9 to 31·4%)	4·3% (2·8 to 6·4%)

Supplemental Results Table 1. All-age deaths, death PAFs, DALYs, DALY PAFs, and percent change for each risk factor and outcome for both sexes combined in 2005 and 2015 for South Asia.

Data in parenthesis are 95% uncertainty intervals. PAFs=population attributable fractions. DALYs=disability-adjusted life-years.

	2005 Deaths (in thousands)	2005 Deaths PAF	2015 Deaths (in thousands)	2015 Deaths PAF	Percent change deaths 2005-2015	Percent change deaths PAF 2005-2015	2005 DALYs (in thousands)	2005 DALYs PAF	2015 DALYs (in thousands)	2015 DALYs PAF	Percent change DALYs 2005-2015	Percent change DALYs PAF 2005-2015
Hemorrhagic stroke	257·9 (205·2 to 307·9)	51·5% (42·0 to 60·4%)	319·9 (256·6 to 378·5)	53·8% (44·3 to 62·9%)	24·1% (16·7 to 34·3%)	4·5% (2·7 to 6·8%)	6,913·8 (5,504·0 to 8,237·8)	50·1% (40·4 to 58·5%)	8,418·5 (6,780·6 to 10,022·3)	53·4% (43·5 to 61·8%)	21·8% (15·2 to 30·2%)	6·7% (5·2 to 8·6%)
Hypertensive heart disease	167·0 (149·6 to 197·3)	100·0% (100·0 to 100·0%)	210·0 (187·5 to 238·9)	100·0% (100·0 to 100·0%)	25·7% (14·1 to 36·7%)	-	4,089·9 (3,696·1 to 4,738·8)	100·0% (100·0 to 100·0%)	4,912·0 (4,410·7 to 5,558·5)	100·0% (100·0 to 100·0%)	20·1% (10·1 to 29·7%)	-
Cardiomyopathy and myocarditis	3·8 (2·7 to 4·9)	24·8% (18·7 to 31·4%)	5·0 (3·7 to 6·4)	27·7% (21·0 to 34·8%)	34·5% (19·7 to 47·7%)	11·6% (8·3 to 15·2%)	143·6 (95·9 to 200·5)	20·3% (13·8 to 27·2%)	189·0 (131·4 to 255·6)	23·7% (16·3 to 31·5%)	31·6% (17·7 to 44·2%)	16·7% (12·3 to 21·4%)
Atrial fibrillation and flutter	4·1 (3·0 to 5·6)	29·6% (23·5 to 35·9%)	6·3 (4·6 to 8·7)	30·7% (24·4 to 37·4%)	56·1% (48·7 to 63·4%)	3·7% (1·2 to 6·2%)	133·9 (102·5 to 175·8)	35·6% (31·1 to 40·6%)	194·0 (148·4 to 253·8)	37·0% (32·3 to 42·1%)	44·9% (41·7 to 48·4%)	3·9% (2·5 to 5·4%)
Aortic aneurysm	4·0 (3·1 to 5·1)	31·2% (25·4 to 36·7%)	6·4 (5·2 to 7·7)	32·4% (26·3 to 38·2%)	60·5% (43·7 to 76·4%)	3·7% (1·8 to 5·9%)	101·7 (80·4 to 128·9)	31·3% (26·2 to 37·1%)	158·3 (130·8 to 189·7)	33·0% (27·7 to 39·0%)	55·7% (39·4 to 71·1%)	5·3% (3·3 to 7·2%)
Peripheral vascular disease	0·2 (0·2 to 0·3)	23·0% (17·3 to 29·1%)	0·5 (0·3 to 0·6)	23·8% (17·8 to 30·4%)	107·7% (76·4 to 148·8%)	3·3% (0·6 to 6·4%)	17·5 (9·7 to 29·9)	24·9% (19·2 to 30·8%)	27·6 (16·1 to 45·4)	25·8% (19·9 to 31·7%)	57·7% (50·6 to 71·2%)	3·7% (1·8 to 5·8%)
Endocarditis	1·2 (0·8 to 1·7)	25·2% (18·9 to 31·6%)	1·7 (1·1 to 2·3)	27·8% (21·1 to 34·5%)	43·2% (32·5 to 59·1%)	10·1% (6·1 to 14·9%)	40·5 (22·1 to 60·9)	20·4% (14·0 to 27·6%)	56·8 (32·2 to 84·1)	23·4% (16·1 to 30·9%)	40·3% (30·1 to 53·9%)	15·1% (9·2 to 22·3%)
Other cardiovascular and circulatory diseases	5·3 (4·5 to 6·2)	34·9% (30·3 to 39·6%)	7·8 (6·6 to 9·2)	35·8% (31·0 to 40·5%)	48·8% (38·3 to 60·0%)	2·5% (-0·4 to 5·1%)	126·9 (109·2 to 147·2)	35·5% (31·2 to 40·1%)	180·1 (153·8 to 209·0)	37·2% (33·0 to 41·8%)	42·0% (33·9 to 50·7%)	4·8% (3·0 to 6·7%)
Chronic kidney disease due to diabetes mellitus	25·8 (17·7 to 34·1)	38·3% (26·7 to 48·9%)	38·5 (26·5 to 50·8)	40·5% (28·7 to 51·5%)	48·9% (37·9 to 61·9%)	5·9% (4·3 to 7·8%)	674·7 (459·2 to 898·4)	32·2% (22·0 to 41·7%)	965·5 (655·6 to 1,296·8)	34·8% (24·2 to 44·7%)	43·1% (33·3 to 54·0%)	8·1% (6·4 to 10·1%)
Chronic kidney disease due to hypertension	117·3 (99·4 to 127·4)	100·0% (100·0 to 100·0%)	165·1 (140·6 to 182·6)	100·0% (100·0 to 100·0%)	40·7% (28·7 to 53·0%)	-	3,266·2 (2,757·4 to 3,554·5)	100·0% (100·0 to 100·0%)	4,172·5 (3,523·8 to 4,605·2)	100·0% (100·0 to 100·0%)	27·7% (17·0 to 38·6%)	-
Chronic kidney disease due to glomerulonephritis	21·4 (14·5 to 28·4)	31·4% (22·2 to 41·3%)	32·5 (22·3 to 42·8)	35·5% (25·3 to 46·0%)	52·0% (40·0 to 65·3%)	12·9% (10·7 to 15·6%)	591·3 (399·3 to 802·6)	22·3% (15·3 to 30·0%)	840·0 (563·7 to 1,126·8)	26·5% (18·2 to 35·5%)	42·1% (32·4 to 52·9%)	19·0% (15·6 to 22·5%)
Chronic kidney disease due to other causes	0·8 (0·5 to 1·0)	21·1% (14·4 to 27·4%)	1·2 (0·8 to 1·6)	28·3% (19·7 to 36·3%)	63·6% (50·3 to 78·1%)	34·5% (28·2 to 40·8%)	117·4 (75·1 to 164·9)	21·6% (14·3 to 28·6%)	165·7 (106·7 to 231·4)	25·8% (17·4 to 33·8%)	41·1% (37·3 to 45·5%)	19·4% (15·2 to 23·9%)
**High body-mass index: All causes**	392·5 (229·8 to 584·5)	3·2% (1·9 to 4·8%)	607·5 (370·9 to 887·1)	4·7% (2·9 to 6·9%)	54·8% (44·9 to 69·6%)	48·2% (40·1 to 60·3%)	13,227·6 (7,762·1 to 19,388·3)	1·9% (1·1 to 2·8%)	20,234·9 (12,537·0 to 28,965·4)	3·1% (1·9 to 4·5%)	53·0% (43·7 to 66·9%)	62·6% (53·2 to 76·6%)
Oesophageal cancer	4·5 (1·3 to 8·6)	9·1% (2·7 to 17·1%)	7·1 (2·2 to 13·2)	11·1% (3·5 to 20·7%)	56·8% (34·5 to 84·8%)	22·5% (6·8 to 42·7%)	124·7 (36·6 to 234·4)	9·2% (2·8 to 17·4%)	195·2 (60·4 to 363·6)	11·6% (3·6 to 21·4%)	56·6% (34·3 to 87·0%)	25·9% (9·9 to 47·4%)
Colon and rectum cancer	1·3 (0·6 to 2·1)	2·9% (1·4 to 4·8%)	2·3 (1·2 to 3·7)	3·6% (1·8 to 5·7%)	78·2% (60·8 to 100·7%)	23·8% (16·2 to 35·2%)	35·9 (17·9 to 59·8)	3·0% (1·5 to 5·0%)	63·1 (33·5 to 101·3)	3·8% (2·0 to 6·0%)	75·8% (57·3 to 98·7%)	27·4% (19·0 to 39·9%)
Liver cancer due to hepatitis B	0·8 (0·3 to 1·5)	5·7% (2·1 to 11·3%)	1·2 (0·4 to 2·3)	7·1% (2·7 to 13·6%)	53·7% (35·4 to 80·2%)	23·7% (15·5 to 35·3%)	23·0 (8·4 to 46·2)	5·6% (2·0 to 11·1%)	35·3 (13·1 to 68·8)	7·1% (2·6 to 13·5%)	53·3% (35·0 to 79·7%)	27·0% (18·1 to 39·5%)
Liver cancer due to hepatitis C	0·6 (0·2 to 1·1)	6·2% (2·4 to 11·4%)	0·9 (0·3 to 1·6)	7·7% (3·1 to 13·7%)	52·3% (33·0 to 81·1%)	23·3% (12·4 to 39·6%)	13·8 (5·2 to 26·3)	6·6% (2·6 to 12·1%)	21·1 (8·2 to 38·5)	8·4% (3·4 to 14·7%)	53·1% (31·6 to 84·4%)	26·1% (14·1 to 43·9%)
Liver cancer due to alcohol use	0·4 (0·1 to 0·7)	6·1% (2·1 to 12·4%)	0·6 (0·2 to 1·2)	7·4% (2·7 to 14·7%)	69·2% (46·9 to 98·5%)	21·8% (13·5 to 33·8%)	8·5 (3·0 to 17·7)	6·3% (2·2 to 12·9%)	14·5 (5·3 to 29·2)	7·7% (2·8 to 15·3%)	69·8% (46·9 to 100·3%)	23·7% (15·0 to 36·0%)
Liver cancer due to other causes	0·4 (0·1 to 0·8)	5·8% (2·2 to 10·7%)	0·6 (0·2 to 1·1)	7·1% (2·8 to 12·7%)	50·1% (33·1 to 76·2%)	22·5% (12·7 to 36·0%)	10·4 (3·9 to 20·2)	5·8% (2·2 to 10·7%)	15·5 (6·2 to 28·8)	7·3% (2·9 to 13·1%)	49·8% (31·5 to 75·9%)	26·4% (15·4 to 41·6%)
Gallbladder and biliary tract cancer	0·8 (0·4 to 1·4)	7·4% (3·6 to 12·4%)	1·1 (0·5 to 1·8)	8·5% (4·3 to 14·0%)	36·5% (20·7 to 57·9%)	15·3% (7·5 to 27·0%)	21·6 (10·5 to 36·9)	7·6% (3·7 to 12·7%)	29·2 (14·6 to 47·9)	8·9% (4·5 to 14·6%)	35·1% (18·1 to 57·1%)	17·2% (8·4 to 29·6%)
Pancreatic cancer	0·5 (0·2 to 1·0)	2·1% (0·7 to 4·1%)	0·9 (0·3 to 1·6)	2·5% (0·8 to 4·8%)	64·6% (51·6 to 79·9%)	19·9% (11·7 to 30·3%)	13·0 (4·0 to 24·9)	2·2% (0·7 to 4·2%)	21·3 (6·8 to 40·0)	2·6% (0·9 to 5·0%)	64·2% (50·6 to 80·6%)	22·3% (13·5 to 32·8%)
Breast cancer	0·5 (0·1 to 1·2)	1·0% (0·2 to 2·3%)	0·9 (0·2 to 2·0)	1·2% (0·3 to 2·8%)	62·0% (16·2 to 144·8%)	18·7% (-16·7 to 75·3%)	3·6 (-10·9 to 17·6)	0·2% (-0·6 to 1·1%)	5·3 (-17·4 to 28·9)	0·2% (-0·8 to 1·3%)	47·5% (-675·1 to 624·3%)	9·5% (-531·0 to 444·4%)
Uterine cancer	1·9 (1·0 to 3·0)	18·1% (9·8 to 27·9%)	2·5 (1·5 to 3·8)	21·7% (12·8 to 31·9%)	35·6% (13·9 to 70·8%)	20·2% (9·6 to 40·6%)	50·7 (27·4 to 80·3)	18·7% (10·2 to 28·8%)	69·2 (39·9 to 104·7)	23·0% (13·8 to 33·4%)	36·5% (12·8 to 73·0%)	23·1% (11·6 to 44·9%)
Ovarian cancer	0·2 (0·0 to 0·5)	1·2% (0·0 to 3·1%)	0·3 (0·0 to 0·7)	1·5% (0·0 to 3·6%)	63·7% (46·7 to 87·6%)	19·7% (12·2 to 32·8%)	5·6 (-0·1 to 14·3)	1·2% (0·0 to 3·1%)	9·3 (-0·3 to 23·0)	1·5% (0·0 to 3·7%)	65·5% (47·3 to 92·1%)	22·7% (14·1 to 37·8%)
Kidney cancer	0·4 (0·2 to 0·6)	6·9% (3·7 to 10·7%)	0·7 (0·4 to 1·1)	8·5% (4·9 to 12·9%)	80·3% (57·8 to 111·0%)	23·2% (12·6 to 36·8%)	11·6 (6·3 to 18·4)	6·4% (3·5 to 9·9%)	21·1 (11·8 to 31·9)	8·2% (4·7 to 12·3%)	81·5% (57·7 to 112·5%)	28·2% (16·3 to 43·3%)
Thyroid cancer	0·1 (0·1 to 0·2)	4·2% (2·0 to 7·1%)	0·2 (0·1 to 0·3)	5·1% (2·5 to 8·4%)	46·0% (30·3 to 65·0%)	20·4% (10·9 to 32·6%)	3·1 (1·5 to 5·3)	4·3% (2·1 to 7·3%)	4·7 (2·4 to 8·0)	5·3% (2·7 to 8·9%)	52·6% (35·8 to 72·9%)	23·3% (13·6 to 36·5%)
Ischaemic heart disease	142·6 (81·1 to 217·8)	9·2% (5·2 to 14·0%)	225·2 (133·5 to 335·6)	10·9% (6·4 to 16·0%)	58·0% (47·3 to 74·1%)	17·9% (11·7 to 27·8%)	4,017·8 (2,254·5 to 6,132·0)	11·0% (6·2 to 16·8%)	6,297·3 (3,768·4 to 9,377·7)	13·4% (8·0 to 19·7%)	56·7% (46·0 to 72·5%)	22·0% (15·5 to 32·1%)
Ischaemic stroke	27·9 (15·0 to 43·9)	6·9% (3·8 to 10·6%)	38·8 (22·1 to 59·5)	7·7% (4·5 to 11·7%)	38·9% (26·2 to 55·8%)	12·6% (4·4 to 22·7%)	732·4 (396·7 to 1,137·3)	9·5% (5·4 to 14·6%)	1,025·0 (590·1 to 1,552·9)	11·3% (6·7 to 16·6%)	40·0% (27·8 to 56·5%)	18·2% (10·5 to 28·7%)
Hemorrhagic stroke	73·6 (42·0 to 111·5)	14·7% (8·5 to 22·1%)	101·2 (61·7 to 146·7)	17·0% (10·4 to 24·7%)	37·5% (27·0 to 52·4%)	15·8% (8·8 to 26·4%)	2,463·5 (1,424·2 to 3,688·0)	17·8% (10·4 to 26·5%)	3,397·8 (2,094·2 to 4,883·1)	21·5% (13·4 to 30·6%)	37·9% (27·1 to 52·9%)	20·8% (13·3 to 31·2%)
Hypertensive heart disease	35·0 (19·5 to 54·8)	21·0% (11·8 to 32·6%)	50·6 (29·1 to 76·1)	24·1% (14·2 to 36·5%)	44·4% (29·1 to 62·5%)	14·7% (7·8 to 24·7%)	953·2 (543·2 to 1,436·0)	23·3% (13·4 to 34·7%)	1,356·2 (814·1 to 1,982·9)	27·6% (16·9 to 39·7%)	42·3% (28·3 to 59·6%)	18·4% (12·0 to 28·6%)
Diabetes mellitus	66·3 (40·0 to 95·8)	23·1% (14·0 to 33·7%)	115·3 (72·3 to 165·1)	25·4% (16·1 to 36·0%)	74·0% (60·7 to 91·3%)	10·4% (4·7 to 18·9%)	3,311·4 (2,004·9 to 4,749·8)	29·0% (18·2 to 40·6%)	5,364·2 (3,432·5 to 7,504·2)	33·6% (22·2 to 45·4%)	62·0% (53·2 to 76·4%)	15·6% (10·4 to 24·5%)
Chronic kidney disease due to diabetes mellitus	10·4 (4·3 to 18·4)	15·3% (6·6 to 27·0%)	16·9 (7·3 to 29·4)	17·8% (8·1 to 30·7%)	63·7% (47·6 to 83·5%)	16·4% (8·5 to 27·4%)	304·8 (119·2 to 552·1)	14·6% (5·9 to 26·7%)	493·0 (203·2 to 883·4)	17·8% (7·5 to 31·3%)	61·7% (48·1 to 80·1%)	22·1% (15·1 to 33·5%)
Chronic kidney disease due to hypertension	15·6 (6·6 to 27·3)	13·3% (5·6 to 23·4%)	25·6 (11·2 to 43·3)	15·5% (6·8 to 26·7%)	64·8% (48·2 to 84·8%)	17·1% (9·1 to 28·0%)	399·1 (163·3 to 733·6)	12·2% (4·9 to 22·5%)	644·6 (266·2 to 1,155·8)	15·5% (6·5 to 27·5%)	61·5% (47·1 to 79·4%)	26·4% (19·0 to 37·8%)
Chronic kidney disease due to glomerulonephritis	7·9 (3·0 to 14·5)	11·6% (4·5 to 20·9%)	13·1 (5·1 to 23·6)	14·3% (5·7 to 25·2%)	66·0% (47·9 to 85·9%)	23·2% (14·1 to 35·4%)	253·4 (80·9 to 498·2)	9·5% (3·2 to 18·4%)	409·8 (142·8 to 767·5)	12·9% (4·5 to 24·0%)	61·7% (48·6 to 80·4%)	35·2% (26·7 to 48·6%)
Chronic kidney disease due to other causes	0·2 (0·1 to 0·4)	5·5% (1·7 to 11·0%)	0·3 (0·1 to 0·7)	7·9% (2·1 to 16·1%)	73·8% (35·2 to 100·0%)	42·7% (11·7 to 61·1%)	50·8 (17·1 to 98·9)	9·3% (3·4 to 17·7%)	80·4 (28·3 to 150·2)	12·5% (4·8 to 22·9%)	58·3% (49·4 to 72·3%)	34·0% (25·7 to 47·1%)
Osteoarthritis	-	-	-	-	-	-	153·1 (71·0 to 273·9)	9·4% (4·8 to 15·3%)	250·1 (120·7 to 431·0)	11·2% (6·1 to 17·6%)	63·4% (54·5 to 76·9%)	19·8% (14·1 to 29·2%)
Low back pain	-	-	-	-	-	-	239·4 (114·0 to 419·0)	2·1% (1·1 to 3·3%)	373·9 (186·8 to 643·0)	2·7% (1·5 to 4·1%)	56·1% (48·8 to 68·3%)	28·6% (22·8 to 38·2%)
**Low bone mineral density: All causes**	52·2 (49·0 to 55·9)	0·4% (0·4 to 0·5%)	69·9 (62·7 to 77·6)	0·5% (0·5 to 0·6%)	33·9% (23·3 to 45·9%)	28·2% (19·0 to 38·6%)	1,383·2 (1,215·8 to 1,565·9)	0·2% (0·2 to 0·2%)	1,759·4 (1,518·6 to 2,001·8)	0·3% (0·2 to 0·3%)	27·2% (19·6 to 35·3%)	35·2% (27·9 to 43·7%)
Pedestrian road injuries	10·3 (9·0 to 12·3)	7·4% (6·9 to 8·1%)	12·6 (10·8 to 15·4)	8·6% (8·0 to 9·4%)	22·7% (8·8 to 39·7%)	15·4% (9·1 to 21·4%)	220·7 (189·7 to 262·8)	3·5% (3·2 to 3·8%)	257·9 (218·8 to 313·0)	4·1% (3·7 to 4·5%)	16·9% (3·6 to 34·4%)	17·6% (10·5 to 24·6%)
Cyclist road injuries	0·4 (0·3 to 0·4)	8·1% (7·3 to 9·1%)	0·5 (0·4 to 0·7)	9·6% (8·6 to 10·9%)	46·1% (24·7 to 70·4%)	19·3% (9·4 to 29·7%)	11·4 (9·1 to 13·8)	4·6% (4·0 to 5·4%)	16·0 (12·6 to 19·5)	5·6% (4·9 to 6·5%)	40·1% (28·2 to 53·0%)	21·5% (12·4 to 30·5%)
Motorcyclist road injuries	5·1 (4·2 to 6·2)	6·9% (6·2 to 7·7%)	7·3 (6·1 to 8·5)	7·9% (7·2 to 8·6%)	43·0% (17·3 to 72·1%)	14·9% (6·6 to 23·7%)	138·5 (113·2 to 169·8)	3·8% (3·3 to 4·4%)	192·0 (157·2 to 224·9)	4·5% (3·9 to 5·1%)	38·6% (16·9 to 61·6%)	17·2% (7·9 to 26·3%)
Motor vehicle road injuries	7·0 (5·6 to 8·2)	7·8% (7·0 to 8·4%)	9·1 (7·2 to 10·7)	9·0% (8·1 to 9·7%)	30·6% (2·4 to 52·1%)	15·6% (9·6 to 23·2%)	186·3 (152·4 to 222·8)	4·2% (3·7 to 4·7%)	238·5 (190·7 to 280·2)	5·0% (4·4 to 5·6%)	28·0% (4·0 to 45·6%)	19·4% (12·3 to 28·7%)
Other road injuries	0·5 (0·4 to 0·6)	8·9% (7·6 to 10·7%)	0·6 (0·5 to 0·8)	9·9% (8·5 to 11·7%)	35·6% (13·5 to 65·6%)	12·0% (-6·2 to 29·6%)	13·2 (10·3 to 16·2)	4·6% (3·8 to 5·8%)	17·8 (14·0 to 22·0)	5·3% (4·4 to 6·5%)	35·1% (20·9 to 55·5%)	15·3% (-4·8 to 31·5%)

Supplemental Results Table 1. All-age deaths, death PAFs, DALYs, DALY PAFs, and percent change for each risk factor and outcome for both sexes combined in 2005 and 2015 for South Asia.

Data in parenthesis are 95% uncertainty intervals. PAFs=population attributable fractions. DALYs=disability-adjusted life-years.

	2005 Deaths (in thousands)	2005 Deaths PAF	2015 Deaths (in thousands)	2015 Deaths PAF	Percent change deaths 2005-2015	Percent change deaths PAF 2005-2015	2005 DALYs (in thousands)	2005 DALYs PAF	2015 DALYs (in thousands)	2015 DALYs PAF	Percent change DALYs 2005-2015	Percent change DALYs PAF 2005-2015
Other transport injuries	3·2 (2·5 to 4·4)	8·8% (8·2 to 9·6%)	4·0 (2·9 to 6·3)	9·9% (9·1 to 10·8%)	26·6% (7·7 to 47·7%)	11·6% (5·3 to 18·1%)	84·5 (67·8 to 113·8)	4·9% (4·5 to 5·4%)	104·0 (77·4 to 157·7)	5·6% (5·1 to 6·2%)	23·1% (5·8 to 41·2%)	14·0% (6·8 to 21·7%)
Falls	20·9 (19·0 to 24·7)	27·5% (25·5 to 29·9%)	29·6 (25·1 to 35·3)	32·4% (29·0 to 35·7%)	41·8% (24·5 to 62·2%)	17·7% (7·5 to 28·4%)	567·8 (476·5 to 673·3)	10·7% (9·7 to 11·7%)	751·5 (613·1 to 900·5)	12·7% (11·3 to 14·0%)	32·4% (24·8 to 41·3%)	17·8% (8·7 to 27·4%)
Other exposure to mechanical forces	3·5 (2·4 to 4·0)	8·6% (6·0 to 9·6%)	4·3 (2·8 to 4·9)	10·4% (7·4 to 11·7%)	22·8% (5·1 to 36·7%)	21·5% (7·6 to 32·1%)	105·6 (86·9 to 127·3)	3·7% (3·0 to 4·2%)	122·7 (97·7 to 147·6)	4·5% (3·7 to 5·0%)	16·2% (7·0 to 23·7%)	22·0% (12·9 to 30·0%)
Non-venomous animal contact	0·1 (0·1 to 0·2)	3·8% (3·2 to 4·4%)	0·1 (0·1 to 0·2)	4·9% (4·1 to 5·5%)	6·5% (-19·7 to 76·5%)	26·1% (11·2 to 42·4%)	5·8 (4·3 to 7·7)	1·9% (1·6 to 2·2%)	6·1 (4·3 to 8·5)	2·4% (2·0 to 2·7%)	6·1% (-6·2 to 33·5%)	25·2% (13·6 to 37·1%)
Assault by other means	1·3 (1·0 to 1·5)	3·1% (2·8 to 3·7%)	1·6 (1·2 to 2·0)	3·7% (3·2 to 4·3%)	25·3% (7·4 to 43·8%)	17·4% (7·6 to 26·9%)	35·5 (26·9 to 40·9)	1·6% (1·4 to 1·9%)	42·0 (30·3 to 50·5)	1·9% (1·7 to 2·2%)	18·6% (3·7 to 34·9%)	15·2% (4·9 to 24·7%)
Exposure to forces of nature	-	-	-	-	-	-	14·0 (6·6 to 22·3)	0·3% (0·1 to 0·5%)	10·9 (5·7 to 17·1)	1·4% (0·8 to 2·1%)	-21·9% (-35·7 to 4·2%)	392·1% (269·4 to 590·3%)
**Low glomerular filtration rate: All causes**	465·1 (415·1 to 511·1)	3·8% (3·4 to 4·1%)	637·1 (564·8 to 702·8)	5·0% (4·4 to 5·5%)	37·0% (30·5 to 44·7%)	31·2% (25·6 to 36·9%)	12,856·8 (11,515·1 to 14,038·7)	1·9% (1·7 to 2·0%)	16,342·5 (14,481·1 to 17,961·4)	2·5% (2·2 to 2·8%)	27·1% (20·7 to 34·2%)	35·1% (28·8 to 41·8%)
Ischaemic heart disease	126·6 (96·0 to 159·2)	8·2% (6·2 to 10·3%)	177·0 (134·9 to 223·2)	8·5% (6·4 to 10·7%)	39·9% (34·0 to 46·2%)	4·5% (2·5 to 6·6%)	2,600·1 (1,951·8 to 3,334·1)	7·1% (5·4 to 9·0%)	3,503·9 (2,655·9 to 4,451·8)	7·5% (5·6 to 9·4%)	34·8% (29·1 to 40·9%)	4·9% (3·2 to 6·8%)
Ischaemic stroke	39·3 (26·6 to 54·8)	9·7% (6·7 to 13·0%)	50·7 (34·1 to 70·2)	10·1% (6·9 to 13·8%)	29·1% (21·2 to 39·0%)	4·7% (1·8 to 7·4%)	677·8 (473·4 to 924·9)	8·8% (6·3 to 11·7%)	837·8 (581·1 to 1,141·1)	9·2% (6·5 to 12·3%)	23·6% (15·9 to 32·3%)	4·5% (2·0 to 6·9%)
Hemorrhagic stroke	42·6 (30·1 to 56·8)	8·5% (6·1 to 11·3%)	53·1 (36·7 to 71·3)	8·9% (6·3 to 11·9%)	24·7% (16·9 to 35·3%)	5·0% (2·5 to 7·6%)	985·7 (681·1 to 1,330·2)	7·1% (4·9 to 9·7%)	1,191·9 (817·1 to 1,606·2)	7·6% (5·2 to 10·2%)	20·9% (14·0 to 30·0%)	5·9% (3·5 to 8·7%)
Peripheral vascular disease	0·1 (0·1 to 0·2)	15·1% (9·7 to 21·0%)	0·3 (0·2 to 0·5)	15·5% (9·8 to 21·9%)	107·1% (74·3 to 147·7%)	3·0% (-1·2 to 6·0%)	11·9 (5·9 to 20·8)	16·9% (11·2 to 23·3%)	18·7 (9·6 to 32·0)	17·4% (11·4 to 24·1%)	57·1% (49·5 to 70·2%)	3·3% (1·5 to 4·8%)
Chronic kidney disease due to diabetes mellitus	67·6 (57·3 to 74·6)	100·0% (100·0 to 100·0%)	95·0 (80·6 to 106·1)	100·0% (100·0 to 100·0%)	40·6% (28·7 to 53·3%)	-	2,095·8 (1,807·6 to 2,306·9)	100·0% (100·0 to 100·0%)	2,774·8 (2,361·1 to 3,091·0)	100·0% (100·0 to 100·0%)	32·4% (22·6 to 42·1%)	-
Chronic kidney disease due to hypertension	117·3 (99·4 to 127·4)	100·0% (100·0 to 100·0%)	165·1 (140·6 to 182·6)	100·0% (100·0 to 100·0%)	40·7% (28·7 to 53·0%)	-	3,266·2 (2,757·4 to 3,554·5)	100·0% (100·0 to 100·0%)	4,172·5 (3,523·8 to 4,605·2)	100·0% (100·0 to 100·0%)	27·7% (17·0 to 38·6%)	-
Chronic kidney disease due to glomerulonephritis	67·9 (57·1 to 74·8)	100·0% (100·0 to 100·0%)	91·5 (77·3 to 103·1)	100·0% (100·0 to 100·0%)	34·6% (22·5 to 46·3%)	-	2,655·0 (2,260·0 to 2,906·7)	100·0% (100·0 to 100·0%)	3,170·4 (2,714·4 to 3,550·5)	100·0% (100·0 to 100·0%)	19·4% (10·0 to 28·6%)	-
Chronic kidney disease due to other causes	3·6 (3·1 to 4·0)	100·0% (100·0 to 100·0%)	4·4 (3·8 to 5·0)	100·0% (100·0 to 100·0%)	21·6% (11·1 to 32·0%)	-	542·2 (440·8 to 649·7)	100·0% (100·0 to 100·0%)	641·0 (512·5 to 774·8)	100·0% (100·0 to 100·0%)	18·2% (13·4 to 22·8%)	-
Gout	-	-	-	-	-	-	22·2 (14·9 to 30·7)	15·6% (14·1 to 17·1%)	31·6 (21·0 to 43·5)	16·6% (15·0 to 18·3%)	41·9% (38·0 to 45·9%)	6·9% (5·1 to 8·7%)

Supplemental Results Table 1. All-age deaths, death PAFs, DALYs, DALY PAFs, and percent change for each risk factor and outcome for both sexes combined in 2005 and 2015 for Southeast Asia, East Asia, and Oceania.

Data in parenthesis are 95% uncertainty intervals. PAFs=population attributable fractions. DALYs=disability-adjusted life-years.

	2005 Deaths (in thousands)	2005 Deaths PAF	2015 Deaths (in thousands)	2015 Deaths PAF	Percent change deaths 2005-2015	Percent change deaths PAF 2005-2015	2005 DALYs (in thousands)	2005 DALYs PAF	2015 DALYs (in thousands)	2015 DALYs PAF	Percent change DALYs 2005-2015	Percent change DALYs PAF 2005-2015
**All risk factors: All causes**	8,523·3<br>(8,166·9 to 8,908·1)	62·4%<br>(60·0 to 64·5%)	9,052·0<br>(8,597·6 to 9,519·6)	64·4%<br>(62·3 to 66·4%)	6·2%<br>(1·8 to 10·7%)	3·1%<br>(2·2 to 4·2%)	247,904·1<br>(232,379·3 to 264,040·6)	43·6%<br>(41·7 to 45·7%)	244,480·2<br>(227,333·0 to 263,844·7)	44·5%<br>(42·3 to 46·9%)	-1·4%<br>(-5·3 to 2·4%)	2·0%<br>(0·4 to 3·6%)
**Environmental/occupational risks: All causes**	2,845·8<br>(2,518·1 to 3,159·8)	20·8%<br>(18·4 to 23·2%)	2,594·7<br>(2,315·3 to 2,887·6)	18·5%<br>(16·5 to 20·5%)	-8·8%<br>(-13·2 to -4·4%)	-11·5%<br>(-14·1 to -8·7%)	81,278·6<br>(73,249·6 to 89,075·7)	14·3%<br>(12·8 to 15·8%)	69,941·4<br>(62,749·6 to 77,218·5)	12·7%<br>(11·5 to 14·0%)	-13·9%<br>(-17·8 to -10·1%)	-11·0%<br>(-13·6 to -8·2%)
**Unsafe water, sanitation, and handwashing: All causes**	209·8<br>(179·4 to 240·4)	1·5%<br>(1·3 to 1·8%)	158·0<br>(131·1 to 186·3)	1·1%<br>(0·9 to 1·3%)	-24·7%<br>(-31·9 to -17·6%)	-26·9%<br>(-32·9 to -20·7%)	10,575·4<br>(8,916·5 to 12,267·3)	1·9%<br>(1·5 to 2·2%)	6,461·3<br>(5,381·0 to 7,735·5)	1·2%<br>(1·0 to 1·4%)	-38·9%<br>(-45·2 to -31·3%)	-36·8%<br>(-43·2 to -29·0%)
**Unsafe water source: All causes**	124·1<br>(90·4 to 152·3)	0·9%<br>(0·7 to 1·1%)	88·1<br>(62·9 to 110·8)	0·6%<br>(0·5 to 0·8%)	-29·0%<br>(-37·4 to -20·3%)	-31·0%<br>(-38·7 to -23·2%)	6,749·2<br>(4,953·6 to 8,445·2)	1·2%<br>(0·9 to 1·5%)	4,237·4<br>(3,025·1 to 5,498·6)	0·8%<br>(0·6 to 1·0%)	-37·2%<br>(-44·9 to -28·3%)	-35·0%<br>(-42·8 to -25·7%)
Diarrhoeal diseases	101·1<br>(74·6 to 121·6)	80·3%<br>(59·4 to 92·3%)	70·1<br>(50·5 to 85·3)	79·6%<br>(58·2 to 91·7%)	-30·7%<br>(-39·3 to -21·0%)	-0·9%<br>(-3·5 to 1·1%)	5,165·2<br>(3,804·3 to 6,292·9)	80·8%<br>(60·1 to 92·4%)	3,025·7<br>(2,165·8 to 3,708·2)	79·8%<br>(58·7 to 91·8%)	-41·4%<br>(-49·4 to -31·8%)	-1·2%<br>(-3·7 to 0·3%)
Typhoid fever	18·8<br>(9·4 to 33·7)	80·0%<br>(59·0 to 92·0%)	15·0<br>(7·6 to 26·2)	79·3%<br>(58·0 to 91·5%)	-20·4%<br>(-29·0 to -9·5%)	-0·9%<br>(-3·1 to 0·7%)	1,298·9<br>(656·7 to 2,303·6)	80·1%<br>(59·1 to 92·0%)	1,007·6<br>(513·7 to 1,759·2)	79·3%<br>(58·1 to 91·5%)	-22·4%<br>(-31·7 to -10·3%)	-0·9%<br>(-3·1 to 0·8%)
Paratyphoid fever	4·1<br>(1·7 to 8·4)	80·2%<br>(59·3 to 92·0%)	3·0<br>(1·3 to 6·2)	79·2%<br>(57·8 to 91·4%)	-26·8%<br>(-34·4 to -17·3%)	-1·3%<br>(-3·6 to 0·0%)	285·2<br>(117·0 to 576·3)	80·2%<br>(59·3 to 92·0%)	204·0<br>(84·6 to 413·1)	79·2%<br>(57·8 to 91·4%)	-28·5%<br>(-36·5 to -18·1%)	-1·3%<br>(-3·5 to 0·0%)
**Unsafe sanitation: All causes**	77·2<br>(65·8 to 90·1)	0·6%<br>(0·5 to 0·7%)	42·6<br>(34·9 to 52·5)	0·3%<br>(0·2 to 0·4%)	-44·8%<br>(-54·1 to -35·0%)	-46·4%<br>(-54·8 to -37·3%)	4,142·7<br>(3,463·9 to 4,920·2)	0·7%<br>(0·6 to 0·9%)	2,026·4<br>(1,615·4 to 2,521·5)	0·4%<br>(0·3 to 0·5%)	-51·1%<br>(-59·1 to -41·9%)	-49·4%<br>(-57·5 to -39·9%)
Diarrhoeal diseases	63·4<br>(55·2 to 72·0)	50·4%<br>(46·2 to 54·3%)	34·1<br>(27·9 to 41·0)	38·7%<br>(32·7 to 44·5%)	-46·3%<br>(-55·6 to -35·9%)	-23·2%<br>(-33·3 to -13·6%)	3,193·3<br>(2,731·8 to 3,714·0)	49·9%<br>(46·0 to 53·8%)	1,449·8<br>(1,186·6 to 1,738·0)	38·3%<br>(32·8 to 43·6%)	-54·6%<br>(-62·4 to -45·6%)	-23·4%<br>(-32·2 to -15·1%)
Typhoid fever	11·2<br>(5·9 to 19·5)	47·7%<br>(43·6 to 51·3%)	7·1<br>(3·8 to 12·1)	37·3%<br>(32·6 to 42·1%)	-37·1%<br>(-46·6 to -26·0%)	-21·7%<br>(-29·5 to -13·6%)	773·2<br>(412·4 to 1,340·7)	47·7%<br>(43·5 to 51·3%)	474·4<br>(258·3 to 810·7)	37·4%<br>(32·6 to 42·2%)	-38·7%<br>(-48·6 to -26·9%)	-21·6%<br>(-29·5 to -13·4%)
Paratyphoid fever	2·5<br>(1·1 to 5·0)	49·5%<br>(45·9 to 53·0%)	1·5<br>(0·7 to 2·9)	39·5%<br>(34·9 to 43·9%)	-40·7%<br>(-48·4 to -31·3%)	-20·1%<br>(-26·6 to -14·0%)	176·2<br>(79·5 to 345·3)	49·6%<br>(46·0 to 53·1%)	102·2<br>(46·4 to 194·0)	39·7%<br>(34·9 to 44·1%)	-42·0%<br>(-49·7 to -32·2%)	-20·0%<br>(-26·6 to -13·7%)
**No handwashing with soap: All causes**	119·4<br>(92·0 to 143·8)	0·9%<br>(0·7 to 1·1%)	93·1<br>(71·2 to 114·4)	0·7%<br>(0·5 to 0·8%)	-22·0%<br>(-28·0 to -16·1%)	-24·3%<br>(-29·5 to -18·9%)	5,679·7<br>(4,410·4 to 6,805·1)	1·0%<br>(0·8 to 1·2%)	3,349·9<br>(2,617·6 to 4,107·3)	0·6%<br>(0·5 to 0·8%)	-41·0%<br>(-46·1 to -34·9%)	-39·0%<br>(-44·1 to -32·8%)
Diarrhoeal diseases	44·3<br>(34·7 to 54·9)	35·1%<br>(27·7 to 41·9%)	29·0<br>(21·9 to 36·3)	32·9%<br>(25·5 to 39·6%)	-34·5%<br>(-42·8 to -25·7%)	-6·3%<br>(-8·3 to -4·5%)	2,252·5<br>(1,727·9 to 2,800·6)	35·2%<br>(27·8 to 42·0%)	1,251·4<br>(951·8 to 1,576·7)	33·0%<br>(25·6 to 39·7%)	-44·4%<br>(-52·2 to -35·3%)	-6·3%<br>(-8·3 to -4·5%)
Typhoid fever	8·2<br>(4·2 to 14·6)	34·7%<br>(26·8 to 41·7%)	6·2<br>(3·2 to 10·9)	32·8%<br>(24·9 to 39·8%)	-24·2%<br>(-32·3 to -13·5%)	-5·6%<br>(-7·5 to -3·9%)	563·0<br>(294·9 to 1,008·6)	34·7%<br>(26·8 to 41·7%)	415·7<br>(218·3 to 725·8)	32·8%<br>(24·9 to 39·8%)	-26·1%<br>(-34·8 to -14·4%)	-5·6%<br>(-7·5 to -3·9%)
Paratyphoid fever	1·8<br>(0·8 to 3·6)	34·9%<br>(27·2 to 41·9%)	1·3<br>(0·6 to 2·5)	33·0%<br>(25·7 to 40·1%)	-29·7%<br>(-37·1 to -20·8%)	-5·3%<br>(-7·1 to -3·7%)	124·1<br>(54·6 to 250·5)	34·9%<br>(27·2 to 41·9%)	85·2<br>(38·4 to 168·6)	33·0%<br>(25·7 to 40·1%)	-31·3%<br>(-38·9 to -21·4%)	-5·3%<br>(-7·1 to -3·7%)
**Air pollution: All causes**	2,324·8<br>(1,984·8 to 2,673·9)	17·0%<br>(14·5 to 19·6%)	2,070·8<br>(1,790·0 to 2,383·6)	14·7%<br>(12·7 to 16·8%)	-10·9%<br>(-15·5 to -6·0%)	-13·5%<br>(-16·7 to -10·1%)	53,518·3<br>(46,084·9 to 61,454·9)	9·4%<br>(7·9 to 11·0%)	43,454·1<br>(37,522·9 to 49,597·5)	7·9%<br>(6·6 to 9·3%)	-18·8%<br>(-23·3 to -14·3%)	-16·0%<br>(-19·8 to -12·2%)
**Ambient particulate matter pollution: All causes**	1,391·6<br>(1,201·9 to 1,597·5)	10·2%<br>(8·8 to 11·7%)	1,399·6<br>(1,202·8 to 1,599·3)	10·0%<br>(8·6 to 11·3%)	0·6%<br>(-4·6 to 6·1%)	-2·3%<br>(-5·4 to 1·1%)	31,654·3<br>(27,711·2 to 35,547·1)	5·6%<br>(4·8 to 6·4%)	29,029·4<br>(25,308·7 to 32,716·0)	5·3%<br>(4·5 to 6·1%)	-8·3%<br>(-12·7 to -3·3%)	-5·1%<br>(-8·5 to -1·2%)
Lower respiratory infections	126·1<br>(90·2 to 166·0)	26·0%<br>(18·7 to 33·8%)	115·8<br>(80·7 to 154·0)	25·2%<br>(18·1 to 33·1%)	-8·2%<br>(-13·2 to -3·1%)	-3·2%<br>(-6·4 to -1·3%)	5,091·5<br>(3,637·1 to 6,778·9)	25·4%<br>(18·1 to 33·3%)	3,089·2<br>(2,180·1 to 4,147·1)	24·3%<br>(17·1 to 32·4%)	-39·3%<br>(-43·7 to -35·1%)	-4·3%<br>(-8·4 to -1·5%)
Tracheal, bronchus, and lung cancer	132·2<br>(87·2 to 177·4)	23·3%<br>(15·4 to 31·3%)	165·0<br>(108·1 to 221·5)	23·4%<br>(15·5 to 31·4%)	24·8%<br>(17·0 to 35·0%)	0·4%<br>(-0·6 to 1·7%)	3,078·7<br>(2,031·0 to 4,140·9)	23·1%<br>(15·3 to 31·1%)	3,639·0<br>(2,390·9 to 4,900·1)	23·3%<br>(15·4 to 31·2%)	18·2%<br>(10·1 to 29·1%)	0·5%<br>(-0·6 to 1·9%)
Ischaemic heart disease	321·5<br>(269·2 to 382·5)	19·4%<br>(16·3 to 23·1%)	391·5<br>(321·0 to 472·5)	18·8%<br>(15·5 to 22·8%)	21·7%<br>(14·4 to 28·8%)	-3·1%<br>(-6·3 to 0·2%)	7,273·2<br>(6,211·6 to 8,365·9)	21·5%<br>(18·5 to 24·6%)	8,151·9<br>(6,820·0 to 9,472·2)	20·7%<br>(17·4 to 23·8%)	12·1%<br>(4·9 to 19·1%)	-3·6%<br>(-6·3 to -1·1%)
Ischaemic stroke	136·3<br>(106·5 to 164·2)	15·2%<br>(11·9 to 18·3%)	149·4<br>(113·0 to 183·6)	14·9%<br>(11·5 to 18·2%)	9·6%<br>(2·1 to 16·8%)	-2·4%<br>(-6·7 to 1·4%)	2,543·5<br>(2,083·0 to 2,977·5)	16·5%<br>(13·7 to 19·5%)	2,640·7<br>(2,148·4 to 3,124·0)	16·2%<br>(13·2 to 19·0%)	3·8%<br>(-3·1 to 10·9%)	-2·2%<br>(-5·4 to 1·0%)
Hemorrhagic stroke	275·2<br>(226·9 to 324·4)	16·8%<br>(13·9 to 19·6%)	257·2<br>(209·2 to 303·9)	16·2%<br>(13·3 to 19·1%)	-6·5%<br>(-12·7 to 0·1%)	-3·3%<br>(-6·5 to -0·3%)	6,552·8<br>(5,481·9 to 7,611·6)	18·3%<br>(15·6 to 21·2%)	5,899·3<br>(4,893·1 to 7,030·7)	17·8%<br>(14·9 to 20·7%)	-10·0%<br>(-15·9 to -3·5%)	-2·7%<br>(-5·7 to 0·2%)
Chronic obstructive pulmonary disease	400·2<br>(252·2 to 565·8)	29·7%<br>(18·6 to 42·0%)	320·7<br>(197·6 to 456·8)	29·0%<br>(18·1 to 41·0%)	-19·9%<br>(-25·6 to -14·3%)	-2·3%<br>(-4·0 to -1·4%)	7,114·6<br>(4,474·3 to 10,057·1)	28·5%<br>(17·7 to 40·3%)	5,609·3<br>(3,467·2 to 7,961·6)	27·8%<br>(17·2 to 39·2%)	-21·2%<br>(-26·3 to -16·2%)	-2·5%<br>(-4·5 to -1·3%)
**Household air pollution from solid fuels: All causes**	1,181·9<br>(832·2 to 1,565·8)	8·7%<br>(6·1 to 11·4%)	818·0<br>(566·8 to 1,103·9)	5·8%<br>(4·0 to 7·9%)	-30·8%<br>(-36·5 to -25·5%)	-32·8%<br>(-37·5 to -28·4%)	28,410·3<br>(20,105·4 to 37,261·7)	5·0%<br>(3·5 to 6·6%)	18,184·3<br>(12,685·8 to 24,522·4)	3·3%<br>(2·3 to 4·5%)	-36·0%<br>(-41·5 to -30·8%)	-33·7%<br>(-39·1 to -28·9%)
Lower respiratory infections	129·1<br>(78·5 to 186·2)	26·7%<br>(16·3 to 38·6%)	85·2<br>(49·5 to 128·8)	18·6%<br>(10·9 to 28·2%)	-34·0%<br>(-40·6 to -27·6%)	-30·4%<br>(-36·7 to -24·3%)	5,824·6<br>(3,539·6 to 8,452·0)	29·0%<br>(17·8 to 41·9%)	2,701·5<br>(1,561·4 to 4,068·5)	21·2%<br>(12·5 to 31·8%)	-53·6%<br>(-58·6 to -48·4%)	-26·9%<br>(-33·1 to -20·7%)
Tracheal, bronchus, and lung cancer	107·3<br>(53·6 to 171·9)	18·9%<br>(9·4 to 30·7%)	89·2<br>(41·6 to 151·8)	12·6%<br>(6·0 to 21·6%)	-16·9%<br>(-25·9 to -8·3%)	-33·1%<br>(-38·7 to -28·5%)	2,523·2<br>(1,260·4 to 4,036·4)	19·0%<br>(9·4 to 30·8%)	2,001·4<br>(942·6 to 3,401·5)	12·8%<br>(6·1 to 21·8%)	-20·7%<br>(-29·4 to -12·1%)	-32·6%<br>(-38·1 to -27·9%)
Ischaemic heart disease	258·0<br>(199·6 to 328·6)	15·6%<br>(12·1 to 19·7%)	216·2<br>(161·2 to 286·2)	10·4%<br>(7·8 to 13·8%)	-16·2%<br>(-24·1 to -8·8%)	-33·3%<br>(-38·6 to -28·4%)	5,942·2<br>(4,694·1 to 7,322·2)	17·6%<br>(13·7 to 21·7%)	4,709·9<br>(3,534·5 to 6,042·1)	12·0%<br>(8·9 to 15·2%)	-20·7%<br>(-28·7 to -12·6%)	-31·9%<br>(-37·9 to -26·3%)
Ischaemic stroke	111·3<br>(76·3 to 153·1)	12·4%<br>(8·7 to 17·1%)	80·4<br>(54·2 to 112·7)	8·0%<br>(5·4 to 11·2%)	-27·7%<br>(-34·6 to -20·6%)	-35·6%<br>(-41·1 to -30·8%)	2,099·2<br>(1,460·1 to 2,782·2)	13·6%<br>(9·5 to 18·0%)	1,463·2<br>(992·5 to 2,000·8)	9·0%<br>(6·2 to 12·3%)	-30·3%<br>(-36·9 to -23·4%)	-34·3%<br>(-39·8 to -29·5%)
Hemorrhagic stroke	230·3<br>(163·0 to 306·9)	14·1%<br>(9·9 to 18·6%)	148·4<br>(104·3 to 203·7)	9·4%<br>(6·6 to 12·8%)	-35·6%<br>(-42·0 to -29·2%)	-33·3%<br>(-38·6 to -28·2%)	5,567·6<br>(4,034·2 to 7,316·9)	15·6%<br>(11·3 to 20·3%)	3,515·1<br>(2,476·0 to 4,838·3)	10·6%<br>(7·4 to 14·3%)	-36·9%<br>(-43·1 to -30·2%)	-31·8%<br>(-37·3 to -26·2%)
Chronic obstructive pulmonary disease	345·9<br>(176·1 to 535·7)	25·7%<br>(13·2 to 39·7%)	198·6<br>(94·7 to 319·4)	18·0%<br>(8·7 to 29·5%)	-42·6%<br>(-48·7 to -36·9%)	-30·0%<br>(-36·1 to -24·9%)	6,221·7<br>(3,160·2 to 9,581·2)	24·9%<br>(12·7 to 38·6%)	3,580·6<br>(1,699·9 to 5,751·2)	17·7%<br>(8·5 to 29·1%)	-42·4%<br>(-48·1 to -36·9%)	-28·8%<br>(-34·6 to -23·6%)
Cataract	-	-	-	-	-	-	231·8<br>(157·2 to 319·1)	28·7%<br>(24·7 to 32·4%)	212·7<br>(143·5 to 297·1)	21·6%<br>(18·0 to 24·9%)	-8·2%<br>(-13·9 to -2·7%)	-24·6%<br>(-29·1 to -20·5%)
**Ambient ozone pollution: All causes**	82·2<br>(30·5 to 142·9)	0·6%<br>(0·2 to 1·1%)	81·4<br>(31·5 to 137·3)	0·6%<br>(0·2 to 1·0%)	-0·9%<br>(-8·6 to 7·9%)	-3·8%<br>(-10·3 to 3·9%)	1,256·0<br>(464·7 to 2,171·7)	0·2%<br>(0·1 to 0·4%)	1,150·4<br>(441·4 to 1,933·5)	0·2%<br>(0·1 to 0·4%)	-8·4%<br>(-16·1 to 0·7%)	-5·2%<br>(-12·4 to 3·3%)
Chronic obstructive pulmonary disease	82·2<br>(30·5 to 142·9)	6·1%<br>(2·3 to 10·6%)	81·4<br>(31·5 to 137·3)	7·4%<br>(2·8 to 12·5%)	-0·9%<br>(-8·6 to 7·9%)	20·7%<br>(16·9 to 26·4%)	1,256·0<br>(464·7 to 2,171·7)	5·0%<br>(1·9 to 8·6%)	1,150·4<br>(441·4 to 1,933·5)	5·7%<br>(2·1 to 9·4%)	-8·4%<br>(-16·1 to 0·7%)	13·3%<br>(8·2 to 19·6%)
**Other environmental risks: All causes**	110·3<br>(48·4 to 191·7)	0·8%<br>(0·4 to 1·4%)	119·4<br>(51·4 to 210·0)	0·8%<br>(0·4 to 1·5%)	8·2%<br>(2·3 to 14·5%)	5·1%<br>(0·2 to 9·8%)	2,302·1<br>(975·9 to 4,064·2)	0·4%<br>(0·2 to 0·7%)	2,340·1<br>(976·5 to 4,193·0)	0·4%<br>(0·2 to 0·8%)	1·6%<br>(-3·9 to 6·9%)	5·2%<br>(0·0 to 9·3%)
**Residential radon: All causes**	11·9<br>(7·8 to 16·7)	0·1%<br>(0·1 to 0·1%)	14·7<br>(9·6 to 20·8)	0·1%<br>(0·1 to 0·1%)	23·8%<br>(13·1 to 35·5%)	20·3%<br>(11·1 to 30·1%)	279·2<br>(187·3 to 389·7)	0·0%<br>(0·0 to 0·1%)	327·7<br>(216·8 to 460·7)	0·1%<br>(0·0 to 0·1%)	17·4%<br>(6·7 to 29·2%)	21·5%<br>(11·5 to 33·3%)
Tracheal, bronchus, and lung cancer	11·9<br>(7·8 to 16·7)	2·1%<br>(1·4 to 2·9%)	14·7<br>(9·6 to 20·8)	2·1%<br>(1·3 to 2·9%)	23·8%<br>(13·1 to 35·5%)	-0·3%<br>(-6·3 to 6·1%)	279·2<br>(187·3 to 389·7)	2·1%<br>(1·4 to 2·9%)	327·7<br>(216·8 to 460·7)	2·1%<br>(1·4 to 2·9%)	17·4%<br>(6·7 to 29·2%)	-0·2%<br>(-6·8 to 5·9%)
**Lead exposure: All causes**	98·5<br>(37·2 to 178·7)	0·7%<br>(0·3 to 1·3%)	104·7<br>(38·1 to 193·7)	0·7%<br>(0·3 to 1·4%)	6·4%<br>(-1·8 to 12·7%)	3·3%<br>(-4·0 to 8·2%)	2,022·9<br>(728·7 to 3,792·3)	0·4%<br>(0·1 to 0·7%)	2,012·3<br>(692·3 to 3,813·4)	0·4%<br>(0·1 to 0·7%)	-0·5%<br>(-8·5 to 5·0%)	3·0%<br>(-4·8 to 7·4%)
Rheumatic heart disease	0·9<br>(0·2 to 1·8)	0·8%<br>(0·2 to 1·6%)	0·6<br>(0·2 to 1·4)	0·7%<br>(0·2 to 1·6%)	-25·1%<br>(-32·0 to -17·9%)	-6·0%<br>(-12·7 to 1·0%)	22·6<br>(4·9 to 54·0)	0·6%<br>(0·1 to 1·5%)	15·5<br>(3·3 to 36·5)	0·6%<br>(0·1 to 1·4%)	-31·3%<br>(-36·9 to -25·5%)	-6·1%<br>(-12·0 to -0·3%)
Ischaemic heart disease	32·2<br>(11·8 to 59·9)	1·9%<br>(0·7 to 3·6%)	37·4<br>(12·9 to 70·7)	1·8%<br>(0·6 to 3·4%)	16·2%<br>(6·4 to 23·3%)	-7·5%<br>(-14·3 to -3·4%)	601·8<br>(195·3 to 1,173·1)	1·8%<br>(0·6 to 3·5%)	654·8<br>(199·6 to 1,297·3)	1·7%<br>(0·5 to 3·3%)	8·8%<br>(-0·7 to 15·4%)	-6·4%<br>(-13·6 to -2·9%)
Ischaemic stroke	16·1<br>(6·1 to 30·2)	1·8%<br>(0·7 to 3·4%)	16·8<br>(6·2 to 32·0)	1·7%<br>(0·6 to 3·2%)	4·5%<br>(-4·8 to 13·1%)	-6·9%<br>(-13·0 to -1·2%)	272·5<br>(94·0 to 521·7)	1·8%<br>(0·6 to 3·4%)	271·5<br>(91·1 to 527·5)	1·7%<br>(0·6 to 3·2%)	-0·3%<br>(-9·0 to 6·0%)	-6·1%<br>(-13·1 to -2·2%)
Hemorrhagic stroke	33·9<br>(11·7 to 64·8)	2·1%<br>(0·7 to 3·9%)	31·2<br>(10·3 to 60·2)	2·0%<br>(0·7 to 3·8%)	-8·0%<br>(-15·8 to -1·5%)	-4·8%<br>(-11·4 to -0·5%)	703·0<br>(214·8 to 1,423·7)	2·0%<br>(0·6 to 4·0%)	628·6<br>(189·4 to 1,269·2)	1·9%<br>(0·6 to 3·9%)	-10·6%<br>(-17·9 to -5·1%)	-3·3%<br>(-9·9 to 0·2%)
Hypertensive heart disease	9·0<br>(2·0 to 23·2)	3·7%<br>(0·8 to 9·5%)	11·3<br>(2·4 to 30·2)	3·4%<br>(0·7 to 9·3%)	25·3%<br>(2·3 to 40·3%)	-6·4%<br>(-15·5 to 1·1%)	154·5<br>(37·4 to 381·9)	3·4%<br>(0·8 to 8·5%)	174·9<br>(38·7 to 453·3)	3·2%<br>(0·7 to 8·2%)	13·1%<br>(-2·8 to 23·7%)	-6·5%<br>(-14·3 to -1·1%)
Cardiomyopathy and myocarditis	0·4<br>(0·1 to 0·8)	1·0%<br>(0·3 to 2·0%)	0·5<br>(0·2 to 1·0)	1·0%<br>(0·3 to 2·0%)	16·3%<br>(4·8 to 28·3%)	-1·1%<br>(-7·9 to 5·0%)	10·7<br>(2·8 to 23·4)	0·7%<br>(0·2 to 1·6%)	11·3<br>(2·9 to 25·1)	0·8%<br>(0·2 to 1·7%)	5·6%<br>(-5·6 to 17·5%)	5·9%<br>(-2·1 to 12·5%)

Supplemental Results Table 1. All-age deaths, death PAFs, DALYs, DALY PAFs, and percent change for each risk factor and outcome for both sexes combined in 2005 and 2015 for Southeast Asia, East Asia, and Oceania.

Data in parenthesis are 95% uncertainty intervals. PAFs=population attributable fractions. DALYs=disability-adjusted life-years.

	2005 Deaths (in thousands)	2005 Deaths PAF	2015 Deaths (in thousands)	2015 Deaths PAF	Percent change deaths 2005-2015	Percent change deaths PAF 2005-2015	2005 DALYs (in thousands)	2005 DALYs PAF	2015 DALYs (in thousands)	2015 DALYs PAF	Percent change DALYs 2005-2015	Percent change DALYs PAF 2005-2015
Atrial fibrillation and flutter	0·4 (0·2 to 0·8)	1·3% (0·6 to 2·3%)	0·5 (0·2 to 1·0)	1·2% (0·5 to 2·2%)	27·0% (15·4 to 34·6%)	-9·7% (-16·8 to -5·9%)	11·7 (4·2 to 22·6)	1·4% (0·5 to 2·5%)	14·2 (4·8 to 28·4)	1·3% (0·4 to 2·4%)	21·6% (11·7 to 27·0%)	-9·3% (-16·4 to -6·3%)
Aortic aneurysm	0·2 (0·1 to 0·4)	1·2% (0·4 to 2·3%)	0·3 (0·1 to 0·5)	1·1% (0·4 to 2·1%)	22·5% (8·4 to 32·7%)	-7·2% (-14·5 to -3·1%)	4·6 (1·5 to 9·4)	1·1% (0·4 to 2·3%)	5·4 (1·6 to 11·1)	1·1% (0·3 to 2·1%)	16·4% (1·8 to 26·0%)	-6·1% (-13·7 to -2·5%)
Peripheral vascular disease	0·0 (0·0 to 0·0)	0·9% (0·3 to 1·6%)	0·0 (0·0 to 0·0)	0·8% (0·3 to 1·5%)	60·8% (39·6 to 80·5%)	-9·4% (-17·6 to -3·9%)	1·4 (0·4 to 3·1)	0·9% (0·3 to 1·7%)	1·9 (0·5 to 4·2)	0·8% (0·3 to 1·6%)	32·5% (18·9 to 40·4%)	-11·3% (-20·2 to -7·0%)
Endocarditis	0·2 (0·0 to 0·3)	1·0% (0·3 to 2·0%)	0·2 (0·1 to 0·4)	1·0% (0·3 to 2·0%)	18·4% (5·4 to 30·8%)	-3·9% (-11·4 to 2·1%)	3·8 (0·9 to 8·7)	0·8% (0·2 to 1·7%)	4·0 (1·0 to 9·4)	0·8% (0·2 to 1·8%)	6·9% (-5·6 to 18·6%)	1·4% (-8·0 to 8·8%)
Other cardiovascular and circulatory diseases	1·3 (0·5 to 2·3)	1·3% (0·5 to 2·3%)	1·3 (0·5 to 2·5)	1·2% (0·4 to 2·2%)	2·8% (-7·5 to 10·9%)	-6·8% (-14·3 to -2·6%)	39·4 (11·7 to 79·6)	1·0% (0·3 to 1·9%)	42·5 (12·0 to 88·9)	1·0% (0·3 to 2·0%)	8·0% (-1·9 to 13·9%)	-0·1% (-7·5 to 3·6%)
Idiopathic developmental intellectual disability	-	-	-	-	-	-	104·7 (50·7 to 175·7)	11·6% (8·2 to 14·7%)	87·1 (42·1 to 146·2)	9·4% (6·3 to 12·1%)	-16·8% (-22·5 to -11·4%)	-19·0% (-24·7 to -13·9%)
Chronic kidney disease due to diabetes mellitus	1·2 (0·4 to 2·4)	1·7% (0·6 to 3·2%)	1·5 (0·5 to 3·0)	1·6% (0·5 to 3·1%)	21·8% (8·4 to 31·1%)	-6·0% (-14·7 to -2·2%)	30·3 (8·9 to 66·2)	1·2% (0·4 to 2·6%)	35·7 (10·1 to 78·9)	1·2% (0·3 to 2·6%)	18·0% (6·4 to 25·7%)	-3·2% (-11·7 to 0·1%)
Chronic kidney disease due to hypertension	1·5 (0·5 to 2·8)	1·7% (0·6 to 3·2%)	1·8 (0·6 to 3·6)	1·6% (0·5 to 3·1%)	24·8% (10·0 to 36·1%)	-5·5% (-14·2 to -1·4%)	29·0 (8·7 to 60·3)	1·2% (0·4 to 2·5%)	33·6 (9·5 to 71·5)	1·2% (0·3 to 2·5%)	15·9% (3·9 to 24·6%)	-2·1% (-10·4 to 1·5%)
Chronic kidney disease due to glomerulonephritis	1·2 (0·4 to 2·2)	1·4% (0·4 to 2·6%)	1·2 (0·4 to 2·3)	1·4% (0·5 to 2·8%)	1·2% (-5·6 to 8·6%)	4·4% (0·8 to 8·9%)	26·3 (7·0 to 55·7)	0·9% (0·3 to 2·0%)	23·6 (6·4 to 50·1)	1·0% (0·3 to 2·1%)	-10·3% (-16·1 to -4·2%)	4·2% (0·4 to 9·4%)
Chronic kidney disease due to other causes	0·2 (0·1 to 0·3)	1·6% (0·6 to 3·0%)	0·2 (0·1 to 0·4)	1·7% (0·6 to 3·1%)	22·2% (2·4 to 43·0%)	3·0% (-10·2 to 14·6%)	6·7 (2·0 to 16·0)	0·9% (0·3 to 2·0%)	7·7 (2·2 to 18·2)	1·0% (0·3 to 2·1%)	14·6% (5·3 to 22·1%)	4·2% (-3·4 to 9·7%)
**Occupational risks: All causes**	401·2 (371·9 to 432·6)	2·9% (2·7 to 3·2%)	427·5 (395·7 to 461·7)	3·0% (2·8 to 3·2%)	6·5% (1·0 to 12·6%)	3·5% (0·1 to 7·4%)	19,950·4 (17,036·4 to 23,214·3)	3·5% (3·2 to 3·8%)	21,757·2 (18,502·8 to 25,593·9)	3·9% (3·7 to 4·3%)	9·1% (5·9 to 12·2%)	12·8% (10·2 to 15·6%)
**Occupational carcinogens: All causes**	194·2 (182·6 to 205·3)	1·4% (1·3 to 1·5%)	240·1 (224·0 to 258·0)	1·7% (1·6 to 1·8%)	23·7% (15·4 to 33·9%)	20·1% (14·0 to 27·8%)	4,340·5 (4,089·4 to 4,588·9)	0·8% (0·7 to 0·8%)	5,260·6 (4,902·2 to 5,659·1)	1·0% (0·9 to 1·1%)	21·2% (12·9 to 31·6%)	25·4% (18·3 to 34·8%)
**Occupational exposure to asbestos: All causes**	21·2 (16·7 to 26·6)	0·2% (0·1 to 0·2%)	35·8 (28·1 to 44·6)	0·3% (0·2 to 0·3%)	69·2% (49·0 to 94·3%)	64·4% (47·8 to 84·3%)	384·1 (300·8 to 483·9)	0·1% (0·1 to 0·1%)	641·8 (496·9 to 809·6)	0·1% (0·1 to 0·1%)	67·1% (44·5 to 95·6%)	73·0% (51·2 to 99·6%)
Larynx cancer	0·2 (0·1 to 0·3)	0·9% (0·6 to 1·4%)	0·3 (0·2 to 0·5)	1·2% (0·7 to 1·8%)	59·2% (36·5 to 87·5%)	29·4% (14·7 to 45·7%)	3·6 (2·1 to 5·4)	0·7% (0·4 to 1·0%)	5·8 (3·3 to 8·8)	0·9% (0·5 to 1·4%)	60·0% (35·1 to 90·5%)	34·5% (18·1 to 53·7%)
Tracheal, bronchus, and lung cancer	18·8 (14·4 to 24·2)	3·3% (2·6 to 4·2%)	31·6 (24·2 to 40·3)	4·5% (3·5 to 5·6%)	68·2% (47·9 to 93·6%)	35·4% (23·1 to 49·0%)	330·4 (249·6 to 430·4)	2·5% (1·9 to 3·2%)	548·3 (411·1 to 710·9)	3·5% (2·7 to 4·4%)	65·9% (42·7 to 94·8%)	41·1% (26·3 to 57·9%)
Ovarian cancer	0·3 (0·1 to 0·5)	1·1% (0·6 to 1·7%)	0·4 (0·2 to 0·7)	1·3% (0·6 to 2·2%)	46·7% (20·0 to 79·4%)	20·2% (1·1 to 43·3%)	5·7 (2·8 to 9·3)	0·7% (0·3 to 1·1%)	8·1 (3·9 to 13·7)	0·8% (0·4 to 1·4%)	41·6% (11·9 to 77·8%)	20·2% (-1·1 to 46·5%)
Mesothelioma	1·9 (1·3 to 2·5)	46·6% (31·7 to 61·5%)	3·5 (2·5 to 4·5)	54·4% (38·6 to 68·9%)	84·0% (62·2 to 112·2%)	16·6% (10·2 to 25·6%)	44·3 (27·8 to 61·8)	36·3% (23·2 to 50·5%)	79·5 (53·4 to 107·1)	44·9% (30·4 to 59·6%)	79·6% (55·7 to 111·9%)	23·7% (15·0 to 36·4%)
**Occupational exposure to arsenic: All causes**	6·4 (5·0 to 8·1)	0·0% (0·0 to 0·1%)	6·2 (4·7 to 7·9)	0·0% (0·0 to 0·1%)	-3·0% (-10·0 to 5·4%)	-5·8% (-11·2 to 1·2%)	142·4 (111·1 to 175·0)	0·0% (0·0 to 0·0%)	136·6 (104·9 to 171·2)	0·0% (0·0 to 0·0%)	-4·1% (-11·4 to 4·9%)	-0·7% (-7·3 to 7·5%)
Tracheal, bronchus, and lung cancer	6·4 (5·0 to 8·1)	1·1% (0·9 to 1·4%)	6·2 (4·7 to 7·9)	0·9% (0·7 to 1·1%)	-3·0% (-10·0 to 5·4%)	-22·0% (-24·4 to -19·4%)	142·4 (111·1 to 175·0)	1·1% (0·8 to 1·3%)	136·6 (104·9 to 171·2)	0·9% (0·7 to 1·1%)	-4·1% (-11·4 to 4·9%)	-18·4% (-21·1 to -15·6%)
**Occupational exposure to benzene: All causes**	2·2 (1·9 to 2·5)	0·0% (0·0 to 0·0%)	2·7 (2·3 to 3·2)	0·0% (0·0 to 0·0%)	24·4% (16·4 to 32·7%)	20·8% (14·9 to 26·3%)	84·6 (71·3 to 99·3)	0·0% (0·0 to 0·0%)	103·4 (86·6 to 121·6)	0·0% (0·0 to 0·0%)	22·3% (14·0 to 31·2%)	26·6% (18·8 to 33·9%)
Leukaemia	2·2 (1·9 to 2·5)	2·3% (2·0 to 2·7%)	2·7 (2·3 to 3·2)	2·7% (2·3 to 3·1%)	24·4% (16·4 to 32·7%)	15·6% (12·0 to 19·2%)	84·6 (71·3 to 99·3)	2·0% (1·7 to 2·3%)	103·4 (86·6 to 121·6)	2·6% (2·2 to 3·0%)	22·3% (14·0 to 31·2%)	27·8% (22·7 to 32·9%)
**Occupational exposure to beryllium: All causes**	0·4 (0·3 to 0·5)	0·0% (0·0 to 0·0%)	0·4 (0·3 to 0·5)	0·0% (0·0 to 0·0%)	-1·8% (-8·8 to 6·8%)	-4·6% (-10·3 to 2·9%)	9·6 (7·7 to 11·6)	0·0% (0·0 to 0·0%)	9·3 (7·4 to 11·3)	0·0% (0·0 to 0·0%)	-2·8% (-9·9 to 6·3%)	0·6% (-6·0 to 9·2%)
Tracheal, bronchus, and lung cancer	0·4 (0·3 to 0·5)	0·1% (0·1 to 0·1%)	0·4 (0·3 to 0·5)	0·1% (0·0 to 0·1%)	-1·8% (-8·8 to 6·8%)	-21·0% (-23·5 to -18·1%)	9·6 (7·7 to 11·6)	0·1% (0·1 to 0·1%)	9·3 (7·4 to 11·3)	0·1% (0·0 to 0·1%)	-2·8% (-9·9 to 6·3%)	-17·4% (-20·1 to -14·6%)
**Occupational exposure to cadmium: All causes**	1·4 (1·2 to 1·7)	0·0% (0·0 to 0·0%)	1·5 (1·2 to 1·8)	0·0% (0·0 to 0·0%)	7·7% (-0·1 to 16·9%)	4·6% (-1·6 to 12·5%)	31·7 (26·1 to 37·5)	0·0% (0·0 to 0·0%)	33·8 (27·5 to 40·2)	0·0% (0·0 to 0·0%)	6·6% (-1·3 to 16·1%)	10·4% (3·2 to 19·4%)
Tracheal, bronchus, and lung cancer	1·4 (1·2 to 1·7)	0·3% (0·2 to 0·3%)	1·5 (1·2 to 1·8)	0·2% (0·2 to 0·3%)	7·7% (-0·1 to 16·9%)	-13·3% (-16·0 to -10·4%)	31·7 (26·1 to 37·5)	0·2% (0·2 to 0·3%)	33·8 (27·5 to 40·2)	0·2% (0·2 to 0·3%)	6·6% (-1·3 to 16·1%)	-9·3% (-12·0 to -6·6%)
**Occupational exposure to chromium: All causes**	5·1 (4·4 to 5·7)	0·0% (0·0 to 0·0%)	5·2 (4·5 to 6·0)	0·0% (0·0 to 0·0%)	3·0% (-4·2 to 11·8%)	0·0% (-5·8 to 7·6%)	112·1 (99·0 to 126·4)	0·0% (0·0 to 0·0%)	114·3 (99·4 to 130·6)	0·0% (0·0 to 0·0%)	1·9% (-5·4 to 11·1%)	5·5% (-1·2 to 14·1%)
Tracheal, bronchus, and lung cancer	5·1 (4·4 to 5·7)	0·9% (0·8 to 1·0%)	5·2 (4·5 to 6·0)	0·7% (0·6 to 0·8%)	3·0% (-4·2 to 11·8%)	-17·1% (-19·5 to -14·7%)	112·1 (99·0 to 126·4)	0·8% (0·7 to 0·9%)	114·3 (99·4 to 130·6)	0·7% (0·6 to 0·8%)	1·9% (-5·4 to 11·1%)	-13·3% (-15·8 to -11·1%)
**Occupational exposure to diesel engine exhaust: All causes**	63·3 (55·8 to 71·0)	0·5% (0·4 to 0·5%)	81·3 (71·8 to 92·2)	0·6% (0·5 to 0·6%)	28·4% (19·6 to 38·7%)	24·7% (18·2 to 33·3%)	1,408·0 (1,251·1 to 1,580·4)	0·2% (0·2 to 0·3%)	1,790·9 (1,581·8 to 2,029·1)	0·3% (0·3 to 0·4%)	27·2% (18·4 to 37·8%)	31·6% (23·8 to 41·5%)
Tracheal, bronchus, and lung cancer	63·3 (55·8 to 71·0)	11·1% (9·9 to 12·4%)	81·3 (71·8 to 92·2)	11·5% (10·3 to 12·9%)	28·4% (19·6 to 38·7%)	3·4% (0·4 to 6·3%)	1,408·0 (1,251·1 to 1,580·4)	10·6% (9·5 to 11·8%)	1,790·9 (1,581·8 to 2,029·1)	11·4% (10·2 to 12·8%)	27·2% (18·4 to 37·8%)	8·2% (5·1 to 10·9%)
**Occupational exposure to second-hand smoke: All causes**	52·4 (48·7 to 56·0)	0·4% (0·4 to 0·4%)	56·8 (52·2 to 61·5)	0·4% (0·4 to 0·4%)	8·5% (1·5 to 17·4%)	5·4% (0·1 to 12·6%)	1,154·5 (1,073·1 to 1,234·1)	0·2% (0·2 to 0·2%)	1,238·6 (1,137·0 to 1,342·5)	0·2% (0·2 to 0·3%)	7·3% (0·3 to 16·4%)	11·0% (4·9 to 19·3%)
Tracheal, bronchus, and lung cancer	52·4 (48·7 to 56·0)	9·2% (8·7 to 9·8%)	56·8 (52·2 to 61·5)	8·1% (7·6 to 8·6%)	8·5% (1·5 to 17·4%)	-12·7% (-14·7 to -10·2%)	1,154·5 (1,073·1 to 1,234·1)	8·7% (8·2 to 9·2%)	1,238·6 (1,137·0 to 1,342·5)	7·9% (7·4 to 8·4%)	7·3% (0·3 to 16·4%)	-8·8% (-11·0 to -6·5%)
**Occupational exposure to formaldehyde: All causes**	1·4 (1·1 to 1·8)	0·0% (0·0 to 0·0%)	1·3 (1·0 to 1·7)	0·0% (0·0 to 0·0%)	-5·9% (-14·4 to 2·6%)	-8·6% (-15·8 to -1·9%)	51·1 (39·4 to 64·1)	0·0% (0·0 to 0·0%)	46·8 (36·6 to 59·4)	0·0% (0·0 to 0·0%)	-8·5% (-16·6 to 0·0%)	-5·2% (-12·8 to 2·4%)
Nasopharynx cancer	0·8 (0·5 to 1·2)	2·4% (1·7 to 3·3%)	0·7 (0·5 to 1·1)	2·0% (1·3 to 2·7%)	-11·7% (-23·4 to -0·3%)	-17·1% (-23·2 to -10·7%)	30·1 (19·2 to 42·5)	2·5% (1·7 to 3·5%)	25·5 (16·2 to 36·8)	2·2% (1·5 to 3·1%)	-15·2% (-27·0 to -3·1%)	-13·7% (-20·0 to -6·8%)
**Occupational exposure to nickel: All causes**	25·1 (18·5 to 32·3)	0·2% (0·1 to 0·2%)	25·7 (18·7 to 33·8)	0·2% (0·1 to 0·2%)	2·6% (-5·0 to 11·4%)	-0·4% (-6·5 to 7·9%)	556·5 (415·7 to 707·3)	0·1% (0·1 to 0·1%)	564·9 (415·1 to 736·3)	0·1% (0·1 to 0·1%)	1·5% (-6·2 to 11·0%)	5·1% (-2·1 to 14·0%)
Tracheal, bronchus, and lung cancer	25·1 (18·5 to 32·3)	4·4% (3·2 to 5·7%)	25·7 (18·7 to 33·8)	3·6% (2·7 to 4·8%)	2·6% (-5·0 to 11·4%)	-17·4% (-20·8 to -14·0%)	556·5 (415·7 to 707·3)	4·2% (3·1 to 5·4%)	564·9 (415·1 to 736·3)	3·6% (2·6 to 4·7%)	1·5% (-6·2 to 11·0%)	-13·7% (-17·3 to -10·0%)
**Occupational exposure to polycyclic aromatic hydrocarbons: All causes**	11·1 (9·1 to 13·1)	0·1% (0·1 to 0·1%)	12·7 (10·4 to 15·2)	0·1% (0·1 to 0·1%)	14·7% (6·7 to 24·6%)	11·4% (5·2 to 19·6%)	245·0 (203·8 to 287·2)	0·0% (0·0 to 0·1%)	278·5 (230·5 to 329·3)	0·1% (0·0 to 0·1%)	13·6% (5·6 to 23·8%)	17·6% (10·6 to 26·8%)
Tracheal, bronchus, and lung cancer	11·1 (9·1 to 13·1)	1·9% (1·6 to 2·3%)	12·7 (10·4 to 15·2)	1·8% (1·5 to 2·1%)	14·7% (6·7 to 24·6%)	-7·7% (-10·3 to -4·8%)	245·0 (203·8 to 287·2)	1·8% (1·5 to 2·2%)	278·5 (230·5 to 329·3)	1·8% (1·5 to 2·1%)	13·6% (5·6 to 23·8%)	-3·4% (-6·0 to -0·5%)
**Occupational exposure to silica: All causes**	47·1 (42·4 to 51·4)	0·3% (0·3 to 0·4%)	62·7 (56·6 to 68·8)	0·4% (0·4 to 0·5%)	33·2% (23·6 to 44·3%)	29·3% (22·0 to 38·7%)	1,038·2 (937·4 to 1,135·4)	0·2% (0·2 to 0·2%)	1,357·9 (1,234·3 to 1,496·8)	0·2% (0·2 to 0·3%)	30·8% (21·3 to 42·5%)	35·4% (26·8 to 46·2%)
Tracheal, bronchus, and lung cancer	47·1 (42·4 to 51·4)	8·3% (7·6 to 9·0%)	62·7 (56·6 to 68·8)	8·9% (8·2 to 9·7%)	33·2% (23·6 to 44·3%)	7·2% (3·0 to 11·1%)	1,038·2 (937·4 to 1,135·4)	7·8% (7·2 to 8·4%)	1,357·9 (1,234·3 to 1,496·8)	8·7% (8·0 to 9·4%)	30·8% (21·3 to 42·5%)	11·2% (7·0 to 14·9%)
**Occupational exposure to sulfphuric acid: All causes**	2·7 (2·1 to 3·4)	0·0% (0·0 to 0·0%)	3·1 (2·3 to 3·8)	0·0% (0·0 to 0·0%)	12·3% (3·9 to 21·2%)	9·1% (3·0 to 15·7%)	65·8 (51·8 to 81·3)	0·0% (0·0 to 0·0%)	74·0 (57·0 to 92·6)	0·0% (0·0 to 0·0%)	12·5% (4·1 to 21·8%)	16·5% (8·9 to 24·6%)
Larynx cancer	2·7 (2·1 to 3·4)	13·1% (10·1 to 16·3%)	3·1 (2·3 to 3·8)	11·9% (9·1 to 14·9%)	12·3% (3·9 to 21·2%)	-8·7% (-12·1 to -5·3%)	65·8 (51·8 to 81·3)	12·4% (9·8 to 15·3%)	74·0 (57·0 to 92·6)	11·8% (9·2 to 14·7%)	12·5% (4·1 to 21·8%)	-5·4% (-8·8 to -1·8%)
**Occupational exposure to trichloroethylene: All causes**	0·1 (0·0 to 0·1)	0·0% (0·0 to 0·0%)	0·1 (0·0 to 0·1)	0·0% (0·0 to 0·0%)	26·2% (17·1 to 35·3%)	22·5% (14·2 to 29·3%)	1·4 (0·3 to 2·6)	0·0% (0·0 to 0·0%)	1·7 (0·4 to 3·3)	0·0% (0·0 to 0·0%)	27·2% (17·9 to 36·7%)	31·6% (22·9 to 39·7%)
Kidney cancer	0·1 (0·0 to 0·1)	0·3% (0·1 to 0·6%)	0·1 (0·0 to 0·1)	0·3% (0·1 to 0·5%)	26·2% (17·1 to 35·3%)	-14·3% (-16·8 to -11·3%)	1·4 (0·3 to 2·6)	0·3% (0·1 to 0·5%)	1·7 (0·4 to 3·3)	0·3% (0·1 to 0·5%)	27·2% (17·9 to 36·7%)	-7·0% (-10·2 to -3·4%)

Supplemental Results Table 1. All-age deaths, death PAFs, DALYs, DALY PAFs, and percent change for each risk factor and outcome for both sexes combined in 2005 and 2015 for Southeast Asia, East Asia, and Oceania.

Data in parenthesis are 95% uncertainty intervals. PAFs=population attributable fractions. DALYs=disability-adjusted life-years.

	2005 Deaths (in thousands)	2005 Deaths PAF	2015 Deaths (in thousands)	2015 Deaths PAF	Percent change deaths 2005-2015	Percent change deaths PAF 2005-2015	2005 DALYs (in thousands)	2005 DALYs PAF	2015 DALYs (in thousands)	2015 DALYs PAF	Percent change DALYs 2005-2015	Percent change DALYs PAF 2005-2015
**Occupational asthmagens: All causes**	14·4 (12·1 to 17·1)	0·1% (0·1 to 0·1%)	12·8 (10·7 to 15·2)	0·1% (0·1 to 0·1%)	-10·9% (-21·8 to 1·0%)	-13·5% (-23·4 to -2·8%)	823·6 (661·2 to 1,009·8)	0·1% (0·1 to 0·2%)	785·0 (624·1 to 968·8)	0·1% (0·1 to 0·2%)	-4·7% (-11·6 to 2·3%)	-1·4% (-7·5 to 4·7%)
Asthma	14·4 (12·1 to 17·1)	12·2% (10·6 to 13·9%)	12·8 (10·7 to 15·2)	11·6% (10·0 to 13·2%)	-10·9% (-21·8 to 1·0%)	-5·3% (-11·4 to 1·2%)	823·6 (661·2 to 1,009·8)	13·8% (12·2 to 15·4%)	785·0 (624·1 to 968·8)	13·5% (12·0 to 15·1%)	-4·7% (-11·6 to 2·3%)	-1·8% (-5·2 to 1·9%)
**Occupational particulate matter, gases, and fumes: All causes**	148·4 (122·2 to 174·8)	1·1% (0·9 to 1·3%)	125·9 (101·5 to 152·4)	0·9% (0·7 to 1·1%)	-15·1% (-22·0 to -8·0%)	-17·6% (-23·2 to -12·2%)	3,461·5 (3,017·8 to 3,929·2)	0·6% (0·5 to 0·7%)	2,906·0 (2,481·7 to 3,348·1)	0·5% (0·4 to 0·6%)	-16·0% (-21·7 to -10·3%)	-13·1% (-17·8 to -7·8%)
Chronic obstructive pulmonary disease	147·0 (120·8 to 173·5)	10·9% (9·0 to 12·9%)	124·5 (100·3 to 150·9)	11·3% (9·1 to 13·5%)	-15·3% (-22·0 to -8·2%)	3·2% (-2·5 to 8·8%)	3,431·5 (2,984·9 to 3,898·5)	13·7% (11·9 to 15·5%)	2,877·6 (2,455·0 to 3,314·0)	14·2% (12·3 to 16·2%)	-16·1% (-21·7 to -10·4%)	3·7% (0·3 to 7·5%)
**Occupational noise: All causes**	-	-	-	-	-	-	3,092·2 (2,106·8 to 4,303·5)	0·5% (0·4 to 0·7%)	3,747·3 (2,543·0 to 5,235·5)	0·7% (0·5 to 0·9%)	21·2% (17·2 to 25·2%)	25·3% (20·2 to 31·2%)
Age-related and other hearing loss	-	-	-	-	-	-	3,092·2 (2,106·8 to 4,303·5)	31·7% (29·4 to 34·8%)	3,747·3 (2,543·0 to 5,235·5)	30·3% (28·0 to 33·1%)	21·2% (17·2 to 25·2%)	-4·5% (-6·3 to -2·4%)
**Occupational injuries: All causes**	44·3 (41·5 to 47·4)	0·3% (0·3 to 0·3%)	48·8 (46·3 to 51·5)	0·3% (0·3 to 0·4%)	10·2% (2·1 to 18·8%)	7·1% (-1·6 to 16·5%)	2,654·8 (2,438·9 to 2,895·4)	0·5% (0·4 to 0·5%)	3,012·5 (2,750·2 to 3,320·4)	0·5% (0·5 to 0·6%)	13·5% (4·7 to 22·8%)	17·4% (8·2 to 27·9%)
Pedestrian road injuries	7·5 (7·0 to 8·3)	3·1% (2·9 to 3·3%)	10·8 (10·1 to 12·1)	4·9% (4·5 to 5·7%)	44·5% (34·7 to 54·4%)	59·3% (42·9 to 79·0%)	375·7 (350·0 to 417·7)	3·4% (3·2 to 3·6%)	532·8 (499·6 to 591·9)	5·7% (5·2 to 6·6%)	41·8% (32·1 to 51·5%)	69·8% (52·2 to 91·8%)
Cyclist road injuries	1·3 (1·2 to 1·5)	3·4% (3·1 to 3·8%)	1·7 (1·6 to 2·0)	5·2% (4·7 to 6·1%)	31·8% (16·4 to 46·8%)	54·3% (34·7 to 76·4%)	82·4 (72·7 to 93·6)	3·8% (3·5 to 4·3%)	122·8 (105·6 to 144·2)	6·6% (5·9 to 7·6%)	48·9% (31·6 to 65·0%)	71·0% (50·0 to 94·6%)
Motorcyclist road injuries	6·9 (5·9 to 7·9)	6·1% (5·4 to 7·1%)	6·8 (6·0 to 8·0)	6·8% (5·8 to 8·0%)	-0·7% (-16·7 to 21·5%)	10·9% (-8·2 to 34·0%)	394·4 (342·0 to 454·5)	6·2% (5·5 to 7·2%)	400·4 (349·2 to 465·2)	7·2% (6·2 to 8·4%)	1·5% (-13·8 to 22·2%)	15·7% (-3·9 to 38·4%)
Motor vehicle road injuries	7·4 (6·6 to 8·5)	7·2% (6·6 to 7·9%)	7·3 (6·5 to 8·2)	8·3% (7·5 to 9·4%)	-2·1% (-11·8 to 10·1%)	15·2% (2·4 to 29·5%)	433·1 (383·0 to 488·9)	7·6% (6·9 to 8·4%)	450·5 (403·4 to 508·4)	9·4% (8·6 to 10·6%)	4·0% (-5·8 to 15·1%)	23·6% (9·8 to 38·5%)
Other road injuries	0·4 (0·3 to 0·5)	6·4% (5·4 to 7·6%)	0·4 (0·3 to 0·5)	7·1% (6·0 to 8·7%)	10·5% (-17·1 to 46·1%)	11·9% (-10·7 to 39·5%)	23·7 (18·4 to 29·1)	6·9% (5·9 to 8·2%)	29·6 (23·7 to 36·4)	8·4% (7·1 to 10·0%)	25·1% (-0·1 to 54·0%)	21·4% (-0·6 to 48·0%)
Other transport injuries	0·9 (0·8 to 1·2)	3·5% (3·2 to 3·8%)	1·3 (1·2 to 1·7)	5·2% (4·7 to 5·9%)	43·4% (28·1 to 61·1%)	49·1% (34·4 to 66·6%)	50·5 (45·8 to 62·2)	3·7% (3·4 to 4·0%)	69·8 (63·1 to 91·0)	5·8% (5·3 to 6·6%)	38·2% (24·4 to 53·4%)	57·2% (41·8 to 75·2%)
Falls	5·6 (4·8 to 6·2)	4·1% (3·7 to 4·7%)	7·4 (6·0 to 8·2)	4·4% (3·9 to 5·4%)	31·1% (14·1 to 50·3%)	7·4% (-5·6 to 22·2%)	400·9 (341·0 to 463·7)	6·6% (6·1 to 7·3%)	571·2 (476·1 to 689·9)	8·3% (7·4 to 9·7%)	42·5% (26·3 to 60·7%)	24·7% (11·1 to 42·1%)
Drowning	5·3 (4·7 to 5·8)	3·8% (3·5 to 4·2%)	4·6 (4·2 to 5·1)	4·7% (4·3 to 5·2%)	-13·7% (-22·9 to -1·6%)	23·2% (10·3 to 38·7%)	283·5 (253·2 to 311·6)	3·4% (3·1 to 3·8%)	234·1 (214·9 to 260·3)	4·5% (4·1 to 5·0%)	-17·4% (-26·1 to -5·9%)	32·4% (18·0 to 48·7%)
Fire, heat, and hot substances	0·9 (0·8 to 1·1)	4·3% (3·9 to 4·9%)	0·9 (0·7 to 1·0)	4·6% (4·1 to 5·4%)	-8·0% (-18·9 to 10·1%)	8·2% (-4·4 to 22·0%)	67·7 (57·5 to 79·3)	5·6% (5·0 to 6·4%)	66·6 (55·7 to 80·1)	7·4% (6·5 to 8·6%)	-1·6% (-13·8 to 14·2%)	31·0% (15·5 to 47·7%)
Poisonings	1·9 (1·4 to 2·1)	5·3% (4·7 to 6·0%)	1·2 (0·9 to 1·3)	4·4% (3·9 to 5·1%)	-36·8% (-44·4 to -27·0%)	-17·7% (-29·0 to -3·6%)	97·5 (72·0 to 111·4)	6·0% (5·3 to 6·8%)	60·7 (46·0 to 69·2)	5·3% (4·7 to 6·3%)	-37·7% (-45·2 to -28·2%)	-12·1% (-24·8 to 5·0%)
Unintentional firearm injuries	0·1 (0·1 to 0·1)	3·0% (2·5 to 3·6%)	0·1 (0·1 to 0·1)	2·9% (2·4 to 3·6%)	-25·5% (-39·9 to -7·3%)	-1·1% (-19·2 to 23·0%)	6·0 (4·7 to 7·7)	3·5% (2·9 to 4·1%)	4·5 (3·5 to 5·9)	3·8% (3·1 to 4·6%)	-25·0% (-38·8 to -7·4%)	10·8% (-9·1 to 37·1%)
Unintentional suffocation	0·0 (0·0 to 0·0)	0·3% (0·3 to 0·4%)	0·1 (0·0 to 0·1)	0·6% (0·5 to 0·7%)	100·3% (6·3 to 142·6%)	99·1% (56·7 to 144·7%)	3·6 (2·8 to 4·4)	0·4% (0·3 to 0·5%)	5·4 (3·6 to 6·7)	0·6% (0·5 to 0·8%)	51·6% (8·7 to 79·5%)	72·6% (40·3 to 109·3%)
Other exposure to mechanical forces	2·7 (2·0 to 3·1)	6·6% (6·1 to 7·2%)	3·1 (1·7 to 3·6)	7·9% (7·1 to 8·9%)	13·3% (-16·6 to 28·9%)	18·7% (4·9 to 33·6%)	166·7 (126·6 to 191·1)	7·0% (6·5 to 7·6%)	192·6 (127·5 to 227·3)	9·0% (8·2 to 10·2%)	15·5% (-4·5 to 28·6%)	29·0% (13·8 to 46·3%)
Venomous animal contact	0·2 (0·1 to 0·2)	2·8% (2·3 to 3·5%)	0·1 (0·1 to 0·1)	2·6% (2·1 to 3·3%)	-30·9% (-45·5 to -9·5%)	-7·6% (-26·7 to 19·0%)	8·6 (6·7 to 10·9)	3·2% (2·6 to 3·9%)	6·1 (4·8 to 7·6)	3·2% (2·5 to 3·9%)	-29·2% (-44·0 to -8·8%)	0·6% (-20·9 to 29·2%)
Non-venomous animal contact	0·1 (0·1 to 0·1)	2·9% (2·4 to 3·6%)	0·1 (0·1 to 0·1)	2·5% (1·9 to 3·1%)	-42·3% (-55·3 to -24·1%)	-16·6% (-34·6 to 7·2%)	6·1 (4·8 to 7·8)	3·1% (2·6 to 3·8%)	3·6 (2·8 to 4·8)	2·9% (2·3 to 3·7%)	-41·4% (-54·3 to -23·7%)	-6·9% (-26·9 to 19·7%)
Pulmonary aspiration and foreign body in airway	0·4 (0·3 to 0·5)	2·2% (1·8 to 2·7%)	0·4 (0·3 to 0·5)	2·5% (2·1 to 3·1%)	18·5% (-5·2 to 44·2%)	15·2% (-5·3 to 42·5%)	31·9 (24·7 to 39·7)	2·8% (2·2 to 3·4%)	34·9 (27·7 to 42·9)	3·5% (2·8 to 4·2%)	9·6% (-7·5 to 28·4%)	26·1% (2·7 to 56·9%)
Foreign body in eyes	-	-	-	-	-	-	0·6 (0·3 to 1·1)	6·2% (5·5 to 7·1%)	1·0 (0·4 to 1·8)	8·0% (7·0 to 9·4%)	55·3% (33·0 to 80·9%)	28·6% (10·4 to 49·2%)
Foreign body in other body part	0·2 (0·1 to 0·3)	3·9% (3·2 to 4·8%)	0·2 (0·1 to 0·2)	4·3% (3·5 to 5·3%)	-9·1% (-29·4 to 18·7%)	10·4% (-14·4 to 43·0%)	12·5 (9·6 to 16·2)	4·7% (3·8 to 5·8%)	11·2 (8·7 to 14·9)	6·1% (4·9 to 7·4%)	-10·4% (-29·0 to 14·7%)	28·2% (-0·2 to 65·7%)
Other unintentional injuries	2·5 (2·3 to 2·8)	6·5% (5·9 to 7·1%)	2·5 (2·2 to 2·9)	7·3% (6·6 to 8·2%)	-1·1% (-14·7 to 14·8%)	13·3% (-0·5 to 29·3%)	209·3 (175·3 to 248·0)	6·7% (6·1 to 7·4%)	214·8 (177·5 to 264·6)	7·7% (7·0 to 8·7%)	2·7% (-9·5 to 17·2%)	16·0% (2·7 to 31·9%)
**Occupational ergonomic factors: All causes**	-	-	-	-	-	-	5,639·9 (3,928·4 to 7,833·1)	1·0% (0·7 to 1·3%)	6,114·3 (4,239·7 to 8,492·6)	1·1% (0·8 to 1·4%)	8·4% (5·6 to 11·2%)	12·1% (8·0 to 16·8%)
Low back pain	-	-	-	-	-	-	5,639·9 (3,928·4 to 7,833·1)	43·4% (41·0 to 45·7%)	6,114·3 (4,239·7 to 8,492·6)	40·6% (38·3 to 42·8%)	8·4% (5·6 to 11·2%)	-6·4% (-7·8 to -5·1%)
**Behavioural risks: All causes**	6,632·5 (6,029·8 to 7,268·7)	48·6% (44·2 to 53·0%)	7,017·5 (6,401·1 to 7,641·0)	49·9% (45·9 to 54·0%)	5·8% (0·8 to 11·2%)	2·8% (0·9 to 5·0%)	190,718·1 (175,848·2 to 206,612·9)	33·5% (31·1 to 36·3%)	184,652·6 (168,906·3 to 201,648·9)	33·6% (31·0 to 36·5%)	-3·2% (-7·3 to 1·1%)	0·2% (-2·2 to 2·7%)
**Child and maternal malnutrition: All causes**	201·3 (180·1 to 221·6)	1·5% (1·3 to 1·6%)	86·2 (74·8 to 99·2)	0·6% (0·5 to 0·7%)	-57·2% (-62·6 to -51·2%)	-58·4% (-63·8 to -52·3%)	27,143·4 (23,490·2 to 31,996·6)	4·8% (4·3 to 5·2%)	16,172·1 (13,146·7 to 20,359·8)	2·9% (2·6 to 3·4%)	-40·4% (-46·3 to -35·0%)	-38·4% (-44·0 to -33·0%)
**Suboptimal breastfeeding: All causes**	77·0 (38·0 to 114·4)	0·6% (0·3 to 0·8%)	33·4 (16·3 to 49·4)	0·2% (0·1 to 0·4%)	-56·6% (-63·0 to -48·9%)	-57·8% (-64·0 to -50·5%)	6,687·9 (3,305·2 to 9,939·1)	1·2% (0·6 to 1·7%)	2,935·1 (1,447·1 to 4,318·0)	0·5% (0·3 to 0·8%)	-56·1% (-62·4 to -48·5%)	-54·6% (-61·0 to -46·8%)
**Non-exclusive breastfeeding : All causes**	74·4 (35·5 to 111·9)	0·5% (0·3 to 0·8%)	32·3 (15·1 to 48·3)	0·2% (0·1 to 0·3%)	-56·7% (-63·1 to -49·1%)	-57·9% (-64·0 to -50·5%)	6,435·0 (3,087·8 to 9,661·4)	1·1% (0·5 to 1·7%)	2,797·7 (1,311·2 to 4,179·1)	0·5% (0·2 to 0·8%)	-56·5% (-62·9 to -48·9%)	-55·0% (-61·5 to -47·1%)
Diarrhoeal diseases	15·1 (8·8 to 21·4)	12·0% (7·3 to 16·5%)	6·2 (3·7 to 9·3)	7·0% (4·1 to 10·4%)	-58·9% (-70·6 to -43·0%)	-41·1% (-55·6 to -21·0%)	1,315·7 (772·5 to 1,855·4)	20·5% (12·7 to 27·8%)	549·4 (333·1 to 809·3)	14·5% (8·8 to 20·4%)	-58·2% (-69·9 to -42·6%)	-29·5% (-43·3 to -11·8%)
Lower respiratory infections	59·4 (21·8 to 96·6)	12·3% (4·5 to 20·2%)	26·1 (9·3 to 41·8)	5·7% (2·0 to 9·2%)	-56·1% (-62·9 to -48·1%)	-53·7% (-60·2 to -46·3%)	5,119·3 (1,887·1 to 8,324·1)	25·5% (9·6 to 41·1%)	2,248·3 (807·2 to 3,602·5)	17·6% (6·3 to 28·0%)	-56·1% (-62·8 to -48·1%)	-30·7% (-38·6 to -21·7%)
**Discontinued breastfeeding : All causes**	4·0 (1·3 to 7·6)	0·0% (0·0 to 0·1%)	1·8 (0·6 to 3·5)	0·0% (0·0 to 0·0%)	-54·5% (-66·0 to -38·8%)	-55·8% (-67·0 to -41·2%)	383·4 (126·5 to 710·1)	0·1% (0·0 to 0·1%)	195·8 (69·0 to 359·3)	0·0% (0·0 to 0·1%)	-48·9% (-60·7 to -33·7%)	-47·2% (-59·1 to -31·5%)
Diarrhoeal diseases	4·0 (1·3 to 7·6)	3·2% (1·0 to 5·9%)	1·8 (0·6 to 3·5)	2·1% (0·7 to 3·8%)	-54·5% (-66·0 to -38·8%)	-34·9% (-49·8 to -14·7%)	383·4 (126·5 to 710·1)	6·0% (2·0 to 10·7%)	195·8 (69·0 to 359·3)	5·2% (1·8 to 9·3%)	-48·9% (-60·7 to -33·7%)	-13·8% (-27·8 to 2·7%)
**Childhood undernutrition: All causes**	179·9 (156·8 to 202·3)	1·3% (1·2 to 1·5%)	75·0 (63·1 to 87·9)	0·5% (0·4 to 0·6%)	-58·3% (-63·7 to -52·2%)	-59·5% (-65·1 to -53·2%)	16,122·2 (14,123·6 to 18,091·2)	2·8% (2·4 to 3·2%)	7,065·6 (6,020·8 to 8,173·7)	1·3% (1·1 to 1·5%)	-56·2% (-61·3 to -50·3%)	-54·6% (-60·1 to -48·4%)
**Childhood underweight: All causes**	38·5 (28·3 to 58·1)	0·3% (0·2 to 0·4%)	13·2 (9·3 to 21·2)	0·1% (0·1 to 0·2%)	-65·7% (-73·7 to -53·6%)	-66·7% (-74·5 to -54·4%)	3,750·9 (2,850·0 to 5,427·0)	0·7% (0·5 to 1·0%)	1,527·0 (1,159·4 to 2,208·2)	0·3% (0·2 to 0·4%)	-59·3% (-67·8 to -48·3%)	-57·9% (-66·6 to -46·4%)
Diarrhoeal diseases	5·1 (3·7 to 7·0)	4·0% (3·0 to 5·3%)	1·6 (1·1 to 2·2)	1·8% (1·3 to 2·5%)	-68·6% (-76·8 to -56·2%)	-55·0% (-66·5 to -40·6%)	468·6 (345·1 to 639·6)	7·3% (5·8 to 9·3%)	161·8 (117·8 to 223·0)	4·3% (3·3 to 5·6%)	-65·5% (-73·7 to -53·8%)	-41·7% (-50·8 to -30·7%)
Lower respiratory infections	21·2 (13·6 to 37·8)	4·4% (2·8 to 7·8%)	7·1 (4·5 to 13·7)	1·5% (1·0 to 3·0%)	-66·5% (-71·8 to -60·4%)	-64·7% (-69·8 to -59·2%)	1,818·8 (1,170·9 to 3,250·8)	9·1% (5·9 to 16·4%)	609·4 (387·0 to 1,182·1)	4·8% (3·1 to 9·1%)	-66·5% (-71·8 to -60·3%)	-47·2% (-53·5 to -40·7%)
Measles	5·9 (0·7 to 22·4)	25·3% (6·9 to 49·5%)	2·0 (0·2 to 8·5)	18·8% (4·4 to 42·6%)	-66·3% (-95·4 to 75·0%)	-25·5% (-55·6 to -10·4%)	499·9 (62·4 to 1,905·8)	25·8% (7·0 to 50·3%)	168·5 (12·9 to 725·4)	19·2% (4·4 to 43·4%)	-66·3% (-95·4 to 74·7%)	-25·6% (-55·7 to -10·5%)
Protein-energy malnutrition	6·4 (5·0 to 8·8)	18·9% (15·1 to 24·4%)	2·6 (2·0 to 3·4)	8·3% (6·3 to 10·8%)	-60·0% (-73·0 to -45·6%)	-56·0% (-69·2 to -40·3%)	963·6 (757·0 to 1,233·4)	65·0% (60·0 to 70·4%)	587·3 (439·8 to 767·9)	57·3% (50·4 to 64·8%)	-39·1% (-53·2 to -25·0%)	-11·8% (-20·7 to -2·9%)
**Childhood wasting: All causes**	157·0 (117·2 to 187·8)	1·1% (0·9 to 1·4%)	66·4 (47·7 to 82·4)	0·5% (0·3 to 0·6%)	-57·7% (-63·1 to -51·2%)	-58·9% (-64·2 to -52·7%)	14,148·4 (10,575·9 to 16,801·9)	2·5% (1·9 to 3·0%)	6,317·4 (4,687·9 to 7,685·3)	1·2% (0·9 to 1·4%)	-55·3% (-60·4 to -49·3%)	-53·8% (-59·3 to -47·5%)

Supplemental Results Table 1. All-age deaths, death PAFs, DALYs, DALY PAFs, and percent change for each risk factor and outcome for both sexes combined in 2005 and 2015 for Southeast Asia, East Asia, and Oceania.

Data in parenthesis are 95% uncertainty intervals. PAFs=population attributable fractions. DALYs=disability-adjusted life-years.

	2005 Deaths (in thousands)	2005 Deaths PAF	2015 Deaths (in thousands)	2015 Deaths PAF	Percent change deaths 2005-2015	Percent change deaths PAF 2005-2015	2005 DALYs (in thousands)	2005 DALYs PAF	2015 DALYs (in thousands)	2015 DALYs PAF	Percent change DALYs 2005-2015	Percent change DALYs PAF 2005-2015
Diarrhoeal diseases	32·8<br>(23·7 to 41·1)	26·0%<br>(19·6 to 31·0%)	13·7<br>(9·4 to 17·9)	15·6%<br>(11·0 to 19·8%)	-58·1%<br>(-69·6 to -42·4%)	-39·9%<br>(-54·8 to -21·9%)	3,052·4<br>(2,220·6 to 3,772·4)	47·6%<br>(37·7 to 53·7%)	1,413·4<br>(994·6 to 1,779·2)	37·2%<br>(28·1 to 43·4%)	-53·7%<br>(-64·9 to -38·8%)	-21·9%<br>(-32·7 to -9·8%)
Lower respiratory infections	110·2<br>(70·0 to 137·6)	22·8%<br>(14·6 to 28·5%)	46·8<br>(29·0 to 59·4)	10·2%<br>(6·4 to 13·4%)	-57·5%<br>(-63·4 to -51·0%)	-55·1%<br>(-60·9 to -48·8%)	9,482·8<br>(6,029·7 to 11,841·3)	47·2%<br>(30·5 to 57·7%)	4,037·4<br>(2,504·2 to 5,118·4)	31·7%<br>(19·9 to 39·7%)	-57·4%<br>(-63·3 to -50·9%)	-32·8%<br>(-39·3 to -26·3%)
Measles	7·6<br>(0·7 to 32·1)	32·5%<br>(6·8 to 75·1%)	3·3<br>(0·2 to 15·6)	30·9%<br>(5·1 to 74·8%)	-57·0%<br>(-94·7 to 150·8%)	-5·1%<br>(-38·4 to 25·4%)	649·5<br>(57·4 to 2,731·5)	33·2%<br>(7·0 to 76·5%)	279·4<br>(18·0 to 1,331·9)	31·5%<br>(5·2 to 76·3%)	-57·0%<br>(-94·7 to 150·5%)	-5·1%<br>(-38·4 to 25·6%)
Protein-energy malnutrition	6·4<br>(5·0 to 8·8)	18·9%<br>(15·1 to 24·4%)	2·6<br>(2·0 to 3·4)	8·3%<br>(6·3 to 10·8%)	-60·0%<br>(-73·0 to -45·6%)	-56·0%<br>(-69·2 to -40·3%)	963·6<br>(757·0 to 1,233·4)	65·0%<br>(60·0 to 70·4%)	587·3<br>(439·8 to 767·9)	57·3%<br>(50·4 to 64·8%)	-39·1%<br>(-53·2 to -25·0%)	-11·8%<br>(-20·7 to -2·9%)
**Childhood stunting: All causes**	43·4<br>(16·4 to 85·6)	0·3%<br>(0·1 to 0·6%)	15·1<br>(5·5 to 32·0)	0·1%<br>(0·0 to 0·2%)	-65·1%<br>(-73·3 to -56·4%)	-66·1%<br>(-74·5 to -57·5%)	3,774·1<br>(1,460·1 to 7,404·3)	0·7%<br>(0·3 to 1·3%)	1,340·5<br>(505·6 to 2,797·8)	0·2%<br>(0·1 to 0·5%)	-64·5%<br>(-72·5 to -55·5%)	-63·2%<br>(-71·5 to -54·2%)
Diarrhoeal diseases	6·8<br>(2·4 to 12·4)	5·4%<br>(2·0 to 9·6%)	2·3<br>(0·8 to 4·5)	2·6%<br>(0·9 to 5·0%)	-65·6%<br>(-74·8 to -51·1%)	-50·7%<br>(-62·9 to -34·2%)	626·8<br>(228·1 to 1,146·3)	9·8%<br>(3·7 to 17·4%)	237·5<br>(79·9 to 452·6)	6·3%<br>(2·2 to 11·7%)	-62·1%<br>(-71·2 to -48·1%)	-35·9%<br>(-45·6 to -23·9%)
Lower respiratory infections	32·1<br>(2·6 to 76·0)	6·6%<br>(0·5 to 15·8%)	11·2<br>(0·7 to 28·2)	2·4%<br>(0·2 to 6·1%)	-65·2%<br>(-71·1 to -55·2%)	-63·2%<br>(-69·2 to -54·0%)	2,758·4<br>(225·4 to 6,544·4)	13·7%<br>(1·1 to 32·4%)	962·2<br>(60·8 to 2,424·1)	7·6%<br>(0·5 to 19·0%)	-65·1%<br>(-71·1 to -55·2%)	-45·0%<br>(-52·7 to -34·5%)
Measles	4·6<br>(0·3 to 17·8)	19·8%<br>(2·0 to 51·5%)	1·7<br>(0·1 to 7·3)	16·5%<br>(1·4 to 45·3%)	-63·8%<br>(-94·1 to 97·2%)	-16·5%<br>(-33·6 to -2·3%)	388·9<br>(24·7 to 1,520·7)	20·1%<br>(2·1 to 52·4%)	140·8<br>(6·3 to 618·3)	16·8%<br>(1·4 to 46·2%)	-63·8%<br>(-94·1 to 97·0%)	-16·5%<br>(-33·6 to -2·7%)
**Iron deficiency: All causes**	4·3<br>(2·3 to 6·8)	0·0%<br>(0·0 to 0·0%)	2·6<br>(1·5 to 3·9)	0·0%<br>(0·0 to 0·0%)	-39·0%<br>(-49·7 to -23·8%)	-40·8%<br>(-51·2 to -26·5%)	9,494·2<br>(6,357·3 to 13,647·7)	1·7%<br>(1·2 to 2·2%)	8,319·1<br>(5,535·1 to 12,116·1)	1·5%<br>(1·1 to 2·0%)	-12·4%<br>(-14·3 to -10·6%)	-9·4%<br>(-12·4 to -6·0%)
Maternal haemorrhage	2·8<br>(1·1 to 4·8)	24·3%<br>(9·2 to 40·1%)	1·3<br>(0·5 to 2·3)	23·3%<br>(8·5 to 37·7%)	-51·6%<br>(-62·4 to -37·7%)	-4·0%<br>(-15·2 to 6·4%)	158·0<br>(60·2 to 275·1)	24·2%<br>(9·1 to 40·0%)	77·6<br>(28·6 to 133·3)	23·2%<br>(8·5 to 37·5%)	-50·9%<br>(-61·5 to -37·7%)	-4·2%<br>(-15·3 to 6·1%)
Maternal sepsis and other pregnancy related infections	0·4<br>(0·1 to 0·7)	23·4%<br>(8·6 to 38·9%)	0·2<br>(0·1 to 0·4)	21·1%<br>(7·6 to 34·4%)	-41·7%<br>(-55·4 to -24·6%)	-9·6%<br>(-19·3 to -0·2%)	23·6<br>(8·6 to 42·6)	23·3%<br>(8·5 to 38·7%)	14·2<br>(4·9 to 25·8)	21·2%<br>(7·7 to 34·6%)	-39·9%<br>(-53·5 to -22·6%)	-9·0%<br>(-18·6 to 0·5%)
Iron-deficiency anaemia	1·2<br>(0·6 to 1·6)	100·0%<br>(100·0 to 100·0%)	1·1<br>(0·6 to 1·5)	100·0%<br>(100·0 to 100·0%)	-8·5%<br>(-23·4 to 10·6%)	-	9,312·6<br>(6,211·8 to 13,478·9)	100·0%<br>(100·0 to 100·0%)	8,227·4<br>(5,431·2 to 12,016·5)	100·0%<br>(100·0 to 100·0%)	-11·7%<br>(-13·3 to -9·9%)	-
**Vitamin A deficiency: All causes**	8·8<br>(4·0 to 19·8)	0·1%<br>(0·0 to 0·1%)	3·2<br>(1·4 to 7·9)	0·0%<br>(0·0 to 0·1%)	-63·9%<br>(-87·2 to 2·4%)	-64·9%<br>(-87·5 to -1·7%)	811·8<br>(400·7 to 1,750·6)	0·1%<br>(0·1 to 0·3%)	329·0<br>(167·3 to 728·3)	0·1%<br>(0·0 to 0·1%)	-59·5%<br>(-83·3 to 0·9%)	-58·1%<br>(-82·9 to 4·0%)
Diarrhoeal diseases	3·9<br>(2·0 to 6·4)	3·1%<br>(1·6 to 4·9%)	1·4<br>(0·7 to 2·3)	1·6%<br>(0·8 to 2·5%)	-64·5%<br>(-75·8 to -47·2%)	-49·1%<br>(-64·0 to -29·9%)	365·5<br>(184·4 to 589·9)	5·7%<br>(3·0 to 8·7%)	144·0<br>(73·6 to 229·2)	3·8%<br>(2·0 to 5·8%)	-60·6%<br>(-71·9 to -44·4%)	-33·5%<br>(-46·5 to -17·2%)
Measles	4·8<br>(0·8 to 16·7)	20·2%<br>(12·2 to 28·8%)	1·8<br>(0·2 to 6·7)	16·4%<br>(9·2 to 24·4%)	-63·4%<br>(-95·7 to 142·0%)	-19·0%<br>(-43·4 to 9·5%)	411·1<br>(72·2 to 1,425·7)	20·6%<br>(12·4 to 29·4%)	150·5<br>(20·1 to 567·3)	16·7%<br>(9·4 to 24·9%)	-63·4%<br>(-95·6 to 140·9%)	-19·0%<br>(-43·7 to 9·9%)
Vitamin A deficiency	-	-	-	-	-	-	35·2<br>(21·4 to 53·2)	100·0%<br>(100·0 to 100·0%)	34·5<br>(21·0 to 51·9)	100·0%<br>(100·0 to 100·0%)	-1·9%<br>(-8·6 to 5·8%)	-
**Zinc deficiency: All causes**	4·9<br>(0·2 to 13·1)	0·0%<br>(0·0 to 0·1%)	2·0<br>(0·1 to 5·5)	0·0%<br>(0·0 to 0·0%)	-58·6%<br>(-74·1 to -29·9%)	-59·8%<br>(-75·0 to -31·4%)	449·5<br>(51·7 to 1,142·3)	0·1%<br>(0·0 to 0·2%)	205·6<br>(40·0 to 493·9)	0·0%<br>(0·0 to 0·1%)	-54·3%<br>(-66·9 to -13·9%)	-52·7%<br>(-65·8 to -10·0%)
Diarrhoeal diseases	1·8<br>(0·0 to 4·7)	1·4%<br>(0·0 to 3·6%)	0·8<br>(0·0 to 2·0)	0·9%<br>(0·0 to 2·2%)	-57·1%<br>(-77·0 to 0·0%)	-38·6%<br>(-64·1 to 3·4%)	184·8<br>(29·7 to 423·9)	2·9%<br>(0·5 to 6·3%)	97·4<br>(29·1 to 202·5)	2·6%<br>(0·8 to 5·2%)	-47·3%<br>(-66·0 to 3·3%)	-11·1%<br>(-38·5 to 72·0%)
Lower respiratory infections	3·1<br>(0·0 to 11·0)	0·6%<br>(0·0 to 2·2%)	1·3<br>(0·0 to 4·6)	0·3%<br>(0·0 to 1·0%)	-59·4%<br>(-70·6 to 0·0%)	-57·2%<br>(-68·6 to 0·0%)	264·7<br>(1·3 to 928·1)	1·3%<br>(0·0 to 4·5%)	108·2<br>(1·0 to 388·9)	0·8%<br>(0·0 to 3·1%)	-59·1%<br>(-69·8 to -19·3%)	-35·6%<br>(-50·4 to 30·4%)
**Tobacco smoke: All causes**	2,706·1<br>(2,141·0 to 3,302·9)	19·8%<br>(15·7 to 24·0%)	2,735·1<br>(2,208·4 to 3,300·9)	19·4%<br>(15·8 to 23·3%)	1·1%<br>(-5·5 to 8·8%)	-1·8%<br>(-5·5 to 3·1%)	64,406·9<br>(52,807·8 to 77,493·0)	11·3%<br>(9·1 to 13·6%)	61,810·2<br>(51,404·3 to 73,555·2)	11·3%<br>(9·2 to 13·6%)	-4·0%<br>(-10·5 to 3·4%)	-0·7%<br>(-5·5 to 5·2%)
**Smoking: All causes**	2,435·3<br>(1,823·2 to 3,063·4)	17·8%<br>(13·3 to 22·3%)	2,462·6<br>(1,892·8 to 3,042·7)	17·5%<br>(13·5 to 21·5%)	1·1%<br>(-6·0 to 10·0%)	-1·8%<br>(-6·1 to 4·2%)	56,334·4<br>(43,752·5 to 69,733·5)	9·9%<br>(7·7 to 12·4%)	55,233·7<br>(44,151·8 to 67,329·7)	10·1%<br>(8·0 to 12·4%)	-2·0%<br>(-9·2 to 6·9%)	1·5%<br>(-4·2 to 9·2%)
Tuberculosis	41·9<br>(21·1 to 68·5)	14·0%<br>(7·2 to 20·8%)	31·4<br>(15·4 to 54·9)	13·9%<br>(7·0 to 20·9%)	-25·0%<br>(-37·6 to -12·4%)	-0·5%<br>(-7·5 to 6·1%)	1,275·6<br>(643·4 to 2,031·0)	12·8%<br>(6·6 to 19·1%)	981·4<br>(491·7 to 1,637·5)	13·1%<br>(6·7 to 19·7%)	-23·1%<br>(-34·4 to -10·6%)	2·4%<br>(-4·5 to 8·7%)
Lower respiratory infections	91·7<br>(73·1 to 112·0)	18·9%<br>(15·4 to 22·6%)	106·8<br>(83·4 to 130·9)	23·2%<br>(19·1 to 27·9%)	16·4%<br>(8·2 to 25·4%)	22·7%<br>(15·7 to 30·2%)	1,755·7<br>(1,400·5 to 2,152·5)	8·7%<br>(7·0 to 10·5%)	1,892·7<br>(1,456·9 to 2,288·7)	14·9%<br>(12·0 to 17·8%)	7·8%<br>(0·3 to 16·3%)	69·9%<br>(54·7 to 86·5%)
Lip and oral cavity cancer	10·0<br>(6·8 to 13·3)	42·0%<br>(29·4 to 56·2%)	15·2<br>(10·0 to 21·0)	40·9%<br>(27·1 to 56·5%)	52·5%<br>(34·0 to 70·1%)	-2·7%<br>(-10·9 to 4·0%)	260·9<br>(178·5 to 354·1)	41·2%<br>(28·5 to 55·3%)	368·3<br>(236·8 to 518·1)	39·1%<br>(25·7 to 54·6%)	41·2%<br>(20·8 to 60·8%)	-5·1%<br>(-15·0 to 3·0%)
Nasopharynx cancer	12·9<br>(6·9 to 19·7)	36·2%<br>(19·8 to 54·3%)	13·0<br>(7·1 to 20·0)	34·1%<br>(19·2 to 51·5%)	0·4%<br>(-14·2 to 17·7%)	-5·7%<br>(-15·8 to 6·4%)	411·5<br>(213·1 to 643·1)	34·6%<br>(18·0 to 53·0%)	374·2<br>(202·5 to 584·2)	32·1%<br>(17·8 to 49·1%)	-9·1%<br>(-24·8 to 10·2%)	-7·4%<br>(-20·1 to 8·9%)
Oesophageal cancer	89·6<br>(33·8 to 154·7)	32·0%<br>(12·0 to 55·4%)	70·6<br>(29·2 to 120·9)	31·6%<br>(12·9 to 53·4%)	-21·2%<br>(-32·7 to -3·9%)	-1·2%<br>(-10·2 to 16·0%)	1,988·9<br>(754·2 to 3,436·7)	31·2%<br>(11·8 to 54·1%)	1,445·2<br>(573·3 to 2,487·7)	29·9%<br>(11·8 to 51·2%)	-27·3%<br>(-39·6 to -9·2%)	-4·2%<br>(-14·7 to 13·5%)
Stomach cancer	35·8<br>(13·4 to 75·1)	8·6%<br>(3·2 to 18·1%)	32·5<br>(12·6 to 66·4)	8·4%<br>(3·3 to 17·5%)	-9·4%<br>(-22·3 to 8·5%)	-1·9%<br>(-12·4 to 14·4%)	834·5<br>(311·3 to 1,756·5)	8·4%<br>(3·1 to 17·8%)	674·4<br>(268·6 to 1,400·4)	7·8%<br>(3·2 to 16·5%)	-19·2%<br>(-32·1 to 0·3%)	-6·8%<br>(-18·9 to 12·5%)
Colon and rectum cancer	8·6<br>(4·3 to 16·3)	4·7%<br>(2·4 to 8·8%)	10·8<br>(5·5 to 19·5)	4·6%<br>(2·4 to 8·3%)	25·0%<br>(8·6 to 49·0%)	-2·3%<br>(-13·0 to 14·3%)	197·5<br>(96·1 to 378·2)	4·5%<br>(2·2 to 8·5%)	215·9<br>(111·0 to 390·8)	4·1%<br>(2·1 to 7·4%)	9·3%<br>(-7·8 to 35·3%)	-9·5%<br>(-21·5 to 9·0%)
Liver cancer due to hepatitis B	28·7<br>(10·0 to 61·4)	14·2%<br>(4·9 to 29·4%)	24·8<br>(9·2 to 51·2)	13·1%<br>(4·8 to 26·6%)	-13·5%<br>(-30·6 to 7·7%)	-7·6%<br>(-21·5 to 13·6%)	937·8<br>(328·4 to 1,970·2)	14·4%<br>(5·0 to 29·6%)	734·4<br>(268·5 to 1,535·2)	12·8%<br>(4·7 to 26·2%)	-21·7%<br>(-40·1 to 1·7%)	-10·9%<br>(-28·1 to 15·8%)
Liver cancer due to hepatitis C	6·6<br>(3·2 to 11·8)	14·2%<br>(6·9 to 25·1%)	7·4<br>(3·7 to 12·8)	14·4%<br>(7·3 to 24·6%)	11·8%<br>(-4·7 to 31·8%)	1·4%<br>(-9·9 to 18·6%)	163·8<br>(79·8 to 293·6)	14·7%<br>(7·0 to 25·9%)	165·3<br>(81·8 to 289·8)	14·1%<br>(7·1 to 24·4%)	0·9%<br>(-16·7 to 23·0%)	-4·1%<br>(-16·9 to 15·8%)
Liver cancer due to alcohol use	19·1<br>(7·3 to 38·4)	15·6%<br>(6·1 to 30·9%)	23·2<br>(9·1 to 45·6)	14·8%<br>(5·8 to 28·8%)	21·5%<br>(2·7 to 45·1%)	-5·1%<br>(-15·3 to 9·3%)	505·4<br>(190·6 to 1,015·0)	16·0%<br>(6·1 to 31·7%)	572·0<br>(222·5 to 1,141·7)	14·6%<br>(5·7 to 28·9%)	13·2%<br>(-7·4 to 38·0%)	-8·8%<br>(-20·8 to 7·1%)
Liver cancer due to other causes	11·9<br>(4·7 to 24·0)	12·2%<br>(4·7 to 24·2%)	10·6<br>(4·4 to 21·0)	11·8%<br>(4·9 to 22·8%)	-10·5%<br>(-24·9 to 9·5%)	-2·9%<br>(-15·0 to 18·8%)	337·1<br>(131·5 to 691·0)	12·5%<br>(4·8 to 25·0%)	267·6<br>(110·9 to 533·7)	11·6%<br>(4·8 to 22·8%)	-20·6%<br>(-36·0 to 2·7%)	-7·2%<br>(-22·6 to 18·4%)
Pancreatic cancer	9·5<br>(4·9 to 16·7)	13·7%<br>(6·9 to 23·9%)	12·2<br>(6·3 to 21·5)	12·7%<br>(6·6 to 22·3%)	28·5%<br>(13·6 to 46·6%)	-7·2%<br>(-15·3 to 3·4%)	218·9<br>(112·2 to 384·4)	13·5%<br>(6·9 to 23·5%)	259·4<br>(134·2 to 461·3)	11·8%<br>(6·2 to 21·0%)	18·5%<br>(2·2 to 38·2%)	-12·0%<br>(-22·4 to 0·4%)
Tracheal, bronchus, and lung cancer	348·1<br>(217·2 to 454·3)	61·3%<br>(38·2 to 79·2%)	416·5<br>(252·9 to 553·4)	59·0%<br>(36·4 to 77·6%)	19·7%<br>(7·8 to 33·9%)	-3·7%<br>(-8·8 to 0·8%)	7,870·0<br>(4,735·6 to 10,399·4)	59·1%<br>(35·7 to 77·7%)	8,755·8<br>(5,105·9 to 11,994·8)	55·9%<br>(33·3 to 75·6%)	11·3%<br>(-2·3 to 27·3%)	-5·4%<br>(-12·6 to 0·0%)
Cervical cancer	3·5<br>(1·0 to 7·6)	5·8%<br>(1·7 to 12·2%)	3·1<br>(0·9 to 6·4)	4·9%<br>(1·5 to 10·1%)	-11·9%<br>(-32·7 to 22·3%)	-15·2%<br>(-30·9 to 11·9%)	111·9<br>(31·1 to 246·7)	5·8%<br>(1·6 to 12·4%)	87·9<br>(25·4 to 185·7)	4·6%<br>(1·4 to 9·5%)	-21·4%<br>(-42·7 to 15·7%)	-20·9%<br>(-38·8 to 10·5%)
Kidney cancer	1·9<br>(0·9 to 3·4)	11·1%<br>(5·5 to 19·3%)	3·0<br>(1·6 to 5·3)	11·9%<br>(6·1 to 20·2%)	58·2%<br>(35·7 to 88·3%)	7·4%<br>(-4·3 to 25·2%)	49·5<br>(23·6 to 87·4)	9·9%<br>(4·8 to 17·4%)	71·6<br>(36·5 to 124·4)	10·5%<br>(5·4 to 17·9%)	44·5%<br>(21·9 to 75·8%)	5·6%<br>(-8·3 to 27·1%)
Bladder cancer	6·0<br>(3·1 to 10·0)	21·1%<br>(11·0 to 34·6%)	7·7<br>(4·1 to 12·9)	21·3%<br>(11·5 to 34·9%)	27·7%<br>(13·9 to 43·2%)	1·0%<br>(-6·3 to 10·8%)	117·4<br>(61·6 to 198·1)	20·0%<br>(10·5 to 33·3%)	138·0<br>(73·9 to 232·2)	19·3%<br>(10·5 to 32·0%)	17·6%<br>(3·6 to 35·4%)	-3·6%<br>(-11·5 to 7·3%)
Ischaemic heart disease	365·8<br>(302·2 to 426·4)	22·1%<br>(18·4 to 25·6%)	429·9<br>(353·1 to 506·9)	20·7%<br>(17·0 to 24·2%)	17·5%<br>(9·6 to 26·4%)	-6·4%<br>(-10·4 to -2·6%)	9,506·5<br>(7,859·5 to 11,053·7)	28·1%<br>(23·4 to 32·4%)	10,693·3<br>(8,769·8 to 12,601·4)	27·2%<br>(22·4 to 31·7%)	12·5%<br>(4·4 to 21·1%)	-3·3%<br>(-6·8 to 0·1%)
Ischaemic stroke	167·2<br>(138·7 to 194·0)	18·7%<br>(15·7 to 21·6%)	176·4<br>(144·7 to 208·1)	17·6%<br>(14·6 to 20·5%)	5·5%<br>(-1·9 to 13·8%)	-6·0%<br>(-10·5 to -1·0%)	3,580·1<br>(2,981·6 to 4,172·0)	23·2%<br>(19·6 to 26·8%)	3,688·5<br>(3,050·5 to 4,358·2)	22·6%<br>(18·9 to 26·3%)	3·0%<br>(-4·3 to 10·5%)	-2·9%<br>(-7·0 to 1·2%)
Hemorrhagic stroke	368·7<br>(308·4 to 427·4)	22·5%<br>(18·9 to 25·8%)	345·7<br>(287·6 to 407·8)	21·8%<br>(18·0 to 25·5%)	-6·2%<br>(-12·6 to 1·2%)	-3·0%<br>(-6·9 to 1·2%)	9,593·8<br>(8,068·1 to 11,103·2)	26·8%<br>(22·7 to 30·7%)	8,861·7<br>(7,343·7 to 10,430·8)	26·8%<br>(22·3 to 31·1%)	-7·6%<br>(-14·0 to -0·6%)	-0·2%<br>(-4·0 to 3·8%)
Hypertensive heart disease	43·6<br>(34·4 to 52·9)	17·9%<br>(14·1 to 21·6%)	55·1<br>(43·4 to 66·8)	16·9%<br>(13·3 to 20·8%)	26·3%<br>(14·2 to 39·2%)	-5·4%<br>(-11·5 to 6·1%)	1,018·1<br>(810·8 to 1,226·3)	22·3%<br>(17·8 to 26·5%)	1,201·9<br>(960·0 to 1,455·2)	21·8%<br>(17·3 to 26·3%)	18·1%<br>(7·4 to 29·9%)	-2·3%<br>(-8·1 to 6·2%)
Atrial fibrillation and flutter	3·8<br>(2·7 to 5·2)	12·6%<br>(9·8 to 15·4%)	4·9<br>(3·5 to 6·5)	11·5%<br>(8·9 to 14·1%)	28·4%<br>(19·5 to 38·9%)	-8·6%<br>(-13·6 to -2·9%)	173·7<br>(122·6 to 236·0)	20·8%<br>(16·5 to 24·9%)	216·3<br>(150·9 to 296·3)	19·3%<br>(15·2 to 23·2%)	24·5%<br>(20·2 to 28·5%)	-7·2%<br>(-9·7 to -4·9%)
Aortic aneurysm	4·5<br>(3·6 to 5·4)	23·7%<br>(19·2 to 28·3%)	5·4<br>(4·1 to 6·6)	21·7%<br>(17·3 to 26·0%)	20·6%<br>(6·7 to 32·6%)	-8·7%<br>(-13·6 to -3·8%)	117·1<br>(94·0 to 140·1)	28·7%<br>(23·3 to 33·8%)	137·5<br>(106·0 to 168·3)	27·1%<br>(21·8 to 32·3%)	17·4%<br>(2·7 to 30·2%)	-5·3%<br>(-10·3 to -0·2%)

Supplemental Results Table 1. All-age deaths, death PAFs, DALYs, DALY PAFs, and percent change for each risk factor and outcome for both sexes combined in 2005 and 2015 for Southeast Asia, East Asia, and Oceania.

Data in parenthesis are 95% uncertainty intervals. PAFs=population attributable fractions. DALYs=disability-adjusted life-years.

	2005 Deaths (in thousands)	2005 Deaths PAF	2015 Deaths (in thousands)	2015 Deaths PAF	Percent change deaths 2005-2015	Percent change deaths PAF 2005-2015	2005 DALYs (in thousands)	2005 DALYs PAF	2015 DALYs (in thousands)	2015 DALYs PAF	Percent change DALYs 2005-2015	Percent change DALYs PAF 2005-2015
Peripheral vascular disease	0·2 (0·2 to 0·3)	18·3% (14·7 to 22·0%)	0·4 (0·3 to 0·5)	16·4% (12·9 to 19·8%)	59·2% (41·5 to 79·0%)	-10·4% (-16·4 to -0·9%)	28·8 (15·5 to 48·7)	18·8% (15·0 to 22·7%)	40·0 (21·8 to 67·9)	17·6% (13·9 to 21·3%)	39·2% (33·9 to 45·1%)	-6·9% (-9·8 to -4·0%)
Other cardiovascular and circulatory diseases	18·2 (14·5 to 21·9)	17·9% (14·3 to 21·3%)	19·5 (15·5 to 23·6)	17·4% (13·9 to 20·9%)	7·5% (-1·7 to 17·9%)	-2·5% (-8·3 to 4·2%)	718·8 (567·0 to 885·3)	17·6% (14·2 to 20·7%)	801·0 (623·1 to 1,009·5)	18·1% (14·5 to 21·6%)	11·4% (4·4 to 18·4%)	3·1% (-1·2 to 7·8%)
Chronic obstructive pulmonary disease	644·5 (291·2 to 958·1)	47·9% (21·7 to 70·7%)	538·3 (271·3 to 776·1)	48·7% (24·6 to 70·4%)	-16·5% (-24·5 to -2·3%)	1·7% (-4·0 to 18·1%)	10,950·5 (5,240·0 to 16,320·0)	43·8% (21·2 to 65·1%)	8,765·9 (4,695·9 to 12,632·7)	43·4% (23·6 to 62·8%)	-19·9% (-28·1 to -5·4%)	-1·0% (-7·6 to 15·6%)
Silicosis	0·8 (0·3 to 1·6)	12·5% (4·6 to 24·4%)	0·8 (0·3 to 1·7)	11·8% (4·1 to 24·1%)	-0·3% (-24·7 to 26·2%)	-5·9% (-22·3 to 9·2%)	23·0 (7·9 to 47·5)	12·4% (4·4 to 24·4%)	19·9 (6·8 to 42·3)	10·6% (3·7 to 22·0%)	-13·3% (-38·7 to 15·7%)	-14·2% (-34·2 to 6·5%)
Asbestosis	0·0 (0·0 to 0·1)	10·4% (4·8 to 19·4%)	0·0 (0·0 to 0·1)	10·9% (5·8 to 18·9%)	5·2% (-25·5 to 52·2%)	5·1% (-13·3 to 40·9%)	1·6 (0·7 to 3·2)	9·3% (3·8 to 17·8%)	1·5 (0·7 to 3·0)	8·3% (3·8 to 15·9%)	-6·8% (-29·4 to 24·8%)	-10·8% (-27·9 to 15·0%)
Coal workers pneumoconiosis	0·2 (0·1 to 0·3)	12·8% (5·3 to 24·4%)	0·2 (0·1 to 0·4)	13·3% (5·8 to 24·6%)	6·3% (-26·4 to 53·4%)	4·3% (-13·0 to 35·3%)	4·0 (1·7 to 7·4)	13·2% (5·5 to 24·4%)	3·8 (1·5 to 7·2)	13·1% (6·0 to 23·7%)	-5·5% (-37·4 to 43·9%)	-1·0% (-22·9 to 37·6%)
Other pneumoconiosis	0·4 (0·2 to 0·7)	11·1% (6·7 to 17·1%)	0·4 (0·3 to 0·8)	11·8% (7·3 to 17·9%)	19·1% (-15·1 to 72·7%)	6·2% (-18·1 to 45·1%)	13·9 (6·9 to 25·5)	8·6% (4·4 to 15·0%)	13·4 (7·3 to 23·2)	7·7% (4·3 to 13·1%)	-3·6% (-22·1 to 26·6%)	-10·0% (-25·6 to 14·6%)
Asthma	22·8 (17·7 to 28·3)	19·4% (15·4 to 23·2%)	20·9 (15·9 to 26·1)	18·9% (14·9 to 22·7%)	-8·1% (-19·9 to 4·4%)	-2·4% (-9·3 to 5·4%)	767·9 (581·8 to 946·6)	12·9% (10·2 to 15·7%)	743·7 (560·0 to 930·8)	12·9% (10·1 to 15·8%)	-3·2% (-12·0 to 5·7%)	-0·1% (-6·2 to 6·0%)
Interstitial lung disease and pulmonary sarcoidosis	0·8 (0·5 to 1·4)	11·1% (6·6 to 18·2%)	1·4 (0·8 to 2·3)	11·4% (6·9 to 18·4%)	67·9% (29·1 to 109·2%)	2·6% (-9·3 to 16·2%)	19·3 (10·7 to 32·0)	10·0% (5·8 to 16·2%)	28·0 (16·6 to 45·9)	9·8% (6·0 to 15·7%)	45·5% (16·2 to 79·5%)	-1·8% (-12·9 to 13·3%)
Other chronic respiratory diseases	0·6 (0·4 to 1·0)	7·4% (4·8 to 10·5%)	1·0 (0·5 to 1·8)	9·3% (5·4 to 14·0%)	60·7% (6·3 to 149·8%)	26·4% (-12·7 to 75·7%)	48·3 (35·4 to 63·4)	5·9% (4·4 to 7·8%)	52·0 (36·8 to 70·0)	6·6% (4·9 to 8·9%)	7·6% (-12·7 to 32·3%)	11·8% (-9·1 to 39·3%)
Diabetes mellitus	21·1 (6·1 to 36·4)	7·2% (2·1 to 12·3%)	25·6 (7·1 to 44·4)	7·1% (2·0 to 12·4%)	21·6% (8·7 to 34·8%)	-0·8% (-8·8 to 7·2%)	1,175·5 (329·9 to 2,053·9)	8·1% (2·2 to 13·9%)	1,393·2 (399·3 to 2,468·1)	7·8% (2·2 to 13·5%)	18·5% (11·3 to 25·8%)	-3·2% (-7·5 to 1·2%)
**Second-hand smoke: All causes**	330·8 (253·7 to 420·5)	2·4% (1·9 to 3·1%)	333·8 (256·9 to 419·4)	2·4% (1·8 to 3·0%)	0·9% (-4·5 to 6·6%)	-2·0% (-6·8 to 2·9%)	9,416·0 (7,247·7 to 11,696·0)	1·7% (1·3 to 2·1%)	7,935·2 (6,131·5 to 9,757·4)	1·4% (1·1 to 1·8%)	-15·7% (-21·4 to -9·8%)	-12·8% (-18·1 to -7·2%)
Lower respiratory infections	52·6 (33·9 to 73·3)	10·9% (6·9 to 15·0%)	37·3 (23·3 to 52·3)	8·1% (5·1 to 11·3%)	-29·1% (-34·4 to -24·0%)	-25·2% (-28·6 to -21·9%)	3,182·5 (2,023·4 to 4,401·8)	15·8% (10·0 to 21·6%)	1,594·1 (996·0 to 2,229·6)	12·5% (7·9 to 17·3%)	-49·9% (-54·7 to -44·9%)	-21·0% (-24·4 to -17·6%)
Otitis media	0·1 (0·0 to 0·1)	9·9% (6·8 to 12·8%)	0·0 (0·0 to 0·0)	8·3% (5·7 to 10·7%)	-47·9% (-56·4 to -36·9%)	-15·8% (-24·9 to -6·1%)	71·5 (39·9 to 114·0)	8·8% (6·0 to 11·5%)	59·9 (33·2 to 96·6)	8·3% (5·6 to 10·8%)	-16·2% (-20·9 to -11·7%)	-5·9% (-10·2 to -1·8%)
Tracheal, bronchus, and lung cancer	15·2 (6·8 to 27·9)	2·7% (1·2 to 4·9%)	20·9 (9·6 to 38·0)	3·0% (1·4 to 5·3%)	37·3% (28·3 to 44·8%)	10·5% (2·7 to 19·7%)	394·8 (175·7 to 719·5)	3·0% (1·3 to 5·4%)	523·2 (245·6 to 943·6)	3·3% (1·6 to 5·9%)	32·5% (22·1 to 41·7%)	12·7% (3·6 to 24·1%)
Ischaemic heart disease	107·9 (83·4 to 136·1)	6·5% (5·1 to 8·3%)	132·0 (100·7 to 167·7)	6·4% (4·8 to 8·1%)	22·4% (14·3 to 30·0%)	-2·5% (-6·8 to 2·0%)	2,409·2 (1,952·0 to 2,922·0)	7·1% (5·8 to 8·7%)	2,718·0 (2,157·4 to 3,314·5)	6·9% (5·5 to 8·4%)	12·8% (4·6 to 20·8%)	-3·0% (-7·6 to 2·0%)
Ischaemic stroke	31·0 (23·3 to 40·1)	3·5% (2·6 to 4·5%)	34·2 (25·2 to 44·6)	3·4% (2·5 to 4·4%)	10·5% (2·8 to 17·9%)	-1·5% (-6·3 to 3·1%)	583·8 (453·1 to 738·0)	3·8% (3·0 to 4·7%)	610·9 (461·3 to 769·8)	3·7% (2·9 to 4·7%)	4·6% (-2·6 to 11·7%)	-1·4% (-6·1 to 2·7%)
Hemorrhagic stroke	64·4 (49·8 to 81·3)	3·9% (3·0 to 4·9%)	61·1 (47·2 to 78·2)	3·9% (3·0 to 4·9%)	-5·1% (-11·4 to 2·3%)	-1·7% (-6·3 to 2·6%)	1,584·6 (1,245·9 to 1,977·0)	4·4% (3·5 to 5·5%)	1,438·0 (1,130·0 to 1,808·3)	4·3% (3·4 to 5·5%)	-9·3% (-15·9 to -1·5%)	-1·9% (-6·8 to 3·3%)
**Alcohol and drug use: All causes**	821·5 (761·8 to 877·9)	6·0% (5·6 to 6·4%)	899·4 (826·4 to 972·2)	6·4% (5·9 to 6·9%)	9·5% (3·1 to 16·2%)	6·3% (2·5 to 10·1%)	32,380·5 (29,870·4 to 34,683·2)	5·7% (5·2 to 6·1%)	33,416·1 (30,744·1 to 36,147·6)	6·1% (5·5 to 6·6%)	3·2% (-1·9 to 8·5%)	6·8% (3·1 to 10·6%)
**Alcohol use: All causes**	726·3 (669·4 to 779·6)	5·3% (4·9 to 5·7%)	779·1 (708·6 to 850·6)	5·5% (5·1 to 6·0%)	7·3% (0·7 to 14·2%)	4·2% (-0·3 to 8·3%)	26,511·5 (24,362·3 to 28,661·9)	4·7% (4·2 to 5·1%)	26,521·0 (24,325·7 to 28,911·8)	4·8% (4·3 to 5·3%)	0·0% (-5·6 to 6·0%)	3·5% (-0·8 to 8·0%)
Tuberculosis	26·2 (20·9 to 35·0)	8·7% (7·5 to 9·9%)	20·7 (15·6 to 29·7)	9·2% (7·2 to 11·0%)	-20·8% (-32·7 to -9·0%)	5·4% (-8·8 to 19·4%)	896·7 (740·0 to 1,154·4)	9·0% (8·0 to 10·1%)	726·9 (570·5 to 981·9)	9·8% (7·9 to 11·4%)	-18·9% (-29·5 to -8·6%)	8·1% (-4·6 to 20·6%)
Lower respiratory infections	13·6 (11·1 to 15·8)	2·8% (2·4 to 3·2%)	17·6 (13·9 to 21·0)	3·8% (3·2 to 4·5%)	29·6% (16·3 to 43·7%)	36·7% (24·2 to 50·9%)	277·6 (230·1 to 321·2)	1·4% (1·2 to 1·6%)	326·7 (260·0 to 379·2)	2·6% (2·1 to 2·9%)	17·7% (5·7 to 30·5%)	85·5% (63·5 to 108·0%)
Lip and oral cavity cancer	5·9 (5·4 to 6·5)	25·1% (22·6 to 27·0%)	10·8 (9·4 to 12·0)	29·0% (25·5 to 31·8%)	81·3% (65·1 to 100·3%)	15·7% (8·8 to 22·0%)	174·3 (158·8 to 189·1)	27·6% (25·3 to 29·4%)	294·9 (257·5 to 327·4)	31·3% (27·9 to 33·9%)	69·1% (53·4 to 87·4%)	13·6% (7·5 to 19·3%)
Nasopharynx cancer	9·9 (6·4 to 10·9)	27·7% (23·5 to 29·7%)	11·5 (7·7 to 13·2)	30·3% (26·3 to 33·0%)	16·4% (2·7 to 31·1%)	9·4% (3·1 to 16·5%)	336·4 (219·8 to 371·8)	28·2% (24·1 to 30·2%)	365·3 (245·7 to 419·4)	31·2% (27·3 to 33·9%)	8·6% (-4·7 to 24·1%)	10·6% (4·7 to 18·2%)
Other pharynx cancer	2·1 (1·9 to 2·4)	24·6% (22·0 to 26·8%)	3·2 (2·7 to 3·7)	28·1% (24·2 to 31·3%)	49·8% (32·4 to 68·9%)	14·2% (6·3 to 22·1%)	64·2 (57·0 to 72·0)	26·3% (23·6 to 28·6%)	93·0 (77·9 to 107·9)	29·8% (25·8 to 33·1%)	44·8% (27·5 to 63·6%)	13·2% (5·3 to 20·9%)
Oesophageal cancer	44·5 (39·9 to 49·1)	15·9% (14·5 to 17·2%)	40·8 (34·5 to 46·9)	18·3% (15·9 to 20·1%)	-8·2% (-19·5 to 4·9%)	15·1% (6·5 to 22·6%)	1,111·5 (997·5 to 1,219·5)	17·4% (16·0 to 18·8%)	972·6 (829·0 to 1,109·0)	20·1% (17·7 to 22·0%)	-12·5% (-23·5 to 0·2%)	15·4% (7·6 to 22·2%)
Colon and rectum cancer	6·3 (5·6 to 6·9)	3·4% (3·1 to 3·8%)	8·6 (7·5 to 9·6)	3·7% (3·2 to 4·1%)	37·2% (27·2 to 47·9%)	7·2% (1·8 to 12·2%)	159·5 (144·4 to 174·6)	3·6% (3·3 to 3·9%)	208·7 (184·4 to 230·7)	3·9% (3·5 to 4·3%)	30·8% (20·4 to 41·9%)	8·3% (2·9 to 13·3%)
Liver cancer due to hepatitis B	1·1 (0·7 to 1·5)	0·5% (0·4 to 0·7%)	0·7 (0·4 to 1·0)	0·4% (0·2 to 0·5%)	-36·6% (-51·8 to -15·9%)	-32·2% (-47·1 to -17·0%)	59·0 (39·9 to 79·2)	0·9% (0·6 to 1·2%)	38·3 (23·9 to 55·3)	0·7% (0·4 to 0·9%)	-35·1% (-49·9 to -14·8%)	-26·0% (-41·7 to -11·1%)
Liver cancer due to hepatitis C	0·0 (0·0 to 0·1)	0·1% (0·1 to 0·1%)	0·0 (0·0 to 0·0)	0·1% (0·0 to 0·1%)	-32·4% (-52·5 to -6·7%)	-38·6% (-55·9 to -17·6%)	2·4 (1·4 to 3·5)	0·2% (0·1 to 0·3%)	1·6 (0·9 to 2·5)	0·1% (0·1 to 0·2%)	-30·8% (-50·7 to -5·5%)	-34·1% (-52·1 to -14·3%)
Liver cancer due to alcohol use	122·0 (99·2 to 132·2)	100·0% (100·0 to 100·0%)	156·2 (141·2 to 173·5)	100·0% (100·0 to 100·0%)	28·0% (17·2 to 46·9%)	-	3,153·3 (2,537·6 to 3,415·5)	100·0% (100·0 to 100·0%)	3,911·0 (3,523·4 to 4,384·8)	100·0% (100·0 to 100·0%)	24·0% (12·9 to 45·3%)	-
Liver cancer due to other causes	0·3 (0·2 to 0·4)	0·3% (0·2 to 0·4%)	0·2 (0·1 to 0·2)	0·2% (0·1 to 0·3%)	-40·4% (-54·5 to -22·5%)	-35·2% (-49·9 to -19·7%)	15·4 (10·3 to 20·8)	0·6% (0·4 to 0·8%)	9·4 (6·1 to 13·7)	0·4% (0·3 to 0·6%)	-39·1% (-53·0 to -20·5%)	-28·7% (-44·1 to -13·6%)
Larynx cancer	3·7 (3·4 to 4·1)	17·9% (16·2 to 19·4%)	5·0 (4·3 to 5·7)	19·6% (17·0 to 21·6%)	34·5% (22·9 to 47·1%)	9·3% (2·3 to 15·1%)	101·0 (92·4 to 109·7)	19·1% (17·5 to 20·5%)	131·1 (113·7 to 146·4)	20·8% (18·4 to 22·7%)	29·8% (18·1 to 41·7%)	9·1% (2·6 to 14·4%)
Breast cancer	3·0 (2·5 to 3·5)	3·1% (2·6 to 3·5%)	3·8 (3·0 to 4·5)	3·2% (2·7 to 3·6%)	26·4% (4·8 to 45·9%)	2·9% (-3·3 to 8·6%)	103·1 (85·7 to 119·8)	3·1% (2·6 to 3·5%)	125·6 (98·5 to 150·7)	3·2% (2·7 to 3·7%)	21·7% (0·5 to 40·6%)	3·0% (-3·3 to 8·7%)
Ischaemic heart disease	-5·9 (-9·6 to -2·2)	-0·4% (-0·6 to -0·1%)	-6·2 (-11·8 to -0·8)	-0·3% (-0·6 to 0·0%)	5·5% (-66·3 to 43·4%)	-16·0% (-72·7 to 13·3%)	-211·1 (-280·2 to -147·2)	-0·6% (-0·8 to -0·4%)	-190·1 (-287·0 to -104·5)	-0·5% (-0·7 to -0·3%)	-10·0% (-35·2 to 11·2%)	-22·6% (-43·6 to -4·9%)
Ischaemic stroke	3·2 (0·0 to 6·6)	0·4% (0·0 to 0·7%)	6·7 (2·9 to 10·7)	0·7% (0·3 to 1·1%)	107·2% (32·9 to 597·0%)	84·5% (19·0 to 509·4%)	98·6 (51·3 to 150·4)	0·6% (0·3 to 1·0%)	177·2 (122·9 to 237·7)	1·1% (0·8 to 1·4%)	79·7% (45·2 to 157·7%)	69·3% (39·3 to 141·8%)
Hemorrhagic stroke	115·3 (102·3 to 128·4)	7·0% (6·3 to 7·8%)	123·1 (105·7 to 140·6)	7·8% (6·6 to 8·8%)	6·7% (-2·3 to 16·3%)	10·4% (2·2 to 17·9%)	2,757·8 (2,487·7 to 3,022·5)	7·7% (7·0 to 8·3%)	2,840·2 (2,457·8 to 3,188·2)	8·6% (7·4 to 9·5%)	3·0% (-5·6 to 11·9%)	11·3% (3·7 to 17·8%)
Hypertensive heart disease	14·6 (11·9 to 17·5)	6·0% (4·8 to 7·2%)	22·9 (17·2 to 28·5)	7·1% (5·3 to 8·8%)	56·8% (34·9 to 77·4%)	17·4% (2·1 to 39·7%)	328·0 (280·1 to 375·6)	7·2% (6·1 to 8·2%)	468·9 (372·3 to 554·9)	8·5% (6·8 to 10·0%)	43·0% (26·4 to 59·4%)	18·3% (6·6 to 33·5%)
Atrial fibrillation and flutter	1·3 (1·0 to 1·7)	4·2% (3·6 to 4·9%)	2·0 (1·5 to 2·6)	4·7% (3·8 to 5·6%)	56·1% (41·9 to 69·3%)	11·2% (2·3 to 18·9%)	46·8 (35·7 to 61·4)	5·6% (5·1 to 6·2%)	68·6 (51·7 to 90·6)	6·1% (5·3 to 6·9%)	46·5% (37·5 to 54·0%)	9·3% (3·2 to 14·5%)
Cirrhosis and other chronic liver diseases due to hepatitis B	41·2 (36·0 to 46·6)	29·5% (26·0 to 32·5%)	39·5 (32·0 to 46·7)	29·1% (24·2 to 33·3%)	-3·9% (-13·8 to 5·9%)	-1·2% (-9·9 to 5·6%)	1,237·8 (1,080·6 to 1,403·0)	29·6% (26·3 to 32·4%)	1,114·1 (903·1 to 1,317·7)	29·0% (24·2 to 33·2%)	-10·0% (-20·1 to -0·9%)	-1·9% (-10·8 to 5·2%)
Cirrhosis and other chronic liver diseases due to hepatitis C	12·2 (10·3 to 14·0)	23·4% (20·3 to 26·3%)	11·6 (9·1 to 14·1)	22·3% (17·8 to 26·2%)	-4·3% (-15·5 to 6·2%)	-5·0% (-15·4 to 4·6%)	378·1 (317·8 to 439·8)	23·2% (20·0 to 26·1%)	343·3 (265·9 to 418·8)	21·7% (17·3 to 25·8%)	-9·2% (-20·5 to 1·1%)	-6·1% (-17·4 to 4·6%)
Cirrhosis and other chronic liver diseases due to alcohol use	63·2 (56·3 to 71·2)	100·0% (100·0 to 100·0%)	72·3 (63·7 to 81·9)	100·0% (100·0 to 100·0%)	14·4% (5·5 to 24·0%)	-	2,127·9 (1,879·6 to 2,403·9)	100·0% (100·0 to 100·0%)	2,301·3 (2,010·8 to 2,645·3)	100·0% (100·0 to 100·0%)	8·1% (-1·0 to 18·0%)	-
Cirrhosis and other chronic liver diseases due to other causes	13·2 (11·4 to 15·1)	22·6% (19·9 to 25·3%)	12·9 (10·5 to 15·4)	22·6% (18·5 to 26·2%)	-2·5% (-13·3 to 7·7%)	-0·3% (-10·2 to 8·3%)	382·4 (329·2 to 435·4)	20·6% (18·1 to 22·9%)	343·2 (278·0 to 409·7)	20·8% (17·0 to 24·1%)	-10·3% (-20·4 to -0·6%)	0·9% (-9·4 to 10·1%)
Pancreatitis	1·6 (1·4 to 1·8)	8·5% (7·5 to 9·4%)	2·2 (1·8 to 2·7)	9·5% (7·8 to 11·0%)	37·8% (16·8 to 56·1%)	11·1% (-0·4 to 21·2%)	56·6 (49·9 to 65·8)	8·9% (8·0 to 9·9%)	71·9 (58·5 to 87·8)	9·9% (8·2 to 11·6%)	27·2% (8·1 to 42·6%)	11·3% (-0·7 to 21·9%)

Supplemental Results Table 1. All-age deaths, death PAFs, DALYs, DALY PAFs, and percent change for each risk factor and outcome for both sexes combined in 2005 and 2015 for Southeast Asia, East Asia, and Oceania.

Data in parenthesis are 95% uncertainty intervals. PAFs=population attributable fractions. DALYs=disability-adjusted life-years.

	2005 Deaths (in thousands)	2005 Deaths PAF	2015 Deaths (in thousands)	2015 Deaths PAF	Percent change deaths 2005-2015	Percent change deaths PAF 2005-2015	2005 DALYs (in thousands)	2005 DALYs PAF	2015 DALYs (in thousands)	2015 DALYs PAF	Percent change DALYs 2005-2015	Percent change DALYs PAF 2005-2015
Epilepsy	3·0<br>(2·7 to 3·2)	10·4%<br>(9·6 to 11·3%)	2·9<br>(2·4 to 3·2)	12·0%<br>(10·2 to 13·4%)	-3·3%<br>(-13·1 to 7·0%)	14·7%<br>(5·4 to 23·0%)	259·2<br>(215·2 to 306·1)	8·6%<br>(7·9 to 9·3%)	234·0<br>(188·9 to 283·9)	9·7%<br>(8·4 to 10·8%)	-9·7%<br>(-17·4 to -2·3%)	13·3%<br>(5·4 to 20·6%)
Alcohol use disorders	28·8<br>(26·9 to 30·8)	100·0%<br>(100·0 to 100·0%)	27·8<br>(25·4 to 30·7)	100·0%<br>(100·0 to 100·0%)	-3·2%<br>(-11·9 to 6·0%)	-	2,735·5<br>(2,164·6 to 3,385·6)	100·0%<br>(100·0 to 100·0%)	2,824·6<br>(2,216·0 to 3,570·0)	100·0%<br>(100·0 to 100·0%)	3·3%<br>(-1·5 to 8·0%)	-
Diabetes mellitus	-5·3<br>(-6·0 to -4·4)	-1·8%<br>(-2·1 to -1·5%)	-5·7<br>(-6·7 to -4·8)	-1·6%<br>(-1·8 to -1·3%)	8·9%<br>(-1·3 to 20·5%)	-11·2%<br>(-18·6 to -3·1%)	-274·1<br>(-344·3 to -214·1)	-1·9%<br>(-2·1 to -1·7%)	-308·5<br>(-394·9 to -231·6)	-1·7%<br>(-1·9 to -1·5%)	12·6%<br>(6·0 to 19·4%)	-8·1%<br>(-13·1 to -2·8%)
Pedestrian road injuries	53·5<br>(43·6 to 64·5)	21·9%<br>(18·5 to 25·8%)	49·6<br>(40·8 to 59·2)	22·4%<br>(19·0 to 26·0%)	-7·1%<br>(-19·0 to 2·6%)	2·3%<br>(-0·9 to 5·8%)	2,668·6<br>(2,184·8 to 3,229·2)	23·9%<br>(20·1 to 28·0%)	2,334·6<br>(1,922·8 to 2,785·8)	25·0%<br>(21·3 to 29·0%)	-12·5%<br>(-23·3 to -3·6%)	4·7%<br>(1·4 to 7·9%)
Cyclist road injuries	7·9<br>(6·3 to 9·7)	20·5%<br>(17·1 to 24·2%)	6·7<br>(5·5 to 8·2)	20·3%<br>(17·1 to 23·9%)	-15·3%<br>(-25·9 to -3·2%)	-0·9%<br>(-5·9 to 3·9%)	427·7<br>(345·6 to 518·6)	19·9%<br>(16·6 to 23·6%)	357·6<br>(295·4 to 428·7)	19·1%<br>(16·2 to 22·5%)	-16·4%<br>(-25·8 to -5·9%)	-4·0%<br>(-8·6 to 1·0%)
Motorcyclist road injuries	37·5<br>(29·3 to 43·9)	33·3%<br>(29·1 to 37·7%)	33·0<br>(27·7 to 38·3)	32·8%<br>(28·7 to 36·8%)	-12·0%<br>(-22·7 to 4·3%)	-1·7%<br>(-8·8 to 5·6%)	2,072·6<br>(1,661·1 to 2,422·1)	32·7%<br>(28·5 to 36·9%)	1,789·4<br>(1,515·8 to 2,065·5)	32·2%<br>(28·2 to 36·0%)	-13·7%<br>(-23·4 to 2·8%)	-1·6%<br>(-8·1 to 5·2%)
Motor vehicle road injuries	36·5<br>(30·8 to 44·3)	35·2%<br>(31·7 to 39·0%)	32·1<br>(25·9 to 38·3)	36·4%<br>(33·2 to 39·8%)	-12·1%<br>(-23·6 to 0·3%)	3·3%<br>(0·0 to 6·8%)	1,949·5<br>(1,653·2 to 2,343·2)	34·2%<br>(30·8 to 38·1%)	1,694·2<br>(1,419·5 to 1,992·2)	35·4%<br>(32·2 to 38·9%)	-13·1%<br>(-22·8 to -2·4%)	3·3%<br>(0·1 to 6·6%)
Falls	13·4<br>(9·9 to 16·6)	9·7%<br>(8·1 to 11·7%)	18·2<br>(11·9 to 22·5)	10·7%<br>(8·8 to 12·9%)	35·8%<br>(12·8 to 51·0%)	10·8%<br>(2·3 to 19·1%)	646·9<br>(513·3 to 795·2)	10·7%<br>(9·1 to 12·8%)	823·8<br>(621·8 to 1,007·6)	11·9%<br>(10·2 to 14·0%)	27·4%<br>(11·7 to 39·3%)	11·3%<br>(5·9 to 16·7%)
Drowning	8·8<br>(7·3 to 10·6)	6·3%<br>(5·3 to 7·8%)	7·9<br>(6·7 to 9·6)	8·1%<br>(7·0 to 9·7%)	-10·4%<br>(-20·3 to 0·2%)	27·8%<br>(18·5 to 37·6%)	403·5<br>(330·4 to 492·6)	4·8%<br>(3·9 to 6·0%)	325·2<br>(272·0 to 399·0)	6·3%<br>(5·3 to 7·6%)	-19·4%<br>(-28·1 to -10·2%)	29·1%<br>(18·4 to 40·5%)
Fire, heat, and hot substances	1·6<br>(1·2 to 2·1)	7·6%<br>(6·2 to 9·3%)	1·7<br>(1·2 to 2·0)	9·0%<br>(7·4 to 10·8%)	0·7%<br>(-14·7 to 13·4%)	18·2%<br>(6·3 to 28·4%)	80·8<br>(64·2 to 101·4)	6·7%<br>(5·5 to 8·2%)	74·3<br>(59·0 to 92·1)	8·2%<br>(7·0 to 9·7%)	-8·0%<br>(-18·4 to 1·5%)	22·5%<br>(12·7 to 32·7%)
Poisonings	3·4<br>(2·0 to 4·3)	9·6%<br>(7·8 to 11·8%)	2·9<br>(1·7 to 3·6)	10·6%<br>(8·8 to 13·1%)	-15·0%<br>(-24·5 to 0·0%)	10·6%<br>(2·7 to 22·0%)	140·5<br>(88·3 to 179·2)	8·7%<br>(7·0 to 10·7%)	110·9<br>(69·3 to 139·1)	9·7%<br>(8·0 to 12·0%)	-21·1%<br>(-29·5 to -6·9%)	11·4%<br>(2·0 to 25·8%)
Unintentional firearm injuries	0·4<br>(0·3 to 0·5)	10·0%<br>(8·5 to 11·9%)	0·3<br>(0·2 to 0·4)	9·9%<br>(8·4 to 12·0%)	-25·1%<br>(-35·8 to -12·4%)	-0·8%<br>(-8·6 to 7·4%)	16·7<br>(12·6 to 22·5)	9·5%<br>(8·0 to 11·3%)	11·9<br>(9·2 to 16·7)	10·0%<br>(8·5 to 11·8%)	-29·0%<br>(-38·4 to -17·4%)	4·6%<br>(-2·8 to 12·6%)
Unintentional suffocation	0·2<br>(0·2 to 0·3)	1·9%<br>(1·5 to 2·6%)	0·5<br>(0·2 to 0·6)	3·9%<br>(2·8 to 4·9%)	110·7%<br>(0·1 to 159·1%)	106·5%<br>(35·5 to 155·7%)	15·4<br>(11·9 to 19·7)	1·5%<br>(1·1 to 2·1%)	22·9<br>(12·7 to 29·6)	2·5%<br>(2·0 to 3·2%)	48·4%<br>(-5·3 to 77·6%)	66·6%<br>(25·6 to 103·5%)
Other exposure to mechanical forces	5·8<br>(3·6 to 7·4)	13·9%<br>(11·4 to 16·7%)	6·2<br>(2·9 to 8·3)	15·4%<br>(12·4 to 18·3%)	6·7%<br>(-25·9 to 25·8%)	10·6%<br>(2·7 to 16·1%)	307·6<br>(202·4 to 390·9)	12·9%<br>(10·6 to 15·4%)	314·2<br>(177·0 to 413·2)	14·6%<br>(12·1 to 17·2%)	2·1%<br>(-21·3 to 17·4%)	13·2%<br>(7·7 to 18·4%)
Self-harm	24·3<br>(20·1 to 29·1)	10·4%<br>(8·8 to 12·5%)	23·1<br>(19·5 to 28·2)	12·0%<br>(10·3 to 14·1%)	-4·7%<br>(-16·1 to 15·7%)	15·8%<br>(3·2 to 25·1%)	950·2<br>(788·7 to 1,149·8)	10·1%<br>(8·6 to 12·2%)	840·4<br>(703·9 to 1,036·4)	12·0%<br>(10·3 to 14·0%)	-11·6%<br>(-22·1 to 6·8%)	17·9%<br>(5·0 to 27·4%)
Assault by firearm	2·0<br>(1·7 to 2·3)	12·0%<br>(10·7 to 13·5%)	1·6<br>(1·4 to 2·0)	11·2%<br>(9·9 to 12·8%)	-16·9%<br>(-27·1 to -4·3%)	-6·6%<br>(-10·2 to -3·2%)	103·3<br>(89·1 to 122·2)	12·7%<br>(11·3 to 14·3%)	83·2<br>(69·8 to 101·0)	12·0%<br>(10·6 to 13·6%)	-19·4%<br>(-29·6 to -7·2%)	-5·8%<br>(-9·5 to -2·5%)
Assault by sharp object	3·5<br>(2·9 to 4·2)	12·1%<br>(10·3 to 14·4%)	2·3<br>(2·0 to 2·9)	11·8%<br>(10·2 to 13·8%)	-32·6%<br>(-39·6 to -23·4%)	-2·8%<br>(-6·5 to 2·2%)	196·8<br>(164·4 to 240·1)	12·7%<br>(10·8 to 15·1%)	129·7<br>(108·9 to 159·0)	12·5%<br>(10·9 to 14·7%)	-34·1%<br>(-40·9 to -25·1%)	-1·2%<br>(-5·0 to 3·7%)
Assault by other means	2·5<br>(2·1 to 3·1)	10·5%<br>(8·8 to 12·6%)	1·9<br>(1·5 to 2·5)	10·6%<br>(9·1 to 12·6%)	-25·6%<br>(-33·3 to -10·8%)	1·7%<br>(-2·5 to 7·4%)	153·4<br>(124·9 to 192·1)	10·9%<br>(9·1 to 13·1%)	114·8<br>(94·3 to 147·1)	11·4%<br>(9·8 to 13·5%)	-25·2%<br>(-32·3 to -12·4%)	4·7%<br>(0·8 to 9·6%)
**Drug use: All causes**	105·3<br>(91·2 to 119·8)	0·8%<br>(0·7 to 0·9%)	130·4<br>(115·8 to 145·3)	0·9%<br>(0·8 to 1·0%)	23·8%<br>(14·4 to 34·2%)	20·2%<br>(13·4 to 29·0%)	6,216·9<br>(5,356·2 to 7,148·5)	1·1%<br>(1·0 to 1·2%)	7,217·3<br>(6,248·2 to 8,239·0)	1·3%<br>(1·2 to 1·5%)	16·1%<br>(9·9 to 22·4%)	20·1%<br>(14·6 to 26·2%)
HIV/AIDS - Tuberculosis	2·7<br>(1·6 to 4·4)	20·5%<br>(16·9 to 25·1%)	3·4<br>(2·1 to 5·0)	19·5%<br>(15·8 to 23·3%)	28·4%<br>(-9·7 to 81·6%)	-4·8%<br>(-20·8 to 13·4%)	128·2<br>(80·6 to 207·5)	19·5%<br>(16·1 to 23·8%)	159·3<br>(104·5 to 227·7)	18·6%<br>(15·2 to 22·3%)	24·2%<br>(-10·7 to 73·3%)	-4·9%<br>(-20·0 to 12·5%)
HIV/AIDS resulting in other diseases	9·8<br>(6·8 to 20·9)	19·9%<br>(17·1 to 26·3%)	16·0<br>(11·7 to 27·2)	19·0%<br>(16·1 to 24·0%)	62·1%<br>(10·3 to 120·2%)	-4·5%<br>(-15·3 to 11·2%)	463·2<br>(320·1 to 985·7)	19·0%<br>(16·3 to 25·3%)	706·2<br>(512·9 to 1,204·5)	18·2%<br>(15·3 to 23·1%)	52·5%<br>(5·8 to 108·2%)	-4·3%<br>(-15·3 to 10·7%)
Hepatitis B	0·2<br>(0·1 to 0·3)	1·0%<br>(0·4 to 1·6%)	0·2<br>(0·1 to 0·3)	1·0%<br>(0·5 to 1·6%)	-23·8%<br>(-34·1 to -11·0%)	4·3%<br>(-6·4 to 17·6%)	7·6<br>(3·2 to 12·6)	1·0%<br>(0·4 to 1·6%)	5·6<br>(2·4 to 9·0)	1·0%<br>(0·4 to 1·6%)	-26·6%<br>(-36·6 to -14·9%)	0·4%<br>(-9·0 to 11·4%)
Hepatitis C	0·5<br>(0·1 to 1·1)	60·3%<br>(49·7 to 69·3%)	0·4<br>(0·1 to 0·9)	61·3%<br>(51·6 to 69·8%)	-20·4%<br>(-37·7 to 2·3%)	1·7%<br>(-7·2 to 11·6%)	16·8<br>(4·0 to 38·7)	62·5%<br>(52·9 to 70·2%)	13·0<br>(3·4 to 29·6)	62·2%<br>(52·1 to 70·1%)	-22·8%<br>(-39·4 to -0·6%)	-0·5%<br>(-7·6 to 7·4%)
Liver cancer due to hepatitis B	2·6<br>(1·1 to 4·4)	1·3%<br>(0·5 to 2·2%)	2·9<br>(1·2 to 4·7)	1·5%<br>(0·7 to 2·4%)	9·0%<br>(-5·6 to 35·0%)	16·6%<br>(5·9 to 31·0%)	89·8<br>(37·5 to 148·9)	1·4%<br>(0·6 to 2·3%)	90·3<br>(40·1 to 148·8)	1·6%<br>(0·7 to 2·5%)	0·6%<br>(-13·2 to 26·4%)	14·7%<br>(4·5 to 27·9%)
Liver cancer due to hepatitis C	25·3<br>(18·4 to 31·0)	54·3%<br>(41·5 to 65·1%)	31·4<br>(25·1 to 37·1)	61·3%<br>(50·6 to 70·2%)	24·3%<br>(11·8 to 43·9%)	12·8%<br>(5·1 to 25·1%)	689·4<br>(530·2 to 814·9)	62·0%<br>(50·7 to 71·1%)	788·7<br>(649·2 to 917·4)	67·5%<br>(58·5 to 75·2%)	14·4%<br>(2·6 to 32·9%)	8·9%<br>(2·8 to 18·3%)
Cirrhosis and other chronic liver diseases due to hepatitis B	1·5<br>(0·6 to 2·4)	1·1%<br>(0·4 to 1·8%)	1·7<br>(0·7 to 2·7)	1·2%<br>(0·5 to 2·0%)	13·0%<br>(0·3 to 28·8%)	16·2%<br>(7·0 to 29·5%)	48·1<br>(20·1 to 79·2)	1·1%<br>(0·5 to 1·9%)	49·7<br>(21·1 to 79·5)	1·3%<br>(0·6 to 2·1%)	3·4%<br>(-8·3 to 17·8%)	12·7%<br>(3·8 to 25·1%)
Cirrhosis and other chronic liver diseases due to hepatitis C	32·2<br>(26·2 to 38·5)	62·0%<br>(51·4 to 70·1%)	34·2<br>(27·7 to 41·4)	65·2%<br>(55·4 to 72·6%)	6·0%<br>(-5·2 to 18·5%)	5·1%<br>(0·7 to 11·3%)	1,124·6<br>(932·1 to 1,331·9)	68·8%<br>(59·1 to 75·7%)	1,120·7<br>(919·9 to 1,353·5)	70·8%<br>(62·0 to 77·0%)	-0·3%<br>(-11·5 to 12·5%)	2·9%<br>(-0·7 to 7·7%)
Opioid use disorders	16·4<br>(14·0 to 17·7)	100·0%<br>(100·0 to 100·0%)	24·5<br>(18·6 to 27·3)	100·0%<br>(100·0 to 100·0%)	49·5%<br>(25·4 to 67·9%)	-	1,986·5<br>(1,571·7 to 2,375·5)	100·0%<br>(100·0 to 100·0%)	2,410·1<br>(1,922·0 to 2,896·5)	100·0%<br>(100·0 to 100·0%)	21·3%<br>(15·4 to 27·2%)	-
Cocaine use disorders	1·0<br>(0·8 to 1·5)	100·0%<br>(100·0 to 100·0%)	1·4<br>(1·0 to 2·3)	100·0%<br>(100·0 to 100·0%)	40·7%<br>(4·2 to 93·3%)	-	139·4<br>(102·1 to 184·9)	100·0%<br>(100·0 to 100·0%)	169·2<br>(124·1 to 230·1)	100·0%<br>(100·0 to 100·0%)	21·4%<br>(9·0 to 43·9%)	-
Amphetamine use disorders	2·0<br>(1·7 to 2·3)	100·0%<br>(100·0 to 100·0%)	3·3<br>(2·7 to 4·0)	100·0%<br>(100·0 to 100·0%)	68·9%<br>(35·1 to 113·3%)	-	489·0<br>(319·3 to 694·4)	100·0%<br>(100·0 to 100·0%)	644·0<br>(430·8 to 898·7)	100·0%<br>(100·0 to 100·0%)	31·7%<br>(23·7 to 41·4%)	-
Cannabis use disorders	-	-	-	-	-	-	202·5<br>(129·2 to 292·3)	100·0%<br>(100·0 to 100·0%)	199·8<br>(128·0 to 286·3)	100·0%<br>(100·0 to 100·0%)	-1·3%<br>(-4·1 to 1·6%)	-
Other drug use disorders	4·7<br>(4·2 to 6·0)	100·0%<br>(100·0 to 100·0%)	5·6<br>(4·8 to 7·3)	100·0%<br>(100·0 to 100·0%)	17·0%<br>(-2·8 to 42·5%)	-	512·4<br>(378·7 to 670·5)	100·0%<br>(100·0 to 100·0%)	596·4<br>(432·1 to 797·7)	100·0%<br>(100·0 to 100·0%)	16·4%<br>(6·6 to 26·8%)	-
Self-harm	6·4<br>(4·1 to 9·8)	2·7%<br>(1·8 to 4·2%)	5·6<br>(3·5 to 8·7)	2·9%<br>(1·9 to 4·4%)	-12·6%<br>(-21·7 to 3·6%)	6·2%<br>(1·4 to 11·0%)	319·3<br>(198·6 to 511·1)	3·4%<br>(2·1 to 5·4%)	264·2<br>(158·9 to 423·9)	3·8%<br>(2·4 to 5·9%)	-17·3%<br>(-25·9 to -2·1%)	10·2%<br>(5·0 to 15·3%)
**Dietary risks: All causes**	3,817·0<br>(3,401·2 to 4,248·5)	28·0%<br>(24·9 to 31·0%)	4,247·1<br>(3,774·3 to 4,745·6)	30·2%<br>(26·9 to 33·4%)	11·3%<br>(6·5 to 16·5%)	8·1%<br>(5·8 to 10·2%)	88,780·5<br>(79,865·8 to 97,557·3)	15·6%<br>(13·9 to 17·5%)	94,130·5<br>(83,909·9 to 103,852·0)	17·1%<br>(15·1 to 19·3%)	6·0%<br>(0·8 to 11·3%)	9·7%<br>(6·5 to 13·0%)
**Diet low in fruits: All causes**	1,023·1<br>(714·5 to 1,353·1)	7·5%<br>(5·2 to 9·9%)	1,058·3<br>(724·9 to 1,411·7)	7·5%<br>(5·2 to 10·0%)	3·4%<br>(-2·8 to 9·5%)	0·5%<br>(-3·5 to 4·5%)	26,514·9<br>(18,851·3 to 34,918·9)	4·7%<br>(3·2 to 6·2%)	26,383·3<br>(18,199·7 to 35,151·4)	4·8%<br>(3·3 to 6·4%)	-0·5%<br>(-6·3 to 5·3%)	3·0%<br>(-1·4 to 7·2%)
Lip and oral cavity cancer	1·7<br>(0·0 to 3·7)	7·2%<br>(0·0 to 15·4%)	2·7<br>(0·0 to 5·8)	7·2%<br>(0·0 to 15·6%)	57·1%<br>(0·0 to 67·9%)	0·4%<br>(-0·8 to 1·6%)	45·1<br>(0·0 to 98·6)	7·1%<br>(0·0 to 15·4%)	67·3<br>(0·0 to 147·0)	7·2%<br>(0·0 to 15·6%)	49·2%<br>(0·0 to 60·4%)	0·3%<br>(-1·2 to 1·6%)
Nasopharynx cancer	2·8<br>(0·0 to 5·8)	7·7%<br>(0·0 to 16·3%)	2·9<br>(0·0 to 6·1)	7·6%<br>(0·0 to 16·1%)	4·5%<br>(-5·7 to 13·4%)	-2·0%<br>(-3·5 to 0·0%)	90·8<br>(0·0 to 191·9)	7·7%<br>(0·0 to 16·2%)	87·7<br>(0·0 to 186·0)	7·5%<br>(0·0 to 16·0%)	-3·4%<br>(-13·5 to 5·5%)	-1·7%<br>(-3·4 to 0·0%)
Other pharynx cancer	0·6<br>(0·0 to 1·4)	7·2%<br>(0·0 to 16·1%)	0·8<br>(0·0 to 1·8)	7·1%<br>(0·0 to 16·0%)	29·8%<br>(0·0 to 42·4%)	-1·0%<br>(-2·3 to 0·2%)	17·5<br>(0·0 to 39·2)	7·2%<br>(0·0 to 16·1%)	22·2<br>(0·0 to 49·3)	7·1%<br>(0·0 to 15·9%)	26·4%<br>(0·0 to 39·9%)	-1·0%<br>(-2·5 to 0·3%)
Oesophageal cancer	67·2<br>(16·9 to 119·0)	24·0%<br>(6·1 to 41·8%)	52·5<br>(12·6 to 92·5)	23·5%<br>(5·9 to 41·2%)	-21·8%<br>(-28·9 to -14·1%)	-1·9%<br>(-3·3 to -1·0%)	1,536·7<br>(387·9 to 2,710·2)	24·1%<br>(6·1 to 42·0%)	1,133·9<br>(273·6 to 2,001·1)	23·5%<br>(5·9 to 41·2%)	-26·2%<br>(-33·6 to -18·1%)	-2·7%<br>(-4·5 to -1·6%)
Larynx cancer	1·6<br>(0·0 to 3·6)	7·6%<br>(0·0 to 16·9%)	1·9<br>(0·0 to 4·3)	7·5%<br>(0·0 to 16·7%)	21·3%<br>(0·0 to 29·7%)	-1·4%<br>(-2·6 to 0·0%)	40·3<br>(0·0 to 90·6)	7·6%<br>(0·0 to 16·9%)	47·0<br>(0·0 to 106·1)	7·5%<br>(0·0 to 16·6%)	16·7%<br>(0·0 to 25·2%)	-1·9%<br>(-3·4 to 0·0%)
Tracheal, bronchus, and lung cancer	74·8<br>(30·9 to 124·9)	13·2%<br>(5·4 to 22·0%)	91·3<br>(37·5 to 152·4)	13·0%<br>(5·3 to 21·6%)	22·1%<br>(14·6 to 31·5%)	-1·7%<br>(-3·1 to -0·9%)	1,765·0<br>(729·7 to 2,944·2)	13·3%<br>(5·4 to 22·1%)	2,025·0<br>(831·3 to 3,372·1)	12·9%<br>(5·3 to 21·6%)	14·7%<br>(6·9 to 25·0%)	-2·5%<br>(-4·1 to -1·4%)
Ischaemic heart disease	222·4<br>(80·1 to 366·7)	13·4%<br>(4·9 to 22·3%)	269·5<br>(97·5 to 451·3)	13·0%<br>(4·7 to 21·6%)	21·2%<br>(14·8 to 27·2%)	-3·5%<br>(-4·8 to -2·5%)	5,375·6<br>(1,973·9 to 8,790·3)	15·9%<br>(5·9 to 26·0%)	5,997·4<br>(2,206·6 to 9,961·8)	15·2%<br>(5·6 to 25·1%)	11·6%<br>(4·4 to 18·1%)	-4·1%<br>(-5·6 to -2·9%)
Ischaemic stroke	184·1<br>(98·0 to 275·3)	20·6%<br>(11·2 to 31·1%)	194·1<br>(101·3 to 296·1)	19·3%<br>(10·4 to 29·5%)	5·4%<br>(-0·9 to 11·6%)	-6·1%<br>(-8·2 to -4·5%)	4,144·8<br>(2,278·9 to 6,079·7)	26·9%<br>(15·0 to 39·6%)	4,221·7<br>(2,307·2 to 6,300·8)	25·8%<br>(14·3 to 38·3%)	1·9%<br>(-4·4 to 7·6%)	-4·0%<br>(-6·1 to -2·6%)

Supplemental Results Table 1. All-age deaths, death PAFs, DALYs, DALY PAFs, and percent change for each risk factor and outcome for both sexes combined in 2005 and 2015 for Southeast Asia, East Asia, and Oceania.

Data in parenthesis are 95% uncertainty intervals. PAFs=population attributable fractions. DALYs=disability-adjusted life-years.

	2005 Deaths (in thousands)	2005 Deaths PAF	2015 Deaths (in thousands)	2015 Deaths PAF	Percent change deaths 2005-2015	Percent change deaths PAF 2005-2015	2005 DALYs (in thousands)	2005 DALYs PAF	2015 DALYs (in thousands)	2015 DALYs PAF	Percent change DALYs 2005-2015	Percent change DALYs PAF 2005-2015
Hemorrhagic stroke	434·9 (238·1 to 643·3)	26·5% (14·5 to 39·0%)	403·4 (221·8 to 597·8)	25·5% (14·0 to 37·8%)	-7·2% (-12·6 to -1·2%)	-4·0% (-6·0 to -2·6%)	11,483·0 (6,495·6 to 16,559·2)	32·1% (18·1 to 46·2%)	10,374·4 (5,849·6 to 15,070·6)	31·3% (17·6 to 45·5%)	-9·7% (-15·3 to -3·7%)	-2·3% (-4·1 to -1·1%)
**Diet low in vegetables: All causes**	443·6 (226·3 to 712·6)	3·2% (1·7 to 5·2%)	460·4 (236·1 to 751·7)	3·3% (1·7 to 5·3%)	3·8% (-3·6 to 11·7%)	0·8% (-5·1 to 6·2%)	10,993·8 (5,628·5 to 17,411·6)	1·9% (1·0 to 3·2%)	10,980·2 (5,758·1 to 17,837·9)	2·0% (1·0 to 3·3%)	-0·1% (-7·1 to 7·8%)	3·4% (-2·6 to 9·8%)
Ischaemic heart disease	230·6 (86·3 to 391·3)	13·9% (5·3 to 23·6%)	263·4 (99·8 to 452·0)	12·7% (4·8 to 21·5%)	14·2% (7·7 to 20·6%)	-9·0% (-11·7 to -6·9%)	5,494·9 (2,115·7 to 9,207·0)	16·2% (6·2 to 27·2%)	5,956·1 (2,290·8 to 10,085·5)	15·1% (5·8 to 25·4%)	8·4% (1·5 to 15·9%)	-6·8% (-9·6 to -4·2%)
Ischaemic stroke	62·6 (14·0 to 118·9)	7·0% (1·6 to 13·3%)	61·1 (13·6 to 116·2)	6·1% (1·4 to 11·6%)	-2·4% (-8·6 to 3·6%)	-13·1% (-16·2 to -10·5%)	1,424·2 (328·5 to 2,681·2)	9·3% (2·1 to 17·4%)	1,359·4 (311·8 to 2,560·1)	8·3% (1·9 to 15·6%)	-4·5% (-10·6 to 1·4%)	-10·1% (-13·2 to -7·5%)
Hemorrhagic stroke	150·3 (40·6 to 291·4)	9·2% (2·4 to 17·7%)	135·9 (37·2 to 262·8)	8·6% (2·4 to 16·6%)	-9·6% (-15·0 to -3·5%)	-6·5% (-9·5 to -3·3%)	4,074·7 (1,104·8 to 7,737·3)	11·4% (3·1 to 21·7%)	3,664·7 (1,030·4 to 6,970·6)	11·1% (3·1 to 21·1%)	-10·1% (-15·8 to -3·3%)	-2·8% (-5·6 to 0·7%)
**Diet low in whole grains: All causes**	1,020·0 (770·6 to 1,300·9)	7·5% (5·6 to 9·5%)	1,098·6 (814·8 to 1,410·6)	7·8% (5·8 to 10·0%)	7·7% (2·2 to 13·4%)	4·6% (1·2 to 7·7%)	27,381·7 (20,791·2 to 34,653·1)	4·8% (3·6 to 6·1%)	28,505·9 (21,397·3 to 36,350·4)	5·2% (3·9 to 6·6%)	4·1% (-1·4 to 9·8%)	7·7% (3·5 to 11·6%)
Ischaemic heart disease	381·0 (230·9 to 543·5)	23·0% (14·1 to 32·7%)	465·8 (281·6 to 666·2)	22·4% (13·7 to 31·8%)	22·3% (16·3 to 28·4%)	-2·6% (-3·5 to -2·0%)	9,006·7 (5,544·8 to 12,624·9)	26·6% (16·6 to 37·3%)	10,188·2 (6,225·6 to 14,417·9)	25·9% (16·1 to 36·3%)	13·1% (6·5 to 19·7%)	-2·7% (-3·7 to -2·0%)
Ischaemic stroke	176·7 (122·0 to 234·2)	19·7% (13·8 to 26·3%)	186·2 (126·6 to 249·1)	18·5% (12·8 to 24·9%)	5·4% (-0·9 to 11·4%)	-6·1% (-7·9 to -4·5%)	4,076·5 (2,844·3 to 5,342·5)	26·5% (18·8 to 34·6%)	4,183·6 (2,886·2 to 5,530·0)	25·6% (18·0 to 33·7%)	2·6% (-3·3 to 8·4%)	-3·3% (-4·7 to -2·0%)
Hemorrhagic stroke	399·9 (283·6 to 531·5)	24·4% (17·5 to 32·2%)	372·6 (266·8 to 492·6)	23·5% (16·7 to 31·3%)	-6·8% (-11·8 to -0·9%)	-3·6% (-5·1 to -2·4%)	10,573·5 (7,618·0 to 13,847·0)	29·6% (21·4 to 38·6%)	9,631·1 (6,935·8 to 12,616·0)	29·1% (21·0 to 38·2%)	-8·9% (-14·1 to -3·3%)	-1·5% (-2·6 to -0·6%)
Diabetes mellitus	62·4 (34·9 to 93·8)	21·3% (11·9 to 31·9%)	74·1 (41·7 to 111·9)	20·6% (11·6 to 31·0%)	18·7% (10·3 to 27·9%)	-3·1% (-4·3 to -2·0%)	3,725·0 (2,071·5 to 5,726·9)	25·6% (14·7 to 37·6%)	4,502·9 (2,503·0 to 6,948·7)	25·2% (14·4 to 37·3%)	20·9% (16·0 to 25·5%)	-1·3% (-2·0 to -0·8%)
**Diet low in nuts and seeds: All causes**	421·5 (269·9 to 591·1)	3·1% (2·0 to 4·3%)	513·7 (325·3 to 727·9)	3·7% (2·3 to 5·1%)	21·9% (16·0 to 28·1%)	18·4% (14·4 to 21·4%)	11,026·9 (7,537·1 to 15,054·8)	1·9% (1·3 to 2·7%)	12,638·9 (8,459·5 to 17,311·0)	2·3% (1·5 to 3·2%)	14·6% (8·4 to 20·6%)	18·6% (13·7 to 22·7%)
Ischaemic heart disease	387·8 (239·8 to 550·1)	23·4% (14·8 to 33·2%)	473·5 (292·8 to 673·4)	22·8% (14·3 to 32·3%)	22·1% (16·3 to 28·2%)	-2·8% (-3·6 to -2·1%)	9,023·8 (5,793·9 to 12,513·0)	26·7% (17·0 to 37·1%)	10,207·2 (6,525·6 to 14,347·5)	25·9% (16·5 to 35·9%)	13·1% (6·4 to 19·9%)	-2·8% (-3·8 to -1·9%)
Diabetes mellitus	33·7 (17·4 to 52·6)	11·5% (6·0 to 18·0%)	40·2 (20·4 to 63·1)	11·2% (5·8 to 17·5%)	19·5% (10·6 to 29·3%)	-2·6% (-4·0 to -1·1%)	2,003·1 (950·2 to 3,187·5)	13·7% (7·2 to 21·3%)	2,431·6 (1,134·1 to 3,964·6)	13·6% (7·1 to 21·1%)	21·4% (16·5 to 26·3%)	-0·9% (-1·5 to -0·4%)
**Diet low in milk: All causes**	32·0 (11·3 to 53·2)	0·2% (0·1 to 0·4%)	41·1 (14·9 to 68·3)	0·3% (0·1 to 0·5%)	28·3% (21·8 to 36·2%)	24·6% (20·1 to 29·4%)	764·9 (271·2 to 1,275·7)	0·1% (0·0 to 0·2%)	928·7 (334·5 to 1,547·2)	0·2% (0·1 to 0·3%)	21·4% (14·2 to 29·5%)	25·7% (19·4 to 32·4%)
Colon and rectum cancer	32·0 (11·3 to 53·2)	17·5% (6·3 to 29·1%)	41·1 (14·9 to 68·3)	17·6% (6·3 to 29·2%)	28·3% (21·8 to 36·2%)	0·3% (0·2 to 0·4%)	764·9 (271·2 to 1,275·7)	17·4% (6·3 to 28·8%)	928·7 (334·5 to 1,547·2)	17·5% (6·3 to 29·0%)	21·4% (14·2 to 29·5%)	0·5% (0·3 to 0·6%)
**Diet high in red meat: All causes**	9·2 (4·1 to 14·9)	0·1% (0·0 to 0·1%)	10·9 (4·9 to 17·6)	0·1% (0·0 to 0·1%)	17·6% (8·5 to 28·0%)	14·2% (6·1 to 22·7%)	459·1 (157·8 to 809·9)	0·1% (0·0 to 0·1%)	538·1 (186·3 to 933·6)	0·1% (0·0 to 0·2%)	17·2% (12·6 to 23·0%)	21·2% (16·9 to 26·9%)
Colon and rectum cancer	4·6 (1·0 to 8·2)	2·5% (0·5 to 4·5%)	5·9 (1·2 to 10·9)	2·5% (0·5 to 4·5%)	27·8% (19·0 to 37·5%)	-0·2% (-3·3 to 2·9%)	117·0 (24·9 to 207·2)	2·7% (0·6 to 4·8%)	143·4 (30·0 to 261·6)	2·7% (0·6 to 4·9%)	22·5% (13·3 to 33·2%)	1·4% (-1·9 to 4·6%)
Diabetes mellitus	4·6 (0·6 to 8·8)	1·6% (0·2 to 2·9%)	4·9 (0·6 to 9·2)	1·4% (0·2 to 2·6%)	7·4% (0·0 to 15·2%)	-12·4% (-17·5 to -6·7%)	342·1 (40·5 to 656·5)	2·3% (0·3 to 4·3%)	394·7 (47·0 to 761·6)	2·2% (0·3 to 4·1%)	15·4% (10·5 to 19·6%)	-5·8% (-9·5 to -1·9%)
**Diet high in processed meat: All causes**	93·3 (27·7 to 160·3)	0·7% (0·2 to 1·2%)	120·2 (37·3 to 205·0)	0·9% (0·3 to 1·5%)	28·9% (22·2 to 38·1%)	25·2% (20·5 to 31·4%)	3,054·3 (1,325·4 to 4,838·8)	0·5% (0·2 to 0·9%)	3,820·4 (1,754·0 to 5,984·0)	0·7% (0·3 to 1·1%)	25·1% (18·2 to 34·0%)	29·4% (23·8 to 37·5%)
Colon and rectum cancer	5·3 (2·7 to 8·3)	2·9% (1·5 to 4·5%)	7·4 (3·9 to 11·5)	3·2% (1·7 to 4·9%)	38·7% (29·9 to 48·1%)	8·3% (4·8 to 12·9%)	131·4 (67·5 to 206·1)	3·0% (1·6 to 4·6%)	172·5 (89·9 to 267·8)	3·2% (1·7 to 5·0%)	31·3% (22·0 to 42·2%)	8·6% (5·0 to 13·0%)
Ischaemic heart disease	67·8 (2·8 to 129·9)	4·1% (0·2 to 7·9%)	86·5 (3·6 to 164·6)	4·2% (0·2 to 8·0%)	27·6% (17·7 to 35·6%)	1·6% (-1·2 to 5·2%)	1,665·0 (68·4 to 3,189·1)	4·9% (0·2 to 9·4%)	1,986·2 (83·1 to 3,762·1)	5·0% (0·2 to 9·6%)	19·3% (9·2 to 27·4%)	2·6% (-0·2 to 5·9%)
Diabetes mellitus	20·1 (9·6 to 30·8)	6·9% (3·3 to 10·4%)	26·3 (13·1 to 39·6)	7·3% (3·6 to 11·0%)	30·5% (21·0 to 42·3%)	6·5% (3·5 to 10·3%)	1,258·0 (592·5 to 2,010·5)	8·6% (4·2 to 13·1%)	1,661·7 (776·8 to 2,609·2)	9·3% (4·6 to 14·0%)	32·1% (26·5 to 38·9%)	7·9% (5·1 to 11·3%)
**Diet high in sugar-sweetened beverages: All causes**	4·3 (2·9 to 6·2)	0·0% (0·0 to 0·0%)	5·3 (3·6 to 7·7)	0·0% (0·0 to 0·1%)	24·5% (12·7 to 37·8%)	20·9% (10·9 to 32·2%)	169·9 (115·8 to 242·7)	0·0% (0·0 to 0·0%)	213·7 (143·9 to 310·6)	0·0% (0·0 to 0·1%)	25·8% (14·9 to 38·0%)	30·1% (20·5 to 41·0%)
Oesophageal cancer	0·1 (0·0 to 0·1)	0·0% (0·0 to 0·0%)	0·1 (0·0 to 0·1)	0·0% (0·0 to 0·1%)	5·6% (-8·5 to 19·6%)	32·6% (15·1 to 49·8%)	1·6 (0·6 to 3·0)	0·0% (0·0 to 0·0%)	1·7 (0·5 to 3·1)	0·0% (0·0 to 0·1%)	3·3% (-10·6 to 17·2%)	36·4% (17·5 to 54·0%)
Colon and rectum cancer	0·0 (0·0 to 0·1)	0·0% (0·0 to 0·0%)	0·0 (0·0 to 0·1)	0·0% (0·0 to 0·0%)	43·5% (31·4 to 56·6%)	12·1% (3·2 to 21·7%)	1·0 (0·7 to 1·5)	0·0% (0·0 to 0·0%)	1·4 (0·9 to 2·0)	0·0% (0·0 to 0·0%)	37·6% (25·5 to 51·0%)	13·9% (3·7 to 24·4%)
Liver cancer due to hepatitis B	0·1 (0·0 to 0·1)	0·0% (0·0 to 0·0%)	0·1 (0·0 to 0·1)	0·0% (0·0 to 0·1%)	9·3% (-3·3 to 26·1%)	17·0% (5·6 to 29·2%)	2·0 (0·8 to 3·5)	0·0% (0·0 to 0·1%)	2·0 (0·8 to 3·6)	0·0% (0·0 to 0·1%)	3·5% (-9·2 to 20·1%)	17·9% (6·2 to 30·6%)
Liver cancer due to hepatitis C	0·0 (0·0 to 0·0)	0·0% (0·0 to 0·1%)	0·0 (0·0 to 0·0)	0·1% (0·0 to 0·1%)	26·0% (10·9 to 42·0%)	14·3% (3·6 to 26·4%)	0·6 (0·3 to 1·1)	0·1% (0·0 to 0·1%)	0·7 (0·3 to 1·3)	0·1% (0·0 to 0·1%)	20·3% (5·1 to 37·6%)	14·4% (2·9 to 27·4%)
Liver cancer due to alcohol use	0·0 (0·0 to 0·1)	0·0% (0·0 to 0·1%)	0·1 (0·0 to 0·1)	0·0% (0·0 to 0·1%)	35·3% (18·4 to 55·2%)	5·7% (-5·6 to 19·2%)	1·1 (0·4 to 1·9)	0·0% (0·0 to 0·1%)	1·4 (0·6 to 2·6)	0·0% (0·0 to 0·1%)	31·0% (13·8 to 52·3%)	5·7% (-6·2 to 19·1%)
Liver cancer due to other causes	0·0 (0·0 to 0·0)	0·0% (0·0 to 0·1%)	0·0 (0·0 to 0·1)	0·0% (0·0 to 0·1%)	12·8% (0·6 to 26·5%)	22·6% (10·9 to 35·8%)	0·9 (0·4 to 1·6)	0·0% (0·0 to 0·1%)	1·0 (0·4 to 1·7)	0·0% (0·0 to 0·1%)	7·1% (-5·4 to 21·5%)	25·2% (12·7 to 39·5%)
Gallbladder and biliary tract cancer	0·0 (0·0 to 0·0)	0·0% (0·0 to 0·1%)	0·0 (0·0 to 0·0)	0·0% (0·0 to 0·1%)	19·1% (2·6 to 35·7%)	2·5% (-8·3 to 13·8%)	0·4 (0·2 to 0·6)	0·1% (0·0 to 0·1%)	0·4 (0·2 to 0·7)	0·1% (0·0 to 0·1%)	14·2% (-2·6 to 30·5%)	3·5% (-8·1 to 14·8%)
Pancreatic cancer	0·0 (0·0 to 0·0)	0·0% (0·0 to 0·0%)	0·0 (0·0 to 0·0)	0·0% (0·0 to 0·0%)	44·0% (29·3 to 58·6%)	4·1% (-5·3 to 13·1%)	0·2 (0·1 to 0·5)	0·0% (0·0 to 0·0%)	0·3 (0·1 to 0·6)	0·0% (0·0 to 0·0%)	41·1% (25·8 to 56·5%)	4·9% (-5·4 to 14·2%)
Breast cancer	0·1 (0·0 to 0·1)	0·1% (0·0 to 0·1%)	0·1 (0·1 to 0·1)	0·1% (0·0 to 0·1%)	37·5% (14·7 to 62·2%)	12·2% (-0·4 to 26·8%)	2·4 (1·4 to 3·7)	0·1% (0·0 to 0·1%)	3·3 (2·0 to 5·0)	0·1% (0·1 to 0·1%)	35·9% (12·9 to 61·5%)	15·4% (2·8 to 30·3%)
Uterine cancer	0·0 (0·0 to 0·0)	0·1% (0·1 to 0·1%)	0·0 (0·0 to 0·0)	0·1% (0·1 to 0·2%)	10·6% (-4·8 to 27·7%)	15·0% (2·2 to 29·0%)	0·8 (0·5 to 1·1)	0·1% (0·1 to 0·2%)	0·9 (0·5 to 1·2)	0·1% (0·1 to 0·2%)	10·0% (-5·3 to 27·2%)	17·1% (3·2 to 32·6%)
Ovarian cancer	0·0 (0·0 to 0·0)	0·0% (0·0 to 0·0%)	0·0 (0·0 to 0·0)	0·0% (0·0 to 0·0%)	37·6% (0·0 to 59·0%)	12·9% (0·0 to 24·7%)	0·1 (0·0 to 0·2)	0·0% (0·0 to 0·0%)	0·1 (0·0 to 0·3)	0·0% (0·0 to 0·0%)	34·9% (0·0 to 56·5%)	14·5% (0·0 to 26·8%)
Kidney cancer	0·0 (0·0 to 0·0)	0·1% (0·0 to 0·1%)	0·0 (0·0 to 0·0)	0·1% (0·0 to 0·1%)	64·9% (47·7 to 83·9%)	11·9% (1·9 to 21·7%)	0·3 (0·2 to 0·4)	0·1% (0·0 to 0·1%)	0·4 (0·3 to 0·7)	0·1% (0·0 to 0·1%)	60·2% (43·0 to 79·4%)	17·1% (6·2 to 28·2%)
Thyroid cancer	0·0 (0·0 to 0·0)	0·0% (0·0 to 0·1%)	0·0 (0·0 to 0·0)	0·0% (0·0 to 0·1%)	38·1% (20·9 to 54·9%)	2·6% (-8·0 to 17·8%)	0·1 (0·1 to 0·2)	0·0% (0·0 to 0·1%)	0·1 (0·1 to 0·2)	0·0% (0·0 to 0·1%)	39·0% (21·4 to 55·7%)	5·8% (-4·9 to 21·1%)
Ischaemic heart disease	1·0 (0·7 to 1·5)	0·1% (0·0 to 0·1%)	1·3 (0·9 to 1·9)	0·1% (0·0 to 0·1%)	24·5% (12·4 to 37·9%)	-0·9% (-8·9 to 8·1%)	34·2 (23·4 to 49·9)	0·1% (0·1 to 0·1%)	41·7 (28·7 to 60·3)	0·1% (0·1 to 0·2%)	22·0% (9·9 to 36·6%)	4·9% (-3·8 to 15·0%)
Ischaemic stroke	0·3 (0·2 to 0·4)	0·0% (0·0 to 0·0%)	0·3 (0·2 to 0·5)	0·0% (0·0 to 0·0%)	18·2% (5·8 to 31·3%)	5·3% (-4·4 to 16·0%)	7·6 (5·2 to 11·3)	0·0% (0·0 to 0·1%)	9·0 (6·0 to 13·2)	0·1% (0·0 to 0·1%)	17·8% (5·5 to 31·2%)	11·1% (1·5 to 21·3%)
Hemorrhagic stroke	1·2 (0·8 to 1·8)	0·1% (0·1 to 0·1%)	1·4 (1·0 to 2·1)	0·1% (0·1 to 0·1%)	16·6% (4·3 to 30·8%)	20·6% (9·5 to 33·4%)	44·2 (29·7 to 64·1)	0·1% (0·1 to 0·2%)	50·4 (33·8 to 73·5)	0·2% (0·1 to 0·2%)	14·1% (1·7 to 28·6%)	23·3% (12·0 to 36·4%)
Hypertensive heart disease	0·3 (0·2 to 0·4)	0·1% (0·1 to 0·2%)	0·3 (0·2 to 0·5)	0·1% (0·1 to 0·1%)	23·5% (9·7 to 39·0%)	-7·4% (-17·3 to 5·0%)	7·4 (4·7 to 10·8)	0·2% (0·1 to 0·2%)	8·8 (5·7 to 13·1)	0·2% (0·1 to 0·2%)	19·5% (5·8 to 34·7%)	-1·1% (-10·8 to 10·7%)
Cardiomyopathy and myocarditis	-	-	-	-	-	-	-	-	-	-	-	-
Atrial fibrillation and flutter	-	-	-	-	-	-	-	-	-	-	-	-
Peripheral vascular disease	-	-	-	-	-	-	-	-	-	-	-	-

Supplemental Results Table 1. All-age deaths, death PAFs, DALYs, DALY PAFs, and percent change for each risk factor and outcome for both sexes combined in 2005 and 2015 for Southeast Asia, East Asia, and Oceania.

Data in parenthesis are 95% uncertainty intervals. PAFs=population attributable fractions. DALYs=disability-adjusted life-years.

	2005 Deaths (in thousands)	2005 Deaths PAF	2015 Deaths (in thousands)	2015 Deaths PAF	Percent change deaths 2005-2015	Percent change deaths PAF 2005-2015	2005 DALYs (in thousands)	2005 DALYs PAF	2015 DALYs (in thousands)	2015 DALYs PAF	Percent change DALYs 2005-2015	Percent change DALYs PAF 2005-2015
Endocarditis	-	-	-	-	-	-	-	-	-	-	-	-
Other cardiovascular and circulatory diseases	-	-	-	-	-	-	-	-	-	-	-	-
Diabetes mellitus	0·8 (0·5 to 1·1)	0·3% (0·2 to 0·4%)	1·1 (0·7 to 1·5)	0·3% (0·2 to 0·4%)	34·8% (19·5 to 53·3%)	10·0% (1·5 to 20·4%)	49·2 (31·5 to 73·3)	0·3% (0·2 to 0·5%)	68·9 (43·7 to 104·7)	0·4% (0·3 to 0·5%)	40·1% (29·7 to 52·9%)	14·4% (7·6 to 22·6%)
Chronic kidney disease due to diabetes mellitus	0·1 (0·1 to 0·2)	0·2% (0·1 to 0·3%)	0·2 (0·1 to 0·3)	0·2% (0·1 to 0·3%)	44·8% (30·3 to 61·1%)	11·8% (3·3 to 22·0%)	3·9 (1·4 to 7·6)	0·2% (0·1 to 0·3%)	5·6 (2·1 to 10·4)	0·2% (0·1 to 0·4%)	41·5% (28·2 to 58·2%)	16·1% (7·4 to 26·4%)
Chronic kidney disease due to hypertension	0·1 (0·1 to 0·2)	0·1% (0·1 to 0·3%)	0·2 (0·1 to 0·3)	0·1% (0·1 to 0·3%)	31·9% (17·2 to 46·6%)	-0·2% (-8·6 to 9·1%)	3·9 (1·6 to 7·4)	0·2% (0·1 to 0·3%)	5·1 (2·0 to 9·5)	0·2% (0·1 to 0·3%)	29·6% (15·9 to 43·7%)	9·4% (0·8 to 19·0%)
Chronic kidney disease due to glomerulonephritis	0·0 (0·0 to 0·1)	0·1% (0·0 to 0·1%)	0·1 (0·0 to 0·1)	0·1% (0·0 to 0·1%)	27·7% (15·0 to 42·7%)	31·7% (19·6 to 46·2%)	1·8 (0·5 to 3·5)	0·1% (0·0 to 0·1%)	2·2 (0·7 to 4·3)	0·1% (0·0 to 0·2%)	24·3% (13·5 to 38·2%)	44·5% (32·8 to 60·7%)
Chronic kidney disease due to other causes	0·0 (0·0 to 0·0)	0·1% (0·1 to 0·3%)	0·0 (0·0 to 0·0)	0·2% (0·1 to 0·3%)	34·3% (15·3 to 59·0%)	13·2% (2·1 to 27·6%)	0·8 (0·2 to 1·8)	0·1% (0·0 to 0·2%)	1·0 (0·3 to 2·2)	0·1% (0·0 to 0·3%)	31·7% (17·5 to 48·5%)	19·5% (9·3 to 32·8%)
**Diet low in fibre: All causes**	52·7 (26·7 to 90·4)	0·4% (0·2 to 0·7%)	66·8 (34·0 to 114·4)	0·5% (0·2 to 0·8%)	26·8% (17·6 to 36·1%)	23·2% (16·0 to 31·2%)	1,410·9 (718·0 to 2,386·8)	0·2% (0·1 to 0·4%)	1,692·3 (875·6 to 2,860·5)	0·3% (0·2 to 0·5%)	19·9% (10·5 to 31·1%)	24·1% (15·7 to 34·0%)
Colon and rectum cancer	5·6 (2·6 to 10·0)	3·1% (1·4 to 5·5%)	8·0 (3·7 to 14·0)	3·4% (1·6 to 6·0%)	41·0% (30·0 to 54·7%)	10·1% (2·1 to 19·1%)	130·8 (59·2 to 235·7)	3·0% (1·4 to 5·3%)	172·9 (81·6 to 306·1)	3·3% (1·6 to 5·8%)	32·2% (21·6 to 46·2%)	9·4% (1·4 to 19·1%)
Ischaemic heart disease	47·0 (23·4 to 81·2)	2·8% (1·4 to 5·0%)	58·8 (29·6 to 102·9)	2·8% (1·4 to 4·9%)	25·1% (15·7 to 34·7%)	-0·4% (-6·0 to 5·8%)	1,280·1 (649·3 to 2,173·1)	3·8% (1·9 to 6·4%)	1,519·3 (777·6 to 2,579·9)	3·9% (2·0 to 6·5%)	18·7% (8·9 to 29·7%)	2·0% (-4·4 to 9·6%)
**Diet low in calcium: All causes**	41·8 (26·4 to 58·4)	0·3% (0·2 to 0·4%)	53·7 (34·1 to 75·3)	0·4% (0·2 to 0·5%)	28·5% (22·1 to 35·9%)	24·8% (20·3 to 29·6%)	993·6 (629·2 to 1,390·7)	0·2% (0·1 to 0·2%)	1,207·0 (770·1 to 1,695·0)	0·2% (0·1 to 0·3%)	21·5% (14·1 to 29·6%)	25·7% (19·5 to 32·5%)
Colon and rectum cancer	41·8 (26·4 to 58·4)	22·9% (14·5 to 31·9%)	53·7 (34·1 to 75·3)	23·0% (14·6 to 32·0%)	28·5% (22·1 to 35·9%)	0·4% (-0·6 to 1·5%)	993·6 (629·2 to 1,390·7)	22·6% (14·3 to 31·5%)	1,207·0 (770·1 to 1,695·0)	22·7% (14·4 to 31·7%)	21·5% (14·1 to 29·6%)	0·6% (-0·6 to 1·7%)
Prostate cancer	-	-	-	-	-	-	-	-	-	-	-	-
**Diet low in seafood omega-3 fatty acids: All causes**	298·8 (126·5 to 478·0)	2·2% (0·9 to 3·5%)	361·8 (154·8 to 580·7)	2·6% (1·1 to 4·1%)	21·1% (14·9 to 27·6%)	17·6% (13·3 to 20·9%)	6,910·6 (2,968·5 to 10,925·8)	1·2% (0·5 to 1·9%)	7,763·3 (3,377·3 to 12,319·5)	1·4% (0·6 to 2·3%)	12·3% (5·2 to 19·5%)	16·3% (9·9 to 21·3%)
Ischaemic heart disease	298·8 (126·5 to 478·0)	18·0% (7·7 to 28·9%)	361·8 (154·8 to 580·7)	17·4% (7·4 to 28·0%)	21·1% (14·9 to 27·6%)	-3·6% (-4·7 to -2·6%)	6,910·6 (2,968·5 to 10,925·8)	20·4% (8·8 to 32·2%)	7,763·3 (3,377·3 to 12,319·5)	19·7% (8·5 to 31·2%)	12·3% (5·2 to 19·5%)	-3·4% (-4·7 to -2·4%)
**Diet low in polyunsaturated fatty acids: All causes**	53·7 (22·7 to 87·7)	0·4% (0·2 to 0·6%)	72·9 (30·9 to 120·8)	0·5% (0·2 to 0·9%)	35·7% (18·0 to 59·3%)	31·8% (15·5 to 53·6%)	1,450·0 (631·9 to 2,385·5)	0·3% (0·1 to 0·4%)	1,930·5 (809·4 to 3,200·6)	0·4% (0·1 to 0·6%)	33·1% (15·0 to 57·1%)	37·8% (19·5 to 62·1%)
Ischaemic heart disease	53·7 (22·7 to 87·7)	3·2% (1·4 to 5·3%)	72·9 (30·9 to 120·8)	3·5% (1·5 to 5·9%)	35·7% (18·0 to 59·3%)	8·1% (-5·0 to 26·0%)	1,450·0 (631·9 to 2,385·5)	4·3% (1·9 to 7·0%)	1,930·5 (809·4 to 3,200·6)	4·9% (2·1 to 8·1%)	33·1% (15·0 to 57·1%)	14·4% (-0·3 to 34·2%)
**Diet high in trans fatty acids: All causes**	46·6 (9·2 to 110·6)	0·3% (0·1 to 0·8%)	54·3 (10·3 to 133·1)	0·4% (0·1 to 0·9%)	16·5% (7·2 to 23·7%)	13·2% (5·5 to 18·6%)	1,333·2 (288·7 to 3,067·7)	0·2% (0·1 to 0·5%)	1,468·4 (314·3 to 3,416·9)	0·3% (0·1 to 0·6%)	10·1% (1·8 to 18·0%)	14·0% (6·6 to 20·7%)
Ischaemic heart disease	46·6 (9·2 to 110·6)	2·8% (0·6 to 6·8%)	54·3 (10·3 to 133·1)	2·6% (0·5 to 6·4%)	16·5% (7·2 to 23·7%)	-7·2% (-13·1 to -3·6%)	1,333·2 (288·7 to 3,067·7)	3·9% (0·9 to 9·0%)	1,468·4 (314·3 to 3,416·9)	3·7% (0·8 to 8·7%)	10·1% (1·8 to 18·0%)	-5·2% (-10·2 to -1·7%)
**Diet high in sodium: All causes**	2,061·6 (1,479·5 to 2,718·1)	15·1% (10·8 to 19·8%)	2,300·7 (1,664·7 to 3,029·6)	16·4% (11·9 to 21·5%)	11·6% (5·3 to 18·6%)	8·4% (3·8 to 13·6%)	44,057·1 (31,489·2 to 58,056·6)	7·8% (5·6 to 10·3%)	46,163·1 (33,710·1 to 60,361·1)	8·4% (6·1 to 11·2%)	4·8% (-1·4 to 11·7%)	8·4% (3·3 to 14·0%)
Stomach cancer	287·4 (206·8 to 354·7)	69·0% (49·9 to 84·5%)	263·2 (181·8 to 326·6)	68·4% (47·6 to 84·7%)	-8·4% (-16·3 to -0·7%)	-0·8% (-6·8 to 4·1%)	6,773·4 (4,952·4 to 8,313·2)	68·4% (50·5 to 83·3%)	5,826·3 (4,213·8 to 7,225·0)	67·9% (48·2 to 83·3%)	-14·0% (-22·0 to -6·3%)	-0·8% (-8·5 to 4·1%)
Rheumatic heart disease	18·7 (10·5 to 31·6)	16·5% (9·2 to 28·1%)	15·5 (8·6 to 26·6)	17·1% (9·5 to 29·5%)	-17·2% (-25·5 to -8·3%)	3·8% (-3·5 to 12·7%)	517·9 (291·1 to 850·9)	14·4% (8·2 to 23·6%)	397·5 (230·0 to 642·8)	15·1% (8·8 to 24·7%)	-23·2% (-30·0 to -15·6%)	4·7% (-2·3 to 13·2%)
Ischaemic heart disease	571·9 (368·0 to 801·4)	34·5% (22·2 to 48·4%)	743·5 (487·5 to 1,033·9)	35·8% (23·4 to 49·7%)	30·0% (21·3 to 40·0%)	3·5% (-0·8 to 9·1%)	11,530·9 (7,579·9 to 16,081·8)	34·1% (22·6 to 47·2%)	13,929·8 (9,361·1 to 19,062·0)	35·4% (24·1 to 48·4%)	20·8% (12·2 to 30·6%)	3·9% (-0·3 to 9·7%)
Ischaemic stroke	304·0 (195·4 to 431·1)	34·0% (21·8 to 47·8%)	343·5 (223·5 to 488·9)	34·2% (22·2 to 48·0%)	13·0% (4·5 to 22·8%)	0·7% (-3·9 to 7·4%)	5,631·5 (3,706·8 to 7,871·7)	36·6% (24·3 to 50·5%)	6,103·2 (4,114·6 to 8,442·0)	37·3% (25·3 to 50·9%)	8·4% (1·0 to 16·8%)	2·1% (-1·4 to 8·1%)
Hemorrhagic stroke	645·1 (415·6 to 894·7)	39·3% (25·4 to 54·7%)	622·3 (405·3 to 860·4)	39·3% (25·7 to 54·1%)	-3·5% (-10·1 to 4·4%)	-0·2% (-4·6 to 5·6%)	14,148·2 (9,332·7 to 19,233·1)	39·5% (26·0 to 53·9%)	13,176·3 (8,759·2 to 17,891·7)	39·8% (26·6 to 53·7%)	-6·9% (-13·6 to 0·6%)	0·7% (-3·4 to 6·2%)
Hypertensive heart disease	127·6 (54·8 to 206·3)	52·4% (22·6 to 84·1%)	178·0 (72·5 to 287·9)	54·6% (23·1 to 86·2%)	39·4% (17·8 to 55·6%)	4·2% (-2·8 to 12·1%)	2,374·9 (1,263·6 to 3,629·2)	52·0% (27·6 to 79·6%)	2,986·6 (1,554·6 to 4,591·4)	54·1% (28·6 to 81·9%)	25·8% (10·9 to 38·2%)	3·9% (-1·0 to 9·8%)
Cardiomyopathy and myocarditis	7·1 (4·3 to 10·6)	17·4% (10·8 to 25·8%)	9·0 (5·6 to 13·4)	18·8% (11·8 to 27·5%)	27·1% (15·0 to 40·5%)	7·9% (1·4 to 16·2%)	194·7 (116·5 to 294·4)	13·5% (8·1 to 20·2%)	228·9 (138·0 to 337·3)	15·9% (9·8 to 23·3%)	17·6% (5·6 to 32·0%)	17·6% (10·2 to 27·5%)
Atrial fibrillation and flutter	6·4 (3·7 to 9·8)	21·2% (13·3 to 30·4%)	9·3 (5·5 to 14·2)	22·0% (14·1 to 31·5%)	45·7% (37·1 to 56·4%)	3·8% (-0·3 to 9·6%)	224·1 (137·0 to 336·0)	26·9% (17·7 to 36·9%)	311·7 (193·7 to 463·7)	27·9% (19·1 to 37·5%)	39·1% (33·3 to 46·8%)	3·7% (0·3 to 9·2%)
Aortic aneurysm	4·0 (2·5 to 5·9)	21·3% (13·3 to 31·0%)	5·3 (3·3 to 7·6)	21·1% (13·3 to 30·8%)	30·8% (19·3 to 42·0%)	-0·8% (-5·3 to 4·5%)	89·2 (56·7 to 128·0)	21·9% (13·9 to 31·2%)	112·3 (71·1 to 161·2)	22·2% (14·3 to 31·6%)	25·9% (13·8 to 37·6%)	1·6% (-2·8 to 7·3%)
Peripheral vascular disease	0·2 (0·1 to 0·3)	17·1% (10·3 to 25·7%)	0·4 (0·3 to 0·6)	17·5% (10·6 to 26·1%)	81·8% (60·7 to 108·6%)	2·3% (-4·5 to 10·7%)	27·7 (12·7 to 53·1)	18·1% (10·8 to 27·0%)	43·2 (20·5 to 80·9)	18·9% (11·5 to 27·5%)	55·9% (49·5 to 65·3%)	4·3% (0·7 to 9·9%)
Endocarditis	2·3 (1·3 to 3·6)	15·1% (8·9 to 23·2%)	3·0 (1·8 to 4·7)	16·2% (9·8 to 24·6%)	32·6% (21·1 to 45·5%)	7·6% (1·3 to 15·2%)	56·8 (31·1 to 93·8)	11·5% (6·5 to 18·3%)	69·1 (38·3 to 110·2)	13·3% (7·6 to 20·6%)	21·7% (11·1 to 33·6%)	15·2% (6·8 to 25·8%)
Other cardiovascular and circulatory diseases	23·2 (14·9 to 32·7)	22·9% (14·6 to 32·2%)	26·7 (17·4 to 37·0)	23·8% (15·6 to 33·0%)	14·9% (5·9 to 25·9%)	4·2% (-1·2 to 10·8%)	798·2 (493·4 to 1,135·9)	19·5% (12·6 to 27·4%)	986·1 (622·1 to 1,407·3)	22·3% (14·8 to 30·7%)	23·5% (16·0 to 32·3%)	14·2% (9·7 to 21·0%)
Chronic kidney disease due to diabetes mellitus	18·1 (10·1 to 27·4)	25·1% (14·0 to 37·6%)	24·3 (13·7 to 36·3)	26·0% (14·8 to 38·5%)	34·5% (25·5 to 45·4%)	3·8% (-0·4 to 8·7%)	516·8 (280·3 to 802·2)	21·2% (11·8 to 31·6%)	669·3 (368·6 to 1,014·2)	22·5% (12·8 to 33·2%)	29·5% (21·9 to 39·3%)	6·2% (1·8 to 12·0%)
Chronic kidney disease due to hypertension	20·4 (10·8 to 32·0)	23·2% (12·4 to 36·2%)	29·8 (16·4 to 46·1)	25·7% (14·2 to 39·1%)	46·5% (34·6 to 60·2%)	10·9% (5·9 to 17·3%)	461·7 (253·3 to 734·3)	19·7% (10·7 to 30·8%)	616·1 (342·9 to 961·0)	22·2% (12·3 to 34·0%)	33·4% (24·6 to 44·8%)	12·7% (7·3 to 19·7%)
Chronic kidney disease due to glomerulonephritis	24·0 (14·0 to 35·8)	28·1% (16·6 to 40·9%)	25·1 (15·3 to 36·8)	30·3% (18·5 to 43·9%)	4·7% (-4·0 to 15·2%)	7·9% (3·5 to 15·0%)	605·0 (352·5 to 905·5)	21·8% (12·8 to 32·5%)	568·2 (338·6 to 844·6)	23·8% (14·5 to 34·9%)	-6·1% (-13·4 to 3·0%)	9·2% (4·0 to 16·7%)
Chronic kidney disease due to other causes	1·3 (0·6 to 2·3)	13·1% (6·4 to 22·3%)	1·8 (0·9 to 3·2)	15·6% (7·8 to 25·8%)	41·4% (25·6 to 59·6%)	19·1% (11·8 to 29·5%)	106·0 (53·9 to 173·7)	14·5% (7·7 to 22·6%)	138·5 (72·9 to 223·5)	17·1% (9·6 to 26·1%)	30·7% (23·4 to 39·4%)	18·7% (12·4 to 27·0%)
**Sexual abuse and violence: All causes**	51·7 (34·8 to 72·5)	0·4% (0·3 to 0·5%)	40·4 (26·7 to 58·3)	0·3% (0·2 to 0·4%)	-21·9% (-30·9 to -3·9%)	-24·2% (-32·2 to -8·0%)	3,222·4 (2,330·8 to 4,316·3)	0·6% (0·4 to 0·8%)	2,819·9 (2,041·8 to 3,785·7)	0·5% (0·4 to 0·7%)	-12·5% (-21·1 to -1·6%)	-9·5% (-17·3 to 1·9%)
**Childhood sexual abuse: All causes**	22·8 (6·0 to 46·4)	0·2% (0·0 to 0·3%)	17·8 (4·9 to 36·4)	0·1% (0·0 to 0·3%)	-21·8% (-29·7 to -8·2%)	-24·0% (-30·9 to -11·1%)	1,382·2 (567·2 to 2,474·9)	0·2% (0·1 to 0·4%)	1,153·9 (484·2 to 2,023·3)	0·2% (0·1 to 0·4%)	-16·5% (-27·6 to -3·1%)	-13·6% (-24·2 to 0·5%)
Alcohol use disorders	1·0 (0·0 to 2·3)	3·5% (0·0 to 8·2%)	0·9 (0·0 to 2·2)	3·4% (0·0 to 7·7%)	-7·7% (-17·4 to 3·6%)	-4·8% (-9·9 to 0·9%)	102·4 (0·0 to 238·8)	3·7% (0·0 to 8·6%)	101·2 (0·0 to 233·2)	3·6% (0·0 to 8·1%)	-1·2% (-7·7 to 4·9%)	-4·4% (-8·8 to 0·5%)
Major depressive disorder	-	-	-	-	-	-	264·7 (28·5 to 596·0)	3·0% (0·4 to 6·4%)	285·9 (31·0 to 644·3)	2·9% (0·3 to 6·0%)	8·0% (4·0 to 11·7%)	-5·6% (-8·2 to -2·7%)
Dysthymia	-	-	-	-	-	-	82·9 (8·7 to 186·7)	3·0% (0·3 to 6·3%)	91·4 (9·6 to 205·0)	2·8% (0·3 to 5·9%)	10·3% (6·3 to 14·0%)	-6·6% (-9·2 to -3·5%)
Self-harm	21·7 (5·1 to 45·3)	9·3% (2·1 to 19·3%)	16·9 (3·9 to 35·3)	8·8% (2·0 to 18·2%)	-22·5% (-30·6 to -8·5%)	-5·7% (-9·8 to -1·9%)	932·2 (219·1 to 1,915·4)	10·0% (2·3 to 20·5%)	675·3 (158·9 to 1,418·9)	9·6% (2·3 to 19·8%)	-27·6% (-35·0 to -14·7%)	-3·3% (-7·7 to 0·5%)
**Intimate partner violence: All causes**	30·6 (23·4 to 37·9)	0·2% (0·2 to 0·3%)	23·7 (18·2 to 30·0)	0·2% (0·1 to 0·2%)	-22·6% (-34·1 to 2·0%)	-24·9% (-35·7 to -1·4%)	1,926·2 (1,517·1 to 2,355·7)	0·3% (0·3 to 0·4%)	1,727·8 (1,330·6 to 2,176·5)	0·3% (0·3 to 0·4%)	-10·3% (-20·4 to 3·5%)	-7·3% (-17·5 to 7·8%)

Supplemental Results Table 1. All-age deaths, death PAFs, DALYs, DALY PAFs, and percent change for each risk factor and outcome for both sexes combined in 2005 and 2015 for Southeast Asia, East Asia, and Oceania.

Data in parenthesis are 95% uncertainty intervals. PAFs=population attributable fractions. DALYs=disability-adjusted life-years.

	2005 Deaths (in thousands)	2005 Deaths PAF	2015 Deaths (in thousands)	2015 Deaths PAF	Percent change deaths 2005-2015	Percent change deaths PAF 2005-2015	2005 DALYs (in thousands)	2005 DALYs PAF	2015 DALYs (in thousands)	2015 DALYs PAF	Percent change DALYs 2005-2015	Percent change DALYs PAF 2005-2015
HIV/AIDS - Tuberculosis	0·2 (0·1 to 0·4)	1·4% (0·7 to 2·2%)	0·2 (0·1 to 0·4)	1·2% (0·6 to 1·9%)	15·6% (-38·7 to 102·1%)	-14·2% (-30·5 to 13·9%)	8·8 (3·6 to 19·1)	1·3% (0·7 to 2·1%)	10·9 (4·4 to 20·6)	1·2% (0·6 to 2·0%)	24·0% (-34·6 to 111·2%)	-5·4% (-24·3 to 25·2%)
HIV/AIDS resulting in other diseases	0·7 (0·3 to 1·9)	1·5% (0·7 to 2·4%)	1·0 (0·4 to 1·8)	1·2% (0·6 to 1·9%)	35·6% (-30·4 to 126·7%)	-19·4% (-36·6 to -1·8%)	33·9 (14·1 to 86·1)	1·4% (0·7 to 2·3%)	44·6 (20·1 to 87·6)	1·1% (0·6 to 1·8%)	31·6% (-33·4 to 129·2%)	-17·0% (-34·6 to 3·6%)
Maternal abortion, miscarriage, and ectopic pregnancy	0·0 (0·0 to 0·0)	0·0% (0·0 to 0·0%)	0·0 (0·0 to 0·0)	0·0% (0·0 to 0·0%)	-41·4% (-57·7 to -16·1%)	0·2% (-21·0 to 22·4%)	0·0 (0·0 to 0·0)	0·0% (0·0 to 0·0%)	0·0 (0·0 to 0·0)	0·0% (0·0 to 0·0%)	-40·9% (-56·0 to -16·7%)	0·3% (-19·2 to 22·0%)
Major depressive disorder	-	-	-	-	-	-	491·2 (313·7 to 710·9)	5·6% (4·4 to 6·9%)	605·0 (386·8 to 879·7)	6·1% (4·7 to 7·4%)	23·2% (17·9 to 29·4%)	7·6% (3·2 to 12·5%)
Dysthymia	-	-	-	-	-	-	163·7 (101·1 to 244·4)	5·9% (4·6 to 7·3%)	207·3 (126·3 to 310·0)	6·3% (4·9 to 7·7%)	26·6% (20·4 to 33·5%)	7·2% (2·4 to 12·3%)
Self-harm	24·2 (17·0 to 31·5)	10·4% (7·7 to 13·3%)	18·7 (13·2 to 24·8)	9·7% (7·0 to 12·6%)	-22·8% (-36·0 to 9·5%)	-6·0% (-18·0 to 15·6%)	918·8 (648·4 to 1,216·0)	9·8% (7·1 to 12·8%)	653·8 (462·8 to 891·3)	9·3% (6·7 to 12·1%)	-28·8% (-41·0 to 3·0%)	-5·1% (-17·6 to 18·9%)
Assault by firearm	0·7 (0·6 to 0·9)	4·5% (3·7 to 5·4%)	0·6 (0·5 to 0·8)	4·4% (3·4 to 5·4%)	-13·4% (-29·5 to 4·3%)	-2·6% (-21·6 to 19·4%)	35·7 (30·5 to 43·0)	4·4% (3·6 to 5·3%)	29·4 (23·9 to 35·9)	4·2% (3·3 to 5·3%)	-17·6% (-32·1 to 0·6%)	-3·6% (-22·2 to 18·7%)
Assault by sharp object	2·0 (1·8 to 2·3)	7·1% (6·1 to 7·9%)	1·3 (1·2 to 1·5)	6·7% (5·8 to 7·6%)	-35·0% (-43·5 to -24·8%)	-6·1% (-18·0 to 6·3%)	108·3 (96·3 to 122·3)	7·0% (6·1 to 7·8%)	66·6 (58·2 to 76·0)	6·5% (5·6 to 7·4%)	-38·5% (-46·5 to -28·5%)	-7·6% (-19·5 to 4·2%)
Assault by other means	2·7 (2·4 to 3·0)	11·3% (10·1 to 12·6%)	1·8 (1·6 to 2·1)	10·4% (8·4 to 11·6%)	-32·9% (-39·9 to -23·3%)	-8·0% (-19·4 to 2·5%)	165·8 (148·5 to 182·7)	11·7% (10·6 to 13·0%)	110·2 (96·6 to 126·4)	10·9% (9·2 to 12·0%)	-33·6% (-40·0 to -25·1%)	-6·9% (-16·7 to 1·8%)
**Unsafe sex: All causes**	119·5 (100·4 to 172·8)	0·9% (0·7 to 1·3%)	146·1 (125·0 to 195·3)	1·0% (0·9 to 1·4%)	22·3% (-4·2 to 51·4%)	18·8% (-6·7 to 47·4%)	5,536·2 (4,569·2 to 8,239·8)	1·0% (0·8 to 1·4%)	6,314·3 (5,285·5 to 8,696·7)	1·1% (0·9 to 1·6%)	14·1% (-11·2 to 42·7%)	18·1% (-8·0 to 48·0%)
HIV/AIDS - Tuberculosis	10·2 (6·1 to 18·4)	77·3% (72·3 to 81·6%)	14·1 (8·2 to 22·1)	78·9% (74·7 to 82·8%)	37·3% (-18·2 to 118·1%)	2·1% (-3·2 to 7·2%)	520·5 (321·6 to 897·4)	78·1% (73·5 to 82·1%)	693·2 (423·5 to 1,063·8)	79·7% (75·7 to 83·4%)	33·2% (-17·5 to 103·1%)	2·1% (-2·8 to 6·7%)
HIV/AIDS resulting in other diseases	38·1 (27·3 to 85·5)	76·8% (71·0 to 80·9%)	64·9 (50·2 to 107·7)	77·4% (72·9 to 81·0%)	70·5% (-1·5 to 152·6%)	0·8% (-4·1 to 4·8%)	1,894·2 (1,372·2 to 4,102·6)	77·6% (71·9 to 81·6%)	3,037·6 (2,347·8 to 5,052·5)	78·1% (73·6 to 81·7%)	60·4% (-5·2 to 135·5%)	0·7% (-3·8 to 4·5%)
Syphilis	10·8 (6·4 to 17·0)	100·0% (100·0 to 100·0%)	4·4 (2·8 to 6·8)	100·0% (100·0 to 100·0%)	-59·4% (-65·5 to -51·8%)	-	883·4 (506·8 to 1,406·7)	100·0% (100·0 to 100·0%)	345·2 (209·4 to 551·3)	100·0% (100·0 to 100·0%)	-60·9% (-66·9 to -53·5%)	-
Chlamydial infection	0·0 (0·0 to 0·0)	100·0% (100·0 to 100·0%)	0·0 (0·0 to 0·0)	100·0% (100·0 to 100·0%)	-13·8% (-27·5 to 5·8%)	-	181·1 (98·0 to 302·1)	100·0% (100·0 to 100·0%)	183·1 (100·5 to 305·6)	100·0% (100·0 to 100·0%)	1·1% (-3·6 to 6·5%)	-
Gonococcal infection	0·1 (0·1 to 0·1)	100·0% (100·0 to 100·0%)	0·1 (0·1 to 0·1)	100·0% (100·0 to 100·0%)	-18·2% (-32·1 to -0·5%)	-	12·5 (7·7 to 23·0)	100·0% (100·0 to 100·0%)	12·7 (7·0 to 24·9)	100·0% (100·0 to 100·0%)	1·4% (-14·6 to 16·2%)	-
Trichomoniasis	-	-	-	-	-	-	41·4 (16·6 to 88·1)	100·0% (100·0 to 100·0%)	44·3 (17·7 to 93·4)	100·0% (100·0 to 100·0%)	6·8% (4·1 to 9·7%)	-
Genital herpes	-	-	-	-	-	-	53·0 (16·3 to 126·1)	100·0% (100·0 to 100·0%)	61·1 (18·7 to 144·8)	100·0% (100·0 to 100·0%)	15·3% (10·2 to 19·7%)	-
Other sexually transmitted diseases	0·0 (0·0 to 0·1)	100·0% (100·0 to 100·0%)	0·0 (0·0 to 0·0)	100·0% (100·0 to 100·0%)	-21·4% (-34·2 to -6·0%)	-	29·4 (20·4 to 41·3)	100·0% (100·0 to 100·0%)	27·5 (18·6 to 39·5)	100·0% (100·0 to 100·0%)	-6·6% (-11·2 to -2·1%)	-
Cervical cancer	60·1 (54·1 to 66·2)	100·0% (100·0 to 100·0%)	62·6 (57·8 to 68·3)	100·0% (100·0 to 100·0%)	4·0% (-6·7 to 17·9%)	-	1,920·7 (1,714·7 to 2,130·0)	100·0% (100·0 to 100·0%)	1,909·6 (1,758·3 to 2,093·1)	100·0% (100·0 to 100·0%)	-0·6% (-11·3 to 12·9%)	-
**Low physical activity: All causes**	316·1 (244·2 to 392·3)	2·3% (1·8 to 2·9%)	386·6 (298·3 to 473·3)	2·7% (2·1 to 3·4%)	22·3% (16·5 to 28·4%)	18·8% (15·4 to 22·5%)	7,432·3 (5,730·4 to 9,264·9)	1·3% (1·0 to 1·6%)	8,774·3 (6,785·9 to 10,781·1)	1·6% (1·3 to 1·9%)	18·1% (12·2 to 24·2%)	22·2% (18·1 to 26·7%)
Colon and rectum cancer	21·4 (14·4 to 28·9)	11·7% (7·9 to 15·8%)	28·2 (18·8 to 38·1)	12·0% (8·1 to 16·1%)	31·4% (24·9 to 38·8%)	2·6% (1·3 to 4·2%)	467·0 (310·0 to 643·1)	10·6% (7·0 to 14·4%)	583·1 (383·4 to 799·4)	11·0% (7·2 to 14·9%)	24·8% (18·0 to 33·1%)	3·3% (1·8 to 4·8%)
Breast cancer	6·5 (4·4 to 8·6)	6·6% (4·7 to 8·5%)	8·3 (5·7 to 10·9)	6·9% (4·9 to 8·9%)	27·3% (7·5 to 43·9%)	3·7% (2·1 to 5·5%)	204·4 (139·0 to 274·2)	6·1% (4·3 to 7·9%)	251·7 (174·9 to 333·6)	6·4% (4·5 to 8·3%)	23·1% (3·1 to 39·8%)	4·3% (2·5 to 6·2%)
Ischaemic heart disease	149·4 (95·4 to 205·1)	9·0% (5·9 to 12·4%)	187·7 (121·7 to 256·2)	9·0% (5·9 to 12·3%)	25·7% (19·0 to 32·6%)	0·1% (-1·7 to 2·1%)	3,249·0 (2,036·5 to 4,477·3)	9·6% (6·1 to 13·3%)	3,816·7 (2,401·4 to 5,253·3)	9·7% (6·1 to 13·4%)	17·5% (9·6 to 25·5%)	1·0% (-1·3 to 3·5%)
Ischaemic stroke	96·8 (52·1 to 144·0)	10·8% (5·8 to 16·0%)	108·7 (60·9 to 160·3)	10·8% (6·0 to 15·8%)	12·3% (6·0 to 18·9%)	0·0% (-1·3 to 1·8%)	1,746·4 (916·9 to 2,618·7)	11·3% (5·9 to 16·9%)	1,866·1 (988·3 to 2,819·1)	11·4% (6·1 to 16·9%)	6·9% (0·8 to 13·0%)	0·7% (-0·7 to 2·3%)
Diabetes mellitus	41·9 (31·7 to 53·3)	14·3% (10·8 to 18·1%)	53·7 (40·2 to 68·6)	14·9% (11·3 to 18·9%)	28·0% (18·2 to 39·2%)	4·4% (2·4 to 6·8%)	1,765·4 (1,239·0 to 2,391·1)	12·1% (9·0 to 15·6%)	2,256·7 (1,592·2 to 3,057·4)	12·7% (9·4 to 16·2%)	27·8% (21·7 to 34·4%)	4·4% (2·8 to 6·2%)
**Metabolic risks: All causes**	4,085·5 (3,841·9 to 4,341·3)	29·9% (28·3 to 31·7%)	4,859·4 (4,560·5 to 5,186·2)	34·6% (32·7 to 36·4%)	18·9% (14·2 to 24·1%)	15·5% (13·4 to 17·7%)	99,410·1 (92,765·5 to 106,669·3)	17·5% (16·3 to 18·6%)	113,004·6 (104,587·5 to 122,328·2)	20·6% (19·2 to 22·0%)	13·7% (8·8 to 18·7%)	17·6% (14·6 to 20·5%)
**High fasting plasma glucose: All causes**	1,131·9 (981·8 to 1,341·6)	8·3% (7·2 to 9·8%)	1,381·1 (1,198·0 to 1,648·0)	9·8% (8·5 to 11·7%)	22·0% (16·2 to 28·9%)	18·5% (14·7 to 22·4%)	33,964·4 (29,807·8 to 38,985·1)	6·0% (5·4 to 6·7%)	40,386·5 (34,995·4 to 46,381·9)	7·3% (6·7 to 8·2%)	18·9% (14·2 to 24·2%)	23·0% (19·8 to 26·5%)
Tuberculosis	33·9 (20·5 to 49·7)	11·3% (7·1 to 16·2%)	29·1 (17·3 to 44·7)	12·9% (8·3 to 18·3%)	-14·1% (-26·8 to -0·6%)	14·1% (9·5 to 19·2%)	1,066·6 (681·0 to 1,509·9)	10·7% (6·9 to 14·9%)	931·6 (576·2 to 1,360·5)	12·5% (8·1 to 17·0%)	-12·7% (-24·5 to 0·0%)	16·2% (11·6 to 21·2%)
Ischaemic heart disease	277·7 (185·5 to 397·7)	16·8% (11·3 to 24·0%)	383·2 (255·1 to 545·9)	18·4% (12·3 to 26·4%)	38·0% (29·7 to 46·5%)	9·9% (5·6 to 14·5%)	5,814·3 (4,043·0 to 7,954·8)	17·2% (12·0 to 23·3%)	7,557·6 (5,308·9 to 10,356·6)	19·2% (13·4 to 26·0%)	30·0% (22·0 to 38·3%)	11·8% (8·4 to 14·8%)
Ischaemic stroke	140·0 (79·3 to 238·9)	15·7% (8·8 to 27·5%)	169·1 (96·3 to 293·7)	16·9% (9·5 to 29·5%)	20·8% (12·7 to 30·0%)	7·7% (3·4 to 14·7%)	2,585·6 (1,559·0 to 4,059·9)	16·8% (10·1 to 26·6%)	3,025·4 (1,846·1 to 4,717·5)	18·5% (11·2 to 28·9%)	17·0% (10·2 to 23·9%)	10·3% (8·1 to 13·3%)
Hemorrhagic stroke	255·5 (175·9 to 355·0)	15·6% (10·6 to 21·5%)	270·3 (184·6 to 374·1)	17·1% (11·7 to 23·4%)	5·8% (-0·2 to 13·3%)	9·4% (7·0 to 12·5%)	5,833·0 (3,979·8 to 8,158·2)	16·3% (11·1 to 22·8%)	6,073·7 (4,150·4 to 8,413·0)	18·3% (12·6 to 25·2%)	4·1% (-1·9 to 11·1%)	12·6% (9·6 to 15·1%)
Diabetes mellitus	293·6 (280·9 to 306·4)	100·0% (100·0 to 100·0%)	359·8 (338·2 to 385·3)	100·0% (100·0 to 100·0%)	22·6% (14·0 to 31·8%)	-	14,549·8 (11,984·4 to 17,619·1)	100·0% (100·0 to 100·0%)	17,817·5 (14,428·6 to 21,586·5)	100·0% (100·0 to 100·0%)	22·5% (17·7 to 27·3%)	-
Chronic kidney disease due to diabetes mellitus	72·1 (64·7 to 79·7)	100·0% (100·0 to 100·0%)	93·5 (82·7 to 102·9)	100·0% (100·0 to 100·0%)	29·6% (21·8 to 37·6%)	-	2,435·6 (2,180·5 to 2,727·0)	100·0% (100·0 to 100·0%)	2,969·5 (2,661·6 to 3,346·8)	100·0% (100·0 to 100·0%)	21·9% (16·3 to 28·0%)	-
Chronic kidney disease due to hypertension	29·3 (20·1 to 38·4)	33·3% (23·2 to 43·4%)	42·5 (29·8 to 55·2)	36·7% (25·8 to 47·2%)	45·4% (34·8 to 56·1%)	10·0% (8·1 to 12·5%)	706·3 (481·9 to 937·7)	30·1% (20·9 to 39·2%)	957·2 (663·8 to 1,251·7)	34·4% (24·0 to 44·2%)	35·5% (27·7 to 44·1%)	14·4% (12·2 to 17·0%)
Chronic kidney disease due to glomerulonephritis	27·1 (19·0 to 36·0)	31·8% (22·0 to 41·4%)	29·6 (20·5 to 39·0)	35·8% (25·1 to 46·0%)	9·1% (2·4 to 16·7%)	12·6% (10·7 to 14·7%)	768·2 (527·4 to 1,029·5)	27·7% (19·1 to 36·3%)	772·7 (532·5 to 1,020·6)	32·4% (22·7 to 42·0%)	0·6% (-5·0 to 7·3%)	16·9% (14·6 to 19·6%)
Chronic kidney disease due to other causes	2·5 (1·5 to 3·8)	25·0% (17·1 to 33·1%)	3·5 (2·1 to 5·1)	29·5% (20·4 to 38·8%)	39·7% (23·3 to 56·9%)	17·7% (12·3 to 24·1%)	165·7 (102·7 to 237·0)	22·6% (15·4 to 30·0%)	224·6 (141·0 to 319·4)	27·8% (19·2 to 36·2%)	35·6% (27·9 to 43·1%)	23·0% (18·8 to 27·9%)
**High total cholesterol: All causes**	857·0 (666·1 to 1,118·0)	6·3% (4·9 to 8·2%)	1,033·1 (784·2 to 1,341·8)	7·3% (5·6 to 9·7%)	20·6% (13·8 to 27·4%)	17·1% (12·0 to 21·7%)	19,875·3 (16,562·8 to 23,896·5)	3·5% (2·9 to 4·2%)	22,334·1 (18,559·8 to 26,923·6)	4·1% (3·4 to 5·0%)	12·4% (6·0 to 18·7%)	16·3% (11·1 to 20·6%)
Ischaemic heart disease	691·7 (547·6 to 853·5)	41·8% (33·3 to 51·2%)	849·3 (660·3 to 1,062·4)	40·8% (31·8 to 50·7%)	22·8% (15·4 to 29·8%)	-2·2% (-6·0 to 0·8%)	16,436·1 (13,808·7 to 19,241·7)	48·6% (41·1 to 56·8%)	18,743·6 (15,806·5 to 22,105·0)	47·6% (40·0 to 56·0%)	14·0% (7·0 to 20·7%)	-2·0% (-3·5 to -0·7%)
Ischaemic stroke	165·3 (75·6 to 325·7)	18·5% (8·4 to 35·9%)	183·9 (78·2 to 359·4)	18·3% (7·9 to 35·6%)	11·2% (0·8 to 19·9%)	-0·9% (-7·7 to 4·2%)	3,439·2 (2,123·9 to 5,574·4)	22·3% (13·9 to 36·3%)	3,590·5 (2,179·5 to 5,918·7)	22·0% (13·4 to 36·4%)	4·4% (-2·3 to 10·2%)	-1·6% (-4·7 to 0·8%)
**High systolic blood pressure: All causes**	2,689·7 (2,413·3 to 2,969·9)	19·7% (17·7 to 21·6%)	3,306·3 (2,973·7 to 3,654·6)	23·5% (21·3 to 25·7%)	22·9% (17·3 to 28·8%)	19·4% (16·2 to 22·5%)	57,619·1 (51,776·6 to 63,367·2)	10·1% (8·9 to 11·3%)	67,052·3 (60,857·6 to 73,623·1)	12·2% (10·8 to 13·7%)	16·4% (10·6 to 22·5%)	20·4% (16·2 to 24·5%)
Rheumatic heart disease	27·1 (18·3 to 41·0)	23·9% (16·2 to 36·5%)	25·1 (16·9 to 38·3)	27·7% (18·7 to 43·0%)	-7·5% (-14·7 to 0·4%)	15·9% (10·5 to 22·8%)	771·2 (524·7 to 1,162·6)	21·5% (14·6 to 32·4%)	681·0 (463·5 to 1,022·4)	25·9% (18·0 to 38·0%)	-11·7% (-17·9 to -4·4%)	20·5% (15·5 to 26·7%)
Ischaemic heart disease	859·0 (703·9 to 999·4)	51·9% (42·8 to 60·8%)	1,174·7 (958·9 to 1,390·3)	56·5% (46·4 to 66·0%)	36·8% (29·7 to 44·0%)	8·9% (6·4 to 11·4%)	17,687·0 (15,148·0 to 20,148·2)	52·3% (44·8 to 59·5%)	22,684·6 (19,463·8 to 25,779·2)	57·6% (49·7 to 64·9%)	28·3% (20·6 to 35·7%)	10·3% (8·2 to 12·7%)
Ischaemic stroke	430·0 (336·4 to 519·0)	48·1% (37·7 to 57·6%)	526·0 (412·8 to 632·6)	52·4% (41·8 to 62·5%)	22·3% (14·4 to 30·3%)	8·9% (5·9 to 13·2%)	7,831·7 (6,168·2 to 9,243·2)	50·8% (40·5 to 59·6%)	9,172·8 (7,308·2 to 10,746·8)	56·1% (45·3 to 64·7%)	17·1% (10·2 to 23·6%)	10·4% (8·0 to 13·1%)

Supplemental Results Table 1. All-age deaths, death PAFs, DALYs, DALY PAFs, and percent change for each risk factor and outcome for both sexes combined in 2005 and 2015 for Southeast Asia, East Asia, and Oceania.

Data in parenthesis are 95% uncertainty intervals. PAFs=population attributable fractions. DALYs=disability-adjusted life-years.

	2005 Deaths (in thousands)	2005 Deaths PAF	2015 Deaths (in thousands)	2015 Deaths PAF	Percent change deaths 2005-2015	Percent change deaths PAF 2005-2015	2005 DALYs (in thousands)	2005 DALYs PAF	2015 DALYs (in thousands)	2015 DALYs PAF	Percent change DALYs 2005-2015	Percent change DALYs PAF 2005-2015
Hemorrhagic stroke	904·4 (730·2 to 1,067·9)	55·2% (44·8 to 65·6%)	958·1 (775·5 to 1,141·3)	60·5% (49·7 to 70·8%)	5·9% (-0·2 to 13·1%)	9·6% (7·0 to 12·8%)	20,266·3 (16,581·8 to 23,493·2)	56·6% (46·3 to 65·5%)	20,786·9 (17,424·6 to 24,149·6)	62·8% (53·0 to 71·3%)	2·6% (-3·5 to 9·1%)	10·9% (8·6 to 13·6%)
Hypertensive heart disease	243·8 (230·1 to 267·8)	100·0% (100·0 to 100·0%)	325·8 (277·5 to 348·8)	100·0% (100·0 to 100·0%)	33·6% (12·2 to 45·0%)	-	4,567·9 (4,312·8 to 4,978·6)	100·0% (100·0 to 100·0%)	5,521·9 (4,937·9 to 5,880·9)	100·0% (100·0 to 100·0%)	20·9% (6·3 to 30·0%)	-
Cardiomyopathy and myocarditis	12·3 (9·7 to 15·1)	30·1% (24·0 to 36·2%)	17·0 (13·2 to 20·7)	35·5% (28·6 to 42·7%)	38·9% (27·2 to 52·2%)	18·0% (13·6 to 22·7%)	343·0 (267·5 to 431·7)	23·8% (18·4 to 29·2%)	449·6 (344·7 to 547·8)	31·2% (24·8 to 37·1%)	31·1% (18·9 to 45·4%)	31·2% (24·9 to 38·0%)
Atrial fibrillation and flutter	10·1 (7·4 to 13·7)	33·4% (26·8 to 40·2%)	15·5 (11·4 to 21·0)	36·5% (29·3 to 44·2%)	53·8% (46·4 to 61·8%)	9·5% (7·0 to 12·1%)	329·7 (249·1 to 433·4)	39·6% (34·8 to 44·5%)	490·8 (373·3 to 641·0)	43·9% (39·0 to 49·0%)	48·8% (45·0 to 52·9%)	11·0% (9·2 to 13·0%)
Aortic aneurysm	6·5 (5·3 to 7·8)	34·5% (28·0 to 41·1%)	9·4 (7·5 to 11·3)	37·8% (31·0 to 44·4%)	44·4% (32·4 to 55·0%)	9·4% (7·0 to 12·2%)	143·6 (119·3 to 168·6)	35·2% (29·5 to 41·3%)	201·0 (164·1 to 237·1)	39·7% (33·7 to 45·9%)	40·0% (27·6 to 51·8%)	12·9% (10·5 to 15·6%)
Peripheral vascular disease	0·3 (0·2 to 0·4)	24·7% (18·3 to 31·5%)	0·7 (0·5 to 0·9)	27·6% (20·3 to 35·5%)	98·7% (77·5 to 121·7%)	11·8% (7·2 to 16·7%)	42·6 (22·2 to 75·9)	27·8% (21·6 to 34·2%)	70·5 (37·1 to 124·2)	30·8% (24·1 to 37·5%)	65·4% (60·6 to 71·6%)	10·7% (8·6 to 13·3%)
Endocarditis	4·7 (3·5 to 6·0)	31·2% (25·0 to 37·8%)	6·5 (4·8 to 8·3)	35·3% (28·2 to 42·4%)	39·3% (28·1 to 52·7%)	13·0% (8·8 to 17·3%)	127·8 (94·0 to 169·4)	26·0% (19·8 to 32·3%)	166·7 (122·8 to 218·1)	32·1% (25·4 to 38·7%)	30·4% (18·5 to 43·7%)	23·4% (16·5 to 30·8%)
Other cardiovascular and circulatory diseases	37·5 (32·7 to 42·7)	36·9% (32·4 to 41·6%)	45·9 (39·8 to 52·3)	41·0% (36·1 to 45·7%)	22·5% (13·7 to 31·9%)	11·1% (8·2 to 13·8%)	1,279·1 (1,067·2 to 1,536·7)	31·2% (27·7 to 35·1%)	1,684·3 (1,375·2 to 2,048·1)	38·0% (34·2 to 42·2%)	31·7% (23·6 to 38·6%)	21·8% (18·7 to 25·0%)
Chronic kidney disease due to diabetes mellitus	30·8 (21·9 to 40·2)	42·7% (30·6 to 54·0%)	43·6 (31·1 to 56·3)	46·6% (33·9 to 58·3%)	41·4% (32·4 to 51·2%)	9·2% (7·3 to 11·5%)	871·3 (598·9 to 1,160·9)	35·8% (25·0 to 46·1%)	1,213·9 (843·3 to 1,595·9)	40·9% (29·0 to 51·7%)	39·3% (31·9 to 47·5%)	14·3% (11·9 to 17·3%)
Chronic kidney disease due to hypertension	87·7 (80·0 to 95·3)	100·0% (100·0 to 100·0%)	115·9 (103·3 to 126·2)	100·0% (100·0 to 100·0%)	32·1% (23·0 to 41·2%)	-	2,348·1 (2,122·7 to 2,565·5)	100·0% (100·0 to 100·0%)	2,781·3 (2,470·6 to 3,057·0)	100·0% (100·0 to 100·0%)	18·5% (11·8 to 25·3%)	-
Chronic kidney disease due to glomerulonephritis	32·0 (22·6 to 41·6)	37·5% (26·8 to 48·3%)	37·4 (27·1 to 47·8)	45·3% (33·0 to 56·8%)	16·9% (8·7 to 25·9%)	20·6% (17·1 to 24·6%)	821·1 (564·2 to 1,082·9)	29·6% (20·6 to 38·9%)	885·8 (628·9 to 1,148·7)	37·1% (26·6 to 47·6%)	7·9% (0·9 to 16·0%)	25·4% (21·2 to 29·9%)
Chronic kidney disease due to other causes	3·4 (2·1 to 5·1)	34·6% (24·2 to 44·6%)	4·7 (2·9 to 6·8)	40·0% (28·2 to 51·1%)	37·2% (21·0 to 55·3%)	15·6% (10·1 to 21·8%)	188·6 (119·8 to 268·2)	25·7% (17·5 to 33·6%)	261·2 (167·1 to 364·6)	32·3% (22·6 to 41·8%)	38·5% (30·2 to 47·0%)	25·8% (20·9 to 31·9%)
**High body-mass index: All causes**	761·3 (415·5 to 1,154·6)	5·6% (3·0 to 8·4%)	934·2 (543·5 to 1,382·0)	6·6% (3·9 to 9·8%)	22·7% (13·8 to 34·2%)	19·1% (12·1 to 29·7%)	24,671·3 (13,833·5 to 36,515·4)	4·3% (2·5 to 6·4%)	30,752·4 (18,612·1 to 43,820·4)	5·6% (3·4 to 7·9%)	24·6% (16·1 to 37·3%)	28·9% (21·6 to 41·4%)
Oesophageal cancer	33·7 (9·5 to 65·5)	12·0% (3·4 to 23·4%)	29·0 (7·8 to 57·1)	13·0% (3·6 to 25·5%)	-13·8% (-27·6 to 1·8%)	8·1% (-6·2 to 23·1%)	784·2 (213·9 to 1,525·4)	12·3% (3·3 to 24·0%)	662·7 (178·5 to 1,315·0)	13·7% (3·8 to 27·1%)	-15·5% (-30·1 to 1·6%)	11·4% (-5·3 to 28·3%)
Colon and rectum cancer	7·2 (3·3 to 12·1)	3·9% (1·8 to 6·6%)	10·8 (5·2 to 17·7)	4·6% (2·3 to 7·5%)	49·9% (38·9 to 64·5%)	17·1% (11·5 to 25·5%)	181·1 (84·0 to 303·2)	4·1% (1·9 to 6·9%)	266·3 (129·2 to 436·9)	5·0% (2·5 to 8·1%)	47·0% (35·1 to 63·6%)	21·6% (15·5 to 31·6%)
Liver cancer due to hepatitis B	17·1 (5·6 to 35·1)	8·5% (2·9 to 17·4%)	18·8 (6·7 to 37·8)	9·9% (3·6 to 19·7%)	9·7% (-2·1 to 32·5%)	17·2% (9·7 to 29·3%)	556·6 (182·2 to 1,147·5)	8·5% (2·9 to 17·5%)	591·4 (212·7 to 1,185·1)	10·3% (3·8 to 20·3%)	6·3% (-6·2 to 31·1%)	21·0% (12·8 to 34·3%)
Liver cancer due to hepatitis C	3·4 (1·2 to 6·9)	7·3% (2·7 to 14·1%)	4·4 (1·6 to 8·6)	8·7% (3·3 to 16·5%)	30·7% (12·3 to 52·5%)	18·6% (4·0 to 34·2%)	85·6 (30·4 to 173·9)	7·7% (2·8 to 14·9%)	109·6 (39·5 to 215·4)	9·4% (3·5 to 17·9%)	28·1% (8·9 to 51·5%)	21·8% (6·0 to 37·8%)
Liver cancer due to alcohol use	10·3 (3·3 to 22·0)	8·5% (2·8 to 17·7%)	15·2 (5·1 to 31·5)	9·7% (3·2 to 19·7%)	46·8% (31·2 to 75·1%)	14·6% (8·3 to 25·1%)	274·9 (87·7 to 588·4)	8·7% (2·8 to 18·3%)	400·9 (133·6 to 827·8)	10·2% (3·3 to 20·8%)	45·9% (29·6 to 77·8%)	17·5% (10·7 to 29·1%)
Liver cancer due to other causes	7·6 (2·7 to 14·7)	7·8% (2·8 to 14·9%)	8·1 (3·1 to 15·3)	9·0% (3·5 to 16·7%)	6·2% (-5·9 to 24·2%)	15·3% (5·3 to 27·6%)	216·6 (78·1 to 418·1)	8·0% (2·9 to 15·4%)	221·3 (83·8 to 424·2)	9·6% (3·6 to 18·0%)	2·1% (-10·0 to 21·9%)	19·4% (8·4 to 33·2%)
Gallbladder and biliary tract cancer	2·6 (1·2 to 4·4)	8·6% (4·0 to 14·7%)	3·4 (1·6 to 5·6)	9·6% (4·7 to 15·9%)	29·8% (14·7 to 47·3%)	11·9% (3·2 to 22·4%)	59·5 (27·5 to 99·9)	9·0% (4·2 to 15·2%)	74·5 (36·4 to 122·4)	10·2% (5·1 to 16·8%)	25·2% (10·5 to 42·7%)	13·5% (4·6 to 24·9%)
Pancreatic cancer	2·0 (0·6 to 3·9)	2·8% (0·8 to 5·6%)	3·1 (0·9 to 6·0)	3·2% (1·0 to 6·3%)	57·1% (40·9 to 73·4%)	13·5% (3·5 to 22·5%)	47·9 (14·0 to 95·1)	2·9% (0·9 to 5·8%)	74·6 (21·8 to 144·3)	3·4% (1·0 to 6·7%)	55·8% (38·5 to 73·0%)	15·7% (4·5 to 25·9%)
Breast cancer	10·1 (4·5 to 18·1)	10·3% (4·5 to 18·4%)	14·7 (7·1 to 25·4)	12·2% (6·0 to 20·8%)	44·6% (19·2 to 74·6%)	17·7% (10·1 to 34·3%)	325·2 (144·7 to 581·6)	9·7% (4·4 to 17·2%)	462·2 (225·2 to 794·3)	11·7% (5·9 to 19·7%)	42·1% (16·9 to 72·6%)	20·2% (11·8 to 38·1%)
Uterine cancer	4·6 (2·3 to 7·4)	19·9% (10·0 to 31·7%)	5·2 (2·8 to 8·0)	23·1% (12·7 to 35·1%)	12·0% (-1·2 to 31·4%)	16·3% (9·7 to 30·1%)	140·1 (69·2 to 225·7)	20·3% (10·1 to 32·3%)	155·5 (85·0 to 236·7)	23·9% (13·2 to 36·1%)	11·0% (-3·1 to 31·1%)	17·9% (11·1 to 32·6%)
Ovarian cancer	0·4 (0·0 to 1·1)	1·5% (0·0 to 4·0%)	0·6 (0·0 to 1·5)	1·8% (-0·1 to 4·5%)	44·6% (29·6 to 68·0%)	18·5% (11·8 to 32·8%)	12·7 (-0·3 to 33·3)	1·5% (0·0 to 4·0%)	18·1 (-0·5 to 45·3)	1·9% (-0·1 to 4·6%)	42·0% (26·7 to 66·6%)	20·5% (13·2 to 35·7%)
Kidney cancer	1·4 (0·7 to 2·3)	8·3% (4·1 to 13·1%)	2·4 (1·3 to 3·8)	9·6% (5·1 to 14·9%)	70·6% (55·7 to 88·0%)	15·8% (7·1 to 25·4%)	38·3 (19·5 to 60·5)	7·7% (3·9 to 12·1%)	64·6 (34·7 to 101·3)	9·5% (5·1 to 14·6%)	68·7% (53·2 to 87·8%)	23·3% (14·2 to 34·9%)
Thyroid cancer	0·4 (0·2 to 0·7)	5·3% (2·5 to 9·4%)	0·7 (0·3 to 1·2)	6·5% (3·1 to 11·4%)	63·8% (29·1 to 92·2%)	21·2% (6·7 to 35·5%)	11·8 (5·3 to 20·9)	5·6% (2·6 to 9·8%)	19·4 (8·7 to 34·7)	7·0% (3·3 to 12·2%)	64·7% (28·4 to 94·0%)	24·8% (9·8 to 39·3%)
Ischaemic heart disease	168·6 (91·4 to 261·7)	10·2% (5·5 to 15·7%)	228·3 (129·6 to 344·8)	11·0% (6·3 to 16·5%)	35·4% (25·2 to 48·3%)	7·8% (1·5 to 16·6%)	4,508·5 (2,459·6 to 6,965·3)	13·3% (7·3 to 20·4%)	5,912·9 (3,397·7 to 8,770·9)	15·0% (8·8 to 22·1%)	31·1% (20·9 to 44·7%)	12·7% (6·7 to 22·2%)
Ischaemic stroke	73·4 (38·6 to 115·5)	8·2% (4·3 to 12·8%)	83·5 (45·1 to 130·3)	8·3% (4·5 to 12·9%)	13·9% (3·4 to 25·8%)	1·4% (-6·7 to 9·9%)	1,803·9 (977·0 to 2,783·0)	11·7% (6·4 to 17·8%)	2,084·7 (1,174·7 to 3,117·3)	12·7% (7·3 to 18·9%)	15·6% (5·8 to 27·4%)	8·9% (2·2 to 17·8%)
Hemorrhagic stroke	239·1 (130·1 to 362·3)	14·6% (8·0 to 22·1%)	253·4 (148·2 to 374·1)	16·0% (9·4 to 23·5%)	6·0% (-3·4 to 18·6%)	9·6% (2·4 to 20·2%)	7,124·1 (3,986·5 to 10,540·8)	19·9% (11·0 to 29·5%)	7,590·2 (4,558·2 to 10,896·4)	22·9% (13·9 to 32·8%)	6·5% (-3·1 to 20·0%)	15·1% (8·3 to 26·7%)
Hypertensive heart disease	53·3 (28·0 to 86·5)	21·9% (11·5 to 35·3%)	77·1 (40·2 to 126·0)	23·7% (12·5 to 38·6%)	44·7% (19·3 to 69·6%)	8·3% (-5·1 to 22·8%)	1,175·4 (659·8 to 1,781·0)	25·7% (14·5 to 38·7%)	1,578·6 (919·5 to 2,336·9)	28·6% (17·1 to 42·1%)	34·3% (17·8 to 52·5%)	11·1% (2·7 to 22·7%)
Diabetes mellitus	80·5 (48·4 to 114·1)	27·4% (16·7 to 39·4%)	111·9 (73·6 to 153·8)	31·1% (20·4 to 42·5%)	38·9% (25·2 to 58·1%)	13·3% (5·8 to 25·4%)	5,083·4 (3,054·9 to 7,364·9)	34·9% (21·7 to 48·2%)	7,274·9 (4,714·5 to 10,188·4)	40·8% (28·1 to 53·4%)	43·1% (33·2 to 58·7%)	16·9% (10·3 to 29·3%)
Chronic kidney disease due to diabetes mellitus	13·8 (6·0 to 23·8)	19·1% (8·3 to 32·3%)	21·0 (9·4 to 34·9)	22·4% (10·3 to 37·2%)	52·3% (37·8 to 74·6%)	17·5% (7·5 to 31·8%)	449·8 (172·7 to 808·5)	18·5% (7·2 to 32·7%)	674·2 (269·7 to 1,155·1)	22·7% (9·6 to 38·6%)	49·9% (36·9 to 70·4%)	22·9% (14·3 to 38·0%)
Chronic kidney disease due to hypertension	14·2 (6·2 to 24·6)	16·2% (7·0 to 28·0%)	22·3 (10·0 to 37·7)	19·2% (8·6 to 32·5%)	56·8% (36·4 to 81·8%)	18·7% (4·8 to 37·5%)	403·2 (163·9 to 729·5)	17·2% (6·9 to 30·8%)	599·5 (263·1 to 1,025·4)	21·6% (9·4 to 36·2%)	48·7% (34·6 to 69·9%)	25·5% (15·9 to 42·2%)
Chronic kidney disease due to glomerulonephritis	13·9 (5·4 to 25·3)	16·3% (6·2 to 29·1%)	15·2 (6·2 to 26·5)	18·4% (7·6 to 31·5%)	8·9% (-7·2 to 31·9%)	12·4% (-2·4 to 33·7%)	419·1 (139·6 to 800·6)	15·1% (4·9 to 28·9%)	444·4 (161·1 to 801·8)	18·6% (6·8 to 33·2%)	6·1% (-5·2 to 25·9%)	23·2% (12·6 to 45·1%)
Chronic kidney disease due to other causes	0·9 (0·3 to 2·0)	9·2% (3·3 to 17·9%)	1·5 (0·5 to 3·1)	12·9% (4·3 to 24·2%)	66·1% (33·1 to 109·7%)	39·9% (14·4 to 73·6%)	84·5 (26·9 to 169·3)	11·5% (4·0 to 21·5%)	127·2 (43·8 to 244·6)	15·7% (5·9 to 27·8%)	50·6% (37·4 to 72·8%)	36·5% (25·7 to 56·7%)
Osteoarthritis	-	-	-	-	-	-	391·8 (173·6 to 727·5)	12·1% (5·9 to 19·9%)	640·7 (303·1 to 1,146·2)	13·9% (7·3 to 22·2%)	63·5% (56·0 to 77·1%)	15·4% (10·6 to 24·9%)
Low back pain	-	-	-	-	-	-	406·8 (186·7 to 724·5)	3·1% (1·6 to 5·0%)	588·9 (287·5 to 1,011·6)	3·9% (2·1 to 6·0%)	44·8% (37·8 to 57·8%)	25·0% (19·5 to 35·5%)
**Low bone mineral density: All causes**	87·1 (67·5 to 94·0)	0·6% (0·5 to 0·7%)	110·8 (79·6 to 122·4)	0·8% (0·6 to 0·9%)	27·2% (15·7 to 38·8%)	23·5% (12·7 to 33·9%)	2,114·7 (1,832·8 to 2,386·8)	0·4% (0·3 to 0·4%)	2,651·6 (2,195·6 to 3,056·5)	0·5% (0·4 to 0·5%)	25·4% (17·4 to 32·2%)	29·7% (22·6 to 36·3%)
Pedestrian road injuries	17·8 (16·2 to 19·4)	7·3% (6·9 to 7·7%)	20·2 (18·0 to 22·3)	9·1% (8·5 to 9·7%)	13·5% (1·4 to 25·0%)	25·0% (19·3 to 31·2%)	426·1 (380·0 to 470·4)	3·8% (3·5 to 4·1%)	478·3 (414·4 to 533·9)	5·1% (4·7 to 5·5%)	12·3% (0·0 to 23·5%)	34·3% (27·6 to 41·0%)
Cyclist road injuries	2·5 (2·2 to 2·9)	6·5% (6·1 to 7·0%)	2·7 (2·3 to 3·1)	8·1% (7·5 to 8·8%)	6·2% (-7·4 to 21·5%)	24·2% (16·7 to 32·3%)	128·7 (104·7 to 155·4)	6·0% (5·2 to 6·8%)	159·7 (126·4 to 199·5)	8·5% (7·4 to 9·8%)	24·1% (13·8 to 33·4%)	42·3% (33·1 to 51·9%)
Motorcyclist road injuries	4·1 (3·3 to 4·6)	3·6% (3·2 to 4·0%)	5·0 (4·1 to 5·8)	4·9% (4·3 to 5·5%)	21·4% (5·8 to 40·9%)	35·5% (23·8 to 46·6%)	193·8 (159·4 to 228·2)	3·1% (2·6 to 3·5%)	250·4 (203·6 to 299·7)	4·5% (3·8 to 5·3%)	29·2% (17·4 to 42·4%)	47·2% (32·2 to 58·8%)
Motor vehicle road injuries	5·8 (5·0 to 6·7)	5·6% (5·1 to 6·0%)	6·7 (5·6 to 7·6)	7·6% (7·0 to 8·1%)	15·4% (1·4 to 30·7%)	35·7% (27·8 to 43·8%)	243·5 (205·1 to 287·9)	4·3% (3·7 to 4·9%)	303·2 (247·2 to 365·3)	6·3% (5·5 to 7·3%)	24·5% (13·2 to 35·1%)	47·8% (39·0 to 56·3%)
Other road injuries	0·4 (0·3 to 0·5)	6·9% (5·8 to 8·7%)	0·5 (0·4 to 0·6)	8·5% (7·0 to 10·9%)	21·9% (-8·7 to 55·1%)	23·5% (3·2 to 45·3%)	18·8 (14·5 to 24·2)	5·5% (4·4 to 6·9%)	28·3 (21·1 to 37·0)	8·0% (6·2 to 10·2%)	50·6% (31·4 to 63·6%)	45·9% (22·6 to 71·5%)

Supplemental Results Table 1. All-age deaths, death PAFs, DALYs, DALY PAFs, and percent change for each risk factor and outcome for both sexes combined in 2005 and 2015 for Southeast Asia, East Asia, and Oceania.

Data in parenthesis are 95% uncertainty intervals. PAFs=population attributable fractions. DALYs=disability-adjusted life-years.

	2005 Deaths (in thousands)	2005 Deaths PAF	2015 Deaths (in thousands)	2015 Deaths PAF	Percent change deaths 2005-2015	Percent change deaths PAF 2005-2015	2005 DALYs (in thousands)	2005 DALYs PAF	2015 DALYs (in thousands)	2015 DALYs PAF	Percent change DALYs 2005-2015	Percent change DALYs PAF 2005-2015
Other transport injuries	2·1 (1·9 to 2·5)	8·1% (7·5 to 8·7%)	2·6 (2·2 to 3·1)	10·2% (9·5 to 11·0%)	21·4% (4·6 to 37·5%)	26·2% (18·4 to 33·7%)	54·7 (47·8 to 63·5)	4·0% (3·7 to 4·3%)	61·4 (53·0 to 71·8)	5·1% (4·7 to 5·5%)	12·2% (-2·0 to 25·1%)	27·6% (20·4 to 35·4%)
Falls	51·2 (31·4 to 57·4)	36·9% (29·8 to 39·6%)	69·5 (40·7 to 79·6)	40·9% (35·0 to 45·0%)	35·6% (19·9 to 53·3%)	10·8% (1·8 to 21·0%)	925·2 (699·6 to 1,057·0)	15·3% (13·1 to 16·2%)	1,226·4 (908·3 to 1,433·1)	17·7% (16·2 to 19·0%)	32·6% (23·3 to 41·5%)	16·0% (8·4 to 25·8%)
Other exposure to mechanical forces	2·0 (1·4 to 2·2)	4·7% (4·3 to 5·2%)	2·6 (1·3 to 3·1)	6·5% (5·9 to 7·6%)	31·9% (-6·5 to 50·2%)	37·3% (28·9 to 47·6%)	70·5 (52·8 to 84·9)	3·0% (2·6 to 3·3%)	86·8 (55·8 to 110·1)	4·1% (3·6 to 4·6%)	23·2% (-0·2 to 37·4%)	37·2% (29·5 to 45·9%)
Non-venomous animal contact	0·2 (0·2 to 0·3)	6·0% (5·2 to 6·5%)	0·2 (0·2 to 0·2)	7·5% (6·5 to 8·1%)	-14·1% (-24·7 to 2·4%)	24·0% (15·3 to 31·7%)	5·4 (4·2 to 6·1)	2·8% (2·3 to 3·1%)	4·3 (3·6 to 5·0)	3·5% (3·0 to 3·9%)	-19·6% (-28·6 to -4·8%)	27·7% (18·2 to 36·8%)
Assault by other means	0·9 (0·8 to 1·0)	3·8% (3·5 to 4·1%)	0·9 (0·8 to 1·1)	5·3% (4·8 to 5·8%)	0·5% (-8·3 to 14·3%)	37·7% (27·0 to 50·1%)	37·9 (31·1 to 46·1)	2·7% (2·3 to 3·1%)	40·1 (31·9 to 50·0)	4·0% (3·2 to 4·6%)	5·7% (-1·0 to 12·9%)	48·1% (36·4 to 58·3%)
Exposure to forces of nature	-	-	-	-	-	-	10·2 (3·1 to 18·9)	2·1% (0·8 to 3·5%)	12·6 (4·6 to 23·3)	4·9% (3·4 to 6·7%)	23·6% (1·1 to 72·7%)	133·5% (63·3 to 379·3%)
**Low glomerular filtration rate: All causes**	487·6 (431·3 to 553·4)	3·6% (3·2 to 4·1%)	561·4 (501·8 to 631·3)	4·0% (3·6 to 4·5%)	15·1% (9·9 to 20·8%)	11·8% (8·6 to 15·5%)	12,434·3 (11,301·6 to 13,758·2)	2·2% (2·0 to 2·4%)	13,267·6 (12,108·4 to 14,603·5)	2·4% (2·2 to 2·7%)	6·7% (2·2 to 11·8%)	10·4% (7·1 to 13·7%)
Ischaemic heart disease	87·4 (68·3 to 106·3)	5·3% (4·1 to 6·4%)	109·0 (83·6 to 132·9)	5·2% (4·1 to 6·4%)	24·7% (18·6 to 30·8%)	-0·7% (-3·3 to 1·9%)	1,533·4 (1,212·4 to 1,849·1)	4·5% (3·6 to 5·5%)	1,779·8 (1,412·2 to 2,143·2)	4·5% (3·6 to 5·5%)	16·1% (9·7 to 22·5%)	-0·2% (-2·5 to 2·4%)
Ischaemic stroke	54·3 (35·7 to 77·1)	6·1% (4·0 to 8·6%)	60·7 (40·3 to 86·5)	6·0% (4·0 to 8·6%)	11·9% (5·5 to 18·6%)	-0·3% (-3·2 to 3·0%)	847·8 (566·6 to 1,170·6)	5·5% (3·7 to 7·6%)	891·3 (604·8 to 1,220·0)	5·5% (3·7 to 7·5%)	5·1% (-0·6 to 11·4%)	-0·9% (-3·4 to 2·3%)
Hemorrhagic stroke	90·7 (60·0 to 126·7)	5·5% (3·7 to 7·7%)	87·6 (61·1 to 120·4)	5·5% (3·8 to 7·5%)	-3·4% (-8·9 to 5·0%)	0·0% (-3·3 to 5·5%)	1,713·4 (1,179·7 to 2,350·4)	4·8% (3·3 to 6·5%)	1,584·2 (1,126·7 to 2,133·6)	4·8% (3·4 to 6·4%)	-7·5% (-13·2 to 0·1%)	0·0% (-3·8 to 5·7%)
Peripheral vascular disease	0·1 (0·1 to 0·2)	9·1% (6·3 to 12·3%)	0·2 (0·1 to 0·3)	8·7% (5·9 to 11·9%)	70·8% (51·9 to 90·6%)	-3·9% (-7·9 to -0·7%)	15·8 (7·8 to 28·8)	10·3% (7·3 to 13·6%)	22·8 (11·2 to 40·6)	9·9% (7·0 to 13·2%)	44·4% (40·3 to 49·0%)	-3·4% (-5·3 to -1·8%)
Chronic kidney disease due to diabetes mellitus	72·1 (64·7 to 79·7)	100·0% (100·0 to 100·0%)	93·5 (82·7 to 102·9)	100·0% (100·0 to 100·0%)	29·6% (21·8 to 37·6%)	-	2,435·6 (2,180·5 to 2,727·0)	100·0% (100·0 to 100·0%)	2,969·5 (2,661·6 to 3,346·8)	100·0% (100·0 to 100·0%)	21·9% (16·3 to 28·0%)	-
Chronic kidney disease due to hypertension	87·7 (80·0 to 95·3)	100·0% (100·0 to 100·0%)	115·9 (103·3 to 126·2)	100·0% (100·0 to 100·0%)	32·1% (23·0 to 41·2%)	-	2,348·1 (2,122·7 to 2,565·5)	100·0% (100·0 to 100·0%)	2,781·3 (2,470·6 to 3,057·0)	100·0% (100·0 to 100·0%)	18·5% (11·8 to 25·3%)	-
Chronic kidney disease due to glomerulonephritis	85·3 (77·0 to 92·7)	100·0% (100·0 to 100·0%)	82·7 (72·9 to 90·1)	100·0% (100·0 to 100·0%)	-3·1% (-9·0 to 3·2%)	-	2,771·5 (2,494·8 to 3,003·1)	100·0% (100·0 to 100·0%)	2,384·6 (2,149·3 to 2,612·0)	100·0% (100·0 to 100·0%)	-14·0% (-18·6 to -8·5%)	-
Chronic kidney disease due to other causes	9·9 (7·0 to 13·4)	100·0% (100·0 to 100·0%)	11·8 (8·4 to 15·8)	100·0% (100·0 to 100·0%)	18·7% (6·1 to 32·1%)	-	734·6 (574·9 to 915·1)	100·0% (100·0 to 100·0%)	809·3 (623·8 to 1,020·4)	100·0% (100·0 to 100·0%)	10·2% (3·5 to 16·8%)	-
Gout	-	-	-	-	-	-	34·2 (22·5 to 47·9)	9·2% (8·2 to 10·4%)	44·7 (29·2 to 63·4)	9·4% (8·4 to 10·5%)	30·6% (27·1 to 34·3%)	2·2% (0·1 to 4·3%)

Supplemental Results Table 1. All-age deaths, death PAFs, DALYs, DALY PAFs, and percent change for each risk factor and outcome for both sexes combined in 2005 and 2015 for Sub-Saharan Africa.

Data in parenthesis are 95% uncertainty intervals. PAFs=population attributable fractions. DALYs=disability-adjusted life-years.

	2005 Deaths (in thousands)	2005 Deaths PAF	2015 Deaths (in thousands)	2015 Deaths PAF	Percent change deaths 2005-2015	Percent change deaths PAF 2005-2015	2005 DALYs (in thousands)	2005 DALYs PAF	2015 DALYs (in thousands)	2015 DALYs PAF	Percent change DALYs 2005-2015	Percent change DALYs PAF 2005-2015
**All risk factors: All causes**	4,479·1<br>(4,289·6 to 4,683·2)	51·6%<br>(50·0 to 53·3%)	3,949·1<br>(3,694·6 to 4,251·3)	49·3%<br>(47·7 to 50·9%)	-11·8%<br>(-17·4 to -5·8%)	-4·5%<br>(-7·2 to -1·8%)	254,755·5<br>(241,134·4 to 270,114·1)	43·8%<br>(41·8 to 45·9%)	212,280·9<br>(197,840·1 to 226,956·7)	40·1%<br>(38·2 to 42·0%)	-16·7%<br>(-21·0 to -11·9%)	-8·4%<br>(-11·8 to -4·9%)
**Environmental/occupational risks: All causes**	1,567·3<br>(1,436·9 to 1,707·5)	18·1%<br>(16·6 to 19·6%)	1,486·5<br>(1,336·4 to 1,647·0)	18·5%<br>(17·1 to 20·0%)	-5·2%<br>(-13·1 to 3·9%)	2·7%<br>(-3·3 to 9·1%)	92,117·7<br>(82,624·7 to 101,918·8)	15·8%<br>(14·2 to 17·5%)	81,107·3<br>(73,607·7 to 88,849·3)	15·3%<br>(13·9 to 16·8%)	-12·0%<br>(-19·7 to -2·9%)	-3·2%<br>(-10·7 to 5·3%)
**Unsafe water, sanitation, and handwashing: All causes**	805·6<br>(721·6 to 894·4)	9·3%<br>(8·4 to 10·2%)	688·4<br>(615·2 to 767·3)	8·6%<br>(7·8 to 9·4%)	-14·5%<br>(-23·0 to -4·1%)	-7·4%<br>(-15·3 to 1·5%)	53,880·3<br>(47,331·4 to 60,909·3)	9·3%<br>(8·1 to 10·5%)	44,000·7<br>(39,120·1 to 49,260·9)	8·3%<br>(7·4 to 9·3%)	-18·3%<br>(-27·3 to -7·3%)	-10·2%<br>(-19·4 to 1·4%)
**Unsafe water source: All causes**	612·9<br>(501·4 to 710·2)	7·1%<br>(5·8 to 8·1%)	512·5<br>(421·4 to 593·6)	6·4%<br>(5·2 to 7·3%)	-16·4%<br>(-25·3 to -5·2%)	-9·4%<br>(-18·4 to 0·3%)	41,574·0<br>(33,772·7 to 48,877·2)	7·1%<br>(5·8 to 8·4%)	33,589·7<br>(27,258·8 to 39,146·4)	6·3%<br>(5·2 to 7·4%)	-19·2%<br>(-28·9 to -7·0%)	-11·2%<br>(-21·5 to 1·5%)
Diarrhoeal diseases	596·3<br>(489·1 to 690·6)	87·0%<br>(71·0 to 95·2%)	492·7<br>(406·2 to 570·0)	86·6%<br>(70·5 to 94·9%)	-17·4%<br>(-26·3 to -6·0%)	-0·5%<br>(-0·9 to -0·3%)	40,334·5<br>(32,632·9 to 47,576·7)	87·1%<br>(71·1 to 95·2%)	32,102·6<br>(26,060·2 to 37,483·4)	86·7%<br>(70·7 to 95·0%)	-20·4%<br>(-30·1 to -8·3%)	-0·5%<br>(-0·8 to -0·2%)
Typhoid fever	15·3<br>(7·3 to 29·0)	87·0%<br>(71·0 to 95·2%)	18·3<br>(8·8 to 34·5)	86·6%<br>(70·5 to 94·9%)	19·5%<br>(7·1 to 32·7%)	-0·5%<br>(-0·9 to -0·3%)	1,138·9<br>(545·9 to 2,168·2)	87·0%<br>(71·0 to 95·2%)	1,366·5<br>(663·9 to 2,605·3)	86·6%<br>(70·5 to 94·9%)	20·0%<br>(7·6 to 33·0%)	-0·5%<br>(-0·9 to -0·3%)
Paratyphoid fever	1·3<br>(0·5 to 3·0)	87·4%<br>(71·7 to 95·3%)	1·6<br>(0·6 to 3·6)	87·1%<br>(71·5 to 95·1%)	18·9%<br>(1·0 to 41·0%)	-0·4%<br>(-0·7 to -0·2%)	100·5<br>(36·7 to 225·8)	87·4%<br>(71·7 to 95·3%)	120·7<br>(43·7 to 275·4)	87·1%<br>(71·5 to 95·1%)	20·0%<br>(1·9 to 41·3%)	-0·4%<br>(-0·7 to -0·2%)
**Unsafe sanitation: All causes**	455·0<br>(406·9 to 511·1)	5·2%<br>(4·7 to 5·9%)	369·4<br>(328·1 to 418·1)	4·6%<br>(4·1 to 5·1%)	-18·8%<br>(-27·3 to -7·8%)	-12·1%<br>(-20·8 to -2·1%)	30,829·7<br>(26,931·1 to 35,229·8)	5·3%<br>(4·6 to 6·0%)	24,142·5<br>(21,161·5 to 27,392·5)	4·6%<br>(4·0 to 5·2%)	-21·7%<br>(-31·2 to -9·9%)	-13·9%<br>(-24·0 to -1·6%)
Diarrhoeal diseases	442·5<br>(395·9 to 495·1)	64·6%<br>(61·3 to 68·1%)	354·9<br>(315·1 to 402·3)	62·4%<br>(58·5 to 65·9%)	-19·8%<br>(-28·5 to -8·7%)	-3·5%<br>(-5·0 to -2·1%)	29,900·8<br>(26,191·5 to 34,093·9)	64·6%<br>(61·3 to 68·1%)	23,061·0<br>(20,339·0 to 26,128·0)	62·3%<br>(58·4 to 65·8%)	-22·9%<br>(-32·3 to -11·0%)	-3·6%<br>(-5·3 to -1·9%)
Typhoid fever	11·5<br>(5·5 to 21·9)	65·3%<br>(61·4 to 68·6%)	13·3<br>(6·6 to 25·5)	63·1%<br>(59·4 to 66·4%)	16·1%<br>(4·1 to 28·5%)	-3·4%<br>(-4·8 to -2·2%)	854·7<br>(419·7 to 1,652·1)	65·3%<br>(61·4 to 68·6%)	996·4<br>(499·5 to 1,922·1)	63·1%<br>(59·4 to 66·4%)	16·6%<br>(4·6 to 29·0%)	-3·4%<br>(-4·8 to -2·2%)
Paratyphoid fever	1·0<br>(0·4 to 2·2)	64·5%<br>(60·8 to 67·7%)	1·1<br>(0·4 to 2·6)	61·4%<br>(57·5 to 65·0%)	13·7%<br>(-3·8 to 34·8%)	-4·8%<br>(-7·7 to -2·3%)	74·2<br>(28·1 to 165·4)	64·5%<br>(60·8 to 67·7%)	85·1<br>(32·7 to 192·7)	61·4%<br>(57·5 to 65·1%)	14·7%<br>(-2·5 to 34·7%)	-4·8%<br>(-7·7 to -2·3%)
**No handwashing with soap: All causes**	399·4<br>(323·9 to 472·8)	4·6%<br>(3·7 to 5·5%)	347·6<br>(286·0 to 408·4)	4·3%<br>(3·6 to 5·1%)	-13·0%<br>(-20·8 to -3·4%)	-5·7%<br>(-12·9 to 2·2%)	26,347·4<br>(21,357·0 to 31,608·0)	4·5%<br>(3·7 to 5·5%)	21,686·2<br>(17,802·0 to 25,595·0)	4·1%<br>(3·3 to 4·8%)	-17·7%<br>(-26·0 to -7·6%)	-9·5%<br>(-17·8 to 0·7%)
Diarrhoeal diseases	270·8<br>(212·8 to 327·9)	39·5%<br>(32·1 to 46·4%)	224·0<br>(177·0 to 272·1)	39·4%<br>(31·9 to 46·3%)	-17·3%<br>(-26·3 to -6·0%)	-0·4%<br>(-0·8 to -0·2%)	18,307·6<br>(14,342·0 to 22,463·7)	39·5%<br>(32·1 to 46·4%)	14,576·5<br>(11,364·1 to 17,727·6)	39·4%<br>(31·9 to 46·3%)	-20·4%<br>(-30·2 to -8·3%)	-0·4%<br>(-0·8 to -0·2%)
Typhoid fever	6·9<br>(3·3 to 13·7)	39·4%<br>(31·2 to 46·2%)	8·3<br>(3·9 to 16·0)	39·2%<br>(31·1 to 46·0%)	19·7%<br>(7·1 to 32·8%)	-0·4%<br>(-0·8 to -0·2%)	515·7<br>(244·1 to 1,019·0)	39·4%<br>(31·2 to 46·2%)	619·8<br>(295·6 to 1,205·5)	39·2%<br>(31·1 to 46·0%)	20·2%<br>(7·8 to 33·1%)	-0·4%<br>(-0·8 to -0·2%)
Paratyphoid fever	0·6<br>(0·2 to 1·4)	39·2%<br>(31·1 to 46·4%)	0·7<br>(0·3 to 1·7)	39·0%<br>(30·9 to 46·1%)	18·8%<br>(0·6 to 40·3%)	-0·6%<br>(-1·1 to -0·3%)	45·1<br>(16·4 to 103·2)	39·2%<br>(31·1 to 46·4%)	54·1<br>(20·2 to 127·2)	39·0%<br>(30·9 to 46·1%)	19·9%<br>(1·9 to 40·8%)	-0·6%<br>(-1·1 to -0·3%)
**Air pollution: All causes**	788·1<br>(677·3 to 895·1)	9·1%<br>(7·8 to 10·3%)	803·9<br>(678·5 to 935·4)	10·0%<br>(8·6 to 11·4%)	2·0%<br>(-6·7 to 12·2%)	10·5%<br>(4·5 to 16·4%)	37,913·3<br>(31,828·1 to 43,635·5)	6·5%<br>(5·4 to 7·5%)	34,391·8<br>(29,010·8 to 39,952·9)	6·5%<br>(5·4 to 7·6%)	-9·3%<br>(-17·0 to 0·2%)	-0·2%<br>(-8·0 to 8·6%)
**Ambient particulate matter pollution: All causes**	331·9<br>(272·6 to 397·6)	3·8%<br>(3·2 to 4·6%)	330·5<br>(270·4 to 401·0)	4·1%<br>(3·4 to 4·9%)	-0·4%<br>(-9·4 to 10·2%)	7·9%<br>(0·8 to 14·8%)	15,733·2<br>(12,296·9 to 19,553·4)	2·7%<br>(2·1 to 3·4%)	13,828·4<br>(10,808·4 to 17,296·8)	2·6%<br>(2·0 to 3·3%)	-12·1%<br>(-20·3 to -1·8%)	-3·3%<br>(-11·6 to 6·5%)
Lower respiratory infections	201·5<br>(147·2 to 262·9)	26·2%<br>(19·4 to 33·7%)	177·4<br>(123·8 to 238·4)	24·2%<br>(17·1 to 32·5%)	-11·9%<br>(-21·4 to -2·1%)	-7·6%<br>(-14·2 to -3·4%)	12,483·2<br>(9,177·1 to 16,331·5)	26·3%<br>(19·5 to 33·7%)	10,033·5<br>(7,042·3 to 13,459·0)	24·5%<br>(17·4 to 32·6%)	-19·6%<br>(-29·2 to -8·8%)	-7·1%<br>(-13·3 to -3·1%)
Tracheal, bronchus, and lung cancer	4·9<br>(3·1 to 6·9)	18·8%<br>(11·9 to 26·1%)	6·2<br>(3·8 to 8·7)	17·0%<br>(10·4 to 24·3%)	25·1%<br>(10·5 to 41·3%)	-9·6%<br>(-13·8 to -6·3%)	123·0<br>(76·2 to 171·9)	18·7%<br>(11·8 to 25·9%)	152·3<br>(93·6 to 215·6)	16·9%<br>(10·3 to 24·1%)	23·8%<br>(8·3 to 41·0%)	-9·6%<br>(-14·0 to -6·2%)
Ischaemic heart disease	57·3<br>(47·1 to 68·1)	18·7%<br>(15·7 to 21·8%)	68·1<br>(54·9 to 83·5)	18·1%<br>(15·1 to 21·1%)	18·8%<br>(5·2 to 34·2%)	-3·5%<br>(-5·7 to -1·7%)	1,391·6<br>(1,149·2 to 1,652·0)	20·2%<br>(17·0 to 23·2%)	1,645·5<br>(1,324·0 to 2,028·0)	19·5%<br>(16·3 to 22·5%)	18·2%<br>(3·3 to 35·8%)	-3·1%<br>(-5·1 to -1·0%)
Ischaemic stroke	18·4<br>(13·7 to 23·3)	13·9%<br>(10·8 to 16·8%)	21·0<br>(15·3 to 27·4)	13·2%<br>(10·1 to 16·0%)	14·4%<br>(1·6 to 28·8%)	-5·2%<br>(-8·5 to -2·5%)	377·0<br>(286·2 to 472·2)	14·8%<br>(12·0 to 17·6%)	423·6<br>(316·7 to 546·7)	14·0%<br>(11·2 to 16·8%)	12·4%<br>(-0·6 to 27·7%)	-5·1%<br>(-7·8 to -2·5%)
Hemorrhagic stroke	30·8<br>(24·9 to 37·6)	15·5%<br>(12·8 to 18·3%)	35·5<br>(27·6 to 44·3)	14·9%<br>(12·2 to 17·7%)	15·1%<br>(1·7 to 31·1%)	-3·8%<br>(-6·4 to -1·4%)	879·9<br>(704·4 to 1,077·8)	15·4%<br>(12·7 to 18·0%)	1,008·9<br>(777·7 to 1,268·0)	15·0%<br>(12·3 to 17·6%)	14·7%<br>(0·8 to 32·0%)	-2·7%<br>(-5·6 to 0·4%)
Chronic obstructive pulmonary disease	19·0<br>(10·8 to 27·9)	23·2%<br>(13·5 to 33·5%)	22·3<br>(12·3 to 33·2)	22·4%<br>(12·5 to 32·8%)	17·7%<br>(4·8 to 31·9%)	-3·5%<br>(-7·7 to -1·0%)	478·5<br>(276·0 to 707·1)	20·0%<br>(11·5 to 28·9%)	564·7<br>(313·7 to 845·0)	19·5%<br>(11·0 to 28·6%)	18·0%<br>(5·9 to 30·9%)	-2·6%<br>(-7·7 to 2·0%)
**Household air pollution from solid fuels: All causes**	598·4<br>(470·7 to 713·1)	6·9%<br>(5·4 to 8·3%)	605·9<br>(472·4 to 739·1)	7·6%<br>(5·9 to 9·1%)	1·2%<br>(-7·6 to 11·5%)	9·6%<br>(3·3 to 16·0%)	29,715·0<br>(22,986·4 to 35,966·6)	5·1%<br>(3·9 to 6·2%)	26,864·0<br>(20,708·4 to 32,554·8)	5·1%<br>(3·8 to 6·2%)	-9·6%<br>(-17·3 to 0·0%)	-0·6%<br>(-8·2 to 8·3%)
Lower respiratory infections	389·3<br>(289·4 to 479·7)	50·7%<br>(38·0 to 60·8%)	359·3<br>(262·5 to 449·9)	49·1%<br>(36·4 to 59·7%)	-7·7%<br>(-15·5 to 1·8%)	-3·2%<br>(-5·3 to -1·4%)	24,404·5<br>(18,042·0 to 29,925·5)	51·5%<br>(38·7 to 61·6%)	20,596·6<br>(15,281·4 to 25,582·1)	50·2%<br>(37·4 to 60·8%)	-15·6%<br>(-23·8 to -5·7%)	-2·5%<br>(-4·5 to -0·8%)
Tracheal, bronchus, and lung cancer	10·2<br>(6·3 to 13·8)	38·9%<br>(24·4 to 52·4%)	13·2<br>(8·2 to 18·5)	36·5%<br>(22·7 to 49·6%)	30·0%<br>(13·8 to 47·2%)	-6·2%<br>(-9·9 to -2·4%)	254·8<br>(158·4 to 345·6)	38·7%<br>(24·2 to 52·0%)	329·6<br>(203·3 to 462·5)	36·5%<br>(22·7 to 49·7%)	29·4%<br>(12·4 to 47·6%)	-5·6%<br>(-9·4 to -1·8%)
Ischaemic heart disease	80·4<br>(64·6 to 98·1)	26·3%<br>(21·4 to 31·3%)	95·0<br>(73·8 to 120·2)	25·2%<br>(20·6 to 30·1%)	18·2%<br>(3·8 to 33·8%)	-4·0%<br>(-7·1 to -1·0%)	1,957·8<br>(1,600·5 to 2,379·0)	28·4%<br>(23·2 to 33·5%)	2,310·0<br>(1,810·0 to 2,898·8)	27·4%<br>(22·4 to 32·5%)	18·0%<br>(2·6 to 35·9%)	-3·3%<br>(-6·6 to -0·1%)
Ischaemic stroke	31·2<br>(22·4 to 42·1)	23·6%<br>(18·0 to 30·5%)	36·1<br>(25·9 to 49·8)	22·6%<br>(17·1 to 29·2%)	15·5%<br>(1·4 to 30·9%)	-4·4%<br>(-7·4 to -1·2%)	646·7<br>(464·5 to 858·0)	25·3%<br>(19·1 to 32·3%)	736·7<br>(526·5 to 989·9)	24·4%<br>(18·3 to 31·0%)	13·9%<br>(-0·7 to 29·4%)	-3·8%<br>(-6·8 to -0·5%)
Hemorrhagic stroke	52·8<br>(39·9 to 67·3)	26·5%<br>(20·5 to 33·1%)	61·4<br>(44·9 to 79·8)	25·8%<br>(19·8 to 32·4%)	16·3%<br>(2·1 to 33·0%)	-2·8%<br>(-5·8 to 0·4%)	1,509·8<br>(1,142·7 to 1,926·1)	26·4%<br>(20·2 to 32·6%)	1,754·7<br>(1,284·1 to 2,277·1)	26·0%<br>(19·8 to 32·5%)	16·2%<br>(1·9 to 33·8%)	-1·4%<br>(-4·8 to 2·4%)
Chronic obstructive pulmonary disease	34·5<br>(21·3 to 51·4)	42·2%<br>(26·9 to 61·8%)	40·9<br>(25·4 to 60·2)	41·0%<br>(26·0 to 60·4%)	18·3%<br>(5·2 to 34·1%)	-3·0%<br>(-6·1 to 0·0%)	867·8<br>(543·4 to 1,276·2)	36·2%<br>(23·2 to 53·3%)	1,038·1<br>(650·0 to 1,519·6)	35·8%<br>(22·7 to 52·6%)	19·6%<br>(7·8 to 33·7%)	-1·2%<br>(-5·5 to 3·4%)
Cataract	-	-	-	-	-	-	73·7<br>(50·4 to 101·3)	32·5%<br>(28·9 to 35·9%)	98·4<br>(67·5 to 136·4)	31·7%<br>(28·0 to 35·0%)	33·5%<br>(30·1 to 36·9%)	-2·5%<br>(-4·3 to -0·4%)
**Ambient ozone pollution: All causes**	4·0<br>(1·4 to 7·0)	0·0%<br>(0·0 to 0·1%)	5·1<br>(1·8 to 8·9)	0·1%<br>(0·0 to 0·1%)	27·7%<br>(12·2 to 46·6%)	38·3%<br>(24·9 to 53·1%)	81·8<br>(28·5 to 141·9)	0·0%<br>(0·0 to 0·0%)	103·2<br>(35·7 to 179·4)	0·0%<br>(0·0 to 0·0%)	26·2%<br>(8·6 to 47·3%)	38·8%<br>(20·6 to 58·6%)
Chronic obstructive pulmonary disease	4·0<br>(1·4 to 7·0)	4·9%<br>(1·8 to 8·6%)	5·1<br>(1·8 to 8·9)	5·1%<br>(1·9 to 9·0%)	27·7%<br>(12·2 to 46·6%)	4·7%<br>(-1·2 to 10·9%)	81·8<br>(28·5 to 141·9)	3·4%<br>(1·2 to 5·9%)	103·2<br>(35·7 to 179·4)	3·6%<br>(1·3 to 6·1%)	26·2%<br>(8·6 to 47·3%)	4·3%<br>(-4·7 to 13·8%)
**Other environmental risks: All causes**	20·7<br>(8·8 to 36·4)	0·2%<br>(0·1 to 0·4%)	25·8<br>(11·1 to 45·8)	0·3%<br>(0·1 to 0·6%)	24·6%<br>(11·2 to 40·7%)	34·8%<br>(25·8 to 47·2%)	538·2<br>(247·1 to 932·3)	0·1%<br>(0·0 to 0·2%)	644·7<br>(296·3 to 1,124·2)	0·1%<br>(0·1 to 0·2%)	19·8%<br>(7·5 to 33·4%)	31·6%<br>(20·5 to 44·8%)
**Residential radon: All causes**	2·4<br>(1·6 to 3·5)	0·0%<br>(0·0 to 0·0%)	3·2<br>(2·2 to 4·5)	0·0%<br>(0·0 to 0·1%)	35·0%<br>(15·2 to 57·2%)	46·1%<br>(28·2 to 64·1%)	60·1<br>(41·5 to 85·4)	0·0%<br>(0·0 to 0·0%)	80·4<br>(54·6 to 110·3)	0·0%<br>(0·0 to 0·0%)	33·8%<br>(14·1 to 56·6%)	47·1%<br>(27·3 to 68·0%)
Tracheal, bronchus, and lung cancer	2·4<br>(1·6 to 3·5)	9·1%<br>(6·2 to 13·1%)	3·2<br>(2·2 to 4·5)	8·9%<br>(6·0 to 12·1%)	35·0%<br>(15·2 to 57·2%)	-2·5%<br>(-11·2 to 4·1%)	60·1<br>(41·5 to 85·4)	9·1%<br>(6·3 to 12·8%)	80·4<br>(54·6 to 110·3)	8·9%<br>(6·2 to 11·9%)	33·8%<br>(14·1 to 56·6%)	-2·3%<br>(-10·7 to 4·6%)
**Lead exposure: All causes**	18·4<br>(6·6 to 33·9)	0·2%<br>(0·1 to 0·4%)	22·6<br>(8·2 to 42·3)	0·3%<br>(0·1 to 0·5%)	23·2%<br>(9·4 to 40·3%)	33·3%<br>(23·8 to 47·1%)	478·2<br>(193·2 to 863·8)	0·1%<br>(0·0 to 0·1%)	564·3<br>(221·5 to 1,037·7)	0·1%<br>(0·0 to 0·2%)	18·0%<br>(5·6 to 31·7%)	29·7%<br>(17·7 to 43·0%)
Rheumatic heart disease	0·1<br>(0·0 to 0·3)	0·7%<br>(0·2 to 1·6%)	0·1<br>(0·0 to 0·3)	0·8%<br>(0·2 to 1·6%)	12·7%<br>(-5·2 to 35·2%)	3·6%<br>(-4·9 to 17·2%)	4·4<br>(1·0 to 10·6)	0·5%<br>(0·1 to 1·2%)	5·0<br>(1·1 to 11·9)	0·5%<br>(0·1 to 1·3%)	13·4%<br>(-2·8 to 33·2%)	2·6%<br>(-7·6 to 16·0%)
Ischaemic heart disease	6·3<br>(2·2 to 11·6)	2·1%<br>(0·7 to 3·8%)	7·8<br>(2·8 to 14·3)	2·1%<br>(0·8 to 3·8%)	23·8%<br>(10·9 to 39·7%)	0·5%<br>(-4·9 to 8·6%)	125·6<br>(37·7 to 244·9)	1·8%<br>(0·6 to 3·6%)	152·5<br>(47·0 to 301·8)	1·8%<br>(0·6 to 3·5%)	21·4%<br>(7·3 to 38·0%)	-0·6%<br>(-7·2 to 7·7%)
Ischaemic stroke	2·7<br>(0·9 to 5·0)	2·0%<br>(0·7 to 3·7%)	3·3<br>(1·2 to 6·1)	2·0%<br>(0·8 to 3·7%)	21·5%<br>(7·1 to 38·1%)	0·5%<br>(-4·9 to 9·7%)	49·1<br>(15·8 to 95·3)	1·9%<br>(0·6 to 3·6%)	58·4<br>(18·5 to 112·6)	1·9%<br>(0·6 to 3·6%)	18·7%<br>(3·8 to 35·4%)	0·1%<br>(-6·1 to 9·5%)
Hemorrhagic stroke	4·2<br>(1·4 to 8·2)	2·1%<br>(0·7 to 4·1%)	5·1<br>(1·7 to 10·0)	2·2%<br>(0·7 to 4·1%)	21·6%<br>(6·4 to 40·6%)	1·6%<br>(-5·6 to 13·0%)	102·5<br>(29·0 to 215·1)	1·8%<br>(0·5 to 3·7%)	123·4<br>(34·2 to 255·2)	1·8%<br>(0·5 to 3·8%)	20·4%<br>(3·3 to 41·0%)	2·1%<br>(-6·4 to 14·7%)
Hypertensive heart disease	3·1<br>(0·7 to 7·5)	3·9%<br>(0·9 to 9·7%)	3·8<br>(0·8 to 9·2)	4·0%<br>(1·0 to 10·0%)	23·0%<br>(4·4 to 43·7%)	2·6%<br>(-5·1 to 13·9%)	62·0<br>(14·4 to 149·7)	3·5%<br>(0·9 to 8·3%)	74·0<br>(16·5 to 178·5)	3·5%<br>(0·9 to 8·4%)	19·4%<br>(1·3 to 41·2%)	1·5%<br>(-6·0 to 14·0%)
Cardiomyopathy and myocarditis	0·2<br>(0·1 to 0·4)	1·0%<br>(0·3 to 2·0%)	0·2<br>(0·1 to 0·5)	1·1%<br>(0·3 to 2·1%)	17·6%<br>(2·7 to 34·2%)	0·7%<br>(-5·3 to 7·9%)	5·0<br>(1·3 to 11·1)	0·7%<br>(0·2 to 1·6%)	5·9<br>(1·5 to 13·2)	0·7%<br>(0·2 to 1·6%)	17·1%<br>(1·3 to 35·7%)	2·4%<br>(-5·8 to 13·1%)

Supplemental Results Table 1. All-age deaths, death PAFs, DALYs, DALY PAFs, and percent change for each risk factor and outcome for both sexes combined in 2005 and 2015 for Sub-Saharan Africa.

Data in parenthesis are 95% uncertainty intervals. PAFs=population attributable fractions. DALYs=disability-adjusted life-years.

	2005 Deaths (in thousands)	2005 Deaths PAF	2015 Deaths (in thousands)	2015 Deaths PAF	Percent change deaths 2005-2015	Percent change deaths PAF 2005-2015	2005 DALYs (in thousands)	2005 DALYs PAF	2015 DALYs (in thousands)	2015 DALYs PAF	Percent change DALYs 2005-2015	Percent change DALYs PAF 2005-2015
Atrial fibrillation and flutter	0·0 (0·0 to 0·1)	1·4% (0·6 to 2·5%)	0·1 (0·0 to 0·1)	1·5% (0·6 to 2·5%)	39·6% (25·1 to 57·1%)	2·0% (-3·3 to 10·2%)	1·2 (0·4 to 2·3)	1·4% (0·5 to 2·6%)	1·6 (0·5 to 3·1)	1·4% (0·5 to 2·6%)	32·4% (25·7 to 40·5%)	-1·1% (-4·3 to 2·9%)
Aortic aneurysm	0·1 (0·0 to 0·1)	1·3% (0·4 to 2·4%)	0·1 (0·0 to 0·2)	1·3% (0·4 to 2·4%)	37·3% (18·8 to 57·4%)	-0·3% (-6·2 to 7·1%)	1·4 (0·4 to 3·1)	1·2% (0·3 to 2·3%)	2·0 (0·6 to 4·1)	1·1% (0·3 to 2·3%)	36·8% (15·3 to 60·4%)	-0·6% (-8·0 to 8·7%)
Peripheral vascular disease	0·0 (0·0 to 0·0)	1·1% (0·4 to 1·9%)	0·0 (0·0 to 0·0)	1·1% (0·4 to 1·9%)	53·9% (33·8 to 74·3%)	-2·7% (-6·7 to 0·3%)	0·4 (0·1 to 0·8)	1·0% (0·3 to 1·8%)	0·5 (0·2 to 1·1)	0·9% (0·3 to 1·7%)	38·1% (28·4 to 49·2%)	-2·7% (-6·1 to -0·5%)
Endocarditis	0·1 (0·0 to 0·2)	0·8% (0·2 to 1·7%)	0·1 (0·0 to 0·2)	0·9% (0·2 to 1·7%)	34·7% (16·0 to 57·4%)	2·4% (-6·3 to 15·3%)	1·7 (0·4 to 4·3)	0·5% (0·1 to 1·3%)	2·3 (0·5 to 5·8)	0·5% (0·1 to 1·3%)	33·1% (12·2 to 57·3%)	3·7% (-9·1 to 20·2%)
Other cardiovascular and circulatory diseases	1·0 (0·3 to 1·9)	1·3% (0·4 to 2·4%)	1·3 (0·4 to 2·4)	1·3% (0·5 to 2·4%)	28·4% (9·8 to 52·2%)	2·1% (-5·7 to 13·8%)	29·4 (8·4 to 59·2)	1·0% (0·3 to 2·0%)	37·5 (11·0 to 77·0)	1·0% (0·3 to 2·0%)	27·5% (11·8 to 44·9%)	3·1% (-4·9 to 13·3%)
Idiopathic developmental intellectual disability	-	-	-	-	-	-	80·1 (35·2 to 141·0)	22·7% (17·1 to 27·6%)	81·9 (35·1 to 145·4)	17·9% (12·5 to 22·5%)	2·3% (-9·6 to 12·1%)	-21·0% (-30·3 to -13·4%)
Chronic kidney disease due to diabetes mellitus	0·1 (0·0 to 0·2)	1·3% (0·5 to 2·4%)	0·1 (0·0 to 0·2)	1·4% (0·5 to 2·6%)	44·3% (26·5 to 66·3%)	8·4% (0·4 to 20·0%)	2·7 (0·8 to 6·2)	0·6% (0·2 to 1·4%)	3·6 (1·0 to 8·2)	0·7% (0·2 to 1·5%)	34·1% (22·5 to 49·0%)	5·3% (-1·7 to 15·3%)
Chronic kidney disease due to hypertension	0·4 (0·1 to 0·8)	1·4% (0·4 to 2·7%)	0·5 (0·2 to 1·0)	1·4% (0·4 to 2·7%)	29·1% (18·4 to 41·2%)	0·3% (-5·9 to 7·4%)	8·4 (2·2 to 18·2)	0·9% (0·2 to 1·9%)	10·6 (2·7 to 23·2)	0·9% (0·2 to 1·9%)	26·3% (15·3 to 39·0%)	-0·1% (-7·3 to 6·7%)
Chronic kidney disease due to glomerulonephritis	0·1 (0·0 to 0·2)	1·0% (0·3 to 2·0%)	0·1 (0·0 to 0·2)	1·0% (0·4 to 2·0%)	14·5% (-0·4 to 32·0%)	1·0% (-5·8 to 12·2%)	3·0 (0·7 to 6·7)	0·6% (0·1 to 1·2%)	3·5 (0·9 to 8·0)	0·5% (0·1 to 1·2%)	16·9% (5·2 to 29·1%)	-1·1% (-7·5 to 6·9%)
Chronic kidney disease due to other causes	0·0 (0·0 to 0·0)	0·8% (0·3 to 1·7%)	0·0 (0·0 to 0·0)	0·9% (0·3 to 1·8%)	40·3% (19·3 to 67·9%)	11·1% (-0·4 to 27·1%)	1·4 (0·4 to 3·5)	0·6% (0·2 to 1·5%)	1·9 (0·5 to 4·5)	0·7% (0·2 to 1·5%)	29·6% (23·7 to 35·9%)	2·0% (-2·8 to 7·7%)
**Occupational risks: All causes**	47·1 (43·3 to 51·3)	0·5% (0·5 to 0·6%)	60·9 (55·5 to 67·2)	0·8% (0·7 to 0·8%)	29·2% (18·2 to 41·3%)	40·0% (31·7 to 48·5%)	5,027·0 (4,076·5 to 6,141·3)	0·9% (0·7 to 1·0%)	6,640·1 (5,372·5 to 8,137·2)	1·3% (1·1 to 1·5%)	32·1% (27·3 to 36·9%)	45·2% (39·1 to 52·0%)
**Occupational carcinogens: All causes**	7·8 (7·0 to 8·7)	0·1% (0·1 to 0·1%)	11·9 (10·6 to 13·6)	0·1% (0·1 to 0·2%)	53·6% (36·2 to 74·1%)	66·3% (52·0 to 81·7%)	183·3 (164·4 to 206·0)	0·0% (0·0 to 0·0%)	278·5 (245·5 to 320·2)	0·1% (0·0 to 0·1%)	51·9% (33·8 to 73·7%)	67·1% (49·7 to 86·3%)
**Occupational exposure to asbestos: All causes**	2·4 (2·0 to 3·0)	0·0% (0·0 to 0·0%)	3·3 (2·7 to 4·0)	0·0% (0·0 to 0·0%)	34·9% (15·7 to 56·8%)	46·1% (28·4 to 66·1%)	50·5 (40·3 to 62·8)	0·0% (0·0 to 0·0%)	65·6 (53·1 to 82·1)	0·0% (0·0 to 0·0%)	29·9% (9·3 to 53·3%)	42·8% (22·0 to 66·3%)
Larynx cancer	0·1 (0·0 to 0·1)	1·7% (1·1 to 2·4%)	0·1 (0·1 to 0·1)	1·8% (1·1 to 2·4%)	36·3% (13·2 to 64·4%)	4·0% (-11·4 to 24·4%)	1·4 (0·9 to 2·0)	1·3% (0·8 to 1·8%)	1·9 (1·2 to 2·6)	1·3% (0·8 to 1·8%)	32·7% (10·5 to 60·7%)	2·0% (-12·4 to 20·8%)
Tracheal, bronchus, and lung cancer	1·8 (1·4 to 2·3)	6·8% (5·4 to 8·4%)	2·5 (2·0 to 3·2)	7·0% (5·6 to 8·6%)	42·5% (21·3 to 65·8%)	3·0% (-8·3 to 15·9%)	35·2 (26·6 to 45·6)	5·3% (4·1 to 6·7%)	48·7 (38·0 to 63·0)	5·4% (4·3 to 6·7%)	38·4% (16·4 to 64·2%)	1·1% (-10·7 to 14·5%)
Ovarian cancer	0·1 (0·1 to 0·2)	1·5% (0·7 to 2·4%)	0·1 (0·1 to 0·2)	1·2% (0·6 to 2·0%)	19·1% (-9·5 to 60·3%)	-15·8% (-28·1 to -0·5%)	2·4 (1·1 to 4·0)	1·0% (0·5 to 1·6%)	2·8 (1·3 to 5·0)	0·8% (0·4 to 1·4%)	20·3% (-12·3 to 66·8%)	-15·6% (-29·5 to 2·6%)
Mesothelioma	0·5 (0·4 to 0·6)	53·4% (39·5 to 66·0%)	0·5 (0·4 to 0·7)	51·8% (39·2 to 64·0%)	10·2% (-5·7 to 30·1%)	-3·0% (-7·0 to 2·6%)	11·6 (8·1 to 15·5)	42·9% (30·4 to 55·2%)	12·2 (8·6 to 16·4)	40·4% (28·9 to 51·9%)	5·6% (-11·6 to 28·4%)	-5·9% (-11·3 to 1·0%)
**Occupational exposure to arsenic: All causes**	0·2 (0·1 to 0·2)	0·0% (0·0 to 0·0%)	0·2 (0·2 to 0·3)	0·0% (0·0 to 0·0%)	43·6% (27·0 to 63·3%)	55·6% (41·4 to 71·7%)	3·7 (2·8 to 4·6)	0·0% (0·0 to 0·0%)	5·2 (3·9 to 6·7)	0·0% (0·0 to 0·0%)	41·3% (24·3 to 61·3%)	55·4% (39·4 to 74·5%)
Tracheal, bronchus, and lung cancer	0·2 (0·1 to 0·2)	0·6% (0·5 to 0·7%)	0·2 (0·2 to 0·3)	0·6% (0·5 to 0·8%)	43·6% (27·0 to 63·3%)	3·8% (-2·0 to 10·1%)	3·7 (2·8 to 4·6)	0·6% (0·4 to 0·7%)	5·2 (3·9 to 6·7)	0·6% (0·4 to 0·7%)	41·3% (24·3 to 61·3%)	3·2% (-2·7 to 9·4%)
**Occupational exposure to benzene: All causes**	0·2 (0·2 to 0·3)	0·0% (0·0 to 0·0%)	0·4 (0·3 to 0·5)	0·0% (0·0 to 0·0%)	74·2% (50·6 to 105·4%)	88·5% (69·4 to 114·6%)	8·1 (6·6 to 9·8)	0·0% (0·0 to 0·0%)	14·2 (11·3 to 17·8)	0·0% (0·0 to 0·0%)	75·3% (50·2 to 108·5%)	92·7% (67·7 to 124·7%)
Leukaemia	0·2 (0·2 to 0·3)	1·5% (1·2 to 1·7%)	0·4 (0·3 to 0·5)	1·8% (1·4 to 2·1%)	74·2% (50·6 to 105·4%)	20·2% (9·5 to 33·6%)	8·1 (6·6 to 9·8)	1·1% (0·9 to 1·3%)	14·2 (11·3 to 17·8)	1·3% (1·0 to 1·5%)	75·3% (50·2 to 108·5%)	17·5% (3·4 to 34·4%)
**Occupational exposure to beryllium: All causes**	0·0 (0·0 to 0·0)	0·0% (0·0 to 0·0%)	0·0 (0·0 to 0·0)	0·0% (0·0 to 0·0%)	42·2% (24·6 to 64·6%)	54·0% (38·4 to 73·4%)	0·2 (0·1 to 0·2)	0·0% (0·0 to 0·0%)	0·2 (0·2 to 0·3)	0·0% (0·0 to 0·0%)	38·9% (21·0 to 61·6%)	52·8% (34·9 to 74·2%)
Tracheal, bronchus, and lung cancer	0·0 (0·0 to 0·0)	0·0% (0·0 to 0·0%)	0·0 (0·0 to 0·0)	0·0% (0·0 to 0·0%)	42·2% (24·6 to 64·6%)	2·7% (-5·5 to 11·7%)	0·2 (0·1 to 0·2)	0·0% (0·0 to 0·0%)	0·2 (0·2 to 0·3)	0·0% (0·0 to 0·0%)	38·9% (21·0 to 61·6%)	1·4% (-6·4 to 10·7%)
**Occupational exposure to cadmium: All causes**	0·0 (0·0 to 0·0)	0·0% (0·0 to 0·0%)	0·0 (0·0 to 0·1)	0·0% (0·0 to 0·0%)	57·1% (37·4 to 81·6%)	70·1% (52·5 to 91·3%)	0·7 (0·5 to 0·8)	0·0% (0·0 to 0·0%)	1·0 (0·8 to 1·3)	0·0% (0·0 to 0·0%)	53·7% (33·6 to 78·0%)	69·0% (49·3 to 92·1%)
Tracheal, bronchus, and lung cancer	0·0 (0·0 to 0·0)	0·1% (0·1 to 0·1%)	0·0 (0·0 to 0·1)	0·1% (0·1 to 0·1%)	57·1% (37·4 to 81·6%)	13·5% (4·6 to 22·5%)	0·7 (0·5 to 0·8)	0·1% (0·1 to 0·1%)	1·0 (0·8 to 1·3)	0·1% (0·1 to 0·1%)	53·7% (33·6 to 78·0%)	12·2% (3·5 to 21·5%)
**Occupational exposure to chromium: All causes**	0·1 (0·1 to 0·1)	0·0% (0·0 to 0·0%)	0·1 (0·1 to 0·2)	0·0% (0·0 to 0·0%)	52·0% (33·1 to 75·0%)	64·6% (48·5 to 83·2%)	2·3 (1·9 to 2·7)	0·0% (0·0 to 0·0%)	3·4 (2·8 to 4·1)	0·0% (0·0 to 0·0%)	48·7% (30·1 to 72·5%)	63·5% (45·4 to 85·6%)
Tracheal, bronchus, and lung cancer	0·1 (0·1 to 0·1)	0·4% (0·3 to 0·4%)	0·1 (0·1 to 0·2)	0·4% (0·3 to 0·5%)	52·0% (33·1 to 75·0%)	9·8% (1·8 to 18·6%)	2·3 (1·9 to 2·7)	0·3% (0·3 to 0·4%)	3·4 (2·8 to 4·1)	0·4% (0·3 to 0·4%)	48·7% (30·1 to 72·5%)	8·6% (0·6 to 17·6%)
**Occupational exposure to diesel engine exhaust: All causes**	1·8 (1·5 to 2·1)	0·0% (0·0 to 0·0%)	3·1 (2·6 to 3·7)	0·0% (0·0 to 0·0%)	75·4% (55·1 to 100·2%)	89·9% (73·1 to 110·1%)	42·0 (35·5 to 49·5)	0·0% (0·0 to 0·0%)	72·3 (60·4 to 86·8)	0·0% (0·0 to 0·0%)	72·2% (51·7 to 97·9%)	89·4% (69·5 to 113·2%)
Tracheal, bronchus, and lung cancer	1·8 (1·5 to 2·1)	6·7% (5·8 to 7·6%)	3·1 (2·6 to 3·7)	8·5% (7·3 to 9·7%)	75·4% (55·1 to 100·2%)	26·6% (19·5 to 34·1%)	42·0 (35·5 to 49·5)	6·4% (5·5 to 7·2%)	72·3 (60·4 to 86·8)	8·0% (7·0 to 9·1%)	72·2% (51·7 to 97·9%)	25·7% (18·2 to 33·5%)
**Occupational exposure to second-hand smoke: All causes**	2·0 (1·8 to 2·2)	0·0% (0·0 to 0·0%)	3·5 (3·0 to 4·0)	0·0% (0·0 to 0·0%)	73·5% (52·7 to 99·7%)	87·9% (71·0 to 108·4%)	47·2 (42·0 to 53·1)	0·0% (0·0 to 0·0%)	80·2 (69·1 to 93·4)	0·0% (0·0 to 0·0%)	70·0% (48·9 to 95·6%)	86·9% (67·4 to 110·9%)
Tracheal, bronchus, and lung cancer	2·0 (1·8 to 2·2)	7·6% (7·0 to 8·2%)	3·5 (3·0 to 4·0)	9·5% (8·7 to 10·4%)	73·5% (52·7 to 99·7%)	25·3% (18·3 to 34·0%)	47·2 (42·0 to 53·1)	7·2% (6·6 to 7·7%)	80·2 (69·1 to 93·4)	8·9% (8·1 to 9·7%)	70·0% (48·9 to 95·6%)	24·0% (17·2 to 32·8%)
**Occupational exposure to formaldehyde: All causes**	0·1 (0·0 to 0·1)	0·0% (0·0 to 0·0%)	0·1 (0·1 to 0·1)	0·0% (0·0 to 0·0%)	48·3% (27·9 to 74·9%)	60·5% (43·6 to 82·8%)	2·0 (1·7 to 2·5)	0·0% (0·0 to 0·0%)	3·0 (2·4 to 3·8)	0·0% (0·0 to 0·0%)	49·2% (27·8 to 77·5%)	64·0% (43·5 to 90·4%)
Nasopharynx cancer	0·0 (0·0 to 0·0)	1·3% (0·9 to 1·7%)	0·0 (0·0 to 0·0)	1·3% (0·9 to 1·9%)	45·7% (19·7 to 81·9%)	5·7% (-2·6 to 16·8%)	0·7 (0·5 to 1·0)	1·3% (0·9 to 1·8%)	1·0 (0·7 to 1·5)	1·3% (0·9 to 1·8%)	46·7% (19·5 to 83·7%)	4·6% (-3·9 to 15·8%)
**Occupational exposure to nickel: All causes**	0·4 (0·3 to 0·6)	0·0% (0·0 to 0·0%)	0·6 (0·4 to 0·9)	0·0% (0·0 to 0·0%)	41·2% (23·4 to 63·1%)	52·9% (36·0 to 71·0%)	10·7 (7·6 to 14·2)	0·0% (0·0 to 0·0%)	14·7 (10·1 to 19·7)	0·0% (0·0 to 0·0%)	37·6% (20·0 to 59·7%)	51·3% (33·6 to 72·7%)
Tracheal, bronchus, and lung cancer	0·4 (0·3 to 0·6)	1·7% (1·2 to 2·3%)	0·6 (0·4 to 0·9)	1·7% (1·2 to 2·3%)	41·2% (23·4 to 63·1%)	2·0% (-6·6 to 10·4%)	10·7 (7·6 to 14·2)	1·6% (1·2 to 2·1%)	14·7 (10·1 to 19·7)	1·6% (1·1 to 2·2%)	37·6% (20·0 to 59·7%)	0·4% (-8·2 to 9·4%)
**Occupational exposure to polycyclic aromatic hydrocarbons: All causes**	0·2 (0·2 to 0·3)	0·0% (0·0 to 0·0%)	0·4 (0·3 to 0·5)	0·0% (0·0 to 0·0%)	67·2% (46·4 to 93·3%)	81·1% (63·2 to 101·1%)	5·7 (4·5 to 6·9)	0·0% (0·0 to 0·0%)	9·3 (7·3 to 11·4)	0·0% (0·0 to 0·0%)	63·7% (42·9 to 89·9%)	80·0% (60·0 to 104·3%)
Tracheal, bronchus, and lung cancer	0·2 (0·2 to 0·3)	0·9% (0·7 to 1·1%)	0·4 (0·3 to 0·5)	1·1% (0·9 to 1·3%)	67·2% (46·4 to 93·3%)	20·8% (12·5 to 29·5%)	5·7 (4·5 to 6·9)	0·9% (0·7 to 1·0%)	9·3 (7·3 to 11·4)	1·0% (0·8 to 1·2%)	63·7% (42·9 to 89·9%)	19·5% (11·3 to 28·2%)
**Occupational exposure to silica: All causes**	1·0 (0·9 to 1·2)	0·0% (0·0 to 0·0%)	1·7 (1·5 to 2·0)	0·0% (0·0 to 0·0%)	67·1% (45·6 to 93·8%)	80·9% (61·5 to 104·7%)	24·8 (21·6 to 28·6)	0·0% (0·0 to 0·0%)	40·4 (34·3 to 48·3)	0·0% (0·0 to 0·0%)	62·9% (41·3 to 89·6%)	79·1% (57·5 to 105·4%)
Tracheal, bronchus, and lung cancer	1·0 (0·9 to 1·2)	3·9% (3·5 to 4·4%)	1·7 (1·5 to 2·0)	4·7% (4·2 to 5·4%)	67·1% (45·6 to 93·8%)	20·7% (10·7 to 31·1%)	24·8 (21·6 to 28·6)	3·8% (3·4 to 4·2%)	40·4 (34·3 to 48·3)	4·5% (4·0 to 5·1%)	62·9% (41·3 to 89·6%)	18·9% (9·0 to 29·6%)
**Occupational exposure to sulfphuric acid: All causes**	0·3 (0·2 to 0·4)	0·0% (0·0 to 0·0%)	0·5 (0·3 to 0·7)	0·0% (0·0 to 0·0%)	47·0% (26·3 to 71·9%)	59·2% (41·2 to 81·9%)	8·6 (6·3 to 11·5)	0·0% (0·0 to 0·0%)	12·5 (9·1 to 17·0)	0·0% (0·0 to 0·0%)	45·1% (25·1 to 69·3%)	59·5% (39·3 to 84·1%)
Larynx cancer	0·3 (0·2 to 0·4)	8·3% (6·1 to 10·8%)	0·5 (0·3 to 0·7)	9·3% (6·8 to 12·1%)	47·0% (26·3 to 71·9%)	12·1% (5·4 to 20·3%)	8·6 (6·3 to 11·5)	7·8% (5·8 to 10·2%)	12·5 (9·1 to 17·0)	8·7% (6·4 to 11·4%)	45·1% (25·1 to 69·3%)	11·4% (4·8 to 19·8%)
**Occupational exposure to trichloroethylene: All causes**	0·0 (0·0 to 0·0)	0·0% (0·0 to 0·0%)	0·0 (0·0 to 0·0)	0·0% (0·0 to 0·0%)	70·9% (48·2 to 99·3%)	85·1% (64·7 to 107·9%)	0·1 (0·0 to 0·3)	0·0% (0·0 to 0·0%)	0·2 (0·1 to 0·5)	0·0% (0·0 to 0·0%)	68·8% (46·2 to 97·4%)	85·6% (63·0 to 112·7%)
Kidney cancer	0·0 (0·0 to 0·0)	0·1% (0·0 to 0·2%)	0·0 (0·0 to 0·0)	0·1% (0·0 to 0·3%)	70·9% (48·2 to 99·3%)	17·3% (1·5 to 38·3%)	0·1 (0·0 to 0·3)	0·1% (0·0 to 0·1%)	0·2 (0·1 to 0·5)	0·1% (0·0 to 0·2%)	68·8% (46·2 to 97·4%)	14·7% (-8·0 to 53·3%)

Supplemental Results Table 1. All-age deaths, death PAFs, DALYs, DALY PAFs, and percent change for each risk factor and outcome for both sexes combined in 2005 and 2015 for Sub-Saharan Africa.

Data in parenthesis are 95% uncertainty intervals. PAFs=population attributable fractions. DALYs=disability-adjusted life-years.

	2005 Deaths (in thousands)	2005 Deaths PAF	2015 Deaths (in thousands)	2015 Deaths PAF	Percent change deaths 2005-2015	Percent change deaths PAF 2005-2015	2005 DALYs (in thousands)	2005 DALYs PAF	2015 DALYs (in thousands)	2015 DALYs PAF	Percent change DALYs 2005-2015	Percent change DALYs PAF 2005-2015
**Occupational asthmagens: All causes**	8·5 (6·8 to 10·6)	0·1% (0·1 to 0·1%)	9·1 (7·3 to 11·4)	0·1% (0·1 to 0·1%)	8·0% (-6·7 to 25·3%)	16·9% (5·2 to 31·6%)	454·2 (364·8 to 554·0)	0·1% (0·1 to 0·1%)	550·3 (439·5 to 681·2)	0·1% (0·1 to 0·1%)	21·2% (9·9 to 33·1%)	33·2% (23·0 to 44·2%)
Asthma	8·5 (6·8 to 10·6)	14·5% (12·5 to 16·4%)	9·1 (7·3 to 11·4)	15·1% (13·2 to 17·2%)	8·0% (-6·7 to 25·3%)	4·6% (-1·0 to 11·0%)	454·2 (364·8 to 554·0)	11·3% (9·5 to 13·1%)	550·3 (439·5 to 681·2)	11·7% (9·9 to 13·7%)	21·2% (9·9 to 33·1%)	3·9% (-1·2 to 10·1%)
**Occupational particulate matter, gases, and fumes: All causes**	12·5 (10·4 to 14·9)	0·1% (0·1 to 0·2%)	16·0 (13·2 to 19·5)	0·2% (0·2 to 0·2%)	28·1% (14·1 to 44·3%)	38·7% (27·5 to 51·2%)	366·6 (316·4 to 426·1)	0·1% (0·1 to 0·1%)	474·2 (401·1 to 555·9)	0·1% (0·1 to 0·1%)	29·4% (17·1 to 42·8%)	42·2% (30·6 to 54·2%)
Chronic obstructive pulmonary disease	12·5 (10·4 to 14·9)	15·3% (13·1 to 17·4%)	16·0 (13·1 to 19·4)	16·0% (13·7 to 18·3%)	28·1% (14·1 to 44·3%)	5·1% (1·0 to 9·4%)	365·5 (315·4 to 425·1)	15·3% (13·4 to 17·1%)	472·9 (399·9 to 554·8)	16·3% (14·4 to 18·3%)	29·4% (17·2 to 42·9%)	6·9% (2·4 to 11·7%)
**Occupational noise: All causes**	-	-	-	-	-	-	1,107·8 (755·2 to 1,514·4)	0·2% (0·1 to 0·2%)	1,519·7 (1,033·9 to 2,077·6)	0·3% (0·2 to 0·4%)	37·2% (35·1 to 39·1%)	50·8% (44·8 to 56·9%)
Age-related and other hearing loss	-	-	-	-	-	-	1,107·8 (755·2 to 1,514·4)	36·5% (33·0 to 40·8%)	1,519·7 (1,033·9 to 2,077·6)	37·6% (34·0 to 42·1%)	37·2% (35·1 to 39·1%)	2·9% (1·8 to 3·9%)
**Occupational injuries: All causes**	18·8 (17·1 to 21·1)	0·2% (0·2 to 0·2%)	25·0 (22·7 to 28·1)	0·3% (0·3 to 0·4%)	33·1% (17·4 to 51·7%)	44·3% (25·9 to 64·8%)	1,300·4 (1,133·7 to 1,495·2)	0·2% (0·2 to 0·3%)	1,852·6 (1,612·5 to 2,152·1)	0·4% (0·3 to 0·4%)	42·5% (24·6 to 61·8%)	56·7% (35·7 to 79·2%)
Pedestrian road injuries	1·2 (1·1 to 1·4)	2·5% (2·2 to 3·0%)	2·1 (1·8 to 2·4)	3·6% (3·0 to 4·2%)	67·3% (44·6 to 96·2%)	41·7% (18·3 to 70·3%)	65·7 (55·6 to 76·3)	2·5% (2·1 to 2·9%)	110·5 (94·3 to 128·2)	3·6% (3·0 to 4·3%)	68·1% (44·4 to 97·5%)	46·5% (20·3 to 77·4%)
Cyclist road injuries	0·1 (0·1 to 0·1)	2·5% (2·1 to 3·0%)	0·2 (0·1 to 0·2)	3·4% (2·8 to 4·1%)	79·0% (51·8 to 113·3%)	36·8% (12·9 to 67·7%)	5·9 (4·9 to 6·9)	2·6% (2·2 to 3·2%)	10·9 (9·2 to 13·0)	3·8% (3·1 to 4·6%)	84·6% (57·2 to 119·6%)	42·4% (16·7 to 74·8%)
Motorcyclist road injuries	1·0 (0·8 to 1·2)	9·3% (7·7 to 11·3%)	1·3 (1·1 to 1·7)	10·2% (8·2 to 12·5%)	36·8% (14·0 to 67·1%)	9·4% (-12·2 to 34·9%)	57·5 (48·0 to 71·2)	9·1% (7·4 to 11·3%)	79·5 (66·1 to 99·4)	10·2% (8·2 to 12·4%)	38·3% (15·8 to 68·4%)	11·2% (-10·8 to 36·8%)
Motor vehicle road injuries	4·8 (4·3 to 5·6)	8·1% (7·2 to 9·3%)	6·4 (5·7 to 7·4)	9·5% (8·2 to 10·9%)	32·5% (16·2 to 51·2%)	16·9% (-0·8 to 36·5%)	274·2 (243·4 to 314·7)	8·2% (7·2 to 9·4%)	366·3 (325·5 to 420·8)	9·8% (8·5 to 11·2%)	33·6% (17·2 to 52·1%)	19·7% (1·3 to 41·3%)
Other road injuries	0·1 (0·1 to 0·2)	8·5% (6·9 to 10·2%)	0·2 (0·1 to 0·2)	10·0% (8·1 to 12·5%)	41·0% (16·8 to 82·8%)	17·9% (-3·3 to 42·4%)	7·2 (5·7 to 9·1)	8·7% (6·8 to 10·9%)	10·5 (8·0 to 13·7)	10·5% (8·4 to 13·1%)	45·6% (22·3 to 84·0%)	20·9% (-2·2 to 49·1%)
Other transport injuries	0·3 (0·2 to 0·3)	3·0% (2·5 to 3·6%)	0·4 (0·3 to 0·5)	4·2% (3·3 to 5·5%)	51·2% (17·4 to 105·6%)	39·6% (10·3 to 84·3%)	15·0 (12·1 to 18·0)	2·9% (2·3 to 3·5%)	23·5 (18·4 to 30·8)	4·2% (3·3 to 5·5%)	57·1% (24·7 to 109·9%)	44·7% (12·9 to 92·3%)
Falls	1·9 (1·6 to 2·2)	4·5% (3·9 to 5·2%)	2·7 (2·3 to 3·1)	5·0% (4·2 to 5·9%)	43·2% (22·1 to 67·8%)	12·1% (-6·8 to 32·7%)	187·7 (148·8 to 237·4)	7·9% (6·4 to 9·5%)	309·8 (236·8 to 396·7)	10·0% (7·9 to 12·2%)	65·1% (39·4 to 92·5%)	27·2% (1·2 to 56·7%)
Drowning	2·0 (1·7 to 2·5)	4·0% (3·3 to 5·1%)	2·4 (2·1 to 2·9)	4·8% (3·9 to 5·8%)	19·6% (1·2 to 41·6%)	18·2% (-5·9 to 48·6%)	112·0 (95·8 to 134·0)	3·2% (2·5 to 4·2%)	134·7 (118·7 to 160·2)	3·9% (3·2 to 4·8%)	20·3% (1·7 to 41·8%)	21·6% (-5·9 to 56·1%)
Fire, heat, and hot substances	1·8 (1·5 to 2·2)	5·0% (4·2 to 6·0%)	2·4 (2·0 to 2·9)	6·0% (5·1 to 7·1%)	32·6% (10·8 to 62·5%)	20·8% (-0·1 to 46·0%)	123·3 (100·7 to 149·8)	5·2% (4·3 to 6·5%)	178·8 (147·8 to 215·1)	7·0% (5·7 to 8·4%)	45·0% (23·3 to 72·7%)	33·8% (10·3 to 62·7%)
Poisonings	0·7 (0·6 to 0·9)	3·6% (2·0 to 6·0%)	0·8 (0·6 to 1·0)	3·8% (2·3 to 6·5%)	12·8% (-7·9 to 37·6%)	7·3% (-34·3 to 83·7%)	44·4 (35·7 to 57·6)	3·2% (1·7 to 5·7%)	54·6 (44·0 to 69·0)	3·7% (2·1 to 6·8%)	23·0% (0·6 to 50·5%)	16·8% (-32·6 to 114·3%)
Unintentional firearm injuries	0·5 (0·4 to 0·6)	6·6% (5·6 to 8·2%)	0·6 (0·4 to 0·7)	7·2% (6·0 to 8·8%)	26·0% (6·2 to 50·0%)	8·3% (-11·4 to 30·4%)	26·9 (20·0 to 33·9)	6·4% (5·1 to 8·1%)	34·3 (24·7 to 41·7)	7·0% (5·7 to 8·6%)	27·4% (7·3 to 51·5%)	9·2% (-11·5 to 32·0%)
Unintentional suffocation	0·0 (0·0 to 0·0)	0·3% (0·2 to 0·5%)	0·0 (0·0 to 0·0)	0·3% (0·2 to 0·5%)	28·4% (3·0 to 61·8%)	9·8% (-22·8 to 60·8%)	1·4 (1·1 to 2·0)	0·3% (0·2 to 0·5%)	2·1 (1·5 to 3·0)	0·4% (0·2 to 0·6%)	43·4% (10·1 to 85·4%)	21·9% (-15·8 to 82·5%)
Other exposure to mechanical forces	1·2 (1·0 to 1·4)	6·4% (5·4 to 7·8%)	1·7 (1·4 to 2·0)	7·6% (6·5 to 9·0%)	39·0% (16·3 to 70·1%)	18·1% (-3·8 to 44·6%)	113·7 (92·7 to 139·1)	7·4% (6·1 to 8·9%)	171·4 (136·7 to 213·0)	9·4% (7·8 to 11·3%)	50·8% (27·9 to 76·1%)	27·8% (3·2 to 54·7%)
Venomous animal contact	0·7 (0·5 to 0·9)	4·8% (3·8 to 6·2%)	0·8 (0·6 to 1·0)	5·0% (4·0 to 6·3%)	10·0% (-13·8 to 44·4%)	4·8% (-19·6 to 35·1%)	46·7 (34·4 to 60·6)	5·3% (4·1 to 7·0%)	56·3 (43·2 to 71·9)	6·1% (4·8 to 7·8%)	20·4% (-4·9 to 51·5%)	14·6% (-13·2 to 48·7%)
Non-venomous animal contact	0·2 (0·1 to 0·2)	3·1% (2·4 to 4·3%)	0·2 (0·1 to 0·3)	3·4% (2·6 to 4·5%)	9·2% (-16·9 to 46·6%)	8·6% (-20·0 to 44·8%)	15·3 (11·1 to 20·7)	3·5% (2·6 to 4·9%)	18·4 (13·8 to 25·0)	4·1% (3·1 to 5·7%)	20·0% (-6·3 to 55·1%)	18·9% (-12·9 to 59·9%)
Pulmonary aspiration and foreign body in airway	0·5 (0·4 to 0·8)	2·9% (2·1 to 4·6%)	0·8 (0·6 to 1·0)	3·4% (2·6 to 4·9%)	41·4% (18·4 to 68·8%)	19·4% (-5·4 to 51·8%)	38·0 (28·9 to 49·9)	2·7% (1·8 to 4·6%)	56·5 (45·1 to 70·4)	3·4% (2·4 to 5·2%)	48·5% (25·9 to 74·7%)	25·7% (-2·4 to 64·1%)
Foreign body in eyes	-	-	-	-	-	-	0·5 (0·2 to 1·0)	9·1% (6·9 to 11·5%)	0·8 (0·3 to 1·6)	11·2% (8·3 to 14·4%)	61·8% (30·3 to 97·0%)	23·3% (-0·3 to 50·7%)
Foreign body in other body part	0·8 (0·5 to 1·3)	6·8% (5·2 to 8·8%)	1·0 (0·6 to 1·4)	7·8% (6·3 to 9·8%)	19·1% (-5·6 to 48·5%)	14·2% (-9·9 to 40·9%)	62·6 (42·2 to 90·2)	7·0% (5·2 to 9·6%)	82·1 (59·1 to 107·1)	8·6% (6·7 to 11·1%)	31·3% (5·9 to 59·1%)	22·9% (-3·8 to 53·7%)
Other unintentional injuries	0·9 (0·7 to 1·0)	7·1% (6·1 to 8·4%)	1·1 (1·0 to 1·3)	8·1% (7·0 to 9·4%)	27·9% (10·4 to 50·0%)	14·4% (-4·3 to 35·0%)	102·2 (80·7 to 127·6)	8·2% (6·9 to 9·7%)	151·4 (116·4 to 192·8)	10·5% (8·7 to 12·4%)	48·2% (25·7 to 71·1%)	27·8% (5·2 to 52·6%)
**Occupational ergonomic factors: All causes**	-	-	-	-	-	-	1,669·1 (1,175·8 to 2,318·8)	0·3% (0·2 to 0·4%)	2,136·3 (1,496·6 to 2,966·0)	0·4% (0·3 to 0·5%)	28·0% (24·3 to 31·7%)	40·7% (34·0 to 48·0%)
Low back pain	-	-	-	-	-	-	1,669·1 (1,175·8 to 2,318·8)	42·5% (39·7 to 45·2%)	2,136·3 (1,496·6 to 2,966·0)	41·2% (38·7 to 43·8%)	28·0% (24·3 to 31·7%)	-3·1% (-5·7 to -0·3%)
**Behavioural risks: All causes**	3,487·8 (3,319·6 to 3,658·4)	40·2% (38·4 to 41·9%)	2,832·9 (2,642·4 to 3,045·2)	35·4% (33·5 to 37·1%)	-18·8% (-23·7 to -13·5%)	-12·0% (-15·4 to -8·7%)	216,834·9 (204,521·4 to 230,486·1)	37·3% (35·3 to 39·4%)	169,970·4 (158,430·4 to 181,892·6)	32·1% (30·4 to 34·0%)	-21·6% (-26·0 to -17·0%)	-13·8% (-17·6 to -10·0%)
**Child and maternal malnutrition: All causes**	1,122·1 (997·0 to 1,262·5)	12·9% (11·5 to 14·5%)	826·2 (737·3 to 923·8)	10·3% (9·1 to 11·6%)	-26·4% (-33·7 to -18·7%)	-20·2% (-28·0 to -11·6%)	103,543·3 (92,817·5 to 115,412·5)	17·8% (16·1 to 19·7%)	79,156·3 (71,055·1 to 88,283·4)	15·0% (13·6 to 16·4%)	-23·6% (-30·9 to -16·6%)	-15·9% (-22·9 to -8·5%)
**Suboptimal breastfeeding: All causes**	241·4 (164·3 to 336·6)	2·8% (1·9 to 3·9%)	198·3 (131·1 to 275·8)	2·5% (1·6 to 3·5%)	-17·9% (-28·9 to -4·7%)	-11·0% (-23·7 to 3·7%)	20,883·3 (14,237·6 to 29,110·5)	3·6% (2·4 to 5·0%)	17,175·0 (11,361·2 to 23,867·8)	3·2% (2·2 to 4·6%)	-17·8% (-28·7 to -4·6%)	-9·6% (-21·5 to 4·5%)
**Non-exclusive breastfeeding : All causes**	218·1 (144·2 to 306·2)	2·5% (1·7 to 3·5%)	181·0 (115·3 to 255·2)	2·3% (1·5 to 3·2%)	-17·0% (-28·1 to -3·9%)	-10·1% (-23·1 to 4·4%)	18,840·2 (12,484·9 to 26,433·1)	3·2% (2·1 to 4·6%)	15,643·1 (9,972·4 to 22,019·5)	3·0% (1·9 to 4·2%)	-17·0% (-28·1 to -3·9%)	-8·7% (-21·0 to 5·5%)
Diarrhoeal diseases	109·8 (67·1 to 152·5)	16·0% (10·1 to 21·9%)	88·9 (54·9 to 123·8)	15·6% (9·8 to 21·4%)	-19·0% (-33·7 to 0·5%)	-2·5% (-16·4 to 15·1%)	9,493·8 (5,811·6 to 13,166·2)	20·5% (12·8 to 27·9%)	7,696·5 (4,767·8 to 10,690·4)	20·8% (13·2 to 28·4%)	-18·9% (-33·6 to 0·5%)	1·3% (-11·9 to 18·2%)
Lower respiratory infections	108·4 (45·1 to 185·7)	14·1% (6·0 to 24·6%)	92·1 (36·8 to 162·3)	12·6% (5·1 to 22·1%)	-15·0% (-28·1 to -0·7%)	-10·8% (-23·2 to 3·8%)	9,346·3 (3,898·4 to 16,004·4)	19·7% (8·4 to 34·1%)	7,946·5 (3,176·6 to 13,992·1)	19·4% (7·8 to 33·6%)	-15·0% (-28·1 to -0·7%)	-1·8% (-13·9 to 12·1%)
**Discontinued breastfeeding : All causes**	28·7 (9·1 to 59·2)	0·3% (0·1 to 0·7%)	21·9 (7·0 to 42·9)	0·3% (0·1 to 0·5%)	-23·7% (-39·8 to -2·0%)	-17·3% (-34·8 to 5·6%)	2,512·3 (792·7 to 5,181·4)	0·4% (0·1 to 0·9%)	1,936·8 (615·2 to 3,814·1)	0·4% (0·1 to 0·7%)	-22·9% (-38·8 to -1·8%)	-15·2% (-32·1 to 6·9%)
Diarrhoeal diseases	28·7 (9·1 to 59·2)	4·2% (1·4 to 8·1%)	21·9 (7·0 to 42·9)	3·8% (1·3 to 7·5%)	-23·7% (-39·8 to -2·0%)	-8·0% (-22·0 to 9·8%)	2,512·3 (792·7 to 5,181·4)	5·4% (1·7 to 10·6%)	1,936·8 (615·2 to 3,814·1)	5·2% (1·7 to 10·0%)	-22·9% (-38·8 to -1·8%)	-3·5% (-16·2 to 12·0%)
**Childhood undernutrition: All causes**	1,021·5 (901·5 to 1,148·7)	11·8% (10·4 to 13·2%)	730·7 (647·3 to 823·5)	9·1% (8·0 to 10·4%)	-28·5% (-35·9 to -20·7%)	-22·5% (-30·8 to -13·7%)	88,770·6 (78,529·5 to 99,433·9)	15·3% (13·5 to 17·2%)	64,040·6 (56,989·5 to 71,888·5)	12·1% (10·6 to 13·6%)	-27·9% (-35·2 to -20·3%)	-20·7% (-28·0 to -12·8%)
**Childhood underweight: All causes**	356·2 (280·1 to 452·6)	4·1% (3·3 to 5·3%)	243·4 (194·3 to 304·2)	3·0% (2·4 to 3·8%)	-31·7% (-44·1 to -16·2%)	-25·9% (-40·3 to -9·7%)	31,207·3 (24,722·2 to 39,314·4)	5·4% (4·2 to 6·8%)	21,669·7 (17,475·1 to 26,967·7)	4·1% (3·3 to 5·1%)	-30·6% (-42·9 to -15·6%)	-23·6% (-37·7 to -7·9%)
Diarrhoeal diseases	59·3 (45·8 to 77·5)	8·6% (7·1 to 10·8%)	37·8 (29·5 to 48·5)	6·6% (5·4 to 8·5%)	-36·3% (-47·3 to -22·3%)	-23·2% (-31·9 to -13·1%)	5,183·7 (4,010·1 to 6,785·5)	11·2% (9·3 to 13·9%)	3,324·2 (2,606·7 to 4,277·5)	9·0% (7·4 to 11·4%)	-35·9% (-46·6 to -22·2%)	-19·8% (-26·4 to -12·4%)
Lower respiratory infections	74·9 (47·6 to 134·4)	9·8% (6·3 to 17·0%)	49·3 (30·8 to 88·4)	6·7% (4·3 to 11·9%)	-34·1% (-43·4 to -23·3%)	-30·8% (-38·6 to -22·4%)	6,424·0 (4,084·2 to 11,526·2)	13·6% (8·9 to 23·6%)	4,233·6 (2,644·3 to 7,587·6)	10·3% (6·7 to 18·2%)	-34·1% (-43·3 to -23·3%)	-23·9% (-30·1 to -17·8%)
Measles	44·3 (7·6 to 118·2)	31·0% (9·1 to 57·4%)	10·0 (1·5 to 30·2)	25·7% (6·9 to 51·3%)	-77·4% (-90·4 to -53·2%)	-17·2% (-35·1 to -6·7%)	3,763·3 (645·4 to 10,043·2)	31·4% (9·2 to 58·1%)	848·7 (124·9 to 2,568·2)	26·0% (7·0 to 51·9%)	-77·4% (-90·4 to -53·2%)	-17·2% (-35·0 to -6·7%)
Protein-energy malnutrition	177·7 (127·6 to 239·3)	81·9% (76·2 to 86·7%)	146·3 (105·8 to 195·6)	78·9% (72·9 to 83·9%)	-17·7% (-39·6 to 11·7%)	-3·7% (-9·4 to 2·1%)	15,836·2 (11,587·9 to 21,121·0)	91·4% (88·5 to 93·8%)	13,263·3 (9,803·6 to 17,533·3)	90·3% (87·1 to 92·9%)	-16·2% (-37·7 to 11·5%)	-1·2% (-3·8 to 1·5%)
**Childhood wasting: All causes**	925·7 (776·1 to 1,072·2)	10·7% (8·9 to 12·4%)	679·6 (572·5 to 776·3)	8·5% (7·1 to 9·8%)	-26·6% (-34·9 to -17·8%)	-20·4% (-29·7 to -10·5%)	80,550·7 (68,008·4 to 93,145·5)	13·8% (11·7 to 16·0%)	59,631·9 (50,305·7 to 68,215·3)	11·3% (9·5 to 13·0%)	-26·0% (-34·2 to -17·3%)	-18·6% (-27·4 to -9·3%)

Supplemental Results Table 1. All-age deaths, death PAFs, DALYs, DALY PAFs, and percent change for each risk factor and outcome for both sexes combined in 2005 and 2015 for Sub-Saharan Africa.

Data in parenthesis are 95% uncertainty intervals. PAFs=population attributable fractions. DALYs=disability-adjusted life-years.

	2005 Deaths (in thousands)	2005 Deaths PAF	2015 Deaths (in thousands)	2015 Deaths PAF	Percent change deaths 2005-2015	Percent change deaths PAF 2005-2015	2005 DALYs (in thousands)	2005 DALYs PAF	2015 DALYs (in thousands)	2015 DALYs PAF	Percent change DALYs 2005-2015	Percent change DALYs PAF 2005-2015
Diarrhoeal diseases	352·4 (281·1 to 424·0)	51·4% (42·8 to 56·8%)	260·4 (209·0 to 306·3)	45·7% (37·1 to 51·4%)	-26·1% (-38·2 to -11·0%)	-11·0% (-19·7 to -2·1%)	30,823·8 (24,618·5 to 36,827·5)	66·5% (55·5 to 71·6%)	22,955·7 (18,437·2 to 26,876·1)	61·9% (51·2 to 67·5%)	-25·5% (-37·3 to -10·8%)	-6·9% (-12·3 to -1·6%)
Lower respiratory infections	335·6 (238·8 to 404·6)	43·7% (31·2 to 52·0%)	258·0 (180·2 to 314·2)	35·3% (24·7 to 43·3%)	-23·1% (-33·4 to -12·7%)	-19·3% (-26·8 to -11·4%)	28,790·9 (20,477·5 to 34,691·3)	60·8% (43·5 to 70·3%)	22,152·5 (15,475·7 to 26,965·5)	54·0% (38·3 to 63·8%)	-23·1% (-33·3 to -12·7%)	-11·1% (-16·4 to -6·3%)
Measles	60·0 (14·0 to 187·3)	42·2% (14·8 to 82·0%)	14·8 (2·7 to 51·4)	38·5% (11·6 to 80·3%)	-75·3% (-89·1 to -48·6%)	-8·7% (-32·1 to 4·7%)	5,099·8 (1,190·8 to 15,921·0)	42·7% (15·1 to 82·9%)	1,260·4 (225·5 to 4,366·1)	39·0% (11·8 to 81·3%)	-75·3% (-89·1 to -48·6%)	-8·7% (-32·2 to 4·6%)
Protein-energy malnutrition	177·7 (127·6 to 239·3)	81·9% (76·2 to 86·7%)	146·3 (105·8 to 195·6)	78·9% (72·9 to 83·9%)	-17·7% (-39·6 to 11·7%)	-3·7% (-9·4 to 2·1%)	15,836·2 (11,587·9 to 21,121·0)	91·4% (88·5 to 93·8%)	13,263·3 (9,803·6 to 17,533·3)	90·3% (87·1 to 92·9%)	-16·2% (-37·7 to 11·5%)	-1·2% (-3·8 to 1·5%)
**Childhood stunting: All causes**	228·5 (113·5 to 379·8)	2·6% (1·3 to 4·4%)	136·5 (62·4 to 236·3)	1·7% (0·8 to 2·9%)	-40·3% (-52·8 to -30·2%)	-35·3% (-49·7 to -24·1%)	19,716·4 (9,799·2 to 32,682·3)	3·4% (1·7 to 5·5%)	11,830·4 (5,529·5 to 20,375·8)	2·2% (1·0 to 3·8%)	-40·0% (-52·4 to -30·0%)	-34·0% (-48·3 to -23·4%)
Diarrhoeal diseases	81·6 (29·9 to 144·3)	11·9% (4·5 to 20·4%)	51·8 (18·4 to 94·8)	9·1% (3·3 to 16·1%)	-36·5% (-47·4 to -22·5%)	-23·5% (-31·5 to -14·2%)	7,134·1 (2,623·0 to 12,630·9)	15·4% (5·8 to 26·6%)	4,570·9 (1,627·4 to 8,366·9)	12·3% (4·5 to 21·8%)	-35·9% (-46·7 to -22·3%)	-19·9% (-26·0 to -13·7%)
Lower respiratory infections	113·1 (9·6 to 246·6)	14·7% (1·3 to 32·0%)	76·8 (5·7 to 172·0)	10·5% (0·8 to 23·6%)	-32·0% (-41·9 to -17·7%)	-28·6% (-37·0 to -16·9%)	9,699·4 (826·5 to 21,161·5)	20·5% (1·9 to 44·6%)	6,596·6 (492·5 to 14,763·5)	16·1% (1·3 to 36·6%)	-32·0% (-41·8 to -17·6%)	-21·4% (-28·4 to -11·5%)
Measles	33·9 (3·1 to 102·6)	24·0% (2·9 to 56·3%)	7·8 (0·6 to 26·0)	20·4% (2·3 to 52·1%)	-77·0% (-89·7 to -45·4%)	-14·9% (-28·4 to -4·0%)	2,882·9 (267·7 to 8,716·4)	24·3% (2·9 to 57·1%)	662·9 (51·4 to 2,210·0)	20·7% (2·3 to 52·8%)	-77·0% (-89·7 to -45·4%)	-14·9% (-28·3 to -4·0%)
**Iron deficiency: All causes**	58·6 (41·9 to 77·7)	0·7% (0·5 to 0·9%)	62·1 (44·7 to 84·5)	0·8% (0·6 to 1·0%)	5·9% (-9·3 to 22·2%)	14·6% (1·9 to 28·9%)	11,111·2 (8,264·9 to 14,651·8)	1·9% (1·5 to 2·4%)	12,164·1 (9,034·3 to 16,229·3)	2·3% (1·8 to 2·9%)	9·5% (4·1 to 14·8%)	20·3% (14·4 to 26·6%)
Maternal haemorrhage	16·6 (6·3 to 27·8)	28·8% (10·8 to 45·9%)	15·5 (5·7 to 26·3)	28·0% (10·4 to 45·0%)	-7·2% (-26·1 to 14·2%)	-2·9% (-13·3 to 5·8%)	918·4 (348·3 to 1,519·9)	28·9% (10·9 to 46·1%)	846·6 (312·5 to 1,450·9)	28·1% (10·4 to 45·2%)	-7·8% (-25·9 to 13·5%)	-3·0% (-13·2 to 5·6%)
Maternal sepsis and other pregnancy related infections	2·9 (1·1 to 5·0)	30·7% (11·9 to 49·2%)	2·3 (0·8 to 4·3)	29·3% (10·9 to 47·3%)	-20·0% (-48·6 to 25·0%)	-4·4% (-13·4 to 4·1%)	164·4 (64·1 to 285·9)	30·7% (11·9 to 49·2%)	131·4 (45·7 to 243·9)	29·3% (10·8 to 47·3%)	-20·1% (-48·1 to 24·4%)	-4·4% (-13·3 to 4·2%)
Iron-deficiency anaemia	39·1 (27·0 to 52·7)	100·0% (100·0 to 100·0%)	44·3 (30·6 to 61·3)	100·0% (100·0 to 100·0%)	13·3% (-2·8 to 31·2%)	-	10,028·4 (7,289·2 to 13,459·5)	100·0% (100·0 to 100·0%)	11,186·1 (8,201·9 to 15,083·9)	100·0% (100·0 to 100·0%)	11·5% (6·4 to 16·4%)	-
**Vitamin A deficiency: All causes**	96·4 (57·1 to 145·6)	1·1% (0·7 to 1·7%)	47·4 (27·9 to 69·8)	0·6% (0·3 to 0·9%)	-50·9% (-64·1 to -37·8%)	-46·8% (-61·5 to -32·3%)	8,388·0 (4,971·9 to 12,650·2)	1·4% (0·9 to 2·2%)	4,203·9 (2,519·8 to 6,141·2)	0·8% (0·5 to 1·2%)	-49·9% (-63·1 to -37·0%)	-44·9% (-59·4 to -30·5%)
Diarrhoeal diseases	58·3 (32·4 to 87·7)	8·5% (4·9 to 12·5%)	38·0 (21·0 to 56·8)	6·7% (3·7 to 9·7%)	-34·8% (-45·6 to -21·4%)	-21·4% (-29·8 to -12·8%)	5,117·5 (2,860·8 to 7,659·2)	11·0% (6·3 to 16·1%)	3,368·3 (1,848·7 to 4,984·6)	9·1% (5·0 to 13·2%)	-34·2% (-44·7 to -21·2%)	-17·6% (-24·1 to -11·6%)
Measles	38·1 (12·4 to 86·6)	26·9% (17·6 to 36·2%)	9·3 (2·6 to 23·3)	24·3% (15·3 to 33·7%)	-75·5% (-88·4 to -49·3%)	-9·5% (-18·4 to -0·4%)	3,234·2 (1,056·7 to 7,342·2)	27·2% (17·8 to 36·7%)	791·7 (224·6 to 1,975·0)	24·6% (15·5 to 34·1%)	-75·5% (-88·4 to -49·4%)	-9·5% (-18·4 to -0·3%)
Vitamin A deficiency	-	-	-	-	-	-	36·3 (22·7 to 54·2)	100·0% (100·0 to 100·0%)	43·8 (27·5 to 66·0)	100·0% (100·0 to 100·0%)	20·7% (15·4 to 26·0%)	-
**Zinc deficiency: All causes**	49·1 (2·8 to 106·3)	0·6% (0·0 to 1·2%)	33·6 (2·0 to 74·9)	0·4% (0·0 to 0·9%)	-31·5% (-44·7 to -14·5%)	-25·7% (-40·6 to -7·1%)	4,269·5 (363·8 to 9,099·9)	0·7% (0·1 to 1·6%)	2,954·3 (273·4 to 6,447·0)	0·6% (0·1 to 1·2%)	-30·8% (-43·5 to -13·1%)	-23·9% (-37·9 to -5·0%)
Diarrhoeal diseases	30·9 (0·0 to 73·1)	4·5% (0·0 to 10·4%)	20·8 (0·0 to 49·2)	3·6% (0·0 to 8·5%)	-32·9% (-47·1 to 0·0%)	-19·2% (-32·6 to 0·0%)	2,727·6 (111·2 to 6,306·1)	5·9% (0·2 to 13·4%)	1,861·2 (101·6 to 4,253·7)	5·0% (0·3 to 11·3%)	-31·8% (-45·6 to -6·6%)	-14·7% (-26·9 to 18·3%)
Lower respiratory infections	18·2 (0·0 to 64·9)	2·4% (0·0 to 8·2%)	12·9 (0·0 to 45·2)	1·8% (0·0 to 6·2%)	-29·2% (-42·7 to 0·0%)	-25·7% (-37·5 to 0·0%)	1,541·9 (3·2 to 5,484·0)	3·2% (0·0 to 11·2%)	1,093·1 (3·3 to 3,825·2)	2·7% (0·0 to 9·1%)	-29·1% (-42·5 to 5·8%)	-18·1% (-29·6 to 26·7%)
**Tobacco smoke: All causes**	271·2 (237·2 to 309·3)	3·1% (2·8 to 3·5%)	302·4 (256·6 to 355·5)	3·8% (3·3 to 4·3%)	11·5% (-0·3 to 25·4%)	20·7% (11·7 to 31·0%)	10,481·4 (8,566·1 to 12,527·5)	1·8% (1·5 to 2·1%)	10,922·8 (8,977·6 to 13,123·6)	2·1% (1·7 to 2·4%)	4·2% (-5·6 to 16·3%)	14·6% (5·2 to 26·2%)
**Smoking: All causes**	196·5 (172·1 to 221·3)	2·3% (2·0 to 2·5%)	229·4 (192·2 to 273·1)	2·9% (2·5 to 3·3%)	16·7% (1·0 to 34·4%)	26·4% (13·9 to 40·5%)	5,731·1 (5,004·9 to 6,522·1)	1·0% (0·9 to 1·1%)	6,679·7 (5,579·1 to 8,039·9)	1·3% (1·1 to 1·5%)	16·6% (0·4 to 34·8%)	28·1% (12·7 to 45·4%)
Tuberculosis	13·7 (6·5 to 22·3)	5·0% (2·5 to 7·7%)	12·8 (5·6 to 21·2)	4·8% (2·3 to 7·7%)	-6·7% (-22·4 to 13·0%)	-3·6% (-14·1 to 6·7%)	471·4 (221·1 to 766·1)	4·1% (2·1 to 6·5%)	447·6 (193·4 to 741·5)	4·1% (2·0 to 6·6%)	-5·1% (-21·5 to 15·9%)	-0·7% (-13·1 to 10·8%)
Lower respiratory infections	37·7 (27·4 to 48·2)	4·9% (3·8 to 6·2%)	40·0 (29·4 to 52·7)	5·5% (4·2 to 7·1%)	6·1% (-9·4 to 21·9%)	11·3% (-2·9 to 25·4%)	1,031·4 (750·6 to 1,314·5)	2·2% (1·6 to 2·8%)	1,086·0 (792·8 to 1,447·5)	2·6% (2·0 to 3·5%)	5·3% (-11·7 to 22·7%)	21·5% (1·1 to 42·4%)
Lip and oral cavity cancer	1·3 (1·0 to 1·7)	23·9% (19·2 to 29·0%)	2·0 (1·5 to 2·6)	25·6% (20·0 to 31·4%)	49·7% (20·2 to 86·6%)	7·0% (-7·4 to 23·7%)	38·0 (29·6 to 48·2)	23·9% (19·3 to 29·0%)	56·1 (40·9 to 74·0)	25·2% (19·7 to 31·2%)	47·8% (17·6 to 87·3%)	5·5% (-8·5 to 22·8%)
Nasopharynx cancer	0·3 (0·2 to 0·4)	20·8% (15·5 to 26·4%)	0·5 (0·3 to 0·7)	23·4% (16·6 to 30·4%)	55·3% (13·9 to 116·4%)	12·5% (-6·7 to 36·8%)	10·1 (6·9 to 14·0)	18·1% (13·0 to 23·6%)	16·0 (9·6 to 24·1)	20·5% (14·1 to 27·3%)	58·3% (14·5 to 126·0%)	12·7% (-9·2 to 40·6%)
Oesophageal cancer	6·5 (4·6 to 8·7)	23·9% (17·4 to 30·6%)	8·7 (5·9 to 12·3)	25·5% (17·9 to 33·2%)	34·6% (4·1 to 73·6%)	6·6% (-10·7 to 25·2%)	175·1 (124·8 to 233·2)	24·7% (17·9 to 31·8%)	235·9 (156·6 to 338·3)	26·5% (18·7 to 34·7%)	34·7% (2·1 to 77·3%)	7·2% (-11·2 to 26·5%)
Stomach cancer	1·4 (0·8 to 2·2)	4·5% (2·7 to 6·7%)	2·2 (1·2 to 3·4)	5·5% (3·2 to 8·1%)	49·8% (11·7 to 102·4%)	21·2% (-2·1 to 52·5%)	40·0 (22·8 to 61·7)	4·9% (2·9 to 7·3%)	59·4 (32·4 to 93·8)	5·9% (3·4 to 8·8%)	48·7% (9·3 to 107·1%)	21·7% (-3·0 to 56·2%)
Colon and rectum cancer	0·6 (0·4 to 0·9)	2·6% (1·7 to 3·7%)	1·0 (0·6 to 1·5)	3·0% (2·0 to 4·2%)	61·8% (21·5 to 116·3%)	13·5% (-7·5 to 39·7%)	16·3 (10·1 to 24·2)	2·6% (1·7 to 3·8%)	26·7 (16·3 to 42·2)	3·1% (1·9 to 4·5%)	63·6% (19·2 to 123·9%)	15·4% (-7·8 to 46·3%)
Liver cancer due to hepatitis B	1·3 (0·6 to 2·2)	7·3% (3·6 to 11·7%)	1·9 (0·9 to 3·4)	8·9% (4·4 to 14·4%)	53·2% (5·3 to 131·2%)	21·7% (-8·8 to 65·2%)	46·2 (20·0 to 83·0)	6·9% (3·2 to 11·6%)	71·2 (31·2 to 127·5)	8·4% (4·1 to 14·1%)	54·0% (4·8 to 145·3%)	21·4% (-10·4 to 74·9%)
Liver cancer due to hepatitis C	0·6 (0·3 to 1·0)	5·8% (3·2 to 9·3%)	0·9 (0·5 to 1·7)	7·3% (3·7 to 12·0%)	58·7% (10·8 to 133·1%)	24·2% (-6·7 to 65·6%)	16·2 (8·2 to 27·8)	6·3% (3·3 to 10·1%)	26·0 (12·3 to 47·0)	7·9% (4·1 to 12·9%)	60·6% (9·5 to 138·4%)	25·6% (-6·6 to 68·7%)
Liver cancer due to alcohol use	1·3 (0·6 to 2·3)	8·5% (4·4 to 13·6%)	2·0 (0·9 to 3·6)	10·2% (5·2 to 16·5%)	51·1% (0·4 to 125·7%)	20·5% (-8·1 to 56·8%)	37·7 (18·0 to 66·3)	9·0% (4·6 to 14·5%)	57·6 (26·9 to 106·8)	10·9% (5·5 to 17·8%)	52·8% (-0·7 to 135·7%)	21·0% (-9·7 to 61·9%)
Liver cancer due to other causes	0·4 (0·2 to 0·7)	5·9% (3·1 to 9·2%)	0·6 (0·3 to 1·1)	7·3% (3·7 to 11·5%)	53·1% (8·5 to 118·4%)	23·6% (-5·4 to 63·6%)	13·4 (6·5 to 22·0)	5·8% (2·9 to 9·2%)	20·8 (9·6 to 35·8)	7·2% (3·6 to 11·5%)	55·7% (9·4 to 132·8%)	24·8% (-5·9 to 72·0%)
Pancreatic cancer	1·1 (0·8 to 1·5)	7·6% (5·7 to 10·0%)	1·9 (1·3 to 2·7)	8·8% (6·5 to 11·8%)	69·5% (32·2 to 123·6%)	15·8% (-2·7 to 38·6%)	27·8 (19·4 to 38·0)	8·0% (5·9 to 10·6%)	47·2 (31·5 to 69·8)	9·3% (6·7 to 12·4%)	69·8% (31·5 to 128·3%)	15·1% (-4·0 to 40·9%)
Tracheal, bronchus, and lung cancer	13·7 (11·4 to 16·2)	52·0% (46·0 to 57·4%)	19·7 (16·2 to 24·1)	54·2% (48·4 to 60·2%)	44·4% (19·4 to 75·2%)	4·2% (-3·9 to 13·9%)	341·8 (283·1 to 409·1)	51·8% (45·7 to 57·3%)	488·0 (396·1 to 603·4)	53·9% (47·8 to 60·0%)	42·8% (17·0 to 75·7%)	4·1% (-4·3 to 14·2%)
Cervical cancer	1·2 (0·4 to 2·5)	2·8% (0·8 to 5·3%)	1·5 (0·4 to 3·1)	3·0% (0·9 to 6·1%)	19·3% (-28·1 to 101·7%)	6·2% (-28·0 to 60·4%)	38·1 (10·8 to 79·2)	2·8% (0·8 to 5·4%)	45·9 (12·4 to 102·4)	3·0% (0·9 to 6·3%)	20·6% (-31·7 to 116·4%)	7·2% (-32·2 to 70·1%)
Kidney cancer	0·2 (0·1 to 0·4)	4·7% (2·8 to 7·1%)	0·4 (0·2 to 0·6)	5·8% (3·4 to 8·8%)	79·3% (33·9 to 146·4%)	23·9% (-5·2 to 64·9%)	6·7 (3·9 to 10·6)	3·1% (1·8 to 5·0%)	11·9 (6·5 to 19·3)	3·8% (2·0 to 6·1%)	77·2% (28·2 to 151·7%)	22·2% (-13·0 to 79·6%)
Bladder cancer	0·9 (0·6 to 1·3)	11·1% (7·5 to 15·3%)	1·4 (0·9 to 2·0)	12·7% (8·8 to 17·4%)	53·6% (15·2 to 106·4%)	14·7% (-5·5 to 40·8%)	22·1 (14·5 to 31·1)	11·2% (7·5 to 15·3%)	34·2 (22·4 to 50·1)	13·0% (8·9 to 18·1%)	54·9% (14·9 to 110·1%)	16·2% (-5·2 to 43·2%)
Ischaemic heart disease	32·7 (26·1 to 39·4)	10·7% (8·6 to 12·8%)	36·8 (27·9 to 47·5)	9·8% (7·7 to 11·9%)	12·5% (-1·6 to 28·0%)	-8·6% (-15·8 to -1·3%)	947·6 (753·5 to 1,159·4)	13·7% (11·1 to 16·5%)	1,077·2 (816·1 to 1,406·6)	12·8% (10·0 to 15·8%)	13·7% (-1·3 to 30·4%)	-6·8% (-14·1 to 0·4%)
Ischaemic stroke	9·8 (7·7 to 12·1)	7·4% (6·0 to 8·9%)	10·5 (8·1 to 13·2)	6·6% (5·2 to 8·1%)	6·6% (-6·9 to 20·6%)	-11·7% (-19·7 to -3·2%)	238·3 (188·4 to 295·7)	9·3% (7·5 to 11·2%)	255·0 (195·8 to 325·1)	8·4% (6·7 to 10·3%)	7·0% (-6·4 to 20·8%)	-9·6% (-17·7 to -1·1%)
Hemorrhagic stroke	20·6 (16·5 to 25·0)	10·4% (8·4 to 12·4%)	22·5 (17·3 to 28·7)	9·5% (7·5 to 11·5%)	9·3% (-4·8 to 26·2%)	-8·6% (-17·4 to -0·4%)	638·5 (512·7 to 777·9)	11·2% (9·1 to 13·3%)	704·0 (539·3 to 903·4)	10·4% (8·3 to 12·8%)	10·3% (-4·7 to 28·0%)	-6·4% (-15·6 to 2·4%)
Hypertensive heart disease	6·3 (4·5 to 9·1)	8·1% (5·6 to 10·7%)	6·8 (4·8 to 10·0)	7·3% (5·0 to 9·7%)	7·7% (-6·7 to 26·0%)	-9·8% (-20·1 to 3·0%)	182·9 (131·9 to 259·5)	10·3% (7·5 to 13·6%)	196·9 (139·4 to 285·7)	9·5% (6·6 to 12·6%)	7·7% (-7·6 to 27·0%)	-8·0% (-18·7 to 5·4%)
Atrial fibrillation and flutter	0·1 (0·1 to 0·2)	5·5% (4·0 to 7·1%)	0·2 (0·1 to 0·2)	4·9% (3·5 to 6·3%)	20·5% (6·9 to 35·9%)	-11·9% (-19·3 to -3·9%)	8·5 (5·7 to 12·0)	10·4% (7·7 to 13·1%)	10·2 (6·8 to 14·2)	9·3% (6·9 to 11·8%)	19·2% (12·8 to 26·0%)	-11·0% (-15·3 to -6·9%)
Aortic aneurysm	0·7 (0·5 to 0·9)	12·9% (9·9 to 16·0%)	0·8 (0·6 to 1·1)	11·8% (8·9 to 15·0%)	25·3% (4·8 to 47·2%)	-8·9% (-16·0 to -1·3%)	19·3 (13·9 to 27·6)	15·5% (11·9 to 19·4%)	24·6 (17·6 to 34·1)	14·4% (10·9 to 18·4%)	27·2% (4·3 to 53·1%)	-7·5% (-14·9 to 0·9%)

Supplemental Results Table 1. All-age deaths, death PAFs, DALYs, DALY PAFs, and percent change for each risk factor and outcome for both sexes combined in 2005 and 2015 for Sub-Saharan Africa.

Data in parenthesis are 95% uncertainty intervals. PAFs=population attributable fractions. DALYs=disability-adjusted life-years.

	2005 Deaths (in thousands)	2005 Deaths PAF	2015 Deaths (in thousands)	2015 Deaths PAF	Percent change deaths 2005-2015	Percent change deaths PAF 2005-2015	2005 DALYs (in thousands)	2005 DALYs PAF	2015 DALYs (in thousands)	2015 DALYs PAF	Percent change DALYs 2005-2015	Percent change DALYs PAF 2005-2015
Peripheral vascular disease	0·1 (0·1 to 0·2)	13·7% (10·4 to 16·9%)	0·2 (0·1 to 0·2)	11·8% (8·7 to 14·9%)	36·7% (17·5 to 59·3%)	-13·6% (-21·5 to -4·5%)	5·3 (3·6 to 7·4)	13·1% (9·6 to 16·5%)	6·7 (4·5 to 9·5)	11·6% (8·5 to 14·9%)	26·2% (13·8 to 42·6%)	-11·2% (-17·3 to -4·7%)
Other cardiovascular and circulatory diseases	6·2 (4·7 to 8·0)	8·1% (6·1 to 10·2%)	7·5 (5·3 to 10·0)	7·8% (5·7 to 10·0%)	20·4% (0·8 to 42·0%)	-4·0% (-14·3 to 8·5%)	230·7 (169·7 to 298·7)	7·7% (5·7 to 9·6%)	279·2 (198·1 to 371·8)	7·5% (5·5 to 9·5%)	21·0% (3·1 to 40·8%)	-2·0% (-12·0 to 9·5%)
Chronic obstructive pulmonary disease	24·2 (19·1 to 29·8)	29·6% (24·0 to 35·2%)	31·4 (24·1 to 40·1)	31·4% (25·3 to 37·5%)	29·8% (6·8 to 60·1%)	6·4% (-6·8 to 20·6%)	617·9 (489·5 to 768·2)	25·8% (20·9 to 30·8%)	805·8 (617·7 to 1,018·8)	27·8% (22·1 to 33·5%)	30·4% (7·2 to 59·5%)	7·6% (-6·2 to 24·1%)
Silicosis	0·0 (0·0 to 0·0)	10·6% (6·9 to 15·5%)	0·0 (0·0 to 0·0)	10·2% (6·9 to 14·8%)	25·1% (-10·6 to 77·7%)	-3·9% (-22·9 to 21·5%)	0·4 (0·3 to 0·6)	10·2% (7·0 to 14·1%)	0·5 (0·3 to 0·8)	9·4% (6·5 to 12·9%)	19·9% (-10·6 to 63·8%)	-7·7% (-25·3 to 13·4%)
Asbestosis	0·0 (0·0 to 0·0)	13·2% (9·9 to 17·2%)	0·0 (0·0 to 0·1)	14·2% (10·8 to 18·3%)	46·6% (16·9 to 82·0%)	7·6% (-4·9 to 22·6%)	0·8 (0·6 to 1·0)	12·9% (9·6 to 16·9%)	1·1 (0·8 to 1·4)	13·0% (9·7 to 16·9%)	38·7% (10·4 to 72·4%)	0·9% (-12·0 to 16·5%)
Coal workers pneumoconiosis	0·0 (0·0 to 0·0)	10·0% (6·6 to 13·9%)	0·0 (0·0 to 0·0)	9·8% (6·8 to 13·5%)	21·7% (-12·9 to 71·9%)	-1·9% (-23·3 to 29·1%)	0·1 (0·1 to 0·2)	13·3% (9·3 to 17·8%)	0·2 (0·1 to 0·2)	13·2% (9·6 to 17·5%)	17·5% (-7·8 to 50·1%)	-1·2% (-17·4 to 18·4%)
Other pneumoconiosis	0·0 (0·0 to 0·1)	3·2% (2·1 to 4·8%)	0·1 (0·0 to 0·1)	3·6% (2·3 to 5·7%)	52·5% (-1·4 to 137·1%)	14·1% (-18·7 to 62·6%)	1·7 (1·0 to 2·7)	3·0% (1·9 to 4·3%)	2·6 (1·4 to 4·2)	3·3% (2·1 to 5·2%)	48·0% (4·9 to 112·3%)	11·6% (-15·7 to 51·0%)
Asthma	4·1 (3·0 to 5·2)	7·0% (5·4 to 8·8%)	3·8 (2·7 to 5·1)	6·4% (4·7 to 8·1%)	-6·6% (-19·0 to 7·5%)	-9·5% (-18·9 to 0·1%)	155·3 (116·7 to 197·1)	3·9% (3·0 to 4·9%)	158·2 (113·0 to 206·8)	3·4% (2·5 to 4·4%)	1·9% (-9·9 to 13·6%)	-12·5% (-20·6 to -4·4%)
Interstitial lung disease and pulmonary sarcoidosis	0·2 (0·2 to 0·3)	10·7% (7·8 to 14·2%)	0·3 (0·2 to 0·5)	11·3% (8·3 to 14·9%)	46·7% (12·9 to 95·8%)	5·0% (-10·9 to 23·3%)	5·8 (3·9 to 8·3)	10·3% (7·5 to 13·6%)	8·3 (5·8 to 11·7)	10·5% (7·6 to 13·8%)	43·0% (9·4 to 88·9%)	1·9% (-14·5 to 22·2%)
Other chronic respiratory diseases	0·2 (0·1 to 0·4)	3·0% (1·8 to 4·6%)	0·4 (0·2 to 0·7)	4·0% (2·3 to 6·3%)	71·4% (11·0 to 169·9%)	31·9% (-9·6 to 92·9%)	10·5 (5·3 to 19·0)	1·9% (1·0 to 3·3%)	16·6 (7·9 to 29·7)	2·4% (1·3 to 4·3%)	59·1% (12·7 to 142·7%)	26·8% (-8·5 to 86·8%)
Diabetes mellitus	3·5 (1·1 to 6·4)	3·2% (1·0 to 5·6%)	4·4 (1·2 to 8·0)	3·0% (0·9 to 5·5%)	24·7% (7·3 to 42·5%)	-4·7% (-16·4 to 6·0%)	141·0 (42·4 to 252·5)	3·4% (1·0 to 6·1%)	178·3 (47·7 to 325·2)	3·2% (0·9 to 5·9%)	26·5% (11·9 to 39·9%)	-5·6% (-15·5 to 1·5%)
**Second-hand smoke: All causes**	78·7 (53·7 to 105·3)	0·9% (0·6 to 1·2%)	77·3 (54·6 to 104·0)	1·0% (0·7 to 1·3%)	-1·8% (-9·3 to 7·4%)	6·4% (-1·0 to 14·7%)	4,865·4 (3,128·4 to 6,718·8)	0·8% (0·5 to 1·2%)	4,369·0 (2,906·9 to 6,025·6)	0·8% (0·6 to 1·1%)	-10·2% (-18·3 to -0·8%)	-1·2% (-9·6 to 8·7%)
Lower respiratory infections	57·7 (35·0 to 82·1)	7·5% (4·7 to 10·7%)	51·8 (31·7 to 73·5)	7·1% (4·5 to 10·0%)	-10·3% (-18·2 to -1·7%)	-5·8% (-10·9 to -0·7%)	4,279·7 (2,604·0 to 6,107·7)	9·0% (5·7 to 12·8%)	3,655·2 (2,256·2 to 5,217·3)	8·9% (5·6 to 12·5%)	-14·6% (-23·0 to -5·3%)	-1·4% (-5·5 to 3·3%)
Otitis media	0·1 (0·1 to 0·1)	7·1% (4·8 to 9·6%)	0·1 (0·0 to 0·1)	6·5% (4·2 to 8·9%)	-10·4% (-27·1 to 12·4%)	-9·3% (-22·3 to 4·3%)	43·8 (24·9 to 68·7)	6·9% (4·6 to 9·3%)	49·4 (27·6 to 78·5)	6·7% (4·4 to 8·9%)	12·9% (7·8 to 17·3%)	-3·6% (-5·7 to -1·5%)
Tracheal, bronchus, and lung cancer	0·4 (0·2 to 0·6)	1·4% (0·8 to 2·3%)	0·5 (0·3 to 0·8)	1·3% (0·8 to 2·2%)	30·0% (24·8 to 35·5%)	-6·1% (-14·7 to 3·5%)	9·8 (5·7 to 15·7)	1·5% (0·9 to 2·4%)	12·8 (7·5 to 20·2)	1·4% (0·8 to 2·3%)	30·2% (23·9 to 37·1%)	-4·9% (-14·3 to 5·2%)
Ischaemic heart disease	11·4 (8·7 to 14·5)	3·7% (2·9 to 4·7%)	14·0 (10·6 to 17·8)	3·7% (2·8 to 4·7%)	22·8% (10·7 to 37·1%)	-0·2% (-6·2 to 5·3%)	286·3 (219·3 to 364·5)	4·2% (3·2 to 5·2%)	354·1 (269·1 to 446·6)	4·2% (3·2 to 5·3%)	23·7% (9·7 to 40·4%)	1·5% (-5·1 to 7·8%)
Ischaemic stroke	2·4 (1·8 to 3·3)	1·9% (1·4 to 2·4%)	2·9 (2·1 to 3·9)	1·8% (1·3 to 2·4%)	19·1% (7·0 to 32·9%)	-1·3% (-6·9 to 4·0%)	51·8 (38·4 to 67·9)	2·0% (1·5 to 2·6%)	61·7 (45·4 to 80·8)	2·0% (1·5 to 2·6%)	19·0% (6·2 to 35·1%)	0·6% (-5·2 to 6·1%)
Hemorrhagic stroke	4·5 (3·4 to 5·8)	2·3% (1·7 to 2·9%)	5·5 (4·1 to 7·2)	2·3% (1·8 to 2·9%)	21·7% (7·6 to 39·8%)	1·8% (-4·7 to 7·9%)	135·7 (100·9 to 177·1)	2·4% (1·8 to 3·0%)	166·2 (123·3 to 220·1)	2·5% (1·9 to 3·1%)	22·5% (7·2 to 42·1%)	4·0% (-3·1 to 11·0%)
**Alcohol and drug use: All causes**	252·7 (226·9 to 279·5)	2·9% (2·6 to 3·2%)	284·6 (244·8 to 333·7)	3·5% (3·1 to 4·0%)	12·6% (-0·3 to 28·1%)	21·9% (12·2 to 33·0%)	10,739·7 (9,747·4 to 11,811·0)	1·8% (1·7 to 2·0%)	12,096·7 (10,602·5 to 13,927·9)	2·3% (2·0 to 2·6%)	12·6% (0·7 to 26·6%)	23·8% (13·2 to 36·0%)
**Alcohol use: All causes**	211·3 (186·9 to 237·1)	2·4% (2·2 to 2·7%)	245·7 (208·6 to 290·8)	3·1% (2·7 to 3·5%)	16·3% (2·2 to 33·5%)	25·9% (15·0 to 38·5%)	8,351·1 (7,443·2 to 9,343·3)	1·4% (1·3 to 1·6%)	9,781·9 (8,461·6 to 11,477·7)	1·8% (1·6 to 2·1%)	17·1% (3·7 to 33·7%)	28·8% (16·3 to 43·5%)
Tuberculosis	33·8 (26·1 to 43·4)	12·3% (10·2 to 14·0%)	33·6 (23·8 to 46·0)	12·6% (9·8 to 15·1%)	-0·6% (-18·2 to 20·2%)	2·7% (-7·7 to 12·0%)	1,303·9 (1,020·4 to 1,651·8)	11·4% (9·8 to 12·9%)	1,313·7 (950·5 to 1,770·8)	12·0% (9·6 to 14·1%)	0·7% (-16·5 to 21·8%)	5·4% (-5·3 to 16·2%)
Lower respiratory infections	16·2 (12·8 to 19·5)	2·1% (1·8 to 2·5%)	18·8 (14·5 to 23·3)	2·6% (2·1 to 3·0%)	16·4% (-1·5 to 39·3%)	21·9% (5·5 to 40·7%)	455·4 (358·2 to 545·6)	1·0% (0·8 to 1·2%)	524·3 (403·7 to 660·1)	1·3% (1·0 to 1·6%)	15·1% (-3·9 to 39·2%)	32·9% (9·8 to 60·3%)
Lip and oral cavity cancer	1·4 (1·2 to 1·6)	24·7% (21·8 to 27·0%)	1·9 (1·6 to 2·3)	24·8% (21·4 to 27·9%)	40·8% (22·4 to 62·3%)	0·7% (-6·7 to 7·5%)	41·2 (36·0 to 46·5)	25·9% (23·2 to 28·1%)	58·0 (48·4 to 69·5)	26·1% (22·7 to 29·0%)	40·9% (22·1 to 63·1%)	0·7% (-6·6 to 7·8%)
Nasopharynx cancer	0·4 (0·3 to 0·5)	26·2% (23·5 to 28·6%)	0·6 (0·4 to 0·7)	26·9% (23·2 to 30·2%)	41·5% (18·1 to 69·6%)	2·8% (-4·9 to 10·0%)	14·3 (11·0 to 16·7)	25·8% (23·3 to 28·0%)	20·6 (14·7 to 26·4)	26·5% (22·9 to 29·6%)	43·9% (19·1 to 74·9%)	2·7% (-5·1 to 10·4%)
Other pharynx cancer	0·4 (0·4 to 0·5)	23·8% (20·9 to 26·2%)	0·6 (0·5 to 0·7)	24·8% (21·2 to 28·1%)	42·5% (22·1 to 66·9%)	4·3% (-3·1 to 11·6%)	13·0 (11·0 to 15·0)	24·7% (22·0 to 27·1%)	18·6 (15·0 to 22·5)	25·9% (22·3 to 29·1%)	43·6% (22·6 to 69·0%)	4·6% (-2·8 to 12·1%)
Oesophageal cancer	4·0 (3·3 to 4·5)	14·6% (12·7 to 16·2%)	5·2 (4·1 to 6·4)	15·1% (12·6 to 17·3%)	30·9% (11·6 to 55·5%)	3·8% (-4·8 to 12·1%)	109·8 (93·2 to 127·0)	15·5% (13·6 to 17·1%)	143·8 (115·2 to 178·4)	16·2% (13·7 to 18·4%)	31·0% (10·4 to 57·0%)	4·3% (-4·5 to 12·7%)
Colon and rectum cancer	0·8 (0·7 to 0·9)	3·5% (3·2 to 3·8%)	1·2 (1·0 to 1·4)	3·5% (3·1 to 3·9%)	42·9% (24·2 to 63·8%)	0·4% (-4·9 to 5·7%)	22·5 (19·7 to 25·6)	3·7% (3·3 to 4·0%)	32·1 (26·8 to 38·5)	3·7% (3·3 to 4·1%)	42·6% (21·7 to 66·6%)	0·8% (-4·9 to 6·5%)
Liver cancer due to hepatitis B	0·1 (0·1 to 0·2)	0·6% (0·4 to 0·9%)	0·1 (0·1 to 0·2)	0·6% (0·3 to 0·9%)	25·7% (-11·4 to 83·1%)	0·3% (-25·5 to 33·4%)	6·1 (3·5 to 10·5)	0·9% (0·6 to 1·3%)	7·7 (4·4 to 13·0)	0·9% (0·6 to 1·3%)	25·6% (-11·3 to 83·1%)	-0·5% (-25·0 to 30·2%)
Liver cancer due to hepatitis C	0·0 (0·0 to 0·0)	0·1% (0·0 to 0·1%)	0·0 (0·0 to 0·0)	0·1% (0·0 to 0·1%)	28·9% (-3·5 to 72·4%)	1·4% (-22·1 to 31·4%)	0·3 (0·2 to 0·5)	0·1% (0·1 to 0·2%)	0·4 (0·2 to 0·7)	0·1% (0·1 to 0·2%)	29·0% (-3·3 to 70·3%)	1·4% (-21·8 to 29·9%)
Liver cancer due to alcohol use	15·4 (12·5 to 18·2)	100·0% (100·0 to 100·0%)	19·2 (15·6 to 23·5)	100·0% (100·0 to 100·0%)	24·9% (5·8 to 48·7%)	-	416·7 (338·3 to 497·5)	100·0% (100·0 to 100·0%)	523·3 (422·4 to 651·2)	100·0% (100·0 to 100·0%)	25·6% (4·8 to 50·5%)	-
Liver cancer due to other causes	0·0 (0·0 to 0·0)	0·3% (0·2 to 0·4%)	0·0 (0·0 to 0·0)	0·3% (0·2 to 0·4%)	24·8% (-3·2 to 61·8%)	1·2% (-18·9 to 24·7%)	1·3 (0·9 to 2·0)	0·6% (0·4 to 0·8%)	1·7 (1·1 to 2·5)	0·6% (0·4 to 0·8%)	24·8% (-3·2 to 60·8%)	0·5% (-18·8 to 23·0%)
Larynx cancer	0·7 (0·6 to 0·8)	18·2% (15·7 to 20·4%)	1·0 (0·8 to 1·2)	18·2% (15·1 to 20·7%)	30·7% (12·5 to 52·0%)	-0·4% (-7·7 to 6·0%)	20·9 (17·8 to 24·1)	18·8% (16·4 to 20·9%)	27·2 (21·8 to 33·2)	18·7% (15·8 to 21·2%)	30·0% (12·6 to 51·3%)	-0·3% (-7·2 to 5·7%)
Breast cancer	1·6 (1·3 to 1·9)	6·5% (5·7 to 7·1%)	2·1 (1·7 to 2·7)	6·4% (5·5 to 7·2%)	34·1% (6·7 to 72·3%)	-0·7% (-8·1 to 6·4%)	53·1 (44·2 to 64·3)	6·5% (5·8 to 7·2%)	72·0 (56·5 to 91·5)	6·5% (5·6 to 7·3%)	35·5% (7·0 to 75·0%)	-0·6% (-8·1 to 6·5%)
Ischaemic heart disease	1·2 (-0·1 to 2·6)	0·4% (0·0 to 0·9%)	0·7 (-1·1 to 2·6)	0·2% (-0·3 to 0·7%)	-41·0% (-387·4 to 282·0%)	-52·0% (-331·0 to 218·3%)	21·0 (-8·6 to 51·0)	0·3% (-0·1 to 0·7%)	6·7 (-33·7 to 48·7)	0·1% (-0·4 to 0·6%)	-67·9% (-770·7 to 546·4%)	-73·2% (-634·3 to 420·6%)
Ischaemic stroke	-0·7 (-1·6 to 0·0)	-0·5% (-1·2 to 0·0%)	-0·8 (-1·8 to 0·1)	-0·5% (-1·1 to 0·1%)	14·7% (-90·7 to 190·5%)	-4·9% (-91·7 to 125·9%)	-7·1 (-22·0 to 6·3)	-0·3% (-0·9 to 0·3%)	-6·2 (-24·8 to 10·6)	-0·2% (-0·8 to 0·4%)	-12·4% (-520·0 to 381·7%)	-26·1% (-448·9 to 294·5%)
Hemorrhagic stroke	16·7 (14·1 to 19·5)	8·4% (7·3 to 9·4%)	19·5 (15·6 to 23·9)	8·2% (6·8 to 9·5%)	16·6% (0·7 to 35·0%)	-2·5% (-11·1 to 5·4%)	459·8 (391·6 to 532·5)	8·0% (7·1 to 8·9%)	536·2 (427·1 to 658·6)	8·0% (6·7 to 9·1%)	16·6% (0·4 to 36·1%)	-1·0% (-9·5 to 6·9%)
Hypertensive heart disease	6·1 (4·1 to 8·9)	7·8% (5·4 to 10·0%)	6·9 (4·2 to 10·6)	7·4% (4·6 to 9·9%)	13·1% (-9·4 to 37·1%)	-5·3% (-23·0 to 8·7%)	161·2 (115·3 to 220·3)	9·1% (6·9 to 11·0%)	182·7 (121·0 to 264·9)	8·8% (6·0 to 11·1%)	13·4% (-7·7 to 36·3%)	-3·2% (-18·8 to 8·4%)
Atrial fibrillation and flutter	0·1 (0·1 to 0·2)	4·5% (3·8 to 5·4%)	0·2 (0·1 to 0·2)	4·5% (3·7 to 5·4%)	36·0% (21·0 to 52·6%)	-0·5% (-8·1 to 7·4%)	5·0 (3·7 to 6·5)	6·1% (5·4 to 6·8%)	6·7 (4·8 to 8·9)	6·1% (5·2 to 6·9%)	34·5% (25·7 to 43·7%)	0·5% (-5·4 to 5·9%)
Cirrhosis and other chronic liver diseases due to hepatitis B	5·7 (3·9 to 7·9)	17·5% (12·9 to 21·9%)	6·9 (4·3 to 10·1)	17·1% (11·8 to 22·2%)	20·8% (-6·5 to 55·5%)	-2·5% (-19·7 to 12·8%)	179·1 (121·6 to 245·7)	17·1% (12·7 to 21·4%)	219·7 (134·4 to 320·7)	16·8% (11·5 to 22·0%)	22·7% (-5·7 to 60·1%)	-1·8% (-19·4 to 14·1%)
Cirrhosis and other chronic liver diseases due to hepatitis C	3·1 (2·0 to 4·3)	15·6% (11·1 to 20·2%)	3·8 (2·2 to 5·7)	15·3% (9·8 to 20·9%)	22·8% (-4·5 to 53·0%)	-2·1% (-20·5 to 13·5%)	94·9 (60·8 to 135·6)	15·7% (11·1 to 20·4%)	118·0 (67·8 to 178·9)	15·5% (9·8 to 21·1%)	24·3% (-4·3 to 56·5%)	-1·4% (-20·6 to 14·9%)
Cirrhosis and other chronic liver diseases due to alcohol use	44·3 (38·8 to 50·2)	100·0% (100·0 to 100·0%)	53·5 (44·9 to 64·0)	100·0% (100·0 to 100·0%)	20·8% (4·0 to 41·3%)	-	1,471·2 (1,285·8 to 1,688·7)	100·0% (100·0 to 100·0%)	1,786·7 (1,484·2 to 2,166·0)	100·0% (100·0 to 100·0%)	21·5% (3·5 to 44·1%)	-
Cirrhosis and other chronic liver diseases due to other causes	3·3 (2·5 to 4·1)	13·4% (10·4 to 16·4%)	3·8 (2·6 to 5·2)	13·2% (9·5 to 16·9%)	15·3% (-7·5 to 43·8%)	-1·2% (-17·5 to 13·7%)	113·9 (87·6 to 140·1)	10·2% (8·0 to 12·5%)	130·7 (90·5 to 176·5)	10·3% (7·3 to 13·2%)	14·8% (-8·5 to 43·8%)	0·9% (-16·5 to 18·9%)
Pancreatitis	0·8 (0·6 to 1·0)	12·4% (10·8 to 13·9%)	0·9 (0·7 to 1·3)	12·5% (10·4 to 14·7%)	22·3% (-4·9 to 65·0%)	0·8% (-11·7 to 15·7%)	30·1 (23·7 to 37·8)	13·0% (11·5 to 14·6%)	37·1 (28·4 to 51·1)	13·1% (11·1 to 15·5%)	23·4% (-5·0 to 67·3%)	0·9% (-11·2 to 15·3%)

Supplemental Results Table 1. All-age deaths, death PAFs, DALYs, DALY PAFs, and percent change for each risk factor and outcome for both sexes combined in 2005 and 2015 for Sub-Saharan Africa.

Data in parenthesis are 95% uncertainty intervals. PAFs=population attributable fractions. DALYs=disability-adjusted life-years.

	2005 Deaths (in thousands)	2005 Deaths PAF	2015 Deaths (in thousands)	2015 Deaths PAF	Percent change deaths 2005-2015	Percent change deaths PAF 2005-2015	2005 DALYs (in thousands)	2005 DALYs PAF	2015 DALYs (in thousands)	2015 DALYs PAF	Percent change DALYs 2005-2015	Percent change DALYs PAF 2005-2015
Epilepsy	3·3 (2·9 to 3·7)	10·6% (9·1 to 12·3%)	4·3 (3·5 to 5·3)	11·1% (9·4 to 13·1%)	31·4% (8·7 to 58·8%)	4·8% (-4·8 to 16·3%)	186·3 (163·1 to 213·1)	8·2% (7·0 to 9·6%)	243·0 (201·9 to 295·5)	8·6% (7·3 to 10·2%)	30·5% (11·0 to 53·0%)	4·8% (-5·1 to 16·1%)
Alcohol use disorders	8·5 (7·1 to 10·3)	100·0% (100·0 to 100·0%)	10·2 (8·1 to 12·8)	100·0% (100·0 to 100·0%)	18·9% (0·2 to 41·6%)	-	758·7 (597·8 to 944·3)	100·0% (100·0 to 100·0%)	967·6 (752·6 to 1,224·3)	100·0% (100·0 to 100·0%)	27·5% (17·5 to 38·5%)	-
Diabetes mellitus	-5·7 (-6·6 to -4·9)	-5·2% (-5·7 to -4·6%)	-6·9 (-8·5 to -5·6)	-4·7% (-5·5 to -4·1%)	20·6% (0·3 to 44·8%)	-8·0% (-18·6 to 3·8%)	-194·9 (-227·7 to -167·3)	-4·7% (-5·1 to -4·3%)	-244·5 (-294·9 to -199·5)	-4·4% (-4·9 to -3·9%)	25·5% (9·7 to 43·6%)	-6·4% (-14·8 to 2·4%)
Pedestrian road injuries	6·8 (5·3 to 8·4)	13·9% (11·5 to 16·5%)	8·2 (6·4 to 10·2)	14·1% (11·8 to 16·6%)	20·3% (4·0 to 40·4%)	1·7% (-4·9 to 8·4%)	368·8 (285·0 to 458·8)	13·9% (11·5 to 16·6%)	439·8 (342·4 to 544·8)	14·4% (12·0 to 17·1%)	19·2% (3·1 to 39·2%)	3·7% (-4·7 to 12·6%)
Cyclist road injuries	0·6 (0·5 to 0·8)	16·1% (13·3 to 19·2%)	0·8 (0·6 to 1·1)	16·1% (13·3 to 19·4%)	31·4% (7·1 to 65·6%)	0·1% (-9·0 to 11·4%)	35·2 (26·7 to 43·9)	15·7% (12·8 to 18·8%)	46·3 (35·3 to 59·9)	15·9% (12·9 to 19·2%)	31·4% (8·5 to 63·1%)	1·1% (-9·7 to 14·9%)
Motorcyclist road injuries	2·9 (2·2 to 3·7)	27·0% (23·2 to 31·2%)	3·5 (2·6 to 4·6)	26·2% (22·8 to 29·9%)	21·3% (1·5 to 47·5%)	-3·3% (-10·7 to 4·5%)	161·3 (123·5 to 207·1)	25·5% (21·4 to 29·8%)	195·4 (146·2 to 257·0)	24·8% (21·3 to 28·7%)	21·1% (2·0 to 46·4%)	-2·8% (-11·4 to 6·5%)
Motor vehicle road injuries	16·9 (14·3 to 19·5)	28·1% (25·2 to 31·1%)	18·6 (15·7 to 22·0)	27·4% (24·5 to 30·3%)	10·5% (-1·9 to 25·1%)	-2·6% (-8·0 to 3·6%)	898·0 (768·9 to 1,043·5)	26·7% (23·7 to 29·8%)	983·4 (836·6 to 1,162·0)	26·2% (23·4 to 29·1%)	9·5% (-2·4 to 23·6%)	-2·0% (-8·7 to 5·1%)
Falls	3·2 (2·5 to 4·1)	7·7% (6·1 to 9·5%)	4·0 (3·0 to 5·2)	7·5% (6·0 to 9·2%)	24·8% (4·6 to 52·7%)	-2·5% (-13·2 to 12·6%)	153·1 (121·1 to 193·6)	6·4% (5·1 to 7·8%)	198·4 (156·1 to 250·8)	6·4% (5·4 to 7·6%)	29·6% (15·8 to 47·8%)	-0·2% (-11·7 to 16·5%)
Drowning	1·9 (1·6 to 2·4)	3·8% (3·0 to 5·0%)	2·1 (1·7 to 2·7)	4·2% (3·3 to 5·2%)	10·8% (-5·4 to 32·0%)	9·4% (-10·1 to 30·7%)	86·7 (70·5 to 111·0)	2·5% (1·9 to 3·4%)	96·3 (77·2 to 123·1)	2·8% (2·2 to 3·6%)	11·1% (-5·9 to 32·6%)	12·2% (-11·2 to 39·3%)
Fire, heat, and hot substances	1·9 (1·5 to 2·3)	5·1% (4·1 to 6·3%)	2·2 (1·7 to 2·7)	5·4% (4·5 to 6·6%)	16·9% (1·1 to 36·3%)	6·3% (-6·1 to 20·1%)	85·1 (69·4 to 104·3)	3·6% (2·9 to 4·5%)	101·6 (81·7 to 126·5)	4·0% (3·3 to 4·8%)	19·3% (5·6 to 35·7%)	10·0% (-4·5 to 25·3%)
Poisonings	0·8 (0·6 to 1·2)	4·2% (2·4 to 7·0%)	0·9 (0·7 to 1·3)	4·4% (2·5 to 7·1%)	9·3% (-6·4 to 29·0%)	3·9% (-33·8 to 71·0%)	39·2 (30·1 to 55·2)	2·8% (1·4 to 5·1%)	44·0 (33·1 to 61·5)	3·0% (1·6 to 5·4%)	12·1% (-2·1 to 29·7%)	6·5% (-37·0 to 88·4%)
Unintentional firearm injuries	0·6 (0·4 to 0·8)	7·8% (6·4 to 9·7%)	0·6 (0·4 to 0·9)	7·4% (6·2 to 9·3%)	11·3% (-8·8 to 41·1%)	-4·7% (-15·1 to 9·0%)	27·7 (18·5 to 38·1)	6·5% (5·2 to 8·4%)	31·0 (19·4 to 42·6)	6·2% (5·1 to 7·9%)	12·1% (-7·7 to 40·6%)	-4·2% (-16·9 to 11·9%)
Unintentional suffocation	0·1 (0·1 to 0·2)	2·5% (1·5 to 3·8%)	0·2 (0·1 to 0·2)	2·5% (1·6 to 3·7%)	18·6% (2·3 to 40·3%)	1·3% (-29·7 to 45·0%)	10·3 (8·2 to 13·2)	2·1% (1·3 to 3·2%)	12·4 (10·0 to 15·7)	2·2% (1·4 to 3·1%)	20·9% (8·4 to 36·4%)	2·6% (-27·9 to 45·7%)
Other exposure to mechanical forces	1·3 (1·0 to 1·6)	6·7% (5·5 to 8·2%)	1·6 (1·1 to 2·1)	7·1% (5·9 to 8·6%)	24·2% (2·0 to 56·7%)	5·3% (-6·0 to 18·5%)	88·8 (69·6 to 111·1)	5·8% (4·7 to 7·0%)	110·7 (84·7 to 141·1)	6·1% (5·1 to 7·2%)	24·8% (10·5 to 45·3%)	5·6% (-4·3 to 17·0%)
Self-harm	6·0 (4·9 to 7·4)	11·7% (10·1 to 13·7%)	7·7 (6·3 to 9·9)	11·5% (10·0 to 13·4%)	28·4% (8·9 to 53·4%)	-1·6% (-7·4 to 4·9%)	258·6 (213·8 to 315·8)	11·4% (10·0 to 13·3%)	331·3 (272·0 to 420·6)	11·3% (9·9 to 13·0%)	28·1% (8·5 to 53·3%)	-1·5% (-7·2 to 4·8%)
Assault by firearm	1·7 (1·3 to 2·1)	13·7% (12·0 to 15·8%)	1·8 (1·4 to 2·2)	12·9% (11·2 to 14·9%)	6·1% (-11·7 to 30·4%)	-6·0% (-10·9 to 0·0%)	96·2 (77·3 to 119·2)	14·1% (12·4 to 16·2%)	102·8 (82·5 to 128·7)	13·3% (11·6 to 15·3%)	6·9% (-10·7 to 30·9%)	-6·0% (-11·0 to 0·1%)
Assault by sharp object	2·4 (2·0 to 2·9)	16·0% (14·4 to 17·8%)	2·6 (2·2 to 3·1)	16·0% (14·5 to 17·9%)	8·3% (-6·6 to 27·4%)	0·2% (-3·1 to 3·8%)	139·3 (114·6 to 168·7)	16·6% (15·0 to 18·5%)	149·9 (125·9 to 178·9)	16·6% (15·0 to 18·6%)	7·6% (-7·1 to 26·4%)	0·1% (-3·2 to 3·7%)
Assault by other means	2·8 (2·3 to 3·5)	11·0% (9·7 to 12·7%)	3·6 (2·9 to 4·7)	11·6% (10·1 to 13·3%)	29·2% (7·0 to 56·3%)	4·8% (-1·5 to 12·1%)	165·1 (138·5 to 202·9)	11·0% (9·6 to 12·6%)	210·8 (167·1 to 270·9)	11·6% (10·1 to 13·4%)	27·7% (5·8 to 53·6%)	6·0% (-0·8 to 13·6%)
**Drug use: All causes**	42·2 (36·2 to 48·3)	0·5% (0·4 to 0·6%)	40·1 (33·2 to 48·7)	0·5% (0·4 to 0·6%)	-5·0% (-17·6 to 10·1%)	2·9% (-8·5 to 16·2%)	2,421·1 (2,108·0 to 2,730·0)	0·4% (0·4 to 0·5%)	2,361·8 (2,001·6 to 2,786·2)	0·4% (0·4 to 0·5%)	-2·5% (-12·9 to 9·8%)	7·3% (-3·5 to 19·0%)
HIV/AIDS - Tuberculosis	3·6 (2·7 to 4·6)	1·3% (1·1 to 1·6%)	2·0 (1·5 to 2·7)	1·3% (1·0 to 1·7%)	-43·0% (-48·5 to -35·9%)	-0·7% (-4·8 to 5·3%)	185·3 (142·2 to 238·6)	1·2% (1·0 to 1·5%)	109·0 (78·8 to 145·4)	1·2% (1·0 to 1·6%)	-41·2% (-46·6 to -34·1%)	1·2% (-2·9 to 7·5%)
HIV/AIDS resulting in other diseases	23·3 (19·2 to 27·8)	2·0% (1·7 to 2·4%)	15·7 (12·5 to 19·7)	2·2% (1·8 to 2·7%)	-32·8% (-43·1 to -22·0%)	10·7% (-3·4 to 25·6%)	1,246·4 (1,031·6 to 1,495·1)	1·9% (1·6 to 2·3%)	869·4 (694·0 to 1,083·0)	2·1% (1·7 to 2·6%)	-30·2% (-40·5 to -19·4%)	10·0% (-3·7 to 23·8%)
Hepatitis B	0·0 (0·0 to 0·0)	0·3% (0·1 to 0·5%)	0·0 (0·0 to 0·1)	0·3% (0·1 to 0·5%)	35·1% (13·0 to 63·0%)	22·8% (10·3 to 37·1%)	1·0 (0·4 to 1·7)	0·3% (0·1 to 0·4%)	1·3 (0·5 to 2·1)	0·3% (0·1 to 0·5%)	32·8% (11·1 to 60·8%)	19·4% (7·6 to 32·8%)
Hepatitis C	0·1 (0·0 to 0·3)	23·9% (15·3 to 33·3%)	0·2 (0·0 to 0·4)	29·0% (19·0 to 40·4%)	31·4% (-1·3 to 75·9%)	21·5% (2·5 to 45·9%)	5·2 (1·1 to 12·4)	24·3% (15·7 to 34·0%)	6·7 (1·5 to 15·8)	28·3% (18·1 to 38·9%)	28·3% (-2·7 to 70·0%)	16·2% (-1·1 to 37·8%)
Liver cancer due to hepatitis B	0·1 (0·0 to 0·1)	0·3% (0·1 to 0·6%)	0·1 (0·0 to 0·1)	0·4% (0·2 to 0·6%)	50·2% (23·2 to 85·0%)	19·9% (9·0 to 33·3%)	2·2 (0·9 to 3·8)	0·3% (0·1 to 0·6%)	3·3 (1·3 to 5·5)	0·4% (0·2 to 0·6%)	46·9% (19·5 to 81·8%)	16·5% (6·1 to 28·3%)
Liver cancer due to hepatitis C	1·7 (1·0 to 2·6)	16·9% (10·3 to 24·6%)	2·8 (1·7 to 4·2)	21·7% (13·8 to 31·3%)	64·1% (35·8 to 98·7%)	28·9% (14·6 to 47·1%)	56·4 (34·6 to 84·2)	21·9% (13·8 to 31·2%)	88·8 (54·4 to 132·0)	27·1% (17·3 to 38·1%)	57·5% (30·3 to 91·4%)	23·6% (10·8 to 39·5%)
Cirrhosis and other chronic liver diseases due to hepatitis B	0·1 (0·0 to 0·2)	0·3% (0·1 to 0·6%)	0·2 (0·1 to 0·3)	0·4% (0·2 to 0·7%)	53·7% (26·8 to 90·1%)	24·0% (11·6 to 41·0%)	3·7 (1·5 to 6·3)	0·4% (0·1 to 0·6%)	5·5 (2·3 to 9·4)	0·4% (0·2 to 0·7%)	51·1% (24·2 to 86·9%)	20·9% (9·4 to 34·7%)
Cirrhosis and other chronic liver diseases due to hepatitis C	4·9 (2·9 to 7·6)	25·3% (15·9 to 36·2%)	7·5 (4·6 to 11·5)	30·6% (19·3 to 43·1%)	51·8% (27·5 to 83·1%)	21·0% (10·8 to 34·8%)	188·7 (114·1 to 285·4)	31·4% (20·1 to 44·3%)	278·5 (167·7 to 421·2)	36·8% (23·6 to 51·2%)	47·6% (23·4 to 77·7%)	17·0% (8·3 to 28·6%)
Opioid use disorders	7·1 (5·1 to 9·0)	100·0% (100·0 to 100·0%)	9·8 (7·2 to 14·3)	100·0% (100·0 to 100·0%)	37·8% (6·6 to 86·5%)	-	627·8 (502·2 to 751·2)	100·0% (100·0 to 100·0%)	849·8 (660·5 to 1,088·7)	100·0% (100·0 to 100·0%)	35·4% (16·9 to 65·2%)	-
Cocaine use disorders	0·0 (0·0 to 0·1)	100·0% (100·0 to 100·0%)	0·1 (0·1 to 0·3)	100·0% (100·0 to 100·0%)	171·8% (80·3 to 293·6%)	-	8·0 (5·3 to 11·1)	100·0% (100·0 to 100·0%)	14·8 (9·0 to 22·6)	100·0% (100·0 to 100·0%)	85·6% (53·9 to 140·7%)	-
Amphetamine use disorders	0·1 (0·0 to 0·1)	100·0% (100·0 to 100·0%)	0·2 (0·1 to 0·3)	100·0% (100·0 to 100·0%)	226·5% (127·2 to 419·7%)	-	5·4 (3·7 to 7·7)	100·0% (100·0 to 100·0%)	12·6 (7·8 to 18·2)	100·0% (100·0 to 100·0%)	131·4% (82·3 to 223·5%)	-
Cannabis use disorders	-	-	-	-	-	-	31·7 (20·0 to 46·5)	100·0% (100·0 to 100·0%)	42·3 (26·7 to 61·9)	100·0% (100·0 to 100·0%)	33·4% (28·0 to 38·7%)	-
Other drug use disorders	0·4 (0·3 to 0·7)	100·0% (100·0 to 100·0%)	0·7 (0·4 to 1·2)	100·0% (100·0 to 100·0%)	48·8% (15·8 to 107·1%)	-	23·3 (16·7 to 33·4)	100·0% (100·0 to 100·0%)	35·2 (24·1 to 57·5)	100·0% (100·0 to 100·0%)	51·3% (22·0 to 103·8%)	-
Self-harm	0·7 (0·4 to 1·1)	1·4% (0·9 to 2·1%)	0·9 (0·6 to 1·4)	1·4% (0·8 to 2·0%)	26·0% (7·7 to 50·2%)	-3·3% (-11·4 to 5·9%)	36·0 (21·8 to 55·3)	1·6% (1·0 to 2·4%)	44·6 (27·4 to 68·5)	1·5% (0·9 to 2·2%)	23·9% (5·7 to 48·7%)	-4·7% (-12·7 to 4·6%)
**Dietary risks: All causes**	453·0 (379·1 to 538·7)	5·2% (4·4 to 6·2%)	558·6 (453·4 to 680·2)	7·0% (5·8 to 8·3%)	23·3% (10·2 to 38·1%)	33·5% (25·2 to 42·3%)	12,118·5 (10,242·5 to 14,283·9)	2·1% (1·8 to 2·4%)	15,023·3 (12,465·5 to 18,275·4)	2·8% (2·4 to 3·4%)	24·0% (11·3 to 39·1%)	36·3% (24·2 to 49·4%)
**Diet low in fruits: All causes**	142·9 (95·3 to 196·8)	1·6% (1·1 to 2·2%)	173·7 (113·4 to 242·6)	2·2% (1·5 to 2·9%)	21·6% (8·3 to 37·5%)	31·6% (22·7 to 41·3%)	4,100·3 (2,784·8 to 5,647·1)	0·7% (0·5 to 1·0%)	5,022·7 (3,336·9 to 6,976·6)	0·9% (0·6 to 1·3%)	22·5% (8·9 to 38·3%)	34·7% (22·1 to 48·2%)
Lip and oral cavity cancer	0·4 (0·0 to 0·9)	7·0% (0·0 to 15·2%)	0·5 (0·0 to 1·2)	7·0% (0·0 to 15·1%)	39·0% (0·0 to 58·0%)	-0·5% (-3·0 to 2·1%)	10·9 (0·0 to 23·6)	6·9% (0·0 to 14·9%)	15·1 (0·0 to 33·6)	6·8% (0·0 to 14·7%)	38·7% (0·0 to 58·7%)	-0·8% (-3·5 to 1·9%)
Nasopharynx cancer	0·1 (0·0 to 0·2)	6·7% (0·0 to 14·3%)	0·1 (0·0 to 0·3)	6·7% (0·0 to 14·1%)	36·9% (0·0 to 63·0%)	-0·5% (-4·7 to 3·8%)	3·4 (0·0 to 7·3)	6·2% (0·0 to 13·3%)	4·8 (0·0 to 10·3)	6·2% (0·0 to 13·1%)	39·1% (0·0 to 67·5%)	-0·5% (-5·3 to 4·3%)
Other pharynx cancer	0·1 (0·0 to 0·3)	6·9% (0·0 to 15·5%)	0·2 (0·0 to 0·4)	7·0% (0·0 to 15·5%)	37·2% (0·0 to 59·7%)	0·6% (-2·4 to 3·7%)	3·6 (0·0 to 8·1)	6·9% (0·0 to 15·4%)	5·0 (0·0 to 11·1)	6·9% (0·0 to 15·4%)	37·6% (0·0 to 60·5%)	0·5% (-2·5 to 3·5%)
Oesophageal cancer	6·0 (1·5 to 10·8)	22·2% (5·6 to 39·0%)	7·5 (1·9 to 13·6)	22·0% (5·5 to 38·8%)	24·9% (9·2 to 45·8%)	-0·8% (-4·2 to 2·2%)	156·6 (38·6 to 281·8)	22·1% (5·5 to 38·8%)	194·3 (48·1 to 347·9)	21·9% (5·5 to 38·6%)	24·1% (7·1 to 46·8%)	-1·0% (-4·5 to 2·1%)
Larynx cancer	0·3 (0·0 to 0·6)	7·0% (0·0 to 15·6%)	0·4 (0·0 to 0·8)	7·0% (0·0 to 15·5%)	30·9% (0·0 to 49·1%)	-0·2% (-3·0 to 2·7%)	7·8 (0·0 to 17·3)	7·0% (0·0 to 15·5%)	10·1 (0·0 to 22·7)	7·0% (0·0 to 15·4%)	29·8% (0·0 to 48·1%)	-0·4% (-3·1 to 2·6%)
Tracheal, bronchus, and lung cancer	3·2 (1·3 to 5·5)	12·4% (5·0 to 20·8%)	4·5 (1·8 to 7·7)	12·3% (5·0 to 20·8%)	38·1% (24·3 to 54·0%)	-0·2% (-2·2 to 2·0%)	81·0 (33·2 to 138·4)	12·3% (5·0 to 20·7%)	110·6 (44·3 to 189·8)	12·3% (5·0 to 20·7%)	36·6% (21·6 to 53·8%)	-0·2% (-2·5 to 2·0%)
Ischaemic heart disease	39·8 (14·5 to 67·7)	13·0% (4·7 to 21·8%)	48·9 (17·9 to 83·1)	13·0% (4·7 to 21·6%)	22·7% (8·3 to 38·5%)	-0·4% (-2·9 to 2·4%)	1,022·0 (378·3 to 1,720·5)	14·8% (5·4 to 24·4%)	1,251·1 (460·1 to 2,108·9)	14·9% (5·4 to 24·5%)	22·4% (7·0 to 40·1%)	0·3% (-2·3 to 3·1%)
Ischaemic stroke	27·0 (14·6 to 41·9)	20·4% (11·0 to 31·0%)	31·9 (16·9 to 50·0)	20·0% (10·8 to 30·6%)	18·0% (4·1 to 33·1%)	-2·2% (-5·1 to 1·0%)	649·0 (356·1 to 977·0)	25·4% (14·0 to 37·7%)	765·0 (414·8 to 1,171·8)	25·3% (14·0 to 37·7%)	17·9% (3·8 to 33·2%)	-0·4% (-2·9 to 2·3%)

Supplemental Results Table 1. All-age deaths, death PAFs, DALYs, DALY PAFs, and percent change for each risk factor and outcome for both sexes combined in 2005 and 2015 for Sub-Saharan Africa.

Data in parenthesis are 95% uncertainty intervals. PAFs=population attributable fractions. DALYs=disability-adjusted life-years.

	2005 Deaths (in thousands)	2005 Deaths PAF	2015 Deaths (in thousands)	2015 Deaths PAF	Percent change deaths 2005-2015	Percent change deaths PAF 2005-2015	2005 DALYs (in thousands)	2005 DALYs PAF	2015 DALYs (in thousands)	2015 DALYs PAF	Percent change DALYs 2005-2015	Percent change DALYs PAF 2005-2015
Hemorrhagic stroke	53·2 (29·6 to 80·5)	26·8% (14·8 to 39·7%)	63·4 (33·4 to 97·3)	26·7% (14·6 to 39·6%)	19·1% (4·4 to 35·7%)	-0·4% (-3·3 to 2·3%)	1,630·5 (926·9 to 2,416·5)	28·5% (16·2 to 41·2%)	1,948·7 (1,056·4 to 2,947·9)	28·9% (16·3 to 41·9%)	19·5% (4·9 to 36·6%)	1·4% (-1·6 to 4·5%)
**Diet low in vegetables: All causes**	102·2 (54·3 to 157·0)	1·2% (0·6 to 1·8%)	123·5 (67·1 to 190·9)	1·5% (0·8 to 2·4%)	20·8% (7·5 to 35·8%)	30·9% (22·3 to 40·1%)	2,715·5 (1,471·3 to 4,160·7)	0·5% (0·3 to 0·7%)	3,277·0 (1,775·7 to 5,022·8)	0·6% (0·3 to 1·0%)	20·7% (6·8 to 36·8%)	32·7% (19·4 to 46·8%)
Ischaemic heart disease	58·6 (23·9 to 95·9)	19·1% (7·5 to 31·2%)	71·8 (28·2 to 120·5)	19·1% (7·5 to 31·0%)	22·6% (9·6 to 37·8%)	-0·5% (-1·8 to 1·1%)	1,465·6 (609·4 to 2,394·7)	21·2% (8·5 to 34·3%)	1,790·5 (721·0 to 2,977·9)	21·3% (8·5 to 34·3%)	22·2% (7·9 to 39·0%)	0·2% (-1·5 to 1·9%)
Ischaemic stroke	14·7 (3·1 to 26·5)	11·1% (2·5 to 19·9%)	17·3 (3·6 to 31·9)	10·8% (2·5 to 19·4%)	17·9% (4·2 to 32·5%)	-2·4% (-4·6 to 0·1%)	355·6 (79·1 to 636·9)	13·9% (3·3 to 24·5%)	418·9 (91·4 to 757·0)	13·9% (3·2 to 24·4%)	17·8% (3·6 to 33·3%)	-0·4% (-2·5 to 1·9%)
Hemorrhagic stroke	29·0 (8·5 to 52·5)	14·6% (4·2 to 26·3%)	34·4 (9·9 to 62·4)	14·5% (4·2 to 26·2%)	18·9% (4·8 to 35·1%)	-0·4% (-2·8 to 1·8%)	894·3 (268·4 to 1,604·5)	15·6% (4·6 to 27·8%)	1,067·5 (311·8 to 1,919·9)	15·9% (4·6 to 28·0%)	19·4% (5·0 to 36·7%)	1·5% (-1·2 to 4·3%)
**Diet low in whole grains: All causes**	110·0 (71·8 to 155·6)	1·3% (0·8 to 1·8%)	134·0 (85·6 to 191·7)	1·7% (1·1 to 2·4%)	21·7% (8·6 to 36·8%)	31·8% (22·9 to 41·4%)	3,336·9 (2,218·5 to 4,667·7)	0·6% (0·4 to 0·8%)	4,120·0 (2,709·7 to 5,812·4)	0·8% (0·5 to 1·1%)	23·5% (10·8 to 38·9%)	35·8% (23·8 to 49·0%)
Ischaemic heart disease	43·9 (23·2 to 67·9)	14·4% (7·6 to 22·2%)	54·0 (28·4 to 85·5)	14·3% (7·6 to 22·1%)	23·0% (9·4 to 38·5%)	-0·2% (-1·5 to 1·2%)	1,156·5 (622·0 to 1,757·7)	16·8% (9·0 to 25·6%)	1,421·2 (752·8 to 2,229·2)	16·9% (9·1 to 25·7%)	22·9% (7·9 to 40·2%)	0·6% (-0·7 to 2·1%)
Ischaemic stroke	17·2 (9·8 to 26·0)	13·0% (7·7 to 18·9%)	20·1 (11·9 to 30·9)	12·6% (7·5 to 18·3%)	17·4% (3·6 to 32·7%)	-2·7% (-5·3 to -0·1%)	442·1 (264·4 to 655·8)	17·3% (10·5 to 24·7%)	520·7 (312·9 to 782·9)	17·2% (10·5 to 24·4%)	17·8% (3·9 to 33·8%)	-0·5% (-2·8 to 1·8%)
Hemorrhagic stroke	33·7 (21·3 to 49·1)	16·9% (10·8 to 24·5%)	40·1 (24·9 to 59·7)	16·9% (10·8 to 24·4%)	19·1% (4·6 to 35·5%)	-0·5% (-2·5 to 1·6%)	1,074·0 (686·8 to 1,550·8)	18·8% (12·2 to 26·7%)	1,285·5 (815·4 to 1,889·4)	19·1% (12·4 to 27·2%)	19·7% (4·9 to 36·9%)	1·6% (-0·7 to 4·0%)
Diabetes mellitus	15·3 (7·4 to 25·0)	13·8% (6·9 to 22·4%)	19·7 (9·7 to 32·7)	13·6% (6·8 to 22·1%)	28·8% (14·6 to 45·9%)	-1·6% (-2·9 to -0·3%)	664·3 (333·4 to 1,076·4)	16·0% (8·2 to 25·6%)	892·5 (452·5 to 1,453·3)	16·1% (8·2 to 25·6%)	34·4% (23·4 to 47·0%)	0·3% (-0·5 to 1·1%)
**Diet low in nuts and seeds: All causes**	82·3 (52·4 to 113·8)	0·9% (0·6 to 1·3%)	102·7 (64·1 to 147·1)	1·3% (0·8 to 1·8%)	24·7% (12·0 to 39·1%)	35·0% (25·9 to 43·9%)	2,256·5 (1,494·7 to 3,107·5)	0·4% (0·3 to 0·5%)	2,846·4 (1,866·9 to 3,988·2)	0·5% (0·4 to 0·7%)	26·1% (13·6 to 40·5%)	38·7% (26·5 to 51·6%)
Ischaemic heart disease	69·7 (41·8 to 99·0)	22·8% (14·1 to 32·6%)	86·2 (50·0 to 125·5)	22·9% (14·2 to 32·8%)	23·8% (10·8 to 38·5%)	0·5% (-1·0 to 1·9%)	1,725·8 (1,062·9 to 2,419·8)	25·0% (15·7 to 35·2%)	2,129·5 (1,272·4 to 3,065·7)	25·3% (15·9 to 35·6%)	23·4% (8·9 to 39·6%)	1·1% (-0·6 to 2·8%)
Diabetes mellitus	12·6 (6·3 to 20·0)	11·4% (5·9 to 18·0%)	16·4 (8·4 to 26·4)	11·3% (5·8 to 17·9%)	30·0% (16·0 to 46·5%)	-0·7% (-2·6 to 1·3%)	530·7 (255·4 to 850·4)	12·8% (6·7 to 20·0%)	716·9 (346·9 to 1,143·0)	12·9% (6·7 to 20·2%)	35·1% (24·6 to 46·8%)	0·8% (-0·7 to 2·2%)
**Diet low in milk: All causes**	4·0 (1·4 to 6·8)	0·0% (0·0 to 0·1%)	5·7 (2·0 to 9·6)	0·1% (0·0 to 0·1%)	42·0% (26·0 to 59·5%)	53·8% (43·1 to 66·1%)	101·8 (35·7 to 171·9)	0·0% (0·0 to 0·0%)	143·6 (49·4 to 242·1)	0·0% (0·0 to 0·0%)	41·1% (23·5 to 61·3%)	55·2% (38·1 to 73·5%)
Colon and rectum cancer	4·0 (1·4 to 6·8)	17·0% (6·1 to 28·2%)	5·7 (2·0 to 9·6)	16·9% (6·1 to 28·1%)	42·0% (26·0 to 59·5%)	-0·1% (-0·7 to 0·3%)	101·8 (35·7 to 171·9)	16·5% (5·9 to 27·4%)	143·6 (49·4 to 242·1)	16·5% (5·9 to 27·3%)	41·1% (23·5 to 61·3%)	-0·2% (-1·2 to 0·6%)
**Diet high in red meat: All causes**	1·3 (0·3 to 2·4)	0·0% (0·0 to 0·0%)	1·7 (0·5 to 3·1)	0·0% (0·0 to 0·0%)	30·8% (16·0 to 47·7%)	41·7% (28·5 to 58·3%)	55·5 (11·9 to 103·5)	0·0% (0·0 to 0·0%)	75·1 (16·6 to 139·2)	0·0% (0·0 to 0·0%)	35·3% (23·7 to 48·3%)	48·7% (37·6 to 62·5%)
Colon and rectum cancer	0·2 (0·0 to 0·4)	0·8% (0·2 to 1·5%)	0·3 (0·1 to 0·5)	0·8% (0·2 to 1·6%)	44·2% (26·4 to 60·0%)	1·7% (-6·8 to 10·2%)	5·2 (1·0 to 9·7)	0·8% (0·2 to 1·6%)	7·6 (1·5 to 14·1)	0·9% (0·2 to 1·7%)	45·2% (24·5 to 62·9%)	3·0% (-6·3 to 12·6%)
Diabetes mellitus	1·1 (0·1 to 2·2)	1·0% (0·1 to 2·0%)	1·5 (0·2 to 2·8)	1·0% (0·1 to 2·0%)	28·4% (12·4 to 45·3%)	-1·7% (-9·6 to 6·5%)	50·3 (5·9 to 98·6)	1·2% (0·1 to 2·4%)	67·6 (7·8 to 132·2)	1·2% (0·1 to 2·4%)	34·2% (21·9 to 46·6%)	0·3% (-5·4 to 6·0%)
**Diet high in processed meat: All causes**	26·2 (11·3 to 42·1)	0·3% (0·1 to 0·5%)	33·9 (14·5 to 54·7)	0·4% (0·2 to 0·7%)	29·3% (15·3 to 45·7%)	40·0% (30·1 to 51·1%)	856·1 (423·6 to 1,321·7)	0·1% (0·1 to 0·2%)	1,137·8 (574·5 to 1,751·4)	0·2% (0·1 to 0·3%)	32·9% (19·6 to 47·3%)	46·1% (34·1 to 58·9%)
Colon and rectum cancer	0·9 (0·5 to 1·4)	3·7% (2·0 to 5·6%)	1·3 (0·7 to 2·0)	3·8% (2·1 to 5·8%)	47·3% (30·6 to 66·1%)	3·5% (1·3 to 6·0%)	22·7 (11·9 to 35·2)	3·7% (2·0 to 5·6%)	33·3 (17·8 to 52·0)	3·8% (2·1 to 5·8%)	46·8% (29·2 to 67·9%)	3·7% (1·2 to 6·3%)
Ischaemic heart disease	15·4 (0·6 to 29·2)	5·0% (0·2 to 9·5%)	19·3 (0·8 to 37·3)	5·1% (0·2 to 9·7%)	25·5% (8·2 to 41·2%)	1·9% (0·0 to 4·1%)	395·3 (16·0 to 750·3)	5·7% (0·2 to 10·8%)	495·8 (19·7 to 952·4)	5·9% (0·3 to 11·1%)	25·4% (6·3 to 42·4%)	2·7% (0·1 to 5·1%)
Diabetes mellitus	9·9 (4·9 to 15·0)	9·0% (4·4 to 13·3%)	13·3 (6·5 to 20·2)	9·1% (4·5 to 13·5%)	33·6% (18·9 to 51·5%)	2·0% (-0·9 to 5·3%)	438·1 (207·0 to 668·5)	10·6% (5·1 to 15·6%)	608·8 (285·8 to 930·0)	11·0% (5·4 to 16·2%)	39·0% (28·1 to 52·2%)	3·7% (1·5 to 6·1%)
**Diet high in sugar-sweetened beverages: All causes**	2·3 (1·6 to 3·3)	0·0% (0·0 to 0·0%)	2·9 (2·1 to 4·1)	0·0% (0·0 to 0·1%)	26·2% (12·5 to 42·6%)	36·7% (27·3 to 46·8%)	89·8 (62·4 to 127·3)	0·0% (0·0 to 0·0%)	116·7 (81·5 to 160·7)	0·0% (0·0 to 0·0%)	30·0% (17·5 to 44·7%)	42·9% (31·7 to 54·6%)
Oesophageal cancer	0·0 (0·0 to 0·1)	0·2% (0·1 to 0·3%)	0·1 (0·0 to 0·1)	0·2% (0·1 to 0·3%)	28·9% (8·8 to 54·0%)	2·5% (-10·3 to 13·9%)	1·5 (0·5 to 2·6)	0·2% (0·1 to 0·4%)	1·9 (0·6 to 3·4)	0·2% (0·1 to 0·4%)	28·8% (7·7 to 55·8%)	2·9% (-10·1 to 14·1%)
Colon and rectum cancer	0·0 (0·0 to 0·0)	0·1% (0·0 to 0·1%)	0·0 (0·0 to 0·0)	0·1% (0·0 to 0·1%)	52·6% (32·9 to 76·6%)	7·3% (0·5 to 15·4%)	0·4 (0·3 to 0·6)	0·1% (0·0 to 0·1%)	0·7 (0·4 to 1·0)	0·1% (0·1 to 0·1%)	52·4% (31·4 to 78·4%)	7·8% (0·3 to 15·9%)
Liver cancer due to hepatitis B	0·0 (0·0 to 0·0)	0·2% (0·1 to 0·3%)	0·0 (0·0 to 0·1)	0·2% (0·1 to 0·3%)	33·5% (10·7 to 64·0%)	6·4% (-0·1 to 14·9%)	1·1 (0·5 to 2·0)	0·2% (0·1 to 0·3%)	1·5 (0·6 to 2·7)	0·2% (0·1 to 0·3%)	34·2% (10·2 to 66·7%)	6·2% (-0·3 to 14·3%)
Liver cancer due to hepatitis C	0·0 (0·0 to 0·0)	0·1% (0·1 to 0·2%)	0·0 (0·0 to 0·0)	0·1% (0·1 to 0·2%)	34·5% (13·9 to 58·8%)	5·8% (-0·7 to 15·8%)	0·3 (0·2 to 0·6)	0·1% (0·1 to 0·2%)	0·5 (0·2 to 0·8)	0·1% (0·1 to 0·2%)	35·2% (14·1 to 60·2%)	6·2% (-0·5 to 16·4%)
Liver cancer due to alcohol use	0·0 (0·0 to 0·0)	0·1% (0·1 to 0·2%)	0·0 (0·0 to 0·0)	0·1% (0·1 to 0·2%)	32·0% (8·1 to 61·1%)	5·7% (-1·0 to 13·9%)	0·7 (0·3 to 1·1)	0·2% (0·1 to 0·3%)	0·9 (0·4 to 1·6)	0·2% (0·1 to 0·3%)	32·9% (7·0 to 64·5%)	5·9% (-0·8 to 14·0%)
Liver cancer due to other causes	0·0 (0·0 to 0·0)	0·1% (0·1 to 0·2%)	0·0 (0·0 to 0·0)	0·1% (0·1 to 0·2%)	32·3% (14·1 to 54·1%)	7·1% (0·4 to 16·4%)	0·3 (0·2 to 0·6)	0·1% (0·1 to 0·2%)	0·4 (0·2 to 0·8)	0·1% (0·1 to 0·2%)	33·5% (14·2 to 57·6%)	7·4% (0·5 to 16·8%)
Gallbladder and biliary tract cancer	0·0 (0·0 to 0·0)	0·1% (0·1 to 0·2%)	0·0 (0·0 to 0·0)	0·1% (0·1 to 0·2%)	17·8% (-2·4 to 46·6%)	1·5% (-3·9 to 6·5%)	0·2 (0·1 to 0·3)	0·2% (0·1 to 0·2%)	0·2 (0·1 to 0·4)	0·2% (0·1 to 0·2%)	17·8% (-3·0 to 47·9%)	2·3% (-3·1 to 7·3%)
Pancreatic cancer	0·0 (0·0 to 0·0)	0·0% (0·0 to 0·1%)	0·0 (0·0 to 0·0)	0·0% (0·0 to 0·1%)	51·7% (31·7 to 76·3%)	3·9% (-3·9 to 10·0%)	0·2 (0·1 to 0·3)	0·0% (0·0 to 0·1%)	0·3 (0·1 to 0·5)	0·1% (0·0 to 0·1%)	53·1% (32·2 to 79·6%)	4·2% (-4·3 to 10·4%)
Breast cancer	0·0 (0·0 to 0·0)	0·0% (0·0 to 0·0%)	0·0 (0·0 to 0·0)	0·0% (0·0 to 0·0%)	38·9% (13·5 to 68·7%)	2·9% (-5·0 to 10·8%)	0·2 (0·1 to 0·3)	0·0% (0·0 to 0·0%)	0·3 (0·2 to 0·5)	0·0% (0·0 to 0·0%)	41·0% (15·6 to 70·1%)	3·5% (-5·6 to 12·5%)
Uterine cancer	0·0 (0·0 to 0·0)	0·3% (0·2 to 0·3%)	0·0 (0·0 to 0·0)	0·3% (0·2 to 0·4%)	22·7% (0·1 to 53·0%)	4·8% (0·6 to 9·8%)	0·5 (0·3 to 0·7)	0·3% (0·2 to 0·4%)	0·6 (0·4 to 0·9)	0·3% (0·2 to 0·4%)	23·0% (-0·4 to 55·2%)	5·8% (1·1 to 11·2%)
Ovarian cancer	0·0 (0·0 to 0·0)	0·0% (0·0 to 0·0%)	0·0 (0·0 to 0·0)	0·0% (0·0 to 0·1%)	48·4% (0·0 to 80·8%)	4·8% (0·0 to 9·9%)	0·1 (0·0 to 0·1)	0·0% (0·0 to 0·1%)	0·1 (0·0 to 0·2)	0·0% (0·0 to 0·1%)	49·7% (0·0 to 84·3%)	5·3% (0·0 to 10·7%)
Kidney cancer	0·0 (0·0 to 0·0)	0·1% (0·1 to 0·2%)	0·0 (0·0 to 0·0)	0·1% (0·1 to 0·2%)	48·8% (29·8 to 73·8%)	2·8% (-10·2 to 21·3%)	0·2 (0·1 to 0·2)	0·1% (0·0 to 0·1%)	0·2 (0·2 to 0·3)	0·1% (0·0 to 0·1%)	49·1% (29·2 to 73·2%)	2·6% (-17·9 to 35·3%)
Thyroid cancer	0·0 (0·0 to 0·0)	0·1% (0·0 to 0·1%)	0·0 (0·0 to 0·0)	0·1% (0·0 to 0·1%)	33·4% (14·5 to 54·7%)	5·0% (-0·4 to 12·2%)	0·0 (0·0 to 0·0)	0·1% (0·1 to 0·1%)	0·0 (0·0 to 0·1)	0·1% (0·1 to 0·1%)	37·4% (18·8 to 58·2%)	4·4% (-1·1 to 11·1%)
Ischaemic heart disease	0·5 (0·3 to 0·7)	0·2% (0·1 to 0·2%)	0·6 (0·4 to 0·9)	0·2% (0·1 to 0·2%)	26·4% (11·4 to 43·7%)	2·7% (-1·4 to 7·6%)	16·0 (11·1 to 22·9)	0·2% (0·2 to 0·3%)	20·4 (13·9 to 28·6)	0·2% (0·2 to 0·3%)	27·6% (11·9 to 45·6%)	4·7% (0·8 to 9·1%)
Ischaemic stroke	0·2 (0·1 to 0·2)	0·1% (0·1 to 0·2%)	0·2 (0·1 to 0·3)	0·1% (0·1 to 0·2%)	20·8% (6·1 to 37·6%)	0·1% (-4·3 to 5·4%)	4·7 (3·2 to 6·7)	0·2% (0·1 to 0·3%)	5·8 (3·9 to 8·1)	0·2% (0·1 to 0·3%)	22·4% (8·3 to 39·6%)	3·4% (-0·7 to 7·8%)
Hemorrhagic stroke	0·6 (0·4 to 0·8)	0·3% (0·2 to 0·4%)	0·7 (0·5 to 1·0)	0·3% (0·2 to 0·4%)	23·7% (7·9 to 41·7%)	3·4% (-0·9 to 8·3%)	21·2 (14·5 to 30·0)	0·4% (0·3 to 0·5%)	26·5 (18·0 to 37·8)	0·4% (0·3 to 0·6%)	24·8% (9·2 to 43·1%)	6·0% (1·8 to 10·8%)
Hypertensive heart disease	0·3 (0·2 to 0·4)	0·3% (0·2 to 0·5%)	0·3 (0·2 to 0·5)	0·4% (0·2 to 0·5%)	21·1% (5·9 to 39·5%)	1·4% (-3·1 to 6·4%)	8·1 (5·2 to 12·0)	0·5% (0·3 to 0·6%)	9·8 (6·3 to 14·2)	0·5% (0·3 to 0·7%)	20·9% (5·3 to 40·0%)	3·3% (-0·3 to 7·6%)
Cardiomyopathy and myocarditis	-	-	-	-	-	-	-	-	-	-	-	-
Atrial fibrillation and flutter	-	-	-	-	-	-	-	-	-	-	-	-
Peripheral vascular disease	-	-	-	-	-	-	-	-	-	-	-	-

Supplemental Results Table 1. All-age deaths, death PAFs, DALYs, DALY PAFs, and percent change for each risk factor and outcome for both sexes combined in 2005 and 2015 for Sub-Saharan Africa.

Data in parenthesis are 95% uncertainty intervals. PAFs=population attributable fractions. DALYs=disability-adjusted life-years.

	2005 Deaths (in thousands)	2005 Deaths PAF	2015 Deaths (in thousands)	2015 Deaths PAF	Percent change deaths 2005-2015	Percent change deaths PAF 2005-2015	2005 DALYs (in thousands)	2005 DALYs PAF	2015 DALYs (in thousands)	2015 DALYs PAF	Percent change DALYs 2005-2015	Percent change DALYs PAF 2005-2015
Endocarditis	-	-	-	-	-	-	-	-	-	-	-	-
Other cardiovascular and circulatory diseases	-	-	-	-	-	-	-	-	-	-	-	-
Diabetes mellitus	0·5 (0·4 to 0·7)	0·5% (0·3 to 0·7%)	0·7 (0·5 to 0·9)	0·5% (0·3 to 0·6%)	29·7% (14·4 to 47·8%)	-0·9% (-4·5 to 3·1%)	26·3 (17·8 to 37·7)	0·6% (0·4 to 0·9%)	36·1 (24·6 to 51·6)	0·6% (0·5 to 0·9%)	37·3% (26·2 to 49·4%)	2·5% (-0·2 to 5·3%)
Chronic kidney disease due to diabetes mellitus	0·0 (0·0 to 0·0)	0·1% (0·1 to 0·3%)	0·0 (0·0 to 0·0)	0·2% (0·1 to 0·3%)	44·5% (24·9 to 66·3%)	8·7% (1·5 to 16·2%)	0·6 (0·2 to 1·2)	0·2% (0·1 to 0·3%)	0·9 (0·3 to 1·7)	0·2% (0·1 to 0·3%)	40·3% (29·3 to 52·4%)	10·2% (4·4 to 16·9%)
Chronic kidney disease due to hypertension	0·1 (0·0 to 0·1)	0·3% (0·1 to 0·4%)	0·1 (0·0 to 0·2)	0·3% (0·1 to 0·5%)	33·0% (18·6 to 50·9%)	3·5% (-0·5 to 7·9%)	2·3 (0·9 to 4·2)	0·2% (0·1 to 0·4%)	3·1 (1·3 to 5·6)	0·3% (0·1 to 0·5%)	32·9% (18·6 to 50·8%)	5·3% (0·5 to 10·6%)
Chronic kidney disease due to glomerulonephritis	0·0 (0·0 to 0·0)	0·2% (0·1 to 0·3%)	0·0 (0·0 to 0·0)	0·2% (0·1 to 0·3%)	8·5% (-11·3 to 27·8%)	-4·3% (-12·4 to 3·9%)	0·9 (0·2 to 1·7)	0·2% (0·0 to 0·3%)	1·1 (0·3 to 2·1)	0·2% (0·0 to 0·3%)	20·1% (5·6 to 32·6%)	1·7% (-5·5 to 8·1%)
Chronic kidney disease due to other causes	0·0 (0·0 to 0·0)	0·1% (0·0 to 0·2%)	0·0 (0·0 to 0·0)	0·1% (0·0 to 0·2%)	39·2% (13·9 to 67·8%)	10·2% (-2·0 to 23·0%)	0·4 (0·1 to 0·7)	0·2% (0·1 to 0·3%)	0·5 (0·2 to 1·0)	0·2% (0·1 to 0·3%)	37·5% (30·5 to 44·4%)	8·3% (3·0 to 13·4%)
**Diet low in fibre: All causes**	3·5 (1·1 to 7·3)	0·0% (0·0 to 0·1%)	4·5 (1·4 to 9·4)	0·1% (0·0 to 0·1%)	29·2% (13·8 to 46·5%)	40·0% (25·1 to 56·4%)	99·4 (31·6 to 207·5)	0·0% (0·0 to 0·0%)	127·9 (40·5 to 270·9)	0·0% (0·0 to 0·1%)	28·6% (10·4 to 49·0%)	41·4% (22·7 to 62·8%)
Colon and rectum cancer	0·3 (0·1 to 0·6)	1·2% (0·3 to 2·6%)	0·4 (0·1 to 0·9)	1·3% (0·4 to 2·8%)	47·8% (31·9 to 67·0%)	4·1% (-7·8 to 15·3%)	7·3 (2·1 to 16·0)	1·2% (0·3 to 2·6%)	10·8 (3·1 to 23·6)	1·2% (0·4 to 2·8%)	47·4% (28·9 to 68·5%)	4·3% (-8·5 to 16·9%)
Ischaemic heart disease	3·2 (1·0 to 6·8)	1·1% (0·3 to 2·2%)	4·1 (1·3 to 8·7)	1·1% (0·3 to 2·3%)	27·6% (12·5 to 45·2%)	3·7% (-8·2 to 15·6%)	92·1 (28·7 to 195·1)	1·3% (0·4 to 2·8%)	117·1 (36·0 to 251·2)	1·4% (0·4 to 2·9%)	27·1% (9·2 to 47·9%)	4·3% (-9·0 to 18·4%)
**Diet low in calcium: All causes**	5·7 (3·7 to 8·0)	0·1% (0·0 to 0·1%)	8·1 (5·3 to 11·4)	0·1% (0·1 to 0·1%)	41·8% (26·0 to 59·6%)	53·5% (42·7 to 65·6%)	143·8 (92·9 to 201·7)	0·0% (0·0 to 0·0%)	202·4 (132·1 to 283·6)	0·0% (0·0 to 0·1%)	40·7% (23·5 to 60·9%)	54·7% (38·0 to 72·8%)
Colon and rectum cancer	5·7 (3·7 to 8·0)	24·1% (15·8 to 33·0%)	8·1 (5·3 to 11·4)	24·0% (15·7 to 33·0%)	41·8% (26·0 to 59·6%)	-0·2% (-1·3 to 0·6%)	143·8 (92·9 to 201·7)	23·3% (15·2 to 32·0%)	202·4 (132·1 to 283·6)	23·2% (15·2 to 31·8%)	40·7% (23·5 to 60·9%)	-0·4% (-1·7 to 0·6%)
Prostate cancer	-	-	-	-	-	-	-	-	-	-	-	-
**Diet low in seafood omega-3 fatty acids: All causes**	59·2 (25·8 to 95·3)	0·7% (0·3 to 1·1%)	72·6 (31·2 to 118·5)	0·9% (0·4 to 1·5%)	22·7% (8·8 to 38·6%)	32·8% (22·7 to 43·5%)	1,446·0 (633·3 to 2,306·2)	0·2% (0·1 to 0·4%)	1,766·3 (772·0 to 2,857·7)	0·3% (0·1 to 0·5%)	22·2% (6·4 to 40·5%)	34·3% (19·2 to 50·2%)
Ischaemic heart disease	59·2 (25·8 to 95·3)	19·3% (8·3 to 30·8%)	72·6 (31·2 to 118·5)	19·3% (8·3 to 30·7%)	22·7% (8·8 to 38·6%)	-0·4% (-2·0 to 1·2%)	1,446·0 (633·3 to 2,306·2)	21·0% (9·1 to 32·9%)	1,766·3 (772·0 to 2,857·7)	21·0% (9·1 to 33·0%)	22·2% (6·4 to 40·5%)	0·1% (-1·8 to 2·0%)
**Diet low in polyunsaturated fatty acids: All causes**	12·6 (5·3 to 19·9)	0·1% (0·1 to 0·2%)	14·4 (5·9 to 23·6)	0·2% (0·1 to 0·3%)	14·3% (-4·7 to 40·0%)	23·7% (5·1 to 46·9%)	344·0 (149·6 to 538·4)	0·1% (0·0 to 0·1%)	396·8 (166·3 to 634·8)	0·1% (0·0 to 0·1%)	15·3% (-4·5 to 41·5%)	26·8% (6·6 to 53·7%)
Ischaemic heart disease	12·6 (5·3 to 19·9)	4·1% (1·8 to 6·6%)	14·4 (5·9 to 23·6)	3·8% (1·6 to 6·1%)	14·3% (-4·7 to 40·0%)	-7·2% (-20·2 to 8·4%)	344·0 (149·6 to 538·4)	5·0% (2·2 to 7·8%)	396·8 (166·3 to 634·8)	4·7% (2·1 to 7·5%)	15·3% (-4·5 to 41·5%)	-5·4% (-18·7 to 9·8%)
**Diet high in trans fatty acids: All causes**	13·6 (4·4 to 28·1)	0·2% (0·1 to 0·3%)	16·7 (5·2 to 35·0)	0·2% (0·1 to 0·4%)	22·7% (7·9 to 40·1%)	32·7% (20·3 to 45·0%)	395·6 (135·6 to 792·6)	0·1% (0·0 to 0·1%)	487·2 (160·9 to 997·7)	0·1% (0·0 to 0·2%)	23·2% (7·2 to 40·9%)	35·4% (19·5 to 52·1%)
Ischaemic heart disease	13·6 (4·4 to 28·1)	4·4% (1·4 to 8·9%)	16·7 (5·2 to 35·0)	4·4% (1·4 to 8·9%)	22·7% (7·9 to 40·1%)	-0·5% (-5·7 to 4·4%)	395·6 (135·6 to 792·6)	5·7% (2·0 to 11·2%)	487·2 (160·9 to 997·7)	5·8% (1·9 to 11·3%)	23·2% (7·2 to 40·9%)	0·9% (-4·3 to 6·1%)
**Diet high in sodium: All causes**	80·0 (19·8 to 195·2)	0·9% (0·2 to 2·2%)	101·0 (25·2 to 238·9)	1·3% (0·3 to 3·0%)	26·3% (8·0 to 60·2%)	36·7% (21·7 to 71·3%)	2,040·4 (518·9 to 4,950·6)	0·4% (0·1 to 0·8%)	2,558·2 (651·1 to 6,152·7)	0·5% (0·1 to 1·1%)	25·4% (7·0 to 58·0%)	37·8% (20·1 to 72·3%)
Stomach cancer	4·4 (1·1 to 10·2)	13·7% (3·4 to 32·3%)	5·4 (1·4 to 12·7)	13·7% (3·5 to 32·0%)	23·6% (4·9 to 49·2%)	0·1% (-9·5 to 16·5%)	110·1 (27·8 to 260·7)	13·4% (3·3 to 31·5%)	134·3 (34·1 to 318·7)	13·4% (3·4 to 31·5%)	22·0% (2·8 to 48·9%)	-0·1% (-10·4 to 16·6%)
Rheumatic heart disease	0·6 (0·1 to 1·6)	3·2% (0·6 to 8·8%)	0·7 (0·1 to 1·8)	3·4% (0·7 to 9·2%)	14·4% (-5·4 to 50·2%)	5·2% (-6·9 to 33·2%)	23·7 (4·4 to 65·7)	2·8% (0·5 to 7·6%)	27·8 (5·2 to 79·2)	3·0% (0·6 to 8·2%)	17·7% (1·8 to 46·2%)	6·5% (-2·6 to 29·6%)
Ischaemic heart disease	26·3 (6·5 to 66·5)	8·6% (2·1 to 21·4%)	33·7 (8·5 to 82·9)	8·9% (2·3 to 21·9%)	28·1% (9·2 to 63·6%)	4·0% (-6·3 to 28·4%)	623·2 (158·5 to 1,552·2)	9·0% (2·4 to 22·2%)	792·5 (204·0 to 1,909·9)	9·4% (2·5 to 22·4%)	27·2% (6·7 to 63·0%)	4·2% (-6·4 to 29·2%)
Ischaemic stroke	9·8 (2·2 to 25·0)	7·4% (1·7 to 18·7%)	12·2 (2·8 to 30·5)	7·6% (1·8 to 18·8%)	24·1% (5·8 to 56·5%)	2·7% (-7·3 to 25·8%)	208·7 (48·1 to 526·9)	8·2% (1·9 to 20·2%)	255·6 (58·8 to 637·2)	8·4% (2·0 to 20·6%)	22·5% (4·3 to 52·2%)	3·4% (-6·4 to 25·4%)
Hemorrhagic stroke	18·3 (4·1 to 45·9)	9·2% (2·1 to 23·4%)	22·7 (5·2 to 57·4)	9·5% (2·2 to 24·2%)	24·1% (5·0 to 56·6%)	3·7% (-6·8 to 26·5%)	507·7 (113·9 to 1,288·3)	8·9% (2·0 to 22·4%)	627·0 (142·1 to 1,546·7)	9·3% (2·1 to 23·3%)	23·5% (4·6 to 56·6%)	4·8% (-6·3 to 28·5%)
Hypertensive heart disease	12·0 (2·2 to 35·5)	15·4% (3·0 to 44·9%)	15·0 (2·9 to 44·5)	15·9% (3·3 to 45·4%)	24·4% (3·8 to 63·3%)	3·7% (-7·0 to 32·0%)	285·9 (62·0 to 797·0)	16·1% (3·5 to 43·9%)	349·3 (75·0 to 973·0)	16·7% (4·0 to 44·3%)	22·2% (1·7 to 61·1%)	3·9% (-6·7 to 32·0%)
Cardiomyopathy and myocarditis	0·9 (0·2 to 2·2)	4·5% (1·0 to 11·9%)	1·1 (0·2 to 2·8)	4·8% (1·1 to 12·4%)	25·4% (6·4 to 63·9%)	7·3% (-2·2 to 35·3%)	26·3 (5·2 to 71·9)	3·7% (0·8 to 10·2%)	32·9 (6·8 to 88·6)	4·1% (0·9 to 10·9%)	24·9% (5·3 to 61·2%)	9·1% (-2·1 to 37·1%)
Atrial fibrillation and flutter	0·1 (0·0 to 0·3)	4·8% (1·2 to 12·6%)	0·2 (0·0 to 0·4)	4·9% (1·2 to 12·6%)	40·2% (21·4 to 73·8%)	2·4% (-7·8 to 22·4%)	5·3 (1·3 to 13·2)	6·5% (1·7 to 16·2%)	7·2 (1·8 to 17·8)	6·6% (1·8 to 16·2%)	35·5% (28·1 to 47·2%)	1·2% (-2·6 to 9·3%)
Aortic aneurysm	0·3 (0·1 to 0·8)	5·7% (1·4 to 14·2%)	0·4 (0·1 to 1·1)	6·1% (1·6 to 14·7%)	45·7% (17·6 to 99·1%)	6·0% (-6·1 to 37·7%)	7·4 (1·7 to 18·8)	6·0% (1·5 to 14·9%)	10·8 (2·6 to 26·3)	6·3% (1·6 to 15·2%)	45·7% (15·2 to 102·3%)	6·1% (-7·7 to 40·2%)
Peripheral vascular disease	0·0 (0·0 to 0·1)	3·3% (0·7 to 9·1%)	0·1 (0·0 to 0·1)	3·5% (0·7 to 9·4%)	65·1% (42·2 to 103·0%)	4·4% (-3·8 to 25·7%)	1·6 (0·3 to 4·8)	3·9% (0·9 to 10·4%)	2·4 (0·5 to 6·7)	4·0% (0·9 to 10·4%)	44·6% (36·0 to 61·1%)	2·0% (-1·7 to 10·1%)
Endocarditis	0·3 (0·1 to 0·9)	4·1% (0·9 to 11·0%)	0·5 (0·1 to 1·3)	4·4% (1·0 to 11·7%)	42·4% (15·9 to 89·8%)	8·0% (-6·8 to 42·1%)	10·4 (2·1 to 29·0)	3·0% (0·6 to 8·7%)	14·8 (2·9 to 41·8)	3·4% (0·7 to 9·2%)	42·9% (13·7 to 93·0%)	11·2% (-7·5 to 48·9%)
Other cardiovascular and circulatory diseases	4·7 (1·2 to 11·3)	6·1% (1·5 to 14·7%)	6·2 (1·6 to 15·0)	6·4% (1·7 to 15·4%)	33·6% (8·5 to 78·6%)	6·4% (-7·1 to 36·0%)	154·0 (38·8 to 385·1)	5·1% (1·3 to 12·4%)	205·1 (49·1 to 493·2)	5·5% (1·4 to 13·0%)	33·1% (13·1 to 71·0%)	7·7% (-4·1 to 33·6%)
Chronic kidney disease due to diabetes mellitus	0·3 (0·1 to 0·8)	4·9% (1·2 to 12·2%)	0·5 (0·1 to 1·2)	5·4% (1·3 to 13·2%)	46·0% (20·5 to 87·8%)	9·7% (-5·2 to 37·6%)	13·9 (3·4 to 35·9)	3·3% (0·8 to 8·6%)	19·2 (4·6 to 49·8)	3·6% (0·9 to 9·2%)	38·1% (23·0 to 63·4%)	8·5% (-1·8 to 26·8%)
Chronic kidney disease due to hypertension	1·5 (0·3 to 4·1)	5·2% (1·0 to 14·2%)	1·9 (0·4 to 5·4)	5·3% (1·0 to 14·5%)	31·6% (17·2 to 53·5%)	2·4% (-5·4 to 17·7%)	37·9 (7·2 to 107·1)	4·0% (0·8 to 11·0%)	49·2 (9·3 to 137·3)	4·1% (0·8 to 11·3%)	29·9% (15·6 to 50·8%)	2·9% (-5·7 to 17·3%)
Chronic kidney disease due to glomerulonephritis	0·4 (0·1 to 1·1)	4·4% (0·9 to 11·7%)	0·5 (0·1 to 1·2)	4·5% (1·0 to 11·5%)	13·8% (-6·5 to 43·5%)	0·7% (-8·8 to 20·2%)	16·5 (3·5 to 43·4)	3·1% (0·7 to 8·0%)	20·0 (4·3 to 52·6)	3·1% (0·7 to 8·2%)	20·9% (5·8 to 42·8%)	2·5% (-5·0 to 18·0%)
Chronic kidney disease due to other causes	0·1 (0·0 to 0·2)	3·3% (0·8 to 8·6%)	0·1 (0·0 to 0·2)	3·8% (0·9 to 9·7%)	42·1% (7·3 to 104·9%)	12·3% (-10·4 to 53·9%)	7·6 (1·7 to 19·7)	3·4% (0·8 to 8·9%)	10·3 (2·3 to 26·2)	3·6% (0·9 to 9·3%)	34·7% (26·8 to 50·3%)	6·1% (0·8 to 16·7%)
**Sexual abuse and violence: All causes**	110·0 (66·2 to 152·7)	1·3% (0·8 to 1·8%)	68·8 (45·3 to 91·5)	0·9% (0·6 to 1·1%)	-37·5% (-44·0 to -27·1%)	-32·2% (-39·2 to -21·9%)	6,327·2 (4,177·8 to 8,470·0)	1·1% (0·7 to 1·5%)	4,697·7 (3,346·0 to 6,083·2)	0·9% (0·6 to 1·1%)	-25·8% (-33·8 to -13·8%)	-18·4% (-27·1 to -6·2%)
**Childhood sexual abuse: All causes**	9·7 (2·8 to 18·0)	0·1% (0·0 to 0·2%)	12·0 (3·2 to 22·9)	0·1% (0·0 to 0·3%)	23·6% (4·5 to 44·1%)	33·8% (17·0 to 49·8%)	862·0 (359·5 to 1,451·4)	0·1% (0·1 to 0·2%)	1,075·8 (454·3 to 1,830·0)	0·2% (0·1 to 0·3%)	24·8% (13·7 to 36·9%)	37·2% (26·2 to 48·0%)
Alcohol use disorders	0·6 (0·0 to 1·3)	6·7% (0·0 to 15·1%)	0·6 (0·0 to 1·5)	6·3% (0·0 to 14·2%)	11·7% (-6·5 to 36·0%)	-6·0% (-10·6 to 0·0%)	54·2 (0·0 to 121·2)	7·2% (0·0 to 15·9%)	65·1 (0·0 to 147·1)	6·7% (0·0 to 15·1%)	20·0% (0·0 to 30·9%)	-5·9% (-9·1 to 0·0%)
Major depressive disorder	-	-	-	-	-	-	342·3 (39·1 to 738·6)	7·6% (0·9 to 15·7%)	430·9 (49·1 to 940·8)	7·2% (0·9 to 15·0%)	25·9% (22·2 to 29·0%)	-5·3% (-7·5 to -3·2%)
Dysthymia	-	-	-	-	-	-	55·0 (6·0 to 120·5)	7·5% (0·9 to 15·5%)	70·1 (7·6 to 154·2)	7·1% (0·9 to 14·7%)	27·5% (24·2 to 30·4%)	-5·0% (-7·1 to -2·9%)
Self-harm	9·1 (2·3 to 17·5)	17·7% (4·4 to 34·2%)	11·3 (2·7 to 22·2)	16·9% (4·1 to 32·9%)	24·3% (5·2 to 45·6%)	-4·7% (-8·1 to -1·8%)	410·5 (103·1 to 789·2)	18·1% (4·5 to 34·8%)	509·7 (123·1 to 993·4)	17·3% (4·3 to 33·5%)	24·2% (4·5 to 46·0%)	-4·5% (-8·1 to -1·5%)
**Intimate partner violence: All causes**	101·6 (59·2 to 144·4)	1·2% (0·7 to 1·6%)	58·2 (36·2 to 79·8)	0·7% (0·4 to 1·0%)	-42·7% (-47·3 to -35·9%)	-37·9% (-43·0 to -31·0%)	5,560·5 (3,452·6 to 7,691·2)	1·0% (0·6 to 1·3%)	3,738·1 (2,549·8 to 4,921·1)	0·7% (0·5 to 0·9%)	-32·8% (-38·9 to -22·9%)	-26·1% (-32·7 to -16·4%)

Supplemental Results Table 1. All-age deaths, death PAFs, DALYs, DALY PAFs, and percent change for each risk factor and outcome for both sexes combined in 2005 and 2015 for Sub-Saharan Africa.

Data in parenthesis are 95% uncertainty intervals. PAFs=population attributable fractions. DALYs=disability-adjusted life-years.

	2005 Deaths (in thousands)	2005 Deaths PAF	2015 Deaths (in thousands)	2015 Deaths PAF	Percent change deaths 2005-2015	Percent change deaths PAF 2005-2015	2005 DALYs (in thousands)	2005 DALYs PAF	2015 DALYs (in thousands)	2015 DALYs PAF	Percent change DALYs 2005-2015	Percent change DALYs PAF 2005-2015
HIV/AIDS - Tuberculosis	16·4 (8·7 to 25·2)	6·0% (3·2 to 8·7%)	7·9 (4·0 to 12·4)	5·0% (2·6 to 7·4%)	-51·7% (-56·7 to -45·7%)	-16·0% (-20·7 to -11·2%)	754·1 (397·8 to 1,165·0)	4·9% (2·6 to 7·2%)	384·6 (193·8 to 599·4)	4·3% (2·2 to 6·3%)	-49·0% (-53·6 to -43·3%)	-12·3% (-17·1 to -7·5%)
HIV/AIDS resulting in other diseases	73·6 (38·1 to 108·1)	6·4% (3·4 to 9·5%)	37·9 (19·5 to 55·9)	5·4% (2·8 to 8·0%)	-48·5% (-53·2 to -43·3%)	-15·1% (-21·2 to -8·4%)	3,684·2 (1,924·3 to 5,493·3)	5·7% (3·0 to 8·4%)	2,007·1 (1,015·6 to 2,943·4)	4·9% (2·5 to 7·2%)	-45·5% (-50·4 to -39·9%)	-14·1% (-19·8 to -7·7%)
Maternal abortion, miscarriage, and ectopic pregnancy	0·0 (0·0 to 0·0)	0·0% (0·0 to 0·0%)	0·0 (0·0 to 0·0)	0·0% (0·0 to 0·0%)	-7·4% (-32·2 to 24·5%)	0·2% (-14·9 to 16·2%)	0·0 (0·0 to 0·0)	0·0% (0·0 to 0·0%)	0·0 (0·0 to 0·0)	0·0% (0·0 to 0·0%)	-8·2% (-31·9 to 22·5%)	0·1% (-13·6 to 17·6%)
Major depressive disorder	-	-	-	-	-	-	486·8 (311·5 to 698·2)	10·8% (8·8 to 12·7%)	657·4 (418·8 to 941·2)	10·9% (8·9 to 12·9%)	35·0% (31·7 to 38·4%)	1·5% (-0·5 to 3·6%)
Dysthymia	-	-	-	-	-	-	86·9 (55·7 to 129·1)	11·8% (9·8 to 13·9%)	118·8 (75·7 to 176·2)	12·0% (9·9 to 14·2%)	36·7% (33·9 to 39·6%)	1·8% (-0·1 to 3·8%)
Self-harm	7·0 (5·3 to 9·1)	13·6% (10·6 to 16·9%)	8·0 (5·8 to 10·5)	12·0% (8·7 to 15·4%)	14·7% (-9·7 to 50·4%)	-11·8% (-28·7 to 10·7%)	273·3 (203·0 to 363·5)	12·1% (9·2 to 15·2%)	311·3 (225·6 to 422·2)	10·6% (7·7 to 13·8%)	13·9% (-10·9 to 51·0%)	-12·1% (-30·3 to 11·5%)
Assault by firearm	0·8 (0·5 to 1·0)	6·5% (4·4 to 8·3%)	0·7 (0·5 to 1·0)	5·2% (3·7 to 7·4%)	-10·1% (-34·4 to 32·2%)	-20·5% (-40·7 to 14·5%)	43·6 (28·9 to 54·7)	6·4% (4·3 to 8·2%)	39·3 (28·5 to 57·1)	5·1% (3·6 to 7·3%)	-9·9% (-35·5 to 33·1%)	-21·0% (-41·5 to 14·4%)
Assault by sharp object	1·2 (1·0 to 1·5)	8·1% (6·9 to 9·8%)	1·1 (0·9 to 1·4)	6·8% (5·3 to 8·6%)	-8·8% (-29·2 to 19·2%)	-15·5% (-33·8 to 8·1%)	67·3 (55·9 to 83·7)	8·0% (6·8 to 9·7%)	61·2 (48·5 to 76·9)	6·8% (5·2 to 8·5%)	-9·0% (-29·4 to 20·3%)	-15·3% (-34·1 to 9·3%)
Assault by other means	2·6 (2·3 to 3·0)	10·3% (8·6 to 11·8%)	2·6 (2·0 to 3·3)	8·2% (6·3 to 10·1%)	-2·1% (-21·3 to 23·8%)	-20·6% (-36·4 to -4·2%)	164·4 (142·8 to 188·4)	11·0% (9·2 to 12·5%)	158·4 (124·2 to 205·8)	8·8% (6·7 to 10·7%)	-3·6% (-22·0 to 20·9%)	-20·0% (-35·4 to -4·3%)
**Unsafe sex: All causes**	1,487·8 (1,406·0 to 1,569·5)	17·1% (16·1 to 18·2%)	948·5 (889·3 to 1,017·0)	11·8% (11·0 to 12·8%)	-36·3% (-39·3 to -32·9%)	-30·9% (-35·1 to -26·5%)	84,619·4 (79,928·4 to 89,519·7)	14·5% (13·6 to 15·5%)	56,237·8 (52,237·7 to 60,714·2)	10·6% (9·8 to 11·6%)	-33·5% (-36·6 to -30·3%)	-26·9% (-30·6 to -22·9%)
HIV/AIDS - Tuberculosis	266·4 (209·0 to 305·9)	97·0% (96·3 to 97·5%)	153·0 (112·2 to 181·4)	96·9% (96·2 to 97·5%)	-42·6% (-48·4 to -35·7%)	-0·1% (-0·4 to 0·1%)	14,942·0 (11,743·5 to 16,976·8)	97·0% (96·3 to 97·5%)	8,685·1 (6,426·0 to 10,248·5)	96·9% (96·2 to 97·5%)	-41·9% (-47·6 to -35·0%)	-0·1% (-0·4 to 0·1%)
HIV/AIDS resulting in other diseases	1,107·5 (1,028·2 to 1,192·5)	95·9% (95·2 to 96·4%)	669·8 (612·3 to 727·8)	95·5% (94·6 to 96·2%)	-39·5% (-43·4 to -35·5%)	-0·4% (-1·0 to 0·1%)	62,241·7 (58,074·8 to 66,650·4)	95·9% (95·3 to 96·5%)	39,326·4 (36,159·5 to 42,676·0)	95·6% (94·7 to 96·3%)	-36·8% (-40·4 to -33·2%)	-0·3% (-1·0 to 0·1%)
Syphilis	69·6 (40·9 to 107·5)	100·0% (100·0 to 100·0%)	76·1 (43·9 to 120·4)	100·0% (100·0 to 100·0%)	9·3% (-2·9 to 22·2%)	-	5,863·3 (3,414·8 to 9,104·3)	100·0% (100·0 to 100·0%)	6,422·9 (3,704·7 to 10,211·7)	100·0% (100·0 to 100·0%)	9·5% (-2·8 to 22·8%)	-
Chlamydial infection	0·0 (0·0 to 0·1)	100·0% (100·0 to 100·0%)	0·0 (0·0 to 0·1)	100·0% (100·0 to 100·0%)	-3·5% (-26·3 to 29·5%)	-	55·3 (32·3 to 87·5)	100·0% (100·0 to 100·0%)	71·5 (41·3 to 114·1)	100·0% (100·0 to 100·0%)	29·5% (24·6 to 33·7%)	-
Gonococcal infection	0·2 (0·2 to 0·3)	100·0% (100·0 to 100·0%)	0·2 (0·2 to 0·3)	100·0% (100·0 to 100·0%)	-1·1% (-20·8 to 26·2%)	-	85·7 (52·0 to 129·5)	100·0% (100·0 to 100·0%)	114·3 (64·2 to 186·8)	100·0% (100·0 to 100·0%)	33·4% (17·1 to 47·8%)	-
Trichomoniasis	-	-	-	-	-	-	37·3 (15·1 to 80·1)	100·0% (100·0 to 100·0%)	48·9 (19·9 to 104·8)	100·0% (100·0 to 100·0%)	31·2% (28·7 to 33·3%)	-
Genital herpes	-	-	-	-	-	-	29·9 (9·6 to 69·9)	100·0% (100·0 to 100·0%)	38·9 (12·3 to 91·1)	100·0% (100·0 to 100·0%)	30·0% (25·6 to 33·9%)	-
Other sexually transmitted diseases	0·1 (0·1 to 0·2)	100·0% (100·0 to 100·0%)	0·1 (0·1 to 0·2)	100·0% (100·0 to 100·0%)	-1·5% (-20·9 to 27·7%)	-	12·8 (10·2 to 16·0)	100·0% (100·0 to 100·0%)	15·7 (12·1 to 20·0)	100·0% (100·0 to 100·0%)	22·8% (10·0 to 36·0%)	-
Cervical cancer	44·0 (36·8 to 50·4)	100·0% (100·0 to 100·0%)	49·2 (38·7 to 59·9)	100·0% (100·0 to 100·0%)	11·9% (-7·0 to 37·7%)	-	1,351·3 (1,114·9 to 1,566·4)	100·0% (100·0 to 100·0%)	1,514·0 (1,187·9 to 1,852·6)	100·0% (100·0 to 100·0%)	12·0% (-7·5 to 38·6%)	-
**Low physical activity: All causes**	59·2 (45·9 to 73·4)	0·7% (0·5 to 0·8%)	74·2 (57·7 to 93·5)	0·9% (0·7 to 1·1%)	25·5% (13·8 to 40·0%)	35·9% (27·8 to 45·0%)	1,486·8 (1,157·5 to 1,850·7)	0·3% (0·2 to 0·3%)	1,880·8 (1,457·5 to 2,350·6)	0·4% (0·3 to 0·4%)	26·5% (15·1 to 40·6%)	39·1% (28·3 to 50·8%)
Colon and rectum cancer	2·7 (1·8 to 3·6)	11·2% (7·5 to 15·1%)	3·8 (2·5 to 5·2)	11·3% (7·5 to 15·3%)	42·8% (28·2 to 59·3%)	0·4% (-2·6 to 3·2%)	62·5 (40·5 to 85·9)	10·2% (6·7 to 13·8%)	88·6 (56·6 to 122·4)	10·2% (6·7 to 13·9%)	41·7% (25·2 to 60·6%)	0·2% (-3·3 to 3·4%)
Breast cancer	1·8 (1·2 to 2·4)	7·2% (5·1 to 9·2%)	2·4 (1·6 to 3·2)	7·1% (5·1 to 9·3%)	33·5% (10·3 to 61·7%)	-1·0% (-6·9 to 3·6%)	54·1 (38·1 to 73·0)	6·7% (4·7 to 8·7%)	72·7 (49·3 to 99·0)	6·6% (4·7 to 8·6%)	34·4% (10·1 to 63·6%)	-1·2% (-7·5 to 3·6%)
Ischaemic heart disease	25·7 (15·6 to 36·3)	8·4% (5·3 to 11·7%)	31·7 (19·2 to 44·8)	8·4% (5·3 to 11·8%)	23·2% (11·0 to 37·5%)	0·1% (-3·4 to 3·7%)	599·2 (353·7 to 849·0)	8·7% (5·4 to 12·2%)	734·9 (432·0 to 1,060·1)	8·7% (5·3 to 12·4%)	22·6% (9·9 to 38·7%)	0·6% (-3·2 to 4·6%)
Ischaemic stroke	13·5 (7·1 to 20·7)	10·2% (5·1 to 15·5%)	16·2 (8·4 to 25·2)	10·2% (5·2 to 15·4%)	20·3% (8·6 to 34·7%)	-0·2% (-3·3 to 2·9%)	269·5 (139·5 to 416·5)	10·6% (5·2 to 16·1%)	320·2 (161·6 to 503·3)	10·6% (5·3 to 16·1%)	18·8% (6·4 to 33·9%)	0·4% (-2·7 to 3·6%)
Diabetes mellitus	15·5 (11·5 to 19·9)	14·0% (10·6 to 17·7%)	20·2 (14·9 to 26·3)	13·9% (10·5 to 17·5%)	29·8% (17·0 to 46·8%)	-0·8% (-3·9 to 1·8%)	501·6 (370·7 to 658·9)	12·1% (9·0 to 15·4%)	664·5 (478·7 to 887·8)	12·0% (9·0 to 15·2%)	32·5% (22·2 to 45·1%)	-1·1% (-3·4 to 0·9%)
**Metabolic risks: All causes**	802·5 (737·6 to 874·0)	9·2% (8·6 to 9·9%)	992·8 (890·8 to 1,117·7)	12·4% (11·5 to 13·3%)	23·7% (11·7 to 37·9%)	34·0% (26·9 to 42·1%)	22,186·9 (20,345·3 to 24,295·5)	3·8% (3·5 to 4·1%)	27,598·7 (24,663·1 to 31,045·5)	5·2% (4·8 to 5·7%)	24·4% (13·4 to 37·7%)	36·8% (27·1 to 47·7%)
**High fasting plasma glucose: All causes**	221·0 (195·5 to 251·8)	2·5% (2·3 to 2·9%)	287·3 (249·4 to 332·6)	3·6% (3·2 to 4·1%)	30·0% (17·6 to 45·3%)	40·8% (32·5 to 50·7%)	7,173·7 (6,335·5 to 8,115·8)	1·2% (1·1 to 1·4%)	9,416·0 (8,174·8 to 10,840·9)	1·8% (1·6 to 2·0%)	31·3% (20·5 to 43·8%)	44·3% (35·0 to 54·7%)
Tuberculosis	20·1 (11·8 to 29·6)	7·3% (4·6 to 10·4%)	21·5 (12·3 to 33·4)	8·1% (5·0 to 11·3%)	7·0% (-9·5 to 27·0%)	10·5% (4·5 to 16·0%)	647·3 (386·5 to 936·3)	5·7% (3·7 to 7·8%)	693·0 (408·4 to 1,038·3)	6·4% (4·2 to 8·7%)	7·1% (-9·3 to 27·8%)	11·9% (3·9 to 19·3%)
Ischaemic heart disease	37·5 (24·7 to 54·7)	12·3% (8·1 to 17·6%)	50·7 (33·4 to 74·5)	13·5% (8·9 to 19·1%)	35·3% (21·5 to 51·5%)	9·9% (6·5 to 13·8%)	819·5 (557·8 to 1,158·9)	11·9% (8·0 to 16·5%)	1,106·7 (732·4 to 1,563·3)	13·2% (9·0 to 18·1%)	35·0% (20·0 to 53·5%)	10·7% (7·3 to 14·4%)
Ischaemic stroke	15·2 (8·4 to 27·3)	11·5% (6·5 to 20·3%)	20·0 (11·1 to 35·8)	12·5% (7·1 to 22·1%)	31·2% (17·3 to 47·8%)	8·8% (5·1 to 13·7%)	294·0 (172·6 to 488·8)	11·5% (6·8 to 18·7%)	382·6 (229·2 to 635·8)	12·7% (7·6 to 20·5%)	30·2% (15·9 to 47·1%)	10·0% (6·6 to 14·3%)
Hemorrhagic stroke	21·3 (14·0 to 30·0)	10·7% (7·2 to 15·0%)	28·0 (18·5 to 39·4)	11·8% (8·1 to 16·4%)	31·4% (15·8 to 49·5%)	9·9% (6·1 to 14·2%)	560·9 (356·9 to 828·5)	9·8% (6·3 to 14·2%)	738·3 (477·9 to 1,063·0)	11·0% (7·1 to 15·8%)	31·6% (15·8 to 51·8%)	11·8% (7·4 to 16·8%)
Diabetes mellitus	110·9 (102·0 to 120·9)	100·0% (100·0 to 100·0%)	145·2 (129·9 to 164·8)	100·0% (100·0 to 100·0%)	31·0% (17·6 to 47·4%)	-	4,146·3 (3,632·1 to 4,728·0)	100·0% (100·0 to 100·0%)	5,556·6 (4,753·2 to 6,442·9)	100·0% (100·0 to 100·0%)	34·0% (24·1 to 46·1%)	-
Chronic kidney disease due to diabetes mellitus	6·8 (5·9 to 7·8)	100·0% (100·0 to 100·0%)	9·0 (7·7 to 10·8)	100·0% (100·0 to 100·0%)	32·9% (19·9 to 48·0%)	-	417·6 (363·3 to 484·0)	100·0% (100·0 to 100·0%)	531·5 (457·4 to 620·6)	100·0% (100·0 to 100·0%)	27·3% (18·5 to 37·4%)	-
Chronic kidney disease due to hypertension	7·0 (4·7 to 9·4)	24·6% (16·9 to 32·6%)	10·1 (6·8 to 13·6)	27·6% (19·1 to 36·0%)	43·9% (30·6 to 60·6%)	12·0% (8·6 to 16·2%)	180·1 (120·7 to 244·7)	18·8% (12·7 to 25·3%)	259·0 (173·1 to 354·8)	21·4% (14·7 to 28·4%)	43·8% (29·1 to 62·6%)	13·9% (9·0 to 18·9%)
Chronic kidney disease due to glomerulonephritis	1·8 (1·2 to 2·4)	18·6% (12·7 to 25·3%)	2·1 (1·4 to 2·9)	19·7% (13·5 to 26·5%)	20·0% (1·4 to 38·8%)	5·9% (-0·3 to 12·7%)	69·2 (45·4 to 97·1)	12·9% (8·6 to 17·8%)	90·0 (58·2 to 125·8)	14·2% (9·5 to 19·3%)	30·1% (15·2 to 45·0%)	10·2% (3·3 to 16·9%)
Chronic kidney disease due to other causes	0·2 (0·1 to 0·3)	10·9% (7·2 to 15·2%)	0·3 (0·2 to 0·4)	13·0% (8·6 to 18·1%)	50·5% (26·6 to 81·0%)	19·2% (8·4 to 33·0%)	28·6 (17·8 to 42·1)	12·8% (8·4 to 17·6%)	42·7 (26·7 to 61·9)	15·0% (10·1 to 20·2%)	48·9% (42·7 to 56·0%)	17·3% (12·6 to 22·4%)
**High total cholesterol: All causes**	114·8 (83·9 to 153·3)	1·3% (1·0 to 1·8%)	135·0 (96·7 to 184·8)	1·7% (1·2 to 2·3%)	17·6% (4·2 to 32·7%)	27·4% (17·0 to 37·2%)	2,926·5 (2,254·9 to 3,748·5)	0·5% (0·4 to 0·6%)	3,373·2 (2,495·4 to 4,459·3)	0·6% (0·5 to 0·8%)	15·3% (1·5 to 32·0%)	26·7% (13·0 to 41·6%)
Ischaemic heart disease	96·5 (72·3 to 124·1)	31·5% (24·1 to 40·1%)	113·8 (82·6 to 150·4)	30·2% (23·0 to 38·9%)	18·0% (4·2 to 33·5%)	-4·2% (-7·9 to -0·8%)	2,500·2 (1,958·4 to 3,142·9)	36·2% (29·0 to 44·4%)	2,897·0 (2,154·3 to 3,760·6)	34·4% (27·1 to 42·9%)	15·9% (2·0 to 32·8%)	-5·0% (-8·3 to -2·1%)
Ischaemic stroke	18·3 (8·6 to 36·9)	13·8% (6·9 to 26·9%)	21·2 (9·3 to 43·9)	13·3% (6·2 to 26·8%)	15·7% (-0·3 to 31·4%)	-4·1% (-12·3 to 1·5%)	426·3 (251·5 to 735·9)	16·7% (10·1 to 27·6%)	476·2 (274·1 to 820·8)	15·7% (9·3 to 26·2%)	11·7% (-2·7 to 27·5%)	-5·6% (-11·2 to -1·7%)
**High systolic blood pressure: All causes**	519·6 (464·9 to 579·5)	6·0% (5·4 to 6·6%)	637·9 (558·3 to 730·5)	8·0% (7·1 to 8·8%)	22·8% (10·6 to 36·8%)	32·9% (25·8 to 41·3%)	12,971·7 (11,545·4 to 14,533·4)	2·2% (2·0 to 2·5%)	15,888·4 (13,922·5 to 18,257·1)	3·0% (2·6 to 3·4%)	22·5% (10·2 to 36·9%)	34·7% (23·4 to 46·7%)
Rheumatic heart disease	4·6 (3·1 to 6·8)	25·4% (17·6 to 38·0%)	5·1 (3·5 to 7·7)	26·3% (18·4 to 39·4%)	12·4% (-2·7 to 29·6%)	3·6% (0·9 to 6·5%)	174·8 (114·8 to 262·6)	20·5% (13·8 to 30·0%)	202·6 (131·9 to 296·8)	21·6% (14·3 to 31·4%)	15·9% (3·3 to 30·3%)	5·0% (0·9 to 9·4%)
Ischaemic heart disease	173·0 (143·6 to 203·6)	56·5% (48·0 to 64·2%)	214·3 (173·4 to 256·0)	56·9% (48·3 to 64·8%)	23·9% (11·5 to 37·9%)	0·7% (-0·6 to 2·0%)	3,883·5 (3,291·4 to 4,506·9)	56·3% (48·9 to 62·9%)	4,794·5 (3,954·8 to 5,675·6)	57·0% (49·5 to 63·6%)	23·5% (9·6 to 39·0%)	1·3% (0·0 to 2·7%)
Ischaemic stroke	70·4 (53·2 to 86·5)	53·2% (41·8 to 62·4%)	84·8 (64·3 to 104·5)	53·1% (42·1 to 62·4%)	20·6% (7·8 to 34·5%)	-0·1% (-1·9 to 1·7%)	1,395·6 (1,089·2 to 1,689·8)	54·6% (44·1 to 62·9%)	1,664·7 (1,295·5 to 2,027·8)	55·1% (44·8 to 63·5%)	19·3% (6·3 to 33·5%)	0·8% (-0·5 to 2·3%)

Supplemental Results Table 1. All-age deaths, death PAFs, DALYs, DALY PAFs, and percent change for each risk factor and outcome for both sexes combined in 2005 and 2015 for Sub-Saharan Africa.

Data in parenthesis are 95% uncertainty intervals. PAFs=population attributable fractions. DALYs=disability-adjusted life-years.

	2005 Deaths (in thousands)	2005 Deaths PAF	2015 Deaths (in thousands)	2015 Deaths PAF	Percent change deaths 2005-2015	Percent change deaths PAF 2005-2015	2005 DALYs (in thousands)	2005 DALYs PAF	2015 DALYs (in thousands)	2015 DALYs PAF	Percent change DALYs 2005-2015	Percent change DALYs PAF 2005-2015
Hemorrhagic stroke	116·6 (94·7 to 137·7)	58·7% (49·3 to 67·1%)	141·5 (113·2 to 171·5)	59·5% (50·1 to 67·8%)	21·3% (7·8 to 36·4%)	1·4% (-0·2 to 3·0%)	3,130·6 (2,569·2 to 3,668·3)	54·7% (45·2 to 62·0%)	3,791·0 (3,022·0 to 4,648·4)	56·2% (46·9 to 63·8%)	21·1% (7·1 to 37·1%)	2·8% (0·5 to 5·2%)
Hypertensive heart disease	78·5 (60·2 to 108·9)	100·0% (100·0 to 100·0%)	93·9 (71·9 to 134·6)	100·0% (100·0 to 100·0%)	19·5% (5·3 to 37·9%)	-	1,781·9 (1,411·0 to 2,335·5)	100·0% (100·0 to 100·0%)	2,088·0 (1,646·5 to 2,809·6)	100·0% (100·0 to 100·0%)	17·2% (2·8 to 35·7%)	-
Cardiomyopathy and myocarditis	6·5 (5·1 to 7·8)	34·3% (27·7 to 40·5%)	7·8 (6·0 to 9·8)	35·2% (28·3 to 41·7%)	19·7% (5·5 to 36·6%)	2·6% (0·0 to 5·2%)	194·1 (147·8 to 241·7)	27·5% (21·6 to 33·6%)	233·4 (176·2 to 297·9)	28·9% (22·8 to 34·7%)	20·3% (5·0 to 38·6%)	5·2% (-0·1 to 10·8%)
Atrial fibrillation and flutter	0·9 (0·7 to 1·3)	37·1% (30·2 to 44·0%)	1·3 (0·9 to 1·7)	37·1% (30·2 to 44·1%)	36·7% (25·5 to 49·7%)	-0·1% (-2·1 to 1·8%)	36·1 (27·1 to 47·3)	44·2% (39·4 to 49·2%)	48·9 (36·6 to 64·3)	44·7% (40·0 to 49·7%)	35·5% (30·4 to 40·9%)	1·2% (0·2 to 2·3%)
Aortic aneurysm	2·0 (1·6 to 2·7)	39·2% (32·3 to 45·5%)	2·8 (2·2 to 3·6)	39·6% (32·6 to 46·0%)	39·1% (21·0 to 58·6%)	1·0% (-0·4 to 2·5%)	49·0 (37·8 to 64·5)	39·4% (33·7 to 45·7%)	68·4 (53·8 to 85·8)	40·1% (34·3 to 46·4%)	39·8% (19·4 to 62·4%)	1·6% (0·0 to 3·4%)
Peripheral vascular disease	0·3 (0·2 to 0·4)	29·7% (23·1 to 36·8%)	0·4 (0·3 to 0·6)	30·1% (23·2 to 37·2%)	59·7% (40·1 to 82·0%)	1·0% (-0·9 to 2·7%)	12·6 (8·0 to 19·7)	30·8% (24·0 to 37·4%)	18·2 (11·8 to 28·1)	31·3% (24·5 to 38·0%)	44·4% (35·8 to 55·9%)	1·7% (0·8 to 3·0%)
Endocarditis	2·4 (1·5 to 3·2)	29·3% (23·0 to 35·8%)	3·3 (2·1 to 4·4)	30·6% (24·1 to 36·8%)	37·0% (19·7 to 59·8%)	4·3% (-0·3 to 10·0%)	70·7 (46·0 to 95·4)	20·6% (14·6 to 27·7%)	97·4 (65·4 to 134·0)	22·1% (15·9 to 29·1%)	37·8% (18·8 to 61·6%)	7·6% (-0·8 to 18·0%)
Other cardiovascular and circulatory diseases	30·5 (26·1 to 35·1)	39·6% (35·5 to 43·9%)	39·2 (32·0 to 46·9)	40·6% (36·1 to 44·9%)	28·5% (11·3 to 47·8%)	2·4% (0·1 to 4·8%)	1,026·3 (869·3 to 1,217·9)	34·0% (30·4 to 37·8%)	1,337·7 (1,099·2 to 1,606·3)	35·9% (32·0 to 40·1%)	30·3% (17·0 to 44·9%)	5·5% (2·1 to 9·6%)
Chronic kidney disease due to diabetes mellitus	2·2 (1·5 to 2·9)	32·0% (22·8 to 40·7%)	3·1 (2·1 to 4·2)	34·2% (24·6 to 43·2%)	42·1% (26·2 to 59·5%)	6·9% (2·9 to 11·6%)	91·1 (60·9 to 122·9)	21·8% (15·0 to 28·6%)	124·9 (83·5 to 168·6)	23·5% (16·3 to 30·6%)	37·0% (27·7 to 47·6%)	7·7% (3·0 to 13·6%)
Chronic kidney disease due to hypertension	28·4 (25·8 to 31·9)	100·0% (100·0 to 100·0%)	36·5 (32·3 to 41·8)	100·0% (100·0 to 100·0%)	28·5% (16·8 to 43·1%)	-	958·1 (860·4 to 1,075·1)	100·0% (100·0 to 100·0%)	1,209·7 (1,052·3 to 1,396·2)	100·0% (100·0 to 100·0%)	26·3% (14·9 to 41·6%)	-
Chronic kidney disease due to glomerulonephritis	2·9 (2·1 to 3·9)	31·3% (22·9 to 39·8%)	3·3 (2·3 to 4·4)	31·0% (22·6 to 39·6%)	12·2% (-5·9 to 29·4%)	-1·0% (-6·0 to 4·1%)	115·5 (79·7 to 153·4)	21·4% (15·1 to 28·1%)	139·0 (94·5 to 187·6)	21·9% (15·6 to 28·6%)	20·4% (7·2 to 32·7%)	2·0% (-3·3 to 7·0%)
Chronic kidney disease due to other causes	0·4 (0·3 to 0·5)	22·5% (15·6 to 29·0%)	0·5 (0·4 to 0·7)	24·7% (17·1 to 32·3%)	38·7% (19·0 to 62·2%)	9·8% (1·4 to 19·0%)	51·9 (33·8 to 71·4)	23·1% (15·8 to 30·3%)	69·8 (45·7 to 95·6)	24·5% (16·9 to 32·1%)	34·6% (29·7 to 39·9%)	6·0% (2·4 to 9·9%)
**High body-mass index: All causes**	207·2 (146·9 to 276·0)	2·4% (1·7 to 3·1%)	266·8 (189·1 to 353·1)	3·3% (2·4 to 4·4%)	28·7% (14·6 to 46·1%)	39·5% (29·1 to 51·6%)	6,674·7 (4,794·4 to 8,829·7)	1·1% (0·8 to 1·5%)	8,751·3 (6,344·7 to 11,519·4)	1·7% (1·2 to 2·1%)	31·1% (18·2 to 47·1%)	44·2% (32·2 to 58·6%)
Oesophageal cancer	4·1 (1·3 to 7·3)	15·2% (4·9 to 26·8%)	5·4 (1·8 to 9·8)	15·8% (5·2 to 28·1%)	30·8% (11·4 to 59·0%)	4·1% (-11·1 to 20·0%)	107·5 (33·9 to 189·8)	15·2% (4·9 to 26·9%)	139·1 (46·2 to 255·1)	15·7% (5·1 to 28·3%)	29·5% (8·8 to 58·5%)	3·6% (-12·4 to 19·4%)
Colon and rectum cancer	1·1 (0·7 to 1·8)	4·8% (2·8 to 7·3%)	1·9 (1·1 to 2·8)	5·5% (3·2 to 8·2%)	61·4% (41·0 to 85·0%)	13·7% (6·4 to 23·6%)	30·0 (17·4 to 45·9)	4·9% (2·9 to 7·4%)	47·8 (28·1 to 72·2)	5·5% (3·2 to 8·2%)	59·5% (38·3 to 84·8%)	12·9% (5·1 to 22·7%)
Liver cancer due to hepatitis B	1·6 (0·6 to 3·1)	9·3% (3·9 to 17·0%)	2·2 (0·9 to 4·1)	10·3% (4·3 to 18·7%)	38·5% (15·4 to 70·5%)	10·6% (1·2 to 20·2%)	58·6 (22·4 to 113·4)	8·8% (3·6 to 16·1%)	81·3 (32·3 to 154·7)	9·7% (3·9 to 17·6%)	38·9% (13·5 to 73·7%)	10·1% (0·3 to 20·1%)
Liver cancer due to hepatitis C	0·9 (0·4 to 1·7)	9·0% (3·7 to 16·0%)	1·3 (0·5 to 2·3)	9·9% (4·1 to 17·2%)	39·1% (18·3 to 64·9%)	9·6% (0·7 to 20·7%)	23·4 (9·5 to 42·3)	9·1% (3·8 to 16·0%)	32·5 (13·5 to 58·4)	9·9% (4·2 to 17·3%)	38·7% (17·4 to 65·0%)	9·2% (0·0 to 20·3%)
Liver cancer due to alcohol use	1·6 (0·6 to 3·0)	10·5% (4·3 to 19·0%)	2·2 (0·9 to 4·0)	11·4% (4·6 to 20·7%)	35·2% (10·8 to 66·9%)	8·4% (-2·2 to 19·1%)	45·0 (17·6 to 86·4)	10·8% (4·3 to 19·5%)	60·6 (23·7 to 112·4)	11·6% (4·6 to 21·1%)	34·7% (9·6 to 69·7%)	7·5% (-2·9 to 18·0%)
Liver cancer due to other causes	0·6 (0·2 to 1·1)	8·8% (3·6 to 15·4%)	0·8 (0·4 to 1·5)	9·8% (4·1 to 16·9%)	38·1% (19·1 to 62·1%)	12·0% (3·2 to 23·8%)	19·0 (7·7 to 34·3)	8·2% (3·4 to 14·5%)	26·3 (11·0 to 45·7)	9·2% (4·0 to 16·0%)	38·7% (17·8 to 64·5%)	11·7% (2·6 to 23·4%)
Gallbladder and biliary tract cancer	0·9 (0·5 to 1·3)	15·7% (9·0 to 23·9%)	1·1 (0·6 to 1·7)	16·7% (9·9 to 25·4%)	23·6% (2·3 to 55·9%)	6·6% (-1·0 to 15·3%)	20·6 (11·3 to 32·6)	15·7% (9·0 to 23·9%)	25·2 (14·1 to 39·9)	16·7% (9·9 to 25·5%)	22·4% (0·0 to 56·8%)	6·3% (-1·6 to 15·0%)
Pancreatic cancer	0·6 (0·2 to 1·1)	4·3% (1·7 to 7·5%)	1·0 (0·4 to 1·7)	4·8% (1·9 to 8·1%)	59·9% (39·4 to 89·9%)	9·6% (0·4 to 18·3%)	15·0 (5·8 to 26·2)	4·3% (1·6 to 7·6%)	23·9 (9·5 to 40·9)	4·7% (1·8 to 8·1%)	59·9% (37·8 to 92·1%)	8·8% (-1·2 to 18·3%)
Breast cancer	0·5 (0·3 to 0·9)	2·2% (1·0 to 3·5%)	0·8 (0·4 to 1·3)	2·3% (1·1 to 3·8%)	44·7% (18·1 to 77·9%)	7·4% (-11·6 to 31·9%)	3·8 (-2·6 to 10·0)	0·5% (-0·3 to 1·3%)	5·0 (-4·7 to 13·9)	0·5% (-0·4 to 1·3%)	30·9% (-461·4 to 347·1%)	-3·9% (-357·4 to 226·7%)
Uterine cancer	1·9 (1·3 to 2·7)	28·4% (18·4 to 39·0%)	2·5 (1·6 to 3·6)	31·2% (20·9 to 42·0%)	28·7% (6·4 to 58·3%)	10·0% (3·5 to 19·1%)	50·8 (32·9 to 71·7)	28·1% (18·3 to 38·7%)	65·0 (41·2 to 94·0)	31·1% (20·8 to 41·8%)	28·1% (4·7 to 60·4%)	10·3% (3·2 to 19·7%)
Ovarian cancer	0·2 (0·0 to 0·4)	2·3% (-0·1 to 5·2%)	0·3 (0·0 to 0·7)	2·6% (-0·1 to 5·9%)	57·9% (32·5 to 88·5%)	11·9% (3·3 to 22·0%)	5·2 (-0·2 to 11·9)	2·2% (-0·1 to 5·0%)	8·2 (-0·3 to 19·2)	2·4% (-0·1 to 5·6%)	58·2% (31·3 to 91·7%)	11·6% (2·4 to 21·5%)
Kidney cancer	0·4 (0·3 to 0·6)	9·2% (5·7 to 13·9%)	0·7 (0·4 to 1·0)	9·8% (6·1 to 14·3%)	53·4% (30·7 to 82·5%)	6·0% (-8·1 to 26·0%)	12·7 (8·1 to 18·2)	5·9% (3·3 to 9·9%)	19·5 (12·5 to 28·1)	6·2% (3·6 to 9·8%)	52·8% (29·3 to 83·1%)	5·2% (-16·0 to 40·1%)
Thyroid cancer	0·1 (0·0 to 0·1)	7·6% (4·4 to 11·8%)	0·1 (0·1 to 0·2)	8·4% (4·8 to 12·8%)	39·2% (19·1 to 60·6%)	9·6% (2·1 to 18·7%)	2·3 (1·3 to 3·7)	7·6% (4·3 to 11·7%)	3·3 (1·9 to 5·3)	8·2% (4·7 to 12·6%)	42·5% (21·2 to 64·7%)	8·3% (0·9 to 17·0%)
Ischaemic heart disease	45·8 (30·4 to 62·7)	15·0% (10·0 to 20·7%)	59·4 (39·3 to 82·8)	15·8% (10·7 to 21·5%)	29·7% (15·1 to 48·1%)	5·5% (-1·0 to 12·5%)	1,209·5 (808·9 to 1,661·9)	17·5% (11·9 to 23·9%)	1,564·1 (1,035·8 to 2,161·8)	18·6% (12·8 to 25·3%)	29·3% (13·9 to 48·6%)	6·1% (-0·3 to 12·9%)
Ischaemic stroke	16·6 (10·7 to 23·8)	12·6% (8·3 to 17·5%)	20·7 (13·4 to 29·1)	13·0% (8·6 to 17·9%)	24·5% (8·8 to 43·1%)	3·1% (-3·5 to 11·0%)	416·4 (272·4 to 585·1)	16·3% (11·0 to 22·0%)	519·5 (339·6 to 715·8)	17·2% (11·6 to 23·1%)	24·8% (9·5 to 43·6%)	5·4% (-0·8 to 12·5%)
Hemorrhagic stroke	45·9 (32·0 to 62·4)	23·1% (16·0 to 30·9%)	57·1 (38·8 to 78·3)	24·1% (16·9 to 32·2%)	24·4% (8·4 to 43·6%)	4·1% (-2·6 to 11·2%)	1,475·7 (1,040·2 to 2,002·8)	25·8% (18·3 to 34·1%)	1,847·5 (1,270·0 to 2,499·5)	27·4% (19·4 to 36·1%)	25·2% (9·1 to 45·5%)	6·3% (0·1 to 13·6%)
Hypertensive heart disease	26·4 (16·6 to 38·2)	33·8% (22·3 to 46·6%)	32·7 (21·0 to 49·2)	35·0% (23·2 to 48·4%)	24·0% (8·0 to 42·5%)	3·8% (-2·7 to 11·2%)	656·1 (439·9 to 915·6)	36·9% (25·8 to 49·0%)	795·8 (529·4 to 1,105·8)	38·3% (27·0 to 50·6%)	21·3% (5·5 to 41·3%)	3·6% (-1·8 to 10·0%)
Diabetes mellitus	47·3 (35·6 to 59·8)	42·7% (32·7 to 53·0%)	62·5 (47·7 to 79·7)	43·1% (33·1 to 53·5%)	32·0% (16·4 to 51·6%)	0·8% (-4·3 to 5·6%)	1,935·6 (1,449·5 to 2,463·2)	46·7% (36·4 to 56·6%)	2,662·3 (1,997·9 to 3,361·8)	47·9% (37·6 to 58·1%)	37·5% (25·1 to 52·0%)	2·7% (-1·3 to 6·3%)
Chronic kidney disease due to diabetes mellitus	0·9 (0·4 to 1·6)	13·2% (6·1 to 22·4%)	1·4 (0·6 to 2·4)	15·0% (6·8 to 25·4%)	50·9% (30·1 to 74·0%)	13·6% (2·4 to 25·2%)	48·0 (19·4 to 83·7)	11·5% (4·8 to 19·4%)	69·2 (28·2 to 116·8)	13·0% (5·6 to 21·6%)	44·2% (33·4 to 56·8%)	13·3% (6·2 to 21·4%)
Chronic kidney disease due to hypertension	7·3 (3·7 to 11·2)	25·8% (12·9 to 39·1%)	10·0 (5·1 to 15·2)	27·5% (14·3 to 40·9%)	37·0% (21·0 to 56·2%)	6·7% (0·4 to 13·6%)	202·2 (94·8 to 325·4)	21·1% (9·8 to 33·3%)	274·3 (130·6 to 434·9)	22·7% (10·9 to 35·5%)	35·7% (20·6 to 55·2%)	7·6% (1·4 to 15·0%)
Chronic kidney disease due to glomerulonephritis	1·6 (0·7 to 2·6)	17·1% (7·7 to 27·3%)	1·8 (0·7 to 2·9)	16·7% (7·1 to 26·6%)	11·0% (-12·0 to 32·1%)	-2·0% (-15·1 to 8·7%)	68·5 (22·4 to 116·8)	12·7% (4·3 to 21·8%)	83·1 (26·4 to 141·7)	13·1% (4·3 to 21·9%)	21·3% (5·1 to 35·0%)	2·7% (-6·5 to 11·1%)
Chronic kidney disease due to other causes	0·2 (0·1 to 0·3)	9·4% (3·9 to 16·6%)	0·2 (0·1 to 0·4)	11·2% (4·3 to 19·2%)	49·4% (14·6 to 81·4%)	18·3% (-1·2 to 37·6%)	29·1 (10·8 to 51·5)	13·0% (5·1 to 22·2%)	41·3 (15·6 to 72·3)	14·5% (5·7 to 24·1%)	42·0% (34·5 to 50·5%)	11·8% (6·4 to 19·0%)
Osteoarthritis	-	-	-	-	-	-	84·1 (48·6 to 129·1)	16·4% (11·0 to 21·9%)	127·2 (74·4 to 192·8)	17·9% (12·3 to 23·6%)	51·3% (46·9 to 57·3%)	9·1% (6·1 to 13·3%)
Low back pain	-	-	-	-	-	-	140·0 (77·6 to 227·6)	3·6% (2·2 to 5·1%)	205·2 (115·7 to 330·7)	4·0% (2·5 to 5·6%)	46·6% (42·2 to 52·8%)	11·0% (8·0 to 15·7%)
**Low bone mineral density: All causes**	22·5 (19·8 to 25·2)	0·3% (0·2 to 0·3%)	28·1 (24·5 to 31·9)	0·4% (0·3 to 0·4%)	25·1% (11·8 to 42·4%)	35·4% (24·8 to 49·2%)	526·7 (462·5 to 591·7)	0·1% (0·1 to 0·1%)	657·3 (566·0 to 760·1)	0·1% (0·1 to 0·1%)	24·8% (14·4 to 37·5%)	37·2% (27·9 to 48·5%)
Pedestrian road injuries	2·9 (2·5 to 3·2)	5·8% (5·2 to 6·4%)	3·4 (3·0 to 4·0)	5·9% (5·3 to 6·5%)	20·8% (6·9 to 39·4%)	2·0% (-5·6 to 10·4%)	63·1 (55·0 to 71·5)	2·4% (2·1 to 2·7%)	76·0 (64·3 to 89·7)	2·5% (2·2 to 2·8%)	20·3% (5·7 to 40·0%)	4·6% (-5·2 to 16·5%)
Cyclist road injuries	0·2 (0·2 to 0·2)	5·4% (4·7 to 6·1%)	0·3 (0·2 to 0·3)	5·3% (4·7 to 6·1%)	30·4% (10·1 to 55·3%)	-0·6% (-10·0 to 10·6%)	6·7 (5·6 to 7·9)	3·0% (2·6 to 3·5%)	8·8 (7·3 to 10·6)	3·0% (2·6 to 3·5%)	31·4% (16·7 to 49·8%)	1·1% (-8·7 to 13·0%)
Motorcyclist road injuries	0·3 (0·3 to 0·4)	3·3% (2·8 to 3·9%)	0·4 (0·3 to 0·5)	3·2% (2·8 to 3·7%)	23·8% (6·8 to 45·5%)	-1·3% (-12·5 to 11·2%)	11·4 (9·4 to 14·2)	1·8% (1·5 to 2·2%)	14·4 (11·6 to 17·8)	1·8% (1·5 to 2·2%)	25·8% (10·9 to 45·0%)	0·9% (-11·2 to 14·9%)
Motor vehicle road injuries	3·2 (2·7 to 3·6)	5·4% (4·7 to 5·9%)	3·8 (3·1 to 4·5)	5·6% (5·0 to 6·1%)	18·4% (5·7 to 33·8%)	4·3% (-2·8 to 12·4%)	89·3 (74·5 to 101·8)	2·7% (2·3 to 3·0%)	105·8 (87·2 to 125·0)	2·8% (2·5 to 3·2%)	18·4% (6·3 to 32·8%)	5·9% (-2·8 to 15·8%)
Other road injuries	0·1 (0·1 to 0·1)	6·6% (5·1 to 8·3%)	0·1 (0·1 to 0·1)	6·4% (5·1 to 7·7%)	16·3% (-7·2 to 46·3%)	-3·1% (-19·0 to 16·9%)	2·6 (2·0 to 3·1)	3·1% (2·3 to 4·0%)	3·1 (2·4 to 3·9)	3·1% (2·5 to 3·9%)	22·6% (7·1 to 42·7%)	1·7% (-15·4 to 21·8%)

Supplemental Results Table 1. All-age deaths, death PAFs, DALYs, DALY PAFs, and percent change for each risk factor and outcome for both sexes combined in 2005 and 2015 for Sub-Saharan Africa.

Data in parenthesis are 95% uncertainty intervals. PAFs=population attributable fractions. DALYs=disability-adjusted life-years.

	2005 Deaths (in thousands)	2005 Deaths PAF	2015 Deaths (in thousands)	2015 Deaths PAF	Percent change deaths 2005-2015	Percent change deaths PAF 2005-2015	2005 DALYs (in thousands)	2005 DALYs PAF	2015 DALYs (in thousands)	2015 DALYs PAF	Percent change DALYs 2005-2015	Percent change DALYs PAF 2005-2015
Other transport injuries	0·5<br>(0·4 to 0·6)	5·4%<br>(4·6 to 6·7%)	0·5<br>(0·4 to 0·6)	5·5%<br>(4·6 to 6·7%)	10·1%<br>(-9·4 to 35·5%)	1·4%<br>(-12·9 to 19·7%)	10·8<br>(8·7 to 12·9)	2·1%<br>(1·7 to 2·6%)	11·8<br>(9·2 to 14·6)	2·1%<br>(1·7 to 2·6%)	9·0%<br>(-8·5 to 31·8%)	0·2%<br>(-14·4 to 19·1%)
Falls	13·9<br>(11·9 to 16·0)	33·3%<br>(29·2 to 37·0%)	17·8<br>(15·2 to 20·7)	33·5%<br>(30·3 to 36·3%)	28·3%<br>(12·2 to 50·4%)	0·4%<br>(-9·5 to 13·3%)	290·2<br>(248·9 to 336·4)	12·2%<br>(10·4 to 13·8%)	373·1<br>(317·5 to 441·7)	12·1%<br>(10·7 to 13·3%)	28·6%<br>(17·2 to 42·8%)	-1·1%<br>(-12·5 to 14·3%)
Other exposure to mechanical forces	0·8<br>(0·6 to 0·9)	4·3%<br>(3·7 to 5·1%)	1·0<br>(0·8 to 1·2)	4·4%<br>(3·8 to 5·0%)	21·1%<br>(5·4 to 40·6%)	2·8%<br>(-9·9 to 17·2%)	33·4<br>(26·5 to 41·4)	2·2%<br>(1·8 to 2·6%)	40·9<br>(32·2 to 51·5)	2·2%<br>(1·9 to 2·6%)	22·5%<br>(13·4 to 34·5%)	3·8%<br>(-6·6 to 15·8%)
Non-venomous animal contact	0·1<br>(0·1 to 0·2)	2·4%<br>(2·0 to 3·6%)	0·2<br>(0·1 to 0·2)	2·7%<br>(2·3 to 3·8%)	13·3%<br>(-6·3 to 49·5%)	12·0%<br>(-4·4 to 29·9%)	4·4<br>(3·2 to 5·6)	1·0%<br>(0·8 to 1·5%)	5·0<br>(3·8 to 6·4)	1·1%<br>(0·9 to 1·5%)	13·2%<br>(-0·5 to 36·0%)	12·0%<br>(-5·2 to 30·7%)
Assault by other means	0·5<br>(0·4 to 0·6)	1·8%<br>(1·6 to 2·0%)	0·6<br>(0·5 to 0·7)	1·9%<br>(1·6 to 2·1%)	25·3%<br>(9·9 to 43·3%)	1·8%<br>(-7·9 to 11·3%)	14·4<br>(12·0 to 17·4)	1·0%<br>(0·8 to 1·1%)	18·0<br>(14·8 to 22·0)	1·0%<br>(0·8 to 1·2%)	24·8%<br>(10·0 to 43·4%)	3·8%<br>(-6·3 to 13·7%)
Exposure to forces of nature	-	-	-	-	-	-	0·3<br>(0·2 to 0·6)	0·8%<br>(0·4 to 1·3%)	0·4<br>(0·3 to 0·8)	0·9%<br>(0·5 to 1·7%)	37·0%<br>(16·9 to 63·6%)	20·2%<br>(-18·3 to 77·0%)
**Low glomerular filtration rate: All causes**	105·1<br>(95·4 to 117·1)	1·2%<br>(1·1 to 1·3%)	128·7<br>(114·0 to 145·5)	1·6%<br>(1·5 to 1·8%)	22·4%<br>(12·0 to 34·4%)	32·6%<br>(25·9 to 39·1%)	3,305·0<br>(3,010·3 to 3,644·6)	0·6%<br>(0·5 to 0·6%)	4,036·6<br>(3,590·5 to 4,536·4)	0·8%<br>(0·7 to 0·8%)	22·1%<br>(12·5 to 32·7%)	34·3%<br>(25·9 to 43·2%)
Ischaemic heart disease	27·5<br>(23·9 to 31·2)	9·0%<br>(8·0 to 10·1%)	33·1<br>(28·3 to 38·5)	8·8%<br>(7·9 to 9·8%)	20·4%<br>(9·0 to 33·4%)	-2·2%<br>(-5·1 to 0·5%)	523·0<br>(455·6 to 595·7)	7·6%<br>(6·8 to 8·5%)	620·0<br>(527·0 to 723·3)	7·4%<br>(6·6 to 8·2%)	18·5%<br>(6·8 to 32·3%)	-2·8%<br>(-5·8 to 0·2%)
Ischaemic stroke	14·0<br>(11·2 to 16·7)	10·6%<br>(9·1 to 12·1%)	16·6<br>(13·4 to 20·1)	10·4%<br>(9·1 to 11·9%)	19·2%<br>(7·1 to 32·9%)	-1·2%<br>(-4·3 to 2·0%)	239·6<br>(195·8 to 286·9)	9·4%<br>(8·2 to 10·6%)	279·0<br>(226·1 to 337·7)	9·2%<br>(8·1 to 10·5%)	16·4%<br>(4·0 to 30·2%)	-1·6%<br>(-4·9 to 1·7%)
Hemorrhagic stroke	17·2<br>(14·6 to 20·2)	8·7%<br>(7·6 to 9·8%)	20·4<br>(16·7 to 24·3)	8·6%<br>(7·6 to 9·6%)	18·4%<br>(5·5 to 33·0%)	-1·0%<br>(-4·2 to 2·5%)	386·5<br>(330·3 to 454·5)	6·8%<br>(5·9 to 7·7%)	452·2<br>(375·8 to 542·6)	6·7%<br>(6·0 to 7·6%)	17·0%<br>(3·6 to 32·8%)	-0·7%<br>(-4·6 to 3·4%)
Peripheral vascular disease	0·2<br>(0·1 to 0·2)	19·2%<br>(13·6 to 24·7%)	0·3<br>(0·2 to 0·4)	18·7%<br>(13·3 to 24·4%)	54·4%<br>(35·2 to 76·3%)	-2·3%<br>(-4·9 to 0·4%)	7·7<br>(4·9 to 11·8)	18·9%<br>(15·2 to 22·7%)	10·8<br>(7·1 to 16·2)	18·6%<br>(14·9 to 22·3%)	39·9%<br>(31·2 to 50·8%)	-1·5%<br>(-3·1 to 0·3%)
Chronic kidney disease due to diabetes mellitus	6·8<br>(5·9 to 7·8)	100·0%<br>(100·0 to 100·0%)	9·0<br>(7·7 to 10·8)	100·0%<br>(100·0 to 100·0%)	32·9%<br>(19·9 to 48·0%)	-	417·6<br>(363·3 to 484·0)	100·0%<br>(100·0 to 100·0%)	531·5<br>(457·4 to 620·6)	100·0%<br>(100·0 to 100·0%)	27·3%<br>(18·5 to 37·4%)	-
Chronic kidney disease due to hypertension	28·4<br>(25·8 to 31·9)	100·0%<br>(100·0 to 100·0%)	36·5<br>(32·3 to 41·8)	100·0%<br>(100·0 to 100·0%)	28·5%<br>(16·8 to 43·1%)	-	958·1<br>(860·4 to 1,075·1)	100·0%<br>(100·0 to 100·0%)	1,209·7<br>(1,052·3 to 1,396·2)	100·0%<br>(100·0 to 100·0%)	26·3%<br>(14·9 to 41·6%)	-
Chronic kidney disease due to glomerulonephritis	9·4<br>(8·3 to 10·8)	100·0%<br>(100·0 to 100·0%)	10·7<br>(9·1 to 12·7)	100·0%<br>(100·0 to 100·0%)	13·3%<br>(-1·3 to 27·8%)	-	538·4<br>(474·5 to 612·5)	100·0%<br>(100·0 to 100·0%)	635·9<br>(547·9 to 736·4)	100·0%<br>(100·0 to 100·0%)	18·1%<br>(7·9 to 28·5%)	-
Chronic kidney disease due to other causes	1·7<br>(1·4 to 2·0)	100·0%<br>(100·0 to 100·0%)	2·2<br>(1·8 to 2·6)	100·0%<br>(100·0 to 100·0%)	26·1%<br>(12·4 to 42·1%)	-	223·8<br>(184·6 to 266·1)	100·0%<br>(100·0 to 100·0%)	284·3<br>(234·2 to 339·6)	100·0%<br>(100·0 to 100·0%)	27·0%<br>(21·0 to 33·0%)	-
Gout	-	-	-	-	-	-	10·1<br>(6·7 to 14·0)	14·7%<br>(13·2 to 16·2%)	13·3<br>(8·9 to 18·4)	14·3%<br>(12·8 to 15·8%)	31·7%<br>(27·9 to 35·5%)	-2·4%<br>(-4·3 to -0·3%)

Supplemental Results Table 1. All-age deaths, death PAFs, DALYs, DALY PAFs, and percent change for each risk factor and outcome for both sexes combined in 2005 and 2015 for Andean Latin America.

Data in parenthesis are 95% uncertainty intervals. PAFs=population attributable fractions. DALYs=disability-adjusted life-years.

	2005 Deaths (in thousands)	2005 Deaths PAF	2015 Deaths (in thousands)	2015 Deaths PAF	Percent change deaths 2005-2015	Percent change deaths PAF 2005-2015	2005 DALYs (in thousands)	2005 DALYs PAF	2015 DALYs (in thousands)	2015 DALYs PAF	Percent change DALYs 2005-2015	Percent change DALYs PAF 2005-2015
**All risk factors: All causes**	116·2<br>(111·3 to 121·2)	48·4%<br>(47·1 to 49·8%)	138·3<br>(129·7 to 147·8)	49·4%<br>(48·0 to 51·0%)	19·0%<br>(11·7 to 26·9%)	2·1%<br>(0·6 to 3·9%)	4,489·6<br>(4,133·6 to 4,911·4)	34·6%<br>(33·4 to 35·9%)	4,502·9<br>(4,053·6 to 5,020·6)	34·2%<br>(32·8 to 35·8%)	0·3%<br>(-4·7 to 5·0%)	-1·0%<br>(-3·4 to 1·4%)
**Environmental/occupational risks: All causes**	35·8<br>(31·9 to 39·7)	14·9%<br>(13·4 to 16·4%)	34·9<br>(30·2 to 39·8)	12·5%<br>(10·9 to 14·1%)	-2·6%<br>(-11·0 to 6·4%)	-16·4%<br>(-21·3 to -10·7%)	1,412·9<br>(1,264·1 to 1,566·3)	10·9%<br>(9·8 to 12·0%)	1,140·8<br>(995·7 to 1,308·8)	8·7%<br>(7·8 to 9·7%)	-19·3%<br>(-25·7 to -11·4%)	-20·3%<br>(-25·7 to -13·7%)
**Unsafe water, sanitation, and handwashing: All causes**	7·3<br>(5·9 to 8·7)	3·0%<br>(2·4 to 3·6%)	5·7<br>(4·2 to 7·2)	2·0%<br>(1·5 to 2·6%)	-22·2%<br>(-32·5 to -12·4%)	-33·2%<br>(-40·8 to -26·4%)	428·1<br>(363·6 to 495·2)	3·3%<br>(2·8 to 3·8%)	233·8<br>(186·4 to 282·6)	1·8%<br>(1·5 to 2·1%)	-45·4%<br>(-51·9 to -38·8%)	-46·1%<br>(-52·2 to -39·9%)
**Unsafe water source: All causes**	2·8<br>(1·9 to 3·5)	1·2%<br>(0·8 to 1·5%)	1·3<br>(0·9 to 1·7)	0·5%<br>(0·3 to 0·6%)	-53·7%<br>(-60·8 to -45·0%)	-60·2%<br>(-66·4 to -52·7%)	240·2<br>(159·6 to 303·3)	1·8%<br>(1·3 to 2·3%)	112·0<br>(70·6 to 148·4)	0·9%<br>(0·6 to 1·1%)	-53·4%<br>(-60·6 to -45·0%)	-54·0%<br>(-60·6 to -45·9%)
Diarrhoeal diseases	2·8<br>(1·9 to 3·5)	74·7%<br>(51·4 to 89·1%)	1·3<br>(0·8 to 1·7)	71·2%<br>(46·6 to 87·1%)	-53·8%<br>(-60·9 to -45·1%)	-4·7%<br>(-9·7 to -1·8%)	239·7<br>(159·3 to 302·7)	74·7%<br>(51·3 to 89·1%)	111·6<br>(70·4 to 147·8)	71·1%<br>(46·5 to 87·1%)	-53·4%<br>(-60·7 to -45·1%)	-4·7%<br>(-9·8 to -1·9%)
Typhoid fever	0·0<br>(0·0 to 0·0)	73·2%<br>(49·2 to 88·3%)	0·0<br>(0·0 to 0·0)	70·5%<br>(45·5 to 86·9%)	-14·9%<br>(-21·9 to -8·1%)	-3·6%<br>(-8·3 to -0·9%)	0·5<br>(0·2 to 0·9)	73·2%<br>(49·3 to 88·3%)	0·4<br>(0·2 to 0·7)	70·5%<br>(45·5 to 86·9%)	-21·3%<br>(-28·5 to -14·3%)	-3·6%<br>(-8·3 to -1·0%)
Paratyphoid fever	0·0<br>(0·0 to 0·0)	73·2%<br>(49·3 to 88·3%)	0·0<br>(0·0 to 0·0)	70·5%<br>(45·5 to 86·9%)	4·6%<br>(-9·6 to 23·5%)	-3·6%<br>(-8·3 to -0·9%)	0·0<br>(0·0 to 0·0)	73·2%<br>(49·3 to 88·3%)	0·0<br>(0·0 to 0·0)	70·5%<br>(45·6 to 86·9%)	-2·5%<br>(-17·3 to 16·9%)	-3·6%<br>(-8·3 to -1·0%)
**Unsafe sanitation: All causes**	1·7<br>(1·4 to 2·0)	0·7%<br>(0·6 to 0·8%)	0·7<br>(0·5 to 0·8)	0·2%<br>(0·2 to 0·3%)	-61·1%<br>(-68·5 to -51·9%)	-66·6%<br>(-73·1 to -58·6%)	146·1<br>(122·7 to 171·8)	1·1%<br>(1·0 to 1·3%)	57·1<br>(44·2 to 72·0)	0·4%<br>(0·3 to 0·5%)	-60·9%<br>(-68·4 to -51·7%)	-61·4%<br>(-68·6 to -52·3%)
Diarrhoeal diseases	1·7<br>(1·4 to 2·0)	45·1%<br>(41·4 to 48·9%)	0·7<br>(0·5 to 0·8)	36·1%<br>(30·9 to 40·8%)	-61·2%<br>(-68·6 to -52·0%)	-20·1%<br>(-28·6 to -11·6%)	145·7<br>(122·5 to 171·6)	45·4%<br>(41·7 to 49·3%)	56·9<br>(44·0 to 71·8)	36·2%<br>(31·1 to 40·9%)	-61·0%<br>(-68·5 to -51·8%)	-20·3%<br>(-29·0 to -11·8%)
Typhoid fever	0·0<br>(0·0 to 0·0)	43·8%<br>(40·0 to 47·4%)	0·0<br>(0·0 to 0·0)	35·1%<br>(30·1 to 40·0%)	-29·2%<br>(-38·5 to -18·5%)	-19·9%<br>(-29·0 to -11·1%)	0·3<br>(0·1 to 0·5)	43·8%<br>(40·1 to 47·4%)	0·2<br>(0·1 to 0·4)	35·1%<br>(30·0 to 40·0%)	-34·6%<br>(-43·6 to -24·7%)	-19·9%<br>(-29·1 to -11·2%)
Paratyphoid fever	0·0<br>(0·0 to 0·0)	43·9%<br>(40·6 to 47·2%)	0·0<br>(0·0 to 0·0)	35·2%<br>(30·4 to 39·8%)	-13·0%<br>(-28·8 to 5·5%)	-19·8%<br>(-28·9 to -11·1%)	0·0<br>(0·0 to 0·0)	43·9%<br>(40·6 to 47·2%)	0·0<br>(0·0 to 0·0)	35·2%<br>(30·3 to 39·8%)	-18·9%<br>(-34·6 to -1·2%)	-19·9%<br>(-29·0 to -11·1%)
**No handwashing with soap: All causes**	5·2<br>(3·8 to 6·6)	2·2%<br>(1·6 to 2·8%)	4·7<br>(3·2 to 6·2)	1·7%<br>(1·1 to 2·2%)	-9·9%<br>(-20·0 to -0·2%)	-22·7%<br>(-30·0 to -16·0%)	248·8<br>(193·2 to 303·6)	1·9%<br>(1·5 to 2·3%)	148·3<br>(109·4 to 187·2)	1·1%<br>(0·8 to 1·4%)	-40·4%<br>(-46·4 to -34·1%)	-41·2%<br>(-46·8 to -35·8%)
Diarrhoeal diseases	1·3<br>(1·0 to 1·7)	35·2%<br>(27·6 to 42·0%)	0·6<br>(0·4 to 0·8)	33·4%<br>(25·9 to 40·2%)	-54·0%<br>(-61·0 to -45·3%)	-5·1%<br>(-7·2 to -3·2%)	112·9<br>(85·3 to 143·3)	35·2%<br>(27·6 to 42·0%)	52·4<br>(38·5 to 68·7)	33·4%<br>(25·9 to 40·2%)	-53·6%<br>(-60·6 to -45·1%)	-5·1%<br>(-7·2 to -3·2%)
Typhoid fever	0·0<br>(0·0 to 0·0)	34·7%<br>(26·6 to 41·9%)	0·0<br>(0·0 to 0·0)	33·0%<br>(25·0 to 40·2%)	-16·1%<br>(-22·4 to -9·7%)	-5·0%<br>(-7·1 to -3·2%)	0·2<br>(0·1 to 0·4)	34·7%<br>(26·6 to 41·9%)	0·2<br>(0·1 to 0·3)	33·0%<br>(25·0 to 40·2%)	-22·4%<br>(-29·3 to -15·5%)	-5·0%<br>(-7·1 to -3·2%)
Paratyphoid fever	0·0<br>(0·0 to 0·0)	34·6%<br>(27·1 to 41·7%)	0·0<br>(0·0 to 0·0)	32·9%<br>(25·4 to 40·2%)	3·2%<br>(-10·0 to 20·6%)	-5·0%<br>(-7·1 to -3·3%)	0·0<br>(0·0 to 0·0)	34·6%<br>(27·1 to 41·7%)	0·0<br>(0·0 to 0·0)	32·9%<br>(25·4 to 40·2%)	-3·8%<br>(-18·0 to 14·5%)	-5·0%<br>(-7·1 to -3·2%)
**Air pollution: All causes**	24·7<br>(20·9 to 28·7)	10·3%<br>(8·7 to 11·9%)	23·1<br>(19·0 to 28·0)	8·3%<br>(6·8 to 9·9%)	-6·5%<br>(-16·5 to 4·0%)	-19·8%<br>(-26·2 to -12·8%)	684·4<br>(568·8 to 803·2)	5·3%<br>(4·3 to 6·4%)	482·0<br>(393·3 to 580·2)	3·7%<br>(2·9 to 4·5%)	-29·6%<br>(-36·9 to -21·5%)	-30·4%<br>(-36·7 to -24·0%)
**Ambient particulate matter pollution: All causes**	16·0<br>(13·5 to 18·8)	6·7%<br>(5·6 to 7·8%)	15·5<br>(12·4 to 19·2)	5·5%<br>(4·4 to 6·8%)	-3·1%<br>(-13·3 to 7·0%)	-16·8%<br>(-23·3 to -10·1%)	433·3<br>(353·7 to 527·7)	3·3%<br>(2·7 to 4·1%)	318·9<br>(249·9 to 398·7)	2·4%<br>(1·9 to 3·1%)	-26·4%<br>(-33·9 to -18·7%)	-27·3%<br>(-34·1 to -21·1%)
Lower respiratory infections	7·3<br>(5·0 to 9·9)	25·0%<br>(17·6 to 33·3%)	6·3<br>(3·6 to 9·5)	19·3%<br>(11·3 to 29·0%)	-13·0%<br>(-32·3 to 4·1%)	-22·8%<br>(-38·1 to -11·0%)	250·7<br>(172·3 to 338·1)	24·6%<br>(17·3 to 33·1%)	145·6<br>(83·9 to 220·9)	18·9%<br>(10·9 to 28·5%)	-41·9%<br>(-55·6 to -31·5%)	-23·3%<br>(-38·7 to -11·2%)
Tracheal, bronchus, and lung cancer	0·6<br>(0·3 to 0·8)	17·3%<br>(10·6 to 24·5%)	0·6<br>(0·3 to 0·9)	12·9%<br>(7·2 to 19·4%)	7·6%<br>(-6·6 to 21·4%)	-25·3%<br>(-32·1 to -20·1%)	11·5<br>(6·9 to 16·3)	17·2%<br>(10·6 to 24·3%)	11·6<br>(6·5 to 17·8)	12·8%<br>(7·2 to 19·2%)	1·3%<br>(-12·1 to 14·1%)	-25·4%<br>(-32·1 to -20·2%)
Ischaemic heart disease	4·7<br>(3·8 to 5·7)	16·8%<br>(13·6 to 20·2%)	4·9<br>(3·7 to 6·2)	14·4%<br>(11·0 to 18·0%)	5·3%<br>(-5·1 to 16·4%)	-14·3%<br>(-19·7 to -9·7%)	97·2<br>(82·0 to 112·4)	19·0%<br>(16·0 to 21·8%)	92·0<br>(76·2 to 109·9)	16·2%<br>(13·5 to 18·9%)	-5·3%<br>(-14·2 to 4·4%)	-14·5%<br>(-18·2 to -11·4%)
Ischaemic stroke	0·9<br>(0·7 to 1·2)	12·6%<br>(9·4 to 15·9%)	1·0<br>(0·7 to 1·4)	10·5%<br>(7·4 to 14·3%)	9·0%<br>(-5·4 to 23·7%)	-16·7%<br>(-25·6 to -9·9%)	16·9<br>(13·7 to 20·3)	14·1%<br>(11·5 to 16·6%)	16·7<br>(13·0 to 21·1)	11·7%<br>(9·2 to 14·4%)	-1·1%<br>(-12·0 to 10·0%)	-16·7%<br>(-22·0 to -11·9%)
Hemorrhagic stroke	1·1<br>(0·9 to 1·3)	14·3%<br>(11·8 to 16·8%)	1·1<br>(0·9 to 1·4)	12·1%<br>(9·5 to 14·7%)	-1·9%<br>(-12·5 to 9·9%)	-15·3%<br>(-20·7 to -10·0%)	32·3<br>(26·6 to 38·7)	14·1%<br>(11·7 to 16·6%)	29·0<br>(23·1 to 36·1)	12·8%<br>(10·3 to 15·4%)	-10·1%<br>(-19·2 to -0·5%)	-9·4%<br>(-14·2 to -4·6%)
Chronic obstructive pulmonary disease	1·4<br>(0·8 to 2·1)	22·8%<br>(12·6 to 33·6%)	1·5<br>(0·7 to 2·4)	18·8%<br>(9·2 to 29·3%)	6·8%<br>(-12·9 to 22·4%)	-17·7%<br>(-30·4 to -11·5%)	24·7<br>(13·8 to 36·5)	20·7%<br>(11·6 to 30·3%)	23·9<br>(11·6 to 38·0)	17·6%<br>(8·7 to 27·3%)	-3·5%<br>(-20·7 to 8·6%)	-15·3%<br>(-28·6 to -8·4%)
**Household air pollution from solid fuels: All causes**	11·5<br>(8·1 to 15·4)	4·8%<br>(3·4 to 6·4%)	9·3<br>(6·3 to 13·5)	3·3%<br>(2·2 to 4·8%)	-19·3%<br>(-35·3 to -2·4%)	-30·8%<br>(-44·2 to -16·8%)	335·4<br>(231·5 to 451·6)	2·6%<br>(1·8 to 3·6%)	201·0<br>(136·2 to 291·5)	1·5%<br>(1·0 to 2·3%)	-40·1%<br>(-52·1 to -28·1%)	-40·8%<br>(-52·2 to -29·0%)
Lower respiratory infections	6·5<br>(4·0 to 9·3)	22·1%<br>(14·2 to 31·9%)	5·1<br>(3·0 to 8·2)	15·5%<br>(9·3 to 24·0%)	-20·7%<br>(-37·1 to -2·9%)	-29·7%<br>(-42·3 to -17·1%)	218·5<br>(136·5 to 315·6)	21·5%<br>(13·8 to 31·1%)	114·0<br>(67·8 to 181·5)	14·8%<br>(8·9 to 22·8%)	-47·8%<br>(-58·3 to -36·8%)	-31·1%<br>(-43·3 to -18·8%)
Tracheal, bronchus, and lung cancer	0·5<br>(0·3 to 0·8)	15·8%<br>(8·6 to 25·2%)	0·5<br>(0·2 to 0·8)	10·9%<br>(5·5 to 18·0%)	-1·1%<br>(-21·8 to 21·7%)	-31·2%<br>(-43·5 to -17·9%)	10·6<br>(5·8 to 17·0)	15·9%<br>(8·6 to 25·4%)	9·8<br>(4·9 to 16·4)	10·8%<br>(5·5 to 17·9%)	-7·6%<br>(-27·3 to 14·3%)	-32·0%<br>(-44·1 to -19·0%)
Ischaemic heart disease	2·4<br>(1·8 to 3·2)	8·6%<br>(6·5 to 11·5%)	1·8<br>(1·3 to 2·7)	5·4%<br>(3·8 to 7·8%)	-22·5%<br>(-38·9 to -3·6%)	-36·9%<br>(-49·3 to -23·6%)	51·5<br>(40·3 to 66·3)	10·0%<br>(7·8 to 12·7%)	35·6<br>(25·5 to 48·4)	6·3%<br>(4·6 to 8·5%)	-31·0%<br>(-45·2 to -15·4%)	-37·7%<br>(-49·7 to -25·1%)
Ischaemic stroke	0·5<br>(0·4 to 0·7)	7·0%<br>(4·9 to 9·8%)	0·4<br>(0·3 to 0·6)	4·4%<br>(2·8 to 6·6%)	-18·5%<br>(-36·4 to 2·9%)	-37·7%<br>(-50·5 to -21·9%)	9·8<br>(7·1 to 13·4)	8·2%<br>(5·9 to 10·9%)	7·2<br>(4·8 to 10·5)	5·0%<br>(3·4 to 7·3%)	-26·9%<br>(-42·4 to -7·9%)	-38·4%<br>(-50·3 to -23·2%)
Hemorrhagic stroke	0·7<br>(0·5 to 0·9)	8·5%<br>(6·2 to 11·3%)	0·5<br>(0·3 to 0·7)	5·4%<br>(3·7 to 7·7%)	-26·8%<br>(-43·0 to -8·1%)	-36·8%<br>(-49·1 to -21·9%)	20·0<br>(14·6 to 26·8)	8·7%<br>(6·3 to 11·6%)	13·5<br>(9·0 to 19·3)	5·9%<br>(4·0 to 8·3%)	-32·3%<br>(-46·9 to -16·4%)	-31·8%<br>(-45·0 to -16·9%)
Chronic obstructive pulmonary disease	1·0<br>(0·5 to 1·7)	16·6%<br>(8·4 to 27·9%)	0·9<br>(0·4 to 1·7)	11·8%<br>(5·5 to 20·9%)	-7·5%<br>(-28·4 to 15·1%)	-28·7%<br>(-42·8 to -14·4%)	18·8<br>(9·5 to 31·5)	15·7%<br>(7·9 to 26·1%)	15·3<br>(7·0 to 27·5)	11·2%<br>(5·2 to 19·6%)	-18·6%<br>(-35·3 to -1·0%)	-28·5%<br>(-42·1 to -15·0%)
Cataract	-	-	-	-	-	-	6·2<br>(4·2 to 8·5)	17·7%<br>(14·7 to 20·8%)	5·7<br>(3·7 to 8·2)	12·6%<br>(9·6 to 16·1%)	-9·2%<br>(-23·7 to 6·5%)	-28·6%<br>(-40·4 to -16·4%)
**Ambient ozone pollution: All causes**	0·1<br>(0·0 to 0·2)	0·0%<br>(0·0 to 0·1%)	0·2<br>(0·1 to 0·5)	0·1%<br>(0·0 to 0·2%)	134·5%<br>(97·2 to 194·3%)	101·3%<br>(70·0 to 150·9%)	1·6<br>(0·5 to 3·1)	0·0%<br>(0·0 to 0·0%)	3·1<br>(1·0 to 6·0)	0·0%<br>(0·0 to 0·0%)	101·7%<br>(71·1 to 145·8%)	99·2%<br>(70·6 to 141·2%)
Chronic obstructive pulmonary disease	0·1<br>(0·0 to 0·2)	1·6%<br>(0·5 to 3·2%)	0·2<br>(0·1 to 0·5)	2·9%<br>(1·0 to 5·5%)	134·5%<br>(97·2 to 194·3%)	80·8%<br>(57·3 to 117·0%)	1·6<br>(0·5 to 3·1)	1·3%<br>(0·4 to 2·6%)	3·1<br>(1·0 to 6·0)	2·3%<br>(0·8 to 4·3%)	101·7%<br>(71·1 to 145·8%)	77·1%<br>(55·3 to 110·8%)
**Other environmental risks: All causes**	2·6<br>(1·5 to 3·7)	1·1%<br>(0·6 to 1·5%)	3·3<br>(2·0 to 4·8)	1·2%<br>(0·7 to 1·7%)	28·1%<br>(15·4 to 42·5%)	10·0%<br>(1·1 to 20·9%)	49·8<br>(27·4 to 73·7)	0·4%<br>(0·2 to 0·6%)	57·1<br>(31·8 to 85·0)	0·4%<br>(0·2 to 0·7%)	14·5%<br>(5·0 to 25·1%)	13·1%<br>(4·8 to 21·5%)
**Residential radon: All causes**	0·1<br>(0·1 to 0·2)	0·1%<br>(0·0 to 0·1%)	0·2<br>(0·1 to 0·3)	0·1%<br>(0·0 to 0·1%)	43·5%<br>(28·8 to 58·2%)	23·2%<br>(14·4 to 32·4%)	2·8<br>(2·1 to 3·7)	0·0%<br>(0·0 to 0·0%)	3·9<br>(2·7 to 5·1)	0·0%<br>(0·0 to 0·0%)	36·3%<br>(23·8 to 48·8%)	34·6%<br>(24·1 to 45·3%)
Tracheal, bronchus, and lung cancer	0·1<br>(0·1 to 0·2)	4·3%<br>(2·9 to 5·8%)	0·2<br>(0·1 to 0·3)	4·3%<br>(2·9 to 5·8%)	43·5%<br>(28·8 to 58·2%)	-0·2%<br>(-4·9 to 4·6%)	2·8<br>(2·1 to 3·7)	4·2%<br>(3·1 to 5·5%)	3·9<br>(2·7 to 5·1)	4·2%<br>(3·0 to 5·5%)	36·3%<br>(23·8 to 48·8%)	0·5%<br>(-3·5 to 3·9%)
**Lead exposure: All causes**	2·4<br>(1·4 to 3·6)	1·0%<br>(0·6 to 1·5%)	3·1<br>(1·8 to 4·6)	1·1%<br>(0·6 to 1·6%)	27·2%<br>(14·0 to 42·3%)	9·2%<br>(-0·6 to 20·9%)	47·0<br>(24·7 to 70·9)	0·4%<br>(0·2 to 0·5%)	53·2<br>(28·1 to 80·9)	0·4%<br>(0·2 to 0·6%)	13·2%<br>(3·1 to 23·7%)	11·8%<br>(3·2 to 20·2%)
Rheumatic heart disease	0·0<br>(0·0 to 0·0)	1·6%<br>(0·7 to 3·1%)	0·0<br>(0·0 to 0·0)	1·6%<br>(0·7 to 3·3%)	3·6%<br>(-10·4 to 22·4%)	1·6%<br>(-7·7 to 15·2%)	0·3<br>(0·1 to 0·6)	1·1%<br>(0·4 to 2·1%)	0·3<br>(0·1 to 0·5)	1·2%<br>(0·4 to 2·2%)	-8·8%<br>(-19·3 to 2·9%)	6·9%<br>(-2·9 to 18·1%)
Ischaemic heart disease	1·2<br>(0·6 to 1·8)	4·2%<br>(2·3 to 6·5%)	1·4<br>(0·8 to 2·2)	4·1%<br>(2·2 to 6·4%)	19·8%<br>(4·5 to 35·7%)	-2·4%<br>(-11·2 to 7·6%)	19·5<br>(9·9 to 30·3)	3·8%<br>(1·9 to 5·8%)	21·0<br>(10·7 to 32·8)	3·7%<br>(1·9 to 5·8%)	7·8%<br>(-2·9 to 18·9%)	-2·7%<br>(-8·0 to 2·8%)
Ischaemic stroke	0·3<br>(0·2 to 0·5)	3·9%<br>(2·1 to 6·0%)	0·4<br>(0·2 to 0·6)	3·7%<br>(2·0 to 5·9%)	24·7%<br>(9·0 to 43·5%)	-4·7%<br>(-13·8 to 5·5%)	4·6<br>(2·3 to 7·1)	3·8%<br>(1·9 to 5·9%)	5·2<br>(2·6 to 8·2)	3·6%<br>(1·8 to 5·7%)	13·4%<br>(1·7 to 26·3%)	-4·4%<br>(-10·3 to 1·9%)
Hemorrhagic stroke	0·3<br>(0·2 to 0·5)	4·1%<br>(2·1 to 6·4%)	0·4<br>(0·2 to 0·6)	4·1%<br>(2·0 to 6·4%)	14·6%<br>(1·1 to 31·5%)	-1·1%<br>(-8·1 to 8·3%)	7·6<br>(3·3 to 12·7)	3·3%<br>(1·4 to 5·5%)	7·8<br>(3·3 to 13·4)	3·4%<br>(1·5 to 5·7%)	2·2%<br>(-7·7 to 13·9%)	3·0%<br>(-3·5 to 9·4%)
Hypertensive heart disease	0·2<br>(0·0 to 0·4)	8·0%<br>(2·4 to 18·2%)	0·3<br>(0·1 to 0·6)	7·8%<br>(2·2 to 18·1%)	59·4%<br>(28·5 to 93·8%)	-2·7%<br>(-16·0 to 13·3%)	2·6<br>(0·9 to 5·5)	6·9%<br>(2·4 to 14·5%)	3·8<br>(1·2 to 8·2)	6·8%<br>(2·2 to 14·5%)	45·6%<br>(23·2 to 66·6%)	-2·3%<br>(-12·5 to 7·8%)
Cardiomyopathy and myocarditis	0·0<br>(0·0 to 0·0)	1·8%<br>(0·9 to 2·7%)	0·0<br>(0·0 to 0·0)	2·0%<br>(1·0 to 3·2%)	19·9%<br>(4·8 to 39·3%)	15·1%<br>(4·0 to 28·2%)	0·4<br>(0·2 to 0·6)	0·9%<br>(0·4 to 1·6%)	0·4<br>(0·2 to 0·7)	1·3%<br>(0·6 to 2·2%)	8·6%<br>(-4·5 to 23·3%)	39·9%<br>(21·2 to 60·6%)

Supplemental Results Table 1. All-age deaths, death PAFs, DALYs, DALY PAFs, and percent change for each risk factor and outcome for both sexes combined in 2005 and 2015 for Andean Latin America.

Data in parenthesis are 95% uncertainty intervals. PAFs=population attributable fractions. DALYs=disability-adjusted life-years.

	2005 Deaths (in thousands)	2005 Deaths PAF	2015 Deaths (in thousands)	2015 Deaths PAF	Percent change deaths 2005-2015	Percent change deaths PAF 2005-2015	2005 DALYs (in thousands)	2005 DALYs PAF	2015 DALYs (in thousands)	2015 DALYs PAF	Percent change DALYs 2005-2015	Percent change DALYs PAF 2005-2015
Atrial fibrillation and flutter	0·0 (0·0 to 0·0)	2·6% (1·4 to 3·9%)	0·0 (0·0 to 0·0)	2·5% (1·4 to 3·7%)	42·1% (19·7 to 66·5%)	-3·5% (-17·0 to 11·1%)	0·4 (0·2 to 0·6)	2·9% (1·6 to 4·2%)	0·5 (0·3 to 0·8)	2·7% (1·5 to 4·0%)	31·6% (20·4 to 42·7%)	-5·7% (-12·8 to 0·9%)
Aortic aneurysm	0·0 (0·0 to 0·0)	2·5% (1·3 to 3·8%)	0·0 (0·0 to 0·0)	2·3% (1·2 to 3·6%)	42·5% (22·6 to 63·9%)	-5·6% (-13·1 to 3·2%)	0·2 (0·1 to 0·3)	2·4% (1·2 to 3·8%)	0·3 (0·1 to 0·5)	2·2% (1·1 to 3·6%)	30·9% (13·9 to 48·9%)	-5·8% (-11·7 to -0·9%)
Peripheral vascular disease	0·0 (0·0 to 0·0)	1·8% (1·0 to 2·9%)	0·0 (0·0 to 0·0)	1·8% (0·9 to 2·9%)	95·0% (55·1 to 143·3%)	-3·8% (-15·5 to 9·4%)	0·0 (0·0 to 0·1)	2·0% (1·1 to 3·0%)	0·0 (0·0 to 0·1)	1·8% (1·0 to 2·9%)	41·2% (28·7 to 54·5%)	-6·2% (-13·4 to 0·5%)
Endocarditis	0·0 (0·0 to 0·0)	1·8% (0·9 to 2·8%)	0·0 (0·0 to 0·0)	2·0% (1·0 to 3·1%)	30·0% (11·2 to 53·2%)	10·5% (0·8 to 23·8%)	0·2 (0·1 to 0·4)	0·9% (0·4 to 1·6%)	0·3 (0·1 to 0·5)	1·2% (0·5 to 2·1%)	14·0% (-0·4 to 30·4%)	27·3% (10·6 to 46·3%)
Other cardiovascular and circulatory diseases	0·1 (0·0 to 0·1)	2·4% (1·3 to 3·6%)	0·1 (0·0 to 0·1)	2·5% (1·4 to 3·7%)	27·5% (9·4 to 50·8%)	3·5% (-4·8 to 13·8%)	1·6 (0·7 to 2·6)	1·5% (0·7 to 2·4%)	1·9 (0·9 to 3·2)	1·7% (0·8 to 2·8%)	18·4% (7·9 to 30·0%)	14·4% (6·9 to 23·2%)
Idiopathic developmental intellectual disability	-	-	-	-	-	-	2·6 (1·0 to 4·9)	18·3% (11·3 to 24·6%)	2·5 (0·9 to 4·9)	15·1% (8·3 to 21·3%)	-3·9% (-51·3 to 70·8%)	-17·7% (-58·0 to 44·9%)
Chronic kidney disease due to diabetes mellitus	0·2 (0·1 to 0·3)	3·6% (1·8 to 5·9%)	0·3 (0·1 to 0·4)	3·5% (1·8 to 5·8%)	43·3% (27·7 to 60·0%)	-1·7% (-8·8 to 4·9%)	3·5 (1·6 to 6·1)	2·8% (1·4 to 4·9%)	4·6 (2·1 to 7·9)	2·8% (1·3 to 4·8%)	31·3% (18·5 to 44·4%)	-1·3% (-6·9 to 3·0%)
Chronic kidney disease due to hypertension	0·2 (0·1 to 0·3)	3·5% (1·8 to 5·6%)	0·3 (0·1 to 0·4)	3·7% (2·0 to 5·8%)	57·7% (36·6 to 82·2%)	4·9% (-6·3 to 16·9%)	2·5 (1·2 to 4·3)	2·3% (1·0 to 3·8%)	3·5 (1·6 to 5·9)	2·6% (1·2 to 4·2%)	36·8% (22·8 to 51·9%)	12·7% (5·8 to 21·4%)
Chronic kidney disease due to glomerulonephritis	0·0 (0·0 to 0·0)	2·9% (1·5 to 4·5%)	0·0 (0·0 to 0·0)	3·3% (1·7 to 5·2%)	48·6% (27·9 to 72·0%)	14·0% (1·3 to 28·5%)	0·6 (0·2 to 1·1)	1·8% (0·8 to 3·1%)	0·7 (0·3 to 1·4)	2·0% (0·8 to 3·4%)	26·7% (18·4 to 34·3%)	9·4% (2·6 to 15·7%)
Chronic kidney disease due to other causes	0·0 (0·0 to 0·0)	2·8% (1·5 to 4·5%)	0·0 (0·0 to 0·0)	3·4% (1·9 to 5·3%)	55·4% (28·5 to 88·3%)	20·7% (4·6 to 41·4%)	0·4 (0·2 to 0·8)	2·2% (1·0 to 3·8%)	0·5 (0·2 to 1·0)	2·2% (1·0 to 3·9%)	29·5% (20·9 to 37·3%)	3·3% (-3·0 to 9·0%)
**Occupational risks: All causes**	3·9 (3·4 to 4·9)	1·6% (1·4 to 2·1%)	5·2 (4·4 to 6·8)	1·9% (1·6 to 2·4%)	34·1% (4·9 to 80·1%)	15·1% (-11·4 to 54·7%)	328·0 (263·2 to 411·9)	2·5% (2·2 to 3·1%)	418·8 (332·2 to 538·2)	3·2% (2·7 to 3·9%)	27·7% (6·8 to 57·8%)	26·0% (4·7 to 56·3%)
**Occupational carcinogens: All causes**	1·3 (1·2 to 1·4)	0·6% (0·5 to 0·6%)	1·8 (1·6 to 2·0)	0·6% (0·6 to 0·7%)	33·6% (17·9 to 50·4%)	14·7% (3·7 to 24·9%)	27·5 (25·6 to 29·4)	0·2% (0·2 to 0·2%)	36·2 (32·2 to 40·5)	0·3% (0·2 to 0·3%)	31·8% (16·4 to 48·3%)	30·1% (16·4 to 43·9%)
**Occupational exposure to asbestos: All causes**	0·2 (0·2 to 0·3)	0·1% (0·1 to 0·1%)	0·3 (0·2 to 0·4)	0·1% (0·1 to 0·1%)	19·3% (-2·4 to 47·1%)	2·3% (-13·9 to 22·0%)	3·3 (2·7 to 4·1)	0·0% (0·0 to 0·0%)	3·6 (2·7 to 4·6)	0·0% (0·0 to 0·0%)	7·3% (-11·5 to 29·6%)	5·9% (-11·0 to 26·6%)
Larynx cancer	0·0 (0·0 to 0·0)	1·3% (0·8 to 2·0%)	0·0 (0·0 to 0·0)	1·1% (0·7 to 1·7%)	4·2% (-22·4 to 39·9%)	-14·3% (-31·3 to 8·0%)	0·0 (0·0 to 0·1)	0·8% (0·4 to 1·1%)	0·0 (0·0 to 0·1)	0·6% (0·4 to 1·0%)	-4·1% (-25·9 to 25·1%)	-16·5% (-30·7 to 1·6%)
Tracheal, bronchus, and lung cancer	0·2 (0·1 to 0·2)	5·2% (4·0 to 6·5%)	0·2 (0·2 to 0·3)	4·5% (3·5 to 5·8%)	24·8% (0·4 to 55·5%)	-13·3% (-26·5 to 2·5%)	2·2 (1·7 to 2·8)	3·3% (2·5 to 4·1%)	2·5 (1·9 to 3·3)	2·7% (2·1 to 3·4%)	13·6% (-7·2 to 40·1%)	-16·3% (-27·3 to -2·7%)
Ovarian cancer	0·0 (0·0 to 0·0)	1·7% (0·8 to 2·7%)	0·0 (0·0 to 0·0)	1·4% (0·7 to 2·3%)	18·8% (-12·8 to 62·4%)	-15·8% (-32·3 to 5·8%)	0·2 (0·1 to 0·3)	1·0% (0·5 to 1·7%)	0·2 (0·1 to 0·3)	0·8% (0·4 to 1·4%)	9·1% (-18·3 to 46·4%)	-19·9% (-35·1 to -0·5%)
Mesothelioma	0·0 (0·0 to 0·1)	52·2% (36·0 to 66·9%)	0·0 (0·0 to 0·1)	49·2% (33·5 to 64·9%)	1·7% (-16·3 to 22·1%)	-5·7% (-13·9 to 1·9%)	0·9 (0·5 to 1·2)	37·9% (23·9 to 52·2%)	0·8 (0·5 to 1·2)	34·4% (21·1 to 48·9%)	-8·0% (-25·2 to 10·6%)	-9·3% (-18·8 to -1·0%)
**Occupational exposure to arsenic: All causes**	0·0 (0·0 to 0·0)	0·0% (0·0 to 0·0%)	0·0 (0·0 to 0·0)	0·0% (0·0 to 0·0%)	15·0% (-1·1 to 32·9%)	-1·3% (-13·0 to 11·5%)	0·6 (0·4 to 0·7)	0·0% (0·0 to 0·0%)	0·6 (0·5 to 0·8)	0·0% (0·0 to 0·0%)	14·0% (-2·0 to 32·1%)	12·6% (-2·1 to 29·0%)
Tracheal, bronchus, and lung cancer	0·0 (0·0 to 0·0)	0·8% (0·6 to 1·1%)	0·0 (0·0 to 0·0)	0·7% (0·5 to 0·9%)	15·0% (-1·1 to 32·9%)	-20·0% (-28·0 to -10·6%)	0·6 (0·4 to 0·7)	0·8% (0·7 to 1·0%)	0·6 (0·5 to 0·8)	0·7% (0·5 to 0·9%)	14·0% (-2·0 to 32·1%)	-15·9% (-24·4 to -6·0%)
**Occupational exposure to benzene: All causes**	0·0 (0·0 to 0·1)	0·0% (0·0 to 0·0%)	0·1 (0·1 to 0·1)	0·0% (0·0 to 0·0%)	35·9% (18·5 to 55·8%)	16·7% (3·7 to 30·7%)	1·7 (1·4 to 2·0)	0·0% (0·0 to 0·0%)	2·3 (1·8 to 2·8)	0·0% (0·0 to 0·0%)	36·4% (18·6 to 56·6%)	34·7% (18·2 to 51·7%)
Leukaemia	0·0 (0·0 to 0·1)	2·4% (2·0 to 2·8%)	0·1 (0·1 to 0·1)	2·7% (2·2 to 3·2%)	35·9% (18·5 to 55·8%)	12·4% (-0·1 to 24·3%)	1·7 (1·4 to 2·0)	1·8% (1·5 to 2·1%)	2·3 (1·8 to 2·8)	2·2% (1·8 to 2·7%)	36·4% (18·6 to 56·6%)	23·9% (8·2 to 39·6%)
**Occupational exposure to beryllium: All causes**	0·0 (0·0 to 0·0)	0·0% (0·0 to 0·0%)	0·0 (0·0 to 0·0)	0·0% (0·0 to 0·0%)	12·1% (-5·8 to 32·2%)	-3·8% (-17·9 to 11·5%)	0·0 (0·0 to 0·0)	0·0% (0·0 to 0·0%)	0·0 (0·0 to 0·1)	0·0% (0·0 to 0·0%)	11·2% (-6·5 to 31·0%)	9·8% (-6·5 to 28·3%)
Tracheal, bronchus, and lung cancer	0·0 (0·0 to 0·0)	0·1% (0·0 to 0·1%)	0·0 (0·0 to 0·0)	0·0% (0·0 to 0·1%)	12·1% (-5·8 to 32·2%)	-22·0% (-32·1 to -10·0%)	0·0 (0·0 to 0·0)	0·1% (0·0 to 0·1%)	0·0 (0·0 to 0·1)	0·0% (0·0 to 0·1%)	11·2% (-6·5 to 31·0%)	-18·0% (-28·2 to -5·6%)
**Occupational exposure to cadmium: All causes**	0·0 (0·0 to 0·0)	0·0% (0·0 to 0·0%)	0·0 (0·0 to 0·0)	0·0% (0·0 to 0·0%)	23·9% (6·6 to 43·0%)	6·4% (-6·3 to 21·2%)	0·1 (0·1 to 0·2)	0·0% (0·0 to 0·0%)	0·2 (0·1 to 0·2)	0·0% (0·0 to 0·0%)	22·9% (6·1 to 42·2%)	21·4% (6·1 to 38·9%)
Tracheal, bronchus, and lung cancer	0·0 (0·0 to 0·0)	0·2% (0·2 to 0·3%)	0·0 (0·0 to 0·0)	0·2% (0·1 to 0·2%)	23·9% (6·6 to 43·0%)	-13·8% (-22·7 to -3·3%)	0·1 (0·1 to 0·2)	0·2% (0·2 to 0·3%)	0·2 (0·1 to 0·2)	0·2% (0·2 to 0·2%)	22·9% (6·1 to 42·2%)	-9·3% (-18·5 to 1·1%)
**Occupational exposure to chromium: All causes**	0·0 (0·0 to 0·0)	0·0% (0·0 to 0·0%)	0·0 (0·0 to 0·0)	0·0% (0·0 to 0·0%)	20·5% (3·6 to 37·7%)	3·4% (-9·7 to 16·6%)	0·5 (0·4 to 0·6)	0·0% (0·0 to 0·0%)	0·6 (0·5 to 0·7)	0·0% (0·0 to 0·0%)	19·5% (3·1 to 37·2%)	18·0% (3·3 to 33·9%)
Tracheal, bronchus, and lung cancer	0·0 (0·0 to 0·0)	0·7% (0·6 to 0·8%)	0·0 (0·0 to 0·0)	0·6% (0·5 to 0·7%)	20·5% (3·6 to 37·7%)	-16·2% (-25·3 to -6·1%)	0·5 (0·4 to 0·6)	0·7% (0·6 to 0·8%)	0·6 (0·5 to 0·7)	0·7% (0·6 to 0·8%)	19·5% (3·1 to 37·2%)	-11·9% (-20·9 to -1·9%)
**Occupational exposure to diesel engine exhaust: All causes**	0·4 (0·4 to 0·5)	0·2% (0·1 to 0·2%)	0·6 (0·5 to 0·7)	0·2% (0·2 to 0·3%)	50·7% (25·2 to 83·1%)	29·3% (9·2 to 52·5%)	8·3 (7·3 to 9·4)	0·1% (0·1 to 0·1%)	12·4 (10·2 to 15·0)	0·1% (0·1 to 0·1%)	49·4% (24·6 to 81·6%)	47·6% (24·0 to 77·4%)
Tracheal, bronchus, and lung cancer	0·4 (0·4 to 0·5)	12·6% (11·2 to 13·9%)	0·6 (0·5 to 0·7)	13·2% (11·0 to 15·6%)	50·7% (25·2 to 83·1%)	4·8% (-10·4 to 21·6%)	8·3 (7·3 to 9·4)	12·4% (11·1 to 13·8%)	12·4 (10·2 to 15·0)	13·7% (11·5 to 16·2%)	49·4% (24·6 to 81·6%)	10·2% (-5·6 to 27·5%)
**Occupational exposure to second-hand smoke: All causes**	0·5 (0·5 to 0·6)	0·2% (0·2 to 0·2%)	0·7 (0·6 to 0·8)	0·2% (0·2 to 0·3%)	26·1% (8·8 to 43·9%)	8·2% (-5·2 to 21·3%)	11·3 (10·5 to 12·2)	0·1% (0·1 to 0·1%)	14·2 (12·3 to 16·1)	0·1% (0·1 to 0·1%)	25·4% (8·2 to 43·2%)	23·8% (7·9 to 40·4%)
Tracheal, bronchus, and lung cancer	0·5 (0·5 to 0·6)	17·1% (16·2 to 18·0%)	0·7 (0·6 to 0·8)	15·0% (13·3 to 16·5%)	26·1% (8·8 to 43·9%)	-12·3% (-21·8 to -2·9%)	11·3 (10·5 to 12·2)	16·9% (16·1 to 17·8%)	14·2 (12·3 to 16·1)	15·7% (13·9 to 17·3%)	25·4% (8·2 to 43·2%)	-7·6% (-17·2 to 2·4%)
**Occupational exposure to formaldehyde: All causes**	0·0 (0·0 to 0·0)	0·0% (0·0 to 0·0%)	0·0 (0·0 to 0·0)	0·0% (0·0 to 0·0%)	17·5% (2·4 to 35·0%)	0·8% (-10·0 to 13·5%)	0·3 (0·3 to 0·4)	0·0% (0·0 to 0·0%)	0·4 (0·3 to 0·4)	0·0% (0·0 to 0·0%)	18·0% (2·3 to 36·0%)	16·5% (2·8 to 32·3%)
Nasopharynx cancer	0·0 (0·0 to 0·0)	1·6% (1·1 to 2·2%)	0·0 (0·0 to 0·0)	1·4% (1·0 to 2·0%)	12·5% (-4·7 to 32·8%)	-9·3% (-18·8 to 1·9%)	0·0 (0·0 to 0·0)	1·6% (1·1 to 2·2%)	0·0 (0·0 to 0·0)	1·5% (1·0 to 2·1%)	12·0% (-5·2 to 32·2%)	-4·0% (-14·5 to 8·3%)
**Occupational exposure to nickel: All causes**	0·1 (0·1 to 0·1)	0·0% (0·0 to 0·1%)	0·1 (0·1 to 0·1)	0·0% (0·0 to 0·1%)	12·4% (-6·2 to 32·8%)	-3·5% (-17·8 to 12·2%)	2·0 (1·4 to 2·6)	0·0% (0·0 to 0·0%)	2·2 (1·6 to 3·0)	0·0% (0·0 to 0·0%)	11·4% (-6·6 to 31·1%)	10·0% (-6·7 to 28·9%)
Tracheal, bronchus, and lung cancer	0·1 (0·1 to 0·1)	3·0% (2·2 to 3·9%)	0·1 (0·1 to 0·1)	2·3% (1·7 to 3·2%)	12·4% (-6·2 to 32·8%)	-21·8% (-31·9 to -10·2%)	2·0 (1·4 to 2·6)	3·0% (2·2 to 3·8%)	2·2 (1·6 to 3·0)	2·4% (1·8 to 3·2%)	11·4% (-6·6 to 31·1%)	-17·8% (-28·2 to -5·9%)
**Occupational exposure to polycyclic aromatic hydrocarbons: All causes**	0·1 (0·0 to 0·1)	0·0% (0·0 to 0·0%)	0·1 (0·1 to 0·1)	0·0% (0·0 to 0·0%)	32·9% (15·3 to 51·8%)	14·1% (0·7 to 27·2%)	1·2 (1·0 to 1·4)	0·0% (0·0 to 0·0%)	1·6 (1·2 to 1·9)	0·0% (0·0 to 0·0%)	31·9% (13·9 to 51·4%)	30·2% (14·3 to 46·7%)
Tracheal, bronchus, and lung cancer	0·1 (0·0 to 0·1)	1·8% (1·5 to 2·1%)	0·1 (0·1 to 0·1)	1·6% (1·3 to 2·0%)	32·9% (15·3 to 51·8%)	-7·5% (-16·7 to 2·1%)	1·2 (1·0 to 1·4)	1·8% (1·5 to 2·1%)	1·6 (1·2 to 1·9)	1·7% (1·4 to 2·1%)	31·9% (13·9 to 51·4%)	-2·8% (-12·0 to 7·0%)
**Occupational exposure to silica: All causes**	0·2 (0·2 to 0·2)	0·1% (0·1 to 0·1%)	0·3 (0·2 to 0·4)	0·1% (0·1 to 0·1%)	45·6% (17·3 to 80·6%)	25·0% (2·7 to 54·1%)	4·0 (3·5 to 4·5)	0·0% (0·0 to 0·0%)	5·8 (4·6 to 7·2)	0·0% (0·0 to 0·1%)	44·3% (16·9 to 79·1%)	42·5% (17·2 to 75·3%)
Tracheal, bronchus, and lung cancer	0·2 (0·2 to 0·2)	6·1% (5·4 to 6·8%)	0·3 (0·2 to 0·4)	6·1% (5·1 to 7·6%)	45·6% (17·3 to 80·6%)	1·3% (-15·6 to 23·5%)	4·0 (3·5 to 4·5)	6·0% (5·4 to 6·7%)	5·8 (4·6 to 7·2)	6·4% (5·3 to 7·8%)	44·3% (16·9 to 79·1%)	6·4% (-11·2 to 29·8%)
**Occupational exposure to sulfphuric acid: All causes**	0·0 (0·0 to 0·1)	0·0% (0·0 to 0·0%)	0·0 (0·0 to 0·1)	0·0% (0·0 to 0·0%)	9·5% (-6·7 to 29·7%)	-6·0% (-18·9 to 8·8%)	0·9 (0·7 to 1·1)	0·0% (0·0 to 0·0%)	1·0 (0·7 to 1·2)	0·0% (0·0 to 0·0%)	9·1% (-6·8 to 27·6%)	7·7% (-6·9 to 24·1%)
Larynx cancer	0·0 (0·0 to 0·1)	14·2% (11·0 to 17·8%)	0·0 (0·0 to 0·1)	12·8% (9·7 to 16·3%)	9·5% (-6·7 to 29·7%)	-9·8% (-18·4 to -0·9%)	0·9 (0·7 to 1·1)	13·9% (10·9 to 17·1%)	1·0 (0·7 to 1·2)	13·2% (10·2 to 16·6%)	9·1% (-6·8 to 27·6%)	-5·0% (-13·5 to 3·5%)
**Occupational exposure to trichloroethylene: All causes**	0·0 (0·0 to 0·0)	0·0% (0·0 to 0·0%)	0·0 (0·0 to 0·0)	0·0% (0·0 to 0·0%)	42·0% (19·1 to 66·7%)	21·8% (4·6 to 39·6%)	0·0 (0·0 to 0·1)	0·0% (0·0 to 0·0%)	0·1 (0·0 to 0·1)	0·0% (0·0 to 0·0%)	41·6% (19·9 to 64·9%)	39·8% (19·4 to 61·4%)
Kidney cancer	0·0 (0·0 to 0·0)	0·4% (0·1 to 0·7%)	0·0 (0·0 to 0·0)	0·3% (0·1 to 0·6%)	42·0% (19·1 to 66·7%)	-6·5% (-15·0 to 3·7%)	0·0 (0·0 to 0·1)	0·3% (0·1 to 0·6%)	0·1 (0·0 to 0·1)	0·3% (0·1 to 0·6%)	41·6% (19·9 to 64·9%)	1·5% (-8·0 to 12·3%)

Supplemental Results Table 1. All-age deaths, death PAFs, DALYs, DALY PAFs, and percent change for each risk factor and outcome for both sexes combined in 2005 and 2015 for Andean Latin America.

Data in parenthesis are 95% uncertainty intervals. PAFs=population attributable fractions. DALYs=disability-adjusted life-years.

	2005 Deaths (in thousands)	2005 Deaths PAF	2015 Deaths (in thousands)	2015 Deaths PAF	Percent change deaths 2005-2015	Percent change deaths PAF 2005-2015	2005 DALYs (in thousands)	2005 DALYs PAF	2015 DALYs (in thousands)	2015 DALYs PAF	Percent change DALYs 2005-2015	Percent change DALYs PAF 2005-2015
**Occupational asthmagens: All causes**	0·0 (0·0 to 0·0)	0·0% (0·0 to 0·0%)	0·0 (0·0 to 0·0)	0·0% (0·0 to 0·0%)	-14·0% (-26·6 to 0·1%)	-26·2% (-36·0 to -15·5%)	8·8 (5·9 to 12·3)	0·1% (0·0 to 0·1%)	10·4 (6·8 to 14·6)	0·1% (0·1 to 0·1%)	17·7% (6·8 to 28·9%)	16·0% (5·1 to 28·3%)
Asthma	0·0 (0·0 to 0·0)	6·8% (5·9 to 7·7%)	0·0 (0·0 to 0·0)	6·3% (5·4 to 7·3%)	-14·0% (-26·6 to 0·1%)	-7·3% (-20·4 to 6·5%)	8·8 (5·9 to 12·3)	4·9% (4·1 to 5·7%)	10·4 (6·8 to 14·6)	5·5% (4·6 to 6·4%)	17·7% (6·8 to 28·9%)	12·1% (2·1 to 22·8%)
**Occupational particulate matter, gases, and fumes: All causes**	0·8 (0·7 to 1·0)	0·3% (0·3 to 0·4%)	1·1 (0·9 to 1·4)	0·4% (0·3 to 0·5%)	34·8% (18·6 to 54·5%)	15·8% (2·9 to 29·5%)	17·5 (15·3 to 19·9)	0·1% (0·1 to 0·2%)	21·3 (18·0 to 25·0)	0·2% (0·1 to 0·2%)	21·8% (9·7 to 34·6%)	20·3% (9·1 to 31·3%)
Chronic obstructive pulmonary disease	0·8 (0·7 to 1·0)	13·4% (10·8 to 16·4%)	1·1 (0·9 to 1·4)	13·9% (10·9 to 17·4%)	34·9% (18·7 to 54·5%)	4·0% (-3·7 to 12·3%)	17·4 (15·2 to 19·8)	14·6% (12·9 to 16·3%)	21·2 (17·9 to 24·9)	15·6% (13·6 to 17·7%)	21·9% (9·8 to 34·6%)	7·1% (0·3 to 14·0%)
**Occupational noise: All causes**	-	-	-	-	-	-	59·5 (40·8 to 81·9)	0·5% (0·3 to 0·6%)	79·2 (54·0 to 110·1)	0·6% (0·5 to 0·7%)	33·1% (29·6 to 36·5%)	31·3% (25·8 to 37·8%)
Age-related and other hearing loss	-	-	-	-	-	-	59·5 (40·8 to 81·9)	28·4% (26·1 to 31·3%)	79·2 (54·0 to 110·1)	28·9% (26·6 to 31·7%)	33·1% (29·6 to 36·5%)	1·7% (-0·7 to 4·2%)
**Occupational injuries: All causes**	1·7 (1·2 to 3·0)	0·7% (0·5 to 1·2%)	2·3 (1·6 to 4·1)	0·8% (0·6 to 1·5%)	35·2% (-23·6 to 157·1%)	16·1% (-35·3 to 122·7%)	102·1 (74·6 to 173·6)	0·8% (0·6 to 1·3%)	135·7 (96·0 to 233·9)	1·0% (0·7 to 1·8%)	32·9% (-23·2 to 145·8%)	31·4% (-24·9 to 143·4%)
Pedestrian road injuries	0·4 (0·3 to 0·8)	9·4% (6·4 to 17·0%)	0·6 (0·4 to 1·1)	11·5% (7·6 to 21·4%)	35·1% (-30·9 to 173·6%)	22·6% (-37·1 to 153·6%)	20·3 (13·7 to 37·6)	9·5% (6·4 to 17·1%)	26·6 (17·6 to 50·5)	12·8% (8·4 to 23·5%)	30·9% (-32·5 to 163·6%)	34·1% (-31·1 to 179·0%)
Cyclist road injuries	0·0 (0·0 to 0·0)	11·4% (7·5 to 22·3%)	0·0 (0·0 to 0·1)	13·6% (8·6 to 27·7%)	81·5% (-17·6 to 309·8%)	18·8% (-43·5 to 159·2%)	1·3 (0·8 to 2·6)	11·7% (7·7 to 22·5%)	2·3 (1·5 to 4·6)	14·5% (9·3 to 29·3%)	75·4% (-18·5 to 295·2%)	24·2% (-40·4 to 165·7%)
Motorcyclist road injuries	0·1 (0·1 to 0·1)	14·9% (10·3 to 26·5%)	0·1 (0·1 to 0·3)	17·6% (11·6 to 33·9%)	56·2% (-15·9 to 220·4%)	17·8% (-39·3 to 148·3%)	5·0 (3·4 to 8·8)	14·6% (10·0 to 25·9%)	7·7 (5·1 to 15·4)	17·6% (11·7 to 34·0%)	54·8% (-16·2 to 217·2%)	21·2% (-37·6 to 153·0%)
Motor vehicle road injuries	0·5 (0·3 to 0·8)	13·8% (10·0 to 22·9%)	0·6 (0·5 to 1·1)	17·1% (12·0 to 28·8%)	41·5% (-18·1 to 154·8%)	23·5% (-29·5 to 122·7%)	25·1 (18·3 to 42·4)	13·7% (9·9 to 22·7%)	34·9 (24·5 to 59·3)	17·9% (12·6 to 30·2%)	39·2% (-19·1 to 150·3%)	30·8% (-24·5 to 135·0%)
Other road injuries	0·0 (0·0 to 0·0)	12·7% (9·1 to 20·8%)	0·0 (0·0 to 0·0)	15·8% (10·8 to 26·8%)	87·6% (7·8 to 251·1%)	24·0% (-26·1 to 128·6%)	0·7 (0·5 to 1·2)	13·0% (9·3 to 21·4%)	1·4 (0·9 to 2·3)	17·2% (11·8 to 29·3%)	84·4% (9·0 to 236·7%)	32·0% (-21·4 to 140·0%)
Other transport injuries	0·0 (0·0 to 0·1)	12·1% (7·7 to 23·5%)	0·1 (0·0 to 0·1)	13·9% (8·7 to 27·3%)	46·6% (-28·8 to 220·2%)	14·9% (-44·4 to 145·6%)	2·7 (1·7 to 5·0)	11·6% (7·2 to 22·4%)	3·8 (2·4 to 7·6)	14·3% (8·7 to 28·1%)	43·0% (-29·5 to 211·1%)	23·5% (-39·3 to 166·0%)
Falls	0·1 (0·1 to 0·2)	7·7% (5·4 to 13·7%)	0·2 (0·1 to 0·3)	8·5% (5·8 to 15·3%)	44·3% (-20·2 to 191·4%)	10·4% (-40·5 to 120·3%)	10·8 (7·6 to 18·5)	11·6% (8·5 to 19·3%)	15·6 (10·5 to 26·5)	14·2% (10·0 to 23·9%)	45·5% (-13·2 to 164·7%)	22·4% (-28·2 to 128·0%)
Drowning	0·2 (0·1 to 0·3)	8·1% (5·7 to 14·3%)	0·2 (0·1 to 0·4)	11·2% (7·5 to 20·8%)	17·9% (-36·8 to 127·6%)	37·3% (-30·0 to 168·4%)	9·4 (6·7 to 16·9)	7·1% (4·9 to 12·5%)	10·7 (7·2 to 20·1)	10·6% (6·9 to 20·1%)	14·4% (-38·8 to 121·3%)	48·6% (-25·5 to 190·8%)
Fire, heat, and hot substances	0·1 (0·0 to 0·1)	6·9% (5·2 to 10·6%)	0·1 (0·1 to 0·1)	9·1% (6·5 to 14·7%)	25·3% (-21·9 to 108·4%)	33·0% (-17·2 to 123·5%)	4·6 (3·4 to 7·2)	7·7% (5·7 to 11·7%)	5·7 (4·0 to 8·8)	11·7% (8·4 to 18·4%)	23·7% (-20·7 to 100·8%)	51·3% (-4·3 to 149·5%)
Poisonings	0·0 (0·0 to 0·0)	3·2% (2·3 to 4·3%)	0·0 (0·0 to 0·0)	3·5% (2·1 to 5·2%)	-7·9% (-37·4 to 32·8%)	7·2% (-35·8 to 68·1%)	1·5 (1·2 to 1·9)	3·0% (2·0 to 4·2%)	1·4 (1·0 to 2·0)	3·6% (1·9 to 5·5%)	-6·8% (-36·8 to 34·5%)	16·9% (-35·8 to 93·6%)
Unintentional firearm injuries	0·1 (0·0 to 0·1)	7·3% (5·7 to 9·2%)	0·1 (0·0 to 0·1)	7·9% (6·0 to 10·4%)	7·9% (-22·4 to 49·2%)	9·1% (-21·0 to 47·8%)	2·9 (1·9 to 3·8)	7·4% (5·9 to 9·5%)	3·1 (2·0 to 4·2)	8·7% (6·6 to 11·3%)	8·3% (-20·9 to 49·2%)	17·1% (-14·8 to 58·3%)
Unintentional suffocation	0·0 (0·0 to 0·0)	0·3% (0·2 to 0·4%)	0·0 (0·0 to 0·0)	0·4% (0·2 to 0·5%)	2·4% (-20·5 to 34·7%)	38·2% (-9·7 to 110·6%)	0·1 (0·1 to 0·1)	0·3% (0·2 to 0·4%)	0·1 (0·1 to 0·1)	0·4% (0·3 to 0·6%)	7·4% (-16·0 to 38·9%)	42·5% (-7·9 to 110·5%)
Other exposure to mechanical forces	0·1 (0·1 to 0·1)	12·1% (8·8 to 20·7%)	0·1 (0·1 to 0·2)	15·3% (10·5 to 28·1%)	19·0% (-33·5 to 118·6%)	26·7% (-31·6 to 134·4%)	6·2 (4·4 to 10·7)	12·7% (9·3 to 21·5%)	7·7 (5·2 to 13·4)	17·1% (11·8 to 29·9%)	22·7% (-29·6 to 114·7%)	33·9% (-27·2 to 138·7%)
Venomous animal contact	0·0 (0·0 to 0·0)	1·8% (1·2 to 2·6%)	0·0 (0·0 to 0·0)	1·9% (0·7 to 3·8%)	-18·4% (-69·4 to 65·7%)	6·9% (-61·1 to 112·0%)	0·2 (0·1 to 0·2)	1·8% (1·2 to 2·6%)	0·1 (0·0 to 0·3)	2·1% (0·8 to 4·1%)	-16·6% (-68·4 to 65·8%)	18·2% (-55·5 to 131·3%)
Non-venomous animal contact	0·0 (0·0 to 0·0)	0·8% (0·6 to 1·3%)	0·0 (0·0 to 0·0)	0·9% (0·3 to 1·8%)	-3·5% (-62·8 to 95·2%)	5·1% (-58·8 to 107·4%)	0·0 (0·0 to 0·1)	0·9% (0·6 to 1·3%)	0·0 (0·0 to 0·1)	1·0% (0·4 to 2·1%)	-6·4% (-63·7 to 90·5%)	18·3% (-53·8 to 134·3%)
Pulmonary aspiration and foreign body in airway	0·1 (0·0 to 0·1)	2·7% (1·7 to 6·8%)	0·1 (0·0 to 0·2)	4·2% (2·2 to 10·6%)	52·8% (-45·5 to 306·3%)	51·4% (-46·2 to 312·6%)	3·8 (2·3 to 9·0)	2·4% (1·5 to 5·7%)	5·3 (2·9 to 12·6)	3·7% (2·0 to 8·9%)	40·3% (-47·1 to 256·5%)	56·7% (-40·9 to 301·7%)
Foreign body in eyes	-	-	-	-	-	-	0·0 (0·0 to 0·0)	6·3% (3·8 to 14·2%)	0·0 (0·0 to 0·1)	8·0% (4·5 to 17·8%)	55·2% (-38·0 to 264·9%)	27·8% (-48·2 to 200·3%)
Foreign body in other body part	0·0 (0·0 to 0·0)	4·8% (3·6 to 7·5%)	0·0 (0·0 to 0·1)	6·7% (4·1 to 12·4%)	31·6% (-26·3 to 143·0%)	38·7% (-24·5 to 148·4%)	1·2 (0·8 to 1·9)	4·9% (3·6 to 7·6%)	1·5 (0·9 to 2·8)	7·8% (4·8 to 14·0%)	28·2% (-28·5 to 136·7%)	59·6% (-12·9 to 185·0%)
Other unintentional injuries	0·1 (0·0 to 0·1)	10·8% (7·8 to 18·1%)	0·1 (0·1 to 0·1)	14·5% (9·8 to 25·9%)	15·0% (-34·9 to 124·3%)	33·7% (-26·9 to 154·2%)	6·3 (4·5 to 10·1)	10·9% (8·1 to 17·4%)	7·6 (5·1 to 13·0)	15·1% (10·5 to 25·8%)	19·8% (-29·6 to 117·6%)	38·2% (-20·1 to 149·2%)
**Occupational ergonomic factors: All causes**	-	-	-	-	-	-	114·2 (78·3 to 159·7)	0·9% (0·6 to 1·1%)	138·3 (96·5 to 193·4)	1·0% (0·8 to 1·3%)	21·1% (13·9 to 28·6%)	19·5% (11·1 to 28·6%)
Low back pain	-	-	-	-	-	-	114·2 (78·3 to 159·7)	32·1% (29·5 to 34·9%)	138·3 (96·5 to 193·4)	30·9% (28·8 to 33·3%)	21·1% (13·9 to 28·6%)	-3·5% (-9·1 to 2·2%)
**Behavioural risks: All causes**	72·0 (66·6 to 78·1)	30·0% (28·0 to 32·3%)	80·7 (72·7 to 89·8)	28·8% (26·6 to 31·4%)	12·0% (4·2 to 20·4%)	-3·9% (-6·4 to -1·1%)	3,131·9 (2,855·9 to 3,427·1)	24·1% (22·8 to 25·5%)	2,919·4 (2,589·0 to 3,296·8)	22·2% (20·8 to 23·7%)	-6·8% (-12·1 to -1·8%)	-8·0% (-10·7 to -5·2%)
**Child and maternal malnutrition: All causes**	8·7 (7·6 to 9·8)	3·6% (3·2 to 4·1%)	3·9 (3·1 to 4·7)	1·4% (1·1 to 1·7%)	-55·4% (-62·0 to -48·3%)	-61·7% (-67·4 to -55·1%)	1,188·4 (1,012·7 to 1,392·0)	9·1% (8·2 to 10·1%)	788·6 (617·9 to 989·2)	6·0% (5·1 to 6·9%)	-33·6% (-41·2 to -27·0%)	-34·6% (-41·3 to -28·8%)
**Suboptimal breastfeeding: All causes**	2·8 (1·5 to 4·7)	1·2% (0·6 to 2·0%)	1·2 (0·6 to 2·1)	0·4% (0·2 to 0·8%)	-57·0% (-65·6 to -47·5%)	-63·1% (-70·6 to -54·7%)	248·8 (136·6 to 411·4)	1·9% (1·0 to 3·2%)	109·1 (58·4 to 188·7)	0·8% (0·4 to 1·4%)	-56·1% (-64·4 to -46·8%)	-56·7% (-64·6 to -47·9%)
**Non-exclusive breastfeeding : All causes**	2·6 (1·3 to 4·5)	1·1% (0·6 to 1·9%)	1·1 (0·6 to 2·1)	0·4% (0·2 to 0·7%)	-56·4% (-65·2 to -46·7%)	-62·6% (-70·2 to -54·2%)	228·4 (116·3 to 391·6)	1·8% (0·9 to 3·0%)	100·2 (50·1 to 179·0)	0·8% (0·4 to 1·4%)	-56·1% (-64·8 to -46·5%)	-56·7% (-65·1 to -47·1%)
Diarrhoeal diseases	0·7 (0·4 to 1·1)	18·8% (10·4 to 28·0%)	0·2 (0·1 to 0·4)	13·0% (6·5 to 21·0%)	-66·5% (-76·9 to -50·9%)	-31·1% (-48·3 to -8·4%)	62·2 (33·7 to 97·3)	19·4% (10·7 to 28·7%)	21·5 (10·9 to 36·3)	13·6% (7·1 to 21·8%)	-65·5% (-76·1 to -50·3%)	-29·6% (-45·9 to -7·9%)
Lower respiratory infections	1·9 (0·7 to 3·8)	6·6% (2·4 to 13·1%)	0·9 (0·3 to 1·8)	2·8% (1·0 to 5·5%)	-52·7% (-61·6 to -41·8%)	-58·0% (-66·0 to -47·8%)	166·2 (60·4 to 328·8)	16·3% (6·0 to 32·0%)	78·7 (29·0 to 154·9)	10·2% (3·8 to 20·2%)	-52·6% (-61·4 to -41·7%)	-37·4% (-47·3 to -25·2%)
**Discontinued breastfeeding : All causes**	0·2 (0·1 to 0·5)	0·1% (0·0 to 0·2%)	0·1 (0·0 to 0·2)	0·0% (0·0 to 0·1%)	-65·4% (-74·6 to -53·3%)	-70·3% (-78·4 to -59·9%)	25·2 (8·7 to 47·7)	0·2% (0·1 to 0·4%)	10·5 (3·4 to 20·2)	0·1% (0·0 to 0·2%)	-58·3% (-68·5 to -47·0%)	-58·9% (-68·8 to -47·5%)
Diarrhoeal diseases	0·2 (0·1 to 0·5)	6·6% (2·4 to 12·4%)	0·1 (0·0 to 0·2)	4·7% (1·6 to 8·9%)	-65·4% (-74·6 to -53·3%)	-28·7% (-42·8 to -13·1%)	25·2 (8·7 to 47·7)	7·8% (2·7 to 14·7%)	10·5 (3·4 to 20·2)	6·7% (2·2 to 12·8%)	-58·3% (-68·5 to -47·0%)	-14·9% (-29·5 to -1·4%)
**Childhood undernutrition: All causes**	7·6 (6·4 to 8·7)	3·2% (2·6 to 3·6%)	3·3 (2·5 to 4·2)	1·2% (0·9 to 1·5%)	-56·5% (-63·2 to -49·3%)	-62·6% (-68·6 to -55·9%)	683·2 (573·2 to 777·2)	5·3% (4·3 to 6·2%)	307·5 (236·2 to 380·0)	2·3% (1·8 to 2·9%)	-55·0% (-61·5 to -48·2%)	-55·6% (-61·5 to -49·0%)
**Childhood underweight: All causes**	1·6 (1·3 to 2·2)	0·7% (0·5 to 0·9%)	0·7 (0·5 to 1·0)	0·2% (0·2 to 0·4%)	-57·4% (-67·9 to -44·3%)	-63·4% (-72·6 to -51·2%)	143·5 (115·6 to 195·3)	1·1% (0·9 to 1·5%)	63·2 (46·8 to 94·0)	0·5% (0·4 to 0·7%)	-55·9% (-66·0 to -43·0%)	-56·5% (-66·3 to -43·9%)
Diarrhoeal diseases	0·1 (0·1 to 0·2)	3·7% (2·8 to 4·8%)	0·0 (0·0 to 0·1)	2·2% (1·5 to 3·0%)	-70·9% (-78·1 to -60·7%)	-40·0% (-50·7 to -29·3%)	13·8 (9·9 to 18·6)	4·3% (3·2 to 5·6%)	4·8 (3·2 to 7·0)	3·1% (2·2 to 4·0%)	-65·3% (-72·2 to -56·1%)	-29·2% (-37·5 to -21·3%)
Lower respiratory infections	0·5 (0·3 to 1·0)	1·6% (1·0 to 3·4%)	0·2 (0·1 to 0·4)	0·6% (0·3 to 1·4%)	-57·0% (-65·1 to -48·8%)	-61·7% (-68·9 to -54·3%)	41·3 (24·5 to 84·0)	4·1% (2·4 to 8·2%)	17·8 (9·4 to 38·1)	2·3% (1·3 to 5·1%)	-56·9% (-64·9 to -48·8%)	-43·0% (-51·6 to -34·5%)
Measles	0·0 (0·0 to 0·0)	8·5% (0·0 to ·%)	0·0 (0·0 to 0·0)	6·5% (0·0 to ·%)	-22·6% (-100·0 to 16,905·0%)	-22·9% (-100·0 to 13·1%)	0·0 (0·0 to 0·0)	9·5% (0·0 to ·%)	0·0 (0·0 to 0·0)	7·2% (0·0 to 27·5%)	-21·4% (-100·0 to 108,668·5%)	-23·6% (-100·0 to 11·0%)
Protein-energy malnutrition	1·0 (0·8 to 1·3)	27·7% (23·3 to 33·3%)	0·4 (0·3 to 0·7)	13·5% (9·4 to 19·2%)	-55·8% (-70·3 to -35·2%)	-51·3% (-65·5 to -30·7%)	88·3 (69·7 to 119·2)	65·2% (60·5 to 70·5%)	40·6 (28·5 to 63·8)	49·5% (41·3 to 59·6%)	-54·0% (-68·1 to -34·0%)	-24·1% (-36·4 to -9·6%)
**Childhood wasting: All causes**	6·5 (4·4 to 8·2)	2·7% (1·8 to 3·4%)	2·9 (1·7 to 3·9)	1·0% (0·6 to 1·4%)	-56·1% (-63·4 to -48·5%)	-62·3% (-68·8 to -55·5%)	588·5 (407·2 to 728·7)	4·5% (3·1 to 5·7%)	267·8 (169·1 to 356·6)	2·0% (1·3 to 2·8%)	-54·5% (-61·4 to -47·3%)	-55·1% (-61·3 to -48·2%)

Supplemental Results Table 1. All-age deaths, death PAFs, DALYs, DALY PAFs, and percent change for each risk factor and outcome for both sexes combined in 2005 and 2015 for Andean Latin America.

Data in parenthesis are 95% uncertainty intervals. PAFs=population attributable fractions. DALYs=disability-adjusted life-years.

	2005 Deaths (in thousands)	2005 Deaths PAF	2015 Deaths (in thousands)	2015 Deaths PAF	Percent change deaths 2005-2015	Percent change deaths PAF 2005-2015	2005 DALYs (in thousands)	2005 DALYs PAF	2015 DALYs (in thousands)	2015 DALYs PAF	Percent change DALYs 2005-2015	Percent change DALYs PAF 2005-2015
Diarrhoeal diseases	1·7 (1·0 to 2·2)	45·8% (27·2 to 54·7%)	0·6 (0·3 to 0·8)	31·4% (17·1 to 40·6%)	-66·7% (-75·0 to -55·6%)	-31·4% (-43·4 to -19·4%)	169·7 (97·9 to 214·1)	52·9% (31·1 to 62·8%)	66·4 (35·0 to 91·2)	42·2% (23·3 to 51·9%)	-60·9% (-68·7 to -51·0%)	-20·1% (-28·6 to -12·8%)
Lower respiratory infections	3·8 (1·8 to 5·3)	13·2% (6·1 to 18·9%)	1·9 (0·8 to 2·7)	5·7% (2·5 to 8·8%)	-51·4% (-59·3 to -42·9%)	-56·8% (-63·9 to -49·1%)	330·4 (155·5 to 456·7)	32·5% (15·3 to 45·1%)	160·8 (70·4 to 235·9)	20·9% (9·3 to 30·6%)	-51·3% (-59·2 to -42·8%)	-35·7% (-43·9 to -27·2%)
Measles	0·0 (0·0 to 0·0)	15·1% (0·0 to ·%)	0·0 (0·0 to 0·0)	13·1% (0·0 to ·%)	-20·0% (-100·0 to 13,870·6%)	-13·4% (-100·0 to 167·7%)	0·0 (0·0 to 0·0)	16·7% (0·0 to ·%)	0·0 (0·0 to 0·0)	14·6% (0·0 to 63·8%)	-18·8% (-100·0 to 155,736·3%)	-12·7% (-100·0 to 227·2%)
Protein-energy malnutrition	1·0 (0·8 to 1·3)	27·7% (23·3 to 33·3%)	0·4 (0·3 to 0·7)	13·5% (9·4 to 19·2%)	-55·8% (-70·3 to -35·2%)	-51·3% (-65·5 to -30·7%)	88·3 (69·7 to 119·2)	65·2% (60·5 to 70·5%)	40·6 (28·5 to 63·8)	49·5% (41·3 to 59·6%)	-54·0% (-68·1 to -34·0%)	-24·1% (-36·4 to -9·6%)
**Childhood stunting: All causes**	1·8 (0·6 to 3·7)	0·7% (0·2 to 1·6%)	0·7 (0·2 to 1·6)	0·2% (0·1 to 0·6%)	-62·8% (-73·1 to -55·1%)	-68·1% (-77·1 to -61·2%)	159·5 (54·5 to 326·3)	1·2% (0·4 to 2·5%)	60·9 (17·6 to 138·9)	0·5% (0·1 to 1·0%)	-61·8% (-69·5 to -54·5%)	-62·3% (-70·2 to -55·2%)
Diarrhoeal diseases	0·4 (0·1 to 0·7)	10·7% (3·9 to 19·4%)	0·1 (0·0 to 0·2)	6·1% (2·0 to 11·4%)	-72·3% (-79·4 to -61·2%)	-43·1% (-52·9 to -32·4%)	39·9 (13·6 to 72·8)	12·4% (4·5 to 22·6%)	13·0 (4·3 to 25·3)	8·3% (2·8 to 15·5%)	-67·4% (-74·3 to -57·7%)	-33·4% (-40·8 to -25·5%)
Lower respiratory infections	1·4 (0·1 to 3·3)	4·8% (0·4 to 11·8%)	0·6 (0·0 to 1·4)	1·7% (0·1 to 4·5%)	-60·1% (-68·0 to -46·6%)	-64·6% (-71·6 to -52·4%)	119·6 (9·2 to 285·6)	11·8% (0·9 to 28·2%)	47·9 (2·8 to 123·8)	6·2% (0·4 to 16·3%)	-60·0% (-67·9 to -46·5%)	-47·2% (-56·4 to -34·0%)
Measles	0·0 (0·0 to 0·0)	15·5% (1·7 to ·%)	0·0 (0·0 to 0·0)	12·3% (1·1 to ·%)	-23·5% (-100·0 to 16,885·5%)	-21·0% (-100·0 to 0·0%)	0·0 (0·0 to 0·0)	17·4% (1·6 to ·%)	0·0 (0·0 to 0·0)	14·0% (0·9 to 44·0%)	-22·2% (-99·6 to 172,187·6%)	-19·7% (-48·6 to 0·0%)
**Iron deficiency: All causes**	0·2 (0·1 to 0·2)	0·1% (0·0 to 0·1%)	0·1 (0·0 to 0·2)	0·0% (0·0 to 0·1%)	-29·2% (-42·0 to -12·3%)	-39·3% (-49·7 to -26·6%)	417·7 (277·4 to 600·9)	3·2% (2·3 to 4·2%)	435·9 (288·9 to 622·3)	3·3% (2·4 to 4·3%)	4·4% (0·9 to 8·1%)	2·9% (-1·9 to 8·2%)
Maternal haemorrhage	0·1 (0·0 to 0·1)	26·2% (9·9 to 41·6%)	0·1 (0·0 to 0·1)	25·8% (9·7 to 41·4%)	-33·0% (-46·8 to -14·7%)	-1·7% (-7·5 to 4·2%)	4·8 (1·8 to 8·0)	26·1% (9·9 to 41·5%)	3·2 (1·2 to 5·4)	25·7% (9·6 to 41·3%)	-33·1% (-46·5 to -16·1%)	-1·8% (-7·6 to 4·0%)
Maternal sepsis and other pregnancy related infections	0·1 (0·0 to 0·1)	25·8% (9·6 to 40·8%)	0·0 (0·0 to 0·1)	24·9% (9·2 to 40·5%)	-28·2% (-43·7 to -9·1%)	-3·3% (-9·1 to 2·5%)	3·5 (1·3 to 5·7)	25·7% (9·6 to 40·9%)	2·5 (0·9 to 4·3)	24·9% (9·2 to 40·3%)	-28·3% (-42·5 to -9·5%)	-3·3% (-9·0 to 2·3%)
Iron-deficiency anaemia	0·0 (0·0 to 0·0)	100·0% (100·0 to 100·0%)	0·0 (0·0 to 0·0)	100·0% (100·0 to 100·0%)	9·7% (-7·7 to 29·7%)	-	409·4 (272·3 to 592·2)	100·0% (100·0 to 100·0%)	430·2 (284·8 to 617·1)	100·0% (100·0 to 100·0%)	5·1% (1·6 to 8·8%)	-
**Vitamin A deficiency: All causes**	0·1 (0·1 to 0·2)	0·0% (0·0 to 0·1%)	0·0 (0·0 to 0·0)	0·0% (0·0 to 0·0%)	-72·7% (-78·9 to -64·8%)	-76·6% (-81·9 to -69·4%)	13·1 (7·7 to 19·5)	0·1% (0·1 to 0·1%)	5·7 (3·5 to 8·0)	0·0% (0·0 to 0·1%)	-56·7% (-64·1 to -46·2%)	-57·3% (-64·4 to -47·5%)
Diarrhoeal diseases	0·1 (0·1 to 0·2)	2·8% (1·5 to 4·3%)	0·0 (0·0 to 0·0)	1·6% (0·8 to 2·5%)	-72·7% (-78·9 to -64·8%)	-44·0% (-52·1 to -34·9%)	11·1 (5·7 to 17·1)	3·4% (1·8 to 5·4%)	3·7 (1·8 to 5·8)	2·3% (1·2 to 3·7%)	-66·8% (-72·7 to -59·9%)	-32·4% (-38·7 to -25·3%)
Measles	0·0 (0·0 to 0·0)	9·4% (4·9 to ·%)	0·0 (0·0 to 0·0)	7·4% (4·1 to ·%)	-26·0% (-100·0 to 31,895·9%)	-21·5% (-100·0 to 37·9%)	0·0 (0·0 to 0·0)	10·3% (4·8 to ·%)	0·0 (0·0 to 0·0)	8·7% (4·5 to 13·8%)	-24·7% (-99·6 to 191,110·1%)	-16·3% (-45·9 to 36·7%)
Vitamin A deficiency	-	-	-	-	-	-	2·1 (1·2 to 3·2)	100·0% (100·0 to 100·0%)	2·0 (1·2 to 3·2)	100·0% (100·0 to 100·0%)	-2·2% (-10·0 to 5·8%)	-
**Zinc deficiency: All causes**	0·2 (0·0 to 0·7)	0·1% (0·0 to 0·3%)	0·1 (0·0 to 0·3)	0·0% (0·0 to 0·1%)	-54·9% (-74·5 to -36·7%)	-61·3% (-78·3 to -44·9%)	23·7 (3·8 to 59·4)	0·2% (0·0 to 0·5%)	11·5 (2·5 to 30·1)	0·1% (0·0 to 0·2%)	-51·3% (-65·5 to -32·4%)	-51·9% (-65·8 to -32·6%)
Diarrhoeal diseases	0·1 (0·0 to 0·3)	2·7% (0·0 to 7·2%)	0·0 (0·0 to 0·1)	1·9% (0·0 to 4·8%)	-66·6% (-76·8 to 0·0%)	-31·1% (-49·4 to 0·0%)	11·7 (2·8 to 25·9)	3·6% (0·9 to 8·1%)	5·1 (1·8 to 10·1)	3·2% (1·2 to 6·1%)	-56·7% (-68·5 to -27·3%)	-11·5% (-34·3 to 53·3%)
Lower respiratory infections	0·1 (0·0 to 0·6)	0·5% (0·0 to 1·9%)	0·1 (0·0 to 0·3)	0·2% (0·0 to 0·9%)	-46·4% (-58·4 to 0·0%)	-52·5% (-63·0 to 0·0%)	12·0 (0·1 to 47·7)	1·2% (0·0 to 4·6%)	6·5 (0·1 to 25·3)	0·8% (0·0 to 3·3%)	-46·1% (-57·5 to 0·5%)	-28·9% (-43·2 to 31·2%)
**Tobacco smoke: All causes**	15·8 (14·1 to 17·5)	6·6% (5·9 to 7·2%)	18·2 (15·8 to 20·9)	6·5% (5·8 to 7·3%)	15·6% (2·5 to 31·0%)	-0·8% (-8·9 to 8·6%)	399·7 (353·7 to 450·0)	3·1% (2·6 to 3·5%)	394·6 (345·8 to 450·9)	3·0% (2·5 to 3·5%)	-1·3% (-11·6 to 11·3%)	-2·5% (-11·2 to 7·3%)
**Smoking: All causes**	14·0 (12·3 to 15·7)	5·8% (5·2 to 6·4%)	16·5 (14·1 to 19·2)	5·9% (5·2 to 6·6%)	17·7% (3·5 to 34·2%)	1·0% (-8·6 to 12·0%)	326·5 (290·4 to 364·2)	2·5% (2·2 to 2·9%)	342·8 (295·4 to 396·3)	2·6% (2·2 to 3·1%)	5·0% (-6·9 to 19·4%)	3·6% (-6·7 to 15·2%)
Tuberculosis	0·3 (0·1 to 0·6)	6·5% (3·3 to 9·9%)	0·2 (0·1 to 0·4)	5·9% (3·0 to 9·1%)	-27·8% (-38·7 to -15·4%)	-10·1% (-20·6 to 1·8%)	9·4 (4·7 to 18·1)	5·5% (2·8 to 8·5%)	6·5 (3·1 to 12·5)	5·2% (2·6 to 8·2%)	-31·3% (-42·3 to -19·1%)	-5·0% (-17·3 to 8·8%)
Lower respiratory infections	2·5 (1·8 to 3·2)	8·4% (6·6 to 10·7%)	2·6 (1·8 to 3·4)	7·8% (6·0 to 10·0%)	4·4% (-13·9 to 26·9%)	-7·4% (-21·7 to 9·1%)	48·9 (35·8 to 62·2)	4·8% (3·6 to 6·0%)	46·3 (33·9 to 59·2)	6·0% (4·7 to 7·5%)	-5·4% (-19·2 to 10·2%)	24·9% (7·7 to 44·3%)
Lip and oral cavity cancer	0·1 (0·1 to 0·1)	23·7% (17·2 to 30·4%)	0·1 (0·1 to 0·2)	25·2% (18·4 to 32·7%)	42·6% (13·4 to 80·4%)	6·4% (-9·5 to 24·8%)	1·8 (1·2 to 2·3)	20·8% (14·6 to 27·1%)	2·3 (1·5 to 3·1)	21·1% (14·9 to 28·0%)	28·8% (0·2 to 64·2%)	1·4% (-15·0 to 19·8%)
Nasopharynx cancer	0·0 (0·0 to 0·0)	21·3% (15·1 to 27·4%)	0·0 (0·0 to 0·0)	22·1% (15·6 to 28·9%)	28·7% (1·0 to 65·2%)	3·7% (-12·2 to 22·0%)	0·3 (0·2 to 0·5)	16·7% (11·0 to 22·2%)	0·4 (0·2 to 0·6)	17·0% (11·1 to 23·4%)	19·0% (-11·6 to 55·8%)	1·9% (-17·3 to 23·4%)
Oesophageal cancer	0·1 (0·1 to 0·2)	23·8% (15·8 to 32·8%)	0·2 (0·1 to 0·3)	24·7% (16·7 to 33·7%)	36·1% (8·5 to 70·3%)	3·6% (-10·5 to 19·5%)	2·4 (1·5 to 3·3)	21·2% (13·9 to 29·5%)	3·0 (1·9 to 4·4)	21·5% (14·2 to 30·3%)	25·7% (-1·9 to 59·9%)	1·0% (-13·9 to 18·3%)
Stomach cancer	0·3 (0·2 to 0·4)	4·3% (2·5 to 6·4%)	0·4 (0·2 to 0·6)	4·6% (2·7 to 7·0%)	29·9% (0·8 to 66·9%)	7·7% (-11·1 to 29·9%)	5·5 (3·1 to 8·3)	3·9% (2·2 to 5·8%)	6·4 (3·6 to 10·0)	4·0% (2·2 to 6·1%)	16·6% (-11·3 to 49·6%)	3·1% (-15·8 to 24·7%)
Colon and rectum cancer	0·1 (0·0 to 0·1)	2·1% (1·4 to 3·0%)	0·1 (0·1 to 0·1)	2·4% (1·6 to 3·4%)	70·5% (25·5 to 130·2%)	14·4% (-11·4 to 45·5%)	1·0 (0·6 to 1·5)	1·9% (1·2 to 2·7%)	1·5 (0·9 to 2·2)	2·0% (1·3 to 2·9%)	48·7% (12·7 to 94·3%)	6·2% (-15·1 to 30·6%)
Liver cancer due to hepatitis B	0·1 (0·0 to 0·1)	5·5% (2·9 to 8·6%)	0·1 (0·0 to 0·1)	5·9% (3·1 to 9·1%)	37·3% (7·5 to 74·3%)	8·0% (-10·4 to 29·0%)	1·4 (0·7 to 2·3)	4·7% (2·4 to 7·7%)	1·7 (0·8 to 2·9)	4·9% (2·4 to 8·0%)	24·0% (-7·1 to 61·6%)	5·0% (-16·4 to 29·3%)
Liver cancer due to hepatitis C	0·0 (0·0 to 0·0)	5·3% (2·9 to 8·2%)	0·0 (0·0 to 0·0)	6·1% (3·2 to 9·7%)	51·6% (14·9 to 98·4%)	14·5% (-8·5 to 41·4%)	0·3 (0·1 to 0·4)	5·0% (2·7 to 7·7%)	0·3 (0·2 to 0·6)	5·4% (2·9 to 8·6%)	34·9% (3·2 to 74·6%)	9·1% (-11·5 to 31·2%)
Liver cancer due to alcohol use	0·0 (0·0 to 0·1)	6·7% (3·3 to 10·7%)	0·1 (0·0 to 0·1)	6·8% (3·5 to 11·1%)	51·2% (15·0 to 97·9%)	2·0% (-16·6 to 22·8%)	0·6 (0·3 to 1·0)	6·2% (3·0 to 10·0%)	0·9 (0·5 to 1·5)	6·1% (3·1 to 10·0%)	42·6% (6·7 to 88·3%)	-0·7% (-19·4 to 22·0%)
Liver cancer due to other causes	0·0 (0·0 to 0·0)	5·2% (2·8 to 8·1%)	0·0 (0·0 to 0·1)	5·9% (3·1 to 9·5%)	45·0% (10·8 to 89·0%)	14·1% (-8·2 to 40·4%)	0·5 (0·3 to 0·8)	4·6% (2·5 to 7·2%)	0·6 (0·3 to 1·0)	5·0% (2·6 to 7·9%)	26·6% (-3·1 to 63·3%)	9·5% (-11·6 to 32·3%)
Pancreatic cancer	0·1 (0·1 to 0·1)	6·8% (4·8 to 8·8%)	0·2 (0·1 to 0·2)	7·4% (5·2 to 9·8%)	57·2% (20·4 to 103·0%)	9·1% (-11·0 to 31·3%)	1·8 (1·3 to 2·5)	6·2% (4·4 to 8·2%)	2·6 (1·8 to 3·8)	6·4% (4·4 to 8·6%)	43·0% (9·1 to 86·7%)	3·2% (-14·7 to 24·8%)
Tracheal, bronchus, and lung cancer	1·5 (1·3 to 1·8)	48·1% (40·8 to 53·8%)	2·3 (1·8 to 2·8)	49·5% (42·1 to 56·0%)	48·4% (20·3 to 79·9%)	3·1% (-7·3 to 14·2%)	29·5 (23·9 to 34·6)	44·0% (36·5 to 50·2%)	40·3 (31·2 to 49·8)	44·4% (36·5 to 51·4%)	36·8% (10·1 to 68·0%)	0·7% (-10·8 to 12·7%)
Cervical cancer	0·1 (0·0 to 0·2)	3·6% (1·0 to 6·9%)	0·1 (0·0 to 0·3)	4·0% (1·2 to 7·7%)	29·5% (-18·2 to 92·6%)	10·7% (-20·7 to 48·0%)	2·8 (0·8 to 5·5)	3·4% (1·0 to 6·7%)	3·1 (0·9 to 6·6)	3·4% (1·0 to 7·0%)	10·3% (-30·3 to 71·6%)	0·8% (-28·8 to 42·9%)
Kidney cancer	0·0 (0·0 to 0·0)	4·6% (2·6 to 6·9%)	0·0 (0·0 to 0·1)	5·0% (2·9 to 7·7%)	65·5% (22·8 to 118·5%)	9·1% (-11·8 to 33·1%)	0·5 (0·3 to 0·8)	3·7% (2·1 to 5·6%)	0·8 (0·4 to 1·3)	4·0% (2·2 to 6·3%)	51·3% (10·3 to 106·9%)	8·5% (-15·0 to 37·6%)
Bladder cancer	0·0 (0·0 to 0·1)	11·0% (7·2 to 15·2%)	0·1 (0·0 to 0·1)	11·9% (7·7 to 16·6%)	58·5% (22·6 to 103·5%)	8·6% (-9·8 to 29·6%)	0·8 (0·5 to 1·1)	9·9% (6·5 to 13·7%)	1·1 (0·7 to 1·6)	10·3% (6·8 to 14·5%)	42·5% (10·9 to 82·1%)	4·4% (-12·3 to 23·9%)
Ischaemic heart disease	3·1 (2·5 to 3·8)	11·3% (9·0 to 13·8%)	3·1 (2·4 to 3·8)	9·0% (7·2 to 11·0%)	-2·3% (-14·2 to 12·5%)	-20·5% (-29·1 to -11·0%)	83·3 (66·8 to 101·0)	16·2% (13·0 to 19·7%)	76·8 (59·6 to 94·2)	13·5% (10·8 to 16·3%)	-7·8% (-18·5 to 5·7%)	-16·8% (-24·6 to -8·8%)
Ischaemic stroke	0·7 (0·6 to 0·9)	9·6% (7·9 to 11·6%)	0·8 (0·6 to 0·9)	7·8% (6·2 to 9·6%)	6·2% (-8·5 to 23·8%)	-18·8% (-29·4 to -6·8%)	16·7 (13·6 to 20·1)	13·9% (11·4 to 16·5%)	16·6 (13·3 to 20·5)	11·6% (9·5 to 14·0%)	-0·4% (-12·5 to 14·8%)	-16·1% (-24·4 to -6·9%)
Hemorrhagic stroke	1·1 (0·9 to 1·3)	13·5% (11·1 to 16·1%)	1·0 (0·8 to 1·3)	11·2% (9·1 to 13·5%)	-4·1% (-15·9 to 9·5%)	-17·2% (-25·0 to -8·8%)	34·2 (27·9 to 41·3)	14·9% (12·3 to 17·8%)	31·3 (24·9 to 38·5)	13·8% (11·2 to 16·5%)	-8·7% (-19·3 to 3·8%)	-8·0% (-16·3 to 1·3%)
Hypertensive heart disease	0·2 (0·1 to 0·2)	8·0% (5·7 to 10·2%)	0·2 (0·2 to 0·3)	6·4% (4·5 to 8·4%)	31·6% (8·0 to 60·3%)	-19·7% (-32·6 to -4·4%)	4·2 (3·1 to 5·4)	11·4% (8·3 to 14·4%)	5·0 (3·6 to 6·5)	9·1% (6·6 to 11·9%)	18·7% (1·3 to 38·1%)	-20·3% (-30·0 to -9·5%)
Atrial fibrillation and flutter	0·0 (0·0 to 0·1)	5·9% (4·2 to 7·8%)	0·1 (0·0 to 0·1)	4·9% (3·4 to 6·7%)	23·0% (-2·9 to 55·4%)	-16·3% (-34·2 to 4·7%)	1·5 (1·0 to 2·0)	10·7% (8·0 to 13·6%)	1·7 (1·2 to 2·4)	8·9% (6·5 to 11·4%)	15·8% (1·6 to 31·2%)	-17·1% (-27·0 to -6·4%)
Aortic aneurysm	0·1 (0·0 to 0·1)	11·6% (8·7 to 14·5%)	0·1 (0·0 to 0·1)	9·1% (6·7 to 11·6%)	18·4% (-2·3 to 42·1%)	-21·7% (-31·6 to -10·4%)	1·4 (1·0 to 1·8)	15·5% (11·6 to 19·4%)	1·6 (1·1 to 2·0)	12·4% (9·1 to 15·6%)	11·4% (-7·6 to 33·1%)	-19·9% (-28·6 to -10·7%)

Supplemental Results Table 1. All-age deaths, death PAFs, DALYs, DALY PAFs, and percent change for each risk factor and outcome for both sexes combined in 2005 and 2015 for Andean Latin America.

Data in parenthesis are 95% uncertainty intervals. PAFs=population attributable fractions. DALYs=disability-adjusted life-years.

	2005 Deaths (in thousands)	2005 Deaths PAF	2015 Deaths (in thousands)	2015 Deaths PAF	Percent change deaths 2005-2015	Percent change deaths PAF 2005-2015	2005 DALYs (in thousands)	2005 DALYs PAF	2015 DALYs (in thousands)	2015 DALYs PAF	Percent change DALYs 2005-2015	Percent change DALYs PAF 2005-2015
Peripheral vascular disease	0·0 (0·0 to 0·0)	7·3% (5·4 to 9·5%)	0·0 (0·0 to 0·0)	5·6% (3·9 to 7·4%)	54·1% (21·0 to 91·2%)	-24·0% (-39·2 to -6·4%)	0·2 (0·1 to 0·3)	10·1% (7·4 to 13·0%)	0·2 (0·1 to 0·3)	8·3% (6·0 to 10·6%)	24·2% (8·5 to 43·0%)	-17·6% (-27·7 to -6·5%)
Other cardiovascular and circulatory diseases	0·2 (0·2 to 0·3)	9·5% (7·1 to 12·0%)	0·3 (0·2 to 0·4)	8·3% (6·1 to 10·7%)	7·7% (-8·9 to 27·6%)	-12·6% (-24·7 to 1·1%)	8·9 (6·6 to 11·5)	8·5% (6·3 to 10·6%)	9·4 (6·9 to 12·1)	8·6% (6·3 to 11·0%)	4·8% (-8·4 to 19·9%)	1·2% (-9·1 to 13·1%)
Chronic obstructive pulmonary disease	2·5 (2·0 to 3·0)	40·9% (32·7 to 48·1%)	3·6 (2·7 to 4·4)	44·6% (35·4 to 52·6%)	41·6% (15·7 to 73·6%)	9·0% (-5·6 to 25·6%)	37·6 (29·7 to 45·4)	31·5% (24·9 to 37·5%)	46·9 (36·1 to 58·4)	34·5% (27·3 to 41·1%)	24·6% (3·1 to 49·2%)	9·4% (-3·8 to 24·1%)
Silicosis	0·0 (0·0 to 0·0)	6·1% (3·9 to 9·1%)	0·0 (0·0 to 0·0)	6·6% (4·0 to 10·2%)	22·3% (-24·3 to 108·9%)	8·2% (-19·2 to 48·2%)	0·1 (0·0 to 0·1)	5·3% (3·1 to 8·1%)	0·1 (0·0 to 0·2)	5·5% (3·2 to 8·9%)	10·9% (-31·9 to 86·0%)	2·5% (-27·2 to 49·0%)
Asbestosis	0·0 (0·0 to 0·0)	4·0% (2·4 to 6·0%)	0·0 (0·0 to 0·0)	4·3% (2·5 to 6·5%)	30·6% (-15·6 to 96·5%)	9·1% (-18·3 to 46·8%)	0·0 (0·0 to 0·0)	3·1% (1·4 to 5·5%)	0·0 (0·0 to 0·0)	3·2% (1·5 to 5·8%)	19·4% (-23·6 to 88·0%)	5·8% (-28·9 to 51·8%)
Coal workers pneumoconiosis	0·0 (0·0 to 0·0)	5·0% (3·2 to 7·1%)	0·0 (0·0 to 0·0)	6·0% (3·9 to 8·7%)	50·5% (-15·7 to 171·5%)	18·8% (-14·6 to 66·3%)	0·0 (0·0 to 0·0)	4·5% (2·6 to 6·8%)	0·0 (0·0 to 0·0)	4·9% (3·1 to 7·1%)	15·0% (-29·2 to 92·2%)	9·3% (-20·6 to 52·1%)
Other pneumoconiosis	0·0 (0·0 to 0·0)	5·6% (3·8 to 7·6%)	0·0 (0·0 to 0·0)	7·1% (4·5 to 10·0%)	76·9% (12·4 to 178·4%)	26·6% (-7·7 to 75·0%)	0·2 (0·1 to 0·3)	4·5% (3·1 to 6·0%)	0·3 (0·2 to 0·4)	5·1% (3·3 to 7·1%)	42·6% (1·8 to 97·5%)	15·0% (-11·5 to 49·6%)
Asthma	0·0 (0·0 to 0·0)	8·0% (6·1 to 10·1%)	0·0 (0·0 to 0·0)	7·4% (5·5 to 9·6%)	-14·1% (-30·5 to 6·8%)	-7·5% (-23·2 to 11·1%)	3·9 (2·6 to 5·4)	2·2% (1·6 to 2·8%)	4·3 (2·8 to 6·0)	2·3% (1·7 to 2·9%)	9·2% (-3·6 to 23·3%)	4·0% (-9·0 to 19·1%)
Interstitial lung disease and pulmonary sarcoidosis	0·1 (0·1 to 0·1)	5·5% (3·8 to 7·3%)	0·2 (0·1 to 0·4)	6·5% (4·3 to 8·9%)	134·2% (57·1 to 234·1%)	17·5% (-11·4 to 52·1%)	1·6 (1·1 to 2·1)	4·8% (3·4 to 6·4%)	3·0 (1·9 to 4·3)	5·4% (3·7 to 7·2%)	92·1% (35·8 to 157·1%)	11·2% (-11·9 to 38·5%)
Other chronic respiratory diseases	0·0 (0·0 to 0·0)	3·7% (2·4 to 5·2%)	0·0 (0·0 to 0·0)	4·9% (3·2 to 6·9%)	29·2% (-11·4 to 84·2%)	32·9% (2·3 to 72·7%)	0·4 (0·2 to 0·7)	1·8% (1·1 to 2·8%)	0·5 (0·3 to 0·7)	2·5% (1·5 to 3·9%)	6·5% (-20·4 to 41·4%)	42·1% (9·1 to 83·0%)
Diabetes mellitus	0·2 (0·1 to 0·4)	3·6% (1·0 to 6·6%)	0·3 (0·1 to 0·6)	3·0% (0·9 to 5·5%)	26·9% (6·9 to 52·9%)	-17·7% (-29·4 to -3·5%)	11·1 (3·1 to 20·7)	4·0% (1·2 to 7·3%)	13·5 (3·7 to 25·5)	3·4% (1·0 to 6·1%)	21·6% (7·3 to 36·4%)	-16·7% (-25·8 to -7·0%)
**Second-hand smoke: All causes**	1·9 (1·3 to 2·6)	0·8% (0·5 to 1·1%)	1·9 (1·3 to 2·6)	0·7% (0·5 to 0·9%)	-0·4% (-8·1 to 7·7%)	-14·4% (-19·5 to -9·1%)	77·1 (47·8 to 110·9)	0·6% (0·4 to 0·9%)	55·6 (36·3 to 77·9)	0·4% (0·3 to 0·6%)	-27·9% (-34·6 to -20·3%)	-28·8% (-34·8 to -22·0%)
Lower respiratory infections	1·0 (0·6 to 1·6)	3·6% (1·9 to 5·6%)	0·9 (0·4 to 1·4)	2·7% (1·4 to 4·2%)	-15·4% (-24·9 to -5·6%)	-24·9% (-31·5 to -19·6%)	57·2 (30·5 to 89·1)	5·6% (3·0 to 8·8%)	34·4 (17·6 to 54·3)	4·5% (2·3 to 7·0%)	-39·9% (-47·2 to -32·1%)	-20·5% (-27·2 to -13·9%)
Otitis media	0·0 (0·0 to 0·0)	6·4% (4·2 to 8·7%)	0·0 (0·0 to 0·0)	4·9% (3·2 to 6·7%)	-37·9% (-52·8 to -20·1%)	-24·4% (-35·4 to -11·5%)	1·7 (0·9 to 2·8)	6·7% (4·5 to 9·0%)	1·5 (0·8 to 2·5)	5·9% (3·9 to 8·0%)	-11·7% (-17·1 to -6·3%)	-12·2% (-16·4 to -7·8%)
Tracheal, bronchus, and lung cancer	0·0 (0·0 to 0·0)	0·9% (0·5 to 1·5%)	0·0 (0·0 to 0·1)	0·8% (0·4 to 1·3%)	26·8% (21·9 to 31·3%)	-11·7% (-20·3 to -2·6%)	0·6 (0·4 to 1·1)	1·0% (0·5 to 1·6%)	0·8 (0·4 to 1·3)	0·9% (0·5 to 1·5%)	23·6% (17·7 to 28·7%)	-8·7% (-18·0 to 0·7%)
Ischaemic heart disease	0·6 (0·4 to 0·8)	2·1% (1·6 to 2·8%)	0·7 (0·5 to 0·9)	2·0% (1·4 to 2·7%)	16·5% (6·1 to 27·3%)	-5·1% (-10·3 to -0·3%)	12·0 (9·3 to 15·2)	2·3% (1·8 to 3·0%)	12·8 (9·8 to 16·2)	2·3% (1·7 to 2·9%)	6·7% (-3·5 to 17·3%)	-3·7% (-8·7 to 1·3%)
Ischaemic stroke	0·1 (0·1 to 0·1)	1·1% (0·7 to 1·5%)	0·1 (0·1 to 0·1)	1·0% (0·7 to 1·4%)	26·4% (12·4 to 40·7%)	-3·4% (-10·5 to 2·6%)	1·4 (1·0 to 1·8)	1·1% (0·8 to 1·4%)	1·6 (1·1 to 2·1)	1·1% (0·8 to 1·4%)	17·1% (5·5 to 29·2%)	-1·3% (-6·9 to 3·9%)
Hemorrhagic stroke	0·1 (0·1 to 0·1)	1·2% (0·9 to 1·5%)	0·1 (0·1 to 0·1)	1·2% (0·9 to 1·5%)	15·6% (3·2 to 29·5%)	-0·2% (-6·2 to 5·5%)	2·6 (2·0 to 3·4)	1·1% (0·9 to 1·5%)	2·8 (2·1 to 3·6)	1·2% (0·9 to 1·6%)	6·5% (-4·2 to 18·1%)	7·4% (1·3 to 13·7%)
**Alcohol and drug use: All causes**	13·0 (12·0 to 14·0)	5·4% (5·0 to 5·8%)	15·1 (13·4 to 16·9)	5·4% (4·9 to 5·9%)	15·9% (4·3 to 27·5%)	-0·5% (-6·9 to 5·7%)	572·3 (527·9 to 617·4)	4·4% (4·0 to 4·8%)	627·8 (562·2 to 696·6)	4·8% (4·3 to 5·3%)	9·7% (1·1 to 19·2%)	8·3% (2·0 to 15·1%)
**Alcohol use: All causes**	11·7 (10·8 to 12·7)	4·9% (4·5 to 5·2%)	13·4 (11·8 to 15·0)	4·8% (4·3 to 5·2%)	13·9% (2·1 to 26·1%)	-2·2% (-8·9 to 4·4%)	473·5 (437·6 to 511·2)	3·6% (3·3 to 4·0%)	504·1 (451·4 to 560·9)	3·8% (3·4 to 4·3%)	6·4% (-2·9 to 16·8%)	5·1% (-2·3 to 12·9%)
Tuberculosis	0·7 (0·5 to 1·3)	15·0% (12·8 to 17·8%)	0·6 (0·4 to 1·1)	15·4% (12·5 to 18·3%)	-17·3% (-30·6 to -2·7%)	2·7% (-9·5 to 15·4%)	26·9 (21·0 to 49·0)	15·6% (13·8 to 18·1%)	21·3 (15·6 to 40·5)	17·0% (14·1 to 19·7%)	-20·7% (-33·7 to -6·2%)	9·4% (-3·8 to 22·4%)
Lower respiratory infections	1·0 (0·7 to 1·3)	3·4% (2·8 to 4·2%)	1·3 (0·9 to 1·7)	4·0% (3·1 to 5·0%)	29·4% (0·8 to 65·3%)	14·8% (-8·5 to 44·4%)	21·1 (15·5 to 25·5)	2·1% (1·6 to 2·5%)	24·5 (17·3 to 30·8)	3·2% (2·4 to 3·9%)	16·1% (-6·8 to 46·8%)	53·2% (24·0 to 91·8%)
Lip and oral cavity cancer	0·1 (0·1 to 0·1)	18·4% (16·3 to 20·5%)	0·1 (0·1 to 0·1)	18·6% (15·8 to 21·4%)	35·6% (17·2 to 56·5%)	1·3% (-9·8 to 13·0%)	1·8 (1·6 to 2·0)	21·0% (19·0 to 22·9%)	2·3 (2·0 to 2·7)	21·6% (18·8 to 24·1%)	30·0% (12·6 to 49·3%)	2·5% (-8·0 to 13·7%)
Nasopharynx cancer	0·0 (0·0 to 0·0)	24·0% (21·0 to 26·5%)	0·0 (0·0 to 0·0)	25·0% (21·4 to 28·1%)	29·3% (9·7 to 51·6%)	4·2% (-5·3 to 14·3%)	0·5 (0·3 to 0·5)	23·6% (20·9 to 25·8%)	0·6 (0·4 to 0·7)	25·4% (22·1 to 28·4%)	25·6% (5·9 to 47·9%)	7·7% (-2·4 to 18·8%)
Other pharynx cancer	0·0 (0·0 to 0·0)	10·7% (9·3 to 12·3%)	0·0 (0·0 to 0·0)	11·6% (9·6 to 13·8%)	24·2% (9·5 to 41·3%)	8·6% (-4·8 to 23·6%)	0·8 (0·7 to 0·9)	12·3% (10·8 to 13·9%)	1·0 (0·9 to 1·2)	13·8% (11·6 to 16·2%)	22·0% (7·5 to 39·5%)	12·1% (-0·8 to 26·7%)
Oesophageal cancer	0·1 (0·1 to 0·1)	15·7% (13·6 to 17·6%)	0·1 (0·1 to 0·1)	15·4% (12·8 to 17·8%)	28·7% (9·7 to 50·4%)	-1·9% (-9·9 to 5·9%)	2·0 (1·8 to 2·3)	18·3% (16·2 to 20·0%)	2·5 (2·1 to 3·0)	18·3% (15·6 to 20·4%)	24·2% (6·4 to 44·5%)	0·0% (-7·3 to 6·7%)
Colon and rectum cancer	0·1 (0·1 to 0·1)	3·2% (2·8 to 3·6%)	0·1 (0·1 to 0·1)	3·2% (2·8 to 3·7%)	47·4% (29·0 to 66·9%)	-0·9% (-9·2 to 8·0%)	1·9 (1·7 to 2·1)	3·6% (3·2 to 4·0%)	2·7 (2·3 to 3·1)	3·6% (3·2 to 4·0%)	39·9% (22·4 to 59·7%)	0·0% (-8·5 to 9·3%)
Liver cancer due to hepatitis B	0·0 (0·0 to 0·0)	0·7% (0·5 to 0·9%)	0·0 (0·0 to 0·0)	0·6% (0·4 to 0·8%)	1·5% (-29·8 to 42·0%)	-20·2% (-44·7 to 11·1%)	0·5 (0·3 to 0·6)	1·6% (1·2 to 2·0%)	0·5 (0·3 to 0·7)	1·4% (1·0 to 1·9%)	1·6% (-28·0 to 41·0%)	-13·9% (-39·1 to 16·1%)
Liver cancer due to hepatitis C	0·0 (0·0 to 0·0)	0·1% (0·0 to 0·1%)	0·0 (0·0 to 0·0)	0·0% (0·0 to 0·1%)	-0·2% (-37·1 to 52·2%)	-24·6% (-53·9 to 18·3%)	0·0 (0·0 to 0·0)	0·2% (0·1 to 0·3%)	0·0 (0·0 to 0·0)	0·2% (0·1 to 0·2%)	0·8% (-34·4 to 49·6%)	-18·3% (-47·5 to 22·6%)
Liver cancer due to alcohol use	0·5 (0·4 to 0·6)	100·0% (100·0 to 100·0%)	0·8 (0·6 to 0·9)	100·0% (100·0 to 100·0%)	48·0% (31·5 to 67·2%)	-	10·2 (8·8 to 11·9)	100·0% (100·0 to 100·0%)	14·7 (12·3 to 17·1)	100·0% (100·0 to 100·0%)	43·3% (27·4 to 63·2%)	-
Liver cancer due to other causes	0·0 (0·0 to 0·0)	0·3% (0·2 to 0·4%)	0·0 (0·0 to 0·0)	0·2% (0·1 to 0·3%)	-5·1% (-33·3 to 29·5%)	-25·3% (-47·8 to 4·7%)	0·1 (0·1 to 0·1)	0·8% (0·5 to 1·0%)	0·1 (0·0 to 0·1)	0·6% (0·4 to 0·9%)	-5·1% (-31·4 to 28·2%)	-17·8% (-42·0 to 10·5%)
Larynx cancer	0·1 (0·0 to 0·1)	17·4% (15·2 to 19·5%)	0·1 (0·0 to 0·1)	16·9% (14·2 to 19·4%)	17·8% (-0·6 to 39·5%)	-3·0% (-11·7 to 6·1%)	1·3 (1·1 to 1·4)	19·7% (17·6 to 21·5%)	1·4 (1·2 to 1·7)	19·5% (16·9 to 21·7%)	13·6% (-2·7 to 32·5%)	-1·0% (-8·6 to 6·8%)
Breast cancer	0·1 (0·0 to 0·1)	2·7% (2·3 to 3·1%)	0·1 (0·1 to 0·1)	2·7% (2·2 to 3·1%)	34·0% (13·5 to 60·0%)	-0·9% (-8·9 to 8·3%)	1·6 (1·3 to 1·9)	2·7% (2·3 to 3·2%)	2·0 (1·5 to 2·5)	2·7% (2·3 to 3·2%)	28·8% (8·8 to 54·6%)	-0·6% (-9·4 to 8·4%)
Ischaemic heart disease	-0·2 (-0·5 to 0·0)	-0·7% (-1·7 to 0·1%)	-0·2 (-0·6 to 0·1)	-0·6% (-1·7 to 0·3%)	4·5% (-189·2 to 156·8%)	-14·9% (-177·1 to 110·2%)	-6·2 (-10·6 to -2·1)	-1·2% (-2·1 to -0·4%)	-6·0 (-10·8 to -1·6)	-1·0% (-1·9 to -0·3%)	-3·7% (-36·8 to 25·2%)	-13·2% (-41·4 to 9·0%)
Ischaemic stroke	0·0 (-0·1 to 0·1)	-0·4% (-1·5 to 0·8%)	0·0 (-0·2 to 0·1)	-0·4% (-1·7 to 0·9%)	36·2% (-399·7 to 428·7%)	3·8% (-318·5 to 300·7%)	-0·2 (-1·2 to 0·8)	-0·2% (-1·0 to 0·7%)	-0·1 (-1·3 to 1·1)	0·0% (-0·9 to 0·8%)	-71·0% (-534·2 to 612·7%)	-75·9% (-483·5 to 512·3%)
Hemorrhagic stroke	0·5 (0·4 to 0·6)	6·1% (5·2 to 6·9%)	0·5 (0·4 to 0·7)	6·0% (5·0 to 7·0%)	15·2% (-2·9 to 35·4%)	-0·5% (-14·3 to 14·5%)	14·7 (12·9 to 16·7)	6·4% (5·7 to 7·1%)	15·8 (13·4 to 18·9)	7·0% (5·9 to 8·0%)	7·5% (-7·9 to 25·0%)	8·4% (-4·3 to 22·4%)
Hypertensive heart disease	0·0 (0·0 to 0·1)	1·8% (0·7 to 2·5%)	0·1 (0·0 to 0·1)	1·9% (0·9 to 2·7%)	72·8% (0·7 to 197·0%)	5·2% (-39·0 to 91·7%)	1·2 (0·9 to 1·5)	3·3% (2·2 to 4·1%)	1·9 (1·4 to 2·5)	3·5% (2·5 to 4·5%)	55·6% (13·7 to 107·7%)	4·3% (-25·3 to 44·5%)
Atrial fibrillation and flutter	0·0 (0·0 to 0·0)	4·3% (3·4 to 5·3%)	0·0 (0·0 to 0·1)	4·2% (3·3 to 5·3%)	44·6% (20·3 to 75·8%)	-1·7% (-17·1 to 17·2%)	0·8 (0·6 to 1·1)	5·9% (5·1 to 6·8%)	1·1 (0·8 to 1·5)	5·9% (4·9 to 6·9%)	38·1% (21·5 to 57·0%)	-1·1% (-12·0 to 11·6%)
Cirrhosis and other chronic liver diseases due to hepatitis B	0·6 (0·4 to 0·7)	25·1% (19·6 to 29·7%)	0·6 (0·4 to 0·8)	22·9% (16·2 to 28·4%)	12·2% (-7·8 to 32·8%)	-8·6% (-22·6 to 4·6%)	15·9 (12·1 to 19·7)	27·9% (22·7 to 32·2%)	17·1 (11·9 to 22·2)	26·1% (19·8 to 30·9%)	7·4% (-10·0 to 26·8%)	-6·4% (-18·5 to 4·7%)
Cirrhosis and other chronic liver diseases due to hepatitis C	0·6 (0·4 to 0·7)	25·8% (20·2 to 30·3%)	0·6 (0·4 to 0·8)	23·4% (16·8 to 28·6%)	8·1% (-10·9 to 28·4%)	-9·2% (-22·6 to 3·5%)	17·1 (12·8 to 21·0)	28·4% (23·2 to 32·9%)	17·8 (12·4 to 23·2)	26·5% (20·2 to 31·5%)	3·9% (-13·1 to 22·3%)	-7·0% (-18·9 to 4·5%)
Cirrhosis and other chronic liver diseases due to alcohol use	2·8 (2·3 to 3·1)	100·0% (100·0 to 100·0%)	3·5 (3·0 to 4·1)	100·0% (100·0 to 100·0%)	28·8% (17·6 to 41·2%)	-	79·8 (67·9 to 89·5)	100·0% (100·0 to 100·0%)	97·3 (82·0 to 110·8)	100·0% (100·0 to 100·0%)	21·9% (10·4 to 33·9%)	-
Cirrhosis and other chronic liver diseases due to other causes	0·4 (0·3 to 0·5)	18·0% (13·7 to 22·3%)	0·5 (0·3 to 0·6)	16·6% (11·0 to 21·4%)	10·6% (-15·3 to 38·1%)	-8·1% (-28·8 to 13·1%)	11·6 (9·1 to 14·3)	18·1% (14·7 to 21·5%)	11·8 (8·4 to 15·2)	17·6% (13·0 to 21·7%)	1·4% (-15·7 to 19·9%)	-2·7% (-17·2 to 12·0%)
Pancreatitis	0·1 (0·1 to 0·1)	10·3% (9·1 to 11·6%)	0·1 (0·1 to 0·1)	10·3% (8·4 to 12·1%)	13·9% (-3·5 to 33·1%)	-0·2% (-11·4 to 11·2%)	3·7 (3·2 to 4·4)	11·9% (10·6 to 13·1%)	4·1 (3·3 to 5·1)	12·4% (10·4 to 14·2%)	9·4% (-8·0 to 28·0%)	4·4% (-6·9 to 15·5%)

Supplemental Results Table 1. All-age deaths, death PAFs, DALYs, DALY PAFs, and percent change for each risk factor and outcome for both sexes combined in 2005 and 2015 for Andean Latin America.

Data in parenthesis are 95% uncertainty intervals. PAFs=population attributable fractions. DALYs=disability-adjusted life-years.

	2005 Deaths (in thousands)	2005 Deaths PAF	2015 Deaths (in thousands)	2015 Deaths PAF	Percent change deaths 2005-2015	Percent change deaths PAF 2005-2015	2005 DALYs (in thousands)	2005 DALYs PAF	2015 DALYs (in thousands)	2015 DALYs PAF	Percent change DALYs 2005-2015	Percent change DALYs PAF 2005-2015
Epilepsy	0·1 (0·1 to 0·1)	12·1% (11·1 to 13·1%)	0·1 (0·1 to 0·1)	12·4% (10·9 to 13·9%)	-10·5% (-23·6 to 4·4%)	2·7% (-8·2 to 14·4%)	10·6 (8·0 to 13·6)	10·1% (9·2 to 11·0%)	9·5 (7·1 to 12·6)	10·4% (9·2 to 11·6%)	-9·7% (-28·6 to 13·6%)	3·0% (-7·5 to 13·7%)
Alcohol use disorders	1·2 (1·1 to 1·3)	100·0% (100·0 to 100·0%)	0·9 (0·8 to 1·0)	100·0% (100·0 to 100·0%)	-21·1% (-30·9 to -10·3%)	-	86·5 (71·7 to 105·3)	100·0% (100·0 to 100·0%)	80·0 (61·8 to 102·8)	100·0% (100·0 to 100·0%)	-7·6% (-18·2 to 1·5%)	-
Diabetes mellitus	-0·3 (-0·4 to -0·3)	-5·3% (-6·3 to -4·4%)	-0·5 (-0·7 to -0·4)	-5·3% (-6·5 to -4·2%)	55·2% (17·5 to 105·0%)	0·6% (-22·6 to 31·5%)	-16·3 (-20·7 to -12·6)	-5·9% (-6·8 to -5·1%)	-23·3 (-29·7 to -17·7)	-5·8% (-6·7 to -5·0%)	42·8% (17·6 to 73·4%)	-2·1% (-17·7 to 18·2%)
Pedestrian road injuries	0·8 (0·7 to 0·9)	17·7% (15·3 to 20·3%)	0·8 (0·7 to 1·0)	17·5% (15·2 to 20·0%)	9·0% (-4·1 to 24·8%)	-1·1% (-5·5 to 3·3%)	40·3 (33·6 to 47·2)	18·9% (16·3 to 21·8%)	41·3 (34·1 to 50·2)	19·8% (17·2 to 22·7%)	2·5% (-10·5 to 17·1%)	4·9% (-0·8 to 10·6%)
Cyclist road injuries	0·0 (0·0 to 0·0)	20·3% (17·5 to 23·2%)	0·1 (0·0 to 0·1)	20·4% (17·6 to 23·3%)	53·3% (28·0 to 86·3%)	0·4% (-5·2 to 6·8%)	2·2 (1·8 to 2·6)	19·7% (17·0 to 22·5%)	3·2 (2·6 to 4·0)	20·3% (17·6 to 23·4%)	46·1% (23·1 to 77·1%)	3·4% (-3·0 to 10·7%)
Motorcyclist road injuries	0·2 (0·2 to 0·3)	36·8% (34·1 to 39·6%)	0·3 (0·2 to 0·4)	38·4% (35·5 to 40·9%)	38·3% (14·3 to 67·1%)	4·2% (0·1 to 8·8%)	12·1 (10·2 to 14·5)	35·2% (32·4 to 38·1%)	16·4 (13·3 to 20·2)	37·3% (34·2 to 40·2%)	35·4% (12·5 to 62·6%)	5·9% (0·6 to 12·0%)
Motor vehicle road injuries	1·1 (0·9 to 1·2)	31·8% (29·3 to 34·6%)	1·3 (1·0 to 1·5)	33·2% (30·5 to 35·7%)	19·7% (-0·7 to 37·9%)	4·3% (-0·7 to 9·7%)	56·2 (48·4 to 64·0)	30·6% (27·9 to 33·7%)	64·8 (51·3 to 75·0)	33·1% (30·2 to 36·0%)	15·4% (-2·4 to 31·9%)	8·2% (1·7 to 15·1%)
Falls	0·2 (0·1 to 0·2)	9·2% (8·1 to 10·3%)	0·2 (0·2 to 0·2)	9·1% (7·8 to 10·4%)	29·5% (12·6 to 49·4%)	-0·9% (-10·2 to 8·3%)	8·9 (7·5 to 10·4)	9·6% (8·7 to 10·6%)	11·0 (9·2 to 13·3)	10·0% (9·1 to 11·1%)	24·6% (13·5 to 36·8%)	4·7% (-1·3 to 11·6%)
Drowning	0·2 (0·2 to 0·2)	7·9% (6·9 to 8·9%)	0·2 (0·2 to 0·2)	9·3% (8·0 to 10·5%)	1·2% (-12·3 to 17·5%)	17·5% (3·8 to 33·0%)	8·1 (7·1 to 9·2)	6·2% (5·2 to 7·2%)	7·9 (6·7 to 9·3)	7·8% (6·3 to 9·0%)	-3·0% (-16·2 to 13·1%)	25·7% (5·8 to 47·7%)
Fire, heat, and hot substances	0·0 (0·0 to 0·1)	5·3% (4·4 to 6·4%)	0·1 (0·0 to 0·1)	6·2% (4·9 to 7·5%)	9·1% (-5·3 to 26·8%)	15·8% (2·1 to 30·1%)	2·8 (2·3 to 3·3)	4·6% (3·9 to 5·4%)	2·8 (2·3 to 3·4)	5·8% (4·6 to 6·9%)	2·6% (-8·2 to 15·6%)	25·5% (10·2 to 40·4%)
Poisonings	0·0 (0·0 to 0·1)	6·4% (4·4 to 8·9%)	0·0 (0·0 to 0·1)	7·2% (4·8 to 9·8%)	-3·0% (-17·6 to 14·7%)	12·7% (-21·9 to 60·7%)	2·5 (1·9 to 3·5)	5·1% (3·3 to 7·4%)	2·4 (1·8 to 3·4)	6·1% (3·5 to 8·7%)	-4·7% (-17·5 to 10·7%)	19·2% (-24·7 to 88·7%)
Unintentional firearm injuries	0·1 (0·0 to 0·1)	10·6% (7·9 to 12·2%)	0·1 (0·0 to 0·1)	11·4% (7·3 to 13·4%)	6·8% (-20·1 to 34·3%)	7·3% (-9·2 to 21·0%)	4·0 (2·3 to 4·9)	10·4% (8·0 to 12·0%)	4·2 (2·0 to 5·4)	11·8% (8·1 to 13·9%)	5·9% (-18·2 to 32·4%)	13·8% (0·7 to 27·1%)
Unintentional suffocation	0·0 (0·0 to 0·0)	1·1% (0·8 to 1·6%)	0·0 (0·0 to 0·0)	1·7% (1·1 to 2·3%)	10·0% (-8·5 to 34·3%)	48·1% (2·0 to 116·4%)	0·4 (0·3 to 0·4)	1·1% (0·8 to 1·6%)	0·4 (0·3 to 0·5)	1·6% (1·0 to 2·3%)	10·7% (-1·9 to 25·9%)	46·6% (2·2 to 106·1%)
Other exposure to mechanical forces	0·1 (0·1 to 0·1)	11·3% (10·0 to 13·4%)	0·1 (0·1 to 0·1)	12·4% (11·1 to 14·3%)	3·2% (-13·3 to 25·4%)	9·7% (1·4 to 19·8%)	5·0 (4·1 to 6·5)	10·3% (9·2 to 11·9%)	5·2 (4·2 to 6·8)	11·6% (10·5 to 13·2%)	4·2% (-7·1 to 18·4%)	13·4% (5·9 to 21·5%)
Self-harm	0·4 (0·3 to 0·4)	11·7% (10·6 to 12·9%)	0·4 (0·3 to 0·5)	11·8% (10·5 to 13·3%)	13·3% (-3·6 to 31·0%)	1·4% (-6·0 to 9·2%)	16·7 (14·4 to 18·9)	11·6% (10·4 to 12·8%)	18·5 (15·2 to 22·4)	12·0% (10·6 to 13·5%)	10·9% (-5·6 to 28·4%)	3·7% (-3·9 to 11·9%)
Assault by firearm	0·3 (0·3 to 0·3)	11·4% (10·4 to 12·5%)	0·3 (0·2 to 0·4)	11·2% (10·3 to 12·4%)	-2·8% (-15·6 to 12·8%)	-1·0% (-4·4 to 2·8%)	17·2 (14·2 to 19·6)	11·9% (10·9 to 13·1%)	16·3 (13·5 to 19·4)	12·0% (11·0 to 13·2%)	-4·9% (-17·6 to 10·4%)	0·4% (-2·9 to 4·0%)
Assault by sharp object	0·1 (0·1 to 0·1)	11·0% (10·1 to 12·2%)	0·1 (0·1 to 0·1)	10·8% (9·9 to 11·9%)	-6·6% (-21·0 to 9·8%)	-1·9% (-6·6 to 2·9%)	5·3 (4·5 to 6·2)	11·7% (10·8 to 12·8%)	4·9 (4·0 to 5·8)	11·7% (10·7 to 12·9%)	-8·8% (-23·3 to 7·3%)	0·2% (-4·3 to 5·0%)
Assault by other means	0·1 (0·1 to 0·1)	10·1% (9·2 to 11·3%)	0·1 (0·1 to 0·1)	10·1% (9·1 to 11·2%)	4·6% (-10·4 to 22·8%)	-0·4% (-5·7 to 5·6%)	3·9 (3·3 to 4·8)	10·3% (9·4 to 11·5%)	3·9 (3·1 to 4·7)	10·7% (9·6 to 11·8%)	-0·1% (-14·0 to 17·0%)	3·5% (-2·7 to 10·4%)
**Drug use: All causes**	1·6 (1·2 to 2·0)	0·7% (0·5 to 0·8%)	2·1 (1·6 to 2·6)	0·7% (0·6 to 0·9%)	32·4% (18·2 to 48·6%)	13·6% (4·5 to 26·5%)	109·1 (90·4 to 128·2)	0·8% (0·7 to 1·0%)	135·8 (112·4 to 160·8)	1·0% (0·9 to 1·2%)	24·5% (15·8 to 33·7%)	22·9% (15·7 to 31·8%)
HIV/AIDS - Tuberculosis	0·0 (0·0 to 0·0)	1·1% (0·9 to 1·3%)	0·0 (0·0 to 0·0)	1·1% (0·9 to 1·3%)	-7·7% (-18·0 to 9·1%)	3·0% (1·5 to 4·5%)	0·3 (0·2 to 0·4)	1·0% (0·8 to 1·2%)	0·2 (0·2 to 0·4)	1·0% (0·9 to 1·2%)	-11·9% (-21·0 to 4·4%)	4·2% (2·4 to 6·1%)
HIV/AIDS resulting in other diseases	0·0 (0·0 to 0·0)	1·1% (0·9 to 1·3%)	0·0 (0·0 to 0·0)	1·1% (0·9 to 1·3%)	22·4% (2·2 to 47·3%)	2·9% (1·4 to 5·0%)	0·5 (0·4 to 0·7)	1·0% (0·8 to 1·2%)	0·6 (0·5 to 0·9)	1·0% (0·9 to 1·3%)	18·9% (0·7 to 41·1%)	4·1% (2·0 to 6·5%)
Hepatitis B	0·0 (0·0 to 0·0)	0·5% (0·2 to 0·9%)	0·0 (0·0 to 0·0)	0·7% (0·3 to 1·1%)	13·8% (-2·8 to 34·5%)	22·0% (11·9 to 34·0%)	0·0 (0·0 to 0·1)	0·6% (0·2 to 1·0%)	0·0 (0·0 to 0·1)	0·7% (0·3 to 1·1%)	6·9% (-7·8 to 24·4%)	20·8% (9·9 to 34·0%)
Hepatitis C	0·0 (0·0 to 0·0)	30·4% (20·7 to 40·7%)	0·0 (0·0 to 0·0)	35·0% (23·9 to 46·2%)	6·4% (-18·2 to 38·9%)	15·0% (-5·0 to 36·2%)	0·2 (0·0 to 0·3)	32·8% (22·5 to 43·5%)	0·2 (0·0 to 0·3)	38·0% (27·1 to 49·0%)	-2·8% (-23·2 to 25·3%)	15·9% (-1·2 to 38·5%)
Liver cancer due to hepatitis B	0·0 (0·0 to 0·0)	0·5% (0·2 to 0·8%)	0·0 (0·0 to 0·0)	0·6% (0·2 to 0·9%)	57·6% (38·0 to 82·1%)	24·1% (12·9 to 38·6%)	0·2 (0·1 to 0·3)	0·5% (0·2 to 0·9%)	0·2 (0·1 to 0·4)	0·7% (0·3 to 1·1%)	43·9% (25·1 to 66·6%)	22·0% (11·4 to 35·6%)
Liver cancer due to hepatitis C	0·0 (0·0 to 0·1)	14·0% (7·8 to 22·2%)	0·1 (0·0 to 0·1)	18·6% (11·2 to 28·4%)	75·4% (53·1 to 104·4%)	32·5% (19·0 to 51·5%)	0·9 (0·6 to 1·4)	18·6% (10·9 to 28·1%)	1·5 (0·9 to 2·2)	23·7% (14·7 to 34·6%)	57·9% (38·2 to 84·0%)	27·7% (14·9 to 45·6%)
Cirrhosis and other chronic liver diseases due to hepatitis B	0·0 (0·0 to 0·0)	0·6% (0·2 to 1·0%)	0·0 (0·0 to 0·0)	0·7% (0·3 to 1·2%)	48·8% (29·4 to 72·2%)	21·2% (11·3 to 35·6%)	0·4 (0·2 to 0·7)	0·7% (0·3 to 1·2%)	0·6 (0·2 to 0·9)	0·9% (0·4 to 1·4%)	36·6% (17·9 to 58·1%)	19·0% (9·0 to 33·0%)
Cirrhosis and other chronic liver diseases due to hepatitis C	0·8 (0·5 to 1·1)	34·7% (21·6 to 48·4%)	1·1 (0·7 to 1·5)	39·8% (26·2 to 53·4%)	36·9% (19·4 to 55·8%)	14·9% (6·6 to 26·9%)	27·0 (17·9 to 36·6)	45·0% (29·6 to 59·4%)	33·7 (22·7 to 45·5)	50·3% (34·6 to 64·2%)	24·9% (8·4 to 42·9%)	11·9% (4·4 to 22·2%)
Opioid use disorders	0·3 (0·2 to 0·4)	100·0% (100·0 to 100·0%)	0·4 (0·3 to 0·4)	100·0% (100·0 to 100·0%)	20·6% (1·9 to 49·1%)	-	42·0 (32·5 to 51·4)	100·0% (100·0 to 100·0%)	50·0 (38·4 to 61·9)	100·0% (100·0 to 100·0%)	19·1% (9·4 to 30·0%)	-
Cocaine use disorders	0·1 (0·1 to 0·1)	100·0% (100·0 to 100·0%)	0·2 (0·1 to 0·2)	100·0% (100·0 to 100·0%)	37·4% (9·1 to 104·6%)	-	9·4 (6·9 to 12·0)	100·0% (100·0 to 100·0%)	12·8 (9·2 to 16·6)	100·0% (100·0 to 100·0%)	35·0% (18·3 to 65·0%)	-
Amphetamine use disorders	0·0 (0·0 to 0·0)	100·0% (100·0 to 100·0%)	0·0 (0·0 to 0·1)	100·0% (100·0 to 100·0%)	59·8% (21·8 to 121·0%)	-	7·5 (4·8 to 11·0)	100·0% (100·0 to 100·0%)	10·5 (6·8 to 15·1)	100·0% (100·0 to 100·0%)	40·8% (23·6 to 59·9%)	-
Cannabis use disorders	-	-	-	-	-	-	2·9 (1·9 to 4·2)	100·0% (100·0 to 100·0%)	3·4 (2·2 to 4·9)	100·0% (100·0 to 100·0%)	17·0% (6·0 to 29·1%)	-
Other drug use disorders	0·1 (0·1 to 0·2)	100·0% (100·0 to 100·0%)	0·2 (0·1 to 0·2)	100·0% (100·0 to 100·0%)	18·5% (-0·9 to 43·1%)	-	12·6 (9·4 to 16·3)	100·0% (100·0 to 100·0%)	15·8 (11·4 to 21·1)	100·0% (100·0 to 100·0%)	25·8% (8·9 to 43·9%)	-
Self-harm	0·1 (0·1 to 0·2)	3·3% (2·1 to 4·9%)	0·1 (0·1 to 0·2)	3·6% (2·3 to 5·4%)	22·3% (6·2 to 39·5%)	9·6% (2·8 to 16·5%)	5·2 (3·2 to 7·9)	3·6% (2·3 to 5·4%)	6·2 (3·8 to 9·5)	4·0% (2·5 to 6·2%)	19·7% (3·0 to 37·4%)	12·0% (4·6 to 20·1%)
**Dietary risks: All causes**	31·1 (26·1 to 37·0)	13·0% (10·9 to 15·4%)	39·0 (32·4 to 46·9)	13·9% (11·7 to 16·7%)	25·4% (16·8 to 35·0%)	7·6% (4·3 to 11·1%)	728·3 (623·6 to 856·2)	5·6% (4·7 to 6·6%)	862·7 (723·3 to 1,021·6)	6·6% (5·5 to 7·8%)	18·4% (10·5 to 26·9%)	16·9% (11·7 to 22·2%)
**Diet low in fruits: All causes**	7·3 (4·5 to 10·2)	3·0% (1·9 to 4·2%)	8·9 (5·6 to 12·6)	3·2% (2·0 to 4·5%)	21·8% (12·5 to 31·9%)	4·5% (0·2 to 9·4%)	198·4 (127·8 to 273·3)	1·5% (1·0 to 2·1%)	228·0 (148·3 to 318·2)	1·7% (1·1 to 2·4%)	14·9% (5·8 to 25·2%)	13·4% (5·7 to 20·9%)
Lip and oral cavity cancer	0·0 (0·0 to 0·0)	6·2% (0·0 to 13·8%)	0·0 (0·0 to 0·1)	6·4% (0·0 to 13·9%)	36·7% (0·0 to 53·4%)	2·1% (-1·7 to 6·2%)	0·5 (0·0 to 1·1)	6·1% (0·0 to 13·5%)	0·7 (0·0 to 1·5)	6·2% (0·0 to 13·6%)	29·1% (0·0 to 43·1%)	1·8% (-1·6 to 5·3%)
Nasopharynx cancer	0·0 (0·0 to 0·0)	6·5% (0·0 to 13·9%)	0·0 (0·0 to 0·0)	6·8% (0·0 to 14·4%)	28·7% (0·0 to 46·8%)	4·0% (0·0 to 7·8%)	0·1 (0·0 to 0·2)	5·8% (0·0 to 12·4%)	0·1 (0·0 to 0·3)	6·1% (0·0 to 13·0%)	23·4% (0·0 to 39·8%)	6·0% (0·0 to 11·5%)
Other pharynx cancer	0·0 (0·0 to 0·0)	5·3% (0·0 to 12·1%)	0·0 (0·0 to 0·0)	5·4% (0·0 to 12·3%)	15·9% (0·0 to 31·9%)	1·1% (-2·9 to 5·3%)	0·4 (0·0 to 0·9)	5·4% (0·0 to 12·1%)	0·4 (0·0 to 0·9)	5·4% (0·0 to 12·3%)	10·3% (-1·8 to 23·8%)	1·2% (-2·8 to 5·7%)
Oesophageal cancer	0·1 (0·0 to 0·2)	23·2% (5·8 to 40·4%)	0·2 (0·0 to 0·3)	23·4% (5·9 to 40·8%)	32·3% (16·7 to 49·5%)	0·9% (-1·3 to 3·0%)	2·5 (0·6 to 4·4)	22·6% (5·7 to 39·6%)	3·2 (0·8 to 5·6)	22·9% (5·8 to 39·9%)	25·6% (10·3 to 41·9%)	1·2% (-0·6 to 3·1%)
Larynx cancer	0·0 (0·0 to 0·0)	7·5% (0·0 to 16·6%)	0·0 (0·0 to 0·1)	7·6% (0·0 to 16·7%)	22·6% (0·0 to 39·8%)	1·0% (-1·6 to 3·7%)	0·5 (0·0 to 1·0)	7·2% (0·0 to 16·0%)	0·5 (0·0 to 1·2)	7·3% (0·0 to 16·2%)	16·2% (0·0 to 30·1%)	1·2% (-1·1 to 3·6%)
Tracheal, bronchus, and lung cancer	0·4 (0·2 to 0·6)	11·7% (4·8 to 19·7%)	0·5 (0·2 to 0·9)	11·7% (4·7 to 19·6%)	43·4% (29·2 to 58·7%)	-0·3% (-3·3 to 2·7%)	7·7 (3·1 to 13·0)	11·4% (4·7 to 19·3%)	10·4 (4·2 to 17·6)	11·4% (4·6 to 19·3%)	35·5% (21·9 to 49·7%)	-0·1% (-2·8 to 2·8%)
Ischaemic heart disease	3·1 (1·1 to 5·3)	11·3% (4·0 to 19·0%)	3·7 (1·3 to 6·3)	11·0% (4·0 to 18·4%)	18·8% (9·7 to 29·3%)	-3·2% (-6·2 to -0·1%)	70·4 (25·5 to 117·8)	13·7% (5·0 to 22·7%)	76·1 (27·8 to 126·4)	13·4% (4·9 to 22·2%)	8·0% (-1·4 to 18·6%)	-2·5% (-5·6 to 0·4%)
Ischaemic stroke	1·2 (0·6 to 1·8)	16·3% (8·8 to 25·0%)	1·5 (0·8 to 2·3)	15·3% (8·1 to 23·8%)	22·2% (10·8 to 34·0%)	-6·7% (-11·0 to -2·2%)	28·3 (15·7 to 41·7)	23·5% (13·1 to 34·7%)	32·1 (18·0 to 48·4)	22·4% (12·4 to 33·5%)	13·5% (3·2 to 24·6%)	-4·4% (-8·6 to -0·2%)

Supplemental Results Table 1. All-age deaths, death PAFs, DALYs, DALY PAFs, and percent change for each risk factor and outcome for both sexes combined in 2005 and 2015 for Andean Latin America.

Data in parenthesis are 95% uncertainty intervals. PAFs=population attributable fractions. DALYs=disability-adjusted life-years.

	2005 Deaths (in thousands)	2005 Deaths PAF	2015 Deaths (in thousands)	2015 Deaths PAF	Percent change deaths 2005-2015	Percent change deaths PAF 2005-2015	2005 DALYs (in thousands)	2005 DALYs PAF	2015 DALYs (in thousands)	2015 DALYs PAF	Percent change DALYs 2005-2015	Percent change DALYs PAF 2005-2015
Hemorrhagic stroke	1·8 (1·0 to 2·7)	22·8% (12·8 to 33·7%)	2·0 (1·1 to 3·1)	22·0% (12·0 to 33·1%)	11·9% (1·4 to 22·5%)	-3·4% (-7·9 to 0·5%)	58·2 (33·3 to 85·6)	25·4% (14·7 to 36·7%)	61·0 (34·0 to 91·7)	26·8% (15·1 to 39·3%)	4·9% (-4·6 to 15·3%)	5·8% (1·7 to 9·8%)
**Diet low in vegetables: All causes**	7·4 (3·8 to 11·3)	3·1% (1·6 to 4·7%)	8·8 (4·5 to 13·5)	3·1% (1·6 to 4·8%)	18·1% (9·9 to 27·4%)	1·4% (-2·1 to 4·9%)	173·1 (95·0 to 257·7)	1·3% (0·7 to 2·0%)	186·7 (102·1 to 281·1)	1·4% (0·8 to 2·1%)	7·9% (-0·1 to 16·3%)	6·5% (0·8 to 12·5%)
Ischaemic heart disease	5·5 (2·2 to 8·8)	19·7% (7·9 to 31·6%)	6·5 (2·6 to 10·5)	19·2% (7·7 to 30·9%)	19·2% (10·8 to 28·8%)	-2·9% (-4·1 to -1·8%)	116·7 (48·0 to 184·3)	22·7% (9·4 to 35·9%)	126·4 (52·1 to 200·9)	22·3% (9·1 to 35·4%)	8·3% (0·1 to 17·1%)	-2·2% (-3·8 to -0·8%)
Ischaemic stroke	0·8 (0·2 to 1·4)	10·6% (2·4 to 18·6%)	1·0 (0·2 to 1·7)	9·8% (2·2 to 17·4%)	20·9% (10·6 to 32·0%)	-7·6% (-10·9 to -4·3%)	18·5 (4·5 to 31·5)	15·3% (3·7 to 26·2%)	20·7 (5·1 to 36·0)	14·5% (3·4 to 25·1%)	12·3% (2·4 to 22·3%)	-5·3% (-8·6 to -2·2%)
Hemorrhagic stroke	1·2 (0·3 to 2·0)	14·9% (4·4 to 26·1%)	1·3 (0·4 to 2·3)	14·3% (4·3 to 25·2%)	11·2% (1·9 to 21·4%)	-3·9% (-7·4 to -0·8%)	38·0 (11·3 to 65·6)	16·6% (5·0 to 28·3%)	39·6 (11·9 to 68·8)	17·4% (5·3 to 29·9%)	4·2% (-4·6 to 13·9%)	5·1% (2·2 to 8·0%)
**Diet low in whole grains: All causes**	5·4 (2·9 to 8·2)	2·3% (1·2 to 3·4%)	6·6 (3·6 to 10·2)	2·4% (1·3 to 3·6%)	21·3% (11·8 to 31·1%)	4·1% (-0·5 to 8·8%)	151·9 (80·2 to 231·2)	1·2% (0·6 to 1·8%)	175·4 (93·1 to 266·2)	1·3% (0·7 to 2·0%)	15·5% (6·7 to 24·7%)	14·0% (7·2 to 20·6%)
Ischaemic heart disease	3·1 (1·5 to 5·1)	11·1% (5·4 to 18·5%)	3·7 (1·8 to 6·2)	10·7% (5·2 to 18·0%)	19·2% (9·1 to 29·7%)	-3·0% (-6·8 to 0·4%)	67·4 (32·4 to 109·2)	13·1% (6·2 to 21·2%)	72·6 (34·7 to 117·9)	12·8% (6·1 to 20·7%)	7·7% (-1·3 to 17·6%)	-2·8% (-6·7 to 0·7%)
Ischaemic stroke	0·7 (0·4 to 1·1)	9·3% (4·8 to 14·7%)	0·8 (0·4 to 1·3)	8·5% (4·3 to 13·5%)	19·3% (8·1 to 30·1%)	-8·8% (-13·7 to -4·7%)	17·3 (9·0 to 26·7)	14·3% (7·6 to 21·9%)	19·2 (10·0 to 29·8)	13·4% (7·1 to 20·6%)	10·9% (0·3 to 21·7%)	-6·5% (-11·4 to -2·2%)
Hemorrhagic stroke	1·0 (0·5 to 1·5)	12·7% (6·8 to 19·8%)	1·1 (0·6 to 1·7)	12·1% (6·6 to 18·9%)	10·4% (0·0 to 21·3%)	-4·7% (-9·4 to -0·2%)	33·1 (16·7 to 51·5)	14·4% (7·3 to 22·5%)	34·3 (17·7 to 54·1)	15·1% (7·7 to 23·5%)	3·7% (-6·1 to 13·6%)	4·6% (-0·1 to 9·8%)
Diabetes mellitus	0·7 (0·3 to 1·2)	10·1% (4·4 to 18·0%)	1·0 (0·4 to 1·8)	9·8% (4·3 to 17·4%)	49·3% (36·9 to 62·7%)	-3·3% (-6·6 to -0·4%)	34·1 (14·9 to 61·0)	12·4% (5·4 to 21·8%)	49·4 (21·7 to 88·8)	12·3% (5·4 to 21·6%)	44·6% (37·0 to 52·9%)	-0·8% (-3·8 to 1·7%)
**Diet low in nuts and seeds: All causes**	7·0 (4·6 to 9·9)	2·9% (1·9 to 4·1%)	8·6 (5·5 to 12·1)	3·1% (2·0 to 4·3%)	21·9% (13·1 to 31·8%)	4·6% (0·9 to 8·7%)	169·5 (114·0 to 231·6)	1·3% (0·9 to 1·8%)	195·8 (134·7 to 267·9)	1·5% (1·0 to 2·0%)	15·5% (7·3 to 24·7%)	14·0% (8·0 to 20·4%)
Ischaemic heart disease	6·3 (4·0 to 8·9)	22·7% (14·4 to 32·3%)	7·5 (4·6 to 10·8)	22·0% (13·7 to 31·3%)	18·7% (10·1 to 28·1%)	-3·3% (-4·8 to -2·1%)	132·7 (86·7 to 184·5)	25·9% (16·6 to 35·7%)	142·6 (90·8 to 200·1)	25·1% (16·0 to 34·8%)	7·4% (-0·8 to 16·4%)	-3·1% (-5·1 to -1·4%)
Diabetes mellitus	0·7 (0·4 to 1·1)	11·1% (5·8 to 17·3%)	1·1 (0·6 to 1·7)	10·7% (5·7 to 16·7%)	49·0% (36·8 to 63·0%)	-3·3% (-5·3 to -1·2%)	36·8 (17·9 to 58·6)	13·4% (7·0 to 20·7%)	53·2 (25·5 to 86·4)	13·3% (7·0 to 20·5%)	44·8% (37·7 to 52·8%)	-0·7% (-1·7 to 0·3%)
**Diet low in milk: All causes**	0·4 (0·1 to 0·7)	0·2% (0·1 to 0·3%)	0·6 (0·2 to 1·0)	0·2% (0·1 to 0·4%)	49·0% (35·1 to 64·3%)	27·9% (19·7 to 36·2%)	8·2 (2·9 to 13·9)	0·1% (0·0 to 0·1%)	11·5 (4·1 to 19·7)	0·1% (0·0 to 0·2%)	40·1% (27·7 to 53·7%)	38·3% (28·9 to 49·0%)
Colon and rectum cancer	0·4 (0·1 to 0·7)	15·6% (5·5 to 26·2%)	0·6 (0·2 to 1·0)	15·6% (5·5 to 26·3%)	49·0% (35·1 to 64·3%)	0·1% (-0·7 to 0·9%)	8·2 (2·9 to 13·9)	15·5% (5·4 to 26·1%)	11·5 (4·1 to 19·7)	15·5% (5·5 to 26·1%)	40·1% (27·7 to 53·7%)	0·1% (-0·7 to 0·9%)
**Diet high in red meat: All causes**	0·2 (0·1 to 0·3)	0·1% (0·0 to 0·1%)	0·3 (0·1 to 0·4)	0·1% (0·0 to 0·2%)	44·4% (31·5 to 58·9%)	23·9% (14·8 to 34·1%)	8·4 (2·1 to 14·7)	0·1% (0·0 to 0·1%)	11·8 (3·0 to 21·0)	0·1% (0·0 to 0·2%)	41·3% (32·1 to 51·1%)	39·4% (31·5 to 48·5%)
Colon and rectum cancer	0·0 (0·0 to 0·1)	1·9% (0·4 to 3·4%)	0·1 (0·0 to 0·1)	1·9% (0·4 to 3·4%)	46·5% (29·8 to 62·1%)	-1·5% (-10·0 to 6·9%)	1·1 (0·2 to 2·0)	2·1% (0·4 to 3·8%)	1·6 (0·3 to 2·9)	2·1% (0·4 to 3·8%)	40·7% (24·8 to 56·2%)	0·6% (-7·5 to 8·9%)
Diabetes mellitus	0·1 (0·0 to 0·2)	2·0% (0·2 to 3·6%)	0·2 (0·0 to 0·4)	1·8% (0·2 to 3·4%)	43·7% (27·8 to 58·6%)	-7·0% (-13·3 to 0·3%)	7·2 (0·8 to 13·5)	2·6% (0·3 to 4·9%)	10·2 (1·2 to 19·4)	2·6% (0·3 to 4·7%)	41·4% (31·6 to 50·8%)	-3·1% (-7·4 to 1·5%)
**Diet high in processed meat: All causes**	2·0 (0·7 to 3·3)	0·8% (0·3 to 1·4%)	2·5 (1·0 to 4·0)	0·9% (0·4 to 1·4%)	26·7% (16·6 to 44·5%)	8·9% (3·5 to 22·1%)	62·2 (29·9 to 94·7)	0·5% (0·2 to 0·7%)	78·0 (40·7 to 118·1)	0·6% (0·3 to 0·9%)	25·4% (14·5 to 41·3%)	23·8% (15·0 to 38·4%)
Colon and rectum cancer	0·1 (0·1 to 0·1)	3·7% (2·0 to 5·7%)	0·1 (0·1 to 0·2)	3·7% (2·0 to 5·6%)	47·2% (34·1 to 61·1%)	-1·0% (-4·1 to 1·7%)	2·1 (1·1 to 3·2)	3·9% (2·1 to 5·9%)	2·9 (1·6 to 4·4)	3·9% (2·1 to 5·9%)	39·1% (26·7 to 52·1%)	-0·5% (-3·4 to 2·3%)
Ischaemic heart disease	1·3 (0·1 to 2·5)	4·8% (0·2 to 9·0%)	1·6 (0·1 to 2·9)	4·6% (0·2 to 8·6%)	17·3% (6·7 to 26·8%)	-4·4% (-7·0 to -1·0%)	29·9 (1·3 to 55·7)	5·8% (0·3 to 10·9%)	32·1 (1·4 to 60·0)	5·7% (0·2 to 10·6%)	7·4% (-1·1 to 16·2%)	-2·9% (-5·5 to 0·0%)
Diabetes mellitus	0·6 (0·3 to 0·8)	8·4% (4·2 to 12·3%)	0·8 (0·4 to 1·2)	7·9% (3·9 to 11·7%)	46·0% (33·4 to 58·9%)	-5·4% (-9·0 to -2·1%)	30·2 (14·3 to 47·2)	11·0% (5·4 to 16·1%)	43·0 (20·2 to 66·5)	10·8% (5·3 to 15·8%)	42·2% (34·5 to 49·9%)	-2·5% (-5·1 to 0·1%)
**Diet high in sugar-sweetened beverages: All causes**	0·6 (0·4 to 0·8)	0·2% (0·2 to 0·3%)	0·8 (0·5 to 1·1)	0·3% (0·2 to 0·4%)	36·6% (25·2 to 48·7%)	17·3% (11·1 to 23·1%)	20·4 (13·8 to 29·2)	0·2% (0·1 to 0·2%)	27·4 (18·4 to 40·0)	0·2% (0·1 to 0·3%)	34·2% (23·6 to 43·9%)	32·4% (23·9 to 39·5%)
Oesophageal cancer	0·0 (0·0 to 0·0)	0·7% (0·2 to 1·3%)	0·0 (0·0 to 0·0)	0·7% (0·2 to 1·4%)	38·6% (21·0 to 57·5%)	5·7% (-1·2 to 12·5%)	0·1 (0·0 to 0·2)	0·8% (0·2 to 1·4%)	0·1 (0·0 to 0·2)	0·8% (0·3 to 1·5%)	32·8% (15·7 to 51·1%)	7·0% (-0·1 to 13·4%)
Colon and rectum cancer	0·0 (0·0 to 0·0)	0·2% (0·2 to 0·3%)	0·0 (0·0 to 0·0)	0·3% (0·2 to 0·4%)	58·4% (41·5 to 76·3%)	6·4% (-1·5 to 14·7%)	0·1 (0·1 to 0·2)	0·3% (0·2 to 0·4%)	0·2 (0·1 to 0·3)	0·3% (0·2 to 0·4%)	51·8% (34·9 to 70·3%)	8·5% (0·8 to 16·2%)
Liver cancer due to hepatitis B	0·0 (0·0 to 0·0)	0·5% (0·2 to 0·8%)	0·0 (0·0 to 0·0)	0·5% (0·2 to 0·8%)	36·6% (20·4 to 54·9%)	7·5% (0·3 to 16·2%)	0·1 (0·1 to 0·2)	0·5% (0·2 to 0·8%)	0·2 (0·1 to 0·3)	0·5% (0·3 to 0·9%)	31·1% (14·7 to 50·5%)	11·2% (3·2 to 20·7%)
Liver cancer due to hepatitis C	0·0 (0·0 to 0·0)	0·4% (0·2 to 0·7%)	0·0 (0·0 to 0·0)	0·4% (0·2 to 0·7%)	39·9% (23·1 to 60·2%)	5·7% (-1·2 to 14·7%)	0·0 (0·0 to 0·0)	0·4% (0·2 to 0·7%)	0·0 (0·0 to 0·1)	0·5% (0·2 to 0·8%)	32·9% (16·5 to 52·8%)	7·6% (0·8 to 18·0%)
Liver cancer due to alcohol use	0·0 (0·0 to 0·0)	0·5% (0·2 to 0·9%)	0·0 (0·0 to 0·0)	0·5% (0·3 to 0·9%)	59·0% (36·8 to 83·9%)	7·5% (-0·2 to 15·3%)	0·1 (0·0 to 0·1)	0·6% (0·3 to 0·9%)	0·1 (0·0 to 0·2)	0·6% (0·3 to 1·0%)	55·8% (33·9 to 81·8%)	8·7% (1·2 to 16·6%)
Liver cancer due to other causes	0·0 (0·0 to 0·0)	0·4% (0·2 to 0·7%)	0·0 (0·0 to 0·0)	0·4% (0·2 to 0·7%)	33·9% (17·8 to 52·9%)	5·3% (-1·4 to 13·4%)	0·0 (0·0 to 0·1)	0·4% (0·2 to 0·7%)	0·1 (0·0 to 0·1)	0·5% (0·2 to 0·8%)	25·9% (10·5 to 43·9%)	8·9% (1·8 to 18·0%)
Gallbladder and biliary tract cancer	0·0 (0·0 to 0·0)	0·5% (0·3 to 0·8%)	0·0 (0·0 to 0·0)	0·6% (0·3 to 0·9%)	27·6% (7·2 to 51·4%)	3·3% (-3·6 to 10·1%)	0·2 (0·1 to 0·3)	0·6% (0·4 to 0·9%)	0·2 (0·1 to 0·4)	0·6% (0·4 to 1·0%)	21·3% (1·6 to 42·6%)	4·5% (-2·4 to 11·3%)
Pancreatic cancer	0·0 (0·0 to 0·0)	0·2% (0·1 to 0·3%)	0·0 (0·0 to 0·0)	0·2% (0·1 to 0·3%)	52·8% (35·2 to 72·5%)	6·3% (-1·2 to 13·3%)	0·1 (0·0 to 0·1)	0·2% (0·1 to 0·3%)	0·1 (0·0 to 0·2)	0·2% (0·1 to 0·4%)	48·3% (30·4 to 67·9%)	7·3% (-0·6 to 13·9%)
Breast cancer	0·0 (0·0 to 0·0)	0·1% (0·1 to 0·2%)	0·0 (0·0 to 0·0)	0·2% (0·1 to 0·2%)	49·6% (25·7 to 76·4%)	10·7% (2·1 to 19·6%)	0·1 (0·0 to 0·1)	0·1% (0·1 to 0·2%)	0·1 (0·1 to 0·2)	0·1% (0·1 to 0·2%)	48·1% (24·1 to 73·9%)	14·3% (4·2 to 25·3%)
Uterine cancer	0·0 (0·0 to 0·0)	1·1% (0·7 to 1·5%)	0·0 (0·0 to 0·0)	1·1% (0·8 to 1·6%)	19·8% (2·2 to 39·2%)	4·6% (-1·0 to 10·5%)	0·2 (0·1 to 0·3)	1·2% (0·8 to 1·7%)	0·3 (0·2 to 0·4)	1·3% (0·8 to 1·8%)	16·8% (0·3 to 35·9%)	5·7% (0·5 to 10·9%)
Ovarian cancer	0·0 (0·0 to 0·0)	0·1% (0·0 to 0·2%)	0·0 (0·0 to 0·0)	0·1% (0·0 to 0·2%)	49·6% (0·0 to 75·6%)	6·2% (0·0 to 11·5%)	0·0 (0·0 to 0·0)	0·1% (0·0 to 0·2%)	0·0 (0·0 to 0·1)	0·1% (0·0 to 0·2%)	45·6% (0·0 to 70·8%)	7·0% (0·0 to 12·0%)
Kidney cancer	0·0 (0·0 to 0·0)	0·5% (0·4 to 0·8%)	0·0 (0·0 to 0·0)	0·6% (0·4 to 0·8%)	62·7% (42·5 to 84·6%)	7·3% (0·9 to 13·6%)	0·1 (0·0 to 0·1)	0·5% (0·3 to 0·8%)	0·1 (0·1 to 0·2)	0·6% (0·4 to 0·9%)	56·5% (37·5 to 77·3%)	12·4% (4·6 to 19·0%)
Thyroid cancer	0·0 (0·0 to 0·0)	0·3% (0·2 to 0·5%)	0·0 (0·0 to 0·0)	0·3% (0·2 to 0·6%)	48·3% (29·1 to 67·4%)	6·4% (-0·3 to 13·0%)	0·0 (0·0 to 0·0)	0·4% (0·2 to 0·6%)	0·0 (0·0 to 0·1)	0·4% (0·2 to 0·6%)	48·5% (30·1 to 67·7%)	7·7% (1·4 to 13·6%)
Ischaemic heart disease	0·2 (0·1 to 0·2)	0·6% (0·4 to 0·8%)	0·2 (0·1 to 0·3)	0·6% (0·4 to 0·8%)	20·8% (9·6 to 32·7%)	-1·6% (-7·6 to 3·8%)	4·4 (3·0 to 6·2)	0·9% (0·6 to 1·2%)	5·0 (3·4 to 7·2)	0·9% (0·6 to 1·3%)	13·6% (2·6 to 24·9%)	2·5% (-3·5 to 7·7%)
Ischaemic stroke	0·0 (0·0 to 0·0)	0·5% (0·3 to 0·7%)	0·0 (0·0 to 0·1)	0·4% (0·3 to 0·6%)	24·1% (10·6 to 38·0%)	-5·2% (-13·1 to 1·6%)	0·9 (0·6 to 1·3)	0·8% (0·5 to 1·1%)	1·1 (0·8 to 1·6)	0·8% (0·5 to 1·1%)	18·8% (6·6 to 30·8%)	0·1% (-6·1 to 5·7%)
Hemorrhagic stroke	0·1 (0·1 to 0·1)	1·1% (0·7 to 1·6%)	0·1 (0·1 to 0·1)	1·1% (0·7 to 1·6%)	14·3% (3·3 to 26·1%)	-1·3% (-8·4 to 5·2%)	3·1 (2·1 to 4·4)	1·4% (0·9 to 2·0%)	3·4 (2·3 to 5·0)	1·5% (1·0 to 2·2%)	10·2% (-0·4 to 21·3%)	11·1% (4·0 to 17·2%)
Hypertensive heart disease	0·0 (0·0 to 0·0)	1·2% (0·7 to 1·9%)	0·0 (0·0 to 0·1)	1·3% (0·7 to 2·0%)	70·2% (46·6 to 92·9%)	3·8% (-5·2 to 11·3%)	0·6 (0·4 to 0·9)	1·6% (1·1 to 2·4%)	1·0 (0·6 to 1·5)	1·7% (1·1 to 2·5%)	56·1% (35·9 to 75·2%)	4·7% (-2·2 to 11·4%)
Cardiomyopathy and myocarditis	-	-	-	-	-	-	-	-	-	-	-	-
Atrial fibrillation and flutter	-	-	-	-	-	-	-	-	-	-	-	-
Peripheral vascular disease	-	-	-	-	-	-	-	-	-	-	-	-

Supplemental Results Table 1. All-age deaths, death PAFs, DALYs, DALY PAFs, and percent change for each risk factor and outcome for both sexes combined in 2005 and 2015 for Andean Latin America.

Data in parenthesis are 95% uncertainty intervals. PAFs=population attributable fractions. DALYs=disability-adjusted life-years.

	2005 Deaths (in thousands)	2005 Deaths PAF	2015 Deaths (in thousands)	2015 Deaths PAF	Percent change deaths 2005-2015	Percent change deaths PAF 2005-2015	2005 DALYs (in thousands)	2005 DALYs PAF	2015 DALYs (in thousands)	2015 DALYs PAF	Percent change DALYs 2005-2015	Percent change DALYs PAF 2005-2015
Endocarditis	-	-	-	-	-	-	-	-	-	-	-	-
Other cardiovascular and circulatory diseases	-	-	-	-	-	-	-	-	-	-	-	-
Diabetes mellitus	0·1 (0·1 to 0·1)	1·5% (1·1 to 2·2%)	0·2 (0·1 to 0·2)	1·6% (1·1 to 2·2%)	57·1% (42·2 to 73·9%)	1·8% (-4·3 to 7·7%)	6·0 (3·9 to 8·8)	2·2% (1·5 to 3·1%)	9·2 (5·8 to 13·9)	2·3% (1·6 to 3·3%)	54·3% (42·6 to 64·6%)	5·8% (0·4 to 10·4%)
Chronic kidney disease due to diabetes mellitus	0·1 (0·0 to 0·1)	1·2% (0·6 to 1·9%)	0·1 (0·0 to 0·2)	1·2% (0·6 to 2·0%)	51·9% (34·6 to 70·5%)	4·1% (-3·2 to 12·3%)	1·5 (0·7 to 2·7)	1·3% (0·6 to 2·2%)	2·3 (1·1 to 3·9)	1·4% (0·7 to 2·4%)	45·5% (29·8 to 62·2%)	9·4% (2·4 to 17·8%)
Chronic kidney disease due to hypertension	0·0 (0·0 to 0·1)	0·9% (0·4 to 1·5%)	0·1 (0·0 to 0·1)	0·9% (0·4 to 1·6%)	58·4% (35·2 to 83·0%)	5·4% (-7·0 to 16·6%)	1·0 (0·5 to 1·7)	0·9% (0·4 to 1·5%)	1·4 (0·7 to 2·5)	1·1% (0·5 to 1·9%)	44·7% (27·9 to 65·0%)	19·2% (10·3 to 30·3%)
Chronic kidney disease due to glomerulonephritis	0·0 (0·0 to 0·0)	0·7% (0·3 to 1·3%)	0·0 (0·0 to 0·0)	0·8% (0·4 to 1·5%)	49·7% (27·5 to 72·2%)	14·8% (1·3 to 27·2%)	0·3 (0·1 to 0·5)	0·8% (0·3 to 1·6%)	0·4 (0·1 to 0·8)	1·0% (0·4 to 2·0%)	43·1% (32·5 to 55·1%)	23·4% (15·7 to 32·4%)
Chronic kidney disease due to other causes	0·0 (0·0 to 0·0)	0·6% (0·2 to 1·0%)	0·0 (0·0 to 0·0)	0·7% (0·3 to 1·3%)	53·7% (24·4 to 79·3%)	19·4% (0·0 to 35·2%)	0·2 (0·1 to 0·3)	0·9% (0·4 to 1·6%)	0·3 (0·1 to 0·5)	1·1% (0·5 to 1·9%)	46·1% (35·2 to 57·9%)	16·5% (9·0 to 24·7%)
**Diet low in fibre: All causes**	1·0 (0·5 to 1·9)	0·4% (0·2 to 0·8%)	1·3 (0·6 to 2·3)	0·5% (0·2 to 0·8%)	22·8% (12·1 to 34·4%)	5·5% (-3·0 to 14·4%)	23·5 (10·4 to 42·8)	0·2% (0·1 to 0·3%)	26·2 (11·7 to 47·2)	0·2% (0·1 to 0·4%)	11·4% (0·6 to 23·5%)	10·1% (0·9 to 21·0%)
Colon and rectum cancer	0·1 (0·0 to 0·2)	4·1% (1·6 to 7·7%)	0·2 (0·1 to 0·3)	4·2% (1·6 to 8·2%)	55·0% (38·1 to 73·1%)	4·2% (-5·3 to 13·8%)	2·1 (0·8 to 3·9)	3·9% (1·5 to 7·4%)	3·0 (1·1 to 5·7)	4·1% (1·6 to 7·8%)	46·1% (31·8 to 63·1%)	4·6% (-4·3 to 14·6%)
Ischaemic heart disease	0·9 (0·4 to 1·7)	3·4% (1·5 to 6·2%)	1·1 (0·5 to 2·1)	3·3% (1·4 to 6·0%)	19·3% (8·6 to 30·3%)	-2·8% (-9·9 to 5·0%)	21·4 (9·3 to 39·1)	4·2% (1·8 to 7·6%)	23·2 (10·1 to 42·4)	4·1% (1·7 to 7·5%)	8·1% (-2·2 to 19·5%)	-2·3% (-9·6 to 5·6%)
**Diet low in calcium: All causes**	0·5 (0·3 to 0·7)	0·2% (0·1 to 0·3%)	0·7 (0·4 to 1·1)	0·3% (0·2 to 0·4%)	50·0% (36·4 to 65·4%)	28·8% (20·7 to 37·5%)	10·1 (5·9 to 14·5)	0·1% (0·0 to 0·1%)	14·2 (8·5 to 20·8)	0·1% (0·1 to 0·2%)	40·7% (28·3 to 54·9%)	38·9% (29·3 to 49·7%)
Colon and rectum cancer	0·5 (0·3 to 0·7)	19·4% (11·4 to 28·1%)	0·7 (0·4 to 1·1)	19·5% (11·6 to 28·5%)	50·0% (36·4 to 65·4%)	0·9% (-1·2 to 3·3%)	10·1 (5·9 to 14·5)	19·0% (11·1 to 27·6%)	14·2 (8·5 to 20·8)	19·1% (11·3 to 27·8%)	40·7% (28·3 to 54·9%)	0·6% (-1·6 to 3·1%)
Prostate cancer	-	-	-	-	-	-	-	-	-	-	-	-
**Diet low in seafood omega-3 fatty acids: All causes**	4·0 (1·7 to 6·7)	1·7% (0·7 to 2·8%)	4·7 (1·9 to 7·9)	1·7% (0·7 to 2·8%)	17·4% (8·4 to 27·2%)	0·8% (-3·4 to 5·1%)	85·6 (35·5 to 139·4)	0·7% (0·3 to 1·1%)	90·3 (36·5 to 150·1)	0·7% (0·3 to 1·2%)	5·5% (-3·5 to 15·0%)	4·2% (-2·6 to 11·0%)
Ischaemic heart disease	4·0 (1·7 to 6·7)	14·5% (6·0 to 24·0%)	4·7 (1·9 to 7·9)	13·9% (5·7 to 22·9%)	17·4% (8·4 to 27·2%)	-4·4% (-6·2 to -2·8%)	85·6 (35·5 to 139·4)	16·7% (7·0 to 27·1%)	90·3 (36·5 to 150·1)	15·9% (6·6 to 26·0%)	5·5% (-3·5 to 15·0%)	-4·8% (-7·0 to -2·8%)
**Diet low in polyunsaturated fatty acids: All causes**	0·5 (0·2 to 0·7)	0·2% (0·1 to 0·3%)	0·6 (0·2 to 0·9)	0·2% (0·1 to 0·3%)	27·5% (5·9 to 52·6%)	9·5% (-7·5 to 30·4%)	8·5 (3·4 to 14·1)	0·1% (0·0 to 0·1%)	10·5 (4·1 to 17·6)	0·1% (0·0 to 0·1%)	22·8% (-3·9 to 57·0%)	21·3% (-5·3 to 53·0%)
Ischaemic heart disease	0·5 (0·2 to 0·7)	1·6% (0·6 to 2·7%)	0·6 (0·2 to 0·9)	1·7% (0·7 to 2·8%)	27·5% (5·9 to 52·6%)	3·9% (-12·4 to 23·8%)	8·5 (3·4 to 14·1)	1·7% (0·7 to 2·8%)	10·5 (4·1 to 17·6)	1·8% (0·7 to 3·1%)	22·8% (-3·9 to 57·0%)	10·9% (-12·6 to 39·9%)
**Diet high in trans fatty acids: All causes**	1·9 (0·9 to 3·2)	0·8% (0·4 to 1·3%)	2·1 (0·9 to 3·7)	0·8% (0·3 to 1·3%)	11·9% (-0·2 to 23·3%)	-4·0% (-11·5 to 1·8%)	50·9 (25·7 to 82·1)	0·4% (0·2 to 0·6%)	52·3 (25·7 to 86·4)	0·4% (0·2 to 0·7%)	2·7% (-8·7 to 13·8%)	1·4% (-8·4 to 9·6%)
Ischaemic heart disease	1·9 (0·9 to 3·2)	6·8% (3·2 to 11·5%)	2·1 (0·9 to 3·7)	6·2% (2·8 to 10·7%)	11·9% (-0·2 to 23·3%)	-8·9% (-15·8 to -4·1%)	50·9 (25·7 to 82·1)	9·9% (5·1 to 15·9%)	52·3 (25·7 to 86·4)	9·2% (4·6 to 15·1%)	2·7% (-8·7 to 13·8%)	-7·4% (-13·6 to -2·8%)
**Diet high in sodium: All causes**	6·3 (1·8 to 13·6)	2·6% (0·8 to 5·7%)	8·1 (2·3 to 17·6)	2·9% (0·8 to 6·3%)	29·2% (19·5 to 39·7%)	10·9% (5·5 to 15·8%)	129·3 (38·0 to 273·5)	1·0% (0·3 to 2·1%)	155·9 (44·7 to 327·6)	1·2% (0·3 to 2·6%)	20·6% (11·3 to 30·5%)	19·1% (12·1 to 26·3%)
Stomach cancer	1·5 (0·5 to 3·1)	22·7% (6·8 to 45·8%)	1·8 (0·5 to 3·7)	22·6% (6·6 to 46·1%)	20·3% (7·6 to 32·3%)	-0·1% (-6·8 to 5·6%)	32·3 (10·2 to 63·3)	22·7% (7·2 to 44·2%)	36·6 (11·6 to 71·6)	22·9% (7·3 to 44·4%)	13·5% (2·4 to 25·1%)	0·7% (-4·0 to 5·1%)
Rheumatic heart disease	0·0 (0·0 to 0·1)	3·0% (0·7 to 7·6%)	0·0 (0·0 to 0·1)	3·2% (0·7 to 8·2%)	9·7% (-2·8 to 25·0%)	7·5% (-0·7 to 15·6%)	0·6 (0·1 to 1·6)	2·3% (0·5 to 6·0%)	0·6 (0·1 to 1·6)	2·8% (0·6 to 7·1%)	1·9% (-8·1 to 14·5%)	19·3% (11·0 to 29·7%)
Ischaemic heart disease	2·3 (0·6 to 5·4)	8·3% (2·1 to 19·4%)	2·8 (0·7 to 6·7)	8·3% (2·1 to 19·5%)	23·7% (12·9 to 35·1%)	0·7% (-4·3 to 5·0%)	42·7 (11·4 to 98·3)	8·3% (2·2 to 19·3%)	49·2 (13·2 to 111·2)	8·7% (2·4 to 19·9%)	15·2% (5·7 to 26·1%)	3·9% (0·4 to 8·6%)
Ischaemic stroke	0·5 (0·1 to 1·3)	7·3% (1·8 to 17·5%)	0·7 (0·2 to 1·7)	7·2% (1·8 to 17·1%)	29·6% (16·5 to 43·1%)	-1·1% (-6·2 to 4·0%)	9·6 (2·5 to 22·1)	8·0% (2·1 to 18·6%)	11·6 (3·0 to 27·1)	8·1% (2·1 to 18·8%)	21·4% (10·2 to 33·1%)	2·3% (-1·3 to 6·8%)
Hemorrhagic stroke	0·7 (0·2 to 1·6)	8·6% (2·2 to 20·1%)	0·8 (0·2 to 1·9)	9·0% (2·4 to 20·9%)	21·1% (9·2 to 36·4%)	4·5% (-1·0 to 11·0%)	17·4 (4·4 to 39·7)	7·6% (1·9 to 17·3%)	19·6 (5·0 to 44·7)	8·6% (2·2 to 19·3%)	12·6% (2·7 to 26·3%)	13·6% (8·7 to 20·6%)
Hypertensive heart disease	0·3 (0·1 to 0·9)	15·1% (2·5 to 44·5%)	0·5 (0·1 to 1·6)	15·6% (2·5 to 45·7%)	68·9% (46·7 to 92·2%)	3·2% (-4·5 to 8·6%)	5·5 (1·1 to 14·2)	14·7% (3·0 to 37·7%)	8·7 (1·8 to 23·6)	15·6% (3·1 to 40·7%)	58·4% (37·4 to 78·0%)	6·3% (-1·7 to 13·5%)
Cardiomyopathy and myocarditis	0·0 (0·0 to 0·1)	3·6% (0·9 to 8·3%)	0·0 (0·0 to 0·1)	4·4% (1·1 to 10·2%)	27·7% (13·4 to 44·8%)	22·3% (13·2 to 32·2%)	0·8 (0·2 to 1·9)	2·1% (0·5 to 4·9%)	1·0 (0·2 to 2·3)	3·2% (0·8 to 7·4%)	20·7% (7·6 to 36·6%)	55·2% (36·5 to 75·7%)
Atrial fibrillation and flutter	0·0 (0·0 to 0·1)	4·6% (1·1 to 11·0%)	0·0 (0·0 to 0·1)	4·6% (1·1 to 10·8%)	45·5% (31·7 to 58·8%)	-1·2% (-5·3 to 2·5%)	0·8 (0·2 to 1·9)	5·9% (1·6 to 13·5%)	1·1 (0·3 to 2·6)	5·9% (1·6 to 13·5%)	39·6% (31·8 to 47·6%)	-0·1% (-3·0 to 3·5%)
Aortic aneurysm	0·0 (0·0 to 0·1)	5·1% (1·3 to 11·7%)	0·0 (0·0 to 0·1)	5·0% (1·3 to 11·8%)	50·2% (29·9 to 69·2%)	-0·6% (-5·2 to 3·7%)	0·5 (0·1 to 1·1)	5·3% (1·4 to 12·3%)	0·7 (0·2 to 1·6)	5·5% (1·4 to 12·7%)	42·5% (23·6 to 61·2%)	2·5% (-1·3 to 6·9%)
Peripheral vascular disease	0·0 (0·0 to 0·0)	3·4% (0·8 to 8·7%)	0·0 (0·0 to 0·0)	3·4% (0·8 to 8·6%)	99·9% (65·7 to 142·1%)	-1·6% (-10·0 to 5·3%)	0·1 (0·0 to 0·2)	4·1% (1·0 to 9·7%)	0·1 (0·0 to 0·3)	4·0% (1·0 to 9·7%)	50·4% (42·6 to 61·6%)	-0·1% (-3·1 to 4·2%)
Endocarditis	0·0 (0·0 to 0·1)	3·6% (0·9 to 8·5%)	0·0 (0·0 to 0·1)	4·2% (1·0 to 9·8%)	36·6% (18·4 to 56·6%)	15·9% (6·6 to 26·1%)	0·5 (0·1 to 1·2)	2·1% (0·5 to 5·1%)	0·6 (0·2 to 1·5)	3·0% (0·7 to 6·9%)	25·5% (10·4 to 43·4%)	39·8% (23·2 to 59·8%)
Other cardiovascular and circulatory diseases	0·1 (0·0 to 0·3)	4·8% (1·2 to 10·8%)	0·2 (0·0 to 0·4)	5·1% (1·3 to 11·4%)	32·5% (17·9 to 50·9%)	7·4% (1·4 to 13·4%)	3·6 (0·9 to 8·4)	3·4% (0·9 to 7·9%)	4·6 (1·1 to 10·5)	4·2% (1·1 to 9·6%)	28·9% (18·8 to 40·1%)	24·4% (17·5 to 33·3%)
Chronic kidney disease due to diabetes mellitus	0·3 (0·1 to 0·8)	7·1% (1·6 to 16·6%)	0·5 (0·1 to 1·3)	7·3% (1·7 to 17·1%)	51·2% (37·8 to 66·0%)	3·6% (0·5 to 7·3%)	7·5 (1·7 to 18·2)	6·1% (1·4 to 14·3%)	10·7 (2·4 to 26·2)	6·5% (1·5 to 15·2%)	42·4% (30·4 to 56·1%)	7·1% (3·9 to 11·1%)
Chronic kidney disease due to hypertension	0·3 (0·1 to 0·7)	6·6% (1·6 to 15·7%)	0·5 (0·1 to 1·1)	7·1% (1·7 to 16·8%)	61·8% (45·1 to 78·4%)	7·7% (2·9 to 12·2%)	5·3 (1·3 to 12·8)	4·7% (1·2 to 11·3%)	7·7 (1·8 to 18·2)	5·7% (1·4 to 13·4%)	44·8% (31·4 to 59·8%)	19·3% (13·7 to 25·8%)
Chronic kidney disease due to glomerulonephritis	0·0 (0·0 to 0·1)	5·4% (1·2 to 12·9%)	0·0 (0·0 to 0·1)	6·3% (1·5 to 15·0%)	52·8% (37·6 to 70·0%)	17·3% (11·7 to 23·1%)	1·3 (0·3 to 3·2)	3·9% (0·9 to 9·6%)	1·8 (0·4 to 4·4)	4·7% (1·1 to 11·3%)	37·9% (30·7 to 45·8%)	19·0% (13·7 to 25·4%)
Chronic kidney disease due to other causes	0·0 (0·0 to 0·0)	5·3% (1·3 to 12·7%)	0·0 (0·0 to 0·0)	6·5% (1·6 to 15·5%)	57·0% (37·2 to 76·7%)	21·9% (15·1 to 29·3%)	0·9 (0·2 to 2·3)	4·7% (1·1 to 11·1%)	1·3 (0·3 to 3·2)	5·2% (1·2 to 12·5%)	39·9% (33·0 to 47·8%)	11·6% (7·4 to 17·1%)
**Sexual abuse and violence: All causes**	1·3 (0·9 to 1·7)	0·5% (0·4 to 0·7%)	1·3 (0·9 to 1·7)	0·4% (0·3 to 0·6%)	-0·9% (-12·4 to 10·7%)	-14·9% (-23·0 to -6·8%)	126·9 (91·9 to 167·1)	1·0% (0·7 to 1·2%)	129·0 (92·3 to 174·4)	1·0% (0·7 to 1·3%)	1·6% (-5·9 to 8·2%)	0·3% (-6·2 to 6·4%)
**Childhood sexual abuse: All causes**	0·5 (0·2 to 1·0)	0·2% (0·1 to 0·4%)	0·5 (0·2 to 1·0)	0·2% (0·1 to 0·4%)	-1·7% (-14·5 to 10·5%)	-15·6% (-25·9 to -7·3%)	54·6 (23·0 to 94·8)	0·4% (0·2 to 0·7%)	56·4 (22·5 to 100·4)	0·4% (0·2 to 0·7%)	3·3% (-6·1 to 10·4%)	1·9% (-6·7 to 8·8%)
Alcohol use disorders	0·1 (0·0 to 0·1)	4·7% (0·0 to 10·8%)	0·0 (0·0 to 0·1)	4·2% (0·0 to 9·7%)	-29·2% (-37·9 to 0·0%)	-10·1% (-14·5 to 0·0%)	4·5 (0·0 to 10·3)	5·2% (0·0 to 11·9%)	3·8 (0·0 to 9·1)	4·8% (0·0 to 10·8%)	-14·9% (-25·7 to 0·0%)	-8·0% (-13·3 to 0·0%)
Major depressive disorder	-	-	-	-	-	-	22·8 (2·6 to 49·7)	6·8% (0·8 to 14·4%)	25·2 (2·8 to 55·3)	6·3% (0·8 to 13·2%)	10·5% (4·7 to 15·9%)	-8·0% (-11·7 to -4·2%)
Dysthymia	-	-	-	-	-	-	4·0 (0·4 to 8·8)	6·8% (0·8 to 14·2%)	4·6 (0·5 to 10·0)	6·2% (0·7 to 13·0%)	13·7% (8·3 to 18·7%)	-8·3% (-11·8 to -4·7%)
Self-harm	0·5 (0·1 to 0·9)	15·6% (3·7 to 30·6%)	0·5 (0·1 to 1·0)	14·1% (3·3 to 28·2%)	1·5% (-10·2 to 13·1%)	-9·1% (-13·5 to -5·3%)	23·2 (5·7 to 45·8)	16·1% (3·9 to 31·6%)	22·7 (5·4 to 46·8)	14·8% (3·5 to 29·3%)	-2·1% (-13·8 to 10·0%)	-8·4% (-13·1 to -4·2%)
**Intimate partner violence: All causes**	0·8 (0·7 to 0·9)	0·3% (0·3 to 0·4%)	0·8 (0·6 to 1·0)	0·3% (0·2 to 0·3%)	-0·9% (-14·5 to 13·8%)	-15·0% (-25·1 to -3·7%)	79·7 (64·8 to 97·4)	0·6% (0·5 to 0·7%)	79·5 (63·1 to 98·6)	0·6% (0·5 to 0·7%)	-0·3% (-8·5 to 8·3%)	-1·6% (-9·2 to 7·0%)

Supplemental Results Table 1. All-age deaths, death PAFs, DALYs, DALY PAFs, and percent change for each risk factor and outcome for both sexes combined in 2005 and 2015 for Andean Latin America.

Data in parenthesis are 95% uncertainty intervals. PAFs=population attributable fractions. DALYs=disability-adjusted life-years.

	2005 Deaths (in thousands)	2005 Deaths PAF	2015 Deaths (in thousands)	2015 Deaths PAF	Percent change deaths 2005-2015	Percent change deaths PAF 2005-2015	2005 DALYs (in thousands)	2005 DALYs PAF	2015 DALYs (in thousands)	2015 DALYs PAF	Percent change DALYs 2005-2015	Percent change DALYs PAF 2005-2015
HIV/AIDS - Tuberculosis	0·0 (0·0 to 0·0)	2·8% (1·5 to 4·3%)	0·0 (0·0 to 0·0)	2·4% (1·2 to 3·7%)	-23·6% (-33·9 to -6·9%)	-14·8% (-21·2 to -6·5%)	0·7 (0·4 to 1·2)	2·7% (1·4 to 4·2%)	0·6 (0·3 to 1·0)	2·5% (1·3 to 3·7%)	-24·5% (-34·7 to -8·8%)	-10·6% (-17·3 to -2·0%)
HIV/AIDS resulting in other diseases	0·0 (0·0 to 0·0)	2·7% (1·4 to 4·3%)	0·0 (0·0 to 0·1)	2·3% (1·2 to 3·7%)	1·7% (-17·5 to 28·1%)	-14·4% (-24·2 to -3·2%)	1·4 (0·7 to 2·3)	2·6% (1·4 to 4·1%)	1·5 (0·7 to 2·5)	2·4% (1·2 to 3·7%)	2·4% (-15·8 to 25·4%)	-10·3% (-19·6 to 0·3%)
Maternal abortion, miscarriage, and ectopic pregnancy	0·0 (0·0 to 0·0)	0·0% (0·0 to 0·0%)	0·0 (0·0 to 0·0)	0·0% (0·0 to 0·0%)	-27·0% (-46·4 to 0·0%)	0·3% (-20·7 to 24·0%)	0·0 (0·0 to 0·0)	0·0% (0·0 to 0·0%)	0·0 (0·0 to 0·0)	0·0% (0·0 to 0·0%)	-25·8% (-44·0 to 0·0%)	0·5% (-19·3 to 22·7%)
Major depressive disorder	-	-	-	-	-	-	32·0 (20·4 to 46·1)	9·6% (7·8 to 11·3%)	34·6 (22·4 to 49·6)	8·6% (7·0 to 10·2%)	8·1% (0·7 to 16·0%)	-9·9% (-15·6 to -4·2%)
Dysthymia	-	-	-	-	-	-	5·9 (3·8 to 8·9)	10·0% (8·3 to 11·8%)	6·6 (4·2 to 9·8)	9·0% (7·5 to 10·7%)	11·7% (5·1 to 18·5%)	-9·9% (-14·8 to -4·7%)
Self-harm	0·4 (0·3 to 0·5)	13·4% (10·1 to 16·5%)	0·4 (0·3 to 0·6)	12·5% (8·8 to 15·9%)	3·9% (-14·7 to 25·3%)	-6·9% (-22·1 to 10·5%)	17·9 (13·0 to 22·6)	12·4% (9·2 to 15·8%)	17·0 (11·9 to 23·2)	11·1% (7·8 to 14·7%)	-4·8% (-22·9 to 16·2%)	-10·9% (-26·4 to 6·3%)
Assault by firearm	0·2 (0·1 to 0·2)	6·1% (4·8 to 7·4%)	0·2 (0·1 to 0·2)	5·8% (4·5 to 7·7%)	-5·8% (-22·0 to 13·4%)	-3·9% (-21·3 to 17·6%)	9·0 (6·6 to 10·8)	6·3% (5·0 to 7·7%)	8·0 (6·2 to 10·4)	5·9% (4·5 to 7·9%)	-11·2% (-27·3 to 8·1%)	-6·1% (-24·4 to 15·7%)
Assault by sharp object	0·1 (0·1 to 0·1)	10·8% (8·8 to 13·4%)	0·1 (0·1 to 0·1)	10·8% (8·1 to 13·7%)	-5·1% (-21·1 to 14·1%)	-0·2% (-19·3 to 22·5%)	5·2 (4·1 to 6·3)	11·4% (9·2 to 14·0%)	4·6 (3·3 to 5·7)	11·2% (8·4 to 14·1%)	-11·0% (-27·2 to 7·7%)	-2·0% (-21·4 to 19·7%)
Assault by other means	0·1 (0·1 to 0·1)	18·6% (15·0 to 21·9%)	0·1 (0·1 to 0·1)	16·8% (12·9 to 20·3%)	-5·1% (-23·9 to 13·4%)	-9·6% (-23·2 to 5·7%)	7·5 (6·0 to 9·0)	19·9% (16·3 to 23·4%)	6·6 (4·6 to 8·1)	18·1% (14·0 to 21·9%)	-12·3% (-28·9 to 6·0%)	-9·1% (-23·1 to 6·4%)
**Unsafe sex: All causes**	5·4 (4·9 to 6·1)	2·2% (2·0 to 2·5%)	5·3 (4·8 to 6·0)	1·9% (1·7 to 2·1%)	-1·1% (-13·0 to 11·3%)	-15·1% (-24·0 to -5·2%)	250·0 (209·8 to 305·6)	1·9% (1·6 to 2·4%)	205·4 (182·9 to 234·7)	1·6% (1·3 to 1·8%)	-17·8% (-29·6 to -5·3%)	-18·9% (-30·0 to -6·5%)
HIV/AIDS - Tuberculosis	0·5 (0·5 to 0·7)	95·8% (95·1 to 96·3%)	0·5 (0·4 to 0·7)	95·9% (95·3 to 96·4%)	-10·3% (-20·1 to 6·4%)	0·1% (0·1 to 0·2%)	25·4 (22·1 to 33·7)	95·7% (95·0 to 96·2%)	21·5 (18·1 to 33·1)	95·8% (95·2 to 96·3%)	-15·3% (-24·1 to 0·1%)	0·1% (0·1 to 0·2%)
HIV/AIDS resulting in other diseases	1·1 (0·9 to 1·3)	95·8% (95·1 to 96·3%)	1·3 (0·9 to 1·7)	95·9% (95·2 to 96·4%)	18·9% (0·2 to 42·2%)	0·1% (0·0 to 0·2%)	51·8 (41·9 to 62·0)	95·7% (95·0 to 96·3%)	59·2 (44·0 to 76·7)	95·8% (95·1 to 96·4%)	14·3% (-2·6 to 34·3%)	0·1% (0·1 to 0·2%)
Syphilis	1·0 (0·5 to 1·6)	100·0% (100·0 to 100·0%)	0·3 (0·2 to 0·5)	100·0% (100·0 to 100·0%)	-67·3% (-75·3 to -57·0%)	-	82·1 (44·3 to 132·6)	100·0% (100·0 to 100·0%)	25·9 (14·2 to 44·3)	100·0% (100·0 to 100·0%)	-68·5% (-76·5 to -58·2%)	-
Chlamydial infection	0·0 (0·0 to 0·0)	100·0% (100·0 to 100·0%)	0·0 (0·0 to 0·0)	100·0% (100·0 to 100·0%)	1·9% (-22·3 to 33·6%)	-	2·2 (1·3 to 3·7)	100·0% (100·0 to 100·0%)	2·4 (1·3 to 4·1)	100·0% (100·0 to 100·0%)	8·9% (-2·4 to 19·9%)	-
Gonococcal infection	0·0 (0·0 to 0·0)	100·0% (100·0 to 100·0%)	0·0 (0·0 to 0·0)	100·0% (100·0 to 100·0%)	-9·0% (-27·7 to 14·1%)	-	0·8 (0·5 to 1·1)	100·0% (100·0 to 100·0%)	0·9 (0·5 to 1·3)	100·0% (100·0 to 100·0%)	9·5% (-9·0 to 30·1%)	-
Trichomoniasis	-	-	-	-	-	-	1·1 (0·4 to 2·3)	100·0% (100·0 to 100·0%)	1·3 (0·5 to 2·7)	100·0% (100·0 to 100·0%)	20·7% (15·0 to 26·4%)	-
Genital herpes	-	-	-	-	-	-	2·1 (0·7 to 4·9)	100·0% (100·0 to 100·0%)	2·5 (0·8 to 6·0)	100·0% (100·0 to 100·0%)	20·5% (11·8 to 28·8%)	-
Other sexually transmitted diseases	0·0 (0·0 to 0·0)	100·0% (100·0 to 100·0%)	0·0 (0·0 to 0·0)	100·0% (100·0 to 100·0%)	-14·4% (-32·1 to 7·7%)	-	0·7 (0·5 to 0·9)	100·0% (100·0 to 100·0%)	0·6 (0·4 to 0·8)	100·0% (100·0 to 100·0%)	-10·6% (-22·2 to 2·7%)	-
Cervical cancer	2·8 (2·6 to 3·1)	100·0% (100·0 to 100·0%)	3·3 (2·8 to 3·7)	100·0% (100·0 to 100·0%)	16·3% (-0·5 to 35·5%)	-	83·8 (77·4 to 91·5)	100·0% (100·0 to 100·0%)	91·1 (78·8 to 105·1)	100·0% (100·0 to 100·0%)	8·8% (-6·7 to 25·8%)	-
**Low physical activity: All causes**	5·3 (4·2 to 6·5)	2·2% (1·8 to 2·7%)	7·3 (5·9 to 8·7)	2·6% (2·1 to 3·1%)	37·1% (27·6 to 48·2%)	17·7% (13·4 to 22·9%)	124·2 (97·8 to 150·8)	1·0% (0·8 to 1·2%)	163·7 (132·1 to 195·1)	1·2% (1·0 to 1·5%)	31·8% (22·8 to 42·4%)	30·1% (24·1 to 37·2%)
Colon and rectum cancer	0·4 (0·2 to 0·5)	14·2% (9·9 to 18·6%)	0·6 (0·4 to 0·7)	14·8% (10·4 to 19·2%)	55·0% (41·1 to 71·6%)	4·3% (2·3 to 7·2%)	7·0 (4·8 to 9·3)	13·2% (9·1 to 17·6%)	10·2 (7·0 to 13·5)	13·8% (9·6 to 18·1%)	46·3% (33·6 to 61·6%)	4·7% (2·5 to 7·9%)
Breast cancer	0·2 (0·1 to 0·2)	8·2% (5·9 to 10·5%)	0·2 (0·1 to 0·3)	8·4% (6·0 to 10·8%)	38·9% (20·8 to 59·3%)	2·8% (1·2 to 4·6%)	4·5 (3·2 to 5·9)	7·8% (5·5 to 10·1%)	6·0 (4·1 to 8·0)	8·0% (5·7 to 10·4%)	32·7% (14·9 to 52·4%)	2·4% (1·0 to 4·0%)
Ischaemic heart disease	2·8 (1·9 to 3·8)	10·2% (6·9 to 13·8%)	3·6 (2·4 to 4·7)	10·5% (7·2 to 13·9%)	26·1% (16·6 to 37·2%)	2·7% (0·1 to 6·5%)	57·0 (37·3 to 78·2)	11·1% (7·3 to 15·1%)	66·1 (43·7 to 88·2)	11·6% (7·8 to 15·6%)	16·0% (6·5 to 27·6%)	4·7% (2·0 to 9·1%)
Ischaemic stroke	0·9 (0·5 to 1·3)	12·0% (6·9 to 17·4%)	1·2 (0·7 to 1·7)	12·3% (7·3 to 17·5%)	33·6% (21·7 to 47·0%)	2·1% (-0·3 to 6·7%)	15·7 (8·7 to 23·1)	13·0% (7·1 to 18·9%)	19·2 (10·9 to 27·6)	13·5% (7·8 to 19·3%)	22·9% (12·1 to 34·8%)	3·6% (0·9 to 8·5%)
Diabetes mellitus	1·1 (0·8 to 1·4)	16·5% (12·8 to 20·6%)	1·8 (1·4 to 2·2)	17·4% (13·6 to 21·5%)	62·5% (49·1 to 77·9%)	5·3% (3·4 to 7·9%)	40·1 (29·1 to 52·7)	14·6% (11·1 to 18·4%)	62·2 (45·1 to 82·9)	15·6% (11·9 to 19·4%)	55·1% (47·4 to 64·2%)	6·4% (4·6 to 8·9%)
**Metabolic risks: All causes**	56·0 (52·8 to 59·3)	23·3% (22·2 to 24·5%)	76·1 (70·6 to 82·5)	27·2% (25·8 to 28·6%)	35·8% (27·2 to 45·2%)	16·6% (13·8 to 19·5%)	1,378·9 (1,276·9 to 1,493·4)	10·6% (10·0 to 11·3%)	1,752·3 (1,585·7 to 1,931·9)	13·3% (12·4 to 14·2%)	27·1% (19·8 to 34·7%)	25·4% (21·4 to 29·9%)
**High fasting plasma glucose: All causes**	21·2 (18·9 to 24·4)	8·8% (7·9 to 10·1%)	30·7 (27·3 to 35·2)	11·0% (9·9 to 12·5%)	44·9% (34·7 to 55·3%)	24·4% (20·1 to 28·8%)	604·0 (535·1 to 680·4)	4·6% (4·3 to 5·0%)	826·2 (722·0 to 945·3)	6·3% (5·8 to 6·9%)	36·8% (29·4 to 44·8%)	35·0% (30·4 to 40·1%)
Tuberculosis	0·3 (0·2 to 0·5)	6·4% (4·0 to 9·4%)	0·3 (0·2 to 0·5)	7·7% (4·7 to 11·2%)	-4·1% (-16·3 to 8·1%)	19·0% (10·6 to 28·1%)	8·6 (5·0 to 14·2)	5·0% (3·2 to 7·0%)	7·7 (4·3 to 13·7)	6·2% (4·0 to 8·7%)	-10·0% (-20·8 to 1·2%)	24·1% (15·4 to 34·5%)
Ischaemic heart disease	5·1 (3·3 to 7·5)	18·4% (11·8 to 26·7%)	6·8 (4·3 to 10·1)	19·9% (12·5 to 28·9%)	32·7% (20·6 to 45·3%)	8·0% (2·2 to 14·2%)	95·7 (67·0 to 130·9)	18·7% (13·2 to 25·2%)	117·2 (82·4 to 160·1)	20·6% (14·6 to 27·9%)	22·5% (12·3 to 33·5%)	10·5% (6·1 to 15·6%)
Ischaemic stroke	1·2 (0·7 to 2·2)	16·7% (9·6 to 29·9%)	1·7 (1·0 to 3·2)	17·6% (10·0 to 31·9%)	38·4% (23·4 to 54·4%)	5·9% (-0·4 to 13·6%)	21·6 (14·2 to 33·8)	17·9% (11·7 to 28·0%)	27·8 (18·2 to 44·2)	19·4% (12·6 to 30·6%)	28·8% (16·9 to 42·4%)	8·6% (3·3 to 14·4%)
Hemorrhagic stroke	1·3 (0·9 to 1·8)	16·4% (11·4 to 23·0%)	1·7 (1·2 to 2·3)	18·2% (12·8 to 25·3%)	28·0% (15·3 to 43·0%)	10·6% (4·6 to 17·1%)	35·0 (22·4 to 50·8)	15·3% (9·8 to 22·4%)	41·9 (28·3 to 59·4)	18·4% (12·4 to 26·2%)	19·8% (8·1 to 33·1%)	20·8% (13·7 to 29·2%)
Diabetes mellitus	6·6 (6·3 to 7·0)	100·0% (100·0 to 100·0%)	10·2 (9·4 to 11·1)	100·0% (100·0 to 100·0%)	54·3% (42·1 to 68·4%)	-	274·0 (228·0 to 328·4)	100·0% (100·0 to 100·0%)	399·6 (328·1 to 479·8)	100·0% (100·0 to 100·0%)	45·8% (39·0 to 53·1%)	-
Chronic kidney disease due to diabetes mellitus	4·9 (4·2 to 5·3)	100·0% (100·0 to 100·0%)	7·2 (6·0 to 7·9)	100·0% (100·0 to 100·0%)	45·9% (33·4 to 59·2%)	-	123·8 (107·6 to 134·1)	100·0% (100·0 to 100·0%)	164·7 (139·8 to 181·6)	100·0% (100·0 to 100·0%)	33·0% (22·8 to 44·1%)	-
Chronic kidney disease due to hypertension	1·6 (1·1 to 2·1)	35·2% (24·8 to 45·3%)	2·7 (1·9 to 3·5)	39·4% (28·1 to 50·3%)	68·2% (51·6 to 85·6%)	11·9% (8·9 to 15·8%)	31·3 (21·8 to 41·0)	28·0% (19·7 to 36·4%)	46·7 (32·6 to 60·9)	34·5% (24·6 to 44·3%)	49·5% (36·4 to 64·1%)	23·0% (19·4 to 27·3%)
Chronic kidney disease due to glomerulonephritis	0·1 (0·1 to 0·2)	29·7% (20·8 to 38·4%)	0·2 (0·1 to 0·3)	35·9% (25·6 to 45·9%)	57·5% (42·1 to 74·3%)	20·9% (16·9 to 25·6%)	8·3 (5·4 to 11·4)	25·2% (17·5 to 33·1%)	12·0 (8·0 to 16·4)	31·6% (22·5 to 40·7%)	45·4% (38·2 to 54·0%)	25·4% (21·3 to 30·8%)
Chronic kidney disease due to other causes	0·0 (0·0 to 0·0)	27·6% (19·1 to 35·9%)	0·0 (0·0 to 0·0)	34·8% (24·8 to 44·7%)	62·6% (43·1 to 83·8%)	26·3% (20·1 to 33·4%)	5·5 (3·4 to 7·9)	28·5% (19·8 to 37·2%)	8·1 (5·2 to 11·7)	33·7% (23·8 to 43·5%)	48·2% (41·2 to 56·5%)	18·3% (14·7 to 22·6%)
**High total cholesterol: All causes**	14·9 (11·6 to 18·7)	6·2% (4·8 to 7·8%)	17·5 (13·1 to 22·4)	6·2% (4·7 to 8·0%)	17·1% (7·3 to 27·7%)	0·5% (-5·4 to 6·3%)	307·8 (263·1 to 360·4)	2·4% (2·0 to 2·8%)	326·3 (270·3 to 390·8)	2·5% (2·0 to 3·0%)	6·0% (-2·4 to 15·0%)	4·7% (-1·6 to 10·9%)
Ischaemic heart disease	13·2 (10·1 to 16·3)	47·5% (36·9 to 57·6%)	15·3 (11·4 to 19·7)	45·0% (33·5 to 55·8%)	16·3% (5·9 to 27·2%)	-5·3% (-10·2 to -1·1%)	273·8 (233·7 to 318·0)	53·4% (46·2 to 61·2%)	288·8 (239·8 to 347·2)	50·8% (43·0 to 58·9%)	5·5% (-3·3 to 14·5%)	-4·8% (-7·8 to -2·2%)
Ischaemic stroke	1·7 (0·8 to 3·3)	23·4% (10·1 to 43·8%)	2·1 (0·8 to 4·2)	22·0% (8·5 to 43·6%)	22·8% (7·0 to 37·6%)	-6·0% (-17·1 to 0·9%)	34·0 (22·2 to 53·0)	28·2% (18·2 to 43·8%)	37·5 (22·8 to 60·4)	26·3% (16·1 to 42·3%)	10·3% (-1·7 to 22·5%)	-7·0% (-14·1 to -1·5%)
**High systolic blood pressure: All causes**	30·4 (26·7 to 34·1)	12·7% (11·2 to 14·1%)	42·4 (36·9 to 47·9)	15·2% (13·5 to 16·8%)	39·4% (30·2 to 49·6%)	19·6% (15·5 to 24·0%)	610·0 (543·7 to 679·1)	4·7% (4·1 to 5·3%)	779·6 (685·1 to 872·7)	5·9% (5·1 to 6·8%)	27·8% (18·9 to 37·4%)	26·2% (20·1 to 32·7%)
Rheumatic heart disease	0·2 (0·1 to 0·3)	19·1% (12·2 to 32·1%)	0·2 (0·1 to 0·3)	22·3% (14·4 to 37·6%)	19·5% (5·4 to 36·1%)	17·0% (10·7 to 24·6%)	3·6 (2·4 to 5·6)	13·5% (9·1 to 20·7%)	4·1 (2·7 to 6·1)	17·8% (12·2 to 27·2%)	12·6% (1·3 to 25·6%)	31·8% (22·9 to 42·4%)
Ischaemic heart disease	13·1 (10·4 to 16·1)	47·4% (37·2 to 57·6%)	17·3 (13·2 to 21·5)	50·7% (39·6 to 62·0%)	31·5% (20·8 to 43·0%)	7·0% (3·9 to 10·3%)	227·9 (189·9 to 268·8)	44·4% (37·1 to 52·0%)	280·3 (229·7 to 329·4)	49·3% (41·3 to 57·1%)	23·0% (13·6 to 34·7%)	11·0% (7·9 to 14·4%)
Ischaemic stroke	3·2 (2·5 to 4·1)	43·7% (33·7 to 54·4%)	4·4 (3·3 to 5·7)	45·7% (35·1 to 57·5%)	37·0% (24·1 to 51·4%)	4·7% (0·9 to 8·7%)	52·4 (41·6 to 63·6)	43·5% (35·1 to 51·6%)	67·2 (52·9 to 81·7)	47·0% (37·9 to 55·4%)	28·4% (17·4 to 40·5%)	8·2% (5·0 to 11·4%)

Supplemental Results Table 1. All-age deaths, death PAFs, DALYs, DALY PAFs, and percent change for each risk factor and outcome for both sexes combined in 2005 and 2015 for Andean Latin America.

Data in parenthesis are 95% uncertainty intervals. PAFs=population attributable fractions. DALYs=disability-adjusted life-years.

	2005 Deaths (in thousands)	2005 Deaths PAF	2015 Deaths (in thousands)	2015 Deaths PAF	Percent change deaths 2005-2015	Percent change deaths PAF 2005-2015	2005 DALYs (in thousands)	2005 DALYs PAF	2015 DALYs (in thousands)	2015 DALYs PAF	Percent change DALYs 2005-2015	Percent change DALYs PAF 2005-2015
Hemorrhagic stroke	3·6 (2·9 to 4·5)	46·4% (37·2 to 55·8%)	4·7 (3·6 to 5·9)	51·5% (41·1 to 61·7%)	28·5% (16·1 to 42·5%)	10·9% (6·8 to 15·6%)	89·5 (69·9 to 109·4)	39·0% (31·1 to 47·1%)	107·3 (85·3 to 130·1)	47·2% (38·2 to 55·6%)	20·0% (8·9 to 32·8%)	20·9% (16·0 to 26·8%)
Hypertensive heart disease	2·1 (1·9 to 2·3)	100·0% (100·0 to 100·0%)	3·4 (3·0 to 3·8)	100·0% (100·0 to 100·0%)	63·9% (42·5 to 84·5%)	-	37·3 (34·7 to 40·5)	100·0% (100·0 to 100·0%)	55·6 (49·0 to 61·2)	100·0% (100·0 to 100·0%)	49·0% (32·8 to 64·3%)	-
Cardiomyopathy and myocarditis	0·2 (0·1 to 0·3)	21·6% (16·6 to 26·8%)	0·3 (0·2 to 0·4)	28·4% (21·9 to 35·4%)	37·3% (21·9 to 55·3%)	31·7% (22·6 to 41·3%)	4·6 (3·5 to 6·0)	11·9% (8·8 to 15·5%)	6·1 (4·5 to 8·4)	20·1% (15·4 to 24·8%)	32·1% (17·4 to 48·9%)	70·0% (49·5 to 92·2%)
Atrial fibrillation and flutter	0·2 (0·2 to 0·3)	31·9% (24·9 to 39·4%)	0·4 (0·2 to 0·5)	33·2% (25·8 to 41·1%)	53·0% (38·2 to 68·4%)	4·0% (-0·3 to 8·5%)	4·9 (3·8 to 6·3)	35·6% (30·3 to 40·8%)	7·2 (5·5 to 9·4)	37·7% (32·1 to 43·2%)	47·8% (39·7 to 56·1%)	5·8% (3·0 to 9·0%)
Aortic aneurysm	0·1 (0·1 to 0·2)	30·0% (23·2 to 36·2%)	0·2 (0·2 to 0·3)	32·1% (25·1 to 38·9%)	61·8% (41·1 to 83·7%)	7·1% (3·3 to 11·0%)	2·7 (2·1 to 3·2)	29·4% (23·9 to 35·3%)	4·1 (3·3 to 5·0)	32·8% (26·9 to 38·6%)	55·3% (36·7 to 75·8%)	11·8% (8·3 to 15·8%)
Peripheral vascular disease	0·0 (0·0 to 0·0)	23·4% (16·5 to 31·2%)	0·0 (0·0 to 0·0)	24·7% (17·0 to 33·4%)	115·1% (75·2 to 162·3%)	5·9% (-0·2 to 11·5%)	0·4 (0·2 to 0·7)	25·1% (18·8 to 31·2%)	0·6 (0·4 to 1·1)	26·7% (20·2 to 33·2%)	59·9% (50·9 to 73·1%)	6·3% (2·9 to 10·2%)
Endocarditis	0·1 (0·1 to 0·2)	22·1% (17·2 to 27·7%)	0·2 (0·1 to 0·3)	27·7% (21·4 to 35·8%)	47·5% (28·3 to 69·6%)	25·3% (17·0 to 34·8%)	2·9 (2·1 to 3·8)	12·2% (9·2 to 15·1%)	4·0 (2·9 to 5·3)	18·6% (14·3 to 23·0%)	37·2% (20·4 to 55·9%)	53·0% (34·9 to 73·0%)
Other cardiovascular and circulatory diseases	0·8 (0·6 to 0·9)	29·3% (24·8 to 33·7%)	1·1 (0·9 to 1·3)	33·9% (28·5 to 39·3%)	42·7% (26·8 to 63·7%)	15·7% (10·8 to 20·5%)	20·0 (16·6 to 23·9)	19·0% (16·2 to 22·1%)	27·9 (23·1 to 33·3)	25·6% (22·2 to 29·3%)	39·8% (28·9 to 52·5%)	35·0% (27·3 to 43·2%)
Chronic kidney disease due to diabetes mellitus	2·0 (1·4 to 2·5)	39·7% (28·2 to 50·7%)	3·1 (2·2 to 4·0)	43·6% (31·1 to 54·9%)	60·0% (45·9 to 75·3%)	9·7% (6·9 to 13·0%)	40·3 (27·3 to 53·2)	32·5% (22·4 to 42·3%)	61·5 (41·8 to 80·8)	37·3% (26·2 to 47·6%)	52·5% (40·0 to 66·4%)	14·7% (11·3 to 18·4%)
Chronic kidney disease due to hypertension	4·5 (3·9 to 4·9)	100·0% (100·0 to 100·0%)	6·8 (5·8 to 7·6)	100·0% (100·0 to 100·0%)	50·3% (35·9 to 65·3%)	-	111·6 (96·9 to 120·1)	100·0% (100·0 to 100·0%)	135·5 (117·9 to 149·9)	100·0% (100·0 to 100·0%)	21·5% (11·4 to 32·4%)	-
Chronic kidney disease due to glomerulonephritis	0·1 (0·1 to 0·2)	32·4% (22·9 to 42·0%)	0·2 (0·2 to 0·3)	40·3% (28·9 to 50·9%)	62·0% (45·8 to 80·2%)	24·3% (19·6 to 30·0%)	7·1 (4·6 to 10·1)	21·7% (14·8 to 29·0%)	10·7 (7·1 to 14·6)	28·1% (19·3 to 36·8%)	50·0% (42·5 to 58·5%)	29·4% (24·0 to 36·2%)
Chronic kidney disease due to other causes	0·0 (0·0 to 0·0)	32·0% (22·5 to 40·4%)	0·0 (0·0 to 0·1)	41·0% (29·2 to 51·5%)	65·0% (45·5 to 87·8%)	28·2% (21·6 to 35·5%)	5·0 (3·1 to 7·1)	25·9% (17·4 to 34·1%)	7·5 (4·8 to 10·6)	31·2% (21·4 to 40·5%)	51·1% (44·0 to 59·7%)	20·6% (16·4 to 25·7%)
**High body-mass index: All causes**	17·6 (13·0 to 22·4)	7·3% (5·4 to 9·3%)	25·3 (19·2 to 32·0)	9·0% (6·9 to 11·4%)	43·5% (31·8 to 57·2%)	23·2% (17·4 to 31·0%)	593·5 (448·8 to 743·6)	4·6% (3·5 to 5·6%)	810·1 (632·8 to 1,004·0)	6·2% (4·8 to 7·4%)	36·5% (27·2 to 46·7%)	34·7% (28·4 to 42·6%)
Oesophageal cancer	0·1 (0·0 to 0·2)	21·6% (6·7 to 38·6%)	0·2 (0·1 to 0·3)	24·1% (8·1 to 42·0%)	46·0% (29·0 to 69·0%)	11·4% (4·5 to 22·4%)	2·6 (0·8 to 4·5)	23·3% (7·4 to 40·8%)	3·6 (1·2 to 6·1)	25·8% (8·7 to 44·0%)	37·6% (21·4 to 58·9%)	11·0% (4·0 to 20·7%)
Colon and rectum cancer	0·2 (0·1 to 0·3)	7·7% (4·7 to 11·2%)	0·3 (0·2 to 0·5)	8·7% (5·4 to 12·4%)	70·0% (52·8 to 91·4%)	14·3% (7·1 to 23·7%)	4·5 (2·8 to 6·5)	8·5% (5·3 to 12·2%)	7·2 (4·6 to 10·2)	9·8% (6·2 to 13·6%)	60·4% (43·9 to 78·7%)	14·7% (8·4 to 23·1%)
Liver cancer due to hepatitis B	0·2 (0·1 to 0·3)	15·1% (6·8 to 25·0%)	0·3 (0·1 to 0·4)	17·3% (8·0 to 27·5%)	44·8% (27·5 to 65·1%)	14·0% (7·1 to 22·4%)	4·5 (2·1 to 7·5)	15·4% (7·0 to 25·3%)	6·2 (2·9 to 9·9)	17·9% (8·3 to 28·2%)	36·6% (19·7 to 55·6%)	15·9% (9·3 to 25·0%)
Liver cancer due to hepatitis C	0·0 (0·0 to 0·1)	13·7% (5·4 to 24·5%)	0·1 (0·0 to 0·1)	15·7% (6·4 to 27·3%)	51·5% (32·8 to 75·2%)	14·5% (7·6 to 24·8%)	0·8 (0·3 to 1·4)	15·2% (6·1 to 26·5%)	1·1 (0·5 to 1·9)	17·4% (7·3 to 29·5%)	41·1% (23·8 to 62·1%)	14·3% (8·0 to 24·6%)
Liver cancer due to alcohol use	0·1 (0·0 to 0·1)	16·3% (7·2 to 27·4%)	0·1 (0·1 to 0·2)	18·5% (8·5 to 30·1%)	67·8% (46·7 to 94·4%)	13·5% (6·1 to 24·1%)	1·8 (0·8 to 3·0)	17·5% (8·0 to 29·1%)	2·9 (1·3 to 4·8)	19·9% (9·3 to 31·9%)	62·6% (41·9 to 90·1%)	13·5% (6·2 to 23·5%)
Liver cancer due to other causes	0·1 (0·0 to 0·1)	13·8% (5·6 to 24·4%)	0·1 (0·0 to 0·2)	15·8% (6·5 to 27·3%)	44·9% (27·9 to 66·9%)	14·1% (6·6 to 22·9%)	1·5 (0·6 to 2·6)	14·7% (6·0 to 25·4%)	2·0 (0·9 to 3·4)	17·0% (7·2 to 28·6%)	33·4% (17·9 to 52·5%)	15·5% (9·2 to 24·4%)
Gallbladder and biliary tract cancer	0·3 (0·2 to 0·5)	19·6% (11·7 to 28·8%)	0·4 (0·3 to 0·7)	21·8% (13·5 to 31·5%)	37·6% (14·3 to 63·4%)	11·4% (3·0 to 19·8%)	6·8 (4·1 to 10·0)	21·3% (13·0 to 30·8%)	8·7 (5·3 to 13·0)	23·6% (14·8 to 33·7%)	28·3% (7·7 to 53·3%)	10·5% (2·8 to 18·1%)
Pancreatic cancer	0·1 (0·0 to 0·1)	6·1% (2·3 to 10·6%)	0·1 (0·1 to 0·2)	7·0% (2·8 to 11·8%)	65·9% (47·8 to 89·4%)	15·4% (8·5 to 24·6%)	2·0 (0·8 to 3·3)	6·6% (2·6 to 11·3%)	3·1 (1·3 to 5·2)	7·6% (3·0 to 12·7%)	58·4% (40·9 to 80·5%)	14·7% (7·1 to 23·2%)
Breast cancer	0·0 (0·0 to 0·1)	2·2% (0·2 to 4·7%)	0·1 (0·0 to 0·2)	3·4% (0·9 to 6·3%)	109·1% (46·7 to 322·9%)	54·9% (14·4 to 227·3%)	-0·2 (-1·5 to 1·0)	-0·3% (-2·5 to 1·6%)	0·5 (-1·3 to 2·3)	0·7% (-1·7 to 3·0%)	-378·1% (-1,937·8 to 2,544·5%)	-319·8% (-1,528·9 to 2,030·0%)
[truncated: 1,078,143 more chars]
