# Supplementary material for: Global, regional, and national comparative risk assessment of 79 behavioural, environmental and occupational, and metabolic risks or clusters of risks, 1990–2015: a systematic analysis for the Global Burden of Disease Study 2015
Source: Lancet. 2016 Oct 8;388(10053):1659–724. doi: 10.1016/S0140-6736(16)31679-8 (PMC5388856; doi:10.1016/S0140-6736(16)31679-8)
Supplement: Supplementary appendix [file mmc1.pdf]

THE LANCET

Supplementary appendix

This appendix formed part of the original submission and has been peer reviewed. We post it as supplied by the authors.

Supplement to: GBD 2015 Risk Factors Collaborators. Global, regional, and national comparative risk assessment of 79 behavioural, environmental and occupational, and metabolic risks or clusters of risks, 1990–2015: a systematic analysis for the Global Burden of Disease Study 2015. *Lancet* 2016; **388:** 1659–724.

Methods appendix to Global, regional, and national comparative risk assessment of 79 behavioural, environmental and occupational, and metabolic risks or clusters of risks: 1990-2015: a systematic analysis for the Global Burden of Disease Study 2015

This appendix provides further methodological detail, supplemental figures, and more detailed results for risk factors. The appendix is organized into broad sections following the structure of the main paper.

Table of Contents

Preamble…… 7

Section 1. GBD overview…… 8

Geographic units of the analysis…… 8

GBD risk factor hierarchy…… 8

Time periods of the analysis…… 8

List of abbreviations…… 8

Section 2. Risk factor estimation overview…… 11

Overview…… 11

Step 1. Effect size estimation…… 12

Step 2 Exposure estimation…… 15

DisMod-MR 2.1 estimation…… 18

Spatiotemporal Gaussian process regression…… 21

Step 3. Estimate summary exposure values…… 25

Step 4. Theoretical minimum-risk exposure level…… 26

Step 5. Estimate population attributable fractions…… 27

Step 6. Mediation…… 28

Step 7. Estimate attributable burden…… 36

Other analysis: Decomposition of deaths and DALYs…… 36

Other analysis: Socio-demographic Index (SDI) analysis and epidemiological transition…… 38

References for Sections 1 and 2…… 40

Section 3. Risk-specific estimation…… 44

Unsafe water…… 45

Unsafe sanitation…… 48

Unsafe hygiene…… 51

Ambient particulate matter pollution…… 54

Household air pollution…… 64

Ambient ozone pollution…… 66

Radon…….. 68
Lead…… 70
Occupational risk factors…… 73
Suboptimal breastfeeding…… 80
Childhood undernutrition…….. 82
Iron deficiency…… 85
Vitamin A deficiency…….. 87
Zinc deficiency…… 89
Smoking…… 91
Second-hand smoke…… 96
Alcohol……. 99
Drug use…….. 106
Dietary risk factors…….. 112
Childhood sexual abuse…… 117
Intimate partner violence…… 118
Unsafe sex……. 123
Low physical activity…….. 125
High fasting plasma glucose…… 129
High cholesterol……. 134
High systolic blood pressure…… 139
High body mass index…… 144
Low bone mineral density…….. 148
Low glomerular filtration rate……. 156

Section 4. Appendix figures and tables

Appendix figures

Appendix Figure 1. A more general causal web of the causes of health outcomes with the categories of causes included in this analysis shown in blue…… 161

Appendix Figure 2. Analytical flowchart of the comparative risk assessment for the estimation of population attributable fractions by geography, age, sex, and year for GBD 2015. Ovals represent data inputs, rectangular boxes represent analytical steps, cylinders represent databases, and parallelograms represent intermediate and final results. GBD=Global Burden of Disease. SEVs=Summary exposure values. TMREL=Theoretical

minimum-risk exposure level. PAFs=Population attributable fractions. YLLs=years of life lost. YLDs=years lived with disability. DALYs=disability-adjusted life-years…………………………………… 162

Appendix Figure 3. Types of Comparative Risk Assessments (CRA) based on the time perspective and the nature of the counterfactual level or distribution of exposure. The shaded box represents the type of CRA currently undertaken in GBD 2015. GBD=Global Burden of Disease……… 163

Appendix Figure 4. Global decomposition of changes in level 3 cause-specific DALYs for all risk factors combined from 1990 to 2015 due to population growth, population ageing, risk exposure and the risk-deleted DALY rate. Causes are reported in order of percent change in the number of DALYs from 1990 to 2015. This figure excludes cervical cancer, HIV/AIDS, and sexually transmitted diseases DALYs because they are not estimated based on exposure and relative risk. DALYs=disability-adjusted life-years………………………………………………………………… 164

Appendix Figure 5. Global age-standardised percent change in SEVs for high and high-middle Socio-demographic Index (SDI) geographies versus middle, low-middle, and low SDI geographies, for males (A) and females (B), 1990 to 2015. Socio-demographic Index (SDI) is calculated for each geography as a function of lag dependent income per capita, average educational attainment in the population over age 15, and the total fertility rate (TFR). SDI units are interpretable; a zero represents the lowest level of income per capita and educational attainment and highest TFR observed from 1980 to 2015, and a one represents the highest income per capita and educational attainment and lowest TFR observed in the same period. Cut-offs on the SDI scale for the quintiles have been selected based on examining the entire distribution of geographies from 1980 to 2015. Annualised rate of change in the age-standardised SEV 1990-2015 in high SDI geographies compared to all other geographies. SEV=summary exposure value…………………………………………………………………………… 166

Appendix Figure 6. Diagram showing the proportion of all-cause DALYs to behavioural, environmental and occupational, and metabolic risk factors and their overlaps for all ages in 2015. DALYs=disability-adjusted life-years……………………………………………………………………………… 169

Appendix Figure 7. DALYs attributable to level 2 risk factors for the low Socio-demographic Index (SDI) quintile (A) and for the high SDI quintile (B), for both sexes combined, 2015. Socio-demographic Index (SDI) is calculated for each geography as a function of lag dependent income per capita, average educational attainment in the population over age 15, and the total fertility rate (TFR). SDI units are interpretable; a zero represents the lowest level of income per capita, educational attainment, and highest TFR observed 1980-2015 and a one represents the highest income per capita, educational attainment and lowest TFR observed in the same period. Cut-offs on the SDI scale for the quintiles have been selected based on examining the entire distribution of geographies 1980-2015. Annualized rate of change in the age-standardized SEV 1990-2015 in high SDI geographies compared to all other geographies. DALYs=disability-adjusted life-years………………………………………………………………… 170

Appendix Figure 8. Leading 10 level 3 global risk factors for DALYs in 2015 by age group. Each cause is colored by the percent change in age specific DALYs from 2005 to 2015. DALYs=disability-adjusted life-years……………………………………………………………………………………… 172

Appendix Figure 9. Observed SEVs compared to the value expected on the basis of SDI alone, across SDI quintiles for 61 risk factors included in the GBD 2015. Each SDI quintile is coloured-coded, and coloured lines represent expected levels, on the basis of SDI, for risk-specific SEVs. To enhance readability, SEVs in this figure have been scaled such that the lowest observed SEV for a given risk equals 0 and the highest observed SEV equals 1. Each circular symbol represents observed SEVs at the country level in 2015, with colours aligning with SDI quintile. Each risk factor corresponds with a vertical line. The ordering of risk factors was determined by the difference in expected SEVs for low SDI (the red line) and high SDI (the purple line). Risks proceed clockwise from those with the largest decline in SEV to those with the largest increase in SEV as SDI increases. SDI = Socio-demographic index. SEV=summary exposure value…… 173

Appendix Figure 10. Global map for Level 3 risk factors in 2013 of attributable DALYs for males (A) and females (B). DALYs=disability-adjusted life-years. ATG = Antigua and Barbuda. VCT = Saint Vincent and the Grenadines. BRB = Barbados. COM = Comoros. DMA = Dominica. GRD = Grenada. MDV = Maldives. MUS = Mauritius. LCA = Saint Lucia. TTO = Trinidad and Tobago. SYC = Seychelles. MLT = Malta. SGP = Singapore. MHL = Marshall Islands. KIR = Kiribati. SLB = Solomon Islands. FSM = Federated States of Micronesia. VUT = Vanuatu. WSM = Samoa. FJI = Fiji. TON = Tonga…… 174

Appendix Figure 11. Map of socio-demographic index (SDI) classifications by location. ATG = Antigua and Barbuda. VCT = Saint Vincent and the Grenadines. BRB = Barbados. COM = Comoros. DMA = Dominica. GRD = Grenada. MDV = Maldives. MUS = Mauritius. LCA = Saint Lucia. TTO = Trinidad and Tobago. SYC = Seychelles. MLT = Malta. SGP = Singapore. MHL = Marshall Islands. KIR = Kiribati. SLB = Solomon Islands. FSM = Federated States of Micronesia. VUT = Vanuatu. WSM = Samoa. FJI = Fiji. TON = Tonga…… 176

Appendix tables

Appendix Table 1. Guidelines for Accurate and Transparent Health Estimates Reporting (GATHER) 18-items checklist with description of compliance and location of information for GBD 2015 risk factors capstone…… 177

Appendix Table 2. GBD 2015 geography hierarchy with levels…… 180

Appendix Table 3. GBD 2015 risk factor hierarchy with levels, modeling strategies, and the main type of data sources used to estimate exposure levels…… 193

Appendix Table 4. Socio-demographic Index (SDI) groupings by geography, based on 2015 values…… 195

Appendix Table 5. Socio-demographic Index (SDI) values for all estimated GBD locations, 1980 to 2015…… 207

Appendix Table 6a. Relative risks used by age and sex and for each outcome for all risk factors except for ambient air pollution and alcohol…… 215

Appendix Table 6b. Relative risks used by age and sex and for each outcome for the particulate matter integrated exposure response curve…… 237

Appendix Table 6c. Relative risks used by age and sex and for each outcome for alcohol use 238

Appendix Table 7. Supplemental information for Table 2: Epidemiological evidence supporting causality between risk-outcome pairs included in the Global Burden of Disease 2015 study including 7A. Citations and 7B. Additional information………………………………………… 245

Preamble

This appendix provides methodological detail, supplemental figures and more detailed results for risk factors. The appendix is organized into broad sections following the structure of the main paper. This study complies with the Guidelines for Accurate and Transparent Health Estimates Reporting (GATHER) recommendations, and this appendix is more comprehensive and encyclopedic than previous Global Burden of Disease appendices. It includes detailed tables, figures, cause modeling write-ups and flowcharts, and information on data in an effort to maximize transparency in our estimation processes and provide a comprehensive description of analytical steps. Components of this document are the same as described in the appendix to our GBD 2013 risk factors paper; substantial components of this appendix are new text. We intend this to be a living document, to be updated with each annual iteration of the Global Burden of Disease.

Section 1. GBD overview

Geographic units of the analysis

In the GBD framework, geographies have been arranged as a set of hierarchical categories: seven super-regions; 21 regions nested within the seven super-regions; and 195 countries and territories nested in the 21 regions. High-quality vital registration data made it possible to expand the geographies considered in the comparative risk assessment of GBD 2015 to include American Samoa, Bermuda, Greenland, Guam, Northern Mariana Islands, Puerto Rico, and the US Virgin Islands. These territories were not previously included in the national totals of the United States (US), United Kingdom (UK), and Denmark, and instead were included only in regional totals in GBD 2013. Additionally, GBD collaborator interest and availability of data resulted in an expansion of countries for which we disaggregate our estimates at the subnational level, including 26 states and one district for Brazil; 34 provinces and municipalities for China; 31 states and union territory groupings for India that include 62 rural and urban units; 47 prefectures for Japan; 47 counties for Kenya; 32 federal entities for Mexico; 13 provinces for Saudi Arabia; nine provinces for South Africa; two regions for Sweden; 13 regions for the UK (Northern Ireland, Scotland, Wales, England, and nine subregions of England); and 51 states and districts for the US. At the first level of subnational division, 256 geographic units are now included in GBD 2015. For this paper we present results for the 195 national and territory-level geographies.

GBD risk factor hierarchy

In this analysis, we focus on three groups of risk factors: behavioural, environmental and occupational, and metabolic. The GBD 2015 risk factors hierarchy and levels are summarized in Appendix Table 3.

Time periods of the analysis

We produced a complete set of age-, sex-, cause-, and location-specific estimates of risk factor exposure and attributable burden for 1990, 1995, 2000, 2005, 2010, and 2015 for included risk factors. Online data visualizations at http://vizhub.healthdata.org/gbd-compare provide access to results for all GBD metrics, including risk factor results, for all years for which estimates were computed from 1990 through 2015.

List of abbreviations

BMI: body-mass index

BMD: bone mineral density

CKD: chronic kidney disease

COD: causes of death

CODEm: cause of death ensemble modeling

COPD: chronic obstructive pulmonary disease

CSA: childhood sexual abuse

CRA: comparative risk assessment

CVD: cardiovascular disease

DALY: disability-adjusted life-year

DRI: data representativeness index

FAO: Food and Agriculture Organization

GATHER: Guidelines for Accurate and Transparent Health Estimates Reporting

GBD: Global Burden of Disease

IER: integrated exposure response

IHD: ischemic heart disease

ILO: International Labour Organization

IPV: intimate partner violence

LDI: lag distributed income per capita

LRI: lower respiratory infection

MDG: Millennium Development Goal

NCD: non-communicable disease

PAF: population attributable fraction

$PM_{2.5}$: particulate matter <2.5μm in diameter

RCT: randomised controlled trial

RMSE: root mean square error

SBP: systolic blood pressure

SD: standard deviation

SDG: sustainable development goal

SDI: Socio-demographic Index

SEER: Surveillance, Epidemiology, and End Results Program

SEV: summary exposure value

SIR: smoking impact ratio

SSB: sugar-sweetened beverages

ST-GPR: spatiotemporal Gaussian process regression

TB: tuberculosis

UI: uncertainty interval

WHO: World Health Organization

YLD: years lived with disability

YLL: years of life lost

Section 2. Risk factor estimation overview

Overview

The CRA conceptual framework was developed by Murray and Lopez,[1] who established a causal web of hierarchically organised risks or causes that contribute to health outcomes (Figure 1), which allows for quantification of risks or causes at any level in the framework. In GBD 2015, as in previous iterations of the GBD study, we evaluated a set of behavioural, environmental and occupational, and metabolic risks, where risk-outcome pairs were included based on evidence rules (see appendix p 7). These risks were organised in four hierarchical levels, where level 1 represents the overarching categories (behavioural, environmental and occupational, and metabolic) nested within level 1 risks; level 2 contains both single risks and risk clusters (such as child and maternal malnutrition); level 3 contains the disaggregated single risks from within level 2 risk clusters; and level 4 details risks with the most granular disaggregation, such as for specific occupational carcinogens, the subcomponents of childhood undernutrition (stunting, wasting, underweight), and suboptimal breastfeeding (discontinued and non-exclusive breastfeeding). At each level of risk, we evaluated whether risk combinations were additive, multiplicative, or shared common pathways for intervention. This approach allows the quantification of the proportion of risk-attributable burden shared with another risk or combination of risks and the measurement of potential overlaps between behavioural, environmental and occupational, and metabolic risks. To date, we have not quantified in the GBD the contribution of other classes of risk factors illustrated in Figure 1; we provide through an analysis of the relationship between risk exposures and development measured using the socio-demographic index (see below for details) some insights into the potential magnitude of distal social, cultural and economic factors.

Two types of risk assessments are possible within the CRA framework: attributable burden and avoidable burden. Attributable burden is the reduction in current disease burden that would have been possible if past population exposure had shifted to an alternative or counterfactual distribution of risk exposure. Avoidable burden is the potential reduction in future disease burden that could be achieved by changing the current distribution of exposure to a counterfactual distribution of exposure. Murray and Lopez identified four types of counterfactual exposure distributions: (1) theoretical minimum risk; (2) plausible minimum risk; (3) feasible minimum risk; and (4) cost-effective minimum risk.[2] The theoretical minimum risk level (TMREL) is the level of risk exposure that minimises risk at the population level, or the level of risk that captures the maximum attributable burden. Other possible forms of risk quantification include plausible minimum risk – which reflects the distribution of risk that is conceivably possible and would minimise population-level risk if achieved – while feasible minimum risk describes the lowest risk distribution that has been attained within a population, and the cost-effective minimum risk is the lowest risk distribution for a population that can be attained in a cost-effective manner. Because no robust set of forecasts for all components of the GBD is available, in this study we focus on quantifying attributable burden using the theoretical minimum risk counterfactual distribution. Figure 2 shows the eight possible types of risk quantification within the CRA framework, with the hatched box representing the type of CRA currently undertaken by the GBD study. As per the definition of avoidable burden, risk reversibility would be incorporated into this type of assessment, as it would involve reducing risk to the counterfactual for the index year, given a history of past risk exposure. Given the focus in this study on attributable burden, risk reversibility is not a criteria used in estimation here.

In general, this analysis follows the CRA methods used in GBD 2013.[3] The methods described here provide a high-level overview of the analytical logic with a focus on areas of notable change from the methods employed in GBD 2013. Key methodological refinements include improved spatial calibration of satellite measures of atmospheric particulate matter <2.5μm in diameter (PM2.5) to ground measurements; an updated integrated exposure response (IER) curve for all outcomes of PM2.5; the development of age-specific relative risks for diet risks based on high systolic blood pressure and cholesterol age curves; a lower TMREL for total cholesterol and for high body mass index (BMI); the incorporation of new data to improve estimation of tourism consumption for alcohol; improvements in exposure data standardization such as age-splitting and severity-splitting for several risks; and the selection of the maximum level of relative risk from dose-response studies for diet and metabolic risks. Here we aim to provide sufficient detail on these methodological improvements to understand the overall structure of the estimation process – greater detail of inputs, analytical processes and outputs, and methods specific to each risk-outcome pairing are now maintained as a single source available as an appendix. This study complies with the Guidelines for Accurate and Transparent Health Estimates Reporting (GATHER) recommendations proposed by the World Health Organization (WHO) and others, which include recommendations on documentation of data sources, estimation methods, and statistical analysis (Table 1).[4]

Step 1. Effect size estimation

1a. Collate relative risk data

Criteria for inclusion of risk-outcome pairs

In this study, as in GBD 2013, we have included risk-outcome pairs that we have assessed as meeting the World Cancer Research Fund (WCRF) grades of convincing or probable evidence.[5] In this framework, convincing evidence consists of biologically plausible associations between exposure and disease established from multiple epidemiological studies in different populations. Evidentiary studies must be substantial, include prospective observational studies, and where relevant, randomised controlled trials (RCTs) of sufficient size, duration, and quality, and showing consistent effects. Probable evidence is similarly based on epidemiological studies with consistent associations between exposure and disease, but for which shortcomings in the evidence exist, such as insufficient trials (or prospective observational studies) available.

The World Cancer Research Fund grading system

Convincing evidence

Evidence based on epidemiological studies showing consistent associations between exposure and disease, with little or no evidence to the contrary. The available evidence is based on a substantial number of studies including prospective observational studies and where relevant, randomized controlled trials of sufficient size, duration, and quality showing consistent effects. The association should be biologically plausible.

Probable evidence

Evidence based on epidemiological studies showing fairly consistent associations between exposure and disease, but for which there are perceived shortcomings in the available evidence or some evidence to the contrary, which precludes a more definite judgment. Shortcomings in the evidence may be any of

the following: insufficient duration of trials (or studies); insufficient trials (or studies) available; inadequate sample sizes; or incomplete follow-up. Laboratory evidence is usually supportive. The association should be biologically plausible.

Possible evidence
Evidence based mainly on findings from case-control and cross-sectional studies. Insufficient randomized controlled trials, observational studies, or non-randomized controlled trials are available. Evidence based on non-epidemiological studies, such as clinical and laboratory investigations, is supportive. More trials are needed to support the tentative associations, which should be biologically plausible.

Insufficient evidence
Evidence based on findings of a few studies which are suggestive, but insufficient to establish an association between exposure and disease. Little or no evidence is available from randomized controlled trials. More well-designed research is needed to support the tentative association.

1b. Determine relative risks

Effect size estimation

The relative risk by level of exposure, or by cause, for mortality or morbidity can be found in published and unpublished primary studies or in secondary studies that summarize relative risks. In Step 1a of the analytical process (Figure 3 in manuscript), we collated information from randomized controlled trials, cohort, pooled cohort, and case control studies, and in Step 1b, used these data to determine the relative risk for the risk-outcome pairs included in GBD 2015. For most risks, data from pooled cohorts, or meta-analyses of cohorts, were used; in the case of the risk of cataracts from household air pollution cohort data were not available, and instead we used case control data. We estimated relative risks of mortality and morbidity for 59 risk factors for which we determined attributable burden using relative risk and exposure. We incorporated relative risks from studies that controlled for confounding but not for factors along the causal pathway between exposure and outcome. For risk-outcome pairs with evidence available for only one of mortality or morbidity, we generally assumed that the estimated relative risks applied equally to both. Given evidence of statistically different relative risks for mortality and morbidity, we incorporated different relative risks for each. We did not find that relative risks were consistently higher or lower for mortality compared with morbidity. Details and citation information for the data sources used for relative risks are provided in searchable form through a new web-tool (http://ghdx.healthdata.org/).

Available data sources for determining relative risks varied across risks. Relative risks for metabolic risks were established from pooled cohorts that were similar across definitions, study design, and control of major confounding factors.[6] Meta-analyses were used in determining the relative risk for zinc deficiency (zinc supplementation trials) and diarrhoea, and meta-analyses of cohort studies were used for the relative risk for cancers from BMI. For both asthmagens and child sexual abuse, one cohort study with strong controls for confounding or biases was available and was used (see data source tool for citation details, http://ghdx.healthdata.org/). We re-estimated the integrated exposure-response curves for six outcomes (lower respiratory infections, lung cancer, ischaemic heart disease, ischaemic stroke, hemorrhagic stroke, and chronic obstructive pulmonary disease [COPD]) from the risk of exposure to

ambient particulate matter pollution (PM2.5). For four outcomes (breast cancer, diabetes, ischaemic heart disease, ischaemic stroke) paired with the risk of low physical activity, we updated relative risk using recently published studies. Relative risks by age and sex for each risk factor and outcome pair are provided in Appendix Table 6.

For the following seven risk factors and a portion of their related outcomes, evidence of the direct relationship between a risk factor and a disease outcome was sparse or lacking and instead we interpolated relative risks from analogous or related outcomes. Suitable studies for the risk from unsafe water, unsafe sanitation, and unsafe handwashing for enteric diseases (eg, salmonella pathogens, typhoid, and paratyphoid fevers) were not located; because of the similar fecal-oral pathway of transmission, we substituted the effect size for diarrhoea. For certain other risks a single effect size was applied to groups of related outcomes where specific relative risks were unavailable. For the risk of atrial fibrillation and peripheral vascular disease associated with high systolic blood pressure we substituted the relative risk from "other cardiovascular diseases" (other CVD), and for the risk for endocarditis, cardiomyopathy, and myocarditis paired with high BMI, we used the relative risk of inflammatory heart disease. For BMI and colon and rectum cancers, we combined the relative risks proportional to the incidence of each cancer in the Surveillance, Epidemiology, and End Results Program (SEER) cancer registry data into a single relative risk for colorectal cancer.

For all outcomes related to unsafe sex, the relative risk and exposure framework was not used to estimate attributable burden. For unsafe sex and HIV, we used a direct attribution approach to address the lack of data on unsafe sexual practices in most populations. The proportion of HIV attributable to unsafe sex was modelled directly using DisMod-MR 2.1 from data on the fraction of cases identified as being through sexual transmission, intravenous drug use, or blood transfusion.

For risks estimated from a continuous exposure distribution where the effect size is reported by categories in pooled or meta-analysis studies, we converted those categories to relative risk per unit increase in exposure. This implies a linear increase in the log of the relative risk and exposure; various studies have suggested this is a reasonable approximation of the dose-response curve for many risks. An example of this is high systolic blood pressure, where data from the Prospective Cohort Study (PSC) and the Asia-Pacific Cohort Studies Collaboration (APCSC) were well described by a linear increase in the logarithm of the relative risk by a 10-unit increase in high systolic blood pressure. This approximately log-linear relationship suggests that the proportional difference in the age-specific risk of stroke death associated with a given absolute difference in exposure is about the same at all levels of risk. Many meta-analyses convert relative risks to per unit increase for convenience, particularly when studies choose different categories that could not otherwise be compared. The log-linear approximation appears plausible[7] even where there is limited consensus on the appropriate TMREL. Where there were insufficient samples in the primary studies at high levels of exposure to inform the shape of the tail of the distribution, we applied a cap to the maximum relative risk using the midpoint of the last category for which a relative risk was reported.

Step 2. Exposure estimation

2a. Collate exposure data

Systematic reviews

GBD 2010 collaborators undertook initial systematic reviews for the majority of risk factors; for GBD2013, updates to these reviews were conducted for all risk factors at IHME using data available through January of 2014. For the GBD 2015 study no data or studies were extracted after January 2016. Household surveys including the Demographic and Health Surveys, Multiple Indicator Cluster Surveys, Living Standards Measurement Surveys, Reproductive Health Surveys, and various national health surveys included in the Global Health Data Exchange (ghdx.healthdata.org) were systematically screened for data relevant to sequelae. For some risk factors, only a small fraction of the existing data appear in the published literature and other sources predominate such as survey data and satellite data. The new source tool in GHDx offers a comprehensive view of data sources used in GBD 2015.

Search terms

Search terms for updates of systematic reviews for GBD 2015 are shown by risk in Section 3 of this appendix.

Survey data preparation

For GBD 2015, survey data constitutes a substantial part of the underlying data used in the estimation process. During extraction, we concentrate on demographic variables (such as location, gender, age), survey design variables (such as sampling strategy and sampling weights), and the variables used to define the population estimate (such a prevalence or a proportion) and a measure of uncertainty (standard error, confidence interval or sample size and number of cases).

2b. Adjust exposure data

A number of adjustments were applied to extracted exposure sources in order to make the data more consistent and suitable for modelling. Commonly applied adjustments included age-sex splitting, adding study-level covariates, and bias correction. Age-sex splitting was applied to literature data reported by age or sex but not by age and sex assuring that the total number of cases remained as reported. If a source did not report sample size by age or sex we applied the age-sex distribution of the population for the same location and year to the reported total sample size. We relied on the metaregression component of DisMod-MR 2.1 for most of the bias correction of data for variations in study attributes such as case definitions and measurement method. DisMod-MR 2.1 calculates a single adjustment that is applied regardless of age, sex, or location. If enough data were available to differentiate these adjustments by age, sex, or location, or if detailed survey data were available to make more precise adjustments between different thresholds on a biochemical measure, we applied bias corrections to the data before entry into DisMod-MR 2.1.

2c. Estimate exposure

Mean exposure estimation

In Step 2a of the estimation process, we used systematic literature reviews to identify risk factor exposure studies published or identified since GBD 2013 and combined these with existing data from household and health examination surveys, census, morbidity, or satellite imagery and ground sensor data (used for PM2.5 estimation). Certain risks, such as diet and alcohol consumption, also incorporated

administrative record systems. Sources of data used in estimating risk factor exposure can be accessed through the data source tool at http://ghdx.healthdata.org/.

A geographic and temporal data representativeness index (DRI) for risk factor exposure estimation was determined for each risk factor as the fraction of countries for which we have identified any data for the risk factor. The DRI is a minimalist measure which does not take into account the quality of the available data, only whether any data for an interval are available. For aggregate causes, the DRI score reflects the availability of any data from any component cause. Table 3 provides the DRI for each risk factor in the GBD hierarchy for three time periods: prior to 2005, 2005 to 2015, and the total for all years. Overall, DRI ranged from a low of 16.2% for diet low in whole grains – indicating the lack of available data for this risk factor from most geographies included in the GBD – to 100% for each of ambient ozone pollution and ambient particulate matter pollution. The DRI for PM2.5 is 100% because data are available for all countries and all years. Once data were collected and compiled, step 2b of the analytical flowchart describes the adjustments applied, where necessary, to correct for bias. Examples of these adjustments include: use of urban studies for lead; crosswalks between different measurements, methods, and definitions, such as for self-report of obesity and glycated hemoglobin (HbA1C) for diabetes; and age-sex splitting of data, such as for fasting plasma glucose, cholesterol, and systolic blood pressure that may be reported from broad age-groups.

For the GBD we developed two modelling approaches, a Bayesian meta-regression model (DisMod-MR 2.1) and a spatiotemporal Gaussian process regression model (ST-GPR), to pool data from different sources, control and adjust for bias in data, and incorporate other types of information such as country-level covariates. DisMod-MR 2.1 and ST-GPR are mixed effect models that borrow information across age, time, and geographies to synthesise multiple sources of data into unified estimates of levels and trends. DisMod-MR 2.1 is an improved version of the method used in GBD 2013 (DisMod-MR 2.0). Key updates from the previous version include improvements in how country covariates differentiate estimates with sparse data, and consolidation of the code into a single language, among other computational efficiencies.[8] A detailed description of the likelihood used for estimation, and a full description of improvements made for DisMod-MR 2.1, are detailed by Vos and colleagues[8] with additional detail in the appendix to that paper. The ST-GPR model has three main hyper-parameters that control for smoothing across time, age, and geography. Values for these hyper-parameters were selected based on cross-validation. Cross-validation tests were conducted for different combinations of the hyper-parameters. In each test, 30% of the data were held out and the performance of each combination of hyper-parameters evaluated on the held out data. For each hyper-parameter combination, 25 cross-validation tests were conducted. The performance of each model in predicting the withheld 30% of the data was evaluated using a combined measure based on root mean square error (RMSE) and uncertainty interval coverage.

The main difference between these methods is their power to include unstructured types of data by sex and age group and in their degree of flexibility. Step 2c in Figure 3 outlines the use of DisMod-MR 2.1 for 23 risk factors where data were available by different age intervals or mixed sex groups; DisMod-MR 2.1 is the preferred tool in these cases because of its ability to integrate over age and adjust for different exposure definitions in the data; however, the use of Bayesian Markov Chain Monte Carlo (MCMC) simulations with large volumes of data renders the analysis computationally intensive and reduces the number of iterations that are possible. If large volumes of standard age-group data are available – as is generally the case for metabolic risks – using ST-GPR becomes the preferred approach.

In some cases, we adapted our methods of modelling exposure to risks where necessary to account for complexities in the risk-outcome relationship or the need for particular handling of data. For dietary risks, we first used ST-GPR to model the national availability of foods and nutrients in all countries based on Food and Agriculture (FAO) data from the United Nations. Then, we used DisMod-MR 2.1 to model the intake of each food group and nutrient and to conduct crosswalks between different methods of dietary assessment (food frequency questionnaire, 24-hour diet recall, and diet record), various definitions of food groups or nutrients, and different levels of dietary assessment (national availability, household availability, individual level intake); additional details on crosswalk methodology is supplied for individual risks in the Appendix, Section 3.

For the GBD 2015 study, our estimates for exposure to ambient air pollution incorporated an updated database of 6,003 ground measurements based on the recently released WHO Air Pollution in Cities database[9] and updated satellite-based estimates.[10] These estimates combine aerosol optical depth retrievals from multiple satellites with the chemical transport model, GEOS Chem.[11] For GBD 2010 and GBD 2013 a single function was used to calibrate available ground measurements to the mean of gridded satellite-based and chemical transport model values. This use of a single, global calibration led to underestimation of ground measurements in some locations[12] and we therefore applied a Bayesian hierarchical modelling approach, which allowed the calibration to vary spatially and to enable the inclusion of information on land use and other factors related to air quality. The within-sample model fit and out-of-sample assessment of predictive ability were used to inform modelling. Predictive validity was assessed using 25 sets of training data, where holdouts were determined by randomly selecting 20% of sites based on sampling probabilities for super-regions and tabulated PM2.5 categories – returning a validation set with the same distribution of PM2.5 exposure and super-regions as the training dataset. Improvements were seen for countries and regions with limited ground monitoring. This process resulted in an improvement in both within-sample fit; with an increase in R^2 from 0.64 (reported in GBD 2013) to 0.91, and out-of-sample predictive ability; with a population-weighted RMSE of 12.1 $\mu g/m^3$ compared to 23.1 $\mu g/m^3$ when using the GBD 2013 model.

To evaluate exposure to smoking for cancers and COPD, we calculated a smoking impact ratio (SIR)[13] using the lung cancer mortality rate estimated for every population compared to the mortality rate in nonsmokers and never-smokers from the few cohorts available; for example, the Cancer Prevention Study II.[14] Estimating exposure directly from smoking prevalence would be preferable but at present is hindered by the variation in tobacco content in cigarettes and other products, filtering, cigarettes per smoker, and other factors which contribute to the effect of tobacco on cancers. The SIR based on observed lung cancer death rates is meant to capture the lifetime cumulative effect of smoking.

In modelling exposure to alcohol consumption, we extracted exposure data from general population surveys reported in both unpublished and published literature; however, these surveys tend to underestimate the amount of alcohol consumption due to self-report.[15,16] To correct for the underreporting of alcohol consumption in surveys, we estimated total alcohol consumed in every country using data of alcohol sales and FAO data of available alcohol for drinking and then adjusted for sales to tourists visiting each country and the estimated volume of illicit production from survey data.[17] Survey-based estimates of consumption by age and sex have been scaled up to match our estimates of total consumption. A complete list of risks and the analytical method used is reported in Appendix Table 3. Additional details for adjustments or adaptations to particular risk models are located in Appendix section 3.

Exposure distributions

In order to select an appropriate distribution for risk factors measured on a continuous scale we used mean and standard deviation (SD) for our models, because these statistics are available in nearly all published studies. We found strong predictive validity (smaller RMSE) between the mean and SD using out-of-sample cross validation compared with the alternative of modelling the coefficient of variation. A correlation coefficient of at least 96% (R^2) was found between the SD and mean of dietary and metabolic risks from survey populations.

In analyses conducted for GBD 2013, we tested normal, beta, lognormal, and gamma distributions for their fit to metabolic and diet risks using individual record datasets (such as the National Health and Nutrition Examination Study [NHANES], the Cebu Longitudinal Health and Nutrition Survey [CLHNS], or the National Income Dynamics Study [NIDS]; see data source tool for details), and found that the lognormal distribution fit the available data best for all but three risk factors: iron deficiency and low bone mineral density, high BMI, and high systolic blood pressure. For iron deficiency and low bone mineral density, the best fit was provided by the normal distribution. For high BMI, GBD authors Ng and colleagues[18] demonstrated that the best fit was provided by a beta distribution fit to the mean prevalence of overweight and prevalence of obesity, constrained such that skewness could not be negative.[19] For high systolic blood pressure, relative risks were corrected for regression dilution bias;[6,20] exposure SD was corrected for a measure of inter-temporal variance in blood pressure observed in cohort studies to ensure the estimates reflected "usual" systolic blood pressure. We did not use a relative risk per unit increase in exposure where the relative risk substantially deviated from a log-linear approximation. For example, for the integrated exposure response curve (PM2.5 exposure and relative risk of outcomes) we fit a nonlinear curve and estimated the relative risk for every level of PM2.5.

DisMod-MR 2.1 Estimation

An estimation method used for modeling the exposure to many risk factors is the Bayesian meta-regression method DisMod-MR 2.1.

DisMod-MR 2.1 description

Until GBD 2010, non-fatal estimates were based on a single data source on prevalence, incidence, remission or a mortality risk selected by the researcher as most relevant to a particular geography and time. For GBD 2010, we set a more ambitious goal: to evaluate all available information on a disease that passes a minimum quality standard. That required a different analytical tool that would be able to pool disparate information presented in varying age groupings and from data sources using different methods. The DisMod-MR 1.0 tool used in GBD 2010 evaluated and pooled all available data, adjusted data for systematic bias associated with methods that varied from the reference and produced estimates by world regions with uncertainty intervals using Bayesian statistical methods. For GBD 2013, the improved DisMod-MR 2.0 had increased computational speed allowing computations that were consistent between all disease parameters at the country rather than region level. The hundred-fold increase in speed of DisMod-MR 2.0 was partly due to a more efficient re-write of the code in C++ but also by changing to a model specification using log rates rather than a negative binomial model used in DisMod-MR 1.0. In cross-validation tests, the log rates specification worked as well or better than the negative binomial specification.[21] For GBD2015, the computational engine (DisMod-MR 2.1) remained substantively unchanged but we re-wrote the 'wrapper' code that organizes the flow of data and settings at each level

of the analytical cascade. The sequence of estimation occurs at 5 levels: global, super-region, region, country and where applicable subnational geographical unit. The super-region priors are generated at the global level with mixed-effects, non-linear regression using all available data; the super-region fit, in turn, informs the region fit, and so on down the cascade. The wrapper gives analysts the choice to branch the cascade in terms of time and sex at different levels depending on data density. The default used in most models is to branch by sex after the global fit but to retain all years of data until the lowest level in the cascade. For GBD 2015, we generated fits for the years 1990, 1995, 2000, 2005, 2010, and 2015.

In updating the 'wrapper,' we consolidated the code base into a single language, Python, to make the code more transparent and efficient and to better deal with subnational estimation. The computational engine is limited to three levels of random effects; we differentiate estimates at the super-region, region and country level. In GBD 2013, the subnational units of China, the UK and Mexico were treated as 'countries' such that a random effect was estimated for every geography with contributing data. However, the lack of a hierarchy between country and subnational units meant that the fit to country data contributed as much to the estimation of a subnational unit as the fits for all other countries in the region. We found inconsistency between the country fit and the aggregation of subnational estimates when the country's epidemiology varied from the average of the region. Adding an additional level of random effects required a prohibitively comprehensive rewrite of the underlying DisMod-MR engine. Instead, we added a fifth layer to the cascade, with subnational estimation informed by the country fit and country covariates, plus an adjustment based on the average of the residuals between the subnational unit's available data and its prior. This mimicked the impact of a random effect on estimates between sub nationals.

For GBD 2015 we improved how country covariates differentiate non-fatal estimates for diseases with sparse data. The coefficients for country covariates are re-estimated at each level of the cascade. For a given geography, country coefficients are calculated using both data and prior information available for that geography. In the absence of data, the coefficient of its parent geography is used, in order to utilize the predictive power of our covariates in data sparse situations.

DisMod-MR 2.1 likelihood estimation

Analysts have the choice of using a Gaussian, log-Guassian, Laplace or Log-Laplace likelihood function in DisMod-MR 2.0. The default log-Gaussian equation for the data likelihood is:

$$-log\left[p\left(y_j|\Phi\right)\right] = \log\left(\sqrt{2\pi}\right) + \log\left(\delta_j + s_j\right) + \frac{1}{2}\left(\frac{\log\left(a_j + \eta_j\right) - \log\left(m_j + \eta_j\right)}{\delta_j + s_j}\right)^2$$

where, yj is a 'measurement value' (i.e. data point); Φ denotes all model random variables; ηj is the offset value, eta, for a particular 'integrand' (prevalence, incidence, remission, excess mortality rate, with-condition mortality rate, cause-specific mortality rate, relative risk or standardized mortality ratio) and aj is the adjusted measurement for data point j, defined by:

$$a_j = e^{(-u_j - c_j)} y_j$$

where u_j is the total 'area effect' (i.e. the sum of the random effects at three levels of the cascade: super-region, region and country) and c_j is the total covariate effect (i.e. the mean combined fixed effects for sex, study level and country level covariates), defined by:

$$c_j = \sum_{k=0}^{K[I(j)]-1} \beta_{I(j),k} \hat{X}_{k,j}$$

with standard deviation

$$s_j = \sum_{l=0}^{L[I(j)]-1} \zeta_{I(j),l} \hat{Z}_{k,j}$$

where k denotes the mean value of each data point in relation to a covariate (also called x-covariate); I(j) denotes a data point for a particular integrand, j; $\beta_{I(j),k}$ is the multiplier of the kth x-covariate for the ith integrand; $\hat{X}_{k,j}$ is the covariate value corresponding to the data point j for covariate k; l denotes the standard deviation of each data point in relation to a covariate (also called z-covariate); $\zeta_{I(j),k}$ is the multiplier of the lth z-covariate for the ith integrand; and δ_j is the standard deviation for adjusted measurement j, defined by:

$$\delta_j = log\left[y_j + e^{(-u_j - c_j)}\eta_j + c_j\right] - log\left[y_j + e^{(-u_j - c_j)}\eta_j\right]$$

Where m_j denotes the model for the jth measurement, not counting effects or measurement noise and defined by:

$$m_j = \frac{1}{B(j) - A(j)} \int_{A(j)}^{B(j)} I_j(\mathrm{a})\, \mathrm{da}$$

where A(j) is the lower bound of the age range for a data point; B(j) is the upper bound of the age range for a data point; and I_j denotes the function of age corresponding to the integrand for data point j.

Modelling Dietary Risks in DisMod-MR 2.1

We used DisMod-MR 2.1 to estimate the intake of each dietary component by age, sex, and year in each country and subnational unit. For each dietary factor, we included in our models study level covariates that provided information about the method of dietary assessment (i.e., 24-hour diet recall, food frequency questionnaire, household budget surveys, FAO Food Balance Sheets), definition of the dietary factor (whether it is consistent with the definition of GBD or not), and representativeness of survey (whether it is representative of the geographical unit or not). We considered data from representative 24-hour diet recall as optimal and adjusted all other data sources accordingly. For some dietary risks, we used relevant country level covariates to help improve our estimates where we had missing data. For example, we used national availability of red meat and pig meat as covariates for processed meat; national availability of hydrogenated oil as a covariate for trans fatty acids; and national availability of sugar for sugar-sweetened beverages.

Spaciotemporal Gaussian process regression

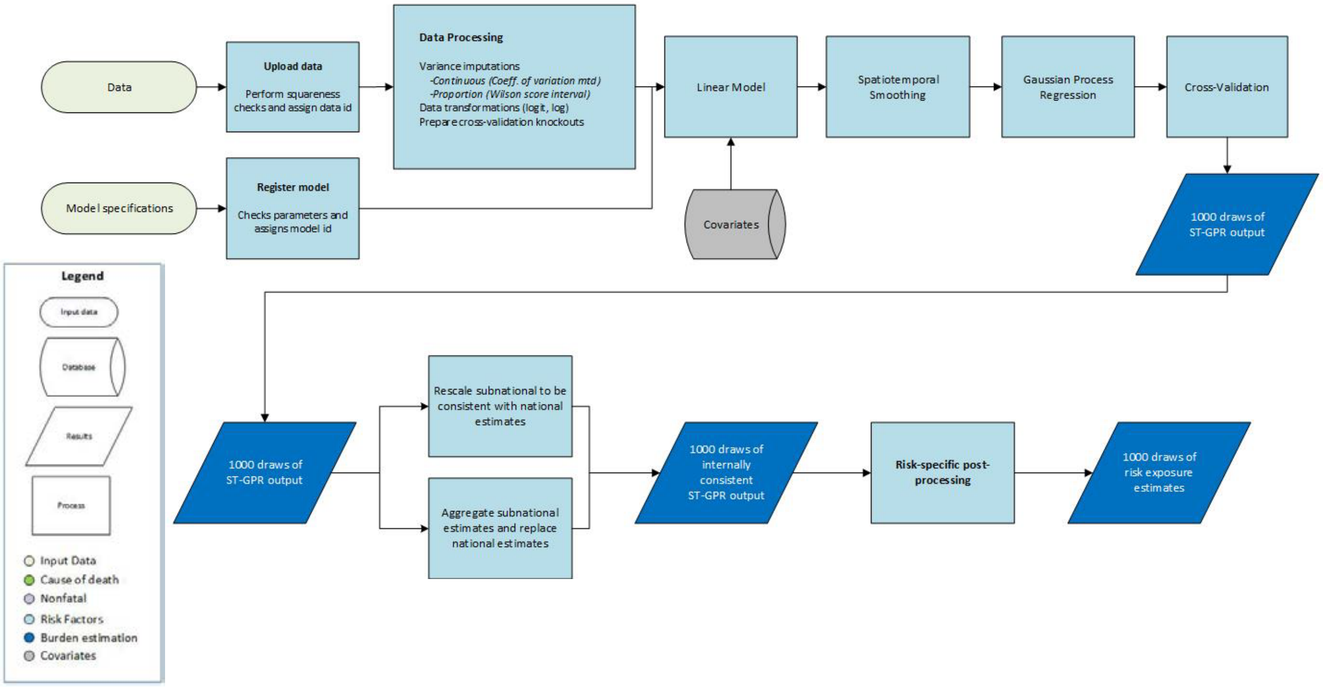

Spaciotemporal Gaussian process regression (ST-GPR) has been used for risk factors where the data density is sufficient to estimate a very flexible time trend. The approach is a stochastic modeling technique that is designed to detect signals amidst noisy data. It also serves as a powerful tool for interpolating non-linear trends .[22,23] Unlike classical linear models that assume that the trend underlying data follows a definitive functional form, GPR assumes that the specific trend of interest follows a Gaussian Process, which is defined by a mean function $m(\cdot)$ and a covariance function $Cov(\cdot)$. For example, let $p_{c,a,s,t}$ be the exposure, in normal, log, or logit space, observed in country *c*, for age group *a*, and sex *s* at time t:

$$(p_{c,a,s,t}) = g_{c,a,s}(t) + \epsilon_{c,a,s,t}$$

where

$$\epsilon_{c,a,s,t} \sim Normal(0, \sigma_p^2\ ),$$

$$g_{c,a,s}(t) \sim GP\left(m_{c,a,s}(t), Cov\left(g_{c,a,s}(t)\right)\right).$$

The derivation of the mean and covariance functions, $m_{c,a,s}(t)$ and $Cov\left(g_{c,a,s}(t)\right)$, along with a more detailed description of the error variance (σ_p^2), is described below.

Estimating mean functions

We estimated mean functions using a two-step approach. To be more specific, $m_{c,a,s}(t)$ can be expressed, depending on the exposure transformation, as:

$$log(p_{c,a,s}(t)) = X_{c,a,s}\beta + h(r_{c,a,s,t})$$

$$logit(p_{c,a,s}(t)) = X_{c,a,s}\beta + h(r_{c,a,s,t})$$

$$p_{c,a,s}(t) = X_{c,a,s}\beta + h(r_{c,a,s,t})$$

where $X\beta$ is the summation of the components of a hierarchical mixed-effects linear regression, including the intercept and the product of covariates with their corresponding fixed effect coefficients. For a majority of models, predictions were not made using the random effects component of the linear model. The second part of the equation, $h(r_{c,a,s,t})$, is a smoothing function for the residuals, $r_{c,a,s,t}$, derived from the linear model .[24] Descriptions of exposure transformations and which covariates were used in linear models can be found in Section 3. Risk-specific estimation.

While the linear component captures the general trend in exposures over time, much of the data varaibility may still not be adequately accounted for. To address this, we fit a locally weighted polynomial regression (LOESS) function $h(r_{c,a,s,t})$ to systematically estimate this residual variability by borrowing strength across time, age, and space patterns (the spatio-temporal component of ST-GPR). The time adjustment parameter, defined by λ , aims to borrow strength from neighboring time points (i.e. the exposure in this year is highly correlated with exposure in the previous year but less so further back in time). The age adjustment parameter, defined by ω, borrows strength from data in neighboring age groups. The space adjustment parameter, defined by ξ, aims to borrow strength across the hierarchy of geographical locations.

Let $w_{c,a,s,t}$ be the final weight assigned to observation $r_{c,a,s,t}$ with reference to a focal observation r_{c_0,a_0,s_0,t_0}. We first generated a preliminary weight $w'_{c,a,s,t}$ for smoothing over time, which was based on the scaled distance along the time dimension of the two observations:

$$w'_{c,a,s,t} = \left(1 - \left(\frac{|t-t_0|}{1+\max|t-t_0|}\right)^{\lambda}\right)^3$$

Next, we calculated the weight $w''_{c,a,s,t}$ to smooth over age, which is based on a distance along the age dimension of two observations. For a point between the age a of the observation $r_{c,a,s,t}$ and a focal observation r_{c_0,a_0,s_0,t_0}, the weight is defined as follows:

$$w''_{c,a,s,t} = \frac{1}{e^{\omega|a-a_0|}}$$

Finally, these combined weights were multiplied and further adjusted to account for geographic patterns.

Specifically, we defined a geospatial relationship by categorizing data based on the GBD location hierarchy. We adapted the weighting strategy used in previous studies estimating time series of global indicators to be more flexible with respect to estimating subnational locations and to borrow strength from all levels. [24,25] A vector of spatial weights corresponding to each level of the location hierarchy was derived as $[\xi, \xi * (1-\xi)^{n_1-1}, \ldots, \xi * (1-\xi)^{n_i-1}, (1-\xi)^{n_i}]$, where the vector is expanded to include the number, n_i, levels in the location hierarchy between the location being estimated and global, which

recieves a pre-rescaling weight of $(1-\xi)^{n_i}$. For example, estimating a country would use the following weighting scheme:

- Country data: ξ
- Regional data not from the country being estimated: $\xi * (1-\xi)$
- Data from other regions in the same super region: $\xi * (1-\xi)^2$
- Global data from other super regions: $(1-\xi)^3$

A full derivation of weights for each category follow, assuming the location being estimated was a country, follows:

1) If the observation $r_{c,t}$ belongs to the same country c_0 of the focal observation r_{c_0,t_0}:

$$w_{c,a,s,t} = \frac{\xi(w'_{c,a,s,t}w''_{c,a,s,t})}{\sum_{c=c_0}(w'_{c,a,s,t}w''_{c,a,s,t})} \qquad \forall c = c_0$$

2) If the observation $r_{c,t}$ belongs to a different country than the focal observation r_{c_0,t_0}, but both belong to the same region R:

$$w_{c,a,s,t} = \frac{\xi * (1-\xi)\,(w'_{c,a,s,t}w''_{c,a,s,t})}{\sum_{c\neq c_0}(w'_{c,a,s,t}w''_{c,a,s,t})} \qquad \forall c \neq c_0 \cap R[c] = R[c_0]$$

3) If the observation $r_{c,t}$ belongs to the same super region SR but to a both different country c_0 and region $R[c_0]$ than the focal observation r_{c_0,t_0}:

$$w_{c,a,s,t} = \frac{\xi * (1-\xi)^2\,(w'_{c,a,s,t}w''_{c,a,s,t})}{\sum_{c\neq c_0}(w'_{c,a,s,t}w''_{c,a,s,t})} \qquad \forall c \neq c_0 \cap R[c] \neq R[c_0] \cap SR[c] = SR[c_0]$$

4) If the observation $r_{c,t}$ is from a different super region than the focal observation r_{c_0,t_0}(ie. all other data currently not receiving a weight):

$$w_{c,a,s,t} = \frac{(1-\xi)^3\,(w'_{c,a,s,t}w''_{c,a,s,t})}{\sum_{c\neq c_0}(w'_{c,a,s,t}w''_{c,a,s,t})} \qquad \forall c \neq c_0 \cap R[c] \neq R[c_0] \cap SR[c] \neq SR[c_0]$$

To allow additional flexibility and specificity in weighting schemes, we allowed for two different ξ to be defined. The higher ξ was applied when at least one age-sex group in the country of estimation had at least five unique data points. The lower ξ was applied when estimating data-scarce countries.

Observations could be downweighted by a factor of 0.1, usually because they were not geographically representative at the unit of estimation. Details of reasons for downweighting can be found in risk-specific modeling summaries. The final weights were then normalized such that the sum of weights across age, time, and geographic hierarchy for a reference group was 1.

Estimating error variance

σ_p^2 represents the error variance in normal or transformed space including sampling variance of the estimates and predication error from any crosswalks performed. First, variance was systematically

imputed if the data extraction did not include any measure of uncertainty. When some sample sizes for data were available, missing sample sizes were imputed as the 5th percentile of available sample sizes. Missing variances were then calculated as $\sigma_p^2 = \frac{p*(1-p)}{n}$ for proportions and using the global coefficient of variation for continuous exposures. When sample sizes were entirely missing and could not be imputed, the 95th percentile of available variances at the most granular geographic level (ie. first country, then region, etc.) were used to impute missing variances. For proportions where *p*n* or *(1-p)*n* is < 20, variance was replaced using the Wilson Interval Score method.

Next, if the exposure was modeled as a log transformation, the error variance was transformed into log-space using the delta method approximation as follows,

$$\sigma_p^2 \cong \frac{\sigma_{p\prime}^2}{p_{c,a,s,t}^2}$$

where $\sigma_{p\prime}^2$ represents the error variance in normal space. If the exposure was modeled as a logit transformation, the error variance was transformed into logit-space using the delta method approximation as follows,

$$\sigma_p^2 \cong \frac{\sigma_{p\prime}^2}{(p_{c,a,s,t} * (1 - p_{c,a,s,t}))^2}$$

Finally, prior to GPR, an approximation of non-sampling variance was added to the error variance. Calculations of non-sampling variance were performed on normal-space variances, and before GPR variances were again transformed using the delta method approximation, if necessary. Non-sampling variance was calculated as the variance of inverse-variance weighted residuals from ST at a given location level hierarchy. If there were fewer than 5 data points at a given level of the location hierarchy the non-sampling variance was replaced with that of the next highest geography level with more than 5 data points.

Estimating the covariance function

The final input into GPR is the covariance function, which defines the shape and distribution of the trends. Here, we have chosen the Matern-Euclidian covariance function, which offers the flexibility to model a wide spectrum of trends with varying degrees of smoothness. The function is defined as follows:

$$M(t, t') = \sigma^2 \frac{2^{1-v}}{\Gamma(\text{v})} \left(\frac{d(t, t')\sqrt{2v}}{l} \right)^v K_v \left(\frac{d(t, t')\sqrt{2v}}{l} \right)$$

where $d(\cdot)$is a distance function; σ^2, v, l, and K_v are hyperparameters of the covariance function—specifically σ^2 is the marginal variance, v is the smoothness parameter that defines the differentiability of the function, l is the length scale, which roughly defines the distance between which two points become uncorrelated, and K_v is the Bessel function. Based on previous applications of ST-GPR, we approximated σ^2 by $MADN(r'_{c,t})$, which is the normalized absolute deviation of the residuals from the smoothing step for each country, region, or super-region depending on the data coverage at a given location hierarchy level. Here, we have used the parameter specifications $v = 2$ and $l = 20$.

Prediction using GPR

Based on the specifications stated above, we integrated over $g_{c,t}(t_*)$ to predict the full time series for a given exposure for country c, age a, sex s, and the prediction time t_*:

$$log\left(p_{c,a,s}(t_*)\right) \sim N\left(m_{c,a,s,t}(t_*), \sigma_p^2 I + Cov\left(g_{c,a,s,t}(t_*)\right)\right)$$

$$logit\left(p_{c,a,s}(t_*)\right) \sim N\left(m_{c,a,s,t}(t_*), \sigma_p^2 I + Cov\left(g_{c,a,s,t}(t_*)\right)\right)$$

$$p_{c,a,s}(t_*) \sim N\left(m_{c,a,s,t}(t_*), \sigma_p^2 I + Cov\left(g_{c,a,s,t}(t_*)\right)\right)$$

Random draws of 1000 samples were obtained from the distributions above for every country for a given indicator. The final estimated mean for each country was the mean of the draws. In addition, 95% uncertainty intervals were calculated by taking the 2.5 and 97.5 percentile of the sample distribution. The entire modeling process was performed in log space and back-transformed to obtain final estimates in the original scale. The linear modeling process was implemented using the lmer4 package in R, and the ST-GPR analysis was implemented through the PyMC2 package in Python.

Subnational Scaling and Aggregation

To ensure consistency of the estimates between countries and their respective subnational locations, national estimates were either created by population-weighted aggregation or subnational estimates were adjusted by population-weighted scaling to the national estimates, depending on the data coverage of a given country compared to that of its subnational locations. For example, if there was better data coverage at the national level, relative to its corresponding subnational locations, for a given country and risk across age, sex, and time, estimates were raked to the national level. Conversely, if there was better data coverage at the subnational level, estimates for its parent country were created through population-weighted aggregation.

3. Estimate summary exposure values

In prior GBD studies, we did not report comparable exposure metrics for the risk factors included because of the complexity of quantifying polytomous and continuous risks. Because of this challenge, prior GBD studies have largely reported attributable deaths or DALYs in rates or numbers. For dichotomous exposures (tobacco or obesity), we previously published separate analyses of the trends in exposure to these risks.[24] Because of substantial interest in this type of exposure trend analysis, for the present analysis we generated a summary measure of exposure for each risk at step 3 of our analytical process (Figure 3). This summary measure, called the summary exposure value (SEV), is the risk-weighted prevalence of exposure. More formally, it is defined:

$$SEV = \frac{\sum_{i=1}^{n} Pr_i \, RR_i - 1}{RR_{max} - 1} \quad (3)$$

where Pr_i is prevalence of category i exposure; RR_i is relative risk of the category i; and RR_{max} is the maximum relative risk observed (between categories). This quantity is estimated for each age, sex, location, and year. In the case of dichotomous exposure, SEV is equal to prevalence. For continuous risks:

$$SEV = \frac{\int_{x=l}^{u} RR(x)\, P(x)\, dx - 1}{RR_{max} - 1} \quad (4)$$

where $P(x)$ is the density of exposure at level x of exposure; $RR(x)$ is relative risk of the level x; and RR_{max} is the highest relative risk that is supported by data and reflects a level where more than 1% of the population are exposed globally.

SEV takes the value zero when there is no excess risk for a population and takes the value 1 when the population is at the highest level of risk; we report SEV on a scale from 0% to 100% to emphasise that it is risk-weighted prevalence. Because risk exposure distributions can include individuals with extremely high levels of exposure that are often inflated by measurement error, and because few cohort studies provide valid relative risks at the highest level of exposure, we computed RR_{max} as the level for exposure with the highest relative risk supported by cohort or trial data and for which at least 1% or more of the global population is exposed. For comparison purposes, we have also computed age-standardised SEVs for every risk factor from the most detailed level using the GBD population standard.

4. Theoretical minimum-risk exposure level

In this and all previous GBD studies, the counterfactual level of risk exposure used is the risk exposure that is both theoretically possible and minimizes risk in the exposed population that consequently captures the maximum population attributable burden.[2] For each risk evaluated in GBD 2015, Step 4 of the analytical flowchart describes the use of the best available epidemiological evidence from published and unpublished relative risks by level of exposure and the lowest observed level of exposure from cohorts, used to select a single level of risk exposure that minimises risk from all causes of DALYs combined to establish the TMREL. In principle, the TMREL for a given risk may vary by age, sex, and location if supported by clear evidence. Based on the available evidence, the TMREL itself can be uncertain, which is reflected in the 95% uncertainty intervals (UIs) in Table 3. An estimation of uncertainty was derived by resampling from a uniform distribution of TMRELs where evidence supporting the selection of the TMREL was uncertain (for example, elevated systolic blood pressure or cholesterol).

Following substantive debate over the appropriate selection of TMREL for sodium, the UIs for the TMREL for sodium intake were widened for GBD 2013[26,27] but were not adjusted further for the present study. For other dietary risks, we used a two-step approach to determine the TMREL. First, we estimated the level of intake associated with the lowest risk for each outcome based on the published reports from cohorts and RCTs evaluating that risk-outcome pair. Then, we calculated the TMREL as the weighted average of these estimates. The weight was estimated by dividing the number of deaths due to each outcome by the total number of deaths from all the outcomes related to the exposure at the global level. Sufficient evidence has accumulated to justify adjusting the TMREL for bone mineral density (BMD) with age;[28] we used the 99th percentile of age-sex subgroups of the NHANES data to capture the decrease in bone density with age while also including the excess risk of fracture resulting from lower BMD in older age groups. For GBD 2015, we altered the TMREL for total cholesterol in light of new evidence from statin trials at low levels of cholesterol; a recent meta-analysis found that cardiovascular outcomes could be improved even at low levels of LDL-cholesterol, below 1.3 mmol/litre.[29] We used the strong correlation between LDL-cholesterol and total cholesterol to map the proposed LDL-cholesterol

TMREL of 0.7-1.3 mmol/litre to a TMREL for total cholesterol of 2.8-3.4 mmol/litre. We also revised the TMREL for PM2.5, previously set as the lowest 5th percentile of observed values in cohorts evaluated for GBD 2010 and GBD 2013; the publication of new cohorts led us to decrease the TMREL for particulate matter air pollution to 2.4-5.9 µg/m^3 from the value previously used (5.9-8.7 µg/m^3). There is insufficient evidence that risk exists below this new TMREL or that a lower level is achievable or even theoretically possible.

5. Estimate population attributable fractions

Risks are categorised on the basis of how exposure was measured: dichotomous, polytomous, and continuous. High total cholesterol is an example of a risk measured on a continuous scale. The population attributable fraction (PAF), which represents the proportion of risk that would be reduced in a given year if the exposure to a risk factor in the past were reduced to an ideal exposure scenario, is defined for a continuous risk factor as:[30]

$$PAF_{joasgt} = \frac{\int_{x=l}^{u} RR_{joasg}(x)P_{jasgt}(x)dx - RR_{joasg}(TMREL_{jas})}{\int_{x=l}^{u} RR_{joasg}(x)P_{jasgt}(x)dx}$$

Where PAF_{joasgt} is the population attributable fraction for cause o due to risk factor j for age group a, sex s, geography g, and year t. $RR_{joasg}(x)$ is the relative risk as a function of exposure level x for risk factor j for cause o, age group a, sex s, and geography g with the lowest level of observed exposure as l and the highest as u; $P_{jasgt}(x)$ is the distribution of exposure at x for age group a, sex s, geography g, and year t; $TMREL_{jas}$ is the TMREL for risk factor *j*, age group *a*, and sex *s*.

The PAF_{joasgt} for dichotomous and polytomous risk factors for every country is defined as:

$$PAF_{joasgt} = \frac{\sum_{x=1}^{u} RR_{joast}(x)P_{jasgt}(x) - RR_{joasg}(TMRE_{jas})}{\sum_{x=1}^{u} RR_{joas}(x)P_{jasgt}(x)}$$

Where PAF_{joasgt} is the population attributable fraction for cause o due to risk factor j for age group a, sex s, geography g, and year t. $RR_{joasg}(x)$ is the relative risk as a function of exposure level x for risk factor j for cause o, age group a, sex s, and geography g on a plausible range of exposure levels from l to u $P_{jasgt}(x)$ is the proportion of population in risk group (prevalence), for age group a, sex s, geography g, and year t; $TMREL_{jas}$ is the TMREL for risk factor *j*, age group *a*, and sex *s*.

6. Mediation

Summary

The portion of the burden of disease that is attributable to various combinations of risk factors or to all risk factors combined has been a topic of broad interest.[31] Assumptions about how one risk factor is mediated through other risk factors are needed in order to estimate the joint risk factor burden for combinations of metabolic risks and behavioural or environmental risks. To accomplish this, in Step 6 of the estimation process, for every two risk factors for an outcome, we used published studies to estimate the fraction of risk that was mediated through the other risk. This resulted in a matrix of parameters containing each possible pairing of risk factors included in the GBD 2015. Using this matrix, we computed the aggregated burden of disease at each level of the GBD 2015 hierarchy and for all risk factors using the following formula:

$$PAF_{Joasgt} = 1 - \prod_{j=1}^{J}\left(1 - PAF_{joasgt}\prod_{i=1}^{J}(1 - MF_{jio})\right) \quad (5)$$

where J is a set of risk factors for the aggregation; PAF_{joasgt} is the PAF for risk j for age group a, sex s, geography g, and year t; and MF_{jio} is the mediation factor for risk j mediated through i for cause o.

Additional detail

In GBD 2010 we only aggregated the burden of risk factors for some clusters of risks including access to improved water and sanitation, child and maternal malnutrition, tobacco smoking, alcohol use, dietary risk factors, occupational risk factors, and sexual abuse and violence. We did not aggregate air pollution and metabolic risk factors. In GBD 2013 and GBD 2015, we aggregated all risk factors into three large categories: behavioral, environmental and occupational, and metabolic risks -- as well as aggregating all GBD risk factors into a single attributable fraction for each diseases and eventually for all-causes of burden.

Aggregating risk factors at different levels share three essential challenges:

1. Risk factor coexistence or aggregation: for example, metabolic risk factors often occur together or high-risk behaviors are related such as drug abuse and unsafe sex.
2. Mediation: a risk factor may effect another risk factor that lies in the physiological pathway to a disease outcome. It can be inside a cluster of risk factors such as the effect of obesity through an increase in fasting plasma glucose (FPG) and later cardiovascular disease outcomes, or between clusters of risk factors such as the effect of fiber on cholesterol.
3. The formula to calculate the aggregated PAF.

The aggregation method is conceptually applicable to other aggregations such as socioeconomic factors, education, homelessness and refugee status that are being considered for inclusion in future GBD iterations. In the next section, we explain our approach to deal with these challenges.

There are three patterns of associations between risk factors to take into consideration. The first concerns confounding; risk B affects risk A and outcome C (Pattern 1 in *Figure. Patterns of associations between risk factors*). In these cases the relative risk (RR) for A should be adjusted for B, for example the fruit RR is adjusted for smoking. If part of the effect of A is through B, a mediator, we do not adjust the

effect of A for B. For example, we do not adjust the RR of body mass index (BMI) for cholesterol as cholesterol lies in the biological pathway between BMI and cardiovascular outcomes (Pattern 2 in in *Figure. Patterns of associations between risk factors*). The third pattern occurs when risks A and B are proxies of a third variable Z and aggregation aims to estimate the total effect of a latent variable Z, on C. An example is childhood undernutrition, which is measured by stunting, wasting, and underweight as proxies.

Figure. Patterns of associations between risk factors

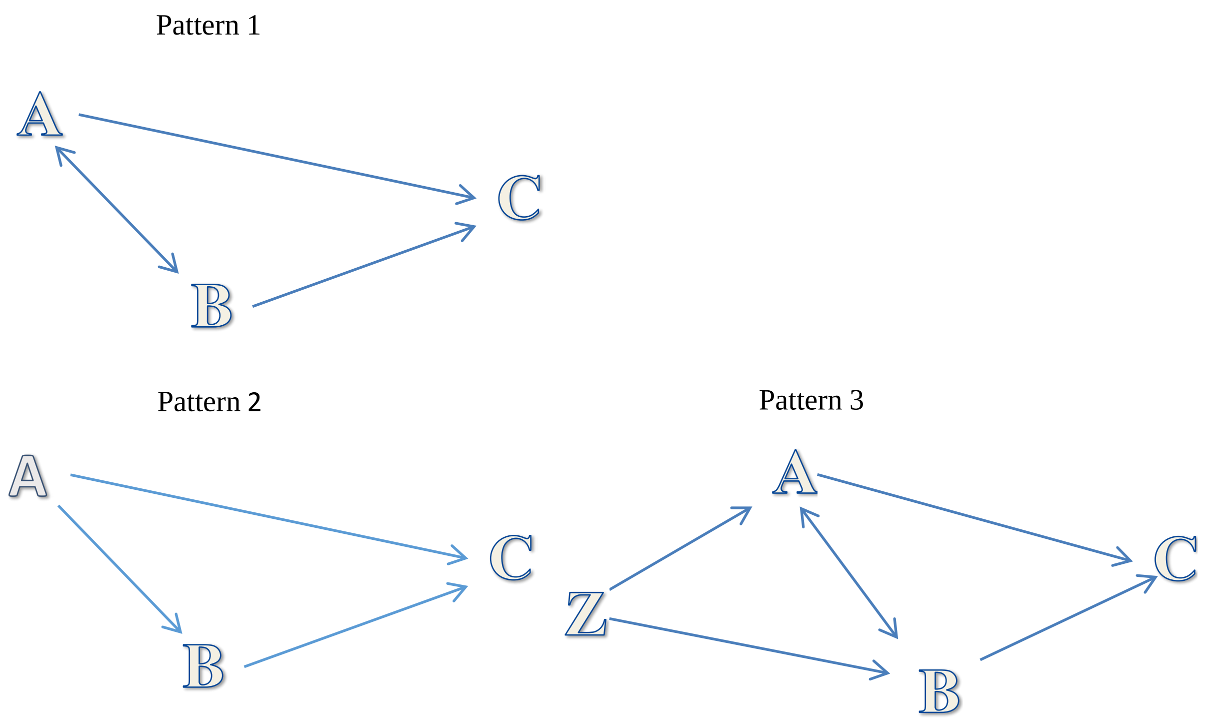

Calculating burden of multiple risk factors

Validation studies have reported congruency between the true risk associated with multiple risk factors affecting the same outcome and a multiplicative aggregation of the population attributable fractions of the individual risk factors (formula below).[32]

$$PAF_{1..i} = 1 - \prod_{i=1}^{n} (1 - PAF_i)$$

Where *PAF* is the population attributable fraction and *i* is each individual risk factor. The same validation studies also found that the overestimation from ignoring the covariance between risk factors is small. This was important to note as there are few data sources from which we can draw information on covariance.

We endeavored to evaluate RRs that were controlled for confounders. However, as we had to rely on the literature for many RRs we did not always have full control over the choice of confounders controlled for in each study.

Adjusting for mediation

When aggregating the effects of multiple risk factors, we included a mediation factor if a part of the effect of one risk factor was included in the effect estimated for in the mediator. First we prepared a list of possible mediations especially between metabolic risk factors and other risk factors. We found limited data primarily for these categories. We did not assume any mediation effect between risk factors for cancers except for sugar sweetened beverages and BMI.

Danaei and colleagues assumed that part of the effect of BMI on ischemic heart disease (IHD) is through high systolic blood pressure (SBP), cholesterol and FPG.[33] The proportion of the BMI effect that can be explained by other metabolic risk factors is the amount of mediation. The difference between the crude RR of BMI on IHD with the RR adjusted for SBP, FPG, and cholesterol reflects the amount of BMI effect on IHD that is mediated and already included in SBP, FPG, and cholesterol:

$$MF = \frac{RR_{crude} - RR_{adjusted}}{RR_{crude} - 1}$$

We used this approach for estimating mediation factors to adjust PAFs before aggregation.

$$MF = \frac{R_c^+ - R_a^+}{R_c^+ - R_c^-}$$

$$\text{So: } R_a^+ = R_c^+ - MF * (R_c^+ - R_c^-)$$

$$PAF_c = \frac{p * (R_c^+ - R_c^-)}{p * R_c^+ + (1-p) * R_c^-} = \frac{p * (R_c^+ - R_c^-)}{R_T}$$

If R_c^+: crude risk of outcome in exposed population

R_c^-: crude risk of outcome in non-exposed population

R_a^+: adjusted risk of outcome in exposed population

R_a^-: adjusted risk of outcome in non-exposed population

R_T is the overall rate of the outcome in the population. Since we are interested in the part which is from BMI but through cholesterol, the total risk in the population will be the same for the adjusted RR, so the unmediated part of the risk factor would be:

$$PAF_a = \frac{p*(R_a^+ - R_a^-)}{R_T} = \frac{p*(R_c^+ - MF*(R_c^+ - R_c^-) - R_c^-)}{R_T} = \frac{p*(R_c^+ - R_c^-)*(1-MF)}{R_T} = PAF_c * (1 - MF)$$

So for aggregating the PAF of multiple risk factors, we first calculated the part of the effect of every risk factor that is not mediated and then aggregated these assuming they are independent.

Therefore the aggregated PAF would be:

If MF is mediation factor of R2 through R1:

$$PAF_{1,2} = 1 - (1 - PAF_1) * \left(1 - PAF_2 * (1 - MF_{2/1})\right)$$

and a generalization for multiple pathways of R1 through other RFs:

$$PAF_{1..i} = 1 - \prod_{i=1}^{n}\left(1 - PAF_i * \left(1 - \prod_{j=1}^{n}(1 - MF_{i/j})\right)\right)$$

For every risk factor outcome pair, the matrix of possible mediations was calculated and used. In the example the matrix of mediation when we aggregate BMI, cholesterol, FPG, and SBP would be:

Table. Example mediation matrix for BMI, cholesterol, FPG, and SBP

	BMI	Cholesterol	FPG	SBP
**BMI**	0	0.111	0.148	0.296
**Cholesterol**	0	0	0	0
**FPG**	0	0	0	0
**SBP**	0	0	0	0

Calculating mediation factor

1 – Comparing crude RR versus mediator-adjusted RR

The best example is the mediation of BMI through SBP, FPG, and cholesterol reported by Danaei et al.[33] In their meta-analysis, they report the adjusted and unadjusted RR of BMI on IHD and stroke based on combined data from individual cohorts. They calculated the mediation factor using equation 4 and we used it directly as mediation factor in risk factor aggregation.

For some risk factor aggregations we just simply added PAFs. For example, the total burden of smoking including smoking and secondhand smoke is the sum of the estimates of the individual risks because we estimate the burden of secondhand smoke in non-smokers only.

2 – Estimating the mediation factor by pathway of the effect

For many other risk factors there are no data available to use the first method. Instead, we searched studies to estimate the effect of the risk factor (for example fruit) on the mediator (SBP) and finally the expected increase in IHD risk. We pooled available studies to calculate the unit increase in the mediator per unit increase in the risk factor to calculate the size of the IHD RR.

Figure. Example of pathway between fruit, high systolic blood pressure, and cardiovascular diseases

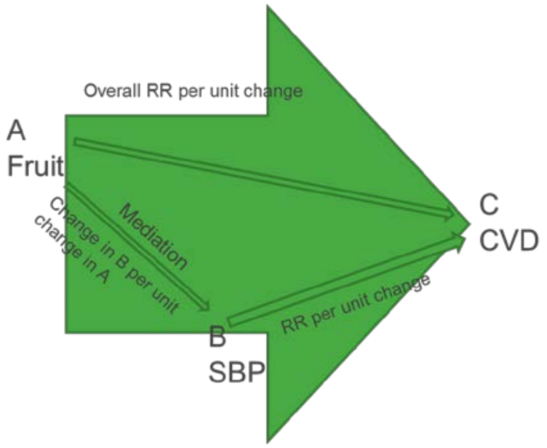

We have RRs for the effect of A on C and B on C in GBD from a meta-analysis of studies in the literature. The effect of A on B was estimated by analysis of diet trials.

$$RR_{ABC} = RR_{BC}{}^{\Delta_{AB}}$$

RR_{ABC} is expected effect of A through B on C

RR_{BC} is relative risk of each unit increase in mediator on outcome C

Δ_{AB} is change in mediator level B per each unit change in A

If RR_{AB} is the overall effect of A on B then:

The mediation factor would be

$$MF = \frac{RR_{ABC} - 1}{RR_{AB} - 1}$$

We kept uncertainty of each parameter by generating and following 1,000 draws of the estimates to calculate 1,000 draws of the posterior distribution of the mediation factor. We did not include risk-mediator pairs if the mediation factor was not significant at 5% level (more than 50 out of 1,000 draws were negative). We truncated the mediation factor distribution at 1 where the whole effect of the risk factor on the outcome would be assumed to be through the mediator pathway.

Some mediation factors equal 1 where the whole effect was calculated through other risk factor e.g. the effect of sugar-sweetened beverages through BMI or salt through SBP or when we assumed other risk factors are sources of the exposure, for example fiber is provided by consuming fruit, vegetable, and whole grains.

Dietary risk factors

We searched for diet trials that reported change in SBP, cholesterol, and FPG by change in dietary risk factors, for example fruit and vegetables. We did a systematic search to find clinical trials that reported the baseline values or change in diet levels. We also searched for a list of important hypothetical mediations primarily through metabolic risk factors because of the great burden of metabolic risk factors and a need to aggregate and control for double-counting of the burden, especially for cardiovascular diseases (CVD).

Considering that outcome of metabolic changes such as SBP and cholesterol are measured objectively (compared with subjective measurements that might be affected by patient or physician knowledge about the intervention group), and there are no issues with blinding and analytical concerns like type of analysis (intention-to-treat or per-protocol), we think they provide a sufficient data on the short-term effect of diet on metabolic risk factors.

Long-term effects are more difficult to capture, given that there is little data available on long-term effects in the literature. Future analysis of cohort studies will be necessary to understand the long-term effects of diet on metabolic risk factors.

We modeled change in a given mediator (e.g. cholesterol) per unit change in diet components. The best possible approach would be controlling for other dietary changes, but it is not possible because of few data points and uncertainty levels for both diet and metabolic risk change. With a limited number of studies providing data points for the analysis and no access to micro-data from diet trials, it is not possible to control for other diet components.

In cases in which there were very few data points, such as for unsaturated fatty acids and trans fats, or if we could not find trials, mediations were excluded. Also, BMI was excluded because our diet analyses are adjusted for a 2,000 calorie diet, thereby addressing mediation through BMI and obesity.

We did not include possible mediation/interaction of diet with many other risk factors and outcomes besides metabolic risks. Fruit and vegetables could have interaction with smoking and possibly air pollution on cancers, but we did not identify sufficient evidence for such an analysis. We assumed all effects of fiber are captured in fruit, vegetables, whole grain and nuts and seeds, so we assumed complete mediation.

In the case of fibre, the mediation is counted as one mechanism of producing covariance between risk factors, and the calculation depends on the concept and direction of mediation. To be consistent with the methodology employed in the GBD, we must aggregate and avoid double-counting of burden and we should control covariance. Covariance might be with or without interaction and mediation is one way of subsequent double-counting of the burden. We think that through mediation analysis we are able to quantify non-random and biologically plausible covariance and improve risk factor aggregation.

Physical activity

We found cohort studies on the effect of physical activity on FPG. The data was more on the effect of physical activity on diabetes incidence, so we calculated the shift in FPG using the provided RR value. We used this to calculate the mediated part of effect of physical activity on CVD.[34–40]

Air pollution

We looked for cohort and time series studies but the data were limited. We found only one study with the effect of last year average of particle pollution (PM) 2.5 on SBP, FPG and cholesterol.[41] However, the effects through FPG and cholesterol were bigger than the effect expected for that level of PM2.5, indicating significant overestimation of the mediation. We found time series studies with different PM2.5 lag (by day) that show very short-term and confounded effects. So we decided to add this when stronger evidence is available.

Assumed mediations

For the risk factors with PAFs of 100% such as FPG and diabetes, low estimated glomerular filtration rate and chronic kidney disease, hypertension and hypertensive heart disease, alcohol and alcohol disorders, childhood underweight and protein-energy malnutrition, and childhood wasting and protein-energy malnutrition, and drug use and drug use disorders, no mediation is needed.

3 – Piecewise aggregation (Pattern 3)

There are three anthropometric indicators that are highly correlated: childhood underweight, stunting, and wasting, as demonstrated in *Figure. Venn diagram demonstrating the correlation between childhood underweight, stunting, and wasting*. Available RRs for each indicator are not adjusted for the other two because there is a high correlation between these indicators and also interaction where the majority of the burden occurs. Estimating the total burden due to undernutrition, a latent variable, is difficult. The three anthropometric indicators are not independent, so the covariance between them should be considered. This was the main reason that GBD 2010 only included childhood underweight. If covariance between these indicators is significant (as is shown in the Figure below), aggregating these indicators assuming independence would overestimate the total burden significantly.

To use the best available data, we adjusted observed RRs reported by Olofin et al for underweight, stunting and wasting by simulating the joint distribution of the three indicators using the distribution of each indicator and covariance between indicators in the countries included in the meta-analysis (extracted from Demographic and Health Survey (DHS) micro-data).[42] Based on the analysis done by McDonald et al, we assumed there is an interaction between the three indicators, and extracted the interaction terms from the corresponding analysis.[43] We calculated the adjusted RRs by minimizing the error between observed crude RRs (from meta-analysis) and expected crude RRs derived from adjusted RRs. To use the best available data, we adjusted observed RRs reported by Olofin et al for underweight, stunting and wasting by simulating the joint distribution of the three indicators using the distribution of each indicator and covariance between indicators in the countries included in the meta-analysis (extracted from Demographic and Health Survey (DHS) micro-data). [42] Based on the analysis done by McDonald et al, we assumed there is an interaction between the three indicators, and extracted the interaction terms from the corresponding analysis. [43] We calculated the adjusted RRs by minimizing the error between observed crude RRs (from meta-analysis) and expected crude RRs from adjusted RRs, interaction terms, and joint distribution of the risk factors.

Figure. Venn diagram demonstrating the correlation between childhood underweight, stunting, and wasting

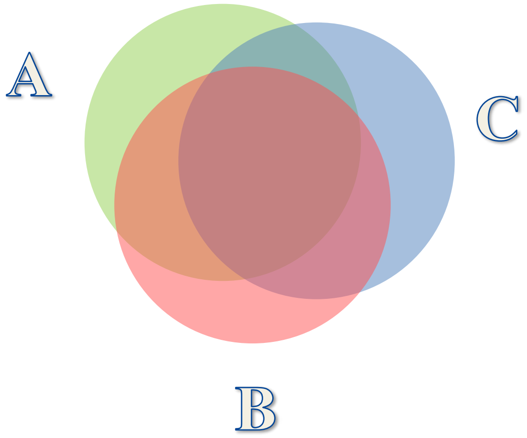

After adjusting for the three risk factors, we calculated the PAFs and aggregated underweight, stunting and wasting burden.

Uncertainty of aggregated and mediated PAFs

We generated 1000 draws of posterior distribution of mediation factor calculated by different methods to use beside draws of other inputs to the PAF aggregation.

Important assumptions in aggregating risk factors and including mediation

1 – The mediation factors or PAF adjustments are similar across countries, age, sex, and years. While it is quite likely that the size of mediation is different in different populations, there is little data to inform the covariance between different risk factors or the mediation factor amount by age and countries. For example in some countries, the size of the mediated BMI-IHD PAF through cholesterol, calculated by the mediation factor, was even bigger than the total burden of cholesterol, indicating that less effect of BMI is mediated through cholesterol and mediation factors are not similar across countries.

2 – For many risk-mediator-outcome pairs, there are no data available, so we assumed the mediation is zero.

3 – Since the covariance between undernutrition indicators is different by countries (and across time, results were not reported), and there is an interaction between these indicators, the total burden might be underestimated.

4 – It is assumed that there is no significant covariance between PAFs, which might not be true between some risk factors such as between metabolic risk factors. While this overestimation is controlled by using adjusted RRs, using crude RRs for BMI and other metabolic risk factors may cause significant overestimation of aggregated metabolic risks burden.

7. Estimate attributable burden

Four key components are included in estimation of the burden attributable to a given risk factor: the metric of burden being assessed (the number of deaths, years of life lost [YLLs], years lived with disability [YLDs], or DALYs [the sum of YLLs and YLDs]); the exposure levels for a risk factor; the relative risk of a given outcome due to exposure; and the counterfactual level of risk factor exposure. Estimates of attributable burden as DALYs for risk-outcome pairs were generated using the following model:

$$AB_{jasgt} = \sum_{o=1}^{w} DALY_{joasgt}\, PAF_{joasgt}$$

where AB_{jasgt} is the attributable burden for risk factor j for age group a, sex s, geography g, and year t; $DALY_{joasgt}$ is total DALYs for cause o (of w relevant outcomes for risk factor j) for age group a, sex s, geography g, and year t; PAF_{joasgt} is the population attributable fraction (PAF) for cause o due to risk factor j for age group a, sex s, geography g, and year t. The proportion of deaths, YLLs, or YLDs attributable to a given risk factor or risk factor cluster were analogously computed by sequentially substituting each metric in place of DALYs in the equation above.

Other analysis: Decomposition of deaths and DALYs

We conducted two related decomposition analyses of changes in DALYs from 1990 to 2015: (1) decomposing changes in cause-specific DALYs due to changes in population growth, population age structure, exposure to all risks for a disease, and risk-deleted death and DALY rates; and (2) decomposing changes in risk-attributable all-cause DALYs due to changes in population growth, population age structure, risk exposure to the single risk factor, and risk-deleted DALY rates. In this case, risk-deleted rates are the rates after removing the effect of a risk factor or combination of risk factors; in other words, observed DALY rates multiplied by one minus the PAF for the risk or set of risks. Our decomposition analyses draw from methods developed by Das Gupta[44] to provide a computationally tractable solution to isolating drivers of burden changes whereby all combinations of possible pathways are averaged across factors. Both the total burden and the attributable burden are determined, following the methods of Das Gupta, as a product of four factors such that:

$$T_{asgt} = \left(A_{sgt}\, B_{asgt}\, C_{asgt}\, D_{asgt}\right)$$

where T_{asgt} represents either the total burden or the attributable burden at year t; $A_{sgt}$is the all-age population size for a given sex s and geography g at year t; $B_{asgt}$is the proportion of the population in the age group for a given age group a, sex s and geography g at year t; C_{asgt} is the underlying rate of the outcome unrelated to the risk factor or observed rate, multiplied by $1 - PAF$ for a given age group a, sex s and geography g at year t; and where D_{asgt} is the ratio of total burden (or attributable burden) to the underlying rate, which reflects the risk effect for a given age group a, sex s, and geography g at year t defined as $1/(1 - PAF)$ in the case of total burden or as $PAF/(1 - PAF)$ in the case of decomposing attributable burden to a risk. The contribution of each factor to total change in either total burden or attributable burden was determined by changing the level of one factor from time t_0 to t_1 – here 1990 to 2015 – with all other factors held constant. Thus, the effect of any of the four factors, for example A_{sgt}on the change of total burden between 1990 (A_{90}) and 2015 (A_{15}) is calculated as:

$$E_A = (A_{15} - A_{90})\left(\frac{B_{90}C_{90}D_{90}+B_{15}C_{15}D_{15}}{4} + \frac{B_{90}C_{90}D_{15}+B_{90}C_{15}D_{90}+B_{15}C_{90}D_{90}+B_{15}C_{15}D_{90}+B_{15}C_{90}D_{15}+B_{90}C_{15}D_{15}}{12}\right)$$

Where E_A is the proportion of change due to factor A, and the subscripts for each factor in the equation denote the year for each estimate. Since the effect depends on the order of entry of the factor, we calculated the average of all combinations of the four factors.[44]

This four factor decomposition method does not work for risks where the PAF, by definition, is 100% (such as high fasting plasma glucose and diabetes) or where the PAF is directly estimated (such as for unsafe sex and HIV). In the cases of childhood underweight and protein-energy malnutrition, childhood wasting and protein-energy malnutrition, vitamin A deficiency and vitamin A deficiency, alcohol use and cirrhosis and other chronic liver diseases due to alcohol use, alcohol use and alcohol use disorders, alcohol use and liver cancer due to alcohol use, drug use and drug use disorders, iron deficiency and iron-deficiency anemia, and low glomerular filtration rate and chronic kidney disease, we used a three factor decomposition method, which examines the contribution of population, ageing, and risk exposure. Effectively, we assume trends in these cases are driven by exposure, not change in the risk deleted rates. For FPG and diabetes, we used GBD estimates of the prevalence of diabetes and the excess DALY rate for each prevalent case of diabetes to decompose trends in diabetes into the contribution of the four factors. We were not able to include three outcomes in this analysis: cervical cancer, sexually transmitted diseases excluding HIV, and HIV/AIDS.

Other analysis: Socio-demographic Index (SDI) analysis & Epidemiological Transition

a. Development of revised SDI indicator

We began exploring the relationship between a composite indicator of socio-demographic development in GBD 2013 DALYs. We used lag distributed income per capita (LDI), average educational attainment over the age 15 years, total fertility rate (TFR), and mean population age and called it SDS, socio-demographic status. In response to feedback, we excluded mean population age due its strong relationship to mortality rates. We renamed the indicator Socio-demographic Index (SDI). SDI has an interpretable scale: zero represents the lowest income per capita, lowest educational attainment, and highest TFR observed across all GBD geographies from 1980 to 2015 and one represents the highest income per capita, highest educational attainment, and lowest TFR.

SDI was calculated using the Human Development Index (HDI) methodology, wherein an index value was determined for each covariate input (log LDI, average educational attainment in the population over age 15, and TFR):

$$I_{Cly} = \frac{C_{ly} - \min(C)}{\max(C) - \min(C)} \; {}^{(C_{ly} - \min(C))}\!\Big/_{(\max(C) - \min(C))}$$

Where I_{cly} – the index for covariate *C*, location *l*, and year *y* – is equal to the difference between the value of that covariate in that location-year and the minimum observed value of the covariate (min(C)) in any location over the 1980-2015 time interval divided by the observed range (max(C)-min(C)). An additional innovation for GBD 2015 was to incorporate subnational locations where estimated (resulting in 519 unique administrative units) for the entire estimation period of 1980-2015. The Socio-Demographic Index is then the geometric mean of these three indices:

$$SDI = \sqrt[3]{I_{lnLDI} I_{educ} I_{TFR}}$$

In our mortality analyses, for LDI and TFR, we noted depreciating gains in life expectancy at birth and 5q0 at the higher and lower terminals, respectively. Due to the significance of these values in indexing, we aimed to identify the point at which increasing income or reducing fertility no longer resulted in improved child mortality or life expectancy. We tested various restrictions, and found that capping LDI at $60,000 and setting a TFR floor at 1 resulted in improved correlations with the resultant health indicators.

We further aimed to validate the use of SDI by regressing it in a variety of forms against life expectancy at birth, 5q0, 35q15, and 20q50. We found that SDI generally is as capable of predicting these demographic indicators as the previous SDS, and also as the inputs. We also found that in incorporating year, we did not substantially reduce the coefficients for SDI. Additionally, in testing lags of 2-10 years, we found the version with no lag to be the most predictive. Appendix Table 5 has SDI values by GBD geography over time and illustrates these results more in-depth.

b. Age-sex-specific relationships between SDI and SEVs

In order to evaluate the relationship between SDI and SEVs, we fit a simple least-squares regression using a smoothing spline on SDI for every cause in levels 1, 2, and 3 of the GBD cause hierarchy:

$$\ln(y_{l,y,a,s,c}) = \sum_{i=0}^{d+k} \beta_i B_{i,d}(SDI) + \gamma_U + \gamma_E + \gamma_C + \gamma_O + \varepsilon_{l,y,a,s,c}$$

where:

- $\ln(y_{l,y,a,s,c})$ is the logit SEV in location *l* and year *y*, and for age *a*, sex *s*, and cause *c*
- $\sum_{i=0}^{d+k} \beta_i B_{i,d}(SDI)$ is resultant parametric curve, of degree *d* and interior knots *k*, of a linear combination of basis splines $B_{i,d}(SDI)$
- γ_U is a dummy variable for the United States
- γ_E is a dummy variable for the GBD region Eastern Europe
- γ_C is a dummy variable for the GBD region Central Asia
- γ_O is a dummy variable for the GBD region Oceania
- $\varepsilon_{l,y,a,s,c}$ is the error term for location *l,* year *y*, age *a*, sex *s*, and cause *c*

Regressions were run separately by age, sex, and cause, using all location-years. Dummy variables were included for locations that were identified in modeling to skew fit due to significant deviation from levels of morbidity observed elsewhere at similar levels of SDI. In the case of the United States because of the inclusion of 50 states, the US collectively had an undue influence on the shape of the relationship which is why a separate dummy variable was included for the US. Because of the mortality crisis in Eastern Europe and Central Asia after the collapse of the Soviet Union, we included a dummy variable to adjust for the mean difference in these regions.

Having a complete set of age specific SEVs, we were then able to produce a full set of age-standardized rates for every SDI level. We evaluated this relationship at each centile value of SDI (i.e., by increments of 0.01). The SDI ranged from 0.060 in Mozambique in 1987 to 0.978 in the District of Columbia, United States in 2015.

We used the same modeling set up but used the logit of the share of population in each age-group as the dependent variable to estimate a smoothed relationship between population age-structure and SDI. Predictions for each age-group at each level of SDI were rescaled to sum to 100%.

References

1 Murray CJ, Lopez AD. Global mortality, disability, and the contribution of risk factors: Global Burden of Disease Study. *Lancet* 1997; **349**: 1436–42.

2 Murray CJ, Lopez AD. On the comparable quantification of health risks: lessons from the Global Burden of Disease Study. *Epidemiol Camb Mass* 1999; **10**: 594–605.

3 GBD 2013 Risk Factors Collaborators, Forouzanfar MH, Alexander L, *et al.* Global, regional, and national comparative risk assessment of 79 behavioural, environmental and occupational, and metabolic risks or clusters of risks in 188 countries, 1990-2013: a systematic analysis for the Global Burden of Disease Study 2013. *Lancet Lond Engl* 2015; **386**: 2287–323.

4 Stevens GA, Alkema L, Black RE, *et al.* Guidelines for Accurate and Transparent Health Estimates Reporting: the GATHER statement. *Lancet Lond Engl* 2016; published online June 28.

5 Food, nutrition, physical activity and the prevention of cancer: a global perspective. Washington, D.C: World Cancer Research Fund & American Institute for Cancer Research, 2007.

6 Singh GM, Danaei G, Farzadfar F, *et al.* The age-specific quantitative effects of metabolic risk factors on cardiovascular diseases and diabetes: a pooled analysis. *PloS One* 2013; **8**: e65174.

7 Law MR, Morris JK, Wald NJ. Use of blood pressure lowering drugs in the prevention of cardiovascular disease: meta-analysis of 147 randomised trials in the context of expectations from prospective epidemiological studies. *The BMJ* 2009; **338**. DOI:10.1136/bmj.b1665.

8 GBD 2015 Diseases and Injury Incidence and prevalence Collaborators. Global, regional, and national incidence, prevalence, and years lived with disability (YLDs) for 310 acute and chronic diseases and injuries, 1990-2015: a systematic analysis for the Global Burden of Disease Study 2015. *Lancet Rev*.

9 World Health Organization. WHO Global Urban Ambient Air Pollution Database (update 2016). 2016. http://www.who.int/phe/health_topics/outdoorair/databases/cities/en/.

10 van Donkelaar A, Martin RV, Brauer M, *et al.* Global Estimates of Fine Particulate Matter using a Combined Geophysical-Statistical Method with Information from Satellites, Models, and Monitors. *Environ Sci Technol* 2016; **50**: 3762–72.

11 GEOS-Chem Model. http://acmg.seas.harvard.edu/geos/.

12 Brauer M, Freedman G, Frostad J, *et al.* Ambient Air Pollution Exposure Estimation for the Global Burden of Disease 2013. *Environ Sci Technol* 2016; **50**: 79–88.

13 Ezzati M, Lopez AD. Regional, disease specific patterns of smoking-attributable mortality in 2000. *Tob Control* 2004; **13**: 388–95.

14 American Cancer Society. Cancer Prevention Study II (CPS II). http://www.cancer.org/research/researchtopreventcancer/currentcancerpreventionstudies/cancer-prevention-study.

15 Gmel G, Rehm J. Measuring Alcohol Consumption. *Contemp Drug Probl* 2004; **31**: 467.

16 Rehm J, Klotsche J, Patra J. Comparative quantification of alcohol exposure as risk factor for global burden of disease. *Int J Methods Psychiatr Res* 2007; **16**: 66–76.

17 World Health Organization. Global Information System on Alcohol and Health. http://apps.who.int/gho/data/?showonly=GISAH&theme=main.

18 Ng M, Liu P, Thomson B, Murray CJL. A novel method for estimating distributions of body mass index. *Popul Health Metr* 2016; **14**: 6.

19 Ng M, Fleming T, Robinson M, *et al.* Global, regional, and national prevalence of overweight and obesity in children and adults during 1980–2013: a systematic analysis for the Global Burden of Disease Study 2013. *The Lancet* 2014; **384**: 766–81.

20 Clarke R, Shipley M, Lewington S, *et al.* Underestimation of risk associations due to regression dilution in long-term follow-up of prospective studies. *Am J Epidemiol* 1999; **150**: 341–53.

21 Kiyono P. An Integrative Metaregression Framework for Descriptive Epidemiology, 1 edition. Seattle: University of Washington Press, 2015.

22 Rasmussen CE, Williams CKI. Gaussian Processes for Machine Learning. MIT Press, 2006.

23 Vasudevan S, Ramos F, Nettleton E, Durrant-Whyte H. Gaussian process modeling of large-scale terrain. *J Field Robot* 2009; **26**: 812–40.

24 Ng M, Freeman MK, Fleming TD, *et al.* Smoking prevalence and cigarette consumption in 187 countries, 1980-2012. *JAMA* 2014; **311**: 183–92.

25 Ng M, Fleming T, Robinson M, *et al.* Global, regional, and national prevalence of overweight and obesity in children and adults during 1980-2013: a systematic analysis for the Global Burden of Disease Study 2013. *Lancet Lond Engl* 2014; **384**: 766–81.

26 Committee on the Consequences of Sodium Reduction in Populations, Food and Nutrition Board, Board on Population Health and Public Health Practice, Institute of Medicine. Sodium Intake in Populations: Assessment of Evidence. Washington (DC): National Academies Press (US), 2013.

27 O’Donnell M, Mente A, Rangarajan S, *et al.* Urinary sodium and potassium excretion, mortality, and cardiovascular events. *N Engl J Med* 2014; **371**: 612–23.

28 Warming L, Hassager C, Christiansen C. Changes in bone mineral density with age in men and women: a longitudinal study. *Osteoporos Int J Establ Result Coop Eur Found Osteoporos Natl Osteoporos Found USA* 2002; **13**: 105–12.

29 Boekholdt SM, Hovingh GK, Mora S, *et al.* Very low levels of atherogenic lipoproteins and the risk for cardiovascular events: a meta-analysis of statin trials. *J Am Coll Cardiol* 2014; **64**: 485–94.

30 Vander Hoorn S, Ezzati M, Rodgers A, Lopez AD, Murray CJL. Estimating attributable burden of disease from exposure and hazard data. In: Comparative Quanitifcation of Health Risks: Global and regional burden of disease attribution to selected major risk factors. World Health Organisation, 2004: 2129–40.

31 Preston SH. Causes and Consequences of Mortality Declines in Less Developed Countries during the Twentieth Century. In: Population and economic change in developing countries. Chicago: Univ. of Chicago Pr, 1980: 289–360.

32 Carnahan E, Lim SS, Nelson EC, *et al.* Validation of a new predictive risk model: measuring the impact of major modifiable risks of death for patients and populations. *The Lancet* 2013; **381**: S26.

33 Danaei G, Singh GM, Paciorek CJ, *et al.* The global cardiovascular risk transition: associations of four metabolic risk factors with national income, urbanization, and Western diet in 1980 and 2008. *Circulation* 2013; **127**: 1493–502, 1502-8.

34 Nieman DC, Brock DW, Butterworth D, Utter AC, Nieman CC. Reducing diet and/or exercise training decreases the lipid and lipoprotein risk factors of moderately obese women. *J Am Coll Nutr* 2002; **21**: 344–50.

35 Tjønna AE, Lee SJ, Rognmo Ø, *et al.* Aerobic interval training versus continuous moderate exercise as a treatment for the metabolic syndrome: a pilot study. *Circulation* 2008; **118**: 346–54.

36 Snyder KA, Donnelly JE, Jabobsen DJ, Hertner G, Jakicic JM. The effects of long-term, moderate intensity, intermittent exercise on aerobic capacity, body composition, blood lipids, insulin and glucose in overweight females. *Int J Obes Relat Metab Disord J Int Assoc Study Obes* 1997; **21**: 1180–9.

37 Nordby P, Auerbach PL, Rosenkilde M, *et al.* Endurance training per se increases metabolic health in young, moderately overweight men. *Obes Silver Spring Md* 2012; **20**: 2202–12.

38 Christiansen T, Paulsen SK, Bruun JM, Pedersen SB, Richelsen B. Exercise training versus diet-induced weight-loss on metabolic risk factors and inflammatory markers in obese subjects: a 12-week randomized intervention study. *Am J Physiol Endocrinol Metab* 2010; **298**: E824-831.

39 Coggan AR, Kohrt WM, Spina RJ, Bier DM, Holloszy JO. Endurance training decreases plasma glucose turnover and oxidation during moderate-intensity exercise in men. *J Appl Physiol Bethesda Md 1985* 1990; **68**: 990–6.

40 Arsenault BJ, Côté M, Cartier A, *et al.* Effect of exercise training on cardiometabolic risk markers among sedentary, but metabolically healthy overweight or obese post-menopausal women with elevated blood pressure. *Atherosclerosis* 2009; **207**: 530–3.

41 Chuang K-J, Yan Y-H, Chiu S-Y, Cheng T-J. Long-term air pollution exposure and risk factors for cardiovascular diseases among the elderly in Taiwan. *Occup Environ Med* 2011; **68**: 64–8.

42 Olofin I, McDonald CM, Ezzati M, *et al.* Associations of suboptimal growth with all-cause and cause-specific mortality in children under five years: a pooled analysis of ten prospective studies. *PloS One* 2013; **8**: e64636.

43 McDonald CM, Olofin I, Flaxman S, *et al.* The effect of multiple anthropometric deficits on child mortality: meta-analysis of individual data in 10 prospective studies from developing countries. *Am J Clin Nutr* 2013; **97**: 896–901.

44 Das Gupta P. Standardization and Decomposition of Rates: A User's Manual. Washington D.C.: U.S. Bureau of the Census, 1993.

Section 3. Risk-specific estimation

The risk-specific modeling write-ups follow the order of the risk factor hierarchy for GBD 2015. In some cases, multiple risk factors are addressed in a single write-up, for example childhood underweight, wasting, and stunting are all included in a single detailed write-up.

Unsafe Water Capstone Appendix

Flowchart

Unsafe Drinking Water

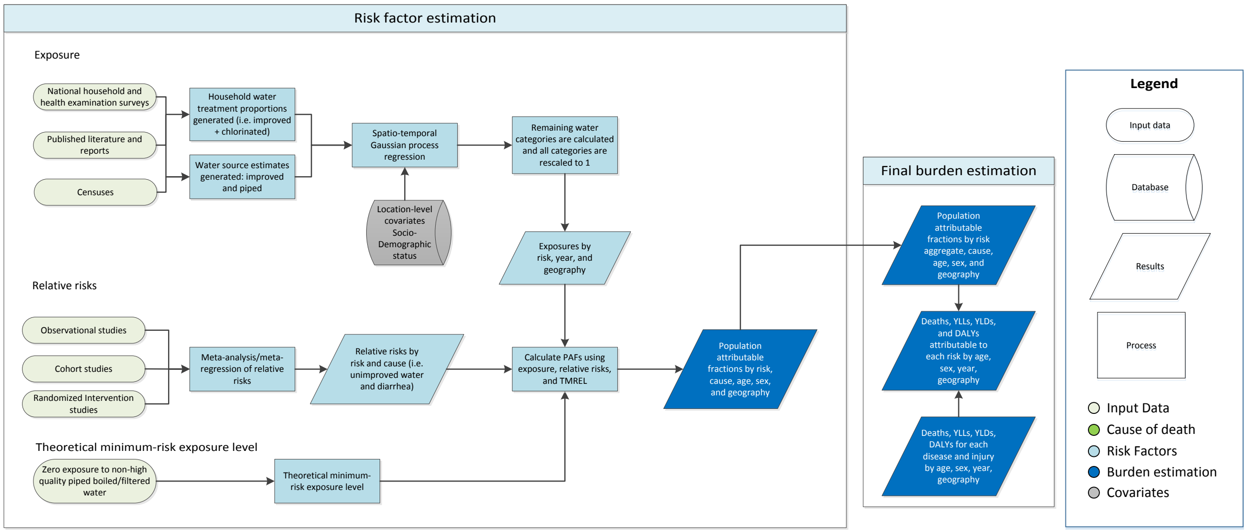

Input Data & Methodological Summary

Exposure

Case Definition

For GBD 2015, exposure to unsafe water is defined based on reported primary water source used by the household and use of household water treatment (HWT) to improve the quality of drinking water before consumption. Water sources were defined as improved based on the JMP designation (The WHO), which includes piped water as improved water, and households with access to piped water connection to the house, yard, or plot were defined as having access to piped water supply. Solar treatment, chlorine treatment, boiling, or the use of filters were all assumed to be effective point-of-use household water treatments, and based on effect sizes published by Wolf et al. (2014) boiling or filtering was the most effective form of water treatment.

Input Data

The search for usable household surveys and censuses was conducted using the Global Health Data Exchange (GHDx) database. All surveys through December 2015 that provide household level micro-data on water source were added. Tabulated and report data was lower priority and was only updated when time permitted. HWT input data was limited to two large survey series (DHS and MICS) due to time constraints. An update to HWT input data is a top priority for estimating exposure to unsafe water in future iterations.

Modeling

Water source data is modeled in two distinct categories: household prevalence of improved water and household proportion of piped water within improved population in order to prevent the population with

access to piped water from exceeding the population with access to improved water (which includes piped). HWT is modeled in 6 distinct categories based on the 3 water treatment categories (filtered/boiled, solar/chlorine, or untreated) and 2 water source categories (piped or improved). We have made no substantive changes in the modeling strategy from GBD 2013. By year and geography, each of the above categories are modeled using a 3-step modeling scheme of mixed effect linear regression followed by spatio-temporal Gaussian process regression (ST-GPR), which outputs full time series estimates for each GBD 2015 location. Socio-demographic status (SDS), an index metric that includes a measure of education and income level, was used as a fixed effect in the linear regression since it proved to have significant coefficients. Random effects were placed at GBD 2015 region and super-region levels.

The process of vetting and validating models was accomplished primarily through an examination of ST-GPR scatter plots by GBD 2015 location from 1990-2015. Any unfitting data points were re-inspected for error at the level of extraction and survey implementation, and subsequently excluded from analysis if deemed appropriate. In addition to SDS, a number of different potential fixed effects were considered, including lag-distributed income and urbanicity, but SDS proved to be the strongest predictor of unsafe water. Uncertainty in the estimates was initially formed based on standard deviation by survey, then propagated through ST-GPR modeling by means of confidence intervals around each data point that reflect the point-estimate specific variance.

Once models are fully vetted, full time series outputs from ST-GPR modeling are then converted from proportion to prevalence by year and geography and then rescaled to form 9 mutually exclusive categories that sum up to 1. The table below provides the final result of this rescaling.

Category	*Definition*
Unimproved, no HWT	Proportion of households that use unimproved source, and *do not* use any HWT to purify their drinking water.
Unimproved, chlorine/solar	Proportion of households that use unimproved source, and solar or chlorine treatment to purify their drinking water.
Unimproved, boil/filter	Proportion of households that use unimproved source, and boil or filter to purify their drinking water.
Improved water except piped, no HWT	Proportion of households that use improved sources other than piped water supply, and *do not* use any HWT to purify their drinking water.
Improved water except piped, chlorine/solar	Proportion of households that use improved sources other than piped water supply, and use solar or chlorine treatment to purify their drinking water.
Improved water except piped, boil/filter	Proportion of households that use improved sources other than piped water supply, and boil/filter their drinking water.
Piped water, no boil/filter	Proportion of households that use piped water supply, and *do not* use any HWT to purify their drinking water
Piped water, chlorine/solar	Proportion of households that use piped water supply, and *use* solar or chlorine water treatment to purify their drinking water.

Piped water, boil/filter	Proportion of households that use piped water supply, and boil or filter to purify their drinking water

Due to the nature of modeling piped water exposure as a proportion of total improved water access, we are limited in only using sources for piped water that also include total improved water values. It should be noted that high-income countries are assumed to have risk of unsafe water which could lead to an underestimate of unsafe water health burden in these countries. Another limitation in our analysis is the paucity of data on HWT. The inclusion of more location-specific data on water treatment utilization at the household level can greatly improve our estimates in future iterations. High-income countries were assumed to have 0 risk of unsafe water, and the TMREL was applied to these countries.

Theoretical minimum-risk exposure level

The theoretical minimum-risk exposure level for unsafe water is defined as all households have access to high quality piped water that has been boiled or filtered before drinking. This exposure level is applied to all households in high-income countries, as well as households in countries in Southern Latin America region or Eastern Europe region that report piped water source and filtered or boiled water treatment.

Relative risks

GBD 2015 employ the same relative risks for unsafe water as was done for GBD 2013. There are 3 adverse health outcomes paired with unsafe water that comprise of diarrheal diseases, typhoid fever, and paratyphoid fever. A meta-analysis by Wolf et al. 2014 provides relative risk evidence for the relationship between unsafe water and diarrheal diseases. Wolf et al. 2014 publish relative risk values for water-source interventions and point-of-use treatment interventions separately so the combined effect of a source intervention and point-of-use intervention is assumed to be multiplicative in order to match GBD 2015 exposure definitions. In the absence of better data, the relative risk for typhoid and paratyphoid fevers were assumed to be the same as the relative risk for diarrheal disease. Furthermore, it is assumed that there is a difference in piped water quality between Eastern Europe and Southern Latin America compared to rest of the developing world. As a result, we use effect sizes that are region-specific. The implication of this assumption is that no household in developing countries have access to high-quality piped water (TMREL). Please refer to appendix tables for more information on relative risk values and citations.

References

1. "Improved and Unimproved Water Sources and Sanitation Facilities."*WHO / UNICEF Joint Monitoring Programme: Wat/san Categories*. The WHO/UNICEF, n.d. Web. 08 June 2016
2. Wolf, Jennyfer, Annette Prüss-Ustün, Oliver Cumming, Jamie Bartram, Sophie Bonjour, Sandy Cairncross, Thomas Clasen, John M. Colford, Valerie Curtis, Jennifer De France, Lorna Fewtrell, Matthew C. Freeman, Bruce Gordon, Paul R. Hunter, Aurelie Jeandron, Richard B. Johnston, Daniel Mäusezahl, Colin Mathers, Maria Neira, and Julian P. T. Higgins. "Systematic Review: Assessing the Impact of Drinking Water and Sanitation on Diarrhoeal Disease in Low- and Middle-income Settings: Systematic Review and Meta-regression." Trop Med Int Health Tropical Medicine & International Health 19.8 (2014): 928-42. Web.

Unsafe Sanitation Capstone Appendix

Flowchart

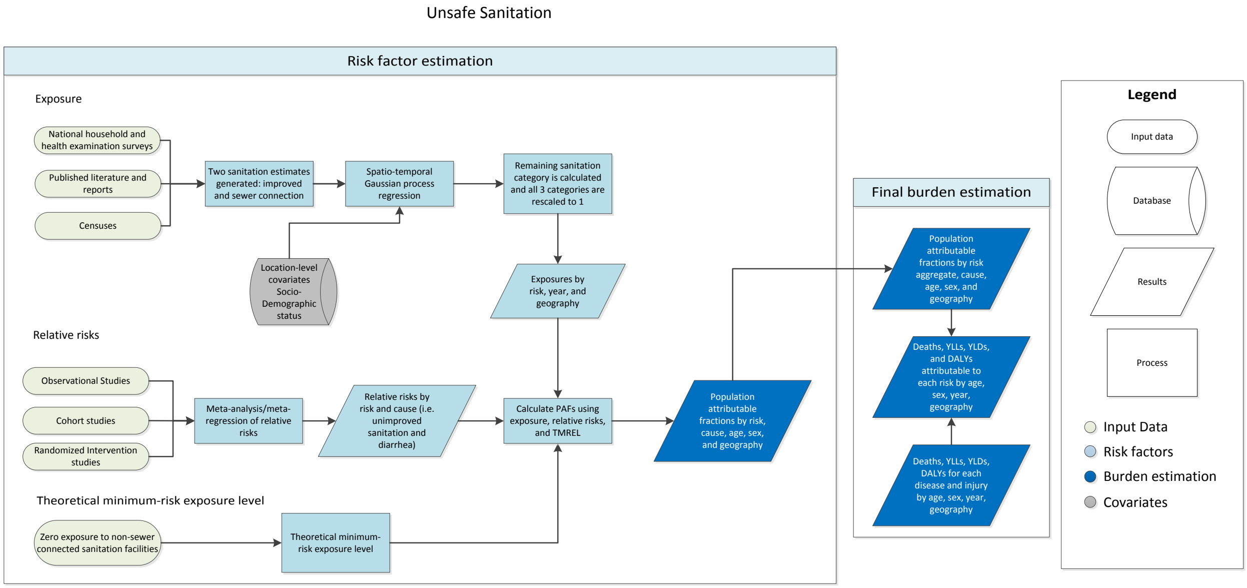

Input Data & Methodological Summary

Exposure

Case Definition

Exposure to unsafe sanitation were defined based on the primary toilet type used by households. Improved facilities are defined as such based on JMP designation (The WHO). Sewer connection toilets included flush toilets or any toilet with connection to the sewer or septic tank.

Input Data

The search for usable household surveys and censuses was conducted using the Global Health Data Exchange (GHDx) database. Searches were conducted from October 2015 to December 2015, with the final search household level micro-data on toilet type conducted on December 15, 2015. Due to the organized nature of the GHDx, the only search term used was "unsafe sanitation", which yielded just under 1400 results, of which 795 were extracted and used as inputs for modeling. Tabulated and report data was lower priority and was only updated when time permitted.

Modeling

There were no substantive changes in the modeling process from GBD 2015. Two distinct models are produced from sanitation data: prevalence of households with improved sanitation and the proportion of households with a sewer connection over the total improved sanitation population. Prevalence of households with a sewer connection is modeling with improved sanitation prevalence as the denominator in order to prevent the population with access to sewer connection from exceeding the population with access to improved sanitation. By each geography-year, both models are generated using a 3-step modeling scheme of mixed effect linear regression followed by spatio-temporal Gaussian process

regression (ST-GPR), which outputs full time series estimates for each GBD 2015 location. Socio-demographic status (SDS), an index metric that includes a measure of education and income level, was used as a fixed effect in the linear regression since it proved to have significant coefficients. Random effects were placed at GBD 2015 region and super-region levels.

The process of vetting and validating models was accomplished primarily through an examination of ST-GPR scatter plots by GBD 2015 location from 1990-2015. Any unfitting data points were re-inspected for error at the level of extraction and survey implementation, and subsequently excluded from analysis if deemed appropriate. In addition to SDS, a number of different potential fixed effects were considered, including lag-distributed income and urbanicity, but SDS proved to be the strongest predictor of unsafe sanitation. Uncertainty in the estimates was initially formed based on standard deviation by survey, then propagated through ST-GPR modeling by means of confidence intervals around each data point that reflect the point-estimate specific variance.

Once models are fully vetted, full time series outputs from ST-GPR modeling are then converted from proportion to prevalence by year and geography and then rescaled to form 3 mutually exclusive categories that sum up to 1. The table below provides the final result of this rescaling.

Category	*Definition*
Unimproved sanitation	Proportion of households that use unimproved sanitation facilities.
Improved sanitation, excluding sewer	Proportion of households that use improved sanitation facilities except those with sewer connection.
Sanitation facilities with sewer connection	Proportion of households that use toilet facilities with sewer connection.

Due to the nature of modeling sanitation with sewer connection as a proportion of total improved sanitation access, we are limited in only using sources for sewer connection that also include total improved sanitation values. It should be noted that high-income countries are assumed to have risk of unsafe sanitation which could lead to an underestimate of unsafe sanitation health burden in these countries. Another limitation that extends to the other two risk factors that comprise WaSH (unsafe water and unsafe hygiene) and can be improved upon in future iterations is taking into account covariance of access to water, sanitation and handwashing facilities. Currently, all three components of WaSH are modeled independently, which may lead to an overestimation of the burden of WaSH factors. High-income countries were assumed to have 0 risk of unsafe sanitation and the TMREL was applied to these countries.

Theoretical minimum-risk exposure level

The theoretical minimum-risk exposure level for unsafe sanitation was defined as all households have access to a sanitation facility with sewer connection. Since it is assumed that all households in high-income countries have access to sewer-connected sanitation, this counterfactual exposure level is applied to all households in high-income countries.

Relative risks

GBD 2015 employ the same relative risks for unsafe water as was done for GBD 2013. Three adverse health outcomes are paired with unsafe sanitation, which comprise of diarrheal diseases, typhoid fever, and paratyphoid fever. A meta-analysis by Wolf et al. 2014 provides relative risk evidence for the relationship between unsafe sanitation and diarrheal diseases. In the absence of better data, the relative risk for typhoid and paratyphoid fevers were assumed to be the same as the relative risk for diarrheal disease. Please refer to appendix tables for more information on relative risk values and citations.

References

1. "Improved and Unimproved Water Sources and Sanitation Facilities."*WHO / UNICEF Joint Monitoring Programme: Wat/san Categories*. The WHO/UNICEF, n.d. Web. 08 June 2016
2. Wolf, Jennyfer, Annette Prüss-Ustün, Oliver Cumming, Jamie Bartram, Sophie Bonjour, Sandy Cairncross, Thomas Clasen, John M. Colford, Valerie Curtis, Jennifer De France, Lorna Fewtrell, Matthew C. Freeman, Bruce Gordon, Paul R. Hunter, Aurelie Jeandron, Richard B. Johnston, Daniel Mäusezahl, Colin Mathers, Maria Neira, and Julian P. T. Higgins. "Systematic Review: Assessing the Impact of Drinking Water and Sanitation on Diarrhoeal Disease in Low- and Middle-income Settings: Systematic Review and Meta-regression." Trop Med Int Health Tropical Medicine & International Health 19.8 (2014): 928-42. Web.

Unsafe Hygiene Capstone Appendix

Flowchart

Unsafe Handwashing

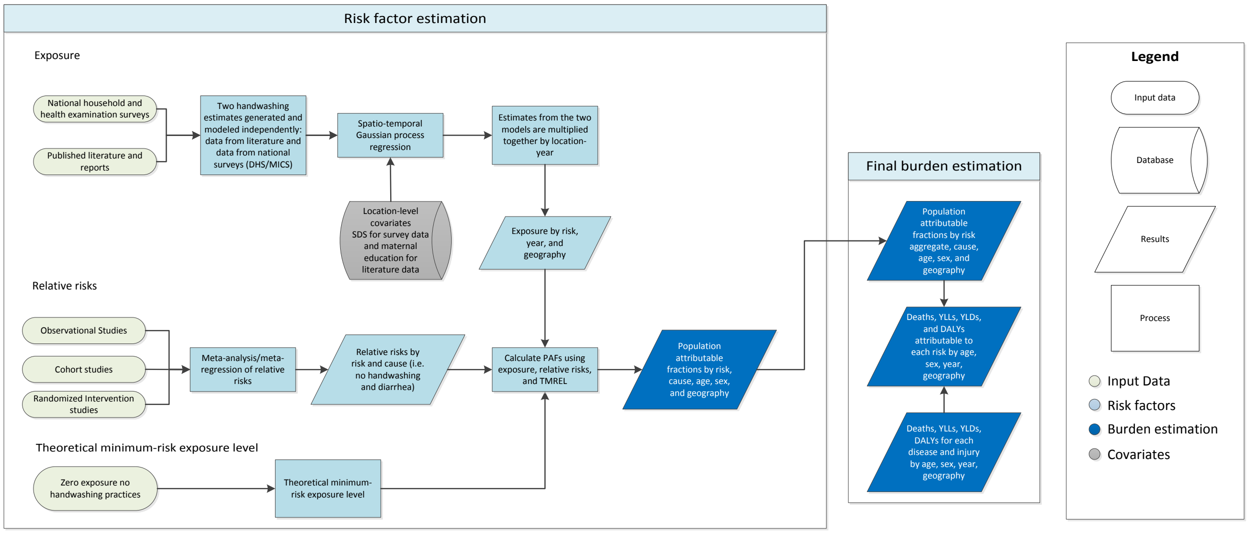

Input Data & Methodological Summary

Exposure

Case Definition

Unsafe hygiene is composed of global handwashing practices. Handwashing is defined as the observed prevalence of handwashing with soap and water after using a toilet or after contact with excreta, including children's excreta. We estimate the burden of unsafe handwashing in both developed and developing settings.

Input Data

There were two main sources that were used in our estimation of handwashing practices, estimates from scientific literature and estimates from household survey series. Relevant literature on handwashing prevalence was gathered from a meta-analysis published recently by Freeman et al. (2014). Since water and soap availability data is very limited, only country-specific Demographic Health Surveys (DHS) and Malaria Indicator Survey Series (MICS) conducted after 2006 were able to be used as input data.

Modeling Strategy

Input data from scientific literature and input data from household survey series were modeled independently. Data from literature primarily measured a population's handwashing practices under ideal conditions, such as when water and soap was readily available. Additionally, these estimates from literature would likely be susceptible to acquiescence bias. Alternatively, data from DHS and MICS only provide insight into the availability of water, soap, and washing stations, which, alone, does not indicate how often a person may wash their hands after contact with excreta. Thus, after modeling data from

literature and data from surveys independently, these values were multiplied together by location-year in order to gain a more accurate representation of true handwashing prevalence.

For GBD 2015, there was a shift away from the ST-GPR modeling used in 2013 toward a more basic one-step modeling approach. This change came in light of the data scarcity and concern that spatial and temporal smoothing within ST-GPR may capture spurious trends in hygiene prevalence. For modeling the act of handwashing under ideal conditions, a variance-weighted linear regression with a fixed effect on average years of education per capita was employed. For modeling the availability of water, soap, and wash station, a multi-level logistic regression with a fixed effect on lag-distributed income per capita and random effect at the GBD 2015 region level was chosen to be the most appropriate method. The fixed effects used in both models, education and LDI per capita, proved to be significant and in the expected direction. Uncertainty intervals were produced by generating a 1000 draws from a normal distribution of the beta, intercept, and random effect, if appropriate, from the variance-covariance matrix of the regression.

The process of vetting and validating models was accomplished primarily through an examination of ST-GPR scatter plots by GBD 2015 location from 1990-2015. Any unfitting data points were re-inspected for error at the level of extraction and survey implementation, and subsequently excluded from analysis if deemed appropriate. In addition to LDI per capita and education per capita, a number of different potential fixed effects were considered, including socio-demographic status and urbanicity, however LDI and education proved to be the strongest predictors of handwashing practices for their respective models. Once models were sufficiently vetted, full time series outputs from each of the models were multiplied together at each location-year.

A considerable limitation for when estimating handwashing practices for over 190 independent locations around the world is data sparseness. Even when data is published on handwashing prevalence, the definition is often altered from the GBD 2015 standard definition or it may only pertain to certain populations (such as hospital patients) and lacks representativeness at the geographic scale we require. The incorporation of questions about soap and water availability in DHS and MICS has added much-needed information but there remains a large data gap that must be filled if we are to become more certain in handwashing estimates.

Theoretical minimum-risk exposure level

The theoretical minimum-risk exposure level for unsafe hygiene is defined as all households engaging in handwashing with soap practices after any contact with excreta, including children’s excreta.

Relative risks

GBD 2015 use the same relative risks for unsafe hygiene as was done for GBD 2013. There are 3 adverse health outcomes paired with unsafe hygiene that include diarrheal diseases, typhoid fever, and paratyphoid fever. A meta-analysis by Freeman et al. 2014 provides relative risk evidence for the relationship between unsafe hygiene and diarrheal diseases. In the absence of adequate data, the relative risk for typhoid and paratyphoid fevers were assumed to be the same as the relative risk for diarrheal disease based on analogous transmission pathways (feco-oral pathway). Please refer to appendix tables for more information on relative risk values and citations.

References

1. Freeman, M. C., Stocks, M. E., Cumming, O., Jeandron, A., Higgins, J. P., Wolf, J., Curtis, V. (2014). Systematic review: Hygiene and health: Systematic review of handwashing practices worldwide and update of health effects. *Trop Med Int Health Tropical Medicine & International Health, 19*(8), 906-916. doi:10.1111/tmi.12339

Ambient Particulate Matter Pollution Capstone Appendix

Flowchart

Ambient PM2.5

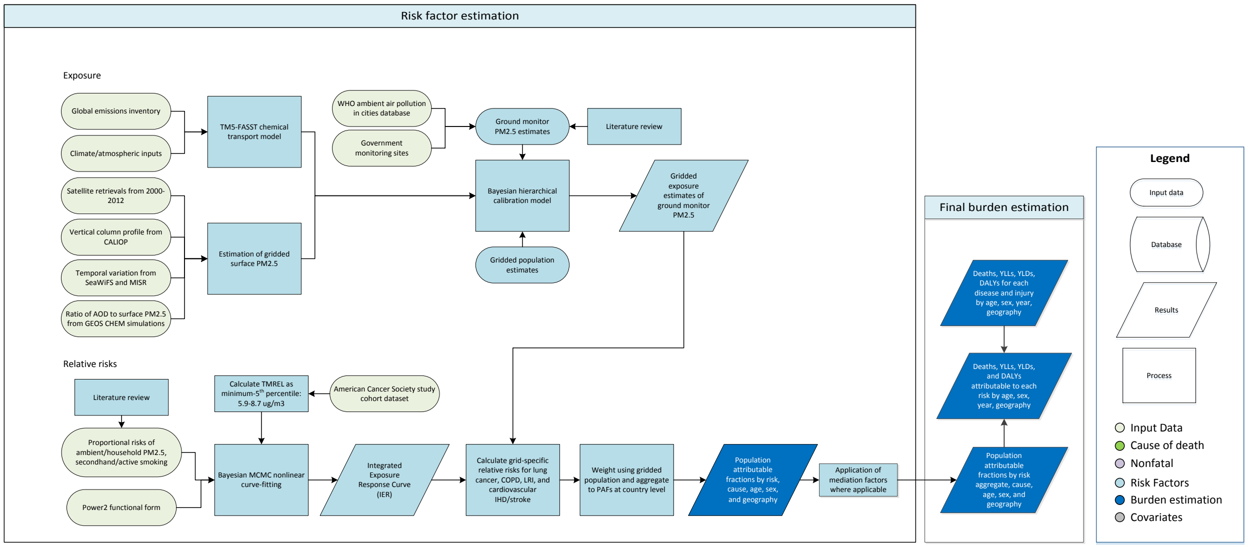

Input data & Modelling strategy

Exposure

Definition

Exposure to ambient air pollution is defined as the population-weighted annual average mass concentration of particles with an aerodynamic diameter less than 2.5 micrometers ($PM_{2.5}$) in a cubic meter of air. This measurement is reported in µg/m^3.

Input data

The data to estimate exposure to ambient air pollution is drawn from estimates of annual concentration of $PM_{2.5}$ – generated using satellite observations of aerosols in the atmosphere. To correct for bias in the satellite modeling approach, a spatially-varying flexible framework is used to combine modeled concentrations with observations from ground-level monitoring of particles in more than 75 countries. All input data for GBD2015 was updated as follows:

Updated $PM_{2.5}$ ground measurement database

For the GBD2015 update we updated the database of annual average PM measurements to include more recent data and to incorporate additional locations where measurement data have become available. To facilitate this we collaborated with WHO and contributed to their recently released WHO Air Pollution in Cities database. We then used disaggregated (monitor-specific values and not the city averages that are reported by WHO) measurements from this database with additional site-specific information (e.g. all monitors in a city, monitor geo coordinates, monitor site type) such as that included in the GBD2013 database. In total measurements of concentrations of PM_{10} and $PM_{2.5}$ were retrieved from 6,003 ground

monitors with the majority contributing measurements from 2014 (as there is a lag in reporting measurements, little data from 2015 were available). Where data were not available for 2014 (2760 monitors), data was used from 2015 (18 monitors), 2013 (2155), 2012 (564), 2011 (60), 2010 (375), 2009 (49), 2008 (21) and 2006 (1). For locations with only PM_{10} measurements, $PM_{2.5}$ measurements were estimated from PM_{10}. This was done by a locally derived conversion factor ($PM_{2.5}/PM_{10}$ ratio) estimated as population-weighted averages of location-specific conversion factors for the country. Location-specific conversion factors were estimated as the mean ratio of $PM_{2.5}$ to PM_{10} of stations for the same year. If national conversion factors were not available, regional ones were used, which were obtained by averaging country-specific conversion factors.

Updated satellite-based estimates

The updated satellite-based estimates are described in detail in van Donkelaar et al. 2016[1]. These estimates (~11 x 11 km resolution at the equator) combine aerosol optical depth retrievals from multiple satellites with the GEOS Chem chemical transport model and land use information.

Updated population data

A comprehensive set of population data on a high-resolution grid was obtained from the Gridded Population of the World (GPW v4) database. These data are provided on a $0.0417^o \times 0.0417^o$ resolution. To aggregate these estimates of population to each $0.1^o \times 0.1^o$ grid cell, the central 3 × 3 population cells were summed. As this accounted for a resolution higher than necessary, the same was done four other times, offset by one cell in a North, South, East and West direction. The average of five quantities was used as the aggregated population estimate for each cell. Estimates of population for 2000, 2005, 2010, 2015 and 2020 were extracted from GPW version 4 and estimates for 1990 and 1995 were extracted from GPW version 3 as described previously for GBD2013[3].

Modelling strategy

The methodology used to estimate the burden of ambient particulate matter pollution has seen significant changes since GBD2013.

The GBD2010 and GBD2013 estimates both used a single global function to calibrate the mean of the chemical transport model and satellite-based estimates to available ground measurements. In both instances the approach taken was recognized at the time to be a compromise between what could be easily implemented under tight timeframes and one that most efficiently utilized all of the data sources. In particular, the GBD2013 exposure estimates were known to underestimate ground measurements in specific locations (see discussion in Brauer et al., 2015[2]) such that it would be desirable to allow measurements to make a larger contribution to the final estimates where they were available. Therefore, for GBD2015 we implemented a Bayesian Hierarchical modelling approach using Integrated Nested Laplace Approximations (INLA) in which the satellite-based estimates, ground measurements and land use information are combined in a spatially varying flexible framework. Formal external evaluation using ground measurements was conducted and shown to lead to improved predictions of ground measurements in all super regions compared to GBD2013 estimates and an alternative geographically-weighted regression approach. Further, based on the external evaluation analyses, addition of the TM5

chemical transport model estimates of PM2.5 annual average did not improve the estimates and these were therefore not included.

Bayesian hierarchical models (BHM) provide an extremely useful and flexible framework in which to model complex relationships and dependencies in data. Uncertainty can also be propagated through the model allowing uncertainty arising from different components, both data sources and models, to be propagated through the models into estimates of uncertainty associated with the final estimates. In the hierarchical modeling approach coefficients associated with satellite-based estimates were estimated for each country. Where data were insufficient within a country, information can be `borrowed' from a higher aggregation (region) and if enough information is still not available from an even higher level (super-region). Individual country level estimates were therefore based on a combination of information from the country, its region and super-region.

All modelling was performed on the log-scale with the unit of measurement being a grid cell. The model was constructed with the inclusion of all variables assessed statistically, based on model fit (DIC, a measure of the relative quality of a model and closely related to that of AIC but for Bayesian models) and predictive ability. The hierarchical structure was applied to the intercept and slope terms with all modelling on the log scale. The model was of the form

$$\log(PM2.5_i) = \beta_0 + \beta_1 \log SAT_i + other\ variables + \varepsilon_i$$

where *i* denotes the grid cell. The following sets of variables were considering in developing the models:

Continuous explanatory variables:

- (SAT) Estimate of $PM_{2.5}$ (in μgm^{-3}) for 2014 from satellite remote sensing on the log-scale.
- (CTM) Estimate of $PM_{2.5}$ (in μgm^{-3}) for 2014 from chemical transport models on the log-scale.
- Estimate of population for 2014 on the log-scale.
- (SNAOC) Estimate of the sum of sulfate, nitrate, ammonium and organic carbon as estimated from GEOS Chem
- (DST) Estimate of compositional concentrations for mineral dust from GEOS Chem
- (EDxDU) The log of the elevation difference between the elevation at the ground measurement location and the mean elevation within the GEOS Chem simulation grid cell multiplied by the inverse distance to the nearest urban land surface

Discrete explanatory variables:

- Binary variable indicting whether exact location of ground measurement is known
- Binary variable indicting whether exact type of ground monitor is known
- Binary variable indicting whether ground measurement is $PM_{2.5}$ or converted from PM_{10}

Random Effects:

- Grid cell random effects on the intercept to allow for multiple ground monitors in a grid cell.
- Country-region-super-region hierarchical random effects for the intercept
- Country-region-super-region hierarchical random effects for the satellite remote sensing term.
- Country-region-super-region hierarchical random effects for the coefficient associated with the difference between estimates from CTM and SAT.
- Country-region-super-region hierarchical random effects for the coefficient log(POP)
- Country level random effects for intercept, satellite and difference between CTM and SAT are independent and identically distributed.
- Country level random effects for population uses a neighbourhood structure allowing specific borrowing of information from neighbouring countries.
- All region random effects are assumed to be independent and identically distributed.
- All super-region random effects are assumed to be independent and identically distributed.

Interactions*:*

- Interactions between the binary variables and the effects of log(SAT) and log(CTM/SAT)

Due to both the complexity of the models and the size of the data, notably the number of spatial predictions that are required in this setting, recently developed techniques that perform 'approximate' Bayesian inference based on integrated nested Laplace approximations (INLA) have been developed as a computationally attractive alternative to Markov Chain Monte Carlo methods. Computation was performed using the R interface to the INLA computational engine (R-INLA) with the size of the task of fitting the models and performing predictions for each of the ca. 1.4 million grid cells requiring the use of a high performance computing cluster (HPC) with high memory nodes. As in GBD2010 and GBD2013 the spatial model was built combining the different data sources for a single year (2014, corresponds to the most recent measurement data). The spatially-varying functions from this model were then applied to the satellite-based estimates from all other years - in other words assuming that the spatial relationship between the different data sources does not change over time. This is undoubtedly a simplification but to do otherwise would require assembling multi-year measurement databases which is not feasible given current data availability and computational constraints. As the spatial model was built using the most recently available (2014) measurement and satellite-based estimates, 2015 estimates were based on extrapolation. Instead of extrapolating using an exponential model based on a 1-year trend as in GBD2013, splines based on a 5 year trend (2010-2014) were fit and applied to the 2014 grid-cell values to estimate levels for 2015. This reduced the likelihood of 2015 estimates being overly influenced by meteorological events in a specific year and to better represent the duration of exposure relevant to the epidemiologic studies included in the integrated exposure-response functions.

Model Evaluation

Model evaluation and comparison was performed by fitting models on a training set and predicting exposures at locations for which measurements were known (the validation set). The selection of the training (20%) and validation (80%) set consisted of taking a random sample of the total number of sites measuring PM2.5 (or having a value converted from PM10 measurements). Sampling was performed

using sampling probabilities based on the cross-tabulation of PM2.5 categories (0-24.9, 25-49.9, 50-74.9, 75-99.9, 100+ µg/m3) and super-regions. The resulting hold-out evaluation data set was a sample of 20% of the sites that have the same distribution over PM2.5 categories and super-regions as the entire set of sites.

This process was used to generate multiple training and validation set combinations, allowing for example cross-validation to be performed. In the evaluation, 25 sets of training/validation data were used. The following models were considered in the evaluation phase:

(A) The GBD2013 model, using a simple linear regression with a fused estimate of SAT and CTM together with interactions with three binary variables representing whether the measurement was converted from PM10 and whether the exact site type and location is known.
(B) A hierarchical model with SAT, the TM5 CTM estimates, population and the three binary variables described above
(C) A hierarchical model with SAT, population, SNAOC, DST, EDxDU, population and the three binary variables
    - Estimate of population for 2014 on the log-scale.
    - Estimate of the sum of sulfate, nitrate, ammonium and organic carbon as estimated from GEOS Chem
    - Estimate of compositional concentrations for mineral dust from GEOS Chem
    - The log of the elevation difference between the elevation at the ground measurement location and the mean elevation within the GEOS Chem simulation grid cell multiplied by the inverse distance to the nearest urban land surface

For each training/evaluation set combination, model fit and prediction accuracy were evaluated for each of the 25 training/evaluation set combinations with the following metrics:

Model fit
- R^2
- DIC

Predictive accuracy
- R^2 arising from a linear regression of predicted vs actual measurements at each location
- RMSE – root mean squared error
- WRMSE – weighted (by population) root mean squared error
- MSE – mean square error
- MAE – mean absolute error

This evaluation indicated the final model improved predictions of ground measurements in all super regions compared to GBD2013 estimates (median global R^2 [population-weighted RMSE] 0.82 (12.1 µg/m^3), 0.60 [13.5 µg.m^3] for GBD2015 and GBD2013, respectively).

Error! Reference source not found. shows the RMSE (median from the 25 runs) for each of the three models, by super-region. The accuracy of the prediction varies between super-regions, with lower errors being observed in areas where there are more monitoring sites. In each of the super-regions, the largest errors are seen for model A which are considerably higher than those for models B and C, with model C showing a small improvement over B (except in super-region 5, North Afirca/Middle East).

Figure 2 shows scatter plots of the observed and predicted measurements using the three models for each super-region. The predicted measurements are the median values over those obtained from the 25 training sets. Predictions from the two Bayesian hierarchical models (B&C) match the observed values more closely than the linear model (A) with much less spread around a straight line (with slope one and zero intercept, shown in red). In Central Europe and Sub-Saharan Africa it is noticeable that, in addition to reduced spread, models B&C are much better at predicting higher values. The same patterns of results in predictive ability were seen when looking at regions and individual countries. In all cases, model C performed better than model B with both being considerable better than model A.

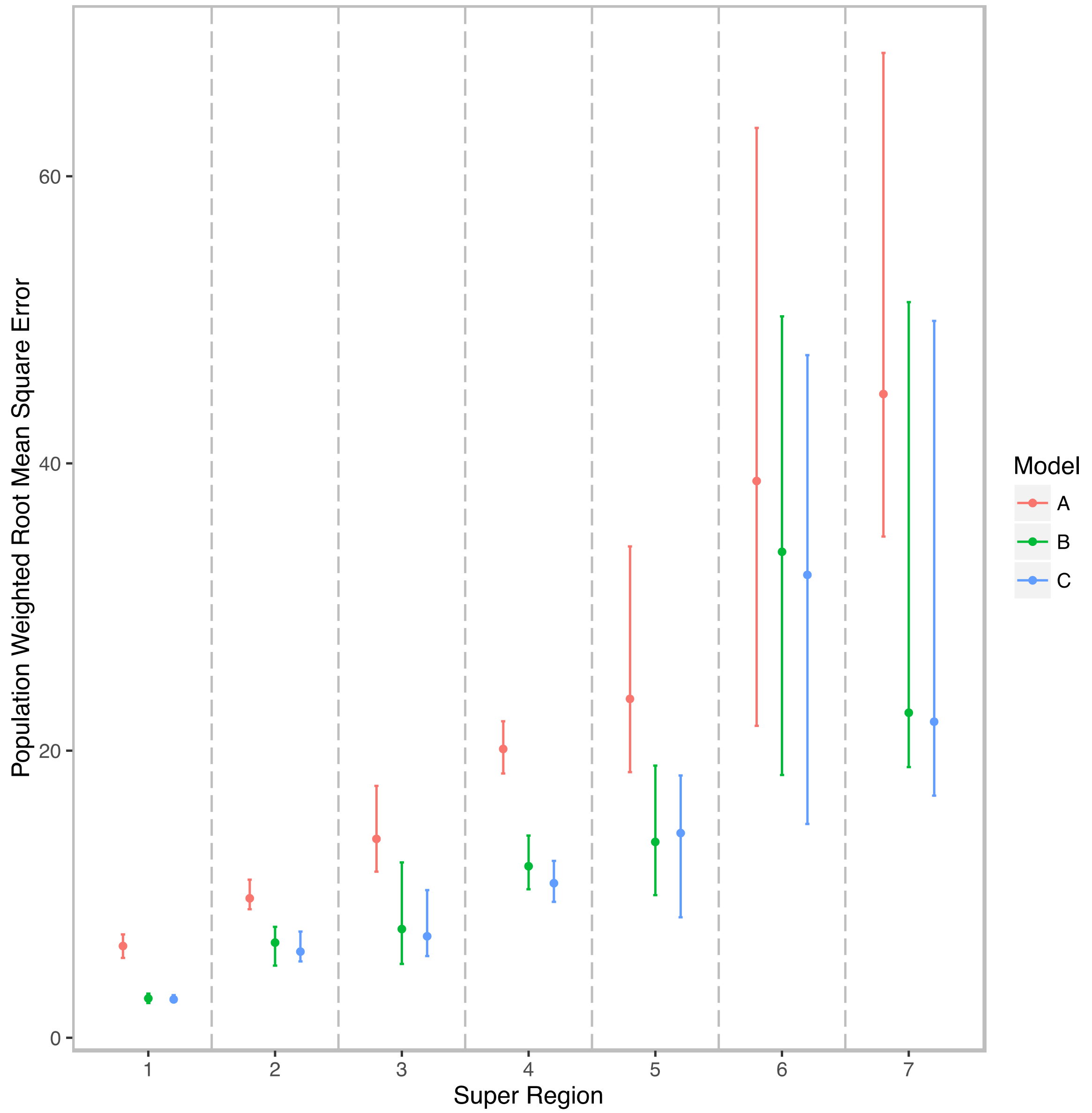

Figure 1: Comparison of RMSE from three models by super-region. Dots denote the median of the distribution from 25 training/evaluation sets and the vertical lines the range of values. Super-regions are

1: high income, 2: Central Europe, Eastern Europe, Central Asia, 3: Latin America and Caribbean, 4: Southeast Asia, East Asia and Oceania, 5: North Africa / Middle East, 6: Sub-Saharan Africa, 7: South Asia.

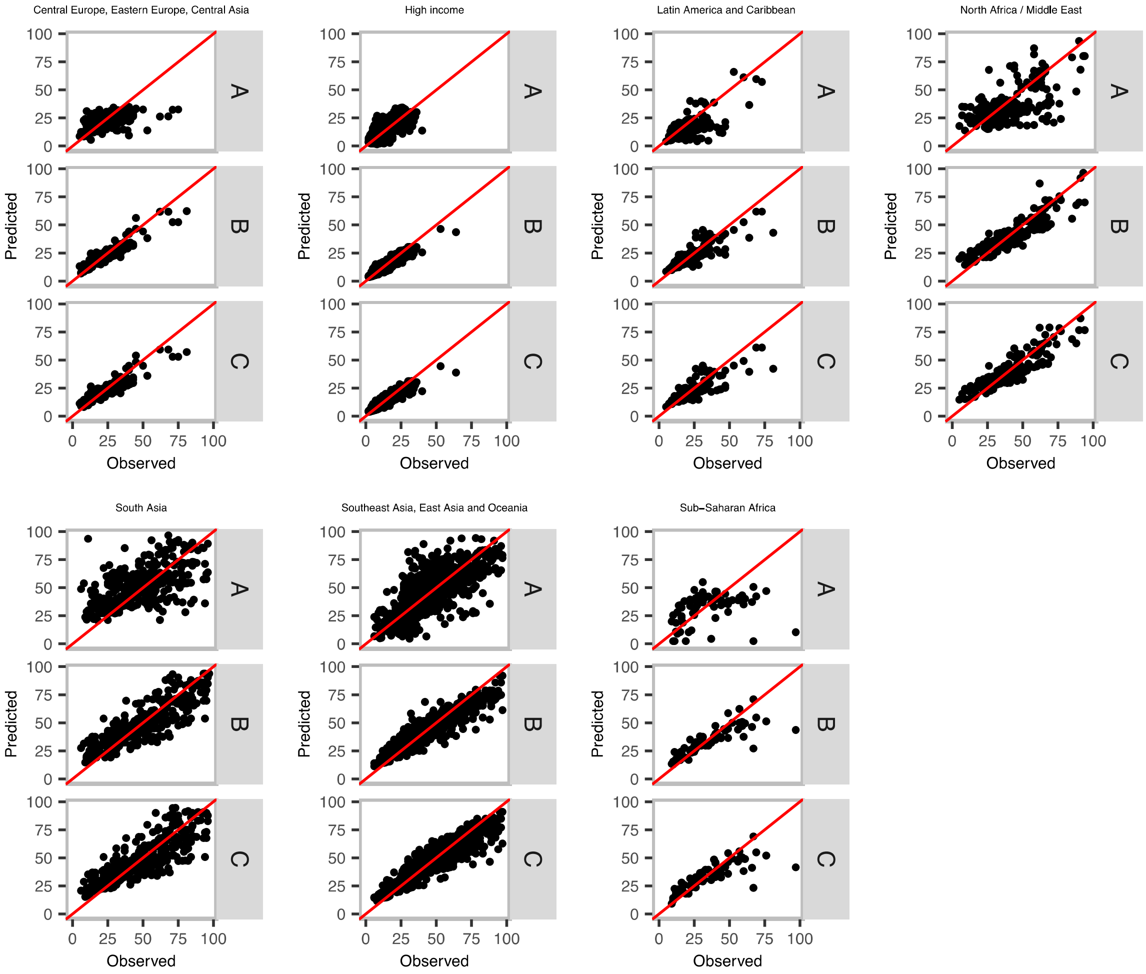

Figure 2: Comparison of observed and predicted measurements using three different models, by super-region. The red line has slope one and intercept zero.

Overall, the best model in terms of model fit and predictive ability was one with the following components:

- Estimates of $PM_{2.5}$ (in μgm^{-3}) from satellite remote sensing (SAT), population, and information on the GEOS Chem simulated chemical composition, elevation and distance to urban land use (SNAOC, DST and EDxDU).
- Binary variables indicting whether exact location and type of ground measurement is known, and whether the measurement was $PM_{2.5}$ or converted from PM_{10}.
- Interactions between the binary variables and the effects of estimates from satellite remote sensing.
- Grid cell random effects on the intercept to allow for multiple ground monitors in a grid cell.

- Country-region-super-region hierarchical random effects for intercepts, satellite remote sensing and population terms.
- Country level random effects for population using a neighbourhood structure allowing specific borrowing of information from neighbouring countries.

Theoretical minimum-risk exposure level

The TMREL for ambient PM is estimated using a uniform distribution between the minimum and 5th percentile of exposure observed in the studies used to generate the GBD estimates. This estimate was updated for GBD2015 as new studies were added to the analysis and studies used previously were updated through continued follow-up. The newer estimates included several large studies that included exposure at lower levels of PM2.5. As a result, the TMREL for GBD2015 was ~U(2.4, 5.9), lower than GBD2013's distribution ~U(5.9, 8.7), which had the effect, all things being equal, of increasing the estimated attributable burden relative to the GBD 2013 estimates.

Relative Risk

Relative risks are generated using integrated exposure-response functions (IER) that are fit to available epidemiologic data using a Bayesian MCMC approach and a modified power function. The IER are estimated based on published relative risks for long-term exposure to ambient PM2.5, household air pollution, second-hand smoking, and active (cigarette) smoking. The concentration of particulate matter for each type of exposure is estimated based on literature values and used to map the relative risks to a curve generated for the entire range of exposure from these sources. The input data for this curve fitting process has been updated since GBD2013, adding new studies that estimate exposure at finer spatial scales, including studies of within-city exposure that focus on traffic-related air pollution. In addition, changes were made to the curve-fitting process. In order to account for differences in study design, temporal patterns of exposure and other differences among the studies of the different sources of PM2.5, a source-specific heterogeneity parameter was added to the IER. This resulted in much wider, and, in our view, more realistic, uncertainty intervals for the burden estimates, by propagating through the entire process the current uncertainty regarding the mechanisms and magnitude of health impacts of exposure to PM2.5 from diverse sources.

IER Functional Form

Data Likelihood

$$\log(RR_i) \sim \mathcal{N}(\mu_i, \sqrt{\sigma_i^2 + \delta_{source_i}})$$

Model

$$\mu_i = \log\left(\frac{1+\alpha \times \left(1 - e^{-\beta \times (exposure_i - TMREL)^\gamma}\right)}{1+\alpha \times \left(1 - e^{-\beta \times (counterfactual_i - TMREL)^\gamma}\right)}\right)$$

Data

RR_i : measured relative risk for data point i
σ_i : variance of data point i based on study information
$source_i$: exposure source type (outdoor/household air pollution, secondhand/active smoking)
$TMREL$: theoretical minimum risk exposure level
$exposure_i$: measured exposure for data point i
$counterfactual_i$: counterfactual exposure for data point i

Priors

$$\alpha \sim \Gamma(1.0, 0.01)$$
$$\beta \sim \Gamma(1.0, 0.01)$$
$$\gamma \sim \Gamma(1.0, 0.01)$$
$$\delta \sim \Gamma(1.0, 0.01)$$

We also modified the way in which age-specific IER for IHD and stroke were estimated. In accordance with previously published work on other cardiovascular risk factors, the impact of air pollution on cardiovascular health is known to vary with age. To account for this phenomenon, age-specific RRs were based on a log-linear model of RR as a function of age, where the intercept (RR=1) is forced to age 110. In GBD2010 and GBD2013 the age for a relative risk estimate from a given study was estimated as the median age at death or disease incidence in that study. However, this may not accurately represent the age distribution of the entire study population so we re-estimated this variable as the mean age at enrollment + half of the average follow-up time to better represent the average age of the study population during the period of follow-up.. When compared to GBD2013, this change produced RRs that were generally lower for younger age groups, given that median age at event tends to produce a higher anchor age than average age during follow-up.

The relative risks are generated on the grid-level based on estimated exposure, and then applied to generate PAFs. These PAFs are aggregated using the grid-level population to create population-weighted national estimates of attributable burden, using the following formula:

PM2.5 Aggregation Formula

$$PAF_{A,\ C,\ L} = \frac{\sum\ ((RR_{A,\ C} - 1)\ *\ Pop_i)}{\sum(\ RR_{A,\ C}\ *\ Pop_i)}$$

A = age group, C = cause, L = location, i = grid, $RR_{A,\ C}$ = *grid-level RR based on* $PM_{2.5}$ *and given age/cause IER curve*

References

1. van Donkelaar, A.; Martin, R. V; Brauer, M.; Hsu, N. C.; Kahn, R. A.; Levy, R. C.; Lyapustin, A.; Sayer, A. M.; Winker, D. M. Global Estimates of Fine Particulate Matter using a Combined Geophysical-Statistical Method with Information from Satellites, Models, and Monitors. *Environ. Sci. Technol.* 2016, *50* (7), 3762–3772.

2. Brauer, M.; Freedman, G.; Frostad, J.; van Donkelaar, A.; Martin, R. V; Dentener, F.; Van Dingenen, R.; Estep, K.; Amini, H.; Apte, J. S.; et al. Ambient Air Pollution Exposure Estimation for the Global Burden of Disease 2013. *Environ. Sci. Technol.* 2015, *50* (1), 79–88.

3. Brauer, M.; Amann, M.; Burnett, R. T.; Cohen, A.; Dentener, F.; Ezzati, M.; Henderson, S. B.; Krzyzanowski, M.; Martin, R. V; Van Dingenen, R.; et al. Exposure assessment for estimation of the global burden of disease attributable to outdoor air pollution. *Environ. Sci. Technol.* 2012, *46* (2), 652–660.

Household Air Pollution Capstone Appendix

Flowchart

Household Air Pollution from Solid Fuels

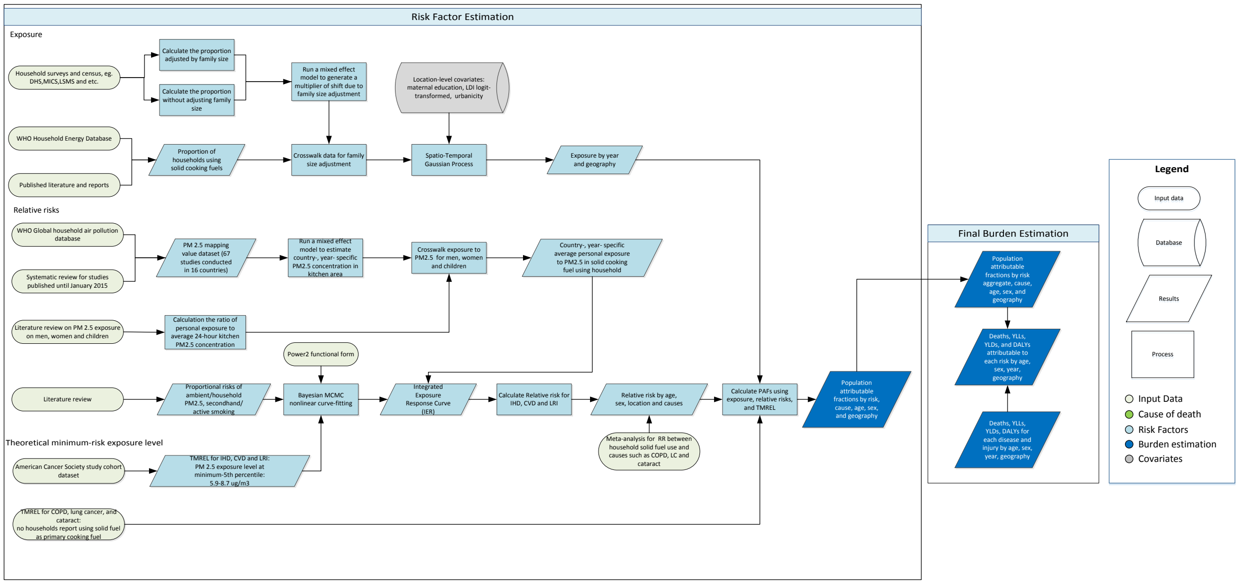

Input Data & Methodological Summary

Exposure

Case Definition

Exposure to household air pollution from solid fuels (HAP) is defined as the proportion of households using solid cooking fuels. The definition of solid fuel in our analysis includes coal, wood, charcoal, dung, and agricultural residues.

Input data

Data were extracted from the standard multi-country survey series such as Demographic and Health Surveys (DHS), Living Standards Measurement Surveys (LSMS), Multiple Indicator Cluster Surveys (MICS), and World Health Surveys (WHS), as well as country-specific survey series such as Kenya Welfare Monitoring Survey and South Africa General Household Survey. To fill the gaps of data in surveys and censuses, we also downloaded and updated HAP estimates from WHO Energy Database and extracted from literature through systematic review done in IHME. Each nationally or sub-nationally representative data point provided an estimate for the percentage of households using solid cooking fuels. Estimates for the usage of solid fuels for non-cooking purpose were excluded, i.e. primary fuels for lighting. The database, with estimates from 1980 to 2015, contained 685 studies from 150 countries. Updates to systematic reviews are performed on an ongoing schedule across all GBD causes and risk factors, an update for household air pollution will be performed in the next 1-2 iterations.

Modeling strategy

Household air pollution was modeled at household level using a three-step modeling strategy ST-GPR that uses linear regression, spatiotemporal regression and Gaussian Process Regression (GPR). The first step is a mixed-effect linear regression of logit-transformed proportion of households using solid cooking fuels. The linear model contains maternal education and proportion of population living in urban areas as covariates and has nested random effect by country, GBD region, and GBD super region respectively. The full ST-GPR process is specified elsewhere in this appendix.

Compared with GBD 2013, we have made changes in terms of the covariates utilized in the linear model. A variety of combinations of socioeconomic and environmental covariates in different transformation format were tested by running mixed-effect models with exposure data. The final list of covariates included in the exposure model are maternal education and the proportion of population living in urban area.

Theoretical minimum-risk exposure level

For outcomes where we extracted RR based on direct epidemiological evidence i.e. COPD, lung cancer, and cataract, TMREL was defined such that no households would report using solid fuel as their primary cooking fuel. For outcomes that utilize evidence based on the Integrated Exposure Response (IER), the TMREL is defined as uniform distribution between 33.3 and 41.9 ug/m^3. TMREL for household air pollution did not change from GBD 2013.

Relative risks

The disease-outcomes paired with household air pollution has not changed since GBD 2013. The list of outcomes paired with household air pollution has not changed since GBD 2013, which included lower respiratory infections (LRI), stroke, Ischemic Heart Disease (IHD), chronic obstructive pulmonary disease (COPD), lung cancer and cataract. The relative risks of all outcomes but not cataract were generated by using the integrated exposure-response functions (IER). The relative risks for cataract were extracted from a meta-analysis paper (1). The IER curves are updated to reflect the newly updated data and utilization of a new method that specified elsewhere.

PM2.5 mapping value

The relative risk estimates describing the association of HAP with outcomes including Ischemic Heart Disease (IHD), cardiovascular disease (CVD), and lower respiratory infections (LRI) were derived from the IER curves. This is done by first estimating the crosswalk values that map household use of solid fuel to PM2.5 exposure because the IER curve measures exposure using PM2.5. This step of the analysis relied on 67 studies conducted in 16 countries to generate the PM2.5 mapping values, which remain the same sources as GBD 2013. The PM2.5 exposure was then cross-walked to men, women and children by generating the ratio of personal exposure to average 24-hour kitchen PM2.5 concentration based on a study after the literature review in GBD 2013.

References

1. Smith KR, Bruce N, Balakrishnan K, Adair-Rohani H, Balmes J, Chafe Z, et al. Millions Dead: How Do We Know and What Does It Mean? Methods Used in the Comparative Risk Assessment of Household Air Pollution. Annu Rev Public Health. 2014;35(1):185–206.

Ambient Ozone Pollution

Flowchart

Ambient ozone

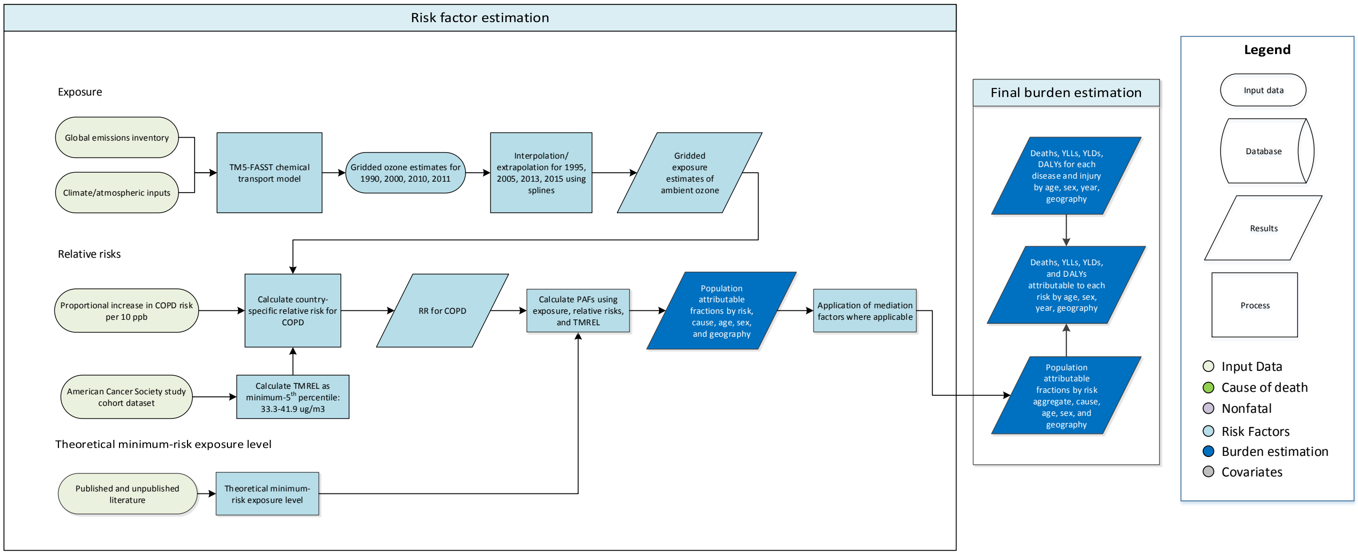

Input data and Methodological Summary

Exposure

Case Definition

For GBD 2015, exposure to ozone pollution is defined as the number of parts-per-billion (ppb) of ozone (O^3).

Input data

Data for estimating ozone exposure is derived from the TM5-FASST chemical transport model, which generates a 3-month running average of daily 1 hour maximum ozone values at the 0.1° × 0.1° for the years 1990, 2000, and 2010.[1]

Modeling Strategy

The process for modeling ozone exposure has remained stable since GBD2010 and GBD2013. Natural cubic splines were used to interpolate for the years 1995, 2005, and 2011. Annualized rate of change was used to predict for the years 2013 and 2015. The uncertainty for exposure at the grid-level was assumed to be ±6% of the estimated concentration, in accordance with previous work. Uncertainty for ozone was calculated by assuming a +/- 6% uncertainty interval around the estimation concentration.

Theoretical minimum-risk exposure level

The TMREL of ozone was defined based on the exposure distribution from American Cancer Society CPS-II study, which was the source of the GBD 2015 ozone mortality RR estimate. As with PM2.5, a uniform distribution was drawn around the minimum and 5th percentile values experienced by the cohort. This value was not updated for GBD 2015, and continues to be defined as ~U(33.3, 41.9), in ppb.

No other significant changes were made from GBD 2013 to GBD 2015.

Relative Risks

The relative risk of ozone exposure for respiratory COPD was extracted from literature and was not updated for GBD 2015. The relative risk is applied linearly per 10 ppb of ozone exposure and is defined as 1.029 (1.010-1048).[2]

References

1. Brauer M, et al. Ambient Air Pollution Exposure Estimation for the Global Burden of Disease 2013. Environ Sci Technol 2016; 50: 79-88.
2. Jerrett M, Burnett RT, Pope CA, et al. Long-term ozone exposure and mortality. N Engl J Med 2009; 360: 1085–95.

Radon Exposure Capstone Appendix

Flowchart

Radon Exposure

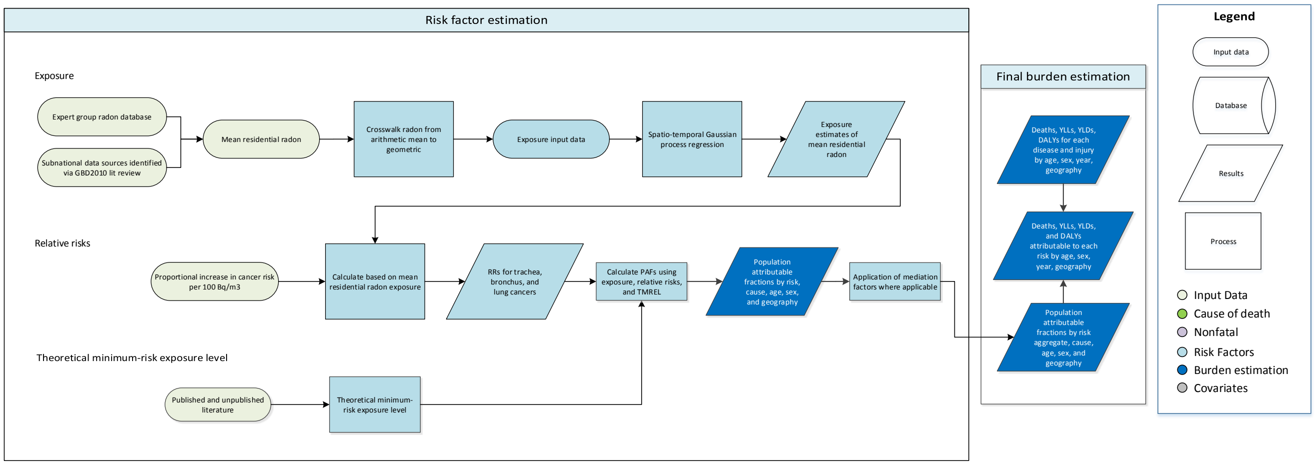

Input Data & Methodological Summary

Exposure

Case definition

Radon is a radioactive gas that is produced as a byproduct of the decay chain of uranium, occurring naturally within the Earth's crust. Some fraction of this natural radon production escapes into the atmosphere, where it forms a low concentration unless build-up is caused by enclosed spaces like homes, mines, or caves. Radon exposure is expressed as average daily exposure to indoor air radon gas levels measured in Becquerels (disintegrations per second) per cubic meter (Bq/m^3).

Input Data

Exposure to radon is determined using values curated by an expert group. These values are taken from a variety of sources including literature, government agencies, and monitoring stations. Their methodology is then inspected to determine if they are robust enough to be considered as country-level averages. This dataset was last updated for GBD2013 by adding new datapoints across time and space. No new datapoints were added for GBD2015.

Modelling Strategy

There has been minor change to the methodology to estimate radon exposure. The modelling process was previously updated by shifting it from a nested random effects model to spatial-temporal GPR. For GBD2015, the spatial-temporal GPR modelling methodology was updated as detailed in the appendix specific to this analytical technique, which is common to a variety of risk factors. Radon is naturally occurring, and is not considered to have much temporal fluctuation[1]. As such, we did not model radon over time, opting instead to use all datapoints for a single year, predict across space using our radon

database, and use the results for that year for the entire GBD time series. This eliminated any spurious time trends that might arise using the traditional ST-GPR approach. The only study level covariate was whether a datapoint was reported as geometric or arithmetic mean. Given the distribution of environmental measurements like radon tends to be skewed, the geometric mean is the preferred measurement. As such, measurements of the arithmetic mean were crosswalked during the linear regression. Uncertainty was extracted as measurement error from the data inputs and propagated through the modelling during the GPR stage. The final estimates of burden uncertainty also incorporate the reported uncertainty of the relative risk.

Theoretical minimum-risk exposure level

The TMREL was also taken directly from literature values that were not updated for GBD2015. Given that radon is naturally occurring, zero exposure would be impossible. As such, we continue to use a TMREL of 10 Bq/m^3, which is equivalent to the outdoor concentration of radon[3].

Relative Risks

The relative risk for radon exposure was extracted from literature values, a 2005 meta-analysis of case-control studies showing the association of radon with lung cancer[2]. This value was used in GBD2010 and was not updated for GBD2013 or GBD2015.

References

1. Steck DJ. Annual average indoor radon variations over two decades. Health Phys. 2009;96(1):37-47.
2. Darby S, Hill D, Auvinen A, et al. Radon in homes and risk of lung cancer: collaborative analysis of individual data from 13 European case-control studies. BMJ. 2005;330(7485):223.
3. Menzler S, Piller G, Gruson M, Rosario AS, Wichmann HE, Kreienbrock L. Population attributable fraction for lung cancer due to residential radon in Switzerland and Germany. Health Phys. 2008;95(2):179-89.

Lead Exposure Capstone Appendix

Flowchart

Lead Exposure

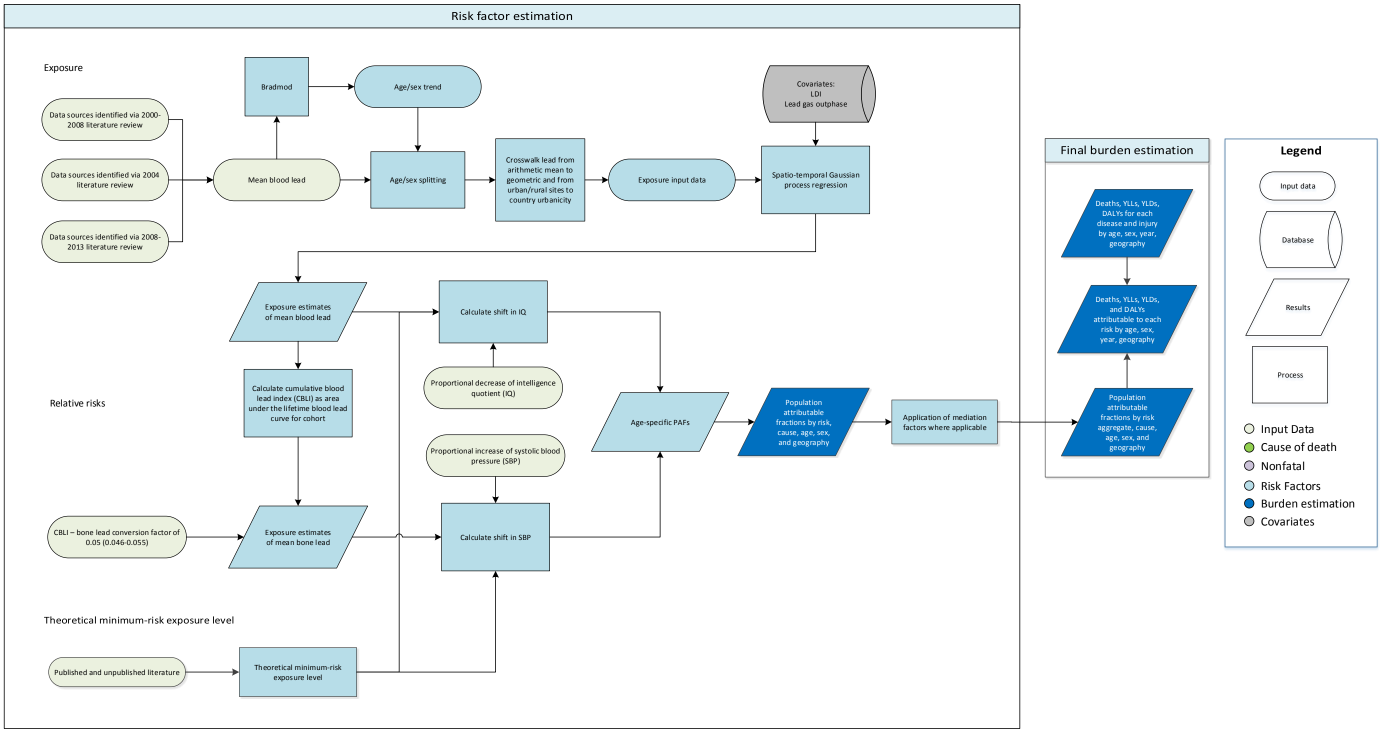

Input Data & Methodological Summary

Exposure

Case definition

Exposure to lead is defined in two different ways according to the currently known pathways of health loss. Acute lead exposure, relevant to disease burden in children, is measured as the micrograms of lead per deciliter of blood (µg/dL). Long-term lead exposure, relevant to disease burden in adults given the manifestation of health impact through increased systolic blood pressure and hence a decline of cardiovascular health, is measured as the accumulation of lead in the bone as micrograms of lead per gram of bone (µg/g).

Input data

The input data for lead exposure is derived from values extracted from literature regarding blood lead. Typically, these values are produced by studies that take blood samples and analyze them using various techniques to determine the level of lead present. The blood lead database for GBD2010 was augmented with an updated literature review for the years 2008-2013. This combined approach yielded 1,573 usable

data points from 332 different studies, which spanned the years 1964 to 2013. More than 400 new data points were added, including 337 for children and 102 country-years. The update for children is particularly relevant since blood lead impacts child IQ. The database of literature values was modelled for data-sparse countries using spatio-temporal GPR (ST-GPR). These values were used as blood lead exposure. The second pathway of burden is related to bone lead exposure, which was estimated by calculating a cumulative blood lead index for cohorts using estimated blood lead over their lifetime. The cumulative blood lead index is then used to estimate bone lead using a scalar defined by the literature[1].

Modelling Strategy

There methodology to estimate lead exposure last underwent significant change in GBD2013. A literature review was conducted to update the exposure dataset, to include new studies and those missed by previous reviews. Global exposure was previously modelled using age-integrating Bayesian hierarchal modelling (DisMod-MR). The modelling process was previously updated for GBD 2013 by shifting to spatial-temporal GPR methodology. This allowed for estimates of all country-age-sex-year groups for single years instead of five year periods. This approach improved the granularity of estimates for bone lead, which requires back-estimation of previous blood lead to calculate a cumulative blood lead index.

For GBD2015, the spatial-temporal GPR modelling methodology was updated as detailed in the appendix specific to this analytical technique, which is common to a variety of risk factors. In order to predict blood lead in country-years with insufficient data, covariates that have been produced across the time and space relevant to this analysis were used. For blood lead exposure, the covariates determined to have predictive ability were lag distributed income per capita (in log) and a binary covariate indicating whether lead in gasoline had been phased out for that country-year. ST-GPR was used to produce estimates of blood lead for all age groups, for both sexes, and for all GBD countries from 1970 to 2015. Next, to calculate blood lead over the lifetime of a given cohort, blood lead was assumed to grow linearly from 2.0 ug/dL in 1920 (see TMREL) to the value for that cohort in 1970. Using that database of blood lead over time and space, cohorts were constructed such that the lifetime blood lead could be expressed as a curve over each year of their life. The area under this curve was the cumulative blood lead index, which could be used to estimate bone lead in a given year with the aforementioned scalar.

Theoretical minimum-risk exposure level

The TMREL is taken from literature estimates of pre-industrial blood lead in humans[4]. This value is estimated at 2.0 ug/dL. The decision was made that the TMREL of blood lead could not be 0 given the ambient sources of lead that would be impossible to eliminate[5].

Relative Risks

The blood lead relative risks were taken from a 2005 pooled analysis that was updated for GBD2010[2]. The bone lead relative risks were taken from a 2008 meta-analysis that was updated for GBD2010[3]. Neither of these effect sizes were modified for GBD2015.

References

1. Hu H, Shih R, Rothenberg S, Schwartz BS. The epidemiology of lead toxicity in adults: measuring dose and consideration of other methodologic issues. Environ Health Perspect. 2007;115(3):455-62.
2. Lanphear BP, Hornung R, Khoury J, et al. Low-level environmental lead exposure and children's intellectual function: an international pooled analysis. Environ Health Perspect. 2005;113(7):894-9.
3. Navas-acien A, Schwartz BS, Rothenberg SJ, Hu H, Silbergeld EK, Guallar E. Bone lead levels and blood pressure endpoints: a meta-analysis. Epidemiology. 2008;19(3):496-504.
4. Flegal AR, Smith DR. Lead levels in preindustrial humans. N Engl J Med. 1992;326(19):1293-4.
5. Pruss-Astun A, Fewtrell L, Landrigan PJ, Ayuso-Mateos JL. Lead Exposure. In: Ezzati M, Lopez AD, Rodgers A, Murray CJ, eds. Comparative quantifications of health risks: Global and regional burden of disease attrituable to selected major risk factors. Geneva, World Health Organization, 2004: 1496-542

Occupational Risk Factors

Flowchart

Occupational Risk Factors
(except abestos and injuries)

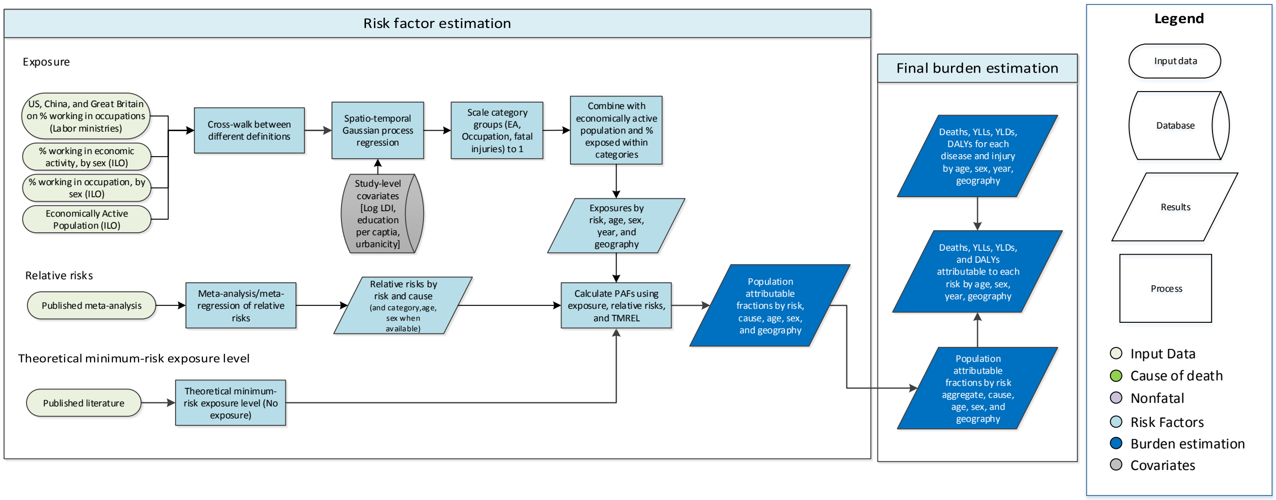

Occupational Risk Factors (Injuries)

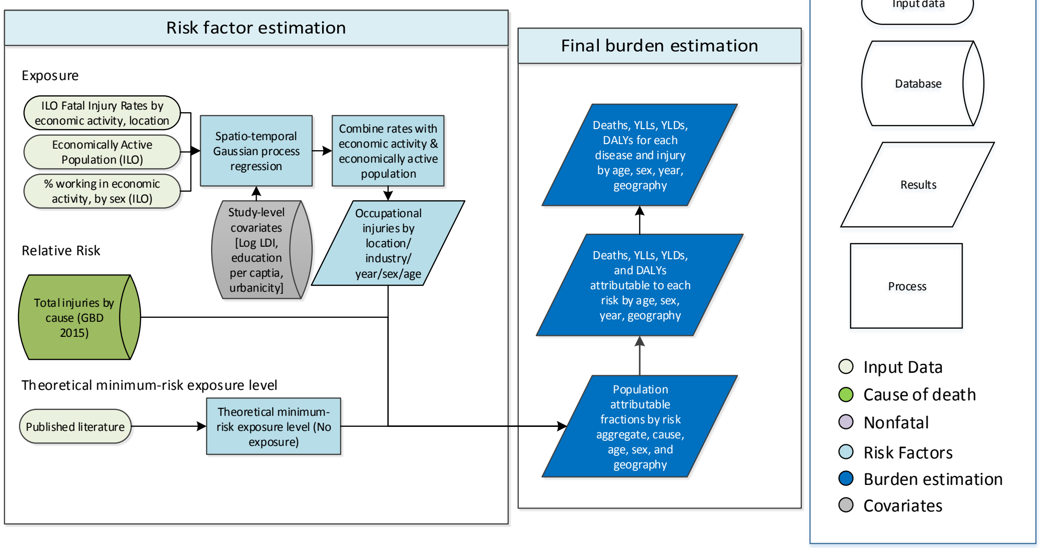

Occupational Risk Factors (abestos)

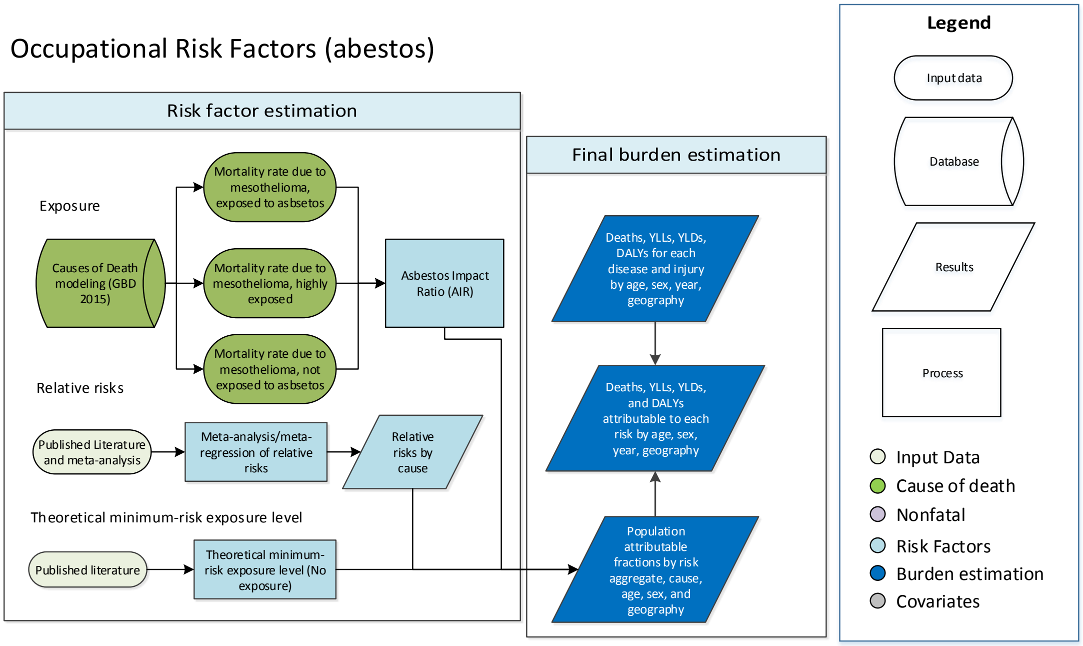

Input Data and Methodological Summary

Exposure

Case Definition

The following definitions were used for occupational risk factor exposures. All exposures were estimated only for ages 15+

Occupational Asbestos	Cumulative exposure to occupational asbestos using mesothelioma death rate as an analogue.
Occupational Asthmagens	Proportion of working population exposed to asthmagens based on distribution of the population in seven occupational groups
Occupational Carcinogens (arsenic, acid, benzene, beryllium, cadmium, chromium, diesel, formaldehyde, nickel, polycyclic aromatic hydrocarbons, second-hand smoke, silica, trichloroethylene)	Proportion of working population ever exposed to carcinogens in high or low exposures groups, based on distribution of the population in nine economic activity groups
Occupational Injuries	Proportion of fatal injuries attributed to occupational work in nine economic activities, based on fatal injury rates in those economic activities.
Occupational Ergonomic Factors	Proportion of working population exposed to lower back pain, based on distribution of the population in seven occupational groups.

Occupational Noise	Proportion of working population exposed to 85+ decibels of noise, based on distribution in nine economic activities.
Occupational Particulates	Proportion of working population exposed based on distribution in nine economic activities

Estimates of the proportion of population involved in economic activities and occupations were coded into the following categories:

**Economic Activities**	**Occupations**
Agriculture, hunting, forestry and fishing	Agriculture, animal husbandry, forestry workers, fishermen, and hunters
Mining and quarrying	Production, transport equipment operators and laborers, and related workers
Wholesale and retail trade, restaurants, and hotels	Professional, technical, and related workers
Manufacturing	Sales workers
Electricity, gas, and water	Administrative and managerial workers
Transport, storage, and communication	Clerical and related workers
Construction	Service workers
Financing, insurance, real estate, and business services	
Community, social, and personal services	

Input data

Primary inputs were obtained from the ILO [1-4], using raw data on economic activity proportions, occupation proportions, fatal injury rates, and economically active population estimates. For different ISIC classifications, estimates were recoded to one of the nine economic activities or occupations. Subnational estimates for UK and China were added to the datasets for economic activities and occupations [5-6].

For occupational asbestos, primary inputs were obtained through GBD 2015 cause of death estimates and published studies. [7,13-14]

Modeling strategy

A spatial-temporal Gaussian process regression was used to generate estimates for all year/locations for the primary inputs (see app section 2). Parameters were chosen by maximizing out-of-sample cross-validation and minimizing RMSE. For economic activity and occupation proportions, estimates from ST-GPR were then re-scaled to sum to 1 across categories by dividing each estimate by the sum of all the estimates.

The following sections describe the modeling approaches for each occupational risk's prevalence exposure.

Occupational carcinogens, occupational noise, occupational particulates

Prevalence of exposure to these risks was determined using the following equation:

$$Prevalence\ of\ Exposure_{c,y,s,a,r,l} = \sum_{EA} Proportion_{EA,c,y} * EAP_{c,y,s,a} * Exposure\ rate_{EA,r,l,d}$$

where:

EAP = Economically active population	c = country	r = risk
EA = economic activity	d = duration	s = sex
a = age	l = level of exposure	y =year

Exposure rate was provided by expert group recommendations and literature [8-11] (see table 1). Duration was only considered for occupational carcinogens, through application of occupational turnover factors [12].

Occupational ergonomic factors and asthmagens

Prevalence of exposure to these risks was determined using the following equation:

$$Prevalence\ of\ Exposure_{c,y,s,a,r} = \sum_{EA} Proportion_{OCC,c,y} * EAP_{c,y,s,a}$$

where:

EAP = Economically active population	c = country	r = risk
OCC = occupation	a = age	s = sex
		y = year

Occupational injuries

Occupational injury counts were estimated using the following equation:

$$Occupational\ fatal\ injuries_{c,y,a,s} = \sum_{EA} Injury\ rate_{EA,c,y,s} * Population_{c,y,a,s} * EAP_{c,y,s,a} * Proportion_{EA,c,y}$$

where:

EAP = Economically active population	c = country	y = year
EA = economic activity	a = age	s = sex

Occupational asbestos

Prevalence of exposure to asbestos was estimated using the asbestos impact ratio (AIR), which is equivalent to the excess deaths due to mesothelioma observed in a population divided by excess deaths due to mesothelioma in a population heavily exposed to asbestos. Formally, this is defined using the following equation:

$$AIR = \frac{Mort_{c,y,s} - N_{c,y,s}}{Mort^{*}_{c,ys,} - N_{c,y,s}}$$

where:

Mort = Mortality rate due to mesothelioma
Mort* = Mortality rate due to mesothelioma in population highly exposed to asbestos
N = Mortality rate due to mesothelioma in population not exposed to asbestos

c = country
y = year
s = sex

Mortality rate due to mesothelioma was estimated from GBD 2015 causes of death [7]. Mortality rate due to mesothelioma in population not exposed to asbestos was calculated using the model in Lin et al. [13], while the mortality rate due to high exposure to asbestos was estimated in Goodman et al. [14]

Theoretical minimum-risk exposure level

For all occupational risks, with the exception of occupational asbestos, the theoretical minimum-risk exposure level was assumed to be no exposure to that risk.

Relative risk

Relative risks were obtained for all occupational risks by conducting a systematic review of published meta-analysis. The estimates used, as well as the associated studies, are reported by category group in appendix table 5.

Population Attributable Fraction

For all occupational risks, with the exception injuries outlined below, PAFs were calculated using the prevalences estimated above, using the PAF formula in appendix section 2.

Occupational injuries PAF

The PAF for occupational injuries was calculated using the following formula:

$$PAF_{c,y,a,s} = \frac{Occupational\ fatal\ injuries_{c,y,a,s} - TMREL}{Fatal\ injuries_{c,y,a,s}}$$

where:

c = country
y = year

a = age
s = sex

Fatal injuries total was obtained from GBD 2015 causes of death [7].

Citations

1. International Labour Organization (ILO). International Labour Organization Database (ILOSTAT) - Employment by Sex and Economic Activity. International Labour Organization (ILO).

2. International Labour Organization (ILO). International Labour Organization Database (ILOSTAT) - Employment by Sex and Occupation. International Labour Organization (ILO).

3. International Labour Organization (ILO). International Labour Organization Database (ILOSTAT) - Fatal Injuries by Sex and Economic Activity. International Labour Organization (ILO).

4. International Labour Organization (ILO). International Labour Organization LABORSTA Economically Active Population, Estimates and Projections, October 2011. International Labour Organization (ILO), 2011.

5. Office for National Statistics (United Kingdom). Nomis Official Labor Market Statistics - Annual Population Survey. Newport, United Kingdom: Office for National Statistics (United Kingdom).

6. National Bureau of Statistics of China. China 1% National Population Sample Survey 1995. Ann Arbor, United States: China Data Center, University of Michigan.

7. GBD 2015 Mortality and Causes of Death Collaborators. Global, regional, and national life expectancy, all-cause and cause-specific mortality for 249 causes of death, 1980–2015: a systematic analysis for the Global Burden of Disease Study 2015. *Lancet Rev*.

8. Wilson DH, Walsh PG, Sanchez L, *et al.* The epidemiology of hearing impairment in an Australian adult population. *Int J Epidemiol* 1999; 28: 247–52

9. Kauppinen T, Toikkanen J, Pederson D, Young R, Kogevinas M, Ahrens W, et al. Occupational Exposure to Carcinogens in the European Union in 1990-93. Helsinki, Finland: Finnish Institute of Occupational Health; 1998.

10. Kauppinen T, Toikkanen J, Pedersen D, Young R, Ahrens W, Boffetta P, et al. Occupational exposure to carcinogens in the European Union. *Occup Environ Med* 2000; 57(1): 10–18.

11. Driscoll T, et al. The global burden of non-malignant respiratory disease due to occupational airborne exposures. *American Journal of Industrial Medicine 2005; 48*(6): 432-445.

12. Nelson, D. I., Concha-Barrientos, M., Driscoll, T., Steenland, K., Fingerhut, M., Punnett, L. & Corvalan, C. (2005). The global burden of selected occupational diseases and injury risks: Methodology and summary. *American journal of industrial medicine*, *48*(6), 400-418

13. Lin R-T, Takahashi K, Karjalainen A, *et al.* Ecological association between asbestos-related diseases and historical asbestos consumption: an international analysis. *Lancet* 2007; **369**: 844–9.

14. Goodman M, Morgan RW, Ray R, Malloy CD, Zhao K. Cancer in asbestos-exposed occupational cohorts: a meta-analysis. *Cancer Causes Control* 1999; **10**: 453–65.

Table 1 – Exposure rate by economic activity (per 100k workers)

Risk Factor	Agriculture, Hunting, Forestry and Fishing	Mining and Quarrying	Manufacturing	Electricity, Gas, and Water	Construction	Wholesale and Retail Trade and Restaurants and Hotels	Transport, Storage, and Communication	Financing, Insurance, Real Estate and Business Services	Community, Social and Personal Services
Arsenic	54	72	399	148	134	6	-	2	11
Asbestos	1,248	10248	589	1702	5203	292	684	16	286
Benzene	59	197	308	91	75	1037	520	41	2330
Beryllium	-	55	207	70	4	2	11	-	3
Cadmium	-	-	486	287	291	2	65	-	48
Chromium VI	-	346	2061	409	237	17	369	-	227
Diesel engine exhaust	646	21970	1192	3359	5816	485	13432	-	920
Second-hand smoke	2,082	163	5249	6172	4830	9278	6965	4584	3633
Formaldehyde	186	255	2103	28	545	53	23	22	594
Nickel	-	2025	1663	352	47	7	3	-	43
Polycyclic aromatic hydrocarbons	-	1021	1650	3066	1328	106	905	-	388
Silica	372	23049	2316	1415	18860	17	476	2	60
Sulfuric acid	-	366	1488	928	577	264	255	81	189
Noise, 90+ dB, high exposure	26100	57200	23300	27400	36200	100	18000	400	15900
Noise, 85-90 dB, high exposure	16700	25400	32200	13800	21000	23100	28700	23000	17600
Noise, 90+ dB, low exposure	18000	39300	10600	20400	25100	0	7900	0	900
Noise, 85-90 dB, low exposure	14400	29400	24500	12300	19400	1800	20200	3100	13100
Particulates, developed, high exposure	10000	10000	10000	10000	10000	0	10000	0	0
Particulates, developed, low exposure	5000	7000	7000	5000	7000	500	5000	500	500
Particulates, developing, high exposure	10000	40000	40000	10000	40000	0	10000	0	0
Particulates, developing, low exposure	70000	40000	40000	70000	40000	10000	70000	10000	10000

Suboptimal Breastfeeding Capstone Appendix

Flowchart

Breastfeeding

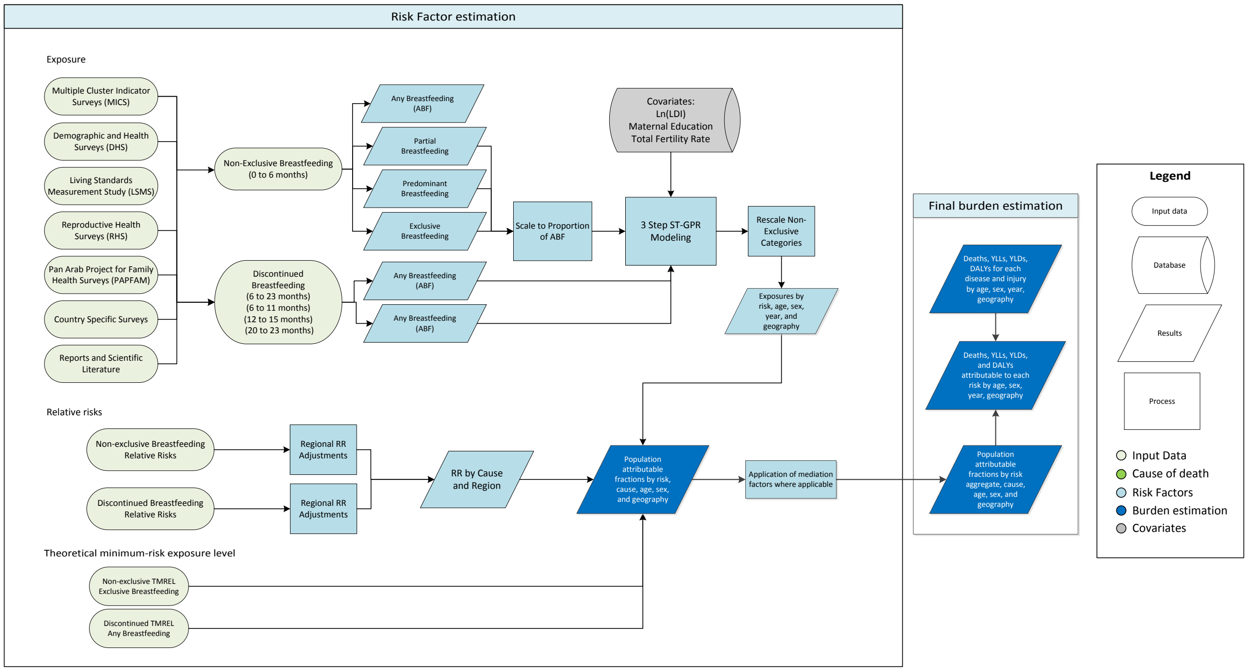

Input Data & Methodological Summary

Exposure

Definition

Exposure to suboptimal breastfeeding is composed of two distinct categories: nonexclusive breastfeeding and discontinued breastfeeding. Non-exclusive breastfeeding is defined as the proportion of children under 6 months who are not exclusively breastfed. Those not exclusively breastfed are then parsed into three categories – predominate, partial, and no breastfeeding. Discontinued breastfeeding is defined as the proportion of children between 6 to 23 months who receive no breast milk.

Input data

The data used in this analysis consists mostly of processed micro data from surveys and tabulated data from scientific literature and reports. The data was primarily sourced from the micro data of surveys. The data updates were focused on the extraction of the larger surveys at the subnational level, especially for those subnational locations added into GBD 2015. Tabulated data was only used when micro data was not available.

Modeling

A complete time series from 1980 to 2015 for the prevalence of breastfeeding patterns for children 0 to 6 months and 6 to 23 months were generated. This was accomplished by carrying the processed micro and tabulated data through a three-step modeling process. First, a robust linear regression incorporating the covariates of log-transformed lag-distributed income, total fertility rate, and the mean years of education of women of reproductive age. This is followed by a spatial-temporal regression that uses the residuals of the predictions from the linear regression to perform a locally-weighted regression that provides a greater weighting factor to those nearer in space and time. The predicted residuals from this step are added to those created in the linear regression. The final of the three steps is the Gaussian Process Regression. This step incorporates the variance of the input data as well as that of the model predictions. It uses predictions from the spatial-temporal regression as the mean function and generates draws from a multinomial distribution, based on the data uncertainty in the prior, to generate the final prevalence estimates and their confidence intervals.

Relative risks

Relative risks used for suboptimal breastfeeding are generated based on two published meta-analyses. Non-exclusive breastfeeding exposure was paired with diarrhea and LRI as disease outcomes. Discontinued breastfeeding was paired with diarrhea only. The outcomes URI and otitis media that were included by analogy to LRI for this round of GBD.

In the case of developed regions, there is assumed to be no risk of diarrheal diseases. We have also applied a novel adjustment to the existing relative risks in order to make them representative to their larger GBD age groups (post neo-natal in the case of nonexclusive breastfeeding and 1 to 4 years in the case of discontinued breastfeeding.

Theoretical minimum-risk exposure level

For non-exclusive breastfeeding, those children that receive no source of nourishment other than breastmilk are considered to be at the lowest risk of any of the disease outcomes. For discontinued breastfeeding, we assume that children aged 6 to 23 months who receive any breastmilk as a source of nourishment to be at the lowest risk of disease outcome.

Childhood Undernutrition Capstone Appendix

Flowchart

Childhood Undernutrition

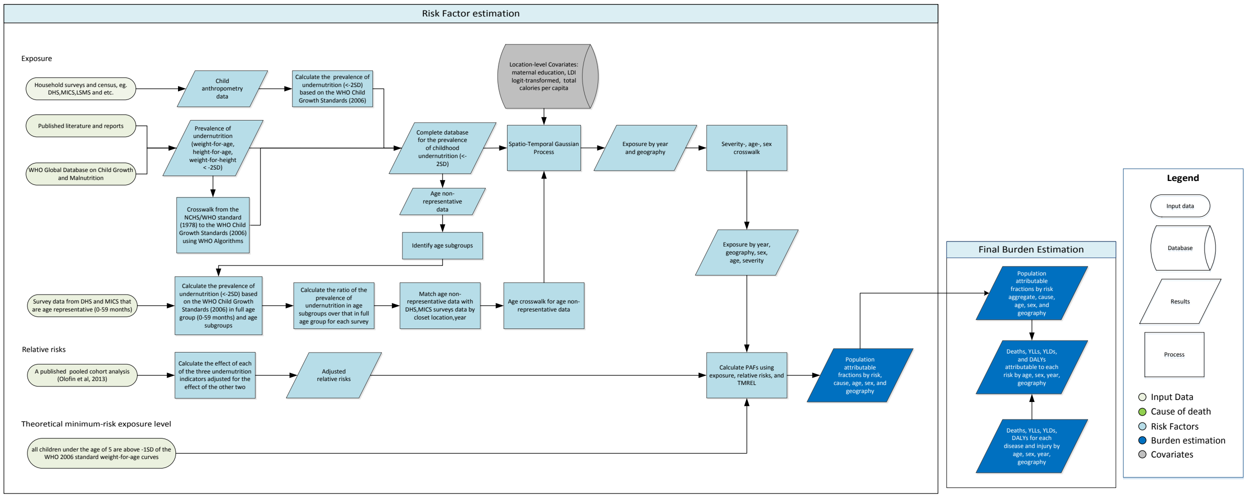

Input Data & Methodological Summary

Exposure

Case Definition

The exposure of childhood undernutrition was modeled by evaluating three anthropometric indicators which include underweight, wasting, and stunting. The definition of the three indicators are as follows:

Childhood underweight: Proportion of children aged 0 to 59 months in a given population who fall below 2 standard deviations (SD) of the WHO 2006 standard weight-for-age (wfa) curve. (1)

Childhood stunting: Proportion of children aged 0 to 59 months in a given population who fall below 2 standard deviations (SD) of the WHO 2006 height-for-age (hfa) curve.

Childhood wasting: Proportion of children {Citation}aged 0 to 59 months in a given population who fall below 2 standard deviations (SD) of the WHO 2006 weight-for-height (wfh) curve.

Input data

There are two main inputs in the GBD 2015 undernutrition database—survey dataset and tabulated dataset. Survey dataset includes the standard multi-country or country-specific survey series such as: Reproductive and Health Surveys (RHS), Multiple Indicator Cluster Surveys (MICS), Demographic and Health Surveys (DHS), Living Standards Measurement Surveys (LSMS), China Health and Nutrition Survey (CHNS) and etc. In the absence of survey data we used tabulated data from survey reports or published literature that have been extracted at IHME, downloaded from external databases or obtained from personal communication with external collaborators. The last update for tabulated dataset was

conducted for GBD 2010. Tabulated data include survey reports or published literature from databases from UNICEF(2), the United Nations (UN) Statistics Division (3), and the WHO Global Database on Child Growth and Malnutrition(4).

Tabulated data based on the National Center for Health Statistics (NCHS)/WHO international growth reference (the NCHS reference) (5) were converted into data based on the World Health Organization (WHO) Child Growth Standards (the WHO 2006 standard) using WHO algorithms (6). Estimates that were not representative of all children under the age of 5 were adjusted based on age groups.

Modeling strategy

Exposure Estimate

To generate a complete time series of prevalence of childhood underweight, wasting, and stunting, we employed a three-step ST-GPR modeling strategy that uses linear regression, spatiotemporal regression and Gaussian Process Regression (GPR) which is specified in the main text of this manuscript. Identical strategies and covariates were used for each undernutrition indicator. A variety of combinations of socioeconomic and environmental covariates in different transformation format were tested by running mixed-effect models with exposure data to decide the inclusion and exclusion. The final list of covariates included in the childhood undernutrition models are mean years of education of women of reproductive age, log transformed lagged-distributed income and total caloric availability (kcal per capita), which remained the same as GBD 2013. Uncertainty in the estimates was based on the data variance, then calculated through ST-GPR.

The final step of exposure estimate is to calculate the distribution of undernutrition prevalence across different levels of severity and age- sex- groups. The levels of severity are defined as follows:

Severe: individuals less than 3SD below the median (<-3SD);

Moderate: individuals between 3SD and 2SD below the median (-3SD to -2SD);

Mild: individuals between 2SD and 1SD below the median (-2SD to -1SD).

In GBD 2013, prevalence of undernutrition in each of severity categories was predicted by applying a linear regression model of the prevalence of undernutrition in each of severity categories against the prevalence of undernutrition below -2SD of the reference median at global level using microdata from 179 DHS surveys. We assumed no difference in the prevalence of undernutrition at any severity level across age and sex among children under 5.

This strategy has experienced a major change in GBD 2015. We estimated the prevalence of undernutrition by GBD age-sex groups, assuming the distribution of undernutrition of different severity categories are difference across age and sex among children under 5. Using available microdata, we first created a pooled global database that consisted of binary indicators of undernutrition by GBD age-sex groups at individual level. Then we ran a logit regression model to predict the proportion of

undernutrition outcome in most-detailed severity category (e.g. <-3SD) among the broader severity category (e.g. <-2SD) against the effects of age group and sex. We also took into account the covariance of the proportions among different age-sex groups by using variance-covariance matrix. Last, we applied the proportions by GBD age-sex group generated above onto our GPR estimates.

Theoretical minimum-risk exposure level

Theoretical minimum risk exposure levels (TMREL) for underweight, stunting, and wasting where all children under the age of 5 are above -1SD of the WHO 2006 standard weight-for-age, height-for-age, and weight-for-height curves respectively.

Relative risks

Relative risks (RRs) of risk-outcome pairs were extracted based on a study that conducted a pooled cohort analysis (7), which remained the same as GBD 2013. The final list of outcomes paired with childhood undernutrition risks included lower respiratory infections (LRI), diarrhea, measles, and protein energy malnutrition (PEM). Originally in GBD 2013, URI and otitis media were considered as analogies for LRI considering the similar pathological pathways they share. However, they were dropped from analysis in GBD 2015 due to the lack of evidence on the causal relationships with undernutrition risks. We also attributed 100% of PEM to childhood wasting and underweight but not stunting. A literature search was conducted for GBD 2015 searching for meta-analysis on the association of risk-outcome pairs published after January 1st, 2013, no updated results was found.

The RRs were adjusted using an optimization algorithm we developed at IHME for GBD 2013 that takes into account covariance between the three undernutrition indicators.

Iron Deficiency Risk Factors Appendix

Flowchart

Iron deficiency

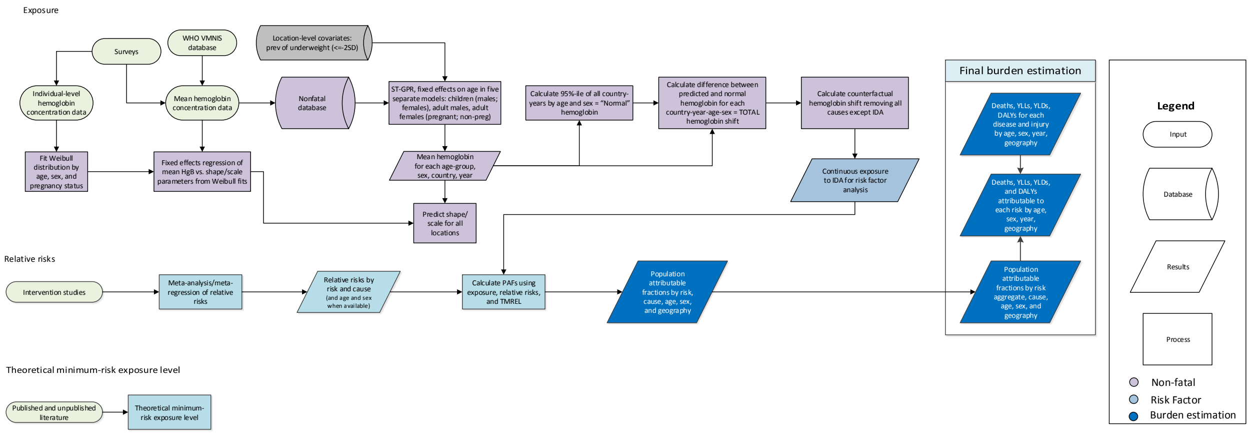

Input Data and Methodological Summary

Exposure

Definition

To estimate anemia in GBD 2015, we employed the same method used in GBD 2013 and largely similar to GBD 2010. Our analytic strategy began with calculation of an anemia envelope – a determination of mean hemoglobin, as well as sum total of anemia prevalence, by severity for each country, age group, and both sexes for each year from 1990 through 2015. The envelope approach avoids double counting while capturing potentially different disease profiles within each population group. We defined a population group as a specific geography, sex, age-group, and year.

Input data

Iron-deficiency anemia (IDA) estimates include acute and chronic hemorrhagic states for which supplementation may be helpful, but poor nutritional intake is not the only underlying problem. A few causes in this category – hookworm, schistosomiasis, upper gastrointestinal bleeding, and gynecologic diseases – were considered separately from IDA because there was enough data from GBD prevalence estimation processes to do so. Distribution of anemia burden to IDA only after assignment to "known" causes avoided double counting of these cases.[1, 2]

Modeling strategy

We estimated the mean hemoglobin in g/dL among pregnant women aged 15 to 49 years of age and the implied mean hemoglobin among pregnant women in the absence of iron deficiency anemia, as the risk exposure for maternal iron deficiency anemia.

Theoretical minimum-risk exposure level

The implied mean hemoglobin in the absence of iron deficiency anemia is the theoretical minimum risk exposure level. This was calculated by adding the iron deficiency shift back onto the observed hemoglobin concentration for each demographic. For example, if the observed hemoglobin concentration among 30-34 year old pregnant women in Ethiopia was 132.9 g/L, and the shift was 1.6 g/L in that demographic, then the counteractual was 134.5 g/L. The GBD 2015 anemia modeling strategy provides details on how the iron deficiency shifts were calculated.

Relative risk

We attribute 100% of iron-deficiency anemia to iron deficiency. The other outcomes are maternal hemorrhage and maternal sepsis and other maternal infections. Sources of evidence for these relative risks are unchanged from GBD 2013.

References

1. Centers for Disease Control and Prevention (CDC). Iron deficiency--United States, 1999-2000. MMWR Morb Mortal Wkly Rep 2002; 51: 897–9.
2. Looker AC, Dallman PR, Carroll MD, Gunter EW, Johnson CL. Prevalence of iron deficiency in the united states. JAMA 1997; 277: 973–6.

Vitamin A Deficiency Capstone Appendix

Flowchart

Vitamin A deficiency

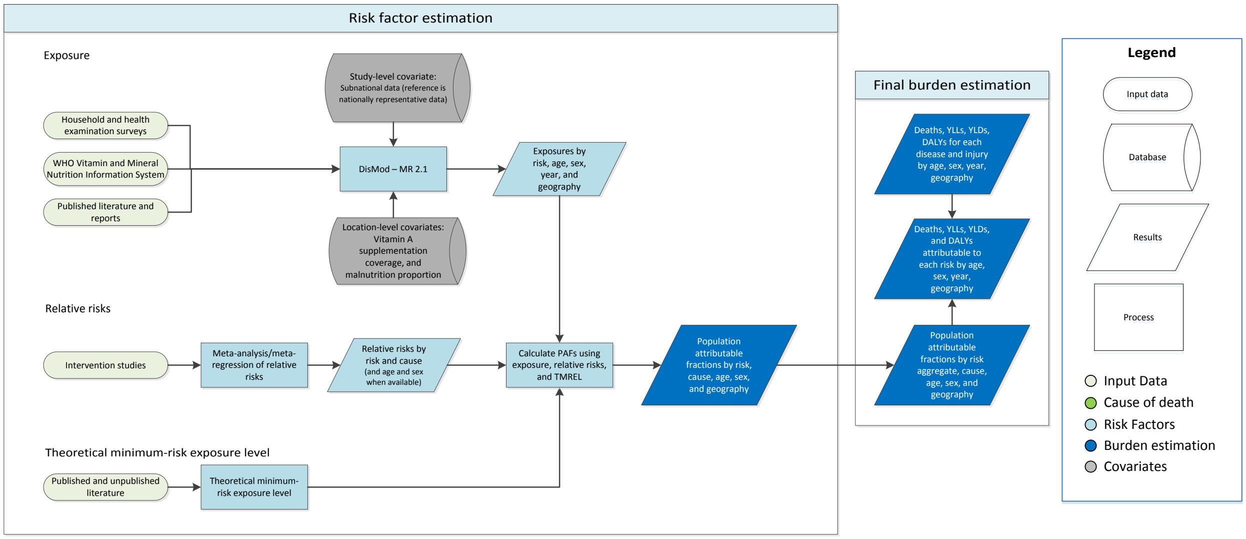

Input Data & Methodological Summary

Exposure

Definition

For GBD 2015, vitamin A deficiency is defined as serum retinol <70 µmol/L. We examined vitamin A deficiency as a risk factor in children aged 6 months to 5 years.

Input data

For GBD 2015, we used data from the WHO Vitamin and Mineral Nutrition Information System, Demographic and Health Surveys, and studies identified through literature review. A systematic review was conducted for GBD 2013.

The PubMed search terms were: ((vitamin A deficiency[Title/Abstract] AND prevalence[Title/Abstract]) AND ("2009"[Date – Publication] : "2013"[Date – Publication]))

The exclusion criteria were:

1. Studies that were not population-based, e.g., hospital or clinic-based studies
2. Studies that did not provide primary data on epidemiological parameters, e.g. commentaries
3. Review articles

4. Case series
5. Self-reported cases

Updates to systematic reviews are performed on an ongoing schedule across all GBD causes and risk factors, an update for vitamin A deficiency will be performed in the next 1-2 iterations.

Modeling Strategy

We used DisMod MR-2.0 to model prevalence of vitamin A deficiency. We used a study level covariate to indicate national and subnational observations, where nationally representative studies were set as the reference category. We used vitamin A supplementation coverage and malnutrition proportion as location-level covariates to inform variation over year and geography, especially in location-years with no or sparse data. We have made no substantive changes in the modeling strategy from GBD 2013.

Theoretical minimum-risk exposure level

The theoretical minimum risk exposure is that the prevalence of vitamin A deficiency is zero.

Relative risks

The relative risks have not changed from GBD 2013.

Zinc Deficiency Capstone Appendix

Flowchart

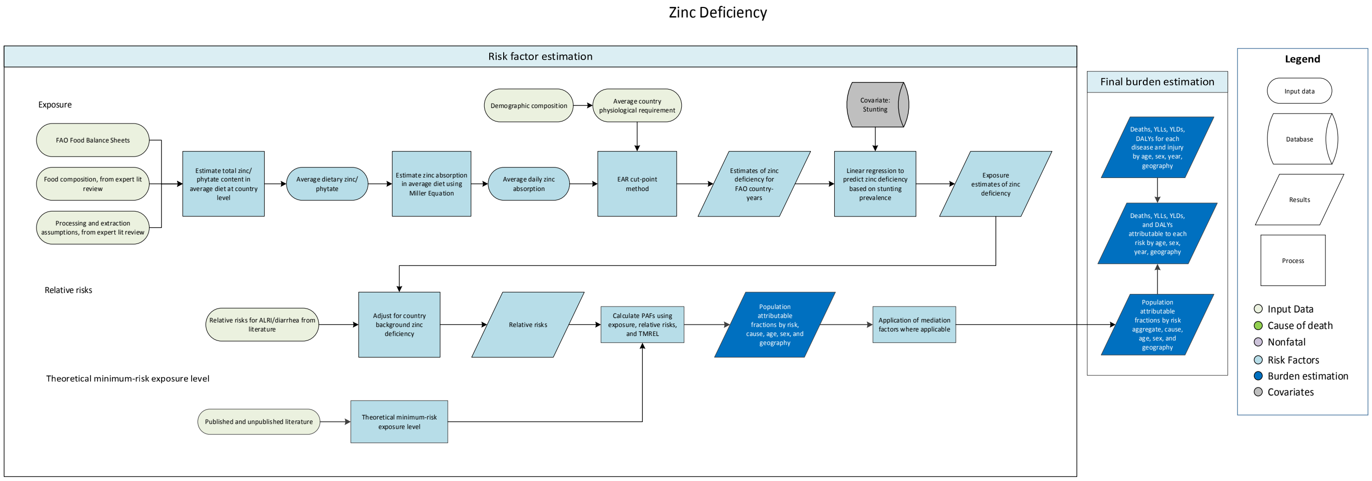

Input Data & Modelling Strategy

Exposure

Case Definition

Exposure to zinc deficiency is a measured of total absorbed zinc which is a function of both zinc and phytate consumption.

Input data

The Food and Agriculture Organization's (FAO) Food Balance Sheets are used to determine the total absorbed zinc per person for each country-year that they publish.

Modeling strategy

For GBD 2015, first, available zinc and phytate in each country-year were calculated using FAO's Food Balance Sheets. The availability of each of these nutrients was determined using composition indices provided by our expert group. We extract phytate as well as zinc due to its functioning to inhibit zinc absorption. Then, using an equation defined by literature, the average total absorbed zinc was estimated based on the ratio of zinc to phytate in available foods. A normal distribution with a standard deviation of .25 was assumed to estimate the proportion of each population that would fall below the recommended zinc intake. Then, a complete time series from 1980 to 2015 for the proportion zinc deficient children 1 to 5 years was generated. This was accomplished by the FAO-based data through a three-step modeling process. First, a robust linear regression incorporating the covariates of log-transformed lag-distributed income as well as the proportion of malnourished individuals for each country-year. This is followed by a spatiotemporal regression that uses the residuals of the predictions from the linear regression to perform a locally-weighted regression that provides a greater weighting factor to those nearer in space and time. The predicted residuals from this step are added to those created in the linear regression. The final of the three steps is the Gaussian Process Regression. This step incorporates the variance of the input data as well as that of the model predictions. It uses predictions from the spatial-temporal regression as the

mean function and generates draws from a multinomial distribution, based on the data uncertainty in the prior, to generate the final prevalence estimates and their confidence intervals.

Theoretical minimum-risk exposure level

The theoretical minimum-risk exposure level for proportion zinc deficient is zero percent deficient.

Relative risks

Relative risks used for zinc deficiency is based on the results of clinical trials that measured the effect of zinc supplementation that were adjusted for background zinc estimates that come from the GBD estimation process.

Smoking Capstone Appendix

Flowchart

Smoking

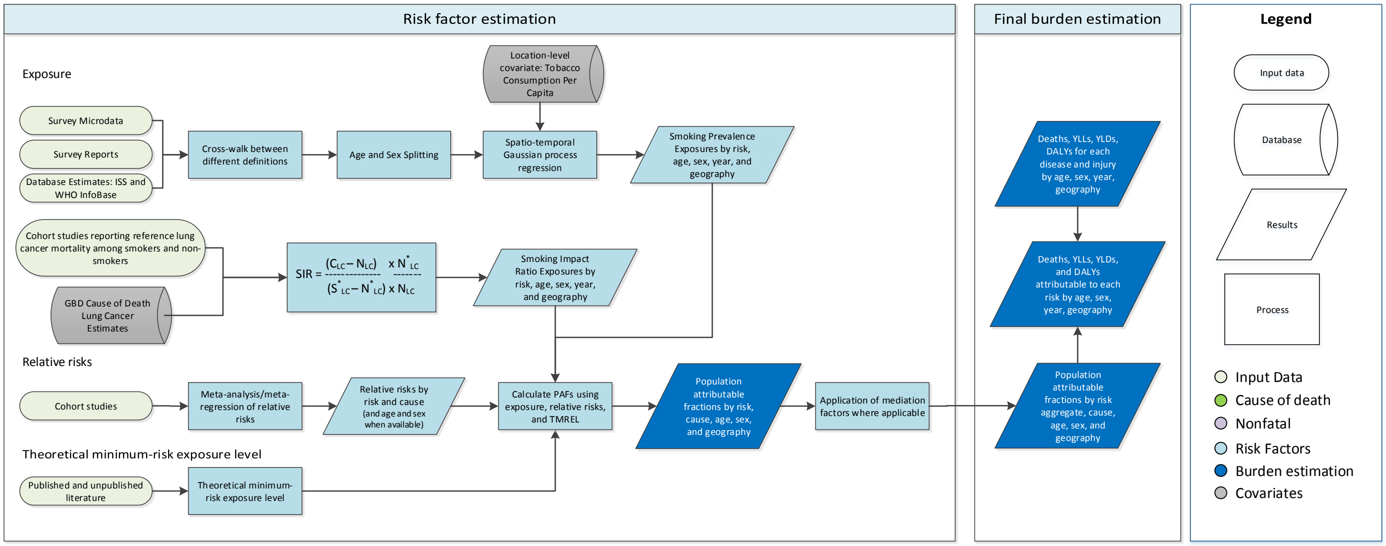

Input Data & Methodological Summary

Exposure

Case definition

We used the Smoking Impact Ratio (SIR) for modeling burden attributable to smoking for cancers, chronic obstructive pulmonary disease (COPD), interstitial lung disease, other chronic respiratory diseases, and pneumoconiosis. SIR is the population lung cancer mortality in excess of lung cancer mortality among never-smokers, relative to excess lung-cancer mortality observed in a known reference group of smokers. Currently, SIR is adjusted to account for differences in baseline never-smoker lung cancer mortality across geography, age, and sex, but not for differences across time.

We used 5-year lagged smoking prevalence, for modeling burden attributable to smoking for cardiovascular diseases, TB, diabetes, lower respiratory infections, asthma, cataracts, macular degeneration, fractures, rheumatoid arthritis, and peptic ulcer disease. Smoking is a dichotomous exposure defined as current daily use of smoked tobacco.

A full list of outcomes included in GBD 2015 and their exposure definition is available in the table below.

Outcome	Exposure
Atrial fibrillation and flutter	5-year lagged smoking prevalence
Aortic aneurysm	5-year lagged smoking prevalence
Hypertensive heart disease	5-year lagged smoking prevalence
Ischemic heart disease	5-year lagged smoking prevalence

Other cardiovascular and circulatory diseases	5-year lagged smoking prevalence
Peripheral vascular disease	5-year lagged smoking prevalence
Hemorrhagic stroke	5-year lagged smoking prevalence
Ischemic stroke	5-year lagged smoking prevalence
Diabetes	5-year lagged smoking prevalence
Lower respiratory infections	5-year lagged smoking prevalence
Asthma	5-year lagged smoking prevalence
Tuberculosis	5-year lagged smoking prevalence
Peptic ulcer disease*	5-year lagged smoking prevalence
Rheumatoid arthritis*	5-year lagged smoking prevalence
Cataract*	5-year lagged smoking prevalence
Macular degeneration*	5-year lagged smoking prevalence
Hip fracture*	5-year lagged smoking prevalence
Non-hip fracture*	5-year lagged smoking prevalence
Bladder cancer	Smoking Impact Ratio (SIR)
Colon and rectum cancer	Smoking Impact Ratio (SIR)
Esophageal cancer	Smoking Impact Ratio (SIR)
Kidney cancer	Smoking Impact Ratio (SIR)
Leukemia	Smoking Impact Ratio (SIR)
Liver cancer	Smoking Impact Ratio (SIR)
Tracheal, bronchus, and lung cancer	Smoking Impact Ratio (SIR)
Lip and oral cavity cancer	Smoking Impact Ratio (SIR)
Nasopharynx cancer	Smoking Impact Ratio (SIR)
Pancreatic cancer	Smoking Impact Ratio (SIR)
Stomach cancer	Smoking Impact Ratio (SIR)
Larynx cancer*	Smoking Impact Ratio (SIR)
Chronic obstructive pulmonary disease	Smoking Impact Ratio (SIR)
Interstitial lung disease and pulmonary sarcoidosis	Smoking Impact Ratio (SIR)
Other chronic respiratory diseases	Smoking Impact Ratio (SIR)
Pneumoconiosis	Smoking Impact Ratio (SIR)

* New outcome in GBD 2015

Input data

Consistent with GBD 2013, we used nationally representative survey data to estimate smoking prevalence. Survey and report data identified in the Global Health Data Exchange (GHDx), the WHO InfoBase, and the International Smoking Statistics (ISS) Database.

Inclusion Criteria

- Nationally representative
- Report current use of any of the following frequency-type combinations:
  - Daily use of smoked tobacco
  - Any use (both daily and occasional) of smoked tobacco
  - Daily use of cigarettes
  - Any use (both daily and occasional) of cigarettes
  - Daily use of any tobacco (both smoked and smokeless)
  - Any use (both daily and occasional) of any tobacco (both smoked and smokeless)
  - Daily use of any tobacco excluding cigarettes

- Report data within the time period of January 1, 1980 – December 31, 2015 for any geography estimated in the GBD framework
- Smoking prevalence reported among individuals ages 10+

Global Health Data Exchange (GHDx)
Sources were identified through a systematic search of the GHDx.

- Search Terms (Keywords): Tobacco Use
- Time Period: January 1, 1980 – December 31, 2015
- Data Type: Survey OR Report
- Search Date: February 16, 2016

Out of 3,912 sources identified in the GHDx, 2,818 sources were included.

WHO InfoBase and International Smoking Statistics (ISS) Database
An effort was made to replace database-derived estimates used in GBD 2013 with original extractions from primary data sources. In GBD 2013, [851] sources were derived from the WHO InfoBase or the ISS Database. In GBD 2015, we replaced [257] sources with extractions from primary data sources and continued to use [594] sources from the WHO InfoBase (n=[281]) and the ISS Databse (n=[313]).

Outliers
Throughout the modeling process, data were assessed for bias and outliers were flagged. A data point was flagged as a candidate outlier if it was not consistent with the majority of other data points in a country with respect to level, age-pattern, sex-pattern, or temporal trend. In data-scarce countries, data points were also compared to data from other countries in a region. Candidate outliers were scrutinized for potential sources of bias and were ultimately excluded if the point or source was deemed to not be representative.

Modeling strategy

Data Extraction

When possible, we extracted individual smoking status for all available frequency-type categories (listed above) from person-level microdata and collapsed these data to produce prevalence estimates in the standard GBD 5-year age-sex groups. If microdata were unavailable we extracted the most granular age-sex groups available from survey reports. Any available measures of uncertainty were extracted, including standard error, confidence or uncertainty intervals, and sample size.

Data Preparation: Crosswalking
Regressions to crosswalk other frequency-type categories to the gold-standard definition of daily use of smoked tobacco were estimated in the form:

$$\mathrm{p}_{\mathrm{daily-smoked,k}} = \beta_1 \mathrm{p}_{\mathrm{i,k}} + \epsilon_{\mathrm{k}}$$

where $p_{daily\text{-}smoked,k}$ is the prevalence of daily smoking reported in survey *k*, and $p_{i,k}$ is the prevalence of an alternative frequency-type combination *i* also reported in survey *k*. Consistent with previous GBD smoking crosswalks, the intercept was omitted from the regression. The estimated regression coefficient β_1 was used to crosswalk alternative frequency-type categories to the gold-standard daily smoking definition in

sources only providing the alternative category. Predication error at the data-point level was used to propagate uncertainty and was calculated using the following equation:

$$\mathrm{PE_k} = \sigma_\epsilon^2 + \mathrm{X_k^2}\mathrm{var}(\hat{\beta})$$

Compared to the separate frequency and type crosswalks used in GBD 2013, the combined frequency-type crosswalk used in GBD 2015 represents an improvement because patterns in frequency that may vary by type and patterns in type that may vary by frequency are captured.

Data Preparation: Age and Sex Splitting

Report data provided in age groups wider than the standard GBD 5-year age groups or as both sexes combined were split using the approach used in Ng et al. Briefly, age-sex patterns were identified using sources with data on multiple age-sex groups and these patterns were applied to split aggregated report data. Uncertainty in the age-sex split was propagated by multiplying the standard error of the data (including the predication error of the crosswalk) by the square root of the number of splits performed.

Modeling: Linear Model

After data preparation, the dataset consisted of prevalence estimates of daily smoked tobacco use in standard GBD country-year-age-sex groups. The mean function used in ST-GPR was estimated using the following hierarchical mixed-effects linear regression, run separately by sex:

$$\mathrm{logit}(\mathrm{p_{c,a,t}}) = \beta_0 + \beta_1 \mathrm{CPC_{c,t}} + \sum_{\mathrm{k}=2}^{16} \beta_\mathrm{k} \mathrm{I_{A[a]}} + \alpha_\mathrm{s} + \alpha_\mathrm{r} + \alpha_\mathrm{c} + \epsilon_{\mathrm{c,a,t}}$$

where $CPC_{c,t}$ is the annual tobacco consumption per capita covariate, $I_{A[a]}$ is a dummy variable indicating specific age group A that the prevalence point $p_{c,a,t}$ is capturing, and α_s, α_r, and α_c are super region, region, and country-specific random effects.

Modeling: Spatio-Temporal Gaussian Process Regression (ST-GPR)

The estimated mean function was then propagated through the ST-GPR framework to obtain 1,000 draws of smoking prevalence estimates for each location, year, age, and sex. Parameter selection for the ST-GPR hyper-parameters were selected through out-of-sample cross-validation using the strategy described elsewhere in this appendix.

Smoking Impact Ratio Estimation

We have made no substantive changes in the SIR estimation strategy from GBD 2013. The only change in input data for estimating never-smoker lung-cancer mortality was to update data from the China Kadoorie Biobank prospective cohort to include follow-up through 2014. Country-year-age-sex specific lung cancer mortality rates are derived from GBD 2015 Cause of Death estimation and detailed in that Capstone's appendix. The formula for calculating SIR is:

$$SIR = \frac{C_{LC} - N_{LC}}{S_{LC}^* - N_{LC}^*} \times \frac{N_{LC}^*}{N_{LC}}$$

C_{LC}: age-sex-specific lung cancer mortality rate in the population of interest
N_{LC}: age-sex-specific lung cancer mortality rate of never-smokers in the population of interest
S^*_{LC}: age-sex-specific lung cancer mortality rate for life-long smokers in a reference population
N^*_{LC}: age-sex-specific lung cancer mortality rate for never smokers in the reference population

Theoretical minimum-risk exposure level

The theoretical minimum-risk exposure level is that no one in the population smokes tobacco; that is, the smoking impact ratio is zero and smoking prevalence is zero.

Relative risk

We have made no substantive updates to relative risks for outcomes included in GBD 2013. The following outcomes using 5-year lagged smoking prevalence as the exposure were added in GBD 2015: peptic ulcer disease, rheumatoid arthritis, cataracts, macular degeneration, hip fracture, and non-hip fracture. Larynx cancer was the only new outcome added using SIR as the exposure. Relative risks for rheumatoid arthritis, cataracts, and macular degeneration were derived from recent published meta-analyses. We performed out own meta-analyses of prospective cohort studies to derive relative risks for peptic ulcer disease, hip fracture, and non-hip fracture. We used Kontis et al.'s re-analysis of CPS-II smokers for the relative risk of larynx cancer.

Second-hand Smoke Capstone Appendix

Flowchart

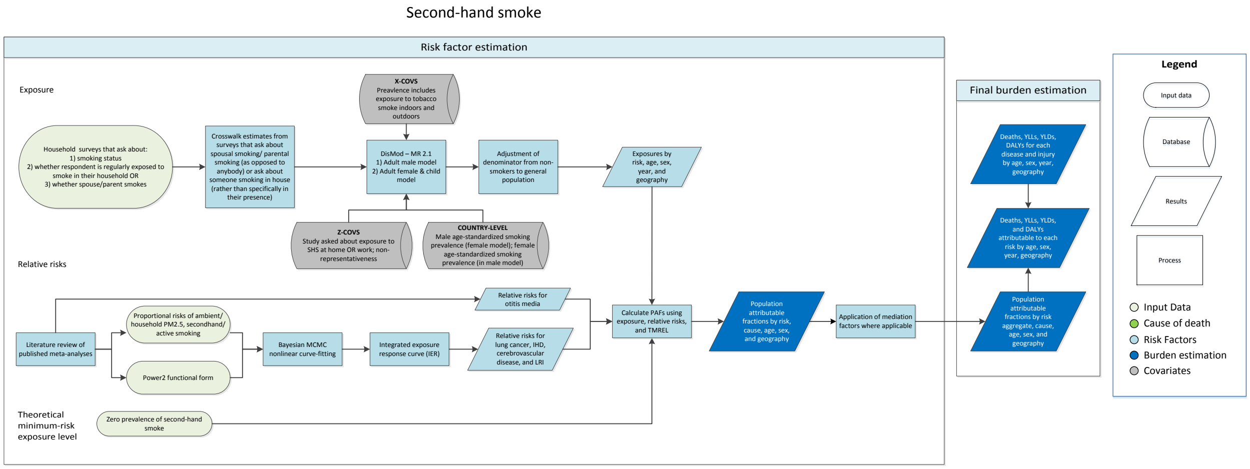

Input Data and Methodological Summary

Exposure

Case Definition

We measure exposure to any tobacco smoke inside the home among non-smokers. Ex-smokers and occasional smokers are considered non-smokers for the purposes of this analysis. Exposure was evaluated for both children and adults.

Input data

We included surveys that had at least one question about smoking status and also asked about either exposure to tobacco smoke inside the home, whether or not the respondent lives with any smokers or whether their spouse smokes. For children we also used surveys that asked about parental smoking. Some main sources include Global Adult Tobacco Survey (GATS), Global Youth Tobacco Survey (GYTS), DHS, NHANES, BRFSS, Eurobarometer, etc.).

Updates to systematic reviews are performed on an ongoing schedule across all GBD causes and risk factors, an update for second-hand smoke will be performed in the next 1-2 iterations.

Many new surveys were added for GBD 2015, which were identified and accessed using GHDx. We cross-referenced with available sources used for smoking in order to evaluate whether these sources were also useful for second-hand smoke. Some of the big new survey series that were added included the National Adult Tobacco Survey and National Youth Tobacco survey series from the U.S., VIGITEL and Risk Factor Chronic Disease Surveillance data from Brazil, and the Chronic Disease Risk Factor Surveillance from China. All new Global Youth Tobacco Surveys (GYTS), Global Adult Tobacco Surveys (GATS), Global school-based student health surveys (GSHS) and Eurobarometer were added as well, in addition to other one-off surveys that evaluated second-hand smoke in the household.

Modeling strategy

We used the traditional PAF equation to estimate burden based on exposure and relative risks. Prevalence of secondhand smoke exposure among nonsmokers is modeled in Dismod-MR and all crosswalks/adjustments are done both within and outside of DisMod to account for alternative case definitions.

In GBD 2015, a new modelling change we implemented was to crosswalk surveys asking about spousal smoking or parental smoking (depending on adults versus children) to our gold standard of any exposure to second-hand smoke in the household by anyone. A sizable group of the DHS surveys do not ask directly about smoke exposure in the household, and thus exposure is ascertained indirectly through looking at the smoking status of each partner in the couple's module to see if there is a "mixed-status" relationship in which one partner is exposed to the other's smoke.

Another adjustment that we made prior to DisMod was for the act of smoking. In some surveys, such as the Global Youth Tobacco Survey, the survey only asks whether their parent smokes, not whether the child being interviewed is actively exposed to smoke on a regular basis (which we define as at least once a week). Thus, in addition to adjusting for spouse/parent versus anybody, we also adjusted for whether the survey asked the person whether they were directly exposed to smoke or just whether people smoked who lived in their home. The two-by-two table below helps illustrate the different potential combinations of alternate definitions that we adjusted for.

	Spouse/Parent	Anybody
Act of smoking	A	B
Non-act	C	D

We used a mixed effects regression to crosswalk these alternative definitions, with interactions between anybody smoking and sex, fixed effects on act of smoking, and nested random effects at the super-region, region and country level. Previously, this crosswalk was done in DisMod.

Once we had crosswalked these alternative definitions, we modeled second-hand smoke prevalence as a single parameter prevalence model in DisMod-MR. Another modelling change that we made in GBD 2015 was to run separate models for male and female secondhand smoke exposure, with children included in the female model. This decision was made because the sex effect being estimated with the combined gender model was underestimating the sizably higher impact of second-hand smoke on women as compared to men. Thus, we decided to model them separately.

In the female model, we used with age mesh points at 0 5 10 15 18 20 30 40 50 60 80 & 100, while in the male model we used age mesh points at 0 15 18 20 30 40 50 60 80 & 100. The difference in age mesh points was due to the fact that all children were modeled as female due to similar rates of exposure, while the male model was limited to adult males greater than 15.

We use the age-standardized smoking prevalence among females as a country-level covariate in the male model, and the age-standardized smoking prevalence among males as a country-level covariate in the female model. This was a modelling change from GBD 2013, in which we only had one second-hand

smoke model and used the age-standardized smoking prevalence rate among men. In addition, we used one study level fixed effects to account for the different case definitions in our dataset:

- Study level fixed effects on integrand value (x-cov)
  - Prevalence figure includes exposure to tobacco smoke outdoors as well as indoors
- Study level fixed effect on integrand variance (z-cov)
  - Study asked about exposure to second-hand smoke at home and/or work (rather than exposure inside the home only)
  - Study was not nationally representative

All raw input CSA data points had a measure of uncertainty going into DisMod – standard error, confidence interval or effective sample size – and the uncertainty around final estimates also takes into account uncertainty from study-level covariate fixed effects on variance, as well as geographic random effects.

Theoretical minimum-risk exposure level

The theoretical minimum-risk exposure level for second-hand smoke is zero exposure among non-smokers to second-hand smoke in the home.

Relative risks

For children under 5 years of age, we estimate the burden of lower respiratory infections (LRI) and otitis media attributable to second-hand smoke exposure. For adults greater or equal to 25 years of age we estimate the burden of lung cancer, ischemic heart disease, cerebrovascular disease and lower respiratory infections (LRI) attributable to second-hand smoke exposure. For GBD 2010 all of these pooled relative risks came from published meta-analyses, but for GBD 2015 we used country-specific relative risks that were created using integrated exposure response curves (IER). The relative risk for otitis media still comes from a published meta-analysis, as opposed to the IER approach.

Alcohol Capstone Appendix

Flowchart

Alcohol Use

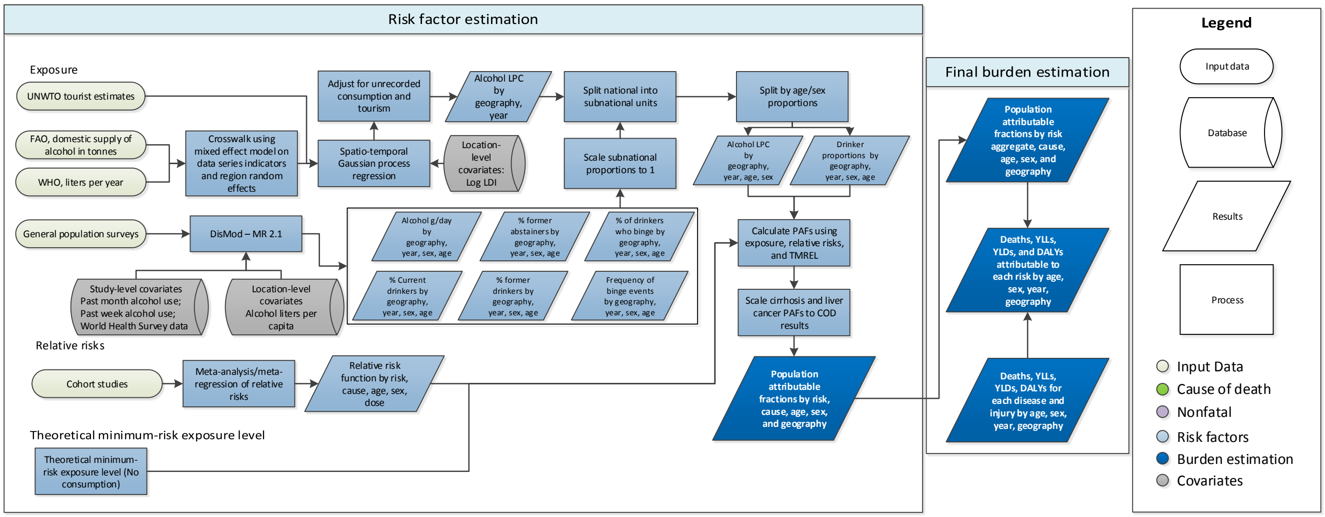

Input Data and Methodological Summary

Exposure

Case definition

The impact of alcohol consumption on morbidity and mortality can be largely described by two separate but related dimensions. The 1st dimension is the individual level drinking and consists of four indicators;

1. Current drinkers, defined as the proportion of individuals who have consumed at least one alcoholic beverage (or some approximation) in the last 12 months.
2. Former drinkers, defined as the proportion of individuals who have ever consumed an alcoholic beverage, but not in the last 12 months.
3. Lifetime abstainers, defined as the proportion of individuals who have never consumed an alcoholic beverage.
4. Alcohol consumption (in grams per day), defined as grams of alcohol consumed by current drinkers, per day, over a 12 month period.

The 2nd dimension of alcohol consumption relates to the pattern of drinking and consists of two indicators;

5. Binge drinkers, defined as the proportion of drinkers who have had a binge event in the past 12 months. A binge event was defined as consuming 60 grams of alcohol (approximately five drinks or more) in a single occasion for males and 48 grams of alcohol in a single occasion for females.
6. Binge times, defined as the proportion of drinking events that are binge amongst binge drinkers i.e. the proportion of days that a binger has a binge event.

Input data

For GBD 2013, a systematic review of the literature was conducted to capture population survey data on all six alcohol use indicators. In summary, the search was conducted in three stages involving electronic searches of the peer-reviewed literature via PubMed, the grey literature and, expert consultation. Updates to systematic reviews via PubMed are performed on an ongoing schedule across all GBD causes and risk factors, an update for alcohol use will be performed in the next 1-2 iterations. For GBD 2015, stages two and three of the literature review were conducted, prioritizing countries for which subnational estimates were generated. The Global Health Exchange (GHDx), IHME's online database of health-related data, was searched for population survey data containing participant-level information from which we could formulate the required alcohol use indicators. Data-sources were included if they captured a sample representative of the geographic location under study and contained variables that could be used to formulate any of the six alcohol use indicators. Relevant survey variables from each data-source were documented in a Microsoft Excel codebook and extracted using STATA 13.1. A total of 629 potential data-sources were available in GhDx across countries with subnational locations, out of which 127 data-sources (66,108 data-points) were included across all six indicators.

To generate estimates of alcohol consumption in grams per day, data from population surveys were used in combination with estimates of per capita consumption from the Food and Agriculture Organization (FAO) [1] and the Global Information System on Alcohol and Health (GISAH database [2]) Per capita consumption is an aggregate measure of recorded, unrecorded, and tourist per capita consumption of alcohol (UNWTO database [3]) derived from sales, production, and other economic statistics. While population-based surveys provide accurate estimates of the prevalence of lifetime abstainers, former drinkers and current drinkers, they typically underestimate real alcohol consumption levels. As a result, the all-age, both-sex per capita consumption figures from the FAO and GISAH are considered to be a better estimate of overall volume of consumption. Per capita consumption, however, does not provide age- and sex-specific consumption estimates needed to compute alcohol-attributable burden of disease. Therefore, we use the age-sex pattern of consumption among drinkers modeled from the population survey data and the overall volume of consumption from FAO and GISAH to determine the total amount of alcohol consumed by country.

To generate estimates of alcohol consumption in liter per capita, raw inputs were obtained from FAOSTAT [1] and WHO GISAH database [2]. To provide more stable time trends in the model, FAO sales data was transformed to a lagged 5-year average. FAO data was used when WHO data wasn't available. Otherwise, FAO and WHO data was adjusted (crosswalked) by running a mixed effect model on the log average of the data with indicators for the FAO and WHO data series, as well as random effects on super region, region, country, and time. Each data point was adjusted by the predicted betas on super-region and region.

$$Log\ Average\ Data = D + (Super\ Region\ |D, Region\ |\ D, Country\ |D, Year|\ D)$$

$$Transformed\ data = \ data\ *\ e^{\widehat{\beta_1} + \widehat{\beta_3}}$$

Where D = Indicator variable for data source

To generate uncertainty, a Lowess model was run on the adjusted data and the standard deviation between the difference of the Lowess smoothed model and the adjusted data points was used for data points missing uncertainty.

Unrecorded consumption was incorporated into the alcohol LPC data using estimates provided by the WHO [4]. WHO estimates were only reported for the years 1990, 2005, and 2010 so for missing years, estimates were interpolated. For years outside this range, unrecorded estimates were carried forward or backwards from the closest year. Unrecorded consumption estimates were reported in liters per capita so estimates were added to adjusted data points to account for unrecorded consumption.

Tourism data was obtained through the UNWTO [4]. A crosswalk was applied across different tourist categories, similar to the one used for FAO and WHO data, to estimate tourist proportions for a given country. Tourism consumption was incorporated after modeling unadjusted alcohol LPC as outlined below.

Modeling strategy

DisMod-MR 2.1 was used to estimate country-, year-, age- and sex-specific proportions of current drinkers, former drinkers, lifetime abstainers, binge drinkers, and binge times; and alcohol consumption as a continuous variable in grams per day. We have made no substantive changes in the modeling strategy from GBD 2013. We ran single-parameter models for each alcohol use indicator and included a combination of location- and study-level covariates in each model. An alcohol liters per capita location-level covariate was used for all six indicators to assist in the predictive power of the models. Additionally, study-level covariates were used to accommodate for known sources of variability in the raw data. In the current drinkers, former drinkers, binge drinkers and binge times models, we included two covariates which adjusted estimates derived in the past week and past month towards those derived in the past year respectively. Estimates derived in the past year were considered to be the gold standard given the previously outlined definition for each indicator.

In the alcohol consumption model, we included a separate study-level covariate flagging data points derived from The World Health Organization's World Health Surveys (WHS) conducted across multiple countries. There was considerable variability in estimates derived from the WHS which may have been influenced by methodological differences in how alcohol use was captured. This study-level covariate looked for unsystematic bias between data-points and added more uncertainty onto those from the WHS. If other data-points causing higher or lower modelled output were identified during the modelling process for a given indicator, the plausibility of these data points was assessed and the study methodology reviewed. Data points with methodological limitations, for instance those derived from survey items not entirely representatively of the alcohol use indicators required, with small sample sizes, or derived from samples not entirely representative of the general population were excluded.

A spatial-temporal Gaussian process regression was used to model total alcohol in liters per capita (see appendix, section 2). Parameters and a random effect model for the prior were chosen using out-of-sample cross validation. This produced estimates of alcohol LPC for a complete time series for the years 1980-2015 by country.

Alcohol LPC was adjusted for each country hosting tourists using the following equations:

$$Alcohol\ LPC_H = Unadjusted\ Alcohol\ LPC_H + Alcohol\ LPC_{Consumption\ abroad} - Alcohol\ LPC_{Tourist\ consumption}$$

$$Alcohol\ LPC_{Consumption\ abroad} = \frac{\sum_V Proportion\ of\ tourists_{H,V} * Unadjusted\ Alcohol\ LPC_H * \frac{Average\ length\ of\ stay_{H,V}}{365} * Tourist\ Population_V}{Population_H}$$

$$Alcohol\ LPC_{Tourist\ consumption} = \frac{\sum_V Proportion\ of\ tourists_V * Unadjusted\ Alcohol\ LPC_V * \frac{Average\ length\ of\ stay_V}{365} * Tourist\ Population_H}{Population_H}$$

Where H = Host country, V = Visiting country

Or, in other words, alcohol LPC was adjusted by adding in the per capita rate of consumption abroad and subtracting the per capita rate of tourist consumption domestically.

After adjusting alcohol LPC by tourist consumption and unrecorded consumption for all location/years reported, sex-specific and age-specific estimates were generated by incorporating estimates modeled in Dismod for percentage of current drinkers within a location/year/sex/age, as well as consumption trends modeled in Dismod g/day by location/year/sex/age, using the following equations.

$$Proportion\ of\ total\ consumption_{l,y,s,a} = \frac{Alcohol\ \ g/day_{l,y,s,a} * Population_{l,y,s,a} * \%\ Current\ drinkers_{l,y,s,a}}{\sum_{s,a} Alcohol\ \ g/day_{l,y,s,a} * Population_{l,y,s,a} * \%\ Current\ drinkers_{l,y,s,a}}$$

$$Alcohol\ LPC_{l,y,s,a} = \frac{Alcohol\ LPC_{l,y} * Population_{l,y} * Proportion\ of\ total\ consumption_{l,y,s,a}}{Population_{l,y,s,a}}$$

Where L = location, Y = Year, S = Sex, A = Age

A similar scalar was applied so that total subnational consumption equaled national consumption.

Theoretical minimum-risk exposure level

For alcohol use, the theoretical minimum-risk exposure level (TMREL) was assumed to be no alcohol use, i.e. 0 g/day of alcohol consumption. This diverges from the definition of other theoretical minimum-risk exposure level of risks because, for some alcohol-use relative risks, there’s a preventative effect for low levels of consumption. However, due to the modeling of alcohol relative risks outlined below, it was found that 0 g/day provided the most consistency between the definition of alcohol-use TMREL and other GBD risk’s TMREL. This is an area of improvement for future GBD iterations. Current research suggests that the preventative effect noted in studies may be due to issues in estimating abstainer populations. [5-7] If this is the case, a TMREL of 0 would still be valid.

Relative Risks

Relative risks were derived for each GBD cause by mapping functions to the dose-response relationships found in meta-analysis. [11-22] Due to data availability, for high levels of consumption, uncertainty in the relative risk functions increases greatly. To minimize the uncertainty of these measures, relative risks were estimated up to the 90th percentile of exposures in men (85 g/day) and the 95th percentile of exposures in women (60 g/day). For exposures beyond this, the associated relative risk was carried forward from these chosen percentile exposure levels. Though a dose-response relationship is evident at higher levels of exposure, the shape of the relative risk function is highly uncertain for higher levels of exposure both due to a lack of observations at these exposure levels, as well as confounding variables affecting estimation of the relative risk of these populations. Thusly, our relative risk estimates are likely an underestimate for the top 10% of male exposures and 5% of female exposures. For exact relative risks used, see appendix section 4.

Population Attributable Fraction

For chronic conditions, PAF was defined as

$$PAF(x) = \frac{P_A + P_F * RR_F + \int_0^{150} P(x) * RR_C(x)\,dx - 1}{P_A + P_F * RR_F + \int_0^{150} P(x) * RR_C(x)\,dx} \qquad P(x) = P_C * \frac{\Gamma(k,\theta)}{\int_{0.1}^{150} \Gamma(k,\theta)}$$

where:

x = alcohol consumption in g/day
P_A = Prevalence of lifetime abstainers
P_F = Prevalence of former drinkers
P(x) = Prevalence of alcohol consumption
RR_F = Relative risk of former drinkers
RR_C (x) = Relative risk function for drinkers

$$k = \frac{\overline{x}^2}{\sigma(\overline{x})^2}$$

$$\theta = \frac{\sigma(\overline{x})^2}{\overline{x}^2}$$

A thousand draws were taken of PAFs to generate uncertainty. The gamma distribution was used to estimate individual level variation within drinking populations [8-9]. Binge drinkers were not taken into account for chronic causes since the pattern of drinking has not been found to be an indicator of most outcomes [10].

For non-chronic conditions, such as injuries, binge drinking was accounted for in the model since patterns of drinking is significant.

$$PAF(x) = \frac{P_A + P_F + P_C + P_{C+B} * RR_{C+B}(x) - 1}{P_A + P_F + P_C + P_{C+B} * RR_{C+B}(x)} \qquad RR_{C+B}(x) = P_D * P_{D+B} * (RR_{crude}(x) - 1) + 1$$

where:

P_{C+B} = Prevalence of current drinkers who binge
RR_{C+B} = Relative risk of current drinkers who binge

P_D = Proportion of a day that is a binge event

RR_{crude} = Relative risk for a given mean level of consumption

P_{D+B} = Proportion of all days where a binge event occurs

The estimated PAF draws were then used to estimate YLL, YLDs, and DALYs, as per the other risk factors (see appendix section 2).

References

1. Food and Agriculture Organization of the United Nations. FAOSTAT Statistics Database.
2. World Health Organization (WHO). WHO Global Health Observatory - Recorded adult per capita alcohol consumption, Total per country. Geneva, Switzerland: World Health Organization (WHO).
3. UN World Tourism Organization (UNWTO). UN World Tourism Organization Compendium of Tourism Statistics 2015 [Electronic]. Madrid, Spain: UN World Tourism Organization (UNWTO), 2016.
4. World Health Organization (WHO). WHO Global Health Observatory – Unrecorded consumption by country. Geneva, Switzerland: World Health Organization (WHO).
5. Rehm, J., et al. "Are lifetime abstainers the best control group in alcohol epidemiology? On the stability and validity of reported lifetime abstention."American journal of epidemiology 168.8 (2008): 866-871.
6. Chikritzhs, Tanya, Kaye Fillmore, and T. I. M. Stockwell. "A healthy dose of scepticism: four good reasons to think again about protective effects of alcohol on coronary heart disease." Drug and alcohol review 28, no. 4 (2009): 441-444.
7. Jackson, Rod, Joanna Broad, Jennie Connor, and Susan Wells. "Alcohol and ischaemic heart disease: probably no free lunch." The Lancet 366, no. 9501 (2005): 1911-1912.
8. Kehoe, Tara, Gerrit Gmel, Kevin D. Shield, Gerhard Gmel, and Jürgen Rehm. "Determining the best population-level alcohol consumption model and its impact on estimates of alcohol-attributable harms." Population health metrics 10, no. 1 (2012): 1.
9. Rehm, Jürgen, Tara Kehoe, Gerrit Gmel, Fred Stinson, Bridget Grant, and Gerhard Gmel. "Statistical modeling of volume of alcohol exposure for epidemiological studies of population health: the US example." Population Health Metrics 8, no. 1 (2010): 1.
10. Rehm, Jürgen, Robin Room, Kathryn Graham, Maristela Monteiro, Gerhard Gmel, and Christopher T. Sempos. "The relationship of average volume of alcohol consumption and patterns of drinking to burden of disease: an overview." Addiction 98, no. 9 (2003): 1209-1228.
11. Roerecke M, Rehm J. Alcohol consumption and the risk for morbidity and mortality of ischemic heart disease - A systemic review and meta-analysis. Toronto, Canada: Centre for Addiction and Mental Health; 2011
12. Bagnardi V, Blangiardo M, La Vecchia C, Corrao G. A meta-analysis of alcohol drinking and cancer risk. Br J Cancer. 2001; 85(11): 1700-5.

13. Corrao G, Bagnardi V, Zambon A, La Vecchia C. A meta-analysis of alcohol consumption and the risk of 15 diseases. Prev Med. 2004; 38(5): 613-9.

14. Samokhvalov AV, Irving HM, Rehm J. Alcohol consumption as a risk factor for pneumonia: a systematic review and meta-analysis. Epidemiol Infect. 2010; 138(12): 1789-95.

15. Samokhvalov AV, Irving H, Mohapatra S, Rehm J. Alcohol consumption, unprovoked seizures, and epilepsy: a systematic review and meta-analysis. Epilepsia. 2010; 51(7): 1177-84.

16. Samokhvalov AV, Irving HM, Rehm J. Alcohol consumption as a risk factor for atrial fibrillation: a systematic review and meta-analysis. Eur J Cardiovasc Prev Rehabil. 2010; 17(6): 706-12.

17. Rehm J, Samokhvalov AV, Neuman MG, Room R, Parry C, Lönnroth K, Patra J, Poznyak V, Popova S. The association between alcohol use, alcohol use disorders and tuberculosis (TB). A systematic review. BMC Public Health. 2009; 450.

18. Lönnroth K, Williams BG, Stadlin S, Jaramillo E, Dye C. Alcohol use as a risk factor for tuberculosis - a systematic review. BMC Public Health. 2008; 289.

19. Roerecke M, Rehm J. Alcohol consumption and the risk for morbidity and mortality of ischemic heart disease - A systemic review and meta-analysis. Toronto, Canada: Centre for Addiction and Mental Health; 2011.

20. Rehm J, Taylor B, Mohapatra S, Irving H, Baliunas D, Patra J, Roerecke M.. Alcohol as a risk factor for liver cirrhosis: a systematic review and meta-analysis. Drug Alcohol Rev. 2010; 29(4): 437-45.

21. Patra J, Taylor B, Irving H, Roerecke M, Baliunas D, Mohapatra S, Rehm J. Alcohol consumption and the risk of morbidity and mortality for different stroke types--a systematic review and meta-analysis. BMC Public Health. 2010; 258.

22. Taylor B, Irving HM, Kanteres F, Room R, Borges G, Cherpitel C, Greenfield T, Rehm J. The more you drink, the harder you fall: a systematic review and meta-analysis of how acute alcohol consumption and injury or collision risk increase together. Drug Alcohol Depend. 2010; 110(1-2): 108-16.

Injecting Drug Use Capstone Appendix

Flowchart

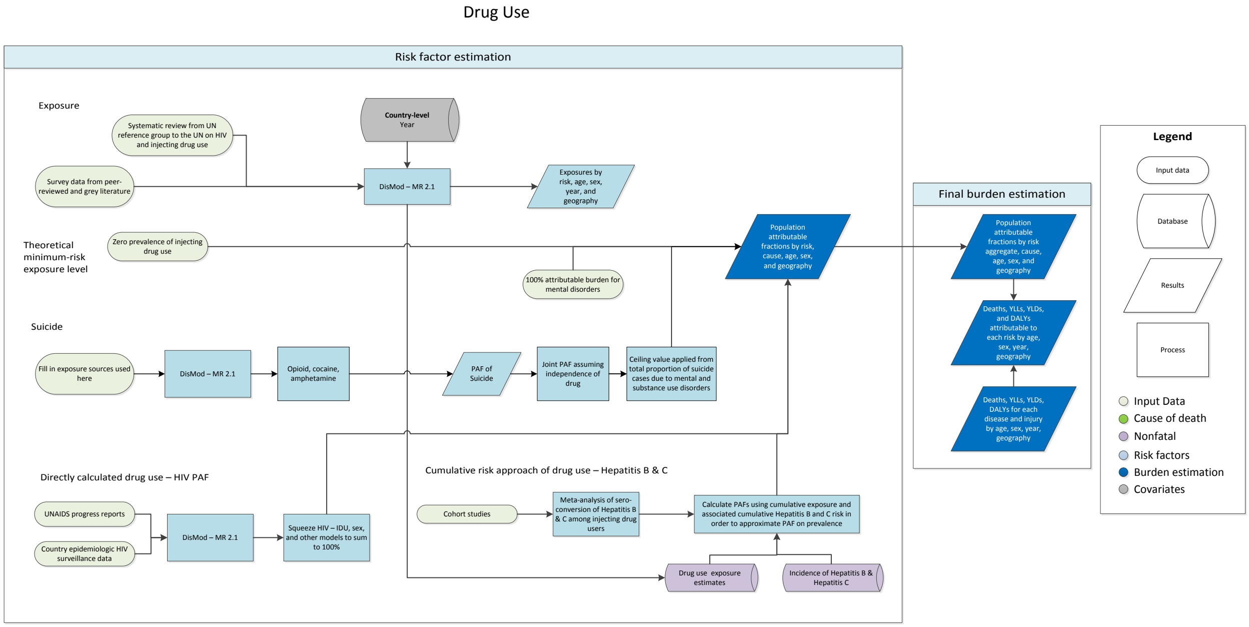

Input Data & Methodological Summary

Exposure

Case definition

Injecting drug users (IDU) are at high risk from blood-borne infections, including human immunodeficiency virus (HIV) and Hepatitis B and C viruses (HBV and HCV, respectively), through the use of shared needles and injection equipment. In GBD 2010, based on the available epidemiological literature and the availability of exposure estimates[2,3] we measure the burden of disease attributable to HIV, HBV and HCV due to injecting drug use. An injecting drug user was defined as a current or recent user aged 15-64 years old.

Input data

The major burden of mortality from viral hepatitis is due to cirrhosis and liver cancer resulting from chronic hepatitis infection. Cirrhosis mortality was modelled with vital registration data using CODEm. Etiologic proportion models, estimated using DisMod-MR 2.0, were used to split the overarching cirrhosis mortality estimates into cases of cirrhosis attributable to hepatitis B, hepatitis C, alcohol, and other causes.(1-4)

Liver cancer mortality was modelled using cancer registry data. The incidence numbers were transformed into mortality estimates using mortality to incidence ratios. The mortality estimates from cancer registries were then combined with vital registration system data as input data into CODEm, which produced the final mortality estimates for liver cancer. As with cirrhosis mortality, etiologic proportions for liver cancer due to hepatitis B, and C, alcohol, and other causes were generated using DisMod-MR 2.0.

To estimate the burden of HIV cases attributable to IDU, we extracted data on the proportion of notified HIV cases by transmission route – sexual intercourse, injecting drug use, commercial sex work and other -- from a number of agencies that conduct surveillance of HIV across the globe.(6-13) This produced 728 data points from 81 countries.

The prevalence of current injecting drug use was estimated using data and estimates from a review conducted by the Reference Group to the UN on HIV and injecting drug use(15). This review used a multistage process of systematic review adhering to international guidelines. It involved multiple stages of peer and expert review, with searches of the peer-reviewed literature in addition to an extensive review of online grey literature databases in the drug and alcohol and HIV fields. Additional data on the age and sex distribution of injecting drug use were sourced for this modelling exercise.

In order to generate a pooled incidence rate/absolute relative risk for viral hepatitis among people who inject drugs, we conducted a meta-analysis of longitudinal epidemiological studies that reported a hepatitis B (16-20) or hepatitis C(16-31) incidence rate among PWID. We calculated confidence intervals for the incidence rate (where no CI was reported) from a Poisson distribution around the number of cases.

We excluded studies that focused on non-representative subgroups, such as recent injectors or adolescents or because hepatitis incidence is far higher in those groups than for all people who inject drugs (e.g.(32)). We did not vary incidence among active injectors according the availability of blood borne virus prevention strategies (e.g. NSPs, opioid substitution therapy) because too few studies have examined different levels of incidence according to variable coverage, and we were not able to estimate coverage by country over time. In any case, in most countries, coverage of virus prevention strategies remains very low among people who inject drugs,(33) and would have been negligible in most countries until recent years.

Modeling strategy

As part of the GBD 2013 study, we measured the burden of hepatitis B and hepatitis C (including attributable cirrhosis and liver cancer) and HIV at the country, regional, and global level for each age-sex group for the years 1990 to 2013. For HIV, hepatitis B and hepatitis C, disease-specific natural history models were used to estimate deaths and YLDs, because the three-state model in DisMod-MR 2.0 (susceptible, cases, dead) did not capture the complexity of the disease processes.

Mortality estimation

Mortality due to overall acute hepatitis was modelled with vital registration data using the Cause of Death Ensemble Modelling tool (CODEm), an analytical tool that tests the predictive power of hundreds of models to estimate trends in causes of death.(5) Due to poor coverage of cause of death data for each of the acute hepatitis varieties, four natural history models for hepatitis B and C were used to estimate mortality by deriving incidence from measurements of seroprevalence and then multiplying incidence by case fatality to estimate the number of deaths. These four models were then squeezed so as to fit the parent cause of death model.

We estimated HIV mortality using a modified UNAIDS Spectrum model.(2) This is a compartmental HIV progression model estimates age-specific incidence, prevalence and death rates using methods described elsewhere.(2) This modelling approach was adapted according to epidemic type, including concentrated and generalized epidemics. For concentrated epidemics, the Spectrum models were corrected for misclassification of HIV deaths and then calibrated to align with vital registration data. For generalised HIV

epidemics, we minimised a loss function to select epidemic curves that were most consistent with the prevalence and all-cause mortality data.(2)

Estimation of Years Lived with Disability

For non-fatal estimation, we estimated the incidence of hepatitis B and C using seroprevalence data in DisMod-MR 2.0. For both hepatitis B and C, we use data on the seroprevalence of the hepatitis surface antigen (a marker of chronic infection in hepatitis B and a marker of ever-infection in hepatitis C), excess mortality, and remission, to estimate incidence of both hepatitis infections. Incidence of cirrhosis was also estimated in DisMod using cirrhosis hospital data and cause-specific mortality rate (CSMR) data.

Incidence of liver cancer was derived by dividing mortality by the mortality to incidence ratios, which were then used to predict liver cancer survival. Finally, we estimated prevalence as a function of incidence and survival by splitting prevalence into four phases. Each phase had different disability weights, which were used to generate YLDs for that phase.

Finally, incidence of HIV was also estimated using the UNAIDS Spectrum modelling approach described above in the mortality estimation section.

Burden of HIV attributable to injecting drug use

We then estimated the proportion of HIV cases attributable to three transmission categories (sex, IDU and other) for all country-time periods using DisMod-MR 2.0. The only covariate used in the model was one that added variance to the data points derived from data sources that attributed a portion of HIV cases to “unknown” transmission sources. We scaled the proportions from each of the three transmission models (sex, IDU and other) to ensure that they fit the total HIV transmission envelope by country, year, age and sex.

Burden of hepatitis B and hepatitis C attributable to injecting drug use

To estimate the relative contribution of IDU to hepatitis B and C disease burden at the country, regional and global level, we used a cohort method. We re-calibrated individuals according to history of injecting drug use, and their accumulated risk of incident hepatitis B and C due to IDU. We made use of data on prevalence of current injecting drug use, pooled in DisMod-MR 2.0; a meta-analysis of incidence rates of hepatitis B and hepatitis C among people who inject drugs; and estimates of population-level incidence of hepatitis B and C between 1990 and 2013. We used back extrapolations to estimate incidence before 1990. These steps are detailed below.

To estimate the lifetime risk of being infected with hepatitis B or C, we undertook a cohort analysis for each country, year, age, and sex category and estimated the probability of an individual having been infected in each preceding year. One of the main inputs to this cohort method was the probability of having injected drugs in a specific age cohort in a given calendar year. For example, for a cohort of 40-year-olds in 2015, the relevant probability in 2005 is the estimated prevalence of injecting drug use among 30-year-olds.

In addition to a global time series of estimated prevalence of injecting drug use, we also used the incidence of hepatitis B or C and the sero-conversion rate of hepatitis B and hepatitis C among people who inject drugs for each age-sex-country-year from 1960 to 2013 by 5-year age groups.

1. Incidence rate of Hepatitis B and C in the general population

We modelled the annual incidence rate of hepatitis B and hepatitis C using sero-prevalence data in DisMod-MR 2.0. We assumed a low remission (mean 0.015 and standard error 0.0075)(14) in the hepatitis B model to reflect the small proportion of cases who spontaneously clear the infection. We assumed zero remission for hepatitis C.

2. Prevalence of ever-injecting drug use

DisMod-MR 2.0 was used to estimate the prevalence of injecting drug use with year as a covariate to estimate the trends over time. DisMod makes an average estimate of the change in drug use over the time period from 1990-2015 and we took draws from a normal distribution of the coefficient to project IDU prevalence backward in time to 1960 from baseline level in 1990.

3. Pooled seroconversion hazard of hepatitis C and hepatitis B among people who ever injected drugs

This pooled sero-conversion hazard for both hepatitis C and hepatitis B was derived from a meta-analysis of longitudinal epidemiologic studies described above in the input data section.

Theoretical minimum-risk exposure level

The theoretical minimum-risk exposure level is defined as zero exposure to injecting drug use.

Relative risks

For drug use, there were not substantial changes made to the effect sizes from GBD 2013. We used a pooled absolute risk of Hepatitis C and Hepatitis B among those who have ever used injecting drugs.

In addition to assessing IDU as a risk factor for blood-borne infections, the broader category of mental and substance use disorders is assessed as risk factors for suicide. The suicide burden attributable to mental and substance use disorders is estimated by comparing the current health status with a theoretical-minimum-risk exposure defined as the counterfactual status of the absence of mental and substance use disorders (Ferrari, Norman et al 2014).

References

1. Fitzmaurice C, Dicker D, Pain A, Hamavid H, Moradi-Lakeh M, MacIntyre MF, et al. The Global Burden of Cancer 2013. JAMA oncology. 2015;1(4):505-27.
2. Murray CJ, Ortblad KF, Guinovart C, Lim SS, Wolock TM, Roberts DA, et al. Global, regional, and national incidence and mortality for HIV, tuberculosis, and malaria during 1990-2013: a systematic analysis for the Global Burden of Disease Study 2013. Lancet. 2014;384(9947):1005-70.
3. GBD 2013 YLDs Collaborators. Global, regional, and national incidence, prevalence and YLDs for 301 acute and chronic diseases and injuries for 188 countries, 1990-2013: A systematic analysis for the Global Burden of Disease Study 2013. The Lancet. 2015;386:743-800.
4. Naghavi M, Wang H, Lozano R, Davis A, Liang X, Zhou M, et al. Global, regional, and national age-sex specific all-cause and cause-specific mortality for 240 causes of death, 1990-2013: a systematic analysis for the Global Burden of Disease Study 2013. Lancet. 2015;385(9963):117-71.

5. Foreman KJ, Lozano R, Lopez AD, Murray C. Modeling causes of death: an integrated approach using CODEm. Population Health Metrics. 2012;10(1).
6. European Centre for Disease Prevention. HIV/AIDS surveillance in Europe 2014 Solna, Sweden. http://ecdc.europa.eu/en/publications/surveillance_reports/HIV_STI_and_blood_borne_viruses/Pages/HIV_STI_and_blood_borne_viruses.aspx: ECDC, 2014.
7. Family Health International, Bureau of AIDS TB and STIs Department of Disease Control. The Asian Epidemic Model (AEM) Projections for HIV/AIDS in Thailand:2005-2025. Bangkok: Family Health International (FHI) and Bureau of AIDS, TB and STIs, Department of Disease Control, Ministry of Public Health, Thailand, 2008.
8. Kirby Institute. 2015 Annual Surveillance Report of HIV, viral hepatitis, STIs. Sydney, New South Wales. https://kirby.unsw.edu.au/surveillance/2015-annual-surveillance-report-hiv-viral-hepatitis-stis: Kirby Institute, UNSW Australia, 2015.
9. Kirby Institute. Australian NSP survey national data report 2015. Sydney, New South Wales: Kirby Institute, University of New South Wales, 2015.
10. Country reports for Global AIDS Response Progress Reporting [Internet]. UNAIDS. 2014.
11. UNAIDS. UNAIDS Country reports. Geneva: Joint United Nations Programme on HIV/AIDS. http://www.unaids.org/en/regionscountries/countries, 2015.
12. United States Center for Disease Control and Prevention. HIV/AIDS Statistics. Atlanta, Georgia: US CDC. http://www.cdc.gov/hiv/statistics/index.html, 2015.
13. Gouws E, White PJ, Stover J, Brown T. Short term estimates of adult HIV incidence by mode of transmission: Kenya and Thailand as examples. Sex Transm Infect. 2006;82 Suppl 3:iii51-5.
14. McMahon B. The natural history of chronic hepatitis B virus infection. Hepatology. 2009;49(5 Suppl):S45-S55.
15. Mathers BM, Degenhardt L, Phillips B, Wiessing L, Hickman M, Strathdee SA, et al. Global epidemiology of injecting drug use and HIV among people who inject drugs: a systematic review. Lancet. 2008;372(9651):1733-45.
16. Jackson JB, Wei L, Liping F, Aramrattana A, Celentano DD, Walshe L, et al. Prevalence and Seroincidence of Hepatitis B and Hepatitis C Infection in High Risk People Who Inject Drugs in China and Thailand. Hepatitis research and treatment. 2014;2014.
17. Månsson A-S, Moestrup T, Nordenfelt E, Widell A. Continued transmission of hepatitis B and C viruses, but no transmission of human immunodeficiency virus among intravenous drug users participating in a syringe/needle exchange program. Scandinavian Journal of Infectious Diseases. 2000;32(3):253-8.
18. Blomé MA, Björkman P, Flamholc L, Jacobsson H, Molnegren V, Widell A. Minimal transmission of HIV despite persistently high transmission of hepatitis C virus in a Swedish needle exchange program. Journal of viral hepatitis. 2011;18(12):831-9.
19. Hagan H, McGough JP, Thiede H, Weiss NS, Hopkins S, Alexander ER. Syringe exchange and risk of infection with hepatitis B and C viruses. American journal of epidemiology. 1999;149(3):203-13.
20. Crofts N, Aitken CK. Incidence of bloodborne virus infection and risk behaviours in a cohort of injecting drug users in Victoria in 1990-1995. Medical Journal of Australia. 1997;167(1):17-20.

21. Roy K, Goldberg D, Taylor A, Hutchinson S, MacDonald L, Wilson K, et al. A method to detect the incidence of hepatitis C infection among injecting drug users in Glasgow 1993–98. Journal of Infection. 2001;43(3):200-5.
22. Abou-Saleh M, Davis P, Rice P, Checinski K, Drummond C, Maxwell D, et al. The effectiveness of behavioural interventions in the primary prevention of hepatitis C amongst injecting drug users: a randomised controlled trial and lessons learned. Harm reduction journal. 2008;5(1):1.
23. Turner KM, Hutchinson S, Vickerman P, Hope V, Craine N, Palmateer N, et al. The impact of needle and syringe provision and opiate substitution therapy on the incidence of hepatitis C virus in injecting drug users: pooling of UK evidence. Addiction. 2011;106(11):1978-88.
24. Grebely J, Lima VD, Marshall BD, Milloy M, DeBeck K, Montaner J, et al. Declining incidence of hepatitis C virus infection among people who inject drugs in a Canadian setting, 1996-2012. PloS one. 2014;9(6):e97726.
25. Foley S, Abou-Saleh MT. Risk behaviors and transmission of hepatitis C in injecting drug users. Addictive Disorders & Their Treatment. 2009;8(1):13-21.
26. Craine N, Hickman M, Parry J, Smith J, Walker A, Russell D, et al. Incidence of hepatitis C in drug injectors: the role of homelessness, opiate substitution treatment, equipment sharing, and community size. Epidemiology and Infection. 2009;137(09):1255-65.
27. Villano SA, Vlahov D, Nelson KE, Lyles CM, Cohn S, Thomas DL. Incidence and risk factors for hepatitis C among injection drug users in Baltimore, Maryland. Journal of clinical microbiology. 1997;35(12):3274-7.
28. Maher L, Jalaludin B, Chant KG, Jayasuriya R, Sladden T, Kaldor JM, et al. Incidence and risk factors for hepatitis C seroconversion in injecting drug users in Australia. Addiction. 2006;101(10):1499-508.
29. Lucidarme D, Bruandet A, Ilef D, Harbonnier J, Jacob C, Decoster A, et al. Incidence and risk factors of HCV and HIV infections in a cohort of intravenous drug users in the North and East of France. Epidemiology and infection. 2004;132(04):699-708.
30. Partanen A, Malin K, Perälä R, Harju O, Holopainen A, Holmström P, et al. Riski-tutkimus 2000-2003. Pistämällä huumeita käyttävien seurantatutkimus. A-Klinikkasäätiön Raporttisarja nro 52. Helsinki: A-Klinikkasäätiön, 2006.
31. Van Den Berg C, Smit C, Van Brussel G, Coutinho R, Prins M. Full participation in harm reduction programmes is associated with decreased risk for human immunodeficiency virus and hepatitis C virus: evidence from the Amsterdam Cohort Studies among drug users. Addiction. 2007;102(9):1454-62.
32. Larney S, Kopinski H, Beckwith CG, Zaller ND, Jarlais DD, Hagan H, et al. Incidence and prevalence of hepatitis C in prisons and other closed settings: results of a systematic review and meta-analysis. Hepatology. 2013;58(4):1215-24.
33. Degenhardt L, Mathers B, Vickerman P, Rhodes T, Latkin C, Hickman M. Prevention of HIV infection for people who inject drugs: Why individual, structural, and combination approaches are needed. The Lancet. 2010;376:285-301.

Dietary Risks Capstone Appendix

Flowchart

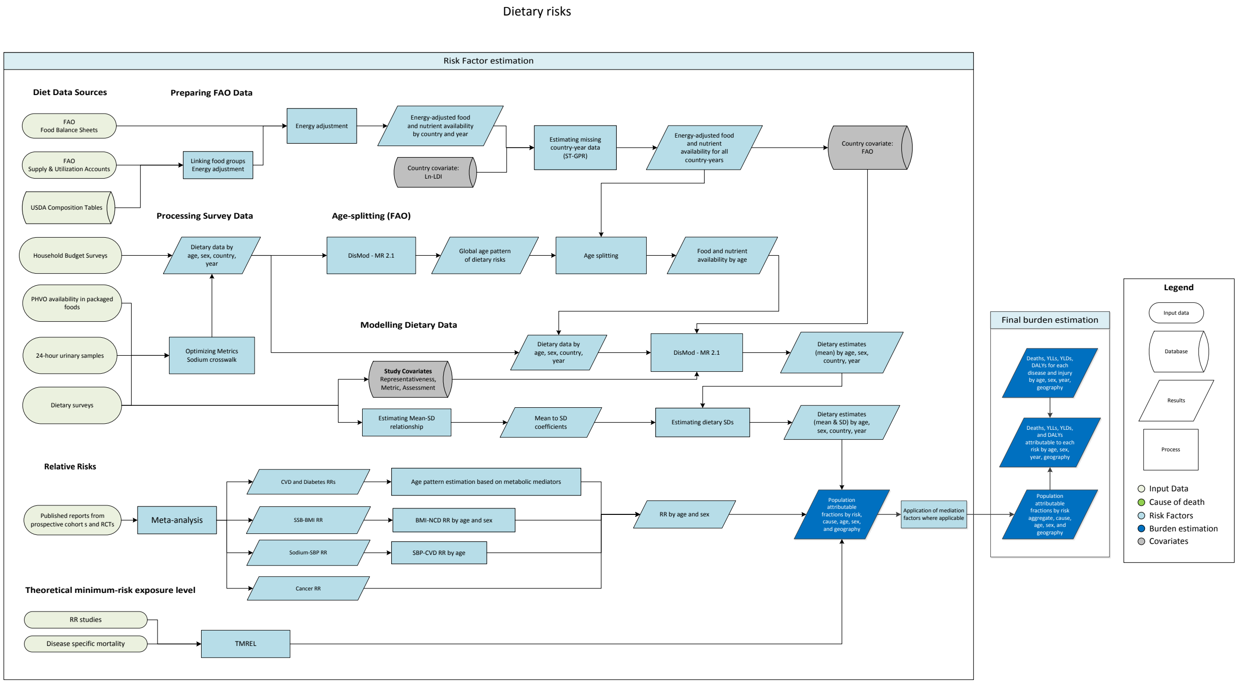

Input data & Methodological summary

Exposure

Case definition

For GBD 2015, risk factors associated with diet include: diet low in fruits, vegetables, whole grains, nuts and seeds, fiber, seafood omega-3 fatty acids, polyunsaturated fatty acids, calcium; and diet high in red meat, processed meat, sugar sweetened beverages, trans fatty acids, and sodium. Exposure to diet low in fruits is defined as average daily consumption of less than 250 grams per day of fruits (fresh, frozen, cooked, canned, or dried, excluding fruit juices and salted or pickled fruits). Exposure to diet low in vegetables is defined as average daily consumption of less than 420 grams per day of vegetables (fresh, frozen, cooked, canned or dried vegetables including legumes but excluding salted or pickled vegetables, juices, nuts and seeds, and starchy vegetables such as potatoes or corn). Exposure to diet low in whole grains is defined as average daily consumption of less than 125 grams per day of whole grains (bran, germ, and endosperm in their natural proportion) from breakfast cereals, bread, rice, pasta, biscuits, muffins, tortillas, pancakes and other sources. Exposure to diet low in nuts and seeds is defined as average daily consumption of less than 20 grams per day of nuts and seeds. Exposure to diet low in milk is defined as average daily consumption of less than 435 grams per day of milk including non-fat, low-fat, and full-fat milk, excluding soy milk and other plant derivatives. Exposure to diet low in calcium is defined as average daily consumption of less than 1.25 grams per day of calcium from all sources, including milk,

yogurt, and cheese. Exposure to diet low in fiber is defined as average daily consumption of less 23 grams per day of than fiber from all sources including fruits, vegetables, grains, legumes and pulses. Exposure to diet low in seafood omega-3 fatty acids is defined as average daily consumption of less than 250 milligrams per day of eicosapentaenoic acid and docosahexaenoic acid. Exposure to diet low in polyunsaturated fatty acids is defined as average daily consumption of less than 11% of total energy intake from polyunsaturated fatty acids as a replacement for high intake of saturated fatty acids (> 7% of total energy intake). Exposure to diet high in red meat is defined as average daily consumption of greater than 23 grams per day of red meat (beef, pork, lamb, and goat but excluding poultry, fish, eggs, and all processed meats). Exposure to diet high in processed meat is defined as average daily consumption of greater than 2 grams of meat preserved by smoking, curing, salting, or addition of chemical preservatives. Exposure to diet high in sugar sweetened beverages is defined as average daily consumption of greater than 2.5 grams per day of beverages with ≥50 kcal per 226.8 gram serving, including carbonated beverages, sodas, energy drinks, fruit drinks, but excluding 100% fruit and vegetable juices. Exposure to diet high in trans fatty acids is defined as average daily consumption of greater than 0.5% of trans fat from all sources, mainly partially hydrogenated vegetable oils and ruminant products. Exposure to diet high in sodium is defined as average 24 hour urinary sodium greater than 3 grams per day.

Input data

We used dietary data from multiple sources including nationally and sub-nationally representative nutrition surveys, household budget surveys, and United Nations FAO Food Balance Sheets and Supply and Utilization Accounts. Additionally, for sodium and trans fatty acids, we used data on 24-hour urinary sodium and availability of partially hydrogenated vegetable oil in packaged foods, respectively. All dietary data (other than sodium and sugar-sweetened beverages) were standardized to 2000 kcal/day. We modelled missing country-year data from FAO using a space-time Gaussian process regression and lag-distributed country income as the covariate. For each dietary factor, we estimated the global age pattern of consumption based on nutrition surveys (i.e., 24-hour diet recall) and applied that age pattern to the FAO data. Substantive changes in input data compared to GBD 2013 are as follows: (a) using data from United Nations Supply and Utilization Accounts to estimate the intakes of fiber, calcium, seafood omega-3 fatty acids, polyunsaturated fatty acids, and saturated fatty acids; (b) using data from United Nations FAO Food Balance Sheets to estimate the intake of fruits; (c) excluding data from United Nations FAO Food Balance Sheets in estimating the whole grain intake.

Modeling strategy

We used DisMod-MR 2.1 to estimate the intake of each dietary factor by age, sex, country, and year. In GBD 2015, for all dietary factors other than sodium, we considered data from 24-hour diet recall as the gold standard, and cross-walked other methods of assessment to the gold standard method. For sodium, the 24-hour urinary sodium was considered as the gold standard. To estimate the 24-hour urinary sodium based on dietary sodium, we performed a crosswalk between these two types of data in a subset of countries with sodium data from both urinary and dietary surveys.

Table 1 summarizes the study-level and country-level covariates used in modeling of each dietary factor.

Table 1. Covariates used in modeling of each dietary factor.

	Sex	Suboptimal metric	Nationally Representativeness	Data from FFQ[1]	Data from HBS[2]	Data from FAO	Country level covariate
Diet low in fruits	●	●	●	●	●	●	-
Diet low in vegetables	●	●	●	●	●	●	-
Diet low in whole grains	●	●	●	●	●	-	-
Diet low in nuts and seeds	●	●	●	●	●	●	-
Diet low in milk	●	●	●	●	●	●	-
Diet high in red meat	●	●	●	●	●	●	-
Diet high in processed meat	●	●	●	●	●	-	National availability of red meat (grams/person/day)<br>National availability of pig meat (% of energy/person/day)
Diet high in sugar-sweetened beverages	●	●	●	●	●	-	National availability of sugar (Kcal/person/day)
Diet low in fiber	●	●	●	●	●	●	-
Diet suboptimal in calcium	●	●	●	●	●	●	-
Diet low in seafood omega-3 fatty acids	●	●	●	●	●	●	Landlocked nation (Yes,/No)
Diet low in polyunsaturated fatty acids	●	●	●	●	●	●	-
Diet high in trans fatty acids	●	●	●	●	●	-	National availability of hydrogenated oil (% of energy/person/day)
Diet high in sodium	●		●	-	-	-	-

[1] Food Frequency Questionnaire
[2] Household Budge Survey

To characterize the distribution of each dietary factor at population level, we used the following equation to model the relationship between the standard deviation and mean of intake in nationally representative nutrition surveys using multiple 24-hour diet recalls:

$$ln\ (Standard\ deviation) = \beta_0 + \beta_1 \times ln\ (Mean_i) + \beta_{risk} \times I_{risk}$$

Then we applied the coefficients of this regression to the outputs of DisMod-MR 2.1 to calculate the standard deviation of intake by age, sex, year, and country.

Theoretical minimum-risk exposure level

In GBD 2015, to estimate the TMREL for each dietary factor, we first calculated the level of intake associated with the lowest risk of mortality from each disease endpoint based on the studies included in the meta-analyses of the dietary relative risks. Then, we calculated the TMREL as the weighted average of these numbers using the global number of deaths from each of outcome as the weight (Table 2).

Table 2. Theoretical minimum-risk exposure level for dietary factors in GBD 2013 and GBD 2015.

Dietary Factor	GBD 2013	GBD 2015
Fruits	200-400 gr/day	200-300 gr/day
Vegetables	350-450 gr/day	340-500 gr/day
Whole grains	100-150 gr/day	100-150 gr/day
Nuts	12-20 gr/day	16-25 gr/day
Red meats	11.4 -17.1 gr/day	18-27 gr/day
Processed meats	0-14.3 gr/day	0-4 gr/day
Milk	425-475 gr/day	350-520 gr/day
Sugar sweetened beverages	0-64.3 gr/day	0-5 gr/day
Polyunsaturated fatty acids	10-15% of total daily energy	9-13% of total daily energy
Seafood omega-3 fatty acids	200-300 mg/day	200-300 mg/day
Trans fatty acids	0-0.8% of total daily energy	0-1%E
Dietary fiber	28-32 gr/day	19-28 gr/day
Dietary calcium	1.0-1.3 gr/day	1-1.5 gr/day

Relative Risk

We obtained the relative risk of each disease endpoint per serving of the dietary components from the most recent dose-response meta-analyses of prospective observational studies, and where available randomized controlled trials. In GBD 2015, we specifically updated the relative risks for the following risk outcome pairs: diet low in fruits-ischemic heart disease; diet low in fruits-ischemic stroke; diet low in fruits-hemorrhagic stroke; diet low in vegetables-ischemic heart disease; diet low in vegetables -ischemic

stroke; diet low in vegetables-hemorrhagic stroke; diet low in whole grains-ischemic heart disease; diet low in whole grains-ischemic stroke; diet low in whole grains- hemorrhagic stroke; and diet low in fiber-ischemic heart disease. We also included diabetes as an outcome for diet low in fruits based on the evidence from a most recent meta-analysis of prospective observational studies. Considering the well-established age trend of the relative risks of metabolic risk factors for cardiovascular disease and diabetes, we conducted a literature review to identify the most important metabolic mediators for each dietary factor and used the age trend of the relative risk of that mediator(s) and the disease endpoint to estimate the age-specific relative risk for each dietary factors (Table 3).

Table 3. Metabolic mediators used to determine the age trend of the effect of dietary factors on cardiometabolic outcomes.

	Body Mass Index	Total Serum Cholesterol	Fasting Plasma Glucose	Systolic Blood Pressure
Diet low in fruits	●	●	●	●
Diet low in vegetables	●	●	●	●
Diet low in whole grains	●	●	●	-
Diet low in nuts and seeds	●	●	●	●
Diet high in red meats	●	-	●	-
Diet high in processed meats	●	-	●	●
Diet low in fiber	-	●	-	-
Diet low in seafood omega-3 fatty acids	●	-	-	●
Diet low in polyunsaturated fatty acids	-	●	●	-
Diet high in trans fatty acids	●	●	-	-

Childhood Sexual Abuse Capstone Appendix

Flowchart

Childhood sexual abuse

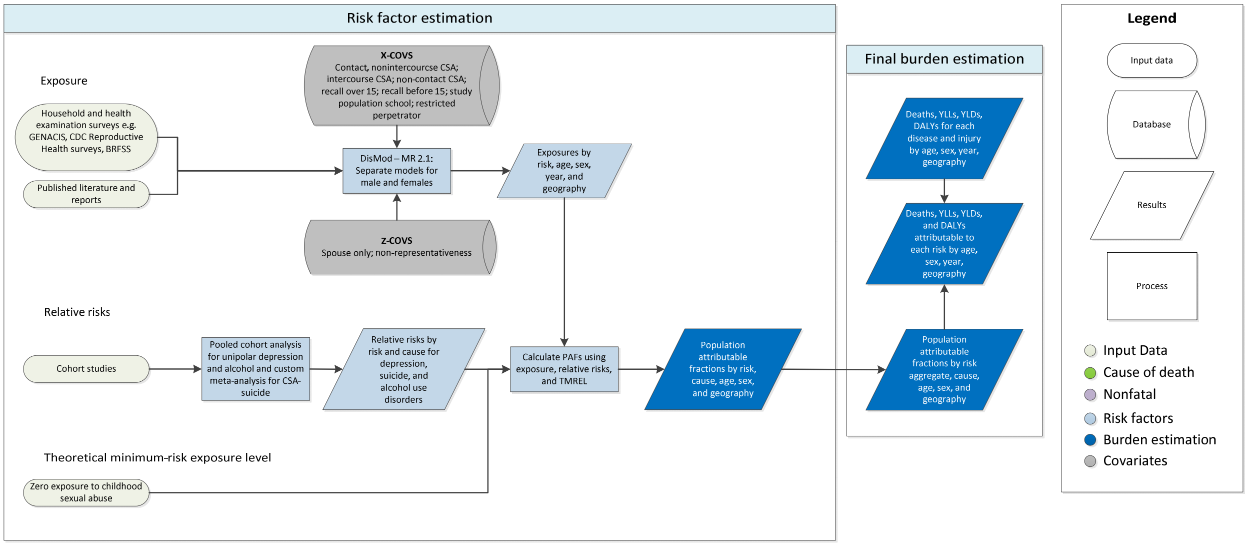

Input Data & Methodological Summary

Exposure

Case Definition

The case definition for childhood sexual abuse (CSA) is ever having had the experience of any contact abuse (i.e. fondling and other sexual touching) or intercourse when aged 15 years or younger, and the perpetrator or partner was older than the victim.

Input data

Currently, we use self-reported survey data to measure CSA prevalence, not data from Child Protection Services (CPS) or other crime data. The reliability and comprehensiveness of CPS and crime statistics varies too much geographically to warrant including it.

An updated systematic review of CSA prevalence literature was conducted for sources published between January 2011 and September 2015. The following search terms were used:

(((("health surveys"[MeSH Terms] AND prevalence[Title/Abstract]) OR ("sentinel surveillance"[MeSH Terms] AND prevalence[Title/Abstract]) OR ("prevalence"[Title/Abstract] AND cross sectional studies[MeSH Terms])) AND (("child abuse"[MeSH Terms] OR "child abuse, sexual"[MeSH Terms]) OR ("sex offenses"[MeSH Terms] OR "child abuse, sexual"[MeSH Terms]) OR (child*[Title/Abstract] AND sexual[Title/Abstract] AND abuse[Title/Abstract]))) NOT ("comment"[Publication Type] OR "letter"[Publication Type] OR "editorial"[Publication Type]))

Intimate Partner Violence Capstone Appendix

Flowchart

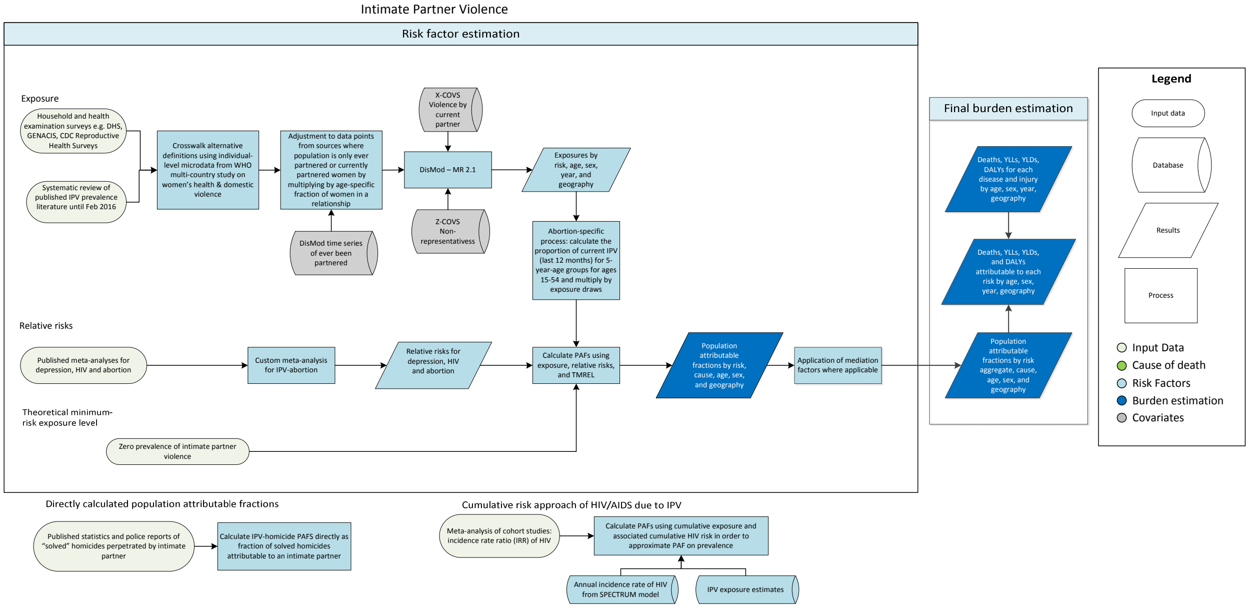

Input Data & Methodological Summary

Exposure

Case Definition

The case definition for intimate partner violence (IPV) is ever experienced one or more acts of physical and/or sexual violence by a current or former intimate partner since the age of 15 years. Estimated in females only because IPV is more common in females and there is more evidence quantifying the associated risk for health outcomes.

- Physical violence is defined as: being slapped or having something thrown at you that could hurt you, being pushed or shoved, being hit with a fist or something else that could hurt, being kicked, dragged, or beaten up, being choked or burnt on purpose, and/or being threatened with or actually having a gun, knife, or other weapon used on you.
- Sexual violence is defined as: being physically forced to have intercourse when you did not want to, having sexual intercourse because you were afraid of what your partner might do, and/or being forced to do something that you found humiliating or degrading (the definition of humiliating and degrading may vary across studies depending on the regional and cultural setting).
- Intimate partner is defined as: a partner to whom you are married or with whom you cohabit. In countries where people date, dating partners will also be considered (a partner with whom you have an intimate (sexual) relationship with but are not married to or cohabiting).

Input data

A systematic review of the intimate partner violence prevalence literature was conducted in Pubmed for anything published between November 2014 and February 2016. The following search terms were used to conduct the systematic review:

((("health surveys"[MeSH Terms] AND prevalence[Title/Abstract]) OR ("sentinel surveillance"[MeSH Terms] AND prevalence[Title/Abstract]) OR ("prevalence"[Title/Abstract] AND cross sectional studies[MeSH Terms])) AND (abuse, sexual[MeSH Terms] OR domestic violence[MeSH Terms] OR abuse, partner[MeSH Terms] OR abuse, spousal[MeSH Terms] OR rape[MeSH Terms]) NOT ("comment"[Publication Type] OR "letter"[Publication Type] OR "editorial"[Publication Type]))

This query produced 92 results, and of these, 33 data points were extracted for 13 different countries. In addition to literature, we supplemented this data with surveys tagged with "intimate partner violence" in the GHDx. Some of the big survey series that were updated or newly added include: all new Demographic and Health surveys, the National Youth Risk Behavior Survey, the Gender, Alcohol and Culture International Study (GENACIS), the CDC Reproductive Health Surveys, Mexican National Addiction Survey, USA Collaborative Psychiatric Epidemiology Surveys, and the Brazil National Alcohol and Drug Survey.

We get the proportion of solved homicides that were perpetrated by an intimate partner from crime statistics and police reports. For GBD 2013, the main source of these crime statistics and police reports came from an IPV-homicide systematic review in the Lancet in 2013.

In GBD 2015, an updated systematic review was done for IPV homicide sources in PubMed through April 2016. The query used for this Pubmed search was:

((IPV[All Fields] OR ("intimate partner violence"[MeSH Terms] OR ("intimate"[All Fields] AND "partner"[All Fields] AND "violence"[All Fields]) OR "intimate partner violence"[All Fields])) AND (("homicide"[MeSH Terms] OR "homicide"[All Fields]) OR femicide[All Fields])) AND ("2013/01/01"[PDAT] : "3000/12/31"[PDAT])

These literature sources were supplemented with sources from the GHDx that were tagged with Intimate partner violence AND Homicide.

Modeling strategy

For GBD 2015, we use three distinct approaches to estimate burden attributable to IPV, including 1) the traditional exposure and relative risk to PAF method for depression, suicide and abortion; 2) the direct PAF approach for estimating the proportion of homicides that are perpetrated by an intimate partner; and 3) a cumulative risk approach for estimating the burden of HIV/AIDS attributable to IPV.

Estimating attributable burden to IPV for depression, suicide and abortion

Before upload to DisMod, we first adjust data with variable recall periods (previous 12 months versus lifetime), type of violence (sexual, physical, or both) and severity (severe only versus all levels). To convert data to our gold standard definition of ever having experienced any IPV, we use data from the WHO multi-country violence against women surveys to construct crosswalk regressions. The dependent variable in each of these regression was ever any IPV (gold standard), while the key independent variable was one of the 11 alternative metrics of IPV that were represented in our dataset:

1. Physical IPV in the past 12 months
2. Sexual IPV in the past 12 months
3. Severe IPV in the past 12 months
4. Severe physical IPV in the past 12 months
5. Severe sexual IPV in the past 12 months
6. Any IPV (physical and/or sexual) in the past 12 months
7. Ever any physical IPV
8. Ever any sexual IPV
9. Ever any severe IPV
10. Ever severe physical IPV
11. Ever severe sexual IPV

For alternate metrics 1-6 there is likely to be a relationship between current exposure and age. For these metrics we included a series of age dummies:

$$logit(GSait) = \beta + \beta 1 logit(ALTait) + \beta 2 Ia + \varepsilon$$

For alternate metrics 7-11, we ran the following regression:

$$logit(GSit) = \beta 0 + \beta 1 logit(ALTit) + \varepsilon$$

where GS refers to the gold standard metric of IPV prevalence, ALT is the alternate metric of IPV prevalence, *Ia* refers to the complete set of age-group indicators, *a* refers to an age-group, *i* refers to a country, and *t* refers to year. We included age-group indicators in the first six regressions because we expected the prevalence of recent IPV to vary by age. Using the intercepts, coefficients, and variance-covariance matrix from each of these eleven regressions, we were able to convert all of the alternate metrics of IPV prevalence in our dataset to estimates of "ever any IPV". We eliminated observations based on alternate metrics of IPV which came from studies that also provided estimates of IPV based on the gold standard definition (i.e. duplicates).

After applying crosswalks to the alternate metrics of IPV in the manner described above, we made an additional adjustment to the subset of our data that was based on only ever-partnered, currently partnered women currently married women or ever married women. To adjust these values so that they reflected IPV prevalence in the entire female population, regardless of partnered status, we multiplied estimates from these studies by the age-specific fraction of women who had ever been partnered.

An updated time series was generated in GBD 2015 using MICS and DHS data in a single parameter DisMod model to reflect the most recent data on proportion of women that have ever been partnered. This revised time series was used to adjust values for surveys with restricted partner status to reflect the prevalence among all women in the population.

After these pre-DisMod crosswalks and adjustments, a single-parameter prevalence model was run in DisMod with age mesh points at 0 14 15 20 30 40 50 60 80 & 100. A study-level covariate fixed effect (x-cov) was used to adjust data points where the survey question used to calculate prevalence only asked about violence perpetrated by the woman's spouse. A study-level fixed effect on integrand variance (z-

cov) to indicate whether a study was nationally representative or not was used to account for the heterogeneity introduced by studies that are not generalizable to the entire population.

We tried using alcohol liters per capita, prevalence of binge drinking, and prevalence of male binge drinking in the GBD 2015 model as national-level fixed effects, but they were not significant so they were ultimately dropped.

Direct PAF for female homicides

The burden of homicides attributable to intimate partner violence is modeled as a direct PAF.

Input data all fed into a single-parameter proportion DisMod model, which has age mesh points at 0 10 20 45 & 100. The model has a study-level covariate fixed effect on integrand value (x-cov) for sources just including police reported homicides. We also included a study-level fixed effect on integrant variance (z-cov) to indicate whether a study was nationally representative or not.

In GBD 2015, we added prevalence of binge drinking to the model as a country-level covariate.

Cumulative risk approach for PAF of HIV/AIDS due to IPV

The third and final modelling approach that we used to assess burden attributable to intimate partner violence was a cumulative risk approach to measure the burden of HIV/AIDS attributable to IPV.

The approach itself remained the same in GBD 2015, but included updated intimate partner violence exposure numbers from the DisMod model described above, as well as revised HIV incidence numbers.

From the literature we have information on the incidence rate ratio (IRR) of HIV incidence from two cohort studies (Jewkes et al, Lancet 2010 & Kouyoumdijian, et al AIDS 2013). As we measure burden based on deaths and prevalence, we need to be able to quantify attributable fractions on prevalence and death rather than incidence. To get a PAF on prevalence we need to consider the history of exposure to IPV and the accumulated associated risk of incident HIV due to IPV, relative to the overall risk of HIV at the population level. The ratio of cumulative IPV-attributable HIV incidence to total HIV incidence is an approximation of the relevant PAF on HIV prevalence and we will assume this PAF can also applied to mortality.

$$\frac{Cumulative\ HIV\ incidence\ due\ to\ IPV}{Cumulative\ HIV\ incidence\ overall} = \frac{1-\prod_{a=o}^{a=n}\left(1-PAF_{ay}*I_{ay}\right)}{1-\prod_{a=o}^{a=n}(1-I_{ay})}$$

Where:

I = annual incidence rate of HIV

a = age (15-84)

y = year (1980-2013)

$$PAF_{HIV\ incidence} = \frac{[Prevalence\ of\ IPV]_{ay}*(IRR-1)}{[Prevalence\ of\ IPV]_{ay}*(IRR-1)+1}$$

Theoretical minimum-risk exposure level

The theoretical minimum-risk exposure level is zero exposure to intimate partner violence, as defined above.

Relative risks

We estimate burden attributable to IPV for abortion, depression, suicide, interpersonal violence (i.e. homicide) and HIV incidence. We have added HIV as an outcome for GBD 2013 in response to bolstered causal evidence from a second prospective study published in 2013 (Kouyoumdjian, 2013). We use a pooled incidence rate ratio (IRR) of 1.59 (95% CI 1.3-1.94) from a meta-analysis of the two available prospective studies as of date.

The relative risks for depression and suicide come from a systematic review of longitudinal studies assessing intimate partner violence and incident depressive symptoms and suicide attempts. For the relative risk for IPV-abortion, we ran a custom meta-analysis in GBD 2013 that we continued to use in GBD 2015. An important methodological note with IPV-abortion is that we must apply the pooled relative risk for abortion to the current prevalence of IPV (in the previous 12 months), rather than lifetime prevalence. This is because the relevant exposure for abortion would be recent IPV, and because the case definition for all but one of the RR component studies was physical or sexual IPV in the past year.

Unsafe Sex Capstone Appendix

Flowchart

Unsafe sex

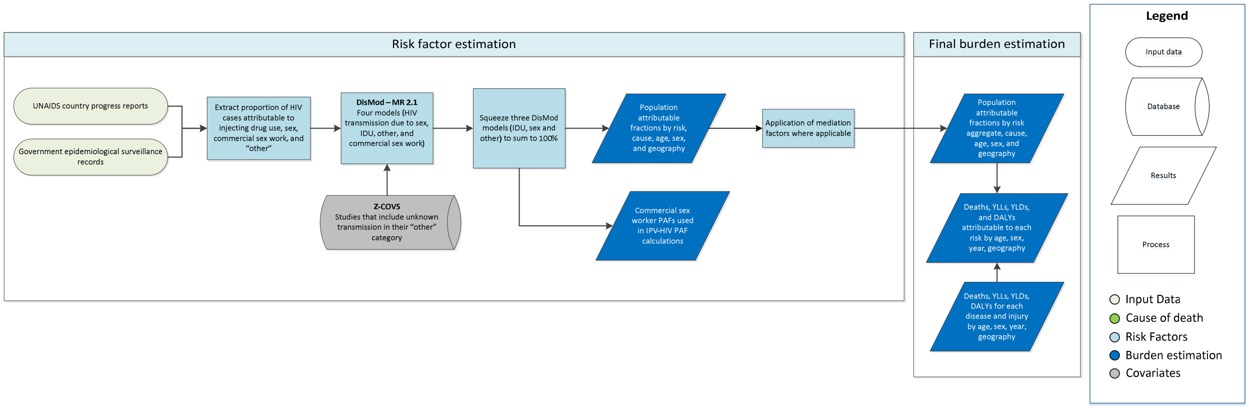

Input Data & Methodological Summary

Exposure

Case Definition

For GBD 2015, unsafe sex is defined as the risk of disease due to sexual transmission.

Input data

To be used in our models, sources must report HIV cases attributable to various modes of transmission. We cannot use data on the prevalence of HIV in the population in general or among specific populations like drug users or CSW. We screened all UNAIDS country progress reports and searched government epidemiological surveillance records for these data. The primary data sources we used were UNAIDS, the European CDC, and the US CDC.

For GBD 2015, we extracted all new European CDC, UNAIDS, and US CDC reports that had been published since the previous iteration of GBD. We also extracted state-level HIV surveillance reports where available. These were found through the US CDC: National Center for HIV/AIDS, Viral Hepatitis, STD, and TB Prevention, Division of HIV/AIDS Prevention's website.

Barring the time for a full systematic review, these ECDC, US CDC and UNAIDS reports are the main sources for breakdown of HIV transmission. Updates to systematic reviews are performed on an ongoing schedule across all GBD causes and risk factors, an update for unsafe sex will be performed in the next 1-2 iterations.

We excluded all extractions where the "other" category for HIV transmissions accounted for greater than 25 percent of all cases. We believe that this indicates issues with the reporting system used to report HIV cases in that location.

Modeling strategy

There were no substantial changes in the modelling approach for unsafe sex from GBD 2013. We model the proportion of HIV cases attributable to unsafe sex for GBD. To do this we collect and clean data, run three DisMod models (HIV attributable to sex, HIV attributable to IDU, HIV attributable to other routes of transmission), squeeze the results of the three DisMod models, and prepare PAFs. Additionally, we run a fourth DisMod model that is the proportion of sexually transmitted HIV cases that are due to commercial sex work (CSW) (defined as in sex workers vs in the 2nd or 3rd contacts of sex workers), that is used in the intimate partner violence calculations.

We attribute burden to unsafe sex for all ages 0-100, for both sexes, and all GBD locations for years 1990 to 2015.

All of the DisMod models include a study-level covariate fixed effect on integrand variance (x-cov) for sources that include unknown cases in their “other” category. We assumed that the inclusion of unknown cases in the other category would impact the uncertainty around the point estimates. We used age mesh points 0 and 100 for all models, since almost all of the data was for large age ranges, rather than age-specific. No study-level x-covs or country level covariates were included in the models.

All raw input unsafe sex data points had a measure of uncertainty going into DisMod – standard error, confidence interval or effective sample size – and the uncertainty around final estimates also takes into account uncertainty from study-level covariate fixed effects on variance, as well as geographic random effects.

After the 3 main HIV transmission models (sexual, IDU, other) are run, the results of all 3 must be squeezed so they sum to 100% for a given country-year-age-sex group.

Theoretical minimum-risk exposure level

The theoretical minimum level used for unsafe sex is the absence of disease transmission due to sexual contact.

Population attributable fraction calculations

The outcomes associated with unsafe sex that we report on include HIV, cervical cancer, and all sexually transmitted diseases (STDs) except for those in neonates from vertical transmission, including HIV, opthalmia neonatorum and neonatal syphilis.

Based on evidence in the literature, we attribute 100% of cervical cancer to unsafe sex. These sources state that HPV infection is necessary for cervical cancer to develop and that HPV is spread through sexual contact. The proportion of STDs attributable to unsafe sex is also 100%.

For HIV, the results from the singe parameter proportion DisMod model for HIV transmission due to sex are used directly as the population attributable fraction.

Low Physical Activity Capstone Appendix

Flowchart

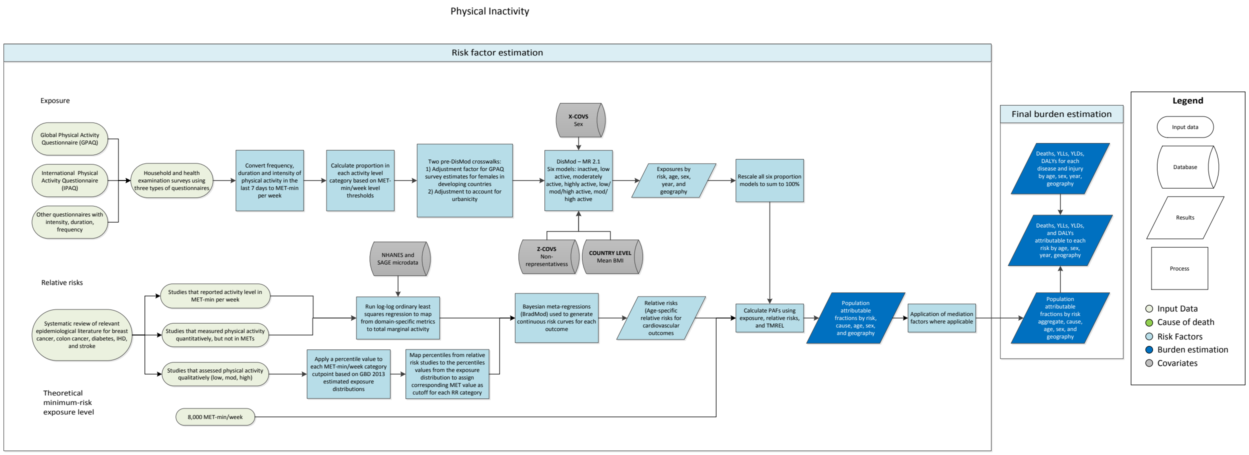

Input Data and Methodological Summary

Exposure

Case Definition

We measure physical activity performed by adults greater than or equal to 25 years of age, for durations of at least ten minutes at a time, across all domains of life (leisure/recreation, work/household and transport). We use frequency, duration and intensity of activity to calculate total metabolic equivalent-minutes per week. MET (Metabolic Equivalent) is the ratio of the working metabolic rate to the resting metabolic rate. One MET is equivalent to 1 kcal/kg/hour and is equal to the energy cost of sitting quietly. A MET is also defined as the oxygen uptake in ml/kg/min with one MET equal to the oxygen cost of sitting quietly, around 3.5 ml/kl/min.

Input data

We included surveys of the general adult population that captured self-reported physical activity in all domains of life (leisure/recreation, work/household and transport), where random sampling was used.

Data were primarily derived from two standardized questionnaires: The Global Physical Activity Questionnaire (GPAQ) and the International Physical Activity Questionnaire (IPAQ), although we included any other survey instrument that asked about intensity, frequency and duration of physical activities performed across all activity domains.

Due to a lack of a consistent relationship on the individual level between activity performed in each domain and total activity, we were not able to use studies that included only recreational/leisure activities.

Physical activity level is categorized by total MET-minutes per week using four categories based on rounded values closest to the quartiles of the global distribution of total MET-minutes/week. The lower limit for the Level 1 category (600 MET-min/week) is the recommended minimum amount of physical

activity to get any health benefit. We used four categories with higher thresholds rather than the GPAQ and IPAQ recommended 3 categories to better capture any additional protective effects from higher activity levels.

- Level 0: < 600 MET-min/week (inactive)
- Level 1: 600-3999 MET-min/week (low-active)
- Level 2: 4000-7,999 MET-min/week (moderately-active)
- Level 3: ≤ 8,000 MET-min/week (highly active)

The GHDx was used to locate all surveys that use the GPAQ or IPAQ questionnaire. Although there were many other surveys that focused specifically on leisure activity, we were unable to use these sources because they did not comprise all three domains (work, transport and leisure). In addition, we excluded any surveys that did not report frequency, duration, and intensity of activity.

Modeling strategy

Pre-DisMod crosswalks

We conducted two crosswalks prior to DisMod to adjust the raw data to our "gold standard" definition. In GBD 2010, our gold standard definition was GPAQ, but for GBD 2013 and into 2015, we shifted to IPAQ due to concern that GPAQ was not accurately capturing "domestic" (house/yard) activities and was thus greatly underestimating activity level. In an empirical comparison between the World Health Survey (IPAQ) and the WHO Study on global AGEing and adult health (SAGE) (GPAQ) showed significantly lower activity levels assessed using GPAQ as compared to IPAQ for females in low income countries.

We calculated an adjustment factor to apply to GPAQ surveys for females only (since the difference between questionnaire activity level estimates were not significantly different for men). A regression was fitted on data from nationally representative surveys that used either GPAQ or IPAQ for each activity category, where the dependent variable was the logit of the proportion in the relevant activity level and the main independent variable was an interaction between super region and survey (1=GPAQ, 0=IPAQ), with fixed effects for age categories and a country level random effect.

We also adjusted non-nationally-representative urban and rural data points. We constructed an urbanicity covariate that is equal to 1 for urban data points, 0 for rural data points and the proportion urban for the country for nationally representative data points. The dependent variable was the logit of the proportion in the relevant activity level and the main independent variable is an interaction between sex and urbanicity, with fixed effects for age categories and a country level random effect.

A new adjustment that we implemented in GBD 2015 prior to DisMod was to shift data points from the Behavioral Risk Factor Surveillance System (BRFSS), which asks about recreation/leisure, transport and household chores, but does not ask about activity on the job. We used data from the National Health and Nutrition Examination Survey (NHANES) to create age-sex level average proportions in each activity category, which we used to adjust the state-level US BRFSS estimates.

DisMod modeling

Once the raw data had been adjusted to meet our gold standard definition of physical activity, we modeled activity as a single parameter proportion model in DisMod. We estimated the proportion of each country/year/age/sex subpopulation in each of the above four activity levels using six separate Dismod models. We use six models rather than four to accommodate the different MET-minute/week cutoffs presented in tabulated data sources where individual unit record data was not available. Since the accepted threshold/definition for inactivity is consistently <600 MET-minutes/week, the vast majority of tabulated data was broken down into proportion inactive (model A) and proportion low, moderate or highly active (model B).

	Label	MET-min/week	Name of sequelae in online visualization tool
A	inactive	<600	Physical inactivity and low physical activity, inactive
B	low/moderately/highly active	≥600	Physical inactivity and low physical activity, low/moderately/highly active
C	low active	600-3999	Physical inactivity and low physical activity, low active
D	moderately/highly active	>4000	Physical inactivity and low physical activity, moderately/highly active
E	moderately active	4000-7999	Physical inactivity and low physical activity, moderately active
F	highly active	≥8,000	Physical inactivity and low physical activity, highly active

These models have mesh points at 0 15 25 35 45 55 65 100, and a study-level fixed effect on integrand variance (Z-cov) for whether a study was nationally representative or not, to account for the heterogeneity introduced by studies that are not generalizable to the entire population. They also have a national level fixed effects on mean BMI.

After DisMod, we rescale these 6 models so that the proportions sum to one. Since we have the most data for models A and B, we rescale the sum of the proportion in each category to be equal to one. Next we rescale the sum of model C and D to be equal to the rescaled value from model B. Then we rescale the sum of models E and F to be equal to the rescaled value from model D. After these three rescales we are left with a proportion for each of the four categories that all sum to 1.

We have made few substantive changes in the modeling strategy from GBD 2013.

Theoretical minimum-risk exposure level

The theoretical minimum-risk exposure level for physical inactivity is 600 MET-min per week, which is the WHO recommended level for physical activity for health.[3]

Relative risks

We estimate burden attributable to physical inactivity for breast cancer, colon cancer, diabetes, ischemic heart disease and stroke. A systematic review of relevant epidemiological literature was conducted for each health outcome up to February 27, 2016. Due to considerable heterogeneity in the literature with respect to physical activity metrics and domain(s) covered, a methodologically intensive strategy was required to standardize the relative risk units to match those of the exposures.[4]

In addition to updates to the literature review, the main other change that has been implemented since GBD 2013 is that we previously used a non-increasing prior in the Bayesian meta-regression to estimate the association between PA and each of the five outcomes. For GBD 2015, we no longer use any prior in the Bayesian meta-regression analysis.

References

1. Ainsworth BE, Haskell WL, Whitt MC, Irwin ML, Swartz AM, Strath SJ, O Brien WL, Bassett DR, Schmitz KH, Emplaincourt PO, Jacobs DR. Compendium of physical activities: an update of activity codes and MET intensities. Medicine and science in sports and exercise. 2000 Sep 1;32(9; SUPP/1):S498-504.

2. IPAQ Research Committee. Guidelines for data processing and analysis of the International Physical Activity Questionnaire (IPAQ)–short and long forms. Retrieved September. 2005;17:2008.

3. World Health Organization. Global Physical Activity Questionnaire (GPAQ) Analysis Guide. 2011. Geneva, Switzerland: WHO Google Scholar. 2013

4. Kyu HH, Bachman VF, Alexander LT, Mumford JE, Afshin A, Estep K, Veerman JL, Delwiche K, Iannarone ML, Moyer ML, Cercy K. Physical activity and risk of breast cancer, colon cancer, diabetes, ischemic heart disease, and ischemic stroke events: systematic review and dose-response meta-analysis for the Global Burden of Disease Study 2013. bmj. 2016 Aug 9;354:i3857.

High Fasting Plasma Glucose Capstone Appendix

Flowchart

High fasting plasma glucose

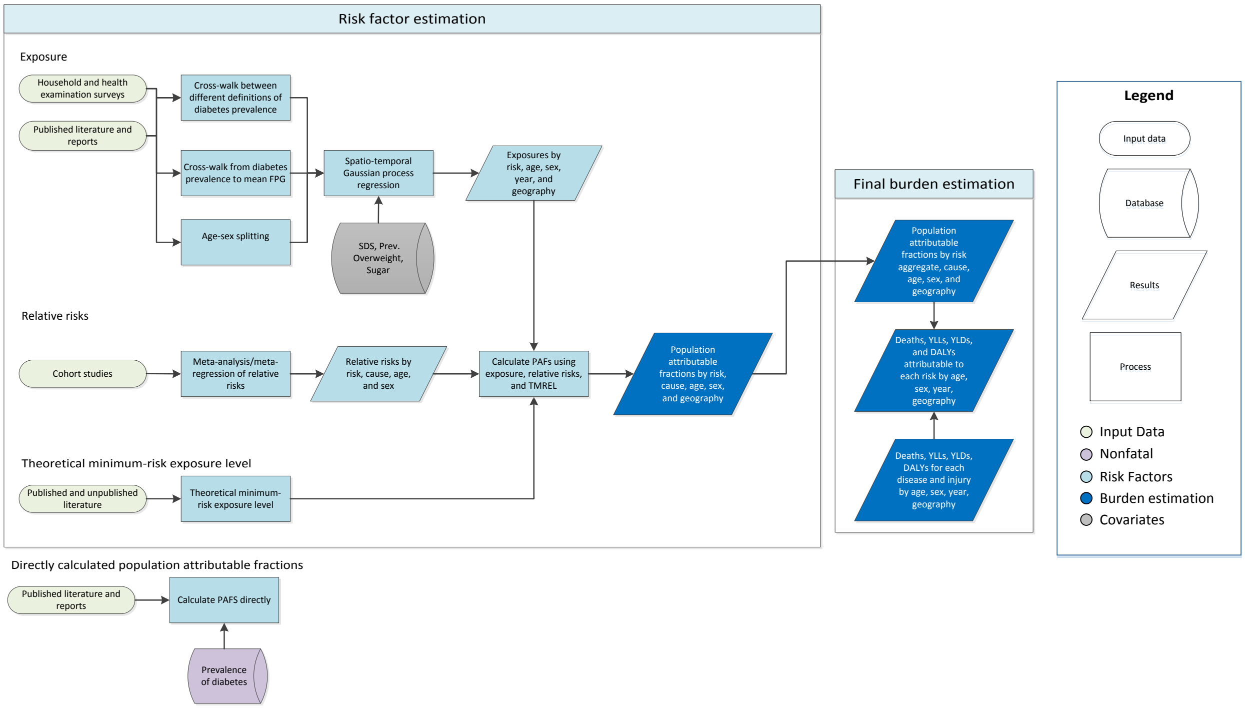

Input Data & Methodological Summary

Exposure

Case Definition

We measure fasting plasma glucose as a continuous exposure in units of mmol/L and define diabetes according to the American Diabetes Association (ADA) and World Health Organization (WHO) diagnostic guidelines as FPG $\geq$ 7.0 mmol/L and/or currently taking diabetes.[1,2]

Input Data

Consistent with GBD 2013, we utilized data on mean fasting plasma glucose from literature and from household survey microdata and reports (e.g. STEPS, NHANES). Please see appendix for a full list of included sources. In GBD 2013, a systematic review of the literature was completed to capture population survey data on mean fasting plasma glucose. For GBD 2015, we updated the systematic review using the same strategy, drawing from the GHDx and Medline via PubMed. In total, we have utilized 717 sources corresponding to 24,926 unique data points.

Global Health Data Exchange Database

We systematically searched the Global Health Data Exchange (GHDx) for multi-country survey programs, national surveys, and longitudinal studies which provide measured individual level data on fasting

plasma glucose. The search was completed for systolic blood pressure, fasting plasma glucose, and blood cholesterol simultaneously, as many sources studying the other metabolic risks will often report mean fasting plasma glucose or diabetes prevalence.

Search Terms (Keywords): Blood pressure OR Blood glucose OR Glucose tests OR Cholesterol OR Cholesterol tests OR Hypercholesterolemia
Data Type: Survey OR Report
Search date: 2/6/2016

Literature Review

We systematically searched PubMed for articles published between 15 July 2009 and 31 December 2015 which provided national or subnational estimates of mean fasting plasma glucose. As above, the literature review was completed for systolic blood pressure, fasting plasma glucose, and blood cholesterol simultaneously for the reasons previously stated.

Search terms:

(("hypertension"[Mesh:NoExp] OR "blood pressure"[Mesh:NoExp] OR "Hyperlipidemias"[Mesh:NoExp] OR "Hypercholesterolemia"[Mesh] OR "Cholesterol"[Mesh] OR “diabetes mellitus”[Mesh:NoExp] OR “diabetes mellitus, type 2”[Mesh] OR “glucose”[Mesh] OR “hyperglycemia”[Mesh] OR “prediabetic state”[Mesh]) AND "Geographic Locations"[Mesh] NOT "United States"[Mesh]) AND ("humans"[Mesh] AND "adult"[MeSH]) AND ("Data Collection"[Mesh] OR "Health Services Research"[Mesh] OR "Population Surveillance"[Mesh] OR "Vital statistics"[Mesh] OR "Population"[Mesh] OR "Epidemiology"[Mesh] OR "surve*"[TiAb]) NOT Comment[ptyp] NOT Case Reports[ptyp] AND ("2009/07/15"[PDAT] : "2015/12/31"[PDAT]) NOT "hospital"[TiAb]

Search date: 1/26/2016

Expert Groups

To capture any remaining sources not identified in the GHDx or in PubMed, we looked to other leaders in the field to ensure our datasets were as comprehensive as possible. These included the IDF Atlas Database and a recent publication on diabetes from the NCD Risk Factor Collaboration. [3,4]

Inclusion Criteria

Studies were included if they were population-based and measured glucose using a blood test (as FPG, HbA1c). We accepted data on diabetes prevalence only if the study performed an objective blood measurement and/or individuals reported self-report of taking anti-diabetic medication. Studies that included self-report of diabetes were excluded. We assumed the data is representative of the location if the geography was not related to the diseases (a mining area) and if it is not an outlier compared to other data in the country or region.

Outliers

Data was utilized in the modeling process unless an assessment of data showed that the data is biased. A data point was considered to be an outlier candidate if the level is not consistent with other (sources)

country data, or - if there are no other data points - not consistent with other country in the region. A candidate outlier source was scrutinized and validated and the data point was excluded if the quality of study did not warrant a valid estimate because of selection (specific populations), different definitions, other biases, or if the study did not provide methodological details for evaluation.

Data Extraction

Where possible, individual level data on fasting plasma glucose was extracted from survey microdata and these were collapsed across demographic groupings to produce mean estimates in the standard GBD 5-year age-sex groups. If microdata were unavailable, information from survey reports or from literature were extracted along with any available measure of uncertainty including standard error, uncertainty intervals, and sample size.

Survey reports and literature often only report information on diabetes prevalence in the population studied. If the study was otherwise representative, we extracted data on the prevalence of diabetes and, using all available data with both estimates of mean fasting plasma glucose and prevalence of diabetes, crosswalked this to estimates of mean fasting plasma glucose.

Crosswalk from Prevalence of Diabetes and HbA1c

We used a mixed-effects regression to crosswalk estimates of diabetes prevalence to the mean fasting plasma glucose of a given population. A separate regression was run for a given diagnostic criteria using the form:

$$\log\left(FPG_{c,a,s,t,k}\right) = \beta_0 + \beta_1 \mathrm{logit}(p_{c,a,s,t,k}) + \beta_2 \mathrm{male} + \sum_{k=10}^{21} \beta_h I_{A[a]} + \alpha_s + \epsilon_{c,a,s,t,k}$$

Where $FPG_{c,a,s,t,k}$ is the outcome of interest—the mean fasting plasma glucose of a given country-, age-, sex-, time-, from survey k; $p_{c,a,s,t,k}$ is the prevalence of diabetes for a given definition or the mean HbA1c level; $I_{A[a]}$ is a dummy variable indicating a specific age group A; and α_s is a super-region specific random effect.

Age and Sex Splitting

Prior to modeling, data provided in age groups wider than the GBD 5-year age groups were split using the approach outlined in Ng et al.[5] Briefly, age-sex patterns were identified using sources of data with multiple age-sex groups and these patterns were applied to split aggregated report data. Uncertainty in the age-sex split was propagated by multiplying the standard error of the data performed by the square root of the number of splits performed.

Modeling

Exposure estimates were produced from 1980 to 2015 for each national and subnational location, sex, and for each 5-year age group starting from 25+. As in GBD 2013, we used a Spatio-Temporal Gaussian Process Regression (ST-GPR) framework to model the mean fasting plasma glucose at the location-, year-, age-, sex- level. Updates to the ST-GR modeling framework for GBD 2015 are detailed in the appendix.
The FPG mean function was estimated using a mixed-effects linear regression, run separately by sex:

$$\text{logit}(\text{FPG}_{c,a,t}) = \beta_0 + \beta_1 \text{SDS}_{c,t} + \beta_2 p_{\text{overweight}_{c,a,t}} + \beta_2 \log(\text{sugar}_{c,,t}) + \sum_{k=2}^{16} \beta_k I_{A[a]} + \alpha_s + \alpha_r + \alpha_c + \epsilon_{c,a,t}$$

where $\text{SDS}_{c,t}$ is socio-demographic status (SDS), an index metric that includes a measure of education and income, $p_{\text{overweight}_{c,a,t}}$ is the prevalence of overweight, $\text{sugar}_{c,,t}$ is the diet adjusted mean consumption of sugar in grams per capita per day, $I_{A[a]}$ is a dummy variable for a fixed effect on a given 5-year age group, and α_s α_r α_c are random effects at the super-region, region, and country level, respectively.

The estimates were then propagated through the ST-GPR framework to obtain 1000 draws for each location, year, age, and sex.

Theoretical minimum-risk exposure level

As in GBD 2013, the theoretical minimum risk exposure level for fasting plasma glucose is between 4.9 and 5.3 mmol/l (uniformly distributed) with a standard deviation 0.3mmol/l. This SD is the lowest reported in population data, after correction for the effects of one-time measurement. We used the same TMREL at all ages because FPG does not rise sharply with age in populations with low blood glucose.

Relative risks

We used Dismod-MR 2.1 to pool effect sizes from included studies and generate a dose-response curve for each of the outcomes associated with high fasting plasma glucose. The tool enabled us to incorporate random effects across studies and include data with different age ranges. RRs were used universally for all countries and the meta-regression only helped to pool the three major sources and produce RRs with uncertainty and covariance across ages taking into account the uncertainty of the data points

As in GBD 2013, RRs for IHD, ischemic, and hemorrhagic stroke are obtained from meta-regressions of pooled epidemiological studies: the Asia Pacific Cohort Studies Collaboration (APCSC), the Prospective Studies Collaboration (PSC), and the Emerging Risk Factor Collaboration (ERFC).6 These studies have shown that relative risks associated with high fasting plasma glucose decline with the log (RR) having an approximately linear relationship with age, approaching a value of 1 between the ages 100 and 110. Thus we estimated age-specific RRs of using DisMod-MR 2.1 with log (RR) as the dependent variable and median age at event as the independent variable with an intercept at age 110. Morbidity and mortality directly caused by diabetes was considered directly attributable to FPG.

In GBD 2015, we have added peripheral vascular disease as an outcome of diabetes using evidence from the CALIBER study, a recent health record linkage cohort study from the UK. In addition, we have updated the relative risks for tuberculosis as an outcome of diabetes using evidence from recent health record linkage studies from the UK, Australia, and Taiwan, as well as other prospective cohort studies. Please see the citation list for a full list of studies utilized.

References

1 Association AD. Classification and Diagnosis of Diabetes. *Diabetes Care* 2015; **38**: S8–16.

2 World Health Organization, International Diabetes Federation. Definition and diagnosis of diabetes mellitus and intermediate hyperglycaemia: report of a WHO/IDF consultation. 2006 http://www.who.int/diabetes/publications/diagnosis%5Fdiabetes2006/en/ (accessed July 24, 2016).

3 IDF diabetes atlas - Home. http://www.diabetesatlas.org/ (accessed July 24, 2016).

4 Worldwide trends in diabetes since 1980: a pooled analysis of 751 population-based studies with 4·4 million participants. *The Lancet* 2016; **387**: 1513–30.

5 Ng M, Fleming T, Robinson M, *et al.* Global, regional, and national prevalence of overweight and obesity in children and adults during 1980–2013: a systematic analysis for the Global Burden of Disease Study 2013. *The Lancet* 2014; **384**: 766–81.

6 Singh GM, Danaei G, Farzadfar F, *et al.* The age-specific quantitative effects of metabolic risk factors on cardiovascular diseases and diabetes: a pooled analysis. *PloS One* 2013; **8**: e65174.

High Total Blood Cholesterol Capstone Appendix

Flowchart

High total cholesterol

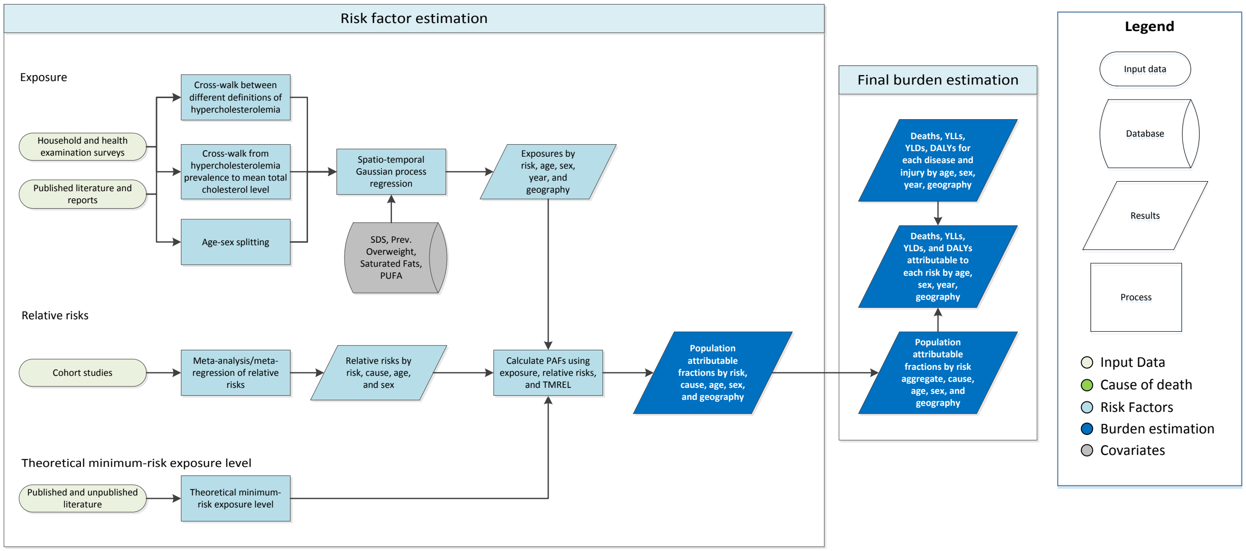

Input Data & Methodological Summary

Exposure

Case Definition

We measure total blood cholesterol as a continuous exposure in units of mmol/L and define hypercholesterolemia according to the World Health Organization (WHO) standard definition as total blood cholesterol $\geq$ 6.2 mmol/L and/or currently on cholesterol-lowering medication.[1]

Input Data

Consistent with GBD 2013, we utilized data on mean total blood cholesterol from literature and from household survey microdata and reports (e.g. STEPS, NHANES). Please see the appendix for a full list of included sources. In GBD 2013, a systematic review of the literature was completed to capture population survey data on mean total blood cholesterol. For GBD 2015, we updated the systematic review using the same strategy, drawing from the GHDx and Medline via PubMed. In total, we have utilized 537 sources corresponding to 36,727 unique data points.

Global Health Data Exchange Database

We systematically searched the Global Health Data Exchange (GHDx) for multi-country survey programs, national surveys, and longitudinal studies which provide measured individual level data on total blood cholesterol. The search was completed for systolic blood pressure, fasting plasma glucose, and blood cholesterol simultaneously, as many sources studying the other metabolic risks will often report mean cholesterol level or hypercholesterolemia prevalence.

Search Terms (Keywords): Blood pressure OR Blood glucose OR Glucose tests OR Cholesterol OR Cholesterol tests OR Hypercholesterolemia
Data Type: Survey OR Report
Search date: 2/6/2016

Literature Review
We systematically searched PubMed for articles published between 15 July 2009 and 31 December 2015 which provided national or subnational estimates of mean total blood cholesterol. As above, the literature review was completed for systolic blood pressure, fasting plasma glucose, and blood cholesterol simultaneously for the reasons previously stated.

Search terms:

(("hypertension"[Mesh:NoExp] OR "blood pressure"[Mesh:NoExp] OR "Hyperlipidemias"[Mesh:NoExp] OR "Hypercholesterolemia"[Mesh] OR "Cholesterol"[Mesh] OR “diabetes mellitus”[Mesh:NoExp] OR “diabetes mellitus, type 2”[Mesh] OR “glucose”[Mesh] OR “hyperglycemia”[Mesh] OR “prediabetic state”[Mesh]) AND "Geographic Locations"[Mesh] NOT "United States"[Mesh]) AND ("humans"[Mesh] AND "adult"[MeSH]) AND ("Data Collection"[Mesh] OR "Health Services Research"[Mesh] OR "Population Surveillance"[Mesh] OR "Vital statistics"[Mesh] OR "Population"[Mesh] OR "Epidemiology"[Mesh] OR "surve*"[TiAb]) NOT Comment[ptyp] NOT Case Reports[ptyp] AND ("2009/07/15"[PDAT] : "2015/12/31"[PDAT]) NOT "hospital"[TiAb]

Search date: 1/26/2016

Inclusion Criteria
Studies were included if they were population-based and measured total blood cholesterol using a blood test. We accepted data on hypercholesterolemia only if the study performed an objective blood measurement and/or individuals reported taking cholesterol-lowering medication. Studies that included self-report of high cholesterol were excluded. We assumed the data is representative of the location if the geography was not related to the diseases (a mining area) and if it is not an outlier compared to other data in the country or region.

Outliers
Data was utilized in the modeling process unless an assessment of data showed that the data is biased. A data point was considered to be an outlier candidate if the level is not consistent with other (sources) country data, or - if there are no other data points - not consistent with other country in the region. A candidate outlier source was scrutinized and validated and the data point was excluded if the quality of study did not warrant a valid estimate because of selection (specific populations), different definitions, other biases, or if the study did not provide methodological details for evaluation.

Data Extraction
Where possible, individual level data on blood pressure estimates were extracted from survey microdata and these were collapsed across individuals and collapsed across demographic groupings to produce mean estimates in the standard GBD 5-year age-sex groups. If microdata were unavailable, information from survey reports or from literature were extracted along with any available measure of uncertainty including standard error, uncertainty intervals, and sample size.

Survey reports and literature often only report information about the prevalence of hypercholesterolemia in the population studied. If the study was otherwise representative, we extracted data on the prevalence of hypercholesterolemia and, using all available data with both estimates of mean total cholesterol and prevalence of hypercholesterolemia, crosswalked this to estimates of mean cholesterol levels.

Crosswalk from Prevalence of Hypercholesterolemia

We used a mixed-effects regression to crosswalk estimates of hypercholesterolemia prevalence to the mean total cholesterol of a given population. A separate regression was run for a given diagnostic criteria using the form:

$$\log(\mathrm{TC}_{c,a,s,t,k}) = \beta_0 + \beta_1 \mathrm{logit}(p_{c,a,s,t,k}) + \beta_2 \mathrm{male} + \sum_{k=10}^{21} \beta_h I_{A[a]} + \alpha_s + \epsilon_{c,a,s,t,k}$$

Where $\mathrm{TC}_{c,a,s,t,k}$ is the outcome of interest—the mean total cholesterol of a given country-, age-, sex-, time-, from survey k; $p_{c,a,s,t,k}$ is the prevalence of hypercholesterolemia for a given definition; $I_{A[a]}$ is a dummy variable indicating a specific age group A; and α_s is a super-region specific random effect.

Age and Sex Splitting

Prior to modeling, data provided in age groups wider than the GBD 5-year age groups were split using the approach outlined in Ng et al.[2] Briefly, age-sex patterns were identified using sources of data with multiple age-sex groups and these patterns were applied to split aggregated report data. Uncertainty in the age-sex split was propagated by multiplying the standard error of the data performed by the square root of the number of splits performed.

Modeling

Exposure estimates were produced from 1980 to 2015 for each national and subnational location, sex, and for each 5-year age group starting from 25+. As in GBD 2013, we used *a* Spatio-Temporal Gaussian Process Regression (ST-GPR) framework to model the mean total blood cholesterol at the location-, year-, age-, sex- level. Updates to the ST-GR modeling framework for GBD 2015 are detailed in the appendix.

The total cholesterol mean function was estimated using a mixed-effects linear regression, run separately by sex:

$$\mathrm{logit}(\mathrm{TC}_{c,a,t}) = \beta_0 + \beta_1 \mathrm{SDS}_{c,t} + \beta_2 p_{\mathrm{overweight}_{c,a,t}} + \beta_3 \mathrm{sat\_fats}_{c,a,t} + \beta_4 \mathrm{PUFA}_{c,a,t} + \sum_{k=2}^{16} \beta_k I_{A[a]} + \alpha_s + \alpha_r + \alpha_c + \epsilon_{c,a,t}$$

where $\mathrm{SDS}_{c,t}$ is socio-demographic status (SDS), an index metric that includes a measure of education and income, $p_{\mathrm{overweight}_{c,a,t}}$ is the prevalence of overweight, $\mathrm{sat\_fats}_{c,a,t}$ is the diet adjusted mean intake of saturated fats per capita per day, $\mathrm{PUFA}_{c,a,t}$ is the diet adjusted mean intake of PUFA per

capita per day, $I_{A[a]}$ is a dummy variable for a fixed effect on a given 5-year age group, and α_s α_r α_c are random effects at the super-region, region, and country level, respectively.

The estimates were then propagated through the ST-GPR framework to obtain 1000 draws for each location, year, age, and sex.

Theoretical minimum-risk exposure level

For GBD 2015, we altered the TMREL for total cholesterol in light of new evidence from statin trials at low levels of cholesterol; a recent meta-analysis found that cardiovascular outcomes could be improved even at low levels of LDL-cholesterol, below 1.3 mmol/l.[3] We used the strong correlation between LDL-cholesterol and total cholesterol to map the proposed LDL-cholesterol TMREL of 0.7-1.3 mmol/l to a TMREL for total cholesterol of 2.8-3.4 mmol/l.

Relative Risks

We used Dismod-MR 2.1 to pool effect sizes from included studies and generate a dose-response curve for each of the outcomes associated with high total cholesterol. The tool enabled us to incorporate random effects across studies and include data with different age ranges. RRs were used universally for all countries and the meta-regression only helped to pool the three major sources and produce RRs with uncertainty and covariance across ages taking into account the uncertainty of the data points

As in GBD 2013, RRs for IHD and ischemic stroke are obtained from meta-regressions of pooled epidemiological studies: the Asia Pacific Cohort Studies Collaboration (APCSC) and the Prospective Studies Collaboration (PSC).[4] RRs for IHD were modeled using with log (RR) as the dependent variable and median age at event as the independent variable with an age intercept (RR equals 1) at age 110. For total cholesterol and ischemic stroke, a similar approach was used, except that there was no age intercept at age 110, due to the fact that there was no statistically significant relationship between total cholesterol and stroke after age 70 with a mean RR less than one. We assumed that there is not a protective effect of high cholesterol and therefore did not include an RR for ages 80+.

References

1 Roth GA, Fihn SD, Mokdad AH, Aekplakorn W, Hasegawa T, Lim SS. High total serum cholesterol, medication coverage and therapeutic control: an analysis of national health examination survey data from eight countries. *Bull World Health Organ* 2011; **89**: 92–101.

2 Ng M, Fleming T, Robinson M, *et al.* Global, regional, and national prevalence of overweight and obesity in children and adults during 1980–2013: a systematic analysis for the Global Burden of Disease Study 2013. *The Lancet* 2014; **384**: 766–81.

3 Boekholdt SM, Hovingh GK, Mora S, *et al.* Very Low Levels of Atherogenic Lipoproteins and the Risk for Cardiovascular EventsA Meta-Analysis of Statin Trials. *J Am Coll Cardiol* 2014; **64**: 485–94.

4 Singh GM, Danaei G, Farzadfar F, *et al.* The age-specific quantitative effects of metabolic risk factors on cardiovascular diseases and diabetes: a pooled analysis. *PloS One* 2013; **8**: e65174.

High Systolic Blood Pressure Capstone Appendix

Flowchart

High Systolic Blood Pressure

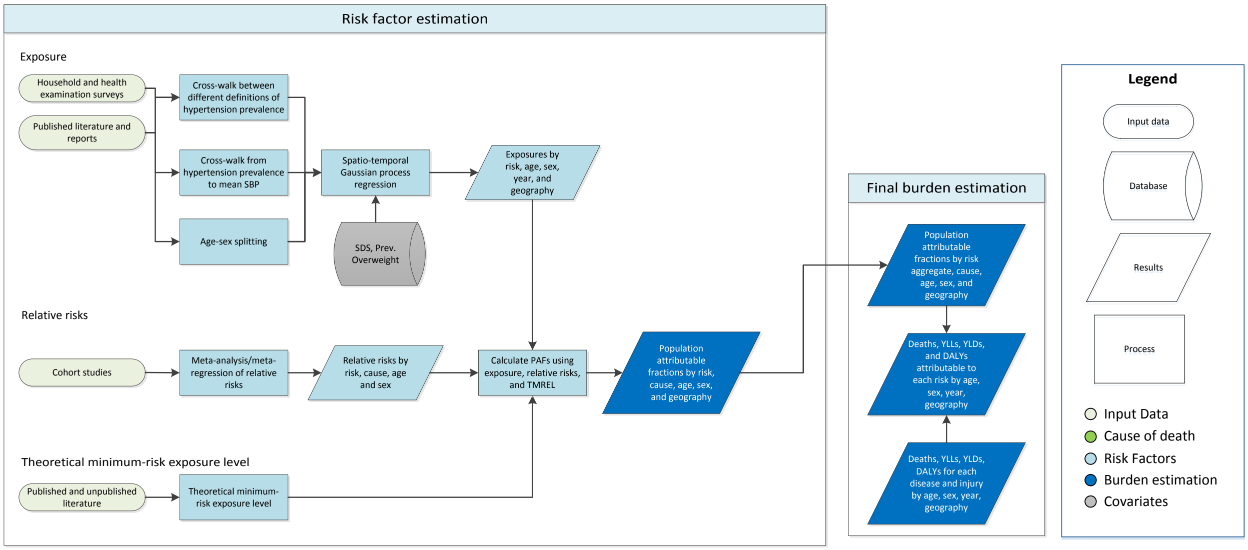

Input Data & Methodological Summary

Exposure

Case Definition

We measure systolic blood pressure as a continuous exposure in units of mmHg and define hypertension according to the World Health Organization (WHO) standard definition as SBP ≥ 140 mmHg and/or DBP ≥ 90 mmHg and/or currently taking anti-hypertensive medication.[1]

Input Data

Consistent with GBD 2013, we utilized data on mean systolic blood pressure from literature and from household survey microdata and reports (e.g. STEPS, NHANES). Please see the appendix for a full list of included sources. In GBD 2013, a systematic review of the literature was completed to capture population survey data on mean systolic blood pressure. For GBD 2015, we updated the systematic review using the same strategy, drawing from the GHDx and Medline via PubMed. In total, we have utilized 844 sources corresponding to 36,727 unique data points.

Global Health Data Exchange Database

We systematically searched the Global Health Data Exchange (GHDx) for multi-country survey programs, national surveys, and longitudinal studies which provide measured individual level data on systolic blood pressure. The search was completed for systolic blood pressure, fasting plasma glucose, and blood cholesterol simultaneously, as many sources studying the other metabolic risks will often report mean blood pressure or hypertension prevalence.

Search Terms (Keywords): Blood pressure OR Blood glucose OR Glucose tests OR Cholesterol OR Cholesterol tests OR Hypercholesterolemia
Data Type: Survey OR Report
Search date: 2/6/2016

Literature Review

We systematically searched PubMed for articles published between 15 July 2009 and 31 December 2015 which provided national or subnational estimates of mean systolic blood pressure. As above, the literature review was completed for systolic blood pressure, fasting plasma glucose, and blood cholesterol simultaneously for the reasons previously stated.

Search terms:

(("hypertension"[Mesh:NoExp] OR "blood pressure"[Mesh:NoExp] OR "Hyperlipidemias"[Mesh:NoExp] OR "Hypercholesterolemia"[Mesh] OR "Cholesterol"[Mesh] OR “diabetes mellitus”[Mesh:NoExp] OR “diabetes mellitus, type 2”[Mesh] OR “glucose”[Mesh] OR “hyperglycemia”[Mesh] OR “prediabetic state”[Mesh]) AND "Geographic Locations"[Mesh] NOT "United States"[Mesh]) AND ("humans"[Mesh] AND "adult"[MeSH]) AND ("Data Collection"[Mesh] OR "Health Services Research"[Mesh] OR "Population Surveillance"[Mesh] OR "Vital statistics"[Mesh] OR "Population"[Mesh] OR "Epidemiology"[Mesh] OR "surve*"[TiAb]) NOT Comment[ptyp] NOT Case Reports[ptyp] AND ("2009/07/15"[PDAT] : "2015/12/31"[PDAT]) NOT "hospital"[TiAb]

Search date: 1/26/2016

Inclusion Criteria
Studies were included if they were population-based and measured SBP using a sphygmomanometer (either manual or electronic). Almost all studies reported an average of repeated measurements of SBP done in a visit. We assumed the data is representative of the location if the geography was not related to the diseases (a mining area) and if it is not an outlier compared to other data in the country or region.

Outliers
Data was utilized in the modeling process unless an assessment of data showed that the data is biased. A data point was considered to be an outlier candidate if the level is not consistent with other (sources) country data, or - if there are no other data points - not consistent with other country in the region. A candidate outlier source was scrutinized and validated and the data point was excluded if the quality of study did not warrant a valid estimate because of selection (specific populations), different definitions, other biases, or if the study did not provide methodological details for evaluation.

Data Extraction
Where possible, individual level data on blood pressure estimates were extracted from survey microdata and these were collapsed across individuals (if multiple measurements were taken for a given individual) and collapsed across demographic groupings to produce mean estimates in the standard GBD 5-year age-sex groups. If microdata were unavailable, information from survey reports or from literature were extracted along with any available measure of uncertainty including standard error, uncertainty intervals, and sample size.

Survey reports and literature often only report information about the prevalence of hypertension in the population studied. If the study was otherwise representative, we extracted data on the prevalence of hypertension and, using all available data with both estimates of mean SBP and prevalence of hypertension, crosswalked this to estimates of mean SBP.

Crosswalk from Prevalence of Hypertension
We used a mixed-effects regression to crosswalk estimates of hypertension prevalence to the mean SBP of a given population. A separate regression was run for a given diagnostic criteria using the form:

$$\log\left(SBP_{c,a,s,t,k}\right) = \beta_0 + \beta_1 \text{logit}(p_{c,a,s,t,k}) + \beta_2 \text{male} + \sum_{k=10}^{21} \beta_h I_{A[a]} + \alpha_s + \epsilon_{c,a,s,t,k}$$

Where $SBP_{c,a,s,t,k}$ is the outcome of interest—the mean SBP of a given country-, age-, sex-, time-, from survey k; $p_{c,a,s,t,k}$ is the prevalence of hypertension for a given definition; $I_{A[a]}$ is a dummy variable indicating a specific age group A; and α_s is a super-region specific random effect.

Age and Sex Splitting
Prior to modeling, data provided in age groups wider than the GBD 5-year age groups were split using the approach outlined in Ng et al.[2] Briefly, age-sex patterns were identified using sources of data with multiple age-sex groups and these patterns were applied to split aggregated report data. Uncertainty in the age-sex split was propagated by multiplying the standard error of the data performed by the square root of the number of splits performed.

Modeling

Exposure estimates were produced from 1980 to 2015 for each national and subnational location, sex, and for each 5-year age group starting from 25+. As in GBD 2013, we used a Spatio-Temporal Gaussian Process Regression (ST-GPR) framework to model the mean systolic blood pressure at the location-, year-, age-, sex- level. Updates to the ST-GR modeling framework for GBD 2015 are detailed in the appendix.
The SBP mean function was estimated using a mixed-effects linear regression, run separately by sex:

$$\text{logit}\left(SBP_{c,a,t}\right) = \beta_0 + \beta_1 SDS_{c,t} + \beta_2 p_{overweight_{c,a,t}} + \sum_{k=2}^{16} \beta_k I_{A[a]} + \alpha_s + \alpha_r + \alpha_c + \epsilon_{c,a,t}$$

where $SDS_{c,t}$ is socio-demographic status (SDS), an index metric that includes a measure of education and income, $p_{overweight_{c,a,t}}$ is the prevalence of overweight, $I_{A[a]}$ is a dummy variable for a fixed effect on a given 5-year age group, and α_s α_r α_c are random effects at the super-region, region, and country level, respectively.

The estimates were then propagated through the ST-GPR framework to obtain 1000 draws for each location, year, age, and sex. Parameter selection for the ST-GPR hyper-parameters were selected through cross-validation using the strategy described in the appendix.

Estimate of Standard Deviation

The standard deviation of SBP was estimated for every age, sex, country, and year by estimating the relationship between the mean of SBP and the standard deviation in available studies. To account for in-person variation, person-level microdata were extracted as means across multiple measurements if possible. To further account for regression dilution bias, we estimated the proportion of the variance of SBP accounted for by measurement error and temporal and inter-individual variation and corrected survey estimates of the standard deviation based on an analysis of multiple cohort studies in the United States, China, Indonesia, South Africa, and Brazil.

Theoretical minimum-risk exposure level

We estimated that the TMREL of SBP ranges from 110 to 115 mm Hg based on pooled prospective cohort studies that show risk of mortality increases for SBP above that level.[3,4] Our selection of a TMREL of 110-115 mmHg is consistent with the GBD study approach of estimating all attributable health loss that could be prevented even if current interventions do not exist that can achieve such a change in exposure level, for example a tobacco smoking prevalence of zero percent. Recent randomized clinical trial results, including the Systolic Blood Pressure Intervention Trial (SPRINT) and the Heart Outcomes Prevention Evaluation (HOPE-3), show that lifestyle modification early in life is likely to be a major component for lowering SBP to near this level given the variable range of benefit observed in these studies when blood pressure was lowered with anti-hypertensive medications alone.[5,6] To include the uncertainty in the TMREL, we took a random draw from the uniform distribution of the interval between 110 mm and 115 mm Hg each time the population attributable burden was calculated.

Relative risks

As with GBD 2013, RRs for chronic kidney disease are from the Renal Risk Collaboration meta-analysis of 2.7 million individuals in 106 cohorts. For other outcomes, we used data from two pooled epidemiological studies: the Asia Pacific Cohort Studies Collaboration (APCSC) and the Prospective Studies Collaboration (PSC).[4,7] In GBD 2015, we have added additional estimates of RR for cardiovascular outcomes from the CALIBER study, a health-record linkage cohort study from the UK.[8]

For cardiovascular disease, epidemiological studies have shown that the RR associated with SBP declines with age, with the log (RR) having an approximately linear relationship with age and reaching a value of 1 between the ages of 100 and 120. RRs were reported per 10 mm Hg increase in SBP above TMREL value (115 mm Hg) as in the equation below:

$$RR_x = RR^{(x-TMREL)}$$

We used Dismod-MR 2.1 to pool effect sizes from included studies and generate a dose-response curve for each of the outcomes associated with high SBP. The tool enabled us to incorporate random effects across studies and include data with different age ranges. RRs were used universally for all countries and the meta-regression only helped to pool the three major sources and produce RRs with uncertainty and covariance across ages taking into account the uncertainty of the data points

References

1 Bangalore S, Gong Y, Cooper-DeHoff RM, Pepine CJ, Messerli FH. 2014 Eighth Joint National Committee panel recommendation for blood pressure targets revisited: results from the INVEST study. *J Am Coll Cardiol* 2014; **64**: 784–93.

2 Ng M, Fleming T, Robinson M, *et al.* Global, regional, and national prevalence of overweight and obesity in children and adults during 1980–2013: a systematic analysis for the Global Burden of Disease Study 2013. *The Lancet* 2014; **384**: 766–81.

3 Singh GM, Danaei G, Farzadfar F, *et al.* The age-specific quantitative effects of metabolic risk factors on cardiovascular diseases and diabetes: a pooled analysis. *PloS One* 2013; **8**: e65174.

4 Collaboration APCS, others. Blood pressure and cardiovascular disease in the Asia Pacific region. *J Hypertens* 2003; **21**: 707–16.

5 Wright JT, Williamson JD, Whelton PK, Snyder JK, Sink KM, Rocco MV, Reboussin DM, Rahman M, Oparil S, Lewis CE, Kimmel PL. A Randomized Trial of Intensive versus Standard Blood-Pressure Control. *N Engl J Med* 2015; **373**: 2103–16.

6 Lonn EM, Bosch J, López-Jaramillo P, *et al.* Blood-Pressure Lowering in Intermediate-Risk Persons without Cardiovascular Disease. *N Engl J Med* 2016; **0**: null.

7 Prospective Studies Collaboration. Age-specific relevance of usual blood pressure to vascular mortality: a meta-analysis of individual data for one million adults in 61 prospective studies. *The Lancet* 2002; **360**: 1903–13.

8 Rapsomaniki E, Timmis A, George J, *et al.* Blood pressure and incidence of twelve cardiovascular diseases: lifetime risks, healthy life-years lost, and age-specific associations in 1·25 million people. *Lancet Lond Engl* 2014; **383**: 1899–911.

Body Mass Index Capstone Appendix

Flowchart

BMI: Data and Model Flow Chart

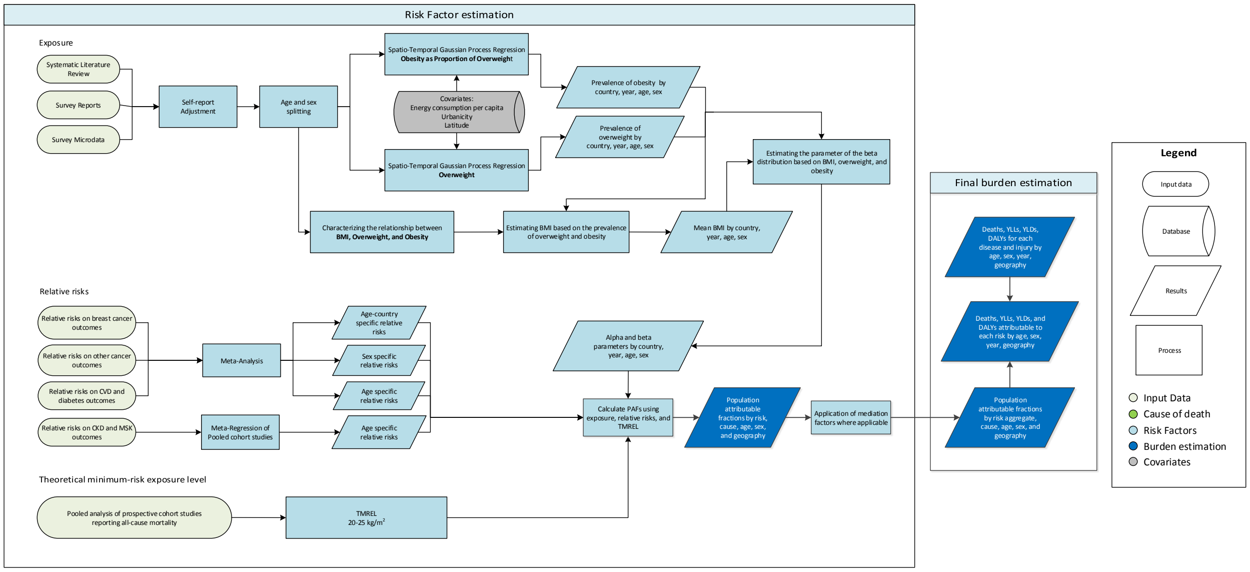

Input Data & Methodological summary

Exposure

Case definition

Exposure to high body mass index (BMI) is defined using metrics related to national and subnational estimates of BMI. If a person has a BMI of 22.5 kg/m^2 or greater, he/she is considered at risk for a range of diseases including cardiovascular diseases, musculoskeletal disorders, and cancers.

Input Data

We used data from multi-country survey programs, national surveys, and longitudinal studies which were available in the Global Health Data Exchange (GHDx) and provided either self-report or measured data on height and weight. A complete description of the data seeking and update process for the GHDx is provided elsewhere (See Section 2 of appendix).

Additionally, to include articles published after our search period for GBD 2013, we systematically searched Medline for studies published between 1 January 2014 and 31 December 2015 providing national or subnational estimates of BMI, overweight, or obesity. The search was conducted on 26 January 2016 using the following search terms: (("Body Mass Index"[Mesh] OR "Overweight"[Mesh] OR "Obesity"[Mesh]) AND "Geographic Locations"[Mesh] NOT "United States"[Mesh]) AND ("humans"[Mesh] AND "adult"[MeSH]) AND ("Data Collection"[Mesh] OR "Health Services Research"[Mesh] OR "Population Surveillance"[Mesh] OR "Vital statistics"[Mesh] OR "Population"[Mesh] OR "Epidemiology"[Mesh] OR

"surve*"[TiAb]) NOT Comment[ptyp] NOT Case Reports[ptyp] NOT "hospital"[TiAb]. Of the 2,036 articles identified through Medline search, 162 articles met inclusion criteria[1] and were selected for data extraction.

Data Preparation

We adjusted self-reported data for overweight prevalence, obesity prevalence, and mean BMI using the following nested hierarchical mixed-effects regression models, fit using maximum likelihood separately by sex:

$$\mathrm{logit(overweight)_{c,a,t}} = \beta_0 + \beta_1 \mathrm{m} + \sum_{k=2}^{15} \beta_k \mathrm{I_{A[a]}} + \sum_{l=16}^{41} \beta_l \, \mathrm{I_{A[a]} I_{M[m]}} + \alpha_s + \alpha_s \mathrm{m} + \alpha_r + \alpha_r \mathrm{m} + \alpha_c + \alpha_c \mathrm{m} + \alpha_t + \alpha_t \mathrm{m} + \epsilon_{c,a,t}$$

$$\mathrm{logit(obesity)_{c,a,t}} = \beta_0 + \beta_1 \mathrm{m} + \sum_{k=2}^{15} \beta_k \mathrm{I_{A[a]}} + \sum_{l=16}^{41} \beta_l \, \mathrm{I_{A[a]} I_{M[m]}} + \alpha_s + \alpha_s \mathrm{m} + \alpha_r + \alpha_r \mathrm{m} + \alpha_c + \alpha_c \mathrm{m} + \alpha_t + \alpha_t \mathrm{m} + \epsilon_{c,a,t}$$

$$\mathrm{log(BMI)_{c,a,t}} = \beta_0 + \beta_1 \mathrm{m} + \sum_{k=2}^{15} \beta_k \mathrm{I_{A[a]}} + \sum_{l=16}^{41} \beta_l \, \mathrm{I_{A[a]} I_{M[m]}} + \alpha_s + \alpha_s \mathrm{m} + \alpha_r + \alpha_r \mathrm{m} + \alpha_c + \alpha_c \mathrm{m} + \alpha_t + \alpha_t \mathrm{m} + \epsilon_{c,a,t}$$

Models included a fixed effect on measurement (m; binary, either measured (1) or self-report (0)), fixed effects on age group (a), interactions between measurement and age group, random intercepts at each level of the geographic hierarchy (α_s, α_r, α_c) and by time-period (α_t; categorical: 1980-1991, 1992-2003, 2004-2015), and random slopes on measurement at each level of the geographic hierarchy and by time-period. Random effects at the country and time-period level were used to fit the model, but were taken as noise and were not used in adjustment. We propagated the uncertainty in the model by adding the variance of each of the regression coefficients to the data variance in delta-transformed space.

After adjusting for self-report bias any report or literature data provided in age groups wider than the standard GBD 5-year age groups or as both sexes combined were split using the approach used by Ng et al.[1] Briefly, age-sex patterns were identified using sources with data on multiple age-sex groups and these patterns were applied to split aggregated report data. Uncertainty in the age-sex split was propagated by multiplying the standard error of the data by the square root of the number of splits performed.

Modeling strategy

We used Spatio-Temporal Gaussian Process Regression (ST-GPR) to estimate the prevalence of overweight and obesity. Consistent with the approach used in GBD2013, the mean functions used in ST-GPR were estimated using the following linear regressions, run separately by sex:

$$logit\left(ow_{c,a,t}\right) = \beta_0 + \beta_1 energy_{c,t} + \beta_2 \, lat_c + \beta_3 urbanicity_{c,t} + \sum_{k=4}^{16} \beta_k I_{A[a]} + \epsilon_{c,a,t};$$

$$logit\left(\left(\frac{ob}{ow}\right)_{c,a,t}\right) = \beta_0 + \beta_1 energy_{c,t} + \beta_2 \, lat_c + \beta_3 urbanicity_{c,t} + \sum_{k=4}^{16} \beta_k I_{A[a]} + \epsilon_{c,a,t}$$

where $energy_{c,t}$ is a 10-year lag distributed energy intake per capita in country *c* at year *t*, lat_c is the absolute latitude of country c, $urbanicity_{c,t}$ is the proportion of people living in urban areas in country c in time t, and $I_{A[a]}$ is an indicator variable for specific age group A that the overweight prevalence point ($ow_{c,a,t}$) or obese as a proportion of overweight point $(ob/ow)_{c,a,t}$ is capturing. The estimated mean

functions were then propagated through the ST-GPR framework to obtain 1,000 draws of overweight prevalence estimates and obesity as a proportion of overweight estimates. Based on the results of out-of-sample cross validation, we used different space-weight parameters for locations with low data coverage (less than 15 years covered by data in at least one age-sex group) versus locations with high data coverage (more than 15 years covered by data in at least one age-sex group).

To estimate the mean BMI for each country, age, sex, and time period estimated in GBD, we first used the following nested hierarchical mixed-effects model, fit using data from sources containing estimates of all three indicators, to characterize the relationship between overweight, obesity, and mean BMI:

$$\log(\mathrm{BMI}_{\mathrm{c,a,t}}) = \beta_0 + \beta_1 \mathrm{ow}_{c,a,t} + \beta_2 \mathrm{ob}_{c,\mathrm{a,t}} + \beta_3 \mathrm{sex} + \sum_{\mathrm{k}=4}^{17} \beta_{\mathrm{k}} \mathrm{I}_{\mathrm{A}[a]} + \alpha_{\mathrm{s}} + \alpha_{\mathrm{s}} \mathrm{ow}_{c,a,t} + \alpha_{\mathrm{s}} \mathrm{ob}_{c,\mathrm{a,t}} + \alpha_{\mathrm{r}} + \alpha_{\mathrm{r}} \mathrm{ow}_{c,a,t} + \alpha_{\mathrm{r}} \mathrm{ob}_{c,\mathrm{a,t}} + \alpha_{\mathrm{c}} + \alpha_{\mathrm{c}} \mathrm{ow}_{c,a,t} + \alpha_{\mathrm{c}} \mathrm{ob}_{c,\mathrm{a,t}} + \epsilon_{c,a,t}$$

Then, we applied the regression coefficients to the 1,000 draws of overweight prevalence and obesity prevalence produced through ST-GPR to estimate 1,000 draws of mean BMI for each country, year, age, and sex. This allowed overweight prevalence, obesity prevalence, and mean BMI to be correlated at the draw level, a methodological improvement compared to GBD2013. The estimated mean BMI, overweight prevalence, and obesity prevalence were used to compute the parameters of a beta distribution for BMI in each location, year, age, and sex. The details of this approach have been described by Ng et al. elsewhere.[2] We updated the constraints on the minimum and maximum of the distribution based on biological plausibility.[3,4]

Relative Risks

The relative risk of change in BMI for each disease endpoint was obtained from meta-analyses, and where available, pooled analyses of prospective observational studies. For most outcomes, we have made no substantive updates to relative risks. We dropped the following outcomes, previously included in GBD 2013, due to a lack of conclusive evidence supporting a causal relationship: other cardiovascular diseases, atrial fibrillation and flutter, cardiomyopathy and myocarditis, peripheral vascular disease, and endocarditis. We updated relative risks for osteoarthritis of the hip and osteoarthritis of the knee using recently published meta-analyses.[5,6]

TMREL

The TMREL of BMI was determined based on the BMI level that was associated with the lowest risk of all-cause mortality in prospective cohort studies. In GBD 2015, based on the findings of the most recent pooled analysis of prospective cohorts[7], we changed the TMREL of BMI from 21-23 to 20-25 kg/m^2.

1 Ng M, Fleming T, Robinson M, *et al.* Global, regional, and national prevalence of overweight and obesity in children and adults during 1980–2013: a systematic analysis for the Global Burden of Disease Study 2013. *The Lancet* 2014; published online May. DOI:10.1016/S0140-6736(14)60460-8.

2 Ng M, Liu P, Thomson B, Murray CJL. A novel method for estimating distributions of body mass index. *Population Health Metrics* 2016; **14**: 6.

3 Lawman HG, Ogden CL, Hassink S, Mallya G, Veur SV, Foster GD. Comparing Methods for Identifying Biologically Implausible Values in Height, Weight, and Body Mass Index Among Youth. *Am J Epidemiol* 2015; : kwv057.

4 Freedman DS, Lawman HG, Skinner AC, McGuire LC, Allison DB, Ogden CL. Validity of the WHO cutoffs for biologically implausible values of weight, height, and BMI in children and adolescents in NHANES from 1999 through 2012. *Am J Clin Nutr* 2015; **102**: 1000–6.

5 Jiang L, Rong J, Wang Y, *et al.* The relationship between body mass index and hip osteoarthritis: a systematic review and meta-analysis. *Joint Bone Spine* 2011; **78**: 150–5.

6 Jiang L, Tian W, Wang Y, *et al.* Body mass index and susceptibility to knee osteoarthritis: a systematic review and meta-analysis. *Joint Bone Spine* 2012; **79**: 291–7.

7 Body-mass index and all-cause mortality: individual-participant-data meta-analysis of 239 prospective studies in four continents. *The Lancet* 2016; **388**: 776–86.

Bone Mineral Density Capstone Appendix

Flowchart

Bone Mineral Density

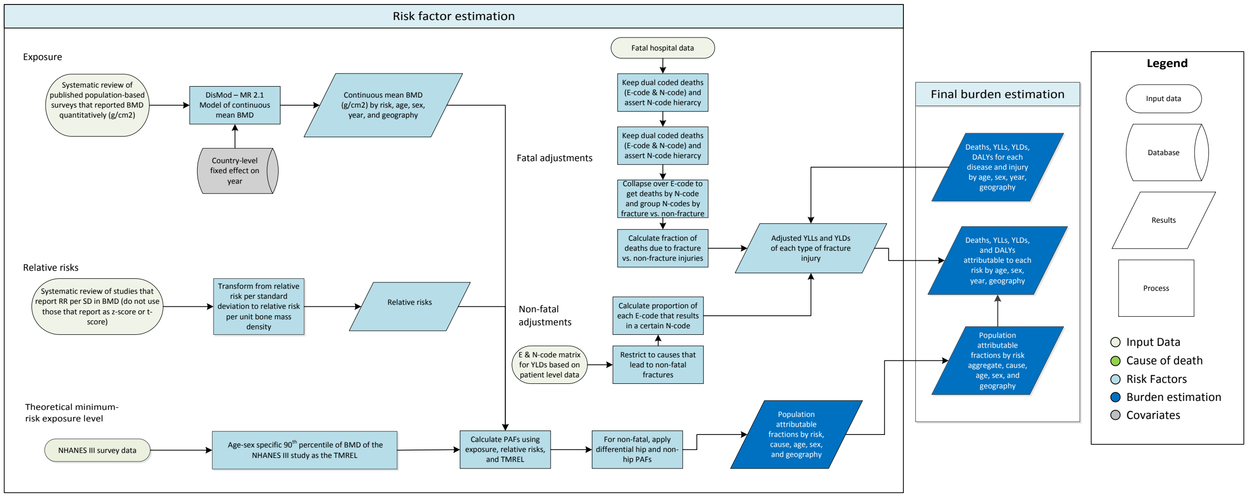

Input Data & Methodological Summary

Exposure

Case Definition

Bone mineral density (BMD) is a continuous variable measured by dual-x-ray-absorptiometry (DXA) at the femoral neck (FN) and is presenting in g/cm2 after standardizing for the brand of densitometer. The burden attributed to low bone mineral density is estimated for adults greater than or equal to 20 years of age.

For estimating burden, we need to estimate:

- Exposure: Mean and standard deviation of standardized BMD according to the brand of densitometer (sBMD) for each country and all subnational levels for which we do GBD estimation.
- Risk of fractures in people exposed to low BMD relative to people who have BMD equal or greater than the TMREL. We consider fatal outcomes for hip and vertebral fractures and non-fatal outcomes for hip and other osteoporosis-associated non-hip fractures. These osteoporotic non-hip fractures include fractures of vertebrae, clavicle, scapula, humerus, skull, sternum, rib, face bone, radius or ulna, femur, patella, tibia, fibula, ankle, pelvis, vertebral and other extremities.

Input data

For GBD 2015, a systematic review was conducted to update the GBD 2013 dataset. Inclusion criteria that informed the search included:

- o Representative, population-based surveys
- o Reporting of quantitative BMD
    - ▪ measured by DXA

- performed at the femoral neck region
- measured in grams/centimeters squared

Mean BMD for was occasionally reported in stratified groups (e.g. by fracture status) so that a total sample BMD mean was not available. In these cases, the stratified means were aggregated to obtain a total mean BMD per study group.

See the search query below for the exact terms used to conduct the systematic review. In GBD 2015, 144 new data points were added, from the following super-regions. The table below indicates the geographic spread of these values.

Super region	GBD 2015 new data points
Central Europe, Eastern Europe, and Central Asia	2
High-income	93
North Africa and Middle East	11
South Asia	30
Southeast Asia, East Asia, and Oceania	8

Modeling strategy

We model mean BMD in DisMod-MR. Mean and standard deviation are correlated for BMD. We used a mixed effects model to predict coefficient of variation (standard deviation over mean BMD) with fixed effects on health system access and Ln_LDI (Lag Distributed Income) and random effects on super-region, region and country.

We model continuous mean BMD using a single parameter model for ages 20 to 100, both sexes, and all GBD locations for years 1990 to 2015. The model has age mesh points at 0 10 20 25 30 40 50 60 70 80 90 & 100, a time window of 10 years for fitting data, and a minimum coefficient of variation of 0.4 for global, 0.2 super region and 0.1 for the region level.

The country covariates of BMI, smoking (different variables), alcohol consumption and milk consumption did not have a significant effect on BMD and some even had a significant effect in the opposite direction to what we know about the pathophysiology. Therefore, we excluded them from our final model.

Some of the data points from the newly added data were outliered during the modeling process.

On both the fatal and non-fatal side, there are various modelling steps that must happen after DisMod modelling of exposure. First, we must calculate the proportion of deaths that are due to fracture. This proportion of death caused by fracture is the envelope that we use to attribute death to bone mineral density. In order to do this, we first used evidence to create a list of all fractures that can be fatal (most are not fatal). Hip fracture and some non-hip fractures (spinal cord and sternum) are considered potentially fatal fractures.

Then, we use available hospital data to estimate what proportion of deaths are due to fracture. However, most hospital data are based just on “E-code”, meaning it is reported that that person died because of a car accident but we do not know the “N-code”, or the nature of the injury (internal hemorrhage, fracture, etc.). First, we restrict to the cases that are dual-coded with both an “E-code” and an “N-code”. Second, many cases have multiple forms of trauma, and therefore, we must apply a severity hierarchy to the fatal

hospital data to decipher what proportion of the deaths are due to one of the fracture types and not to a more severe fatal trauma. Then, once each observation has just one E-code and N-code we collapse over E-code to find the number of deaths attributable to fracture versus non-fracture injuries. We apply this fracture to the YLL of each of these types of fracture injury.

On the non-fatal side, we first restrict to a list of causes such as transportation injuries, falls, homicide, and disasters that cause that cause non-fatal fractures. Then, we use an E to N-code matrix generated from dual-coded (E-code/N-code) patient level data in order to calculate the proportion of each E-code that results in a certain N-code. This proportion is then applied to the YLDs. The hip and non-hip fracture population attributable fractions are then applied to get the YLD population attributable fractions.

Theoretical minimum-risk exposure level

The theoretical minimum of risk exposure level or TMREL is the age-sex specific 90th percentile of BMD of the NHANES III study as the reference population.

Relative risks

For relative risks, we use a systematic review of bone mineral density that was also used in GBD 2013. Relative risks must be reported per standard deviation or per unit bone mass density in order for us to use the data. Many studies report relative risk based on a z-score or the relative risks in the osteoporotic group versus the non-osteoporotic group; neither of these relative risks are usable.

Updates to systematic reviews are performed on an ongoing schedule across all GBD causes and risk factors, an update for bone mineral density will be performed in the next 1-2 iterations.

The table below illustrates the GBD 2015 search queries.

Search	Query	Items found	Time
#11	Search (#8 AND #10) Filters: Humans	326	12:37:09
#10	Search ("Cross-Sectional Studies"[Mesh] OR "cross-sectional"[title/abstract] OR "Health Surveys"[Mesh] OR Survey[title/abstract] OR cohort[title/abstract] OR "Diet Surveys"[Mesh] OR "Longitudinal Studies"[Mesh] OR "Nutrition Surveys"[Mesh] OR "Surveys and Questionnaires"[Mesh]) Filters: Humans	1324376	12:36:29
#8	Search (#7 AND #6) Filters: Humans	622	12:33:16
#7	Search ("Absorptiometry, Photon"[Mesh] OR "dual-energy x-ray absorptiometry" OR "dual energy x-ray absorptiometry") Filters: Humans	21368	12:32:34
#6	Search (#5 AND ("2010"[Date - Publication] : "3000"[Date - Publication])) Filters: Humans	1387	12:30:26
#5	Search ((#1 OR #2) AND #3) Filters: Humans	3702	12:29:47
#4	Search ((#1 OR #2) AND #3)	4015	12:29:33
#3	Search (((("bone mineral density"[title/abstract] OR "bone mineral densities"[title/abstract]) OR "Bone Density"[Mesh]) AND (mean[title/abstract] OR average[title/abstract])))	12892	12:29:00

#2	Search ((((multinational[TIAB] OR international[TIAB] OR national[TIAB] OR nationwide[TIAB] OR nation-wide[TIAB] OR equitorial[TIAB] OR equator[TIAB] OR global[TIAB] OR globe[TIAB] OR world[TIAB] OR worldwide[TIAB] OR world-wide[TIAB] OR countrywide[TIAB] OR countries[TIAB] OR continental[TIAB] OR continent[TIAB] OR continents[TIAB] OR global burden[TIAB] OR burden of disease[TIAB] OR disease burden[TIAB] OR tropic[TIAB] OR tropics[TIAB] OR tropical[TIAB] OR Oceania[TIAB] OR South America[TIAB] OR Central America[TIAB] OR Mesoamerica[TIAB] OR Americas[TIAB] OR Latin America[TIAB] OR paho[TIAB] OR pan-american[TIAB] OR panamerican[TIAB] OR pan-america[TIAB] OR Caribbean[TIAB] OR Indies[TIAB] OR Australasia[TIAB] OR Australasian[TIAB] OR developing countries[TIAB] OR developing nations[TIAB] OR developed countries[TIAB] OR developed nations[TIAB] OR commonwealth[TIAB] OR industrialized[TIAB] OR nonindustrialized[TIAB] OR non-industrialized[TIAB] OR underdeveloped countries[TIAB] OR underdeveloped nation[TIAB] OR underdeveloped nations[TIAB] OR under-developed country[TIAB] OR under-developed countries[TIAB] OR under-developed nation[TIAB] OR under-developed nations[TIAB] OR low-income country[TIAB] OR low-income countries[TIAB] OR low-income nation[TIAB] OR low-income nations[TIAB] OR nondeveloped country[TIAB] OR nondeveloped countries[TIAB] OR nondeveloped nation[TIAB] OR nondeveloped nations[TIAB] OR non-developed country[TIAB] OR non-developed countries[TIAB] OR non-developed nation[TIAB] OR non-developed nations[TIAB] OR International Cooperation[TIAB] OR World Health Organization[TIAB] OR Asia[TIAB] OR Far East[TIAB] OR Near East[TIAB] OR Middle East[TIAB] OR Scandinavia[TIAB] OR Europe[TIAB] OR European[TIAB] OR Eastern Hemisphere[TIAB] OR Western Hemisphere[TIAB] OR Northern Hemisphere[TIAB] OR Southern Hemisphere[TIAB] OR North America[TIAB] OR island[TIAB] OR islands[TIAB] OR United Nations[TIAB] OR unesco[TIAB] OR unicef[TIAB] OR Worldbank[TIAB] OR Benelux[TIAB] OR sub-Saharan[TIAB] OR subsaharan[TIAB] OR Sahara[TIAB] OR sub-Sahara[TIAB] OR Amazon[TIAB] OR Amazonian[TIAB] OR valley[TIAB] OR river[TIAB] OR mountain[TIAB] OR mountains[TIAB] OR forest[TIAB] OR forests[TIAB] OR rainforest[TIAB] OR rainforests[TIAB] OR jungle[TIAB] OR jungles[TIAB] OR archipelago[TIAB] OR archipelagos[TIAB] OR archipelagoes[TIAB] OR patagonia[TIAB] OR andes[TIAB] OR mediterranean region[TIAB] OR Africa[TIAB] OR registry[TIAB] OR North Korea[TIAB] OR Timor[TIAB] OR Palestine[TIAB] OR Syrian Arab Republic[TIAB] OR Baltic[TIAB] OR Atlantic Islands[TIAB] OR Indian Ocean[TIAB] OR Pacific[TIAB] OR multicenter[TIAB] OR multi-center[TIAB] OR registry[TIAB] OR registries[TIAB] OR Algeria[TIAB] OR Egypt[TIAB] OR Libya[TIAB] OR Morocco[TIAB] OR Tunisia[TIAB] OR Cameroon[TIAB] OR Central African Republic[TIAB] OR Chad[TIAB] OR Congo[TIAB] OR Congo[TIAB] OR Equatorial Guinea[TIAB] OR Gabon[TIAB] OR Burundi[TIAB] OR Djibouti[TIAB] OR Eritrea[TIAB] OR Ethiopia[TIAB] OR Kenya[TIAB] OR Rwanda[TIAB] OR Somalia[TIAB] OR Sudan[TIAB] OR Tanzania[TIAB] OR Uganda[TIAB] OR Angola[TIAB] OR Botswana[TIAB] OR Lesotho[TIAB] OR Malawi[TIAB] OR Mozambique[TIAB] OR Namibia[TIAB] OR South Africa[TIAB] OR Swaziland[TIAB] OR Zambia[TIAB] OR Zimbabwe[TIAB] OR Benin[TIAB] OR Burkina Faso[TIAB] OR Cote d'Ivoire[TIAB] OR Gambia[TIAB] OR Ghana[TIAB] OR Guinea[TIAB] OR Guinea-Bissau[TIAB] OR Liberia[TIAB] OR Mali[TIAB] OR Mauritania[TIAB] OR Niger[TIAB] OR Nigeria[TIAB] OR Senegal[TIAB] OR Sierra Leone[TIAB] OR Togo[TIAB] OR Antigua[TIAB] OR Bahamas[TIAB] OR Barbados[TIAB] OR Cuba[TIAB] OR Dominica[TIAB] OR Dominican Republic[TIAB] OR Grenada[TIAB] OR Guadeloupe[TIAB] OR Haiti[TIAB] OR Jamaica[TIAB] OR Martinique[TIAB] OR Netherlands Antilles[TIAB] OR Puerto Rico[TIAB] OR Saint Kitts and Nevis[TIAB] OR Saint Lucia[TIAB] OR Saint Vincent[TIAB] OR Grenadines[TIAB] OR Trinidad and Tobago[TIAB] OR Virgin Islands[TIAB] OR Belize[TIAB] OR Costa Rica[TIAB] OR El Salvador[TIAB] OR Guatemala[TIAB] OR Honduras[TIAB] OR Nicaragua[TIAB] OR Panama[TIAB] OR Mexico[TIAB] OR Argentina[TIAB] OR Bolivia[TIAB] OR Brazil[TIAB] OR Chile[TIAB] OR Colombia[TIAB] OR Ecuador[TIAB] OR French Guiana[TIAB] OR French Guiana[TIAB] OR Paraguay[TIAB] OR Peru[TIAB] OR Suriname[TIAB] OR Uruguay[TIAB] OR Venezuela[TIAB] OR Kazakhstan[TIAB] OR Kyrgyzstan[TIAB] OR Tajikistan[TIAB] OR Turkmenistan[TIAB] OR Uzbekistan[TIAB] OR Borneo[TIAB] OR Cambodia[TIAB] OR Timor[TIAB] OR Indonesia[TIAB] OR Laos[TIAB] OR Malaysia[TIAB] OR Mekong Valley[TIAB] OR Myanmar[TIAB] OR Philippines[TIAB] OR Thailand[TIAB] OR Vietnam[TIAB] OR Viet Nam[TIAB] OR Bangladesh[TIAB] OR Bhutan[TIAB] OR India[TIAB] OR Afghanistan[TIAB] OR Bahrain[TIAB] OR Iran[TIAB] OR Iraq[TIAB] OR Jordan[TIAB] OR Kuwait[TIAB] OR Lebanon[TIAB] OR Oman[TIAB] OR Qatar[TIAB] OR Saudi Arabia[TIAB] OR Syria[TIAB] OR Turkey[TIAB] OR United Arab Emirates[TIAB] OR Yemen[TIAB] OR Nepal[TIAB] OR	3585538	12:28:22

	Pakistan[TIAB] OR Sri Lanka[TIAB] OR China[TIAB] OR Macao[TIAB] OR Mongolia[TIAB] OR Taiwan[TIAB] OR Azores[TIAB] OR Bermuda[TIAB] OR Falkland Islands[TIAB] OR Albania[TIAB] OR Estonia[TIAB] OR Latvia[TIAB] OR Lithuania[TIAB] OR Bosnia-Herzegovina[TIAB] OR Bulgaria[TIAB] OR Byelarus[TIAB] OR Croatia[TIAB] OR Czech Republic[TIAB] OR Hungary[TIAB] OR Macedonia[TIAB] OR Moldova[TIAB] OR Montenegro[TIAB] OR Poland[TIAB] OR Romania[TIAB] OR Russia[TIAB] OR Slovakia[TIAB] OR Slovenia[TIAB] OR Ukraine[TIAB] OR Yugoslavia[TIAB] OR Armenia[TIAB] OR Azerbaijan[TIAB] OR Georgia[TIAB] OR Comoros[TIAB] OR Madagascar[TIAB] OR Mauritius[TIAB] OR Reunion[TIAB] OR Seychelles[TIAB] OR Fiji[TIAB] OR New Caledonia[TIAB] OR Papua New Guinea[TIAB] OR Vanuatu[TIAB] OR Guam[TIAB] OR Palau[TIAB] OR Pitcairn Island[TIAB] OR Samoa[TIAB] OR Tonga[TIAB] OR Czechoslovakia[TIAB] OR East Germany[TIAB] OR New Guinea[TIAB] OR USSR[TIAB] OR Yugoslavia[TIAB] OR Ivory Coast[TIAB] OR Hong Kong[TIAB] OR china[TIAB] OR North Korea[TIAB] OR Palestine[TIAB] OR Syrian Arab Republic[TIAB]) AND (hasabstract[text] AND Humans[Mesh] AND middle age[MeSH])) OR ((International Cooperation[Mesh:noexp] OR developing countries[Mesh] OR developed countries[Mesh] OR WORLD HEALTH[Mesh] OR WORLD HEALTH ORGANIZATION[Mesh] OR AFRICA[Mesh] OR Americas[Mesh:noexp] OR Caribbean Region[Mesh] OR West Indies[Mesh] OR Central America[Mesh] OR Latin America[Mesh:noexp] OR North America[Mesh:noexp] OR South America[Mesh] OR Antarctic Regions[Mesh:noexp] OR Arctic Regions[Mesh:noexp] OR Asia[Mesh:noexp] OR Asia, Central[Mesh] OR Asia, Southeastern[Mesh:noexp] OR Asia, Western[Mesh:noexp] OR Middle East[Mesh:noexp] OR Far East[Mesh:noexp] OR Atlantic Islands[Mesh] OR Europe[Mesh:noexp] OR Europe, Eastern[Mesh] OR Scandinavia[Mesh:noexp] OR Transcaucasia[Mesh] OR Indian Ocean Islands[Mesh] OR Oceania[Mesh:noexp] OR Australasia[Mesh:noexp] OR Pacific Islands[Mesh:noexp] OR Melanesia[Mesh:noexp] OR Micronesia[Mesh:noexp] OR Polynesia[Mesh:noexp] OR Mexico[Mesh] OR Borneo[Mesh] OR Cambodia[Mesh] OR East Timor[Mesh] OR Indonesia[Mesh] OR Laos[Mesh] OR Malaysia[Mesh] OR Mekong Valley[Mesh] OR Myanmar[Mesh] OR Philippines[Mesh] OR Thailand[Mesh] OR Vietnam[Mesh] OR Bangladesh[Mesh] OR Bhutan[Mesh] OR India[Mesh] OR Afghanistan[Mesh] OR Bahrain[Mesh] OR Iran[Mesh] OR Iraq[Mesh] OR Jordan[Mesh] OR Kuwait[Mesh] OR Lebanon[Mesh] OR Oman[Mesh] OR Qatar[Mesh] OR Saudi Arabia[Mesh] OR Syria[Mesh] OR Turkey[Mesh] OR United Arab Emirates[Mesh] OR Yemen[Mesh] OR Nepal[Mesh] OR Pakistan[Mesh] OR Sri Lanka[Mesh] OR China[Mesh] OR Macao[Mesh] OR Mongolia[Mesh] OR Taiwan[Mesh] OR Multicenter Studies As Topic[Mesh] OR Multicenter Study[PT] OR Algeria[PL] OR Egypt[PL] OR Libya[PL] OR Morocco[PL] OR Tunisia[PL] OR Cameroon[PL] OR Central African Republic[PL] OR Chad[PL] OR Congo[PL] OR Congo[PL] OR Equatorial Guinea[PL] OR Gabon[PL] OR Burundi[PL] OR Djibouti[PL] OR Eritrea[PL] OR Ethiopia[PL] OR Kenya[PL] OR Rwanda[PL] OR Somalia[PL] OR Sudan[PL] OR Tanzania[PL] OR Uganda[PL] OR Angola[PL] OR Botswana[PL] OR Lesotho[PL] OR Malawi[PL] OR Mozambique[PL] OR Namibia[PL] OR South Africa[PL] OR Swaziland[PL] OR Zambia[PL] OR Zimbabwe[PL] OR Benin[PL] OR Burkina Faso[PL] OR Cote d'Ivoire[PL] OR Gambia[PL] OR Ghana[PL] OR Guinea[PL] OR Guinea-Bissau[PL] OR Liberia[PL] OR Mali[PL] OR Mauritania[PL] OR Niger[PL] OR Nigeria[PL] OR Senegal[PL] OR Sierra Leone[PL] OR Togo[PL] OR Antigua[PL] OR Bahamas[PL] OR Barbados[PL] OR Cuba[PL] OR Dominica[PL] OR Dominican Republic[PL] OR Grenada[PL] OR Guadeloupe[PL] OR Haiti[PL] OR Jamaica[PL] OR Martinique[PL] OR Netherlands Antilles[PL] OR Puerto Rico[PL] OR Saint Kitts and Nevis[PL] OR Saint Lucia[PL] OR Saint Vincent[PL] OR Grenadines[PL] OR Trinidad and Tobago[PL] OR Virgin Islands[PL] OR Belize[PL] OR Costa Rica[PL] OR El Salvador[PL] OR Guatemala[PL] OR Honduras[PL] OR Nicaragua[PL] OR Panama[PL] OR Mexico[PL] OR Argentina[PL] OR Bolivia[PL] OR Brazil[PL] OR Chile[PL] OR Colombia[PL] OR Ecuador[PL] OR French Guiana[PL] OR French Guiana[PL] OR Paraguay[PL] OR Peru[PL] OR Suriname[PL] OR Uruguay[PL] OR Venezuela[PL] OR Kazakhstan[PL] OR Kyrgyzstan[PL] OR Tajikistan[PL] OR Turkmenistan[PL] OR Uzbekistan[PL] OR Borneo[PL] OR Cambodia[PL] OR East Timor[PL] OR Indonesia[PL] OR Laos[PL] OR Malaysia[PL] OR Mekong Valley[PL] OR Myanmar[PL] OR Philippines[PL] OR Thailand[PL] OR Vietnam[PL] OR Bangladesh[PL] OR Bhutan[PL] OR India[PL] OR Afghanistan[PL] OR Bahrain[PL] OR Iran[PL] OR Iraq[PL] OR Jordan[PL] OR Kuwait[PL] OR Lebanon[PL] OR Oman[PL] OR Qatar[PL] OR Saudi Arabia[PL] OR Syria[PL] OR Turkey[PL] OR United Arab Emirates[PL] OR Yemen[PL] OR Nepal[PL] OR Pakistan[PL] OR Sri Lanka[PL] OR China[PL] OR Macao[PL] OR Mongolia[PL] OR Taiwan[PL] OR Azores[PL] OR Bermuda[PL] OR Falkland Islands[PL] OR Albania[PL] OR Estonia[PL]		

	OR Latvia[PL] OR Lithuania[PL] OR Bosnia-Herzegovina[PL] OR Bulgaria[PL] OR Byelarus[PL] OR Croatia[PL] OR Czech Republic[PL] OR Hungary[PL] OR Macedonia[PL] OR Moldova[PL] OR Montenegro[PL] OR Poland[PL] OR Romania[PL] OR Russia[PL] OR Slovakia[PL] OR Slovenia[PL] OR Ukraine[PL] OR Armenia[PL] OR Azerbaijan[PL] OR Georgia[PL] OR Comoros[PL] OR Madagascar[PL] OR Mauritius[PL] OR Reunion[PL] OR Seychelles[PL] OR Fiji[PL] OR New Caledonia[PL] OR Papua New Guinea[PL] OR Vanuatu[PL] OR Guam[PL] OR Palau[PL] OR Pitcairn Island[PL] OR Samoa[PL] OR Tonga[PL] OR Czechoslovakia[PL] OR Germany, East[PL] OR New Guinea[PL] OR USSR[PL] OR Yugoslavia[PL] OR Ivory Coast[PL] OR Hong Kong[PL] OR republic of china[PL])))		

#1	Search ((Canada[Mesh] OR Greenland[Mesh] OR United States[Mesh] OR Brunei[Mesh] OR Singapore[Mesh] OR Israel[Mesh] OR Japan[Mesh] OR Korea[Mesh] OR Australia[Mesh] OR Andorra[Mesh] OR Austria[Mesh] OR Belgium[Mesh] OR Finland[Mesh] OR France[Mesh] OR Germany[Mesh] OR Gibraltar[Mesh] OR Great Britain[Mesh] OR Greece[Mesh] OR Iceland[Mesh] OR Ireland[Mesh] OR Italy[Mesh] OR Liechtenstein[Mesh] OR Luxembourg[Mesh] OR Mediterranean Region[Mesh] OR Monaco[Mesh] OR Netherlands[Mesh] OR Portugal[Mesh] OR San Marino[Mesh] OR Scandinavia[Mesh] OR Spain[Mesh] OR Switzerland[Mesh] OR Vatican City[Mesh] OR Australia[Mesh] OR New Zealand[Mesh] OR Brunei[TIAB] OR Japan[TIAB] OR South Korea[TIAB] OR Singapore[TIAB] OR Andorra[TIAB] OR Austria[TIAB] OR Belgium[TIAB] OR Cyprus[TIAB] OR Denmark[TIAB] OR Finland[TIAB] OR France[TIAB] OR Germany[TIAB] OR Gibraltar[TIAB] OR Greece[TIAB] OR Greenland[TIAB] OR Vatican[TIAB] OR Iceland[TIAB] OR Ireland[TIAB] OR Israel[TIAB] OR Italy[TIAB] OR Liechtenstein[TIAB] OR Luxembourg[TIAB] OR Malta[TIAB] OR Monaco[TIAB] OR Netherlands[TIAB] OR Norway[TIAB] OR Portugal[TIAB] OR San Marino[TIAB] OR Spain[TIAB] OR Sweden[TIAB] OR Switzerland[TIAB] OR United Kingdom[TIAB] OR England[TIAB] OR Wales[TIAB] OR Scotland[TIAB] OR Canada[TIAB] OR United States[TIAB] OR Australia[TIAB] OR New Zealand[TIAB]))	3182449	12:27:20

Glomerular Filtration Rate Capstone Appendix

Flowchart

Low glomerular filtration rate

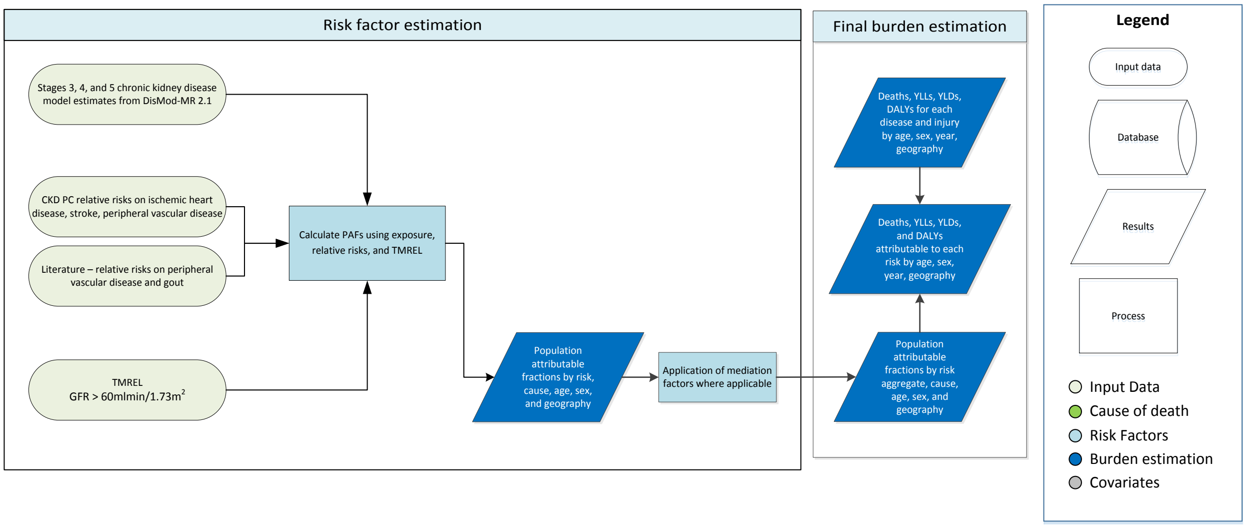

Input Data & Methodological Summary

Exposure

Case Definition

For GBD 2015, the reduced glomerular filtration rate (GFR) risk factor, exposure is defined as the three categories of reduced renal function included in the Global Burden of Disease Study (GBD): chronic kidney disease (CKD) stage 3 (GFR of 30-60ml/min/1.73m^2), stage 4 (GFR of 15-30ml/min/1.73m^2), and stage 5 (GFR <15ml/min/1.73m^2, not yet on renal replacement therapy). These exposure categories were each modeled for the GBD 2015 YLD capstone manuscript, and the modeling approach is described in detail there.

Input data

For GBD 2010, a systematic review of the prevalence of CKD throughout the world was conducted. This search was updated for GBD 2013. For GBD 2015, this literature search was repeated using PubMed search terms: ((chronic kidney disease[Title/Abstract]) AND prevalence[Title/Abstract]) AND ('2012/01/01'[Date - Publication] : '3000'[Date - Publication]) (humans).

Disease	Number of sources	Super-regions with Data
CKD Stage III	64	All Seven super-regions
CKD Stage IV	49	
CKD Stage V	43	

Included surveys were identified by querying the IHME global health data index for any surveys including the term “glomerular filtration rate”. Five total surveys were included of the seventy-six survey results that were identified by the search. Exclusion criteria included surveys that were not population-representative.

Modeling strategy

The reduced GFR modeling strategy involved determining the population-attributable burden of cardiovascular outcomes of ischemic heart disease, stroke, peripheral arterial disease, and musculoskeletal outcome gout, to reduced GFR (Equation 1). This was achieved by determining the relative risk of these outcomes based on CKD stage. The CKD stages exposure was obtained from the GBD 2015 analysis, which includes stage-specific prevalence estimates at the country level across twenty age-groups for both genders. CKD stage models included country-level covariates diabetes mellitus and systolic blood pressure. The data informing the model included a cross-walk adjusting data points estimated using the CKD-Epi equation to the MDRD equation, which is our gold standard CKD estimating equation for CKD stages 3-5 for GBD 2015.

The relative risks were calculated by the Chronic Kidney Disease Prognosis Consortium, a consortium composed of population-level cohorts with prospective data collection from several countries (details below). Cardiovascular and gout population prevalences at the country level were obtained from the GBD 2015 Study for the same geographic, time-period, and age-groups as detailed above.

Theoretical minimum-risk exposure level

The theoretical minimum risk is a diagnosis of CKD stages 3, 4, or 5 as an eGFR<60ml/min/1.73m^2 has been demonstrated in the literature to be the GFR below which increased cardiovascular and gout events occur secondary to reduced GFR. (1-10)

Relative risk

A two-stage pooled meta-analysis was used to calculate relative risks for ischemic heart disease and stroke. The relative risk of ischemic heart disease and stroke was first determined within each cohort, and then a pooled analysis of cohort-level relative risks was performed using a random effects modeling approach. Uncertainty intervals overlapped in a separate analysis of the relative risk of fatal and nonfatal cardiovascular events from GFR exposure. Thus we decided to use the relative risks from the combined analysis for fatal and nonfatal cardiovascular outcomes. The relative risk for peripheral vascular disease by stage of reduced renal function was determined from the Atherosclerotic Risk in the Communities (ARIC) cohort.(11) Gout relative risk was determined by meta-analysis of a literature review performed for GBD 2013. Search terms included “gout” and “chronic kidney disease”. Exclusion criteria for search results included special populations, reversal of exposure and outcome categories, unclear exposure category definition. This search resulted in four articles.

The relative risks have not changed between GBD 2013 and GBD 2015 analyses.

Population Attributable Fraction

We calculated the cardiovascular and gout fatal and nonfatal burden attributable to the categorical exposure of low GFR stages using the following equation:

$$PAF = \frac{\sum_{i=1}^{n} P_i(RR_i - 1)}{\sum_{i=1}^{n} P_i(RR_i - 1) + 1}$$

Equation 1. PAF based on categorical exposure

where RR_i is the relative risk for exposure level *i*, P_i is the proportion of the population in that exposure category, and *n* is the number of exposure categories.(12) *P* is obtained from GBD 2015 CKD stage estimates, and *n* refers to the three CKD stages.

References

1. Go AS, Chertow GM, Fan D, McCulloch CE, Hsu CY. Chronic kidney disease and the risks of death, cardiovascular events, and hospitalization. N Engl J Med. 2004;351(13):1296-305.
2. Ninomiya T, Kiyohara Y, Kubo M, Tanizaki Y, Doi Y, Okubo K, et al. Chronic kidney disease and cardiovascular disease in a general Japanese population: the Hisayama Study. Kidney international. 2005;68(1):228-36.
3. Shara NM, Wang H, Mete M, Al-Balha YR, Azalddin N, Lee ET, et al. Estimated GFR and incident cardiovascular disease events in American Indians: the Strong Heart Study. American journal of kidney diseases : the official journal of the National Kidney Foundation. 2012;60(5):795-803.
4. Mann JF, Gerstein HC, Pogue J, Bosch J, Yusuf S. Renal insufficiency as a predictor of cardiovascular outcomes and the impact of ramipril: the HOPE randomized trial. Annals of internal medicine. 2001;134(8):629-36.
5. Chronic Kidney Disease Prognosis C, Matsushita K, van der Velde M, Astor BC, Woodward M, Levey AS, et al. Association of estimated glomerular filtration rate and albuminuria with all-cause and cardiovascular mortality in general population cohorts: a collaborative meta-analysis. Lancet. 2010;375(9731):2073-81.
6. De Graauw J, Chonchol M, Poppert H, Etgen T, Sander D. Relationship between kidney function and risk of asymptomatic peripheral arterial disease in elderly subjects. Nephrology, dialysis, transplantation : official publication of the European Dialysis and Transplant Association - European Renal Association. 2011;26(3):927-32.
7. Wattanakit K, Folsom AR, Selvin E, Coresh J, Hirsch AT, Weatherley BD. Kidney function and risk of peripheral arterial disease: results from the Atherosclerosis Risk in Communities (ARIC) Study. Journal of the American Society of Nephrology : JASN. 2007;18(2):629-36.
8. O'Hare AM, Vittinghoff E, Hsia J, Shlipak MG. Renal insufficiency and the risk of lower extremity peripheral arterial disease: results from the Heart and Estrogen/Progestin Replacement Study (HERS). Journal of the American Society of Nephrology : JASN. 2004;15(4):1046-51.

9. Manjunath G, Tighiouart H, Coresh J, Macleod B, Salem DN, Griffith JL, et al. Level of kidney function as a risk factor for cardiovascular outcomes in the elderly. Kidney international. 2003;63(3):1121-9.
10. Manjunath G, Tighiouart H, Ibrahim H, MacLeod B, Salem DN, Griffith JL, et al. Level of kidney function as a risk factor for atherosclerotic cardiovascular outcomes in the community. Journal of the American College of Cardiology. 2003;41(1):47-55.
11. The Atherosclerosis Risk in Communities (ARIC) Study: design and objectives. The ARIC investigators. American journal of epidemiology. 1989;129(4):687-702.
12. Miettinen OS. Proportion of disease caused or prevented by a given exposure, trait or intervention. American journal of epidemiology. 1974;99(5):325-32.

Section 4. Methods appendix figures and tables

Appendix Figure 1. A more general causal web of the causes of health outcomes with the categories of causes included in this analysis shown in blue.

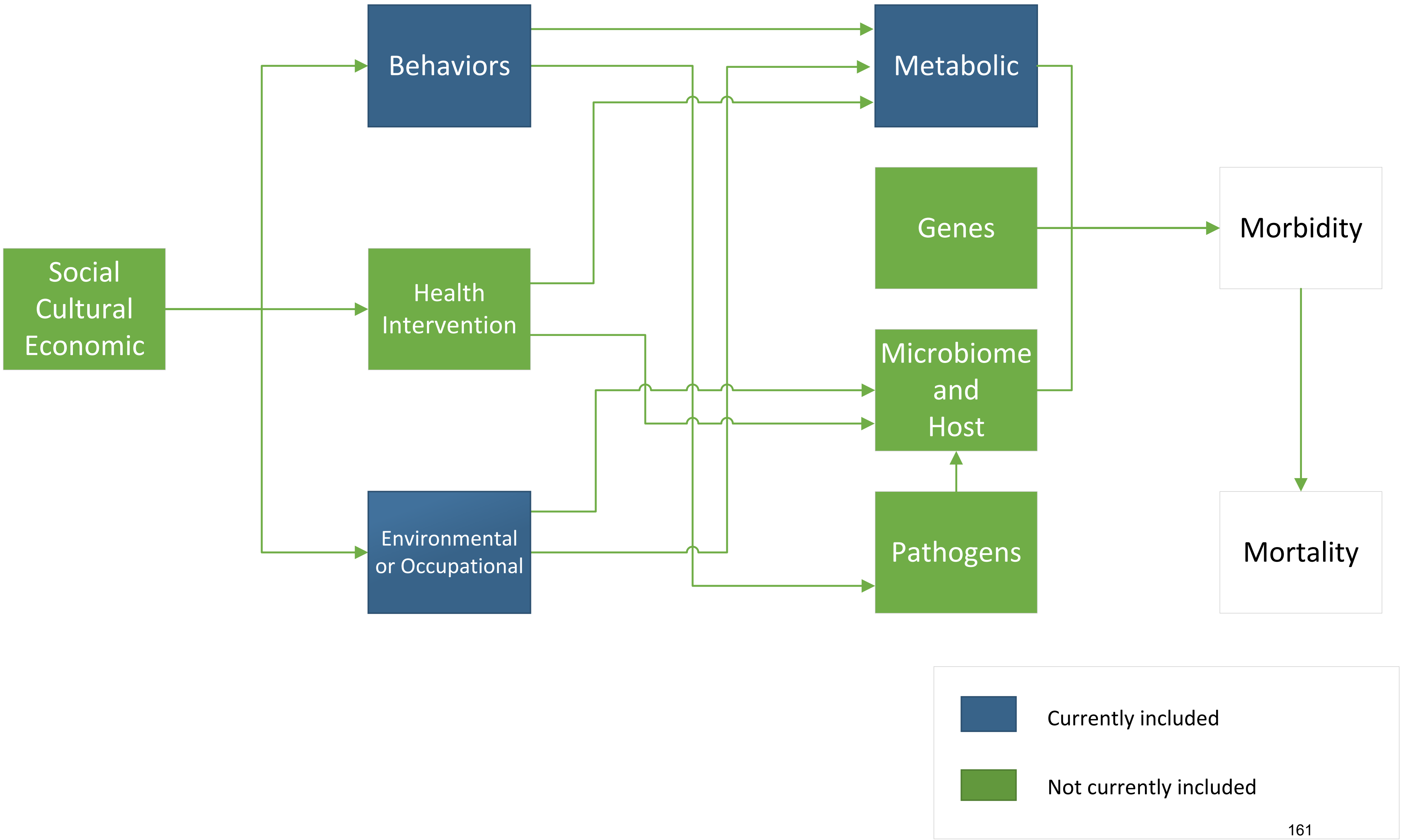

Appendix Figure 2. Analytical flowchart of the comparative risk assessment for the estimation of population attributable fractions by geography, age, sex, and year for GBD 2015. Ovals represent data inputs, rectangular boxes represent analytical steps, cylinders represent databases, and parallelograms represent intermediate and final results. GBD=Global Burden of Disease. SEVs=Summary exposure values. TMREL=Theoretical minimum-risk exposure level. PAFs=Population attributable fractions. YLLs=years of life lost. YLDs=years lived with disability. DALYs=disability-adjusted life-years.

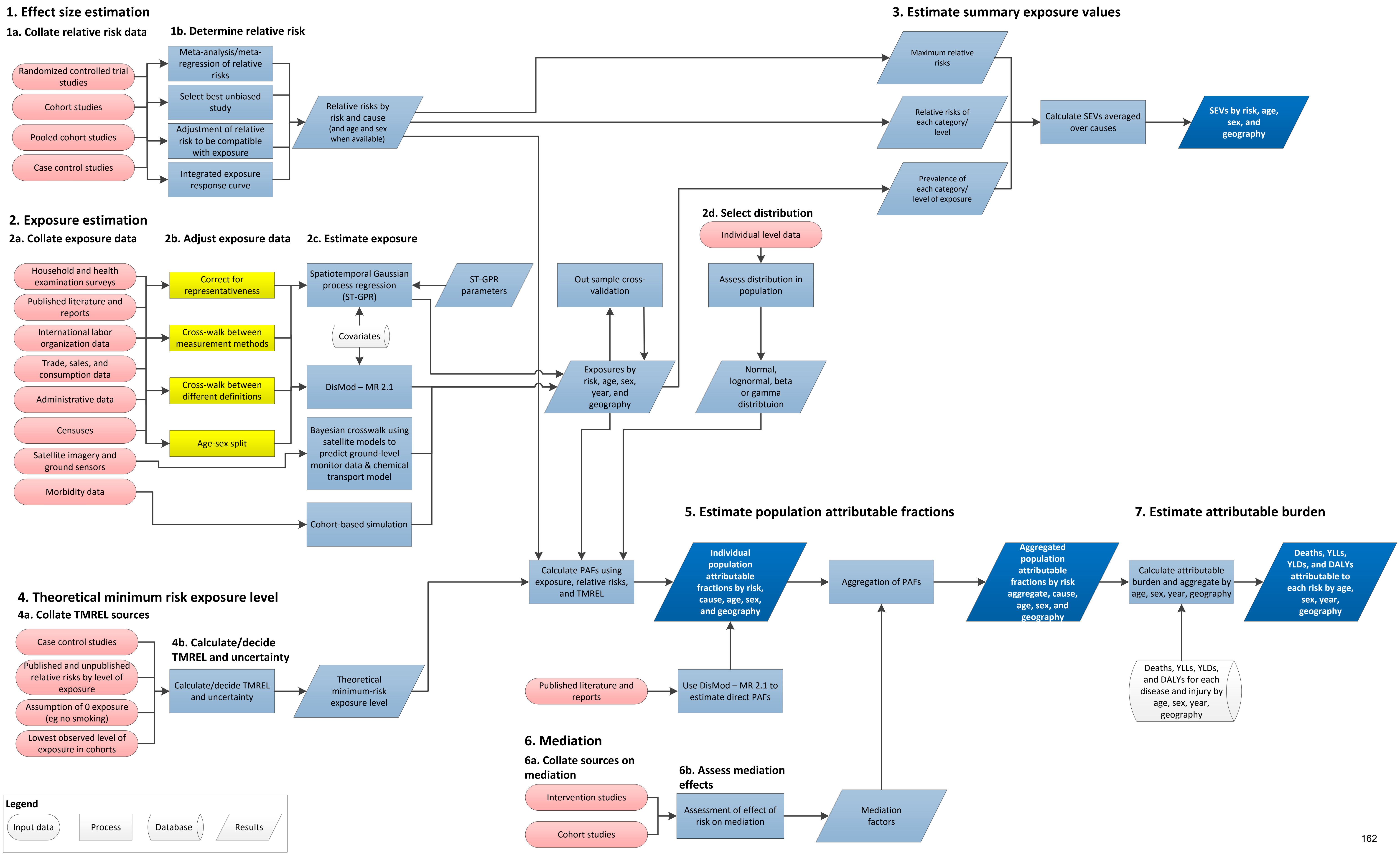

Appendix Figure 3. Types of Comparative Risk Assessments based on the time perspective and the nature of the counterfactual level or distribution of exposure. The shaded box represents the type of CRA currently undertaken in GBD 2015. GBD=Global Burden of Disease.

	**Counterfactual distributions of exposure**			
Construct	Theoretical minimum risk: level of risk with the lowest level of burden	Plausible minimum risk: level of risk with the lowest level of burden that could be imagined with current technology and knowledge	Feasible minimum risk: level of risk with the lowest level of burden that has been achieved in any population	Cost-effective minimum risk: lowest level of risk that can be achieved cost-effectively in a given population
**Attributable burden:** burden of disease today that would be avoided if each individual in the past had been exposed to the counterfactual level of exposure	**Currently in GBD**			
**Avoidable burden:** burden of disease in the future that would be avoided if each individual today was shifted to the counterfactual level of exposure				

Appendix Figure 4. Global decomposition of changes in level 3 cause-specific DALYs for all risk factors combined from 1990 to 2015 due to population growth, population ageing, risk exposure and the risk-deleted DALY rate. Causes are reported in order of percent change in the number of DALYs from 1990 to 2015. This figure excludes cervical cancer, HIV/AIDS, and sexually transmitted diseases DALYs becasue the associated risks are not estimated based on exposure and relative risk. DALYs=disability-adjusted life-years.

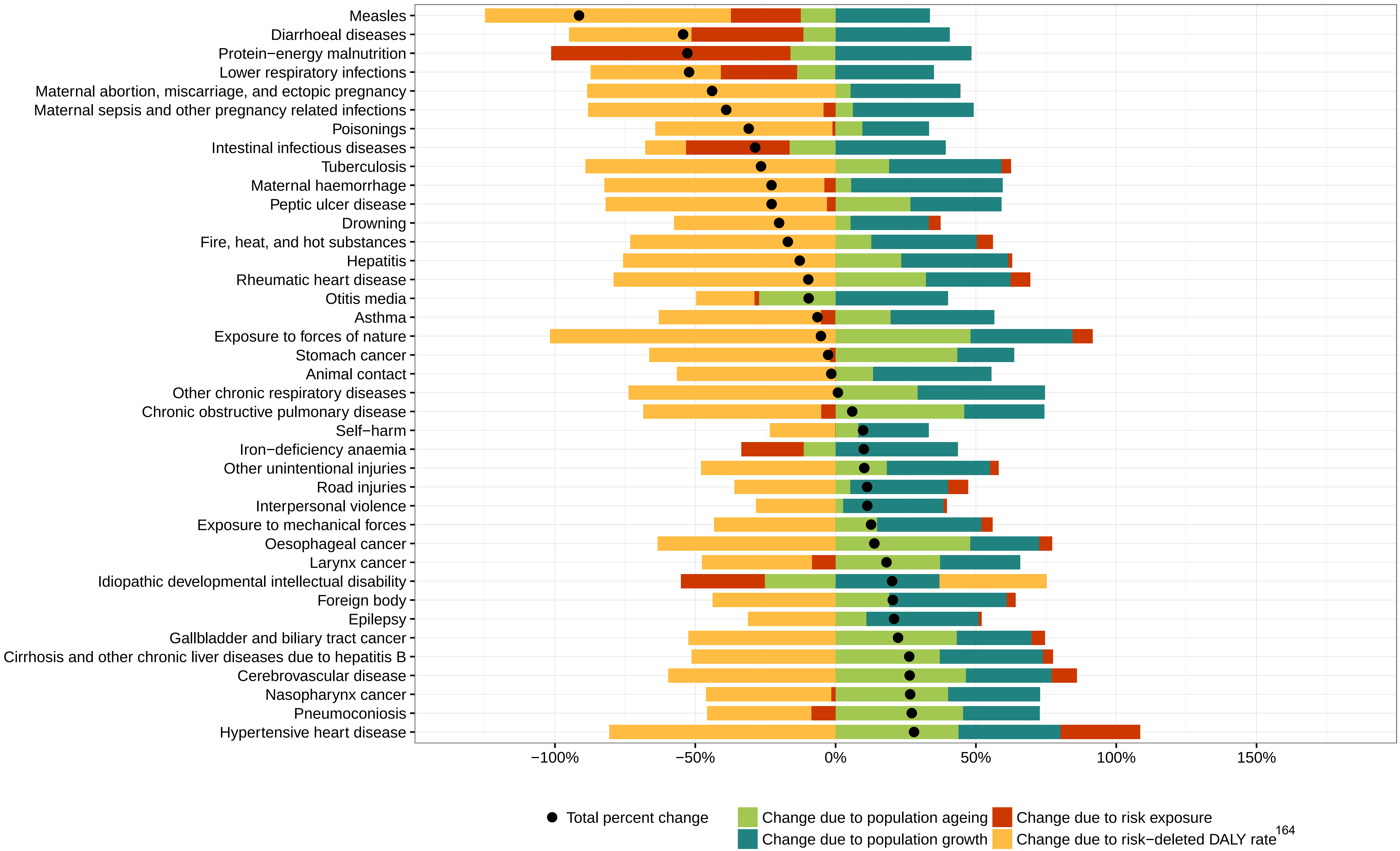

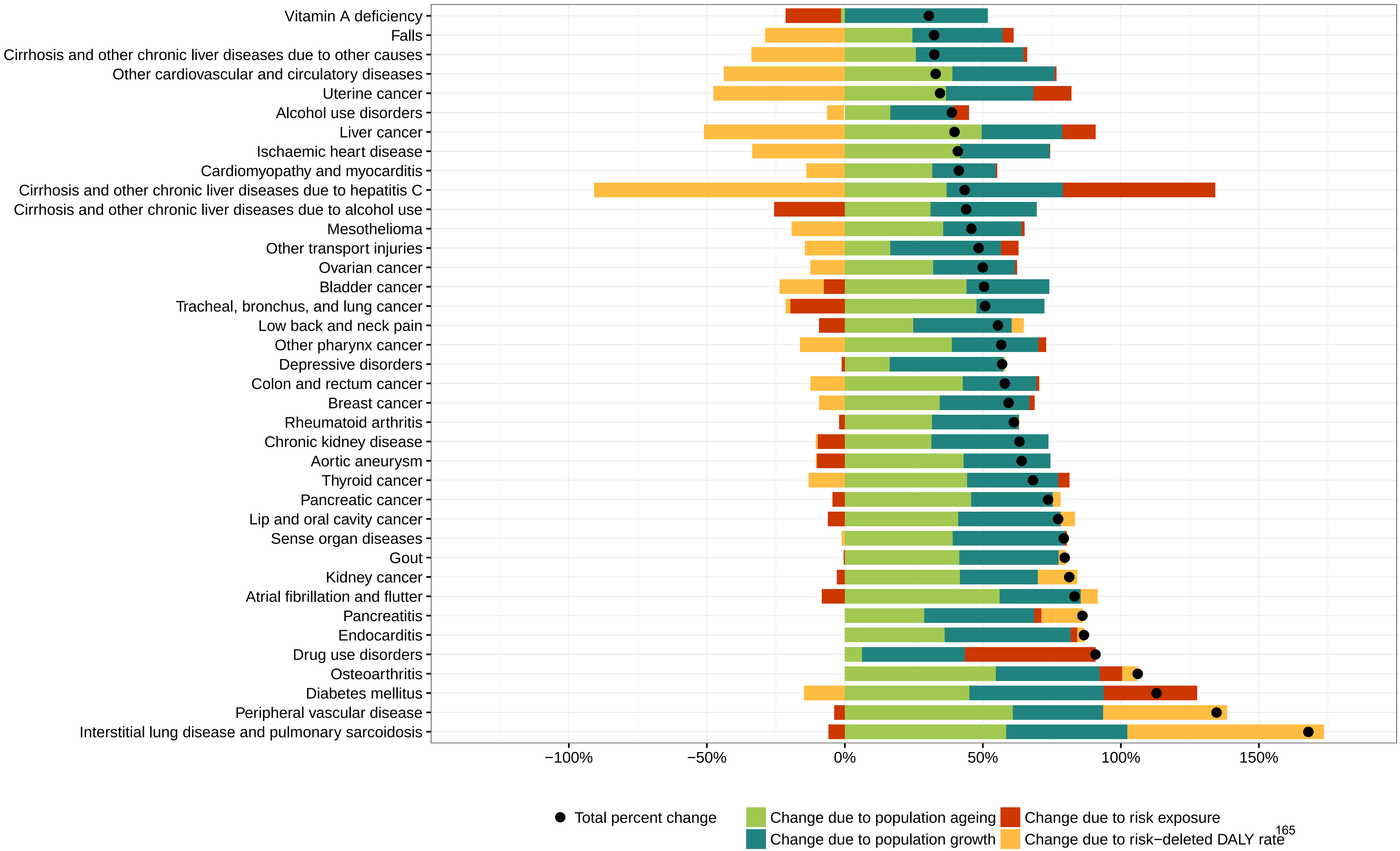
Vitamin A deficiency
Falls
Cirrhosis and other chronic liver diseases due to other causes
Other cardiovascular and circulatory diseases
Uterine cancer
Alcohol use disorders
Liver cancer
Ischaemic heart disease
Cardiomyopathy and myocarditis
Cirrhosis and other chronic liver diseases due to hepatitis C
Cirrhosis and other chronic liver diseases due to alcohol use
Mesothelioma
Other transport injuries
Ovarian cancer
Bladder cancer
Tracheal, bronchus, and lung cancer
Low back and neck pain
Other pharynx cancer
Depressive disorders
Colon and rectum cancer
Breast cancer
Rheumatoid arthritis
Chronic kidney disease
Aortic aneurysm
Thyroid cancer
Pancreatic cancer
Lip and oral cavity cancer
Sense organ diseases
Gout
Kidney cancer
Atrial fibrillation and flutter
Pancreatitis
Endocarditis
Drug use disorders
Osteoarthritis
Diabetes mellitus
Peripheral vascular disease
Interstitial lung disease and pulmonary sarcoidosis
−100%
−50%
0%
50%
100%
150%
Total percent change
Change due to population ageing
Change due to population growth
Change due to risk exposure
Change due to risk−deleted DALY rate

Appendix Figure 5. Global age-standardised percent change in SEVs for high and high-middle Socio-demographic Index (SDI) geographies versus middle, low-middle, and low SDI geographies, for (A) males and (B) females, 1990 to 2015. Socio-demographic Index (SDI) is calculated for each geography as a function of lag dependent income per capita, average educational attainment in the population over age 15, and the total fertility rate (TFR). SDI units are interpretable; a zero represents the lowest level of income per capita, educational attainment, and highest TFR observed 1980-2015 and a one represents the highest income per capita, educational attainment and lowest TFR observed in the same period. Cut-offs on the SDI scale for the quintiles have been selected based on examining the entire distribution of geographies 1980-2015. Annualized rate of change in the age-standardized SEV 1990-2015 in high SDI geographies compared to all other geographies. SEV=summary exposure value. Water=Unsafe water. Sanitation =Unsafe sanitation. Handwashing=No handwashing with soap. Ambient PM=Ambient particulate matter pollution. Household air=Household air pollution. Ozone=Ambient ozone pollution. Radon=Residential radon. Lead=Lead exposure. Asbestos=Occupational exposure to asbestos. Arsenic=Occupational exposure to arsenic. Beryllium=Occupational exposure to beryllium. Cadmium=Occupational exposure to cadmium. Chromium=Occupational exposure to chromium. Occ SHS=Occupational exposure to second-hand smoke. Formaldehyde=Occupational exposure to formaldehyde. Nickel=Occupational exposure to nickel. PAH=Occupational exposure to polycyclic aromatic hydrocarbons. Sulfphuric acid=Occupational exposure to sulfphuric acid. Trichloroethylene=Occupational exposure to trichloroethylene. Asthmagens=Occupational asthmagens. PM, gases, and fumes=Occupational particulate matter, gases, and fumes. Noise=Occupational noise. Ergonomic=Occupational ergonomic factors. Non-excl breast=Non-exclusive breastfeeding. Disc breast=Discontinued breastfeeding. Underweight=Childhood underweight. Wasting=Childhood wasting. Stunting=Childhood stunting. Iron def=Iron deficiency. Vitamin A=Vitamin A deficiency. Zinc=Zinc deficiency. Smoking=Smoking. SHS=Second-hand smoke. Alcohol=Alcohol use. Drug use=Drug use. Fruits=Diet low in fruits. Vegetables=Diet low in vegetables. Whole grains=Diet low in whole grains. Nuts and seeds=Diet low in nuts and seeds. Milk=Diet low in milk. Red meat=Diet high in red meat. Processed meat=Diet high in processed meat. Sugar-sweet bvgs=Diet high in sugar-sweetened beverages. Fibre=Diet low in fibre. Calcium=Diet low in calcium. Omega 3=Diet low in seafood omega-3 fatty acids. PUFA=Diet low in polyunsaturated fatty acids. Trans fatty acids=Diet high in trans fatty acids. Sodium=Diet high in sodium. Child sexual abuse=Childhood sexual abuse. IPV=Intimate partner violence. Physical activity=Low physical activity. FPG=High fasting plasma glucose. Cholesterol=High total cholesterol. SBP=High systolic blood pressure. BMI=High body-mass index. BMD=Low bone mineral density. GFR=Low glomerular filtration rate.

(A) Males

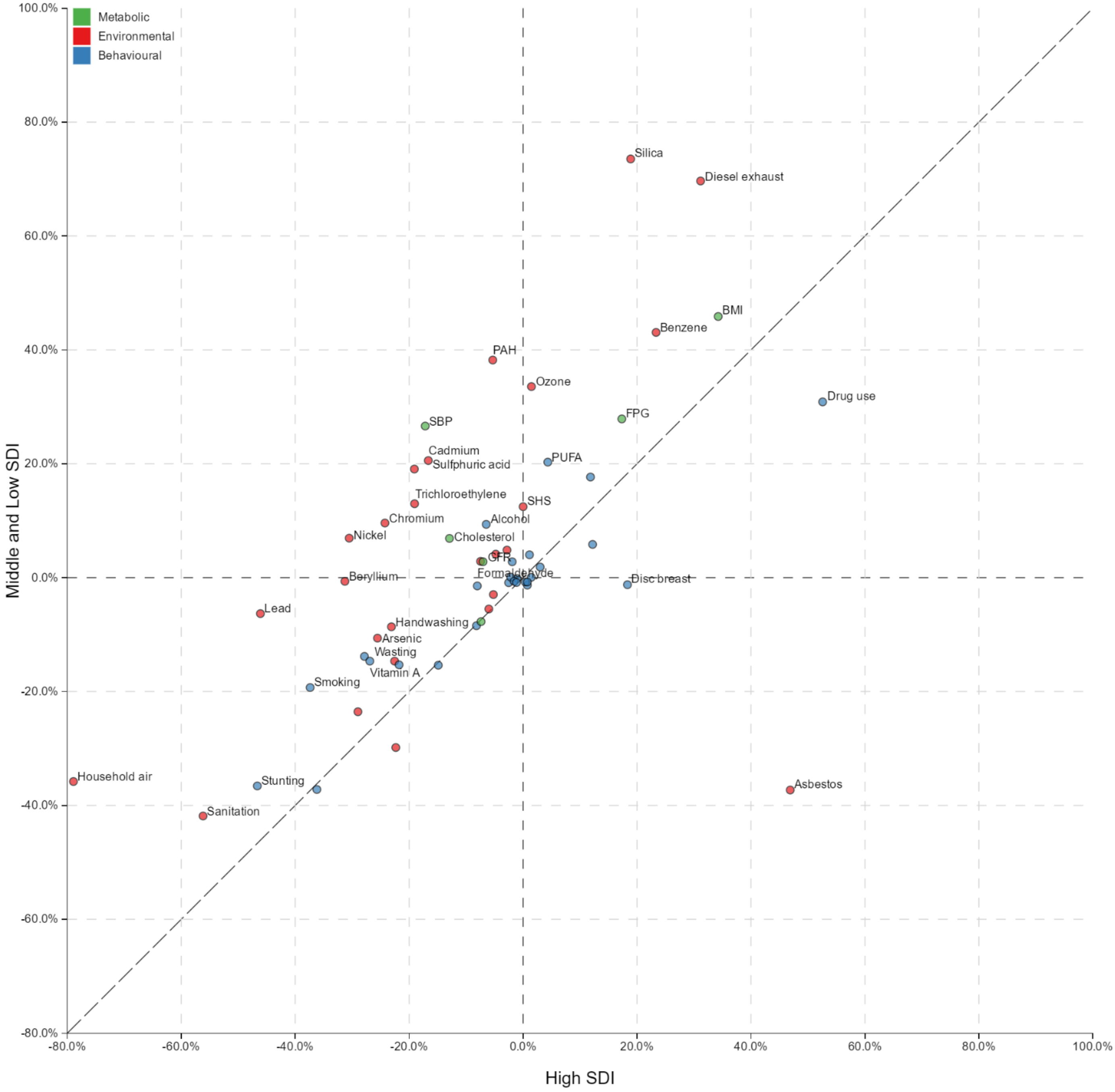

Metabolic
Environmental
Behavioural
100.0%
80.0%
60.0%
40.0%
20.0%
0.0%
-20.0%
-40.0%
-60.0%
-80.0%
Middle and Low SDI
-80.0%
-60.0%
-40.0%
-20.0%
0.0%
20.0%
40.0%
60.0%
80.0%
100.0%
High SDI
Silica
Diesel exhaust
BMI
Benzene
PAH
Ozone
Drug use
FPG
SBP
Cadmium
Sulfphuric acid
PUFA
Trichloroethylene
SHS
Chromium
Alcohol
Nickel
Cholesterol
GFR
Formaldehyde
Beryllium
Disc breast
Lead
Handwashing
Arsenic
Wasting
Vitamin A
Smoking
Household air
Stunting
Asbestos
Sanitation

(B) Females

This figure excludes occupational exposure to occupational exposure to diesel engine exhaust which had SEV increases greater than 100%.

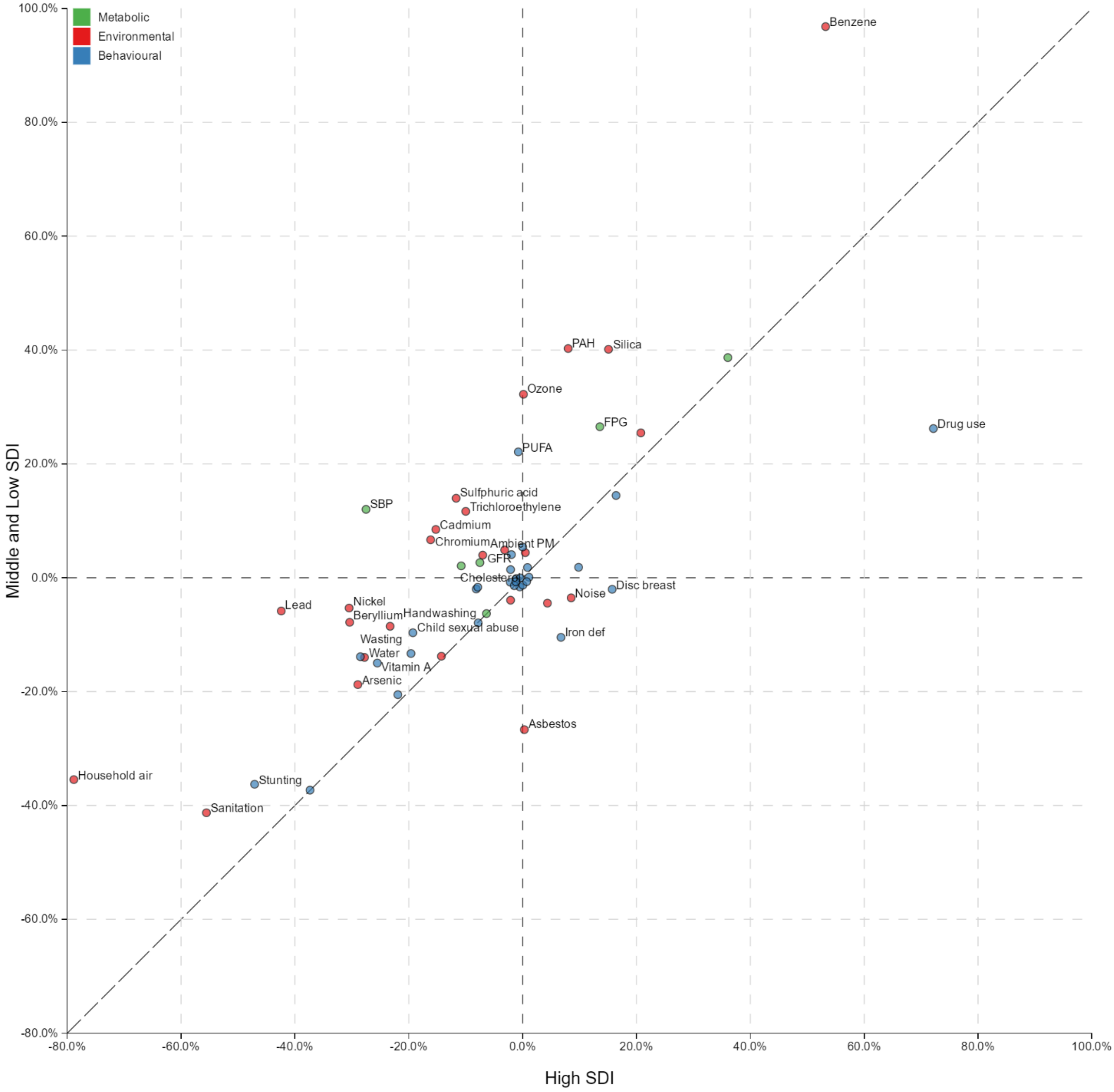

Appendix Figure 6. Diagram showing the proportion of all-cause DALYs to behavioural, environmental and occupational, and metabolic risk factors and their overlaps for all ages in 2015. DALYs=disability-adjusted life-years.

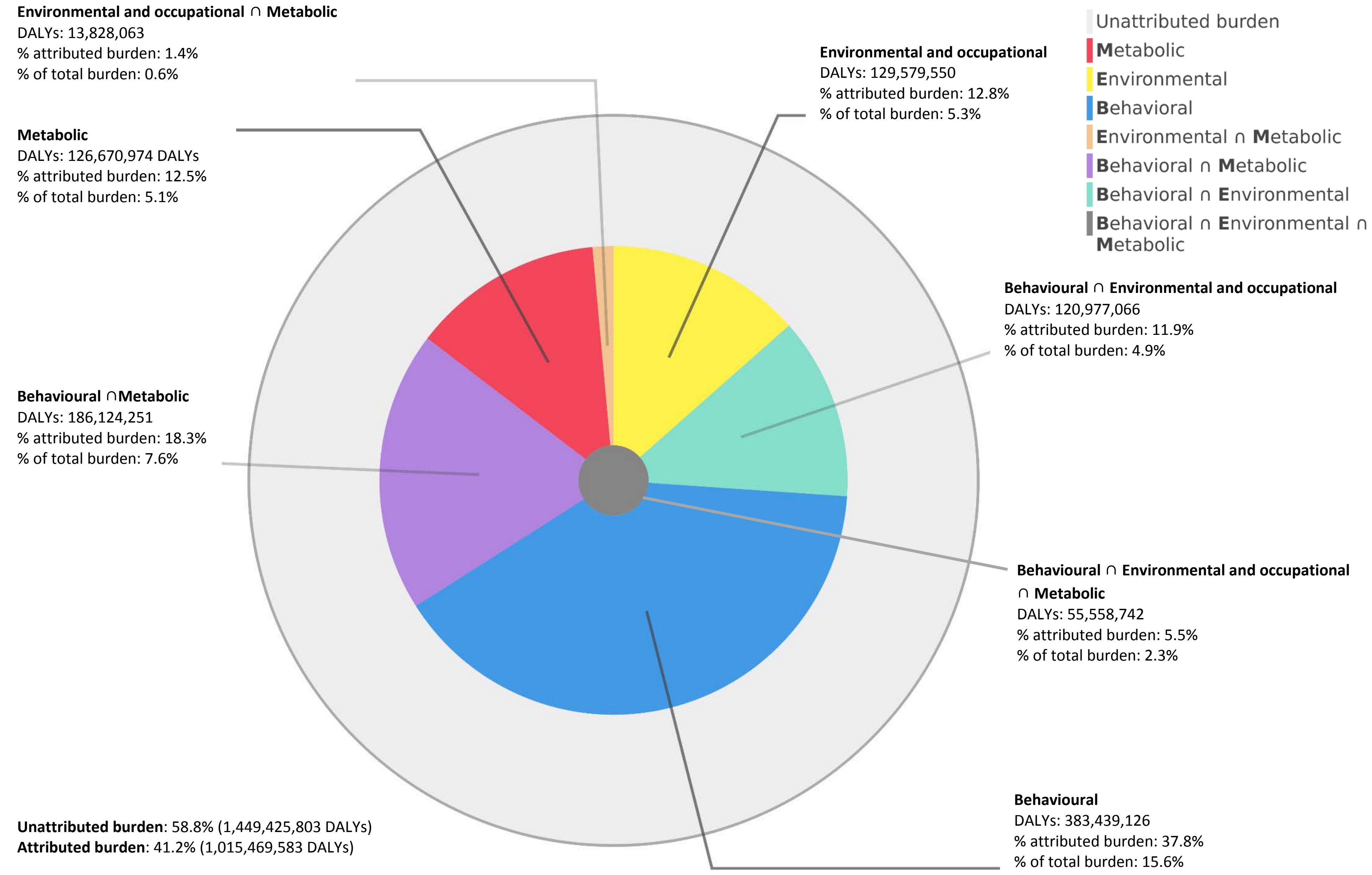

Appendix Figure 7. DALYs attributable to level 2 risk factors for the low Socio-demographic Index (SDI) quintile (A) and for the high SDI quintile (B), for both sexes combined, 2015.
(A) Low SDI. DALYs from different causes attributable to each risk factor are shown in different colours. Socio-demographic Index (SDI) is calculated for each geography as a function of lag dependent income per capita, average educational attainment in the population over age 15, and the total fertility rate (TFR). SDI units are interpretable; a zero represents the lowest level of income per capita, educational attainment, and highest TFR observed 1980-2015 and a one represents the highest income per capita, educational attainment and lowest TFR observed in the same period. Cut-offs on the SDI scale for the quintiles have been selected based on examining the entire distribution of geographies 1980-2015. DALYs=disability-adjusted life-years.

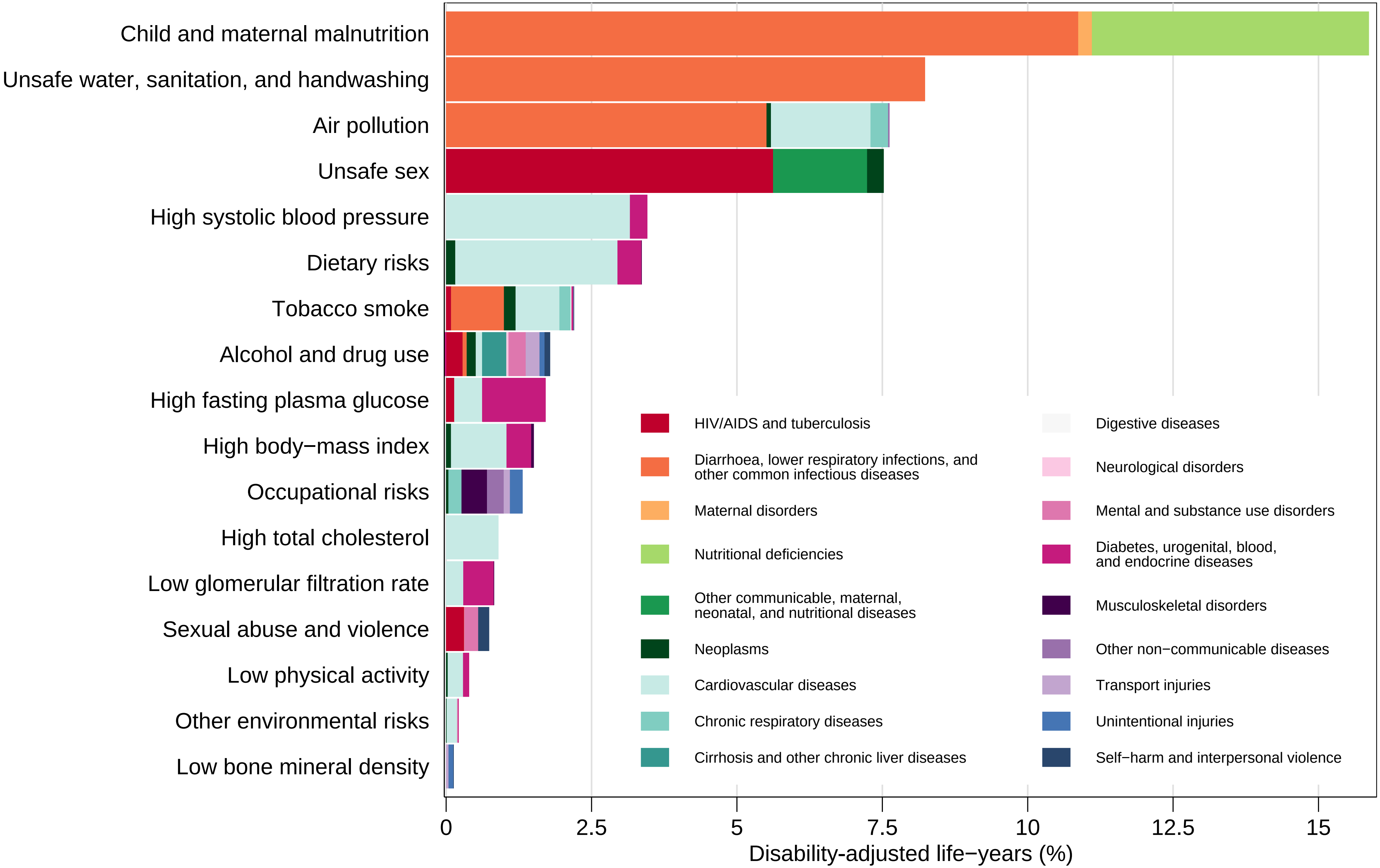

(B) High SDI. DALYs from different causes attributable to each risk factor are shown in different colours. Socio-demographic Index (SDI) is calculated for each geography as a function of lag dependent income per capita, average educational attainment in the population over age 15, and the total fertility rate (TFR). SDI units are interpretable; a zero represents the lowest level of income per capita, educational attainment, and highest TFR observed 1980-2015 and a one represents the highest income per capita, educational attainment and lowest TFR observed in the same period. Cut-offs on the SDI scale for the quintiles have been selected based on examining the entire distribution of geographies 1980-2015. DALYs=disability-adjusted life-years.

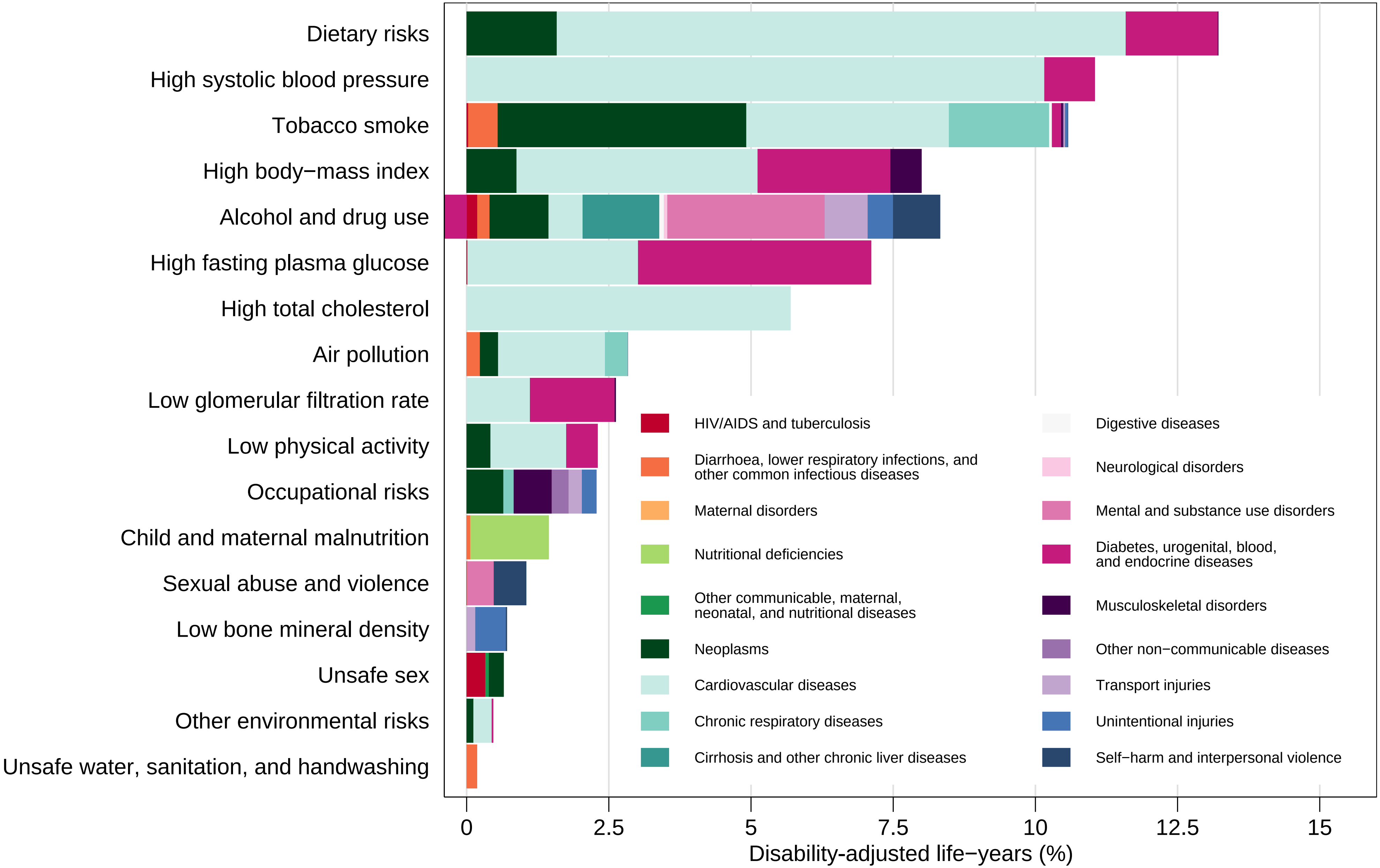

Appendix Figure 8. Leading 10 level 3 global risk factors for DALYs in 2015 by age group. Each cause is coloured by the percent change in age-specific DALYs from 2005 to 2015. DALYs=disability-adjusted life-years.

	1	2	3	4	5	6	7	8	9	10
**Early Neonatal**	Childhood undernutrition	Household air pollution	Ambient particulate matter	Unsafe sex	Handwashing	Unsafe water	Second-hand smoke	Unsafe sanitation	Iron deficiency	Alcohol use
**Late Neonatal**	Childhood undernutrition	Suboptimal breastfeeding	Household air pollution	Unsafe water	Handwashing	Ambient particulate matter	Unsafe sanitation	Unsafe sex	Second-hand smoke	Iron deficiency
**Post Neonatal**	Childhood undernutrition	Suboptimal breastfeeding	Unsafe water	Handwashing	Unsafe sanitation	Household air pollution	Ambient particulate matter	Unsafe sex	Second-hand smoke	Iron deficiency
**1 to 4**	Childhood undernutrition	Unsafe water	Unsafe sanitation	Handwashing	Household air pollution	Iron deficiency	Zinc deficiency	Ambient particulate matter	Unsafe sex	Vitamin A deficiency
**5 to 9**	Iron deficiency	Unsafe water	Unsafe sex	Unsafe sanitation	Handwashing	Household air pollution	Ambient particulate matter	Second-hand smoke	Low glomerular filtration	Alcohol use
**10 to 14**	Iron deficiency	Unsafe sex	Unsafe water	Unsafe sanitation	Handwashing	Household air pollution	Ambient particulate matter	Low glomerular filtration	Alcohol use	High fasting plasma glucose
**15 to 19**	Iron deficiency	Alcohol use	Unsafe water	Unsafe sex	Unsafe sanitation	Handwashing	Occupational injury	Drug use	Low glomerular filtration	Childhood sexual abuse
**20 to 24**	Alcohol use	Unsafe sex	Iron deficiency	Drug use	Unsafe water	Occupational injury	Unsafe sanitation	High fasting plasma glucose	Handwashing	Low glomerular filtration
**25 to 29**	Unsafe sex	Alcohol use	Drug use	High fasting plasma glucose	High blood pressure	Iron deficiency	High body-mass index	Low whole grains	Low fruit	Ambient particulate matter
**30 to 34**	Unsafe sex	Alcohol use	High blood pressure	High fasting plasma glucose	High body-mass index	Smoking	Low whole grains	Drug use	Iron deficiency	Low fruit
**35 to 39**	Unsafe sex	Alcohol use	High blood pressure	High body-mass index	High fasting plasma glucose	Smoking	Low whole grains	High total cholesterol	Low fruit	Ambient particulate matter
**40 to 44**	High blood pressure	Smoking	High body-mass index	Alcohol use	High fasting plasma glucose	Unsafe sex	High total cholesterol	Low whole grains	Low fruit	Ambient particulate matter
**45 to 49**	High blood pressure	Smoking	High body-mass index	High fasting plasma glucose	Alcohol use	High total cholesterol	Low whole grains	Low fruit	Ambient particulate matter	Unsafe sex
**50 to 54**	High blood pressure	Smoking	High body-mass index	High fasting plasma glucose	High total cholesterol	Low whole grains	Alcohol use	Low fruit	Ambient particulate matter	High sodium
**55 to 59**	High blood pressure	Smoking	High fasting plasma glucose	High body-mass index	High total cholesterol	Low whole grains	High sodium	Ambient particulate matter	Low fruit	Alcohol use
**60 to 64**	High blood pressure	Smoking	High fasting plasma glucose	High body-mass index	High sodium	High total cholesterol	Ambient particulate matter	Low whole grains	Low fruit	Household air pollution
**65 to 69**	High blood pressure	Smoking	High fasting plasma glucose	High body-mass index	High sodium	Ambient particulate matter	Low whole grains	Low fruit	High total cholesterol	Household air pollution
**70 to 74**	High blood pressure	Smoking	High fasting plasma glucose	High body-mass index	High sodium	Ambient particulate matter	Low whole grains	Low fruit	High total cholesterol	Household air pollution
**75 to 79**	High blood pressure	High fasting plasma glucose	Smoking	High sodium	Ambient particulate matter	High body-mass index	High total cholesterol	Low whole grains	Low glomerular filtration	Low fruit
**80 plus**	High blood pressure	High fasting plasma glucose	Smoking	High total cholesterol	High sodium	Ambient particulate matter	Low glomerular filtration	High body-mass index	Low whole grains	Low nuts and seeds

Percent change in age-specific DALYs from 2005 to 2015

-59·40 to -37·90	-37·90 to -28·55	-28·55 to -16·79	-16·79 to -7·78	-7·78 to -3·27	-3·27 to 1·05	1·05 to 6·64	6·64 to 12·12	12·12 to 29·56	29·56 to 50·61

Appendix Figure 9. Observed SEVs compared to the value expected on the basis of SDI alone, across SDI quintiles for 60 risk factors included in the GBD 2015.

Each SDI quintile is coloured-coded, and coloured lines represent expected levels, on the basis of SDI, for risk-specific SEVs. To enhance readability, SEVs in this figure have been scaled such that the lowest observed SEV for a given risk equals 0 and the highest observed SEV equals 1. Each circular symbol represents observed SEVs at the country level in 2015, with colours aligning with SDI quintile. Each risk factor corresponds with a vertical line. The ordering of risk factors was determined by the difference in expected SEVs for low SDI (the red line) and high SDI (the purple line). Risks proceed clockwise from those with the largest decline in SEV to those with the largest increase in SEV as SDI increases. SDI = Socio-demographic index. SEV=summary exposure value.

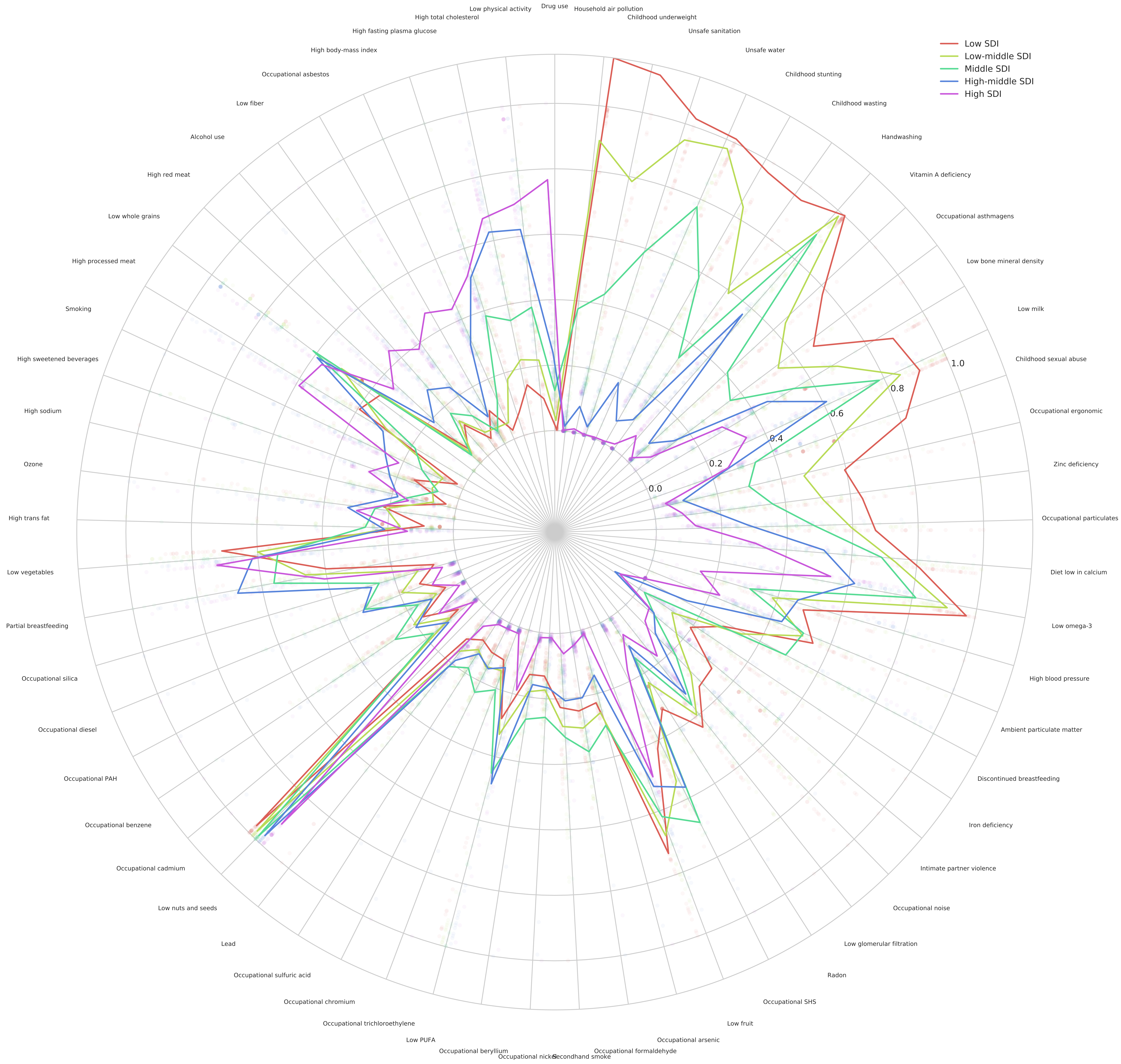

Appendix Figure 10. Global map for leading level 3 risk factors in terms of attributable DALYs for males (A) and females (B), 2015.
(A) Males. DALYs=disability−adjusted life−years. ATG = Antigua and Barbuda. VCT = Saint Vincent and the Grenadines. BRB = Barbados. COM = Comoros. DMA = Dominica. GRD = Grenada. MDV = Maldives. MUS = Mauritius. LCA = Saint Lucia. TTO = Trinidad and Tobago. SYC = Seychelles. MLT = Malta. SGP = Singapore. MHL = Marshall Islands. KIR = Kiribati. SLB = Solomon Islands. FSM = Federated States of Micronesia. VUT = Vanuatu. WSM = Samoa. FJI = Fiji. TON = Tonga.

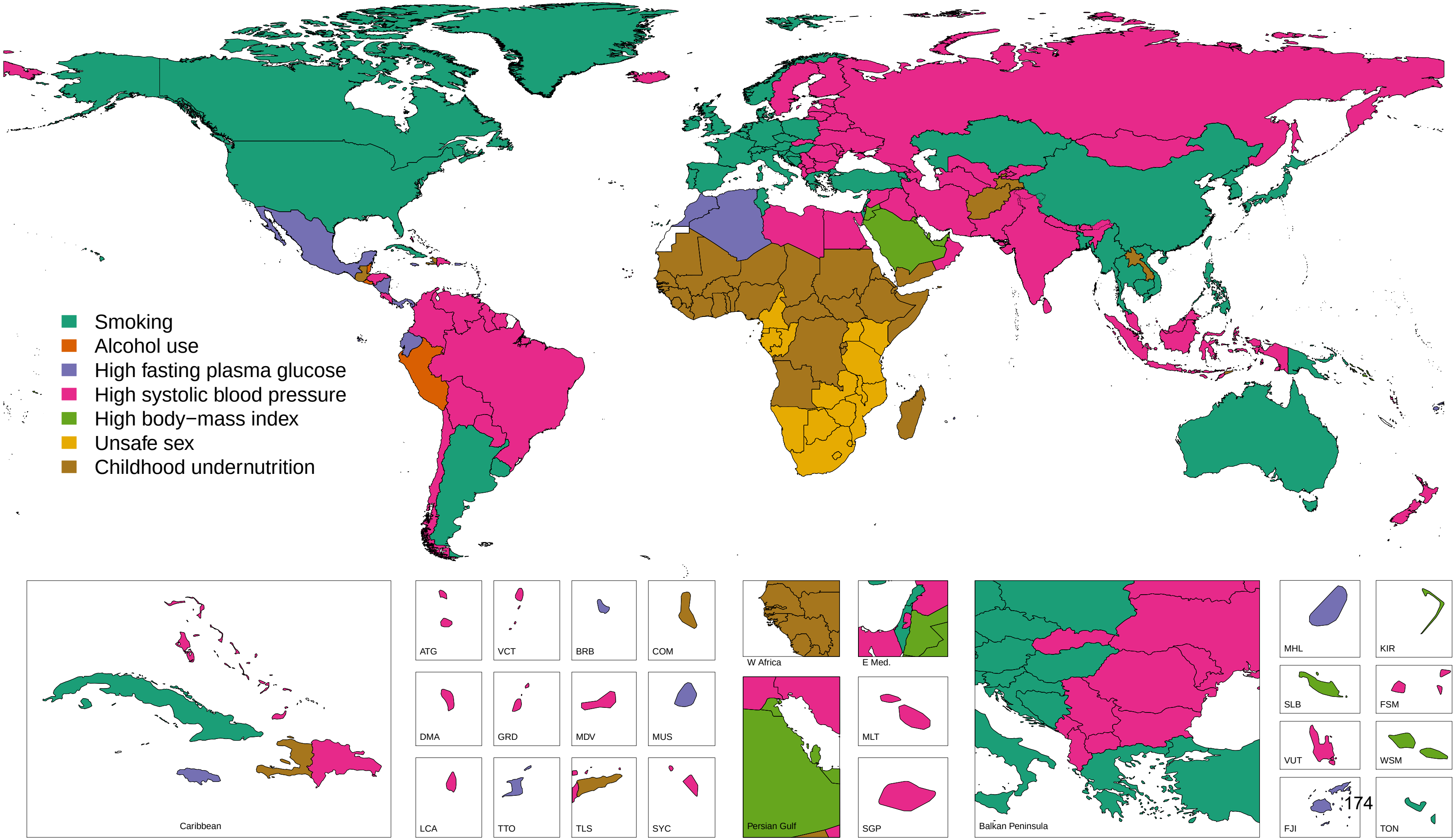

(B) Females. DALYs=disability−adjusted life−years. ATG = Antigua and Barbuda. VCT = Saint Vincent and the Grenadines. BRB = Barbados. COM = Comoros. DMA = Dominica. GRD = Grenada. MDV = Maldives. MUS = Mauritius. LCA = Saint Lucia. TTO = Trinidad and Tobago. SYC = Seychelles. MLT = Malta. SGP = Singapore. MHL = Marshall Islands. KIR = Kiribati. SLB = Solomon Islands. FSM = Federated States of Micronesia. VUT = Vanuatu. WSM = Samoa. FJI = Fiji. TON = Tonga.

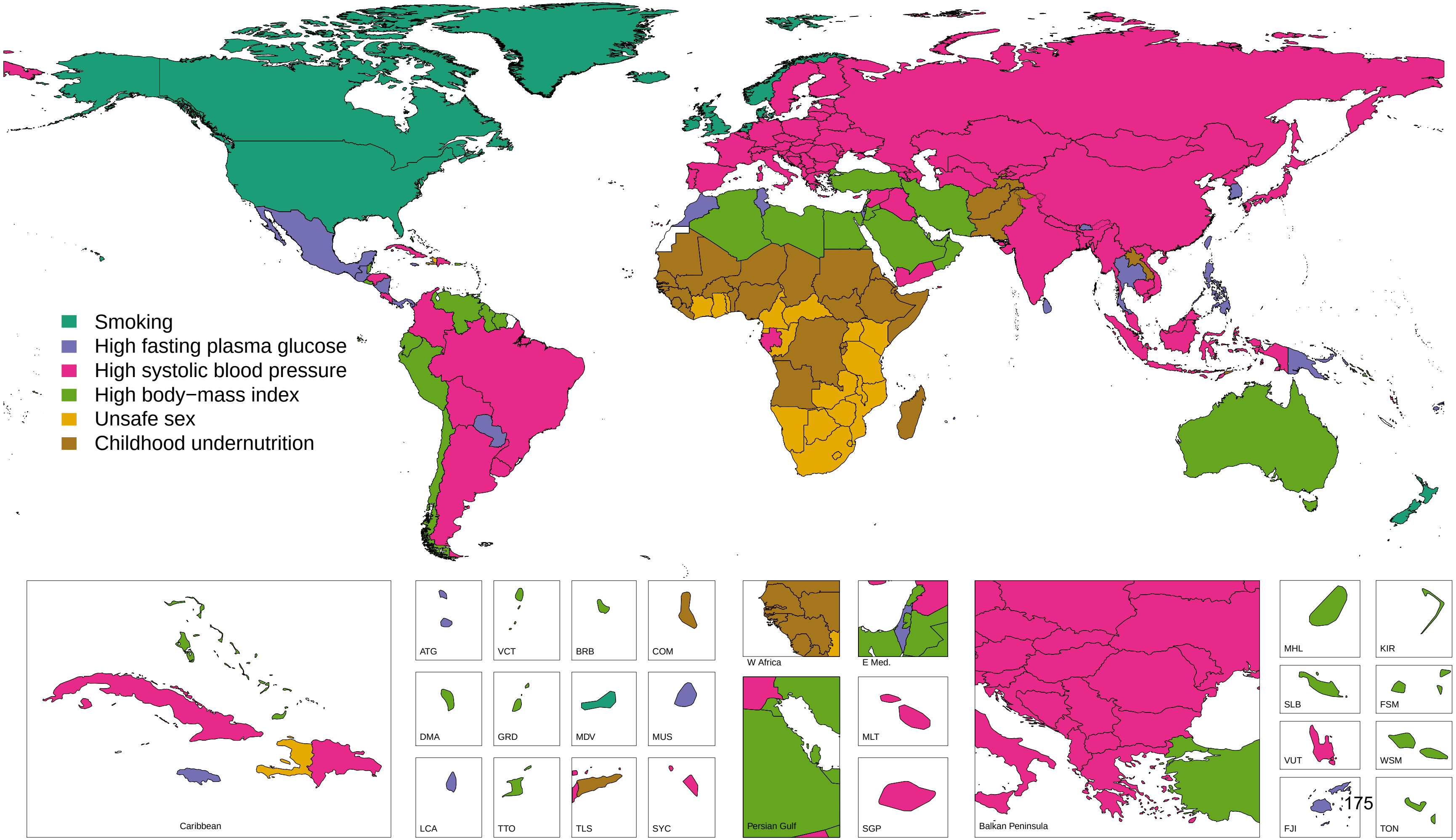

Appendix Figure 11. Socio−demographic Index (SDI) quintiles by GBD subnational level 1 geography, 2015. ATG = Antigua and Barbuda. VCT = Saint Vincent and the Grenadines. BRB = Barbados. COM = Comoros. DMA = Dominica. GRD = Grenada. MDV = Maldives. MUS = Mauritius. LCA = Saint Lucia. TTO = Trinidad and Tobago. SYC = Seychelles. MLT = Malta. SGP = Singapore. MHL = Marshall Islands. KIR = Kiribati. SLB = Solomon Islands. FSM = Federated States of Micronesia. VUT = Vanuatu. WSM = Samoa. FJI = Fiji. TON = Tonga.

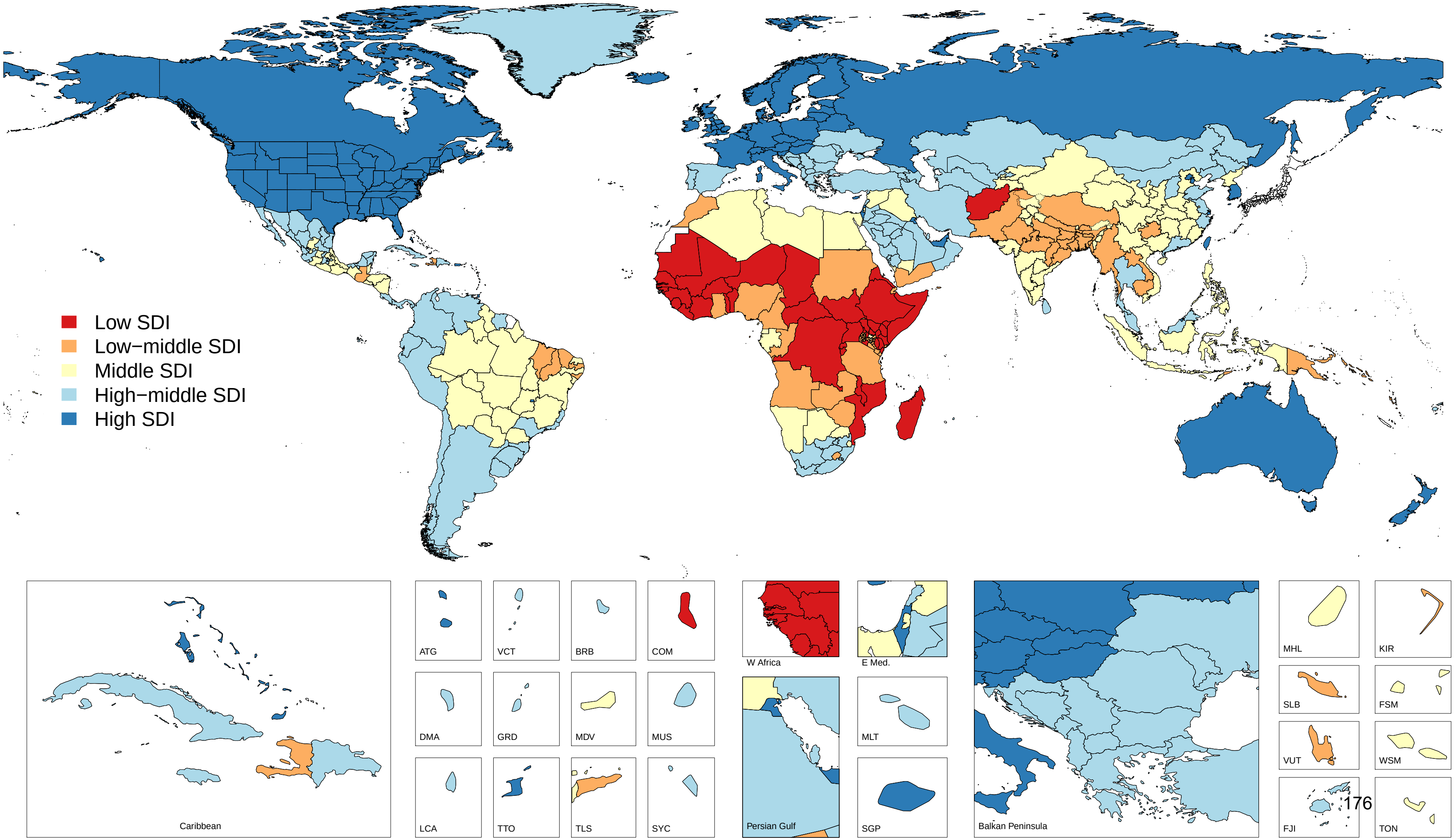

Appendix Table 1. Guidelines for Accurate and Transparent Health Estimates Reporting (GATHER) 18-items checklist with description of compliance and location of information for GBD 2015 risk factors capstone

#	GATHER checklist item	Description of compliance	Reference
**Objectives and funding**			
1	Define the indicators, populations, and time periods for which estimates were made.	Narrative provided in paper and appendix describing indicators, definitions, and populations.	Main text; Tables & Figures; and Appendix, Section 1. GBD Overview
2	List the funding sources for the work.	Funding sources listed in paper.	Main text, Summary, Funding.
**Data Inputs**			
For all data inputs from multiple sources that are synthesized as part of the study:			
3	Describe how the data were identified and how the data were accessed.	Narrative description of data seeking methodology provided.	Main text, Methods, Estimation process, Effect size estimation and Exposure estimation; and Appendix, Section 2. Risk factor estimation, Step 1. Effect size estimation, 1a. Collate relative risk data and Step 2. Exposure estimation, 2a collate exposure data; and Section 3. Risk-specific estimation
4	Specify the inclusion and exclusion criteria. Identify all ad-hoc exclusions.	Narrative about inclusion and exclusion criteria by data type provided.	Main text, Methods, Estimation process, Effect size estimation and Exposure estimation; and Appendix, Section 2. Risk factor estimation, Step 1. Effect size estimation, 1a. Collate relative risk data and Step 2. Exposure estimation, 2a collate exposure data; and Section 3. Risk-specific estimation
5	Provide information on all included data sources and their main characteristics. For each data source used, report reference information or contact name/institution, population represented, data collection method, year(s) of data collection, sex and age range, diagnostic criteria or measurement method, and sample size, as relevant.	Interactive, online data source tool that provides metadata for data sources by component, geography, cause, risk, or impairment has been developed.	Online data tool: http://ghdx.healthdata.org/global-burden-disease-study-2015
6	Identify and describe any categories of input data that have potentially important biases (e.g., based on characteristics listed in item 5).	Summary of known biases by risk included in methodological appendix.	Appendix, Section 3. Risk-specific estimation

For data inputs that contribute to the analysis but were not synthesized as part of the study:			
7	Describe and give sources for any other data inputs.	Interactive, online data source tool that provides metadata for data sources by component, geography, cause, risk, or impairment has been developed.	Online data tools: http://ghdx.healthdata.org/global-burden-disease-study-2015
For all data inputs:			
8	Provide all data inputs in a file format from which data can be efficiently extracted (e.g., a spreadsheet as opposed to a PDF), including all relevant meta-data listed in item 5. For any data inputs that cannot be shared due to ethical or legal reasons, such as third-party ownership, provide a contact name or the name of the institution that retains the right to the data.	Downloads of input data will be available through online tools, including data visualization tools and data query tools. Input data not available in tools will be made available upon request.	Online data tools: http://ghdx.healthdata.org/global-burden-disease-study-2015
**Data analysis**			
9	Provide a conceptual overview of the data analysis method. A diagram may be helpful.	Flow diagrams of the overall methodological processes, as well as risk-specific modelling processes have been provided.	Main text, Methods; Appendix, Section 2, DisMod-MR 2.1 Estimation and spatiotemporal Gaussian process regression estimation; Appendix, Section 3. Risk-specific estimation; and Appendix Figure 2. Analytical flowchart of the comparative risk assessment for the estimation of population attributable fractions by geography, age, sex, and year for GBD 2015
10	Provide a detailed description of all steps of the analysis, including mathematical formulae. This description should cover, as relevant, data cleaning, data pre-processing, data adjustments and weighting of data sources, and mathematical or statistical model(s).	Flow diagrams and corresponding methodological write-ups for each risk and modelling processes have been provided.	Appendix, Section 2, DisMod-MR 2.1 Estimation and spatiotemporal Gaussian process regression estimation; and Appendix, Section 3. Risk-specific estimation
11	Describe how candidate models were evaluated and how the final model(s) were selected.	Provided in the methodological write-ups.	Appendix, Section 3. Risk-specific estimation
12	Provide the results of an evaluation of model performance, if done, as well as the results of any relevant sensitivity analysis.	Provided in the methodological write-ups.	Appendix, Section 3. Risk-specific estimation
13	Describe methods for calculating uncertainty of the estimates. State which sources of uncertainty were, and were not, accounted for in the uncertainty analysis.	Provided in the methodological write-ups.	Appendix, Section 3. Risk-specific estimation
14	State how analytic or statistical source code used to generate estimates can be accessed.	Access statement provided.	Available online at http://ghdx.healthdata.org/global-burden-disease-study-2015
**Results and Discussion**			

15	Provide published estimates in a file format from which data can be efficiently extracted.	GBD 2015 results will be made available through online data visualization tools, the Global Health Data Exchange, and the online data query tool (these tools are already available for GBD 2013 results).	Main text; Appendix, Section 4. Supplemental Appendix Materials and Detailed Results for Risk Factors; and online data tools: http://ghdx.healthdata.org/global-burden-disease-study-2015
16	Report a quantitative measure of the uncertainty of the estimates (e.g. uncertainty intervals).	Uncertainty intervals are provided with all results.	Main text; Appendix, Section 3. Risk-specific estimation; and online data tools: http://ghdx.healthdata.org/global-burden-disease-study-2015
17	Interpret results in light of existing evidence. If updating a previous set of estimates, describe the reasons for changes in estimates.	Discussion of methodological changes between GBD rounds provided in the narrative of the paper and appendix.	Main text, Methods; Appendix, Section 2, Step 1. Effect size estimation, Step 2 Exposure estimation; and Appendix, Section 3. Risk-specific estimation
18	Discuss limitations of the estimates. Include a discussion of any modelling assumptions or data limitations that affect interpretation of the estimates.	Discussion of limitations provided in the narrative of the main paper as well as in the methodological write-ups in the appendix.	Main text, Limitations; and Appendix, Section 3. Risk-specific estimation

Appendix Table 2. GBD 2015 geography hierarchy with levels	
**Location**	**Level**
Global	0
High SDI	1
High-middle SDI	1
Middle SDI	1
Low-middle SDI	1
Low SDI	1
Southeast Asia, East Asia, and Oceania	1
East Asia	2
China	3
Anhui	4
Beijing	4
Chongqing	4
Fujian	4
Gansu	4
Guangdong	4
Guangxi	4
Guizhou	4
Hainan	4
Hebei	4
Heilongjiang	4
Henan	4
Hong Kong Special Administrative Region of China	4
Hubei	4
Hunan	4
Inner Mongolia	4
Jiangsu	4
Jiangxi	4
Jilin	4
Liaoning	4
Macao Special Administrative Region of China	4
Ningxia	4
Qinghai	4
Shaanxi	4
Shandong	4
Shanghai	4
Shanxi	4
Sichuan	4
Tianjin	4
Tibet	4
Xinjiang	4
Yunnan	4
Zhejiang	4
North Korea	3
Taiwan	3
Southeast Asia	2
Cambodia	3

Location	Level
Indonesia	3
Laos	3
Malaysia	3
Maldives	3
Mauritius	3
Myanmar	3
Philippines	3
Sri Lanka	3
Seychelles	3
Thailand	3
Timor-Leste	3
Vietnam	3
Oceania	2
American Samoa	3
Federated States of Micronesia	3
Fiji	3
Guam	3
Kiribati	3
Marshall Islands	3
Northern Mariana Islands	3
Papua New Guinea	3
Samoa	3
Solomon Islands	3
Tonga	3
Vanuatu	3
Central Europe, Eastern Europe, and Central Asia	1
Central Asia	2
Armenia	3
Azerbaijan	3
Georgia	3
Kazakhstan	3
Kyrgyzstan	3
Mongolia	3
Tajikistan	3
Turkmenistan	3
Uzbekistan	3
Central Europe	2
Albania	3
Bosnia and Herzegovina	3
Bulgaria	3
Croatia	3
Czech Republic	3
Hungary	3
Macedonia	3
Montenegro	3
Poland	3
Romania	3
Serbia	3

Location	Level
Slovakia	3
Slovenia	3
Eastern Europe	2
Belarus	3
Estonia	3
Latvia	3
Lithuania	3
Moldova	3
Russia	3
Ukraine	3
High-income	1
High-income Asia Pacific	2
Brunei	3
Japan	3
Aichi	4
Akita	4
Aomori	4
Chiba	4
Ehime	4
Fukui	4
Fukuoka	4
Fukushima	4
Gifu	4
Gunma	4
Hiroshima	4
Hokkaidō	4
Hyōgo	4
Ibaraki	4
Ishikawa	4
Iwate	4
Kagawa	4
Kagoshima	4
Kanagawa	4
Kōchi	4
Kumamoto	4
Kyōto	4
Mie	4
Miyagi	4
Miyazaki	4
Nagano	4
Nagasaki	4
Nara	4
Niigata	4
Ôita	4
Okayama	4
Okinawa	4
Ōsaka	4
Saga	4

Location	Level
Saitama	4
Shiga	4
Shimane	4
Shizuoka	4
Tochigi	4
Tokushima	4
Tōkyō	4
Tottori	4
Toyama	4
Wakayama	4
Yamagata	4
Yamaguchi	4
Yamanashi	4
South Korea	3
Singapore	3
Australasia	2
Australia	3
New Zealand	3
Western Europe	2
Andorra	3
Austria	3
Belgium	3
Cyprus	3
Denmark	3
Finland	3
France	3
Germany	3
Greece	3
Iceland	3
Ireland	3
Israel	3
Italy	3
Luxembourg	3
Malta	3
Netherlands	3
Norway	3
Portugal	3
Spain	3
Sweden	3
Stockholm	4
Sweden except Stockholm	4
Switzerland	3
United Kingdom	3
England	4
East Midlands	5
East of England	5
Greater London	5
North East England	5

Location	Level
North West England	5
South East England	5
South West England	5
West Midlands	5
Yorkshire and the Humber	5
Northern Ireland	4
Scotland	4
Wales	4
Southern Latin America	2
Argentina	3
Chile	3
Uruguay	3
High-income North America	2
Canada	3
Greenland	3
United States	3
Alabama	4
Alaska	4
Arizona	4
Arkansas	4
California	4
Colorado	4
Connecticut	4
Delaware	4
District of Columbia	4
Florida	4
Georgia	4
Hawaii	4
Idaho	4
Illinois	4
Indiana	4
Iowa	4
Kansas	4
Kentucky	4
Louisiana	4
Maine	4
Maryland	4
Massachusetts	4
Michigan	4
Minnesota	4
Mississippi	4
Missouri	4
Montana	4
Nebraska	4
Nevada	4
New Hampshire	4
New Jersey	4
New Mexico	4

Location	Level
New York	4
North Carolina	4
North Dakota	4
Ohio	4
Oklahoma	4
Oregon	4
Pennsylvania	4
Rhode Island	4
South Carolina	4
South Dakota	4
Tennessee	4
Texas	4
Utah	4
Vermont	4
Virginia	4
Washington	4
West Virginia	4
Wisconsin	4
Wyoming	4
Latin America and Caribbean	1
Caribbean	2
Antigua and Barbuda	3
The Bahamas	3
Barbados	3
Belize	3
Bermuda	3
Cuba	3
Dominica	3
Dominican Republic	3
Grenada	3
Guyana	3
Haiti	3
Jamaica	3
Puerto Rico	3
Saint Lucia	3
Saint Vincent and the Grenadines	3
Suriname	3
Trinidad and Tobago	3
Virgin Islands, U.S.	3
Andean Latin America	2
Bolivia	3
Ecuador	3
Peru	3
Central Latin America	2
Colombia	3
Costa Rica	3
El Salvador	3
Guatemala	3

Location	Level
Honduras	3
Mexico	3
Aguascalientes	4
Baja California	4
Baja California Sur	4
Campeche	4
Chiapas	4
Chihuahua	4
Coahuila	4
Colima	4
Distrito Federal	4
Durango	4
Guanajuato	4
Guerrero	4
Hidalgo	4
Jalisco	4
México	4
Michoacán de Ocampo	4
Morelos	4
Nayarit	4
Nuevo León	4
Oaxaca	4
Puebla	4
Querétaro	4
Quintana Roo	4
San Luis Potosí	4
Sinaloa	4
Sonora	4
Tabasco	4
Tamaulipas	4
Tlaxcala	4
Veracruz de Ignacio de la Llave	4
Yucatán	4
Zacatecas	4
Nicaragua	3
Panama	3
Venezuela	3
Tropical Latin America	2
Brazil	3
Acre	4
Alagoas	4
Amapá	4
Amazonas	4
Bahia	4
Ceará	4
Distrito Federal	4
Espírito Santo	4
Goiás	4

Location	Level
Maranhão	4
Mato Grosso	4
Mato Grosso do Sul	4
Minas Gerais	4
Pará	4
Paraíba	4
Paraná	4
Pernambuco	4
Piaui	4
Rio de Janeiro	4
Rio Grande do Norte	4
Rio Grande do Sul	4
Rondônia	4
Roraima	4
Santa Catarina	4
São Paulo	4
Sergipe	4
Tocantins	4
Paraguay	3
North Africa and Middle East	1
North Africa and Middle East	2
Afghanistan	3
Algeria	3
Bahrain	3
Egypt	3
Iran	3
Iraq	3
Jordan	3
Kuwait	3
Lebanon	3
Libya	3
Morocco	3
Palestine	3
Oman	3
Qatar	3
Saudi Arabia	3
'Asir	4
Bahah	4
Eastern Province	4
Ha'il	4
Jawf	4
Jizan	4
Madinah	4
Makkah	4
Najran	4
Northern Borders	4
Qassim	4
Riyadh	4

Location	Level
Tabuk	4
Sudan	3
Syria	3
Tunisia	3
Turkey	3
United Arab Emirates	3
Yemen	3
South Asia	1
South Asia	2
Bangladesh	3
Bhutan	3
India	3
Andhra Pradesh	4
Andhra Pradesh, Rural	5
Andhra Pradesh, Urban	5
Arunāchal Pradesh	4
Arunāchal Pradesh, Rural	5
Arunāchal Pradesh, Urban	5
Assam	4
Assam, Rural	5
Assam, Urban	5
Bihār	4
Bihār, Rural	5
Bihār, Urban	5
Chhattīsgarh	4
Chhattīsgarh, Rural	5
Chhattīsgarh, Urban	5
Delhi	4
Delhi, Rural	5
Delhi, Urban	5
Goa	4
Goa, Rural	5
Goa, Urban	5
Gujarāt	4
Gujarāt, Rural	5
Gujarāt, Urban	5
Haryāna	4
Haryāna, Rural	5
Haryāna, Urban	5
Himachal Pradesh	4
Himachal Pradesh, Rural	5
Himachal Pradesh, Urban	5
Jammu and Kashmīr	4
Jammu and Kashmīr, Rural	5
Jammu and Kashmīr, Urban	5
Jharkhand	4
Jharkhand, Rural	5
Jharkhand, Urban	5

Location	Level
Karnātaka	4
Karnātaka, Rural	5
Karnātaka, Urban	5
Kerala	4
Kerala, Rural	5
Kerala, Urban	5
Madhya Pradesh	4
Madhya Pradesh, Rural	5
Madhya Pradesh, Urban	5
Mahārāshtra	4
Mahārāshtra, Rural	5
Mahārāshtra, Urban	5
Manipur	4
Manipur, Rural	5
Manipur, Urban	5
Meghālaya	4
Meghālaya, Rural	5
Meghālaya, Urban	5
Mizoram	4
Mizoram, Rural	5
Mizoram, Urban	5
Nāgāland	4
Nāgāland, Rural	5
Nāgāland, Urban	5
Orissa	4
Orissa, Rural	5
Orissa, Urban	5
Punjab	4
Punjab, Rural	5
Punjab, Urban	5
Rājasthān	4
Rājasthān, Rural	5
Rājasthān, Urban	5
Sikkim	4
Sikkim, Rural	5
Sikkim, Urban	5
Tamil Nādu	4
Tamil Nādu, Rural	5
Tamil Nādu, Urban	5
Telangana	4
Telangana, Rural	5
Telangana, Urban	5
Tripura	4
Tripura, Rural	5
Tripura, Urban	5
Uttar Pradesh	4
Uttar Pradesh, Rural	5
Uttar Pradesh, Urban	5

Location	Level
Uttarakhand	4
Uttarakhand, Rural	5
Uttarakhand, Urban	5
West Bengal	4
West Bengal, Rural	5
West Bengal, Urban	5
The Six Minor Territories	4
The Six Minor Territories, Rural	5
The Six Minor Territories, Urban	5
Nepal	3
Pakistan	3
Sub-Saharan Africa	1
Central Sub-Saharan Africa	2
Angola	3
Central African Republic	3
Congo	3
Democratic Republic of the Congo	3
Equatorial Guinea	3
Gabon	3
Eastern Sub-Saharan Africa	2
Burundi	3
Comoros	3
Djibouti	3
Eritrea	3
Ethiopia	3
Kenya	3
Baringo	4
Bomet	4
Bungoma	4
Busia	4
Elgeyo-Marakwet	4
Embu	4
Garissa	4
HomaBay	4
Isiolo	4
Kajiado	4
Kakamega	4
Kericho	4
Kiambu	4
Kilifi	4
Kirinyaga	4
Kisii	4
Kisumu	4
Kitui	4
Kwale	4
Laikipia	4
Lamu	4
Machakos	4

Location	Level
Makueni	4
Mandera	4
Marsabit	4
Meru	4
Migori	4
Mombasa	4
Murang’a	4
Nairobi	4
Nakuru	4
Nandi	4
Narok	4
Nyamira	4
Nyandarua	4
Nyeri	4
Samburu	4
Siaya	4
TaitaTaveta	4
TanaRiver	4
TharakaNithi	4
TransNzoia	4
Turkana	4
UasinGishu	4
Vihiga	4
Wajir	4
WestPokot	4
Madagascar	3
Malawi	3
Mozambique	3
Rwanda	3
Somalia	3
South Sudan	3
Tanzania	3
Uganda	3
Zambia	3
Southern Sub-Saharan Africa	2
Botswana	3
Lesotho	3
Namibia	3
South Africa	3
Eastern Cape	4
Free State	4
Gauteng	4
KwaZulu-Natal	4
Limpopo	4
Mpumalanga	4
North-West	4
Northern Cape	4
Western Cape	4

Location	Level
Swaziland	3
Zimbabwe	3
Western Sub-Saharan Africa	2
Benin	3
Burkina Faso	3
Cameroon	3
Cape Verde	3
Chad	3
Cote d'Ivoire	3
The Gambia	3
Ghana	3
Guinea	3
Guinea-Bissau	3
Liberia	3
Mali	3
Mauritania	3
Niger	3
Nigeria	3
Sao Tome and Principe	3
Senegal	3
Sierra Leone	3
Togo	3

Appendix Table 3. GBD 2015 risk factor hierarchy with levels, modeling strategies, and the main type of data sources used to estimate exposure levels
GBD=Global Burden of Disease.

Risk factor	Level	Model type	Main data source for exposure
**All risk factors**	**0**		
**Environmental/occupational risks**	**1**		
**Unsafe water, sanitation, and handwashing**	**2**		
Unsafe water source	3	Spatiotemporal Gauissian process regression (ST-GPR)	Population surveys and censuses
Unsafe sanitation	3	ST-GPR	Population surveys and censuses
No handwashing with soap	3	ST-GPR	Population surveys, censuses, and epidemiological studies
**Air pollution**	**2**		
Ambient particulate matter pollution	3	Regression crosswalk between grid-level fusion of satellite/chemical transport models and ground level monitoring data	Atmospheric chemical transport models, satellite measurements of aerosols in the atmosphere, data from ground-level monitoring sites
Household air pollution from solid fuels	3	ST-GPR	Population surveys and censuses
Ambient ozone pollution	3	Chemical transport model	Atmospheric chemical transport models
**Other environmental risks**	**2**		
Residential radon	3	ST-GPR	Literature review
Lead exposure	3	ST-GPR	Literature review
**Occupational risks**	**2**		
Occupational carcinogens	3		
Occupational exposure to asbestos	4	Asbestos Impact Ratio approach	GBD cause-specific mortality data for mesothelioma, epidemiological studies
Occupational exposure to arsenic	4	ST-GPR	Labor force surveys, censuses, and international information system on occupational exposure to carcinogens
Occupational exposure to benzene	4	ST-GPR	Labor force surveys, censuses, and international information system on occupational exposure to carcinogens
Occupational exposure to beryllium	4	ST-GPR	Labor force surveys, censuses, and international information system on occupational exposure to carcinogens
Occupational exposure to cadmium	4	ST-GPR	Labor force surveys, censuses, and international information system on occupational exposure to carcinogens
Occupational exposure to chromium	4	ST-GPR	Labor force surveys, censuses, and international information system on occupational exposure to carcinogens
Occupational exposure to diesel engine exhaust	4	ST-GPR	Labor force surveys, censuses, and international information system on occupational exposure to carcinogens
Occupational exposure to second-hand smoke	4	ST-GPR	Labor force surveys, censuses, and international information system on occupational exposure to carcinogens
Occupational exposure to formaldehyde	4	ST-GPR	Labor force surveys, censuses, and international information system on occupational exposure to carcinogens
Occupational exposure to nickel	4	ST-GPR	Labor force surveys, censuses, and international information system on occupational exposure to carcinogens
Occupational exposure to polycyclic aromatic hydrocarbons	4	ST-GPR	Labor force surveys, censuses, and international information system on occupational exposure to carcinogens
Occupational exposure to silica	4	ST-GPR	Labor force surveys, censuses, and international information system on occupational exposure to carcinogens
Occupational exposure to sulfphuric acid	4	ST-GPR	Labor force surveys, censuses, and international information system on occupational exposure to carcinogens
Occupational exposure to trichloroethylene	4	ST-GPR	Labor force surveys, censuses, and international information system on occupational exposure to carcinogens
Occupational asthmagens	3	ST-GPR	Labor force surveys and censuses
Occupational particulate matter, gases, and fumes	3	ST-GPR	Labor force surveys and censuses
Occupational noise	3	ST-GPR	Labor force surveys and censuses, industry-based surveys of noise exposure
Occupational injuries	3	ST-GPR	International Labor Organization injury database
Occupational ergonomic factors	3	ST-GPR	Labor force surveys and censuses
**Behavioural risks**	**1**		
**Child and maternal malnutrition**	**2**		
Suboptimal breastfeeding	3		
Non-exclusive breastfeeding	4	ST-GPR	Population surveys
Discontinued breastfeeding	4	ST-GPR	Population surveys
Childhood undernutrition	3		
Childhood underweight	4	ST-GPR	Examination surveys and epidemiological studies
Childhood wasting	4	ST-GPR	Examination surveys and epidemiological studies
Childhood stunting	4	ST-GPR	Examination surveys and epidemiological studies
Iron deficiency	3	Mixed effect regression	Examination surveys and epidemiological studies
Vitamin A deficiency	3	DisMod-MR 2.1	Examination surveys and epidemiological studies
Zinc deficiency	3	Mixed effect regression based on stunting prevalence and dietary composition	FAO food balance sheets
**Tobacco smoke**	**2**		
Smoking	3	• Smoking Impact Ratio (SIR) calculated from lung cancer mortality rates<br>• Smoking prevalence estimated using ST-GPR	SIR input data: mortality and cause of death data including vital registration and verbal autopsy<br>Smoking prevalence input data: nationally representative survey and report data
Second-hand smoke	3	DisMod-MR 2.1	Household surveys and national health surveys
**Alcohol and drug use**	**2**		
Alcohol use	3	• Alcohol consumption per capita obtained from the FAO and the WHO Global Information System on Alcohol and Health (GISAH)<br>• ST-GPR used to integrate the data and to derive coherent time series for each country<br>• Prevalence of current alcohol drinkers, lifetime abstainers, former drinkers, and binge drinkers estimated using DisMod-MR 2.1<br>• DisMod-MR 2.1 used to estimate the relative sex- and age-specific pattern of alcohol consumption in current drinkers	Population surveys, alcohol sales, production, and other economic statistics

Risk factor	Level	Model type	Main data source for exposure
Drug use	3	DisMod-MR 2.1	Systematic review of published literature, reports from governments and international organizations, which include data from: school surveys, population surveys, registration data, and indirect estimates of prevalence
**Dietary risks**	**2**		
Diet low in fruits	3	DisMod-MR 2.1	Nutrition and health surveys, FAO food balance sheets
Diet low in vegetables	3	DisMod-MR 2.1	Nutrition and health surveys, FAO food balance sheets
Diet low in whole grains	3	DisMod-MR 2.1	Nutrition and health surveys
Diet low in nuts and seeds	3	DisMod-MR 2.1	Nutrition and health surveys, FAO food balance sheets
Diet low in milk	3	DisMod-MR 2.1	Nutrition and health surveys, FAO food balance sheets
Diet high in red meat	3	DisMod-MR 2.1	Nutrition and health surveys, FAO food balance sheets
Diet high in processed meat	3	DisMod-MR 2.1	Nutrition and health surveys
Diet high in sugar-sweetened beverages	3	DisMod-MR 2.1	Nutrition and health surveys
Diet low in fibre	3	DisMod-MR 2.1	Nutrition and health surveys, FAO SUA/USDA
Diet suboptimal in calcium	3	DisMod-MR 2.1	Nutrition and health surveys, FAO SUA/USDA
Diet low in seafood omega-3 fatty acids	3	DisMod-MR 2.1	Nutrition and health surveys, FAO SUA/USDA
Diet low in polyunsaturated fatty acids	3	DisMod-MR 2.1	Nutrition and health surveys, FAO SUA/USDA
Diet high in trans fatty acids	3	DisMod-MR 2.1	Nutrition and health surveys
Diet high in sodium	3	DisMod-MR 2.1	Nutrition and health surveys
**Sexual abuse and violence**	**2**		
Childhood sexual abuse	3	DisMod-MR 2.1	Systematic review of published literature, national health surveys, violence-specific surveys
Intimate partner violence	3	DisMod-MR 2.1	Systematic review of published literature, national health surveys, violence-specific surveys
**Unsafe sex**	2	DisMod-MR 2.1	UNAIDS country progress reports, disease surveillance reports
**Low physical activity**	2	DisMod-MR 2.1	Surveys of the adult population that capture reported frequency, duration and intensity of physical activity undertaken in the past seven days across all domains of life (work, transport, recreation or house/yard work)
**Metabolic risks**	**1**		
High fasting plasma glucose	2	ST-GPR	Examination surveys and epidemiological studies
High total cholesterol	2	ST-GPR	Examination surveys and epidemiological studies
High systolic blood pressure	2	ST-GPR	Examination surveys and epidemiological studies
High body-mass index	2	ST-GPR	Examination surveys and epidemiological studies
Low bone mineral density	2	DisMod-MR 2.1	Examination surveys and epidemiological studies
Low glomerular filtration rate	2	DisMod-MR 2.1	Examination surveys and epidemiological studies

Appendix Table 4. Socio-demographic Index (SDI) groupings by geography, based on 2015 values	
**Location**	**SDI level**
Aichi	High SDI
Akita	High SDI
Alabama	High SDI
Alaska	High SDI
Andorra	High SDI
Antigua and Barbuda	High SDI
Aomori	High SDI
Arizona	High SDI
Arkansas	High SDI
Australia	High SDI
Austria	High SDI
Beijing	High SDI
Belarus	High SDI
Belgium	High SDI
Bermuda	High SDI
Brunei	High SDI
California	High SDI
Canada	High SDI
Chiba	High SDI
Colorado	High SDI
Connecticut	High SDI
Cyprus	High SDI
Czech Republic	High SDI
Delaware	High SDI
Denmark	High SDI
District of Columbia	High SDI
Distrito Federal	High SDI
Distrito Federal	High SDI
East Midlands	High SDI
East of England	High SDI
Ehime	High SDI
Estonia	High SDI
Finland	High SDI
Florida	High SDI
France	High SDI
Fukui	High SDI
Fukuoka	High SDI
Fukushima	High SDI
Georgia	High SDI
Germany	High SDI
Gifu	High SDI
Greater London	High SDI
Guam	High SDI
Gunma	High SDI

Appendix Table 4. Socio-demographic index (SDI) groupings by geography, based on 2015 values

Location	SDI level
Hawaii	High SDI
Hiroshima	High SDI
Hokkaidō	High SDI
Hong Kong Special Administrative Region of China	High SDI
Hungary	High SDI
Hyōgo	High SDI
Ibaraki	High SDI
Iceland	High SDI
Idaho	High SDI
Illinois	High SDI
Indiana	High SDI
Iowa	High SDI
Ireland	High SDI
Ishikawa	High SDI
Israel	High SDI
Italy	High SDI
Iwate	High SDI
Kagawa	High SDI
Kagoshima	High SDI
Kanagawa	High SDI
Kansas	High SDI
Kentucky	High SDI
Kōchi	High SDI
Kumamoto	High SDI
Kuwait	High SDI
Kyōto	High SDI
Latvia	High SDI
Lithuania	High SDI
Louisiana	High SDI
Luxembourg	High SDI
Macao Special Administrative Region of China	High SDI
Maine	High SDI
Maryland	High SDI
Massachusetts	High SDI
Michigan	High SDI
Mie	High SDI
Minnesota	High SDI
Mississippi	High SDI
Missouri	High SDI
Miyagi	High SDI
Miyazaki	High SDI
Montana	High SDI
Nagano	High SDI
Nagasaki	High SDI

Appendix Table 4. Socio-demographic index (SDI) groupings by geography, based on 2015 values	
**Location**	**SDI level**
Nara	High SDI
Nebraska	High SDI
Netherlands	High SDI
Nevada	High SDI
New Hampshire	High SDI
New Jersey	High SDI
New Mexico	High SDI
New York	High SDI
New Zealand	High SDI
Niigata	High SDI
North Carolina	High SDI
North Dakota	High SDI
North West England	High SDI
Northern Mariana Islands	High SDI
Norway	High SDI
Ohio	High SDI
Ôita	High SDI
Okayama	High SDI
Okinawa	High SDI
Oklahoma	High SDI
Oregon	High SDI
Ōsaka	High SDI
Pennsylvania	High SDI
Poland	High SDI
Puerto Rico	High SDI
Rhode Island	High SDI
Russia	High SDI
Saga	High SDI
Saitama	High SDI
Scotland	High SDI
Shanghai	High SDI
Shiga	High SDI
Shimane	High SDI
Shizuoka	High SDI
Singapore	High SDI
Slovakia	High SDI
Slovenia	High SDI
South Carolina	High SDI
South Dakota	High SDI
South East England	High SDI
South Korea	High SDI
South West England	High SDI
Stockholm	High SDI
Sweden except Stockholm	High SDI

Appendix Table 4. Socio-demographic index (SDI) groupings by geography, based on 2015 values

Location	SDI level
Switzerland	High SDI
Taiwan	High SDI
Tennessee	High SDI
Texas	High SDI
The Bahamas	High SDI
Tianjin	High SDI
Tochigi	High SDI
Tokushima	High SDI
Tōkyō	High SDI
Tottori	High SDI
Toyama	High SDI
Trinidad and Tobago	High SDI
United Arab Emirates	High SDI
Utah	High SDI
Vermont	High SDI
Virgin Islands, U.S.	High SDI
Virginia	High SDI
Wakayama	High SDI
Wales	High SDI
Washington	High SDI
West Midlands	High SDI
West Virginia	High SDI
Wisconsin	High SDI
Wyoming	High SDI
Yamagata	High SDI
Yamaguchi	High SDI
Yamanashi	High SDI
Yorkshire and the Humber	High SDI
'Asir	High-middle SDI
Aguascalientes	High-middle SDI
Albania	High-middle SDI
American Samoa	High-middle SDI
Andhra Pradesh, Urban	High-middle SDI
Argentina	High-middle SDI
Armenia	High-middle SDI
Azerbaijan	High-middle SDI
Bahah	High-middle SDI
Bahrain	High-middle SDI
Baja California	High-middle SDI
Baja California Sur	High-middle SDI
Barbados	High-middle SDI
Bosnia and Herzegovina	High-middle SDI
Bulgaria	High-middle SDI
Campeche	High-middle SDI

Appendix Table 4. Socio-demographic index (SDI) groupings by geography, based on 2015 values	
**Location**	**SDI level**
Chihuahua	High-middle SDI
Chile	High-middle SDI
Coahuila	High-middle SDI
Colima	High-middle SDI
Colombia	High-middle SDI
Costa Rica	High-middle SDI
Croatia	High-middle SDI
Cuba	High-middle SDI
Delhi, Rural	High-middle SDI
Delhi, Urban	High-middle SDI
Dominica	High-middle SDI
Dominican Republic	High-middle SDI
Durango	High-middle SDI
Eastern Cape	High-middle SDI
Eastern Province	High-middle SDI
Ecuador	High-middle SDI
Espírito Santo	High-middle SDI
Fiji	High-middle SDI
Free State	High-middle SDI
Gauteng	High-middle SDI
Georgia	High-middle SDI
Goa, Rural	High-middle SDI
Goa, Urban	High-middle SDI
Greece	High-middle SDI
Greenland	High-middle SDI
Grenada	High-middle SDI
Guangdong	High-middle SDI
Ha'il	High-middle SDI
Haryāna, Urban	High-middle SDI
Heilongjiang	High-middle SDI
Himachal Pradesh, Urban	High-middle SDI
Inner Mongolia	High-middle SDI
Iran	High-middle SDI
Jalisco	High-middle SDI
Jamaica	High-middle SDI
Jawf	High-middle SDI
Jiangsu	High-middle SDI
Jilin	High-middle SDI
Jordan	High-middle SDI
Karnātaka, Urban	High-middle SDI
Kazakhstan	High-middle SDI
KwaZulu-Natal	High-middle SDI
Lebanon	High-middle SDI
Liaoning	High-middle SDI

Appendix Table 4. Socio-demographic index (SDI) groupings by geography, based on 2015 values	
**Location**	**SDI level**
Macedonia	High-middle SDI
Madinah	High-middle SDI
Mahārāshtra, Urban	High-middle SDI
Makkah	High-middle SDI
Malaysia	High-middle SDI
Malta	High-middle SDI
Mauritius	High-middle SDI
México	High-middle SDI
Moldova	High-middle SDI
Mongolia	High-middle SDI
Montenegro	High-middle SDI
Morelos	High-middle SDI
Mpumalanga	High-middle SDI
Nairobi	High-middle SDI
Nayarit	High-middle SDI
North East England	High-middle SDI
North-West	High-middle SDI
Northern Borders	High-middle SDI
Northern Cape	High-middle SDI
Northern Ireland	High-middle SDI
Nuevo León	High-middle SDI
Oman	High-middle SDI
Panama	High-middle SDI
Peru	High-middle SDI
Portugal	High-middle SDI
Punjab, Urban	High-middle SDI
Qassim	High-middle SDI
Qatar	High-middle SDI
Querétaro	High-middle SDI
Quintana Roo	High-middle SDI
Rio de Janeiro	High-middle SDI
Rio Grande do Sul	High-middle SDI
Riyadh	High-middle SDI
Romania	High-middle SDI
Saint Lucia	High-middle SDI
Saint Vincent and the Grenadines	High-middle SDI
San Luis Potosí	High-middle SDI
Santa Catarina	High-middle SDI
São Paulo	High-middle SDI
Serbia	High-middle SDI
Seychelles	High-middle SDI
Shandong	High-middle SDI
Shanxi	High-middle SDI
Sikkim, Urban	High-middle SDI

Appendix Table 4. Socio-demographic index (SDI) groupings by geography, based on 2015 values

Location	SDI level
Sinaloa	High-middle SDI
Sonora	High-middle SDI
Spain	High-middle SDI
Sri Lanka	High-middle SDI
Suriname	High-middle SDI
Tabasco	High-middle SDI
Tabuk	High-middle SDI
Tamaulipas	High-middle SDI
Tamil Nādu, Urban	High-middle SDI
Thailand	High-middle SDI
The Six Minor Territories, Urban	High-middle SDI
Tlaxcala	High-middle SDI
Turkey	High-middle SDI
Turkmenistan	High-middle SDI
Ukraine	High-middle SDI
Uruguay	High-middle SDI
Uttarakhand, Urban	High-middle SDI
Uzbekistan	High-middle SDI
Venezuela	High-middle SDI
Western Cape	High-middle SDI
Yucatán	High-middle SDI
Zhejiang	High-middle SDI
Acre	Middle SDI
Algeria	Middle SDI
Amapá	Middle SDI
Amazonas	Middle SDI
Andhra Pradesh, Rural	Middle SDI
Anhui	Middle SDI
Arunāchal Pradesh, Urban	Middle SDI
Assam, Urban	Middle SDI
Bahia	Middle SDI
Belize	Middle SDI
Bihār, Urban	Middle SDI
Bolivia	Middle SDI
Botswana	Middle SDI
Chhattīsgarh, Urban	Middle SDI
Chiapas	Middle SDI
Chongqing	Middle SDI
Egypt	Middle SDI
El Salvador	Middle SDI
Equatorial Guinea	Middle SDI
Federated States of Micronesia	Middle SDI
Fujian	Middle SDI
Gabon	Middle SDI

Appendix Table 4. Socio-demographic index (SDI) groupings by geography, based on 2015 values

Location	SDI level
Gansu	Middle SDI
Goiás	Middle SDI
Guanajuato	Middle SDI
Guangxi	Middle SDI
Guerrero	Middle SDI
Gujarāt, Urban	Middle SDI
Guyana	Middle SDI
Hainan	Middle SDI
Haryāna, Rural	Middle SDI
Hebei	Middle SDI
Henan	Middle SDI
Hidalgo	Middle SDI
Himachal Pradesh, Rural	Middle SDI
Honduras	Middle SDI
Hubei	Middle SDI
Hunan	Middle SDI
Indonesia	Middle SDI
Iraq	Middle SDI
Jammu and Kashmīr, Urban	Middle SDI
Jharkhand, Urban	Middle SDI
Jiangxi	Middle SDI
Jizan	Middle SDI
Kerala, Rural	Middle SDI
Kerala, Urban	Middle SDI
Kiambu	Middle SDI
Kyrgyzstan	Middle SDI
Laikipia	Middle SDI
Libya	Middle SDI
Limpopo	Middle SDI
Madhya Pradesh, Urban	Middle SDI
Mahārāshtra, Rural	Middle SDI
Maldives	Middle SDI
Manipur, Urban	Middle SDI
Marshall Islands	Middle SDI
Mato Grosso	Middle SDI
Mato Grosso do Sul	Middle SDI
Meghālaya, Urban	Middle SDI
Michoacán de Ocampo	Middle SDI
Minas Gerais	Middle SDI
Mizoram, Urban	Middle SDI
Mombasa	Middle SDI
Nāgāland, Rural	Middle SDI
Nāgāland, Urban	Middle SDI
Najran	Middle SDI

Appendix Table 4. Socio-demographic index (SDI) groupings by geography, based on 2015 values

Location	SDI level
Namibia	Middle SDI
Nicaragua	Middle SDI
Ningxia	Middle SDI
North Korea	Middle SDI
Nyeri	Middle SDI
Oaxaca	Middle SDI
Orissa, Urban	Middle SDI
Palestine	Middle SDI
Pará	Middle SDI
Paraguay	Middle SDI
Paraná	Middle SDI
Pernambuco	Middle SDI
Philippines	Middle SDI
Puebla	Middle SDI
Punjab, Rural	Middle SDI
Qinghai	Middle SDI
Rājasthān, Urban	Middle SDI
Rio Grande do Norte	Middle SDI
Rondônia	Middle SDI
Roraima	Middle SDI
Samoa	Middle SDI
Sergipe	Middle SDI
Shaanxi	Middle SDI
Sichuan	Middle SDI
Sikkim, Rural	Middle SDI
Swaziland	Middle SDI
Syria	Middle SDI
Tajikistan	Middle SDI
Tamil Nādu, Rural	Middle SDI
Telangana, Urban	Middle SDI
The Six Minor Territories, Rural	Middle SDI
Tocantins	Middle SDI
Tonga	Middle SDI
Tripura, Urban	Middle SDI
Tunisia	Middle SDI
Uttar Pradesh, Urban	Middle SDI
Uttarakhand, Rural	Middle SDI
Veracruz de Ignacio de la Llave	Middle SDI
Vietnam	Middle SDI
West Bengal, Urban	Middle SDI
Xinjiang	Middle SDI
Yunnan	Middle SDI
Zacatecas	Middle SDI
Alagoas	Low-middle SDI

Appendix Table 4. Socio-demographic index (SDI) groupings by geography, based on 2015 values

Location	SDI level
Angola	Low-middle SDI
Arunāchal Pradesh, Rural	Low-middle SDI
Assam, Rural	Low-middle SDI
Bangladesh	Low-middle SDI
Bhutan	Low-middle SDI
Bihār, Rural	Low-middle SDI
Bomet	Low-middle SDI
Bungoma	Low-middle SDI
Cambodia	Low-middle SDI
Cameroon	Low-middle SDI
Cape Verde	Low-middle SDI
Ceará	Low-middle SDI
Chhattīsgarh, Rural	Low-middle SDI
Congo	Low-middle SDI
Djibouti	Low-middle SDI
Elgeyo-Marakwet	Low-middle SDI
Embu	Low-middle SDI
Ghana	Low-middle SDI
Guatemala	Low-middle SDI
Guizhou	Low-middle SDI
Gujarāt, Rural	Low-middle SDI
Haiti	Low-middle SDI
HomaBay	Low-middle SDI
Jammu and Kashmīr, Rural	Low-middle SDI
Jharkhand, Rural	Low-middle SDI
Kajiado	Low-middle SDI
Kakamega	Low-middle SDI
Karnātaka, Rural	Low-middle SDI
Kericho	Low-middle SDI
Kiribati	Low-middle SDI
Kirinyaga	Low-middle SDI
Kisii	Low-middle SDI
Kisumu	Low-middle SDI
Kwale	Low-middle SDI
Lamu	Low-middle SDI
Laos	Low-middle SDI
Lesotho	Low-middle SDI
Machakos	Low-middle SDI
Madhya Pradesh, Rural	Low-middle SDI
Makueni	Low-middle SDI
Manipur, Rural	Low-middle SDI
Maranhão	Low-middle SDI
Meghālaya, Rural	Low-middle SDI
Meru	Low-middle SDI

Appendix Table 4. Socio-demographic index (SDI) groupings by geography, based on 2015 values	
**Location**	**SDI level**
Migori	Low-middle SDI
Mizoram, Rural	Low-middle SDI
Morocco	Low-middle SDI
Murang’a	Low-middle SDI
Myanmar	Low-middle SDI
Nakuru	Low-middle SDI
Nandi	Low-middle SDI
Nepal	Low-middle SDI
Nigeria	Low-middle SDI
Nyamira	Low-middle SDI
Nyandarua	Low-middle SDI
Orissa, Rural	Low-middle SDI
Pakistan	Low-middle SDI
Papua New Guinea	Low-middle SDI
Paraíba	Low-middle SDI
Piaui	Low-middle SDI
Rājasthān, Rural	Low-middle SDI
Sao Tome and Principe	Low-middle SDI
Siaya	Low-middle SDI
Solomon Islands	Low-middle SDI
Sudan	Low-middle SDI
TaitaTaveta	Low-middle SDI
Tanzania	Low-middle SDI
Telangana, Rural	Low-middle SDI
TharakaNithi	Low-middle SDI
Tibet	Low-middle SDI
Timor-Leste	Low-middle SDI
TransNzoia	Low-middle SDI
Tripura, Rural	Low-middle SDI
UasinGishu	Low-middle SDI
Uttar Pradesh, Rural	Low-middle SDI
Vanuatu	Low-middle SDI
Vihiga	Low-middle SDI
West Bengal, Rural	Low-middle SDI
Yemen	Low-middle SDI
Zambia	Low-middle SDI
Zimbabwe	Low-middle SDI
Afghanistan	Low SDI
Baringo	Low SDI
Benin	Low SDI
Burkina Faso	Low SDI
Burundi	Low SDI
Busia	Low SDI
Central African Republic	Low SDI

Appendix Table 4. Socio-demographic index (SDI) groupings by geography, based on 2015 values	
**Location**	**SDI level**
Chad	Low SDI
Comoros	Low SDI
Cote d'Ivoire	Low SDI
Democratic Republic of the Congo	Low SDI
Eritrea	Low SDI
Ethiopia	Low SDI
Garissa	Low SDI
Guinea	Low SDI
Guinea-Bissau	Low SDI
Isiolo	Low SDI
Kilifi	Low SDI
Kitui	Low SDI
Liberia	Low SDI
Madagascar	Low SDI
Malawi	Low SDI
Mali	Low SDI
Mandera	Low SDI
Marsabit	Low SDI
Mauritania	Low SDI
Mozambique	Low SDI
Narok	Low SDI
Niger	Low SDI
Rwanda	Low SDI
Samburu	Low SDI
Senegal	Low SDI
Sierra Leone	Low SDI
Somalia	Low SDI
South Sudan	Low SDI
TanaRiver	Low SDI
The Gambia	Low SDI
Togo	Low SDI
Turkana	Low SDI
Uganda	Low SDI
Wajir	Low SDI
WestPokot	Low SDI

Appendix Table 5. Socio-demographic Index (SDI) values for all estimated GBD 2015 locations, 1980–2015
GBD=Global Burden of Disease

Location	1980	1981	1982	1983	1984	1985	1986	1987	1988	1989	1990	1991	1992	1993	1994	1995	1996	1997	1998	1999	2000	2001	2002	2003	2004	2005	2006	2007	2008	2009	2010	2011	2012	2013	2014	2015
Global	0.4199	0.4256	0.4311	0.4365	0.4424	0.4489	0.4551	0.4615	0.4681	0.4745	0.4811	0.4877	0.4944	0.5011	0.5078	0.5146	0.5214	0.5279	0.5340	0.5398	0.5456	0.5512	0.5568	0.5625	0.5685	0.5748	0.5815	0.5886	0.5953	0.6015	0.6079	0.6143	0.6204	0.6265	0.6324	0.6381
Southeast Asia, East Asia, and Oceania	0.3345	0.3444	0.3544	0.3636	0.3744	0.3865	0.3973	0.4081	0.4187	0.4284	0.4380	0.4483	0.4596	0.4715	0.4836	0.4957	0.5074	0.5185	0.5281	0.5370	0.5454	0.5535	0.5618	0.5698	0.5782	0.5869	0.5958	0.6052	0.6138	0.6219	0.6301	0.6380	0.6458	0.6533	0.6605	0.6672
East Asia	0.3249	0.3346	0.3447	0.3538	0.3650	0.3783	0.3901	0.4019	0.4134	0.4237	0.4338	0.4449	0.4572	0.4703	0.4835	0.4967	0.5092	0.5211	0.5316	0.5413	0.5504	0.5592	0.5681	0.5768	0.5858	0.5951	0.6047	0.6149	0.6240	0.6327	0.6413	0.6495	0.6576	0.6653	0.6725	0.6792
China	0.3160	0.3258	0.3361	0.3452	0.3565	0.3700	0.3819	0.3939	0.4057	0.4161	0.4265	0.4377	0.4502	0.4636	0.4773	0.4909	0.5038	0.5161	0.5268	0.5368	0.5463	0.5553	0.5644	0.5733	0.5826	0.5921	0.6019	0.6124	0.6216	0.6305	0.6393	0.6477	0.6561	0.6639	0.6712	0.6780
Anhui	0.2770	0.2948	0.3045	0.3140	0.3279	0.3402	0.3532	0.3665	0.3721	0.3790	0.3881	0.3984	0.4118	0.4258	0.4382	0.4502	0.4615	0.4709	0.4798	0.4888	0.4985	0.5075	0.5177	0.5250	0.5318	0.5395	0.5472	0.5558	0.5627	0.5700	0.5793	0.5868	0.5916	0.5983	0.6047	0.6110
Beijing	0.5106	0.5177	0.5194	0.5281	0.5328	0.5418	0.5529	0.5649	0.5813	0.5979	0.6083	0.6240	0.6331	0.6429	0.6529	0.6633	0.6733	0.6828	0.6917	0.7003	0.7089	0.7183	0.7280	0.7380	0.7482	0.7587	0.7697	0.7809	0.7915	0.8019	0.8122	0.8225	0.8325	0.8423	0.8516	0.8605
Chongqing	0.2739	0.2896	0.3005	0.2931	0.2987	0.3053	0.3373	0.3653	0.3735	0.3864	0.4016	0.4080	0.4194	0.4338	0.4490	0.4630	0.4773	0.4894	0.5020	0.5151	0.5268	0.5359	0.5440	0.5492	0.5581	0.5688	0.5753	0.5837	0.5914	0.5994	0.6089	0.6116	0.6144	0.6203	0.6257	0.6309
Fujian	0.3017	0.3122	0.3218	0.3318	0.3412	0.3577	0.3780	0.3979	0.4094	0.4139	0.4253	0.4411	0.4563	0.4734	0.4892	0.5050	0.5228	0.5366	0.5497	0.5604	0.5701	0.5781	0.5880	0.5969	0.6063	0.6158	0.6247	0.6361	0.6452	0.6548	0.6663	0.6742	0.6797	0.6807	0.6798	0.6770
Gansu	0.2973	0.3077	0.3142	0.3208	0.3279	0.3406	0.3522	0.3642	0.3728	0.3795	0.3927	0.3988	0.4061	0.4140	0.4231	0.4325	0.4469	0.4572	0.4666	0.4751	0.4832	0.4907	0.4978	0.5055	0.5131	0.5200	0.5264	0.5335	0.5402	0.5464	0.5577	0.5630	0.5697	0.5771	0.5841	0.5908
Guangdong	0.2644	0.2774	0.2982	0.2651	0.2380	0.3139	0.3240	0.3388	0.3700	0.3936	0.4041	0.4200	0.4441	0.4702	0.4917	0.5086	0.5274	0.5458	0.5609	0.5765	0.5877	0.5956	0.6066	0.6133	0.6244	0.6359	0.6454	0.6556	0.6671	0.6772	0.6857	0.6996	0.7046	0.7165	0.7284	0.7402
Guangxi	0.2408	0.2522	0.2642	0.2819	0.2830	0.2995	0.3177	0.3364	0.3511	0.3688	0.3816	0.3823	0.3958	0.4095	0.4244	0.4398	0.4541	0.4663	0.4763	0.4870	0.4960	0.5046	0.5142	0.5211	0.5327	0.5416	0.5503	0.5615	0.5702	0.5798	0.5917	0.6011	0.6098	0.6195	0.6291	0.6385
Guizhou	0.1644	0.1827	0.1954	0.2076	0.2265	0.2523	0.2667	0.2805	0.2909	0.3098	0.3175	0.3234	0.3325	0.3439	0.3561	0.3701	0.3821	0.3915	0.4010	0.4090	0.4191	0.4277	0.4350	0.4468	0.4569	0.4679	0.4773	0.4882	0.4949	0.5012	0.5108	0.5187	0.5262	0.5339	0.5415	0.5489
Hainan	0.3372	0.3483	0.3562	0.3515	0.3557	0.3606	0.3839	0.4058	0.4126	0.4153	0.4243	0.4325	0.4456	0.4585	0.4715	0.4845	0.4970	0.5090	0.5205	0.5320	0.5416	0.5511	0.5594	0.5689	0.5778	0.5865	0.5953	0.6050	0.6137	0.6224	0.6311	0.6389	0.6493	0.6588	0.6683	0.6774
Hebei	0.3012	0.3000	0.3102	0.3194	0.3271	0.3358	0.3373	0.3391	0.3550	0.3712	0.3842	0.4054	0.4212	0.4335	0.4505	0.4678	0.4816	0.4948	0.5050	0.5128	0.5236	0.5346	0.5400	0.5481	0.5540	0.5568	0.5649	0.5715	0.5827	0.5923	0.5971	0.6056	0.6192	0.6289	0.6388	0.6485
Heilongjiang	0.4107	0.4211	0.4243	0.4322	0.4416	0.4501	0.4553	0.4604	0.4713	0.4766	0.4880	0.4964	0.5026	0.5125	0.5239	0.5369	0.5479	0.5567	0.5652	0.5746	0.5823	0.5898	0.5974	0.6046	0.6116	0.6186	0.6261	0.6340	0.6412	0.6486	0.6557	0.6627	0.6693	0.6756	0.6816	0.6870
Henan	0.2599	0.2737	0.2863	0.3021	0.3261	0.3410	0.3521	0.3619	0.3732	0.3806	0.3907	0.4066	0.4202	0.4378	0.4525	0.4675	0.4809	0.4928	0.5030	0.5126	0.5218	0.5308	0.5417	0.5516	0.5630	0.5746	0.5849	0.5976	0.6073	0.6169	0.6265	0.6349	0.6438	0.6433	0.6401	0.6338
Hong Kong Special Administrative Region of China	0.6812	0.6920	0.7014	0.7095	0.7179	0.7255	0.7341	0.7432	0.7521	0.7599	0.7669	0.7746	0.7823	0.7901	0.7980	0.8055	0.8115	0.8161	0.8194	0.8224	0.8257	0.8294	0.8329	0.8364	0.8404	0.8449	0.8495	0.8545	0.8589	0.8622	0.8654	0.8680	0.8702	0.8723	0.8743	0.8762
Hubei	0.3301	0.3433	0.3583	0.3660	0.3777	0.3887	0.3979	0.4074	0.4212	0.4283	0.4388	0.4451	0.4578	0.4668	0.4832	0.4988	0.5107	0.5228	0.5367	0.5471	0.5583	0.5668	0.5752	0.5840	0.5929	0.6019	0.6099	0.6191	0.6275	0.6346	0.6394	0.6459	0.6519	0.6592	0.6662	0.6730
Hunan	0.3095	0.3186	0.3209	0.3331	0.3484	0.3595	0.3692	0.3792	0.3883	0.4020	0.4120	0.4233	0.4406	0.4574	0.4695	0.4825	0.4944	0.5045	0.5142	0.5235	0.5308	0.5375	0.5456	0.5525	0.5611	0.5718	0.5806	0.5903	0.5970	0.6040	0.6108	0.6169	0.6251	0.6340	0.6426	0.6510
Inner Mongolia	0.3320	0.3421	0.3502	0.3683	0.3758	0.3954	0.4090	0.4226	0.4305	0.4430	0.4500	0.4648	0.4738	0.4834	0.4960	0.5122	0.5276	0.5410	0.5537	0.5660	0.5779	0.5894	0.6015	0.6104	0.6177	0.6237	0.6323	0.6407	0.6506	0.6598	0.6685	0.6772	0.6852	0.6945	0.7033	0.7115
Jiangsu	0.3724	0.3806	0.3916	0.4044	0.4194	0.4297	0.4429	0.4563	0.4640	0.4652	0.4762	0.4889	0.5012	0.5168	0.5296	0.5446	0.5569	0.5683	0.5787	0.5881	0.5974	0.6069	0.6157	0.6252	0.6342	0.6447	0.6545	0.6655	0.6756	0.6844	0.6925	0.7013	0.7117	0.7207	0.7293	0.7375
Jiangxi	0.3010	0.3100	0.3222	0.3338	0.3432	0.3554	0.3685	0.3821	0.3922	0.3962	0.4046	0.4160	0.4287	0.4382	0.4528	0.4652	0.4801	0.4900	0.5007	0.5096	0.5184	0.5265	0.5353	0.5443	0.5539	0.5615	0.5698	0.5790	0.5873	0.5954	0.6036	0.6111	0.6202	0.6295	0.6386	0.6476
Jilin	0.3815	0.3897	0.4007	0.4116	0.4225	0.4323	0.4430	0.4538	0.4615	0.4699	0.4803	0.4902	0.5021	0.5143	0.5288	0.5427	0.5550	0.5658	0.5763	0.5871	0.5964	0.6055	0.6147	0.6237	0.6326	0.6417	0.6513	0.6615	0.6709	0.6802	0.6882	0.6982	0.7068	0.7150	0.7228	0.7300
Liaoning	0.4291	0.4406	0.4443	0.4539	0.4686	0.4730	0.4822	0.4919	0.5010	0.5128	0.5194	0.5332	0.5398	0.5498	0.5604	0.5703	0.5804	0.5896	0.5987	0.6083	0.6172	0.6255	0.6340	0.6423	0.6507	0.6591	0.6680	0.6772	0.6860	0.6945	0.7030	0.7113	0.7194	0.7272	0.7346	0.7415
Macao Special Administrative Region of China	0.6366	0.6438	0.6507	0.6569	0.6640	0.6715	0.6798	0.6902	0.7014	0.7122	0.7228	0.7317	0.7412	0.7505	0.7593	0.7678	0.7755	0.7827	0.7888	0.7943	0.7986	0.8027	0.8072	0.8129	0.8211	0.8294	0.8386	0.8483	0.8569	0.8635	0.8709	0.8726	0.8742	0.8757	0.8771	0.8785
Ningxia	0.3169	0.3372	0.3447	0.3577	0.3627	0.3754	0.3860	0.3976	0.4056	0.4217	0.4325	0.4415	0.4545	0.4671	0.4784	0.4900	0.5019	0.5111	0.5214	0.5296	0.5377	0.5449	0.5519	0.5610	0.5689	0.5772	0.5865	0.5979	0.6075	0.6149	0.6229	0.6310	0.6407	0.6482	0.6556	0.6630
Qinghai	0.3005	0.3151	0.3245	0.3308	0.3373	0.3501	0.3575	0.3657	0.3808	0.3903	0.3924	0.3970	0.4055	0.4188	0.4253	0.4341	0.4436	0.4505	0.4583	0.4649	0.4711	0.4764	0.4834	0.4914	0.5000	0.5083	0.5162	0.5246	0.5326	0.5388	0.5431	0.5495	0.5567	0.5594	0.5619	0.5644
Shaanxi	0.3076	0.3161	0.3242	0.3336	0.3413	0.3534	0.3679	0.3827	0.3923	0.4008	0.4093	0.4216	0.4327	0.4473	0.4601	0.4755	0.4897	0.5023	0.5129	0.5240	0.5343	0.5450	0.5541	0.5625	0.5724	0.5834	0.5927	0.6034	0.6131	0.6227	0.6339	0.6423	0.6508	0.6560	0.6604	0.6641
Shandong	0.3279	0.3385	0.3532	0.3664	0.3777	0.3940	0.4014	0.4073	0.4303	0.4439	0.4522	0.4640	0.4828	0.4969	0.5113	0.5235	0.5346	0.5440	0.5533	0.5632	0.5711	0.5789	0.5874	0.5958	0.6027	0.6136	0.6254	0.6382	0.6479	0.6559	0.6651	0.6740	0.6828	0.6926	0.7020	0.7110
Shanghai	0.5528	0.5550	0.5590	0.5615	0.5661	0.5713	0.5809	0.5913	0.6010	0.6109	0.6271	0.6383	0.6463	0.6547	0.6631	0.6717	0.6800	0.6879	0.6952	0.7023	0.7095	0.7178	0.7263	0.7355	0.7449	0.7546	0.7645	0.7744	0.7839	0.7931	0.8023	0.8117	0.8209	0.8299	0.8387	0.8472
Shanxi	0.3492	0.3544	0.3617	0.3735	0.3846	0.3945	0.4062	0.4185	0.4257	0.4374	0.4429	0.4487	0.4620	0.4783	0.4905	0.5036	0.5154	0.5259	0.5356	0.5441	0.5547	0.5652	0.5737	0.5834	0.5922	0.6019	0.6124	0.6230	0.6321	0.6423	0.6503	0.6589	0.6676	0.6765	0.6851	0.6935
Sichuan	0.2353	0.2335	0.2437	0.2598	0.2784	0.2689	0.2859	0.3047	0.3123	0.3280	0.3413	0.3550	0.3577	0.3641	0.3741	0.3819	0.3948	0.4293	0.4408	0.4492	0.4589	0.4677	0.4757	0.4884	0.4970	0.5042	0.5158	0.5247	0.5301	0.5395	0.5488	0.5467	0.5539	0.5565	0.5587	0.5611
Tianjin	0.4934	0.4956	0.4993	0.5020	0.5099	0.5204	0.5346	0.5491	0.5582	0.5692	0.5793	0.5931	0.6000	0.6153	0.6257	0.6381	0.6491	0.6584	0.6672	0.6758	0.6843	0.6940	0.7039	0.7139	0.7240	0.7342	0.7448	0.7558	0.7662	0.7764	0.7865	0.7958	0.8062	0.8159	0.8250	0.8337
Tibet	0.2045	0.2114	0.2176	0.2074	0.2150	0.2190	0.2354	0.2509	0.2546	0.2631	0.2732	0.2768	0.2830	0.2855	0.2955	0.3038	0.3118	0.3184	0.3242	0.3296	0.3374	0.3443	0.3484	0.3561	0.3624	0.3675	0.3742	0.3824	0.3897	0.3952	0.3984	0.4034	0.4084	0.4142	0.4199	0.4253
Xinjiang	0.3267	0.3382	0.3462	0.3477	0.3527	0.3596	0.3721	0.3856	0.4132	0.4071	0.4195	0.4282	0.4417	0.4573	0.4723	0.4883	0.4999	0.5103	0.5209	0.5324	0.5422	0.5508	0.5597	0.5672	0.5748	0.5802	0.5890	0.5946	0.6047	0.6127	0.6204	0.6304	0.6384	0.6460	0.6534	0.6605
Yunnan	0.2386	0.2436	0.2558	0.2544	0.2714	0.2919	0.3050	0.3186	0.3271	0.3417	0.3530	0.3612	0.3719	0.3815	0.3944	0.4080	0.4197	0.4294	0.4401	0.4491	0.4569	0.4632	0.4709	0.4796	0.4911	0.5011	0.5131	0.5226	0.5320	0.5399	0.5456	0.5532	0.5616	0.5690	0.5763	0.5835
Zhejiang	0.3510	0.3602	0.3709	0.3797	0.3988	0.4124	0.4268	0.4412	0.4544	0.4667	0.4789	0.4874	0.4971	0.5122	0.5265	0.5407	0.5548	0.5669	0.5777	0.5876	0.5969	0.6064	0.6161	0.6267	0.6344	0.6428	0.6558	0.6671	0.6783	0.6883	0.6966	0.7087	0.7173	0.7274	0.7372	0.7466
North Korea	0.5315	0.5362	0.5411	0.5463	0.5521	0.5581	0.5636	0.5689	0.5734	0.5776	0.5817	0.5862	0.5892	0.5923	0.5913	0.5880	0.5828	0.5767	0.5704	0.5641	0.5579	0.5524	0.5479	0.5449	0.5442	0.5449	0.5466	0.5486	0.5507	0.5521	0.5538	0.5558	0.5580	0.5603	0.5627	0.5652
Taiwan	0.6114	0.6199	0.6299	0.6413	0.6516	0.6633	0.6772	0.6848	0.6884	0.7015	0.7059	0.7168	0.7246	0.7314	0.7391	0.7456	0.7526	0.7590	0.7749	0.7782	0.7799	0.7939	0.8011	0.8094	0.8159	0.8228	0.8275	0.8328	0.8384	0.8430	0.8485	0.8512	0.8496	0.8590	0.8613	0.8648
Southeast Asia	0.3610	0.3711	0.3807	0.3902	0.3995	0.4084	0.4169	0.4252	0.4331	0.4413	0.4496	0.4580	0.4667	0.4756	0.4849	0.4945	0.5042	0.5133	0.5209	0.5279	0.5349	0.5417	0.5484	0.5552	0.5623	0.5696	0.5773	0.5851	0.5928	0.6000	0.6072	0.6146	0.6220	0.6294	0.6365	0.6436
Cambodia	0.2063	0.2041	0.2026	0.2025	0.2042	0.2076	0.2105	0.2169	0.2253	0.2337	0.2412	0.2502	0.2591	0.2669	0.2744	0.2824	0.2899	0.2978	0.3060	0.3159	0.3265	0.3372	0.3481	0.3590	0.3703	0.3822	0.3946	0.4069	0.4187	0.4287	0.4383	0.4482	0.4582	0.4682	0.4775	0.4861
Indonesia	0.3612	0.3726	0.3834	0.3940	0.4049	0.4154	0.4257	0.4359	0.4459	0.4562	0.4668	0.4777	0.4886	0.4993	0.5101	0.5208	0.5313	0.5409	0.5480	0.5540	0.5594	0.5644	0.5692	0.5737	0.5786	0.5839	0.5900	0.5965	0.6033	0.6099	0.6167	0.6237	0.6309	0.6381	0.6452	0.6523
Laos	0.2058	0.2109	0.2163	0.2217	0.2270	0.2328	0.2385	0.2435	0.2482	0.2539	0.2602	0.2668	0.2745	0.2835	0.2936	0.3044	0.3151	0.3263	0.3377	0.3490	0.3598	0.3702	0.3801	0.3896	0.3989	0.4081	0.4175	0.4273	0.4370	0.4471	0.4572	0.4674	0.4776	0.4880	0.4980	0.5077
Malaysia	0.4959	0.5045	0.5125	0.5196	0.5269	0.5331	0.5391	0.5451	0.5520	0.5590	0.5660	0.5738	0.5815	0.5895	0.5979	0.6071	0.6172	0.6278	0.6371	0.6468	0.6570	0.6664	0.6757	0.6849	0.6940	0.7026	0.7103	0.7179	0.7255	0.7319	0.7383	0.7445	0.7504	0.7560	0.7615	0.7669
Maldives	0.2582	0.2627	0.2678	0.2724	0.2797	0.2899	0.3003	0.3133	0.3273	0.3421	0.3571	0.3728	0.3885	0.4044	0.4198	0.4346	0.4490	0.4631	0.4768	0.4896	0.5016	0.5134	0.5243	0.5357	0.5457	0.5534	0.5621	0.5709	0.5796	0.5862	0.5929	0.5997	0.6060	0.6116	0.6174	0.6229
Mauritius	0.4827	0.4912	0.4994	0.5070	0.5139	0.5205	0.5275	0.5352	0.5428	0.5505	0.5588	0.5676	0.5765	0.5852	0.5938	0.6025	0.6106	0.6183	0.6260	0.6330	0.6400	0.6467	0.6529	0.6590	0.6652	0.6711	0.6772	0.6839	0.6907	0.6974	0.7038	0.7106	0.7172	0.7234	0.7295	0.7353
Myanmar	0.1835	0.1927	0.2022	0.2116	0.2211	0.2301	0.2376	0.2431	0.2451	0.2471	0.2490	0.2500	0.2531	0.2576	0.2639	0.2717	0.2807	0.2903	0.2999	0.3110	0.3240	0.3376	0.3520	0.3672	0.3830	0.3991	0.4152	0.4309	0.4448	0.4574	0.4690	0.4801	0.4905	0.5006	0.5104	0.5200
Philippines	0.4486	0.4568	0.4647	0.4720	0.4776	0.4817	0.4858	0.4901	0.4952	0.5006	0.5062	0.5114	0.5163	0.5210	0.5261	0.5314	0.5370	0.5421	0.5461	0.5500	0.5543	0.5587	0.5635	0.5686	0.5743	0.5802	0.5864	0.5929	0.5992	0.6052	0.6115	0.6178	0.6244	0.6315	0.6385	0.6454
Sri Lanka	0.4658	0.4711	0.4769	0.4831	0.4902	0.4982	0.5066	0.5146	0.5220	0.5288	0.5355	0.5421	0.5481	0.5545	0.5610	0.5675	0.5752	0.5827	0.5901	0.5966	0.6029	0.6083	0.6133	0.6184	0.6235	0.6290	0.6351	0.6418	0.6489	0.6562	0.6641	0.6723	0.6810	0.6893	0.6975	0.7054
Seychelles	0.5098	0.5209	0.5313	0.5417	0.5518	0.5622	0.5703	0.5781	0.5859	0.5944	0.6037	0.6149	0.6263	0.6375	0.6471	0.6553	0.6625	0.6700	0.6769	0.6829	0.6883	0.6949	0.7005	0.7048	0.7082	0.7120	0.7148	0.7186	0.7218	0.7251	0.7291	0.7343	0.7402	0.7465	0.7526	0.7585
Thailand	0.4398	0.4509	0.4616	0.4717	0.4816	0.4910	0.5000	0.5090	0.5184	0.5281	0.5378	0.5475	0.5571	0.5668	0.5764	0.5861	0.5956	0.6032	0.6086	0.6136	0.6183	0.6235	0.6289	0.6347	0.6407	0.6468	0.6530	0.6594	0.6652	0.6704	0.6760	0.6817	0.6878	0.6938	0.6994	0.7050
Timor-Leste	0.2015	0.2077	0.2135	0.2196	0.2264	0.2337	0.2398	0.2463	0.2529	0.2594	0.2652	0.2709	0.2764	0.2811	0.2852	0.2888	0.2851	0.2780	0.2674	0.2519	0.2402	0.2521	0.2656	0.2783	0.3014	0.3270	0.3551	0.3739	0.3893	0.4005	0.4099	0.4209	0.4307	0.4380	0.4436	0.4499
Vietnam	0.2937	0.3056	0.3153	0.3261	0.3368	0.3466	0.3558	0.3642	0.3725	0.3810	0.3898	0.3992	0.4097	0.4209	0.4328	0.4455	0.4586	0.4716	0.4836	0.4946	0.5047	0.5142	0.5232	0.5317	0.5402	0.5490	0.5575	0.5660	0.5744	0.5824	0.5905	0.5986	0.6064	0.6139	0.6212	0.6283
Oceania	0.3305	0.3348	0.3389	0.3440	0.3490	0.3538	0.3589	0.3637	0.3688	0.3733	0.3771	0.3816	0.3865	0.3928	0.3991	0.4044	0.4097	0.4138	0.4176	0.4213	0.4244	0.4270	0.4290	0.4312	0.4335	0.4362	0.4394	0.4434	0.4481	0.4533	0.4591	0.4657	0.4726	0.4795	0.4867	0.4944
American Samoa	0.6156	0.6188	0.6306	0.6395	0.6266	0.6177	0.6314	0.6285	0.6431	0.6426	0.6427	0.6528	0.6432	0.6435	0.6605	0.6753	0.6894	0.7027	0.7026	0.7031	0.7089	0.7214	0.7116	0.7122	0.7128	0.7128	0.7124	0.7115	0.7117	0.7131	0.7131	0.7130	0.7135	0.7137	0.7137	0.7136
Federated States of Micronesia	0.4218	0.4280	0.4338	0.4401	0.4474	0.4555	0.4623	0.4692	0.4760	0.4826	0.4889	0.4956	0.5017	0.5079	0.5131	0.5189	0.5254	0.5304	0.5356	0.5412	0.5480	0.5534	0.5597	0.5664	0.5725	0.5789	0.5852	0.5906	0.5952	0.5994	0.6039	0.6093	0.6143	0.6180	0.6212	0.6242
Fiji	0.5149	0.5218	0.5286	0.5344	0.5405	0.5456	0.5497	0.5525	0.5559	0.5603	0.5657	0.5706	0.5763	0.5815	0.5872	0.5932	0.5989	0.6043	0.6096	0.6153	0.6207	0.6265	0.6318	0.6367	0.6417	0.6464	0.6520	0.6573	0.6625	0.6672	0.6720	0.6756	0.6792	0.6835	0.6883	0.6934
Guam	0.7046	0.7126	0.7197	0.7259	0.7315	0.7368	0.7421	0.7477	0.7539	0.7607	0.7681	0.7749	0.7815	0.7876	0.7933	0.7985	0.8030	0.8076	0.8124	0.8175	0.8228	0.8276	0.8323	0.8367	0.8415	0.8464	0.8510	0.8552	0.8593	0.8634	0.8672	0.8709	0.8746	0.8780	0.8812	0.8841
Kiribati	0.4170	0.4140	0.4110	0.4071	0.4031	0.3991	0.3978	0.3962	0.3970	0.3980	0.4002	0.4010	0.4018	0.4032	0.4055	0.4080	0.4117	0.4161	0.4218	0.4268	0.4327	0.4374	0.4418	0.4466	0.4504	0.4539	0.4573	0.4601	0.4623	0.4642	0.4660	0.4668	0.4687	0.4713	0.4744	0.4778
Marshall Islands	0.3554	0.3625	0.3693	0.3770	0.3841	0.3896	0.3974	0.4059	0.4057	0.4211	0.4312	0.4697	0.4836	0.4860	0.4808	0.4800	0.4828	0.4768	0.4768	0.4830	0.4958	0.5011	0.5057	0.5107	0.5159	0.5214	0.5273	0.5344	0.5416	0.5480	0.5548	0.5616	0.5695	0.5774	0.5847	0.5916
Northern Mariana Islands	0.6463	0.6647	0.6810	0.6957	0.7090	0.7217	0.7332	0.7446	0.7552	0.7652	0.7748	0.7792	0.7835	0.7879	0.7926	0.7974	0.8056	0.8133	0.8202	0.8269	0.8334	0.8385	0.8434	0.8474	0.8506	0.8519	0.8523	0.8521	0.8516	0.8495	0.8473	0.8442	0.8417	0.8401	0.8397	0.8406
Papua New Guinea	0.2716	0.2752	0.2782	0.2829	0.2875	0.2925	0.2976	0.3032	0.3090	0.3138	0.3173	0.3214	0.3264	0.3333	0.3402	0.3455	0.3511	0.3552	0.3593	0.3632	0.3664	0.3689	0.3712	0.3738	0.3764	0.3797	0.3832	0.3877	0.3930	0.3991	0.4059	0.4138	0.4221	0.4303	0.4388	0.4481
Samoa	0.4295	0.4383	0.4462	0.4532	0.4596	0.4659	0.4742	0.4817	0.4883	0.4946	0.4998	0.5065	0.5121	0.5193	0.5243	0.5288	0.5354	0.5414	0.5457	0.5496	0.5541	0.5613	0.5680	0.5742	0.5796	0.5848	0.5905	0.5964	0.6025	0.6078	0.6131	0.6182	0.6234	0.6280	0.6324	0.6367
Solomon Islands	0.2539	0.2615	0.2679	0.2735	0.2786	0.2832	0.2874	0.2920	0.2970	0.3029	0.3098	0.3173	0.3269	0.3368	0.3475	0.3587	0.3684	0.3767	0.3839	0.3900	0.3929	0.3938	0.3933	0.3931	0.3935	0.3954	0.3990	0.4042	0.4109	0.4165	0.4235	0.4318	0.4401	0.4478	0.4548	0.4614
Tonga	0.3910	0.3983	0.4080	0.4183	0.4332	0.4481	0.4635	0.4777	0.4890	0.4981	0.5056	0.5127	0.5176	0.5224	0.5269	0.5315	0.5367	0.5413	0.5455	0.5492	0.5522	0.5592	0.5655	0.5709	0.5757	0.5801	0.5847	0.5890	0.5936	0.5985	0.6038	0.6078	0.6116	0.6149	0.6185	0.6224
Vanuatu	0.3452	0.3506	0.3559	0.3615	0.3676	0.3731	0.3786	0.3834	0.3872	0.3909	0.3957	0.4012	0.4065	0.4117	0.4175	0.4230	0.4281	0.4336	0.4394	0.4452	0.4518	0.4579	0.4632	0.4688	0.4743	0.4799	0.4865	0.4929	0.4997	0.5062	0.5123	0.5176	0.5228	0.5277	0.5323	0.5360
Central Europe, Eastern Europe, and Central Asia	0.6545	0.6590	0.6633	0.6675	0.6716	0.6758	0.6807	0.6860	0.6919	0.6981	0.7040	0.7095	0.7137	0.7165	0.7186	0.7204	0.7218	0.7231	0.7245	0.7262	0.7292	0.7329	0.7374	0.7430	0.7493	0.7559	0.7630	0.7707	0.7784	0.7839	0.7893	0.7950	0.8005	0.8058	0.8109	0.8158
Central Asia	0.5516	0.5571	0.5622	0.5670	0.5716	0.5762	0.5813	0.5866	0.5925	0.5985	0.6043	0.6094	0.6122	0.6140	0.6145	0.6144	0.6145	0.6147	0.6149	0.6159	0.6181	0.6218	0.6269	0.6331	0.6401	0.6477	0.6560	0.6647	0.6733	0.6807	0.6883	0.6959	0.7034	0.7106	0.7176	0.7240
Armenia	0.5513	0.5561	0.5604	0.5643	0.5678	0.5712	0.5758	0.5806	0.5857	0.5910	0.5955	0.5991	0.5969	0.5933	0.5909	0.5898	0.5911	0.5929	0.5961	0.6000	0.6052	0.6126	0.6227	0.6344	0.6464	0.6595	0.6731	0.6873	0.7002	0.7084	0.7163	0.7246	0.7333	0.7415	0.7488	0.7552
Azerbaijan	0.5867	0.5946	0.6021	0.6092	0.6155	0.6208	0.6264	0.6314	0.6361	0.6408	0.6446	0.6505	0.6533	0.6541	0.6530	0.6502	0.6473	0.6443	0.6415	0.6398	0.6403	0.6432	0.6485	0.6556	0.6637	0.6757	0.6918	0.7092	0.7250	0.7377	0.7493	0.7589	0.7673	0.7751	0.7820	0.7883
Georgia	0.6430	0.6483	0.6533	0.6582	0.6627	0.6671	0.6724	0.6778	0.6834	0.6890	0.6938	0.6959	0.6920	0.6850	0.6766	0.6679	0.6599	0.6534	0.6481	0.6451	0.6451	0.6489	0.6558	0.6646	0.6733	0.6824	0.6918	0.7022	0.7117	0.7193	0.7267	0.7344	0.7419	0.7488	0.7552	0.7611
Kazakhstan	0.6312	0.6361	0.6405	0.6446	0.6486	0.6527	0.6576	0.6630	0.6691	0.6755	0.6818	0.6875	0.6936	0.6980	0.7012	0.7035	0.7062	0.7087	0.7106	0.7125	0.7156	0.7202	0.7253	0.7310	0.7369	0.7431	0.7494	0.7559	0.7623	0.7676	0.7738	0.7805	0.7873	0.7941	0.8006	0.8067
Kyrgyzstan	0.5515	0.5549	0.5581	0.5615	0.5651	0.5691	0.5733	0.5778	0.5827	0.5876	0.5925	0.5972	0.5997	0.6003	0.5987	0.5961	0.5938	0.5918	0.5889	0.5865	0.5853	0.5854	0.5861	0.5888	0.5927	0.5955	0.5974	0.6004	0.6045	0.6081	0.6108	0.6143	0.6174	0.6217	0.6261	0.6307
Mongolia	0.3804	0.3917	0.4030	0.4145	0.4265	0.4390	0.4524	0.4668	0.4824	0.4977	0.5133	0.5257	0.5361	0.5442	0.5518	0.5598	0.5672	0.5744	0.5810	0.5878	0.5944	0.6011	0.6077	0.6144	0.6216	0.6289	0.6365	0.6441	0.6511	0.6563	0.6620	0.6693	0.6777	0.6868	0.6960	0.7047
Tajikistan	0.4511	0.4567	0.4614	0.4655	0.4696	0.4739	0.4792	0.4853	0.4918	0.4987	0.5054	0.5106	0.5111	0.5104	0.5071	0.5022	0.4952	0.4878	0.4799	0.4718	0.4662	0.4647	0.4689	0.4768	0.4872	0.4973	0.5074	0.5170	0.5255	0.5331	0.5407	0.5473	0.5538	0.5606	0.5674	0.5741
Turkmenistan	0.5304	0.5378	0.5446	0.5511	0.5573	0.5632	0.5688	0.5744	0.5807	0.5875	0.5947	0.6019	0.6082	0.6161	0.6213	0.6255	0.6292	0.6311	0.6331	0.6364	0.6421	0.6481	0.6547	0.6618	0.6701	0.6808	0.6919	0.7031	0.7139	0.7240	0.7340	0.7446	0.7547	0.7641	0.7727	0.7805
Uzbekistan	0.4818	0.4885	0.4947	0.5007	0.5064	0.5118	0.5178	0.5238	0.5306	0.5376	0.5446	0.5508	0.5551	0.5599	0.5634	0.5669	0.5708	0.5752	0.5797	0.5849	0.5902	0.5956	0.6012	0.6071	0.6134	0.6201	0.6273	0.6346	0.6426	0.6506	0.6589	0.6674	0.6758	0.6840	0.6918	0.6992
Central Europe	0.6428	0.6483	0.6534	0.6584	0.6634	0.6682	0.6733	0.6785	0.6840	0.6896	0.6946	0.6986	0.7021	0.7057	0.7099	0.7148	0.7209	0.7272	0.7337	0.7400	0.7464	0.7529	0.7592	0.7652	0.7710	0.7767	0.7828	0.7892	0.7958	0.8013	0.8067	0.8123	0.8175	0.8226	0.8276	0.8327
Albania	0.5091	0.5167	0.5238	0.5303	0.5363	0.5419	0.5481	0.5541	0.5598	0.5663	0.5719	0.5713	0.5693	0.5688	0.5707	0.5750	0.5830	0.5893	0.5969	0.6068	0.6176	0.6288	0.6395	0.6500	0.6604	0.6703	0.6798	0.6885	0.6965	0.7030	0.7083	0.7146	0.7205	0.7259	0.7312	0.7364
Bosnia and Herzegovina	0.4259	0.4345	0.4424	0.4494	0.4562	0.4629	0.4697	0.4760	0.4814	0.4866	0.4891	0.4903	0.4909	0.4918	0.4953	0.5041	0.5280	0.5555	0.5817	0.6043	0.6226	0.6382	0.6516	0.6628	0.6724	0.6805	0.6881	0.6954	0.7026	0.7086	0.7144	0.7199	0.7248	0.7297	0.7343	0.7387
Bulgaria	0.6353	0.6425	0.6499	0.6570	0.6639	0.6699	0.6759	0.6826	0.6900	0.6968	0.7019	0.7067	0.7101	0.7129	0.7158	0.7186	0.7209	0.7229	0.7254	0.7275	0.7304	0.7340	0.7384	0.7430	0.7479	0.7530	0.7589	0.7657	0.7727	0.7785	0.7840	0.7894	0.7944	0.7992	0.8037	0.8079

Location	1980	1981	1982	1983	1984	1985	1986	1987	1988	1989	1990	1991	1992	1993	1994	1995	1996	1997	1998	1999	2000	2001	2002	2003	2004	2005	2006	2007	2008	2009	2010	2011	2012	2013	2014	2015
Croatia	0.6084	0.6157	0.6225	0.6287	0.6349	0.6410	0.6473	0.6535	0.6596	0.6654	0.6702	0.6725	0.6735	0.6734	0.6736	0.6745	0.6775	0.6817	0.6870	0.6926	0.6995	0.7066	0.7140	0.7214	0.7283	0.7347	0.7411	0.7477	0.7541	0.7591	0.7637	0.7684	0.7726	0.7766	0.7805	0.7842
Czech Republic	0.7264	0.7341	0.7412	0.7481	0.7547	0.7606	0.7656	0.7701	0.7749	0.7801	0.7857	0.7901	0.7949	0.8000	0.8057	0.8121	0.8187	0.8245	0.8297	0.8343	0.8387	0.8429	0.8465	0.8497	0.8529	0.8563	0.8602	0.8648	0.8690	0.8724	0.8758	0.8792	0.8823	0.8854	0.8885	0.8918
Hungary	0.6771	0.6845	0.6915	0.6979	0.7033	0.7076	0.7113	0.7153	0.7193	0.7234	0.7274	0.7304	0.7335	0.7369	0.7411	0.7458	0.7509	0.7562	0.7616	0.7670	0.7729	0.7792	0.7855	0.7914	0.7973	0.8031	0.8091	0.8146	0.8198	0.8238	0.8278	0.8319	0.8357	0.8399	0.8443	0.8491
Macedonia	0.5956	0.6023	0.6085	0.6139	0.6193	0.6244	0.6299	0.6349	0.6391	0.6435	0.6465	0.6495	0.6519	0.6537	0.6558	0.6582	0.6610	0.6645	0.6686	0.6735	0.6792	0.6843	0.6896	0.6950	0.7009	0.7073	0.7141	0.7212	0.7280	0.7338	0.7393	0.7446	0.7491	0.7535	0.7578	0.7622
Montenegro	0.6382	0.6443	0.6500	0.6555	0.6609	0.6660	0.6704	0.6747	0.6788	0.6829	0.6870	0.6907	0.6921	0.6903	0.6885	0.6877	0.6896	0.6927	0.6967	0.7000	0.7056	0.7119	0.7178	0.7239	0.7300	0.7361	0.7431	0.7513	0.7597	0.7664	0.7727	0.7790	0.7844	0.7896	0.7944	0.7989
Poland	0.6632	0.6656	0.6676	0.6705	0.6740	0.6782	0.6831	0.6884	0.6941	0.7005	0.7057	0.7103	0.7153	0.7211	0.7277	0.7353	0.7437	0.7527	0.7618	0.7708	0.7795	0.7871	0.7936	0.7994	0.8047	0.8096	0.8144	0.8198	0.8253	0.8308	0.8368	0.8431	0.8492	0.8553	0.8614	0.8675
Romania	0.6376	0.6439	0.6497	0.6552	0.6604	0.6654	0.6710	0.6767	0.6825	0.6879	0.6931	0.6964	0.6984	0.7001	0.7018	0.7037	0.7065	0.7086	0.7104	0.7128	0.7158	0.7199	0.7247	0.7298	0.7349	0.7407	0.7476	0.7550	0.7631	0.7691	0.7744	0.7800	0.7851	0.7901	0.7947	0.7991
Serbia	0.6039	0.6091	0.6140	0.6187	0.6233	0.6279	0.6326	0.6375	0.6426	0.6478	0.6531	0.6579	0.6601	0.6596	0.6589	0.6585	0.6602	0.6632	0.6670	0.6697	0.6741	0.6807	0.6885	0.6966	0.7051	0.7136	0.7218	0.7300	0.7383	0.7448	0.7503	0.7558	0.7605	0.7649	0.7686	0.7718
Slovakia	0.6493	0.6564	0.6632	0.6697	0.6762	0.6823	0.6881	0.6938	0.6997	0.7060	0.7126	0.7173	0.7218	0.7267	0.7323	0.7387	0.7454	0.7526	0.7598	0.7665	0.7729	0.7793	0.7855	0.7915	0.7974	0.8032	0.8096	0.8168	0.8241	0.8297	0.8355	0.8411	0.8465	0.8516	0.8566	0.8615
Slovenia	0.6788	0.6874	0.6957	0.7037	0.7115	0.7191	0.7266	0.7338	0.7406	0.7472	0.7529	0.7570	0.7604	0.7641	0.7682	0.7725	0.7774	0.7827	0.7882	0.7941	0.8004	0.8064	0.8123	0.8176	0.8227	0.8273	0.8320	0.8367	0.8412	0.8440	0.8465	0.8487	0.8505	0.8521	0.8540	0.8561
Eastern Europe	0.6892	0.6933	0.6972	0.7012	0.7051	0.7092	0.7143	0.7201	0.7264	0.7334	0.7403	0.7469	0.7521	0.7552	0.7570	0.7580	0.7575	0.7568	0.7560	0.7559	0.7575	0.7601	0.7639	0.7696	0.7764	0.7836	0.7916	0.8001	0.8086	0.8140	0.8192	0.8246	0.8297	0.8346	0.8391	0.8431
Belarus	0.6315	0.6366	0.6415	0.6462	0.6509	0.6559	0.6616	0.6674	0.6738	0.6806	0.6868	0.6932	0.6985	0.7026	0.7043	0.7051	0.7065	0.7088	0.7122	0.7163	0.7214	0.7274	0.7341	0.7418	0.7506	0.7600	0.7700	0.7802	0.7910	0.8002	0.8094	0.8185	0.8267	0.8340	0.8408	0.8468
Estonia	0.6590	0.6635	0.6677	0.6717	0.6757	0.6799	0.6849	0.6906	0.6973	0.7048	0.7123	0.7195	0.7240	0.7274	0.7307	0.7348	0.7388	0.7444	0.7506	0.7565	0.7633	0.7702	0.7771	0.7840	0.7907	0.7978	0.8056	0.8140	0.8214	0.8266	0.8320	0.8381	0.8444	0.8504	0.8558	0.8608
Latvia	0.6867	0.6904	0.6938	0.6972	0.7004	0.7040	0.7086	0.7142	0.7206	0.7277	0.7348	0.7401	0.7414	0.7422	0.7434	0.7447	0.7459	0.7481	0.7515	0.7555	0.7603	0.7662	0.7731	0.7803	0.7878	0.7959	0.8050	0.8149	0.8235	0.8292	0.8343	0.8400	0.8458	0.8514	0.8566	0.8614
Lithuania	0.6683	0.6734	0.6783	0.6832	0.6878	0.6922	0.6970	0.7019	0.7071	0.7125	0.7175	0.7220	0.7243	0.7251	0.7253	0.7260	0.7274	0.7301	0.7344	0.7392	0.7449	0.7518	0.7597	0.7682	0.7764	0.7843	0.7922	0.8008	0.8088	0.8137	0.8173	0.8215	0.8256	0.8296	0.8334	0.8369
Moldova	0.5747	0.5781	0.5813	0.5844	0.5875	0.5907	0.5949	0.5995	0.6046	0.6101	0.6156	0.6203	0.6210	0.6214	0.6190	0.6155	0.6112	0.6071	0.6030	0.5990	0.5970	0.5983	0.6032	0.6100	0.6185	0.6278	0.6367	0.6450	0.6539	0.6605	0.6678	0.6759	0.6829	0.6905	0.6974	0.7033
Russia	0.7150	0.7185	0.7217	0.7250	0.7281	0.7315	0.7360	0.7414	0.7475	0.7544	0.7613	0.7683	0.7736	0.7769	0.7792	0.7805	0.7799	0.7792	0.7780	0.7777	0.7793	0.7816	0.7849	0.7899	0.7957	0.8020	0.8091	0.8168	0.8246	0.8296	0.8343	0.8391	0.8438	0.8482	0.8524	0.8564
Ukraine	0.6403	0.6461	0.6518	0.6575	0.6632	0.6691	0.6757	0.6823	0.6894	0.6968	0.7038	0.7099	0.7150	0.7185	0.7195	0.7192	0.7181	0.7166	0.7152	0.7142	0.7143	0.7166	0.7205	0.7270	0.7361	0.7453	0.7551	0.7656	0.7756	0.7814	0.7871	0.7933	0.7987	0.8041	0.8080	0.8111
High-income	0.7386	0.7442	0.7490	0.7537	0.7585	0.7634	0.7684	0.7733	0.7784	0.7835	0.7887	0.7938	0.7988	0.8034	0.8082	0.8128	0.8177	0.8226	0.8272	0.8317	0.8361	0.8402	0.8440	0.8474	0.8509	0.8543	0.8579	0.8618	0.8655	0.8686	0.8720	0.8755	0.8789	0.8822	0.8853	0.8884
High-income Asia Pacific	0.7119	0.7188	0.7251	0.7312	0.7376	0.7442	0.7510	0.7579	0.7651	0.7727	0.7802	0.7877	0.7946	0.8012	0.8075	0.8138	0.8202	0.8264	0.8317	0.8366	0.8413	0.8460	0.8503	0.8543	0.8581	0.8613	0.8645	0.8680	0.8709	0.8732	0.8759	0.8784	0.8810	0.8836	0.8862	0.8887
Brunei	0.7178	0.7254	0.7320	0.7384	0.7433	0.7469	0.7542	0.7613	0.7687	0.7762	0.7839	0.7900	0.7973	0.8050	0.8137	0.8233	0.8325	0.8424	0.8527	0.8625	0.8714	0.8767	0.8815	0.8858	0.8898	0.8934	0.8963	0.8991	0.9021	0.9053	0.9089	0.9121	0.9150	0.9179	0.9206	0.9232
Japan	0.7495	0.7553	0.7603	0.7648	0.7695	0.7746	0.7798	0.7853	0.7916	0.7984	0.8054	0.8124	0.8190	0.8250	0.8305	0.8357	0.8411	0.8462	0.8505	0.8544	0.8580	0.8615	0.8648	0.8678	0.8706	0.8730	0.8755	0.8784	0.8807	0.8824	0.8845	0.8865	0.8886	0.8910	0.8932	0.8955
Aichi	0.7613	0.7674	0.7736	0.7764	0.7808	0.7857	0.7918	0.7960	0.8034	0.8107	0.8177	0.8252	0.8312	0.8374	0.8431	0.8482	0.8534	0.8583	0.8617	0.8656	0.8692	0.8731	0.8766	0.8794	0.8815	0.8841	0.8859	0.8885	0.8901	0.8917	0.8938	0.8954	0.8978	0.9007	0.9030	0.9053
Akita	0.7333	0.7401	0.7444	0.7493	0.7529	0.7604	0.7637	0.7682	0.7747	0.7791	0.7869	0.7936	0.7982	0.8040	0.8082	0.8138	0.8207	0.8248	0.8305	0.8337	0.8380	0.8410	0.8455	0.8491	0.8515	0.8529	0.8560	0.8598	0.8622	0.8651	0.8691	0.8698	0.8715	0.8756	0.8781	0.8807
Aomori	0.7357	0.7421	0.7474	0.7512	0.7562	0.7618	0.7659	0.7719	0.7788	0.7843	0.7923	0.7967	0.8038	0.8085	0.8138	0.8188	0.8245	0.8296	0.8339	0.8377	0.8421	0.8444	0.8482	0.8523	0.8548	0.8589	0.8618	0.8657	0.8673	0.8706	0.8708	0.8720	0.8745	0.8758	0.8778	0.8799
Chiba	0.7415	0.7448	0.7507	0.7560	0.7607	0.7664	0.7733	0.7787	0.7853	0.7923	0.7997	0.8066	0.8142	0.8197	0.8248	0.8305	0.8356	0.8408	0.8452	0.8487	0.8518	0.8550	0.8574	0.8605	0.8625	0.8649	0.8675	0.8700	0.8718	0.8727	0.8748	0.8769	0.8797	0.8821	0.8842	0.8865
Ehime	0.7371	0.7422	0.7478	0.7526	0.7573	0.7627	0.7665	0.7729	0.7786	0.7845	0.7920	0.7991	0.8047	0.8109	0.8172	0.8210	0.8275	0.8319	0.8369	0.8416	0.8449	0.8482	0.8525	0.8538	0.8578	0.8598	0.8623	0.8646	0.8675	0.8680	0.8701	0.8712	0.8732	0.8770	0.8794	0.8819
Fukui	0.7448	0.7532	0.7567	0.7611	0.7643	0.7699	0.7752	0.7788	0.7864	0.7911	0.7993	0.8071	0.8142	0.8191	0.8240	0.8294	0.8353	0.8411	0.8450	0.8493	0.8535	0.8567	0.8607	0.8645	0.8680	0.8688	0.8709	0.8739	0.8763	0.8772	0.8805	0.8823	0.8836	0.8874	0.8897	0.8920
Fukuoka	0.7455	0.7510	0.7557	0.7605	0.7650	0.7704	0.7752	0.7824	0.7888	0.7944	0.8019	0.8081	0.8155	0.8215	0.8268	0.8321	0.8374	0.8425	0.8472	0.8514	0.8552	0.8585	0.8618	0.8652	0.8675	0.8699	0.8718	0.8736	0.8758	0.8772	0.8787	0.8805	0.8826	0.8845	0.8863	0.8882
Fukushima	0.7312	0.7378	0.7419	0.7463	0.7506	0.7567	0.7604	0.7670	0.7720	0.7796	0.7861	0.7920	0.7987	0.8052	0.8112	0.8158	0.8216	0.8279	0.8322	0.8352	0.8395	0.8429	0.8469	0.8501	0.8537	0.8557	0.8592	0.8628	0.8655	0.8677	0.8702	0.8745	0.8804	0.8790	0.8817	0.8843
Gifu	0.7408	0.7469	0.7527	0.7560	0.7608	0.7652	0.7725	0.7763	0.7829	0.7900	0.7970	0.8048	0.8101	0.8162	0.8225	0.8268	0.8321	0.8381	0.8415	0.8449	0.8486	0.8528	0.8552	0.8578	0.8619	0.8637	0.8660	0.8692	0.8717	0.8725	0.8754	0.8762	0.8784	0.8817	0.8840	0.8864
Gunma	0.7454	0.7512	0.7560	0.7604	0.7655	0.7699	0.7766	0.7806	0.7870	0.7932	0.8011	0.8072	0.8139	0.8204	0.8253	0.8309	0.8363	0.8415	0.8465	0.8501	0.8533	0.8570	0.8602	0.8631	0.8668	0.8685	0.8721	0.8753	0.8767	0.8793	0.8817	0.8838	0.8873	0.8896	0.8921	0.8946
Hiroshima	0.7456	0.7525	0.7563	0.7613	0.7661	0.7710	0.7753	0.7819	0.7887	0.7949	0.8012	0.8094	0.8156	0.8217	0.8280	0.8335	0.8388	0.8437	0.8486	0.8529	0.8565	0.8592	0.8630	0.8649	0.8679	0.8698	0.8722	0.8734	0.8761	0.8762	0.8780	0.8788	0.8813	0.8832	0.8849	0.8867
Hokkaidō	0.7460	0.7521	0.7561	0.7606	0.7656	0.7714	0.7759	0.7817	0.7877	0.7946	0.8005	0.8074	0.8136	0.8203	0.8257	0.8308	0.8364	0.8412	0.8453	0.8497	0.8532	0.8562	0.8588	0.8614	0.8644	0.8672	0.8697	0.8728	0.8753	0.8767	0.8784	0.8801	0.8819	0.8839	0.8859	0.8879
Hyōgo	0.7410	0.7471	0.7518	0.7563	0.7613	0.7665	0.7716	0.7780	0.7844	0.7914	0.7975	0.8051	0.8114	0.8177	0.8225	0.8285	0.8338	0.8384	0.8425	0.8461	0.8499	0.8540	0.8567	0.8598	0.8626	0.8652	0.8672	0.8699	0.8717	0.8734	0.8749	0.8763	0.8786	0.8812	0.8834	0.8857
Ibaraki	0.7456	0.7511	0.7559	0.7611	0.7657	0.7721	0.7770	0.7825	0.7899	0.7962	0.8032	0.8101	0.8164	0.8232	0.8292	0.8348	0.8400	0.8453	0.8496	0.8530	0.8571	0.8603	0.8636	0.8665	0.8692	0.8728	0.8746	0.8777	0.8804	0.8817	0.8840	0.8864	0.8883	0.8912	0.8934	0.8958
Ishikawa	0.7422	0.7495	0.7535	0.7587	0.7639	0.7699	0.7741	0.7812	0.7861	0.7931	0.7998	0.8082	0.8139	0.8212	0.8248	0.8318	0.8364	0.8417	0.8452	0.8495	0.8529	0.8560	0.8604	0.8617	0.8656	0.8679	0.8707	0.8727	0.8752	0.8774	0.8805	0.8816	0.8828	0.8851	0.8873	0.8896
Iwate	0.7280	0.7351	0.7396	0.7436	0.7494	0.7550	0.7595	0.7661	0.7707	0.7768	0.7829	0.7889	0.7955	0.8015	0.8079	0.8127	0.8194	0.8252	0.8282	0.8321	0.8359	0.8389	0.8429	0.8464	0.8491	0.8519	0.8553	0.8587	0.8613	0.8634	0.8657	0.8688	0.8697	0.8714	0.8735	0.8758
Kagawa	0.7423	0.7493	0.7534	0.7578	0.7622	0.7675	0.7717	0.7777	0.7840	0.7912	0.7983	0.8064	0.8122	0.8193	0.8240	0.8284	0.8347	0.8383	0.8424	0.8454	0.8488	0.8531	0.8557	0.8589	0.8615	0.8635	0.8670	0.8686	0.8712	0.8728	0.8741	0.8753	0.8774	0.8797	0.8819	0.8841
Kagoshima	0.7243	0.7293	0.7337	0.7396	0.7448	0.7508	0.7597	0.7581	0.7666	0.7731	0.7807	0.7883	0.7944	0.8001	0.8059	0.8120	0.8174	0.8230	0.8284	0.8328	0.8348	0.8388	0.8423	0.8448	0.8482	0.8495	0.8527	0.8553	0.8566	0.8592	0.8605	0.8607	0.8635	0.8661	0.8679	0.8697
Kanagawa	0.7491	0.7549	0.7605	0.7650	0.7703	0.7748	0.7809	0.7862	0.7917	0.7997	0.8066	0.8133	0.8205	0.8264	0.8319	0.8373	0.8427	0.8475	0.8516	0.8553	0.8590	0.8623	0.8652	0.8677	0.8705	0.8729	0.8750	0.8777	0.8801	0.8814	0.8836	0.8861	0.8882	0.8911	0.8936	0.8961
Kōchi	0.7268	0.7264	0.7327	0.7367	0.7417	0.7467	0.7515	0.7582	0.7642	0.7706	0.7790	0.7840	0.7910	0.7939	0.8016	0.8067	0.8111	0.8183	0.8233	0.8269	0.8298	0.8329	0.8372	0.8410	0.8445	0.8460	0.8485	0.8531	0.8542	0.8577	0.8581	0.8615	0.8623	0.8639	0.8660	0.8684
Kumamoto	0.7277	0.7324	0.7384	0.7428	0.7464	0.7524	0.7568	0.7625	0.7690	0.7751	0.7825	0.7887	0.7953	0.8007	0.8071	0.8112	0.8168	0.8226	0.8272	0.8306	0.8340	0.8377	0.8408	0.8440	0.8463	0.8485	0.8507	0.8531	0.8551	0.8566	0.8587	0.8599	0.8616	0.8634	0.8651	0.8668
Kyōto	0.7522	0.7590	0.7633	0.7671	0.7713	0.7773	0.7810	0.7885	0.7942	0.8008	0.8080	0.8168	0.8224	0.8293	0.8347	0.8401	0.8448	0.8503	0.8539	0.8579	0.8618	0.8658	0.8695	0.8722	0.8751	0.8768	0.8792	0.8828	0.8844	0.8866	0.8881	0.8904	0.8932	0.8953	0.8976	0.9002
Mie	0.7469	0.7542	0.7585	0.7616	0.7666	0.7729	0.7770	0.7828	0.7890	0.7956	0.8029	0.8115	0.8172	0.8236	0.8293	0.8345	0.8399	0.8447	0.8490	0.8530	0.8560	0.8605	0.8628	0.8664	0.8693	0.8722	0.8743	0.8770	0.8793	0.8800	0.8827	0.8843	0.8867	0.8893	0.8915	0.8940
Miyagi	0.7379	0.7441	0.7484	0.7537	0.7594	0.7652	0.7701	0.7757	0.7831	0.7888	0.7964	0.8036	0.8101	0.8179	0.8233	0.8275	0.8340	0.8390	0.8432	0.8470	0.8507	0.8544	0.8576	0.8609	0.8645	0.8670	0.8700	0.8725	0.8748	0.8778	0.8797	0.8835	0.8833	0.8846	0.8866	0.8887
Miyazaki	0.7269	0.7317	0.7358	0.7421	0.7466	0.7531	0.7566	0.7628	0.7690	0.7742	0.7832	0.7891	0.7944	0.8002	0.8048	0.8094	0.8149	0.8203	0.8255	0.8298	0.8330	0.8357	0.8399	0.8438	0.8455	0.8481	0.8499	0.8514	0.8539	0.8551	0.8567	0.8575	0.8601	0.8614	0.8627	0.8641
Nagano	0.7380	0.7467	0.7518	0.7558	0.7604	0.7651	0.7684	0.7751	0.7807	0.7861	0.7934	0.8008	0.8061	0.8140	0.8185	0.8238	0.8300	0.8351	0.8395	0.8443	0.8476	0.8510	0.8549	0.8579	0.8612	0.8632	0.8659	0.8686	0.8720	0.8748	0.8764	0.8782	0.8799	0.8827	0.8850	0.8875
Nagasaki	0.7282	0.7327	0.7381	0.7424	0.7474	0.7533	0.7566	0.7626	0.7688	0.7747	0.7823	0.7876	0.7947	0.7997	0.8060	0.8122	0.8163	0.8229	0.8262	0.8304	0.8340	0.8376	0.8419	0.8444	0.8463	0.8492	0.8515	0.8547	0.8567	0.8583	0.8589	0.8605	0.8609	0.8628	0.8642	0.8657
Nara	0.7242	0.7298	0.7334	0.7378	0.7431	0.7501	0.7541	0.7619	0.7665	0.7737	0.7808	0.7890	0.7948	0.8001	0.8077	0.8122	0.8170	0.8221	0.8256	0.8303	0.8338	0.8378	0.8408	0.8431	0.8463	0.8484	0.8505	0.8534	0.8564	0.8575	0.8592	0.8610	0.8612	0.8643	0.8663	0.8685
Niigata	0.7390	0.7450	0.7505	0.7547	0.7586	0.7640	0.7682	0.7744	0.7802	0.7852	0.7934	0.7999	0.8070	0.8118	0.8178	0.8238	0.8295	0.8347	0.8390	0.8437	0.8475	0.8515	0.8567	0.8596	0.8622	0.8648	0.8674	0.8707	0.8740	0.8758	0.8774	0.8794	0.8812	0.8841	0.8864	0.8888
Ôita	0.7377	0.7443	0.7498	0.7538	0.7596	0.7642	0.7684	0.7741	0.7803	0.7862	0.7941	0.7995	0.8062	0.8117	0.8167	0.8220	0.8270	0.8323	0.8368	0.8413	0.8443	0.8472	0.8521	0.8549	0.8582	0.8595	0.8615	0.8642	0.8659	0.8685	0.8700	0.8713	0.8746	0.8767	0.8787	0.8808
Okayama	0.7406	0.7473	0.7512	0.7549	0.7596	0.7648	0.7698	0.7756	0.7829	0.7892	0.7959	0.8043	0.8103	0.8171	0.8229	0.8273	0.8323	0.8370	0.8419	0.8459	0.8495	0.8530	0.8565	0.8602	0.8629	0.8660	0.8681	0.8712	0.8738	0.8765	0.8774	0.8788	0.8815	0.8836	0.8859	0.8882
Okinawa	0.7094	0.7174	0.7230	0.7291	0.7338	0.7376	0.7414	0.7489	0.7559	0.7625	0.7728	0.7778	0.7846	0.7908	0.7985	0.8035	0.8086	0.8146	0.8183	0.8217	0.8256	0.8273	0.8328	0.8358	0.8383	0.8393	0.8425	0.8450	0.8472	0.8479	0.8487	0.8504	0.8512	0.8528	0.8544	0.8560
Ōsaka	0.7637	0.7704	0.7748	0.7791	0.7838	0.7880	0.7931	0.7991	0.8051	0.8120	0.8190	0.8264	0.8327	0.8395	0.8448	0.8494	0.8536	0.8581	0.8620	0.8656	0.8697	0.8731	0.8763	0.8791	0.8817	0.8838	0.8864	0.8893	0.8911	0.8929	0.8948	0.8964	0.8982	0.9006	0.9028	0.9050
Saga	0.7276	0.7344	0.7380	0.7435	0.7483	0.7524	0.7569	0.7641	0.7703	0.7783	0.7826	0.7899	0.7967	0.8025	0.8081	0.8137	0.8183	0.8226	0.8282	0.8318	0.8343	0.8383	0.8434	0.8471	0.8497	0.8520	0.8550	0.8573	0.8588	0.8618	0.8629	0.8639	0.8664	0.8694	0.8714	0.8735
Saitama	0.7362	0.7413	0.7468	0.7520	0.7571	0.7616	0.7686	0.7739	0.7793	0.7861	0.7934	0.7994	0.8068	0.8120	0.8178	0.8233	0.8287	0.8340	0.8389	0.8427	0.8463	0.8493	0.8520	0.8544	0.8572	0.8589	0.8616	0.8645	0.8668	0.8681	0.8702	0.8722	0.8745	0.8759	0.8780	0.8802
Shiga	0.7484	0.7550	0.7603	0.7633	0.7680	0.7742	0.7790	0.7849	0.7913	0.7988	0.8051	0.8146	0.8209	0.8281	0.8330	0.8392	0.8439	0.8500	0.8543	0.8578	0.8625	0.8660	0.8694	0.8727	0.8754	0.8786	0.8804	0.8835	0.8854	0.8875	0.8886	0.8899	0.8919	0.8951	0.8974	0.8997
Shimane	0.7241	0.7294	0.7361	0.7389	0.7449	0.7493	0.7526	0.7576	0.7644	0.7701	0.7771	0.7844	0.7920	0.7974	0.8037	0.8084	0.8143	0.8214	0.8266	0.8302	0.8333	0.8352	0.8406	0.8443	0.8465	0.8500	0.8509	0.8541	0.8589	0.8600	0.8602	0.8624	0.8633	0.8652	0.8669	0.8686
Shizuoka	0.7539	0.7584	0.7637	0.7679	0.7722	0.7774	0.7835	0.7871	0.7940	0.8016	0.8092	0.8156	0.8236	0.8285	0.8342	0.8403	0.8452	0.8502	0.8543	0.8577	0.8611	0.8645	0.8669	0.8701	0.8728	0.8753	0.8772	0.8788	0.8818	0.8833	0.8850	0.8869	0.8885	0.8911	0.8931	0.8952
Tochigi	0.7480	0.7536	0.7587	0.7632	0.7683	0.7723	0.7784	0.7821	0.7902	0.7973	0.8042	0.8114	0.8188	0.8253	0.8309	0.8369	0.8420	0.8478	0.8522	0.8560	0.8588	0.8618	0.8659	0.8683	0.8715	0.8728	0.8757	0.8793	0.8816	0.8827	0.8864	0.8891	0.8902	0.8933	0.8957	0.8981
Tokushima	0.7421	0.7469	0.7517	0.7559	0.7609	0.7658	0.7710	0.7777	0.7816	0.7891	0.7954	0.8034	0.8109	0.8160	0.8230	0.8252	0.8319	0.8378	0.8420	0.8455	0.8489	0.8529	0.8569	0.8605	0.8631	0.8678	0.8685	0.8727	0.8754	0.8758	0.8773	0.8779	0.8805	0.8828	0.8849	0.8871
Tōkyō	0.8040	0.8096	0.8152	0.8201	0.8245	0.8293	0.8346	0.8400	0.8466	0.8539	0.8606	0.8675	0.8742	0.8802	0.8857	0.8910	0.8965	0.9012	0.9057	0.9094	0.9129	0.9170	0.9196	0.9227	0.9254	0.9279	0.9305	0.9334	0.9359	0.9376	0.9403	0.9432	0.9454	0.9473	0.9496	0.9517
Tottori	0.7244	0.7299	0.7347	0.7365	0.7427	0.7495	0.7529	0.7592	0.7639	0.7713	0.7766	0.7848	0.7921	0.7981	0.8033	0.8084	0.8163	0.8207	0.8255	0.8309	0.8323	0.8358	0.8409	0.8419	0.8464	0.8492	0.8509	0.8555	0.8590	0.8597	0.8622	0.8615	0.8643	0.8662	0.8680	0.8701
Toyama	0.7486	0.7540	0.7590	0.7634	0.7671	0.7723	0.7770	0.7843	0.7888	0.7963	0.8033	0.8108	0.8173	0.8237	0.8287	0.8327	0.8379	0.8432	0.8482	0.8511	0.8550	0.8585	0.8615	0.8655	0.8675	0.8697	0.8733	0.8767	0.8789	0.8810	0.8840	0.8872	0.8881	0.8909	0.8935	0.8963
Wakayama	0.7379	0.7440	0.7479	0.7522	0.7577	0.7631	0.7672	0.7740	0.7808	0.7872	0.7943	0.8011	0.8081	0.8143	0.8197	0.8240	0.8285	0.8342	0.8381	0.8421	0.8458	0.8488	0.8529	0.8559	0.8599	0.8613	0.8640	0.8675	0.8682	0.8706	0.8714	0.8727	0.8736	0.8773	0.8795	0.8819
Yamagata	0.7250	0.7317	0.7373	0.7421	0.7464	0.7517	0.7544	0.7604	0.7644	0.7714	0.7786	0.7849	0.7921	0.7973	0.8026	0.8078	0.8131	0.8193	0.8248	0.8282	0.8315	0.8343	0.8390	0.8427	0.8459	0.8487	0.8517	0.8562	0.8584	0.8615	0.8636	0.8649	0.8681	0.8699	0.8723	0.8747
Yamaguchi	0.7465	0.7519	0.7568	0.7612	0.7655	0.7701	0.7750	0.7812	0.7881	0.7938	0.8016	0.8073	0.8146	0.8193	0.8247	0.8299	0.8355	0.8403	0.8447	0.8479	0.8518	0.8547	0.8586	0.8617	0.8642	0.8661	0.8688	0.8711	0.8740	0.8753	0.8757	0.8777	0.8807	0.8825	0.8844	0.8863
Yamanashi	0.7419	0.7479	0.7527	0.7569	0.7621	0.7652	0.7740	0.7738	0.7831	0.7891	0.7969	0.8017	0.8085	0.8147	0.8200	0.8262	0.8315	0.8368	0.8425	0.8467	0.8503	0.8543	0.8579	0.8605	0.8639	0.8663	0.8699	0.8729	0.8753	0.8777	0.8788	0.8814	0.8829	0.8856	0.8879	0.8905
South Korea	0.6008	0.6119	0.6229	0.6344	0.6462	0.6577	0.6696	0.6815	0.6929	0.7031	0.7127	0.7219	0.7302	0.7383	0.7469	0.7562	0.7656	0.7750	0.7828	0.7910	0.7990	0.8064	0.8137	0.8202	0.8262	0.8317	0.8369	0.8419	0.8464	0.8503	0.8545	0.8583	0.8618	0.8652	0.8684	0.8713
Singapore	0.6401	0.6491	0.6577	0.6689	0.6763	0.6827	0.6943	0.6960	0.6916	0.7058	0.7125	0.7237	0.7316	0.7391	0.7482	0.7575	0.7657	0.7754	0.7866	0.7931	0.7951	0.8071	0.8136	0.8232	0.8298	0.8362	0.8423	0.8464	0.8520	0.8588	0.8660	0.8658	0.8708	0.8743	0.8777	0.8811
Australasia	0.7541	0.7600	0.7653	0.7701	0.7750	0.7799	0.7847	0.7899	0.7952	0.8003	0.8051	0.8095	0.8136	0.8178	0.8225	0.8277	0.8332	0.8387	0.8440	0.8493	0.8542	0.8587	0.8629	0.8665	0.8697	0.8726	0.8754	0.8786	0.8820	0.8856	0.8894	0.8935	0.8978	0.9020	0.9062	0.9103
Australia	0.7558	0.7619	0.7670	0.7717	0.7766	0.7817	0.7867	0.7921	0.7977	0.8032	0.8081	0.8125	0.8167	0.8210	0.8258	0.8309	0.8364	0.8419	0.8474	0.8529	0.8580	0.8626	0.8668	0.8704	0.8736	0.8765	0.8793	0.8825	0.8861	0.8898	0.8938	0.8980	0.9023	0.9066	0.9109	0.9151
New Zealand	0.7458	0.7514	0.7568	0.7621	0.7671	0.7711	0.7749	0.7789	0.7827	0.7861	0.7901	0.7945	0.7981	0.8020	0.8067	0.8120	0.8175	0.8227	0.8271	0.8314	0.8354	0.8392	0.8433	0.8473	0.8507	0.8535	0.8562	0.8590	0.8614	0.8640	0.8670	0.8703	0.8739	0.8774	0.8810	0.8842
Western Europe	0.7000	0.7065	0.7124	0.7179	0.7235	0.7290	0.7347	0.7404	0.7464	0.7526	0.7588	0.7649	0.7707	0.7760	0.7811	0.7861	0.7910	0.7960	0.8010	0.8060	0.8111	0.8159	0.8204	0.8246	0.8285	0.8323	0.8363	0.8404	0.8443	0.8475	0.8509	0.8544	0.8577	0.8609	0.8640	0.8671
Andorra	0.7740	0.7800	0.7857	0.7914	0.7969	0.8015	0.8055	0.8097	0.8140	0.8185	0.8240	0.8274	0.8299	0.8360	0.8413	0.8438	0.8473	0.8497	0.8506	0.8510	0.8620	0.8643	0.8702	0.8776	0.8766	0.8804	0.8835	0.8861	0.8894	0.8930	0.8972	0.9017	0.9062	0.9105	0.9148	0.9186
Austria	0.7409	0.7462	0.7512	0.7561	0.7608	0.7655	0.7701	0.7745	0.7787	0.7831	0.7879	0.7929	0.7977	0.8020	0.8062	0.8102	0.8148	0.8194	0.8241	0.8289	0.8339	0.8385	0.8430	0.8472	0.8514	0.8555	0.8596	0.8638	0.8678	0.8709	0.8742	0.8774	0.8805	0.8833	0.8859	0.8883
Belgium	0.7294	0.7354	0.7409	0.7459	0.7508	0.7556	0.7603	0.7649	0.7698	0.7747	0.7797	0.7847	0.7896	0.7941	0.7986	0.8030	0.8079	0.8130	0.8178	0.8224	0.8271	0.8313	0.8354	0.8389	0.8423	0.8457	0.8492	0.8530	0.8568	0.8604	0.8641	0.8679	0.8715	0.8750	0.8783	0.8815
Cyprus	0.6425	0.6509	0.6594	0.6677	0.6763	0.6846	0.6924	0.7002	0.7082	0.7164	0.7246	0.7318	0.7393	0.7459	0.7526	0.7596	0.7657	0.7715	0.7776	0.7838	0.7908	0.7978	0.8050	0.8118	0.8188	0.8259	0.8334	0.8416	0.8499	0.8574	0.8641	0.8703	0.8749	0.8771	0.8788	0.8805
Denmark	0.7747	0.7808	0.7865	0.7919	0.7971	0.8021	0.8069	0.8111	0.8146	0.8178	0.8211	0.8245	0.8282	0.8316	0.8354	0.8398	0.8444	0.8494	0.8544	0.8594	0.8643	0.8685	0.8721	0.8751	0.8781	0.8809	0.8840	0.8872	0.8908	0.8939	0.8972	0.9005	0.9034	0.9059	0.9079	0.9096
Finland	0.7278	0.7338	0.7398	0.7458	0.7519	0.7578	0.7634	0.7689	0.7746	0.7802	0.7851	0.7887	0.7918	0.7948	0.7983	0.8025	0.8072	0.8125	0.8183	0.8243	0.8304	0.8360	0.8411	0.8456	0.8500	0.8541	0.8584	0.8632	0.8682	0.8720	0.8760	0.8802	0.8839	0.8872	0.8901	0.8929
France	0.6884	0.6935	0.6982	0.7026	0.7070	0.7114	0.7159	0.7206	0.7255	0.7307	0.7359	0.7407	0.7453	0.7492	0.7529	0.7562	0.7600	0.7638	0.7679	0.7719	0.7762	0.7804	0.7845	0.7882	0.7920	0.7959	0.8000	0.8042	0.8080	0.8114	0.8152	0.8191	0.8230	0.8268	0.8306	0.8343
Germany	0.7265	0.7334	0.7397	0.7457	0.7515	0.7575	0.7638	0.7703	0.7771	0.7844	0.7919	0.7994	0.8066	0.8129	0.8187	0.8240	0.8288	0.8334	0.8379	0.8425	0.8472	0.8521	0.8568	0.8611	0.8651	0.8689	0.8729	0.8771	0.8811	0.8840	0.8874	0.8910	0.8943	0.8972	0.9000	0.9026
Greece	0.6386	0.6464	0.6539	0.6609	0.6679	0.6749	0.6821	0.6888	0.6957	0.7025	0.7089	0.7154	0.7215	0.7270	0.7324	0.7378	0.7433	0.7492	0.7551	0.7610	0.7667	0.7722	0.7774	0.7824	0.7874	0.7920	0.7971	0.8024	0.8078	0.8126	0.8168	0.8194	0.8211	0.8223	0.8235	0.8246

Location	1980	1981	1982	1983	1984	1985	1986	1987	1988	1989	1990	1991	1992	1993	1994	1995	1996	1997	1998	1999	2000	2001	2002	2003	2004	2005	2006	2007	2008	2009	2010	2011	2012	2013	2014	2015
Iceland	0.7224	0.7306	0.7388	0.7455	0.7522	0.7583	0.7642	0.7710	0.7774	0.7826	0.7866	0.7911	0.7953	0.7992	0.8031	0.8068	0.8109	0.8162	0.8226	0.8292	0.8356	0.8415	0.8457	0.8487	0.8522	0.8565	0.8615	0.8673	0.8730	0.8765	0.8786	0.8812	0.8841	0.8871	0.8901	0.8933
Ireland	0.6418	0.6519	0.6617	0.6713	0.6811	0.6908	0.7003	0.7094	0.7180	0.7266	0.7350	0.7429	0.7506	0.7577	0.7645	0.7717	0.7788	0.7862	0.7936	0.8012	0.8091	0.8168	0.8240	0.8300	0.8355	0.8414	0.8479	0.8543	0.8587	0.8607	0.8631	0.8663	0.8699	0.8742	0.8794	0.8852
Israel	0.7086	0.7143	0.7193	0.7238	0.7276	0.7312	0.7348	0.7386	0.7425	0.7463	0.7509	0.7558	0.7611	0.7661	0.7709	0.7758	0.7809	0.7855	0.7898	0.7940	0.7985	0.8027	0.8063	0.8092	0.8120	0.8147	0.8179	0.8212	0.8240	0.8263	0.8284	0.8307	0.8331	0.8358	0.8389	0.8424
Italy	0.7129	0.7210	0.7276	0.7336	0.7394	0.7448	0.7500	0.7551	0.7603	0.7654	0.7703	0.7751	0.7794	0.7832	0.7868	0.7903	0.7942	0.7982	0.8021	0.8058	0.8095	0.8129	0.8161	0.8193	0.8228	0.8258	0.8289	0.8326	0.8363	0.8393	0.8424	0.8457	0.8486	0.8513	0.8536	0.8559
Luxembourg	0.7312	0.7371	0.7427	0.7482	0.7540	0.7599	0.7667	0.7732	0.7797	0.7866	0.7932	0.8000	0.8062	0.8123	0.8181	0.8238	0.8294	0.8352	0.8411	0.8478	0.8547	0.8603	0.8645	0.8679	0.8707	0.8736	0.8787	0.8841	0.8882	0.8920	0.8955	0.8989	0.9021	0.9052	0.9082	0.9111
Malta	0.6110	0.6195	0.6273	0.6337	0.6390	0.6435	0.6478	0.6518	0.6561	0.6607	0.6652	0.6696	0.6738	0.6778	0.6819	0.6864	0.6934	0.7013	0.7099	0.7189	0.7283	0.7363	0.7438	0.7504	0.7560	0.7611	0.7666	0.7719	0.7769	0.7809	0.7848	0.7891	0.7933	0.7974	0.8017	0.8062
Netherlands	0.7434	0.7486	0.7529	0.7569	0.7611	0.7653	0.7696	0.7738	0.7782	0.7830	0.7882	0.7933	0.7983	0.8031	0.8079	0.8127	0.8173	0.8221	0.8269	0.8317	0.8366	0.8415	0.8458	0.8497	0.8535	0.8572	0.8613	0.8657	0.8703	0.8743	0.8782	0.8819	0.8852	0.8882	0.8909	0.8936
Norway	0.7712	0.7783	0.7844	0.7901	0.7959	0.8015	0.8068	0.8118	0.8163	0.8206	0.8248	0.8292	0.8340	0.8390	0.8443	0.8498	0.8558	0.8623	0.8687	0.8750	0.8812	0.8865	0.8912	0.8950	0.8986	0.9021	0.9057	0.9096	0.9140	0.9178	0.9216	0.9252	0.9287	0.9317	0.9343	0.9365
Portugal	0.5133	0.5215	0.5297	0.5375	0.5449	0.5522	0.5596	0.5673	0.5752	0.5832	0.5913	0.5997	0.6080	0.6157	0.6230	0.6302	0.6374	0.6445	0.6517	0.6588	0.6657	0.6723	0.6791	0.6857	0.6921	0.6983	0.7045	0.7107	0.7167	0.7221	0.7277	0.7330	0.7378	0.7425	0.7473	0.7522
Spain	0.5816	0.5902	0.5987	0.6067	0.6146	0.6222	0.6299	0.6377	0.6456	0.6537	0.6620	0.6704	0.6785	0.6860	0.6934	0.7007	0.7074	0.7141	0.7206	0.7271	0.7338	0.7404	0.7469	0.7532	0.7592	0.7653	0.7717	0.7783	0.7848	0.7899	0.7950	0.8001	0.8050	0.8096	0.8143	0.8192
Sweden	0.7422	0.7473	0.7518	0.7557	0.7596	0.7632	0.7669	0.7706	0.7744	0.7785	0.7832	0.7881	0.7934	0.7990	0.8057	0.8129	0.8198	0.8266	0.8330	0.8390	0.8445	0.8488	0.8524	0.8554	0.8584	0.8611	0.8642	0.8677	0.8708	0.8733	0.8766	0.8799	0.8831	0.8861	0.8891	0.8921
Stockholm	0.7792	0.7861	0.7910	0.7957	0.7990	0.8034	0.8059	0.8091	0.8126	0.8168	0.8219	0.8277	0.8311	0.8358	0.8406	0.8481	0.8543	0.8608	0.8665	0.8723	0.8770	0.8797	0.8817	0.8836	0.8850	0.8874	0.8905	0.8940	0.8970	0.8996	0.9028	0.9064	0.9098	0.9111	0.9148	0.9175
Sweden except Stockholm	0.7339	0.7385	0.7428	0.7465	0.7505	0.7539	0.7577	0.7615	0.7653	0.7695	0.7740	0.7786	0.7844	0.7902	0.7973	0.8043	0.8114	0.8181	0.8246	0.8305	0.8361	0.8408	0.8447	0.8480	0.8514	0.8541	0.8572	0.8606	0.8637	0.8661	0.8692	0.8724	0.8754	0.8789	0.8816	0.8846
Switzerland	0.7779	0.7835	0.7886	0.7935	0.7985	0.8037	0.8090	0.8144	0.8198	0.8255	0.8314	0.8375	0.8430	0.8477	0.8521	0.8563	0.8603	0.8643	0.8685	0.8728	0.8774	0.8821	0.8865	0.8902	0.8935	0.8966	0.8997	0.9033	0.9073	0.9106	0.9138	0.9170	0.9200	0.9229	0.9256	0.9282
United Kingdom	0.7364	0.7414	0.7460	0.7507	0.7556	0.7609	0.7663	0.7721	0.7783	0.7846	0.7909	0.7965	0.8018	0.8069	0.8125	0.8180	0.8236	0.8294	0.8354	0.8416	0.8479	0.8537	0.8586	0.8628	0.8667	0.8701	0.8731	0.8759	0.8781	0.8796	0.8814	0.8835	0.8856	0.8878	0.8903	0.8930
England	0.7408	0.7458	0.7498	0.7544	0.7592	0.7644	0.7698	0.7753	0.7814	0.7874	0.7936	0.7993	0.8045	0.8096	0.8149	0.8205	0.8260	0.8319	0.8378	0.8439	0.8501	0.8559	0.8606	0.8647	0.8685	0.8720	0.8749	0.8777	0.8801	0.8814	0.8831	0.8850	0.8870	0.8890	0.8914	0.8939
East Midlands	0.7368	0.7418	0.7461	0.7509	0.7558	0.7613	0.7668	0.7718	0.7782	0.7835	0.7897	0.7955	0.8008	0.8064	0.8113	0.8172	0.8231	0.8292	0.8344	0.8404	0.8460	0.8521	0.8566	0.8610	0.8653	0.8681	0.8714	0.8738	0.8756	0.8770	0.8781	0.8794	0.8816	0.8837	0.8860	0.8886
East of England	0.6853	0.6901	0.6957	0.7003	0.7047	0.7107	0.7159	0.7212	0.7269	0.7332	0.7399	0.7461	0.7511	0.7559	0.7615	0.7683	0.7749	0.7808	0.7871	0.7935	0.7990	0.8049	0.8096	0.8142	0.8184	0.8220	0.8251	0.8272	0.8289	0.8301	0.8319	0.8337	0.8348	0.8359	0.8372	0.8386
Greater London	0.8159	0.8208	0.8237	0.8281	0.8325	0.8373	0.8425	0.8473	0.8525	0.8587	0.8655	0.8708	0.8745	0.8793	0.8834	0.8879	0.8928	0.8977	0.9032	0.9088	0.9140	0.9191	0.9227	0.9258	0.9293	0.9320	0.9344	0.9368	0.9407	0.9420	0.9439	0.9471	0.9494	0.9525	0.9557	0.9590
North East England	0.6542	0.6591	0.6635	0.6688	0.6742	0.6788	0.6851	0.6928	0.7008	0.7085	0.7147	0.7197	0.7263	0.7325	0.7395	0.7450	0.7520	0.7587	0.7653	0.7722	0.7795	0.7858	0.7904	0.7957	0.7999	0.8031	0.8065	0.8106	0.8133	0.8153	0.8159	0.8183	0.8211	0.8238	0.8267	0.8298
North West England	0.7298	0.7346	0.7382	0.7424	0.7476	0.7525	0.7583	0.7648	0.7709	0.7774	0.7829	0.7885	0.7951	0.8007	0.8069	0.8127	0.8174	0.8237	0.8300	0.8358	0.8421	0.8479	0.8532	0.8576	0.8606	0.8641	0.8675	0.8707	0.8728	0.8741	0.8758	0.8779	0.8800	0.8820	0.8843	0.8869
South East England	0.7633	0.7682	0.7736	0.7780	0.7830	0.7878	0.7932	0.7975	0.8031	0.8086	0.8146	0.8204	0.8249	0.8294	0.8342	0.8399	0.8457	0.8512	0.8561	0.8620	0.8682	0.8740	0.8788	0.8826	0.8868	0.8905	0.8925	0.8951	0.8970	0.8980	0.8994	0.9006	0.9024	0.9045	0.9068	0.9093
South West England	0.7435	0.7487	0.7528	0.7581	0.7628	0.7681	0.7732	0.7786	0.7847	0.7899	0.7966	0.8030	0.8080	0.8126	0.8177	0.8240	0.8288	0.8348	0.8407	0.8469	0.8539	0.8599	0.8641	0.8683	0.8730	0.8769	0.8798	0.8820	0.8838	0.8849	0.8858	0.8869	0.8890	0.8911	0.8935	0.8961
West Midlands	0.7326	0.7374	0.7401	0.7446	0.7494	0.7547	0.7601	0.7659	0.7729	0.7778	0.7837	0.7893	0.7953	0.8005	0.8057	0.8110	0.8157	0.8211	0.8276	0.8333	0.8392	0.8446	0.8495	0.8537	0.8569	0.8606	0.8643	0.8666	0.8693	0.8707	0.8728	0.8737	0.8752	0.8768	0.8786	0.8806
Yorkshire and the Humber	0.7297	0.7347	0.7392	0.7432	0.7485	0.7538	0.7587	0.7651	0.7719	0.7784	0.7841	0.7896	0.7950	0.8000	0.8059	0.8102	0.8161	0.8226	0.8282	0.8340	0.8406	0.8461	0.8516	0.8561	0.8597	0.8633	0.8662	0.8697	0.8715	0.8721	0.8745	0.8768	0.8783	0.8797	0.8814	0.8833
Northern Ireland	0.6186	0.6278	0.6354	0.6409	0.6461	0.6551	0.6604	0.6706	0.6796	0.6916	0.6995	0.7069	0.7149	0.7220	0.7297	0.7357	0.7412	0.7488	0.7565	0.7639	0.7731	0.7773	0.7846	0.7912	0.7955	0.7997	0.8029	0.8053	0.8070	0.8096	0.8113	0.8125	0.8144	0.8165	0.8190	0.8216
Scotland	0.7442	0.7488	0.7572	0.7629	0.7683	0.7735	0.7794	0.7858	0.7922	0.7996	0.8051	0.8090	0.8142	0.8201	0.8261	0.8311	0.8371	0.8417	0.8483	0.8546	0.8606	0.8656	0.8710	0.8757	0.8792	0.8821	0.8855	0.8877	0.8890	0.8911	0.8947	0.8983	0.9015	0.9047	0.9082	0.9118
Wales	0.7128	0.7179	0.7215	0.7271	0.7322	0.7376	0.7433	0.7500	0.7567	0.7641	0.7707	0.7772	0.7825	0.7878	0.7946	0.8000	0.8052	0.8113	0.8187	0.8261	0.8329	0.8394	0.8458	0.8504	0.8548	0.8582	0.8623	0.8655	0.8676	0.8700	0.8708	0.8731	0.8763	0.8796	0.8831	0.8869
Southern Latin America	0.5788	0.5846	0.5894	0.5941	0.5986	0.6023	0.6074	0.6124	0.6171	0.6216	0.6261	0.6318	0.6389	0.6464	0.6543	0.6619	0.6703	0.6786	0.6862	0.6924	0.6979	0.7025	0.7059	0.7096	0.7140	0.7191	0.7248	0.7312	0.7375	0.7434	0.7497	0.7566	0.7631	0.7691	0.7746	0.7797
Argentina	0.5768	0.5819	0.5868	0.5920	0.5969	0.6006	0.6062	0.6114	0.6159	0.6196	0.6234	0.6285	0.6352	0.6425	0.6504	0.6576	0.6660	0.6743	0.6820	0.6881	0.6934	0.6975	0.7001	0.7032	0.7070	0.7117	0.7172	0.7234	0.7298	0.7357	0.7422	0.7493	0.7557	0.7617	0.7671	0.7720
Chile	0.5872	0.5941	0.5986	0.6023	0.6062	0.6100	0.6141	0.6187	0.6241	0.6306	0.6371	0.6444	0.6528	0.6613	0.6697	0.6786	0.6876	0.6960	0.7036	0.7101	0.7164	0.7223	0.7278	0.7332	0.7391	0.7453	0.7519	0.7587	0.7649	0.7703	0.7762	0.7823	0.7885	0.7944	0.7998	0.8048
Uruguay	0.5662	0.5734	0.5787	0.5826	0.5859	0.5887	0.5925	0.5968	0.6008	0.6047	0.6086	0.6128	0.6183	0.6239	0.6301	0.6355	0.6414	0.6476	0.6538	0.6591	0.6636	0.6671	0.6694	0.6719	0.6748	0.6786	0.6831	0.6885	0.6948	0.7013	0.7086	0.7164	0.7238	0.7312	0.7383	0.7450
High-income North America	0.8377	0.8410	0.8431	0.8452	0.8479	0.8508	0.8534	0.8557	0.8579	0.8602	0.8623	0.8641	0.8662	0.8683	0.8706	0.8731	0.8760	0.8790	0.8820	0.8850	0.8881	0.8907	0.8931	0.8954	0.8978	0.9005	0.9035	0.9068	0.9101	0.9131	0.9162	0.9194	0.9227	0.9258	0.9288	0.9317
Canada	0.8144	0.8201	0.8247	0.8290	0.8334	0.8379	0.8421	0.8461	0.8504	0.8546	0.8581	0.8607	0.8633	0.8660	0.8694	0.8733	0.8773	0.8815	0.8857	0.8902	0.8949	0.8990	0.9025	0.9055	0.9082	0.9107	0.9131	0.9156	0.9182	0.9202	0.9226	0.9255	0.9284	0.9314	0.9345	0.9375
Greenland	0.5788	0.5883	0.5985	0.6169	0.6158	0.6079	0.6281	0.6275	0.6094	0.6155	0.6059	0.6154	0.5999	0.6086	0.6148	0.6207	0.6275	0.6089	0.6406	0.6481	0.6620	0.6459	0.6420	0.6688	0.6648	0.6729	0.6906	0.6907	0.7003	0.6893	0.7030	0.7252	0.7439	0.7412	0.7506	0.7575
United States	0.8403	0.8433	0.8451	0.8470	0.8496	0.8522	0.8547	0.8568	0.8588	0.8608	0.8628	0.8646	0.8666	0.8686	0.8708	0.8732	0.8759	0.8788	0.8816	0.8845	0.8874	0.8898	0.8921	0.8943	0.8967	0.8994	0.9025	0.9059	0.9093	0.9123	0.9155	0.9188	0.9221	0.9252	0.9282	0.9311
Alabama	0.8073	0.8121	0.8154	0.8175	0.8202	0.8240	0.8268	0.8299	0.8323	0.8346	0.8384	0.8414	0.8447	0.8476	0.8509	0.8537	0.8566	0.8588	0.8618	0.8646	0.8674	0.8723	0.8760	0.8790	0.8824	0.8851	0.8877	0.8904	0.8930	0.8966	0.9005	0.9034	0.9071	0.9096	0.9124	0.9153
Alaska	0.8805	0.8768	0.8723	0.8714	0.8702	0.8701	0.8738	0.8740	0.8784	0.8770	0.8757	0.8747	0.8724	0.8757	0.8779	0.8801	0.8819	0.8827	0.8849	0.8866	0.8901	0.8899	0.8923	0.8951	0.8964	0.8994	0.9017	0.9062	0.9055	0.9095	0.9113	0.9162	0.9233	0.9243	0.9285	0.9324
Arizona	0.8369	0.8392	0.8412	0.8412	0.8440	0.8443	0.8466	0.8481	0.8498	0.8524	0.8527	0.8548	0.8556	0.8569	0.8586	0.8599	0.8604	0.8646	0.8671	0.8684	0.8698	0.8721	0.8730	0.8743	0.8758	0.8790	0.8817	0.8873	0.8931	0.8995	0.9043	0.9080	0.9101	0.9132	0.9156	0.9179
Arkansas	0.8287	0.8335	0.8355	0.8364	0.8388	0.8412	0.8438	0.8450	0.8459	0.8467	0.8483	0.8508	0.8532	0.8557	0.8564	0.8570	0.8571	0.8593	0.8620	0.8650	0.8666	0.8692	0.8699	0.8725	0.8739	0.8761	0.8783	0.8818	0.8853	0.8881	0.8915	0.8937	0.8980	0.9019	0.9058	0.9095
California	0.8541	0.8553	0.8572	0.8580	0.8594	0.8606	0.8616	0.8619	0.8618	0.8612	0.8591	0.8591	0.8605	0.8617	0.8636	0.8654	0.8686	0.8735	0.8784	0.8824	0.8858	0.8888	0.8909	0.8929	0.8952	0.8976	0.9010	0.9045	0.9085	0.9128	0.9159	0.9195	0.9227	0.9271	0.9296	0.9321
Colorado	0.8725	0.8731	0.8739	0.8746	0.8771	0.8792	0.8794	0.8819	0.8835	0.8866	0.8876	0.8876	0.8878	0.8903	0.8927	0.8947	0.8960	0.8983	0.8992	0.9004	0.9025	0.9035	0.9040	0.9059	0.9090	0.9113	0.9150	0.9196	0.9229	0.9257	0.9291	0.9335	0.9372	0.9409	0.9444	0.9478
Connecticut	0.8718	0.8742	0.8767	0.8782	0.8808	0.8837	0.8862	0.8880	0.8919	0.8955	0.8991	0.9016	0.9034	0.9048	0.9068	0.9094	0.9110	0.9149	0.9166	0.9198	0.9245	0.9261	0.9287	0.9300	0.9333	0.9355	0.9387	0.9415	0.9450	0.9485	0.9511	0.9537	0.9578	0.9608	0.9635	0.9660
Delaware	0.8633	0.8667	0.8684	0.8688	0.8712	0.8729	0.8748	0.8770	0.8778	0.8805	0.8822	0.8831	0.8874	0.8883	0.8898	0.8920	0.8946	0.8957	0.8963	0.8979	0.8993	0.9033	0.9030	0.9054	0.9086	0.9105	0.9139	0.9166	0.9187	0.9229	0.9243	0.9272	0.9318	0.9357	0.9380	0.9403
District of Columbia	0.8673	0.8706	0.8720	0.8731	0.8737	0.8761	0.8778	0.8795	0.8810	0.8770	0.8798	0.8803	0.8876	0.8912	0.8987	0.9065	0.9140	0.9210	0.9283	0.9343	0.9377	0.9406	0.9443	0.9459	0.9454	0.9478	0.9488	0.9507	0.9518	0.9559	0.9571	0.9601	0.9638	0.9688	0.9734	0.9779
Florida	0.8414	0.8439	0.8456	0.8467	0.8488	0.8514	0.8540	0.8558	0.8574	0.8600	0.8628	0.8670	0.8697	0.8706	0.8734	0.8763	0.8791	0.8812	0.8845	0.8876	0.8900	0.8919	0.8951	0.8970	0.8994	0.9016	0.9050	0.9089	0.9132	0.9169	0.9198	0.9229	0.9261	0.9278	0.9291	0.9304
Georgia	0.8166	0.8218	0.8248	0.8271	0.8304	0.8338	0.8371	0.8394	0.8426	0.8454	0.8492	0.8534	0.8558	0.8588	0.8621	0.8646	0.8680	0.8697	0.8730	0.8751	0.8777	0.8799	0.8830	0.8854	0.8868	0.8885	0.8912	0.8948	0.8991	0.9024	0.9064	0.9089	0.9126	0.9152	0.9182	0.9213
Hawaii	0.8516	0.8533	0.8527	0.8510	0.8539	0.8588	0.8600	0.8615	0.8633	0.8667	0.8674	0.8715	0.8732	0.8750	0.8760	0.8806	0.8822	0.8883	0.8892	0.8920	0.8922	0.8946	0.8943	0.8948	0.8979	0.9035	0.9049	0.9083	0.9096	0.9138	0.9147	0.9179	0.9212	0.9241	0.9272	0.9299
Idaho	0.8154	0.8195	0.8203	0.8230	0.8276	0.8320	0.8364	0.8392	0.8422	0.8455	0.8472	0.8485	0.8486	0.8519	0.8554	0.8568	0.8583	0.8624	0.8639	0.8661	0.8700	0.8705	0.8720	0.8732	0.8743	0.8773	0.8806	0.8834	0.8854	0.8909	0.8928	0.8979	0.8981	0.9028	0.9053	0.9076
Illinois	0.8466	0.8505	0.8526	0.8543	0.8563	0.8591	0.8621	0.8631	0.8649	0.8667	0.8687	0.8701	0.8726	0.8737	0.8756	0.8782	0.8820	0.8855	0.8886	0.8918	0.8950	0.8967	0.8997	0.9017	0.9044	0.9076	0.9119	0.9158	0.9194	0.9225	0.9253	0.9288	0.9323	0.9354	0.9385	0.9414
Indiana	0.8272	0.8318	0.8336	0.8358	0.8386	0.8414	0.8443	0.8477	0.8487	0.8515	0.8537	0.8556	0.8587	0.8605	0.8637	0.8653	0.8676	0.8701	0.8725	0.8745	0.8775	0.8800	0.8828	0.8849	0.8866	0.8892	0.8928	0.8951	0.8974	0.8992	0.9016	0.9036	0.9068	0.9094	0.9126	0.9158
Iowa	0.8414	0.8459	0.8482	0.8494	0.8520	0.8557	0.8594	0.8618	0.8633	0.8642	0.8663	0.8671	0.8689	0.8699	0.8722	0.8737	0.8754	0.8785	0.8805	0.8823	0.8847	0.8866	0.8882	0.8900	0.8922	0.8935	0.8962	0.9003	0.9045	0.9070	0.9104	0.9144	0.9172	0.9195	0.9243	0.9284
Kansas	0.8630	0.8649	0.8679	0.8688	0.8714	0.8746	0.8758	0.8784	0.8797	0.8816	0.8826	0.8836	0.8830	0.8834	0.8834	0.8837	0.8859	0.8858	0.8863	0.8874	0.8891	0.8911	0.8912	0.8939	0.8955	0.8975	0.9004	0.9030	0.9062	0.9085	0.9111	0.9160	0.9181	0.9240	0.9275	0.9308
Kentucky	0.7920	0.7969	0.7997	0.8029	0.8071	0.8114	0.8146	0.8180	0.8222	0.8240	0.8276	0.8303	0.8339	0.8378	0.8407	0.8442	0.8476	0.8504	0.8539	0.8577	0.8609	0.8652	0.8685	0.8713	0.8738	0.8764	0.8793	0.8819	0.8852	0.8869	0.8903	0.8931	0.8952	0.8975	0.9003	0.9030
Louisiana	0.8195	0.8240	0.8258	0.8287	0.8312	0.8337	0.8363	0.8391	0.8390	0.8415	0.8441	0.8434	0.8461	0.8479	0.8509	0.8547	0.8575	0.8588	0.8612	0.8631	0.8656	0.8697	0.8722	0.8755	0.8781	0.8867	0.8840	0.8871	0.8926	0.8956	0.9012	0.9051	0.9081	0.9105	0.9144	0.9181
Maine	0.8257	0.8283	0.8300	0.8314	0.8345	0.8386	0.8424	0.8464	0.8508	0.8553	0.8604	0.8628	0.8659	0.8698	0.8729	0.8759	0.8785	0.8811	0.8838	0.8874	0.8919	0.8933	0.8970	0.8997	0.9026	0.9049	0.9094	0.9130	0.9171	0.9184	0.9213	0.9248	0.9269	0.9290	0.9322	0.9352
Maryland	0.8691	0.8706	0.8705	0.8715	0.8735	0.8758	0.8780	0.8794	0.8817	0.8847	0.8875	0.8892	0.8911	0.8939	0.8956	0.8975	0.9000	0.9028	0.9039	0.9064	0.9085	0.9117	0.9142	0.9165	0.9201	0.9233	0.9263	0.9297	0.9325	0.9362	0.9390	0.9425	0.9460	0.9496	0.9514	0.9532
Massachusetts	0.8636	0.8665	0.8686	0.8710	0.8743	0.8777	0.8815	0.8847	0.8876	0.8904	0.8938	0.8974	0.8990	0.9014	0.9034	0.9065	0.9099	0.9122	0.9145	0.9177	0.9217	0.9242	0.9262	0.9293	0.9324	0.9358	0.9395	0.9428	0.9456	0.9487	0.9517	0.9541	0.9583	0.9619	0.9646	0.9670
Michigan	0.8536	0.8567	0.8580	0.8596	0.8598	0.8628	0.8650	0.8661	0.8693	0.8683	0.8690	0.8706	0.8737	0.8756	0.8773	0.8800	0.8828	0.8843	0.8871	0.8894	0.8920	0.8944	0.8973	0.8990	0.9012	0.9041	0.9075	0.9107	0.9129	0.9140	0.9151	0.9169	0.9200	0.9221	0.9260	0.9299
Minnesota	0.8368	0.8406	0.8445	0.8491	0.8521	0.8569	0.8617	0.8662	0.8692	0.8733	0.8771	0.8793	0.8821	0.8842	0.8862	0.8889	0.8914	0.8931	0.8964	0.8991	0.9026	0.9046	0.9062	0.9075	0.9096	0.9123	0.9146	0.9190	0.9226	0.9251	0.9279	0.9304	0.9333	0.9357	0.9389	0.9420
Mississippi	0.8027	0.8080	0.8105	0.8136	0.8158	0.8195	0.8230	0.8254	0.8265	0.8281	0.8308	0.8323	0.8345	0.8369	0.8398	0.8433	0.8472	0.8492	0.8508	0.8543	0.8558	0.8600	0.8630	0.8646	0.8669	0.8709	0.8687	0.8723	0.8770	0.8818	0.8879	0.8904	0.8955	0.8980	0.9016	0.9050
Missouri	0.8267	0.8311	0.8334	0.8356	0.8396	0.8421	0.8461	0.8494	0.8518	0.8542	0.8568	0.8581	0.8614	0.8635	0.8670	0.8691	0.8711	0.8737	0.8760	0.8789	0.8825	0.8851	0.8875	0.8893	0.8915	0.8938	0.8965	0.9001	0.9029	0.9057	0.9084	0.9109	0.9144	0.9169	0.9206	0.9241
Montana	0.8313	0.8326	0.8328	0.8351	0.8356	0.8404	0.8431	0.8454	0.8490	0.8513	0.8536	0.8544	0.8558	0.8583	0.8623	0.8638	0.8683	0.8703	0.8738	0.8764	0.8795	0.8803	0.8818	0.8836	0.8876	0.8912	0.8916	0.8977	0.9002	0.9037	0.9065	0.9097	0.9133	0.9154	0.9211	0.9268
Nebraska	0.8381	0.8413	0.8438	0.8456	0.8481	0.8527	0.8562	0.8593	0.8618	0.8638	0.8665	0.8680	0.8709	0.8718	0.8729	0.8740	0.8769	0.8790	0.8819	0.8834	0.8853	0.8856	0.8853	0.8876	0.8890	0.8929	0.8965	0.9005	0.9029	0.9044	0.9095	0.9131	0.9163	0.9191	0.9221	0.9246
Nevada	0.8819	0.8822	0.8832	0.8845	0.8844	0.8854	0.8842	0.8840	0.8826	0.8818	0.8798	0.8818	0.8829	0.8843	0.8829	0.8827	0.8840	0.8872	0.8877	0.8908	0.8928	0.8939	0.8932	0.8949	0.8962	0.8972	0.8990	0.9027	0.9083	0.9119	0.9147	0.9167	0.9198	0.9214	0.9208	0.9202
New Hampshire	0.8504	0.8556	0.8570	0.8610	0.8644	0.8661	0.8698	0.8714	0.8764	0.8805	0.8853	0.8891	0.8902	0.8919	0.8935	0.8962	0.8991	0.9018	0.9047	0.9094	0.9116	0.9135	0.9165	0.9200	0.9217	0.9250	0.9298	0.9341	0.9379	0.9395	0.9426	0.9446	0.9504	0.9529	0.9564	0.9599
New Jersey	0.8832	0.8862	0.8875	0.8886	0.8907	0.8925	0.8932	0.8939	0.8955	0.8973	0.9008	0.9009	0.9020	0.9029	0.9027	0.9044	0.9061	0.9081	0.9099	0.9120	0.9147	0.9151	0.9170	0.9179	0.9207	0.9235	0.9269	0.9294	0.9332	0.9355	0.9384	0.9411	0.9450	0.9486	0.9523	0.9559
New Mexico	0.8184	0.8200	0.8203	0.8219	0.8253	0.8281	0.8309	0.8328	0.8358	0.8378	0.8406	0.8397	0.8409	0.8424	0.8462	0.8508	0.8529	0.8563	0.8589	0.8618	0.8658	0.8674	0.8685	0.8725	0.8745	0.8770	0.8799	0.8829	0.8862	0.8908	0.8952	0.8993	0.9029	0.9069	0.9090	0.9109
New York	0.8478	0.8501	0.8518	0.8535	0.8568	0.8597	0.8620	0.8640	0.8670	0.8698	0.8735	0.8760	0.8787	0.8810	0.8832	0.8860	0.8903	0.8946	0.8981	0.9016	0.9048	0.9075	0.9098	0.9121	0.9152	0.9186	0.9223	0.9256	0.9289	0.9310	0.9334	0.9373	0.9408	0.9449	0.9482	0.9513
North Carolina	0.8117	0.8155	0.8171	0.8204	0.8234	0.8269	0.8302	0.8328	0.8355	0.8385	0.8425	0.8469	0.8489	0.8539	0.8575	0.8610	0.8637	0.8665	0.8691	0.8723	0.8734	0.8773	0.8803	0.8835	0.8857	0.8875	0.8908	0.8942	0.8971	0.9009	0.9043	0.9075	0.9107	0.9133	0.9155	0.9176
North Dakota	0.8192	0.8200	0.8214	0.8237	0.8295	0.8323	0.8379	0.8416	0.8431	0.8480	0.8528	0.8547	0.8562	0.8579	0.8607	0.8629	0.8677	0.8695	0.8766	0.8810	0.8846	0.8862	0.8869	0.8893	0.8913	0.8933	0.8970	0.9015	0.9052	0.9080	0.9119	0.9143	0.9186	0.9227	0.9302	0.9361
Ohio	0.8313	0.8342	0.8364	0.8388	0.8413	0.8442	0.8471	0.8498	0.8521	0.8546	0.8574	0.8586	0.8612	0.8634	0.8660	0.8681	0.8715	0.8735	0.8766	0.8793	0.8820	0.8852	0.8884	0.8911	0.8938	0.8965	0.8997	0.9028	0.9049	0.9070	0.9099	0.9125	0.9150	0.9174	0.9214	0.9253
Oklahoma	0.8329	0.8355	0.8346	0.8387	0.8431	0.8468	0.8498	0.8528	0.8534	0.8550	0.8563	0.8555	0.8561	0.8581	0.8595	0.8600	0.8614	0.8603	0.8620	0.8657	0.8687	0.8698	0.8719	0.8749	0.8774	0.8805	0.8839	0.8874	0.8911	0.8930	0.8965	0.9013	0.9051	0.9080	0.9116	0.9149
Oregon	0.8594	0.8610	0.8640	0.8640	0.8654	0.8672	0.8682	0.8696	0.8699	0.8711	0.8721	0.8744	0.8763	0.8788	0.8801	0.8805	0.8824	0.8854	0.8870	0.8905	0.8940	0.8965	0.8990	0.9009	0.9033	0.9059	0.9072	0.9106	0.9133	0.9174	0.9205	0.9234	0.9267	0.9292	0.9336	0.9380
Pennsylvania	0.8506	0.8527	0.8541	0.8558	0.8583	0.8601	0.8615	0.8632	0.8653	0.8677	0.8700	0.8719	0.8742	0.8761	0.8784	0.8812	0.8844	0.8877	0.8895	0.8918	0.8949	0.8973	0.8992	0.9009	0.9041	0.9067	0.9099	0.9131	0.9160	0.9186	0.9212	0.9240	0.9277	0.9312	0.9350	0.9387
Rhode Island	0.8363	0.8392	0.8421	0.8439	0.8480	0.8520	0.8543	0.8567	0.8612	0.8644	0.8673	0.8709	0.8729	0.8761	0.8799	0.8847	0.8879	0.8913	0.8945	0.8986	0.9021	0.9032	0.9050	0.9073	0.9124	0.9151	0.9207	0.9237	0.9269	0.9311	0.9330	0.9362	0.9395	0.9424	0.9470	0.9513
South Carolina	0.8022	0.8059	0.8087	0.8112	0.8149	0.8184	0.8217	0.8246	0.8264	0.8292	0.8332	0.8374	0.8414	0.8464	0.8515	0.8556	0.8589	0.8613	0.8642	0.8668	0.8704	0.8734	0.8778	0.8805	0.8829	0.8852	0.8857	0.8899	0.8925	0.8969	0.9004	0.9035	0.9067	0.9096	0.9126	0.9156
South Dakota	0.8102	0.8154	0.8165	0.8182	0.8212	0.8263	0.8300	0.8324	0.8366	0.8399	0.8437	0.8450	0.8457	0.8497	0.8542	0.8562	0.8599	0.8647	0.8677	0.8693	0.8763	0.8765	0.8771	0.8790	0.8807	0.8838	0.8860	0.8894	0.8942	0.8970	0.9004	0.9036	0.9065	0.9090	0.9137	0.9174
Tennessee	0.8022	0.8071	0.8097	0.8124	0.8164	0.8198	0.8237	0.8268	0.8293	0.8325	0.8363	0.8397	0.8438	0.8479	0.8517	0.8555	0.8595	0.8630	0.8658	0.8697	0.8733	0.8767	0.8801	0.8827	0.8853	0.8869	0.8901	0.8922	0.8950	0.8988	0.9019	0.9040	0.9066	0.9095	0.9127	0.9158
Texas	0.8223	0.8258	0.8276	0.8309	0.8333	0.8357	0.8379	0.8405	0.8421	0.8447	0.8460	0.8465	0.8469	0.8478	0.8501	0.8518	0.8531	0.8561	0.8596	0.8623	0.8651	0.8677	0.8690	0.8721	0.8745	0.8774	0.8817	0.8855	0.8894	0.8919	0.8970	0.9025	0.9060	0.9088	0.9117	0.9143
Utah	0.8021	0.8073	0.8108	0.8164	0.8225	0.8289	0.8327	0.8367	0.8367	0.8417	0.8446	0.8480	0.8482	0.8514	0.8524	0.8526	0.8517	0.8542	0.8552	0.8572	0.8613	0.8623	0.8627	0.8657	0.8675	0.8703	0.8747	0.8786	0.8812	0.8857	0.8894	0.8938	0.8966	0.9007	0.9046	0.9084
Vermont	0.8500	0.8525	0.8543	0.8558	0.8581	0.8622	0.8638	0.8675	0.8722	0.8739	0.8794	0.8814	0.8830	0.8850	0.8860	0.8917	0.8929	0.8957	0.8984	0.9008	0.9051	0.9074	0.9098	0.9103	0.9129	0.9186	0.9213	0.9250	0.9287	0.9322	0.9319	0.9359	0.9395	0.9429	0.9467	0.9502
Virginia	0.8601	0.8630	0.8642	0.8662	0.8683	0.8706	0.8730	0.8748	0.8775	0.8796	0.8820	0.8840	0.8851	0.8874	0.8883	0.8909	0.8931	0.8954	0.8965	0.8986	0.9008	0.9028	0.9046	0.9074	0.9091	0.9127	0.9164	0.9205	0.9246	0.9275	0.9306	0.9338	0.9371	0.9405	0.9434	0.9461
Washington	0.8626	0.8647	0.8674	0.8686	0.8707	0.8729	0.8754	0.8771	0.8787	0.8805	0.8815	0.8828	0.8850	0.8870	0.8897	0.8913	0.8929	0.8956	0.8986	0.9020	0.9056	0.9088	0.9115	0.9134	0.9154	0.9177	0.9199	0.9230	0.9245	0.9269	0.9302	0.9325	0.9360	0.9398	0.9432	0.9467

Location	1980	1981	1982	1983	1984	1985	1986	1987	1988	1989	1990	1991	1992	1993	1994	1995	1996	1997	1998	1999	2000	2001	2002	2003	2004	2005	2006	2007	2008	2009	2010	2011	2012	2013	2014	2015
West Virginia	0.7910	0.7966	0.7995	0.8026	0.8069	0.8105	0.8135	0.8170	0.8208	0.8231	0.8261	0.8288	0.8326	0.8362	0.8401	0.8429	0.8472	0.8500	0.8535	0.8561	0.8595	0.8625	0.8644	0.8676	0.8707	0.8738	0.8785	0.8788	0.8823	0.8845	0.8883	0.8902	0.8929	0.8951	0.8990	0.9027
Wisconsin	0.8274	0.8311	0.8335	0.8359	0.8384	0.8420	0.8455	0.8500	0.8542	0.8576	0.8610	0.8636	0.8669	0.8695	0.8732	0.8760	0.8795	0.8827	0.8855	0.8877	0.8911	0.8928	0.8955	0.8974	0.9000	0.9020	0.9055	0.9090	0.9113	0.9136	0.9167	0.9193	0.9225	0.9254	0.9276	0.9295
Wyoming	0.8565	0.8587	0.8590	0.8622	0.8640	0.8666	0.8697	0.8752	0.8769	0.8797	0.8795	0.8809	0.8799	0.8816	0.8834	0.8855	0.8862	0.8859	0.8903	0.8941	0.8966	0.8991	0.8971	0.8995	0.9026	0.9022	0.9060	0.9111	0.9141	0.9184	0.9233	0.9282	0.9314	0.9343	0.9373	0.9400
Latin America and Caribbean	0.4475	0.4566	0.4652	0.4732	0.4810	0.4888	0.4967	0.5044	0.5119	0.5189	0.5256	0.5322	0.5387	0.5451	0.5515	0.5578	0.5642	0.5706	0.5768	0.5827	0.5885	0.5946	0.6004	0.6060	0.6119	0.6180	0.6246	0.6313	0.6380	0.6439	0.6498	0.6559	0.6618	0.6675	0.6731	0.6781
Caribbean	0.5033	0.5099	0.5158	0.5213	0.5268	0.5322	0.5375	0.5429	0.5483	0.5534	0.5580	0.5621	0.5657	0.5687	0.5715	0.5745	0.5780	0.5818	0.5858	0.5903	0.5951	0.6000	0.6049	0.6095	0.6139	0.6186	0.6239	0.6293	0.6343	0.6391	0.6434	0.6480	0.6524	0.6568	0.6612	0.6655
Antigua and Barbuda	0.6574	0.6643	0.6704	0.6773	0.6850	0.6935	0.7022	0.7111	0.7210	0.7299	0.7374	0.7445	0.7500	0.7546	0.7589	0.7616	0.7665	0.7713	0.7760	0.7803	0.7842	0.7874	0.7908	0.7945	0.7989	0.8039	0.8097	0.8164	0.8227	0.8270	0.8297	0.8319	0.8342	0.8363	0.8386	0.8410
The Bahamas	0.6491	0.6558	0.6623	0.6691	0.6772	0.6860	0.6941	0.7019	0.7088	0.7151	0.7205	0.7251	0.7289	0.7328	0.7373	0.7426	0.7488	0.7558	0.7636	0.7721	0.7805	0.7889	0.7966	0.8027	0.8075	0.8116	0.8149	0.8178	0.8201	0.8217	0.8236	0.8254	0.8276	0.8299	0.8323	0.8348
Barbados	0.6178	0.6249	0.6310	0.6363	0.6422	0.6482	0.6548	0.6618	0.6691	0.6765	0.6829	0.6882	0.6924	0.6964	0.7004	0.7044	0.7090	0.7141	0.7191	0.7239	0.7288	0.7338	0.7384	0.7431	0.7474	0.7521	0.7569	0.7614	0.7652	0.7683	0.7708	0.7734	0.7758	0.7780	0.7802	0.7824
Belize	0.3743	0.3863	0.3971	0.4073	0.4171	0.4266	0.4347	0.4442	0.4540	0.4641	0.4745	0.4833	0.4918	0.5000	0.5077	0.5153	0.5257	0.5356	0.5449	0.5537	0.5626	0.5714	0.5801	0.5891	0.5981	0.6068	0.6143	0.6213	0.6276	0.6332	0.6385	0.6443	0.6498	0.6549	0.6600	0.6652
Bermuda	0.7780	0.7853	0.7922	0.7989	0.8051	0.8112	0.8175	0.8237	0.8297	0.8352	0.8393	0.8405	0.8448	0.8542	0.8562	0.8590	0.8615	0.8632	0.8679	0.8707	0.8740	0.8789	0.8828	0.8864	0.8902	0.8933	0.8995	0.9033	0.9069	0.9094	0.9114	0.9135	0.9151	0.9166	0.9182	0.9164
Cuba	0.5976	0.6063	0.6142	0.6216	0.6290	0.6358	0.6422	0.6481	0.6541	0.6597	0.6647	0.6680	0.6695	0.6690	0.6683	0.6677	0.6681	0.6689	0.6702	0.6730	0.6771	0.6821	0.6873	0.6927	0.6983	0.7049	0.7126	0.7203	0.7275	0.7339	0.7398	0.7458	0.7515	0.7570	0.7619	0.7662
Dominica	0.5220	0.5210	0.5397	0.5468	0.5547	0.5629	0.5712	0.5781	0.5866	0.5933	0.5992	0.6140	0.6062	0.6141	0.6257	0.6372	0.6462	0.6550	0.6635	0.6712	0.6784	0.6836	0.6884	0.6938	0.6991	0.7039	0.7087	0.7140	0.7195	0.7247	0.7300	0.7351	0.7394	0.7434	0.7479	0.7526
Dominican Republic	0.4344	0.4437	0.4523	0.4607	0.4685	0.4756	0.4826	0.4899	0.4971	0.5039	0.5096	0.5151	0.5213	0.5281	0.5348	0.5418	0.5491	0.5568	0.5647	0.5726	0.5803	0.5875	0.5946	0.6007	0.6065	0.6130	0.6202	0.6280	0.6353	0.6422	0.6495	0.6565	0.6632	0.6699	0.6768	0.6837
Grenada	0.4761	0.4858	0.4958	0.5055	0.5149	0.5242	0.5279	0.5322	0.5362	0.5411	0.5468	0.5614	0.5762	0.5898	0.6024	0.6136	0.6236	0.6329	0.6423	0.6521	0.6618	0.6706	0.6791	0.6879	0.6954	0.7035	0.7102	0.7169	0.7231	0.7278	0.7323	0.7365	0.7404	0.7444	0.7488	0.7532
Guyana	0.4605	0.4661	0.4696	0.4715	0.4735	0.4762	0.4795	0.4832	0.4866	0.4892	0.4917	0.4946	0.4996	0.5066	0.5149	0.5239	0.5335	0.5432	0.5512	0.5582	0.5640	0.5699	0.5751	0.5794	0.5841	0.5885	0.5939	0.6000	0.6058	0.6112	0.6165	0.6243	0.6320	0.6397	0.6472	0.6546
Haiti	0.2545	0.2580	0.2617	0.2661	0.2710	0.2766	0.2820	0.2876	0.2933	0.2986	0.3035	0.3085	0.3127	0.3163	0.3185	0.3218	0.3254	0.3295	0.3341	0.3392	0.3444	0.3494	0.3542	0.3590	0.3629	0.3666	0.3705	0.3748	0.3790	0.3836	0.3871	0.3914	0.3960	0.4012	0.4065	0.4118
Jamaica	0.5301	0.5345	0.5393	0.5447	0.5507	0.5565	0.5622	0.5690	0.5755	0.5821	0.5888	0.5961	0.6032	0.6104	0.6172	0.6240	0.6300	0.6355	0.6405	0.6455	0.6504	0.6554	0.6603	0.6652	0.6699	0.6744	0.6800	0.6855	0.6908	0.6954	0.6998	0.7039	0.7078	0.7116	0.7153	0.7189
Puerto Rico	0.7052	0.7130	0.7197	0.7258	0.7320	0.7380	0.7444	0.7505	0.7567	0.7627	0.7684	0.7742	0.7801	0.7860	0.7919	0.7978	0.8039	0.8097	0.8157	0.8214	0.8265	0.8326	0.8382	0.8433	0.8482	0.8528	0.8571	0.8608	0.8641	0.8670	0.8696	0.8719	0.8740	0.8763	0.8790	0.8820
Saint Lucia	0.4802	0.4929	0.5042	0.5139	0.5212	0.5284	0.5378	0.5466	0.5556	0.5645	0.5736	0.5837	0.5938	0.6030	0.6116	0.6199	0.6293	0.6381	0.6473	0.6562	0.6642	0.6710	0.6772	0.6836	0.6903	0.6962	0.7006	0.7052	0.7099	0.7147	0.7188	0.7240	0.7287	0.7329	0.7369	0.7408
Saint Vincent and the Grenadines	0.4918	0.5023	0.5125	0.5226	0.5329	0.5430	0.5525	0.5609	0.5702	0.5782	0.5855	0.5940	0.6031	0.6113	0.6186	0.6264	0.6340	0.6415	0.6488	0.6561	0.6631	0.6704	0.6776	0.6847	0.6916	0.6979	0.7049	0.7114	0.7176	0.7231	0.7279	0.7321	0.7361	0.7401	0.7437	0.7473
Suriname	0.4876	0.4958	0.5025	0.5082	0.5132	0.5186	0.5242	0.5292	0.5353	0.5416	0.5476	0.5529	0.5581	0.5625	0.5667	0.5710	0.5767	0.5829	0.5891	0.5943	0.5996	0.6058	0.6121	0.6189	0.6262	0.6335	0.6408	0.6482	0.6557	0.6630	0.6703	0.6776	0.6847	0.6914	0.6977	0.7037
Trinidad and Tobago	0.6293	0.6364	0.6430	0.6485	0.6539	0.6596	0.6643	0.6688	0.6731	0.6770	0.6813	0.6863	0.6909	0.6953	0.7002	0.7053	0.7111	0.7173	0.7237	0.7308	0.7385	0.7457	0.7531	0.7615	0.7698	0.7775	0.7860	0.7939	0.8014	0.8068	0.8115	0.8164	0.8209	0.8251	0.8290	0.8327
Virgin Islands, U.S.	0.6970	0.7072	0.7156	0.7225	0.7282	0.7336	0.7384	0.7437	0.7496	0.7559	0.7625	0.7716	0.7810	0.7903	0.7996	0.8088	0.8173	0.8256	0.8331	0.8393	0.8441	0.8490	0.8527	0.8554	0.8577	0.8607	0.8632	0.8663	0.8685	0.8707	0.8735	0.8767	0.8798	0.8823	0.8843	0.8861
Andean Latin America	0.4513	0.4600	0.4683	0.4758	0.4830	0.4899	0.4967	0.5036	0.5099	0.5148	0.5197	0.5247	0.5298	0.5352	0.5415	0.5482	0.5550	0.5622	0.5690	0.5753	0.5815	0.5874	0.5933	0.5992	0.6054	0.6119	0.6190	0.6262	0.6338	0.6406	0.6477	0.6550	0.6622	0.6694	0.6761	0.6824
Bolivia	0.3764	0.3825	0.3884	0.3939	0.3995	0.4050	0.4098	0.4146	0.4193	0.4244	0.4300	0.4363	0.4426	0.4494	0.4565	0.4641	0.4719	0.4798	0.4879	0.4955	0.5028	0.5100	0.5169	0.5237	0.5306	0.5377	0.5451	0.5524	0.5600	0.5673	0.5746	0.5822	0.5898	0.5975	0.6049	0.6120
Ecuador	0.4627	0.4724	0.4811	0.4888	0.4962	0.5034	0.5101	0.5158	0.5222	0.5283	0.5347	0.5411	0.5476	0.5539	0.5602	0.5662	0.5722	0.5783	0.5842	0.5893	0.5944	0.5995	0.6049	0.6102	0.6163	0.6227	0.6295	0.6359	0.6425	0.6485	0.6544	0.6607	0.6671	0.6735	0.6797	0.6852
Peru	0.4702	0.4792	0.4880	0.4959	0.5035	0.5106	0.5181	0.5260	0.5328	0.5371	0.5410	0.5449	0.5488	0.5534	0.5594	0.5663	0.5731	0.5806	0.5875	0.5942	0.6007	0.6066	0.6126	0.6185	0.6246	0.6311	0.6384	0.6461	0.6543	0.6614	0.6690	0.6768	0.6844	0.6918	0.6987	0.7050
Central Latin America	0.4575	0.4682	0.4778	0.4865	0.4949	0.5029	0.5106	0.5180	0.5250	0.5318	0.5387	0.5457	0.5530	0.5601	0.5673	0.5738	0.5802	0.5869	0.5934	0.5996	0.6060	0.6120	0.6176	0.6230	0.6288	0.6349	0.6416	0.6484	0.6550	0.6606	0.6662	0.6720	0.6778	0.6834	0.6887	0.6938
Colombia	0.4681	0.4766	0.4849	0.4929	0.5007	0.5083	0.5160	0.5234	0.5305	0.5374	0.5442	0.5510	0.5578	0.5647	0.5718	0.5791	0.5860	0.5927	0.5988	0.6039	0.6088	0.6136	0.6183	0.6231	0.6282	0.6339	0.6402	0.6471	0.6542	0.6608	0.6673	0.6742	0.6809	0.6874	0.6938	0.6998
Costa Rica	0.5156	0.5205	0.5246	0.5284	0.5325	0.5364	0.5406	0.5447	0.5490	0.5538	0.5587	0.5641	0.5704	0.5770	0.5837	0.5904	0.5973	0.6047	0.6126	0.6210	0.6289	0.6357	0.6422	0.6486	0.6547	0.6608	0.6673	0.6742	0.6807	0.6868	0.6930	0.6992	0.7056	0.7117	0.7176	0.7232
El Salvador	0.3758	0.3824	0.3882	0.3939	0.3995	0.4050	0.4109	0.4168	0.4228	0.4288	0.4353	0.4424	0.4502	0.4585	0.4671	0.4759	0.4843	0.4929	0.5016	0.5102	0.5186	0.5269	0.5349	0.5425	0.5499	0.5572	0.5646	0.5721	0.5791	0.5852	0.5910	0.5969	0.6026	0.6082	0.6135	0.6187
Guatemala	0.3117	0.3167	0.3218	0.3274	0.3333	0.3394	0.3452	0.3510	0.3568	0.3625	0.3680	0.3739	0.3798	0.3859	0.3924	0.3993	0.4063	0.4136	0.4212	0.4289	0.4366	0.4443	0.4521	0.4597	0.4671	0.4745	0.4820	0.4896	0.4969	0.5038	0.5104	0.5170	0.5235	0.5299	0.5363	0.5427
Honduras	0.3092	0.3170	0.3247	0.3321	0.3395	0.3468	0.3535	0.3602	0.3669	0.3736	0.3800	0.3863	0.3930	0.4001	0.4068	0.4140	0.4210	0.4285	0.4362	0.4433	0.4511	0.4590	0.4670	0.4751	0.4834	0.4921	0.5012	0.5104	0.5193	0.5273	0.5350	0.5424	0.5495	0.5563	0.5625	0.5684
Mexico	0.4661	0.4800	0.4921	0.5027	0.5127	0.5223	0.5311	0.5394	0.5473	0.5549	0.5626	0.5705	0.5783	0.5860	0.5936	0.5999	0.6063	0.6130	0.6198	0.6265	0.6335	0.6400	0.6460	0.6519	0.6578	0.6638	0.6704	0.6767	0.6827	0.6875	0.6924	0.6975	0.7028	0.7078	0.7127	0.7176
Aguascalientes	0.4838	0.4985	0.5112	0.5222	0.5326	0.5429	0.5528	0.5620	0.5706	0.5791	0.5867	0.5939	0.6011	0.6082	0.6151	0.6208	0.6268	0.6334	0.6401	0.6467	0.6538	0.6611	0.6684	0.6754	0.6825	0.6896	0.6972	0.7045	0.7113	0.7170	0.7227	0.7285	0.7343	0.7400	0.7455	0.7510
Baja California	0.5383	0.5522	0.5643	0.5748	0.5846	0.5929	0.5992	0.6051	0.6105	0.6160	0.6217	0.6280	0.6343	0.6406	0.6469	0.6519	0.6579	0.6649	0.6721	0.6793	0.6862	0.6931	0.7004	0.7073	0.7141	0.7209	0.7282	0.7352	0.7418	0.7472	0.7528	0.7586	0.7645	0.7702	0.7758	0.7815
Baja California Sur	0.5165	0.5329	0.5473	0.5599	0.5716	0.5837	0.5959	0.6075	0.6186	0.6294	0.6385	0.6459	0.6532	0.6604	0.6676	0.6735	0.6789	0.6839	0.6889	0.6937	0.6991	0.7050	0.7108	0.7165	0.7224	0.7284	0.7351	0.7417	0.7479	0.7531	0.7584	0.7640	0.7698	0.7753	0.7807	0.7862
Campeche	0.4470	0.4607	0.4726	0.4829	0.4926	0.5029	0.5133	0.5229	0.5321	0.5410	0.5492	0.5568	0.5645	0.5720	0.5794	0.5855	0.5913	0.5970	0.6029	0.6085	0.6150	0.6222	0.6295	0.6363	0.6432	0.6499	0.6571	0.6639	0.6702	0.6753	0.6804	0.6856	0.6910	0.6960	0.7010	0.7059
Chiapas	0.3174	0.3315	0.3437	0.3543	0.3643	0.3747	0.3852	0.3952	0.4048	0.4141	0.4231	0.4320	0.4407	0.4490	0.4572	0.4641	0.4711	0.4786	0.4861	0.4934	0.5011	0.5083	0.5149	0.5212	0.5275	0.5336	0.5401	0.5462	0.5518	0.5561	0.5603	0.5647	0.5691	0.5731	0.5771	0.5810
Chihuahua	0.5067	0.5201	0.5319	0.5421	0.5517	0.5603	0.5676	0.5744	0.5807	0.5870	0.5931	0.5992	0.6054	0.6115	0.6176	0.6225	0.6279	0.6343	0.6407	0.6469	0.6533	0.6596	0.6659	0.6721	0.6785	0.6850	0.6922	0.6992	0.7058	0.7112	0.7169	0.7227	0.7286	0.7344	0.7401	0.7457
Coahuila	0.5324	0.5474	0.5606	0.5720	0.5827	0.5928	0.6021	0.6108	0.6191	0.6273	0.6347	0.6416	0.6485	0.6553	0.6621	0.6677	0.6732	0.6791	0.6850	0.6909	0.6973	0.7038	0.7100	0.7161	0.7224	0.7289	0.7360	0.7430	0.7496	0.7552	0.7609	0.7668	0.7729	0.7788	0.7846	0.7904
Colima	0.4874	0.5020	0.5149	0.5261	0.5366	0.5479	0.5594	0.5702	0.5803	0.5902	0.5986	0.6059	0.6132	0.6205	0.6277	0.6336	0.6395	0.6455	0.6516	0.6578	0.6643	0.6702	0.6755	0.6806	0.6860	0.6915	0.6976	0.7036	0.7093	0.7138	0.7185	0.7235	0.7287	0.7337	0.7385	0.7434
Distrito Federal	0.6447	0.6559	0.6656	0.6739	0.6817	0.6882	0.6930	0.6974	0.7014	0.7053	0.7116	0.7205	0.7294	0.7384	0.7474	0.7552	0.7627	0.7701	0.7777	0.7852	0.7925	0.7980	0.8023	0.8068	0.8118	0.8172	0.8234	0.8298	0.8362	0.8417	0.8476	0.8539	0.8604	0.8669	0.8734	0.8799
Durango	0.4585	0.4729	0.4854	0.4962	0.5064	0.5168	0.5268	0.5364	0.5453	0.5542	0.5623	0.5700	0.5777	0.5853	0.5928	0.5989	0.6047	0.6101	0.6156	0.6210	0.6276	0.6350	0.6422	0.6490	0.6558	0.6625	0.6697	0.6766	0.6829	0.6880	0.6933	0.6987	0.7042	0.7095	0.7147	0.7199
Guanajuato	0.3999	0.4149	0.4281	0.4397	0.4506	0.4616	0.4726	0.4831	0.4930	0.5025	0.5114	0.5199	0.5282	0.5361	0.5440	0.5505	0.5568	0.5631	0.5692	0.5753	0.5822	0.5897	0.5970	0.6036	0.6101	0.6165	0.6231	0.6293	0.6351	0.6396	0.6441	0.6488	0.6535	0.6579	0.6622	0.6665
Guerrero	0.3573	0.3706	0.3823	0.3924	0.4021	0.4119	0.4216	0.4308	0.4395	0.4479	0.4554	0.4624	0.4692	0.4758	0.4822	0.4872	0.4930	0.4996	0.5061	0.5123	0.5195	0.5272	0.5345	0.5417	0.5488	0.5559	0.5633	0.5702	0.5764	0.5811	0.5859	0.5907	0.5955	0.5998	0.6041	0.6082
Hidalgo	0.4023	0.4176	0.4310	0.4428	0.4539	0.4655	0.4770	0.4878	0.4980	0.5078	0.5164	0.5242	0.5320	0.5395	0.5469	0.5530	0.5590	0.5651	0.5714	0.5776	0.5845	0.5910	0.5966	0.6021	0.6077	0.6134	0.6195	0.6254	0.6308	0.6351	0.6394	0.6439	0.6484	0.6527	0.6568	0.6609
Jalisco	0.4703	0.4847	0.4973	0.5082	0.5185	0.5283	0.5373	0.5458	0.5537	0.5615	0.5693	0.5774	0.5855	0.5935	0.6014	0.6079	0.6147	0.6218	0.6290	0.6361	0.6434	0.6504	0.6572	0.6636	0.6702	0.6766	0.6835	0.6902	0.6965	0.7015	0.7067	0.7121	0.7175	0.7228	0.7280	0.7331
México	0.4501	0.4678	0.4832	0.4965	0.5090	0.5193	0.5274	0.5349	0.5419	0.5487	0.5564	0.5651	0.5739	0.5825	0.5910	0.5981	0.6055	0.6134	0.6214	0.6292	0.6366	0.6422	0.6472	0.6520	0.6570	0.6622	0.6680	0.6737	0.6791	0.6832	0.6876	0.6922	0.6970	0.7016	0.7061	0.7105
Michoacán de Ocampo	0.3912	0.4048	0.4166	0.4269	0.4367	0.4473	0.4583	0.4687	0.4785	0.4880	0.4968	0.5049	0.5130	0.5209	0.5286	0.5350	0.5413	0.5477	0.5542	0.5607	0.5680	0.5753	0.5820	0.5881	0.5943	0.6003	0.6065	0.6125	0.6180	0.6222	0.6265	0.6309	0.6354	0.6397	0.6439	0.6480
Morelos	0.4707	0.4850	0.4976	0.5086	0.5189	0.5294	0.5397	0.5493	0.5584	0.5672	0.5748	0.5812	0.5878	0.5942	0.6006	0.6057	0.6110	0.6168	0.6225	0.6281	0.6343	0.6402	0.6458	0.6512	0.6569	0.6626	0.6690	0.6753	0.6811	0.6857	0.6905	0.6956	0.7007	0.7056	0.7105	0.7152
Nayarit	0.4403	0.4548	0.4674	0.4784	0.4888	0.4993	0.5095	0.5193	0.5285	0.5376	0.5460	0.5539	0.5618	0.5695	0.5772	0.5835	0.5893	0.5947	0.6002	0.6056	0.6121	0.6187	0.6245	0.6301	0.6358	0.6417	0.6481	0.6542	0.6600	0.6645	0.6692	0.6741	0.6791	0.6839	0.6886	0.6932
Nuevo León	0.5741	0.5898	0.6036	0.6156	0.6270	0.6367	0.6446	0.6519	0.6588	0.6655	0.6724	0.6796	0.6869	0.6942	0.7014	0.7073	0.7129	0.7183	0.7236	0.7288	0.7347	0.7410	0.7472	0.7532	0.7594	0.7657	0.7727	0.7795	0.7861	0.7916	0.7974	0.8034	0.8096	0.8157	0.8218	0.8278
Oaxaca	0.3500	0.3620	0.3725	0.3815	0.3900	0.3995	0.4095	0.4189	0.4279	0.4367	0.4449	0.4528	0.4607	0.4683	0.4758	0.4819	0.4885	0.4956	0.5029	0.5102	0.5182	0.5256	0.5322	0.5384	0.5445	0.5506	0.5571	0.5632	0.5688	0.5731	0.5774	0.5819	0.5863	0.5905	0.5945	0.5985
Puebla	0.4101	0.4239	0.4358	0.4461	0.4558	0.4655	0.4749	0.4836	0.4918	0.4999	0.5079	0.5161	0.5243	0.5323	0.5401	0.5466	0.5536	0.5614	0.5691	0.5767	0.5845	0.5917	0.5983	0.6045	0.6109	0.6173	0.6241	0.6307	0.6367	0.6415	0.6464	0.6514	0.6565	0.6613	0.6660	0.6706
Querétaro	0.4258	0.4435	0.4589	0.4724	0.4851	0.4984	0.5119	0.5244	0.5362	0.5477	0.5579	0.5672	0.5764	0.5854	0.5941	0.6015	0.6089	0.6167	0.6245	0.6322	0.6400	0.6473	0.6541	0.6605	0.6669	0.6730	0.6796	0.6858	0.6916	0.6960	0.7006	0.7052	0.7098	0.7142	0.7185	0.7227
Quintana Roo	0.4308	0.4538	0.4733	0.4900	0.5052	0.5198	0.5337	0.5468	0.5592	0.5712	0.5813	0.5899	0.5982	0.6062	0.6141	0.6207	0.6276	0.6350	0.6423	0.6494	0.6565	0.6629	0.6689	0.6746	0.6805	0.6864	0.6929	0.6991	0.7048	0.7094	0.7141	0.7189	0.7236	0.7282	0.7326	0.7369
San Luis Potosí	0.4188	0.4331	0.4456	0.4566	0.4671	0.4781	0.4895	0.5003	0.5104	0.5203	0.5290	0.5367	0.5444	0.5518	0.5591	0.5650	0.5712	0.5778	0.5843	0.5906	0.5977	0.6063	0.6154	0.6236	0.6310	0.6380	0.6451	0.6516	0.6575	0.6619	0.6664	0.6710	0.6755	0.6799	0.6843	0.6885
Sinaloa	0.4717	0.4873	0.5011	0.5131	0.5245	0.5350	0.5445	0.5534	0.5618	0.5700	0.5778	0.5853	0.5928	0.6001	0.6074	0.6133	0.6192	0.6252	0.6311	0.6369	0.6432	0.6497	0.6561	0.6620	0.6681	0.6741	0.6807	0.6870	0.6929	0.6975	0.7023	0.7072	0.7123	0.7171	0.7219	0.7265
Sonora	0.5285	0.5428	0.5553	0.5662	0.5764	0.5860	0.5945	0.6025	0.6101	0.6177	0.6248	0.6317	0.6386	0.6455	0.6523	0.6578	0.6632	0.6685	0.6737	0.6786	0.6843	0.6905	0.6967	0.7029	0.7092	0.7156	0.7227	0.7296	0.7362	0.7416	0.7472	0.7530	0.7589	0.7647	0.7704	0.7761
Tabasco	0.4356	0.4535	0.4692	0.4829	0.4959	0.5095	0.5235	0.5368	0.5493	0.5614	0.5724	0.5825	0.5924	0.6019	0.6113	0.6193	0.6271	0.6349	0.6428	0.6507	0.6586	0.6654	0.6713	0.6768	0.6825	0.6882	0.6943	0.7003	0.7059	0.7103	0.7149	0.7196	0.7244	0.7289	0.7333	0.7376
Tamaulipas	0.5234	0.5380	0.5508	0.5620	0.5725	0.5824	0.5914	0.5998	0.6078	0.6157	0.6227	0.6292	0.6357	0.6421	0.6486	0.6537	0.6589	0.6644	0.6700	0.6755	0.6817	0.6879	0.6937	0.6994	0.7053	0.7113	0.7180	0.7246	0.7309	0.7362	0.7416	0.7473	0.7531	0.7588	0.7644	0.7699
Tlaxcala	0.4191	0.4335	0.4461	0.4570	0.4673	0.4788	0.4910	0.5025	0.5132	0.5236	0.5327	0.5408	0.5488	0.5567	0.5644	0.5707	0.5772	0.5844	0.5916	0.5988	0.6061	0.6125	0.6182	0.6236	0.6293	0.6350	0.6414	0.6475	0.6532	0.6576	0.6623	0.6671	0.6720	0.6766	0.6812	0.6856
Veracruz de Ignacio de la Llave	0.4391	0.4526	0.4644	0.4746	0.4844	0.4936	0.5019	0.5099	0.5173	0.5247	0.5321	0.5398	0.5474	0.5549	0.5624	0.5685	0.5748	0.5816	0.5886	0.5955	0.6025	0.6087	0.6143	0.6195	0.6249	0.6303	0.6362	0.6420	0.6474	0.6516	0.6559	0.6605	0.6652	0.6696	0.6740	0.6782
Yucatán	0.4533	0.4661	0.4773	0.4869	0.4960	0.5048	0.5130	0.5207	0.5279	0.5351	0.5428	0.5511	0.5595	0.5678	0.5759	0.5827	0.5898	0.5974	0.6051	0.6128	0.6202	0.6265	0.6321	0.6376	0.6433	0.6492	0.6557	0.6620	0.6680	0.6728	0.6777	0.6830	0.6883	0.6934	0.6985	0.7035
Zacatecas	0.4020	0.4163	0.4287	0.4395	0.4496	0.4617	0.4751	0.4878	0.4999	0.5115	0.5211	0.5290	0.5368	0.5443	0.5518	0.5579	0.5633	0.5682	0.5730	0.5778	0.5841	0.5907	0.5965	0.6021	0.6079	0.6138	0.6201	0.6263	0.6321	0.6367	0.6414	0.6463	0.6513	0.6560	0.6607	0.6652
Nicaragua	0.3450	0.3499	0.3547	0.3606	0.3666	0.3726	0.3786	0.3848	0.3896	0.3945	0.3993	0.4043	0.4092	0.4145	0.4202	0.4265	0.4335	0.4409	0.4485	0.4567	0.4649	0.4729	0.4802	0.4870	0.4940	0.5007	0.5074	0.5141	0.5206	0.5260	0.5315	0.5376	0.5439	0.5503	0.5567	0.5631
Panama	0.5293	0.5382	0.5467	0.5541	0.5611	0.5680	0.5749	0.5813	0.5857	0.5898	0.5940	0.5990	0.6045	0.6100	0.6152	0.6198	0.6250	0.6307	0.6370	0.6432	0.6490	0.6544	0.6595	0.6648	0.6701	0.6757	0.6817	0.6883	0.6952	0.7017	0.7084	0.7157	0.7236	0.7316	0.7393	0.7467
Venezuela	0.5327	0.5400	0.5468	0.5527	0.5583	0.5634	0.5687	0.5743	0.5802	0.5852	0.5909	0.5974	0.6043	0.6108	0.6166	0.6224	0.6278	0.6333	0.6384	0.6426	0.6472	0.6518	0.6553	0.6580	0.6627	0.6688	0.6759	0.6836	0.6915	0.6977	0.7035	0.7095	0.7155	0.7210	0.7254	0.7283
Tropical Latin America	0.4223	0.4306	0.4389	0.4468	0.4549	0.4633	0.4723	0.4813	0.4899	0.4982	0.5055	0.5127	0.5193	0.5257	0.5322	0.5389	0.5457	0.5522	0.5583	0.5640	0.5694	0.5757	0.5818	0.5879	0.5942	0.6005	0.6070	0.6137	0.6206	0.6266	0.6328	0.6392	0.6449	0.6506	0.6565	0.6611
Brazil	0.4225	0.4309	0.4392	0.4471	0.4553	0.4638	0.4729	0.4819	0.4906	0.4989	0.5063	0.5135	0.5201	0.5266	0.5331	0.5397	0.5465	0.5530	0.5590	0.5647	0.5700	0.5764	0.5825	0.5886	0.5949	0.6013	0.6077	0.6145	0.6214	0.6274	0.6336	0.6399	0.6457	0.6513	0.6571	0.6616
Acre	0.2930	0.3030	0.3132	0.3231	0.3332	0.3434	0.3556	0.3674	0.3785	0.3890	0.3983	0.4057	0.4123	0.4186	0.4248	0.4310	0.4381	0.4449	0.4512	0.4573	0.4636	0.4682	0.4690	0.4756	0.4804	0.4858	0.4969	0.5052	0.5062	0.5172	0.5258	0.5286	0.5398	0.5448	0.5512	0.5557
Alagoas	0.2781	0.2867	0.2953	0.3035	0.3120	0.3209	0.3309	0.3408	0.3503	0.3593	0.3674	0.3740	0.3800	0.3859	0.3917	0.3977	0.4044	0.4107	0.4165	0.4219	0.4278	0.4289	0.4370	0.4437	0.4530	0.4578	0.4650	0.4704	0.4768	0.4835	0.4899	0.4961	0.5025	0.5073	0.5076	0.5116
Amapá	0.3376	0.3492	0.3612	0.3730	0.3849	0.3970	0.4115	0.4256	0.4386	0.4508	0.4616	0.4718	0.4808	0.4894	0.4977	0.5060	0.5134	0.5203	0.5268	0.5330	0.5285	0.5352	0.5462	0.5509	0.5655	0.5736	0.5774	0.5869	0.5935	0.6044	0.6083	0.6172	0.6255	0.6289	0.6364	0.6416
Amazonas	0.3448	0.3559	0.3672	0.3783	0.3897	0.4011	0.4157	0.4298	0.4429	0.4551	0.4660	0.4724	0.4780	0.4833	0.4884	0.4934	0.5014	0.5090	0.5162	0.5231	0.5381	0.5419	0.5479	0.5558	0.5633	0.5695	0.5735	0.5832	0.5914	0.5971	0.6059	0.6129	0.6187	0.6242	0.6304	0.6355
Bahia	0.3123	0.3205	0.3289	0.3369	0.3453	0.3541	0.3659	0.3775	0.3883	0.3986	0.4078	0.4151	0.4217	0.4282	0.4347	0.4413	0.4490	0.4563	0.4631	0.4695	0.4808	0.4849	0.4894	0.4944	0.5011	0.5076	0.5143	0.5196	0.5261	0.5315	0.5378	0.5435	0.5493	0.5552	0.5550	0.5590
Ceará	0.3161	0.3232	0.3303	0.3372	0.3444	0.3520	0.3608	0.3696	0.3781	0.3862	0.3935	0.4008	0.4074	0.4139	0.4204	0.4270	0.4339	0.4405	0.4466	0.4522	0.4649	0.4663	0.4737	0.4805	0.4875	0.4928	0.4993	0.5054	0.5125	0.5182	0.5247	0.5313	0.5371	0.5428	0.5441	0.5483
Distrito Federal	0.5742	0.5837	0.5931	0.6022	0.6115	0.6212	0.6324	0.6435	0.6541	0.6643	0.6735	0.6811	0.6881	0.6949	0.7018	0.7089	0.7160	0.7229	0.7294	0.7356	0.7302	0.7350	0.7432	0.7503	0.7592	0.7675	0.7754	0.7849	0.7949	0.8025	0.8102	0.8210	0.8288	0.8352	0.8486	0.8546
Espírito Santo	0.4432	0.4525	0.4617	0.4704	0.4794	0.4886	0.4979	0.5072	0.5160	0.5244	0.5320	0.5398	0.5470	0.5540	0.5610	0.5682	0.5750	0.5815	0.5875	0.5931	0.5948	0.6010	0.6081	0.6154	0.6218	0.6292	0.6348	0.6410	0.6476	0.6531	0.6586	0.6646	0.6704	0.6747	0.6809	0.6851
Goiás	0.3900	0.4024	0.4144	0.4257	0.4368	0.4479	0.4570	0.4661	0.4746	0.4829	0.4903	0.4972	0.5036	0.5099	0.5163	0.5228	0.5292	0.5352	0.5407	0.5458	0.5514	0.5560	0.5603	0.5667	0.5730	0.5790	0.5859	0.5926	0.5985	0.6033	0.6089	0.6144	0.6179	0.6223	0.6292	0.6330
Maranhão	0.2667	0.2716	0.2769	0.2820	0.2877	0.2940	0.3051	0.3161	0.3265	0.3365	0.3454	0.3533	0.3605	0.3674	0.3742	0.3810	0.3890	0.3966	0.4037	0.4104	0.4359	0.4355	0.4364	0.4376	0.4447	0.4498	0.4558	0.4618	0.4688	0.4756	0.4828	0.4888	0.4959	0.5014	0.4980	0.5020
Mato Grosso	0.3947	0.4063	0.4179	0.4289	0.4400	0.4512	0.4613	0.4712	0.4807	0.4899	0.4980	0.5058	0.5129	0.5198	0.5266	0.5334	0.5410	0.5483	0.5551	0.5615	0.5684	0.5742	0.5793	0.5839	0.5869	0.5923	0.6008	0.6092	0.6141	0.6206	0.6260	0.6307	0.6362	0.6397	0.6469	0.6509
Mato Grosso do Sul	0.4227	0.4327	0.4425	0.4517	0.4612	0.4708	0.4794	0.4881	0.4963	0.5043	0.5113	0.5182	0.5244	0.5304	0.5366	0.5428	0.5496	0.5560	0.5619	0.5675	0.5739	0.5779	0.5831	0.5899	0.5926	0.5997	0.6076	0.6150	0.6192	0.6258	0.6322	0.6369	0.6427	0.6485	0.6560	0.6601
Minas Gerais	0.4267	0.4352	0.4437	0.4517	0.4599	0.4685	0.4769	0.4853	0.4933	0.5010	0.5078	0.5149	0.5213	0.5276	0.5340	0.5406	0.5475	0.5540	0.5601	0.5658	0.5734	0.5776	0.5848	0.5899	0.5970	0.6031	0.6097	0.6165	0.6234	0.6297	0.6350	0.6410	0.6464	0.6520	0.6551	0.6595

Location	1980	1981	1982	1983	1984	1985	1986	1987	1988	1989	1990	1991	1992	1993	1994	1995	1996	1997	1998	1999	2000	2001	2002	2003	2004	2005	2006	2007	2008	2009	2010	2011	2012	2013	2014	2015
Pará	0.3317	0.3398	0.3481	0.3562	0.3647	0.3735	0.3846	0.3955	0.4059	0.4157	0.4243	0.4318	0.4385	0.4449	0.4513	0.4578	0.4647	0.4712	0.4772	0.4830	0.5059	0.5042	0.5091	0.5110	0.5190	0.5253	0.5287	0.5353	0.5436	0.5527	0.5604	0.5679	0.5758	0.5814	0.5819	0.5868
Paraíba	0.3029	0.3109	0.3188	0.3264	0.3342	0.3424	0.3521	0.3618	0.3710	0.3799	0.3878	0.3948	0.4011	0.4074	0.4136	0.4201	0.4276	0.4349	0.4416	0.4478	0.4615	0.4592	0.4648	0.4701	0.4773	0.4817	0.4893	0.4953	0.5004	0.5064	0.5127	0.5190	0.5253	0.5300	0.5311	0.5349
Paraná	0.4400	0.4500	0.4599	0.4693	0.4787	0.4883	0.4965	0.5046	0.5123	0.5198	0.5265	0.5333	0.5395	0.5457	0.5518	0.5581	0.5650	0.5715	0.5775	0.5832	0.5853	0.5928	0.5979	0.6058	0.6106	0.6163	0.6231	0.6306	0.6365	0.6419	0.6466	0.6531	0.6582	0.6628	0.6704	0.6746
Pernambuco	0.3480	0.3565	0.3650	0.3730	0.3814	0.3901	0.3997	0.4093	0.4184	0.4272	0.4350	0.4423	0.4490	0.4555	0.4621	0.4688	0.4755	0.4818	0.4875	0.4929	0.4974	0.5005	0.5083	0.5152	0.5218	0.5272	0.5339	0.5405	0.5470	0.5530	0.5604	0.5660	0.5709	0.5760	0.5806	0.5848
Piauí	0.2732	0.2807	0.2883	0.2955	0.3030	0.3108	0.3210	0.3312	0.3407	0.3499	0.3581	0.3650	0.3713	0.3775	0.3837	0.3900	0.3972	0.4040	0.4103	0.4162	0.4231	0.4267	0.4338	0.4402	0.4461	0.4501	0.4556	0.4625	0.4694	0.4749	0.4810	0.4862	0.4923	0.4978	0.4988	0.5026
Rio de Janeiro	0.5202	0.5285	0.5366	0.5442	0.5520	0.5602	0.5680	0.5759	0.5833	0.5906	0.5969	0.6033	0.6091	0.6149	0.6207	0.6268	0.6331	0.6390	0.6444	0.6494	0.6485	0.6573	0.6646	0.6699	0.6767	0.6850	0.6911	0.6976	0.7059	0.7112	0.7180	0.7245	0.7302	0.7362	0.7477	0.7529
Rio Grande do Norte	0.3393	0.3472	0.3550	0.3626	0.3706	0.3789	0.3883	0.3977	0.4067	0.4153	0.4230	0.4308	0.4379	0.4448	0.4518	0.4589	0.4660	0.4727	0.4789	0.4847	0.4898	0.4957	0.5011	0.5075	0.5151	0.5198	0.5285	0.5342	0.5398	0.5455	0.5522	0.5584	0.5646	0.5699	0.5709	0.5749
Rio Grande do Sul	0.5034	0.5100	0.5164	0.5225	0.5289	0.5357	0.5430	0.5504	0.5574	0.5642	0.5702	0.5769	0.5830	0.5891	0.5953	0.6017	0.6081	0.6141	0.6197	0.6250	0.6256	0.6344	0.6409	0.6484	0.6528	0.6613	0.6683	0.6771	0.6841	0.6899	0.6963	0.7022	0.7080	0.7130	0.7212	0.7262
Rondônia	0.3377	0.3512	0.3646	0.3771	0.3895	0.4017	0.4119	0.4219	0.4313	0.4402	0.4481	0.4555	0.4621	0.4685	0.4748	0.4811	0.4887	0.4959	0.5027	0.5091	0.5110	0.5231	0.5261	0.5328	0.5389	0.5473	0.5584	0.5677	0.5680	0.5743	0.5806	0.5843	0.5917	0.5960	0.5997	0.6040
Roraima	0.3680	0.3800	0.3920	0.4035	0.4151	0.4268	0.4364	0.4460	0.4551	0.4638	0.4715	0.4813	0.4881	0.4947	0.5013	0.5079	0.5140	0.5196	0.5249	0.5299	0.5189	0.5300	0.5445	0.5410	0.5549	0.5653	0.5732	0.5813	0.5874	0.5979	0.6047	0.6125	0.6150	0.6206	0.6329	0.6381
Santa Catarina	0.4770	0.4857	0.4942	0.5023	0.5106	0.5192	0.5272	0.5352	0.5428	0.5502	0.5568	0.5641	0.5707	0.5773	0.5839	0.5906	0.5975	0.6041	0.6102	0.6159	0.6185	0.6281	0.6345	0.6417	0.6464	0.6535	0.6590	0.6664	0.6723	0.6789	0.6847	0.6908	0.6960	0.7016	0.7103	0.7149
São Paulo	0.5197	0.5274	0.5351	0.5423	0.5499	0.5578	0.5664	0.5751	0.5834	0.5914	0.5985	0.6060	0.6128	0.6196	0.6265	0.6335	0.6400	0.6461	0.6518	0.6571	0.6561	0.6657	0.6716	0.6785	0.6841	0.6908	0.6974	0.7042	0.7116	0.7173	0.7233	0.7302	0.7356	0.7421	0.7518	0.7567
Sergipe	0.3167	0.3270	0.3372	0.3471	0.3572	0.3675	0.3780	0.3885	0.3985	0.4080	0.4166	0.4241	0.4309	0.4375	0.4442	0.4509	0.4578	0.4644	0.4706	0.4763	0.4799	0.4845	0.4942	0.5002	0.5080	0.5122	0.5175	0.5250	0.5313	0.5383	0.5454	0.5510	0.5574	0.5624	0.5666	0.5709
Tocantins	0.3736	0.3802	0.3868	0.3932	0.3999	0.4071	0.4129	0.4188	0.4245	0.4299	0.4345	0.4425	0.4497	0.4567	0.4635	0.4705	0.4784	0.4860	0.4931	0.4999	0.5044	0.5085	0.5168	0.5241	0.5323	0.5381	0.5445	0.5534	0.5600	0.5673	0.5746	0.5809	0.5881	0.5944	0.5960	0.6006
Paraguay	0.4137	0.4214	0.4283	0.4344	0.4404	0.4464	0.4522	0.4582	0.4646	0.4712	0.4779	0.4842	0.4903	0.4966	0.5032	0.5102	0.5176	0.5254	0.5330	0.5404	0.5470	0.5531	0.5589	0.5649	0.5708	0.5762	0.5819	0.5880	0.5946	0.6004	0.6077	0.6150	0.6215	0.6293	0.6367	0.6436
North Africa and Middle East	0.3300	0.3378	0.3462	0.3550	0.3641	0.3737	0.3829	0.3924	0.4025	0.4124	0.4225	0.4325	0.4421	0.4514	0.4602	0.4689	0.4779	0.4868	0.4956	0.5040	0.5121	0.5195	0.5268	0.5337	0.5408	0.5479	0.5550	0.5620	0.5685	0.5745	0.5803	0.5858	0.5908	0.5956	0.6001	0.6045
North Africa and Middle East	0.3300	0.3378	0.3462	0.3550	0.3641	0.3737	0.3829	0.3924	0.4025	0.4124	0.4225	0.4325	0.4421	0.4514	0.4602	0.4689	0.4779	0.4868	0.4956	0.5040	0.5121	0.5195	0.5268	0.5337	0.5408	0.5479	0.5550	0.5620	0.5685	0.5745	0.5803	0.5858	0.5908	0.5956	0.6001	0.6045
Afghanistan	0.1291	0.1314	0.1336	0.1355	0.1373	0.1392	0.1407	0.1413	0.1418	0.1427	0.1440	0.1473	0.1514	0.1536	0.1530	0.1526	0.1499	0.1464	0.1432	0.1415	0.1416	0.1439	0.1524	0.1625	0.1716	0.1812	0.1892	0.1984	0.2072	0.2186	0.2303	0.2428	0.2560	0.2682	0.2791	0.2888
Algeria	0.3173	0.3301	0.3435	0.3563	0.3694	0.3823	0.3947	0.4067	0.4188	0.4311	0.4434	0.4558	0.4682	0.4805	0.4924	0.5042	0.5142	0.5236	0.5324	0.5404	0.5474	0.5537	0.5592	0.5641	0.5680	0.5714	0.5735	0.5750	0.5762	0.5772	0.5784	0.5800	0.5822	0.5846	0.5872	0.5900
Bahrain	0.5059	0.5172	0.5267	0.5350	0.5429	0.5498	0.5568	0.5651	0.5750	0.5856	0.5969	0.6062	0.6165	0.6278	0.6392	0.6503	0.6585	0.6664	0.6735	0.6792	0.6849	0.6920	0.6981	0.7042	0.7110	0.7187	0.7301	0.7409	0.7501	0.7569	0.7621	0.7642	0.7662	0.7687	0.7721	0.7764
Egypt	0.3470	0.3541	0.3616	0.3694	0.3777	0.3865	0.3961	0.4063	0.4176	0.4292	0.4409	0.4526	0.4643	0.4754	0.4864	0.4970	0.5078	0.5177	0.5269	0.5357	0.5439	0.5517	0.5590	0.5660	0.5726	0.5788	0.5843	0.5895	0.5942	0.5980	0.6012	0.6045	0.6075	0.6107	0.6144	0.6191
Iran	0.3456	0.3485	0.3535	0.3606	0.3698	0.3814	0.3940	0.4088	0.4249	0.4418	0.4600	0.4783	0.4958	0.5133	0.5300	0.5461	0.5617	0.5764	0.5897	0.6020	0.6134	0.6238	0.6343	0.6446	0.6543	0.6636	0.6720	0.6802	0.6868	0.6923	0.6977	0.7029	0.7066	0.7095	0.7126	0.7154
Iraq	0.3234	0.3327	0.3420	0.3507	0.3589	0.3664	0.3726	0.3792	0.3857	0.3921	0.3997	0.4041	0.4093	0.4162	0.4227	0.4282	0.4345	0.4424	0.4544	0.4676	0.4800	0.4913	0.5002	0.5047	0.5116	0.5172	0.5223	0.5263	0.5296	0.5331	0.5376	0.5438	0.5517	0.5606	0.5685	0.5756
Jordan	0.3625	0.3818	0.4007	0.4194	0.4374	0.4531	0.4633	0.4727	0.4814	0.4891	0.4967	0.5130	0.5287	0.5426	0.5549	0.5657	0.5714	0.5770	0.5826	0.5887	0.5952	0.6021	0.6090	0.6157	0.6228	0.6302	0.6379	0.6456	0.6535	0.6606	0.6669	0.6728	0.6784	0.6839	0.6894	0.6949
Kuwait	0.5264	0.5424	0.5599	0.5785	0.5979	0.6181	0.6346	0.6524	0.6680	0.6820	0.6911	0.6972	0.7048	0.7121	0.7173	0.7216	0.7260	0.7333	0.7438	0.7548	0.7652	0.7710	0.7751	0.7793	0.7840	0.7890	0.7973	0.8066	0.8165	0.8264	0.8359	0.8420	0.8479	0.8534	0.8582	0.8624
Lebanon	0.5012	0.5084	0.5126	0.5184	0.5278	0.5394	0.5494	0.5596	0.5656	0.5677	0.5698	0.5763	0.5829	0.5894	0.5959	0.6026	0.6093	0.6166	0.6249	0.6335	0.6423	0.6503	0.6593	0.6686	0.6781	0.6870	0.6941	0.7005	0.7067	0.7135	0.7202	0.7277	0.7353	0.7424	0.7489	0.7547
Libya	0.3584	0.3715	0.3843	0.3958	0.4069	0.4182	0.4294	0.4404	0.4509	0.4623	0.4747	0.4879	0.5007	0.5140	0.5259	0.5358	0.5452	0.5537	0.5616	0.5688	0.5756	0.5817	0.5873	0.5935	0.5997	0.6068	0.6138	0.6211	0.6282	0.6347	0.6413	0.6399	0.6429	0.6444	0.6439	0.6430
Morocco	0.2522	0.2591	0.2669	0.2749	0.2831	0.2916	0.3005	0.3088	0.3180	0.3265	0.3347	0.3434	0.3512	0.3587	0.3669	0.3743	0.3830	0.3908	0.3986	0.4058	0.4123	0.4188	0.4248	0.4307	0.4362	0.4411	0.4468	0.4521	0.4574	0.4626	0.4677	0.4734	0.4789	0.4846	0.4902	0.4959
Palestine	0.3528	0.3610	0.3696	0.3795	0.3884	0.3959	0.4023	0.4075	0.4123	0.4173	0.4229	0.4298	0.4381	0.4475	0.4586	0.4713	0.4854	0.5008	0.5156	0.5294	0.5410	0.5481	0.5511	0.5519	0.5518	0.5512	0.5503	0.5490	0.5476	0.5466	0.5471	0.5494	0.5531	0.5575	0.5622	0.5670
Oman	0.2905	0.2932	0.2995	0.3084	0.3186	0.3306	0.3421	0.3555	0.3716	0.3893	0.4089	0.4336	0.4575	0.4798	0.5005	0.5201	0.5386	0.5576	0.5757	0.5930	0.6098	0.6219	0.6332	0.6430	0.6514	0.6583	0.6663	0.6720	0.6771	0.6812	0.6863	0.6953	0.7049	0.7142	0.7226	0.7301
Qatar	0.4963	0.5107	0.5244	0.5375	0.5503	0.5630	0.5742	0.5862	0.5977	0.6078	0.6162	0.6283	0.6375	0.6453	0.6512	0.6571	0.6606	0.6668	0.6728	0.6789	0.6850	0.6973	0.7081	0.7175	0.7258	0.7330	0.7434	0.7528	0.7615	0.7692	0.7760	0.7820	0.7879	0.7937	0.7992	0.8045
Saudi Arabia	0.4005	0.4179	0.4355	0.4522	0.4684	0.4832	0.4909	0.4985	0.5065	0.5149	0.5245	0.5333	0.5426	0.5524	0.5624	0.5726	0.5883	0.6034	0.6176	0.6308	0.6433	0.6530	0.6623	0.6716	0.6809	0.6901	0.6991	0.7075	0.7158	0.7227	0.7299	0.7369	0.7435	0.7495	0.7547	0.7593
'Asir	0.3632	0.3824	0.4015	0.4189	0.4355	0.4507	0.4588	0.4670	0.4750	0.4833	0.4929	0.5017	0.5112	0.5208	0.5309	0.5413	0.5581	0.5740	0.5885	0.6017	0.6139	0.6239	0.6333	0.6426	0.6517	0.6605	0.6694	0.6777	0.6862	0.6938	0.7021	0.7118	0.7210	0.7295	0.7342	0.7383
Bahah	0.3956	0.4101	0.4240	0.4366	0.4486	0.4595	0.4654	0.4712	0.4770	0.4828	0.4899	0.4968	0.5047	0.5127	0.5211	0.5298	0.5435	0.5566	0.5683	0.5790	0.5891	0.5977	0.6058	0.6142	0.6227	0.6310	0.6397	0.6480	0.6567	0.6644	0.6729	0.6828	0.6923	0.7011	0.7061	0.7104
Eastern Province	0.4236	0.4407	0.4579	0.4743	0.4901	0.5045	0.5124	0.5207	0.5293	0.5383	0.5486	0.5578	0.5677	0.5772	0.5868	0.5966	0.6117	0.6264	0.6404	0.6535	0.6659	0.6756	0.6848	0.6939	0.7031	0.7112	0.7193	0.7268	0.7344	0.7411	0.7483	0.7573	0.7660	0.7738	0.7790	0.7837
Ha'il	0.4064	0.4227	0.4388	0.4535	0.4676	0.4806	0.4878	0.4950	0.5022	0.5096	0.5182	0.5265	0.5356	0.5447	0.5541	0.5639	0.5793	0.5938	0.6071	0.6191	0.6304	0.6399	0.6488	0.6577	0.6666	0.6753	0.6841	0.6924	0.7010	0.7086	0.7169	0.7258	0.7343	0.7420	0.7469	0.7512
Jawf	0.2983	0.3293	0.3592	0.3855	0.4098	0.4315	0.4426	0.4540	0.4650	0.4764	0.4893	0.5010	0.5135	0.5258	0.5384	0.5515	0.5721	0.5914	0.6089	0.6248	0.6393	0.6507	0.6614	0.6719	0.6821	0.6927	0.7034	0.7135	0.7238	0.7331	0.7430	0.7549	0.7663	0.7766	0.7825	0.7879
Jizan	0.3172	0.3320	0.3464	0.3590	0.3709	0.3816	0.3870	0.3923	0.3974	0.4027	0.4092	0.4155	0.4227	0.4304	0.4387	0.4473	0.4613	0.4746	0.4865	0.4971	0.5072	0.5154	0.5232	0.5310	0.5391	0.5475	0.5562	0.5643	0.5727	0.5797	0.5873	0.5969	0.6062	0.6147	0.6190	0.6228
Madinah	0.3047	0.3317	0.3586	0.3832	0.4062	0.4269	0.4374	0.4483	0.4591	0.4702	0.4827	0.4940	0.5059	0.5176	0.5296	0.5419	0.5610	0.5791	0.5957	0.6110	0.6252	0.6362	0.6467	0.6571	0.6673	0.6763	0.6852	0.6933	0.7015	0.7086	0.7162	0.7246	0.7326	0.7398	0.7452	0.7500
Makkah	0.4182	0.4341	0.4500	0.4651	0.4796	0.4929	0.4999	0.5071	0.5146	0.5225	0.5317	0.5403	0.5496	0.5588	0.5683	0.5780	0.5928	0.6071	0.6205	0.6329	0.6446	0.6541	0.6630	0.6720	0.6811	0.6884	0.6958	0.7025	0.7095	0.7153	0.7217	0.7286	0.7353	0.7414	0.7468	0.7518
Najran	0.2531	0.2835	0.3118	0.3362	0.3581	0.3770	0.3868	0.3968	0.4065	0.4162	0.4271	0.4358	0.4452	0.4547	0.4648	0.4753	0.4938	0.5114	0.5276	0.5422	0.5556	0.5657	0.5752	0.5844	0.5933	0.6019	0.6106	0.6186	0.6266	0.6336	0.6413	0.6509	0.6601	0.6686	0.6732	0.6771
Northern Borders	0.4254	0.4370	0.4491	0.4609	0.4724	0.4828	0.4865	0.4909	0.4958	0.5014	0.5083	0.5144	0.5212	0.5301	0.5399	0.5498	0.5655	0.5808	0.5952	0.6086	0.6214	0.6311	0.6403	0.6494	0.6586	0.6748	0.6922	0.7074	0.7187	0.7235	0.7246	0.7215	0.7177	0.7128	0.7183	0.7233
Qassim	0.3603	0.3804	0.4016	0.4221	0.4422	0.4613	0.4727	0.4846	0.4963	0.5084	0.5212	0.5320	0.5433	0.5543	0.5656	0.5772	0.5951	0.6121	0.6278	0.6423	0.6558	0.6664	0.6765	0.6863	0.6960	0.7051	0.7130	0.7203	0.7274	0.7349	0.7429	0.7517	0.7601	0.7679	0.7727	0.7771
Riyadh	0.5005	0.5099	0.5209	0.5331	0.5457	0.5578	0.5632	0.5672	0.5723	0.5781	0.5854	0.5918	0.5988	0.6081	0.6182	0.6281	0.6422	0.6562	0.6698	0.6829	0.6955	0.7051	0.7143	0.7235	0.7327	0.7418	0.7502	0.7576	0.7644	0.7692	0.7734	0.7775	0.7815	0.7850	0.7902	0.7944
Tabuk	0.3509	0.3679	0.3870	0.4065	0.4252	0.4420	0.4479	0.4552	0.4631	0.4717	0.4819	0.4893	0.4974	0.5088	0.5215	0.5344	0.5559	0.5766	0.5961	0.6141	0.6306	0.6426	0.6540	0.6649	0.6755	0.7008	0.7254	0.7448	0.7571	0.7609	0.7594	0.7546	0.7490	0.7423	0.7480	0.7531
Sudan	0.2318	0.2363	0.2416	0.2459	0.2493	0.2519	0.2543	0.2565	0.2600	0.2634	0.2667	0.2710	0.2756	0.2803	0.2847	0.2889	0.2921	0.2972	0.3033	0.3105	0.3188	0.3270	0.3351	0.3428	0.3506	0.3582	0.3663	0.3745	0.3823	0.3898	0.3971	0.4046	0.4107	0.4166	0.4224	0.4282
Syria	0.2946	0.3037	0.3130	0.3226	0.3316	0.3410	0.3499	0.3588	0.3692	0.3785	0.3881	0.3980	0.4084	0.4190	0.4293	0.4394	0.4487	0.4575	0.4664	0.4746	0.4830	0.4903	0.4980	0.5054	0.5143	0.5253	0.5376	0.5498	0.5613	0.5716	0.5807	0.5844	0.5836	0.5819	0.5801	0.5790
Tunisia	0.3408	0.3516	0.3622	0.3726	0.3831	0.3936	0.4045	0.4157	0.4269	0.4384	0.4503	0.4629	0.4761	0.4891	0.5017	0.5140	0.5253	0.5359	0.5457	0.5552	0.5642	0.5726	0.5802	0.5875	0.5944	0.6007	0.6072	0.6135	0.6194	0.6248	0.6298	0.6342	0.6388	0.6432	0.6475	0.6515
Turkey	0.4416	0.4494	0.4571	0.4649	0.4730	0.4811	0.4897	0.4988	0.5077	0.5159	0.5242	0.5318	0.5393	0.5468	0.5530	0.5594	0.5665	0.5738	0.5807	0.5869	0.5935	0.5993	0.6053	0.6112	0.6178	0.6247	0.6320	0.6392	0.6460	0.6518	0.6581	0.6650	0.6716	0.6780	0.6842	0.6900
United Arab Emirates	0.5343	0.5449	0.5553	0.5657	0.5765	0.5874	0.5944	0.6019	0.6104	0.6205	0.6324	0.6509	0.6704	0.6902	0.7094	0.7273	0.7391	0.7506	0.7622	0.7725	0.7813	0.7904	0.7982	0.8064	0.8149	0.8237	0.8335	0.8440	0.8538	0.8622	0.8691	0.8724	0.8746	0.8742	0.8742	0.8747
Yemen	0.0960	0.0975	0.0993	0.1022	0.1053	0.1082	0.1103	0.1126	0.1172	0.1236	0.1329	0.1464	0.1599	0.1723	0.1845	0.1964	0.2094	0.2226	0.2354	0.2476	0.2595	0.2713	0.2826	0.2946	0.3067	0.3196	0.3323	0.3441	0.3555	0.3662	0.3766	0.3845	0.3918	0.3989	0.4052	0.4080
South Asia	0.2421	0.2471	0.2522	0.2580	0.2640	0.2704	0.2771	0.2842	0.2922	0.3008	0.3096	0.3179	0.3262	0.3346	0.3431	0.3519	0.3609	0.3697	0.3784	0.3874	0.3963	0.4050	0.4136	0.4224	0.4314	0.4407	0.4506	0.4608	0.4707	0.4806	0.4907	0.5005	0.5099	0.5190	0.5277	0.5362
South Asia	0.2421	0.2471	0.2522	0.2580	0.2640	0.2704	0.2771	0.2842	0.2922	0.3008	0.3096	0.3179	0.3262	0.3346	0.3431	0.3519	0.3609	0.3697	0.3784	0.3874	0.3963	0.4050	0.4136	0.4224	0.4314	0.4407	0.4506	0.4608	0.4707	0.4806	0.4907	0.5005	0.5099	0.5190	0.5277	0.5362
Bangladesh	0.1990	0.2050	0.2116	0.2190	0.2268	0.2349	0.2427	0.2505	0.2582	0.2663	0.2746	0.2825	0.2905	0.2983	0.3060	0.3139	0.3216	0.3292	0.3367	0.3439	0.3512	0.3585	0.3659	0.3734	0.3810	0.3889	0.3971	0.4056	0.4141	0.4225	0.4308	0.4391	0.4474	0.4555	0.4636	0.4716
Bhutan	0.2037	0.2081	0.2125	0.2177	0.2233	0.2296	0.2375	0.2479	0.2591	0.2704	0.2818	0.2909	0.2994	0.3078	0.3166	0.3266	0.3382	0.3504	0.3624	0.3744	0.3861	0.3978	0.4094	0.4203	0.4306	0.4405	0.4498	0.4602	0.4703	0.4802	0.4901	0.4995	0.5084	0.5167	0.5245	0.5321
India	0.2518	0.2567	0.2617	0.2674	0.2733	0.2795	0.2863	0.2935	0.3018	0.3105	0.3195	0.3280	0.3364	0.3448	0.3535	0.3625	0.3716	0.3806	0.3895	0.3989	0.4081	0.4171	0.4260	0.4352	0.4447	0.4544	0.4650	0.4759	0.4865	0.4970	0.5080	0.5186	0.5286	0.5383	0.5476	0.5564
Andhra Pradesh	0.2137	0.2194	0.2250	0.2314	0.2373	0.2435	0.2498	0.2575	0.2674	0.2778	0.2890	0.3002	0.3104	0.3222	0.3345	0.3476	0.3606	0.3724	0.3857	0.3989	0.4128	0.4263	0.4391	0.4520	0.4647	0.4768	0.4903	0.5054	0.5195	0.5327	0.5479	0.5619	0.5748	0.5870	0.5979	0.6079
Andhra Pradesh, Rural	0.1747	0.1796	0.1845	0.1902	0.1954	0.2009	0.2066	0.2136	0.2227	0.2323	0.2428	0.2534	0.2633	0.2748	0.2870	0.3001	0.3134	0.3256	0.3395	0.3534	0.3680	0.3822	0.3956	0.4090	0.4220	0.4342	0.4476	0.4623	0.4758	0.4882	0.5024	0.5154	0.5277	0.5393	0.5501	0.5598
Andhra Pradesh, Urban	0.3473	0.3527	0.3579	0.3639	0.3691	0.3746	0.3800	0.3870	0.3963	0.4061	0.4167	0.4272	0.4364	0.4474	0.4588	0.4711	0.4831	0.4938	0.5062	0.5183	0.5310	0.5428	0.5534	0.5638	0.5738	0.5831	0.5939	0.6065	0.6182	0.6293	0.6429	0.6559	0.6685	0.6810	0.6932	0.7046
Arunāchal Pradesh	0.1910	0.1991	0.2079	0.2167	0.2263	0.2371	0.2481	0.2586	0.2695	0.2796	0.2906	0.3017	0.3134	0.3254	0.3366	0.3491	0.3606	0.3723	0.3830	0.3933	0.4037	0.4144	0.4238	0.4337	0.4448	0.4557	0.4662	0.4771	0.4880	0.5028	0.5169	0.5301	0.5420	0.5534	0.5623	0.5707
Arunāchal Pradesh, Rural	0.1836	0.1910	0.1991	0.2072	0.2160	0.2259	0.2361	0.2456	0.2555	0.2647	0.2747	0.2847	0.2953	0.3063	0.3165	0.3279	0.3384	0.3491	0.3590	0.3683	0.3779	0.3879	0.3968	0.4062	0.4171	0.4277	0.4379	0.4485	0.4589	0.4732	0.4865	0.4985	0.5090	0.5186	0.5266	0.5340
Arunāchal Pradesh, Urban	0.2973	0.3085	0.3201	0.3315	0.3433	0.3561	0.3691	0.3809	0.3929	0.4036	0.4149	0.4261	0.4375	0.4490	0.4592	0.4706	0.4806	0.4905	0.4991	0.5071	0.5154	0.5243	0.5318	0.5400	0.5498	0.5594	0.5688	0.5787	0.5886	0.6031	0.6168	0.6296	0.6413	0.6524	0.6626	0.6724
Assam	0.2347	0.2416	0.2488	0.2569	0.2661	0.2750	0.2836	0.2923	0.3002	0.3091	0.3181	0.3252	0.3319	0.3383	0.3448	0.3516	0.3580	0.3641	0.3712	0.3811	0.3908	0.4001	0.4096	0.4185	0.4274	0.4357	0.4447	0.4534	0.4614	0.4694	0.4784	0.4869	0.4946	0.5024	0.5103	0.5182
Assam, Rural	0.2190	0.2257	0.2327	0.2407	0.2496	0.2583	0.2668	0.2753	0.2831	0.2919	0.3008	0.3078	0.3144	0.3207	0.3270	0.3337	0.3400	0.3460	0.3530	0.3628	0.3725	0.3818	0.3913	0.4002	0.4091	0.4175	0.4264	0.4351	0.4430	0.4508	0.4595	0.4676	0.4748	0.4821	0.4894	0.4967
Assam, Urban	0.3788	0.3861	0.3936	0.4019	0.4113	0.4201	0.4284	0.4366	0.4437	0.4518	0.4598	0.4657	0.4710	0.4760	0.4810	0.4862	0.4908	0.4951	0.5002	0.5083	0.5163	0.5238	0.5316	0.5389	0.5463	0.5535	0.5616	0.5697	0.5773	0.5853	0.5946	0.6040	0.6130	0.6226	0.6329	0.6437
Bihār	0.1901	0.1939	0.1983	0.2029	0.2089	0.2150	0.2221	0.2288	0.2369	0.2444	0.2524	0.2594	0.2659	0.2693	0.2726	0.2742	0.2776	0.2811	0.2801	0.2813	0.2851	0.2888	0.2947	0.2997	0.3067	0.3133	0.3250	0.3375	0.3515	0.3637	0.3774	0.3912	0.4051	0.4183	0.4308	0.4421
Bihār, Rural	0.1722	0.1757	0.1797	0.1838	0.1894	0.1950	0.2015	0.2077	0.2153	0.2223	0.2300	0.2369	0.2435	0.2473	0.2511	0.2532	0.2572	0.2612	0.2608	0.2624	0.2667	0.2708	0.2771	0.2823	0.2894	0.2961	0.3080	0.3205	0.3345	0.3467	0.3604	0.3740	0.3878	0.4008	0.4132	0.4243
Bihār, Urban	0.3349	0.3396	0.3447	0.3500	0.3568	0.3634	0.3710	0.3781	0.3865	0.3942	0.4024	0.4096	0.4163	0.4198	0.4233	0.4251	0.4289	0.4327	0.4323	0.4338	0.4373	0.4402	0.4448	0.4482	0.4532	0.4579	0.4674	0.4778	0.4901	0.5009	0.5135	0.5264	0.5398	0.5527	0.5651	0.5767
Chhattīsgarh	0.2506	0.2560	0.2610	0.2661	0.2710	0.2758	0.2810	0.2865	0.2927	0.2992	0.3061	0.3123	0.3190	0.3257	0.3321	0.3388	0.3458	0.3533	0.3609	0.3686	0.3750	0.3831	0.3911	0.4009	0.4107	0.4214	0.4337	0.4469	0.4598	0.4707	0.4806	0.4908	0.4999	0.5085	0.5162	0.5235
Chhattīsgarh, Rural	0.2194	0.2246	0.2295	0.2343	0.2391	0.2438	0.2488	0.2541	0.2601	0.2664	0.2731	0.2792	0.2858	0.2923	0.2986	0.3052	0.3122	0.3197	0.3272	0.3349	0.3412	0.3491	0.3569	0.3665	0.3759	0.3863	0.3983	0.4111	0.4237	0.4342	0.4437	0.4536	0.4623	0.4705	0.4779	0.4848
Chhattīsgarh, Urban	0.3942	0.3992	0.4039	0.4086	0.4132	0.4177	0.4227	0.4280	0.4340	0.4404	0.4470	0.4530	0.4594	0.4656	0.4716	0.4778	0.4843	0.4912	0.4980	0.5050	0.5104	0.5174	0.5242	0.5327	0.5411	0.5505	0.5615	0.5734	0.5853	0.5952	0.6043	0.6140	0.6229	0.6316	0.6405	0.6492
Delhi	0.4357	0.4416	0.4479	0.4533	0.4588	0.4656	0.4730	0.4806	0.4886	0.4967	0.5044	0.5131	0.5216	0.5300	0.5385	0.5463	0.5536	0.5630	0.5717	0.5802	0.5891	0.5974	0.6058	0.6146	0.6239	0.6338	0.6441	0.6549	0.6661	0.6785	0.6907	0.7034	0.7158	0.7282	0.7405	0.7529
Delhi, Rural	0.3536	0.3602	0.3674	0.3740	0.3808	0.3890	0.3979	0.4073	0.4172	0.4274	0.4373	0.4481	0.4584	0.4686	0.4789	0.4885	0.4978	0.5091	0.5197	0.5302	0.5410	0.5511	0.5610	0.5708	0.5809	0.5913	0.6016	0.6122	0.6228	0.6342	0.6450	0.6557	0.6656	0.6751	0.6840	0.6927
Delhi, Urban	0.4436	0.4492	0.4553	0.4605	0.4659	0.4726	0.4799	0.4875	0.4955	0.5038	0.5115	0.5201	0.5281	0.5359	0.5438	0.5511	0.5580	0.5671	0.5756	0.5839	0.5926	0.6008	0.6089	0.6173	0.6264	0.6361	0.6462	0.6568	0.6678	0.6801	0.6922	0.7049	0.7173	0.7298	0.7424	0.7550
Goa	0.4344	0.4383	0.4435	0.4478	0.4538	0.4580	0.4637	0.4694	0.4755	0.4822	0.4886	0.4956	0.5037	0.5128	0.5215	0.5311	0.5409	0.5505	0.5633	0.5743	0.5850	0.5946	0.6041	0.6123	0.6210	0.6307	0.6399	0.6494	0.6612	0.6723	0.6826	0.6944	0.7032	0.7116	0.7207	0.7298
Goa, Rural	0.4210	0.4242	0.4287	0.4324	0.4376	0.4411	0.4460	0.4510	0.4563	0.4624	0.4681	0.4744	0.4819	0.4904	0.4986	0.5077	0.5171	0.5263	0.5387	0.5492	0.5594	0.5686	0.5776	0.5852	0.5934	0.6023	0.6109	0.6196	0.6306	0.6408	0.6502	0.6611	0.6689	0.6763	0.6849	0.6932
Goa, Urban	0.4665	0.4705	0.4758	0.4802	0.4861	0.4903	0.4959	0.5015	0.5074	0.5140	0.5202	0.5268	0.5346	0.5432	0.5515	0.5606	0.5699	0.5789	0.5912	0.6010	0.6117	0.6208	0.6297	0.6372	0.6453	0.6543	0.6629	0.6718	0.6831	0.6937	0.7036	0.7150	0.7234	0.7315	0.7410	0.7504
Gujarāt	0.2808	0.2873	0.2935	0.3017	0.3092	0.3160	0.3239	0.3304	0.3401	0.3496	0.3588	0.3663	0.3764	0.3861	0.3978	0.4091	0.4205	0.4310	0.4415	0.4506	0.4584	0.4664	0.4747	0.4838	0.4927	0.5030	0.5131	0.5235	0.5329	0.5431	0.5534	0.5632	0.5719	0.5801	0.5880	0.5957
Gujarāt, Rural	0.2355	0.2414	0.2470	0.2546	0.2615	0.2679	0.2754	0.2816	0.2909	0.3001	0.3089	0.3163	0.3261	0.3356	0.3470	0.3581	0.3694	0.3798	0.3903	0.3994	0.4072	0.4151	0.4230	0.4317	0.4401	0.4498	0.4594	0.4692	0.4781	0.4877	0.4973	0.5062	0.5141	0.5212	0.5283	0.5349
Gujarāt, Urban	0.3817	0.3882	0.3942	0.4024	0.4095	0.4158	0.4232	0.4289	0.4380	0.4469	0.4552	0.4617	0.4709	0.4797	0.4906	0.5011	0.5117	0.5213	0.5309	0.5390	0.5457	0.5524	0.5592	0.5668	0.5741	0.5830	0.5920	0.6015	0.6101	0.6200	0.6302	0.6401	0.6492	0.6581	0.6674	0.6766
Haryāna	0.2694	0.2765	0.2844	0.2921	0.3000	0.3093	0.3183	0.3276	0.3390	0.3503	0.3625	0.3744	0.3852	0.3967	0.4086	0.4204	0.4331	0.4451	0.4568	0.4688	0.4813	0.4936	0.5053	0.5168	0.5281	0.5396	0.5513	0.5633	0.5750	0.5864	0.5976	0.6081	0.6178	0.6268	0.6349	0.6427
Haryāna, Rural	0.2385	0.2452	0.2527	0.2600	0.2675	0.2763	0.2848	0.2936	0.3045	0.3154	0.3271	0.3387	0.3493	0.3606	0.3725	0.3843	0.3972	0.4094	0.4214	0.4336	0.4462	0.4584	0.4699	0.4809	0.4917	0.5027	0.5141	0.5258	0.5374	0.5488	0.5598	0.5700	0.5788	0.5867	0.5938	0.6003
Haryāna, Urban	0.3938	0.4001	0.4071	0.4137	0.4203	0.4284	0.4360	0.4438	0.4538	0.4636	0.4743	0.4847	0.4937	0.5034	0.5134	0.5233	0.5339	0.5434	0.5525	0.5617	0.5713	0.5804	0.5888	0.5969	0.6050	0.6137	0.6233	0.6338	0.6446	0.6559	0.6673	0.6782	0.6881	0.6973	0.7067	0.7159
Himachal Pradesh	0.2884	0.2939	0.2990	0.3046	0.3090	0.3152	0.3220	0.3294	0.3392	0.3493	0.3595	0.3693	0.3795	0.3898	0.4007	0.4116	0.4246	0.4373	0.4506	0.4653	0.4798	0.4932	0.5054	0.5169	0.5279	0.5383	0.5486	0.5587	0.5687	0.5785	0.5894	0.5998	0.6095	0.6187	0.6275	0.6360

Location	1980	1981	1982	1983	1984	1985	1986	1987	1988	1989	1990	1991	1992	1993	1994	1995	1996	1997	1998	1999	2000	2001	2002	2003	2004	2005	2006	2007	2008	2009	2010	2011	2012	2013	2014	2015
Himachal Pradesh, Rural	0.2777	0.2831	0.2881	0.2935	0.2979	0.3039	0.3107	0.3180	0.3277	0.3377	0.3479	0.3577	0.3679	0.3782	0.3890	0.4000	0.4131	0.4259	0.4392	0.4540	0.4685	0.4821	0.4945	0.5060	0.5172	0.5276	0.5380	0.5480	0.5579	0.5676	0.5782	0.5883	0.5974	0.6061	0.6143	0.6221
Himachal Pradesh, Urban	0.4217	0.4273	0.4322	0.4374	0.4411	0.4466	0.4525	0.4589	0.4677	0.4766	0.4853	0.4935	0.5019	0.5103	0.5190	0.5278	0.5387	0.5492	0.5603	0.5729	0.5853	0.5967	0.6073	0.6172	0.6271	0.6367	0.6466	0.6567	0.6673	0.6783	0.6911	0.7040	0.7169	0.7301	0.7437	0.7578
Jammu and Kashmīr	0.2588	0.2639	0.2696	0.2757	0.2824	0.2892	0.2965	0.3020	0.3093	0.3160	0.3221	0.3273	0.3326	0.3438	0.3544	0.3656	0.3767	0.3929	0.4072	0.4246	0.4398	0.4531	0.4652	0.4753	0.4849	0.4930	0.5008	0.5079	0.5158	0.5234	0.5313	0.5398	0.5483	0.5568	0.5657	0.5745
Jammu and Kashmīr, Rural	0.2289	0.2336	0.2389	0.2446	0.2508	0.2573	0.2643	0.2695	0.2765	0.2831	0.2891	0.2941	0.2994	0.3106	0.3212	0.3324	0.3436	0.3599	0.3745	0.3920	0.4076	0.4211	0.4333	0.4436	0.4533	0.4613	0.4691	0.4761	0.4837	0.4909	0.4984	0.5063	0.5140	0.5217	0.5300	0.5381
Jammu and Kashmīr, Urban	0.3757	0.3810	0.3870	0.3932	0.3999	0.4067	0.4139	0.4190	0.4258	0.4318	0.4370	0.4411	0.4452	0.4553	0.4645	0.4741	0.4834	0.4977	0.5100	0.5254	0.5386	0.5499	0.5600	0.5685	0.5766	0.5835	0.5904	0.5967	0.6041	0.6114	0.6194	0.6283	0.6375	0.6472	0.6574	0.6678
Jharkhand	0.2816	0.2855	0.2890	0.2925	0.2960	0.2996	0.3037	0.3083	0.3137	0.3195	0.3256	0.3309	0.3365	0.3419	0.3470	0.3519	0.3562	0.3635	0.3703	0.3765	0.3807	0.3860	0.3917	0.3979	0.4064	0.4149	0.4231	0.4321	0.4395	0.4482	0.4580	0.4660	0.4731	0.4801	0.4876	0.4949
Jharkhand, Rural	0.2662	0.2698	0.2729	0.2760	0.2791	0.2822	0.2858	0.2900	0.2949	0.3003	0.3061	0.3112	0.3168	0.3223	0.3277	0.3328	0.3374	0.3451	0.3522	0.3587	0.3631	0.3686	0.3744	0.3806	0.3890	0.3974	0.4054	0.4143	0.4213	0.4298	0.4392	0.4468	0.4536	0.4601	0.4673	0.4743
Jharkhand, Urban	0.4063	0.4110	0.4153	0.4196	0.4238	0.4279	0.4326	0.4377	0.4435	0.4498	0.4564	0.4624	0.4690	0.4755	0.4821	0.4886	0.4946	0.5037	0.5122	0.5202	0.5259	0.5322	0.5386	0.5450	0.5535	0.5619	0.5700	0.5791	0.5869	0.5964	0.6070	0.6160	0.6242	0.6324	0.6410	0.6497
Karnātaka	0.2598	0.2646	0.2698	0.2762	0.2830	0.2894	0.2970	0.3051	0.3139	0.3232	0.3325	0.3432	0.3532	0.3636	0.3744	0.3854	0.3966	0.4076	0.4202	0.4324	0.4444	0.4550	0.4651	0.4746	0.4851	0.4966	0.5078	0.5195	0.5305	0.5403	0.5515	0.5616	0.5709	0.5795	0.5877	0.5957
Karnātaka, Rural	0.2158	0.2201	0.2247	0.2305	0.2368	0.2428	0.2499	0.2576	0.2659	0.2748	0.2838	0.2940	0.3036	0.3136	0.3239	0.3346	0.3456	0.3564	0.3690	0.3813	0.3937	0.4050	0.4159	0.4263	0.4376	0.4496	0.4608	0.4721	0.4821	0.4907	0.5003	0.5090	0.5172	0.5250	0.5327	0.5402
Karnātaka, Urban	0.3750	0.3796	0.3846	0.3908	0.3974	0.4035	0.4107	0.4183	0.4265	0.4351	0.4436	0.4533	0.4620	0.4710	0.4801	0.4896	0.4992	0.5087	0.5201	0.5311	0.5421	0.5517	0.5609	0.5695	0.5791	0.5897	0.5995	0.6099	0.6191	0.6272	0.6370	0.6464	0.6558	0.6655	0.6756	0.6857
Kerala	0.3417	0.3455	0.3507	0.3569	0.3636	0.3699	0.3774	0.3851	0.3927	0.4009	0.4091	0.4178	0.4262	0.4353	0.4450	0.4559	0.4664	0.4765	0.4872	0.4982	0.5083	0.5170	0.5253	0.5332	0.5415	0.5509	0.5600	0.5695	0.5789	0.5890	0.5987	0.6089	0.6187	0.6282	0.6382	0.6484
Kerala, Rural	0.3316	0.3353	0.3403	0.3464	0.3530	0.3591	0.3666	0.3742	0.3819	0.3900	0.3982	0.4069	0.4153	0.4245	0.4342	0.4453	0.4560	0.4664	0.4774	0.4888	0.4994	0.5082	0.5166	0.5243	0.5324	0.5413	0.5497	0.5584	0.5668	0.5757	0.5841	0.5931	0.6018	0.6104	0.6197	0.6291
Kerala, Urban	0.3798	0.3832	0.3879	0.3936	0.3996	0.4052	0.4120	0.4189	0.4258	0.4331	0.4404	0.4482	0.4557	0.4639	0.4728	0.4830	0.4928	0.5023	0.5125	0.5231	0.5329	0.5412	0.5491	0.5565	0.5644	0.5732	0.5816	0.5903	0.5988	0.6081	0.6172	0.6273	0.6374	0.6478	0.6595	0.6716
Madhya Pradesh	0.2066	0.2114	0.2168	0.2228	0.2282	0.2345	0.2406	0.2486	0.2575	0.2666	0.2770	0.2857	0.2943	0.3025	0.3105	0.3188	0.3273	0.3357	0.3433	0.3519	0.3595	0.3683	0.3759	0.3853	0.3941	0.4025	0.4117	0.4211	0.4307	0.4416	0.4519	0.4626	0.4735	0.4851	0.4958	0.5058
Madhya Pradesh, Rural	0.1639	0.1679	0.1723	0.1773	0.1819	0.1872	0.1924	0.1994	0.2073	0.2157	0.2253	0.2335	0.2419	0.2500	0.2580	0.2663	0.2748	0.2829	0.2902	0.2982	0.3051	0.3132	0.3202	0.3291	0.3375	0.3457	0.3551	0.3649	0.3753	0.3870	0.3980	0.4091	0.4198	0.4306	0.4407	0.4498
Madhya Pradesh, Urban	0.3873	0.3916	0.3965	0.4021	0.4067	0.4123	0.4173	0.4247	0.4329	0.4413	0.4511	0.4589	0.4666	0.4738	0.4807	0.4878	0.4949	0.5013	0.5064	0.5121	0.5163	0.5216	0.5253	0.5310	0.5363	0.5418	0.5490	0.5571	0.5666	0.5781	0.5892	0.6007	0.6120	0.6235	0.6350	0.6458
Mahārāshtra	0.3133	0.3186	0.3238	0.3295	0.3351	0.3415	0.3480	0.3554	0.3640	0.3743	0.3844	0.3935	0.4040	0.4149	0.4253	0.4365	0.4473	0.4568	0.4657	0.4752	0.4836	0.4922	0.5009	0.5099	0.5193	0.5295	0.5402	0.5514	0.5615	0.5715	0.5821	0.5919	0.6007	0.6091	0.6172	0.6251
Mahārāshtra, Rural	0.2509	0.2557	0.2605	0.2657	0.2710	0.2770	0.2833	0.2905	0.2987	0.3086	0.3184	0.3273	0.3376	0.3483	0.3586	0.3699	0.3809	0.3911	0.4008	0.4113	0.4209	0.4306	0.4405	0.4504	0.4607	0.4715	0.4825	0.4938	0.5038	0.5134	0.5233	0.5323	0.5401	0.5472	0.5540	0.5605
Mahārāshtra, Urban	0.4320	0.4367	0.4411	0.4459	0.4505	0.4559	0.4611	0.4674	0.4746	0.4835	0.4919	0.4990	0.5072	0.5157	0.5235	0.5323	0.5406	0.5479	0.5546	0.5622	0.5688	0.5755	0.5823	0.5893	0.5969	0.6055	0.6147	0.6247	0.6339	0.6434	0.6538	0.6640	0.6735	0.6829	0.6928	0.7027
Manipur	0.2974	0.3036	0.3091	0.3157	0.3229	0.3297	0.3378	0.3484	0.3586	0.3674	0.3758	0.3841	0.3918	0.4000	0.4071	0.4145	0.4221	0.4300	0.4384	0.4497	0.4590	0.4685	0.4764	0.4839	0.4925	0.5019	0.5096	0.5168	0.5228	0.5300	0.5364	0.5444	0.5520	0.5597	0.5675	0.5757
Manipur, Rural	0.2835	0.2892	0.2942	0.3002	0.3068	0.3130	0.3205	0.3305	0.3401	0.3484	0.3564	0.3643	0.3719	0.3800	0.3872	0.3947	0.4025	0.4105	0.4191	0.4305	0.4399	0.4494	0.4573	0.4646	0.4729	0.4820	0.4893	0.4961	0.5015	0.5080	0.5136	0.5208	0.5275	0.5341	0.5411	0.5485
Manipur, Urban	0.3634	0.3692	0.3742	0.3801	0.3865	0.3925	0.3996	0.4091	0.4182	0.4258	0.4331	0.4404	0.4474	0.4549	0.4616	0.4686	0.4758	0.4833	0.4914	0.5023	0.5110	0.5200	0.5274	0.5343	0.5424	0.5514	0.5587	0.5656	0.5715	0.5786	0.5850	0.5931	0.6009	0.6088	0.6174	0.6266
Meghālaya	0.2307	0.2341	0.2376	0.2416	0.2463	0.2516	0.2577	0.2648	0.2715	0.2807	0.2897	0.2978	0.3052	0.3133	0.3205	0.3290	0.3368	0.3453	0.3541	0.3634	0.3733	0.3832	0.3918	0.3998	0.4078	0.4161	0.4243	0.4324	0.4403	0.4487	0.4563	0.4641	0.4704	0.4767	0.4836	0.4905
Meghālaya, Rural	0.2008	0.2036	0.2066	0.2100	0.2142	0.2189	0.2244	0.2309	0.2371	0.2456	0.2542	0.2617	0.2688	0.2766	0.2836	0.2918	0.2994	0.3077	0.3162	0.3253	0.3351	0.3448	0.3533	0.3612	0.3691	0.3771	0.3851	0.3930	0.4005	0.4084	0.4154	0.4224	0.4276	0.4327	0.4387	0.4444
Meghālaya, Urban	0.3849	0.3892	0.3935	0.3983	0.4038	0.4097	0.4164	0.4242	0.4312	0.4411	0.4507	0.4588	0.4662	0.4742	0.4810	0.4893	0.4966	0.5048	0.5133	0.5223	0.5318	0.5413	0.5495	0.5571	0.5651	0.5736	0.5823	0.5911	0.6000	0.6096	0.6189	0.6289	0.6374	0.6466	0.6566	0.6670
Mizoram	0.2869	0.2902	0.2938	0.2989	0.3072	0.3195	0.3325	0.3479	0.3585	0.3674	0.3746	0.3835	0.3918	0.4007	0.4071	0.4154	0.4258	0.4328	0.4392	0.4466	0.4556	0.4641	0.4723	0.4793	0.4856	0.4914	0.4968	0.5027	0.5088	0.5164	0.5245	0.5316	0.5395	0.5472	0.5557	0.5642
Mizoram, Rural	0.2380	0.2405	0.2433	0.2477	0.2553	0.2670	0.2792	0.2939	0.3039	0.3122	0.3190	0.3274	0.3353	0.3437	0.3498	0.3578	0.3678	0.3746	0.3808	0.3880	0.3967	0.4048	0.4127	0.4192	0.4250	0.4302	0.4350	0.4403	0.4455	0.4522	0.4591	0.4650	0.4714	0.4775	0.4846	0.4916
Mizoram, Urban	0.3512	0.3548	0.3587	0.3640	0.3724	0.3849	0.3980	0.4137	0.4244	0.4331	0.4401	0.4488	0.4568	0.4653	0.4712	0.4791	0.4891	0.4954	0.5011	0.5080	0.5165	0.5245	0.5325	0.5392	0.5453	0.5511	0.5564	0.5625	0.5686	0.5766	0.5851	0.5927	0.6012	0.6097	0.6193	0.6290
Nāgāland	0.3069	0.3125	0.3196	0.3267	0.3344	0.3416	0.3489	0.3585	0.3687	0.3783	0.3879	0.3965	0.4048	0.4159	0.4257	0.4351	0.4439	0.4527	0.4587	0.4643	0.4734	0.4823	0.4916	0.4994	0.5074	0.5151	0.5222	0.5312	0.5405	0.5493	0.5575	0.5667	0.5750	0.5829	0.5907	0.5987
Nāgāland, Rural	0.2973	0.3024	0.3092	0.3158	0.3230	0.3298	0.3367	0.3459	0.3556	0.3648	0.3741	0.3824	0.3906	0.4015	0.4113	0.4207	0.4295	0.4383	0.4443	0.4499	0.4588	0.4673	0.4760	0.4830	0.4900	0.4965	0.5022	0.5098	0.5174	0.5244	0.5308	0.5382	0.5446	0.5507	0.5574	0.5644
Nāgāland, Urban	0.3805	0.3865	0.3941	0.4014	0.4091	0.4162	0.4234	0.4327	0.4425	0.4517	0.4609	0.4690	0.4768	0.4873	0.4965	0.5054	0.5135	0.5217	0.5271	0.5323	0.5410	0.5497	0.5589	0.5667	0.5750	0.5830	0.5906	0.6004	0.6104	0.6201	0.6293	0.6396	0.6490	0.6583	0.6682	0.6786
Orissa	0.2042	0.2086	0.2127	0.2192	0.2242	0.2308	0.2377	0.2435	0.2516	0.2604	0.2668	0.2747	0.2820	0.2899	0.2988	0.3093	0.3170	0.3263	0.3367	0.3480	0.3579	0.3676	0.3763	0.3872	0.3989	0.4095	0.4224	0.4368	0.4493	0.4603	0.4720	0.4819	0.4916	0.5005	0.5088	0.5167
Orissa, Rural	0.1857	0.1897	0.1934	0.1994	0.2039	0.2100	0.2164	0.2217	0.2292	0.2375	0.2434	0.2509	0.2578	0.2653	0.2738	0.2840	0.2916	0.3007	0.3111	0.3223	0.3324	0.3423	0.3512	0.3623	0.3742	0.3849	0.3978	0.4120	0.4242	0.4348	0.4459	0.4553	0.4646	0.4731	0.4813	0.4889
Orissa, Urban	0.3645	0.3690	0.3731	0.3797	0.3844	0.3908	0.3973	0.4023	0.4098	0.4179	0.4233	0.4302	0.4364	0.4432	0.4510	0.4604	0.4668	0.4748	0.4840	0.4940	0.5027	0.5110	0.5183	0.5279	0.5384	0.5477	0.5595	0.5729	0.5845	0.5947	0.6059	0.6156	0.6255	0.6350	0.6447	0.6542
Punjab	0.3218	0.3285	0.3355	0.3425	0.3502	0.3584	0.3668	0.3763	0.3861	0.3970	0.4073	0.4175	0.4279	0.4382	0.4481	0.4581	0.4682	0.4779	0.4873	0.4973	0.5075	0.5172	0.5257	0.5341	0.5420	0.5505	0.5592	0.5691	0.5788	0.5886	0.5981	0.6074	0.6160	0.6241	0.6319	0.6397
Punjab, Rural	0.2883	0.2947	0.3015	0.3083	0.3158	0.3239	0.3322	0.3416	0.3512	0.3621	0.3723	0.3823	0.3926	0.4025	0.4123	0.4221	0.4321	0.4419	0.4515	0.4617	0.4724	0.4826	0.4918	0.5010	0.5096	0.5188	0.5278	0.5379	0.5476	0.5570	0.5660	0.5747	0.5826	0.5900	0.5971	0.6039
Punjab, Urban	0.4152	0.4215	0.4281	0.4345	0.4415	0.4490	0.4566	0.4651	0.4739	0.4839	0.4930	0.5019	0.5109	0.5196	0.5278	0.5361	0.5444	0.5523	0.5599	0.5682	0.5767	0.5846	0.5914	0.5981	0.6044	0.6114	0.6185	0.6271	0.6357	0.6445	0.6533	0.6625	0.6714	0.6804	0.6898	0.6994
Rājasthān	0.1817	0.1862	0.1912	0.1981	0.2035	0.2092	0.2156	0.2219	0.2312	0.2400	0.2511	0.2604	0.2703	0.2795	0.2906	0.3014	0.3131	0.3237	0.3337	0.3454	0.3558	0.3669	0.3760	0.3876	0.3985	0.4091	0.4211	0.4333	0.4451	0.4560	0.4695	0.4827	0.4943	0.5046	0.5140	0.5226
Rājasthān, Rural	0.1452	0.1488	0.1530	0.1590	0.1636	0.1684	0.1739	0.1793	0.1878	0.1957	0.2060	0.2147	0.2241	0.2329	0.2437	0.2542	0.2656	0.2761	0.2857	0.2972	0.3075	0.3185	0.3280	0.3400	0.3513	0.3623	0.3746	0.3869	0.3987	0.4095	0.4228	0.4357	0.4470	0.4568	0.4660	0.4743
Rājasthān, Urban	0.3283	0.3336	0.3395	0.3474	0.3537	0.3600	0.3672	0.3740	0.3841	0.3934	0.4051	0.4149	0.4252	0.4348	0.4463	0.4575	0.4695	0.4806	0.4908	0.5028	0.5132	0.5237	0.5317	0.5419	0.5511	0.5599	0.5700	0.5804	0.5905	0.6000	0.6124	0.6249	0.6363	0.6469	0.6572	0.6671
Sikkim	0.2633	0.2674	0.2727	0.2784	0.2866	0.2959	0.3065	0.3169	0.3264	0.3353	0.3440	0.3511	0.3567	0.3654	0.3735	0.3818	0.3908	0.4001	0.4094	0.4184	0.4284	0.4384	0.4487	0.4589	0.4691	0.4792	0.4890	0.4988	0.5091	0.5311	0.5494	0.5657	0.5798	0.5927	0.6033	0.6125
Sikkim, Rural	0.2537	0.2577	0.2627	0.2681	0.2760	0.2850	0.2953	0.3054	0.3146	0.3233	0.3317	0.3385	0.3438	0.3522	0.3600	0.3679	0.3766	0.3855	0.3944	0.4030	0.4124	0.4218	0.4313	0.4406	0.4498	0.4587	0.4672	0.4756	0.4844	0.5045	0.5210	0.5355	0.5479	0.5591	0.5690	0.5775
Sikkim, Urban	0.3666	0.3713	0.3771	0.3832	0.3920	0.4020	0.4134	0.4245	0.4345	0.4437	0.4525	0.4596	0.4648	0.4734	0.4813	0.4893	0.4981	0.5072	0.5162	0.5249	0.5346	0.5442	0.5540	0.5636	0.5733	0.5829	0.5921	0.6015	0.6116	0.6348	0.6543	0.6722	0.6882	0.7034	0.7174	0.7303
Tamil Nādu	0.2800	0.2857	0.2903	0.2957	0.3026	0.3102	0.3187	0.3283	0.3378	0.3483	0.3587	0.3688	0.3794	0.3915	0.4037	0.4157	0.4276	0.4395	0.4525	0.4647	0.4769	0.4872	0.4964	0.5051	0.5144	0.5250	0.5360	0.5466	0.5564	0.5682	0.5800	0.5911	0.6010	0.6107	0.6201	0.6290
Tamil Nādu, Rural	0.2369	0.2423	0.2467	0.2520	0.2589	0.2666	0.2751	0.2847	0.2943	0.3046	0.3148	0.3244	0.3343	0.3454	0.3565	0.3674	0.3784	0.3897	0.4024	0.4148	0.4276	0.4389	0.4495	0.4597	0.4705	0.4822	0.4939	0.5047	0.5140	0.5249	0.5354	0.5454	0.5545	0.5636	0.5725	0.5809
Tamil Nādu, Urban	0.3685	0.3744	0.3791	0.3847	0.3918	0.3996	0.4081	0.4177	0.4269	0.4367	0.4460	0.4543	0.4625	0.4718	0.4810	0.4897	0.4986	0.5078	0.5184	0.5287	0.5395	0.5487	0.5572	0.5654	0.5741	0.5839	0.5938	0.6029	0.6109	0.6207	0.6308	0.6408	0.6507	0.6612	0.6721	0.6829
Telangana	0.2724	0.2780	0.2833	0.2887	0.2942	0.2997	0.3059	0.3125	0.3199	0.3277	0.3360	0.3441	0.3526	0.3613	0.3705	0.3805	0.3909	0.4014	0.4121	0.4231	0.4341	0.4449	0.4553	0.4658	0.4759	0.4866	0.4968	0.5068	0.5163	0.5238	0.5313	0.5381	0.5435	0.5479	0.5522	0.5568
Telangana, Rural	0.2282	0.2331	0.2378	0.2427	0.2476	0.2527	0.2584	0.2645	0.2714	0.2788	0.2867	0.2945	0.3030	0.3117	0.3210	0.3313	0.3422	0.3533	0.3648	0.3767	0.3886	0.4002	0.4114	0.4224	0.4330	0.4439	0.4541	0.4639	0.4729	0.4796	0.4864	0.4925	0.4974	0.5015	0.5057	0.5103
Telangana, Urban	0.4243	0.4288	0.4328	0.4368	0.4407	0.4446	0.4491	0.4540	0.4597	0.4660	0.4726	0.4789	0.4858	0.4927	0.5001	0.5085	0.5174	0.5263	0.5356	0.5450	0.5543	0.5630	0.5708	0.5785	0.5856	0.5933	0.6006	0.6077	0.6146	0.6196	0.6252	0.6306	0.6353	0.6397	0.6447	0.6505
Tripura	0.2541	0.2592	0.2646	0.2696	0.2746	0.2795	0.2849	0.2907	0.2993	0.3073	0.3142	0.3198	0.3244	0.3316	0.3372	0.3448	0.3531	0.3622	0.3710	0.3878	0.4043	0.4200	0.4329	0.4450	0.4561	0.4660	0.4747	0.4822	0.4901	0.4975	0.5048	0.5127	0.5198	0.5269	0.5339	0.5412
Tripura, Rural	0.2381	0.2428	0.2480	0.2527	0.2574	0.2621	0.2672	0.2729	0.2812	0.2890	0.2958	0.3012	0.3058	0.3129	0.3186	0.3261	0.3344	0.3435	0.3522	0.3689	0.3852	0.4004	0.4127	0.4241	0.4343	0.4433	0.4509	0.4572	0.4638	0.4700	0.4760	0.4826	0.4885	0.4944	0.5006	0.5071
Tripura, Urban	0.3649	0.3696	0.3748	0.3792	0.3837	0.3880	0.3927	0.3978	0.4057	0.4128	0.4187	0.4231	0.4265	0.4326	0.4371	0.4435	0.4506	0.4584	0.4660	0.4818	0.4974	0.5123	0.5243	0.5355	0.5457	0.5549	0.5628	0.5695	0.5768	0.5839	0.5911	0.5994	0.6072	0.6155	0.6248	0.6347
Uttar Pradesh	0.2103	0.2137	0.2182	0.2226	0.2274	0.2329	0.2390	0.2455	0.2533	0.2618	0.2709	0.2795	0.2876	0.2951	0.3028	0.3105	0.3191	0.3271	0.3351	0.3433	0.3518	0.3606	0.3704	0.3809	0.3917	0.4031	0.4144	0.4260	0.4373	0.4488	0.4602	0.4713	0.4818	0.4921	0.5017	0.5109
Uttar Pradesh, Rural	0.1855	0.1883	0.1921	0.1958	0.1999	0.2047	0.2101	0.2159	0.2230	0.2308	0.2393	0.2474	0.2550	0.2622	0.2695	0.2770	0.2854	0.2931	0.3009	0.3090	0.3175	0.3265	0.3367	0.3477	0.3590	0.3709	0.3826	0.3946	0.4061	0.4179	0.4295	0.4407	0.4513	0.4614	0.4711	0.4802
Uttar Pradesh, Urban	0.3361	0.3401	0.3453	0.3503	0.3556	0.3616	0.3681	0.3749	0.3829	0.3915	0.4007	0.4093	0.4173	0.4248	0.4324	0.4401	0.4489	0.4570	0.4653	0.4736	0.4817	0.4892	0.4969	0.5046	0.5124	0.5208	0.5293	0.5385	0.5478	0.5578	0.5679	0.5780	0.5877	0.5975	0.6072	0.6168
Uttarakhand	0.2942	0.3009	0.3071	0.3133	0.3195	0.3255	0.3320	0.3388	0.3463	0.3542	0.3622	0.3695	0.3772	0.3847	0.3935	0.4021	0.4104	0.4185	0.4264	0.4356	0.4464	0.4568	0.4681	0.4792	0.4912	0.5044	0.5179	0.5317	0.5447	0.5616	0.5767	0.5905	0.6022	0.6130	0.6225	0.6314
Uttarakhand, Rural	0.2691	0.2756	0.2817	0.2877	0.2937	0.2996	0.3059	0.3125	0.3198	0.3275	0.3354	0.3425	0.3500	0.3573	0.3659	0.3743	0.3824	0.3903	0.3980	0.4071	0.4177	0.4280	0.4391	0.4500	0.4619	0.4749	0.4882	0.5018	0.5144	0.5311	0.5457	0.5592	0.5704	0.5806	0.5896	0.5980
Uttarakhand, Urban	0.4130	0.4179	0.4224	0.4268	0.4311	0.4353	0.4400	0.4451	0.4508	0.4571	0.4635	0.4692	0.4753	0.4812	0.4885	0.4956	0.5023	0.5088	0.5151	0.5227	0.5319	0.5409	0.5507	0.5604	0.5710	0.5829	0.5952	0.6078	0.6197	0.6357	0.6500	0.6632	0.6744	0.6848	0.6951	0.7049
West Bengal	0.2703	0.2748	0.2796	0.2850	0.2913	0.2970	0.3029	0.3101	0.3167	0.3235	0.3302	0.3363	0.3416	0.3474	0.3538	0.3615	0.3692	0.3775	0.3875	0.3976	0.4077	0.4178	0.4272	0.4369	0.4460	0.4548	0.4635	0.4728	0.4816	0.4905	0.4991	0.5079	0.5165	0.5250	0.5334	0.5417
West Bengal, Rural	0.2218	0.2260	0.2304	0.2356	0.2416	0.2471	0.2528	0.2599	0.2664	0.2732	0.2799	0.2861	0.2917	0.2977	0.3045	0.3124	0.3204	0.3291	0.3392	0.3495	0.3597	0.3697	0.3790	0.3884	0.3973	0.4058	0.4144	0.4235	0.4323	0.4412	0.4498	0.4583	0.4665	0.4744	0.4820	0.4895
West Bengal, Urban	0.4086	0.4129	0.4175	0.4227	0.4288	0.4341	0.4396	0.4463	0.4522	0.4583	0.4642	0.4694	0.4738	0.4786	0.4842	0.4910	0.4979	0.5054	0.5146	0.5237	0.5326	0.5409	0.5481	0.5552	0.5616	0.5677	0.5742	0.5815	0.5889	0.5969	0.6051	0.6139	0.6230	0.6324	0.6424	0.6527
The Six Minor Territories	0.4068	0.4123	0.4173	0.4222	0.4270	0.4318	0.4370	0.4426	0.4487	0.4552	0.4618	0.4678	0.4742	0.4803	0.4875	0.4956	0.5047	0.5161	0.5268	0.5374	0.5481	0.5586	0.5693	0.5801	0.5905	0.6018	0.6130	0.6240	0.6348	0.6457	0.6551	0.6644	0.6732	0.6818	0.6903	0.6988
The Six Minor Territories, Rural	0.3487	0.3530	0.3567	0.3603	0.3638	0.3673	0.3711	0.3753	0.3802	0.3855	0.3913	0.3967	0.4028	0.4089	0.4164	0.4249	0.4346	0.4466	0.4579	0.4691	0.4803	0.4912	0.5022	0.5132	0.5235	0.5346	0.5454	0.5559	0.5658	0.5754	0.5833	0.5906	0.5969	0.6027	0.6085	0.6140
The Six Minor Territories, Urban	0.4757	0.4795	0.4826	0.4855	0.4882	0.4908	0.4937	0.4970	0.5009	0.5053	0.5100	0.5143	0.5193	0.5243	0.5306	0.5380	0.5464	0.5572	0.5671	0.5769	0.5867	0.5961	0.6058	0.6154	0.6246	0.6347	0.6448	0.6549	0.6648	0.6749	0.6836	0.6924	0.7006	0.7088	0.7177	0.7268
Nepal	0.1738	0.1776	0.1818	0.1856	0.1903	0.1952	0.2003	0.2053	0.2111	0.2173	0.2238	0.2308	0.2380	0.2455	0.2539	0.2622	0.2707	0.2792	0.2873	0.2953	0.3036	0.3115	0.3186	0.3256	0.3326	0.3396	0.3473	0.3551	0.3634	0.3718	0.3806	0.3896	0.3985	0.4070	0.4152	0.4227
Pakistan	0.2139	0.2191	0.2245	0.2301	0.2359	0.2421	0.2482	0.2549	0.2622	0.2702	0.2786	0.2868	0.2956	0.3043	0.3129	0.3218	0.3308	0.3396	0.3483	0.3571	0.3656	0.3736	0.3811	0.3885	0.3957	0.4029	0.4102	0.4171	0.4238	0.4301	0.4363	0.4424	0.4487	0.4550	0.4613	0.4676
Sub-Saharan Africa	0.2327	0.2359	0.2390	0.2422	0.2455	0.2490	0.2519	0.2549	0.2587	0.2626	0.2666	0.2699	0.2728	0.2757	0.2783	0.2810	0.2837	0.2870	0.2900	0.2932	0.2966	0.2999	0.3033	0.3072	0.3122	0.3180	0.3243	0.3315	0.3387	0.3458	0.3534	0.3610	0.3686	0.3763	0.3839	0.3914
Central Sub-Saharan Africa	0.2324	0.2333	0.2343	0.2357	0.2386	0.2410	0.2421	0.2424	0.2438	0.2458	0.2468	0.2471	0.2472	0.2459	0.2442	0.2423	0.2391	0.2355	0.2311	0.2270	0.2217	0.2151	0.2088	0.2048	0.2032	0.2039	0.2094	0.2184	0.2277	0.2359	0.2466	0.2574	0.2681	0.2788	0.2897	0.3007
Angola	0.2266	0.2286	0.2313	0.2345	0.2381	0.2415	0.2432	0.2455	0.2481	0.2513	0.2553	0.2593	0.2639	0.2671	0.2706	0.2746	0.2776	0.2811	0.2844	0.2891	0.2935	0.2972	0.3028	0.3088	0.3154	0.3234	0.3320	0.3421	0.3531	0.3629	0.3732	0.3827	0.3921	0.4015	0.4105	0.4191
Central African Republic	0.1917	0.1946	0.1973	0.1992	0.2020	0.2053	0.2078	0.2096	0.2118	0.2148	0.2177	0.2208	0.2231	0.2251	0.2280	0.2311	0.2321	0.2341	0.2368	0.2402	0.2437	0.2467	0.2502	0.2526	0.2557	0.2590	0.2629	0.2673	0.2726	0.2774	0.2824	0.2876	0.2931	0.2895	0.2853	0.2817
Congo	0.3246	0.3329	0.3418	0.3512	0.3609	0.3699	0.3768	0.3835	0.3903	0.3976	0.4042	0.4103	0.4159	0.4209	0.4245	0.4283	0.4324	0.4361	0.4396	0.4428	0.4473	0.4506	0.4543	0.4579	0.4623	0.4679	0.4745	0.4801	0.4861	0.4918	0.4983	0.5035	0.5087	0.5142	0.5204	0.5265
Democratic Republic of the Congo	0.2261	0.2258	0.2254	0.2255	0.2276	0.2291	0.2294	0.2284	0.2290	0.2301	0.2295	0.2280	0.2262	0.2226	0.2184	0.2136	0.2073	0.1997	0.1912	0.1826	0.1724	0.1604	0.1486	0.1401	0.1348	0.1324	0.1369	0.1462	0.1556	0.1639	0.1756	0.1879	0.2000	0.2125	0.2256	0.2388
Equatorial Guinea	0.2270	0.2422	0.2539	0.2637	0.2703	0.2738	0.2710	0.2675	0.2649	0.2631	0.2611	0.2588	0.2626	0.2683	0.2752	0.2860	0.3075	0.3487	0.3758	0.3990	0.4234	0.4488	0.4705	0.4879	0.5062	0.5222	0.5344	0.5458	0.5572	0.5663	0.5743	0.5818	0.5895	0.5965	0.6030	0.6086
Gabon	0.4257	0.4290	0.4325	0.4366	0.4410	0.4457	0.4501	0.4543	0.4590	0.4650	0.4715	0.4785	0.4854	0.4925	0.5001	0.5080	0.5162	0.5245	0.5323	0.5392	0.5461	0.5525	0.5587	0.5648	0.5709	0.5776	0.5835	0.5895	0.5957	0.6013	0.6079	0.6144	0.6213	0.6285	0.6359	0.6435
Eastern Sub-Saharan Africa	0.1682	0.1710	0.1738	0.1768	0.1799	0.1829	0.1859	0.1899	0.1947	0.1994	0.2041	0.2073	0.2098	0.2126	0.2151	0.2181	0.2212	0.2258	0.2301	0.2346	0.2392	0.2441	0.2490	0.2537	0.2594	0.2660	0.2732	0.2814	0.2901	0.2988	0.3078	0.3169	0.3257	0.3345	0.3431	0.3515
Burundi	0.1371	0.1399	0.1420	0.1440	0.1458	0.1485	0.1508	0.1536	0.1568	0.1607	0.1649	0.1675	0.1705	0.1730	0.1752	0.1762	0.1753	0.1748	0.1739	0.1732	0.1728	0.1753	0.1782	0.1819	0.1860	0.1897	0.1933	0.1970	0.2028	0.2077	0.2129	0.2181	0.2235	0.2295	0.2357	0.2395
Comoros	0.2041	0.2071	0.2104	0.2143	0.2187	0.2237	0.2290	0.2347	0.2408	0.2465	0.2522	0.2574	0.2630	0.2689	0.2734	0.2782	0.2825	0.2870	0.2914	0.2959	0.3007	0.3056	0.3109	0.3159	0.3207	0.3254	0.3300	0.3342	0.3381	0.3419	0.3455	0.3491	0.3529	0.3570	0.3611	0.3650
Djibouti	0.2970	0.2976	0.2963	0.2942	0.2934	0.2945	0.3001	0.3064	0.3125	0.3178	0.3228	0.3252	0.3287	0.3332	0.3378	0.3435	0.3494	0.3553	0.3608	0.3661	0.3710	0.3754	0.3796	0.3842	0.3896	0.3951	0.4014	0.4085	0.4164	0.4234	0.4301	0.4366	0.4430	0.4491	0.4553	0.4615
Eritrea	0.1461	0.1486	0.1508	0.1532	0.1561	0.1596	0.1631	0.1675	0.1724	0.1775	0.1825	0.1865	0.1920	0.1995	0.2093	0.2202	0.2342	0.2478	0.2600	0.2703	0.2786	0.2860	0.2928	0.2983	0.3025	0.3059	0.3071	0.3082	0.3076	0.3077	0.3084	0.3106	0.3139	0.3173	0.3207	0.3238
Ethiopia	0.1078	0.1093	0.1114	0.1148	0.1178	0.1197	0.1217	0.1236	0.1263	0.1286	0.1309	0.1314	0.1304	0.1305	0.1304	0.1313	0.1334	0.1366	0.1386	0.1419	0.1467	0.1526	0.1581	0.1623	0.1686	0.1774	0.1876	0.1994	0.2120	0.2249	0.2380	0.2513	0.2642	0.2771	0.2899	0.3022
Kenya	0.2295	0.2395	0.2499	0.2599	0.2698	0.2801	0.2908	0.3021	0.3140	0.3261	0.3378	0.3488	0.3586	0.3673	0.3753	0.3820	0.3866	0.3901	0.3932	0.3962	0.3989	0.4011	0.4032	0.4056	0.4084	0.4120	0.4163	0.4215	0.4263	0.4312	0.4375	0.4443	0.4510	0.4578	0.4646	0.4716
Baringo	0.1624	0.1673	0.1722	0.1769	0.1816	0.1866	0.1920	0.1978	0.2041	0.2106	0.2178	0.2245	0.2301	0.2346	0.2388	0.2421	0.2441	0.2453	0.2463	0.2472	0.2473	0.2467	0.2459	0.2455	0.2459	0.2472	0.2495	0.2529	0.2561	0.2596	0.2640	0.2688	0.2738	0.2789	0.2842	0.2901
Bomet	0.1949	0.2070	0.2186	0.2300	0.2412	0.2529	0.2647	0.2773	0.2901	0.3024	0.3150	0.3265	0.3369	0.3462	0.3544	0.3610	0.3650	0.3680	0.3712	0.3749	0.3787	0.3826	0.3871	0.3924	0.3980	0.4041	0.4107	0.4174	0.4227	0.4282	0.4347	0.4417	0.4487	0.4560	0.4634	0.4708

Location	1980	1981	1982	1983	1984	1985	1986	1987	1988	1989	1990	1991	1992	1993	1994	1995	1996	1997
Bungoma	0.1602	0.1724	0.1843	0.1959	0.2074	0.2195	0.2317	0.2448	0.2588	0.2733	0.2879	0.3011	0.3130	0.3234	0.3328	0.3406	0.3458	0.3501
Busia	0.1866	0.1957	0.2047	0.2135	0.2223	0.2314	0.2407	0.2506	0.2610	0.2714	0.2823	0.2924	0.3012	0.3085	0.3149	0.3200	0.3232	0.3251
Elgeyo-Marakwet	0.2278	0.2352	0.2426	0.2500	0.2575	0.2656	0.2742	0.2835	0.2936	0.3043	0.3156	0.3261	0.3354	0.3433	0.3505	0.3565	0.3603	0.3634
Embu	0.2885	0.2964	0.3042	0.3118	0.3195	0.3274	0.3359	0.3447	0.3542	0.3638	0.3739	0.3834	0.3923	0.4010	0.4097	0.4173	0.4230	0.4276
Garissa	0.2424	0.2473	0.2522	0.2570	0.2618	0.2668	0.2721	0.2777	0.2837	0.2896	0.2969	0.3038	0.3100	0.3155	0.3205	0.3245	0.3271	0.3289
HomaBay	0.1562	0.1677	0.1788	0.1895	0.2002	0.2112	0.2224	0.2344	0.2466	0.2583	0.2704	0.2817	0.2918	0.3008	0.3088	0.3153	0.3194	0.3224
Isiolo	0.1255	0.1289	0.1321	0.1349	0.1376	0.1403	0.1435	0.1467	0.1503	0.1538	0.1580	0.1618	0.1646	0.1668	0.1691	0.1707	0.1716	0.1718
Kajiado	0.2513	0.2616	0.2718	0.2819	0.2920	0.3024	0.3131	0.3243	0.3359	0.3474	0.3589	0.3698	0.3795	0.3879	0.3954	0.4017	0.4059	0.4088
Kakamega	0.1987	0.2073	0.2159	0.2245	0.2332	0.2424	0.2520	0.2624	0.2736	0.2853	0.2975	0.3088	0.3188	0.3277	0.3358	0.3427	0.3473	0.3503
Kericho	0.1863	0.1976	0.2087	0.2195	0.2304	0.2419	0.2536	0.2661	0.2795	0.2933	0.3077	0.3208	0.3326	0.3436	0.3537	0.3623	0.3683	0.3731
Kiambu	0.3041	0.3158	0.3275	0.3389	0.3504	0.3621	0.3741	0.3865	0.3994	0.4123	0.4252	0.4374	0.4485	0.4585	0.4679	0.4760	0.4821	0.4871
Kilifi	0.2235	0.2301	0.2367	0.2433	0.2499	0.2569	0.2644	0.2724	0.2808	0.2893	0.2986	0.3073	0.3153	0.3225	0.3292	0.3346	0.3380	0.3407
Kirinyaga	0.2748	0.2838	0.2928	0.3017	0.3107	0.3199	0.3295	0.3394	0.3499	0.3606	0.3712	0.3812	0.3906	0.3997	0.4087	0.4166	0.4227	0.4276
Kisii	0.1756	0.1934	0.2099	0.2253	0.2400	0.2545	0.2688	0.2833	0.2978	0.3117	0.3258	0.3389	0.3507	0.3611	0.3703	0.3779	0.3832	0.3872
Kisumu	0.2338	0.2460	0.2580	0.2698	0.2815	0.2936	0.3059	0.3188	0.3323	0.3459	0.3603	0.3737	0.3853	0.3945	0.4021	0.4081	0.4118	0.4145
Kitui	0.1946	0.2011	0.2077	0.2142	0.2207	0.2277	0.2351	0.2431	0.2517	0.2600	0.2686	0.2765	0.2839	0.2913	0.2986	0.3048	0.3090	0.3123
Kwale	0.2364	0.2433	0.2503	0.2570	0.2638	0.2709	0.2784	0.2862	0.2945	0.3028	0.3119	0.3204	0.3275	0.3330	0.3374	0.3407	0.3424	0.3434
Laikipia	0.3290	0.3371	0.3453	0.3535	0.3617	0.3704	0.3795	0.3892	0.3994	0.4094	0.4202	0.4303	0.4402	0.4504	0.4605	0.4696	0.4764	0.4814
Lamu	0.2833	0.2917	0.3001	0.3084	0.3168	0.3254	0.3343	0.3436	0.3530	0.3616	0.3697	0.3775	0.3845	0.3909	0.3968	0.4018	0.4051	0.4078
Machakos	0.2535	0.2622	0.2711	0.2798	0.2887	0.2980	0.3077	0.3179	0.3290	0.3407	0.3531	0.3647	0.3753	0.3848	0.3937	0.4013	0.4068	0.4109
Makueni	0.2029	0.2124	0.2219	0.2312	0.2404	0.2500	0.2600	0.2704	0.2811	0.2915	0.3022	0.3122	0.3213	0.3295	0.3370	0.3432	0.3475	0.3510
Mandera	0.1477	0.1511	0.1543	0.1572	0.1600	0.1628	0.1660	0.1694	0.1732	0.1773	0.1817	0.1857	0.1888	0.1913	0.1936	0.1951	0.1958	0.1958
Marsabit	0.1771	0.1805	0.1839	0.1870	0.1901	0.1932	0.1967	0.2003	0.2041	0.2076	0.2123	0.2166	0.2202	0.2232	0.2261	0.2285	0.2300	0.2309
Meru	0.2487	0.2574	0.2661	0.2748	0.2834	0.2922	0.3015	0.3111	0.3213	0.3320	0.3440	0.3554	0.3660	0.3757	0.3846	0.3922	0.3977	0.4020
Migori	0.0898	0.1091	0.1407	0.1646	0.1852	0.2045	0.2226	0.2407	0.2583	0.2748	0.2912	0.3062	0.3199	0.3320	0.3426	0.3513	0.3569	0.3615
Mombasa	0.3803	0.3875	0.3947	0.4017	0.4086	0.4158	0.4234	0.4314	0.4400	0.4490	0.4589	0.4682	0.4765	0.4838	0.4906	0.4964	0.5004	0.5033
Murang'a	0.2624	0.2703	0.2783	0.2862	0.2942	0.3026	0.3115	0.3209	0.3312	0.3421	0.3531	0.3634	0.3726	0.3810	0.3889	0.3958	0.4009	0.4050
Nairobi	0.3892	0.4039	0.4185	0.4329	0.4472	0.4615	0.4759	0.4905	0.5050	0.5193	0.5332	0.5463	0.5580	0.5683	0.5776	0.5856	0.5915	0.5963
Nakuru	0.2941	0.3035	0.3129	0.3224	0.3319	0.3418	0.3521	0.3629	0.3741	0.3851	0.3964	0.4072	0.4171	0.4261	0.4345	0.4416	0.4467	0.4508
Nandi	0.1993	0.2076	0.2159	0.2242	0.2326	0.2415	0.2509	0.2609	0.2716	0.2825	0.2938	0.3042	0.3139	0.3231	0.3319	0.3393	0.3446	0.3484
Narok	0.1503	0.1587	0.1671	0.1753	0.1834	0.1919	0.2007	0.2100	0.2197	0.2291	0.2384	0.2470	0.2545	0.2610	0.2669	0.2716	0.2746	0.2766
Nyamira	0.2612	0.2693	0.2773	0.2852	0.2930	0.3012	0.3099	0.3190	0.3284	0.3377	0.3476	0.3568	0.3651	0.3725	0.3795	0.3853	0.3894	0.3914
Nyandarua	0.2608	0.2695	0.2782	0.2868	0.2955	0.3046	0.3141	0.3241	0.3350	0.3465	0.3578	0.3682	0.3774	0.3854	0.3928	0.3991	0.4035	0.4069
Nyeri	0.2890	0.3000	0.3110	0.3219	0.3327	0.3437	0.3551	0.3668	0.3794	0.3930	0.4065	0.4191	0.4303	0.4401	0.4493	0.4573	0.4635	0.4686
Samburu	0.1781	0.1842	0.1902	0.1961	0.2020	0.2081	0.2146	0.2213	0.2281	0.2341	0.2400	0.2455	0.2503	0.2544	0.2584	0.2616	0.2637	0.2652
Siaya	0.2090	0.2156	0.2223	0.2288	0.2353	0.2424	0.2499	0.2580	0.2669	0.2761	0.2861	0.2954	0.3036	0.3110	0.3177	0.3233	0.3270	0.3292
TaitaTaveta	0.2894	0.2979	0.3064	0.3148	0.3232	0.3320	0.3413	0.3510	0.3615	0.3722	0.3834	0.3939	0.4035	0.4123	0.4207	0.4280	0.4334	0.4375
TanaRiver	0.1967	0.2025	0.2085	0.2143	0.2202	0.2265	0.2332	0.2403	0.2478	0.2551	0.2631	0.2705	0.2771	0.2830	0.2883	0.2926	0.2952	0.2971
TharakaNithi	0.3002	0.3063	0.3123	0.3182	0.3242	0.3304	0.3371	0.3442	0.3518	0.3592	0.3676	0.3756	0.3828	0.3893	0.3954	0.4007	0.4044	0.4073
TransNzoia	0.2135	0.2225	0.2314	0.2401	0.2490	0.2584	0.2681	0.2786	0.2900	0.3022	0.3149	0.3266	0.3369	0.3459	0.3540	0.3608	0.3653	0.3685
Turkana	0.0625	0.0644	0.0663	0.0678	0.0691	0.0703	0.0718	0.0731	0.0746	0.0759	0.0795	0.0827	0.0851	0.0869	0.0889	0.0904	0.0915	0.0921
UasinGishu	0.2419	0.2510	0.2603	0.2695	0.2787	0.2884	0.2984	0.3089	0.3200	0.3311	0.3424	0.3530	0.3626	0.3711	0.3788	0.3852	0.3894	0.3931
Vihiga	0.2198	0.2281	0.2363	0.2444	0.2525	0.2610	0.2698	0.2792	0.2889	0.2983	0.3079	0.3171	0.3253	0.3328	0.3398	0.3456	0.3498	0.3531
Wajir	0.1230	0.1256	0.1280	0.1302	0.1322	0.1343	0.1368	0.1393	0.1423	0.1456	0.1504	0.1549	0.1585	0.1615	0.1644	0.1665	0.1680	0.1687
WestPokot	0.1638	0.1694	0.1750	0.1806	0.1861	0.1920	0.1981	0.2047	0.2118	0.2191	0.2269	0.2342	0.2403	0.2450	0.2492	0.2523	0.2542	0.2550
Madagascar	0.2353	0.2402	0.2443	0.2478	0.2505	0.2524	0.2541	0.2552	0.2565	0.2585	0.2614	0.2632	0.2655	0.2688	0.2718	0.2747	0.2779	0.2815
Malawi	0.1568	0.1564	0.1554	0.1547	0.1557	0.1594	0.1673	0.1759	0.1835	0.1895	0.1938	0.1942	0.1921	0.1921	0.1919	0.1951	0.1997	0.2064
Mozambique	0.0886	0.0901	0.0901	0.0860	0.0809	0.0729	0.0617	0.0604	0.0608	0.0614	0.0622	0.0645	0.0669	0.0691	0.0710	0.0727	0.0740	0.0893
Rwanda	0.1339	0.1359	0.1368	0.1388	0.1425	0.1489	0.1600	0.1731	0.1858	0.1964	0.2050	0.2083	0.2110	0.2127	0.2093	0.2112	0.2144	0.2210
Somalia	0.1098	0.1101	0.1094	0.1070	0.1052	0.1050	0.1056	0.1080	0.1108	0.1137	0.1158	0.1157	0.1137	0.1115	0.1083	0.1059	0.1046	0.1040
South Sudan	0.1117	0.1119	0.1123	0.1127	0.1149	0.1202	0.1249	0.1297	0.1345	0.1392	0.1435	0.1461	0.1480	0.1494	0.1513	0.1541	0.1587	0.1648
Tanzania	0.2463	0.2487	0.2513	0.2541	0.2572	0.2609	0.2629	0.2646	0.2678	0.2713	0.2757	0.2798	0.2836	0.2873	0.2907	0.2939	0.2967	0.2997
Uganda	0.1963	0.1946	0.1945	0.1954	0.1960	0.1962	0.1959	0.1965	0.1987	0.2016	0.2049	0.2071	0.2096	0.2129	0.2187	0.2253	0.2311	0.2370
Zambia	0.2811	0.2850	0.2884	0.2942	0.2998	0.3053	0.3092	0.3137	0.3177	0.3213	0.3257	0.3283	0.3311	0.3336	0.3348	0.3368	0.3387	0.3413
Southern Sub-Saharan Africa	0.4784	0.4852	0.4918	0.4981	0.5045	0.5106	0.5163	0.5220	0.5281	0.5343	0.5401	0.5456	0.5503	0.5547	0.5579	0.5611	0.5666	0.5718
Botswana	0.2931	0.3028	0.3131	0.3246	0.3365	0.3492	0.3626	0.3766	0.3910	0.4058	0.4202	0.4345	0.4478	0.4609	0.4733	0.4854	0.4952	0.5051
Lesotho	0.2808	0.2858	0.2906	0.2949	0.2997	0.3047	0.3087	0.3125	0.3175	0.3232	0.3289	0.3363	0.3439	0.3515	0.3589	0.3657	0.3731	0.3796
Namibia	0.3574	0.3640	0.3701	0.3759	0.3820	0.3887	0.3997	0.4110	0.4219	0.4320	0.4408	0.4478	0.4552	0.4621	0.4695	0.4772	0.4852	0.4935
South Africa	0.5446	0.5511	0.5569	0.5622	0.5675	0.5724	0.5767	0.5810	0.5855	0.5898	0.5937	0.5971	0.6001	0.6026	0.6032	0.6040	0.6084	0.6124
Eastern Cape	0.5165	0.5232	0.5290	0.5340	0.5392	0.5436	0.5473	0.5511	0.5550	0.5589	0.5620	0.5645	0.5663	0.5677	0.5674	0.5678	0.5719	0.5756
Free State	0.5479	0.5538	0.5593	0.5643	0.5693	0.5738	0.5779	0.5821	0.5862	0.5903	0.5940	0.5973	0.6003	0.6031	0.6035	0.6023	0.6054	0.6113
Gauteng	0.6152	0.6207	0.6260	0.6309	0.6358	0.6403	0.6446	0.6488	0.6530	0.6571	0.6609	0.6644	0.6675	0.6697	0.6704	0.6711	0.6740	0.6767
KwaZulu-Natal	0.5319	0.5382	0.5438	0.5489	0.5541	0.5588	0.5630	0.5672	0.5715	0.5757	0.5794	0.5826	0.5855	0.5881	0.5889	0.5900	0.5947	0.5987
Limpopo	0.4966	0.5030	0.5085	0.5133	0.5182	0.5223	0.5258	0.5293	0.5329	0.5364	0.5393	0.5415	0.5432	0.5445	0.5435	0.5428	0.5452	0.5463
Mpumalanga	0.5533	0.5591	0.5644	0.5692	0.5740	0.5783	0.5822	0.5861	0.5901	0.5940	0.5974	0.6004	0.6031	0.6051	0.6043	0.6037	0.6074	0.6099
North-West	0.5397	0.5457	0.5511	0.5560	0.5610	0.5654	0.5694	0.5733	0.5774	0.5812	0.5847	0.5877	0.5902	0.5921	0.5921	0.5927	0.5971	0.6011
Northern Cape	0.5448	0.5505	0.5557	0.5605	0.5653	0.5697	0.5737	0.5776	0.5816	0.5855	0.5890	0.5921	0.5948	0.5966	0.5960	0.5960	0.6015	0.6066
Western Cape	0.6019	0.6078	0.6133	0.6185	0.6236	0.6284	0.6329	0.6373	0.6417	0.6461	0.6501	0.6538	0.6569	0.6593	0.6600	0.6614	0.6674	0.6736
Swaziland	0.3211	0.3274	0.3328	0.3381	0.3441	0.3512	0.3615	0.3743	0.3878	0.4017	0.4159	0.4262	0.4364	0.4470	0.4577	0.4693	0.4810	0.4922
Zimbabwe	0.3064	0.3168	0.3283	0.3412	0.3533	0.3657	0.3773	0.3884	0.4007	0.4129	0.4245	0.4350	0.4434	0.4514	0.4602	0.4681	0.4761	0.4838
Western Sub-Saharan Africa	0.2265	0.2296	0.2327	0.2356	0.2386	0.2423	0.2450	0.2476	0.2507	0.2542	0.2581	0.2618	0.2656	0.2696	0.2736	0.2777	0.2817	0.2861
Benin	0.1635	0.1668	0.1704	0.1740	0.1779	0.1820	0.1856	0.1887	0.1919	0.1948	0.1984	0.2028	0.2074	0.2125	0.2172	0.2223	0.2269	0.2319
Burkina Faso	0.0719	0.0721	0.0723	0.0724	0.0724	0.0732	0.0740	0.0754	0.0804	0.0852	0.0897	0.0943	0.0986	0.1030	0.1070	0.1113	0.1160	0.1211
Cameroon	0.2646	0.2693	0.2744	0.2800	0.2864	0.2933	0.2996	0.3058	0.3116	0.3173	0.3227	0.3273	0.3316	0.3355	0.3393	0.3435	0.3478	0.3526
Cape Verde	0.2256	0.2356	0.2452	0.2554	0.2647	0.2738	0.2810	0.2876	0.2936	0.2998	0.3056	0.3142	0.3232	0.3325	0.3427	0.3532	0.3638	0.3746
Chad	0.1235	0.1238	0.1243	0.1257	0.1272	0.1302	0.1322	0.1343	0.1379	0.1414	0.1447	0.1480	0.1507	0.1527	0.1552	0.1576	0.1592	0.1612
Cote d'Ivoire	0.2075	0.2131	0.2191	0.2249	0.2305	0.2361	0.2408	0.2453	0.2500	0.2552	0.2602	0.2649	0.2696	0.2745	0.2793	0.2844	0.2896	0.2951
The Gambia	0.2015	0.2024	0.2049	0.2083	0.2116	0.2151	0.2196	0.2244	0.2291	0.2340	0.2388	0.2400	0.2412	0.2428	0.2446	0.2469	0.2496	0.2530
Ghana	0.3018	0.3061	0.3101	0.3138	0.3180	0.3225	0.3268	0.3313	0.3367	0.3424	0.3486	0.3555	0.3626	0.3696	0.3765	0.3829	0.3887	0.3945
Guinea	0.1425	0.1454	0.1486	0.1519	0.1549	0.1578	0.1598	0.1616	0.1638	0.1666	0.1700	0.1745	0.1794	0.1846	0.1894	0.1941	0.1982	0.2018
Guinea-Bissau	0.1478	0.1490	0.1505	0.1520	0.1541	0.1563	0.1587	0.1618	0.1656	0.1698	0.1744	0.1794	0.1842	0.1895	0.1952	0.2013	0.2074	0.2142
Liberia	0.1925	0.1949	0.1982	0.2016	0.2045	0.2073	0.2087	0.2106	0.2134	0.2164	0.2152	0.2122	0.2056	0.1957	0.1833	0.1682	0.1498	0.1409
Mali	0.0768	0.0807	0.0846	0.0885	0.0923	0.0965	0.0997	0.1026	0.1052	0.1085	0.1122	0.1170	0.1217	0.1268	0.1317	0.1365	0.1409	0.1455
Mauritania	0.2279	0.2324	0.2364	0.2404	0.2440	0.2476	0.2509	0.2543	0.2579	0.2617	0.2654	0.2693	0.2733	0.2778	0.2819	0.2864	0.2914	0.2957

Location	1998	1999	2000	2001	2002	2003	2004	2005	2006	2007	2008	2009	2010	2011	2012	2013	2014	2015
Bungoma	0.3544	0.3584	0.3624	0.3662	0.3698	0.3733	0.3771	0.3818	0.3873	0.3935	0.3991	0.4046	0.4106	0.4171	0.4237	0.4305	0.4373	0.4444
Busia	0.3263	0.3273	0.3284	0.3285	0.3288	0.3298	0.3315	0.3340	0.3377	0.3432	0.3486	0.3541	0.3601	0.3665	0.3729	0.3796	0.3863	0.3933
Elgeyo-Marakwet	0.3663	0.3686	0.3702	0.3711	0.3721	0.3738	0.3762	0.3798	0.3847	0.3911	0.3970	0.4026	0.4086	0.4151	0.4217	0.4286	0.4354	0.4427
Embu	0.4317	0.4358	0.4398	0.4437	0.4471	0.4499	0.4529	0.4568	0.4617	0.4676	0.4733	0.4788	0.4852	0.4919	0.4987	0.5056	0.5125	0.5194
Garissa	0.3309	0.3327	0.3336	0.3336	0.3337	0.3343	0.3355	0.3376	0.3399	0.3431	0.3463	0.3495	0.3529	0.3565	0.3602	0.3641	0.3680	0.3731
HomaBay	0.3259	0.3299	0.3333	0.3352	0.3367	0.3377	0.3391	0.3419	0.3462	0.3527	0.3597	0.3674	0.3756	0.3838	0.3910	0.3980	0.4051	0.4125
Isiolo	0.1719	0.1720	0.1715	0.1705	0.1694	0.1686	0.1685	0.1696	0.1721	0.1760	0.1791	0.1820	0.1859	0.1902	0.1946	0.1991	0.2036	0.2087
Kajiado	0.4110	0.4132	0.4159	0.4187	0.4218	0.4256	0.4300	0.4353	0.4417	0.4494	0.4565	0.4628	0.4693	0.4763	0.4835	0.4908	0.4980	0.5052
Kakamega	0.3522	0.3541	0.3556	0.3563	0.3569	0.3582	0.3604	0.3637	0.3680	0.3741	0.3804	0.3862	0.3920	0.3985	0.4050	0.4116	0.4184	0.4254
Kericho	0.3777	0.3820	0.3850	0.3865	0.3880	0.3902	0.3931	0.3970	0.4015	0.4074	0.4138	0.4200	0.4263	0.4330	0.4399	0.4469	0.4541	0.4613
Kiambu	0.4915	0.4955	0.4991	0.5022	0.5054	0.5093	0.5137	0.5189	0.5246	0.5306	0.5364	0.5432	0.5508	0.5588	0.5668	0.5749	0.5831	0.5908
Kilifi	0.3435	0.3457	0.3473	0.3483	0.3497	0.3517	0.3544	0.3578	0.3617	0.3662	0.3704	0.3747	0.3792	0.3842	0.3891	0.3941	0.3991	0.4049
Kirinyaga	0.4317	0.4353	0.4387	0.4422	0.4459	0.4501	0.4549	0.4603	0.4661	0.4722	0.4776	0.4831	0.4894	0.4962	0.5029	0.5098	0.5167	0.5235
Kisii	0.3905	0.3945	0.3985	0.4018	0.4050	0.4082	0.4117	0.4162	0.4217	0.4284	0.4349	0.4419	0.4497	0.4579	0.4659	0.4741	0.4822	0.4898
Kisumu	0.4170	0.4198	0.4229	0.4250	0.4265	0.4275	0.4287	0.4312	0.4352	0.4419	0.4502	0.4590	0.4676	0.4764	0.4848	0.4931	0.5015	0.5095
Kitui	0.3155	0.3183	0.3206	0.3227	0.3252	0.3287	0.3330	0.3380	0.3435	0.3495	0.3547	0.3593	0.3643	0.3699	0.3756	0.3815	0.3874	0.3938
Kwale	0.3445	0.3461	0.3478	0.3488	0.3498	0.3512	0.3534	0.3565	0.3604	0.3653	0.3700	0.3750	0.3801	0.3858	0.3915	0.3974	0.4034	0.4099
Laikipia	0.4848	0.4877	0.4905	0.4928	0.4954	0.4988	0.5029	0.5079	0.5134	0.5203	0.5273	0.5337	0.5400	0.5468	0.5537	0.5607	0.5678	0.5750
Lamu	0.4108	0.4139	0.4173	0.4202	0.4234	0.4271	0.4312	0.4360	0.4407	0.4455	0.4501	0.4553	0.4609	0.4670	0.4732	0.4795	0.4858	0.4924
Machakos	0.4145	0.4181	0.4213	0.4242	0.4278	0.4326	0.4385	0.4451	0.4524	0.4607	0.4682	0.4747	0.4813	0.4884	0.4956	0.5029	0.5102	0.5175
Makueni	0.3542	0.3565	0.3569	0.3561	0.3569	0.3601	0.3648	0.3702	0.3762	0.3829	0.3891	0.3952	0.4018	0.4090	0.4162	0.4236	0.4310	0.4384
Mandera	0.1956	0.1952	0.1949	0.1941	0.1929	0.1920	0.1919	0.1927	0.1940	0.1960	0.1978	0.2002	0.2028	0.2057	0.2087	0.2118	0.2151	0.2195
Marsabit	0.2317	0.2322	0.2332	0.2342	0.2356	0.2375	0.2402	0.2434	0.2471	0.2516	0.2552	0.2585	0.2618	0.2654	0.2691	0.2729	0.2767	0.2814
Meru	0.4057	0.4090	0.4118	0.4144	0.4171	0.4201	0.4236	0.4277	0.4324	0.4373	0.4414	0.4462	0.4521	0.4584	0.4647	0.4713	0.4779	0.4845
Migori	0.3663	0.3723	0.3792	0.3851	0.3889	0.3902	0.3910	0.3933	0.3970	0.4024	0.4081	0.4148	0.4224	0.4307	0.4389	0.4472	0.4555	0.4635
Mombasa	0.5055	0.5077	0.5102	0.5129	0.5160	0.5195	0.5236	0.5282	0.5334	0.5388	0.5434	0.5484	0.5545	0.5611	0.5678	0.5746	0.5814	0.5885
Murang'a	0.4087	0.4120	0.4150	0.4181	0.4216	0.4260	0.4312	0.4372	0.4437	0.4506	0.4564	0.4619	0.4682	0.4749	0.4816	0.4884	0.4952	0.5021
Nairobi	0.6005	0.6049	0.6102	0.6157	0.6209	0.6259	0.6311	0.6370	0.6434	0.6504	0.6574	0.6653	0.6737	0.6827	0.6916	0.7008	0.7099	0.7182
Nakuru	0.4547	0.4583	0.4612	0.4633	0.4654	0.4679	0.4711	0.4751	0.4801	0.4865	0.4930	0.4993	0.5061	0.5134	0.5207	0.5282	0.5357	0.5432
Nandi	0.3514	0.3542	0.3575	0.3608	0.3638	0.3666	0.3695	0.3733	0.3776	0.3825	0.3870	0.3920	0.3976	0.4036	0.4096	0.4159	0.4222	0.4289
Narok	0.2785	0.2802	0.2817	0.2825	0.2834	0.2848	0.2868	0.2897	0.2930	0.2965	0.2996	0.3038	0.3090	0.3146	0.3202	0.3261	0.3321	0.3386
Nyamira	0.3925	0.3954	0.4005	0.4062	0.4107	0.4129	0.4142	0.4164	0.4195	0.4240	0.4292	0.4360	0.4439	0.4516	0.4590	0.4666	0.4742	0.4817
Nyandarua	0.4101	0.4133	0.4160	0.4183	0.4210	0.4243	0.4283	0.4331	0.4382	0.4438	0.4489	0.4540	0.4598	0.4659	0.4721	0.4785	0.4849	0.4915
Nyeri	0.4730	0.4770	0.4805	0.4839	0.4873	0.4910	0.4952	0.5000	0.5058	0.5126	0.5191	0.5255	0.5325	0.5399	0.5473	0.5547	0.5622	0.5694
Samburu	0.2665	0.2671	0.2672	0.2670	0.2671	0.2679	0.2691	0.2709	0.2732	0.2765	0.2798	0.2840	0.2890	0.2945	0.3000	0.3056	0.3113	0.3174
Siaya	0.3307	0.3320	0.3338	0.3351	0.3367	0.3391	0.3424	0.3466	0.3516	0.3575	0.3635	0.3701	0.3772	0.3845	0.3909	0.3971	0.4034	0.4102
TaitaTaveta	0.4412	0.4448	0.4483	0.4518	0.4559	0.4607	0.4662	0.4725	0.4789	0.4852	0.4909	0.4969	0.5036	0.5108	0.5181	0.5255	0.5328	0.5401
TanaRiver	0.2984	0.2984	0.2970	0.2945	0.2927	0.2926	0.2933	0.2945	0.2961	0.2984	0.3009	0.3044	0.3087	0.3135	0.3184	0.3235	0.3287	0.3348
TharakaNithi	0.4100	0.4121	0.4135	0.4148	0.4168	0.4202	0.4248	0.4301	0.4361	0.4431	0.4494	0.4548	0.4606	0.4669	0.4732	0.4797	0.4862	0.4930
TransNzoia	0.3711	0.3737	0.3760	0.3772	0.3783	0.3799	0.3822	0.3853	0.3894	0.3945	0.3997	0.4053	0.4115	0.4181	0.4248	0.4318	0.4387	0.4459
Turkana	0.0925	0.0924	0.0918	0.0911	0.0903	0.0898	0.0898	0.0908	0.0930	0.0969	0.1001	0.1030	0.1063	0.1099	0.1134	0.1171	0.1207	0.1249
UasinGishu	0.3974	0.4021	0.4074	0.4127	0.4169	0.4197	0.4223	0.4256	0.4300	0.4362	0.4424	0.4484	0.4548	0.4616	0.4686	0.4757	0.4828	0.4900
Vihiga	0.3562	0.3588	0.3607	0.3620	0.3631	0.3646	0.3667	0.3698	0.3742	0.3808	0.3874	0.3931	0.3991	0.4056	0.4122	0.4190	0.4259	0.4328
Wajir	0.1697	0.1707	0.1707	0.1699	0.1689	0.1681	0.1679	0.1685	0.1699	0.1726	0.1748	0.1769	0.1796	0.1826	0.1856	0.1887	0.1920	0.1962
WestPokot	0.2554	0.2557	0.2560	0.2557	0.2555	0.2557	0.2563	0.2576	0.2599	0.2641	0.2683	0.2725	0.2772	0.2823	0.2875	0.2929	0.2983	0.3044
Madagascar	0.2856	0.2905	0.2961	0.3024	0.3065	0.3116	0.3170	0.3225	0.3281	0.3338	0.3399	0.3445	0.3489	0.3531	0.3574	0.3615	0.3656	0.3698
Malawi	0.2121	0.2173	0.2218	0.2233	0.2248	0.2270	0.2299	0.2331	0.2374	0.2444	0.2522	0.2610	0.2703	0.2792	0.2871	0.2947	0.3019	0.3086
Mozambique	0.1065	0.1206	0.1310	0.1424	0.1540	0.1650	0.1755	0.1860	0.1960	0.2058	0.2152	0.2244	0.2334	0.2422	0.2510	0.2600	0.2690	0.2780
Rwanda	0.2273	0.2317	0.2347	0.2391	0.2451	0.2509	0.2583	0.2664	0.2762	0.2864	0.2978	0.3092	0.3204	0.3315	0.3426	0.3528	0.3624	0.3713
Somalia	0.1036	0.1035	0.1034	0.1031	0.1036	0.1054	0.1078	0.1108	0.1142	0.1183	0.1226	0.1270	0.1315	0.1356	0.1397	0.1439	0.1475	0.1506
South Sudan	0.1713	0.1775	0.1830	0.1877	0.1921	0.1966	0.2015	0.2066	0.2120	0.2176	0.2234	0.2293	0.2352	0.2405	0.2459	0.2513	0.2565	0.2616
Tanzania	0.3030	0.3066	0.3106	0.3147	0.3193	0.3244	0.3300	0.3364	0.3424	0.3493	0.3567	0.3642	0.3719	0.3798	0.3874	0.3952	0.4032	0.4114
Uganda	0.2433	0.2500	0.2566	0.2631	0.2699	0.2769	0.2841	0.2920	0.3000	0.3084	0.3174	0.3268	0.3361	0.3451	0.3534	0.3614	0.3692	0.3768
Zambia	0.3440	0.3473	0.3511	0.3544	0.3580	0.3627	0.3681	0.3743	0.3814	0.3902	0.3998	0.4096	0.4204	0.4307	0.4407	0.4501	0.4590	0.4670
Southern Sub-Saharan Africa	0.5763	0.5814	0.5868	0.5922	0.5978	0.6030	0.6084	0.6138	0.6192	0.6249	0.6298	0.6346	0.6398	0.6456	0.6516	0.6577	0.6636	0.6688
Botswana	0.5148	0.5250	0.5346	0.5437	0.5527	0.5612	0.5696	0.5774	0.5854	0.5931	0.6006	0.6053	0.6107	0.6162	0.6217	0.6281	0.6346	0.6411
Lesotho	0.3865	0.3936	0.4014	0.4092	0.4167	0.4246	0.4323	0.4399	0.4480	0.4563	0.4643	0.4719	0.4803	0.4883	0.4965	0.5050	0.5135	0.5217
Namibia	0.5024	0.5109	0.5190	0.5256	0.5322	0.5385	0.5456	0.5526	0.5596	0.5664	0.5727	0.5782	0.5837	0.5897	0.5959	0.6026	0.6096	0.6170
South Africa	0.6156	0.6198	0.6245	0.6298	0.6357	0.6416	0.6478	0.6545	0.6614	0.6686	0.6757	0.6819	0.6881	0.6945	0.7007	0.7065	0.7119	0.7164
Eastern Cape	0.5784	0.5822	0.5869	0.5922	0.5982	0.6044	0.6111	0.6184	0.6263	0.6346	0.6426	0.6492	0.6558	0.6627	0.6692	0.6752	0.6806	0.6850
Free State	0.6160	0.6203	0.6250	0.6299	0.6353	0.6407	0.6464	0.6525	0.6589	0.6656	0.6721	0.6778	0.6836	0.6896	0.6952	0.7005	0.7055	0.7096
Gauteng	0.6798	0.6841	0.6886	0.6934	0.6982	0.7031	0.7082	0.7136	0.7192	0.7250	0.7307	0.7359	0.7412	0.7466	0.7519	0.7569	0.7618	0.7659
KwaZulu-Natal	0.6016	0.6055	0.6100	0.6151	0.6207	0.6264	0.6325	0.6390	0.6459	0.6530	0.6600	0.6660	0.6721	0.6784	0.6843	0.6899	0.6950	0.6992
Limpopo	0.5476	0.5514	0.5557	0.5604	0.5658	0.5713	0.5773	0.5838	0.5908	0.5981	0.6050	0.6107	0.6163	0.6225	0.6283	0.6335	0.6382	0.6419
Mpumalanga	0.6119	0.6158	0.6201	0.6247	0.6297	0.6349	0.6403	0.6461	0.6523	0.6587	0.6648	0.6702	0.6756	0.6812	0.6866	0.6916	0.6962	0.7000
North-West	0.6039	0.6072	0.6111	0.6156	0.6207	0.6259	0.6314	0.6373	0.6435	0.6500	0.6563	0.6618	0.6674	0.6732	0.6786	0.6838	0.6885	0.6923
Northern Cape	0.6089	0.6113	0.6145	0.6188	0.6240	0.6292	0.6348	0.6406	0.6467	0.6532	0.6594	0.6650	0.6706	0.6765	0.6819	0.6872	0.6920	0.6961
Western Cape	0.6769	0.6794	0.6829	0.6876	0.6929	0.6982	0.7038	0.7096	0.7157	0.7221	0.7283	0.7340	0.7397	0.7455	0.7512	0.7566	0.7618	0.7663
Swaziland	0.5028	0.5125	0.5210	0.5275	0.5333	0.5364	0.5396	0.5455	0.5523	0.5599	0.5686	0.5772	0.5857	0.5935	0.6014	0.6089	0.6162	0.6232
Zimbabwe	0.4909	0.4976	0.5031	0.5072	0.5095	0.5101	0.5102	0.5091	0.5071	0.5060	0.5021	0.5018	0.5036	0.5076	0.5141	0.5219	0.5302	0.5384
Western Sub-Saharan Africa	0.2906	0.2953	0.3003	0.3053	0.3106	0.3166	0.3239	0.3316	0.3386	0.3459	0.3529	0.3598	0.3668	0.3736	0.3804	0.3872	0.3941	0.4009
Benin	0.2370	0.2427	0.2488	0.2555	0.2625	0.2696	0.2767	0.2835	0.2899	0.2964	0.3028	0.3089	0.3146	0.3201	0.3257	0.3318	0.3381	0.3446
Burkina Faso	0.1268	0.1328	0.1386	0.1443	0.1501	0.1563	0.1625	0.1691	0.1755	0.1821	0.1887	0.1952	0.2023	0.2093	0.2165	0.2236	0.2306	0.2374
Cameroon	0.3581	0.3639	0.3700	0.3759	0.3820	0.3881	0.3944	0.4005	0.4063	0.4123	0.4186	0.4248	0.4311	0.4372	0.4436	0.4501	0.4570	0.4641
Cape Verde	0.3862	0.3990	0.4120	0.4249	0.4373	0.4494	0.4609	0.4715	0.4819	0.4923	0.5019	0.5100	0.5176	0.5251	0.5318	0.5379	0.5434	0.5485
Chad	0.1638	0.1667	0.1696	0.1729	0.1772	0.1832	0.1931	0.2037	0.2133	0.2222	0.2307	0.2388	0.2478	0.2554	0.2634	0.2713	0.2792	0.2871
Cote d'Ivoire	0.3012	0.3072	0.3126	0.3169	0.3209	0.3245	0.3280	0.3317	0.3355	0.3393	0.3432	0.3475	0.3519	0.3558	0.3607	0.3668	0.3735	0.3807
The Gambia	0.2570	0.2619	0.2672	0.2713	0.2747	0.2787	0.2836	0.2878	0.2909	0.2941	0.2979	0.3019	0.3064	0.3095	0.3134	0.3176	0.3218	0.3266
Ghana	0.3999	0.4056	0.4111	0.4163	0.4214	0.4272	0.4332	0.4393	0.4452	0.4512	0.4577	0.4641	0.4710	0.4788	0.4869	0.4951	0.5031	0.5110
Guinea	0.2050	0.2085	0.2121	0.2158	0.2197	0.2235	0.2273	0.2313	0.2353	0.2397	0.2447	0.2493	0.2539	0.2586	0.2636	0.2685	0.2733	0.2777
Guinea-Bissau	0.2181	0.2224	0.2267	0.2310	0.2347	0.2387	0.2427	0.2469	0.2508	0.2552	0.2597	0.2646	0.2695	0.2749	0.2798	0.2845	0.2893	0.2943
Liberia	0.1404	0.1520	0.1764	0.1944	0.2093	0.2113	0.2134	0.2160	0.2200	0.2251	0.2306	0.2359	0.2415	0.2485	0.2570	0.2672	0.2756	0.2827
Mali	0.1501	0.1547	0.1585	0.1629	0.1673	0.1718	0.1763	0.1810	0.1854	0.1901	0.1954	0.2008	0.2064	0.2112	0.2159	0.2205	0.2255	0.2309
Mauritania	0.2999	0.3046	0.3090	0.3135	0.3180	0.3230	0.3280	0.3335	0.3412	0.3483	0.3548	0.3608	0.3673	0.3740	0.3808	0.3877	0.3947	0.4014

Location	1980	1981	1982	1983	1984	1985	1986	1987	1988	1989	1990	1991	1992	1993	1994	1995	1996	1997	1998	1999	2000	2001	2002	2003	2004	2005	2006	2007	2008	2009	2010	2011	2012	2013	2014	2015
Niger	0.0804	0.0802	0.0801	0.0800	0.0790	0.0782	0.0769	0.0787	0.0807	0.0826	0.0846	0.0864	0.0882	0.0902	0.0924	0.0943	0.0957	0.0971	0.0991	0.1010	0.1026	0.1045	0.1068	0.1094	0.1116	0.1141	0.1164	0.1187	0.1215	0.1239	0.1270	0.1297	0.1335	0.1375	0.1420	0.1465
Nigeria	0.2793	0.2821	0.2847	0.2870	0.2894	0.2928	0.2947	0.2959	0.2978	0.3002	0.3036	0.3066	0.3099	0.3138	0.3179	0.3221	0.3266	0.3315	0.3364	0.3412	0.3467	0.3523	0.3583	0.3659	0.3760	0.3867	0.3963	0.4062	0.4156	0.4246	0.4336	0.4421	0.4503	0.4582	0.4661	0.4740
Sao Tome and Principe	0.2622	0.2666	0.2720	0.2769	0.2809	0.2849	0.2895	0.2926	0.2952	0.2987	0.3028	0.3067	0.3112	0.3162	0.3209	0.3252	0.3301	0.3347	0.3394	0.3443	0.3496	0.3551	0.3609	0.3671	0.3735	0.3799	0.3872	0.3940	0.4015	0.4086	0.4153	0.4224	0.4291	0.4352	0.4415	0.4481
Senegal	0.1600	0.1633	0.1677	0.1722	0.1770	0.1821	0.1873	0.1928	0.1984	0.2042	0.2099	0.2154	0.2209	0.2266	0.2319	0.2372	0.2420	0.2467	0.2519	0.2574	0.2629	0.2685	0.2738	0.2793	0.2848	0.2902	0.2950	0.2996	0.3037	0.3076	0.3114	0.3152	0.3193	0.3237	0.3286	0.3341
Sierra Leone	0.1459	0.1486	0.1513	0.1535	0.1560	0.1587	0.1617	0.1657	0.1702	0.1748	0.1796	0.1832	0.1851	0.1880	0.1917	0.1952	0.1971	0.1982	0.1995	0.1997	0.2018	0.2057	0.2133	0.2217	0.2299	0.2375	0.2446	0.2521	0.2597	0.2676	0.2757	0.2836	0.2932	0.3051	0.3167	0.3230
Togo	0.2028	0.2067	0.2107	0.2146	0.2187	0.2228	0.2265	0.2298	0.2341	0.2390	0.2447	0.2500	0.2550	0.2579	0.2620	0.2673	0.2727	0.2788	0.2835	0.2882	0.2922	0.2957	0.2990	0.3026	0.3061	0.3093	0.3132	0.3174	0.3213	0.3257	0.3307	0.3361	0.3421	0.3484	0.3550	0.3617

Appendix Table 6a. Relative risks used by age and sex and for each outcome for all risk factors except for ambient air pollution and alcohol.

Risk - Outcome	Category / Units	Morbidity / Mortality	Sex	All ages	0-6 Days	7-27 Days	28-364 Days	1-4 years	5-9 years	10-14 years	15-19 years	20-24 years	25-29 years	30-34 years	35-39 years	40-44 years
					Age											
**Unsafe water source**																
Diarrhoeal diseases	Unimproved & untreated	Both	Both		11·238 (3·643 to 25·544)	11·238 (3·643 to 25·544)	11·238 (3·643 to 25·544)	11·238 (3·643 to 25·544)	11·238 (3·643 to 25·544)	11·238 (3·643 to 25·544)	11·238 (3·643 to 25·544)	11·238 (3·643 to 25·544)	11·238 (3·643 to 25·544)	11·238 (3·643 to 25·544)	11·238 (3·643 to 25·544)	11·238 (3·643 to 25·544)
Diarrhoeal diseases	Unimproved & chlorinated	Both	Both		9·151 (3·165 to 20·449)	9·151 (3·165 to 20·449)	9·151 (3·165 to 20·449)	9·151 (3·165 to 20·449)	9·151 (3·165 to 20·449)	9·151 (3·165 to 20·449)	9·151 (3·165 to 20·449)	9·151 (3·165 to 20·449)	9·151 (3·165 to 20·449)	9·151 (3·165 to 20·449)	9·151 (3·165 to 20·449)	9·151 (3·165 to 20·449)
Diarrhoeal diseases	Unimproved & filtered	Both	Both		5·857 (2·065 to 13·038)	5·857 (2·065 to 13·038)	5·857 (2·065 to 13·038)	5·857 (2·065 to 13·038)	5·857 (2·065 to 13·038)	5·857 (2·065 to 13·038)	5·857 (2·065 to 13·038)	5·857 (2·065 to 13·038)	5·857 (2·065 to 13·038)	5·857 (2·065 to 13·038)	5·857 (2·065 to 13·038)	5·857 (2·065 to 13·038)
Diarrhoeal diseases	Improved & untreated	Both	Both		9·705 (3·700 to 20·399)	9·705 (3·700 to 20·399)	9·705 (3·700 to 20·399)	9·705 (3·700 to 20·399)	9·705 (3·700 to 20·399)	9·705 (3·700 to 20·399)	9·705 (3·700 to 20·399)	9·705 (3·700 to 20·399)	9·705 (3·700 to 20·399)	9·705 (3·700 to 20·399)	9·705 (3·700 to 20·399)	9·705 (3·700 to 20·399)
Diarrhoeal diseases	Improved & chlorinated	Both	Both		7·904 (3·202 to 16·211)	7·904 (3·202 to 16·211)	7·904 (3·202 to 16·211)	7·904 (3·202 to 16·211)	7·904 (3·202 to 16·211)	7·904 (3·202 to 16·211)	7·904 (3·202 to 16·211)	7·904 (3·202 to 16·211)	7·904 (3·202 to 16·211)	7·904 (3·202 to 16·211)	7·904 (3·202 to 16·211)	7·904 (3·202 to 16·211)
Diarrhoeal diseases	Improved & filtered	Both	Both		5·059 (2·094 to 10·265)	5·059 (2·094 to 10·265)	5·059 (2·094 to 10·265)	5·059 (2·094 to 10·265)	5·059 (2·094 to 10·265)	5·059 (2·094 to 10·265)	5·059 (2·094 to 10·265)	5·059 (2·094 to 10·265)	5·059 (2·094 to 10·265)	5·059 (2·094 to 10·265)	5·059 (2·094 to 10·265)	5·059 (2·094 to 10·265)
Diarrhoeal diseases	Piped & untreated, all locations except Eastern Europe and Southern Latin America	Both	Both		8·331 (3·355 to 16·932)	8·331 (3·355 to 16·932)	8·331 (3·355 to 16·932)	8·331 (3·355 to 16·932)	8·331 (3·355 to 16·932)	8·331 (3·355 to 16·932)	8·331 (3·355 to 16·932)	8·331 (3·355 to 16·932)	8·331 (3·355 to 16·932)	8·331 (3·355 to 16·932)	8·331 (3·355 to 16·932)	8·331 (3·355 to 16·932)
Diarrhoeal diseases	Piped & chlorinated, all locations except Eastern Europe and Southern Latin America	Both	Both		6·785 (2·895 to 13·410)	6·785 (2·895 to 13·410)	6·785 (2·895 to 13·410)	6·785 (2·895 to 13·410)	6·785 (2·895 to 13·410)	6·785 (2·895 to 13·410)	6·785 (2·895 to 13·410)	6·785 (2·895 to 13·410)	6·785 (2·895 to 13·410)	6·785 (2·895 to 13·410)	6·785 (2·895 to 13·410)	6·785 (2·895 to 13·410)
Diarrhoeal diseases	Piped & filtered, all locations except Eastern Europe and Southern Latin America	Both	Both		4·343 (1·905 to 8·494)	4·343 (1·905 to 8·494)	4·343 (1·905 to 8·494)	4·343 (1·905 to 8·494)	4·343 (1·905 to 8·494)	4·343 (1·905 to 8·494)	4·343 (1·905 to 8·494)	4·343 (1·905 to 8·494)	4·343 (1·905 to 8·494)	4·343 (1·905 to 8·494)	4·343 (1·905 to 8·494)	4·343 (1·905 to 8·494)
Diarrhoeal diseases	High quality piped & untreated, Eastern Europe and Southern Latin America	Both	Both		1·919 (1·467 to 2·474)	1·919 (1·467 to 2·474)	1·919 (1·467 to 2·474)	1·919 (1·467 to 2·474)	1·919 (1·467 to 2·474)	1·919 (1·467 to 2·474)	1·919 (1·467 to 2·474)	1·919 (1·467 to 2·474)	1·919 (1·467 to 2·474)	1·919 (1·467 to 2·474)	1·919 (1·467 to 2·474)	1·919 (1·467 to 2·474)
Diarrhoeal diseases	High quality piped & chlorinated, Eastern Europe and Southern Latin America	Both	Both		1·562 (1·426 to 1·704)	1·562 (1·426 to 1·704)	1·562 (1·426 to 1·704)	1·562 (1·426 to 1·704)	1·562 (1·426 to 1·704)	1·562 (1·426 to 1·704)	1·562 (1·426 to 1·704)	1·562 (1·426 to 1·704)	1·562 (1·426 to 1·704)	1·562 (1·426 to 1·704)	1·562 (1·426 to 1·704)	1·562 (1·426 to 1·704)
Diarrhoeal diseases	High quality piped & filtered, (TMREL) applied to all high income countries except Southern Latin America	Both	Both		1·000 (1·000 to 1·000)	1·000 (1·000 to 1·000)	1·000 (1·000 to 1·000)	1·000 (1·000 to 1·000)	1·000 (1·000 to 1·000)	1·000 (1·000 to 1·000)	1·000 (1·000 to 1·000)	1·000 (1·000 to 1·000)	1·000 (1·000 to 1·000)	1·000 (1·000 to 1·000)	1·000 (1·000 to 1·000)	1·000 (1·000 to 1·000)
Typhoid fever	Unimproved & untreated	Both	Both		11·238 (3·643 to 25·544)	11·238 (3·643 to 25·544)	11·238 (3·643 to 25·544)	11·238 (3·643 to 25·544)	11·238 (3·643 to 25·544)	11·238 (3·643 to 25·544)	11·238 (3·643 to 25·544)	11·238 (3·643 to 25·544)	11·238 (3·643 to 25·544)	11·238 (3·643 to 25·544)	11·238 (3·643 to 25·544)	11·238 (3·643 to 25·544)
Typhoid fever	Unimproved & chlorinated	Both	Both		9·151 (3·165 to 20·449)	9·151 (3·165 to 20·449)	9·151 (3·165 to 20·449)	9·151 (3·165 to 20·449)	9·151 (3·165 to 20·449)	9·151 (3·165 to 20·449)	9·151 (3·165 to 20·449)	9·151 (3·165 to 20·449)	9·151 (3·165 to 20·449)	9·151 (3·165 to 20·449)	9·151 (3·165 to 20·449)	9·151 (3·165 to 20·449)
Typhoid fever	Unimproved & filtered	Both	Both		5·857 (2·065 to 13·038)	5·857 (2·065 to 13·038)	5·857 (2·065 to 13·038)	5·857 (2·065 to 13·038)	5·857 (2·065 to 13·038)	5·857 (2·065 to 13·038)	5·857 (2·065 to 13·038)	5·857 (2·065 to 13·038)	5·857 (2·065 to 13·038)	5·857 (2·065 to 13·038)	5·857 (2·065 to 13·038)	5·857 (2·065 to 13·038)
Typhoid fever	Improved & untreated	Both	Both		9·705 (3·700 to 20·399)	9·705 (3·700 to 20·399)	9·705 (3·700 to 20·399)	9·705 (3·700 to 20·399)	9·705 (3·700 to 20·399)	9·705 (3·700 to 20·399)	9·705 (3·700 to 20·399)	9·705 (3·700 to 20·399)	9·705 (3·700 to 20·399)	9·705 (3·700 to 20·399)	9·705 (3·700 to 20·399)	9·705 (3·700 to 20·399)
Typhoid fever	Improved & chlorinated	Both	Both		7·904 (3·202 to 16·211)	7·904 (3·202 to 16·211)	7·904 (3·202 to 16·211)	7·904 (3·202 to 16·211)	7·904 (3·202 to 16·211)	7·904 (3·202 to 16·211)	7·904 (3·202 to 16·211)	7·904 (3·202 to 16·211)	7·904 (3·202 to 16·211)	7·904 (3·202 to 16·211)	7·904 (3·202 to 16·211)	7·904 (3·202 to 16·211)
Typhoid fever	Improved & filtered	Both	Both		5·059 (2·094 to 10·265)	5·059 (2·094 to 10·265)	5·059 (2·094 to 10·265)	5·059 (2·094 to 10·265)	5·059 (2·094 to 10·265)	5·059 (2·094 to 10·265)	5·059 (2·094 to 10·265)	5·059 (2·094 to 10·265)	5·059 (2·094 to 10·265)	5·059 (2·094 to 10·265)	5·059 (2·094 to 10·265)	5·059 (2·094 to 10·265)
Typhoid fever	Piped & untreated, all locations except Eastern Europe and Southern Latin America	Both	Both		8·331 (3·355 to 16·932)	8·331 (3·355 to 16·932)	8·331 (3·355 to 16·932)	8·331 (3·355 to 16·932)	8·331 (3·355 to 16·932)	8·331 (3·355 to 16·932)	8·331 (3·355 to 16·932)	8·331 (3·355 to 16·932)	8·331 (3·355 to 16·932)	8·331 (3·355 to 16·932)	8·331 (3·355 to 16·932)	8·331 (3·355 to 16·932)
Typhoid fever	Piped & chlorinated, all locations except Eastern Europe and Southern Latin America	Both	Both		6·785 (2·895 to 13·410)	6·785 (2·895 to 13·410)	6·785 (2·895 to 13·410)	6·785 (2·895 to 13·410)	6·785 (2·895 to 13·410)	6·785 (2·895 to 13·410)	6·785 (2·895 to 13·410)	6·785 (2·895 to 13·410)	6·785 (2·895 to 13·410)	6·785 (2·895 to 13·410)	6·785 (2·895 to 13·410)	6·785 (2·895 to 13·410)
Typhoid fever	Piped & filtered, all locations except Eastern Europe and Southern Latin America	Both	Both		4·343 (1·905 to 8·494)	4·343 (1·905 to 8·494)	4·343 (1·905 to 8·494)	4·343 (1·905 to 8·494)	4·343 (1·905 to 8·494)	4·343 (1·905 to 8·494)	4·343 (1·905 to 8·494)	4·343 (1·905 to 8·494)	4·343 (1·905 to 8·494)	4·343 (1·905 to 8·494)	4·343 (1·905 to 8·494)	4·343 (1·905 to 8·494)
Typhoid fever	High quality piped & untreated, Eastern Europe and Southern Latin America	Both	Both		1·919 (1·467 to 2·474)	1·919 (1·467 to 2·474)	1·919 (1·467 to 2·474)	1·919 (1·467 to 2·474)	1·919 (1·467 to 2·474)	1·919 (1·467 to 2·474)	1·919 (1·467 to 2·474)	1·919 (1·467 to 2·474)	1·919 (1·467 to 2·474)	1·919 (1·467 to 2·474)	1·919 (1·467 to 2·474)	1·919 (1·467 to 2·474)
Typhoid fever	High quality piped & chlorinated, Eastern Europe and Southern Latin America	Both	Both		1·562 (1·426 to 1·704)	1·562 (1·426 to 1·704)	1·562 (1·426 to 1·704)	1·562 (1·426 to 1·704)	1·562 (1·426 to 1·704)	1·562 (1·426 to 1·704)	1·562 (1·426 to 1·704)	1·562 (1·426 to 1·704)	1·562 (1·426 to 1·704)	1·562 (1·426 to 1·704)	1·562 (1·426 to 1·704)	1·562 (1·426 to 1·704)
Typhoid fever	High quality piped & filtered, (TMREL) applied to all high income countries except Southern Latin America	Both	Both		1·000 (1·000 to 1·000)	1·000 (1·000 to 1·000)	1·000 (1·000 to 1·000)	1·000 (1·000 to 1·000)	1·000 (1·000 to 1·000)	1·000 (1·000 to 1·000)	1·000 (1·000 to 1·000)	1·000 (1·000 to 1·000)	1·000 (1·000 to 1·000)	1·000 (1·000 to 1·000)	1·000 (1·000 to 1·000)	1·000 (1·000 to 1·000)
Paratyphoid fever	Unimproved & untreated	Both	Both		11·238 (3·643 to 25·544)	11·238 (3·643 to 25·544)	11·238 (3·643 to 25·544)	11·238 (3·643 to 25·544)	11·238 (3·643 to 25·544)	11·238 (3·643 to 25·544)	11·238 (3·643 to 25·544)	11·238 (3·643 to 25·544)	11·238 (3·643 to 25·544)	11·238 (3·643 to 25·544)	11·238 (3·643 to 25·544)	11·238 (3·643 to 25·544)
Paratyphoid fever	Unimproved & chlorinated	Both	Both		9·151 (3·165 to 20·449)	9·151 (3·165 to 20·449)	9·151 (3·165 to 20·449)	9·151 (3·165 to 20·449)	9·151 (3·165 to 20·449)	9·151 (3·165 to 20·449)	9·151 (3·165 to 20·449)	9·151 (3·165 to 20·449)	9·151 (3·165 to 20·449)	9·151 (3·165 to 20·449)	9·151 (3·165 to 20·449)	9·151 (3·165 to 20·449)
Paratyphoid fever	Unimproved & filtered	Both	Both		5·857 (2·065 to 13·038)	5·857 (2·065 to 13·038)	5·857 (2·065 to 13·038)	5·857 (2·065 to 13·038)	5·857 (2·065 to 13·038)	5·857 (2·065 to 13·038)	5·857 (2·065 to 13·038)	5·857 (2·065 to 13·038)	5·857 (2·065 to 13·038)	5·857 (2·065 to 13·038)	5·857 (2·065 to 13·038)	5·857 (2·065 to 13·038)
Paratyphoid fever	Improved & untreated	Both	Both		9·705 (3·700 to 20·399)	9·705 (3·700 to 20·399)	9·705 (3·700 to 20·399)	9·705 (3·700 to 20·399)	9·705 (3·700 to 20·399)	9·705 (3·700 to 20·399)	9·705 (3·700 to 20·399)	9·705 (3·700 to 20·399)	9·705 (3·700 to 20·399)	9·705 (3·700 to 20·399)	9·705 (3·700 to 20·399)	9·705 (3·700 to 20·399)
Paratyphoid fever	Improved & chlorinated	Both	Both		7·904 (3·202 to 16·211)	7·904 (3·202 to 16·211)	7·904 (3·202 to 16·211)	7·904 (3·202 to 16·211)	7·904 (3·202 to 16·211)	7·904 (3·202 to 16·211)	7·904 (3·202 to 16·211)	7·904 (3·202 to 16·211)	7·904 (3·202 to 16·211)	7·904 (3·202 to 16·211)	7·904 (3·202 to 16·211)	7·904 (3·202 to 16·211)
Paratyphoid fever	Improved & filtered	Both	Both		5·059 (2·094 to 10·265)	5·059 (2·094 to 10·265)	5·059 (2·094 to 10·265)	5·059 (2·094 to 10·265)	5·059 (2·094 to 10·265)	5·059 (2·094 to 10·265)	5·059 (2·094 to 10·265)	5·059 (2·094 to 10·265)	5·059 (2·094 to 10·265)	5·059 (2·094 to 10·265)	5·059 (2·094 to 10·265)	5·059 (2·094 to 10·265)
Paratyphoid fever	Piped & untreated, all locations except Eastern Europe and Southern Latin America	Both	Both		8·331 (3·355 to 16·932)	8·331 (3·355 to 16·932)	8·331 (3·355 to 16·932)	8·331 (3·355 to 16·932)	8·331 (3·355 to 16·932)	8·331 (3·355 to 16·932)	8·331 (3·355 to 16·932)	8·331 (3·355 to 16·932)	8·331 (3·355 to 16·932)	8·331 (3·355 to 16·932)	8·331 (3·355 to 16·932)	8·331 (3·355 to 16·932)
Paratyphoid fever	Piped & chlorinated, all locations except Eastern Europe and Southern Latin America	Both	Both		6·785 (2·895 to 13·410)	6·785 (2·895 to 13·410)	6·785 (2·895 to 13·410)	6·785 (2·895 to 13·410)	6·785 (2·895 to 13·410)	6·785 (2·895 to 13·410)	6·785 (2·895 to 13·410)	6·785 (2·895 to 13·410)	6·785 (2·895 to 13·410)	6·785 (2·895 to 13·410)	6·785 (2·895 to 13·410)	6·785 (2·895 to 13·410)
Paratyphoid fever	Piped & filtered, all locations except Eastern Europe and Southern Latin America	Both	Both		4·343 (1·905 to 8·494)	4·343 (1·905 to 8·494)	4·343 (1·905 to 8·494)	4·343 (1·905 to 8·494)	4·343 (1·905 to 8·494)	4·343 (1·905 to 8·494)	4·343 (1·905 to 8·494)	4·343 (1·905 to 8·494)	4·343 (1·905 to 8·494)	4·343 (1·905 to 8·494)	4·343 (1·905 to 8·494)	4·343 (1·905 to 8·494)
Paratyphoid fever	High quality piped & untreated, Eastern Europe and Southern Latin America	Both	Both		1·919 (1·467 to 2·474)	1·919 (1·467 to 2·474)	1·919 (1·467 to 2·474)	1·919 (1·467 to 2·474)	1·919 (1·467 to 2·474)	1·919 (1·467 to 2·474)	1·919 (1·467 to 2·474)	1·919 (1·467 to 2·474)	1·919 (1·467 to 2·474)	1·919 (1·467 to 2·474)	1·919 (1·467 to 2·474)	1·919 (1·467 to 2·474)
Paratyphoid fever	High quality piped & chlorinated, Eastern Europe and Southern Latin America	Both	Both		1·562 (1·426 to 1·704)	1·562 (1·426 to 1·704)	1·562 (1·426 to 1·704)	1·562 (1·426 to 1·704)	1·562 (1·426 to 1·704)	1·562 (1·426 to 1·704)	1·562 (1·426 to 1·704)	1·562 (1·426 to 1·704)	1·562 (1·426 to 1·704)	1·562 (1·426 to 1·704)	1·562 (1·426 to 1·704)	1·562 (1·426 to 1·704)
Paratyphoid fever	High quality piped & filtered, (TMREL) applied to all high income countries except Southern Latin America	Both	Both		1·000 (1·000 to 1·000)	1·000 (1·000 to 1·000)	1·000 (1·000 to 1·000)	1·000 (1·000 to 1·000)	1·000 (1·000 to 1·000)	1·000 (1·000 to 1·000)	1·000 (1·000 to 1·000)	1·000 (1·000 to 1·000)	1·000 (1·000 to 1·000)	1·000 (1·000 to 1·000)	1·000 (1·000 to 1·000)	1·000 (1·000 to 1·000)
**Unsafe sanitation**																
Diarrhoeal diseases	Unimproved & untreated	Both	Both		3·229 (2·818 to 3·712)	3·229 (2·818 to 3·712)	3·229 (2·818 to 3·712)	3·229 (2·818 to 3·712)	3·229 (2·818 to 3·712)	3·229 (2·818 to 3·712)	3·229 (2·818 to 3·712)	3·229 (2·818 to 3·712)	3·229 (2·818 to 3·712)	3·229 (2·818 to 3·712)	3·229 (2·818 to 3·712)	3·229 (2·818 to 3·712)
Diarrhoeal diseases	Improved	Both	Both		2·711 (2·550 to 2·879)	2·711 (2·550 to 2·879)	2·711 (2·550 to 2·879)	2·711 (2·550 to 2·879)	2·711 (2·550 to 2·879)	2·711 (2·550 to 2·879)	2·711 (2·550 to 2·879)	2·711 (2·550 to 2·879)	2·711 (2·550 to 2·879)	2·711 (2·550 to 2·879)	2·711 (2·550 to 2·879)	2·711 (2·550 to 2·879)
Diarrhoeal diseases	Sewer	Both	Both		1·000 (1·000 to 1·000)	1·000 (1·000 to 1·000)	1·000 (1·000 to 1·000)	1·000 (1·000 to 1·000)	1·000 (1·000 to 1·000)	1·000 (1·000 to 1·000)	1·000 (1·000 to 1·000)	1·000 (1·000 to 1·000)	1·000 (1·000 to 1·000)	1·000 (1·000 to 1·000)	1·000 (1·000 to 1·000)	1·000 (1·000 to 1·000)
Typhoid fever	Unimproved & untreated	Both	Both		3·234 (2·772 to 3·701)	3·234 (2·772 to 3·701)	3·234 (2·772 to 3·701)	3·234 (2·772 to 3·701)	3·234 (2·772 to 3·701)	3·234 (2·772 to 3·701)	3·234 (2·772 to 3·701)	3·234 (2·772 to 3·701)	3·234 (2·772 to 3·701)	3·234 (2·772 to 3·701)	3·234 (2·772 to 3·701)	3·234 (2·772 to 3·701)
Typhoid fever	Improved	Both	Both		2·705 (2·540 to 2·877)	2·705 (2·540 to 2·877)	2·705 (2·540 to 2·877)	2·705 (2·540 to 2·877)	2·705 (2·540 to 2·877)	2·705 (2·540 to 2·877)	2·705 (2·540 to 2·877)	2·705 (2·540 to 2·877)	2·705 (2·540 to 2·877)	2·705 (2·540 to 2·877)	2·705 (2·540 to 2·877)	2·705 (2·540 to 2·877)

Appendix Table 6a. Relative risks used by age and sex and for each outcome for all risk factors except for ambient air pollution and alcohol.

Risk - Outcome	Category / Units	Morbidity / Mortality	Sex	45-49 years	50-54 years	55-59 years	60-64 years	65-69 years	70-74 years	75-79 years	80+ years
**Unsafe water source**											
Diarrhoeal diseases	Unimproved & untreated	Both	Both	11·238 (3·643 to 25·544)	11·238 (3·643 to 25·544)	11·238 (3·643 to 25·544)	11·238 (3·643 to 25·544)	11·238 (3·643 to 25·544)	11·238 (3·643 to 25·544)	11·238 (3·643 to 25·544)	11·238 (3·643 to 25·544)
Diarrhoeal diseases	Unimproved & chlorinated	Both	Both	9·151 (3·165 to 20·449)	9·151 (3·165 to 20·449)	9·151 (3·165 to 20·449)	9·151 (3·165 to 20·449)	9·151 (3·165 to 20·449)	9·151 (3·165 to 20·449)	9·151 (3·165 to 20·449)	9·151 (3·165 to 20·449)
Diarrhoeal diseases	Unimproved & filtered	Both	Both	5·857 (2·065 to 13·038)	5·857 (2·065 to 13·038)	5·857 (2·065 to 13·038)	5·857 (2·065 to 13·038)	5·857 (2·065 to 13·038)	5·857 (2·065 to 13·038)	5·857 (2·065 to 13·038)	5·857 (2·065 to 13·038)
Diarrhoeal diseases	Improved & untreated	Both	Both	9·705 (3·700 to 20·399)	9·705 (3·700 to 20·399)	9·705 (3·700 to 20·399)	9·705 (3·700 to 20·399)	9·705 (3·700 to 20·399)	9·705 (3·700 to 20·399)	9·705 (3·700 to 20·399)	9·705 (3·700 to 20·399)
Diarrhoeal diseases	Improved & chlorinated	Both	Both	7·904 (3·202 to 16·211)	7·904 (3·202 to 16·211)	7·904 (3·202 to 16·211)	7·904 (3·202 to 16·211)	7·904 (3·202 to 16·211)	7·904 (3·202 to 16·211)	7·904 (3·202 to 16·211)	7·904 (3·202 to 16·211)
Diarrhoeal diseases	Improved & filtered	Both	Both	5·059 (2·094 to 10·265)	5·059 (2·094 to 10·265)	5·059 (2·094 to 10·265)	5·059 (2·094 to 10·265)	5·059 (2·094 to 10·265)	5·059 (2·094 to 10·265)	5·059 (2·094 to 10·265)	5·059 (2·094 to 10·265)
Diarrhoeal diseases	Piped & untreated, all locations except Eastern Europe and Southern Latin America	Both	Both	8·331 (3·355 to 16·932)	8·331 (3·355 to 16·932)	8·331 (3·355 to 16·932)	8·331 (3·355 to 16·932)	8·331 (3·355 to 16·932)	8·331 (3·355 to 16·932)	8·331 (3·355 to 16·932)	8·331 (3·355 to 16·932)
Diarrhoeal diseases	Piped & chlorinated, all locations except Eastern Europe and Southern Latin America	Both	Both	6·785 (2·895 to 13·410)	6·785 (2·895 to 13·410)	6·785 (2·895 to 13·410)	6·785 (2·895 to 13·410)	6·785 (2·895 to 13·410)	6·785 (2·895 to 13·410)	6·785 (2·895 to 13·410)	6·785 (2·895 to 13·410)
Diarrhoeal diseases	Piped & filtered, all locations except Eastern Europe and Southern Latin America	Both	Both	4·343 (1·905 to 8·494)	4·343 (1·905 to 8·494)	4·343 (1·905 to 8·494)	4·343 (1·905 to 8·494)	4·343 (1·905 to 8·494)	4·343 (1·905 to 8·494)	4·343 (1·905 to 8·494)	4·343 (1·905 to 8·494)
Diarrhoeal diseases	High quality piped & untreated, Eastern Europe and Southern Latin America	Both	Both	1·919 (1·467 to 2·474)	1·919 (1·467 to 2·474)	1·919 (1·467 to 2·474)	1·919 (1·467 to 2·474)	1·919 (1·467 to 2·474)	1·919 (1·467 to 2·474)	1·919 (1·467 to 2·474)	1·919 (1·467 to 2·474)
Diarrhoeal diseases	High quality piped & chlorinated, Eastern Europe and Southern Latin America	Both	Both	1·562 (1·426 to 1·704)	1·562 (1·426 to 1·704)	1·562 (1·426 to 1·704)	1·562 (1·426 to 1·704)	1·562 (1·426 to 1·704)	1·562 (1·426 to 1·704)	1·562 (1·426 to 1·704)	1·562 (1·426 to 1·704)
Diarrhoeal diseases	High quality piped & filtered, (TMREL) applied to all high income countries except Southern Latin America	Both	Both	1·000 (1·000 to 1·000)	1·000 (1·000 to 1·000)	1·000 (1·000 to 1·000)	1·000 (1·000 to 1·000)	1·000 (1·000 to 1·000)	1·000 (1·000 to 1·000)	1·000 (1·000 to 1·000)	1·000 (1·000 to 1·000)
Typhoid fever	Unimproved & untreated	Both	Both	11·238 (3·643 to 25·544)	11·238 (3·643 to 25·544)	11·238 (3·643 to 25·544)	11·238 (3·643 to 25·544)	11·238 (3·643 to 25·544)	11·238 (3·643 to 25·544)	11·238 (3·643 to 25·544)	11·238 (3·643 to 25·544)
Typhoid fever	Unimproved & chlorinated	Both	Both	9·151 (3·165 to 20·449)	9·151 (3·165 to 20·449)	9·151 (3·165 to 20·449)	9·151 (3·165 to 20·449)	9·151 (3·165 to 20·449)	9·151 (3·165 to 20·449)	9·151 (3·165 to 20·449)	9·151 (3·165 to 20·449)
Typhoid fever	Unimproved & filtered	Both	Both	5·857 (2·065 to 13·038)	5·857 (2·065 to 13·038)	5·857 (2·065 to 13·038)	5·857 (2·065 to 13·038)	5·857 (2·065 to 13·038)	5·857 (2·065 to 13·038)	5·857 (2·065 to 13·038)	5·857 (2·065 to 13·038)
Typhoid fever	Improved & untreated	Both	Both	9·705 (3·700 to 20·399)	9·705 (3·700 to 20·399)	9·705 (3·700 to 20·399)	9·705 (3·700 to 20·399)	9·705 (3·700 to 20·399)	9·705 (3·700 to 20·399)	9·705 (3·700 to 20·399)	9·705 (3·700 to 20·399)
Typhoid fever	Improved & chlorinated	Both	Both	7·904 (3·202 to 16·211)	7·904 (3·202 to 16·211)	7·904 (3·202 to 16·211)	7·904 (3·202 to 16·211)	7·904 (3·202 to 16·211)	7·904 (3·202 to 16·211)	7·904 (3·202 to 16·211)	7·904 (3·202 to 16·211)
Typhoid fever	Improved & filtered	Both	Both	5·059 (2·094 to 10·265)	5·059 (2·094 to 10·265)	5·059 (2·094 to 10·265)	5·059 (2·094 to 10·265)	5·059 (2·094 to 10·265)	5·059 (2·094 to 10·265)	5·059 (2·094 to 10·265)	5·059 (2·094 to 10·265)
Typhoid fever	Piped & untreated, all locations except Eastern Europe and Southern Latin America	Both	Both	8·331 (3·355 to 16·932)	8·331 (3·355 to 16·932)	8·331 (3·355 to 16·932)	8·331 (3·355 to 16·932)	8·331 (3·355 to 16·932)	8·331 (3·355 to 16·932)	8·331 (3·355 to 16·932)	8·331 (3·355 to 16·932)
Typhoid fever	Piped & chlorinated, all locations except Eastern Europe and Southern Latin America	Both	Both	6·785 (2·895 to 13·410)	6·785 (2·895 to 13·410)	6·785 (2·895 to 13·410)	6·785 (2·895 to 13·410)	6·785 (2·895 to 13·410)	6·785 (2·895 to 13·410)	6·785 (2·895 to 13·410)	6·785 (2·895 to 13·410)
Typhoid fever	Piped & filtered, all locations except Eastern Europe and Southern Latin America	Both	Both	4·343 (1·905 to 8·494)	4·343 (1·905 to 8·494)	4·343 (1·905 to 8·494)	4·343 (1·905 to 8·494)	4·343 (1·905 to 8·494)	4·343 (1·905 to 8·494)	4·343 (1·905 to 8·494)	4·343 (1·905 to 8·494)
Typhoid fever	High quality piped & untreated, Eastern Europe and Southern Latin America	Both	Both	1·919 (1·467 to 2·474)	1·919 (1·467 to 2·474)	1·919 (1·467 to 2·474)	1·919 (1·467 to 2·474)	1·919 (1·467 to 2·474)	1·919 (1·467 to 2·474)	1·919 (1·467 to 2·474)	1·919 (1·467 to 2·474)
Typhoid fever	High quality piped & chlorinated, Eastern Europe and Southern Latin America	Both	Both	1·562 (1·426 to 1·704)	1·562 (1·426 to 1·704)	1·562 (1·426 to 1·704)	1·562 (1·426 to 1·704)	1·562 (1·426 to 1·704)	1·562 (1·426 to 1·704)	1·562 (1·426 to 1·704)	1·562 (1·426 to 1·704)
Typhoid fever	High quality piped & filtered, (TMREL) applied to all high income countries except Southern Latin America	Both	Both	1·000 (1·000 to 1·000)	1·000 (1·000 to 1·000)	1·000 (1·000 to 1·000)	1·000 (1·000 to 1·000)	1·000 (1·000 to 1·000)	1·000 (1·000 to 1·000)	1·000 (1·000 to 1·000)	1·000 (1·000 to 1·000)
Paratyphoid fever	Unimproved & untreated	Both	Both	11·238 (3·643 to 25·544)	11·238 (3·643 to 25·544)	11·238 (3·643 to 25·544)	11·238 (3·643 to 25·544)	11·238 (3·643 to 25·544)	11·238 (3·643 to 25·544)	11·238 (3·643 to 25·544)	11·238 (3·643 to 25·544)
Paratyphoid fever	Unimproved & chlorinated	Both	Both	9·151 (3·165 to 20·449)	9·151 (3·165 to 20·449)	9·151 (3·165 to 20·449)	9·151 (3·165 to 20·449)	9·151 (3·165 to 20·449)	9·151 (3·165 to 20·449)	9·151 (3·165 to 20·449)	9·151 (3·165 to 20·449)
Paratyphoid fever	Unimproved & filtered	Both	Both	5·857 (2·065 to 13·038)	5·857 (2·065 to 13·038)	5·857 (2·065 to 13·038)	5·857 (2·065 to 13·038)	5·857 (2·065 to 13·038)	5·857 (2·065 to 13·038)	5·857 (2·065 to 13·038)	5·857 (2·065 to 13·038)
Paratyphoid fever	Improved & untreated	Both	Both	9·705 (3·700 to 20·399)	9·705 (3·700 to 20·399)	9·705 (3·700 to 20·399)	9·705 (3·700 to 20·399)	9·705 (3·700 to 20·399)	9·705 (3·700 to 20·399)	9·705 (3·700 to 20·399)	9·705 (3·700 to 20·399)
Paratyphoid fever	Improved & chlorinated	Both	Both	7·904 (3·202 to 16·211)	7·904 (3·202 to 16·211)	7·904 (3·202 to 16·211)	7·904 (3·202 to 16·211)	7·904 (3·202 to 16·211)	7·904 (3·202 to 16·211)	7·904 (3·202 to 16·211)	7·904 (3·202 to 16·211)
Paratyphoid fever	Improved & filtered	Both	Both	5·059 (2·094 to 10·265)	5·059 (2·094 to 10·265)	5·059 (2·094 to 10·265)	5·059 (2·094 to 10·265)	5·059 (2·094 to 10·265)	5·059 (2·094 to 10·265)	5·059 (2·094 to 10·265)	5·059 (2·094 to 10·265)
Paratyphoid fever	Piped & untreated, all locations except Eastern Europe and Southern Latin America	Both	Both	8·331 (3·355 to 16·932)	8·331 (3·355 to 16·932)	8·331 (3·355 to 16·932)	8·331 (3·355 to 16·932)	8·331 (3·355 to 16·932)	8·331 (3·355 to 16·932)	8·331 (3·355 to 16·932)	8·331 (3·355 to 16·932)
Paratyphoid fever	Piped & chlorinated, all locations except Eastern Europe and Southern Latin America	Both	Both	6·785 (2·895 to 13·410)	6·785 (2·895 to 13·410)	6·785 (2·895 to 13·410)	6·785 (2·895 to 13·410)	6·785 (2·895 to 13·410)	6·785 (2·895 to 13·410)	6·785 (2·895 to 13·410)	6·785 (2·895 to 13·410)
Paratyphoid fever	Piped & filtered, all locations except Eastern Europe and Southern Latin America	Both	Both	4·343 (1·905 to 8·494)	4·343 (1·905 to 8·494)	4·343 (1·905 to 8·494)	4·343 (1·905 to 8·494)	4·343 (1·905 to 8·494)	4·343 (1·905 to 8·494)	4·343 (1·905 to 8·494)	4·343 (1·905 to 8·494)
Paratyphoid fever	High quality piped & untreated, Eastern Europe and Southern Latin America	Both	Both	1·919 (1·467 to 2·474)	1·919 (1·467 to 2·474)	1·919 (1·467 to 2·474)	1·919 (1·467 to 2·474)	1·919 (1·467 to 2·474)	1·919 (1·467 to 2·474)	1·919 (1·467 to 2·474)	1·919 (1·467 to 2·474)
Paratyphoid fever	High quality piped & chlorinated, Eastern Europe and Southern Latin America	Both	Both	1·562 (1·426 to 1·704)	1·562 (1·426 to 1·704)	1·562 (1·426 to 1·704)	1·562 (1·426 to 1·704)	1·562 (1·426 to 1·704)	1·562 (1·426 to 1·704)	1·562 (1·426 to 1·704)	1·562 (1·426 to 1·704)
Paratyphoid fever	High quality piped & filtered, (TMREL) applied to all high income countries except Southern Latin America	Both	Both	1·000 (1·000 to 1·000)	1·000 (1·000 to 1·000)	1·000 (1·000 to 1·000)	1·000 (1·000 to 1·000)	1·000 (1·000 to 1·000)	1·000 (1·000 to 1·000)	1·000 (1·000 to 1·000)	1·000 (1·000 to 1·000)
**Unsafe sanitation**											
Diarrhoeal diseases	Unimproved & untreated	Both	Both	3·229 (2·818 to 3·712)	3·229 (2·818 to 3·712)	3·229 (2·818 to 3·712)	3·229 (2·818 to 3·712)	3·229 (2·818 to 3·712)	3·229 (2·818 to 3·712)	3·229 (2·818 to 3·712)	3·229 (2·818 to 3·712)
Diarrhoeal diseases	Improved	Both	Both	2·711 (2·550 to 2·879)	2·711 (2·550 to 2·879)	2·711 (2·550 to 2·879)	2·711 (2·550 to 2·879)	2·711 (2·550 to 2·879)	2·711 (2·550 to 2·879)	2·711 (2·550 to 2·879)	2·711 (2·550 to 2·879)
Diarrhoeal diseases	Sewer	Both	Both	1·000 (1·000 to 1·000)	1·000 (1·000 to 1·000)	1·000 (1·000 to 1·000)	1·000 (1·000 to 1·000)	1·000 (1·000 to 1·000)	1·000 (1·000 to 1·000)	1·000 (1·000 to 1·000)	1·000 (1·000 to 1·000)
Typhoid fever	Unimproved & untreated	Both	Both	3·234 (2·772 to 3·701)	3·234 (2·772 to 3·701)	3·234 (2·772 to 3·701)	3·234 (2·772 to 3·701)	3·234 (2·772 to 3·701)	3·234 (2·772 to 3·701)	3·234 (2·772 to 3·701)	3·234 (2·772 to 3·701)
Typhoid fever	Improved	Both	Both	2·705 (2·540 to 2·877)	2·705 (2·540 to 2·877)	2·705 (2·540 to 2·877)	2·705 (2·540 to 2·877)	2·705 (2·540 to 2·877)	2·705 (2·540 to 2·877)	2·705 (2·540 to 2·877)	2·705 (2·540 to 2·877)

Risk - Outcome	Category / Units	Morbidity / Mortality	Sex	All ages	0-6 Days	7-27 Days	28-364 Days	1-4 years	5-9 years	10-14 years	15-19 years	20-24 years	25-29 years	30-34 years	35-39 years	40-44 years
Typhoid fever	Sewer	Both	Both		1·000 (1·000 to 1·000)	1·000 (1·000 to 1·000)	1·000 (1·000 to 1·000)	1·000 (1·000 to 1·000)	1·000 (1·000 to 1·000)	1·000 (1·000 to 1·000)	1·000 (1·000 to 1·000)	1·000 (1·000 to 1·000)	1·000 (1·000 to 1·000)	1·000 (1·000 to 1·000)	1·000 (1·000 to 1·000)	1·000 (1·000 to 1·000)
Paratyphoid fever	Unimproved & untreated	Both	Both		3·236 (2·778 to 3·725)	3·236 (2·778 to 3·725)	3·236 (2·778 to 3·725)	3·236 (2·778 to 3·725)	3·236 (2·778 to 3·725)	3·236 (2·778 to 3·725)	3·236 (2·778 to 3·725)	3·236 (2·778 to 3·725)	3·236 (2·778 to 3·725)	3·236 (2·778 to 3·725)	3·236 (2·778 to 3·725)	3·236 (2·778 to 3·725)
Paratyphoid fever	Improved	Both	Both		2·715 (2·560 to 2·870)	2·715 (2·560 to 2·870)	2·715 (2·560 to 2·870)	2·715 (2·560 to 2·870)	2·715 (2·560 to 2·870)	2·715 (2·560 to 2·870)	2·715 (2·560 to 2·870)	2·715 (2·560 to 2·870)	2·715 (2·560 to 2·870)	2·715 (2·560 to 2·870)	2·715 (2·560 to 2·870)	2·715 (2·560 to 2·870)
Paratyphoid fever	Sewer	Both	Both		1·000 (1·000 to 1·000)	1·000 (1·000 to 1·000)	1·000 (1·000 to 1·000)	1·000 (1·000 to 1·000)	1·000 (1·000 to 1·000)	1·000 (1·000 to 1·000)	1·000 (1·000 to 1·000)	1·000 (1·000 to 1·000)	1·000 (1·000 to 1·000)	1·000 (1·000 to 1·000)	1·000 (1·000 to 1·000)	1·000 (1·000 to 1·000)
**No handwashing with soap**																
Diarrhoeal diseases	No handwashing w/soap & water	Both	Both	1·673 (1·483 to 1·878)	1·673 (1·483 to 1·878)	1·673 (1·483 to 1·878)	1·673 (1·483 to 1·878)	1·673 (1·483 to 1·878)	1·673 (1·483 to 1·878)	1·673 (1·483 to 1·878)	1·673 (1·483 to 1·878)	1·673 (1·483 to 1·878)	1·673 (1·483 to 1·878)	1·673 (1·483 to 1·878)	1·673 (1·483 to 1·878)	1·673 (1·483 to 1·878)
Diarrhoeal diseases	Handwashing w/soap & water	Both	Both	1·000 (1·000 to 1·000)	1·000 (1·000 to 1·000)	1·000 (1·000 to 1·000)	1·000 (1·000 to 1·000)	1·000 (1·000 to 1·000)	1·000 (1·000 to 1·000)	1·000 (1·000 to 1·000)	1·000 (1·000 to 1·000)	1·000 (1·000 to 1·000)	1·000 (1·000 to 1·000)	1·000 (1·000 to 1·000)	1·000 (1·000 to 1·000)	1·000 (1·000 to 1·000)
Typhoid fever	No handwashing w/soap & water	Both	Both	1·667 (1·461 to 1·875)	1·667 (1·461 to 1·875)	1·667 (1·461 to 1·875)	1·667 (1·461 to 1·875)	1·667 (1·461 to 1·875)	1·667 (1·461 to 1·875)	1·667 (1·461 to 1·875)	1·667 (1·461 to 1·875)	1·667 (1·461 to 1·875)	1·667 (1·461 to 1·875)	1·667 (1·461 to 1·875)	1·667 (1·461 to 1·875)	1·667 (1·461 to 1·875)
Typhoid fever	Handwashing w/soap & water	Both	Both	1·000 (1·000 to 1·000)	1·000 (1·000 to 1·000)	1·000 (1·000 to 1·000)	1·000 (1·000 to 1·000)	1·000 (1·000 to 1·000)	1·000 (1·000 to 1·000)	1·000 (1·000 to 1·000)	1·000 (1·000 to 1·000)	1·000 (1·000 to 1·000)	1·000 (1·000 to 1·000)	1·000 (1·000 to 1·000)	1·000 (1·000 to 1·000)	1·000 (1·000 to 1·000)
Paratyphoid fever	No handwashing w/soap & water	Both	Both	1·665 (1·459 to 1·888)	1·665 (1·459 to 1·888)	1·665 (1·459 to 1·888)	1·665 (1·459 to 1·888)	1·665 (1·459 to 1·888)	1·665 (1·459 to 1·888)	1·665 (1·459 to 1·888)	1·665 (1·459 to 1·888)	1·665 (1·459 to 1·888)	1·665 (1·459 to 1·888)	1·665 (1·459 to 1·888)	1·665 (1·459 to 1·888)	1·665 (1·459 to 1·888)
Paratyphoid fever	Handwashing w/soap & water	Both	Both	1·000 (1·000 to 1·000)	1·000 (1·000 to 1·000)	1·000 (1·000 to 1·000)	1·000 (1·000 to 1·000)	1·000 (1·000 to 1·000)	1·000 (1·000 to 1·000)	1·000 (1·000 to 1·000)	1·000 (1·000 to 1·000)	1·000 (1·000 to 1·000)	1·000 (1·000 to 1·000)	1·000 (1·000 to 1·000)	1·000 (1·000 to 1·000)	1·000 (1·000 to 1·000)
Lower respiratory infections	No handwashing w/soap & water	Both	Both	1·192 (1·124 to 1·266)	1·192 (1·124 to 1·266)	1·192 (1·124 to 1·266)	1·192 (1·124 to 1·266)	1·192 (1·124 to 1·266)	1·192 (1·124 to 1·266)	1·192 (1·124 to 1·266)	1·192 (1·124 to 1·266)	1·192 (1·124 to 1·266)	1·192 (1·124 to 1·266)	1·192 (1·124 to 1·266)	1·192 (1·124 to 1·266)	1·192 (1·124 to 1·266)
Lower respiratory infections	Handwashing w/soap & water	Both	Both	1·000 (1·000 to 1·000)	1·000 (1·000 to 1·000)	1·000 (1·000 to 1·000)	1·000 (1·000 to 1·000)	1·000 (1·000 to 1·000)	1·000 (1·000 to 1·000)	1·000 (1·000 to 1·000)	1·000 (1·000 to 1·000)	1·000 (1·000 to 1·000)	1·000 (1·000 to 1·000)	1·000 (1·000 to 1·000)	1·000 (1·000 to 1·000)	1·000 (1·000 to 1·000)
**Household air pollution from solid fuels**																
Cataract	Exposed	Morbidity	Both								2·522 (1·599 to 3·739)	2·496 (1·595 to 3·701)	2·513 (1·707 to 3·724)	2·512 (1·578 to 3·800)	2·513 (1·622 to 3·687)	2·511 (1·624 to 3·731)
Cataract	Not exposed	Morbidity	Both								1·000 (1·000 to 1·000)	1·000 (1·000 to 1·000)	1·000 (1·000 to 1·000)	1·000 (1·000 to 1·000)	1·000 (1·000 to 1·000)	1·000 (1·000 to 1·000)
**Ambient ozone pollution**																
Chronic obstructive pulmonary disease	10 ppb	Mortality	Both										1·029 (1·010 to 1·048)	1·029 (1·010 to 1·048)	1·029 (1·009 to 1·048)	1·029 (1·010 to 1·048)
**Residential radon**																
Tracheal, bronchus, and lung cancer	Bq/m3	Both	Both		1·002 (1·000 to 1·003)	1·002 (1·000 to 1·003)	1·002 (1·000 to 1·003)	1·002 (1·000 to 1·003)	1·002 (1·000 to 1·003)	1·002 (1·000 to 1·003)	1·002 (1·000 to 1·003)	1·002 (1·000 to 1·003)	1·002 (1·000 to 1·003)	1·002 (1·000 to 1·003)	1·002 (1·000 to 1·003)	1·002 (1·000 to 1·003)
**Lead exposure**																
Rheumatic heart disease	10 µg/g	Morbidity	Both										1·000 (1·000 to 1·000)	1·000 (1·000 to 1·000)	1·000 (1·000 to 1·000)	1·000 (1·000 to 1·000)
Rheumatic heart disease	10 µg/g	Mortality	Both										1·040 (0·991 to 1·088)	1·035 (0·996 to 1·086)	1·028 (1·001 to 1·080)	1·023 (0·998 to 1·079)
Ischaemic heart disease	10 µg/g	Both	Both										1·049 (1·010 to 1·083)	1·042 (1·013 to 1·071)	1·033 (1·022 to 1·046)	1·028 (1·017 to 1·039)
Ischaemic stroke	10 µg/g	Both	Both										1·056 (1·022 to 1·088)	1·052 (1·027 to 1·079)	1·047 (1·039 to 1·056)	1·042 (1·034 to 1·050)
Hemorrhagic stroke	10 µg/g	Both	Both										1·062 (1·026 to 1·093)	1·058 (1·031 to 1·086)	1·052 (1·037 to 1·067)	1·046 (1·034 to 1·058)
Hypertensive heart disease	10 µg/g	Both	Both										1·049 (1·010 to 1·083)	1·042 (1·013 to 1·071)	1·033 (1·022 to 1·046)	1·028 (1·017 to 1·039)
Cardiomyopathy and myocarditis	10 µg/g	Morbidity	Both										1·000 (1·000 to 1·000)	1·000 (1·000 to 1·000)	1·000 (1·000 to 1·000)	1·000 (1·000 to 1·000)
Cardiomyopathy and myocarditis	10 µg/g	Mortality	Both										1·045 (0·992 to 1·089)	1·041 (1·002 to 1·088)	1·034 (1·008 to 1·084)	1·029 (1·005 to 1·081)
Atrial fibrillation and flutter	10 µg/g	Both	Both										1·040 (1·005 to 1·072)	1·033 (1·006 to 1·060)	1·026 (1·018 to 1·033)	1·021 (1·013 to 1·028)
Aortic aneurysm	10 µg/g	Both	Both										1·053 (0·998 to 1·092)	1·048 (1·007 to 1·090)	1·041 (1·013 to 1·090)	1·036 (1·010 to 1·090)
Peripheral vascular disease	10 µg/g	Both	Both										1·040 (1·005 to 1·072)	1·033 (1·006 to 1·060)	1·026 (1·018 to 1·033)	1·021 (1·013 to 1·028)
Endocarditis	10 µg/g	Morbidity	Both										1·000 (1·000 to 1·000)	1·000 (1·000 to 1·000)	1·000 (1·000 to 1·000)	1·000 (1·000 to 1·000)
Endocarditis	10 µg/g	Mortality	Both										1·045 (0·992 to 1·089)	1·041 (1·002 to 1·088)	1·034 (1·008 to 1·084)	1·029 (1·005 to 1·081)
Other cardiovascular and circulatory diseases	10 µg/g	Both	Both										1·040 (1·005 to 1·072)	1·033 (1·006 to 1·060)	1·026 (1·018 to 1·033)	1·021 (1·013 to 1·028)
Chronic kidney disease due to diabetes mellitus	10 µg/g	Both	Both										1·013 (1·009 to 1·017)	1·013 (1·009 to 1·017)	1·013 (1·009 to 1·017)	1·013 (1·009 to 1·017)
Chronic kidney disease due to hypertension	10 µg/g	Both	Both										1·013 (1·009 to 1·017)	1·013 (1·009 to 1·017)	1·013 (1·009 to 1·017)	1·013 (1·009 to 1·017)
Chronic kidney disease due to glomerulonephritis	10 µg/g	Both	Both										1·013 (1·009 to 1·017)	1·013 (1·009 to 1·017)	1·013 (1·009 to 1·017)	1·013 (1·009 to 1·017)
Chronic kidney disease due to other causes	10 µg/g	Both	Both										1·013 (1·009 to 1·017)	1·013 (1·009 to 1·017)	1·013 (1·009 to 1·017)	1·013 (1·009 to 1·017)
**Occupational exposure to asbestos**																
Larynx cancer	High exposure	Both	Male								1·380 (1·188 to 1·612)	1·380 (1·188 to 1·612)	1·380 (1·188 to 1·612)	1·380 (1·188 to 1·612)	1·380 (1·188 to 1·612)	1·380 (1·188 to 1·612)
Larynx cancer	Low exposure	Both	Male								1·000 (1·000 to 1·000)	1·000 (1·000 to 1·000)	1·000 (1·000 to 1·000)	1·000 (1·000 to 1·000)	1·000 (1·000 to 1·000)	1·000 (1·000 to 1·000)
Larynx cancer	No exposure	Both	Male								1·000 (1·000 to 1·000)	1·000 (1·000 to 1·000)	1·000 (1·000 to 1·000)	1·000 (1·000 to 1·000)	1·000 (1·000 to 1·000)	1·000 (1·000 to 1·000)
Larynx cancer	High exposure	Both	Female								1·385 (1·187 to 1·598)	1·385 (1·187 to 1·598)	1·385 (1·187 to 1·598)	1·385 (1·187 to 1·598)	1·385 (1·187 to 1·598)	1·385 (1·187 to 1·598)
Larynx cancer	Low exposure	Both	Female								1·000 (1·000 to 1·000)	1·000 (1·000 to 1·000)	1·000 (1·000 to 1·000)	1·000 (1·000 to 1·000)	1·000 (1·000 to 1·000)	1·000 (1·000 to 1·000)
Larynx cancer	No exposure	Both	Female								1·000 (1·000 to 1·000)	1·000 (1·000 to 1·000)	1·000 (1·000 to 1·000)	1·000 (1·000 to 1·000)	1·000 (1·000 to 1·000)	1·000 (1·000 to 1·000)
Tracheal, bronchus, and lung cancer	High exposure	Both	Male								2·279 (1·740 to 2·936)	2·279 (1·740 to 2·936)	2·279 (1·740 to 2·936)	2·279 (1·740 to 2·936)	2·279 (1·740 to 2·936)	2·279 (1·740 to 2·936)
Tracheal, bronchus, and lung cancer	Low exposure	Both	Male								1·655 (1·501 to 1·809)	1·655 (1·501 to 1·809)	1·655 (1·501 to 1·809)	1·655 (1·501 to 1·809)	1·655 (1·501 to 1·809)	1·655 (1·501 to 1·809)
Tracheal, bronchus, and lung cancer	No exposure	Both	Male								1·000 (1·000 to 1·000)	1·000 (1·000 to 1·000)	1·000 (1·000 to 1·000)	1·000 (1·000 to 1·000)	1·000 (1·000 to 1·000)	1·000 (1·000 to 1·000)
Tracheal, bronchus, and lung cancer	High exposure	Both	Female								1·875 (1·588 to 2·213)	1·875 (1·588 to 2·213)	1·875 (1·588 to 2·213)	1·875 (1·588 to 2·213)	1·875 (1·588 to 2·213)	1·875 (1·588 to 2·213)
Tracheal, bronchus, and lung cancer	Low exposure	Both	Female								1·520 (1·461 to 1·580)	1·520 (1·461 to 1·580)	1·520 (1·461 to 1·580)	1·520 (1·461 to 1·580)	1·520 (1·461 to 1·580)	1·520 (1·461 to 1·580)
Tracheal, bronchus, and lung cancer	No exposure	Both	Female								1·000 (1·000 to 1·000)	1·000 (1·000 to 1·000)	1·000 (1·000 to 1·000)	1·000 (1·000 to 1·000)	1·000 (1·000 to 1·000)	1·000 (1·000 to 1·000)
Ovarian cancer	High exposure	Both	Both								1·811 (1·385 to 2·306)	1·811 (1·385 to 2·306)	1·811 (1·385 to 2·306)	1·811 (1·385 to 2·306)	1·811 (1·385 to 2·306)	1·811 (1·385 to 2·306)
Ovarian cancer	Low exposure	Both	Both								1·000 (1·000 to 1·000)	1·000 (1·000 to 1·000)	1·000 (1·000 to 1·000)	1·000 (1·000 to 1·000)	1·000 (1·000 to 1·000)	1·000 (1·000 to 1·000)
Ovarian cancer	No exposure	Both	Both								1·000 (1·000 to 1·000)	1·000 (1·000 to 1·000)	1·000 (1·000 to 1·000)	1·000 (1·000 to 1·000)	1·000 (1·000 to 1·000)	1·000 (1·000 to 1·000)
Mesothelioma	High exposure	Both	Both								44·272 (13·126 to 119·191)	44·272 (13·126 to 119·191)	44·272 (13·126 to 119·191)	44·272 (13·126 to 119·191)	44·272 (13·126 to 119·191)	44·272 (13·126 to 119·191)
Mesothelioma	Low exposure	Both	Both								24·712 (6·454 to 65·792)	24·712 (6·454 to 65·792)	24·712 (6·454 to 65·792)	24·712 (6·454 to 65·792)	24·712 (6·454 to 65·792)	24·712 (6·454 to 65·792)
Mesothelioma	No exposure	Both	Both								1·000 (1·000 to 1·000)	1·000 (1·000 to 1·000)	1·000 (1·000 to 1·000)	1·000 (1·000 to 1·000)	1·000 (1·000 to 1·000)	1·000 (1·000 to 1·000)

Risk - Outcome	Category / Units	Morbidity / Mortality	Sex	45-49 years	50-54 years	55-59 years	60-64 years	65-69 years	70-74 years	75-79 years	80+ years
Typhoid fever	Sewer	Both	Both	1·000 (1·000 to 1·000)	1·000 (1·000 to 1·000)	1·000 (1·000 to 1·000)	1·000 (1·000 to 1·000)	1·000 (1·000 to 1·000)	1·000 (1·000 to 1·000)	1·000 (1·000 to 1·000)	1·000 (1·000 to 1·000)
Paratyphoid fever	Unimproved & untreated	Both	Both	3·236 (2·778 to 3·725)	3·236 (2·778 to 3·725)	3·236 (2·778 to 3·725)	3·236 (2·778 to 3·725)	3·236 (2·778 to 3·725)	3·236 (2·778 to 3·725)	3·236 (2·778 to 3·725)	3·236 (2·778 to 3·725)
Paratyphoid fever	Improved	Both	Both	2·715 (2·560 to 2·870)	2·715 (2·560 to 2·870)	2·715 (2·560 to 2·870)	2·715 (2·560 to 2·870)	2·715 (2·560 to 2·870)	2·715 (2·560 to 2·870)	2·715 (2·560 to 2·870)	2·715 (2·560 to 2·870)
Paratyphoid fever	Sewer	Both	Both	1·000 (1·000 to 1·000)	1·000 (1·000 to 1·000)	1·000 (1·000 to 1·000)	1·000 (1·000 to 1·000)	1·000 (1·000 to 1·000)	1·000 (1·000 to 1·000)	1·000 (1·000 to 1·000)	1·000 (1·000 to 1·000)
**No handwashing with soap**											
Diarrhoeal diseases	No handwashing w/soap & water	Both	Both	1·673 (1·483 to 1·878)	1·673 (1·483 to 1·878)	1·673 (1·483 to 1·878)	1·673 (1·483 to 1·878)	1·673 (1·483 to 1·878)	1·673 (1·483 to 1·878)	1·673 (1·483 to 1·878)	1·673 (1·483 to 1·878)
Diarrhoeal diseases	Handwashing w/soap & water	Both	Both	1·000 (1·000 to 1·000)	1·000 (1·000 to 1·000)	1·000 (1·000 to 1·000)	1·000 (1·000 to 1·000)	1·000 (1·000 to 1·000)	1·000 (1·000 to 1·000)	1·000 (1·000 to 1·000)	1·000 (1·000 to 1·000)
Typhoid fever	No handwashing w/soap & water	Both	Both	1·667 (1·461 to 1·875)	1·667 (1·461 to 1·875)	1·667 (1·461 to 1·875)	1·667 (1·461 to 1·875)	1·667 (1·461 to 1·875)	1·667 (1·461 to 1·875)	1·667 (1·461 to 1·875)	1·667 (1·461 to 1·875)
Typhoid fever	Handwashing w/soap & water	Both	Both	1·000 (1·000 to 1·000)	1·000 (1·000 to 1·000)	1·000 (1·000 to 1·000)	1·000 (1·000 to 1·000)	1·000 (1·000 to 1·000)	1·000 (1·000 to 1·000)	1·000 (1·000 to 1·000)	1·000 (1·000 to 1·000)
Paratyphoid fever	No handwashing w/soap & water	Both	Both	1·665 (1·459 to 1·888)	1·665 (1·459 to 1·888)	1·665 (1·459 to 1·888)	1·665 (1·459 to 1·888)	1·665 (1·459 to 1·888)	1·665 (1·459 to 1·888)	1·665 (1·459 to 1·888)	1·665 (1·459 to 1·888)
Paratyphoid fever	Handwashing w/soap & water	Both	Both	1·000 (1·000 to 1·000)	1·000 (1·000 to 1·000)	1·000 (1·000 to 1·000)	1·000 (1·000 to 1·000)	1·000 (1·000 to 1·000)	1·000 (1·000 to 1·000)	1·000 (1·000 to 1·000)	1·000 (1·000 to 1·000)
Lower respiratory infections	No handwashing w/soap & water	Both	Both	1·192 (1·124 to 1·266)	1·192 (1·124 to 1·266)	1·192 (1·124 to 1·266)	1·192 (1·124 to 1·266)	1·192 (1·124 to 1·266)	1·192 (1·124 to 1·266)	1·192 (1·124 to 1·266)	1·192 (1·124 to 1·266)
Lower respiratory infections	Handwashing w/soap & water	Both	Both	1·000 (1·000 to 1·000)	1·000 (1·000 to 1·000)	1·000 (1·000 to 1·000)	1·000 (1·000 to 1·000)	1·000 (1·000 to 1·000)	1·000 (1·000 to 1·000)	1·000 (1·000 to 1·000)	1·000 (1·000 to 1·000)
**Household air pollution from solid fuels**											
Cataract	Exposed	Morbidity	Both	2·518 (1·622 to 3·726)	2·545 (1·680 to 3·785)	2·532 (1·612 to 3·645)	2·522 (1·651 to 3·680)	2·539 (1·633 to 3·745)	2·518 (1·622 to 3·796)	2·509 (1·638 to 3·589)	2·497 (1·610 to 3·634)
Cataract	Not exposed	Morbidity	Both	1·000 (1·000 to 1·000)	1·000 (1·000 to 1·000)	1·000 (1·000 to 1·000)	1·000 (1·000 to 1·000)	1·000 (1·000 to 1·000)	1·000 (1·000 to 1·000)	1·000 (1·000 to 1·000)	1·000 (1·000 to 1·000)
**Ambient ozone pollution**											
Chronic obstructive pulmonary disease	10 ppb	Mortality	Both	1·028 (1·008 to 1·048)	1·030 (1·011 to 1·049)	1·029 (1·011 to 1·047)	1·029 (1·010 to 1·048)	1·029 (1·010 to 1·048)	1·029 (1·012 to 1·048)	1·029 (1·010 to 1·049)	1·029 (1·010 to 1·049)
**Residential radon**											
Tracheal, bronchus, and lung cancer	Bq/m3	Both	Both	1·002 (1·000 to 1·003)	1·002 (1·000 to 1·003)	1·002 (1·000 to 1·003)	1·002 (1·000 to 1·003)	1·002 (1·000 to 1·003)	1·002 (1·000 to 1·003)	1·002 (1·000 to 1·003)	1·002 (1·000 to 1·003)
**Lead exposure**											
Rheumatic heart disease	10 µg/g	Morbidity	Both	1·000 (1·000 to 1·000)	1·000 (1·000 to 1·000)	1·000 (1·000 to 1·000)	1·000 (1·000 to 1·000)	1·000 (1·000 to 1·000)	1·000 (1·000 to 1·000)	1·000 (1·000 to 1·000)	1·000 (1·000 to 1·000)
Rheumatic heart disease	10 µg/g	Mortality	Both	1·022 (0·998 to 1·074)	1·021 (0·998 to 1·074)	1·020 (0·998 to 1·073)	1·018 (0·996 to 1·074)	1·017 (0·993 to 1·072)	1·016 (0·994 to 1·074)	1·017 (0·992 to 1·077)	1·016 (0·993 to 1·075)
Ischaemic heart disease	10 µg/g	Both	Both	1·027 (1·019 to 1·037)	1·026 (1·018 to 1·035)	1·024 (1·017 to 1·033)	1·022 (1·013 to 1·031)	1·019 (1·011 to 1·027)	1·017 (1·009 to 1·025)	1·015 (1·007 to 1·023)	1·012 (1·004 to 1·021)
Ischaemic stroke	10 µg/g	Both	Both	1·039 (1·034 to 1·043)	1·035 (1·031 to 1·040)	1·031 (1·028 to 1·035)	1·028 (1·024 to 1·032)	1·024 (1·021 to 1·028)	1·021 (1·018 to 1·024)	1·017 (1·013 to 1·021)	1·008 (1·004 to 1·013)
Hemorrhagic stroke	10 µg/g	Both	Both	1·042 (1·031 to 1·055)	1·038 (1·026 to 1·051)	1·034 (1·023 to 1·047)	1·030 (1·018 to 1·043)	1·026 (1·015 to 1·037)	1·022 (1·010 to 1·035)	1·018 (1·006 to 1·030)	1·010 (0·999 to 1·024)
Hypertensive heart disease	10 µg/g	Both	Both	1·027 (1·019 to 1·037)	1·026 (1·018 to 1·035)	1·024 (1·017 to 1·033)	1·022 (1·013 to 1·031)	1·019 (1·011 to 1·027)	1·017 (1·009 to 1·025)	1·015 (1·007 to 1·023)	1·012 (1·004 to 1·021)
Cardiomyopathy and myocarditis	10 µg/g	Morbidity	Both	1·000 (1·000 to 1·000)	1·000 (1·000 to 1·000)	1·000 (1·000 to 1·000)	1·000 (1·000 to 1·000)	1·000 (1·000 to 1·000)	1·000 (1·000 to 1·000)	1·000 (1·000 to 1·000)	1·000 (1·000 to 1·000)
Cardiomyopathy and myocarditis	10 µg/g	Mortality	Both	1·027 (1·004 to 1·080)	1·026 (1·004 to 1·076)	1·025 (1·003 to 1·074)	1·023 (1·001 to 1·076)	1·022 (1·001 to 1·073)	1·020 (1·000 to 1·073)	1·018 (0·995 to 1·069)	1·018 (0·995 to 1·074)
Atrial fibrillation and flutter	10 µg/g	Both	Both	1·021 (1·016 to 1·025)	1·020 (1·015 to 1·023)	1·018 (1·015 to 1·021)	1·016 (1·013 to 1·019)	1·015 (1·012 to 1·017)	1·013 (1·010 to 1·015)	1·011 (1·009 to 1·014)	1·008 (1·005 to 1·011)
Aortic aneurysm	10 µg/g	Both	Both	1·034 (1·010 to 1·087)	1·032 (1·007 to 1·088)	1·030 (1·008 to 1·084)	1·028 (1·006 to 1·081)	1·026 (1·004 to 1·080)	1·025 (1·002 to 1·080)	1·024 (1·004 to 1·077)	1·022 (1·000 to 1·078)
Peripheral vascular disease	10 µg/g	Both	Both	1·021 (1·016 to 1·025)	1·020 (1·015 to 1·023)	1·018 (1·015 to 1·021)	1·016 (1·013 to 1·019)	1·015 (1·012 to 1·017)	1·013 (1·010 to 1·015)	1·011 (1·009 to 1·014)	1·008 (1·005 to 1·011)
Endocarditis	10 µg/g	Morbidity	Both	1·000 (1·000 to 1·000)	1·000 (1·000 to 1·000)	1·000 (1·000 to 1·000)	1·000 (1·000 to 1·000)	1·000 (1·000 to 1·000)	1·000 (1·000 to 1·000)	1·000 (1·000 to 1·000)	1·000 (1·000 to 1·000)
Endocarditis	10 µg/g	Mortality	Both	1·027 (1·004 to 1·080)	1·026 (1·004 to 1·076)	1·025 (1·003 to 1·074)	1·023 (1·001 to 1·076)	1·022 (1·001 to 1·073)	1·020 (1·000 to 1·073)	1·018 (0·995 to 1·069)	1·018 (0·995 to 1·074)
Other cardiovascular and circulatory diseases	10 µg/g	Both	Both	1·021 (1·016 to 1·025)	1·020 (1·015 to 1·023)	1·018 (1·015 to 1·021)	1·016 (1·013 to 1·019)	1·015 (1·012 to 1·017)	1·013 (1·010 to 1·015)	1·011 (1·009 to 1·014)	1·008 (1·005 to 1·011)
Chronic kidney disease due to diabetes mellitus	10 µg/g	Both	Both	1·013 (1·009 to 1·017)	1·013 (1·009 to 1·017)	1·013 (1·009 to 1·017)	1·013 (1·009 to 1·017)	1·013 (1·009 to 1·017)	1·013 (1·009 to 1·017)	1·013 (1·009 to 1·017)	1·013 (1·009 to 1·017)
Chronic kidney disease due to hypertension	10 µg/g	Both	Both	1·013 (1·009 to 1·017)	1·013 (1·009 to 1·017)	1·013 (1·009 to 1·017)	1·013 (1·009 to 1·017)	1·013 (1·009 to 1·017)	1·013 (1·009 to 1·017)	1·013 (1·009 to 1·017)	1·013 (1·009 to 1·017)
Chronic kidney disease due to glomerulonephritis	10 µg/g	Both	Both	1·013 (1·009 to 1·017)	1·013 (1·009 to 1·017)	1·013 (1·009 to 1·017)	1·013 (1·009 to 1·017)	1·013 (1·009 to 1·017)	1·013 (1·009 to 1·017)	1·013 (1·009 to 1·017)	1·013 (1·009 to 1·017)
Chronic kidney disease due to other causes	10 µg/g	Both	Both	1·013 (1·009 to 1·017)	1·013 (1·009 to 1·017)	1·013 (1·009 to 1·017)	1·013 (1·009 to 1·017)	1·013 (1·009 to 1·017)	1·013 (1·009 to 1·017)	1·013 (1·009 to 1·017)	1·013 (1·009 to 1·017)
**Occupational exposure to asbestos**											
Larynx cancer	High exposure	Both	Male	1·380 (1·188 to 1·612)	1·380 (1·188 to 1·612)	1·380 (1·188 to 1·612)	1·380 (1·188 to 1·612)	1·380 (1·188 to 1·612)	1·380 (1·188 to 1·612)	1·380 (1·188 to 1·612)	1·380 (1·188 to 1·612)
Larynx cancer	Low exposure	Both	Male	1·000 (1·000 to 1·000)	1·000 (1·000 to 1·000)	1·000 (1·000 to 1·000)	1·000 (1·000 to 1·000)	1·000 (1·000 to 1·000)	1·000 (1·000 to 1·000)	1·000 (1·000 to 1·000)	1·000 (1·000 to 1·000)
Larynx cancer	No exposure	Both	Male	1·000 (1·000 to 1·000)	1·000 (1·000 to 1·000)	1·000 (1·000 to 1·000)	1·000 (1·000 to 1·000)	1·000 (1·000 to 1·000)	1·000 (1·000 to 1·000)	1·000 (1·000 to 1·000)	1·000 (1·000 to 1·000)
Larynx cancer	High exposure	Both	Female	1·385 (1·187 to 1·598)	1·385 (1·187 to 1·598)	1·385 (1·187 to 1·598)	1·385 (1·187 to 1·598)	1·385 (1·187 to 1·598)	1·385 (1·187 to 1·598)	1·385 (1·187 to 1·598)	1·385 (1·187 to 1·598)
Larynx cancer	Low exposure	Both	Female	1·000 (1·000 to 1·000)	1·000 (1·000 to 1·000)	1·000 (1·000 to 1·000)	1·000 (1·000 to 1·000)	1·000 (1·000 to 1·000)	1·000 (1·000 to 1·000)	1·000 (1·000 to 1·000)	1·000 (1·000 to 1·000)
Larynx cancer	No exposure	Both	Female	1·000 (1·000 to 1·000)	1·000 (1·000 to 1·000)	1·000 (1·000 to 1·000)	1·000 (1·000 to 1·000)	1·000 (1·000 to 1·000)	1·000 (1·000 to 1·000)	1·000 (1·000 to 1·000)	1·000 (1·000 to 1·000)
Tracheal, bronchus, and lung cancer	High exposure	Both	Male	2·279 (1·740 to 2·936)	2·279 (1·740 to 2·936)	2·279 (1·740 to 2·936)	2·279 (1·740 to 2·936)	2·279 (1·740 to 2·936)	2·279 (1·740 to 2·936)	2·279 (1·740 to 2·936)	2·279 (1·740 to 2·936)
Tracheal, bronchus, and lung cancer	Low exposure	Both	Male	1·655 (1·501 to 1·809)	1·655 (1·501 to 1·809)	1·655 (1·501 to 1·809)	1·655 (1·501 to 1·809)	1·655 (1·501 to 1·809)	1·655 (1·501 to 1·809)	1·655 (1·501 to 1·809)	1·655 (1·501 to 1·809)
Tracheal, bronchus, and lung cancer	No exposure	Both	Male	1·000 (1·000 to 1·000)	1·000 (1·000 to 1·000)	1·000 (1·000 to 1·000)	1·000 (1·000 to 1·000)	1·000 (1·000 to 1·000)	1·000 (1·000 to 1·000)	1·000 (1·000 to 1·000)	1·000 (1·000 to 1·000)
Tracheal, bronchus, and lung cancer	High exposure	Both	Female	1·875 (1·588 to 2·213)	1·875 (1·588 to 2·213)	1·875 (1·588 to 2·213)	1·875 (1·588 to 2·213)	1·875 (1·588 to 2·213)	1·875 (1·588 to 2·213)	1·875 (1·588 to 2·213)	1·875 (1·588 to 2·213)
Tracheal, bronchus, and lung cancer	Low exposure	Both	Female	1·520 (1·461 to 1·580)	1·520 (1·461 to 1·580)	1·520 (1·461 to 1·580)	1·520 (1·461 to 1·580)	1·520 (1·461 to 1·580)	1·520 (1·461 to 1·580)	1·520 (1·461 to 1·580)	1·520 (1·461 to 1·580)
Tracheal, bronchus, and lung cancer	No exposure	Both	Female	1·000 (1·000 to 1·000)	1·000 (1·000 to 1·000)	1·000 (1·000 to 1·000)	1·000 (1·000 to 1·000)	1·000 (1·000 to 1·000)	1·000 (1·000 to 1·000)	1·000 (1·000 to 1·000)	1·000 (1·000 to 1·000)
Ovarian cancer	High exposure	Both	Both	1·811 (1·385 to 2·306)	1·811 (1·385 to 2·306)	1·811 (1·385 to 2·306)	1·811 (1·385 to 2·306)	1·811 (1·385 to 2·306)	1·811 (1·385 to 2·306)	1·811 (1·385 to 2·306)	1·811 (1·385 to 2·306)
Ovarian cancer	Low exposure	Both	Both	1·000 (1·000 to 1·000)	1·000 (1·000 to 1·000)	1·000 (1·000 to 1·000)	1·000 (1·000 to 1·000)	1·000 (1·000 to 1·000)	1·000 (1·000 to 1·000)	1·000 (1·000 to 1·000)	1·000 (1·000 to 1·000)
Ovarian cancer	No exposure	Both	Both	1·000 (1·000 to 1·000)	1·000 (1·000 to 1·000)	1·000 (1·000 to 1·000)	1·000 (1·000 to 1·000)	1·000 (1·000 to 1·000)	1·000 (1·000 to 1·000)	1·000 (1·000 to 1·000)	1·000 (1·000 to 1·000)
Mesothelioma	High exposure	Both	Both	44·272 (13·126 to 119·191)	44·272 (13·126 to 119·191)	44·272 (13·126 to 119·191)	44·272 (13·126 to 119·191)	44·272 (13·126 to 119·191)	44·272 (13·126 to 119·191)	44·272 (13·126 to 119·191)	44·272 (13·126 to 119·191)
Mesothelioma	Low exposure	Both	Both	24·712 (6·454 to 65·792)	24·712 (6·454 to 65·792)	24·712 (6·454 to 65·792)	24·712 (6·454 to 65·792)	24·712 (6·454 to 65·792)	24·712 (6·454 to 65·792)	24·712 (6·454 to 65·792)	24·712 (6·454 to 65·792)
Mesothelioma	No exposure	Both	Both	1·000 (1·000 to 1·000)	1·000 (1·000 to 1·000)	1·000 (1·000 to 1·000)	1·000 (1·000 to 1·000)	1·000 (1·000 to 1·000)	1·000 (1·000 to 1·000)	1·000 (1·000 to 1·000)	1·000 (1·000 to 1·000)

Risk - Outcome	Category / Units	Morbidity / Mortality	Sex	All ages	0-6 Days	7-27 Days	28-364 Days	1-4 years	5-9 years	10-14 years	15-19 years	20-24 years	25-29 years	30-34 years	35-39 years	40-44 years
**Occupational exposure to arsenic**																
Tracheal, bronchus, and lung cancer	High exposure	Both	Male								2·085 (1·446 to 2·904)	2·090 (1·411 to 2·922)	2·093 (1·449 to 2·870)	2·079 (1·433 to 2·914)	2·067 (1·437 to 2·864)	2·053 (1·436 to 2·879)
Tracheal, bronchus, and lung cancer	Low exposure	Both	Male								1·000 (1·000 to 1·000)	1·000 (1·000 to 1·000)	1·000 (1·000 to 1·000)	1·000 (1·000 to 1·000)	1·000 (1·000 to 1·000)	1·000 (1·000 to 1·000)
Tracheal, bronchus, and lung cancer	No exposure	Both	Male								1·000 (1·000 to 1·000)	1·000 (1·000 to 1·000)	1·000 (1·000 to 1·000)	1·000 (1·000 to 1·000)	1·000 (1·000 to 1·000)	1·000 (1·000 to 1·000)
Tracheal, bronchus, and lung cancer	High exposure	Both	Female								2·085 (1·435 to 2·911)	2·089 (1·467 to 2·874)	2·084 (1·428 to 2·990)	2·086 (1·459 to 2·832)	2·090 (1·407 to 2·867)	2·081 (1·438 to 2·915)
Tracheal, bronchus, and lung cancer	Low exposure	Both	Female								1·000 (1·000 to 1·000)	1·000 (1·000 to 1·000)	1·000 (1·000 to 1·000)	1·000 (1·000 to 1·000)	1·000 (1·000 to 1·000)	1·000 (1·000 to 1·000)
Tracheal, bronchus, and lung cancer	No exposure	Both	Female								1·000 (1·000 to 1·000)	1·000 (1·000 to 1·000)	1·000 (1·000 to 1·000)	1·000 (1·000 to 1·000)	1·000 (1·000 to 1·000)	1·000 (1·000 to 1·000)
**Occupational exposure to benzene**																
Leukaemia	High exposure	Both	Male								2·686 (1·570 to 4·190)	2·701 (1·617 to 4·452)	2·730 (1·585 to 4·471)	2·711 (1·555 to 4·443)	2·741 (1·597 to 4·549)	2·690 (1·594 to 4·317)
Leukaemia	Low exposure	Both	Male								1·676 (1·127 to 2·432)	1·682 (1·130 to 2·400)	1·666 (1·109 to 2·402)	1·657 (1·096 to 2·361)	1·667 (1·100 to 2·421)	1·676 (1·087 to 2·393)
Leukaemia	No exposure	Both	Male								1·000 (1·000 to 1·000)	1·000 (1·000 to 1·000)	1·000 (1·000 to 1·000)	1·000 (1·000 to 1·000)	1·000 (1·000 to 1·000)	1·000 (1·000 to 1·000)
Leukaemia	High exposure	Both	Female								2·722 (1·565 to 4·362)	2·701 (1·577 to 4·369)	2·687 (1·586 to 4·289)	2·738 (1·606 to 4·557)	2·719 (1·585 to 4·469)	2·749 (1·640 to 4·474)
Leukaemia	Low exposure	Both	Female								1·677 (1·114 to 2·486)	1·694 (1·121 to 2·410)	1·655 (1·078 to 2·416)	1·691 (1·124 to 2·439)	1·660 (1·079 to 2·376)	1·667 (1·135 to 2·446)
Leukaemia	No exposure	Both	Female								1·000 (1·000 to 1·000)	1·000 (1·000 to 1·000)	1·000 (1·000 to 1·000)	1·000 (1·000 to 1·000)	1·000 (1·000 to 1·000)	1·000 (1·000 to 1·000)
**Occupational exposure to beryllium**																
Tracheal, bronchus, and lung cancer	High exposure	Both	Male								1·174 (1·086 to 1·270)	1·169 (1·064 to 1·277)	1·170 (1·073 to 1·274)	1·169 (1·073 to 1·269)	1·172 (1·080 to 1·271)	1·168 (1·070 to 1·276)
Tracheal, bronchus, and lung cancer	Low exposure	Both	Male								1·000 (1·000 to 1·000)	1·000 (1·000 to 1·000)	1·000 (1·000 to 1·000)	1·000 (1·000 to 1·000)	1·000 (1·000 to 1·000)	1·000 (1·000 to 1·000)
Tracheal, bronchus, and lung cancer	No exposure	Both	Male								1·000 (1·000 to 1·000)	1·000 (1·000 to 1·000)	1·000 (1·000 to 1·000)	1·000 (1·000 to 1·000)	1·000 (1·000 to 1·000)	1·000 (1·000 to 1·000)
Tracheal, bronchus, and lung cancer	High exposure	Both	Female								1·170 (1·081 to 1·262)	1·170 (1·076 to 1·277)	1·169 (1·072 to 1·278)	1·170 (1·077 to 1·276)	1·172 (1·075 to 1·274)	1·171 (1·077 to 1·276)
Tracheal, bronchus, and lung cancer	Low exposure	Both	Female								1·000 (1·000 to 1·000)	1·000 (1·000 to 1·000)	1·000 (1·000 to 1·000)	1·000 (1·000 to 1·000)	1·000 (1·000 to 1·000)	1·000 (1·000 to 1·000)
Tracheal, bronchus, and lung cancer	No exposure	Both	Female								1·000 (1·000 to 1·000)	1·000 (1·000 to 1·000)	1·000 (1·000 to 1·000)	1·000 (1·000 to 1·000)	1·000 (1·000 to 1·000)	1·000 (1·000 to 1·000)
**Occupational exposure to cadmium**																
Tracheal, bronchus, and lung cancer	High exposure	Both	Male								1·192 (1·097 to 1·293)	1·188 (1·083 to 1·288)	1·190 (1·101 to 1·295)	1·191 (1·091 to 1·305)	1·190 (1·090 to 1·298)	1·190 (1·092 to 1·299)
Tracheal, bronchus, and lung cancer	Low exposure	Both	Male								1·000 (1·000 to 1·000)	1·000 (1·000 to 1·000)	1·000 (1·000 to 1·000)	1·000 (1·000 to 1·000)	1·000 (1·000 to 1·000)	1·000 (1·000 to 1·000)
Tracheal, bronchus, and lung cancer	No exposure	Both	Male								1·000 (1·000 to 1·000)	1·000 (1·000 to 1·000)	1·000 (1·000 to 1·000)	1·000 (1·000 to 1·000)	1·000 (1·000 to 1·000)	1·000 (1·000 to 1·000)
Tracheal, bronchus, and lung cancer	High exposure	Both	Female								1·191 (1·087 to 1·296)	1·191 (1·099 to 1·291)	1·188 (1·093 to 1·294)	1·190 (1·092 to 1·302)	1·191 (1·088 to 1·292)	1·194 (1·095 to 1·297)
Tracheal, bronchus, and lung cancer	Low exposure	Both	Female								1·000 (1·000 to 1·000)	1·000 (1·000 to 1·000)	1·000 (1·000 to 1·000)	1·000 (1·000 to 1·000)	1·000 (1·000 to 1·000)	1·000 (1·000 to 1·000)
Tracheal, bronchus, and lung cancer	No exposure	Both	Female								1·000 (1·000 to 1·000)	1·000 (1·000 to 1·000)	1·000 (1·000 to 1·000)	1·000 (1·000 to 1·000)	1·000 (1·000 to 1·000)	1·000 (1·000 to 1·000)
**Occupational exposure to chromium**																
Tracheal, bronchus, and lung cancer	High exposure	Both	Male								1·179 (1·114 to 1·246)	1·181 (1·117 to 1·250)	1·181 (1·117 to 1·250)	1·180 (1·116 to 1·244)	1·183 (1·117 to 1·249)	1·180 (1·117 to 1·240)
Tracheal, bronchus, and lung cancer	Low exposure	Both	Male								1·000 (1·000 to 1·000)	1·000 (1·000 to 1·000)	1·000 (1·000 to 1·000)	1·000 (1·000 to 1·000)	1·000 (1·000 to 1·000)	1·000 (1·000 to 1·000)
Tracheal, bronchus, and lung cancer	No exposure	Both	Male								1·000 (1·000 to 1·000)	1·000 (1·000 to 1·000)	1·000 (1·000 to 1·000)	1·000 (1·000 to 1·000)	1·000 (1·000 to 1·000)	1·000 (1·000 to 1·000)
Tracheal, bronchus, and lung cancer	High exposure	Both	Female								1·179 (1·116 to 1·248)	1·180 (1·115 to 1·248)	1·180 (1·115 to 1·244)	1·179 (1·117 to 1·243)	1·180 (1·115 to 1·248)	1·181 (1·115 to 1·246)
Tracheal, bronchus, and lung cancer	Low exposure	Both	Female								1·000 (1·000 to 1·000)	1·000 (1·000 to 1·000)	1·000 (1·000 to 1·000)	1·000 (1·000 to 1·000)	1·000 (1·000 to 1·000)	1·000 (1·000 to 1·000)
Tracheal, bronchus, and lung cancer	No exposure	Both	Female								1·000 (1·000 to 1·000)	1·000 (1·000 to 1·000)	1·000 (1·000 to 1·000)	1·000 (1·000 to 1·000)	1·000 (1·000 to 1·000)	1·000 (1·000 to 1·000)
**Occupational exposure to diesel engine exhaust**																
Tracheal, bronchus, and lung cancer	High exposure	Both	Male								1·469 (1·294 to 1·658)	1·477 (1·300 to 1·665)	1·473 (1·290 to 1·669)	1·474 (1·292 to 1·676)	1·472 (1·301 to 1·670)	1·470 (1·294 to 1·663)
Tracheal, bronchus, and lung cancer	Low exposure	Both	Male								1·000 (1·000 to 1·000)	1·000 (1·000 to 1·000)	1·000 (1·000 to 1·000)	1·000 (1·000 to 1·000)	1·000 (1·000 to 1·000)	1·000 (1·000 to 1·000)
Tracheal, bronchus, and lung cancer	No exposure	Both	Male								1·000 (1·000 to 1·000)	1·000 (1·000 to 1·000)	1·000 (1·000 to 1·000)	1·000 (1·000 to 1·000)	1·000 (1·000 to 1·000)	1·000 (1·000 to 1·000)
Tracheal, bronchus, and lung cancer	High exposure	Both	Female								1·473 (1·286 to 1·683)	1·476 (1·302 to 1·686)	1·469 (1·288 to 1·670)	1·467 (1·281 to 1·671)	1·473 (1·288 to 1·661)	1·475 (1·293 to 1·682)
Tracheal, bronchus, and lung cancer	Low exposure	Both	Female								1·000 (1·000 to 1·000)	1·000 (1·000 to 1·000)	1·000 (1·000 to 1·000)	1·000 (1·000 to 1·000)	1·000 (1·000 to 1·000)	1·000 (1·000 to 1·000)
Tracheal, bronchus, and lung cancer	No exposure	Both	Female								1·000 (1·000 to 1·000)	1·000 (1·000 to 1·000)	1·000 (1·000 to 1·000)	1·000 (1·000 to 1·000)	1·000 (1·000 to 1·000)	1·000 (1·000 to 1·000)
**Occupational exposure to second-hand smoke**																
Tracheal, bronchus, and lung cancer	High exposure	Both	Male								1·241 (1·186 to 1·301)	1·240 (1·187 to 1·298)	1·242 (1·183 to 1·296)	1·240 (1·185 to 1·292)	1·243 (1·189 to 1·296)	1·242 (1·187 to 1·298)
Tracheal, bronchus, and lung cancer	Low exposure	Both	Male								1·000 (1·000 to 1·000)	1·000 (1·000 to 1·000)	1·000 (1·000 to 1·000)	1·000 (1·000 to 1·000)	1·000 (1·000 to 1·000)	1·000 (1·000 to 1·000)
Tracheal, bronchus, and lung cancer	No exposure	Both	Male								1·000 (1·000 to 1·000)	1·000 (1·000 to 1·000)	1·000 (1·000 to 1·000)	1·000 (1·000 to 1·000)	1·000 (1·000 to 1·000)	1·000 (1·000 to 1·000)
Tracheal, bronchus, and lung cancer	High exposure	Both	Female								1·240 (1·184 to 1·300)	1·240 (1·189 to 1·294)	1·241 (1·190 to 1·297)	1·240 (1·182 to 1·298)	1·240 (1·188 to 1·296)	1·240 (1·186 to 1·298)
Tracheal, bronchus, and lung cancer	Low exposure	Both	Female								1·000 (1·000 to 1·000)	1·000 (1·000 to 1·000)	1·000 (1·000 to 1·000)	1·000 (1·000 to 1·000)	1·000 (1·000 to 1·000)	1·000 (1·000 to 1·000)
Tracheal, bronchus, and lung cancer	No exposure	Both	Female								1·000 (1·000 to 1·000)	1·000 (1·000 to 1·000)	1·000 (1·000 to 1·000)	1·000 (1·000 to 1·000)	1·000 (1·000 to 1·000)	1·000 (1·000 to 1·000)
**Occupational exposure to formaldehyde**																
Nasopharynx cancer	High exposure	Both	Male								2·222 (1·023 to 4·247)	2·294 (1·054 to 4·415)	2·204 (1·022 to 4·044)	2·211 (0·993 to 4·228)	2·230 (1·026 to 4·147)	2·241 (1·076 to 4·078)
Nasopharynx cancer	Low exposure	Both	Male								1·000 (1·000 to 1·000)	1·000 (1·000 to 1·000)	1·000 (1·000 to 1·000)	1·000 (1·000 to 1·000)	1·000 (1·000 to 1·000)	1·000 (1·000 to 1·000)
Nasopharynx cancer	No exposure	Both	Male								1·000 (1·000 to 1·000)	1·000 (1·000 to 1·000)	1·000 (1·000 to 1·000)	1·000 (1·000 to 1·000)	1·000 (1·000 to 1·000)	1·000 (1·000 to 1·000)
Nasopharynx cancer	High exposure	Both	Female								2·202 (1·040 to 4·060)	2·246 (1·036 to 4·201)	2·227 (1·046 to 4·220)	2·269 (1·082 to 4·189)	2·264 (1·064 to 4·394)	2·217 (1·039 to 4·233)
Nasopharynx cancer	Low exposure	Both	Female								1·000 (1·000 to 1·000)	1·000 (1·000 to 1·000)	1·000 (1·000 to 1·000)	1·000 (1·000 to 1·000)	1·000 (1·000 to 1·000)	1·000 (1·000 to 1·000)
Nasopharynx cancer	No exposure	Both	Female								1·000 (1·000 to 1·000)	1·000 (1·000 to 1·000)	1·000 (1·000 to 1·000)	1·000 (1·000 to 1·000)	1·000 (1·000 to 1·000)	1·000 (1·000 to 1·000)
Leukaemia	High exposure	Both	Male								1·483 (1·191 to 1·818)	1·479 (1·183 to 1·833)	1·474 (1·182 to 1·816)	1·480 (1·192 to 1·831)	1·479 (1·195 to 1·806)	1·467 (1·174 to 1·844)
Leukaemia	Low exposure	Both	Male								1·000 (1·000 to 1·000)	1·000 (1·000 to 1·000)	1·000 (1·000 to 1·000)	1·000 (1·000 to 1·000)	1·000 (1·000 to 1·000)	1·000 (1·000 to 1·000)

Risk - Outcome	Category / Units	Morbidity / Mortality	Sex	45-49 years	50-54 years	55-59 years	60-64 years	65-69 years	70-74 years	75-79 years	80+ years
**Occupational exposure to arsenic**											
Tracheal, bronchus, and lung cancer	High exposure	Both	Male	2·074 (1·474 to 2·925)	2·078 (1·437 to 2·926)	2·074 (1·460 to 2·849)	2·064 (1·456 to 2·811)	2·092 (1·474 to 2·863)	2·096 (1·457 to 2·966)	2·097 (1·477 to 2·826)	2·090 (1·471 to 2·858)
Tracheal, bronchus, and lung cancer	Low exposure	Both	Male	1·000 (1·000 to 1·000)	1·000 (1·000 to 1·000)	1·000 (1·000 to 1·000)	1·000 (1·000 to 1·000)	1·000 (1·000 to 1·000)	1·000 (1·000 to 1·000)	1·000 (1·000 to 1·000)	1·000 (1·000 to 1·000)
Tracheal, bronchus, and lung cancer	No exposure	Both	Male	1·000 (1·000 to 1·000)	1·000 (1·000 to 1·000)	1·000 (1·000 to 1·000)	1·000 (1·000 to 1·000)	1·000 (1·000 to 1·000)	1·000 (1·000 to 1·000)	1·000 (1·000 to 1·000)	1·000 (1·000 to 1·000)
Tracheal, bronchus, and lung cancer	High exposure	Both	Female	2·069 (1·430 to 2·824)	2·109 (1·475 to 2·977)	2·079 (1·429 to 2·883)	2·076 (1·467 to 2·944)	2·070 (1·482 to 2·888)	2·098 (1·457 to 2·936)	2·089 (1·429 to 2·918)	2·096 (1·488 to 2·901)
Tracheal, bronchus, and lung cancer	Low exposure	Both	Female	1·000 (1·000 to 1·000)	1·000 (1·000 to 1·000)	1·000 (1·000 to 1·000)	1·000 (1·000 to 1·000)	1·000 (1·000 to 1·000)	1·000 (1·000 to 1·000)	1·000 (1·000 to 1·000)	1·000 (1·000 to 1·000)
Tracheal, bronchus, and lung cancer	No exposure	Both	Female	1·000 (1·000 to 1·000)	1·000 (1·000 to 1·000)	1·000 (1·000 to 1·000)	1·000 (1·000 to 1·000)	1·000 (1·000 to 1·000)	1·000 (1·000 to 1·000)	1·000 (1·000 to 1·000)	1·000 (1·000 to 1·000)
**Occupational exposure to benzene**											
Leukaemia	High exposure	Both	Male	2·713 (1·623 to 4·233)	2·713 (1·616 to 4·421)	2·710 (1·580 to 4·459)	2·672 (1·591 to 4·408)	2·699 (1·535 to 4·498)	2·700 (1·545 to 4·405)	2·719 (1·586 to 4·416)	2·712 (1·507 to 4·505)
Leukaemia	Low exposure	Both	Male	1·678 (1·133 to 2·455)	1·664 (1·144 to 2·403)	1·689 (1·110 to 2·422)	1·658 (1·102 to 2·428)	1·684 (1·118 to 2·469)	1·656 (1·101 to 2·342)	1·684 (1·134 to 2·465)	1·689 (1·139 to 2·431)
Leukaemia	No exposure	Both	Male	1·000 (1·000 to 1·000)	1·000 (1·000 to 1·000)	1·000 (1·000 to 1·000)	1·000 (1·000 to 1·000)	1·000 (1·000 to 1·000)	1·000 (1·000 to 1·000)	1·000 (1·000 to 1·000)	1·000 (1·000 to 1·000)
Leukaemia	High exposure	Both	Female	2·699 (1·524 to 4·392)	2·682 (1·578 to 4·337)	2·737 (1·533 to 4·538)	2·716 (1·576 to 4·449)	2·723 (1·567 to 4·550)	2·736 (1·597 to 4·459)	2·740 (1·620 to 4·458)	2·732 (1·517 to 4·526)
Leukaemia	Low exposure	Both	Female	1·661 (1·090 to 2·424)	1·684 (1·148 to 2·402)	1·668 (1·094 to 2·424)	1·686 (1·122 to 2·434)	1·675 (1·106 to 2·418)	1·697 (1·151 to 2·482)	1·662 (1·102 to 2·368)	1·680 (1·110 to 2·436)
Leukaemia	No exposure	Both	Female	1·000 (1·000 to 1·000)	1·000 (1·000 to 1·000)	1·000 (1·000 to 1·000)	1·000 (1·000 to 1·000)	1·000 (1·000 to 1·000)	1·000 (1·000 to 1·000)	1·000 (1·000 to 1·000)	1·000 (1·000 to 1·000)
**Occupational exposure to beryllium**											
Tracheal, bronchus, and lung cancer	High exposure	Both	Male	1·171 (1·070 to 1·274)	1·171 (1·074 to 1·273)	1·170 (1·073 to 1·271)	1·171 (1·071 to 1·271)	1·172 (1·080 to 1·276)	1·171 (1·076 to 1·270)	1·174 (1·078 to 1·276)	1·171 (1·072 to 1·273)
Tracheal, bronchus, and lung cancer	Low exposure	Both	Male	1·000 (1·000 to 1·000)	1·000 (1·000 to 1·000)	1·000 (1·000 to 1·000)	1·000 (1·000 to 1·000)	1·000 (1·000 to 1·000)	1·000 (1·000 to 1·000)	1·000 (1·000 to 1·000)	1·000 (1·000 to 1·000)
Tracheal, bronchus, and lung cancer	No exposure	Both	Male	1·000 (1·000 to 1·000)	1·000 (1·000 to 1·000)	1·000 (1·000 to 1·000)	1·000 (1·000 to 1·000)	1·000 (1·000 to 1·000)	1·000 (1·000 to 1·000)	1·000 (1·000 to 1·000)	1·000 (1·000 to 1·000)
Tracheal, bronchus, and lung cancer	High exposure	Both	Female	1·173 (1·074 to 1·276)	1·172 (1·074 to 1·274)	1·173 (1·081 to 1·277)	1·169 (1·074 to 1·269)	1·173 (1·077 to 1·282)	1·167 (1·069 to 1·269)	1·169 (1·073 to 1·275)	1·171 (1·067 to 1·279)
Tracheal, bronchus, and lung cancer	Low exposure	Both	Female	1·000 (1·000 to 1·000)	1·000 (1·000 to 1·000)	1·000 (1·000 to 1·000)	1·000 (1·000 to 1·000)	1·000 (1·000 to 1·000)	1·000 (1·000 to 1·000)	1·000 (1·000 to 1·000)	1·000 (1·000 to 1·000)
Tracheal, bronchus, and lung cancer	No exposure	Both	Female	1·000 (1·000 to 1·000)	1·000 (1·000 to 1·000)	1·000 (1·000 to 1·000)	1·000 (1·000 to 1·000)	1·000 (1·000 to 1·000)	1·000 (1·000 to 1·000)	1·000 (1·000 to 1·000)	1·000 (1·000 to 1·000)
**Occupational exposure to cadmium**											
Tracheal, bronchus, and lung cancer	High exposure	Both	Male	1·192 (1·095 to 1·299)	1·193 (1·095 to 1·296)	1·190 (1·089 to 1·293)	1·192 (1·088 to 1·298)	1·193 (1·088 to 1·300)	1·187 (1·095 to 1·291)	1·191 (1·094 to 1·289)	1·190 (1·088 to 1·296)
Tracheal, bronchus, and lung cancer	Low exposure	Both	Male	1·000 (1·000 to 1·000)	1·000 (1·000 to 1·000)	1·000 (1·000 to 1·000)	1·000 (1·000 to 1·000)	1·000 (1·000 to 1·000)	1·000 (1·000 to 1·000)	1·000 (1·000 to 1·000)	1·000 (1·000 to 1·000)
Tracheal, bronchus, and lung cancer	No exposure	Both	Male	1·000 (1·000 to 1·000)	1·000 (1·000 to 1·000)	1·000 (1·000 to 1·000)	1·000 (1·000 to 1·000)	1·000 (1·000 to 1·000)	1·000 (1·000 to 1·000)	1·000 (1·000 to 1·000)	1·000 (1·000 to 1·000)
Tracheal, bronchus, and lung cancer	High exposure	Both	Female	1·188 (1·091 to 1·300)	1·190 (1·095 to 1·291)	1·190 (1·095 to 1·295)	1·189 (1·095 to 1·290)	1·192 (1·094 to 1·296)	1·192 (1·089 to 1·299)	1·193 (1·096 to 1·297)	1·193 (1·099 to 1·301)
Tracheal, bronchus, and lung cancer	Low exposure	Both	Female	1·000 (1·000 to 1·000)	1·000 (1·000 to 1·000)	1·000 (1·000 to 1·000)	1·000 (1·000 to 1·000)	1·000 (1·000 to 1·000)	1·000 (1·000 to 1·000)	1·000 (1·000 to 1·000)	1·000 (1·000 to 1·000)
Tracheal, bronchus, and lung cancer	No exposure	Both	Female	1·000 (1·000 to 1·000)	1·000 (1·000 to 1·000)	1·000 (1·000 to 1·000)	1·000 (1·000 to 1·000)	1·000 (1·000 to 1·000)	1·000 (1·000 to 1·000)	1·000 (1·000 to 1·000)	1·000 (1·000 to 1·000)
**Occupational exposure to chromium**											
Tracheal, bronchus, and lung cancer	High exposure	Both	Male	1·180 (1·117 to 1·249)	1·182 (1·120 to 1·246)	1·181 (1·118 to 1·246)	1·179 (1·115 to 1·243)	1·182 (1·121 to 1·247)	1·180 (1·118 to 1·248)	1·180 (1·113 to 1·246)	1·181 (1·117 to 1·245)
Tracheal, bronchus, and lung cancer	Low exposure	Both	Male	1·000 (1·000 to 1·000)	1·000 (1·000 to 1·000)	1·000 (1·000 to 1·000)	1·000 (1·000 to 1·000)	1·000 (1·000 to 1·000)	1·000 (1·000 to 1·000)	1·000 (1·000 to 1·000)	1·000 (1·000 to 1·000)
Tracheal, bronchus, and lung cancer	No exposure	Both	Male	1·000 (1·000 to 1·000)	1·000 (1·000 to 1·000)	1·000 (1·000 to 1·000)	1·000 (1·000 to 1·000)	1·000 (1·000 to 1·000)	1·000 (1·000 to 1·000)	1·000 (1·000 to 1·000)	1·000 (1·000 to 1·000)
Tracheal, bronchus, and lung cancer	High exposure	Both	Female	1·181 (1·118 to 1·250)	1·181 (1·118 to 1·245)	1·181 (1·117 to 1·248)	1·181 (1·117 to 1·245)	1·179 (1·119 to 1·245)	1·181 (1·116 to 1·255)	1·181 (1·116 to 1·246)	1·179 (1·113 to 1·247)
Tracheal, bronchus, and lung cancer	Low exposure	Both	Female	1·000 (1·000 to 1·000)	1·000 (1·000 to 1·000)	1·000 (1·000 to 1·000)	1·000 (1·000 to 1·000)	1·000 (1·000 to 1·000)	1·000 (1·000 to 1·000)	1·000 (1·000 to 1·000)	1·000 (1·000 to 1·000)
Tracheal, bronchus, and lung cancer	No exposure	Both	Female	1·000 (1·000 to 1·000)	1·000 (1·000 to 1·000)	1·000 (1·000 to 1·000)	1·000 (1·000 to 1·000)	1·000 (1·000 to 1·000)	1·000 (1·000 to 1·000)	1·000 (1·000 to 1·000)	1·000 (1·000 to 1·000)
**Occupational exposure to diesel engine exhaust**											
Tracheal, bronchus, and lung cancer	High exposure	Both	Male	1·475 (1·300 to 1·669)	1·469 (1·299 to 1·645)	1·474 (1·295 to 1·677)	1·477 (1·291 to 1·671)	1·477 (1·311 to 1·680)	1·476 (1·300 to 1·669)	1·473 (1·286 to 1·669)	1·477 (1·299 to 1·669)
Tracheal, bronchus, and lung cancer	Low exposure	Both	Male	1·000 (1·000 to 1·000)	1·000 (1·000 to 1·000)	1·000 (1·000 to 1·000)	1·000 (1·000 to 1·000)	1·000 (1·000 to 1·000)	1·000 (1·000 to 1·000)	1·000 (1·000 to 1·000)	1·000 (1·000 to 1·000)
Tracheal, bronchus, and lung cancer	No exposure	Both	Male	1·000 (1·000 to 1·000)	1·000 (1·000 to 1·000)	1·000 (1·000 to 1·000)	1·000 (1·000 to 1·000)	1·000 (1·000 to 1·000)	1·000 (1·000 to 1·000)	1·000 (1·000 to 1·000)	1·000 (1·000 to 1·000)
Tracheal, bronchus, and lung cancer	High exposure	Both	Female	1·477 (1·299 to 1·681)	1·475 (1·302 to 1·675)	1·476 (1·290 to 1·681)	1·481 (1·308 to 1·687)	1·478 (1·293 to 1·684)	1·473 (1·290 to 1·662)	1·473 (1·289 to 1·677)	1·476 (1·289 to 1·663)
Tracheal, bronchus, and lung cancer	Low exposure	Both	Female	1·000 (1·000 to 1·000)	1·000 (1·000 to 1·000)	1·000 (1·000 to 1·000)	1·000 (1·000 to 1·000)	1·000 (1·000 to 1·000)	1·000 (1·000 to 1·000)	1·000 (1·000 to 1·000)	1·000 (1·000 to 1·000)
Tracheal, bronchus, and lung cancer	No exposure	Both	Female	1·000 (1·000 to 1·000)	1·000 (1·000 to 1·000)	1·000 (1·000 to 1·000)	1·000 (1·000 to 1·000)	1·000 (1·000 to 1·000)	1·000 (1·000 to 1·000)	1·000 (1·000 to 1·000)	1·000 (1·000 to 1·000)
**Occupational exposure to second-hand smoke**											
Tracheal, bronchus, and lung cancer	High exposure	Both	Male	1·241 (1·185 to 1·293)	1·239 (1·187 to 1·298)	1·240 (1·187 to 1·292)	1·241 (1·187 to 1·298)	1·241 (1·190 to 1·299)	1·239 (1·186 to 1·298)	1·241 (1·187 to 1·297)	1·240 (1·187 to 1·295)
Tracheal, bronchus, and lung cancer	Low exposure	Both	Male	1·000 (1·000 to 1·000)	1·000 (1·000 to 1·000)	1·000 (1·000 to 1·000)	1·000 (1·000 to 1·000)	1·000 (1·000 to 1·000)	1·000 (1·000 to 1·000)	1·000 (1·000 to 1·000)	1·000 (1·000 to 1·000)
Tracheal, bronchus, and lung cancer	No exposure	Both	Male	1·000 (1·000 to 1·000)	1·000 (1·000 to 1·000)	1·000 (1·000 to 1·000)	1·000 (1·000 to 1·000)	1·000 (1·000 to 1·000)	1·000 (1·000 to 1·000)	1·000 (1·000 to 1·000)	1·000 (1·000 to 1·000)
Tracheal, bronchus, and lung cancer	High exposure	Both	Female	1·240 (1·186 to 1·294)	1·239 (1·185 to 1·295)	1·239 (1·188 to 1·296)	1·241 (1·188 to 1·299)	1·239 (1·189 to 1·293)	1·240 (1·187 to 1·294)	1·241 (1·186 to 1·294)	1·240 (1·184 to 1·297)
Tracheal, bronchus, and lung cancer	Low exposure	Both	Female	1·000 (1·000 to 1·000)	1·000 (1·000 to 1·000)	1·000 (1·000 to 1·000)	1·000 (1·000 to 1·000)	1·000 (1·000 to 1·000)	1·000 (1·000 to 1·000)	1·000 (1·000 to 1·000)	1·000 (1·000 to 1·000)
Tracheal, bronchus, and lung cancer	No exposure	Both	Female	1·000 (1·000 to 1·000)	1·000 (1·000 to 1·000)	1·000 (1·000 to 1·000)	1·000 (1·000 to 1·000)	1·000 (1·000 to 1·000)	1·000 (1·000 to 1·000)	1·000 (1·000 to 1·000)	1·000 (1·000 to 1·000)
**Occupational exposure to formaldehyde**											
Nasopharynx cancer	High exposure	Both	Male	2·265 (1·055 to 4·357)	2·198 (1·036 to 4·100)	2·232 (1·101 to 4·097)	2·215 (1·021 to 4·197)	2·251 (1·056 to 4·239)	2·233 (1·022 to 4·334)	2·234 (1·017 to 4·329)	2·204 (1·061 to 4·177)
Nasopharynx cancer	Low exposure	Both	Male	1·000 (1·000 to 1·000)	1·000 (1·000 to 1·000)	1·000 (1·000 to 1·000)	1·000 (1·000 to 1·000)	1·000 (1·000 to 1·000)	1·000 (1·000 to 1·000)	1·000 (1·000 to 1·000)	1·000 (1·000 to 1·000)
Nasopharynx cancer	No exposure	Both	Male	1·000 (1·000 to 1·000)	1·000 (1·000 to 1·000)	1·000 (1·000 to 1·000)	1·000 (1·000 to 1·000)	1·000 (1·000 to 1·000)	1·000 (1·000 to 1·000)	1·000 (1·000 to 1·000)	1·000 (1·000 to 1·000)
Nasopharynx cancer	High exposure	Both	Female	2·201 (1·032 to 4·163)	2·221 (1·034 to 4·255)	2·225 (1·021 to 4·230)	2·276 (1·067 to 4·207)	2·261 (1·034 to 4·216)	2·258 (1·040 to 4·377)	2·241 (1·027 to 4·168)	2·229 (1·019 to 4·249)
Nasopharynx cancer	Low exposure	Both	Female	1·000 (1·000 to 1·000)	1·000 (1·000 to 1·000)	1·000 (1·000 to 1·000)	1·000 (1·000 to 1·000)	1·000 (1·000 to 1·000)	1·000 (1·000 to 1·000)	1·000 (1·000 to 1·000)	1·000 (1·000 to 1·000)
Nasopharynx cancer	No exposure	Both	Female	1·000 (1·000 to 1·000)	1·000 (1·000 to 1·000)	1·000 (1·000 to 1·000)	1·000 (1·000 to 1·000)	1·000 (1·000 to 1·000)	1·000 (1·000 to 1·000)	1·000 (1·000 to 1·000)	1·000 (1·000 to 1·000)
Leukaemia	High exposure	Both	Male	1·481 (1·178 to 1·840)	1·490 (1·197 to 1·835)	1·470 (1·196 to 1·842)	1·487 (1·198 to 1·847)	1·485 (1·188 to 1·807)	1·480 (1·180 to 1·836)	1·482 (1·201 to 1·840)	1·488 (1·198 to 1·831)
Leukaemia	Low exposure	Both	Male	1·000 (1·000 to 1·000)	1·000 (1·000 to 1·000)	1·000 (1·000 to 1·000)	1·000 (1·000 to 1·000)	1·000 (1·000 to 1·000)	1·000 (1·000 to 1·000)	1·000 (1·000 to 1·000)	1·000 (1·000 to 1·000)

Risk - Outcome	Category / Units	Morbidity / Mortality	Sex	All ages	0-6 Days	7-27 Days	28-364 Days	1-4 years	5-9 years	10-14 years	15-19 years	20-24 years	25-29 years	30-34 years	35-39 years	40-44 years
Leukaemia	No exposure	Both	Male								1·000 (1·000 to 1·000)	1·000 (1·000 to 1·000)	1·000 (1·000 to 1·000)	1·000 (1·000 to 1·000)	1·000 (1·000 to 1·000)	1·000 (1·000 to 1·000)
Leukaemia	High exposure	Both	Female								1·485 (1·197 to 1·846)	1·485 (1·183 to 1·864)	1·479 (1·184 to 1·846)	1·471 (1·181 to 1·777)	1·469 (1·182 to 1·819)	1·486 (1·196 to 1·825)
Leukaemia	Low exposure	Both	Female								1·000 (1·000 to 1·000)	1·000 (1·000 to 1·000)	1·000 (1·000 to 1·000)	1·000 (1·000 to 1·000)	1·000 (1·000 to 1·000)	1·000 (1·000 to 1·000)
Leukaemia	No exposure	Both	Female								1·000 (1·000 to 1·000)	1·000 (1·000 to 1·000)	1·000 (1·000 to 1·000)	1·000 (1·000 to 1·000)	1·000 (1·000 to 1·000)	1·000 (1·000 to 1·000)
**Occupational exposure to nickel**																
Tracheal, bronchus, and lung cancer	High exposure	Both	Male								2·148 (1·313 to 3·270)	2·190 (1·331 to 3·416)	2·169 (1·322 to 3·362)	2·151 (1·304 to 3·329)	2·137 (1·336 to 3·228)	2·152 (1·378 to 3·267)
Tracheal, bronchus, and lung cancer	Low exposure	Both	Male								1·000 (1·000 to 1·000)	1·000 (1·000 to 1·000)	1·000 (1·000 to 1·000)	1·000 (1·000 to 1·000)	1·000 (1·000 to 1·000)	1·000 (1·000 to 1·000)
Tracheal, bronchus, and lung cancer	No exposure	Both	Male								1·000 (1·000 to 1·000)	1·000 (1·000 to 1·000)	1·000 (1·000 to 1·000)	1·000 (1·000 to 1·000)	1·000 (1·000 to 1·000)	1·000 (1·000 to 1·000)
Tracheal, bronchus, and lung cancer	High exposure	Both	Female								2·147 (1·372 to 3·280)	2·161 (1·337 to 3·310)	2·158 (1·338 to 3·266)	2·154 (1·394 to 3·347)	2·160 (1·271 to 3·437)	2·160 (1·348 to 3·411)
Tracheal, bronchus, and lung cancer	Low exposure	Both	Female								1·000 (1·000 to 1·000)	1·000 (1·000 to 1·000)	1·000 (1·000 to 1·000)	1·000 (1·000 to 1·000)	1·000 (1·000 to 1·000)	1·000 (1·000 to 1·000)
Tracheal, bronchus, and lung cancer	No exposure	Both	Female								1·000 (1·000 to 1·000)	1·000 (1·000 to 1·000)	1·000 (1·000 to 1·000)	1·000 (1·000 to 1·000)	1·000 (1·000 to 1·000)	1·000 (1·000 to 1·000)
**Occupational exposure to polycyclic aromatic hydrocarbons**																
Tracheal, bronchus, and lung cancer	High exposure	Both	Male								1·310 (1·165 to 1·471)	1·304 (1·145 to 1·466)	1·313 (1·162 to 1·486)	1·314 (1·166 to 1·478)	1·315 (1·169 to 1·475)	1·314 (1·159 to 1·476)
Tracheal, bronchus, and lung cancer	Low exposure	Both	Male								1·000 (1·000 to 1·000)	1·000 (1·000 to 1·000)	1·000 (1·000 to 1·000)	1·000 (1·000 to 1·000)	1·000 (1·000 to 1·000)	1·000 (1·000 to 1·000)
Tracheal, bronchus, and lung cancer	No exposure	Both	Male								1·000 (1·000 to 1·000)	1·000 (1·000 to 1·000)	1·000 (1·000 to 1·000)	1·000 (1·000 to 1·000)	1·000 (1·000 to 1·000)	1·000 (1·000 to 1·000)
Tracheal, bronchus, and lung cancer	High exposure	Both	Female								1·310 (1·154 to 1·487)	1·311 (1·157 to 1·470)	1·313 (1·153 to 1·473)	1·313 (1·155 to 1·469)	1·316 (1·162 to 1·483)	1·313 (1·156 to 1·477)
Tracheal, bronchus, and lung cancer	Low exposure	Both	Female								1·000 (1·000 to 1·000)	1·000 (1·000 to 1·000)	1·000 (1·000 to 1·000)	1·000 (1·000 to 1·000)	1·000 (1·000 to 1·000)	1·000 (1·000 to 1·000)
Tracheal, bronchus, and lung cancer	No exposure	Both	Female								1·000 (1·000 to 1·000)	1·000 (1·000 to 1·000)	1·000 (1·000 to 1·000)	1·000 (1·000 to 1·000)	1·000 (1·000 to 1·000)	1·000 (1·000 to 1·000)
**Occupational exposure to silica**																
Tracheal, bronchus, and lung cancer	High exposure	Both	Male								1·323 (1·243 to 1·411)	1·319 (1·233 to 1·413)	1·321 (1·237 to 1·406)	1·322 (1·237 to 1·411)	1·319 (1·235 to 1·404)	1·319 (1·230 to 1·400)
Tracheal, bronchus, and lung cancer	Low exposure	Both	Male								1·000 (1·000 to 1·000)	1·000 (1·000 to 1·000)	1·000 (1·000 to 1·000)	1·000 (1·000 to 1·000)	1·000 (1·000 to 1·000)	1·000 (1·000 to 1·000)
Tracheal, bronchus, and lung cancer	No exposure	Both	Male								1·000 (1·000 to 1·000)	1·000 (1·000 to 1·000)	1·000 (1·000 to 1·000)	1·000 (1·000 to 1·000)	1·000 (1·000 to 1·000)	1·000 (1·000 to 1·000)
Tracheal, bronchus, and lung cancer	High exposure	Both	Female								1·321 (1·239 to 1·407)	1·316 (1·229 to 1·407)	1·321 (1·237 to 1·408)	1·320 (1·237 to 1·406)	1·320 (1·235 to 1·407)	1·321 (1·234 to 1·407)
Tracheal, bronchus, and lung cancer	Low exposure	Both	Female								1·000 (1·000 to 1·000)	1·000 (1·000 to 1·000)	1·000 (1·000 to 1·000)	1·000 (1·000 to 1·000)	1·000 (1·000 to 1·000)	1·000 (1·000 to 1·000)
Tracheal, bronchus, and lung cancer	No exposure	Both	Female								1·000 (1·000 to 1·000)	1·000 (1·000 to 1·000)	1·000 (1·000 to 1·000)	1·000 (1·000 to 1·000)	1·000 (1·000 to 1·000)	1·000 (1·000 to 1·000)
**Occupational exposure to sulfphuric acid**																
Larynx cancer	High exposure	Both	Male								4·566 (2·192 to 8·629)	4·540 (2·143 to 8·509)	4·531 (2·156 to 8·611)	4·582 (2·030 to 9·128)	4·562 (2·149 to 8·787)	4·567 (2·148 to 8·480)
Larynx cancer	Low exposure	Both	Male								2·044 (0·958 to 3·828)	2·047 (0·958 to 3·834)	2·033 (0·965 to 3·834)	2·045 (0·975 to 3·769)	2·053 (0·907 to 3·852)	2·007 (0·959 to 3·574)
Larynx cancer	No exposure	Both	Male								1·000 (1·000 to 1·000)	1·000 (1·000 to 1·000)	1·000 (1·000 to 1·000)	1·000 (1·000 to 1·000)	1·000 (1·000 to 1·000)	1·000 (1·000 to 1·000)
Larynx cancer	High exposure	Both	Female								4·619 (2·057 to 8·763)	4·539 (2·217 to 8·582)	4·623 (2·099 to 8·849)	4·510 (2·167 to 8·101)	4·608 (2·105 to 8·521)	4·630 (2·153 to 8·590)
Larynx cancer	Low exposure	Both	Female								2·030 (0·956 to 3·788)	2·052 (0·994 to 3·887)	1·990 (0·970 to 3·845)	2·023 (0·974 to 3·834)	2·061 (0·962 to 3·928)	2·007 (0·950 to 3·826)
Larynx cancer	No exposure	Both	Female								1·000 (1·000 to 1·000)	1·000 (1·000 to 1·000)	1·000 (1·000 to 1·000)	1·000 (1·000 to 1·000)	1·000 (1·000 to 1·000)	1·000 (1·000 to 1·000)
**Occupational exposure to trichloroethylene**																
Kidney cancer	High exposure	Both	Both								1·245 (1·054 to 1·458)	1·245 (1·054 to 1·458)	1·245 (1·054 to 1·458)	1·245 (1·054 to 1·458)	1·245 (1·054 to 1·458)	1·245 (1·054 to 1·458)
Kidney cancer	Low esposure	Both	Both								1·000 (1·000 to 1·000)	1·000 (1·000 to 1·000)	1·000 (1·000 to 1·000)	1·000 (1·000 to 1·000)	1·000 (1·000 to 1·000)	1·000 (1·000 to 1·000)
Kidney cancer	No exposure	Both	Both								1·000 (1·000 to 1·000)	1·000 (1·000 to 1·000)	1·000 (1·000 to 1·000)	1·000 (1·000 to 1·000)	1·000 (1·000 to 1·000)	1·000 (1·000 to 1·000)
**Occupational asthmagens**																
Asthma	Admin	Both	Male								1·000 (1·000 to 1·000)	1·000 (1·000 to 1·000)	1·000 (1·000 to 1·000)	1·000 (1·000 to 1·000)	1·000 (1·000 to 1·000)	1·000 (1·000 to 1·000)
Asthma	Technical	Both	Male								1·050 (0·977 to 1·126)	1·051 (0·979 to 1·122)	1·051 (0·981 to 1·124)	1·051 (0·983 to 1·118)	1·050 (0·977 to 1·120)	1·050 (0·981 to 1·122)
Asthma	Sales	Both	Male								1·140 (1·047 to 1·237)	1·140 (1·048 to 1·235)	1·144 (1·055 to 1·233)	1·142 (1·048 to 1·234)	1·140 (1·045 to 1·234)	1·141 (1·053 to 1·235)
Asthma	Agriculture	Both	Male								1·519 (1·097 to 2·030)	1·527 (1·119 to 2·069)	1·524 (1·102 to 2·022)	1·513 (1·122 to 2·000)	1·523 (1·108 to 2·023)	1·516 (1·081 to 2·045)
Asthma	Mining	Both	Male								1·959 (1·575 to 2·431)	1·959 (1·567 to 2·382)	1·971 (1·602 to 2·422)	1·966 (1·571 to 2·396)	1·963 (1·601 to 2·396)	1·959 (1·588 to 2·388)
Asthma	Transport	Both	Male								1·313 (1·220 to 1·402)	1·311 (1·221 to 1·406)	1·312 (1·225 to 1·399)	1·310 (1·218 to 1·398)	1·312 (1·225 to 1·404)	1·311 (1·225 to 1·401)
Asthma	Manufact	Both	Male								1·559 (1·476 to 1·647)	1·561 (1·474 to 1·652)	1·560 (1·474 to 1·657)	1·562 (1·472 to 1·658)	1·561 (1·472 to 1·650)	1·561 (1·471 to 1·656)
Asthma	Services	Both	Male								1·531 (1·415 to 1·646)	1·531 (1·414 to 1·652)	1·529 (1·409 to 1·649)	1·532 (1·413 to 1·654)	1·529 (1·423 to 1·650)	1·529 (1·415 to 1·646)
Asthma	Other	Both	Male								1·000 (1·000 to 1·000)	1·000 (1·000 to 1·000)	1·000 (1·000 to 1·000)	1·000 (1·000 to 1·000)	1·000 (1·000 to 1·000)	1·000 (1·000 to 1·000)
Asthma	Admin	Both	Female								1·000 (1·000 to 1·000)	1·000 (1·000 to 1·000)	1·000 (1·000 to 1·000)	1·000 (1·000 to 1·000)	1·000 (1·000 to 1·000)	1·000 (1·000 to 1·000)
Asthma	Technical	Both	Female								1·060 (1·028 to 1·095)	1·060 (1·024 to 1·099)	1·059 (1·025 to 1·095)	1·061 (1·025 to 1·099)	1·060 (1·027 to 1·094)	1·060 (1·026 to 1·094)
Asthma	Sales	Both	Female								1·131 (1·082 to 1·182)	1·130 (1·083 to 1·178)	1·129 (1·083 to 1·178)	1·130 (1·081 to 1·182)	1·131 (1·084 to 1·181)	1·130 (1·080 to 1·182)
Asthma	Agriculture	Both	Female								1·520 (1·124 to 2·025)	1·506 (1·099 to 1·999)	1·520 (1·115 to 2·050)	1·514 (1·108 to 2·050)	1·509 (1·098 to 1·975)	1·513 (1·127 to 2·029)
Asthma	Mining	Both	Female								1·956 (1·580 to 2·412)	1·961 (1·571 to 2·397)	1·967 (1·586 to 2·417)	1·952 (1·565 to 2·378)	1·959 (1·573 to 2·422)	1·959 (1·567 to 2·422)
Asthma	Transport	Both	Female								1·221 (1·132 to 1·312)	1·220 (1·137 to 1·312)	1·217 (1·136 to 1·303)	1·221 (1·138 to 1·314)	1·221 (1·137 to 1·317)	1·221 (1·132 to 1·314)
Asthma	Manufact	Both	Female								1·330 (1·272 to 1·393)	1·331 (1·270 to 1·391)	1·330 (1·271 to 1·390)	1·329 (1·271 to 1·388)	1·331 (1·268 to 1·392)	1·329 (1·269 to 1·391)
Asthma	Services	Both	Female								1·410 (1·352 to 1·468)	1·410 (1·356 to 1·469)	1·412 (1·357 to 1·467)	1·410 (1·356 to 1·465)	1·409 (1·353 to 1·463)	1·409 (1·354 to 1·467)
Asthma	Other	Both	Female								1·000 (1·000 to 1·000)	1·000 (1·000 to 1·000)	1·000 (1·000 to 1·000)	1·000 (1·000 to 1·000)	1·000 (1·000 to 1·000)	1·000 (1·000 to 1·000)
**Occupational particulate matter, gases, and fumes**																
Chronic obstructive pulmonary disease	High	Both	Male								2·364 (1·454 to 3·643)	2·341 (1·425 to 3·556)	2·370 (1·409 to 3·615)	2·391 (1·452 to 3·761)	2·387 (1·407 to 3·797)	2·379 (1·399 to 3·681)
Chronic obstructive pulmonary disease	Low	Both	Male								1·462 (1·057 to 1·916)	1·457 (1·051 to 1·936)	1·464 (1·084 to 1·914)	1·450 (1·066 to 1·949)	1·452 (1·058 to 1·965)	1·454 (1·075 to 1·961)

Risk - Outcome	Category / Units	Morbidity / Mortality	Sex	45-49 years	50-54 years	55-59 years	60-64 years	65-69 years	70-74 years	75-79 years	80+ years
Leukaemia	No exposure	Both	Male	1·000 (1·000 to 1·000)	1·000 (1·000 to 1·000)	1·000 (1·000 to 1·000)	1·000 (1·000 to 1·000)	1·000 (1·000 to 1·000)	1·000 (1·000 to 1·000)	1·000 (1·000 to 1·000)	1·000 (1·000 to 1·000)
Leukaemia	High exposure	Both	Female	1·485 (1·190 to 1·851)	1·481 (1·191 to 1·857)	1·480 (1·199 to 1·817)	1·470 (1·183 to 1·815)	1·473 (1·190 to 1·815)	1·471 (1·193 to 1·795)	1·490 (1·209 to 1·860)	1·464 (1·179 to 1·789)
Leukaemia	Low exposure	Both	Female	1·000 (1·000 to 1·000)	1·000 (1·000 to 1·000)	1·000 (1·000 to 1·000)	1·000 (1·000 to 1·000)	1·000 (1·000 to 1·000)	1·000 (1·000 to 1·000)	1·000 (1·000 to 1·000)	1·000 (1·000 to 1·000)
Leukaemia	No exposure	Both	Female	1·000 (1·000 to 1·000)	1·000 (1·000 to 1·000)	1·000 (1·000 to 1·000)	1·000 (1·000 to 1·000)	1·000 (1·000 to 1·000)	1·000 (1·000 to 1·000)	1·000 (1·000 to 1·000)	1·000 (1·000 to 1·000)
**Occupational exposure to nickel**											
Tracheal, bronchus, and lung cancer	High exposure	Both	Male	2·175 (1·293 to 3·302)	2·168 (1·350 to 3·355)	2·162 (1·346 to 3·318)	2·142 (1·266 to 3·314)	2·159 (1·353 to 3·226)	2·165 (1·331 to 3·345)	2·153 (1·352 to 3·197)	2·134 (1·328 to 3·329)
Tracheal, bronchus, and lung cancer	Low exposure	Both	Male	1·000 (1·000 to 1·000)	1·000 (1·000 to 1·000)	1·000 (1·000 to 1·000)	1·000 (1·000 to 1·000)	1·000 (1·000 to 1·000)	1·000 (1·000 to 1·000)	1·000 (1·000 to 1·000)	1·000 (1·000 to 1·000)
Tracheal, bronchus, and lung cancer	No exposure	Both	Male	1·000 (1·000 to 1·000)	1·000 (1·000 to 1·000)	1·000 (1·000 to 1·000)	1·000 (1·000 to 1·000)	1·000 (1·000 to 1·000)	1·000 (1·000 to 1·000)	1·000 (1·000 to 1·000)	1·000 (1·000 to 1·000)
Tracheal, bronchus, and lung cancer	High exposure	Both	Female	2·127 (1·327 to 3·223)	2·173 (1·385 to 3·279)	2·175 (1·349 to 3·325)	2·153 (1·345 to 3·328)	2·188 (1·373 to 3·305)	2·166 (1·363 to 3·280)	2·142 (1·353 to 3·123)	2·153 (1·348 to 3·270)
Tracheal, bronchus, and lung cancer	Low exposure	Both	Female	1·000 (1·000 to 1·000)	1·000 (1·000 to 1·000)	1·000 (1·000 to 1·000)	1·000 (1·000 to 1·000)	1·000 (1·000 to 1·000)	1·000 (1·000 to 1·000)	1·000 (1·000 to 1·000)	1·000 (1·000 to 1·000)
Tracheal, bronchus, and lung cancer	No exposure	Both	Female	1·000 (1·000 to 1·000)	1·000 (1·000 to 1·000)	1·000 (1·000 to 1·000)	1·000 (1·000 to 1·000)	1·000 (1·000 to 1·000)	1·000 (1·000 to 1·000)	1·000 (1·000 to 1·000)	1·000 (1·000 to 1·000)
**Occupational exposure to polycyclic aromatic hydrocarbons**											
Tracheal, bronchus, and lung cancer	High exposure	Both	Male	1·315 (1·153 to 1·479)	1·312 (1·159 to 1·478)	1·318 (1·165 to 1·484)	1·311 (1·158 to 1·479)	1·313 (1·166 to 1·486)	1·314 (1·154 to 1·484)	1·313 (1·160 to 1·479)	1·309 (1·176 to 1·478)
Tracheal, bronchus, and lung cancer	Low exposure	Both	Male	1·000 (1·000 to 1·000)	1·000 (1·000 to 1·000)	1·000 (1·000 to 1·000)	1·000 (1·000 to 1·000)	1·000 (1·000 to 1·000)	1·000 (1·000 to 1·000)	1·000 (1·000 to 1·000)	1·000 (1·000 to 1·000)
Tracheal, bronchus, and lung cancer	No exposure	Both	Male	1·000 (1·000 to 1·000)	1·000 (1·000 to 1·000)	1·000 (1·000 to 1·000)	1·000 (1·000 to 1·000)	1·000 (1·000 to 1·000)	1·000 (1·000 to 1·000)	1·000 (1·000 to 1·000)	1·000 (1·000 to 1·000)
Tracheal, bronchus, and lung cancer	High exposure	Both	Female	1·314 (1·154 to 1·485)	1·314 (1·170 to 1·484)	1·311 (1·146 to 1·479)	1·312 (1·163 to 1·482)	1·309 (1·147 to 1·492)	1·316 (1·168 to 1·475)	1·315 (1·150 to 1·480)	1·315 (1·166 to 1·482)
Tracheal, bronchus, and lung cancer	Low exposure	Both	Female	1·000 (1·000 to 1·000)	1·000 (1·000 to 1·000)	1·000 (1·000 to 1·000)	1·000 (1·000 to 1·000)	1·000 (1·000 to 1·000)	1·000 (1·000 to 1·000)	1·000 (1·000 to 1·000)	1·000 (1·000 to 1·000)
Tracheal, bronchus, and lung cancer	No exposure	Both	Female	1·000 (1·000 to 1·000)	1·000 (1·000 to 1·000)	1·000 (1·000 to 1·000)	1·000 (1·000 to 1·000)	1·000 (1·000 to 1·000)	1·000 (1·000 to 1·000)	1·000 (1·000 to 1·000)	1·000 (1·000 to 1·000)
**Occupational exposure to silica**											
Tracheal, bronchus, and lung cancer	High exposure	Both	Male	1·321 (1·242 to 1·410)	1·318 (1·233 to 1·405)	1·321 (1·237 to 1·412)	1·320 (1·238 to 1·410)	1·323 (1·241 to 1·410)	1·323 (1·243 to 1·408)	1·322 (1·240 to 1·413)	1·320 (1·233 to 1·402)
Tracheal, bronchus, and lung cancer	Low exposure	Both	Male	1·000 (1·000 to 1·000)	1·000 (1·000 to 1·000)	1·000 (1·000 to 1·000)	1·000 (1·000 to 1·000)	1·000 (1·000 to 1·000)	1·000 (1·000 to 1·000)	1·000 (1·000 to 1·000)	1·000 (1·000 to 1·000)
Tracheal, bronchus, and lung cancer	No exposure	Both	Male	1·000 (1·000 to 1·000)	1·000 (1·000 to 1·000)	1·000 (1·000 to 1·000)	1·000 (1·000 to 1·000)	1·000 (1·000 to 1·000)	1·000 (1·000 to 1·000)	1·000 (1·000 to 1·000)	1·000 (1·000 to 1·000)
Tracheal, bronchus, and lung cancer	High exposure	Both	Female	1·321 (1·237 to 1·405)	1·320 (1·231 to 1·409)	1·321 (1·241 to 1·403)	1·318 (1·232 to 1·404)	1·317 (1·234 to 1·405)	1·319 (1·238 to 1·406)	1·321 (1·236 to 1·406)	1·320 (1·238 to 1·409)
Tracheal, bronchus, and lung cancer	Low exposure	Both	Female	1·000 (1·000 to 1·000)	1·000 (1·000 to 1·000)	1·000 (1·000 to 1·000)	1·000 (1·000 to 1·000)	1·000 (1·000 to 1·000)	1·000 (1·000 to 1·000)	1·000 (1·000 to 1·000)	1·000 (1·000 to 1·000)
Tracheal, bronchus, and lung cancer	No exposure	Both	Female	1·000 (1·000 to 1·000)	1·000 (1·000 to 1·000)	1·000 (1·000 to 1·000)	1·000 (1·000 to 1·000)	1·000 (1·000 to 1·000)	1·000 (1·000 to 1·000)	1·000 (1·000 to 1·000)	1·000 (1·000 to 1·000)
**Occupational exposure to sulfphuric acid**											
Larynx cancer	High exposure	Both	Male	4·496 (2·098 to 8·418)	4·512 (2·183 to 8·441)	4·572 (2·199 to 8·827)	4·589 (2·114 to 9·193)	4·584 (2·029 to 8·556)	4·586 (1·965 to 8·424)	4·596 (2·061 to 8·303)	4·582 (2·134 to 8·669)
Larynx cancer	Low exposure	Both	Male	2·003 (0·949 to 3·619)	2·000 (0·970 to 3·799)	2·037 (0·998 to 3·698)	2·030 (0·968 to 3·844)	2·009 (1·017 to 3·774)	2·028 (0·959 to 3·916)	2·011 (0·965 to 3·754)	2·062 (1·005 to 3·853)
Larynx cancer	No exposure	Both	Male	1·000 (1·000 to 1·000)	1·000 (1·000 to 1·000)	1·000 (1·000 to 1·000)	1·000 (1·000 to 1·000)	1·000 (1·000 to 1·000)	1·000 (1·000 to 1·000)	1·000 (1·000 to 1·000)	1·000 (1·000 to 1·000)
Larynx cancer	High exposure	Both	Female	4·567 (2·141 to 8·593)	4·530 (2·178 to 8·637)	4·573 (2·094 to 9·248)	4·441 (2·095 to 8·542)	4·538 (2·106 to 8·372)	4·518 (2·234 to 8·377)	4·552 (2·082 to 8·545)	4·582 (2·170 to 8·433)
Larynx cancer	Low exposure	Both	Female	2·030 (0·952 to 3·897)	2·028 (0·987 to 3·725)	2·035 (0·940 to 3·768)	2·017 (0·939 to 3·698)	2·015 (0·953 to 3·752)	2·043 (0·965 to 3·838)	2·016 (0·955 to 4·004)	2·016 (0·937 to 3·961)
Larynx cancer	No exposure	Both	Female	1·000 (1·000 to 1·000)	1·000 (1·000 to 1·000)	1·000 (1·000 to 1·000)	1·000 (1·000 to 1·000)	1·000 (1·000 to 1·000)	1·000 (1·000 to 1·000)	1·000 (1·000 to 1·000)	1·000 (1·000 to 1·000)
**Occupational exposure to trichloroethylene**											
Kidney cancer	High exposure	Both	Both	1·245 (1·054 to 1·458)	1·245 (1·054 to 1·458)	1·245 (1·054 to 1·458)	1·245 (1·054 to 1·458)	1·245 (1·054 to 1·458)	1·245 (1·054 to 1·458)	1·245 (1·054 to 1·458)	1·245 (1·054 to 1·458)
Kidney cancer	Low eposure	Both	Both	1·000 (1·000 to 1·000)	1·000 (1·000 to 1·000)	1·000 (1·000 to 1·000)	1·000 (1·000 to 1·000)	1·000 (1·000 to 1·000)	1·000 (1·000 to 1·000)	1·000 (1·000 to 1·000)	1·000 (1·000 to 1·000)
Kidney cancer	No exposure	Both	Both	1·000 (1·000 to 1·000)	1·000 (1·000 to 1·000)	1·000 (1·000 to 1·000)	1·000 (1·000 to 1·000)	1·000 (1·000 to 1·000)	1·000 (1·000 to 1·000)	1·000 (1·000 to 1·000)	1·000 (1·000 to 1·000)
**Occupational asthmagens**											
Asthma	Admin	Both	Male	1·000 (1·000 to 1·000)	1·000 (1·000 to 1·000)	1·000 (1·000 to 1·000)	1·000 (1·000 to 1·000)	1·000 (1·000 to 1·000)	1·000 (1·000 to 1·000)	1·000 (1·000 to 1·000)	
Asthma	Technical	Both	Male	1·051 (0·980 to 1·121)	1·051 (0·986 to 1·122)	1·050 (0·976 to 1·126)	1·050 (0·987 to 1·118)	1·051 (0·984 to 1·121)	1·051 (0·981 to 1·122)	1·051 (0·981 to 1·122)	
Asthma	Sales	Both	Male	1·143 (1·052 to 1·238)	1·140 (1·046 to 1·236)	1·141 (1·056 to 1·235)	1·138 (1·046 to 1·236)	1·139 (1·056 to 1·226)	1·141 (1·052 to 1·243)	1·140 (1·058 to 1·240)	
Asthma	Agriculture	Both	Male	1·522 (1·105 to 2·038)	1·520 (1·120 to 2·032)	1·519 (1·102 to 2·020)	1·531 (1·122 to 2·055)	1·520 (1·105 to 2·042)	1·508 (1·080 to 2·022)	1·498 (1·079 to 1·965)	
Asthma	Mining	Both	Male	1·956 (1·594 to 2·414)	1·964 (1·569 to 2·398)	1·969 (1·615 to 2·417)	1·954 (1·567 to 2·408)	1·965 (1·556 to 2·403)	1·955 (1·572 to 2·393)	1·953 (1·550 to 2·420)	
Asthma	Transport	Both	Male	1·313 (1·226 to 1·402)	1·310 (1·222 to 1·398)	1·314 (1·221 to 1·397)	1·313 (1·224 to 1·408)	1·311 (1·227 to 1·398)	1·312 (1·225 to 1·409)	1·311 (1·226 to 1·407)	
Asthma	Manufact	Both	Male	1·560 (1·471 to 1·650)	1·562 (1·473 to 1·655)	1·558 (1·460 to 1·656)	1·562 (1·479 to 1·655)	1·559 (1·475 to 1·653)	1·562 (1·474 to 1·657)	1·561 (1·472 to 1·654)	
Asthma	Services	Both	Male	1·533 (1·418 to 1·647)	1·535 (1·426 to 1·649)	1·531 (1·414 to 1·656)	1·528 (1·411 to 1·652)	1·530 (1·416 to 1·653)	1·530 (1·411 to 1·659)	1·531 (1·413 to 1·655)	
Asthma	Other	Both	Male	1·000 (1·000 to 1·000)	1·000 (1·000 to 1·000)	1·000 (1·000 to 1·000)	1·000 (1·000 to 1·000)	1·000 (1·000 to 1·000)	1·000 (1·000 to 1·000)	1·000 (1·000 to 1·000)	
Asthma	Admin	Both	Female	1·000 (1·000 to 1·000)	1·000 (1·000 to 1·000)	1·000 (1·000 to 1·000)	1·000 (1·000 to 1·000)	1·000 (1·000 to 1·000)	1·000 (1·000 to 1·000)	1·000 (1·000 to 1·000)	
Asthma	Technical	Both	Female	1·059 (1·024 to 1·093)	1·060 (1·028 to 1·094)	1·060 (1·027 to 1·092)	1·061 (1·027 to 1·095)	1·060 (1·027 to 1·097)	1·061 (1·023 to 1·096)	1·060 (1·027 to 1·096)	
Asthma	Sales	Both	Female	1·130 (1·083 to 1·181)	1·130 (1·079 to 1·179)	1·131 (1·077 to 1·184)	1·130 (1·081 to 1·182)	1·129 (1·082 to 1·181)	1·131 (1·082 to 1·184)	1·131 (1·083 to 1·183)	
Asthma	Agriculture	Both	Female	1·508 (1·106 to 2·002)	1·526 (1·101 to 2·076)	1·519 (1·114 to 1·984)	1·530 (1·105 to 2·024)	1·519 (1·105 to 2·025)	1·518 (1·115 to 2·019)	1·502 (1·094 to 2·030)	
Asthma	Mining	Both	Female	1·962 (1·585 to 2·387)	1·955 (1·589 to 2·395)	1·948 (1·570 to 2·381)	1·964 (1·596 to 2·409)	1·965 (1·589 to 2·419)	1·959 (1·586 to 2·391)	1·973 (1·583 to 2·395)	
Asthma	Transport	Both	Female	1·220 (1·132 to 1·312)	1·220 (1·134 to 1·313)	1·221 (1·139 to 1·315)	1·223 (1·138 to 1·309)	1·221 (1·133 to 1·312)	1·224 (1·134 to 1·316)	1·220 (1·133 to 1·313)	
Asthma	Manufact	Both	Female	1·330 (1·269 to 1·391)	1·332 (1·272 to 1·394)	1·331 (1·273 to 1·389)	1·331 (1·272 to 1·392)	1·330 (1·268 to 1·393)	1·330 (1·268 to 1·394)	1·331 (1·272 to 1·390)	
Asthma	Services	Both	Female	1·410 (1·354 to 1·467)	1·411 (1·357 to 1·464)	1·409 (1·357 to 1·460)	1·411 (1·355 to 1·466)	1·410 (1·353 to 1·465)	1·410 (1·357 to 1·467)	1·411 (1·357 to 1·465)	
Asthma	Other	Both	Female	1·000 (1·000 to 1·000)	1·000 (1·000 to 1·000)	1·000 (1·000 to 1·000)	1·000 (1·000 to 1·000)	1·000 (1·000 to 1·000)	1·000 (1·000 to 1·000)	1·000 (1·000 to 1·000)	
**Occupational particulate matter, gases, and fumes**											
Chronic obstructive pulmonary disease	High	Both	Male	2·409 (1·436 to 3·838)	2·361 (1·446 to 3·689)	2·367 (1·452 to 3·666)	2·373 (1·367 to 3·649)	2·357 (1·433 to 3·691)	2·402 (1·430 to 3·769)	2·398 (1·439 to 3·814)	2·359 (1·422 to 3·698)
Chronic obstructive pulmonary disease	Low	Both	Male	1·446 (1·055 to 1·932)	1·457 (1·075 to 1·934)	1·453 (1·051 to 1·910)	1·460 (1·067 to 1·954)	1·443 (1·056 to 1·917)	1·462 (1·096 to 1·956)	1·464 (1·077 to 1·952)	1·462 (1·096 to 1·967)

Risk - Outcome	Category / Units	Morbidity / Mortality	Sex	All ages	0-6 Days	7-27 Days	28-364 Days	1-4 years	5-9 years	10-14 years	15-19 years	20-24 years	25-29 years	30-34 years	35-39 years	40-44 years
Chronic obstructive pulmonary disease	None	Both	Male								1·000 (1·000 to 1·000)	1·000 (1·000 to 1·000)	1·000 (1·000 to 1·000)	1·000 (1·000 to 1·000)	1·000 (1·000 to 1·000)	1·000 (1·000 to 1·000)
Chronic obstructive pulmonary disease	High	Both	Female								2·371 (1·456 to 3·727)	2·395 (1·470 to 3·694)	2·364 (1·466 to 3·706)	2·377 (1·430 to 3·781)	2·375 (1·439 to 3·699)	2·326 (1·436 to 3·577)
Chronic obstructive pulmonary disease	Low	Both	Female								1·446 (1·058 to 1·911)	1·459 (1·089 to 1·962)	1·453 (1·054 to 2·003)	1·459 (1·071 to 1·932)	1·456 (1·054 to 1·969)	1·470 (1·089 to 1·965)
Chronic obstructive pulmonary disease	None	Both	Female								1·000 (1·000 to 1·000)	1·000 (1·000 to 1·000)	1·000 (1·000 to 1·000)	1·000 (1·000 to 1·000)	1·000 (1·000 to 1·000)	1·000 (1·000 to 1·000)
**Occupational noise**																
Rheumatoid arthritis	High exposure, >90dB	Morbidity	Both								8·249 (4·719 to 13·212)	8·292 (4·728 to 13·169)	6·707 (4·723 to 9·362)	6·687 (4·735 to 9·187)	6·075 (4·296 to 8·400)	5·983 (4·239 to 8·224)
Rheumatoid arthritis	Low exposure, 85-90dB	Morbidity	Both								3·023 (1·794 to 4·974)	2·972 (1·757 to 4·886)	3·444 (2·425 to 4·757)	3·482 (2·460 to 4·765)	3·837 (2·719 to 5·267)	3·867 (2·708 to 5·359)
Rheumatoid arthritis	No exposure	Morbidity	Both								1·000 (1·000 to 1·000)	1·000 (1·000 to 1·000)	1·000 (1·000 to 1·000)	1·000 (1·000 to 1·000)	1·000 (1·000 to 1·000)	1·000 (1·000 to 1·000)
Osteoarthritis	High exposure, >90dB	Morbidity	Both								8·249 (4·719 to 13·212)	8·292 (4·728 to 13·169)	6·707 (4·723 to 9·362)	6·687 (4·735 to 9·187)	6·075 (4·296 to 8·400)	5·983 (4·239 to 8·224)
Osteoarthritis	Low exposure, 85-90dB	Morbidity	Both								3·023 (1·794 to 4·974)	2·972 (1·757 to 4·886)	3·444 (2·425 to 4·757)	3·482 (2·460 to 4·765)	3·837 (2·719 to 5·267)	3·867 (2·708 to 5·359)
Osteoarthritis	No exposure	Morbidity	Both								1·000 (1·000 to 1·000)	1·000 (1·000 to 1·000)	1·000 (1·000 to 1·000)	1·000 (1·000 to 1·000)	1·000 (1·000 to 1·000)	1·000 (1·000 to 1·000)
Low back pain	High exposure, >90dB	Morbidity	Both								8·330 (4·769 to 13·642)	8·437 (4·937 to 13·546)	6·690 (4·737 to 9·276)	6·734 (4·792 to 9·240)	5·974 (4·166 to 8·162)	5·981 (4·271 to 8·414)
Low back pain	Low exposure, 85-90dB	Morbidity	Both								3·023 (1·718 to 5·041)	3·018 (1·734 to 4·918)	3·455 (2·437 to 4·683)	3·446 (2·448 to 4·732)	3·875 (2·753 to 5·336)	3·848 (2·721 to 5·146)
Low back pain	No exposure	Morbidity	Both								1·000 (1·000 to 1·000)	1·000 (1·000 to 1·000)	1·000 (1·000 to 1·000)	1·000 (1·000 to 1·000)	1·000 (1·000 to 1·000)	1·000 (1·000 to 1·000)
Neck pain	High exposure, >90dB	Morbidity	Both								8·242 (4·849 to 13·312)	8·371 (4·697 to 13·338)	6·771 (4·776 to 9·412)	6·703 (4·711 to 9·423)	5·932 (4·203 to 8·019)	5·941 (4·152 to 8·223)
Neck pain	Low exposure, 85-90dB	Morbidity	Both								3·004 (1·775 to 4·916)	2·986 (1·801 to 4·892)	3·482 (2·472 to 4·718)	3·478 (2·437 to 4·724)	3·858 (2·702 to 5·288)	3·813 (2·739 to 5·347)
Neck pain	No exposure	Morbidity	Both								1·000 (1·000 to 1·000)	1·000 (1·000 to 1·000)	1·000 (1·000 to 1·000)	1·000 (1·000 to 1·000)	1·000 (1·000 to 1·000)	1·000 (1·000 to 1·000)
Gout	High exposure, >90dB	Morbidity	Both								8·242 (4·849 to 13·312)	8·371 (4·697 to 13·338)	6·771 (4·776 to 9·412)	6·703 (4·711 to 9·423)	5·932 (4·203 to 8·019)	5·941 (4·152 to 8·223)
Gout	Low exposure, 85-90dB	Morbidity	Both								3·004 (1·775 to 4·916)	2·986 (1·801 to 4·892)	3·482 (2·472 to 4·718)	3·478 (2·437 to 4·724)	3·858 (2·702 to 5·288)	3·813 (2·739 to 5·347)
Gout	No exposure	Morbidity	Both								1·000 (1·000 to 1·000)	1·000 (1·000 to 1·000)	1·000 (1·000 to 1·000)	1·000 (1·000 to 1·000)	1·000 (1·000 to 1·000)	1·000 (1·000 to 1·000)
**Occupational ergonomic factors**																
Low back pain	Professional, technical and related workers	Morbidity	Both								1·173 (1·065 to 1·282)	1·172 (1·061 to 1·285)	1·169 (1·065 to 1·283)	1·170 (1·062 to 1·285)	1·170 (1·061 to 1·287)	1·172 (1·062 to 1·283)
Low back pain	Administrative and managerial workers	Morbidity	Both								1·211 (0·963 to 1·508)	1·210 (0·964 to 1·497)	1·209 (0·963 to 1·490)	1·209 (0·963 to 1·527)	1·207 (0·975 to 1·498)	1·207 (0·964 to 1·500)
Low back pain	Clerical and related workers	Morbidity	Both								1·000 (1·000 to 1·000)	1·000 (1·000 to 1·000)	1·000 (1·000 to 1·000)	1·000 (1·000 to 1·000)	1·000 (1·000 to 1·000)	1·000 (1·000 to 1·000)
Low back pain	Sales workers	Morbidity	Both								1·220 (1·028 to 1·435)	1·210 (1·016 to 1·418)	1·213 (1·027 to 1·435)	1·214 (1·004 to 1·451)	1·207 (1·017 to 1·446)	1·218 (1·015 to 1·457)
Low back pain	Service workers	Morbidity	Both								1·472 (1·385 to 1·568)	1·472 (1·383 to 1·569)	1·471 (1·371 to 1·564)	1·472 (1·382 to 1·571)	1·469 (1·377 to 1·568)	1·472 (1·377 to 1·571)
Low back pain	Agriculture, animal husbandry and forestry workers, fishermen and hunters	Morbidity	Both								3·789 (2·575 to 5·379)	3·762 (2·620 to 5·285)	3·869 (2·631 to 5·492)	3·775 (2·560 to 5·374)	3·774 (2·604 to 5·318)	3·771 (2·530 to 5·319)
Low back pain	Production and related workers, transport equipment operators and labourers	Morbidity	Both								1·543 (1·408 to 1·679)	1·540 (1·403 to 1·676)	1·542 (1·414 to 1·677)	1·543 (1·412 to 1·695)	1·542 (1·415 to 1·685)	1·543 (1·418 to 1·685)
Low back pain	Background	Morbidity	Both								1·000 (1·000 to 1·000)	1·000 (1·000 to 1·000)	1·000 (1·000 to 1·000)	1·000 (1·000 to 1·000)	1·000 (1·000 to 1·000)	1·000 (1·000 to 1·000)
**Non-exclusive breastfeeding**																
Diarrhoeal diseases	None	Morbidity	Both			2·737 (1·717 to 4·014)	2·684 (1·698 to 4·059)									
Diarrhoeal diseases	Partial	Morbidity	Both			1·732 (1·037 to 2·723)	1·746 (0·995 to 2·812)									
Diarrhoeal diseases	Predominant	Morbidity	Both			1·274 (0·806 to 1·909)	1·311 (0·806 to 2·024)									
Diarrhoeal diseases	Exclusive	Morbidity	Both			1·000 (1·000 to 1·000)	1·000 (1·000 to 1·000)									
Diarrhoeal diseases	None	Mortality	Both			13·497 (2·999 to 40·902)	13·154 (2·899 to 38·934)									
Diarrhoeal diseases	Partial	Mortality	Both			5·120 (1·841 to 11·303)	5·098 (1·751 to 11·169)									
Diarrhoeal diseases	Predominant	Mortality	Both			2·645 (0·869 to 6·136)	2·586 (0·850 to 6·036)									
Diarrhoeal diseases	Exclusive	Mortality	Both			1·000 (1·000 to 1·000)	1·000 (1·000 to 1·000)									
Lower respiratory infections	None	Morbidity	Both			4·347 (0·189 to 22·450)	4·732 (0·186 to 21·417)									
Lower respiratory infections	Partial	Morbidity	Both			4·868 (0·250 to 26·546)	5·534 (0·281 to 25·017)									
Lower respiratory infections	Predominant	Morbidity	Both			1·793 (1·276 to 2·497)	1·814 (1·298 to 2·543)									
Lower respiratory infections	Exclusive	Morbidity	Both			1·000 (1·000 to 1·000)	1·000 (1·000 to 1·000)									
Lower respiratory infections	None	Mortality	Both			41·580 (0·671 to 259·590)	50·178 (0·566 to 326·944)									
Lower respiratory infections	Partial	Mortality	Both			2·793 (1·013 to 6·601)	2·757 (1·006 to 6·172)									
Lower respiratory infections	Predominant	Mortality	Both			1·941 (0·516 to 4·983)	1·944 (0·559 to 4·769)									
Lower respiratory infections	Exclusive	Mortality	Both			1·000 (1·000 to 1·000)	1·000 (1·000 to 1·000)									
**Discontinued breastfeeding**																
Diarrhoeal diseases	Not continued	Both	Both				2·313 (1·108 to 4·143)	2·308 (1·138 to 4·442)								
Diarrhoeal diseases	Continued	Both	Both				1·000 (1·000 to 1·000)	1·000 (1·000 to 1·000)								
**Childhood underweight**																
Diarrhoeal diseases	<-3 sd	Both	Both		2·332 (2·075 to 2·806)	2·332 (2·075 to 2·806)	2·332 (2·075 to 2·806)	2·332 (2·075 to 2·806)								
Diarrhoeal diseases	-3 to -2 sd	Both	Both		1·230 (1·162 to 1·314)	1·230 (1·162 to 1·314)	1·230 (1·162 to 1·314)	1·230 (1·162 to 1·314)								
Diarrhoeal diseases	-2 to -1 sd	Both	Both		1·088 (1·046 to 1·134)	1·088 (1·046 to 1·134)	1·088 (1·046 to 1·134)	1·088 (1·046 to 1·134)								
Diarrhoeal diseases	-1 sd and above	Both	Both		1·000 (1·000 to 1·000)	1·000 (1·000 to 1·000)	1·000 (1·000 to 1·000)	1·000 (1·000 to 1·000)								
Lower respiratory infections	<-3 sd	Both	Both		2·593 (1·907 to 4·400)	2·593 (1·907 to 4·400)	2·593 (1·907 to 4·400)	2·593 (1·907 to 4·400)								
Lower respiratory infections	-3 to -2 sd	Both	Both		1·365 (1·215 to 1·756)	1·365 (1·215 to 1·756)	1·365 (1·215 to 1·756)	1·365 (1·215 to 1·756)								
Lower respiratory infections	-2 to -1 sd	Both	Both		1·145 (1·044 to 1·365)	1·145 (1·044 to 1·365)	1·145 (1·044 to 1·365)	1·145 (1·044 to 1·365)								
Lower respiratory infections	-1 sd and above	Both	Both		1·000 (1·000 to 1·000)	1·000 (1·000 to 1·000)	1·000 (1·000 to 1·000)	1·000 (1·000 to 1·000)								

Risk - Outcome	Category / Units	Morbidity / Mortality	Sex	45-49 years	50-54 years	55-59 years	60-64 years	65-69 years	70-74 years	75-79 years	80+ years
Chronic obstructive pulmonary disease	None	Both	Male	1·000 (1·000 to 1·000)	1·000 (1·000 to 1·000)	1·000 (1·000 to 1·000)	1·000 (1·000 to 1·000)	1·000 (1·000 to 1·000)	1·000 (1·000 to 1·000)	1·000 (1·000 to 1·000)	1·000 (1·000 to 1·000)
Chronic obstructive pulmonary disease	High	Both	Female	2·350 (1·431 to 3·704)	2·350 (1·421 to 3·614)	2·364 (1·451 to 3·678)	2·364 (1·474 to 3·652)	2·395 (1·467 to 3·719)	2·336 (1·426 to 3·685)	2·363 (1·431 to 3·743)	2·404 (1·454 to 3·632)
Chronic obstructive pulmonary disease	Low	Both	Female	1·440 (1·045 to 1·933)	1·457 (1·097 to 1·926)	1·455 (1·060 to 1·921)	1·451 (1·082 to 1·912)	1·459 (1·102 to 1·951)	1·448 (1·077 to 1·931)	1·467 (1·072 to 1·974)	1·456 (1·056 to 1·928)
Chronic obstructive pulmonary disease	None	Both	Female	1·000 (1·000 to 1·000)	1·000 (1·000 to 1·000)	1·000 (1·000 to 1·000)	1·000 (1·000 to 1·000)	1·000 (1·000 to 1·000)	1·000 (1·000 to 1·000)	1·000 (1·000 to 1·000)	1·000 (1·000 to 1·000)
**Occupational noise**											
Rheumatoid arthritis	High exposure, >90dB	Morbidity	Both	5·660 (4·032 to 7·902)	5·610 (3·944 to 7·536)	3·620 (2·540 to 5·017)	3·583 (2·509 to 5·122)	2·138 (1·604 to 2·804)	2·173 (1·644 to 2·819)	1·294 (1·058 to 1·571)	
Rheumatoid arthritis	Low exposure, 85-90dB	Morbidity	Both	3·980 (2·741 to 5·532)	3·943 (2·746 to 5·559)	2·711 (1·904 to 3·809)	2·693 (1·925 to 3·739)	1·830 (1·384 to 2·422)	1·825 (1·346 to 2·414)	1·218 (0·998 to 1·475)	
Rheumatoid arthritis	No exposure	Morbidity	Both	1·000 (1·000 to 1·000)	1·000 (1·000 to 1·000)	1·000 (1·000 to 1·000)	1·000 (1·000 to 1·000)	1·000 (1·000 to 1·000)	1·000 (1·000 to 1·000)	1·000 (1·000 to 1·000)	
Osteoarthritis	High exposure, >90dB	Morbidity	Both	5·660 (4·032 to 7·902)	5·610 (3·944 to 7·536)	3·620 (2·540 to 5·017)	3·583 (2·509 to 5·122)	2·138 (1·604 to 2·804)	2·173 (1·644 to 2·819)	1·294 (1·058 to 1·571)	
Osteoarthritis	Low exposure, 85-90dB	Morbidity	Both	3·980 (2·741 to 5·532)	3·943 (2·746 to 5·559)	2·711 (1·904 to 3·809)	2·693 (1·925 to 3·739)	1·830 (1·384 to 2·422)	1·825 (1·346 to 2·414)	1·218 (0·998 to 1·475)	
Osteoarthritis	No exposure	Morbidity	Both	1·000 (1·000 to 1·000)	1·000 (1·000 to 1·000)	1·000 (1·000 to 1·000)	1·000 (1·000 to 1·000)	1·000 (1·000 to 1·000)	1·000 (1·000 to 1·000)	1·000 (1·000 to 1·000)	
Low back pain	High exposure, >90dB	Morbidity	Both	5·629 (3·964 to 7·886)	5·589 (3·837 to 7·704)	3·591 (2·488 to 5·123)	3·607 (2·476 to 5·058)	2·144 (1·616 to 2·831)	2·173 (1·637 to 2·827)	1·294 (1·080 to 1·563)	
Low back pain	Low exposure, 85-90dB	Morbidity	Both	3·943 (2·697 to 5·394)	3·944 (2·766 to 5·399)	2·697 (1·933 to 3·791)	2·690 (1·880 to 3·733)	1·833 (1·372 to 2·410)	1·813 (1·339 to 2·387)	1·222 (0·997 to 1·480)	
Low back pain	No exposure	Morbidity	Both	1·000 (1·000 to 1·000)	1·000 (1·000 to 1·000)	1·000 (1·000 to 1·000)	1·000 (1·000 to 1·000)	1·000 (1·000 to 1·000)	1·000 (1·000 to 1·000)	1·000 (1·000 to 1·000)	
Neck pain	High exposure, >90dB	Morbidity	Both	5·620 (3·955 to 7·928)	5·628 (3·992 to 7·790)	3·625 (2·498 to 5·133)	3·625 (2·558 to 5·047)	2·170 (1·613 to 2·805)	2·170 (1·596 to 2·890)	1·291 (1·059 to 1·548)	
Neck pain	Low exposure, 85-90dB	Morbidity	Both	3·917 (2·804 to 5·385)	3·985 (2·824 to 5·487)	2·701 (1·872 to 3·674)	2·695 (1·885 to 3·734)	1·812 (1·374 to 2·372)	1·824 (1·355 to 2·407)	1·222 (1·015 to 1·477)	
Neck pain	No exposure	Morbidity	Both	1·000 (1·000 to 1·000)	1·000 (1·000 to 1·000)	1·000 (1·000 to 1·000)	1·000 (1·000 to 1·000)	1·000 (1·000 to 1·000)	1·000 (1·000 to 1·000)	1·000 (1·000 to 1·000)	
Gout	High exposure, >90dB	Morbidity	Both	5·620 (3·955 to 7·928)	5·628 (3·992 to 7·790)	3·625 (2·498 to 5·133)	3·625 (2·558 to 5·047)	2·170 (1·613 to 2·805)	2·170 (1·596 to 2·890)	1·291 (1·059 to 1·548)	
Gout	Low exposure, 85-90dB	Morbidity	Both	3·917 (2·804 to 5·385)	3·985 (2·824 to 5·487)	2·701 (1·872 to 3·674)	2·695 (1·885 to 3·734)	1·812 (1·374 to 2·372)	1·824 (1·355 to 2·407)	1·222 (1·015 to 1·477)	
Gout	No exposure	Morbidity	Both	1·000 (1·000 to 1·000)	1·000 (1·000 to 1·000)	1·000 (1·000 to 1·000)	1·000 (1·000 to 1·000)	1·000 (1·000 to 1·000)	1·000 (1·000 to 1·000)	1·000 (1·000 to 1·000)	
**Occupational ergonomic factors**											
Low back pain	Professional, technical and related workers	Morbidity	Both	1·171 (1·071 to 1·270)	1·169 (1·063 to 1·289)	1·171 (1·057 to 1·281)	1·170 (1·057 to 1·287)	1·170 (1·070 to 1·279)	1·172 (1·064 to 1·287)	1·172 (1·070 to 1·283)	
Low back pain	Administrative and managerial workers	Morbidity	Both	1·205 (0·946 to 1·489)	1·205 (0·965 to 1·473)	1·205 (0·961 to 1·512)	1·203 (0·948 to 1·516)	1·209 (0·975 to 1·482)	1·210 (0·963 to 1·491)	1·203 (0·959 to 1·502)	
Low back pain	Clerical and related workers	Morbidity	Both	1·000 (1·000 to 1·000)	1·000 (1·000 to 1·000)	1·000 (1·000 to 1·000)	1·000 (1·000 to 1·000)	1·000 (1·000 to 1·000)	1·000 (1·000 to 1·000)	1·000 (1·000 to 1·000)	
Low back pain	Sales workers	Morbidity	Both	1·212 (1·012 to 1·447)	1·216 (1·010 to 1·449)	1·219 (1·019 to 1·451)	1·211 (1·011 to 1·444)	1·213 (1·014 to 1·455)	1·210 (1·007 to 1·425)	1·214 (1·014 to 1·449)	
Low back pain	Service workers	Morbidity	Both	1·469 (1·374 to 1·568)	1·470 (1·378 to 1·570)	1·472 (1·379 to 1·576)	1·472 (1·381 to 1·572)	1·474 (1·386 to 1·572)	1·470 (1·377 to 1·568)	1·472 (1·379 to 1·571)	
Low back pain	Agriculture, animal husbandry and forestry workers, fishermen and hunters	Morbidity	Both	3·793 (2·631 to 5·371)	3·785 (2·551 to 5·345)	3·776 (2·642 to 5·173)	3·792 (2·535 to 5·426)	3·802 (2·679 to 5·433)	3·746 (2·606 to 5·175)	3·770 (2·627 to 5·158)	
Low back pain	Production and related workers, transport equipment operators and labourers	Morbidity	Both	1·541 (1·402 to 1·684)	1·542 (1·410 to 1·684)	1·541 (1·404 to 1·684)	1·540 (1·414 to 1·679)	1·540 (1·408 to 1·683)	1·538 (1·408 to 1·673)	1·541 (1·408 to 1·677)	
Low back pain	Background	Morbidity	Both	1·000 (1·000 to 1·000)	1·000 (1·000 to 1·000)	1·000 (1·000 to 1·000)	1·000 (1·000 to 1·000)	1·000 (1·000 to 1·000)	1·000 (1·000 to 1·000)	1·000 (1·000 to 1·000)	
**Non-exclusive breastfeeding**											
Diarrhoeal diseases	None	Morbidity	Both								
Diarrhoeal diseases	Partial	Morbidity	Both								
Diarrhoeal diseases	Predominant	Morbidity	Both								
Diarrhoeal diseases	Exclusive	Morbidity	Both								
Diarrhoeal diseases	None	Mortality	Both								
Diarrhoeal diseases	Partial	Mortality	Both								
Diarrhoeal diseases	Predominant	Mortality	Both								
Diarrhoeal diseases	Exclusive	Mortality	Both								
Lower respiratory infections	None	Morbidity	Both								
Lower respiratory infections	Partial	Morbidity	Both								
Lower respiratory infections	Predominant	Morbidity	Both								
Lower respiratory infections	Exclusive	Morbidity	Both								
Lower respiratory infections	None	Mortality	Both								
Lower respiratory infections	Partial	Mortality	Both								
Lower respiratory infections	Predominant	Mortality	Both								
Lower respiratory infections	Exclusive	Mortality	Both								
**Discontinued breastfeeding**											
Diarrhoeal diseases	Not continued	Both	Both								
Diarrhoeal diseases	Continued	Both	Both								
**Childhood underweight**											
Diarrhoeal diseases	<-3 sd	Both	Both								
Diarrhoeal diseases	-3 to -2 sd	Both	Both								
Diarrhoeal diseases	-2 to -1 sd	Both	Both								
Diarrhoeal diseases	-1 sd and above	Both	Both								
Lower respiratory infections	<-3 sd	Both	Both								
Lower respiratory infections	-3 to -2 sd	Both	Both								
Lower respiratory infections	-2 to -1 sd	Both	Both								
Lower respiratory infections	-1 sd and above	Both	Both								

Risk - Outcome	Category / Units	Morbidity / Mortality	Sex	All ages	0-6 Days	7-27 Days	28-364 Days	1-4 years	5-9 years	10-14 years	15-19 years	20-24 years	25-29 years	30-34 years	35-39 years	40-44 years
Measles	<-3 sd	Both	Both		5·668 (1·766 to 12·426)	5·668 (1·766 to 12·426)	5·668 (1·766 to 12·426)	5·668 (1·766 to 12·426)								
Measles	-3 to -2 sd	Both	Both		2·458 (1·260 to 5·144)	2·458 (1·260 to 5·144)	2·458 (1·260 to 5·144)	2·458 (1·260 to 5·144)								
Measles	-2 to -1 sd	Both	Both		0·995 (0·499 to 1·729)	0·995 (0·499 to 1·729)	0·995 (0·499 to 1·729)	0·995 (0·499 to 1·729)								
Measles	-1 sd and above	Both	Both		1·000 (1·000 to 1·000)	1·000 (1·000 to 1·000)	1·000 (1·000 to 1·000)	1·000 (1·000 to 1·000)								
**Childhood wasting**																
Diarrhoeal diseases	<-3 sd	Both	Both		105·759 (42·172 to 158·035)	105·759 (42·172 to 158·035)	105·759 (42·172 to 158·035)	105·759 (42·172 to 158·035)								
Diarrhoeal diseases	-3 to -2 sd	Both	Both		23·261 (8·903 to 35·861)	23·261 (8·903 to 35·861)	23·261 (8·903 to 35·861)	23·261 (8·903 to 35·861)								
Diarrhoeal diseases	-2 to -1 sd	Both	Both		6·601 (2·157 to 11·254)	6·601 (2·157 to 11·254)	6·601 (2·157 to 11·254)	6·601 (2·157 to 11·254)								
Diarrhoeal diseases	-1 sd and above	Both	Both		1·000 (1·000 to 1·000)	1·000 (1·000 to 1·000)	1·000 (1·000 to 1·000)	1·000 (1·000 to 1·000)								
Lower respiratory infections	<-3 sd	Both	Both		47·670 (15·810 to 95·045)	47·670 (15·810 to 95·045)	47·670 (15·810 to 95·045)	47·670 (15·810 to 95·045)								
Lower respiratory infections	-3 to -2 sd	Both	Both		20·455 (7·048 to 37·934)	20·455 (7·048 to 37·934)	20·455 (7·048 to 37·934)	20·455 (7·048 to 37·934)								
Lower respiratory infections	-2 to -1 sd	Both	Both		5·941 (1·971 to 12·111)	5·941 (1·971 to 12·111)	5·941 (1·971 to 12·111)	5·941 (1·971 to 12·111)								
Lower respiratory infections	-1 sd and above	Both	Both		1·000 (1·000 to 1·000)	1·000 (1·000 to 1·000)	1·000 (1·000 to 1·000)	1·000 (1·000 to 1·000)								
Measles	<-3 sd	Both	Both		37·936 (5·069 to 200·729)	37·936 (5·069 to 200·729)	37·936 (5·069 to 200·729)	37·936 (5·069 to 200·729)								
Measles	-3 to -2 sd	Both	Both		8·477 (1·330 to 42·943)	8·477 (1·330 to 42·943)	8·477 (1·330 to 42·943)	8·477 (1·330 to 42·943)								
Measles	-2 to -1 sd	Both	Both		1·833 (0·568 to 9·018)	1·833 (0·568 to 9·018)	1·833 (0·568 to 9·018)	1·833 (0·568 to 9·018)								
Measles	-1 sd and above	Both	Both		1·000 (1·000 to 1·000)	1·000 (1·000 to 1·000)	1·000 (1·000 to 1·000)	1·000 (1·000 to 1·000)								
**Childhood stunting**																
Diarrhoeal diseases	<-3 sd	Both	Both		1·851 (1·280 to 2·701)	1·851 (1·280 to 2·701)	1·851 (1·280 to 2·701)	1·851 (1·280 to 2·701)								
Diarrhoeal diseases	-3 to -2 sd	Both	Both		1·222 (1·067 to 1·501)	1·222 (1·067 to 1·501)	1·222 (1·067 to 1·501)	1·222 (1·067 to 1·501)								
Diarrhoeal diseases	-2 to -1 sd	Both	Both		1·111 (1·022 to 1·274)	1·111 (1·022 to 1·274)	1·111 (1·022 to 1·274)	1·111 (1·022 to 1·274)								
Diarrhoeal diseases	-1 sd and above	Both	Both		1·000 (1·000 to 1·000)	1·000 (1·000 to 1·000)	1·000 (1·000 to 1·000)	1·000 (1·000 to 1·000)								
Lower respiratory infections	<-3 sd	Both	Both		2·355 (1·096 to 5·155)	2·355 (1·096 to 5·155)	2·355 (1·096 to 5·155)	2·355 (1·096 to 5·155)								
Lower respiratory infections	-3 to -2 sd	Both	Both		1·318 (1·011 to 2·167)	1·318 (1·011 to 2·167)	1·318 (1·011 to 2·167)	1·318 (1·011 to 2·167)								
Lower respiratory infections	-2 to -1 sd	Both	Both		1·158 (0·998 to 1·664)	1·158 (0·998 to 1·664)	1·158 (0·998 to 1·664)	1·158 (0·998 to 1·664)								
Lower respiratory infections	-1 sd and above	Both	Both		1·000 (1·000 to 1·000)	1·000 (1·000 to 1·000)	1·000 (1·000 to 1·000)	1·000 (1·000 to 1·000)								
Measles	<-3 sd	Both	Both		2·487 (1·127 to 6·555)	2·487 (1·127 to 6·555)	2·487 (1·127 to 6·555)	2·487 (1·127 to 6·555)								
Measles	-3 to -2 sd	Both	Both		1·540 (1·028 to 3·295)	1·540 (1·028 to 3·295)	1·540 (1·028 to 3·295)	1·540 (1·028 to 3·295)								
Measles	-2 to -1 sd	Both	Both		1·103 (0·861 to 1·762)	1·103 (0·861 to 1·762)	1·103 (0·861 to 1·762)	1·103 (0·861 to 1·762)								
Measles	-1 sd and above	Both	Both		1·000 (1·000 to 1·000)	1·000 (1·000 to 1·000)	1·000 (1·000 to 1·000)	1·000 (1·000 to 1·000)								
**Iron deficiency**																
Maternal haemorrhage	1 g/dL	Both	Both								1·252 (1·087 to 1·425)	1·252 (1·087 to 1·425)	1·252 (1·087 to 1·425)	1·252 (1·087 to 1·425)	1·252 (1·087 to 1·425)	1·252 (1·087 to 1·425)
Maternal sepsis and other pregnancy related infections	1 g/dL	Both	Both								1·252 (1·087 to 1·425)	1·252 (1·087 to 1·425)	1·252 (1·087 to 1·425)	1·252 (1·087 to 1·425)	1·252 (1·087 to 1·425)	1·252 (1·087 to 1·425)
**Vitamin A deficiency**																
Diarrhoeal diseases	Vitamin A deficient	Both	Both				1·323 (1·109 to 1·578)	1·595 (1·213 to 2·025)								
Diarrhoeal diseases	Not deficient	Both	Both				1·000 (1·000 to 1·000)	1·000 (1·000 to 1·000)								
Measles	Vitamin A deficient	Both	Both				1·766 (1·327 to 2·327)	2·402 (1·605 to 3·485)								
Measles	Not deficient	Both	Both				1·000 (1·000 to 1·000)	1·000 (1·000 to 1·000)								
**Zinc deficiency**																
Diarrhoeal diseases	Not deficient	Both	Both					1·000 (1·000 to 1·000)								
Diarrhoeal diseases	Zinc deficient	Morbidity	Both					1·903 (1·515 to 2·337)								
Diarrhoeal diseases	Zinc deficient	Mortality	Both					1·951 (0·903 to 3·914)								
Lower respiratory infections	Not deficient	Both	Both					1·000 (1·000 to 1·000)								
Lower respiratory infections	Zinc deficient	Morbidity	Both					1·837 (1·273 to 2·530)								
Lower respiratory infections	Zinc deficient	Mortality	Both					1·672 (0·456 to 4·155)								
**Smoking (prevalence approach)**																
Tuberculosis	Smoker (5 year lag)	Both	Male											1·588 (1·242 to 2·039)	1·588 (1·242 to 2·039)	1·588 (1·242 to 2·039)
Tuberculosis	Nonsmoker (5 year lag)	Both	Male											1·000 (1·000 to 1·000)	1·000 (1·000 to 1·000)	1·000 (1·000 to 1·000)
Tuberculosis	Smoker (5 year lag)	Both	Female											1·599 (1·258 to 2·024)	1·599 (1·258 to 2·024)	1·599 (1·258 to 2·024)
Tuberculosis	Nonsmoker (5 year lag)	Both	Female											1·000 (1·000 to 1·000)	1·000 (1·000 to 1·000)	1·000 (1·000 to 1·000)
Ischaemic heart disease	Smoker (5 year lag)	Both	Male											4·316 (3·127 to 5·810)	3·924 (2·905 to 5·186)	3·569 (2·699 to 4·630)
Ischaemic heart disease	Nonsmoker (5 year lag)	Both	Male											1·000 (1·000 to 1·000)	1·000 (1·000 to 1·000)	1·000 (1·000 to 1·000)
Ischaemic heart disease	Smoker (5 year lag)	Both	Female											6·145 (5·060 to 7·413)	5·464 (4·557 to 6·515)	4·859 (4·105 to 5·725)
Ischaemic heart disease	Nonsmoker (5 year lag)	Both	Female											1·000 (1·000 to 1·000)	1·000 (1·000 to 1·000)	1·000 (1·000 to 1·000)
Ischaemic stroke	Smoker (5 year lag)	Both	Male											4·175 (3·165 to 5·452)	3·805 (2·939 to 4·887)	3·468 (2·728 to 4·381)
Ischaemic stroke	Nonsmoker (5 year lag)	Both	Male											1·000 (1·000 to 1·000)	1·000 (1·000 to 1·000)	1·000 (1·000 to 1·000)
Ischaemic stroke	Smoker (5 year lag)	Both	Female											6·020 (4·248 to 8·410)	5·357 (3·869 to 7·331)	4·767 (3·525 to 6·390)
Ischaemic stroke	Nonsmoker (5 year lag)	Both	Female											1·000 (1·000 to 1·000)	1·000 (1·000 to 1·000)	1·000 (1·000 to 1·000)

Risk - Outcome	Category / Units	Morbidity / Mortality	Sex	45-49 years	50-54 years	55-59 years	60-64 years	65-69 years	70-74 years	75-79 years	80+ years
Measles	<-3 sd	Both	Both								
Measles	-3 to -2 sd	Both	Both								
Measles	-2 to -1 sd	Both	Both								
Measles	-1 sd and above	Both	Both								
**Childhood wasting**											
Diarrhoeal diseases	<-3 sd	Both	Both								
Diarrhoeal diseases	-3 to -2 sd	Both	Both								
Diarrhoeal diseases	-2 to -1 sd	Both	Both								
Diarrhoeal diseases	-1 sd and above	Both	Both								
Lower respiratory infections	<-3 sd	Both	Both								
Lower respiratory infections	-3 to -2 sd	Both	Both								
Lower respiratory infections	-2 to -1 sd	Both	Both								
Lower respiratory infections	-1 sd and above	Both	Both								
Measles	<-3 sd	Both	Both								
Measles	-3 to -2 sd	Both	Both								
Measles	-2 to -1 sd	Both	Both								
Measles	-1 sd and above	Both	Both								
**Childhood stunting**											
Diarrhoeal diseases	<-3 sd	Both	Both								
Diarrhoeal diseases	-3 to -2 sd	Both	Both								
Diarrhoeal diseases	-2 to -1 sd	Both	Both								
Diarrhoeal diseases	-1 sd and above	Both	Both								
Lower respiratory infections	<-3 sd	Both	Both								
Lower respiratory infections	-3 to -2 sd	Both	Both								
Lower respiratory infections	-2 to -1 sd	Both	Both								
Lower respiratory infections	-1 sd and above	Both	Both								
Measles	<-3 sd	Both	Both								
Measles	-3 to -2 sd	Both	Both								
Measles	-2 to -1 sd	Both	Both								
Measles	-1 sd and above	Both	Both								
**Iron deficiency**											
Maternal haemorrhage	1 g/dL	Both	Both	1·252 (1·087 to 1·425)							
Maternal sepsis and other pregnancy related infections	1 g/dL	Both	Both	1·252 (1·087 to 1·425)							
**Vitamin A deficiency**											
Diarrhoeal diseases	Vitamin A deficient	Both	Both								
Diarrhoeal diseases	Not deficient	Both	Both								
Measles	Vitamin A deficient	Both	Both								
Measles	Not deficient	Both	Both								
**Zinc deficiency**											
Diarrhoeal diseases	Not deficient	Both	Both								
Diarrhoeal diseases	Zinc deficient	Morbidity	Both								
Diarrhoeal diseases	Zinc deficient	Mortality	Both								
Lower respiratory infections	Not deficient	Both	Both								
Lower respiratory infections	Zinc deficient	Morbidity	Both								
Lower respiratory infections	Zinc deficient	Mortality	Both								
**Smoking (prevalence approach)**											
Tuberculosis	Smoker (5 year lag)	Both	Male	1·588 (1·242 to 2·039)	1·588 (1·242 to 2·039)	1·588 (1·242 to 2·039)	1·588 (1·242 to 2·039)	1·588 (1·242 to 2·039)	1·588 (1·242 to 2·039)	1·588 (1·242 to 2·039)	1·588 (1·242 to 2·039)
Tuberculosis	Nonsmoker (5 year lag)	Both	Male	1·000 (1·000 to 1·000)	1·000 (1·000 to 1·000)	1·000 (1·000 to 1·000)	1·000 (1·000 to 1·000)	1·000 (1·000 to 1·000)	1·000 (1·000 to 1·000)	1·000 (1·000 to 1·000)	1·000 (1·000 to 1·000)
Tuberculosis	Smoker (5 year lag)	Both	Female	1·599 (1·258 to 2·024)	1·599 (1·258 to 2·024)	1·599 (1·258 to 2·024)	1·599 (1·258 to 2·024)	1·599 (1·258 to 2·024)	1·599 (1·258 to 2·024)	1·599 (1·258 to 2·024)	1·599 (1·258 to 2·024)
Tuberculosis	Nonsmoker (5 year lag)	Both	Female	1·000 (1·000 to 1·000)	1·000 (1·000 to 1·000)	1·000 (1·000 to 1·000)	1·000 (1·000 to 1·000)	1·000 (1·000 to 1·000)	1·000 (1·000 to 1·000)	1·000 (1·000 to 1·000)	1·000 (1·000 to 1·000)
Ischaemic heart disease	Smoker (5 year lag)	Both	Male	3·246 (2·508 to 4·133)	2·952 (2·330 to 3·689)	2·685 (2·165 to 3·293)	2·443 (2·011 to 2·940)	2·223 (1·869 to 2·624)	2·023 (1·736 to 2·343)	1·841 (1·613 to 2·091)	1·598 (1·445 to 1·764)
Ischaemic heart disease	Nonsmoker (5 year lag)	Both	Male	1·000 (1·000 to 1·000)	1·000 (1·000 to 1·000)	1·000 (1·000 to 1·000)	1·000 (1·000 to 1·000)	1·000 (1·000 to 1·000)	1·000 (1·000 to 1·000)	1·000 (1·000 to 1·000)	1·000 (1·000 to 1·000)
Ischaemic heart disease	Smoker (5 year lag)	Both	Female	4·321 (3·697 to 5·031)	3·843 (3·330 to 4·421)	3·417 (2·999 to 3·885)	3·039 (2·701 to 3·414)	2·703 (2·433 to 3·000)	2·404 (2·191 to 2·636)	2·139 (1·974 to 2·317)	1·794 (1·687 to 1·908)
Ischaemic heart disease	Nonsmoker (5 year lag)	Both	Female	1·000 (1·000 to 1·000)	1·000 (1·000 to 1·000)	1·000 (1·000 to 1·000)	1·000 (1·000 to 1·000)	1·000 (1·000 to 1·000)	1·000 (1·000 to 1·000)	1·000 (1·000 to 1·000)	1·000 (1·000 to 1·000)
Ischaemic stroke	Smoker (5 year lag)	Both	Male	3·161 (2·533 to 3·927)	2·882 (2·351 to 3·520)	2·627 (2·183 to 3·155)	2·395 (2·026 to 2·828)	2·184 (1·881 to 2·535)	1·992 (1·746 to 2·272)	1·816 (1·621 to 2·036)	1·582 (1·450 to 1·728)
Ischaemic stroke	Nonsmoker (5 year lag)	Both	Male	1·000 (1·000 to 1·000)	1·000 (1·000 to 1·000)	1·000 (1·000 to 1·000)	1·000 (1·000 to 1·000)	1·000 (1·000 to 1·000)	1·000 (1·000 to 1·000)	1·000 (1·000 to 1·000)	1·000 (1·000 to 1·000)
Ischaemic stroke	Smoker (5 year lag)	Both	Female	4·243 (3·211 to 5·569)	3·777 (2·925 to 4·855)	3·363 (2·664 to 4·231)	2·994 (2·427 to 3·688)	2·666 (2·210 to 3·215)	2·375 (2·014 to 2·802)	2·115 (1·834 to 2·442)	1·778 (1·595 to 1·988)
Ischaemic stroke	Nonsmoker (5 year lag)	Both	Female	1·000 (1·000 to 1·000)	1·000 (1·000 to 1·000)	1·000 (1·000 to 1·000)	1·000 (1·000 to 1·000)	1·000 (1·000 to 1·000)	1·000 (1·000 to 1·000)	1·000 (1·000 to 1·000)	1·000 (1·000 to 1·000)

Risk - Outcome	Category / Units	Morbidity / Mortality	Sex	All ages	0-6 Days	7-27 Days	28-364 Days	1-4 years	5-9 years	10-14 years	15-19 years	20-24 years	25-29 years	30-34 years	35-39 years	40-44 years
Hemorrhagic stroke	Smoker (5 year lag)	Both	Male											4·175 (3·165 to 5·452)	3·805 (2·939 to 4·887)	3·468 (2·728 to 4·381)
Hemorrhagic stroke	Nonsmoker (5 year lag)	Both	Male											1·000 (1·000 to 1·000)	1·000 (1·000 to 1·000)	1·000 (1·000 to 1·000)
Hemorrhagic stroke	Smoker (5 year lag)	Both	Female											6·020 (4·248 to 8·410)	5·357 (3·869 to 7·331)	4·767 (3·525 to 6·390)
Hemorrhagic stroke	Nonsmoker (5 year lag)	Both	Female											1·000 (1·000 to 1·000)	1·000 (1·000 to 1·000)	1·000 (1·000 to 1·000)
Hypertensive heart disease	Smoker (5 year lag)	Both	Male											4·153 (2·995 to 5·659)	3·785 (2·790 to 5·061)	3·451 (2·600 to 4·525)
Hypertensive heart disease	Nonsmoker (5 year lag)	Both	Male											1·000 (1·000 to 1·000)	1·000 (1·000 to 1·000)	1·000 (1·000 to 1·000)
Hypertensive heart disease	Smoker (5 year lag)	Both	Female											4·110 (2·053 to 7·209)	3·740 (1·960 to 6·346)	3·405 (1·871 to 5·587)
Hypertensive heart disease	Nonsmoker (5 year lag)	Both	Female											1·000 (1·000 to 1·000)	1·000 (1·000 to 1·000)	1·000 (1·000 to 1·000)
Atrial fibrillation and flutter	Smoker (5 year lag)	Both	Male											4·153 (2·995 to 5·659)	3·785 (2·790 to 5·061)	3·451 (2·600 to 4·525)
Atrial fibrillation and flutter	Nonsmoker (5 year lag)	Both	Male											1·000 (1·000 to 1·000)	1·000 (1·000 to 1·000)	1·000 (1·000 to 1·000)
Atrial fibrillation and flutter	Smoker (5 year lag)	Both	Female											4·110 (2·053 to 7·209)	3·740 (1·960 to 6·346)	3·405 (1·871 to 5·587)
Atrial fibrillation and flutter	Nonsmoker (5 year lag)	Both	Female											1·000 (1·000 to 1·000)	1·000 (1·000 to 1·000)	1·000 (1·000 to 1·000)
Aortic aneurysm	Smoker (5 year lag)	Both	Male											4·153 (2·995 to 5·659)	3·785 (2·790 to 5·061)	3·451 (2·600 to 4·525)
Aortic aneurysm	Nonsmoker (5 year lag)	Both	Male											1·000 (1·000 to 1·000)	1·000 (1·000 to 1·000)	1·000 (1·000 to 1·000)
Aortic aneurysm	Smoker (5 year lag)	Both	Female											4·110 (2·053 to 7·209)	3·740 (1·960 to 6·346)	3·405 (1·871 to 5·587)
Aortic aneurysm	Nonsmoker (5 year lag)	Both	Female											1·000 (1·000 to 1·000)	1·000 (1·000 to 1·000)	1·000 (1·000 to 1·000)
Peripheral vascular disease	Smoker (5 year lag)	Both	Male											4·153 (2·995 to 5·659)	3·785 (2·790 to 5·061)	3·451 (2·600 to 4·525)
Peripheral vascular disease	Nonsmoker (5 year lag)	Both	Male											1·000 (1·000 to 1·000)	1·000 (1·000 to 1·000)	1·000 (1·000 to 1·000)
Peripheral vascular disease	Smoker (5 year lag)	Both	Female											4·110 (2·053 to 7·209)	3·740 (1·960 to 6·346)	3·405 (1·871 to 5·587)
Peripheral vascular disease	Nonsmoker (5 year lag)	Both	Female											1·000 (1·000 to 1·000)	1·000 (1·000 to 1·000)	1·000 (1·000 to 1·000)
Other cardiovascular and circulatory diseases	Smoker (5 year lag)	Both	Male											4·153 (2·995 to 5·659)	3·785 (2·790 to 5·061)	3·451 (2·600 to 4·525)
Other cardiovascular and circulatory diseases	Nonsmoker (5 year lag)	Both	Male											1·000 (1·000 to 1·000)	1·000 (1·000 to 1·000)	1·000 (1·000 to 1·000)
Other cardiovascular and circulatory diseases	Smoker (5 year lag)	Both	Female											4·110 (2·053 to 7·209)	3·740 (1·960 to 6·346)	3·405 (1·871 to 5·587)
Other cardiovascular and circulatory diseases	Nonsmoker (5 year lag)	Both	Female											1·000 (1·000 to 1·000)	1·000 (1·000 to 1·000)	1·000 (1·000 to 1·000)
Asthma	Smoker (5 year lag)	Both	Male											2·098 (1·761 to 2·460)	2·098 (1·761 to 2·460)	2·098 (1·761 to 2·460)
Asthma	Nonsmoker (5 year lag)	Both	Male											1·000 (1·000 to 1·000)	1·000 (1·000 to 1·000)	1·000 (1·000 to 1·000)
Asthma	Smoker (5 year lag)	Both	Female											1·976 (1·788 to 2·181)	1·976 (1·788 to 2·181)	1·976 (1·788 to 2·181)
Asthma	Nonsmoker (5 year lag)	Both	Female											1·000 (1·000 to 1·000)	1·000 (1·000 to 1·000)	1·000 (1·000 to 1·000)
Peptic ulcer disease	Smoker (5 year lag)	Both	Both							2·040 (1·684 to 2·483)	2·040 (1·684 to 2·483)	2·040 (1·684 to 2·483)	2·040 (1·684 to 2·483)	2·040 (1·684 to 2·483)	2·040 (1·684 to 2·483)	2·040 (1·684 to 2·483)
Peptic ulcer disease	Nonsmoker (5 year lag)	Both	Both							1·000 (1·000 to 1·000)	1·000 (1·000 to 1·000)	1·000 (1·000 to 1·000)	1·000 (1·000 to 1·000)	1·000 (1·000 to 1·000)	1·000 (1·000 to 1·000)	1·000 (1·000 to 1·000)
Diabetes mellitus	Smoker (5 year lag)	Both	Male											1·426 (1·094 to 1·842)	1·426 (1·094 to 1·842)	1·426 (1·094 to 1·842)
Diabetes mellitus	Nonsmoker (5 year lag)	Both	Male											1·000 (1·000 to 1·000)	1·000 (1·000 to 1·000)	1·000 (1·000 to 1·000)
Diabetes mellitus	Smoker (5 year lag)	Both	Female											1·102 (0·953 to 1·275)	1·102 (0·953 to 1·275)	1·102 (0·953 to 1·275)
Diabetes mellitus	Nonsmoker (5 year lag)	Both	Female											1·000 (1·000 to 1·000)	1·000 (1·000 to 1·000)	1·000 (1·000 to 1·000)
Rheumatoid arthritis	Smoker (5 year lag)	Both	Both							1·375 (1·142 to 1·652)	1·375 (1·142 to 1·652)	1·375 (1·142 to 1·652)	1·375 (1·142 to 1·652)	1·375 (1·142 to 1·652)	1·375 (1·142 to 1·652)	1·375 (1·142 to 1·652)
Rheumatoid arthritis	Nonsmoker (5 year lag)	Both	Both							1·000 (1·000 to 1·000)	1·000 (1·000 to 1·000)	1·000 (1·000 to 1·000)	1·000 (1·000 to 1·000)	1·000 (1·000 to 1·000)	1·000 (1·000 to 1·000)	1·000 (1·000 to 1·000)
Cataract	Smoker (5 year lag)	Both	Both							1·671 (1·479 to 1·875)	1·671 (1·479 to 1·875)	1·671 (1·479 to 1·875)	1·671 (1·479 to 1·875)	1·671 (1·479 to 1·875)	1·671 (1·479 to 1·875)	1·671 (1·479 to 1·875)
Cataract	Nonsmoker (5 year lag)	Both	Both							1·000 (1·000 to 1·000)	1·000 (1·000 to 1·000)	1·000 (1·000 to 1·000)	1·000 (1·000 to 1·000)	1·000 (1·000 to 1·000)	1·000 (1·000 to 1·000)	1·000 (1·000 to 1·000)
Macular degeneration	Smoker (5 year lag)	Both	Both							1·911 (1·265 to 2·740)	1·911 (1·265 to 2·740)	1·911 (1·265 to 2·740)	1·911 (1·265 to 2·740)	1·911 (1·265 to 2·740)	1·911 (1·265 to 2·740)	1·911 (1·265 to 2·740)
Macular degeneration	Nonsmoker (5 year lag)	Both	Both							1·000 (1·000 to 1·000)	1·000 (1·000 to 1·000)	1·000 (1·000 to 1·000)	1·000 (1·000 to 1·000)	1·000 (1·000 to 1·000)	1·000 (1·000 to 1·000)	1·000 (1·000 to 1·000)
**Smoking (SIR approach)**																
Lip and oral cavity cancer	SIR	Both	Male											8·162 (5·617 to 11·378)	8·162 (5·617 to 11·378)	8·162 (5·617 to 11·378)
Lip and oral cavity cancer	1-SIR	Both	Male											1·000 (1·000 to 1·000)	1·000 (1·000 to 1·000)	1·000 (1·000 to 1·000)
Lip and oral cavity cancer	SIR	Both	Female											6·056 (4·232 to 8·541)	6·056 (4·232 to 8·541)	6·056 (4·232 to 8·541)
Lip and oral cavity cancer	1-SIR	Both	Female											1·000 (1·000 to 1·000)	1·000 (1·000 to 1·000)	1·000 (1·000 to 1·000)
Nasopharynx cancer	SIR	Both	Male											8·227 (5·677 to 11·505)	8·227 (5·677 to 11·505)	8·227 (5·677 to 11·505)
Nasopharynx cancer	1-SIR	Both	Male											1·000 (1·000 to 1·000)	1·000 (1·000 to 1·000)	1·000 (1·000 to 1·000)
Nasopharynx cancer	SIR	Both	Female											6·089 (4·288 to 8·470)	6·089 (4·288 to 8·470)	6·089 (4·288 to 8·470)
Nasopharynx cancer	1-SIR	Both	Female											1·000 (1·000 to 1·000)	1·000 (1·000 to 1·000)	1·000 (1·000 to 1·000)
Oesophageal cancer	SIR	Both	Male											6·676 (4·136 to 10·250)	6·676 (4·136 to 10·250)	6·676 (4·136 to 10·250)
Oesophageal cancer	1-SIR	Both	Male											1·000 (1·000 to 1·000)	1·000 (1·000 to 1·000)	1·000 (1·000 to 1·000)
Oesophageal cancer	SIR	Both	Female											6·357 (4·442 to 8·634)	6·357 (4·442 to 8·634)	6·357 (4·442 to 8·634)
Oesophageal cancer	1-SIR	Both	Female											1·000 (1·000 to 1·000)	1·000 (1·000 to 1·000)	1·000 (1·000 to 1·000)
Stomach cancer	SIR	Both	Male											1·927 (1·443 to 2·535)	1·927 (1·443 to 2·535)	1·927 (1·443 to 2·535)
Stomach cancer	1-SIR	Both	Male											1·000 (1·000 to 1·000)	1·000 (1·000 to 1·000)	1·000 (1·000 to 1·000)
Stomach cancer	SIR	Both	Female											1·570 (1·246 to 1·925)	1·570 (1·246 to 1·925)	1·570 (1·246 to 1·925)
Stomach cancer	1-SIR	Both	Female											1·000 (1·000 to 1·000)	1·000 (1·000 to 1·000)	1·000 (1·000 to 1·000)
Colon and rectum cancer	SIR	Both	Male											1·325 (1·195 to 1·471)	1·325 (1·195 to 1·471)	1·325 (1·195 to 1·471)

Risk - Outcome	Category / Units	Morbidity / Mortality	Sex	45-49 years	50-54 years	55-59 years	60-64 years	65-69 years	70-74 years	75-79 years	80+ years
Hemorrhagic stroke	Smoker (5 year lag)	Both	Male	3·161 (2·533 to 3·927)	2·882 (2·351 to 3·520)	2·627 (2·183 to 3·155)	2·395 (2·026 to 2·828)	2·184 (1·881 to 2·535)	1·992 (1·746 to 2·272)	1·816 (1·621 to 2·036)	1·582 (1·450 to 1·728)
Hemorrhagic stroke	Nonsmoker (5 year lag)	Both	Male	1·000 (1·000 to 1·000)	1·000 (1·000 to 1·000)	1·000 (1·000 to 1·000)	1·000 (1·000 to 1·000)	1·000 (1·000 to 1·000)	1·000 (1·000 to 1·000)	1·000 (1·000 to 1·000)	1·000 (1·000 to 1·000)
Hemorrhagic stroke	Smoker (5 year lag)	Both	Female	4·243 (3·211 to 5·569)	3·777 (2·925 to 4·855)	3·363 (2·664 to 4·231)	2·994 (2·427 to 3·688)	2·666 (2·210 to 3·215)	2·375 (2·014 to 2·802)	2·115 (1·834 to 2·442)	1·778 (1·595 to 1·988)
Hemorrhagic stroke	Nonsmoker (5 year lag)	Both	Female	1·000 (1·000 to 1·000)	1·000 (1·000 to 1·000)	1·000 (1·000 to 1·000)	1·000 (1·000 to 1·000)	1·000 (1·000 to 1·000)	1·000 (1·000 to 1·000)	1·000 (1·000 to 1·000)	1·000 (1·000 to 1·000)
Hypertensive heart disease	Smoker (5 year lag)	Both	Male	3·146 (2·422 to 4·046)	2·868 (2·257 to 3·618)	2·616 (2·102 to 3·236)	2·386 (1·959 to 2·893)	2·176 (1·825 to 2·587)	1·985 (1·700 to 2·313)	1·811 (1·584 to 2·069)	1·578 (1·425 to 1·749)
Hypertensive heart disease	Nonsmoker (5 year lag)	Both	Male	1·000 (1·000 to 1·000)	1·000 (1·000 to 1·000)	1·000 (1·000 to 1·000)	1·000 (1·000 to 1·000)	1·000 (1·000 to 1·000)	1·000 (1·000 to 1·000)	1·000 (1·000 to 1·000)	1·000 (1·000 to 1·000)
Hypertensive heart disease	Smoker (5 year lag)	Both	Female	3·102 (1·786 to 4·919)	2·826 (1·705 to 4·330)	2·576 (1·628 to 3·812)	2·350 (1·554 to 3·356)	2·144 (1·484 to 2·954)	1·957 (1·416 to 2·601)	1·787 (1·352 to 2·290)	1·560 (1·261 to 1·891)
Hypertensive heart disease	Nonsmoker (5 year lag)	Both	Female	1·000 (1·000 to 1·000)	1·000 (1·000 to 1·000)	1·000 (1·000 to 1·000)	1·000 (1·000 to 1·000)	1·000 (1·000 to 1·000)	1·000 (1·000 to 1·000)	1·000 (1·000 to 1·000)	1·000 (1·000 to 1·000)
Atrial fibrillation and flutter	Smoker (5 year lag)	Both	Male	3·146 (2·422 to 4·046)	2·868 (2·257 to 3·618)	2·616 (2·102 to 3·236)	2·386 (1·959 to 2·893)	2·176 (1·825 to 2·587)	1·985 (1·700 to 2·313)	1·811 (1·584 to 2·069)	1·578 (1·425 to 1·749)
Atrial fibrillation and flutter	Nonsmoker (5 year lag)	Both	Male	1·000 (1·000 to 1·000)	1·000 (1·000 to 1·000)	1·000 (1·000 to 1·000)	1·000 (1·000 to 1·000)	1·000 (1·000 to 1·000)	1·000 (1·000 to 1·000)	1·000 (1·000 to 1·000)	1·000 (1·000 to 1·000)
Atrial fibrillation and flutter	Smoker (5 year lag)	Both	Female	3·102 (1·786 to 4·919)	2·826 (1·705 to 4·330)	2·576 (1·628 to 3·812)	2·350 (1·554 to 3·356)	2·144 (1·484 to 2·954)	1·957 (1·416 to 2·601)	1·787 (1·352 to 2·290)	1·560 (1·261 to 1·891)
Atrial fibrillation and flutter	Nonsmoker (5 year lag)	Both	Female	1·000 (1·000 to 1·000)	1·000 (1·000 to 1·000)	1·000 (1·000 to 1·000)	1·000 (1·000 to 1·000)	1·000 (1·000 to 1·000)	1·000 (1·000 to 1·000)	1·000 (1·000 to 1·000)	1·000 (1·000 to 1·000)
Aortic aneurysm	Smoker (5 year lag)	Both	Male	3·146 (2·422 to 4·046)	2·868 (2·257 to 3·618)	2·616 (2·102 to 3·236)	2·386 (1·959 to 2·893)	2·176 (1·825 to 2·587)	1·985 (1·700 to 2·313)	1·811 (1·584 to 2·069)	1·578 (1·425 to 1·749)
Aortic aneurysm	Nonsmoker (5 year lag)	Both	Male	1·000 (1·000 to 1·000)	1·000 (1·000 to 1·000)	1·000 (1·000 to 1·000)	1·000 (1·000 to 1·000)	1·000 (1·000 to 1·000)	1·000 (1·000 to 1·000)	1·000 (1·000 to 1·000)	1·000 (1·000 to 1·000)
Aortic aneurysm	Smoker (5 year lag)	Both	Female	3·102 (1·786 to 4·919)	2·826 (1·705 to 4·330)	2·576 (1·628 to 3·812)	2·350 (1·554 to 3·356)	2·144 (1·484 to 2·954)	1·957 (1·416 to 2·601)	1·787 (1·352 to 2·290)	1·560 (1·261 to 1·891)
Aortic aneurysm	Nonsmoker (5 year lag)	Both	Female	1·000 (1·000 to 1·000)	1·000 (1·000 to 1·000)	1·000 (1·000 to 1·000)	1·000 (1·000 to 1·000)	1·000 (1·000 to 1·000)	1·000 (1·000 to 1·000)	1·000 (1·000 to 1·000)	1·000 (1·000 to 1·000)
Peripheral vascular disease	Smoker (5 year lag)	Both	Male	3·146 (2·422 to 4·046)	2·868 (2·257 to 3·618)	2·616 (2·102 to 3·236)	2·386 (1·959 to 2·893)	2·176 (1·825 to 2·587)	1·985 (1·700 to 2·313)	1·811 (1·584 to 2·069)	1·578 (1·425 to 1·749)
Peripheral vascular disease	Nonsmoker (5 year lag)	Both	Male	1·000 (1·000 to 1·000)	1·000 (1·000 to 1·000)	1·000 (1·000 to 1·000)	1·000 (1·000 to 1·000)	1·000 (1·000 to 1·000)	1·000 (1·000 to 1·000)	1·000 (1·000 to 1·000)	1·000 (1·000 to 1·000)
Peripheral vascular disease	Smoker (5 year lag)	Both	Female	3·102 (1·786 to 4·919)	2·826 (1·705 to 4·330)	2·576 (1·628 to 3·812)	2·350 (1·554 to 3·356)	2·144 (1·484 to 2·954)	1·957 (1·416 to 2·601)	1·787 (1·352 to 2·290)	1·560 (1·261 to 1·891)
Peripheral vascular disease	Nonsmoker (5 year lag)	Both	Female	1·000 (1·000 to 1·000)	1·000 (1·000 to 1·000)	1·000 (1·000 to 1·000)	1·000 (1·000 to 1·000)	1·000 (1·000 to 1·000)	1·000 (1·000 to 1·000)	1·000 (1·000 to 1·000)	1·000 (1·000 to 1·000)
Other cardiovascular and circulatory diseases	Smoker (5 year lag)	Both	Male	3·146 (2·422 to 4·046)	2·868 (2·257 to 3·618)	2·616 (2·102 to 3·236)	2·386 (1·959 to 2·893)	2·176 (1·825 to 2·587)	1·985 (1·700 to 2·313)	1·811 (1·584 to 2·069)	1·578 (1·425 to 1·749)
Other cardiovascular and circulatory diseases	Nonsmoker (5 year lag)	Both	Male	1·000 (1·000 to 1·000)	1·000 (1·000 to 1·000)	1·000 (1·000 to 1·000)	1·000 (1·000 to 1·000)	1·000 (1·000 to 1·000)	1·000 (1·000 to 1·000)	1·000 (1·000 to 1·000)	1·000 (1·000 to 1·000)
Other cardiovascular and circulatory diseases	Smoker (5 year lag)	Both	Female	3·102 (1·786 to 4·919)	2·826 (1·705 to 4·330)	2·576 (1·628 to 3·812)	2·350 (1·554 to 3·356)	2·144 (1·484 to 2·954)	1·957 (1·416 to 2·601)	1·787 (1·352 to 2·290)	1·560 (1·261 to 1·891)
Other cardiovascular and circulatory diseases	Nonsmoker (5 year lag)	Both	Female	1·000 (1·000 to 1·000)	1·000 (1·000 to 1·000)	1·000 (1·000 to 1·000)	1·000 (1·000 to 1·000)	1·000 (1·000 to 1·000)	1·000 (1·000 to 1·000)	1·000 (1·000 to 1·000)	1·000 (1·000 to 1·000)
Asthma	Smoker (5 year lag)	Both	Male	2·098 (1·761 to 2·460)	2·098 (1·761 to 2·460)	2·098 (1·761 to 2·460)	2·098 (1·761 to 2·460)	2·098 (1·761 to 2·460)	2·098 (1·761 to 2·460)	2·098 (1·761 to 2·460)	2·098 (1·761 to 2·460)
Asthma	Nonsmoker (5 year lag)	Both	Male	1·000 (1·000 to 1·000)	1·000 (1·000 to 1·000)	1·000 (1·000 to 1·000)	1·000 (1·000 to 1·000)	1·000 (1·000 to 1·000)	1·000 (1·000 to 1·000)	1·000 (1·000 to 1·000)	1·000 (1·000 to 1·000)
Asthma	Smoker (5 year lag)	Both	Female	1·976 (1·788 to 2·181)	1·976 (1·788 to 2·181)	1·976 (1·788 to 2·181)	1·976 (1·788 to 2·181)	1·976 (1·788 to 2·181)	1·976 (1·788 to 2·181)	1·976 (1·788 to 2·181)	1·976 (1·788 to 2·181)
Asthma	Nonsmoker (5 year lag)	Both	Female	1·000 (1·000 to 1·000)	1·000 (1·000 to 1·000)	1·000 (1·000 to 1·000)	1·000 (1·000 to 1·000)	1·000 (1·000 to 1·000)	1·000 (1·000 to 1·000)	1·000 (1·000 to 1·000)	1·000 (1·000 to 1·000)
Peptic ulcer disease	Smoker (5 year lag)	Both	Both	2·040 (1·684 to 2·483)	2·040 (1·684 to 2·483)	2·040 (1·684 to 2·483)	2·040 (1·684 to 2·483)	2·040 (1·684 to 2·483)	2·040 (1·684 to 2·483)	2·040 (1·684 to 2·483)	2·040 (1·684 to 2·483)
Peptic ulcer disease	Nonsmoker (5 year lag)	Both	Both	1·000 (1·000 to 1·000)	1·000 (1·000 to 1·000)	1·000 (1·000 to 1·000)	1·000 (1·000 to 1·000)	1·000 (1·000 to 1·000)	1·000 (1·000 to 1·000)	1·000 (1·000 to 1·000)	1·000 (1·000 to 1·000)
Diabetes mellitus	Smoker (5 year lag)	Both	Male	1·426 (1·094 to 1·842)	1·426 (1·094 to 1·842)	1·426 (1·094 to 1·842)	1·426 (1·094 to 1·842)	1·426 (1·094 to 1·842)	1·426 (1·094 to 1·842)	1·426 (1·094 to 1·842)	1·426 (1·094 to 1·842)
Diabetes mellitus	Nonsmoker (5 year lag)	Both	Male	1·000 (1·000 to 1·000)	1·000 (1·000 to 1·000)	1·000 (1·000 to 1·000)	1·000 (1·000 to 1·000)	1·000 (1·000 to 1·000)	1·000 (1·000 to 1·000)	1·000 (1·000 to 1·000)	1·000 (1·000 to 1·000)
Diabetes mellitus	Smoker (5 year lag)	Both	Female	1·102 (0·953 to 1·275)	1·102 (0·953 to 1·275)	1·102 (0·953 to 1·275)	1·102 (0·953 to 1·275)	1·102 (0·953 to 1·275)	1·102 (0·953 to 1·275)	1·102 (0·953 to 1·275)	1·102 (0·953 to 1·275)
Diabetes mellitus	Nonsmoker (5 year lag)	Both	Female	1·000 (1·000 to 1·000)	1·000 (1·000 to 1·000)	1·000 (1·000 to 1·000)	1·000 (1·000 to 1·000)	1·000 (1·000 to 1·000)	1·000 (1·000 to 1·000)	1·000 (1·000 to 1·000)	1·000 (1·000 to 1·000)
Rheumatoid arthritis	Smoker (5 year lag)	Both	Both	1·375 (1·142 to 1·652)	1·375 (1·142 to 1·652)	1·375 (1·142 to 1·652)	1·375 (1·142 to 1·652)	1·375 (1·142 to 1·652)	1·375 (1·142 to 1·652)	1·375 (1·142 to 1·652)	1·375 (1·142 to 1·652)
Rheumatoid arthritis	Nonsmoker (5 year lag)	Both	Both	1·000 (1·000 to 1·000)	1·000 (1·000 to 1·000)	1·000 (1·000 to 1·000)	1·000 (1·000 to 1·000)	1·000 (1·000 to 1·000)	1·000 (1·000 to 1·000)	1·000 (1·000 to 1·000)	1·000 (1·000 to 1·000)
Cataract	Smoker (5 year lag)	Both	Both	1·671 (1·479 to 1·875)	1·671 (1·479 to 1·875)	1·671 (1·479 to 1·875)	1·671 (1·479 to 1·875)	1·671 (1·479 to 1·875)	1·671 (1·479 to 1·875)	1·671 (1·479 to 1·875)	1·671 (1·479 to 1·875)
Cataract	Nonsmoker (5 year lag)	Both	Both	1·000 (1·000 to 1·000)	1·000 (1·000 to 1·000)	1·000 (1·000 to 1·000)	1·000 (1·000 to 1·000)	1·000 (1·000 to 1·000)	1·000 (1·000 to 1·000)	1·000 (1·000 to 1·000)	1·000 (1·000 to 1·000)
Macular degeneration	Smoker (5 year lag)	Both	Both	1·911 (1·265 to 2·740)	1·911 (1·265 to 2·740)	1·911 (1·265 to 2·740)	1·911 (1·265 to 2·740)	1·911 (1·265 to 2·740)	1·911 (1·265 to 2·740)	1·911 (1·265 to 2·740)	1·911 (1·265 to 2·740)
Macular degeneration	Nonsmoker (5 year lag)	Both	Both	1·000 (1·000 to 1·000)	1·000 (1·000 to 1·000)	1·000 (1·000 to 1·000)	1·000 (1·000 to 1·000)	1·000 (1·000 to 1·000)	1·000 (1·000 to 1·000)	1·000 (1·000 to 1·000)	1·000 (1·000 to 1·000)
**Smoking (SIR approach)**											
Lip and oral cavity cancer	SIR	Both	Male	8·162 (5·617 to 11·378)	8·162 (5·617 to 11·378)	8·162 (5·617 to 11·378)	8·162 (5·617 to 11·378)	8·162 (5·617 to 11·378)	8·162 (5·617 to 11·378)	8·162 (5·617 to 11·378)	8·162 (5·617 to 11·378)
Lip and oral cavity cancer	1-SIR	Both	Male	1·000 (1·000 to 1·000)	1·000 (1·000 to 1·000)	1·000 (1·000 to 1·000)	1·000 (1·000 to 1·000)	1·000 (1·000 to 1·000)	1·000 (1·000 to 1·000)	1·000 (1·000 to 1·000)	1·000 (1·000 to 1·000)
Lip and oral cavity cancer	SIR	Both	Female	6·056 (4·232 to 8·541)	6·056 (4·232 to 8·541)	6·056 (4·232 to 8·541)	6·056 (4·232 to 8·541)	6·056 (4·232 to 8·541)	6·056 (4·232 to 8·541)	6·056 (4·232 to 8·541)	6·056 (4·232 to 8·541)
Lip and oral cavity cancer	1-SIR	Both	Female	1·000 (1·000 to 1·000)	1·000 (1·000 to 1·000)	1·000 (1·000 to 1·000)	1·000 (1·000 to 1·000)	1·000 (1·000 to 1·000)	1·000 (1·000 to 1·000)	1·000 (1·000 to 1·000)	1·000 (1·000 to 1·000)
Nasopharynx cancer	SIR	Both	Male	8·227 (5·677 to 11·505)	8·227 (5·677 to 11·505)	8·227 (5·677 to 11·505)	8·227 (5·677 to 11·505)	8·227 (5·677 to 11·505)	8·227 (5·677 to 11·505)	8·227 (5·677 to 11·505)	8·227 (5·677 to 11·505)
Nasopharynx cancer	1-SIR	Both	Male	1·000 (1·000 to 1·000)	1·000 (1·000 to 1·000)	1·000 (1·000 to 1·000)	1·000 (1·000 to 1·000)	1·000 (1·000 to 1·000)	1·000 (1·000 to 1·000)	1·000 (1·000 to 1·000)	1·000 (1·000 to 1·000)
Nasopharynx cancer	SIR	Both	Female	6·089 (4·288 to 8·470)	6·089 (4·288 to 8·470)	6·089 (4·288 to 8·470)	6·089 (4·288 to 8·470)	6·089 (4·288 to 8·470)	6·089 (4·288 to 8·470)	6·089 (4·288 to 8·470)	6·089 (4·288 to 8·470)
Nasopharynx cancer	1-SIR	Both	Female	1·000 (1·000 to 1·000)	1·000 (1·000 to 1·000)	1·000 (1·000 to 1·000)	1·000 (1·000 to 1·000)	1·000 (1·000 to 1·000)	1·000 (1·000 to 1·000)	1·000 (1·000 to 1·000)	1·000 (1·000 to 1·000)
Oesophageal cancer	SIR	Both	Male	6·676 (4·136 to 10·250)	6·676 (4·136 to 10·250)	6·676 (4·136 to 10·250)	6·676 (4·136 to 10·250)	6·676 (4·136 to 10·250)	6·676 (4·136 to 10·250)	6·676 (4·136 to 10·250)	6·676 (4·136 to 10·250)
Oesophageal cancer	1-SIR	Both	Male	1·000 (1·000 to 1·000)	1·000 (1·000 to 1·000)	1·000 (1·000 to 1·000)	1·000 (1·000 to 1·000)	1·000 (1·000 to 1·000)	1·000 (1·000 to 1·000)	1·000 (1·000 to 1·000)	1·000 (1·000 to 1·000)
Oesophageal cancer	SIR	Both	Female	6·357 (4·442 to 8·634)	6·357 (4·442 to 8·634)	6·357 (4·442 to 8·634)	6·357 (4·442 to 8·634)	6·357 (4·442 to 8·634)	6·357 (4·442 to 8·634)	6·357 (4·442 to 8·634)	6·357 (4·442 to 8·634)
Oesophageal cancer	1-SIR	Both	Female	1·000 (1·000 to 1·000)	1·000 (1·000 to 1·000)	1·000 (1·000 to 1·000)	1·000 (1·000 to 1·000)	1·000 (1·000 to 1·000)	1·000 (1·000 to 1·000)	1·000 (1·000 to 1·000)	1·000 (1·000 to 1·000)
Stomach cancer	SIR	Both	Male	1·927 (1·443 to 2·535)	1·927 (1·443 to 2·535)	1·927 (1·443 to 2·535)	1·927 (1·443 to 2·535)	1·927 (1·443 to 2·535)	1·927 (1·443 to 2·535)	1·927 (1·443 to 2·535)	1·927 (1·443 to 2·535)
Stomach cancer	1-SIR	Both	Male	1·000 (1·000 to 1·000)	1·000 (1·000 to 1·000)	1·000 (1·000 to 1·000)	1·000 (1·000 to 1·000)	1·000 (1·000 to 1·000)	1·000 (1·000 to 1·000)	1·000 (1·000 to 1·000)	1·000 (1·000 to 1·000)
Stomach cancer	SIR	Both	Female	1·570 (1·246 to 1·925)	1·570 (1·246 to 1·925)	1·570 (1·246 to 1·925)	1·570 (1·246 to 1·925)	1·570 (1·246 to 1·925)	1·570 (1·246 to 1·925)	1·570 (1·246 to 1·925)	1·570 (1·246 to 1·925)
Stomach cancer	1-SIR	Both	Female	1·000 (1·000 to 1·000)	1·000 (1·000 to 1·000)	1·000 (1·000 to 1·000)	1·000 (1·000 to 1·000)	1·000 (1·000 to 1·000)	1·000 (1·000 to 1·000)	1·000 (1·000 to 1·000)	1·000 (1·000 to 1·000)
Colon and rectum cancer	SIR	Both	Male	1·325 (1·195 to 1·471)	1·325 (1·195 to 1·471)	1·325 (1·195 to 1·471)	1·325 (1·195 to 1·471)	1·325 (1·195 to 1·471)	1·325 (1·195 to 1·471)	1·325 (1·195 to 1·471)	1·325 (1·195 to 1·471)

Risk - Outcome	Category / Units	Morbidity / Mortality	Sex	All ages	0-6 Days	7-27 Days	28-364 Days	1-4 years	5-9 years	10-14 years	15-19 years	20-24 years	25-29 years	30-34 years	35-39 years	40-44 years
Colon and rectum cancer	1-SIR	Both	Male											1·000 (1·000 to 1·000)	1·000 (1·000 to 1·000)	1·000 (1·000 to 1·000)
Colon and rectum cancer	SIR	Both	Female											1·418 (1·278 to 1·571)	1·418 (1·278 to 1·571)	1·418 (1·278 to 1·571)
Colon and rectum cancer	1-SIR	Both	Female											1·000 (1·000 to 1·000)	1·000 (1·000 to 1·000)	1·000 (1·000 to 1·000)
Pancreatic cancer	SIR	Both	Male											2·506 (1·962 to 3·111)	2·506 (1·962 to 3·111)	2·506 (1·962 to 3·111)
Pancreatic cancer	1-SIR	Both	Male											1·000 (1·000 to 1·000)	1·000 (1·000 to 1·000)	1·000 (1·000 to 1·000)
Pancreatic cancer	SIR	Both	Female											2·098 (1·838 to 2·371)	2·098 (1·838 to 2·371)	2·098 (1·838 to 2·371)
Pancreatic cancer	1-SIR	Both	Female											1·000 (1·000 to 1·000)	1·000 (1·000 to 1·000)	1·000 (1·000 to 1·000)
Larynx cancer	SIR	Both	Male							14·602 (8·528 to 23·334)	14·602 (8·528 to 23·334)	14·602 (8·528 to 23·334)	14·602 (8·528 to 23·334)	14·602 (8·528 to 23·334)	14·602 (8·528 to 23·334)	14·602 (8·528 to 23·334)
Larynx cancer	1-SIR	Both	Male							1·000 (1·000 to 1·000)	1·000 (1·000 to 1·000)	1·000 (1·000 to 1·000)	1·000 (1·000 to 1·000)	1·000 (1·000 to 1·000)	1·000 (1·000 to 1·000)	1·000 (1·000 to 1·000)
Larynx cancer	SIR	Both	Female							135·959 (23·287 to 465·991)	135·959 (23·287 to 465·991)	135·959 (23·287 to 465·991)	135·959 (23·287 to 465·991)	135·959 (23·287 to 465·991)	135·959 (23·287 to 465·991)	135·959 (23·287 to 465·991)
Larynx cancer	1-SIR	Both	Female							1·000 (1·000 to 1·000)	1·000 (1·000 to 1·000)	1·000 (1·000 to 1·000)	1·000 (1·000 to 1·000)	1·000 (1·000 to 1·000)	1·000 (1·000 to 1·000)	1·000 (1·000 to 1·000)
Tracheal, bronchus, and lung cancer	SIR	Both	Male											22·511 (19·062 to 26·715)	22·511 (19·062 to 26·715)	22·511 (19·062 to 26·715)
Tracheal, bronchus, and lung cancer	1-SIR	Both	Male											1·000 (1·000 to 1·000)	1·000 (1·000 to 1·000)	1·000 (1·000 to 1·000)
Tracheal, bronchus, and lung cancer	SIR	Both	Female											14·095 (13·045 to 15·359)	14·095 (13·045 to 15·359)	14·095 (13·045 to 15·359)
Tracheal, bronchus, and lung cancer	1-SIR	Both	Female											1·000 (1·000 to 1·000)	1·000 (1·000 to 1·000)	1·000 (1·000 to 1·000)
Cervical cancer	SIR	Both	Both											1·679 (1·207 to 2·240)	1·679 (1·207 to 2·240)	1·679 (1·207 to 2·240)
Cervical cancer	1-SIR	Both	Both											1·000 (1·000 to 1·000)	1·000 (1·000 to 1·000)	1·000 (1·000 to 1·000)
Kidney cancer	SIR	Both	Male											2·293 (1·677 to 3·039)	2·293 (1·677 to 3·039)	2·293 (1·677 to 3·039)
Kidney cancer	1-SIR	Both	Male											1·000 (1·000 to 1·000)	1·000 (1·000 to 1·000)	1·000 (1·000 to 1·000)
Kidney cancer	SIR	Both	Female											1·518 (1·204 to 1·874)	1·518 (1·204 to 1·874)	1·518 (1·204 to 1·874)
Kidney cancer	1-SIR	Both	Female											1·000 (1·000 to 1·000)	1·000 (1·000 to 1·000)	1·000 (1·000 to 1·000)
Bladder cancer	SIR	Both	Male											3·332 (2·364 to 4·558)	3·332 (2·364 to 4·558)	3·332 (2·364 to 4·558)
Bladder cancer	1-SIR	Both	Male											1·000 (1·000 to 1·000)	1·000 (1·000 to 1·000)	1·000 (1·000 to 1·000)
Bladder cancer	SIR	Both	Female											2·582 (1·923 to 3·420)	2·582 (1·923 to 3·420)	2·582 (1·923 to 3·420)
Bladder cancer	1-SIR	Both	Female											1·000 (1·000 to 1·000)	1·000 (1·000 to 1·000)	1·000 (1·000 to 1·000)
Leukaemia	SIR	Both	Male											2·013 (1·390 to 2·873)	2·013 (1·390 to 2·873)	2·013 (1·390 to 2·873)
Leukaemia	1-SIR	Both	Male											1·000 (1·000 to 1·000)	1·000 (1·000 to 1·000)	1·000 (1·000 to 1·000)
Leukaemia	SIR	Both	Female											1·163 (0·894 to 1·479)	1·163 (0·894 to 1·479)	1·163 (0·894 to 1·479)
Leukaemia	1-SIR	Both	Female											1·000 (1·000 to 1·000)	1·000 (1·000 to 1·000)	1·000 (1·000 to 1·000)
Chronic obstructive pulmonary disease	SIR	Both	Male											11·546 (8·894 to 14·932)	11·546 (8·894 to 14·932)	11·546 (8·894 to 14·932)
Chronic obstructive pulmonary disease	1-SIR	Both	Male											1·000 (1·000 to 1·000)	1·000 (1·000 to 1·000)	1·000 (1·000 to 1·000)
Chronic obstructive pulmonary disease	SIR	Both	Female											15·257 (13·637 to 17·152)	15·257 (13·637 to 17·152)	15·257 (13·637 to 17·152)
Chronic obstructive pulmonary disease	1-SIR	Both	Female											1·000 (1·000 to 1·000)	1·000 (1·000 to 1·000)	1·000 (1·000 to 1·000)
Interstitial lung disease and pulmonary sarcoidosis	SIR	Both	Male											2·086 (1·774 to 2·441)	2·086 (1·774 to 2·441)	2·086 (1·774 to 2·441)
Interstitial lung disease and pulmonary sarcoidosis	1-SIR	Both	Male											1·000 (1·000 to 1·000)	1·000 (1·000 to 1·000)	1·000 (1·000 to 1·000)
Interstitial lung disease and pulmonary sarcoidosis	SIR	Both	Female											1·967 (1·768 to 2·176)	1·967 (1·768 to 2·176)	1·967 (1·768 to 2·176)
Interstitial lung disease and pulmonary sarcoidosis	1-SIR	Both	Female											1·000 (1·000 to 1·000)	1·000 (1·000 to 1·000)	1·000 (1·000 to 1·000)
Other chronic respiratory diseases	SIR	Both	Male											2·100 (1·774 to 2·462)	2·100 (1·774 to 2·462)	2·100 (1·774 to 2·462)
Other chronic respiratory diseases	1-SIR	Both	Male											1·000 (1·000 to 1·000)	1·000 (1·000 to 1·000)	1·000 (1·000 to 1·000)
Other chronic respiratory diseases	SIR	Both	Female											1·982 (1·800 to 2·172)	1·982 (1·800 to 2·172)	1·982 (1·800 to 2·172)
Other chronic respiratory diseases	1-SIR	Both	Female											1·000 (1·000 to 1·000)	1·000 (1·000 to 1·000)	1·000 (1·000 to 1·000)
**Second-hand smoke**																
Otitis media	Exposed	Morbidity	Both		1·372 (1·244 to 1·498)	1·372 (1·244 to 1·498)	1·372 (1·244 to 1·498)	1·372 (1·244 to 1·498)	1·372 (1·244 to 1·498)	1·372 (1·244 to 1·498)						
Otitis media	Not exposed	Morbidity	Both		1·000 (1·000 to 1·000)	1·000 (1·000 to 1·000)	1·000 (1·000 to 1·000)	1·000 (1·000 to 1·000)	1·000 (1·000 to 1·000)	1·000 (1·000 to 1·000)						
Otitis media	Exposed	Mortality	Both		1·372 (1·244 to 1·498)	1·372 (1·244 to 1·498)	1·372 (1·244 to 1·498)	1·372 (1·244 to 1·498)	1·372 (1·244 to 1·498)	1·372 (1·244 to 1·498)						
Otitis media	Not exposed	Mortality	Both		1·000 (1·000 to 1·000)	1·000 (1·000 to 1·000)	1·000 (1·000 to 1·000)	1·000 (1·000 to 1·000)	1·000 (1·000 to 1·000)	1·000 (1·000 to 1·000)						
**Diet low in fruits**																
Lip and oral cavity cancer	100 g/day	Both	Both										1·042 (0·994 to 1·091)	1·042 (0·994 to 1·091)	1·042 (0·994 to 1·091)	1·042 (0·994 to 1·091)
Nasopharynx cancer	100 g/day	Both	Both										1·043 (0·991 to 1·092)	1·043 (0·991 to 1·092)	1·043 (0·991 to 1·092)	1·043 (0·991 to 1·092)
Other pharynx cancer	100 g/day	Both	Both										1·042 (0·996 to 1·095)	1·042 (0·996 to 1·095)	1·042 (0·996 to 1·095)	1·042 (0·996 to 1·095)
Oesophageal cancer	100 g/day	Both	Both										1·153 (1·033 to 1·288)	1·153 (1·033 to 1·288)	1·153 (1·033 to 1·288)	1·153 (1·033 to 1·288)
Larynx cancer	100 g/day	Both	Both										1·042 (0·995 to 1·095)	1·042 (0·995 to 1·095)	1·042 (0·995 to 1·095)	1·042 (0·995 to 1·095)
Tracheal, bronchus, and lung cancer	100 g/day	Both	Both										1·076 (1·031 to 1·123)	1·076 (1·031 to 1·123)	1·076 (1·031 to 1·123)	1·076 (1·031 to 1·123)
Ischaemic heart disease	100 g/day	Both	Both										1·254 (1·081 to 1·444)	1·209 (1·068 to 1·363)	1·159 (1·052 to 1·273)	1·131 (1·043 to 1·222)
Ischaemic stroke	100 g/day	Both	Both										2·024 (1·464 to 2·828)	1·834 (1·389 to 2·451)	1·621 (1·300 to 2·047)	1·480 (1·238 to 1·791)
Hemorrhagic stroke	100 g/day	Both	Both										1·688 (1·318 to 2·182)	1·576 (1·272 to 1·973)	1·444 (1·215 to 1·733)	1·365 (1·180 to 1·595)
Diabetes mellitus	100 g/day	Both	Both										1·125 (1·027 to 1·238)	1·122 (1·026 to 1·233)	1·119 (1·025 to 1·226)	1·113 (1·024 to 1·214)
Ischaemic heart disease	100 g/day	Both	Both										1·249 (1·089 to 1·449)	1·205 (1·074 to 1·364)	1·154 (1·057 to 1·271)	1·126 (1·047 to 1·220)

Risk - Outcome	Category / Units	Morbidity / Mortality	Sex	45-49 years	50-54 years	55-59 years	60-64 years	65-69 years	70-74 years	75-79 years	80+ years
Colon and rectum cancer	1-SIR	Both	Male	1·000 (1·000 to 1·000)	1·000 (1·000 to 1·000)	1·000 (1·000 to 1·000)	1·000 (1·000 to 1·000)	1·000 (1·000 to 1·000)	1·000 (1·000 to 1·000)	1·000 (1·000 to 1·000)	1·000 (1·000 to 1·000)
Colon and rectum cancer	SIR	Both	Female	1·418 (1·278 to 1·571)	1·418 (1·278 to 1·571)	1·418 (1·278 to 1·571)	1·418 (1·278 to 1·571)	1·418 (1·278 to 1·571)	1·418 (1·278 to 1·571)	1·418 (1·278 to 1·571)	1·418 (1·278 to 1·571)
Colon and rectum cancer	1-SIR	Both	Female	1·000 (1·000 to 1·000)	1·000 (1·000 to 1·000)	1·000 (1·000 to 1·000)	1·000 (1·000 to 1·000)	1·000 (1·000 to 1·000)	1·000 (1·000 to 1·000)	1·000 (1·000 to 1·000)	1·000 (1·000 to 1·000)
Pancreatic cancer	SIR	Both	Male	2·506 (1·962 to 3·111)	2·506 (1·962 to 3·111)	2·506 (1·962 to 3·111)	2·506 (1·962 to 3·111)	2·506 (1·962 to 3·111)	2·506 (1·962 to 3·111)	2·506 (1·962 to 3·111)	2·506 (1·962 to 3·111)
Pancreatic cancer	1-SIR	Both	Male	1·000 (1·000 to 1·000)	1·000 (1·000 to 1·000)	1·000 (1·000 to 1·000)	1·000 (1·000 to 1·000)	1·000 (1·000 to 1·000)	1·000 (1·000 to 1·000)	1·000 (1·000 to 1·000)	1·000 (1·000 to 1·000)
Pancreatic cancer	SIR	Both	Female	2·098 (1·838 to 2·371)	2·098 (1·838 to 2·371)	2·098 (1·838 to 2·371)	2·098 (1·838 to 2·371)	2·098 (1·838 to 2·371)	2·098 (1·838 to 2·371)	2·098 (1·838 to 2·371)	2·098 (1·838 to 2·371)
Pancreatic cancer	1-SIR	Both	Female	1·000 (1·000 to 1·000)	1·000 (1·000 to 1·000)	1·000 (1·000 to 1·000)	1·000 (1·000 to 1·000)	1·000 (1·000 to 1·000)	1·000 (1·000 to 1·000)	1·000 (1·000 to 1·000)	1·000 (1·000 to 1·000)
Larynx cancer	SIR	Both	Male	14·602 (8·528 to 23·334)	14·602 (8·528 to 23·334)	14·602 (8·528 to 23·334)	14·602 (8·528 to 23·334)	14·602 (8·528 to 23·334)	14·602 (8·528 to 23·334)	14·602 (8·528 to 23·334)	14·602 (8·528 to 23·334)
Larynx cancer	1-SIR	Both	Male	1·000 (1·000 to 1·000)	1·000 (1·000 to 1·000)	1·000 (1·000 to 1·000)	1·000 (1·000 to 1·000)	1·000 (1·000 to 1·000)	1·000 (1·000 to 1·000)	1·000 (1·000 to 1·000)	1·000 (1·000 to 1·000)
Larynx cancer	SIR	Both	Female	135·959 (23·287 to 465·991)	135·959 (23·287 to 465·991)	135·959 (23·287 to 465·991)	135·959 (23·287 to 465·991)	135·959 (23·287 to 465·991)	135·959 (23·287 to 465·991)	135·959 (23·287 to 465·991)	135·959 (23·287 to 465·991)
Larynx cancer	1-SIR	Both	Female	1·000 (1·000 to 1·000)	1·000 (1·000 to 1·000)	1·000 (1·000 to 1·000)	1·000 (1·000 to 1·000)	1·000 (1·000 to 1·000)	1·000 (1·000 to 1·000)	1·000 (1·000 to 1·000)	1·000 (1·000 to 1·000)
Tracheal, bronchus, and lung cancer	SIR	Both	Male	22·511 (19·062 to 26·715)	22·511 (19·062 to 26·715)	22·511 (19·062 to 26·715)	22·511 (19·062 to 26·715)	22·511 (19·062 to 26·715)	22·511 (19·062 to 26·715)	22·511 (19·062 to 26·715)	22·511 (19·062 to 26·715)
Tracheal, bronchus, and lung cancer	1-SIR	Both	Male	1·000 (1·000 to 1·000)	1·000 (1·000 to 1·000)	1·000 (1·000 to 1·000)	1·000 (1·000 to 1·000)	1·000 (1·000 to 1·000)	1·000 (1·000 to 1·000)	1·000 (1·000 to 1·000)	1·000 (1·000 to 1·000)
Tracheal, bronchus, and lung cancer	SIR	Both	Female	14·095 (13·045 to 15·359)	14·095 (13·045 to 15·359)	14·095 (13·045 to 15·359)	14·095 (13·045 to 15·359)	14·095 (13·045 to 15·359)	14·095 (13·045 to 15·359)	14·095 (13·045 to 15·359)	14·095 (13·045 to 15·359)
Tracheal, bronchus, and lung cancer	1-SIR	Both	Female	1·000 (1·000 to 1·000)	1·000 (1·000 to 1·000)	1·000 (1·000 to 1·000)	1·000 (1·000 to 1·000)	1·000 (1·000 to 1·000)	1·000 (1·000 to 1·000)	1·000 (1·000 to 1·000)	1·000 (1·000 to 1·000)
Cervical cancer	SIR	Both	Both	1·679 (1·207 to 2·240)	1·679 (1·207 to 2·240)	1·679 (1·207 to 2·240)	1·679 (1·207 to 2·240)	1·679 (1·207 to 2·240)	1·679 (1·207 to 2·240)	1·679 (1·207 to 2·240)	1·679 (1·207 to 2·240)
Cervical cancer	1-SIR	Both	Both	1·000 (1·000 to 1·000)	1·000 (1·000 to 1·000)	1·000 (1·000 to 1·000)	1·000 (1·000 to 1·000)	1·000 (1·000 to 1·000)	1·000 (1·000 to 1·000)	1·000 (1·000 to 1·000)	1·000 (1·000 to 1·000)
Kidney cancer	SIR	Both	Male	2·293 (1·677 to 3·039)	2·293 (1·677 to 3·039)	2·293 (1·677 to 3·039)	2·293 (1·677 to 3·039)	2·293 (1·677 to 3·039)	2·293 (1·677 to 3·039)	2·293 (1·677 to 3·039)	2·293 (1·677 to 3·039)
Kidney cancer	1-SIR	Both	Male	1·000 (1·000 to 1·000)	1·000 (1·000 to 1·000)	1·000 (1·000 to 1·000)	1·000 (1·000 to 1·000)	1·000 (1·000 to 1·000)	1·000 (1·000 to 1·000)	1·000 (1·000 to 1·000)	1·000 (1·000 to 1·000)
Kidney cancer	SIR	Both	Female	1·518 (1·204 to 1·874)	1·518 (1·204 to 1·874)	1·518 (1·204 to 1·874)	1·518 (1·204 to 1·874)	1·518 (1·204 to 1·874)	1·518 (1·204 to 1·874)	1·518 (1·204 to 1·874)	1·518 (1·204 to 1·874)
Kidney cancer	1-SIR	Both	Female	1·000 (1·000 to 1·000)	1·000 (1·000 to 1·000)	1·000 (1·000 to 1·000)	1·000 (1·000 to 1·000)	1·000 (1·000 to 1·000)	1·000 (1·000 to 1·000)	1·000 (1·000 to 1·000)	1·000 (1·000 to 1·000)
Bladder cancer	SIR	Both	Male	3·332 (2·364 to 4·558)	3·332 (2·364 to 4·558)	3·332 (2·364 to 4·558)	3·332 (2·364 to 4·558)	3·332 (2·364 to 4·558)	3·332 (2·364 to 4·558)	3·332 (2·364 to 4·558)	3·332 (2·364 to 4·558)
Bladder cancer	1-SIR	Both	Male	1·000 (1·000 to 1·000)	1·000 (1·000 to 1·000)	1·000 (1·000 to 1·000)	1·000 (1·000 to 1·000)	1·000 (1·000 to 1·000)	1·000 (1·000 to 1·000)	1·000 (1·000 to 1·000)	1·000 (1·000 to 1·000)
Bladder cancer	SIR	Both	Female	2·582 (1·923 to 3·420)	2·582 (1·923 to 3·420)	2·582 (1·923 to 3·420)	2·582 (1·923 to 3·420)	2·582 (1·923 to 3·420)	2·582 (1·923 to 3·420)	2·582 (1·923 to 3·420)	2·582 (1·923 to 3·420)
Bladder cancer	1-SIR	Both	Female	1·000 (1·000 to 1·000)	1·000 (1·000 to 1·000)	1·000 (1·000 to 1·000)	1·000 (1·000 to 1·000)	1·000 (1·000 to 1·000)	1·000 (1·000 to 1·000)	1·000 (1·000 to 1·000)	1·000 (1·000 to 1·000)
Leukaemia	SIR	Both	Male	2·013 (1·390 to 2·873)	2·013 (1·390 to 2·873)	2·013 (1·390 to 2·873)	2·013 (1·390 to 2·873)	2·013 (1·390 to 2·873)	2·013 (1·390 to 2·873)	2·013 (1·390 to 2·873)	2·013 (1·390 to 2·873)
Leukaemia	1-SIR	Both	Male	1·000 (1·000 to 1·000)	1·000 (1·000 to 1·000)	1·000 (1·000 to 1·000)	1·000 (1·000 to 1·000)	1·000 (1·000 to 1·000)	1·000 (1·000 to 1·000)	1·000 (1·000 to 1·000)	1·000 (1·000 to 1·000)
Leukaemia	SIR	Both	Female	1·163 (0·894 to 1·479)	1·163 (0·894 to 1·479)	1·163 (0·894 to 1·479)	1·163 (0·894 to 1·479)	1·163 (0·894 to 1·479)	1·163 (0·894 to 1·479)	1·163 (0·894 to 1·479)	1·163 (0·894 to 1·479)
Leukaemia	1-SIR	Both	Female	1·000 (1·000 to 1·000)	1·000 (1·000 to 1·000)	1·000 (1·000 to 1·000)	1·000 (1·000 to 1·000)	1·000 (1·000 to 1·000)	1·000 (1·000 to 1·000)	1·000 (1·000 to 1·000)	1·000 (1·000 to 1·000)
Chronic obstructive pulmonary disease	SIR	Both	Male	11·546 (8·894 to 14·932)	11·546 (8·894 to 14·932)	11·546 (8·894 to 14·932)	11·546 (8·894 to 14·932)	11·546 (8·894 to 14·932)	11·546 (8·894 to 14·932)	11·546 (8·894 to 14·932)	11·546 (8·894 to 14·932)
Chronic obstructive pulmonary disease	1-SIR	Both	Male	1·000 (1·000 to 1·000)	1·000 (1·000 to 1·000)	1·000 (1·000 to 1·000)	1·000 (1·000 to 1·000)	1·000 (1·000 to 1·000)	1·000 (1·000 to 1·000)	1·000 (1·000 to 1·000)	1·000 (1·000 to 1·000)
Chronic obstructive pulmonary disease	SIR	Both	Female	15·257 (13·637 to 17·152)	15·257 (13·637 to 17·152)	15·257 (13·637 to 17·152)	15·257 (13·637 to 17·152)	15·257 (13·637 to 17·152)	15·257 (13·637 to 17·152)	15·257 (13·637 to 17·152)	15·257 (13·637 to 17·152)
Chronic obstructive pulmonary disease	1-SIR	Both	Female	1·000 (1·000 to 1·000)	1·000 (1·000 to 1·000)	1·000 (1·000 to 1·000)	1·000 (1·000 to 1·000)	1·000 (1·000 to 1·000)	1·000 (1·000 to 1·000)	1·000 (1·000 to 1·000)	1·000 (1·000 to 1·000)
Interstitial lung disease and pulmonary sarcoidosis	SIR	Both	Male	2·086 (1·774 to 2·441)	2·086 (1·774 to 2·441)	2·086 (1·774 to 2·441)	2·086 (1·774 to 2·441)	2·086 (1·774 to 2·441)	2·086 (1·774 to 2·441)	2·086 (1·774 to 2·441)	2·086 (1·774 to 2·441)
Interstitial lung disease and pulmonary sarcoidosis	1-SIR	Both	Male	1·000 (1·000 to 1·000)	1·000 (1·000 to 1·000)	1·000 (1·000 to 1·000)	1·000 (1·000 to 1·000)	1·000 (1·000 to 1·000)	1·000 (1·000 to 1·000)	1·000 (1·000 to 1·000)	1·000 (1·000 to 1·000)
Interstitial lung disease and pulmonary sarcoidosis	SIR	Both	Female	1·967 (1·768 to 2·176)	1·967 (1·768 to 2·176)	1·967 (1·768 to 2·176)	1·967 (1·768 to 2·176)	1·967 (1·768 to 2·176)	1·967 (1·768 to 2·176)	1·967 (1·768 to 2·176)	1·967 (1·768 to 2·176)
Interstitial lung disease and pulmonary sarcoidosis	1-SIR	Both	Female	1·000 (1·000 to 1·000)	1·000 (1·000 to 1·000)	1·000 (1·000 to 1·000)	1·000 (1·000 to 1·000)	1·000 (1·000 to 1·000)	1·000 (1·000 to 1·000)	1·000 (1·000 to 1·000)	1·000 (1·000 to 1·000)
Other chronic respiratory diseases	SIR	Both	Male	2·100 (1·774 to 2·462)	2·100 (1·774 to 2·462)	2·100 (1·774 to 2·462)	2·100 (1·774 to 2·462)	2·100 (1·774 to 2·462)	2·100 (1·774 to 2·462)	2·100 (1·774 to 2·462)	2·100 (1·774 to 2·462)
Other chronic respiratory diseases	1-SIR	Both	Male	1·000 (1·000 to 1·000)	1·000 (1·000 to 1·000)	1·000 (1·000 to 1·000)	1·000 (1·000 to 1·000)	1·000 (1·000 to 1·000)	1·000 (1·000 to 1·000)	1·000 (1·000 to 1·000)	1·000 (1·000 to 1·000)
Other chronic respiratory diseases	SIR	Both	Female	1·982 (1·800 to 2·172)	1·982 (1·800 to 2·172)	1·982 (1·800 to 2·172)	1·982 (1·800 to 2·172)	1·982 (1·800 to 2·172)	1·982 (1·800 to 2·172)	1·982 (1·800 to 2·172)	1·982 (1·800 to 2·172)
Other chronic respiratory diseases	1-SIR	Both	Female	1·000 (1·000 to 1·000)	1·000 (1·000 to 1·000)	1·000 (1·000 to 1·000)	1·000 (1·000 to 1·000)	1·000 (1·000 to 1·000)	1·000 (1·000 to 1·000)	1·000 (1·000 to 1·000)	1·000 (1·000 to 1·000)
**Second-hand smoke**											
Otitis media	Exposed	Morbidity	Both								
Otitis media	Not exposed	Morbidity	Both								
Otitis media	Exposed	Mortality	Both								
Otitis media	Not exposed	Mortality	Both								
**Diet low in fruits**											
Lip and oral cavity cancer	100 g/day	Both	Both	1·042 (0·994 to 1·091)	1·042 (0·994 to 1·091)	1·042 (0·994 to 1·091)	1·042 (0·994 to 1·091)	1·042 (0·994 to 1·091)	1·042 (0·994 to 1·091)	1·042 (0·994 to 1·091)	1·042 (0·994 to 1·091)
Nasopharynx cancer	100 g/day	Both	Both	1·043 (0·991 to 1·092)	1·043 (0·991 to 1·092)	1·043 (0·991 to 1·092)	1·043 (0·991 to 1·092)	1·043 (0·991 to 1·092)	1·043 (0·991 to 1·092)	1·043 (0·991 to 1·092)	1·043 (0·991 to 1·092)
Other pharynx cancer	100 g/day	Both	Both	1·042 (0·996 to 1·095)	1·042 (0·996 to 1·095)	1·042 (0·996 to 1·095)	1·042 (0·996 to 1·095)	1·042 (0·996 to 1·095)	1·042 (0·996 to 1·095)	1·042 (0·996 to 1·095)	1·042 (0·996 to 1·095)
Oesophageal cancer	100 g/day	Both	Both	1·153 (1·033 to 1·288)	1·153 (1·033 to 1·288)	1·153 (1·033 to 1·288)	1·153 (1·033 to 1·288)	1·153 (1·033 to 1·288)	1·153 (1·033 to 1·288)	1·153 (1·033 to 1·288)	1·153 (1·033 to 1·288)
Larynx cancer	100 g/day	Both	Both	1·042 (0·995 to 1·095)	1·042 (0·995 to 1·095)	1·042 (0·995 to 1·095)	1·042 (0·995 to 1·095)	1·042 (0·995 to 1·095)	1·042 (0·995 to 1·095)	1·042 (0·995 to 1·095)	1·042 (0·995 to 1·095)
Tracheal, bronchus, and lung cancer	100 g/day	Both	Both	1·076 (1·031 to 1·123)	1·076 (1·031 to 1·123)	1·076 (1·031 to 1·123)	1·076 (1·031 to 1·123)	1·076 (1·031 to 1·123)	1·076 (1·031 to 1·123)	1·076 (1·031 to 1·123)	1·076 (1·031 to 1·123)
Ischaemic heart disease	100 g/day	Both	Both	1·125 (1·041 to 1·212)	1·114 (1·038 to 1·194)	1·099 (1·033 to 1·168)	1·087 (1·029 to 1·147)	1·078 (1·026 to 1·131)	1·070 (1·024 to 1·118)	1·064 (1·022 to 1·107)	1·057 (1·020 to 1·096)
Ischaemic stroke	100 g/day	Both	Both	1·403 (1·203 to 1·655)	1·333 (1·170 to 1·535)	1·272 (1·141 to 1·433)	1·222 (1·116 to 1·349)	1·181 (1·096 to 1·284)	1·145 (1·077 to 1·226)	1·114 (1·061 to 1·176)	1·054 (1·029 to 1·082)
Hemorrhagic stroke	100 g/day	Both	Both	1·336 (1·166 to 1·544)	1·300 (1·150 to 1·483)	1·260 (1·131 to 1·415)	1·226 (1·115 to 1·359)	1·193 (1·099 to 1·305)	1·164 (1·084 to 1·257)	1·133 (1·069 to 1·207)	1·065 (1·034 to 1·100)
Diabetes mellitus	100 g/day	Both	Both	1·102 (1·022 to 1·194)	1·093 (1·020 to 1·177)	1·085 (1·019 to 1·161)	1·076 (1·017 to 1·144)	1·068 (1·015 to 1·128)	1·061 (1·013 to 1·114)	1·052 (1·012 to 1·098)	1·036 (1·008 to 1·066)
Ischaemic heart disease	100 g/day	Both	Both	1·121 (1·045 to 1·211)	1·111 (1·042 to 1·194)	1·098 (1·037 to 1·169)	1·086 (1·032 to 1·148)	1·077 (1·029 to 1·133)	1·070 (1·027 to 1·120)	1·064 (1·024 to 1·109)	1·057 (1·022 to 1·097)

Risk - Outcome	Category / Units	Morbidity / Mortality	Sex	All ages	0-6 Days	7-27 Days	28-364 Days	1-4 years	5-9 years	10-14 years	15-19 years	20-24 years	25-29 years	30-34 years	35-39 years	40-44 years
Ischaemic stroke	100 g/day	Both	Both										1·249 (1·048 to 1·463)	1·211 (1·041 to 1·389)	1·165 (1·033 to 1·300)	1·132 (1·026 to 1·238)
Hemorrhagic stroke	100 g/day	Both	Both										1·177 (1·046 to 1·326)	1·153 (1·040 to 1·278)	1·122 (1·032 to 1·220)	1·102 (1·027 to 1·184)
**Diet low in whole grains**																
Ischaemic heart disease	50 g/day	Both	Both										1·478 (1·273 to 1·722)	1·387 (1·224 to 1·578)	1·285 (1·168 to 1·419)	1·228 (1·136 to 1·333)
Ischaemic stroke	50 g/day	Both	Both										2·075 (1·669 to 2·519)	1·863 (1·548 to 2·200)	1·624 (1·406 to 1·850)	1·466 (1·309 to 1·625)
Hemorrhagic stroke	50 g/day	Both	Both										1·596 (1·406 to 1·825)	1·484 (1·333 to 1·662)	1·349 (1·244 to 1·471)	1·276 (1·194 to 1·369)
Diabetes mellitus	50 g/day	Both	Both										1·231 (1·124 to 1·349)	1·226 (1·121 to 1·341)	1·220 (1·118 to 1·331)	1·208 (1·112 to 1·313)
**Diet low in nuts and seeds**																
Ischaemic heart disease	4.05 g/day	Morbidity	Both										1·176 (1·053 to 1·322)	1·143 (1·044 to 1·260)	1·105 (1·033 to 1·188)	1·084 (1·026 to 1·150)
Ischaemic heart disease	4.05 g/day	Mortality	Both										1·209 (1·128 to 1·296)	1·169 (1·105 to 1·239)	1·124 (1·077 to 1·174)	1·099 (1·062 to 1·138)
Diabetes mellitus	4.05 g/day	Both	Both										1·050 (1·025 to 1·075)	1·049 (1·025 to 1·073)	1·048 (1·024 to 1·071)	1·045 (1·023 to 1·068)
**Diet low in milk**																
Colon and rectum cancer	226.8 g/day	Both	Both										1·113 (1·038 to 1·203)	1·113 (1·038 to 1·203)	1·113 (1·038 to 1·203)	1·113 (1·038 to 1·203)
**Diet high in red meat**																
Colon and rectum cancer	100 g/day	Both	Both										1·167 (1·033 to 1·309)	1·167 (1·033 to 1·309)	1·167 (1·033 to 1·309)	1·167 (1·033 to 1·309)
Diabetes mellitus	100 g/day	Both	Both										1·322 (1·036 to 1·604)	1·314 (1·035 to 1·588)	1·305 (1·034 to 1·570)	1·288 (1·033 to 1·536)
**Diet high in processed meat**																
Colon and rectum cancer	50 g/day	Both	Both										1·179 (1·092 to 1·267)	1·179 (1·092 to 1·267)	1·179 (1·092 to 1·267)	1·179 (1·092 to 1·267)
Ischaemic heart disease	50 g/day	Both	Both										2·568 (1·045 to 4·695)	2·124 (1·036 to 3·501)	1·720 (1·026 to 2·501)	1·545 (1·021 to 2·101)
Diabetes mellitus	50 g/day	Both	Both										1·940 (1·388 to 2·558)	1·913 (1·379 to 2·508)	1·881 (1·368 to 2·451)	1·824 (1·347 to 2·348)
**Diet high in sugar-sweetened beverages***																
	BMI < 25	Both	Both										0·090 (0·050 to 0·140)	0·090 (0·050 to 0·140)	0·090 (0·050 to 0·140)	0·090 (0·050 to 0·140)
	BMI > 25	Both	Both										0·230 (0·140 to 0·320)	0·230 (0·140 to 0·320)	0·230 (0·140 to 0·320)	0·230 (0·140 to 0·320)
**Diet low in fibre**																
Colon and rectum cancer	20 g/day	Both	Both										1·236 (1·133 to 1·350)	1·236 (1·133 to 1·350)	1·236 (1·133 to 1·350)	1·236 (1·133 to 1·350)
Ischaemic heart disease	20 g/day	Both	Both										1·688 (1·414 to 2·029)	1·622 (1·377 to 1·923)	1·529 (1·325 to 1·777)	1·450 (1·279 to 1·654)
**Diet low in calcium**																
Colon and rectum cancer	g/day	Both	Both										1·372 (1·267 to 1·485)	1·372 (1·267 to 1·485)	1·372 (1·267 to 1·485)	1·372 (1·267 to 1·485)
**Diet low in seafood omega-3 fatty acids**																
Ischaemic heart disease	100 mg/day	Morbidity	Both										1·000 (1·000 to 1·000)	1·000 (1·000 to 1·000)	1·000 (1·000 to 1·000)	1·000 (1·000 to 1·000)
Ischaemic heart disease	100 mg/day	Mortality	Both										1·291 (1·109 to 1·506)	1·249 (1·094 to 1·429)	1·199 (1·076 to 1·339)	1·173 (1·067 to 1·293)
**Diet low in polyunsaturated fatty acids**																
Ischaemic heart disease	5% energy/day	Both	Both										1·267 (1·097 to 1·452)	1·211 (1·078 to 1·352)	1·148 (1·056 to 1·244)	1·114 (1·043 to 1·186)
**Diet high in trans fatty acids**																
Ischaemic heart disease	2% energy/day	Both	Both										1·901 (1·590 to 2·276)	1·775 (1·514 to 2·085)	1·615 (1·414 to 1·849)	1·517 (1·352 to 1·707)
**Diet high in sodium****																
	Non-Black, Non-Hypertensive	Both	Both										-1·366 (-1·937 to -0·795)	-1·882 (-2·434 to -1·330)	-2·397 (-2·967 to -1·828)	-2·913 (-3·533 to -2·292)
	Non-Black, Hypertensive	Both	Both										-3·300 (-4·147 to -2·454)	-3·816 (-4·547 to -3·085)	-4·331 (-4·959 to -3·704)	-4·847 (-5·389 to -4·305)
	Black, Non-Hypertensive	Both	Both										-3·910 (-5·065 to -2·755)	-4·426 (-5·564 to -3·287)	-4·941 (-6·081 to -3·802)	-5·457 (-6·616 to -4·298)
	Black, Hypertensive	Both	Both										-5·844 (-7·222 to -4·467)	-6·360 (-7·663 to -5·057)	-6·876 (-8·117 to -5·635)	-7·391 (-8·584 to -6·198)
**Childhood sexual abuse**																
Alcohol use disorders	Exposed	Both	Male		1·583 (0·993 to 2·376)	1·583 (0·993 to 2·376)	1·583 (0·993 to 2·376)	1·583 (0·993 to 2·376)	1·583 (0·993 to 2·376)	1·583 (0·993 to 2·376)	1·583 (0·993 to 2·376)	1·583 (0·993 to 2·376)	1·583 (0·993 to 2·376)	1·583 (0·993 to 2·376)	1·583 (0·993 to 2·376)	1·583 (0·993 to 2·376)
Alcohol use disorders	Not exposed	Both	Male		1·000 (1·000 to 1·000)	1·000 (1·000 to 1·000)	1·000 (1·000 to 1·000)	1·000 (1·000 to 1·000)	1·000 (1·000 to 1·000)	1·000 (1·000 to 1·000)	1·000 (1·000 to 1·000)	1·000 (1·000 to 1·000)	1·000 (1·000 to 1·000)	1·000 (1·000 to 1·000)	1·000 (1·000 to 1·000)	1·000 (1·000 to 1·000)
Alcohol use disorders	Exposed	Both	Female		1·583 (0·993 to 2·376)	1·583 (0·993 to 2·376)	1·583 (0·993 to 2·376)	1·583 (0·993 to 2·376)	1·583 (0·993 to 2·376)	1·583 (0·993 to 2·376)	1·583 (0·993 to 2·376)	1·583 (0·993 to 2·376)	1·583 (0·993 to 2·376)	1·583 (0·993 to 2·376)	1·583 (0·993 to 2·376)	1·583 (0·993 to 2·376)
Alcohol use disorders	Not exposed	Both	Female		1·000 (1·000 to 1·000)	1·000 (1·000 to 1·000)	1·000 (1·000 to 1·000)	1·000 (1·000 to 1·000)	1·000 (1·000 to 1·000)	1·000 (1·000 to 1·000)	1·000 (1·000 to 1·000)	1·000 (1·000 to 1·000)	1·000 (1·000 to 1·000)	1·000 (1·000 to 1·000)	1·000 (1·000 to 1·000)	1·000 (1·000 to 1·000)
Self-harm	Exposed	Both	Male		2·874 (1·369 to 5·430)	2·874 (1·369 to 5·430)	2·874 (1·369 to 5·430)	2·874 (1·369 to 5·430)	2·874 (1·369 to 5·430)	2·874 (1·369 to 5·430)	2·874 (1·369 to 5·430)	2·874 (1·369 to 5·430)	2·874 (1·369 to 5·430)	2·874 (1·369 to 5·430)	2·874 (1·369 to 5·430)	2·874 (1·369 to 5·430)
Self-harm	Not exposed	Both	Male		1·000 (1·000 to 1·000)	1·000 (1·000 to 1·000)	1·000 (1·000 to 1·000)	1·000 (1·000 to 1·000)	1·000 (1·000 to 1·000)	1·000 (1·000 to 1·000)	1·000 (1·000 to 1·000)	1·000 (1·000 to 1·000)	1·000 (1·000 to 1·000)	1·000 (1·000 to 1·000)	1·000 (1·000 to 1·000)	1·000 (1·000 to 1·000)
Self-harm	Exposed	Both	Female		2·874 (1·369 to 5·430)	2·874 (1·369 to 5·430)	2·874 (1·369 to 5·430)	2·874 (1·369 to 5·430)	2·874 (1·369 to 5·430)	2·874 (1·369 to 5·430)	2·874 (1·369 to 5·430)	2·874 (1·369 to 5·430)	2·874 (1·369 to 5·430)	2·874 (1·369 to 5·430)	2·874 (1·369 to 5·430)	2·874 (1·369 to 5·430)
Self-harm	Not exposed	Both	Female		1·000 (1·000 to 1·000)	1·000 (1·000 to 1·000)	1·000 (1·000 to 1·000)	1·000 (1·000 to 1·000)	1·000 (1·000 to 1·000)	1·000 (1·000 to 1·000)	1·000 (1·000 to 1·000)	1·000 (1·000 to 1·000)	1·000 (1·000 to 1·000)	1·000 (1·000 to 1·000)	1·000 (1·000 to 1·000)	1·000 (1·000 to 1·000)
**Intimate partner violence (exposure approach)**																
Maternal abortion, miscarriage, and ectopic	Exposed	Both	Both								2·090 (1·613 to 2·674)	2·076 (1·630 to 2·621)	2·092 (1·624 to 2·676)	2·083 (1·633 to 2·628)	2·099 (1·618 to 2·636)	2·094 (1·648 to 2·651)
Maternal abortion, miscarriage, and ectopic	Not exposed	Both	Both								1·000 (1·000 to 1·000)	1·000 (1·000 to 1·000)	1·000 (1·000 to 1·000)	1·000 (1·000 to 1·000)	1·000 (1·000 to 1·000)	1·000 (1·000 to 1·000)
Self-harm	Exposed	Both	Both								5·086 (1·784 to 11·744)	5·017 (1·762 to 11·534)	4·979 (1·669 to 11·483)	5·042 (1·782 to 11·936)	5·036 (1·702 to 11·893)	5·135 (1·754 to 11·833)
Self-harm	Not exposed	Both	Both								1·000 (1·000 to 1·000)	1·000 (1·000 to 1·000)	1·000 (1·000 to 1·000)	1·000 (1·000 to 1·000)	1·000 (1·000 to 1·000)	1·000 (1·000 to 1·000)
**Low physical activity**																
Colon and rectum cancer	<600 METs	Both	Both										1·293 (1·211 to 1·381)	1·293 (1·211 to 1·381)	1·293 (1·211 to 1·381)	1·293 (1·211 to 1·381)
Colon and rectum cancer	600-3,999 METs	Both	Both										1·172 (1·094 to 1·260)	1·172 (1·094 to 1·260)	1·172 (1·094 to 1·260)	1·172 (1·094 to 1·260)
Colon and rectum cancer	4,000-7,999 METs	Both	Both										1·067 (0·965 to 1·181)	1·067 (0·965 to 1·181)	1·067 (0·965 to 1·181)	1·067 (0·965 to 1·181)

Risk - Outcome	Category / Units	Morbidity / Mortality	Sex	45-49 years	50-54 years	55-59 years	60-64 years	65-69 years	70-74 years	75-79 years	80+ years
Ischaemic stroke	100 g/day	Both	Both	1·113 (1·023 to 1·203)	1·095 (1·019 to 1·170)	1·079 (1·016 to 1·141)	1·065 (1·013 to 1·116)	1·054 (1·011 to 1·096)	1·044 (1·009 to 1·077)	1·035 (1·007 to 1·061)	1·017 (1·004 to 1·029)
Hemorrhagic stroke	100 g/day	Both	Both	1·095 (1·025 to 1·170)	1·086 (1·023 to 1·153)	1·075 (1·020 to 1·134)	1·066 (1·018 to 1·117)	1·057 (1·015 to 1·101)	1·049 (1·013 to 1·086)	1·040 (1·011 to 1·071)	1·020 (1·005 to 1·035)
**Diet low in whole grains**											
Ischaemic heart disease	50 g/day	Both	Both	1·216 (1·129 to 1·314)	1·194 (1·116 to 1·282)	1·165 (1·099 to 1·238)	1·141 (1·085 to 1·203)	1·125 (1·076 to 1·179)	1·112 (1·068 to 1·160)	1·102 (1·062 to 1·145)	1·097 (1·059 to 1·138)
Ischaemic stroke	50 g/day	Both	Both	1·380 (1·255 to 1·506)	1·304 (1·206 to 1·402)	1·241 (1·165 to 1·316)	1·189 (1·130 to 1·247)	1·150 (1·104 to 1·195)	1·117 (1·081 to 1·152)	1·090 (1·063 to 1·116)	1·041 (1·029 to 1·053)
Hemorrhagic stroke	50 g/day	Both	Both	1·258 (1·182 to 1·344)	1·232 (1·165 to 1·309)	1·201 (1·143 to 1·267)	1·176 (1·126 to 1·233)	1·150 (1·108 to 1·198)	1·128 (1·092 to 1·169)	1·106 (1·076 to 1·139)	1·050 (1·036 to 1·065)
Diabetes mellitus	50 g/day	Both	Both	1·189 (1·102 to 1·283)	1·172 (1·093 to 1·256)	1·156 (1·085 to 1·232)	1·139 (1·076 to 1·207)	1·125 (1·068 to 1·185)	1·111 (1·061 to 1·163)	1·095 (1·053 to 1·140)	1·064 (1·036 to 1·094)
**Diet low in nuts and seeds**											
Ischaemic heart disease	4.05 g/day	Morbidity	Both	1·081 (1·025 to 1·144)	1·074 (1·023 to 1·132)	1·064 (1·020 to 1·114)	1·056 (1·018 to 1·099)	1·050 (1·016 to 1·089)	1·046 (1·015 to 1·081)	1·042 (1·013 to 1·075)	1·039 (1·013 to 1·069)
Ischaemic heart disease	4.05 g/day	Mortality	Both	1·095 (1·060 to 1·133)	1·088 (1·055 to 1·122)	1·076 (1·048 to 1·105)	1·066 (1·042 to 1·092)	1·059 (1·037 to 1·082)	1·054 (1·034 to 1·075)	1·050 (1·032 to 1·069)	1·046 (1·029 to 1·064)
Diabetes mellitus	4.05 g/day	Both	Both	1·041 (1·021 to 1·062)	1·038 (1·019 to 1·057)	1·035 (1·017 to 1·052)	1·031 (1·016 to 1·046)	1·028 (1·014 to 1·042)	1·025 (1·013 to 1·037)	1·022 (1·011 to 1·032)	1·015 (1·007 to 1·022)
**Diet low in milk**											
Colon and rectum cancer	226.8 g/day	Both	Both	1·113 (1·038 to 1·203)	1·113 (1·038 to 1·203)	1·113 (1·038 to 1·203)	1·113 (1·038 to 1·203)	1·113 (1·038 to 1·203)	1·113 (1·038 to 1·203)	1·113 (1·038 to 1·203)	1·113 (1·038 to 1·203)
**Diet high in red meat**											
Colon and rectum cancer	100 g/day	Both	Both	1·167 (1·033 to 1·309)	1·167 (1·033 to 1·309)	1·167 (1·033 to 1·309)	1·167 (1·033 to 1·309)	1·167 (1·033 to 1·309)	1·167 (1·033 to 1·309)	1·167 (1·033 to 1·309)	1·167 (1·033 to 1·309)
Diabetes mellitus	100 g/day	Both	Both	1·260 (1·030 to 1·481)	1·236 (1·027 to 1·434)	1·213 (1·025 to 1·390)	1·190 (1·023 to 1·346)	1·169 (1·020 to 1·306)	1·150 (1·018 to 1·270)	1·128 (1·016 to 1·230)	1·086 (1·011 to 1·152)
**Diet high in processed meat**											
Colon and rectum cancer	50 g/day	Both	Both	1·179 (1·092 to 1·267)	1·179 (1·092 to 1·267)	1·179 (1·092 to 1·267)	1·179 (1·092 to 1·267)	1·179 (1·092 to 1·267)	1·179 (1·092 to 1·267)	1·179 (1·092 to 1·267)	1·179 (1·092 to 1·267)
Ischaemic heart disease	50 g/day	Both	Both	1·547 (1·021 to 2·106)	1·520 (1·020 to 2·045)	1·467 (1·019 to 1·928)	1·422 (1·017 to 1·832)	1·386 (1·016 to 1·755)	1·354 (1·015 to 1·687)	1·325 (1·014 to 1·626)	1·252 (1·011 to 1·478)
Diabetes mellitus	50 g/day	Both	Both	1·731 (1·313 to 2·182)	1·653 (1·284 to 2·045)	1·583 (1·257 to 1·924)	1·512 (1·229 to 1·804)	1·450 (1·204 to 1·700)	1·393 (1·180 to 1·607)	1·332 (1·154 to 1·508)	1·216 (1·103 to 1·325)
**Diet high in sugar-sweetened beverages***											
	BMI < 25	Both	Both	0·090 (0·050 to 0·140)	0·090 (0·050 to 0·140)	0·090 (0·050 to 0·140)	0·090 (0·050 to 0·140)	0·090 (0·050 to 0·140)	0·090 (0·050 to 0·140)	0·090 (0·050 to 0·140)	0·090 (0·050 to 0·140)
	BMI > 25	Both	Both	0·230 (0·140 to 0·320)	0·230 (0·140 to 0·320)	0·230 (0·140 to 0·320)	0·230 (0·140 to 0·320)	0·230 (0·140 to 0·320)	0·230 (0·140 to 0·320)	0·230 (0·140 to 0·320)	0·230 (0·140 to 0·320)
**Diet low in fibre**											
Colon and rectum cancer	20 g/day	Both	Both	1·236 (1·133 to 1·350)	1·236 (1·133 to 1·350)	1·236 (1·133 to 1·350)	1·236 (1·133 to 1·350)	1·236 (1·133 to 1·350)	1·236 (1·133 to 1·350)	1·236 (1·133 to 1·350)	1·236 (1·133 to 1·350)
Ischaemic heart disease	20 g/day	Both	Both	1·387 (1·243 to 1·559)	1·318 (1·201 to 1·455)	1·242 (1·155 to 1·343)	1·184 (1·119 to 1·258)	1·147 (1·096 to 1·205)	1·118 (1·077 to 1·164)	1·097 (1·064 to 1·135)	1·133 (1·087 to 1·185)
**Diet low in calcium**											
Colon and rectum cancer	g/day	Both	Both	1·372 (1·267 to 1·485)	1·372 (1·267 to 1·485)	1·372 (1·267 to 1·485)	1·372 (1·267 to 1·485)	1·372 (1·267 to 1·485)	1·372 (1·267 to 1·485)	1·372 (1·267 to 1·485)	1·372 (1·267 to 1·485)
**Diet low in seafood omega-3 fatty acids**											
Ischaemic heart disease	100 mg/day	Morbidity	Both	1·000 (1·000 to 1·000)	1·000 (1·000 to 1·000)	1·000 (1·000 to 1·000)	1·000 (1·000 to 1·000)	1·000 (1·000 to 1·000)	1·000 (1·000 to 1·000)	1·000 (1·000 to 1·000)	1·000 (1·000 to 1·000)
Ischaemic heart disease	100 mg/day	Mortality	Both	1·165 (1·064 to 1·280)	1·154 (1·060 to 1·260)	1·140 (1·055 to 1·236)	1·126 (1·050 to 1·212)	1·113 (1·045 to 1·189)	1·101 (1·040 to 1·168)	1·088 (1·035 to 1·145)	1·062 (1·025 to 1·102)
**Diet low in polyunsaturated fatty acids**											
Ischaemic heart disease	5% energy/day	Both	Both	1·111 (1·042 to 1·181)	1·101 (1·039 to 1·165)	1·086 (1·033 to 1·140)	1·075 (1·029 to 1·121)	1·068 (1·026 to 1·110)	1·063 (1·024 to 1·102)	1·060 (1·023 to 1·097)	1·063 (1·024 to 1·102)
**Diet high in trans fatty acids**											
Ischaemic heart disease	2% energy/day	Both	Both	1·461 (1·316 to 1·627)	1·396 (1·273 to 1·535)	1·323 (1·225 to 1·433)	1·264 (1·185 to 1·352)	1·222 (1·156 to 1·294)	1·186 (1·132 to 1·246)	1·158 (1·112 to 1·207)	1·150 (1·107 to 1·197)
**Diet high in sodium****											
	Non-Black, Non-Hypertensive	Both	Both	-3·428 (-4·126 to -2·730)	-3·944 (-4·738 to -3·150)	-4·459 (-5·362 to -3·556)	-4·975 (-5·995 to -3·954)	-5·490 (-6·634 to -4·347)	-5·490 (-6·634 to -4·347)	-5·490 (-6·634 to -4·347)	-5·490 (-6·634 to -4·347)
	Non-Black, Hypertensive	Both	Both	-5·363 (-5·848 to -4·877)	-5·878 (-6·346 to -5·411)	-6·394 (-6·886 to -5·901)	-6·909 (-7·464 to -6·354)	-7·425 (-8·069 to -6·781)	-7·425 (-8·069 to -6·781)	-7·425 (-8·069 to -6·781)	-7·425 (-8·069 to -6·781)
	Black, Non-Hypertensive	Both	Both	-5·972 (-7·168 to -4·777)	-6·488 (-7·735 to -5·241)	-7·004 (-8·316 to -5·691)	-7·519 (-8·909 to -6·129)	-8·035 (-9·512 to -6·557)	-8·035 (-9·512 to -6·557)	-8·035 (-9·512 to -6·557)	-8·035 (-9·512 to -6·557)
	Black, Hypertensive	Both	Both	-7·907 (-9·068 to -6·745)	-8·422 (-9·569 to -7·275)	-8·938 (-10·088 to -7·788)	-9·453 (-10·624 to -8·282)	-9·969 (-11·178 to -8·760)	-9·969 (-11·178 to -8·760)	-9·969 (-11·178 to -8·760)	-9·969 (-11·178 to -8·760)
**Childhood sexual abuse**											
Alcohol use disorders	Exposed	Both	Male	1·583 (0·993 to 2·376)	1·583 (0·993 to 2·376)	1·583 (0·993 to 2·376)	1·583 (0·993 to 2·376)	1·583 (0·993 to 2·376)	1·583 (0·993 to 2·376)	1·583 (0·993 to 2·376)	1·583 (0·993 to 2·376)
Alcohol use disorders	Not exposed	Both	Male	1·000 (1·000 to 1·000)	1·000 (1·000 to 1·000)	1·000 (1·000 to 1·000)	1·000 (1·000 to 1·000)	1·000 (1·000 to 1·000)	1·000 (1·000 to 1·000)	1·000 (1·000 to 1·000)	1·000 (1·000 to 1·000)
Alcohol use disorders	Exposed	Both	Female	1·583 (0·993 to 2·376)	1·583 (0·993 to 2·376)	1·583 (0·993 to 2·376)	1·583 (0·993 to 2·376)	1·583 (0·993 to 2·376)	1·583 (0·993 to 2·376)	1·583 (0·993 to 2·376)	1·583 (0·993 to 2·376)
Alcohol use disorders	Not exposed	Both	Female	1·000 (1·000 to 1·000)	1·000 (1·000 to 1·000)	1·000 (1·000 to 1·000)	1·000 (1·000 to 1·000)	1·000 (1·000 to 1·000)	1·000 (1·000 to 1·000)	1·000 (1·000 to 1·000)	1·000 (1·000 to 1·000)
Self-harm	Exposed	Both	Male	2·874 (1·369 to 5·430)	2·874 (1·369 to 5·430)	2·874 (1·369 to 5·430)	2·874 (1·369 to 5·430)	2·874 (1·369 to 5·430)	2·874 (1·369 to 5·430)	2·874 (1·369 to 5·430)	2·874 (1·369 to 5·430)
Self-harm	Not exposed	Both	Male	1·000 (1·000 to 1·000)	1·000 (1·000 to 1·000)	1·000 (1·000 to 1·000)	1·000 (1·000 to 1·000)	1·000 (1·000 to 1·000)	1·000 (1·000 to 1·000)	1·000 (1·000 to 1·000)	1·000 (1·000 to 1·000)
Self-harm	Exposed	Both	Female	2·874 (1·369 to 5·430)	2·874 (1·369 to 5·430)	2·874 (1·369 to 5·430)	2·874 (1·369 to 5·430)	2·874 (1·369 to 5·430)	2·874 (1·369 to 5·430)	2·874 (1·369 to 5·430)	2·874 (1·369 to 5·430)
Self-harm	Not exposed	Both	Female	1·000 (1·000 to 1·000)	1·000 (1·000 to 1·000)	1·000 (1·000 to 1·000)	1·000 (1·000 to 1·000)	1·000 (1·000 to 1·000)	1·000 (1·000 to 1·000)	1·000 (1·000 to 1·000)	1·000 (1·000 to 1·000)
**Intimate partner violence (exposure approach)**											
Maternal abortion, miscarriage, and ectopic	Exposed	Both	Both	2·087 (1·649 to 2·638)							
Maternal abortion, miscarriage, and ectopic	Not exposed	Both	Both	1·000 (1·000 to 1·000)							
Self-harm	Exposed	Both	Both	5·208 (1·716 to 11·294)	5·074 (1·751 to 11·401)	4·996 (1·820 to 11·124)	5·002 (1·757 to 10·765)	5·190 (1·830 to 11·750)	5·037 (1·783 to 11·163)	5·106 (1·859 to 11·258)	5·081 (1·848 to 11·578)
Self-harm	Not exposed	Both	Both	1·000 (1·000 to 1·000)	1·000 (1·000 to 1·000)	1·000 (1·000 to 1·000)	1·000 (1·000 to 1·000)	1·000 (1·000 to 1·000)	1·000 (1·000 to 1·000)	1·000 (1·000 to 1·000)	1·000 (1·000 to 1·000)
**Low physical activity**											
Colon and rectum cancer	<600 METs	Both	Both	1·293 (1·211 to 1·381)	1·293 (1·211 to 1·381)	1·293 (1·211 to 1·381)	1·293 (1·211 to 1·381)	1·293 (1·211 to 1·381)	1·293 (1·211 to 1·381)	1·293 (1·211 to 1·381)	1·293 (1·211 to 1·381)
Colon and rectum cancer	600-3,999 METs	Both	Both	1·172 (1·094 to 1·260)	1·172 (1·094 to 1·260)	1·172 (1·094 to 1·260)	1·172 (1·094 to 1·260)	1·172 (1·094 to 1·260)	1·172 (1·094 to 1·260)	1·172 (1·094 to 1·260)	1·172 (1·094 to 1·260)
Colon and rectum cancer	4,000-7,999 METs	Both	Both	1·067 (0·965 to 1·181)	1·067 (0·965 to 1·181)	1·067 (0·965 to 1·181)	1·067 (0·965 to 1·181)	1·067 (0·965 to 1·181)	1·067 (0·965 to 1·181)	1·067 (0·965 to 1·181)	1·067 (0·965 to 1·181)

Risk - Outcome	Category / Units	Morbidity / Mortality	Sex	All ages	0-6 Days	7-27 Days	28-364 Days	1-4 years	5-9 years	10-14 years	15-19 years	20-24 years	25-29 years	30-34 years	35-39 years	40-44 years
Colon and rectum cancer	≥8,000 METs	Both	Both										1·000 (1·000 to 1·000)	1·000 (1·000 to 1·000)	1·000 (1·000 to 1·000)	1·000 (1·000 to 1·000)
Breast cancer	<600 METs	Both	Both										1·159 (1·111 to 1·207)	1·159 (1·111 to 1·207)	1·159 (1·111 to 1·207)	1·159 (1·111 to 1·207)
Breast cancer	600-3,999 METs	Both	Both										1·120 (1·081 to 1·162)	1·120 (1·081 to 1·162)	1·120 (1·081 to 1·162)	1·120 (1·081 to 1·162)
Breast cancer	4,000-7,999 METs	Both	Both										1·090 (1·047 to 1·135)	1·090 (1·047 to 1·135)	1·090 (1·047 to 1·135)	1·090 (1·047 to 1·135)
Breast cancer	≥8,000 METs	Both	Both										1·000 (1·000 to 1·000)	1·000 (1·000 to 1·000)	1·000 (1·000 to 1·000)	1·000 (1·000 to 1·000)
Ischaemic heart disease	<600 METs	Both	Both										1·565 (1·397 to 1·742)	1·524 (1·370 to 1·686)	1·484 (1·343 to 1·632)	1·445 (1·317 to 1·579)
Ischaemic heart disease	600-3,999 METs	Both	Both										1·181 (1·063 to 1·310)	1·170 (1·059 to 1·289)	1·158 (1·055 to 1·269)	1·147 (1·051 to 1·249)
Ischaemic heart disease	4,000-7,999 METs	Both	Both										1·034 (0·878 to 1·206)	1·032 (0·885 to 1·193)	1·030 (0·891 to 1·180)	1·028 (0·898 to 1·167)
Ischaemic heart disease	≥8,000 METs	Both	Both										1·000 (1·000 to 1·000)	1·000 (1·000 to 1·000)	1·000 (1·000 to 1·000)	1·000 (1·000 to 1·000)
Ischaemic stroke	<600 METs	Both	Both										1·666 (1·412 to 1·990)	1·617 (1·383 to 1·911)	1·569 (1·356 to 1·835)	1·522 (1·328 to 1·762)
Ischaemic stroke	600-3,999 METs	Both	Both										1·255 (1·056 to 1·510)	1·238 (1·053 to 1·474)	1·221 (1·049 to 1·439)	1·205 (1·046 to 1·404)
Ischaemic stroke	4,000-7,999 METs	Both	Both										1·177 (0·879 to 1·531)	1·166 (0·885 to 1·493)	1·154 (0·892 to 1·456)	1·142 (0·899 to 1·420)
Ischaemic stroke	≥8,000 METs	Both	Both										1·000 (1·000 to 1·000)	1·000 (1·000 to 1·000)	1·000 (1·000 to 1·000)	1·000 (1·000 to 1·000)
Diabetes mellitus	<600 METs	Both	Both										1·387 (1·301 to 1·476)	1·387 (1·301 to 1·476)	1·387 (1·301 to 1·476)	1·387 (1·301 to 1·476)
Diabetes mellitus	600-3,999 METs	Both	Both										1·189 (1·120 to 1·264)	1·189 (1·120 to 1·264)	1·189 (1·120 to 1·264)	1·189 (1·120 to 1·264)
Diabetes mellitus	4,000-7,999 METs	Both	Both										1·037 (0·960 to 1·119)	1·037 (0·960 to 1·119)	1·037 (0·960 to 1·119)	1·037 (0·960 to 1·119)
Diabetes mellitus	≥8,000 METs	Both	Both										1·000 (1·000 to 1·000)	1·000 (1·000 to 1·000)	1·000 (1·000 to 1·000)	1·000 (1·000 to 1·000)
**High fasting plasma glucose (continuous)**																
Ischaemic heart disease	mmol/L	Both	Both										1·471 (1·145 to 2·100)	1·373 (1·153 to 1·742)	1·274 (1·130 to 1·451)	1·220 (1·085 to 1·365)
Ischaemic stroke	mmol/L	Both	Both										1·526 (1·110 to 2·228)	1·400 (1·101 to 1·856)	1·275 (1·077 to 1·561)	1·210 (1·043 to 1·441)
Hemorrhagic stroke	mmol/L	Both	Both										1·506 (1·111 to 2·226)	1·382 (1·109 to 1·846)	1·258 (1·085 to 1·488)	1·196 (1·053 to 1·372)
Chronic kidney disease due to hypertension	mmol/L	Both	Both										1·388 (1·272 to 1·512)	1·388 (1·272 to 1·512)	1·388 (1·272 to 1·512)	1·388 (1·272 to 1·512)
Chronic kidney disease due to glomerulonephritis	mmol/L	Both	Both										1·388 (1·272 to 1·512)	1·388 (1·272 to 1·512)	1·388 (1·272 to 1·512)	1·388 (1·272 to 1·512)
Chronic kidney disease due to other causes	mmol/L	Both	Both										1·388 (1·272 to 1·512)	1·388 (1·272 to 1·512)	1·388 (1·272 to 1·512)	1·388 (1·272 to 1·512)
**High fasting plasma glucose (categorical)**																
Tuberculosis	Diabetic	Both	Both										2·730 (1·972 to 3·604)	2·801 (2·053 to 3·672)	2·871 (2·039 to 3·710)	2·798 (1·963 to 3·630)
Tuberculosis	Not diabetic	Both	Both										1·000 (1·000 to 1·000)	1·000 (1·000 to 1·000)	1·000 (1·000 to 1·000)	1·000 (1·000 to 1·000)
Peripheral vascular disease	Diabetic	Both	Both										8·264 (5·990 to 9·304)	6·651 (5·371 to 7·454)	5·039 (4·433 to 5·676)	4·138 (3·560 to 4·767)
Peripheral vascular disease	Not diabetic	Both	Both										1·000 (1·000 to 1·000)	1·000 (1·000 to 1·000)	1·000 (1·000 to 1·000)	1·000 (1·000 to 1·000)
**High total cholesterol**																
Ischaemic heart disease	mmol/L	Both	Both										2·016 (1·682 to 2·544)	2·027 (1·768 to 2·354)	2·038 (1·829 to 2·278)	1·971 (1·774 to 2·192)
Ischaemic stroke	mmol/L	Both	Both										1·670 (1·333 to 2·341)	1·626 (1·352 to 2·042)	1·583 (1·342 to 1·850)	1·518 (1·285 to 1·760)
**High systolic blood pressure**																
Rheumatic heart disease	10 mmHg	Both	Both										1·631 (1·173 to 2·307)	1·474 (1·170 to 1·900)	1·317 (1·141 to 1·575)	1·229 (1·089 to 1·424)
Ischaemic heart disease	10 mmHg	Both	Both										1·972 (1·436 to 2·598)	1·818 (1·458 to 2·207)	1·665 (1·458 to 1·911)	1·568 (1·398 to 1·799)
Ischaemic stroke	10 mmHg	Both	Both										1·854 (1·394 to 2·590)	1·774 (1·426 to 2·253)	1·694 (1·404 to 2·036)	1·628 (1·353 to 1·950)
Hemorrhagic stroke	10 mmHg	Both	Both										2·134 (1·554 to 2·919)	2·050 (1·593 to 2·661)	1·966 (1·588 to 2·465)	1·874 (1·491 to 2·303)
Cardiomyopathy and myocarditis	10 mmHg	Both	Both										1·755 (1·265 to 2·424)	1·605 (1·290 to 2·012)	1·455 (1·277 to 1·642)	1·365 (1·229 to 1·511)
Atrial fibrillation and flutter	10 mmHg	Both	Both										1·760 (1·336 to 2·430)	1·631 (1·377 to 2·027)	1·503 (1·396 to 1·644)	1·423 (1·340 to 1·505)
Aortic aneurysm	10 mmHg	Both	Both										1·544 (1·258 to 2·169)	1·469 (1·290 to 1·818)	1·394 (1·299 to 1·538)	1·345 (1·226 to 1·452)
Peripheral vascular disease	10 mmHg	Both	Both										1·728 (1·203 to 2·430)	1·491 (1·206 to 1·871)	1·254 (1·182 to 1·330)	1·138 (1·019 to 1·263)
Endocarditis	10 mmHg	Both	Both										1·755 (1·265 to 2·424)	1·605 (1·290 to 2·012)	1·455 (1·277 to 1·642)	1·365 (1·229 to 1·511)
Other cardiovascular and circulatory diseases	10 mmHg	Both	Both										1·744 (1·339 to 2·397)	1·624 (1·382 to 2·006)	1·504 (1·405 to 1·626)	1·427 (1·354 to 1·499)
Chronic kidney disease due to diabetes mellitus	10 mmHg	Both	Both										1·283 (1·186 to 1·397)	1·283 (1·186 to 1·397)	1·283 (1·186 to 1·397)	1·283 (1·186 to 1·397)
Chronic kidney disease due to glomerulonephritis	10 mmHg	Both	Both										1·281 (1·181 to 1·383)	1·281 (1·181 to 1·383)	1·281 (1·181 to 1·383)	1·281 (1·181 to 1·383)
Chronic kidney disease due to other causes	10 mmHg	Both	Both										1·282 (1·181 to 1·396)	1·282 (1·181 to 1·396)	1·282 (1·181 to 1·396)	1·282 (1·181 to 1·396)
**High body-mass index**																
Oesophageal cancer	5 kg/m²	Both	Male										1·391 (1·076 to 1·758)	1·391 (1·076 to 1·758)	1·391 (1·076 to 1·758)	1·391 (1·076 to 1·758)
Oesophageal cancer	5 kg/m²	Both	Female										1·351 (1·012 to 1·745)	1·351 (1·012 to 1·745)	1·351 (1·012 to 1·745)	1·351 (1·012 to 1·745)
Colon and rectum cancer	5 kg/m²	Both	Male										1·177 (1·145 to 1·208)	1·177 (1·145 to 1·208)	1·177 (1·145 to 1·208)	1·177 (1·145 to 1·208)
Colon and rectum cancer	5 kg/m²	Both	Female										1·059 (1·031 to 1·083)	1·059 (1·031 to 1·083)	1·059 (1·031 to 1·083)	1·059 (1·031 to 1·083)
Gallbladder and biliary tract cancer	5 kg/m²	Both	Male										1·155 (1·033 to 1·282)	1·155 (1·033 to 1·282)	1·155 (1·033 to 1·282)	1·155 (1·033 to 1·282)
Gallbladder and biliary tract cancer	5 kg/m²	Both	Female										1·344 (1·223 to 1·478)	1·344 (1·223 to 1·478)	1·344 (1·223 to 1·478)	1·344 (1·223 to 1·478)
Pancreatic cancer	5 kg/m²	Both	Male										1·071 (0·999 to 1·154)	1·071 (0·999 to 1·154)	1·071 (0·999 to 1·154)	1·071 (0·999 to 1·154)
Pancreatic cancer	5 kg/m²	Both	Female										1·092 (1·037 to 1·144)	1·092 (1·037 to 1·144)	1·092 (1·037 to 1·144)	1·092 (1·037 to 1·144)
Breast cancer, post-menopausal	5 kg/m²	Both	Both										1·149 (1·036 to 1·268)	1·149 (1·036 to 1·268)	1·149 (1·036 to 1·268)	1·149 (1·036 to 1·268)
Uterine cancer	5 kg/m²	Both	Both										1·613 (1·543 to 1·681)	1·613 (1·543 to 1·681)	1·613 (1·543 to 1·681)	1·613 (1·543 to 1·681)
Ovarian cancer	5 kg/m²	Both	Both										1·038 (0·998 to 1·077)	1·038 (0·998 to 1·077)	1·038 (0·998 to 1·077)	1·038 (0·998 to 1·077)

Risk - Outcome	Category / Units	Morbidity / Mortality	Sex	45-49 years	50-54 years	55-59 years	60-64 years	65-69 years	70-74 years	75-79 years	80+ years
Colon and rectum cancer	≥8,000 METs	Both	Both	1·000 (1·000 to 1·000)	1·000 (1·000 to 1·000)	1·000 (1·000 to 1·000)	1·000 (1·000 to 1·000)	1·000 (1·000 to 1·000)	1·000 (1·000 to 1·000)	1·000 (1·000 to 1·000)	1·000 (1·000 to 1·000)
Breast cancer	<600 METs	Both	Both	1·159 (1·111 to 1·207)	1·159 (1·111 to 1·207)	1·159 (1·111 to 1·207)	1·159 (1·111 to 1·207)	1·159 (1·111 to 1·207)	1·159 (1·111 to 1·207)	1·159 (1·111 to 1·207)	1·159 (1·111 to 1·207)
Breast cancer	600-3,999 METs	Both	Both	1·120 (1·081 to 1·162)	1·120 (1·081 to 1·162)	1·120 (1·081 to 1·162)	1·120 (1·081 to 1·162)	1·120 (1·081 to 1·162)	1·120 (1·081 to 1·162)	1·120 (1·081 to 1·162)	1·120 (1·081 to 1·162)
Breast cancer	4,000-7,999 METs	Both	Both	1·090 (1·047 to 1·135)	1·090 (1·047 to 1·135)	1·090 (1·047 to 1·135)	1·090 (1·047 to 1·135)	1·090 (1·047 to 1·135)	1·090 (1·047 to 1·135)	1·090 (1·047 to 1·135)	1·090 (1·047 to 1·135)
Breast cancer	≥8,000 METs	Both	Both	1·000 (1·000 to 1·000)	1·000 (1·000 to 1·000)	1·000 (1·000 to 1·000)	1·000 (1·000 to 1·000)	1·000 (1·000 to 1·000)	1·000 (1·000 to 1·000)	1·000 (1·000 to 1·000)	1·000 (1·000 to 1·000)
Ischaemic heart disease	<600 METs	Both	Both	1·408 (1·292 to 1·529)	1·371 (1·266 to 1·479)	1·336 (1·242 to 1·432)	1·301 (1·218 to 1·386)	1·267 (1·194 to 1·341)	1·234 (1·171 to 1·298)	1·202 (1·148 to 1·257)	1·171 (1·125 to 1·216)
Ischaemic heart disease	600-3,999 METs	Both	Both	1·136 (1·047 to 1·229)	1·125 (1·044 to 1·210)	1·114 (1·040 to 1·191)	1·103 (1·036 to 1·172)	1·092 (1·033 to 1·154)	1·081 (1·029 to 1·135)	1·071 (1·025 to 1·117)	1·060 (1·022 to 1·100)
Ischaemic heart disease	4,000-7,999 METs	Both	Both	1·025 (0·905 to 1·154)	1·023 (0·912 to 1·141)	1·021 (0·919 to 1·129)	1·019 (0·926 to 1·116)	1·017 (0·933 to 1·104)	1·015 (0·940 to 1·092)	1·013 (0·948 to 1·080)	1·011 (0·955 to 1·068)
Ischaemic heart disease	≥8,000 METs	Both	Both	1·000 (1·000 to 1·000)	1·000 (1·000 to 1·000)	1·000 (1·000 to 1·000)	1·000 (1·000 to 1·000)	1·000 (1·000 to 1·000)	1·000 (1·000 to 1·000)	1·000 (1·000 to 1·000)	1·000 (1·000 to 1·000)
Ischaemic stroke	<600 METs	Both	Both	1·477 (1·302 to 1·693)	1·433 (1·276 to 1·625)	1·390 (1·250 to 1·561)	1·349 (1·225 to 1·499)	1·309 (1·200 to 1·440)	1·270 (1·176 to 1·382)	1·233 (1·153 to 1·328)	1·196 (1·129 to 1·275)
Ischaemic stroke	600-3,999 METs	Both	Both	1·189 (1·043 to 1·371)	1·173 (1·039 to 1·338)	1·157 (1·036 to 1·306)	1·142 (1·033 to 1·275)	1·127 (1·029 to 1·244)	1·112 (1·026 to 1·214)	1·097 (1·023 to 1·185)	1·082 (1·019 to 1·157)
Ischaemic stroke	4,000-7,999 METs	Both	Both	1·131 (0·906 to 1·385)	1·120 (0·913 to 1·351)	1·109 (0·920 to 1·317)	1·098 (0·927 to 1·285)	1·088 (0·934 to 1·253)	1·077 (0·941 to 1·222)	1·067 (0·948 to 1·192)	1·057 (0·955 to 1·162)
Ischaemic stroke	≥8,000 METs	Both	Both	1·000 (1·000 to 1·000)	1·000 (1·000 to 1·000)	1·000 (1·000 to 1·000)	1·000 (1·000 to 1·000)	1·000 (1·000 to 1·000)	1·000 (1·000 to 1·000)	1·000 (1·000 to 1·000)	1·000 (1·000 to 1·000)
Diabetes mellitus	<600 METs	Both	Both	1·387 (1·301 to 1·476)	1·387 (1·301 to 1·476)	1·387 (1·301 to 1·476)	1·387 (1·301 to 1·476)	1·387 (1·301 to 1·476)	1·387 (1·301 to 1·476)	1·387 (1·301 to 1·476)	1·387 (1·301 to 1·476)
Diabetes mellitus	600-3,999 METs	Both	Both	1·189 (1·120 to 1·264)	1·189 (1·120 to 1·264)	1·189 (1·120 to 1·264)	1·189 (1·120 to 1·264)	1·189 (1·120 to 1·264)	1·189 (1·120 to 1·264)	1·189 (1·120 to 1·264)	1·189 (1·120 to 1·264)
Diabetes mellitus	4,000-7,999 METs	Both	Both	1·037 (0·960 to 1·119)	1·037 (0·960 to 1·119)	1·037 (0·960 to 1·119)	1·037 (0·960 to 1·119)	1·037 (0·960 to 1·119)	1·037 (0·960 to 1·119)	1·037 (0·960 to 1·119)	1·037 (0·960 to 1·119)
Diabetes mellitus	≥8,000 METs	Both	Both	1·000 (1·000 to 1·000)	1·000 (1·000 to 1·000)	1·000 (1·000 to 1·000)	1·000 (1·000 to 1·000)	1·000 (1·000 to 1·000)	1·000 (1·000 to 1·000)	1·000 (1·000 to 1·000)	1·000 (1·000 to 1·000)
**High fasting plasma glucose (continuous)**											
Ischaemic heart disease	mmol/L	Both	Both	1·211 (1·111 to 1·327)	1·201 (1·120 to 1·297)	1·192 (1·123 to 1·270)	1·182 (1·111 to 1·267)	1·173 (1·085 to 1·271)	1·168 (1·080 to 1·273)	1·168 (1·097 to 1·265)	1·169 (1·071 to 1·318)
Ischaemic stroke	mmol/L	Both	Both	1·204 (1·074 to 1·384)	1·199 (1·094 to 1·335)	1·194 (1·107 to 1·303)	1·188 (1·101 to 1·297)	1·183 (1·069 to 1·309)	1·174 (1·056 to 1·310)	1·162 (1·075 to 1·295)	1·133 (1·055 to 1·333)
Hemorrhagic stroke	mmol/L	Both	Both	1·193 (1·082 to 1·334)	1·189 (1·106 to 1·298)	1·189 (1·115 to 1·264)	1·187 (1·115 to 1·253)	1·191 (1·095 to 1·265)	1·175 (1·087 to 1·264)	1·158 (1·089 to 1·233)	1·116 (1·056 to 1·250)
Chronic kidney disease due to hypertension	mmol/L	Both	Both	1·388 (1·272 to 1·512)	1·388 (1·272 to 1·512)	1·388 (1·272 to 1·512)	1·388 (1·272 to 1·512)	1·388 (1·272 to 1·512)	1·388 (1·272 to 1·512)	1·388 (1·272 to 1·512)	1·388 (1·272 to 1·512)
Chronic kidney disease due to glomerulonephritis	mmol/L	Both	Both	1·388 (1·272 to 1·512)	1·388 (1·272 to 1·512)	1·388 (1·272 to 1·512)	1·388 (1·272 to 1·512)	1·388 (1·272 to 1·512)	1·388 (1·272 to 1·512)	1·388 (1·272 to 1·512)	1·388 (1·272 to 1·512)
Chronic kidney disease due to other causes	mmol/L	Both	Both	1·388 (1·272 to 1·512)	1·388 (1·272 to 1·512)	1·388 (1·272 to 1·512)	1·388 (1·272 to 1·512)	1·388 (1·272 to 1·512)	1·388 (1·272 to 1·512)	1·388 (1·272 to 1·512)	1·388 (1·272 to 1·512)
**High fasting plasma glucose (categorical)**											
Tuberculosis	Diabetic	Both	Both	2·581 (1·906 to 3·275)	2·364 (1·813 to 2·946)	2·147 (1·684 to 2·677)	1·930 (1·485 to 2·441)	1·713 (1·231 to 2·326)	1·598 (1·123 to 2·242)	1·587 (1·182 to 2·116)	1·559 (1·180 to 2·179)
Tuberculosis	Not diabetic	Both	Both	1·000 (1·000 to 1·000)	1·000 (1·000 to 1·000)	1·000 (1·000 to 1·000)	1·000 (1·000 to 1·000)	1·000 (1·000 to 1·000)	1·000 (1·000 to 1·000)	1·000 (1·000 to 1·000)	1·000 (1·000 to 1·000)
Peripheral vascular disease	Diabetic	Both	Both	3·947 (3·446 to 4·506)	3·756 (3·345 to 4·236)	3·565 (3·211 to 3·973)	3·374 (3·078 to 3·716)	3·183 (2·918 to 3·467)	2·992 (2·758 to 3·250)	2·801 (2·556 to 3·051)	2·324 (1·976 to 2·672)
Peripheral vascular disease	Not diabetic	Both	Both	1·000 (1·000 to 1·000)	1·000 (1·000 to 1·000)	1·000 (1·000 to 1·000)	1·000 (1·000 to 1·000)	1·000 (1·000 to 1·000)	1·000 (1·000 to 1·000)	1·000 (1·000 to 1·000)	1·000 (1·000 to 1·000)
**High total cholesterol**											
Ischaemic heart disease	mmol/L	Both	Both	1·828 (1·675 to 2·006)	1·685 (1·560 to 1·818)	1·541 (1·446 to 1·648)	1·398 (1·306 to 1·495)	1·254 (1·141 to 1·373)	1·193 (1·088 to 1·312)	1·213 (1·124 to 1·321)	1·262 (1·109 to 1·467)
Ischaemic stroke	mmol/L	Both	Both	1·434 (1·241 to 1·638)	1·350 (1·210 to 1·515)	1·265 (1·164 to 1·391)	1·181 (1·109 to 1·300)	1·096 (1·043 to 1·225)	1·062 (1·008 to 1·193)	1·077 (1·012 to 1·216)	1·116 (1·014 to 1·353)
**High systolic blood pressure**											
Rheumatic heart disease	10 mmHg	Both	Both	1·211 (1·100 to 1·370)	1·193 (1·107 to 1·328)	1·175 (1·101 to 1·289)	1·157 (1·086 to 1·266)	1·139 (1·055 to 1·249)	1·127 (1·048 to 1·241)	1·120 (1·060 to 1·239)	1·104 (1·040 to 1·284)
Ischaemic heart disease	10 mmHg	Both	Both	1·527 (1·393 to 1·706)	1·487 (1·385 to 1·620)	1·446 (1·368 to 1·536)	1·405 (1·332 to 1·489)	1·364 (1·255 to 1·456)	1·330 (1·222 to 1·424)	1·303 (1·225 to 1·404)	1·266 (1·133 to 1·437)
Ischaemic stroke	10 mmHg	Both	Both	1·574 (1·359 to 1·825)	1·521 (1·360 to 1·700)	1·468 (1·344 to 1·598)	1·414 (1·301 to 1·524)	1·361 (1·214 to 1·490)	1·318 (1·168 to 1·452)	1·284 (1·177 to 1·390)	1·201 (1·108 to 1·370)
Hemorrhagic stroke	10 mmHg	Both	Both	1·775 (1·481 to 2·117)	1·676 (1·446 to 1·934)	1·577 (1·399 to 1·755)	1·478 (1·330 to 1·619)	1·379 (1·207 to 1·540)	1·323 (1·162 to 1·495)	1·311 (1·192 to 1·450)	1·279 (1·126 to 1·519)
Cardiomyopathy and myocarditis	10 mmHg	Both	Both	1·335 (1·222 to 1·449)	1·306 (1·218 to 1·394)	1·276 (1·211 to 1·342)	1·247 (1·182 to 1·304)	1·217 (1·131 to 1·285)	1·193 (1·116 to 1·264)	1·175 (1·119 to 1·238)	1·128 (1·071 to 1·237)
Atrial fibrillation and flutter	10 mmHg	Both	Both	1·392 (1·328 to 1·458)	1·361 (1·313 to 1·411)	1·330 (1·293 to 1·369)	1·299 (1·266 to 1·333)	1·268 (1·233 to 1·309)	1·237 (1·201 to 1·277)	1·208 (1·176 to 1·238)	1·134 (1·092 to 1·185)
Aortic aneurysm	10 mmHg	Both	Both	1·321 (1·232 to 1·405)	1·296 (1·229 to 1·362)	1·272 (1·218 to 1·327)	1·248 (1·191 to 1·299)	1·223 (1·160 to 1·286)	1·200 (1·137 to 1·262)	1·177 (1·126 to 1·230)	1·119 (1·071 to 1·185)
Peripheral vascular disease	10 mmHg	Both	Both	1·142 (1·047 to 1·243)	1·146 (1·071 to 1·224)	1·150 (1·094 to 1·209)	1·154 (1·110 to 1·199)	1·159 (1·113 to 1·207)	1·152 (1·104 to 1·201)	1·136 (1·097 to 1·176)	1·095 (1·054 to 1·155)
Endocarditis	10 mmHg	Both	Both	1·335 (1·222 to 1·449)	1·306 (1·218 to 1·394)	1·276 (1·211 to 1·342)	1·247 (1·182 to 1·304)	1·217 (1·131 to 1·285)	1·193 (1·116 to 1·264)	1·175 (1·119 to 1·238)	1·128 (1·071 to 1·237)
Other cardiovascular and circulatory diseases	10 mmHg	Both	Both	1·395 (1·336 to 1·452)	1·363 (1·317 to 1·406)	1·330 (1·296 to 1·365)	1·298 (1·266 to 1·332)	1·265 (1·231 to 1·303)	1·235 (1·201 to 1·271)	1·207 (1·176 to 1·235)	1·137 (1·095 to 1·188)
Chronic kidney disease due to diabetes mellitus	10 mmHg	Both	Both	1·283 (1·186 to 1·397)	1·283 (1·186 to 1·397)	1·283 (1·186 to 1·397)	1·283 (1·186 to 1·397)	1·283 (1·186 to 1·397)	1·283 (1·186 to 1·397)	1·283 (1·186 to 1·397)	1·283 (1·186 to 1·397)
Chronic kidney disease due to glomerulonephritis	10 mmHg	Both	Both	1·281 (1·181 to 1·383)	1·281 (1·181 to 1·383)	1·281 (1·181 to 1·383)	1·281 (1·181 to 1·383)	1·281 (1·181 to 1·383)	1·281 (1·181 to 1·383)	1·281 (1·181 to 1·383)	1·281 (1·181 to 1·383)
Chronic kidney disease due to other causes	10 mmHg	Both	Both	1·282 (1·181 to 1·396)	1·282 (1·181 to 1·396)	1·282 (1·181 to 1·396)	1·282 (1·181 to 1·396)	1·282 (1·181 to 1·396)	1·282 (1·181 to 1·396)	1·282 (1·181 to 1·396)	1·282 (1·181 to 1·396)
**High body-mass index**											
Oesophageal cancer	5 kg/m²	Both	Male	1·391 (1·076 to 1·758)	1·391 (1·076 to 1·758)	1·391 (1·076 to 1·758)	1·391 (1·076 to 1·758)	1·391 (1·076 to 1·758)	1·391 (1·076 to 1·758)	1·391 (1·076 to 1·758)	1·391 (1·076 to 1·758)
Oesophageal cancer	5 kg/m²	Both	Female	1·351 (1·012 to 1·745)	1·351 (1·012 to 1·745)	1·351 (1·012 to 1·745)	1·351 (1·012 to 1·745)	1·351 (1·012 to 1·745)	1·351 (1·012 to 1·745)	1·351 (1·012 to 1·745)	1·351 (1·012 to 1·745)
Colon and rectum cancer	5 kg/m²	Both	Male	1·177 (1·145 to 1·208)	1·177 (1·145 to 1·208)	1·177 (1·145 to 1·208)	1·177 (1·145 to 1·208)	1·177 (1·145 to 1·208)	1·177 (1·145 to 1·208)	1·177 (1·145 to 1·208)	1·177 (1·145 to 1·208)
Colon and rectum cancer	5 kg/m²	Both	Female	1·059 (1·031 to 1·083)	1·059 (1·031 to 1·083)	1·059 (1·031 to 1·083)	1·059 (1·031 to 1·083)	1·059 (1·031 to 1·083)	1·059 (1·031 to 1·083)	1·059 (1·031 to 1·083)	1·059 (1·031 to 1·083)
Gallbladder and biliary tract cancer	5 kg/m²	Both	Male	1·155 (1·033 to 1·282)	1·155 (1·033 to 1·282)	1·155 (1·033 to 1·282)	1·155 (1·033 to 1·282)	1·155 (1·033 to 1·282)	1·155 (1·033 to 1·282)	1·155 (1·033 to 1·282)	1·155 (1·033 to 1·282)
Gallbladder and biliary tract cancer	5 kg/m²	Both	Female	1·344 (1·223 to 1·478)	1·344 (1·223 to 1·478)	1·344 (1·223 to 1·478)	1·344 (1·223 to 1·478)	1·344 (1·223 to 1·478)	1·344 (1·223 to 1·478)	1·344 (1·223 to 1·478)	1·344 (1·223 to 1·478)
Pancreatic cancer	5 kg/m²	Both	Male	1·071 (0·999 to 1·154)	1·071 (0·999 to 1·154)	1·071 (0·999 to 1·154)	1·071 (0·999 to 1·154)	1·071 (0·999 to 1·154)	1·071 (0·999 to 1·154)	1·071 (0·999 to 1·154)	1·071 (0·999 to 1·154)
Pancreatic cancer	5 kg/m²	Both	Female	1·092 (1·037 to 1·144)	1·092 (1·037 to 1·144)	1·092 (1·037 to 1·144)	1·092 (1·037 to 1·144)	1·092 (1·037 to 1·144)	1·092 (1·037 to 1·144)	1·092 (1·037 to 1·144)	1·092 (1·037 to 1·144)
Breast cancer, post-menopausal	5 kg/m²	Both	Both	1·149 (1·036 to 1·268)	1·348 (1·128 to 1·615)	1·348 (1·128 to 1·615)	1·348 (1·128 to 1·615)	1·348 (1·128 to 1·615)	1·348 (1·128 to 1·615)	1·348 (1·128 to 1·615)	1·348 (1·128 to 1·615)
Uterine cancer	5 kg/m²	Both	Both	1·613 (1·543 to 1·681)	1·613 (1·543 to 1·681)	1·613 (1·543 to 1·681)	1·613 (1·543 to 1·681)	1·613 (1·543 to 1·681)	1·613 (1·543 to 1·681)	1·613 (1·543 to 1·681)	1·613 (1·543 to 1·681)
Ovarian cancer	5 kg/m²	Both	Both	1·038 (0·998 to 1·077)	1·038 (0·998 to 1·077)	1·038 (0·998 to 1·077)	1·038 (0·998 to 1·077)	1·038 (0·998 to 1·077)	1·038 (0·998 to 1·077)	1·038 (0·998 to 1·077)	1·038 (0·998 to 1·077)

Risk - Outcome	Category / Units	Morbidity / Mortality	Sex	All ages	0-6 Days	7-27 Days	28-364 Days	1-4 years	5-9 years	10-14 years	15-19 years	20-24 years	25-29 years	30-34 years	35-39 years	40-44 years
Kidney cancer	5 kg/m²	Both	Male										1·240 (1·171 to 1·313)	1·240 (1·171 to 1·313)	1·240 (1·171 to 1·313)	1·240 (1·171 to 1·313)
Kidney cancer	5 kg/m²	Both	Female										1·320 (1·254 to 1·395)	1·320 (1·254 to 1·395)	1·320 (1·254 to 1·395)	1·320 (1·254 to 1·395)
Thyroid cancer	5 kg/m²	Both	Male										1·221 (1·067 to 1·382)	1·221 (1·067 to 1·382)	1·221 (1·067 to 1·382)	1·221 (1·067 to 1·382)
Thyroid cancer	5 kg/m²	Both	Female										1·136 (1·094 to 1·178)	1·136 (1·094 to 1·178)	1·136 (1·094 to 1·178)	1·136 (1·094 to 1·178)
Leukaemia	5 kg/m²	Both	Male										1·086 (1·053 to 1·119)	1·086 (1·053 to 1·119)	1·086 (1·053 to 1·119)	1·086 (1·053 to 1·119)
Leukaemia	5 kg/m²	Both	Female										1·131 (1·061 to 1·208)	1·131 (1·061 to 1·208)	1·131 (1·061 to 1·208)	1·131 (1·061 to 1·208)
Ischaemic heart disease	5 kg/m²	Both	Both										2·274 (1·257 to 3·686)	2·018 (1·296 to 3·109)	1·724 (1·532 to 1·932)	1·599 (1·418 to 1·785)
Ischaemic stroke	5 kg/m²	Both	Both										2·472 (1·399 to 3·980)	2·235 (1·454 to 3·334)	1·979 (1·694 to 2·313)	1·826 (1·600 to 2·076)
Hemorrhagic stroke	5 kg/m²	Both	Both										3·066 (1·750 to 5·337)	2·913 (1·860 to 4·399)	2·597 (1·974 to 3·387)	2·389 (1·869 to 3·002)
Hypertensive heart disease	5 kg/m²	Both	Both										3·122 (1·588 to 5·502)	3·000 (1·748 to 4·912)	2·769 (1·814 to 4·217)	2·573 (1·741 to 3·647)
Diabetes mellitus	5 kg/m²	Both	Both										3·547 (2·308 to 5·228)	3·455 (2·509 to 4·693)	3·349 (2·803 to 3·919)	3·160 (2·694 to 3·700)
Chronic kidney disease due to diabetes mellitus	5 kg/m²	Both	Both												1·746 (1·053 to 2·748)	1·746 (1·053 to 2·748)
Chronic kidney disease due to hypertension	5 kg/m²	Both	Both												1·763 (1·088 to 2·760)	1·763 (1·088 to 2·760)
Chronic kidney disease due to glomerulonephritis	5 kg/m²	Both	Both												1·742 (1·019 to 2·791)	1·742 (1·019 to 2·791)
Chronic kidney disease due to other causes	5 kg/m²	Both	Both												1·732 (1·047 to 2·684)	1·732 (1·047 to 2·684)
Low back pain	5 kg/m²	Morbidity	Both										1·100 (1·073 to 1·126)	1·100 (1·073 to 1·127)	1·101 (1·076 to 1·128)	1·100 (1·074 to 1·126)
**Low bone mineral density**																
Hip	0.1 g/cm²	Both	Male													2·945 (2·121 to 3·924)
Hip	0.1 g/cm²	Both	Female													3·255 (2·261 to 4·515)
Non-hip	0.1 g/cm²	Both	Male													1·077 (1·073 to 1·080)
Non-hip	0.1 g/cm²	Both	Female													1·083 (1·080 to 1·087)
**Low glomerular filtration rate**																
Ischaemic heart disease	Stage 5 CKD	Both	Both										13·688 (5·178 to 29·689)	11·878 (4·932 to 24·189)	10·330 (4·752 to 19·709)	9·003 (4·534 to 16·152)
Ischaemic heart disease	Stage 4 CKD	Both	Both										10·439 (4·599 to 20·249)	9·175 (4·393 to 16·959)	8·077 (4·181 to 14·156)	7·121 (3·958 to 11·702)
Ischaemic heart disease	Stage 3 CKD	Both	Both										1·322 (0·937 to 1·848)	1·314 (0·963 to 1·772)	1·306 (0·990 to 1·703)	1·299 (1·021 to 1·640)
Ischaemic heart disease	None	Both	Both										1·000 (1·000 to 1·000)	1·000 (1·000 to 1·000)	1·000 (1·000 to 1·000)	1·000 (1·000 to 1·000)
Peripheral vascular disease	Stage 5 CKD	Both	Both										18·258 (4·656 to 52·637)	14·983 (4·377 to 40·559)	12·337 (4·062 to 31·134)	10·192 (3·753 to 23·666)
Peripheral vascular disease	Stage 4 CKD	Both	Both										15·408 (2·104 to 57·225)	12·683 (2·020 to 42·954)	10·487 (1·918 to 32·532)	8·710 (1·892 to 25·455)
Peripheral vascular disease	Stage 3 CKD	Both	Both										3·856 (1·870 to 7·052)	3·533 (1·829 to 6·154)	3·240 (1·805 to 5·393)	2·974 (1·772 to 4·698)
Peripheral vascular disease	None	Both	Both										1·000 (1·000 to 1·000)	1·000 (1·000 to 1·000)	1·000 (1·000 to 1·000)	1·000 (1·000 to 1·000)
Gout	Stage 5 CKD	Both	Both										2·741 (2·462 to 3·045)	2·751 (2·484 to 3·064)	2·747 (2·482 to 3·034)	2·743 (2·461 to 3·043)
Gout	Stage 4 CKD	Both	Both										2·745 (2·481 to 3·043)	2·743 (2·454 to 3·047)	2·740 (2·457 to 3·030)	2·736 (2·454 to 3·033)
Gout	Stage 3 CKD	Both	Both										2·751 (2·482 to 3·044)	2·745 (2·487 to 3·033)	2·745 (2·482 to 3·046)	2·736 (2·444 to 3·007)
Gout	None	Both	Both										1·000 (1·000 to 1·000)	1·000 (1·000 to 1·000)	1·000 (1·000 to 1·000)	1·000 (1·000 to 1·000)

* Shifts are reported for diet high in sugar-sweetened beverages as the estimation is based on mediation through body-mass index level.
** Shifts are reported for diet high in sodium as the estimation is based on mediation through high systolic blood pressure.

Risk-outcome pairs with 100% attribution

Alcohol use
Liver cancer due to alcohol use
Cirrhosis due to alcohol use
Alcohol use disorders
Childhood underweight
Protein-energy malnutrition
Childhood wasting
Protein-energy malnutrition
Drug use
Opioid use disorders
Cocaine use disorders
Amphetamine use disorders
Cannabis use disorders
Other drug use disorders

High fasting plasma glucose
Diabetes mellitus
Chronic kidney disease due to diabetes mellitus
High systolic blood pressure
Hypertensive heart disease
Chronic kidney disease due to hypertension
Iron deficiency
Iron-deficiency anemia
Low glomerular filtration rate
Chronic kidney disease due to diabetes mellitus
Chronic kidney disease due to hypertension
Chronic kidney disease due to glomerulonephritis
Chronic kidney disease due to other causes

Occupational particulate matter, gases, and fumes
Coal workers pneumoconiosis
Unsafe sex
Syphilis
Chlamydial infection
Gonococcal infection
Trichomoniasis
Genital herpes
Other sexually transmitted diseases
Cervical cancer
Sexually transmitted diseases excluding HIV
Vitamin A deficiency
Vitamin A deficiency

Risk - Outcome	Category / Units	Morbidity / Mortality	Sex	45-49 years	50-54 years	55-59 years	60-64 years	65-69 years	70-74 years	75-79 years	80+ years
Kidney cancer	5 kg/m²	Both	Male	1·240 (1·171 to 1·313)	1·240 (1·171 to 1·313)	1·240 (1·171 to 1·313)	1·240 (1·171 to 1·313)	1·240 (1·171 to 1·313)	1·240 (1·171 to 1·313)	1·240 (1·171 to 1·313)	1·240 (1·171 to 1·313)
Kidney cancer	5 kg/m²	Both	Female	1·320 (1·254 to 1·395)	1·320 (1·254 to 1·395)	1·320 (1·254 to 1·395)	1·320 (1·254 to 1·395)	1·320 (1·254 to 1·395)	1·320 (1·254 to 1·395)	1·320 (1·254 to 1·395)	1·320 (1·254 to 1·395)
Thyroid cancer	5 kg/m²	Both	Male	1·221 (1·067 to 1·382)	1·221 (1·067 to 1·382)	1·221 (1·067 to 1·382)	1·221 (1·067 to 1·382)	1·221 (1·067 to 1·382)	1·221 (1·067 to 1·382)	1·221 (1·067 to 1·382)	1·221 (1·067 to 1·382)
Thyroid cancer	5 kg/m²	Both	Female	1·136 (1·094 to 1·178)	1·136 (1·094 to 1·178)	1·136 (1·094 to 1·178)	1·136 (1·094 to 1·178)	1·136 (1·094 to 1·178)	1·136 (1·094 to 1·178)	1·136 (1·094 to 1·178)	1·136 (1·094 to 1·178)
Leukaemia	5 kg/m²	Both	Male	1·086 (1·053 to 1·119)	1·086 (1·053 to 1·119)	1·086 (1·053 to 1·119)	1·086 (1·053 to 1·119)	1·086 (1·053 to 1·119)	1·086 (1·053 to 1·119)	1·086 (1·053 to 1·119)	1·086 (1·053 to 1·119)
Leukaemia	5 kg/m²	Both	Female	1·131 (1·061 to 1·208)	1·131 (1·061 to 1·208)	1·131 (1·061 to 1·208)	1·131 (1·061 to 1·208)	1·131 (1·061 to 1·208)	1·131 (1·061 to 1·208)	1·131 (1·061 to 1·208)	1·131 (1·061 to 1·208)
Ischaemic heart disease	5 kg/m²	Both	Both	1·567 (1·457 to 1·680)	1·520 (1·417 to 1·631)	1·466 (1·372 to 1·557)	1·414 (1·324 to 1·504)	1·364 (1·287 to 1·448)	1·319 (1·242 to 1·400)	1·274 (1·187 to 1·365)	1·170 (1·091 to 1·253)
Ischaemic stroke	5 kg/m²	Both	Both	1·733 (1·581 to 1·898)	1·635 (1·479 to 1·796)	1·543 (1·441 to 1·653)	1·455 (1·345 to 1·566)	1·380 (1·310 to 1·458)	1·304 (1·233 to 1·376)	1·228 (1·159 to 1·305)	1·068 (0·992 to 1·143)
Hemorrhagic stroke	5 kg/m²	Both	Both	2·199 (1·821 to 2·673)	1·996 (1·625 to 2·419)	1·805 (1·573 to 2·060)	1·665 (1·437 to 1·933)	1·523 (1·377 to 1·684)	1·410 (1·265 to 1·571)	1·295 (1·162 to 1·439)	1·070 (0·928 to 1·220)
Hypertensive heart disease	5 kg/m²	Both	Both	2·407 (1·716 to 3·296)	2·281 (1·597 to 3·189)	2·159 (1·499 to 3·039)	2·035 (1·451 to 2·822)	1·955 (1·342 to 2·700)	1·860 (1·296 to 2·617)	1·792 (1·169 to 2·553)	1·697 (1·067 to 2·620)
Diabetes mellitus	5 kg/m²	Both	Both	2·864 (2·450 to 3·314)	2·624 (2·224 to 3·038)	2·417 (2·086 to 2·779)	2·215 (1·865 to 2·608)	2·046 (1·724 to 2·382)	1·896 (1·596 to 2·229)	1·740 (1·444 to 2·079)	1·461 (1·207 to 1·760)
Chronic kidney disease due to diabetes mellitus	5 kg/m²	Both	Both	1·746 (1·053 to 2·748)	1·746 (1·053 to 2·748)	1·746 (1·053 to 2·748)	2·036 (1·298 to 3·056)	2·036 (1·298 to 3·056)	1·621 (1·061 to 2·380)	1·621 (1·061 to 2·380)	1·431 (0·800 to 2·404)
Chronic kidney disease due to hypertension	5 kg/m²	Both	Both	1·763 (1·088 to 2·760)	1·763 (1·088 to 2·760)	1·763 (1·088 to 2·760)	2·044 (1·302 to 3·089)	2·044 (1·302 to 3·089)	1·605 (1·066 to 2·327)	1·605 (1·066 to 2·327)	1·437 (0·828 to 2·426)
Chronic kidney disease due to glomerulonephritis	5 kg/m²	Both	Both	1·742 (1·019 to 2·791)	1·742 (1·019 to 2·791)	1·742 (1·019 to 2·791)	2·044 (1·254 to 3·155)	2·044 (1·254 to 3·155)	1·604 (1·108 to 2·255)	1·604 (1·108 to 2·255)	1·452 (0·851 to 2·350)
Chronic kidney disease due to other causes	5 kg/m²	Both	Both	1·732 (1·047 to 2·684)	1·732 (1·047 to 2·684)	1·732 (1·047 to 2·684)	2·032 (1·214 to 3·105)	2·032 (1·214 to 3·105)	1·625 (1·068 to 2·368)	1·625 (1·068 to 2·368)	1·433 (0·776 to 2·345)
Low back pain	5 kg/m²	Morbidity	Both	1·099 (1·075 to 1·123)	1·100 (1·075 to 1·128)	1·100 (1·075 to 1·126)	1·101 (1·077 to 1·126)	1·100 (1·075 to 1·126)	1·100 (1·076 to 1·124)	1·100 (1·075 to 1·124)	1·100 (1·074 to 1·125)
**Low bone mineral density**											
Hip	0.1 g/cm²	Both	Male	2·850 (2·127 to 3·822)	2·614 (2·017 to 3·328)	2·439 (1·995 to 2·966)	2·286 (1·962 to 2·665)	2·184 (1·911 to 2·477)	2·102 (1·888 to 2·323)	1·921 (1·785 to 2·084)	1·732 (1·628 to 1·840)
Hip	0.1 g/cm²	Both	Female	2·940 (2·145 to 3·909)	2·713 (2·069 to 3·442)	2·643 (2·094 to 3·273)	2·474 (2·061 to 2·951)	2·412 (2·057 to 2·772)	2·320 (2·075 to 2·573)	2·118 (1·938 to 2·300)	1·876 (1·747 to 2·003)
Non-hip	0.1 g/cm²	Both	Male	1·114 (1·112 to 1·115)	1·151 (1·057 to 1·259)	1·182 (1·100 to 1·265)	1·214 (1·147 to 1·285)	1·247 (1·186 to 1·310)	1·297 (1·240 to 1·354)	1·339 (1·278 to 1·399)	1·370 (1·297 to 1·448)
Non-hip	0.1 g/cm²	Both	Female	1·118 (1·116 to 1·120)	1·163 (1·063 to 1·273)	1·203 (1·118 to 1·295)	1·239 (1·161 to 1·317)	1·287 (1·215 to 1·361)	1·343 (1·273 to 1·418)	1·401 (1·329 to 1·481)	1·437 (1·352 to 1·526)
**Low glomerular filtration rate**											
Ischaemic heart disease	Stage 5 CKD	Both	Both	7·863 (4·236 to 13·224)	6·883 (3·952 to 11·130)	6·038 (3·578 to 9·583)	5·308 (3·223 to 8·376)	4·677 (2·861 to 7·293)	4·130 (2·510 to 6·520)	3·655 (2·136 to 5·963)	3·103 (1·711 to 5·328)
Ischaemic heart disease	Stage 4 CKD	Both	Both	6·288 (3·775 to 9·766)	5·562 (3·537 to 8·223)	4·927 (3·304 to 6·891)	4·372 (3·053 to 5·895)	3·885 (2·789 to 5·120)	3·459 (2·518 to 4·547)	3·084 (2·192 to 4·166)	2·642 (1·818 to 3·740)
Ischaemic heart disease	Stage 3 CKD	Both	Both	1·292 (1·050 to 1·574)	1·286 (1·073 to 1·519)	1·281 (1·098 to 1·478)	1·276 (1·115 to 1·453)	1·271 (1·123 to 1·419)	1·267 (1·122 to 1·415)	1·263 (1·107 to 1·432)	1·259 (1·081 to 1·464)
Ischaemic heart disease	None	Both	Both	1·000 (1·000 to 1·000)	1·000 (1·000 to 1·000)	1·000 (1·000 to 1·000)	1·000 (1·000 to 1·000)	1·000 (1·000 to 1·000)	1·000 (1·000 to 1·000)	1·000 (1·000 to 1·000)	1·000 (1·000 to 1·000)
Peripheral vascular disease	Stage 5 CKD	Both	Both	8·448 (3·379 to 18·625)	7·027 (3·067 to 14·209)	5·864 (2·752 to 11·271)	4·910 (2·466 to 9·029)	4·125 (2·146 to 7·333)	3·478 (1·874 to 6·000)	2·942 (1·592 to 5·042)	2·352 (1·225 to 4·184)
Peripheral vascular disease	Stage 4 CKD	Both	Both	7·267 (1·864 to 19·329)	6·091 (1·808 to 15·142)	5·129 (1·731 to 11·931)	4·339 (1·679 to 9·354)	3·688 (1·552 to 7·294)	3·150 (1·402 to 6·087)	2·703 (1·275 to 5·102)	2·209 (1·042 to 4·151)
Peripheral vascular disease	Stage 3 CKD	Both	Both	2·733 (1·727 to 4·077)	2·515 (1·678 to 3·596)	2·316 (1·621 to 3·186)	2·135 (1·553 to 2·816)	1·970 (1·474 to 2·515)	1·820 (1·395 to 2·283)	1·683 (1·306 to 2·135)	1·515 (1·132 to 1·983)
Peripheral vascular disease	None	Both	Both	1·000 (1·000 to 1·000)	1·000 (1·000 to 1·000)	1·000 (1·000 to 1·000)	1·000 (1·000 to 1·000)	1·000 (1·000 to 1·000)	1·000 (1·000 to 1·000)	1·000 (1·000 to 1·000)	1·000 (1·000 to 1·000)
Gout	Stage 5 CKD	Both	Both	2·751 (2·466 to 3·036)	2·738 (2·485 to 3·032)	2·745 (2·475 to 3·048)	2·745 (2·490 to 3·043)	2·740 (2·477 to 3·041)	2·743 (2·475 to 3·036)	2·745 (2·488 to 3·041)	2·741 (2·472 to 3·028)
Gout	Stage 4 CKD	Both	Both	2·742 (2·478 to 3·032)	2·737 (2·462 to 3·043)	2·732 (2·471 to 3·037)	2·747 (2·462 to 3·043)	2·747 (2·494 to 3·019)	2·747 (2·469 to 3·027)	2·738 (2·453 to 3·048)	2·736 (2·458 to 3·024)
Gout	Stage 3 CKD	Both	Both	2·747 (2·478 to 3·061)	2·741 (2·475 to 3·015)	2·740 (2·473 to 3·025)	2·749 (2·463 to 3·026)	2·749 (2·482 to 3·044)	2·744 (2·461 to 3·035)	2·739 (2·479 to 3·016)	2·737 (2·473 to 3·037)
Gout	None	Both	Both	1·000 (1·000 to 1·000)	1·000 (1·000 to 1·000)	1·000 (1·000 to 1·000)	1·000 (1·000 to 1·000)	1·000 (1·000 to 1·000)	1·000 (1·000 to 1·000)	1·000 (1·000 to 1·000)	1·000 (1·000 to 1·000)

Appendix Table 6b. Relative risks used by age and sex and for each outcome for the particulate matter integrated exposure response curve.

Risk - Outcome	Category / Units	Morbidity / Mortality	Sex	Age												
				All ages	25-29 years	30-34 years	35-39 years	40-44 years	45-49 years	50-54 years	55-59 years	60-64 years	65-69 years	70-74 years	75-79 years	80+ years
**Ambient particulate matter pollution (PM2.5)**																
Lower respiratory infections	600 μg/m^3	Both	Both	2·550 (2·020 to 3·110)												
Lower respiratory infections	500 μg/m^3	Both	Both	2·500 (1·980 to 3·060)												
Lower respiratory infections	400 μg/m^3	Both	Both	2·440 (1·920 to 2·980)												
Lower respiratory infections	300 μg/m^3	Both	Both	2·330 (1·840 to 2·860)												
Lower respiratory infections	200 μg/m^3	Both	Both	2·140 (1·720 to 2·650)												
Lower respiratory infections	150 μg/m^3	Both	Both	1·990 (1·640 to 2·440)												
Lower respiratory infections	135 μg/m^3	Both	Both	1·940 (1·620 to 2·380)												
Lower respiratory infections	120 μg/m^3	Both	Both	1·870 (1·580 to 2·270)												
Lower respiratory infections	105 μg/m^3	Both	Both	1·800 (1·540 to 2·140)												
Lower respiratory infections	90 μg/m^3	Both	Both	1·720 (1·490 to 2·010)												
Lower respiratory infections	75 μg/m^3	Both	Both	1·630 (1·430 to 1·880)												
Lower respiratory infections	60 μg/m^3	Both	Both	1·540 (1·360 to 1·720)												
Lower respiratory infections	45 μg/m^3	Both	Both	1·420 (1·280 to 1·590)												
Lower respiratory infections	30 μg/m^3	Both	Both	1·300 (1·170 to 1·480)												
Lower respiratory infections	25 μg/m^3	Both	Both	1·250 (1·130 to 1·420)												
Lower respiratory infections	20 μg/m^3	Both	Both	1·210 (1·090 to 1·370)												
Lower respiratory infections	15 μg/m^3	Both	Both	1·150 (1·050 to 1·310)												
Lower respiratory infections	10 μg/m^3	Both	Both	1·100 (1·020 to 1·240)												
Lower respiratory infections	5 μg/m^3	Both	Both	1·030 (1·000 to 1·130)												
Lower respiratory infections	0 μg/m^3	Both	Both	1·000 (1·000 to 1·000)												
Tracheal, bronchus, and lung cancer	600 μg/m^3	Both	Both	3·160 (2·660 to 3·710)												
Tracheal, bronchus, and lung cancer	500 μg/m^3	Both	Both	2·880 (2·420 to 3·380)												
Tracheal, bronchus, and lung cancer	400 μg/m^3	Both	Both	2·580 (2·180 to 3·050)												
Tracheal, bronchus, and lung cancer	300 μg/m^3	Both	Both	2·270 (1·920 to 2·660)												
Tracheal, bronchus, and lung cancer	200 μg/m^3	Both	Both	1·920 (1·650 to 2·250)												
Tracheal, bronchus, and lung cancer	150 μg/m^3	Both	Both	1·740 (1·500 to 2·010)												
Tracheal, bronchus, and lung cancer	135 μg/m^3	Both	Both	1·680 (1·460 to 1·940)												
Tracheal, bronchus, and lung cancer	120 μg/m^3	Both	Both	1·620 (1·410 to 1·870)												
Tracheal, bronchus, and lung cancer	105 μg/m^3	Both	Both	1·560 (1·360 to 1·790)												
Tracheal, bronchus, and lung cancer	90 μg/m^3	Both	Both	1·490 (1·310 to 1·710)												
Tracheal, bronchus, and lung cancer	75 μg/m^3	Both	Both	1·420 (1·260 to 1·620)												
Tracheal, bronchus, and lung cancer	60 μg/m^3	Both	Both	1·350 (1·220 to 1·530)												
Tracheal, bronchus, and lung cancer	45 μg/m^3	Both	Both	1·280 (1·160 to 1·430)												
Tracheal, bronchus, and lung cancer	30 μg/m^3	Both	Both	1·200 (1·110 to 1·310)												
Tracheal, bronchus, and lung cancer	25 μg/m^3	Both	Both	1·170 (1·090 to 1·270)												
Tracheal, bronchus, and lung cancer	20 μg/m^3	Both	Both	1·130 (1·070 to 1·220)												
Tracheal, bronchus, and lung cancer	15 μg/m^3	Both	Both	1·100 (1·050 to 1·170)												
Tracheal, bronchus, and lung cancer	10 μg/m^3	Both	Both	1·060 (1·030 to 1·120)												
Tracheal, bronchus, and lung cancer	5 μg/m^3	Both	Both	1·010 (1·000 to 1·050)												
Tracheal, bronchus, and lung cancer	0 μg/m^3	Both	Both	1·000 (1·000 to 1·000)												
Ischaemic heart disease	600 μg/m^3	Both	Both		2·660 (2·020 to 4·300)	2·460 (1·920 to 3·750)	2·320 (1·880 to 3·520)	2·190 (1·740 to 3·380)	2·070 (1·670 to 3·130)	1·940 (1·650 to 2·800)	1·810 (1·550 to 2·480)	1·690 (1·480 to 2·070)	1·620 (1·420 to 2·070)	1·520 (1·350 to 1·740)	1·440 (1·300 to 1·790)	1·370 (1·240 to 1·660)
Ischaemic heart disease	500 μg/m^3	Both	Both		2·570 (1·940 to 4·270)	2·380 (1·840 to 3·670)	2·250 (1·820 to 3·440)	2·130 (1·690 to 3·320)	2·010 (1·620 to 3·130)	1·890 (1·610 to 2·760)	1·760 (1·510 to 2·460)	1·660 (1·440 to 2·020)	1·590 (1·390 to 2·050)	1·490 (1·330 to 1·700)	1·420 (1·280 to 1·770)	1·350 (1·230 to 1·650)
Ischaemic heart disease	400 μg/m^3	Both	Both		2·460 (1·860 to 4·160)	2·280 (1·760 to 3·640)	2·160 (1·740 to 3·320)	2·060 (1·630 to 3·210)	1·950 (1·570 to 3·070)	1·830 (1·560 to 2·730)	1·710 (1·460 to 2·430)	1·620 (1·410 to 1·970)	1·550 (1·360 to 2·040)	1·460 (1·300 to 1·670)	1·400 (1·260 to 1·750)	1·330 (1·210 to 1·650)
Ischaemic heart disease	300 μg/m^3	Both	Both		2·330 (1·750 to 3·980)	2·160 (1·660 to 3·520)	2·060 (1·650 to 3·180)	1·970 (1·550 to 3·150)	1·870 (1·500 to 2·980)	1·760 (1·500 to 2·630)	1·650 (1·410 to 2·360)	1·560 (1·360 to 1·900)	1·510 (1·320 to 2·000)	1·420 (1·270 to 1·620)	1·370 (1·220 to 1·720)	1·310 (1·180 to 1·620)
Ischaemic heart disease	200 μg/m^3	Both	Both		2·150 (1·620 to 3·680)	2·010 (1·540 to 3·250)	1·920 (1·540 to 2·940)	1·850 (1·450 to 2·970)	1·770 (1·420 to 2·810)	1·670 (1·420 to 2·500)	1·580 (1·340 to 2·240)	1·500 (1·300 to 1·830)	1·450 (1·260 to 1·920)	1·380 (1·220 to 1·560)	1·330 (1·190 to 1·650)	1·270 (1·160 to 1·580)
Ischaemic heart disease	150 μg/m^3	Both	Both		2·040 (1·540 to 3·410)	1·910 (1·470 to 3·020)	1·840 (1·470 to 2·730)	1·770 (1·390 to 2·820)	1·700 (1·370 to 2·650)	1·610 (1·380 to 2·370)	1·520 (1·310 to 2·130)	1·460 (1·260 to 1·780)	1·410 (1·230 to 1·860)	1·350 (1·200 to 1·530)	1·300 (1·170 to 1·600)	1·250 (1·140 to 1·530)
Ischaemic heart disease	135 μg/m^3	Both	Both		2·000 (1·510 to 3·300)	1·880 (1·450 to 2·930)	1·810 (1·450 to 2·650)	1·740 (1·380 to 2·760)	1·680 (1·360 to 2·590)	1·590 (1·360 to 2·320)	1·510 (1·290 to 2·070)	1·440 (1·250 to 1·760)	1·400 (1·220 to 1·830)	1·340 (1·190 to 1·510)	1·290 (1·160 to 1·580)	1·250 (1·130 to 1·510)
Ischaemic heart disease	120 μg/m^3	Both	Both		1·950 (1·480 to 3·180)	1·840 (1·420 to 2·840)	1·770 (1·420 to 2·570)	1·710 (1·350 to 2·690)	1·650 (1·340 to 2·520)	1·560 (1·340 to 2·260)	1·490 (1·270 to 2·010)	1·430 (1·240 to 1·740)	1·390 (1·210 to 1·800)	1·320 (1·180 to 1·500)	1·280 (1·150 to 1·550)	1·240 (1·130 to 1·490)
Ischaemic heart disease	105 μg/m^3	Both	Both		1·910 (1·450 to 3·040)	1·800 (1·400 to 2·740)	1·730 (1·400 to 2·470)	1·680 (1·330 to 2·590)	1·620 (1·320 to 2·460)	1·540 (1·330 to 2·180)	1·470 (1·260 to 1·960)	1·410 (1·220 to 1·700)	1·370 (1·190 to 1·770)	1·310 (1·170 to 1·490)	1·270 (1·140 to 1·520)	1·230 (1·120 to 1·470)
Ischaemic heart disease	90 μg/m^3	Both	Both		1·850 (1·420 to 2·880)	1·750 (1·370 to 2·620)	1·690 (1·370 to 2·370)	1·640 (1·300 to 2·470)	1·590 (1·300 to 2·370)	1·510 (1·310 to 2·100)	1·440 (1·240 to 1·900)	1·390 (1·210 to 1·660)	1·350 (1·180 to 1·720)	1·300 (1·160 to 1·470)	1·260 (1·130 to 1·490)	1·220 (1·110 to 1·440)
Ischaemic heart disease	75 μg/m^3	Both	Both		1·790 (1·380 to 2·700)	1·700 (1·330 to 2·490)	1·650 (1·340 to 2·250)	1·600 (1·280 to 2·330)	1·550 (1·270 to 2·260)	1·480 (1·280 to 2·000)	1·410 (1·220 to 1·840)	1·370 (1·190 to 1·620)	1·330 (1·160 to 1·670)	1·280 (1·150 to 1·450)	1·240 (1·120 to 1·450)	1·200 (1·100 to 1·410)
Ischaemic heart disease	60 μg/m^3	Both	Both		1·720 (1·340 to 2·480)	1·640 (1·300 to 2·320)	1·590 (1·300 to 2·100)	1·550 (1·240 to 2·170)	1·500 (1·250 to 2·110)	1·440 (1·250 to 1·880)	1·380 (1·200 to 1·760)	1·340 (1·170 to 1·580)	1·310 (1·150 to 1·600)	1·260 (1·130 to 1·420)	1·220 (1·110 to 1·410)	1·190 (1·090 to 1·380)
Ischaemic heart disease	45 μg/m^3	Both	Both		1·640 (1·290 to 2·220)	1·570 (1·260 to 2·110)	1·530 (1·260 to 1·960)	1·490 (1·210 to 1·960)	1·450 (1·210 to 1·930)	1·390 (1·220 to 1·740)	1·340 (1·170 to 1·650)	1·310 (1·150 to 1·530)	1·280 (1·130 to 1·510)	1·240 (1·120 to 1·390)	1·200 (1·100 to 1·350)	1·170 (1·080 to 1·320)
Ischaemic heart disease	30 μg/m^3	Both	Both		1·530 (1·230 to 1·920)	1·480 (1·200 to 1·870)	1·450 (1·210 to 1·780)	1·410 (1·160 to 1·720)	1·380 (1·170 to 1·710)	1·330 (1·180 to 1·570)	1·290 (1·130 to 1·530)	1·270 (1·120 to 1·460)	1·240 (1·110 to 1·430)	1·210 (1·100 to 1·350)	1·180 (1·080 to 1·290)	1·150 (1·070 to 1·250)
Ischaemic heart disease	25 μg/m^3	Both	Both		1·490 (1·210 to 1·840)	1·440 (1·170 to 1·770)	1·410 (1·190 to 1·720)	1·380 (1·150 to 1·630)	1·350 (1·150 to 1·630)	1·310 (1·160 to 1·520)	1·270 (1·120 to 1·480)	1·250 (1·110 to 1·430)	1·220 (1·100 to 1·400)	1·190 (1·090 to 1·330)	1·160 (1·070 to 1·270)	1·140 (1·060 to 1·230)
Ischaemic heart disease	20 μg/m^3	Both	Both		1·440 (1·180 to 1·750)	1·400 (1·140 to 1·700)	1·370 (1·170 to 1·670)	1·340 (1·130 to 1·580)	1·320 (1·130 to 1·550)	1·280 (1·140 to 1·460)	1·250 (1·110 to 1·430)	1·230 (1·090 to 1·400)	1·210 (1·090 to 1·360)	1·180 (1·080 to 1·300)	1·150 (1·060 to 1·250)	1·130 (1·050 to 1·210)
Ischaemic heart disease	15 μg/m^3	Both	Both		1·370 (1·150 to 1·640)	1·340 (1·120 to 1·610)	1·330 (1·140 to 1·600)	1·300 (1·100 to 1·520)	1·280 (1·110 to 1·490)	1·250 (1·120 to 1·420)	1·220 (1·090 to 1·390)	1·200 (1·070 to 1·360)	1·180 (1·070 to 1·330)	1·160 (1·060 to 1·280)	1·130 (1·050 to 1·230)	1·110 (1·050 to 1·190)
Ischaemic heart disease	10 μg/m^3	Both	Both		1·290 (1·100 to 1·530)	1·270 (1·080 to 1·520)	1·260 (1·100 to 1·510)	1·240 (1·080 to 1·430)	1·220 (1·080 to 1·410)	1·200 (1·090 to 1·350)	1·180 (1·060 to 1·340)	1·160 (1·050 to 1·300)	1·150 (1·050 to 1·280)	1·130 (1·050 to 1·250)	1·110 (1·040 to 1·200)	1·090 (1·030 to 1·170)
Ischaemic heart disease	5 μg/m^3	Both	Both		1·110 (1·000 to 1·360)	1·100 (1·000 to 1·340)	1·110 (1·000 to 1·350)	1·100 (1·000 to 1·290)	1·090 (1·000 to 1·280)	1·080 (1·000 to 1·240)	1·080 (1·000 to 1·230)	1·070 (1·000 to 1·220)	1·070 (1·000 to 1·190)	1·060 (1·000 to 1·160)	1·050 (1·000 to 1·130)	1·040 (1·000 to 1·110)
Ischaemic heart disease	0 μg/m^3	Both	Both		1·000 (1·000 to 1·000)	1·000 (1·000 to 1·000)	1·000 (1·000 to 1·000)	1·000 (1·000 to 1·000)	1·000 (1·000 to 1·000)	1·000 (1·000 to 1·000)	1·000 (1·000 to 1·000)	1·000 (1·000 to 1·000)	1·000 (1·000 to 1·000)	1·000 (1·000 to 1·000)	1·000 (1·000 to 1·000)	1·000 (1·000 to 1·000)
Chronic obstructive pulmonary disease	600 μg/m^3	Both	Both	2·970 (2·210 to 5·740)												
Chronic obstructive pulmonary disease	500 μg/m^3	Both	Both	2·780 (2·090 to 5·310)												
Chronic obstructive pulmonary disease	400 μg/m^3	Both	Both	2·570 (1·950 to 4·820)												
Chronic obstructive pulmonary disease	300 μg/m^3	Both	Both	2·330 (1·780 to 4·120)												
Chronic obstructive pulmonary disease	200 μg/m^3	Both	Both	2·040 (1·610 to 3·230)												
Chronic obstructive pulmonary disease	150 μg/m^3	Both	Both	1·870 (1·510 to 2·700)												
Chronic obstructive pulmonary disease	135 μg/m^3	Both	Both	1·810 (1·480 to 2·540)												
Chronic obstructive pulmonary disease	120 μg/m^3	Both	Both	1·750 (1·440 to 2·390)												
Chronic obstructive pulmonary disease	105 μg/m^3	Both	Both	1·690 (1·400 to 2·260)												
Chronic obstructive pulmonary disease	90 μg/m^3	Both	Both	1·630 (1·360 to 2·120)												
Chronic obstructive pulmonary disease	75 μg/m^3	Both	Both	1·560 (1·310 to 1·950)												
Chronic obstructive pulmonary disease	60 μg/m^3	Both	Both	1·480 (1·270 to 1·810)												
Chronic obstructive pulmonary disease	45 μg/m^3	Both	Both	1·400 (1·210 to 1·650)												
Chronic obstructive pulmonary disease	30 μg/m^3	Both	Both	1·300 (1·150 to 1·480)												
Chronic obstructive pulmonary disease	25 μg/m^3	Both	Both	1·270 (1·130 to 1·420)												
Chronic obstructive pulmonary disease	20 μg/m^3	Both	Both	1·230 (1·100 to 1·370)												
Chronic obstructive pulmonary disease	15 μg/m^3	Both	Both	1·180 (1·080 to 1·310)												
Chronic obstructive pulmonary disease	10 μg/m^3	Both	Both	1·120 (1·040 to 1·240)												
Chronic obstructive pulmonary disease	5 μg/m^3	Both	Both	1·030 (1·000 to 1·120)												
Chronic obstructive pulmonary disease	0 μg/m^3	Both	Both	1·000 (1·000 to 1·000)												

Risk - Outcome	Category / Units	Morbidity / Mortality	Location (Global unless otherwise specified)	Sex	Age														
					All ages	15-19 years	20-24 years	25-29 years	30-34 years	35-39 years	40-44 years	45-49 years	50-54 years	55-59 years	60-64 years	65-69 years	70-74 years	75-79 years	80+ years
**Alcohol use**																			
Tuberculosis	Former	Both	Global	Male	1·210 (1·095 to 1·313)														
Tuberculosis	85 g/day	Both	Global	Male	2·960 (2·302 to 3·835)														
Tuberculosis	80 g/day	Both	Global	Male	2·960 (2·302 to 3·835)														
Tuberculosis	70 g/day	Both	Global	Male	2·960 (2·302 to 3·835)														
Tuberculosis	60 g/day	Both	Global	Male	2·960 (2·302 to 3·835)														
Tuberculosis	50 g/day	Both	Global	Male	2·960 (2·302 to 3·835)														
Tuberculosis	40 g/day	Both	Global	Male	2·960 (2·302 to 3·835)														
Tuberculosis	30 g/day	Both	Global	Male	1·000 (1·000 to 1·000)														
Tuberculosis	20 g/day	Both	Global	Male	1·000 (1·000 to 1·000)														
Tuberculosis	10 g/day	Both	Global	Male	1·000 (1·000 to 1·000)														
Tuberculosis	0 g/day	Both	Global	Male	1·000 (1·000 to 1·000)														
Tuberculosis	Former	Both	Global	Female	1·440 (1·280 to 1·608)														
Tuberculosis	60 g/day	Both	Global	Female	2·960 (2·253 to 3·851)														
Tuberculosis	50 g/day	Both	Global	Female	2·960 (2·253 to 3·851)														
Tuberculosis	40 g/day	Both	Global	Female	2·960 (2·253 to 3·851)														
Tuberculosis	30 g/day	Both	Global	Female	1·000 (1·000 to 1·000)														
Tuberculosis	20 g/day	Both	Global	Female	1·000 (1·000 to 1·000)														
Tuberculosis	10 g/day	Both	Global	Female	1·000 (1·000 to 1·000)														
Tuberculosis	0 g/day	Both	Global	Female	1·000 (1·000 to 1·000)														
Tuberculosis	Former	Both	Russia	Male	1·210 (1·108 to 1·324)														
Tuberculosis	85 g/day	Both	Russia	Male	4·140 (3·416 to 4·960)														
Tuberculosis	80 g/day	Both	Russia	Male	4·140 (3·416 to 4·960)														
Tuberculosis	70 g/day	Both	Russia	Male	1·970 (1·629 to 2·366)														
Tuberculosis	60 g/day	Both	Russia	Male	1·970 (1·629 to 2·366)														
Tuberculosis	50 g/day	Both	Russia	Male	1·970 (1·629 to 2·366)														
Tuberculosis	40 g/day	Both	Russia	Male	1·970 (1·629 to 2·366)														
Tuberculosis	30 g/day	Both	Russia	Male	1·970 (1·629 to 2·366)														
Tuberculosis	20 g/day	Both	Russia	Male	1·010 (0·834 to 1·223)														
Tuberculosis	10 g/day	Both	Russia	Male	1·010 (0·834 to 1·223)														
Tuberculosis	0 g/day	Both	Russia	Male	1·010 (0·834 to 1·223)														
Tuberculosis	Former	Both	Russia	Female	1·440 (1·286 to 1·623)														
Tuberculosis	60 g/day	Both	Russia	Female	4·060 (3·089 to 5·531)														
Tuberculosis	50 g/day	Both	Russia	Female	4·060 (3·089 to 5·531)														
Tuberculosis	40 g/day	Both	Russia	Female	4·060 (3·089 to 5·531)														
Tuberculosis	30 g/day	Both	Russia	Female	4·060 (3·089 to 5·531)														
Tuberculosis	20 g/day	Both	Russia	Female	0·930 (0·628 to 1·365)														
Tuberculosis	10 g/day	Both	Russia	Female	0·930 (0·628 to 1·365)														
Tuberculosis	0 g/day	Both	Russia	Female	0·930 (0·628 to 1·365)														
Lower respiratory infections	Former	Both	Global	Male	1·210 (1·108 to 1·325)														
Lower respiratory infections	85 g/day	Both	Global	Male	1·500 (1·109 to 1·993)														
Lower respiratory infections	80 g/day	Both	Global	Male	1·464 (1·102 to 1·914)														
Lower respiratory infections	70 g/day	Both	Global	Male	1·396 (1·089 to 1·765)														
Lower respiratory infections	60 g/day	Both	Global	Male	1·331 (1·076 to 1·627)														
Lower respiratory infections	50 g/day	Both	Global	Male	1·269 (1·063 to 1·500)														
Lower respiratory infections	40 g/day	Both	Global	Male	1·210 (1·050 to 1·384)														
Lower respiratory infections	30 g/day	Both	Global	Male	1·154 (1·037 to 1·276)														
Lower respiratory infections	20 g/day	Both	Global	Male	1·100 (1·025 to 1·176)														
Lower respiratory infections	10 g/day	Both	Global	Male	1·049 (1·012 to 1·085)														
Lower respiratory infections	0 g/day	Both	Global	Male	1·000 (1·000 to 1·000)														
Lower respiratory infections	Former	Both	Global	Female	1·440 (1·282 to 1·609)														
Lower respiratory infections	60 g/day	Both	Global	Female	1·331 (1·064 to 1·661)														
Lower respiratory infections	50 g/day	Both	Global	Female	1·269 (1·053 to 1·526)														
Lower respiratory infections	40 g/day	Both	Global	Female	1·210 (1·042 to 1·403)														
Lower respiratory infections	30 g/day	Both	Global	Female	1·154 (1·031 to 1·289)														
Lower respiratory infections	20 g/day	Both	Global	Female	1·100 (1·021 to 1·185)														
Lower respiratory infections	10 g/day	Both	Global	Female	1·049 (1·010 to 1·089)														
Lower respiratory infections	0 g/day	Both	Global	Female	1·000 (1·000 to 1·000)														
Lower respiratory infections	Former	Both	Russia	Male	1·210 (1·100 to 1·326)														
Lower respiratory infections	85 g/day	Both	Russia	Male	3·290 (2·520 to 4·241)														
Lower respiratory infections	80 g/day	Both	Russia	Male	3·290 (2·520 to 4·241)														
Lower respiratory infections	70 g/day	Both	Russia	Male	1·920 (1·520 to 2·433)														
Lower respiratory infections	60 g/day	Both	Russia	Male	1·920 (1·520 to 2·433)														
Lower respiratory infections	50 g/day	Both	Russia	Male	1·920 (1·520 to 2·433)														
Lower respiratory infections	40 g/day	Both	Russia	Male	1·920 (1·520 to 2·433)														
Lower respiratory infections	30 g/day	Both	Russia	Male	1·920 (1·520 to 2·433)														
Lower respiratory infections	20 g/day	Both	Russia	Male	0·950 (0·817 to 1·106)														
Lower respiratory infections	10 g/day	Both	Russia	Male	0·950 (0·817 to 1·106)														
Lower respiratory infections	0 g/day	Both	Russia	Male	0·950 (0·817 to 1·106)														
Lower respiratory infections	Former	Both	Russia	Female	1·440 (1·292 to 1·611)														
Lower respiratory infections	60 g/day	Both	Russia	Female	3·210 (2·582 to 4·066)														
Lower respiratory infections	50 g/day	Both	Russia	Female	3·210 (2·582 to 4·066)														
Lower respiratory infections	40 g/day	Both	Russia	Female	3·210 (2·582 to 4·066)														
Lower respiratory infections	30 g/day	Both	Russia	Female	3·210 (2·582 to 4·066)														
Lower respiratory infections	20 g/day	Both	Russia	Female	2·100 (1·755 to 2·509)														
Lower respiratory infections	10 g/day	Both	Russia	Female	2·100 (1·755 to 2·509)														
Lower respiratory infections	0 g/day	Both	Russia	Female	2·100 (1·755 to 2·509)														
Lip and oral cavity cancer	Former	Both	Global	Male	1·210 (1·098 to 1·325)														
Lip and oral cavity cancer	85 g/day	Both	Global	Male	5·393 (4·849 to 6·030)														
Lip and oral cavity cancer	80 g/day	Both	Global	Male	5·042 (4·553 to 5·624)														
Lip and oral cavity cancer	70 g/day	Both	Global	Male	4·358 (3·965 to 4·837)														
Lip and oral cavity cancer	60 g/day	Both	Global	Male	3·710 (3·393 to 4·090)														
Lip and oral cavity cancer	50 g/day	Both	Global	Male	3·110 (2·865 to 3·393)														
Lip and oral cavity cancer	40 g/day	Both	Global	Male	2·564 (2·388 to 2·771)														
Lip and oral cavity cancer	30 g/day	Both	Global	Male	2·080 (1·962 to 2·214)														
Lip and oral cavity cancer	20 g/day	Both	Global	Male	1·658 (1·588 to 1·734)														
Lip and oral cavity cancer	10 g/day	Both	Global	Male	1·299 (1·269 to 1·332)														
Lip and oral cavity cancer	0 g/day	Both	Global	Male	1·000 (1·000 to 1·000)														
Lip and oral cavity cancer	Former	Both	Global	Female	1·440 (1·289 to 1·606)														
Lip and oral cavity cancer	60 g/day	Both	Global	Female	3·710 (3·353 to 4·078)														
Lip and oral cavity cancer	50 g/day	Both	Global	Female	3·110 (2·840 to 3·393)														
Lip and oral cavity cancer	40 g/day	Both	Global	Female	2·564 (2·367 to 2·769)														
Lip and oral cavity cancer	30 g/day	Both	Global	Female	2·080 (1·951 to 2·215)														
Lip and oral cavity cancer	20 g/day	Both	Global	Female	1·658 (1·583 to 1·736)														
Lip and oral cavity cancer	10 g/day	Both	Global	Female	1·299 (1·267 to 1·332)														
Lip and oral cavity cancer	0 g/day	Both	Global	Female	1·000 (1·000 to 1·000)														
Nasopharynx cancer	Former	Both	Global	Male	1·210 (1·098 to 1·325)														
Nasopharynx cancer	85 g/day	Both	Global	Male	5·393 (4·849 to 6·030)														
Nasopharynx cancer	80 g/day	Both	Global	Male	5·042 (4·553 to 5·624)														
Nasopharynx cancer	70 g/day	Both	Global	Male	4·358 (3·965 to 4·837)														
Nasopharynx cancer	60 g/day	Both	Global	Male	3·710 (3·393 to 4·090)														

Risk - Outcome	Category / Units	Morbidity / Mortality	(Global unless otherwise specified)	Sex	All ages	15-19 years	20-24 years	25-29 years	30-34 years	35-39 years	40-44 years	45-49 years	50-54 years	55-59 years	60-64 years	65-69 years	70-74 years	75-79 years	80+ years
Nasopharynx cancer	50 g/day	Both	Global	Male	3·110 (2·865 to 3·393)														
Nasopharynx cancer	40 g/day	Both	Global	Male	2·564 (2·388 to 2·771)														
Nasopharynx cancer	30 g/day	Both	Global	Male	2·080 (1·962 to 2·214)														
Nasopharynx cancer	20 g/day	Both	Global	Male	1·658 (1·588 to 1·734)														
Nasopharynx cancer	10 g/day	Both	Global	Male	1·299 (1·269 to 1·332)														
Nasopharynx cancer	0 g/day	Both	Global	Male	1·000 (1·000 to 1·000)														
Nasopharynx cancer	Former	Both	Global	Female	1·440 (1·289 to 1·606)														
Nasopharynx cancer	60 g/day	Both	Global	Female	3·710 (3·353 to 4·078)														
Nasopharynx cancer	50 g/day	Both	Global	Female	3·110 (2·840 to 3·393)														
Nasopharynx cancer	40 g/day	Both	Global	Female	2·564 (2·367 to 2·769)														
Nasopharynx cancer	30 g/day	Both	Global	Female	2·080 (1·951 to 2·215)														
Nasopharynx cancer	20 g/day	Both	Global	Female	1·658 (1·583 to 1·736)														
Nasopharynx cancer	10 g/day	Both	Global	Female	1·299 (1·267 to 1·332)														
Nasopharynx cancer	0 g/day	Both	Global	Female	1·000 (1·000 to 1·000)														
Other pharynx cancer	Former	Both	Global	Male	1·210 (1·098 to 1·325)														
Other pharynx cancer	85 g/day	Both	Global	Male	5·393 (4·849 to 6·030)														
Other pharynx cancer	80 g/day	Both	Global	Male	5·042 (4·553 to 5·624)														
Other pharynx cancer	70 g/day	Both	Global	Male	4·358 (3·965 to 4·837)														
Other pharynx cancer	60 g/day	Both	Global	Male	3·710 (3·393 to 4·090)														
Other pharynx cancer	50 g/day	Both	Global	Male	3·110 (2·865 to 3·393)														
Other pharynx cancer	40 g/day	Both	Global	Male	2·564 (2·388 to 2·771)														
Other pharynx cancer	30 g/day	Both	Global	Male	2·080 (1·962 to 2·214)														
Other pharynx cancer	20 g/day	Both	Global	Male	1·658 (1·588 to 1·734)														
Other pharynx cancer	10 g/day	Both	Global	Male	1·299 (1·269 to 1·332)														
Other pharynx cancer	0 g/day	Both	Global	Male	1·000 (1·000 to 1·000)														
Other pharynx cancer	Former	Both	Global	Female	1·440 (1·289 to 1·606)														
Other pharynx cancer	60 g/day	Both	Global	Female	3·710 (3·353 to 4·078)														
Other pharynx cancer	50 g/day	Both	Global	Female	3·110 (2·840 to 3·393)														
Other pharynx cancer	40 g/day	Both	Global	Female	2·564 (2·367 to 2·769)														
Other pharynx cancer	30 g/day	Both	Global	Female	2·080 (1·951 to 2·215)														
Other pharynx cancer	20 g/day	Both	Global	Female	1·658 (1·583 to 1·736)														
Other pharynx cancer	10 g/day	Both	Global	Female	1·299 (1·267 to 1·332)														
Other pharynx cancer	0 g/day	Both	Global	Female	1·000 (1·000 to 1·000)														
Oesophageal cancer	Former	Both	Global	Male	1·210 (1·103 to 1·329)														
Oesophageal cancer	85 g/day	Both	Global	Male	2·995 (2·816 to 3·181)														
Oesophageal cancer	80 g/day	Both	Global	Male	2·816 (2·656 to 2·980)														
Oesophageal cancer	70 g/day	Both	Global	Male	2·485 (2·361 to 2·613)														
Oesophageal cancer	60 g/day	Both	Global	Male	2·189 (2·095 to 2·286)														
Oesophageal cancer	50 g/day	Both	Global	Male	1·925 (1·856 to 1·997)														
Oesophageal cancer	40 g/day	Both	Global	Male	1·691 (1·643 to 1·742)														
Oesophageal cancer	30 g/day	Both	Global	Male	1·484 (1·452 to 1·518)														
Oesophageal cancer	20 g/day	Both	Global	Male	1·302 (1·283 to 1·321)														
Oesophageal cancer	10 g/day	Both	Global	Male	1·141 (1·133 to 1·150)														
Oesophageal cancer	0 g/day	Both	Global	Male	1·000 (1·000 to 1·000)														
Oesophageal cancer	Former	Both	Global	Female	1·440 (1·282 to 1·618)														
Oesophageal cancer	60 g/day	Both	Global	Female	2·189 (2·091 to 2·294)														
Oesophageal cancer	50 g/day	Both	Global	Female	1·925 (1·853 to 2·002)														
Oesophageal cancer	40 g/day	Both	Global	Female	1·691 (1·640 to 1·745)														
Oesophageal cancer	30 g/day	Both	Global	Female	1·484 (1·451 to 1·520)														
Oesophageal cancer	20 g/day	Both	Global	Female	1·302 (1·282 to 1·322)														
Oesophageal cancer	10 g/day	Both	Global	Female	1·141 (1·132 to 1·150)														
Oesophageal cancer	0 g/day	Both	Global	Female	1·000 (1·000 to 1·000)														
Colon and rectum cancer	Former	Both	Global	Male	1·210 (1·103 to 1·330)														
Colon and rectum cancer	85 g/day	Both	Global	Male	1·175 (1·037 to 1·319)														
Colon and rectum cancer	80 g/day	Both	Global	Male	1·164 (1·035 to 1·298)														
Colon and rectum cancer	70 g/day	Both	Global	Male	1·142 (1·031 to 1·256)														
Colon and rectum cancer	60 g/day	Both	Global	Male	1·121 (1·026 to 1·216)														
Colon and rectum cancer	50 g/day	Both	Global	Male	1·100 (1·022 to 1·177)														
Colon and rectum cancer	40 g/day	Both	Global	Male	1·079 (1·017 to 1·139)														
Colon and rectum cancer	30 g/day	Both	Global	Male	1·059 (1·013 to 1·103)														
Colon and rectum cancer	20 g/day	Both	Global	Male	1·039 (1·009 to 1·067)														
Colon and rectum cancer	10 g/day	Both	Global	Male	1·019 (1·004 to 1·033)														
Colon and rectum cancer	0 g/day	Both	Global	Male	1·000 (1·000 to 1·000)														
Colon and rectum cancer	Former	Both	Global	Female	1·440 (1·280 to 1·604)														
Colon and rectum cancer	60 g/day	Both	Global	Female	1·121 (1·031 to 1·225)														
Colon and rectum cancer	50 g/day	Both	Global	Female	1·100 (1·026 to 1·184)														
Colon and rectum cancer	40 g/day	Both	Global	Female	1·079 (1·021 to 1·145)														
Colon and rectum cancer	30 g/day	Both	Global	Female	1·059 (1·015 to 1·107)														
Colon and rectum cancer	20 g/day	Both	Global	Female	1·039 (1·010 to 1·070)														
Colon and rectum cancer	10 g/day	Both	Global	Female	1·019 (1·005 to 1·034)														
Colon and rectum cancer	0 g/day	Both	Global	Female	1·000 (1·000 to 1·000)														
Larynx cancer	Former	Both	Global	Male	1·210 (1·105 to 1·324)														
Larynx cancer	85 g/day	Both	Global	Male	3·202 (2·873 to 3·535)														
Larynx cancer	80 g/day	Both	Global	Male	3·005 (2·712 to 3·303)														
Larynx cancer	70 g/day	Both	Global	Male	2·639 (2·409 to 2·872)														
Larynx cancer	60 g/day	Both	Global	Male	2·310 (2·134 to 2·486)														
Larynx cancer	50 g/day	Both	Global	Male	2·018 (1·887 to 2·146)														
Larynx cancer	40 g/day	Both	Global	Male	1·758 (1·666 to 1·848)														
Larynx cancer	30 g/day	Both	Global	Male	1·529 (1·468 to 1·588)														
Larynx cancer	20 g/day	Both	Global	Male	1·328 (1·293 to 1·363)														
Larynx cancer	10 g/day	Both	Global	Male	1·153 (1·137 to 1·168)														
Larynx cancer	0 g/day	Both	Global	Male	1·000 (1·000 to 1·000)														
Larynx cancer	Former	Both	Global	Female	1·440 (1·286 to 1·616)														
Larynx cancer	60 g/day	Both	Global	Female	2·310 (2·135 to 2·496)														
Larynx cancer	50 g/day	Both	Global	Female	2·018 (1·888 to 2·153)														
Larynx cancer	40 g/day	Both	Global	Female	1·758 (1·666 to 1·852)														
Larynx cancer	30 g/day	Both	Global	Female	1·529 (1·468 to 1·591)														
Larynx cancer	20 g/day	Both	Global	Female	1·328 (1·293 to 1·364)														
Larynx cancer	10 g/day	Both	Global	Female	1·153 (1·137 to 1·168)														
Larynx cancer	0 g/day	Both	Global	Female	1·000 (1·000 to 1·000)														
Breast cancer	Former	Both	Global	Female	1·440 (1·268 to 1·623)														
Breast cancer	60 g/day	Both	Global	Female	1·695 (1·548 to 1·850)														
Breast cancer	50 g/day	Both	Global	Female	1·552 (1·439 to 1·670)														
Breast cancer	40 g/day	Both	Global	Female	1·421 (1·338 to 1·507)														
Breast cancer	30 g/day	Both	Global	Female	1·302 (1·244 to 1·360)														
Breast cancer	20 g/day	Both	Global	Female	1·192 (1·157 to 1·228)														
Breast cancer	10 g/day	Both	Global	Female	1·092 (1·076 to 1·108)														
Breast cancer	0 g/day	Both	Global	Female	1·000 (1·000 to 1·000)														
Ischaemic heart disease	Former	Morbidity	Global	Male		1·210 (1·103 to 1·325)	1·210 (1·103 to 1·325)	1·210 (1·103 to 1·325)	1·210 (1·103 to 1·325)	1·210 (1·104 to 1·324)	1·210 (1·104 to 1·324)	1·210 (1·104 to 1·324)	1·210 (1·104 to 1·324)	1·210 (1·104 to 1·324)	1·210 (1·104 to 1·324)	1·210 (1·106 to 1·320)	1·210 (1·106 to 1·320)	1·210 (1·106 to 1·320)	1·210 (1·106 to 1·320)
Ischaemic heart disease	85 g/day	Morbidity	Global	Male		1·000 (0·772 to 1·203)	1·000 (0·772 to 1·203)	1·000 (0·772 to 1·203)	1·000 (0·772 to 1·203)	1·000 (0·771 to 1·201)	1·000 (0·771 to 1·201)	1·000 (0·771 to 1·201)	1·000 (0·771 to 1·201)	1·000 (0·771 to 1·201)	1·000 (0·771 to 1·201)	1·000 (0·846 to 1·143)	1·000 (0·846 to 1·143)	1·000 (0·846 to 1·143)	1·000 (0·846 to 1·143)
Ischaemic heart disease	80 g/day	Morbidity	Global	Male		1·000 (0·772 to 1·203)	1·000 (0·772 to 1·203)	1·000 (0·772 to 1·203)	1·000 (0·772 to 1·203)	1·000 (0·771 to 1·201)	1·000 (0·771 to 1·201)	1·000 (0·771 to 1·201)	1·000 (0·771 to 1·201)	1·000 (0·771 to 1·201)	1·000 (0·771 to 1·201)	1·000 (0·846 to 1·143)	1·000 (0·846 to 1·143)	1·000 (0·846 to 1·143)	1·000 (0·846 to 1·143)
Ischaemic heart disease	70 g/day	Morbidity	Global	Male		1·000 (0·772 to 1·203)	1·000 (0·772 to 1·203)	1·000 (0·772 to 1·203)	1·000 (0·772 to 1·203)	1·000 (0·771 to 1·201)	1·000 (0·771 to 1·201)	1·000 (0·771 to 1·201)	1·000 (0·771 to 1·201)	1·000 (0·771 to 1·201)	1·000 (0·771 to 1·201)	1·000 (0·846 to 1·143)	1·000 (0·846 to 1·143)	1·000 (0·846 to 1·143)	1·000 (0·846 to 1·143)
Ischaemic heart disease	60 g/day	Morbidity	Global	Male		1·000 (0·772 to 1·203)	1·000 (0·772 to 1·203)	1·000 (0·772 to 1·203)	1·000 (0·772 to 1·203)	1·000 (0·771 to 1·201)	1·000 (0·771 to 1·201)	1·000 (0·771 to 1·201)	1·000 (0·771 to 1·201)	1·000 (0·771 to 1·201)	1·000 (0·771 to 1·201)	1·000 (0·846 to 1·143)	1·000 (0·846 to 1·143)	1·000 (0·846 to 1·143)	1·000 (0·846 to 1·143)

Risk - Outcome	Category / Units	Morbidity / Mortality	(Global unless otherwise specified)	Sex	All ages	15-19 years	20-24 years	25-29 years	30-34 years	35-39 years	40-44 years	45-49 years	50-54 years	55-59 years	60-64 years	65-69 years	70-74 years	75-79 years	80+ years
Ischaemic heart disease	50 g/day	Morbidity	Global	Male		0·846 (0·610 to 1·030)	0·846 (0·610 to 1·030)	0·846 (0·610 to 1·030)	0·846 (0·610 to 1·030)	0·856 (0·628 to 1·043)	0·856 (0·628 to 1·043)	0·856 (0·628 to 1·043)	0·856 (0·628 to 1·043)	0·856 (0·628 to 1·043)	0·856 (0·628 to 1·043)	0·892 (0·749 to 0·994)	0·892 (0·749 to 0·994)	0·892 (0·749 to 0·994)	0·892 (0·749 to 0·994)
Ischaemic heart disease	40 g/day	Morbidity	Global	Male		0·793 (0·559 to 1·046)	0·793 (0·559 to 1·046)	0·793 (0·559 to 1·046)	0·793 (0·559 to 1·046)	0·806 (0·571 to 1·060)	0·806 (0·571 to 1·060)	0·806 (0·571 to 1·060)	0·806 (0·571 to 1·060)	0·806 (0·571 to 1·060)	0·806 (0·571 to 1·060)	0·854 (0·705 to 0·978)	0·854 (0·705 to 0·978)	0·854 (0·705 to 0·978)	0·854 (0·705 to 0·978)
Ischaemic heart disease	30 g/day	Morbidity	Global	Male		0·779 (0·550 to 1·050)	0·779 (0·550 to 1·050)	0·779 (0·550 to 1·050)	0·779 (0·550 to 1·050)	0·792 (0·560 to 1·064)	0·792 (0·560 to 1·064)	0·792 (0·560 to 1·064)	0·792 (0·560 to 1·064)	0·792 (0·560 to 1·064)	0·792 (0·560 to 1·064)	0·843 (0·706 to 0·972)	0·843 (0·706 to 0·972)	0·843 (0·706 to 0·972)	0·843 (0·706 to 0·972)
Ischaemic heart disease	20 g/day	Morbidity	Global	Male		0·796 (0·582 to 1·045)	0·796 (0·582 to 1·045)	0·796 (0·582 to 1·045)	0·796 (0·582 to 1·045)	0·808 (0·591 to 1·059)	0·808 (0·591 to 1·059)	0·808 (0·591 to 1·059)	0·808 (0·591 to 1·059)	0·808 (0·591 to 1·059)	0·808 (0·591 to 1·059)	0·856 (0·730 to 0·975)	0·856 (0·730 to 0·975)	0·856 (0·730 to 0·975)	0·856 (0·730 to 0·975)
Ischaemic heart disease	10 g/day	Morbidity	Global	Male		0·844 (0·672 to 1·034)	0·844 (0·672 to 1·034)	0·844 (0·672 to 1·034)	0·844 (0·672 to 1·034)	0·854 (0·678 to 1·043)	0·854 (0·678 to 1·043)	0·854 (0·678 to 1·043)	0·854 (0·678 to 1·043)	0·854 (0·678 to 1·043)	0·854 (0·678 to 1·043)	0·891 (0·794 to 0·981)	0·891 (0·794 to 0·981)	0·891 (0·794 to 0·981)	0·891 (0·794 to 0·981)
Ischaemic heart disease	0 g/day	Morbidity	Global	Male		0·995 (0·987 to 1·001)	0·995 (0·987 to 1·001)	0·995 (0·987 to 1·001)	0·995 (0·987 to 1·001)	0·995 (0·988 to 1·001)	0·995 (0·988 to 1·001)	0·995 (0·988 to 1·001)	0·995 (0·988 to 1·001)	0·995 (0·988 to 1·001)	0·995 (0·988 to 1·001)	0·996 (0·993 to 0·999)	0·996 (0·993 to 0·999)	0·996 (0·993 to 0·999)	0·996 (0·993 to 0·999)
Ischaemic heart disease	Former	Morbidity	Global	Female		1·360 (1·159 to 1·604)	1·360 (1·159 to 1·604)	1·360 (1·159 to 1·604)	1·360 (1·159 to 1·604)	1·360 (1·167 to 1·609)	1·360 (1·167 to 1·609)	1·360 (1·167 to 1·609)	1·360 (1·167 to 1·609)	1·360 (1·167 to 1·609)	1·360 (1·167 to 1·609)	1·360 (1·165 to 1·597)	1·360 (1·165 to 1·597)	1·360 (1·165 to 1·597)	1·360 (1·165 to 1·597)
Ischaemic heart disease	60 g/day	Morbidity	Global	Female		1·345 (0·910 to 2·640)	1·345 (0·910 to 2·640)	1·345 (0·910 to 2·640)	1·345 (0·910 to 2·640)	1·317 (0·855 to 2·119)	1·317 (0·855 to 2·119)	1·317 (0·855 to 2·119)	1·317 (0·855 to 2·119)	1·317 (0·855 to 2·119)	1·317 (0·855 to 2·119)	1·223 (0·937 to 1·699)	1·223 (0·937 to 1·699)	1·223 (0·937 to 1·699)	1·223 (0·937 to 1·699)
Ischaemic heart disease	50 g/day	Morbidity	Global	Female		1·217 (0·846 to 2·391)	1·217 (0·846 to 2·391)	1·217 (0·846 to 2·391)	1·217 (0·846 to 2·391)	1·200 (0·796 to 1·913)	1·200 (0·796 to 1·913)	1·200 (0·796 to 1·913)	1·200 (0·796 to 1·913)	1·200 (0·796 to 1·913)	1·200 (0·796 to 1·913)	1·143 (0·877 to 1·571)	1·143 (0·877 to 1·571)	1·143 (0·877 to 1·571)	1·143 (0·877 to 1·571)
Ischaemic heart disease	40 g/day	Morbidity	Global	Female		1·101 (0·773 to 2·087)	1·101 (0·773 to 2·087)	1·101 (0·773 to 2·087)	1·101 (0·773 to 2·087)	1·094 (0·731 to 1·770)	1·094 (0·731 to 1·770)	1·094 (0·731 to 1·770)	1·094 (0·731 to 1·770)	1·094 (0·731 to 1·770)	1·094 (0·731 to 1·770)	1·068 (0·821 to 1·459)	1·068 (0·821 to 1·459)	1·068 (0·821 to 1·459)	1·068 (0·821 to 1·459)
Ischaemic heart disease	30 g/day	Morbidity	Global	Female		0·994 (0·641 to 1·622)	0·994 (0·641 to 1·622)	0·994 (0·641 to 1·622)	0·994 (0·641 to 1·622)	0·994 (0·599 to 1·449)	0·994 (0·599 to 1·449)	0·994 (0·599 to 1·449)	0·994 (0·599 to 1·449)	0·994 (0·599 to 1·449)	0·994 (0·599 to 1·449)	0·996 (0·737 to 1·299)	0·996 (0·737 to 1·299)	0·996 (0·737 to 1·299)	0·996 (0·737 to 1·299)
Ischaemic heart disease	20 g/day	Morbidity	Global	Female		0·867 (0·559 to 1·192)	0·867 (0·559 to 1·192)	0·867 (0·559 to 1·192)	0·867 (0·559 to 1·192)	0·875 (0·530 to 1·152)	0·875 (0·530 to 1·152)	0·875 (0·530 to 1·152)	0·875 (0·530 to 1·152)	0·875 (0·530 to 1·152)	0·875 (0·530 to 1·152)	0·907 (0·683 to 1·102)	0·907 (0·683 to 1·102)	0·907 (0·683 to 1·102)	0·907 (0·683 to 1·102)
Ischaemic heart disease	10 g/day	Morbidity	Global	Female		0·827 (0·587 to 1·040)	0·827 (0·587 to 1·040)	0·827 (0·587 to 1·040)	0·827 (0·587 to 1·040)	0·838 (0·555 to 1·038)	0·838 (0·555 to 1·038)	0·838 (0·555 to 1·038)	0·838 (0·555 to 1·038)	0·838 (0·555 to 1·038)	0·838 (0·555 to 1·038)	0·879 (0·699 to 1·000)	0·879 (0·699 to 1·000)	0·879 (0·699 to 1·000)	0·879 (0·699 to 1·000)
Ischaemic heart disease	0 g/day	Morbidity	Global	Female		0·999 (0·997 to 1·000)	0·999 (0·997 to 1·000)	0·999 (0·997 to 1·000)	0·999 (0·997 to 1·000)	0·999 (0·997 to 1·000)	0·999 (0·997 to 1·000)	0·999 (0·997 to 1·000)	0·999 (0·997 to 1·000)	0·999 (0·997 to 1·000)	0·999 (0·997 to 1·000)	0·999 (0·998 to 1·000)	0·999 (0·998 to 1·000)	0·999 (0·998 to 1·000)	0·999 (0·998 to 1·000)
Ischaemic heart disease	Former	Mortality	Global	Male		1·210 (1·108 to 1·338)	1·210 (1·108 to 1·338)	1·210 (1·108 to 1·338)	1·210 (1·108 to 1·338)	1·210 (1·111 to 1·320)	1·210 (1·111 to 1·320)	1·210 (1·111 to 1·320)	1·210 (1·111 to 1·320)	1·210 (1·111 to 1·320)	1·210 (1·111 to 1·320)	1·210 (1·104 to 1·333)	1·210 (1·104 to 1·333)	1·210 (1·104 to 1·333)	1·210 (1·104 to 1·333)
Ischaemic heart disease	85 g/day	Mortality	Global	Male		1·000 (0·759 to 1·239)	1·000 (0·759 to 1·239)	1·000 (0·759 to 1·239)	1·000 (0·759 to 1·239)	1·000 (0·801 to 1·198)	1·000 (0·801 to 1·198)	1·000 (0·801 to 1·198)	1·000 (0·801 to 1·198)	1·000 (0·801 to 1·198)	1·000 (0·801 to 1·198)	1·000 (0·853 to 1·141)	1·000 (0·853 to 1·141)	1·000 (0·853 to 1·141)	1·000 (0·853 to 1·141)
Ischaemic heart disease	80 g/day	Mortality	Global	Male		1·000 (0·759 to 1·239)	1·000 (0·759 to 1·239)	1·000 (0·759 to 1·239)	1·000 (0·759 to 1·239)	1·000 (0·801 to 1·198)	1·000 (0·801 to 1·198)	1·000 (0·801 to 1·198)	1·000 (0·801 to 1·198)	1·000 (0·801 to 1·198)	1·000 (0·801 to 1·198)	1·000 (0·853 to 1·141)	1·000 (0·853 to 1·141)	1·000 (0·853 to 1·141)	1·000 (0·853 to 1·141)
Ischaemic heart disease	70 g/day	Mortality	Global	Male		1·000 (0·759 to 1·239)	1·000 (0·759 to 1·239)	1·000 (0·759 to 1·239)	1·000 (0·759 to 1·239)	1·000 (0·801 to 1·198)	1·000 (0·801 to 1·198)	1·000 (0·801 to 1·198)	1·000 (0·801 to 1·198)	1·000 (0·801 to 1·198)	1·000 (0·801 to 1·198)	1·000 (0·853 to 1·141)	1·000 (0·853 to 1·141)	1·000 (0·853 to 1·141)	1·000 (0·853 to 1·141)
Ischaemic heart disease	60 g/day	Mortality	Global	Male		1·000 (0·759 to 1·239)	1·000 (0·759 to 1·239)	1·000 (0·759 to 1·239)	1·000 (0·759 to 1·239)	1·000 (0·801 to 1·198)	1·000 (0·801 to 1·198)	1·000 (0·801 to 1·198)	1·000 (0·801 to 1·198)	1·000 (0·801 to 1·198)	1·000 (0·801 to 1·198)	1·000 (0·853 to 1·141)	1·000 (0·853 to 1·141)	1·000 (0·853 to 1·141)	1·000 (0·853 to 1·141)
Ischaemic heart disease	50 g/day	Mortality	Global	Male		0·846 (0·592 to 1·033)	0·846 (0·592 to 1·033)	0·846 (0·592 to 1·033)	0·846 (0·592 to 1·033)	0·856 (0·649 to 1·043)	0·856 (0·649 to 1·043)	0·856 (0·649 to 1·043)	0·856 (0·649 to 1·043)	0·856 (0·649 to 1·043)	0·856 (0·649 to 1·043)	0·892 (0·745 to 0·995)	0·892 (0·745 to 0·995)	0·892 (0·745 to 0·995)	0·892 (0·745 to 0·995)
Ischaemic heart disease	40 g/day	Mortality	Global	Male		0·793 (0·546 to 1·048)	0·793 (0·546 to 1·048)	0·793 (0·546 to 1·048)	0·793 (0·546 to 1·048)	0·806 (0·599 to 1·063)	0·806 (0·599 to 1·063)	0·806 (0·599 to 1·063)	0·806 (0·599 to 1·063)	0·806 (0·599 to 1·063)	0·806 (0·599 to 1·063)	0·854 (0·704 to 0·975)	0·854 (0·704 to 0·975)	0·854 (0·704 to 0·975)	0·854 (0·704 to 0·975)
Ischaemic heart disease	30 g/day	Mortality	Global	Male		0·779 (0·539 to 1·053)	0·779 (0·539 to 1·053)	0·779 (0·539 to 1·053)	0·779 (0·539 to 1·053)	0·792 (0·589 to 1·068)	0·792 (0·589 to 1·068)	0·792 (0·589 to 1·068)	0·792 (0·589 to 1·068)	0·792 (0·589 to 1·068)	0·792 (0·589 to 1·068)	0·843 (0·699 to 0·973)	0·843 (0·699 to 0·973)	0·843 (0·699 to 0·973)	0·843 (0·699 to 0·973)
Ischaemic heart disease	20 g/day	Mortality	Global	Male		0·796 (0·576 to 1·048)	0·796 (0·576 to 1·048)	0·796 (0·576 to 1·048)	0·796 (0·576 to 1·048)	0·808 (0·615 to 1·060)	0·808 (0·615 to 1·060)	0·808 (0·615 to 1·060)	0·808 (0·615 to 1·060)	0·808 (0·615 to 1·060)	0·808 (0·615 to 1·060)	0·856 (0·726 to 0·975)	0·856 (0·726 to 0·975)	0·856 (0·726 to 0·975)	0·856 (0·726 to 0·975)
Ischaemic heart disease	10 g/day	Mortality	Global	Male		0·844 (0·665 to 1·035)	0·844 (0·665 to 1·035)	0·844 (0·665 to 1·035)	0·844 (0·665 to 1·035)	0·854 (0·698 to 1·044)	0·854 (0·698 to 1·044)	0·854 (0·698 to 1·044)	0·854 (0·698 to 1·044)	0·854 (0·698 to 1·044)	0·854 (0·698 to 1·044)	0·891 (0·789 to 0·981)	0·891 (0·789 to 0·981)	0·891 (0·789 to 0·981)	0·891 (0·789 to 0·981)
Ischaemic heart disease	0 g/day	Mortality	Global	Male		0·995 (0·987 to 1·001)	0·995 (0·987 to 1·001)	0·995 (0·987 to 1·001)	0·995 (0·987 to 1·001)	0·995 (0·989 to 1·001)	0·995 (0·989 to 1·001)	0·995 (0·989 to 1·001)	0·995 (0·989 to 1·001)	0·995 (0·989 to 1·001)	0·995 (0·989 to 1·001)	0·996 (0·992 to 0·999)	0·996 (0·992 to 0·999)	0·996 (0·992 to 0·999)	0·996 (0·992 to 0·999)
Ischaemic heart disease	Former	Mortality	Global	Female		1·360 (1·136 to 1·585)	1·360 (1·136 to 1·585)	1·360 (1·136 to 1·585)	1·360 (1·136 to 1·585)	1·360 (1·167 to 1·613)	1·360 (1·167 to 1·613)	1·360 (1·167 to 1·613)	1·360 (1·167 to 1·613)	1·360 (1·167 to 1·613)	1·360 (1·167 to 1·613)	1·360 (1·152 to 1·617)	1·360 (1·152 to 1·617)	1·360 (1·152 to 1·617)	1·360 (1·152 to 1·617)
Ischaemic heart disease	60 g/day	Mortality	Global	Female		1·345 (0·888 to 2·508)	1·345 (0·888 to 2·508)	1·345 (0·888 to 2·508)	1·345 (0·888 to 2·508)	1·317 (0·885 to 2·351)	1·317 (0·885 to 2·351)	1·317 (0·885 to 2·351)	1·317 (0·885 to 2·351)	1·317 (0·885 to 2·351)	1·317 (0·885 to 2·351)	1·223 (0·959 to 1·806)	1·223 (0·959 to 1·806)	1·223 (0·959 to 1·806)	1·223 (0·959 to 1·806)
Ischaemic heart disease	50 g/day	Mortality	Global	Female		1·217 (0·835 to 2·229)	1·217 (0·835 to 2·229)	1·217 (0·835 to 2·229)	1·217 (0·835 to 2·229)	1·200 (0·830 to 2·110)	1·200 (0·830 to 2·110)	1·200 (0·830 to 2·110)	1·200 (0·830 to 2·110)	1·200 (0·830 to 2·110)	1·200 (0·830 to 2·110)	1·143 (0·897 to 1·680)	1·143 (0·897 to 1·680)	1·143 (0·897 to 1·680)	1·143 (0·897 to 1·680)
Ischaemic heart disease	40 g/day	Mortality	Global	Female		1·101 (0·767 to 2·005)	1·101 (0·767 to 2·005)	1·101 (0·767 to 2·005)	1·101 (0·767 to 2·005)	1·094 (0·763 to 1·905)	1·094 (0·763 to 1·905)	1·094 (0·763 to 1·905)	1·094 (0·763 to 1·905)	1·094 (0·763 to 1·905)	1·094 (0·763 to 1·905)	1·068 (0·837 to 1·567)	1·068 (0·837 to 1·567)	1·068 (0·837 to 1·567)	1·068 (0·837 to 1·567)
Ischaemic heart disease	30 g/day	Mortality	Global	Female		0·994 (0·643 to 1·546)	0·994 (0·643 to 1·546)	0·994 (0·643 to 1·546)	0·994 (0·643 to 1·546)	0·994 (0·648 to 1·532)	0·994 (0·648 to 1·532)	0·994 (0·648 to 1·532)	0·994 (0·648 to 1·532)	0·994 (0·648 to 1·532)	0·994 (0·648 to 1·532)	0·996 (0·752 to 1·373)	0·996 (0·752 to 1·373)	0·996 (0·752 to 1·373)	0·996 (0·752 to 1·373)
Ischaemic heart disease	20 g/day	Mortality	Global	Female		0·867 (0·562 to 1·198)	0·867 (0·562 to 1·198)	0·867 (0·562 to 1·198)	0·867 (0·562 to 1·198)	0·875 (0·573 to 1·142)	0·875 (0·573 to 1·142)	0·875 (0·573 to 1·142)	0·875 (0·573 to 1·142)	0·875 (0·573 to 1·142)	0·875 (0·573 to 1·142)	0·907 (0·712 to 1·128)	0·907 (0·712 to 1·128)	0·907 (0·712 to 1·128)	0·907 (0·712 to 1·128)
Ischaemic heart disease	10 g/day	Mortality	Global	Female		0·827 (0·575 to 1·048)	0·827 (0·575 to 1·048)	0·827 (0·575 to 1·048)	0·827 (0·575 to 1·048)	0·838 (0·584 to 1·044)	0·838 (0·584 to 1·044)	0·838 (0·584 to 1·044)	0·838 (0·584 to 1·044)	0·838 (0·584 to 1·044)	0·838 (0·584 to 1·044)	0·879 (0·725 to 1·013)	0·879 (0·725 to 1·013)	0·879 (0·725 to 1·013)	0·879 (0·725 to 1·013)
Ischaemic heart disease	0 g/day	Mortality	Global	Female		0·999 (0·997 to 1·000)	0·999 (0·997 to 1·000)	0·999 (0·997 to 1·000)	0·999 (0·997 to 1·000)	0·999 (0·997 to 1·000)	0·999 (0·997 to 1·000)	0·999 (0·997 to 1·000)	0·999 (0·997 to 1·000)	0·999 (0·997 to 1·000)	0·999 (0·997 to 1·000)	0·999 (0·998 to 1·000)	0·999 (0·998 to 1·000)	0·999 (0·998 to 1·000)	0·999 (0·998 to 1·000)
Ischaemic heart disease	Former	Both	Russia	Male												1·210 (1·106 to 1·326)	1·210 (1·106 to 1·326)	1·210 (1·106 to 1·326)	1·210 (1·106 to 1·326)
Ischaemic heart disease	85 g/day	Both	Russia	Male												1·740 (1·226 to 2·518)	1·740 (1·226 to 2·518)	1·740 (1·226 to 2·518)	1·740 (1·226 to 2·518)
Ischaemic heart disease	80 g/day	Both	Russia	Male												1·740 (1·226 to 2·518)	1·740 (1·226 to 2·518)	1·740 (1·226 to 2·518)	1·740 (1·226 to 2·518)
Ischaemic heart disease	70 g/day	Both	Russia	Male												1·067 (0·735 to 1·574)	1·067 (0·735 to 1·574)	1·067 (0·735 to 1·574)	1·067 (0·735 to 1·574)
Ischaemic heart disease	60 g/day	Both	Russia	Male												1·067 (0·735 to 1·574)	1·067 (0·735 to 1·574)	1·067 (0·735 to 1·574)	1·067 (0·735 to 1·574)
Ischaemic heart disease	50 g/day	Both	Russia	Male												1·067 (0·735 to 1·574)	1·067 (0·735 to 1·574)	1·067 (0·735 to 1·574)	1·067 (0·735 to 1·574)
Ischaemic heart disease	40 g/day	Both	Russia	Male												1·067 (0·735 to 1·574)	1·067 (0·735 to 1·574)	1·067 (0·735 to 1·574)	1·067 (0·735 to 1·574)
Ischaemic heart disease	30 g/day	Both	Russia	Male												1·067 (0·735 to 1·574)	1·067 (0·735 to 1·574)	1·067 (0·735 to 1·574)	1·067 (0·735 to 1·574)
Ischaemic heart disease	20 g/day	Both	Russia	Male												0·779 (0·527 to 1·166)	0·779 (0·527 to 1·166)	0·779 (0·527 to 1·166)	0·779 (0·527 to 1·166)
Ischaemic heart disease	10 g/day	Both	Russia	Male												0·779 (0·527 to 1·166)	0·779 (0·527 to 1·166)	0·779 (0·527 to 1·166)	0·779 (0·527 to 1·166)
Ischaemic heart disease	0 g/day	Both	Russia	Male												0·779 (0·527 to 1·166)	0·779 (0·527 to 1·166)	0·779 (0·527 to 1·166)	0·779 (0·527 to 1·166)
Ischaemic heart disease	Former	Both	Russia	Female												1·360 (1·161 to 1·594)	1·360 (1·161 to 1·594)	1·360 (1·161 to 1·594)	1·360 (1·161 to 1·594)
Ischaemic heart disease	60 g/day	Both	Russia	Female												2·451 (1·704 to 3·641)	2·451 (1·704 to 3·641)	2·451 (1·704 to 3·641)	2·451 (1·704 to 3·641)
Ischaemic heart disease	50 g/day	Both	Russia	Female												2·451 (1·704 to 3·641)	2·451 (1·704 to 3·641)	2·451 (1·704 to 3·641)	2·451 (1·704 to 3·641)
Ischaemic heart disease	40 g/day	Both	Russia	Female												2·451 (1·704 to 3·641)	2·451 (1·704 to 3·641)	2·451 (1·704 to 3·641)	2·451 (1·704 to 3·641)
Ischaemic heart disease	30 g/day	Both	Russia	Female												2·451 (1·704 to 3·641)	2·451 (1·704 to 3·641)	2·451 (1·704 to 3·641)	2·451 (1·704 to 3·641)
Ischaemic heart disease	20 g/day	Both	Russia	Female												1·080 (0·755 to 1·563)	1·080 (0·755 to 1·563)	1·080 (0·755 to 1·563)	1·080 (0·755 to 1·563)
Ischaemic heart disease	10 g/day	Both	Russia	Female												1·080 (0·755 to 1·563)	1·080 (0·755 to 1·563)	1·080 (0·755 to 1·563)	1·080 (0·755 to 1·563)
Ischaemic heart disease	0 g/day	Both	Russia	Female												1·080 (0·755 to 1·563)	1·080 (0·755 to 1·563)	1·080 (0·755 to 1·563)	1·080 (0·755 to 1·563)
Ischaemic stroke	Former	Morbidity	Global	Male		1·330 (0·900 to 1·926)	1·330 (0·900 to 1·926)	1·330 (0·900 to 1·926)	1·330 (0·900 to 1·926)	1·330 (0·912 to 1·953)	1·330 (0·912 to 1·953)	1·330 (0·912 to 1·953)	1·330 (0·912 to 1·953)	1·330 (0·912 to 1·953)	1·330 (0·912 to 1·953)	1·330 (0·887 to 2·035)	1·330 (0·887 to 2·035)	1·330 (0·887 to 2·035)	1·330 (0·887 to 2·035)
Ischaemic stroke	85 g/day	Morbidity	Global	Male		1·417 (0·936 to 2·241)	1·417 (0·936 to 2·241)	1·417 (0·936 to 2·241)	1·417 (0·936 to 2·241)	1·384 (0·932 to 2·154)	1·384 (0·932 to 2·154)	1·384 (0·932 to 2·154)	1·384 (0·932 to 2·154)	1·384 (0·932 to 2·154)	1·384 (0·932 to 2·154)	1·268 (1·031 to 1·584)	1·268 (1·031 to 1·584)	1·268 (1·031 to 1·584)	1·268 (1·031 to 1·584)
Ischaemic stroke	80 g/day	Morbidity	Global	Male		1·370 (0·941 to 2·068)	1·370 (0·941 to 2·068)	1·370 (0·941 to 2·068)	1·370 (0·941 to 2·068)	1·341 (0·940 to 1·999)	1·341 (0·940 to 1·999)	1·341 (0·940 to 1·999)	1·341 (0·940 to 1·999)	1·341 (0·940 to 1·999)	1·341 (0·940 to 1·999)	1·239 (1·028 to 1·524)	1·239 (1·028 to 1·524)	1·239 (1·028 to 1·524)	1·239 (1·028 to 1·524)
Ischaemic stroke	70 g/day	Morbidity	Global	Male		1·279 (0·952 to 1·790)	1·279 (0·952 to 1·790)	1·279 (0·952 to 1·790)	1·279 (0·952 to 1·790)	1·258 (0·955 to 1·748)	1·258 (0·955 to 1·748)	1·258 (0·955 to 1·748)	1·258 (0·955 to 1·748)	1·258 (0·955 to 1·748)	1·258 (0·955 to 1·748)	1·183 (1·022 to 1·406)	1·183 (1·022 to 1·406)	1·183 (1·022 to 1·406)	1·183 (1·022 to 1·406)
Ischaemic stroke	60 g/day	Morbidity	Global	Male		1·193 (0·966 to 1·545)	1·193 (0·966 to 1·545)	1·193 (0·966 to 1·545)	1·193 (0·966 to 1·545)	1·179 (0·968 to 1·499)	1·179 (0·968 to 1·499)	1·179 (0·968 to 1·499)	1·179 (0·968 to 1·499)	1·179 (0·968 to 1·499)	1·179 (0·968 to 1·499)	1·128 (1·017 to 1·288)	1·128 (1·017 to 1·288)	1·128 (1·017 to 1·288)	1·128 (1·017 to 1·288)
Ischaemic stroke	50 g/day	Morbidity	Global	Male		1·111 (0·980 to 1·343)	1·111 (0·980 to 1·343)	1·111 (0·980 to 1·343)	1·111 (0·980 to 1·343)	1·103 (0·984 to 1·323)	1·103 (0·984 to 1·323)	1·103 (0·984 to 1·323)	1·103 (0·984 to 1·323)	1·103 (0·984 to 1·323)	1·103 (0·984 to 1·323)	1·075 (1·004 to 1·188)	1·075 (1·004 to 1·188)	1·075 (1·004 to 1·188)	1·075 (1·004 to 1·188)
Ischaemic stroke	40 g/day	Morbidity	Global	Male		1·034 (0·960 to 1·172)	1·034 (0·960 to 1·172)	1·034 (0·960 to 1·172)	1·034 (0·960 to 1·172)	1·032 (0·955 to 1·164)	1·032 (0·955 to 1·164)	1·032 (0·955 to 1·164)	1·032 (0·955 to 1·164)	1·032 (0·955 to 1·164)	1·032 (0·955 to 1·164)	1·023 (0·967 to 1·096)	1·023 (0·967 to 1·096)	1·023 (0·967 to 1·096)	1·023 (0·967 to 1·096)
Ischaemic stroke	30 g/day	Morbidity	Global	Male		0·962 (0·854 to 1·049)	0·962 (0·854 to 1·049)	0·962 (0·854 to 1·049)	0·962 (0·854 to 1·049)	0·964 (0·851 to 1·048)	0·964 (0·851 to 1·048)	0·964 (0·851 to 1·048)	0·964 (0·851 to 1·048)	0·964 (0·851 to 1·048)	0·964 (0·851 to 1·048)	0·974 (0·909 to 1·030)	0·974 (0·909 to 1·030)	0·974 (0·909 to 1·030)	0·974 (0·909 to 1·030)
Ischaemic stroke	20 g/day	Morbidity	Global	Male		0·896 (0·744 to 1·020)	0·896 (0·744 to 1·020)	0·896 (0·744 to 1·020)	0·896 (0·744 to 1·020)	0·903 (0·754 to 1·029)	0·903 (0·754 to 1·029)	0·903 (0·754 to 1·029)	0·903 (0·754 to 1·029)	0·903 (0·754 to 1·029)	0·903 (0·754 to 1·029)	0·928 (0·844 to 0·996)	0·928 (0·844 to 0·996)	0·928 (0·844 to 0·996)	0·928 (0·844 to 0·996)
Ischaemic stroke	10 g/day	Morbidity	Global	Male		0·842 (0·664 to 1·029)	0·842 (0·664 to 1·029)	0·842 (0·664 to 1·029)	0·842 (0·664 to 1·029)	0·852 (0·667 to 1·041)	0·852 (0·667 to 1·041)	0·852 (0·667 to 1·041)	0·852 (0·667 to 1·041)	0·852 (0·667 to 1·041)	0·852 (0·667 to 1·041)	0·889 (0·783 to 0·986)	0·889 (0·783 to 0·986)	0·889 (0·783 to 0·986)	0·889 (0·783 to 0·986)
Ischaemic stroke	0 g/day	Morbidity	Global	Male		1·000 (1·000 to 1·000)	1·000 (1·000 to 1·000)	1·000 (1·000 to 1·000)	1·000 (1·000 to 1·000)	1·000 (1·000 to 1·000)	1·000 (1·000 to 1·000)	1·000 (1·000 to 1·000)	1·000 (1·000 to 1·000)	1·000 (1·000 to 1·000)	1·000 (1·000 to 1·000)	1·000 (1·000 to 1·000)	1·000 (1·000 to 1·000)	1·000 (1·000 to 1·000)	1·000 (1·000 to 1·000)
Ischaemic stroke	Former	Morbidity	Global	Female		1·150 (0·714 to 1·976)	1·150 (0·714 to 1·976)	1·150 (0·714 to 1·976)	1·150 (0·714 to 1·976)	1·150 (0·702 to 1·861)	1·150 (0·702 to 1·861)	1·150 (0·702 to 1·861)	1·150 (0·702 to 1·861)	1·150 (0·702 to 1·861)	1·150 (0·702 to 1·861)	1·150 (0·681 to 1·840)	1·150 (0·681 to 1·840)	1·150 (0·681 to 1·840)	1·150 (0·681 to 1·840)
Ischaemic stroke	60 g/day	Morbidity	Global	Female		1·393 (0·942 to 2·328)	1·393 (0·942 to 2·328)	1·393 (0·942 to 2·328)	1·393 (0·942 to 2·328)	1·362 (0·931 to 2·346)	1·362 (0·931 to 2·346)	1·362 (0·931 to 2·346)	1·362 (0·931 to 2·346)	1·362 (0·931 to 2·346)	1·362 (0·931 to 2·346)	1·253 (1·028 to 1·621)	1·253 (1·028 to 1·621)	1·253 (1·028 to 1·621)	1·253 (1·028 to 1·621)
Ischaemic stroke	50 g/day	Morbidity	Global	Female		1·112 (0·937 to 1·447)	1·112 (0·937 to 1·447)	1·112 (0·937 to 1·447)	1·112 (0·937 to 1·447)	1·104 (0·943 to 1·468)	1·104 (0·943 to 1·468)	1·104 (0·943 to 1·468)	1·104 (0·943 to 1·468)	1·104 (0·943 to 1·468)	1·104 (0·943 to 1·468)	1·075 (0·965 to 1·252)	1·075 (0·965 to 1·252)	1·075 (0·965 to 1·252)	1·075 (0·965 to 1·252)
Ischaemic stroke	40 g/day	Morbidity	Global	Female		0·905 (0·671 to 1·074)	0·905 (0·671 to 1·074)	0·905 (0·671 to 1·074)	0·905 (0·671 to 1·074)	0·911 (0·687 to 1·072)	0·911 (0·687 to 1·072)	0·911 (0·687 to 1·072)	0·911 (0·687 to 1·072)	0·911 (0·687 to 1·072)	0·911 (0·687 to 1·072)	0·934 (0·784 to 1·045)	0·934 (0·784 to 1·045)	0·934 (0·784 to 1·045)	0·934 (0·784 to 1·045)
Ischaemic stroke	30 g/day	Morbidity	Global	Female		0·757 (0·475 to 1·054)	0·757 (0·475 to 1·054)	0·757 (0·475 to 1·054)	0·757 (0·475 to 1·054)	0·772 (0·498 to 1·070)	0·772 (0·498 to 1·070)	0·772 (0·498 to 1·070)	0·772 (0·498 to 1·070)	0·772 (0·498 to 1·070)	0·772 (0·498 to 1·070)	0·828 (0·634 to 0·982)	0·828 (0·634 to 0·982)	0·828 (0·634 to 0·982)	0·828 (0·634 to 0·982)
Ischaemic stroke	20 g/day	Morbidity	Global	Female		0·662 (0·355 to 1·082)	0·662 (0·355 to 1·082)	0·662 (0·355 to 1·082)	0·662 (0·355 to 1·082)	0·681 (0·382 to 1·103)	0·681 (0·382 to 1·103)	0·681 (0·382 to 1·103)	0·681 (0·382 to 1·103)	0·681 (0·382 to 1·103)	0·681 (0·382 to 1·103)	0·755 (0·547 to 0·967)	0·755 (0·547 to 0·967)	0·755 (0·547 to 0·967)	0·755 (0·547 to 0·967)
Ischaemic stroke	10 g/day	Morbidity	Global	Female		0·630 (0·323 to 1·094)	0·630 (0·323 to 1·094)	0·630 (0·323 to 1·094)	0·630 (0·323 to 1·094)	0·650 (0·349 to 1·119)	0·650 (0·349 to 1·119)	0·650 (0·349 to 1·119)	0·650 (0·349 to 1·119)	0·650 (0·349 to 1·119)	0·650 (0·349 to 1·119)	0·730 (0·524 to 0·960)	0·730 (0·524 to 0·960)	0·730 (0·524 to 0·960)	0·730 (0·524 to 0·960)
Ischaemic stroke	0 g/day	Morbidity	Global	Female		1·000 (1·000 to 1·000)	1·000 (1·000 to 1·000)	1·000 (1·000 to 1·000)	1·000 (1·000 to 1·000)	1·000 (1·000 to 1·000)	1·000 (1·000 to 1·000)	1·000 (1·000 to 1·000)	1·000 (1·000 to 1·000)	1·000 (1·000 to 1·000)	1·000 (1·000 to 1·000)	1·000 (1·000 to 1·000)	1·000 (1·000 to 1·000)	1·000 (1·000 to 1·000)	1·000 (1·000 to 1·000)
Ischaemic stroke	Former	Mortality	Global	Male		1·330 (0·933 to 1·957)	1·330 (0·933 to 1·957)	1·330 (0·933 to 1·957)	1·330 (0·933 to 1·957)	1·330 (0·897 to 1·925)	1·330 (0·897 to 1·925)	1·330 (0·897 to 1·925)	1·330 (0·897 to 1·925)	1·330 (0·897 to 1·925)	1·330 (0·897 to 1·925)	1·330 (0·898 to 1·984)	1·330 (0·898 to 1·984)	1·330 (0·898 to 1·984)	1·330 (0·898 to 1·984)
Ischaemic stroke	85 g/day	Mortality	Global	Male		1·417 (0·964 to 2·155)	1·417 (0·964 to 2·155)	1·417 (0·964 to 2·155)	1·417 (0·964 to 2·155)	1·384 (0·901 to 2·291)	1·384 (0·901 to 2·291)	1·384 (0·901 to 2·291)	1·384 (0·901 to 2·291)	1·384 (0·901 to 2·291)	1·384 (0·901 to 2·291)	1·325 (1·032 to 1·731)	1·325 (1·032 to 1·731)	1·325 (1·032 to 1·731)	1·325 (1·032 to 1·731)
Ischaemic stroke	80 g/day	Mortality	Global	Male		1·370 (0·967 to 2·006)	1·370 (0·967 to 2·006)	1·370 (0·967 to 2·006)	1·370 (0·967 to 2·006)	1·341 (0·911 to 2·128)	1·341 (0·911 to 2·128)	1·341 (0·911 to 2·128)	1·341 (0·911 to 2·128)	1·341 (0·911 to 2·128)	1·341 (0·911 to 2·128)	1·239 (1·025 to 1·515)	1·239 (1·025 to 1·515)	1·239 (1·025 to 1·515)	1·239 (1·025 to 1·515)
Ischaemic stroke	70 g/day	Mortality	Global	Male		1·279 (0·975 to 1·736)	1·279 (0·975 to 1·736)	1·279 (0·975 to 1·736)	1·279 (0·975 to 1·736)	1·258 (0·930 to 1·831)	1·258 (0·930 to 1·831)	1·258 (0·930 to 1·831)	1·258 (0·930 to 1·831)	1·258 (0·930 to 1·831)	1·258 (0·930 to 1·831)	1·183 (1·020 to 1·398)	1·183 (1·020 to 1·398)	1·183 (1·020 to 1·398)	1·183 (1·020 to 1·398)
Ischaemic stroke	60 g/day	Mortality	Global	Male		1·193 (0·983 to 1·502)	1·193 (0·983 to 1·502)	1·193 (0·983 to 1·502)	1·193 (0·983 to 1·502)	1·179 (0·951 to 1·571)	1·179 (0·951 to 1·571)	1·179 (0·951 to 1·571)	1·179 (0·951 to 1·571)	1·179 (0·951 to 1·571)	1·179 (0·951 to 1·571)	1·128 (1·012 to 1·280)	1·128 (1·012 to 1·280)	1·128 (1·012 to 1·280)	1·128 (1·012 to 1·280)
Ischaemic stroke	50 g/day	Mortality	Global	Male		1·111 (0·989 to 1·312)	1·111 (0·989 to 1·312)	1·111 (0·989 to 1·312)	1·111 (0·989 to 1·312)	1·103 (0·974 to 1·339)	1·103 (0·974 to 1·339)	1·103 (0·974 to 1·339)	1·103 (0·974 to 1·339)	1·103 (0·974 to 1·339)	1·103 (0·974 to 1·339)	1·075 (1·002 to 1·182)	1·075 (1·002 to 1·182)	1·075 (1·002 to 1·182)	1·075 (1·002 to 1·182)
Ischaemic stroke	40 g/day	Mortality	Global	Male		1·034 (0·954 to 1·146)	1·034 (0·954 to 1·146)	1·034 (0·954 to 1·146)	1·034 (0·954 to 1·146)	1·032 (0·954 to 1·167)	1·032 (0·954 to 1·167)	1·032 (0·954 to 1·167)	1·032 (0·954 to 1·167)	1·032 (0·954 to 1·167)	1·032 (0·954 to 1·167)	1·023 (0·973 to 1·101)	1·023 (0·973 to 1·101)	1·023 (0·973 to 1·101)	1·023 (0·973 to 1·101)
Ischaemic stroke	30 g/day	Mortality	Global	Male		0·962 (0·853 to 1·038)	0·962 (0·853 to 1·038)	0·962 (0·853 to 1·038)	0·962 (0·853 to 1·038)	0·964 (0·864 to 1·044)	0·964 (0·864 to 1·044)	0·964 (0·864 to 1·044)	0·964 (0·864 to 1·044)	0·964 (0·864 to 1·044)	0·964 (0·864 to 1·044)	0·974 (0·906 to 1·023)	0·974 (0·906 to 1·023)	0·974 (0·906 to 1·023)	0·974 (0·906 to 1·023)
Ischaemic stroke	20 g/day	Mortality	Global	Male		0·896 (0·748 to 1·009)	0·896 (0·748 to 1·009)	0·896 (0·748 to 1·009)	0·896 (0·748 to 1·009)	0·903 (0·752 to 1·030)	0·903 (0·752 to 1·030)	0·903 (0·752 to 1·030)	0·903 (0·752 to 1·030)	0·903 (0·752 to 1·030)	0·903 (0·752 to 1·030)	0·928 (0·842 to 0·996)	0·928 (0·842 to 0·996)	0·928 (0·842 to 0·996)	0·928 (0·842 to 0·996)
Ischaemic stroke	10 g/day	Mortality	Global	Male		0·842 (0·657 to 1·015)	0·842 (0·657 to 1·015)	0·842 (0·657 to 1·015)	0·842 (0·657 to 1·015)	0·852 (0·657 to 1·049)	0·852 (0·657 to 1·049)	0·852 (0·657 to 1·049)	0·852 (0·657 to 1·049)	0·852 (0·657 to 1·049)	0·852 (0·657 to 1·049)	0·889 (0·783 to 0·989)	0·889 (0·783 to 0·989)	0·889 (0·783 to 0·989)	0·889 (0·783 to 0·989)
Ischaemic stroke	0 g/day	Mortality	Global	Male		1·000 (1·000 to 1·000)	1·000 (1·000 to 1·000)	1·000 (1·000 to 1·000)	1·000 (1·000 to 1·000)	1·000 (1·000 to 1·000)	1·000 (1·000 to 1·000)	1·000 (1·000 to 1·000)	1·000 (1·000 to 1·000)	1·000 (1·000 to 1·000)	1·000 (1·000 to 1·000)	1·000 (1·000 to 1·000)	1·000 (1·000 to 1·000)	1·000 (1·000 to 1·000)	1·000 (1·000 to 1·000)
Ischaemic stroke	Former	Mortality	Global	Female		1·150 (0·726 to 1·880)	1·150 (0·726 to 1·880)	1·150 (0·726 to 1·880)	1·150 (0·726 to 1·880)	1·150 (0·727 to 1·887)	1·150 (0·727 to 1·887)	1·150 (0·727 to 1·887)	1·150 (0·727 to 1·887)	1·150 (0·727 to 1·887)	1·150 (0·727 to 1·887)	1·150 (0·721 to 1·909)	1·150 (0·721 to 1·909)	1·150 (0·721 to 1·909)	1·150 (0·721 to 1·909)
Ischaemic stroke	60 g/day	Mortality	Global	Female		1·393 (0·946 to 2·358)	1·393 (0·946 to 2·358)	1·393 (0·946 to 2·358)	1·393 (0·946 to 2·358)	1·362 (0·943 to 2·219)	1·362 (0·943 to 2·219)	1·362 (0·943 to 2·219)	1·362 (0·943 to 2·219)	1·362 (0·943 to 2·219)	1·362 (0·943 to 2·219)	1·253 (1·031 to 1·634)	1·253 (1·031 to 1·634)	1·253 (1·031 to 1·634)	1·253 (1·031 to 1·634)
Ischaemic stroke	50 g/day	Mortality	Global	Female		1·112 (0·932 to 1·482)	1·112 (0·932 to 1·482)	1·112 (0·932 to 1·482)	1·112 (0·932 to 1·482)	1·104 (0·937 to 1·444)	1·104 (0·937 to 1·444)	1·104 (0·937 to 1·444)	1·104 (0·937 to 1·444)	1·104 (0·937 to 1·444)	1·104 (0·937 to 1·444)	1·075 (0·968 to 1·260)	1·075 (0·968 to 1·260)	1·075 (0·968 to 1·260)	1·075 (0·968 to 1·260)
Ischaemic stroke	40 g/day	Mortality	Global	Female		0·905 (0·670 to 1·071)	0·905 (0·670 to 1·071)	0·905 (0·670 to 1·071)	0·905 (0·670 to 1·071)	0·911 (0·703 to 1·075)	0·911 (0·703 to 1·075)	0·911 (0·703 to 1·075)	0·911 (0·703 to 1·075)	0·911 (0·703 to 1·075)	0·911 (0·703 to 1·075)	0·934 (0·804 to 1·044)	0·934 (0·804 to 1·044)	0·934 (0·804 to 1·044)	0·934 (0·804 to 1·044)
Ischaemic stroke	30 g/day	Mortality	Global	Female		0·757 (0·468 to 1·045)	0·757 (0·468 to 1·045)	0·757 (0·468 to 1·045)	0·757 (0·468 to 1·045)	0·772 (0·506 to 1·062)	0·772 (0·506 to 1·062)	0·772 (0·506 to 1·062)	0·772 (0·506 to 1·062)	0·772 (0·506 to 1·062)	0·772 (0·506 to 1·062)	0·828 (0·647 to 0·978)	0·828 (0·647 to 0·978)	0·828 (0·647 to 0·978)	0·828 (0·647 to 0·978)
Ischaemic stroke	20 g/day	Mortality	Global	Female		0·662 (0·356 to 1·072)	0·662 (0·356 to 1·072)	0·662 (0·356 to 1·072)	0·662 (0·356 to 1·072)	0·681 (0·389 to 1·089)	0·681 (0·389 to 1·089)	0·681 (0·389 to 1·089)	0·681 (0·389 to 1·089)	0·681 (0·389 to 1·089)	0·681 (0·389 to 1·089)	0·755 (0·556 to 0·961)	0·755 (0·556 to 0·961)	0·755 (0·556 to 0·961)	0·755 (0·556 to 0·961)
Ischaemic stroke	10 g/day	Mortality	Global	Female		0·630 (0·320 to 1·083)	0·630 (0·320 to 1·083)	0·630 (0·320 to 1·083)	0·630 (0·320 to 1·083)	0·650 (0·356 to 1·100)	0·650 (0·356 to 1·100)	0·650 (0·356 to 1·100)	0·650 (0·356 to 1·100)	0·650 (0·356 to 1·100)	0·650 (0·356 to 1·100)	0·730 (0·526 to 0·948)	0·730 (0·526 to 0·948)	0·730 (0·526 to 0·948)	0·730 (0·526 to 0·948)
Ischaemic stroke	0 g/day	Mortality	Global	Female		1·000 (1·000 to 1·000)	1·000 (1·000 to 1·000)	1·000 (1·000 to 1·000)	1·000 (1·000 to 1·000)	1·000 (1·000 to 1·000)	1·000 (1·000 to 1·000)	1·000 (1·000 to 1·000)	1·000 (1·000 to 1·000)	1·000 (1·000 to 1·000)	1·000 (1·000 to 1·000)	1·000 (1·000 to 1·000)	1·000 (1·000 to 1·000)	1·000 (1·000 to 1·000)	1·000 (1·000 to 1·000)
Ischaemic stroke	Former	Both	Russia	Male												1·330 (0·884 to 1·983)	1·330 (0·884 to 1·983)	1·330 (0·884 to 1·983)	1·330 (0·884 to 1·983)
Ischaemic stroke	85 g/day	Both	Russia	Male												1·280 (1·160 to 1·419)	1·280 (1·160 to 1·419)	1·280 (1·160 to 1·419)	1·280 (1·160 to 1·419)
Ischaemic stroke	80 g/day	Both	Russia	Male												1·280 (1·160 to 1·419)	1·280 (1·160 to 1·419)	1·280 (1·160 to 1·419)	1·280 (1·160 to 1·419)
Ischaemic stroke	70 g/day	Both	Russia	Male												1·140 (1·025 to 1·262)	1·140 (1·025 to 1·262)	1·140 (1·025 to 1·262)	1·140 (1·025 to 1·262)
Ischaemic stroke	60 g/day	Both	Russia	Male												1·140 (1·025 to 1·262)	1·140 (1·025 to 1·262)	1·140 (1·025 to 1·262)	1·140 (1·025 to 1·262)
Ischaemic stroke	50 g/day	Both	Russia	Male												1·140 (1·025 to 1·262)	1·140 (1·025 to 1·262)	1·140 (1·025 to 1·262)	1·140 (1·025 to 1·262)
Ischaemic stroke	40 g/day	Both	Russia	Male												1·140 (1·025 to 1·262)	1·140 (1·025 to 1·262)	1·140 (1·025 to 1·262)	1·140 (1·025 to 1·262)
Ischaemic stroke	30 g/day	Both	Russia	Male												1·140 (1·025 to 1·262)	1·140 (1·025 to 1·262)	1·140 (1·025 to 1·262)	1·140 (1·025 to 1·262)
Ischaemic stroke	20 g/day	Both	Russia	Male												1·060 (0·965 to 1·170)	1·060 (0·965 to 1·170)	1·060 (0·965 to 1·170)	1·060 (0·965 to 1·170)
Ischaemic stroke	10 g/day	Both	Russia	Male												1·060 (0·965 to 1·170)	1·060 (0·965 to 1·170)	1·060 (0·965 to 1·170)	1·060 (0·965 to 1·170)
Ischaemic stroke	0 g/day	Both	Russia	Male												1·060 (0·965 to 1·170)	1·060 (0·965 to 1·170)	1·060 (0·965 to 1·170)	1·060 (0·965 to 1·170)
Ischaemic stroke	Former	Both	Russia	Female												1·150 (0·664 to 1·933)	1·150 (0·664 to 1·933)	1·150 (0·664 to 1·933)	1·150 (0·664 to 1·933)
Ischaemic stroke	60 g/day	Both	Russia	Female												1·360 (1·191 to 1·553)	1·360 (1·191 to 1·553)	1·360 (1·191 to 1·553)	1·360 (1·191 to 1·553)

Risk - Outcome	Category / Units	Morbidity / Mortality	(Global unless otherwise specified)	Sex	All ages	15-19 years	20-24 years	25-29 years	30-34 years	35-39 years	40-44 years	45-49 years	50-54 years	55-59 years	60-64 years	65-69 years	70-74 years	75-79 years	80+ years
Ischaemic stroke	50 g/day	Both	Russia	Female												1·360 (1·191 to 1·553)	1·360 (1·191 to 1·553)	1·360 (1·191 to 1·553)	1·360 (1·191 to 1·553)
Ischaemic stroke	40 g/day	Both	Russia	Female												1·360 (1·191 to 1·553)	1·360 (1·191 to 1·553)	1·360 (1·191 to 1·553)	1·360 (1·191 to 1·553)
Ischaemic stroke	30 g/day	Both	Russia	Female												1·360 (1·191 to 1·553)	1·360 (1·191 to 1·553)	1·360 (1·191 to 1·553)	1·360 (1·191 to 1·553)
Ischaemic stroke	20 g/day	Both	Russia	Female												1·380 (1·272 to 1·500)	1·380 (1·272 to 1·500)	1·380 (1·272 to 1·500)	1·380 (1·272 to 1·500)
Ischaemic stroke	10 g/day	Both	Russia	Female												1·380 (1·272 to 1·500)	1·380 (1·272 to 1·500)	1·380 (1·272 to 1·500)	1·380 (1·272 to 1·500)
Ischaemic stroke	0 g/day	Both	Russia	Female												1·380 (1·272 to 1·500)	1·380 (1·272 to 1·500)	1·380 (1·272 to 1·500)	1·380 (1·272 to 1·500)
Hemorrhagic stroke	Former	Morbidity	Global	Male	1·330 (0·911 to 1·982)														
Hemorrhagic stroke	85 g/day	Morbidity	Global	Male	1·923 (1·483 to 2·469)														
Hemorrhagic stroke	80 g/day	Morbidity	Global	Male	1·851 (1·449 to 2·341)														
Hemorrhagic stroke	70 g/day	Morbidity	Global	Male	1·714 (1·383 to 2·105)														
Hemorrhagic stroke	60 g/day	Morbidity	Global	Male	1·587 (1·321 to 1·892)														
Hemorrhagic stroke	50 g/day	Morbidity	Global	Male	1·469 (1·261 to 1·702)														
Hemorrhagic stroke	40 g/day	Morbidity	Global	Male	1·360 (1·204 to 1·530)														
Hemorrhagic stroke	30 g/day	Morbidity	Global	Male	1·260 (1·149 to 1·376)														
Hemorrhagic stroke	20 g/day	Morbidity	Global	Male	1·166 (1·097 to 1·237)														
Hemorrhagic stroke	10 g/day	Morbidity	Global	Male	1·080 (1·047 to 1·112)														
Hemorrhagic stroke	0 g/day	Morbidity	Global	Male	1·000 (1·000 to 1·000)														
Hemorrhagic stroke	Former	Morbidity	Global	Female	1·150 (0·708 to 1·834)														
Hemorrhagic stroke	60 g/day	Morbidity	Global	Female	1·425 (1·009 to 2·055)														
Hemorrhagic stroke	50 g/day	Morbidity	Global	Female	1·223 (0·896 to 1·715)														
Hemorrhagic stroke	40 g/day	Morbidity	Global	Female	1·048 (0·792 to 1·429)														
Hemorrhagic stroke	30 g/day	Morbidity	Global	Female	0·898 (0·683 to 1·200)														
Hemorrhagic stroke	20 g/day	Morbidity	Global	Female	0·772 (0·602 to 1·010)														
Hemorrhagic stroke	10 g/day	Morbidity	Global	Female	0·677 (0·543 to 0·862)														
Hemorrhagic stroke	0 g/day	Morbidity	Global	Female	1·000 (1·000 to 1·000)														
Hemorrhagic stroke	Former	Mortality	Global	Male	1·330 (0·919 to 2·000)														
Hemorrhagic stroke	85 g/day	Mortality	Global	Male	1·798 (1·499 to 2·192)														
Hemorrhagic stroke	80 g/day	Mortality	Global	Male	1·737 (1·464 to 2·093)														
Hemorrhagic stroke	70 g/day	Mortality	Global	Male	1·621 (1·396 to 1·909)														
Hemorrhagic stroke	60 g/day	Mortality	Global	Male	1·513 (1·331 to 1·740)														
Hemorrhagic stroke	50 g/day	Mortality	Global	Male	1·412 (1·269 to 1·587)														
Hemorrhagic stroke	40 g/day	Mortality	Global	Male	1·318 (1·210 to 1·447)														
Hemorrhagic stroke	30 g/day	Mortality	Global	Male	1·230 (1·154 to 1·319)														
Hemorrhagic stroke	20 g/day	Mortality	Global	Male	1·148 (1·100 to 1·203)														
Hemorrhagic stroke	10 g/day	Mortality	Global	Male	1·071 (1·049 to 1·097)														
Hemorrhagic stroke	0 g/day	Mortality	Global	Male	1·000 (1·000 to 1·000)														
Hemorrhagic stroke	Former	Mortality	Global	Female	1·150 (0·708 to 1·814)														
Hemorrhagic stroke	60 g/day	Mortality	Global	Female	2·411 (1·571 to 3·572)														
Hemorrhagic stroke	50 g/day	Mortality	Global	Female	2·082 (1·457 to 2·889)														
Hemorrhagic stroke	40 g/day	Mortality	Global	Female	1·798 (1·351 to 2·337)														
Hemorrhagic stroke	30 g/day	Mortality	Global	Female	1·553 (1·253 to 1·890)														
Hemorrhagic stroke	20 g/day	Mortality	Global	Female	1·341 (1·163 to 1·529)														
Hemorrhagic stroke	10 g/day	Mortality	Global	Female	1·158 (1·078 to 1·236)														
Hemorrhagic stroke	0 g/day	Mortality	Global	Female	1·000 (1·000 to 1·000)														
Hemorrhagic stroke	Former	Both	Russia	Male	1·330 (0·915 to 1·901)														
Hemorrhagic stroke	85 g/day	Both	Russia	Male	1·280 (1·151 to 1·427)														
Hemorrhagic stroke	80 g/day	Both	Russia	Male	1·280 (1·151 to 1·427)														
Hemorrhagic stroke	70 g/day	Both	Russia	Male	1·140 (1·031 to 1·262)														
Hemorrhagic stroke	60 g/day	Both	Russia	Male	1·140 (1·031 to 1·262)														
Hemorrhagic stroke	50 g/day	Both	Russia	Male	1·140 (1·031 to 1·262)														
Hemorrhagic stroke	40 g/day	Both	Russia	Male	1·140 (1·031 to 1·262)														
Hemorrhagic stroke	30 g/day	Both	Russia	Male	1·140 (1·031 to 1·262)														
Hemorrhagic stroke	20 g/day	Both	Russia	Male	1·060 (0·955 to 1·171)														
Hemorrhagic stroke	10 g/day	Both	Russia	Male	1·060 (0·955 to 1·171)														
Hemorrhagic stroke	0 g/day	Both	Russia	Male	1·060 (0·955 to 1·171)														
Hemorrhagic stroke	Former	Both	Russia	Female	1·150 (0·722 to 1·865)														
Hemorrhagic stroke	60 g/day	Both	Russia	Female	1·360 (1·201 to 1·557)														
Hemorrhagic stroke	50 g/day	Both	Russia	Female	1·360 (1·201 to 1·557)														
Hemorrhagic stroke	40 g/day	Both	Russia	Female	1·360 (1·201 to 1·557)														
Hemorrhagic stroke	30 g/day	Both	Russia	Female	1·360 (1·201 to 1·557)														
Hemorrhagic stroke	20 g/day	Both	Russia	Female	1·380 (1·269 to 1·491)														
Hemorrhagic stroke	10 g/day	Both	Russia	Female	1·380 (1·269 to 1·491)														
Hemorrhagic stroke	0 g/day	Both	Russia	Female	1·380 (1·269 to 1·491)														
Hypertensive heart disease	Former	Both	Global	Male	1·000 (1·000 to 1·000)														
Hypertensive heart disease	85 g/day	Both	Global	Male	2·162 (1·836 to 2·548)														
Hypertensive heart disease	80 g/day	Both	Global	Male	2·066 (1·772 to 2·412)														
Hypertensive heart disease	70 g/day	Both	Global	Male	1·887 (1·650 to 2·161)														
Hypertensive heart disease	60 g/day	Both	Global	Male	1·723 (1·536 to 1·936)														
Hypertensive heart disease	50 g/day	Both	Global	Male	1·574 (1·430 to 1·734)														
Hypertensive heart disease	40 g/day	Both	Global	Male	1·438 (1·331 to 1·554)														
Hypertensive heart disease	30 g/day	Both	Global	Male	1·313 (1·239 to 1·392)														
Hypertensive heart disease	20 g/day	Both	Global	Male	1·199 (1·154 to 1·247)														
Hypertensive heart disease	10 g/day	Both	Global	Male	1·095 (1·074 to 1·117)														
Hypertensive heart disease	0 g/day	Both	Global	Male	1·000 (1·000 to 1·001)														
Hypertensive heart disease	Former	Both	Global	Female	1·000 (1·000 to 1·000)														
Hypertensive heart disease	60 g/day	Both	Global	Female	3·589 (2·274 to 5·784)														
Hypertensive heart disease	50 g/day	Both	Global	Female	2·775 (1·819 to 4·280)														
Hypertensive heart disease	40 g/day	Both	Global	Female	2·124 (1·462 to 3·098)														
Hypertensive heart disease	30 g/day	Both	Global	Female	1·607 (1·164 to 2·223)														
Hypertensive heart disease	20 g/day	Both	Global	Female	1·200 (0·932 to 1·565)														
Hypertensive heart disease	10 g/day	Both	Global	Female	0·888 (0·737 to 1·072)														
Hypertensive heart disease	0 g/day	Both	Global	Female	1·000 (1·000 to 1·000)														
Atrial fibrillation and flutter	Former	Both	Global	Male	1·210 (1·112 to 1·326)														
Atrial fibrillation and flutter	85 g/day	Both	Global	Male	1·631 (1·379 to 1·925)														
Atrial fibrillation and flutter	80 g/day	Both	Global	Male	1·585 (1·354 to 1·852)														
Atrial fibrillation and flutter	70 g/day	Both	Global	Male	1·496 (1·303 to 1·715)														
Atrial fibrillation and flutter	60 g/day	Both	Global	Male	1·413 (1·255 to 1·588)														
Atrial fibrillation and flutter	50 g/day	Both	Global	Male	1·334 (1·208 to 1·470)														
Atrial fibrillation and flutter	40 g/day	Both	Global	Male	1·259 (1·164 to 1·361)														
Atrial fibrillation and flutter	30 g/day	Both	Global	Male	1·189 (1·120 to 1·260)														
Atrial fibrillation and flutter	20 g/day	Both	Global	Male	1·122 (1·079 to 1·167)														
Atrial fibrillation and flutter	10 g/day	Both	Global	Male	1·060 (1·039 to 1·080)														
Atrial fibrillation and flutter	0 g/day	Both	Global	Male	1·000 (1·000 to 1·000)														
Atrial fibrillation and flutter	Former	Both	Global	Female	1·440 (1·279 to 1·612)														
Atrial fibrillation and flutter	60 g/day	Both	Global	Female	1·413 (1·262 to 1·602)														
Atrial fibrillation and flutter	50 g/day	Both	Global	Female	1·334 (1·214 to 1·481)														
Atrial fibrillation and flutter	40 g/day	Both	Global	Female	1·259 (1·168 to 1·369)														
Atrial fibrillation and flutter	30 g/day	Both	Global	Female	1·189 (1·124 to 1·266)														
Atrial fibrillation and flutter	20 g/day	Both	Global	Female	1·122 (1·081 to 1·170)														
Atrial fibrillation and flutter	10 g/day	Both	Global	Female	1·060 (1·040 to 1·082)														
Atrial fibrillation and flutter	0 g/day	Both	Global	Female	1·000 (1·000 to 1·000)														
Pancreatitis	Former	Both	Global	Male	1·210 (1·108 to 1·331)														
Pancreatitis	85 g/day	Both	Global	Male	2·970 (2·355 to 3·782)														

Risk - Outcome	Category / Units	Morbidity / Mortality	(Global unless otherwise specified)	Sex	All ages	15-19 years	20-24 years	25-29 years	30-34 years	35-39 years	40-44 years	45-49 years	50-54 years	55-59 years	60-64 years	65-69 years	70-74 years	75-79 years	80+ years
Pancreatitis	80 g/day	Both	Global	Male	2·624 (2·137 to 3·251)														
Pancreatitis	70 g/day	Both	Global	Male	2·095 (1·789 to 2·468)														
Pancreatitis	60 g/day	Both	Global	Male	1·723 (1·534 to 1·944)														
Pancreatitis	50 g/day	Both	Global	Male	1·460 (1·347 to 1·588)														
Pancreatitis	40 g/day	Both	Global	Male	1·275 (1·211 to 1·346)														
Pancreatitis	30 g/day	Both	Global	Male	1·147 (1·114 to 1·183)														
Pancreatitis	20 g/day	Both	Global	Male	1·064 (1·050 to 1·078)														
Pancreatitis	10 g/day	Both	Global	Male	1·016 (1·013 to 1·020)														
Pancreatitis	0 g/day	Both	Global	Male	1·000 (1·000 to 1·000)														
Pancreatitis	Former	Both	Global	Female	1·440 (1·285 to 1·612)														
Pancreatitis	60 g/day	Both	Global	Female	1·723 (1·528 to 1·952)														
Pancreatitis	50 g/day	Both	Global	Female	1·460 (1·343 to 1·592)														
Pancreatitis	40 g/day	Both	Global	Female	1·275 (1·208 to 1·348)														
Pancreatitis	30 g/day	Both	Global	Female	1·147 (1·113 to 1·184)														
Pancreatitis	20 g/day	Both	Global	Female	1·064 (1·049 to 1·079)														
Pancreatitis	10 g/day	Both	Global	Female	1·016 (1·012 to 1·020)														
Pancreatitis	0 g/day	Both	Global	Female	1·000 (1·000 to 1·000)														
Pancreatitis	Former	Both	Russia	Male	1·210 (1·105 to 1·320)														
Pancreatitis	85 g/day	Both	Russia	Male	6·690 (5·078 to 9·051)														
Pancreatitis	80 g/day	Both	Russia	Male	6·690 (5·078 to 9·051)														
Pancreatitis	70 g/day	Both	Russia	Male	2·070 (1·535 to 2·751)														
Pancreatitis	60 g/day	Both	Russia	Male	2·070 (1·535 to 2·751)														
Pancreatitis	50 g/day	Both	Russia	Male	2·070 (1·535 to 2·751)														
Pancreatitis	40 g/day	Both	Russia	Male	2·070 (1·535 to 2·751)														
Pancreatitis	30 g/day	Both	Russia	Male	2·070 (1·535 to 2·751)														
Pancreatitis	20 g/day	Both	Russia	Male	1·430 (1·010 to 2·004)														
Pancreatitis	10 g/day	Both	Russia	Male	1·430 (1·010 to 2·004)														
Pancreatitis	0 g/day	Both	Russia	Male	1·430 (1·010 to 2·004)														
Pancreatitis	Former	Both	Russia	Female	1·440 (1·280 to 1·620)														
Pancreatitis	60 g/day	Both	Russia	Female	5·010 (3·470 to 7·295)														
Pancreatitis	50 g/day	Both	Russia	Female	5·010 (3·470 to 7·295)														
Pancreatitis	40 g/day	Both	Russia	Female	5·010 (3·470 to 7·295)														
Pancreatitis	30 g/day	Both	Russia	Female	5·010 (3·470 to 7·295)														
Pancreatitis	20 g/day	Both	Russia	Female	1·090 (0·701 to 1·705)														
Pancreatitis	10 g/day	Both	Russia	Female	1·090 (0·701 to 1·705)														
Pancreatitis	0 g/day	Both	Russia	Female	1·090 (0·701 to 1·705)														
Epilepsy	Former	Both	Global	Male	1·210 (1·100 to 1·322)														
Epilepsy	85 g/day	Both	Global	Male	2·859 (2·257 to 3·564)														
Epilepsy	80 g/day	Both	Global	Male	2·689 (2·152 to 3·309)														
Epilepsy	70 g/day	Both	Global	Male	2·378 (1·956 to 2·852)														
Epilepsy	60 g/day	Both	Global	Male	2·103 (1·779 to 2·458)														
Epilepsy	50 g/day	Both	Global	Male	1·860 (1·617 to 2·118)														
Epilepsy	40 g/day	Both	Global	Male	1·645 (1·470 to 1·826)														
Epilepsy	30 g/day	Both	Global	Male	1·455 (1·337 to 1·574)														
Epilepsy	20 g/day	Both	Global	Male	1·286 (1·215 to 1·356)														
Epilepsy	10 g/day	Both	Global	Male	1·138 (1·105 to 1·169)														
Epilepsy	0 g/day	Both	Global	Male	1·006 (1·005 to 1·007)														
Epilepsy	Former	Both	Global	Female	1·440 (1·279 to 1·625)														
Epilepsy	60 g/day	Both	Global	Female	2·103 (1·780 to 2·458)														
Epilepsy	50 g/day	Both	Global	Female	1·860 (1·618 to 2·118)														
Epilepsy	40 g/day	Both	Global	Female	1·645 (1·471 to 1·826)														
Epilepsy	30 g/day	Both	Global	Female	1·455 (1·337 to 1·574)														
Epilepsy	20 g/day	Both	Global	Female	1·286 (1·216 to 1·356)														
Epilepsy	10 g/day	Both	Global	Female	1·138 (1·105 to 1·169)														
Epilepsy	0 g/day	Both	Global	Female	1·006 (1·005 to 1·007)														
Diabetes mellitus	Former	Both	Global	Male	1·000 (1·000 to 1·000)														
Diabetes mellitus	85 g/day	Both	Global	Male	1·000 (1·000 to 1·000)														
Diabetes mellitus	80 g/day	Both	Global	Male	1·000 (1·000 to 1·000)														
Diabetes mellitus	70 g/day	Both	Global	Male	1·000 (1·000 to 1·000)														
Diabetes mellitus	60 g/day	Both	Global	Male	1·000 (1·000 to 1·000)														
Diabetes mellitus	50 g/day	Both	Global	Male	1·000 (1·000 to 1·000)														
Diabetes mellitus	40 g/day	Both	Global	Male	1·000 (1·000 to 1·000)														
Diabetes mellitus	30 g/day	Both	Global	Male	1·000 (1·000 to 1·000)														
Diabetes mellitus	20 g/day	Both	Global	Male	1·000 (1·000 to 1·000)														
Diabetes mellitus	10 g/day	Both	Global	Male	1·000 (1·000 to 1·000)														
Diabetes mellitus	0 g/day	Both	Global	Male	1·000 (1·000 to 1·000)														
Diabetes mellitus	Former	Both	Global	Female	1·000 (1·000 to 1·000)														
Diabetes mellitus	60 g/day	Both	Global	Female	0·620 (0·469 to 0·782)														
Diabetes mellitus	50 g/day	Both	Global	Female	0·620 (0·469 to 0·782)														
Diabetes mellitus	40 g/day	Both	Global	Female	0·620 (0·469 to 0·782)														
Diabetes mellitus	30 g/day	Both	Global	Female	0·620 (0·469 to 0·782)														
Diabetes mellitus	20 g/day	Both	Global	Female	0·620 (0·469 to 0·782)														
Diabetes mellitus	10 g/day	Both	Global	Female	0·620 (0·469 to 0·782)														
Diabetes mellitus	0 g/day	Both	Global	Female	0·620 (0·469 to 0·782)														
Motorcyclist road injuries	Former	Morbidity	Global	Male	1·000 (1·000 to 1·000)														
Motorcyclist road injuries	85 g/day	Morbidity	Global	Male	20·873 (14·663 to 29·426)														
Motorcyclist road injuries	80 g/day	Morbidity	Global	Male	15·096 (10·998 to 20·549)														
Motorcyclist road injuries	70 g/day	Morbidity	Global	Male	8·339 (6·499 to 10·637)														
Motorcyclist road injuries	60 g/day	Morbidity	Global	Male	4·961 (4·105 to 5·971)														
Motorcyclist road injuries	50 g/day	Morbidity	Global	Male	3·183 (2·774 to 3·642)														
Motorcyclist road injuries	40 g/day	Morbidity	Global	Male	2·207 (2·009 to 2·420)														
Motorcyclist road injuries	30 g/day	Morbidity	Global	Male	1·656 (1·560 to 1·755)														
Motorcyclist road injuries	20 g/day	Morbidity	Global	Male	1·347 (1·302 to 1·393)														
Motorcyclist road injuries	10 g/day	Morbidity	Global	Male	1·173 (1·152 to 1·193)														
Motorcyclist road injuries	0 g/day	Morbidity	Global	Male	1·070 (1·063 to 1·078)														
Motorcyclist road injuries	Former	Morbidity	Global	Female	1·000 (1·000 to 1·000)														
Motorcyclist road injuries	60 g/day	Morbidity	Global	Female	4·961 (4·093 to 5·972)														
Motorcyclist road injuries	50 g/day	Morbidity	Global	Female	3·183 (2·768 to 3·643)														
Motorcyclist road injuries	40 g/day	Morbidity	Global	Female	2·207 (2·006 to 2·420)														
Motorcyclist road injuries	30 g/day	Morbidity	Global	Female	1·656 (1·559 to 1·755)														
Motorcyclist road injuries	20 g/day	Morbidity	Global	Female	1·347 (1·301 to 1·393)														
Motorcyclist road injuries	10 g/day	Morbidity	Global	Female	1·173 (1·152 to 1·193)														
Motorcyclist road injuries	0 g/day	Morbidity	Global	Female	1·070 (1·063 to 1·078)														
Motor vehicle road injuries	Former	Morbidity	Global	Male	1·000 (1·000 to 1·000)														
Motor vehicle road injuries	85 g/day	Morbidity	Global	Male	20·873 (14·663 to 29·426)														
Motor vehicle road injuries	80 g/day	Morbidity	Global	Male	15·096 (10·998 to 20·549)														
Motor vehicle road injuries	70 g/day	Morbidity	Global	Male	8·339 (6·499 to 10·637)														
Motor vehicle road injuries	60 g/day	Morbidity	Global	Male	4·961 (4·105 to 5·971)														
Motor vehicle road injuries	50 g/day	Morbidity	Global	Male	3·183 (2·774 to 3·642)														
Motor vehicle road injuries	40 g/day	Morbidity	Global	Male	2·207 (2·009 to 2·420)														
Motor vehicle road injuries	30 g/day	Morbidity	Global	Male	1·656 (1·560 to 1·755)														
Motor vehicle road injuries	20 g/day	Morbidity	Global	Male	1·347 (1·302 to 1·393)														
Motor vehicle road injuries	10 g/day	Morbidity	Global	Male	1·173 (1·152 to 1·193)														

Risk - Outcome	Category / Units	Morbidity / Mortality	(Global unless otherwise specified)	Sex	All ages	15-19 years	20-24 years	25-29 years	30-34 years	35-39 years	40-44 years	45-49 years	50-54 years	55-59 years	60-64 years	65-69 years	70-74 years	75-79 years	80+ years
Motor vehicle road injuries	0 g/day	Morbidity	Global	Male	1·070 (1·063 to 1·078)														
Motor vehicle road injuries	Former	Morbidity	Global	Female	1·000 (1·000 to 1·000)														
Motor vehicle road injuries	60 g/day	Morbidity	Global	Female	4·961 (4·093 to 5·972)														
Motor vehicle road injuries	50 g/day	Morbidity	Global	Female	3·183 (2·768 to 3·643)														
Motor vehicle road injuries	40 g/day	Morbidity	Global	Female	2·207 (2·006 to 2·420)														
Motor vehicle road injuries	30 g/day	Morbidity	Global	Female	1·656 (1·559 to 1·755)														
Motor vehicle road injuries	20 g/day	Morbidity	Global	Female	1·347 (1·301 to 1·393)														
Motor vehicle road injuries	10 g/day	Morbidity	Global	Female	1·173 (1·152 to 1·193)														
Motor vehicle road injuries	0 g/day	Morbidity	Global	Female	1·070 (1·063 to 1·078)														
Falls	Former	Morbidity	Global	Male	1·000 (1·000 to 1·000)														
Falls	85 g/day	Morbidity	Global	Male	7·623 (4·850 to 11·882)														
Falls	80 g/day	Morbidity	Global	Male	7·180 (4·628 to 11·054)														
Falls	70 g/day	Morbidity	Global	Male	6·333 (4·193 to 9·501)														
Falls	60 g/day	Morbidity	Global	Male	5·533 (3·771 to 8·075)														
Falls	50 g/day	Morbidity	Global	Male	4·776 (3·357 to 6·766)														
Falls	40 g/day	Morbidity	Global	Male	4·053 (2·947 to 5·561)														
Falls	30 g/day	Morbidity	Global	Male	3·348 (2·528 to 4·435)														
Falls	20 g/day	Morbidity	Global	Male	2·502 (1·976 to 3·194)														
Falls	10 g/day	Morbidity	Global	Male	2·157 (1·773 to 2·644)														
Falls	0 g/day	Morbidity	Global	Male	1·830 (1·572 to 2·142)														
Falls	Former	Morbidity	Global	Female	1·000 (1·000 to 1·000)														
Falls	60 g/day	Morbidity	Global	Female	8·079 (4·984 to 12·651)														
Falls	50 g/day	Morbidity	Global	Female	6·751 (4·336 to 10·191)														
Falls	40 g/day	Morbidity	Global	Female	5·533 (3·714 to 8·026)														
Falls	30 g/day	Morbidity	Global	Female	4·411 (3·111 to 6·118)														
Falls	20 g/day	Morbidity	Global	Female	3·348 (2·501 to 4·414)														
Falls	10 g/day	Morbidity	Global	Female	2·324 (1·856 to 2·899)														
Falls	0 g/day	Morbidity	Global	Female	1·830 (1·563 to 2·136)														
Drowning	Former	Morbidity	Global	Male	1·000 (1·000 to 1·000)														
Drowning	85 g/day	Morbidity	Global	Male	7·623 (4·850 to 11·882)														
Drowning	80 g/day	Morbidity	Global	Male	7·180 (4·628 to 11·054)														
Drowning	70 g/day	Morbidity	Global	Male	6·333 (4·193 to 9·501)														
Drowning	60 g/day	Morbidity	Global	Male	5·533 (3·771 to 8·075)														
Drowning	50 g/day	Morbidity	Global	Male	4·776 (3·357 to 6·766)														
Drowning	40 g/day	Morbidity	Global	Male	4·053 (2·947 to 5·561)														
Drowning	30 g/day	Morbidity	Global	Male	3·348 (2·528 to 4·435)														
Drowning	20 g/day	Morbidity	Global	Male	2·502 (1·976 to 3·194)														
Drowning	10 g/day	Morbidity	Global	Male	2·157 (1·773 to 2·644)														
Drowning	0 g/day	Morbidity	Global	Male	1·830 (1·572 to 2·142)														
Drowning	Former	Morbidity	Global	Female	1·000 (1·000 to 1·000)														
Drowning	60 g/day	Morbidity	Global	Female	8·079 (4·984 to 12·651)														
Drowning	50 g/day	Morbidity	Global	Female	6·751 (4·336 to 10·191)														
Drowning	40 g/day	Morbidity	Global	Female	5·533 (3·714 to 8·026)														
Drowning	30 g/day	Morbidity	Global	Female	4·411 (3·111 to 6·118)														
Drowning	20 g/day	Morbidity	Global	Female	3·348 (2·501 to 4·414)														
Drowning	10 g/day	Morbidity	Global	Female	2·324 (1·856 to 2·899)														
Drowning	0 g/day	Morbidity	Global	Female	1·830 (1·563 to 2·136)														
Fire, heat, and hot substances	Former	Morbidity	Global	Male	1·000 (1·000 to 1·000)														
Fire, heat, and hot substances	85 g/day	Morbidity	Global	Male	7·623 (4·850 to 11·882)														
Fire, heat, and hot substances	80 g/day	Morbidity	Global	Male	7·180 (4·628 to 11·054)														
Fire, heat, and hot substances	70 g/day	Morbidity	Global	Male	6·333 (4·193 to 9·501)														
Fire, heat, and hot substances	60 g/day	Morbidity	Global	Male	5·533 (3·771 to 8·075)														
Fire, heat, and hot substances	50 g/day	Morbidity	Global	Male	4·776 (3·357 to 6·766)														
Fire, heat, and hot substances	40 g/day	Morbidity	Global	Male	4·053 (2·947 to 5·561)														
Fire, heat, and hot substances	30 g/day	Morbidity	Global	Male	3·348 (2·528 to 4·435)														
Fire, heat, and hot substances	20 g/day	Morbidity	Global	Male	2·502 (1·976 to 3·194)														
Fire, heat, and hot substances	10 g/day	Morbidity	Global	Male	2·157 (1·773 to 2·644)														
Fire, heat, and hot substances	0 g/day	Morbidity	Global	Male	1·830 (1·572 to 2·142)														
Fire, heat, and hot substances	Former	Morbidity	Global	Female	1·000 (1·000 to 1·000)														
Fire, heat, and hot substances	60 g/day	Morbidity	Global	Female	8·079 (4·984 to 12·651)														
Fire, heat, and hot substances	50 g/day	Morbidity	Global	Female	6·751 (4·336 to 10·191)														
Fire, heat, and hot substances	40 g/day	Morbidity	Global	Female	5·533 (3·714 to 8·026)														
Fire, heat, and hot substances	30 g/day	Morbidity	Global	Female	4·411 (3·111 to 6·118)														
Fire, heat, and hot substances	20 g/day	Morbidity	Global	Female	3·348 (2·501 to 4·414)														
Fire, heat, and hot substances	10 g/day	Morbidity	Global	Female	2·324 (1·856 to 2·899)														
Fire, heat, and hot substances	0 g/day	Morbidity	Global	Female	1·830 (1·563 to 2·136)														
Poisonings	Former	Morbidity	Global	Male	1·000 (1·000 to 1·000)														
Poisonings	85 g/day	Morbidity	Global	Male	7·623 (4·850 to 11·882)														
Poisonings	80 g/day	Morbidity	Global	Male	7·180 (4·628 to 11·054)														
Poisonings	70 g/day	Morbidity	Global	Male	6·333 (4·193 to 9·501)														
Poisonings	60 g/day	Morbidity	Global	Male	5·533 (3·771 to 8·075)														
Poisonings	50 g/day	Morbidity	Global	Male	4·776 (3·357 to 6·766)														
Poisonings	40 g/day	Morbidity	Global	Male	4·053 (2·947 to 5·561)														
Poisonings	30 g/day	Morbidity	Global	Male	3·348 (2·528 to 4·435)														
Poisonings	20 g/day	Morbidity	Global	Male	2·502 (1·976 to 3·194)														
Poisonings	10 g/day	Morbidity	Global	Male	2·157 (1·773 to 2·644)														
Poisonings	0 g/day	Morbidity	Global	Male	1·830 (1·572 to 2·142)														
Poisonings	Former	Morbidity	Global	Female	1·000 (1·000 to 1·000)														
Poisonings	60 g/day	Morbidity	Global	Female	8·079 (4·984 to 12·651)														
Poisonings	50 g/day	Morbidity	Global	Female	6·751 (4·336 to 10·191)														
Poisonings	40 g/day	Morbidity	Global	Female	5·533 (3·714 to 8·026)														
Poisonings	30 g/day	Morbidity	Global	Female	4·411 (3·111 to 6·118)														
Poisonings	20 g/day	Morbidity	Global	Female	3·348 (2·501 to 4·414)														
Poisonings	10 g/day	Morbidity	Global	Female	2·324 (1·856 to 2·899)														
Poisonings	0 g/day	Morbidity	Global	Female	1·830 (1·563 to 2·136)														
Self-harm	Former	Morbidity	Global	Male	1·000 (1·000 to 1·000)														
Self-harm	85 g/day	Morbidity	Global	Male	7·623 (4·850 to 11·882)														
Self-harm	80 g/day	Morbidity	Global	Male	7·180 (4·628 to 11·054)														
Self-harm	70 g/day	Morbidity	Global	Male	6·333 (4·193 to 9·501)														
Self-harm	60 g/day	Morbidity	Global	Male	5·533 (3·771 to 8·075)														
Self-harm	50 g/day	Morbidity	Global	Male	4·776 (3·357 to 6·766)														
Self-harm	40 g/day	Morbidity	Global	Male	4·053 (2·947 to 5·561)														
Self-harm	30 g/day	Morbidity	Global	Male	3·348 (2·528 to 4·435)														
Self-harm	20 g/day	Morbidity	Global	Male	2·502 (1·976 to 3·194)														
Self-harm	10 g/day	Morbidity	Global	Male	2·157 (1·773 to 2·644)														
Self-harm	0 g/day	Morbidity	Global	Male	1·830 (1·572 to 2·142)														
Self-harm	Former	Morbidity	Global	Female	1·000 (1·000 to 1·000)														
Self-harm	60 g/day	Morbidity	Global	Female	8·079 (4·984 to 12·651)														
Self-harm	50 g/day	Morbidity	Global	Female	6·751 (4·336 to 10·191)														
Self-harm	40 g/day	Morbidity	Global	Female	5·533 (3·714 to 8·026)														
Self-harm	30 g/day	Morbidity	Global	Female	4·411 (3·111 to 6·118)														
Self-harm	20 g/day	Morbidity	Global	Female	3·348 (2·501 to 4·414)														
Self-harm	10 g/day	Morbidity	Global	Female	2·324 (1·856 to 2·899)														

Risk - Outcome	Category / Units	Morbidity / Mortality	(Global unless otherwise specified)	Sex	All ages	15-19 years	20-24 years	25-29 years	30-34 years	35-39 years	40-44 years	45-49 years	50-54 years	55-59 years	60-64 years	65-69 years	70-74 years	75-79 years	80+ years
Self-harm	0 g/day	Morbidity	Global	Female	1·830 (1·563 to 2·136)														

Appendix Table 7. Supplemental information for Table 2: Epidemiological evidence supporting causality between risk-outcome pairs included in the Global Burden of Disease 2015 study including 7A. Citations and 7B. Additional information
7A. Citations

Risk	Outcome	Citation/Note
Unsafe water	Diarrhoeal diseases	Cairncross S, Valdmanis V. Water Supply, Sanitation, and Hygiene Promotion. In: Jamison DT, Breman JG, Measham AR, et al., eds. Disease Control Priorities in Developing Countries, 2nd edn. Washington (DC): World Bank, 2006.
Unsafe water	Diarrhoeal diseases	Wolf J, Prüss-Ustün A, Cumming O, et al. Assessing the impact of drinking water and sanitation on diarrhoeal disease in low- and middle-income settings: systematic review and meta-regression. Trop Med Int Health 2014; 19: 928–42.
Unsafe sanitation - improved sanitation	Diarrhoeal diseases	Wolf J, Prüss-Ustün A, Cumming O, et al. Assessing the impact of drinking water and sanitation on diarrhoeal disease in low- and middle-income settings: systematic review and meta-regression. Trop Med Int Health 2014; 19: 928–42.
Unsafe sanitation - piped	Diarrhoeal diseases	Wolf J, Prüss-Ustün A, Cumming O, et al. Assessing the impact of drinking water and sanitation on diarrhoeal disease in low- and middle-income settings: systematic review and meta-regression. Trop Med Int Health 2014; 19: 928–42.
No handwashing with soap	Diarrhoeal diseases	Ejemot-Nwadiaro RI, Ehiri JE, Arikpo D, Meremikwu MM, Critchley JA. Hand washing promotion for preventing diarrhoea. Cochrane Database Syst Rev 2015; : CD004265.
No handwashing with soap	Lower respiratory infections	Rabie T, Curtis V. Handwashing and risk of respiratory infections: a quantitative systematic review. Trop Med Int Health Tropical Medicine and International Health. 2006;11(3):258–67.
Ambient particulate matter pollution	Lower respiratory infections	Hoek G, Krishnan RM, Beelen R, et al. Long-term air pollution exposure and cardio- respiratory mortality: a review. Environ Health 2013; 12: 43.
Ambient particulate matter pollution	Lower respiratory infections	Mehta S, Shin H, Burnett R, North T, Cohen AJ. Ambient particulate air pollution and acute lower respiratory infections: a systematic review and implications for estimating the global burden of disease. Air Qual Atmos Health 2013; 6: 69–83.
Ambient particulate matter pollution	Tracheal, bronchus and lung cancer	Burnett RT, Pope CA, Ezzati M, et al. An integrated risk function for estimating the global burden of disease attributable to ambient fine particulate matter exposure. Environ Health Perspect 2014; 122: 397–403.
Ambient particulate matter pollution	Ischaemic heart disease	Hoek G, Krishnan RM, Beelen R, et al. Long-term air pollution exposure and cardio- respiratory mortality: a review. Environ Health 2013; 12: 43.
Ambient particulate matter pollution	Ischaemic heart disease	Newby DE, Mannucci PM, Tell GS, et al. Expert position paper on air pollution and cardiovascular disease. Eur Heart J 2015; 36: 83–93b.
Ambient particulate matter pollution	Cerebrovascular disease	Hoek G, Krishnan RM, Beelen R, et al. Long-term air pollution exposure and cardio- respiratory mortality: a review. Environ Health 2013; 12: 43.
Ambient particulate matter pollution	Cerebrovascular disease	Hong Y-C, Lee J-T, Kim H, Kwon H-J. Air pollution: a new risk factor in ischemic stroke mortality. Stroke 2002; 33: 2165–9.
Ambient particulate matter pollution	Cerebrovascular disease	Newby DE, Mannucci PM, Tell GS, et al. Expert position paper on air pollution and cardiovascular disease. Eur Heart J 2015; 36: 83–93b.
Ambient particulate matter pollution	Chronic obstructive pulmonary disease	Hoek G, Krishnan RM, Beelen R, et al. Long-term air pollution exposure and cardio- respiratory mortality: a review. Environ Health 2013; 12: 43.
Ambient particulate matter pollution	Chronic obstructive pulmonary disease	Laumbach RJ, Kipen HM. Respiratory health effects of air pollution: update on biomass smoke and traffic pollution. J Allergy Clin Immunol 2012; 129: 3-11-13.
Ambient particulate matter pollution	Chronic obstructive pulmonary disease	Newby DE, Mannucci PM, Tell GS, et al. Expert position paper on air pollution and cardiovascular disease. Eur Heart J 2015; 36: 83–93b.
Household air pollution from solid fuels	Lower respiratory infections	Dherani M, Pope D, Mascarenhas M, Smith KR, Weber M, Bruce N. Indoor air pollution from unprocessed solid fuel use and pneumonia risk in children aged under five years: a systematic review and meta-analysis. Bull World Health Organ 2008; 86: 390–398C.
Household air pollution from solid fuels	Lower respiratory infections	Marbury MC, Maldonado G, Waller L. The indoor air and children's health study: methods and incidence rates. Epidemiology 1996; 7: 166–74.
Household air pollution from solid fuels	Tracheal, bronchus and lung cancer	Josyula S, Lin J, Xue X, et al. Household air pollution and cancers other than lung: a meta-analysis. Environ Health 2015; 14: 24.
Household air pollution from solid fuels	Tracheal, bronchus and lung cancer	Smith KR, Bruce N, Balakrishnan K, et al. Millions dead: how do we know and what does it mean? Methods used in the comparative risk assessment of household air pollution. Annu Rev Public Health 2014; 35: 185–206.
Household air pollution from solid fuels	Chronic obstructive pulmonary disease	Rivera RM, Cosio MG, Ghezzo H, Salazar M, Pérez-Padilla R. Comparison of lung morphology in COPD secondary to cigarette and biomass smoke. Int J Tuberc Lung Dis 2008; 12: 972–7.
Household air pollution from solid fuels	Chronic obstructive pulmonary disease	Smith KR, Bruce N, Balakrishnan K, et al. Millions dead: how do we know and what does it mean? Methods used in the comparative risk assessment of household air pollution. Annu Rev Public Health 2014; 35: 185–206.
Household air pollution from solid fuels	Cataract	Smith KR, Bruce N, Balakrishnan K, et al. Millions dead: how do we know and what does it mean? Methods used in the comparative risk assessment of household air pollution. Annu Rev Public Health 2014; 35: 185–206.
Ambient ozone pollution	Chronic obstructive pulmonary disease	Jerrett M, Burnett RT, Pope CA, et al. Long-term ozone exposure and mortality. N Engl J Med 2009; 360: 1085–95.
Ambient ozone pollution	Chronic obstructive pulmonary disease	Turner MC, Jerrett M, Pope CA, et al. Long-Term Ozone Exposure and Mortality in a Large Prospective Study. Am J Respir Crit Care Med 2016; 193: 1134–42.
Residential radon	Tracheal, bronchus and lung cancer	Oh S-S, Koh S, Kang H, Lee J. Radon exposure and lung cancer: risk in nonsmokers among cohort studies. Ann Occup Environ Med 2016; 28: 11.
Residential radon	Tracheal, bronchus and lung cancer	Sethi TK, El-Ghamry MN, Kloecker GH. Radon and lung cancer. Clin Adv Hematol Oncol 2012; 10: 157–64.
Residential radon	Tracheal, bronchus and lung cancer	Sheen S, Lee KS, Chung WY, Nam S, Kang DR. An updated review of case-control studies of lung cancer and indoor radon-Is indoor radon the risk factor for lung cancer? Ann Occup Environ Med 2016; 28: 9.

Appendix Table 7. Supplemental information for Table 2: Epidemiological evidence supporting causality between risk-outcome pairs included in the Global Burden of Disease 2015 study including 7A. Citations and 7B. Additional information

7A. Citations

Risk	Outcome	Citation/Note
Lead exposure and high systolic blood pressure	n/a	Navas-Acien A, Schwartz BS, Rothenberg SJ, Hu H, Silbergeld EK, Guallar E. Bone lead levels and blood pressure endpoints: a meta-analysis. Epidemiology 2008; 19: 496–504.
Lead exposure	Rheumatic heart disease	Lanphear BP, Hornung R, Khoury J, et al. Low-level environmental lead exposure and children's intellectual function: an international pooled analysis. Environ Health Perspect 2005; 113: 894–9.
Lead exposure	Rheumatic heart disease	Liu J, Li L, Wang Y, Yan C, Liu X. Impact of low blood lead concentrations on IQ and school performance in Chinese children. PLoS ONE 2013; 8: e65230.
Lead exposure	Idiopathic intellectual disability	Lanphear BP, Hornung R, Khoury J, et al. Low-level environmental lead exposure and children's intellectual function: an international pooled analysis. Environ Health Perspect 2005; 113: 894–9.
Lead exposure	Systolic blood pressure	Lanphear BP, Hornung R, Khoury J, et al. Low-level environmental lead exposure and children's intellectual function: an international pooled analysis. Environ Health Perspect 2005; 113: 894–9.
Lead exposure	Systolic blood pressure	Liu J, Li L, Wang Y, Yan C, Liu X. Impact of low blood lead concentrations on IQ and school performance in Chinese children. PLoS ONE 2013; 8: e65230.
Lead exposure	Systolic blood pressure	Weisskopf MG, Jain N, Nie H, et al. A prospective study of bone lead concentration and death from all causes, cardiovascular diseases, and cancer in the Department of Veterans Affairs Normative Aging Study. Circulation 2009; 120: 1056–64.
Occupational exposure to asbestos	Larynx cancer	Goodman M, Morgan RW, Ray R, Malloy CD, Zhao K. Cancer in asbestos-exposed occupational cohorts: a meta-analysis. Cancer Causes Control 1999; 10: 453–65.
Occupational exposure to asbestos	Tracheal, bronchus and lung cancer	Lenters V, Vermeulen R, Dogger S, et al. A meta-analysis of asbestos and lung cancer: is better quality exposure assessment associated with steeper slopes of the exposure-response relationships? Environ Health Perspect 2011; 119: 1547–55.
Occupational exposure to asbestos	Ovarian cancer	Camargo MC, Stayner LT, Straif K, et al. Occupational exposure to asbestos and ovarian cancer: a meta-analysis. Environ Health Perspect 2011; 119: 1211–7.
Occupational exposure to asbestos	Mesothelioma	Bourdès V, Boffetta P, Pisani P. Environmental exposure to asbestos and risk of pleural mesothelioma: review and meta-analysis. Eur J Epidemiol 2000; 16: 411–7.
Occupational exposure to arsenic	Tracheal, bronchus and lung cancer	Lenters V, Vermeulen R, Dogger S, et al. A meta-analysis of asbestos and lung cancer: is better quality exposure assessment associated with steeper slopes of the exposure-response relationships? Environ Health Perspect 2011; 119: 1547–55.
Occupational exposure to benzene	Leukaemia	Khalade A, Jaakkola MS, Pukkala E, Jaakkola JJK. Exposure to benzene at work and the risk of leukemia: a systematic review and meta-analysis. Environ Health 2010; 9: 31.
Occupational exposure to beryllium	Tracheal, bronchus and lung cancer	Boffetta P, Fryzek JP, Mandel JS. Occupational exposure to beryllium and cancer risk: a review of the epidemiologic evidence. Crit Rev Toxicol 2012; 42: 107–18.
Occupational exposure to cadmium	Tracheal, bronchus and lung cancer	Verougstraete V, Lison D, Hotz P. Cadmium, lung and prostate cancer: a systematic review of recent epidemiological data. J Toxicol Environ Health B Crit Rev 2003; 6: 227–55.
Occupational exposure to chromium	Tracheal, bronchus and lung cancer	Denis Ambroise, Pascal Wild and Jean-Jacques Moulin, Scandinavian Journal of Work, Environment & Health, Vol. 32, No. 1 (February 2006), pp. 22-31
Occupational exposure to diesel engine exhaust	Tracheal, bronchus and lung cancer	Lipsett M, Campleman S. Occupational exposure to diesel exhaust and lung cancer: a meta-analysis. Am J Public Health 1999; 89: 1009–17.
Occupational exposure to second-hand smoke	Tracheal, bronchus and lung cancer	Stayner L, Bena J, Sasco AJ, et al. Lung cancer risk and workplace exposure to environmental tobacco smoke. Am J Public Health 2007; 97: 545–51.
Occupational exposure to formaldehyde	Nasopharynx cancer	Hauptmann M, Lubin JH, Stewart PA, Hayes RB, Blair A. Mortality from solid cancers among workers in formaldehyde industries. Am J Epidemiol 2004; 159: 1117–30.
Occupational exposure to formaldehyde	Leukaemia	Collins JJ, Lineker GA. A review and meta-analysis of formaldehyde exposure and leukemia. Regul Toxicol Pharmacol 2004; 40: 81–91.
Occupational exposure to nickel	Tracheal, bronchus and lung cancer	Grimsrud TK, Berge SR, Haldorsen T, Andersen A. Can lung cancer risk among nickel refinery workers be explained by occupational exposures other than nickel? Epidemiology 2005; 16: 146–54.
Occupational exposure to polycyclic aromatic hydrocarbons	Tracheal, bronchus and lung cancer	Armstrong B, Hutchinson E, Unwin J, Fletcher T. Lung cancer risk after exposure to polycyclic aromatic hydrocarbons: a review and meta-analysis. Environ Health Perspect 2004; 112: 970–8.
Occupational exposure to silica	Tracheal, bronchus and lung cancer	Stayner L, Bena J, Sasco AJ, et al. Lung cancer risk and workplace exposure to environmental tobacco smoke. Am J Public Health 2007; 97: 545–51.
Occupational exposure to sulfuric acid	Larynx cancer	Soskolne CL, Jhangri GS, Siemiatycki J, et al. Occupational exposure to sulfuric acid in southern Ontario, Canada, in association with laryngeal cancer. Scand J Work Environ Health 1992; 18: 225–32.
Occupational exposure to trichloroethylene	Kidney cancer	Kelsh MA, Alexander DD, Mink PJ, Mandel JH. Occupational trichloroethylene exposure and kidney cancer: a meta-analysis. Epidemiology 2010; 21: 95–102.
Occupational asthmagens	Asthma	Karjalainen A, Kurppa K, Martikainen R, Klaukka T, Karjalainen J. Work is related to a substantial portion of adult-onset asthma incidence in the Finnish population. Am J Respir Crit Care Med 2001; 164: 565–8.
Occupational particulate matter, gases, and fumes	Chronic obstructive pulmonary disease	Blanc PD, Iribarren C, Trupin L, et al. Occupational exposures and the risk of COPD: dusty trades revisited. Thorax 2009; 64: 6–12.
Occupational noise	Age-related and other hearing loss	Agrawal Y, Platz EA, Niparko JK. Prevalence of hearing loss and differences by demographic characteristics among US adults: data from the National Health and Nutrition Examination Survey, 1999-2004. Arch Intern Med 2008; 168: 1522–30.
Occupational noise	Age-related and other hearing loss	Davis A. The prevalence of hearing impairment and reported hearing disability among adults in Great Britain. International Journal of Epidemiology 1989,18: 911-917.
Occupational noise	Age-related and other hearing loss	Wilson D, Walsh P, Sanchez L, Davis A, Taylor A, Tucker G, Meagher I. The epidemiology of hearing impairment in an Australian adult population. International Journal of Epidemiology 1999;28:247-252.
Occupational injuries	Injuries	International Labour Organization. Resolution concerning statistics of occupational injuries (resulting from occupational accidents). 1998; published online Oct. http://www.ilo.org/global/statistics-and-databases/standards-and-guidelines/resolutions-adopted-by-international-conferences-of-labour-statisticians/WCMS_087528/lang--en/index.htm.
Occupational injuries	Injuries	Eurostat. Accidents at work statistics. http://ec.europa.eu/eurostat/statistics-explained/index.php/Accidents_at_work_statistics.

Appendix Table 7. Supplemental information for Table 2: Epidemiological evidence supporting causality between risk-outcome pairs included in the Global Burden of Disease 2015 study including 7A. Citations and 7B. Additional information

7A. Citations

Risk	Outcome	Citation/Note
Occupational ergonomic factors	Low back pain	Driscoll T, Jacklyn G, Orchard J, et al. The global burden of occupationally related low back pain: estimates from the Global Burden of Disease 2010 study. Ann Rheum Dis 2014; 73: 975–81.
Non-exclusive breastfeeding	Lower respiratory infections	Horta BL, Victora CG. Short-term effects of breastfeeding: a systematic review on the benefits of breastfeeding on diarrhoea and pneumonia mortality. World Health Organization, 2013 http://allattamento.sip.it/wp-content/uploads/2014/03/WHO_breve-termine.pdf.
Discontinued breastfeeding	Diarrhoeal diseases	Olofin I, McDonald CM, Ezzati M, et al. Associations of suboptimal growth with all-cause and cause-specific mortality in children under five years: a pooled analysis of ten prospective studies. PLoS ONE 2013; 8: e64636.
Childhood underweight	Diarrhoeal diseases	Olofin I, McDonald CM, Ezzati M, et al. Associations of suboptimal growth with all-cause and cause-specific mortality in children under five years: a pooled analysis of ten prospective studies. PLoS ONE 2013; 8: e64636.
Childhood underweight	Lower respiratory infections	Olofin I, McDonald CM, Ezzati M, et al. Associations of suboptimal growth with all-cause and cause-specific mortality in children under five years: a pooled analysis of ten prospective studies. PLoS ONE 2013; 8: e64636.
Childhood underweight	Measles	Olofin I, McDonald CM, Ezzati M, et al. Associations of suboptimal growth with all-cause and cause-specific mortality in children under five years: a pooled analysis of ten prospective studies. PLoS ONE 2013; 8: e64636.
Childhood wasting	Diarrhoeal diseases	Olofin I, McDonald CM, Ezzati M, et al. Associations of suboptimal growth with all-cause and cause-specific mortality in children under five years: a pooled analysis of ten prospective studies. PLoS ONE 2013; 8: e64636.
Childhood wasting	Lower respiratory infections	Olofin I, McDonald CM, Ezzati M, et al. Associations of suboptimal growth with all-cause and cause-specific mortality in children under five years: a pooled analysis of ten prospective studies. PLoS ONE 2013; 8: e64636.
Childhood wasting	Measles	Olofin I, McDonald CM, Ezzati M, et al. Associations of suboptimal growth with all-cause and cause-specific mortality in children under five years: a pooled analysis of ten prospective studies. PLoS ONE 2013; 8: e64636.
Childhood stunting	Diarrhoeal diseases	Olofin I, McDonald CM, Ezzati M, et al. Associations of suboptimal growth with all-cause and cause-specific mortality in children under five years: a pooled analysis of ten prospective studies. PLoS ONE 2013; 8: e64636.
Childhood stunting	Lower respiratory infections	Olofin I, McDonald CM, Ezzati M, et al. Associations of suboptimal growth with all-cause and cause-specific mortality in children under five years: a pooled analysis of ten prospective studies. PLoS ONE 2013; 8: e64636.
Childhood stunting	Measles	Olofin I, McDonald CM, Ezzati M, et al. Associations of suboptimal growth with all-cause and cause-specific mortality in children under five years: a pooled analysis of ten prospective studies. PLoS ONE 2013; 8: e64636.
Iron deficiency	Maternal hemorrhage	Murray-Kolb LE, Chen L, Chen P, Shapiro M, Caulfield L. CHERG Iron Report: Maternal Mortality, Child Mortality, Perinatal Mortality, Child Cognition, and Estimates of Prevalence of Anemia due to Iron Deficiency. Baltimore, USA: CHERG, 2012.
Vitamin A deficiency	Diarrhoeal diseases	Awasthi S, Peto R, Read S, et al. Vitamin A supplementation every 6 months with retinol in 1 million pre-school children in north India: DEVTA, a cluster-randomised trial. Lancet 2013; 381: 1469–77.
Vitamin A deficiency	Diarrhoeal diseases	Diness BR, Christoffersen D, Pedersen UB, Rodrigues A, Fischer TK, Andersen A, Whittle H, Yazdanbakhsh M, Aaby P, Benn CS. The effect of high-dose vitamin A supplementation given with bacille Calmette-Guérin vaccine at birth on infant rotavirus infection and diarrhea: a randomized prospective study from Guinea-Bissau. J Infect Dis. 2010; S243-251.
Vitamin A deficiency	Diarrhoeal diseases	Imdad A, Herzer K, Mayo-Wilson E, Yakoob MY, Bhutta ZA. Vitamin A supplementation for preventing morbidity and mortality in children from 6 months to 5 years of age. Cochrane Database Syst Rev. 2010; CD008524.
Vitamin A deficiency	Diarrhoeal diseases	Imdad A, Yakoob MY, Sudfeld C, Haider BA, Black RE, Bhutta ZA. Impact of vitamin A supplementation on infant and childhood mortality. BMC Public Health. 2011; S20.
Vitamin A deficiency	Measles	Awasthi S, Peto R, Read S, et al. Vitamin A supplementation every 6 months with retinol in 1 million pre-school children in north India: DEVTA, a cluster-randomised trial. Lancet 2013; 381: 1469–77.
Vitamin A deficiency	Measles	Imdad A, Herzer K, Mayo-Wilson E, Yakoob MY, Bhutta ZA. Vitamin A supplementation for preventing morbidity and mortality in children from 6 months to 5 years of age. Cochrane Database Syst Rev. 2010; CD008524.
Vitamin A deficiency	Measles	Imdad A, Yakoob MY, Sudfeld C, Haider BA, Black RE, Bhutta ZA. Impact of vitamin A supplementation on infant and childhood mortality. BMC Public Health. 2011; S20.
Zinc deficiency	Diarrhoeal diseases	Shankar AH, Prasad AS. Zinc and immune function: the biological basis of altered resistance to infection. Am J Clin Nutr 1998; 68: 447S–463S.
Zinc deficiency	Diarrhoeal diseases	Yakoob MY, Theodoratou E, Jabeen A, et al. Preventive zinc supplementation in developing countries: impact on mortality and morbidity due to diarrhea, pneumonia and malaria. BMC Public Health 2011; 11 Suppl 3: S23.
Zinc deficiency	Lower respiratory infections	Shankar AH, Prasad AS. Zinc and immune function: the biological basis of altered resistance to infection. Am J Clin Nutr 1998; 68: 447S–463S.
Zinc deficiency	Lower respiratory infections	Yakoob MY, Theodoratou E, Jabeen A, et al. Preventive zinc supplementation in developing countries: impact on mortality and morbidity due to diarrhea, pneumonia and malaria. BMC Public Health 2011; 11 Suppl 3: S23.
Smoking	Tuberculosis	Bates MN, Khalakdina A, Pai M, Chang L, Lessa F, Smith KR. Risk of tuberculosis from exposure to tobacco smoke: a systematic review and meta-analysis. Arch Intern Med 2007; 167: 335–42.
Smoking	Tuberculosis	Lin H-H, Ezzati M, Murray M. Tobacco smoke, indoor air pollution and tuberculosis: a systematic review and meta-analysis. PLoS Med 2007; 4: e20.

Appendix Table 7. Supplemental information for Table 2: Epidemiological evidence supporting causality between risk-outcome pairs included in the Global Burden of Disease 2015 study including 7A. Citations and 7B. Additional information

7A. Citations

Risk	Outcome	Citation/Note
Smoking	Tuberculosis	Slama K, Chiang C-Y, Enarson DA, et al. Tobacco and tuberculosis: a qualitative systematic review and meta-analysis. Int J Tuberc Lung Dis 2007; 11: 1049–61.
Smoking	Lower respiratory infections	Surgeon General's Report - The Health Consequences of Smoking. U.S. Department of Health & Human Services, 2004 http://www.cdc.gov/tobacco/data_statistics/sgr/2004/.
Smoking	Lip and oral cavity cancer	Surgeon General's Report - The Health Consequences of Smoking. U.S. Department of Health & Human Services, 2004 http://www.cdc.gov/tobacco/data_statistics/sgr/2004/.
Smoking	Nasopharynx cancer	Surgeon General's Report - The Health Consequences of Smoking. U.S. Department of Health & Human Services, 2004 http://www.cdc.gov/tobacco/data_statistics/sgr/2004/.
Smoking	Oesophageal cancer	Surgeon General's Report - The Health Consequences of Smoking. U.S. Department of Health & Human Services, 2004 http://www.cdc.gov/tobacco/data_statistics/sgr/2004/.
Smoking	Stomach cancer	Surgeon General's Report - The Health Consequences of Smoking. U.S. Department of Health & Human Services, 2004 http://www.cdc.gov/tobacco/data_statistics/sgr/2004/.
Smoking	Colon and rectum cancer	Surgeon General's Report - The Health Consequences of Smoking. U.S. Department of Health & Human Services, 2004 http://www.cdc.gov/tobacco/data_statistics/sgr/2004/.
Smoking	Liver cancer	Surgeon General's Report - The Health Consequences of Smoking. U.S. Department of Health & Human Services, 2004 http://www.cdc.gov/tobacco/data_statistics/sgr/2004/.
Smoking	Pancreatic cancer	Surgeon General's Report - The Health Consequences of Smoking. U.S. Department of Health & Human Services, 2004 http://www.cdc.gov/tobacco/data_statistics/sgr/2004/.
Smoking	Larynx cancer	International Agency for Research on Cancer Working Group on the Evaluation of Carcinogenic Risks to Humans. IARC monographs on the evaluation of carcinogenic risks to humans: Tobacco Smoke and Involuntary Smoking. Lyon: IARC, 2004.
Smoking	Tracheal, bronchus and lung cancer	International Agency for Research on Cancer Working Group on the Evaluation of Carcinogenic Risks to Humans. IARC monographs on the evaluation of carcinogenic risks to humans: Tobacco Smoke and Involuntary Smoking. Lyon: IARC, 2004.
Smoking	Cervical cancer	International Agency for Research on Cancer Working Group on the Evaluation of Carcinogenic Risks to Humans. IARC monographs on the evaluation of carcinogenic risks to humans: Tobacco Smoke and Involuntary Smoking. Lyon: IARC, 2004.
Smoking	Kidney cancer	International Agency for Research on Cancer Working Group on the Evaluation of Carcinogenic Risks to Humans. IARC monographs on the evaluation of carcinogenic risks to humans: Tobacco Smoke and Involuntary Smoking. Lyon: IARC, 2004.
Smoking	Bladder cancer	International Agency for Research on Cancer Working Group on the Evaluation of Carcinogenic Risks to Humans. IARC monographs on the evaluation of carcinogenic risks to humans: Tobacco Smoke and Involuntary Smoking. Lyon: IARC, 2004.
Smoking	Leukaemia	Surgeon General's Report - The Health Consequences of Smoking. U.S. Department of Health & Human Services, 2004 http://www.cdc.gov/tobacco/data_statistics/sgr/2004/.
Smoking	Ischaemic heart disease	Huxley RR, Woodward M. Cigarette smoking as a risk factor for coronary heart disease in women compared with men: a systematic review and meta-analysis of prospective cohort studies. Lancet 2011; 378: 1297–305.
Smoking	Cerebrovascular disease	Peters SAE, Huxley RR, Woodward M. Smoking as a risk factor for stroke in women compared with men: a systematic review and meta-analysis of 81 cohorts, including 3,980,359 individuals and 42,401 strokes. Stroke 2013; 44: 2821–8.
Smoking	Hypertensive heart disease	Carter BD, Abnet CC, Feskanich D, et al. Smoking and mortality--beyond established causes. N Engl J Med 2015; 372: 631–40.
Smoking	Atrial fibrillation and flutter	Zhu W, Yuan P, Shen Y, Wan R, Hong K. Association of smoking with the risk of incident atrial fibrillation: A meta-analysis of prospective studies. Int J Cardiol 2016; 218: 259–66.
Smoking	Aortic aneurysm	Lederle FA, Nelson DB, Joseph AM. Smokers' relative risk for aortic aneurysm compared with other smoking-related diseases: a systematic review. J Vasc Surg 2003; 38: 329–34.
Smoking	Peripheral vascular disease	Lu L, Mackay DF, Pell JP. Meta-analysis of the association between cigarette smoking and peripheral arterial disease. Heart 2014; 100: 414–23.
Smoking	Other cardiovascular and circulatory diseases	Carter BD, Abnet CC, Feskanich D, et al. Smoking and mortality--beyond established causes. N Engl J Med 2015; 372: 631–40.
Smoking	Chronic obstructive pulmonary disease	Forey BA, Thornton AJ, Lee PN. Systematic review with meta-analysis of the epidemiological evidence relating smoking to COPD, chronic bronchitis and emphysema. BMC Pulm Med 2011; 11: 36.
Smoking	Asthma	The Health Consequences of Smoking—50 Years of Progress. U.S. Department of Health & Human Services, 2014 http://www.surgeongeneral.gov/library/reports/50-years-of-progress/full-report.pdf.
Smoking	Other chronic respiratory diseases	Carter BD, Abnet CC, Feskanich D, et al. Smoking and mortality--beyond established causes. N Engl J Med 2015; 372: 631–40.
Smoking	Peptic ulcer disease	Kurata JH, Nogawa AN. Meta-analysis of risk factors for peptic ulcer. Nonsteroidal antiinflammatory drugs, Helicobacter pylori, and smoking. J Clin Gastroenterol 1997; 24: 2–17.
Smoking	Diabetes mellitus	The Health Consequences of Smoking—50 Years of Progress. U.S. Department of Health & Human Services, 2014 http://www.surgeongeneral.gov/library/reports/50-years-of-progress/full-report.pdf.
Smoking	Rheumatoid arthritis	Sugiyama D, Nishimura K, Tamaki K, et al. Impact of smoking as a risk factor for developing rheumatoid arthritis: a meta-analysis of observational studies. Ann Rheum Dis 2010; 69: 70–81.
Smoking	Cataract	Ye J, He J, Wang C, et al. Smoking and risk of age-related cataract: a meta-analysis. Invest Ophthalmol Vis Sci 2012; 53: 3885–95.
Smoking	Macular degeneration	Chakravarthy U, Wong TY, Fletcher A, et al. Clinical risk factors for age-related macular degeneration: a systematic review and meta-analysis. BMC Ophthalmol 2010; 10: 31.
Smoking	Injuries	Vestergaard P, Mosekilde L. Fracture risk associated with smoking: a meta-analysis. J Intern Med 2003; 254: 572–83.
Second-hand smoke	Lower respiratory infections	Baker RJ, Hertz-Picciotto I, Dostal M, Keller JA, Nozicka J, Kotesovec F, Dejmek J, Loomis D, Sram RJ. Coal home heating and environmental tobacco smoke in relation to lower respiratory illness in Czech children, from birth to 3 years of age. Environ Health Perspect. 2006; 1126-32.

Appendix Table 7. Supplemental information for Table 2: Epidemiological evidence supporting causality between risk-outcome pairs included in the Global Burden of Disease 2015 study including 7A. Citations and 7B. Additional information

7A. Citations

Risk	Outcome	Citation/Note
Second-hand smoke	Lower respiratory infections	Blizzard L, Ponsonby A-L, Dwyer T, Venn A, Cochrane JA. Parental smoking and infant respiratory infection: how important is not smoking in the same room with the baby?. Am J Public Health. 2003; 482-8.
Second-hand smoke	Lower respiratory infections	Bonu S, Rani M, Jha P, Peters DH, Nguyen SN. Household tobacco and alcohol use, and child health: an exploratory study from India. Health Policy. 2004; 67-83.
Second-hand smoke	Lower respiratory infections	Broor S, Pandey RM, Ghosh M, Maitreyi RS, Lodha R, Singhal T, Kabra SK. Risk factors for severe acute lower respiratory tract infection in under-five children. Indian Pediatr. 2001; 1361-9.
Second-hand smoke	Lower respiratory infections	Chen Y, Li WX, Yu SZ, Qian WH. Chang-Ning epidemiological study of children's health: I: Passive smoking and children's respiratory diseases. Int J Epidemiol. 1988; 348-55.
Second-hand smoke	Lower respiratory infections	Duijts L, Jaddoe VWV, Hofman A, Steegers EAP, Mackenbach JP, de Jongste JC, Moll HA. Maternal smoking in pre-natal and early post-natal life and the risk of respiratory tract infections in infancy. The Generation R study. Eur J Epidemiol. 2008; 547-55.
Second-hand smoke	Lower respiratory infections	Ekwo EE, Weinberger MM, Lachenbruch PA, Huntley WH. Relationship of parental smoking and gas cooking to respiratory disease in children. Chest. 1983; 662-8.
Second-hand smoke	Lower respiratory infections	Etiler N, Velipasaoglu S, Aktekin M. Incidence of acute respiratory infections and the relationship with some factors in infancy in Antalya, Turkey. Pediatr Int 2002; 44: 64–9.
Second-hand smoke	Lower respiratory infections	Ferris BG, Ware JH, Berkey CS, Dockery DW, Spiro A, Speizer FE. Effects of passive smoking on health of children. Environ Health Perspect. 1985; 289-95.
Second-hand smoke	Lower respiratory infections	Forastiere F, Corbo GM, Michelozzi P, Pistelli R, Agabiti N, Brancato G, Ciappi G, Perucci CA. Effects of environment and passive smoking on the respiratory health of children. Int J Epidemiol. 1992; 66-73.
Second-hand smoke	Lower respiratory infections	Gardner G, Frank AL, Taber LH. Effects of social and family factors on viral respiratory infection and illness in the first year of life. J Epidemiol Community Health. 1984; 42-8.
Second-hand smoke	Lower respiratory infections	Hassan MK, Al-Sadoon I. Risk factors for severe pneumonia in children in Basrah. Trop Doct. 2001; 139-41.
Second-hand smoke	Lower respiratory infections	Koch A, Molbak K, Homoe P, Sorensen P, Hjuler T, Olesen ME, Pejl J, Pedersen FK, Olsen OR, Melbye M. Risk factors for acute respiratory tract infections in young Greenlandic children. Am J Epidemiol. 2003; 374-84.
Second-hand smoke	Lower respiratory infections	Kristensen IA, Olsen J. Determinants of acute respiratory infections in Soweto--a population-based birth cohort. S Afr Med J. 2006; 633-40.
Second-hand smoke	Lower respiratory infections	Margolis PA, Keyes LL, Greenberg RA, Bauman KE, LaVange LM. Urinary cotinine and parent history (questionnaire) as indicators of passive smoking and predictors of lower respiratory illness in infants. Pediatr Pulmonol. 1997; 417-23.
Second-hand smoke	Lower respiratory infections	Nuesslein TG, Beckers D, Rieger CH. Cotinine in meconium indicates risk for early respiratory tract infections. Hum Exp Toxicol. 1999; 283-90.
Second-hand smoke	Lower respiratory infections	Ogston SA, Florey CD, Walker CH. The Tayside infant morbidity and mortality study: effect on health of using gas for cooking. BMJ. 1985; 957-60.
Second-hand smoke	Lower respiratory infections	Ogston SA, Florey CD, Walker CH. Association of infant alimentary and respiratory illness with parental smoking and other environmental factors. J Epidemiol Community Health. 1987; 21-5.
Second-hand smoke	Lower respiratory infections	Pedreira FA, Guandolo VL, Feroli EJ, Mella GW, Weiss IP. Involuntary smoking and incidence of respiratory illness during the first year of life. Pediatrics. 1985; 594-7.
Second-hand smoke	Lower respiratory infections	Rylander E, Pershagen G, Eriksson M, Bermann G. Parental smoking, urinary cotinine, and wheezing bronchitis in children. Epidemiology. 1995; 289-93.
Second-hand smoke	Lower respiratory infections	Suzuki M, Thiem VD, Yanai H, Matsubayashi T, Yoshida LM, Tho LH, Minh TT, Anh DD, Kilgore PE, Ariyoshi K. Association of environmental tobacco smoking exposure with an increased risk of hospital admissions for pneumonia in children under 5 years of age in Vietnam. Thorax. 2009; 484-9.
Second-hand smoke	Lower respiratory infections	Taylor B, Wadsworth J. Maternal smoking during pregnancy and lower respiratory tract illness in early life. Arch Dis Child. 1987; 786-91.
Second-hand smoke	Lower respiratory infections	Victora CG, Fuchs SC, Flores JA, Fonseca W, Kirkwood B. Risk factors for pneumonia among children in a Brazilian metropolitan area. Pediatrics. 1994; 977-85.
Second-hand smoke	Otitis media	Jones LL, Hassanien A, Cook DG, Britton J, Leonardi-Bee J. Parental smoking and the risk of middle ear disease in children: a systematic review and meta-analysis. Arch Pediatr Adolesc Med 2012; 166: 18–27.
Second-hand smoke	Tracheal, bronchus, and lung cancer	Akiba S, Kato H, Blot WJ. Passive smoking and lung cancer among Japanese women. Cancer Res 1986; 46: 4804–7.
Second-hand smoke	Tracheal, bronchus, and lung cancer	Boffetta P, Agudo A, Ahrens W, et al. Multicenter case-control study of exposure to environmental tobacco smoke and lung cancer in Europe. J Natl Cancer Inst 1998; 90: 1440–50.
Second-hand smoke	Tracheal, bronchus, and lung cancer	Brownson RC, Alavanja MC, Hock ET, Loy TS. Passive smoking and lung cancer in nonsmoking women. Am J Public Health 1992; 82: 1525–30.
Second-hand smoke	Tracheal, bronchus, and lung cancer	Brownson RC, Reif JS, Keefe TJ, Ferguson SW, Pritzl JA. Risk factors for adenocarcinoma of the lung. Am J Epidemiol 1987; 125: 25–34.
Second-hand smoke	Tracheal, bronchus, and lung cancer	Bulter TL. The relationship of passive smoking to various health outcomes among Seventh-day Adventists in California [dissertation]. Los Angeles, United States: University of California, Los Angeles, 1988.
Second-hand smoke	Tracheal, bronchus, and lung cancer	Cardenas VM, Thun MJ, Austin H, et al. Environmental tobacco smoke and lung cancer mortality in the American Cancer Society's Cancer Prevention Study. II. Cancer Causes Control 1997; 8: 57–64.
Second-hand smoke	Tracheal, bronchus, and lung cancer	Chan WC, Fung SC. Lung cancer in non-smokers in Hong Kong. Geographical Pathology in Cancer Epidemiology. In: Grundmann E, Clemmesen J, Muir CS, eds. Cancer Campaign. Vol. 6. Cancer Epidemiology. Stuttgart, Germany: Gustav Fischer Verlag; 1982. p. 199-202.
Second-hand smoke	Tracheal, bronchus, and lung cancer	Correa P, Pickle LW, Fontham E, Lin Y, Haenszel W. Passive smoking and lung cancer. Lancet 1983; 2: 595–7.
Second-hand smoke	Tracheal, bronchus, and lung cancer	Fontham ET, Correa P, Reynolds P, et al. Environmental tobacco smoke and lung cancer in nonsmoking women. A multicenter study. JAMA 1994; 271: 1752–9.
Second-hand smoke	Tracheal, bronchus, and lung cancer	Gao YT, Blot WJ, Zheng W, et al. Lung cancer among Chinese women. Int J Cancer 1987; 40: 604–9.

Appendix Table 7. Supplemental information for Table 2: Epidemiological evidence supporting causality between risk-outcome pairs included in the Global Burden of Disease 2015 study including 7A. Citations and 7B. Additional information<br>7A. Citations		
**Risk**	**Outcome**	**Citation/Note**
Second-hand smoke	Tracheal, bronchus, and lung cancer	Garfinkel L, Auerbach O, Joubert L. Involuntary smoking and lung cancer: a case-control study. J Natl Cancer Inst 1985; 75: 463–9.
Second-hand smoke	Tracheal, bronchus, and lung cancer	Geng G-Y, Liang ZH, Zhang A-Y, Wu GL. On the relationship between smoking and female lung cancer. In: Aoki M, Hisamichi S, Tominaga S, editors. Smoking and Health 1987; Proceedings of the 6th World Conference on Smoking and Health; Nov 9-12 1987; Tokyo, Japan. Amsterdam (Netherlands): Elsevier Science; 1988. p. 483-6.
Second-hand smoke	Tracheal, bronchus, and lung cancer	Hirayama T. Non-smoking wives of heavy smokers have a higher risk of lung cancer: a study from Japan. 1981. Bull World Health Organ 2000; 78: 940–2.
Second-hand smoke	Tracheal, bronchus, and lung cancer	Hole DJ, Gillis CR, Chopra C, Hawthorne VM. Passive smoking and cardiorespiratory health in a general population in the west of Scotland. BMJ 1989; 299: 423–7.
Second-hand smoke	Tracheal, bronchus, and lung cancer	Humble CG, Samet JM, Pathak DR. Marriage to a smoker and lung cancer risk. Am J Public Health 1987; 77: 598–602.
Second-hand smoke	Tracheal, bronchus, and lung cancer	Inoue R, Hirayama T. Passive smoking and lung cancer in women. In: Aoki M, Hisamichi S, Tominaga S, eds. Smoking and Health 1987; Proceedings of the 6th World Conference on Smoking and Health; Nov 9-12, 1987; Tokyo, Japan. Amsterdam (Netherlands): Elsevier Science; 1988. p. 283-5.
Second-hand smoke	Tracheal, bronchus, and lung cancer	Johnson KC, Hu J, Mao Y, Canadian Cancer Registries Epidemiology Research Group. Lifetime residential and workplace exposure to environmental tobacco smoke and lung cancer in never-smoking women, Canada 1994-97. Int J Cancer 2001; 93: 902–6.
Second-hand smoke	Tracheal, bronchus, and lung cancer	Kabat GC, Stellman SD, Wynder EL. Relation between exposure to environmental tobacco smoke and lung cancer in lifetime nonsmokers. Am J Epidemiol 1995; 142: 141–8.
Second-hand smoke	Tracheal, bronchus, and lung cancer	Kabat GC, Wynder EL. Lung cancer in nonsmokers. Cancer 1984; 53: 1214–21.
Second-hand smoke	Tracheal, bronchus, and lung cancer	Kalandidi A, Katsouyanni K, Voropoulou N, Bastas G, Saracci R, Trichopoulos D. Passive smoking and diet in the etiology of lung cancer among non-smokers. Cancer Causes Control 1990; 1: 15–21.
Second-hand smoke	Tracheal, bronchus, and lung cancer	Ko YC, Lee CH, Chen MJ, et al. Risk factors for primary lung cancer among non-smoking women in Taiwan. Int J Epidemiol 1997; 26: 24–31.
Second-hand smoke	Tracheal, bronchus, and lung cancer	Koo LC, Ho JH, Saw D, Ho CY. Measurements of passive smoking and estimates of lung cancer risk among non-smoking Chinese females. Int J Cancer 1987; 39: 162–9.
Second-hand smoke	Tracheal, bronchus, and lung cancer	Lam TH, Kung IT, Wong CM, et al. Smoking, passive smoking and histological types in lung cancer in Hong Kong Chinese women. Br J Cancer 1987; 56: 673–8.
Second-hand smoke	Tracheal, bronchus, and lung cancer	Lee PN, Chamberlain J, Alderson MR. Relationship of passive smoking to risk of lung cancer and other smoking-associated diseases. Br J Cancer 1986; 54: 97–105.
Second-hand smoke	Tracheal, bronchus, and lung cancer	Liu Q, Sasco AJ, Riboli E, Hu MX. Indoor air pollution and lung cancer in Guangzhou, People's Republic of China. Am J Epidemiol 1993; 137: 145–54.
Second-hand smoke	Tracheal, bronchus, and lung cancer	Liu ZY, He XZ, Chapman RS. Smoking and other risk factors for lung cancer in Xuanwei, China. Int J Epidemiol 1991; 20: 26–31.
Second-hand smoke	Tracheal, bronchus, and lung cancer	Nyberg F, Agrenius V, Svartengren K, Svensson C, Pershagen G. Environmental tobacco smoke and lung cancer in nonsmokers: does time since exposure play a role? Epidemiology 1998; 9: 301–8.
Second-hand smoke	Tracheal, bronchus, and lung cancer	Pershagen G, Hrubec Z, Svensson C. Passive smoking and lung cancer in Swedish women. Am J Epidemiol 1987; 125: 17–24.
Second-hand smoke	Tracheal, bronchus, and lung cancer	Seow A, Poh W-T, Teh M, et al. Diet, reproductive factors and lung cancer risk among Chinese women in Singapore: evidence for a protective effect of soy in nonsmokers. Int J Cancer 2002; 97: 365–71.
Second-hand smoke	Tracheal, bronchus, and lung cancer	Shen XB, Wang GX, Huang YZ, Xiang LS, Wang XH. Analysis and estimates of attributable risk factors for lung cancer in Nanjing, China. Lung Cancer 1996; 14 Suppl 1: S107-112.
Second-hand smoke	Tracheal, bronchus, and lung cancer	Shimizu H, Morishita M, Mizuno K, et al. A case-control study of lung cancer in nonsmoking women. Tohoku J Exp Med 1988; 154: 389–97.
Second-hand smoke	Tracheal, bronchus, and lung cancer	Sobue T. Association of indoor air pollution and lifestyle with lung cancer in Osaka, Japan. Int J Epidemiol 1990; 19 Suppl 1: S62-66.
Second-hand smoke	Tracheal, bronchus, and lung cancer	Stockwell HG, Goldman AL, Lyman GH, et al. Environmental tobacco smoke and lung cancer risk in nonsmoking women. J Natl Cancer Inst 1992; 84: 1417–22.
Second-hand smoke	Tracheal, bronchus, and lung cancer	Svensson C, Pershagen G, Klominek J. Smoking and passive smoking in relation to lung cancer in women. Acta Oncol 1989; 28: 623–9.
Second-hand smoke	Tracheal, bronchus, and lung cancer	Trichopoulos D, Kalandidi A, Sparros L, MacMahon B. Lung cancer and passive smoking. Int J Cancer 1981; 27: 1–4.
Second-hand smoke	Tracheal, bronchus, and lung cancer	Wang FL, Love EJ, Liu N, Dai XD. Childhood and adolescent passive smoking and the risk of female lung cancer. Int J Epidemiol 1994; 23: 223–30.
Second-hand smoke	Tracheal, bronchus, and lung cancer	Wang L, Lubin JH, Zhang SR, et al. Lung cancer and environmental tobacco smoke in a non-industrial area of China. Int J Cancer 2000; 88: 139–45.
Second-hand smoke	Tracheal, bronchus, and lung cancer	Wu AH, Henderson BE, Pike MC, Yu MC. Smoking and other risk factors for lung cancer in women. J Natl Cancer Inst 1985; 74: 747–51.
Second-hand smoke	Tracheal, bronchus, and lung cancer	Wu-Williams AH, Dai XD, Blot W, et al. Lung cancer among women in north-east China. Br J Cancer 1990; 62: 982–7.
Second-hand smoke	Tracheal, bronchus, and lung cancer	Zaridze D, Maximovitch D, Zemlyanaya G, Aitakov ZN, Boffetta P. Exposure to environmental tobacco smoke and risk of lung cancer in non-smoking women from Moscow, Russia. Int J Cancer 1998; 75: 335–8.
Second-hand smoke	Ischaemic heart disease	Ciruzzi M, Pramparo P, Esteban O, et al. Case-control study of passive smoking at home and risk of acute myocardial infarction. Argentine FRICAS Investigators. Factores de Riesgo Coronario en América del Sur. J Am Coll Cardiol 1998; 31: 797–803.
Second-hand smoke	Ischaemic heart disease	He Y, Lam TH, Li LS, et al. Passive smoking at work as a risk factor for coronary heart disease in Chinese women who have never smoked. BMJ 1994; 308: 380–4.
Second-hand smoke	Ischaemic heart disease	Hirayama T. Non-smoking wives of heavy smokers have a higher risk of lung cancer: a study from Japan. Br Med J (Clin Res Ed) 1981; 282: 183–5.

Appendix Table 7. Supplemental information for Table 2: Epidemiological evidence supporting causality between risk-outcome pairs included in the Global Burden of Disease 2015 study including 7A. Citations and 7B. Additional information

7A. Citations

Risk	Outcome	Citation/Note
Second-hand smoke	Ischaemic heart disease	Hole DJ, Gillis CR, Chopra C, Hawthorne VM. Passive smoking and cardiorespiratory health in a general population in the west of Scotland. BMJ 1989; 299: 423–7.
Second-hand smoke	Ischaemic heart disease	La Vecchia C, D'Avanzo B, Franzosi MG, Tognoni G. Passive smoking and the risk of acute myocardial infarction GISSI-EFRIM investigations. Lancet 1993; 341: 505–6.
Second-hand smoke	Ischaemic heart disease	Rosenlund M, Berglind N, Gustavsson A, et al. Environmental tobacco smoke and myocardial infarction among never-smokers in the Stockholm Heart Epidemiology Program (SHEEP). Epidemiology 2001; 12: 558–64.
Second-hand smoke	Ischaemic heart disease	Steenland K, Thun M, Lally C, Heath C. Environmental tobacco smoke and coronary heart disease in the American Cancer Society CPS-II cohort. Circulation 1996; 94: 622–8.
Second-hand smoke	Ischaemic heart disease	Svendsen KH, Kuller LH, Martin MJ, Ockene JK. Effects of passive smoking in the Multiple Risk Factor Intervention Trial. Am J Epidemiol 1987; 126: 783–95.
Second-hand smoke	Ischaemic stroke	Oono IP, Mackay DF, Pell JP. Meta-analysis of the association between secondhand smoke exposure and stroke. J Public Health (Oxf) 2011; 33: 496–502.
Alcohol use	Tuberculosis	Lönnroth K, Williams BG, Stadlin S, Jaramillo E, Dye C. Alcohol use as a risk factor for tuberculosis - a systematic review. BMC Public Health 2008; 8: 289.
Alcohol use	Lower respiratory infections	Samokhvalov AV, Irving HM, Rehm J. Alcohol consumption as a risk factor for pneumonia: a systematic review and meta-analysis. Epidemiol Infect 2010; 138: 1789–95.
Alcohol use	Lip and oral cavity cancer	Bagnardi V, Blangiardo M, La Vecchia C, Corrao G. A meta-analysis of alcohol drinking and cancer risk. Br J Cancer 2001; 85: 1700–5.
Alcohol use	Nasopharynx cancer	Bagnardi V, Blangiardo M, La Vecchia C, Corrao G. A meta-analysis of alcohol drinking and cancer risk. Br J Cancer 2001; 85: 1700–5.
Alcohol use	Other pharynx cancer	Bagnardi V, Blangiardo M, La Vecchia C, Corrao G. A meta-analysis of alcohol drinking and cancer risk. Br J Cancer 2001; 85: 1700–5.
Alcohol use	Oesophageal cancer	Bagnardi V, Blangiardo M, La Vecchia C, Corrao G. A meta-analysis of alcohol drinking and cancer risk. Br J Cancer 2001; 85: 1700–5.
Alcohol use	Colon and rectum cancer	Bagnardi V, Blangiardo M, La Vecchia C, Corrao G. A meta-analysis of alcohol drinking and cancer risk. Br J Cancer 2001; 85: 1700–5.
Alcohol use	Larynx cancer	Bagnardi V, Blangiardo M, La Vecchia C, Corrao G. A meta-analysis of alcohol drinking and cancer risk. Br J Cancer 2001; 85: 1700–5.
Alcohol use	Breast cancer	Bagnardi V, Blangiardo M, La Vecchia C, Corrao G. A meta-analysis of alcohol drinking and cancer risk. Br J Cancer 2001; 85: 1700–5.
Alcohol use	Ischaemic heart disease	Roerecke M, Rehm J. The cardioprotective association of average alcohol consumption and ischaemic heart disease: a systematic review and meta-analysis. Addiction 2012; 107: 1246–60.
Alcohol use	Cerebrovascular disease	Patra J, Taylor B, Irving H, et al. Alcohol consumption and the risk of morbidity and mortality for different stroke types--a systematic review and meta-analysis. BMC Public Health 2010; 10: 258.
Alcohol use	Hypertensive heart disease	Corrao G, Bagnardi V, Zambon A, La Vecchia C. A meta-analysis of alcohol consumption and the risk of 15 diseases. Prev Med 2004; 38: 613–9.
Alcohol use	Atrial fibrillation and flutter	Kodama S, Saito K, Tanaka S, et al. Alcohol consumption and risk of atrial fibrillation: a meta-analysis. J Am Coll Cardiol 2011; 57: 427–36.
Alcohol use	Cirrhosis and other chronic liver diseases	Rehm J, Taylor B, Mohapatra S, et al. Alcohol as a risk factor for liver cirrhosis: a systematic review and meta-analysis. Drug Alcohol Rev 2010; 29: 437–45.
Alcohol use	Pancreatitis	Irving, Samokhvalov, and Rehm, 2012, Alcohol as a risk factor for pancreatitis - A systematic review and meta-analysis, *JOP.; 10(4): 387-392*
Alcohol use	Epilepsy	Samokhvalov AV, Irving H, Mohapatra S, Rehm J. Alcohol consumption, unprovoked seizures, and epilepsy: a systematic review and meta-analysis. Epilepsia 2010; 51: 1177–84.
Alcohol use	Diabetes mellitus	Carlsson S, Hammar N, Grill V. Alcohol consumption and type 2 diabetes Meta-analysis of epidemiological studies indicates a U-shaped relationship. Diabetologia 2005; 48: 1051–4.
Alcohol use	Injuries	Anda RF, Williamson DF, Remington PL. Alcohol and fatal injuries among US adults. Findings from the NHANES I Epidemiologic Follow-up Study. JAMA 1988; 260: 2529–32.
Alcohol use	Injuries	Smith GS, Branas CC, Miller TR. Fatal nontraffic injuries involving alcohol: A metaanalysis. Ann Emerg Med 1999; 33: 659–68.
Alcohol use	Injuries	Taylor B, Irving HM, Kanteres F, et al. The more you drink, the harder you fall: a systematic review and meta-analysis of how acute alcohol consumption and injury or collision risk increase together. Drug Alcohol Depend 2010; 110: 108–16.
Alcohol use	Self-harm	Haw C, Hawton K, Casey D, Bale E, Shepherd A. Alcohol dependence, excessive drinking and deliberate self-harm: trends and patterns in Oxford, 1989-2002. Soc Psychiatry Psychiatr Epidemiol 2005; 40: 964–71.
Alcohol use	Interpersonal violence	Corrao G, Bagnardi V, Zambon A, La Vecchia C. A meta-analysis of alcohol consumption and the risk of 15 diseases. Prev Med 2004; 38: 613–9.
Drug use	Hepatitis B	Blomé MA, Björkman P, Flamholc L, Jacobsson H, Molnegren V, Widell A. Minimal transmission of HIV despite persistently high transmission of hepatitis C virus in a Swedish needle exchange program. J Viral Hepat 2011; 18: 831–9.
Drug use	Hepatitis B	Crofts N, Aitken CK. Incidence of bloodborne virus infection and risk behaviours in a cohort of injecting drug users in Victoria, 1990-1995. Med J Aust 1997; 167: 17–20.
Drug use	Hepatitis B	Hagan H, McGough JP, Thiede H, Weiss NS, Hopkins S, Alexander ER. Syringe Exchange and Risk of Infection with Hepatitis B and C Viruses, Am. J. Epi. 1999; 149(3): 203–213.
Drug use	Hepatitis B	Jackson JB, Wei L, Liping F, et al. Prevalence and seroincidence of hepatitis B and hepatitis C infection in high risk people who inject drugs in china and Thailand. Hepat Res Treat 2014; 2014: 296958.
Drug use	Hepatitis B	Månsson AS, Moestrup T, Nordenfelt E, Widell A. Continued transmission of hepatitis B and C viruses, but no transmission of human immunodeficiency virus among intravenous drug users participating in a syringe/needle exchange program. Scand J Infect Dis 2000; 32: 253–8.

Appendix Table 7. Supplemental information for Table 2: Epidemiological evidence supporting causality between risk-outcome pairs included in the Global Burden of Disease 2015 study including 7A. Citations and 7B. Additional information

7A. Citations

Risk	Outcome	Citation/Note
Drug use	Hepatitis C	Abou-Saleh M, Davis P, Rice P, et al. The effectiveness of behavioural interventions in the primary prevention of hepatitis C amongst injecting drug users: a randomised controlled trial and lessons learned. Harm Reduct J 2008; 5: 25.
Drug use	Hepatitis C	Blomé MA, Björkman P, Flamholc L, Jacobsson H, Molnegren V, Widell A. Minimal transmission of HIV despite persistently high transmission of hepatitis C virus in a Swedish needle exchange program. J Viral Hepat 2011; 18: 831–9.
Drug use	Hepatitis C	Craine N, Hickman M, Parry JV, et al. Incidence of hepatitis C in drug injectors: the role of homelessness, opiate substitution treatment, equipment sharing, and community size. Epidemiol Infect 2009; 137: 1255–65.
Drug use	Hepatitis C	Crofts N, Aitken CK. Incidence of bloodborne virus infection and risk behaviours in a cohort of injecting drug users in Victoria, 1990-1995. Med J Aust 1997; 167: 17–20.
Drug use	Hepatitis C	Foley SB, Abou-Saleh MT. Risk Behaviors and Transmission of Hepatitis C in Injecting Drug Users. Addictive Disorders & Their Treatment 2009; 8: 13–21.
Drug use	Hepatitis C	Grebely J, Lima VD, Marshall BDL, et al. Declining incidence of hepatitis C virus infection among people who inject drugs in a Canadian setting, 1996-2012. PLoS ONE 2014; 9: e97726.
Drug use	Hepatitis C	Hagan H, McGough JP, Thiede H, Weiss NS, Hopkins S, Alexander ER. Syringe exchange and risk of infection with hepatitis B and C viruses. Am J Epidemiol 1999; 149: 203–13.
Drug use	Hepatitis C	Jackson JB, Wei L, Liping F, et al. Prevalence and seroincidence of hepatitis B and hepatitis C infection in high risk people who inject drugs in china and Thailand. Hepat Res Treat 2014; 2014: 296958.
Drug use	Hepatitis C	Lucidarme D, Bruandet A, Ilef D, et al. Incidence and risk factors of HCV and HIV infections in a cohort of intravenous drug users in the North and East of France. Epidemiol Infect 2004; 132: 699–708.
Drug use	Hepatitis C	Maher L, Jalaludin B, Chant KG, et al. Incidence and risk factors for hepatitis C seroconversion in injecting drug users in Australia. Addiction 2006; 101: 1499–508.
Drug use	Hepatitis C	Månsson AS, Moestrup T, Nordenfelt E, Widell A. Continued transmission of hepatitis B and C viruses, but no transmission of human immunodeficiency virus among intravenous drug users participating in a syringe/needle exchange program. Scand J Infect Dis 2000; 32: 253–8.
Drug use	Hepatitis C	Partanen A, Malin K, Perälä R, Harju O, Holopainen A, Holmström P, et al. Riski-tutkimus 2000-2003. Pistämällä huumeita käyttävien seurantatutkimus. A-Klinikkasäätiön Raporttisarja nro 52. Helsinki: A-Klinikkasäätiön, 2006.
Drug use	Hepatitis C	Roy KM, Goldberg D, Taylor A, et al. A method to detect the incidence of hepatitis C infection among injecting drug users in Glasgow 1993-98. J Infect 2001; 43: 200–5.
Drug use	Hepatitis C	Turner KME, Hutchinson S, Vickerman P, et al. The impact of needle and syringe provision and opiate substitution therapy on the incidence of hepatitis C virus in injecting drug users: pooling of UK evidence. Addiction 2011; 106: 1978–88.
Drug use	Hepatitis C	Van Den Berg C, Smit C, Van Brussel G, Coutinho R, Prins M, Amsterdam Cohort. Full participation in harm reduction programmes is associated with decreased risk for human immunodeficiency virus and hepatitis C virus: evidence from the Amsterdam Cohort Studies among drug users. Addiction 2007; 102: 1454–62.
Drug use	Hepatitis C	Villano SA, Vlahov D, Nelson KE, Lyles CM, Cohn S, Thomas DL. Incidence and risk factors for hepatitis C among injection drug users in Baltimore, Maryland. J Clin Microbiol 1997; 35: 3274–7.
Diet low in fruits	Lip and oral cavity cancer	Key TJ. Fruit and vegetables and cancer risk. British Journal of Cancer 2011; 104: 6–11.
Diet low in fruits	Nasopharynx cancer	Key TJ. Fruit and vegetables and cancer risk. British Journal of Cancer 2011; 104: 6–11.
Diet low in fruits	Other pharynx cancer	Key TJ. Fruit and vegetables and cancer risk. British Journal of Cancer 2011; 104: 6–11.
Diet low in fruits	Larynx cancer	Key TJ. Fruit and vegetables and cancer risk. British Journal of Cancer 2011; 104: 6–11.
Diet low in fruits	Oesophageal cancer	Liu J, Wang J, Leng Y, Lv C. Intake of fruit and vegetables and risk of esophageal squamous cell carcinoma: a meta-analysis of observational studies. Int J Cancer 2013; 133: 473–85.
Diet low in fruits	Tracheal, bronchus and lung cancer	Vieira AR, Abar L, Vingeliene S, et al. Fruits, vegetables and lung cancer risk: a systematic review and meta-analysis. Ann Oncol 2016; 27: 81–96.
Diet low in fruits	Ischaemic heart disease	Wang X, Ouyang Y, Liu J, et al. Fruit and vegetable consumption and mortality from all causes, cardiovascular disease, and cancer: systematic review and dose-response meta-analysis of prospective cohort studies. BMJ 2014; 349: g4490.
Diet low in fruits	Ischaemic stroke	Hu D, Huang J, Wang Y, Zhang D, Qu Y. Fruits and vegetables consumption and risk of stroke: a meta-analysis of prospective cohort studies. Stroke 2014; 45: 1613–9.
Diet low in fruits	Hemorrhagic stroke	Hu D, Huang J, Wang Y, Zhang D, Qu Y. Fruits and vegetables consumption and risk of stroke: a meta-analysis of prospective cohort studies. Stroke 2014; 45: 1613–9.
Diet low in fruits	Diabetes	Li M, Fan Y, Zhang X, Hou W, Tang Z. Fruit and vegetable intake and risk of type 2 diabetes mellitus: meta-analysis of prospective cohort studies. BMJ open 2014; 4(11): e005497.
Diet low in vegetables	Oesophageal cancer	Liu J, Wang J, Leng Y, Lv C. Intake of fruit and vegetables and risk of esophageal squamous cell carcinoma: a meta-analysis of observational studies. Int J Cancer 2013; 133: 473–85.
Diet low in vegetables	Ischaemic heart disease	Wang X, Ouyang Y, Liu J, et al. Fruit and vegetable consumption and mortality from all causes, cardiovascular disease, and cancer: systematic review and dose-response meta-analysis of prospective cohort studies. BMJ 2014; 349: g4490.
Diet low in vegetables	Ischaemic stroke	Hu D, Huang J, Wang Y, Zhang D, Qu Y. Fruits and vegetables consumption and risk of stroke: a meta-analysis of prospective cohort studies. Stroke 2014; 45: 1613–9.
Diet low in vegetables	Hemorrhagic stroke	Hu D, Huang J, Wang Y, Zhang D, Qu Y. Fruits and vegetables consumption and risk of stroke: a meta-analysis of prospective cohort studies. Stroke 2014; 45: 1613–9.
Diet low in whole grains	Diabetes	Aune D, Norat T, Romundstad P, Vatten LJ. Whole grain and refined grain consumption and the risk of type 2 diabetes: a systematic review and dose-response meta-analysis of cohort studies. Eur J Epidemiol 2013; 28: 845–58.
Diet low in whole grains	Ischaemic heart disease	Aune D, Keum N, Giovannucci E, et al. Whole grain consumption and risk of cardiovascular disease, cancer, and all cause and cause specific mortality: systematic review and dose-response meta-analysis of prospective studies. BMJ 2016; 353: i2716.

**Appendix Table 7. Supplemental information for Table 2: Epidemiological evidence supporting causality between risk-outcome pairs included in the Global Burden of Disease 2015 study including 7A. Citations and 7B. Additional information<br>7A. Citations**		
**Risk**	**Outcome**	**Citation/Note**
Diet low in nuts and seeds	Ischaemic heart disease	Afshin A, Micha R, Khatibzadeh S, Mozaffarian D. Consumption of nuts and legumes and risk of incident ischemic heart disease, stroke, and diabetes: a systematic review and meta-analysis. Am J Clin Nutr 2014; 100: 278–88.
Diet low in nuts and seeds	Diabetes	Afshin A, Micha R, Khatibzadeh S, Mozaffarian D. Consumption of nuts and legumes and risk of incident ischemic heart disease, stroke, and diabetes: a systematic review and meta-analysis. Am J Clin Nutr 2014; 100: 278–88.
Diet low in milk	Colon and rectum cancer	World Cancer Research Fund, American Institute for Cancer Research, Imperial College London. WCRF/AICR Systematic Literature Review Continuous Update Project Report: The Associations between Food, Nutrition and Physical Activity and the Risk of Colorectal Cancer. Oct 2010.
Diet high in red meat	Colon and rectum cancer	World Cancer Research Fund, American Institute for Cancer Research, Imperial College London. WCRF/AICR Systematic Literature Review Continuous Update Project Report: The Associations between Food, Nutrition and Physical Activity and the Risk of Colorectal Cancer. Oct 2010.
Diet high in red meat	Diabetes	Pan A, Sun Q, Bernstein AM, et al. Red meat consumption and risk of type 2 diabetes: 3 cohorts of US adults and an updated meta-analysis. Am J Clin Nutr 2011; 94: 1088–96.
Diet high in processed meat	Colon and rectum cancer	World Cancer Research Fund, American Institute for Cancer Research, Imperial College London. WCRF/AICR Systematic Literature Review Continuous Update Project Report: The Associations between Food, Nutrition and Physical Activity and the Risk of Colorectal Cancer. Oct 2010.
Diet high in processed meat	Ischaemic heart disease	Micha R, Wallace SK, Mozaffarian D. Red and processed meat consumption and risk of incident coronary heart disease, stroke, and diabetes mellitus: a systematic review and meta-analysis. Circulation 2010; 121: 2271–83.
Diet high in processed meat	Diabetes	Pan A, Sun Q, Bernstein AM, et al. Red meat consumption and risk of type 2 diabetes: 3 cohorts of US adults and an updated meta-analysis. Am J Clin Nutr 2011; 94: 1088–96.
Diet high in sugar-sweetened beverages and high body-mass index	n/a	Malik VS, Pan A, Willett WC, Hu FB. Sugar-sweetened beverages and weight gain in children and adults: a systematic review and meta-analysis. Am J Clin Nutr 2013; 98: 1084–102.
Diet low fibre	Colon and rectum cancer	World Cancer Research Fund, American Institute for Cancer Research, Imperial College London. WCRF/AICR Systematic Literature Review Continuous Update Project Report: The Associations between Food, Nutrition and Physical Activity and the Risk of Colorectal Cancer. Oct 2010.
Diet low fibre	Ischaemic heart disease	Threapleton DE, Greenwood DC, Evans CE, et al. Dietary fibre intake and risk of cardiovascular disease: systematic review and meta-analysis. BMJ (Clinical research ed) 2013; 347: f6879.
Diet low in calcium	Colon and rectum cancer	World Cancer Research Fund, American Institute for Cancer Research, Imperial College London. WCRF/AICR Systematic Literature Review Continuous Update Project Report: The Associations between Food, Nutrition and Physical Activity and the Risk of Colorectal Cancer. Oct 2010.
Diet low in seafood omega-3 fats	Ischaemic heart disease	Chowdhury R, Stevens S, Gorman D, et al. Association between fish consumption, long chain omega 3 fatty acids, and risk of cerebrovascular disease: systematic review and meta-analysis. BMJ (Clinical research ed) 2012; 345: e6698.
Diet low in polyunsaturated fats	Ischaemic heart disease	Farvid MS, Ding M, Pan A, et al. Dietary linoleic acid and risk of coronary heart disease: a systematic review and meta-analysis of prospective cohort studies. Circulation 2014; 130: 1568–78.
Diet low in polyunsaturated fats	Ischaemic heart disease	Mozaffarian D, Micha R, Wallace S. Effects on coronary heart disease of increasing polyunsaturated fat in place of saturated fat: a systematic review and meta-analysis of randomized controlled trials. PLoS Med 2010; 7: e1000252.
Diet high in trans fats	Ischaemic heart disease	Mozaffarian D, Clarke R. Quantitative effects on cardiovascular risk factors and coronary heart disease risk of replacing partially hydrogenated vegetable oils with other fats and oils. Eur J Clin Nutr. 2009; 63(Suppl 2): S22-33.
Diet high in sodium and high systolic blood pressure	n/a	Aburto NJ, Ziolkovska A, Hooper L, Elliott P, Cappuccio FP, Meerpohl JJ. Effect of lower sodium intake on health: systematic review and meta-analyses. BMJ 2013; 346: f1326.
Diet high in sodium	Stomach cancer	World Cancer Research Fund, American Institute for Cancer Research. Food, Nutrition, Physical Activity, and the Prevention of Cancer: a Global Perspective. Washington DC: AICR, 2007.
Childhood sexual abuse	Major depressive disorder	Brown J, Cohen P, Johnson JG, Smailes EM. Childhood abuse and neglect: specificity of effects on adolescent and young adult depression and suicidality. J Am Acad Child Adolesc Psychiatry 1999; 38: 1490–6.
Childhood sexual abuse	Alcohol use disorders	Dinwiddie S, Heath AC, Dunne MP, et al. Early sexual abuse and lifetime psychopathology: a co-twin-control study. Psychol Med 2000; 30: 41–52.
Childhood sexual abuse	Alcohol use disorders	Hamburger ME, Leeb RT, Swahn MH. Childhood maltreatment and early alcohol use among high-risk adolescents. J Stud Alcohol Drugs 2008; 69: 291–5.
Childhood sexual abuse	Alcohol use disorders	Kendler KS, Bulik CM, Silberg J, Hettema JM, Myers J, Prescott CA. Childhood sexual abuse and adult psychiatric and substance use disorders in women: an epidemiological and cotwin control analysis. Arch Gen Psychiatry 2000; 57: 953–9.
Childhood sexual abuse	Alcohol use disorders	MacMillan HL, Fleming JE, Streiner DL, et al. Childhood abuse and lifetime psychopathology in a community sample. Am J Psychiatry 2001; 158: 1878–83.
Childhood sexual abuse	Alcohol use disorders	Sartor CE, Lynskey MT, Bucholz KK, et al. Childhood sexual abuse and the course of alcohol dependence development: findings from a female twin sample. Drug Alcohol Depend 2007; 89: 139–44.
Childhood sexual abuse	Alcohol use disorders	Wilsnack SC, Vogeltanz ND, Klassen AD, Harris TR. Childhood sexual abuse and women's substance abuse: national survey findings. J Stud Alcohol 1997; 58: 264–71.
Childhood sexual abuse	Major depressive disorder	Ernst C, Angst J, Földényi M. The Zurich Study. XVII. Sexual abuse in childhood. Frequency and relevance for adult morbidity data of a longitudinal epidemiological study. Eur Arch Psychiatry Clin Neurosci 1993; 242: 293–300.
Childhood sexual abuse	Major depressive disorder	Fergusson DM, Boden JM, Horwood LJ. Exposure to childhood sexual and physical abuse and adjustment in early adulthood. Child Abuse Negl 2008; 32: 607–19.
Childhood sexual abuse	Major depressive disorder	Jaffee SR, Moffitt TE, Caspi A, Fombonne E, Poulton R, Martin J. Differences in early childhood risk factors for juvenile-onset and adult-onset depression. Arch Gen Psychiatry 2002; 59: 215–22.
Childhood sexual abuse	Major depressive disorder	Kendler KS, Karkowski LM, Prescott CA. Causal relationship between stressful life events and the onset of major depression. Am J Psychiatry 1999; 156: 837–41.

Appendix Table 7. Supplemental information for Table 2: Epidemiological evidence supporting causality between risk-outcome pairs included in the Global Burden of Disease 2015 study including 7A. Citations and 7B. Additional information

7A. Citations

Risk	Outcome	Citation/Note
Childhood sexual abuse	Self-harm	Devries KM, Mak JYT, Child JC, et al. Childhood sexual abuse and suicidal behavior: a meta-analysis. Pediatrics 2014; 133: e1331-1344.
Intimate partner violence	HIV/AIDS	Jewkes RK, Dunkle K, Nduna M, Shai N. Intimate partner violence, relationship power inequity, and incidence of HIV infection in young women in South Africa: a cohort study. Lancet 2010; 376: 41–8.
Intimate partner violence	HIV/AIDS	Kouyoumdjian FG, Calzavara LM, Bondy SJ, et al. Intimate partner violence is associated with incident HIV infection in women in Uganda. AIDS 2013; 27: 1331–8.
Intimate partner violence	Major depressive disorder	Devries KM, Mak JY, Bacchus LJ, et al. Intimate partner violence and incident depressive symptoms and suicide attempts: a systematic review of longitudinal studies. PLoS Med 2013; 10: e1001439.
Intimate partner violence	Maternal abortion, miscarriage, and ectopic pregnancy	Bourassa D, Bérubé J. The prevalence of intimate partner violence among women and teenagers seeking abortion compared with those continuing pregnancy. J Obstet Gynaecol Can 2007; 29: 415–23.
Intimate partner violence	Maternal abortion, miscarriage, and ectopic pregnancy	Leung TW, Leung WC, Chan PL, Ho PC. A comparison of the prevalence of domestic violence between patients seeking termination of pregnancy and other general gynecology patients. Int J Gynaecol Obstet 2002; 77: 47–54.
Intimate partner violence	Maternal abortion, miscarriage, and ectopic pregnancy	Romito P, Escribà-Agüir V, Pomicino L, Lucchetta C, Scrimin F, Molzan Turan J. Violence in the lives of women in Italy who have an elective abortion. Womens Health Issues 2009; 19: 335–43.
Intimate partner violence	Maternal abortion, miscarriage, and ectopic pregnancy	Taft AJ, Watson LF. Termination of pregnancy: associations with partner violence and other factors in a national cohort of young Australian women. Aust N Z J Public Health 2007; 31: 135–42.
Intimate partner violence	Self-harm	Devries KM, Mak JY, Bacchus LJ, et al. Intimate partner violence and incident depressive symptoms and suicide attempts: a systematic review of longitudinal studies. PLoS Med 2013; 10: e1001439.
Low physical activity	Colon and rectum cancer	Bostick RM, Potter JD, Kushi LH, et al. Sugar, meat, and fat intake, and non-dietary risk factors for colon cancer incidence in Iowa women (United States). Cancer Causes Control 1994; 5: 38–52.
Low physical activity	Colon and rectum cancer	Calton BA, Lacey JV, Schatzkin A, et al. Physical activity and the risk of colon cancer among women: a prospective cohort study (United States). Int J Cancer 2006; 119: 385–91.
Low physical activity	Colon and rectum cancer	Chao A, Connell CJ, Jacobs EJ, et al. Amount, type, and timing of recreational physical activity in relation to colon and rectal cancer in older adults: the Cancer Prevention Study II Nutrition Cohort. Cancer Epidemiol Biomarkers Prev 2004; 13: 2187–95.
Low physical activity	Colon and rectum cancer	Colbert LH, Hartman TJ, Malila N, et al. Physical activity in relation to cancer of the colon and rectum in a cohort of male smokers. Cancer Epidemiol Biomarkers Prev 2001; 10: 265–8.
Low physical activity	Colon and rectum cancer	Fraser G, Pearce N. Occupational physical activity and risk of cancer of the colon and rectum in New Zealand males. Cancer Causes Control 1993; 4: 45–50.
Low physical activity	Colon and rectum cancer	Friedenreich C, Norat T, Steindorf K, et al. Physical activity and risk of colon and rectal cancers: the European prospective investigation into cancer and nutrition. Cancer Epidemiol Biomarkers Prev 2006; 15: 2398–407.
Low physical activity	Colon and rectum cancer	Garabrant DH, Peters JM, Mack TM, Bernstein L. Job activity and colon cancer risk. Am J Epidemiol 1984; 119: 1005–14.
Low physical activity	Colon and rectum cancer	Gerhardsson M, Norell SE, Kiviranta H, Pedersen NL, Ahlbom A. Sedentary jobs and colon cancer. Am J Epidemiol 1986; 123: 775–80.
Low physical activity	Colon and rectum cancer	Giovannucci E, Ascherio A, Rimm EB, Colditz GA, Stampfer MJ, Willett WC. Physical activity, obesity, and risk for colon cancer and adenoma in men. Ann Intern Med 1995; 122: 327–34.
Low physical activity	Colon and rectum cancer	Howard RA, Freedman DM, Park Y, Hollenbeck A, Schatzkin A, Leitzmann MF. Physical activity, sedentary behavior, and the risk of colon and rectal cancer in the NIH-AARP Diet and Health Study. Cancer Causes Control 2008; 19: 939–53.
Low physical activity	Colon and rectum cancer	Larsson SC, Rutegård J, Bergkvist L, Wolk A. Physical activity, obesity, and risk of colon and rectal cancer in a cohort of Swedish men. Eur J Cancer 2006; 42: 2590–7.
Low physical activity	Colon and rectum cancer	Lee IM, Manson JE, Ajani U, Paffenbarger RS, Hennekens CH, Buring JE. Physical activity and risk of colon cancer: the Physicians' Health Study (United States). Cancer Causes Control 1997; 8: 568–74.
Low physical activity	Colon and rectum cancer	Lee IM, Paffenbarger RS. Physical activity and its relation to cancer risk: a prospective study of college alumni. Med Sci Sports Exerc 1994; 26: 831–7.
Low physical activity	Colon and rectum cancer	Lee K-J, Inoue M, Otani T, et al. Physical activity and risk of colorectal cancer in Japanese men and women: the Japan Public Health Center-based prospective study. Cancer Causes Control 2007; 18: 199–209.
Low physical activity	Colon and rectum cancer	Mai PL, Sullivan-Halley J, Ursin G, et al. Physical activity and colon cancer risk among women in the California Teachers Study. Cancer Epidemiol Biomarkers Prev 2007; 16: 517–25.
Low physical activity	Colon and rectum cancer	Moradi T, Gridley G, Björk J, et al. Occupational physical activity and risk for cancer of the colon and rectum in Sweden among men and women by anatomic subsite. Eur J Cancer Prev 2008; 17: 201–8.
Low physical activity	Colon and rectum cancer	Nilsen TIL, Romundstad PR, Petersen H, Gunnell D, Vatten LJ. Recreational physical activity and cancer risk in subsites of the colon (the Nord-Trøndelag Health Study). Cancer Epidemiol Biomarkers Prev 2008; 17: 183–8.
Low physical activity	Colon and rectum cancer	Severson RK, Nomura AM, Grove JS, Stemmermann GN. A prospective analysis of physical activity and cancer. Am J Epidemiol 1989; 130: 522–9.
Low physical activity	Colon and rectum cancer	Thune I, Lund E. Physical activity and risk of colorectal cancer in men and women. Br J Cancer 1996; 73: 1134–40.
Low physical activity	Colon and rectum cancer	Wolin KY, Lee I-M, Colditz GA, Glynn RJ, Fuchs C, Giovannucci E. Leisure-time physical activity patterns and risk of colon cancer in women. Int J Cancer 2007; 121: 2776–81.
Low physical activity	Breast cancer	Bardia A, Hartmann LC, Vachon CM, et al. Recreational physical activity and risk of postmenopausal breast cancer based on hormone receptor status. Arch Intern Med 2006; 166: 2478–83.
Low physical activity	Breast cancer	Borch KB, Lund E, Braaten T, Weiderpass E. Physical activity and the risk of postmenopausal breast cancer - the Norwegian Women and Cancer Study. J Negat Results Biomed 2014; 13: 3.
Low physical activity	Breast cancer	Breslow RA, Ballard-Barbash R, Munoz K, Graubard BI. Long-term recreational physical activity and breast cancer in the National Health and Nutrition Examination Survey I epidemiologic follow-up study. Cancer Epidemiol Biomarkers Prev 2001; 10: 805–8.
Low physical activity	Breast cancer	Cerhan JR, Chiu BC, Wallace RB, et al. Physical activity, physical function, and the risk of breast cancer in a prospective study among elderly women. J Gerontol A Biol Sci Med Sci 1998; 53: M251-256.

Appendix Table 7. Supplemental information for Table 2: Epidemiological evidence supporting causality between risk-outcome pairs included in the Global Burden of Disease 2015 study including 7A. Citations and 7B. Additional information<br>7A. Citations		
**Risk**	**Outcome**	**Citation/Note**
Low physical activity	Breast cancer	Chang S-C, Ziegler RG, Dunn B, et al. Association of energy intake and energy balance with postmenopausal breast cancer in the prostate, lung, colorectal, and ovarian cancer screening trial. Cancer Epidemiol Biomarkers Prev 2006; 15: 334–41.
Low physical activity	Breast cancer	Colditz GA, Feskanich D, Chen WY, Hunter DJ, Willett WC. Physical activity and risk of breast cancer in premenopausal women. Br J Cancer 2003; 89: 847–51.
Low physical activity	Breast cancer	Dallal CM, Sullivan-Halley J, Ross RK, et al. Long-term recreational physical activity and risk of invasive and in situ breast cancer: the California teachers study. Arch Intern Med 2007; 167: 408–15.
Low physical activity	Breast cancer	Dorgan JF, Brown C, Barrett M, et al. Physical activity and risk of breast cancer in the Framingham Heart Study. Am J Epidemiol 1994; 139: 662–9.
Low physical activity	Breast cancer	Eliassen AH, Hankinson SE, Rosner B, Holmes MD, Willett WC. Physical activity and risk of breast cancer among postmenopausal women. Arch Intern Med 2010; 170: 1758–64.
Low physical activity	Breast cancer	Frisch RE, Wyshak G, Witschi J, Albright NL, Albright TE, Schiff I. Lower lifetime occurrence of breast cancer and cancers of the reproductive system among former college athletes. Int J Fertil 1987; 32: 217–25.
Low physical activity	Breast cancer	Hastert TA, Beresford SAA, Patterson RE, Kristal AR, White E. Adherence to WCRF/AICR cancer prevention recommendations and risk of postmenopausal breast cancer. Cancer Epidemiol Biomarkers Prev 2013; 22: 1498–508.
Low physical activity	Breast cancer	Hildebrand JS, Gapstur SM, Campbell PT, Gaudet MM, Patel AV. Recreational physical activity and leisure-time sitting in relation to postmenopausal breast cancer risk. Cancer Epidemiol Biomarkers Prev 2013; 22: 1906–12.
Low physical activity	Breast cancer	Howard RA, Leitzmann MF, Linet MS, Freedman DM. Physical activity and breast cancer risk among pre- and postmenopausal women in the U.S. Radiologic Technologists cohort. Cancer Causes Control 2009; 20: 323–33.
Low physical activity	Breast cancer	Leitzmann MF, Moore SC, Peters TM, et al. Prospective study of physical activity and risk of postmenopausal breast cancer. Breast Cancer Res 2008; 10: R92.
Low physical activity	Breast cancer	Luoto R, Latikka P, Pukkala E, Hakulinen T, Vihko V. The effect of physical activity on breast cancer risk: a cohort study of 30,548 women. Eur J Epidemiol 2000; 16: 973–80.
Low physical activity	Breast cancer	Margolis KL, Mucci L, Braaten T, et al. Physical activity in different periods of life and the risk of breast cancer: the Norwegian-Swedish Women's Lifestyle and Health cohort study. Cancer Epidemiol Biomarkers Prev 2005; 14: 27–32.
Low physical activity	Breast cancer	Mertens AJ, Sweeney C, Shahar E, Rosamond WD, Folsom AR. Physical activity and breast cancer incidence in middle-aged women: a prospective cohort study. Breast Cancer Res Treat 2006; 97: 209–14.
Low physical activity	Breast cancer	Ministry of Health (Benin), National Institute of Statistics and Economic Analysis (INSAE) (Benin). Benin Health Statistical Yearbook 2005. Porto-Novo, Benin: Ministry of Health (Benin), 2006.
Low physical activity	Breast cancer	Ministry of Health (Burkina Faso). Burkina Faso Health Statistical Yearbook 2007. Ouagadougou, Burkina Faso: Ministry of Health (Burkina Faso), 2008.
Low physical activity	Breast cancer	Ministry of Health (Burkina Faso). Burkina Faso Health Statistical Yearbook 2008. Ouagadougou, Burkina Faso: Ministry of Health (Burkina Faso), 2009.
Low physical activity	Breast cancer	Moradi T, Adami HO, Bergström R, et al. Occupational physical activity and risk for breast cancer in a nationwide cohort study in Sweden. Cancer Causes Control 1999; 10: 423–30.
Low physical activity	Breast cancer	Moradi T, Adami H-O, Ekbom A, et al. Physical activity and risk for breast cancer a prospective cohort study among Swedish twins. Int J Cancer 2002; 100: 76–81.
Low physical activity	Breast cancer	Peters TM, Schatzkin A, Gierach GL, et al. Physical activity and postmenopausal breast cancer risk in the NIH-AARP diet and health study. Cancer Epidemiol Biomarkers Prev 2009; 18: 289–96.
Low physical activity	Breast cancer	Pronk A, Ji B-T, Shu X-O, et al. Physical activity and breast cancer risk in Chinese women. Br J Cancer 2011; 105: 1443–50.
Low physical activity	Breast cancer	Rintala by PE, Pukkala E, Paakkulainen HT, Vihko VJ. Self-experienced physical workload and risk of breast cancer. Scand J Work Environ Health 2002; 28: 158–62.
Low physical activity	Breast cancer	Rockhill B, Willett WC, Hunter DJ, Manson JE, Hankinson SE, Colditz GA. A prospective study of recreational physical activity and breast cancer risk. Arch Intern Med 1999; 159: 2290–6.
Low physical activity	Breast cancer	Rosenberg L, Palmer JR, Bethea TN, Ban Y, Kipping-Ruane K, Adams-Campbell LL. A prospective study of physical activity and breast cancer incidence in African-American women. Cancer Epidemiol Biomarkers Prev 2014; 23: 2522–31.
Low physical activity	Breast cancer	Sesso HD, Paffenbarger RS, Lee IM. Physical activity and breast cancer risk in the College Alumni Health Study (United States). Cancer Causes Control 1998; 9: 433–9.
Low physical activity	Breast cancer	Silvera SAN, Jain M, Howe GR, Miller AB, Rohan TE. Energy balance and breast cancer risk: a prospective cohort study. Breast Cancer Res Treat 2006; 97: 97–106.
Low physical activity	Breast cancer	Suzuki R, Iwasaki M, Yamamoto S, et al. Leisure-time physical activity and breast cancer risk defined by estrogen and progesterone receptor status--the Japan Public Health Center-based Prospective Study. Prev Med 2011; 52: 227–33.
Low physical activity	Breast cancer	Suzuki S, Kojima M, Tokudome S, et al. Effect of physical activity on breast cancer risk: findings of the Japan collaborative cohort study. Cancer Epidemiol Biomarkers Prev 2008; 17: 3396–401.
Low physical activity	Breast cancer	Thune I, Brenn T, Lund E, Gaard M. Physical activity and the risk of breast cancer. N Engl J Med 1997; 336: 1269–75.
Low physical activity	Breast cancer	Wyrwich KW, Wolinsky FD. Physical activity, disability, and the risk of hospitalization for breast cancer among older women. J Gerontol A Biol Sci Med Sci 2000; 55: M418-421.
Low physical activity	Breast cancer	Wyshak G, Frisch RE. Breast cancer among former college athletes compared to non-athletes: a 15-year follow-up. Br J Cancer 2000; 82: 726–30.
Low physical activity	Ischaemic stroke	Abbott RD, Rodriguez BL, Burchfiel CM, Curb JD. Physical activity in older middle-aged men and reduced risk of stroke: the Honolulu Heart Program. Am J Epidemiol 1994; 139: 881–93.
Low physical activity	Ischaemic stroke	Agnarsson U, Thorgeirsson G, Sigvaldason H, Sigfusson N. Effects of leisure-time physical activity and ventilatory function on risk for stroke in men: the Reykjavík Study. Ann Intern Med 1999; 130: 987–90.

Appendix Table 7. Supplemental information for Table 2: Epidemiological evidence supporting causality between risk-outcome pairs included in the Global Burden of Disease 2015 study including 7A. Citations and 7B. Additional information

7A. Citations

Risk	Outcome	Citation/Note
Low physical activity	Ischaemic stroke	Autenrieth CS, Evenson KR, Yatsuya H, Shahar E, Baggett C, Rosamond WD. Association between physical activity and risk of stroke subtypes: the atherosclerosis risk in communities study. Neuroepidemiology 2013; 40: 109–16.
Low physical activity	Ischaemic stroke	Bijnen FC, Caspersen CJ, Feskens EJ, Saris WH, Mosterd WL, Kromhout D. Physical activity and 10-year mortality from cardiovascular diseases and all causes: The Zutphen Elderly Study. Arch Intern Med 1998; 158: 1499–505.
Low physical activity	Ischaemic stroke	Calling S, Hedblad B, Engström G, Berglund G, Janzon L. Effects of body fatness and physical activity on cardiovascular risk: risk prediction using the bioelectrical impedance method. Scand J Public Health 2006; 34: 568–75.
Low physical activity	Ischaemic stroke	Chiuve SE, Rexrode KM, Spiegelman D, Logroscino G, Manson JE, Rimm EB. Primary prevention of stroke by healthy lifestyle. Circulation 2008; 118: 947–54.
Low physical activity	Ischaemic stroke	Ellekjaer H, Holmen J, Ellekjaer E, Vatten L. Physical activity and stroke mortality in women. Ten-year follow-up of the Nord-Trondelag health survey, 1984-1986. Stroke 2000; 31: 14–8.
Low physical activity	Ischaemic stroke	Gulsvik AK, Thelle DS, Samuelsen SO, Myrstad M, Mowé M, Wyller TB. Ageing, physical activity and mortality--a 42-year follow-up study. Int J Epidemiol 2012; 41: 521–30.
Low physical activity	Ischaemic stroke	Håheim LL, Holme I, Hjermann I, Leren P. Risk factors of stroke incidence and mortality. A 12-year follow-up of the Oslo Study. Stroke 1993; 24: 1484–9.
Low physical activity	Ischaemic stroke	Hu FB, Stampfer MJ, Colditz GA, et al. Physical activity and risk of stroke in women. JAMA 2000; 283: 2961–7.
Low physical activity	Ischaemic stroke	Hu G, Sarti C, Jousilahti P, Silventoinen K, Barengo NC, Tuomilehto J. Leisure time, occupational, and commuting physical activity and the risk of stroke. Stroke 2005; 36: 1994–9.
Low physical activity	Ischaemic stroke	Lapidus L, Bengtsson C. Socioeconomic factors and physical activity in relation to cardiovascular disease and death. A 12 year follow up of participants in a population study of women in Gothenburg, Sweden. Br Heart J 1986; 55: 295–301.
Low physical activity	Ischaemic stroke	Lee IM, Hennekens CH, Berger K, Buring JE, Manson JE. Exercise and risk of stroke in male physicians. Stroke 1999; 30: 1–6.
Low physical activity	Ischaemic stroke	Lee IM, Paffenbarger RS. Physical activity and stroke incidence: the Harvard Alumni Health Study. Stroke 1998; 29: 2049–54.
Low physical activity	Ischaemic stroke	Lindenstrøm E, Boysen G, Nyboe J. Lifestyle factors and risk of cerebrovascular disease in women. The Copenhagen City Heart Study. Stroke 1993; 24: 1468–72.
Low physical activity	Ischaemic stroke	Myint PK, Luben RN, Wareham NJ, et al. Combined work and leisure physical activity and risk of stroke in men and women in the European prospective investigation into Cancer-Norfolk Prospective Population Study. Neuroepidemiology 2006; 27: 122–9.
Low physical activity	Ischaemic stroke	Okada H, Horibe H, Yoshiyuki O, Hayakawa N, Aoki N. A prospective study of cerebrovascular disease in Japanese rural communities, Akabane and Asahi. Part 1: evaluation of risk factors in the occurrence of cerebral hemorrhage and thrombosis. Stroke 1976; 7: 599–607.
Low physical activity	Ischaemic stroke	Paffenbarger RS, Brand RJ, Sholtz RI, Jung DL. Energy expenditure, cigarette smoking, and blood pressure level as related to death from specific diseases. Am J Epidemiol 1978; 108: 12–8.
Low physical activity	Ischaemic stroke	Paganini-Hill A, Perez Barreto M. Stroke risk in older men and women: aspirin, estrogen, exercise, vitamins, and other factors. J Gend Specif Med 2001; 4: 18–28.
Low physical activity	Ischaemic stroke	Salonen JT, Puska P, Tuomilehto J. Physical activity and risk of myocardial infarction, cerebral stroke and death: a longitudinal study in Eastern Finland. Am J Epidemiol 1982; 115: 526–37.
Low physical activity	Ischaemic stroke	Sattelmair JR, Kurth T, Buring JE, Lee I-M. Physical activity and risk of stroke in women. Stroke 2010; 41: 1243–50.
Low physical activity	Ischaemic stroke	Simonsick EM, Lafferty ME, Phillips CL, et al. Risk due to inactivity in physically capable older adults. Am J Public Health 1993; 83: 1443–50.
Low physical activity	Ischaemic stroke	Wannamethee G, Shaper AG. Physical activity and stroke in British middle aged men. BMJ 1992; 304: 597–601.
Low physical activity	Ischaemic stroke	Willey JZ, Moon YP, Paik MC, Boden-Albala B, Sacco RL, Elkind MSV. Physical activity and risk of ischemic stroke in the Northern Manhattan Study. Neurology 2009; 73: 1774–9.
Low physical activity	Ischaemic stroke	Zhang Q, Zhou Y, Gao X, et al. Ideal cardiovascular health metrics and the risks of ischemic and intracerebral hemorrhagic stroke. Stroke 2013; 44: 2451–6.
Low physical activity	Diabetes mellitus	Baan CA, Stolk RP, Grobbee DE, Witteman JC, Feskens EJ. Physical activity in elderly subjects with impaired glucose tolerance and newly diagnosed diabetes mellitus. Am J Epidemiol 1999; 149: 219–27.
Low physical activity	Diabetes mellitus	Bonora E, Kiechl S, Willeit J, et al. Population-based incidence rates and risk factors for type 2 diabetes in white individuals: the Bruneck study. Diabetes 2004; 53: 1782–9.
Low physical activity	Diabetes mellitus	Burchfiel CM, Sharp DS, Curb JD, et al. Physical activity and incidence of diabetes: the Honolulu Heart Program. Am J Epidemiol 1995; 141: 360–8.
Low physical activity	Diabetes mellitus	Carlsson S, Ahlbom A, Lichtenstein P, Andersson T. Shared genetic influence of BMI, physical activity and type 2 diabetes: a twin study. Diabetologia 2013; 56: 1031–5.
Low physical activity	Diabetes mellitus	Carlsson S, Midthjell K, Tesfamarian MY, Grill V. Age, overweight and physical inactivity increase the risk of latent autoimmune diabetes in adults: results from the Nord-Trøndelag health study. Diabetologia 2007; 50: 55–8.
Low physical activity	Diabetes mellitus	Chien K-L, Chen M-F, Hsu H-C, Su T-C, Lee Y-T. Sports activity and risk of type 2 diabetes in Chinese. Diabetes Res Clin Pract 2009; 84: 311–8.
Low physical activity	Diabetes mellitus	Demakakos P, Hamer M, Stamatakis E, Steptoe A. Low-intensity physical activity is associated with reduced risk of incident type 2 diabetes in older adults: evidence from the English Longitudinal Study of Ageing. Diabetologia 2010; 53: 1877–85.
Low physical activity	Diabetes mellitus	Doi Y, Ninomiya T, Hata J, et al. Two risk score models for predicting incident Type 2 diabetes in Japan. Diabet Med 2012; 29: 107–14.
Low physical activity	Diabetes mellitus	Dotevall A, Johansson S, Wilhelmsen L, Rosengren A. Increased levels of triglycerides, BMI and blood pressure and low physical activity increase the risk of diabetes in Swedish women. A prospective 18-year follow-up of the BEDA study. Diabet Med 2004; 21: 615–22.

**Appendix Table 7. Supplemental information for Table 2: Epidemiological evidence supporting causality between risk-outcome pairs included in the Global Burden of Disease 2015 study including 7A. Citations and 7B. Additional information**<br>**7A. Citations**		
**Risk**	**Outcome**	**Citation/Note**
Low physical activity	Diabetes mellitus	Elwood P, Galante J, Pickering J, et al. Healthy lifestyles reduce the incidence of chronic diseases and dementia: evidence from the Caerphilly cohort study. PLoS ONE 2013; 8: e81877.
Low physical activity	Diabetes mellitus	Fan S, Chen J, Huang J, et al. Physical activity level and incident type 2 diabetes among Chinese adults. Med Sci Sports Exerc 2015; 47: 751–6.
Low physical activity	Diabetes mellitus	Folsom AR, Kushi LH, Hong CP. Physical activity and incident diabetes mellitus in postmenopausal women. Am J Public Health 2000; 90: 134–8.
Low physical activity	Diabetes mellitus	Fretts AM, Howard BV, Kriska AM, et al. Physical activity and incident diabetes in American Indians: the Strong Heart Study. Am J Epidemiol 2009; 170: 632–9.
Low physical activity	Diabetes mellitus	Grøntved A, Pan A, Mekary RA, et al. Muscle-strengthening and conditioning activities and risk of type 2 diabetes: a prospective study in two cohorts of US women. PLoS Med 2014; 11: e1001587.
Low physical activity	Diabetes mellitus	Gurwitz JH, Field TS, Glynn RJ, et al. Risk factors for non-insulin-dependent diabetes mellitus requiring treatment in the elderly. J Am Geriatr Soc 1994; 42: 1235–40.
Low physical activity	Diabetes mellitus	Haapanen N, Miilunpalo S, Vuori I, Oja P, Pasanen M. Association of leisure time physical activity with the risk of coronary heart disease, hypertension and diabetes in middle-aged men and women. Int J Epidemiol 1997; 26: 739–47.
Low physical activity	Diabetes mellitus	Helmrich SP, Ragland DR, Paffenbarger RS. Prevention of non-insulin-dependent diabetes mellitus with physical activity. Med Sci Sports Exerc 1994; 26: 824–30.
Low physical activity	Diabetes mellitus	Holme I, Tonstad S, Sogaard AJ, Larsen PGL, Haheim LL. Leisure time physical activity in middle age predicts the metabolic syndrome in old age: results of a 28-year follow-up of men in the Oslo study. BMC Public Health 2007; 7: 154.
Low physical activity	Diabetes mellitus	Hsia J, Wu L, Allen C, et al. Physical activity and diabetes risk in postmenopausal women. Am J Prev Med 2005; 28: 19–25.
Low physical activity	Diabetes mellitus	Hu FB, Leitzmann MF, Stampfer MJ, Colditz GA, Willett WC, Rimm EB. Physical activity and television watching in relation to risk for type 2 diabetes mellitus in men. Arch Intern Med 2001; 161: 1542–8.
Low physical activity	Diabetes mellitus	Hu FB, Sigal RJ, Rich-Edwards JW, et al. Walking compared with vigorous physical activity and risk of type 2 diabetes in women: a prospective study. JAMA 1999; 282: 1433–9.
Low physical activity	Diabetes mellitus	Hu G, Qiao Q, Silventoinen K, et al. Occupational, commuting, and leisure-time physical activity in relation to risk for Type 2 diabetes in middle-aged Finnish men and women. Diabetologia 2003; 46: 322–9.
Low physical activity	Diabetes mellitus	James SA, Jamjoum L, Raghunathan TE, Strogatz DS, Furth ED, Khazanie PG. Physical activity and NIDDM in African-Americans. The Pitt County Study. Diabetes Care 1998; 21: 555–62.
Low physical activity	Diabetes mellitus	Jefferis BJ, Whincup PH, Lennon L, Wannamethee SG. Longitudinal associations between changes in physical activity and onset of type 2 diabetes in older British men: the influence of adiposity. Diabetes Care 2012; 35: 1876–83.
Low physical activity	Diabetes mellitus	Joseph J, Svartberg J, Njølstad I, Schirmer H. Incidence of and risk factors for type-2 diabetes in a general population: the Tromsø Study. Scand J Public Health 2010; 38: 768–75.
Low physical activity	Diabetes mellitus	Koloverou E, Panagiotakos DB, Pitsavos C, et al. 10-year incidence of diabetes and associated risk factors in Greece: the ATTICA study (2002-2012). Rev Diabet Stud 2014; 11: 181–9.
Low physical activity	Diabetes mellitus	Krishnan S, Rosenberg L, Palmer JR. Physical activity and television watching in relation to risk of type 2 diabetes: the Black Women's Health Study. Am J Epidemiol 2009; 169: 428–34.
Low physical activity	Diabetes mellitus	Laaksonen MA, Knekt P, Rissanen H, et al. The relative importance of modifiable potential risk factors of type 2 diabetes: a meta-analysis of two cohorts. Eur J Epidemiol 2010; 25: 115–24.
Low physical activity	Diabetes mellitus	Lee D, Park I, Jun T-W, et al. Physical activity and body mass index and their associations with the development of type 2 diabetes in korean men. Am J Epidemiol 2012; 176: 43–51.
Low physical activity	Diabetes mellitus	Longo-Mbenza B, On'kin JBKL, Okwe AN, Kabangu NK, Fuele SM. Metabolic syndrome, aging, physical inactivity, and incidence of type 2 diabetes in general African population. Diab Vasc Dis Res 2010; 7: 28–39.
Low physical activity	Diabetes mellitus	Lucke J, Waters B, Hockey R, et al. Trends in women's risk factors and chronic conditions: findings from the Australian Longitudinal Study on Women's Health. Womens Health (Lond) 2007; 3: 423–32.
Low physical activity	Diabetes mellitus	Magliano DJ, Barr ELM, Zimmet PZ, et al. Glucose indices, health behaviors, and incidence of diabetes in Australia: the Australian Diabetes, Obesity and Lifestyle Study. Diabetes Care 2008; 31: 267–72.
Low physical activity	Diabetes mellitus	Manson JE, Nathan DM, Krolewski AS, Stampfer MJ, Willett WC, Hennekens CH. A prospective study of exercise and incidence of diabetes among US male physicians. JAMA 1992; 268: 63–7.
Low physical activity	Diabetes mellitus	Manson JE, Rimm EB, Stampfer MJ, et al. Physical activity and incidence of non-insulin-dependent diabetes mellitus in women. Lancet 1991; 338: 774–8.
Low physical activity	Diabetes mellitus	Meisinger C, Löwel H, Thorand B, Döring A. Leisure time physical activity and the risk of type 2 diabetes in men and women from the general population. The MONICA/KORA Augsburg Cohort Study. Diabetologia 2005; 48: 27–34.
Low physical activity	Diabetes mellitus	Mozaffarian D, Kamineni A, Carnethon M, Djoussé L, Mukamal KJ, Siscovick D. Lifestyle risk factors and new-onset diabetes mellitus in older adults: the cardiovascular health study. Arch Intern Med 2009; 169: 798–807.
Low physical activity	Diabetes mellitus	Okada K, Hayashi T, Tsumura K, Suematsu C, Endo G, Fujii S. Leisure-time physical activity at weekends and the risk of Type 2 diabetes mellitus in Japanese men: the Osaka Health Survey. Diabet Med 2000; 17: 53–8.
Low physical activity	Diabetes mellitus	Panagiotakos DB, Pitsavos C, Skoumas Y, Lentzas Y, Stefanadis C. Five-year incidence of type 2 diabetes mellitus among cardiovascular disease-free Greek adults: findings from the ATTICA study. Vasc Health Risk Manag 2008; 4: 691–8.
Low physical activity	Diabetes mellitus	Rathmann W, Strassburger K, Heier M, et al. Incidence of Type 2 diabetes in the elderly German population and the effect of clinical and lifestyle risk factors: KORA S4/F4 cohort study. Diabet Med 2009; 26: 1212–9.
Low physical activity	Diabetes mellitus	Reis JP, Loria CM, Sorlie PD, Park Y, Hollenbeck A, Schatzkin A. Lifestyle factors and risk for new-onset diabetes: a population-based cohort study. Ann Intern Med 2011; 155: 292–9.

Appendix Table 7. Supplemental information for Table 2: Epidemiological evidence supporting causality between risk-outcome pairs included in the Global Burden of Disease 2015 study including 7A. Citations and 7B. Additional information

7A. Citations

Risk	Outcome	Citation/Note
Low physical activity	Diabetes mellitus	Shi L, Shu X-O, Li H, et al. Physical activity, smoking, and alcohol consumption in association with incidence of type 2 diabetes among middle-aged and elderly Chinese men. PLoS ONE 2013; 8: e77919.
Low physical activity	Diabetes mellitus	Siegel LC, Sesso HD, Bowman TS, Lee I-M, Manson JE, Gaziano JM. Physical activity, body mass index, and diabetes risk in men: a prospective study. Am J Med 2009; 122: 1115–21.
Low physical activity	Diabetes mellitus	Simonsick EM, Lafferty ME, Phillips CL, et al. Risk due to inactivity in physically capable older adults. Am J Public Health 1993; 83: 1443–50.
Low physical activity	Diabetes mellitus	Steinbrecher A, Erber E, Grandinetti A, Nigg C, Kolonel LN, Maskarinec G. Physical activity and risk of type 2 diabetes among Native Hawaiians, Japanese Americans, and Caucasians: the Multiethnic Cohort. J Phys Act Health 2012; 9: 634–41.
Low physical activity	Diabetes mellitus	Stringhini S, Tabak AG, Akbaraly TN, et al. Contribution of modifiable risk factors to social inequalities in type 2 diabetes: prospective Whitehall II cohort study. BMJ 2012; 345: e5452.
Low physical activity	Diabetes mellitus	Sun F, Tao Q, Zhan S. An accurate risk score for estimation 5-year risk of type 2 diabetes based on a health screening population in Taiwan. Diabetes Res Clin Pract 2009; 85: 228–34.
Low physical activity	Diabetes mellitus	Tsai AC, Lee S-H. Determinants of new-onset diabetes in older adults—Results of a national cohort study. Clin Nutr 2015; 34: 937–42.
Low physical activity	Diabetes mellitus	Villegas R, Shu X-O, Li H, et al. Physical activity and the incidence of type 2 diabetes in the Shanghai women's health study. Int J Epidemiol 2006; 35: 1553–62.
Low physical activity	Diabetes mellitus	Waki K, Noda M, Sasaki S, et al. Alcohol consumption and other risk factors for self-reported diabetes among middle-aged Japanese: a population-based prospective study in the JPHC study cohort I. Diabet Med 2005; 22: 323–31.
Low physical activity	Diabetes mellitus	Waller K, Kaprio J, Lehtovirta M, Silventoinen K, Koskenvuo M, Kujala UM. Leisure-time physical activity and type 2 diabetes during a 28 year follow-up in twins. Diabetologia 2010; 53: 2531–7.
Low physical activity	Diabetes mellitus	Wannamethee SG, Shaper AG, Alberti KG. Physical activity, metabolic factors, and the incidence of coronary heart disease and type 2 diabetes. Arch Intern Med 2000; 160: 2108–16.
Low physical activity	Diabetes mellitus	Weinstein AR, Sesso HD, Lee IM, et al. Relationship of physical activity vs body mass index with type 2 diabetes in women. JAMA 2004; 292: 1188–94.
Low physical activity	Diabetes mellitus	Williams PT, Thompson PD. Walking versus running for hypertension, cholesterol, and diabetes mellitus risk reduction. Arterioscler Thromb Vasc Biol 2013; 33: 1085–91.
Low physical activity	Diabetes mellitus	Xu F, Ware RS, Tse LA, et al. Joint associations of physical activity and hypertension with the development of type 2 diabetes among urban men and women in Mainland China. PLoS ONE 2014; 9: e88719.
High fasting plasma glucose	Ischaemic heart disease	Boussageon R, Bejan-Angoulvant T, Saadatian-Elahi M, et al. Effect of intensive glucose lowering treatment on all cause mortality, cardiovascular death, and microvascular events in type 2 diabetes: meta-analysis of randomised controlled trials. BMJ 2011; 343: d4169.
High fasting plasma glucose	Ischaemic heart disease	Singh GM, Danaei G, Farzadfar F, et al. The age-specific quantitative effects of metabolic risk factors on cardiovascular diseases and diabetes: a pooled analysis. PLoS ONE 2013; 8: e65174.
High fasting plasma glucose	Ischaemic stroke	Singh GM, Danaei G, Farzadfar F, et al. The age-specific quantitative effects of metabolic risk factors on cardiovascular diseases and diabetes: a pooled analysis. PLoS ONE 2013; 8: e65174.
High fasting plasma glucose	Ischaemic stroke	Zhang C, Zhou Y-H, Xu C-L, Chi F-L, Ju H-N. Efficacy of intensive control of glucose in stroke prevention: a meta-analysis of data from 59,197 participants in 9 randomized controlled trials. PloS One 2013; 8: e54465.
High fasting plasma glucose	Hemorrhagic stroke	Singh GM, Danaei G, Farzadfar F, et al. The age-specific quantitative effects of metabolic risk factors on cardiovascular diseases and diabetes: a pooled analysis. PLoS ONE 2013; 8: e65174.
High fasting plasma glucose	Hemorrhagic stroke	Zhang C, Zhou Y-H, Xu C-L, Chi F-L, Ju H-N. Efficacy of intensive control of glucose in stroke prevention: a meta-analysis of data from 59,197 participants in 9 randomized controlled trials. PloS One 2013; 8: e54465.
High fasting plasma glucose	Chronic kidney disease due to diabetes mellitus	Coca SG, Ismail-Beigi F, Haq N, Krumholz HM, Parikh CR. Role of intensive glucose control in development of renal end points in type 2 diabetes mellitus: systematic review and meta-analysis intensive glucose control in type 2 diabetes. Arch Intern Med 2012; 172: 761–9.
High fasting plasma glucose	Chronic kidney disease due to diabetes mellitus	O'Seaghdha CM, Perkovic V, Lam TH, et al. Blood Pressure Is a Major Risk Factor for Renal Death An Analysis of 560 352 Participants From the Asia-Pacific Region. Hypertension 2009; 54: 509–15.
High fasting plasma glucose	Chronic kidney disease due to hypertension	Coca SG, Ismail-Beigi F, Haq N, Krumholz HM, Parikh CR. Role of intensive glucose control in development of renal end points in type 2 diabetes mellitus: systematic review and meta-analysis intensive glucose control in type 2 diabetes. Arch Intern Med 2012; 172: 761–9.
High fasting plasma glucose	Chronic kidney disease due to hypertension	O'Seaghdha CM, Perkovic V, Lam TH, et al. Blood Pressure Is a Major Risk Factor for Renal Death An Analysis of 560 352 Participants From the Asia-Pacific Region. Hypertension 2009; 54: 509–15.
High fasting plasma glucose	Chronic kidney disease due to glomerulonephritis	Coca SG, Ismail-Beigi F, Haq N, Krumholz HM, Parikh CR. Role of intensive glucose control in development of renal end points in type 2 diabetes mellitus: systematic review and meta-analysis intensive glucose control in type 2 diabetes. Arch Intern Med 2012; 172: 761–9.
High fasting plasma glucose	Chronic kidney disease due to glomerulonephritis	O'Seaghdha CM, Perkovic V, Lam TH, et al. Blood Pressure Is a Major Risk Factor for Renal Death An Analysis of 560 352 Participants From the Asia-Pacific Region. Hypertension 2009; 54: 509–15.
High fasting plasma glucose	Chronic kidney disease due to other causes	Coca SG, Ismail-Beigi F, Haq N, Krumholz HM, Parikh CR. Role of intensive glucose control in development of renal end points in type 2 diabetes mellitus: systematic review and meta-analysis intensive glucose control in type 2 diabetes. Arch Intern Med 2012; 172: 761–9.
High fasting plasma glucose	Chronic kidney disease due to other causes	O'Seaghdha CM, Perkovic V, Lam TH, et al. Blood Pressure Is a Major Risk Factor for Renal Death An Analysis of 560 352 Participants From the Asia-Pacific Region. Hypertension 2009; 54: 509–15.
High total cholesterol	Ischaemic heart disease	Cholesterol Treatment Trialists' (CTT) Collaboration, Baigent C, Blackwell L, et al. Efficacy and safety of more intensive lowering of LDL cholesterol: a meta-analysis of data from 170,000 participants in 26 randomised trials. Lancet Lond Engl 2010; 376: 1670–81.
High total cholesterol	Ischaemic heart disease	Singh GM, Danaei G, Farzadfar F, et al. The age-specific quantitative effects of metabolic risk factors on cardiovascular diseases and diabetes: a pooled analysis. PLoS ONE 2013; 8: e65174.

Appendix Table 7. Supplemental information for Table 2: Epidemiological evidence supporting causality between risk-outcome pairs included in the Global Burden of Disease 2015 study including 7A. Citations and 7B. Additional information

7A. Citations

Risk	Outcome	Citation/Note
High total cholesterol	Ischaemic stroke	Cholesterol Treatment Trialists' (CTT) Collaboration, Baigent C, Blackwell L, et al. Efficacy and safety of more intensive lowering of LDL cholesterol: a meta-analysis of data from 170,000 participants in 26 randomised trials. Lancet Lond Engl 2010; 376: 1670–81.
High total cholesterol	Ischaemic stroke	Singh GM, Danaei G, Farzadfar F, et al. The age-specific quantitative effects of metabolic risk factors on cardiovascular diseases and diabetes: a pooled analysis. PLoS ONE 2013; 8: e65174.
High systolic blood pressure	Rheumatic heart disease	Singh GM, Danaei G, Farzadfar F, et al. The age-specific quantitative effects of metabolic risk factors on cardiovascular diseases and diabetes: a pooled analysis. PLoS ONE 2013; 8: e65174.
High systolic blood pressure	Ischaemic heart disease	Singh GM, Danaei G, Farzadfar F, et al. The age-specific quantitative effects of metabolic risk factors on cardiovascular diseases and diabetes: a pooled analysis. PLoS ONE 2013; 8: e65174.
High systolic blood pressure	Ischaemic heart disease	Thomopoulos C, Parati G, Zanchetti A. Effects of blood pressure lowering on outcome incidence in hypertension. 1. Overview, meta-analyses, and meta-regression analyses of randomized trials. J Hypertens 2014; 32: 2285–95.
High systolic blood pressure	Ischaemic stroke	Singh GM, Danaei G, Farzadfar F, et al. The age-specific quantitative effects of metabolic risk factors on cardiovascular diseases and diabetes: a pooled analysis. PLoS ONE 2013; 8: e65174.
High systolic blood pressure	Ischaemic stroke	Thomopoulos C, Parati G, Zanchetti A. Effects of blood pressure lowering on outcome incidence in hypertension. 1. Overview, meta-analyses, and meta-regression analyses of randomized trials. J Hypertens 2014; 32: 2285–95.
High systolic blood pressure	Hemorrhagic stroke	Singh GM, Danaei G, Farzadfar F, et al. The age-specific quantitative effects of metabolic risk factors on cardiovascular diseases and diabetes: a pooled analysis. PLoS ONE 2013; 8: e65174.
High systolic blood pressure	Hemorrhagic stroke	Thomopoulos C, Parati G, Zanchetti A. Effects of blood pressure lowering on outcome incidence in hypertension. 1. Overview, meta-analyses, and meta-regression analyses of randomized trials. J Hypertens 2014; 32: 2285–95.
High systolic blood pressure	Hypertensive heart disease	Singh GM, Danaei G, Farzadfar F, et al. The age-specific quantitative effects of metabolic risk factors on cardiovascular diseases and diabetes: a pooled analysis. PLoS ONE 2013; 8: e65174.
High systolic blood pressure	Hypertensive heart disease	Thomopoulos C, Parati G, Zanchetti A. Effects of blood pressure lowering on outcome incidence in hypertension. 1. Overview, meta-analyses, and meta-regression analyses of randomized trials. J Hypertens 2014; 32: 2285–95.
High systolic blood pressure	Cardiomyopathy and myocarditis	Singh GM, Danaei G, Farzadfar F, et al. The age-specific quantitative effects of metabolic risk factors on cardiovascular diseases and diabetes: a pooled analysis. PLoS ONE 2013; 8: e65174.
High systolic blood pressure	Atrial fibrillation and flutter	Emdin CA, Callender T, Cao J, Rahimi K. Effect of antihypertensive agents on risk of atrial fibrillation: a meta-analysis of large-scale randomized trials. Eur Eur Pacing Arrhythm Card Electrophysiol J Work Groups Card Pacing Arrhythm Card Cell Electrophysiol Eur Soc Cardiol 2015; 17: 701–10.
High systolic blood pressure	Atrial fibrillation and flutter	Singh GM, Danaei G, Farzadfar F, et al. The age-specific quantitative effects of metabolic risk factors on cardiovascular diseases and diabetes: a pooled analysis. PLoS ONE 2013; 8: e65174.
High systolic blood pressure	Aortic aneurysm	Singh GM, Danaei G, Farzadfar F, et al. The age-specific quantitative effects of metabolic risk factors on cardiovascular diseases and diabetes: a pooled analysis. PLoS ONE 2013; 8: e65174.
High systolic blood pressure	Peripheral vascular disease	Singh GM, Danaei G, Farzadfar F, et al. The age-specific quantitative effects of metabolic risk factors on cardiovascular diseases and diabetes: a pooled analysis. PLoS ONE 2013; 8: e65174.
High systolic blood pressure	Endocarditis	Singh GM, Danaei G, Farzadfar F, et al. The age-specific quantitative effects of metabolic risk factors on cardiovascular diseases and diabetes: a pooled analysis. PLoS ONE 2013; 8: e65174.
High systolic blood pressure	Other cardiovascular and circulatory diseases	Singh GM, Danaei G, Farzadfar F, et al. The age-specific quantitative effects of metabolic risk factors on cardiovascular diseases and diabetes: a pooled analysis. PLoS ONE 2013; 8: e65174.
High systolic blood pressure	Chronic kidney disease due to diabetes mellitus	The Renal Risk Collaboration, Foote C, Lin J, et al. The effect of Blood Pressure on Kidney Failure: a systematic review and meta-analysis in 2.7 million participants (unpublished).
High systolic blood pressure	Chronic kidney disease due to diabetes mellitus	Xie X, Atkins E, Lv J, et al. Effects of intensive blood pressure lowering on cardiovascular and renal outcomes: updated systematic review and meta-analysis. Lancet Lond Engl 2016; 387: 435–43.
High systolic blood pressure	Chronic kidney disease due to hypertension	The Renal Risk Collaboration, Foote C, Lin J, et al. The effect of Blood Pressure on Kidney Failure: a systematic review and meta-analysis in 2.7 million participants (unpublished).
High systolic blood pressure	Chronic kidney disease due to hypertension	Xie X, Atkins E, Lv J, et al. Effects of intensive blood pressure lowering on cardiovascular and renal outcomes: updated systematic review and meta-analysis. Lancet Lond Engl 2016; 387: 435–43.
High systolic blood pressure	Chronic kidney disease due to glomerulonephritis	The Renal Risk Collaboration, Foote C, Lin J, et al. The effect of Blood Pressure on Kidney Failure: a systematic review and meta-analysis in 2.7 million participants (unpublished).
High systolic blood pressure	Chronic kidney disease due to glomerulonephritis	Xie X, Atkins E, Lv J, et al. Effects of intensive blood pressure lowering on cardiovascular and renal outcomes: updated systematic review and meta-analysis. Lancet Lond Engl 2016; 387: 435–43.
High systolic blood pressure	Chronic kidney disease due to other causes	The Renal Risk Collaboration, Foote C, Lin J, et al. The effect of Blood Pressure on Kidney Failure: a systematic review and meta-analysis in 2.7 million participants (unpublished).
High systolic blood pressure	Chronic kidney disease due to other causes	Xie X, Atkins E, Lv J, et al. Effects of intensive blood pressure lowering on cardiovascular and renal outcomes: updated systematic review and meta-analysis. Lancet Lond Engl 2016; 387: 435–43.
High body-mass index	Oesophageal cancer	Renehan AG, Tyson M, Egger M, Heller RF, Zwahlen M. Body-mass index and incidence of cancer: a systematic review and meta-analysis of prospective observational studies. Lancet 2008; 371: 569–78.
High body-mass index	Colon and rectum cancer	Karahalios A, English DR, Simpson JA. Weight change and risk of colorectal cancer: a systematic review and meta-analysis. Am J Epidemiol 2015; 181: 832–45.
High body-mass index	Colon and rectum cancer	Renehan AG, Tyson M, Egger M, Heller RF, Zwahlen M. Body-mass index and incidence of cancer: a systematic review and meta-analysis of prospective observational studies. Lancet 2008; 371: 569–78.
High body-mass index	Colon and rectum cancer	Schlesinger S, Lieb W, Koch M, et al. Body weight gain and risk of colorectal cancer: a systematic review and meta-analysis of observational studies. Obes Rev 2015; 16: 607–19.
High body-mass index	Liver cancer	Chen Y, Wang X, Wang J, Yan Z, Luo J. Excess body weight and the risk of primary liver cancer: an updated meta-analysis of prospective studies. Eur J Cancer 2012; 48: 2137–45.
High body-mass index	Liver cancer	Renehan AG, Tyson M, Egger M, Heller RF, Zwahlen M. Body-mass index and incidence of cancer: a systematic review and meta-analysis of prospective observational studies. Lancet 2008; 371: 569–78.
High body-mass index	Liver cancer	Rui R, Lou J, Zou L, et al. Excess body mass index and risk of liver cancer: a nonlinear dose-response meta-analysis of prospective studies. PLoS ONE 2012; 7: e44522.

Appendix Table 7. Supplemental information for Table 2: Epidemiological evidence supporting causality between risk-outcome pairs included in the Global Burden of Disease 2015 study including 7A. Citations and 7B. Additional information

7A. Citations

Risk	Outcome	Citation/Note
High body-mass index	Liver cancer	Tanaka K, Tsuji I, Tamakoshi A, et al. Obesity and liver cancer risk: an evaluation based on a systematic review of epidemiologic evidence among the Japanese population. Jpn J Clin Oncol 2012; 42: 212–21.
High body-mass index	Liver cancer	Wang Y, Wang B, Shen F, Fan J, Cao H. Body mass index and risk of primary liver cancer: a meta-analysis of prospective studies. Oncologist 2012; 17: 1461–8.
High body-mass index	Gallbladder and biliary tract cancer	Park M, Song DY, Je Y, Lee JE. Body mass index and biliary tract disease: a systematic review and meta-analysis of prospective studies. Prev Med 2014; 65: 13–22.
High body-mass index	Gallbladder and biliary tract cancer	Renehan AG, Tyson M, Egger M, Heller RF, Zwahlen M. Body-mass index and incidence of cancer: a systematic review and meta-analysis of prospective observational studies. Lancet 2008; 371: 569–78.
High body-mass index	Pancreatic cancer	Alsamarrai A, Das SLM, Windsor JA, Petrov MS. Factors that affect risk for pancreatic disease in the general population: a systematic review and meta-analysis of prospective cohort studies. Clin Gastroenterol Hepatol 2014; 12: 1635–1644.e5; quiz e103.
High body-mass index	Pancreatic cancer	Renehan AG, Tyson M, Egger M, Heller RF, Zwahlen M. Body-mass index and incidence of cancer: a systematic review and meta-analysis of prospective observational studies. Lancet 2008; 371: 569–78.
High body-mass index	Breast cancer (Pre-menopause)	Renehan AG, Tyson M, Egger M, Heller RF, Zwahlen M. Body-mass index and incidence of cancer: a systematic review and meta-analysis of prospective observational studies. Lancet 2008; 371: 569–78.
High body-mass index	Breast cancer (Pre-menopause)	Xia X, Chen W, Li J, et al. Body mass index and risk of breast cancer: a nonlinear dose-response meta-analysis of prospective studies. Sci Rep 2014; 4: 7480.
High body-mass index	Breast cancer (Post-menopause)	Renehan AG, Tyson M, Egger M, Heller RF, Zwahlen M. Body-mass index and incidence of cancer: a systematic review and meta-analysis of prospective observational studies. Lancet 2008; 371: 569–78.
High body-mass index	Breast cancer (Post-menopause)	Xia X, Chen W, Li J, et al. Body mass index and risk of breast cancer: a nonlinear dose-response meta-analysis of prospective studies. Sci Rep 2014; 4: 7480.
High body-mass index	Uterine cancer	Aune D, Greenwood DC, Chan DSM, et al. Body mass index, abdominal fatness and pancreatic cancer risk: a systematic review and non-linear dose-response meta-analysis of prospective studies. Ann Oncol 2012; 23: 843–52.
High body-mass index	Uterine cancer	Jenabi E, Poorolajal J. The effect of body mass index on endometrial cancer: a meta-analysis. Public Health 2015; 129: 872–80.
High body-mass index	Uterine cancer	Renehan AG, Tyson M, Egger M, Heller RF, Zwahlen M. Body-mass index and incidence of cancer: a systematic review and meta-analysis of prospective observational studies. Lancet 2008; 371: 569–78.
High body-mass index	Ovarian cancer	Aune D, Navarro Rosenblatt DA, Chan DSM, et al. Anthropometric factors and ovarian cancer risk: a systematic review and nonlinear dose-response meta-analysis of prospective studies. Int J Cancer 2015; 136: 1888–98.
High body-mass index	Ovarian cancer	Collaborative Group on Epidemiological Studies of Ovarian Cancer. Ovarian cancer and body size: individual participant meta-analysis including 25,157 women with ovarian cancer from 47 epidemiological studies. PLoS Med 2012; 9: e1001200.
High body-mass index	Ovarian cancer	Liu Z, Zhang T-T, Zhao J-J, et al. The association between overweight, obesity and ovarian cancer: a meta-analysis. Jpn J Clin Oncol 2015; 45: 1107–15.
High body-mass index	Ovarian cancer	Renehan AG, Tyson M, Egger M, Heller RF, Zwahlen M. Body-mass index and incidence of cancer: a systematic review and meta-analysis of prospective observational studies. Lancet 2008; 371: 569–78.
High body-mass index	Kidney cancer	Renehan AG, Tyson M, Egger M, Heller RF, Zwahlen M. Body-mass index and incidence of cancer: a systematic review and meta-analysis of prospective observational studies. Lancet 2008; 371: 569–78.
High body-mass index	Kidney cancer	Wang F, Xu Y. Body mass index and risk of renal cell cancer: a dose-response meta-analysis of published cohort studies. Int J Cancer 2014; 135: 1673–86.
High body-mass index	Thyroid cancer	Ma J, Huang M, Wang L, Ye W, Tong Y, Wang H. Obesity and risk of thyroid cancer: evidence from a meta-analysis of 21 observational studies. Med Sci Monit 2015; 21: 283–91.
High body-mass index	Thyroid cancer	Renehan AG, Tyson M, Egger M, Heller RF, Zwahlen M. Body-mass index and incidence of cancer: a systematic review and meta-analysis of prospective observational studies. Lancet. 2008; 371(9612): 569-78.
High body-mass index	Leukaemia	Castillo JJ, Reagan JL, Ingham RR, et al. Obesity but not overweight increases the incidence and mortality of leukemia in adults: a meta-analysis of prospective cohort studies. Leuk Res 2012; 36: 868–75.
High body-mass index	Leukaemia	Renehan AG, Tyson M, Egger M, Heller RF, Zwahlen M. Body-mass index and incidence of cancer: a systematic review and meta-analysis of prospective observational studies. Lancet. 2008; 371(9612): 569-78.
High body-mass index	Ischaemic heart disease	Singh GM, Danaei G, Farzadfar F, et al. The age-specific quantitative effects of metabolic risk factors on cardiovascular diseases and diabetes: a pooled analysis. PLoS ONE 2013; 8: e65174.
High body-mass index	Cerebrovascular disease	Singh GM, Danaei G, Farzadfar F, et al. The age-specific quantitative effects of metabolic risk factors on cardiovascular diseases and diabetes: a pooled analysis. PLoS ONE 2013; 8: e65174.
High body-mass index	Hypertensive heart disease	Singh GM, Danaei G, Farzadfar F, et al. The age-specific quantitative effects of metabolic risk factors on cardiovascular diseases and diabetes: a pooled analysis. PLoS ONE 2013; 8: e65174.
High body-mass index	Diabetes mellitus	Singh GM, Danaei G, Farzadfar F, et al. The age-specific quantitative effects of metabolic risk factors on cardiovascular diseases and diabetes: a pooled analysis. PLoS ONE 2013; 8: e65174.
High body-mass index	Chronic kidney disease	Emerging Risk Factors Collaboration, Wormser D, Kaptoge S, et al. Separate and combined associations of body-mass index and abdominal adiposity with cardiovascular disease: collaborative analysis of 58 prospective studies. Lancet 2011; 377: 1085–95.
High body-mass index	Chronic kidney disease	Ni Mhurchu C, Rodgers A, Pan WH, Gu DF, Woodward M, Asia Pacific Cohort Studies Collaboration. Body mass index and cardiovascular disease in the Asia-Pacific Region: an overview of 33 cohorts involving 310 000 participants. Int J Epidemiol 2004; 33: 751–8.
High body-mass index	Chronic kidney disease	Prospective Studies Collaboration, Whitlock G, Lewington S, et al. Body-mass index and cause-specific mortality in 900 000 adults: collaborative analyses of 57 prospective studies. Lancet 2009; 373: 1083–96.
High body-mass index	Osteoarthritis	Jiang L, Rong J, Wang Y, et al. The relationship between body mass index and hip osteoarthritis: a systematic review and meta-analysis. Joint Bone Spine 2011; 78: 150–5.

Appendix Table 7. Supplemental information for Table 2: Epidemiological evidence supporting causality between risk-outcome pairs included in the Global Burden of Disease 2015 study including 7A. Citations and 7B. Additional information

7A. Citations

Risk	Outcome	Citation/Note
High body-mass index	Osteoarthritis	Jiang L, Tian W, Wang Y, et al. Body mass index and susceptibility to knee osteoarthritis: a systematic review and meta-analysis. Joint Bone Spine 2012; 79: 291–7.
High body-mass index	Osteoarthritis	Silverwood V, Blagojevic-Bucknall M, Jinks C, Jordan JL, Protheroe J, Jordan KP. Current evidence on risk factors for knee osteoarthritis in older adults: a systematic review and meta-analysis. Osteoarthr Cartil 2015; 23: 507–15.
High body-mass index	Low back pain	Shiri R, Karppinen J, Leino-Arjas P, Solovieva S, Viikari-Juntura E. The association between obesity and low back pain: a meta-analysis. Am J Epidemiol 2010; 171: 135–54.
Low bone mineral density	Injuries	Johnell O, Kanis JA, Oden A, et al. Predictive value of BMD for hip and other fractures. J Bone Miner Res 2005; 20: 1185–94.
Low glomerular filtration rate	Ischaemic heart disease	Chronic Kidney Disease Prognosis Consortium, Matsushita K, van der Velde M, et al. Association of estimated glomerular filtration rate and albuminuria with all-cause and cardiovascular mortality in general population cohorts: a collaborative meta-analysis. Lancet 2010; 375: 2073–81.
Low glomerular filtration rate	Cerebrovascular disease	Fox CS, Matsushita K, Woodward M, et al. Associations of kidney disease measures with mortality and end-stage renal disease in individuals with and without diabetes: a meta-analysis. Lancet 2012; 380: 1662–73.
Low glomerular filtration rate	Peripheral vascular disease	O'Hare AM, Vittinghoff E, Hsia J, Shlipak MG. Renal insufficiency and the risk of lower extremity peripheral arterial disease: results from the Heart and Estrogen/Progestin Replacement Study (HERS). J Am Soc Nephrol 2004; 15: 1046–51.
Low glomerular filtration rate	Gout	Cea Soriano L, Rothenbacher D, Choi HK, García Rodríguez LA. Contemporary epidemiology of gout in the UK general population. Arthritis Res Ther 2011; 13: R39.
Low glomerular filtration rate	Gout	Krishnan E. Chronic kidney disease and the risk of incident gout among middle-aged men: a seven-year prospective observational study. Arthritis Rheum 2013; 65: 3271–8.
Low glomerular filtration rate	Gout	McAdams-DeMarco MA, Maynard JW, Baer AN, Coresh J. Hypertension and the risk of incident gout in a population-based study: the atherosclerosis risk in communities cohort. J Clin Hypertens (Greenwich) 2012; 14: 675–9.
Low glomerular filtration rate	Gout	Trifirò G, Morabito P, Cavagna L, et al. Epidemiology of gout and hyperuricaemia in Italy during the years 2005-2009: a nationwide population-based study. Ann Rheum Dis 2013; 72: 694–700.

**Appendix Table 7. Supplemental information for Table 2: Epidemiological evidence supporting causality between risk-outcome pairs included in the Global Burden of Disease 2015 study including 7A. Citations and 7B. Additional information**		
**7A. Citations**		
**Risk**	**Outcome**	**Citation/Note**
**7B. Supplemental information**		

"RCTs (Number)" represents the total number of independent randomized controlled trials evaluating the relationship of each risk-outcome pair. "RCTs with significant effect in the opposite direction (%)" represents the percentage of randomized controlled trials showing a significant effect in the opposite direction. "Prospective observational studies (Number)" shows the total number of independent prospective cohort studies or non-randomized interventions evaluating the relationship of the risk-outcome pair. "Prospective observational studies with significant association in the opposite direction (%)" represents the percentage of prospective cohort studies or non-randomized interventions reporting a significant association in the opposite direction. "Lower limit of RR > 1.5" shows whether the lower limit of the 95% confidence interval for the relative risk of the risk-outcome pair is greater than 1.5. "Dose-response relationship" shows whether there is any evidence of linear or non-linear dose-response relationship between the risk and the outcome. "Biologic plausibility" shows whether there is any biologic or mechanistic pathway that could potentially explain the relationship of the risk-outcome pair. "Analogy" shows whether the risk is associated with another outcome from the same category and there is evidence that it can cause the current outcome through the same pathway. The numbers in the table represent the independent RCTs and prospective observational studies evaluated the relationship between each risk-outcome pairs. If there were multiple reports from one study, they were counted as one study. Dose-response relationship was only assessed for continuous risks. To evaluate the magnitude of the effect size for continuous risks, we evaluated the RR comparing the 75th percentile to the 25th percentile of the exposure distribution at the global level .

Unsafe water, sanitation, and handwashing	Typhoid and paratyphoid fever	Typhoid and paratyphoid were included as outcome for unsafe water and sanitation by analogy to diarrhoeal diseases
Household air pollution from solid fuels	Cataract	Evidence on the relationship between household air pollution and cataract was from 6 case-control and 1 cross-sectional studies
Air pollution	--	The relationships of cerebrovascular disease, chronic obstructive pulmonary disease, ischaemic heart disease, and lung cancer with ambient air pollution, second-hand smoke, and active smoking were used to interpolate their relationship with household air pollution. We considered the biological pathway for health impact of all four sources to be PM2.5 exposure, with the effect size being a function of the level of PM2.5. As such, we presented data from cohorts reporting on ambient PM2.5 and the outcome was used to inform the strength of evidence for household air pollution.
Other environmental risks and dietary risks	Cardiovascular diseases and chronic kidney disease.	The health effects of lead and sodium on cardiovascular outcomes and chronic kidney disease were assessed through systolic blood pressure and the health effects of sugar sweetened beverages were assessed through body mass index.
Residential Radon	Tracheal, bronchus, and lung cancer	In evaluation of evidence on the relationship of residential radon and lung cancer, we excluded evidence from cohorts of miners as they were not from a representative population. Evidence on this risk-outcome pair mostly comes from case-control studies
Occupational injuries	Injuries	Evidence from International Labour Organization Safety and Health and Eurostat Safety and Health was used to establish causality between occupational injuries and injuries
Child and maternal malnutrition	--	Evidence on the causal relationship of childhood stunting, underweight, and wasting was from a pooled analysis of 7 prospective cohorts
Child and maternal malnutrition	--	For the following risk-outcome pairs, the risk factor was considered as the necessary cause: childhood underweight and protein-energy malnutrition; childhood wasting and protein-energy malnutrition; vitamin A deficiency and vitamin A deficiency; alcohol use and cirrhosis due to alcohol use; alcohol use and alcohol use disorders; alcohol use and liver cancer due to alcohol use; drug use and amphetamine use disorders; drug use and cannabis use disorders; drug use and cocaine use disorders; drug use and opioid use disorders; drug use and other drug use disorders; iron deficiency and iron-deficiency anemia; unsafe sex and cervical cancer; unsafe sex and syphilis; unsafe sex and chlamydial infection; unsafe sex and gonococcal infection; unsafe sex and trichomoniasis; unsafe sex and genital herpes; unsafe sex and other sexually transmitted diseases; high systolic blood pressure and hypertensive heart disease; high systolic blood pressure and chronic kidney disease due to hypertension; high fasting plasma glucose and chronic kidney disease due to diabetes mellitus; high fasting plasma glucose and diabetes mellitus; low glomerular filtration rate and chronic kidney disease
Iron deficiency	Maternal haemorrhage	Evidence on the relationship of iron deficiency with maternal haemorrhage and maternal sepsis mainly came 10 observational studies evaluating the association between low hemoglobin and maternal mortality using hospital records
Smoking, alcohol use, and high body mass index	--	For smoking, alcohol use, and high body mass index evidence from risk reduction trials has not been included
Smoking, alcohol use, and high body mass index	Liver cancer	Liver cancer included liver cancer due to alcohol use, hepatitis B, hepatitis C, and other causes
Smoking	Lower respiratory infections	Evidence on the relationship between smoking and lower respiratory infections comes 10 case-control or cross-sectional studies
Smoking, alcohol use	Nasopharynx cancer	The evidence on causal relationship of alcohol and smoking with nasopharynx cancer was from the studies evaluating oral cavity and pharyngeal cancers as outcome
Smoking	Bladder cancer	The evidence on causal relationship of smoking and bladder cancer was based on the studies evaluating the lower urinary tract as outcome
Smoking	Asbestosis	Asbestosis, coal workers pneumoconiosis, other pneumoconiosis, silicosis were included as outcomes for smoking as they were included in the other chronic respiratory diseases category
Alcohol use	Ischaemic heart disease, cerebrovascular disease, hypertensive heart disease, and diabetes mellitus	Alcohol was included as both a protective and harmful risk factor for ischaemic heart disease, cerebrovascular disease, hypertensive heart disease, and diabetes mellitus
Alcohol use	Cirrhosis	Cirrhosis included cirrhosis due to alcohol use, hepatitis B, hepatitis C, and other causes
Alcohol use	Self-harm	Self-harm was included as an outcome for alcohol use by analogy to injury
Alcohol use	Injuries	Injuries included pedestrian road injuries, cyclist road injuries, motorcyclist road injuries, motor vehicle road injuries, drowning, falls, fire, heat, hot substances, poisonings, unintentional firearm injuries, unintentional suffocation, other exposure to mechanical forces
Alcohol use	Interpersonal violence	Interpersonal violence included assault by firearm, sharp object, other means
Diet low in nuts and seeds	Ischaemic heart disease and diabetes mellitus	Experimental evidence on the relationship of nuts with ischaemic heart disease and diabetes mellitus come from the PREDIMED trial; a randomized trial consisting of three arms: a Mediterranean diet with extra-virgin olive oil, a Mediterranean diet with nuts, and a control diet. Given that the intake of dietary factors other than nuts changed in the intervention arms of this trial, the observed effect might be fully attributable to nuts.

Appendix Table 7. Supplemental information for Table 2: Epidemiological evidence supporting causality between risk-outcome pairs included in the Global Burden of Disease 2015 study including 7A. Citations and 7B. Additional information

7A. Citations

Risk	Outcome	Citation/Note
Diet high in sugar sweetened beverages and body mass index	--	Evidence on the relationship between sugar-sweetened beverages and body mass index comes from the interventional and prospective observational studies evaluating the relationship of sugar-sweetened beverages with weight change
Diet high in sodium	Cardiovascular diseases	Evidence on the direct effect of sodium on cardiovascular disease mainly comes from prospective cohort studies. Considering that, in GBD, we have only evaluated the effect of sodium mediated through systolic blood pressure, we did not present epidemiologic evidence on the direct effect of sodium on cardiovascular disease in this table. Evidence on the effect of sodium on systolic blood pressure mostly comes from randomized controlled trials. While some cohort studies evaluated the relationship between sodium and systolic blood pressure, we did not identify a systematic evaluation of these studies.
Drug use	Hepatitis B and C	We included liver cancer due to Hepatitis B and Hepatitis C and cirrhosis due to Hepatitis B and Hepatitis C as outcomes for drug use because these were considered secondary outcomes of Hepatitis B and Hepatitis C.
Drug use, unsafe sex	HIV/AIDS	For the following risk-outcome pairs, the risk factor was considered as the sufficient cause: drug use and HIV/AIDS and unsafe sex and HIV/AIDS
Metabolic risks	Chronic kidney disease	Chronic kidney disease included chronic kidney disease due to diabetes mellitus, hypertension, glomerulonephritis, or
High fasting plasma glucose	Cerebrovascular disease, chronic kidney disease, ischaemic heart disease	Evidence on the relationship of high fasting plasma glucose with stroke (DECODE, APCSC, ERFC); chronic kidney disease (APCSC), and ischaemic heart disease (DECODE, APCSC, ERFC) was from pooled analysis of cohorts
High systolic blood pressure	Atrial fibrillation and flutter, peripheral vascular disease	Evidence on the relationship of high systolic blood pressure with atrial fibrillation and peripheral vascular disease was from two pooled cohort analysis (APCSC and PSC)
High systolic blood pressure	Rheumatic heart disease, cardiomyopathy and myocarditis, aortic aneurysm, endocarditis, and other cardiovascular diseases	Evidence on the relationship of high systolic blood pressure with rheumatic heart disease, cardiomyopathy and myocarditis, aortic aneurysm, endocarditis, and other cardiovascular diseases came from a pooled cohort analysis (PSC)
High body-mass index	Ischaemic heart disease	Evidence on the relationship of high body-mass index with ischaemic heart disease (APCSC, ERFC, PSC) and stroke (ischaemic: APCSC, ERFC, PSC; hemorrhagic: PSC and ERFC) came from three pooled cohort analysis
High body-mass index	Diabetes mellitus, hypertensive heart disease	Evidence on the relationship of high body-mass index with diabetes mellitus and hypertensive heart disease came from two pooled cohort analysis (APCSC and PSC)
High body-mass index	Chronic kidney disease	Evidence on the relationship of high body-mass index with chronic kidney disease was from a pooled cohort analysis (PSC)
High total cholesterol	Ischaemic heart disease, ischaemic stroke	Evidence on the relationship of high total cholesterol with ischaemic heart disease and ischaemic stroke came from two pooled cohort analysis (APCSC and PSC)
